# AMPing Up the Search: A Structural and Functional Repository of Antimicrobial Peptides for Biofilm Studies, and a Case Study of Its Application to *Corynebacterium striatum*, an Emerging Pathogen

**DOI:** 10.3389/fcimb.2021.803774

**Published:** 2021-12-16

**Authors:** Shreeya Mhade, Stutee Panse, Gandhar Tendulkar, Rohit Awate, Yatindrapravanan Narasimhan, Snehal Kadam, Ragothaman M. Yennamalli, Karishma S. Kaushik

**Affiliations:** ^1^ Department of Bioinformatics, Guru Nanak Khalsa College of Arts, Science and Commerce (Autonomous), Mumbai, India; ^2^ Huck Institutes of Life Sciences, The Pennsylvania State University, University Park, College State, PA, United States; ^3^ Department of Bioinformatics, Sir Sitaram and Lady Shantabai Patkar College of Arts and Science and V P Varde College of Commerce and Economics (Autonomous), Mumbai, India; ^4^ Khoury College of Computer Sciences, Northeastern University, Boston, MA, United States; ^5^ Department of Bioinformatics, School of Chemical and Biotechnology, Shanmugha Arts, Science, Technology & Research Academy (SASTRA), Deemed to Be University, Thanjavur, India; ^6^ Hull York Medical School, University of Hull, Hull, United Kingdom; ^7^ Department of Biotechnology, Savitribai Phule Pune University, Pune, India

**Keywords:** antimicrobial peptides, biofilms, structural repository, functional annotation, curated database, molecular modeling, molecular docking

## Abstract

Antimicrobial peptides (AMPs) have been recognized for their ability to target processes important for biofilm formation. Given the vast array of AMPs, identifying potential anti-biofilm candidates remains a significant challenge, and prompts the need for preliminary *in silico* investigations prior to extensive *in vitro* and *in vivo* studies. We have developed Biofilm-AMP (B-AMP), a curated 3D structural and functional repository of AMPs relevant to biofilm studies. In its current version, B-AMP contains predicted 3D structural models of 5544 AMPs (from the DRAMP database) developed using a suite of molecular modeling tools. The repository supports a user-friendly search, using source, name, DRAMP ID, and PepID (unique to B-AMP). Further, AMPs are annotated to existing biofilm literature, consisting of a vast library of over 10,000 articles, enhancing the functional capabilities of B-AMP. To provide an example of the usability of B-AMP, we use the sortase C biofilm target of the emerging pathogen *Corynebacterium striatum* as a case study. For this, 100 structural AMP models from B-AMP were subject to *in silico* protein-peptide molecular docking against the catalytic site residues of the *C. striatum* sortase C protein. Based on docking scores and interacting residues, we suggest a preference scale using which candidate AMPs could be taken up for further *in silico*, *in vitro* and *in vivo* testing. The 3D protein-peptide interaction models and preference scale are available in B-AMP. B-AMP is a comprehensive structural and functional repository of AMPs, and will serve as a starting point for future studies exploring AMPs for biofilm studies. B-AMP is freely available to the community at https://b-amp.karishmakaushiklab.com and will be regularly updated with AMP structures, interaction models with potential biofilm targets, and annotations to biofilm literature.

## Introduction

Antimicrobial peptides (AMPs) are a diverse class of peptides with a wide range of inhibitory effects on bacteria and fungi (Mahlapuu et al., 2016). In addition to disrupting membrane integrity, AMPs can also target specific intracellular components, and thereby inhibit specific bacterial processes (Chung and Khanum, 2017; Le et al., 2017; Zhejun et al., 2017; Jianfeng et al., 2018; Muhammad et al., 2018; Benfield and Henriques, 2020). Biofilms or multicellular microbial aggregates are associated with serious infection states and display increased tolerance to conventional antibiotics, prompting the need to expand therapeutic options (Hoiby et al., 2010). Biofilm formation is initiated by the attachment of individual cells or small aggregates on a biotic or abiotic surface (Palmer et al., 2007). This initial attachment is typically mediated by microbial adhesions, including cell-surface pili. Following attachment, microbial cells secrete an extracellular matrix, and proliferate to form the three-dimensional, heterogeneous biofilm structure (Garrett et al., 2008). Mature biofilms disperse by the release of single cells or small clusters of cells, which can seed new surfaces (Kaplan, 2010). The evaluation of AMPs as potential anti-biofilm approaches across the different stages of biofilm formation holds promise (Muhammad et al., 2018; Di Somma et al., 2020; Hancock et al., 2021). However, the vast and expanding array of AMPs makes the identification of potential candidates for anti-biofilm testing a challenge (Bahar and Ren, 2013; Muhammad et al., 2018; Galdiero et al., 2019). This is particularly relevant with respect to *in vitro* and *in vivo* biofilm studies, which are time-, labor-, and resource-intensive. Given this, preliminary *in silico* investigations can help identify candidate AMPs with anti-biofilm potential, and thereby enable a steady pipeline of AMPs for *in vitro* and *in vivo* testing (Lüthy et al., 1992; Agrawal et al., 2018; Oyama et al., 2019; Atanaki et al., 2020; An et al., 2021; Singh et al., 2021). For this, building organized sets of resources relevant to *in silico* AMP studies against biofilms is both necessary and important.

We present Biofilm-AMP (B-AMP), a manually-curated structural and functional repository of AMPs, with a special focus on AMPs for biofilm studies. B-AMP contains predicted 3D structural models of a diverse array of 5544 AMPs (from the DRAMP database) built using a combination of *in silico* peptide modeling tools. To enhance the functional capabilities of B-AMP, AMPs are annotated to a vast library of existing biofilm literature which includes information on source or synthesis of the AMP, experimental testing, and degree and nature of anti-biofilm activity. To provide an example of the feasibility of B-AMP for *in silico* evaluation of AMPs with anti-biofilm potential, we present a case study using the emerging pathogen *Corynebacterium striatum*. *C. striatum* is a multidrug resistant, biofilm-forming pathogen, increasingly associated with a range of wound, skin and eye infections (Watkins et al., 1993; Wong et al., 2010; Alibi et al., 2017; McMullen et al., 2017; Nudel et al., 2018; Datta et al., 2021). In *C. striatum*, the sortase-pilin machinery is known to be important for biofilm formation, encoding the pilus-specific sortase C enzyme (de Souza et al., 2015). From the B-AMP structural library, select AMPs with known anti-Gram positive activity were evaluated for their ability to interact with catalytic site residues of the sortase C protein using *in silico* molecular docking. Docking scores and interacting residues were used to develop a preference scale to categorize AMPs for future *in vitro* and *in vivo* testing against *C. striatum* biofilms. While our study focuses on *C. striatum*, the approach we present, considerations described, and resources available in the B-AMP repository can be leveraged for similar investigations across other biofilm targets and biofilm-forming pathogens.

## Methods

### 3D Predictive Modeling of AMP Sequences From the DRAMP Database

From DRAMP V3.0 (Kang et al., 2019; Shi et al., 2021), we downloaded a dataset of 5562 AMP sequences, with known antibacterial, antifungal and antibiofilm activities. These AMPs were listed as Pep2 to Pep5563, where Pep1 is the LPXTG motif of the pilin subunit of *C. striatum* (GenPept ID: WP_170219081.1) (SpaH/EbpB family LPXTG-anchored major pilin [Corynebacterium striatum] - Protein - NCBI). FASTA files were generated using an in-house Python script. The Python script reads the.csv file from the DRAMP database and generates FASTA files that were used for predictive modeling using PEP-FOLD3, I-TASSER, and Rosetta algorithms. For modeling AMPs with less than 50 amino acid residues, we used PEP-FOLD3 (Lamiable et al., 2016), which predicts 3D structures from the sequence information using a *de novo* approach. The FASTA file for the structure to be generated was uploaded in the query form, following which a structure for the given sequence was generated and saved in the pdb format. For modeling AMPs with more than 50 amino acids, we used the deep-learning-based method TrRosetta (Yang et al., 2020) available under Robetta (Robetta: Homepage). The FASTA files of the AMP were uploaded in the query submission interface and the resulting model was obtained in pdb format. For AMPs with unusual or unknown (X) amino acids, we used the *ab-initio* modeling feature of I-TASSER (I-TASSER server for protein structure and function prediction; Roy et al., 2010). The FASTA files of the AMP sequence were uploaded into the query submission form using the default parameters, and the resulting models were obtained in pdb format. Predicted and chosen models were selected based on cluster ranking and scoring.

### AMP Annotations to Existing Literature Sources

We mined PubMed using keyword-based searches *via* an automated script for application programming interface (API) mode of data retrieval (https://b-amp.karishmakaushiklab.com/code.html). The search was designed to find the keyword ‘biofilm’ in either the title or the abstract of the scientific paper. This includes papers with terminologies such as ‘anti biofilm’, ‘anti-biofilm’, ‘against biofilm’, and ‘biofilm control’. Relevant literature was extracted and compiled as part of the B-AMP repository. The literature data was further culled to identify the most relevant literature hits for each AMP.

### Building a Structural and Functional Repository of AMPs for Biofilm Studies

To enable data sharing and open-science, we developed a user-friendly and easily searchable repository of predicted 3D AMP structures, annotations to relevant literature, and predicted AMP interactions with biofilm targets (B-AMP) (https://b-amp.karishmakaushiklab.com/). The repository was built using HTML/CSS/JavaScript delivered over GitHub pages. The scripts and other resources employed can be found here (https://github.com/KarishmaKaushikLab/B-AMP).

### Homology Modeling of the *C. striatum* Sortase C Protein

Given that the experimentally derived 3D structure of the sortase C protein from *C. striatum* is not available in the RCSB Protein Data Bank (PDB) (RCSB PDB: Homepage), we used homology modeling to develop a molecular model of the protein. The amino acid sequence of the sortase C protein was retrieved from the GenPept Database (ID: WP_034656562) (Class C sortase [Corynebacterium striatum] - Protein - NCBI). The physicochemical properties of the protein such as molecular weight, isoelectric point (pI), amino acid composition, estimated half-life, and instability index were determined using the ProtParam tool (ExPASy - ProtParam tool). The secondary structure of the sortase C protein was predicted by the PSIPRED server (PSIPRED Workbench). The 3D structure of the C sortase enzyme was constructed using I-TASSER (Roy et al., 2010), by submitting the FASTA sequence without any user-specific restraints. To mimic the semi-open lid conformation of the *C. striatum* sortase C protein, we generated a model that included a double mutation D93A/W95A (replacing Aspartate (93) and Tryptophan (95) with Alanine) in the protein (Jacobitz et al., 2017; Khare and Narayana, 2017).

### Validation of the 3D Models of the *C. striatum* Sortase C Protein

The predicted sortase C protein models, including the modified protein model with semi-open lid conformation, were verified using SAVES 6.0 (Bowie et al., 1991; Lüthy et al., 1992; Colovos and Yeates, 1993). The PDB file generated from I-TASSER was submitted as an input to SAVES 6.0. The quality and reliability of the generated model were checked by using the default quality parameters of the ERRAT and VERIFY3D software. In addition to the above, PROCHECK software was used to validate the generated models by generating Ramachandran plots.

### *In Silico* Molecular Docking of AMP Structural Models to the *C. striatum* Sortase C Protein

AMPs with annotated anti-Gram positive activity (based on information in DRAMP; (Kang et al., 2019; Shi et al., 2021) were filtered from B-AMP using an in-house developed Python script (https://github.com/KarishmaKaushikLab/B-AMP). The Python script employs a conditional statement to automatically scan the.csv file for the term “Anti-Gram+” from the activity column in the sheet. From this filtered subset, 88 AMPs ranging 2 to 8 residues in length, and 12 AMPs ranging 9 to 20 residues, were selected for *in silico* molecular docking against the semi-open lid conformation of the sortase C protein (D93A/W95A). Prior to molecular docking, the 3D AMP structures were subject to standard energy minimization in the Swiss-PDBViewer SPDBV v4.1.0 (Guex and Peitsch, 1997) using the partial implementation of the GROMOS96 43B1 force field (GROMOS – Group for Computer-Aided Chemistry | ETH Zurich; van Gunsteren, 1996). We also performed the molecular docking of the standard LPMTG pilin subunit motif against the semi-open lid conformation of the sortase C protein as a positive control. This would enable establishing the veracity of the docking method and parameters employed, and also help identify good binders among the various AMPs. AutoDockTool1.5.6 (Morris et al., 2009) was used to generate the necessary input PDBQT files for docking and AutoDock Vina (Trott and Olson, 2010) was used to perform the *in silico* molecular docking. Specifically, we used a 3D grid centered on the active site of the sortase C protein with a dimension of 28x28x28 Å^3^. AutoDock Vina uses a heuristic method of determining the time spent on finding the optimal confirmation of a ligand docked to a rigid protein molecule. Therefore, depending on the number of atoms, the number of rotatable bonds of the ligand, the algorithm identifies the most probable conformation of the AMP that would bind to the active site of the sortase C protein.

## Results

### B-AMP as a Structural and Functional Repository for AMPs

**Figure 1:**
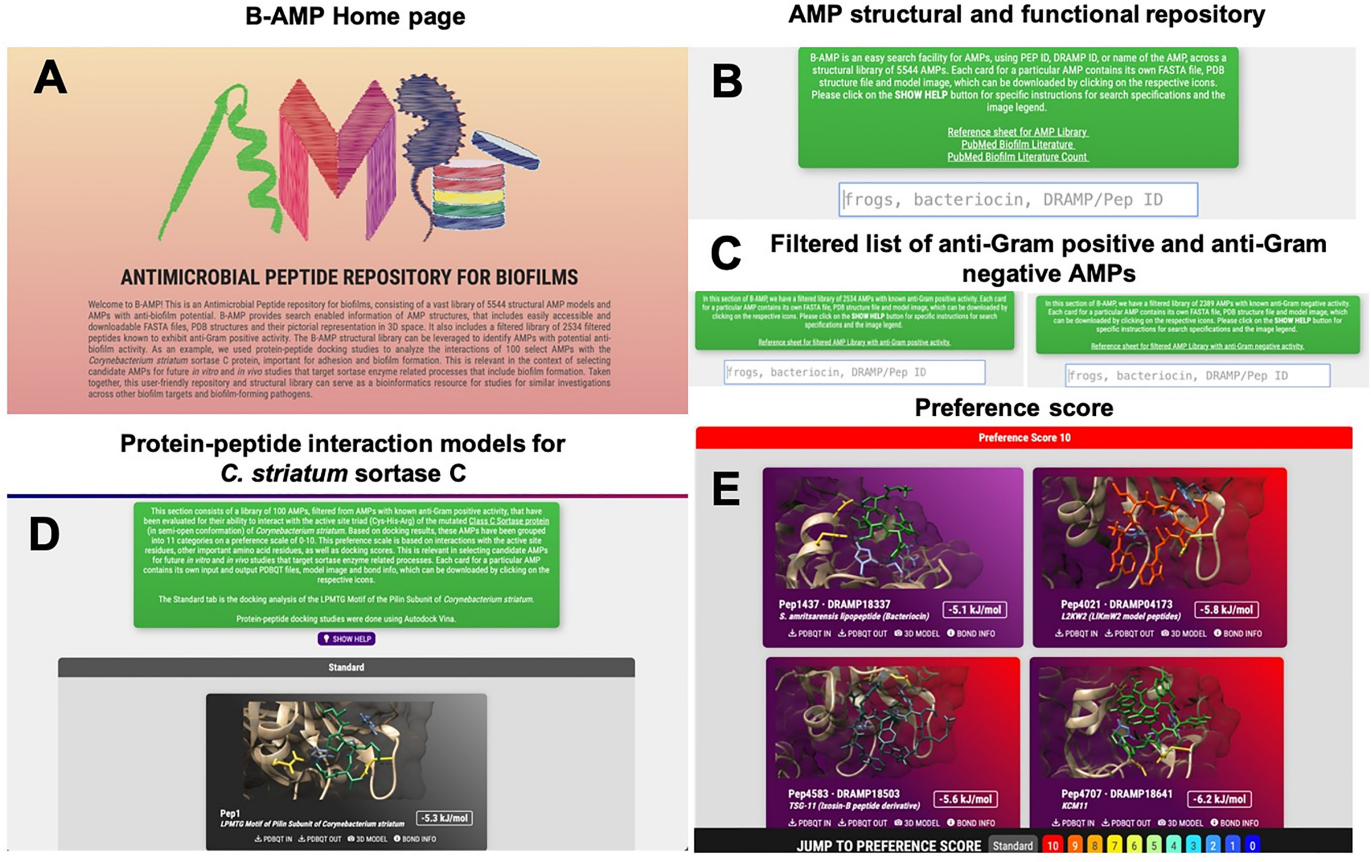
Features and usage of B-AMP **(A)** B-AMP is a manually curated structural and functional repository of AMPs for biofilm studies **(B)** This user-friendly, search enabled repository includes predicted structures for 5544 AMPs, searchable by their Pep ID, DRAMP ID, name and source, and a comprehensive list of AMP annotations to existing biofilm literature sources **(C)** B-AMP also includes a separate subset of AMPs filtered by known anti-Gram positive and anti-Gram negative activity. For each peptide, the repository includes FASTA files, PDB files, and predicted and chosen 3D models **(D, E)** B-AMP hosts protein–peptide interaction models of AMPs docked to biofilm targets, using the sortase-pilin machinery of *C*. striatum as a case study. For the 100 AMPs selected for *in silico* molecular docking, the repository hosts protein-peptide docking models, input & output PDBQT files, images, and AMPs categorized in order of preference, based on docking scores and interacting residues. B-AMP will be continually updated with new structural AMP models, AMP interaction models with potential biofilm targets and annotations to relevant biofilm literature. Artwork in **(A)** was done by Shreeya Mhade.

B-AMP is hosted at b-amp.karishmakaushiklab.com/(version1), with a simple and user-friendly interface (**Figure 1A**). Multi-option search is enabled with DRAMP ID, PepID, name or source for the entire database (**Figure 1B** and **Supplementary Table 1**), and also for the filtered list of 2534 AMPs with anti-Gram positive activity and 2389 AMPs with anti-Gram negative activity (**Figure 1C** and **Supplementary Table 2**). For each AMP, the FASTA files, PDB files, and images of the predicted and chosen 3D models are available. The Python scripts used for auto-generating FASTA files and filtering out AMPs are available in a GitHub repository (https://github.com/KarishmaKaushikLab/B-AMP). B-AMP was last updated on October 1, 2021 and the next update is scheduled on January 1, 2022. In addition, AMPs are annotated to existing biofilm literature sources, consisting of a library of 613 AMPs with a total of 11,611 literature references (**Figure 1B** and **Supplementary Tables 3**, **4**). This is available as compiled lists with PepID (unique to B-AMP), DRAMP ID, the query used to find the literature, AMP name, PMID and article title. Predicted protein-peptide interaction models of 100 AMPs to the sortase C protein of *C. striatum* are also available (**Figures 1D, E**). Kindly refer to the sub-section ‘Sortase C as an anti-biofilm target’ for the detailed description.

### Distribution of AMPs in B-AMP by Length

**Figure 2:**
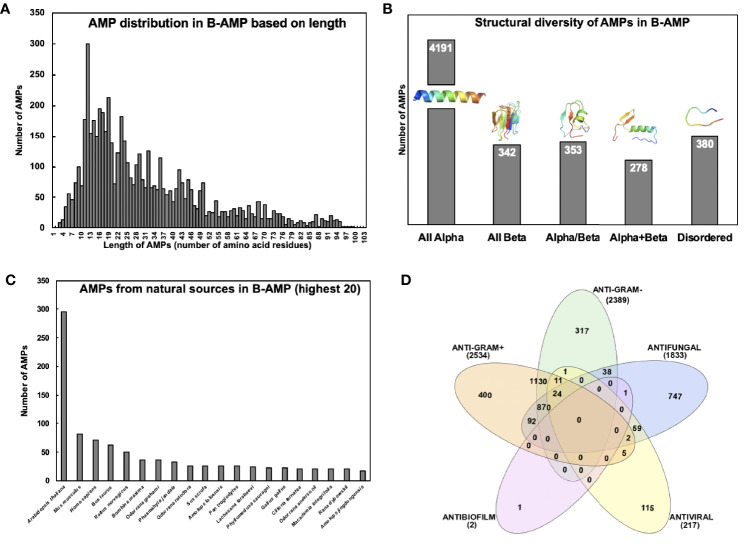
Diversity of AMPs in B-AMP **(A)** AMPs in B-AMP display a varied distribution in lengths, with the shortest peptide being 2 amino acids in length, and the longest peptide being 102 amino acids in length. However, the vast majority of AMPs are between 5-80 amino acids in length **(B)** AMPs possess a vast diversity in secondary structures, such as α-helices, β-sheets, coils, loops, and combinations of these structures. The representative structures for α AMP is Pep502-DRAMP02038, all β is Pep2068-DRAMP00932, for α/β is Pep2069-DRAMP00934, for α+β is Pep2070-DRAMP00936, and for disordered is Pep539-DRAMP18376 **(C)** B-AMP contains AMPs derived from a range of natural and synthetic sources, based on sequences in the DRAMP database. The distribution of natural AMPs based on the highest 20 natural sources reveals plant, animal and human sources **(D)** AMPs in B-AMP possess a range of antimicrobial activities including antibacterial, antifungal, antiviral and antibiofilm activity. **(B)** made using PyMol and **(D)** made using InteraciVenn.

AMPs in B-AMP display varied distribution in lengths, with the shortest peptide being 2 amino acids in length, and the longest peptide being 102 amino acids in length (**Figure 2A**). However, the vast majority of AMPs were observed to be between 5-80 amino acids in length. It is important to note that 2110 AMPs were oligopeptides, in the range of 2-20 amino acids in length. This is of value given that oligopeptides are increasingly being recognized for their diverse range of antimicrobial activity, including the ability to target specific bacterial proteins (Saido-Sakanaka et al., 1999; Wakieć et al., 2008; Jianfeng et al., 2018; Li et al., 2021).

### Structural Diversity of AMPs in B-AMP

Analysis of the structural diversity of the 5544 3D predicted AMP models in B-AMP revealed AMPs with a range of secondary structures such as α-helices, β-sheets, coils or combinations (**Figure 2B**). The majority of the AMP structural models were observed to be exclusively α-helical peptides (n=4191, also known as all α-fold class). Also, AMPs from other structural fold classes (as defined by SCOP), such as all β, α/β (β-α-β), and α+β (α and β are segregated) are also represented in B-AMP (Murzin et al., 1995). Specifically, there are 342 all β AMPs, 353 α/β AMPs, and 278 α+β AMPs (**Figure 2B**). AMPs that do not have a specific secondary structure assigned after modeling are classified in B-AMP as disordered (n=380). These peptides are believed to gain their functional 3D structure on interaction with cellular targets.

### Distribution of AMPs in B-AMP by Source

B-AMP contains AMPs derived from a range of natural and synthetic sources, based on sequences in the DRAMP database. Taken together, AMPs in B-AMP represent >1000 sources such as bacteria, fungi, plants, fish, animals, and insects. As shown in **Figure 2C**, the distribution of natural AMPs, based on the highest twenty natural sources, reveals plant, animal, and human sources. The largest source of AMPs in B-AMP is *Arabidopsis thaliana* (n=295), which is followed by *Mus musculus* and *Homo sapiens* with 82 and 71 AMPs each. Apart from the natural sources, 1360 AMPs in B-AMP are synthetic or artificial in origin, modified from existing AMPs or designed computationally, followed by synthesis.

### Antimicrobial Activities of AMPs in B-AMP

Based on annotation in the DRAMP database, AMPs in B-AMP possess a range of antimicrobial activities including antibacterial, antifungal, antiviral and antibiofilm activity. DRAMP annotates activity information using the PubMed ID of the corresponding reference for activity data of an AMP. As shown in **Figure 2D**, 4925 AMPs had antibacterial activity; 2389 had known anti-Gram negative activity and 2534 had known activity against anti-Gram positive bacteria. A filtered list of AMPs with annotated anti-Gram positive activity and anti-Gram negative activity is available in B-AMP. It is important to note that 1130 AMPs had both anti-Gram negative and anti-Gram positive activity. Further, 1833 AMPs had annotated antifungal activity and 217 with antiviral activity. Based on information in the DRAMP database, only 2 AMPs were annotated to possess antibiofilm activity, of which one of them also had antifungal activity.

### Feasibility of B-AMP for *In Silico* Evaluation of AMPs With Anti-Biofilm Potential Using the Sortase-Pilin Machinery of *C. striatum* as a Case Study

#### Sequence-Based Prediction of Physicochemical Properties and Secondary Structure of the *C. striatum* Sortase C Protein

The amino acid sequence of the *C. striatum* sortase C protein was retrieved from the GenPept Database (ID: WP_034656562) (Class C sortase [Corynebacterium striatum] - Protein - NCBI). Using the sequence, the physicochemical properties of the protein were predicted using ProtParam analysis. As shown in **Table 1**, the analysis revealed a molecular weight of 35134.72 Da, and a theoretical isoelectric point of 5.38 and 5.46 respectively, indicating a negatively-charged protein. In particular, the sortase C protein contained 46 negatively charged residues (Aspartic acid and Glutamic acid) and 33 positively charged residues (Arginine and Lysine). The computed instability index of 33.54 indicates that the protein is stable, as the obtained value is less than the cut-off of 40 (Gasteiger et al., 2005). Using the PSIPRED server (PSIPRED Workbench), the secondary structure of the sortase C protein was observed to contain a combination of α-helices (41%), coils (44%), as well as β-sheets (15%) (**Supplementary Figure 1A**).

**Table 1:** Physicochemical properties of the *C. striatum* sortase C protein (wild-type and double mutant D93A/W95A) using ProtParam analysis.

Physicochemical Property		Value		
		Sortase C	Mutant Sortase C (D93A/W95A)	
Number of amino acids		312	312	
Molecular weight		35134.72	34975.57	
Theoretical pI (isoelectric point)		5.38	5.46	
**Amino acid Composition**				
*Amino acid name*	*Number of residues*	*Percentage*	*Number of residues*	*Percentage*
Ala (A)	25	8.0%	27	8.7%
Arg (R)	12	3.8%	12	3.8%
Asn (N)	15	4.8%	15	4.8%
Asp (D)	24	7.7%	23	7.4%
Cys (C)	1	0.3%	1	0.3%
Gln (Q)	12	3.8%	12	3.8%
Glu (E)	22	7.1%	22	7.1%
Gly (G)	19	6.1%	19	6.1%
His (H)	12	3.8%	12	3.8%
Ile (I)	10	3.2%	10	3.2%
Leu (L)	37	11.9%	37	11.9%
Lys (K)	21	6.7%	21	6.7%
Met (M)	7	2.2%	7	2.2%
Phe (F)	6	1.9%	6	1.9%
Pro (P)	16	5.1%	16	5.1%
Ser (S)	10	3.2%	10	3.2%
Thr (T)	25	8.0%	25	8.0%
Trp (W)	7	2.2%	6	1.9%
Tyr (Y)	10	2.2%	10	2.2%
Val (V)	21	6.7%	21	6.7%
Pyl (O)	0	0.0%	0	0.0%
Sec (U)	0	0.0%	0	0.0%
Total number of negatively charged residues (Asp + Glu):		46	45	
Total number of positively charged residues (Arg + Lys):		33	33	
**Atomic composition**				
Carbon	C	1565	1556	
Hydrogen	H	2449	2444	
Nitrogen	N	427	426	
Oxygen	O	477	475	
Sulfur	S	8	8	

#### Homology Model of the *C. striatum* Sortase C Protein

**Figure 3:**
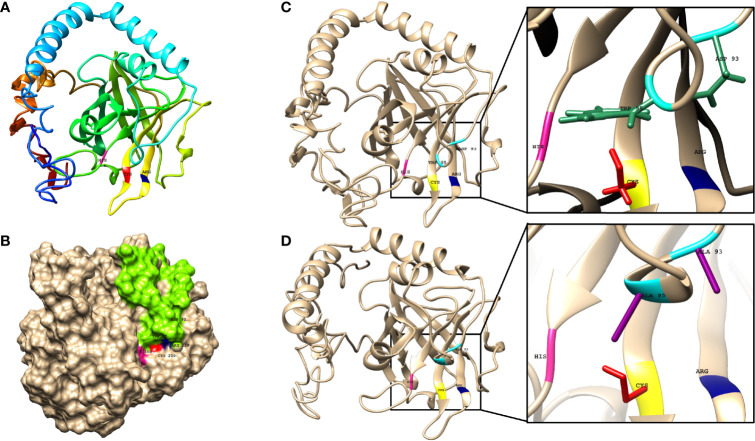
The predicted structure of the *C. striatum* sortase C protein showing the presence of the putative active site triad essential for catalytic activity and a ‘lid’. **(A)** The sortase C protein in ribbon form, showing the overall organization of the protein backbone in 3D space. The modeled structure reveals the presence of the putative Cys-His-Arg triad essential for catalytic activity. The Cys230 residue is shown in red, the His168 residue in pink, and the Arg239 residue in blue. The default ribbon style is smooth (rounded) **(B)** Surface representation of the class C sortase protein showing the sortase ‘lid’ (green) occluding the active site residues. Homology modeling was done using the I-TASSER server using the amino acid sequence of the sortase C protein retrieved from GenPept (ID: WP_034656562) **(C)** In the wild-type sortase C protein, the sortase ‘lid’ is predicted as a loop-like extension, in close proximity with the putative active site Cys-His-Arg (yellow-pink-blue) residues. The closer version of the region, predicts interactions between the aromatic ring of the Trp95 residue (green) and the Asp93 residue (green) of the ‘lid’ with Cys230 and Arg239 residues respectively **(D)** In the mutant sortase C protein (D93A/W95A), the lid is predicted to be displaced from the triad residues. In the closer version, a cavity is predicted between the mutated alanine residues (purple) and the putative active site triad. This ‘semi-open’ lid conformation of the sortase C protein was used for protein-peptide *in silico* molecular docking studies. Images made using UCSF Chimera.

The 3D structure of the sortase C protein of *C. striatum* is not available in the RCSB Protein Data Bank (PDB) (RCSB PDB: Homepage). To model the structure of the enzyme, I-TASSER selected the following crystal structures as templates: class C sortases of *Streptococcus pneumoniae* (PDB ID: 2W1J, 2W1K, 3G66, and 3O0P), *Streptococcus agalactiae* (PDB ID: 3RBI, 4D7W, and 4G1H), *Actinomyces oris* (PDB ID: 2XWG), *Clostridium perfringens* (PDB ID: 6IXZ) and Class A sortase from *Corynebacterium diphtheriae* (PDB ID: 5K9A). The sequence identity between *C. striatum* sortase C and the selected templates of *Streptococcus pneumoniae*, *Streptococcus agalactiae*, *Actinomyces oris*, *Clostridium perfringens* and *Corynebacterium diphtheriae* was 50% respectively, which is above the threshold of 30% sequence identity to generate good quality models (https://pubmed.ncbi.nlm.nih.gov/16787261/). As seen in **Figure 3A**, I-TASSER model of the sortase C protein predicted the presence of all three secondary structure states (α-helices, coils, β-sheets), correlating with the PSIPRED server results (**Supplementary Figure 1A**). In Gram-positive bacteria, including *Corynebacterium* spp., the sortase C enzyme is known to possess a highly conserved active site cysteine residue (Cys230), responsible for cleavage of pilin motifs. Additionally, histidine and arginine residues (His168 and Arg239), that play essential roles in sortase activity, form the catalytic triad (Spirig et al., 2011). The overall quality factor of the predicted sortase C model was 73.0263 by the ERRAT (**Supplementary Figure 1**). Moreover, the model was validated by VERIFY3D showing 80.45% of the residues with an averaged 3D-1D score ≥ 0.2. Ramachandran plot analysis from PROCHECK identified 95.6% of residues of the protein were in the most favored and generously allowed regions, and only 4.4% residues in outlier regions.

#### Modeling the *C. striatum* Sortase C Protein in Semi-Open Lid Conformation

In addition to the characteristic β-barrel structure and active site Cys230-His168-Arg239 triad, the homology model of the sortase C protein predicted the presence of a ‘lid’ consisting of a structural loop lodged in the putative active site (**Figure 3B**). The ‘lid’ of the sortase C protein is suggested to play a role during polymerization of the pilin subunits, likely *via* the recognition of the sorting signal motif (Jacobitz et al., 2017; Khare and Narayana, 2017). However, there is no evidence to indicate that the pilin substrates are able to access the active site in this ‘closed’ confirmation, suggesting that the lid likely undergoes a conformational change during pilus biogenesis (Jacobitz et al., 2017; Khare and Narayana, 2017). As seen in **Figure 3C**, the ‘lid’ of the sortase C protein carries two important residues, which typically include an aspartate residue (Asp93) and a hydrophobic amino acid (Trp95), two residues after aspartate, that interact with the putative catalytic site. In the ‘closed’ confirmation, the sortase C lid blocks access to the active site, observed as proximity between the Asp93 and Arg239 residues. The presence of the aromatic ring of the Trp95 residue in the lid, which is close to the Cys230 residue, renders the active site inaccessible (Jacobitz et al., 2016). Point mutations in the ‘lid’, such as a double mutation D93A/W95A (**Figure 3D**), replacing Aspartate (93) and Tryptophan (95) with Alanine, are known to result in a ‘semi-open’ lid conformation and increased substrate accessibility (Jacobitz et al., 2016). To mimic the semi-open lid conformation, we generated a model that included a double mutation D93A/W95A in the sortase C protein. As seen in **Figures 3C, D**, I-TASSER modeling of the mutated sortase C protein (D93A/W95A) predicted the presence of a ‘semi-open’ lid conformation, with the lid displaced away from the triad residues and a cavity observed between the alanine residues and the putative active site triad. The mutated sortase C protein displayed similar physicochemical properties and secondary prediction as compared with the wild-type protein (**Table 1**), with a distribution of α-helices (41%), coils (44%), as well as β-sheets (15%) (**Supplementary Figure 1B**). Further, the sortase C protein model with ‘semi-open’ lid conformation generated a 3D score of 82.37% by the Verify3D Program under SAVES v6.0. The ‘semi-open’ conformation of the sortase C protein was used for *in silico* molecular docking studies.

#### *In Silico* Molecular Docking of Candidate AMPs Against the Semi-Open Lid Conformation of the *C. striatum* Sortase C Protein

**Figure 4:**
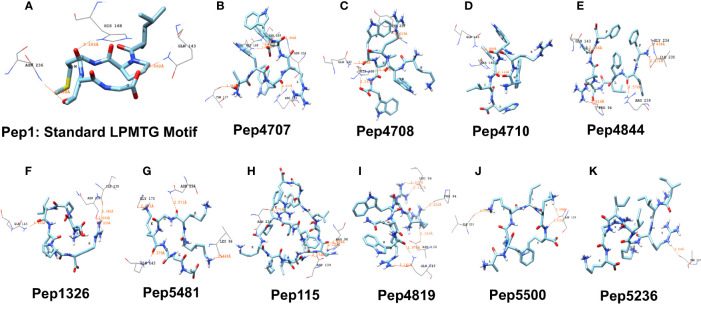
Representative results from *in silico* molecular docking for the standard pilin subunit LPMTG and select AMPs with the semi-open lid conformation of the *C. striatum* sortase C protein. **(A)** The standard LPMTG motif is predicted to form hydrogen bonds with His168, Asn236, Gln143 **(B)** Pep4707 is predicted to interact with HIS168, ARG239, ASN236, THR137, THR169 **(C)** Pep4708 is predicted to interact with HIS168, HIS168, GLN143, TYR233 **(D)** Pep4710 is predicted to interact with HIS168, GLN143 **(E)** Pep4844 is predicted to interact with ARG239, GLN143, PRO94, ILE235, GLY234 **(F)** Pep1326 is predicted to interact with ASN236, ASN236, GLN143, ILE235 **(G)** Pep5481 is predicted to interact with ASN236, GLN143, LEU96, GLY170 **(H)** Pep115 is predicted to interact with ASN236, ASP139, ARG98, ARG98 **(I)** Pep4819 is predicted to interact with GLN143, GLN143, ASP139, LEU96, LEU96, PRO94 **(J)** Pep5500 is predicted to interact with ASP139, ASP139, ILE235 **(K)** Pep5236 is predicted to interact with THR137. Images made using UCSF Chimera.

**Figure 5:**
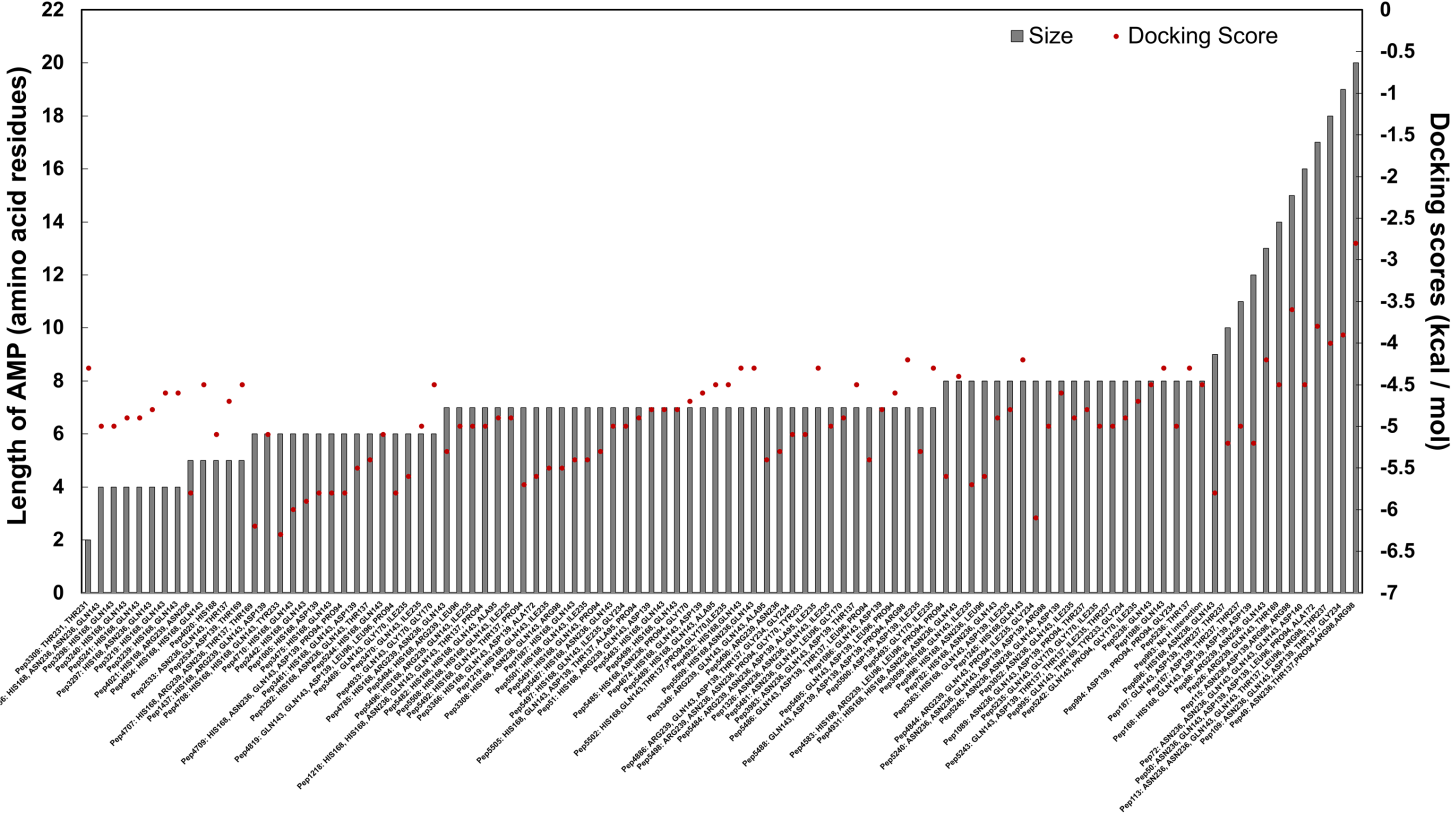
*In silico* molecular docking scores and interacting residues for 100 candidate AMPs and the semi-open conformation of the *C. striatum* sortase C protein. AMPs were predicted to interact with the putative active site triad residues, protruding residues near the triad as well as residues further away from the triad.

To identify candidate AMPs with potential to interact with the putative active site of the sortase C protein of *C. striatum*, we curated a set of 100 predicted 3D AMP structural models from the filtered set of anti-Gram positive AMPs from B-AMP (**Supplementary Table 2**). This included 88 AMPs with a length ranging from 2-8 residues, and 12 representative AMPs each ranging from 9-20 residues in length. These peptides were subject to *in silico* molecular docking using AutoDock Vina, against the ‘semi-open’ conformation of the sortase C protein (Trott and Olson, 2010). It is important to note that AutoDock Vina is typically suited for peptides with less than 32 bonds, and if the number of rotatable bonds exceeds 32, the tool flags select bonds as rigid or non-rotatable to fit parameters. Given this, we chose AMPs of lengths ranging from 2-8 residues, with a few select AMPs of 9-20 residues in length. To validate our molecular docking results, the LPXTG motif (X being any amino acid) of the pilin subunit of *C. striatum* was considered as the positive control or standard (**Supplementary Figure 2**), and designated as Pep1 (GenPept ID: WP_170219081.1). As seen in **Figure 4A**, the LPXTG motif interacted with the semi-open lid conformation of the sortase C protein at the active site, forming three hydrogen bonds with His168, Asn236 and Gln143, with a binding affinity of −5.3 kcal/mol. Of the 100 candidate AMPs, 99 AMPs were predicted to form hydrogen bonds with the semi-open lid conformation of the sortase C protein (**Figures 4B-K**, **5** and **Supplementary Table 5** **)**. It is important to note that, based on our docking results, no interactions were predicted between the LPXTG motif or any of the AMPs with the active site Cys230 residue. This is could be due to the conformation of the sortase C protein that limits access to Cys230 or the chemical structure of cysteine that confers it selective interactions. All protein-peptide interaction models (input and output PDBQT files, model images, and bond information) are available in B-AMP (https://b-amp.karishmakaushiklab.com/docked.html).

#### Preference Scale of Candidate AMPs Based on Docking Results with the Semi-Open Lid Conformation of the *C. striatum* Sortase C Protein

Using interacting residues and docking scores, we categorized 100 candidate AMPs based on an in-house preference scale of 0-10 (**Figure 5** and **Supplementary Table 5**), with 0 being lowest and 10 being the highest. AMPs were categorized, in order of priority, starting with hydrogen interactions with at least two catalytic site residues (His168 and Arg239), which was given a preference score of 10. Since Cys230 was not observed to interact with the positive control (the LPXTG motif of the pilin subunit), we considered any AMP interacting with His168 and Arg239 as a potential candidate. Additionally, in the preference score, exclusive interactions with His168 were given priority over Arg239, as the histidine residue is conserved across sortases, whereas arginine can be replaced with another residue. This was followed by the next preference score 9, which consisted of AMPs with multiple hydrogen interactions with residues in the substrate-binding site that includes His168 from the triad. Similarly, AMPs with only one explicit hydrogen interaction with His168, along with interactions with other amino acid residues, were given a preference score of 8. AMPs interacting with Arg239 (along with other amino acid residues) were designated a preference score of 7. AMPs with multiple explicit interactions with Asn236 near the triad were categorized in preference score 6. AMPs with interactions with Asn236 and Gln143 near the triad were classified in preference score 5. AMPs with only one interaction with Asn236 near the triad were categorized in preference score 4, and AMPs with only one interaction with Gln143 near the triad were categorized in preference score 3. Finally, interactions with Asp139, Thr137, Gly170, Ile235, Thr231 were categorized into preference scores 2 and 1, based on their location further away from the triad. If an AMP showed no hydrogen bond interactions with the sortase C protein a preference score of 0 was assigned (**Supplementary Figure 3**). Finally, in each preference score, candidate AMPs were sorted based on docking binding energy scores (in kcal/mol), into those with the highest affinity (relatively more negative binding energy value) to those with lowest affinity (relatively more positive binding energy values) (**Supplementary Table 5**).

## Discussion

### Features and Functions of B-AMP

**Figure 6:**
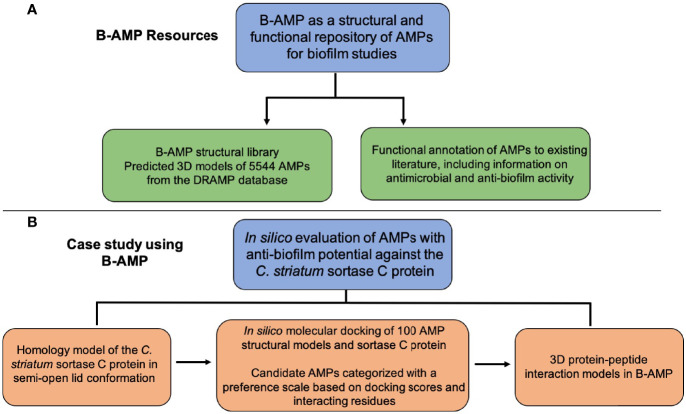
Resources in B-AMP and a case study to demonstrate the usability of B-AMP to identify AMPs with anti-biofilm potential. **(A)** The resources and data generated in this approach are freely available in Biofilm-AMP (B-AMP), a user-friendly, search-enabled repository of AMP structures and AMP interactions with biofilm targets. B-AMP also includes annotations of AMPs to existing biofilm literature sources **(B)** To demonstrate the feasibility of B-AMP for *in silico* evaluation of AMPs with anti-biofilm potential, we present a case study using the emerging pathogen *C. striatum*. For this, we used a combination of homology modeling, predictive peptide modeling and protein-peptide molecular docking, and suggest a preference score based on which candidate AMPs could be taken up for further evaluation.

To the best of our knowledge, B-AMP is a unique structural and functional repository with a vast set of natural and synthetic AMPs, along with annotations of existing biofilm literature sources, and downloadable files that can be leveraged for *in silico* investigations without further modifications. (**Figure 6A**). AMPs in B-AMP are diverse in terms of length, structure, sources, and activity, which is important for several reasons. The length of AMPs is known to influence a range of activities, including antimicrobial effects, hemolytic activity and membrane binding and accumulation (Bahar and Ren, 2013; Li et al., 2017; Clark et al., 2021). Further, AMPs possess a vast diversity in secondary structures, such as α-helices, β-sheets, coils, loops, and combinations of these structures; AMPs from all structural groups have been observed to exhibit antimicrobial activity, and certain AMPs are also known to change conformation on interaction with target structures (Epand and Vogel, 1999; Bahar and Ren, 2013). Consequently, the structural motifs of AMPs influence the mechanisms of action, including interactions with the microbial cell membrane and specific targets.

In the section ‘AMP Library’, the structural library of 5544 AMPs can be searched using DRAMP ID (e.g.: DRAMP00005), PepID (eg: Pep2), name (eg: bacteriocin) or source (eg: frog), which self-populates results of relevant AMPs as color-coded tiles. Every match has three links, FASTA sequence of the AMP, the 3D predicted model of the AMP, and a thumbnail image of the 3D model. The ‘show help’ tab below the search bar provides the legend for the color-coded tiles, in which purple alone represent an AMP with anti-Gram positive activity, red indicates an AMP with anti-Gram negative activity and grey indicates an AMP with antiviral or antifungal activity. Tiles colored both purple and red indicate that the AMP is known to possess both anti-Gram positive and anti-Gram negative activity. The ‘AMP Library’ tab also has a comprehensive list of all AMPs in B-AMP in a reference sheet, which includes the PepID, DRAMP ID, name and activity. Further, the vast functional library of AMPs annotated to existing biofilm literature is also available in comprehensive sheets with AMP name, query words, PMID number and article title.

In the ‘Anti-Gram positive’ and ‘Anti-Gram negative’ tabs, a curated list of AMPs with known anti-Gram positive and anti-Gram negative activity (based on the DRAMP database) are available for search. Similar to the earlier page ‘AMP Library’ AMPs in this filtered list can be searched for using DRAMP ID, PepID, name or source, where the AMO tiles self-populate as the search term is keyed in. Similarly, the results are displayed as individual cards with same functionalities, i.e., sequence, structure, and thumbnail of the AMP. Every match has three links, FASTA sequence of the AMP, the 3D predicted model of the AMP, and a thumbnail image of the 3D model.

The tab ‘AMPs docked to biofilm targets’ has *in silico* protein-peptide interaction models of 100 AMPs docked to the *C. striatum* sortase C protein (semi-open lid conformation). The specific case study has been described in detail in the following sub-section ‘*In silico* evaluation of AMPs with anti-biofilm potential against the sortase-pilin machinery of the emerging pathogen *C. striatum*’. The ‘show help’ tab below the search bar provides the legend for the color scheme of interacting residues within the images, where shades of blue indicate the His168-Cys230-Arg239 catalytic site triad, and shades of orange indicate other interacting residues. Based on docking scores and interacting residues, the tab lists the *in silico* models in order of a preference score ranging from 10 to 0. For each docking result, the user can access the input file (in PDBQT formal), the output file (in PDBQT format), the 3D protein-peptide complex, and interactions. The predicted binding energy is highlighted in the thumbnail of the docked complex.

Finally, in the ‘Code’ tab B-AMP also hosts the code used to generate FASTA files, filter AMPs, update AMP lists, and to retrieve biofilm related literature for the AMPs. Relevant references and resources used in the study are listed in the ‘References’ tab.

### B-AMP in the Context of Existing AMP Databases for Biofilm Studies

While there are several databases dedicated to curating AMP sequences and activities, there is an increasing focus on building AMP resources and prediction tools specific for biofilm studies BaAMPs is an open-access, manually-curated database with information on AMPs tested against microbial biofilms (Di Luca et al., 2015). The database provides information on microbial species activity, testing conditions and assay guidelines, and relevant literature. BaAMPs curates 221 AMPs, 1022 descriptions of experimental data, and 116 target organisms (BaAMPs - Home). The resource aBiofilm contains biological, chemical and structural information on a wide range of anti-biofilm agents including AMPs, with data visualization modules to summarize the biofilm stages targeted (Rajput et al., 2018). The web-servers dPABBs and BIPEP facilitate the prediction and design of anti-biofilm peptides, and dPABBs also enables the generation of peptide variations with improved activity and physicochemical properties (Antimicrobial Peptide Database - DBAASP; BIPEP: Homepage; Sharma et al., 2016; Atanaki et al., 2020). The BIOFIN platform allows the user to predict the biofilm inhibitory nature of the peptides as well as do a similarity search against experimentally validated biofilm inhibitory peptides from the BaAMP database (BIOFIN: Homepage). Given this, the resources in B-AMP complement existing AMP databases (BaAMPs - Home; BIOFIN: Homepage; Di Luca et al., 2015; Rajput et al., 2018), as well as serve as a potential one-stop structural and functional repository of AMPs for biofilm studies.

### Limitations of B-AMP

While a comprehensive database, B-AMP does have certain limitations with respect to integration of the structural and functional features of the repository. In the future, based on literature evidence, we plan to map potential host targets to each AMP in B-AMP, nd provide links to relevant biofilm literature sources for each B-AMP in the search tile itself. Further, currently, the B-AMP database has relatively more AMPs with reported anti-Gram positive activity (2534 B-AMPs) than anti-Gram negative activity (2389 B-AMPs), which is a reflection of the AMP sequences in the DRAMP database. To overcome this, we plan to integrate AMP sequences from additional databases and build and host their 3D structural models in B-AMP (3D Structure of Antimicrobial Peptides; dPABBs: A webserver for Designing of Peptides Against Bacterial Biofilms; Wang et al., 2016; Kang et al., 2019; Pirtskhalava et al., 2021; Shi et al., 2021).

### Potential Applications of Resources in B-AMP

The structural library of AMPs in B-AMP can be leveraged for *in silico* studies such as similar structural homolog identification and annotation, automated virtual screening of AMPs using molecular docking techniques, and understanding the mechanism of action of AMPs with molecular dynamics simulations. This would enable the development of an *in silico* screening pipeline for AMPs against biofilm targets, based on which candidate AMPs could be taken up for further *in vitro* and *in vivo* experimental studies. In addition to its biological and functional applications, B-AMP can also serve as a resource for deriving sequence and structural features of AMPs to predict antimicrobial activity using machine learning tools.

Further, the vast structural and functional library of AMPs in B-AMP can be leveraged for biofilms studies across a range of microbes, including bacterial and fungal biofilm targets. The filtered list of AMP structural models with known anti-Gram positive and anti-Gram negative activity, and AMPs with known antifungal activity, can facilitate these studies. For example, the filtered list of AMP models with known anti-Gram positive can be subject to *in silico* studies against biofilm targets across a range of Gram positive biofilm-forming pathogens, including *S. aureus* and *Streptococcus* spp. Similarly, biofilm targets in Gram negative pathogens such as lipopolysaccharide (LPS) and flagella, can be evaluated using *in silico* approaches with known anti-Gram negative AMPs such as *E. coli*, *Salmonella* spp and *Shigella* spp.

### Sortase C as a Potential Biofilm Target for *C. striatum*

To demonstrate the feasibility of B-AMP for *in silico* evaluation of AMPs with anti-biofilm potential, we present a case study using the emerging skin, wound and ocular pathogen *C. striatum* (**Figure 6B**). Sortase enzymes have been identified as promising antimicrobial targets (Cossart and Jonquières, 2000; Spirig et al., 2011; Zhang et al., 2014; Jacobitz et al., 2017), and based on its relevance to *C. striatum* pilus biogenesis and biofilm formation, the sortase C protein was selected as a potential biofilm target. In *C. striatum*, the sortase-pilin machinery includes the *spaDEF* operon that encodes a set of pilus proteins and their respective sortases (Nudel et al., 2018; Ramos et al., 2018). In *Corynebacterium* spp., the sortase C enzyme functions as a pilus-specific sortase, recognizing and cleaving the sorting signal (LPMTG) in the pilin subunits, which is followed by their surface display (Spirig et al., 2011; Jacobitz et al., 2017). Targeting the sortase C enzyme could disrupt biofilm formation in *C. striatum* by influencing important processes in biofilm development. For example, inhibition or blockage of sortase C mediated assembly of cell-surface pili could impair the initial attachment of *C. striatum* cells to biotic and abiotic surfaces (de Souza et al., 2015), and thereby prevent or retard the formation of biofilms. On the other hand, AMPs targeting sortase C mediated pilus assembly may not be able to promote dispersal of pre-established biofilms and the activity of the AMP maybe limited by factors such as penetration into the biofilm matrix. Therefore, while *in silico* screening approaches enable the narrowing down on potential candidate AMPs, elucidating mechanisms and limitations of anti-biofilm activity necessitate additional *in vitro* and *in vivo* testing.

### Further Evaluation of Candidate AMPs for Anti-Biofilm Potential Against the *C. striatum* Sortase C Protein Based on *In Silico* Docking Results

Using *in silico* molecular docking, we identified AMPs interacting with active site residues of the *C. striatum* sortase C protein, as well as residues in the same spatial location to the triad. It is important to note that *in silico* docking was done using a computationally derived model of the *C. striatum* sortase C protein, that needs to be validated with experimental evidence. While this is beyond the scope of this paper, our work does provide a computational model that can be used for further validation. Based on binding energy scores and interacting residues from molecular docking, we suggest a preference scale (ranging from 0 to 10), based on which candidate AMPs could be taken up for subsequent testing against *C. striatum* biofilms. This includes but not limited to additional *in silico* approaches such as molecular dynamics simulations and RMSD analyses, in addition to standard *in vitro* and *in vivo* anti-biofilm testing, such as assays for metabolic activity, biofilm eradication and inhibitory concentrations, advanced microscopy, and animal models of infection (Jianfeng et al., 2018). Further evaluations can also focus on bioactivity, stability, and toxicity testing of candidate AMPs. To improve biocompatibility and minimize toxicity, candidate AMPs can also be subject to peptide design and engineering to produce suitable modifications (Antimicrobial Peptide Database - DBAASP; Di Luca et al., 2015; Sharma et al., 2016; Dostert et al., 2019). Further, it is well known that a range of host and microbial factors can influence the activity of AMPs, such as susceptibility to degradation by host (proteolytic) enzymes, inhibition by salts, proteins and ions in the host environment, as well as strain-specific modifications of the sortase C active site, AMP solubility in and affinity to the biofilm matrix (Bahar and Ren, 2013). Evaluating these in *in vitro* and *in vivo* systems that mimic host environments, would be important in taking these candidate AMPs further. Given this, while certainly not inclusive of all aspects critical to the evaluation of candidate AMPs as potential anti-biofilm agents, our approach provides a starting point for subsequent *in silico*, as well as *in vitro* and *in vivo* evaluation of AMPs for anti-biofilm potential. While our study focused on the sortase-pilin machinery of *C. striatum*, the approach used and resources developed, can be leveraged for similar studies against other biofilm targets and biofilm-forming pathogens.

## Conclusions

With the ever-increasing resistance of microbial pathogens to conventional antibiotics, AMPs hold potential as alternative and adjunct antimicrobial approaches. This is particularly relevant in the context of biofilms, which are recalcitrant to antibiotics and often require long term and repeated antibiotic usage. Towards this, B-AMP is a comprehensive structural and functional repository of AMPs relevant to biofilm studies, with pre-determined 3D structures, associated literature evidence, and protein-peptide interaction models. We believe B-AMP will be valuable to the research community, with scope for multiple applications in the study of AMPs for biofilms.

## Preprint

This manuscript is submitted as a preprint to bioRxiv: https://www.biorxiv.org/content/10.1101/2021.08.16.456477v2.

## Data Availability Statement

The datasets presented in this study can be found in online repositories. The names of the repository/repositories and accession number(s) can be found in the article/**Supplementary Material**.

## Author Contributions

SM: Conceptualization, Methodology, Investigation, Validation, Formal analysis, Data curation, Visualization, Writing the original draft, Editing draft. SP: Conceptualization, Methodology, Investigation, Validation, Formal analysis, Data curation, Visualization, Writing the original draft, Editing draft. GT: Conceptualization, Methodology, Investigation, Validation, Formal analysis, Data curation, Visualization, Writing the original draft, Editing draft. RA: Methodology, Investigation. YN: Data analysis, Data visualization, Investigation. SK: Conceptualization, Investigation, Editing draft. RY: Data Analysis, Data visualization, Editing draft. KK: Conceptualization, Data Analysis, Data visualization, Project administration, Supervision, Writing the original draft, Editing draft. All authors contributed to the article and approved the submitted version.

## Funding

KK’s academic appointment is funded by the Ramalingaswami Re-entry Fellowship (BT/HRD/35/02/2006). Snehal Kadam was supported for a select duration of this project (June 2020-December 2020) on the Ramalingaswami Re-entry Fellowship (to KSK).

## Conflict of Interest

The authors declare that the research was conducted in the absence of any commercial or financial relationships that could be construed as a potential conflict of interest.

## Publisher’s Note

All claims expressed in this article are solely those of the authors and do not necessarily represent those of their affiliated organizations, or those of the publisher, the editors and the reviewers. Any product that may be evaluated in this article, or claim that may be made by its manufacturer, is not guaranteed or endorsed by the publisher.

## Supplementary Material

Supplementary Figure 1 Secondary structures of the wild-type and mutated sortase C protein based on PSIPRED analysis. **(A)** The secondary structure of the wild-type sortase C protein contains a combination of α-helices (41%), coils (44%), and β-strands (15%). The cysteine (Cys230) and histidine (His168) residues are found to be in between two β-strands, constituting a turn connecting the strands and acting as a major groove for hydrophobic interaction. The arginine (Arg239) residue was predicted to initiate the second β-strand of the hydrophobic groove. The image is a snapshot of the results obtained from the PSIPRED analysis program **(B)** The secondary structure of the mutated sortase C protein with Asp93 and Trp95 replaced with Alanine (D93A/W95A). The image is a snapshot of the results obtained from the PSIPRED analysis program.

Supplementary Figure 2 Predicted and chosen model of the *C. striatum* pilin subunit showing the presence of the cell sorting LPMTG motif. The *C. striatum* pilin subunit in ribbon form, showing the overall organization of the protein backbone in 3D space. The modeled structure reveals the presence of the cell sorting LPXTG motif (X is represented by M) at the C terminus of the protein. This motif is followed by a hydrophobic domain seen here as a helix. Homology modeling was done using the I-TASSER Server using the amino acid sequence of the pilin subunit retrieved from GenPept (ID: WP_170219081.1). Image made with UCSF Chimera.

Supplementary Figure 3 Visual representation of predicted interacting residues for 100 AMPs docked to the semi-open lid conformation of the *C. striatum* sortase C protein. AMPs were predicted to interact with the putative active site triad residues (except Cys230), protruding residues near the triad, as well as residues further away from the triad. Triad residues are colored purple, other interacting residues are colored forest green. Based on interacting residues and docking scores, AMPs were categorized into a preference score (10-0, highest to lowest) for further *in silico*, *in vitro* and *in vivo* testing.
