# Supplementary figures and images for: AMPing Up the Search: A Structural and Functional Repository of Antimicrobial Peptides for Biofilm Studies, and a Case Study of Its Application to Corynebacterium striatum, an Emerging Pathogen
Source: Front Cell Infect Microbiol. 2021 Dec 16;11:803774. doi: 10.3389/fcimb.2021.803774 (PMC8716830; doi:10.3389/fcimb.2021.803774)

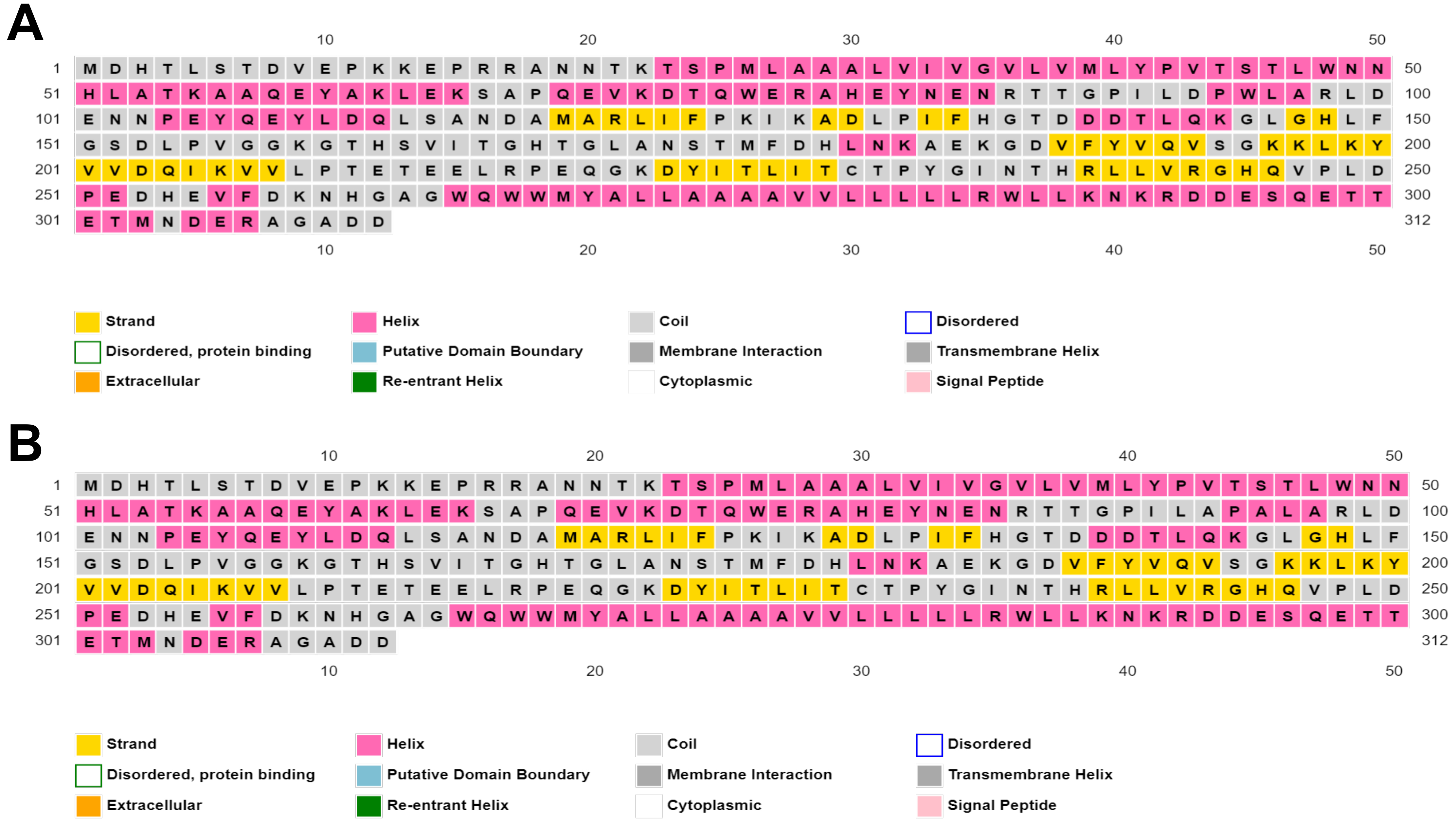

Supplement: Supplementary Figure 1 — Secondary structures of the wild-type and mutated sortase C protein based on PSIPRED analysis. (A) The secondary structure of the wild-type sortase C protein contains a combination of α-helices (41%), coils (44%), and β-strands (15%). The cysteine (Cys230) and histidine (His168) residues are found to be in between two β-strands, constituting a turn connecting the strands and acting as a major groove for hydrophobic interaction. The arginine (Arg239) residue was predicted to initiate the second β-strand of the hydrophobic groove. The image is a snapshot of the results obtained from the PSIPRED analysis program (B) The secondary structure of the mutated sortase C protein with Asp93 and Trp95 replaced with Alanine (D93A/W95A). The image is a snapshot of the results obtained from the PSIPRED analysis program. [file Image_1.jpeg]

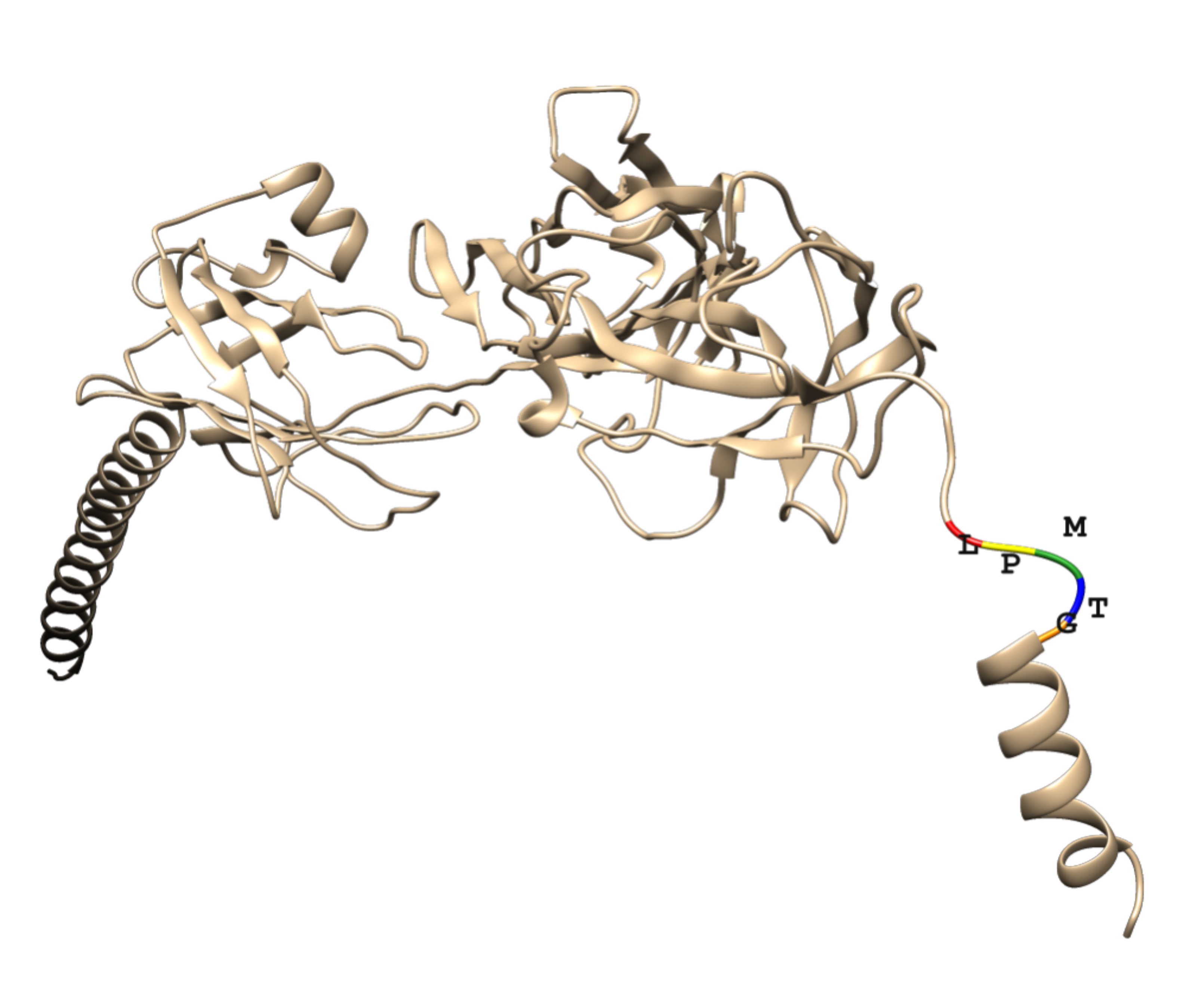

Supplement: Supplementary Figure 2 — Predicted and chosen model of the C. striatum pilin subunit showing the presence of the cell sorting LPMTG motif. The C. striatum pilin subunit in ribbon form, showing the overall organization of the protein backbone in 3D space. The modeled structure reveals the presence of the cell sorting LPXTG motif (X is represented by M) at the C terminus of the protein. This motif is followed by a hydrophobic domain seen here as a helix. Homology modeling was done using the I-TASSER Server using the amino acid sequence of the pilin subunit retrieved from GenPept (ID: WP_170219081.1). Image made with UCSF Chimera. [file Image_2.jpeg]

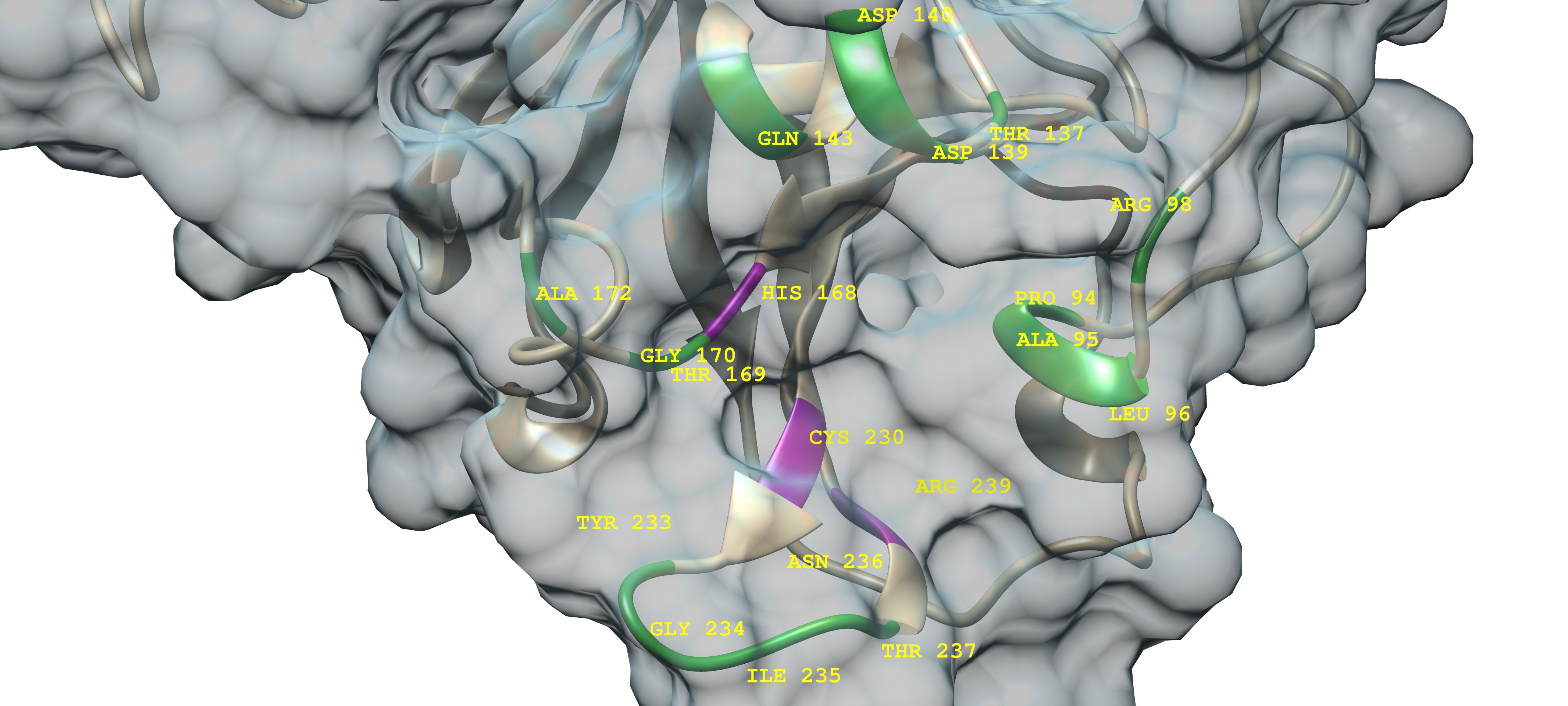

Supplement: Supplementary Figure 3 — Visual representation of predicted interacting residues for 100 AMPs docked to the semi-open lid conformation of the C. striatum sortase C protein. AMPs were predicted to interact with the putative active site triad residues (except Cys230), protruding residues near the triad, as well as residues further away from the triad. Triad residues are colored purple, other interacting residues are colored forest green. Based on interacting residues and docking scores, AMPs were categorized into a preference score (10-0, highest to lowest) for further in silico, in vitro and in vivo testing. [file Image_3.jpg]
