# Supplementary material for: AMPing Up the Search: A Structural and Functional Repository of Antimicrobial Peptides for Biofilm Studies, and a Case Study of Its Application to Corynebacterium striatum, an Emerging Pathogen
Source: Front Cell Infect Microbiol. 2021 Dec 16;11:803774. doi: 10.3389/fcimb.2021.803774 (PMC8716830; doi:10.3389/fcimb.2021.803774)
Supplement: Supplementary file 4 [file Table_1.pdf]

PepID	DRAMP_ID	Name	Activity
0	-	Pilin Subunit of *Corynebacterium striatum*	-
1	-	LPMTG Motif of Pilin Subunit of *Corynebacterium striatum*	-
2	DRAMP00005	Epicidin 280 (Bacteriocin)	Antibacterial, Anti-Gram+, Antimicrobial
3	DRAMP00017	Microbisporicin A1 (Bacteriocin)	Antibacterial, Antimicrobial
4	DRAMP00032	Ruminococcin A (RumA; Bacteriocin)	Antibacterial, Anti-Gram+, Antimicrobial
5	DRAMP00063	Lantibiotic michiganin-A (Bacteriocin)	Antibacterial, Antimicrobial
6	DRAMP00068	Aureocin A53 (Bacteriocin)	Antibacterial, Anti-Gram+, Antimicrobial
7	DRAMP00069	Garvieacin Q (GarQ; Bacteriocin)	Antibacterial, Anti-Gram+, Antimicrobial
8	DRAMP00074	Enterocin P (Pediocin-like peptide; Bacteriocin)	Antibacterial, Anti-Gram+, Antimicrobial
9	DRAMP00089	Bacteriocin E50-52 (Preclinical)	Antibacterial, Anti-Gram+, Anti-Gram-, Antimicrobial
10	DRAMP00090	Carnobacteriocin B2 (CbnB2; Bacteriocin)	Antibacterial , Anti-Gram+, Antimicrobial
11	DRAMP00105	Enterocin X alpha (Two-peptide bacteriocin)	Antibacterial, Anti-Gram+, Antimicrobial
12	DRAMP00106	Enterocin X beta (Two-peptide bacteriocin)	Antibacterial, Anti-Gram+, Antimicrobial
13	DRAMP00107	Bacteriocin L-1077	Antibacterial, Anti-Gram+, Anti-Gram-, Antimicrobial
14	DRAMP00126	Plantaricin E (PlnE; Bacteriocin)	Antibacterial, Anti-Gram+, Antimicrobial
15	DRAMP00127	Plantaricin F (PlnF; Bacteriocin)	Antibacterial, Anti-Gram+, Antimicrobial
16	DRAMP00128	Plantaricin J (PlnJ; Bacteriocin)	Antibacterial, Anti-Gram+, Antimicrobial
17	DRAMP00129	Plantaricin K (PlnK; Bacteriocin)	Antibacterial, Anti-Gram+, Antimicrobial
18	DRAMP00136	Enterocin E-760 (Bacteriocin)	Antibacterial, Anti-Gram+, Anti-Gram-, Antimicrobial
19	DRAMP00171	Lactocyclicin Q (Bacteriocin)	Antibacterial, Anti-Gram+, Anti-Gram-, Antimicrobial
20	DRAMP00173	Leucocyclicin Q (Bacteriocin)	Antibacterial, Anti-Gram+, Antimicrobial
21	DRAMP00177	Enterocin B (EntB; Bacteriocin)	Antibacterial, Anti-Gram+, Antimicrobial
22	DRAMP00178	Enterocin EJ97 (EntEJ97; Bacteriocin)	Antibacterial, Anti-Gram+, Antimicrobial
23	DRAMP00189	Leucocin Q (Bacteriocin)	Antibacterial, Anti-Gram+, Antimicrobial
24	DRAMP00190	Leucocin N (Bacteriocin)	Antibacterial, Anti-Gram+, Antimicrobial
25	DRAMP00191	Microcin J25 (MccJ25; Bacteriocin)	Antibacterial, Anti-Gram-, Antimicrobial
26	DRAMP00201	Amythiamicin A/B (Bacteriocin)	Antibacterial ,Anti-Gram+, Antimicrobial
27	DRAMP00204	Thiocillin GE37468 (Antibiotic GE37468; Bacteriocin)	Antibacterial, Anti-Gram+, Antimicrobial
28	DRAMP00218	Plantazolicin (PZN; Bacteriocin)	Antibacterial, Anti-Gram+, Antimicrobial
29	DRAMP00222	Microcin E492 (MccE492; Bacteriocin)	Antibacterial, Anti-Gram-, Antimicrobial
30	DRAMP00232	Cypemycin (Bacteriocin)	Antibacterial, Antimicrobial
31	DRAMP00244	Hominicin (Bacteriocin)	Antibacterial, Anti-Gram+, Antimicrobial
32	DRAMP00254	Propionicin-F (Bacteriocin)	Antibacterial, Anti-Gram+, Antimicrobial
33	DRAMP00275	Snakin-1 (StSN1; Cys-rich; Plant defensin)	Antibacterial, Antifungal, Anti-Gram+, Antimicrobial
34	DRAMP00336	ChaC7 (Chassatide C7; uncyclotides; Plant defensin)	Antibacterial, Anti-Gram+, Anti-Gram-, Antimicrobial
35	DRAMP00337	ChaC8 (Chassatide C8; uncyclotides; Plant defensin)	Antibacterial, Anti-Gram+, Anti-Gram-, Antimicrobial
36	DRAMP00338	ChaC11 (Chassatide C11; uncyclotides; Plant defensin)	Antibacterial, Anti-Gram+, Anti-Gram-, Antimicrobial
37	DRAMP00384	Cc-GRP (Gly-rich; Plants)	Antifungal, Antimicrobial
38	DRAMP00385	Panitide L2 (plants)	Antibacterial, Anti-Gram-, Antimicrobial
39	DRAMP00416	Raphanus sativus Antifungal Protein 3 (Rs-AFP3; Plant defensin)	Antifungal, Antimicrobial
40	DRAMP00417	Raphanus sativus Antifungal Protein 4 (Rs-AFP4; Plant defensin)	Antifungal, Antimicrobial
41	DRAMP00422	Defensin-like protein 1 (Sa-AFP1; Plant defensin)	Antifungal, Antimicrobial
42	DRAMP00423	Defensin-like protein 2 (Sa-AFP2; Plant defensin)	Antifungal, Antimicrobial
43	DRAMP00425	Tn-AFP1 (Trapa natans antifungal peptide; Plant defensin)	Antifungal, Antimicrobial
44	DRAMP00431	Defensin-like protein 2 (Cp-thionin II; Cp-thionin-2; Gamma-thionin II; Plant defensin)	Antibacterial, Anti-Gram+, Anti-Gram-, Antimicrobial
45	DRAMP00436	Pisum sativum defensin 1 (Psd1; Plant defensin)	Antifungal, Antimicrobial
46	DRAMP00437	Pisum sativum defensin 2 (Psd2; Plant defensin)	Antifungal, Antimicrobial
47	DRAMP00450	Defensin-like protein 2A (AFP2A; M2A; Plant defensin)	Antifungal, Antimicrobial
48	DRAMP00454	Petunia hybrida defensin 1 (PhD1; Cys-rich; Plant defensin)	Antifungal, Antimicrobial
49	DRAMP00764	Piceain 1 (Plants)	Antibacterial, Antifungal, Anti-Gram+, Anti-Gram-, Antimicrobial
50	DRAMP00765	Piceain 2 (Plants)	Antibacterial, Antifungal, Anti-Gram+, Anti-Gram-, Antimicrobial
51	DRAMP00766	JCpep7 (Plants)	Antibacterial, Anti-Gram+, Anti-Gram-, Antimicrobial

52	DRAMP00774	Hedyotide B1 (hB1; Plants)	Antibacterial, Anti-Gram+, Anti-Gram-, Antimicrobial
53	DRAMP00795	Cliotide T1 (cT1; Plant defensin)	Antibacterial, Anticancer, Anti-Gram+, Anti-Gram-, Antimicrobial
54	DRAMP00798	Cliotide T4 (cT4; Plant defensin)	Antibacterial, Anticancer, Anti-Gram+, Anti-Gram-, Antimicrobial
55	DRAMP00856	Kalata-B1 (Plant defensin)	Antibacterial, Antifungal, Insecticidal, Anti-Gram+, Anti-Gram-, Antimicrobial
56	DRAMP00877	Circulin-A (CIRA; Plant defensin)	Antibacterial, Antifungal, Antiviral, Anti-Gram+, Anti-Gram-, Antimicrobial
57	DRAMP00878	Circulin-B (CIRB; Plant defensin)	Antibacterial, Antifungal, Antiviral, Anti-Gram+, Anti-Gram-, Antimicrobial
58	DRAMP01374	Odorranain-D1 (OdD1; Frogs, amphibians, animals)	Antimicrobial, Antibacterial, Antifungal, Anti-Gram+, Anti-Gram-,
59	DRAMP01373	Odorranain-C1 (OdC1; Frogs, amphibians, animals)	Antimicrobial, Antibacterial, Antifungal, Anti-Gram+, Anti-Gram-,
60	DRAMP01372	Odorranain-B1 (Frogs, amphibians, animals)	Antimicrobial, Antibacterial, Antifungal, Anti-Gram+, Anti-Gram-,
61	DRAMP00933	Antimicrobial peptide 1 (AMP1; MiAMP1; Plant defensin)	Antibacterial, Antifungal, Antimicrobial
62	DRAMP01004	Cucurmoschin (Plants)	Antifungal, Antimicrobial
63	DRAMP01012	Pg-AMP (Gly-rich; Plants)	Antibacterial, Anti-Gram-, Antimicrobial
64	DRAMP01016	Antimicrobial peptide 1 (MJ-AMP1; Plant defensin)	Antibacterial, Antifungal, Anti-Gram+, Antimicrobial
65	DRAMP01017	Antimicrobial peptide 2 (MJ-AMP2; Plant defensin)	Antibacterial, Antifungal, Anti-Gram+, Antimicrobial
66	DRAMP01018	Cyclopsychotride-A (CPT; Plant defensin)	Antibacterial, Antifungal, Anti-Gram+, Anti-Gram-, Antimicrobial
67	DRAMP01061	Antifungal peptide (Cm-p1; Plants)	Antifungal, Antimicrobial
68	DRAMP18193	Cathelicidin-related peptide crotalicidin	Antibacterial, Anti-Gram+, Anti-Gram-, Antimicrobial
69	DRAMP01064	Anticancerous peptide 1 (Cr-ACP1; Plants)	Anticancer, Antibacterial, Anti-Gram+, Anti-Gram-, Antimicrobial
70	DRAMP01066	Kunitz-type serine protease inhibitor 1 (Xb-KTI; Plants)	Antibacterial, Anti-Gram-, Antimicrobial
71	DRAMP01081	Alyteserin-1a (toads, amphibians, animals)	Antibacterial, Anti-Gram-, Antimicrobial
72	DRAMP01082	Alyteserin-2a (toads, amphibians, animals)	Antibacterial, Cytotoxicity, Anti-Gram+, Antimicrobial
73	DRAMP01083	Alyteserin-1b (toads, amphibians, animals)	Antibacterial, Anti-Gram-, Antimicrobial
74	DRAMP01085	Alyteserin-1c (toads, amphibians, animals)	Antibacterial, Anti-Gram-, Antimicrobial
75	DRAMP01088	Alyteserin-1Ma (toads, amphibians, animals)	Antibacterial, Antifungal, Anti-Gram+, Anti-Gram-, Antimicrobial
76	DRAMP01089	Alyteserin-1Mb (toads, amphibians, animals)	Antibacterial, Antifungal, Anti-Gram+, Anti-Gram-, Antimicrobial
77	DRAMP01090	Alyteserin-2Ma (toads, amphibians, animals)	Antibacterial, Antifungal, Anti-Gram+, Anti-Gram-, Antimicrobial
78	DRAMP01091	Alyteserin-2Mb (toads, amphibians, animals)	Antibacterial, Antifungal, Anti-Gram+, Antimicrobial
79	DRAMP01096	Bombinin-like peptide 2 (BLP-2; toads, amphibians, animals)	Antibacterial, Antifungal, Anti-Gram-, Antimicrobial
80	DRAMP01097	Bombinin-like peptide 1 (toads, amphibians, animals)	Antibacterial, Anti-Gram-, Antimicrobial
81	DRAMP01098	Bombinin-like peptide 3 (BLP-3; toads, amphibians, animals)	Antibacterial, Anti-Gram-, Antimicrobial
82	DRAMP01105	Maximin-S4 (chain of Maximins-S type B/C; toads, amphibians, animals)	Antibacterial, Antimicrobial
83	DRAMP02090	Brevinin-1Lb (Frogs, amphibians, animals)	Antibacterial, Anti-Gram+, Anti-Gram-, Antimicrobial
84	DRAMP02091	Brevinin-1Ba (Frogs, amphibians, animals)	Antibacterial, Anti-Gram+, Antimicrobial
85	DRAMP01829	Temporin-1Lc (Temporin 1Lc; Frogs, amphibians, animals)	Antibacterial, Anti-Gram+, Antimicrobial
86	DRAMP01828	Temporin-1Lb (Temporin 1Lb; Frogs, amphibians, animals)	Antibacterial, Anti-Gram+, Antimicrobial
87	DRAMP01518	Esculentin-2L (Frogs, amphibians, animals)	Antimicrobial, Antibacterial, Antifungal, Anti-Gram+, Anti-Gram-,
88	DRAMP01827	Temporin-1La (Temporin 1La; Frogs, amphibians, animals)	Antibacterial, Anti-Gram+, Antimicrobial
89	DRAMP01517	Esculentin-2P (Frogs, amphibians, animals)	Antibacterial, Antiviral , Anti-Gram-, Antimicrobial
90	DRAMP01516	Esculentin-2B (Frogs, amphibians, animals)	Antimicrobial, Antibacterial, Antifungal, Anti-Gram+, Anti-Gram-,
91	DRAMP02077	Brevinin-1Pb (Frogs, amphibians, animals)	Antimicrobial, Antibacterial, Antifungal, Anti-Gram+, Anti-Gram-,
92	DRAMP01830	Temporin-1P (Temporin-1M; Temporin-1CSa; Frogs, amphibians, animals)	Antibacterial,, Anti-Gram+, Antimicrobial
93	DRAMP01151	Uperin-3.5 (toads, amphibians, animals)	Antibacterial, Anti-Gram+, Antimicrobial
94	DRAMP01152	Uperin-3.6 (toads, amphibians, animals)	Antibacterial, Anti-Gram+, Antimicrobial

95	DRAMP01153	Ala4-uperin 3.6 (toads, amphibians, animals)	Antibacterial, Anti-Gram+, Antimicrobial
96	DRAMP01154	Ala7-uperin 3.6 (toads, amphibians, animals)	Antibacterial, Anti-Gram+, Antimicrobial
97	DRAMP01155	Ala14-uperin 3.6 (toads, amphibians, animals)	Antibacterial, Anti-Gram+, Antimicrobial
98	DRAMP01162	Buforin-1 (Buforin I; Fragment of Histone H2A; toads, amphibians, animals)	Antibacterial, Antifungal, Anti-Gram+, Anti-Gram-, Antimicrobial
99	DRAMP01163	Buforin-2 (Buforin II; Fragment of Histone H2A; toads, amphibians, animals)	Antibacterial, Antifungal, Anti-Gram+, Anti-Gram-, Antimicrobial
100	DRAMP01164	Bombinin (toads, amphibians, animals)	Antibacterial, Anti-Gram+, Anti-Gram-, Antimicrobial
101	DRAMP01165	Preprotemporin-1SKa (Frogs, amphibians, animals)	Antibacterial, Antifungal, Anti-Gram-, Antimicrobial
102	DRAMP01167	Hylaseptin-P1 (HSP1)	Antibacterial, Anti-Gram+, Anti-Gram-, Antimicrobial
103	DRAMP01170	Distinctin 2 (Frogs, amphibians, animals)	Antibacterial, Anti-Gram+, Anti-Gram-, Antimicrobial
104	DRAMP01174	Ocellatin-4 (Frogs, amphibians, animals)	Antibacterial, Anti-Gram+, Anti-Gram-, Antimicrobial
105	DRAMP01177	Ocellatin-F1 (Fallaxin; Frogs, amphibians, animals)	Antibacterial, Anti-Gram-, Antimicrobial
106	DRAMP01182	Ocellatin-P1 (Pentadactylin; Frogs, amphibians, animals)	Antibacterial, Anti-Gram+, Anti-Gram-, Antimicrobial
107	DRAMP01184	SPX(1-22)(truncated peptide of Syphaxin; Frogs, amphibians, animals)	Antibacterial, Anti-Gram+, Anti-Gram-, Antimicrobial
108	DRAMP01185	SPX(1-16)(truncated peptide of Syphaxin; Frogs, amphibians, animals)	Antibacterial, Anti-Gram+, Anti-Gram-, Antimicrobial
109	DRAMP01188	Chensinin-1ZHa (Frogs, amphibians, animals)	Antibacterial, Antifungal, Anti-Gram+, Anti-Gram-, Antimicrobial
110	DRAMP01189	Andersonin-W1 (Frogs, amphibians, animals)	Antibacterial, Antifungal, Anti-Gram+, Anti-Gram-, Antimicrobial
111	DRAMP01190	Andersonin-W2 (Frogs, amphibians, animals)	Antibacterial, Antifungal, Anti-Gram+, Anti-Gram-, Antimicrobial
112	DRAMP01191	Andersonin-X1 (Frogs, amphibians, animals)	Antibacterial, Antifungal, Anti-Gram+, Anti-Gram-, Antimicrobial
113	DRAMP01192	Andersonin-Y1 (Frogs, amphibians, animals)	Antibacterial, Antifungal, Anti-Gram+, Anti-Gram-, Antimicrobial
114	DRAMP01194	Andersonin-C1 (Frogs, amphibians, animals)	Antibacterial, Antifungal, Anti-Gram+, Anti-Gram-, Antimicrobial
115	DRAMP01195	Andersonin-D1 (Frogs, amphibians, animals)	Antibacterial, Antifungal, Anti-Gram+, Anti-Gram-, Antimicrobial
116	DRAMP01199	Hejiangin-A1 (Frogs, amphibians, animals)	Antibacterial, Antifungal, Anti-Gram+, Anti-Gram-, Antimicrobial
117	DRAMP01200	Hejiangin-F1 (frog, amphibians, animals)	Antibacterial, Antifungal, Anti-Gram+, Anti-Gram-, Antimicrobial
118	DRAMP01201	Schmackerin-C1 (Frogs, amphibians, animals)	Antibacterial, Antifungal, Anti-Gram+, Anti-Gram-, Antimicrobial
119	DRAMP01202	Fallaxidin 3.2 (Frogs, amphibians, animals)	Antibacterial, Anti-Gram+, Antimicrobial
120	DRAMP01203	Fallaxidin 3.1 (Frogs, amphibians, animals)	Antibacterial, Anti-Gram+, Antimicrobial
121	DRAMP01204	Fallaxidin 4.1 (Frogs, amphibians, animals)	Antibacterial, Anti-Gram+, Antimicrobial
122	DRAMP01208	Pleurain-A1 (Pleurain A1; Frogs, amphibians, animals)	Antibacterial, Antifungal, Anti-Gram+, Anti-Gram-, Antimicrobial
123	DRAMP01209	Pleurain-A2 (Pleurain A2; Frogs, amphibians, animals)	Antibacterial, Antifungal, Anti-Gram+, Anti-Gram-, Antimicrobial
124	DRAMP01214	Kassinatuerin-2Ma (Frogs, amphibians, animals)	Antibacterial, Anti-Gram+, Anti-Gram-, Antimicrobial
125	DRAMP01218	Kassinatuerin-1 (Frogs, amphibians, animals)	Antibacterial, Antifungal, Anti-Gram+, Anti-Gram-, Antimicrobial
126	DRAMP01219	Palustrin-2LTa (Frogs, amphibians, animals)	Antibacterial, Anti-Gram+, Antimicrobial
127	DRAMP01222	Palustrin-2AJ1 (PL2AJ1; Frogs, amphibians, animals)	Antibacterial, Anti-Gram+, Anti-Gram-, Antimicrobial
128	DRAMP01227	Palustrin-1b (Frogs, amphibians, animals)	Antibacterial, Anti-Gram-, Antimicrobial
129	DRAMP01228	Palustrin-1c (Frogs, amphibians, animals)	Antibacterial, Anti-Gram-, Antimicrobial
130	DRAMP01229	Palustrin-1d (Frogs, amphibians, animals)	Antibacterial, Anti-Gram-, Antimicrobial
131	DRAMP01231	Palustrin-2b (Frogs, amphibians, animals)	Antibacterial, Anti-Gram-, Antimicrobial
132	DRAMP01232	Palustrin-2ISc (Frogs, amphibians, animals)	Antibacterial, Anti-Gram+, Anti-Gram-, Antimicrobial
133	DRAMP01233	Palustrin-2c (Frogs, amphibians, animals)	Antibacterial, Anti-Gram-, Antimicrobial
134	DRAMP01234	Palustrin-3a (Frogs, amphibians, animals)	Antibacterial, Anti-Gram-, Antimicrobial
135	DRAMP01235	Palustrin-3b (Frogs, amphibians, animals)	Antibacterial, Anti-Gram-, Antimicrobial
136	DRAMP01237	Palustrin-2ISa (Frogs, amphibians, animals)	Antibacterial, Antifungal, Anti-Gram+, Anti-Gram-, Antimicrobial
137	DRAMP01238	Palustrin-2SIb (Frogs, amphibians, animals)	Antibacterial, Anti-Gram+, Anti-Gram-, Antimicrobial

138	DRAMP01244	Japonicin-1 (Frogs, amphibians, animals)	Antibacterial, Anti-Gram+, Anti-Gram-, Antimicrobial
139	DRAMP01245	Japonicin-1CDYa (Frogs, amphibians, animals)	Antibacterial, Anti-Gram+, Anti-Gram-, Antimicrobial
140	DRAMP01246	Japonicin-2 (Frogs, amphibians, animals)	Antibacterial, Anti-Gram+, Anti-Gram-, Antimicrobial
141	DRAMP01248	Dybowskin-1 (Frogs, amphibians, animals)	Antibacterial, Anti-Gram+, Antimicrobial
142	DRAMP01249	Dybowskin-2 (Frogs, amphibians, animals)	Antibacterial, Anti-Gram+, Anti-Gram-, Antimicrobial
143	DRAMP01250	Dybowskin-3 (Frogs, amphibians, animals)	Antibacterial, Anti-Gram+, Anti-Gram-, Antimicrobial
144	DRAMP01251	Dybowskin-4 (Frogs, amphibians, animals)	Antibacterial, Antifungal, Anti-Gram+, Anti-Gram-, Antimicrobial
145	DRAMP01252	Dybowskin-5 (Frogs, amphibians, animals)	Antibacterial, Antifungal, Anti-Gram+, Anti-Gram-, Antimicrobial
146	DRAMP01253	Dybowskin-6 (Frogs, amphibians, animals)	Antibacterial, Antifungal, Anti-Gram+, Antimicrobial
147	DRAMP01254	Dybowskin-1CDYa (Frogs, amphibians, animals)	Antibacterial, Anti-Gram+, Anti-Gram-, Antimicrobial
148	DRAMP01255	Dybowskin-2CDYa (Chensinin-1; Frogs, amphibians, animals)	Antibacterial, Anti-Gram+, Anti-Gram-, Antimicrobial
149	DRAMP01257	Dermadistinctin-K (DD K; Frogs, amphibians, animals)	Antibacterial, Antifungal, Antiprotozoal, Anti-Gram+, Anti-Gram-, Antimicrobial
150	DRAMP01258	Dermadistinctin-L (DD L; Frogs, amphibians, animals)	Antibacterial, Antifungal, Antiprotozoal, Anti-Gram+, Anti-Gram-, Antimicrobial
151	DRAMP01259	Dermadistinctin-M (DD M; Frogs, amphibians, animals)	Antibacterial, Anti-Gram+, Anti-Gram-, Antimicrobial
152	DRAMP01260	Dermadistinctin-Q1 (DD Q1; Frogs, amphibians, animals)	Antibacterial, Anti-Gram+, Anti-Gram-, Antimicrobial
153	DRAMP01261	Dermadistinctin-Q2 (DD Q2; Frogs, amphibians, animals)	Antibacterial, Anti-Gram+, Anti-Gram-, Antimicrobial
154	DRAMP01288	Phylloseptin-1 (PSN-1; Frogs, amphibians, animals)	Antibacterial, Antifungal, Antimicrobial
155	DRAMP01301	Phylloseptin-1 (PS-1; Frogs, amphibians, animals)	Antibacterial, Antifungal, Antiprotozoal, Anti-Gram+, Anti-Gram-, Antimicrobial
156	DRAMP01302	Phylloseptin-2 (PS-2; Frogs, amphibians, animals)	Antibacterial, Antifungal, Anti-Gram+, Anti-Gram-, Antimicrobial
157	DRAMP01303	Phylloseptin-3 (PS-3; Frogs, amphibians, animals)	Antibacterial, Antifungal, Anti-Gram+, Anti-Gram-, Antimicrobial
158	DRAMP01305	Phylloseptin-7 (PS-7; Frogs, amphibians, animals)	Antibacterial, Anti-Gram+, Anti-Gram-, Antimicrobial
159	DRAMP01306	Phylloseptin-7 (PS-7; Frogs, amphibians, animals)	Antibacterial, Anti-Gram+, Anti-Gram-, Antimicrobial
160	DRAMP01314	Phylloseptin 12 (PS-12; Frogs, amphibians, animals)	Antibacterial, Anti-Gram+, Antimicrobial
161	DRAMP01319	Cathelicidin-AL (Gly-rich; Frogs, amphibians, animals)	Antibacterial, Antifungal, Anti-Gram+, Anti-Gram-, Antimicrobial
162	DRAMP01320	Ranacyclin-B-AL1 (Frogs, amphibians, animals)	Antibacterial, Anti-Gram+, Antimicrobial
163	DRAMP01339	Amolopin-2a (Frogs, amphibians, animals)	Antibacterial, Antifungal, Anti-Gram+, Anti-Gram-, Antimicrobial
164	DRAMP01341	Amolopin-1b (Frogs, amphibians, animals)	Antibacterial, Antifungal, Anti-Gram+, Anti-Gram-, Antimicrobial
165	DRAMP01346	Prepromelittin-related peptide (Frogs, amphibians, animals)	Antibacterial, Antifungal, Anti-Gram+, Anti-Gram-, Antimicrobial
166	DRAMP01347	Prepromelittin-related peptide (Frogs, amphibians, animals)	Antibacterial, Antifungal, Anti-Gram+, Anti-Gram-, Antimicrobial
167	DRAMP01350	Tigerinin-1 (Frogs, amphibians, animals)	Antibacterial, Antifungal, Anti-Gram+, Anti-Gram-, Antimicrobial
168	DRAMP01351	Tigerinin-2 (Frogs, amphibians, animals)	Antibacterial, Antifungal, Anti-Gram+, Anti-Gram-, Antimicrobial
169	DRAMP01352	Tigerinin-3 (Frogs, amphibians, animals)	Antibacterial, Antifungal, Anti-Gram+, Anti-Gram-, Antimicrobial
170	DRAMP01353	Tigerinin-4 (Frogs, amphibians, animals)	Antibacterial, Antifungal, Anti-Gram+, Anti-Gram-, Antimicrobial
171	DRAMP01354	Peptide leucine arginine (pLR; Frogs, amphibians, animals)	Antibacterial, Antifungal, Anti-Gram+, Antimicrobial
172	DRAMP01355	Ranalexin (Frogs, amphibians, animals)	Antibacterial, Antifungal, Anti-Gram+, Anti-Gram-, Antimicrobial
173	DRAMP01393	Odorranain-W1 (OdW1; Frogs, amphibians, animals)	Antimicrobial, Antibacterial, Antifungal, Anti-Gram+, Anti-Gram-,
174	DRAMP01358	Ranalexin-Vb (Frogs, amphibians, animals)	Antibacterial, Anti-Gram+, Anti-Gram-, Antimicrobial
175	DRAMP01359	Ranalexin-1G (Frogs, amphibians, animals)	Antibacterial, Anti-Gram+, Anti-Gram-, Antimicrobial
176	DRAMP01360	Frenatin-1 (Frogs, amphibians, animals)	Antibacterial, Anti-Gram+, Antimicrobial
177	DRAMP01361	Frenatin-2 (Frogs, amphibians, animals)	Antibacterial, Anti-Gram+, Antimicrobial
178	DRAMP01362	Frenatin-3 (Frogs, amphibians, animals)	Antibacterial, Anti-Gram+, Anti-Gram-, Antimicrobial
179	DRAMP01364	Maculatin-1.1 (Frogs, amphibians, animals)	Antibacterial, Antifungal, Antiviral, Anti-Gram+, Anti-Gram-, Antimicrobial

180	DRAMP01367	Maculatin-1.4 (frog, amphibia, animals)	Antibacterial, Anti-Gram+, Antimicrobial
181	DRAMP01368	Maculatin-2.1 (Frogs, amphibians, animals)	Antibacterial, Anti-Gram+, Antimicrobial
182	DRAMP01370	Oh-defensin (O. hainana defensin; spiders, animals)	Antibacterial, Antifungal, Anti-Gram+, Anti-Gram-, Antimicrobial
183	DRAMP01371	Odorranain-NR (Frogs, amphibians, animals)	Antibacterial, Antifungal, Anti-Gram+, Anti-Gram-, Antimicrobial
184	DRAMP00931	Antimicrobial peptide 3 (Cn-AMP3; Plant defensin)	Antibacterial, Anti-Gram+, Anti-Gram-, Antimicrobial
185	DRAMP00930	Antimicrobial peptide 2 (Cn-AMP2; Plant defensin)	Antibacterial, Anti-Gram+, Anti-Gram-, Antimicrobial
186	DRAMP00929	Antimicrobial peptide 1 (Cn-AMP1; Plant defensin)	Antibacterial, Anti-Gram+, Anti-Gram-, Antimicrobial
187	DRAMP03542	Neurokinin A (NKA; chicken, animals)	Neuropeptide,Antibacterial, Anti-Gram+, Anti-Gram-, Antimicrobial
188	DRAMP04532	Myxinidin (Hagfish, animals)	Antimicrobial, Antibacterial, Antifungal, Anti-Gram+, Anti-Gram-,
189	DRAMP02344	40S ribosomal protein S30 (Fish, chordates, animals)	Antibacterial, Anti-Gram+, Antimicrobial
190	DRAMP02993	Abaecin (Pro-rich; insects, arthropods, invertebrates, animals)	Antibacterial, Anti-Gram+, Anti-Gram-, Antimicrobial
191	DRAMP02997	Apidaecin-1B (Apidaecin IB; Insects, animals)	Antibacterial, Anti-Gram+, Anti-Gram-, Antimicrobial
192	DRAMP18322	Hominicin (Bacteriocin)	Antibacterial, Anti-Gram+, Antimicrobial
193	DRAMP02840	Lactoferricin B (Lfcin B; mammals, animals)	Antibacterial, Anti-Gram+, Anti-Gram-, Antimicrobial
194	DRAMP02244	Ranatuerin-2Ca (Ranatuerin 2Ca; Frogs, amphibians, animals)	Antimicrobial, Antibacterial, Antifungal,
195	DRAMP01821	Temporin-1Cc (Temporin 1Cc; Frogs, amphibians, animals)	Antibacterial, Antimicrobial
196	DRAMP01819	Temporin-1Ca (Temporin 1Ca; Frogs, amphibians, animals)	Antibacterial, Antimicrobial
197	DRAMP01357	Ranalexin-1Cb (Ranatuerin 1Cb; Frogs, amphibians, animals)	Antimicrobial, Antibacterial, Antifungal,
198	DRAMP02246	Ranatuerin-1C (Ranatuerin 1C; Frogs, amphibians, animals)	Antimicrobial, Antibacterial, Antifungal, Anti-Gram+, Anti-Gram-,
199	DRAMP01394	Odorranain-W2 (Frogs, amphibians, animals)	Antibacterial, Antifungal, Anti-Gram+, Anti-Gram-, Antimicrobial
200	DRAMP01395	Odorranain-A-OA1 (Frogs, amphibians, animals)	Antibacterial, Antifungal , Anti-Gram+, Anti-Gram-, Antimicrobial
201	DRAMP01396	Odorranain-F-OA1 (Frogs, amphibians, animals)	Antibacterial, Antifungal , Anti-Gram+, Anti-Gram-, Antimicrobial
202	DRAMP01397	Odorranain-F-OA2 (Frogs, amphibians, animals)	Antibacterial, Antifungal , Anti-Gram+, Anti-Gram-, Antimicrobial
203	DRAMP01398	Odorranain-F-OA3 (Frogs, amphibians, animals)	Antibacterial, Antifungal , Anti-Gram+, Anti-Gram-, Antimicrobial
204	DRAMP01399	Odorranain-F-OA4 (Frogs, amphibians, animals)	Antibacterial, Antifungal , Anti-Gram+, Anti-Gram-, Antimicrobial
205	DRAMP18395	moroPC-NH2 (moronecidin-like peptide; fish, animals)	Antibacterial, antifungal, Antimicrobial
206	DRAMP01401	Odorranain-F-OW1 (Frogs, amphibians, animals)	Antibacterial, Antifungal , Anti-Gram+, Anti-Gram-, Antimicrobial
207	DRAMP01402	Odorranain-J-OA1 (Frogs, amphibians, animals)	Antibacterial, Antifungal , Anti-Gram+, Anti-Gram-, Antimicrobial
208	DRAMP01403	Odorranain-J-OA2 (Frogs, amphibians, animals)	Antibacterial, Antifungal , Anti-Gram+, Anti-Gram-, Antimicrobial
209	DRAMP01409	Nigrocin-OR1 (Frogs, amphibians, animals)	Antibacterial, Antifungal, Anti-Gram+, Anti-Gram-, Antimicrobial
210	DRAMP01410	Nigrocin-OR2 (Frogs, amphibians, animals)	Antibacterial, Antifungal, Anti-Gram+, Anti-Gram-, Antimicrobial
211	DRAMP01411	Nigrocin-OR3 (Frogs, amphibians, animals)	Antibacterial, Antifungal, Anti-Gram+, Anti-Gram-, Antimicrobial
212	DRAMP01412	Nigrocin-2HSa (Frogs, amphibians, animals)	Antibacterial, Anti-Gram+, Anti-Gram-, Antimicrobial
213	DRAMP01413	Nigrocin-2HSb (Frogs, amphibians, animals)	Antibacterial, Anti-Gram+, Anti-Gram-, Antimicrobial
214	DRAMP01414	Nigrocin-2ISa (Frogs, amphibians, animals)	Antibacterial, Antifungal, Anti-Gram+, Anti-Gram-, Antimicrobial
215	DRAMP01415	Nigrocin-2ISb (Frogs, amphibians, animals)	Antibacterial, Antifungal, Anti-Gram+, Anti-Gram-, Antimicrobial
216	DRAMP01416	Nigrocin-2ISc (Frogs, amphibians, animals)	Antibacterial, Antifungal, Anti-Gram+, Anti-Gram-, Antimicrobial
217	DRAMP01417	Nigrocin-2GRa (Frogs, amphibians, animals)	Antibacterial, Anti-Gram-, Antimicrobial
218	DRAMP01418	Nigrocin-2GRb (Frogs, amphibians, animals)	Antibacterial, Antifungal, Anti-Gram+, Anti-Gram-, Antimicrobial
219	DRAMP01419	Nigrocin-2GRc (Frogs, amphibians, animals)	Antibacterial, Anti-Gram-, Antimicrobial

220	DRAMP01420	Nigrocin-OG4 (Frogs, amphibians, animals)	Antibacterial, Antifungal , Anti-Gram+, Anti-Gram-, Antimicrobial
221	DRAMP01421	Nigrocin-OG5 (Frogs, amphibians, animals)	Antibacterial, Antifungal , Anti-Gram+, Anti-Gram-, Antimicrobial
222	DRAMP01422	Nigrosin-OG21 (Frogs, amphibians, animals)	Antibacterial, Antifungal , Anti-Gram+, Anti-Gram-, Antimicrobial
223	DRAMP01423	Nigrosin-OG13 (Frogs, amphibians, animals)	Antibacterial, Antifungal , Anti-Gram+, Anti-Gram-, Antimicrobial
224	DRAMP01426	Nigrocin-1-OA1 (Frogs, amphibians, animals)	Antibacterial, Antifungal, Anti-Gram+, Anti-Gram-, Antimicrobial
225	DRAMP01427	Nigrocin-1-OA2 (Frogs, amphibians, animals)	Antibacterial, Antifungal, Anti-Gram+, Anti-Gram-, Antimicrobial
226	DRAMP01428	Nigrocin-1-OA3 (Frogs, amphibians, animals)	Antibacterial, Antifungal, Anti-Gram+, Anti-Gram-, Antimicrobial
227	DRAMP01429	Nigrocin-1-OR1 (Frogs, amphibians, animals)	Antibacterial, Antifungal, Anti-Gram+, Anti-Gram-, Antimicrobial
228	DRAMP01430	Nigrocin-1-OR2 (Frogs, amphibians, animals)	Antibacterial, Antifungal, Anti-Gram+, Anti-Gram-, Antimicrobial
229	DRAMP01431	Nigrocin-1-OR3 (Frogs, amphibians, animals)	Antibacterial, Antifungal, Anti-Gram+, Anti-Gram-, Antimicrobial
230	DRAMP01432	Nigrocin-1-OW2 (Frogs, amphibians, animals)	Antibacterial, Antifungal, Anti-Gram+, Anti-Gram-, Antimicrobial
231	DRAMP01433	Nigrocin-1-OW3 (Frogs, amphibians, animals)	Antibacterial, Antifungal, Anti-Gram+, Anti-Gram-, Antimicrobial
232	DRAMP01434	Nigrocin-1-OW4 (Frogs, amphibians, animals)	Antibacterial, Antifungal, Anti-Gram+, Anti-Gram-, Antimicrobial
233	DRAMP01435	Nigrocin-1-OW5 (Frogs, amphibians, animals)	Antibacterial, Antifungal, Anti-Gram+, Anti-Gram-, Antimicrobial
234	DRAMP01436	Nigrocin-1-OW1 (Frogs, amphibians, animals)	Antibacterial, Antifungal, Anti-Gram+, Anti-Gram-, Antimicrobial
235	DRAMP18394	NCR335 (nodule-specific cysteine-rich peptides; plants)	Antibacterial, Antimicrobial
236	DRAMP01438	Nigrocin-2JDa (Frogs, amphibians, animals)	Antibacterial, Antifungal , Anti-Gram+, Anti-Gram-, Antimicrobial
237	DRAMP01439	Nigrocin-2JDb (Odorranain-H2; Frogs, amphibians, animals)	Antibacterial, Antifungal , Anti-Gram+, Anti-Gram-, Antimicrobial
238	DRAMP01440	Nigrocin-2LVb (Frogs, amphibians, animals)	Antibacterial, Anti-Gram+, Anti-Gram-, Antimicrobial
239	DRAMP01441	Nigrocin-2VB (Frogs, amphibians, animals)	Antibacterial, Anti-Gram-, Antimicrobial
240	DRAMP01442	Nigrocin-2SCa (Frogs, amphibians, animals)	Antibacterial, Anti-Gram-, Antimicrobial
241	DRAMP01443	Nigrocin-2SCc (Frogs, amphibians, animals)	Antibacterial, Anti-Gram-, Antimicrobial
242	DRAMP01447	Esculentin-2CHa (Frogs, amphibians, animals)	Antibacterial, Antifungal, Anti-Gram+, Anti-Gram-, Antimicrobial
243	DRAMP01452	Esculentin-1LTa (Frogs, amphibians, animals)	Antibacterial, Antifungal, Anti-Gram+, Anti-Gram-, Antimicrobial
244	DRAMP01453	Esculentin-2LTa (Frogs, amphibians, animals)	Antibacterial, Anti-Gram+, Anti-Gram-, Antimicrobial
245	DRAMP01454	Esculentin-2JDa (Frogs, amphibians, animals)	Antibacterial, Anti-Gram+, Anti-Gram-, Antimicrobial
246	DRAMP01456	Esculentin-2PLa (Frogs, amphibians, animals)	Antibacterial, Antifungal, Anti-Gram+, Anti-Gram-, Antimicrobial
247	DRAMP01457	Esculentin-1V (Frogs, amphibians, animals)	Antibacterial, Anti-Gram+, Anti-Gram-, Antimicrobial
248	DRAMP01458	Esculentin-2V (Frogs, amphibians, animals)	Antibacterial, Anti-Gram+, Anti-Gram-, Antimicrobial
249	DRAMP01461	Esculentin-1S (Frogs, amphibians, animals)	Antibacterial, Anti-Gram+, Anti-Gram-, Antimicrobial
250	DRAMP01462	Esculentin-2S (Frogs, amphibians, animals)	Antibacterial , Anti-Gram+, Anti-Gram-, Antimicrobial
251	DRAMP01469	Esculentin-2-Ala (Frogs, amphibians, animals)	Antibacterial, Anti-Gram+, Anti-Gram-, Antimicrobial
252	DRAMP01470	Esculentin-2-ALb (Frogs, amphibians, animals)	Antibacterial, Anti-Gram+, Anti-Gram-, Antimicrobial
253	DRAMP01471	Esculentin-1PLa (Frogs, amphibians, animals)	Antibacterial, Anti-Gram+, Anti-Gram-, Antimicrobial
254	DRAMP01472	Esculentin-1PLb (Frogs, amphibians, animals)	Antibacterial, Anti-Gram+, Anti-Gram-, Antimicrobial
255	DRAMP18393	VK6 (histone derived; reptiles; animals)	Antibacterial, Antimicrobial
256	DRAMP01474	Esculentin-1ARa (Frogs, amphibians, animals)	Antibacterial, Anti-Gram+, Anti-Gram-, Antimicrobial
257	DRAMP01475	Esculentin-1ARb (Frogs, amphibians, animals)	Antibacterial, Anti-Gram+, Anti-Gram-, Antimicrobial
258	DRAMP01476	Esculentin-2HSa (Frogs, amphibians, animals)	Antibacterial, Anti-Gram+, Anti-Gram-, Antimicrobial
259	DRAMP01477	Esculentin-1HSa (Frogs, amphibians, animals)	Antibacterial, Anti-Gram+, Anti-Gram-, Antimicrobial
260	DRAMP01479	Esculentin-1CPa (Frogs, amphibians, animals)	Antibacterial, Antifungal, Anti-Gram+, Anti-Gram-, Antimicrobial

261	DRAMP01480	Esculentin-2CPa (Frogs, amphibians, animals)	Antibacterial, Antifungal, Anti-Gram+, Anti-Gram-, Antimicrobial
262	DRAMP01482	Esculentin-1ISa (Frogs, amphibians, animals)	Antibacterial, Anti-Gram+, Anti-Gram-, Antimicrobial
263	DRAMP01483	Esculentin-1ISb (Frogs, amphibians, animals)	Antibacterial, Antifungal, Anti-Gram+, Anti-Gram-, Antimicrobial
264	DRAMP01484	Esculentin-2ISa (Frogs, amphibians, animals)	Antibacterial, Antifungal, Anti-Gram+, Anti-Gram-, Antimicrobial
265	DRAMP01486	Esculentin-1GRa (Frogs, amphibians, animals)	Antibacterial, Antifungal, Anti-Gram+, Anti-Gram-, Antimicrobial
266	DRAMP01490	Esculentin-2A (Frogs, amphibians, animals)	Antibacterial, Anti-Gram+, Anti-Gram-, Antimicrobial
267	DRAMP01491	Esculentin-1B (Frogs, amphibians, animals)	Antibacterial, Anti-Gram+, Anti-Gram-, Antimicrobial
268	DRAMP01493	Esculentin-1-OA1 (Frogs, amphibians, animals)	Antibacterial, Antifungal, Anti-Gram+, Anti-Gram-, Antimicrobial
269	DRAMP01494	Esculentin-1-OA2 (Frogs, amphibians, animals)	Antibacterial, Antifungal, Anti-Gram+, Anti-Gram-, Antimicrobial
270	DRAMP01495	Esculentin-1-OA3 (Frogs, amphibians, animals)	Antibacterial, Antifungal, Anti-Gram+, Anti-Gram-, Antimicrobial
271	DRAMP01496	Esculentin-1-OA4 (Frogs, amphibians, animals)	Antibacterial, Antifungal, Anti-Gram+, Anti-Gram-, Antimicrobial
272	DRAMP01497	Esculentin-1-OA5 (Frogs, amphibians, animals)	Antibacterial, Antifungal, Anti-Gram+, Anti-Gram-, Antimicrobial
273	DRAMP01499	Esculentin-1-OR1 (Frogs, amphibians, animals)	Antibacterial, Antifungal, Anti-Gram+, Anti-Gram-, Antimicrobial
274	DRAMP01501	Esculentin-1-OR3 (Frogs, amphibians, animals)	Antibacterial, Antifungal, Anti-Gram+, Anti-Gram-, Antimicrobial
275	DRAMP01502	Esculentin-1-OR4 (Frogs, amphibians, animals)	Antibacterial, Antifungal, Anti-Gram+, Anti-Gram-, Antimicrobial
276	DRAMP01503	Esculentin-1-OR5 (Frogs, amphibians, animals)	Antibacterial, Antifungal, Anti-Gram+, Anti-Gram-, Antimicrobial
277	DRAMP01504	Esculentin-2-OA1 (Frogs, amphibians, animals)	Antibacterial, Antifungal, Anti-Gram+, Anti-Gram-, Antimicrobial
278	DRAMP01505	Esculentin-2-OA2 (Frogs, amphibians, animals)	Antibacterial, Antifungal, Anti-Gram+, Anti-Gram-, Antimicrobial
279	DRAMP18392	VK7 (histone derived; reptiles; animals)	Antibacterial, Antimicrobial
280	DRAMP01507	Esculentin-2-OR1 (Frogs, amphibians, animals)	Antibacterial, Antifungal, Anti-Gram+, Anti-Gram-, Antimicrobial
281	DRAMP01508	Esculentin-2-OR2 (Frogs, amphibians, animals)	Antibacterial, Antifungal, Anti-Gram+, Anti-Gram-, Antimicrobial
282	DRAMP01509	Esculentin-2-OR3 (Frogs, amphibians, animals)	Antibacterial, Antifungal, Anti-Gram+, Anti-Gram-, Antimicrobial
283	DRAMP01510	Esculentin-2-OR4 (Frogs, amphibians, animals)	Antibacterial, Antifungal, Anti-Gram+, Anti-Gram-, Antimicrobial
284	DRAMP01511	Esculentin-2-OR5 (Frogs, amphibians, animals)	Antibacterial, Antifungal, Anti-Gram+, Anti-Gram-, Antimicrobial
285	DRAMP01513	Esculentin-1 (Frogs, amphibians, animals)	Antibacterial, Antifungal, Anti-Gram+, Anti-Gram-, Antimicrobial
286	DRAMP01520	Rugosin-A (Frogs, amphibians, animals)	Antibacterial, Anti-Gram+, Anti-Gram-, Antimicrobial
287	DRAMP01521	Rugosin-B (Frogs, amphibians, animals)	Antibacterial, Anti-Gram+, Anti-Gram-, Antimicrobial
288	DRAMP01524	Rugosin-RN1 (Frogs, amphibians, animals)	Antibacterial, Antifungal, Anti-Gram+, Anti-Gram-, Antimicrobial
289	DRAMP01525	Rugosin-RN3 (Frogs, amphibians, animals)	Antibacterial, Antifungal, Anti-Gram+, Anti-Gram-, Antimicrobial
290	DRAMP01526	Rugosin-RN5 (Frogs, amphibians, animals)	Antibacterial, Antifungal, Anti-Gram+, Anti-Gram-, Antimicrobial
291	DRAMP01533	Nigroain-B1 (Frogs, amphibians, animals)	Antibacterial, Anti-Gram+, Anti-Gram-, Antimicrobial
292	DRAMP01539	Nigroain-C2 (Frogs, amphibians, animals)	Antibacterial, Antifungal, Anti-Gram+, Anti-Gram-, Antimicrobial
293	DRAMP01542	Nigroain-D3 (Frogs, amphibians, animals)	Antibacterial, Anti-Gram+, Antimicrobial
294	DRAMP01543	Nigroain-E1 (Frogs, amphibians, animals)	Antibacterial, Antifungal, Anti-Gram+, Antimicrobial
295	DRAMP01546	Nigroain-K1 (Frogs, amphibians, animals)	Antibacterial, Antifungal, Anti-Gram+, Anti-Gram-, Antimicrobial
296	DRAMP01547	Nigroain-K2 (Frogs, amphibians, animals)	Antibacterial, Antifungal, Anti-Gram+, Antimicrobial

297	DRAMP01549	Caerin-1.1 (Frogs, amphibians, animals)	Antibacterial, Antiviral, Anti-Gram+, Anti-Gram-, Antimicrobial
298	DRAMP01550	Caerin-1.11 (Frogs, amphibians, animals)	Antibacterial, Anti-Gram+, Anti-Gram-, Antimicrobial
299	DRAMP01552	Caerin-1.3 (Frogs, amphibians, animals)	Antibacterial, Anti-Gram+, Anti-Gram-, Antimicrobial
300	DRAMP01553	Caerin-1.4 (Frogs, amphibians, animals)	Antibacterial, Anti-Gram+, Anti-Gram-, Antimicrobial
301	DRAMP01555	Caerin-1.5 (Frogs, amphibians, animals)	Antibacterial, Anti-Gram+, Anti-Gram-, Antimicrobial
302	DRAMP01560	Caerin-1.9 (Frogs, amphibians, animals)	Antibacterial, Antifungal, Antiviral, Anti-Gram+, Anti-Gram-, Antimicrobial
303	DRAMP01562	Caerin-2.1 (Frogs, amphibians, animals)	Antibacterial , Anti-Gram-, Antimicrobial
304	DRAMP01563	Caerin-2.2 (Frogs, amphibians, animals)	Antibacterial , Anti-Gram+, Anti-Gram-, Antimicrobial
305	DRAMP01567	Caerin-2.6 (Frogs, amphibians, animals)	Antibacterial, Anti-Gram+, Antimicrobial
306	DRAMP01568	Caerin-2.7 (Frogs, amphibians, animals)	Antibacterial, Anti-Gram+, Antimicrobial
307	DRAMP01570	Caerin-3.2 (Frogs, amphibians, animals)	Antibacterial, Anti-Gram+, Antimicrobial
308	DRAMP01573	Caerin-3.5 (Frogs, amphibians, animals)	Antibacterial, Anti-Gram+, Antimicrobial
309	DRAMP01574	Caerin-4.1 (Frogs, amphibians, animals)	Antibacterial, Antiviral, Anti-Gram+, Anti-Gram-, Antimicrobial
310	DRAMP01576	Caerin-4.3 (Frogs, amphibians, animals)	Antibacterial, Anti-Gram+, Anti-Gram-, Antimicrobial
311	DRAMP01577	Caerin-1.10 (Frogs, amphibians, animals)	Antibacterial, Anti-Gram+, Anti-Gram-, Antimicrobial
312	DRAMP18389	VK12 (histone derived; animals)	Antibacterial, Antimicrobial
313	DRAMP18390	VK11 (histone derived; reptiles; animals)	Antibacterial, Antimicrobial
314	DRAMP18391	VK10 (histone derived; reptiles; animals)	Antibacterial, Antimicrobial
315	DRAMP01584	Caerin-1.17 (Frogs, amphibians, animals)	Antibacterial, Anti-Gram+, Antimicrobial
316	DRAMP01585	Caerin-1.18 (Frogs, amphibians, animals)	Antibacterial, Anti-Gram+, Anti-Gram-, Antimicrobial
317	DRAMP01586	Caerin-1.19 (Frogs, amphibians, animals)	Antibacterial, Anti-Gram+, Anti-Gram-, Antimicrobial
318	DRAMP01587	Citropin-1.1 (Frogs, amphibians, animals)	Antibacterial, Anti-Gram+, Antimicrobial
319	DRAMP01588	Citropin-1.1 sm1 (Frogs, amphibians, animals)	Antibacterial, Anti-Gram+, Antimicrobial
320	DRAMP01589	Citropin-1.1 sm2 (Frogs, amphibians, animals)	Antibacterial, Anti-Gram+, Antimicrobial
321	DRAMP01590	Citropin 1.1 M14 (Frogs, amphibians, animals)	Antibacterial, Anti-Gram+, Anti-Gram-, Antimicrobial
322	DRAMP01591	Citropin 1.1 M15 (Frogs, amphibians, animals)	Antibacterial, Anti-Gram+, Anti-Gram-, Antimicrobial
323	DRAMP01594	Citropin-1.2 (Frogs, amphibians, animals)	Antibacterial, Anti-Gram+, Antimicrobial
324	DRAMP01600	Citropin-1.3 (Frogs, amphibians, animals)	Antibacterial, Anti-Gram+, Antimicrobial
325	DRAMP01601	Citropin-2.1 (Frogs, amphibians, animals)	Antibacterial, Anti-Gram+, Antimicrobial
326	DRAMP01602	Citropin-2.1.3 (Frogs, amphibians, animals)	Antibacterial, Anti-Gram+, Antimicrobial
327	DRAMP01603	Citropin 1.1.3 (Frogs, amphibians, animals)	Antibacterial, Anti-Gram+, Antimicrobial
328	DRAMP01606	Aurein-1.1 (Frogs, amphibians, animals)	Antibacterial, Anti-Gram+, Antimicrobial
329	DRAMP01607	Aurein-1.2 (Frogs, amphibians, animals)	Antibacterial, Anticancer, Anti-Gram+, Anti-Gram-, Antimicrobial
330	DRAMP01608	Aurein-2.1 (Frogs, amphibians, animals)	Antimicrobial, Anticancer, Anti-Gram+,
331	DRAMP01612	Aurein-2.5 (Frogs, amphibians, animals)	Antibacterial, Anticancer, Anti-Gram+, Antimicrobial
332	DRAMP01613	Aurein-2.6 (Frogs, amphibians, animals)	Antibacterial, Anticancer, Anti-Gram+, Antimicrobial
333	DRAMP01614	Aurein-3.1 (Frogs, amphibians, animals)	Antibacterial, Anticancer, Anti-Gram+, Antimicrobial
334	DRAMP01617	Aurein-3.2 (Frogs, amphibians, animals)	Antibacterial, Anticancer, Anti-Gram+, Antimicrobial
335	DRAMP01618	Aurein-3.3 (Frogs, amphibians, animals)	Antibacterial, Anticancer, Anti-Gram+, Anti-Gram-, Antimicrobial
336	DRAMP01620	Aurein-5.2 (Frogs, amphibians, animals)	Antibacterial, Anticancer, Anti-Gram+, Antimicrobial
337	DRAMP01621	Bombinin-H1 (Frogs, amphibians, animals)	Antibacterial, Anti-Gram+, Anti-Gram-, Antimicrobial
338	DRAMP01623	Bombinin-H4 (bombinin H isomers; Frogs, amphibians, animals)	Antibacterial, Anti-Gram+, Anti-Gram-, Antimicrobial
339	DRAMP01626	Bombinin-H5 (Frogs, amphibians, animals)	Antibacterial, Anti-Gram+, Anti-Gram-, Antimicrobial
340	DRAMP01627	Skin peptide tyrosine-tyrosine (Skin-PYY; SPYY; Frogs, amphibians, animals)	Antibacterial, Antifungal, Anti-Gram+, Anti-Gram-, Antimicrobial
341	DRAMP01628	Phylloxin (Frogs, amphibians, animals)	Antibacterial, Anti-Gram+, Anti-Gram-, Antimicrobial
342	DRAMP01638	Dermaseptin-L1 (Frogs, amphibians, animals)	Antibacterial, Antifungal, Anti-Gram-, Antimicrobial
343	DRAMP01639	Dermaseptin-1 (DShypo01, DPh-1; Frogs, amphibians, animals)	Antibacterial, Antiprotozoal, Anti-Gram+, Anti-Gram-, Antimicrobial
344	DRAMP01643	Dermaseptin-5 (DShypo05, DS 01; Frogs, amphibians, animals)	Antibacterial, Antiprotozoal, Anti-Gram+, Anti-Gram-, Antimicrobial
345	DRAMP01646	Adenoregulin (Dermaseptin BII; Dermaseptin B2; Frogs, amphibians, animals)	Antibacterial, Antifungal, Anti-Gram+, Anti-Gram-, Antimicrobial

346	DRAMP01647	DRP-PBN1 (Frogs, amphibians, animals)	Antibacterial, Anti-Gram+, Antimicrobial
347	DRAMP01648	Dermaseptin-like PBN2 (DRP-PBN2; Plasticin-B1a; Frogs, amphibians, animals)	Antibacterial, Antifungal, Anti-Gram+, Anti-Gram-, Antimicrobial
348	DRAMP01649	Dermaseptin-BI (Dermaseptin B1; Frogs, amphibians, animals)	Antibacterial, Antifungal, Anti-Gram+, Anti-Gram-, Antimicrobial
349	DRAMP01650	Dermaseptin-B3 (Dermaseptin BIII; Frogs, amphibians, animals)	Antibacterial, Anti-Gram+, Anti-Gram-, Antimicrobial
350	DRAMP01651	Dermaseptin-B4 (Dermaseptin BIV; Frogs, amphibians, animals)	Antibacterial, Anti-Gram+, Anti-Gram-, Antimicrobial
351	DRAMP01657	Dermaseptin DRG3 (Dermaseptin-3; Frogs, amphibians, animals)	Antibacterial, Antimicrobial
352	DRAMP01668	Dermaseptin-1 (DS I; Dermaseptin-S1, DS1; Frogs, amphibians, animals)	Antibacterial, Antifungal, Antiprotozoal, Anti-Gram+, Anti-Gram-, Antimicrobial
353	DRAMP18386	VK25 (histone derived; reptiles; animals)	Antibacterial, Antimicrobial
354	DRAMP18387	VK14 (histone derived;reptiles; animals)	Antibacterial, Antimicrobial
355	DRAMP18388	VK13 (histone derived; reptiles; animals)	Antibacterial, Antimicrobial
356	DRAMP18385	cOT2 (reptiles; animals)	Antibacterial, Antimicrobial
357	DRAMP01702	Dermaseptin-H5 (Dermaseptin-like peptide 5, DMS5; Frogs, amphibians, animals)	Antibacterial , Anti-Gram+, Anti-Gram-, Antimicrobial
358	DRAMP01721	[T5k]temporin-DRa (Frogs, amphibians, animals)	Antibacterial, Antimicrobial
359	DRAMP01730	Temporin-A (Frogs, amphibians, animals)	Antibacterial, Antifungal, Anti-Gram+, Anti-Gram-, Antimicrobial
360	DRAMP01731	Temporin-ALd (Frogs, amphibians, animals)	Antibacterial, Anti-Gram+, Anti-Gram-, Antimicrobial
361	DRAMP01732	Temporin-ALe (Frogs, amphibians, animals)	Antibacterial, Anti-Gram+, Anti-Gram-, Antimicrobial
362	DRAMP01733	Temporin-ALf (Frogs, amphibians, animals)	Antibacterial, Anti-Gram+, Anti-Gram-, Antimicrobial
363	DRAMP01734	Temporin-ALg (Frogs, amphibians, animals)	Antibacterial, Anti-Gram+, Anti-Gram-, Antimicrobial
364	DRAMP01735	Temporin-ALh (Frogs, amphibians, animals)	Antibacterial, Anti-Gram+, Anti-Gram-, Antimicrobial
365	DRAMP01736	Temporin-ALi (Frogs, amphibians, animals)	Antibacterial, Anti-Gram+, Anti-Gram-, Antimicrobial
366	DRAMP01737	Temporin-ALj (Frogs, amphibians, animals)	Antibacterial, Anti-Gram+, Anti-Gram-, Antimicrobial
367	DRAMP01738	Temporin-ALk (Frogs, amphibians, animals)	Antibacterial, Anti-Gram+, Anti-Gram-, Antimicrobial
368	DRAMP01739	Temporin-B (Frogs, amphibians, animals)	Antibacterial, Antifungal, Anti-Gram+, Anti-Gram-, Antimicrobial
369	DRAMP01750	Temporin-1PLa (Frogs, amphibians, animals)	Antibacterial, Anti-Gram+, Antimicrobial
370	DRAMP01751	Temporin-LT1 (Frogs, amphibians, animals)	Antibacterial, Anti-Gram+, Antimicrobial
371	DRAMP01752	Temporin-LT2 (Frogs, amphibians, animals)	Antibacterial, Anti-Gram+, Antimicrobial
372	DRAMP01753	Temporin-1CEa (Frogs, amphibians, animals)	Antibacterial, Anti-Gram+, Anti-Gram-, Antimicrobial
373	DRAMP01754	Temporin-1CEb (Frogs, amphibians, animals)	Antibacterial, Anti-Gram+, Anti-Gram-, Antimicrobial
374	DRAMP01755	Temporin-1TSa (Frogs, amphibians, animals)	Antibacterial, Anti-Gram+, Anti-Gram-, Antimicrobial
375	DRAMP01759	Temporin-1SPb (Frogs, amphibians, animals)	Antibacterial, Anti-Gram+, Antimicrobial
376	DRAMP01764	Temporin-1TGa (Frogs, amphibians, animals)	Antibacterial, Antifungal, Anti-Gram+, Anti-Gram-, Antimicrobial
377	DRAMP01765	Temporin-1TGb (Frogs, amphibians, animals)	Antibacterial, Anti-Gram+, Anti-Gram-, Antimicrobial
378	DRAMP01766	Temporin-1TGc (Frogs, amphibians, animals)	Antibacterial, Anti-Gram+, Anti-Gram-, Antimicrobial
379	DRAMP01768	Temporin-1SKa (Frogs, amphibians, animals)	Antibacterial, Anti-Gram+, Antimicrobial
380	DRAMP18384	sOT2 (reptiles; animals)	Antibacterial, Antimicrobial
381	DRAMP01771	Temporin-1Oa (Frogs, amphibians, animals)	Antibacterial, Anti-Gram+, Anti-Gram-, Antimicrobial
382	DRAMP01773	Temporin-1Oc (Frogs, amphibians, animals)	Antibacterial, Anti-Gram+, Antimicrobial
383	DRAMP01775	Temporin-1Sa (Frogs, amphibians, animals)	Antibacterial, Anti-Gram+, Anti-Gram-, Antimicrobial
384	DRAMP01776	Temporin-1Sb (Temporin-SHb; Frogs, amphibians, animals)	Antibacterial, Antifungal, Anti-Gram+, Anti-Gram-, Antimicrobial
385	DRAMP01777	Temporin-1Sc (Temporin-SHc; Frogs, amphibians, animals)	Antibacterial, Antifungal, Anti-Gram+, Anti-Gram-, Antimicrobial
386	DRAMP01779	Temporin-SHf (Frogs, amphibians, animals)	Antibacterial, Anti-Gram+, Anti-Gram-, Antimicrobial
387	DRAMP01780	Temporin-SHa (Temporin-1Sa; Frogs, amphibians, animals)	Antibacterial, Antifungal, Anti-Gram+, Anti-Gram-, Antimicrobial
388	DRAMP01782	Temporin-LTa (Frogs, amphibians, animals)	Antibacterial, Anti-Gram+, Antimicrobial
389	DRAMP01783	Temporin-LTb (Frogs, amphibians, animals)	Antibacterial, Anti-Gram+, Antimicrobial
390	DRAMP01784	Temporin-LTc (Frogs, amphibians, animals)	Antibacterial, Antiviral, Anti-Gram+, Anti-Gram-, Antimicrobial
391	DRAMP01785	Temporin-CPa (Frogs, amphibians, animals)	Antibacterial, Anti-Gram+, Anti-Gram-, Antimicrobial

392	DRAMP01787	Temporin-HN1 (Frogs, amphibians, animals)	Antibacterial, Antifungal, Anti-Gram+, Anti-Gram-, Antimicrobial
393	DRAMP01788	Temporin-HN2 (Frogs, amphibians, animals)	Antibacterial, Antifungal, Anti-Gram+, Anti-Gram-, Antimicrobial
394	DRAMP01789	Temporin-1Va (Temporin 1Va; Frogs, amphibians, animals)	Antibacterial, Antifungal, Anti-Gram+, Anti-Gram-, Antimicrobial
395	DRAMP01790	Temporin-1Vb (Temporin 1Vb; Frogs, amphibians, animals)	Antibacterial, Anti-Gram+, Antimicrobial
396	DRAMP01791	Temporin-1Vc (Temporin 1Vc; Frogs, amphibians, animals)	Antibacterial, Anti-Gram+, Anti-Gram-, Antimicrobial
397	DRAMP01807	Temporin-RN1 (Frogs, amphibians, animals)	Antibacterial, Antifungal, Anti-Gram+, Anti-Gram-, Antimicrobial
398	DRAMP01808	Temporin-RN3 (Frogs, amphibians, animals)	Antibacterial, Antifungal, Anti-Gram+, Anti-Gram-, Antimicrobial
399	DRAMP01811	Temporin-Ra (Frogs, amphibians, animals)	Antibacterial, Anti-Gram+, Anti-Gram-, Antimicrobial
400	DRAMP01812	Temporin-Rb (Frogs, amphibians, animals)	Antibacterial, Anti-Gram+, Anti-Gram-, Antimicrobial
401	DRAMP01815	Temporin-GH (AMP-5; Frogs, amphibians, animals)	Antibacterial, Anti-Gram+, Antimicrobial
402	DRAMP01816	Temporin-1CSb (Frogs, amphibians, animals)	Antibacterial, Anti-Gram+, Anti-Gram-, Antimicrobial
403	DRAMP01817	Temporin-1CSc (Frogs, amphibians, animals)	Antibacterial, Anti-Gram+, Anti-Gram-, Antimicrobial
404	DRAMP01818	Temporin-1CSd (Temporin-1DRb; Frogs, amphibians, animals)	Antibacterial, Antifungal, Anti-Gram+, Anti-Gram-, Antimicrobial
405	DRAMP01392	Odorranain-V1 (OdV1; Frogs, amphibians, animals)	Antimicrobial, Antibacterial, Antifungal, Anti-Gram+, Anti-Gram-,
406	DRAMP01391	Odorranain-U1 (OdU1; Frogs, amphibians, animals)	Antimicrobial, Antibacterial, Antifungal, Anti-Gram+, Anti-Gram-,
407	DRAMP01832	Temporin-Eca (Frogs, amphibians, animals)	Antibacterial, Anti-Gram+, Anti-Gram-, Antimicrobial
408	DRAMP01833	Buforin-EC (Frogs, amphibians, animals)	Antibacterial, Anti-Gram+, Anti-Gram-, Antimicrobial
409	DRAMP01834	Cyanophlyctin (Frogs, amphibians, animals)	Antibacterial, Anti-Gram+, Anti-Gram-, Antimicrobial
410	DRAMP01840	Ascaphin-1 (Frogs, amphibians, animals)	Antibacterial, Anti-Gram+, Anti-Gram-, Antimicrobial
411	DRAMP01842	Ascaphin-3 (Frogs, amphibians, animals)	Antibacterial, Anti-Gram+, Anti-Gram-, Antimicrobial
412	DRAMP01844	Ascaphin-5 (Frogs, amphibians, animals)	Antibacterial, Antifungal, Anti-Gram+, Anti-Gram-, Antimicrobial
413	DRAMP01846	Ascaphin-7 (Frogs, amphibians, animals)	Antibacterial, Anti-Gram+, Anti-Gram-, Antimicrobial
414	DRAMP01847	Ascaphin-8 (Frogs, amphibians, animals)	Antibacterial, Antiviral, Anti-Gram+, Anti-Gram-, Antimicrobial
415	DRAMP01849	Jindongenin-1a (JD1a; Frogs, amphibians, animals)	Antibacterial, Antifungal, Anti-Gram+, Anti-Gram-, Antimicrobial
416	DRAMP01869	Brevinin-1SPa (Frogs, amphibians, animals)	Antibacterial, Antifungal, Anti-Gram+, Anti-Gram-, Antimicrobial
417	DRAMP01870	Brevinin-1SPb (Frogs, amphibians, animals)	Antibacterial, Antifungal, Anti-Gram+, Anti-Gram-, Antimicrobial
418	DRAMP01872	Brevinin-1SPd (Frogs, amphibians, animals)	Antibacterial, Antifungal, Anti-Gram+, Anti-Gram-, Antimicrobial
419	DRAMP01873	Brevinin-2-related peptide (Frogs, amphibians, animals)	Antibacterial, Antifungal, Anti-Gram+, Anti-Gram-, Antimicrobial
420	DRAMP01875	Brevinin-2PRa (Frogs, amphibians, animals)	Antibacterial, Anti-Gram+, Anti-Gram-, Antimicrobial
421	DRAMP01876	Brevinin-2PRb (Frogs, amphibians, animals)	Antibacterial, Anti-Gram+, Anti-Gram-, Antimicrobial
422	DRAMP01877	Brevinin-2PRd (Frogs, amphibians, animals)	Antibacterial, Anti-Gram+, Anti-Gram-, Antimicrobial
423	DRAMP01878	Brevinin-2PRe (Frogs, amphibians, animals)	Antibacterial, Anti-Gram+, Anti-Gram-, Antimicrobial
424	DRAMP01879	Brevinin-2LTa (Frogs, amphibians, animals)	Antibacterial, Anti-Gram+, Anti-Gram-, Antimicrobial
425	DRAMP01880	Brevinin-2LTb (Frogs, amphibians, animals)	Antibacterial, Anti-Gram+, Anti-Gram-, Antimicrobial
426	DRAMP01881	Brevinin-2LTc (Frogs, amphibians, animals)	Antibacterial, Anti-Gram+, Anti-Gram-, Antimicrobial
427	DRAMP01885	Brevinin-1TEa (Frogs, amphibians, animals)	Antibacterial, Anti-Gram+, Anti-Gram-, Antimicrobial
428	DRAMP01886	Brevinin-2TEa (Frogs, amphibians, animals)	Antibacterial, Anti-Gram+, Anti-Gram-, Antimicrobial
429	DRAMP01887	Brevinin-2TEb (Frogs, amphibians, animals)	Antibacterial, Anti-Gram+, Anti-Gram-, Antimicrobial
430	DRAMP01888	Brevinin-1CHc (Frogs, amphibians, animals)	Antibacterial, Antifungal, Anti-Gram+, Anti-Gram-, Antimicrobial
431	DRAMP01889	Brevinin-1TOa (Frogs, amphibians, animals)	Antibacterial, Antifungal, Anti-Gram+, Anti-Gram-, Antimicrobial
432	DRAMP01890	Brevinin-1VLa (Frogs, amphibians, animals)	Antibacterial, Antifungal, Anti-Gram+, Anti-Gram-, Antimicrobial
433	DRAMP01891	Brevinin-1VLc (Frogs, amphibians, animals)	Antibacterial, Antifungal, Anti-Gram+, Anti-Gram-, Antimicrobial

434	DRAMP01892	Brevinin-1VLd (Frogs, amphibians, animals)	Antibacterial, Antifungal, Anti-Gram+, Anti-Gram-, Antimicrobial
435	DRAMP01893	Brevinin-1VLe (Frogs, amphibians, animals)	Antibacterial, Antifungal, Anti-Gram+, Anti-Gram-, Antimicrobial
436	DRAMP01896	Brevinin-1CG1 (Frogs, amphibians, animals)	Antibacterial, Antifungal, Anti-Gram+, Anti-Gram-, Antimicrobial
437	DRAMP01897	Brevinin-1CG2 (Frogs, amphibians, animals)	Antibacterial, Antifungal, Anti-Gram+, Anti-Gram-, Antimicrobial
438	DRAMP01898	Brevinin-1CG3 (Frogs, amphibians, animals)	Antibacterial, Antifungal, Anti-Gram+, Anti-Gram-, Antimicrobial
439	DRAMP01899	Brevinin-1CG4 (Frogs, amphibians, animals)	Antibacterial, Antifungal, Anti-Gram+, Anti-Gram-, Antimicrobial
440	DRAMP01900	Brevinin-1CG5 (Frogs, amphibians, animals)	Antibacterial, Antifungal, Anti-Gram+, Anti-Gram-, Antimicrobial
441	DRAMP01909	Brevinin-2GHa (AMP-1; Frogs, amphibians, animals)	Antibacterial, Anti-Gram+, Antimicrobial
442	DRAMP01910	Brevinin-2GHb (AMP-2; Frogs, amphibians, animals)	Antibacterial, Anti-Gram+, Anti-Gram-, Antimicrobial
443	DRAMP01911	Brevinin-2GHc (AMP-4; Frogs, amphibians, animals)	Antibacterial, Anti-Gram+, Anti-Gram-, Antimicrobial
444	DRAMP01913	Brevinin-1GRa (Frogs, amphibians, animals)	Antibacterial, Anti-Gram+, Anti-Gram-, Antimicrobial
445	DRAMP01914	Brevinin-2GRa (Frogs, amphibians, animals)	Antibacterial, Antifungal, Anti-Gram+, Anti-Gram-, Antimicrobial
446	DRAMP01918	Brevinin-1PLb (Frogs, amphibians, animals)	Antibacterial, Antifungal, Anti-Gram+, Anti-Gram-, Antimicrobial
447	DRAMP01919	Brevinin-1PLc (Frogs, amphibians, animals)	Antibacterial, Antifungal, Anti-Gram+, Anti-Gram-, Antimicrobial
448	DRAMP01920	Brevinin-1CSa (Frogs, amphibians, animals)	Antibacterial, Anti-Gram+, Anti-Gram-, Antimicrobial
449	DRAMP18383	XLAsp-P2 (X. laevis antibacterial peptide-P2; frog, amphibians, animals)	Antibacterial, Antimicrobial
450	DRAMP01922	Brevinin-2SKb (Frogs, amphibians, animals)	Antibacterial, Anti-Gram+, Anti-Gram-, Antimicrobial
451	DRAMP01933	Brevinin-2Ef (Frogs, amphibians, animals)	Antibacterial, Anti-Gram+, Anti-Gram-, Antimicrobial
452	DRAMP01934	Brevinin-2Ei (Frogs, amphibians, animals)	Antibacterial, Anti-Gram-, Antimicrobial
453	DRAMP01935	Brevinin-2Ej (Frogs, amphibians, animals)	Antibacterial, Anti-Gram-, Antimicrobial
454	DRAMP01937	CPRF-Ea (caerulein precursor-related fragment; Frogs, amphibians, animals)	Antibacterial, Anti-Gram-, Antimicrobial
455	DRAMP01938	CPRF-Eb (caerulein precursor-related fragment; Frogs, amphibians, animals)	Antibacterial, Anti-Gram-, Antimicrobial
456	DRAMP01939	CPRF-Ec (caerulein precursor-related fragment; Frogs, amphibians, animals)	Antibacterial, Anti-Gram-, Antimicrobial
457	DRAMP01940	Brevinin-1CHa (Frogs, amphibians, animals)	Antibacterial, Antifungal, Anti-Gram+, Anti-Gram-, Antimicrobial
458	DRAMP01941	Brevinin-1CHb (Frogs, amphibians, animals)	Antibacterial, Antifungal, Anti-Gram+, Anti-Gram-, Antimicrobial
459	DRAMP01942	Brevinin-1Sa (Frogs, amphibians, animals)	Antibacterial, Anti-Gram-, Antimicrobial
460	DRAMP01943	Brevinin-1Sb (Frogs, amphibians, animals)	Antibacterial, Anti-Gram-, Antimicrobial
461	DRAMP01944	Brevinin-1Sc (Frogs, amphibians, animals)	Antibacterial, Anti-Gram-, Antimicrobial
462	DRAMP01949	Brevinin-1HSa (Frogs, amphibians, animals)	Antibacterial, Anti-Gram+, Anti-Gram-, Antimicrobial
463	DRAMP01950	Brevinin-1HSb (Brevinin-1JDb; Frogs, amphibians, animals)	Antibacterial, Anti-Gram+, Anti-Gram-, Antimicrobial
464	DRAMP01951	Brevinin-1PTa (Frogs, amphibians, animals)	Antibacterial, Anti-Gram+, Anti-Gram-, Antimicrobial
465	DRAMP01953	Brevinin-2HSa (Frogs, amphibians, animals)	Antibacterial, Anti-Gram+, Anti-Gram-, Antimicrobial
466	DRAMP01955	Brevinin-2PTa (Frogs, amphibians, animals)	Antibacterial, Anti-Gram+, Anti-Gram-, Antimicrobial
467	DRAMP01956	Brevinin-2PTb (Frogs, amphibians, animals)	Antibacterial, Anti-Gram+, Anti-Gram-, Antimicrobial
468	DRAMP01957	Brevinin-2PTc (Frogs, amphibians, animals)	Antibacterial, Anti-Gram+, Anti-Gram-, Antimicrobial
469	DRAMP01959	Brevinin-2PTe (Frogs, amphibians, animals)	Antibacterial, Anti-Gram+, Anti-Gram-, Antimicrobial
470	DRAMP01963	Brevinin-1BLa (Frogs, amphibians, animals)	Antibacterial, Antifungal, Anti-Gram+, Anti-Gram-, Antimicrobial
471	DRAMP01965	Brevinin-1BLc (Frogs, amphibians, animals)	Antibacterial, Antifungal, Anti-Gram+, Anti-Gram-, Antimicrobial
472	DRAMP01968	Brevinin-1Yc (Frogs, amphibians, animals)	Antibacterial, Antifungal, Anti-Gram+, Anti-Gram-, Antimicrobial
473	DRAMP01969	Brevinin-1Ja (Frogs, amphibians, animals)	Antibacterial, Anti-Gram+, Anti-Gram-, Antimicrobial
474	DRAMP01970	Brevinin-1ZHa (Frogs, amphibians, animals)	Antibacterial, Antifungal, Anti-Gram+, Anti-Gram-, Antimicrobial

475	DRAMP01971	Brevinin-1ZHb (Frogs, amphibians, animals)	Antibacterial, Antifungal, Anti-Gram+, Anti-Gram-, Antimicrobial
476	DRAMP01974	Brevinin-2ZHa (Frogs, amphibians, animals)	Antibacterial, Antifungal, Anti-Gram+, Anti-Gram-, Antimicrobial
477	DRAMP01986	Brevinin-2HS2 (Frogs, amphibians, animals)	Antibacterial, Antifungal, Anti-Gram+, Anti-Gram-, Antimicrobial
478	DRAMP01990	Brevinin-1LT1 (Frogs, amphibians, animals)	Antibacterial, Anti-Gram+, Anti-Gram-, Antimicrobial
479	DRAMP01994	Brevinin-2ISa (Frogs, amphibians, animals)	Antibacterial, Anti-Gram+, Anti-Gram-, Antimicrobial
480	DRAMP01995	Brevinin-2ISb (Frogs, amphibians, animals)	Antibacterial, Antifungal, Anti-Gram+, Anti-Gram-, Antimicrobial
481	DRAMP01996	Brevinin-2ISc (Frogs, amphibians, animals)	Antibacterial, Anti-Gram+, Anti-Gram-, Antimicrobial
482	DRAMP02001	Brevinin-1HN1 (Frogs, amphibians, animals)	Antibacterial, Antifungal, Anti-Gram+, Anti-Gram-, Antimicrobial
483	DRAMP02004	Brevinin-1V (Frogs, amphibians, animals)	Antibacterial, Antifungal, Anti-Gram+, Anti-Gram-, Antimicrobial
484	DRAMP02006	Brevinin-2Va (Frogs, amphibians, animals)	Antibacterial, Anti-Gram-, Antimicrobial
485	DRAMP02009	Brevinin-1 (Frogs, amphibians, animals)	Antibacterial, Anti-Gram+, Anti-Gram-, Antimicrobial
486	DRAMP02010	Brevinin-2 (Frogs, amphibians, animals)	Antibacterial, Anti-Gram+, Anti-Gram-, Antimicrobial
487	DRAMP02019	Brevinin-2DYb (Frogs, amphibians, animals)	Antibacterial, Anti-Gram+, Anti-Gram-, Antimicrobial
488	DRAMP18381	H4-(86-100) (histone-derived)	Antibacterial, Antimicrobial
489	DRAMP18382	HNr (histone-derived)	Antibacterial, Antimicrobial
490	DRAMP02023	Brevinin-2DYd (Frogs, amphibians, animals)	Antibacterial, Anti-Gram+, Anti-Gram-, Antimicrobial
491	DRAMP18380	Acipensin 1 (Ac1)	Antibacterial, antifungal, Antimicrobial
492	DRAMP02025	Brevinin-2DYe (Brevinin-2CDYa; Frogs, amphibians, animals)	Antibacterial, Anti-Gram+, Anti-Gram-, Antimicrobial
493	DRAMP02026	Brevinin-1CDYa (Frogs, amphibians, animals)	Antibacterial, Anti-Gram+, Anti-Gram-, Antimicrobial
494	DRAMP18379	Acipensin 2 (Ac2)	Antibacterial, antifungal, Antimicrobial
495	DRAMP02031	Brevinin-1Da (Frogs, amphibians, animals)	Antibacterial, Anti-Gram+, Anti-Gram-, Antimicrobial
496	DRAMP02032	Brevinin-1TSa (Frogs, amphibians, animals)	Antibacterial, Antifungal, Anti-Gram+, Anti-Gram-, Antimicrobial
497	DRAMP02033	Brevinin-2TSa (Frogs, amphibians, animals)	Antibacterial, Antifungal, Anti-Gram+, Anti-Gram-, Antimicrobial
498	DRAMP02034	Brevinin-1AUa (Frogs, amphibians, animals)	Antibacterial, Antifungal, Anti-Gram+, Anti-Gram-, Antimicrobial
499	DRAMP02035	Brevinin-1AUb (Frogs, amphibians, animals)	Antibacterial, Antifungal, Anti-Gram+, Anti-Gram-, Antimicrobial
500	DRAMP02036	Brevinin-2-RN1 (Frogs, amphibians, animals)	Antibacterial, Antifungal, Anti-Gram+, Anti-Gram-, Antimicrobial
501	DRAMP02037	Brevinin-2-RN2 (Frogs, amphibians, animals)	Antibacterial, Antifungal, Anti-Gram+, Anti-Gram-, Antimicrobial
502	DRAMP02038	Brevinin-1-OA1 (Frogs, amphibians, animals)	Antibacterial, Antifungal, Anti-Gram+, Anti-Gram-, Antimicrobial
503	DRAMP18378	Acipensin 6 (Ac6)	Antibacterial, Antimicrobial
504	DRAMP02040	Brevinin-1-OA12 (Frogs, amphibians, animals)	Antibacterial, Antifungal, Anti-Gram+, Anti-Gram-, Antimicrobial
505	DRAMP02041	Brevinin-1-OR1 (Frogs, amphibians, animals)	Antibacterial, Antifungal, Anti-Gram+, Anti-Gram-, Antimicrobial
506	DRAMP02042	Brevinin-1-OR3 (Frogs, amphibians, animals)	Antibacterial, Antifungal, Anti-Gram+, Anti-Gram-, Antimicrobial
507	DRAMP02043	Brevinin-1-OR4 (Frogs, amphibians, animals)	Antibacterial, Antifungal, Anti-Gram+, Anti-Gram-, Antimicrobial
508	DRAMP02044	Brevinin-1-OR5 (Frogs, amphibians, animals)	Antibacterial, Antifungal, Anti-Gram+, Anti-Gram-, Antimicrobial
509	DRAMP02045	Brevinin-1-OR6 (Frogs, amphibians, animals)	Antibacterial, Antifungal, Anti-Gram+, Anti-Gram-, Antimicrobial
510	DRAMP02046	Brevinin-1-OR7 (Frogs, amphibians, animals)	Antibacterial, Antifungal, Anti-Gram+, Anti-Gram-, Antimicrobial
511	DRAMP02047	Brevinin-1-OR8 (Frogs, amphibians, animals)	Antibacterial, Antifungal, Anti-Gram+, Anti-Gram-, Antimicrobial
512	DRAMP02048	Brevinin-1-OR9 (Frogs, amphibians, animals)	Antibacterial, Antifungal, Anti-Gram+, Anti-Gram-, Antimicrobial
513	DRAMP02049	Brevinin-1-OR10 (Frogs, amphibians, animals)	Antibacterial, Antifungal, Anti-Gram+, Anti-Gram-, Antimicrobial

514	DRAMP02050	Brevinin-1-OR11 (Frogs, amphibians, animals)	Antibacterial, Antifungal, Anti-Gram+, Anti-Gram-, Antimicrobial
515	DRAMP02051	Lividin-1 (Brevinin-1-OR2; Frogs, amphibians, animals)	Antibacterial, Antifungal, Anti-Gram+, Anti-Gram-, Antimicrobial
516	DRAMP02052	Lividin-2 (Brevinin-2-OR8; Frogs, amphibians, animals)	Antibacterial, Antifungal, Anti-Gram+, Anti-Gram-, Antimicrobial
517	DRAMP02053	Lividin-3 (Brevinin-2-OR1; Frogs, amphibians, animals)	Antibacterial, Antifungal, Anti-Gram+, Anti-Gram-, Antimicrobial
518	DRAMP02054	Brevinin-2-OA1 (Frogs, amphibians, animals)	Antibacterial, Antifungal, Anti-Gram+, Anti-Gram-, Antimicrobial
519	DRAMP02055	Brevinin-2-OA2 (Brevinin-2E-OG1; Frogs, amphibians, animals)	Antibacterial, Antifungal, Anti-Gram+, Anti-Gram-, Antimicrobial
520	DRAMP02056	Brevinin-2-OA3 (Frogs, amphibians, animals)	Antibacterial, Antifungal, Anti-Gram+, Anti-Gram-, Antimicrobial
521	DRAMP02057	Brevinin-2-OA4 (Frogs, amphibians, animals)	Antibacterial, Antifungal, Anti-Gram+, Anti-Gram-, Antimicrobial
522	DRAMP02058	Brevinin-2-OA5 (Frogs, amphibians, animals)	Antibacterial, Antifungal, Anti-Gram+, Anti-Gram-, Antimicrobial
523	DRAMP02059	Brevinin-2-OA6 (Frogs, amphibians, animals)	Antibacterial, Antifungal, Anti-Gram+, Anti-Gram-, Antimicrobial
524	DRAMP02060	Brevinin-2-OA7 (Frogs, amphibians, animals)	Antibacterial, Antifungal, Anti-Gram+, Anti-Gram-, Antimicrobial
525	DRAMP02061	Brevinin-2-OA8 (Frogs, amphibians, animals)	Antibacterial, Antifungal, Anti-Gram+, Anti-Gram-, Antimicrobial
526	DRAMP02062	Brevinin-2-OR2 (Frogs, amphibians, animals)	Antibacterial, Antifungal, Anti-Gram+, Anti-Gram-, Antimicrobial
527	DRAMP02063	Brevinin-2-OR3 (Frogs, amphibians, animals)	Antibacterial, Antifungal, Anti-Gram+, Anti-Gram-, Antimicrobial
528	DRAMP02064	Brevinin-2-OR4 (Frogs, amphibians, animals)	Antibacterial, Antifungal, Anti-Gram+, Anti-Gram-, Antimicrobial
529	DRAMP02065	Brevinin-2-OR5 (Frogs, amphibians, animals)	Antibacterial, Antifungal, Anti-Gram+, Anti-Gram-, Antimicrobial
530	DRAMP02066	Brevinin-2-OR6 (Frogs, amphibians, animals)	Antibacterial, Antifungal, Anti-Gram+, Anti-Gram-, Antimicrobial
531	DRAMP02067	Brevinin-2-OR7 (Frogs, amphibians, animals)	Antibacterial, Antifungal, Anti-Gram+, Anti-Gram-, Antimicrobial
532	DRAMP02068	Brevinin-2-OR9 (Frogs, amphibians, animals)	Antibacterial, Antifungal, Anti-Gram+, Anti-Gram-, Antimicrobial
533	DRAMP02069	Brevinin-2-OR10 (Frogs, amphibians, animals)	Antibacterial, Antifungal, Anti-Gram+, Anti-Gram-, Antimicrobial
534	DRAMP02070	Brevinin-2-OW1 (Frogs, amphibians, animals)	Antibacterial, Antifungal, Anti-Gram+, Anti-Gram-, Antimicrobial
535	DRAMP02071	Brevinin-2-OW2 (Frogs, amphibians, animals)	Antibacterial, Antifungal, Anti-Gram+, Anti-Gram-, Antimicrobial
536	DRAMP02072	Brevinin-2-OW3 (Frogs, amphibians, animals)	Antibacterial, Antifungal, Anti-Gram+, Anti-Gram-, Antimicrobial
537	DRAMP02073	Brevinin-1JDa (Frogs, amphibians, animals)	Antibacterial, Antifungal, Anti-Gram+, Anti-Gram-, Antimicrobial
538	DRAMP02075	Brevinin-1JDc (Frogs, amphibians, animals)	Antibacterial, Anti-Gram+, Anti-Gram-, Antimicrobial
539	DRAMP18376	Peptide fraction II (Gly-rich; insects, arthropods, invertebrates, animals)	Antibacterial, Antimicrobial
540	DRAMP18377	Sphistin (histone-derived, Crustaceans, arthropods, invertebrates, animals)	Antibacterial, Antimicrobial
541	DRAMP01745	Temporin-K (Frogs, amphibians, animals)	Antibacterial, Antimicrobial
542	DRAMP01746	Temporin-L (Temporin-1Tl; temporin-Tl; TL; Frogs, amphibians, animals)	Antibacterial, Antiparasitic, Anticancer., Antimicrobial
543	DRAMP02078	Brevinin-1SY (Frogs, amphibians, animals)	Antibacterial, Anti-Gram+, Anti-Gram-, Antimicrobial
544	DRAMP02081	Brevinin-1E (Frogs, amphibians, animals)	Antibacterial, Anti-Gram+, Anti-Gram-, Antimicrobial
545	DRAMP02084	Brevinin-2E (Frogs, amphibians, animals)	Antibacterial, Antifungal, Anti-Gram+, Anti-Gram-, Antimicrobial
546	DRAMP02101	Brevinin-1RTa (Frogs, amphibians, animals)	Antibacterial, Anti-Gram+, Anti-Gram-, Antimicrobial
547	DRAMP02102	Brevinin-1RTb (Frogs, amphibians, animals)	Antibacterial, Anti-Gram+, Anti-Gram-, Antimicrobial

548	DRAMP02104	Brevinin-2RTa (Frogs, amphibians, animals)	Antibacterial, Antifungal, Anti-Gram+, Anti-Gram-, Antimicrobial
549	DRAMP02105	Brevinin-2RTb (Frogs, amphibians, animals)	Antibacterial, Anti-Gram+, Anti-Gram-, Antimicrobial
550	DRAMP02114	Raniseptin-1 (Rsp-1; Frogs, amphibians, animals)	Antibacterial, Anti-Gram+, Anti-Gram-, Antimicrobial
551	DRAMP02125	Hylin-a1 (Hy-a1; Frogs, amphibians, animals)	Antibacterial, Antifungal, Anti-Gram+, Anti-Gram-, Antimicrobial
552	DRAMP02127	Leptoglycin (Gly-rich; Frogs, amphibians, animals)	Antibacterial, Anti-Gram-, Antimicrobial
553	DRAMP02128	Kassorin-S (PreproKassorin-S; Frogs, amphibians, animals)	Antibacterial, Antifungal, Anti-Gram+, Antimicrobial
554	DRAMP02129	Kasstasin (Frogs, amphibians, animals)	Antibacterial, Anti-Gram+, Anti-Gram-, Antimicrobial
555	DRAMP02130	Antimicrobial peptide 1 (XT-1; Frogs, amphibians, animals)	Antibacterial, Antifungal, Anti-Gram+, Anti-Gram-, Antimicrobial
556	DRAMP02131	Antimicrobial peptide 2 (XT-2; Frogs, amphibians, animals)	Antibacterial, Anti-Gram+, Anti-Gram-, Antimicrobial
557	DRAMP02133	Antimicrobial peptide 4 (XT-4; Frogs, amphibians, animals)	Antibacterial, Antifungal, Anti-Gram+, Anti-Gram-, Antimicrobial
558	DRAMP02135	Antimicrobial peptide 6 (XT-6; Frogs, amphibians, animals)	Antibacterial, Antifungal, Anti-Gram+, Anti-Gram-, Antimicrobial
559	DRAMP02136	Antimicrobial peptide 7 (XT-7; Frogs, amphibians, animals)	Antibacterial, Antifungal, Anti-Gram+, Anti-Gram-, Antimicrobial
560	DRAMP02142	CPF-SP1 (Frogs, amphibians, animals)	Antibacterial, Antimicrobial
561	DRAMP02219	Ranatuerin-2AUa (Frogs, amphibians, animals)	Antimicrobial, Antibacterial, Antifungal, Anti-Gram+, Anti-Gram-,
562	DRAMP02220	Ranatuerin-2PLa (Frogs, amphibians, animals)	Antibacterial, Anti-Gram-, Antimicrobial
563	DRAMP02221	Ranatuerin-2PLb (Frogs, amphibians, animals)	Antibacterial, Anti-Gram-, Antimicrobial
564	DRAMP02222	Ranatuerin-2PLc (Frogs, amphibians, animals)	Antibacterial, Antifungal, Anti-Gram-, Antimicrobial
565	DRAMP02223	Ranatuerin-2PLd (Frogs, amphibians, animals)	Antibacterial, Antifungal, Anti-Gram-, Antimicrobial
566	DRAMP02224	Ranatuerin-2PLe (Frogs, amphibians, animals)	Antibacterial, Anti-Gram-, Antimicrobial
567	DRAMP02225	Ranatuerin-2PLf (Frogs, amphibians, animals)	Antibacterial, Antifungal, Anti-Gram-, Antimicrobial
568	DRAMP02228	Ranatuerin-1 (Frogs, amphibians, animals)	Antibacterial, Antifungal, Anti-Gram+, Anti-Gram-, Antimicrobial
569	DRAMP02229	Ranatuerin-2 (Frogs, amphibians, animals)	Antibacterial, Anti-Gram+, Antimicrobial
570	DRAMP02230	Ranatuerin-3 (Frogs, amphibians, animals)	Antibacterial, Anti-Gram+, Antimicrobial
571	DRAMP02231	Ranatuerin-4 (Frogs, amphibians, animals)	Antibacterial, Anti-Gram+, Antimicrobial
572	DRAMP02233	Ranatuerin-6 (Frogs, amphibians, animals)	Antibacterial, Antiviral, Anti-Gram+, Antimicrobial
573	DRAMP02234	Ranatuerin-7 (Frogs, amphibians, animals)	Antibacterial, Anti-Gram+, Antimicrobial
574	DRAMP02235	Ranatuerin-8 (Frogs, amphibians, animals)	Antibacterial, Anti-Gram+, Antimicrobial
575	DRAMP02236	Ranatuerin-9 (Frogs, amphibians, animals)	Antibacterial, Antiviral, Anti-Gram+, Antimicrobial
576	DRAMP02237	Ranatuerin-2Ya (Frogs, amphibians, animals)	Cytolytic,Antibacterial, Anti-Gram+, Anti-Gram-, Antimicrobial
577	DRAMP02238	Ranatuerin-2ZHa (Frogs, amphibians, animals)	Antibacterial, Antifungal, Anti-Gram+, Anti-Gram-, Antimicrobial
578	DRAMP02239	Ranatuerin-1Ga (Frogs, amphibians, animals)	Antibacterial, Antifungal, Anti-Gram+, Anti-Gram-, Antimicrobial
579	DRAMP02241	Ranatuerin-2G (Frogs, amphibians, animals)	Antibacterial, Antifungal, Anti-Gram+, Anti-Gram-, Antimicrobial
580	DRAMP01390	Odorranain-T1 (OdT1; Frogs, amphibians, animals)	Antimicrobial, Antibacterial, Antifungal, Anti-Gram+, Anti-Gram-,
581	DRAMP01389	Odorranain-S1 (OdS1; Frogs, amphibians, animals)	Antimicrobial, Antibacterial, Antifungal, Anti-Gram+, Anti-Gram-,
582	DRAMP02251	Ranatuerin-2CSa (Frogs, amphibians, animals)	Antibacterial, Anti-Gram+, Anti-Gram-, Antimicrobial
583	DRAMP02252	Ranatuerin 2SKa (Frogs, amphibians, animals)	Antibacterial, Antifungal, Anti-Gram+, Anti-Gram-, Antimicrobial
584	DRAMP01108	Maximin-2 (Toads, amphibians, animals)	Antimicrobial, Antibacterial, Antifungal, Anti-Gram+, Anti-Gram-,
585	DRAMP03998	PAF26 (Trp-rich; combinatorial library)	Antimicrobial, Antibacterial, Antifungal, Anti-Gram-,
586	DRAMP01107	Maximin-1 (Toads, amphibians, animals)	Antimicrobial, Antibacterial, Antifungal, Antiviral, Anticancer, Anti-Gram+, Anti-Gram-,
587	DRAMP02268	Xenopsin precursor fragment (XPF; Frogs, amphibians, animals)	Antibacterial, Antifungal, Anti-Gram+, Anti-Gram-, Antimicrobial
588	DRAMP02269	Antimicrobial peptide PGQ (PGQ; Frogs, amphibians, animals)	Antibacterial, Antifungal, Anti-Gram+, Anti-Gram-, Antimicrobial
589	DRAMP02271	Magainin-2 (Magainin II; chain of Magainins; Frogs, amphibians, animals)	Antibacterial, Antifungal, Antiprotozoal, Anti-Gram+, Anti-Gram-, Antimicrobial

590	DRAMP02272	PGLa (chain of PYLa/PGLa A; Frogs, amphibians, animals)	Antibacterial, Antifungal, Anti-Gram+, Anti-Gram-, Antimicrobial
591	DRAMP02273	PGLa-H (chain of PYLa/PGLa A; Frogs, amphibians, animals)	Antibacterial, Anti-Gram+, Anti-Gram-, Antimicrobial
592	DRAMP02274	Ranacyclin-E (Frogs, amphibians, animals)	Antibacterial, Antifungal, Anti-Gram+, Anti-Gram-, Antimicrobial
593	DRAMP02275	Ranacyclin-T (Frogs, amphibians, animals)	Antibacterial, Antifungal, Anti-Gram+, Anti-Gram-, Antimicrobial
594	DRAMP02276	Ranacyclin B3 (Frogs, amphibians, animals)	Antibacterial, Antifungal, Anti-Gram+, Antimicrobial
595	DRAMP02277	Ranacyclin B5 (Frogs, amphibians, animals)	Antibacterial, Antifungal, Anti-Gram+, Antimicrobial
596	DRAMP02278	Ranacyclin-B-RL1 (Frogs, amphibians, animals)	Antibacterial, Antifungal, Anti-Gram+, Anti-Gram-, Antimicrobial
597	DRAMP02279	Ranacyclin-B-RN1 (Frogs, amphibians, animals)	Antibacterial, Anti-Gram+, Antimicrobial
598	DRAMP02280	Ranacyclin-B-RN2 (Frogs, amphibians, animals)	Antibacterial, Anti-Gram+, Antimicrobial
599	DRAMP02281	Ranacyclin-B-RN6 (Frogs, amphibians, animals)	Antibacterial, Antifungal, Anti-Gram+, Antimicrobial
600	DRAMP02282	Ranacyclin-B-LK1 (Frogs, amphibians, animals)	Antibacterial, Anti-Gram+, Antimicrobial
601	DRAMP02283	Ranacyclin-B-LK2 (Frogs, amphibians, animals)	Antibacterial, Anti-Gram+, Antimicrobial
602	DRAMP02288	Gaegurin-RN1 (Frogs, amphibians, animals)	Antibacterial, Antifungal, Anti-Gram+, Anti-Gram-, Antimicrobial
603	DRAMP02289	Gaegurin-RN4 (Frogs, amphibians, animals)	Antibacterial, Antifungal, Anti-Gram+, Antimicrobial
604	DRAMP02290	Gaegurin-RN5 (Frogs, amphibians, animals)	Antibacterial, Antifungal, Anti-Gram+, Anti-Gram-, Antimicrobial
605	DRAMP02291	Gaegurin-1 (Gaegurin 1; GGN1; Frogs, amphibians, animals)	Antibacterial, Antifungal, Anti-Gram+, Anti-Gram-, Antimicrobial
606	DRAMP02292	Gaegurin-2 (Gaegurin 2; GGN2; Frogs, amphibians, animals)	Antibacterial, Antifungal, Anti-Gram+, Anti-Gram-, Antimicrobial
607	DRAMP02293	Gaegurin-3 (Gaegurin 3; GGN3; Frogs, amphibians, animals)	Antibacterial, Antifungal, Anti-Gram+, Anti-Gram-, Antimicrobial
608	DRAMP02294	Gaegurin-4 (Gaegurin 4; GGN4; Frogs, amphibians, animals)	Antibacterial, Antifungal, Antiprotozoal, Anti-Gram+, Anti-Gram-, Antimicrobial
609	DRAMP02295	Gaegurin-5 (Gaegurin 5; GGN5; Brevinin-1EMa; Frogs, amphibians, animals)	Antibacterial, Antifungal, Antiprotozoal, Anti-Gram+, Anti-Gram-, Antimicrobial
610	DRAMP02296	Gaegurin-6 (Gaegurin 6; GGN6; Frogs, amphibians, animals)	Antibacterial, Antifungal, Anti-Gram+, Anti-Gram-, Antimicrobial
611	DRAMP02300	Guentherin (AMP-3; Frogs, amphibians, animals)	Antibacterial, Anti-Gram+, Antimicrobial
612	DRAMP02306	Riparin-2.1 (Frogs, amphibians, animals)	Antibacterial, Anti-Gram+, Antimicrobial
613	DRAMP02307	Deserticolin-1 (Frogs, amphibians, animals)	Antibacterial, Anti-Gram+, Antimicrobial
614	DRAMP02308	Signiferin-2.1 (Frogs, amphibians, animals)	Antibacterial, Anti-Gram+, Antimicrobial
615	DRAMP02312	Hipposin (fish, chordates, animals)	Antibacterial, Antimicrobial
616	DRAMP02314	Hepcidin (fish, chordates, animals)	Antibacterial, Antifungal, Anti-Gram+, Anti-Gram-, Antimicrobial
617	DRAMP02315	Chrysophsin-1 (fish, chordates, animals)	Antibacterial, Anti-Gram+, Anti-Gram-, Antimicrobial
618	DRAMP02316	Chrysophsin-2 (fish, chordates, animals)	Antibacterial, Anti-Gram+, Anti-Gram-, Antimicrobial
619	DRAMP02317	Chrysophsin-3 (fish, chordates, animals)	Antibacterial, Anti-Gram+, Anti-Gram-, Antimicrobial
620	DRAMP02318	Grammistin Pp1 (Group II grammistin; fish, chordates, animals)	Antibacterial, Anti-Gram+, Anti-Gram-, Antimicrobial
621	DRAMP02320	Grammistin PpIIb (Group II grammistin; fish, chordates, animals)	Antibacterial, Anti-Gram+, Anti-Gram-, Antimicrobial
622	DRAMP02321	Grammistin Pp3 (Group III grammistin; fish, chordates, animals)	Antibacterial, Anti-Gram+, Anti-Gram-, Antimicrobial
623	DRAMP02324	SAMP H1 (fish, chordates, animals)	Antibacterial, Anti-Gram+, Anti-Gram-, Antimicrobial
624	DRAMP02330	Piscidin-1 (Pis-1; Piscidin 1; fish, chordates, animals)	Antibacterial, Antifungal, Anti-Gram+, Anti-Gram-, Antimicrobial
625	DRAMP02331	Piscidin-2 (Pis-2; fish, chordates, animals)	Antibacterial, Antifungal, Anti-Gram+, Anti-Gram-, Antimicrobial
626	DRAMP02336	Oncorhyncin II (Oncorhyncin 2; fish, chordates, animals)	Antibacterial, Anti-Gram+, Anti-Gram-, Antimicrobial
627	DRAMP02337	Oncorhyncin III (Oncorhyncin 3; fish, chordates, animals)	Antibacterial, Anti-Gram+, Anti-Gram-, Antimicrobial
628	DRAMP02347	NRC-1 (fish, chordates, animals)	Antibacterial, Antifungal, Anti-Gram+, Anti-Gram-, Antimicrobial
629	DRAMP02348	NRC-2 (fish, chordates, animals)	Antibacterial, Antifungal, Anti-Gram+, Anti-Gram-, Antimicrobial
630	DRAMP02349	NRC-3 (fish, chordates, animals)	Antibacterial, Antifungal, Anti-Gram+, Anti-Gram-, Antimicrobial
631	DRAMP02350	Pleurocidin (NRC-4; fish, chordates, animals)	Antibacterial, Antifungal, Anti-Gram+, Anti-Gram-, Antimicrobial

632	DRAMP02351	NRC-10 (fish, chordates, animals)	Antibacterial, Antifungal, Anti-Gram+, Anti-Gram-, Antimicrobial
633	DRAMP02352	NRC-16 (fish, chordates, animals)	Antibacterial, Antifungal, Anti-Gram+, Anti-Gram-, Antimicrobial
634	DRAMP02354	Pleurocidin-like peptide WFY (fish, chordates, animals)	Antibacterial, Antifungal, Anti-Gram+, Anti-Gram-, Antimicrobial
635	DRAMP02357	Pleurocidin-like peptide WF3 (NRC-5; fish, chordates, animals)	Antibacterial, Antifungal, Anti-Gram+, Anti-Gram-, Antimicrobial
636	DRAMP02358	Pleurocidin-like peptide WF4 (NRC-6; fish, chordates, animals)	Antibacterial, Antifungal, Anti-Gram+, Anti-Gram-, Antimicrobial
637	DRAMP02359	Pleurocidin-like peptide YT2 (NRC-7; fish, chordates, animals; Predicted)	Antibacterial, Antifungal, Anti-Gram+, Anti-Gram-, Antimicrobial
638	DRAMP02360	Pleurocidin-like peptide AP1 (NRC-11; fish, chordates, animals; Predicted)	Antibacterial, Antifungal, Anti-Gram+, Anti-Gram-, Antimicrobial
639	DRAMP02361	Pleurocidin-like peptide AP2 (NRC-12; fish, chordates, animals; Predicted)	Antibacterial, Antifungal, Anti-Gram+, Anti-Gram-, Antimicrobial
640	DRAMP02362	Pleurocidin-like peptide AP3 (NRC-13; fish, chordates, animals; Predicted)	Antibacterial, Antifungal, Anti-Gram+, Anti-Gram-, Antimicrobial
641	DRAMP02363	Pleurocidin-like peptide GcSc4C5 (NRC-14; fish, chordates, animals)	Antibacterial, Antifungal, Anti-Gram+, Anti-Gram-, Antimicrobial
642	DRAMP02364	Pleurocidin-like peptide GcSc4B7 (NRC-15; fish, chordates, animals; Predicted)	Antibacterial, Antifungal, Anti-Gram+, Anti-Gram-, Antimicrobial
643	DRAMP02365	Pleurocidin-like peptide GC3.8 (NRC-17; fish, chordates, animals; Predicted)	Antibacterial, Antifungal, Anti-Gram+, Anti-Gram-, Antimicrobial
644	DRAMP02366	Pleurocidin-like peptide GC3.2 (NRC-18; fish, chordates, animals; Predicted)	Antibacterial, Antifungal, Anti-Gram+, Anti-Gram-, Antimicrobial
645	DRAMP02367	Pleurocidin-like peptide Hb26 (NRC-19; fish, chordates, animals; Predicted)	Antibacterial, Antifungal, Anti-Gram+, Anti-Gram-, Antimicrobial
646	DRAMP02368	Pleurocidin-like peptide Hb18 (NRC-20; fish, chordates, animals; Predicted)	Antibacterial, Antifungal, Anti-Gram+, Anti-Gram-, Antimicrobial
647	DRAMP02374	Bass hepcidin (fish, chordates, animals)	Antibacterial, Antifungal, Anti-Gram-, Antimicrobial
648	DRAMP02376	Grammistin Gs 1 (Grammistin Gs F; Group I grammistin; soapfish, chordates, animals)	Antibacterial, Anti-Gram+, Anti-Gram-, Antimicrobial
649	DRAMP02377	Grammistin Gs 2 (Grammistin Gs G; Group I grammistin; soapfish, chordates, animals)	Antibacterial, Anti-Gram+, Anti-Gram-, Antimicrobial
650	DRAMP02378	Grammistin Gs A (Group III grammistin; soapfish, chordates, animals)	Antibacterial, Anti-Gram+, Anti-Gram-, Antimicrobial
651	DRAMP02379	Grammistin Gs B (Group II grammistin; soapfish, chordates, animals)	Antibacterial, Anti-Gram+, Anti-Gram-, Antimicrobial
652	DRAMP02380	Grammistin Gs C (Group III grammistin; soapfish, chordates, animals)	Antibacterial, Anti-Gram+, Anti-Gram-, Antimicrobial
653	DRAMP18375	hBD-5 (human beta-defensin 5)	Antibacterial, Antimicrobial
654	DRAMP18373	mBD-6 (Murine beta-defensin 6)	Antibacterial, Anti-Gram-, Antimicrobial
655	DRAMP18374	hBD-6 (human beta-defensin 6)	Antibacterial, Chemotactic, Anti-Gram-, Antimicrobial
656	DRAMP02386	Parasin I (histone-H2A-derived; catfishes, chordates, animals)	Antibacterial, Antimicrobial
657	DRAMP02390	Astacidin 2 (crayfish, Arthropods, animals)	Antibacterial, Anti-Gram+, Anti-Gram-, Antimicrobial
658	DRAMP02391	Hematopoietic antimicrobial peptide-37 (MgCath37; hagfishes, chordates, animals)	Antibacterial, Antifungal, Anti-Gram+, Anti-Gram-, Antimicrobial
659	DRAMP02393	HFIAP-1 (HFIAP-2; hagfishes, chordates, animals)	Antibacterial, Antifungal, Anti-Gram+, Anti-Gram-, Antimicrobial
660	DRAMP02394	HFIAP-3 (hagfishes, chordates, animals)	Antibacterial, Anti-Gram+, Anti-Gram-, Antimicrobial
661	DRAMP02395	Aurelin (jellyfish, chordates, animals)	Antibacterial, Anti-Gram+, Anti-Gram-, Antimicrobial
662	DRAMP02397	Big defensin (RPD-1)	Anticancer, Antibacterial, Anti-Gram+, Anti-Gram-, Antimicrobial
663	DRAMP02402	Antimicrobial peptide scolopin-1	Antibacterial, Antifungal, Anti-Gram+, Anti-Gram-, Antimicrobial
664	DRAMP02403	Antimicrobial peptide scolopin-2	Antibacterial, Antifungal, Anti-Gram+, Anti-Gram-, Antimicrobial
665	DRAMP02409	M-theraphotoxin-Gr1a (M-TRTX-Gr1a; GsMTx-4)	Antibacterial, Anti-Gram+, Anti-Gram-, Antimicrobial
666	DRAMP02410	Antimicrobial peptide lumbricin-1	Antibacterial, Antifungal, Anti-Gram+, Anti-Gram-, Antimicrobial
667	DRAMP02411	Armadillidin (Glyc-rich)	Antibacterial, Anti-Gram+, Anti-Gram-, Antimicrobial
668	DRAMP02412	Panusin (Defensin-like peptide 7, PaD7)	Antibacterial, Anti-Gram+, Anti-Gram-, Antimicrobial

669	DRAMP02419	Amblyomma defensin peptide 2 (ADP-2; Ticks, Arthropods, animals)	Antibacterial, Antimicrobial
670	DRAMP02421	Hlgut-defensin (H. longicornis midgut defensin; Ticks, Arthropods, animals)	Antibacterial, Anti-Gram+, Anti-Gram-, Antimicrobial
671	DRAMP02422	Hlsal-defensin (H. longicornis salivary gland defensin; Ticks, Arthropods, animals)	Antibacterial, Anti-Gram+, Anti-Gram-, Antimicrobial
672	DRAMP02423	HlMS-defensin (Ticks, Arthropods, animals)	Antibacterial, Antifungal, Anti-Gram+, Anti-Gram-, Antimicrobial
673	DRAMP02425	Ixosin-B (Ticks, Arthropods, animals)	Antibacterial, Antifungal, Anti-Gram+, Anti-Gram-, Antimicrobial
674	DRAMP02427	Ixodes ricinus defensin def1 (Ticks, Arthropods, animals)	Antibacterial, Anti-Gram+, Antimicrobial
675	DRAMP02428	Ixodes ricinus defensin def2 (Ticks, Arthropods, animals)	Antibacterial, Anti-Gram+, Antimicrobial
676	DRAMP02429	Chymotrypsin-elastase inhibitor ixodidin (Ticks, Arthropods, animals)	Antibacterial, Antimicrobial
677	DRAMP02430	Ixosin (Ticks, Arthropods, animals)	Antibacterial, Antifungal, Anti-Gram-, Antimicrobial
678	DRAMP02432	Antimicrobial peptide ISAMP (Ticks, Arthropods, animals)	Antibacterial, Anti-Gram+, Anti-Gram-, Antimicrobial
679	DRAMP02433	Antimicrobial peptide microplusin (Microplusin; Ticks, Arthropods, animals)	Antibacterial, Antifungal, Antimicrobial
680	DRAMP02434	Antimicrobial peptide lumbricin-PG (Lumbricin-PG)	Antibacterial, Anti-Gram+, Anti-Gram-, Antimicrobial
681	DRAMP02439	U-theraphotoxin-Aju1a (U-TRTX-Aju1a; Juruin)	Antifungal, Antimicrobial
682	DRAMP02441	Gramicidin S (GS)	Antibacterial, Antimicrobial
683	DRAMP02445	Antimicrobial protein BL-A60	Antibacterial, Anti-Gram+, Anti-Gram-, Antimicrobial
684	DRAMP02446	Antimicrobial protein 1 (Antimicrobial protein AN5-1)	Antibacterial, Anti-Gram+, Anti-Gram-, Antimicrobial
685	DRAMP02449	Lectin	Antifungal, Antimicrobial
686	DRAMP02456	L-amino-acid oxidase (LAAO; LAO; Dactylomelin-P)	Antibacterial, Anti-Gram+, Antimicrobial
687	DRAMP02470	Nosiheptide (NOS; Antibiotic 9671-RP)	Antibacterial, Anti-Gram+, Anti-Gram-, Antimicrobial
688	DRAMP02473	Cathelicidin-BF (Cathelicidin-related protein; Snakes, reptiles, animals)	Antibacterial, Antifungal, Anti-Gram+, Anti-Gram-, Antimicrobial
689	DRAMP02474	cathelicidin-BF15 (Snakes, reptiles, animals)	Antibacterial, Antifungal, Anti-Gram+, Anti-Gram-, Antimicrobial
690	DRAMP02478	L-amino-acid oxidase (Bm-LAO; LAAO; LAO; Snakes, reptiles, animals)	Antibacterial, Antiparasitic, Anti-Gram+, Anti-Gram-, Antimicrobial
691	DRAMP02520	OH-CATH (Snakes, reptiles, animals)	Antibacterial, Anti-Gram-, Antimicrobial
692	DRAMP02522	L-amino-acid oxidase (LAAO, LAO, Oh-LAAO; Snakes, reptiles, animals)	Antibacterial, Anti-Gram+, Anti-Gram-, Antimicrobial
693	DRAMP02573	Penaeidin-3a (Pen-3a; shrimps, Arthropods, animals)	Antibacterial, Antifungal, Anti-Gram+, Anti-Gram-, Antimicrobial
694	DRAMP02574	[T8A]-Penaeidin-3a ([T8A]-Pen-3a; shrimps, Arthropods, animals)	Antibacterial, Antifungal, Anti-Gram+, Anti-Gram-, Antimicrobial
695	DRAMP18370	Crinicepsin-2 (insects, arthropods, invertebrates, animals)	Antibacterial, Anti-Gram+, Antimicrobial
696	DRAMP18371	Crinicepsin-1 (insects, arthropods, invertebrates, animals)	Antibacterial, Anti-Gram+, Antimicrobial
697	DRAMP02584	Penaeidin-4a (Pen-4a; shrimps, Arthropods, animals)	Antibacterial, Antifungal, Anti-Gram+, Anti-Gram-, Antimicrobial
698	DRAMP02586	Penaeidin-2d (Pen-2d; shrimps, Arthropods, animals)	Antibacterial, Antifungal, Anti-Gram+, Anti-Gram-, Antimicrobial
699	DRAMP02591	Penaeidin-4d (Pen-4d; shrimps, Arthropods, animals)	Antibacterial, Antifungal, Anti-Gram+, Antimicrobial
700	DRAMP02603	Putative antimicrobial peptide A Northern Europe Heligoland (chordates, animals)	Antibacterial, Antifungal, Anti-Gram+, Anti-Gram-, Antimicrobial
701	DRAMP02609	CjaRL-37 (cathelicidin; primates, mammals, animals)	Antibacterial, Antifungal, Antimicrobial
702	DRAMP02650	Sperm associated antigen 11 isoform C (primates, mammals, animals)	Antibacterial, Anti-Gram-, Antimicrobial
703	DRAMP02727	HmdSL-37 (cathelicidin; primates, mammals, animals)	Antibacterial, Antifungal, Antimicrobial
704	DRAMP18369	ccBD(channel catfish beta defensin)	Antibacterial, Antimicrobial
705	DRAMP02740	TBD-1 (Turtle beta-defensin 1; Reptiles, animals)	Antibacterial, Antifungal, Anti-Gram+, Anti-Gram-, Antimicrobial
706	DRAMP02768	Pilosulin-1 (Myr b I; ants, insects, animals)	Antibacterial, Antifungal, Anti-Gram+, Anti-Gram-, Antimicrobial
707	DRAMP02774	Antimicrobial peptide Alo-1 (Alo-1; knottin-type peptide; Insects, animals)	Antifungal, Antimicrobial
708	DRAMP02776	Antimicrobial peptide Alo-3 (Alo-3; knottin-type peptide; Insects, animals)	Antifungal, Antimicrobial
709	DRAMP02777	Rhinocerosin (Insects, animals)	Antibacterial, Anti-Gram+, Anti-Gram-, Antimicrobial

710	DRAMP02778	Defensin (Insects, animals)	Antibacterial, Anti-Gram+, Anti-Gram-, Antimicrobial
711	DRAMP02779	Defensin-A (Defensin A; Insects, animals)	Antibacterial , Anti-Gram+, Anti-Gram-, Antimicrobial
712	DRAMP02780	Defensin-B (Defensin B; Insects, animals)	Antibacterial, Anti-Gram+, Anti-Gram-, Antimicrobial
713	DRAMP02802	Paneth cell-specific alpha-defensin 1 (DEFA1; horse defensin; houses, mammals, animals)	Antibacterial, Antifungal, Anti-Gram+, Anti-Gram-, Antimicrobial
714	DRAMP02809	Myticin-B (Myt B; Cys-rich; molluscas, animals)	Antibacterial, Antifungal, Anti-Gram+, Anti-Gram-, Antimicrobial
715	DRAMP02811	Defensin MGD-1 (molluscas, animals)	Antibacterial, Anti-Gram+, Anti-Gram-, Antimicrobial
716	DRAMP02813	Conolysin-Mt1	Antibacterial, Anti-Gram+, Antimicrobial
717	DRAMP02817	Pyrrhocoricin	Antibacterial, Anti-Gram+, Anti-Gram-, Antimicrobial
718	DRAMP02835	Enkelytin (one chain of Proenkephalin-A; mammals, animals)	Antibacterial, Anti-Gram+, Antimicrobial
719	DRAMP01381	Odorranain-K1 (OdK1; Frogs, amphibians, animals)	Antimicrobial, Antibacterial, Antifungal, Anti-Gram+, Anti-Gram-,
720	DRAMP01383	Odorranain-M1 (OdM1; Frogs, amphibians, animals)	Antimicrobial, Antibacterial, Antifungal, Anti-Gram+, Anti-Gram-,
721	DRAMP02841	Lumbricin I(6-34)	Antibacterial, Antifungal, Anti-Gram+, Anti-Gram-, Antimicrobial
722	DRAMP02843	chain a, Structure Of An Indolicidin Peptide Derivative	Antibacterial, Anti-Gram+, Anti-Gram-, Antimicrobial
723	DRAMP02844	CP10A (Indolicidin peptide derivative; mammals, animals)	Antibacterial, Anti-Gram+, Antimicrobial
724	DRAMP02845	CP-11 (cathelicidin; mammals, animals)	Antibacterial, Anti-Gram+, Anti-Gram-, Antimicrobial
725	DRAMP02849	Bactenecin 5 (mammals, animals)	Antibacterial, Anti-Gram-, Antimicrobial
726	DRAMP02851	Cathelicidin-1 (Bactenecin-1, Bac1; Cyclic dodecapeptide; mammals, animals)	Antibacterial, Anti-Gram+, Anti-Gram-, Antimicrobial
727	DRAMP02854	Cathelicidin-5 (Antibacterial peptide BMAP-28)	Antibacterial, Antifungal , Anti-Gram+, Anti-Gram-, Antimicrobial
728	DRAMP02855	Cathelicidin-6 (Antibacterial peptide BMAP-27)	Antibacterial, Antifungal, Anti-Gram+, Anti-Gram-, Antimicrobial
729	DRAMP02858	Bovine Beta-defensin 1 (bBD-1; BNBD-1; BNDB-1; mammals, animals)	Antibacterial, Anti-Gram-, Antimicrobial
730	DRAMP02859	Bovine Beta-defensin 2 (bBD-2; BNBD-2; BNDB-2; mammals, animals)	Antibacterial, Anti-Gram+, Anti-Gram-, Antimicrobial
731	DRAMP02860	Bovine Beta-defensin 3 (bBD-3; BNBD-3; BNDB-3; mammals, animals)	Antibacterial, Anti-Gram+, Anti-Gram-, Antimicrobial
732	DRAMP02861	Bovine Beta-defensin 4 (bBD-4; BNBD-4; BNDB-4; mammals, animals)	Antibacterial, Anti-Gram+, Anti-Gram-, Antimicrobial
733	DRAMP02862	Bovine Beta-defensin 5 (bBD-5; BNBD-5; BNDB-5; mammals, animals)	Antibacterial, Anti-Gram-, Antimicrobial
734	DRAMP02863	Bovine Beta-defensin 6 (bBD-6; BNBD-6; BNDB-6; mammals, animals)	Antibacterial, Anti-Gram+, Anti-Gram-, Antimicrobial
735	DRAMP02865	Bovine Beta-defensin 8 (bBD-8; BNBD-8; BNDB-8; mammals, animals)	Antibacterial, Anti-Gram+, Anti-Gram-, Antimicrobial
736	DRAMP02866	Bovine Beta-defensin 9 (bBD-9; BNBD-9; BNDB-9; mammals, animals)	Antibacterial, Anti-Gram+, Anti-Gram-, Antimicrobial
737	DRAMP02867	Bovine Beta-defensin 10 (bBD-10; BNBD-10; BNDB-10; mammals, animals)	Antibacterial, Anti-Gram+, Anti-Gram-, Antimicrobial
738	DRAMP02868	Bovine Beta-defensin 11 (bBD-11; BNBD-11; BNDB-11; mammals, animals)	Antibacterial, Anti-Gram+, Anti-Gram-, Antimicrobial
739	DRAMP02869	Bovine Beta-defensin 12 (bBD-12; BNBD-12; BNDB-12; mammals, animals)	Antibacterial, Anti-Gram+, Anti-Gram-, Antimicrobial
740	DRAMP02870	Bovine Beta-defensin 13 (bBD-13; BNBD-13; BNDB-13; mammals, animals)	Antibacterial, Anti-Gram+, Anti-Gram-, Antimicrobial
741	DRAMP02872	Myeloid antimicrobial peptide BMAP-27 (1-18) (mammals, animals)	Antibacterial, Antifungal, Anti-Gram+, Anti-Gram-, Antimicrobial
742	DRAMP02873	Myeloid antimicrobial peptide BMAP-28 (1-18) (mammals, animals)	Antibacterial, Antifungal, Anti-Gram+, Anti-Gram-, Antimicrobial
743	DRAMP02875	Vasostatin-1 (VS-1; N-terminal fragment of Chromogranin-A; mammals, animals)	Antibacterial, Antifungal, Anti-Gram+, Antimicrobial
744	DRAMP02877	mBMAP28 (mammals, animals)	Antibacterial, Anti-Gram+, Anti-Gram-, Antimicrobial
745	DRAMP02878	Tracheal antimicrobial peptide (TAP; mammals, animals)	Antibacterial, Antifungal, Anti-Gram+, Anti-Gram-, Antimicrobial
746	DRAMP02903	Bombin H7	Antibacterial, Anti-Gram+, Anti-Gram-, Antimicrobial
747	DRAMP02910	Ovispirin-2 (OV-2; mammals, animals)	Antibacterial, Anti-Gram-, Antimicrobial

748	DRAMP02911	Ovispirin-3 (OV-3; mammals, animals)	Antibacterial, Anti-Gram-, Antimicrobial
749	DRAMP02912	SMAP-29 (Cathelin-related peptide SC5; Myeloid antibacterial peptide MAP-29; mammals, animals)	Antibacterial, Antifungal, Antimicrobial
750	DRAMP02913	SMAP-34 (cathelicidin; mammals, animals)	Antibacterial, Antimicrobial
751	DRAMP02922	Canine beta-defensin (dogs, mammals, animals)	Antibacterial, Antifungal, Anti-Gram+, Anti-Gram-, Antimicrobial
752	DRAMP02923	cBD-1 (Canine beta-defensin 1; dogs, mammals, animals)	Antibacterial, Antifungal, Anti-Gram+, Anti-Gram-, Antimicrobial
753	DRAMP18367	Chaxapeptin (a class 2 lasso peptide; class 1 microcin, bacteriocins)	Antibacterial, Cancer cells, Anti-Gram+, Antimicrobial
754	DRAMP18368	Bacteriocin TSU4 (bacteria, prokaryotes)	Antibacterial, Antimicrobial
755	DRAMP02925	Cathelicidin (dogs, mammals, animals)	Antibacterial, Antifungal, Anti-Gram+, Anti-Gram-, Antimicrobial
756	DRAMP02931	Arasin-likeSp (crabs, Arthropods, animals)	Antibacterial, Anti-Gram+, Anti-Gram-, Antimicrobial
757	DRAMP02932	GRPSp (crabs, Arthropods, animals)	Antibacterial, Anti-Gram+, Antimicrobial
758	DRAMP02933	Polyphemusin-1 (PM1; crabs, Arthropods, animals)	Antibacterial, Antifungal, Anti-Gram+, Anti-Gram-, Antimicrobial
759	DRAMP02934	PM1-S (linear derivative of PM1)	Antibacterial, Antifungal, Anti-Gram+, Anti-Gram-, Antimicrobial
760	DRAMP02950	PtALF5 (Portunus trituberculatus anti-lipopolysaccharide factor isoform 5; crabs, Arthropods, animals)	Antibacterial, Anti-Gram-, Antimicrobial
761	DRAMP02951	PtALF6 (Portunus trituberculatus anti-lipopolysaccharide factor isoform 6; crabs, Arthropods, animals)	Antibacterial, Antifungal, Anti-Gram+, Anti-Gram-, Antimicrobial
762	DRAMP02952	PtALF7 (Portunus trituberculatus anti-lipopolysaccharide factor isoform 7; crabs, Arthropods, animals)	Antibacterial, Anti-Gram+, Anti-Gram-, Antimicrobial
763	DRAMP02953	Arasin-1 (Pro-rich, Arg-rich; crabs, Arthropods, animals)	Antibacterial, Anti-Gram+, Anti-Gram-, Antimicrobial
764	DRAMP02956	Dolabellanin B2	Antibacterial, Antifungal, Anti-Gram+, Anti-Gram-, Antimicrobial
765	DRAMP02959	Antibacterial protein PR-39 (pigs, mammals, animals)	Antibacterial, Anti-Gram+, Anti-Gram-, Antimicrobial
766	DRAMP02960	Antibacterial peptide PMAP-23 (Myeloid antibacterial peptide 23; pigs, mammals, animals)	Antibacterial, Anti-Gram+, Anti-Gram-, Antimicrobial
767	DRAMP02961	Antibacterial peptide PMAP-37 (Myeloid antibacterial peptide 37; pigs, mammals, animals)	Antibacterial, Anti-Gram+, Anti-Gram-, Antimicrobial
768	DRAMP02962	Antibacterial peptide PMAP-36 (Myeloid antibacterial peptide 36; pigs, mammals, animals)	Antibacterial, Anti-Gram+, Anti-Gram-, Antimicrobial
769	DRAMP02963	PMAP-36(1-20)	Antibacterial, Antifungal, Anti-Gram+, Anti-Gram-, Antimicrobial
770	DRAMP02964	PMAP-36(1-34)	Antibacterial, Antifungal, Anti-Gram+, Anti-Gram-, Antimicrobial
771	DRAMP02965	PMAP-36(1-35)2	Antibacterial, Antifungal, Anti-Gram+, Anti-Gram-, Antimicrobial
772	DRAMP02966	DBI(32-86) (pigs, mammals, animals)	Antibacterial, Anti-Gram+, Anti-Gram-, Antimicrobial
773	DRAMP02970	Protegrin-1 (Protegrin 1; PG-1; pigs, mammals, animals)	Antibacterial, Anti-Gram+, Anti-Gram-, Antimicrobial
774	DRAMP02975	Tritrpticin (Trp-rich; pigs, mammals, animals)	Antibacterial, Antifungal, Anti-Gram+, Anti-Gram-, Antimicrobial
775	DRAMP02980	Antimicrobial peptide NK-lysin (NKL; pigs, mammals, animals)	Antibacterial, Antifungal, Antitumor, Antimicrobial
776	DRAMP01376	Odorranain-F1 (OdF1; Frogs, amphibians, animals)	Antimicrobial, Antibacterial, Antifungal, Anti-Gram+, Anti-Gram-,
777	DRAMP01377	Odorranain-G1 (OdG1; Frogs, amphibians, animals)	Antimicrobial, Antibacterial, Antifungal, Anti-Gram+, Anti-Gram-,
778	DRAMP02995	Hymenoptaecin (Insects, animals)	Antibacterial, Anti-Gram+, Anti-Gram-, Antimicrobial
779	DRAMP02996	Apidaecin-2 (Apidaecin II; Insects, animals)	Antibacterial, Anti-Gram+, Anti-Gram-, Antimicrobial
780	DRAMP01378	Odorranain-H1 (OdH1; Frogs, amphibians, animals)	Antimicrobial, Antibacterial, Antifungal, Anti-Gram+, Anti-Gram-,
781	DRAMP02998	Apidaecin-1A (Apidaecin IA; Insects, animals)	Antibacterial, Anti-Gram+, Anti-Gram-, Antimicrobial
782	DRAMP02999	Jellein-1 (Jelleine-I; chain of Major royal jelly protein 1; Insects, animals)	Antibacterial, Antifungal, Anti-Gram+, Anti-Gram-, Antimicrobial
783	DRAMP03000	Jellein-2 (Jelleine-II; chain of Major royal jelly protein 1; Insects, animals)	Antibacterial, Antifungal, Anti-Gram+, Anti-Gram-, Antimicrobial
784	DRAMP03001	Jellein-3 (Jelleine-III; Insects, animals)	Antibacterial, Antifungal, Anti-Gram+, Anti-Gram-, Antimicrobial
785	DRAMP03002	Melittin (Allergen Api m 3; Allergen Api m III; Insects, animals)	Antibacterial, Antifungal, Anti-Gram+, Anti-Gram-, Antimicrobial

786	DRAMP03003	Melectin (MEP; Insects, animals)	Antibacterial, Anti-Gram+, Anti-Gram-, Antimicrobial
787	DRAMP03007	Osmin (Insects, animals)	Antibacterial, Antifungal, Anti-Gram+, Anti-Gram-, Antimicrobial
788	DRAMP03019	Mastoparan PDD-B	Antibacterial, Anti-Gram+, Anti-Gram-, Antimicrobial
789	DRAMP03020	Mastoparan PDD-A	Antibacterial, Anti-Gram+, Anti-Gram-, Antimicrobial
790	DRAMP03021	Mastoparan PMM	Antibacterial, Anti-Gram+, Anti-Gram-, Antimicrobial
791	DRAMP03022	Mastoparan MP	Antibacterial, Anti-Gram+, Anti-Gram-, Antimicrobial
792	DRAMP03028	Mastoparan-1 (MP-1; Venom protein MP-1; Insects, animals)	Antibacterial, Anti-Gram+, Anti-Gram-, Antimicrobial
793	DRAMP03033	Mastoparan-like peptide 12a (Insects, animals)	Antibacterial, Antifungal, Anti-Gram+, Anti-Gram-, Antimicrobial
794	DRAMP03034	Mastoparan-like peptide 12b (Insects, animals)	Antibacterial, Antifungal, Anti-Gram+, Anti-Gram-, Antimicrobial
795	DRAMP03035	Mastoparan-like peptide 12c (Insects, animals)	Antibacterial, Antifungal, Anti-Gram+, Anti-Gram-, Antimicrobial
796	DRAMP03036	Mastoparan-like peptide 12d (Insects, animals)	Antibacterial, Antifungal, Anti-Gram+, Anti-Gram-, Antimicrobial
797	DRAMP03037	Eumenitin (Er-12; Insects, animals)	Antibacterial, Anti-Gram+, Anti-Gram-, Antimicrobial
798	DRAMP03038	Eumenitin-R (Insects, animals)	Antibacterial, Antifungal, Anti-Gram+, Anti-Gram-, Antimicrobial
799	DRAMP03039	Eumenitin-F (Insects, animals)	Antibacterial, Antifungal, Anti-Gram+, Anti-Gram-, Antimicrobial
800	DRAMP03040	Eumenine mastoparan-EF (EMP-EF; Insects, animals)	Antibacterial, Antifungal, Anti-Gram+, Anti-Gram-, Antimicrobial
801	DRAMP03041	Eumenine mastoparan-ER (EMP-ER; Insects, animals)	Antibacterial, Antifungal, Anti-Gram+, Anti-Gram-, Antimicrobial
802	DRAMP03042	Eumenine mastoparan-AF (EMP-AF; Af-113; Insects, animals)	Antibacterial, Anti-Gram+, Anti-Gram-, Antimicrobial
803	DRAMP03043	Agelaia-mastoparan (Agelaia-MP; Insects, animals)	Antibacterial, Anti-Gram+, Anti-Gram-, Antimicrobial
804	DRAMP03044	Protonectin (Agelaia-chemotactic peptide, Agelaia-CP; Insects, animals)	Antibacterial, Anti-Gram+, Anti-Gram-, Antimicrobial
805	DRAMP03045	Defensin-NV (Insects, animals)	Antibacterial, Antifungal, Anti-Gram+, Anti-Gram-, Antimicrobial
806	DRAMP03046	Orancis-protonectin (chain of Venom peptide 2-long; Venom peptide 2, OdVP2; Insects, animals)	Antifungal, Anti-Gram+, Antimicrobial
807	DRAMP03047	Venom peptide 2-long (OdVP2L; analog of OdVP2; Insects, animals)	Antibacterial, Antifungal, Anti-Gram+, Anti-Gram-, Antimicrobial
808	DRAMP03050	Dominulin-A (Insects, animals)	Antibacterial, Anti-Gram+, Anti-Gram-, Antimicrobial
809	DRAMP03051	Dominulin-B (Insects, animals)	Antibacterial, Anti-Gram+, Anti-Gram-, Antimicrobial
810	DRAMP03052	PP13 (Insects, animals)	Antibacterial, Anti-Gram+, Antimicrobial
811	DRAMP03053	PP102 (Insects, animals)	Antibacterial, Anti-Gram+, Antimicrobial
812	DRAMP03054	PP113 (Insects, animals)	Antibacterial, Anti-Gram+, Antimicrobial
813	DRAMP03055	PP30 (Pro-rich; abaecin-like; Insects, animals)	Antibacterial, Anti-Gram+, Anti-Gram-, Antimicrobial
814	DRAMP03056	Decoralin (Insects, animals)	Antibacterial, Antifungal, Anti-Gram+, Anti-Gram-, Antimicrobial
815	DRAMP03057	Thanatin (Insects, animals)	Antibacterial, Antifungal, Anti-Gram+, Anti-Gram-, Antimicrobial
816	DRAMP03063	Defensin ARD1 (Heliomicin analogs; Insects, animals)	Antifungal, Antimicrobial
817	DRAMP03069	Diptericin (Insects, animals)	Antibacterial, Antimicrobial
818	DRAMP03075	Cecropin-D	Antibacterial, Anti-Gram+, Anti-Gram-, Antimicrobial
819	DRAMP03089	Drosophila cecropin-A1/A2 (Insects, animals)	Antibacterial, Anti-Gram+, Anti-Gram-, Antimicrobial
820	DRAMP03090	Drosophila cecropin B (CecB; Insects, animals)	Antibacterial, Antifungal, Anti-Gram+, Anti-Gram-, Antimicrobial
821	DRAMP03095	Andropin (Insects, animals)	Antibacterial, Anti-Gram+, Anti-Gram-, Antimicrobial
822	DRAMP03096	Metchnikowin-2 (Insects, animals)	Antibacterial, Antifungal, Anti-Gram+, Antimicrobial
823	DRAMP18495	Gomesin (Gm; Spiders, arachnids, Chelicerata, arthropods, invertebrates, animals)	Antimicrobial, Antibacterial, Antifungal, Antiparasitic, Antimalarial, Antic, Anti-Gram+, Anti-Gram-,
824	DRAMP03098	Metchnikowin (Pro-rich; Insects, animals)	Antibacterial, Antifungal, Antimicrobial
825	DRAMP03104	Sapecin (defensins; Insects, animals)	Antibacterial, Anti-Gram+, Anti-Gram-, Antimicrobial
826	DRAMP03113	SK84 (Gly-rich; Insects, animals)	Antibacterial, Antifungal, Antiviral, In, Anti-Gram+, Antimicrobial
827	DRAMP03116	Ceratotoxin-C (Insects, animals)	Antibacterial, Anti-Gram+, Anti-Gram-, Antimicrobial
828	DRAMP03117	Drosophila cecropin-A1 (Insects, animals)	Antibacterial, Anti-Gram+, Anti-Gram-, Antimicrobial

829	DRAMP03137	Defensin-A (AaeDefA; Insects, animals)	Antibacterial, Anti-Gram+, Antimicrobial
830	DRAMP03138	Cecropin-A (Insects, animals)	Antibacterial, Antifungal, Anti-Gram+, Anti-Gram-, Antimicrobial
831	DRAMP03140	Anopheles cecropin-A amidated isoform (Insects, animals)	Antibacterial, Antifungal, Anti-Gram+, Anti-Gram-, Antimicrobial
832	DRAMP03150	Gambicin (Insects, animals)	Antibacterial, Antifungal, Antiparasitic, Anti-Gram+, Anti-Gram-, Antimicrobial
833	DRAMP03153	27 kDa antibacterial protein	Antibacterial, Anti-Gram+, Anti-Gram-, Antimicrobial
834	DRAMP03162	Phlebotomus duboscqi defensin (PduDef; defensins; Insects, animals)	Antibacterial, Antifungal, Antiparasitic, Anti-Gram+, Antimicrobial
835	DRAMP03166	P15 (deer beta-defensin; ruminant, animals)	Antibacterial, Antifungal, Anti-Gram+, Anti-Gram-, Antimicrobial
836	DRAMP03169	Protease-activated antimicrobial peptide	Antibacterial, Antimicrobial
837	DRAMP03173	Arenicin-1 (Ar-1; marine polychaeta, animals)	Antibacterial, Antifungal, Cytotoxicity, Anti-Gram+, Anti-Gram-, Antimicrobial
838	DRAMP03176	Cecropin-P1 (CP1; nematodes, animals)	Antibacterial, Antifungal, Antimicrobial
839	DRAMP03181	Spinigerin (Insects, animals)	Antibacterial, Antifungal, Anti-Gram+, Anti-Gram-, Antimicrobial
840	DRAMP03186	Spheniscin-2 (Sphe-2; penguin avian beta-defensin 103b; birds , animals)	Antibacterial, Antifungal, Anti-Gram+, Anti-Gram-, Antimicrobial
841	DRAMP03187	Beta defensin 1(BD-1; mammals, animals)	Antibacterial, Antifungal, Anti-Gram+, Anti-Gram-, Antimicrobial
842	DRAMP03190	PobRL-37 (cathelicidin; primates, mammals, animals)	Antibacterial, Antifungal, Antimicrobial
843	DRAMP03196	PhTD-1 (PhTD1; primates, mammals, animals)	Antibacterial, Antifungal, Antimicrobial
844	DRAMP03197	PhTD-3 (PhTD3; primates, mammals, animals)	Antibacterial, Antifungal, Antimicrobial
845	DRAMP03198	Alpha-defensin PhD-4 (primates, mammals, animals)	Antibacterial, Antifungal, Anti-Gram+, Anti-Gram-, Antimicrobial
846	DRAMP03215	Gomesin (Gm; spiders, Arthropods, animals)	Antibacterial, Antifungal, Anti-Gram+, Anti-Gram-, Antimicrobial
847	DRAMP03216	Oxyopinin-4a (Oxt-4a; spiders, Arthropods, animals)	Antibacterial, Anti-Gram+, Anti-Gram-, Antimicrobial
848	DRAMP03217	M-oxotoxin-Ot1a (Oxyopinin-1, Oxki1; spiders, Arthropods, animals)	Antibacterial, Insecticidal, Anti-Gram+, Anti-Gram-, Antimicrobial
849	DRAMP03222	M-ctenitoxin-Cs1a (M-CNTX-Cs1a; Cupiennin-1a; spiders, Arthropods, animals)	Antibacterial, Insecticidal, Anti-Gram+, Anti-Gram-, Antimicrobial
850	DRAMP03225	M-ctenitoxin-Cs1d (M-CNTX-Cs1d; Cupiennin-1d; spiders, Arthropods, animals)	Antibacterial, Anti-Gram+, Anti-Gram-, Antimicrobial
851	DRAMP03226	M-zodatoxin-Lt1a (M-ZDTX-Lt1a; Latarcin-1, Ltc-1, Ltc1; spiders, Arthropods, animals)	Antibacterial, Antifungal, Anti-Gram+, Anti-Gram-, Antimicrobial
852	DRAMP03227	M-zodatoxin-Lt2a (M-ZDTX-Lt2a; Latarcin-2a, Ltc-2a, Ltc2a; spiders, Arthropods, animals)	Antibacterial, Antifungal, Anti-Gram+, Anti-Gram-, Antimicrobial
853	DRAMP03229	M-zodatoxin-Lt3a (M-ZDTX-Lt3a; Latarcin-3a, Ltc-3a; spiders, Arthropods, animals)	Antibacterial, Antifungal, Anti-Gram+, Anti-Gram-, Antimicrobial
854	DRAMP03230	M-zodatoxin-Lt3b (M-ZDTX-Lt3b; Latarcin-3b, Ltc-3b; spiders, Arthropods, animals)	Antibacterial, Antifungal, Anti-Gram+, Anti-Gram-, Antimicrobial
855	DRAMP03231	M-zodatoxin-Lt4a (M-ZDTX-Lt4a; Latarcin-4a, Ltc-4a; spiders, Arthropods, animals)	Antibacterial, Antifungal, Anti-Gram+, Anti-Gram-, Antimicrobial
856	DRAMP03232	M-zodatoxin-Lt4b (M-ZDTX-Lt4b; Latarcin-4b, Ltc-4b; spiders, Arthropods, animals)	Antibacterial, Antifungal, Anti-Gram+, Anti-Gram-, Antimicrobial
857	DRAMP03233	M-zodatoxin-Lt5a (M-ZDTX-Lt5a; Latarcin-5, Ltc-5; spiders, Arthropods, animals)	Antibacterial, Antifungal, Anti-Gram+, Anti-Gram-, Antimicrobial
858	DRAMP03236	M-zodatoxin-Lt8a (M-ZDTX-Lt8a; Cytoinsectotoxin-1a, CIT-1a; spiders, Arthropods, animals)	Antibacterial, Insecticidal, Anti-Gram+, Anti-Gram-, Antimicrobial
859	DRAMP03253	M-lycotoxin-Ls3a (M-LCTX-Ls3a; Lycocitin-1; spiders, Arthropods, animals)	Antibacterial, Antifungal, Anti-Gram+, Anti-Gram-, Antimicrobial
860	DRAMP03254	M-lycotoxin-Ls3b (M-LCTX-Ls3b; Lycocitin-2; spiders, Arthropods, animals)	Antibacterial, Antifungal, Anti-Gram+, Anti-Gram-, Antimicrobial
861	DRAMP03278	M-lycotoxin-Hc1a (M-LCTX-Hc1a; Lycotoxin I; spiders, Arthropods, animals)	Antibacterial, Antifungal, Anti-Gram+, Anti-Gram-, Antimicrobial
862	DRAMP03279	M-lycotoxin-Hc2a (M-LCTX-Hc2a; Lycotoxin-2; Lycotoxin II; spiders, Arthropods, animals)	Antibacterial, Antifungal, Anti-Gram+, Anti-Gram-, Antimicrobial
863	DRAMP03280	AcAMP (A. clavatus antimicrobial peptide)	Antibacterial, Antifungal, Antiviral, Anti-Gram+, Anti-Gram-, Antimicrobial
864	DRAMP03285	Ostricacin-1 (Beta-defensin 2; Birds, animals)	Antibacterial, Anti-Gram+, Anti-Gram-, Antimicrobial

865	DRAMP03286	Ostricacin-2 (Beta-defensin 1; Birds, animals)	Antibacterial, Antifungal, Anti-Gram+, Anti-Gram-, Antimicrobial
866	DRAMP03287	Ostricacin-3 (Beta-defensin 7; Birds, animals)	Antibacterial, Anti-Gram+, Anti-Gram-, Antimicrobial
867	DRAMP03288	Ostricacin-4 (Beta-defensin 8; Birds, animals)	Antibacterial, Anti-Gram+, Anti-Gram-, Antimicrobial
868	DRAMP03311	Stomoxyn (Insects, animals)	Antibacterial, Anti-Gram+, Anti-Gram-, Antimicrobial
869	DRAMP03312	Metalnikowin-1 (Metalnikowin I; Insects, animals)	Antibacterial, Anti-Gram-, Antimicrobial
870	DRAMP03320	Pore-forming peptide ameobapore A (EH-APP; saposin-like protein)	Antibacterial, Anti-Gram+, Antimicrobial
871	DRAMP03321	Colutellin-A	Antifungal, Antimicrobial
872	DRAMP03370	Beta-defensin 6 (BD-6, mBD-6; Defensin, beta 6; Rodents, mammals, animals)	Antibacterial, Anti-Gram-, Antimicrobial
873	DRAMP03405	mCRAMP-1 (mouse cathelin-related antimicrobial peptide 1; cathelicidin; Rodents, mammals, animals)	Antibacterial, Antifungal, Anti-Gram+, Anti-Gram-, Antimicrobial
874	DRAMP03406	mCRAMP-2 (mouse cathelin-related antimicrobial peptide 2; cathelicidin; Rodents, mammals, animals)	Antibacterial, Antifungal, Anti-Gram+, Anti-Gram-, Antimicrobial
875	DRAMP03419	Neutrophil antibiotic peptide NP-1 (RatNP-1; Rodents, mammals, animals)	Antibacterial, Antifungal, Anti-Gram+, Anti-Gram-, Antimicrobial
876	DRAMP03422	Neutrophil antibiotic peptide NP-4 (RatNP-4; Rodents, mammals, animals)	Antibacterial, Antifungal, Antiviral, Anti-Gram+, Anti-Gram-, Antimicrobial
877	DRAMP03463	rCRAMP (rat cathelin-related antimicrobial peptide; Rodents, mammals, animals)	Antibacterial, Antimicrobial
878	DRAMP03464	Cryptonin (Insects, animals)	Antibacterial, Antifungal, Anti-Gram+, Anti-Gram-, Antimicrobial
879	DRAMP03465	Cicadin (Insects, animals)	Antifungal, Antiviral, Antimicrobial
880	DRAMP03467	Antibacterial napin (Plants)	Antibacterial, Anti-Gram+, Anti-Gram-, Antimicrobial
881	DRAMP03471	Recombinant Crassostrea Gigas Defensin (Cg-Def; molluscs, animals)	Antibacterial, Antifungal, Anti-Gram+, Anti-Gram-, Antimicrobial
882	DRAMP03472	cgUbiquitin	Antibacterial, Antifungal, Anti-Gram+, Anti-Gram-, Antimicrobial
883	DRAMP03474	Pleurostatin (Fungus)	Antifungal, Antimicrobial
884	DRAMP03477	Polybia-CP (Polybia chemotactic peptide; Venom protein CP; Insects, animals)	Antibacterial, Anti-Gram+, Antimicrobial
885	DRAMP03486	Manduca Sexta Moricin (MS moricin; Insects, animals)	Antibacterial, Anti-Gram+, Anti-Gram-, Antimicrobial
886	DRAMP03493	Defensin heliomicin (Insects, animals)	Antifungal, Antimicrobial
887	DRAMP03507	Cecropin-B (Insects, animals)	Antibacterial, Antifungal, Anti-Gram+, Anti-Gram-, Antimicrobial
888	DRAMP03513	G. mellonella moricin-like peptide A (Gm-mlpA; Insects, animals; Predicted)	Antibacterial, Antifungal, Anti-Gram+, Anti-Gram-, Antimicrobial
889	DRAMP03514	G. mellonella moricin-like peptide B (Gm-mlpB; Insects, animals; Predicted)	Antibacterial, Antifungal, Anti-Gram+, Anti-Gram-, Antimicrobial
890	DRAMP03515	Moricin-like peptide C1 (Gm-mlpC1; Insects, animals; Predicted)	Antibacterial, Antifungal, Anti-Gram+, Anti-Gram-, Antimicrobial
891	DRAMP03516	Moricin-like peptide C2 (Gm-mlpC2; Insects, animals; Predicted)	Antibacterial, Antifungal, Anti-Gram+, Anti-Gram-, Antimicrobial
892	DRAMP03517	Moricin-like peptide C3 (Gm-mlpC3; Insects, animals; Predicted)	Antibacterial, Antifungal, Anti-Gram+, Anti-Gram-, Antimicrobial
893	DRAMP03518	Moricin-like peptide C4/C5 (Gm-mlpC4/C5; Insects, animals; Predicted)	Antibacterial, Antifungal, Anti-Gram-, Antimicrobial
894	DRAMP03519	Moricin-like peptide D (Gm-mlpD; Insects, animals; Predicted)	Antibacterial, Antifungal, Anti-Gram-, Antimicrobial
895	DRAMP03520	Cecropin-D-like peptide (Insects, animals)	Antibacterial, Antifungal, Anti-Gram+, Antimicrobial
896	DRAMP03521	Proline-rich antimicrobial peptide 1 (Insects, animals)	Antibacterial, Antifungal, Anti-Gram+, Antimicrobial
897	DRAMP03523	Anionic antimicrobial peptide 2 (Insects, animals)	Antibacterial, Antifungal, Anti-Gram+, Antimicrobial
898	DRAMP03524	Lebocin-like anionic peptide 1 (Insects, animals)	Antibacterial, Antifungal, Anti-Gram+, Antimicrobial
899	DRAMP03525	Apolipophorin-3 (Apolipophorin-III; Insects, animals)	Antibacterial, Anti-Gram+, Antimicrobial
900	DRAMP03526	Proline-rich antimicrobial peptide 2 (Insects, animals)	Antibacterial, Anti-Gram+, Antimicrobial
901	DRAMP03527	Gm defensin-like peptide (Insects, animals)	Antibacterial, Antifungal, Anti-Gram+, Antimicrobial
902	DRAMP03528	Defensin (Galiomicin; Insects, animals)	Antifungal, Antimicrobial
903	DRAMP03532	Moricin-1 (Insects, animals)	Antibacterial, Anti-Gram+, Anti-Gram-, Antimicrobial
904	DRAMP03539	Antifungal protein (Psc-AFP)	Antifungal, Antimicrobial
905	DRAMP03540	Human drosomycin-like defensin (DLD; Human, mammals, animals)	Antifungal, Antimicrobial
906	DRAMP03562	P1 (Human, mammals, animals)	Antifungal, Antimicrobial

907	DRAMP03563	P2 (Human, mammals, animals)	Antifungal, Antimicrobial
908	DRAMP03567	KR-20 (Derived from LL-37)	Antibacterial, Antifungal, Anti-Gram+, Anti-Gram-, Antimicrobial
909	DRAMP03568	RK-31 (Derived from LL-37)	Antibacterial, Antifungal, Anti-Gram+, Anti-Gram-, Antimicrobial
910	DRAMP03569	KS-30 (Derived from LL-37)	Antibacterial, Antifungal, Anti-Gram+, Anti-Gram-, Antimicrobial
911	DRAMP03570	LL-23 (Derived from LL-37)	Antibacterial, Antifungal, Anti-Gram+, Anti-Gram-, Antimicrobial
912	DRAMP03571	Antibacterial protein LL-37 (one chain of hCAP-18; Human, mammals, animals)	Antibacterial, Anticancer, Anti-Gram+, Anti-Gram-, Antimicrobial
913	DRAMP03573	LL-37(13-37)(C-terminal fragment of LL-37; Human, mammals, animals)	Antibacterial, Anticancer, Anti-Gram-, Antimicrobial
914	DRAMP03574	LL-37(17-32)(C-terminal fragment of LL-37; Human, mammals, animals)	Antibacterial, Anticancer, Anti-Gram-, Antimicrobial
915	DRAMP03598	Human beta-defensin 2 (hBD-2; Defensin, beta 2; Beta-defensin 4A; Human, mammals, animals)	Antibacterial, Antiviral, Anti-Gram+, Anti-Gram-, Antimicrobial
916	DRAMP03599	Human beta-defensin 3 (BD-3, hBD-3; Hbd3; Beta-defensin 103; Human, mammals, animals)	Antibacterial, Antifungal, Anti-Gram+, Anti-Gram-, Antimicrobial
917	DRAMP03600	Human beta-defensin 4 (hBD-4, BD-4; Beta-defensin 104; Human, mammals, animals)	Antibacterial, Anti-Gram+, Anti-Gram-, Antimicrobial
918	DRAMP03603	Human beta-defensin 28 (hBD-28; hBD28; Human, mammals, animals)	Antibacterial, Anti-Gram+, Anti-Gram-, Antimicrobial
919	DRAMP03638	VpBD (V.philippinarum beta defensin; big defensin)	Antibacterial, Anti-Gram+, Anti-Gram-, Antimicrobial
920	DRAMP03642	Chicken heterophil peptides 1 (Antimicrobial peptide CHP1; Birds, animals)	Antibacterial, Antifungal, Anti-Gram+, Anti-Gram-, Antimicrobial
921	DRAMP03645	Cathelicidin-2 (CATH-2; Fowlicidin-2; Birds, animals)	Antibacterial, Anti-Gram+, Anti-Gram-, Antimicrobial
922	DRAMP03646	Cathelicidin-3 (CATH-3; Fowlicidin-3; Birds, animals)	Antibacterial, Anti-Gram+, Anti-Gram-, Antimicrobial
923	DRAMP03647	Cathelicidin-B1 (CATH-B1; cathelicidin; Birds, animals)	Antibacterial, Anti-Gram+, Anti-Gram-, Antimicrobial
924	DRAMP03659	Gallinacin-11 (Gal-11; Beta-defensin 11; Birds, animals)	Antibacterial, Anti-Gram-, Antimicrobial
925	DRAMP03676	GLFcin (Lactoferrin fragment)	Antibacterial, Anti-Gram+, Anti-Gram-, Antimicrobial
926	DRAMP03677	GLFcin II (Lactoferrin fragment)	Antibacterial, Anti-Gram+, Anti-Gram-, Antimicrobial
927	DRAMP03679	Cathelicidin-2 (Bactenecin-5, Bac5; ChBac5; ruminant, animals)	Antibacterial, Anti-Gram+, Anti-Gram-, Antimicrobial
928	DRAMP03682	Vespid chemotactic peptide 5e (VCP 5e; Insects, animals)	Antibacterial, Antifungal, Anti-Gram+, Anti-Gram-, Antimicrobial
929	DRAMP03683	Vespid chemotactic peptide 5g (VCP 5g; Insects, animals)	Antibacterial, Antifungal, Anti-Gram+, Anti-Gram-, Antimicrobial
930	DRAMP03684	Vespid chemotactic peptide 5f (VCP 5f; Insects, animals)	Antibacterial, Antifungal, Anti-Gram+, Anti-Gram-, Antimicrobial
931	DRAMP03687	TsAP-1 (T. serrulatus antimicrobial peptide 1; scorpions, arachnids, invertebrates, animals)	Antibacterial, Anticancer, Anti-Gram+, Anti-Gram-, Antimicrobial
932	DRAMP03688	TsAP-2 (T. serrulatus antimicrobial peptide 2; scorpions, arachnids, invertebrates, animals)	Antibacterial, Antifungal, Anticancer, Anti-Gram+, Antimicrobial
933	DRAMP03691	Im-1 (Arthropods, animals)	Antibacterial, Anti-Gram+, Anti-Gram-, Antimicrobial
934	DRAMP03693	Bactridin-1 (Bact1; Bactridine 1; Arthropods, animals)	Antibacterial, Anti-Gram+, Anti-Gram-, Antimicrobial
935	DRAMP03694	Bactridin-2 (Bact2, Bactridine 2; P-Mice-Antm-beta* NaTx14.8; Arthropods, animals)	Antibacterial, Anti-Gram+, Anti-Gram-, Antimicrobial
936	DRAMP03700	Antimicrobial peptide ctriporin (Riporin; Arthropods, animals)	Antibacterial, Anti-Gram+, Antimicrobial
937	DRAMP03702	Mucroporin (Antimicrobial peptide 36.21; Arthropods, animals)	Antibacterial, Anti-Gram+, Anti-Gram-, Antimicrobial
938	DRAMP03706	Antimicrobial peptide 1 (AamAP1; Arthropods, animals)	Antibacterial, Antifungal, Anti-Gram+, Anti-Gram-, Antimicrobial
939	DRAMP03707	Antimicrobial peptide 2 (AamAP2; Arthropods, animals)	Antibacterial, Antifungal, Anti-Gram+, Anti-Gram-, Antimicrobial
940	DRAMP03714	Amphiphatic peptide CT1 (VmCT1; Non-disulfide-bridged peptide 5.13, NDBP-5.13; Arthropods, animals)	Antibacterial, Anti-Gram+, Anti-Gram-, Antimicrobial
941	DRAMP03715	Amphiphatic peptide CT2 (VmCT2; Non-disulfide-bridged peptide 5.14, NDBP-5.14; Arthropods, animals)	Antibacterial, Anti-Gram+, Anti-Gram-, Antimicrobial
942	DRAMP03721	Cytotoxic linear peptide IsCT (IsCT; NDBP-5.2; Arthropods, animals)	Antibacterial, Anti-Gram+, Anti-Gram-, Antimicrobial
943	DRAMP03723	Pandinin-1 (Pin1; Arthropods, animals)	Antibacterial, Anti-Gram+, Anti-Gram-, Antimicrobial
944	DRAMP03724	Pandinin-2 (Pin2; Arthropods, animals)	Antibacterial, Hemolytic activity, Anti-Gram+, Anti-Gram-, Antimicrobial

945	DRAMP03729	Opiscorpine-1 (Arthropods, animals)	Antibacterial, Antifungal, Anti-Gram-, Antimicrobial
946	DRAMP03734	Parabutoporin (PP; Non-disulfide-bridged peptide 3.2, NDBP-3.2; Arthropods, animals)	Antibacterial, Antifungal, Anti-Gram+, Anti-Gram-, Antimicrobial
947	DRAMP03735	Opistoporin-1 (OP1; Non-disulfide-bridged peptide 3.5; Opistoporin-3, OP3; Arthropods, animals)	Antibacterial, Antifungal, Anti-Gram+, Anti-Gram-, Antimicrobial
948	DRAMP03738	Scorpine (defensins; Arthropods, animals)	Antibacterial, Anti-Gram+, Anti-Gram-, Antimicrobial
949	DRAMP02828	BMAP-34 (BMAP 34, bovine cathelicidin, cattle, ruminant, mammals, animals)	Antimicrobial, Antibacterial, Antifungal, Anti-Gram+, Anti-Gram-,
950	DRAMP02926	Tachyplesin I (Tac; TP1; Horseshoe Crab, arachnids, Chelicerata, arthropods, invertebrates, animals)	Antibacterial, Antiviral, Anti-HIV, Anticancer, Antimicrobial
951	DRAMP03746	Peptide BmKn2 (Biologically active peptide 4; NDBP-5.1; Arthropods, animals)	Antibacterial, Anti-Gram+, Anti-Gram-, Antimicrobial
952	DRAMP03748	Bradykinin-potentiating peptide BmK3 (Bpp BmK3; NDBP-3.3; Arthropods, animals)	Antibacterial, Antifungal, Anti-Gram+, Anti-Gram-, Antimicrobial
953	DRAMP03750	Venom antimicrobial peptide-6 (Meucin-13; NDBP-5; Arthropods, animals)	Antibacterial, Antifungal, Anti-Gram+, Anti-Gram-, Antimicrobial
954	DRAMP03751	Venom antimicrobial peptide-9 (Meucin-18; NDBP-5; Arthropods, animals)	Antibacterial, Antifungal, Anti-Gram+, Anti-Gram-, Antimicrobial
955	DRAMP03752	Peptide BmKb1 (Non-disulfide-bridged peptide 4.2, NDBP-4.2; Arthropods, animals)	Antibacterial, Anti-Gram+, Anti-Gram-, Antimicrobial
956	DRAMP03753	Amphipathic peptide CT1 (StCT1; Non-disulfide-bridged peptide 5, NDBP-5; Arthropods, animals)	Antibacterial, Anti-Gram+, Anti-Gram-, Antimicrobial
957	DRAMP03754	Amphipathic peptide CT2 (StCT2; Non-disulfide-bridged peptide 5, NDBP-5; Arthropods, animals)	Antibacterial, Anti-Gram+, Anti-Gram-, Antimicrobial
958	DRAMP03774	UyCT1 (Arthropods, animals)	Antibacterial, Anti-Gram+, Anti-Gram-, Antimicrobial
959	DRAMP03775	UyCT2 (Arthropods, animals)	Antibacterial, Anti-Gram-, Antimicrobial
960	DRAMP03776	UyCT3 (Arthropods, animals)	Antibacterial, Anti-Gram+, Anti-Gram-, Antimicrobial
961	DRAMP03777	UyCT5 (Arthropods, animals)	Antibacterial, Anti-Gram+, Anti-Gram-, Antimicrobial
962	DRAMP03797	CAP7 (C-terminal fragment of CAP18; lagomorphs, mammals, animals)	Antibacterial, Antimicrobial
963	DRAMP03814	D16W (GGN4 analogue peptide with single substitution)	Antibacterial, Anti-Gram+, Anti-Gram-, Antimicrobial
964	DRAMP03815	D16W-N23 (single amino acid substitution)	Antibacterial, Anti-Gram+, Anti-Gram-, Antimicrobial
965	DRAMP03816	D16F-N23 (single amino acid substitution)	Antibacterial, Anti-Gram+, Anti-Gram-, Antimicrobial
966	DRAMP03823	Dermaseptin derivative K4-S4-(1-13)	Antibacterial, Anti-Gram+, Anti-Gram-, Antimicrobial
967	DRAMP03824	CNBr-cleaved lactoferricin Subfragment 1	Antibacterial, Anti-Gram+, Anti-Gram-, Antimicrobial
968	DRAMP03825	CNBr-cleaved lactoferricin Subfragment 2	Antibacterial, Anti-Gram+, Anti-Gram-, Antimicrobial
969	DRAMP03826	Ovispirin-1 (OV-1; N-terminal 18 amino acids of SMAP-29)	Antibacterial, Cytotoxicity, Anti-Gram+, Anti-Gram-, Antimicrobial
970	DRAMP03827	Novispirin G-10 (mutation of Ovispirin-1)	Antibacterial, Cytotoxicity, Anti-Gram+, Anti-Gram-, Antimicrobial
971	DRAMP03828	Novispirin T-7 (mutation of Ovispirin-1)	Antibacterial, Cytotoxicity, Anti-Gram+, Anti-Gram-, Antimicrobial
972	DRAMP03829	GLK-19	Antibacterial, Anticancer, Anti-Gram-, Antimicrobial
973	DRAMP03830	Palustrin-2ISb + 3aa	Antibacterial, Anti-Gram+, Anti-Gram-, Antimicrobial
974	DRAMP03831	Palustrin-2ISb-des-C7	Antibacterial, Antifungal, Anti-Gram+, Anti-Gram-, Antimicrobial
975	DRAMP03832	Palustrin-2ISb-des-C7-4D	Antibacterial, Antifungal, Anti-Gram+, Anti-Gram-, Antimicrobial
976	DRAMP03833	Palustrin-2ISb-des-C7-12N	Antibacterial, Antifungal, Anti-Gram+, Anti-Gram-, Antimicrobial
977	DRAMP03834	Palustrin-2ISb-des-C7-23,29S	Antibacterial, Antifungal, Anti-Gram+, Anti-Gram-, Antimicrobial
978	DRAMP03835	Palustrin-2ISb-des-C7-des-N9	Antifungal, Antimicrobial
979	DRAMP03852	G1 (Bac2A variant through single amino acid substitution)	Antibacterial, Antifungal, Anti-Gram+, Anti-Gram-, Antimicrobial
980	DRAMP03853	G2 (Bac2A variant through single amino acid substitution)	Antibacterial, Antifungal, Anti-Gram+, Anti-Gram-, Antimicrobial
981	DRAMP03854	R2 (Bac2A variant through single amino acid substitution)	Antibacterial, Antifungal, Anti-Gram+, Anti-Gram-, Antimicrobial
982	DRAMP03855	R3 (Bac2A variant through single amino acid substitution)	Antibacterial, Antifungal, Anti-Gram+, Anti-Gram-, Antimicrobial

983	DRAMP03856	W3 (Bac2A variant through single amino acid substitution)	Antibacterial, Antifungal, Anti-Gram+, Anti-Gram-, Antimicrobial
984	DRAMP03857	R5 (Bac2A variant through single amino acid substitution)	Antibacterial, Antifungal, Anti-Gram+, Anti-Gram-, Antimicrobial
985	DRAMP03858	K7 (Bac2A variant through single amino acid substitution)	Antibacterial, Antifungal, Anti-Gram+, Anti-Gram-, Antimicrobial
986	DRAMP03859	W10 (Bac2A variant through single amino acid substitution)	Antibacterial, Antifungal, Anti-Gram+, Anti-Gram-, Antimicrobial
987	DRAMP03860	R11 (Bac2A variant through single amino acid substitution)	Antibacterial, Antifungal, Anti-Gram+, Anti-Gram-, Antimicrobial
988	DRAMP03861	G12 (Bac2A variant through single amino acid substitution)	Antibacterial, Antifungal, Anti-Gram+, Anti-Gram-, Antimicrobial
989	DRAMP03862	Sub2 (Bac2A variant through two amino acids substitution)	Antibacterial, Antifungal, Anti-Gram+, Anti-Gram-, Antimicrobial
990	DRAMP03863	Sub3 (Bac2A variant through three amino acids substitution)	Antibacterial, Antifungal, Anti-Gram+, Anti-Gram-, Antimicrobial
991	DRAMP03864	Sub5 (Bac2A variant through five amino acids substitution)	Antibacterial, Antifungal, Anti-Gram+, Anti-Gram-, Antimicrobial
992	DRAMP03865	Sub6 (Bac2A variant through six amino acids substitution)	Antibacterial, Antifungal, Anti-Gram+, Anti-Gram-, Antimicrobial
993	DRAMP03866	Bac8a (Bac2A variant)	Antibacterial, Antifungal, Anti-Gram+, Anti-Gram-, Antimicrobial
994	DRAMP03867	Bac8b (Bac2A variant)	Antibacterial, Antifungal, Anti-Gram+, Anti-Gram-, Antimicrobial
995	DRAMP03868	Bac8c (Bac2A variant)	Antibacterial, Antifungal, Anti-Gram+, Anti-Gram-, Antimicrobial
996	DRAMP03869	Bac8d (Bac2A variant)	Antibacterial, Antifungal, Anti-Gram+, Anti-Gram-, Antimicrobial
997	DRAMP03870	Bac2A (a linear variant of bovine dodecapeptide)	Antibacterial, Antifungal, Anti-Gram+, Anti-Gram-, Antimicrobial
998	DRAMP03871	cLf 20-29 (fragment of caprine lalctoferricin, residues 20-29)	Antibacterial, Anti-Gram+, Anti-Gram-, Antimicrobial
999	DRAMP03875	bLf 20-29 (fragment of bovine lactoferricin, residues 20-29)	Antibacterial, Anti-Gram+, Anti-Gram-, Antimicrobial
1000	DRAMP03876	LFB-RW (derivative of bovine lactoferrin with residues substitution)	Antibacterial, Anti-Gram+, Anti-Gram-, Antimicrobial
1001	DRAMP03877	LFB-KW (derivative of bovine lactoferrin with residues substitution)	Antibacterial, Anti-Gram+, Anti-Gram-, Antimicrobial
1002	DRAMP03878	LFB-Rwa (derivative of bovine lactoferrin with residues substitution)	Antibacterial, Anti-Gram+, Anti-Gram-, Antimicrobial
1003	DRAMP03879	LFB-RF (derivative of bovine lactoferrin with residues substitution)	Antibacterial, Anti-Gram+, Anti-Gram-, Antimicrobial
1004	DRAMP03880	LFB-RI (derivative of bovine lactoferrin with residues substitution)	Antibacterial, Anti-Gram+, Anti-Gram-, Antimicrobial
1005	DRAMP03881	LFB-6RW (derivative of bovine lactoferrin with residues substitution)	Antibacterial, Anti-Gram+, Anti-Gram-, Antimicrobial
1006	DRAMP03882	LFC (fragment of mature caprine lactoferrin, residues 17 to 31)	Antibacterial, Anti-Gram+, Anti-Gram-, Antimicrobial
1007	DRAMP03883	LFH W8 (tryptophan-modified human lactoferricin derivative)	Antibacterial, Anti-Gram+, Anti-Gram-, Antimicrobial
1008	DRAMP03884	LFC W8 (tryptophan-modified caprine lactoferricin derivative)	Antibacterial, Anti-Gram+, Anti-Gram-, Antimicrobial
1009	DRAMP03885	LFP W8 (tryptophan-modified porcine lactoferricin derivative)	Antibacterial, Anti-Gram+, Anti-Gram-, Antimicrobial
1010	DRAMP03886	LFB (fragment of bovine lalctoferricin, residues 17 to 31)	Antibacterial, Anti-Gram+, Anti-Gram-, Antimicrobial
1011	DRAMP03887	LFB A1 (derivative of LFB, residue substitution with alanine at position 1)	Antibacterial, Anti-Gram+, Anti-Gram-, Antimicrobial
1012	DRAMP03888	LFB A2 (derivative of LFB, residue substitution with alanine at position 2)	Antibacterial, Anti-Gram+, Anti-Gram-, Antimicrobial
1013	DRAMP03889	LFB A3 (derivative of LFB, residue substitution with alanine at position 3)	Antibacterial, Anti-Gram+, Anti-Gram-, Antimicrobial
1014	DRAMP03890	LFB A4 (derivative of LFB, residue substitution with alanine at position 4)	Antibacterial, Anti-Gram+, Anti-Gram-, Antimicrobial
1015	DRAMP03891	LFB A5 (derivative of LFB, residue substitution with alanine at position 5)	Antibacterial, Anti-Gram+, Anti-Gram-, Antimicrobial
1016	DRAMP03892	LFB A7 (derivative of LFB, residue substitution with alanine at position 7)	Antibacterial, Anti-Gram+, Anti-Gram-, Antimicrobial
1017	DRAMP03893	LFB A9 (derivative of LFB, residue substitution with alanine at position 9)	Antibacterial, Anti-Gram+, Anti-Gram-, Antimicrobial

1018	DRAMP03894	LFB A10 (derivative of LFB, residue substitution with alanine at position 10)	Antibacterial, Anti-Gram+, Anti-Gram-, Antimicrobial
1019	DRAMP03895	LFB A11 (derivative of LFB, residue substitution with alanine at position 11)	Antibacterial, Anti-Gram+, Anti-Gram-, Antimicrobial
1020	DRAMP03896	LFB A12 (derivative of LFB, residue substitution with alanine at position 12)	Antibacterial, Anti-Gram+, Anti-Gram-, Antimicrobial
1021	DRAMP03897	LFB A13 (derivative of LFB, residue substitution with alanine at position 13)	Antibacterial, Anti-Gram+, Anti-Gram-, Antimicrobial
1022	DRAMP03898	LFB A14 (derivative of LFB, residue substitution with alanine at position 14)	Antibacterial, Anti-Gram+, Anti-Gram-, Antimicrobial
1023	DRAMP03899	LFM A1 W8 (LFM W8 derivative with residues substitution)	Antibacterial, Anti-Gram-, Antimicrobial
1024	DRAMP03900	LFM A1,9 W8 (LFM W8 derivative with residues substitution)	Antibacterial, Anti-Gram-, Antimicrobial
1025	DRAMP03901	LFM R1 W8 (LFM W8 derivative with residues substitution)	Antibacterial, Anti-Gram+, Anti-Gram-, Antimicrobial
1026	DRAMP03902	LFM R9 W8 (LFM W8 derivative with residues substitution)	Antibacterial, Anti-Gram-, Antimicrobial
1027	DRAMP03903	LFM A1 R9 W8 (LFM W8 derivative with residues substitution)	Antibacterial, Anti-Gram+, Anti-Gram-, Antimicrobial
1028	DRAMP03904	LFM A9 R1 W8 (LFM W8 derivative with residues substitution)	Antibacterial, Anti-Gram+, Anti-Gram-, Antimicrobial
1029	DRAMP03905	LFM R1,9 W8 (LFM W8 derivative with residues substitution)	Antibacterial, Anti-Gram+, Anti-Gram-, Antimicrobial
1030	DRAMP03906	LFM A1 W8 Y13 (LFM W8 derivative with residues substitution)	Antibacterial, Anti-Gram-, Antimicrobial
1031	DRAMP03907	LFM A1,9 W8 Y13 (LFM W8 derivative with residues substitution)	Antibacterial, Anti-Gram-, Antimicrobial
1032	DRAMP03908	LFM R1 W8 Y13 (LFM W8 derivative with residues substitution)	Antibacterial, Anti-Gram+, Anti-Gram-, Antimicrobial
1033	DRAMP03909	LFM R9 W8 Y13 (LFM W8 derivative with residues substitution)	Antibacterial, Anti-Gram-, Antimicrobial
1034	DRAMP03910	LFM A1 R9 W8 Y13 (LFM W8 derivative with residues substitution)	Antibacterial, Anti-Gram+, Anti-Gram-, Antimicrobial
1035	DRAMP03911	LFM A9 R1 W8 Y13 (LFM W8 derivative with residues substitution)	Antibacterial, Anti-Gram+, Anti-Gram-, Antimicrobial
1036	DRAMP03912	LFM R1,9 W8 Y13 (LFM W8 derivative with residues substitution)	Antibacterial, Anti-Gram+, Anti-Gram-, Antimicrobial
1037	DRAMP03920	Cecropin A (1-8)-melittin (1-13)hybrid peptide	Antibacterial, Anti-Gram+, Anti-Gram-, Antimicrobial
1038	DRAMP03921	Cecropin A (1-8)-melittin (1-18)hybrid peptide	Antibacterial, Anti-Gram+, Anti-Gram-, Antimicrobial
1039	DRAMP03922	Cecropin A (1-8)-melittin (1-12)hybrid peptide	Antibacterial, Anti-Gram+, Anti-Gram-, Antimicrobial
1040	DRAMP03923	Cecropin A (1-8)-melittin (1-10)hybrid peptide	Antibacterial, Anti-Gram+, Anti-Gram-, Antimicrobial
1041	DRAMP03924	Cecropin A (1-7)-melittin (1-8)hybrid peptide	Antibacterial, Anti-Gram+, Anti-Gram-, Antimicrobial
1042	DRAMP03925	Cecropin A (1-7)-melittin (3-10)hybrid peptide	Antibacterial, Anti-Gram+, Anti-Gram-, Antimicrobial
1043	DRAMP03927	Cecropin A (1-7)-melittin (2-9)hybrid peptide	Antibacterial, Anti-Gram+, Anti-Gram-, Antimicrobial
1044	DRAMP03928	Cecropin A (1-7)-melittin (4-11)hybrid peptide (CAM)	Antibacterial, Anti-Gram+, Anti-Gram-, Antimicrobial
1045	DRAMP03929	Cecropin A (1-7)-melittin (5-12)hybrid peptide	Antibacterial, Anti-Gram+, Anti-Gram-, Antimicrobial
1046	DRAMP03930	Cecropin A (1-7)-melittin (6-13)hybrid peptide	Antibacterial, Antifungal, Anti-Gram+, Anti-Gram-, Antimicrobial
1047	DRAMP03931	hPAB-beta (a hBD-2 variant; beta-defensins)	Antibacterial, Antimicrobial
1048	DRAMP03933	I14M (truncated isoform of thanatin, residue 8-21)	Antibacterial, Antifungal, Anti-Gram+, Anti-Gram-, Antimicrobial
1049	DRAMP03934	Y12M (truncated isoform of thanatin, residue 10-21)	Antibacterial, Antifungal, Anti-Gram+, Antimicrobial
1050	DRAMP03935	V16M (truncated isoform of thanatin, residue 6-21)	Antibacterial, Antifungal, Anti-Gram+, Anti-Gram-, Antimicrobial
1051	DRAMP03936	K18M (truncated isoform of thanatin, residue 4-21)	Antibacterial, Antifungal, Anti-Gram+, Anti-Gram-, Antimicrobial
1052	DRAMP03937	G18C (truncated isoform of thanatin, residue 1-18)	Antibacterial, Antifungal, Anti-Gram+, Antimicrobial
1053	DRAMP03938	G19Q (truncated isoform of thanatin, residue 1-19)	Antibacterial, Antifungal, Anti-Gram+, Antimicrobial
1054	DRAMP03939	G20R (truncated isoform of thanatin, residue 1-20)	Antibacterial, Antifungal, Anti-Gram+, Antimicrobial
1055	DRAMP03945	Del 1-4 (Ranalexin analog)	Antibacterial, Anti-Gram+, Anti-Gram-, Antimicrobial
1056	DRAMP03947	Del 1-2 (Ranalexin analog)	Antibacterial, Anti-Gram+, Anti-Gram-, Antimicrobial
1057	DRAMP03948	Del 1 (Ranalexin analog)	Antibacterial, Anti-Gram+, Anti-Gram-, Antimicrobial
1058	DRAMP03949	Del 20 (Ranalexin analog)	Antibacterial, Anti-Gram+, Anti-Gram-, Antimicrobial
1059	DRAMP03954	Rat CGA7	Antifungal, Antimicrobial
1060	DRAMP03955	Human recombinant Ser-Thr-Ala-CGA1-78 peptide (hrVS-1)	Antibacterial, Antifungal, Anti-Gram+, Antimicrobial
1061	DRAMP03957	CGA47-60 (hrVS-1-derived peptide)	Antifungal, Antimicrobial
1062	DRAMP03958	CGA41-60 (hrVS-1-derived peptide)	Antifungal, Antimicrobial

1063	DRAMP03959	CGA41-70 (hrVS-1-derived peptide)	Antifungal, Antimicrobial
1064	DRAMP03960	CGA47-70 (hrVS-1-derived peptide)	Antifungal, Antimicrobial
1065	DRAMP03967	P18 (Cecropin A(1-8)-Magainin 2(1-12) hybrid peptide analogue)	Antibacterial, Antitumour, Anti-Gram+, Anti-Gram-, Antimicrobial
1066	DRAMP03968	[L9]-P18 (analog of P18)	Antibacterial, Antitumour, Anti-Gram+, Anti-Gram-, Antimicrobial
1067	DRAMP03969	[S9]-P18 (analog of P18)	Antibacterial, Antitumour, Anti-Gram+, Anti-Gram-, Antimicrobial
1068	DRAMP03970	N-1 (analog of P18)	Antibacterial, Antitumour, Anti-Gram+, Anti-Gram-, Antimicrobial
1069	DRAMP03971	N-2 (analog of P18)	Antibacterial, Antitumour, Anti-Gram+, Anti-Gram-, Antimicrobial
1070	DRAMP03972	N-3 (analog of P18)	Antibacterial, Antitumour, Anti-Gram+, Anti-Gram-, Antimicrobial
1071	DRAMP03973	N-4 (analog of P18)	Antibacterial, Antitumour, Anti-Gram+, Anti-Gram-, Antimicrobial
1072	DRAMP03974	N-5 (analog of P18)	Antibacterial, Antitumour, Anti-Gram+, Anti-Gram-, Antimicrobial
1073	DRAMP03975	N-3L (analog of P18)	Antibacterial, Antitumour, Anti-Gram+, Anti-Gram-, Antimicrobial
1074	DRAMP03976	N-4L (analog of P18)	Antibacterial, Antitumour, Anti-Gram+, Anti-Gram-, Antimicrobial
1075	DRAMP03977	N-5L (analog of P18)	Antibacterial, Antitumour, Anti-Gram+, Anti-Gram-, Antimicrobial
1076	DRAMP03978	C-1 (analog of P18)	Antibacterial, Antitumour, Anti-Gram+, Anti-Gram-, Antimicrobial
1077	DRAMP03979	C-2 (analog of P18)	Antibacterial, Antitumour, Anti-Gram+, Anti-Gram-, Antimicrobial
1078	DRAMP03980	C-3 (analog of P18)	Antibacterial, Antitumour, Anti-Gram+, Anti-Gram-, Antimicrobial
1079	DRAMP03981	C-4 (analog of P18)	Antibacterial, Antitumour, Anti-Gram+, Anti-Gram-, Antimicrobial
1080	DRAMP03982	C-5 (analog of P18)	Antibacterial, Antitumour, Anti-Gram+, Anti-Gram-, Antimicrobial
1081	DRAMP03983	C-6 (analog of P18)	Antibacterial, Antitumour, Anti-Gram+, Anti-Gram-, Antimicrobial
1082	DRAMP03984	C-7 (analog of P18)	Antibacterial, Antitumour, Anti-Gram+, Anti-Gram-, Antimicrobial
1083	DRAMP03985	C-8 (analog of P18)	Antibacterial, Antitumour, Anti-Gram+, Anti-Gram-, Antimicrobial
1084	DRAMP03986	C-9 (analog of P18)	Antibacterial, Antitumour, Anti-Gram+, Anti-Gram-, Antimicrobial
1085	DRAMP03987	C-10 (analog of P18)	Antibacterial, Antitumour, Anti-Gram+, Anti-Gram-, Antimicrobial
1086	DRAMP03988	L3K3W4 (LlKmWn model peptide)	Antibacterial, Anti-Gram+, Antimicrobial
1087	DRAMP03989	L4K2W4 (LlKmWn model peptide)	Antibacterial, Anti-Gram+, Antimicrobial
1088	DRAMP03990	L4K3W4 (LlKmWn model peptide)	Antibacterial, Anti-Gram+, Anti-Gram-, Antimicrobial
1089	DRAMP03991	L4K3W5 (LlKmWn model peptide)	Antibacterial, Anti-Gram+, Antimicrobial
1090	DRAMP03992	L5K3W5 (LlKmWn model peptide)	Antibacterial, Anti-Gram+, Anti-Gram-, Antimicrobial
1091	DRAMP03993	L5K5W6 (LlKmWn model peptide)	Antibacterial, Anti-Gram+, Anti-Gram-, Antimicrobial
1092	DRAMP03994	L6K4W6 (LlKmWn model peptide)	Antibacterial, Anti-Gram+, Anti-Gram-, Antimicrobial
1093	DRAMP03995	L7K3W6 (LlKmWn model peptide)	Antibacterial, Anti-Gram+, Anti-Gram-, Antimicrobial
1094	DRAMP03996	L7K5W7 (LlKmWn model peptide)	Antibacterial, Anti-Gram+, Antimicrobial
1095	DRAMP03997	L8K4W7 (LlKmWn model peptide)	Antibacterial, Anti-Gram+, Antimicrobial
1096	DRAMP03999	[A6]-IsCT (Mutant: W6A; IsCT analog)	Antibacterial, Anti-Gram+, Anti-Gram-, Antimicrobial
1097	DRAMP04000	[L6]-IsCT (Mutant: W6L; IsCT analog)	Antibacterial, Anti-Gram+, Anti-Gram-, Antimicrobial
1098	DRAMP04001	[K7]-IsCT (Mutant: E7K; IsCT analog)	Antibacterial, Anti-Gram+, Anti-Gram-, Antimicrobial
1099	DRAMP04002	[L6, K11]-IsCT (IsCT analog through amino acids substitution)	Antibacterial, Anti-Gram+, Anti-Gram-, Antimicrobial
1100	DRAMP04003	[K7, P8, K11]-IsCT (IsCT analog through amino acids substitution)	Antibacterial, Anti-Gram+, Anti-Gram-, Antimicrobial
1101	DRAMP04004	Gramicidin analogue ([Scr2]-GS)	Antibacterial, Anti-Gram+, Anti-Gram-, Antimicrobial
1102	DRAMP04005	Gramicidin analogue ([Ser2,2']-GS)	Antibacterial, Anti-Gram+, Anti-Gram-, Antimicrobial

1103	DRAMP04011	Plasticin PD36 KF (analog of PD36)	Antibacterial, Anti-Gram+, Anti-Gram-, Antimicrobial
1104	DRAMP04012	Plasticin PD36 K (analog of PD36)	Antibacterial, Anti-Gram+, Anti-Gram-, Antimicrobial
1105	DRAMP04013	Plasticin ANC KF (analog of natural peptide ANC)	Antibacterial, Anti-Gram+, Anti-Gram-, Antimicrobial
1106	DRAMP04014	LL-37A9 (LL-37 variants)	Antibacterial, Anti-Gram-, Antimicrobial
1107	DRAMP04015	LL-37V9 (LL-37 variants)	Antibacterial, Anti-Gram-, Antimicrobial
1108	DRAMP04016	LL-23A9 (LL-23 variants)	Antibacterial, Anti-Gram+, Antimicrobial
1109	DRAMP04017	LL-23V9 (LL-23 variants)	Antibacterial, Anti-Gram+, Anti-Gram-, Antimicrobial
1110	DRAMP04019	Bac014 (Scrambled Variants of Bac2A)	Antibacterial, Antifungal, Anti-Gram+, Anti-Gram-, Antimicrobial
1111	DRAMP04020	Bac020 (Scrambled Variants of Bac2A)	Antibacterial, Antifungal, Anti-Gram+, Anti-Gram-, Antimicrobial
1112	DRAMP04021	Bac034 (Scrambled Variants of Bac2A)	Antibacterial, Antifungal, Anti-Gram+, Anti-Gram-, Antimicrobial
1113	DRAMP04022	F3 (single amino acid substitution of Bac034, which is a scrambled Variant of Bac2A)	Antibacterial, Antifungal, Anti-Gram+, Anti-Gram-, Antimicrobial
1114	DRAMP04023	W3 (single amino acid substitution of Bac034, which is a scrambled Variant of Bac2A)	Antibacterial, Antifungal, Anti-Gram+, Anti-Gram-, Antimicrobial
1115	DRAMP04024	W4 (single amino acid substitution of Bac034, which is a scrambled Variant of Bac2A)	Antibacterial, Antifungal, Anti-Gram+, Anti-Gram-, Antimicrobial
1116	DRAMP04025	R10 (single amino acid substitution of Bac034, which is a scrambled Variant of Bac2A)	Antibacterial, Antifungal, Anti-Gram+, Anti-Gram-, Antimicrobial
1117	DRAMP04026	K12 (single amino acid substitution of Bac034, which is a scrambled Variant of Bac2A)	Antibacterial, Antifungal, Anti-Gram+, Anti-Gram-, Antimicrobial
1118	DRAMP04027	opt1 (multiple amino acid substitution of Bac034, which is a scrambled Variant of Bac2A)	Antibacterial, Antifungal, Anti-Gram+, Anti-Gram-, Antimicrobial
1119	DRAMP04028	opt2 (multiple amino acid substitution of Bac034, which is a scrambled Variant of Bac2A)	Antibacterial, Antifungal, Anti-Gram+, Anti-Gram-, Antimicrobial
1120	DRAMP04029	opt3 (multiple amino acid substitution of Bac034, which is a scrambled Variant of Bac2A)	Antibacterial, Antifungal, Anti-Gram+, Anti-Gram-, Antimicrobial
1121	DRAMP04030	opt4 (multiple amino acid substitution of Bac034, which is a scrambled Variant of Bac2A)	Antibacterial, Antifungal, Anti-Gram+, Anti-Gram-, Antimicrobial
1122	DRAMP04031	opt5 (multiple amino acid substitution of Bac034, which is a scrambled Variant of Bac2A)	Antibacterial, Antifungal, Anti-Gram+, Anti-Gram-, Antimicrobial
1123	DRAMP04032	Modified defensin	Antibacterial, Anti-Gram+, Anti-Gram-, Antimicrobial
1124	DRAMP04033	Modified defensin	Antibacterial, Anti-Gram+, Anti-Gram-, Antimicrobial
1125	DRAMP04034	Modified defensin	Antibacterial, Anti-Gram+, Anti-Gram-, Antimicrobial
1126	DRAMP04035	Modified defensin	Antibacterial, Anti-Gram+, Anti-Gram-, Antimicrobial
1127	DRAMP04036	Modified defensin	Antibacterial, Anti-Gram+, Anti-Gram-, Antimicrobial
1128	DRAMP04048	BacR (cyclic derivative of bactenecin)	Antibacterial, Anti-Gram+, Anti-Gram-, Antimicrobial
1129	DRAMP04049	BacP3R (cyclic derivative of bactenecin)	Antibacterial, Anti-Gram+, Anti-Gram-, Antimicrobial
1130	DRAMP04050	BacP3R-V (cyclic derivative of bactenecin)	Antibacterial, Anti-Gram+, Anti-Gram-, Antimicrobial
1131	DRAMP04051	Bac2I-NH2 (cyclic derivative of bactenecin)	Antibacterial, Anti-Gram+, Anti-Gram-, Antimicrobial
1132	DRAMP04052	BacP2R-NH2 (cyclic derivative of bactenecin)	Antibacterial, Anti-Gram+, Anti-Gram-, Antimicrobial
1133	DRAMP04053	BacP1 (cyclic derivative of bactenecin)	Antibacterial, Anti-Gram+, Anti-Gram-, Antimicrobial
1134	DRAMP04054	BacW (cyclic derivative of bactenecin)	Antibacterial, Anti-Gram+, Anti-Gram-, Antimicrobial
1135	DRAMP04055	BacW2R (cyclic derivative of bactenecin)	Antibacterial, Anti-Gram+, Anti-Gram-, Antimicrobial
1136	DRAMP04056	Lin Bac2S-NH2 (linear derivative of bactenecin)	Antibacterial, Anti-Gram+, Anti-Gram-, Antimicrobial
1137	DRAMP04057	Lin BacS-NH2 (linear derivative of bactenecin)	Antibacterial, Anti-Gram+, Anti-Gram-, Antimicrobial
1138	DRAMP18364	Thuricin 4A-4 (bacteriocin)	Antibacterial, Antimicrobial
1139	DRAMP18365	Thusin (ThsA1, ThsA2; a two-chain lantibiotic, type 2, class 1 bacteriocins)	Antibacterial, Antimicrobial
1140	DRAMP18363	Sviceucin (bacteriocin)	Antibacterial, Anti-Gram+, Antimicrobial
1141	DRAMP18361	YD1 (bacteriocin)	Antibacterial, Antimicrobial
1142	DRAMP18362	Formicin (bacteriocin)	Antibacterial, Anti-Gram+, Antimicrobial
1143	DRAMP18360	Paenicidin B (bacteriocin)	Antibacterial, Antimicrobial
1144	DRAMP18359	Sh-lantibiotic-alpha (bacteriocin)	Antibacterial, Anti-Gram+, Antimicrobial
1145	DRAMP18358	Sh-lantibiotic-beta (bacteriocin)	Antibacterial, Anti-Gram+, Antimicrobial
1146	DRAMP04064	Cyclic cationic V1 peptide	Antibacterial, Anti-Gram-, Antimicrobial
1147	DRAMP04065	Cyclic cationic V2 peptide	Antibacterial, Anti-Gram-, Antimicrobial
1148	DRAMP04066	Cyclic cationic V3 peptide	Antibacterial, Anti-Gram-, Antimicrobial

1149	DRAMP04067	Cyclic cationic V4 peptide	Antibacterial, Anti-Gram-, Antimicrobial
1150	DRAMP04068	Cyclic cationic V5 peptide	Antibacterial, Anti-Gram-, Antimicrobial
1151	DRAMP04069	Cyclic cationic V6 peptide	Antibacterial, Anti-Gram-, Antimicrobial
1152	DRAMP04070	Cyclic cationic V7 peptide	Antibacterial, Anti-Gram-, Antimicrobial
1153	DRAMP04075	Antimicrobial peptide HP (2-20)	Antibacterial, Antifungal, Anti-Gram+, Anti-Gram-, Antimicrobial
1154	DRAMP04076	Anal 1 (antimicrobial peptide HP (2-20)analogue)	Antibacterial, Antifungal, Anti-Gram+, Anti-Gram-, Antimicrobial
1155	DRAMP04077	Anal 2 (antimicrobial peptide HP (2-20)analogue)	Antibacterial, Antifungal, Anti-Gram+, Anti-Gram-, Antimicrobial
1156	DRAMP04078	Anal 3 (antimicrobial peptide HP (2-20)analogue)	Antibacterial, Antifungal, Anti-Gram+, Anti-Gram-, Antimicrobial
1157	DRAMP04079	Anal 4 (antimicrobial peptide HP (2-20)analogue)	Antibacterial, Antifungal, Anti-Gram+, Anti-Gram-, Antimicrobial
1158	DRAMP04080	Anal 5 (antimicrobial peptide HP (2-20)analogue)	Antibacterial, Antifungal, Anti-Gram+, Anti-Gram-, Antimicrobial
1159	DRAMP04081	Anal 6 (antimicrobial peptide HP (2-20)analogue)	Antibacterial, Antifungal, Anti-Gram+, Anti-Gram-, Antimicrobial
1160	DRAMP04082	Anal 7 (antimicrobial peptide HP (2-20)analogue)	Antibacterial, Antifungal, Anti-Gram+, Anti-Gram-, Antimicrobial
1161	DRAMP04083	D-amino-acid pexiganan (MSI-214)	Antibacterial, Anti-Gram+, Anti-Gram-, Antimicrobial
1162	DRAMP04095	Cupiennin-1D (spiders, Arthropods, animals)	Antibacterial, Anti-Gram+, Anti-Gram-, Antimicrobial
1163	DRAMP04096	2IQ2	Antibacterial, Antifungal, Anti-Gram+, Anti-Gram-, Antimicrobial
1164	DRAMP04097	2IQ3	Antibacterial, Antifungal, Anti-Gram+, Anti-Gram-, Antimicrobial
1165	DRAMP04098	3IQ1	Antibacterial, Antifungal, Anti-Gram+, Anti-Gram-, Antimicrobial
1166	DRAMP04099	3IQ2	Antibacterial, Antifungal, Anti-Gram+, Anti-Gram-, Antimicrobial
1167	DRAMP04100	3IQ3	Antibacterial, Antifungal, Anti-Gram+, Anti-Gram-, Antimicrobial
1168	DRAMP04101	3IQ4	Antibacterial, Antifungal, Anti-Gram+, Anti-Gram-, Antimicrobial
1169	DRAMP04102	CP-P	Antibacterial, Anti-Gram-, Antimicrobial
1170	DRAMP04103	S16 (derivative of CP-P)	Antibacterial, Anti-Gram-, Antimicrobial
1171	DRAMP04104	F2 (derivative of CP-P)	Antibacterial, Anti-Gram-, Antimicrobial
1172	DRAMP04105	N3 (derivative of CP-P)	Antibacterial, Anti-Gram-, Antimicrobial
1173	DRAMP04106	K6 (derivative of CP-P)	Antibacterial, Anti-Gram-, Antimicrobial
1174	DRAMP04107	N7 (derivative of CP-P)	Antibacterial, Anti-Gram-, Antimicrobial
1175	DRAMP04108	A9 (derivative of CP-P)	Antibacterial, Anti-Gram-, Antimicrobial
1176	DRAMP04111	S9 (derivative of CP-P)	Antibacterial, Anti-Gram-, Antimicrobial
1177	DRAMP04112	L10 (derivative of CP-P)	Antibacterial, Anti-Gram-, Antimicrobial
1178	DRAMP04113	A10 (derivative of CP-P)	Antibacterial, Anti-Gram-, Antimicrobial
1179	DRAMP04114	D11 (derivative of CP-P)	Antibacterial, Anti-Gram-, Antimicrobial
1180	DRAMP04115	K11 (derivative of CP-P)	Antibacterial, Anti-Gram+, Anti-Gram-, Antimicrobial
1181	DRAMP04117	A13 (derivative of CP-P)	Antibacterial, Anti-Gram-, Antimicrobial
1182	DRAMP04119	K17 (derivative of CP-P)	Antibacterial, Anti-Gram-, Antimicrobial
1183	DRAMP04120	D18 (derivative of CP-P)	Antibacterial, Anti-Gram-, Antimicrobial
1184	DRAMP04121	N18 (derivative of CP-P)	Antibacterial, Anti-Gram-, Antimicrobial
1185	DRAMP04122	N20 (derivative of CP-P)	Antibacterial, Anti-Gram-, Antimicrobial
1186	DRAMP04123	D0-NH2	Antibacterial, Anti-Gram+, Anti-Gram-, Antimicrobial
1187	DRAMP04124	D1-NH2	Antibacterial, Anti-Gram+, Anti-Gram-, Antimicrobial
1188	DRAMP04125	D2-NH2	Antibacterial, Anti-Gram+, Anti-Gram-, Antimicrobial
1189	DRAMP04126	D3-NH2	Antibacterial, Anti-Gram+, Anti-Gram-, Antimicrobial
1190	DRAMP04127	D4-NH2	Antibacterial, Anti-Gram+, Anti-Gram-, Antimicrobial
1191	DRAMP04128	D5-NH2	Antibacterial, Anti-Gram+, Anti-Gram-, Antimicrobial
1192	DRAMP04129	D6-NH2	Antibacterial, Anti-Gram+, Anti-Gram-, Antimicrobial
1193	DRAMP04136	LRR-1	Antibacterial, Anti-Gram+, Anti-Gram-, Antimicrobial
1194	DRAMP04137	LRR-2	Antibacterial, Anti-Gram+, Anti-Gram-, Antimicrobial

1195	DRAMP04138	L1 (first 8 N-terminal residues of bovine LF)	Antibacterial, Anti-Gram-, Antimicrobial
1196	DRAMP04139	L2	Antibacterial, Anti-Gram-, Antimicrobial
1197	DRAMP04140	L3	Antibacterial, Anti-Gram-, Antimicrobial
1198	DRAMP04141	L4	Antibacterial, Anti-Gram-, Antimicrobial
1199	DRAMP04142	L5	Antibacterial, Anti-Gram-, Antimicrobial
1200	DRAMP04143	L6	Antibacterial, Anti-Gram-, Antimicrobial
1201	DRAMP04144	L7	Antibacterial, Anti-Gram-, Antimicrobial
1202	DRAMP04145	L8	Antibacterial, Anti-Gram-, Antimicrobial
1203	DRAMP04146	L9	Antibacterial, Anti-Gram-, Antimicrobial
1204	DRAMP04147	L10	Antibacterial, Antifungal, Anti-Gram-, Antimicrobial
1205	DRAMP04159	LR2 (homologue of Pc-CATH1)	Antibacterial, Antifungal, Anti-Gram+, Anti-Gram-, Antimicrobial
1206	DRAMP04160	LR3 (homologue of Pc-CATH1)	Antibacterial, Antifungal, Anti-Gram+, Anti-Gram-, Antimicrobial
1207	DRAMP04161	LR4 (homologue of Pc-CATH1)	Antibacterial, Antifungal, Anti-Gram+, Anti-Gram-, Antimicrobial
1208	DRAMP04162	LR5 (homologue of Pc-CATH1)	Antibacterial, Antifungal, Anti-Gram+, Anti-Gram-, Antimicrobial
1209	DRAMP04163	LR6 (homologue of Pc-CATH1)	Antibacterial, Antifungal, Anti-Gram+, Anti-Gram-, Antimicrobial
1210	DRAMP04164	LR7 (homologue of Pc-CATH1)	Antibacterial, Antifungal, Anti-Gram+, Anti-Gram-, Antimicrobial
1211	DRAMP04165	LR8 (homologue of Pc-CATH1)	Antibacterial, Antifungal, Anti-Gram+, Anti-Gram-, Antimicrobial
1212	DRAMP04166	LR9 (homologue of Pc-CATH1)	Antibacterial, Antifungal, Anti-Gram+, Anti-Gram-, Antimicrobial
1213	DRAMP04167	LR10 (homologue of Pc-CATH1)	Antifungal, Anti-Gram+, Anti-Gram-, Antimicrobial
1214	DRAMP04168	LR11 (homologue of Pc-CATH1)	Antifungal, Anti-Gram+, Anti-Gram-, Antimicrobial
1215	DRAMP04169	LR13 (homologue of Pc-CATH1)	Antifungal, Anti-Gram+, Anti-Gram-, Antimicrobial
1216	DRAMP04170	LR15 (homologue of Pc-CATH1)	Antifungal, Anti-Gram+, Anti-Gram-, Antimicrobial
1217	DRAMP04171	LR16 (homologue of Pc-CATH1)	Antifungal, Anti-Gram+, Anti-Gram-, Antimicrobial
1218	DRAMP04174	L2K3W2 (LlKmW2 model peptides)	Antibacterial, Anti-Gram+, Anti-Gram-, Antimicrobial
1219	DRAMP04175	L3K2W2 (LlKmW2 model peptides)	Antibacterial, Anti-Gram+, Anti-Gram-, Antimicrobial
1220	DRAMP04176	L2K5W2 (LlKmW2 model peptides)	Antibacterial, Anti-Gram+, Anti-Gram-, Antimicrobial
1221	DRAMP04177	L3K4W2 (LlKmW2 model peptides)	Antibacterial, Anti-Gram+, Anti-Gram-, Antimicrobial
1222	DRAMP04178	L4K3W2 (LlKmW2 model peptides)	Antibacterial, Anti-Gram+, Anti-Gram-, Antimicrobial
1223	DRAMP04179	L5K2W2 (LlKmW2 model peptides)	Antibacterial, Anti-Gram+, Anti-Gram-, Antimicrobial
1224	DRAMP04180	L3K6W2 (LlKmW2 model peptides)	Antibacterial, Anti-Gram+, Anti-Gram-, Antimicrobial
1225	DRAMP04181	L4K5W2 (LlKmW2 model peptides)	Antibacterial, Anti-Gram+, Anti-Gram-, Antimicrobial
1226	DRAMP04182	L5K4W2 (LlKmW2 model peptides)	Antibacterial, Anti-Gram+, Anti-Gram-, Antimicrobial
1227	DRAMP04183	L6K3W2 (LlKmW2 model peptides)	Antibacterial, Anti-Gram+, Anti-Gram-, Antimicrobial
1228	DRAMP04184	DFTamP1	Antibacterial, Anti-Gram+, Antimicrobial
1229	DRAMP04185	DFTamP1-p	Antibacterial, Anti-Gram+, Anti-Gram-, Antimicrobial
1230	DRAMP04186	L5K5W1 (L5K5Wn model peptide)	Antibacterial, Anti-Gram+, Anti-Gram-, Antimicrobial
1231	DRAMP04187	L5K5W2 (L5K5Wn model peptide)	Antibacterial, Anti-Gram+, Anti-Gram-, Antimicrobial
1232	DRAMP04188	L5K5W3 (L5K5Wn model peptide)	Antibacterial, Anti-Gram+, Anti-Gram-, Antimicrobial
1233	DRAMP04189	L5K5W4 (L5K5Wn model peptide)	Antibacterial, Anti-Gram+, Anti-Gram-, Antimicrobial
1234	DRAMP04190	L5K5W5 (L5K5Wn model peptide)	Antibacterial, Anti-Gram+, Anti-Gram-, Antimicrobial
1235	DRAMP18357	Warnerin (bacteriocin)	Antibacterial, Antimicrobial
1236	DRAMP04192	L5K5W7 (L5K5Wn model peptide)	Antibacterial, Anti-Gram+, Anti-Gram-, Antimicrobial
1237	DRAMP04193	L5K5W8 (L5K5Wn model peptide)	Antibacterial, Anti-Gram+, Anti-Gram-, Antimicrobial
1238	DRAMP04194	L5K5W9 (L5K5Wn model peptide)	Antibacterial, Anti-Gram+, Anti-Gram-, Antimicrobial
1239	DRAMP04195	L5K5W10 (L5K5Wn model peptide)	Antibacterial, Anti-Gram+, Anti-Gram-, Antimicrobial
1240	DRAMP04196	L5K5W11 (L5K5Wn model peptide)	Antibacterial, Anti-Gram+, Anti-Gram-, Antimicrobial
1241	DRAMP04233	D28 (Rational design peptide)	Antibacterial, Anti-Gram+, Antimicrobial
1242	DRAMP04234	D51 (Rational design peptide)	Antibacterial, Anti-Gram+, Antimicrobial
1243	DRAMP04235	D22 (Rational design peptide)	Antibacterial, Anti-Gram+, Antimicrobial
1244	DRAMP04237	Antibacterial peptide A4	Antibacterial, Anti-Gram-, Antimicrobial
1245	DRAMP04240	Synthetic 1	Antibacterial, Anti-Gram+, Anti-Gram-, Antimicrobial

1246	DRAMP04241	Synthetic 2	Antibacterial, Anti-Gram+, Anti-Gram-, Antimicrobial
1247	DRAMP04242	Synthetic 3	Antibacterial, Anti-Gram+, Anti-Gram-, Antimicrobial
1248	DRAMP04243	Synthetic 4	Antibacterial, Anti-Gram+, Anti-Gram-, Antimicrobial
1249	DRAMP04244	Synthetic 5	Antibacterial, Anti-Gram+, Anti-Gram-, Antimicrobial
1250	DRAMP04264	CP26	Antibacterial, Anti-Gram-, Antimicrobial
1251	DRAMP04265	CP29	Antibacterial, Anti-Gram-, Antimicrobial
1252	DRAMP04279	CP11CN	Antibacterial, Anti-Gram+, Antimicrobial
1253	DRAMP04359	PDD-A-1 (PDD-A analog)	Antibacterial, Anti-Gram+, Anti-Gram-, Antimicrobial
1254	DRAMP04360	PDD-A-2 (PDD-A analog)	Antibacterial, Anti-Gram+, Anti-Gram-, Antimicrobial
1255	DRAMP04361	PDD-A-3 (PDD-A analog)	Antibacterial, Anti-Gram+, Anti-Gram-, Antimicrobial
1256	DRAMP04362	PDD-A-4 (PDD-A analog)	Antibacterial, Anti-Gram+, Anti-Gram-, Antimicrobial
1257	DRAMP04363	PDD-A-5 (PDD-A analog)	Antibacterial, Anti-Gram+, Anti-Gram-, Antimicrobial
1258	DRAMP04364	PDD-A-6 (PDD-A analog)	Antibacterial, Anti-Gram+, Anti-Gram-, Antimicrobial
1259	DRAMP04365	PDD-A-7 (PDD-A analog)	Antibacterial, Anti-Gram+, Anti-Gram-, Antimicrobial
1260	DRAMP04367	PDD-A-9 (PDD-A analog)	Antibacterial, Anti-Gram+, Anti-Gram-, Antimicrobial
1261	DRAMP04368	PDD-A-10 (PDD-A analog)	Antibacterial, Anti-Gram+, Anti-Gram-, Antimicrobial
1262	DRAMP04369	PDD-A-11 (PDD-A analog)	Antibacterial, Anti-Gram+, Anti-Gram-, Antimicrobial
1263	DRAMP04370	PDD-A-12 (PDD-A analog)	Antibacterial, Anti-Gram+, Anti-Gram-, Antimicrobial
1264	DRAMP04371	PDD-B-1 (PDD-B analog)	Antibacterial, Anti-Gram+, Anti-Gram-, Antimicrobial
1265	DRAMP04372	PDD-B-2 (PDD-B analog)	Antibacterial, Anti-Gram+, Anti-Gram-, Antimicrobial
1266	DRAMP04373	PDD-B-3 (PDD-B analog)	Antibacterial, Anti-Gram+, Anti-Gram-, Antimicrobial
1267	DRAMP04374	PDD-B-4 (PDD-B analog)	Antibacterial, Anti-Gram+, Anti-Gram-, Antimicrobial
1268	DRAMP04376	MP-1 (MP analog)	Antibacterial, Anti-Gram+, Anti-Gram-, Antimicrobial
1269	DRAMP04377	MP-2 (MP analog)	Antibacterial, Anti-Gram+, Anti-Gram-, Antimicrobial
1270	DRAMP04378	MP-5 (MP analog)	Antibacterial, Anti-Gram+, Anti-Gram-, Antimicrobial
1271	DRAMP04379	MP-6 (MP analog)	Antibacterial, Anti-Gram+, Anti-Gram-, Antimicrobial
1272	DRAMP04380	PMM-1 (PMM analog)	Antibacterial, Anti-Gram+, Anti-Gram-, Antimicrobial
1273	DRAMP04381	PMM-2 (PMM analog)	Antibacterial, Anti-Gram+, Anti-Gram-, Antimicrobial
1274	DRAMP04382	PMM-3 (PMM analog)	Antibacterial, Anti-Gram+, Anti-Gram-, Antimicrobial
1275	DRAMP04383	PMM-4 (PMM analog)	Antibacterial, Anti-Gram+, Anti-Gram-, Antimicrobial
1276	DRAMP04385	PMM-6 (PMM analog)	Antibacterial, Anti-Gram+, Anti-Gram-, Antimicrobial
1277	DRAMP04386	PMM-7 (PMM analog)	Antibacterial, Anti-Gram+, Anti-Gram-, Antimicrobial
1278	DRAMP04387	PMM-8 (PMM analog)	Antibacterial, Anti-Gram+, Anti-Gram-, Antimicrobial
1279	DRAMP04389	PMM-10 (PMM analog)	Antibacterial, Anti-Gram+, Anti-Gram-, Antimicrobial
1280	DRAMP04390	PMM-11 (PMM analog)	Antibacterial, Anti-Gram+, Anti-Gram-, Antimicrobial
1281	DRAMP04391	PMM-12 (PMM analog)	Antibacterial, Anti-Gram+, Anti-Gram-, Antimicrobial
1282	DRAMP04392	PMM-13 (PMM analog)	Antibacterial, Anti-Gram+, Anti-Gram-, Antimicrobial
1283	DRAMP04393	PMM-14 (PMM analog)	Antibacterial, Anti-Gram+, Anti-Gram-, Antimicrobial
1284	DRAMP04528	CrusEs (cDNA encoding crustin-like peptide)	Antibacterial, Antifungal, Antiviral, Anti-Gram+, Antimicrobial
1285	DRAMP04542	Polybia-MP-I (insects, vertebrates, animals)	Antibacterial, Anti-Gram+, Anti-Gram-, Antimicrobial
1286	DRAMP04543	Polybia-MP-II (insects, vertebrates, animals)	Antibacterial, Cytotoxicity, Anti-Gram+, Anti-Gram-, Antimicrobial
1287	DRAMP04544	Polybia-MP-III (insects, vertebrates, animals)	Antibacterial, Cytotoxicity, Anti-Gram+, Anti-Gram-, Antimicrobial
1288	DRAMP04545	Limnonectin-1Fa (Frogs, amphibians, animals)	Antibacterial, Anti-Gram-, Antimicrobial
1289	DRAMP04546	Limnonectin-1Fb (Frogs, amphibians, animals)	Antibacterial, Anti-Gram-, Antimicrobial
1290	DRAMP04553	H. erythraea B2RP	Antimicrobial, Antibacterial, Antifungal,, Anti-Gram-,
1291	DRAMP04640	PGLa-AN2	Antibacterial, Anti-Gram+, Anti-Gram-, Antimicrobial
1292	DRAMP04665	Px-cec1	Antibacterial, Antifungal, Anti-Gram+, Anti-Gram-, Antimicrobial
1293	DRAMP04670	PBD1-42	Antibacterial, Anti-Gram+, Anti-Gram-, Antimicrobial
1294	DRAMP04671	Myticusin-1	Antibacterial, Antifungal, Anti-Gram+, Anti-Gram-, Antimicrobial
1295	DRAMP04676	Brevinin-2HS2A	Antibacterial, Antifungal, Anti-Gram+, Anti-Gram-, Antimicrobial
1296	DRAMP04677	Brevinin-2HS2B	Antibacterial, Antifungal, Anti-Gram+, Anti-Gram-, Antimicrobial
1297	DRAMP04679	Senegalin	Antibacterial, Antifungal, Anti-Gram+, Antimicrobial

1298	DRAMP00001	Variacin (Bacteriocin)	Antibacterial, Anti-Gram+, Antimicrobial
1299	DRAMP00002	Entianin (Bacteriocin)	Antibacterial, Antimicrobial
1300	DRAMP00003	Bovicin HJ50 (Bacteriocin; Predicted)	Antibacterial, Anti-Gram+, Antimicrobial
1301	DRAMP00004	Lantibiotic (Bacteriocin)	Antibacterial, Antimicrobial
1302	DRAMP00006	Butyrivibriocin OR79 (Bacteriocin)	Antibacterial, Anti-Gram+, Antimicrobial
1303	DRAMP18350	Siamycin II(Bacteriocin)	Antiviral, Anti-HIV, Antimicrobial
1304	DRAMP00008	Lacticin 3147 A1 (LtnA1; Bacteriocin; Preclinical)	Antibacterial, Anti-Gram+, Antimicrobial
1305	DRAMP00009	Bacteriocin lacticin 3147 A2 (LtnA2; Bacteriocin; Preclinical)	Antibacterial, Anti-Gram+, Antimicrobial
1306	DRAMP00010	Plantaricin W alpha (Plw-alpha; Bacteriocin)	Antibacterial, Anti-Gram+, Antimicrobial
1307	DRAMP00011	Plantaricin W beta (Plw-beta; Bacteriocin)	Antibacterial, Anti-Gram+, Antimicrobial
1308	DRAMP00012	Lantibiotic lichenicidin VK21 A1 (LchA1; Lchalpha; Bacteriocin)	Antibacterial, Antimicrobial
1309	DRAMP00013	Lantibiotic lichenicidin VK21 A2 (LchA2; Lchbeta; Bacteriocin)	Antibacterial, Antimicrobial
1310	DRAMP00014	Geobacillin I (nisin analog; Bacteriocin)	Antibacterial, Anti-Gram+, Antimicrobial
1311	DRAMP00015	Geobacillin II (nisin analog; Bacteriocin)	Antibacterial, Anti-Gram+, Antimicrobial
1312	DRAMP00016	Salivaricin 9 (Sal9; Bacteriocin)	Antibacterial, Anti-Gram+, Antimicrobial
1313	DRAMP00018	SmbA1 (Bacteriocin)	Antibacterial, Antimicrobial
1314	DRAMP00019	SmbA2 (Bacteriocin)	Antibacterial, Antimicrobial
1315	DRAMP00020	HalA1 (one chain of haloduracin; Bacteriocin)	Antibacterial, Antimicrobial
1316	DRAMP00021	HalA2 (one chain of haloduracin; Bacteriocin)	Antibacterial, Antimicrobial
1317	DRAMP00022	Staphylococcin C55alpha (SacAalpha; chain alpha of Staphylococcin C55; Bacteriocin)	Antibacterial, Anti-Gram+, Antimicrobial
1318	DRAMP00023	Staphylococcin C55beta (SacAbeta; chain beta of Staphylococcin C55; Bacteriocin)	Antibacterial, Anti-Gram+, Antimicrobial
1319	DRAMP00024	CylLS (a structural subunit of cytolysin; Bacteriocin)	Antibacterial, Antimicrobial
1320	DRAMP00025	CylLL (a structural subunit of cytolysin; Bacteriocin)	Antibacterial, Antimicrobial
1321	DRAMP00026	Salivaricin A (SalA; Bacteriocin; Preclinical)	Antibacterial, Antimicrobial
1322	DRAMP00027	Salivaricin B (SboB; Bacteriocin; Preclinical)	Antibacterial, Antimicrobial
1323	DRAMP00028	Lantibiotic epidermin (Bacteriocin)	Antibacterial, Antimicrobial
1324	DRAMP00029	Streptococcin A-FF22 (Antibacterial peptide SA-FF22; Bacteriocin)	Antibacterial, Antimicrobial
1325	DRAMP18349	Siamycin I (Bacteriocin)	Antiviral, Anti-HIV, Antimicrobial
1326	DRAMP00031	Lantibiotic carnocin-UI49 (Bacteriocin)	Antibacterial, Anti-Gram+, Antimicrobial
1327	DRAMP00033	Lantibiotic epilancin 15X (Bacteriocin)	Antibacterial, Anti-Gram+, Antimicrobial
1328	DRAMP00034	Lantibiotic epilancin K7 (Bacteriocin)	Antibacterial, Antimicrobial
1329	DRAMP00035	Lantibiotic paenibacillin (Bacteriocin)	Antibacterial, Anti-Gram+, Antimicrobial
1330	DRAMP00036	Nisin A (Bacteriocin; Preclinical)	Antibacterial, Anti-Gram+, Antimicrobial
1331	DRAMP00037	Nisin Z (Bacteriocin; Preclinical)	Antibacterial, Anti-Gram+, Antimicrobial
1332	DRAMP00038	Nisin U (Bacteriocin)	Antibacterial, Anti-Gram+, Antimicrobial
1333	DRAMP00039	Pep5 (Bacteriocin)	Antibacterial, Antimicrobial
1334	DRAMP00040	Gallidermin (Bacteriocin; Preclinical)	Antibacterial, Antimicrobial
1335	DRAMP00041	Mutacin-1140 (Mutacin III; Bacteriocin)	Antibacterial, Anti-Gram+, Antimicrobial
1336	DRAMP00042	Bacteriocin mutacin B-Ny266 (Preclinical)	Antibacterial, Anti-Gram+, Antimicrobial
1337	DRAMP00043	Bacteriocin nukacin (Nukacin KQ-1; Nukacin KQU-131)	Antibacterial, Antimicrobial
1338	DRAMP00044	Bacteriocin nukacin (Nukacin 3299; Simulancin 3299)	Antibacterial, Antimicrobial
1339	DRAMP18347	Siamycin(Bacteriocin)	Antiviral, Anti-HIV, Antimicrobial
1340	DRAMP00046	Lantibiotic streptin (Bacteriocin)	Antibacterial, Anti-Gram+, Antimicrobial
1341	DRAMP00047	Streptin 1 (Bacteriocin)	Antibacterial, Antimicrobial
1342	DRAMP00048	Lantibiotic subtilin (Bacteriocin)	Antibacterial, Antimicrobial
1343	DRAMP00049	Bacteriocin lacticin-481 (Lactococcin-DR)	Antibacterial, Anti-Gram+, Antimicrobial
1344	DRAMP18345	Tricyclic peptide RP 71955 (Bacteriocin)	Antiviral, Anti-HIV, Antimicrobial
1345	DRAMP00051	Mutacin I (Bacteriocin)	Antibacterial, Anti-Gram+, Antimicrobial
1346	DRAMP00052	Mutacin-2 (Mutacin II mutacin H-29B; Bacteriocin)	Antibacterial, Anti-Gram+, Anti-Gram-, Antimicrobial
1347	DRAMP00053	Nisin F (Bacteriocin; Perclinical)	Antibacterial, Anti-Gram+, Antimicrobial
1348	DRAMP00054	Nisin Q (Bacteriocin)	Antibacterial, Anti-Gram+, Antimicrobial
1349	DRAMP00055	Bacteriocin 97518	Antibacterial, Anti-Gram-, Antimicrobial
1350	DRAMP00059	Lantibiotic duramycin C (Bacteriocin)	Antibacterial, Antimicrobial

1351	DRAMP00060	Bacteriocin cinnamycin (Lanthiopeptin Ro 09-0198)	Antibacterial, Antiviral, Antimicrobial
1352	DRAMP00061	Actagardine (Gardimycin; Bacteriocin)	Antibacterial, Anti-Gram+, Antimicrobial
1353	DRAMP00062	Mersacidin (Bacteriocin; Preclinical)	Antibacterial, Anti-Gram+, Antimicrobial
1354	DRAMP00064	Enterocin 96 (Bacteriocin)	Antibacterial, Anti-Gram+, Anti-Gram-, Antimicrobial
1355	DRAMP00065	Plantaricin 1.25 beta (thermostable Bacteriocin)	Antibacterial, Antimicrobial
1356	DRAMP00066	Lacticin Q (Bacteriocin)	Antibacterial, Anti-Gram+, Antimicrobial
1357	DRAMP00067	Leucocin C-TA33a (Bacteriocin)	Antibacterial, Antimicrobial
1358	DRAMP00070	Laterosporulin (Bacteriocin)	Antibacterial, Anti-Gram+, Anti-Gram-, Antimicrobial
1359	DRAMP00071	Ubericin-A (Bacteriocin)	Antibacterial, Anti-Gram+, Antimicrobial
1360	DRAMP00072	Bacteriocin curvaticin	Antibacterial, Anti-Gram+, Antimicrobial
1361	DRAMP00073	Weissellin-A (Bacteriocin)	Antibacterial, Anti-Gram+, Antimicrobial
1362	DRAMP00075	Enterocin M (Bacteriocin)	Antibacterial, Antimicrobial
1363	DRAMP00076	Mundticin ATO6 (Bacteriocin)	Antibacterial, Anti-Gram+, Antimicrobial
1364	DRAMP00077	Mundticin KS (Bacteriocin)	Antibacterial, Anti-Gram+, Antimicrobial
1365	DRAMP00078	Leucocin C (Leu C; Pediocin-like peptide; Bacteriocin)	Antibacterial, Antimicrobial
1366	DRAMP00079	Bacteriocin hiracin-JM79 (HirJM79; Bacteriocin)	Antibacterial, Anti-Gram+, Antimicrobial
1367	DRAMP00080	Curvacin A (Bacteriocin)	Antibacterial, Anti-Gram+, Antimicrobial
1368	DRAMP00081	Leucocin-A (Leucocin A-UAL 187; Leu A; Bacteriocin)	Antibacterial, Anti-Gram+, Antimicrobial
1369	DRAMP00082	Bavaricin-MN (Bacteriocin)	Antibacterial, Anti-Gram+, Antimicrobial
1370	DRAMP00083	Bavaricin-A (Bacteriocin)	Antibacterial, Anti-Gram+, Antimicrobial
1371	DRAMP00084	Mutacin F-59.1 (Bacteriocin)	Antibacterial, Antimicrobial
1372	DRAMP00085	Bacteriocin	Antibacterial, Anti-Gram+, Anti-Gram-, Antimicrobial
1373	DRAMP00086	Divergicin M35 (Pediocin-like peptide; Bacteriocin)	Antibacterial, Anti-Gram+, Antimicrobial
1374	DRAMP18344	Tricyclic peptide RP 71955 (Bacteriocin)	Antiviral, Anti-HIV, Antimicrobial
1375	DRAMP00088	Enterocin-HF (Bacteriocin)	Antibacterial, Antimicrobial
1376	DRAMP00091	Carnobacteriocin BM1 (Carnobacteriocin B1; Bacteriocin)	Antibacterial, Antimicrobial
1377	DRAMP00092	Bacteriocin SRCAM 602 (Preclinical)	Antibacterial, Anti-Gram+, Antimicrobial
1378	DRAMP00093	Bacteriocin SRCAM 37	Antibacterial, Anti-Gram+, Antimicrobial
1379	DRAMP00094	Bacteriocin SRCAM 1580	Antibacterial, Anti-Gram+, Antimicrobial
1380	DRAMP00095	Mesentericin Y105 (MesY105; Bacteriocin)	Antibacterial, Antimicrobial
1381	DRAMP00096	Pediocin PA-1 (Pediocin ACH; Bacteriocin)	Antibacterial, Anti-Gram+, Antimicrobial
1382	DRAMP00097	Lactococcin MMFII (Pediocin-like peptide; Bacteriocin)	Antibacterial, Antimicrobial
1383	DRAMP00099	Sakacin 5X (Sak5X; Pediocin-like peptide; Bacteriocin)	Antibacterial, Anti-Gram+, Antimicrobial
1384	DRAMP00100	Sakacin G (SakG; Pediocin-like peptide; Bacteriocin)	Antibacterial, Antilisterial, Anti-Gram+, Antimicrobial
1385	DRAMP00101	Sakacin P (Sakacin 674; Pediocin-like peptide; Bacteriocin)	Antibacterial, Anti-Gram+, Antimicrobial
1386	DRAMP00102	Piscicolin-126 (Pisc126; Bacteriocin)	Antibacterial, Antimicrobial
1387	DRAMP00103	Bacteriocin OR-7 (Preclinical)	Antibacterial, Antimicrobial
1388	DRAMP00108	Leucocin C (Pediocin-like peptide; Bacteriocin)	Antibacterial, Antimicrobial
1389	DRAMP00109	Plantaricin C19 (Pediocin-like peptide; Bacteriocin)	Antibacterial, Anti-Gram+, Antimicrobial
1390	DRAMP00110	Plantaricin 423 (Pediocin-like peptide; Bacteriocin)	Antibacterial, Anti-Gram+, Antimicrobial
1391	DRAMP00111	Penocin A (PenA; Bacteriocin)	Antibacterial, Anti-Gram+, Antimicrobial
1392	DRAMP00113	Enterocin A (EntA; Pediocin-like peptide; Bacteriocin)	Antibacterial, Antimicrobial
1393	DRAMP00114	Listeriocin 743A (Pediocin-like peptide; Bacteriocin)	Antibacterial, Antimicrobial
1394	DRAMP00116	Bacteriocin 32 (Bac 32; Bacteriocin)	Antibacterial, Anti-Gram+, Antimicrobial
1395	DRAMP18340	Daptomycin(Bacteriocin)	Antibacterial, Anti-Gram+, Antimicrobial
1396	DRAMP00119	Listeriocin 743A	Antibacterial, Anti-Gram+, Antimicrobial
1397	DRAMP00130	Lactococcin Q alpha (Qalpha; Bacteriocin)	Antibacterial, Anti-Gram+, Antimicrobial
1398	DRAMP00131	Lactococcin Q beta (Qbeta; Bacteriocin)	Antibacterial, Anti-Gram+, Antimicrobial
1399	DRAMP00132	Lactococcin G subunit alpha (Galpha; Bacteriocin)	Antibacterial, Antimicrobial
1400	DRAMP00133	Lactococcin G subunit beta (Gbeta ; Bacteriocin)	Antibacterial, Antimicrobial
1401	DRAMP00134	Plantaricin NC8 beta peptide (PLNC8 beta; Bacteriocin)	Antibacterial, Antimicrobial
1402	DRAMP00135	Plantaricin NC8 alpha peptide (PLNC8 alpha; Bacteriocin)	Antibacterial, Antimicrobial
1403	DRAMP00137	Plantaricin S beta (Bacteriocin)	Antibacterial, Antimicrobial
1404	DRAMP00138	Acidocin J1132 alpha peptide (Bacteriocin)	Antibacterial, Antimicrobial
1405	DRAMP00139	Acidocin J1132 beta peptide (Bacteriocin)	Antibacterial, Antimicrobial
1406	DRAMP00140	Lactacin-F subunit LafA (Bacteriocin)	Antibacterial, Antimicrobial
1407	DRAMP00141	Lactacin-F subunit LafX (Bacteriocin)	Antibacterial, Antimicrobial

1408	DRAMP00142	Gassericin T (gassericin K7 B; Bacteriocin)	Antibacterial, Antimicrobial
1409	DRAMP00143	Plantaricin-A (PlnA; Bacteriocin)	Antibacterial, Antimicrobial
1410	DRAMP00144	Amylovorin-L (Lactobin-A; Amylovorin-L471; Bacteriocin)	Antibacterial, Antimicrobial
1411	DRAMP00145	Lactocin-705 (Bacteriocin)	Antibacterial, Antimicrobial
1412	DRAMP00146	Abp118 alpha (Salivaricin CRL1328 alpha peptide)	Antibacterial, Antimicrobial
1413	DRAMP00147	Abp118 beta (Salivaricin CRL1328 beta peptide)	Antibacterial, Antimicrobial
1414	DRAMP00148	Thermophilin 9 (BlpDst; Bacteriocin)	Antibacterial, Antimicrobial
1415	DRAMP00150	Sln2 (chain b of Salivaricin P; Bacteriocin)	Antibacterial, Antimicrobial
1416	DRAMP00151	Enterocin 1071A (Ent1071A; Bacteriocin)	Antibacterial, Antimicrobial
1417	DRAMP00152	Enterocin 1071B (Ent1071B; Bacteriocin)	Antibacterial, Antimicrobial
1418	DRAMP00153	NlmA (chain a of Mutacin IV; Bacteriocin)	Antibacterial, Antimicrobial
1419	DRAMP00154	NlmB (chain b of Mutacin IV; Bacteriocin)	Antibacterial, Antimicrobial
1420	DRAMP00155	BrcA (chain a of Brochocin C; Bacteriocin)	Antibacterial, Antimicrobial
1421	DRAMP00156	BrcB (NKR-5-3A; chain b of Brochocin C; Bacteriocin)	Antibacterial, Antimicrobial
1422	DRAMP00157	Plantaricin S alpha (Bacteriocin)	Antibacterial, Anti-Gram+, Antimicrobial
1423	DRAMP00158	Lactocin 705alpha (lac705alpha; chain a of Lactocin 705; Bacteriocin)	Antibacterial, Antimicrobial
1424	DRAMP00159	Lactocin 705beta (lac705beta; chain b of Lactocin 705; Bacteriocin)	Antibacterial, Antimicrobial
1425	DRAMP00160	ThmA (chain a of Thermophilin 13; Bacteriocin)	Antibacterial, Anti-Gram+, Antimicrobial
1426	DRAMP00161	ThmB (chain b of Thermophilin 13; Bacteriocin)	Antibacterial, Anti-Gram+, Antimicrobial
1427	DRAMP00162	Thuricin CDalpha (Trn-alpha; one peptide of Thuricin CD; Bacteriocin)	Antibacterial, Anti-Gram+, Antimicrobial
1428	DRAMP00163	Thuricin CDdbeta (Trn-beta; one peptide of Thuricin CD; Bacteriocin)	Antibacterial, Anti-Gram+, Antimicrobial
1429	DRAMP00164	Bacteriocin uberolysin (Bacteriocin)	Antibacterial, Antimicrobial
1430	DRAMP00165	Gassericin A (GaaA; Bacteriocin)	Antibacterial, Anti-Gram+, Antimicrobial
1431	DRAMP00166	Butyrivibriocin AR10 (Bacteriocin)	Antibacterial, Anti-Gram+, Antimicrobial
1432	DRAMP00167	Subtilosin A (Antilisterial bacteriocin subtilosin; D-amino acid; Bacteriocin; Preclinical)	Antibacterial, Anti-Gram+, Antimicrobial
1433	DRAMP00168	Divergicin A (Bacteriocin)	Antibacterial, Antimicrobial
1434	DRAMP00169	Enterocin AS-48 (AS-48; Bacteriocin)	Antibacterial, Anti-Gram+, Anti-Gram-, Antimicrobial
1435	DRAMP00170	Carnocyclin A (CclA; Bacteriocin)	Antibacterial, Anti-Gram+, Antimicrobial
1436	DRAMP00172	Garvicin ML (Bacteriocin)	Antibacterial, Anti-Gram+, Antimicrobial
1437	DRAMP18337	S. amritsarensis lipopeptide (Bacteriocin)	Antibacterial, Anti-Gram+, Antimicrobial
1438	DRAMP18338	Thiocillin GE37468 (Bacteriocin)	Antibacterial, Anti-Gram+, Anti-Gram-, Antimicrobial
1439	DRAMP00175	Enterocin L50A (EntL50A; Bacteriocin)	Antibacterial, Anti-Gram+, Antimicrobial
1440	DRAMP00176	Enterocin L50B (EntL51B; Bacteriocin)	Antibacterial, Anti-Gram+, Antimicrobial
1441	DRAMP00179	Enterocin Q (EntQ; Bacteriocin)	Antibacterial, Antimicrobial
1442	DRAMP00180	Lactococcin-B (LCN-B; Bacteriocin)	Antibacterial, Antimicrobial
1443	DRAMP00181	Lactococcin-A (LCN-A; Bacteriocin)	Antibacterial, Antimicrobial
1444	DRAMP00182	Thuricin-S (Bacteriocin)	Antibacterial, Anti-Gram+, Anti-Gram-, Antimicrobial
1445	DRAMP00183	Thuricin-17 (Thurincin H; Bacteriocin)	Antibacterial, Anti-Gram+, Antimicrobial
1446	DRAMP00184	Lactococcin 972 (Lcn972; homodimer; Bacteriocin)	Antibacterial, Antimicrobial
1447	DRAMP00185	Leucocin-B (Leu B; Leucocin B-TA33a; Bacteriocin)	Antibacterial, Antimicrobial
1448	DRAMP00186	LSEI_2163 (m2163; Bacteriocin)	Antibacterial, Anti-Gram-, Antimicrobial
1449	DRAMP00187	LSEI_2386 (m2386; Bacteriocin)	Antibacterial, Anti-Gram-, Antimicrobial
1450	DRAMP00188	Enterocin RJ-11 (EntRJ-11; Bacteriocin)	Antibacterial, Anti-Gram+, Antimicrobial
1451	DRAMP00192	Microcin C7 (MccC7; Microcin C51, MccC51; Bacteriocin)	Antibacterial, Anti-Gram-, Antimicrobial
1452	DRAMP00193	Microcin B17 (MccB17; Bacteriocin)	Antibacterial, Antimicrobial
1453	DRAMP00194	Capistruin (Bacteriocin)	Antibacterial, Antimicrobial
1454	DRAMP00195	Colicin-V (Microcin-V; Bacteriocin)	Antibacterial, Anti-Gram-, Antimicrobial
1455	DRAMP00196	Microcin L (MccL; Bacteriocin)	Antibacterial, Anti-Gram-, Antimicrobial
1456	DRAMP00197	Microcin 24 (Mcc24; Bacteriocin)	Antibacterial, Anti-Gram-, Antimicrobial
1457	DRAMP00198	Microcin H47 (MccH47; Bacteriocin)	Antibacterial, Antimicrobial
1458	DRAMP00199	Microcin I47 (MccI47; Bacteriocin)	Antibacterial, Antimicrobial
1459	DRAMP00200	Microcin M (MccM; Bacteriocin)	Antibacterial, Antimicrobial
1460	DRAMP00202	Thiocillin (Bacteriocin)	Antibacterial, Antimicrobial

1461	DRAMP00203	Thiocillin GE2270 (Antibiotic GE2270; Bacteriocin)	Antibacterial , Antimicrobial
1462	DRAMP00205	Tricyclic peptide RP 71955 (Bacteriocin)	Antiviral, Antimicrobial
1463	DRAMP00206	Acidocin A (Bacteriocin)	Antibacterial, Anti-Gram+, Antimicrobial
1464	DRAMP00207	Acidocin B (Bacteriocin)	Antibacterial, Anti-Gram+, Antimicrobial
1465	DRAMP00208	Acidocin 8912 (Bacteriocin)	Antibacterial, Antimicrobial
1466	DRAMP00209	Fulvocin C (Bacteriocin)	Antibacterial, Antimicrobial
1467	DRAMP18336	Thermophilin 1277 (Bacteriocin)	Antibacterial, Anti-Gram+, Antimicrobial
1468	DRAMP00211	Sublancin-168 (Bacteriocin)	Antibacterial, Antimicrobial
1469	DRAMP00212	Rhamnosin A (Bacteriocin)	Antibacterial, Anti-Gram+, Antimicrobial
1470	DRAMP00213	Lactocin S (Bacteriocin)	Antibacterial, Antimicrobial
1471	DRAMP00214	Lactococcin K (Bacteriocin)	Antibacterial, Antimicrobial
1472	DRAMP00215	Enterocin E-760 (Bacteriocin)	Antibacterial, Anti-Gram+, Antimicrobial
1473	DRAMP00216	Antimicrobial peptide LCI (Bacteriocin)	Antibacterial, Antimicrobial
1474	DRAMP00217	Bacteriocin	Antibacterial, Antimicrobial
1475	DRAMP00219	PlnA-22 (Bacteriocin)	Antibacterial, Anti-Gram+, Antimicrobial
1476	DRAMP00220	PlnA-17 (Bacteriocin)	Antibacterial, Anti-Gram+, Antimicrobial
1477	DRAMP00221	Carnobacteriocin-A (Piscicolin-61; Bacteriocin)	Antibacterial, Anti-Gram+, Antimicrobial
1478	DRAMP18335	Salivaricin G32(Bacteriocin)	Antibacterial, Anti-Gram+, Antimicrobial
1479	DRAMP00224	BTL (Bacteriocin)	Antifungal, Antimicrobial
1480	DRAMP00225	Bacteriocin UviB	Antibacterial, Antimicrobial
1481	DRAMP00226	Bioactive peptide 3 (BAP3; Curvalicin-28c; Bacteriocin)	Antibacterial, Anti-Gram+, Antimicrobial
1482	DRAMP00227	Bioactive peptide 2 (BAP2;Curvalicin-28b; Bacteriocin)	Antibacterial, Anti-Gram+, Antimicrobial
1483	DRAMP00228	Bioactive peptide 1 (BAP1; Curvalicin-28a; Bacteriocin)	Antibacterial, Anti-Gram+, Antimicrobial
1484	DRAMP00229	Bacteriocin plantarican ASM1 (PASM1; Bacteriocin)	Antibacterial, Anti-Gram+, Antimicrobial
1485	DRAMP00230	Lariatins A (lasso peptide; Bacteriocin)	Antibacterial, Anti-Gram+, Antimicrobial
1486	DRAMP00231	Lariatins B (lasso peptide; Bacteriocin)	Antibacterial, Anti-Gram+, Antimicrobial
1487	DRAMP00233	Lichenin (Bacteriocin-like)	Antibacterial, Antimicrobial
1488	DRAMP00234	Trifolitoxin (TFX; Bacteriocin)	Antibacterial, Antimicrobial
1489	DRAMP00235	AFP1 (Bacteriocin)	Antifungal, Antimicrobial
1490	DRAMP00236	Halocin-S8 (HalS8; Bacteriocin)	Antibacterial, Anti-Gram+, Antimicrobial
1491	DRAMP00237	Halocin-C8 (HalC8; Bacteriocin)	Antibacterial, Antimicrobial
1492	DRAMP00238	Curvaticin FS47 (Bacteriocin)	Antibacterial, Anti-Gram+, Antimicrobial
1493	DRAMP00239	Enterocin NKR-5-3D (Bacteriocin)	Antibacterial, Antimicrobial
1494	DRAMP00240	Pep27 (Bacteriocin)	Antibacterial, Anticancer, Antimicrobial
1495	DRAMP00242	Subpeptin JM4-B (Bacteriocin)	Antibacterial, Anti-Gram+, Antimicrobial
1496	DRAMP00243	Subpeptin JM4-A (Bacteriocin)	Antibacterial, Anti-Gram+, Antimicrobial
1497	DRAMP00245	Gramicidin A (GA; Nonribosomally synthesized bacteriocin)	Antibacterial, Antiviral, Antimicrobial
1498	DRAMP00246	Gramicidin B (GB; Bacteriocin)	Antibacterial, Antiviral, Antimicrobial
1499	DRAMP00247	Gramicidin C (GC; Bacteriocin)	Antibacterial, Antiviral, Antimicrobial
1500	DRAMP00248	Glycocin F (GccF; S-glycosylated bacteriocin)	Antibacterial, Anti-Gram+, Antimicrobial
1501	DRAMP00249	Pln149 (Plantaricin 149; Bacteriocin; Derivatives: Pln149a)	Antibacterial, Antifungal, Antimicrobial
1502	DRAMP00250	Bacteriocin serracin-P 43 kDa subunit (Bacteriocin)	Antibacterial, Anti-Gram-, Antimicrobial
1503	DRAMP00251	Bacteriocin serracin-P 23 kDa subunit (Bacteriocin)	Antibacterial, Anti-Gram-, Antimicrobial
1504	DRAMP00252	AdDLP (A. dehalogenans defensin-like peptide; Bacteriocin)	Antibacterial, Antifungal, Antiparasitic, Antimicrobial
1505	DRAMP00253	Ipomicin (Bacteriocin)	Antibacterial, Antimicrobial
1506	DRAMP00261	Antiviral lectin scytovirin (SVN)	Antiviral, Antimicrobial
1507	DRAMP00262	Cyanovirin-N (CV-N)	Antiviral, Antimicrobial
1508	DRAMP00264	Coconut antifungal peptide (Plants)	Antifungal, Antiviral, Antimicrobial
1509	DRAMP00265	Putative antimicrobial protein 1 (Ls-AMP1; Plant defensin)	Antifungal, Antimicrobial
1510	DRAMP00266	Putative antimicrobial protein 2 (Ls-AMP2; Plant defensin)	Antifungal, Antimicrobial
1511	DRAMP00267	Antifungal lectin AMML (AMML; Plant defensin)	Antifungal, Antimicrobial
1512	DRAMP00268	Antifungal protein Pr-2 (Pr-2; Plant defensin)	Antifungal, Antimicrobial
1513	DRAMP00269	Antifungal protein 1 (GAFP-1; Plant defensin)	Antifungal, Antimicrobial
1514	DRAMP00270	Osmotin (CpOsm; Plant defensin)	Antifungal, Antimicrobial
1515	DRAMP00271	Osmotin-like protein (Thaumatin-like protein; Plant defensin)	Antifungal, Antimicrobial
1516	DRAMP00272	Thaumatin-like protein (Plants)	Antifungal, Antiviral, Antimicrobial
1517	DRAMP00273	Thaumatin-like protein (Plants)	Antifungal, Antimicrobial

1518	DRAMP00274	Amaryllin (Plant defensin)	Antifungal, Antimicrobial
1519	DRAMP00276	Snakin-2 (StSN2; Cys-rich; Plant defensin)	Antibacterial, Antifungal, Anti-Gram+, Antimicrobial
1520	DRAMP00277	Potamin-1 (PT-1; Plants)	Antifungal, Antibacterial, Anti-Gram+, Antimicrobial
1521	DRAMP00278	Antifungal protein J (AFP-J; Plants)	Antifungal, Antimicrobial
1522	DRAMP00279	Plastocyanin (Plants)	Antiviral, Antimicrobial
1523	DRAMP00280	Trypsin inhibitor (FtTI; Plant defensin)	Antifungal, Antimicrobial
1524	DRAMP00281	Defensin-like protein 230 (Disease resistance response protein 230; Plant defensin)	Antifungal, Antimicrobial
1525	DRAMP00282	Defensin-like protein P322 (Probable protease inhibitor P322; Plant defensin)	Antifungal, Antimicrobial
1526	DRAMP00283	Defensin Ec-AMP-D1 (Plant defensin)	Antifungal, Antimicrobial
1527	DRAMP00284	Defensin-like protein 1 (SI alpha-1; Plant defensin)	Antifungal, Antimicrobial
1528	DRAMP00285	Defensin-like protein 2 (SI alpha-2; Plant defensin)	Antifungal, Antimicrobial
1529	DRAMP00286	Defensin-like protein 3 (SI alpha-3; Plant defensin)	Antifungal, Antimicrobial
1530	DRAMP00287	Defensin-like protein 21 (SI alpha-2.1; Plant defensin)	Antifungal, Antimicrobial
1531	DRAMP00288	Defensin Ec-AMP-D2 (Plant defensin)	Antifungal, Antimicrobial
1532	DRAMP00289	Defensin-like protein (Gamma-thionin; Plant defensin)	Antifungal, Antimicrobial
1533	DRAMP00290	Defensin Lc-def (Plant defensin)	Antifungal, Antimicrobial
1534	DRAMP00291	Defensin-like protein (Gamma-thionin homolog; Plant defensin)	Antifungal, Antimicrobial
1535	DRAMP00292	Defensin-like protein (8.4 kDa sulfur-rich protein; Plant defensin)	Antifungal, Antimicrobial
1536	DRAMP00293	Gamma1-hordothionin (Gamma 1-H; Plant defensin)	Antifungal, Antimicrobial
1537	DRAMP00294	Glycine-rich protein GWK (Plants)	Antifungal, Antimicrobial
1538	DRAMP00295	Soybean toxin 27 kDa chain (SBTX 27 kDa chain; Plant defensin)	Antifungal, Antimicrobial
1539	DRAMP00296	Soybean toxin 17 kDa chain (SBTX 17 kDa chain; Plant defensin)	Antifungal, Cytotoxicity, Antimicrobial
1540	DRAMP00299	Ribonuclease (Plants)	Antiviral, Antimicrobial
1541	DRAMP00300	Antifungal protein 1 (Pa-AFP1; Plant defensin)	Antifungal, Antimicrobial
1542	DRAMP00303	Basic endochitinase CH1 (Plant defensin)	Antifungal, Antimicrobial
1543	DRAMP00304	Endochitinase 1 (Plant defensin)	Antifungal, Antimicrobial
1544	DRAMP00305	Endochitinase 1 (Plant defensin)	Antifungal, Antimicrobial
1545	DRAMP00306	Endochitinase 1 (Plant defensin)	Antifungal, Antimicrobial
1546	DRAMP00307	Endochitinase A1 (Plant defensin)	Antifungal, Antimicrobial
1547	DRAMP00308	Endochitinase 1 (Plant defensin)	Antifungal, Antimicrobial
1548	DRAMP00309	Endochitinase 2 (CHIT 2; Plant defensin)	Antifungal, Antimicrobial
1549	DRAMP00310	Endochitinase 2 (Plant defensin)	Antifungal, Antimicrobial
1550	DRAMP00311	Endochitinase 2 (Plant defensin)	Antifungal, Antimicrobial
1551	DRAMP00312	Endochitinase 2 (Plant defensin)	Antifungal, Antimicrobial
1552	DRAMP00313	Endochitinase 3 (CHIT 3; Plant defensin)	Antibacterial, Antifungal, Antimicrobial
1553	DRAMP00314	Endochitinase 3 (Plant defensin)	Antifungal, Antimicrobial
1554	DRAMP00315	Endochitinase 4 (CHIT 4; Plant defensin)	Antibacterial, Antifungal, Antimicrobial
1555	DRAMP00316	Endochitinase 1A (CHIT 1A; Plant defensin)	Antibacterial, Antifungal, Antimicrobial
1556	DRAMP00317	Endochitinase 1B (CHIT 1B; Plant defensin)	Antibacterial, Antifungal, Antimicrobial
1557	DRAMP00318	Endochitinase B (Plant defensin)	Antifungal, Antimicrobial
1558	DRAMP00319	Endochitinase (Plant defensin)	Antifungal, Antimicrobial
1559	DRAMP00320	Acyclotide phyb-K (Plant defensin)	Antimicrobial,
1560	DRAMP00321	Acyclotide phyb-M (Plant defensin)	Antimicrobial,
1561	DRAMP00324	Protein PR-L1 (Plant defensin)	Antibacterial, Antifungal, Antimicrobial
1562	DRAMP00325	Protein PR-L2 (Plant defensin)	Antibacterial, Antifungal, Antimicrobial
1563	DRAMP00326	Protein PR-L3 (Plant defensin)	Antibacterial, Antifungal, Antimicrobial
1564	DRAMP00327	Protein PR-L4 (Plant defensin)	Antibacterial, Antifungal, Antimicrobial
1565	DRAMP00328	Protein PR-L5 (Plant defensin)	Antibacterial, Antifungal, Antimicrobial
1566	DRAMP00329	Protein PR-L6 (Plant defensin)	Antibacterial, Antifungal, Antimicrobial
1567	DRAMP00330	Pathogenesis-related protein (PR-1; Plant defensin)	Antimicrobial,
1568	DRAMP00331	Pathogenesis-related protein (PRP; Plant defensin)	Antimicrobial,
1569	DRAMP00332	Pathogenesis-related protein (Plant defensin)	Antimicrobial,

1570	DRAMP00333	Antiviral protein DAP-32 (Ribosome-inactivating protein; Plant defensin)	Antiviral, Antimicrobial
1571	DRAMP00334	Antiviral protein GAP-31 (Ribosome-inactivating protein; Plant defensin)	Antiviral, Antimicrobial
1572	DRAMP00339	Alpha-basrubrin (Fragment; Plants)	Antifungal, Antiviral, Antimicrobial
1573	DRAMP00340	Beta-basrubin (Plants)	Antifungal, Antiviral, Antimicrobial
1574	DRAMP00341	Antifungal protein ginkbilobin-1 (Ginkbilobin, GNL; Plants)	Antibacterial, Antifungal, Antiviral, Anti-Gram+, Anti-Gram-, Antimicrobial
1575	DRAMP00342	Antifungal protein R (Plant defensin)	Antifungal, Antimicrobial
1576	DRAMP00343	Antifungal protein S (Plant defensin)	Antifungal, Antimicrobial
1577	DRAMP00346	Non-specific lipid-transfer protein Cw18 (LTP Cw-18; PKG2316; Plants)	Antibacterial, Antifungal, Antimicrobial
1578	DRAMP00347	Non-specific lipid-transfer protein 3 (LTP 3; CW-19; CW-20; Plants)	Antibacterial, Antifungal, Antimicrobial
1579	DRAMP00348	Non-specific lipid-transfer protein 4.1 (LTP 4.1; CW-21; Plants)	Antibacterial, Antifungal, Antimicrobial
1580	DRAMP18333	BHT-Ab(Bacteriocin)	Antibacterial, Anti-Gram+, Antimicrobial
1581	DRAMP18334	BHT-B (Bacteriocin)	Antibacterial, Anti-Gram+, Antimicrobial
1582	DRAMP00362	Non-specific lipid-transfer protein 1 (Lc-LTP1; Plants)	Antibacterial, Antimicrobial
1583	DRAMP00363	Non-specific lipid-transfer protein 2 (Lc-LTP2; Plants)	Antibacterial, Antimicrobial
1584	DRAMP00364	Non-specific lipid-transfer protein 4 (Lc-LTP4; Plants)	Antibacterial, Antimicrobial
1585	DRAMP00365	Non-specific lipid-transfer protein 5 (Lc-LTP5; Plants)	Antibacterial, Antimicrobial
1586	DRAMP00366	Non-specific lipid-transfer protein 6 (Lc-LTP6; Plants)	Antibacterial, Antimicrobial
1587	DRAMP00367	Non-specific lipid-transfer protein 7 (Lc-LTP7; Plants)	Antibacterial, Antimicrobial
1588	DRAMP00368	Non-specific lipid-transfer protein 8 (Lc-LTP8; Plants)	Antibacterial, Antimicrobial
1589	DRAMP00369	IWF1 (Bv-LTP1; Plant defensin)	Antifungal, Antimicrobial
1590	DRAMP00370	Antifungal protein 5 (CW-5; Plants)	Antifungal, Antimicrobial
1591	DRAMP00372	Non-specific lipid-transfer protein 3 (Os-LTP3)	Antifungal, Antimicrobial
1592	DRAMP00373	Seed non-specific lipid transfer protein-like (ns-LTP; Plants)	Antifungal, Antimicrobial
1593	DRAMP00375	Antifungal protein 1 small subunit (CW-1; Plants)	Antifungal, Antimicrobial
1594	DRAMP00376	Antifungal protein 1 large subunit (CW-1; Plants)	Antifungal, Antimicrobial
1595	DRAMP00377	Antifungal protein 2 small subunit (CW-2; Plants)	Antifungal, Antimicrobial
1596	DRAMP00378	Antifungal protein 2 large subunit (CW-2; Plants)	Antifungal, Antimicrobial
1597	DRAMP00379	Antifungal protein 3 (CW-3; Plants)	Antibacterial; Antifungal, Antimicrobial
1598	DRAMP00380	Antifungal protein 4 (CW-4; Plants)	Antibacterial; Antifungal, Antimicrobial
1599	DRAMP00381	IWF4 (Plants)	Antifungal, Antimicrobial
1600	DRAMP00382	Datucin (Glycopeptide; Plants)	Antifungal, Antimicrobial
1601	DRAMP00383	Antimicrobial peptide 1 (Mc-AMP1; knottin-type peptide; Plant defensin)	Antibacterial, Antifungal, Anti-Gram+, Antimicrobial
1602	DRAMP00386	EcLTP (E. crus-galli lipid transfer protein; Plants)	Antifungal, Antimicrobial
1603	DRAMP00387	Antimicrobial peptide 1 (EcAMP1; hairpin-like peptides; Plants)	Antifungal, Antimicrobial
1604	DRAMP00388	Antimicrobial peptide 2 (EcAMP2; Plants)	Antifungal, Antimicrobial
1605	DRAMP00389	EcAMP2.1 (truncated EcAMP2 without five C-terminal residues)	Antifungal, Antimicrobial
1606	DRAMP00390	Antimicrobial peptide 3 (ToAMP3; Cys-rich; Plant defensin)	Antibacterial, Antifungal, Antimicrobial
1607	DRAMP00391	Antimicrobial peptide 2 (ToAMP2; Cys-rich; Plant defensin)	Antibacterial, Antifungal, Antimicrobial
1608	DRAMP00392	Antimicrobial peptide 1 (ToAMP1; Cys-rich; Plant defensin)	Antibacterial, Antifungal, Antimicrobial
1609	DRAMP00393	Hedyotide B2 (hB2; Uncyclotides; Plants)	Antifungal, Anti-Gram+, Anti-Gram-, Antimicrobial
1610	DRAMP00395	Cyclotide vitri-A (Vbc6; Plant defensin)	Antimicrobial,
1611	DRAMP00396	Ct-AMP1 (CtAMP1, C. ternatea-antimicrobial peptide 1; Plant defensin)	Antibacterial, Antifungal, Anti-Gram+, Antimicrobial
1612	DRAMP00397	Defensin D1 (Ns-D1; Plant defensin)	Antifungal, Anti-Gram+, Anti-Gram-, Antimicrobial
1613	DRAMP00398	Defensin D2 (Ns-D2; Plant defensin)	Antifungal, Anti-Gram+, Anti-Gram-, Antimicrobial
1614	DRAMP00399	Defensin-like protein 39 (Disease resistance response protein 39; Plant defensin)	Antifungal, Antimicrobial
1615	DRAMP00400	Defensin-like protein 1 (Defensin AMP1, CtAMP1; Plant defensin)	Antifungal, Antimicrobial
1616	DRAMP00401	Defensin-like protein 2 (MTI-2; Trypsin inhibitor 2; Plant defensin)	Antifungal, Antimicrobial
1617	DRAMP00402	Defensin D1 (So-D1; Antimicrobial peptide D1; Plant defensin)	Antibacterial, Anti-Gram+, Anti-Gram-, Antimicrobial

1618	DRAMP00403	Defensin D2 (So-D2; Antimicrobial peptide D2; Plant defensin)	Antibacterial, Antifungal, Anti-Gram+, Anti-Gram-, Antimicrobial
1619	DRAMP00404	Defensin D3 (So-D3; Antimicrobial peptide D3; Plant defensin)	Antibacterial, Antifungal, Antimicrobial
1620	DRAMP00405	Defensin D4 (So-D4; Antimicrobial peptide D4; Plant defensin)	Antibacterial, Antifungal, Antimicrobial
1621	DRAMP00406	Defensin D5 (So-D5; Antimicrobial peptide D5; Plant defensin)	Antibacterial, Antifungal, Anti-Gram+, Anti-Gram-, Antimicrobial
1622	DRAMP00407	Defensin D6 (So-D6; Antimicrobial peptide D6; Plant defensin)	Antibacterial, Antifungal, Anti-Gram+, Anti-Gram-, Antimicrobial
1623	DRAMP00408	Defensin D7 (So-D7; Antimicrobial peptide D7; Plant defensin)	Antibacterial, Antifungal, Antimicrobial
1624	DRAMP00409	Defensin-like protein (Sesquin; Plant defensin)	Antibacterial, Antifungal, Antiviral, Anti-Gram+, Anti-Gram-, Antimicrobial
1625	DRAMP00410	Defensin-like protein 1 (Bn-AFP1; Plant defensin)	Antifungal, Antimicrobial
1626	DRAMP00411	Defensin-like protein 2 (Bn-AFP2; Plant defensin)	Antifungal, Antimicrobial
1627	DRAMP00412	Defensin-like protein 1 (Br-AFP1; Plant defensin)	Antifungal, Antimicrobial
1628	DRAMP00413	Defensin-like protein 2 (Br-AFP2; Plant defensin)	Antibacterial, Antifungal, Antimicrobial
1629	DRAMP18331	Antibacterial peptide A-M49 (Bacteriocin)	Antibacterial, Anti-Gram+, Antimicrobial
1630	DRAMP18332	BHT-Aa(Bacteriocin)	Antibacterial, Anti-Gram+, Antimicrobial
1631	DRAMP00415	Raphanus sativus Antifungal Protein 2 (Rs-AFP2; Plant defensin)	Antifungal, Antimicrobial
1632	DRAMP00418	Hc-AFP4 (Plant defensin)	Antifungal, Antimicrobial
1633	DRAMP00419	Hc-AFP3 (Plant defensin)	Antifungal, Antimicrobial
1634	DRAMP00420	Hc-AFP2 (Plant defensin)	Antifungal, Antimicrobial
1635	DRAMP00421	Hc-AFP1 (Plant defensin)	Antifungal, Antimicrobial
1636	DRAMP00424	Br-AFP1 (Defensin 1.2; Plant defensin)	Antifungal, Antimicrobial
1637	DRAMP00426	Defensin-like protein 1 (Hs-AFP1; Plant defensin)	Antifungal, Antimicrobial
1638	DRAMP00427	Defensin-like protein AX1 (Antifungal protein AX1; Plant defensin)	Antibacterial, Antifungal, Antiviral, Antimicrobial
1639	DRAMP00428	Defensin-like protein AX2 (Antifungal protein AX2; Cys-rich; Plant defensin)	Antibacterial, Antifungal, Antiviral, Antimicrobial
1640	DRAMP00429	Aesculus hippocastanum antimicrobial protein 1 (Ah-AMP1; Cys-rich; Plant defensin)	Antibacterial, Antifungal, Anti-Gram+, Antimicrobial
1641	DRAMP00430	Defensin-like protein 1 (Cp-thionin I; Cp-thionin-1; Gamma-thionin I; Plant defensin)	Unknown, Antimicrobial
1642	DRAMP00432	Defensin-like protein 1 (Gamma-zeathionin-1; Plant defensin)	Antifungal, Antimicrobial
1643	DRAMP00433	Defensin-like protein 2 (Gamma-zeathionin-2; Plant defensin)	Antifungal, Antimicrobial
1644	DRAMP00434	Defensin-like protein 1 (Gamma-1-purothionin; Plant defensin)	Antifungal, Antimicrobial
1645	DRAMP00435	Defensin-like protein 2 (Gamma-2-purothionin; Plant defensin)	Antifungal, Antimicrobial
1646	DRAMP00438	Defensin-like protein 1 (Gamma-thionin 1; Plant defensin)	Antifungal, Antimicrobial
1647	DRAMP00439	Defensin-like protein (Gamma-thionin homolog PPT; Plant defensin)	Antifungal, Antimicrobial
1648	DRAMP00440	Defensin SD2 (Plant defensin)	Antifungal, Antimicrobial
1649	DRAMP00441	Defensin Tk-AMP-D1 (Plant defensin)	Antifungal, Antimicrobial
1650	DRAMP00442	Defensin Tk-AMP-D1.1 (Plant defensin)	Antifungal, Antimicrobial
1651	DRAMP00443	Defensin Tk-AMP-D2 (Plant defensin)	Antifungal, Antimicrobial
1652	DRAMP00444	Defensin Tk-AMP-D3 (Plant defensin)	Antifungal, Antimicrobial
1653	DRAMP00445	Defensin Tk-AMP-D4 (Plant defensin)	Antifungal, Antimicrobial
1654	DRAMP00446	Defensin Tk-AMP-D5 (Plant defensin)	Antifungal, Antimicrobial
1655	DRAMP00447	Defensin Tk-AMP-D6 (Plant defensin)	Antifungal, Antimicrobial
1656	DRAMP00448	Defensin Tk-AMP-D6.1 (Plant defensin)	Antifungal, Antimicrobial
1657	DRAMP00449	Defensin Tm-AMP-D1.2 (Plant defensin)	Antifungal, Antimicrobial
1658	DRAMP00451	Defensin-like protein 2B (AFP2b; M2B; Plant defensin)	Antifungal, Antimicrobial
1659	DRAMP00452	Defensin J1-1 (Plant defensin)	Antifungal, Antimicrobial
1660	DRAMP00453	Defensin J1-2 (Plant defensin)	Antifungal, Antimicrobial
1661	DRAMP00455	Defensin-like protein 2 (Fabatin-2; Plant defensin)	Antibacterial, Anti-Gram+, Anti-Gram-, Antimicrobial
1662	DRAMP00456	Defensin-like protein 1 (Fabatin-1; Plant defensin)	Antibacterial, Anti-Gram+, Anti-Gram-, Antimicrobial
1663	DRAMP00457	Defensin-like protein 1 (LCR68; Plant defensin 2.3; Plant defensin)	Antifungal, Antimicrobial
1664	DRAMP00458	Defensin-like protein 2 (LCR69; Plant defensin 2.2; Plant defensin)	Antifungal, Antimicrobial
1665	DRAMP00459	Defensin-like protein 3 (Protein LCR71; Plant defensin)	Antifungal, Antimicrobial

1666	DRAMP00460	Defensin-like protein 4 (Protein LCR70; Plant defensin 2.1; Plant defensin)	Antifungal, Antimicrobial
1667	DRAMP00461	Defensin-like protein 5 (Protein LCR66; Plant defensin 2.4; Plant defensin)	Antifungal, Antimicrobial
1668	DRAMP00462	Defensin-like protein 6 (Protein LCR74; Plant defensin 2.5; Plant defensin)	Antifungal, Antimicrobial
1669	DRAMP00463	Defensin-like protein 7 (Protein LCR75; LCR79; Plant defensin)	Antifungal, Antimicrobial
1670	DRAMP00464	Defensin-like protein 8 (Protein LCR73; Plant defensin)	Unknown, Antimicrobial
1671	DRAMP00465	Defensin-like protein 9 (Protein LCR76; Plant defensin)	Antifungal, Antimicrobial
1672	DRAMP00466	Defensin-like protein 10 (Protein LCR72; Plant defensin 2.6; Plant defensin)	Antifungal, Antimicrobial
1673	DRAMP00467	Defensin-like protein 11 (Plant defensin)	Antifungal, Antimicrobial
1674	DRAMP00468	Putative defensin-like protein 12 (Plant defensin)	Antifungal, Antimicrobial
1675	DRAMP00469	Defensin-like protein 13 (At-AFP1; Protein LCR67; Plant defensin)	Antifungal, Antimicrobial
1676	DRAMP00470	Defensin-like protein 14 (Plant defensin 1.3; Plant defensin)	Antifungal, Antimicrobial
1677	DRAMP00471	Defensin-like protein 15 (Putative plant defensin 1.2b; Plant defensin)	Antimicrobial,
1678	DRAMP18329	Pneumococin N(Bacteriocin)	Antibacterial, Anti-Gram+, Antimicrobial
1679	DRAMP18328	Pneumococin M(Bacteriocin)	Antibacterial, Anti-Gram+, Antimicrobial
1680	DRAMP00474	Defensin-like protein 18 (Plant defensin 1.5; Plant defensin)	Antifungal, Antimicrobial
1681	DRAMP00475	Defensin-like protein 19 (Protein LCR78; Plant defensin 1.4; Plant defensin)	Antifungal, Antimicrobial
1682	DRAMP00476	Putative defensin-like protein 20 (Plant defensin)	Antifungal, Antimicrobial
1683	DRAMP00477	Defensin-like protein 21 (Plant defensin)	Antifungal, Antimicrobial
1684	DRAMP00478	Defensin-like protein 22 (Plant defensin)	Antifungal, Antimicrobial
1685	DRAMP00479	Putative defensin-like protein 23 (Plant defensin)	Antifungal, Antimicrobial
1686	DRAMP00480	Defensin-like protein 24 (Plant defensin)	Antifungal, Antimicrobial
1687	DRAMP00481	Putative defensin-like protein 25 (Plant defensin)	Antifungal, Antimicrobial
1688	DRAMP00482	Putative defensin-like protein 26 (Plant defensin)	Antifungal, Antimicrobial
1689	DRAMP00483	Putative defensin-like protein 27 (Plant defensin)	Antifungal, Antimicrobial
1690	DRAMP00484	Putative defensin-like protein 28 (Plant defensin)	Antifungal, Antimicrobial
1691	DRAMP00485	Putative defensin-like protein 29 (Plant defensin)	Antifungal, Antimicrobial
1692	DRAMP00486	Putative defensin-like protein 30 (Plant defensin)	Antifungal, Antimicrobial
1693	DRAMP00487	Putative defensin-like protein 31 (Plant defensin)	Antifungal, Antimicrobial
1694	DRAMP00488	Defensin-like protein 32 (Plant defensin)	Antifungal, Antimicrobial
1695	DRAMP00489	Putative defensin-like protein 33 (Plant defensin)	Antifungal, Antimicrobial
1696	DRAMP00490	Defensin-like protein 34 (Plant defensin)	Antifungal, Antimicrobial
1697	DRAMP00491	Defensin-like protein 35 (Plant defensin)	Antifungal, Antimicrobial
1698	DRAMP00492	Putative defensin-like protein 36 (Plant defensin)	Antifungal, Antimicrobial
1699	DRAMP00493	Defensin-like protein 37 (Plant defensin)	Antifungal, Antimicrobial
1700	DRAMP00494	Putative defensin-like protein 38 (Plant defensin)	Antifungal, Antimicrobial
1701	DRAMP00495	Putative defensin-like protein 39 (Plant defensin)	Antifungal, Antimicrobial
1702	DRAMP00496	Putative defensin-like protein 40 (Plant defensin)	Antifungal, Antimicrobial
1703	DRAMP00497	Defensin-like protein 41 (Plant defensin)	Antifungal, Antimicrobial
1704	DRAMP00498	Putative defensin-like protein 42 (Plant defensin)	Antifungal, Antimicrobial
1705	DRAMP00499	Defensin-like protein 43 (Plant defensin)	Antifungal, Antimicrobial
1706	DRAMP00500	Defensin-like protein 44 (Plant defensin)	Antifungal, Antimicrobial
1707	DRAMP00501	Defensin-like protein 45 (Plant defensin)	Antifungal, Antimicrobial
1708	DRAMP00502	Defensin-like protein 46 (Plant defensin)	Antifungal, Antimicrobial
1709	DRAMP00503	Defensin-like protein 47 (Plant defensin)	Antifungal, Antimicrobial
1710	DRAMP00504	Putative defensin-like protein 48 (Plant defensin)	Antifungal, Antimicrobial
1711	DRAMP00505	Defensin-like protein 49 (Plant defensin)	Antifungal, Antimicrobial
1712	DRAMP00506	Defensin-like protein 50 (Protein LCR49; Plant defensin)	Antifungal, Antimicrobial
1713	DRAMP00507	Defensin-like protein 51 (Protein LCR48; Plant defensin)	Antifungal, Antimicrobial
1714	DRAMP00508	Putative defensin-like protein 52 (Plant defensin)	Antifungal, Antimicrobial
1715	DRAMP00509	Putative defensin-like protein 53 (Plant defensin)	Antifungal, Antimicrobial
1716	DRAMP00510	Defensin-like protein 54 (Plant defensin)	Antifungal, Antimicrobial
1717	DRAMP00511	Putative defensin-like protein 55 (Plant defensin)	Antifungal, Antimicrobial

1718	DRAMP00512	Putative defensin-like protein 56 (Plant defensin)	Antifungal, Antimicrobial
1719	DRAMP00513	Putative defensin-like protein 57 (Plant defensin)	Antifungal, Antimicrobial
1720	DRAMP00514	Defensin-like protein 58 (Plant defensin)	Antifungal, Antimicrobial
1721	DRAMP00515	Defensin-like protein 59 (Plant defensin)	Antifungal, Antimicrobial
1722	DRAMP00516	Putative defensin-like protein 60 (Plant defensin)	Antifungal, Antimicrobial
1723	DRAMP00517	Putative defensin-like protein 62 (Plant defensin)	Antifungal, Antimicrobial
1724	DRAMP00518	Putative defensin-like protein 63 (Plant defensin)	Antifungal, Antimicrobial
1725	DRAMP00519	Putative defensin-like protein 64 (Plant defensin)	Antifungal, Antimicrobial
1726	DRAMP00520	Putative defensin-like protein 66 (Plant defensin)	Antifungal, Antimicrobial
1727	DRAMP00521	Putative defensin-like protein 67 (Plant defensin)	Antifungal, Antimicrobial
1728	DRAMP00522	Defensin-like protein 68 (Plant defensin)	Antifungal, Antimicrobial
1729	DRAMP00523	Defensin-like protein 69 (Plant defensin)	Antifungal, Antimicrobial
1730	DRAMP00524	Putative defensin-like protein 70 (Protein LCR83; Plant defensin)	Antifungal, Antimicrobial
1731	DRAMP00525	Defensin-like protein 71 (Plant defensin)	Antifungal, Antimicrobial
1732	DRAMP00526	Putative defensin-like protein 72 (Plant defensin)	Antifungal, Antimicrobial
1733	DRAMP00527	Putative defensin-like protein 73 (Protein LCR44; Plant defensin)	Antifungal, Antimicrobial
1734	DRAMP00528	Defensin-like protein 74 (Protein LCR43; Plant defensin)	Antifungal, Antimicrobial
1735	DRAMP00529	Defensin-like protein 75 (Protein LCR45; Plant defensin)	Antifungal, Antimicrobial
1736	DRAMP00530	Defensin-like protein 76 (Protein LCR86; Plant defensin)	Antifungal, Antimicrobial
1737	DRAMP00531	Defensin-like protein 78 (Plant defensin)	Antifungal, Antimicrobial
1738	DRAMP00532	Putative defensin-like protein 79 (Plant defensin)	Antifungal, Antimicrobial
1739	DRAMP00533	Putative defensin-like protein 80 (Protein LCR81; Plant defensin)	Antifungal, Antimicrobial
1740	DRAMP00534	Defensin-like protein 81 (Plant defensin)	Antifungal, Antimicrobial
1741	DRAMP00535	Defensin-like protein 82 (Plant defensin)	Antifungal, Antimicrobial
1742	DRAMP00536	Putative defensin-like protein 83 (Protein LCR46; Plant defensin)	Antifungal, Antimicrobial
1743	DRAMP00537	Putative defensin-like protein 84 (Plant defensin)	Antifungal, Antimicrobial
1744	DRAMP00538	Defensin-like protein 85 (Plant defensin)	Antifungal, Antimicrobial
1745	DRAMP00539	Putative defensin-like protein 86 (Protein LCR82; Plant defensin)	Antifungal, Antimicrobial
1746	DRAMP00540	Defensin-like protein 87 (Plant defensin)	Antifungal, Antimicrobial
1747	DRAMP00541	Putative defensin-like protein 88 (Plant defensin)	Antifungal, Antimicrobial
1748	DRAMP00542	Putative defensin-like protein 89 (Plant defensin)	Antifungal, Antimicrobial
1749	DRAMP00543	Defensin-like protein 90 (Plant defensin)	Antifungal, Antimicrobial
1750	DRAMP00544	Defensin-like protein 91 (Protein LCR47; Plant defensin)	Antifungal, Antimicrobial
1751	DRAMP00545	Defensin-like protein 95 (Plant defensin)	Antifungal, Antimicrobial
1752	DRAMP00546	Defensin-like protein 96 (Plant defensin)	Antifungal, Antimicrobial
1753	DRAMP00547	Defensin-like protein 97 (Protein LCR85; Plant defensin)	Antifungal, Antimicrobial
1754	DRAMP00548	Defensin-like protein 98 (Plant defensin)	Antifungal, Antimicrobial
1755	DRAMP00549	Defensin-like protein 100 (Plant defensin)	Antifungal, Antimicrobial
1756	DRAMP00550	Putative defensin-like protein 101 (Plant defensin)	Antifungal, Antimicrobial
1757	DRAMP00551	Putative defensin-like protein 102 (Plant defensin)	Antifungal, Antimicrobial
1758	DRAMP00552	Defensin-like protein 103 (Plant defensin)	Antifungal, Antimicrobial
1759	DRAMP00553	Putative defensin-like protein 104 (Plant defensin)	Antifungal, Antimicrobial
1760	DRAMP00554	Putative defensin-like protein 105 (Plant defensin)	Antifungal, Antimicrobial
1761	DRAMP00555	Defensin-like protein 106 (Plant defensin)	Antifungal, Antimicrobial
1762	DRAMP00556	Defensin-like protein 107 (Plant defensin)	Antifungal, Antimicrobial
1763	DRAMP00557	Defensin-like protein 108 (Protein LCR51; Plant defensin)	Antifungal, Antimicrobial
1764	DRAMP00558	Defensin-like protein 109 (Plant defensin)	Antifungal, Antimicrobial
1765	DRAMP00559	Putative defensin-like protein 110 (Plant defensin)	Antifungal, Antimicrobial
1766	DRAMP00560	Putative defensin-like protein 111 (Protein LCR50; Plant defensin)	Antifungal, Antimicrobial
1767	DRAMP00561	Defensin-like protein 112 (Plant defensin)	Antifungal, Antimicrobial
1768	DRAMP00562	Defensin-like protein 113 (Plant defensin)	Antifungal, Antimicrobial
1769	DRAMP00563	Putative defensin-like protein 114 (Plant defensin)	Antifungal, Antimicrobial

1770	DRAMP00564	Defensin-like protein 115 (Plant defensin)	Antifungal, Antimicrobial
1771	DRAMP00565	Defensin-like protein 116 (Plant defensin)	Antifungal, Antimicrobial
1772	DRAMP00566	Defensin-like protein 117 (Plant defensin)	Antifungal, Antimicrobial
1773	DRAMP00567	Putative defensin-like protein 118 (Protein LCR52; Plant defensin)	Antifungal, Antimicrobial
1774	DRAMP00568	Putative defensin-like protein 119 (Protein LCR53; Plant defensin)	Antifungal, Antimicrobial
1775	DRAMP00569	Putative defensin-like protein 120 (Protein LCR56; Plant defensin)	Antifungal, Antimicrobial
1776	DRAMP00570	Putative defensin-like protein 121 (Protein LCR55; Plant defensin)	Antifungal, Antimicrobial
1777	DRAMP00571	Defensin-like protein 122 (Protein LCR30; Plant defensin)	Antifungal, Antimicrobial
1778	DRAMP00572	Defensin-like protein 123 (Plant defensin; Uncertain)	Antifungal, Antimicrobial
1779	DRAMP00573	Defensin-like protein 124 (Protein LCR16; Plant defensin)	Antifungal, Antimicrobial
1780	DRAMP00574	Defensin-like protein 125 (Protein LCR54; Plant defensin)	Antifungal, Antimicrobial
1781	DRAMP00575	Putative defensin-like protein 126 (Protein LCR6; Plant defensin)	Antifungal, Antimicrobial
1782	DRAMP00576	Defensin-like protein 127 (Protein LCR20; Plant defensin)	Antifungal, Antimicrobial
1783	DRAMP00577	Putative defensin-like protein 128 (Protein LCR8; Plant defensin)	Antifungal, Antimicrobial
1784	DRAMP00578	Putative defensin-like protein 129 (Protein LCR13; Plant defensin)	Antifungal, Antimicrobial
1785	DRAMP00579	Defensin-like protein 130 (Protein LCR28; Plant defensin)	Antifungal, Antimicrobial
1786	DRAMP00580	Putative defensin-like protein 131 (Protein LCR29; Plant defensin)	Antifungal, Antimicrobial
1787	DRAMP00581	Putative defensin-like protein 133 (Protein LCR33; Plant defensin)	Antifungal, Antimicrobial
1788	DRAMP00582	Putative defensin-like protein 134 (Plant defensin)	Antifungal, Antimicrobial
1789	DRAMP00583	Putative defensin-like protein 135 (Plant defensin)	Antifungal, Antimicrobial
1790	DRAMP00584	Putative defensin-like protein 137 (Protein LCR14; Plant defensin)	Antifungal, Antimicrobial
1791	DRAMP00585	Putative defensin-like protein 139 (Protein LCR7; Plant defensin)	Antifungal, Antimicrobial
1792	DRAMP00586	Defensin-like protein 140 (Protein LCR15; Plant defensin)	Antifungal, Antimicrobial
1793	DRAMP00587	Defensin-like protein 141 (Protein LCR3; Plant defensin)	Antifungal, Antimicrobial
1794	DRAMP00588	Putative defensin-like protein 142 (Protein LCR34; Plant defensin)	Antifungal, Antimicrobial
1795	DRAMP00589	Defensin-like protein 144 (Protein LCR10; Plant defensin)	Antifungal, Antimicrobial
1796	DRAMP00590	Putative defensin-like protein 145 (Protein LCR2; Plant defensin)	Antifungal, Antimicrobial
1797	DRAMP00591	Putative defensin-like protein 146 (Protein LCR9; Plant defensin)	Antifungal, Antimicrobial
1798	DRAMP00592	Defensin-like protein 147 (Protein LCR1; Plant defensin)	Antifungal, Antimicrobial
1799	DRAMP00593	Putative defensin-like protein 148 (Protein LCR4; Plant defensin)	Antifungal, Antimicrobial
1800	DRAMP00594	Defensin-like protein 149 (Protein LCR5; Plant defensin)	Antifungal, Antimicrobial
1801	DRAMP00595	Putative defensin-like protein 150 (Protein LCR32; Plant defensin)	Antifungal, Antimicrobial
1802	DRAMP00596	Defensin-like protein 151 (Protein LCR17; Plant defensin)	Antifungal, Antimicrobial
1803	DRAMP00597	Putative defensin-like protein 152 (Protein LCR11; Plant defensin)	Antifungal, Antimicrobial
1804	DRAMP00598	Defensin-like protein 153 (Protein LCR31; Plant defensin)	Antifungal, Antimicrobial
1805	DRAMP00599	Putative defensin-like protein 154 (Protein LCR35; Plant defensin)	Antifungal, Antimicrobial
1806	DRAMP00600	Defensin-like protein 155 (Protein LCR36; Plant defensin)	Antifungal, Antimicrobial
1807	DRAMP00601	Defensin-like protein 156 (Protein LCR21; Plant defensin)	Antifungal, Antimicrobial
1808	DRAMP00602	Putative defensin-like protein 157 (Protein LCR22; Plant defensin)	Antifungal, Antimicrobial
1809	DRAMP00603	Putative defensin-like protein 158 (Protein LCR23; Plant defensin)	Antifungal, Antimicrobial
1810	DRAMP00604	Defensin-like protein 159 (Protein LCR25; Plant defensin)	Antifungal, Antimicrobial

1811	DRAMP00605	Putative defensin-like protein 160 (Protein LCR26; Plant defensin)	Antifungal, Antimicrobial
1812	DRAMP00606	Defensin-like protein 161 (Protein LCR27; Plant defensin)	Antifungal, Antimicrobial
1813	DRAMP00607	Putative defensin-like protein 162 (Protein LCR37; Plant defensin)	Antifungal, Antimicrobial
1814	DRAMP00608	Defensin-like protein 163 (Protein LCR24; Plant defensin)	Antifungal, Antimicrobial
1815	DRAMP00609	Defensin-like protein 164 (Protein LCR38; Plant defensin)	Antifungal, Antimicrobial
1816	DRAMP00610	Putative defensin-like protein 165 (Protein LCR12; Plant defensin)	Antifungal, Antimicrobial
1817	DRAMP00611	Defensin-like protein 166 (Plant defensin)	Antifungal, Antimicrobial
1818	DRAMP00612	Putative defensin-like protein 168 (Plant defensin)	Antifungal, Antimicrobial
1819	DRAMP00613	Putative defensin-like protein 169 (Plant defensin)	Antifungal, Antimicrobial
1820	DRAMP00614	Putative defensin-like protein 170 (Protein LCR62; Plant defensin)	Antifungal, Antimicrobial
1821	DRAMP00615	Defensin-like protein 171 (Protein LCR61; Plant defensin)	Antifungal, Antimicrobial
1822	DRAMP00616	Defensin-like protein 172 (Protein LCR60; Plant defensin)	Antifungal, Antimicrobial
1823	DRAMP00617	Defensin-like protein 173 (Protein LCR63; Plant defensin)	Antifungal, Antimicrobial
1824	DRAMP00618	Defensin-like protein 175 (Plant defensin)	Antifungal, Antimicrobial
1825	DRAMP00619	Defensin-like protein 176 (Protein LCR65; Plant defensin)	Antifungal, Antimicrobial
1826	DRAMP00620	Defensin-like protein 178 (Protein LCR64; Plant defensin)	Antifungal, Antimicrobial
1827	DRAMP00621	Putative defensin-like protein 179 (Protein LCR57; Plant defensin)	Antifungal, Antimicrobial
1828	DRAMP00622	Defensin-like protein 181 (Protein LCR80; Plant defensin 3.1; Plant defensin)	Antifungal, Antimicrobial
1829	DRAMP00623	Defensin-like protein 183 (Protein LCR19; Plant defensin)	Antifungal, Antimicrobial
1830	DRAMP00624	Putative defensin-like protein 184 (Protein LCR18; Plant defensin)	Antifungal, Antimicrobial
1831	DRAMP00625	Putative defensin-like protein 185 (Protein LCR39; Plant defensin)	Antifungal, Antimicrobial
1832	DRAMP00626	Putative defensin-like protein 186 (Protein LCR40; Plant defensin)	Antifungal, Antimicrobial
1833	DRAMP00627	Putative defensin-like protein 187 (Protein LCR42; Plant defensin)	Antifungal, Antimicrobial
1834	DRAMP00628	Putative defensin-like protein 188 (Protein LCR41; Plant defensin)	Antifungal, Antimicrobial
1835	DRAMP00629	Putative defensin-like protein 189 (Plant defensin)	Antifungal, Antimicrobial
1836	DRAMP00630	Putative defensin-like protein 190 (Plant defensin)	Antifungal, Antimicrobial
1837	DRAMP00631	Putative defensin-like protein 191 (Plant defensin)	Antifungal, Antimicrobial
1838	DRAMP00632	Defensin-like protein 192 (Trypsin inhibitor ATTI-7; Plant defensin)	Antifungal, Antimicrobial
1839	DRAMP00633	Defensin-like protein 193 (Trypsin inhibitor ATTI-2; Plant defensin)	Antifungal, Antimicrobial
1840	DRAMP00634	Defensin-like protein 194 (Trypsin inhibitor ATTI-3; Plant defensin)	Antifungal, Antimicrobial
1841	DRAMP00635	Defensin-like protein 195 (Trypsin inhibitor ATTI-1; diDi 4T-1; Plant defensin)	Antifungal, Antimicrobial
1842	DRAMP00636	Defensin-like protein 196 (Trypsin inhibitor ATTI-4; Plant defensin)	Antifungal, Antimicrobial
1843	DRAMP00637	Defensin-like protein 197 (Trypsin inhibitor ATTI-6; Plant defensin)	Antifungal, Antimicrobial
1844	DRAMP00638	Defensin-like protein 199 (Plant defensin)	Antifungal, Antimicrobial
1845	DRAMP00639	Defensin-like protein 201 (Plant defensin)	Antifungal, Antimicrobial
1846	DRAMP00640	Putative defensin-like protein 202 (Plant defensin)	Antifungal, Antimicrobial
1847	DRAMP00641	Putative defensin-like protein 203 (Plant defensin)	Antifungal, Antimicrobial
1848	DRAMP00642	Defensin-like protein 204 (Plant defensin)	Antifungal, Antimicrobial
1849	DRAMP00643	Defensin-like protein 205 (His-rich; Plant defensin)	Antifungal, Antimicrobial
1850	DRAMP00644	Defensin-like protein 206 (Plant defensin)	Antifungal, Antimicrobial
1851	DRAMP18327	Sil(Bacteriocin)	Antibacterial, Anti-Gram+, Antimicrobial
1852	DRAMP00646	Defensin-like protein 208 (Plant defensin)	Antifungal, Antimicrobial
1853	DRAMP00647	Defensin-like protein 209 (Plant defensin)	Antifungal, Antimicrobial
1854	DRAMP00648	Defensin-like protein 210 (Plant defensin)	Antifungal, Antimicrobial

1855	DRAMP00649	Putative defensin-like protein 211 (Plant defensin)	Antifungal, Antimicrobial
1856	DRAMP00650	Defensin-like protein 212 (Plant defensin)	Antifungal, Antimicrobial
1857	DRAMP00651	Defensin-like protein 213 (Plant defensin)	Antifungal, Antimicrobial
1858	DRAMP00652	Defensin-like protein 214 (Plant defensin)	Antifungal, Antimicrobial
1859	DRAMP00653	Defensin-like protein 215 (Plant defensin)	Antifungal, Antimicrobial
1860	DRAMP00654	Defensin-like protein 216 (Plant defensin)	Antifungal, Antimicrobial
1861	DRAMP00655	Defensin-like protein 217 (Plant defensin)	Antifungal, Antimicrobial
1862	DRAMP00656	Defensin-like protein 218 (Plant defensin)	Antifungal, Antimicrobial
1863	DRAMP00657	Defensin-like protein 219 (Plant defensin)	Antifungal, Antimicrobial
1864	DRAMP00658	Defensin-like protein 220 (Plant defensin)	Antifungal, Antimicrobial
1865	DRAMP00659	Putative defensin-like protein 221 (Plant defensin)	Antifungal, Antimicrobial
1866	DRAMP00660	Defensin-like protein 222 (Plant defensin)	Antifungal, Antimicrobial
1867	DRAMP00661	Defensin-like protein 223 (Plant defensin)	Antifungal, Antimicrobial
1868	DRAMP00662	Putative defensin-like protein 225 (Protein SCRL1; SCR-like protein 1; Plant defensin)	Antifungal, Antimicrobial
1869	DRAMP00663	Defensin-like protein 226 (Protein SCRL2; SCR-like protein 2; Plant defensin)	Antifungal, Antimicrobial
1870	DRAMP00664	Putative defensin-like protein 227 (Protein SCRL28; SCR-like protein 28; Plant defensin)	Antifungal, Antimicrobial
1871	DRAMP00665	Putative defensin-like protein 228 (Protein SCRL3; SCR-like protein 3; Plant defensin)	Antifungal, Antimicrobial
1872	DRAMP00666	Defensin-like protein 229 (Protein SCRL27; SCR-like protein 27; Plant defensin)	Antifungal, Antimicrobial
1873	DRAMP00667	Putative defensin-like protein 230 (Protein SCRL24; SCR-like protein 24; Plant defensin)	Antifungal, Antimicrobial
1874	DRAMP00668	Putative defensin-like protein 231 (Protein SCRL25; SCR-like protein 25; Plant defensin)	Antifungal, Antimicrobial
1875	DRAMP00669	Defensin-like protein 232 (Protein SCRL23; SCR-like protein 23; Plant defensin)	Antifungal, Antimicrobial
1876	DRAMP00670	Putative defensin-like protein 233 (Protein SCRL22; SCR-like protein 22; Plant defensin)	Antifungal, Antimicrobial
1877	DRAMP00671	Putative defensin-like protein 234 (Protein SCRL14; SCR-like protein 14; Plant defensin)	Antifungal, Antimicrobial
1878	DRAMP00672	Putative defensin-like protein 235 (Protein SCRL26; SCR-like protein 26; Plant defensin)	Antifungal, Antimicrobial
1879	DRAMP00673	Putative defensin-like protein 236 (Protein SCRL20; SCR-like protein 20; Plant defensin)	Antifungal, Antimicrobial
1880	DRAMP00674	Putative defensin-like protein 237 (Protein SCRL21; SCR-like protein 21; Plant defensin)	Antifungal, Antimicrobial
1881	DRAMP00675	Putative defensin-like protein 238 (Protein SCRL16; SCR-like protein 16; Plant defensin)	Antifungal, Antimicrobial
1882	DRAMP00676	Putative defensin-like protein 239 (Protein SCRL17; SCR-like protein 17; Plant defensin)	Antifungal, Antimicrobial
1883	DRAMP00677	Putative defensin-like protein 240 (Protein SCRL18; SCR-like protein 18; Plant defensin)	Antifungal, Antimicrobial
1884	DRAMP00678	Defensin-like protein 241 (Protein SCRL19; SCR-like protein 19; Plant defensin)	Antifungal, Antimicrobial
1885	DRAMP00679	Defensin-like protein 242 (Protein SCRL10; SCR-like protein 10; Plant defensin)	Antifungal, Antimicrobial
1886	DRAMP00680	Putative defensin-like protein 243 (Protein SCRL9; SCR-like protein 9; Plant defensin)	Antifungal, Antimicrobial
1887	DRAMP00681	Putative defensin-like protein 244 (Protein SCRL11; SCR-like protein 11; Plant defensin)	Antifungal, Antimicrobial
1888	DRAMP00682	Defensin-like protein 245 (Protein SCRL4; SCR-like protein 4; Plant defensin)	Antifungal, Antimicrobial
1889	DRAMP00683	Defensin-like protein 246 (Protein SCRL5; SCR-like protein 5; Plant defensin)	Antifungal, Antimicrobial
1890	DRAMP00684	Defensin-like protein 247 (Protein SCRL6; SCR-like protein 6; Plant defensin)	Antifungal, Antimicrobial
1891	DRAMP00685	Defensin-like protein 249 (Protein SCRL7; SCR-like protein 7; Plant defensin)	Antifungal, Antimicrobial
1892	DRAMP00686	Defensin-like protein 250 (Protein SCRL8; CR-like protein 8; Plant defensin)	Antifungal, Antimicrobial

1893	DRAMP00687	Putative defensin-like protein 251 (Protein SCRL12; SCR-like protein 12; Plant defensin)	Antifungal, Antimicrobial
1894	DRAMP00688	Putative defensin-like protein 252 (Protein SCRL13; SCR-like protein 13; Plant defensin)	Antifungal, Antimicrobial
1895	DRAMP00689	Putative defensin-like protein 253 (Protein SCRL15; SCR-like protein 15; Plant defensin)	Antifungal, Antimicrobial
1896	DRAMP00690	Putative defensin-like protein 254 (Plant defensin)	Antifungal, Antimicrobial
1897	DRAMP00691	Defensin-like protein 255 (Plant defensin)	Antifungal, Antimicrobial
1898	DRAMP00692	Putative defensin-like protein 256 (Plant defensin)	Antifungal, Antimicrobial
1899	DRAMP00693	Putative defensin-like protein 257 (Plant defensin)	Antifungal, Antimicrobial
1900	DRAMP00694	Putative defensin-like protein 258 (Plant defensin)	Antifungal, Antimicrobial
1901	DRAMP00695	Defensin-like protein 259 (Plant defensin)	Antifungal, Antimicrobial
1902	DRAMP00696	Defensin-like protein 260 (Plant defensin)	Antifungal, Antimicrobial
1903	DRAMP00697	Putative defensin-like protein 262 (Plant defensin)	Antifungal, Antimicrobial
1904	DRAMP00698	Putative defensin-like protein 263 (Plant defensin)	Antifungal, Antimicrobial
1905	DRAMP00699	Putative defensin-like protein 264 (Plant defensin)	Antifungal, Antimicrobial
1906	DRAMP00700	Putative defensin-like protein 265 (Plant defensin)	Antifungal, Antimicrobial
1907	DRAMP00701	Defensin-like protein 267 (Plant defensin)	Antifungal, Antimicrobial
1908	DRAMP00702	Defensin-like protein 268 (Plant defensin)	Antifungal, Antimicrobial
1909	DRAMP00703	Defensin-like protein 266 (Plant defensin)	Antifungal, Antimicrobial
1910	DRAMP00704	Defensin-like protein 269 (Plant defensin)	Antifungal, Antimicrobial
1911	DRAMP00705	Putative defensin-like protein 270 (Plant defensin)	Antifungal, Antimicrobial
1912	DRAMP00706	Putative defensin-like protein 271 (Plant defensin)	Antifungal, Antimicrobial
1913	DRAMP00707	Defensin-like protein 272 (Plant defensin)	Antifungal, Antimicrobial
1914	DRAMP00708	Putative defensin-like protein 274 (Plant defensin)	Antifungal, Antimicrobial
1915	DRAMP00709	Defensin-like protein 275 (Plant defensin)	Antifungal, Antimicrobial
1916	DRAMP00710	Defensin-like protein 276 (Plant defensin)	Antifungal, Antimicrobial
1917	DRAMP00711	Putative defensin-like protein 277 (Plant defensin)	Antifungal, Antimicrobial
1918	DRAMP00712	Defensin-like protein 278 (Plant defensin)	Antifungal, Antimicrobial
1919	DRAMP00713	Putative defensin-like protein 279 (Plant defensin)	Antifungal, Antimicrobial
1920	DRAMP00714	Putative defensin-like protein 280 (Plant defensin)	Antifungal, Antimicrobial
1921	DRAMP00715	Defensin-like protein 281 (Plant defensin)	Antifungal, Antimicrobial
1922	DRAMP00716	Putative defensin-like protein 282 (Plant defensin)	Antifungal, Antimicrobial
1923	DRAMP00717	Putative defensin-like protein 283 (Plant defensin)	Antifungal, Antimicrobial
1924	DRAMP00718	Defensin-like protein 285 (Plant defensin)	Antifungal, Antimicrobial
1925	DRAMP00719	Defensin-like protein 286 (Plant defensin)	Antifungal, Antimicrobial
1926	DRAMP00720	Defensin-like protein 287 (Plant defensin)	Antifungal, Antimicrobial
1927	DRAMP00721	Putative defensin-like protein 288 (Plant defensin)	Antifungal, Antimicrobial
1928	DRAMP00722	Defensin-like protein 289 (Plant defensin)	Antifungal, Antimicrobial
1929	DRAMP00723	Defensin-like protein 291 (Plant defensin)	Antifungal, Antimicrobial
1930	DRAMP00724	Defensin-like protein 292 (Plant defensin; Uncertain)	Antifungal, Antimicrobial
1931	DRAMP00725	Defensin-like protein 293 (Plant defensin)	Antifungal, Antimicrobial
1932	DRAMP00726	Defensin-like protein 294 (Plant defensin)	Antifungal, Antimicrobial
1933	DRAMP00727	Defensin-like protein 295 (Plant defensin)	Antifungal, Antimicrobial
1934	DRAMP00728	Defensin-like protein 296 (Plant defensin)	Antifungal, Antimicrobial
1935	DRAMP00729	Putative defensin-like protein 298 (Plant defensin)	Antifungal, Antimicrobial
1936	DRAMP00730	Defensin-like protein 301 (Plant defensin)	Antifungal, Antimicrobial
1937	DRAMP00731	Defensin-like protein 302 (Plant defensin)	Antifungal, Antimicrobial
1938	DRAMP00732	Putative defensin-like protein 303 (Plant defensin)	Antifungal, Antimicrobial
1939	DRAMP00733	Putative defensin-like protein 304 (Plant defensin)	Antifungal, Antimicrobial
1940	DRAMP00734	Putative defensin-like protein 305 (Plant defensin)	Antifungal, Antimicrobial
1941	DRAMP00735	Defensin-like protein 306 (Plant defensin)	Antifungal, Antimicrobial
1942	DRAMP00736	Putative defensin-like protein 307 (Plant defensin)	Antifungal, Antimicrobial
1943	DRAMP00737	Defensin-like protein 308 (Plant defensin)	Antifungal, Antimicrobial
1944	DRAMP00738	Putative defensin-like protein 309 (Plant defensin)	Antifungal, Antimicrobial
1945	DRAMP00739	Defensin-like protein 311 (Plant defensin)	Antifungal, Antimicrobial
1946	DRAMP00740	Putative defensin-like protein 312 (Plant defensin)	Antifungal, Antimicrobial
1947	DRAMP00741	Defensin-like protein 316 (Plant defensin)	Antifungal, Antimicrobial

1948	DRAMP00742	Putative defensin-like protein 317 (Plant defensin)	Antifungal, Antimicrobial
1949	DRAMP00743	Defensin-like protein 313 (Plant defensin)	Antifungal, Antimicrobial
1950	DRAMP00744	Putative defensin-like protein 315 (Plant defensin)	Antifungal, Antimicrobial
1951	DRAMP00745	Floral defensin-like protein 2 (PhD2; Plant defensin)	Antifungal, Antimicrobial
1952	DRAMP00746	Flower-specific defensin (NaD1; Plant defensin)	Antifungal, Anti-Gram+, Anti-Gram-, Antimicrobial
1953	DRAMP00747	Defensin-like protein (Flower-specific gamma-thionin; Plant defensin)	Antifungal, Antimicrobial
1954	DRAMP00748	Defensin-like protein(Brazzein; Plants)	Antibacterial, Antifungal, Anti-Gram+, Antimicrobial
1955	DRAMP00749	Defensin-like protein 1 (Dm-AMP1; Plant defensin)	Antifungal, Anti-Gram+, Antimicrobial
1956	DRAMP00750	Defensin-like protein 2 (Dm-AMP2; Plant defensin)	Antifungal, Antimicrobial
1957	DRAMP00751	Defensin-2 (Plant defensin)	Antifungal, Antimicrobial
1958	DRAMP00752	PsDef1 (P. sylvestris defensin 1, p1; Plant defensin)	Antifungal, Antimicrobial
1959	DRAMP00753	Elicitor peptide 1 (AtPep1; Plant defensin)	Antifungal, Antimicrobial
1960	DRAMP00754	Elicitor peptide 2 (AtPep2; Plant defensin)	Antimicrobial,
1961	DRAMP00755	Elicitor peptide 3 (AtPep3; Plant defensin)	Antimicrobial,
1962	DRAMP00756	Elicitor peptide 4 (AtPep4; Plant defensin)	Antimicrobial,
1963	DRAMP00757	Elicitor peptide 5 (AtPep5; Plant defensin)	Antimicrobial,
1964	DRAMP00758	Elicitor peptide 6 (AtPep6; Plant defensin)	Antimicrobial,
1965	DRAMP00759	Elicitor peptide 7 (AtPep7; Plant defensin)	Antimicrobial,
1966	DRAMP00760	NmDef02 (Plants)	Antifungal, Antimicrobial
1967	DRAMP00762	Vaby D (Plant defensin)	Antibacterial, Antifungal, Antiviral, Antimicrobial
1968	DRAMP00763	Vaby A (Plant defensin)	Antibacterial, Antifungal, Antiviral, Antimicrobial
1969	DRAMP00767	ChaC1 (Chassatide C1; Plant defensin)	Antibacterial, Anticancer, Anti-Gram+, Anti-Gram-, Antimicrobial
1970	DRAMP00768	ChaC2 (Chassatide C2; Plant defensin)	Antibacterial, Anticancer, Anti-Gram+, Anti-Gram-, Antimicrobial
1971	DRAMP00769	ChaC4 (Chassatide C4; Plant defensin)	Antibacterial, Anticancer, Anti-Gram+, Anti-Gram-, Antimicrobial
1972	DRAMP00770	ChaC10 (Chassatide C10; Plant defensin)	Antibacterial, Anticancer, Anti-Gram+, Anti-Gram-, Antimicrobial
1973	DRAMP18326	Bovicin 255(Bacteriocin)	Antibacterial, Anti-Gram+, Antimicrobial
1974	DRAMP00792	Vodo peptide N (Plant defensin)	Antimicrobial,
1975	DRAMP00793	Vodo peptide M (Plant defensin)	Antimicrobial,
1976	DRAMP18325	delta-lysin I (Bacteriocin)	Antibacterial, Anti-Gram+, Anti-Gram-, Antimicrobial
1977	DRAMP00796	Cliotide T2 (cT2; Plant defensin)	Antibacterial, Anticancer, Anti-Gram+, Anti-Gram-, Antimicrobial
1978	DRAMP00797	Cliotide T3 (cT3; Plant defensin)	Antibacterial, Anticancer, Anti-Gram+, Anti-Gram-, Antimicrobial
1979	DRAMP00799	Cycloviolin-A (Plant defensin)	Antiviral, Antimicrobial
1980	DRAMP00800	Cycloviolin-B (Plant defensin)	Antiviral, Antimicrobial
1981	DRAMP00801	Cycloviolin-C (Plant defensin)	Antiviral, Antimicrobial
1982	DRAMP00802	Cycloviolin-D (Plant defensin)	Antiviral, Antimicrobial
1983	DRAMP00803	Palicourein (Cyclotides; Plants)	Antiviral, Antimicrobial
1984	DRAMP18324	Warnericin RK (Bacteriocin)	Antibacterial, Anti-Gram-, Antimicrobial
1985	DRAMP18196	Esculentin-1A	Antibacterial,Antibiotic, Antimicrobial
1986	DRAMP00829	Cycloviolacin-H1 (Plant defensin)	Antimicrobial,
1987	DRAMP00830	Cycloviolacin-H2 (Plant defensin)	Antiviral, Antimicrobial
1988	DRAMP00831	Cycloviolacin-H3 (Plant defensin)	Antiviral, Antimicrobial
1989	DRAMP00832	Cycloviolacin-H4 (Plant defensin)	Antiviral, Antimicrobial
1990	DRAMP00833	Cycloviolacin-Y1 (Plants)	Antiviral, Antimicrobial
1991	DRAMP00834	Cycloviolacin-Y2 (Plants)	Antiviral, Antimicrobial
1992	DRAMP00835	Cycloviolacin-Y3 (Plants)	Antiviral, Antimicrobial
1993	DRAMP00836	Cycloviolacin-Y4 (Plants)	Antiviral, Antimicrobial
1994	DRAMP00837	Cycloviolacin-Y5 (Plants)	Antiviral, Antimicrobial
1995	DRAMP00838	Cyclotide phyb-A (Plant defensin)	Antimicrobial,
1996	DRAMP00839	Cyclotide cter-A (Plant defensin)	Antimicrobial,
1997	DRAMP00840	Cyclotide cter-B (Plant defensin)	Antimicrobial,
1998	DRAMP00841	Cyclotide cter-C (Plant defensin)	Antimicrobial,
1999	DRAMP00842	Cyclotide cter-D (Plant defensin)	Antimicrobial,

2000	DRAMP00843	Cyclotide cter-E (Plant defensin)	Antimicrobial,
2001	DRAMP00844	Cyclotide cter-F (Plant defensin)	Antimicrobial,
2002	DRAMP00845	Cyclotide cter-G (Plant defensin)	Antimicrobial,
2003	DRAMP00846	Cyclotide cter-H (Plant defensin)	Antimicrobial,
2004	DRAMP00847	Cyclotide cter-I (Plant defensin)	Antimicrobial,
2005	DRAMP00848	Cyclotide cter-J (Plant defensin)	Antimicrobial,
2006	DRAMP00849	Cyclotide cter-K (Plant defensin)	Antimicrobial,
2007	DRAMP00850	Cyclotide cter-L (Plant defensin)	Antimicrobial,
2008	DRAMP00851	Cyclotide cter-O (Plant defensin)	Antimicrobial,
2009	DRAMP18323	Nukacin ISK-1(Bacteriocin)	Antibacterial, Anti-Gram+, Antimicrobial
2010	DRAMP00853	Cyclotide cter-Q (Plant defensin)	Antimicrobial,
2011	DRAMP00854	Cyclotide cter-R (Plant defensin)	Antimicrobial,
2012	DRAMP00855	Cyclotide cter-N (Plant defensin)	Antimicrobial,
2013	DRAMP00859	Kalata-B4 (Plant defensin)	Antimicrobial,
2014	DRAMP00860	Kalata-B5 (Plant defensin)	Antimicrobial,
2015	DRAMP00862	Kalata-B7 (Plant defensin)	Antimicrobial,
2016	DRAMP00863	Kalata-B8 (Plant defensin)	Antiviral, Antimicrobial
2017	DRAMP00864	Kalata-B9 (Plant defensin)	Antimicrobial,
2018	DRAMP00865	Vicilin-like Antimicrobial peptide 2a (MiAMP2a; Plant defensin)	Antibacterial, Antifungal, Anti-Gram+, Antimicrobial
2019	DRAMP00866	Kalata-B10 (Plant defensin)	Antimicrobial,
2020	DRAMP00867	Kalata-B11 (Plant defensin)	Antimicrobial,
2021	DRAMP00868	Kalata-B12 (Plant defensin)	Antimicrobial,
2022	DRAMP00869	Kalata-B13 (Plant defensin)	Antimicrobial,
2023	DRAMP00870	Kalata-B14 (Plant defensin)	Antimicrobial,
2024	DRAMP00871	Kalata-B15 (Plant defensin)	Antimicrobial,
2025	DRAMP00872	Kalata-B16 (Plant defensin)	Antimicrobial,
2026	DRAMP00873	Kalata-B17 (Plant defensin)	Antimicrobial,
2027	DRAMP00875	Leaf cyclotide 1 (Vhl-1; Plant defensin)	Antiviral, Antimicrobial
2028	DRAMP00876	Leaf cyclotide 2 (Vhl-2; Plant defensin)	Antiviral, Antimicrobial
2029	DRAMP00879	Circulin-C (CIRC; Plant defensin)	Antiviral, Antimicrobial
2030	DRAMP00880	Circulin-D (CIRD; Plant defensin)	Antiviral, Antimicrobial
2031	DRAMP00881	Circulin-E (CIRE; Plant defensin)	Antiviral, Antimicrobial
2032	DRAMP00882	Circulin-F (CIRF; Plant defensin)	Antiviral, Antimicrobial
2033	DRAMP00883	Cyclotide hypa-A (hypa-A; Plant defensin)	Antimicrobial,
2034	DRAMP00884	Cyclotide hyfl-A (Plant defensin)	Antimicrobial,
2035	DRAMP00885	Cyclotide hyfl-B (Plant defensin)	Antimicrobial,
2036	DRAMP00886	Cyclotide hyfl-C (Plant defensin)	Antimicrobial,
2037	DRAMP00887	Cyclotide hyfl-D (Plant defensin)	Antimicrobial,
2038	DRAMP00888	Cyclotide hyfl-E (Plant defensin)	Antimicrobial,
2039	DRAMP00889	Cyclotide hyfl-F (Plant defensin)	Antimicrobial,
2040	DRAMP00890	Cyclotide hyfl-G (Plant defensin)	Antimicrobial,
2041	DRAMP00891	Cyclotide hyfl-H (Plant defensin)	Antimicrobial,
2042	DRAMP00892	Cyclotide hyfl-I (Plant defensin)	Antimicrobial,
2043	DRAMP00893	Cyclotide hyfl-J (Plant defensin)	Antimicrobial,
2044	DRAMP00894	Cyclotide hyfl-K (Plant defensin)	Antimicrobial,
2045	DRAMP00895	Cyclotide hyfl-L (Plant defensin)	Antimicrobial,
2046	DRAMP00896	Cyclotide hyfl-M (Plant defensin)	Antimicrobial,
2047	DRAMP00897	Cyclotide hyfl-N (Plant defensin)	Antimicrobial,
2048	DRAMP00898	Cyclotide hyfl-O (Plant defensin)	Antimicrobial,
2049	DRAMP00899	Cyclotide hyfl-P (Plant defensin)	Antimicrobial,
2050	DRAMP00910	Tricyclon-A (Cyclotides; Plant defensin)	Antimicrobial, Cytolysis,
2051	DRAMP00911	Tricyclon-B (Plant defensin)	Antimicrobial,
2052	DRAMP00912	Root cyclotide 1 (Vhr1; Plant defensin)	Antimicrobial,
2053	DRAMP00913	Cyclotide vibi-A (Plant defensin)	Antimicrobial, Cytotoxicity,
2054	DRAMP00914	Cyclotide vibi-B (Plant defensin)	Antimicrobial, Cytotoxicity,
2055	DRAMP00915	Cyclotide vibi-C (Plant defensin)	Antimicrobial, Cytotoxicity,

2056	DRAMP00916	Cyclotide vibi-D (Plant defensin)	Antimicrobial, Cytotoxicity,
2057	DRAMP00917	Cyclotide vibi-E (Vbc1; Plant defensin)	Antimicrobial, Cytotoxicity,
2058	DRAMP00918	Cyclotide vibi-F (Plant defensin)	Antimicrobial, Cytotoxicity,
2059	DRAMP00919	Cyclotide vibi-G (Plant defensin)	Antimicrobial, Cytotoxicity,
2060	DRAMP00920	Cyclotide vibi-H (Plant defensin)	Antimicrobial, Cytotoxicity,
2061	DRAMP00921	Cyclotide vibi-I (Vbc2; Plant defensin)	Antimicrobial, Cytotoxicity,
2062	DRAMP00922	Cyclotide vibi-J (Vbc3; Plant defensin)	Antimicrobial, Cytotoxicity,
2063	DRAMP00923	Cyclotide vibi-K (Vbc4; Plant defensin)	Antimicrobial, Cytotoxicity,
2064	DRAMP00925	Cyclopeptide F (Plant defensin)	Antimicrobial, Cytotoxicity,
2065	DRAMP00926	Violacin-A (Violacin-1; linearized uncyclotides; Plant defensin)	Antimicrobial,
2066	DRAMP00927	Cyclotide vico-A (Plant defensin)	Antimicrobial,
2067	DRAMP00928	Cyclotide vico-B (Plant defensin)	Antimicrobial,
2068	DRAMP00932	Antimicrobial peptide 1 (PMAP1; Plant defensin)	Antibacterial, Antifungal, Antimicrobial
2069	DRAMP00934	Vv-AMP1 (Vitis vinifera antimicrobial peptide 1; Plant defensin)	Antifungal, Antimicrobial
2070	DRAMP00936	Ribosome-inactivating protein TAP-29 (rRNA N-glycosidase; Plant defensin)	Antiviral, Cytotoxicity, Antimicrobial
2071	DRAMP00937	Tu-AMP1 (Plant defensin)	Antibacterial, Antifungal, Anti-Gram+, Anti-Gram-, Antimicrobial
2072	DRAMP00938	Tu-AMP2 (Plant defensin)	Antibacterial, Antifungal, Anti-Gram+, Anti-Gram-, Antimicrobial
2073	DRAMP00946	Thionin-like protein 2 (Plant defensin)	Antimicrobial,
2074	DRAMP00952	Viscotoxin A1 (Plant defensin)	Antibacterial, Antifungal, Antiviral, Antimicrobial
2075	DRAMP00954	Viscotoxin-A3 (Plant defensin)	Antifungal, Cytotoxicity, Antimicrobial
2076	DRAMP00955	Viscotoxin-B (VtB; Plant defensin)	Antifungal, Cytotoxicity, Antimicrobial
2077	DRAMP00957	Pp-AMP1 (P. pubescens AMP1; Plant defensin)	Antibacterial, Antifungal, Anti-Gram+, Anti-Gram-, Antimicrobial
2078	DRAMP00958	Pp-AMP2 (P. pubescens AMP2; Plant defensin)	Antibacterial, Antifungal, Anti-Gram+, Anti-Gram-, Antimicrobial
2079	DRAMP00962	Pseudothionin Solanum tuberosum 1 (Pth-St1; Plant defensin)	Antibacterial, Antimicrobial
2080	DRAMP18321	Epidermicin NI01(Bacteriocin)	Antibacterial, Anti-Gram+, Antimicrobial
2081	DRAMP01380	Odorranain-J1 (OdJ1; Frogs, amphibians, animals)	Antimicrobial, Antibacterial, Antifungal, Anti-Gram+, Anti-Gram-,
2082	DRAMP00968	Leaf-specific thionin BTH6 (Plant defensin)	Antifungal, Antimicrobial
2083	DRAMP00969	Alpha-hordothionin (alpha-HT; Plant defensin)	Antibacterial, Antifungal, Antimicrobial
2084	DRAMP00970	Beta-hordothionin (beta-HT; Plant defensin)	Antifungal, Antimicrobial
2085	DRAMP00975	SI alpha-2 (SIa2; Plant defensin)	Antifungal, Antimicrobial
2086	DRAMP00976	Antimicrobial peptide 1 (AC-AMP1; Plant defensin)	Antibacterial, Antifungal, Anti-Gram+, Antimicrobial
2087	DRAMP00977	Antimicrobial peptide 2 (AC-AMP2; Plant defensin)	Antibacterial, Antifungal, Anti-Gram+, Antimicrobial
2088	DRAMP00978	Antifungal peptide 1 (EAFP1; Plant defensin)	Antifungal, Antimicrobial
2089	DRAMP00979	Antifungal peptide 2 (EAFP2; Plant defensin)	Antifungal, Antimicrobial
2090	DRAMP00980	Antimicrobial peptide 1a (WAMP-1a; Plant defensin)	Antibacterial, Antifungal, Anti-Gram+, Anti-Gram-, Antimicrobial
2091	DRAMP00981	Antimicrobial peptide 1b (WAMP-1b; Plant defensin)	Antibacterial, Antifungal, Anti-Gram+, Anti-Gram-, Antimicrobial
2092	DRAMP00982	Fa-AMP1 (Fagopyrum antimicrobial peptide 1; hevein-type; Plant defensin)	Antibacterial, Antifungal, Anti-Gram+, Anti-Gram-, Antimicrobial
2093	DRAMP00983	Fa-AMP2 (Fagopyrum antimicrobial peptide 2; hevein-type; Plant defensin)	Antibacterial, Antifungal, Anti-Gram+, Anti-Gram-, Antimicrobial
2094	DRAMP00984	Antimicrobial peptide Ar-AMP (Ar-AMP; hevin-like peptides; Plant defensin)	Antibacterial, Antifungal, Anti-Gram+, Antimicrobial
2095	DRAMP00985	Pn-AMP1 (PnAMP1; Plant defensin)	Antibacterial, Antifungal, Anti-Gram+, Antimicrobial
2096	DRAMP00986	Pn-AMP2 (PnAMP2; Plant defensin)	Antifungal, Anti-Gram+, Antimicrobial
2097	DRAMP00987	Ee-CBPb (Hevein-like antimicrobial peptide; Plants)	Antibacterial, Antifungal, Anti-Gram+, Antimicrobial
2098	DRAMP00988	Ee-CBPl (Hevein-like antimicrobial peptide; Plants)	Antifungal, Antimicrobial
2099	DRAMP00989	Hevein (Hev b 6; Plants)	Antifungal, Antimicrobial
2100	DRAMP00990	Sm-AMP-D1 (Plant defensin)	Antifungal, Antimicrobial
2101	DRAMP00991	Pseudo-hevein (Minor hevein; hevein-like peptides; Plants)	Antifungal, Antimicrobial
2102	DRAMP00992	Sm-AMP-D2 (Plant defensin)	Antifungal, Antimicrobial
2103	DRAMP00993	WjAMP-1 (C-terminal domain of hevein; Plant defensin)	Antibacterial, Antifungal, Anti-Gram-, Antimicrobial

2104	DRAMP00994	IB-AMP1 (IBAMP1; Basic peptide AMP1; Plants)	Antibacterial, Antifungal, Anti-Gram+, Antimicrobial
2105	DRAMP00995	IB-AMP2 (IBAMP2; Basic peptide AMP2; Plants)	Antifungal, Antimicrobial
2106	DRAMP00996	IB-AMP3 (IBAMP3; Basic peptide AMP3; Plants)	Antifungal, Antimicrobial
2107	DRAMP00997	IB-AMP4 (IBAMP4; Basic peptide AMP4; Plants)	Antibacterial, Antifungal, Anti-Gram+, Anti-Gram-, Antimicrobial
2108	DRAMP00998	Antimicrobial peptide MBP-1 (Maize Basic Peptide 1; Plant defensin)	Antibacterial, Antifungal, Anti-Gram+, Anti-Gram-, Antimicrobial
2109	DRAMP00999	Plectasin (fungal defensin)	Antibacterial, Antifungal, Anti-Gram+, Antimicrobial
2110	DRAMP01000	Ascalin (Plants)	Antifungal, Antiviral, Antimicrobial
2111	DRAMP01001	Arietin (Plants)	Antifungal, Antimicrobial
2112	DRAMP01002	Alpha-benincasin (Plants)	Antifungal, Antimicrobial
2113	DRAMP01003	Gymnin (Plants)	Antifungal, Antiviral, Antimicrobial
2114	DRAMP01005	Cicerarin (Plant defensin)	Antifungal, Antiviral, Antimicrobial
2115	DRAMP01006	Cicerin (Plant defensin)	Antifungal, Antimicrobial
2116	DRAMP01007	Non-specific lipid transfer peptide (LTP 1; nsLTP; Plants)	Antibacterial, Antifungal, Anti-Gram+, Antimicrobial
2117	DRAMP01008	Antifungal lectin PVAP (Plant defensin)	Antifungal, Antimicrobial
2118	DRAMP01009	Alliumin (Plants)	Antibacterial, Antifungal, Anti-Gram-, Antimicrobial
2119	DRAMP01010	Lunatusin (Plants)	Antibacterial, Antifungal, Antiviral, Anti-Gram+, Anti-Gram-, Antimicrobial
2120	DRAMP01011	White cloud bean defensin (Plant defensin)	Antibacterial, Antifungal, Antimicrobial
2121	DRAMP01013	PvD1 (P. vulgaris defensin 1; Plants)	Antifungal, Antimicrobial
2122	DRAMP01014	VrD1 (V.radiata defensin 1; Plants)	Antifungal, Antimicrobial
2123	DRAMP01015	VaD1 (Plant defensin)	Antibacterial, Antifungal, Anti-Gram+, Anti-Gram-, Antimicrobial
2124	DRAMP01019	VrD2 (Vigna radiata defensin 2; plant defensin)	Antibacterial, Antifungal, Antimicrobial
2125	DRAMP01020	SIalpha1 (Plant defensin)	Antibacterial, Antimicrobial
2126	DRAMP01022	Cy-AMP1 (Plant defensin)	Antibacterial, Antifungal, Anti-Gram+, Anti-Gram-, Antimicrobial
2127	DRAMP01023	Cy-AMP2 (Plant defensin)	Antibacterial, Antifungal, Anti-Gram+, Anti-Gram-, Antimicrobial
2128	DRAMP01024	Cy-AMP3 (Plant defensin)	Antibacterial, Antifungal, Anti-Gram+, Anti-Gram-, Antimicrobial
2129	DRAMP01025	Vicilin-like Antimicrobial peptide 2a (MiAMP2a; Plant defensin)	Antibacterial, Antifungal, Anti-Gram+, Antimicrobial
2130	DRAMP01026	Vicilin-like Antimicrobial peptide 2b (MiAMP2b; Plant defensin)	Antibacterial, Antifungal, Anti-Gram+, Antimicrobial
2131	DRAMP01027	MiAMP2c (Gln- and Glu-rich; Plant defensin)	Antifungal, Antimicrobial
2132	DRAMP01028	Vicilin-like Antimicrobial peptide 2c-3 (MiAMP2c-3; Plant defensin)	Antibacterial, Antifungal, Anti-Gram+, Antimicrobial
2133	DRAMP01029	Vicilin-like Antimicrobial peptide 2c-2 (MiAMP2c-2; Plant defensin)	Antibacterial, Antifungal, Anti-Gram+, Antimicrobial
2134	DRAMP01030	Vicilin-like Antimicrobial peptide 2c-1 (MiAMP2c-1; Plant defensin)	Antibacterial, Antifungal, Anti-Gram+, Antimicrobial
2135	DRAMP01031	Vicilin-like Antimicrobial peptide 2d (MiAMP2d; Plant defensin)fens	Antibacterial, Antifungal, Anti-Gram+, Antimicrobial
2136	DRAMP01032	Vicilin-like Antimicrobial peptide 2b (MiAMP2b; Plant defensin)	Antibacterial, Antifungal, Anti-Gram+, Antimicrobial
2137	DRAMP01033	Vicilin-like Antimicrobial peptide 2c-3 (MiAMP2c-3; Plant defensin)	Antibacterial, Antifungal, Anti-Gram+, Antimicrobial
2138	DRAMP01034	Vicilin-like Antimicrobial peptide 2c-2 (MiAMP2c-2; Plant defensin)	Antibacterial, Antifungal, Anti-Gram+, Antimicrobial
2139	DRAMP01035	Vicilin-like Antimicrobial peptide 2c-1 (MiAMP2c-1; Plant defensin)	Antibacterial, Antifungal, Anti-Gram+, Antimicrobial
2140	DRAMP01036	Vicilin-like Antimicrobial peptide 2d (MiAMP2d; Plant defensin)	Antibacterial, Antifungal, Anti-Gram+, Antimicrobial
2141	DRAMP01037	Vicilin-like Antimicrobial peptide 2b (MiAMP2b; Plant defensin)	Antibacterial, Antifungal, Anti-Gram+, Antimicrobial
2142	DRAMP01038	Vicilin-like Antimicrobial peptide 2c-3 (MiAMP2c-3; Plant defensin)	Antibacterial, Antifungal, Anti-Gram+, Antimicrobial
2143	DRAMP01039	Vicilin-like Antimicrobial peptide 2c-2 (MiAMP2c-2; Plant defensin)	Antibacterial, Antifungal, Anti-Gram+, Antimicrobial

2144	DRAMP01040	Vicilin-like Antimicrobial peptide 2c-1 (MiAMP2c-1; Plant defensin)	Antibacterial, Antifungal, Anti-Gram+, Antimicrobial
2145	DRAMP01041	Vicilin-like Antimicrobial peptide 2d (MiAMP2d; Plant defensin)	Antibacterial, Antifungal, Anti-Gram+, Antimicrobial
2146	DRAMP01043	TPP3 (Plant defensin)	Antifungal, Antimicrobial
2147	DRAMP01044	Defensin-like protein (Clone PSAS10; Plant defensin)	Antifungal, Antimicrobial
2148	DRAMP01045	IWF2 (Bv-LTP2; Plant defensin)	Antifungal, Antimicrobial
2149	DRAMP01046	Shepherin I (fragement of shep-GRP; Plants)	Antibacterial, Antifungal, Anti-Gram-, Antimicrobial
2150	DRAMP01047	shepherin II (fragement of shep-GRP; Plants)	Antibacterial, Antifungal, Anti-Gram-, Antimicrobial
2151	DRAMP01048	Sd5 (sugarcane defensin 5; Plant defensin)	Antifungal, Antimicrobial
2152	DRAMP01049	Sd3 (sugarcane defensin 3; Plant defensin)	Antifungal, Antimicrobial
2153	DRAMP01050	Sd1 (sugarcane defensin 1; Plant defensin)	Antifungal, Antimicrobial
2154	DRAMP01051	NpThio1 (Plant defensin)	Antifungal, Antimicrobial
2155	DRAMP01052	NeThio2 (Plant defensin)	Antifungal, Antimicrobial
2156	DRAMP01053	Thaumatin-like protein (CdTLP; Plants)	Antifungal, Antimicrobial
2157	DRAMP01054	Antimicrobial protein Ace-AMP1 (Ace-AMP1; Plant defensin)	Antibacterial, Antifungal, Anti-Gram+, Antimicrobial
2158	DRAMP01055	P. americana AMP (Pa-AMP-1; PAFP-S; Cys-rich; Plant defensin)	Antibacterial, Antifungal, Anti-Gram+, Antimicrobial
2159	DRAMP01056	Ha-DEF1 (H. annuus defensin 1; Plants)	Antifungal, Antimicrobial
2160	DRAMP01057	30 kDa antifungal protein (Plants)	Antifungal, Antimicrobial
2161	DRAMP01058	Antifungal protein (Plants)	Antifungal, Antimicrobial
2162	DRAMP01059	Putative antimicrobial protein 1 (Plants)	Antimicrobial,
2163	DRAMP01060	Putative antimicrobial protein 2 (Plants)	Antimicrobial,
2164	DRAMP01062	Putative antimicrobial protein 3 (Cm-p2; Plants)	Antifungal, Antimicrobial
2165	DRAMP01065	Chitin-binding protein HM30 (Plants)	Antifungal, Antimicrobial
2166	DRAMP01067	Coccinin (Plant defensin)	Antifungal, Antiviral, Antimicrobial
2167	DRAMP01068	Non-specific lipid-transfer protein (Plant defensin)	Antimicrobial,
2168	DRAMP01069	Putative plant defensin SPI1B (Plants)	Antimicrobial,
2169	DRAMP01070	Non-specific lipid-transfer protein (Plants)	Antifungal, Antimicrobial
2170	DRAMP01071	Non-specific lipid-transfer protein (Plants)	Antimicrobial,
2171	DRAMP01072	Non-specific lipid-transfer protein (Plants)	Antimicrobial,
2172	DRAMP01073	Non-specific lipid-transfer protein (Plants)	Antimicrobial,
2173	DRAMP01074	Non-specific lipid-transfer protein (Plants)	Antimicrobial,
2174	DRAMP01075	Non-specific lipid-transfer protein (Plants)	Antimicrobial,
2175	DRAMP01076	Non-specific lipid-transfer protein (Plants)	Antimicrobial,
2176	DRAMP01077	Non-specific lipid-transfer protein (Plants)	Antimicrobial,
2177	DRAMP01078	Non-specific lipid-transfer protein (Plants)	Antimicrobial,
2178	DRAMP01079	Non-specific lipid-transfer protein (Plants)	Antimicrobial,
2179	DRAMP01080	Non-specific lipid-transfer protein (Plants)	Antimicrobial,
2180	DRAMP01084	Alyteserin-2b (toads, amphibians, animals)	Antibacterial, Antifungal, Antiviral, Antimicrobial
2181	DRAMP01086	Alyteserin-2c (toads, amphibians, animals)	Antibacterial, Antifungal, Antiviral, Antimicrobial
2182	DRAMP01087	Alyteserin-1d (toads, amphibians, animals)	Antibacterial, Antifungal, Antiviral, Antimicrobial
2183	DRAMP01092	Alytesin (toads, amphibians, animals)	Antimicrobial,
2184	DRAMP01093	Bombinin GH-1L (bombinin H isomers; toads, amphibians, animals)	Antibacterial, Antimicrobial
2185	DRAMP01095	Bombinin-like peptide 2 (Contains: Bombinin H2; toads, amphibians, animals)	Antibacterial, Antimicrobial
2186	DRAMP01099	Bombinin-like peptide 4 (BLP-4; toads, amphibians, animals)	Antibacterial, Anti-Gram-, Antimicrobial
2187	DRAMP01100	Bombinin-like peptide 1 (Contains: Bombinin H; toads, amphibians, animals)	Antibacterial, Anti-Gram+, Anti-Gram-, Antimicrobial
2188	DRAMP01101	Maximin-Ht (Maximin-7; toads, amphibians, animals)	Antibacterial, Antimicrobial
2189	DRAMP01102	Maximin-Hu (Maximin-8; toads, amphibians, animals)	Antibacterial, Antimicrobial
2190	DRAMP01103	Maximin-S2 (chain of Maximins-S type B/C; toads, amphibians, animals)	Antibacterial, Antimicrobial
2191	DRAMP01104	Maximin-S3 (chain of Maximins-S type B/C; toads, amphibians, animals)	Antibacterial, Antimicrobial
2192	DRAMP01106	Maximin-S5 (chain of Maximins-S type B/C; toads, amphibians, animals)	Antibacterial, Antimicrobial
2193	DRAMP01112	Maximin-11 (Maximin-6; toads, amphibians, animals)	Antibacterial, Antifungal, Antimicrobial

2194	DRAMP01113	Maximin-7 (toads, amphibians, animals)	Antibacterial, Antifungal, Antimicrobial
2195	DRAMP01114	Maximin-8 (toads, amphibians, animals)	Antibacterial, Antifungal, Antimicrobial
2196	DRAMP01115	Maximin-9 (toads, amphibians, animals)	Antibacterial, Antifungal, Antimicrobial
2197	DRAMP01116	Maximin-10 (toads, amphibians, animals)	Antibacterial, Antifungal, Antimicrobial
2198	DRAMP01117	Maximin-11 (toads, amphibians, animals)	Antibacterial, Antifungal, Antimicrobial
2199	DRAMP01118	Maximin y type 2 (toads, amphibians, animals)	Antibacterial, Antifungal, Antimicrobial
2200	DRAMP01119	Maximin-y (toads, amphibians, animals)	Antibacterial, Antifungal, Antimicrobial
2201	DRAMP01120	Maximin-Hv (toads, amphibians, animals)	Antibacterial, Antifungal, Antimicrobial
2202	DRAMP01121	Maximin-Hw (toads, amphibians, animals)	Antibacterial, Antifungal, Antimicrobial
2203	DRAMP01122	Maximin-z (toads, amphibians, animals)	Antibacterial, Antifungal, Antimicrobial
2204	DRAMP01127	Maximin-H5 (toads, amphibians, animals)	Antibacterial, Anti-Gram+, Antimicrobial
2205	DRAMP01128	Maximin-H6 (toads, amphibians, animals)	Antibacterial, Antifungal, Antimicrobial
2206	DRAMP01129	Maximin-H7 (toads, amphibians, animals)	Antibacterial, Antifungal, Antimicrobial
2207	DRAMP01130	Maximin-H8 (toads, amphibians, animals)	Antibacterial, Antifungal, Antimicrobial
2208	DRAMP01131	Maximin-H9 (toads, amphibians, animals)	Antibacterial, Antifungal, Antimicrobial
2209	DRAMP01132	Maximin-H10 (toads, amphibians, animals)	Antibacterial, Antifungal, Antimicrobial
2210	DRAMP01133	Maximin-H11 (toads, amphibians, animals)	Antibacterial, Antifungal, Antimicrobial
2211	DRAMP01134	Maximin-H13 (toads, amphibians, animals)	Antibacterial, Antifungal, Antimicrobial
2212	DRAMP01135	Maximin-H12 (toads, amphibians, animals)	Antibacterial, Antifungal, Antimicrobial
2213	DRAMP01136	Maximin-H14 (toads, amphibians, animals)	Antibacterial, Antifungal, Antimicrobial
2214	DRAMP01137	Maximin-H15 (toads, amphibians, animals)	Antibacterial, Antifungal, Antimicrobial
2215	DRAMP01138	Maximin-H16 (toads, amphibians, animals)	Antibacterial, Antifungal, Antimicrobial
2216	DRAMP01139	Uperin-2.1 (toads, amphibians, animals)	Antibacterial, Antimicrobial
2217	DRAMP01140	Uperin-2.2 (toads, amphibians, animals)	Antibacterial, Anti-Gram+, Anti-Gram-, Antimicrobial
2218	DRAMP01141	Uperin-2.3 (toads, amphibians, animals)	Antibacterial, Anti-Gram+, Antimicrobial
2219	DRAMP01142	Uperin-2.4 (toads, amphibians, animals)	Antibacterial, Anti-Gram+, Antimicrobial
2220	DRAMP01143	Uperin-2.5 (toads, amphibians, animals)	Antibacterial, Anti-Gram+, Antimicrobial
2221	DRAMP01144	Uperin-2.6 (toads, amphibians, animals)	Antibacterial, Antimicrobial
2222	DRAMP01145	Uperin-2.7 (toads, amphibians, animals)	Antibacterial, Antimicrobial
2223	DRAMP01146	Uperin-2.8 (toads, amphibians, animals)	Antibacterial, Anti-Gram+, Antimicrobial
2224	DRAMP01147	Uperin-3.1 (toads, amphibians, animals)	Antibacterial, Anti-Gram+, Antimicrobial
2225	DRAMP01148	Uperin-3.2 (toads, amphibians, animals)	Antibacterial, Antimicrobial
2226	DRAMP01149	Uperin-3.3 (toads, amphibians, animals)	Antibacterial, Antimicrobial
2227	DRAMP01150	Uperin-3.4 (toads, amphibians, animals)	Antibacterial, Antimicrobial
2228	DRAMP01156	Uperin-3.7 (toads, amphibians, animals)	Antibacterial, Antimicrobial
2229	DRAMP01157	Uperin-4.1 (toads, amphibians, animals)	Antibacterial, Anti-Gram+, Antimicrobial
2230	DRAMP01158	Uperin-5.1 (toads, amphibians, animals)	Antimicrobial,
2231	DRAMP01159	Uperin-6.1 (toads, amphibians, animals)	Antimicrobial,
2232	DRAMP01160	Uperin-6.2 (toads, amphibians, animals)	Antimicrobial,
2233	DRAMP01161	Uperin-7.1 (Frogs, amphibians, animals)	Antibacterial, Antiviral, Anti-Gram+, Antimicrobial
2234	DRAMP01166	Preprotemporin-1SKd (Frogs, amphibians, animals)	Antimicrobial,
2235	DRAMP01168	Ranakinin-N (bradykinin-like peptide; Frogs, amphibians, animals)	Antimicrobial,
2236	DRAMP01169	Distinctin 1 (Frogs, amphibians, animals)	Antimicrobial,
2237	DRAMP01171	Ocellatin-1 (Frogs, amphibians, animals)	Antibacterial, Anti-Gram-, Antimicrobial
2238	DRAMP01172	Ocellatin-2 (Frogs, amphibians, animals)	Antibacterial, Anti-Gram-, Antimicrobial
2239	DRAMP01173	Ocellatin-3 (Frogs, amphibians, animals)	Antibacterial, Anti-Gram-, Antimicrobial
2240	DRAMP01175	Ocellatin-5 (Frogs, amphibians, animals)	Antibacterial, Anti-Gram-, Antimicrobial
2241	DRAMP01176	Ocellatin-6 (Frogs, amphibians, animals)	Antibacterial, Antimicrobial
2242	DRAMP01178	Ocellatin-K1 (Frogs, amphibians, animals)	Antibacterial, Antimicrobial
2243	DRAMP01179	Ocellatin-V1 (Frogs, amphibians, animals)	Antibacterial, Antimicrobial
2244	DRAMP01180	Ocellatin-V2 (Frogs, amphibians, animals)	Antibacterial, Antimicrobial
2245	DRAMP01181	Ocellatin-V3 (Frogs, amphibians, animals)	Antibacterial, Antimicrobial
2246	DRAMP01183	Syphaxin (SPX; Frogs, amphibians, animals)	Antibacterial, Antimicrobial
2247	DRAMP01186	Chensinin-1CEb (Frogs, amphibians, animals)	Antibacterial, Antimicrobial
2248	DRAMP01187	Chensinin-3CE (Frogs, amphibians, animals)	Antibacterial, Antimicrobial
2249	DRAMP01193	Andersonin-Y2 (Frogs, amphibians, animals)	Antibacterial, Antifungal, Antimicrobial

2250	DRAMP01196	Andersonin-G1 (Frogs, amphibians, animals)	Antibacterial, Antifungal, Anti-Gram+, Anti-Gram-, Antimicrobial
2251	DRAMP01197	Andersonin-N1 (Frogs, amphibians, animals)	Antibacterial, Antifungal, Anti-Gram+, Anti-Gram-, Antimicrobial
2252	DRAMP01198	Andersonin-Q1 (Frogs, amphibians, animals)	Antibacterial, Antifungal, Anti-Gram+, Anti-Gram-, Antimicrobial
2253	DRAMP01205	Dahlein-1.1 (Frogs, amphibians, animals)	Antibacterial, Antimicrobial
2254	DRAMP01206	Dahlein-1.2 (Frogs, amphibians, animals)	Antibacterial, Antimicrobial
2255	DRAMP01207	Galensin (Frogs, amphibians, animals)	Antibacterial, Anti-Gram+, Anti-Gram-, Antimicrobial
2256	DRAMP01210	Pleurain-D1 antimicrobial peptide (Frogs, amphibians, animals)	Antimicrobial,
2257	DRAMP01211	Pleurain-D2 antimicrobial peptide (Frogs, amphibians, animals)	Antimicrobial,
2258	DRAMP01212	Pleurain-A3 (Pleurain A3; Frogs, amphibians, animals)	Antibacterial, Antifungal, Anti-Gram+, Anti-Gram-, Antimicrobial
2259	DRAMP01213	Pleurain-A4 (Pleurain A4; Frogs, amphibians, animals)	Antibacterial, Antifungal, Anti-Gram+, Anti-Gram-, Antimicrobial
2260	DRAMP01215	Kassinatuerin-2Mb (Frogs, amphibians, animals)	Antibacterial, Anti-Gram+, Antimicrobial
2261	DRAMP01216	Kassinatuerin-2Mc (Frogs, amphibians, animals)	Antibacterial, Anti-Gram+, Antimicrobial
2262	DRAMP01217	Kassinatuerin-2Md (Frogs, amphibians, animals)	Antibacterial, Anti-Gram+, Antimicrobial
2263	DRAMP18320	Epilancin 15X(Bacteriocin)	Antibacterial, Anti-Gram+, Antimicrobial
2264	DRAMP01223	Palustrin-2AJ2 (PL2AJ12; Frogs, amphibians, animals)	Antibacterial, Anti-Gram+, Anti-Gram-, Antimicrobial
2265	DRAMP01224	Palustrin-2AR (Palustrin-2ARa; Ranatuerin-2SEa; Frogs, amphibians, animals)	Antibacterial, Anti-Gram+, Anti-Gram-, Antimicrobial
2266	DRAMP01225	Palustrin-3AR (Frogs, amphibians, animals)	Antibacterial, Antiviral, Antimicrobial
2267	DRAMP01226	Palustrin-1a (Frogs, amphibians, animals)	Antibacterial, Anti-Gram-, Antimicrobial
2268	DRAMP01230	Palustrin-2a (Frogs, amphibians, animals)	Antibacterial, Anti-Gram-, Antimicrobial
2269	DRAMP01236	Palustrin-2CE (Frogs, amphibians, animals)	Antibacterial, Antimicrobial
2270	DRAMP01239	Palustrin-2CG1 (Frogs, amphibians, animals)	Antibacterial, Antimicrobial
2271	DRAMP01241	Palustrin-RA2 antimicrobial peptide (Frogs, amphibians, animals)	Antimicrobial,
2272	DRAMP01242	Japonicin-1Npa (Frogs, amphibians, animals)	Antimicrobial,
2273	DRAMP01243	Japonicin-1Npb (Frogs, amphibians, animals)	Antimicrobial,
2274	DRAMP01247	OGG1 antimicrobial peptide (Frogs, amphibians, animals)	Antimicrobial,
2275	DRAMP01256	Dybowskin-2CDYb (Frogs, amphibians, animals)	Antibacterial, Antimicrobial
2276	DRAMP01262	Bradykinin (Frogs, amphibians, animals)	Antimicrobial ,
2277	DRAMP01263	Des-Arg-bradykinin (Frogs, amphibians, animals)	Antimicrobial ,
2278	DRAMP01264	[Thr6]-bradykinin (Frogs, amphibians, animals)	Antimicrobial ,
2279	DRAMP01265	Des-Arg-[Thr6]-bradykinin (Frogs, amphibians, animals)	Antimicrobial ,
2280	DRAMP01266	Phyllokinin (Frogs, amphibians, animals)	Antimicrobial ,
2281	DRAMP01267	[Thr6]-Phyllokinin (Frogs, amphibians, animals)	Antimicrobial ,
2282	DRAMP01268	[Arg4]-Phyllocaerulein (Frogs, amphibians, animals)	Antimicrobial ,
2283	DRAMP01269	Dermorphin (Frogs, amphibians, animals)	Antimicrobial ,
2284	DRAMP01270	[D-Met2]-deltorphin (Frogs, amphibians, animals)	Antimicrobial ,
2285	DRAMP18404	Polybia-MPII (mastoparan; insects, arthropods, invertebrates, animals)	Antibacterial, antifungal, Anti-Gram+, Anti-Gram-, Antimicrobial
2286	DRAMP18403	Tridecaptin A1 (TriA1; lipopeptides; nonribosomally synthesized peptide antibiotic)	Antibacterial, Antimicrobial
2287	DRAMP18402	Tridecaptin B1 (TriB1; lipopeptides; nonribosomally synthesized peptide antibiotic)	Antibacterial, Anti-Gram-, Antimicrobial
2288	DRAMP18401	Uy17 (scorpions, arachnids, Chelicerata, arthropods, invertebrates, animals)	Antibacterial, Antimicrobial
2289	DRAMP01275	Phylloseptin-s1 (Frogs, amphibians, animals)	Antimicrobial ,
2290	DRAMP01276	Phylloseptin-s2 (Frogs, amphibians, animals)	Antimicrobial ,
2291	DRAMP01277	Phylloseptin-s3 (Frogs, amphibians, animals)	Antimicrobial ,
2292	DRAMP18319	BsaA2(Bacteriocin)	Antibacterial, Anti-Gram+, Antimicrobial
2293	DRAMP01279	Phylloseptin Bu-1 (Frogs, amphibians, animals)	Antimicrobial ,
2294	DRAMP01280	Phylloseptin Bu-2 (Frogs, amphibians, animals)	Antimicrobial ,
2295	DRAMP01281	Phylloseptin-J1 (PLS-J1; PS-J1; Frogs, amphibians, animals)	Antimicrobial ,
2296	DRAMP01282	Phylloseptin-J2 (PLS-J2; PS-J2; Frogs, amphibians, animals)	Antimicrobial ,
2297	DRAMP01283	Phylloseptin-J3 (PLS-J3; PS-J3; Frogs, amphibians, animals)	Antimicrobial ,

2298	DRAMP01284	Phylloseptin-J4 (PLS-J4; PS-J4; Frogs, amphibians, animals)	Antimicrobial ,
2299	DRAMP01285	Phylloseptin-J5 (PLS-J5; PS-J5; Frogs, amphibians, animals)	Antimicrobial ,
2300	DRAMP01286	Phylloseptin-J6 (PLS-J6; PS-J6; Frogs, amphibians, animals)	Antimicrobial ,
2301	DRAMP01287	Phylloseptin-J7 (PLS-J7; PS-J7; Frogs, amphibians, animals)	Antimicrobial ,
2302	DRAMP01289	Phylloseptin-1 (PStar 01; Frogs, amphibians, animals)	Antimicrobial ,
2303	DRAMP01290	Phylloseptin-2 (PStar 02; Frogs, amphibians, animals)	Antimicrobial ,
2304	DRAMP01291	Phylloseptin-3 (PStar 03; Frogs, amphibians, animals)	Antimicrobial ,
2305	DRAMP01292	Phylloseptin-4 (PS-4; Frogs, amphibians, animals)	Antiprotozoal , Antimicrobial
2306	DRAMP01293	Phylloseptin-5 (PS-5; Frogs, amphibians, animals)	Antiprotozoal , Antimicrobial
2307	DRAMP01294	Tryptophyllin-5.1 (Frogs, amphibians, animals)	Antimicrobial ,
2308	DRAMP01295	Distinctin-like peptide (Frogs, amphibians, animals)	Antimicrobial,
2309	DRAMP18400	Uy192 (scorpions, arachnids, Chelicerata, arthropods, invertebrates, animals)	Antibacterial, Antimicrobial
2310	DRAMP18399	Uy234 (scorpions, arachnids, Chelicerata, arthropods, invertebrates, animals)	Antibacterial, Antimicrobial
2311	DRAMP01298	Hyposin-J1 (HPS-J1; Frogs, amphibians, animals)	Antimicrobial ,
2312	DRAMP01299	Hyposin-HA4 (Hyposin-4; Frogs, amphibians, animals)	Antimicrobial ,
2313	DRAMP01300	Hyposin-HA3 (Hyposin-3; Frogs, amphibians, animals)	Antimicrobial ,
2314	DRAMP01304	Phylloseptin-6 (PS-6; Frogs, amphibians, animals)	Antimicrobial ,
2315	DRAMP01307	Phylloseptin-8 (PS-8; Frogs, amphibians, animals)	Antimicrobial ,
2316	DRAMP01308	Phylloseptin-8 (PS-8; Frogs, amphibians, animals)	Antimicrobial ,
2317	DRAMP18318	BacCH91(Bacteriocin)	Antibacterial, Anti-Gram+, Antimicrobial
2318	DRAMP01310	Phylloseptin-9 (PS-9; Frogs, amphibians, animals)	Antimicrobial ,
2319	DRAMP01311	Phylloseptin-10 (PS-10; Frogs, amphibians, animals)	Antimicrobial ,
2320	DRAMP01312	Phylloseptin-11 (PS-11; Frogs, amphibians, animals)	Antimicrobial ,
2321	DRAMP01313	Phylloseptin-12 (PS-12; Frogs, amphibians, animals)	Antimicrobial ,
2322	DRAMP01315	Phylloseptin 13 (PS-13; Frogs, amphibians, animals)	Antimicrobial ,
2323	DRAMP01316	Phylloseptin 14 (PS-14; Frogs, amphibians, animals)	Antimicrobial ,
2324	DRAMP01317	Phylloseptin 15 (PS-15; Frogs, amphibians, animals)	Antimicrobial ,
2325	DRAMP01318	Antimicrobial peptide 1 (Frogs, amphibians, animals)	Antibacterial, Anti-Gram+, Antimicrobial
2326	DRAMP01321	Amolopin-n1 antimicrobial peptide (Frogs, amphibians, animals)	Antimicrobial,
2327	DRAMP01322	Amolopin-n2 antimicrobial peptide (Frogs, amphibians, animals)	Antimicrobial,
2328	DRAMP01323	Amolopin-2e antimicrobial peptide (Frogs, amphibians, animals)	Antimicrobial,
2329	DRAMP01324	Amolopin-2f antimicrobial peptide (Frogs, amphibians, animals)	Antimicrobial,
2330	DRAMP01325	Amolopin-2g antimicrobial peptide (Frogs, amphibians, animals)	Antimicrobial,
2331	DRAMP01326	Amolopin-2h antimicrobial peptide (Frogs, amphibians, animals)	Antimicrobial,
2332	DRAMP01327	Amolopin-2i antimicrobial peptide (Frogs, amphibians, animals)	Antimicrobial,
2333	DRAMP01328	Amolopin-2k antimicrobial peptide (Frogs, amphibians, animals)	Antimicrobial,
2334	DRAMP01329	Amolopin-5a antimicrobial peptide (Frogs, amphibians, animals)	Antimicrobial,
2335	DRAMP01330	Amolopin-6a antimicrobial peptide (Frogs, amphibians, animals)	Antimicrobial,
2336	DRAMP01331	Amolopin-6b antimicrobial peptide (Frogs, amphibians, animals)	Antimicrobial,
2337	DRAMP01332	Amolopin-7a antimicrobial peptide (Frogs, amphibians, animals)	Antimicrobial,
2338	DRAMP01333	Amolopin-8a antimicrobial peptide (Frogs, amphibians, animals)	Antimicrobial,
2339	DRAMP01334	Amolopin-9a antimicrobial peptide (Frogs, amphibians, animals)	Antimicrobial,
2340	DRAMP01335	Amolopin-9b antimicrobial peptide (Frogs, amphibians, animals)	Antimicrobial,
2341	DRAMP01336	Amolopin-p1	Antimicrobial,
2342	DRAMP01337	Amolopin-p2	Antimicrobial,
2343	DRAMP01338	Amolopin-1a (Frogs, amphibians, animals)	Antibacterial, Antifungal, Anti-Gram+, Anti-Gram-, Antimicrobial
2344	DRAMP01340	Amolopin-3a (Frogs, amphibians, animals)	Antimicrobial,
2345	DRAMP01342	Amolopin-2b (Frogs, amphibians, animals)	Antibacterial, Antifungal, Anti-Gram+, Anti-Gram-, Antimicrobial
2346	DRAMP01343	Amolopin-1c (Frogs, amphibians, animals)	Antibacterial, Antifungal, Anti-Gram+, Anti-Gram-, Antimicrobial
2347	DRAMP01344	Amolopin-2c (Frogs, amphibians, animals)	Antibacterial, Antifungal, Anti-Gram+, Anti-Gram-, Antimicrobial
2348	DRAMP01345	Amolopin-1d (Frogs, amphibians, animals)	Antibacterial, Antifungal, Anti-Gram+, Anti-Gram-, Antimicrobial
2349	DRAMP01348	Tigerinin-RC1 (Frogs, amphibians, animals)	Antimicrobial,

2350	DRAMP01349	Tigerinin-RC2 (Frogs, amphibians, animals)	Antimicrobial,
2351	DRAMP01388	Odorranain-R1 (OdR1; Frogs, amphibians, animals)	Antifungal, Antimicrobial
2352	DRAMP01363	Frenatin-4 (Frogs, amphibians, animals)	Antimicrobial,
2353	DRAMP01365	Maculatin-1.2 (Frogs, amphibians, animals)	Antibacterial, Anti-Gram+, Antimicrobial
2354	DRAMP01366	Maculatin-1.3 (frog, amphibia, animals)	Antibacterial, Antiviral, Antimicrobial
2355	DRAMP01369	Maculatin-3.1 (Frogs, amphibians, animals)	Antibacterial, Anti-Gram+, Antimicrobial
2356	DRAMP01404	Odorranain-J2 antimicrobial peptide (Frogs, amphibians, animals)	Antimicrobial,
2357	DRAMP01405	Odorranain-C6 antimicrobial peptide (Frogs, amphibians, animals)	Antimicrobial,
2358	DRAMP01406	Antimicrobial peptide odorranain-O3 (Frogs, amphibians, animals)	Antimicrobial,
2359	DRAMP01407	Antimicrobial peptide odorranain B4 (Frogs, amphibians, animals)	Antimicrobial,
2360	DRAMP01408	Antimicrobial peptide odorranain B5 (Frogs, amphibians, animals)	Antimicrobial,
2361	DRAMP18317	Aureocin A70 (AurD)(Bacteriocin)	Antibacterial, Anti-Gram+, Antimicrobial
2362	DRAMP01425	Nigrocin-OG21 (frog, amphibians, animals)	Antibacterial, Antifungal , Antimicrobial
2363	DRAMP01444	Nigrocin-1 (Frogs, amphibians, animals)	Antibacterial, Antifungal, Anti-Gram+, Anti-Gram-, Antimicrobial
2364	DRAMP01445	Nigrocin-2 (Nigrocin-2LVa; Frogs, amphibians, animals)	Antibacterial, Antifungal, Anti-Gram+, Anti-Gram-, Antimicrobial
2365	DRAMP01446	Proteinase inhibitor PSKP-1 (Frogs, amphibians, animals)	Antibacterial, Anti-Gram-, Antimicrobial
2366	DRAMP01449	Esculentin-2-OG3 antimicrobial peptide (Frogs, amphibians, animals)	Antimicrobial,
2367	DRAMP01450	Esculentin-2-OG5 antimicrobial peptide (Frogs, amphibians, animals)	Antimicrobial,
2368	DRAMP01451	Esculentin-2-OG11 antimicrobial peptide (Frogs, amphibians, animals)	Antimicrobial,
2369	DRAMP18316	Aureocin A70 (AurC)(Bacteriocin)	Antibacterial, Anti-Gram+, Antimicrobial
2370	DRAMP18315	Aureocin A70 (AurB)(Bacteriocin)	Antibacterial, Anti-Gram+, Antimicrobial
2371	DRAMP01460	Esculentin-2Vb (2VEb; Frogs, amphibians, animals)	Antimicrobial,
2372	DRAMP01463	Esculentin-1SEa (Frogs, amphibians, animals)	Antibacterial, Anti-Gram+, Anti-Gram-, Antimicrobial
2373	DRAMP01464	Esculentin-1SEb (Frogs, amphibians, animals)	Antibacterial, Anti-Gram+, Anti-Gram-, Antimicrobial
2374	DRAMP01465	Esculentin-1R (Frogs, amphibians, animals)	Antibacterial, Anti-Gram+, Anti-Gram-, Antimicrobial
2375	DRAMP01466	Esculentin-2R (Frogs, amphibians, animals)	Antimicrobial,
2376	DRAMP01467	Esculentin-2Rb (Frogs, amphibians, animals)	Antimicrobial,
2377	DRAMP01468	Esculentin-2Ra (Frogs, amphibians, animals)	Antimicrobial,
2378	DRAMP18314	Aureocin A70 (AurA)(Bacteriocin)	Antibacterial, Anti-Gram+, Antimicrobial
2379	DRAMP01481	Esculentin-2Wa (Frogs, amphibians, animals)	Antibacterial, Antimicrobial
2380	DRAMP18313	Sclerosin(Bacteriocin)	Antibacterial, Antimicrobial
2381	DRAMP01488	Esculentin-1C (Frogs, amphibians, animals)	Antibacterial, Antimicrobial
2382	DRAMP01489	Esculentin-1A (Frogs, amphibians, animals)	Antibacterial, Anti-Gram+, Anti-Gram-, Antimicrobial
2383	DRAMP01492	Esculentin-IIb (Frogs, amphibians, animals)	Antibacterial, Anti-Gram+, Anti-Gram-, Antimicrobial
2384	DRAMP01498	Esculentin-1-OA6 (Frogs, amphibians, animals)	Antibacterial, Antifungal, Antimicrobial
2385	DRAMP01500	Esculentin-1-OR2 (Frogs, amphibians, animals)	Antibacterial, Antifungal, Antimicrobial
2386	DRAMP01512	Esculentin-2-OR6 (Frogs, amphibians, animals)	Antibacterial, Antifungal, Antimicrobial
2387	DRAMP01514	Esculentin-1 (Frogs, amphibians, animals)	Antibacterial, Antimicrobial
2388	DRAMP18312	Propionicin PLG-1(Bacteriocin)	Antibacterial, Antifungal, Anti-Gram+, Anti-Gram-, Antimicrobial
2389	DRAMP01744	Temporin-G (Frogs, amphibians, animals)	Antibacterial, Antimicrobial
2390	DRAMP01743	Temporin-F (Frogs, amphibians, animals)	Antibacterial, Antiparasitic, Antimicrobial
2391	DRAMP01742	Temporin-E (Frogs, amphibians, animals)	Antibacterial, Antimicrobial
2392	DRAMP01519	Esculentin-2PRa (Frogs, amphibians, animals)	Antibacterial, Antifungal, Anti-Gram+, Anti-Gram-, Antimicrobial
2393	DRAMP01522	Rugosin-C (Frogs, amphibians, animals)	Antibacterial, Anti-Gram+, Antimicrobial
2394	DRAMP01523	Rugosin A-like peptide (Frogs, amphibians, animals)	Antibacterial, Antimicrobial
2395	DRAMP01527	Nigroain-L antimicrobial peptide (Frogs, amphibians, animals)	Antimicrobial,
2396	DRAMP01528	Nigroain-H antimicrobial peptide (Frogs, amphibians, animals)	Antimicrobial,
2397	DRAMP01529	Nigroain-H antimicrobial peptide (Frogs, amphibians, animals)	Antimicrobial,

2398	DRAMP01530	Nigroain-E antimicrobial peptide (Frogs, amphibians, animals)	Antimicrobial,
2399	DRAMP01531	Nigroain-D antimicrobial peptide (Frogs, amphibians, animals)	Antimicrobial,
2400	DRAMP01532	Nigroain-B antimicrobial peptide (Frogs, amphibians, animals)	Antimicrobial,
2401	DRAMP01534	Nigroain-B2 (Frogs, amphibians, animals)	Antimicrobial,
2402	DRAMP01535	Nigroain-B3 (Frogs, amphibians, animals)	Antimicrobial,
2403	DRAMP01536	Nigroain-B4 (Frogs, amphibians, animals)	Antimicrobial,
2404	DRAMP01537	Nigroain-C antimicrobial peptide (Frogs, amphibians, animals)	Antimicrobial,
2405	DRAMP01538	Nigroain-C1 (Frogs, amphibians, animals)	Antimicrobial,
2406	DRAMP01540	Nigroain-D1 (Frogs, amphibians, animals)	Antimicrobial,
2407	DRAMP01541	Nigroain-D2 (Frogs, amphibians, animals)	Antimicrobial,
2408	DRAMP01544	Nigroain-E2 (Frogs, amphibians, animals)	Antimicrobial,
2409	DRAMP01545	Nigroain-I (Frogs, amphibians, animals)	Antimicrobial,
2410	DRAMP01548	Caerin-1	Antimicrobial,
2411	DRAMP01551	Caerin-1.2 (Frogs, amphibians, animals)	Antibacterial, Antimicrobial
2412	DRAMP01554	Caerin-1.4.1 (Chain of Caerin-1.4)	Antibacterial, Antimicrobial
2413	DRAMP01556	Caerin-1.6 (Frogs, amphibians, animals)	Antibacterial, Antimicrobial
2414	DRAMP01557	Caerin-1.7 (Frogs, amphibians, animals)	Antibacterial, Antimicrobial
2415	DRAMP01559	Caerin-1.8 (Frogs, amphibians, animals)	Antibacterial, Antifungal, Antiviral, Antimicrobial
2416	DRAMP01561	Caerin-2 (Venom antimicrobial peptide-10)	Antimicrobial,
2417	DRAMP01564	Caerin-2.2.1 (Chain of Caerin-2.2)	Antimicrobial,
2418	DRAMP01565	Caerin-2.4 (Frogs, amphibians, animals)	Antibacterial, Antimicrobial
2419	DRAMP01566	Caerin-2.5 (Frogs, amphibians, animals)	Antibacterial, Antimicrobial
2420	DRAMP01569	Caerin-3.1 (Frogs, amphibians, animals)	Antibacterial, Anti-Gram+, Antimicrobial
2421	DRAMP01571	Caerin-3.3 (Frogs, amphibians, animals)	Antibacterial, Antimicrobial
2422	DRAMP01572	Caerin-3.4 (Frogs, amphibians, animals)	Antibacterial, Antimicrobial
2423	DRAMP01575	Caerin-4.2 (Frogs, amphibians, animals)	Antibacterial, Antimicrobial
2424	DRAMP01579	Caerin 1.12 (Frogs, amphibians, animals)	Antibacterial, Antimicrobial
2425	DRAMP01580	Caerin 1.13 (Frogs, amphibians, animals)	Antibacterial, Antimicrobial
2426	DRAMP01581	Caerin 1.14 (Frogs, amphibians, animals)	Antibacterial, Antimicrobial
2427	DRAMP01582	Caerin 1.15 (Frogs, amphibians, animals)	Antibacterial, Antimicrobial
2428	DRAMP01592	Citropin-1.1.1 (Frogs, amphibians, animals)	Antibacterial, Antimicrobial
2429	DRAMP01593	Citropin-1.1.2 (Frogs, amphibians, animals)	Antibacterial, Antimicrobial
2430	DRAMP01595	Citropin-1.2.1 (Frogs, amphibians, animals)	Antibacterial, Antimicrobial
2431	DRAMP01597	Citropin-1.2.3 (Frogs, amphibians, animals)	Antibacterial, Antimicrobial
2432	DRAMP01598	Citropin-1.2.4 (Frogs, amphibians, animals)	Antimicrobial,
2433	DRAMP01599	Citropin-1.2.5 (Frogs, amphibians, animals)	Antimicrobial,
2434	DRAMP01604	Citropin-1.1.4 (Frogs, amphibians, animals)	Antimicrobial,
2435	DRAMP01605	Citropin-3.1.2 (Frogs, amphibians, animals)	Antimicrobial,
2436	DRAMP01609	Aurein-2.2 (Frogs, amphibians, animals)	Antibacterial, Anticancer, Anti-Gram+, Antimicrobial
2437	DRAMP01610	Aurein-2.3 (Frogs, amphibians, animals)	Antibacterial, Anticancer, Anti-Gram+, Antimicrobial
2438	DRAMP01611	Aurein-2.4 (Frogs, amphibians, animals)	Antibacterial, Anticancer, Anti-Gram+, Antimicrobial
2439	DRAMP01615	Aurein-3.1.1 (Frogs, amphibians, animals)	Antibacterial, Anticancer, Antimicrobial
2440	DRAMP01616	Aurein-3.1.2 (Frogs, amphibians, animals)	Antibacterial, Anticancer, Antimicrobial
2441	DRAMP01619	Aurein-3.3.1 (Frogs, amphibians, animals)	Antibacterial, Anticancer, Antimicrobial
2442	DRAMP18309	Baceridin(Bacteriocin)	Antibacterial, Anti-Gram+, Antimicrobial
2443	DRAMP18308	Planosporicin(Bacteriocin)	Antibacterial, Antimicrobial
2444	DRAMP01629	Phylloxin-S1 (Frogs, amphibians, animals)	Antibacterial, Antifungal, Antiviral, Antimicrobial
2445	DRAMP01630	Dermaseptin-LI1 (Frogs, amphibians, animals)	Antibacterial, Antifungal, Antiviral, Antimicrobial
2446	DRAMP01631	Dermaseptin-S7 (Frogs, amphibians, animals)	Antibacterial, Antifungal, Antiviral, Antimicrobial
2447	DRAMP01632	Dermaseptin-S8 (Frogs, amphibians, animals)	Antibacterial, Antifungal, Antiviral, Antimicrobial
2448	DRAMP01633	Dermaseptin S9 (Frogs, amphibians, animals)	Antimicrobial,
2449	DRAMP01634	Preprodermaseptin S10 (Frogs, amphibians, animals)	Antimicrobial,
2450	DRAMP01635	Preprodermaseptin S11 (Frogs, amphibians, animals)	Antimicrobial,
2451	DRAMP01636	Preprodermaseptin S12 (Frogs, amphibians, animals)	Antimicrobial,
2452	DRAMP01637	Dermaseptin S13 (Frogs, amphibians, animals)	Antimicrobial,
2453	DRAMP01640	Dermaseptin-2 (DShypo02; Frogs, amphibians, animals)	Antimicrobial,
2454	DRAMP01641	Dermaseptin-3 (DShypo03; Frogs, amphibians, animals)	Antimicrobial,

2455	DRAMP01642	Dermaseptin-4 (DShypo04; Frogs, amphibians, animals)	Antimicrobial,
2456	DRAMP01644	Dermaseptin-6 (DShypo06; Frogs, amphibians, animals)	Antimicrobial,
2457	DRAMP01645	Dermaseptin-7 (DShypo07; Frogs, amphibians, animals)	Antimicrobial,
2458	DRAMP01652	Dermaseptin-B5 (Dermaseptin BV; Frogs, amphibians, animals)	Antibacterial, Antimicrobial
2459	DRAMP01653	Dermaseptin-B6 (Dermaseptin BVI; Frogs, amphibians, animals)	Antibacterial, Antimicrobial
2460	DRAMP01654	Dermaseptin-B8 (Frogs, amphibians, animals)	Antibacterial, Antifungal, Antiviral, Antimicrobial
2461	DRAMP01655	Dermaseptin DRG1 (Dermaseptin-1; Dermaseptin-B7, DRS-B7; Frogs, amphibians, animals)	Antimicrobial,
2462	DRAMP01656	Dermaseptin DRG2 (Dermaseptin-2; Frogs, amphibians, animals)	Antimicrobial,
2463	DRAMP01658	Dermaseptin-J1 (DRS-J1; Frogs, amphibians, animals)	Antimicrobial ,
2464	DRAMP01659	Dermaseptin-J2 (DRS-J2; Frogs, amphibians, animals)	Antimicrobial ,
2465	DRAMP01660	Dermaseptin-J3 (DRS-J3; Frogs, amphibians, animals)	Antimicrobial ,
2466	DRAMP01661	Dermaseptin-J4 (DRS-J4; Frogs, amphibians, animals)	Antimicrobial ,
2467	DRAMP01662	Dermaseptin-J5 (DRS-J5; Frogs, amphibians, animals)	Antimicrobial ,
2468	DRAMP01663	Dermaseptin-J6 (DRS-J6; Frogs, amphibians, animals)	Antimicrobial ,
2469	DRAMP18307	LMW peptide (Bacteriocin)	Antibacterial, Anti-Gram+, Antimicrobial
2470	DRAMP01665	Dermaseptin-J8 (DRS-J8; Frogs, amphibians, animals)	Antimicrobial ,
2471	DRAMP01666	Dermaseptin-J9 (DRS-J9; Frogs, amphibians, animals)	Antimicrobial ,
2472	DRAMP01667	Dermaseptin-J10 (DRS-J10; Frogs, amphibians, animals)	Antimicrobial ,
2473	DRAMP01669	Dermaseptin-2 (DS II; Dermaseptin-S2, DS2; Frogs, amphibians, animals)	Antibacterial, Antifungal, Antiprotozoal, Antimicrobial
2474	DRAMP01670	Dermaseptin-3 (DS III; Dermaseptin-S3, DS3; Frogs, amphibians, animals)	Antibacterial, Antifungal, Antiprotozoal, Antimicrobial
2475	DRAMP01671	Dermaseptin-4 (DS IV; Dermaseptin-S4, DS4; Frogs, amphibians, animals)	Antibacterial, Antifungal, Antiviral, Anti-Gram+, Anti-Gram-, Antimicrobial
2476	DRAMP01672	Dermaseptin-5 (DS V; Dermaseptin-S5, DS5; Frogs, amphibians, animals)	Antibacterial, Antifungal, Antimicrobial
2477	DRAMP01673	Dermaseptin-1 (DStar 01; Frogs, amphibians, animals)	Antibacterial, Antifungal, Anti-Gram+, Antimicrobial
2478	DRAMP18306	Pediocin ACCEL(Bacteriocin)	Antibacterial, Anti-Gram+, Antimicrobial
2479	DRAMP01675	Dermaseptin-3 (DStar 03; Frogs, amphibians, animals)	Antibacterial, Antifungal, Anti-Gram+, Antimicrobial
2480	DRAMP01676	Dermaseptin-4 (DStar 04; Frogs, amphibians, animals)	Antibacterial, Anti-Gram+, Antimicrobial
2481	DRAMP01677	Dermaseptin-5 (DStar 05; Frogs, amphibians, animals)	Antibacterial, Antifungal, Anti-Gram+, Antimicrobial
2482	DRAMP01678	Dermaseptin-6 (DStar 06; Frogs, amphibians, animals)	Antibacterial, Anti-Gram+, Antimicrobial
2483	DRAMP01679	Dermaseptin-7 (DStar 07; Frogs, amphibians, animals)	Antibacterial, Anti-Gram+, Antimicrobial
2484	DRAMP01680	Dermaseptin-8 (DStar 08; Frogs, amphibians, animals)	Antibacterial, Anti-Gram+, Antimicrobial
2485	DRAMP01681	Dermaseptin-S6 (DS6; Frogs, amphibians, animals)	Antibacterial, Antimicrobial
2486	DRAMP01682	Dermaseptin-S9 (DS9; Frogs, amphibians, animals)	Antibacterial, Antimicrobial
2487	DRAMP01683	Dermaseptin-S11 (DS11; Frogs, amphibians, animals)	Antibacterial, Antimicrobial
2488	DRAMP01684	Dermaseptin-S12 (DS12; Frogs, amphibians, animals)	Antibacterial, Antimicrobial
2489	DRAMP01685	Dermaseptin-S13 (DS13; Frogs, amphibians, animals)	Antibacterial, Antimicrobial
2490	DRAMP01686	Dermaseptin AA-1-1 (Frogs, amphibians, animals)	Antimicrobial,
2491	DRAMP01687	Dermaseptin AA-3-1 (Frogs, amphibians, animals)	Antibacterial, Antimicrobial
2492	DRAMP01688	Dermaseptin AA-3-3 (Frogs, amphibians, animals)	Antibacterial, Antimicrobial
2493	DRAMP01689	Dermaseptin AA-3-4 (Frogs, amphibians, animals)	Antibacterial, Antimicrobial
2494	DRAMP01690	Dermaseptin AA-3-6 (Frogs, amphibians, animals)	Antibacterial, Antimicrobial
2495	DRAMP01691	Dermaseptin AA-2-5 (Frogs, amphibians, animals)	Antibacterial, Antimicrobial
2496	DRAMP01692	Dermaseptin PD-2-2 (Frogs, amphibians, animals)	Antibacterial, Antimicrobial
2497	DRAMP01693	Dermaseptin PD-3-3 (DRS-DA3; Frogs, amphibians, animals)	Antibacterial, Antimicrobial
2498	DRAMP18305	Paenibacterin (Bacteriocin)	Antibacterial, Antimicrobial
2499	DRAMP01695	Dermaseptin PD-3-6 (Plasticin-DA1; Frogs, amphibians, animals)	Antibacterial, Antimicrobial
2500	DRAMP01696	Dermaseptin PD-3-7 (Frogs, amphibians, animals)	Antibacterial, Antimicrobial
2501	DRAMP01697	Dermaseptin-DA4 (DRS-DA4; Frogs, amphibians, animals)	Antibacterial, Anti-Gram-, Antimicrobial
2502	DRAMP01699	Dermaseptin-H2 (Dermaseptin-like peptide 2, DMS2; Frogs, amphibians, animals)	Antibacterial, Antimicrobial
2503	DRAMP01700	Dermaseptin-H3 (Dermaseptin-like peptide 3, DMS3; Frogs, amphibians, animals)	Antibacterial, Anti-Gram+, Anti-Gram-, Antimicrobial

2504	DRAMP18304	Paenicidin A (Bacteriocin)	Antibacterial, Anti-Gram+, Antimicrobial
2505	DRAMP01704	Dermaseptin-C3 (Frogs, amphibians, animals)	Antimicrobial ,
2506	DRAMP01705	Dermaseptin-like peptide (SmDLP; Frogs, amphibians, animals)	Antibacterial, Anti-Gram+, Antimicrobial
2507	DRAMP01706	Dermaseptin-like DRP-AC-1 (Frogs, amphibians, animals)	Antimicrobia, Antimicrobial
2508	DRAMP01707	Dermaseptin-like DRP-AC-2 (Frogs, amphibians, animals)	Antimicrobia, Antimicrobial
2509	DRAMP01708	Dermaseptin-like DRP-AC-3 (Frogs, amphibians, animals)	Antimicrobia, Antimicrobial
2510	DRAMP01709	Dermaseptin-1 (DStomo01; Frogs, amphibians, animals)	Antimicrobial,
2511	DRAMP01710	Dermaseptin DS VIII-like peptide (Frogs, amphibians, animals)	Antibacterial, Antifungal, Antimicrobial
2512	DRAMP01711	Dermaseptin III-like peptide (Frogs, amphibians, animals)	Antibacterial, Antifungal, Antimicrobial
2513	DRAMP18303	Paenicidin A (Bacteriocin)	Antibacterial, Anti-Gram+, Antimicrobial
2514	DRAMP01713	OGA1 antimicrobial peptide (Frogs, amphibians, animals)	Antibacterial, Antifungal, Antiviral, Antimicrobial
2515	DRAMP01714	OGF2 antimicrobial peptide (Frogs, amphibians, animals)	Antibacterial, Antifungal, Antiviral, Antimicrobial
2516	DRAMP01715	OGG1 antimicrobial peptide (Frogs, amphibians, animals)	Antibacterial, Antifungal, Antiviral, Antimicrobial
2517	DRAMP01716	OGF1 antimicrobial peptide (Frogs, amphibians, animals)	Antibacterial, Antifungal, Antiviral, Antimicrobial
2518	DRAMP01717	Dermatoxin (Frogs, amphibians, animals)	Antibacterial, Anti-Gram+, Anti-Gram-, Antimicrobial
2519	DRAMP01718	Dermatoxin-J1 (DRT-J1; Frogs, amphibians, animals)	Antimicrobial ,
2520	DRAMP01719	Dermatoxin-J2 (DRT-J2; Frogs, amphibians, animals)	Antimicrobial ,
2521	DRAMP01720	Dermatoxin-J3 (DRT-J3; Frogs, amphibians, animals)	Antimicrobial ,
2522	DRAMP01722	Temporin-LTe antimicrobial peptide (Frogs, amphibians, animals)	Antimicrobial ,
2523	DRAMP01723	Temporin-TOa (Frogs, amphibians, animals)	Antimicrobial ,
2524	DRAMP01724	Temporin-TOb (Frogs, amphibians, animals)	Antimicrobial ,
2525	DRAMP01725	Temporin-CG1 antimicrobial peptide (Frogs, amphibians, animals)	Antimicrobial ,
2526	DRAMP01726	Temporin-CG2 antimicrobial peptide (Frogs, amphibians, animals)	Antimicrobial ,
2527	DRAMP01727	Temporin-CG3 antimicrobial peptide (Frogs, amphibians, animals)	Antimicrobial ,
2528	DRAMP01728	Temporin-CG4 antimicrobial peptide (Frogs, amphibians, animals)	Antimicrobial ,
2529	DRAMP01729	Temporin-CG5 antimicrobial peptide (Frogs, amphibians, animals)	Antimicrobial ,
2530	DRAMP02857	Indolicidin (Cathelicidin-4; mammals, animals)	Antibacterial, Anti-Gram+, Anti-Gram-, Antimicrobial
2531	DRAMP01741	Temporin-D (Frogs, amphibians, animals)	Antibacterial, Anti-Gram+, Antimicrobial
2532	DRAMP02819	Anoplin (Insects, arthropods, invertebrates, animals)	Antimicrobial, Antibacterial, Antifungal, Anti-Gram+, Anti-Gram-,
2533	DRAMP04395	EP3 (Earthworm,animals)	Antibacterial, Anti-Gram+, Anti-Gram-, Antimicrobial
2534	DRAMP04394	EP2 (Earthworm,animals)	Antibacterial, Anti-Gram+, Anti-Gram-, Antimicrobial
2535	DRAMP02396	OEP3121 (EP5-1;earthworm,animals)	Antibacterial, Anticancer, Antimicrobial
2536	DRAMP01747	Temporin-1CEe (Frogs, amphibians, animals)	Antibacterial, Antimicrobial
2537	DRAMP01748	Temporin-AJ8 (Frogs, amphibians, animals)	Antibacterial, Antifungal, Antimicrobial
2538	DRAMP01749	Temporin-1ARa (Temporin 1ARa; Frogs, amphibians, animals)	Antibacterial, Antimicrobial
2539	DRAMP01756	Temporin-2-RA2 peptide (Frogs, amphibians, animals)	Antimicrobial ,
2540	DRAMP01757	Temporin-1-RA1 peptide (Frogs, amphibians, animals)	Antimicrobial ,
2541	DRAMP01758	Temporin-1SPa (Frogs, amphibians, animals)	Antibacterial, Anti-Gram+, Antimicrobial
2542	DRAMP01760	Temporin-1AUa (Temporin 1AUa; Frogs, amphibians, animals)	Antibacterial, Antimicrobial
2543	DRAMP01761	Temporin-PRa (Frogs, amphibians, animals)	Antibacterial, Antimicrobial
2544	DRAMP01762	Temporin-PRb (Frogs, amphibians, animals)	Antibacterial, Antimicrobial
2545	DRAMP01763	Temporin-PRc (Frogs, amphibians, animals)	Antibacterial, Antimicrobial
2546	DRAMP01767	Temporin-She (Frogs, amphibians, animals)	Antimicrobial,
2547	DRAMP01770	Temporin-1SKc (Frogs, amphibians, animals)	Antibacterial, Antimicrobial
2548	DRAMP01772	Temporin-1Ob (Frogs, amphibians, animals)	Antibacterial, Antifungal, Anti-Gram+, Anti-Gram-, Antimicrobial
2549	DRAMP01774	Temporin-1Od (Frogs, amphibians, animals)	Antibacterial, Anti-Gram+, Antimicrobial
2550	DRAMP01778	Temporin-1Sd (Frogs, amphibians, animals)	Antimicrobial,
2551	DRAMP18302	GE2270A(Bacteriocin)	Antibacterial, Anti-Gram+, Antimicrobial
2552	DRAMP01786	Temporin-CPb (Frogs, amphibians, animals)	Antibacterial, Antimicrobial
2553	DRAMP01792	Temporin 1HKa (Temporin-1HKa; Frogs, amphibians, animals)	Antibacterial, Antimicrobial
2554	DRAMP01793	Temporin-1OLa (Temporin 1OLa; Frogs, amphibians, animals)	Antimicrobial ,

2555	DRAMP01794	Temporin-1OLb (Temporin 1OLb; Frogs, amphibians, animals)	Antimicrobial ,
2556	DRAMP01795	Temporin-1Ga (Frogs, amphibians, animals)	Antibacterial, Antimicrobial
2557	DRAMP01796	Temporin-1Gb (Frogs, amphibians, animals)	Antibacterial, Anti-Gram+, Antimicrobial
2558	DRAMP01797	Temporin-1Gc (Frogs, amphibians, animals)	Antibacterial, Anti-Gram+, Antimicrobial
2559	DRAMP01798	Temporin-1Gd (Frogs, amphibians, animals)	Antibacterial, Anti-Gram+, Antimicrobial
2560	DRAMP01799	Temporin-1PRa (Temporin 1PRa; Frogs, amphibians, animals)	Antibacterial, Anti-Gram+, Anti-Gram-, Antimicrobial
2561	DRAMP01800	Temporin-1PRb (Temporin 1PRb; Frogs, amphibians, animals)	Antibacterial, Antifungal, Anti-Gram+, Anti-Gram-, Antimicrobial
2562	DRAMP01801	Temporin-1DYa (Frogs, amphibians, animals)	Antibacterial, Anti-Gram+, Anti-Gram-, Antimicrobial
2563	DRAMP01802	Temporin-PTa (Frogs, amphibians, animals)	Antibacterial, Anti-Gram+, Anti-Gram-, Antimicrobial
2564	DRAMP01803	Temporin-CDYa (Frogs, amphibians, animals)	Antibacterial, Antimicrobial
2565	DRAMP01804	Temporin-CDYb (Brevinin-1CDYb; Frogs, amphibians, animals)	Antibacterial, Anti-Gram+, Anti-Gram-, Antimicrobial
2566	DRAMP01805	Temporin-CDYd (Frogs, amphibians, animals)	Antibacterial, Antimicrobial
2567	DRAMP01806	Temporin-CDYe (Frogs, amphibians, animals)	Antibacterial, Antimicrobial
2568	DRAMP01810	Temporin-1BYa (Frogs, amphibians, animals)	Antibacterial, Anti-Gram+, Antimicrobial
2569	DRAMP18352	Durancin GL (bacteriocin)	Antibacterial, Antimicrobial
2570	DRAMP18301	nocathiacin I(Bacteriocin)	Antibacterial, Antimicrobial
2571	DRAMP01387	Odorranain-P2a (OdP2a; Frogs, amphibians, animals)	Antimicrobial, Antibacterial, Antifungal, Anti-Gram+, Anti-Gram-,
2572	DRAMP01386	Odorranain-P1a (OdP1a; Brevinin-1HS1; Brevinin-1-OA2; Frogs, amphibians, animals)	Antimicrobial, Antibacterial, Antifungal, Anti-Gram+, Anti-Gram-,
2573	DRAMP01824	Temporin-1Ja (Frogs, amphibians, animals)	Antibacterial, Anti-Gram+, Anti-Gram-, Antimicrobial
2574	DRAMP01825	Temporin-1DRa (Frogs, amphibians, animals)	Antibacterial, Antifungal, Antimicrobial
2575	DRAMP01740	Temporin-C (Frogs, amphibians, animals)	Antibacterial, Antimicrobial
2576	DRAMP01126	Maximin-H4 (Toads, amphibians, animals)	Antimicrobial, Antibacterial, Antifungal, Anti-Gram+, Anti-Gram-,
2577	DRAMP01125	Maximin-H3 (Toads, amphibians, animals)	Antimicrobial, Antibacterial, Antifungal, Anti-Gram+, Anti-Gram-,
2578	DRAMP18299	Taromycin A (Bacteriocin)	Antibacterial, Anti-Gram+, Antimicrobial
2579	DRAMP01835	Ascaphin-1M (Frogs, amphibians, animals)	Antibacterial, Antimicrobial
2580	DRAMP01836	Ascaphin-3M (Frogs, amphibians, animals)	Antibacterial, Antimicrobial
2581	DRAMP01837	Ascaphin-4M (Frogs, amphibians, animals)	Antibacterial, Antimicrobial
2582	DRAMP01838	Ascaphin-5M (Frogs, amphibians, animals)	Antibacterial, Antimicrobial
2583	DRAMP01839	Ascaphin-7M (Frogs, amphibians, animals)	Antibacterial, Antimicrobial
2584	DRAMP01841	Ascaphin-2 (Frogs, amphibians, animals)	Antibacterial, Anti-Gram-, Antimicrobial
2585	DRAMP01843	Ascaphin-4 (Frogs, amphibians, animals)	Antibacterial, Anti-Gram-, Antimicrobial
2586	DRAMP01845	Ascaphin-6 (Frogs, amphibians, animals)	Antibacterial, Anti-Gram-, Antimicrobial
2587	DRAMP01848	[D4k]ascaphin-8	Antimicrobial,
2588	DRAMP18296	Leucocin H beta(Bacteriocin)	Antibacterial, Anti-Gram+, Antimicrobial
2589	DRAMP01853	Brevinin-1-RAB2 antimicrobial peptide (Frogs, amphibians, animals)	Antimicrobial,
2590	DRAMP01854	Brevinin-2-RA21 antimicrobial peptide (Frogs, amphibians, animals)	Antimicrobial,
2591	DRAMP01855	Brevinin-2E-OG4 antimicrobial peptide (Frogs, amphibians, animals)	Antimicrobial,
2592	DRAMP18295	Leucocin H alpha(Bacteriocin)	Antibacterial, Anti-Gram+, Antimicrobial
2593	DRAMP01857	Brevinin-2-RA20 antimicrobial peptide (Frogs, amphibians, animals)	Antimicrobial,
2594	DRAMP01858	Brevinin-2-RA6 antimicrobial peptide (Frogs, amphibians, animals)	Antimicrobial,
2595	DRAMP01859	Brevinin-2-RA22 antimicrobial peptide (Frogs, amphibians, animals)	Antimicrobial,
2596	DRAMP01860	Brevinin-1RTa antimicrobial peptide (Frogs, amphibians, animals)	Antimicrobial,
2597	DRAMP01861	Brevinin-2RTb antimicrobial peptide (Frogs, amphibians, animals)	Antimicrobial,
2598	DRAMP01862	Brevinin-1RTb antimicrobial peptide (Frogs, amphibians, animals)	Antimicrobial,
2599	DRAMP01863	Brevinin-2RTa antimicrobial peptide (Frogs, amphibians, animals)	Antimicrobial,

2600	DRAMP01864	Brevinin-2-RA13 antimicrobial peptide (Frogs, amphibians, animals)	Antimicrobial,
2601	DRAMP01868	Brevinin-1E-RTa antimicrobial peptide (Frogs, amphibians, animals)	Antibiotic, Antimicrobial,
2602	DRAMP01871	Brevinin-1SPc (Frogs, amphibians, animals)	Antibacterial, Antifungal, Antimicrobial
2603	DRAMP01874	Brevinin-1Eba (Frogs, amphibians, animals)	Antimicrobial,
2604	DRAMP01882	Pleurain-M1 antimicrobial peptide (Frogs, amphibians, animals)	Antimicrobial,
2605	DRAMP01883	Salivary gland antimicrobial peptide 1	Antimicrobial,
2606	DRAMP01884	Nigroain-F antimicrobial peptide (Frogs, amphibians, animals)	Antimicrobial,
2607	DRAMP01894	Brevinin 1Pg (Frogs, amphibians, animals)	Antimicrobial,
2608	DRAMP01895	Brevinin-2CE (Frogs, amphibians, animals)	Antibacterial, Antimicrobial
2609	DRAMP01901	Brevinin-2CG1 (Frogs, amphibians, animals)	Antibacterial, Antifungal, Anti-Gram-, Antimicrobial
2610	DRAMP01902	Brevinin-2E-OG7 (Frogs, amphibians, animals)	Antimicrobial,
2611	DRAMP01903	Brevinin-1E-OG1 (brevinin-1E-OG3; Frogs, amphibians, animals)	Antimicrobial,
2612	DRAMP01904	Brevinin-2E-OG2 (Frogs, amphibians, animals)	Antimicrobial,
2613	DRAMP01905	Brevinin-1E-OG5 (Frogs, amphibians, animals)	Antimicrobial,
2614	DRAMP01906	Brevinin-1E-OG4 (Frogs, amphibians, animals)	Antimicrobial,
2615	DRAMP01907	Antimicrobial peptide brevinin-1E-OG7 (Frogs, amphibians, animals)	Antimicrobial,
2616	DRAMP01908	Brevinin 2Ta (Frogs, amphibians, animals)	Antimicrobial,
2617	DRAMP01915	Brevinin-2GRb (Frogs, amphibians, animals)	Antibacterial, Antimicrobial
2618	DRAMP01916	Brevinin-2GU (Frogs, amphibians, animals)	Antimicrobial,
2619	DRAMP01917	Brevinin-1PLa (Frogs, amphibians, animals)	Antifungal, Antimicrobial
2620	DRAMP01923	Brevinin-1R (Frogs, amphibians, animals)	Antimicrobial ,
2621	DRAMP01924	Brevinin-1Ea (Frogs, amphibians, animals)	Antibacterial, Anti-Gram+, Anti-Gram-, Antimicrobial
2622	DRAMP01925	Brevinin-1Eb (Frogs, amphibians, animals)	Antibacterial, Anti-Gram+, Anti-Gram-, Antimicrobial
2623	DRAMP01926	Brevinin-1Ec (Frogs, amphibians, animals)	Antibacterial, Anti-Gram+, Anti-Gram-, Antimicrobial
2624	DRAMP01927	Brevinin-2Ea (Frogs, amphibians, animals)	Antibacterial, Anti-Gram+, Anti-Gram-, Antimicrobial
2625	DRAMP01928	Brevinin-2Eb (Frogs, amphibians, animals)	Antibacterial, Anti-Gram+, Anti-Gram-, Antimicrobial
2626	DRAMP01929	Brevinin-2Ec (Frogs, amphibians, animals)	Antibacterial, Anti-Gram+, Anti-Gram-, Antimicrobial
2627	DRAMP01930	Brevinin-1Ed (Frogs, amphibians, animals)	Antibacterial, Anti-Gram+, Antimicrobial
2628	DRAMP01931	Brevinin-2Ed (Frogs, amphibians, animals)	Antibacterial, Anti-Gram+, Anti-Gram-, Antimicrobial
2629	DRAMP01932	Brevinin-2Ee (Frogs, amphibians, animals)	Antibacterial, Anti-Gram+, Anti-Gram-, Antimicrobial
2630	DRAMP01936	Brevinin-2Ek (Frogs, amphibians, animals)	Antibacterial, Anti-Gram-, Antimicrobial
2631	DRAMP01945	Brevinin-1SE (Frogs, amphibians, animals)	Antibacterial, Anti-Gram+, Anti-Gram-, Antimicrobial
2632	DRAMP01946	Brevinin-20a (Frogs, amphibians, animals)	Antibacterial, Antifungal, Anti-Gram+, Anti-Gram-, Antimicrobial
2633	DRAMP01947	Brevinin-20b (Frogs, amphibians, animals)	Antibacterial, Antifungal, Anti-Gram+, Anti-Gram-, Antimicrobial
2634	DRAMP18294	lactococcin Z(Bacteriocin)	Antibacterial, Anti-Gram+, Anti-Gram-, Antimicrobial
2635	DRAMP01952	Brevinin-1PTb (Frogs, amphibians, animals)	Antibacterial, Anti-Gram+, Anti-Gram-, Antimicrobial
2636	DRAMP01954	Brevinin-2HSb (Frogs, amphibians, animals)	Antibacterial, Anti-Gram+, Anti-Gram-, Antimicrobial
2637	DRAMP01958	Brevinin-2PTd (Frogs, amphibians, animals)	Antibacterial, Anti-Gram+, Anti-Gram-, Antimicrobial
2638	DRAMP01960	Brevinin-1BYa (Frogs, amphibians, animals)	Antibacterial, Antifungal, Anti-Gram+, Anti-Gram-, Antimicrobial
2639	DRAMP01961	Brevinin-1BYb (Frogs, amphibians, animals)	Antibacterial, Antifungal, Anti-Gram+, Anti-Gram-, Antimicrobial
2640	DRAMP01962	Brevinin-1BYc (Frogs, amphibians, animals)	Antibacterial, Antifungal, Anti-Gram+, Antimicrobial
2641	DRAMP01964	Brevinin-1BLb (Frogs, amphibians, animals)	Antibacterial, Antifungal, Antimicrobial
2642	DRAMP01966	Brevinin-1Ya (Frogs, amphibians, animals)	Antibacterial, Antifungal, Anti-Gram+, Anti-Gram-, Antimicrobial
2643	DRAMP01967	Brevinin-1Yb (Frogs, amphibians, animals)	Antibacterial, Antifungal, Anti-Gram+, Anti-Gram-, Antimicrobial
2644	DRAMP01972	Brevinin-1ZHc (Frogs, amphibians, animals)	Antibacterial, Antifungal, Antimicrobial
2645	DRAMP01973	Brevinin-1ZHd (Frogs, amphibians, animals)	Antibacterial, Antifungal, Antimicrobial
2646	DRAMP01975	Brevinin-1Ra (Frogs, amphibians, animals)	Antimicrobial ,
2647	DRAMP01976	Brevinin-2Eg (Frogs, amphibians, animals)	Antibacterial, Anti-Gram+, Anti-Gram-, Antimicrobial
2648	DRAMP18292	LsbA(Bacteriocin)	Antimicrobial, Anti-Gram+,

2649	DRAMP01978	Brevinin-2R (Frogs, amphibians, animals)	Antibacterial, Anticancer, Antimicrobial
2650	DRAMP01979	Brevinin-2Ra (Frogs, amphibians, animals)	Antimicrobial ,
2651	DRAMP01980	Brevinin-2Rb (Frogs, amphibians, animals)	Antimicrobial ,
2652	DRAMP01981	Brevinin-2Rc (Frogs, amphibians, animals)	Antimicrobial ,
2653	DRAMP01982	Brevinin-2Rd (Frogs, amphibians, animals)	Antimicrobial ,
2654	DRAMP18291	Garviecin LG34(Bacteriocin)	Antibacterial, Anti-Gram+, Anti-Gram-, Antimicrobial
2655	DRAMP01984	Brevinin-1HS2 (Frogs, amphibians, animals)	Antimicrobial ,
2656	DRAMP01985	Brevinin-2HS1 (Frogs, amphibians, animals)	Antibacterial, Antifungal, Antiviral, Antimicrobial
2657	DRAMP01987	Brevinin-2HS3 (Frogs, amphibians, animals)	Antimicrobial ,
2658	DRAMP01988	Brevinin-1LTa (Frogs, amphibians, animals)	Antibacterial, Antifungal, Antimicrobial
2659	DRAMP01989	Brevinin-1LTb (Frogs, amphibians, animals)	Antibacterial, Antifungal, Antimicrobial
2660	DRAMP01991	Brevinin-1LT2 (Frogs, amphibians, animals)	Antibacterial, Antimicrobial
2661	DRAMP01992	Brevinin-1CPa (Frogs, amphibians, animals)	Antibacterial, Antimicrobial
2662	DRAMP01993	Brevinin-1Wa (Frogs, amphibians, animals)	Antimicrobial ,
2663	DRAMP18289	Bactofencin A (Bacteriocin)	Antibacterial, Anti-Gram+, Antimicrobial
2664	DRAMP18290	Garvicin A (Bacteriocin)	Antibacterial, Anti-Gram+, Antimicrobial
2665	DRAMP18287	Blp1b(Bacteriocin)	Antibacterial, Anti-Gram+, Antimicrobial
2666	DRAMP18288	Sln1 (Bacteriocin)	Antibacterial, Antimicrobial
2667	DRAMP18286	Blp1a(Bacteriocin)	Antibacterial, Anti-Gram+, Antimicrobial
2668	DRAMP02002	Brevinin-1P (Frogs, amphibians, animals)	Antibacterial, Antimicrobial
2669	DRAMP02003	Brevinin-1S (Frogs, amphibians, animals)	Antibacterial, Antimicrobial
2670	DRAMP18285	Bacteriocin LS2 (Bacteriocin)	Antibacterial, Anti-Gram+, Antimicrobial
2671	DRAMP18284	Reutericin 6 (Bacteriocin)	Antibacterial, Anti-Gram+, Antimicrobial
2672	DRAMP18283	Plantaricin ZJ5 (Bacteriocin)	Antibacterial, Antimicrobial
2673	DRAMP02011	Brevinin-2Tb (Frogs, amphibians, animals)	Antibacterial, Antimicrobial
2674	DRAMP02012	Brevinin-1T (Brevinin-2T; Frogs, amphibians, animals)	Antibacterial, Anti-Gram+, Anti-Gram-, Antimicrobial
2675	DRAMP02013	Brevinin-1Ta (Frogs, amphibians, animals)	Antibacterial, Anti-Gram+, Anti-Gram-, Antimicrobial
2676	DRAMP02014	Brevinin-2Tc (Frogs, amphibians, animals)	Antibacterial, Antimicrobial
2677	DRAMP02015	Brevinin-2Td (Frogs, amphibians, animals)	Antibacterial, Antimicrobial
2678	DRAMP02016	Brevinin-1DYa (Frogs, amphibians, animals)	Antibacterial, Anti-Gram+, Anti-Gram-, Antimicrobial
2679	DRAMP02017	Brevinin-2DYa (Frogs, amphibians, animals)	Antibacterial, Anti-Gram+, Anti-Gram-, Antimicrobial
2680	DRAMP02018	Brevinin-1DYb (Brevinin-1CDYb; Frogs, amphibians, animals)	Antibacterial, Anti-Gram+, Anti-Gram-, Antimicrobial
2681	DRAMP02020	Brevinin-1DYc (Frogs, amphibians, animals)	Antibacterial, Anti-Gram+, Anti-Gram-, Antimicrobial
2682	DRAMP18282	Plantaricin ZJ008 (Bacteriocin)	Antibacterial, Antimicrobial
2683	DRAMP02027	Brevinin-2CDYb (Frogs, amphibians, animals)	Antimicrobial,
2684	DRAMP18281	Plantaricin KL-1Y (Bacteriocin)	Antibacterial, Anti-Gram+, Anti-Gram-, Antimicrobial
2685	DRAMP02029	Brevinin-1CDYd (Frogs, amphibians, animals)	Antibacterial, Antimicrobial
2686	DRAMP18280	Plantaricin 163 (Bacteriocin)	Antibacterial, Antimicrobial
2687	DRAMP02079	Brevinin-1AVa (Frogs, amphibians, animals)	Antimicrobial,
2688	DRAMP02080	Brevinin-1AVb (Frogs, amphibians, animals)	Antimicrobial,
2689	DRAMP18279	Plantaricin A(Bacteriocin)	Antibacterial, Anti-Gram+, Antimicrobial
2690	DRAMP18277	Halocin C8 (Bacteriocin)	Antibacterial, Antimicrobial
2691	DRAMP02086	Brevinin-1PRa (Frogs, amphibians, animals)	Antibacterial, Antifungal, Antimicrobial
2692	DRAMP02087	Brevinin-1PRb (Frogs, amphibians, animals)	Antibacterial, Antifungal, Antimicrobial
2693	DRAMP02088	Brevinin-1PRc (Frogs, amphibians, animals)	Antibacterial, Antifungal, Antimicrobial
2694	DRAMP02089	Brevinin-1La (Brevinin-1PRd; Frogs, amphibians, animals)	Antibacterial, Anti-Gram+, Anti-Gram-, Antimicrobial
2695	DRAMP01124	Maximin-H2 (Toads, amphibians, animals)	Antimicrobial, Antibacterial, Antifungal, Anti-Gram+, Anti-Gram-,
2696	DRAMP01123	Maximin-H1 (Toads, amphibians, animals)	Antimicrobial, Antibacterial, Antifungal, Anti-Gram+, Anti-Gram-,
2697	DRAMP01111	Maximin-5 (Toads, amphibians, animals)	Antimicrobial, Antibacterial, Antifungal, Antiviral, Anticancer, Anti-Gram+, Anti-Gram-,
2698	DRAMP01110	Maximin-4 (Toads, amphibians, animals)	Antimicrobial, Antibacterial, Antifungal, Antiviral, Anticancer, Anti-Gram+, Anti-Gram-,
2699	DRAMP01109	Maximin-3 (Toads, amphibians, animals)	Antimicrobial, Antibacterial, Antifungal, Antiviral, Anticancer, Anti-Gram+, Anti-Gram-,
2700	DRAMP02100	Brevinin-1Pe (Frogs, amphibians, animals)	Antibacterial, Antifungal, Anti-Gram+, Anti-Gram-, Antimicrobial

2701	DRAMP02103	Brevinin-1RTc (Frogs, amphibians, animals)	Antibacterial, Antimicrobial
2702	DRAMP18353	Durancin L28-1A (bacteriocin)	Antibacterial, Antimicrobial
2703	DRAMP02107	Brevinin-1E-OG2 antimicrobial peptide (Frogs, amphibians, animals)	Antimicrobial,
2704	DRAMP18354	Hyicin 3682 (bacteriocin)	Antibacterial, Antimicrobial
2705	DRAMP18273	Enterocin CRL35 (Bacteriocin)	Antibacterial, Antiviral, Anti-Gram+, Antimicrobial
2706	DRAMP18272	Enterocin AS-48RJ (Bacteriocin)	Antibacterial, Anti-Gram+, Anti-Gram-, Antimicrobial
2707	DRAMP02115	Raniseptin-2 (Rsp-2; Frogs, amphibians, animals)	Antibacterial, Antimicrobial
2708	DRAMP02116	Raniseptin-3 (Rsp-3; Frogs, amphibians, animals)	Antibacterial, Antimicrobial
2709	DRAMP02117	Raniseptin-4 (Rsp-4; Frogs, amphibians, animals)	Antibacterial, Antimicrobial
2710	DRAMP02118	Raniseptin-5 (Rsp-5; Frogs, amphibians, animals)	Antibacterial, Antimicrobial
2711	DRAMP02119	Raniseptin-6 (Rsp-6; Frogs, amphibians, animals)	Antibacterial, Antimicrobial
2712	DRAMP02120	Raniseptin-7 (Rsp-7; Frogs, amphibians, animals)	Antibacterial, Antimicrobial
2713	DRAMP02121	Raniseptin-8 (Rsp-8; Frogs, amphibians, animals)	Antibacterial, Antimicrobial
2714	DRAMP02122	Raniseptin-9 (Rsp-9; Frogs, amphibians, animals)	Antibacterial, Antimicrobial
2715	DRAMP02123	Hylin-b1 (Hy-b1; Frogs, amphibians, animals)	Antimicrobial,
2716	DRAMP02124	Hylin-b2 (Hy-b2; Frogs, amphibians, animals)	Antimicrobial,
2717	DRAMP02126	Melittin-like peptide (MLP; Frogs, amphibians, animals)	Antibacterial, Antimicrobial
2718	DRAMP02132	Antimicrobial peptide 3 (XT-3; Levitide-like peptide; Frogs, amphibians, animals)	Antibacterial, Anti-Gram+, Anti-Gram-, Antimicrobial
2719	DRAMP02134	Antimicrobial peptide 5 (XT-5; PGLa-like peptide; Frogs, amphibians, animals)	Antibacterial, Antifungal, Anti-Gram+, Anti-Gram-, Antimicrobial
2720	DRAMP02137	[G4K]XT-7 (Frogs, amphibians, animals)	Antibacterial, Antimicrobial
2721	DRAMP02138	XTG1 (Frogs, amphibians, animals)	Antibacterial, Antimicrobial
2722	DRAMP02139	XTG2 (Frogs, amphibians, animals)	Antibacterial, Antimicrobial
2723	DRAMP02140	XPF-SP1 (Frogs, amphibians, animals)	Antibacterial, Antimicrobial
2724	DRAMP02141	XPF-SP2 (Frogs, amphibians, animals)	Antibacterial, Antimicrobial
2725	DRAMP02143	CPF-SP2 (Frogs, amphibians, animals)	Antibacterial, Antimicrobial
2726	DRAMP02144	Reactive oxygen species modulator 1 (ROS modulator 1; Frogs, amphibians, animals)	Antibacterial, Antimicrobial
2727	DRAMP02145	Electrin-1 (Frogs, amphibians, animals)	Antimicrobial,
2728	DRAMP02146	Electrin-3 (Frogs, amphibians, animals)	Antimicrobial,
2729	DRAMP02147	Electrin-4 (Frogs, amphibians, animals)	Antimicrobial,
2730	DRAMP02148	Electrin-5 (Frogs, amphibians, animals)	Antimicrobial,
2731	DRAMP02149	Electrin-2.1 (Frogs, amphibians, animals)	Antimicrobial,
2732	DRAMP02150	Electrin-2.2 (Frogs, amphibians, animals)	Antimicrobial,
2733	DRAMP02151	Preprofallaxidin-4 (Frogs, amphibians, animals)	Antimicrobial,
2734	DRAMP02152	Preprofallaxidin-5 (Frogs, amphibians, animals)	Antimicrobial,
2735	DRAMP02153	Hemolytic protein A1 (Frogs, amphibians, animals)	Antimicrobial,
2736	DRAMP02154	Hemolytic protein B9 (Frogs, amphibians, animals)	Antimicrobial,
2737	DRAMP02155	[Leu8]-phyllolitorin (Frogs, amphibians, animals)	Antimicrobial,
2738	DRAMP02156	Litorin (Frogs, amphibians, animals)	Antimicrobial,
2739	DRAMP02157	[Phe8]-phyllolitorin (Frogs, amphibians, animals)	Antimicrobial,
2740	DRAMP02158	Rhodei-litorin (Frogs, amphibians, animals)	Antimicrobial,
2741	DRAMP02159	Melittin-related peptide (Frogs, amphibians, animals)	Antimicrobial,
2742	DRAMP02160	Peroniin-1.1b (Frogs, amphibians, animals)	Antimicrobial,
2743	DRAMP02161	Peroniin-1.2a (Frogs, amphibians, animals)	Antimicrobial,
2744	DRAMP02162	Peroniin-1.3a (Frogs, amphibians, animals)	Antimicrobial,
2745	DRAMP02163	Peroniin-1.5 (Frogs, amphibians, animals)	Antimicrobial,
2746	DRAMP02164	Ranatensin (Frogs, amphibians, animals)	Antimicrobial,
2747	DRAMP02165	Ranatensin-C (Frogs, amphibians, animals)	Antimicrobial,
2748	DRAMP02166	Ranatensin-R (Frogs, amphibians, animals)	Antimicrobial,
2749	DRAMP02167	Tryptophyllin-T2-1 (Pha-T2-1; Tryptophyllin-11; Frogs, amphibians, animals)	Antimicrobial,
2750	DRAMP02168	Tryptophyllin-T2-2 (Pha-T2-2; Tryptophyllin-3; Frogs, amphibians, animals)	Antimicrobial,
2751	DRAMP18397	Um4 (scorpions, arachnids, Chelicerata, arthropods, invertebrates, animals)	Antibacterial, Antimicrobial

2752	DRAMP02170	Tryptophyllin-T2-4 (Pha-T2-4; Tryptophyllin-8; Frogs, amphibians, animals)	Antimicrobial,
2753	DRAMP02171	Tryptophyllin-T2-5 (Pha-T2-5; Tryptophyllin-1; Frogs, amphibians, animals)	Antimicrobial,
2754	DRAMP02172	Tryptophyllin-T2-6 (Pha-T2-6; Tryptophyllin-6; Frogs, amphibians, animals)	Antimicrobial,
2755	DRAMP02173	Tryptophyllin-T2-7 (Pha-T2-7; Tryptophyllin-7; Frogs, amphibians, animals)	Antimicrobial,
2756	DRAMP02174	Tryptophyllin-T2-8 (Pha-T2-8; Tryptophyllin-12; Frogs, amphibians, animals)	Antimicrobial,
2757	DRAMP02175	Tryptophyllin-T2-9 (Pha-T2-9; Tryptophyllin-4; Frogs, amphibians, animals)	Antimicrobial,
2758	DRAMP02176	Tryptophyllin-T3-1 (Pj-T3-1; Frogs, amphibians, animals)	Antimicrobial ,
2759	DRAMP02177	Tryptophyllin-T3-2 (Pj-T3-2; Frogs, amphibians, animals)	Antimicrobial ,
2760	DRAMP02178	Tryptophyllin-T3-3 (Pj-T3-3; Frogs, amphibians, animals)	Antimicrobial ,
2761	DRAMP02179	Tryptophyllin-T3-1 (Pha-T3-1; Tryptophyllin-9; Frogs, amphibians, animals)	Antimicrobial ,
2762	DRAMP02180	Tryptophyllin-T3-2 (Pha-T3-2; Tryptophyllin-10; Frogs, amphibians, animals)	Antimicrobial ,
2763	DRAMP02181	Tryptophyllin-1 (Frogs, amphibians, animals)	Antimicrobial ,
2764	DRAMP02182	Tryptophyllin-2 (Frogs, amphibians, animals)	Antimicrobial ,
2765	DRAMP02183	Tryptophyllin-3 (Frogs, amphibians, animals)	Antimicrobial ,
2766	DRAMP02184	Tryptophyllin-4 (Frogs, amphibians, animals)	Antimicrobial ,
2767	DRAMP02185	Tryptophyllin-13 (Frogs, amphibians, animals)	Antimicrobial ,
2768	DRAMP02186	Tryptophyllin-14 (Frogs, amphibians, animals)	Antimicrobial ,
2769	DRAMP02187	Ranatuerin-2AVa (Frogs, amphibians, animals)	Antibacterial, Anti-Gram+, Antimicrobial
2770	DRAMP02188	Ranatuerin-2AVb (Frogs, amphibians, animals)	Antibacterial, Antimicrobial
2771	DRAMP02189	Ranatuerin-2PLg (Frogs, amphibians, animals)	Antimicrobial,
2772	DRAMP02190	Ranatuerin-2Vb (ranat2Vb; 2VEb; Frogs, amphibians, animals)	Antimicrobial ,
2773	DRAMP18271	NKR-5-3B(Bacteriocin)	Antibacterial, Anti-Gram+, Antimicrobial
2774	DRAMP02192	Ranatuerin-2PTa (Frogs, amphibians, animals)	Antimicrobial ,
2775	DRAMP02193	Ranatuerin-2PTb (Frogs, amphibians, animals)	Antimicrobial ,
2776	DRAMP02194	Ranatuerin-2BLa (Frogs, amphibians, animals)	Antimicrobial ,
2777	DRAMP02195	Ranatuerin-2BLc (Frogs, amphibians, animals)	Antimicrobial ,
2778	DRAMP02196	Ranatuerin-2BLb (Frogs, amphibians, animals)	Antimicrobial ,
2779	DRAMP02197	Ranatuerin-2SRa (Frogs, amphibians, animals)	Antimicrobial ,
2780	DRAMP02198	Ranatuerin-2SRb (Frogs, amphibians, animals)	Antimicrobial ,
2781	DRAMP02199	Ranatuerin-2TGa (Frogs, amphibians, animals)	Antimicrobial ,
2782	DRAMP02200	Ranatuerin-2RC antimicrobial peptide (Frogs, amphibians, animals)	Antimicrobial ,
2783	DRAMP02201	Ranatuerin-2BYa (Frogs, amphibians, animals)	Antimicrobial ,
2784	DRAMP02202	Ranatuerin-2BYb (Frogs, amphibians, animals)	Antimicrobial ,
2785	DRAMP02203	Ranatuerin-5Ca antimicrobial peptide (Frogs, amphibians, animals)	Antimicrobial ,
2786	DRAMP02204	Ranatuerin-5Cb antimicrobial peptide (Frogs, amphibians, animals)	Antimicrobial ,
2787	DRAMP02205	Ranatuerin-2YJ (Frogs, amphibians, animals)	Antimicrobial ,
2788	DRAMP02206	Ranatuerin 2CHa (Frogs, amphibians, animals)	Antimicrobial ,
2789	DRAMP02207	Ranatuerin 2CHb (Frogs, amphibians, animals)	Antimicrobial ,
2790	DRAMP02208	Ranatuerin-1Ca antimicrobial peptide (Frogs, amphibians, animals)	Antimicrobial ,
2791	DRAMP02209	Ranatuerin-1Cb antimicrobial peptide (Frogs, amphibians, animals)	Antimicrobial ,
2792	DRAMP02210	Ranatuerin-2AMa protein (Frogs, amphibians, animals)	Antimicrobial ,
2793	DRAMP02211	Ranatuerin-2AMb protein (Frogs, amphibians, animals)	Antimicrobial ,
2794	DRAMP02212	Antimicrobial peptide ranatuerin-2ZHa (Frogs, amphibians, animals)	Antimicrobial ,
2795	DRAMP02213	Ranatuerin-2TOa (Frogs, amphibians, animals)	Antimicrobial ,
2796	DRAMP02214	Ranatuerin-2TOb (Frogs, amphibians, animals)	Antimicrobial ,
2797	DRAMP02215	Ranatuerin-2TOc (Frogs, amphibians, animals)	Antimicrobial ,

2798	DRAMP02216	Ranatuerin-2TOd (Frogs, amphibians, animals)	Antimicrobial ,
2799	DRAMP02217	Ranatuerin-2Ra (Frogs, amphibians, animals)	Antimicrobial ,
2800	DRAMP02218	Ranatuerin-2R (Frogs, amphibians, animals)	Antimicrobial ,
2801	DRAMP02226	Ranatuerin-2ARa (Frogs, amphibians, animals)	Antibacterial, Antimicrobial
2802	DRAMP02227	Ranatuerin-2Pa (Frogs, amphibians, animals)	Antimicrobial,
2803	DRAMP02232	Ranatuerin-5 (Frogs, amphibians, animals)	Antibacterial, Anti-Gram+, Antimicrobial
2804	DRAMP02240	Ranatuerin-1Gb (Frogs, amphibians, animals)	Antibacterial, Antimicrobial
2805	DRAMP02242	Ranatuerin-2PRa (Frogs, amphibians, animals)	Antibacterial, Antifungal, Antimicrobial
2806	DRAMP02243	Ranatuerin-1T (Brevinin-2T; Frogs, amphibians, animals)	Antibacterial, Anti-Gram+, Anti-Gram-, Antimicrobial
2807	DRAMP01384	Odorranain-N1 (OdN1; Frogs, amphibians, animals)	Antifungal, Antimicrobial
2808	DRAMP18270	Enterocin NKR-5-3Z(Bacteriocin)	Antibacterial, Anti-Gram+, Antimicrobial
2809	DRAMP18269	Enterocin NKR-5-3A(Bacteriocin)	Antibacterial, Anti-Gram+, Antimicrobial
2810	DRAMP02249	Ranatuerin-2SEB (Frogs, amphibians, animals)	Antibacterial, Anti-Gram+, Anti-Gram-, Antimicrobial
2811	DRAMP02250	Ranatuerin-2SEC (Frogs, amphibians, animals)	Antibacterial, Anti-Gram+, Anti-Gram-, Antimicrobial
2812	DRAMP02253	Ranatuerin-IIbYb (Ranatuerin-2bYa; Frogs, amphibians, animals)	Antibacterial, Anti-Gram-, Antimicrobial
2813	DRAMP02258	Ranatuerin-IIbYa (Ranatuerin-2bYa; Frogs, amphibians, animals)	Antibacterial, Anti-Gram+, Anti-Gram-, Antimicrobial
2814	DRAMP02265	Reactive oxygen species modulator 1 (ROS modulator 1; Frogs, amphibians, animals)	Antibacterial, Antimicrobial
2815	DRAMP02266	Prolevitide (Frogs, amphibians, animals)	Antimicrobial ,
2816	DRAMP02267	Antimicrobial amphipathic helix-forming peptide (Frogs, amphibians, animals)	Antibacterial, Antimicrobial
2817	DRAMP02270	Magainin-1 (Magainin I; chain of Magainins; Frogs, amphibians, animals)	Antibacterial, Antimicrobial
2818	DRAMP02284	Pseudin-1 (Pseudin 1; Frogs, amphibians, animals)	Antibacterial, Antifungal, Anti-Gram+, Anti-Gram-, Antimicrobial
2819	DRAMP02285	Pseudin-2 (Pseudin 2; Frogs, amphibians, animals)	Antibacterial, Antifungal, Anti-Gram+, Anti-Gram-, Antimicrobial
2820	DRAMP02286	Pseudin-3 (Pseudin 3; Frogs, amphibians, animals)	Antibacterial, Antifungal, Anti-Gram+, Anti-Gram-, Antimicrobial
2821	DRAMP02287	Pseudin-4 (Pseudin 4; Frogs, amphibians, animals)	Antibacterial, Antifungal, Anti-Gram+, Anti-Gram-, Antimicrobial
2822	DRAMP02297	Gaegurin-6-RN antimicrobial peptide (Frogs, amphibians, animals)	Antimicrobial,
2823	DRAMP02298	Gaegurin-6-RN antimicrobial peptide (Frogs, amphibians, animals)	Antimicrobial,
2824	DRAMP02299	Gaegurin-6-RN antimicrobial peptide (Frogs, amphibians, animals)	Antimicrobial,
2825	DRAMP02301	Riparin-1.5 amide (Frogs, amphibians, animals)	Antimicrobial ,
2826	DRAMP02302	Riparin-1.3 (Frogs, amphibians, animals)	Antimicrobial ,
2827	DRAMP02303	Riparin-1.4 (Frogs, amphibians, animals)	Antimicrobial ,
2828	DRAMP02304	Riparin-1.5 acid (Frogs, amphibians, animals)	Antimicrobial ,
2829	DRAMP02309	Signiferin-2.2 (Frogs, amphibians, animals)	Antibacterial, Anti-Gram-, Antimicrobial
2830	DRAMP02310	HbbetaP-1 (fish, chordates, animals)	Antibacterial, Antiparasitic, Antimicrobial
2831	DRAMP02311	Lysozyme (1,4-beta-N-acetylmuramidase; starfish, chordates, animals)	Antimicrobial,
2832	DRAMP02313	Hepcidin-1 (fish, chordates, animals)	Antimicrobial,
2833	DRAMP02319	Grammistin Pp2a (Group II grammistin; fish, chordates, animals)	Antibacterial, Antimicrobial
2834	DRAMP18268	Enterocin NKR-5-3C(Bacteriocin)	Antibacterial, Anti-Gram+, Antimicrobial
2835	DRAMP02323	Grammistin Pp4b (Group I grammistin; fish, chordates, animals)	Antibacterial, Antimicrobial
2836	DRAMP02325	Moronecidin 1 (fish, chordates, animals)	Antibacterial, Antimicrobial
2837	DRAMP02326	Moronecidin 2 (fish, chordates, animals)	Antibacterial, Antimicrobial
2838	DRAMP02327	Moronecidin 3 (fish, chordates, animals)	Antibacterial, Antimicrobial
2839	DRAMP02328	Moronecidin 4 (fish, chordates, animals)	Antibacterial, Antimicrobial
2840	DRAMP02329	Moronecidin 5 (fish, chordates, animals)	Antibacterial, Antimicrobial
2841	DRAMP02332	Piscidin-3 (Pis-3; fish, chordates, animals)	Antibacterial, Antiviral, Anti-Gram+, Anti-Gram-, Antimicrobial
2842	DRAMP02333	Piscidin-4 (Pis-4; fish, chordates, animals)	Antibacterial, Antiviral, Antimicrobial

2843	DRAMP02334	Hepcidin (fish, chordates, animals)	Antimicrobial,
2844	DRAMP02335	Oncorhyncin I (Oncorhyncin 1; fish, chordates, animals)	Antibacterial, Antimicrobial
2845	DRAMP02338	Beta-defensin 1 (fish, chordates, animals)	Antimicrobial,
2846	DRAMP02339	Histone H6-like protein (fish, chordates, animals)	Antibacterial, Antimicrobial
2847	DRAMP02340	Salmocidin-1 (fish, chordates, animals)	Antibacterial, Antimicrobial
2848	DRAMP02341	Salmocidin-2A (fish, chordates, animals)	Antibacterial, Antimicrobial
2849	DRAMP02342	Salmocidin-IIb (fish, chordates, animals)	Antibacterial, Antimicrobial
2850	DRAMP02343	Salmocidin-3 (fish, chordates, animals)	Antibacterial, Antimicrobial
2851	DRAMP01375	Odorranain-E1 (OdE1; Frogs, amphibians, animals)	Antimicrobial, Antibacterial, Antifungal, Anti-Gram+, Anti-Gram-,
2852	DRAMP02345	Liver-expressed antimicrobial peptide 2A (LEAP-2A; fish, chordates, animals)	Antimicrobial,
2853	DRAMP02346	Liver-expressed antimicrobial peptide 2B	Antimicrobial,
2854	DRAMP02353	Pleurocidin-like peptide WFX (fish, chordates, animals; Predicted)	Antibacterial, Antifungal, Anti-Gram+, Anti-Gram-, Antimicrobial
2855	DRAMP18267	BacFL31(Bacteriocin)	Antilisterial, Antibacterial, Anti-Gram+, Antimicrobial
2856	DRAMP02356	HKPLP (pleurocidin-like peptide; glycine-rich; fish, chordates, animals)	Antibacterial, Antimicrobial
2857	DRAMP02369	Liver-expressed antimicrobial peptide 2 (fish, chordates, animals)	Antimicrobial , Anti-Gram-,
2858	DRAMP02370	Mucus envelope protein (parrotfish, chordates, animals)	Antimicrobial,
2859	DRAMP02371	Reactive oxygen species modulator 1 (ROS modulator 1; zebrafish, chordates, animals)	Antibacterial, Antimicrobial
2860	DRAMP02372	Hepcidin-1 (zebrafish, chordates, animals)	Antimicrobial ,
2861	DRAMP18266	Bacteriocin T8(Bacteriocin)	Antimicrobial, Anti-Gram+,
2862	DRAMP02375	Misgurin (weatherfish, chordates, animals)	Antibacterial, Antifungal, Antimicrobial
2863	DRAMP02383	saBD (seabream beta defensin; fish, chordates, animals)	Antibacterial, Antimicrobial
2864	DRAMP02385	Hepcidin AS-hepc6 (fish, chordates, animals)	Antibacterial, Antimicrobial
2865	DRAMP02387	Histone HIIb-3 (Antibacterial histone-like protein 3, HLP-3; catfishes, chordates, animals)	Antifungal, Antimicrobial
2866	DRAMP02388	Histone HIIb-1 (Antibacterial histone-like protein 1, HLP-1; catfishes, chordates, animals)	Antibacterial, Antifungal, Antimicrobial
2867	DRAMP02392	Hematopoietic antimicrobial peptide-29 (MgCath29; hagfishes, chordates, animals)	Antibacterial, Antifungal, Antimicrobial
2868	DRAMP02245	Ranatuerin-2Cb (Ranatuerin 2Cb; Frogs, amphibians, animals)	Antimicrobial, Antibacterial, Antifungal, Anti-Gram+, Anti-Gram-,
2869	DRAMP02398	Antimicrobial peptide GP-19 (GP-19)	Antibacterial, Antifungal, Anti-Gram+, Anti-Gram-, Antimicrobial
2870	DRAMP02399	Antimicrobial peptide EP-20 (EP-20)	Antifungal, Antimicrobial
2871	DRAMP02400	Antimicrobial peptide AJN-10 (AJN-10)	Antibacterial, Anti-Gram-, Antimicrobial
2872	DRAMP18265	Enterocin RM6 (Bacteriocin)	Antibacterial, Anti-Gram+, Antimicrobial
2873	DRAMP02404	Chitin-binding protein 3 (Mo-CBP3)	Antifungal, Antimicrobial
2874	DRAMP02405	Antifungal protein 1 (Pf-AFP1; Plants)	Antifungal, Antimicrobial
2875	DRAMP02406	Antimicrobial protein 2 (Si-AMP2; Plants)	Antibacterial, Antimicrobial
2876	DRAMP02407	Napin-like polypeptide (Contains: Napin-like polypeptide small chain and large chain)	Antibacterial, Anti-Gram+, Anti-Gram-, Antimicrobial
2877	DRAMP02408	2S albumin (To-A1)	Antifungal, Antimicrobial
2878	DRAMP02413	Ornithodoros defensin B (Ticks, Arthropods, animals)	Antimicrobial,
2879	DRAMP02414	Ornithodoros defensin C (Ticks, Arthropods, animals)	Antimicrobial,
2880	DRAMP02415	Ornithodoros defensin D (Ticks, Arthropods, animals)	Antimicrobial,
2881	DRAMP02416	Orinthodoros defensin A (Ticks, Arthropods, animals)	Antimicrobial,
2882	DRAMP02417	Defensin (Ticks, Arthropods, animals)	Antibacterial, Antimicrobial
2883	DRAMP02418	Longicin (Ticks, Arthropods, animals)	Antiparasitic, Antibacterial,Antifungal, Antimicrobial
2884	DRAMP02420	Amblyomma defensin peptide 1 (ADP-1; Ticks, Arthropods, animals)	Antibacterial, Antimicrobial
2885	DRAMP02424	Defensin (Ticks, Arthropods, animals)	Antibacterial, Antimicrobial
2886	DRAMP02426	Defensin (Varisin A1; Ticks, Arthropods, animals)	Antibacterial, Anti-Gram+, Antimicrobial
2887	DRAMP02431	Antimicrobial peptide microplusin (Ticks, Arthropods, animals)	Antibacterial, Antifungal , Antimicrobial
2888	DRAMP02435	Antifungal protein (PgAFP; Cys-rich)	Antifungal, Antimicrobial
2889	DRAMP02436	Chitinase	Antifungal, Antimicrobial

2890	DRAMP02437	Papillosin	Antibacterial, Anti-Gram+, Anti-Gram-, Antimicrobial
2891	DRAMP02438	Halocyntin	Antibacterial, Anti-Gram+, Anti-Gram-, Antimicrobial
2892	DRAMP02440	Sporulation-killing factor SkfA	Antibacterial, Antimicrobial
2893	DRAMP02442	Ericin S (lantibiotic-like peptide)	Antibacterial, Antimicrobial
2894	DRAMP02443	Ericin A (lantibiotic-like peptide)	Antibacterial, Antimicrobial
2895	DRAMP18264	Enterocin W beta(Bacteriocin)	Antibacterial, Anti-Gram+, Antimicrobial
2896	DRAMP02447	Antimicrobial protein 2 (Antimicrobial protein AN5-2)	Antibacterial, Anti-Gram+, Anti-Gram-, Antimicrobial
2897	DRAMP02448	Antibacterial protein LC3 (Antibacterial protein LCIII)	Antibacterial, Antimicrobial
2898	DRAMP02450	Lysozyme C (1,4-beta-N-acetylmuramidase C)	Antimicrobial,
2899	DRAMP02451	Lysozyme C (1,4-beta-N-acetylmuramidase C)	Antimicrobial,
2900	DRAMP02452	Lysozyme	Antimicrobial,
2901	DRAMP02453	S. litura moricin (Sl moricin; Insects, animals)	Antibacterial, Anti-Gram+, Anti-Gram-, Antimicrobial
2902	DRAMP02454	Theromacin (Arthropods, animals)	Antibacterial, Anti-Gram+, Anti-Gram-, Antimicrobial
2903	DRAMP02455	L-amino-acid oxidase (ACL-LAO; LAAO; LAO)	Antibacterial, Antiparasitic, Antimicrobial
2904	DRAMP02457	L-amino-acid oxidase (Balt-LAAO-I; LAAO; LAO; snakes, reptils, animals)	Antibacterial, Anti-Gram+, Anti-Gram-, Antimicrobial
2905	DRAMP02458	L-amino-acid oxidase (BiLAO; LAAO; LAO; snakes, reptils, animals)	Antibacterial, Antiparasitic, Antimicrobial
2906	DRAMP02459	L-amino-acid oxidase (BjarLAAO-I; LAAO; LAO; snakes, reptils, animals)	Antibacterial, Antiparasitic, Antitumor, Anti-Gram+, Antimicrobial
2907	DRAMP02460	L-amino-acid oxidase (LAAO; LAO; snakes, reptils, animals)	Antibacterial, Antiparasitic, Antimicrobial
2908	DRAMP02461	L-amino-acid oxidase (BmarLAAO; LAAO; LAO; snakes, reptils, animals)	Antibacterial, Antifungal, Antiparasitic, Antimicrobial
2909	DRAMP02462	L-amino-acid oxidase (LAAO; LAO; snakes, reptils, animals)	Antibacterial, Antiparasitic, Anti-Gram+, Anti-Gram-, Antimicrobial
2910	DRAMP02463	L-amino-acid oxidase (LAAO, LAO, LN-AAO; Reptiles, animals)	Antibacterial, Antimicrobial
2911	DRAMP02464	L-amino-acid oxidase (LAAO, LAO; reptilia, animals)	Antibacterial, Antiparasitic, Antimicrobial
2912	DRAMP02465	L-amino-acid oxidase L1 (LAAO; LAAO-L1; LAO; Reptiles, animals)	Antibacterial, Antiparasitic, Antitumor, Antimicrobial
2913	DRAMP02466	L-amino-acid oxidase L2 (LAAO; LAAO-L2; LAO; Reptiles, animals)	Antibacterial, Antiparasitic, Antitumor, Antimicrobial
2914	DRAMP02467	L-amino-acid oxidase (LAAO; LAO; Reptiles, animals)	Antibacterial, Antiparasitic, Antitumor, Antimicrobial
2915	DRAMP02468	Acidic phospholipase A2 PnPLA2 (svPLA2; Reptiles, animals)	Antibacterial, Anti-Gram+, Antimicrobial
2916	DRAMP02469	Alpha-lytic protease L1	Antibacterial, Antimicrobial
2917	DRAMP02471	Thiostrepton (Alaninamide; Bryamycin; Gargon; Thiactin)	Antibacterial, Antimicrobial
2918	DRAMP02475	Nawaprin (Snakes, reptiles, animals)	Antibacterial, Antimicrobial
2919	DRAMP02476	Waprin-Phi3 (Snakes, reptiles, animals)	Antimicrobial,
2920	DRAMP02477	Waprin-Phi2 (Snakes, reptiles, animals)	Antimicrobial,
2921	DRAMP02479	Scuwaprin-a (Snakes, reptiles, animals)	Antibacterial, Antimalarial, Antimicrobial
2922	DRAMP02480	Omwaprin-b (Oxywaprin-b; Snakes, reptiles, animals)	Antibacterial, Antimalarial, Antimicrobial
2923	DRAMP02481	Omwaprin-c (Oxywaprin-c; Snakes, reptiles, animals)	Antibacterial, Antimalarial, Antimicrobial
2924	DRAMP02482	Omwaprin-a (Oxywaprin; Oxywaprin-a; Snakes, reptiles, animals)	Antibacterial, Anti-Gram+, Antimicrobial
2925	DRAMP02483	Cathelicidin-NA (Cathelicidin-related protein; Snakes, reptiles, animals)	Antibacterial, Antifungal, Anti-Gram-, Antimicrobial
2926	DRAMP02484	Waprin-Thr1 (Snakes, reptiles, animals)	Antimicrobial,
2927	DRAMP02485	Waprin-Rha1 (Snakes, reptiles, animals)	Antimicrobial,
2928	DRAMP02486	Waprin-Lio1 (Snakes, reptiles, animals)	Antimicrobial,
2929	DRAMP02487	Waprin-Enh1 (Snakes, reptiles, animals)	Antimicrobial,
2930	DRAMP02488	L-amino-acid oxidase ACTX-8 (LAAO; LAO; Snakes, reptiles, animals)	Antibacterial, Antiparasitic, Antitumor, Antimicrobial
2931	DRAMP02489	Supwaprin-a (Snakes, reptiles, animals)	Antibacterial, Antimalarial, Antimicrobial
2932	DRAMP18263	Enterocin W alpha(Bacteriocin)	Antibacterial, Anti-Gram+, Antimicrobial
2933	DRAMP02491	Stewaprin-a (Snakes, reptiles, animals)	Antibacterial, Antimalarial, Antimicrobial
2934	DRAMP02492	Notewaprin-a (Snakes, reptiles, animals)	Antibacterial, Antimalarial, Antimicrobial
2935	DRAMP02493	Auswaprin-a (Snakes, reptiles, animals)	Antibacterial, Antimalarial, Antimicrobial
2936	DRAMP02494	Porwaprin-a (Snakes, reptiles, animals)	Antibacterial, Antimalarial, Antimicrobial
2937	DRAMP02496	Nigwaprin-a (Snakes, reptiles, animals)	Antibacterial, Antimalarial, Antimicrobial
2938	DRAMP02497	Carwaprin-a (Snakes, reptiles, animals)	Antibacterial, Antimalarial, Antimicrobial

2939	DRAMP18262	Enterocin 7A(Bacteriocin)	Antibacterial, Anti-Gram+, Antimicrobial
2940	DRAMP02499	Veswaprin-b (Snakes, reptiles, animals)	Antibacterial, Antimalarial, Antimicrobial
2941	DRAMP02500	Notewaprin-b (Snakes, reptiles, animals)	Antibacterial, Antimalarial, Antimicrobial
2942	DRAMP02502	Porwaprin-b (Snakes, reptiles, animals)	Antibacterial, Antimalarial, Antimicrobial
2943	DRAMP02503	Nigwaprin-b (Snakes, reptiles, animals)	Antibacterial, Antimalarial, Antimicrobial
2944	DRAMP18261	Enterocin 7B (Bacteriocin)	Antibacterial, Anti-Gram+, Antimicrobial
2945	DRAMP18260	Bacteriocin 31 (Bacteriocin)	Antibacterial, Anti-Gram+, Antimicrobial
2946	DRAMP02506	Veswaprin-c (Snakes, reptiles, animals)	Antibacterial, Antimalarial, Antimicrobial
2947	DRAMP18259	Enterocin O16 (Bacteriocin)	Antibacterial, Anti-Gram+, Antimicrobial
2948	DRAMP18355	Lacticin Z (bacteriocin)	Antibacterial, Antimicrobial
2949	DRAMP18258	Closticin 574(Bacteriocin)	Antibacterial, Anti-Gram+, Antimicrobial
2950	DRAMP18256	Boticin B (Bacteriocin)	Antimicrobial, Anti-Gram+,
2951	DRAMP02511	Crotamine (defensin-like toxin; Snakes, reptiles, animals)	Antibacterial, Antifungal, Cytotoxicity, Anti-Gram+, Anti-Gram-, Antimicrobial
2952	DRAMP02512	L-amino-acid oxidase (Casca LAO, LAAO, LAO; Snakes, reptiles, animals)	Antibacterial, Antiparasitic, Cytotoxicity, Antimicrobial
2953	DRAMP18398	Um2 (scorpions, arachnids, Chelicerata, arthropods, invertebrates, animals)	Antibacterial, Antimicrobial
2954	DRAMP02521	Cathelicidin-OH (Cathelicidin-related protein; Snakes, reptiles, animals)	Antibacterial, Antifungal, Anti-Gram-, Antimicrobial
2955	DRAMP02523	CRISPR-associated endoribonuclease Cas2 1 (Antiviral defensin)	Antiviral, Antimicrobial
2956	DRAMP02524	CRISPR-associated endoribonuclease Cas2 1 (Antiviral defensin)	Antiviral, Antimicrobial
2957	DRAMP02525	CRISPR-associated endoribonuclease Cas2 1 (Antiviral defensin)	Antiviral, Antimicrobial
2958	DRAMP02526	CRISPR-associated endoribonuclease Cas2 1 (Antiviral defensin)	Antiviral, Antimicrobial
2959	DRAMP02527	CRISPR-associated endoribonuclease Cas2 1 (Antiviral defensin)	Antiviral, Antimicrobial
2960	DRAMP02528	CRISPR-associated endoribonuclease Cas2 1 (Antiviral defensin)	Antiviral, Antimicrobial
2961	DRAMP02529	CRISPR-associated endoribonuclease Cas2 1 (Antiviral defensin)	Antiviral, Antimicrobial
2962	DRAMP02530	CRISPR-associated endoribonuclease Cas2 2 (Antiviral defensin)	Antiviral, Antimicrobial
2963	DRAMP02531	CRISPR-associated endoribonuclease Cas2 2 (Antiviral defensin)	Antiviral, Antimicrobial
2964	DRAMP02532	CRISPR-associated endoribonuclease Cas2 2 (Antiviral defensin)	Antiviral, Antimicrobial
2965	DRAMP02533	CRISPR-associated endoribonuclease Cas2 2 (Antiviral defensin)	Antiviral, Antimicrobial
2966	DRAMP02534	CRISPR-associated endoribonuclease Cas2 2 (Antiviral defensin)	Antiviral, Antimicrobial
2967	DRAMP02535	CRISPR-associated endoribonuclease Cas2 2 (Antiviral defensin)	Antiviral, Antimicrobial
2968	DRAMP02536	CRISPR-associated endoribonuclease Cas2 2 (Antiviral defensin)	Antiviral, Antimicrobial
2969	DRAMP02537	CRISPR-associated endonuclease Cas2 2 (Antiviral defensin)	Antiviral, Antimicrobial
2970	DRAMP02538	CRISPR-associated endoribonuclease Cas2 2 (Antiviral defensin)	Antiviral, Antimicrobial
2971	DRAMP02539	CRISPR-associated endoribonuclease Cas2 3 (Antiviral defensin)	Antiviral, Antimicrobial
2972	DRAMP02540	CRISPR-associated endoribonuclease Cas2 3 (Antiviral defensin)	Antiviral, Antimicrobial
2973	DRAMP02541	CRISPR-associated endoribonuclease Cas2 3 (Antiviral defensin)	Antiviral, Antimicrobial
2974	DRAMP02542	CRISPR-associated endoribonuclease Cas2 3 (Antiviral defensin)	Antiviral, Antimicrobial
2975	DRAMP02543	CRISPR-associated endoribonuclease Cas2 3 (Antiviral defensin)	Antiviral, Antimicrobial
2976	DRAMP02544	CRISPR-associated endoribonuclease Cas2 (Antiviral defensin)	Antiviral, Antimicrobial

2977	DRAMP02545	CRISPR-associated endoribonuclease Cas2 (Antiviral defensin)	Antiviral, Antimicrobial
2978	DRAMP02546	CRISPR-associated endonuclease Cas2 (Antiviral defensin)	Antiviral, Antimicrobial
2979	DRAMP02547	CRISPR-associated endoribonuclease Cas2 (Antiviral defensin)	Antiviral, Antimicrobial
2980	DRAMP02548	CRISPR-associated endoribonuclease Cas2 (Antiviral defensin)	Antiviral, Antimicrobial
2981	DRAMP02549	CRISPR-associated endoribonuclease Cas2 (Antiviral defensin)	Antiviral, Antimicrobial
2982	DRAMP02550	CRISPR-associated endoribonuclease Cas2 (Antiviral defensin)	Antiviral, Antimicrobial
2983	DRAMP02551	CRISPR-associated endoribonuclease Cas2 (Antiviral defensin)	Antiviral, Antimicrobial
2984	DRAMP02552	CRISPR-associated endoribonuclease Cas2 (Antiviral defensin)	Antiviral, Antimicrobial
2985	DRAMP02553	CRISPR-associated endoribonuclease Cas2 (Antiviral defensin)	Antiviral, Antimicrobial
2986	DRAMP02554	CRISPR-associated endoribonuclease Cas2 (Antiviral defensin)	Antiviral, Antimicrobial
2987	DRAMP02555	CRISPR-associated endoribonuclease Cas2 (Antiviral defensin)	Antiviral, Antimicrobial
2988	DRAMP02556	CRISPR-associated endoribonuclease Cas2 (Antiviral defensin)	Antiviral, Antimicrobial
2989	DRAMP18356	BacSP222 (bacteriocin)	Antibacterial, Mammalian cells, Antimicrobial
2990	DRAMP18255	Circularin A(Bacteriocin)	Antibacterial, Anti-Gram+, Antimicrobial
2991	DRAMP02558	CRISPR-associated endoribonuclease Cas2 (Antiviral defensin)	Antiviral, Antimicrobial
2992	DRAMP02559	CRISPR-associated endoribonuclease Cas2 (Antiviral defensin)	Antiviral, Antimicrobial
2993	DRAMP02560	CRISPR-associated endoribonuclease Cas2 (Antiviral defensin)	Antiviral, Antimicrobial
2994	DRAMP02561	CRISPR-associated endoribonuclease Cas2 (Antiviral defensin)	Antiviral, Antimicrobial
2995	DRAMP02562	CRISPR-associated endoribonuclease Cas2 (Antiviral defensin)	Antiviral, Antimicrobial
2996	DRAMP02563	CRISPR-associated endoribonuclease Cas2 (Antiviral defensin)	Antiviral, Antimicrobial
2997	DRAMP02564	CRISPR-associated endoribonuclease Cas2 (Antiviral defensin)	Antiviral, Antimicrobial
2998	DRAMP02565	CRISPR-associated endoribonuclease Cas2 (Antiviral defensin)	Antiviral, Antimicrobial
2999	DRAMP18253	Piscicocin CS526(Bacteriocin)	Antibacterial, Anti-Gram+, Anti-Gram-, Antimicrobial
3000	DRAMP02567	CRISPR-associated endoribonuclease Cas2 1 (Antiviral defensin)	Antiviral, Antimicrobial
3001	DRAMP02568	Penaeidin 3-1	Antimicrobial,
3002	DRAMP02569	Penaeidin	Antimicrobial,
3003	DRAMP02570	Penaeidin-1 (Pen-1; shrimps, Arthropods, animals)	Antibacterial, Antifungal, Anti-Gram+, Anti-Gram-, Antimicrobial
3004	DRAMP02571	Penaeidin-2a (Pen-2a; shrimps, Arthropods, animals)	Antibacterial, Antifungal, Anti-Gram+, Anti-Gram-, Antimicrobial
3005	DRAMP02572	Penaeidin-2b (Pen-2b; shrimps, Arthropods, animals)	Antibacterial, Antifungal, Antimicrobial
3006	DRAMP02575	Penaeidin-3b (Pen-3b; shrimps, Arthropods, animals)	Antibacterial, Antifungal, Anti-Gram+, Anti-Gram-, Antimicrobial
3007	DRAMP02576	Penaeidin-3c (Pen-3c; shrimps, Arthropods, animals)	Antibacterial, Antifungal, Anti-Gram+, Anti-Gram-, Antimicrobial
3008	DRAMP02577	Penaeidin-3d (Pen-3d; shrimps, Arthropods, animals)	Antibacterial, Antifungal, Antimicrobial
3009	DRAMP18252	Carnolysin A2(Bacteriocin)	Antibacterial, Anti-Gram+, Antimicrobial
3010	DRAMP02580	Penaeidin-3g (Pen-3g; shrimps, Arthropods, animals)	Antibacterial, Antifungal, Antimicrobial
3011	DRAMP02581	Penaeidin-3h (Pen-3h; shrimps, Arthropods, animals)	Antibacterial, Antifungal, Antimicrobial
3012	DRAMP02582	Penaeidin-3i (Pen-3i; shrimps, Arthropods, animals)	Antibacterial, Antifungal, Antimicrobial
3013	DRAMP02583	Penaeidin-3j (Pen-3j; shrimps, Arthropods, animals)	Antibacterial, Antifungal, Antimicrobial
3014	DRAMP02585	Penaeidin-4c (Pen-4c; shrimps, Arthropods, animals)	Antibacterial, Antifungal, Antimicrobial
3015	DRAMP02587	Penaeidin-3k (Pen-3k; shrimps, Arthropods, animals)	Antibacterial, Antifungal, Antimicrobial
3016	DRAMP02588	Penaeidin-3l (Pen-3l; shrimps, Arthropods, animals)	Antibacterial, Antifungal, Antimicrobial
3017	DRAMP02589	Penaeidin-3m (Pen-3m; shrimps, Arthropods, animals)	Antibacterial, Antifungal, Antimicrobial
3018	DRAMP02590	Penaeidin-3n (Pen-3n; shrimps, Arthropods, animals)	Antibacterial, Antifungal, Antimicrobial
3019	DRAMP02592	Styelin-A (Styelin A; invertebrates, animals)	Antibacterial, Antimicrobial
3020	DRAMP02593	Styelin-B (Styelin B; invertebrates, animals)	Antibacterial, Antimicrobial
3021	DRAMP02594	Styelin-C (Styelin C; chordates, animals)	Antibacterial, Antimicrobial
3022	DRAMP02596	Styelin-E (Styelin E; chordates, animals)	Antibacterial, Antimicrobial
3023	DRAMP02597	Clavanin-A (His-rich; chordates, animals)	Antibacterial, Antifungal, Anti-Gram-, Antimicrobial
3024	DRAMP02598	Clavanin-B (His-rich; chordates, animals)	Antibacterial, Antifungal, Antimicrobial
3025	DRAMP02599	Clavanin-C (His-rich; chordates, animals)	Antibacterial, Antifungal, Anti-Gram-, Antimicrobial
3026	DRAMP02600	Clavanin-D (His-rich; chordates, animals)	Antibacterial, Antifungal, Anti-Gram-, Antimicrobial
3027	DRAMP02601	Clavanin E (His-rich; chordates, animals)	Antibacterial, Antifungal, Anti-Gram-, Antimicrobial
3028	DRAMP02602	Clavaspirin (chordates, animals)	Antibacterial, Anti-Gram+, Anti-Gram-, Antimicrobial
3029	DRAMP18251	Carnolysin A1(Bacteriocin)	Antibacterial, Anti-Gram+, Antimicrobial

3030	DRAMP02606	Antibacterial protein LL-37 (cathelicidin; primates, mammals, animals)	Antibacterial, Antimicrobial
3031	DRAMP02607	Antibacterial protein LL-37 (AFRLL-37; cathelicidin; primates, mammals, animals)	Antibacterial, Antimicrobial
3032	DRAMP02608	Antibacterial protein LL-37 (cathelicidin; primates, mammals, animals)	Antibacterial, Antimicrobial
3033	DRAMP02610	WAP four-disulfide core domain protein 12 (primates, mammals, animals)	Antibacterial, Antimicrobial
3034	DRAMP02611	Beta-defensin 1 (BD-1; Defensin, beta 1; primates, mammals, animals)	Antibacterial, Antimicrobial
3035	DRAMP02613	Mfa-hst 5 (M.fascicularis histatin 5; primates, mammals, animals)	Antifungal, Antimicrobial
3036	DRAMP02614	Beta-defensin 105A (Defensin, beta 105; Defensin, beta 105A; primates, mammals, animals)	Antimicrobial,
3037	DRAMP02615	Beta-defensin 1 (BD-1; Defensin, beta 1; primates, mammals, animals)	Antibacterial, Antimicrobial
3038	DRAMP02616	Beta-defensin 126 (Defensin, beta 126; Epididymal secretory protein 13.2, ESP13.2; primates, mammals, animals)	Antibacterial, Antimicrobial
3039	DRAMP02617	Beta-defensin 107A (Defensin, beta 107; Defensin, beta 107A; primates, mammals, animals)	Antibacterial, Antimicrobial
3040	DRAMP02618	Beta-defensin 118 (Defensin, beta 118; primates, mammals, animals)	Antibacterial, Antimicrobial
3041	DRAMP02619	Beta-defensin 119 (Defensin, beta 119; Beta-defensin 120; Defensin, beta 120; primates, mammals, animals)	Antibacterial, Antimicrobial
3042	DRAMP02620	Beta-defensin 128 (Defensin, beta 128; primates, mammals, animals)	Antibacterial, Antimicrobial
3043	DRAMP02621	Beta-defensin 132 (Defensin, beta 132; primates, mammals, animals)	Antibacterial, Antimicrobial
3044	DRAMP02622	Antibacterial protein LL-37 (primates, mammals, animals)	Antibacterial, Antimicrobial
3045	DRAMP02623	Macaque histatin (His-rich; M-Histatin 1; primates, mammals, animals)	Antibacterial, Antifungal, Antimicrobial
3046	DRAMP02624	WAP four-disulfide core domain protein 12 (primates, mammals, animals)	Antibacterial, Antimicrobial
3047	DRAMP02625	WAP four-disulfide core domain protein 12 (primates, mammals, animals)	Antibacterial, Antimicrobial
3048	DRAMP02626	WAP four-disulfide core domain protein 12 (primates, mammals, animals)	Antibacterial, Antimicrobial
3049	DRAMP02627	Beta-defensin 1 (BD-1; Defensin, beta 1; primates, mammals, animals)	Antibacterial, Antimicrobial
3050	DRAMP18250	Laterosporulin (Bacteriocin)	Antibacterial, Anti-Gram+, Anti-Gram-, Antimicrobial
3051	DRAMP02630	Rhesus monkey beta-defensin 2 (RhBD-2; Defensin, beta 2; primates, mammals, animals)	Antibacterial, Antimicrobial
3052	DRAMP18248	Bifidin I(Bacteriocin)	Antibacterial, Anti-Gram+, Anti-Gram-, Antimicrobial
3053	DRAMP18249	Bac-GM100 (Bacteriocin)	Antibacterial, Antifungal, Anti-Gram+, Anti-Gram-, Antimicrobial
3054	DRAMP02632	Beta-defensin 121 (Defensin, beta 121; primates, mammals, animals)	Antibacterial, Antimicrobial
3055	DRAMP02633	Beta-defensin 122 (Defensin, beta 122; primates, mammals, animals)	Antibacterial, Antimicrobial
3056	DRAMP02634	Beta-defensin 123 (Defensin, beta 123; primates, mammals, animals)	Antibacterial, Antimicrobial
3057	DRAMP02635	Beta-defensin 119 (Defensin, beta 119; Beta-defensin 120; Defensin, beta 120; primates, mammals, animals)	Antibacterial, Antimicrobial
3058	DRAMP18246	Thuricin 439A,439B(Bacteriocin)	Antibacterial, Anti-Gram+, Antimicrobial
3059	DRAMP18247	Bacthuricin F4(Bacteriocin)	Antibacterial, Anti-Gram+, Anti-Gram-, Antimicrobial
3060	DRAMP02637	Antibacterial protein FALL-39 (FALL-39 peptide antibiotic; primates, mammals, animals)	Antibacterial, Antimicrobial
3061	DRAMP02638	Liver-expressed antimicrobial peptide 2 (LEAP-2; primates, mammals, animals)	Antimicrobial,
3062	DRAMP02639	Demidefensin-3 (primates, mammals, animals)	Antibacterial, Antifungal, Antimicrobial
3063	DRAMP02640	Rhesus theta defensin-1/2 subunit B (RTD-1b; primates, mammals, animals)	Antibacterial, Antifungal, Antimicrobial

3064	DRAMP02641	Rhesus theta defensin-1/3 subunit A (RTD-1a; primates, mammals, animals)	Antibacterial, Antifungal, Antimicrobial
3065	DRAMP02642	Rhesus theta-defensin 1 (RTD-1; primates, mammals, animals)	Antibacterial, Antifungal, Antiviral, Anti-Gram+, Anti-Gram-, Antimicrobial
3066	DRAMP02643	Rhesus theta-defensin 2 (RTD-2; primates, mammals, animals)	Antibacterial, Antifungal, Anti-Gram+, Anti-Gram-, Antimicrobial
3067	DRAMP02644	Rhesus theta-defensin 3 (RTD-3; primates, mammals, animals)	Antibacterial, Antiviral, Anti-Gram+, Anti-Gram-, Antimicrobial
3068	DRAMP02645	Rhesus theta-defensin 4 (RTD-4; primates, mammals, animals)	Antibacterial, Antifungal, Antimicrobial
3069	DRAMP02646	Rhesus theta-defensin 5 (RTD-5; primates, mammals, animals)	Antibacterial, Antifungal, Antimicrobial
3070	DRAMP02647	Beta-defensin 2	Antibacterial, Antimicrobial
3071	DRAMP02648	Cationic antimicrobial protein	Antimicrobial,
3072	DRAMP02649	EP2B protein	Antimicrobial,
3073	DRAMP02651	Sperm associated antigen 11 isoform E (primates, mammals, animals)	Antibacterial, Antimicrobial
3074	DRAMP02652	WAP four-disulfide core domain protein 12 (primates, mammals, animals)	Antibacterial, Antimicrobial
3075	DRAMP02653	Neutrophil defensin 1 (RMAD-1; primates, mammals, animals)	Antibacterial, Antifungal, Anti-Gram+, Anti-Gram-, Antimicrobial
3076	DRAMP02654	Neutrophil defensin 2 (RMAD-2; primates, mammals, animals)	Antibacterial, Antifungal, Anti-Gram+, Anti-Gram-, Antimicrobial
3077	DRAMP02655	Alpha defensin (primates, mammals, animals)	Antimicrobial,
3078	DRAMP02656	Alpha-defensin 2 (primates, mammals, animals)	Antimicrobial,
3079	DRAMP02657	Alpha-defensin 1 (primates, mammals, animals)	Antimicrobial,
3080	DRAMP02658	Alpha-defensin 6 (primates, mammals, animals)	Antimicrobial,
3081	DRAMP02659	Neutrophil defensin 3 (RMAD-3; primates, mammals, animals)	Antibacterial, Antifungal, Anti-Gram+, Anti-Gram-, Antimicrobial
3082	DRAMP02660	Neutrophil defensin 4 (RMAD-4; primates, mammals, animals)	Antibacterial, Antifungal, Anti-Gram+, Anti-Gram-, Antimicrobial
3083	DRAMP02661	Neutrophil defensin 5 (RMAD-5; primates, mammals, animals)	Antibacterial, Antifungal, Anti-Gram+, Anti-Gram-, Antimicrobial
3084	DRAMP02662	Neutrophil defensin 6 (RMAD-6; primates, mammals, animals)	Antibacterial, Antifungal , Anti-Gram+, Anti-Gram-, Antimicrobial
3085	DRAMP02663	Neutrophil defensin 7 (RMAD-7; primates, mammals, animals)	Antibacterial, Antifungal, Antimicrobial, Anti-Gram+, Anti-Gram-,
3086	DRAMP02664	Neutrophil defensin 8 (RMAD-8; primates, mammals, animals)	Antibacterial, Antifungal, Antimicrobial
3087	DRAMP02665	Defensin-7 (Defensin, alpha 7; primates, mammals, animals)	Antimicrobial,
3088	DRAMP02666	WAP four-disulfide core domain protein 12 (primates, mammals, animals)	Antibacterial, Antimicrobial
3089	DRAMP02668	Defensin-6 (Defensin, alpha 6; primates, mammals, animals)	Antibacterial, Antimicrobial
3090	DRAMP02669	Neutrophil defensin 1 (Defensin, alpha 1; primates, mammals, animals)	Antibacterial, Antifungal, Antiviral, Antimicrobial
3091	DRAMP02670	Neutrophil defensin 4 (Defensin, alpha 4; primates, mammals, animals)	Antibacterial, Antifungal, Antimicrobial
3092	DRAMP02671	Beta-defensin 1 (BD-1; primates, mammals, animals)	Antibacterial, Antimicrobial
3093	DRAMP02672	Beta-defensin 4A (Beta-defensin 2, BD-2; primates, mammals, animals)	Antimicrobial,
3094	DRAMP02673	Beta-defensin 103A (Beta-defensin 3, BD-3; primates, mammals, animals)	Antibacterial, Antimicrobial
3095	DRAMP18245	Ticin A4(Bacteriocin)	Antibacterial, Anti-Gram+, Antimicrobial
3096	DRAMP02675	Beta-defensin 105A (Beta-defensin 5, BD-5; primates, mammals, animals)	Antimicrobial,
3097	DRAMP02676	Beta-defensin 106A (Beta-defensin 6, BD-6; primates, mammals, animals)	Antimicrobial,
3098	DRAMP02677	Beta-defensin 107A (Beta-defensin 7; BD-7, DEFB-7, cBD-7; primates, mammals, animals)	Antibacterial, Antimicrobial
3099	DRAMP02678	Beta-defensin 108B (Beta-defensin 8; BD-8, DEFB-8, hBD-8; primates, mammals, animals)	Antibacterial, Antimicrobial
3100	DRAMP02679	Beta-defensin109 (Defensin, beta 109; Defensin, beta 109; primates, mammals, animals)	Antibacterial, Antimicrobial
3101	DRAMP02680	Beta-defensin 110 (Defensin, beta 110; Beta-defensin 111; primates, mammals, animals)	Antibacterial, Antimicrobial

3102	DRAMP02681	Beta-defensin 113 (Defensin, beta 113; primates, mammals, animals)	Antibacterial, Antimicrobial
3103	DRAMP02682	Beta-defensin 116 (Defensin, beta 116; primates, mammals, animals)	Antibacterial, Antimicrobial
3104	DRAMP02683	Beta-defensin 121 (Defensin, beta 121; primates, mammals, animals)	Antibacterial, Antimicrobial
3105	DRAMP02684	Beta-defensin 124 (Defensin, beta 124; Defensin, beta 126; primates, mammals, animals)	Antibacterial, Antimicrobial
3106	DRAMP02685	Beta-defensin 133 (Defensin, beta 133; primates, mammals, animals)	Antibacterial, Antimicrobial
3107	DRAMP02686	Beta-defensin 134 (Defensin, beta 134; primates, mammals, animals)	Antibacterial, Antimicrobial
3108	DRAMP02687	Beta-defensin 135 (Defensin, beta 135; primates, mammals, animals)	Antibacterial, Antimicrobial
3109	DRAMP02688	Beta-defensin 136 (Defensin, beta 136; primates, mammals, animals)	Antibacterial, Antimicrobial
3110	DRAMP02689	Beta-defensin 118 (Defensin, beta 118; primates, mammals, animals)	Antibacterial, Antimicrobial
3111	DRAMP02690	Beta-defensin 119 (Defensin, beta 119; Beta-defensin 120; primates, mammals, animals)	Antibacterial, Antimicrobial
3112	DRAMP02691	Beta-defensin 123 (Defensin, beta 123; primates, mammals, animals)	Antibacterial, Antimicrobial
3113	DRAMP02692	Beta-defensin 126 (Defensin, beta 126; primates, mammals, animals)	Antibacterial, Antimicrobial
3114	DRAMP02693	Beta-defensin 127 (Defensin, beta 127; primates, mammals, animals)	Antibacterial, Antimicrobial
3115	DRAMP02694	Beta-defensin 130 (Defensin, beta 130; primates, mammals, animals)	Antibacterial, Antimicrobial
3116	DRAMP02695	Beta-defensin 131 (Defensin, beta 131; primates, mammals, animals)	Antibacterial, Antimicrobial
3117	DRAMP02696	Beta-defensin 132 (Defensin, beta 132; primates, mammals, animals)	Antibacterial, Antimicrobial
3118	DRAMP02697	Defensin-5 (Defensin, alpha 5; primates, mammals, animals)	Antibacterial, Antimicrobial
3119	DRAMP02698	Rhesus macaque oral alpha-defensins (ROADs; primates, mammals, animals)	Antibacterial, Antifungal, Anti-Gram+, Anti-Gram-, Antimicrobial
3120	DRAMP02699	WAP four-disulfide core domain protein 12 (primates, mammals,animals)	Antibacterial, Antimicrobial
3121	DRAMP02700	Beta-defensin 107A (Defensin, beta 107; Defensin, beta 107A; primates, mammals,animals)	Antibacterial, Antimicrobial
3122	DRAMP18244	Ticin A3(Bacteriocin)	Antibacterial, Anti-Gram+, Antimicrobial
3123	DRAMP02702	Beta-defensin 119 (Defensin, beta 119; Beta-defensin 120; primates, mammals,animals)	Antibacterial, Antimicrobial
3124	DRAMP18243	Ticin A1(Bacteriocin)	Antibacterial, Anti-Gram+, Antimicrobial
3125	DRAMP02704	Beta-defensin 126 (Defensin, beta 126; primates, mammals, animals)	Antibacterial, Antimicrobial
3126	DRAMP02705	Beta-defensin 132 (Defensin, beta 132; primates, mammals, animals)	Antibacterial, Antimicrobial
3127	DRAMP02706	Beta-defensin 104A (Defensin, beta 104; Defensin, beta 104A; primates, mammals, animals)	Antimicrobial,
3128	DRAMP18242	Fengycin B2 (Bacteriocin)	Antibacterial, Antifungal, Anti-Gram+, Anti-Gram-, Antimicrobial
3129	DRAMP02709	Antibacterial protein LL-37 (cathelicidin; primates, mammals, animals)	Antibacterial, Antimicrobial
3130	DRAMP18240	Fengycin C(Bacteriocin)	Antibacterial, Antifungal, Anti-Gram+, Anti-Gram-, Antimicrobial
3131	DRAMP18241	Subtilomycin(Bacteriocin)	Antibacterial, Anti-Gram+, Anti-Gram-, Antimicrobial
3132	DRAMP18239	Fengycin A2(Bacteriocin)	Antibacterial, Antifungal, Anti-Gram+, Anti-Gram-, Antimicrobial
3133	DRAMP18238	Fengycin B(Bacteriocin)	Antibacterial, Antifungal, Anti-Gram+, Anti-Gram-, Antimicrobial
3134	DRAMP02713	Hepcidin (primates, mammals, animals)	Antimicrobial ,
3135	DRAMP02714	Antibacterial protein LL-37 (cathelicidin; primates, mammals, animals)	Antibacterial, Antimicrobial

3136	DRAMP02715	Beta-defensin 106A (Defensin, beta 106; Defensin, beta 106A; primates, mammals,animals)	Antimicrobial,
3137	DRAMP02716	Beta-defensin 105A (Defensin, beta 105; Defensin, beta 105A; primates, mammals,animals)	Antimicrobial,
3138	DRAMP02717	Beta-defensin 119 (Defensin, beta 119; Beta-defensin 120; Defensin, beta 120; primates, mammals,animals)	Antibacterial, Antimicrobial
3139	DRAMP02718	Beta-defensin 123 (Defensin, beta 123; primates, mammals, animals)	Antibacterial, Antimicrobial
3140	DRAMP02719	Beta-defensin 126 (Defensin, beta 126; primates, mammals, animals)	Antibacterial, Antimicrobial
3141	DRAMP02720	Beta-defensin 128 (Defensin, beta 128; primates, mammals, animals)	Antibacterial, Antimicrobial
3142	DRAMP02721	Beta-defensin 132 (Defensin, beta 132; primates, mammals, animals)	Antibacterial, Antimicrobial
3143	DRAMP02722	Beta-defensin 1 (BD-1; Defensin, beta 1; primates, mammals, animals)	Antibacterial, Antimicrobial
3144	DRAMP18237	Fengycin A(Bacteriocin)	Antibacterial, Antifungal, Anti-Gram+, Anti-Gram-, Antimicrobial
3145	DRAMP02724	Antibacterial protein LL-37 (cathelicidin; primates, mammals, animals)	Antibacterial, Antimicrobial
3146	DRAMP02725	Antibacterial protein LL-37 (cathelicidin; primates, mammals, animals)	Antibacterial, Antimicrobial
3147	DRAMP02729	Beta-defensin 107A (Defensin, beta 107; Defensin, beta 107A; primates, mammals, animals)	Antibacterial, Antimicrobial
3148	DRAMP02730	Beta-defensin 104A (Defensin, beta 104; Defensin, beta 104A; primates, mammals, animals)	Antimicrobial,
3149	DRAMP02731	Beta-defensin 105A (Defensin, beta 105; Defensin, beta 105A; primates, mammals, animals)	Antimicrobial,
3150	DRAMP02732	Beta-defensin 106A (Defensin, beta 106; Defensin, beta 106A; primates, mammals, animals)	Antimicrobial,
3151	DRAMP18236	NRWC(Bacteriocin)	Antibacterial, Antimicrobial
3152	DRAMP02734	Beta-defensin 119 (Defensin, beta 119; Beta-defensin 120; primates, mammals, animals)	Antibacterial, Antimicrobial
3153	DRAMP02735	Beta-defensin 126 (Defensin, beta 126; primates, mammals, animals)	Antibacterial, Antimicrobial
3154	DRAMP02736	Beta-defensin 128 (Defensin, beta 128; primates, mammals, animals)	Antibacterial, Antimicrobial
3155	DRAMP02737	Beta-defensin 132 (Defensin, beta 132; primates, mammals, animals)	Antibacterial, Antimicrobial
3156	DRAMP02738	Beta-defensin 1 (BD-1; Defensin, beta 1; primates, mammals, animals)	Antibacterial, Antimicrobial
3157	DRAMP02739	TEWP (turtle egg-white protein; Reptiles, animals)	Antibacterial, Antiviral, Anti-Gram+, Anti-Gram-, Antimicrobial
3158	DRAMP02741	Pelovaterin (defensin-like AMP; Gly-rich; Reptiles, animals)	Antibacterial, Anti-Gram+, Anti-Gram-, Antimicrobial
3159	DRAMP02742	Defensin-like turtle egg white protein TEWP (TEWP; Reptiles, animals)	Antibacterial, Antiviral, Anti-Gram+, Anti-Gram-, Antimicrobial
3160	DRAMP02743	Diapause-specific peptide (Dsp)	Antifungal, Antimicrobial
3161	DRAMP02744	Dinoponeratoxin Da-2501 (ants, insects, animals)	Antibacterial, Antimicrobial
3162	DRAMP02745	Dinoponeratoxin Da-1585 (ants, insects, animals)	Antibacterial, Antimicrobial
3163	DRAMP02746	Dinoponeratoxin Da-3105 (ants, insects, animals)	Antibacterial, Antimicrobial
3164	DRAMP02747	Dinoponeratoxin Da-1837 (ants, insects, animals)	Antibacterial, Antimicrobial
3165	DRAMP02748	Dinoponeratoxin Da-1039 (ants, insects, animals)	Antibacterial, Antimicrobial
3166	DRAMP02749	Dinoponeratoxin Da-3177 (ants, insects, animals)	Antibacterial, Antimicrobial
3167	DRAMP02750	Defensin (ants, insects, animals)	Antibacterial, Antimicrobial
3168	DRAMP03743	Androctonin (Arthropods, animals)	Antimicrobial, Antibacterial, Antifungal, Anti-Gram+, Anti-Gram-,
3169	DRAMP02753	Ponericin G1 (ants, insects, animals)	Antibacterial, Antifungal, Antimicrobial
3170	DRAMP02754	Ponericin G2 (ants, insects, animals)	Antibacterial, Antifungal, Antimicrobial
3171	DRAMP02755	Ponericin G3 (ants, insects, animals)	Antibacterial, Antifungal, Anti-Gram+, Anti-Gram-, Antimicrobial
3172	DRAMP02756	Ponericin G4 (ants, insects, animals)	Antibacterial, Antifungal, Anti-Gram+, Anti-Gram-, Antimicrobial
3173	DRAMP02757	Ponericin G5 (ants, insects, animals)	Antibacterial, Antimicrobial

3174	DRAMP02758	Ponericin G6 (ants, insects, animals)	Antibacterial, Antifungal, Anti-Gram+, Anti-Gram-, Antimicrobial
3175	DRAMP02759	Ponericin G7 (ants, insects, animals)	Antibacterial, Antifungal, Anti-Gram+, Antimicrobial
3176	DRAMP02760	Ponericin-L1 (ants, insects, animals)	Antibacterial, Anti-Gram+, Anti-Gram-, Antimicrobial
3177	DRAMP02761	Ponericin-L2 (ants, insects, animals)	Antibacterial, Antiviral, Anti-Gram+, Anti-Gram-, Antimicrobial
3178	DRAMP02762	Ponericin-W1 (ants, insects, animals)	Antibacterial, Antifungal, Insecticidal, Anti-Gram+, Anti-Gram-, Antimicrobial
3179	DRAMP02763	Ponericin-W2 (ants, insects, animals)	Antibacterial, Antifungal, Insecticidal, Antimicrobial
3180	DRAMP02764	Ponericin-W3 (ants, insects, animals)	Antibacterial, Antifungal, Insecticidal, Anti-Gram+, Anti-Gram-, Antimicrobial
3181	DRAMP02765	Ponericin-W4 (ants, insects, animals)	Antibacterial, Antifungal, Insecticidal, Anti-Gram+, Anti-Gram-, Antimicrobial
3182	DRAMP02766	Ponericin-W5 (ants, insects, animals)	Antibacterial, Antifungal, Insecticidal, Anti-Gram+, Anti-Gram-, Antimicrobial
3183	DRAMP02767	Ponericin-W6 (ants, insects, animals)	Antibacterial, Anti-Gram+, Anti-Gram-, Antimicrobial
3184	DRAMP02770	Pilosulin 3 (ants, insects, animals)	Antibacterial, Anti-Gram+, Anti-Gram-, Antimicrobial
3185	DRAMP02771	Pilosulin 4 (ants, insects, animals)	Antibacterial, Anti-Gram+, Anti-Gram-, Antimicrobial
3186	DRAMP02772	Pilosulin-3a (Allergen Myr p II; Pilosulin-2; ants, insects, animals)	Antibacterial, Antifungal, Antimicrobial
3187	DRAMP02773	Pilosulin 5 (Myr b III; ants, insects, animals)	Antibacterial, Antifungal, Antimicrobial
3188	DRAMP02775	Antimicrobial peptide Alo-2 (Alo-2; knottin-type peptide; Insects, animals)	Antifungal, Antimicrobial
3189	DRAMP02781	Coleoptericin (Insects, animals)	Antibacterial, Anti-Gram+, Anti-Gram-, Antimicrobial
3190	DRAMP02782	Defensin, isoforms B and C (Insects, animals)	Antibacterial, Antimicrobial
3191	DRAMP02783	Peptide C (Insects, animals)	Antibacterial, Anti-Gram+, Anti-Gram-, Antimicrobial
3192	DRAMP02784	Acaloleptin-A1 (chain of Acaloleptin A; Insects, animals)	Antibacterial, Anti-Gram-, Antimicrobial
3193	DRAMP02785	Acaloleptin-A2 (chain of Acaloleptin A; Insects, animals)	Antibacterial, Anti-Gram-, Antimicrobial
3194	DRAMP02786	Acaloleptin-A3 (chain of Acaloleptin A; Insects, animals)	Antibacterial, Anti-Gram-, Antimicrobial
3195	DRAMP02787	Acaloleptin-A4 (chain of Acaloleptin A; Insects, animals)	Antibacterial, Anti-Gram-, Antimicrobial
3196	DRAMP02788	Acaloleptin-A5 (chain of Acaloleptin A; Insects, animals)	Antibacterial, Antifungal, Anti-Gram-, Antimicrobial
3197	DRAMP02789	Tenecin-1 (Insects, animals)	Antibacterial, Antimicrobial
3198	DRAMP02790	Tenecin-3 (Insects, animals)	Antifungal, Antimicrobial
3199	DRAMP02791	A.dichotoma defensin (defensins; Insects, animals)	Antibacterial, Anti-Gram+, Antimicrobial
3200	DRAMP02792	Scarabaecin, major form (Insects, animals)	Antibacterial, Antifungal, Antimicrobial
3201	DRAMP02793	Antibacterial protein1 (Gonococcal growth inhibitor I)	Antibacterial, Antimicrobial
3202	DRAMP02794	Antibacterial protein 2 (Gonococcal growth inhibitor II)	Antibacterial, Antimicrobial
3203	DRAMP02795	Antibacterial protein 3 (Gonococcal growth inhibitor III)	Antibacterial, Antimicrobial
3204	DRAMP02796	Defensin (Type 1 invertebrate defensin; Insects, animals)	Antibacterial, Anti-Gram+, Antimicrobial
3205	DRAMP02797	Beta-defensin 103A (Defensin, beta 103; Defensin, beta 103A; houses, mammals, animals)	Antibacterial, Antimicrobial
3206	DRAMP02798	Antimicrobial peptide NK-lysin (houses, mammals, animals)	Antimicrobial,
3207	DRAMP02799	Antimicrobial peptide eNAP-1 (houses, mammals, animals)	Antibacterial, Antimicrobial
3208	DRAMP02800	Antimicrobial peptide eNAP-2 (houses, mammals, animals)	Antibacterial, Antimicrobial
3209	DRAMP02801	Lysozyme C, spleen isozyme (1,4-beta-N-acetylmuramidase C; houses, mammals, animals)	Antimicrobial,
3210	DRAMP02803	Mytilin-A (molluscas, animals)	Antibacterial, Anti-Gram+, Anti-Gram-, Antimicrobial
3211	DRAMP02804	Mytilus defensin-B (molluscas, animals)	Antibacterial, Anti-Gram+, Anti-Gram-, Antimicrobial
3212	DRAMP02805	Mytilin-B (molluscas, animals)	Antibacterial, Antiviral, Antimicrobial
3213	DRAMP02806	Mytilus defensin-A (molluscas, animals)	Antibacterial, Antimicrobial
3214	DRAMP02807	Mytimycin (molluscas, animals)	Antifungal, Antimicrobial
3215	DRAMP02808	Myticin-A (Myt A; Cys-rich; molluscas, animals)	Antibacterial, Anti-Gram+, Antimicrobial
3216	DRAMP02810	Myticin C	Antibacterial, Antifungal, Antiviral, Antimicrobial
3217	DRAMP02812	Endopeptidase L4	Antibacterial, Antimicrobial
3218	DRAMP02814	Conolysin-Mt2	Antibacterial, Antimicrobial
3219	DRAMP18235	Gageopeptide D(Bacteriocin)	Antifungal, Antibacterial, Anti-Gram+, Anti-Gram-, Antimicrobial
3220	DRAMP02816	Lumbrican	Antibacterial, Antifungal, Anti-Gram+, Anti-Gram-, Antimicrobial
3221	DRAMP02818	Dicynthaurin	Antibacterial, Anti-Gram+, Anti-Gram-, Antimicrobial

3222	DRAMP01820	Temporin-1Cb (Temporin 1Cb; Frogs, amphibians, animals)	Antibacterial, Anti-Gram+, Antimicrobial
3223	DRAMP01822	Temporin-1Cd (Temporin 1Cd; Frogs, amphibians, animals)	Antibacterial, Anti-Gram+, Antimicrobial
3224	DRAMP02820	Holotricin-1 (Gly-rich; His-rich; invertebrate defensin; animals)	Antibacterial, Antimicrobial
3225	DRAMP02821	Holotricin-2 (Gly-rich; His-rich; invertebrate defensin; animals)	Antibacterial, Antimicrobial
3226	DRAMP02822	Holotricin-3 (Gly-rich; His-rich; invertebrate defensin; animals)	Antifungal, Antimicrobial
3227	DRAMP02824	Lingual antimicrobial peptide (mammals, animals)	Antibacterial, Antiviral, Antimicrobial
3228	DRAMP02825	Catestatin	Antimicrobial,
3229	DRAMP02826	Isracidin	Antimicrobial,
3230	DRAMP02827	BHP (pepsin-derived bovine hemoglobin fragment)	Antimicrobial,
3231	DRAMP02829	Seminalplasmin	Antimicrobial,
3232	DRAMP02830	Chrombacin	Antimicrobial,
3233	DRAMP02831	Protein S100-A8 (Calgranulin-A; MRP-8; mammals, animals)	Antibacterial, Antifungal, Antimicrobial
3234	DRAMP02832	Reactive oxygen species modulator 1 (ROS modulator 1; mammals, animals)	Antibacterial, Anti-Gram+, Anti-Gram-, Antimicrobial
3235	DRAMP02833	Glycolactin (Antiviral defensin; mammals, animals)	Antiviral, Antimicrobial
3236	DRAMP02834	Seminalplasmin (Calcium transport inhibitor; Peptide YY-2; mammals, animals)	Antibacterial, Antifungal, Anti-Gram-, Antimicrobial
3237	DRAMP02836	Cathelicidin antimicrobial peptide (cathelicidin; mammals, animals)	Antibacterial, Antimicrobial
3238	DRAMP02837	Beta-defensin C7 (BBD(C7); mammals, animals)	Antibacterial, Anti-Gram-, Antimicrobial
3239	DRAMP18233	Gageopeptide B(Bacteriocin)	Antifungal, Antibacterial, Anti-Gram+, Anti-Gram-, Antimicrobial
3240	DRAMP18234	Gageopeptide C(Bacteriocin)	Antifungal, Antibacterial, Anti-Gram+, Anti-Gram-, Antimicrobial
3241	DRAMP02839	Enteric Beta-defensin (mammals, animals)	Antibacterial, Antimicrobial
3242	DRAMP02846	Secretolytin (mammals, animals)	Antibacterial, Antimicrobial
3243	DRAMP02847	Apolipoprotein A-II (Antimicrobial peptide BAMP-1; mammals, animals)	Antibacterial, Antifungal, Antimicrobial,
3244	DRAMP02848	Apolipoprotein A-II(1-76)(mammals, animals)	Antibacterial, Antifungal, Antimicrobial,
3245	DRAMP02850	Casocidin-1 (mammals, animals)	Antibacterial, Antimicrobial
3246	DRAMP02852	Cathelicidin-2 (Bactenecin-5, Bac5; PR-42; mammals, animals)	Antibacterial, Antimicrobial
3247	DRAMP02853	Cathelicidin-3 (Bactenecin-7, Bac7; PR-59; mammals, animals)	Antibacterial, Anti-Gram-, Antimicrobial
3248	DRAMP02856	Cathelicidin-7 (Antibacterial peptide BMAP-34; mammals, animals)	Antimicrobial,
3249	DRAMP01356	Ranalexin-1Ca (Ranatuerin 1Ca; Frogs, amphibians, animals)	Antimicrobial, Antibacterial, Antifungal, Anti-Gram+, Anti-Gram-,
3250	DRAMP02864	Bovine Beta-defensin 7 (bBD-7; BNBD-7; BNDB-7; mammals, animals)	Antibacterial, Anti-Gram+, Anti-Gram-, Antimicrobial
3251	DRAMP02871	Beta-defensin 119 (Defensin, beta 119; mammals, animals)	Antibacterial, Anti-Gram+, Anti-Gram-, Antimicrobial
3252	DRAMP02874	Chromacin (mammals, animals)	Antibacterial, Anti-Gram+, Antimicrobial
3253	DRAMP02876	Alpha-melanocyte-stimulating hormone (Alpha-MSH; mammals, animals)	Antibacterial, Anti-Gram+, Antimicrobial
3254	DRAMP02879	Tracheal antimicrobial peptide (mammals, animals)	Antibacterial, Antifungal, Antimicrobial
3255	DRAMP02881	Antimicrobial protein exons 1-2 (mammals, animals)	Antibacterial, Antifungal, Antimicrobial
3256	DRAMP02882	Liver-expressed antimicrobial peptide 2 (LEAP-2; mammals, animals)	Antimicrobial,
3257	DRAMP02883	Drosomycin-like A (Predicted)	Antifungal, Antimicrobial
3258	DRAMP02884	Drosomycin-like C	Antifungal, Antimicrobial
3259	DRAMP02885	Drosomycin-like 3 (Drosomycin-like E; Drosomycin-like G; Predicted)	Antifungal, Antimicrobial
3260	DRAMP02886	Drosomycin-like 5 (Drosomycin-like E; Drosomycin-like G; Predicted)	Antifungal, Antimicrobial
3261	DRAMP02887	Dro1 protein (Drosomycin-like 1; Drosomycin-like C1; Predicted)	Antifungal, Antimicrobial
3262	DRAMP02888	Dro2 protein (Drosomycin 2; Drosomycin-like 2; Drosomycin-like D; Drosomycin-like H; Predicted)	Antifungal, Antimicrobial
3263	DRAMP02889	Dro4 protein (Drosomycin 4; Drosomycin-like 4; Drosomycin-like F; Predicted)	Antifungal, Antimicrobial
3264	DRAMP02890	Dro6 protein (Drosomycin-like 6; Drosomycin-like D; Drosomycin-like I; Predicted)	Antifungal, Antimicrobial
3265	DRAMP02891	Tracheal antimicrobial peptide	Antimicrobial,

3266	DRAMP02892	Hepcidin antimicrobial peptide 1	Antimicrobial,
3267	DRAMP02893	Hepcidin antimicrobial peptide 2	Antimicrobial,
3268	DRAMP02894	Hepcidin antimicrobial peptide 3	Antimicrobial,
3269	DRAMP02895	Hepcidin antimicrobial peptide 4	Antimicrobial,
3270	DRAMP02896	Brevinin-1-AJ1 antimicrobial peptide	Antibacterial, Antifungal, Antimicrobial
3271	DRAMP02897	Brevinin-1-AJ2 antimicrobial peptide	Antimicrobial,
3272	DRAMP02898	Brevinin-1-AJ3 antimicrobial peptide	Antimicrobial,
3273	DRAMP02899	Brevinin-1MT1 antimicrobial peptide	Antimicrobial,
3274	DRAMP02900	Brevinin-1MT2 antimicrobial peptide	Antimicrobial,
3275	DRAMP02901	Tricyclic peptide MS-271	Antibacterial, Anti-Gram+, Antimicrobial
3276	DRAMP02902	L-amino-acid oxidase (LAAO, LAO; BpirLAAO-I; reptilia, animals)	Antibacterial, Antiparasitic, Anti-Gram-, Antimicrobial
3277	DRAMP02904	Antibacterial substance A	Antimicrobial,
3278	DRAMP02905	Hinnavin II	Antibacterial, Antimicrobial
3279	DRAMP02908	Beta-defensin 1 (BD-1; sBD-1; mammals, animals)	Antibacterial, Antimicrobial
3280	DRAMP02909	Beta-defensin 2 (BD-2; sBD-2; mammals, animals)	Antibacterial, Antimicrobial
3281	DRAMP02914	Cathelicidin-1 (Bactenecin-1, Bac1; Cyclic dodecapeptide; mammals, animals)	Antibacterial, Anti-Gram+, Anti-Gram-, Antimicrobial
3282	DRAMP02915	Cathelicidin-2 (Bactenecin-5, Bac5; OaBac5; mammals, animals)	Antimicrobial,
3283	DRAMP02916	OaBac6	Antimicrobial,
3284	DRAMP02917	Hepcidin (dogs, mammals, animals)	Antimicrobial,
3285	DRAMP02918	Beta-defensin 1	Antimicrobial,
3286	DRAMP02919	Beta-defensin 110 (Defensin, beta 110; dogs, mammals, animals)	Antibacterial, Antimicrobial
3287	DRAMP02920	Beta-defensin 119 (Defensin, beta 119; dogs, mammals, animals)	Antibacterial, Antimicrobial
3288	DRAMP02921	Beta-defensin 138	Antimicrobial,
3289	DRAMP02927	Antibacterial 11.5 kDa protein (crabs, Arthropods, animals)	Antibacterial, Antimicrobial
3290	DRAMP02928	Antibacterial 6.5 kDa protein (crabs, Arthropods, animals)	Antibacterial, Antimicrobial
3291	DRAMP02929	Antimicrobial protein 1	Antibacterial, Anti-Gram+, Anti-Gram-, Antimicrobial
3292	DRAMP02930	Antimicrobial protein 2 (crabs, Arthropods, animals)	Antibacterial, Anti-Gram+, Anti-Gram-, Antimicrobial
3293	DRAMP02935	Polyphemusin-2 (Polyphemusin II; crabs, Arthropods, animals)	Antibacterial, Antifungal, Antimicrobial
3294	DRAMP02936	Big defensin (crabs, Arthropods, animals)	Antibacterial, Antifungal, Anti-Gram+, Anti-Gram-, Antimicrobial
3295	DRAMP02937	Tachycitin (crabs, Arthropods, animals)	Antibacterial, Antifungal, Anti-Gram+, Anti-Gram-, Antimicrobial
3296	DRAMP18230	Gageotetrin B (Bacteriocin)	Antifungal, Antibacterial, Anti-Gram+, Anti-Gram-, Antimicrobial
3297	DRAMP18231	Gageotetrin C (Bacteriocin)	Antifungal, Antibacterial, Anti-Gram+, Anti-Gram-, Antimicrobial
3298	DRAMP18232	Gageopeptide A(Bacteriocin)	Antifungal, Antibacterial, Anti-Gram+, Anti-Gram-, Antimicrobial
3299	DRAMP02939	Tachyplesin-2 (Tachyplesin II; crabs, Arthropods, animals)	Antibacterial, Antifungal, Antimicrobial
3300	DRAMP02940	Tachyplesin-3 (Tachyplesin III; crabs, Arthropods, animals)	Antibacterial, Antimicrobial
3301	DRAMP02941	Tachystatin-A1 (crabs, Arthropods, animals)	Antibacterial, Antifungal, Anti-Gram+, Anti-Gram-, Antimicrobial
3302	DRAMP02942	Tachystatin-A2 (crabs, Arthropods, animals)	Antibacterial, Antifungal, Anti-Gram+, Anti-Gram-, Antimicrobial
3303	DRAMP02943	Tachystatin-B1 (crabs, Arthropods, animals)	Antibacterial, Antifungal, Anti-Gram+, Antimicrobial
3304	DRAMP02944	Tachystatin-B2 (crabs, Arthropods, animals)	Antibacterial, Antifungal, Anti-Gram+, Antimicrobial
3305	DRAMP02945	Tachystatin-C (crabs, Arthropods, animals)	Antibacterial, Antifungal, Anti-Gram+, Anti-Gram-, Antimicrobial
3306	DRAMP02946	PtALF1 (Portunus trituberculatus anti-lipopolysaccharide factor isoform 1; crabs, Arthropods, animals)	Antibacterial, Antimicrobial
3307	DRAMP02947	PtALF2 (Portunus trituberculatus anti-lipopolysaccharide factor isoform 2; crabs, Arthropods, animals)	Antibacterial, Antimicrobial
3308	DRAMP18228	Gageostatin C (Bacteriocin)	Antibacterial, antifungal, Anti-Gram+, Anti-Gram-, Antimicrobial
3309	DRAMP18229	Gageotetrin A (Bacteriocin)	Antifungal, Antibacterial, Anti-Gram+, Anti-Gram-, Antimicrobial

3310	DRAMP02949	PtALF4 (Portunus trituberculatus anti-lipopolysaccharide factor isoform 4; crabs, Arthropods, animals)	Antibacterial, Antimicrobial
3311	DRAMP02954	Arasin 2 (Pro-rich, Arg-rich; crabs, Arthropods, animals)	Antibacterial, Antimicrobial
3312	DRAMP02955	Hedistin (marine annelid, Metazoa)	Antibacterial, Anti-Gram+, Anti-Gram-, Antimicrobial
3313	DRAMP02957	Liver-expressed antimicrobial protein 2 (pigs, mammals, animals)	Antimicrobial,
3314	DRAMP02958	Cathelin (pigs, mammals, animals)	Antimicrobial,
3315	DRAMP02967	Antibacterial peptide 3910 (AP 3910; pigs, mammals, animals)	Antibacterial, Anti-Gram+, Antimicrobial
3316	DRAMP02968	Prophenin-1 (C6, PF-1; Pro-rich; pigs, mammals, animals)	Antibacterial, Anti-Gram+, Anti-Gram-, Antimicrobial
3317	DRAMP02969	Prophenin-2 (C12, PF-2, PR-2; Pro-rich; pigs, mammals, animals)	Antibacterial, Anti-Gram+, Anti-Gram-, Antimicrobial
3318	DRAMP02971	Protegrin-2 (Protegrin 2; PG-2; pigs, mammals, animals)	Antibacterial, Antifungal, Anti-Gram+, Anti-Gram-, Antimicrobial
3319	DRAMP02972	Protegrin-3 (Protegrin 3; PG-3; pigs, mammals, animals)	Antibacterial, Antifungal, Anti-Gram+, Anti-Gram-, Antimicrobial
3320	DRAMP02973	Protegrin-4 (Protegrin 4; PG-4; pigs, mammals, animals)	Antibacterial, Antifungal, Antimicrobial
3321	DRAMP02974	Protegrin-5 (Protegrin 5; PG-5; pigs, mammals, animals)	Antibacterial, Antifungal, Antimicrobial
3322	DRAMP02976	Beta-defensin 1 (BD-1; Defensin, beta 1; pigs, mammals, animals)	Antibacterial, Anti-Gram+, Anti-Gram-, Antimicrobial
3323	DRAMP02977	pBD-1 (porcine beta-defensin 1; pigs, mammals, animals)	Antibacterial, Anti-Gram+, Antimicrobial
3324	DRAMP02978	pBD-2 (porcine beta-defensin 2; pigs, mammals, animals)	Antibacterial, Antimicrobial
3325	DRAMP02979	Hepcidin (pigs, mammals, animals)	Antimicrobial,
3326	DRAMP02981	Liver-expressed antimicrobial peptide 2 (LEAP-2; pigs, mammals, animals)	Antimicrobial ,
3327	DRAMP02982	Reactive oxygen species modulator 1 (ROS modulator 1; pigs, mammals, animals)	Antibacterial, Anti-Gram+, Anti-Gram-, Antimicrobial
3328	DRAMP02983	Liver-expressed antimicrobial peptide 2 (LEAP-2; pigs, mammals, animals)	Antimicrobial ,
3329	DRAMP02984	Neutrophil cationic antibacterial polypeptide of 11 kDa (CAP11; pigs, mammals, animals)	Antibacterial, Anti-Gram+, Anti-Gram-, Antimicrobial
3330	DRAMP02985	Neutrophil cationic peptide 2 (CP-2; GNCP-2; pigs, mammals, animals)	Antibacterial, Antifungal, Antiviral, Anti-Gram+, Anti-Gram-, Antimicrobial
3331	DRAMP02986	Neutrophil cationic peptide 1 (GPNP; Antiviral defensin; pigs, mammals, animals)	Antibacterial, Antifungal, Antiviral, Anti-Gram+, Anti-Gram-, Antimicrobial
3332	DRAMP02987	Tricholongin BII (Fungi)	Antibacterial, Antifungal, Antimicrobial
3333	DRAMP02988	Tricholongin BI (Fungi)	Antibacterial, Antifungal, Antimicrobial
3334	DRAMP02989	Lasioglossin LL-I (Insects, animals)	Antibacterial, Anticancer, Anti-Gram+, Anti-Gram-, Antimicrobial
3335	DRAMP02990	Lasioglossin LL-II (Insects, animals)	Antibacterial, Anticancer, Anti-Gram+, Anti-Gram-, Antimicrobial
3336	DRAMP02991	Lasioglossin LL-III (Insects, animals)	Antibacterial, Anticancer, Anti-Gram+, Anti-Gram-, Antimicrobial
3337	DRAMP02992	Lasiocepsin (Insects, animals)	Antibacterial, Antifungal, Antiviral, Antimicrobial
3338	DRAMP02994	Defensin-1 (Royalisin; Insects, animals)	Antibacterial, Antimicrobial
3339	DRAMP03004	Abaecin (Insects, animals)	Antibacterial, Anti-Gram-, Antimicrobial
3340	DRAMP03005	Apidaecin (Insects, animals)	Antibacterial, Anti-Gram-, Antimicrobial
3341	DRAMP03006	Defensin (Insects, animals)	Antibacterial, Antifungal, Anti-Gram-, Antimicrobial
3342	DRAMP03013	Bombolitin-6 (Insects, animals)	Antibacterial, Antifungal, Antimicrobial
3343	DRAMP03014	Bombolitin-7 (Insects, animals)	Antibacterial, Antifungal, Antimicrobial
3344	DRAMP03015	Bombolitin-8 (Insects, animals)	Antibacterial, Antifungal, Antimicrobial
3345	DRAMP03016	Defensin-1 (Insects, animals)	Antibacterial, Antimicrobial
3346	DRAMP03017	VESP-VB1 (Insects, animals)	Antibacterial, Antifungal, Antimicrobial
3347	DRAMP03018	MP-VB1 (Insects, animals)	Antibacterial, Antifungal, Antimicrobial
3348	DRAMP03023	Mastoparan (Protonectarina-MP; Insects, animals)	Antibacterial, Antifungal, Anti-Gram+, Anti-Gram-, Antimicrobial
3349	DRAMP18227	Gageostatin B (Bacteriocin)	Antibacterial, antifungal, Anti-Gram+, Anti-Gram-, Antimicrobial
3350	DRAMP03029	Mastoparan-V1 (Insects, animals)	Antibacterial, Antimicrobial
3351	DRAMP03030	Mastoparan-V2 (Insects, animals)	Antibacterial, Antimicrobial
3352	DRAMP03031	Mastoparan-T (Insects, animals)	Antibacterial, Antimicrobial
3353	DRAMP03032	Mastoparan-A (Insects, animals)	Antibacterial, Antimicrobial

3354	DRAMP03048	Venom peptide 3 (OdVP3; Insects, animals)	Antibacterial, Antifungal, Antimicrobial
3355	DRAMP03049	Eumenine mastoparan-OD (EMP-OD; Venom peptide 1, OdVP1; Insects, animals)	Antibacterial, Antifungal, Anti-Gram+, Anti-Gram-, Antimicrobial
3356	DRAMP03058	Phormia defensin A (insect defensin A, Ptdefensin A; Insects, animals)	Antibacterial, Antimicrobial
3357	DRAMP03059	Phormia defensin B (insect defensin B; Insects, animals)	Antibacterial, Antimicrobial
3358	DRAMP03060	S.calcitrans defensin 2 (Smd2; defensins; Insects, animals)	Antibacterial, Antimicrobial
3359	DRAMP03061	S.calcitrans defensin 1 (Smd1; defensins; Insects, animals)	Antibacterial, Antimicrobial
3360	DRAMP03062	Defensin-A (DefA; GmDefA; Insects, animals)	Antibacterial, Antiprotozoal, Anti-Gram-, Antimicrobial
3361	DRAMP03064	Alloferon-1 (Insects, animals)	Antiparasitic, Antiviral, Antitumor, Antimicrobial
3362	DRAMP03065	Alloferon-2 (Insects, animals)	Antibacterial, Antifungal, Antiviral, Antimicrobial
3363	DRAMP03066	Lucifensin (Lucifensin II; Insects, animals)	Antibacterial, Anti-Gram+, Antimicrobial
3364	DRAMP03067	Lucifensin (Lucilia defensin; Insects, animals)	Antibacterial, Anti-Gram+, Antimicrobial
3365	DRAMP03068	Antifungal protein 1 (MAF-1; Insects, animals)	Antimicrobial,
3366	DRAMP18226	Gageostatin A (Bacteriocin)	Antibacterial, antifungal, Anti-Gram+, Anti-Gram-, Antimicrobial
3367	DRAMP03071	Diptericin-A (Insects, animals)	Antibacterial, Anti-Gram-, Antimicrobial
3368	DRAMP03072	Diptericin-D (Insects, animals)	Antibacterial, Anti-Gram-, Antimicrobial
3369	DRAMP03073	Sapecin-C (Sapecin C; defensins; Insects, animals)	Antibacterial, Anti-Gram+, Anti-Gram-, Antimicrobial
3370	DRAMP03074	Cecropin (Insects, animals)	Antibacterial, Antimicrobial
3371	DRAMP03076	Cecropin-1 (Cecropin 1; Insects, animals)	Antibacterial, Antimicrobial
3372	DRAMP03077	Cecropin-2 (Cecropin 2; Md-Cec, Mdc; Insects, animals)	Antibacterial, Antifungal, Antimicrobial
3373	DRAMP03078	Cecropin-A2 (Insects, animals)	Antibacterial, Antimicrobial
3374	DRAMP03079	Cecropin-A1 (Insects, animals)	Antibacterial, Antimicrobial
3375	DRAMP03080	Cecropin-B (Insects, animals)	Antibacterial, Antimicrobial
3376	DRAMP03081	Cecropin-C (Insects, animals)	Antibacterial, Antimicrobial
3377	DRAMP03082	Defensin (invertebrate defensin; Insects, animals)	Antibacterial, Antimicrobial
3378	DRAMP03083	Sapecin-B (defensins; Insects, animals)	Antibacterial, Anti-Gram+, Anti-Gram-, Antimicrobial
3379	DRAMP03084	Ceratotoxin-B (Insects, animals)	Antibacterial, Anti-Gram+, Anti-Gram-, Antimicrobial
3380	DRAMP03085	Ceratotoxin-A (Insects, animals)	Antibacterial, Anti-Gram+, Anti-Gram-, Antimicrobial
3381	DRAMP03086	Ceratotoxin-D (Insects, animals)	Antibacterial, Anti-Gram+, Anti-Gram-, Antimicrobial
3382	DRAMP03087	Drosophila diptericin (Insects, animals)	Antibacterial, Antifungal, Antiviral, Antimicrobial
3383	DRAMP03088	Diptericin (Insects, animals)	Antibacterial, Antimicrobial
3384	DRAMP18225	Subtilosin A1 (Bacteriocin)	Antibacterial, Antimicrobial
3385	DRAMP03092	Drosophila defensin (Insects, animals)	Antibacterial, Antimicrobial
3386	DRAMP03093	Drosomycin (Cys-rich; insect defensins; Insects, animals)	Antifungal, Antimicrobial
3387	DRAMP03094	Drosomycin-2 (Insects, animals)	Antifungal, Antiparasitic, Antimicrobial
3388	DRAMP03099	Attacin-C (Insects, animals)	Antibacterial, Antifungal, Antimicrobial
3389	DRAMP18224	Ala-6-fenycin (Bacteriocin)	Antibacterial, Antiviral, Anti-Gram+, Antimicrobial
3390	DRAMP03101	Sarcotoxin-1C (Sarcotoxin IC; Insects, animals)	Antibacterial, Anti-Gram-, Antimicrobial
3391	DRAMP03102	Sarcotoxin-1B (Sarcotoxin IB; Insects, animals)	Antibacterial, Anti-Gram-, Antimicrobial
3392	DRAMP03103	Sarcotoxin-1D (Sarcotoxin ID; Insects, animals)	Antibacterial, Anti-Gram+, Antimicrobial
3393	DRAMP03105	Antifungal protein (AFP; Insects, animals)	Antifungal, Antimicrobial
3394	DRAMP03106	Andropin (Insects, animals)	Antibacterial, Antimicrobial
3395	DRAMP03107	Andropin (Insects, animals)	Antibacterial, Antimicrobial
3396	DRAMP03108	Andropin (Insects, animals)	Antibacterial, Antimicrobial
3397	DRAMP03109	Andropin (Insects, animals)	Antibacterial, Antimicrobial
3398	DRAMP03110	Andropin (Insects, animals)	Antibacterial, Antiprotozoal, Antimicrobial
3399	DRAMP03111	Andropin (Insects, animals)	Antibacterial, Antimicrobial
3400	DRAMP18223	Sonorensin(Bacteriocin)	Antibacterial, Anti-Gram+, Anti-Gram-, Antimicrobial
3401	DRAMP03114	Cecropin-1/3 (Insects, animals)	Antibacterial, Antimicrobial
3402	DRAMP03118	Cecropin-C (AgCecC; Insects, animals)	Antibacterial, Antimicrobial
3403	DRAMP03119	Def-BAT (hybrid defensins; Insects, animals)	Antibacterial, Anti-Gram+, Antimicrobial
3404	DRAMP03120	Def-BBB (hybrid defensins; Insects, animals)	Antibacterial, Anti-Gram+, Antimicrobial
3405	DRAMP03121	Def-ABB (hybrid defensins; Insects, animals)	Antibacterial, Anti-Gram+, Antimicrobial
3406	DRAMP03122	Anopheles gambiae defensin (DEF-AAA; Insects, animals)	Antibacterial, Anti-Gram+, Antimicrobial
3407	DRAMP03123	Cecropin-B (AgCecB; Insects, animals)	Antibacterial, Antimicrobial
3408	DRAMP03124	Def-AcAA (hybrid defensins; Insects, animals)	Antibacterial, Anti-Gram+, Antimicrobial

3409	DRAMP03125	Def-DAA (hybrid defensins; Insects, animals)	Antibacterial, Anti-Gram+, Antimicrobial
3410	DRAMP03126	Antimicrobial peptide defensin 3 (AgDef3; Insects, animals)	Antibacterial, Antimicrobial
3411	DRAMP03127	Cecropin-A (Insects, animals)	Antibacterial, Antimicrobial
3412	DRAMP03128	Cecropin-B1 (Insects, animals)	Antibacterial, Antimicrobial
3413	DRAMP03129	Cecropin-B2 (Insects, animals)	Antibacterial, Antimicrobial
3414	DRAMP03130	Cecropin-A2 (Insects, animals)	Antibacterial, Antimicrobial
3415	DRAMP03131	Cecropin-B (Insects, animals)	Antibacterial, Antimicrobial
3416	DRAMP03132	Cecropin-C (Insects, animals)	Antibacterial, Antimicrobial
3417	DRAMP03133	Cecropin-A1 (AalCecA; Cecropin-A; Insects, animals)	Antibacterial, Antimicrobial
3418	DRAMP03134	Defensin-D (AaeDefD; Insects, animals)	Antibacterial, Anti-Gram+, Anti-Gram-, Antimicrobial
3419	DRAMP03135	Defensin-B (AaeDefB; Insects, animals)	Antibacterial, Antimicrobial
3420	DRAMP03136	Defensin-C (AaeDefC; Insects, animals)	Antibacterial, Antimicrobial
3421	DRAMP03139	AAEL003849-PA	Antimicrobial,
3422	DRAMP03141	Putative defensin 5	Antimicrobial,
3423	DRAMP03142	AGAP004632-PA (Defensin)	Antimicrobial,
3424	DRAMP03143	AGAP008645-PA (Putative infection responsive short peptide)	Antimicrobial,
3425	DRAMP03144	Salivary defensin	Antimicrobial,
3426	DRAMP03145	Defensin	Antimicrobial,
3427	DRAMP03146	Defensin	Antimicrobial,
3428	DRAMP03147	Defensin-A	Antimicrobial,
3429	DRAMP03148	Defensin	Antimicrobial,
3430	DRAMP03149	Defensin D	Antimicrobial,
3431	DRAMP03151	Spodoptera cecropins A (insects, invertebrates, animals)	Antibacterial, Antimicrobial
3432	DRAMP03152	Spodoptera cecropins B (insects, invertebrates, animals)	Antibacterial, Antimicrobial
3433	DRAMP03154	Hadrurin (Non-disulfide-bridged peptide 3.1)	Antibacterial, Anti-Gram-, Antimicrobial
3434	DRAMP03155	Sillucin	Antibacterial, Antimicrobial
3435	DRAMP03156	Cathelicidin-3 (Bactenecin-7, Bac7; PR-59; mammals, animals)	Antibacterial, Antimicrobial
3436	DRAMP03157	Halocidin subunit B	Antibacterial, Anti-Gram+, Anti-Gram-, Antimicrobial
3437	DRAMP03158	Halocidin subunit A (invertebrates, animals; Preclinical)	Antibacterial, Anti-Gram+, Anti-Gram-, Antimicrobial
3438	DRAMP03159	WAP four-disulfide core domain protein 12 (mammals, animals)	Antibacterial, Antimicrobial
3439	DRAMP03160	WAP four-disulfide core domain protein 12 (mammals, animals)	Antibacterial, Antimicrobial
3440	DRAMP03161	Beta-defensin 1 (BD-1; Defensin, beta 1; mammals, animals)	Antibacterial, Antimicrobial
3441	DRAMP03163	R. prolixus defensin A (RprDefA; insect defensin; Insects, animals)	Antibacterial, Insecticidal, Anti-Gram+, Anti-Gram-, Antimicrobial
3442	DRAMP03164	R. prolixus defensin B (RprDefB; insect defensin; Insects, animals)	Antibacterial, Insecticidal, Anti-Gram+, Anti-Gram-, Antimicrobial
3443	DRAMP03165	R. prolixus defensin C (RprDefC; insect defensin; Insects, animals)	Antibacterial, Insecticidal, Anti-Gram+, Anti-Gram-, Antimicrobial
3444	DRAMP03167	P9	Antimicrobial,
3445	DRAMP03168	L-amino-acid oxidase (K-LAO; LAAO; LAO; reptilia, animals)	Antibacterial, Antimicrobial
3446	DRAMP03170	Antibacterial protein 1 homolog (Gram-positive bacteria)	Antibacterial, Antimicrobial
3447	DRAMP18222	PseA(Bacteriocin)	Antibacterial, Anti-Gram+, Antimicrobial
3448	DRAMP03172	Antibacterial protein 3 homolog (Gram-positive bacteria)	Antibacterial, Antimicrobial
3449	DRAMP03174	Arenicin-2 (Ar-2; marine polychaeta, animals)	Antibacterial, Antifungal, Anti-Gram+, Anti-Gram-, Antimicrobial
3450	DRAMP03175	Perinerin	Antibacterial, Antifungal, Anti-Gram+, Anti-Gram-, Antimicrobial
3451	DRAMP03177	Cecropin-P2 (CP2; nematodes, animals)	Antibacterial, Antifungal, Antimicrobial
3452	DRAMP03178	Cecropin-P3 (CP3; nematodes, animals)	Antibacterial, Antifungal, Antimicrobial
3453	DRAMP03179	Cecropin-P4 (CP4; nematodes, animals)	Antibacterial, Antifungal, Antimicrobial
3454	DRAMP03180	ASABF-alpha (ASABF; nematodes, animals)	Antibacterial, Antifungal, Anti-Gram+, Anti-Gram-, Antimicrobial
3455	DRAMP03182	Termicin (Termite defensin; Insects, animals)	Antibacterial, Antifungal, Anti-Gram+, Antimicrobial
3456	DRAMP03183	Naegleriapore A	Antibacterial, Antiprotozoal, Cytotoxicity, Anti-Gram+, Anti-Gram-, Antimicrobial
3457	DRAMP03184	Naegleriapore B	Antibacterial, Antiprotozoal, Cytotoxicity, Anti-Gram+, Anti-Gram-, Antimicrobial
3458	DRAMP03185	Spheniscin-1 (Sphe-1; penguin avian beta-defensin 103a; birds , animals)	Antibacterial, Antifungal, Antimicrobial

3459	DRAMP03188	Antibacterial protein LL-37 (primates, mammals, animals)	Antibacterial, Anti-Gram-, Antimicrobial
3460	DRAMP03189	Antibacterial protein LL-39 (primates, mammals, animals)	Antibacterial, Antimicrobial
3461	DRAMP18221	Fusaricidin D (Bacteriocin)	Antibacterial, Antifungal, Anti-Gram+, Antimicrobial
3462	DRAMP03192	Antibacterial protein LL-37 (cathelicidin; primates, mammals, animals)	Antibacterial, Antimicrobial
3463	DRAMP03194	Theta defensin subunit A (primates, mammals, animals)	Antibacterial, Antifungal, Antimicrobial
3464	DRAMP03195	Theta defensin subunit B (primates, mammals, animals)	Antibacterial, Antifungal, Antimicrobial
3465	DRAMP03199	PhD1 (PhD-1; Defensin-1; primates, mammals, animals)	Antibacterial, Antifungal, Anti-Gram+, Anti-Gram-, Antimicrobial
3466	DRAMP03200	PhD2 (PhD-2; Defensin-2; primates, mammals, animals)	Antibacterial, Antifungal, Anti-Gram+, Anti-Gram-, Antimicrobial
3467	DRAMP03201	PhD3 (PhD-3; Defensin-3; primates, mammals, animals)	Antibacterial, Antifungal, Anti-Gram+, Anti-Gram-, Antimicrobial
3468	DRAMP03202	WAP four-disulfide core domain protein 12 (primates, mammals, animals)	Antibacterial, Antimicrobial
3469	DRAMP18220	Fusaricidin C (Bacteriocin)	Antibacterial, Antifungal, Anti-Gram+, Antimicrobial
3470	DRAMP18219	Fusaricidin B (Bacteriocin)	Antibacterial, Antifungal, Anti-Gram+, Antimicrobial
3471	DRAMP03206	Theta defensin subunit C (BTD-c; BTD-4 subunit 2; primates, mammals, animals)	Antibacterial, Antifungal, Antimicrobial
3472	DRAMP03207	Theta defensin subunit D (BTD-d; BTD-7 subunit 2; primates, mammals, animals)	Antibacterial, Antifungal, Antimicrobial
3473	DRAMP03208	BTD-1 (theta-defensin; primates, mammals, animals)	Antibacterial, Antifungal, Anti-Gram+, Anti-Gram-, Antimicrobial
3474	DRAMP03209	BTD-2 (theta-defensin; primates, mammals, animals)	Antibacterial, Antifungal, Anti-Gram+, Anti-Gram-, Antimicrobial
3475	DRAMP18218	Fusaricidin A (Bacteriocin)	Antibacterial, Antifungal, Anti-Gram+, Antimicrobial
3476	DRAMP03211	BTD-4 (theta-defensin; primates, mammals, animals)	Antibacterial, Antifungal, Anti-Gram+, Anti-Gram-, Antimicrobial
3477	DRAMP03212	BTD-7 (theta-defensin; primates, mammals, animals)	Antibacterial, Antifungal, Anti-Gram+, Anti-Gram-, Antimicrobial
3478	DRAMP18217	Licheniocin 50.2(Bacteriocin)	Antibacterial, Anti-Gram+, Antimicrobial
3479	DRAMP18216	Cerein 7A(Bacteriocin)	Antibacterial, Anti-Gram+, Antimicrobial
3480	DRAMP03218	M-oxotoxin-Ot2a (Oxyopinin-2a, Oxki2a; spiders, Arthropods, animals)	Antibacterial, Insecticidal, Antimicrobial
3481	DRAMP03219	M-oxotoxin-OtIIb (Oxyopinin-IIb, OxkiIIb; spiders, Arthropods, animals)	Antibacterial, Insecticidal, Antimicrobial
3482	DRAMP03220	M-oxotoxin-Ot2c (Oxyopinin-2c, Oxki2c; spiders, Arthropods, animals)	Antibacterial, Insecticidal, Antimicrobial
3483	DRAMP03221	M-oxotoxin-Ot2d (Oxyopinin-2d, Oxki2d; spiders, Arthropods, animals)	Antibacterial, Insecticidal, Antimicrobial
3484	DRAMP03223	M-ctenitoxin-Cs1b (M-CNTX-Cs1b; Cupiennin-1b; spiders, Arthropods, animals)	Antibacterial, Insecticidal, Antimicrobial
3485	DRAMP03224	M-ctenitoxin-Cs1c (M-CNTX-Cs1c; Cupiennin-1c; spiders, Arthropods, animals)	Antibacterial, Anti-Gram+, Anti-Gram-, Antimicrobial
3486	DRAMP03228	M-zodatoxin-Lt2b (M-ZDTX-Lt2b; Latarcin-2b, Ltc-2b; spiders, Arthropods, animals)	Antibacterial, Antifungal, Antimicrobial
3487	DRAMP03235	M-zodatoxin-Lt6b (M-ZDTX-Lt6b; Latarcin 6b, Ltc-6b; spiders, Arthropods, animals)	Antibacterial, Antifungal, Antiviral, Antimicrobial
3488	DRAMP18215	Cerecidin A7(Bacteriocin)	Antibacterial, Anti-Gram+, Antimicrobial
3489	DRAMP03238	M-zodatoxin-Lt8c (M-ZDTX-Lt8c; Cytoinsectotoxin-1c, CIT-1c; spiders, Arthropods, animals)	Antibacterial, Insecticidal, Anti-Gram+, Anti-Gram-, Antimicrobial
3490	DRAMP03239	M-zodatoxin-Lt8d (M-ZDTX-Lt8d; Cytoinsectotoxin-1d, CIT-1d; spiders, Arthropods, animals)	Antibacterial, Insecticidal, Antimicrobial
3491	DRAMP03240	M-zodatoxin-Lt8e (M-ZDTX-Lt8e; Cytoinsectotoxin-1e, CIT-1e; spiders, Arthropods, animals)	Antibacterial, Insecticidal, Antimicrobial
3492	DRAMP03241	M-zodatoxin-Lt8f (M-ZDTX-Lt8f; Cytoinsectotoxin-1f, CIT-1f; spiders, Arthropods, animals)	Antibacterial, Insecticidal, Anti-Gram-, Antimicrobial
3493	DRAMP03242	M-zodatoxin-Lt8g (M-ZDTX-Lt8g; Cytoinsectotoxin-1g, CIT-1g; spiders, Arthropods, animals)	Antibacterial, Insecticidal, Anti-Gram-, Antimicrobial
3494	DRAMP03243	M-zodatoxin-Lt8h (M-ZDTX-Lt8h; Cytoinsectotoxin-1h, CIT-1h; spiders, Arthropods, animals)	Antibacterial, Insecticidal, Anti-Gram-, Antimicrobial
3495	DRAMP03244	M-zodatoxin-Lt8j (M-ZDTX-Lt8j; Cytoinsectotoxin 1-9; spiders, Arthropods, animals)	Antibacterial, Insecticidal, Antimicrobial

3496	DRAMP03245	M-zodatoxin-Lt8k (M-ZDTX-Lt8k; Cytoinsectotoxin 1-10; spiders, Arthropods, animals)	Antibacterial, Insecticidal, Antimicrobial
3497	DRAMP03246	M-zodatoxin-Lt8i (M-ZDTX-Lt8i; Cytoinsectotoxin 1-6; spiders, Arthropods, animals)	Antibacterial, Insecticidal, Antimicrobial
3498	DRAMP03247	M-zodatoxin-Lt8l (M-ZDTX-Lt8l; Cytoinsectotoxin 1-12; spiders, Arthropods, animals)	Antibacterial, Insecticidal, Antimicrobial
3499	DRAMP03248	M-zodatoxin-Lt8m (M-ZDTX-Lt8m; Cytoinsectotoxin 1-13; spiders, Arthropods, animals)	Antibacterial, Insecticidal, Antimicrobial
3500	DRAMP03249	M-zodatoxin-Lt8o (M-ZDTX-Lt8o; Cytoinsectotoxin 1-14; spiders, Arthropods, animals)	Antibacterial, Insecticidal, Antimicrobial
3501	DRAMP03250	M-zodatoxin-Lt8p (M-ZDTX-Lt8p; Cytoinsectotoxin 1-15; spiders, Arthropods, animals)	Antibacterial, Insecticidal, Antimicrobial
3502	DRAMP03251	M-zodatoxin-Lt8q (M-ZDTX-Lt8q; Cytoinsectotoxin 1-16; spiders, Arthropods, animals)	Antibacterial, Insecticidal, Antimicrobial
3503	DRAMP03252	M-lycotoxin-Ls2a (M-LCTX-Ls2a; Lycocitin-3; spiders, Arthropods, animals)	Antibacterial, Antifungal, Antimicrobial
3504	DRAMP03255	M-lycotoxin-Ls4a (M-LCTX-Ls4a; Peptide 2340; spiders, Arthropods, animals)	Antibacterial, Antimicrobial
3505	DRAMP03256	U1-lycotoxin-Ls1a (U1-LCTX-Ls1a; Peptide 2034; spiders, Arthropods, animals)	Antibacterial, Antimicrobial
3506	DRAMP03257	Oligoventin (spiders, Arthropods, animals)	Antibacterial, Antimicrobial
3507	DRAMP03258	Rondonin (spiders, Arthropods, animals)	Antifungal, Antimicrobial
3508	DRAMP03260	U14-lycotoxin-Ls1a (Toxin-like structure LSTX-N1; spiders, Arthropods, animals)	Antibacterial, Antimicrobial
3509	DRAMP03262	U14-lycotoxin-Ls1b (Toxin-like structure LSTX-N3; spiders, Arthropods, animals)	Antibacterial, Antimicrobial
3510	DRAMP18214	Cerecidin A1(Bacteriocin)	Antibacterial, Anti-Gram+, Antimicrobial
3511	DRAMP03265	U14-lycotoxin-Ls1c (Toxin-like structure LSTX-N6; spiders, Arthropods, animals)	Antibacterial, Antimicrobial
3512	DRAMP03266	U15-lycotoxin-Ls1f (Toxin-like structure LSTX-N7; spiders, Arthropods, animals)	Antibacterial, Antimicrobial
3513	DRAMP03267	U15-lycotoxin-Ls1d (Toxin-like structure LSTX-N8; spiders, Arthropods, animals)	Antibacterial, Antimicrobial
3514	DRAMP18213	Gramicidin S(Bacteriocin)	Antibacterial, Antifungal, Anti-Gram+, Anti-Gram-, Antimicrobial
3515	DRAMP03269	U15-lycotoxin-Ls1a (Toxin-like structure LSTX-N10; spiders, Arthropods, animals)	Antibacterial, Antimicrobial
3516	DRAMP03270	U15-lycotoxin-Ls1b (Toxin-like structure LSTX-N11; spiders, Arthropods, animals)	Antibacterial, Antimicrobial
3517	DRAMP03271	U15-lycotoxin-Ls1c (Toxin-like structure LSTX-N12; spiders, Arthropods, animals)	Antibacterial, Antimicrobial
3518	DRAMP18211	Anacyclamide A10(Bacteriocin)	Antibacterial, Antimicrobial
3519	DRAMP18212	Amylocyclicin(Bacteriocin)	Antibacterial, Anti-Gram+, Antimicrobial
3520	DRAMP03273	U15-lycotoxin-Ls1e (Toxin-like structure LSTX-N14; spiders, Arthropods, animals)	Antibacterial, Antimicrobial
3521	DRAMP18210	Amythiamicin C/D(Bacteriocin)	Antibacterial,Antiparasitic, Anti-Gram+, Antimicrobial
3522	DRAMP03276	U15-lycotoxin-Ls1g (Toxin-like structure LSTX-N17; spiders, Arthropods, animals)	Antibacterial, Antimicrobial
3523	DRAMP03277	U15-lycotoxin-Ls1h (Toxin-like structure LSTX-N18; spiders, Arthropods, animals)	Antibacterial, Antimicrobial
3524	DRAMP03281	Turkey Heterophil Peptide 3 (Antimicrobial peptide THP3; Birds, animals)	Antibacterial, Anti-Gram+, Antimicrobial
3525	DRAMP03282	Turkey Heterophil Peptide 1 (Antimicrobial peptide THP1; Birds, animals)	Antibacterial, Anti-Gram+, Anti-Gram-, Antimicrobial
3526	DRAMP03283	Turkey Heterophil Peptide 2 (Antimicrobial peptide THP2, THP2; Birds, animals)	Antibacterial, Anti-Gram+, Antimicrobial
3527	DRAMP03284	Beta-defensin (Birds, animals)	Antibacterial, Antimicrobial
3528	DRAMP03289	Apl-AvBD16 (Beta defensins; Ducks, birds, animals)	Antibacterial, Antiviral, Antimicrobial
3529	DRAMP03290	Anas platyrhynchos avian beta-defensin 2 (Apl_AvBD2; Ducks, birds, animals)	Antibacterial, Antimicrobial
3530	DRAMP03291	Beta defensin-6-like antimicrobial peptide (Ducks, birds, animals; Predicted)	Antimicrobial,
3531	DRAMP03292	Defensin-B1 (DefB1; OaDefB1; mammals, animals)	Antimicrobial,

3532	DRAMP03293	Defensin-B2 (DefB2; OaDefB2; mammals, animals)	Antimicrobial,
3533	DRAMP03294	Defensin-B3 (DefB3; OaDefB3; mammals, animals)	Antimicrobial,
3534	DRAMP03295	Defensin-B4 (DefB4; OaDefB4; mammals, animals)	Antimicrobial,
3535	DRAMP03296	Defensin-B5 (DefB5; OaDefB5; mammals, animals)	Antimicrobial,
3536	DRAMP03297	Defensin-B6 (DefB6; OaDefB6; mammals, animals)	Antimicrobial,
3537	DRAMP03298	Defensin-A1 (DefA1; OaDefA1; mammals, animals)	Antimicrobial,
3538	DRAMP03299	Defensin-A2 (DefA2; OaDefA2; mammals, animals)	Antimicrobial,
3539	DRAMP03300	Defensin-A3 (DefA3; OaDefA3; mammals, animals)	Antimicrobial,
3540	DRAMP03301	Defensin-A4 (DefA4; OaDefA4; mammals, animals)	Antimicrobial,
3541	DRAMP03302	Defensin-BvL (DefB-vL; OaDefB-vL; mammals, animals)	Antimicrobial,
3542	DRAMP03303	Theromyzin (Frogs, amphibians, animals)	Antibacterial, Anti-Gram+, Antimicrobial
3543	DRAMP03304	Hydramacin-1 (Hm-1; annelida, animals)	Antibacterial, Anti-Gram+, Anti-Gram-, Antimicrobial
3544	DRAMP03305	Neuromacin (Annelida, animals)	Antimicrobial,
3545	DRAMP03306	Theromacin (Annelida, animals)	Antibacterial, Anti-Gram+, Antimicrobial
3546	DRAMP03307	Duck AvBD9 (avian beta defensin 9; Birds, animals)	Antibacterial, Antimicrobial
3547	DRAMP03308	Duck AvBD10 (avian beta defensin 10; Birds, animals)	Antibacterial, Antimicrobial
3548	DRAMP18209	NAI-802 (Bacteriocin)	Antibacterial, Anti-Gram+, Antimicrobial
3549	DRAMP03310	Crabrolin (Insects, animals)	Antibacterial, Antimicrobial
3550	DRAMP03313	Metalnikowin-2A (Metalnikowin IIA; Insects, animals)	Antibacterial, Antimicrobial
3551	DRAMP03314	Metalnikowin-2B (Metalnikowin IIB; Insects, animals)	Antibacterial, Antimicrobial
3552	DRAMP03315	Metalnikowin-3 (Metalnikowin III; Insects, animals)	Antibacterial, Antimicrobial
3553	DRAMP03316	Defensin (Insects, animals)	Antibacterial, Antimicrobial
3554	DRAMP03317	Cathelicidin-related antimicrobial peptide (AMPs)	Antibacterial, Anti-Gram+, Anti-Gram-, Antimicrobial
3555	DRAMP03318	Pore-forming peptide ameobapore B (EH-APP; saposin-like protein)	Antibacterial, Anti-Gram+, Antimicrobial
3556	DRAMP03319	Pore-forming peptide ameobapore C (EH-APP; saposin-like protein)	Antibacterial, Anti-Gram+, Antimicrobial
3557	DRAMP03322	PW2	Antifungal, Antimicrobial
3558	DRAMP03323	Testis defensin	Antimicrobial,
3559	DRAMP03324	Testis defensin	Antimicrobial,
3560	DRAMP03325	Hepcidin antimicrobial peptide 2	Antimicrobial,
3561	DRAMP18204	Labyrinthopeptin A1(Bacteriocin)	Antiviral, Anti-HIV, Anti-HSV, Antimicrobial
3562	DRAMP03327	Protein S100-A8 (Calgranulin-A; MRP-8; Rodents, mammals, animals)	Antibacterial, Antifungal, Antimicrobial
3563	DRAMP18206	Deoxyactagardine B(Bacteriocin)	Antibacterial, Anti-Gram+, Antimicrobial
3564	DRAMP18205	Labyrinthopeptin A2 (Bacteriocin)	Antiviral, Antimicrobial
3565	DRAMP03329	Alpha-defensin cryptdin-1 (Crp1; Rodents, mammals, animals)	Antibacterial, Anti-Gram+, Anti-Gram-, Antimicrobial
3566	DRAMP03330	Alpha-defensin cryptdin-2 (Defensin-related cryptdin-2; Rodents, mammals, animals)	Antibacterial, Anti-Gram+, Anti-Gram-, Antimicrobial
3567	DRAMP03331	Alpha-defensin cryptdin-3 (Defensin-related cryptdin-3; Rodents, mammals, animals)	Antibacterial, Anti-Gram+, Anti-Gram-, Antimicrobial
3568	DRAMP03332	Alpha-defensin cryptdin-4 (Defensin-related cryptdin4; Rodents, mammals, animals)	Antibacterial, Anti-Gram+, Anti-Gram-, Antimicrobial
3569	DRAMP03333	Alpha-defensin cryptdin-5 (Defensin-related cryptdin5; Rodents, mammals, animals)	Antibacterial, Anti-Gram+, Anti-Gram-, Antimicrobial
3570	DRAMP03334	Alpha-defensin cryptdin-6/12 (Defensin-related cryptdin-6/12; Rodents, mammals, animals)	Antibacterial, Anti-Gram+, Anti-Gram-, Antimicrobial
3571	DRAMP03335	Alpha-defensin cryptdin-7 (Defensin-related cryptdin-7; Rodents, mammals, animals)	Antibacterial, Antimicrobial
3572	DRAMP03336	Alpha-defensin cryptdin-8 (Defensin-related cryptdin-8; Rodents, mammals, animals)	Antimicrobial,
3573	DRAMP03337	Alpha-defensin cryptdin-9 (Defensin-related cryptdin-9; Rodents, mammals, animals)	Antimicrobial,
3574	DRAMP03338	Alpha-defensin cryptdin-10 (Defensin-related cryptdin-10; Rodents, mammals, animals)	Antimicrobial,
3575	DRAMP03339	Alpha-defensin cryptdin-11 (Defensin-related cryptdin-11; Rodents, mammals, animals)	Antimicrobial,
3576	DRAMP03340	Alpha-defensin cryptdin-13 (Defensin-related cryptdin-13; Rodents, mammals, animals)	Antimicrobial,

3577	DRAMP03341	Alpha-defensin cryptdin-14 (Defensin-related cryptdin-14; Rodents, mammals, animals)	Antimicrobial,
3578	DRAMP03342	Alpha-defensin cryptdin-15 (Defensin-related cryptdin-15; Rodents, mammals, animals)	Antimicrobial,
3579	DRAMP03343	Alpha-defensin cryptdin-16 (Defensin-related cryptdin-16; Rodents, mammals, animals)	Antimicrobial,
3580	DRAMP03344	Alpha-defensin cryptdin-17 (CRYP17; Rodents, mammals, animals)	Antimicrobial,
3581	DRAMP03345	Alpha-defensin cryptdin-20 (Defensin-related cryptdin-20; Rodents, mammals, animals)	Antimicrobial,
3582	DRAMP03346	Alpha-defensin cryptdin-21 (Defensin-related cryptdin-21; Rodents, mammals, animals)	Antimicrobial,
3583	DRAMP03347	Alpha-defensin cryptdin-22 (Defensin-related cryptdin-22; Rodents, mammals, animals)	Antimicrobial,
3584	DRAMP03348	Alpha-defensin cryptdin-23 (Defensin-related cryptdin-23; Rodents, mammals, animals)	Antimicrobial,
3585	DRAMP03349	Alpha-defensin cryptdin-24 (Defensin-related cryptdin-24; Rodents, mammals, animals)	Antimicrobial,
3586	DRAMP03350	Alpha-defensin cryptdin-25 (Defensin-related cryptdin-25; Rodents, mammals, animals)	Antimicrobial,
3587	DRAMP03351	Alpha-defensin cryptdin-26 (Defensin-related cryptdin-26; Rodents, mammals, animals)	Antimicrobial,
3588	DRAMP03352	Alpha-defensin-related sequence 1 (Defensin-related cryptdin 1; Rodents, mammals, animals)	Antimicrobial,
3589	DRAMP03353	Alpha-defensin-related sequence 2 (Defensin-related cryptdin 2; Rodents, mammals, animals)	Antimicrobial,
3590	DRAMP03354	Alpha-defensin-related sequence 7 (Defensin-related cryptdin 3; Rodents, mammals, animals)	Antimicrobial,
3591	DRAMP03355	Alpha-defensin-related sequence 10 (CRS4C-4; Cryptdin-related protein 4C-4; Rodents, mammals, animals)	Antimicrobial,
3592	DRAMP03356	Alpha-defensin-related sequence 12 (Defensin-related cryptdin 12; Rodents, mammals, animals)	Antimicrobial,
3593	DRAMP03357	Cryptdin related sequence peptide (CRS4C-1a; Rodents, mammals, animals)	Antibacterial, Anti-Gram+, Anti-Gram-, Antimicrobial
3594	DRAMP03358	Cryptdin related sequence peptide (CRS4C-1d; Rodents, mammals, animals)	Antibacterial, Anti-Gram+, Anti-Gram-, Antimicrobial
3595	DRAMP03359	Cryptdin related sequence peptide (CRS4C-2; Rodents, mammals, animals)	Antibacterial, Anti-Gram+, Anti-Gram-, Antimicrobial
3596	DRAMP03360	Cryptdin related sequence peptide (CRS4C-2b; Rodents, mammals, animals)	Antibacterial, Anti-Gram+, Anti-Gram-, Antimicrobial
3597	DRAMP03361	CRS4C-3a (Cryptdin related sequence peptide; Rodents, mammals, animals)	Antibacterial, Antimicrobial
3598	DRAMP03362	CRS4C-3c (Cryptdin related sequence peptide; Rodents, mammals, animals)	Antimicrobial , Anti-Gram+, Anti-Gram-,
3599	DRAMP03363	CRS4C-3d (Cryptdin related sequence peptide; Rodents, mammals, animals)	Antimicrobial ,
3600	DRAMP03364	Hepcidin (mammals, rodents, animals)	Antimicrobial ,
3601	DRAMP03365	Hepcidin-2 (mammals, rodents, animals)	Antimicrobial ,
3602	DRAMP03366	Beta-defensin 1 (BD-1; mBD-1; Defensin, beta 1; Rodents, mammals, animals)	Antibacterial, Antifungal, Anti-Gram+, Anti-Gram-, Antimicrobial
3603	DRAMP03367	Beta-defensin 2 (BD-2, mBD-2; Defensin, beta 2; Defb2; Rodents, mammals, animals)	Antibacterial, Antimicrobial
3604	DRAMP03368	Beta-defensin 3 (BD-3, mBD-3; Defensin, beta 3; Rodents, mammals, animals)	Antibacterial, Anti-Gram-, Antimicrobial
3605	DRAMP03369	Beta-defensin 4 (BD-4, mBD-4; Defensin, beta 4; Rodents, mammals, animals)	Antibacterial, Anti-Gram+, Anti-Gram-, Antimicrobial
3606	DRAMP03371	Beta-defensin 5 (BD-5, mBD-5; Defensin, beta 5; Rodents, mammals, animals)	Antibacterial, Antifungal, Antimicrobial
3607	DRAMP03372	Beta-defensin 7 (BD-7, mBD-7; Defensin, beta 7; Rodents, mammals, animals)	Antibacterial, Antimicrobial
3608	DRAMP03373	Beta-defensin 8 (BD-8, mBD-8; Defensin, beta 8; Rodents, mammals, animals)	Antibacterial, Anti-Gram+, Anti-Gram-, Antimicrobial
3609	DRAMP03374	Beta-defensin 9 (BD-9, mBD-9; Defensin, beta 9; Rodents, mammals, animals)	Antibacterial, Antimicrobial

3610	DRAMP03375	Beta-defensin 10 (BD-10, mBD-10; Defensin, beta 10; Rodents, mammals, animals)	Antibacterial, Antimicrobial
3611	DRAMP03376	Beta-defensin 11 (BD-11, mBD-11; Defensin, beta 11; Rodents, mammals, animals)	Antibacterial, Antimicrobial
3612	DRAMP03377	Beta-defensin 12 (BD-12, mBD-12; Defensin, beta 12; Rodents, mammals, animals)	Antibacterial, Antimicrobial
3613	DRAMP03378	Beta-defensin 13 (BD-13, mBD-13; Defensin, beta 13; Rodents, mammals, animals)	Antibacterial, Antimicrobial
3614	DRAMP03379	Beta-defensin 14 (BD-14, mBD-14; Defensin, beta 14; Rodents, mammals, animals)	Antibacterial, Antifungal, Antimicrobial
3615	DRAMP03380	Beta-defensin 15 (BD-15, mBD-15; Defensin, beta 15; Rodents, mammals, animals)	Antibacterial, Antimicrobial
3616	DRAMP03381	Beta-defensin 17 (BD-17, mBD-17; Defensin, beta 17; Rodents, mammals, animals)	Antibacterial, Antimicrobial
3617	DRAMP03382	Beta-defensin 18 (BD-18, mBD-18; Defensin, beta 18; Rodents, mammals, animals)	Antibacterial, Antimicrobial
3618	DRAMP03383	Beta-defensin 19 (BD-19, mBD-19; Defensin, beta 19; Rodents, mammals, animals)	Antibacterial, Antimicrobial
3619	DRAMP03384	Beta-defensin 20 (BD-20, mBD-20; Defensin, beta 20; Rodents, mammals, animals)	Antibacterial, Antimicrobial
3620	DRAMP03385	Beta-defensin 25 (BD-25, mBD-25; Defensin, beta 25; Rodents, mammals, animals)	Antibacterial, Antimicrobial
3621	DRAMP03386	Beta-defensin 29 (BD-29, mBD-29; Defensin, beta 29; Rodents, mammals, animals)	Antibacterial, Antimicrobial
3622	DRAMP03387	Beta-defensin 30 (BD-30, mBD-30; Defensin, beta 30; Rodents, mammals, animals)	Antibacterial, Antimicrobial
3623	DRAMP03388	Beta-defensin 33 (BD-33, mBD-33; Defensin, beta 33; Rodents, mammals, animals)	Antibacterial, Antimicrobial
3624	DRAMP03389	Beta-defensin 34 (BD-34, mBD-34; Defensin, beta 34; Rodents, mammals, animals)	Antibacterial, Antimicrobial
3625	DRAMP03390	Beta-defensin 35 (BD-35, mBD-35; Defensin, beta 35; Rodents, mammals, animals)	Antibacterial, Antimicrobial
3626	DRAMP03391	Beta-defensin 36 (BD-36, mBD-36; Defensin, beta 36; Rodents, mammals, animals)	Antibacterial, Antimicrobial
3627	DRAMP03392	Beta-defensin 37 (BD-37, mBD-37; Defensin, beta 37; Rodents, mammals, animals)	Antibacterial, Antimicrobial
3628	DRAMP03393	Beta-defensin 38 (BD-38, mBD-38; Defensin, beta 38; Rodents, mammals, animals)	Antibacterial, Anti-Gram-, Antimicrobial
3629	DRAMP03394	Beta-defensin 39 (BD-39, mBD-39; Defensin, beta 39; Rodents, mammals, animals)	Antibacterial, Antimicrobial
3630	DRAMP03395	Beta-defensin 40 (BD-40, mBD-40; Defensin, beta 40; Rodents, mammals, animals)	Antibacterial, Antimicrobial
3631	DRAMP03396	Beta-defensin 41 (BD-41, mBD-41; Defensin, beta 41; Rodents, mammals, animals)	Antibacterial, Antimicrobial
3632	DRAMP03397	Beta-defensin 43 (BD-43, mBD-43; Defensin, beta 43; Rodents, mammals, animals)	Antibacterial, Antimicrobial
3633	DRAMP03398	Beta-defensin 50 (BD-50, mBD-50; Defensin, beta 50; Prostate beta-defensin 1; Rodents, mammals, animals)	Antibacterial, Antimicrobial
3634	DRAMP03399	Sperm-associated antigen 11 (Rodents, mammals, animals)	Antibacterial, Anti-Gram-, Antimicrobial
3635	DRAMP03400	WAP four-disulfide core domain protein 12 (Rodents, mammals, animals)	Antibacterial, Anti-Gram+, Anti-Gram-, Antimicrobial
3636	DRAMP03401	WAP four-disulfide core domain protein 15A (Rodents, mammals, animals)	Antibacterial, Antimicrobial
3637	DRAMP03402	WAP four-disulfide core domain protein 15B (Elafin-like protein I; Rodents, mammals, animals)	Antibacterial, Anti-Gram+, Anti-Gram-, Antimicrobial
3638	DRAMP03403	SP-BN (N-terminal region of Surfactant Protein B; SAPLIP; Rodents, mammals, animals)	Antibacterial, Antimicrobial
3639	DRAMP03407	Defr1 (Murine beta-defensin related peptide; Rodents, mammals, animals)	Antibacterial, Anti-Gram+, Anti-Gram-, Antimicrobial
3640	DRAMP03408	Neutrophil defensin 1 (HANP-1; alpha-defensin; Rodents, mammals, animals)	Antibacterial, Antifungal, Anti-Gram+, Anti-Gram-, Antimicrobial
3641	DRAMP03409	Neutrophil defensin 2 (HANP-2; alpha-defensin; Rodents, mammals, animals)	Antibacterial, Antifungal, Anti-Gram+, Anti-Gram-, Antimicrobial

3642	DRAMP03410	Neutrophil defensin 3 (HANP-3; alpha-defensin; Rodents, mammals, animals)	Antibacterial, Antifungal, Anti-Gram+, Anti-Gram-, Antimicrobial
3643	DRAMP03411	Neutrophil defensin 4 (HANP-4; alpha-defensin; Rodents, mammals, animals)	Antibacterial, Antifungal, Anti-Gram+, Anti-Gram-, Antimicrobial
3644	DRAMP03412	Beta-defensin 2	Antimicrobial,
3645	DRAMP03413	Defensin 7	Antimicrobial,
3646	DRAMP03414	Defensin alpha 6 (Protein Defa6)	Antimicrobial,
3647	DRAMP03415	Defensin alpha 7 (Defensin)	Antimicrobial,
3648	DRAMP03416	Defensin alpha 10 (Defensin)	Antimicrobial,
3649	DRAMP03417	Defensin alpha-related sequence 1 (Protein Defa-rs1)	Antimicrobial,
3650	DRAMP03418	Hepcidin (mammals, rodents, animals)	Antimicrobial,
3651	DRAMP03420	Neutrophil antibiotic peptide NP-2 (RatNP-2; Rodents, mammals, animals)	Antibacterial, Antifungal, Anti-Gram+, Anti-Gram-, Antimicrobial
3652	DRAMP03421	Neutrophil antibiotic peptide NP-3 (RatNP-3a, RatNP-3b; Rodents, mammals, animals)	Antibacterial, Antifungal, Antiviral, Anti-Gram+, Anti-Gram-, Antimicrobial
3653	DRAMP03423	BIN1b (Sperm-associated antigen 11; Antimicrobial-like protein Bin-1b; Rodents, mammals, animals)	Antibacterial, Anti-Gram-, Antimicrobial
3654	DRAMP03424	Beta-defensin 1 (BD-1, RBD-1; Defensin, beta 1; Rodents, mammals, animals)	Antibacterial, Anti-Gram-, Antimicrobial
3655	DRAMP03425	Beta-defensin 3 (BD-3, RBD-3; Defensin, beta 3; Rodents, mammals, animals)	Antibacterial, Antimicrobial
3656	DRAMP03426	Beta-defensin 4 (BD-4, RBD-4; Defensin, beta 4; RBD-2; Rodents, mammals, animals)	Antibacterial, Antimicrobial
3657	DRAMP03427	Beta-defensin 5 (BD-5, RBD-5; Defensin, beta 5; Rodents, mammals, animals)	Antibacterial, Antimicrobial
3658	DRAMP03428	Beta-defensin 9 (BD-9, RBD-9; Defensin, beta 9; Rodents, mammals, animals)	Antibacterial, Antimicrobial
3659	DRAMP03429	Beta-defensin 10 (BD-10, RBD-10; Defensin, beta 10; Rodents, mammals, animals)	Antibacterial, Antimicrobial
3660	DRAMP03430	Beta-defensin 11 (BD-11, RBD-11; Defensin, beta 11; Rodents, mammals, animals)	Antibacterial, Antimicrobial
3661	DRAMP03431	Beta-defensin 12 (BD-12, RBD-12; Defensin, beta 12; Rodents, mammals, animals)	Antibacterial, Antimicrobial
3662	DRAMP03432	Beta-defensin 13 (BD-13, RBD-13; Defensin, beta 13; Rodents, mammals, animals)	Antibacterial, Antimicrobial
3663	DRAMP03433	Beta-defensin 14 (BD-14, RBD-14; Defensin, beta 14; Rodents, mammals, animals)	Antibacterial, Antimicrobial
3664	DRAMP03434	Beta-defensin 15 (BD-15, RBD-15; Defensin, beta 15; Rodents, mammals, animals)	Antibacterial, Antimicrobial
3665	DRAMP03435	Beta-defensin 17 (BD-17, RBD-17; Defensin, beta 17; Rodents, mammals, animals)	Antibacterial, Antimicrobial
3666	DRAMP03436	Beta-defensin 18 (BD-18, RBD-18; Defensin, beta 18; Rodents, mammals, animals)	Antibacterial, Antimicrobial
3667	DRAMP03437	Beta-defensin 19 (Rodents, mammals, animals)	Antimicrobial,
3668	DRAMP03438	Beta-defensin 20 (BD-20, RBD-20; Defensin, beta 20; Rodents, mammals, animals)	Antibacterial, Antimicrobial
3669	DRAMP03439	Beta-defensin 21 (DEFB21; Protein Defb21; rodents, mammals, animals)	Antimicrobial,
3670	DRAMP03440	Beta-defensin 24 (DEFB24; Protein Defb24; rodents, mammals, animals)	Antimicrobial,
3671	DRAMP03441	Beta-defensin 25 (BD-25, RBD-25; Defensin, beta 25; Rodents, mammals, animals)	Antibacterial, Antimicrobial
3672	DRAMP03442	Beta-defensin 27 (DEFB27; Protein Defb27; rodents, mammals, animals)	Antimicrobial,
3673	DRAMP03443	Beta-defensin 28 (Rodents, mammals, animals)	Antimicrobial,
3674	DRAMP03444	Beta-defensin 29 (BD-29, RBD-29; Defensin, beta 29; Rodents, mammals, animals)	Antibacterial, Antimicrobial
3675	DRAMP03445	Beta-defensin 30 (BD-30, RBD-30; Defensin, beta 30; Rodents, mammals, animals)	Antibacterial, Anti-Gram-, Antimicrobial
3676	DRAMP03446	Beta-defensin 33 (BD-33, RBD-33; Defensin, beta 33; Rodents, mammals, animals)	Antibacterial, Antimicrobial
3677	DRAMP03447	Beta-defensin 37 (Rodents, mammals, animals)	Antimicrobial,

3678	DRAMP03448	Beta-defensin 38 (BD-38, RBD-38; Defensin, beta 38; Rodents, mammals, animals)	Antibacterial, Antimicrobial
3679	DRAMP03449	Beta-defensin 39 (BD-39, RBD-39; Defensin, beta 39; Rodents, mammals, animals)	Antibacterial, Antimicrobial
3680	DRAMP03450	Beta-defensin 40 (Protein Defb40; rodents, mammals, animals)	Antimicrobial,
3681	DRAMP03451	Beta-defensin 41 (Protein Defb41; rodents, mammals, animals)	Antimicrobial,
3682	DRAMP03452	Beta-defensin 42 (Protein Defb42; rodents, mammals, animals)	Antimicrobial,
3683	DRAMP03453	Beta-defensin 43 (BD-43, RBD-43; Defensin, beta 43; Rodents, mammals, animals)	Antibacterial, Antimicrobial
3684	DRAMP03454	Beta-defensin 44 (Protein Defb44; rodents, mammals, animals)	Antimicrobial,
3685	DRAMP03455	Beta-defensin 49 (Protein Defb49; rodents, mammals, animals)	Antimicrobial,
3686	DRAMP03456	Beta-defensin 50 (BD-50, RBD-50; Defensin, beta 50; Rodents, mammals, animals)	Antibacterial, Antimicrobial
3687	DRAMP03457	Beta-defensin 51 (Protein Defb51; rodents, mammals, animals)	Antimicrobial,
3688	DRAMP03458	Beta-defensin 52 (Protein Defb52; rodents, mammals, animals)	Antimicrobial,
3689	DRAMP03459	DEFB24 (defensin; Rodents, mammals, animals)	Antibacterial, Antimicrobial
3690	DRAMP03460	WAP four-disulfide core domain protein 12 (mammals, rodents, animals)	Antibacterial, Antimicrobial
3691	DRAMP03461	Defensin 5 (Enteric defensin; RD-5; Rodents, mammals, animals)	Antimicrobial,
3692	DRAMP03462	Protein S100-A8 (Calgranulin-A; MRP-8; Rodents, mammals, animals)	Antibacterial, Antifungal, Antimicrobial
3693	DRAMP03466	Ribosome-inactivating protein luffacylin (rRNA N-glycosidase; Plants)	Antifungal, Antimicrobial
3694	DRAMP03468	Alveolarin (Fungus)	Antifungal, Antimicrobial
3695	DRAMP03469	Serine protease inhibitor Cvsi-1 (molluscs, animals)	Antibacterial, Antiparasitic, Antimicrobial
3696	DRAMP03470	Defensin-1 (American oyster defensin, AOD; molluscs, animals)	Antibacterial, Anti-Gram+, Anti-Gram-, Antimicrobial
3697	DRAMP03473	Antimicrobial ribonuclease (Fungus)	Antibacterial, Antifungal, Antimicrobial,
3698	DRAMP03475	Vicilin-like Antimicrobial peptide 2a (MiAMP2a; Plant defensin)	Antibacterial, Antifungal, Anti-Gram+, Antimicrobial
3699	DRAMP03476	Eryngin (mushroom, fungi)	Antifungal, Antimicrobial
3700	DRAMP03478	Ganodermin (Fungus)	Antifungal, Antimicrobial
3701	DRAMP03479	Bactericidin B-2 (Cecropin-like peptide B-2; Insects, animals)	Antibacterial, Antimicrobial
3702	DRAMP03480	Bactericidin B-3 (Cecropin-like peptide B-3; Insects, animals)	Antibacterial, Antimicrobial
3703	DRAMP03481	Bactericidin B-4 (Cecropin-like peptide B-4; Insects, animals)	Antibacterial, Antimicrobial
3704	DRAMP03482	Defensin-like protein 1 (Predicted; Insects, animals; Predicted)	Antimicrobial,
3705	DRAMP03483	Defensin-like protein 2 (Predicted; Insects, animals)	Antimicrobial,
3706	DRAMP03484	Defensin-like protein 3 (Predicted; Insects, animals; Predicted)	Antimicrobial,
3707	DRAMP03485	Bactericidin B-5P (Cecropin-like peptide B-5; Insects, animals)	Antibacterial, Antimicrobial
3708	DRAMP03487	Lebocin Peptide 1A (LP1A; Insects, animals)	Antibacterial, Antimicrobial
3709	DRAMP03488	Defense protein 4 (DFP-4; Insects, animals)	Antimicrobial,
3710	DRAMP03489	Defense protein 6 (DFP-6; Insects, animals)	Antimicrobial,
3711	DRAMP03490	Gloverin (Insects, animals)	Antibacterial, Antimicrobial
3712	DRAMP03491	Viresin (Insects, animals)	Antibacterial, Anti-Gram-, Antimicrobial
3713	DRAMP03492	Defensin heliomicin (Mutation: K23L, R24L)	Antibacterial, Antifungal, Antimicrobial
3714	DRAMP03494	Cecropin (Insects, animals)	Antibacterial, Antifungal, Antimicrobial
3715	DRAMP03495	Psychimicin (Insects, animals)	Antibacterial, Antifungal, Antimicrobial
3716	DRAMP03496	Cecropin-A (Insects, animals)	Antibacterial, Antifungal, Antimicrobial
3717	DRAMP03497	Cecropin-B (Insects, animals)	Antibacterial, Antifungal, Antimicrobial
3718	DRAMP03498	Cecropin-C (Insects, animals)	Antibacterial, Antifungal, Antimicrobial
3719	DRAMP03499	Heliocin (Pro-rich; Insects, animals)	Antibacterial, Antimicrobial
3720	DRAMP03500	Virescein (Insects, animals)	Antibacterial, Antimicrobial
3721	DRAMP03501	La-LTP (LJAFP; Insects, animals)	Antibacterial, Antifungal, Anti-Gram+, Anti-Gram-, Antimicrobial
3722	DRAMP03502	Hyphancin-3D (Hyphancin IIID; Cecropin-A; Insects, animals)	Antibacterial, Antimicrobial
3723	DRAMP03503	Hyphancin-3E (Hyphancin IIIE; Cecropin-A1; Insects, animals)	Antibacterial, Antimicrobial
3724	DRAMP03504	Hyphancin-3F (Hyphancin IIIF; Cecropin-A2; Insects, animals)	Antibacterial, Antimicrobial
3725	DRAMP03505	Hyphancin-3G (Hyphancin IIIG; Cecropin-A3; Insects, animals)	Antibacterial, Antimicrobial
3726	DRAMP03506	Attacin (Insects, animals)	Antibacterial, Antimicrobial

3727	DRAMP03509	Cecropin-A (Insects, animals)	Antibacterial, Antimicrobial
3728	DRAMP03510	Cecropin-A (Insects, animals)	Antibacterial, Antiviral, Anti-Gram+, Anti-Gram-, Antimicrobial
3729	DRAMP03511	Cecropin-B (Immune protein P9; Insects, animals)	Antibacterial, Anti-Gram+, Anti-Gram-, Antimicrobial
3730	DRAMP03512	Cecropin-D (Cecropin D; Insects, animals)	Antibacterial, Anti-Gram+, Anti-Gram-, Antimicrobial
3731	DRAMP03522	Gallerimycin (defensins; Insects, animals)	Antifungal, Antimicrobial
3732	DRAMP03529	Cecropin-A (Insects, animals)	Antibacterial, Antimicrobial
3733	DRAMP03530	Cecropin-B (Lepidopteran-A/B; Insects, animals)	Antibacterial, Antimicrobial
3734	DRAMP03531	Cecropin-D (Insects, animals)	Antibacterial, Antimicrobial
3735	DRAMP18208	NAI-112(Bacteriocin)	Antibacterial, Antinociceptive, Antiallodynic, Anti-Gram+, Antimicrobial
3736	DRAMP18207	Actagardine B(Bacteriocin)	Antibacterial, Anti-Gram+, Antimicrobial
3737	DRAMP03534	Antibacterial peptide enbocin (Moricin; Insects, animals)	Antibacterial, Anti-Gram+, Anti-Gram-, Antimicrobial
3738	DRAMP03535	Lebocin-1/2 (Pro-rich; Insects, animals)	Antibacterial, Anti-Gram+, Anti-Gram-, Antimicrobial
3739	DRAMP03536	Lebocin-3 (LEB 3; Insects, animals)	Antibacterial, Anti-Gram+, Anti-Gram-, Antimicrobial
3740	DRAMP03537	Lebocin-4 (Leb 4; Insects, animals)	Antibacterial, Antimicrobial
3741	DRAMP03538	Cecropin (Antibacterial peptide CM-IV; Insects, animals)	Antibacterial, Antimicrobial
3742	DRAMP03554	CCL20(1-67) (Human, mammals, animals)	Antibacterial, Anti-Gram+, Anti-Gram-, Antimicrobial
3743	DRAMP03555	CCL20(2-70) (Human, mammals, animals)	Antibacterial, Anti-Gram+, Anti-Gram-, Antimicrobial
3744	DRAMP03556	C-C motif chemokine 20 (Human, mammals, animals)	Antibacterial, Anti-Gram+, Anti-Gram-, Antimicrobial
3745	DRAMP03557	Granulysin (Lymphokine LAG-2; Human, mammals, animals)	Antibacterial, Antifungal, Antiparasitic, Antimicrobial
3746	DRAMP03559	CXCL10 (Human, mammals, animals)	Antibacterial, Antiparasitic, Antimicrobial
3747	DRAMP03560	CXC chemokine GRObeta [5-73] (Human, mammals, animals)	Antibacterial, Antiparasitic, Chemotactic, Antimicrobial
3748	DRAMP03561	CXCL6 (C-X-C motif chemokine 6; Human, mammals, animals)	Antibacterial, Antiparasitic, Antimicrobial
3749	DRAMP03564	Human hepcidin-20 (Hepc20; one chain of Hepcidin; Human, mammals, animals)	Antibacterial, Antifungal, Anti-Gram+, Anti-Gram-, Antimicrobial
3750	DRAMP03565	Human hepcidin-25 (Hepc25; one chain of Hepcidin; Human, mammals, animals)	Antibacterial, Antifungal, Anti-Gram+, Anti-Gram-, Antimicrobial
3751	DRAMP03566	Salvic (Human, mammals, animals)	Antibacterial, Anti-Gram+, Anti-Gram-, Antimicrobial
3752	DRAMP03572	Antibacterial protein FALL-39 (one chain of hCAP-18; Human, mammals, animals)	Antibacterial, Antimicrobial
3753	DRAMP03576	Histatin-1 (His-rich; Human, mammals, animals)	Antifungal, Antimicrobial
3754	DRAMP03577	Histatin-2 (His-rich; Human, mammals, animals)	Antifungal, Antimicrobial
3755	DRAMP03578	Histatin-3 (His-rich; Human, mammals, animals)	Antibacterial, Antifungal, Antimicrobial
3756	DRAMP03579	Histatin-4 (His-rich; Human, mammals, animals)	Antifungal, Antimicrobial
3757	DRAMP03580	His3-(20-43)-peptide (Histatin 5; derivatives: Dh-5; Clinical)	Antibacterial, Antifungal, Antimicrobial
3758	DRAMP03581	Histatin 6 (His-rich; Human, mammals, animals)	Antifungal, Antimicrobial
3759	DRAMP03582	Histatin 7 (His-rich; Human, mammals, animals)	Antifungal, Antimicrobial
3760	DRAMP03583	Histatin 8 (His-rich; Human, mammals, animals)	Antibacterial, Antifungal, Antimicrobial
3761	DRAMP03584	Histatin 9 (His-rich; Also detected are Histatin 10; Human, mammals, animals)	Antifungal, Antimicrobial
3762	DRAMP03585	Human TC-1 (Chain of Platelet basic protein; Human, mammals, animals)	Antibacterial, Antifungal, Anti-Gram+, Anti-Gram-, Antimicrobial
3763	DRAMP03586	Human TC-2 (Chain of Platelet basic protein; Human, mammals, animals)	Antibacterial, Antifungal, Anti-Gram+, Anti-Gram-, Antimicrobial
3764	DRAMP03587	DCD-1 (chain of Dermcidin; Human, mammals, animals)	Antibacterial, Antifungal, Proteolytic, Anti-Gram+, Anti-Gram-, Antimicrobial
3765	DRAMP03588	Human MUC7 20-Mer (Human, mammals, animals)	Antibacterial, Antifungal, Anti-Gram+, Anti-Gram-, Antimicrobial
3766	DRAMP03589	WAP four-disulfide core domain protein 12 (Putative protease inhibitor WAP12; Human, mammals, animals)	Antibacterial, Antimicrobial
3767	DRAMP03590	Calcitermin (Human, mammals, animals)	Antibacterial, Antifungal, Anti-Gram-, Antimicrobial
3768	DRAMP03591	Neutrophil defensin 1 (Defensin, alpha 1; HNP-1, HP-1; Human, mammals, animals)	Antimicrobial, Antibacterial, Antifungal, Anti-Gram+, Anti-Gram-,
3769	DRAMP03592	Neutrophil defensin 2 (HNP-2, HP-2, HP2; Human, mammals, animals)	Antifungal, Antiviral, Anti-Gram+, Anti-Gram-, Antimicrobial
3770	DRAMP03593	Neutrophil defensin 3 (Defensin, alpha 3; HNP-3, HP-3, HP3; Human, mammals, animals)	Antibacterial, Antifungal, Antiviral, Anti-Gram+, Anti-Gram-, Antimicrobial
3771	DRAMP03594	Neutrophil defensin 4 (Defensin, alpha 4; HNP-4, HP-4; Human, mammals, animals)	Antibacterial, Antifungal, Antiviral, Anti-Gram+, Anti-Gram-, Antimicrobial

3772	DRAMP03595	Human defensin-5 (HD-5; Defensin, alpha 5; Human, mammals, animals)	Antibacterial, Antifungal, Antiviral, Anti-Gram+, Anti-Gram-, Antimicrobial
3773	DRAMP03596	Human defensin-6 (HD-6; Defensin, alpha 6; Human, mammals, animals)	Antifungal, Antiviral, Anti-Gram+, Anti-Gram-, Antimicrobial
3774	DRAMP03601	Human beta-defensin 26 (hBD-26; hBD26; Human, mammals, animals)	Antibacterial, Anti-Gram-, Antimicrobial
3775	DRAMP03602	Human beta-defensin 27 (hBD-27; hBD27; Human, mammals, animals)	Antibacterial, Anti-Gram-, Antimicrobial
3776	DRAMP18202	WB Piscidin 5 (fish, animals)	Antibacterial & Gram-, Antiparasitic, Antimicrobial
3777	DRAMP18203	Panusin (beta defensins; crustaceans, arthropods, invertebrates, animals)	Antibacterial, Anti-Gram+, Anti-Gram-, Antifungal, Antimicrobial
3778	DRAMP03606	Beta-defensin 107 (Defensin, beta 107; Beta-defensin 7, BD-7; Human, mammals, animals)	Antibacterial, Antimicrobial
3779	DRAMP03607	Putative beta-defensin 108A (Defensin, beta 108A; Human, mammals, animals)	Antibacterial, Antimicrobial
3780	DRAMP03608	Beta-defensin 108B (Beta-defensin 8; hBD-8; Defensin, beta 108B; Human, mammals, animals)	Antibacterial, Antimicrobial
3781	DRAMP03610	Beta-defensin 109 (Defensin, beta 109; Defensin, beta 109; Human, mammals, animals)	Antibacterial, Antimicrobial
3782	DRAMP03611	Beta-defensin 110 (Beta-defensin 10, DEFB-10; Defensin, beta 110; Human, mammals, animals)	Antibacterial, Antimicrobial
3783	DRAMP03612	Beta-defensin 113 (Beta-defensin 13, DEFB-13; Defensin, beta 113; Human, mammals, animals)	Antibacterial, Antimicrobial
3784	DRAMP03613	Beta-defensin 114 (Beta-defensin 14, DEFB-14; Defensin, beta 114; Human, mammals, animals)	Antibacterial, Antimicrobial
3785	DRAMP03614	Beta-defensin 115 (Beta-defensin 15, DEFB-15; Defensin, beta 115; Human, mammals, animals)	Antibacterial, Antimicrobial
3786	DRAMP03616	Beta-defensin 118 (Beta-defensin 18, DEFB-18; Defensin, beta 118; Human, mammals, animals)	Antibacterial, Antimicrobial
3787	DRAMP03617	Beta-defensin 119 (Beta-defensin 19, DEFB-19; Defensin, beta 119; Human, mammals, animals)	Antibacterial, Antimicrobial
3788	DRAMP03618	Beta-defensin 121 (Beta-defensin 21, DEFB-21; Defensin, beta 121; Human, mammals, animals)	Antibacterial, Antimicrobial
3789	DRAMP18120	BnPRP1 (Plant defensin)	Antibacterial, Antifugal, Anti-Gram+, Anti-Gram-, Antimicrobial
3790	DRAMP03620	Beta-defensin 124 (Beta-defensin 24, DEFB-24; Defensin, beta 124; Human, mammals, animals)	Antibacterial, Antimicrobial
3791	DRAMP03621	Beta-defensin 125 (Beta-defensin 25, DEFB-25; Defensin, beta 125; Human, mammals, animals)	Antibacterial, Antimicrobial
3792	DRAMP03624	Beta-defensin 128 (Beta-defensin 28, DEFB-28; Defensin, beta 128; Human, mammals, animals)	Antibacterial, Antimicrobial
3793	DRAMP03626	Beta-defensin 131 (Beta-defensin 31, DEFB-31; Defensin, beta 131; Human, mammals, animals)	Antibacterial, Antimicrobial
3794	DRAMP03627	Beta-defensin 132 (Defensin, beta 132; Beta-defensin 32, BD-32; Human, mammals, animals)	Antibacterial, Antimicrobial
3795	DRAMP03628	Beta-defensin 133 (Defensin, beta 133; Human, mammals, animals)	Antibacterial, Antimicrobial
3796	DRAMP03629	Beta-defensin 134 (Defensin, beta 134; Human, mammals, animals)	Antibacterial, Antimicrobial
3797	DRAMP03630	Beta-defensin 135 (Defensin, beta 135; Human, mammals, animals)	Antibacterial, Antimicrobial
3798	DRAMP03631	Beta-defensin 136 (Defensin, beta 136; Human, mammals, animals)	Antibacterial, Antimicrobial
3799	DRAMP18140	VG16KRKP	Antibacterial, Antifugal, Anti-Gram+, Anti-Gram-, Antimicrobial
3800	DRAMP18195	LsbB (Bacteriocin)	Antibacterial, Antimicrobial
3801	DRAMP03633	Protein S100-A8 (Calgranulin-A; MRP-8; Human, mammals, animals)	Antibacterial, Antifungal, Antimicrobial
3802	DRAMP18194	AAEL000598-PA	Antibacterial, Anti-Gram+, Anti-Gram-, Antimicrobial
3803	DRAMP03635	Human lactoferricin (LfcinH; one chain of Lactotransferrin; Human, mammals, animals)	Antibacterial, Anti-Gram-, Antimicrobial
3804	DRAMP03636	Kaliocin-1 (one chain of Lactotransferrin; Human, mammals, animals)	Antibacterial, Anti-Gram-, Antimicrobial
3805	DRAMP03637	Thaumatin-like protein (Actc2)	Antifungal, Antiviral, Antimicrobial

3806	DRAMP03639	Astexin-1 (lasso peptide)	Antibacterial, Anti-Gram-, Antimicrobial
3807	DRAMP03640	Hcl-hst 5 (N.leucogenys histatin 5)	Antifungal, Antimicrobial
3808	DRAMP03641	Longicornsin (defensin-like; Arthropods, invertebrates, animals)	Antibacterial, Antifungal, Anti-Gram+, Anti-Gram-, Antimicrobial
3809	DRAMP03643	Liver-expressed antimicrobial peptide 2 (LEAP-2; Birds, animals)	Antimicrobial ,
3810	DRAMP03644	Cathelicidin-1 (CATH-1; Fowlicidin-1; Birds, animals)	Antibacterial, Cytolytic, Anti-Gram+, Anti-Gram-, Antimicrobial
3811	DRAMP03648	Gallinacin-1 (Gal-1; Beta-defensin 1; Birds, animals)	Antibacterial, Antifungal, Anti-Gram+, Anti-Gram-, Antimicrobial
3812	DRAMP03649	Gallinacin-1 alpha (Gal-1 alpha; Antimicrobial peptide CHP2; Birds, animals)	Antibacterial, Antifungal, Anti-Gram+, Anti-Gram-, Antimicrobial
3813	DRAMP03650	Gallinacin-2 (Gal-2; Beta-defensin 2; Birds, animals)	Antibacterial, Anti-Gram+, Anti-Gram-, Antimicrobial
3814	DRAMP03651	Gallinacin-3 (Gal-3; Beta-defensin 3; Birds, animals)	Antibacterial, Antimicrobial
3815	DRAMP03652	Gallinacin-4 (Gal-4; Beta-defensin 4; Birds, animals)	Antibacterial, Anti-Gram-, Antimicrobial
3816	DRAMP03653	Gallinacin-5 (Gal-5; Beta-defensin 5; Birds, animals)	Antibacterial, Anti-Gram-, Antimicrobial
3817	DRAMP03654	Gallinacin-6 (Gal 6; Beta-defensin 6; Birds, animals)	Antibacterial, Anti-Gram-, Antimicrobial
3818	DRAMP03655	Gallinacin-7 (Gal 7; Beta-defensin 7; Birds, animals)	Antibacterial, Anti-Gram-, Antimicrobial
3819	DRAMP03656	Gallinacin-8 (Gal-8; Beta-defensin 8; Birds, animals)	Antibacterial, Antimicrobial
3820	DRAMP03657	Gallinacin-9 (Gal-9; Beta-defensin 9; Gallinacin-6, Gal-6; Birds, animals)	Antibacterial, Antimicrobial
3821	DRAMP03658	Gallinacin-10 (Gal-10; Beta-defensin 10; Birds, animals)	Antibacterial, Antimicrobial
3822	DRAMP03660	Gallinacin-12 (Gal-12; Beta-defensin 12; Birds, animals)	Antibacterial, Antimicrobial
3823	DRAMP03661	Gallinacin-13 (Gal-13; Beta-defensin 13; Birds, animals)	Antibacterial, Anti-Gram+, Anti-Gram-, Antimicrobial
3824	DRAMP03662	Gallinacin-14 (Gal-14; Beta-defensin 14; Birds, animals)	Antibacterial, Antimicrobial
3825	DRAMP03663	cLEAP-2 (Chicken LEAP-2; Birds, animals)	Antibacterial, Anti-Gram-, Antimicrobial
3826	DRAMP03664	L-amino-acid oxidase (LAAO, LAO, TM-LAO; reptilia, animals)	Antibacterial, Anti-Gram-, Antimicrobial
3827	DRAMP03665	Lysozyme	Antibacterial, Antimicrobial
3828	DRAMP03666	N-acetylmuramoyl-L-alanine amidase L2	Antibacterial, Antimicrobial
3829	DRAMP03667	Proteinase L5	Antibacterial, Antimicrobial
3830	DRAMP03668	Megourin-1 (arthropod; animals)	Antibacterial, Antifungal, Antimicrobial
3831	DRAMP03669	Megourin-2 (arthropod; animals)	Antibacterial, Antifungal, Antimicrobial
3832	DRAMP03670	Megourin-3 (arthropod; animals)	Antibacterial, Antifungal, Antimicrobial
3833	DRAMP03671	Locustin (Insects, animals)	Antibacterial, Anti-Gram+, Antimicrobial
3834	DRAMP03672	Spodomicin (Insects, animals)	Antifungal, Antimicrobial
3835	DRAMP03673	Dicentracin (Dicentracine)	Antimicrobial,
3836	DRAMP03674	Cystatin-1 (Cystatin-I)	Antibacterial, Anti-Gram+, Anti-Gram-, Antimicrobial
3837	DRAMP18192	Antimicrobial peptide AcrAP2	Antibacterial, Antifungal, Anti-Gram+, Antimicrobial
3838	DRAMP03678	Beta-defensin 1 (GBD-1; Defensin, beta 1; ruminant, animals)	Antibacterial, Antimicrobial
3839	DRAMP03680	Cathelicidin-3.4 (Bactenecin-3.4, Bac3.4; ChBac3.4; ruminant, animals)	Antibacterial, Anti-Gram+, Anti-Gram-, Antimicrobial
3840	DRAMP03681	L-amino-acid oxidase (LAAO; OHAP-1)	Antibacterial, Antimicrobial
3841	DRAMP03689	Scorpine-like (Arthropods, animals)	Antibacterial, Anti-Gram+, Antimicrobial
3842	DRAMP03690	Probable antimicrobial peptide Con10 (Arthropods, animals)	Antimicrobial,
3843	DRAMP03692	Defensin-1 (Cll-dlp; Arthropods, animals)	Antibacterial, Anti-Gram+, Anti-Gram-, Antimicrobial
3844	DRAMP03695	Probable antimicrobial peptide Con13 (Arthropods, animals)	Antibacterial, Antifungal, Antimicrobial
3845	DRAMP03696	Bradykinin-potentiating peptide-like (BPP; Non-disulfide-bridged peptide 3.7; Arthropods, animals)	Antimicrobial,
3846	DRAMP03697	Neurotoxin MeuNaTx-2 (Arthropods, animals)	Antibacterial, Antimicrobial
3847	DRAMP03698	Neurotoxin MeuNaTx-5 (Arthropods, animals)	Antimicrobial,
3848	DRAMP03699	Neurotoxin MeuNaTx-6 (Arthropods, animals)	Antimicrobial,
3849	DRAMP03701	Imcroporin (Arthropods, animals)	Antibacterial, Anti-Gram+, Antimicrobial
3850	DRAMP03703	Mucroporin-like peptide (NDBP13; Arthropods, animals)	Antibacterial, Antimicrobial
3851	DRAMP03704	Antimicrobial peptide 36.4 (Arthropods, animals)	Antibacterial, Antimicrobial
3852	DRAMP03705	Antimicrobial peptide 143 (Arthropods, animals)	Antibacterial, Antimicrobial
3853	DRAMP03708	Tddefensin (Arthropods, animals)	Antibacterial, Antimicrobial
3854	DRAMP03709	Potassium channel toxin MeuTXK-beta-1 (MeuTXKbeta1; Arthropods, animals)	Antibacterial, Antimicrobial

3855	DRAMP03710	Potassium channel toxin MeuTXK-beta-2 (MeuTXKbeta2; Arthropods, animals)	Antibacterial, Antimicrobial
3856	DRAMP03711	Scorpine-like peptide (OcyC7; Arthropods, animals)	Antibacterial, Antimicrobial
3857	DRAMP03712	Peptide Hp1090 (Non-disulfide-bridged peptide 5.9, NDBP-5.9; Arthropods, animals)	Antibacterial, Antiviral, Antimicrobial
3858	DRAMP03713	Peptide Hp1035 (Non-disulfide-bridged peptide 5.10, NDBP-5.10; Arthropods, animals)	Antibacterial, Antimicrobial
3859	DRAMP03716	Potassium channel toxin Hge-beta-KTx (HgebetaKTx; Arthropods, animals)	Antibacterial, Antimicrobial
3860	DRAMP03719	Scorpion defensin (Arthropods, animals)	Antibacterial, Antimicrobial
3861	DRAMP03720	4 kDa defensin (Antibacterial 4 kDa peptide; Arthropods, animals)	Antibacterial, Antimicrobial
3862	DRAMP03722	Cytotoxic linear peptide IsCT2 (Non-disulfide-bridged peptide 5.3, NDBP-5.3; Arthropods, animals)	Antibacterial, Antimicrobial
3863	DRAMP03725	Non-disulfide-bridged peptide 5.5 (NDBP-5.5; Arthropods, animals)	Antibacterial, Antimicrobial
3864	DRAMP03726	Non-disulfide-bridged peptide 5.6 (NDBP-5.6; Arthropods, animals)	Antibacterial, Antimicrobial
3865	DRAMP03727	Hge-scorpine (Hg-scorpine-like 1, HgeScplp1, Hgscplike1; Arthropods, animals)	Antibacterial, Antimicrobial
3866	DRAMP03728	Hg-scorpine-like 2 (Arthropods, animals)	Antibacterial, Antifungal, Antimicrobial
3867	DRAMP03730	Opiscorpine-2 (Arthropods, animals)	Antibacterial, Antifungal, Antimicrobial
3868	DRAMP03731	Opiscorpine-3 (Arthropods, animals)	Antibacterial, Antifungal, Antimicrobial
3869	DRAMP03732	Opiscorpine-4 (Arthropods, animals)	Antibacterial, Antifungal, Antimicrobial
3870	DRAMP03733	Lysozyme (Arthropods, animals)	Antibacterial, Antimicrobial
3871	DRAMP03736	Opistoporin-2 (OP2; Non-disulfide-bridged peptide 3.6, NDBP-3.6; Arthropods, animals)	Antibacterial, Antifungal, Anti-Gram+, Anti-Gram-, Antimicrobial
3872	DRAMP03737	Opistoporin-4 (Non-disulfide-bridged peptides 3.7, NDBP-3.7; Arthropods, animals)	Antibacterial, Antifungal, Anti-Gram+, Anti-Gram-, Antimicrobial
3873	DRAMP03739	Buthinin (Sahara scorpion; Arthropods, animals)	Antibacterial, Anti-Gram+, Anti-Gram-, Antimicrobial
3874	DRAMP03740	Androctonus defensin (4 kDa defensin; Arthropods, animals)	Antibacterial, Anti-Gram+, Anti-Gram-, Antimicrobial
3875	DRAMP03741	Ponericin-W-like 32.1 (Arthropods, animals)	Antibacterial, Antifungal, Insecticidal, Anti-Gram+, Anti-Gram-, Antimicrobial
3876	DRAMP03742	Ponericin-W-like 32.2 (Arthropods, animals)	Antibacterial, Antifungal, Insecticidal, Anti-Gram+, Anti-Gram-, Antimicrobial
3877	DRAMP03745	Peptide BmKn1 (Biologically active peptide 4; NDBP-5.1; Arthropods, animals)	Antibacterial, Antimicrobial
3878	DRAMP03747	Peptide BmKb2 (Arthropods, animals)	Antimicrobial,
3879	DRAMP03749	Peptide BmKa2 (Acidic venom peptide Ka2; NDBP-6.2; Arthropods, animals)	Antibacterial, Antimicrobial
3880	DRAMP03755	Potassium channel toxin alpha-KTx 1.1 (ChTX-Lq1; charybdotoxin; Arthropods, animals)	Antibacterial, Antifungal, Antiviral, Anti-Gram+, Anti-Gram-, Antimicrobial
3881	DRAMP03756	Cytotoxic linear peptide (Non-disulfide bridged protein family 5, NDBP-5; Arthropods, animals)	Antibacterial, Antimicrobial
3882	DRAMP03757	Amphipathic peptide Tx348 (BoiTx348; Arthropods, animals)	Antibacterial, Antimicrobial
3883	DRAMP18191	Antimicrobial peptide AcrAP1	Antibacterial, Antifungal, Anti-Gram+, Antimicrobial
3884	DRAMP03759	Putative antimicrobial peptide clone 5 (Arthropods, animals)	Antibacterial, Antimicrobial
3885	DRAMP03760	Putative antimicrobial peptide clone 6 (Arthropods, animals)	Antibacterial, Antimicrobial
3886	DRAMP03761	Scorpine-like peptide Tco 41.46-2 (Arthropods, animals)	Antibacterial, Antimicrobial
3887	DRAMP03762	Anionic peptide clone 7 (Asp-rich; Arthropods, animals)	Antimicrobial,
3888	DRAMP03763	Anionic peptide clone 8 (Asp-rich; Arthropods, animals)	Antimicrobial,
3889	DRAMP03764	Anionic peptide clone 9 (Asp-rich; Arthropods, animals)	Antimicrobial,
3890	DRAMP03765	Anionic peptide clone 10 (Asp-rich; Arthropods, animals)	Antimicrobial,
3891	DRAMP03766	Heteroscorpine-1 (HS-1; defensins; Arthropods, animals)	Antibacterial, Anti-Gram+, Anti-Gram-, Antimicrobial
3892	DRAMP03768	OcyC1f (one chain of Non-disulfide-bridged peptide 5.7; Arthropods, animals)	Antimicrobial,
3893	DRAMP03769	OcyC2 (one chain of Non-disulfide-bridged peptide 5.8; Arthropods, animals)	Antimicrobial,
3894	DRAMP03770	OcyC2f (one chain of Non-disulfide-bridged peptide 5.8; Arthropods, animals)	Antimicrobial,
3895	DRAMP03771	Non-disulfide-bridged peptide 4.3 (NDBP-4.3; OcyC3; Arthropods, animals)	Antibacterial, Antimicrobial

3896	DRAMP03772	Amphipathic peptide Hj0164 (Non-disulfide bridged protein family 5, NDBP-5; Arthropods, animals)	Antibacterial, Antimicrobial
3897	DRAMP03773	Meucin-49 (Arthropods, animals)	Antimicrobial,
3898	DRAMP03778	Agrocybin (Fungus)	Antibacterial, Antifungal, Antiviral, Antimicrobial
3899	DRAMP03779	Antifungal protein (AFP; Fungi)	Antifungal, Antimicrobial
3900	DRAMP03780	Big defensin (AiBD)	Antibacterial, Antimicrobial
3901	DRAMP03781	CECdir-CECret	Antimicrobial,
3902	DRAMP03782	Corticostatin-related peptide LCRP (alpha-defensin)	Antimicrobial,
3903	DRAMP03783	Neuropeptide-like protein 27 (NLP-27; Gly-rich, Tyr-rich; nematodes, animals; Predicted)	Antibacterial, Antifungal, Antiviral, Antimicrobial
3904	DRAMP03784	Neuropeptide-like protein 28 (NLP-28; Gly-rich, Tyr-rich; nematodes, animals; Predicted)	Antibacterial, Antifungal, Antiviral, Antimicrobial
3905	DRAMP03785	Neuropeptide-like protein 29 (NLP-29; nematodes, animals)	Antibacterial, Antifungal, Anti-Gram-, Antimicrobial
3906	DRAMP03786	Neuropeptide-like protein 30 (NLP-30; Gly-rich, Tyr-rich; nematodes, animals; Predicted)	Antibacterial, Antifungal, Antiviral, Antimicrobial
3907	DRAMP03787	Neuropeptide-like protein 31 (NLP-31; nematodes, animals)	Antifungal, Antibacterial, Anti-Gram+, Anti-Gram-, Antimicrobial
3908	DRAMP03788	Neuropeptide-like protein 33 (NLP-33; nematodes, animals)	Antibacterial, Antimicrobial
3909	DRAMP03789	ABF-1 (nematodes, animals)	Antibacterial, Antifungal, Antimicrobial
3910	DRAMP03790	ABF-2 (nematodes, animals)	Antibacterial, Antifungal, Anti-Gram+, Anti-Gram-, Antimicrobial
3911	DRAMP03791	Caenacin-1 (Gly-rich, Tyr-rich; CNC-1; nematode, invertebrate, animals)	Antibacterial, Antifungal, Antiviral, Antimicrobial
3912	DRAMP03792	Caenacin-2 (Gly-rich, Tyr-rich; CNC-2, nematode, invertebrate, animals)	Antibacterial, Antifungal, Antiviral, Antimicrobial
3913	DRAMP03793	Caenacin-3 (Gly-rich, Tyr-rich; CNC-3, nematode, invertebrate, animals)	Antibacterial, Antifungal, Antiviral, Antimicrobial
3914	DRAMP03794	Caenacin-4 (Gly-rich, Tyr-rich; CNC-4, nematode, invertebrate, animals)	Antibacterial, Antifungal, Antiviral, Antimicrobial
3915	DRAMP03795	Caenacin-5 (Gly-rich, Tyr-rich; CNC-5, nematode, invertebrate, animals)	Antibacterial, Antifungal, Antiviral, Antimicrobial
3916	DRAMP03796	T07C4.4 (SPP-1; saposin-like protein, SAPLIP; roundworm, nematoda, animals)	Antibacterial, Antimicrobial
3917	DRAMP03798	Corticostatin-related peptide RK-1 (RK-1; lagomorphs, mammals, animals)	Antibacterial, Anti-Gram-, Antimicrobial
3918	DRAMP03799	Rabbit kidney defensin RK-2 (alpha-defensins; lagomorphs, mammals, animals)	Antibacterial, Anti-Gram-, Antimicrobial
3919	DRAMP03800	Neutrophil antibiotic peptide NP-5 (Microbicidal peptide NP-5; lagomorphs, mammals, animals)	Antimicrobial,
3920	DRAMP03801	Neutrophil antibiotic peptide NP-4 (Microbicidal peptide NP-4; lagomorphs, mammals, animals)	Antimicrobial,
3921	DRAMP03802	Corticostatin 1 (Antiadrenocorticotropin peptide I; lagomorphs, mammals, animals)	Antibacterial, Antimicrobial
3922	DRAMP03803	Corticostatin-2 (Antiadrenocorticotropin peptide II; Corticostatin II; lagomorphs, mammals, animals)	Antimicrobial,
3923	DRAMP03804	Corticostatin-3 (Corticostatin III; Macrophage antibiotic peptide MCP-1; lagomorphs, mammals, animals)	Antibacterial, Antifungal, Antiviral, Antimicrobial
3924	DRAMP03805	Corticostatin-4 (Corticostatin IV; Macrophage antibiotic peptide MCP-2; lagomorphs, mammals, animals)	Antibacterial, Antifungal, Antiviral, Antimicrobial
3925	DRAMP03806	Corticostatin-6 (Corticostatin VI; Neutrophil antibiotic peptide NP-6; lagomorphs, mammals, animals)	Antibacterial, Antimicrobial
3926	DRAMP03817	cRW2 peptide	Antimicrobial,
3927	DRAMP03818	cRW3 cationic antimicrobial peptide	Antimicrobial,
3928	DRAMP03819	Cyclic hexapeptide RRWWRF	Antimicrobial,
3929	DRAMP03820	Cyclic hexapeptide RRYYRF	Antimicrobial,
3930	DRAMP03821	Cyclic hexapeptide KKWWKF	Antimicrobial,
3931	DRAMP03822	Cyclic hexapeptide RR(NAL)(NAL)RF	Antimicrobial,
3932	DRAMP03836	ETD135 (Mutant of ARD1; Heliomicin analogs)	Antifungal, Antimicrobial
3933	DRAMP03837	ETD179 (Mutant of ARD1; Heliomicin analogs)	Antifungal, Antimicrobial
3934	DRAMP03838	ETD151 (Mutant of ARD1; Heliomicin analogs)	Antifungal, Antimicrobial
3935	DRAMP03839	ETD131 (Mutant of ARD1; Heliomicin analogs)	Antifungal, Antimicrobial
3936	DRAMP03840	ETD130 (Mutant of ARD1; Heliomicin analogs)	Antifungal, Antimicrobial

3937	DRAMP03841	ETD140 (Mutant of ARD1; Heliomicin analogs)	Antifungal, Antimicrobial
3938	DRAMP03842	ETD150 (Mutant of ARD1; Heliomicin analogs)	Antifungal, Antimicrobial
3939	DRAMP03843	ETD152 (Mutant of ARD1; Heliomicin analogs)	Antifungal, Antimicrobial
3940	DRAMP03844	ETD132 (Mutant of ARD1; Heliomicin analogs)	Antifungal, Antimicrobial
3941	DRAMP03845	ETD133 (Mutant of ARD1; Heliomicin analogs)	Antifungal, Antimicrobial
3942	DRAMP03846	ETD134 (Mutant of ARD1; Heliomicin analogs)	Antifungal, Antimicrobial
3943	DRAMP03847	ETD154 (Mutant of ARD1; Heliomicin analogs)	Antifungal, Antimicrobial
3944	DRAMP18190	Pantinin-3 (Non-disulfide-bridged peptide 4.22, NDBP-4.22, Non-disulfide-bridged peptide 5.23)	Antibacterial, Antifungal, Anti-Gram+, Anti-Gram-, Antimicrobial
3945	DRAMP03849	ETD156 (Mutant of ARD1; Heliomicin analogs)	Antifungal, Antimicrobial
3946	DRAMP03850	Antibacterial peptide/melittin homolog	Antibacterial, Anti-Gram-, Antimicrobial
3947	DRAMP03851	Cyclic L27-11 (protegrin-1-mimetic)	Antibacterial, Antimicrobial
3948	DRAMP03872	hLf 21-30 (fragment of human lalctoferricin, residues 21-30)	Antibacterial, Anti-Gram+, Anti-Gram-, Antimicrobial
3949	DRAMP03873	mLf 20-29 (fragment of murine lalctoferricin, residues 20-29)	Antibacterial, Anti-Gram+, Anti-Gram-, Antimicrobial
3950	DRAMP03874	pLf20-29 (fragment of porcine lactoferricin, residues 20-29)	Antibacterial, Anti-Gram+, Anti-Gram-, Antimicrobial
3951	DRAMP03913	(KW)3	Antifungal, Antimicrobial
3952	DRAMP03914	(KW)4	Antifungal, Antimicrobial
3953	DRAMP03915	(KW)5	Antifungal, Antimicrobial
3954	DRAMP03916	(RW)2	Antifungal, Antimicrobial
3955	DRAMP03917	(RW)3	Antifungal, Antimicrobial
3956	DRAMP03918	(RW)4	Antifungal, Antimicrobial
3957	DRAMP03919	(RW)5	Antifungal, Antimicrobial
3958	DRAMP03926	Cecropin B (1-7)-melittin (4-11)hybrid peptide (CBM)	Antibacterial, Antimicrobial
3959	DRAMP03932	Indolicidin derivative	Antimicrobial,
3960	DRAMP03940	Peptide 4 (Trp- and Arg-rich; derivative of Tritrpticin)	Antibacterial, Antifungal, Antimicrobial
3961	DRAMP03941	Peptide 3 (Trp- and Arg-rich; derivative of Tritrpticin)	Antibacterial, Antifungal, Anti-Gram+, Anti-Gram-, Antimicrobial
3962	DRAMP03942	Peptide 2 (Trp- and Arg-rich; derivative of Tritrpticin)	Antibacterial, Antifungal, Anti-Gram+, Anti-Gram-, Antimicrobial
3963	DRAMP03943	Gratisin analogue	Antibacterial, Anti-Gram-, Antimicrobial
3964	DRAMP18396	moroNC-NH2 (moronecidin-like peptide; fish, animals)	Antibacterial, antifungal<br><br>, Antimicrobial
3965	DRAMP03946	Del 1-3 (Ranalexin analog)	Antibacterial, Anti-Gram+, Anti-Gram-, Antimicrobial
3966	DRAMP03950	Rs-AFP2 variant (Mutation: Q5M)	Antifungal, Antimicrobial
3967	DRAMP03951	Rs-AFP2 variant (Mutation: G16M)	Antifungal, Antimicrobial
3968	DRAMP03952	Rs-AFP2 variant (Mutation: G9R)	Antifungal, Antimicrobial
3969	DRAMP03953	Rs-AFP2 variant (Mutation: V39R)	Antifungal, Antimicrobial
3970	DRAMP03961	KR-12	Antibacterial, Anti-Gram+, Anti-Gram-, Antimicrobial
3971	DRAMP03962	Cecropin A(1-8)-Magainin 2(1-12)hybrid peptide (CAMA)	Antibacterial, Antimicrobial
3972	DRAMP03963	Cecropin A(1-8)-Magainin 2(1-12)hybrid peptide analogue (P1)	Antibacterial, Antimicrobial
3973	DRAMP03964	Cecropin A(1-8)-Magainin 2(1-12)hybrid peptide analogue (P2)	Antibacterial, Antimicrobial
3974	DRAMP03965	Cecropin A(1-8)-Magainin 2(1-12)hybrid peptide analogue (P3)	Antibacterial, Antimicrobial
3975	DRAMP03966	Cecropin A(1-8)-Magainin 2(1-12)hybrid peptide analogue (P4)	Antibacterial, Antimicrobial
3976	DRAMP02092	Brevinin-1Bb (Frogs, amphibians, animals)	Antimicrobial, Antibacterial, Antifungal, Anti-Gram+, Anti-Gram-,
3977	DRAMP04006	Plasticin-A1 (PTC-A1; DRP-AA-2-5; frogs, amphibians, animals)	Antibacterial, Antifungal, Antiviral, Antimicrobial
3978	DRAMP18189	Pantinin-2 (Non-disulfide-bridged peptide 4.21, NDBP-4.21, Non-disulfide-bridged peptide 5.22)	Antibacterial, Antifungal, Anti-Gram+, Anti-Gram-, Antimicrobial
3979	DRAMP04008	Plasticin-C1 (PTC-C1; DRP-AC-1; frogs, amphibians, animals)	Antibacterial, Antifungal, Antiviral, Antimicrobial
3980	DRAMP04009	Plasticin-C2 (PTC-C2; DRP-AC-2; frogs, amphibians, animals)	Antibacterial, Antifungal, Antiviral, Antimicrobial
3981	DRAMP04010	Plasticin-S1 (PTC-S1; frogs, amphibians, animals)	Antibacterial, Antifungal, Antiviral, Antimicrobial
3982	DRAMP04018	Rp-1	Antibacterial, Antifungal, Anti-Gram+, Anti-Gram-, Antimicrobial
3983	DRAMP04037	Immobilized peptide E07LKK	Antibacterial, Antifungal, Anti-Gram+, Anti-Gram-, Antimicrobial
3984	DRAMP04038	Immobilized peptide E14LKK/H14LKK	Antibacterial, Antifungal, Anti-Gram+, Anti-Gram-, Antimicrobial

3985	DRAMP04039	Immobilized peptige E16KGL/H16KGL	Antibacterial, Antifungal, Anti-Gram+, Anti-Gram-, Antimicrobial
3986	DRAMP04040	Immobilized peptide E17KGG	Antibacterial, Antifungal, Anti-Gram+, Anti-Gram-, Antimicrobial
3987	DRAMP04041	Immobilized peptide E18KGG	Antibacterial, Antifungal, Anti-Gram+, Anti-Gram-, Antimicrobial
3988	DRAMP04042	Immobilized peptide E16LKL	Antibacterial, Antifungal, Anti-Gram+, Anti-Gram-, Antimicrobial
3989	DRAMP04043	Immobilized peptid E10KKL	Antibacterial, Antifungal, Anti-Gram+, Anti-Gram-, Antimicrobial
3990	DRAMP04044	Immobilized peptid E12LLK	Antibacterial, Antifungal, Anti-Gram+, Anti-Gram-, Antimicrobial
3991	DRAMP04045	Immobilized peptid E14KKL	Antibacterial, Antifungal, Anti-Gram+, Anti-Gram-, Antimicrobial
3992	DRAMP04046	Immobilized peptide E23GIG magainin2	Antibacterial, Antifungal, Anti-Gram+, Anti-Gram-, Antimicrobial
3993	DRAMP04047	Immobilized peptide E17HSA magainin 2 deletion	Antibacterial, Antifungal, Anti-Gram+, Anti-Gram-, Antimicrobial
3994	DRAMP04071	LL-37 pentamide	Antibacterial, Anti-Gram+, Antimicrobial
3995	DRAMP04072	Hinnavin II/MSH hybrid (rhin/MSH)	Antibacterial, Antimicrobial
3996	DRAMP04073	TvD1 (Recombinant peptide; alpha-defensin)	Antimicrobial,
3997	DRAMP04074	PFR peptide (Recombinant peptide)	Antimicrobial,
3998	DRAMP04084	W9F (mutant of Def-DAA defensin)	Antibacterial, Anti-Gram+, Antimicrobial
3999	DRAMP04085	K8I,W9F (mutant of Def-DAA defensin)	Antibacterial, Anti-Gram+, Antimicrobial
4000	DRAMP04086	W9F,W11V (mutant of Def-DAA defensin)	Antibacterial, Anti-Gram+, Antimicrobial
4001	DRAMP04087	K8I,W9F,W11V (mutant of Def-DAA defensin)	Antibacterial, Anti-Gram+, Antimicrobial
4002	DRAMP04088	W9F,T14A (mutant of Def-DAA defensin)	Antibacterial, Anti-Gram+, Antimicrobial
4003	DRAMP04089	K8I,W9F,T14A (mutant of Def-DAA defensin)	Antibacterial, Anti-Gram+, Antimicrobial
4004	DRAMP04090	W11V,T14A (mutant of Def-DAA defensin)	Antibacterial, Anti-Gram+, Antimicrobial
4005	DRAMP04091	K8I,W11V,T14A (mutant of Def-DAA defensin)	Antibacterial, Anti-Gram+, Antimicrobial
4006	DRAMP04092	W11V (mutant of Def-DAA defensin)	Antibacterial, Anti-Gram+, Antimicrobial
4007	DRAMP04093	K8I,W11V (mutant of Def-DAA defensin)	Antibacterial, Anti-Gram+, Antimicrobial
4008	DRAMP04094	K8I (mutant of Def-DAA defensin)	Antibacterial, Anti-Gram+, Antimicrobial
4009	DRAMP04148	Gramicidin S	Antibacterial, Antimicrobial
4010	DRAMP04149	Gramicidin Analogue	Antibacterial, Antimicrobial
4011	DRAMP04150	Gramicidin Analogue 1	Antibacterial, Antimicrobial
4012	DRAMP04151	RL1 (homologue of Pc-CATH1)	Antibacterial, Antifungal, Antimicrobial
4013	DRAMP04152	RL2 (homologue of Pc-CATH1)	Antibacterial, Antifungal, Antimicrobial
4014	DRAMP04153	RL3 (homologue of Pc-CATH1)	Antibacterial, Antifungal, Antimicrobial
4015	DRAMP04154	RL4 (homologue of Pc-CATH1)	Antibacterial, Antifungal, Antimicrobial
4016	DRAMP04155	RL5 (homologue of Pc-CATH1)	Antibacterial, Antifungal, Antimicrobial
4017	DRAMP04156	RL6 (homologue of Pc-CATH1)	Antibacterial, Antifungal, Antimicrobial
4018	DRAMP04157	RL7 (homologue of Pc-CATH1)	Antifungal, Antimicrobial
4019	DRAMP04158	RL8 (homologue of Pc-CATH1)	Antifungal, Antimicrobial
4020	DRAMP04172	LK2W2 (LlKmW2 model peptides)	Antibacterial, Anti-Gram+, Anti-Gram-, Antimicrobial
4021	DRAMP04173	L2KW2 (LlKmW2 model peptides)	Antibacterial, Anti-Gram+, Anti-Gram-, Antimicrobial
4022	DRAMP04197	Penetratin	Antibacterial, Antimicrobial
4023	DRAMP04198	PenArg	Antibacterial, Antimicrobial
4024	DRAMP04199	PenLys	Antibacterial, Antimicrobial
4025	DRAMP04200	PenLeu	Antibacterial, Antimicrobial
4026	DRAMP04201	Pen13	Antibacterial, Antimicrobial
4027	DRAMP04202	Pen13Arg	Antibacterial, Antimicrobial
4028	DRAMP04203	Pen13Lys	Antibacterial, Antimicrobial
4029	DRAMP04204	PenShuf	Antibacterial, Antimicrobial
4030	DRAMP04205	PenShufLeu	Antibacterial, Antimicrobial
4031	DRAMP04206	PenShufLysLeu	Antibacterial, Antimicrobial
4032	DRAMP04207	PenShufArgLeu	Antibacterial, Antimicrobial
4033	DRAMP04208	WR8	Antibacterial, Antimicrobial
4034	DRAMP04209	Tat13	Antibacterial, Antimicrobial

4035	DRAMP04210	ADP1	Antibacterial, Antimicrobial
4036	DRAMP04211	ADP2	Antibacterial, Antimicrobial
4037	DRAMP04212	ADP3	Antibacterial, Antimicrobial
4038	DRAMP04213	BP100	Antibacterial, Antimicrobial
4039	DRAMP04214	R-BP100	Antibacterial, Antimicrobial
4040	DRAMP04215	RW-BP100	Antibacterial, Antimicrobial
4041	DRAMP04216	P1	Antibacterial, Antimicrobial
4042	DRAMP04217	P2	Antibacterial, Antimicrobial
4043	DRAMP04218	Lys-a1	Antibacterial, Antimicrobial
4044	DRAMP04219	CHRG01 (hBD3 derivative)	Antibacterial, Antimicrobial
4045	DRAMP04220	CHRG02 (hBD3 derivative)	Antibacterial, Antimicrobial
4046	DRAMP04221	CHRG04 (hBD3 derivative)	Antibacterial, Antimicrobial
4047	DRAMP04222	CHRG06 (hBD3 derivative)	Antibacterial, Antimicrobial
4048	DRAMP04223	CHRG07 (hBD3 derivative)	Antibacterial, Antimicrobial
4049	DRAMP04224	A3-APO	Antibacterial, Antimicrobial
4050	DRAMP04225	Dros-Pyrr-Dros	Antibacterial, Antimicrobial
4051	DRAMP04226	Pyrr-Pyrr-Dros	Antibacterial, Antimicrobial
4052	DRAMP04227	PGG	Antibacterial, Antimicrobial
4053	DRAMP04228	PGP	Antibacterial, Antimicrobial
4054	DRAMP04229	PGYa	Antibacterial, Antimicrobial
4055	DRAMP04230	PGAa	Antibacterial, Antimicrobial
4056	DRAMP04231	C18A	Antibacterial, Antimicrobial
4057	DRAMP04232	C18Q	Antibacterial, Antimicrobial
4058	DRAMP04236	Antibacterial peptide A2	Antibacterial, Anti-Gram-, Antimicrobial
4059	DRAMP04238	PL-101	Antibacterial, Antimicrobial
4060	DRAMP04239	Neurotensin	Antibacterial, Antimicrobial
4061	DRAMP04245	Dhvar5	Antibacterial, Antimicrobial
4062	DRAMP04246	Dhvar4	Antibacterial, Antimicrobial
4063	DRAMP04247	STRO06	Antibacterial, Antimicrobial
4064	DRAMP04248	(KIGAKI)3-NH2	Antibacterial, Antimicrobial
4065	DRAMP04249	(KIAGKIA)3-NH2	Antibacterial, Antimicrobial
4066	DRAMP04250	(KLAGLAK)3-NH2	Antibacterial, Antimicrobial
4067	DRAMP04251	WLBU2	Antibacterial, Antimicrobial
4068	DRAMP04252	Sushi peptide 1 (truncated fragment)	Antibacterial, Antimicrobial
4069	DRAMP04253	Sushi peptide 3 (truncated fragment)	Antibacterial, Antimicrobial
4070	DRAMP04254	Neuropeptide Y (NPY)	Antibacterial, Antimicrobial
4071	DRAMP04255	Neuropeptide Y (NPY)	Antibacterial, Antimicrobial
4072	DRAMP04256	R	Antibacterial, Antimicrobial
4073	DRAMP04257	4A	Antibacterial, Antimicrobial
4074	DRAMP04258	AA	Antibacterial, Antimicrobial
4075	DRAMP04259	LL	Antibacterial, Antimicrobial
4076	DRAMP04260	CM-1	Antibacterial, Antimicrobial
4077	DRAMP04261	CM-2	Antibacterial, Antimicrobial
4078	DRAMP04262	CM-3	Antibacterial, Antimicrobial
4079	DRAMP04263	CM-4	Antibacterial, Antimicrobial
4080	DRAMP04266	CPα1	Antibacterial, Anti-Gram-, Antimicrobial
4081	DRAMP04267	CPα2	Antibacterial, Anti-Gram-, Antimicrobial
4082	DRAMP04268	CPα3	Antibacterial, Anti-Gram-, Antimicrobial
4083	DRAMP04269	CP201	Antibacterial, Anti-Gram-, Antimicrobial
4084	DRAMP04270	CP202	Antibacterial, Anti-Gram-, Antimicrobial
4085	DRAMP04271	CP203	Antibacterial, Anti-Gram-, Antimicrobial
4086	DRAMP04274	CP206	Antibacterial, Anti-Gram-, Antimicrobial
4087	DRAMP04275	CP207	Antibacterial, Anti-Gram-, Antimicrobial
4088	DRAMP04276	CP208	Antibacterial, Anti-Gram-, Antimicrobial
4089	DRAMP04277	CP209	Antibacterial, Anti-Gram-, Antimicrobial
4090	DRAMP04280	CM1	Antibacterial, Anti-Gram-, Antimicrobial
4091	DRAMP04281	CM2	Antibacterial, Anti-Gram-, Antimicrobial

4092	DRAMP04282	CM3	Antibacterial, Anti-Gram-, Antimicrobial
4093	DRAMP04283	CM4	Antibacterial, Anti-Gram-, Antimicrobial
4094	DRAMP04284	CM5	Antibacterial, Anti-Gram-, Antimicrobial
4095	DRAMP04285	CM6	Antibacterial, Anti-Gram-, Antimicrobial
4096	DRAMP04286	CM7	Antibacterial, Anti-Gram-, Antimicrobial
4097	DRAMP04287	MC-03	Antibacterial, Antimicrobial
4098	DRAMP04288	MC-04	Antibacterial, Antimicrobial
4099	DRAMP04289	MC-05	Antibacterial, Antimicrobial
4100	DRAMP04290	MC-08	Antibacterial, Antimicrobial
4101	DRAMP04291	MC-10	Antibacterial, Antimicrobial
4102	DRAMP04292	MB-00	Antibacterial, Antimicrobial
4103	DRAMP04293	MB-03	Antibacterial, Antimicrobial
4104	DRAMP04294	MB-04	Antibacterial, Antimicrobial
4105	DRAMP04295	MB-10	Antibacterial, Antimicrobial
4106	DRAMP04296	MB-15	Antibacterial, Antimicrobial
4107	DRAMP04297	MB-18	Antibacterial, Antimicrobial
4108	DRAMP04298	MB-21	Antibacterial, Antimicrobial
4109	DRAMP04299	MB-22	Antibacterial, Antimicrobial
4110	DRAMP04300	MB-25	Antibacterial, Antimicrobial
4111	DRAMP04301	MB-31	Antibacterial, Antimicrobial
4112	DRAMP04302	MB-32	Antibacterial, Antimicrobial
4113	DRAMP04303	MB-33	Antibacterial, Antimicrobial
4114	DRAMP04304	MB-34	Antibacterial, Antimicrobial
4115	DRAMP04305	MB-35	Antibacterial, Antimicrobial
4116	DRAMP04306	MB-36	Antibacterial, Antimicrobial
4117	DRAMP04307	MB-37	Antibacterial, Antimicrobial
4118	DRAMP04308	MB-38	Antibacterial, Antimicrobial
4119	DRAMP04309	MB-40	Antibacterial, Antimicrobial
4120	DRAMP04310	MB-41	Antibacterial, Antimicrobial
4121	DRAMP04311	MB-43	Antibacterial, Antimicrobial
4122	DRAMP04312	MB-45	Antibacterial, Antimicrobial
4123	DRAMP04313	MB-46	Antibacterial, Antimicrobial
4124	DRAMP04314	MB-47	Antibacterial, Antimicrobial
4125	DRAMP04315	MB-48	Antibacterial, Antimicrobial
4126	DRAMP04316	MB-50	Antibacterial, Antimicrobial
4127	DRAMP04317	DASamP1	Antibacterial, Antimicrobial
4128	DRAMP04318	DASamP2	Antibacterial, Antimicrobial
4129	DRAMP04319	22A-30R-NH2 fragment 1	Antibacterial, Antimicrobial
4130	DRAMP04320	22A-30R-NH2 fragment 2	Antibacterial, Antimicrobial
4131	DRAMP04321	22A-30R-NH2 fragment 3	Antibacterial, Antimicrobial
4132	DRAMP04322	22A-30R-NH2 fragment 4	Antibacterial, Antimicrobial
4133	DRAMP04323	22A-30R-NH2 fragment 5	Antibacterial, Antimicrobial
4134	DRAMP04324	19L-30R-NH2 fragment 1	Antibacterial, Antimicrobial
4135	DRAMP04325	19L-30R-NH2 fragment 2	Antibacterial, Antimicrobial
4136	DRAMP04326	19L-30R-NH2 fragment 3	Antibacterial, Antimicrobial
4137	DRAMP04327	19L-30R-NH2 fragment 4	Antibacterial, Antimicrobial
4138	DRAMP04328	19L-30R-NH2 fragment 5	Antibacterial, Antimicrobial
4139	DRAMP04329	19L-30R-NH2 fragment 6	Antibacterial, Antimicrobial
4140	DRAMP04330	19L-30R-NH2 fragment 7	Antibacterial, Antimicrobial
4141	DRAMP04331	19L-30R-NH2 fragment 8	Antibacterial, Antimicrobial
4142	DRAMP04332	19L-30R-NH2 fragment 9	Antibacterial, Antimicrobial
4143	DRAMP04333	19L-30R-NH2 fragment 10	Antibacterial, Antimicrobial
4144	DRAMP04334	19L-30R-NH2 fragment 11	Antibacterial, Antimicrobial
4145	DRAMP04335	AamAP-S1	Antibacterial, Antimicrobial
4146	DRAMP04336	IG-19 (residues 13-31 of LL-37)	Antibacterial, Antimicrobial
4147	DRAMP04337	a1 (IG-19 analogs)	Antibacterial, Antimicrobial
4148	DRAMP04338	a2 (IG-19 analogs)	Antibacterial, Antimicrobial

4149	DRAMP04339	a3 (IG-19 analogs)	Antibacterial, Antimicrobial
4150	DRAMP04340	a4 (IG-19 analogs)	Antibacterial, Antimicrobial
4151	DRAMP04341	a5 (IG-19 analogs)	Antibacterial, Antimicrobial
4152	DRAMP04342	a6 (IG-19 analogs)	Antibacterial, Antimicrobial
4153	DRAMP04343	a7 (IG-19 analogs)	Antibacterial, Antimicrobial
4154	DRAMP04344	a8 (IG-19 analogs)	Antibacterial, Antimicrobial
4155	DRAMP04345	a4-W1	Antibacterial, Antimicrobial
4156	DRAMP04346	a4-W2	Antibacterial, Antimicrobial
4157	DRAMP04347	Buforin	Antibacterial, Antimicrobial
4158	DRAMP04348	Buf IIIa	Antibacterial, Antimicrobial
4159	DRAMP04349	Buf IIIb	Antibacterial, Antimicrobial
4160	DRAMP04350	Buf IIIc	Antibacterial, Antimicrobial
4161	DRAMP04351	Buf IIId	Antibacterial, Antimicrobial
4162	DRAMP04352	Asn-2-Polybia-MP	Antibacterial, Antimicrobial
4163	DRAMP04353	MK-578	Antibacterial, Antimicrobial
4164	DRAMP04354	PpTG20	Antibacterial, Antimicrobial
4165	DRAMP04355	P7	Antibacterial, Antimicrobial
4166	DRAMP04356	CAD	Antibacterial, Antimicrobial
4167	DRAMP04357	CEME(MBI-27)	Antibacterial, Anti-Gram-, Antimicrobial
4168	DRAMP04358	CEMA(MBI-28)	Antibacterial, Anti-Gram-, Antimicrobial
4169	DRAMP04366	PDD-A-8 (PDD-A analog)	Antibacterial, Anti-Gram+, Anti-Gram-, Antimicrobial
4170	DRAMP04375	PDD-B-5 (PDD-B analog)	Antibacterial, Anti-Gram+, Anti-Gram-, Antimicrobial
4171	DRAMP04384	PMM-5 (PMM analog)	Antibacterial, Anti-Gram+, Anti-Gram-, Antimicrobial
4172	DRAMP04388	PMM-9 (PMM analog)	Antibacterial, Anti-Gram+, Anti-Gram-, Antimicrobial
4173	DRAMP01823	Temporin-1Ce (Temporin 1Ce; Frogs, amphibians, animals)	Antibacterial, Anti-Gram+, Antimicrobial
4174	DRAMP04396	Antimicrobial peptide OGC1	Antimicrobial,
4175	DRAMP04397	Antimicrobial peptide OGC2	Antimicrobial,
4176	DRAMP04398	Antifungal protein (PAF)	Antifungal, Antiviral, Antimicrobial
4177	DRAMP04399	Odorranain-P1g antimicrobial peptide (Frogs, amphibians, animals)	Antimicrobial,
4178	DRAMP04400	Odorranain-P1c antimicrobial peptide (Frogs, amphibians, animals)	Antimicrobial,
4179	DRAMP04401	Odorranain-P1e antimicrobial peptide (Frogs, amphibians, animals)	Antimicrobial,
4180	DRAMP04402	Odorranain-P1c antimicrobial peptide (Frogs, amphibians, animals)	Antimicrobial,
4181	DRAMP04403	Odorranain-P1h antimicrobial peptide (Frogs, amphibians, animals)	Antimicrobial,
4182	DRAMP04404	Odorranain-P1d antimicrobial peptide (Frogs, amphibians, animals)	Antimicrobial,
4183	DRAMP04405	Granulosusin-E1 antimicrobial peptide (Frogs, amphibians, animals)	Antimicrobial,
4184	DRAMP04406	Granulosusin-D1 antimicrobial peptide (Frogs, amphibians, animals)	Antimicrobial,
4185	DRAMP04407	Taipehensin-A1 antimicrobial peptide (Frogs, amphibians, animals)	Antimicrobial,
4186	DRAMP04408	Carcinin	Antimicrobial,
4187	DRAMP04409	Ranacyclin Ca antimicrobial peptide	Antimicrobial,
4188	DRAMP04410	Lantibiotic cytolysin	Antimicrobial,
4189	DRAMP04411	Defensin like protein 2 (Predicted)	Antimicrobial,
4190	DRAMP04412	Defensin	Antimicrobial,
4191	DRAMP04413	Defensin-like protein (Predicted)	Antimicrobial,
4192	DRAMP04414	Defensin 1 (Def1)	Antimicrobial,
4193	DRAMP04415	Defensin 2 (Def2)	Antimicrobial,
4194	DRAMP04416	Defensin 3 (Def3)	Antimicrobial,
4195	DRAMP04417	Defensin 4 (Def4)	Antimicrobial,
4196	DRAMP04418	Defensin	Antimicrobial,
4197	DRAMP04419	Putative uncharacterized protein	Antimicrobial,
4198	DRAMP04420	Defensin B	Antimicrobial,

4199	DRAMP04421	Defensin	Antimicrobial,
4200	DRAMP04422	Defensin 1	Antimicrobial,
4201	DRAMP04423	Defensin	Antimicrobial,
4202	DRAMP04424	BacA protein	Antimicrobial,
4203	DRAMP04425	Termicin	Antimicrobial,
4204	DRAMP04426	Defensin C	Antimicrobial,
4205	DRAMP04427	Beta defensin-2	Antimicrobial,
4206	DRAMP04428	Beta defensin 1 (Beta-defensin-1)	Antimicrobial,
4207	DRAMP04429	Defensin	Antimicrobial,
4208	DRAMP04430	Putative potassium channel blocker TXKs2	Antimicrobial,
4209	DRAMP04431	Precursor of durancin TW-49M (Prepeptide of durancin TW-49M)	Antimicrobial,
4210	DRAMP04432	Defensin 1	Antimicrobial,
4211	DRAMP04433	Defensin 2	Antimicrobial,
4212	DRAMP04435	Defensin domain protein	Antimicrobial,
4213	DRAMP04436	Beta defensin 1 (Beta-defensin-1)	Antimicrobial,
4214	DRAMP04437	Amphipathic pore-forming peptide	Antimicrobial,
4215	DRAMP04438	Non-specific lipid-transfer protein	Antimicrobial,
4216	DRAMP04439	Defensin-like protein (Putative defensin; Predicted)	Antimicrobial,
4217	DRAMP04440	Leaf thionin Asthi1	Antimicrobial,
4218	DRAMP04441	Leaf thionin Asthi2	Antimicrobial,
4219	DRAMP04442	Leaf thionin Asthi3	Antimicrobial,
4220	DRAMP04443	Thionin Asthi4	Antimicrobial,
4221	DRAMP04444	Thionin Asthi5	Antimicrobial,
4222	DRAMP04445	Lipid binding protein	Antimicrobial,
4223	DRAMP04446	Alcohol dehydrogenase 6	Antimicrobial,
4224	DRAMP04447	Defensin	Antimicrobial,
4225	DRAMP04448	Putative gamma-thionin protein	Antimicrobial,
4226	DRAMP04449	Gamma-thionin 1	Antimicrobial,
4227	DRAMP04450	Defensin	Antimicrobial,
4228	DRAMP04451	Os02g0629800 protein (Putative defensin)	Antimicrobial,
4229	DRAMP04452	Defensin protein 2	Antimicrobial,
4230	DRAMP04453	Defensin protein 1	Antimicrobial,
4231	DRAMP04454	Defensin EGAD1	Antimicrobial,
4232	DRAMP04455	Antifungal protein defensin	Antimicrobial,
4233	DRAMP04456	Defensin	Antimicrobial,
4234	DRAMP04457	PDF1	Antimicrobial,
4235	DRAMP04458	Defensin A	Antimicrobial,
4236	DRAMP04459	Defensin 1	Antimicrobial,
4237	DRAMP04460	Defensin 2a	Antimicrobial,
4238	DRAMP04461	Defensin 1a	Antimicrobial,
4239	DRAMP04462	Putative uncharacterized protein	Antimicrobial,
4240	DRAMP04463	Enterocin 1071A (Enterocin EntC1 peptide)	Antimicrobial,
4241	DRAMP04464	Hepcidin 1	Antimicrobial,
4242	DRAMP04465	Hepcidin 2	Antimicrobial,
4243	DRAMP04466	Defensin TY 2	Antimicrobial,
4244	DRAMP04467	Columbicin A	Antimicrobial,
4245	DRAMP04468	Defensin I	Antimicrobial,
4246	DRAMP04469	Cecropin (Cecropin 1 (Cecropin A)	Antimicrobial,
4247	DRAMP04470	Plantaricin NC8 beta peptide	Antimicrobial,
4248	DRAMP04471	Durancin Q prepeptide (Prepeptide of durancin Q)	Antimicrobial,
4249	DRAMP04472	Pore-forming protein isoform B	Antimicrobial,
4250	DRAMP04473	Propionicin T1	Antimicrobial,
4251	DRAMP04474	Defensin (Spodoptericin)	Antimicrobial,
4252	DRAMP04475	Putative defensin	Antimicrobial,
4253	DRAMP04476	Beta defensin 1 (Beta-defensin-1)	Antimicrobial,
4254	DRAMP04477	Beta defensin-2	Antimicrobial,

4255	DRAMP04478	Defensin TY 1	Antimicrobial,
4256	DRAMP04479	Defensin like 3 (Predicted)	Antimicrobial,
4257	DRAMP04480	Alpha defensin	Antimicrobial,
4258	DRAMP04481	Circularin A	Antimicrobial,
4259	DRAMP04482	Antimicrobial-like peptide PP-1 (Predicted)	Antimicrobial,
4260	DRAMP04483	Acidocin LF221B (Gassericin K7 B)	Antimicrobial,
4261	DRAMP04484	Crustin-like protein fc-2 (Predicted)	Antimicrobial,
4262	DRAMP04485	Retrocyclin	Antimicrobial,
4263	DRAMP04486	Cold-regulated LTCOR12	Antimicrobial,
4264	DRAMP04487	LTCOR11	Antimicrobial,
4265	DRAMP04488	Antimicrobial peptide 2 (Antimicrobial peptide 4)	Antimicrobial,
4266	DRAMP04489	MAP34-A protein (MAP34-B protein)	Antimicrobial,
4267	DRAMP04490	Myeloid antimicrobial peptide	Antibacterial, Antimicrobial
4268	DRAMP04491	Non-specific lipid-transfer protein	Antimicrobial,
4269	DRAMP04492	Persulcatusin	Antimicrobial,
4270	DRAMP04493	Amercin	Antimicrobial,
4271	DRAMP04494	Defensin	Antimicrobial,
4272	DRAMP04495	Preprodefensin	Antimicrobial,
4273	DRAMP04496	Cysteine-rich protein	Antimicrobial,
4274	DRAMP04497	Gasa4-like protein (Predicted)	Antimicrobial,
4275	DRAMP04498	GAST1 protein, putative	Antimicrobial,
4276	DRAMP04499	Hepcidin (Hepcidin type I)	Antimicrobial,
4277	DRAMP04500	GEG protein	Antimicrobial,
4278	DRAMP04501	Antimicrobial peptide Def1-2	Antimicrobial,
4279	DRAMP04502	Antimicrobial peptide Def1-1	Antimicrobial,
4280	DRAMP04503	Pelophylaxin-1 (Frogs, amphibians, animals)	Antibacterial, Antifungal, Antiviral, Antimicrobial
4281	DRAMP04504	Pelophylaxin-2 (Frogs, amphibians, animals)	Antibacterial, Antifungal, Antiviral, Antimicrobial
4282	DRAMP04505	Pelophylaxin-3 (Frogs, amphibians, animals)	Antibacterial, Antifungal, Antiviral, Antimicrobial
4283	DRAMP04506	Pelophylaxin-4 (Frogs, amphibians, animals)	Antibacterial, Antifungal, Antiviral, Antimicrobial
4284	DRAMP04507	Esculentin-2-AJ2 antimicrobial peptide (Frogs, amphibians, animals)	Antimicrobial,
4285	DRAMP04508	Esculentin-2-AJ3 antimicrobial peptide (Frogs, amphibians, animals)	Antimicrobial,
4286	DRAMP04509	Esculentin-2-AJ4 antimicrobial peptide (Frogs, amphibians, animals)	Antimicrobial,
4287	DRAMP04510	Esculentin-2-AJ5 antimicrobial peptide (Frogs, amphibians, animals)	Antimicrobial,
4288	DRAMP04511	Esculentin-2-AJ6 antimicrobial peptide (Frogs, amphibians, animals)	Antimicrobial,
4289	DRAMP04512	Brevinin-2-AJ2 antimicrobial peptide (Frogs, amphibians, animals)	Antimicrobial,
4290	DRAMP04513	Esculentin-2-AJ1 antimicrobial peptide (Frogs, amphibians, animals)	Antimicrobial,
4291	DRAMP04514	Brevinin-2-AJ7 antimicrobial peptide (Frogs, amphibians, animals)	Antimicrobial,
4292	DRAMP04515	Brevinin-2-AJ4 antimicrobial peptide (Frogs, amphibians, animals)	Antimicrobial,
4293	DRAMP04516	Brevinin-2-AJ1 antimicrobial peptide (Frogs, amphibians, animals)	Antimicrobial,
4294	DRAMP04517	Brevinin-2-AJ3 antimicrobial peptide (Frogs, amphibians, animals)	Antimicrobial,
4295	DRAMP04518	Odorranain-P1a antimicrobial peptide (Frogs, amphibians, animals)	Antimicrobial,
4296	DRAMP04519	Odorranain-P1b antimicrobial peptide (Frogs, amphibians, animals)	Antimicrobial,
4297	DRAMP04520	Esculentin-2MT1 antimicrobial peptide (Frogs, amphibians, animals)	Antimicrobial,
4298	DRAMP04521	Esculentin-2MT2 antimicrobial peptide (Frogs, amphibians, animals)	Antimicrobial,
4299	DRAMP04522	Brevinin-2LT2 antimicrobial peptide (Frogs, amphibians, animals)	Antimicrobial,

4300	DRAMP04523	Odorranain-P1b antimicrobial peptide (Frogs, amphibians, animals)	Antimicrobial,
4301	DRAMP04524	Odorranain-P1b antimicrobial peptide (Frogs, amphibians, animals)	Antimicrobial,
4302	DRAMP04527	Luxuriosin	Antibacterial, Antimicrobial
4303	DRAMP04529	LAP-like antimicrobial peptide (Bovine beta-defensins)	Antibacterial, Antimicrobial
4304	DRAMP04530	Urechistachykinins I (UI)	Antimicrobial,
4305	DRAMP04531	Urechistachykinin II (UII)	Antimicrobial,
4306	DRAMP04533	P-113D	Antimicrobial,
4307	DRAMP04534	PG-KI	Antimicrobial,
4308	DRAMP04535	PG-KII	Antimicrobial,
4309	DRAMP04536	PG-KIII	Antimicrobial,
4310	DRAMP04537	PG-SPI	Antimicrobial,
4311	DRAMP04538	PG-SPII	Antimicrobial,
4312	DRAMP04539	PG-L	Antimicrobial,
4313	DRAMP04540	PR-bombesin	Antimicrobial,
4314	DRAMP04541	Bb-AMP4	Antimicrobial,
4315	DRAMP04547	Shuchin 1	Antimicrobial,
4316	DRAMP04548	Shuchin 2	Antimicrobial,
4317	DRAMP04549	17Hc	Antimicrobial,
4318	DRAMP04550	18HcKK	Antimicrobial,
4319	DRAMP04551	K19Hc	Antimicrobial,
4320	DRAMP04552	K19HcKK	Antimicrobial,
4321	DRAMP04554	Oncopeltus antibacterial peptide 4	Antimicrobial,
4322	DRAMP04555	Bactrocerin-1	Antimicrobial,
4323	DRAMP04556	Parkerin	Antimicrobial,
4324	DRAMP04557	Pelteobagrin	Antimicrobial,
4325	DRAMP04558	C18	Antimicrobial,
4326	DRAMP04559	C18AA	Antimicrobial,
4327	DRAMP04560	Spingerin C-4	Antimicrobial,
4328	DRAMP04561	Ci-PAP-A2	Antimicrobial,
4329	DRAMP04562	Ci-MAM-A24	Antimicrobial,
4330	DRAMP04563	2S albumin large chain 25	Antimicrobial,
4331	DRAMP04564	Epinecidin-1 (Epi-1; fish, animals)	Antibacterial, Antifungal, Antiviral, Antimicrobial
4332	DRAMP04565	Laticeptin	Antimicrobial,
4333	DRAMP04566	Kenojeinin I	Antimicrobial,
4334	DRAMP04567	Centrocin 1	Antimicrobial,
4335	DRAMP04568	Centrocin 2	Antimicrobial,
4336	DRAMP04569	Arminin-1a	Antimicrobial,
4337	DRAMP04570	Callinectin	Antimicrobial,
4338	DRAMP04571	bullfrog pepsinogen C-derived antimicrobial peptide (bPcAP)	Antimicrobial,
4339	DRAMP04572	bullfrog pepsinogen A-derived antimicrobial peptide (bPaAP)	Antimicrobial,
4340	DRAMP04573	Psacotheasin	Antimicrobial,
4341	DRAMP04574	Dendrocin	Antimicrobial,
4342	DRAMP04575	Chironomus defensin	Antimicrobial,
4343	DRAMP04576	Cg-Prp	Antimicrobial,
4344	DRAMP04577	Papiliocin	Antimicrobial,
4345	DRAMP04578	ECATH-1	Antimicrobial,
4346	DRAMP04579	ECATH-2	Antimicrobial,
4347	DRAMP04580	ECATH-3	Antimicrobial,
4348	DRAMP04581	Ap	Antimicrobial,
4349	DRAMP04582	Strongylocin 1 (Cys-rich; sea urchin, Echinoidea, animals)	Antibacterial, Antifungal, Antiviral, Antimicrobial
4350	DRAMP04583	Strongylocin 2 (Cys-rich; sea urchin, Echinoidea, animals)	Antimicrobial,
4351	DRAMP04584	SpStrongylocin 1 (S. purpuratus Strongylocin, sea urchin, Echinoidea, animals)	Antibacterial, Antifungal, Antiviral,, Antimicrobial
4352	DRAMP04585	SpStrongylocin 2 (S. purpuratus Strongylocin; sea urchin, Echinoidea, animals)	Antimicrobial,
4353	DRAMP04586	Vejovine	Antimicrobial,

4354	DRAMP04587	aCATH	Antimicrobial,
4355	DRAMP04588	rtCATH 1	Antimicrobial,
4356	DRAMP04589	Cod cathelicidin	Antimicrobial,
4357	DRAMP04590	Rhamp	Antimicrobial,
4358	DRAMP04591	CXCL14	Antimicrobial,
4359	DRAMP04592	Ls-Stylicin 1	Antimicrobial,
4360	DRAMP04593	CrusSp	Antimicrobial,
4361	DRAMP04594	TCP	Antimicrobial,
4362	DRAMP04595	Prolixicin	Antimicrobial,
4363	DRAMP04596	PuroA	Antimicrobial,
4364	DRAMP04597	PuroB	Antimicrobial,
4365	DRAMP04598	Pinb-2v1	Antimicrobial,
4366	DRAMP04599	Pinb-2v3	Antimicrobial,
4367	DRAMP04600	Pinb-B	Antimicrobial,
4368	DRAMP04601	Pinb-D	Antimicrobial,
4369	DRAMP04602	Pinb-Q	Antimicrobial,
4370	DRAMP04603	Pina-M	Antimicrobial,
4371	DRAMP04604	Pina-R39 G	Antimicrobial,
4372	DRAMP04605	Pina-W	Antimicrobial,
4373	DRAMP04606	Hina	Antimicrobial,
4374	DRAMP04607	GSP-5D	Antimicrobial,
4375	DRAMP04608	BCP61	Antimicrobial,
4376	DRAMP04609	Cg-lgPrp	Antimicrobial,
4377	DRAMP04610	Cg-lgPrp P/Q	Antimicrobial,
4378	DRAMP04611	Cg-Defh1	Antimicrobial,
4379	DRAMP04612	Cg-Defh2	Antimicrobial,
4380	DRAMP04613	HV-BBI(3-18) (X)	Antimicrobial,
4381	DRAMP04614	[Ser14,Ile15]OGTI (II)	Antimicrobial,
4382	DRAMP04615	HV-BBI (IX)	Antimicrobial,
4383	DRAMP04616	Cancrin	Antimicrobial,
4384	DRAMP04617	Hfp1	Antimicrobial,
4385	DRAMP04618	Hfp2	Antimicrobial,
4386	DRAMP04619	Hfp3	Antimicrobial,
4387	DRAMP04620	Hfp4	Antimicrobial,
4388	DRAMP04621	Hainanenin-1	Antimicrobial,
4389	DRAMP04622	Hainanenin-5	Antimicrobial,
4390	DRAMP04623	X. clivii-C1	Antimicrobial,
4391	DRAMP04624	X. clivii-C2	Antimicrobial,
4392	DRAMP04625	X. borealis-B1	Antimicrobial,
4393	DRAMP04626	X. borealis-B2	Antimicrobial,
4394	DRAMP04627	X. borealis-B3	Antimicrobial,
4395	DRAMP04628	X. borealis-B4	Antimicrobial,
4396	DRAMP04629	X. amieti-AM1	Antimicrobial,
4397	DRAMP04630	X. amieti-AM3	Antimicrobial,
4398	DRAMP04631	X. amieti-AM4	Antimicrobial,
4399	DRAMP04632	XPF-C1	Antimicrobial,
4400	DRAMP04633	CPF-C1	Antimicrobial,
4401	DRAMP04634	CPF-C2	Antimicrobial,
4402	DRAMP04635	X. laevis 1	Antimicrobial,
4403	DRAMP04636	X. laevis 2	Antimicrobial,
4404	DRAMP04637	X. laevis 3	Antimicrobial,
4405	DRAMP04638	X. laevis 4	Antimicrobial,
4406	DRAMP04639	Magainin-AN2	Antimicrobial,
4407	DRAMP04641	XPF-AN1	Antimicrobial,
4408	DRAMP04642	Magainin-M1	Antimicrobial,
4409	DRAMP04643	CPF-M1	Antimicrobial,
4410	DRAMP04644	PGLa-MW1	Antimicrobial,

4411	DRAMP04645	CPF-MW1	Antimicrobial,
4412	DRAMP04646	CPF-MW2	Antimicrobial,
4413	DRAMP04647	CPF-L1	Antimicrobial,
4414	DRAMP04648	CPF-L2	Antimicrobial,
4415	DRAMP04649	CPF-P2	Antimicrobial,
4416	DRAMP04650	CPF-P3	Antimicrobial,
4417	DRAMP04651	CPF-P4	Antimicrobial,
4418	DRAMP04652	CPF-P5	Antimicrobial,
4419	DRAMP04653	CPF-PG1	Antimicrobial,
4420	DRAMP04654	Hymenochirin-1B	Antimicrobial,
4421	DRAMP04655	Hymenochirin-2B	Antimicrobial,
4422	DRAMP04656	Hymenochirin-3B	Antimicrobial,
4423	DRAMP04657	Hymenochirin-4B	Antimicrobial,
4424	DRAMP04658	Hymenochirin-5B	Antimicrobial, Anti-Gram+, Anti-Gram-,
4425	DRAMP04659	HPA3NT3	Antimicrobial,
4426	DRAMP04660	HPA3NT3-F1A (HPA3NT3 peptide analogs)	Antimicrobial,
4427	DRAMP04661	HPA3NT3-F8A (HPA3NT3 peptide analogs)	Antimicrobial,
4428	DRAMP04662	HPA3NT3-F1AF8A (HPA3NT3 peptide analogs)	Antimicrobial,
4429	DRAMP04663	HPA3NT3-A1 (HPA3NT3 peptide analogs)	Antimicrobial,
4430	DRAMP04664	HPA3NT3-A2 (HPA3NT3 peptide analogs)	Antimicrobial,
4431	DRAMP04666	LtTx-1a	Antimicrobial,
4432	DRAMP04667	Melt	Antimicrobial,
4433	DRAMP04668	Coprisin	Antimicrobial,
4434	DRAMP04669	Protamine y1	Antimicrobial,
4435	DRAMP18188	Pantinin-1 (Non-disulfide-bridged peptide 4.20, NDBP-4.20, Non-disulfide-bridged peptide 5.21)	Antibacterial, Antifungal, Anti-Gram+, Anti-Gram-, Antimicrobial
4436	DRAMP04673	Alamethicin (ALM; fungi)	Antibacterial, Antifungal, Antiparasitic, Antimicrobial
4437	DRAMP04674	Antiviral protein Y3 (Fungi)	Antiviral, Antimicrobial
4438	DRAMP04675	Antimicrobial defensin peptide DRR230-c	Antifungal, Antimicrobial
4439	DRAMP04678	Defensin-like protein 4	Antibacterial, Antifungal, Antimicrobial
4440	DRAMP04681	Antiviral serine protease	Antiviral, Antimicrobial
4441	DRAMP04682	Lactogenin	Antiviral, Antimicrobial
4442	DRAMP04683	NADPH oxidoreductase	Antiviral, Antimicrobial
4443	DRAMP04684	Bacteriocin BAC-IB17	Antibacterial, Anti-Gram+, Anti-Gram-, Antimicrobial
4444	DRAMP04687	Glucan endo-1,3-beta-glucosidase, basic vacuolar isoform (Plant defensin)	Antibacterial, Antifungal, Antimicrobial
4445	DRAMP04688	Glucan endo-1,3-beta-glucosidase	Antifungal, Antimicrobial
4446	DRAMP04689	Allergen Fra a 1	Antifungal, Antimicrobial
4447	DRAMP04690	Guanine nucleotide-binding protein subunit gamma 1 (Ggamma-subunit 1;Heterotrimeric G protein gamma 1)	Antifungal, Antimicrobial
4448	DRAMP04693	Luffin P1c (one chain of ribosome-inactivating protein luffin P1; Plant defensin)	Antiviral, Toxin, Antimicrobial
4449	DRAMP04698	Amphipathic peptide CT1 (VsCT1; Non-disulfide-bridged peptide 5.11, NDBP-5.11)	Antimicrobial,
4450	DRAMP04699	Amphipathic peptide CT2 (VsCT2; Non-disulfide-bridged peptide 5.12, NDBP-5.12)	Antimicrobial,
4451	DRAMP04700	Basic phospholipase A2 BnpTX-1 (BnPTx-I, svPLA2; Phosphatidylcholine 2-acylhydrolase)	Antibacterial, Antiparasitic, Anti-Gram+, Anti-Gram-, Antimicrobial
4452	DRAMP04701	Phospholipase A2 homolog (BmarPLA2, svPLA2 homolog)	Antibacterial, Antiparasitic, Antimicrobial
4453	DRAMP04702	38 kDa autolysin (Beta-glycosidase; Peptidoglycan hydrolase)	Antimicrobial, Toxin,
4454	DRAMP04703	Crotamine CRO1	Antimicrobial, Toxin,
4455	DRAMP04704	Crotamine CRO3	Antimicrobial, Toxin,
4456	DRAMP04705	Myotoxin-1 (Crotamine-1)	Antimicrobial, Toxin,
4457	DRAMP04706	Myotoxin-1 (Myotoxin I)	Antimicrobial, Toxin,
4458	DRAMP04707	Myotoxin-A (Myotoxin-1)	Antimicrobial, Toxin,
4459	DRAMP04708	Myotoxin-2 (Crotamine-2 Fragment)	Antimicrobial, Toxin,
4460	DRAMP04709	Myotoxin-2 (Myotoxin II)	Antimicrobial, Toxin,
4461	DRAMP04710	Myotoxin-2	Antimicrobial, Toxin,
4462	DRAMP04711	Myotoxin-3 (Crotamine-3)	Antimicrobial, Toxin,

4463	DRAMP04712	Myotoxin-3	Antimicrobial, Toxin,
4464	DRAMP04713	Myotoxin-4 (Crotamine-4 Fragment)	Antimicrobial, Toxin,
4465	DRAMP04714	Crotamine-IV-2	Antimicrobial, Toxin,
4466	DRAMP04715	Crotamine-IV-3	Antimicrobial, Toxin,
4467	DRAMP04716	Myotoxin (Crotamine-4; Toxic peptide C)	Antimicrobial, Toxin,
4468	DRAMP04717	Myotoxin (CAM-toxin)	Antimicrobial, Toxin,
4469	DRAMP04718	Crotamine Ile-19 (CRO_Ile-19)	Antimicrobial, Toxin,
4470	DRAMP18108	Asteropin-A (Asteropine-A)	Antibacterial, Antimicrobial
4471	DRAMP18186	Beta-KTx-like peptide LaIT2	Antibacterial, Antimicrobial
4472	DRAMP18187	Toxin LyeTx 1	Antibacterial, Anti-Gram+, Anti-Gram-, Antimicrobial
4473	DRAMP18128	Antimicrobial peptide HsAp4;	Antimicrobial, Antifungal, Anti-Gram+, Anti-Gram-,
4474	DRAMP18129	Antimicrobial peptide HsAp3;	Antimicrobial, Antifungal, Anti-Gram+, Anti-Gram-,
4475	DRAMP18130	Antimicrobial peptide HsAp2	Antimicrobial, Antifungal, Anti-Gram+, Anti-Gram-,
4476	DRAMP18131	Antimicrobial peptide HsAp1 (HsAp)	Antimicrobial, Antifungal, Anti-Gram+, Anti-Gram-,
4477	DRAMP18132	L-amino-acid oxidase Bfon20 (LAAO; LAO)	Antitumor, Antibacterial, Antiparasitic, Antimicrobial
4478	DRAMP18133	Peptide Ctri9819	Antimicrobial, Antiviral,
4479	DRAMP18134	Peptide Ctri9677	Antimicrobial, Antiviral,
4480	DRAMP18135	Peptide Hp1478 (Non-disulfide-bridged peptide 5; NDBP-5)	Antimicrobial,
4481	DRAMP18136	Peptide Hp1412 (Non-disulfide-bridged peptide 5; NDBP-5)	Antimicrobial,
4482	DRAMP18137	Peptide Hp1239 (Non-disulfide-bridged peptide 5; NDBP-5)	Antibacterial, Antiviral, Antimicrobial
4483	DRAMP18138	Peptide Hp1165 (Non-disulfide-bridged peptide 5; NDBP-5)	Antimicrobial,
4484	DRAMP18139	Peptide Hp1036 (Non-disulfide-bridged peptide 5; NDBP-5)	Antibacterial, Antiviral, Antimicrobial
4485	DRAMP18185	Jingdongin-1	Antibacterial, Antifungal, Anti-Gram+, Anti-Gram-, Antimicrobial
4486	DRAMP01296	Omega-conotoxin-like protein 1 (OCLP1)	Antibacterial, Antimicrobial
4487	DRAMP01297	Longipin (Fragment)	Antifungal, Antimicrobial, Anti-Gram+,
4488	DRAMP02595	Styelin-D (Styelin D; chordates)	Antibacterial, Antimicrobial
4489	DRAMP18184	Beta-defensin 42 (BD-42, mBD-42)	Antibacterial, Antimicrobial
4490	DRAMP18405	PDC213 (beta-casein 213-224, human)	Antibacterial, Antimicrobial
4491	DRAMP18406	CaThi (thionin-like peptide 2; plants)	Antifungal, Antimicrobial
4492	DRAMP18407	Thionin-like peptide 1 (plants)	Antibacterial, Antifungal, Antimicrobial
4493	DRAMP18408	Dm-AMP2 (Dahlia defensin, plants)	Antfungal, Antimicrobial
4494	DRAMP18409	Mastoparan V1 (MP-V1, insects, arthropods, invertebrates, animals)	Antibacterial, antifungal, Antimicrobial
4495	DRAMP18410	Cc-LTP2 (Coffea canephora lipid transfer protein 2, plants)	Antibacterial, antifungal, Antimicrobial
4496	DRAMP18411	Thaulin-1 (frog, amphibians, animals; Other sequences reported: Thaulin-2, Thaulin-3, Thaulin-4)	Antibacterial, Antimicrobial
4497	DRAMP18412	Longipin (arachnids, Chelicerata, arthropods, invertebrates, animals)	Antifungal, Antimicrobial
4498	DRAMP18413	Phylloseptin-PBa (frog, amphibians, animals)	Antibacterial, antifungal, Antimicrobial
4499	DRAMP18414	NDBP-5.8 (scorpions, arachnids, Chelicerata, arthropods, invertebrates, animals)	Antifungal, Antimicrobial
4500	DRAMP18415	Con10 (scorpions, arachnids, Chelicerata, arthropods, invertebrates, animals)	Antifungal, Antimicrobial
4501	DRAMP18416	ToAP3 (scorpions, arachnids, Chelicerata, arthropods, invertebrates, animals)	Antifungal, Antimicrobial
4502	DRAMP18417	ToAP2 (scorpions, arachnids, Chelicerata, arthropods, invertebrates, animals)	Antibacterial, Antibiofilm, Antimicrobial
4503	DRAMP18418	ToAP1 (scorpions, arachnids, Chelicerata, arthropods, invertebrates, animals)	Antifungal, Antibiofilm, Antimicrobial
4504	DRAMP18424	TLN-58 (TLN58; human cathelicidin)	Antibacterial, Antimicrobial
4505	DRAMP18427	MrDN (pellino-1 derived, Crustaceans, arthropods, invertebrates, animals)	Antibacterial, Antimicrobial
4506	DRAMP18428	Saha-CATH6 (cathelicidins; mammals, animals)	Antibacterial, antifungal, Antimicrobial
4507	DRAMP18429	Saha-CATH5 (cathelicidins; mammals, animals)	Antibacterial, antifungal, Antimicrobial
4508	DRAMP18431	Armadillidin Q (Gly-rich; Terrestrial Isopod, Crustaceans, arthropods, invertebrates, animals)	Antibacterial, antifungal, Antimicrobial
4509	DRAMP18432	Antapin (ANTP; insects, arthropods, invertebrates, animals)	Antibacterial, Antimicrobial
4510	DRAMP18433	Cremycin-15 (nematode; invertebrates, animals)	Antibacterial, Anti-Gram+, Antimicrobial
4511	DRAMP18434	Cremycin-5 (nematode; invertebrates, animals)	Antifungal, Antimicrobial

4512	DRAMP18435	Labaditin (plants)	Antibacterial, Antimicrobial
4513	DRAMP18436	AtPDF2.3 (flowering plants)	Antifungal, Antimicrobial
4514	DRAMP18437	Oryctes rhinoceros defensin (insects, arthropods, invertebrate, animals)	Antibacterial, Antimicrobial
4515	DRAMP18438	SB Piscidin 6 (fish, animals)	Antibacterial, antiparasitic, Antimicrobial
4516	DRAMP18439	WB Piscidin 6 (fish, animals)	Antibacterial, antiparasitic, Antimicrobial
4517	DRAMP18440	WB Piscidin 5 (fish, animals)	Antibacterial, antiparasitic, Antimicrobial
4518	DRAMP18441	Panusin (beta defensins; crustaceans, arthropods, invertebrates, animals)	Antibacterial, antifungal, Antimicrobial
4519	DRAMP18442	Smp43 (scorpions, arachnids, Chelicerata, arthropods, invertebrates, animals)	Antibacterial, antifungal, Antimicrobial
4520	DRAMP18443	Smp24 (scorpions, arachnids, Chelicerata, arthropods, invertebrates, animals)	Antibacterial, antifungal, Antimicrobial
4521	DRAMP18444	Lacrain (myriapods, arthropods, invertebrates, animals)	Antibacterial, Antimicrobial
4522	DRAMP18445	hdMolluscidin (mollusca/molluscs/mollusks, invertebrates, animals; Lys-rich; Ala-rich)	Antibacterial, Antimicrobial
4523	DRAMP18446	Gm0026x00785(77	Antibacterial, Antimicrobial
4524	DRAMP18447	Gm0025x00667(75	Antibacterial, Antimicrobial
4525	DRAMP18448	Diapausin-1 (insects, arthropods, invertebrates, animals)	Antifungal, Antimicrobial
4526	DRAMP18449	Kunitzin-OS (amphibians, animals)	Antibacterial, Enzyme inhibitor, Anti-Gram-, Antimicrobial
4527	DRAMP18450	Kunitzin-RE (amphibians, animals)	Antibacterial, Enzyme inhibitor, Anti-Gram-, Antimicrobial
4528	DRAMP18451	CZS-3 (cruzioseptin-3; frog, amphibians, animals)	Antibacterial, antifungal, Antimicrobial
4529	DRAMP18452	CZS-2 (cruzioseptin-2; frog, amphibians, animals)	Antibacterial, antifungal, Antimicrobial
4530	DRAMP18453	CZS-1 (cruzioseptin-1; frog, amphibians, animals)	Antibacterial, antifungal, Antimicrobial
4531	DRAMP18454	Tepmporin-1Ee (frog, amphibians, animals)	Antibacterial, Anti-Gram+, Anti-Gram-, Antimicrobial
4532	DRAMP18455	Tomato Snakin-2 (plants)	Antibacterial, antifungal, Antimicrobial
4533	DRAMP18456	Pepcon (peptide consensus sequence, synthetic)	Antibacterial, Anti-Gram+, Anti-Gram-, Antimicrobial
4534	DRAMP18457	Tet213 (synthetic, Trp-rich, Arg-rich)	Antibacterial, Antimicrobial
4535	DRAMP18458	Melimine (a hybrid peptide of melittin and protamine, synthetic)	Antibacterial, Antimicrobial
4536	DRAMP18459	hLF(1-11) (hLF1-11, first 11 residues, human lactoferrin; synthetic)	Antibacterial, Antimicrobial
4537	DRAMP20762	Halocin S8 (HalS8, Halocin-S8; Microhalocin, Archaeocin, Bacteriocin, Archaea, Euryarchaeota, Prokaryotes)	Antibacterial, Antimicrobial
4538	DRAMP18471	D-LAK60	Antibacterial, Anti-Gram-, Antimicrobial
4539	DRAMP18472	D-LAK80	Antibacterial, Anti-Gram-, Antimicrobial
4540	DRAMP18473	D-LAK100	Antibacterial, Anti-Gram-, Antimicrobial
4541	DRAMP18474	D-LAK140	Antibacterial, Anti-Gram-, Antimicrobial
4542	DRAMP18475	D-LAK160	Antibacterial, Anti-Gram-, Antimicrobial
4543	DRAMP18476	LAK80	Antibacterial, Antiplasmodial, Anti-Gram-, Antimicrobial
4544	DRAMP18477	LAK80-P7	Antibacterial, Antiplasmodial, Anti-Gram-, Antimicrobial
4545	DRAMP18478	LAK80-P10	Antibacterial, Antiplasmodial, Anti-Gram-, Antimicrobial
4546	DRAMP18479	LAK80-P12	Antibacterial, Antiplasmodial, Anti-Gram-, Antimicrobial
4547	DRAMP18480	LAK120	Antibacterial, Antiplasmodial, Anti-Gram-, Antimicrobial
4548	DRAMP18481	LAK120-P7	Antibacterial, Antiplasmodial, Anti-Gram-, Antimicrobial
4549	DRAMP18482	LAK120-P10	Antibacterial, Antiplasmodial, Anti-Gram-, Antimicrobial
4550	DRAMP18483	LAK120-P12	Antibacterial, Antiplasmodial, Anti-Gram-, Antimicrobial
4551	DRAMP18484	LAK160	Antibacterial, Antiplasmodial, Anti-Gram-, Antimicrobial
4552	DRAMP18485	LAK160-P7	Antibacterial, Antiplasmodial, Anti-Gram-, Antimicrobial
4553	DRAMP18486	LAK160-P10	Antibacterial, Antiplasmodial, Anti-Gram-, Antimicrobial
4554	DRAMP18487	LAK160-P12	Antibacterial, Antiplasmodial, Anti-Gram-, Antimicrobial
4555	DRAMP18488	Css54 (Css from the species name below; scorpions, arachnids, Chelicerata, arthropods, invertebrates, animals)	Antibacterial, Cytolysis, Hemolysis, Anti-Gram+, Anti-Gram-, Antimicrobial
4556	DRAMP18489	Cathelicidin-PY (Frog, amphibians, animals; BBL)	Antimicrobial, Anti-inflammatory. , Anti-Gram+,
4557	DRAMP18491	CecropinXJ (Insects, arthropods, invertebrates, animals)	Antibacterial, Anticancer, Anti-Gram+, Antimicrobial
4558	DRAMP18492	cgUbiquitin (Oyster, mollusca/molluscs/mollusks, invertebrates, animals)	Antimicrobial, Anti-Gram+,
4559	DRAMP18493	Tilapia piscidin 3 (TP3; His-rich; fish, animals; inactive: TP1, TP2, and TP5)	Antibacterial, wound healing, immunomodulatory, Anti-Gram+, Antimicrobial
4560	DRAMP18494	Tilapia piscidin 4 (TP4; Oreoch-2; MSP-4; fish, animals)	Antimicrobial, Anticancer, wound healing, Anti-Gram+,

4561	DRAMP02751	Formaecin-1 (Pro-rich; ants, insects, animals)	Antibacterial, Anti-Gram-, Antimicrobial
4562	DRAMP02752	Formaecin-2 (Pro-rich; ants, insects, animals)	Antibacterial, Anti-Gram-, Antimicrobial
4563	DRAMP03097	Drosocin (Glycopeptide,insects, arthropods, invertebrates, animals)	Antibacterial, Antimicrobial
4564	DRAMP03575	LL-37(17-29) (C-terminal fragment of LL-37, LL; Human, mammals, animals)	Antibacterial, Anticancer, Anti-Gram+, Anti-Gram-, Antimicrobial
4565	DRAMP02093	Brevinin-1Bc (Frogs, amphibians, animals)	Antibacterial, Anti-Gram+, Antimicrobial
4566	DRAMP02094	Brevinin-1Bd (Frogs, amphibians, animals)	Antimicrobial, Antibacterial, Antifungal, Anti-Gram+, Anti-Gram-,
4567	DRAMP02095	Brevinin-1Be (Frogs, amphibians, animals)	Antibacterial, Anti-Gram+, Anti-Gram-, Antimicrobial
4568	DRAMP02096	Brevinin-1Bf (Frogs, amphibians, animals)	Antibacterial, Anti-Gram+, Anti-Gram-, Antimicrobial
4569	DRAMP02097	Brevinin-1Pa (Frogs, amphibians, animals)	Antimicrobial, Antibacterial, Antifungal, Anti-Gram+, Anti-Gram-,
4570	DRAMP02098	Brevinin-1Pc (Frogs, amphibians, animals)	Antimicrobial, Antibacterial, Antifungal, Anti-Gram+, Anti-Gram-,
4571	DRAMP02099	Brevinin-1Pd (Frogs, amphibians, animals)	Antimicrobial, Antibacterial, Antifungal, Anti-Gram+, Anti-Gram-,
4572	DRAMP02255	Ranatuerin-2Lb (Ranatuerin 2Lb; Ranaturin-2PRd; Frogs, amphibians, animals)	Antimicronial, Antibacterial, Antifungal, Anti-Gram+, Anti-Gram-, Antimicrobial
4573	DRAMP02254	Ranatuerin-2La (Ranatuerin 2La; Ranaturin-2PRa; Frogs, amphibians, animals)	Antibacterial, Anti-Gram+, Anti-Gram-, Antimicrobial
4574	DRAMP02256	Ranatuerin-2B (Ranatuerin 2B, Frog, amphibians, animals)	Antimicronial, Antibacterial, Antifungal, Anti-Gram+, Anti-Gram-, Antimicrobial
4575	DRAMP02257	Ranatuerin-2P (Ranatuerin 2P; Frogs, amphibians, animals)	Antimicronial, Antibacterial, Antifungal, Anti-Gram+, Anti-Gram-, Antimicrobial
4576	DRAMP18496	rNZ2114	Antibacterial, Anti-Gram+, Antimicrobial
4577	DRAMP18497	TSG-6 (Ixosin-B peptide derivative)	Antibacterial, Anti-Gram+, Anti-Gram-, Antimicrobial
4578	DRAMP18498	TSG-7 (Ixosin-B peptide derivative)	Antibacterial, Anti-Gram+, Anti-Gram-, Antimicrobial
4579	DRAMP18499	TSG-8 (Ixosin-B peptide derivative)	Antibacterial, Anti-Gram+, Anti-Gram-, Antimicrobial
4580	DRAMP18500	TSG-8-1 (Ixosin-B peptide derivative)	Antibacterial, Anti-Gram+, Anti-Gram-, Antimicrobial
4581	DRAMP18501	TSG-9 (Ixosin-B peptide derivative)	Antibacterial, Anti-Gram+, Anti-Gram-, Antimicrobial
4582	DRAMP18502	TSG-10 (Ixosin-B peptide derivative)	Antibacterial, Anti-Gram+, Anti-Gram-, Antimicrobial
4583	DRAMP18503	TSG-11 (Ixosin-B peptide derivative)	Antibacterial, Anti-Gram+, Anti-Gram-, Antimicrobial
4584	DRAMP18504	E(AU)2 (Aurein 1.2 peptide derivative)	Antibacterial, Anti-Gram-, Antimicrobial
4585	DRAMP18505	(AU)2K (Aurein 1.2 peptide derivative)	Antibacterial, Anti-Gram-, Antimicrobial
4586	DRAMP18506	OG2 (Palustrin-OG1 peptide derivative)	Antibacterial, Anti-Gram+, Anti-Gram-, Antimicrobial
4587	DRAMP18508	gp41w-FKA (gp41 peptide derivative)	Antibacterial, Anti-Gram+, Anti-Gram-, Antimicrobial
4588	DRAMP18509	Px-cec1 (cecropin1 peptide derivative)	Antimicrobial, Antibacterial, Anitifungal, Anti-Gram+, Anti-Gram-,
4589	DRAMP18510	D-LAK120	Antibacterial, Antiplasmodial, Anti-Gram-, Antimicrobial
4590	DRAMP18511	D-LAK120-P13	Antibacterial, Antiplasmodial, Anti-Gram-, Antimicrobial
4591	DRAMP18512	D-LAK120-A	Antibacterial, Antiplasmodial, Anti-Gram-, Antimicrobial
4592	DRAMP18513	D-LAK120-AP13	Antibacterial, Antiplasmodial, Anti-Gram-, Antimicrobial
4593	DRAMP18514	D-LAK120-H	Antibacterial, Antiplasmodial, Anti-Gram-, Antimicrobial
4594	DRAMP18515	A12L/A20L (V13KL peptide derivative)	Antibacterial, Anti-Gram-, Antimicrobial
4595	DRAMP18533	V13KL (V681 peptide derivative)	Antibacterial, Anti-Gram+, Anti-Gram-, Antimicrobial
4596	DRAMP18534	A23L (V13KL peptide derivative)	Antibacterial, Anti-Gram-, Antimicrobial
4597	DRAMP18535	A12L (V13KL peptide derivative)	Antibacterial, Anti-Gram-, Antimicrobial
4598	DRAMP18536	A20L (V13KL peptide derivative)	Antibacterial, Anti-Gram-, Antimicrobial
4599	DRAMP18537	A12L/A23L (V13KL peptide derivative)	Antibacterial, Anti-Gram-, Antimicrobial
4600	DRAMP18538	V681	Antibacterial, Anti-Gram+, Anti-Gram-, Antimicrobial
4601	DRAMP18539	V13LL (V681 peptide derivative)	Antibacterial, Anti-Gram+, Anti-Gram-, Antimicrobial
4602	DRAMP18540	V13AL (V681 peptide derivative)	Antibacterial, Anti-Gram+, Anti-Gram-, Antimicrobial
4603	DRAMP18541	V13G (V681 peptide derivative)	Antibacterial, Anti-Gram+, Anti-Gram-, Antimicrobial
4604	DRAMP18542	V13SL (V681 peptide derivative)	Antibacterial, Anti-Gram+, Anti-Gram-, Antimicrobial
4605	DRAMP18543	V13LD (V681 peptide derivative)	Antibacterial, Anti-Gram+, Anti-Gram-, Antimicrobial
4606	DRAMP18544	V13VD (V681 peptide derivative)	Antibacterial, Anti-Gram+, Anti-Gram-, Antimicrobial
4607	DRAMP18545	V13AD (V681 peptide derivative)	Antibacterial, Anti-Gram+, Anti-Gram-, Antimicrobial
4608	DRAMP18546	V13SD (V681 peptide derivative)	Antibacterial, Anti-Gram+, Anti-Gram-, Antimicrobial
4609	DRAMP18547	V13KD (V681 peptide derivative)	Antibacterial, Anti-Gram+, Anti-Gram-, Antimicrobial

4610	DRAMP18548	S11LL (V681 peptide derivative)	Antibacterial, Anti-Gram+, Anti-Gram-, Antimicrobial
4611	DRAMP18549	S11VL (V681 peptide derivative)	Antibacterial, Anti-Gram+, Anti-Gram-, Antimicrobial
4612	DRAMP18550	S11AL (V681 peptide derivative)	Antibacterial, Anti-Gram+, Anti-Gram-, Antimicrobial
4613	DRAMP18551	S11G (V681 peptide derivative)	Antibacterial, Anti-Gram+, Anti-Gram-, Antimicrobial
4614	DRAMP18552	S11KL (V681 peptide derivative)	Antibacterial, Anti-Gram+, Anti-Gram-, Antimicrobial
4615	DRAMP18553	S11LD (V681 peptide derivative)	Antibacterial, Anti-Gram+, Anti-Gram-, Antimicrobial
4616	DRAMP18554	S11VD (V681 peptide derivative)	Antibacterial, Anti-Gram+, Anti-Gram-, Antimicrobial
4617	DRAMP18555	S11AD (V681 peptide derivative)	Antibacterial, Anti-Gram+, Anti-Gram-, Antimicrobial
4618	DRAMP18556	S11SD (V681 peptide derivative)	Antibacterial, Anti-Gram+, Anti-Gram-, Antimicrobial
4619	DRAMP18557	S11KD (V681 peptide derivative)	Antibacterial, Anti-Gram+, Anti-Gram-, Antimicrobial
4620	DRAMP18558	Kn2-7 (BmKn2 peptide derivative)	Antibacterial, Anti-Gram+, Anti-Gram-, Antimicrobial
4621	DRAMP18559	HFU3	Antibacterial, Anti-Gram+, Anti-Gram-, Antimicrobial
4622	DRAMP18560	HFU4	Antibacterial, Anti-Gram+, Anti-Gram-, Antimicrobial
4623	DRAMP18561	HFU5	Antibacterial, Anti-Gram+, Anti-Gram-, Antimicrobial
4624	DRAMP18562	MAP-04-01 (Ixosin-B peptide derivative)	Antibacterial, Anti-Gram+, Anti-Gram-, Antimicrobial
4625	DRAMP18563	MAP-04-02 (Ixosin-B peptide derivative)	Antibacterial, Anti-Gram+, Anti-Gram-, Antimicrobial
4626	DRAMP18564	MAP-04-03 (Ixosin-B peptide derivative)	Antibacterial, Anti-Gram+, Anti-Gram-, Antimicrobial
4627	DRAMP18565	MAP-04-04 (Ixosin-B peptide derivative)	Antibacterial, Anti-Gram+, Anti-Gram-, Antimicrobial
4628	DRAMP18566	LL-IIIs-1 (lasioglossin III peptide derivative)	Antimicrobial, Antibacterial, Anitifungal, Anti-Gram+, Anti-Gram-,
4629	DRAMP18567	LL-IIIs-2 (lasioglossin III peptide derivative)	Antimicrobial, Antibacterial, Anitifungal, Anti-Gram+, Anti-Gram-,
4630	DRAMP18568	LL-IIIs-3 (lasioglossin III peptide derivative)	Antimicrobial, Antibacterial, Anitifungal, Anti-Gram+, Anti-Gram-,
4631	DRAMP18569	LL-IIIs-4 (lasioglossin III peptide derivative)	Antibacterial, Anti-Gram+, Anti-Gram-, Antimicrobial
4632	DRAMP18570	LL-IIIs-5 cis (lasioglossin III peptide derivative)	Antibacterial, Anti-Gram+, Anti-Gram-, Antimicrobial
4633	DRAMP18571	LL-IIIs-5 trans (lasioglossin III peptide derivative)	Antibacterial, Anti-Gram+, Anti-Gram-, Antimicrobial
4634	DRAMP18572	LL-IIIs-6a (lasioglossin III peptide derivative)	Antibacterial, Anti-Gram+, Anti-Gram-, Antimicrobial
4635	DRAMP18573	LL-IIIs-6b (lasioglossin III peptide derivative)	Antibacterial, Anti-Gram+, Anti-Gram-, Antimicrobial
4636	DRAMP18574	MEP-N (melectin peptide derivative)	Antimicrobial, Antibacterial, Anitifungal, Anti-Gram+, Anti-Gram-,
4637	DRAMP18575	MEP-Ns-1 (melectin peptide derivative)	Antimicrobial, Antibacterial, Anitifungal, Anti-Gram+, Anti-Gram-,
4638	DRAMP18576	MEP-Ns-2 (melectin peptide derivative)	Antibacterial, Anti-Gram+, Anti-Gram-, Antimicrobial
4639	DRAMP18577	MEP-Ns-3 (melectin peptide derivative)	Antibacterial, Anti-Gram+, Anti-Gram-, Antimicrobial
4640	DRAMP18578	MEP-Ns-4 cis (melectin peptide derivative)	Antibacterial, Anti-Gram+, Anti-Gram-, Antimicrobial
4641	DRAMP18579	MEP-Ns-4 trans (melectin peptide derivative)	Antibacterial, Anti-Gram+, Anti-Gram-, Antimicrobial
4642	DRAMP18580	MEP-Ns-5 (melectin peptide derivative)	Antibacterial, Anti-Gram+, Anti-Gram-, Antimicrobial
4643	DRAMP18581	MEP-Ns-6 (melectin peptide derivative)	Antibacterial, Anti-Gram+, Anti-Gram-, Antimicrobial
4644	DRAMP18582	CDT (Tachyplesin-1 peptide derivative)	Antibacterial, Antimicrobial
4645	DRAMP18583	Tricystine cyclic cystine TP (ccTP, Tachyplesin-1 peptide derivative)	Antimicrobial, Antibacterial, Anitifungal, Anti-Gram+, Anti-Gram-,
4646	DRAMP18584	[Arg13]ccTP (ccTP peptide derivative)	Antimicrobial, Antibacterial, Anitifungal, Anti-Gram+, Anti-Gram-,
4647	DRAMP18585	[Arg4,8]ccTP (ccTP peptide derivative)	Antimicrobial, Antibacterial, Anitifungal, Anti-Gram+, Anti-Gram-,
4648	DRAMP18586	[Arg4,8,13]ccTP (ccTP peptide derivative)	Antimicrobial, Antibacterial, Anitifungal, Anti-Gram+, Anti-Gram-,
4649	DRAMP18587	[Arg4,8,13][Lys18]ccTP (ccTP peptide derivative)	Antimicrobial, Antibacterial, Anitifungal, Anti-Gram+, Anti-Gram-,
4650	DRAMP18588	RTD	Antimicrobial, Antibacterial, Anitifungal, Anti-Gram+, Anti-Gram-,
4651	DRAMP18589	DSE (Ctx-Ha peptide derivative)	Antimicrobial, Antibacterial, Anitifungal, Anti-Gram+, Anti-Gram-,
4652	DRAMP18590	DEP (Ctx-Ha peptide derivative)	Antimicrobial, Antibacterial, Anitifungal, Anti-Gram+, Anti-Gram-,
4653	DRAMP18591	DEA (Ctx-Ha peptide derivative)	Antimicrobial, Antibacterial, Anitifungal, Anti-Gram+, Anti-Gram-,
4654	DRAMP18592	Ctx(Ile21)-Ha (Ctx-Ha peptide derivative)	Antimicrobial, Antibacterial, Anitifungal, Anti-Gram+, Anti-Gram-,

4655	DRAMP18593	Ctx(Ile21)-Ha-VD16 (Ctx-Ha peptide derivative)	Antimicrobial, Antibacterial, Anitifungal, Anti-Gram+, Anti-Gram-,
4656	DRAMP18594	Ctx(Ile21)-Ha-VD5,16 (Ctx-Ha peptide derivative)	Antimicrobial, Antibacterial, Anitifungal, Anti-Gram+, Anti-Gram-,
4657	DRAMP18595	Ctx(Ile21)-Ha-I9K (Ctx-Ha peptide derivative)	Antimicrobial, Antibacterial, Anitifungal, Anti-Gram-,
4658	DRAMP18596	LL-I/1 (Lasioglossin LL-I peptide derivative)	Antibacterial, Anti-Gram+, Anti-Gram-, Antimicrobial
4659	DRAMP18597	LL-I/2 (Lasioglossin LL-I peptide derivative)	Antibacterial, Anti-Gram+, Anti-Gram-, Antimicrobial
4660	DRAMP18598	LL-I/3 (Lasioglossin LL-I peptide derivative)	Antibacterial, Anti-Gram+, Anti-Gram-, Antimicrobial
4661	DRAMP18599	LL-I/4 (Lasioglossin LL-I peptide derivative)	Antibacterial, Anti-Gram+, Anti-Gram-, Antimicrobial
4662	DRAMP18600	LL-II/1 (Lasioglossin LL-II peptide derivative)	Antibacterial, Anti-Gram+, Anti-Gram-, Antimicrobial
4663	DRAMP18601	LL-II/2 (Lasioglossin LL-II peptide derivative)	Antibacterial, Anti-Gram+, Anti-Gram-, Antimicrobial
4664	DRAMP18602	LL-II/3 (Lasioglossin LL-II peptide derivative)	Antibacterial, Anti-Gram+, Anti-Gram-, Antimicrobial
4665	DRAMP18603	LL-II/4 (Lasioglossin LL-II peptide derivative)	Antibacterial, Anti-Gram+, Anti-Gram-, Antimicrobial
4666	DRAMP18604	LL-III/1 (Lasioglossin LL-III peptide derivative)	Antibacterial, Anti-Gram+, Anti-Gram-, Antimicrobial
4667	DRAMP18605	LL-III/2 (Lasioglossin LL-III peptide derivative)	Antibacterial, Anti-Gram+, Anti-Gram-, Antimicrobial
4668	DRAMP18606	LL-III/3 (Lasioglossin LL-III peptide derivative)	Antibacterial, Anti-Gram+, Anti-Gram-, Antimicrobial
4669	DRAMP18607	LL-III/4 (Lasioglossin LL-III peptide derivative)	Antibacterial, Anti-Gram+, Anti-Gram-, Antimicrobial
4670	DRAMP18608	LL-III/5 (Lasioglossin LL-III peptide derivative)	Antibacterial, Anti-Gram+, Anti-Gram-, Antimicrobial
4671	DRAMP18609	LL-III/6 (Lasioglossin LL-III peptide derivative)	Antibacterial, Anti-Gram+, Anti-Gram-, Antimicrobial
4672	DRAMP18610	LL-III/7 (Lasioglossin LL-III peptide derivative)	Antibacterial, Anti-Gram+, Anti-Gram-, Antimicrobial
4673	DRAMP18611	LL-III/8 (Lasioglossin LL-III peptide derivative)	Antibacterial, Anti-Gram+, Antimicrobial
4674	DRAMP18612	LL-III/10 (Lasioglossin LL-III peptide derivative)	Antibacterial, Anti-Gram+, Antimicrobial
4675	DRAMP18613	TPG (Tritrpticin peptide derivative)	Antimicrobial, Antibacterial, Anitifungal, Anti-Gram+, Anti-Gram-,
4676	DRAMP18627	D4-K9L8W (D-amino acid substitution of K9L8W)	Antibacterial, Anti-Gram+, Anti-Gram-, Antimicrobial
4677	DRAMP18507	SolyC (Plant defensin; tomato, plants)	Antibacterial, Anti-Gram+, Anti-Gram-, Antimicrobial
4678	DRAMP20763	Halocin C8 (HalC8, Halocin-C8; Microhalocins, Archaeocin, Bacteriocin, Archaea, Euryarchaeota, Prokaryotes)	Antibacterial, Antimicrobial
4679	DRAMP20764	Halocin R1 (HalR1, Halocin-R1; Microhalocins, Archaeocin, Bacteriocin, Archaea, Euryarchaeota, Prokaryotes)	Antibacterial, Antimicrobial
4680	DRAMP20765	Halocin A4 (HalA4, Halocin-A4, Halocin U1; Microhalocins, Archaeocin, Bacteriocin, Archaea, Euryarchaeota, Prokaryotes)	Antibacterial, Antimicrobial
4681	DRAMP18614	TPA (Tritrpticin peptide derivative)	Antimicrobial, Antibacterial, Anitifungal, Anti-Gram+, Anti-Gram-,
4682	DRAMP18615	TWF (Tritrpticin peptide derivative)	Antimicrobial, Antibacterial, Anitifungal, Anti-Gram+, Anti-Gram-,
4683	DRAMP18616	[K22,25,27]-SMAP-29 (SMAP-29 peptide derivative)	Antimicrobial, Antibacterial, Anitifungal, Anti-Gram+, Anti-Gram-,
4684	DRAMP18617	[A19]-SMAP-29 (SMAP-29 peptide derivative)	Antimicrobial, Antibacterial, Anitifungal, Anti-Gram+, Anti-Gram-,
4685	DRAMP18618	SMAP-29(1-17) (SMAP-29 peptide derivative)	Antimicrobial, Antibacterial, Anitifungal, Anti-Gram+, Anti-Gram-,
4686	DRAMP18619	[K2,7,13]-SMAP-29(1-17) (SMAP-29 peptide derivative)	Antimicrobial, Antibacterial, Anitifungal, Anti-Gram+, Anti-Gram-,
4687	DRAMP18620	Pep-1-K (Pep-1 peptide derivative)	Antibacterial, Anti-Gram+, Anti-Gram-, Antimicrobial
4688	DRAMP18621	Temporin-PEa (Temporin-PE peptide derivative)	Antimicrobial, Antibacterial, Anitifungal, Anti-Gram+,
4689	DRAMP18622	Temporin-PEb (Temporin-PE peptide derivative)	Antimicrobial, Antibacterial, Anitifungal, Anti-Gram+, Anti-Gram-,
4690	DRAMP18623	[I5,R8] Mastoparan-L ([I5,R8] MP-L;Mastoparan-L peptide derivative)	Antimicrobial, Antibacterial, Anitifungal, Anti-Gram+, Anti-Gram-,
4691	DRAMP18624	K9L8W	Antibacterial, Anti-Gram+, Anti-Gram-, Antimicrobial
4692	DRAMP18625	D3-K9L8W-1 (D-amino acid substitution of K9L8W)	Antibacterial, Anti-Gram+, Anti-Gram-, Antimicrobial
4693	DRAMP18626	D3-K9L8W-2 (D-amino acid substitution of K9L8W)	Antibacterial, Anti-Gram+, Anti-Gram-, Antimicrobial
4694	DRAMP18628	D6-K9L8W (D-amino acid substitution of K9L8W)	Antibacterial, Anti-Gram+, Anti-Gram-, Antimicrobial
4695	DRAMP18629	D9-K9L8W-1 (D-amino acid substitution of K9L8W)	Antibacterial, Anti-Gram+, Anti-Gram-, Antimicrobial
4696	DRAMP18630	D9-K9L8W-2 (D-amino acid substitution of K9L8W)	Antibacterial, Anti-Gram+, Anti-Gram-, Antimicrobial
4697	DRAMP18631	H5(61-90) V1 (Histone H5 peptide derivative)	Antibacterial, Anti-Gram+, Anti-Gram-, Antimicrobial
4698	DRAMP18632	Peptide H5 (71-90) (Histone H5 peptide derivative)	Antibacterial, Anti-Gram-, Antimicrobial
4699	DRAMP18633	H5(61-90) V2 (Histone H5 peptide derivative)	Antibacterial, Anti-Gram-, Antimicrobial
4700	DRAMP18634	H5(61-90) V3 (Histone H5 peptide derivative)	Antibacterial, Anti-Gram+, Anti-Gram-, Antimicrobial

4701	DRAMP18635	NCP-0	Antibacterial, Anti-Gram-, Antimicrobial
4702	DRAMP18636	NCP-3a (CTX-1 peptide derivative)	Antimicrobial, Antibacterial, Antifungal, Anti-Gram+, Anti-Gram-,
4703	DRAMP18637	NCP-3b (CTX-1 peptide derivative)	Antimicrobial, Antibacterial, Antifungal, Anti-Gram+, Anti-Gram-,
4704	DRAMP18638	VT18-LV (VT18 peptide derivative)	Antibacterial, Anti-Gram+, Antimicrobial
4705	DRAMP18639	CTO17 (TO17 peptide derivative)	Antibacterial, Anti-Gram-, Antimicrobial
4706	DRAMP18640	TO19 (TO17 peptide derivative)	Antibacterial, Anti-Gram-, Antimicrobial
4707	DRAMP18641	KCM11	Antibacterial, Anti-Gram+, Anti-Gram-, Antimicrobial
4708	DRAMP18642	KCM12	Antimicrobial, Antibacterial, Antifungal, Anti-Gram+, Anti-Gram-,
4709	DRAMP18643	KCM21	Antimicrobial, Antibacterial, Antifungal, Anti-Gram+, Anti-Gram-,
4710	DRAMP18644	KRS22	Antibacterial, Anti-Gram+, Anti-Gram-, Antimicrobial
4711	DRAMP18645	PAF19	Antifungal, Antimicrobial
4712	DRAMP18646	PAF32	Antifungal, Antimicrobial
4713	DRAMP18647	PAF34	Antimicrobial, Antibacterial, Antifungal, Anti-Gram-,
4714	DRAMP18648	[Pro3,DLeu9]TL(3) (Temporin L peptide derivative)	Antimicrobial, Antibacterial, Antifungal, Anti-Gram+, Anti-Gram-,
4715	DRAMP18649	HLP6 (HLP2 peptide derivative)	Antibacterial, Anti-Gram-, Antimicrobial
4716	DRAMP18650	HLP7 (HLP2 peptide derivative)	Antibacterial, Anti-Gram-, Antimicrobial
4717	DRAMP18651	Cm-p3 (Cm-p1 peptide derivative)	Antifungal, Antimicrobial
4718	DRAMP18652	Cm-p4 (Cm-p1 peptide derivative)	Antifungal, Antimicrobial
4719	DRAMP18653	Cm-p5 (Cm-p1 peptide derivative)	Antifungal, Antimicrobial
4720	DRAMP18654	dPSMα1 (PSMα1 peptide derivative; bacteriocin; staphylococcus aureus, bacteria)	Antibacterial, Anti-Gram+, Antimicrobial
4721	DRAMP18655	dPSMα4 (PSMα4 peptide derivative; bacteriocin; staphylococcus aureus, bacteria)	Antibacterial, Anti-Gram+, Antimicrobial
4722	DRAMP18656	Microcin 7 (Bacteriocin; Escherichia coli, Bacteria)	Antibacterial, Anti-Gram-, Antimicrobial
4723	DRAMP18657	Chitinase (Bacteriocin; Streptomyces violaceusniger, Bacteria)	Antifungal, Antimicrobial
4724	DRAMP18658	Temporin-PE (Edible frogs, amphibians, animals)	Antimicrobial, Antibacterial, Anitifungal, Anticancer, Anti-Gram+, Anti-Gram-,
4725	DRAMP18659	YFGAP-OH(Yellowfin tuna GAPDH-related antimicrobial peptide; fish, animals)	Antibacterial, Anti-Gram+, Anti-Gram-, Antimicrobial
4726	DRAMP18660	YFGAP-NH2(Yellowfin tuna GAPDH-related antimicrobial peptide; fish, animals)	Antimicrobial, Antibacterial, Anitifungal, Anti-Gram+, Anti-Gram-,
4727	DRAMP18661	Ctx-Ha (Frogs, amphibians, animals)	Antimicrobial, Antibacterial, Anitifungal, Anti-Gram+, Anti-Gram-,
4728	DRAMP18662	Brevinin 21 (Brevinin-1E truncated peptide 21; Frogs, amphibians, animals)	Antimicrobial, Antibacterial, Anitifungal, Anti-Gram+, Anti-Gram-,
4729	DRAMP18663	Brevinin 18 (Brevinin-1E truncated peptide 18; Frogs, amphibians, animals)	Antimicrobial, Antibacterial, Anitifungal, Anti-Gram+, Anti-Gram-,
4730	DRAMP18664	Brevinin 15 (Brevinin-1E truncated peptide 15; Frogs, amphibians, animals)	Antimicrobial, Antibacterial, Anitifungal, Anti-Gram+, Anti-Gram-,
4731	DRAMP18665	Mastoparan-L (MP-L; insects, arthropods, invertebrates, animals)	Antibacterial, Anti-Gram+, Anti-Gram-, Antimicrobial
4732	DRAMP18666	P1 (Pilosulin-1 1-20; Ant, insects, arthropods, invertebrates, animals)	Antimicrobial, Antibacterial, Anitifungal, Anti-Gram+, Anti-Gram-,
4733	DRAMP18667	Pep-1	Antibacterial, Anti-Gram+, Anti-Gram-, Antimicrobial
4734	DRAMP18668	AI-hemocidins 1 (Hb-1 truncated peptide)	Antibacterial, Anti-Gram-, Antimicrobial
4735	DRAMP18669	AI-hemocidins 3 (Hb-1 truncated peptide)	Antibacterial, Anti-Gram-, Antimicrobial
4736	DRAMP18670	AI-hemocidins 4 (Hb-1 truncated peptide)	Antibacterial, Anti-Gram+, Antimicrobial
4737	DRAMP18671	TO17 (TFPI-1 C-terminal peptide)	Antibacterial, Anti-Gram+, Anti-Gram-, Antimicrobial
4738	DRAMP18672	Peptide 7 (Mollusca/molluscs/mollusks, invertebrates, animals)	Antibacterial, Anti-Gram+, Anti-Gram-, Antimicrobial
4739	DRAMP18673	Peptide 3 (Mollusca/molluscs/mollusks, invertebrates, animals)	Antibacterial, Anti-Gram+, Anti-Gram-, Antimicrobial
4740	DRAMP18674	Peptide 2 (Mollusca/molluscs/mollusks, invertebrates, animals)	Antibacterial, Anti-Gram+, Anti-Gram-, Antimicrobial
4741	DRAMP18675	Peptide 4 (Mollusca/molluscs/mollusks, invertebrates, animals)	Antibacterial, Anti-Gram+, Anti-Gram-, Antimicrobial
4742	DRAMP18676	Peptide 5 (Mollusca/molluscs/mollusks, invertebrates, animals)	Antibacterial, Anti-Gram+, Anti-Gram-, Antimicrobial
4743	DRAMP18677	Peptide 6 (Mollusca/molluscs/mollusks, invertebrates, animals)	Antibacterial, Anti-Gram+, Anti-Gram-, Antimicrobial
4744	DRAMP18678	Peptide 8 (Mollusca/molluscs/mollusks, invertebrates, animals)	Antibacterial, Anti-Gram+, Anti-Gram-, Antimicrobial
4745	DRAMP18679	Peptide 9 (Mollusca/molluscs/mollusks, invertebrates, animals)	Antibacterial, Anti-Gram+, Anti-Gram-, Antimicrobial

4746	DRAMP18680	Ctry2146 (Scorpions, animals)	Antiviral, Antimicrobial
4747	DRAMP18681	Ctry2346 (Scorpions, animals)	Antiviral, Antimicrobial
4748	DRAMP18682	Ctry2606 (Scorpions, animals)	Antiviral, Antimicrobial
4749	DRAMP18683	Ctri9194 (Scorpions, animals)	Antiviral, Antimicrobial
4750	DRAMP18684	Ctri9293 (Scorpions, animals)	Antiviral, Antimicrobial
4751	DRAMP18685	Ctri9594 (Scorpions, animals)	Antiviral, Antimicrobial
4752	DRAMP18686	Ctri9610 (Scorpions, animals)	Antiviral, Antimicrobial
4753	DRAMP18687	Ctri10033 (Scorpions, animals)	Antiviral, Antimicrobial
4754	DRAMP18688	Ctri10036 (Scorpions, animals)	Antiviral, Antimicrobial
4755	DRAMP18689	Ctri10261 (Scorpions, animals)	Antiviral, Antimicrobial
4756	DRAMP18690	Hyposin-HA1 (Frogs, amphibians, animals)	Antimicrobial,
4757	DRAMP18691	Hyposin-HA2 (Frogs, amphibians, animals)	Antimicrobial,
4758	DRAMP18692	Hyposin-HA5 (Frogs, amphibians, animals)	Antimicrobial,
4759	DRAMP18693	Substance P (Mammals, animals)	Antimicrobial, Antibacterial, Antifungal, Anti-Gram+, Anti-Gram-,
4760	DRAMP18694	substance P antagonist (Mammals, animals)	Antimicrobial, Antibacterial, Antifungal, Anti-Gram+, Anti-Gram-,
4761	DRAMP18695	Venom peptide 1 (Scorpions, animals)	Antibacterial, Antimicrobial
4762	DRAMP18696	HLP1 (Lactotransferrin truncated peptide)	Antibacterial, Anti-Gram+, Anti-Gram-, Antimicrobial
4763	DRAMP18697	HLP2 (Lactotransferrin truncated peptide)	Antibacterial, Anti-Gram+, Anti-Gram-, Antimicrobial
4764	DRAMP18698	Histone H2A (Trouts, fish, animals)	Antimicrobial, Antibacterial, Antifungal, Anti-Gram+,
4765	DRAMP18699	Pleurain-B1 (Frogs, amphibians, animals)	Antimicrobial, Antibacterial, Antifungal, Anti-Gram+, Anti-Gram-,
4766	DRAMP18700	Pleurain-C1 (Frogs, amphibians, animals)	Antimicrobial, Antibacterial, Antifungal, Anti-Gram+, Anti-Gram-,
4767	DRAMP18701	Pleurain-D4 (Frogs, amphibians, animals)	Antimicrobial, Antibacterial, Antifungal, Anti-Gram+, Anti-Gram-,
4768	DRAMP18702	Pleurain-E1 (Frogs, amphibians, animals)	Antimicrobial, Antibacterial, Antifungal, Anti-Gram+, Anti-Gram-,
4769	DRAMP18703	Pleurain-G1 (Frogs, amphibians, animals)	Antimicrobial, Antibacterial, Antifungal, Anti-Gram+, Anti-Gram-,
4770	DRAMP18704	Pleurain-J1 (Frogs, amphibians, animals)	Antimicrobial, Antibacterial, Antifungal, Anti-Gram+, Anti-Gram-,
4771	DRAMP18705	Pleurain-N1 (Frogs, amphibians, animals)	Antimicrobial, Antibacterial, Antifungal, Anti-Gram+, Anti-Gram-,
4772	DRAMP18706	Pleurain-R1 (Frogs, amphibians, animals)	Antimicrobial, Antibacterial, Antifungal, Anti-Gram+, Anti-Gram-,
4773	DRAMP18707	BACTENECIN 7 (bac 7, Pro-rich; bovine cathelicidin, cattle, ruminant, mammals, animals)	Antibacterial, Anti-Gram+, Anti-Gram-, Antimicrobial
4774	DRAMP18708	Dermaseptin-S4 (DRS-S4, DS4; frog, amphibians, animals)	Antimicrobial, Antibacterial, Antifungal, Anti-Gram+, Anti-Gram-,
4775	DRAMP18709	Mastoparan (MP; insects, arthropods, invertebrates, animals)	Antibacterial, Cell degranulating, Antimicrobial
4776	DRAMP18710	Royalisin (Insects, arthropods, invertebrates, animals)	Antibacterial, Anti-Gram+, Antimicrobial
4777	DRAMP18711	Chicken Heterophil Peptide 2 (CHP-2, avian beta-defensin, birds, animals)	Antimicrobial, Antibacterial, Antifungal,
4778	DRAMP18712	Styelin A (Tunicate, invertebrates, animals)	Antibacterial, Anti-Gram+, Anti-Gram-, Antimicrobial
4779	DRAMP18713	Styelin B (Tunicate, invertebrates, animals)	Antibacterial, Anti-Gram+, Anti-Gram-, Antimicrobial
4780	DRAMP18714	Cathepsin G(1-5) (Human, primates, mammals, animals)	Antibacterial, Antimicrobial
4781	DRAMP18715	Cathepsin G(77-83) (Human, primates, mammals, animals)	Antibacterial, Antimicrobial
4782	DRAMP18716	Histone H2B-1(HLP-1) (fish, animals)	Antibacterial, Anti-Gram-, Antimicrobial
4783	DRAMP18717	Charybdotoxin (Yellow scorpions, arachnids, Chelicerata, arthropods, invertebrates, animals)	Antimicrobial, Antibacterial, Antifungal, Anti-Gram+, Anti-Gram-,
4784	DRAMP18718	SAAP fraction 2 (Surfactant-associated anionic peptides; Asp-rich; sheep, ruminant, mammals, animals)	Antibacterial, Anti-Gram-, Antimicrobial
4785	DRAMP18719	SAAP fraction 3 (Surfactant-associated anionic peptides; Asp-rich; sheep, ruminant, mammals, animals)	Antibacterial, Anti-Gram+, Anti-Gram-, Antimicrobial
4786	DRAMP18720	SAAP fraction 6 (Surfactant-associated anionic peptides; Asp-rich; sheep, ruminant, mammals, animals)	Antibacterial, Anti-Gram-, Antimicrobial
4787	DRAMP18721	Hinnavin I (Hin I; insects, arthropods, invertebrates, animals)	Antimicrobial, Antibacterial, Antifungal, Anti-Gram+, Anti-Gram-,
4788	DRAMP18722	MA (Magainin 2 (9-21) truncated peptide)	Antibacterial, Anti-Gram-, Antimicrobial

4789	DRAMP18723	CE Cecropin A	Antibacterial, Anti-Gram-, Antimicrobial
4790	DRAMP18724	Oncorhyncin III (Oncorhyncin-3, histone-derived; fish, animals)	Antibacterial, Anti-Gram+, Anti-Gram-, Antimicrobial
4791	DRAMP03024	Mastoparan B (MP-B; insects, arthropods, invertebrates, animals)	Antimicrobial, Antibacterial, Anti-Gram+, Anti-Gram-, Anti Mammalian cells, Anti-cancer
4792	DRAMP00056	Bacteriocin ancovenin	Antimicrobial
4793	DRAMP00057	Bacteriocin duramycin (Leucopeptin; Bacteriocin)	Antimicrobial, Antibacterial
4794	DRAMP00058	Lantibiotic duramycin B (Bacteriocin)	Antimicrobial, Antibacterial
4795	DRAMP00121	Enterocin SE-K4	Antimicrobial
4796	DRAMP00122	Avicin A	Antimicrobial
4797	DRAMP00123	Divercin V41 (Bacteriocin)	Antimicrobial
4798	DRAMP00124	Coagulin (Bacteriocin)	Antimicrobial
4799	DRAMP00125	Bifidocin B (Bacteriocin)	Antimicrobial
4800	DRAMP00241	Brevicin 27 (Bacteriocin)	Antimicrobial
4801	DRAMP00256	Divergicin 750 (Bacteriocin)	Antimicrobial
4802	DRAMP00257	Bacteriocin	Antimicrobial
4803	DRAMP00258	Lacticin Z (Bacteriocin)	Antimicrobial
4804	DRAMP00259	Bacteriocin boticin B	Antimicrobial
4805	DRAMP00260	Bacteriocin (Class IIa sec-dependent bacteriocin)	Antimicrobial
4806	DRAMP00263	Bacteriocin cerein 7B	Antimicrobial
4807	DRAMP00360	Non-specific lipid-transfer protein 1 (LTP 1; Plants)	Antimicrobial, Antifungal
4808	DRAMP00361	Non-specific lipid-transfer protein (LTP; Harmalin; Plants)	Antimicrobial, Antifungal, Antiviral, Anti-cancer
4809	DRAMP00394	Antimicrobial peptide1 (Plant defensin)	Antimicrobial
4810	DRAMP00771	Psyle A (Cyclotides; Plants)	Antimicrobial, Anti-HIV, Anti-cancer
4811	DRAMP18197	Diapausin-1	Antimicrobial, Antifungal
4812	DRAMP00951	Viscotoxin A (Plants)	Antimicrobial
4813	DRAMP01021	Antimicrobial peptide 1a (LAMP-1a; Plant defensin)	Antimicrobial
4814	DRAMP01379	Odorranain-I1 (OdI1; Frogs, amphibians, animals)	Antimicrobial
4815	DRAMP01382	Odorranain-L1 (OdL1; Frogs, amphibians, animals)	Antimicrobial
4816	DRAMP01385	Odorranain-O1 (OdO1; Frogs, amphibians, animals)	Antimicrobial
4817	DRAMP01624	Bombinin-H6 (bombinin H isomers; Frogs, amphibians, animals)	Antimicrobial, Antibacterial
4818	DRAMP01826	RV-23 (Frogs, amphibians, animals)	Antimicrobial, Antibacterial, Anti-Gram+, Anti-Gram-
4819	DRAMP02842	LfcinB(20-25)	Antimicrobial, Antibacterial, Anti-Gram+
4820	DRAMP03025	Mastoparan M	Antimicrobial, Antibacterial, Anti-gram+, Anti-gram-
4821	DRAMP18200	SB Piscidin 6 (fish, animals)	Antimicrobial, Antibacterial, Anti-Gram+, Antiparasitic
4822	DRAMP18201	WB Piscidin 6 (fish, animals)	Antimicrobial, Antibacterial, Anti-Gram+, Antiparasitic
4823	DRAMP18199	Formicin (Bacteriocin;lantibiotic;Gram-positive bacteria, prokaryotes; U	Antimicrobial, Antibacterial, Anti-Gram+
4824	DRAMP18198	Chaxapeptin	Antimicrobial, Antibacterial, Anti-Gram+, Anti-cancer
4825	DRAMP03812	Pardaxin P-4 (Pardaxin P1a; Pardaxin Pa4)	Antimicrobial, Antibacterial, Anti-Gram+, Anti-Gram-, Cytotoxic
4826	DRAMP03010	Bombolitin III (insects, arthropods, invertebrates, animals)	Antimicrobial, Antibacterial, Anti Mammalian Cells
4827	DRAMP03009	Bombolitin II (insects, arthropods, invertebrates, animals)	Antimicrobial, Antibacterial, Anti Mammalian Cells
4828	DRAMP03008	Bombolitin I (insects, arthropods, invertebrates, animals)	Antimicrobial, Antibacterial, Anti Mammalian Cells
4829	DRAMP18423	HEdefensin (arachnids, Chelicerata, arthropods, invertebrates, animals)	Antimicrobial, Antiviral
4830	DRAMP18430	Saha-CATH3 (cathelicidins; mammals, animals)	Antimicrobial, Antifungal
4831	DRAMP03011	Bombolitin IV (insects, arthropods, invertebrates, animals)	Antimicrobial, Antibacterial, Anti Mammalian Cells
4832	DRAMP03012	Bombolitin V (insects, arthropods, invertebrates, animals)	Antimicrobial, Antibacterial, Anti Mammalian Cells
4833	DRAMP18516	K7D (A12L/A20L peptide derivative)	Antimicrobial, Antibacterial, Anti-Gram-, Antiplasmodial
4834	DRAMP02518	NA-CATH	Antimicrobial, Antibacterial, Anti-Gram-
4835	DRAMP20766	Pore-forming peptide ameobapore A(Amoeba peptide, AP-A; Parasite, amoebozoa, protozoa, protists)	Antimicrobial, Antibacterial, Anti-Gram+, Antibiotic
4836	DRAMP20767	Pore-forming peptide ameobapore B (Parasite, amoebozoa, protozoa, protists)	Antimicrobial, Antibacterial, Anti-Gram+, Antibiotic
4837	DRAMP20768	Pore-forming peptide ameobapore C (EH-APP; Parasite, amoebozoa, protozoa, protists)	Antimicrobial, Antibacterial, Anti-Gram+, Antibiotic
4838	DRAMP20769	Naegleriapore A (NP-A; parasite, amoebozoa; protozoa, protists)	Antimicrobial, Antibacterial, Anti-Gram+
4839	DRAMP20771	Acanthaporin (parasite, amoebozoa, protozoa, protists)	Antimicrobial, Antibacterial, Anti-Gram+, Anti-Gram-

4840	DRAMP20772	cPcAMP1/26 (ciliate, Protists)	Antimicrobial, Antibacterial, Anti-Gram+, Anti-Gram-
4841	DRAMP20775	Ctry2459-WT (Ctry2459, scorpions,animals)	Antimicrobial, Antiviral
4842	DRAMP20776	HaA4 (beetles,insects,animals)	Antimicrobial, Antibacterial, Anti-Gram+, Anti-Gram-
4843	DRAMP20777	Cath-BF	Antimicrobial, Antibacterial, Anti-Gram+, Anti-Gram-
4844	DRAMP20778	Temporin-SHf (frogs,amphibians,animals)	Antimicrobial, Antibacterial, Anti-Gram+, Anti-Gram-, Antifungal
4845	DRAMP20779	Halictine 1 (bees,insects,animals)	Antimicrobial, Antibacterial, Anti-Gram+, Anti-Gram-
4846	DRAMP20780	Halictine 2 (bees,insects,animals)	Antimicrobial, Antibacterial, Anti-Gram+, Anti-Gram-
4847	DRAMP20781	Panurgine 1(bees,insects,animals)	Antimicrobial, Antibacterial, Anti-Gram+, Anti-Gram-
4848	DRAMP20782	Pleurain-D1 (Frogs,amphibians,animals)	Antimicrobial, Antibacterial, Anti-Gram+, Anti-Gram-
4849	DRAMP20783	Pleurain-M1 (Frogs,amphibians,animals)	Antimicrobial, Antibacterial, Anti-Gram+, Anti-Gram-
4850	DRAMP20784	Megin 1	Antimicrobial, Antibacterial, Anti-Gram+, Anti-Gram-, Antifungal
4851	DRAMP20785	Megin 2	Antimicrobial, Antibacterial, Anti-Gram+, Anti-Gram-, Antifungal
4852	DRAMP20786	mini-ChBac7.5N alpha	Antimicrobial, Antibacterial, Anti-Gram+, Anti-Gram-, Antifungal
4853	DRAMP20787	mini-ChBac7.5N beta	Antimicrobial, Antibacterial, Anti-Gram+, Anti-Gram-, Antifungal
4854	DRAMP20788	Bovine neutrophil Beta-defensin 3 (BNBD-3; cattle, ruminant, mammals; animals)	Antimicrobial, Antibacterial
4855	DRAMP20789	Bovine neutrophil Beta-defensin 12 (BNBD-12; cattle, ruminant, mammals; animals)	Antimicrobial, Antibacterial
4856	DRAMP20790	Cecropin B (Insects, arthropods, invertebrates, animals)	Antimicrobial, Antibacterial, Anti-Gram+, Anti-Gram-
4857	DRAMP20791	Cecropin A1 (insects, arthropods, invertebrates, animals)	Antimicrobial, Antibacterial
4858	DRAMP20792	Maculatin 1.1 (Frog, amphibians, animals)	Antimicrobial, Antibacterial, Anti-Gram+, Anti-Gram-
4859	DRAMP20793	Maculatin 1.2 (Frog, amphibians, animals)	Antimicrobial, Antibacterial, Anti-Gram+
4860	DRAMP20794	Turkey Heterophil Peptide 2 (THP-2; avian beta-defensin, birds, animals)	Antimicrobial, Antibacterial
4861	DRAMP20795	So-D1 (S. oleracea defensin 1; spinach defensins, plants)	Antimicrobial, Antibacterial
4862	DRAMP20796	So-D6 (S. oleracea defensin 6, spinach defensins; plants)	Antimicrobial, Antibacterial
4863	DRAMP20797	Uperin 3.6 (Toad, amphibians, animals)	Antimicrobial, Antibacterial, Anti-Gram+, Anti-Gram-
4864	DRAMP20798	Lingual antimicrobial peptide (LAP, beta defensin, cattle, ruminant, animals)	Antimicrobial, Antibacterial, Anti-Gram+, Anti-Gram-, Antifungal
4865	DRAMP20799	XT-2 (frog, amphibians, animals)	Antimicrobial, Antibacterial, Anti-Gram-
4866	DRAMP20800	the K4 peptide (synthetic; Phe-rich >25%)	Antimicrobial, Antibacterial, Anti-Gram-
4867	DRAMP20801	Mussel Defensin MGD-1 (Mediterranean mussel defensin 1; mollusca/molluscs/mollusks, invertebrates, animals)	Antimicrobial, Antibacterial, Anti-Gram+, Anti-Gram-
4868	DRAMP20802	CPF-AM1 (caerulein precursor fragment-AM1, frogs, amphibians, animals)	Antimicrobial, Antibacterial, Anti-Gram+, Anti-Gram-
4869	DRAMP20803	moronecidin-like peptide	Antimicrobial, Antibacterial, Anti-Gram+, Anti-Gram-, Antifungal
4870	DRAMP20804	AI-hemocidins 2 (Hb-1 truncated peptide)	Antimicrobial, Antibacterial, Anti-Gram+, Anti-Gram-
4871	DRAMP20805	Apo5 APOC164-88	Antimicrobial, Antibacterial, Anti-Gram+, Anti-Gram-
4872	DRAMP20806	Apo6 APOC167-88	Antimicrobial, Antibacterial, Anti-Gram+, Anti-Gram-
4873	DRAMP20807	A1P394-428	Antimicrobial, Antibacterial, Anti-Gram+, Anti-Gram-
4874	DRAMP20808	RI21 (PMAP-36 peptide derivative)	Antimicrobial, Antibacterial, Anti-Gram+, Anti-Gram-, Antifungal
4875	DRAMP20809	RI18 (PMAP-36 peptide derivative)	Antimicrobial, Antibacterial, Anti-Gram+, Anti-Gram-, Antifungal
4876	DRAMP20810	TI15 (PMAP-36 peptide derivative)	Antimicrobial, Antibacterial, Anti-Gram+, Anti-Gram-, Antifungal
4877	DRAMP20811	RI12 (PMAP-36 peptide derivative)	Antimicrobial, Antibacterial, Anti-Gram+, Anti-Gram-, Antifungal
4878	DRAMP20812	K8	Antimicrobial, Antibacterial, Anti-Gram+, Anti-Gram-
4879	DRAMP20813	L1K8	Antimicrobial, Antibacterial, Anti-Gram+, Anti-Gram-
4880	DRAMP20814	S1K8	Antimicrobial, Antibacterial, Anti-Gram+, Anti-Gram-
4881	DRAMP20815	F1K8	Antimicrobial, Antibacterial, Anti-Gram+, Anti-Gram-
4882	DRAMP20816	K1K8	Antimicrobial, Antibacterial, Anti-Gram+, Anti-Gram-
4883	DRAMP20817	RR12	Antimicrobial, Antibacterial, Anti-Gram+, Anti-Gram-
4884	DRAMP20818	RR12Wpolar	Antimicrobial, Antibacterial, Anti-Gram+, Anti-Gram-

4885	DRAMP20819	RR12Whydro	Antimicrobial, Antibacterial, Anti-Gram+, Anti-Gram-
4886	DRAMP20820	FV7	Antimicrobial, Antibacterial, Anti-Gram+, Anti-Gram-
4887	DRAMP20821	FV-LL (FV7 and LL(LL-37,(17-29)) hybrid peptide)	Antimicrobial, Antibacterial, Anti-Gram+, Anti-Gram-
4888	DRAMP20822	FV-MA (FV7 and MA(Magainin 2 (9-21)) hybrid peptide)	Antimicrobial, Antibacterial, Anti-Gram+, Anti-Gram-
4889	DRAMP20823	FV-CE (FV7 and CE(Cecropin A (1	Antimicrobial, Antibacterial, Anti-Gram+, Anti-Gram-
4890	DRAMP20824	AM-CATH36	Antimicrobial, Antibacterial, Anti-Gram+, Anti-Gram-
4891	DRAMP20825	AM-CATH28	Antimicrobial, Antibacterial, Anti-Gram-
4892	DRAMP20826	AM-CATH21	Antimicrobial, Antibacterial, Anti-Gram+, Anti-Gram-
4893	DRAMP20827	TB_L1FK	Antimicrobial, Antibacterial, Anti-Gram+, Anti-Gram-
4894	DRAMP20828	TB_KKG6A	Antimicrobial, Antibacterial, Anti-Gram+, Anti-Gram-
4895	DRAMP20831	IsCT1L1	Antimicrobial, Antibacterial, Anti-Gram+, Anti-Gram-
4896	DRAMP20832	Polybia-MP1S-D8N	Antimicrobial, Antibacterial, Anti-Gram+, Anti-Gram-
4897	DRAMP20833	[Pro3,DLeu9]TL(1) (Temporin L peptide derivative)	Antimicrobial, Antibacterial, Anti-Gram+, Anti-Gram-
4898	DRAMP20834	PLS	Antimicrobial, Antibacterial, Anti-Gram+, Anti-Gram-
4899	DRAMP20837	Pb-CATH1 Python bivittatus antimicrobial peptides peptide derivative	Antimicrobial, Antibacterial, Anti-Gram+, Anti-Gram-
4900	DRAMP20838	Pb-CATH4 bivittatus antimicrobial peptides peptide derivative	Antimicrobial, Antibacterial, Anti-Gram+, Anti-Gram-
4901	DRAMP20839	Xylopin	Antimicrobial, Antibacterial, Anti-Gram+, Anti-Gram-
4902	DRAMP20841	C1b	Antimicrobial, Antibacterial, Anti-Gram+, Anti-Gram-
4903	DRAMP20842	C1b(1-11)	Antimicrobial, Antibacterial, Anti-Gram+, Anti-Gram-
4904	DRAMP20843	C1b(1-13)	Antimicrobial, Antibacterial, Anti-Gram+, Anti-Gram-
4905	DRAMP20844	C1b(3-13)	Antimicrobial, Antibacterial, Anti-Gram+, Anti-Gram-
4906	DRAMP20845	C1b(3-11)	Antimicrobial, Antibacterial, Anti-Gram+, Anti-Gram-
4907	DRAMP20846	C1b(3-12)	Antimicrobial, Antibacterial, Anti-Gram+, Anti-Gram-
4908	DRAMP20847	C1b(4-13)	Antimicrobial, Antibacterial, Anti-Gram+, Anti-Gram-
4909	DRAMP20848	[K4]C1b(3-11)	Antimicrobial, Antibacterial, Anti-Gram+, Anti-Gram-
4910	DRAMP20849	[R4]C1b(3-11)	Antimicrobial, Antibacterial, Anti-Gram+, Anti-Gram-
4911	DRAMP20850	[K4,K10]C1b(3-13)	Antimicrobial, Antibacterial, Anti-Gram+, Anti-Gram-
4912	DRAMP20851	[R4,R10]C1b(3-13)	Antimicrobial, Antibacterial, Anti-Gram+, Anti-Gram-
4920	DRAMP20859	TT(1-24)	Antimicrobial, Antibacterial, Anti-Gram+, Anti-Gram-
4921	DRAMP20860	TT(1-35)	Antimicrobial, Antibacterial, Anti-Gram+, Anti-Gram-
4923	DRAMP20862	rtCATH2(5-40)	Antimicrobial, Antibacterial, Anti-Gram+, Anti-Gram-
4924	DRAMP20863	rtCATH2(1-40)	Antimicrobial, Antibacterial, Anti-Gram+, Anti-Gram-
4925	DRAMP20864	SF(18-45)	Antimicrobial, Antibacterial, Anti-Gram+, Anti-Gram-
4926	DRAMP20865	the dimeric RRWQWR motif peptide molecule	Antimicrobial, Antibacterial, Anti-Gram+, Anti-Gram-
4927	DRAMP20866	the tetrameric RRWQWR motif peptide molecule	Antimicrobial, Antibacterial, Anti-Gram+, Anti-Gram-
4928	DRAMP20867	the palindromic RRWQWR motif peptide molecule	Antimicrobial, Antibacterial, Anti-Gram+, Anti-Gram-
4929	DRAMP20868	H4	Antimicrobial, Antibacterial, Anti-Gram+, Anti-Gram-
4930	DRAMP20869	Pal-ano-9 (Pal-anoplin peptide derivative)	Antimicrobial, Antibacterial, Anti-Gram+, Anti-Gram-, Antifungal
4931	DRAMP20870	Pal-ano-8 (Pal-anoplin peptide derivative)	Antimicrobial, Antibacterial, Anti-Gram+, Anti-Gram-, Antifungal
4932	DRAMP20871	Pal-ano-7 (Pal-anoplin peptide derivative)	Antimicrobial, Antibacterial, Anti-Gram+, Anti-Gram-, Antifungal
4933	DRAMP20872	Pal-ano-6 (Pal-anoplin peptide derivative)	Antimicrobial, Antibacterial, Anti-Gram+, Anti-Gram-, Antifungal
4934	DRAMP20873	Pal-ano-5 (Pal-anoplin peptide derivative)	Antimicrobial, Antibacterial, Anti-Gram+, Anti-Gram-, Antifungal
4935	DRAMP20874	Chensinin-1b	Antimicrobial, Antibacterial, Anti-Gram+, Anti-Gram-
4936	DRAMP20875	OA-C1b	Antimicrobial, Antibacterial, Anti-Gram+, Anti-Gram-
4937	DRAMP20876	LA-C1b	Antimicrobial, Antibacterial, Anti-Gram+, Anti-Gram-
4938	DRAMP20877	PA-C1b	Antimicrobial, Antibacterial, Anti-Gram+, Anti-Gram-
4939	DRAMP20878	rVpDef	Antimicrobial, Antibacterial, Anti-Gram+, Anti-Gram-
4940	DRAMP20879	DAN1	Antimicrobial, Antibacterial, Anti-Gram+, Anti-Gram-
4941	DRAMP20880	DAN2	Antimicrobial, Antibacterial, Anti-Gram+, Anti-Gram-, Antifungal
4942	DRAMP20881	HOLO1	Antimicrobial, Antibacterial, Anti-Gram+, Antifungal
4943	DRAMP20882	LOUDEF1	Antimicrobial, Antibacterial, Anti-Gram+, Antifungal
4944	DRAMP20883	Cath-A	Antimicrobial, Antibacterial, Anti-Gram+, Anti-Gram-

4945	DRAMP20884	Cath-B	Antimicrobial, Antibacterial, Anti-Gram+, Anti-Gram-
4946	DRAMP20885	Hp1404-T1a	Antimicrobial, Antibacterial, Anti-Gram-
4947	DRAMP20886	Hp1404-T1b	Antimicrobial, Antibacterial, Anti-Gram-
4948	DRAMP20887	NCP-2 (CTX-1 peptide derivative)	Antimicrobial, Antibacterial, Anti-Gram+, Anti-Gram-, Antifungal
4949	DRAMP20888	NCP-3 (CTX-1 peptide derivative)	Antimicrobial, Antibacterial, Anti-Gram+, Anti-Gram-, Antifungal
4950	DRAMP20889	VT18-KKLV (VT18 peptide derivative)	Antimicrobial, Antibacterial, Anti-Gram+
4951	DRAMP20890	VT18-CAKKLV (VT18 peptide derivative)	Antimicrobial, Antibacterial, Anti-Gram+
4952	DRAMP20891	cVT18-CAKKLV (VT18 peptide derivative)	Antimicrobial, Antibacterial, Anti-Gram+
4953	DRAMP20892	H3A/H4A	Antimicrobial, Antibacterial, Anti-Gram+, Antifungal
4954	DRAMP20893	I16A	Antimicrobial, Antibacterial, Anti-Gram+, Anti-Gram-, Antifungal
4955	DRAMP20894	L19H/I20H	Antimicrobial, Antibacterial, Anti-Gram+, Anti-Gram-, Antifungal
4956	DRAMP20895	F1A/I2A	Antimicrobial, Antibacterial, Anti-Gram+, Anti-Gram-, Antifungal
4957	DRAMP20896	L9A/F10A	Antimicrobial, Antifungal
4958	DRAMP20897	I16R	Antimicrobial, Antifungal
4959	DRAMP20898	I16E	Antimicrobial, Antifungal
4960	DRAMP20899	I5A/I6A	Antimicrobial, Antibacterial, Anti-Gram-, Antifungal
4961	DRAMP20900	A12I/A15I	Antimicrobial, Antibacterial, Anti-Gram+, Anti-Gram-, Antifungal
4962	DRAMP20901	A12V/A15H	Antimicrobial, Antibacterial, Anti-Gram+, Anti-Gram-, Antifungal
4963	DRAMP20902	VHSH	Antimicrobial, Antibacterial, Anti-Gram+, Antifungal
4964	DRAMP20903	dC2	Antimicrobial, Antibacterial, Anti-Gram+, Anti-Gram-, Antifungal
4965	DRAMP20904	R18S/R21H	Antimicrobial, Antibacterial, Anti-Gram+, Anti-Gram-, Antifungal
4966	DRAMP20905	dC4	Antimicrobial, Antibacterial, Anti-Gram+, Antifungal
4967	DRAMP20906	dN2	Antimicrobial, Antibacterial, Anti-Gram+, Anti-Gram-, Antifungal
4968	DRAMP20907	dN4	Antimicrobial, Antibacterial, Anti-Gram+, Anti-Gram-, Antifungal
4969	DRAMP20908	Ctry2459-H3 (Ctry2459 peptide derivative, His-rich)	Antimicrobial, Antiviral
4970	DRAMP20909	Ctry2459-H2 (Ctry2459 peptide derivative, His-rich)	Antimicrobial, Antiviral
4971	DRAMP20910	RN7-IN7(designed based on indolicidin and ranalexin)	Antimicrobial, Antibacterial, Anti-Gram+, Anti-Gram-
4972	DRAMP20911	Sm-AMP-X2	Antimicrobial, Antifungal
4973	DRAMP20912	RN7-IN9(designed based on indolicidin and ranalexin)	Antimicrobial, Antibacterial, Anti-Gram+, Anti-Gram-
4974	DRAMP20913	Myxinidin (G1)	Antimicrobial, Antibacterial, Anti-Gram+, Anti-Gram-
4975	DRAMP20914	Myxinidin (I2)	Antimicrobial, Antibacterial, Anti-Gram+, Anti-Gram-
4976	DRAMP20915	Myxinidin (H3)	Antimicrobial, Antibacterial, Anti-Gram+, Anti-Gram-
4977	DRAMP20916	Myxinidin (D4)	Antimicrobial, Antibacterial, Anti-Gram+, Anti-Gram-
4978	DRAMP20917	Myxinidin (I5)	Antimicrobial, Antibacterial, Anti-Gram+, Anti-Gram-
4979	DRAMP20918	Myxinidin (L6)	Antimicrobial, Antibacterial, Anti-Gram+, Anti-Gram-
4980	DRAMP20919	Myxinidin (K7)	Antimicrobial, Antibacterial, Anti-Gram+, Anti-Gram-
4981	DRAMP20920	Myxinidin (Y8)	Antimicrobial, Antibacterial, Anti-Gram+, Anti-Gram-
4982	DRAMP20921	Myxinidin (G9)	Antimicrobial, Antibacterial, Anti-Gram+, Anti-Gram-
4983	DRAMP20922	Myxinidin (K10)	Antimicrobial, Antibacterial, Anti-Gram+, Anti-Gram-
4984	DRAMP20923	Myxinidin (P11)	Antimicrobial, Antibacterial, Anti-Gram+, Anti-Gram-
4985	DRAMP20924	Myxinidin (S12)	Antimicrobial, Antibacterial, Anti-Gram+, Anti-Gram-
4986	DRAMP20925	MH3R	Antimicrobial, Antibacterial, Anti-Gram+, Anti-Gram-
4987	DRAMP20926	IN1(designed based on indolicidin and ranalexin)	Antimicrobial, Antibacterial, Anti-Gram+, Anti-Gram-
4988	DRAMP20927	IN2(designed based on indolicidin and ranalexin)	Antimicrobial, Antibacterial, Anti-Gram+, Anti-Gram-
4989	DRAMP20928	IN3(designed based on indolicidin and ranalexin)	Antimicrobial, Antibacterial, Anti-Gram+, Anti-Gram-
4990	DRAMP20929	RN7-IN6(designed based on indolicidin and ranalexin)	Antimicrobial, Antibacterial, Anti-Gram+, Anti-Gram-
4991	DRAMP20930	BP100-Ala-NH-C16H33	Antimicrobial, Antibacterial, Anti-Gram+, Anti-Gram-
4992	DRAMP20932	Hp1404	Antimicrobial, Antibacterial, Anti-Gram-

4993	DRAMP20933	Stigmurin	Antimicrobial, Antibacterial, Anti-Gram+, Antifungal, Antiparasitic, Antiproliferative
4994	DRAMP20934	Lucilin Peptide	Antimicrobial, Antibacterial, Anti-Gram-, Wound-healing
4995	DRAMP20935	Macropin 1(solitary bee, insects, animals)	Antimicrobial, Antibacterial, Anti-Gram+, Anti-Gram-, Antifungal
4996	DRAMP20936	ΔPb-CATH1	Antimicrobial, Antibacterial, Anti-Gram+, Anti-Gram-
4997	DRAMP20937	Pb-CATH3	Antimicrobial, Antibacterial, Anti-Gram+, Anti-Gram-
4998	DRAMP20938	Cbf-14	Antimicrobial, Antibacterial, Anti-Gram+, Anti-Gram-
4999	DRAMP20939	D-Cbf-14	Antimicrobial, Antibacterial, Anti-Gram+, Anti-Gram-
5000	DRAMP20940	Polybia-MP1S-Q12K	Antimicrobial, Antibacterial, Anti-Gram+
5001	DRAMP20941	[Pro3,DLeu9]TL(8) (Temporin L peptide derivative)	Antimicrobial, Antibacterial, Anti-Gram+, Anti-Gram-, Antifungal
5002	DRAMP20942	[Pro3,DLeu9]TL(9) (Temporin L peptide derivative)	Antimicrobial, Antibacterial, Anti-Gram+, Anti-Gram-, Antifungal
5003	DRAMP20943	[Pro3,DLeu9]TL(10) (Temporin L peptide derivative)	Antimicrobial, Antibacterial, Anti-Gram+, Anti-Gram-, Antifungal
5004	DRAMP20944	[Pro3,DLeu9]TL(11) (Temporin L peptide derivative)	Antimicrobial, Antibacterial, Anti-Gram+, Anti-Gram-, Antifungal
5005	DRAMP20945	Recombinant Cecropin A (1–8)–LL37 (17–30) (C–L)	Antimicrobial, Antibacterial, Anti-Gram+, Anti-Gram-
5006	DRAMP20946	ΔPb-CATH4 bivittatus antimicrobial peptides peptide derivative	Antimicrobial, Antibacterial
5007	DRAMP20947	[K]3-VmCT1-NH2 VmCT1 petide derivative	Antimicrobial, Antibacterial
5008	DRAMP20948	[K]7-VmCT1-NH2 VmCT1 petide derivative	Antimicrobial, Antibacterial
5009	DRAMP20949	[K]11-VmCT1-NH2 VmCT1 petide derivative	Antimicrobial, Antibacterial
5010	DRAMP20950	[E]4-VmCT1-NH2 VmCT1 petide derivative	Antimicrobial, Antibacterial
5011	DRAMP20951	[E]7-VmCT1-NH2 VmCT1 petide derivative	Antimicrobial, Antibacterial
5012	DRAMP20952	[W]9-VmCT1-NH2 VmCT1 petide derivative	Antimicrobial, Antibacterial
5013	DRAMP20953	[E]4[W]9-VmCT1-NH2 VmCT1 petide derivative	Antimicrobial, Antibacterial
5014	DRAMP20955	L31-P113	Antimicrobial, Antibacterial, Anti-Gram+, Anti-Gram-, Antifungal
5015	DRAMP20956	AL32-P113	Antimicrobial, Antibacterial, Anti-Gram+, Anti-Gram-, Antifungal
5016	DRAMP20957	StigA6	Antimicrobial, Antibacterial, Anti-Gram+, Anti-Gram-, Antifungal, Antiparasitic, Antiproliferative
5017	DRAMP20958	StigA16	Antimicrobial, Antibacterial, Anti-Gram+, Anti-Gram-, Antifungal, Antiparasitic, Antiproliferative
5018	DRAMP20959	Hp1404-T1c	Antimicrobial, Antibacterial, Anti-Gram-
5019	DRAMP20960	Hp1404-T1d	Antimicrobial, Antibacterial, Anti-Gram-
5020	DRAMP20961	Hp1404-T1e	Antimicrobial, Antibacterial, Anti-Gram-
5021	DRAMP20963	Cp1 alpha s1-casein peptide derivative	Antimicrobial, Antibacterial, Anti-Gram+, Anti-Gram-, low hemolytic and toxic effects
5022	DRAMP20964	Synthesized Cecropin A (1–8)–LL37 (17–30) (C–L)	Antimicrobial, Antibacterial, Anti-Gram+, Anti-Gram-
5023	DRAMP20965	LPcin-YK3 (bovine cathelicidin, cattle, ruminant, mammals, animals)	Antimicrobial, Antibacterial, Anti-Gram+, Anti-Gram-, Antifungal
5024	DRAMP20966	andricin B (Andrias davidianus, Amphibians, Animals)	Antimicrobial, Antibacterial, Anti-Gram+, Anti-Gram-, Antifungal
5025	DRAMP20967	andricin 01 (Andrias davidianus, Amphibians, Animals)	Antimicrobial, Antibacterial, Anti-Gram+, Anti-Gram-
5026	DRAMP20968	Catesbeianin-1 (Ranidae, Anura, Amphibia, Animals)	Antimicrobial, Antibacterial, Anti-Gram+, Anti-Gram-
5027	DRAMP20969	HJH-1 (bovine cathelicidin, cattle, ruminant, mammals, animals)	Antimicrobial, Antibacterial, Anti-Gram+, Anti-Gram-, Antifungal
5028	DRAMP20970	P3 (bovine cathelicidin, cattle, ruminant, mammals, animals)	Antimicrobial, Antibacterial, Anti-Gram+, Anti-Gram-, Antifungal
5029	DRAMP20971	JH-0 (Derived from P3)	Antimicrobial, Antibacterial, Anti-Gram+, Anti-Gram-
5030	DRAMP20972	JH-1 (Derived from P3)	Antimicrobial, Antibacterial, Anti-Gram+, Anti-Gram-
5031	DRAMP20973	JH-2 (Derived from P3)	Antimicrobial, Antibacterial, Anti-Gram+, Anti-Gram-, Antifungal
5032	DRAMP20974	JH-3 (Derived from P3)	Antimicrobial, Antibacterial, Anti-Gram+, Anti-Gram-, Antifungal
5033	DRAMP20975	OH-CM6 (Derived from OH-CATH30)	Antimicrobial, Antibacterial, Anti-Gram+, Anti-Gram-
5034	DRAMP20976	adevonin (Derived from Adenanthera pavonina trypsin inhibitor (ApTI)) inhibitor (ApTI))	Antimicrobial, Antibacterial, Anti-Gram+, Anti-Gram-

5035	DRAMP20977	Anoplin-1 (Derived from Anoplin)	Antimicrobial, Antibacterial, Anti-Gram+, Anti-Gram-
5036	DRAMP20978	Anoplin-2 (Derived from Anoplin)	Antimicrobial, Antibacterial, Anti-Gram+, Anti-Gram-
5037	DRAMP20979	Anoplin-3 (Derived from Anoplin)	Antimicrobial, Antibacterial, Anti-Gram+, Anti-Gram-
5038	DRAMP20980	Anoplin-4 (Derived from Anoplin)	Antimicrobial, Antibacterial, Anti-Gram+, Anti-Gram-
5039	DRAMP20981	CPF-C1 (Frogs, Amphibians, Animals)	Antimicrobial, Antibacterial, Anti-Gram+, Anti-Gram-
5040	DRAMP20982	CPF-1 (Derived from CPF-C1)	Antimicrobial, Antibacterial, Anti-Gram+, Anti-Gram-
5041	DRAMP20983	CPF-2 (Derived from CPF-C1)	Antimicrobial, Antibacterial, Anti-Gram+, Anti-Gram-
5042	DRAMP20984	CPF-3 (Derived from CPF-C1)	Antimicrobial, Antibacterial, Anti-Gram+, Anti-Gram-
5043	DRAMP20985	CPF-4 (Derived from CPF-C1)	Antimicrobial, Antibacterial, Anti-Gram+, Anti-Gram-
5044	DRAMP20986	CPF-5 (Derived from CPF-C1)	Antimicrobial, Antibacterial, Anti-Gram+, Anti-Gram-
5045	DRAMP20987	CPF-6 (Derived from CPF-C1)	Antimicrobial, Antibacterial, Anti-Gram+, Anti-Gram-
5046	DRAMP20988	CPF-7 (Derived from CPF-C1)	Antimicrobial, Antibacterial, Anti-Gram+, Anti-Gram-
5047	DRAMP20989	CPF-8 (Derived from CPF-C1)	Antimicrobial, Antibacterial, Anti-Gram+, Anti-Gram-
5048	DRAMP20990	CPF-9 (Derived from CPF-C1)	Antimicrobial, Antibacterial, Anti-Gram+, Anti-Gram-
5049	DRAMP20991	CPF-10 (Derived from CPF-C1)	Antimicrobial, Antibacterial, Anti-Gram+, Anti-Gram-
5050	DRAMP20992	CPF-11 (Derived from CPF-C1)	Antimicrobial, Antibacterial, Anti-Gram+, Anti-Gram-
5051	DRAMP20993	CPF-12 (Derived from CPF-C1)	Antimicrobial, Antibacterial, Anti-Gram+, Anti-Gram-
5052	DRAMP20994	anoplin analog 4	Antimicrobial, Antibacterial, Anti-Gram+, Anti-Gram-, Antifungal
5053	DRAMP20995	anoplin analog 5	Antimicrobial, Antibacterial, Anti-Gram+, Anti-Gram-, Antifungal
5054	DRAMP20996	anoplin analog 6	Antimicrobial, Antibacterial, Anti-Gram+, Anti-Gram-, Antifungal
5055	DRAMP20997	anoplin analog 7	Antimicrobial, Antibacterial, Anti-Gram+, Anti-Gram-
5056	DRAMP20998	anoplin analog 8	Antimicrobial, Antibacterial, Anti-Gram+, Anti-Gram-
5057	DRAMP20999	anoplin analog 9	Antimicrobial, Antibacterial, Anti-Gram+, Anti-Gram-
5058	DRAMP21000	cGm (Derived from Gm)	Antimicrobial, Antibacterial, Anti-Gram+, Anti-Gram-, Antifungal, Antitumor
5059	DRAMP21001	[Y7W]cGm (Derived from Gm)	Antimicrobial, Antibacterial, Anti-Gram+, Anti-Gram-, Antitumor
5060	DRAMP21002	[Y14W]cGm (Derived from Gm)	Antimicrobial, Antibacterial, Anti-Gram+, Anti-Gram-, Antitumor
5061	DRAMP21003	[K8R]cGm (Derived from Gm)	Antimicrobial, Antibacterial, Anti-Gram+, Anti-Gram-, Antitumor
5062	DRAMP21004	[Y7W, K8R, Y14W]cGm (Derived from Gm)	Antimicrobial, Antibacterial, Anti-Gram+, Anti-Gram-, Antifungal, Antitumor
5063	DRAMP21005	[R4A, R18A]cGm (Derived from Gm)	Antimicrobial, Antibacterial, Anti-Gram+, Anti-Gram-, Antifungal, Antitumor
5064	DRAMP21006	[G1K, K8R]cGm (Derived from Gm)	Antimicrobial, Antibacterial, Anti-Gram+, Anti-Gram-, Antifungal, Antitumor
5065	DRAMP21007	[C/U]cGm (Derived from Gm)	Antimicrobial, Antibacterial, Anti-Gram+, Anti-Gram-, Antifungal, Antitumor
5066	DRAMP21008	[L5W]cGm (Derived from Gm)	Antimicrobial, Antibacterial, Anti-Gram+, Anti-Gram-, Antitumor
5067	DRAMP21009	[D-P L-P]cGm (Derived from Gm)	Antimicrobial, Antibacterial, Anti-Gram+, Anti-Gram-, Antifungal, Antitumor
5068	DRAMP21010	[G1K, L5Y, K8R]cGm (Derived from Gm)	Antimicrobial, Antibacterial, Anti-Gram+, Anti-Gram-, Antifungal, Antitumor
5069	DRAMP21011	[C/U, G1K, L5Y, K8R]cGm (Derived from Gm)	Antimicrobial, Antibacterial, Anti-Gram+, Anti-Gram-, Antifungal, Antitumor
5070	DRAMP21012	NK-2 (Mammals, Animals)	Antimicrobial, Antibacterial, Anti-Gram+, Anti-Gram-, Antifungal, Antitumor
5071	DRAMP21013	NK-pro (Derived from NK-2)	Antimicrobial, Antibacterial, Anti-Gram+, Anti-Gram-, Antifungal, Antitumor
5072	DRAMP21014	NK-dpro (Derived from NK-2)	Antimicrobial, Antibacterial, Anti-Gram+, Anti-Gram-, Antifungal, Antitumor
5073	DRAMP21015	A (A1R) (Derived from AR-23)	Antimicrobial, Antibacterial, Anti-Gram+, Anti-Gram-
5074	DRAMP21016	A (A8R) (Derived from AR-23)	Antimicrobial, Antibacterial, Anti-Gram+, Anti-Gram-
5075	DRAMP21017	A (I17K) (Derived from AR-23)	Antimicrobial, Antibacterial, Anti-Gram+, Anti-Gram-
5076	DRAMP21018	A (I17R) (Derived from AR-23)	Antimicrobial, Antibacterial, Anti-Gram+, Anti-Gram-
5077	DRAMP21019	A (A1R, A8R) (Derived from AR-23)	Antimicrobial, Antibacterial, Anti-Gram+, Anti-Gram-

5078	DRAMP21020	A (A1R, I17K) (Derived from AR-23)	Antimicrobial, Antibacterial, Anti-Gram+, Anti-Gram-
5079	DRAMP21021	A (A8R, I17K) (Derived from AR-23)	Antimicrobial, Antibacterial, Anti-Gram+, Anti-Gram-
5080	DRAMP21022	A (A1R, A8R, I17K) (Derived from AR-23)	Antimicrobial, Antibacterial, Anti-Gram+, Anti-Gram-
5081	DRAMP21023	A (A1R, A8R, I17R) (Derived from AR-23)	Antimicrobial, Antibacterial, Anti-Gram+, Anti-Gram-
5082	DRAMP21024	Stigmurin (Tityus, Scorpionida, Arachnida)	Antimicrobial, Antibacterial, Anti-Gram+, Anti-Gram-, Antifungal
5083	DRAMP21025	StigA25 (Derived from Stigmurin)	Antimicrobial, Antibacterial, Anti-Gram+, Anti-Gram-, Antifungal
5084	DRAMP21026	StigA31 (Derived from Stigmurin)	Antimicrobial, Antibacterial, Anti-Gram+, Anti-Gram-, Antifungal
5085	DRAMP21027	K5, 17-DPS3 (Derived from dermaseptin-PS3 (DPS3))	Antimicrobial, Antibacterial, Anti-Gram+, Anti-Gram-, Antifungal
5086	DRAMP21028	L10, 11-DPS3 (Derived from dermaseptin-PS3 (DPS3))	Antimicrobial, Antibacterial, Anti-Gram+, Anti-Gram-, Antifungal
5087	DRAMP21029	D5R (Derived from HD5)	Antimicrobial, Antibacterial, Anti-Gram+, Anti-Gram-, Antifungal
5088	DRAMP21030	D5r (Derived from HD5)	Antimicrobial, Antibacterial, Anti-Gram+, Anti-Gram-, Antifungal
5089	DRAMP21031	MyD5R (Derived from HD5)	Antimicrobial, Antibacterial, Anti-Gram+, Anti-Gram-, Antifungal
5090	DRAMP21032	MyD5r (Derived from HD5)	Antimicrobial, Antibacterial, Anti-Gram+, Anti-Gram-, Antifungal
5091	DRAMP21033	LaD5R (Derived from HD5)	Antimicrobial, Antibacterial, Anti-Gram+, Anti-Gram-, Antifungal
5092	DRAMP21034	LaD5r (Derived from HD5)	Antimicrobial, Antibacterial, Anti-Gram+, Anti-Gram-, Antifungal
5093	DRAMP21035	AC-UM-14W (De novo synthesis)	Antimicrobial, Antibacterial, Anti-Gram+, Anti-Gram-
5094	DRAMP21036	PapMA (Derived from Papiliocin and Magainin 2)	Antimicrobial, Antibacterial, Anti-Gram+, Anti-Gram-
5095	DRAMP21037	PapMA-k (Derived from Papiliocin and Magainin 2)	Antimicrobial, Antibacterial, Anti-Gram+, Anti-Gram-
5096	DRAMP21038	analog 1 (Derived from Ib-AMP1)	Antimicrobial, Antibacterial, Anti-Gram+, Anti-Gram-
5097	DRAMP21039	analog 2 (Derived from Ib-AMP2)	Antimicrobial, Antibacterial, Anti-Gram+, Anti-Gram-
5098	DRAMP21040	analog 3 (Derived from Ib-AMP2)	Antimicrobial, Antibacterial, Anti-Gram+, Anti-Gram-
5099	DRAMP21041	analog 4 (Derived from Ib-AMP2)	Antimicrobial, Antibacterial, Anti-Gram+, Anti-Gram-
5100	DRAMP21042	A2 (Derived from Indolicidin (IN))	Antimicrobial, Antibacterial, Anti-Gram+, Anti-Gram-
5101	DRAMP21043	A3 (Derived from Indolicidin (IN))	Antimicrobial, Antibacterial, Anti-Gram+, Anti-Gram-
5102	DRAMP21044	A4 (Derived from Indolicidin (IN))	Antimicrobial, Antibacterial, Anti-Gram+, Anti-Gram-
5103	DRAMP21045	A5 (Derived from Indolicidin (IN))	Antimicrobial, Antibacterial, Anti-Gram+, Anti-Gram-
5104	DRAMP21046	A6 (Derived from Indolicidin (IN))	Antimicrobial, Antibacterial, Anti-Gram+, Anti-Gram-
5105	DRAMP21047	A7 (Derived from Indolicidin (IN))	Antimicrobial, Antibacterial, Anti-Gram+, Anti-Gram-
5106	DRAMP21048	peptide 6 (Derived from seq2)	Antimicrobial, Antibacterial, Anti-Gram+, Anti-Gram-
5107	DRAMP21049	peptide 6.2 (Derived from seq2)	Antimicrobial, Antibacterial, Anti-Gram+, Anti-Gram-
5108	DRAMP21050	TP1[K1A] (Derived from TP1)	Antimicrobial, Antibacterial, Anti-Gram+, Anti-Gram-, Antifungal
5109	DRAMP21051	TP1[W2A] (Derived from TP1)	Antimicrobial, Antibacterial, Anti-Gram+, Anti-Gram-, Antifungal
5110	DRAMP21052	TP1[C3A, C16S] (Derived from TP1)	Antimicrobial, Antibacterial, Anti-Gram+, Anti-Gram-, Antifungal
5111	DRAMP21053	TP1[F4A] (Derived from TP1)	Antimicrobial, Antibacterial, Anti-Gram+, Anti-Gram-, Antifungal
5112	DRAMP21054	TP1[R5A] (Derived from TP1)	Antimicrobial, Antibacterial, Anti-Gram+, Anti-Gram-, Antifungal
5113	DRAMP21055	TP1[V6A] (Derived from TP1)	Antimicrobial, Antibacterial, Anti-Gram+, Anti-Gram-, Antifungal
5114	DRAMP21056	TP1[C7A, C12S] (Derived from TP1)	Antimicrobial, Antibacterial, Anti-Gram+, Anti-Gram-, Antifungal
5115	DRAMP21057	TP1[Y8A] (Derived from TP1)	Antimicrobial, Antibacterial, Anti-Gram+, Anti-Gram-, Antifungal
5116	DRAMP21058	TP1[R9A] (Derived from TP1)	Antimicrobial, Antibacterial, Anti-Gram+, Anti-Gram-, Antifungal
5117	DRAMP21059	TP1[G10A] (Derived from TP1)	Antimicrobial, Antibacterial, Anti-Gram+, Anti-Gram-, Antifungal

5118	DRAMP21060	TP1[I11A] (Derived from TP1)	Antimicrobial, Antibacterial, Anti-Gram+, Anti-Gram-, Antifungal
5119	DRAMP21061	TP1[C7S, C12A] (Derived from TP1)	Antimicrobial, Antibacterial, Anti-Gram+, Anti-Gram-, Antifungal
5120	DRAMP21062	TP1[Y13A] (Derived from TP1)	Antimicrobial, Antibacterial, Anti-Gram+, Anti-Gram-, Antifungal
5121	DRAMP21063	TP1[R14A] (Derived from TP1)	Antimicrobial, Antibacterial, Anti-Gram+, Anti-Gram-, Antifungal
5122	DRAMP21064	TP1[R15A] (Derived from TP1)	Antimicrobial, Antibacterial, Anti-Gram+, Anti-Gram-, Antifungal
5123	DRAMP21065	TP1[C3S, C16A] (Derived from TP1)	Antimicrobial, Antibacterial, Anti-Gram+, Anti-Gram-, Antifungal
5124	DRAMP21066	TP1[R17A] (Derived from TP1)	Antimicrobial, Antibacterial, Anti-Gram+, Anti-Gram-, Antifungal
5125	DRAMP21067	TP1[C3A, C16A] (Derived from TP1)	Antimicrobial, Antibacterial, Anti-Gram+, Anti-Gram-, Antifungal
5126	DRAMP21068	TP1[C7A, C12A] (Derived from TP1)	Antimicrobial, Antibacterial, Anti-Gram+, Anti-Gram-, Antifungal
5127	DRAMP21069	TP1[C3A, C7A, C12A, C16A] (Derived from TP1)	Antimicrobial, Antibacterial, Anti-Gram+, Anti-Gram-, Antifungal
5128	DRAMP21070	TP1[V6R, R9A] (Derived from TP1)	Antimicrobial, Antibacterial, Anti-Gram+, Anti-Gram-, Antifungal
5129	DRAMP21071	TP1[K1R] (Derived from TP1)	Antimicrobial, Antibacterial, Anti-Gram+, Anti-Gram-, Antifungal
5130	DRAMP21072	TP1[F4G] (Derived from TP1)	Antimicrobial, Antibacterial, Anti-Gram+, Anti-Gram-, Antifungal
5131	DRAMP21073	TP1[F4S] (Derived from TP1)	Antimicrobial, Antibacterial, Anti-Gram+, Anti-Gram-, Antifungal
5132	DRAMP21074	TP1[Y8G] (Derived from TP1)	Antimicrobial, Antibacterial, Anti-Gram+, Anti-Gram-, Antifungal
5133	DRAMP21075	TP1[I11G] (Derived from TP1)	Antimicrobial, Antibacterial, Anti-Gram+, Anti-Gram-, Antifungal
5134	DRAMP21076	TP1[F4A, Y8A, I11A] (Derived from TP1)	Antimicrobial, Antibacterial, Anti-Gram+, Anti-Gram-, Antifungal
5135	DRAMP21077	TP1[-R5, R17G] (Derived from TP1)	Antimicrobial, Antibacterial, Anti-Gram+, Anti-Gram-, Antifungal
5136	DRAMP21078	TP1[K1A, F4A] (Derived from TP1)	Antimicrobial, Antibacterial, Anti-Gram+, Anti-Gram-, Antifungal
5137	DRAMP21079	TP1[K1A, Y8A] (Derived from TP1)	Antimicrobial, Antibacterial, Anti-Gram+, Anti-Gram-, Antifungal
5138	DRAMP21080	TP1[K1A, I11A] (Derived from TP1)	Antimicrobial, Antibacterial, Anti-Gram+, Anti-Gram-, Antifungal
5139	DRAMP21081	TP1[R9A, R17A] (Derived from TP1)	Antimicrobial, Antibacterial, Anti-Gram+, Anti-Gram-, Antifungal
5140	DRAMP21082	ccTP 3 (Derived from TP2)	Antimicrobial, Antibacterial, Anti-Gram+, Anti-Gram-, Antifungal
5141	DRAMP21083	ccTP 5 (Derived from TP2)	Antimicrobial, Antibacterial, Anti-Gram+, Anti-Gram-, Antifungal
5142	DRAMP21084	ccTP 6 (Derived from TP2)	Antimicrobial, Antibacterial, Anti-Gram+, Anti-Gram-, Antifungal
5143	DRAMP21085	PRW4 (PR) (Derived from PMAP-36)	Antimicrobial, Antibacterial, Anti-Gram+, Anti-Gram-
5144	DRAMP21086	PR-FO (Derived from PRW4)	Antimicrobial, Antibacterial, Anti-Gram+, Anti-Gram-
5145	DRAMP21087	PR-PG (Derived from PRW4)	Antimicrobial, Antibacterial, Anti-Gram+, Anti-Gram-
5146	DRAMP21088	PR-TR (Derived from PRW4)	Antimicrobial, Antibacterial, Anti-Gram+, Anti-Gram-
5147	DRAMP21089	C4 (Derived from PRW4)	Antimicrobial, Antibacterial, Anti-Gram+, Anti-Gram-
5148	DRAMP21090	D4 (Derived from PRW4)	Antimicrobial, Antibacterial, Anti-Gram+, Anti-Gram-
5149	DRAMP21091	I4 (Derived from PRW4)	Antimicrobial, Antibacterial, Anti-Gram+, Anti-Gram-
5150	DRAMP21092	P4 (Derived from PRW4)	Antimicrobial, Antibacterial, Anti-Gram+, Anti-Gram-
5151	DRAMP21093	PRW4-d (Derived from PRW4)	Antimicrobial, Antibacterial, Anti-Gram+, Anti-Gram-
5152	DRAMP21094	PRW4-R (Derived from PRW4)	Antimicrobial, Antibacterial, Anti-Gram+, Anti-Gram-
5153	DRAMP21095	IR1 (Derived from PG-1)	Antimicrobial, Antibacterial, Anti-Gram+, Anti-Gram-
5154	DRAMP21096	IR2 (Derived from PG-1)	Antimicrobial, Antibacterial, Anti-Gram+, Anti-Gram-
5155	DRAMP21097	FR1 (Derived from PG-1)	Antimicrobial, Antibacterial, Anti-Gram+, Anti-Gram-

5156	DRAMP21098	FR2 (Derived from PG-1)	Antimicrobial, Antibacterial, Anti-Gram+, Anti-Gram-
5157	DRAMP21099	WR1 (Derived from PG-1)	Antimicrobial, Antibacterial, Anti-Gram+, Anti-Gram-
5158	DRAMP21100	WR2 (Derived from PG-1)	Antimicrobial, Antibacterial, Anti-Gram+, Anti-Gram-
5159	DRAMP21101	PR1 (Derived from PG-1)	Antimicrobial, Antibacterial, Anti-Gram+, Anti-Gram-
5160	DRAMP21102	PR2 (Derived from PG-1)	Antimicrobial, Antibacterial, Anti-Gram+, Anti-Gram-
5161	DRAMP21165	HYL-11 (Derived from HYL)	Antimicrobial, Antibacterial, Anti-Gram+, Anti-Gram-, Antifungal
5162	DRAMP21166	HYL-12 (Derived from HYL)	Antimicrobial, Antibacterial, Anti-Gram+, Anti-Gram-, Antifungal
5163	DRAMP21164	HYL-10 (Derived from HYL)	Antimicrobial, Antibacterial, Anti-Gram+, Anti-Gram-, Antifungal
5164	DRAMP21158	HYL-4 (Derived from HYL)	Antimicrobial, Antibacterial, Anti-Gram+, Anti-Gram-, Antifungal
5165	DRAMP21159	HYL-5 (Derived from HYL)	Antimicrobial, Antibacterial, Anti-Gram+, Anti-Gram-, Antifungal
5166	DRAMP21160	HYL-6 (Derived from HYL)	Antimicrobial, Antibacterial, Anti-Gram+, Anti-Gram-, Antifungal
5167	DRAMP21161	HYL-7 (Derived from HYL)	Antimicrobial, Antibacterial, Anti-Gram+, Anti-Gram-, Antifungal
5168	DRAMP21162	HYL-8 (Derived from HYL)	Antimicrobial, Antibacterial, Anti-Gram+, Anti-Gram-, Antifungal
5169	DRAMP21163	HYL-9 (Derived from HYL)	Antimicrobial, Antibacterial, Anti-Gram+, Anti-Gram-, Antifungal
5170	DRAMP21157	HYL-3 (Derived from HYL)	Antimicrobial, Antibacterial, Anti-Gram+, Anti-Gram-, Antifungal
5171	DRAMP21156	HYL-2 (Derived from HYL)	Antimicrobial, Antibacterial, Anti-Gram+, Anti-Gram-, Antifungal
5172	DRAMP21155	HYL-1 (Derived from HYL)	Antimicrobial, Antibacterial, Anti-Gram+, Anti-Gram-, Antifungal
5173	DRAMP21154	HYL (Bee, Insecta, Animals)	Antimicrobial, Antibacterial, Anti-Gram+, Anti-Gram-, Antifungal
5174	DRAMP21153	KR-12-a8 (Derived from KR-12)	Antimicrobial, Antibacterial, Anti-Gram+, Anti-Gram-
5175	DRAMP21151	KR-12-a6 (Derived from KR-12)	Antimicrobial, Antibacterial, Anti-Gram+, Anti-Gram-
5176	DRAMP21150	KR-12-a5 (Derived from KR-12)	Antimicrobial, Antibacterial, Anti-Gram+, Anti-Gram-
5177	DRAMP21152	KR-12-a7 (Derived from KR-12)	Antimicrobial, Antibacterial, Anti-Gram+, Anti-Gram-
5178	DRAMP21149	KR-12-a4 (Derived from KR-12)	Antimicrobial, Antibacterial, Anti-Gram+, Anti-Gram-
5179	DRAMP21146	KR-12-a1 (Derived from KR-12)	Antimicrobial, Antibacterial, Anti-Gram+, Anti-Gram-
5180	DRAMP21148	KR-12-a3 (Derived from KR-12)	Antimicrobial, Antibacterial, Anti-Gram+, Anti-Gram-
5181	DRAMP21147	KR-12-a2 (Derived from KR-12)	Antimicrobial, Antibacterial, Anti-Gram+, Anti-Gram-
5182	DRAMP21145	Myxinidin3 (Derived from Myxinidin)	Antimicrobial, Antibacterial, Anti-Gram+, Anti-Gram-
5183	DRAMP21142	AMP2041 (De novo synthesis)	Antimicrobial, Antibacterial, Anti-Gram+, Anti-Gram-
5184	DRAMP21141	AMP126 (De novo synthesis)	Antimicrobial, Antibacterial, Anti-Gram+, Anti-Gram-
5185	DRAMP21144	Myxinidin2 (Derived from Myxinidin)	Antimicrobial, Antibacterial, Anti-Gram+, Anti-Gram-
5186	DRAMP21143	Myxinidin1 (Derived from Myxinidin)	Antimicrobial, Antibacterial, Anti-Gram+, Anti-Gram-
5187	DRAMP21140	AMP72 (De novo synthesis)	Antimicrobial, Antibacterial, Anti-Gram+, Anti-Gram-
5188	DRAMP21139	GNU7 (De novo synthesis)	Antimicrobial, Antibacterial, Anti-Gram+, Anti-Gram-, Antifungal
5189	DRAMP21138	GNU6 (De novo synthesis)	Antimicrobial, Antibacterial, Anti-Gram+, Anti-Gram-, Antifungal
5190	DRAMP21137	GNU5 (De novo synthesis)	Antimicrobial, Antibacterial, Anti-Gram+, Anti-Gram-, Antifungal
5191	DRAMP21135	P7 (Derived from P5)	Antimicrobial, Antibacterial, Anti-Gram+, Anti-Gram-
5192	DRAMP21136	P8 (Derived from P5)	Antimicrobial, Antibacterial, Anti-Gram+, Anti-Gram-
5193	DRAMP21134	P6 (Derived from P5)	Antimicrobial, Antibacterial, Anti-Gram+, Anti-Gram-
5194	DRAMP21133	P5 (Derived from Octa 2)	Antimicrobial, Antibacterial, Anti-Gram+, Anti-Gram-
5195	DRAMP21132	P4 (Derived from P5)	Antimicrobial, Antibacterial, Anti-Gram+, Anti-Gram-
5196	DRAMP21131	P3 (Derived from P5)	Antimicrobial, Antibacterial, Anti-Gram+, Anti-Gram-
5197	DRAMP21130	P2 (Derived from P5)	Antimicrobial, Antibacterial, Anti-Gram+, Anti-Gram-
5198	DRAMP21129	P1 (Derived from P5)	Antimicrobial, Antibacterial, Anti-Gram+, Anti-Gram-
5199	DRAMP21128	T9F (Derived from RI16)	Antimicrobial, Antibacterial, Anti-Gram+, Anti-Gram-
5200	DRAMP21127	T9K (Derived from RI16)	Antimicrobial, Antibacterial, Anti-Gram+, Anti-Gram-

5201	DRAMP21126	T9I (Derived from RI16)	Antimicrobial, Antibacterial, Anti-Gram+, Anti-Gram-
5202	DRAMP21125	T9W (Derived from RI16)	Antimicrobial, Antibacterial, Anti-Gram+, Anti-Gram-
5203	DRAMP21124	RI16 (Derived from PMAP-36)	Antimicrobial, Antibacterial, Anti-Gram+, Anti-Gram-
5204	DRAMP21123	KR-12-a5 (7-(D)L) (Derived from LL-37)	Antimicrobial, Antibacterial, Anti-Gram+, Anti-Gram-
5205	DRAMP21122	KR-12-a5 (6-(D)L) (Derived from LL-37)	Antimicrobial, Antibacterial, Anti-Gram+, Anti-Gram-
5206	DRAMP21121	KR-12-a5 (5-(D)K) (Derived from LL-37)	Antimicrobial, Antibacterial, Anti-Gram+, Anti-Gram-
5207	DRAMP21119	I11R (Derived from tachyplesin I)	Antimicrobial, Antibacterial, Anti-Gram+, Anti-Gram-
5208	DRAMP21120	KR-12-a5 (Derived from LL-37)	Antimicrobial, Antibacterial, Anti-Gram+, Anti-Gram-
5209	DRAMP21118	I11S (Derived from tachyplesin I)	Antimicrobial, Antibacterial, Anti-Gram+, Anti-Gram-
5210	DRAMP21117	Y8R (Derived from tachyplesin I)	Antimicrobial, Antibacterial, Anti-Gram+, Anti-Gram-
5211	DRAMP21116	Y8S (Derived from tachyplesin I)	Antimicrobial, Antibacterial, Anti-Gram+, Anti-Gram-
5212	DRAMP21115	V6R (Derived from tachyplesin I)	Antimicrobial, Antibacterial, Anti-Gram+, Anti-Gram-
5213	DRAMP21114	V6S (Derived from tachyplesin I)	Antimicrobial, Antibacterial, Anti-Gram+, Anti-Gram-
5214	DRAMP21111	ASA (Derived from SLZP)	Antimicrobial, Antibacterial, Anti-Gram+, Anti-Gram-, Antifungal
5215	DRAMP21112	DLSA (Derived from SLZP)	Antimicrobial, Antibacterial, Anti-Gram+, Anti-Gram-, Antifungal
5216	DRAMP21113	PSA (Derived from SLZP)	Antimicrobial, Antibacterial, Anti-Gram+, Anti-Gram-, Antifungal
5217	DRAMP21103	L-RW (De novo synthesis)	Antimicrobial, Antibacterial, Anti-Gram+, Anti-Gram-
5218	DRAMP21110	SLZP (De novo synthesis)	Antimicrobial, Antibacterial, Anti-Gram+, Anti-Gram-, Antifungal
5219	DRAMP21109	FPA-Bombinin-BO (toads, amphibians, animals)	Antimicrobial, Antibacterial, Anti-Gram+, Anti-Gram-, Antifungal
5220	DRAMP21108	Feleucin-K3 (Derived from Feleucin-BO1)	Antimicrobial, Antibacterial, Anti-Gram+, Anti-Gram-, Antifungal
5221	DRAMP21104	Feleucin-2 (toads, amphibians, animals)	Antimicrobial, Antibacterial, Anti-Gram+, Anti-Gram-, Antifungal
5222	DRAMP21105	Feleucin-BV1 (toads, amphibians, animals)	Antimicrobial, Antibacterial, Anti-Gram+
5223	DRAMP21106	Feleucin-BV2 (toads, amphibians, animals)	Antimicrobial, Antibacterial, Anti-Gram+
5224	DRAMP21107	Feleucin-BO1 (toads, amphibians, animals)	Antimicrobial, Antibacterial, Anti-Gram+, Anti-Gram-, Antifungal
5225	DRAMP21232	Ranatuerin-2PLx (R2PLx; Frogs, Amphibians, Animals)	Antimicrobial, Antibacterial, Anti-Gram+, Anti-Gram-
5226	DRAMP21231	S-6K-F17-3GN (Derived from S-6K-F17)	Antimicrobial, Antibacterial, Anti-Gram-
5227	DRAMP21230	S-6K-F17-3G (Derived from S-6K-F17)	Antimicrobial, Antibacterial, Anti-Gram-
5228	DRAMP21229	S-6K-F17-2G (Derived from S-6K-F17)	Antimicrobial, Antibacterial, Anti-Gram-
5229	DRAMP21227	IsCT-P (Derived from IsCT)	Antimicrobial, Antibacterial, Anti-Gram+, Anti-Gram-
5230	DRAMP21228	IsCT-a (Derived from IsCT-P)	Antimicrobial, Antibacterial, Anti-Gram+, Anti-Gram-
5231	DRAMP21225	STPk (Derived from STP)	Antimicrobial, Antibacterial, Anti-Gram+, Anti-Gram-
5232	DRAMP21226	Ink (Derived from IN)	Antimicrobial, Antibacterial, Anti-Gram+, Anti-Gram-
5233	DRAMP21223	IsCT-p (Derived from IsCT-P)	Antimicrobial, Antibacterial, Anti-Gram+, Anti-Gram-
5234	DRAMP21224	TPk (Derived from TP)	Antimicrobial, Antibacterial, Anti-Gram+, Anti-Gram-
5235	DRAMP21222	Control-4D (Derived from IK12-all L)	Antimicrobial, Antibacterial, Anti-Gram+, Anti-Gram-, Antifungal
5236	DRAMP21221	Control-all D (Derived from IK12-all L)	Antimicrobial, Antibacterial, Anti-Gram+, Anti-Gram-, Antifungal
5237	DRAMP21219	IK12-all D (Derived from IK12-all L)	Antimicrobial, Antibacterial, Anti-Gram+, Anti-Gram-, Antifungal
5238	DRAMP21220	Control-all L (Derived from IK12-all L)	Antimicrobial, Antibacterial, Anti-Gram+, Anti-Gram-, Antifungal
5239	DRAMP21218	IK12-all L (De novo synthesis)	Antimicrobial, Antibacterial, Anti-Gram+, Anti-Gram-, Antifungal
5240	DRAMP21217	IK8-2D (Derived from IK8-all L)	Antimicrobial, Antibacterial, Anti-Gram+, Anti-Gram-, Antifungal
5241	DRAMP21215	IK4-all D (Derived from IK8-all L)	Antimicrobial, Antibacterial, Anti-Gram+, Anti-Gram-, Antifungal
5242	DRAMP21216	IK8-4D (Derived from IK8-all L)	Antimicrobial, Antibacterial, Anti-Gram+, Anti-Gram-, Antifungal
5243	DRAMP21213	IK8-all D (Derived from IK8-all L)	Antimicrobial, Antibacterial, Anti-Gram+, Anti-Gram-, Antifungal

5244	DRAMP21214	IK6-all D (Derived from IK8-all L)	Antimicrobial, Antibacterial, Anti-Gram+, Anti-Gram-, Antifungal
5245	DRAMP21212	IK8-all L (De novo synthesis)	Antimicrobial, Antibacterial, Anti-Gram+, Anti-Gram-, Antifungal
5246	DRAMP21211	AmyI-1-18 (N3L, G12R) (Derived from AmyI-1-18)	Antimicrobial, Antibacterial, Anti-Gram-
5247	DRAMP21208	AmyI-1-18 (E9L) (Derived from AmyI-1-18)	Antimicrobial, Antibacterial, Anti-Gram-
5248	DRAMP21209	AmyI-1-18 (E9L, G12R) (Derived from AmyI-1-18)	Antimicrobial, Antibacterial, Anti-Gram-
5249	DRAMP21205	AmyI-1-18 (G12R) (Derived from AmyI-1-18)	Antimicrobial, Antibacterial, Anti-Gram-, Antifungal
5250	DRAMP21210	AmyI-1-18 (N3L, E9L) (Derived from AmyI-1-18)	Antimicrobial, Antibacterial, Anti-Gram-
5251	DRAMP21207	AmyI-1-18 (N3L) (Derived from AmyI-1-18)	Antimicrobial, Antibacterial, Anti-Gram-, Antifungal
5252	DRAMP21206	AmyI-1-18 (D15R) (Derived from AmyI-1-18)	Antimicrobial, Antibacterial, Anti-Gram-, Antifungal
5253	DRAMP21204	AmyI-1-18 (I11R) (Derived from AmyI-1-18)	Antimicrobial, Antibacterial, Anti-Gram-, Antifungal
5254	DRAMP21203	AmyI-1-18 (Oryza sativa L., Angiospermae, Plants)	Antimicrobial, Antibacterial, Anti-Gram-, Antifungal
5255	DRAMP21202	HPA3NT3-analog (Derived from HPA3NT3)	Antimicrobial, Antibacterial, Anti-Gram+, Anti-Gram-, Antifungal
5256	DRAMP21201	Magainin 2a (M2a; Frogs, Amphibians, Animals)	Antimicrobial, Antibacterial, Anti-Gram+, Anti-Gram-
5257	DRAMP21200	GW-M4 (De novo synthesis)	Antimicrobial, Antibacterial, Anti-Gram+, Anti-Gram-
5258	DRAMP21199	GW-M3 (De novo synthesis)	Antimicrobial, Antibacterial, Anti-Gram+, Anti-Gram-
5259	DRAMP21198	GW-M1 (De novo synthesis)	Antimicrobial, Antibacterial, Anti-Gram+, Anti-Gram-
5260	DRAMP21197	GW-H3 (De novo synthesis)	Antimicrobial, Antibacterial, Anti-Gram+, Anti-Gram-
5261	DRAMP21196	GW-H1 (De novo synthesis)	Antimicrobial, Antibacterial, Anti-Gram+, Anti-Gram-
5262	DRAMP21195	GW-A5 (De novo synthesis)	Antimicrobial, Antibacterial, Anti-Gram+, Anti-Gram-
5263	DRAMP21194	GW-A4 (De novo synthesis)	Antimicrobial, Antibacterial, Anti-Gram+, Anti-Gram-
5264	DRAMP21193	GW-A2 (De novo synthesis)	Antimicrobial, Antibacterial, Anti-Gram+, Anti-Gram-
5265	DRAMP21192	GW-A1 (De novo synthesis)	Antimicrobial, Antibacterial, Anti-Gram+, Anti-Gram-
5266	DRAMP21191	GW-Q6 (De novo synthesis)	Antimicrobial, Antibacterial, Anti-Gram+, Anti-Gram-
5267	DRAMP21190	GW-Q5 (De novo synthesis)	Antimicrobial, Antibacterial, Anti-Gram+, Anti-Gram-
5268	DRAMP21189	GW-Q4 (De novo synthesis)	Antimicrobial, Antibacterial, Anti-Gram+, Anti-Gram-
5269	DRAMP21188	GW-Q3 (De novo synthesis)	Antimicrobial, Antibacterial, Anti-Gram+, Anti-Gram-
5270	DRAMP21187	WRL4 (Derived from leucocin A)	Antimicrobial, Antibacterial, Anti-Gram+, Anti-Gram-, Antifungal
5271	DRAMP21186	WRL3 (Derived from leucocin A)	Antimicrobial, Antibacterial, Anti-Gram+, Anti-Gram-, Antifungal
5272	DRAMP21185	WRL2 (Derived from leucocin A)	Antimicrobial, Antibacterial, Anti-Gram+, Anti-Gram-, Antifungal
5273	DRAMP21184	WR7 (Derived from leucocin A)	Antimicrobial, Antibacterial, Anti-Gram+, Anti-Gram-, Antifungal
5274	DRAMP21183	WR5 (Derived from leucocin A)	Antimicrobial, Antibacterial, Anti-Gram+, Anti-Gram-, Antifungal
5275	DRAMP21182	WR3 (Derived from leucocin A)	Antimicrobial, Antibacterial, Anti-Gram+, Anti-Gram-, Antifungal
5276	DRAMP21181	WR1 (Derived from leucocin A)	Antimicrobial, Antibacterial, Anti-Gram+, Anti-Gram-, Antifungal
5277	DRAMP21180	WG18 (Derived from leucocin A)	Antimicrobial, Antibacterial, Anti-Gram+, Anti-Gram-, Antifungal
5278	DRAMP21179	HYL-26 (Derived from HYL)	Antimicrobial, Antibacterial, Anti-Gram+, Anti-Gram-, Antifungal
5279	DRAMP21178	HYL-25 (Derived from HYL)	Antimicrobial, Antibacterial, Anti-Gram+, Anti-Gram-, Antifungal
5280	DRAMP21177	HYL-24 (Derived from HYL)	Antimicrobial, Antibacterial, Anti-Gram+, Anti-Gram-, Antifungal
5281	DRAMP21176	HYL-23 (Derived from HYL)	Antimicrobial, Antibacterial, Anti-Gram+, Anti-Gram-, Antifungal
5282	DRAMP21175	HYL-22 (Derived from HYL)	Antimicrobial, Antibacterial, Anti-Gram+, Anti-Gram-, Antifungal
5283	DRAMP21174	HYL-21 (Derived from HYL)	Antimicrobial, Antibacterial, Anti-Gram+, Anti-Gram-, Antifungal
5284	DRAMP21173	HYL-20 (Derived from HYL)	Antimicrobial, Antibacterial, Anti-Gram+, Anti-Gram-, Antifungal
5285	DRAMP21172	HYL-19 (Derived from HYL)	Antimicrobial, Antibacterial, Anti-Gram+, Anti-Gram-, Antifungal

5286	DRAMP21168	HYL-15 (Derived from HYL)	Antimicrobial, Antibacterial, Anti-Gram+, Anti-Gram-, Antifungal
5287	DRAMP21169	HYL-16 (Derived from HYL)	Antimicrobial, Antibacterial, Anti-Gram+, Anti-Gram-, Antifungal
5288	DRAMP21170	HYL-17 (Derived from HYL)	Antimicrobial, Antibacterial, Anti-Gram+, Anti-Gram-, Antifungal
5289	DRAMP21171	HYL-18 (Derived from HYL)	Antimicrobial, Antibacterial, Anti-Gram+, Anti-Gram-, Antifungal
5290	DRAMP21243	pardaxin-6 (GE-6) (Derived from pardaxin)	Antimicrobial, Antibacterial, Anti-Gram+, Anti-Gram-
5291	DRAMP21242	Epinecidin-8 (Derived from Epinecidin)	Antimicrobial, Antibacterial, Anti-Gram+, Anti-Gram-
5292	DRAMP21241	Epinecidin-1 (Derived from Epinecidin)	Antimicrobial, Antibacterial, Anti-Gram+, Anti-Gram-
5293	DRAMP21240	FK13-a7 (Derived from FK13)	Antimicrobial, Antibacterial, Anti-Gram+, Anti-Gram-
5294	DRAMP21239	FK13-a6 (Derived from FK13)	Antimicrobial, Antibacterial, Anti-Gram+, Anti-Gram-
5295	DRAMP21238	FK13-a5 (Derived from FK13)	Antimicrobial, Antibacterial, Anti-Gram+, Anti-Gram-
5296	DRAMP21237	FK13-a4 (Derived from FK13)	Antimicrobial, Antibacterial, Anti-Gram+, Anti-Gram-
5297	DRAMP21236	FK13-a3 (Derived from FK13)	Antimicrobial, Antibacterial, Anti-Gram+, Anti-Gram-
5298	DRAMP21235	FK13-a2 (Derived from FK13)	Antimicrobial, Antibacterial, Anti-Gram+, Anti-Gram-
5299	DRAMP21167	HYL-13 (Derived from HYL)	Antimicrobial, Antibacterial, Anti-Gram+, Anti-Gram-, Antifungal
5300	DRAMP21234	FK13-a1 (Derived from FK13)	Antimicrobial, Antibacterial, Anti-Gram+, Anti-Gram-
5301	DRAMP21233	R2PLx-22 (Derived from R2PLx)	Antimicrobial, Antibacterial, Anti-Gram+, Anti-Gram-
5302	DRAMP21244	TsAP-S1 (Derived from TsAP-1)	Antimicrobial, Antibacterial, Anti-Gram+, Anti-Gram-, Antifungal
5303	DRAMP21245	TsAP-S2 (Derived from TsAP-2)	Antimicrobial, Antibacterial, Anti-Gram+, Anti-Gram-, Antifungal
5304	DRAMP21246	pEM-2 (Derived from the venom of the snake Bothrops asper)	Antimicrobial, Antibacterial, Anti-Gram+, Anti-Gram-
5305	DRAMP21247	PV (Derived from pEM-2 and MP-VT1)	Antimicrobial, Antibacterial, Anti-Gram+, Anti-Gram-
5306	DRAMP21248	BVP (Derived from pEM-2 and MP-VT1 and MP-B)	Antimicrobial, Antibacterial, Anti-Gram+, Anti-Gram-
5307	DRAMP21249	PVP (Derived from MP-B and MP-VT1)	Antimicrobial, Antibacterial, Anti-Gram+, Anti-Gram-
5308	DRAMP21250	PV3 (Derived from pEM-2 and MP-VT1)	Antimicrobial, Antibacterial, Anti-Gram+, Anti-Gram-
5309	DRAMP21251	AaeAP1 (Scorpionida, Arachricla, Arthropoda)	Antimicrobial, Antibacterial, Anti-Gram+, Antifungal
5310	DRAMP21252	AaeAP2 (Scorpionida, Arachricla, Arthropoda)	Antimicrobial, Antibacterial, Anti-Gram+, Antifungal
5311	DRAMP21253	AaeAP1a (Derived from AaeAP1)	Antimicrobial, Antibacterial, Anti-Gram+, Anti-Gram-, Antifungal
5312	DRAMP21254	AaeAP2a (Derived from AaeAP2)	Antimicrobial, Antibacterial, Anti-Gram+, Anti-Gram-, Antifungal
5313	DRAMP21255	WL1 (Derived from CP-1)	Antimicrobial, Antibacterial, Anti-Gram+, Anti-Gram-
5314	DRAMP21256	WL2 (Derived from CP-1)	Antimicrobial, Antibacterial, Anti-Gram+, Anti-Gram-
5315	DRAMP21257	WL3 (Derived from CP-1)	Antimicrobial, Antibacterial, Anti-Gram+, Anti-Gram-
5316	DRAMP21258	Cecropin P1 (CP-1) (nematodes, animals)	Antimicrobial, Antibacterial, Anti-Gram+, Anti-Gram-
5317	DRAMP21259	Scolopendin 1 (Centipedes, Arthropoda, Animals)	Antimicrobial, Antibacterial, Anti-Gram+, Anti-Gram-, Antifungal
5318	DRAMP21260	KL0A10 (De novo synthesis)	Antimicrobial, Antibacterial, Anti-Gram+, Anti-Gram-
5319	DRAMP21261	KL4A6 (De novo synthesis)	Antimicrobial, Antibacterial, Anti-Gram+, Anti-Gram-
5320	DRAMP21262	KL6A4 (De novo synthesis)	Antimicrobial, Antibacterial, Anti-Gram+, Anti-Gram-
5321	DRAMP21263	KL10A0 (De novo synthesis)	Antimicrobial, Antibacterial, Anti-Gram+, Anti-Gram-
5322	DRAMP21264	LK (De novo synthesis)	Antimicrobial, Antibacterial, Anti-Gram+, Anti-Gram-
5323	DRAMP21265	LK-L1A (Derived from LK)	Antimicrobial, Antibacterial, Anti-Gram+, Anti-Gram-
5324	DRAMP21266	LK-L4A (Derived from LK)	Antimicrobial, Antibacterial, Anti-Gram+, Anti-Gram-
5325	DRAMP21267	LK-L5A (Derived from LK)	Antimicrobial, Antibacterial, Anti-Gram+, Anti-Gram-
5326	DRAMP21268	LK-L7A (Derived from LK)	Antimicrobial, Antibacterial, Anti-Gram+, Anti-Gram-
5327	DRAMP21269	LK-L8A (Derived from LK)	Antimicrobial, Antibacterial, Anti-Gram+, Anti-Gram-
5328	DRAMP21270	LK-L11A (Derived from LK)	Antimicrobial, Antibacterial, Anti-Gram+, Anti-Gram-
5329	DRAMP21271	LK-L12A (Derived from LK)	Antimicrobial, Antibacterial, Anti-Gram+, Anti-Gram-
5330	DRAMP21272	LK-L14A (Derived from LK)	Antimicrobial, Antibacterial, Anti-Gram+, Anti-Gram-
5331	DRAMP21273	LK-L8G (Derived from LK)	Antimicrobial, Antibacterial, Anti-Gram+, Anti-Gram-
5332	DRAMP21274	LK-L8S (Derived from LK)	Antimicrobial, Antibacterial, Anti-Gram+, Anti-Gram-
5333	DRAMP21275	LK-L8P (Derived from LK)	Antimicrobial, Antibacterial, Anti-Gram+, Anti-Gram-
5334	DRAMP21276	LK-L8N (Derived from LK)	Antimicrobial, Antibacterial, Anti-Gram+, Anti-Gram-

5335	DRAMP21277	LK-L8Q (Derived from LK)	Antimicrobial, Antibacterial, Anti-Gram+, Anti-Gram-
5336	DRAMP21278	LK-L8D (Derived from LK)	Antimicrobial, Antibacterial, Anti-Gram+, Anti-Gram-
5337	DRAMP21279	LK-L8E (Derived from LK)	Antimicrobial, Antibacterial, Anti-Gram+, Anti-Gram-
5338	DRAMP21280	LK-L8K (Derived from LK)	Antimicrobial, Antibacterial, Anti-Gram+, Anti-Gram-
5339	DRAMP21281	LK-L8H (Derived from LK)	Antimicrobial, Antibacterial, Anti-Gram+, Anti-Gram-
5340	DRAMP21282	Lt-F1A (Derived from Lt)	Antimicrobial, Antibacterial, Anti-Gram+, Anti-Gram-
5341	DRAMP21283	Lt-I4A (Derived from Lt)	Antimicrobial, Antibacterial, Anti-Gram+, Anti-Gram-
5342	DRAMP21284	Lt-V5A (Derived from Lt)	Antimicrobial, Antibacterial, Anti-Gram+, Anti-Gram-
5343	DRAMP21285	Lt-I8A (Derived from Lt)	Antimicrobial, Antibacterial, Anti-Gram+, Anti-Gram-
5344	DRAMP21286	Lt-F11A (Derived from Lt)	Antimicrobial, Antibacterial, Anti-Gram+, Anti-Gram-
5345	DRAMP21287	Lt-F12A (Derived from Lt)	Antimicrobial, Antibacterial, Anti-Gram+, Anti-Gram-
5346	DRAMP21288	Lt-I4G (Derived from Lt)	Antimicrobial, Antibacterial, Anti-Gram+, Anti-Gram-
5347	DRAMP21289	Lt-I4S (Derived from Lt)	Antimicrobial, Antibacterial, Anti-Gram+, Anti-Gram-
5348	DRAMP21290	Lt-I4N (Derived from Lt)	Antimicrobial, Antibacterial, Anti-Gram+, Anti-Gram-
5349	DRAMP21291	Lt-I4Q (Derived from Lt)	Antimicrobial, Antibacterial, Anti-Gram+, Anti-Gram-
5350	DRAMP21292	Lt-I4H (Derived from Lt)	Antimicrobial, Antibacterial, Anti-Gram+, Anti-Gram-
5351	DRAMP21293	Lt-V5G (Derived from Lt)	Antimicrobial, Antibacterial, Anti-Gram+, Anti-Gram-
5352	DRAMP21294	Lt-V5S (Derived from Lt)	Antimicrobial, Antibacterial, Anti-Gram+, Anti-Gram-
5353	DRAMP21295	Lt-V5N (Derived from Lt)	Antimicrobial, Antibacterial, Anti-Gram+, Anti-Gram-
5354	DRAMP21296	Lt-V5Q (Derived from Lt)	Antimicrobial, Antibacterial, Anti-Gram+, Anti-Gram-
5355	DRAMP21297	Lt-V5H (Derived from Lt)	Antimicrobial, Antibacterial, Anti-Gram+, Anti-Gram-
5356	DRAMP21298	Lt-F11G (Derived from Lt)	Antimicrobial, Antibacterial, Anti-Gram+, Anti-Gram-
5357	DRAMP21299	Lt-F11S (Derived from Lt)	Antimicrobial, Antibacterial, Anti-Gram+, Anti-Gram-
5358	DRAMP21300	Lt-F11N (Derived from Lt)	Antimicrobial, Antibacterial, Anti-Gram+, Anti-Gram-
5359	DRAMP21301	Lt-F11Q (Derived from Lt)	Antimicrobial, Antibacterial, Anti-Gram+, Anti-Gram-
5360	DRAMP21302	Lt-F11H (Derived from Lt)	Antimicrobial, Antibacterial, Anti-Gram+, Anti-Gram-
5361	DRAMP21303	A7-PMAP-23 (Derived from PMAP-23)	Antimicrobial, Antibacterial, Anti-Gram+, Anti-Gram-
5362	DRAMP21304	A21-PMAP-23 (Derived from PMAP-23)	Antimicrobial, Antibacterial, Anti-Gram+, Anti-Gram-
5363	DRAMP21305	R8 (De novo synthesis)	Antimicrobial, Antibacterial, Anti-Gram+, Anti-Gram-
5364	DRAMP21306	TL-1 (Derived from Temporin-1Tl (TL))	Antimicrobial, Antibacterial, Anti-Gram+, Anti-Gram-
5365	DRAMP21307	TL-2 (Derived from Temporin-2Tl (TL))	Antimicrobial, Antibacterial, Anti-Gram+, Anti-Gram-
5366	DRAMP21308	TL-3 (Derived from Temporin-3Tl (TL))	Antimicrobial, Antibacterial, Anti-Gram+, Anti-Gram-
5367	DRAMP21309	TL-4 (Derived from Temporin-4Tl (TL))	Antimicrobial, Antibacterial, Anti-Gram+, Anti-Gram-
5368	DRAMP21311	2W-1 (Derived from PMAP-36)	Antimicrobial, Antibacterial, Anti-Gram+, Anti-Gram-
5369	DRAMP21312	2W-2 (Derived from PMAP-36)	Antimicrobial, Antibacterial, Anti-Gram+, Anti-Gram-
5370	DRAMP21313	2W-3 (Derived from PMAP-36)	Antimicrobial, Antibacterial, Anti-Gram+, Anti-Gram-
5371	DRAMP21314	3W-1 (Derived from PMAP-36)	Antimicrobial, Antibacterial, Anti-Gram+, Anti-Gram-
5372	DRAMP21315	3W-2 (Derived from PMAP-36)	Antimicrobial, Antibacterial, Anti-Gram+, Anti-Gram-
5373	DRAMP21316	3W-3 (Derived from PMAP-36)	Antimicrobial, Antibacterial, Anti-Gram+, Anti-Gram-
5374	DRAMP21317	3W-4 (Derived from PMAP-36)	Antimicrobial, Antibacterial, Anti-Gram+, Anti-Gram-
5375	DRAMP21318	3W-5 (Derived from PMAP-36)	Antimicrobial, Antibacterial, Anti-Gram+, Anti-Gram-
5376	DRAMP21319	3V (Derived from PMAP-36)	Antimicrobial, Antibacterial, Anti-Gram+, Anti-Gram-
5377	DRAMP21320	3L (Derived from PMAP-36)	Antimicrobial, Antibacterial, Anti-Gram+, Anti-Gram-
5378	DRAMP21321	4W (Derived from PMAP-36)	Antimicrobial, Antibacterial, Anti-Gram+, Anti-Gram-
5379	DRAMP21322	RTV (Derived from PMAP-36)	Antimicrobial, Antibacterial, Anti-Gram+, Anti-Gram-
5380	DRAMP21323	RTI (Derived from PMAP-36)	Antimicrobial, Antibacterial, Anti-Gram+, Anti-Gram-
5381	DRAMP21324	RTF (Derived from PMAP-36)	Antimicrobial, Antibacterial, Anti-Gram+, Anti-Gram-
5382	DRAMP21325	RTL (Derived from PMAP-36)	Antimicrobial, Antibacterial, Anti-Gram+, Anti-Gram-
5383	DRAMP21326	RLR (Derived from PMAP-36)	Antimicrobial, Antibacterial, Anti-Gram+, Anti-Gram-
5384	DRAMP21327	RVR (Derived from PMAP-36)	Antimicrobial, Antibacterial, Anti-Gram+, Anti-Gram-
5385	DRAMP21328	RTR (Derived from PMAP-36)	Antimicrobial, Antibacterial, Anti-Gram+, Anti-Gram-
5386	DRAMP21329	RFR (Derived from PMAP-36)	Antimicrobial, Antibacterial, Anti-Gram+, Anti-Gram-
5387	DRAMP21330	KVK (Derived from PMAP-36)	Antimicrobial, Antibacterial, Anti-Gram+, Anti-Gram-
5388	DRAMP21331	KLK (Derived from PMAP-36)	Antimicrobial, Antibacterial, Anti-Gram+, Anti-Gram-
5389	DRAMP21332	KIK (Derived from PMAP-36)	Antimicrobial, Antibacterial, Anti-Gram+, Anti-Gram-
5390	DRAMP21333	RVK (Derived from PMAP-36)	Antimicrobial, Antibacterial, Anti-Gram+, Anti-Gram-

5391	DRAMP21334	Ranatuerin-2Pb (Frogs, amphibians, animals)	Antimicrobial, Antibacterial, Anti-Gram+, Anti-Gram-, Antifungal
5392	DRAMP21335	RPa (Frogs, amphibians, animals)	Antimicrobial, Antibacterial, Anti-Gram+, Anti-Gram-, Antifungal
5393	DRAMP21336	RPb (Frogs, amphibians, animals)	Antimicrobial, Antibacterial, Anti-Gram+, Anti-Gram-, Antifungal
5394	DRAMP21337	BMAP-27 (Bovine, mammals, animals)	Antimicrobial, Antibacterial, Anti-Gram+, Anti-Gram-
5395	DRAMP21338	[Arg]3-VmCT1-NH2 (Derived from VmCT1)	Antimicrobial, Antibacterial, Anti-Gram+, Anti-Gram-, Antifungal
5396	DRAMP21339	[Arg]7-VmCT1-NH2 (Derived from VmCT1)	Antimicrobial, Antibacterial, Anti-Gram+, Anti-Gram-, Antifungal
5397	DRAMP21340	[Arg]11-VmCT1-NH2 (Derived from VmCT1)	Antimicrobial, Antibacterial, Anti-Gram+, Anti-Gram-, Antifungal
5398	DRAMP21341	[Gly]1-VmCT1-NH2 (Derived from VmCT1)	Antimicrobial, Antibacterial, Anti-Gram+, Anti-Gram-, Antifungal
5399	DRAMP21342	[Pro]8-VmCT1-NH2 (Derived from VmCT1)	Antimicrobial, Antibacterial, Anti-Gram+, Anti-Gram-, Antifungal
5400	DRAMP21343	[Leu]9-VmCT1-NH2 (Derived from VmCT1)	Antimicrobial, Antibacterial, Anti-Gram+, Anti-Gram-, Antifungal
5401	DRAMP21344	[Phe]9-VmCT1-NH2 (Derived from VmCT1)	Antimicrobial, Antibacterial, Anti-Gram+, Anti-Gram-, Antifungal
5402	DRAMP21345	[Leu]12-VmCT1-NH2 (Derived from VmCT1)	Antimicrobial, Antibacterial, Anti-Gram+, Anti-Gram-, Antifungal
5403	DRAMP21346	[Tyr]12-VmCT1-NH2 (Derived from VmCT1)	Antimicrobial, Antibacterial, Anti-Gram+, Anti-Gram-, Antifungal
5404	DRAMP21347	2IH1 (De Novo Synthesis)	Antimicrobial, Antibacterial, Anti-Gram-
5405	DRAMP21348	2IH2 (De Novo Synthesis)	Antimicrobial, Antibacterial, Anti-Gram-
5406	DRAMP21349	2IH3 (De Novo Synthesis)	Antimicrobial, Antibacterial, Anti-Gram-
5407	DRAMP21350	2IH4 (De Novo Synthesis)	Antimicrobial, Antibacterial, Anti-Gram-
5408	DRAMP21351	3IH1 (De Novo Synthesis)	Antimicrobial, Antibacterial, Anti-Gram-
5409	DRAMP21352	3IH2 (De Novo Synthesis)	Antimicrobial, Antibacterial, Anti-Gram-
5410	DRAMP21353	3IH3 (De Novo Synthesis)	Antimicrobial, Antibacterial, Anti-Gram-
5411	DRAMP21354	3IH4 (De Novo Synthesis)	Antimicrobial, Antibacterial, Anti-Gram-
5412	DRAMP21355	5Kamp (De Novo Synthesis)	Antimicrobial, Antibacterial, Anti-Gram-
5413	DRAMP21356	4Kamp (De Novo Synthesis)	Antimicrobial, Antibacterial, Anti-Gram-
5414	DRAMP21357	3Kamp (De Novo Synthesis)	Antimicrobial, Antibacterial, Anti-Gram-
5415	DRAMP21358	2Kamp (De Novo Synthesis)	Antimicrobial, Antibacterial, Anti-Gram-
5416	DRAMP21359	1Kamp (De Novo Synthesis)	Antimicrobial, Antibacterial, Anti-Gram-
5417	DRAMP21360	6K-F17-4L (De Novo Synthesis)	Antimicrobial, Antibacterial, Anti-Gram-
5418	DRAMP21361	5Kamp-4L (De Novo Synthesis)	Antimicrobial, Antibacterial, Anti-Gram-
5419	DRAMP21362	4Kamp-4L (De Novo Synthesis)	Antimicrobial, Antibacterial, Anti-Gram-
5420	DRAMP21363	3Kamp-4L (De Novo Synthesis)	Antimicrobial, Antibacterial, Anti-Gram-
5421	DRAMP21364	2Kamp-4L (De Novo Synthesis)	Antimicrobial, Antibacterial, Anti-Gram-
5422	DRAMP21365	1Kamp-4L (De Novo Synthesis)	Antimicrobial, Antibacterial, Anti-Gram-
5423	DRAMP21366	[Lys]1-VmCT1-NH2 (Derived from VmCT1)	Antimicrobial, Antibacterial, Anti-Gram+, Anti-Gram-, Antifungal
5424	DRAMP21367	[Lys]9-VmCT1-NH2 (Derived from VmCT1)	Antimicrobial, Antibacterial, Anti-Gram+, Antifungal
5425	DRAMP21368	[Lys]1[Lys]12-VmCT1-NH2 (Derived from VmCT1)	Antimicrobial, Antibacterial, Anti-Gram+, Anti-Gram-, Antifungal
5426	DRAMP21369	[Lys]3[Lys]7-VmCT1-NH2 (Derived from VmCT1)	Antimicrobial, Antibacterial, Anti-Gram+, Anti-Gram-, Antifungal
5427	DRAMP21370	[Lys]3[Lys]11-VmCT1-NH2 (Derived from VmCT1)	Antimicrobial, Antibacterial, Anti-Gram+, Anti-Gram-, Antifungal
5428	DRAMP21371	[Lys]7[Lys]11-VmCT1-NH2 (Derived from VmCT1)	Antimicrobial, Antibacterial, Anti-Gram+, Anti-Gram-, Antifungal
5429	DRAMP21372	[Lys]3[Lys]7[Lys]11-VmCT1-NH2 (Derived from VmCT1)	Antimicrobial, Antibacterial, Anti-Gram+, Anti-Gram-, Antifungal
5430	DRAMP21373	SP1 (De Novo Synthesis)	Antimicrobial, Antibacterial, Anti-Gram-
5431	DRAMP21374	SP2 (De Novo Synthesis)	Antimicrobial, Antibacterial, Anti-Gram-
5432	DRAMP21375	SP3 (De Novo Synthesis)	Antimicrobial, Antibacterial, Anti-Gram-
5433	DRAMP21376	SP4 (De Novo Synthesis)	Antimicrobial, Antibacterial, Anti-Gram-

5434	DRAMP21377	SP5 (De Novo Synthesis)	Antimicrobial, Antibacterial, Anti-Gram-
5435	DRAMP21378	SP6 (De Novo Synthesis)	Antimicrobial, Antibacterial, Anti-Gram-
5436	DRAMP21379	SP7 (De Novo Synthesis)	Antimicrobial, Antibacterial, Anti-Gram-
5437	DRAMP21380	SP8 (De Novo Synthesis)	Antimicrobial, Antibacterial, Anti-Gram-
5438	DRAMP21381	SP1D * (De Novo Synthesis)	Antimicrobial, Antibacterial, Anti-Gram-
5439	DRAMP21382	SP9 (De Novo Synthesis)	Antimicrobial, Antibacterial, Anti-Gram-
5440	DRAMP21383	SP10 (De Novo Synthesis)	Antimicrobial, Antibacterial, Anti-Gram-
5441	DRAMP21384	SP11 (De Novo Synthesis)	Antimicrobial, Antibacterial, Anti-Gram-
5442	DRAMP21385	SP12 (De Novo Synthesis)	Antimicrobial, Antibacterial, Anti-Gram-
5443	DRAMP21386	SP13 (De Novo Synthesis)	Antimicrobial, Antibacterial, Anti-Gram-
5444	DRAMP21387	SP14 (De Novo Synthesis)	Antimicrobial, Antibacterial, Anti-Gram-
5445	DRAMP21388	SP15 * (De Novo Synthesis)	Antimicrobial, Antibacterial, Anti-Gram-
5446	DRAMP21389	SP15D * (De Novo Synthesis)	Antimicrobial, Antibacterial, Anti-Gram-
5447	DRAMP21390	K17 (Derived from ATG16)	Antimicrobial, Antibacterial, Anti-Gram+, Anti-Gram-, Antifungal
5448	DRAMP21391	K18 (Derived from ATG16)	Antimicrobial, Antibacterial, Anti-Gram+, Anti-Gram-, Antifungal
5449	DRAMP21392	K22 (Derived from ATG16)	Antimicrobial, Antibacterial, Anti-Gram+, Anti-Gram-, Antifungal
5450	DRAMP21393	K22.2 (Derived from ATG16)	Antimicrobial, Antibacterial, Anti-Gram+, Anti-Gram-, Antifungal
5451	DRAMP21394	K30 (Derived from ATG16)	Antimicrobial, Antibacterial, Anti-Gram+, Anti-Gram-, Antifungal
5452	DRAMP21395	K31 (Derived from ATG16)	Antimicrobial, Antibacterial, Anti-Gram+, Anti-Gram-, Antifungal
5453	DRAMP21396	K33 (Derived from ATG16)	Antimicrobial, Antibacterial, Anti-Gram+, Anti-Gram-, Antifungal
5454	DRAMP21397	K36 (Derived from ATG16)	Antimicrobial, Antibacterial, Anti-Gram+, Anti-Gram-, Antifungal
5455	DRAMP21398	K46 (Derived from ATG16)	Antimicrobial, Antibacterial, Anti-Gram+, Anti-Gram-, Antifungal
5456	DRAMP21399	Pep-H (Human, mammals, animals)	Antimicrobial, Antibacterial, Anti-Gram+
5457	DRAMP21400	NBC2253 (De Novo Synthesis)	Antimicrobial, Antibacterial, Anti-Gram+, Anti-Gram-
5458	DRAMP21401	NBC2254 (De Novo Synthesis)	Antimicrobial, Antibacterial, Anti-Gram+, Anti-Gram-
5459	DRAMP21402	B1 (Derived from LL-37 and BMAP-27)	Antimicrobial, Antibacterial, Anti-Gram+, Anti-Gram-
5460	DRAMP21403	peptide 1 (De Novo Synthesis)	Antimicrobial, Antibacterial, Anti-Gram+, Anti-Gram-
5461	DRAMP21404	peptide 2 (De Novo Synthesis)	Antimicrobial, Antibacterial, Anti-Gram+, Anti-Gram-
5462	DRAMP21405	LGL13K (De Novo Synthesis)	Antimicrobial, Antibacterial, Anti-Gram+, Anti-Gram-
5463	DRAMP21406	DGL13K (De Novo Synthesis)	Antimicrobial, Antibacterial, Anti-Gram+, Anti-Gram-
5464	DRAMP21407	Bac4K (Derived from CAMPs)	Antimicrobial, Antibacterial, Anti-Gram+, Anti-Gram-
5465	DRAMP21408	Bac3W (Derived from CAMPs)	Antimicrobial, Antibacterial, Anti-Gram+, Anti-Gram-
5466	DRAMP21409	dBac (Derived from CAMPs)	Antimicrobial, Antibacterial, Anti-Gram+, Anti-Gram-
5467	DRAMP21410	dBac4K (Derived from CAMPs)	Antimicrobial, Antibacterial, Anti-Gram+, Anti-Gram-
5468	DRAMP21411	dBac3W (Derived from CAMPs)	Antimicrobial, Antibacterial, Anti-Gram+, Anti-Gram-
5469	DRAMP21412	dBacK (Derived from CAMPs)	Antimicrobial, Antibacterial, Anti-Gram+, Anti-Gram-
5470	DRAMP21413	dBacK- (cap) (Derived from CAMPs)	Antimicrobial, Antibacterial, Anti-Gram+, Anti-Gram-
5471	DRAMP21414	CecB Q53 (Derived from CecB E53)	Antimicrobial, Antibacterial, Anti-Gram+, Anti-Gram-, Antifungal
5472	DRAMP21415	α4-short (Derived from α4)	Antimicrobial, Antibacterial, Anti-Gram+, Anti-Gram-
5473	DRAMP21416	WV (De Novo Synthesis)	Antimicrobial, Antibacterial, Anti-Gram+, Anti-Gram-
5474	DRAMP21417	WI (De Novo Synthesis)	Antimicrobial, Antibacterial, Anti-Gram+, Anti-Gram-
5475	DRAMP21418	WF (De Novo Synthesis)	Antimicrobial, Antibacterial, Anti-Gram+, Anti-Gram-
5476	DRAMP21419	WW (De Novo Synthesis)	Antimicrobial, Antibacterial, Anti-Gram+, Anti-Gram-
5477	DRAMP21420	AY1C (Derived from AY1)	Antimicrobial, Antibacterial, Anti-Gram-
5478	DRAMP21421	AY1C-AgNP (Derived from AY1)	Antimicrobial, Antibacterial, Anti-Gram-
5479	DRAMP21422	CAY1 (Derived from AY1)	Antimicrobial, Antibacterial, Anti-Gram-
5480	DRAMP21423	CAY1-AgNP (Derived from AY1)	Antimicrobial, Antibacterial, Anti-Gram-
5481	DRAMP21424	B1 (De Novo Synthesis)	Antimicrobial, Antibacterial, Anti-Gram+, Anti-Gram-
5482	DRAMP21425	peptide 2 (Derived from B1)	Antimicrobial, Antibacterial, Anti-Gram+, Anti-Gram-

5483	DRAMP21426	peptide 3 (Derived from B1)	Antimicrobial, Antibacterial, Anti-Gram+, Anti-Gram-
5484	DRAMP21427	peptide 4 (Derived from B1)	Antimicrobial, Antibacterial, Anti-Gram+, Anti-Gram-
5485	DRAMP21428	peptide 5 (Derived from B1)	Antimicrobial, Antibacterial, Anti-Gram+, Anti-Gram-
5486	DRAMP21429	peptide 6 (Derived from B1)	Antimicrobial, Antibacterial, Anti-Gram+, Anti-Gram-
5487	DRAMP21430	peptide 7 (Derived from B1)	Antimicrobial, Antibacterial, Anti-Gram+, Anti-Gram-
5488	DRAMP21431	peptide 8 (Derived from B1)	Antimicrobial, Antibacterial, Anti-Gram+, Anti-Gram-
5489	DRAMP21432	peptide 9 (Derived from B1)	Antimicrobial, Antibacterial, Anti-Gram+, Anti-Gram-
5490	DRAMP21433	peptide 10 (Derived from B1)	Antimicrobial, Antibacterial, Anti-Gram+, Anti-Gram-
5491	DRAMP21434	peptide 11 (Derived from B1)	Antimicrobial, Antibacterial, Anti-Gram+, Anti-Gram-
5492	DRAMP21435	peptide 12 (Derived from B1)	Antimicrobial, Antibacterial, Anti-Gram+
5493	DRAMP21436	peptide 13 (Derived from B1)	Antimicrobial, Antibacterial, Anti-Gram+
5494	DRAMP21437	peptide 14 (Derived from B1)	Antimicrobial, Antibacterial, Anti-Gram+, Anti-Gram-
5495	DRAMP21438	peptide 15 (Derived from B1)	Antimicrobial, Antibacterial, Anti-Gram+, Anti-Gram-
5496	DRAMP21439	peptide 16 (Derived from B1)	Antimicrobial, Antibacterial, Anti-Gram+, Anti-Gram-
5497	DRAMP21440	peptide 17 (Derived from B1)	Antimicrobial, Antibacterial, Anti-Gram+, Anti-Gram-
5498	DRAMP21441	peptide 18 (Derived from B1)	Antimicrobial, Antibacterial, Anti-Gram+
5499	DRAMP21442	peptide 19 (Derived from B1)	Antimicrobial, Antibacterial, Anti-Gram+
5500	DRAMP21443	peptide 20 (Derived from B1)	Antimicrobial, Antibacterial, Anti-Gram+
5501	DRAMP21444	peptide 21 (Derived from B1)	Antimicrobial, Antibacterial, Anti-Gram+, Anti-Gram-
5502	DRAMP21445	peptide 22 (Derived from B1)	Antimicrobial, Antibacterial, Anti-Gram+
5505	DRAMP21448	peptide 25 (Derived from B1)	Antimicrobial, Antibacterial, Anti-Gram+, Anti-Gram-
5508	DRAMP21451	peptide 28 (Derived from B1)	Antimicrobial, Antibacterial, Anti-Gram+, Anti-Gram-
5509	DRAMP21452	peptide 29 (Derived from B1)	Antimicrobial, Antibacterial, Anti-Gram+, Anti-Gram-
5510	DRAMP21453	Hybrid (Derived from Melittin and thanatin)	Antimicrobial, Antibacterial, Anti-Gram+, Anti-Gram-
5511	DRAMP21454	PLP1 (Insects, animals)	Antimicrobial, Antibacterial, Anti-Gram+, Anti-Gram-, Antifungal
5512	DRAMP21455	PLP2 (Insects, animals)	Antimicrobial, Antibacterial, Anti-Gram+, Anti-Gram-, Antifungal
5513	DRAMP21456	PLP3 (Insects, animals)	Antimicrobial, Antibacterial, Anti-Gram+, Anti-Gram-, Antifungal
5514	DRAMP21457	PLP4 (Insects, animals)	Antimicrobial, Antibacterial, Anti-Gram+, Anti-Gram-, Antifungal
5515	DRAMP21458	PLP5 (Insects, animals)	Antimicrobial, Antibacterial, Anti-Gram+, Anti-Gram-, Antifungal
5516	DRAMP21459	PLP6 (Insects, animals)	Antimicrobial, Antibacterial, Anti-Gram+, Anti-Gram-, Antifungal
5517	DRAMP21460	PQ (De Novo Synthesis)	Antimicrobial, Antibacterial, Anti-Gram+, Anti-Gram-
5518	DRAMP21461	PP (De Novo Synthesis)	Antimicrobial, Antibacterial, Anti-Gram+, Anti-Gram-
5519	DRAMP21462	GG (De Novo Synthesis)	Antimicrobial, Antibacterial, Anti-Gram+, Anti-Gram-
5520	DRAMP21463	Qa (De Novo Synthesis)	Antimicrobial, Antibacterial, Anti-Gram+, Anti-Gram-
5521	DRAMP21464	Qna (De Novo Synthesis)	Antimicrobial, Antibacterial, Anti-Gram+, Anti-Gram-
5522	DRAMP21465	P1-Ll-1577 (De Novo Synthesis)	Antimicrobial, Antibacterial, Anti-Gram+, Anti-Gram-
5523	DRAMP21466	P2-Ll-1298 (De Novo Synthesis)	Antimicrobial, Antibacterial, Anti-Gram+, Anti-Gram-
5524	DRAMP21467	P3-Ll-2085 (De Novo Synthesis)	Antimicrobial, Antibacterial, Anti-Gram+, Anti-Gram-
5525	DRAMP21310	RK12 (Derived from PMAP-36)	Antimicrobial, Antibacterial, Anti-Gram+, Anti-Gram-
5526	DRAMP21577	LP1	Antimicrobial, Antifungal
5527	DRAMP21481	6K-F17	Antimicrobial, Antibacterial, Anti-Gram-
5528	DRAMP21494	MEP-N	Antimicrobial, Antibacterial, Anti-Gram+, Anti-Gram-, Antifungal
5529	DRAMP21579	Val-nHSLP	Antimicrobial, Antibacterial, Anti-Gram+, Anti-Gram-
5530	DRAMP21581	Cap-nHSLP	Antimicrobial, Antibacterial, Anti-Gram+
5531	DRAMP21596	Ac-UM-14W	Non-antimicrobial
5538	DRAMP21616	DRIM	Antimicrobial, Antibacterial, Anti-Gram+, Anti-Gram-
5539	DRAMP21618	WWSP	Antimicrobial, Antibacterial, Anti-Gram+, Anti-Gram-
5540	DRAMP21620	KFGF	Antimicrobial, Antibacterial, Anti-Gram+, Anti-Gram-
5541	DRAMP21622	MAP-1	Antimicrobial, Antibacterial, Anti-Gram+, Anti-Gram-
5542	DRAMP21626	E2EM23W	Antimicrobial, Antibacterial, Anti-Gram+
5543	DRAMP21627	E2EM15W	Antimicrobial, Antibacterial, Anti-Gram+, Anti-Gram-
5544	DRAMP21631	SLAY-P1	Antimicrobial, Antibacterial, Anti-Gram-

5545	DRAMP21632	SLAY-P2	Antimicrobial, Antibacterial, Anti-Gram-
5546	DRAMP21633	SLAY-P3 cyclic	Antimicrobial, Antibacterial, Anti-Gram-
5547	DRAMP21634	SLAY-P4	Antimicrobial, Antibacterial, Anti-Gram-
5548	DRAMP21635	SLAY-P5 cyclic	Antimicrobial, Antibacterial, Anti-Gram-
5549	DRAMP21636	SLAY-P6	Antimicrobial, Antibacterial, Anti-Gram-
5550	DRAMP21637	SLAY-P7	Antimicrobial, Antibacterial, Anti-Gram-
5551	DRAMP21638	SLAY-P8	Antimicrobial, Antibacterial, Anti-Gram-
5552	DRAMP21639	SLAY-P9	Antimicrobial, Antibacterial, Anti-Gram-
5553	DRAMP21640	SLAY-P10	Antimicrobial, Antibacterial, Anti-Gram-
5554	DRAMP21641	SLAY-P11	Antimicrobial, Antibacterial, Anti-Gram-
5555	DRAMP21642	SLAY-P12	Antimicrobial, Antibacterial, Anti-Gram-
5556	DRAMP21643	SLAY-P13	Antimicrobial, Antibacterial, Anti-Gram-
5557	DRAMP21644	SLAY-P14	Antimicrobial, Antibacterial, Anti-Gram-
5558	DRAMP21645	SLAY-P15	Antimicrobial, Antibacterial, Anti-Gram-
5559	DRAMP21646	SLAY-P16	Antimicrobial, Antibacterial, Anti-Gram-
5560	DRAMP21647	SLAY-P17	Antimicrobial, Antibacterial, Anti-Gram-
5561	DRAMP21648	SLAY-P18	Antimicrobial, Antibacterial, Anti-Gram-
5562	DRAMP21649	SLAY-P3	Antimicrobial, Antibacterial, Anti-Gram-
5563	DRAMP21650	SLAY-P5	Antimicrobial, Antibacterial, Anti-Gram-
