# Supplementary material for: AMPing Up the Search: A Structural and Functional Repository of Antimicrobial Peptides for Biofilm Studies, and a Case Study of Its Application to Corynebacterium striatum, an Emerging Pathogen
Source: Front Cell Infect Microbiol. 2021 Dec 16;11:803774. doi: 10.3389/fcimb.2021.803774 (PMC8716830; doi:10.3389/fcimb.2021.803774)
Supplement: Supplementary file 5 [file Table_2.pdf]

PepID	DRAMP_ID	Name
2	DRAMP00005	Epicidin 280 (Bacteriocin)
4	DRAMP00032	Ruminococcin A (RumA; Bacteriocin)
6	DRAMP00068	Aureocin A53 (Bacteriocin)
7	DRAMP00069	Garvieacin Q (GarQ; Bacteriocin)
8	DRAMP00074	Enterocin P (Pediocin-like peptide; Bacteriocin)
9	DRAMP00089	Bacteriocin E50-52 (Preclinical)
10	DRAMP00090	Carnobacteriocin B2 (CbnB2; Bacteriocin)
11	DRAMP00105	Enterocin X alpha (Two-peptide bacteriocin)
12	DRAMP00106	Enterocin X beta (Two-peptide bacteriocin)
13	DRAMP00107	Bacteriocin L-1077
14	DRAMP00126	Plantaricin E (PlnE; Bacteriocin)
15	DRAMP00127	Plantaricin F (PlnF; Bacteriocin)
16	DRAMP00128	Plantaricin J (PlnJ; Bacteriocin)
17	DRAMP00129	Plantaricin K (PlnK; Bacteriocin)
18	DRAMP00136	Enterocin E-760 (Bacteriocin)
19	DRAMP00171	Lactocyclicin Q (Bacteriocin)
20	DRAMP00173	Leucocyclicin Q (Bacteriocin)
21	DRAMP00177	Enterocin B (EntB; Bacteriocin)
22	DRAMP00178	Enterocin EJ97 (EntEJ97; Bacteriocin)
23	DRAMP00189	Leucocin Q (Bacteriocin)
24	DRAMP00190	Leucocin N (Bacteriocin)
26	DRAMP00201	Amythiamicin A/B (Bacteriocin)
27	DRAMP00204	Thiocillin GE37468 (Antibiotic GE37468; Bacteriocin)
28	DRAMP00218	Plantazolicin (PZN; Bacteriocin)
31	DRAMP00244	Hominicin (Bacteriocin)
32	DRAMP00254	Propionicin-F (Bacteriocin)
33	DRAMP00275	Snakin-1 (StSN1; Cys-rich; Plant defensin)
34	DRAMP00336	ChaC7 (Chassatide C7; uncyclotides; Plant defensin)
35	DRAMP00337	ChaC8 (Chassatide C8; uncyclotides; Plant defensin)
36	DRAMP00338	ChaC11 (Chassatide C11; uncyclotides; Plant defensin)
44	DRAMP00431	Defensin-like protein 2 (Cp-thionin II; Cp-thionin-2; Gamma-thionin II; Plant defensin)
49	DRAMP00764	Piceain 1 (Plants)
50	DRAMP00765	Piceain 2 (Plants)
51	DRAMP00766	JCpep7 (Plants)
52	DRAMP00774	Hedyotide B1 (hB1; Plants)
53	DRAMP00795	Cliotide T1 (cT1; Plant defensin)
54	DRAMP00798	Cliotide T4 (cT4; Plant defensin)
55	DRAMP00856	Kalata-B1 (Plant defensin)
56	DRAMP00877	Circulin-A (CIRA; Plant defensin)
57	DRAMP00878	Circulin-B (CIRB; Plant defensin)
58	DRAMP01374	Odorranain-D1 (OdD1; Frogs, amphibians, animals)
59	DRAMP01373	Odorranain-C1 (OdC1; Frogs, amphibians, animals)
60	DRAMP01372	Odorranain-B1 (Frogs, amphibians, animals)
64	DRAMP01016	Antimicrobial peptide 1 (MJ-AMP1; Plant defensin)
65	DRAMP01017	Antimicrobial peptide 2 (MJ-AMP2; Plant defensin)
66	DRAMP01018	Cyclopsychotride-A (CPT; Plant defensin)

68	DRAMP18193	Cathelicidin-related peptide crotalicidin
69	DRAMP01064	Anticancerous peptide 1 (Cr-ACP1; Plants)
72	DRAMP01082	Alyteserin-2a (toads, amphibians, animals)
75	DRAMP01088	Alyteserin-1Ma (toads, amphibians, animals)
76	DRAMP01089	Alyteserin-1Mb (toads, amphibians, animals)
77	DRAMP01090	Alyteserin-2Ma (toads, amphibians, animals)
78	DRAMP01091	Alyteserin-2Mb (toads, amphibians, animals)
83	DRAMP02090	Brevinin-1Lb (Frogs, amphibians, animals)
84	DRAMP02091	Brevinin-1Ba (Frogs, amphibians, animals)
85	DRAMP01829	Temporin-1Lc (Temporin 1Lc; Frogs, amphibians, animals)
86	DRAMP01828	Temporin-1Lb (Temporin 1Lb; Frogs, amphibians, animals)
87	DRAMP01518	Esculentin-2L (Frogs, amphibians, animals)
88	DRAMP01827	Temporin-1La (Temporin 1La; Frogs, amphibians, animals)
90	DRAMP01516	Esculentin-2B (Frogs, amphibians, animals)
91	DRAMP02077	Brevinin-1Pb (Frogs, amphibians, animals)
92	DRAMP01830	Temporin-1P (Temporin-1M; Temporin-1CSa; Frogs, amphibians, animals)
93	DRAMP01151	Uperin-3.5 (toads, amphibians, animals)
94	DRAMP01152	Uperin-3.6 (toads, amphibians, animals)
95	DRAMP01153	Ala4-uperin 3.6 (toads, amphibians, animals)
96	DRAMP01154	Ala7-uperin 3.6 (toads, amphibians, animals)
97	DRAMP01155	Ala14-uperin 3.6 (toads, amphibians, animals)
98	DRAMP01162	Buforin-1 (Buforin I; Fragment of Histone H2A; toads, amphibians, animals)
99	DRAMP01163	Buforin-2 (Buforin II; Fragment of Histone H2A; toads, amphibians, animals)
100	DRAMP01164	Bombinin (toads, amphibians, animals)
102	DRAMP01167	Hylaseptin-P1 (HSP1)
103	DRAMP01170	Distinctin 2 (Frogs, amphibians, animals)
104	DRAMP01174	Ocellatin-4 (Frogs, amphibians, animals)
106	DRAMP01182	Ocellatin-P1 (Pentadactylin; Frogs, amphibians, animals)
107	DRAMP01184	SPX(1-22)(truncated peptide of Syphaxin; Frogs, amphibians, animals)
108	DRAMP01185	SPX(1-16)(truncated peptide of Syphaxin; Frogs, amphibians, animals)
109	DRAMP01188	Chensinin-1ZHa (Frogs, amphibians, animals)
110	DRAMP01189	Andersonin-W1 (Frogs, amphibians, animals)
111	DRAMP01190	Andersonin-W2 (Frogs, amphibians, animals)
112	DRAMP01191	Andersonin-X1 (Frogs, amphibians, animals)
113	DRAMP01192	Andersonin-Y1 (Frogs, amphibians, animals)
114	DRAMP01194	Andersonin-C1 (Frogs, amphibians, animals)
115	DRAMP01195	Andersonin-D1 (Frogs, amphibians, animals)
116	DRAMP01199	Hejiangin-A1 (Frogs, amphibians, animals)
117	DRAMP01200	Hejiangin-F1 (frog, amphibians, animals)
118	DRAMP01201	Schmackerin-C1 (Frogs, amphibians, animals)
119	DRAMP01202	Fallaxidin 3.2 (Frogs, amphibians, animals)
120	DRAMP01203	Fallaxidin 3.1 (Frogs, amphibians, animals)
121	DRAMP01204	Fallaxidin 4.1 (Frogs, amphibians, animals)
122	DRAMP01208	Pleurain-A1 (Pleurain A1; Frogs, amphibians, animals)
123	DRAMP01209	Pleurain-A2 (Pleurain A2; Frogs, amphibians, animals)
124	DRAMP01214	Kassinatuerin-2Ma (Frogs, amphibians, animals)
125	DRAMP01218	Kassinatuerin-1 (Frogs, amphibians, animals)

126	DRAMP01219	Palustrin-2LTa (Frogs, amphibians, animals)
127	DRAMP01222	Palustrin-2AJ1 (PL2AJ1; Frogs, amphibians, animals)
132	DRAMP01232	Palustrin-2ISc (Frogs, amphibians, animals)
136	DRAMP01237	Palustrin-2ISa (Frogs, amphibians, animals)
137	DRAMP01238	Palustrin-2SIb (Frogs, amphibians, animals)
138	DRAMP01244	Japonicin-1 (Frogs, amphibians, animals)
139	DRAMP01245	Japonicin-1CDYa (Frogs, amphibians, animals)
140	DRAMP01246	Japonicin-2 (Frogs, amphibians, animals)
141	DRAMP01248	Dybowskin-1 (Frogs, amphibians, animals)
142	DRAMP01249	Dybowskin-2 (Frogs, amphibians, animals)
143	DRAMP01250	Dybowskin-3 (Frogs, amphibians, animals)
144	DRAMP01251	Dybowskin-4 (Frogs, amphibians, animals)
145	DRAMP01252	Dybowskin-5 (Frogs, amphibians, animals)
146	DRAMP01253	Dybowskin-6 (Frogs, amphibians, animals)
147	DRAMP01254	Dybowskin-1CDYa (Frogs, amphibians, animals)
148	DRAMP01255	Dybowskin-2CDYa (Chensinin-1; Frogs, amphibians, animals)
149	DRAMP01257	Dermadistinctin-K (DD K; Frogs, amphibians, animals)
150	DRAMP01258	Dermadistinctin-L (DD L; Frogs, amphibians, animals)
151	DRAMP01259	Dermadistinctin-M (DD M; Frogs, amphibians, animals)
152	DRAMP01260	Dermadistinctin-Q1 (DD Q1; Frogs, amphibians, animals)
153	DRAMP01261	Dermadistinctin-Q2 (DD Q2; Frogs, amphibians, animals)
155	DRAMP01301	Phylloseptin-1 (PS-1; Frogs, amphibians, animals)
156	DRAMP01302	Phylloseptin-2 (PS-2; Frogs, amphibians, animals)
157	DRAMP01303	Phylloseptin-3 (PS-3; Frogs, amphibians, animals)
158	DRAMP01305	Phylloseptin-7 (PS-7; Frogs, amphibians, animals)
159	DRAMP01306	Phylloseptin-7 (PS-7; Frogs, amphibians, animals)
160	DRAMP01314	Phylloseptin 12 (PS-12; Frogs, amphibians, animals)
161	DRAMP01319	Cathelicidin-AL (Gly-rich; Frogs, amphibians, animals)
162	DRAMP01320	Ranacyclin-B-AL1 (Frogs, amphibians, animals)
163	DRAMP01339	Amolopin-2a (Frogs, amphibians, animals)
164	DRAMP01341	Amolopin-1b (Frogs, amphibians, animals)
165	DRAMP01346	Prepromelittin-related peptide (Frogs, amphibians, animals)
166	DRAMP01347	Prepromelittin-related peptide (Frogs, amphibians, animals)
167	DRAMP01350	Tigerinin-1 (Frogs, amphibians, animals)
168	DRAMP01351	Tigerinin-2 (Frogs, amphibians, animals)
169	DRAMP01352	Tigerinin-3 (Frogs, amphibians, animals)
170	DRAMP01353	Tigerinin-4 (Frogs, amphibians, animals)
171	DRAMP01354	Peptide leucine arginine (pLR; Frogs, amphibians, animals)
172	DRAMP01355	Ranalexin (Frogs, amphibians, animals)
173	DRAMP01393	Odorranain-W1 (OdW1; Frogs, amphibians, animals)
174	DRAMP01358	Ranalexin-Vb (Frogs, amphibians, animals)
175	DRAMP01359	Ranalexin-1G (Frogs, amphibians, animals)
176	DRAMP01360	Frenatin-1 (Frogs, amphibians, animals)
177	DRAMP01361	Frenatin-2 (Frogs, amphibians, animals)
178	DRAMP01362	Frenatin-3 (Frogs, amphibians, animals)
179	DRAMP01364	Maculatin-1.1 (Frogs, amphibians, animals)
180	DRAMP01367	Maculatin-1.4 (frog, amphibia, animals)

181	DRAMP01368	Maculatin-2.1 (Frogs, amphibians, animals)
182	DRAMP01370	Oh-defensin (O. hainana defensin; spiders, animals)
183	DRAMP01371	Odorranain-NR (Frogs, amphibians, animals)
184	DRAMP00931	Antimicrobial peptide 3 (Cn-AMP3; Plant defensin)
185	DRAMP00930	Antimicrobial peptide 2 (Cn-AMP2; Plant defensin)
186	DRAMP00929	Antimicrobial peptide 1 (Cn-AMP1; Plant defensin)
187	DRAMP03542	Neurokinin A (NKA; chicken, animals)
188	DRAMP04532	Myxinidin (Hagfish, animals)
189	DRAMP02344	40S ribosomal protein S30 (Fish, chordates, animals)
190	DRAMP02993	Abaecin (Pro-rich; insects, arthropods, invertebrates, animals)
191	DRAMP02997	Apidaecin-1B (Apidaecin IB; Insects, animals)
192	DRAMP18322	Hominicin (Bacteriocin)
193	DRAMP02840	Lactoferricin B (Lfcin B; mammals, animals)
198	DRAMP02246	Ranatuerin-1C (Ranatuerin 1C; Frogs, amphibians, animals)
199	DRAMP01394	Odorranain-W2 (Frogs, amphibians, animals)
200	DRAMP01395	Odorranain-A-OA1 (Frogs, amphibians, animals)
201	DRAMP01396	Odorranain-F-OA1 (Frogs, amphibians, animals)
202	DRAMP01397	Odorranain-F-OA2 (Frogs, amphibians, animals)
203	DRAMP01398	Odorranain-F-OA3 (Frogs, amphibians, animals)
204	DRAMP01399	Odorranain-F-OA4 (Frogs, amphibians, animals)
206	DRAMP01401	Odorranain-F-OW1 (Frogs, amphibians, animals)
207	DRAMP01402	Odorranain-J-OA1 (Frogs, amphibians, animals)
208	DRAMP01403	Odorranain-J-OA2 (Frogs, amphibians, animals)
209	DRAMP01409	Nigrocin-OR1 (Frogs, amphibians, animals)
210	DRAMP01410	Nigrocin-OR2 (Frogs, amphibians, animals)
211	DRAMP01411	Nigrocin-OR3 (Frogs, amphibians, animals)
212	DRAMP01412	Nigrocin-2HSa (Frogs, amphibians, animals)
213	DRAMP01413	Nigrocin-2HSb (Frogs, amphibians, animals)
214	DRAMP01414	Nigrocin-2ISa (Frogs, amphibians, animals)
215	DRAMP01415	Nigrocin-2ISb (Frogs, amphibians, animals)
216	DRAMP01416	Nigrocin-2ISc (Frogs, amphibians, animals)
218	DRAMP01418	Nigrocin-2GRb (Frogs, amphibians, animals)
220	DRAMP01420	Nigrocin-OG4 (Frogs, amphibians, animals)
221	DRAMP01421	Nigrocin-OG5 (Frogs, amphibians, animals)
222	DRAMP01422	Nigrosin-OG21 (Frogs, amphibians, animals)
223	DRAMP01423	Nigrosin-OG13 (Frogs, amphibians, animals)
224	DRAMP01426	Nigrocin-1-OA1 (Frogs, amphibians, animals)
225	DRAMP01427	Nigrocin-1-OA2 (Frogs, amphibians, animals)
226	DRAMP01428	Nigrocin-1-OA3 (Frogs, amphibians, animals)
227	DRAMP01429	Nigrocin-1-OR1 (Frogs, amphibians, animals)
228	DRAMP01430	Nigrocin-1-OR2 (Frogs, amphibians, animals)
229	DRAMP01431	Nigrocin-1-OR3 (Frogs, amphibians, animals)
230	DRAMP01432	Nigrocin-1-OW2 (Frogs, amphibians, animals)
231	DRAMP01433	Nigrocin-1-OW3 (Frogs, amphibians, animals)
232	DRAMP01434	Nigrocin-1-OW4 (Frogs, amphibians, animals)
233	DRAMP01435	Nigrocin-1-OW5 (Frogs, amphibians, animals)
234	DRAMP01436	Nigrocin-1-OW1 (Frogs, amphibians, animals)

236	DRAMP01438	Nigrocin-2JDa (Frogs, amphibians, animals)
237	DRAMP01439	Nigrocin-2JDb (Odorranain-H2; Frogs, amphibians, animals)
238	DRAMP01440	Nigrocin-2LVb (Frogs, amphibians, animals)
242	DRAMP01447	Esculentin-2CHa (Frogs, amphibians, animals)
243	DRAMP01452	Esculentin-1LTa (Frogs, amphibians, animals)
244	DRAMP01453	Esculentin-2LTa (Frogs, amphibians, animals)
245	DRAMP01454	Esculentin-2JDa (Frogs, amphibians, animals)
246	DRAMP01456	Esculentin-2PLa (Frogs, amphibians, animals)
247	DRAMP01457	Esculentin-1V (Frogs, amphibians, animals)
248	DRAMP01458	Esculentin-2V (Frogs, amphibians, animals)
249	DRAMP01461	Esculentin-1S (Frogs, amphibians, animals)
250	DRAMP01462	Esculentin-2S (Frogs, amphibians, animals)
251	DRAMP01469	Esculentin-2-Ala (Frogs, amphibians, animals)
252	DRAMP01470	Esculentin-2-ALb (Frogs, amphibians, animals)
253	DRAMP01471	Esculentin-1PLa (Frogs, amphibians, animals)
254	DRAMP01472	Esculentin-1PLb (Frogs, amphibians, animals)
256	DRAMP01474	Esculentin-1ARa (Frogs, amphibians, animals)
257	DRAMP01475	Esculentin-1ARb (Frogs, amphibians, animals)
258	DRAMP01476	Esculentin-2HSa (Frogs, amphibians, animals)
259	DRAMP01477	Esculentin-1HSa (Frogs, amphibians, animals)
260	DRAMP01479	Esculentin-1CPa (Frogs, amphibians, animals)
261	DRAMP01480	Esculentin-2CPa (Frogs, amphibians, animals)
262	DRAMP01482	Esculentin-1ISa (Frogs, amphibians, animals)
263	DRAMP01483	Esculentin-1ISb (Frogs, amphibians, animals)
264	DRAMP01484	Esculentin-2ISa (Frogs, amphibians, animals)
265	DRAMP01486	Esculentin-1GRa (Frogs, amphibians, animals)
266	DRAMP01490	Esculentin-2A (Frogs, amphibians, animals)
267	DRAMP01491	Esculentin-1B (Frogs, amphibians, animals)
268	DRAMP01493	Esculentin-1-OA1 (Frogs, amphibians, animals)
269	DRAMP01494	Esculentin-1-OA2 (Frogs, amphibians, animals)
270	DRAMP01495	Esculentin-1-OA3 (Frogs, amphibians, animals)
271	DRAMP01496	Esculentin-1-OA4 (Frogs, amphibians, animals)
272	DRAMP01497	Esculentin-1-OA5 (Frogs, amphibians, animals)
273	DRAMP01499	Esculentin-1-OR1 (Frogs, amphibians, animals)
274	DRAMP01501	Esculentin-1-OR3 (Frogs, amphibians, animals)
275	DRAMP01502	Esculentin-1-OR4 (Frogs, amphibians, animals)
276	DRAMP01503	Esculentin-1-OR5 (Frogs, amphibians, animals)
277	DRAMP01504	Esculentin-2-OA1 (Frogs, amphibians, animals)
278	DRAMP01505	Esculentin-2-OA2 (Frogs, amphibians, animals)
280	DRAMP01507	Esculentin-2-OR1 (Frogs, amphibians, animals)
281	DRAMP01508	Esculentin-2-OR2 (Frogs, amphibians, animals)
282	DRAMP01509	Esculentin-2-OR3 (Frogs, amphibians, animals)
283	DRAMP01510	Esculentin-2-OR4 (Frogs, amphibians, animals)
284	DRAMP01511	Esculentin-2-OR5 (Frogs, amphibians, animals)
285	DRAMP01513	Esculentin-1 (Frogs, amphibians, animals)
286	DRAMP01520	Rugosin-A (Frogs, amphibians, animals)
287	DRAMP01521	Rugosin-B (Frogs, amphibians, animals)

288	DRAMP01524	Rugosin-RN1 (Frogs, amphibians, animals)
289	DRAMP01525	Rugosin-RN3 (Frogs, amphibians, animals)
290	DRAMP01526	Rugosin-RN5 (Frogs, amphibians, animals)
291	DRAMP01533	Nigroain-B1 (Frogs, amphibians, animals)
292	DRAMP01539	Nigroain-C2 (Frogs, amphibians, animals)
293	DRAMP01542	Nigroain-D3 (Frogs, amphibians, animals)
294	DRAMP01543	Nigroain-E1 (Frogs, amphibians, animals)
295	DRAMP01546	Nigroain-K1 (Frogs, amphibians, animals)
296	DRAMP01547	Nigroain-K2 (Frogs, amphibians, animals)
297	DRAMP01549	Caerin-1.1 (Frogs, amphibians, animals)
298	DRAMP01550	Caerin-1.11 (Frogs, amphibians, animals)
299	DRAMP01552	Caerin-1.3 (Frogs, amphibians, animals)
300	DRAMP01553	Caerin-1.4 (Frogs, amphibians, animals)
301	DRAMP01555	Caerin-1.5 (Frogs, amphibians, animals)
302	DRAMP01560	Caerin-1.9 (Frogs, amphibians, animals)
304	DRAMP01563	Caerin-2.2 (Frogs, amphibians, animals)
305	DRAMP01567	Caerin-2.6 (Frogs, amphibians, animals)
306	DRAMP01568	Caerin-2.7 (Frogs, amphibians, animals)
307	DRAMP01570	Caerin-3.2 (Frogs, amphibians, animals)
308	DRAMP01573	Caerin-3.5 (Frogs, amphibians, animals)
309	DRAMP01574	Caerin-4.1 (Frogs, amphibians, animals)
310	DRAMP01576	Caerin-4.3 (Frogs, amphibians, animals)
311	DRAMP01577	Caerin-1.10 (Frogs, amphibians, animals)
315	DRAMP01584	Caerin-1.17 (Frogs, amphibians, animals)
316	DRAMP01585	Caerin-1.18 (Frogs, amphibians, animals)
317	DRAMP01586	Caerin-1.19 (Frogs, amphibians, animals)
318	DRAMP01587	Citropin-1.1 (Frogs, amphibians, animals)
319	DRAMP01588	Citropin-1.1 sm1 (Frogs, amphibians, animals)
320	DRAMP01589	Citropin-1.1 sm2 (Frogs, amphibians, animals)
321	DRAMP01590	Citropin 1.1 M14 (Frogs, amphibians, animals)
322	DRAMP01591	Citropin 1.1 M15 (Frogs, amphibians, animals)
323	DRAMP01594	Citropin-1.2 (Frogs, amphibians, animals)
324	DRAMP01600	Citropin-1.3 (Frogs, amphibians, animals)
325	DRAMP01601	Citropin-2.1 (Frogs, amphibians, animals)
326	DRAMP01602	Citropin-2.1.3 (Frogs, amphibians, animals)
327	DRAMP01603	Citropin 1.1.3 (Frogs, amphibians, animals)
328	DRAMP01606	Aurein-1.1 (Frogs, amphibians, animals)
329	DRAMP01607	Aurein-1.2 (Frogs, amphibians, animals)
330	DRAMP01608	Aurein-2.1 (Frogs, amphibians, animals)
331	DRAMP01612	Aurein-2.5 (Frogs, amphibians, animals)
332	DRAMP01613	Aurein-2.6 (Frogs, amphibians, animals)
333	DRAMP01614	Aurein-3.1 (Frogs, amphibians, animals)
334	DRAMP01617	Aurein-3.2 (Frogs, amphibians, animals)
335	DRAMP01618	Aurein-3.3 (Frogs, amphibians, animals)
336	DRAMP01620	Aurein-5.2 (Frogs, amphibians, animals)
337	DRAMP01621	Bombinin-H1 (Frogs, amphibians, animals)
338	DRAMP01623	Bombinin-H4 (bombinin H isomers; Frogs, amphibians, animals)

339	DRAMP01626	Bombinin-H5 (Frogs, amphibians, animals)
340	DRAMP01627	Skin peptide tyrosine-tyrosine (Skin-PYY; SPYY; Frogs, amphibians, animals)
341	DRAMP01628	Phylloxin (Frogs, amphibians, animals)
343	DRAMP01639	Dermaseptin-1 (DShypo01, DPh-1; Frogs, amphibians, animals)
344	DRAMP01643	Dermaseptin-5 (DShypo05, DS 01; Frogs, amphibians, animals)
345	DRAMP01646	Adenoregulin (Dermaseptin BII; Dermaseptin B2; Frogs, amphibians, animals)
346	DRAMP01647	DRP-PBN1 (Frogs, amphibians, animals)
347	DRAMP01648	Dermaseptin-like PBN2 (DRP-PBN2; Plasticin-B1a; Frogs, amphibians, animals)
348	DRAMP01649	Dermaseptin-BI (Dermaseptin B1; Frogs, amphibians, animals)
349	DRAMP01650	Dermaseptin-B3 (Dermaseptin BIII; Frogs, amphibians, animals)
350	DRAMP01651	Dermaseptin-B4 (Dermaseptin BIV; Frogs, amphibians, animals)
352	DRAMP01668	Dermaseptin-1 (DS I; Dermaseptin-S1, DS1; Frogs, amphibians, animals)
357	DRAMP01702	Dermaseptin-H5 (Dermaseptin-like peptide 5, DMS5; Frogs, amphibians, animals)
359	DRAMP01730	Temporin-A (Frogs, amphibians, animals)
360	DRAMP01731	Temporin-ALd (Frogs, amphibians, animals)
361	DRAMP01732	Temporin-ALe (Frogs, amphibians, animals)
362	DRAMP01733	Temporin-ALf (Frogs, amphibians, animals)
363	DRAMP01734	Temporin-ALg (Frogs, amphibians, animals)
364	DRAMP01735	Temporin-ALh (Frogs, amphibians, animals)
365	DRAMP01736	Temporin-ALi (Frogs, amphibians, animals)
366	DRAMP01737	Temporin-ALj (Frogs, amphibians, animals)
367	DRAMP01738	Temporin-ALk (Frogs, amphibians, animals)
368	DRAMP01739	Temporin-B (Frogs, amphibians, animals)
369	DRAMP01750	Temporin-1PLa (Frogs, amphibians, animals)
370	DRAMP01751	Temporin-LT1 (Frogs, amphibians, animals)
371	DRAMP01752	Temporin-LT2 (Frogs, amphibians, animals)
372	DRAMP01753	Temporin-1CEa (Frogs, amphibians, animals)
373	DRAMP01754	Temporin-1CEb (Frogs, amphibians, animals)
374	DRAMP01755	Temporin-1TSa (Frogs, amphibians, animals)
375	DRAMP01759	Temporin-1SPb (Frogs, amphibians, animals)
376	DRAMP01764	Temporin-1TGa (Frogs, amphibians, animals)
377	DRAMP01765	Temporin-1TGb (Frogs, amphibians, animals)
378	DRAMP01766	Temporin-1TGc (Frogs, amphibians, animals)
379	DRAMP01768	Temporin-1SKa (Frogs, amphibians, animals)
381	DRAMP01771	Temporin-1Oa (Frogs, amphibians, animals)
382	DRAMP01773	Temporin-1Oc (Frogs, amphibians, animals)
383	DRAMP01775	Temporin-1Sa (Frogs, amphibians, animals)
384	DRAMP01776	Temporin-1Sb (Temporin-SHb; Frogs, amphibians, animals)
385	DRAMP01777	Temporin-1Sc (Temporin-SHc; Frogs, amphibians, animals)
386	DRAMP01779	Temporin-SHf (Frogs, amphibians, animals)
387	DRAMP01780	Temporin-SHa (Temporin-1Sa; Frogs, amphibians, animals)
388	DRAMP01782	Temporin-LTa (Frogs, amphibians, animals)
389	DRAMP01783	Temporin-LTb (Frogs, amphibians, animals)
390	DRAMP01784	Temporin-LTc (Frogs, amphibians, animals)
391	DRAMP01785	Temporin-CPa (Frogs, amphibians, animals)
392	DRAMP01787	Temporin-HN1 (Frogs, amphibians, animals)
393	DRAMP01788	Temporin-HN2 (Frogs, amphibians, animals)

394	DRAMP01789	Temporin-1Va (Temporin 1Va; Frogs, amphibians, animals)
395	DRAMP01790	Temporin-1Vb (Temporin 1Vb; Frogs, amphibians, animals)
396	DRAMP01791	Temporin-1Vc (Temporin 1Vc; Frogs, amphibians, animals)
397	DRAMP01807	Temporin-RN1 (Frogs, amphibians, animals)
398	DRAMP01808	Temporin-RN3 (Frogs, amphibians, animals)
399	DRAMP01811	Temporin-Ra (Frogs, amphibians, animals)
400	DRAMP01812	Temporin-Rb (Frogs, amphibians, animals)
401	DRAMP01815	Temporin-GH (AMP-5; Frogs, amphibians, animals)
402	DRAMP01816	Temporin-1CSb (Frogs, amphibians, animals)
403	DRAMP01817	Temporin-1CSc (Frogs, amphibians, animals)
404	DRAMP01818	Temporin-1CSd (Temporin-1DRb; Frogs, amphibians, animals)
405	DRAMP01392	Odorranain-V1 (OdV1; Frogs, amphibians, animals)
406	DRAMP01391	Odorranain-U1 (OdU1; Frogs, amphibians, animals)
407	DRAMP01832	Temporin-Eca (Frogs, amphibians, animals)
408	DRAMP01833	Buforin-EC (Frogs, amphibians, animals)
409	DRAMP01834	Cyanophlyctin (Frogs, amphibians, animals)
410	DRAMP01840	Ascaphin-1 (Frogs, amphibians, animals)
411	DRAMP01842	Ascaphin-3 (Frogs, amphibians, animals)
412	DRAMP01844	Ascaphin-5 (Frogs, amphibians, animals)
413	DRAMP01846	Ascaphin-7 (Frogs, amphibians, animals)
414	DRAMP01847	Ascaphin-8 (Frogs, amphibians, animals)
415	DRAMP01849	Jindongenin-1a (JD1a; Frogs, amphibians, animals)
416	DRAMP01869	Brevinin-1SPa (Frogs, amphibians, animals)
417	DRAMP01870	Brevinin-1SPb (Frogs, amphibians, animals)
418	DRAMP01872	Brevinin-1SPd (Frogs, amphibians, animals)
419	DRAMP01873	Brevinin-2-related peptide (Frogs, amphibians, animals)
420	DRAMP01875	Brevinin-2PRa (Frogs, amphibians, animals)
421	DRAMP01876	Brevinin-2PRb (Frogs, amphibians, animals)
422	DRAMP01877	Brevinin-2PRd (Frogs, amphibians, animals)
423	DRAMP01878	Brevinin-2PRe (Frogs, amphibians, animals)
424	DRAMP01879	Brevinin-2LTa (Frogs, amphibians, animals)
425	DRAMP01880	Brevinin-2LTb (Frogs, amphibians, animals)
426	DRAMP01881	Brevinin-2LTc (Frogs, amphibians, animals)
427	DRAMP01885	Brevinin-1TEa (Frogs, amphibians, animals)
428	DRAMP01886	Brevinin-2TEa (Frogs, amphibians, animals)
429	DRAMP01887	Brevinin-2TEb (Frogs, amphibians, animals)
430	DRAMP01888	Brevinin-1CHc (Frogs, amphibians, animals)
431	DRAMP01889	Brevinin-1TOa (Frogs, amphibians, animals)
432	DRAMP01890	Brevinin-1VLa (Frogs, amphibians, animals)
433	DRAMP01891	Brevinin-1VLc (Frogs, amphibians, animals)
434	DRAMP01892	Brevinin-1VLd (Frogs, amphibians, animals)
435	DRAMP01893	Brevinin-1VLe (Frogs, amphibians, animals)
436	DRAMP01896	Brevinin-1CG1 (Frogs, amphibians, animals)
437	DRAMP01897	Brevinin-1CG2 (Frogs, amphibians, animals)
438	DRAMP01898	Brevinin-1CG3 (Frogs, amphibians, animals)
439	DRAMP01899	Brevinin-1CG4 (Frogs, amphibians, animals)
440	DRAMP01900	Brevinin-1CG5 (Frogs, amphibians, animals)

441	DRAMP01909	Brevinin-2GHa (AMP-1; Frogs, amphibians, animals)
442	DRAMP01910	Brevinin-2GHb (AMP-2; Frogs, amphibians, animals)
443	DRAMP01911	Brevinin-2GHc (AMP-4; Frogs, amphibians, animals)
444	DRAMP01913	Brevinin-1GRa (Frogs, amphibians, animals)
445	DRAMP01914	Brevinin-2GRa (Frogs, amphibians, animals)
446	DRAMP01918	Brevinin-1PLb (Frogs, amphibians, animals)
447	DRAMP01919	Brevinin-1PLc (Frogs, amphibians, animals)
448	DRAMP01920	Brevinin-1CSa (Frogs, amphibians, animals)
450	DRAMP01922	Brevinin-2SKb (Frogs, amphibians, animals)
451	DRAMP01933	Brevinin-2Ef (Frogs, amphibians, animals)
457	DRAMP01940	Brevinin-1CHa (Frogs, amphibians, animals)
458	DRAMP01941	Brevinin-1CHb (Frogs, amphibians, animals)
462	DRAMP01949	Brevinin-1HSa (Frogs, amphibians, animals)
463	DRAMP01950	Brevinin-1HSb (Brevinin-1JDb; Frogs, amphibians, animals)
464	DRAMP01951	Brevinin-1PTa (Frogs, amphibians, animals)
465	DRAMP01953	Brevinin-2HSa (Frogs, amphibians, animals)
466	DRAMP01955	Brevinin-2PTa (Frogs, amphibians, animals)
467	DRAMP01956	Brevinin-2PTb (Frogs, amphibians, animals)
468	DRAMP01957	Brevinin-2PTc (Frogs, amphibians, animals)
469	DRAMP01959	Brevinin-2PTe (Frogs, amphibians, animals)
470	DRAMP01963	Brevinin-1BLa (Frogs, amphibians, animals)
471	DRAMP01965	Brevinin-1BLc (Frogs, amphibians, animals)
472	DRAMP01968	Brevinin-1Yc (Frogs, amphibians, animals)
473	DRAMP01969	Brevinin-1Ja (Frogs, amphibians, animals)
474	DRAMP01970	Brevinin-1ZHa (Frogs, amphibians, animals)
475	DRAMP01971	Brevinin-1ZHb (Frogs, amphibians, animals)
476	DRAMP01974	Brevinin-2ZHa (Frogs, amphibians, animals)
477	DRAMP01986	Brevinin-2HS2 (Frogs, amphibians, animals)
478	DRAMP01990	Brevinin-1LT1 (Frogs, amphibians, animals)
479	DRAMP01994	Brevinin-2ISa (Frogs, amphibians, animals)
480	DRAMP01995	Brevinin-2ISb (Frogs, amphibians, animals)
481	DRAMP01996	Brevinin-2ISc (Frogs, amphibians, animals)
482	DRAMP02001	Brevinin-1HN1 (Frogs, amphibians, animals)
483	DRAMP02004	Brevinin-1V (Frogs, amphibians, animals)
485	DRAMP02009	Brevinin-1 (Frogs, amphibians, animals)
486	DRAMP02010	Brevinin-2 (Frogs, amphibians, animals)
487	DRAMP02019	Brevinin-2DYb (Frogs, amphibians, animals)
490	DRAMP02023	Brevinin-2DYd (Frogs, amphibians, animals)
492	DRAMP02025	Brevinin-2DYe (Brevinin-2CDYa; Frogs, amphibians, animals)
493	DRAMP02026	Brevinin-1CDYa (Frogs, amphibians, animals)
495	DRAMP02031	Brevinin-1Da (Frogs, amphibians, animals)
496	DRAMP02032	Brevinin-1TSa (Frogs, amphibians, animals)
497	DRAMP02033	Brevinin-2TSa (Frogs, amphibians, animals)
498	DRAMP02034	Brevinin-1AUa (Frogs, amphibians, animals)
499	DRAMP02035	Brevinin-1AUb (Frogs, amphibians, animals)
500	DRAMP02036	Brevinin-2-RN1 (Frogs, amphibians, animals)
501	DRAMP02037	Brevinin-2-RN2 (Frogs, amphibians, animals)

502	DRAMP02038	Brevinin-1-OA1 (Frogs, amphibians, animals)
504	DRAMP02040	Brevinin-1-OA12 (Frogs, amphibians, animals)
505	DRAMP02041	Brevinin-1-OR1 (Frogs, amphibians, animals)
506	DRAMP02042	Brevinin-1-OR3 (Frogs, amphibians, animals)
507	DRAMP02043	Brevinin-1-OR4 (Frogs, amphibians, animals)
508	DRAMP02044	Brevinin-1-OR5 (Frogs, amphibians, animals)
509	DRAMP02045	Brevinin-1-OR6 (Frogs, amphibians, animals)
510	DRAMP02046	Brevinin-1-OR7 (Frogs, amphibians, animals)
511	DRAMP02047	Brevinin-1-OR8 (Frogs, amphibians, animals)
512	DRAMP02048	Brevinin-1-OR9 (Frogs, amphibians, animals)
513	DRAMP02049	Brevinin-1-OR10 (Frogs, amphibians, animals)
514	DRAMP02050	Brevinin-1-OR11 (Frogs, amphibians, animals)
515	DRAMP02051	Lividin-1 (Brevinin-1-OR2; Frogs, amphibians, animals)
516	DRAMP02052	Lividin-2 (Brevinin-2-OR8; Frogs, amphibians, animals)
517	DRAMP02053	Lividin-3 (Brevinin-2-OR1; Frogs, amphibians, animals)
518	DRAMP02054	Brevinin-2-OA1 (Frogs, amphibians, animals)
519	DRAMP02055	Brevinin-2-OA2 (Brevinin-2E-OG1; Frogs, amphibians, animals)
520	DRAMP02056	Brevinin-2-OA3 (Frogs, amphibians, animals)
521	DRAMP02057	Brevinin-2-OA4 (Frogs, amphibians, animals)
522	DRAMP02058	Brevinin-2-OA5 (Frogs, amphibians, animals)
523	DRAMP02059	Brevinin-2-OA6 (Frogs, amphibians, animals)
524	DRAMP02060	Brevinin-2-OA7 (Frogs, amphibians, animals)
525	DRAMP02061	Brevinin-2-OA8 (Frogs, amphibians, animals)
526	DRAMP02062	Brevinin-2-OR2 (Frogs, amphibians, animals)
527	DRAMP02063	Brevinin-2-OR3 (Frogs, amphibians, animals)
528	DRAMP02064	Brevinin-2-OR4 (Frogs, amphibians, animals)
529	DRAMP02065	Brevinin-2-OR5 (Frogs, amphibians, animals)
530	DRAMP02066	Brevinin-2-OR6 (Frogs, amphibians, animals)
531	DRAMP02067	Brevinin-2-OR7 (Frogs, amphibians, animals)
532	DRAMP02068	Brevinin-2-OR9 (Frogs, amphibians, animals)
533	DRAMP02069	Brevinin-2-OR10 (Frogs, amphibians, animals)
534	DRAMP02070	Brevinin-2-OW1 (Frogs, amphibians, animals)
535	DRAMP02071	Brevinin-2-OW2 (Frogs, amphibians, animals)
536	DRAMP02072	Brevinin-2-OW3 (Frogs, amphibians, animals)
537	DRAMP02073	Brevinin-1JDa (Frogs, amphibians, animals)
538	DRAMP02075	Brevinin-1JDc (Frogs, amphibians, animals)
543	DRAMP02078	Brevinin-1SY (Frogs, amphibians, animals)
544	DRAMP02081	Brevinin-1E (Frogs, amphibians, animals)
545	DRAMP02084	Brevinin-2E (Frogs, amphibians, animals)
546	DRAMP02101	Brevinin-1RTa (Frogs, amphibians, animals)
547	DRAMP02102	Brevinin-1RTb (Frogs, amphibians, animals)
548	DRAMP02104	Brevinin-2RTa (Frogs, amphibians, animals)
549	DRAMP02105	Brevinin-2RTb (Frogs, amphibians, animals)
550	DRAMP02114	Raniseptin-1 (Rsp-1; Frogs, amphibians, animals)
551	DRAMP02125	Hylin-a1 (Hy-a1; Frogs, amphibians, animals)
553	DRAMP02128	Kassorin-S (PreproKassorin-S; Frogs, amphibians, animals)
554	DRAMP02129	Kasstasin (Frogs, amphibians, animals)

555	DRAMP02130	Antimicrobial peptide 1 (XT-1; Frogs, amphibians, animals)
556	DRAMP02131	Antimicrobial peptide 2 (XT-2; Frogs, amphibians, animals)
557	DRAMP02133	Antimicrobial peptide 4 (XT-4; Frogs, amphibians, animals)
558	DRAMP02135	Antimicrobial peptide 6 (XT-6; Frogs, amphibians, animals)
559	DRAMP02136	Antimicrobial peptide 7 (XT-7; Frogs, amphibians, animals)
561	DRAMP02219	Ranatuerin-2AUa (Frogs, amphibians, animals)
568	DRAMP02228	Ranatuerin-1 (Frogs, amphibians, animals)
569	DRAMP02229	Ranatuerin-2 (Frogs, amphibians, animals)
570	DRAMP02230	Ranatuerin-3 (Frogs, amphibians, animals)
571	DRAMP02231	Ranatuerin-4 (Frogs, amphibians, animals)
572	DRAMP02233	Ranatuerin-6 (Frogs, amphibians, animals)
573	DRAMP02234	Ranatuerin-7 (Frogs, amphibians, animals)
574	DRAMP02235	Ranatuerin-8 (Frogs, amphibians, animals)
575	DRAMP02236	Ranatuerin-9 (Frogs, amphibians, animals)
576	DRAMP02237	Ranatuerin-2Ya (Frogs, amphibians, animals)
577	DRAMP02238	Ranatuerin-2ZHa (Frogs, amphibians, animals)
578	DRAMP02239	Ranatuerin-1Ga (Frogs, amphibians, animals)
579	DRAMP02241	Ranatuerin-2G (Frogs, amphibians, animals)
580	DRAMP01390	Odorranain-T1 (OdT1; Frogs, amphibians, animals)
581	DRAMP01389	Odorranain-S1 (OdS1; Frogs, amphibians, animals)
582	DRAMP02251	Ranatuerin-2CSa (Frogs, amphibians, animals)
583	DRAMP02252	Ranatuerin 2SKa (Frogs, amphibians, animals)
584	DRAMP01108	Maximin-2 (Toads, amphibians, animals)
586	DRAMP01107	Maximin-1 (Toads, amphibians, animals)
587	DRAMP02268	Xenopsin precursor fragment (XPF; Frogs, amphibians, animals)
588	DRAMP02269	Antimicrobial peptide PGQ (PGQ; Frogs, amphibians, animals)
589	DRAMP02271	Magainin-2 (Magainin II; chain of Magainins; Frogs, amphibians, animals)
590	DRAMP02272	PGLa (chain of PYLa/PGLa A; Frogs, amphibians, animals)
591	DRAMP02273	PGLa-H (chain of PYLa/PGLa A; Frogs, amphibians, animals)
592	DRAMP02274	Ranacyclin-E (Frogs, amphibians, animals)
593	DRAMP02275	Ranacyclin-T (Frogs, amphibians, animals)
594	DRAMP02276	Ranacyclin B3 (Frogs, amphibians, animals)
595	DRAMP02277	Ranacyclin B5 (Frogs, amphibians, animals)
596	DRAMP02278	Ranacyclin-B-RL1 (Frogs, amphibians, animals)
597	DRAMP02279	Ranacyclin-B-RN1 (Frogs, amphibians, animals)
598	DRAMP02280	Ranacyclin-B-RN2 (Frogs, amphibians, animals)
599	DRAMP02281	Ranacyclin-B-RN6 (Frogs, amphibians, animals)
600	DRAMP02282	Ranacyclin-B-LK1 (Frogs, amphibians, animals)
601	DRAMP02283	Ranacyclin-B-LK2 (Frogs, amphibians, animals)
602	DRAMP02288	Gaegurin-RN1 (Frogs, amphibians, animals)
603	DRAMP02289	Gaegurin-RN4 (Frogs, amphibians, animals)
604	DRAMP02290	Gaegurin-RN5 (Frogs, amphibians, animals)
605	DRAMP02291	Gaegurin-1 (Gaegurin 1; GGN1; Frogs, amphibians, animals)
606	DRAMP02292	Gaegurin-2 (Gaegurin 2; GGN2; Frogs, amphibians, animals)
607	DRAMP02293	Gaegurin-3 (Gaegurin 3; GGN3; Frogs, amphibians, animals)
608	DRAMP02294	Gaegurin-4 (Gaegurin 4; GGN4; Frogs, amphibians, animals)
609	DRAMP02295	Gaegurin-5 (Gaegurin 5; GGN5; Brevinin-1EMa; Frogs, amphibians, animals)

610	DRAMP02296	Gaegurin-6 (Gaegurin 6; GGN6; Frogs, amphibians, animals)
611	DRAMP02300	Guentherin (AMP-3; Frogs, amphibians, animals)
612	DRAMP02306	Riparin-2.1 (Frogs, amphibians, animals)
613	DRAMP02307	Deserticolin-1 (Frogs, amphibians, animals)
614	DRAMP02308	Signiferin-2.1 (Frogs, amphibians, animals)
616	DRAMP02314	Hepcidin (fish, chordates, animals)
617	DRAMP02315	Chrysophsin-1 (fish, chordates, animals)
618	DRAMP02316	Chrysophsin-2 (fish, chordates, animals)
619	DRAMP02317	Chrysophsin-3 (fish, chordates, animals)
620	DRAMP02318	Grammistin Pp1 (Group II grammistin; fish, chordates, animals)
621	DRAMP02320	Grammistin PpIIb (Group II grammistin; fish, chordates, animals)
622	DRAMP02321	Grammistin Pp3 (Group III grammistin; fish, chordates, animals)
623	DRAMP02324	SAMP H1 (fish, chordates, animals)
624	DRAMP02330	Piscidin-1 (Pis-1; Piscidin 1; fish, chordates, animals)
625	DRAMP02331	Piscidin-2 (Pis-2; fish, chordates, animals)
626	DRAMP02336	Oncorhyncin II (Oncorhyncin 2; fish, chordates, animals)
627	DRAMP02337	Oncorhyncin III (Oncorhyncin 3; fish, chordates, animals)
628	DRAMP02347	NRC-1 (fish, chordates, animals)
629	DRAMP02348	NRC-2 (fish, chordates, animals)
630	DRAMP02349	NRC-3 (fish, chordates, animals)
631	DRAMP02350	Pleurocidin (NRC-4; fish, chordates, animals)
632	DRAMP02351	NRC-10 (fish, chordates, animals)
633	DRAMP02352	NRC-16 (fish, chordates, animals)
634	DRAMP02354	Pleurocidin-like peptide WFY (fish, chordates, animals)
635	DRAMP02357	Pleurocidin-like peptide WF3 (NRC-5; fish, chordates, animals)
636	DRAMP02358	Pleurocidin-like peptide WF4 (NRC-6; fish, chordates, animals)
637	DRAMP02359	Pleurocidin-like peptide YT2 (NRC-7; fish, chordates, animals; Predicted)
638	DRAMP02360	Pleurocidin-like peptide AP1 (NRC-11; fish, chordates, animals; Predicted)
639	DRAMP02361	Pleurocidin-like peptide AP2 (NRC-12; fish, chordates, animals; Predicted)
640	DRAMP02362	Pleurocidin-like peptide AP3 (NRC-13; fish, chordates, animals; Predicted)
641	DRAMP02363	Pleurocidin-like peptide GcSc4C5 (NRC-14; fish, chordates, animals)
642	DRAMP02364	Pleurocidin-like peptide GcSc4B7 (NRC-15; fish, chordates, animals; Predicted)
643	DRAMP02365	Pleurocidin-like peptide GC3.8 (NRC-17; fish, chordates, animals; Predicted)
644	DRAMP02366	Pleurocidin-like peptide GC3.2 (NRC-18; fish, chordates, animals; Predicted)
645	DRAMP02367	Pleurocidin-like peptide Hb26 (NRC-19; fish, chordates, animals; Predicted)
646	DRAMP02368	Pleurocidin-like peptide Hb18 (NRC-20; fish, chordates, animals; Predicted)
648	DRAMP02376	Grammistin Gs 1 (Grammistin Gs F; Group I grammistin; soapfish, chordates, animals)
649	DRAMP02377	Grammistin Gs 2 (Grammistin Gs G; Group I grammistin; soapfish, chordates, animals)
650	DRAMP02378	Grammistin Gs A (Group III grammistin; soapfish, chordates, animals)
651	DRAMP02379	Grammistin Gs B (Group II grammistin; soapfish, chordates, animals)
652	DRAMP02380	Grammistin Gs C (Group III grammistin; soapfish, chordates, animals)
657	DRAMP02390	Astacidin 2 (crayfish, Arthropods, animals)
658	DRAMP02391	Hematopoietic antimicrobial peptide-37 (MgCath37; hagfishes, chordates, animals)
659	DRAMP02393	HFIAP-1 (HFIAP-2; hagfishes, chordates, animals)
660	DRAMP02394	HFIAP-3 (hagfishes, chordates, animals)
661	DRAMP02395	Aurelin (jellyfish, chordates, animals)
662	DRAMP02397	Big defensin (RPD-1)

663	DRAMP02402	Antimicrobial peptide scolopin-1
664	DRAMP02403	Antimicrobial peptide scolopin-2
665	DRAMP02409	M-theraphotoxin-Gr1a (M-TRTX-Gr1a; GsMTx-4)
666	DRAMP02410	Antimicrobial peptide lumbricin-1
667	DRAMP02411	Armadillidin (Glyc-rich)
668	DRAMP02412	Panusin (Defensin-like peptide 7, PaD7)
670	DRAMP02421	Hlgut-defensin (H. longicornis midgut defensin; Ticks, Arthropods, animals)
671	DRAMP02422	Hlsal-defensin (H. longicornis salivary gland defensin; Ticks, Arthropods, animals)
672	DRAMP02423	HlMS-defensin (Ticks, Arthropods, animals)
673	DRAMP02425	Ixosin-B (Ticks, Arthropods, animals)
674	DRAMP02427	Ixodes ricinus defensin def1 (Ticks, Arthropods, animals)
675	DRAMP02428	Ixodes ricinus defensin def2 (Ticks, Arthropods, animals)
678	DRAMP02432	Antimicrobial peptide ISAMP (Ticks, Arthropods, animals)
680	DRAMP02434	Antimicrobial peptide lumbricin-PG (Lumbricin-PG)
683	DRAMP02445	Antimicrobial protein BL-A60
684	DRAMP02446	Antimicrobial protein 1 (Antimicrobial protein AN5-1)
686	DRAMP02456	L-amino-acid oxidase (LAAO; LAO; Dactylomelin-P)
687	DRAMP02470	Nosiheptide (NOS; Antibiotic 9671-RP)
688	DRAMP02473	Cathelicidin-BF (Cathelicidin-related protein; Snakes, reptiles, animals)
689	DRAMP02474	cathelicidin-BF15 (Snakes, reptiles, animals)
690	DRAMP02478	L-amino-acid oxidase (Bm-LAO; LAAO; LAO; Snakes, reptiles, animals)
692	DRAMP02522	L-amino-acid oxidase (LAAO, LAO, Oh-LAAO; Snakes, reptiles, animals)
693	DRAMP02573	Penaeidin-3a (Pen-3a; shrimps, Arthropods, animals)
694	DRAMP02574	[T8A]-Penaeidin-3a ([T8A]-Pen-3a; shrimps, Arthropods, animals)
695	DRAMP18370	Crinicepsin-2 (insects, arthropods, invertebrates, animals)
696	DRAMP18371	Crinicepsin-1 (insects, arthropods, invertebrates, animals)
697	DRAMP02584	Penaeidin-4a (Pen-4a; shrimps, Arthropods, animals)
698	DRAMP02586	Penaeidin-2d (Pen-2d; shrimps, Arthropods, animals)
699	DRAMP02591	Penaeidin-4d (Pen-4d; shrimps, Arthropods, animals)
700	DRAMP02603	Putative antimicrobial peptide A Northern Europe Heligoland (chordates, animals)
705	DRAMP02740	TBD-1 (Turtle beta-defensin 1; Reptiles, animals)
706	DRAMP02768	Pilosulin-1 (Myr b I; ants, insects, animals)
709	DRAMP02777	Rhinocerosin (Insects, animals)
710	DRAMP02778	Defensin (Insects, animals)
711	DRAMP02779	Defensin-A (Defensin A; Insects, animals)
712	DRAMP02780	Defensin-B (Defensin B; Insects, animals)
713	DRAMP02802	Paneth cell-specific alpha-defensin 1 (DEFA1; horse defensin; houses, mammals, animals）
714	DRAMP02809	Myticin-B (Myt B; Cys-rich; molluscas, animals)
715	DRAMP02811	Defensin MGD-1 (molluscas, animals)
716	DRAMP02813	Conolysin-Mt1
717	DRAMP02817	Pyrrhocoricin
718	DRAMP02835	Enkelytin (one chain of Proenkephalin-A; mammals, animals)
719	DRAMP01381	Odorranain-K1 (OdK1; Frogs, amphibians, animals)
720	DRAMP01383	Odorranain-M1 (OdM1; Frogs, amphibians, animals)
721	DRAMP02841	Lumbricin I(6-34)
722	DRAMP02843	chain a, Structure Of An Indolicidin Peptide Derivative
723	DRAMP02844	CP10A (Indolicidin peptide derivative; mammals, animals)

724	DRAMP02845	CP-11 (cathelicidin; mammals, animals)
726	DRAMP02851	Cathelicidin-1 (Bactenecin-1, Bac1; Cyclic dodecapeptide; mammals, animals)
727	DRAMP02854	Cathelicidin-5 (Antibacterial peptide BMAP-28)
728	DRAMP02855	Cathelicidin-6 (Antibacterial peptide BMAP-27)
730	DRAMP02859	Bovine Beta-defensin 2 (bBD-2; BNBD-2; BNDB-2; mammals, animals)
731	DRAMP02860	Bovine Beta-defensin 3 (bBD-3; BNBD-3; BNDB-3; mammals, animals)
732	DRAMP02861	Bovine Beta-defensin 4 (bBD-4; BNBD-4; BNDB-4; mammals, animals)
734	DRAMP02863	Bovine Beta-defensin 6 (bBD-6; BNBD-6; BNDB-6; mammals, animals)
735	DRAMP02865	Bovine Beta-defensin 8 (bBD-8; BNBD-8; BNDB-8; mammals, animals)
736	DRAMP02866	Bovine Beta-defensin 9 (bBD-9; BNBD-9; BNDB-9; mammals, animals)
737	DRAMP02867	Bovine Beta-defensin 10 (bBD-10; BNBD-10; BNDB-10; mammals, animals)
738	DRAMP02868	Bovine Beta-defensin 11 (bBD-11; BNBD-11; BNDB-11; mammals, animals)
739	DRAMP02869	Bovine Beta-defensin 12 (bBD-12; BNBD-12; BNDB-12; mammals, animals)
740	DRAMP02870	Bovine Beta-defensin 13 (bBD-13; BNBD-13; BNDB-13; mammals, animals)
741	DRAMP02872	Myeloid antimicrobial peptide BMAP-27 (1-18) (mammals, animals)
742	DRAMP02873	Myeloid antimicrobial peptide BMAP-28 (1-18) (mammals, animals)
743	DRAMP02875	Vasostatin-1 (VS-1; N-terminal fragment of Chromogranin-A; mammals, animals)
744	DRAMP02877	mBMAP28 (mammals, animals)
745	DRAMP02878	Tracheal antimicrobial peptide (TAP; mammals, animals)
746	DRAMP02903	Bombin H7
751	DRAMP02922	Canine beta-defensin (dogs, mammals, animals)
752	DRAMP02923	cBD-1 (Canine beta-defensin 1; dogs, mammals, animals)
753	DRAMP18367	Chaxapeptin (a class 2 lasso peptide; class 1 microcin, bacteriocins)
755	DRAMP02925	Cathelicidin (dogs, mammals, animals)
756	DRAMP02931	Arasin-likeSp (crabs, Arthropods, animals)
757	DRAMP02932	GRPSp (crabs, Arthropods, animals)
758	DRAMP02933	Polyphemusin-1 (PM1; crabs, Arthropods, animals)
759	DRAMP02934	PM1-S (linear derivative of PM1)
761	DRAMP02951	PtALF6 (Portunus trituberculatus anti-lipopolysaccharide factor isoform 6; crabs, Arthropods, animals)
762	DRAMP02952	PtALF7 (Portunus trituberculatus anti-lipopolysaccharide factor isoform 7; crabs, Arthropods, animals)
763	DRAMP02953	Arasin-1 (Pro-rich, Arg-rich; crabs, Arthropods, animals)
764	DRAMP02956	Dolabellanin B2
765	DRAMP02959	Antibacterial protein PR-39 (pigs, mammals, animals)
766	DRAMP02960	Antibacterial peptide PMAP-23 (Myeloid antibacterial peptide 23; pigs, mammals, animals)
767	DRAMP02961	Antibacterial peptide PMAP-37 (Myeloid antibacterial peptide 37; pigs, mammals, animals)
768	DRAMP02962	Antibacterial peptide PMAP-36 (Myeloid antibacterial peptide 36; pigs, mammals, animals)
769	DRAMP02963	PMAP-36(1-20)
770	DRAMP02964	PMAP-36(1-34)
771	DRAMP02965	PMAP-36(1-35)2
772	DRAMP02966	DBI(32-86) (pigs, mammals, animals)
773	DRAMP02970	Protegrin-1 (Protegrin 1; PG-1; pigs, mammals, animals)
774	DRAMP02975	Tritrpticin (Trp-rich; pigs, mammals, animals)
776	DRAMP01376	Odorranain-F1 (OdF1; Frogs, amphibians, animals)
777	DRAMP01377	Odorranain-G1 (OdG1; Frogs, amphibians, animals)
778	DRAMP02995	Hymenoptaecin (Insects, animals)

779	DRAMP02996	Apidaecin-2 (Apidaecin II; Insects, animals)
780	DRAMP01378	Odorranain-H1 (OdH1; Frogs, amphibians, animals)
781	DRAMP02998	Apidaecin-1A (Apidaecin IA; Insects, animals)
782	DRAMP02999	Jellein-1 (Jelleine-I; chain of Major royal jelly protein 1; Insects, animals)
783	DRAMP03000	Jellein-2 (Jelleine-II; chain of Major royal jelly protein 1; Insects, animals)
784	DRAMP03001	Jellein-3 (Jelleine-III; Insects, animals)
785	DRAMP03002	Melittin (Allergen Api m 3; Allergen Api m III; Insects, animals)
786	DRAMP03003	Melectin (MEP; Insects, animals)
787	DRAMP03007	Osmin (Insects, animals)
788	DRAMP03019	Mastoparan PDD-B
789	DRAMP03020	Mastoparan PDD-A
790	DRAMP03021	Mastoparan PMM
791	DRAMP03022	Mastoparan MP
792	DRAMP03028	Mastoparan-1 (MP-1; Venom protein MP-1; Insects, animals)
793	DRAMP03033	Mastoparan-like peptide 12a (Insects, animals)
794	DRAMP03034	Mastoparan-like peptide 12b (Insects, animals)
795	DRAMP03035	Mastoparan-like peptide 12c (Insects, animals)
796	DRAMP03036	Mastoparan-like peptide 12d (Insects, animals)
797	DRAMP03037	Eumenitin (Er-12; Insects, animals)
798	DRAMP03038	Eumenitin-R (Insects, animals)
799	DRAMP03039	Eumenitin-F (Insects, animals)
800	DRAMP03040	Eumenine mastoparan-EF (EMP-EF; Insects, animals)
801	DRAMP03041	Eumenine mastoparan-ER (EMP-ER; Insects, animals)
802	DRAMP03042	Eumenine mastoparan-AF (EMP-AF; Af-113; Insects, animals)
803	DRAMP03043	Agelaia-mastoparan (Agelaia-MP; Insects, animals)
804	DRAMP03044	Protonectin (Agelaia-chemotactic peptide, Agelaia-CP; Insects, animals)
805	DRAMP03045	Defensin-NV (Insects, animals)
806	DRAMP03046	Orancis-protonectin (chain of Venom peptide 2-long; Venom peptide 2, OdVP2; Insects, animals)
807	DRAMP03047	Venom peptide 2-long (OdVP2L; analog of OdVP2; Insects, animals)
808	DRAMP03050	Dominulin-A (Insects, animals)
809	DRAMP03051	Dominulin-B (Insects, animals)
810	DRAMP03052	PP13 (Insects, animals)
811	DRAMP03053	PP102 (Insects, animals)
812	DRAMP03054	PP113 (Insects, animals)
813	DRAMP03055	PP30 (Pro-rich; abaecin-like; Insects, animals)
814	DRAMP03056	Decoralin (Insects, animals)
815	DRAMP03057	Thanatin (Insects, animals)
818	DRAMP03075	Cecropin-D
819	DRAMP03089	Drosophila cecropin-A1/A2 (Insects, animals)
820	DRAMP03090	Drosophila cecropin B (CecB; Insects, animals)
821	DRAMP03095	Andropin (Insects, animals)
822	DRAMP03096	Metchnikowin-2 (Insects, animals)
823	DRAMP18495	Gomesin (Gm; Spiders, arachnids, Chelicerata, arthropods, invertebrates, animals)
825	DRAMP03104	Sapecin (defensins; Insects, animals)
826	DRAMP03113	SK84 (Gly-rich; Insects, animals)
827	DRAMP03116	Ceratotoxin-C (Insects, animals)
828	DRAMP03117	Drosophila cecropin-A1 (Insects, animals)

829	DRAMP03137	Defensin-A (AaeDefA; Insects, animals)
830	DRAMP03138	Cecropin-A (Insects, animals)
831	DRAMP03140	Anopheles cecropin-A amidated isoform (Insects, animals)
832	DRAMP03150	Gambicin (Insects, animals)
833	DRAMP03153	27 kDa antibacterial protein
834	DRAMP03162	Phlebotomus duboscqi defensin (PduDef; defensins; Insects, animals)
835	DRAMP03166	P15 (deer beta-defensin; ruminant, animals)
837	DRAMP03173	Arenicin-1 (Ar-1; marine polychaeta, animals)
839	DRAMP03181	Spinigerin (Insects, animals)
840	DRAMP03186	Spheniscin-2 (Sphe-2; penguin avian beta-defensin 103b; birds ,animals)
841	DRAMP03187	Beta defensin 1(BD-1; mammals, animals)
845	DRAMP03198	Alpha-defensin PhD-4 (primates, mammals, animals)
846	DRAMP03215	Gomesin (Gm; spiders, Arthropods, animals)
847	DRAMP03216	Oxyopinin-4a (Oxt-4a; spiders, Arthropods, animals)
848	DRAMP03217	M-oxotoxin-Ot1a (Oxyopinin-1, Oxki1; spiders, Arthropods, animals)
849	DRAMP03222	M-ctenitoxin-Cs1a (M-CNTX-Cs1a; Cupiennin-1a; spiders, Arthropods, animals)
850	DRAMP03225	M-ctenitoxin-Cs1d (M-CNTX-Cs1d; Cupiennin-1d; spiders, Arthropods, animals)
851	DRAMP03226	M-zodatoxin-Lt1a (M-ZDTX-Lt1a; Latarcin-1, Ltc-1, Ltc1; spiders, Arthropods, animals)
852	DRAMP03227	M-zodatoxin-Lt2a (M-ZDTX-Lt2a; Latarcin-2a, Ltc-2a, Ltc2a; spiders, Arthropods, animals)
853	DRAMP03229	M-zodatoxin-Lt3a (M-ZDTX-Lt3a; Latarcin-3a, Ltc-3a; spiders, Arthropods, animals)
854	DRAMP03230	M-zodatoxin-Lt3b (M-ZDTX-Lt3b; Latarcin-3b, Ltc-3b; spiders, Arthropods, animals)
855	DRAMP03231	M-zodatoxin-Lt4a (M-ZDTX-Lt4a; Latarcin-4a, Ltc-4a; spiders, Arthropods, animals)
856	DRAMP03232	M-zodatoxin-Lt4b (M-ZDTX-Lt4b; Latarcin-4b, Ltc-4b; spiders, Arthropods, animals)
857	DRAMP03233	M-zodatoxin-Lt5a (M-ZDTX-Lt5a; Latarcin-5, Ltc-5; spiders, Arthropods, animals)
858	DRAMP03236	M-zodatoxin-Lt8a (M-ZDTX-Lt8a; Cytoinsectotoxin-1a, CIT-1a; spiders, Arthropods, animals)
859	DRAMP03253	M-lycotoxin-Ls3a (M-LCTX-Ls3a; Lycocitin-1; spiders, Arthropods, animals)
860	DRAMP03254	M-lycotoxin-Ls3b (M-LCTX-Ls3b; Lycocitin-2; spiders, Arthropods, animals)
861	DRAMP03278	M-lycotoxin-Hc1a (M-LCTX-Hc1a; Lycotoxin I; spiders, Arthropods, animals)
862	DRAMP03279	M-lycotoxin-Hc2a (M-LCTX-Hc2a; Lycotoxin-2; Lycotoxin II; spiders, Arthropods, animals)
863	DRAMP03280	AcAMP (A. clavatus antimicrobial peptide)
864	DRAMP03285	Ostricacin-1 (Beta-defensin 2; Birds, animals)
865	DRAMP03286	Ostricacin-2 (Beta-defensin 1; Birds, animals)
866	DRAMP03287	Ostricacin-3 (Beta-defensin 7; Birds, animals)
867	DRAMP03288	Ostricacin-4 (Beta-defensin 8; Birds, animals)
868	DRAMP03311	Stomoxyn (Insects, animals)
870	DRAMP03320	Pore-forming peptide ameobapore A (EH-APP; saposin-like protein)
873	DRAMP03405	mCRAMP-1 (mouse cathelin-related antimicrobial peptide 1; cathelicidin; Rodents, mammals, animals)
874	DRAMP03406	mCRAMP-2 (mouse cathelin-related antimicrobial peptide 2; cathelicidin; Rodents, mammals, animals)
875	DRAMP03419	Neutrophil antibiotic peptide NP-1 (RatNP-1; Rodents, mammals, animals)
876	DRAMP03422	Neutrophil antibiotic peptide NP-4 (RatNP-4; Rodents, mammals, animals)
878	DRAMP03464	Cryptonin (Insects, animals)
880	DRAMP03467	Antibacterial napin (Plants)
881	DRAMP03471	Recombinant Crassostrea Gigas Defensin (Cg-Def; molluscs, animals)
882	DRAMP03472	cgUbiquitin
884	DRAMP03477	Polybia-CP (Polybia chemotactic peptide; Venom protein CP; Insects, animals)

885	DRAMP03486	Manduca Sexta Moricin (MS moricin; Insects, animals)
887	DRAMP03507	Cecropin-B (Insects, animals)
888	DRAMP03513	G. mellonella moricin-like peptide A (Gm-mlpA; Insects, animals; Predicted)
889	DRAMP03514	G. mellonella moricin-like peptide B (Gm-mlpB; Insects, animals; Predicted)
890	DRAMP03515	Moricin-like peptide C1 (Gm-mlpC1; Insects, animals; Predicted)
891	DRAMP03516	Moricin-like peptide C2 (Gm-mlpC2; Insects, animals; Predicted)
892	DRAMP03517	Moricin-like peptide C3 (Gm-mlpC3; Insects, animals; Predicted)
895	DRAMP03520	Cecropin-D-like peptide (Insects, animals)
896	DRAMP03521	Proline-rich antimicrobial peptide 1 (Insects, animals)
897	DRAMP03523	Anionic antimicrobial peptide 2 (Insects, animals)
898	DRAMP03524	Lebocin-like anionic peptide 1 (Insects, animals)
899	DRAMP03525	Apolipophorin-3 (Apolipophorin-III; Insects, animals)
900	DRAMP03526	Proline-rich antimicrobial peptide 2 (Insects, animals)
901	DRAMP03527	Gm defensin-like peptide (Insects, animals)
903	DRAMP03532	Moricin-1 (Insects, animals)
908	DRAMP03567	KR-20 (Derived from LL-37)
909	DRAMP03568	RK-31 (Derived from LL-37)
910	DRAMP03569	KS-30 (Derived from LL-37)
911	DRAMP03570	LL-23 (Derived from LL-37)
912	DRAMP03571	Antibacterial protein LL-37 (one chain of hCAP-18; Human, mammals, animals)
915	DRAMP03598	Human beta-defensin 2 (hBD-2; Defensin, beta 2; Beta-defensin 4A; Human, mammals, animals)
916	DRAMP03599	Human beta-defensin 3 (BD-3, hBD-3; Hbd3; Beta-defensin 103; Human, mammals, animals)
917	DRAMP03600	Human beta-defensin 4 (hBD-4, BD-4; Beta-defensin 104; Human, mammals, animals)
918	DRAMP03603	Human beta-defensin 28 (hBD-28; hBD28; Human, mammals, animals)
919	DRAMP03638	VpBD (V.philippinarum beta defensin; big defensin)
920	DRAMP03642	Chicken heterophil peptides 1 (Antimicrobial peptide CHP1; Birds, animals)
921	DRAMP03645	Cathelicidin-2 (CATH-2; Fowlicidin-2; Birds, animals)
922	DRAMP03646	Cathelicidin-3 (CATH-3; Fowlicidin-3; Birds, animals)
923	DRAMP03647	Cathelicidin-B1 (CATH-B1; cathelicidin; Birds, animals)
925	DRAMP03676	GLFcin (Lactoferrin fragment)
926	DRAMP03677	GLFcin II (Lactoferrin fragment)
927	DRAMP03679	Cathelicidin-2 (Bactenecin-5, Bac5; ChBac5; ruminant, animals)
928	DRAMP03682	Vespid chemotactic peptide 5e (VCP 5e; Insects, animals)
929	DRAMP03683	Vespid chemotactic peptide 5g (VCP 5g; Insects, animals)
930	DRAMP03684	Vespid chemotactic peptide 5f (VCP 5f; Insects, animals)
931	DRAMP03687	TsAP-1 (T. serrulatus antimicrobial peptide 1; scorpions, arachnids, invertebrates, animals)
932	DRAMP03688	TsAP-2 (T. serrulatus antimicrobial peptide 2; scorpions, arachnids, invertebrates, animals)
933	DRAMP03691	Im-1 (Arthropods, animals)
934	DRAMP03693	Bactridin-1 (Bact1; Bactridine 1; Arthropods, animals)
935	DRAMP03694	Bactridin-2 (Bact2, Bactridine 2; P-Mice-Antm-beta* NaTx14.8; Arthropods, animals)
936	DRAMP03700	Antimicrobial peptide ctriporin (Riporin; Arthropods, animals)
937	DRAMP03702	Mucroporin (Antimicrobial peptide 36.21; Arthropods, animals)
938	DRAMP03706	Antimicrobial peptide 1 (AamAP1; Arthropods, animals)
939	DRAMP03707	Antimicrobial peptide 2 (AamAP2; Arthropods, animals)
940	DRAMP03714	Amphiphatic peptide CT1 (VmCT1; Non-disulfide-bridged peptide 5.13, NDBP-5.13; Arthropods, animals)

941	DRAMP03715	Amphiphatic peptide CT2 (VmCT2; Non-disulfide-bridged peptide 5.14, NDBP-5.14; Arthropods, animals)
942	DRAMP03721	Cytotoxic linear peptide IsCT (IsCT; NDBP-5.2; Arthropods, animals)
943	DRAMP03723	Pandinin-1 (Pin1; Arthropods, animals)
944	DRAMP03724	Pandinin-2 (Pin2; Arthropods, animals)
946	DRAMP03734	Parabutoporin (PP; Non-disulfide-bridged peptide 3.2, NDBP-3.2; Arthropods, animals)
947	DRAMP03735	Opistoporin-1 (OP1; Non-disulfide-bridged peptide 3.5; Opistoporin-3, OP3; Arthropods, animals)
948	DRAMP03738	Scorpine (defensins; Arthropods, animals)
949	DRAMP02828	BMAP-34 (BMAP 34, bovine cathelicidin, cattle, ruminant, mammals, animals)
951	DRAMP03746	Peptide BmKn2 (Biologically active peptide 4; NDBP-5.1; Arthropods, animals)
952	DRAMP03748	Bradykinin-potentiating peptide BmK3 (Bpp BmK3; NDBP-3.3; Arthropods, animals)
953	DRAMP03750	Venom antimicrobial peptide-6 (Meucin-13; NDBP-5; Arthropods, animals)
954	DRAMP03751	Venom antimicrobial peptide-9 (Meucin-18; NDBP-5; Arthropods, animals)
955	DRAMP03752	Peptide BmKb1 (Non-disulfide-bridged peptide 4.2, NDBP-4.2; Arthropods, animals)
956	DRAMP03753	Amphipathic peptide CT1 (StCT1; Non-disulfide-bridged peptide 5, NDBP-5; Arthropods, animals)
957	DRAMP03754	Amphipathic peptide CT2 (StCT2; Non-disulfide-bridged peptide 5, NDBP-5; Arthropods, animals)
958	DRAMP03774	UyCT1 (Arthropods, animals)
960	DRAMP03776	UyCT3 (Arthropods, animals)
961	DRAMP03777	UyCT5 (Arthropods, animals)
963	DRAMP03814	D16W (GGN4 analogue peptide with single substitution)
964	DRAMP03815	D16W-N23 (single amino acid substitution)
965	DRAMP03816	D16F-N23 (single amino acid substitution)
966	DRAMP03823	Dermaseptin derivative K4-S4-(1-13)
967	DRAMP03824	CNBr-cleaved lactoferricin Subfragment 1
968	DRAMP03825	CNBr-cleaved lactoferricin Subfragment 2
969	DRAMP03826	Ovispirin-1 (OV-1; N-terminal 18 amino acids of SMAP-29)
970	DRAMP03827	Novispirin G-10 (mutation of Ovispirin-1)
971	DRAMP03828	Novispirin T-7 (mutation of Ovispirin-1)
973	DRAMP03830	Palustrin-2ISb + 3aa
974	DRAMP03831	Palustrin-2ISb-des-C7
975	DRAMP03832	Palustrin-2ISb-des-C7-4D
976	DRAMP03833	Palustrin-2ISb-des-C7-12N
977	DRAMP03834	Palustrin-2ISb-des-C7-23,29S
979	DRAMP03852	G1 (Bac2A variant through single amino acid substitution)
980	DRAMP03853	G2 (Bac2A variant through single amino acid substitution)
981	DRAMP03854	R2 (Bac2A variant through single amino acid substitution)
982	DRAMP03855	R3 (Bac2A variant through single amino acid substitution)
983	DRAMP03856	W3 (Bac2A variant through single amino acid substitution)
984	DRAMP03857	R5 (Bac2A variant through single amino acid substitution)
985	DRAMP03858	K7 (Bac2A variant through single amino acid substitution)
986	DRAMP03859	W10 (Bac2A variant through single amino acid substitution)
987	DRAMP03860	R11 (Bac2A variant through single amino acid substitution)
988	DRAMP03861	G12 (Bac2A variant through single amino acid substitution)
989	DRAMP03862	Sub2 (Bac2A variant through two amino acids substitution)
990	DRAMP03863	Sub3 (Bac2A variant through three amino acids substitution)
991	DRAMP03864	Sub5 (Bac2A variant through five amino acids substitution)

992	DRAMP03865	Sub6 (Bac2A variant through six amino acids substitution)
993	DRAMP03866	Bac8a (Bac2A variant)
994	DRAMP03867	Bac8b (Bac2A variant)
995	DRAMP03868	Bac8c (Bac2A variant)
996	DRAMP03869	Bac8d (Bac2A variant)
997	DRAMP03870	Bac2A (a linear variant of bovine dodecapeptide)
998	DRAMP03871	cLf 20-29 (fragment of caprine lalctoferricin, residues 20-29)
999	DRAMP03875	bLf 20-29 (fragment of bovine lactoferricin, residues 20-29)
1000	DRAMP03876	LFB-RW (derivative of bovine lactoferrin with residues substitution)
1001	DRAMP03877	LFB-KW (derivative of bovine lactoferrin with residues substitution)
1002	DRAMP03878	LFB-Rwa (derivative of bovine lactoferrin with residues substitution)
1003	DRAMP03879	LFB-RF (derivative of bovine lactoferrin with residues substitution)
1004	DRAMP03880	LFB-RI (derivative of bovine lactoferrin with residues substitution)
1005	DRAMP03881	LFB-6RW (derivative of bovine lactoferrin with residues substitution)
1006	DRAMP03882	LFC (fragment of mature caprine lactoferrin, residues 17 to 31)
1007	DRAMP03883	LFH W8 (tryptophan-modified human lactoferricin derivative)
1008	DRAMP03884	LFC W8 (tryptophan-modified caprine lactoferricin derivative)
1009	DRAMP03885	LFP W8 (tryptophan-modified porcine lactoferricin derivative)
1010	DRAMP03886	LFB (fragment of bovine lalctoferricin, residues 17 to 31)
1011	DRAMP03887	LFB A1 (derivative of LFB, residue substitution with alanine at position 1)
1012	DRAMP03888	LFB A2 (derivative of LFB, residue substitution with alanine at position 2)
1013	DRAMP03889	LFB A3 (derivative of LFB, residue substitution with alanine at position 3)
1014	DRAMP03890	LFB A4 (derivative of LFB, residue substitution with alanine at position 4)
1015	DRAMP03891	LFB A5 (derivative of LFB, residue substitution with alanine at position 5)
1016	DRAMP03892	LFB A7 (derivative of LFB, residue substitution with alanine at position 7)
1017	DRAMP03893	LFB A9 (derivative of LFB, residue substitution with alanine at position 9)
1018	DRAMP03894	LFB A10 (derivative of LFB, residue substitution with alanine at position 10)
1019	DRAMP03895	LFB A11 (derivative of LFB, residue substitution with alanine at position 11)
1020	DRAMP03896	LFB A12 (derivative of LFB, residue substitution with alanine at position 12)
1021	DRAMP03897	LFB A13 (derivative of LFB, residue substitution with alanine at position 13)
1022	DRAMP03898	LFB A14 (derivative of LFB, residue substitution with alanine at position 14)
1025	DRAMP03901	LFM R1 W8 (LFM W8 derivative with residues substitution)
1027	DRAMP03903	LFM A1 R9 W8 (LFM W8 derivative with residues substitution)
1028	DRAMP03904	LFM A9 R1 W8 (LFM W8 derivative with residues substitution)
1029	DRAMP03905	LFM R1,9 W8 (LFM W8 derivative with residues substitution)
1032	DRAMP03908	LFM R1 W8 Y13 (LFM W8 derivative with residues substitution)
1034	DRAMP03910	LFM A1 R9 W8 Y13 (LFM W8 derivative with residues substitution)
1035	DRAMP03911	LFM A9 R1 W8 Y13 (LFM W8 derivative with residues substitution)
1036	DRAMP03912	LFM R1,9 W8 Y13 (LFM W8 derivative with residues substitution)
1037	DRAMP03920	Cecropin A (1-8)-melittin (1-13)hybrid peptide
1038	DRAMP03921	Cecropin A (1-8)-melittin (1-18)hybrid peptide
1039	DRAMP03922	Cecropin A (1-8)-melittin (1-12)hybrid peptide
1040	DRAMP03923	Cecropin A (1-8)-melittin (1-10)hybrid peptide
1041	DRAMP03924	Cecropin A (1-7)-melittin (1-8)hybrid peptide
1042	DRAMP03925	Cecropin A (1-7)-melittin (3-10)hybrid peptide
1043	DRAMP03927	Cecropin A (1-7)-melittin (2-9)hybrid peptide
1044	DRAMP03928	Cecropin A (1-7)-melittin (4-11)hybrid peptide (CAM)

1045	DRAMP03929	Cecropin A (1-7)-melittin (5-12)hybrid peptide
1046	DRAMP03930	Cecropin A (1-7)-melittin (6-13)hybrid peptide
1048	DRAMP03933	I14M (truncated isoform of thanatin, residue 8-21)
1049	DRAMP03934	Y12M (truncated isoform of thanatin, residue 10-21)
1050	DRAMP03935	V16M (truncated isoform of thanatin, residue 6-21)
1051	DRAMP03936	K18M (truncated isoform of thanatin, residue 4-21)
1052	DRAMP03937	G18C (truncated isoform of thanatin, residue 1-18)
1053	DRAMP03938	G19Q (truncated isoform of thanatin, residue 1-19)
1054	DRAMP03939	G20R (truncated isoform of thanatin, residue 1-20)
1055	DRAMP03945	Del 1-4 (Ranalexin analog)
1056	DRAMP03947	Del 1-2 (Ranalexin analog)
1057	DRAMP03948	Del 1 (Ranalexin analog)
1058	DRAMP03949	Del 20 (Ranalexin analog)
1060	DRAMP03955	Human recombinant Ser-Thr-Ala-CGA1-78 peptide (hrVS-1)
1065	DRAMP03967	P18 (Cecropin A(1-8)-Magainin 2(1-12) hybrid peptide analogue)
1066	DRAMP03968	[L9]-P18 (analog of P18)
1067	DRAMP03969	[S9]-P18 (analog of P18)
1068	DRAMP03970	N-1 (analog of P18)
1069	DRAMP03971	N-2 (analog of P18)
1070	DRAMP03972	N-3 (analog of P18)
1071	DRAMP03973	N-4 (analog of P18)
1072	DRAMP03974	N-5 (analog of P18)
1073	DRAMP03975	N-3L (analog of P18)
1074	DRAMP03976	N-4L (analog of P18)
1075	DRAMP03977	N-5L (analog of P18)
1076	DRAMP03978	C-1 (analog of P18)
1077	DRAMP03979	C-2 (analog of P18)
1078	DRAMP03980	C-3 (analog of P18)
1079	DRAMP03981	C-4 (analog of P18)
1080	DRAMP03982	C-5 (analog of P18)
1081	DRAMP03983	C-6 (analog of P18)
1082	DRAMP03984	C-7 (analog of P18)
1083	DRAMP03985	C-8 (analog of P18)
1084	DRAMP03986	C-9 (analog of P18)
1085	DRAMP03987	C-10 (analog of P18)
1086	DRAMP03988	L3K3W4 (LlKmWn model peptide)
1087	DRAMP03989	L4K2W4 (LlKmWn model peptide)
1088	DRAMP03990	L4K3W4 (LlKmWn model peptide)
1089	DRAMP03991	L4K3W5 (LlKmWn model peptide)
1090	DRAMP03992	L5K3W5 (LlKmWn model peptide)
1091	DRAMP03993	L5K5W6 (LlKmWn model peptide)
1092	DRAMP03994	L6K4W6 (LlKmWn model peptide)
1093	DRAMP03995	L7K3W6 (LlKmWn model peptide)
1094	DRAMP03996	L7K5W7 (LlKmWn model peptide)
1095	DRAMP03997	L8K4W7 (LlKmWn model peptide)
1096	DRAMP03999	[A6]-IsCT (Mutant: W6A; IsCT analog)
1097	DRAMP04000	[L6]-IsCT (Mutant: W6L; IsCT analog)

1098	DRAMP04001	[K7]-IsCT (Mutant: E7K; IsCT analog)
1099	DRAMP04002	[L6, K11]-IsCT (IsCT analog through amino acids substitution)
1100	DRAMP04003	[K7, P8, K11]-IsCT (IsCT analog through amino acids substitution)
1101	DRAMP04004	Gramicidin analogue ([Scr2]-GS)
1102	DRAMP04005	Gramicidin analogue ([Ser2,2']-GS)
1103	DRAMP04011	Plasticin PD36 KF (analog of PD36)
1104	DRAMP04012	Plasticin PD36 K (analog of PD36)
1105	DRAMP04013	Plasticin ANC KF (analog of natural peptide ANC)
1108	DRAMP04016	LL-23A9 (LL-23 variants)
1109	DRAMP04017	LL-23V9 (LL-23 variants)
1110	DRAMP04019	Bac014 (Scrambled Variants of Bac2A)
1111	DRAMP04020	Bac020 (Scrambled Variants of Bac2A)
1112	DRAMP04021	Bac034 (Scrambled Variants of Bac2A)
1113	DRAMP04022	F3 (single amino acid substitution of Bac034, which is a scrambled Variant of Bac2A)
1114	DRAMP04023	W3 (single amino acid substitution of Bac034, which is a scrambled Variant of Bac2A)
1115	DRAMP04024	W4 (single amino acid substitution of Bac034, which is a scrambled Variant of Bac2A)
1116	DRAMP04025	R10 (single amino acid substitution of Bac034, which is a scrambled Variant of Bac2A)
1117	DRAMP04026	K12 (single amino acid substitution of Bac034, which is a scrambled Variant of Bac2A)
1118	DRAMP04027	opt1 (multiple amino acid substitution of Bac034, which is a scrambled Variant of Bac2A)
1119	DRAMP04028	opt2 (multiple amino acid substitution of Bac034, which is a scrambled Variant of Bac2A)
1120	DRAMP04029	opt3 (multiple amino acid substitution of Bac034, which is a scrambled Variant of Bac2A)
1121	DRAMP04030	opt4 (multiple amino acid substitution of Bac034, which is a scrambled Variant of Bac2A)
1122	DRAMP04031	opt5 (multiple amino acid substitution of Bac034, which is a scrambled Variant of Bac2A)
1123	DRAMP04032	Modified defensin
1124	DRAMP04033	Modified defensin
1125	DRAMP04034	Modified defensin
1126	DRAMP04035	Modified defensin
1127	DRAMP04036	Modified defensin
1128	DRAMP04048	BacR (cyclic derivative of bactenecin)
1129	DRAMP04049	BacP3R (cyclic derivative of bactenecin)
1130	DRAMP04050	BacP3R-V (cyclic derivative of bactenecin)
1131	DRAMP04051	Bac2I-NH2 (cyclic derivative of bactenecin)
1132	DRAMP04052	BacP2R-NH2 (cyclic derivative of bactenecin)
1133	DRAMP04053	BacP1 (cyclic derivative of bactenecin)
1134	DRAMP04054	BacW (cyclic derivative of bactenecin)
1135	DRAMP04055	BacW2R (cyclic derivative of bactenecin)
1136	DRAMP04056	Lin Bac2S-NH2 (linear derivative of bactenecin)
1137	DRAMP04057	Lin BacS-NH2 (linear derivative of bactenecin)
1140	DRAMP18363	Sviceucin (bacteriocin)
1142	DRAMP18362	Formicin (bacteriocin)
1144	DRAMP18359	Sh-lantibiotic-alpha (bacteriocin)
1145	DRAMP18358	Sh-lantibiotic-beta (bacteriocin)
1153	DRAMP04075	Antimicrobial peptide HP (2-20)
1154	DRAMP04076	Anal 1 (antimicrobial peptide HP (2-20)analogue)
1155	DRAMP04077	Anal 2 (antimicrobial peptide HP (2-20)analogue)
1156	DRAMP04078	Anal 3 (antimicrobial peptide HP (2-20)analogue)
1157	DRAMP04079	Anal 4 (antimicrobial peptide HP (2-20)analogue)

1158	DRAMP04080	Anal 5 (antimicrobial peptide HP (2-20)analogue)
1159	DRAMP04081	Anal 6 (antimicrobial peptide HP (2-20)analogue)
1160	DRAMP04082	Anal 7 (antimicrobial peptide HP (2-20)analogue)
1161	DRAMP04083	D-amino-acid pexiganan (MSI-214)
1162	DRAMP04095	Cupiennin-1D (spiders, Arthropods, animals)
1163	DRAMP04096	2IQ2
1164	DRAMP04097	2IQ3
1165	DRAMP04098	3IQ1
1166	DRAMP04099	3IQ2
1167	DRAMP04100	3IQ3
1168	DRAMP04101	3IQ4
1180	DRAMP04115	K11 (derivative of CP-P)
1186	DRAMP04123	D0-NH2
1187	DRAMP04124	D1-NH2
1188	DRAMP04125	D2-NH2
1189	DRAMP04126	D3-NH2
1190	DRAMP04127	D4-NH2
1191	DRAMP04128	D5-NH2
1192	DRAMP04129	D6-NH2
1193	DRAMP04136	LRR-1
1194	DRAMP04137	LRR-2
1205	DRAMP04159	LR2 (homologue of Pc-CATH1)
1206	DRAMP04160	LR3 (homologue of Pc-CATH1)
1207	DRAMP04161	LR4 (homologue of Pc-CATH1)
1208	DRAMP04162	LR5 (homologue of Pc-CATH1)
1209	DRAMP04163	LR6 (homologue of Pc-CATH1)
1210	DRAMP04164	LR7 (homologue of Pc-CATH1)
1211	DRAMP04165	LR8 (homologue of Pc-CATH1)
1212	DRAMP04166	LR9 (homologue of Pc-CATH1)
1213	DRAMP04167	LR10 (homologue of Pc-CATH1)
1214	DRAMP04168	LR11 (homologue of Pc-CATH1)
1215	DRAMP04169	LR13 (homologue of Pc-CATH1)
1216	DRAMP04170	LR15 (homologue of Pc-CATH1)
1217	DRAMP04171	LR16 (homologue of Pc-CATH1)
1218	DRAMP04174	L2K3W2 (LlKmW2 model peptides)
1219	DRAMP04175	L3K2W2 (LlKmW2 model peptides)
1220	DRAMP04176	L2K5W2 (LlKmW2 model peptides)
1221	DRAMP04177	L3K4W2 (LlKmW2 model peptides)
1222	DRAMP04178	L4K3W2 (LlKmW2 model peptides)
1223	DRAMP04179	L5K2W2 (LlKmW2 model peptides)
1224	DRAMP04180	L3K6W2 (LlKmW2 model peptides)
1225	DRAMP04181	L4K5W2 (LlKmW2 model peptides)
1226	DRAMP04182	L5K4W2 (LlKmW2 model peptides)
1227	DRAMP04183	L6K3W2 (LlKmW2 model peptides)
1228	DRAMP04184	DFTamP1
1229	DRAMP04185	DFTamP1-p
1230	DRAMP04186	L5K5W1 (L5K5Wn model peptide)

1231	DRAMP04187	L5K5W2 (L5K5Wn model peptide)
1232	DRAMP04188	L5K5W3 (L5K5Wn model peptide)
1233	DRAMP04189	L5K5W4 (L5K5Wn model peptide)
1234	DRAMP04190	L5K5W5 (L5K5Wn model peptide)
1236	DRAMP04192	L5K5W7 (L5K5Wn model peptide)
1237	DRAMP04193	L5K5W8 (L5K5Wn model peptide)
1238	DRAMP04194	L5K5W9 (L5K5Wn model peptide)
1239	DRAMP04195	L5K5W10 (L5K5Wn model peptide)
1240	DRAMP04196	L5K5W11 (L5K5Wn model peptide)
1241	DRAMP04233	D28 (Rational design peptide)
1242	DRAMP04234	D51 (Rational design peptide)
1243	DRAMP04235	D22 (Rational design peptide)
1245	DRAMP04240	Synthetic 1
1246	DRAMP04241	Synthetic 2
1247	DRAMP04242	Synthetic 3
1248	DRAMP04243	Synthetic 4
1249	DRAMP04244	Synthetic 5
1252	DRAMP04279	CP11CN
1253	DRAMP04359	PDD-A-1 (PDD-A analog)
1254	DRAMP04360	PDD-A-2 (PDD-A analog)
1255	DRAMP04361	PDD-A-3 (PDD-A analog)
1256	DRAMP04362	PDD-A-4 (PDD-A analog)
1257	DRAMP04363	PDD-A-5 (PDD-A analog)
1258	DRAMP04364	PDD-A-6 (PDD-A analog)
1259	DRAMP04365	PDD-A-7 (PDD-A analog)
1260	DRAMP04367	PDD-A-9 (PDD-A analog)
1261	DRAMP04368	PDD-A-10 (PDD-A analog)
1262	DRAMP04369	PDD-A-11 (PDD-A analog)
1263	DRAMP04370	PDD-A-12 (PDD-A analog)
1264	DRAMP04371	PDD-B-1 (PDD-B analog)
1265	DRAMP04372	PDD-B-2 (PDD-B analog)
1266	DRAMP04373	PDD-B-3 (PDD-B analog)
1267	DRAMP04374	PDD-B-4 (PDD-B analog)
1268	DRAMP04376	MP-1 (MP analog)
1269	DRAMP04377	MP-2 (MP analog)
1270	DRAMP04378	MP-5 (MP analog)
1271	DRAMP04379	MP-6 (MP analog)
1272	DRAMP04380	PMM-1 (PMM analog)
1273	DRAMP04381	PMM-2 (PMM analog)
1274	DRAMP04382	PMM-3 (PMM analog)
1275	DRAMP04383	PMM-4 (PMM analog)
1276	DRAMP04385	PMM-6 (PMM analog)
1277	DRAMP04386	PMM-7 (PMM analog)
1278	DRAMP04387	PMM-8 (PMM analog)
1279	DRAMP04389	PMM-10 (PMM analog)
1280	DRAMP04390	PMM-11 (PMM analog)
1281	DRAMP04391	PMM-12 (PMM analog)

1282	DRAMP04392	PMM-13 (PMM analog)
1283	DRAMP04393	PMM-14 (PMM analog)
1284	DRAMP04528	CrusEs (cDNA encoding crustin-like peptide)
1285	DRAMP04542	Polybia-MP-I (insects, vertebrates, animals)
1286	DRAMP04543	Polybia-MP-II (insects, vertebrates, animals)
1287	DRAMP04544	Polybia-MP-III (insects, vertebrates, animals)
1291	DRAMP04640	PGLa-AN2
1292	DRAMP04665	Px-cec1
1293	DRAMP04670	PBD1-42
1294	DRAMP04671	Myticusin-1
1295	DRAMP04676	Brevinin-2HS2A
1296	DRAMP04677	Brevinin-2HS2B
1297	DRAMP04679	Senegalin
1298	DRAMP00001	Variacin (Bacteriocin)
1300	DRAMP00003	Bovicin HJ50 (Bacteriocin; Predicted)
1302	DRAMP00006	Butyrivibriocin OR79 (Bacteriocin)
1304	DRAMP00008	Lacticin 3147 A1 (LtnA1; Bacteriocin; Preclinical)
1305	DRAMP00009	Bacteriocin lacticin 3147 A2 (LtnA2; Bacteriocin; Preclinical)
1306	DRAMP00010	Plantaricin W alpha (Plw-alpha; Bacteriocin)
1307	DRAMP00011	Plantaricin W beta (Plw-beta; Bacteriocin)
1310	DRAMP00014	Geobacillin I (nisin analog; Bacteriocin)
1311	DRAMP00015	Geobacillin II (nisin analog; Bacteriocin)
1312	DRAMP00016	Salivaricin 9 (Sal9; Bacteriocin)
1317	DRAMP00022	Staphylococcin C55alpha (SacAalpha; chain alpha of Staphylococcin C55; Bacteriocin)
1318	DRAMP00023	Staphylococcin C55beta (SacAbeta; chain beta of Staphylococcin C55; Bacteriocin)
1326	DRAMP00031	Lantibiotic carnocin-UI49 (Bacteriocin)
1327	DRAMP00033	Lantibiotic epilancin 15X (Bacteriocin)
1329	DRAMP00035	Lantibiotic paenibacillin (Bacteriocin)
1330	DRAMP00036	Nisin A (Bacteriocin; Preclinical)
1331	DRAMP00037	Nisin Z (Bacteriocin; Preclinical)
1332	DRAMP00038	Nisin U (Bacteriocin)
1335	DRAMP00041	Mutacin-1140 (Mutacin III; Bacteriocin)
1336	DRAMP00042	Bacteriocin mutacin B-Ny266 (Preclinical)
1340	DRAMP00046	Lantibiotic streptin (Bacteriocin)
1343	DRAMP00049	Bacteriocin lacticin-481 (Lactococcin-DR)
1345	DRAMP00051	Mutacin I (Bacteriocin)
1346	DRAMP00052	Mutacin-2 (Mutacin II mutacin H-29B; Bacteriocin)
1347	DRAMP00053	Nisin F (Bacteriocin; Perclinical)
1348	DRAMP00054	Nisin Q (Bacteriocin)
1352	DRAMP00061	Actagardine (Gardimycin; Bacteriocin)
1353	DRAMP00062	Mersacidin (Bacteriocin; Preclinical)
1354	DRAMP00064	Enterocin 96 (Bacteriocin)
1356	DRAMP00066	Lacticin Q (Bacteriocin)
1358	DRAMP00070	Laterosporulin (Bacteriocin)
1359	DRAMP00071	Ubericin-A (Bacteriocin)
1360	DRAMP00072	Bacteriocin curvaticin
1361	DRAMP00073	Weissellin-A (Bacteriocin)

1363	DRAMP00076	Mundticin ATO6 (Bacteriocin)
1364	DRAMP00077	Mundticin KS (Bacteriocin)
1366	DRAMP00079	Bacteriocin hiracin-JM79 (HirJM79; Bacteriocin)
1367	DRAMP00080	Curvacin A (Bacteriocin)
1368	DRAMP00081	Leucocin-A (Leucocin A-UAL 187; Leu A; Bacteriocin)
1369	DRAMP00082	Bavaricin-MN (Bacteriocin)
1370	DRAMP00083	Bavaricin-A (Bacteriocin)
1372	DRAMP00085	Bacteriocin
1373	DRAMP00086	Divergicin M35 (Pediocin-like peptide; Bacteriocin)
1377	DRAMP00092	Bacteriocin SRCAM 602 (Preclinical)
1378	DRAMP00093	Bacteriocin SRCAM 37
1379	DRAMP00094	Bacteriocin SRCAM 1580
1381	DRAMP00096	Pediocin PA-1 (Pediocin ACH; Bacteriocin)
1383	DRAMP00099	Sakacin 5X (Sak5X; Pediocin-like peptide; Bacteriocin)
1384	DRAMP00100	Sakacin G (SakG; Pediocin-like peptide; Bacteriocin)
1385	DRAMP00101	Sakacin P (Sakacin 674; Pediocin-like peptide; Bacteriocin)
1389	DRAMP00109	Plantaricin C19 (Pediocin-like peptide; Bacteriocin)
1390	DRAMP00110	Plantaricin 423 (Pediocin-like peptide; Bacteriocin)
1391	DRAMP00111	Penocin A (PenA; Bacteriocin)
1394	DRAMP00116	Bacteriocin 32 (Bac 32; Bacteriocin)
1395	DRAMP18340	Daptomycin(Bacteriocin)
1396	DRAMP00119	Listeriocin 743A
1397	DRAMP00130	Lactococcin Q alpha (Qalpha; Bacteriocin)
1398	DRAMP00131	Lactococcin Q beta (Qbeta; Bacteriocin)
1422	DRAMP00157	Plantaricin S alpha (Bacteriocin)
1425	DRAMP00160	ThmA (chain a of Thermophilin 13; Bacteriocin)
1426	DRAMP00161	ThmB (chain b of Thermophilin 13; Bacteriocin)
1427	DRAMP00162	Thuricin CDalpha (Trn-alpha; one peptide of Thuricin CD; Bacteriocin)
1428	DRAMP00163	Thuricin CDdbeta (Trn-beta; one peptide of Thuricin CD; Bacteriocin)
1430	DRAMP00165	Gassericin A (GaaA; Bacteriocin)
1431	DRAMP00166	Butyrivibriocin AR10 (Bacteriocin)
1432	DRAMP00167	Subtilosin A (Antilisterial bacteriocin subtilosin; D-amino acid; Bacteriocin; Preclinical)
1434	DRAMP00169	Enterocin AS-48 (AS-48; Bacteriocin)
1435	DRAMP00170	Carnocyclin A (CclA; Bacteriocin)
1436	DRAMP00172	Garvicin ML (Bacteriocin)
1437	DRAMP18337	S. amritsarensis lipopeptide (Bacteriocin)
1438	DRAMP18338	Thiocillin GE37468 (Bacteriocin)
1439	DRAMP00175	Enterocin L50A (EntL50A; Bacteriocin)
1440	DRAMP00176	Enterocin L50B (EntL51B; Bacteriocin)
1444	DRAMP00182	Thuricin-S (Bacteriocin)
1445	DRAMP00183	Thuricin-17 (Thurincin H; Bacteriocin)
1450	DRAMP00188	Enterocin RJ-11 (EntRJ-11; Bacteriocin)
1463	DRAMP00206	Acidocin A (Bacteriocin)
1464	DRAMP00207	Acidocin B (Bacteriocin)
1467	DRAMP18336	Thermophilin 1277 (Bacteriocin)
1469	DRAMP00212	Rhamnosin A (Bacteriocin)
1472	DRAMP00215	Enterocin E-760 (Bacteriocin)

1475	DRAMP00219	PlnA-22 (Bacteriocin)
1476	DRAMP00220	PlnA-17 (Bacteriocin)
1477	DRAMP00221	Carnobacteriocin-A (Piscicolin-61; Bacteriocin)
1478	DRAMP18335	Salivaricin G32(Bacteriocin)
1481	DRAMP00226	Bioactive peptide 3 (BAP3; Curvalicin-28c; Bacteriocin)
1482	DRAMP00227	Bioactive peptide 2 (BAP2;Curvalicin-28b; Bacteriocin)
1483	DRAMP00228	Bioactive peptide 1 (BAP1; Curvalicin-28a; Bacteriocin)
1484	DRAMP00229	Bacteriocin plantarican ASM1 (PASM1; Bacteriocin)
1485	DRAMP00230	Lariatins A (lasso peptide; Bacteriocin)
1486	DRAMP00231	Lariatins B (lasso peptide; Bacteriocin)
1490	DRAMP00236	Halocin-S8 (HalS8; Bacteriocin)
1492	DRAMP00238	Curvaticin FS47 (Bacteriocin)
1495	DRAMP00242	Subpeptin JM4-B (Bacteriocin)
1496	DRAMP00243	Subpeptin JM4-A (Bacteriocin)
1500	DRAMP00248	Glycocin F (GccF; S-glycosylated bacteriocin)
1519	DRAMP00276	Snakin-2 (StSN2; Cys-rich; Plant defensin)
1520	DRAMP00277	Potamin-1 (PT-1; Plants)
1574	DRAMP00341	Antifungal protein ginkbilobin-1 (Ginkbilobin, GNL; Plants)
1580	DRAMP18333	BHT-Ab(Bacteriocin)
1581	DRAMP18334	BHT-B (Bacteriocin)
1601	DRAMP00383	Antimicrobial peptide 1 (Mc-AMP1; knottin-type peptide; Plant defensin)
1609	DRAMP00393	Hedyotide B2 (hB2; Uncyclotides; Plants)
1611	DRAMP00396	Ct-AMP1 (CtAMP1, C. ternatea-antimicrobial peptide 1; Plant defensin)
1612	DRAMP00397	Defensin D1 (Ns-D1; Plant defensin)
1613	DRAMP00398	Defensin D2 (Ns-D2; Plant defensin)
1617	DRAMP00402	Defensin D1 (So-D1; Antimicrobial peptide D1; Plant defensin)
1618	DRAMP00403	Defensin D2 (So-D2; Antimicrobial peptide D2; Plant defensin)
1621	DRAMP00406	Defensin D5 (So-D5; Antimicrobial peptide D5; Plant defensin)
1622	DRAMP00407	Defensin D6 (So-D6; Antimicrobial peptide D6; Plant defensin)
1624	DRAMP00409	Defensin-like protein (Sesquin; Plant defensin)
1629	DRAMP18331	Antibacterial peptide A-M49 (Bacteriocin)
1630	DRAMP18332	BHT-Aa(Bacteriocin)
1640	DRAMP00429	Aesculus hippocastanum antimicrobial protein 1 (Ah-AMP1; Cys-rich; Plant defensin)
1661	DRAMP00455	Defensin-like protein 2 (Fabatin-2; Plant defensin)
1662	DRAMP00456	Defensin-like protein 1 (Fabatin-1; Plant defensin)
1678	DRAMP18329	Pneumococin N(Bacteriocin)
1679	DRAMP18328	Pneumococin M(Bacteriocin)
1851	DRAMP18327	Sil(Bacteriocin)
1952	DRAMP00746	Flower-specific defensin (NaD1; Plant defensin)
1954	DRAMP00748	Defensin-like protein(Brazzein; Plants)
1955	DRAMP00749	Defensin-like protein 1 (Dm-AMP1; Plant defensin)
1969	DRAMP00767	ChaC1 (Chassatide C1; Plant defensin)
1970	DRAMP00768	ChaC2 (Chassatide C2; Plant defensin)
1971	DRAMP00769	ChaC4 (Chassatide C4; Plant defensin)
1972	DRAMP00770	ChaC10 (Chassatide C10; Plant defensin)
1973	DRAMP18326	Bovicin 255(Bacteriocin)
1976	DRAMP18325	delta-lysin I (Bacteriocin)

1977	DRAMP00796	Cliotide T2 (cT2; Plant defensin)
1978	DRAMP00797	Cliotide T3 (cT3; Plant defensin)
2009	DRAMP18323	Nukacin ISK-1(Bacteriocin)
2018	DRAMP00865	Vicilin-like Antimicrobial peptide 2a (MiAMP2a; Plant defensin)
2071	DRAMP00937	Tu-AMP1 (Plant defensin)
2072	DRAMP00938	Tu-AMP2 (Plant defensin)
2077	DRAMP00957	Pp-AMP1 (P. pubescens AMP1; Plant defensin)
2078	DRAMP00958	Pp-AMP2 (P. pubescens AMP2; Plant defensin)
2080	DRAMP18321	Epidermicin NI01(Bacteriocin)
2081	DRAMP01380	Odorranain-J1 (OdJ1; Frogs, amphibians, animals)
2086	DRAMP00976	Antimicrobial peptide 1 (AC-AMP1; Plant defensin)
2087	DRAMP00977	Antimicrobial peptide 2 (AC-AMP2; Plant defensin)
2090	DRAMP00980	Antimicrobial peptide 1a (WAMP-1a; Plant defensin)
2091	DRAMP00981	Antimicrobial peptide 1b (WAMP-1b; Plant defensin)
2092	DRAMP00982	Fa-AMP1 (Fagopyrum antimicrobial peptide 1; hevein-type; Plant defensin)
2093	DRAMP00983	Fa-AMP2 (Fagopyrum antimicrobial peptide 2; hevein-type; Plant defensin)
2094	DRAMP00984	Antimicrobial peptide Ar-AMP (Ar-AMP; hevin-like peptides; Plant defensin)
2095	DRAMP00985	Pn-AMP1 (PnAMP1; Plant defensin)
2096	DRAMP00986	Pn-AMP2 (PnAMP2; Plant defensin)
2097	DRAMP00987	Ee-CBPb (Hevein-like antimicrobial peptide; Plants)
2104	DRAMP00994	IB-AMP1 (IBAMP1; Basic peptide AMP1; Plants)
2107	DRAMP00997	IB-AMP4 (IBAMP4; Basic peptide AMP4; Plants)
2108	DRAMP00998	Antimicrobial peptide MBP-1 (Maize Basic Peptide 1; Plant defensin)
2109	DRAMP00999	Plectasin (fungal defensin)
2116	DRAMP01007	Non-specific lipid transfer peptide (LTP 1; nsLTP; Plants)
2119	DRAMP01010	Lunatusin (Plants)
2123	DRAMP01015	VaD1 (Plant defensin)
2126	DRAMP01022	Cy-AMP1 (Plant defensin)
2127	DRAMP01023	Cy-AMP2 (Plant defensin)
2128	DRAMP01024	Cy-AMP3 (Plant defensin)
2129	DRAMP01025	Vicilin-like Antimicrobial peptide 2a (MiAMP2a; Plant defensin)
2130	DRAMP01026	Vicilin-like Antimicrobial peptide 2b (MiAMP2b; Plant defensin)
2132	DRAMP01028	Vicilin-like Antimicrobial peptide 2c-3 (MiAMP2c-3; Plant defensin)
2133	DRAMP01029	Vicilin-like Antimicrobial peptide 2c-2 (MiAMP2c-2; Plant defensin)
2134	DRAMP01030	Vicilin-like Antimicrobial peptide 2c-1 (MiAMP2c-1; Plant defensin)
2135	DRAMP01031	Vicilin-like Antimicrobial peptide 2d (MiAMP2d; Plant defensin)fens
2136	DRAMP01032	Vicilin-like Antimicrobial peptide 2b (MiAMP2b; Plant defensin)
2137	DRAMP01033	Vicilin-like Antimicrobial peptide 2c-3 (MiAMP2c-3; Plant defensin)
2138	DRAMP01034	Vicilin-like Antimicrobial peptide 2c-2 (MiAMP2c-2; Plant defensin)
2139	DRAMP01035	Vicilin-like Antimicrobial peptide 2c-1 (MiAMP2c-1; Plant defensin)
2140	DRAMP01036	Vicilin-like Antimicrobial peptide 2d (MiAMP2d; Plant defensin)
2141	DRAMP01037	Vicilin-like Antimicrobial peptide 2b (MiAMP2b; Plant defensin)
2142	DRAMP01038	Vicilin-like Antimicrobial peptide 2c-3 (MiAMP2c-3; Plant defensin)
2143	DRAMP01039	Vicilin-like Antimicrobial peptide 2c-2 (MiAMP2c-2; Plant defensin)
2144	DRAMP01040	Vicilin-like Antimicrobial peptide 2c-1 (MiAMP2c-1; Plant defensin)
2145	DRAMP01041	Vicilin-like Antimicrobial peptide 2d (MiAMP2d; Plant defensin)
2157	DRAMP01054	Antimicrobial protein Ace-AMP1 (Ace-AMP1; Plant defensin)

2158	DRAMP01055	P. americana AMP (Pa-AMP-1; PAFP-S; Cys-rich; Plant defensin)
2187	DRAMP01100	Bombinin-like peptide 1 (Contains: Bombinin H; toads, amphibians, animals)
2204	DRAMP01127	Maximin-H5 (toads, amphibians, animals)
2217	DRAMP01140	Uperin-2.2 (toads, amphibians, animals)
2218	DRAMP01141	Uperin-2.3 (toads, amphibians, animals)
2219	DRAMP01142	Uperin-2.4 (toads, amphibians, animals)
2220	DRAMP01143	Uperin-2.5 (toads, amphibians, animals)
2223	DRAMP01146	Uperin-2.8 (toads, amphibians, animals)
2224	DRAMP01147	Uperin-3.1 (toads, amphibians, animals)
2229	DRAMP01157	Uperin-4.1 (toads, amphibians, animals)
2233	DRAMP01161	Uperin-7.1 (Frogs, amphibians, animals)
2250	DRAMP01196	Andersonin-G1 (Frogs, amphibians, animals)
2251	DRAMP01197	Andersonin-N1 (Frogs, amphibians, animals)
2252	DRAMP01198	Andersonin-Q1 (Frogs, amphibians, animals)
2255	DRAMP01207	Galensin (Frogs, amphibians, animals)
2258	DRAMP01212	Pleurain-A3 (Pleurain A3; Frogs, amphibians, animals)
2259	DRAMP01213	Pleurain-A4 (Pleurain A4; Frogs, amphibians, animals)
2260	DRAMP01215	Kassinatuerin-2Mb (Frogs, amphibians, animals)
2261	DRAMP01216	Kassinatuerin-2Mc (Frogs, amphibians, animals)
2262	DRAMP01217	Kassinatuerin-2Md (Frogs, amphibians, animals)
2263	DRAMP18320	Epilancin 15X(Bacteriocin)
2264	DRAMP01223	Palustrin-2AJ2 (PL2AJ12; Frogs, amphibians, animals)
2265	DRAMP01224	Palustrin-2AR (Palustrin-2ARa; Ranatuerin-2SEa; Frogs, amphibians, animals)
2285	DRAMP18404	Polybia-MPII (mastoparan; insects, arthropods, invertebrates, animals)
2292	DRAMP18319	BsaA2(Bacteriocin)
2317	DRAMP18318	BacCH91(Bacteriocin)
2325	DRAMP01318	Antimicrobial peptide 1 (Frogs, amphibians, animals)
2343	DRAMP01338	Amolopin-1a (Frogs, amphibians, animals)
2345	DRAMP01342	Amolopin-2b (Frogs, amphibians, animals)
2346	DRAMP01343	Amolopin-1c (Frogs, amphibians, animals)
2347	DRAMP01344	Amolopin-2c (Frogs, amphibians, animals)
2348	DRAMP01345	Amolopin-1d (Frogs, amphibians, animals)
2353	DRAMP01365	Maculatin-1.2 (Frogs, amphibians, animals)
2355	DRAMP01369	Maculatin-3.1 (Frogs, amphibians, animals)
2361	DRAMP18317	Aureocin A70 (AurD)(Bacteriocin)
2363	DRAMP01444	Nigrocin-1 (Frogs, amphibians, animals)
2364	DRAMP01445	Nigrocin-2 (Nigrocin-2LVa; Frogs, amphibians, animals)
2369	DRAMP18316	Aureocin A70 (AurC)(Bacteriocin)
2370	DRAMP18315	Aureocin A70 (AurB)(Bacteriocin)
2372	DRAMP01463	Esculentin-1SEa (Frogs, amphibians, animals)
2373	DRAMP01464	Esculentin-1SEb (Frogs, amphibians, animals)
2374	DRAMP01465	Esculentin-1R (Frogs, amphibians, animals)
2378	DRAMP18314	Aureocin A70 (AurA)(Bacteriocin)
2382	DRAMP01489	Esculentin-1A (Frogs, amphibians, animals)
2383	DRAMP01492	Esculentin-IIb (Frogs, amphibians, animals)
2388	DRAMP18312	Propionicin PLG-1(Bacteriocin)
2392	DRAMP01519	Esculentin-2PRa (Frogs, amphibians, animals)

2393	DRAMP01522	Rugosin-C (Frogs, amphibians, animals)
2420	DRAMP01569	Caerin-3.1 (Frogs, amphibians, animals)
2436	DRAMP01609	Aurein-2.2 (Frogs, amphibians, animals)
2437	DRAMP01610	Aurein-2.3 (Frogs, amphibians, animals)
2438	DRAMP01611	Aurein-2.4 (Frogs, amphibians, animals)
2442	DRAMP18309	Baceridin(Bacteriocin)
2469	DRAMP18307	LMW peptide (Bacteriocin)
2475	DRAMP01671	Dermaseptin-4 (DS IV; Dermaseptin-S4, DS4; Frogs, amphibians, animals)
2477	DRAMP01673	Dermaseptin-1 (DStar 01; Frogs, amphibians, animals)
2478	DRAMP18306	Pediocin ACCEL(Bacteriocin)
2479	DRAMP01675	Dermaseptin-3 (DStar 03; Frogs, amphibians, animals)
2480	DRAMP01676	Dermaseptin-4 (DStar 04; Frogs, amphibians, animals)
2481	DRAMP01677	Dermaseptin-5 (DStar 05; Frogs, amphibians, animals)
2482	DRAMP01678	Dermaseptin-6 (DStar 06; Frogs, amphibians, animals)
2483	DRAMP01679	Dermaseptin-7 (DStar 07; Frogs, amphibians, animals)
2484	DRAMP01680	Dermaseptin-8 (DStar 08; Frogs, amphibians, animals)
2503	DRAMP01700	Dermaseptin-H3 (Dermaseptin-like peptide 3, DMS3; Frogs, amphibians, animals)
2504	DRAMP18304	Paenicidin A (Bacteriocin)
2506	DRAMP01705	Dermaseptin-like peptide (SmDLP; Frogs, amphibians, animals)
2513	DRAMP18303	Paenicidin A (Bacteriocin)
2518	DRAMP01717	Dermatoxin (Frogs, amphibians, animals)
2530	DRAMP02857	Indolicidin (Cathelicidin-4; mammals, animals)
2531	DRAMP01741	Temporin-D (Frogs, amphibians, animals)
2532	DRAMP02819	Anoplin (Insects, arthropods, invertebrates, animals)
2533	DRAMP04395	EP3 (Earthworm,animals)
2534	DRAMP04394	EP2 (Earthworm,animals)
2541	DRAMP01758	Temporin-1SPa (Frogs, amphibians, animals)
2548	DRAMP01772	Temporin-1Ob (Frogs, amphibians, animals)
2549	DRAMP01774	Temporin-1Od (Frogs, amphibians, animals)
2551	DRAMP18302	GE2270A(Bacteriocin)
2557	DRAMP01796	Temporin-1Gb (Frogs, amphibians, animals)
2558	DRAMP01797	Temporin-1Gc (Frogs, amphibians, animals)
2559	DRAMP01798	Temporin-1Gd (Frogs, amphibians, animals)
2560	DRAMP01799	Temporin-1PRa (Temporin 1PRa; Frogs, amphibians, animals)
2561	DRAMP01800	Temporin-1PRb (Temporin 1PRb; Frogs, amphibians, animals)
2562	DRAMP01801	Temporin-1DYa (Frogs, amphibians, animals)
2563	DRAMP01802	Temporin-PTa (Frogs, amphibians, animals)
2565	DRAMP01804	Temporin-CDYb (Brevinin-1CDYb; Frogs, amphibians, animals)
2568	DRAMP01810	Temporin-1BYa (Frogs, amphibians, animals)
2571	DRAMP01387	Odorranain-P2a (OdP2a; Frogs, amphibians, animals)
2572	DRAMP01386	Odorranain-P1a (OdP1a; Brevinin-1HS1; Brevinin-1-OA2; Frogs, amphibians, animals)
2573	DRAMP01824	Temporin-1Ja (Frogs, amphibians, animals)
2576	DRAMP01126	Maximin-H4 (Toads, amphibians, animals)
2577	DRAMP01125	Maximin-H3 (Toads, amphibians, animals)
2578	DRAMP18299	Taromycin A (Bacteriocin)
2588	DRAMP18296	Leucocin H beta(Bacteriocin)
2592	DRAMP18295	Leucocin H alpha(Bacteriocin)

2621	DRAMP01924	Brevinin-1Ea (Frogs, amphibians, animals)
2622	DRAMP01925	Brevinin-1Eb (Frogs, amphibians, animals)
2623	DRAMP01926	Brevinin-1Ec (Frogs, amphibians, animals)
2624	DRAMP01927	Brevinin-2Ea (Frogs, amphibians, animals)
2625	DRAMP01928	Brevinin-2Eb (Frogs, amphibians, animals)
2626	DRAMP01929	Brevinin-2Ec (Frogs, amphibians, animals)
2627	DRAMP01930	Brevinin-1Ed (Frogs, amphibians, animals)
2628	DRAMP01931	Brevinin-2Ed (Frogs, amphibians, animals)
2629	DRAMP01932	Brevinin-2Ee (Frogs, amphibians, animals)
2631	DRAMP01945	Brevinin-1SE (Frogs, amphibians, animals)
2632	DRAMP01946	Brevinin-20a (Frogs, amphibians, animals)
2633	DRAMP01947	Brevinin-20b (Frogs, amphibians, animals)
2634	DRAMP18294	lactococcin Z(Bacteriocin)
2635	DRAMP01952	Brevinin-1PTb (Frogs, amphibians, animals)
2636	DRAMP01954	Brevinin-2HSb (Frogs, amphibians, animals)
2637	DRAMP01958	Brevinin-2PTd (Frogs, amphibians, animals)
2638	DRAMP01960	Brevinin-1BYa (Frogs, amphibians, animals)
2639	DRAMP01961	Brevinin-1BYb (Frogs, amphibians, animals)
2640	DRAMP01962	Brevinin-1BYc (Frogs, amphibians, animals)
2642	DRAMP01966	Brevinin-1Ya (Frogs, amphibians, animals)
2643	DRAMP01967	Brevinin-1Yb (Frogs, amphibians, animals)
2647	DRAMP01976	Brevinin-2Eg (Frogs, amphibians, animals)
2648	DRAMP18292	LsbA(Bacteriocin)
2654	DRAMP18291	Garviecin LG34(Bacteriocin)
2663	DRAMP18289	Bactofencin A (Bacteriocin)
2664	DRAMP18290	Garvicin A (Bacteriocin)
2665	DRAMP18287	Blp1b(Bacteriocin)
2667	DRAMP18286	Blp1a(Bacteriocin)
2670	DRAMP18285	Bacteriocin LS2 (Bacteriocin)
2671	DRAMP18284	Reutericin 6 (Bacteriocin)
2674	DRAMP02012	Brevinin-1T (Brevinin-2T; Frogs, amphibians, animals)
2675	DRAMP02013	Brevinin-1Ta (Frogs, amphibians, animals)
2678	DRAMP02016	Brevinin-1DYa (Frogs, amphibians, animals)
2679	DRAMP02017	Brevinin-2DYa (Frogs, amphibians, animals)
2680	DRAMP02018	Brevinin-1DYb (Brevinin-1CDYb; Frogs, amphibians, animals)
2681	DRAMP02020	Brevinin-1DYc (Frogs, amphibians, animals)
2684	DRAMP18281	Plantaricin KL-1Y (Bacteriocin)
2689	DRAMP18279	Plantaricin A(Bacteriocin)
2694	DRAMP02089	Brevinin-1La (Brevinin-1PRd; Frogs, amphibians, animals)
2695	DRAMP01124	Maximin-H2 (Toads, amphibians, animals)
2696	DRAMP01123	Maximin-H1 (Toads, amphibians, animals)
2697	DRAMP01111	Maximin-5 (Toads, amphibians, animals)
2698	DRAMP01110	Maximin-4 (Toads, amphibians, animals)
2699	DRAMP01109	Maximin-3 (Toads, amphibians, animals)
2700	DRAMP02100	Brevinin-1Pe (Frogs, amphibians, animals)
2705	DRAMP18273	Enterocin CRL35 (Bacteriocin)
2706	DRAMP18272	Enterocin AS-48RJ (Bacteriocin)

2718	DRAMP02132	Antimicrobial peptide 3 (XT-3; Levitide-like peptide; Frogs, amphibians, animals)
2719	DRAMP02134	Antimicrobial peptide 5 (XT-5; PGLa-like peptide; Frogs, amphibians, animals)
2769	DRAMP02187	Ranatuerin-2AVa (Frogs, amphibians, animals)
2773	DRAMP18271	NKR-5-3B(Bacteriocin)
2803	DRAMP02232	Ranatuerin-5 (Frogs, amphibians, animals)
2806	DRAMP02243	Ranatuerin-1T (Brevinin-2T; Frogs, amphibians, animals)
2808	DRAMP18270	Enterocin NKR-5-3Z(Bacteriocin)
2809	DRAMP18269	Enterocin NKR-5-3A(Bacteriocin)
2810	DRAMP02249	Ranatuerin-2SEB (Frogs, amphibians, animals)
2811	DRAMP02250	Ranatuerin-2SEC (Frogs, amphibians, animals)
2813	DRAMP02258	Ranatuerin-IIbYa (Ranatuerin-2bYa; Frogs, amphibians, animals)
2818	DRAMP02284	Pseudin-1 (Pseudin 1; Frogs, amphibians, animals)
2819	DRAMP02285	Pseudin-2 (Pseudin 2; Frogs, amphibians, animals)
2820	DRAMP02286	Pseudin-3 (Pseudin 3; Frogs, amphibians, animals)
2821	DRAMP02287	Pseudin-4 (Pseudin 4; Frogs, amphibians, animals)
2834	DRAMP18268	Enterocin NKR-5-3C(Bacteriocin)
2841	DRAMP02332	Piscidin-3 (Pis-3; fish, chordates, animals)
2851	DRAMP01375	Odorranain-E1 (OdE1; Frogs, amphibians, animals)
2854	DRAMP02353	Pleurocidin-like peptide WFX (fish, chordates, animals; Predicted)
2855	DRAMP18267	BacFL31(Bacteriocin)
2861	DRAMP18266	Bacteriocin T8(Bacteriocin)
2868	DRAMP02245	Ranatuerin-2Cb (Ranatuerin 2Cb; Frogs, amphibians, animals)
2869	DRAMP02398	Antimicrobial peptide GP-19 (GP-19)
2872	DRAMP18265	Enterocin RM6 (Bacteriocin)
2876	DRAMP02407	Napin-like polypeptide (Contains: Napin-like polypeptide small chain and large chain)
2886	DRAMP02426	Defensin (Varisin A1; Ticks, Arthropods, animals)
2890	DRAMP02437	Papillosin
2891	DRAMP02438	Halocyntin
2895	DRAMP18264	Enterocin W beta(Bacteriocin)
2896	DRAMP02447	Antimicrobial protein 2 (Antimicrobial protein AN5-2)
2901	DRAMP02453	S. litura moricin (Sl moricin; Insects, animals)
2902	DRAMP02454	Theromacin (Arthropods, animals)
2904	DRAMP02457	L-amino-acid oxidase (Balt-LAAO-I; LAAO; LAO; snakes, reptils, animals)
2906	DRAMP02459	L-amino-acid oxidase (BjarLAAO-I; LAAO; LAO; snakes, reptils, animals)
2909	DRAMP02462	L-amino-acid oxidase (LAAO; LAO; snakes, reptils, animals)
2915	DRAMP02468	Acidic phospholipase A2 PnPLA2 (svPLA2; Reptiles, animals)
2924	DRAMP02482	Omwaprin-a (Oxywaprin; Oxywaprin-a; Snakes, reptiles, animals)
2932	DRAMP18263	Enterocin W alpha(Bacteriocin)
2939	DRAMP18262	Enterocin 7A(Bacteriocin)
2944	DRAMP18261	Enterocin 7B (Bacteriocin)
2945	DRAMP18260	Bacteriocin 31 (Bacteriocin)
2947	DRAMP18259	Enterocin O16 (Bacteriocin)
2949	DRAMP18258	Closticin 574(Bacteriocin)
2950	DRAMP18256	Boticin B (Bacteriocin)
2951	DRAMP02511	Crotamine (defensin-like toxin; Snakes, reptiles, animals)
2990	DRAMP18255	Circularin A(Bacteriocin)
2999	DRAMP18253	Piscicocin CS526(Bacteriocin)

3003	DRAMP02570	Penaeidin-1 (Pen-1; shrimps, Arthropods, animals)
3004	DRAMP02571	Penaeidin-2a (Pen-2a; shrimps, Arthropods, animals)
3006	DRAMP02575	Penaeidin-3b (Pen-3b; shrimps, Arthropods, animals)
3007	DRAMP02576	Penaeidin-3c (Pen-3c; shrimps, Arthropods, animals)
3009	DRAMP18252	Carnolysin A2(Bacteriocin)
3028	DRAMP02602	Clavaspirin (chordates, animals)
3029	DRAMP18251	Carnolysin A1(Bacteriocin)
3050	DRAMP18250	Laterosporulin (Bacteriocin)
3052	DRAMP18248	Bifidin I(Bacteriocin)
3053	DRAMP18249	Bac-GM100 (Bacteriocin)
3058	DRAMP18246	Thuricin 439A,439B(Bacteriocin)
3059	DRAMP18247	Bacthuricin F4(Bacteriocin)
3065	DRAMP02642	Rhesus theta-defensin 1 (RTD-1; primates, mammals, animals)
3066	DRAMP02643	Rhesus theta-defensin 2 (RTD-2; primates, mammals, animals)
3067	DRAMP02644	Rhesus theta-defensin 3 (RTD-3; primates, mammals, animals)
3075	DRAMP02653	Neutrophil defensin 1 (RMAD-1; primates, mammals, animals)
3076	DRAMP02654	Neutrophil defensin 2 (RMAD-2; primates, mammals, animals)
3081	DRAMP02659	Neutrophil defensin 3 (RMAD-3; primates, mammals, animals)
3082	DRAMP02660	Neutrophil defensin 4 (RMAD-4; primates, mammals, animals)
3083	DRAMP02661	Neutrophil defensin 5 (RMAD-5; primates, mammals, animals)
3084	DRAMP02662	Neutrophil defensin 6 (RMAD-6; primates, mammals, animals)
3085	DRAMP02663	Neutrophil defensin 7 (RMAD-7; primates, mammals, animals)
3095	DRAMP18245	Ticin A4(Bacteriocin)
3119	DRAMP02698	Rhesus macaque oral alpha-defensins (ROADs; primates, mammals, animals)
3122	DRAMP18244	Ticin A3(Bacteriocin)
3124	DRAMP18243	Ticin A1(Bacteriocin)
3128	DRAMP18242	Fengycin B2 (Bacteriocin)
3130	DRAMP18240	Fengycin C(Bacteriocin)
3131	DRAMP18241	Subtilomycin(Bacteriocin)
3132	DRAMP18239	Fengycin A2(Bacteriocin)
3133	DRAMP18238	Fengycin B(Bacteriocin)
3144	DRAMP18237	Fengycin A(Bacteriocin)
3157	DRAMP02739	TEWP (turtle egg-white protein; Reptiles, animals)
3158	DRAMP02741	Pelovaterin (defensin-like AMP; Gly-rich; Reptiles, animals)
3159	DRAMP02742	Defensin-like turtle egg white protein TEWP (TEWP; Reptiles, animals)
3168	DRAMP03743	Androctonin (Arthropods, animals)
3171	DRAMP02755	Ponericin G3 (ants, insects, animals)
3172	DRAMP02756	Ponericin G4 (ants, insects, animals)
3174	DRAMP02758	Ponericin G6 (ants, insects, animals)
3175	DRAMP02759	Ponericin G7 (ants, insects, animals)
3176	DRAMP02760	Ponericin-L1 (ants, insects, animals)
3177	DRAMP02761	Ponericin-L2 (ants, insects, animals)
3178	DRAMP02762	Ponericin-W1 (ants, insects, animals)
3180	DRAMP02764	Ponericin-W3 (ants, insects, animals)
3181	DRAMP02765	Ponericin-W4 (ants, insects, animals)
3182	DRAMP02766	Ponericin-W5 (ants, insects, animals)
3183	DRAMP02767	Ponericin-W6 (ants, insects, animals)

3184	DRAMP02770	Pilosulin 3 (ants, insects, animals)
3185	DRAMP02771	Pilosulin 4 (ants, insects, animals)
3189	DRAMP02781	Coleoptericin (Insects, animals)
3191	DRAMP02783	Peptide C (Insects, animals)
3199	DRAMP02791	A.dichotoma defensin (defensins; Insects, animals)
3204	DRAMP02796	Defensin (Type 1 invertebrate defensin; Insects, animals)
3210	DRAMP02803	Mytilin-A (molluscas, animals)
3211	DRAMP02804	Mytilus defensin-B (molluscas, animals)
3215	DRAMP02808	Myticin-A (Myt A; Cys-rich; molluscas, animals)
3219	DRAMP18235	Gageopeptide D(Bacteriocin)
3220	DRAMP02816	Lumbrican
3221	DRAMP02818	Dicynthaurin
3222	DRAMP01820	Temporin-1Cb (Temporin 1Cb; Frogs, amphibians, animals)
3223	DRAMP01822	Temporin-1Cd (Temporin 1Cd; Frogs, amphibians, animals)
3234	DRAMP02832	Reactive oxygen species modulator 1 (ROS modulator 1; mammals, animals)
3239	DRAMP18233	Gageopeptide B(Bacteriocin)
3240	DRAMP18234	Gageopeptide C(Bacteriocin)
3249	DRAMP01356	Ranalexin-1Ca (Ranatuerin 1Ca; Frogs, amphibians, animals)
3250	DRAMP02864	Bovine Beta-defensin 7 (bBD-7; BNBD-7; BNDB-7; mammals, animals)
3251	DRAMP02871	Beta-defensin 119 (Defensin, beta 119; mammals, animals)
3252	DRAMP02874	Chromacin (mammals, animals)
3253	DRAMP02876	Alpha-melanocyte-stimulating hormone (Alpha-MSH; mammals, animals)
3275	DRAMP02901	Tricyclic peptide MS-271
3281	DRAMP02914	Cathelicidin-1 (Bactenecin-1, Bac1; Cyclic dodecapeptide; mammals, animals)
3291	DRAMP02929	Antimicrobial protein 1
3292	DRAMP02930	Antimicrobial protein 2 (crabs, Arthropods, animals)
3294	DRAMP02936	Big defensin (crabs, Arthropods, animals)
3295	DRAMP02937	Tachycitin (crabs, Arthropods, animals)
3296	DRAMP18230	Gageotetrin B (Bacteriocin)
3297	DRAMP18231	Gageotetrin C (Bacteriocin)
3298	DRAMP18232	Gageopeptide A(Bacteriocin)
3301	DRAMP02941	Tachystatin-A1 (crabs, Arthropods, animals)
3302	DRAMP02942	Tachystatin-A2 (crabs, Arthropods, animals)
3303	DRAMP02943	Tachystatin-B1 (crabs, Arthropods, animals)
3304	DRAMP02944	Tachystatin-B2 (crabs, Arthropods, animals)
3305	DRAMP02945	Tachystatin-C (crabs, Arthropods, animals)
3308	DRAMP18228	Gageostatin C (Bacteriocin)
3309	DRAMP18229	Gageotetrin A (Bacteriocin)
3312	DRAMP02955	Hedistin (marine annelid, Metazoa)
3315	DRAMP02967	Antibacterial peptide 3910 (AP 3910; pigs, mammals, animals)
3316	DRAMP02968	Prophenin-1 (C6, PF-1; Pro-rich; pigs, mammals, animals)
3317	DRAMP02969	Prophenin-2 (C12, PF-2, PR-2; Pro-rich; pigs, mammals, animals)
3318	DRAMP02971	Protegrin-2 (Protegrin 2; PG-2; pigs, mammals, animals)
3319	DRAMP02972	Protegrin-3 (Protegrin 3; PG-3; pigs, mammals, animals)
3322	DRAMP02976	Beta-defensin 1 (BD-1; Defensin, beta 1; pigs, mammals, animals)
3323	DRAMP02977	pBD-1 (porcine beta-defensin 1; pigs, mammals, animals)
3327	DRAMP02982	Reactive oxygen species modulator 1 (ROS modulator 1; pigs, mammals, animals)

3329	DRAMP02984	Neutrophil cationic antibacterial polypeptide of 11 kDa (CAP11; pigs, mammals, animals)
3330	DRAMP02985	Neutrophil cationic peptide 2 (CP-2; GNCP-2; pigs, mammals, animals)
3331	DRAMP02986	Neutrophil cationic peptide 1 (GPNP; Antiviral defensin; pigs, mammals, animals)
3334	DRAMP02989	Lasioglossin LL-I (Insects, animals)
3335	DRAMP02990	Lasioglossin LL-II (Insects, animals)
3336	DRAMP02991	Lasioglossin LL-III (Insects, animals)
3348	DRAMP03023	Mastoparan (Protonectarina-MP; Insects, animals)
3349	DRAMP18227	Gageostatin B (Bacteriocin)
3355	DRAMP03049	Eumenine mastoparan-OD (EMP-OD; Venom peptide 1, OdVP1; Insects, animals)
3363	DRAMP03066	Lucifensin (Lucifensin II; Insects, animals)
3364	DRAMP03067	Lucifensin (Lucilia defensin; Insects, animals)
3366	DRAMP18226	Gageostatin A (Bacteriocin)
3369	DRAMP03073	Sapecin-C (Sapecin C; defensins; Insects, animals)
3378	DRAMP03083	Sapecin-B (defensins; Insects, animals)
3379	DRAMP03084	Ceratotoxin-B (Insects, animals)
3380	DRAMP03085	Ceratotoxin-A (Insects, animals)
3381	DRAMP03086	Ceratotoxin-D (Insects, animals)
3389	DRAMP18224	Ala-6-fenycin (Bacteriocin)
3392	DRAMP03103	Sarcotoxin-1D (Sarcotoxin ID; Insects, animals)
3400	DRAMP18223	Sonorensin(Bacteriocin)
3403	DRAMP03119	Def-BAT (hybrid defensins; Insects, animals)
3404	DRAMP03120	Def-BBB (hybrid defensins; Insects, animals)
3405	DRAMP03121	Def-ABB (hybrid defensins; Insects, animals)
3406	DRAMP03122	Anopheles gambiae defensin (DEF-AAA; Insects, animals)
3408	DRAMP03124	Def-AcAA (hybrid defensins; Insects, animals)
3409	DRAMP03125	Def-DAA (hybrid defensins; Insects, animals)
3418	DRAMP03134	Defensin-D (AaeDefD; Insects, animals)
3436	DRAMP03157	Halocidin subunit B
3437	DRAMP03158	Halocidin subunit A (invertebrates, animals; Preclinical)
3441	DRAMP03163	R. prolixus defensin A (RprDefA; insect defensin; Insects, animals)
3442	DRAMP03164	R. prolixus defensin B (RprDefB; insect defensin; Insects, animals)
3443	DRAMP03165	R. prolixus defensin C (RprDefC; insect defensin; Insects, animals)
3447	DRAMP18222	PseA(Bacteriocin)
3449	DRAMP03174	Arenicin-2 (Ar-2; marine polychaeta, animals)
3450	DRAMP03175	Perinerin
3454	DRAMP03180	ASABF-alpha (ASABF; nematodes, animals)
3455	DRAMP03182	Termicin (Termite defensin; Insects, animals)
3456	DRAMP03183	Naegleriapore A
3457	DRAMP03184	Naegleriapore B
3461	DRAMP18221	Fusaricidin D (Bacteriocin)
3465	DRAMP03199	PhD1 (PhD-1; Defensin-1; primates, mammals, animals)
3466	DRAMP03200	PhD2 (PhD-2; Defensin-2; primates, mammals, animals)
3467	DRAMP03201	PhD3 (PhD-3; Defensin-3; primates, mammals, animals)
3469	DRAMP18220	Fusaricidin C (Bacteriocin)
3470	DRAMP18219	Fusaricidin B (Bacteriocin)
3473	DRAMP03208	BTD-1 (theta-defensin; primates, mammals, animals)
3474	DRAMP03209	BTD-2 (theta-defensin; primates, mammals, animals)

3475	DRAMP18218	Fusaricidin A (Bacteriocin)
3476	DRAMP03211	BTD-4 (theta-defensin; primates, mammals, animals)
3477	DRAMP03212	BTD-7 (theta-defensin; primates, mammals, animals)
3478	DRAMP18217	Licheniocin 50.2(Bacteriocin)
3479	DRAMP18216	Cerein 7A(Bacteriocin)
3485	DRAMP03224	M-ctenitoxin-Cs1c (M-CNTX-Cs1c; Cupiennin-1c; spiders, Arthropods, animals)
3488	DRAMP18215	Cerecidin A7(Bacteriocin)
3489	DRAMP03238	M-zodatoxin-Lt8c (M-ZDTX-Lt8c; Cytoinsectotoxin-1c, CIT-1c; spiders, Arthropods, animals)
3510	DRAMP18214	Cerecidin A1(Bacteriocin)
3514	DRAMP18213	Gramicidin S(Bacteriocin)
3519	DRAMP18212	Amylocyclicin(Bacteriocin)
3521	DRAMP18210	Amythiamicin C/D(Bacteriocin)
3524	DRAMP03281	Turkey Heterophil Peptide 3 (Antimicrobial peptide THP3; Birds, animals)
3525	DRAMP03282	Turkey Heterophil Peptide 1 (Antimicrobial peptide THP1; Birds, animals)
3526	DRAMP03283	Turkey Heterophil Peptide 2 (Antimicrobial peptide THP2, THP2; Birds, animals)
3542	DRAMP03303	Theromyzin (Frogs, amphibians, animals)
3543	DRAMP03304	Hydramacin-1 (Hm-1; annelida, animals)
3545	DRAMP03306	Theromacin (Annelida, animals)
3548	DRAMP18209	NAI-802 (Bacteriocin)
3554	DRAMP03317	Cathelicidin-related antimicrobial peptide (AMPs)
3555	DRAMP03318	Pore-forming peptide ameobapore B (EH-APP; saposin-like protein)
3556	DRAMP03319	Pore-forming peptide ameobapore C (EH-APP; saposin-like protein)
3563	DRAMP18206	Deoxyactagardine B(Bacteriocin)
3565	DRAMP03329	Alpha-defensin cryptdin-1 (Crp1; Rodents, mammals, animals)
3566	DRAMP03330	Alpha-defensin cryptdin-2 (Defensin-related cryptdin-2; Rodents, mammals, animals)
3567	DRAMP03331	Alpha-defensin cryptdin-3 (Defensin-related cryptdin-3; Rodents, mammals, animals)
3568	DRAMP03332	Alpha-defensin cryptdin-4 (Defensin-related cryptdin4; Rodents, mammals, animals)
3569	DRAMP03333	Alpha-defensin cryptdin-5 (Defensin-related cryptdin5; Rodents, mammals, animals)
3570	DRAMP03334	Alpha-defensin cryptdin-6/12 (Defensin-related cryptdin-6/12; Rodents, mammals, animals)
3593	DRAMP03357	Cryptdin related sequence peptide (CRS4C-1a; Rodents, mammals, animals)
3594	DRAMP03358	Cryptdin related sequence peptide (CRS4C-1d; Rodents, mammals, animals)
3595	DRAMP03359	Cryptdin related sequence peptide (CRS4C-2; Rodents, mammals, animals)
3596	DRAMP03360	Cryptdin related sequence peptide (CRS4C-2b; Rodents, mammals, animals)
3598	DRAMP03362	CRS4C-3c (Cryptdin related sequence peptide; Rodents, mammals, animals)
3602	DRAMP03366	Beta-defensin 1 (BD-1; mBD-1; Defensin, beta 1; Rodents, mammals, animals)
3605	DRAMP03369	Beta-defensin 4 (BD-4, mBD-4; Defensin, beta 4; Rodents, mammals, animals)
3608	DRAMP03373	Beta-defensin 8 (BD-8, mBD-8; Defensin, beta 8; Rodents, mammals, animals)
3635	DRAMP03400	WAP four-disulfide core domain protein 12 (Rodents, mammals, animals)
3637	DRAMP03402	WAP four-disulfide core domain protein 15B (Elafin-like protein I; Rodents, mammals, animals)
3639	DRAMP03407	Defr1 (Murine beta-defensin related peptide; Rodents, mammals, animals)
3640	DRAMP03408	Neutrophil defensin 1 (HANP-1; alpha-defensin; Rodents, mammals, animals)
3641	DRAMP03409	Neutrophil defensin 2 (HANP-2; alpha-defensin; Rodents, mammals, animals)
3642	DRAMP03410	Neutrophil defensin 3 (HANP-3; alpha-defensin; Rodents, mammals, animals)
3643	DRAMP03411	Neutrophil defensin 4 (HANP-4; alpha-defensin; Rodents, mammals, animals)
3651	DRAMP03420	Neutrophil antibiotic peptide NP-2 (RatNP-2; Rodents, mammals, animals)
3652	DRAMP03421	Neutrophil antibiotic peptide NP-3 (RatNP-3a, RatNP-3b; Rodents, mammals, animals)
3696	DRAMP03470	Defensin-1 (American oyster defensin, AOD; molluscs, animals)

3698	DRAMP03475	Vicilin-like Antimicrobial peptide 2a (MiAMP2a; Plant defensin)
3721	DRAMP03501	La-LTP (LJAFP; Insects, animals)
3728	DRAMP03510	Cecropin-A (Insects, animals)
3729	DRAMP03511	Cecropin-B (Immune protein P9; Insects, animals)
3730	DRAMP03512	Cecropin-D (Cecropin D; Insects, animals)
3735	DRAMP18208	NAI-112(Bacteriocin)
3736	DRAMP18207	Actagardine B(Bacteriocin)
3737	DRAMP03534	Antibacterial peptide enbocin (Moricin; Insects, animals)
3738	DRAMP03535	Lebocin-1/2 (Pro-rich; Insects, animals)
3739	DRAMP03536	Lebocin-3 (LEB 3; Insects, animals)
3742	DRAMP03554	CCL20(1-67) (Human, mammals, animals)
3743	DRAMP03555	CCL20(2-70) (Human, mammals, animals)
3744	DRAMP03556	C-C motif chemokine 20 (Human, mammals, animals)
3749	DRAMP03564	Human hepcidin-20 (Hepc20; one chain of Hepcidin; Human, mammals, animals)
3750	DRAMP03565	Human hepcidin-25 (Hepc25; one chain of Hepcidin; Human, mammals, animals)
3751	DRAMP03566	Salvic (Human, mammals, animals)
3762	DRAMP03585	Human TC-1 (Chain of Platelet basic protein; Human, mammals, animals)
3763	DRAMP03586	Human TC-2 (Chain of Platelet basic protein; Human, mammals, animals)
3764	DRAMP03587	DCD-1 (chain of Dermcidin; Human, mammals, animals)
3765	DRAMP03588	Human MUC7 20-Mer (Human, mammals, animals)
3768	DRAMP03591	Neutrophil defensin 1 (Defensin, alpha 1; HNP-1, HP-1; Human, mammals, animals)
3769	DRAMP03592	Neutrophil defensin 2 (HNP-2, HP-2, HP2; Human, mammals, animals)
3770	DRAMP03593	Neutrophil defensin 3 (Defensin, alpha 3; HNP-3, HP-3, HP3; Human, mammals, animals)
3771	DRAMP03594	Neutrophil defensin 4 (Defensin, alpha 4; HNP-4, HP-4; Human, mammals, animals)
3772	DRAMP03595	Human defensin-5 (HD-5; Defensin, alpha 5; Human, mammals, animals)
3773	DRAMP03596	Human defensin-6 (HD-6; Defensin, alpha 6; Human, mammals, animals)
3777	DRAMP18203	Panusin (beta defensins; crustaceans, arthropods, invertebrates, animals)
3789	DRAMP18120	BnPRP1 (Plant defensin)
3799	DRAMP18140	VG16KRKP
3802	DRAMP18194	AAEL000598-PA
3808	DRAMP03641	Longicornsin (defensin-like; Arthropods, invertebrates, animals)
3810	DRAMP03644	Cathelicidin-1 (CATH-1; Fowlicidin-1; Birds, animals)
3811	DRAMP03648	Gallinacin-1 (Gal-1; Beta-defensin 1; Birds, animals)
3812	DRAMP03649	Gallinacin-1 alpha (Gal-1 alpha; Antimicrobial peptide CHP2; Birds, animals)
3813	DRAMP03650	Gallinacin-2 (Gal-2; Beta-defensin 2; Birds, animals)
3823	DRAMP03661	Gallinacin-13 (Gal-13; Beta-defensin 13; Birds, animals)
3833	DRAMP03671	Locustin (Insects, animals)
3836	DRAMP03674	Cystatin-1 (Cystatin-I)
3837	DRAMP18192	Antimicrobial peptide AcrAP2
3839	DRAMP03680	Cathelicidin-3.4 (Bactenecin-3.4, Bac3.4; ChBac3.4; ruminant, animals)
3841	DRAMP03689	Scorpine-like (Arthropods, animals)
3843	DRAMP03692	Defensin-1 (Cll-dlp; Arthropods, animals)
3849	DRAMP03701	Imcroporin (Arthropods, animals)
3871	DRAMP03736	Opistoporin-2 (OP2; Non-disulfide-bridged peptide 3.6, NDBP-3.6; Arthropods, animals)
3872	DRAMP03737	Opistoporin-4 (Non-disulfide-bridged peptides 3.7, NDBP-3.7; Arthropods, animals)
3873	DRAMP03739	Buthinin (Sahara scorpion; Arthropods, animals)
3874	DRAMP03740	Androctonus defensin (4 kDa defensin; Arthropods, animals)

3875	DRAMP03741	Ponericin-W-like 32.1 (Arthropods, animals)
3876	DRAMP03742	Ponericin-W-like 32.2 (Arthropods, animals)
3880	DRAMP03755	Potassium channel toxin alpha-KTx 1.1 (ChTX-Lq1; charybdotoxin; Arthropods, animals)
3883	DRAMP18191	Antimicrobial peptide AcrAP1
3891	DRAMP03766	Heteroscorpine-1 (HS-1; defensins; Arthropods, animals)
3907	DRAMP03787	Neuropeptide-like protein 31 (NLP-31; nematodes, animals)
3910	DRAMP03790	ABF-2 (nematodes, animals)
3944	DRAMP18190	Pantinin-3 (Non-disulfide-bridged peptide 4.22, NDBP-4.22, Non-disulfide-bridged peptide 5.23)
3948	DRAMP03872	hLf 21-30 (fragment of human lalctoferricin, residues 21-30)
3949	DRAMP03873	mLf 20-29 (fragment of murine lalctoferricin, residues 20-29)
3950	DRAMP03874	pLf20-29 (fragment of porcine lactoferricin, residues 20-29)
3961	DRAMP03941	Peptide 3 (Trp- and Arg-rich; derivative of Tritrpticin)
3962	DRAMP03942	Peptide 2 (Trp- and Arg-rich; derivative of Tritrpticin)
3965	DRAMP03946	Del 1-3 (Ranalexin analog)
3970	DRAMP03961	KR-12
3976	DRAMP02092	Brevinin-1Bb (Frogs, amphibians, animals)
3978	DRAMP18189	Pantinin-2 (Non-disulfide-bridged peptide 4.21, NDBP-4.21, Non-disulfide-bridged peptide 5.22)
3982	DRAMP04018	Rp-1
3983	DRAMP04037	Immobilized peptide E07LKK
3984	DRAMP04038	Immobilized peptide E14LKK/H14LKK
3985	DRAMP04039	Immobilized peptige E16KGL/H16KGL
3986	DRAMP04040	Immobilized peptide E17KGG
3987	DRAMP04041	Immobilized peptide E18KGG
3988	DRAMP04042	Immobilized peptide E16LKL
3989	DRAMP04043	Immobilized peptid E10KKL
3990	DRAMP04044	Immobilized peptid E12LLK
3991	DRAMP04045	Immobilized peptid E14KKL
3992	DRAMP04046	Immobilized peptide E23GIG magainin2
3993	DRAMP04047	Immobilized peptide E17HSA magainin 2 deletion
3994	DRAMP04071	LL-37 pentamide
3998	DRAMP04084	W9F (mutant of Def-DAA defensin)
3999	DRAMP04085	K8I,W9F (mutant of Def-DAA defensin)
4000	DRAMP04086	W9F,W11V (mutant of Def-DAA defensin)
4001	DRAMP04087	K8I,W9F,W11V (mutant of Def-DAA defensin)
4002	DRAMP04088	W9F,T14A (mutant of Def-DAA defensin)
4003	DRAMP04089	K8I,W9F,T14A (mutant of Def-DAA defensin)
4004	DRAMP04090	W11V,T14A (mutant of Def-DAA defensin)
4005	DRAMP04091	K8I,W11V,T14A (mutant of Def-DAA defensin)
4006	DRAMP04092	W11V (mutant of Def-DAA defensin)
4007	DRAMP04093	K8I,W11V (mutant of Def-DAA defensin)
4008	DRAMP04094	K8I (mutant of Def-DAA defensin)
4020	DRAMP04172	LK2W2 (LlKmW2 model peptides)
4021	DRAMP04173	L2KW2 (LlKmW2 model peptides)
4169	DRAMP04366	PDD-A-8 (PDD-A analog)
4170	DRAMP04375	PDD-B-5 (PDD-B analog)
4171	DRAMP04384	PMM-5 (PMM analog)
4172	DRAMP04388	PMM-9 (PMM analog)

4173	DRAMP01823	Temporin-1Ce (Temporin 1Ce; Frogs, amphibians, animals)
4424	DRAMP04658	Hymenochirin-5B
4435	DRAMP18188	Pantinin-1 (Non-disulfide-bridged peptide 4.20, NDBP-4.20, Non-disulfide-bridged peptide 5.21)
4443	DRAMP04684	Bacteriocin BAC-IB17
4451	DRAMP04700	Basic phospholipase A2 BnpTX-1 (BnPTx-I, svPLA2; Phosphatidylcholine 2-acylhydrolase)
4472	DRAMP18187	Toxin LyeTx 1
4473	DRAMP18128	Antimicrobial peptide HsAp4;
4474	DRAMP18129	Antimicrobial peptide HsAp3;
4475	DRAMP18130	Antimicrobial peptide HsAp2
4476	DRAMP18131	Antimicrobial peptide HsAp1 (HsAp)
4485	DRAMP18185	Jingdongin-1
4487	DRAMP01297	Longipin (Fragment)
4510	DRAMP18433	Cremycin-15 (nematode; invertebrates, animals)
4531	DRAMP18454	Tepmporin-1Ee (frog, amphibians, animals)
4533	DRAMP18456	Pepcon (peptide consensus sequence, synthetic)
4555	DRAMP18488	Css54 (Css from the species name below; scorpions, arachnids, Chelicerata, arthropods, invertebrates, animals)
4556	DRAMP18489	Cathelicidin-PY (Frog, amphibians, animals; BBL)
4557	DRAMP18491	CecropinXJ (Insects, arthropods, invertebrates, animals)
4558	DRAMP18492	cgUbiquitin (Oyster, mollusca/molluscs/mollusks, invertebrates, animals)
4559	DRAMP18493	Tilapia piscidin 3 (TP3; His-rich; fish, animals; inactive: TP1, TP2, and TP5)
4560	DRAMP18494	Tilapia piscidin 4 (TP4; Oreoch-2; MSP-4; fish, animals)
4564	DRAMP03575	LL-37(17-29) (C-terminal fragment of LL-37, LL; Human, mammals, animals)
4565	DRAMP02093	Brevinin-1Bc (Frogs, amphibians, animals)
4566	DRAMP02094	Brevinin-1Bd (Frogs, amphibians, animals)
4567	DRAMP02095	Brevinin-1Be (Frogs, amphibians, animals)
4568	DRAMP02096	Brevinin-1Bf (Frogs, amphibians, animals)
4569	DRAMP02097	Brevinin-1Pa (Frogs, amphibians, animals)
4570	DRAMP02098	Brevinin-1Pc (Frogs, amphibians, animals)
4571	DRAMP02099	Brevinin-1Pd (Frogs, amphibians, animals)
4572	DRAMP02255	Ranatuerin-2Lb (Ranatuerin 2Lb; Ranaturin-2PRd; Frogs, amphibians, animals)
4573	DRAMP02254	Ranatuerin-2La (Ranatuerin 2La; Ranatuerin-2PRa; Frogs, amphibians, animals)
4574	DRAMP02256	Ranatuerin-2B (Ranatuerin 2B, Frog, amphibians, animals)
4575	DRAMP02257	Ranatuerin-2P (Ranatuerin 2P; Frogs, amphibians, animals)
4576	DRAMP18496	rNZ2114
4577	DRAMP18497	TSG-6 (Ixosin-B peptide derivative)
4578	DRAMP18498	TSG-7 (Ixosin-B peptide derivative)
4579	DRAMP18499	TSG-8 (Ixosin-B peptide derivative)
4580	DRAMP18500	TSG-8-1 (Ixosin-B peptide derivative)
4581	DRAMP18501	TSG-9 (Ixosin-B peptide derivative)
4582	DRAMP18502	TSG-10 (Ixosin-B peptide derivative)
4583	DRAMP18503	TSG-11 (Ixosin-B peptide derivative)
4586	DRAMP18506	OG2 (Palustrin-OG1 peptide derivative)
4587	DRAMP18508	gp41w-FKA (gp41 peptide derivative)
4588	DRAMP18509	Px-cec1 (cecropin1 peptide derivative)
4595	DRAMP18533	V13KL (V681 peptide derivative)
4600	DRAMP18538	V681

4601	DRAMP18539	V13LL (V681 peptide derivative)
4602	DRAMP18540	V13AL (V681 peptide derivative)
4603	DRAMP18541	V13G (V681 peptide derivative)
4604	DRAMP18542	V13SL (V681 peptide derivative)
4605	DRAMP18543	V13LD (V681 peptide derivative)
4606	DRAMP18544	V13VD (V681 peptide derivative)
4607	DRAMP18545	V13AD (V681 peptide derivative)
4608	DRAMP18546	V13SD (V681 peptide derivative)
4609	DRAMP18547	V13KD (V681 peptide derivative)
4610	DRAMP18548	S11LL (V681 peptide derivative)
4611	DRAMP18549	S11VL (V681 peptide derivative)
4612	DRAMP18550	S11AL (V681 peptide derivative)
4613	DRAMP18551	S11G (V681 peptide derivative)
4614	DRAMP18552	S11KL (V681 peptide derivative)
4615	DRAMP18553	S11LD (V681 peptide derivative)
4616	DRAMP18554	S11VD (V681 peptide derivative)
4617	DRAMP18555	S11AD (V681 peptide derivative)
4618	DRAMP18556	S11SD (V681 peptide derivative)
4619	DRAMP18557	S11KD (V681 peptide derivative)
4620	DRAMP18558	Kn2-7 (BmKn2 peptide derivative)
4621	DRAMP18559	HFU3
4622	DRAMP18560	HFU4
4623	DRAMP18561	HFU5
4624	DRAMP18562	MAP-04-01 (Ixosin-B peptide derivative)
4625	DRAMP18563	MAP-04-02 (Ixosin-B peptide derivative)
4626	DRAMP18564	MAP-04-03 (Ixosin-B peptide derivative)
4627	DRAMP18565	MAP-04-04 (Ixosin-B peptide derivative)
4628	DRAMP18566	LL-IIIs-1 (lasioglossin III peptide derivative)
4629	DRAMP18567	LL-IIIs-2 (lasioglossin III peptide derivative)
4630	DRAMP18568	LL-IIIs-3 (lasioglossin III peptide derivative)
4631	DRAMP18569	LL-IIIs-4 (lasioglossin III peptide derivative)
4632	DRAMP18570	LL-IIIs-5 cis (lasioglossin III peptide derivative)
4633	DRAMP18571	LL-IIIs-5 trans (lasioglossin III peptide derivative)
4634	DRAMP18572	LL-IIIs-6a (lasioglossin III peptide derivative)
4635	DRAMP18573	LL-IIIs-6b (lasioglossin III peptide derivative)
4636	DRAMP18574	MEP-N (melectin peptide derivative)
4637	DRAMP18575	MEP-Ns-1 (melectin peptide derivative)
4638	DRAMP18576	MEP-Ns-2 (melectin peptide derivative)
4639	DRAMP18577	MEP-Ns-3 (melectin peptide derivative)
4640	DRAMP18578	MEP-Ns-4 cis (melectin peptide derivative)
4641	DRAMP18579	MEP-Ns-4 trans (melectin peptide derivative)
4642	DRAMP18580	MEP-Ns-5 (melectin peptide derivative)
4643	DRAMP18581	MEP-Ns-6 (melectin peptide derivative)
4645	DRAMP18583	Tricystine cyclic cystine TP (ccTP, Tachyplesin-1 peptide derivative)
4646	DRAMP18584	[Arg13]ccTP (ccTP peptide derivative)
4647	DRAMP18585	[Arg4,8]ccTP (ccTP peptide derivative)
4648	DRAMP18586	[Arg4,8,13]ccTP (ccTP peptide derivative)

4649	DRAMP18587	[Arg4,8,13][Lys18]ccTP (ccTP peptide derivative)
4650	DRAMP18588	RTD
4651	DRAMP18589	DSE (Ctx-Ha peptide derivative)
4652	DRAMP18590	DEP (Ctx-Ha peptide derivative)
4653	DRAMP18591	DEA (Ctx-Ha peptide derivative)
4654	DRAMP18592	Ctx(Ile21)-Ha (Ctx-Ha peptide derivative)
4655	DRAMP18593	Ctx(Ile21)-Ha-VD16 (Ctx-Ha peptide derivative)
4656	DRAMP18594	Ctx(Ile21)-Ha-VD5,16 (Ctx-Ha peptide derivative)
4658	DRAMP18596	LL-I/1 (Lasioglossin LL-I peptide derivative)
4659	DRAMP18597	LL-I/2 (Lasioglossin LL-I peptide derivative)
4660	DRAMP18598	LL-I/3 (Lasioglossin LL-I peptide derivative)
4661	DRAMP18599	LL-I/4 (Lasioglossin LL-I peptide derivative)
4662	DRAMP18600	LL-II/1 (Lasioglossin LL-II peptide derivative)
4663	DRAMP18601	LL-II/2 (Lasioglossin LL-II peptide derivative)
4664	DRAMP18602	LL-II/3 (Lasioglossin LL-II peptide derivative)
4665	DRAMP18603	LL-II/4 (Lasioglossin LL-II peptide derivative)
4666	DRAMP18604	LL-III/1 (Lasioglossin LL-III peptide derivative)
4667	DRAMP18605	LL-III/2 (Lasioglossin LL-III peptide derivative)
4668	DRAMP18606	LL-III/3 (Lasioglossin LL-III peptide derivative)
4669	DRAMP18607	LL-III/4 (Lasioglossin LL-III peptide derivative)
4670	DRAMP18608	LL-III/5 (Lasioglossin LL-III peptide derivative)
4671	DRAMP18609	LL-III/6 (Lasioglossin LL-III peptide derivative)
4672	DRAMP18610	LL-III/7 (Lasioglossin LL-III peptide derivative)
4673	DRAMP18611	LL-III/8 (Lasioglossin LL-III peptide derivative)
4674	DRAMP18612	LL-III/10 (Lasioglossin LL-III peptide derivative)
4675	DRAMP18613	TPG (Tritrpticin peptide derivative)
4676	DRAMP18627	D4-K9L8W (D-amino acid substitution of K9L8W)
4677	DRAMP18507	SolyC (Plant defensin; tomato, plants)
4681	DRAMP18614	TPA (Tritrpticin peptide derivative)
4682	DRAMP18615	TWF (Tritrpticin peptide derivative)
4683	DRAMP18616	[K22,25,27]-SMAP-29 (SMAP-29 peptide derivative)
4684	DRAMP18617	[A19]-SMAP-29 (SMAP-29 peptide derivative)
4685	DRAMP18618	SMAP-29(1-17) (SMAP-29 peptide derivative)
4686	DRAMP18619	[K2,7,13]-SMAP-29(1-17) (SMAP-29 peptide derivative)
4687	DRAMP18620	Pep-1-K (Pep-1 peptide derivative)
4688	DRAMP18621	Temporin-PEa (Temporin-PE peptide derivative)
4689	DRAMP18622	Temporin-PEb (Temporin-PE peptide derivative)
4690	DRAMP18623	[I5,R8] Mastoparan-L ([I5,R8] MP-L;Mastoparan-L peptide derivative)
4691	DRAMP18624	K9L8W
4692	DRAMP18625	D3-K9L8W-1 (D-amino acid substitution of K9L8W)
4693	DRAMP18626	D3-K9L8W-2 (D-amino acid substitution of K9L8W)
4694	DRAMP18628	D6-K9L8W (D-amino acid substitution of K9L8W)
4695	DRAMP18629	D9-K9L8W-1 (D-amino acid substitution of K9L8W)
4696	DRAMP18630	D9-K9L8W-2 (D-amino acid substitution of K9L8W)
4697	DRAMP18631	H5(61-90) V1 (Histone H5 peptide derivative)
4700	DRAMP18634	H5(61-90) V3 (Histone H5 peptide derivative)
4702	DRAMP18636	NCP-3a (CTX-1 peptide derivative)

4703	DRAMP18637	NCP-3b (CTX-1 peptide derivative)
4704	DRAMP18638	VT18-LV (VT18 peptide derivative)
4707	DRAMP18641	KCM11
4708	DRAMP18642	KCM12
4709	DRAMP18643	KCM21
4710	DRAMP18644	KRS22
4714	DRAMP18648	[Pro3,DLeu9]TL(3) (Temporin L peptide derivative)
4720	DRAMP18654	dPSMα1 (PSMα1 peptide derivative; bacteriocin; staphylococcus aureus, bacteria)
4721	DRAMP18655	dPSMα4 (PSMα4 peptide derivative; bacteriocin; staphylococcus aureus, bacteria)
4724	DRAMP18658	Temporin-PE (Edible frogs, amphibians, animals)
4725	DRAMP18659	YFGAP-OH(Yellowfin tuna GAPDH-related antimicrobial peptide; fish, animals)
4726	DRAMP18660	YFGAP-NH2(Yellowfin tuna GAPDH-related antimicrobial peptide; fish, animals)
4727	DRAMP18661	Ctx-Ha (Frogs, amphibians, animals)
4728	DRAMP18662	Brevinin 21 (Brevinin-1E truncated peptide 21; Frogs, amphibians, animals)
4729	DRAMP18663	Brevinin 18 (Brevinin-1E truncated peptide 18; Frogs, amphibians, animals)
4730	DRAMP18664	Brevinin 15 (Brevinin-1E truncated peptide 15; Frogs, amphibians, animals)
4731	DRAMP18665	Mastoparan-L (MP-L; insects, arthropods, invertebrates, animals)
4732	DRAMP18666	P1 (Pilosulin-1 1-20; Ant, insects, arthropods, invertebrates, animals)
4733	DRAMP18667	Pep-1
4736	DRAMP18670	AI-hemocidins 4 (Hb-1 truncated peptide)
4737	DRAMP18671	TO17 (TFPI-1 C-terminal peptide)
4738	DRAMP18672	Peptide 7 (Mollusca/molluscs/mollusks, invertebrates, animals)
4739	DRAMP18673	Peptide 3 (Mollusca/molluscs/mollusks, invertebrates, animals)
4740	DRAMP18674	Peptide 2 (Mollusca/molluscs/mollusks, invertebrates, animals)
4741	DRAMP18675	Peptide 4 (Mollusca/molluscs/mollusks, invertebrates, animals)
4742	DRAMP18676	Peptide 5 (Mollusca/molluscs/mollusks, invertebrates, animals)
4743	DRAMP18677	Peptide 6 (Mollusca/molluscs/mollusks, invertebrates, animals)
4744	DRAMP18678	Peptide 8 (Mollusca/molluscs/mollusks, invertebrates, animals)
4745	DRAMP18679	Peptide 9 (Mollusca/molluscs/mollusks, invertebrates, animals)
4759	DRAMP18693	Substance P (Mammals, animals)
4760	DRAMP18694	substance P antagonist (Mammals, animals)
4762	DRAMP18696	HLP1 (Lactotransferrin truncated peptide)
4763	DRAMP18697	HLP2 (Lactotransferrin truncated peptide)
4764	DRAMP18698	Histone H2A (Trouts, fish, animals)
4765	DRAMP18699	Pleurain-B1 (Frogs, amphibians, animals)
4766	DRAMP18700	Pleurain-C1 (Frogs, amphibians, animals)
4767	DRAMP18701	Pleurain-D4 (Frogs, amphibians, animals)
4768	DRAMP18702	Pleurain-E1 (Frogs, amphibians, animals)
4769	DRAMP18703	Pleurain-G1 (Frogs, amphibians, animals)
4770	DRAMP18704	Pleurain-J1 (Frogs, amphibians, animals)
4771	DRAMP18705	Pleurain-N1 (Frogs, amphibians, animals)
4772	DRAMP18706	Pleurain-R1 (Frogs, amphibians, animals)
4773	DRAMP18707	BACTENECIN 7 (bac 7, Pro-rich; bovine cathelicidin, cattle, ruminant, mammals, animals)
4774	DRAMP18708	Dermaseptin-S4 (DRS-S4, DS4; frog, amphibians, animals)
4776	DRAMP18710	Royalisin (Insects, arthropods, invertebrates, animals)
4778	DRAMP18712	Styelin A (Tunicate, invertebrates, animals)
4779	DRAMP18713	Styelin B (Tunicate, invertebrates, animals)

4783	DRAMP18717	Charybdotoxin (Yellow scorpions, arachnids, Chelicerata, arthropods, invertebrates, animals)
4785	DRAMP18719	SAAP fraction 3 (Surfactant-associated anionic peptides; Asp-rich; sheep, ruminant, mammals, animals)
4787	DRAMP18721	Hinnavin I (Hin I; insects, arthropods, invertebrates, animals)
4790	DRAMP18724	Oncorhyncin III (Oncorhyncin-3, histone-derived; fish, animals)
4791	DRAMP03024	Mastoparan B (MP-B; insects, arthropods, invertebrates, animals)
4818	DRAMP01826	RV-23 (Frogs, amphibians, animals)
4819	DRAMP02842	LfcinB(20-25)
4820	DRAMP03025	Mastoparan M
4821	DRAMP18200	SB Piscidin 6 (fish, animals)
4822	DRAMP18201	WB Piscidin 6 (fish, animals)
4823	DRAMP18199	Formicin (Bacteriocin;lantibiotic;Gram-positive bacteria, prokaryotes; U
4824	DRAMP18198	Chaxapeptin
4825	DRAMP03812	Pardaxin P-4 (Pardaxin P1a; Pardaxin Pa4)
4835	DRAMP20766	Pore-forming peptide ameobapore A(Amoeba peptide, AP-A; Parasite, amoebozoa, protozoa, protists)
4836	DRAMP20767	Pore-forming peptide ameobapore B (Parasite, amoebozoa, protozoa, protists)
4837	DRAMP20768	Pore-forming peptide ameobapore C (EH-APP; Parasite, amoebozoa, protozoa, protists)
4838	DRAMP20769	Naegleriapore A (NP-A; parasite, amoebozoa; protozoa, protists)
4839	DRAMP20771	Acanthaporin (parasite, amoebozoa, protozoa, protists)
4840	DRAMP20772	cPcAMP1/26 (ciliate, Protists)
4842	DRAMP20776	HaA4 (beetles,insects,animals)
4843	DRAMP20777	Cath-BF
4844	DRAMP20778	Temporin-SHf (frogs,amphibians,animals)
4845	DRAMP20779	Halictine 1 (bees,insects,animals)
4846	DRAMP20780	Halictine 2 (bees,insects,animals)
4847	DRAMP20781	Panurgine 1(bees,insects,animals)
4848	DRAMP20782	Pleurain-D1 (Frogs,amphibians,animals)
4849	DRAMP20783	Pleurain-M1 (Frogs,amphibians,animals)
4850	DRAMP20784	Megin 1
4851	DRAMP20785	Megin 2
4852	DRAMP20786	mini-ChBac7.5N alpha
4853	DRAMP20787	mini-ChBac7.5N beta
4856	DRAMP20790	Cecropin B (Insects, arthropods, invertebrates, animals)
4858	DRAMP20792	Maculatin 1.1 (Frog, amphibians, animals)
4859	DRAMP20793	Maculatin 1.2 (Frog, amphibians, animals)
4863	DRAMP20797	Uperin 3.6 (Toad, amphibians, animals)
4864	DRAMP20798	Lingual antimicrobial peptide (LAP, beta defensin, cattle, ruminant, animals)
4867	DRAMP20801	Mussel Defensin MGD-1 (Mediterranean mussel defensin 1; mollusca/molluscs/mollusks, invertebrates, animals)
4868	DRAMP20802	CPF-AM1 (caerulein precursor fragment-AM1, frogs, amphibians, animals)
4869	DRAMP20803	moronecidin-like peptide
4870	DRAMP20804	AI-hemocidins 2 (Hb-1 truncated peptide)
4871	DRAMP20805	Apo5 APOC164-88
4872	DRAMP20806	Apo6 APOC167-88
4873	DRAMP20807	A1P394-428
4874	DRAMP20808	RI21 (PMAP-36 peptide derivative)
4875	DRAMP20809	RI18 (PMAP-36 peptide derivative)

4876	DRAMP20810	TI15 (PMAP-36 peptide derivative)
4877	DRAMP20811	RI12 (PMAP-36 peptide derivative)
4878	DRAMP20812	K8
4879	DRAMP20813	L1K8
4880	DRAMP20814	S1K8
4881	DRAMP20815	F1K8
4882	DRAMP20816	K1K8
4883	DRAMP20817	RR12
4884	DRAMP20818	RR12Wpolar
4885	DRAMP20819	RR12Whydro
4886	DRAMP20820	FV7
4887	DRAMP20821	FV-LL (FV7 and LL(LL-37,(17-29)) hybrid peptide)
4888	DRAMP20822	FV-MA (FV7 and MA(Magainin 2 (9-21)) hybrid peptide)
4889	DRAMP20823	FV-CE (FV7 and CE(Cecropin A (1
4890	DRAMP20824	AM-CATH36
4892	DRAMP20826	AM-CATH21
4893	DRAMP20827	TB_L1FK
4894	DRAMP20828	TB_KKG6A
4895	DRAMP20831	IsCT1L1
4896	DRAMP20832	Polybia-MP1S-D8N
4897	DRAMP20833	[Pro3,DLeu9]TL(1) (Temporin L peptide derivative)
4898	DRAMP20834	PLS
4899	DRAMP20837	Pb-CATH1 Python bivittatus antimicrobial peptides peptide derivative
4900	DRAMP20838	Pb-CATH4 bivittatus antimicrobial peptides peptide derivative
4901	DRAMP20839	Xylopin
4902	DRAMP20841	C1b
4903	DRAMP20842	C1b(1-11)
4904	DRAMP20843	C1b(1-13)
4905	DRAMP20844	C1b(3-13)
4906	DRAMP20845	C1b(3-11)
4907	DRAMP20846	C1b(3-12)
4908	DRAMP20847	C1b(4-13)
4909	DRAMP20848	[K4]C1b(3-11)
4910	DRAMP20849	[R4]C1b(3-11)
4911	DRAMP20850	[K4,K10]C1b(3-13)
4912	DRAMP20851	[R4,R10]C1b(3-13)
4920	DRAMP20859	TT(1-24)
4921	DRAMP20860	TT(1-35)
4923	DRAMP20862	rtCATH2(5-40)
4924	DRAMP20863	rtCATH2(1-40)
4925	DRAMP20864	SF(18-45)
4926	DRAMP20865	the dimeric RRWQWR motif peptide molecule
4927	DRAMP20866	the tetrameric RRWQWR motif peptide molecule
4928	DRAMP20867	the palindromic RRWQWR motif peptide molecule
4929	DRAMP20868	H4
4930	DRAMP20869	Pal-ano-9 (Pal-anoplin peptide derivative)
4931	DRAMP20870	Pal-ano-8 (Pal-anoplin peptide derivative)

4932	DRAMP20871	Pal-ano-7 (Pal-anoplin peptide derivative)
4933	DRAMP20872	Pal-ano-6 (Pal-anoplin peptide derivative)
4934	DRAMP20873	Pal-ano-5 (Pal-anoplin peptide derivative)
4935	DRAMP20874	Chensinin-1b
4936	DRAMP20875	OA-C1b
4937	DRAMP20876	LA-C1b
4938	DRAMP20877	PA-C1b
4939	DRAMP20878	rVpDef
4940	DRAMP20879	DAN1
4941	DRAMP20880	DAN2
4942	DRAMP20881	HOLO1
4943	DRAMP20882	LOUDEF1
4944	DRAMP20883	Cath-A
4945	DRAMP20884	Cath-B
4948	DRAMP20887	NCP-2 (CTX-1 peptide derivative)
4949	DRAMP20888	NCP-3 (CTX-1 peptide derivative)
4950	DRAMP20889	VT18-KKLV (VT18 peptide derivative)
4951	DRAMP20890	VT18-CAKKLV (VT18 peptide derivative)
4952	DRAMP20891	cVT18-CAKKLV (VT18 peptide derivative)
4953	DRAMP20892	H3A/H4A
4954	DRAMP20893	I16A
4955	DRAMP20894	L19H/I20H
4956	DRAMP20895	F1A/I2A
4961	DRAMP20900	A12I/A15I
4962	DRAMP20901	A12V/A15H
4963	DRAMP20902	VHSH
4964	DRAMP20903	dC2
4965	DRAMP20904	R18S/R21H
4966	DRAMP20905	dC4
4967	DRAMP20906	dN2
4968	DRAMP20907	dN4
4971	DRAMP20910	RN7-IN7(designed based on indolicidin and ranalexin)
4973	DRAMP20912	RN7-IN9(designed based on indolicidin and ranalexin)
4974	DRAMP20913	Myxinidin (G1)
4975	DRAMP20914	Myxinidin (I2)
4976	DRAMP20915	Myxinidin (H3)
4977	DRAMP20916	Myxinidin (D4)
4978	DRAMP20917	Myxinidin (I5)
4979	DRAMP20918	Myxinidin (L6)
4980	DRAMP20919	Myxinidin (K7)
4981	DRAMP20920	Myxinidin (Y8)
4982	DRAMP20921	Myxinidin (G9)
4983	DRAMP20922	Myxinidin (K10)
4984	DRAMP20923	Myxinidin (P11)
4985	DRAMP20924	Myxinidin (S12)
4986	DRAMP20925	MH3R
4987	DRAMP20926	IN1(designed based on indolicidin and ranalexin)

4988	DRAMP20927	IN2(designed based on indolicidin and ranalexin)
4989	DRAMP20928	IN3(designed based on indolicidin and ranalexin)
4990	DRAMP20929	RN7-IN6(designed based on indolicidin and ranalexin)
4991	DRAMP20930	BP100-Ala-NH-C16H33
4993	DRAMP20933	Stigmurin
4995	DRAMP20935	Macropin 1(solitary bee, insects, animals)
4996	DRAMP20936	ΔPb-CATH1
4997	DRAMP20937	Pb-CATH3
4998	DRAMP20938	Cbf-14
4999	DRAMP20939	D-Cbf-14
5000	DRAMP20940	Polybia-MP1S-Q12K
5001	DRAMP20941	[Pro3,DLeu9]TL(8) (Temporin L peptide derivative)
5002	DRAMP20942	[Pro3,DLeu9]TL(9) (Temporin L peptide derivative)
5003	DRAMP20943	[Pro3,DLeu9]TL(10) (Temporin L peptide derivative)
5004	DRAMP20944	[Pro3,DLeu9]TL(11) (Temporin L peptide derivative)
5005	DRAMP20945	Recombinant Cecropin A (1–8)–LL37 (17–30) (C–L)
5014	DRAMP20955	L31-P113
5015	DRAMP20956	AL32-P113
5016	DRAMP20957	StigA6
5017	DRAMP20958	StigA16
5021	DRAMP20963	Cp1 alpha s1-casein peptide derivative
5022	DRAMP20964	Synthesized Cecropin A (1–8)–LL37 (17–30) (C–L)
5023	DRAMP20965	LPcin-YK3 (bovine cathelicidin, cattle, ruminant, mammals, animals)
5024	DRAMP20966	andricin B (Andrias davidianus, Amphibians, Animals)
5025	DRAMP20967	andricin 01 (Andrias davidianus, Amphibians, Animals)
5026	DRAMP20968	Catesbeianin-1 (Ranidae, Anura, Amphibia, Animals)
5027	DRAMP20969	HJH-1 (bovine cathelicidin, cattle, ruminant, mammals, animals)
5028	DRAMP20970	P3 (bovine cathelicidin, cattle, ruminant, mammals, animals)
5029	DRAMP20971	JH-0 (Derived from P3)
5030	DRAMP20972	JH-1 (Derived from P3)
5031	DRAMP20973	JH-2 (Derived from P3)
5032	DRAMP20974	JH-3 (Derived from P3)
5033	DRAMP20975	OH-CM6 (Derived from OH-CATH30)
5034	DRAMP20976	adevonin (Derived from Adenanthera pavonina trypsin inhibitor (ApTI)) inhibitor (ApTI))
5035	DRAMP20977	Anoplin-1 (Derived from Anoplin)
5036	DRAMP20978	Anoplin-2 (Derived from Anoplin)
5037	DRAMP20979	Anoplin-3 (Derived from Anoplin)
5038	DRAMP20980	Anoplin-4 (Derived from Anoplin)
5039	DRAMP20981	CPF-C1 (Frogs, Amphibians, Animals)
5040	DRAMP20982	CPF-1 (Derived from CPF-C1)
5041	DRAMP20983	CPF-2 (Derived from CPF-C1)
5042	DRAMP20984	CPF-3 (Derived from CPF-C1)
5043	DRAMP20985	CPF-4 (Derived from CPF-C1)
5044	DRAMP20986	CPF-5 (Derived from CPF-C1)
5045	DRAMP20987	CPF-6 (Derived from CPF-C1)
5046	DRAMP20988	CPF-7 (Derived from CPF-C1)

5047	DRAMP20989	CPF-8 (Derived from CPF-C1)
5048	DRAMP20990	CPF-9 (Derived from CPF-C1)
5049	DRAMP20991	CPF-10 (Derived from CPF-C1)
5050	DRAMP20992	CPF-11 (Derived from CPF-C1)
5051	DRAMP20993	CPF-12 (Derived from CPF-C1)
5052	DRAMP20994	anoplin analog 4
5053	DRAMP20995	anoplin analog 5
5054	DRAMP20996	anoplin analog 6
5055	DRAMP20997	anoplin analog 7
5056	DRAMP20998	anoplin analog 8
5057	DRAMP20999	anoplin analog 9
5058	DRAMP21000	cGm (Derived from Gm)
5059	DRAMP21001	[Y7W]cGm (Derived from Gm)
5060	DRAMP21002	[Y14W]cGm (Derived from Gm)
5061	DRAMP21003	[K8R]cGm (Derived from Gm)
5062	DRAMP21004	[Y7W, K8R, Y14W]cGm (Derived from Gm)
5063	DRAMP21005	[R4A, R18A]cGm (Derived from Gm)
5064	DRAMP21006	[G1K, K8R]cGm (Derived from Gm)
5065	DRAMP21007	[C/U]cGm (Derived from Gm)
5066	DRAMP21008	[L5W]cGm (Derived from Gm)
5067	DRAMP21009	[D-P L-P]cGm (Derived from Gm)
5068	DRAMP21010	[G1K, L5Y, K8R]cGm (Derived from Gm)
5069	DRAMP21011	[C/U, G1K, L5Y, K8R]cGm (Derived from Gm)
5070	DRAMP21012	NK-2 (Mammals, Animals)
5071	DRAMP21013	NK-pro (Derived from NK-2)
5072	DRAMP21014	NK-dpro (Derived from NK-2)
5073	DRAMP21015	A (A1R) (Derived from AR-23)
5074	DRAMP21016	A (A8R) (Derived from AR-23)
5075	DRAMP21017	A (I17K) (Derived from AR-23)
5076	DRAMP21018	A (I17R) (Derived from AR-23)
5077	DRAMP21019	A (A1R, A8R) (Derived from AR-23)
5078	DRAMP21020	A (A1R, I17K) (Derived from AR-23)
5079	DRAMP21021	A (A8R, I17K) (Derived from AR-23)
5080	DRAMP21022	A (A1R, A8R, I17K) (Derived from AR-23)
5081	DRAMP21023	A (A1R, A8R, I17R) (Derived from AR-23)
5082	DRAMP21024	Stigmurin (Tityus, Scorpionida, Arachnida)
5083	DRAMP21025	StigA25 (Derived from Stigmurin)
5084	DRAMP21026	StigA31 (Derived from Stigmurin)
5085	DRAMP21027	K5, 17-DPS3 (Derived from dermaseptin-PS3 (DPS3))
5086	DRAMP21028	L10, 11-DPS3 (Derived from dermaseptin-PS3 (DPS3))
5087	DRAMP21029	D5R (Derived from HD5)
5088	DRAMP21030	D5r (Derived from HD5)
5089	DRAMP21031	MyD5R (Derived from HD5)
5090	DRAMP21032	MyD5r (Derived from HD5)
5091	DRAMP21033	LaD5R (Derived from HD5)
5092	DRAMP21034	LaD5r (Derived from HD5)
5093	DRAMP21035	AC-UM-14W (De novo synthesis)

5094	DRAMP21036	PapMA (Derived from Papiliocin and Magainin 2)
5095	DRAMP21037	PapMA-k (Derived from Papiliocin and Magainin 2)
5096	DRAMP21038	analog 1 (Derived from Ib-AMP1)
5097	DRAMP21039	analog 2 (Derived from Ib-AMP2)
5098	DRAMP21040	analog 3 (Derived from Ib-AMP2)
5099	DRAMP21041	analog 4 (Derived from Ib-AMP2)
5100	DRAMP21042	A2 (Derived from Indolicidin (IN))
5101	DRAMP21043	A3 (Derived from Indolicidin (IN))
5102	DRAMP21044	A4 (Derived from Indolicidin (IN))
5103	DRAMP21045	A5 (Derived from Indolicidin (IN))
5104	DRAMP21046	A6 (Derived from Indolicidin (IN))
5105	DRAMP21047	A7 (Derived from Indolicidin (IN))
5106	DRAMP21048	peptide 6 (Derived from seq2)
5107	DRAMP21049	peptide 6.2 (Derived from seq2)
5108	DRAMP21050	TP1[K1A] (Derived from TP1)
5109	DRAMP21051	TP1[W2A] (Derived from TP1)
5110	DRAMP21052	TP1[C3A, C16S] (Derived from TP1)
5111	DRAMP21053	TP1[F4A] (Derived from TP1)
5112	DRAMP21054	TP1[R5A] (Derived from TP1)
5113	DRAMP21055	TP1[V6A] (Derived from TP1)
5114	DRAMP21056	TP1[C7A, C12S] (Derived from TP1)
5115	DRAMP21057	TP1[Y8A] (Derived from TP1)
5116	DRAMP21058	TP1[R9A] (Derived from TP1)
5117	DRAMP21059	TP1[G10A] (Derived from TP1)
5118	DRAMP21060	TP1[I11A] (Derived from TP1)
5119	DRAMP21061	TP1[C7S, C12A] (Derived from TP1)
5120	DRAMP21062	TP1[Y13A] (Derived from TP1)
5121	DRAMP21063	TP1[R14A] (Derived from TP1)
5122	DRAMP21064	TP1[R15A] (Derived from TP1)
5123	DRAMP21065	TP1[C3S, C16A] (Derived from TP1)
5124	DRAMP21066	TP1[R17A] (Derived from TP1)
5125	DRAMP21067	TP1[C3A, C16A] (Derived from TP1)
5126	DRAMP21068	TP1[C7A, C12A] (Derived from TP1)
5127	DRAMP21069	TP1[C3A, C7A, C12A, C16A] (Derived from TP1)
5128	DRAMP21070	TP1[V6R, R9A] (Derived from TP1)
5129	DRAMP21071	TP1[K1R] (Derived from TP1)
5130	DRAMP21072	TP1[F4G] (Derived from TP1)
5131	DRAMP21073	TP1[F4S] (Derived from TP1)
5132	DRAMP21074	TP1[Y8G] (Derived from TP1)
5133	DRAMP21075	TP1[I11G] (Derived from TP1)
5134	DRAMP21076	TP1[F4A, Y8A, I11A] (Derived from TP1)
5135	DRAMP21077	TP1[-R5, R17G] (Derived from TP1)
5136	DRAMP21078	TP1[K1A, F4A] (Derived from TP1)
5137	DRAMP21079	TP1[K1A, Y8A] (Derived from TP1)
5138	DRAMP21080	TP1[K1A, I11A] (Derived from TP1)
5139	DRAMP21081	TP1[R9A, R17A] (Derived from TP1)
5140	DRAMP21082	ccTP 3 (Derived from TP2)

5141	DRAMP21083	ccTP 5 (Derived from TP2)
5142	DRAMP21084	ccTP 6 (Derived from TP2)
5143	DRAMP21085	PRW4 (PR) (Derived from PMAP-36)
5144	DRAMP21086	PR-FO (Derived from PRW4)
5145	DRAMP21087	PR-PG (Derived from PRW4)
5146	DRAMP21088	PR-TR (Derived from PRW4)
5147	DRAMP21089	C4 (Derived from PRW4)
5148	DRAMP21090	D4 (Derived from PRW4)
5149	DRAMP21091	I4 (Derived from PRW4)
5150	DRAMP21092	P4 (Derived from PRW4)
5151	DRAMP21093	PRW4-d (Derived from PRW4)
5152	DRAMP21094	PRW4-R (Derived from PRW4)
5153	DRAMP21095	IR1 (Derived from PG-1)
5154	DRAMP21096	IR2 (Derived from PG-1)
5155	DRAMP21097	FR1 (Derived from PG-1)
5156	DRAMP21098	FR2 (Derived from PG-1)
5157	DRAMP21099	WR1 (Derived from PG-1)
5158	DRAMP21100	WR2 (Derived from PG-1)
5159	DRAMP21101	PR1 (Derived from PG-1)
5160	DRAMP21102	PR2 (Derived from PG-1)
5161	DRAMP21165	HYL-11 (Derived from HYL)
5162	DRAMP21166	HYL-12 (Derived from HYL)
5163	DRAMP21164	HYL-10 (Derived from HYL)
5164	DRAMP21158	HYL-4 (Derived from HYL)
5165	DRAMP21159	HYL-5 (Derived from HYL)
5166	DRAMP21160	HYL-6 (Derived from HYL)
5167	DRAMP21161	HYL-7 (Derived from HYL)
5168	DRAMP21162	HYL-8 (Derived from HYL)
5169	DRAMP21163	HYL-9 (Derived from HYL)
5170	DRAMP21157	HYL-3 (Derived from HYL)
5171	DRAMP21156	HYL-2 (Derived from HYL)
5172	DRAMP21155	HYL-1 (Derived from HYL)
5173	DRAMP21154	HYL (Bee, Insecta, Animals)
5174	DRAMP21153	KR-12-a8 (Derived from KR-12)
5175	DRAMP21151	KR-12-a6 (Derived from KR-12)
5176	DRAMP21150	KR-12-a5 (Derived from KR-12)
5177	DRAMP21152	KR-12-a7 (Derived from KR-12)
5178	DRAMP21149	KR-12-a4 (Derived from KR-12)
5179	DRAMP21146	KR-12-a1 (Derived from KR-12)
5180	DRAMP21148	KR-12-a3 (Derived from KR-12)
5181	DRAMP21147	KR-12-a2 (Derived from KR-12)
5182	DRAMP21145	Myxinidin3 (Derived from Myxinidin)
5183	DRAMP21142	AMP2041 (De novo synthesis)
5184	DRAMP21141	AMP126 (De novo synthesis)
5185	DRAMP21144	Myxinidin2 (Derived from Myxinidin)
5186	DRAMP21143	Myxinidin1 (Derived from Myxinidin)
5187	DRAMP21140	AMP72 (De novo synthesis)

5188	DRAMP21139	GNU7 (De novo synthesis)
5189	DRAMP21138	GNU6 (De novo synthesis)
5190	DRAMP21137	GNU5 (De novo synthesis)
5191	DRAMP21135	P7 (Derived from P5)
5192	DRAMP21136	P8 (Derived from P5)
5193	DRAMP21134	P6 (Derived from P5)
5194	DRAMP21133	P5 (Derived from Octa 2)
5195	DRAMP21132	P4 (Derived from P5)
5196	DRAMP21131	P3 (Derived from P5)
5197	DRAMP21130	P2 (Derived from P5)
5198	DRAMP21129	P1 (Derived from P5)
5199	DRAMP21128	T9F (Derived from RI16)
5200	DRAMP21127	T9K (Derived from RI16)
5201	DRAMP21126	T9I (Derived from RI16)
5202	DRAMP21125	T9W (Derived from RI16)
5203	DRAMP21124	RI16 (Derived from PMAP-36)
5204	DRAMP21123	KR-12-a5 (7-(D)L) (Derived from LL-37)
5205	DRAMP21122	KR-12-a5 (6-(D)L) (Derived from LL-37)
5206	DRAMP21121	KR-12-a5 (5-(D)K) (Derived from LL-37)
5207	DRAMP21119	I11R (Derived from tachyplesin I)
5208	DRAMP21120	KR-12-a5 (Derived from LL-37)
5209	DRAMP21118	I11S (Derived from tachyplesin I)
5210	DRAMP21117	Y8R (Derived from tachyplesin I)
5211	DRAMP21116	Y8S (Derived from tachyplesin I)
5212	DRAMP21115	V6R (Derived from tachyplesin I)
5213	DRAMP21114	V6S (Derived from tachyplesin I)
5214	DRAMP21111	ASA (Derived from SLZP)
5215	DRAMP21112	DLSA (Derived from SLZP)
5216	DRAMP21113	PSA (Derived from SLZP)
5217	DRAMP21103	L-RW (De novo synthesis)
5218	DRAMP21110	SLZP (De novo synthesis)
5219	DRAMP21109	FPA-Bombinin-BO (toads, amphibians, animals)
5220	DRAMP21108	Feleucin-K3 (Derived from Feleucin-BO1)
5221	DRAMP21104	Feleucin-2 (toads, amphibians, animals)
5222	DRAMP21105	Feleucin-BV1 (toads, amphibians, animals)
5223	DRAMP21106	Feleucin-BV2 (toads, amphibians, animals)
5224	DRAMP21107	Feleucin-BO1 (toads, amphibians, animals)
5225	DRAMP21232	Ranatuerin-2PLx (R2PLx; Frogs, Amphibians, Animals)
5229	DRAMP21227	IsCT-P (Derived from IsCT)
5230	DRAMP21228	IsCT-a (Derived from IsCT-P)
5231	DRAMP21225	STPk (Derived from STP)
5232	DRAMP21226	Ink (Derived from IN)
5233	DRAMP21223	IsCT-p (Derived from IsCT-P)
5234	DRAMP21224	TPk (Derived from TP)
5235	DRAMP21222	Control-4D (Derived from IK12-all L)
5236	DRAMP21221	Control-all D (Derived from IK12-all L)
5237	DRAMP21219	IK12-all D (Derived from IK12-all L)

5238	DRAMP21220	Control-all L (Derived from IK12-all L)
5239	DRAMP21218	IK12-all L (De novo synthesis)
5240	DRAMP21217	IK8-2D (Derived from IK8-all L)
5241	DRAMP21215	IK4-all D (Derived from IK8-all L)
5242	DRAMP21216	IK8-4D (Derived from IK8-all L)
5243	DRAMP21213	IK8-all D (Derived from IK8-all L)
5244	DRAMP21214	IK6-all D (Derived from IK8-all L)
5245	DRAMP21212	IK8-all L (De novo synthesis)
5255	DRAMP21202	HPA3NT3-analog (Derived from HPA3NT3)
5256	DRAMP21201	Magainin 2a (M2a; Frogs, Amphibians, Animals)
5257	DRAMP21200	GW-M4 (De novo synthesis)
5258	DRAMP21199	GW-M3 (De novo synthesis)
5259	DRAMP21198	GW-M1 (De novo synthesis)
5260	DRAMP21197	GW-H3 (De novo synthesis)
5261	DRAMP21196	GW-H1 (De novo synthesis)
5262	DRAMP21195	GW-A5 (De novo synthesis)
5263	DRAMP21194	GW-A4 (De novo synthesis)
5264	DRAMP21193	GW-A2 (De novo synthesis)
5265	DRAMP21192	GW-A1 (De novo synthesis)
5266	DRAMP21191	GW-Q6 (De novo synthesis)
5267	DRAMP21190	GW-Q5 (De novo synthesis)
5268	DRAMP21189	GW-Q4 (De novo synthesis)
5269	DRAMP21188	GW-Q3 (De novo synthesis)
5270	DRAMP21187	WRL4 (Derived from leucocin A)
5271	DRAMP21186	WRL3 (Derived from leucocin A)
5272	DRAMP21185	WRL2 (Derived from leucocin A)
5273	DRAMP21184	WR7 (Derived from leucocin A)
5274	DRAMP21183	WR5 (Derived from leucocin A)
5275	DRAMP21182	WR3 (Derived from leucocin A)
5276	DRAMP21181	WR1 (Derived from leucocin A)
5277	DRAMP21180	WG18 (Derived from leucocin A)
5278	DRAMP21179	HYL-26 (Derived from HYL)
5279	DRAMP21178	HYL-25 (Derived from HYL)
5280	DRAMP21177	HYL-24 (Derived from HYL)
5281	DRAMP21176	HYL-23 (Derived from HYL)
5282	DRAMP21175	HYL-22 (Derived from HYL)
5283	DRAMP21174	HYL-21 (Derived from HYL)
5284	DRAMP21173	HYL-20 (Derived from HYL)
5285	DRAMP21172	HYL-19 (Derived from HYL)
5286	DRAMP21168	HYL-15 (Derived from HYL)
5287	DRAMP21169	HYL-16 (Derived from HYL)
5288	DRAMP21170	HYL-17 (Derived from HYL)
5289	DRAMP21171	HYL-18 (Derived from HYL)
5290	DRAMP21243	pardaxin-6 (GE-6) (Derived from pardaxin)
5291	DRAMP21242	Epinecidin-8 (Derived from Epinecidin)
5292	DRAMP21241	Epinecidin-1 (Derived from Epinecidin)
5293	DRAMP21240	FK13-a7 (Derived from FK13)

5294	DRAMP21239	FK13-a6 (Derived from FK13)
5295	DRAMP21238	FK13-a5 (Derived from FK13)
5296	DRAMP21237	FK13-a4 (Derived from FK13)
5297	DRAMP21236	FK13-a3 (Derived from FK13)
5298	DRAMP21235	FK13-a2 (Derived from FK13)
5299	DRAMP21167	HYL-13 (Derived from HYL)
5300	DRAMP21234	FK13-a1 (Derived from FK13)
5301	DRAMP21233	R2PLx-22 (Derived from R2PLx)
5302	DRAMP21244	TsAP-S1 (Derived from TsAP-1)
5303	DRAMP21245	TsAP-S2 (Derived from TsAP-2)
5304	DRAMP21246	pEM-2 (Derived from the venom of the snake Bothrops asper)
5305	DRAMP21247	PV (Derived from pEM-2 and MP-VT1)
5306	DRAMP21248	BVP (Derived from pEM-2 and MP-VT1 and MP-B)
5307	DRAMP21249	PVP (Derived from MP-B and MP-VT1)
5308	DRAMP21250	PV3 (Derived from pEM-2 and MP-VT1)
5309	DRAMP21251	AaeAP1 (Scorpionida, Arachricla, Arthropoda)
5310	DRAMP21252	AaeAP2 (Scorpionida, Arachricla, Arthropoda)
5311	DRAMP21253	AaeAP1a (Derived from AaeAP1)
5312	DRAMP21254	AaeAP2a (Derived from AaeAP2)
5313	DRAMP21255	WL1 (Derived from CP-1)
5314	DRAMP21256	WL2 (Derived from CP-1)
5315	DRAMP21257	WL3 (Derived from CP-1)
5316	DRAMP21258	Cecropin P1 (CP-1) (nematodes, animals)
5317	DRAMP21259	Scolopendin 1 (Centipedes, Arthropoda, Animals)
5318	DRAMP21260	KL0A10 (De novo synthesis)
5319	DRAMP21261	KL4A6 (De novo synthesis)
5320	DRAMP21262	KL6A4 (De novo synthesis)
5321	DRAMP21263	KL10A0 (De novo synthesis)
5322	DRAMP21264	LK (De novo synthesis)
5323	DRAMP21265	LK-L1A (Derived from LK)
5324	DRAMP21266	LK-L4A (Derived from LK)
5325	DRAMP21267	LK-L5A (Derived from LK)
5326	DRAMP21268	LK-L7A (Derived from LK)
5327	DRAMP21269	LK-L8A (Derived from LK)
5328	DRAMP21270	LK-L11A (Derived from LK)
5329	DRAMP21271	LK-L12A (Derived from LK)
5330	DRAMP21272	LK-L14A (Derived from LK)
5331	DRAMP21273	LK-L8G (Derived from LK)
5332	DRAMP21274	LK-L8S (Derived from LK)
5333	DRAMP21275	LK-L8P (Derived from LK)
5334	DRAMP21276	LK-L8N (Derived from LK)
5335	DRAMP21277	LK-L8Q (Derived from LK)
5336	DRAMP21278	LK-L8D (Derived from LK)
5337	DRAMP21279	LK-L8E (Derived from LK)
5338	DRAMP21280	LK-L8K (Derived from LK)
5339	DRAMP21281	LK-L8H (Derived from LK)
5340	DRAMP21282	Lt-F1A (Derived from Lt)

5341	DRAMP21283	Lt-I4A (Derived from Lt)
5342	DRAMP21284	Lt-V5A (Derived from Lt)
5343	DRAMP21285	Lt-I8A (Derived from Lt)
5344	DRAMP21286	Lt-F11A (Derived from Lt)
5345	DRAMP21287	Lt-F12A (Derived from Lt)
5346	DRAMP21288	Lt-I4G (Derived from Lt)
5347	DRAMP21289	Lt-I4S (Derived from Lt)
5348	DRAMP21290	Lt-I4N (Derived from Lt)
5349	DRAMP21291	Lt-I4Q (Derived from Lt)
5350	DRAMP21292	Lt-I4H (Derived from Lt)
5351	DRAMP21293	Lt-V5G (Derived from Lt)
5352	DRAMP21294	Lt-V5S (Derived from Lt)
5353	DRAMP21295	Lt-V5N (Derived from Lt)
5354	DRAMP21296	Lt-V5Q (Derived from Lt)
5355	DRAMP21297	Lt-V5H (Derived from Lt)
5356	DRAMP21298	Lt-F11G (Derived from Lt)
5357	DRAMP21299	Lt-F11S (Derived from Lt)
5358	DRAMP21300	Lt-F11N (Derived from Lt)
5359	DRAMP21301	Lt-F11Q (Derived from Lt)
5360	DRAMP21302	Lt-F11H (Derived from Lt)
5361	DRAMP21303	A7-PMAP-23 (Derived from PMAP-23)
5362	DRAMP21304	A21-PMAP-23 (Derived from PMAP-23)
5363	DRAMP21305	R8 (De novo synthesis)
5364	DRAMP21306	TL-1 (Derived from Temporin-1Tl (TL))
5365	DRAMP21307	TL-2 (Derived from Temporin-2Tl (TL))
5366	DRAMP21308	TL-3 (Derived from Temporin-3Tl (TL))
5367	DRAMP21309	TL-4 (Derived from Temporin-4Tl (TL))
5368	DRAMP21311	2W-1 (Derived from PMAP-36)
5369	DRAMP21312	2W-2 (Derived from PMAP-36)
5370	DRAMP21313	2W-3 (Derived from PMAP-36)
5371	DRAMP21314	3W-1 (Derived from PMAP-36)
5372	DRAMP21315	3W-2 (Derived from PMAP-36)
5373	DRAMP21316	3W-3 (Derived from PMAP-36)
5374	DRAMP21317	3W-4 (Derived from PMAP-36)
5375	DRAMP21318	3W-5 (Derived from PMAP-36)
5376	DRAMP21319	3V (Derived from PMAP-36)
5377	DRAMP21320	3L (Derived from PMAP-36)
5378	DRAMP21321	4W (Derived from PMAP-36)
5379	DRAMP21322	RTV (Derived from PMAP-36)
5380	DRAMP21323	RTI (Derived from PMAP-36)
5381	DRAMP21324	RTF (Derived from PMAP-36)
5382	DRAMP21325	RTL (Derived from PMAP-36)
5383	DRAMP21326	RLR (Derived from PMAP-36)
5384	DRAMP21327	RVR (Derived from PMAP-36)
5385	DRAMP21328	RTR (Derived from PMAP-36)
5386	DRAMP21329	RFR (Derived from PMAP-36)
5387	DRAMP21330	KVK (Derived from PMAP-36)

5388	DRAMP21331	KLK (Derived from PMAP-36)
5389	DRAMP21332	KIK (Derived from PMAP-36)
5390	DRAMP21333	RVK (Derived from PMAP-36)
5391	DRAMP21334	Ranatuerin-2Pb (Frogs, amphibians, animals)
5392	DRAMP21335	RPa (Frogs, amphibians, animals)
5393	DRAMP21336	RPb (Frogs, amphibians, animals)
5394	DRAMP21337	BMAP-27 (Bovine, mammals, animals)
5395	DRAMP21338	[Arg]3-VmCT1-NH2 (Derived from VmCT1)
5396	DRAMP21339	[Arg]7-VmCT1-NH2 (Derived from VmCT1)
5397	DRAMP21340	[Arg]11-VmCT1-NH2 (Derived from VmCT1)
5398	DRAMP21341	[Gly]1-VmCT1-NH2 (Derived from VmCT1)
5399	DRAMP21342	[Pro]8-VmCT1-NH2 (Derived from VmCT1)
5400	DRAMP21343	[Leu]9-VmCT1-NH2 (Derived from VmCT1)
5401	DRAMP21344	[Phe]9-VmCT1-NH2 (Derived from VmCT1)
5402	DRAMP21345	[Leu]12-VmCT1-NH2 (Derived from VmCT1)
5403	DRAMP21346	[Tyr]12-VmCT1-NH2 (Derived from VmCT1)
5423	DRAMP21366	[Lys]1-VmCT1-NH2 (Derived from VmCT1)
5424	DRAMP21367	[Lys]9-VmCT1-NH2 (Derived from VmCT1)
5425	DRAMP21368	[Lys]1[Lys]12-VmCT1-NH2 (Derived from VmCT1)
5426	DRAMP21369	[Lys]3[Lys]7-VmCT1-NH2 (Derived from VmCT1)
5427	DRAMP21370	[Lys]3[Lys]11-VmCT1-NH2 (Derived from VmCT1)
5428	DRAMP21371	[Lys]7[Lys]11-VmCT1-NH2 (Derived from VmCT1)
5429	DRAMP21372	[Lys]3[Lys]7[Lys]11-VmCT1-NH2 (Derived from VmCT1)
5447	DRAMP21390	K17 (Derived from ATG16)
5448	DRAMP21391	K18 (Derived from ATG16)
5449	DRAMP21392	K22 (Derived from ATG16)
5450	DRAMP21393	K22.2 (Derived from ATG16)
5451	DRAMP21394	K30 (Derived from ATG16)
5452	DRAMP21395	K31 (Derived from ATG16)
5453	DRAMP21396	K33 (Derived from ATG16)
5454	DRAMP21397	K36 (Derived from ATG16)
5455	DRAMP21398	K46 (Derived from ATG16)
5456	DRAMP21399	Pep-H (Human, mammals, animals)
5457	DRAMP21400	NBC2253 (De Novo Synthesis)
5458	DRAMP21401	NBC2254 (De Novo Synthesis)
5459	DRAMP21402	B1 (Derived from LL-37 and BMAP-27)
5460	DRAMP21403	peptide 1 (De Novo Synthesis)
5461	DRAMP21404	peptide 2 (De Novo Synthesis)
5462	DRAMP21405	LGL13K (De Novo Synthesis)
5463	DRAMP21406	DGL13K (De Novo Synthesis)
5464	DRAMP21407	Bac4K (Derived from CAMPs)
5465	DRAMP21408	Bac3W (Derived from CAMPs)
5466	DRAMP21409	dBac (Derived from CAMPs)
5467	DRAMP21410	dBac4K (Derived from CAMPs)
5468	DRAMP21411	dBac3W (Derived from CAMPs)
5469	DRAMP21412	dBacK (Derived from CAMPs)
5470	DRAMP21413	dBacK- (cap) (Derived from CAMPs)

5471	DRAMP21414	CecB Q53 (Derived from CecB E53)
5472	DRAMP21415	α4-short (Derived from α4)
5473	DRAMP21416	WV (De Novo Synthesis)
5474	DRAMP21417	WI (De Novo Synthesis)
5475	DRAMP21418	WF (De Novo Synthesis)
5476	DRAMP21419	WW (De Novo Synthesis)
5481	DRAMP21424	B1 (De Novo Synthesis)
5482	DRAMP21425	peptide 2 (Derived from B1)
5483	DRAMP21426	peptide 3 (Derived from B1)
5484	DRAMP21427	peptide 4 (Derived from B1)
5485	DRAMP21428	peptide 5 (Derived from B1)
5486	DRAMP21429	peptide 6 (Derived from B1)
5487	DRAMP21430	peptide 7 (Derived from B1)
5488	DRAMP21431	peptide 8 (Derived from B1)
5489	DRAMP21432	peptide 9 (Derived from B1)
5490	DRAMP21433	peptide 10 (Derived from B1)
5491	DRAMP21434	peptide 11 (Derived from B1)
5492	DRAMP21435	peptide 12 (Derived from B1)
5493	DRAMP21436	peptide 13 (Derived from B1)
5494	DRAMP21437	peptide 14 (Derived from B1)
5495	DRAMP21438	peptide 15 (Derived from B1)
5496	DRAMP21439	peptide 16 (Derived from B1)
5497	DRAMP21440	peptide 17 (Derived from B1)
5498	DRAMP21441	peptide 18 (Derived from B1)
5499	DRAMP21442	peptide 19 (Derived from B1)
5500	DRAMP21443	peptide 20 (Derived from B1)
5501	DRAMP21444	peptide 21 (Derived from B1)
5502	DRAMP21445	peptide 22 (Derived from B1)
5505	DRAMP21448	peptide 25 (Derived from B1)
5508	DRAMP21451	peptide 28 (Derived from B1)
5509	DRAMP21452	peptide 29 (Derived from B1)
5510	DRAMP21453	Hybrid (Derived from Melittin and thanatin)
5511	DRAMP21454	PLP1 (Insects, animals)
5512	DRAMP21455	PLP2 (Insects, animals)
5513	DRAMP21456	PLP3 (Insects, animals)
5514	DRAMP21457	PLP4 (Insects, animals)
5515	DRAMP21458	PLP5 (Insects, animals)
5516	DRAMP21459	PLP6 (Insects, animals)
5517	DRAMP21460	PQ (De Novo Synthesis)
5518	DRAMP21461	PP (De Novo Synthesis)
5519	DRAMP21462	GG (De Novo Synthesis)
5520	DRAMP21463	Qa (De Novo Synthesis)
5521	DRAMP21464	Qna (De Novo Synthesis)
5522	DRAMP21465	P1-Ll-1577 (De Novo Synthesis)
5523	DRAMP21466	P2-Ll-1298 (De Novo Synthesis)
5524	DRAMP21467	P3-Ll-2085 (De Novo Synthesis)
5525	DRAMP21310	RK12 (Derived from PMAP-36)

5528	DRAMP21494	MEP-N
5529	DRAMP21579	Val-nHSLP
5530	DRAMP21581	Cap-nHSLP
5538	DRAMP21616	DRIM
5539	DRAMP21618	WWSP
5540	DRAMP21620	KFGF
5541	DRAMP21622	MAP-1
5542	DRAMP21626	E2EM23W
5543	DRAMP21627	E2EM15W

PepID	DRAMP_ID	Name
9	DRAMP00089	Bacteriocin E50-52 (Preclinical)
13	DRAMP00107	Bacteriocin L-1077
18	DRAMP00136	Enterocin E-760 (Bacteriocin)
19	DRAMP00171	Lactocyclicin Q (Bacteriocin)
25	DRAMP00191	Microcin J25 (MccJ25; Bacteriocin)
29	DRAMP00222	Microcin E492 (MccE492; Bacteriocin)
34	DRAMP00336	ChaC7 (Chassatide C7; uncyclotides; Plant defensin)
35	DRAMP00337	ChaC8 (Chassatide C8; uncyclotides; Plant defensin)
36	DRAMP00338	ChaC11 (Chassatide C11; uncyclotides; Plant defensin)
38	DRAMP00385	Panitide L2 (plants)
44	DRAMP00431	Defensin-like protein 2 (Cp-thionin II; Cp-thionin-2; Gamma-thionin II; Plant defensin)
49	DRAMP00764	Piceain 1 (Plants)
50	DRAMP00765	Piceain 2 (Plants)
51	DRAMP00766	JCpep7 (Plants)
52	DRAMP00774	Hedyotide B1 (hB1; Plants)
53	DRAMP00795	Cliotide T1 (cT1; Plant defensin)
54	DRAMP00798	Cliotide T4 (cT4; Plant defensin)
55	DRAMP00856	Kalata-B1 (Plant defensin)
56	DRAMP00877	Circulin-A (CIRA; Plant defensin)
57	DRAMP00878	Circulin-B (CIRB; Plant defensin)
58	DRAMP01374	Odorranain-D1 (OdD1; Frogs, amphibians, animals)
59	DRAMP01373	Odorranain-C1 (OdC1; Frogs, amphibians, animals)
60	DRAMP01372	Odorranain-B1 (Frogs, amphibians, animals)
63	DRAMP01012	Pg-AMP (Gly-rich; Plants)
66	DRAMP01018	Cyclopsychotride-A (CPT; Plant defensin)
68	DRAMP18193	Cathelicidin-related peptide crotalicidin
69	DRAMP01064	Anticancerous peptide 1 (Cr-ACP1; Plants)
70	DRAMP01066	Kunitz-type serine protease inhibitor 1 (Xb-KTI; Plants)
71	DRAMP01081	Alyteserin-1a (toads, amphibians, animals)
73	DRAMP01083	Alyteserin-1b (toads, amphibians, animals)
74	DRAMP01085	Alyteserin-1c (toads, amphibians, animals)
75	DRAMP01088	Alyteserin-1Ma (toads, amphibians, animals)
76	DRAMP01089	Alyteserin-1Mb (toads, amphibians, animals)
77	DRAMP01090	Alyteserin-2Ma (toads, amphibians, animals)
79	DRAMP01096	Bombinin-like peptide 2 (BLP-2; toads, amphibians, animals)
80	DRAMP01097	Bombinin-like peptide 1 (toads, amphibians, animals)
81	DRAMP01098	Bombinin-like peptide 3 (BLP-3; toads, amphibians, animals)
83	DRAMP02090	Brevinin-1Lb (Frogs, amphibians, animals)
87	DRAMP01518	Esculentin-2L (Frogs, amphibians, animals)
89	DRAMP01517	Esculentin-2P (Frogs, amphibians, animals)
90	DRAMP01516	Esculentin-2B (Frogs, amphibians, animals)
91	DRAMP02077	Brevinin-1Pb (Frogs, amphibians, animals)
98	DRAMP01162	Buforin-1 (Buforin I; Fragment of Histone H2A; toads, amphibians, animals)
99	DRAMP01163	Buforin-2 (Buforin II; Fragment of Histone H2A; toads, amphibians, animals)
100	DRAMP01164	Bombinin (toads, amphibians, animals)
101	DRAMP01165	Preprotemporin-1SKa (Frogs, amphibians, animals)
102	DRAMP01167	Hylaseptin-P1 (HSP1)
103	DRAMP01170	Distinctin 2 (Frogs, amphibians, animals)
104	DRAMP01174	Ocellatin-4 (Frogs, amphibians, animals)

105	DRAMP01177	Ocellatin-F1 (Fallaxin; Frogs, amphibians, animals)
106	DRAMP01182	Ocellatin-P1 (Pentadactylin; Frogs, amphibians, animals)
107	DRAMP01184	SPX(1-22)(truncated peptide of Syphaxin; Frogs, amphibians, animals)
108	DRAMP01185	SPX(1-16)(truncated peptide of Syphaxin; Frogs, amphibians, animals)
109	DRAMP01188	Chensinin-1ZHa (Frogs, amphibians, animals)
110	DRAMP01189	Andersonin-W1 (Frogs, amphibians, animals)
111	DRAMP01190	Andersonin-W2 (Frogs, amphibians, animals)
112	DRAMP01191	Andersonin-X1 (Frogs, amphibians, animals)
113	DRAMP01192	Andersonin-Y1 (Frogs, amphibians, animals)
114	DRAMP01194	Andersonin-C1 (Frogs, amphibians, animals)
115	DRAMP01195	Andersonin-D1 (Frogs, amphibians, animals)
116	DRAMP01199	Hejiangin-A1 (Frogs, amphibians, animals)
117	DRAMP01200	Hejiangin-F1 (frog, amphibians, animals)
118	DRAMP01201	Schmackerin-C1 (Frogs, amphibians, animals)
122	DRAMP01208	Pleurain-A1 (Pleurain A1; Frogs, amphibians, animals)
123	DRAMP01209	Pleurain-A2 (Pleurain A2; Frogs, amphibians, animals)
124	DRAMP01214	Kassinatuerin-2Ma (Frogs, amphibians, animals)
125	DRAMP01218	Kassinatuerin-1 (Frogs, amphibians, animals)
127	DRAMP01222	Palustrin-2AJ1 (PL2AJ1; Frogs, amphibians, animals)
128	DRAMP01227	Palustrin-1b (Frogs, amphibians, animals)
129	DRAMP01228	Palustrin-1c (Frogs, amphibians, animals)
130	DRAMP01229	Palustrin-1d (Frogs, amphibians, animals)
131	DRAMP01231	Palustrin-2b (Frogs, amphibians, animals)
132	DRAMP01232	Palustrin-2ISc (Frogs, amphibians, animals)
133	DRAMP01233	Palustrin-2c (Frogs, amphibians, animals)
134	DRAMP01234	Palustrin-3a (Frogs, amphibians, animals)
135	DRAMP01235	Palustrin-3b (Frogs, amphibians, animals)
136	DRAMP01237	Palustrin-2ISa (Frogs, amphibians, animals)
137	DRAMP01238	Palustrin-2SIb (Frogs, amphibians, animals)
138	DRAMP01244	Japonicin-1 (Frogs, amphibians, animals)
139	DRAMP01245	Japonicin-1CDYa (Frogs, amphibians, animals)
140	DRAMP01246	Japonicin-2 (Frogs, amphibians, animals)
142	DRAMP01249	Dybowskin-2 (Frogs, amphibians, animals)
143	DRAMP01250	Dybowskin-3 (Frogs, amphibians, animals)
144	DRAMP01251	Dybowskin-4 (Frogs, amphibians, animals)
145	DRAMP01252	Dybowskin-5 (Frogs, amphibians, animals)
147	DRAMP01254	Dybowskin-1CDYa (Frogs, amphibians, animals)
148	DRAMP01255	Dybowskin-2CDYa (Chensinin-1; Frogs, amphibians, animals)
149	DRAMP01257	Dermadistinctin-K (DD K; Frogs, amphibians, animals)
150	DRAMP01258	Dermadistinctin-L (DD L; Frogs, amphibians, animals)
151	DRAMP01259	Dermadistinctin-M (DD M; Frogs, amphibians, animals)
152	DRAMP01260	Dermadistinctin-Q1 (DD Q1; Frogs, amphibians, animals)
153	DRAMP01261	Dermadistinctin-Q2 (DD Q2; Frogs, amphibians, animals)
155	DRAMP01301	Phylloseptin-1 (PS-1; Frogs, amphibians, animals)
156	DRAMP01302	Phylloseptin-2 (PS-2; Frogs, amphibians, animals)
157	DRAMP01303	Phylloseptin-3 (PS-3; Frogs, amphibians, animals)
158	DRAMP01305	Phylloseptin-7 (PS-7; Frogs, amphibians, animals)
159	DRAMP01306	Phylloseptin-7 (PS-7; Frogs, amphibians, animals)
161	DRAMP01319	Cathelicidin-AL (Gly-rich; Frogs, amphibians, animals)
163	DRAMP01339	Amolopin-2a (Frogs, amphibians, animals)

164	DRAMP01341	Amolopin-1b (Frogs, amphibians, animals)
165	DRAMP01346	Prepromelittin-related peptide (Frogs, amphibians, animals)
166	DRAMP01347	Prepromelittin-related peptide (Frogs, amphibians, animals)
167	DRAMP01350	Tigerinin-1 (Frogs, amphibians, animals)
168	DRAMP01351	Tigerinin-2 (Frogs, amphibians, animals)
169	DRAMP01352	Tigerinin-3 (Frogs, amphibians, animals)
170	DRAMP01353	Tigerinin-4 (Frogs, amphibians, animals)
172	DRAMP01355	Ranalexin (Frogs, amphibians, animals)
173	DRAMP01393	Odorranain-W1 (OdW1; Frogs, amphibians, animals)
174	DRAMP01358	Ranalexin-Vb (Frogs, amphibians, animals)
175	DRAMP01359	Ranalexin-1G (Frogs, amphibians, animals)
178	DRAMP01362	Frenatin-3 (Frogs, amphibians, animals)
179	DRAMP01364	Maculatin-1.1 (Frogs, amphibians, animals)
182	DRAMP01370	Oh-defensin (O. hainana defensin; spiders, animals)
183	DRAMP01371	Odorranain-NR (Frogs, amphibians, animals)
184	DRAMP00931	Antimicrobial peptide 3 (Cn-AMP3; Plant defensin)
185	DRAMP00930	Antimicrobial peptide 2 (Cn-AMP2; Plant defensin)
186	DRAMP00929	Antimicrobial peptide 1 (Cn-AMP1; Plant defensin)
187	DRAMP03542	Neurokinin A (NKA; chicken, animals)
188	DRAMP04532	Myxinidin (Hagfish, animals)
190	DRAMP02993	Abaecin (Pro-rich; insects, arthropods, invertebrates, animals)
191	DRAMP02997	Apidaecin-1B (Apidaecin IB; Insects, animals)
193	DRAMP02840	Lactoferricin B (Lfcin B; mammals, animals)
198	DRAMP02246	Ranatuerin-1C (Ranatuerin 1C; Frogs, amphibians, animals)
199	DRAMP01394	Odorranain-W2 (Frogs, amphibians, animals)
200	DRAMP01395	Odorranain-A-OA1 (Frogs, amphibians, animals)
201	DRAMP01396	Odorranain-F-OA1 (Frogs, amphibians, animals)
202	DRAMP01397	Odorranain-F-OA2 (Frogs, amphibians, animals)
203	DRAMP01398	Odorranain-F-OA3 (Frogs, amphibians, animals)
204	DRAMP01399	Odorranain-F-OA4 (Frogs, amphibians, animals)
206	DRAMP01401	Odorranain-F-OW1 (Frogs, amphibians, animals)
207	DRAMP01402	Odorranain-J-OA1 (Frogs, amphibians, animals)
208	DRAMP01403	Odorranain-J-OA2 (Frogs, amphibians, animals)
209	DRAMP01409	Nigrocin-OR1 (Frogs, amphibians, animals)
210	DRAMP01410	Nigrocin-OR2 (Frogs, amphibians, animals)
211	DRAMP01411	Nigrocin-OR3 (Frogs, amphibians, animals)
212	DRAMP01412	Nigrocin-2HSa (Frogs, amphibians, animals)
213	DRAMP01413	Nigrocin-2HSb (Frogs, amphibians, animals)
214	DRAMP01414	Nigrocin-2ISa (Frogs, amphibians, animals)
215	DRAMP01415	Nigrocin-2ISb (Frogs, amphibians, animals)
216	DRAMP01416	Nigrocin-2ISc (Frogs, amphibians, animals)
217	DRAMP01417	Nigrocin-2GRa (Frogs, amphibians, animals)
218	DRAMP01418	Nigrocin-2GRb (Frogs, amphibians, animals)
219	DRAMP01419	Nigrocin-2GRc (Frogs, amphibians, animals)
220	DRAMP01420	Nigrocin-OG4 (Frogs, amphibians, animals)
221	DRAMP01421	Nigrocin-OG5 (Frogs, amphibians, animals)
222	DRAMP01422	Nigrosin-OG21 (Frogs, amphibians, animals)
223	DRAMP01423	Nigrosin-OG13 (Frogs, amphibians, animals)
224	DRAMP01426	Nigrocin-1-OA1 (Frogs, amphibians, animals)
225	DRAMP01427	Nigrocin-1-OA2 (Frogs, amphibians, animals)

226	DRAMP01428	Nigrocin-1-OA3 (Frogs, amphibians, animals)
227	DRAMP01429	Nigrocin-1-OR1 (Frogs, amphibians, animals)
228	DRAMP01430	Nigrocin-1-OR2 (Frogs, amphibians, animals)
229	DRAMP01431	Nigrocin-1-OR3 (Frogs, amphibians, animals)
230	DRAMP01432	Nigrocin-1-OW2 (Frogs, amphibians, animals)
231	DRAMP01433	Nigrocin-1-OW3 (Frogs, amphibians, animals)
232	DRAMP01434	Nigrocin-1-OW4 (Frogs, amphibians, animals)
233	DRAMP01435	Nigrocin-1-OW5 (Frogs, amphibians, animals)
234	DRAMP01436	Nigrocin-1-OW1 (Frogs, amphibians, animals)
236	DRAMP01438	Nigrocin-2JDa (Frogs, amphibians, animals)
237	DRAMP01439	Nigrocin-2JDb (Odorranain-H2; Frogs, amphibians, animals)
238	DRAMP01440	Nigrocin-2LVb (Frogs, amphibians, animals)
239	DRAMP01441	Nigrocin-2VB (Frogs, amphibians, animals)
240	DRAMP01442	Nigrocin-2SCa (Frogs, amphibians, animals)
241	DRAMP01443	Nigrocin-2SCc (Frogs, amphibians, animals)
242	DRAMP01447	Esculentin-2CHa (Frogs, amphibians, animals)
243	DRAMP01452	Esculentin-1LTa (Frogs, amphibians, animals)
244	DRAMP01453	Esculentin-2LTa (Frogs, amphibians, animals)
245	DRAMP01454	Esculentin-2JDa (Frogs, amphibians, animals)
246	DRAMP01456	Esculentin-2PLa (Frogs, amphibians, animals)
247	DRAMP01457	Esculentin-1V (Frogs, amphibians, animals)
248	DRAMP01458	Esculentin-2V (Frogs, amphibians, animals)
249	DRAMP01461	Esculentin-1S (Frogs, amphibians, animals)
250	DRAMP01462	Esculentin-2S (Frogs, amphibians, animals)
251	DRAMP01469	Esculentin-2-Ala (Frogs, amphibians, animals)
252	DRAMP01470	Esculentin-2-ALb (Frogs, amphibians, animals)
253	DRAMP01471	Esculentin-1PLa (Frogs, amphibians, animals)
254	DRAMP01472	Esculentin-1PLb (Frogs, amphibians, animals)
256	DRAMP01474	Esculentin-1ARa (Frogs, amphibians, animals)
257	DRAMP01475	Esculentin-1ARb (Frogs, amphibians, animals)
258	DRAMP01476	Esculentin-2HSa (Frogs, amphibians, animals)
259	DRAMP01477	Esculentin-1HSa (Frogs, amphibians, animals)
260	DRAMP01479	Esculentin-1CPa (Frogs, amphibians, animals)
261	DRAMP01480	Esculentin-2CPa (Frogs, amphibians, animals)
262	DRAMP01482	Esculentin-1ISa (Frogs, amphibians, animals)
263	DRAMP01483	Esculentin-1ISb (Frogs, amphibians, animals)
264	DRAMP01484	Esculentin-2ISa (Frogs, amphibians, animals)
265	DRAMP01486	Esculentin-1GRa (Frogs, amphibians, animals)
266	DRAMP01490	Esculentin-2A (Frogs, amphibians, animals)
267	DRAMP01491	Esculentin-1B (Frogs, amphibians, animals)
268	DRAMP01493	Esculentin-1-OA1 (Frogs, amphibians, animals)
269	DRAMP01494	Esculentin-1-OA2 (Frogs, amphibians, animals)
270	DRAMP01495	Esculentin-1-OA3 (Frogs, amphibians, animals)
271	DRAMP01496	Esculentin-1-OA4 (Frogs, amphibians, animals)
272	DRAMP01497	Esculentin-1-OA5 (Frogs, amphibians, animals)
273	DRAMP01499	Esculentin-1-OR1 (Frogs, amphibians, animals)
274	DRAMP01501	Esculentin-1-OR3 (Frogs, amphibians, animals)
275	DRAMP01502	Esculentin-1-OR4 (Frogs, amphibians, animals)
276	DRAMP01503	Esculentin-1-OR5 (Frogs, amphibians, animals)
277	DRAMP01504	Esculentin-2-OA1 (Frogs, amphibians, animals)

278	DRAMP01505	Esculentin-2-OA2 (Frogs, amphibians, animals)
280	DRAMP01507	Esculentin-2-OR1 (Frogs, amphibians, animals)
281	DRAMP01508	Esculentin-2-OR2 (Frogs, amphibians, animals)
282	DRAMP01509	Esculentin-2-OR3 (Frogs, amphibians, animals)
283	DRAMP01510	Esculentin-2-OR4 (Frogs, amphibians, animals)
284	DRAMP01511	Esculentin-2-OR5 (Frogs, amphibians, animals)
285	DRAMP01513	Esculentin-1 (Frogs, amphibians, animals)
286	DRAMP01520	Rugosin-A (Frogs, amphibians, animals)
287	DRAMP01521	Rugosin-B (Frogs, amphibians, animals)
288	DRAMP01524	Rugosin-RN1 (Frogs, amphibians, animals)
289	DRAMP01525	Rugosin-RN3 (Frogs, amphibians, animals)
290	DRAMP01526	Rugosin-RN5 (Frogs, amphibians, animals)
291	DRAMP01533	Nigroain-B1 (Frogs, amphibians, animals)
292	DRAMP01539	Nigroain-C2 (Frogs, amphibians, animals)
295	DRAMP01546	Nigroain-K1 (Frogs, amphibians, animals)
297	DRAMP01549	Caerin-1.1 (Frogs, amphibians, animals)
298	DRAMP01550	Caerin-1.11 (Frogs, amphibians, animals)
299	DRAMP01552	Caerin-1.3 (Frogs, amphibians, animals)
300	DRAMP01553	Caerin-1.4 (Frogs, amphibians, animals)
301	DRAMP01555	Caerin-1.5 (Frogs, amphibians, animals)
302	DRAMP01560	Caerin-1.9 (Frogs, amphibians, animals)
303	DRAMP01562	Caerin-2.1 (Frogs, amphibians, animals)
304	DRAMP01563	Caerin-2.2 (Frogs, amphibians, animals)
309	DRAMP01574	Caerin-4.1 (Frogs, amphibians, animals)
310	DRAMP01576	Caerin-4.3 (Frogs, amphibians, animals)
311	DRAMP01577	Caerin-1.10 (Frogs, amphibians, animals)
316	DRAMP01585	Caerin-1.18 (Frogs, amphibians, animals)
317	DRAMP01586	Caerin-1.19 (Frogs, amphibians, animals)
321	DRAMP01590	Citropin 1.1 M14 (Frogs, amphibians, animals)
322	DRAMP01591	Citropin 1.1 M15 (Frogs, amphibians, animals)
329	DRAMP01607	Aurein-1.2 (Frogs, amphibians, animals)
335	DRAMP01618	Aurein-3.3 (Frogs, amphibians, animals)
337	DRAMP01621	Bombinin-H1 (Frogs, amphibians, animals)
338	DRAMP01623	Bombinin-H4 (bombinin H isomers; Frogs, amphibians, animals)
339	DRAMP01626	Bombinin-H5 (Frogs, amphibians, animals)
340	DRAMP01627	Skin peptide tyrosine-tyrosine (Skin-PYY; SPYY; Frogs, amphibians, animals)
341	DRAMP01628	Phylloxin (Frogs, amphibians, animals)
342	DRAMP01638	Dermaseptin-L1 (Frogs, amphibians, animals)
343	DRAMP01639	Dermaseptin-1 (DShypo01, DPh-1; Frogs, amphibians, animals)
344	DRAMP01643	Dermaseptin-5 (DShypo05, DS 01; Frogs, amphibians, animals)
345	DRAMP01646	Adenoregulin (Dermaseptin BII; Dermaseptin B2; Frogs, amphibians, animals)
347	DRAMP01648	Dermaseptin-like PBN2 (DRP-PBN2; Plasticin-B1a; Frogs, amphibians, animals)
348	DRAMP01649	Dermaseptin-BI (Dermaseptin B1; Frogs, amphibians, animals)
349	DRAMP01650	Dermaseptin-B3 (Dermaseptin BIII; Frogs, amphibians, animals)
350	DRAMP01651	Dermaseptin-B4 (Dermaseptin BIV; Frogs, amphibians, animals)
352	DRAMP01668	Dermaseptin-1 (DS I; Dermaseptin-S1, DS1; Frogs, amphibians, animals)
357	DRAMP01702	Dermaseptin-H5 (Dermaseptin-like peptide 5, DMS5; Frogs, amphibians, animals)
359	DRAMP01730	Temporin-A (Frogs, amphibians, animals)
360	DRAMP01731	Temporin-ALd (Frogs, amphibians, animals)
361	DRAMP01732	Temporin-ALe (Frogs, amphibians, animals)

362	DRAMP01733	Temporin-ALf (Frogs, amphibians, animals)
363	DRAMP01734	Temporin-ALg (Frogs, amphibians, animals)
364	DRAMP01735	Temporin-ALh (Frogs, amphibians, animals)
365	DRAMP01736	Temporin-ALi (Frogs, amphibians, animals)
366	DRAMP01737	Temporin-ALj (Frogs, amphibians, animals)
367	DRAMP01738	Temporin-ALk (Frogs, amphibians, animals)
368	DRAMP01739	Temporin-B (Frogs, amphibians, animals)
372	DRAMP01753	Temporin-1CEa (Frogs, amphibians, animals)
373	DRAMP01754	Temporin-1CEb (Frogs, amphibians, animals)
374	DRAMP01755	Temporin-1TSa (Frogs, amphibians, animals)
376	DRAMP01764	Temporin-1TGa (Frogs, amphibians, animals)
377	DRAMP01765	Temporin-1TGb (Frogs, amphibians, animals)
378	DRAMP01766	Temporin-1TGc (Frogs, amphibians, animals)
381	DRAMP01771	Temporin-1Oa (Frogs, amphibians, animals)
383	DRAMP01775	Temporin-1Sa (Frogs, amphibians, animals)
384	DRAMP01776	Temporin-1Sb (Temporin-SHb; Frogs, amphibians, animals)
385	DRAMP01777	Temporin-1Sc (Temporin-SHc; Frogs, amphibians, animals)
386	DRAMP01779	Temporin-SHf (Frogs, amphibians, animals)
387	DRAMP01780	Temporin-SHa (Temporin-1Sa; Frogs, amphibians, animals)
390	DRAMP01784	Temporin-LTc (Frogs, amphibians, animals)
391	DRAMP01785	Temporin-CPa (Frogs, amphibians, animals)
392	DRAMP01787	Temporin-HN1 (Frogs, amphibians, animals)
393	DRAMP01788	Temporin-HN2 (Frogs, amphibians, animals)
394	DRAMP01789	Temporin-1Va (Temporin 1Va; Frogs, amphibians, animals)
396	DRAMP01791	Temporin-1Vc (Temporin 1Vc; Frogs, amphibians, animals)
397	DRAMP01807	Temporin-RN1 (Frogs, amphibians, animals)
398	DRAMP01808	Temporin-RN3 (Frogs, amphibians, animals)
399	DRAMP01811	Temporin-Ra (Frogs, amphibians, animals)
400	DRAMP01812	Temporin-Rb (Frogs, amphibians, animals)
402	DRAMP01816	Temporin-1CSb (Frogs, amphibians, animals)
403	DRAMP01817	Temporin-1CSc (Frogs, amphibians, animals)
404	DRAMP01818	Temporin-1CSd (Temporin-1DRb; Frogs, amphibians, animals)
405	DRAMP01392	Odorranain-V1 (OdV1; Frogs, amphibians, animals)
406	DRAMP01391	Odorranain-U1 (OdU1; Frogs, amphibians, animals)
407	DRAMP01832	Temporin-Eca (Frogs, amphibians, animals)
408	DRAMP01833	Buforin-EC (Frogs, amphibians, animals)
409	DRAMP01834	Cyanophlyctin (Frogs, amphibians, animals)
410	DRAMP01840	Ascaphin-1 (Frogs, amphibians, animals)
411	DRAMP01842	Ascaphin-3 (Frogs, amphibians, animals)
412	DRAMP01844	Ascaphin-5 (Frogs, amphibians, animals)
413	DRAMP01846	Ascaphin-7 (Frogs, amphibians, animals)
414	DRAMP01847	Ascaphin-8 (Frogs, amphibians, animals)
415	DRAMP01849	Jindongenin-1a (JD1a; Frogs, amphibians, animals)
416	DRAMP01869	Brevinin-1SPa (Frogs, amphibians, animals)
417	DRAMP01870	Brevinin-1SPb (Frogs, amphibians, animals)
418	DRAMP01872	Brevinin-1SPd (Frogs, amphibians, animals)
419	DRAMP01873	Brevinin-2-related peptide (Frogs, amphibians, animals)
420	DRAMP01875	Brevinin-2PRa (Frogs, amphibians, animals)
421	DRAMP01876	Brevinin-2PRb (Frogs, amphibians, animals)
422	DRAMP01877	Brevinin-2PRd (Frogs, amphibians, animals)

423	DRAMP01878	Brevinin-2PRe (Frogs, amphibians, animals)
424	DRAMP01879	Brevinin-2LTa (Frogs, amphibians, animals)
425	DRAMP01880	Brevinin-2LTb (Frogs, amphibians, animals)
426	DRAMP01881	Brevinin-2LTc (Frogs, amphibians, animals)
427	DRAMP01885	Brevinin-1TEa (Frogs, amphibians, animals)
428	DRAMP01886	Brevinin-2TEa (Frogs, amphibians, animals)
429	DRAMP01887	Brevinin-2TEb (Frogs, amphibians, animals)
430	DRAMP01888	Brevinin-1CHc (Frogs, amphibians, animals)
431	DRAMP01889	Brevinin-1TOa (Frogs, amphibians, animals)
432	DRAMP01890	Brevinin-1VLa (Frogs, amphibians, animals)
433	DRAMP01891	Brevinin-1VLc (Frogs, amphibians, animals)
434	DRAMP01892	Brevinin-1VLd (Frogs, amphibians, animals)
435	DRAMP01893	Brevinin-1VLe (Frogs, amphibians, animals)
436	DRAMP01896	Brevinin-1CG1 (Frogs, amphibians, animals)
437	DRAMP01897	Brevinin-1CG2 (Frogs, amphibians, animals)
438	DRAMP01898	Brevinin-1CG3 (Frogs, amphibians, animals)
439	DRAMP01899	Brevinin-1CG4 (Frogs, amphibians, animals)
440	DRAMP01900	Brevinin-1CG5 (Frogs, amphibians, animals)
442	DRAMP01910	Brevinin-2GHb (AMP-2; Frogs, amphibians, animals)
443	DRAMP01911	Brevinin-2GHc (AMP-4; Frogs, amphibians, animals)
444	DRAMP01913	Brevinin-1GRa (Frogs, amphibians, animals)
445	DRAMP01914	Brevinin-2GRa (Frogs, amphibians, animals)
446	DRAMP01918	Brevinin-1PLb (Frogs, amphibians, animals)
447	DRAMP01919	Brevinin-1PLc (Frogs, amphibians, animals)
448	DRAMP01920	Brevinin-1CSa (Frogs, amphibians, animals)
450	DRAMP01922	Brevinin-2SKb (Frogs, amphibians, animals)
451	DRAMP01933	Brevinin-2Ef (Frogs, amphibians, animals)
452	DRAMP01934	Brevinin-2Ei (Frogs, amphibians, animals)
453	DRAMP01935	Brevinin-2Ej (Frogs, amphibians, animals)
454	DRAMP01937	CPRF-Ea (caerulein precursor-related fragment; Frogs, amphibians, animals)
455	DRAMP01938	CPRF-Eb (caerulein precursor-related fragment; Frogs, amphibians, animals)
456	DRAMP01939	CPRF-Ec (caerulein precursor-related fragment; Frogs, amphibians, animals)
457	DRAMP01940	Brevinin-1CHa (Frogs, amphibians, animals)
458	DRAMP01941	Brevinin-1CHb (Frogs, amphibians, animals)
459	DRAMP01942	Brevinin-1Sa (Frogs, amphibians, animals)
460	DRAMP01943	Brevinin-1Sb (Frogs, amphibians, animals)
461	DRAMP01944	Brevinin-1Sc (Frogs, amphibians, animals)
462	DRAMP01949	Brevinin-1HSa (Frogs, amphibians, animals)
463	DRAMP01950	Brevinin-1HSb (Brevinin-1JDb; Frogs, amphibians, animals)
464	DRAMP01951	Brevinin-1PTa (Frogs, amphibians, animals)
465	DRAMP01953	Brevinin-2HSa (Frogs, amphibians, animals)
466	DRAMP01955	Brevinin-2PTa (Frogs, amphibians, animals)
467	DRAMP01956	Brevinin-2PTb (Frogs, amphibians, animals)
468	DRAMP01957	Brevinin-2PTc (Frogs, amphibians, animals)
469	DRAMP01959	Brevinin-2PTe (Frogs, amphibians, animals)
470	DRAMP01963	Brevinin-1BLa (Frogs, amphibians, animals)
471	DRAMP01965	Brevinin-1BLc (Frogs, amphibians, animals)
472	DRAMP01968	Brevinin-1Yc (Frogs, amphibians, animals)
473	DRAMP01969	Brevinin-1Ja (Frogs, amphibians, animals)
474	DRAMP01970	Brevinin-1ZHa (Frogs, amphibians, animals)

475	DRAMP01971	Brevinin-1ZHb (Frogs, amphibians, animals)
476	DRAMP01974	Brevinin-2ZHa (Frogs, amphibians, animals)
477	DRAMP01986	Brevinin-2HS2 (Frogs, amphibians, animals)
478	DRAMP01990	Brevinin-1LT1 (Frogs, amphibians, animals)
479	DRAMP01994	Brevinin-2ISa (Frogs, amphibians, animals)
480	DRAMP01995	Brevinin-2ISb (Frogs, amphibians, animals)
481	DRAMP01996	Brevinin-2ISc (Frogs, amphibians, animals)
482	DRAMP02001	Brevinin-1HN1 (Frogs, amphibians, animals)
483	DRAMP02004	Brevinin-1V (Frogs, amphibians, animals)
484	DRAMP02006	Brevinin-2Va (Frogs, amphibians, animals)
485	DRAMP02009	Brevinin-1 (Frogs, amphibians, animals)
486	DRAMP02010	Brevinin-2 (Frogs, amphibians, animals)
487	DRAMP02019	Brevinin-2DYb (Frogs, amphibians, animals)
490	DRAMP02023	Brevinin-2DYd (Frogs, amphibians, animals)
492	DRAMP02025	Brevinin-2DYe (Brevinin-2CDYa; Frogs, amphibians, animals)
493	DRAMP02026	Brevinin-1CDYa (Frogs, amphibians, animals)
495	DRAMP02031	Brevinin-1Da (Frogs, amphibians, animals)
496	DRAMP02032	Brevinin-1TSa (Frogs, amphibians, animals)
497	DRAMP02033	Brevinin-2TSa (Frogs, amphibians, animals)
498	DRAMP02034	Brevinin-1AUa (Frogs, amphibians, animals)
499	DRAMP02035	Brevinin-1AUb (Frogs, amphibians, animals)
500	DRAMP02036	Brevinin-2-RN1 (Frogs, amphibians, animals)
501	DRAMP02037	Brevinin-2-RN2 (Frogs, amphibians, animals)
502	DRAMP02038	Brevinin-1-OA1 (Frogs, amphibians, animals)
504	DRAMP02040	Brevinin-1-OA12 (Frogs, amphibians, animals)
505	DRAMP02041	Brevinin-1-OR1 (Frogs, amphibians, animals)
506	DRAMP02042	Brevinin-1-OR3 (Frogs, amphibians, animals)
507	DRAMP02043	Brevinin-1-OR4 (Frogs, amphibians, animals)
508	DRAMP02044	Brevinin-1-OR5 (Frogs, amphibians, animals)
509	DRAMP02045	Brevinin-1-OR6 (Frogs, amphibians, animals)
510	DRAMP02046	Brevinin-1-OR7 (Frogs, amphibians, animals)
511	DRAMP02047	Brevinin-1-OR8 (Frogs, amphibians, animals)
512	DRAMP02048	Brevinin-1-OR9 (Frogs, amphibians, animals)
513	DRAMP02049	Brevinin-1-OR10 (Frogs, amphibians, animals)
514	DRAMP02050	Brevinin-1-OR11 (Frogs, amphibians, animals)
515	DRAMP02051	Lividin-1 (Brevinin-1-OR2; Frogs, amphibians, animals)
516	DRAMP02052	Lividin-2 (Brevinin-2-OR8; Frogs, amphibians, animals)
517	DRAMP02053	Lividin-3 (Brevinin-2-OR1; Frogs, amphibians, animals)
518	DRAMP02054	Brevinin-2-OA1 (Frogs, amphibians, animals)
519	DRAMP02055	Brevinin-2-OA2 (Brevinin-2E-OG1; Frogs, amphibians, animals)
520	DRAMP02056	Brevinin-2-OA3 (Frogs, amphibians, animals)
521	DRAMP02057	Brevinin-2-OA4 (Frogs, amphibians, animals)
522	DRAMP02058	Brevinin-2-OA5 (Frogs, amphibians, animals)
523	DRAMP02059	Brevinin-2-OA6 (Frogs, amphibians, animals)
524	DRAMP02060	Brevinin-2-OA7 (Frogs, amphibians, animals)
525	DRAMP02061	Brevinin-2-OA8 (Frogs, amphibians, animals)
526	DRAMP02062	Brevinin-2-OR2 (Frogs, amphibians, animals)
527	DRAMP02063	Brevinin-2-OR3 (Frogs, amphibians, animals)
528	DRAMP02064	Brevinin-2-OR4 (Frogs, amphibians, animals)
529	DRAMP02065	Brevinin-2-OR5 (Frogs, amphibians, animals)

530	DRAMP02066	Brevinin-2-OR6 (Frogs, amphibians, animals)
531	DRAMP02067	Brevinin-2-OR7 (Frogs, amphibians, animals)
532	DRAMP02068	Brevinin-2-OR9 (Frogs, amphibians, animals)
533	DRAMP02069	Brevinin-2-OR10 (Frogs, amphibians, animals)
534	DRAMP02070	Brevinin-2-OW1 (Frogs, amphibians, animals)
535	DRAMP02071	Brevinin-2-OW2 (Frogs, amphibians, animals)
536	DRAMP02072	Brevinin-2-OW3 (Frogs, amphibians, animals)
537	DRAMP02073	Brevinin-1JDa (Frogs, amphibians, animals)
538	DRAMP02075	Brevinin-1JDc (Frogs, amphibians, animals)
543	DRAMP02078	Brevinin-1SY (Frogs, amphibians, animals)
544	DRAMP02081	Brevinin-1E (Frogs, amphibians, animals)
545	DRAMP02084	Brevinin-2E (Frogs, amphibians, animals)
546	DRAMP02101	Brevinin-1RTa (Frogs, amphibians, animals)
547	DRAMP02102	Brevinin-1RTb (Frogs, amphibians, animals)
548	DRAMP02104	Brevinin-2RTa (Frogs, amphibians, animals)
549	DRAMP02105	Brevinin-2RTb (Frogs, amphibians, animals)
550	DRAMP02114	Raniseptin-1 (Rsp-1; Frogs, amphibians, animals)
551	DRAMP02125	Hylin-a1 (Hy-a1; Frogs, amphibians, animals)
552	DRAMP02127	Leptoglycin (Gly-rich; Frogs, amphibians, animals)
554	DRAMP02129	Kasstasin (Frogs, amphibians, animals)
555	DRAMP02130	Antimicrobial peptide 1 (XT-1; Frogs, amphibians, animals)
556	DRAMP02131	Antimicrobial peptide 2 (XT-2; Frogs, amphibians, animals)
557	DRAMP02133	Antimicrobial peptide 4 (XT-4; Frogs, amphibians, animals)
558	DRAMP02135	Antimicrobial peptide 6 (XT-6; Frogs, amphibians, animals)
559	DRAMP02136	Antimicrobial peptide 7 (XT-7; Frogs, amphibians, animals)
561	DRAMP02219	Ranatuerin-2AUa (Frogs, amphibians, animals)
562	DRAMP02220	Ranatuerin-2PLa (Frogs, amphibians, animals)
563	DRAMP02221	Ranatuerin-2PLb (Frogs, amphibians, animals)
564	DRAMP02222	Ranatuerin-2PLc (Frogs, amphibians, animals)
565	DRAMP02223	Ranatuerin-2PLd (Frogs, amphibians, animals)
566	DRAMP02224	Ranatuerin-2PLe (Frogs, amphibians, animals)
567	DRAMP02225	Ranatuerin-2PLf (Frogs, amphibians, animals)
568	DRAMP02228	Ranatuerin-1 (Frogs, amphibians, animals)
576	DRAMP02237	Ranatuerin-2Ya (Frogs, amphibians, animals)
577	DRAMP02238	Ranatuerin-2ZHa (Frogs, amphibians, animals)
578	DRAMP02239	Ranatuerin-1Ga (Frogs, amphibians, animals)
579	DRAMP02241	Ranatuerin-2G (Frogs, amphibians, animals)
580	DRAMP01390	Odorranain-T1 (OdT1; Frogs, amphibians, animals)
581	DRAMP01389	Odorranain-S1 (OdS1; Frogs, amphibians, animals)
582	DRAMP02251	Ranatuerin-2CSa (Frogs, amphibians, animals)
583	DRAMP02252	Ranatuerin 2SKa (Frogs, amphibians, animals)
584	DRAMP01108	Maximin-2 (Toads, amphibians, animals)
585	DRAMP03998	PAF26 (Trp-rich; combinatorial library)
586	DRAMP01107	Maximin-1 (Toads, amphibians, animals)
587	DRAMP02268	Xenopsin precursor fragment (XPF; Frogs, amphibians, animals)
588	DRAMP02269	Antimicrobial peptide PGQ (PGQ; Frogs, amphibians, animals)
589	DRAMP02271	Magainin-2 (Magainin II; chain of Magainins; Frogs, amphibians, animals)
590	DRAMP02272	PGLa (chain of PYLa/PGLa A; Frogs, amphibians, animals)
591	DRAMP02273	PGLa-H (chain of PYLa/PGLa A; Frogs, amphibians, animals)
592	DRAMP02274	Ranacyclin-E (Frogs, amphibians, animals)

593	DRAMP02275	Ranacyclin-T (Frogs, amphibians, animals)
596	DRAMP02278	Ranacyclin-B-RL1 (Frogs, amphibians, animals)
602	DRAMP02288	Gaegurin-RN1 (Frogs, amphibians, animals)
604	DRAMP02290	Gaegurin-RN5 (Frogs, amphibians, animals)
605	DRAMP02291	Gaegurin-1 (Gaegurin 1; GGN1; Frogs, amphibians, animals)
606	DRAMP02292	Gaegurin-2 (Gaegurin 2; GGN2; Frogs, amphibians, animals)
607	DRAMP02293	Gaegurin-3 (Gaegurin 3; GGN3; Frogs, amphibians, animals)
608	DRAMP02294	Gaegurin-4 (Gaegurin 4; GGN4; Frogs, amphibians, animals)
609	DRAMP02295	Gaegurin-5 (Gaegurin 5; GGN5; Brevinin-1EMa; Frogs, amphibians, animals)
610	DRAMP02296	Gaegurin-6 (Gaegurin 6; GGN6; Frogs, amphibians, animals)
616	DRAMP02314	Hepcidin (fish, chordates, animals)
617	DRAMP02315	Chrysophsin-1 (fish, chordates, animals)
618	DRAMP02316	Chrysophsin-2 (fish, chordates, animals)
619	DRAMP02317	Chrysophsin-3 (fish, chordates, animals)
620	DRAMP02318	Grammistin Pp1 (Group II grammistin; fish, chordates, animals)
621	DRAMP02320	Grammistin PpIIb (Group II grammistin; fish, chordates, animals)
622	DRAMP02321	Grammistin Pp3 (Group III grammistin; fish, chordates, animals)
623	DRAMP02324	SAMP H1 (fish, chordates, animals)
624	DRAMP02330	Piscidin-1 (Pis-1; Piscidin 1; fish, chordates, animals)
625	DRAMP02331	Piscidin-2 (Pis-2; fish, chordates, animals)
626	DRAMP02336	Oncorhyncin II (Oncorhyncin 2; fish, chordates, animals)
627	DRAMP02337	Oncorhyncin III (Oncorhyncin 3; fish, chordates, animals)
628	DRAMP02347	NRC-1 (fish, chordates, animals)
629	DRAMP02348	NRC-2 (fish, chordates, animals)
630	DRAMP02349	NRC-3 (fish, chordates, animals)
631	DRAMP02350	Pleurocidin (NRC-4; fish, chordates, animals)
632	DRAMP02351	NRC-10 (fish, chordates, animals)
633	DRAMP02352	NRC-16 (fish, chordates, animals)
634	DRAMP02354	Pleurocidin-like peptide WFY (fish, chordates, animals)
635	DRAMP02357	Pleurocidin-like peptide WF3 (NRC-5; fish, chordates, animals)
636	DRAMP02358	Pleurocidin-like peptide WF4 (NRC-6; fish, chordates, animals)
637	DRAMP02359	Pleurocidin-like peptide YT2 (NRC-7; fish, chordates, animals; Predicted)
638	DRAMP02360	Pleurocidin-like peptide AP1 (NRC-11; fish, chordates, animals; Predicted)
639	DRAMP02361	Pleurocidin-like peptide AP2 (NRC-12; fish, chordates, animals; Predicted)
640	DRAMP02362	Pleurocidin-like peptide AP3 (NRC-13; fish, chordates, animals; Predicted)
641	DRAMP02363	Pleurocidin-like peptide GcSc4C5 (NRC-14; fish, chordates, animals)
642	DRAMP02364	Pleurocidin-like peptide GcSc4B7 (NRC-15; fish, chordates, animals; Predicted)
643	DRAMP02365	Pleurocidin-like peptide GC3.8 (NRC-17; fish, chordates, animals; Predicted)
644	DRAMP02366	Pleurocidin-like peptide GC3.2 (NRC-18; fish, chordates, animals; Predicted)
645	DRAMP02367	Pleurocidin-like peptide Hb26 (NRC-19; fish, chordates, animals; Predicted)
646	DRAMP02368	Pleurocidin-like peptide Hb18 (NRC-20; fish, chordates, animals; Predicted)
647	DRAMP02374	Bass hepcidin (fish, chordates, animals)
648	DRAMP02376	Grammistin Gs 1 (Grammistin Gs F; Group I grammistin; soapfish, chordates, animals)
649	DRAMP02377	Grammistin Gs 2 (Grammistin Gs G; Group I grammistin; soapfish, chordates, animals)
650	DRAMP02378	Grammistin Gs A (Group III grammistin; soapfish, chordates, animals)
651	DRAMP02379	Grammistin Gs B (Group II grammistin; soapfish, chordates, animals)
652	DRAMP02380	Grammistin Gs C (Group III grammistin; soapfish, chordates, animals)
654	DRAMP18373	mBD-6 (Murine beta-defensin 6)
655	DRAMP18374	hBD-6 (human beta-defensin 6)
657	DRAMP02390	Astacidin 2 (crayfish, Arthropods, animals)

658	DRAMP02391	Hematopoietic antimicrobial peptide-37 (MgCath37; hagfishes, chordates, animals)
659	DRAMP02393	HFIAP-1 (HFIAP-2; hagfishes, chordates, animals)
660	DRAMP02394	HFIAP-3 (hagfishes, chordates, animals)
661	DRAMP02395	Aurelin (jellyfish, chordates, animals)
662	DRAMP02397	Big defensin (RPD-1)
663	DRAMP02402	Antimicrobial peptide scolopin-1
664	DRAMP02403	Antimicrobial peptide scolopin-2
665	DRAMP02409	M-theraphotoxin-Gr1a (M-TRTX-Gr1a; GsMTx-4)
666	DRAMP02410	Antimicrobial peptide lumbricin-1
667	DRAMP02411	Armadillidin (Glyc-rich)
668	DRAMP02412	Panusin (Defensin-like peptide 7, PaD7)
670	DRAMP02421	Hlgut-defensin (H. longicornis midgut defensin; Ticks, Arthropods, animals)
671	DRAMP02422	Hlsal-defensin (H. longicornis salivary gland defensin; Ticks, Arthropods, animals)
672	DRAMP02423	HlMS-defensin (Ticks, Arthropods, animals)
673	DRAMP02425	Ixosin-B (Ticks, Arthropods, animals)
677	DRAMP02430	Ixosin (Ticks, Arthropods, animals)
678	DRAMP02432	Antimicrobial peptide ISAMP (Ticks, Arthropods, animals)
680	DRAMP02434	Antimicrobial peptide lumbricin-PG (Lumbricin-PG)
683	DRAMP02445	Antimicrobial protein BL-A60
684	DRAMP02446	Antimicrobial protein 1 (Antimicrobial protein AN5-1)
687	DRAMP02470	Nosiheptide (NOS; Antibiotic 9671-RP)
688	DRAMP02473	Cathelicidin-BF (Cathelicidin-related protein; Snakes, reptiles, animals)
689	DRAMP02474	cathelicidin-BF15 (Snakes, reptiles, animals)
690	DRAMP02478	L-amino-acid oxidase (Bm-LAO; LAAO; LAO; Snakes, reptiles, animals)
691	DRAMP02520	OH-CATH (Snakes, reptiles, animals)
692	DRAMP02522	L-amino-acid oxidase (LAAO, LAO, Oh-LAAO; Snakes, reptiles, animals)
693	DRAMP02573	Penaeidin-3a (Pen-3a; shrimps, Arthropods, animals)
694	DRAMP02574	[T8A]-Penaeidin-3a ([T8A]-Pen-3a; shrimps, Arthropods, animals)
697	DRAMP02584	Penaeidin-4a (Pen-4a; shrimps, Arthropods, animals)
698	DRAMP02586	Penaeidin-2d (Pen-2d; shrimps, Arthropods, animals)
700	DRAMP02603	Putative antimicrobial peptide A Northern Europe Heligoland (chordates, animals)
702	DRAMP02650	Sperm associated antigen 11 isoform C (primates, mammals, animals)
705	DRAMP02740	TBD-1 (Turtle beta-defensin 1; Reptiles, animals)
706	DRAMP02768	Pilosulin-1 (Myr b I; ants, insects, animals)
709	DRAMP02777	Rhinocerosin (Insects, animals)
710	DRAMP02778	Defensin (Insects, animals)
711	DRAMP02779	Defensin-A (Defensin A; Insects, animals)
712	DRAMP02780	Defensin-B (Defensin B; Insects, animals)
713	DRAMP02802	Paneth cell-specific alpha-defensin 1 (DEFA1; horse defensin; houses, mammals, animalsï¼‰
714	DRAMP02809	Myticin-B (Myt B; Cys-rich; molluscas, animals)
715	DRAMP02811	Defensin MGD-1 (molluscas, animals)
717	DRAMP02817	Pyrrhocoricin
719	DRAMP01381	Odorranain-K1 (OdK1; Frogs, amphibians, animals)
720	DRAMP01383	Odorranain-M1 (OdM1; Frogs, amphibians, animals)
721	DRAMP02841	Lumbricin I(6-34)
722	DRAMP02843	chain a, Structure Of An Indolicidin Peptide Derivative
724	DRAMP02845	CP-11 (cathelicidin; mammals, animals)
725	DRAMP02849	Bactenecin 5 (mammals, animals)
726	DRAMP02851	Cathelicidin-1 (Bactenecin-1, Bac1; Cyclic dodecapeptide; mammals, animals)
727	DRAMP02854	Cathelicidin-5 (Antibacterial peptide BMAP-28)

728	DRAMP02855	Cathelicidin-6 (Antibacterial peptide BMAP-27)
729	DRAMP02858	Bovine Beta-defensin 1 (bBD-1; BNBD-1; BNDB-1; mammals, animals)
730	DRAMP02859	Bovine Beta-defensin 2 (bBD-2; BNBD-2; BNDB-2; mammals, animals)
731	DRAMP02860	Bovine Beta-defensin 3 (bBD-3; BNBD-3; BNDB-3; mammals, animals)
732	DRAMP02861	Bovine Beta-defensin 4 (bBD-4; BNBD-4; BNDB-4; mammals, animals)
733	DRAMP02862	Bovine Beta-defensin 5 (bBD-5; BNBD-5; BNDB-5; mammals, animals)
734	DRAMP02863	Bovine Beta-defensin 6 (bBD-6; BNBD-6; BNDB-6; mammals, animals)
735	DRAMP02865	Bovine Beta-defensin 8 (bBD-8; BNBD-8; BNDB-8; mammals, animals)
736	DRAMP02866	Bovine Beta-defensin 9 (bBD-9; BNBD-9; BNDB-9; mammals, animals)
737	DRAMP02867	Bovine Beta-defensin 10 (bBD-10; BNBD-10; BNDB-10; mammals, animals)
738	DRAMP02868	Bovine Beta-defensin 11 (bBD-11; BNBD-11; BNDB-11; mammals, animals)
739	DRAMP02869	Bovine Beta-defensin 12 (bBD-12; BNBD-12; BNDB-12; mammals, animals)
740	DRAMP02870	Bovine Beta-defensin 13 (bBD-13; BNBD-13; BNDB-13; mammals, animals)
741	DRAMP02872	Myeloid antimicrobial peptide BMAP-27 (1-18) (mammals, animals)
742	DRAMP02873	Myeloid antimicrobial peptide BMAP-28 (1-18) (mammals, animals)
744	DRAMP02877	mBMAP28 (mammals, animals)
745	DRAMP02878	Tracheal antimicrobial peptide (TAP; mammals, animals)
746	DRAMP02903	Bombin H7
747	DRAMP02910	Ovispirin-2 (OV-2; mammals, animals)
748	DRAMP02911	Ovispirin-3 (OV-3; mammals, animals)
751	DRAMP02922	Canine beta-defensin (dogs, mammals, animals)
752	DRAMP02923	cBD-1 (Canine beta-defensin 1; dogs, mammals, animals)
755	DRAMP02925	Cathelicidin (dogs, mammals, animals)
756	DRAMP02931	Arasin-likeSp (crabs, Arthropods, animals)
758	DRAMP02933	Polyphemusin-1 (PM1; crabs, Arthropods, animals)
759	DRAMP02934	PM1-S (linear derivative of PM1)
760	DRAMP02950	tALF5 (Portunus trituberculatus anti-lipopolysaccharide factor isoform 5; crabs, Arthropods, animal[illegible]
761	DRAMP02951	tALF6 (Portunus trituberculatus anti-lipopolysaccharide factor isoform 6; crabs, Arthropods, animal[illegible]
762	DRAMP02952	tALF7 (Portunus trituberculatus anti-lipopolysaccharide factor isoform 7; crabs, Arthropods, animal[illegible]
763	DRAMP02953	Arasin-1 (Pro-rich, Arg-rich; crabs, Arthropods, animals)
764	DRAMP02956	Dolabellanin B2
765	DRAMP02959	Antibacterial protein PR-39 (pigs, mammals, animals)
766	DRAMP02960	Antibacterial peptide PMAP-23 (Myeloid antibacterial peptide 23; pigs, mammals, animals)
767	DRAMP02961	Antibacterial peptide PMAP-37 (Myeloid antibacterial peptide 37; pigs, mammals, animals)
768	DRAMP02962	Antibacterial peptide PMAP-36 (Myeloid antibacterial peptide 36; pigs, mammals, animals)
769	DRAMP02963	PMAP-36(1-20)
770	DRAMP02964	PMAP-36(1-34)
771	DRAMP02965	PMAP-36(1-35)2
772	DRAMP02966	DBI(32-86) (pigs, mammals, animals)
773	DRAMP02970	Protegrin-1 (Protegrin 1; PG-1; pigs, mammals, animals)
774	DRAMP02975	Tritrpticin (Trp-rich; pigs, mammals, animals)
776	DRAMP01376	Odorranain-F1 (OdF1; Frogs, amphibians, animals)
777	DRAMP01377	Odorranain-G1 (OdG1; Frogs, amphibians, animals)
778	DRAMP02995	Hymenoptaecin (Insects, animals)
779	DRAMP02996	Apidaecin-2 (Apidaecin II; Insects, animals)
780	DRAMP01378	Odorranain-H1 (OdH1; Frogs, amphibians, animals)
781	DRAMP02998	Apidaecin-1A (Apidaecin IA; Insects, animals)
782	DRAMP02999	Jellein-1 (Jelleine-I; chain of Major royal jelly protein 1; Insects, animals)
783	DRAMP03000	Jellein-2 (Jelleine-II; chain of Major royal jelly protein 1; Insects, animals)
784	DRAMP03001	Jellein-3 (Jelleine-III; Insects, animals)

785	DRAMP03002	Melittin (Allergen Api m 3; Allergen Api m III; Insects, animals)
786	DRAMP03003	Melectin (MEP; Insects, animals)
787	DRAMP03007	Osmin (Insects, animals)
788	DRAMP03019	Mastoparan PDD-B
789	DRAMP03020	Mastoparan PDD-A
790	DRAMP03021	Mastoparan PMM
791	DRAMP03022	Mastoparan MP
792	DRAMP03028	Mastoparan-1 (MP-1; Venom protein MP-1; Insects, animals)
793	DRAMP03033	Mastoparan-like peptide 12a (Insects, animals)
794	DRAMP03034	Mastoparan-like peptide 12b (Insects, animals)
795	DRAMP03035	Mastoparan-like peptide 12c (Insects, animals)
796	DRAMP03036	Mastoparan-like peptide 12d (Insects, animals)
797	DRAMP03037	Eumenitin (Er-12; Insects, animals)
798	DRAMP03038	Eumenitin-R (Insects, animals)
799	DRAMP03039	Eumenitin-F (Insects, animals)
800	DRAMP03040	Eumenine mastoparan-EF (EMP-EF; Insects, animals)
801	DRAMP03041	Eumenine mastoparan-ER (EMP-ER; Insects, animals)
802	DRAMP03042	Eumenine mastoparan-AF (EMP-AF; Af-113; Insects, animals)
803	DRAMP03043	Agelaia-mastoparan (Agelaia-MP; Insects, animals)
804	DRAMP03044	Protonectin (Agelaia-chemotactic peptide, Agelaia-CP; Insects, animals)
805	DRAMP03045	Defensin-NV (Insects, animals)
807	DRAMP03047	Venom peptide 2-long (OdVP2L; analog of OdVP2; Insects, animals)
808	DRAMP03050	Dominulin-A (Insects, animals)
809	DRAMP03051	Dominulin-B (Insects, animals)
813	DRAMP03055	PP30 (Pro-rich; abaecin-like; Insects, animals)
814	DRAMP03056	Decoralin (Insects, animals)
815	DRAMP03057	Thanatin (Insects, animals)
818	DRAMP03075	Cecropin-D
819	DRAMP03089	Drosophila cecropin-A1/A2 (Insects, animals)
820	DRAMP03090	Drosophila cecropin B (CecB; Insects, animals)
821	DRAMP03095	Andropin (Insects, animals)
823	DRAMP18495	Gomesin (Gm; Spiders, arachnids, Chelicerata, arthropods, invertebrates, animals)
825	DRAMP03104	Sapecin (defensins; Insects, animals)
827	DRAMP03116	Ceratotoxin-C (Insects, animals)
828	DRAMP03117	Drosophila cecropin-A1 (Insects, animals)
830	DRAMP03138	Cecropin-A (Insects, animals)
831	DRAMP03140	Anopheles cecropin-A amidated isoform (Insects, animals)
832	DRAMP03150	Gambicin (Insects, animals)
833	DRAMP03153	27 kDa antibacterial protein
835	DRAMP03166	P15 (deer beta-defensin; ruminant, animals)
837	DRAMP03173	Arenicin-1 (Ar-1; marine polychaeta, animals)
839	DRAMP03181	Spinigerin (Insects, animals)
840	DRAMP03186	Spheniscin-2 (Sphe-2; penguin avian beta-defensin 103b; birds ,animals)
841	DRAMP03187	Beta defensin 1(BD-1; mammals, animals)
845	DRAMP03198	Alpha-defensin PhD-4 (primates, mammals, animals)
846	DRAMP03215	Gomesin (Gm; spiders, Arthropods, animals)
847	DRAMP03216	Oxyopinin-4a (Oxt-4a; spiders, Arthropods, animals)
848	DRAMP03217	M-oxotoxin-Ot1a (Oxyopinin-1, Oxki1; spiders, Arthropods, animals)
849	DRAMP03222	M-ctenitoxin-Cs1a (M-CNTX-Cs1a; Cupiennin-1a; spiders, Arthropods, animals)
850	DRAMP03225	M-ctenitoxin-Cs1d (M-CNTX-Cs1d; Cupiennin-1d; spiders, Arthropods, animals)

851	DRAMP03226	M-zodatoxin-Lt1a (M-ZDTX-Lt1a; Latarcin-1, Ltc-1, Ltc1; spiders, Arthropods, animals)
852	DRAMP03227	M-zodatoxin-Lt2a (M-ZDTX-Lt2a; Latarcin-2a, Ltc-2a, Ltc2a; spiders, Arthropods, animals)
853	DRAMP03229	M-zodatoxin-Lt3a (M-ZDTX-Lt3a; Latarcin-3a, Ltc-3a; spiders, Arthropods, animals)
854	DRAMP03230	M-zodatoxin-Lt3b (M-ZDTX-Lt3b; Latarcin-3b, Ltc-3b; spiders, Arthropods, animals)
855	DRAMP03231	M-zodatoxin-Lt4a (M-ZDTX-Lt4a; Latarcin-4a, Ltc-4a; spiders, Arthropods, animals)
856	DRAMP03232	M-zodatoxin-Lt4b (M-ZDTX-Lt4b; Latarcin-4b, Ltc-4b; spiders, Arthropods, animals)
857	DRAMP03233	M-zodatoxin-Lt5a (M-ZDTX-Lt5a; Latarcin-5, Ltc-5; spiders, Arthropods, animals)
858	DRAMP03236	M-zodatoxin-Lt8a (M-ZDTX-Lt8a; Cytoinsectotoxin-1a, CIT-1a; spiders, Arthropods, animals)
859	DRAMP03253	M-lycotoxin-Ls3a (M-LCTX-Ls3a; Lycocitin-1; spiders, Arthropods, animals)
860	DRAMP03254	M-lycotoxin-Ls3b (M-LCTX-Ls3b; Lycocitin-2; spiders, Arthropods, animals)
861	DRAMP03278	M-lycotoxin-Hc1a (M-LCTX-Hc1a; Lycotoxin I; spiders, Arthropods, animals)
862	DRAMP03279	M-lycotoxin-Hc2a (M-LCTX-Hc2a; Lycotoxin-2; Lycotoxin II; spiders, Arthropods, animals)
863	DRAMP03280	AcAMP (A. clavatus antimicrobial peptide)
864	DRAMP03285	Ostricacin-1 (Beta-defensin 2; Birds, animals)
865	DRAMP03286	Ostricacin-2 (Beta-defensin 1; Birds, animals)
866	DRAMP03287	Ostricacin-3 (Beta-defensin 7; Birds, animals)
867	DRAMP03288	Ostricacin-4 (Beta-defensin 8; Birds, animals)
868	DRAMP03311	Stomoxyn (Insects, animals)
869	DRAMP03312	Metalnikowin-1 (Metalnikowin I; Insects, animals)
872	DRAMP03370	Beta-defensin 6 (BD-6, mBD-6; Defensin, beta 6; Rodents, mammals, animals)
873	DRAMP03405	CRAMP-1 (mouse cathelin-related antimicrobial peptide 1; cathelicidin; Rodents, mammals, animal
874	DRAMP03406	CRAMP-2 (mouse cathelin-related antimicrobial peptide 2; cathelicidin; Rodents, mammals, animal
875	DRAMP03419	Neutrophil antibiotic peptide NP-1 (RatNP-1; Rodents, mammals, animals)
876	DRAMP03422	Neutrophil antibiotic peptide NP-4 (RatNP-4; Rodents, mammals, animals)
878	DRAMP03464	Cryptonin (Insects, animals)
880	DRAMP03467	Antibacterial napin (Plants)
881	DRAMP03471	Recombinant Crassostrea Gigas Defensin (Cg-Def; molluscs, animals)
882	DRAMP03472	cgUbiquitin
885	DRAMP03486	Manduca Sexta Moricin (MS moricin; Insects, animals)
887	DRAMP03507	Cecropin-B (Insects, animals)
888	DRAMP03513	G. mellonella moricin-like peptide A (Gm-mlpA; Insects, animals; Predicted)
889	DRAMP03514	G. mellonella moricin-like peptide B (Gm-mlpB; Insects, animals; Predicted)
890	DRAMP03515	Moricin-like peptide C1 (Gm-mlpC1; Insects, animals; Predicted)
891	DRAMP03516	Moricin-like peptide C2 (Gm-mlpC2; Insects, animals; Predicted)
892	DRAMP03517	Moricin-like peptide C3 (Gm-mlpC3; Insects, animals; Predicted)
893	DRAMP03518	Moricin-like peptide C4/C5 (Gm-mlpC4/C5; Insects, animals; Predicted)
894	DRAMP03519	Moricin-like peptide D (Gm-mlpD; Insects, animals; Predicted)
903	DRAMP03532	Moricin-1 (Insects, animals)
908	DRAMP03567	KR-20 (Derived from LL-37)
909	DRAMP03568	RK-31 (Derived from LL-37)
910	DRAMP03569	KS-30 (Derived from LL-37)
911	DRAMP03570	LL-23 (Derived from LL-37)
912	DRAMP03571	Antibacterial protein LL-37 (one chain of hCAP-18; Human, mammals, animals)
913	DRAMP03573	LL-37(13-37)(C-terminal fragment of LL-37; Human, mammals, animals)
914	DRAMP03574	LL-37(17-32)(C-terminal fragment of LL-37; Human, mammals, animals)
915	DRAMP03598	Human beta-defensin 2 (hBD-2; Defensin, beta 2; Beta-defensin 4A; Human, mammals, animals)
916	DRAMP03599	Human beta-defensin 3 (BD-3, hBD-3; Hbd3; Beta-defensin 103; Human, mammals, animals)
917	DRAMP03600	Human beta-defensin 4 (hBD-4, BD-4; Beta-defensin 104; Human, mammals, animals)
918	DRAMP03603	Human beta-defensin 28 (hBD-28; hBD28; Human, mammals, animals)
919	DRAMP03638	VpBD (V.philippinarum beta defensin; big defensin)

920	DRAMP03642	Chicken heterophil peptides 1 (Antimicrobial peptide CHP1; Birds, animals)
921	DRAMP03645	Cathelicidin-2 (CATH-2; Fowlicidin-2; Birds, animals)
922	DRAMP03646	Cathelicidin-3 (CATH-3; Fowlicidin-3; Birds, animals)
923	DRAMP03647	Cathelicidin-B1 (CATH-B1; cathelicidin; Birds, animals)
924	DRAMP03659	Gallinacin-11 (Gal-11; Beta-defensin 11; Birds, animals)
925	DRAMP03676	GLFcin (Lactoferrin fragment)
926	DRAMP03677	GLFcin II (Lactoferrin fragment)
927	DRAMP03679	Cathelicidin-2 (Bactenecin-5, Bac5; ChBac5; ruminant, animals)
928	DRAMP03682	Vespid chemotactic peptide 5e (VCP 5e; Insects, animals)
929	DRAMP03683	Vespid chemotactic peptide 5g (VCP 5g; Insects, animals)
930	DRAMP03684	Vespid chemotactic peptide 5f (VCP 5f; Insects, animals)
931	DRAMP03687	TsAP-1 (T. serrulatus antimicrobial peptide 1; scorpions, arachnids, invertebrates, animals)
933	DRAMP03691	Im-1 (Arthropods, animals)
934	DRAMP03693	Bactridin-1 (Bact1; Bactridine 1; Arthropods, animals)
935	DRAMP03694	Bactridin-2 (Bact2, Bactridine 2; P-Mice-Antm-beta* NaTx14.8; Arthropods, animals)
937	DRAMP03702	Mucroporin (Antimicrobial peptide 36.21; Arthropods, animals)
938	DRAMP03706	Antimicrobial peptide 1 (AamAP1; Arthropods, animals)
939	DRAMP03707	Antimicrobial peptide 2 (AamAP2; Arthropods, animals)
940	DRAMP03714	phiphatic peptide CT1 (VmCT1; Non-disulfide-bridged peptide 5.13, NDBP-5.13; Arthropods, anim
941	DRAMP03715	phiphatic peptide CT2 (VmCT2; Non-disulfide-bridged peptide 5.14, NDBP-5.14; Arthropods, anim
942	DRAMP03721	Cytotoxic linear peptide IsCT (IsCT; NDBP-5.2; Arthropods, animals)
943	DRAMP03723	Pandinin-1 (Pin1; Arthropods, animals)
944	DRAMP03724	Pandinin-2 (Pin2; Arthropods, animals)
945	DRAMP03729	Opiscorpine-1 (Arthropods, animals)
946	DRAMP03734	Parabutoporin (PP; Non-disulfide-bridged peptide 3.2, NDBP-3.2; Arthropods, animals)
947	DRAMP03735	Opistoporin-1 (OP1; Non-disulfide-bridged peptide 3.5; Opistoporin-3, OP3; Arthropods, animals)
948	DRAMP03738	Scorpine (defensins; Arthropods, animals)
949	DRAMP02828	BMAP-34 (BMAP 34, bovine cathelicidin, cattle, ruminant, mammals, animals)
951	DRAMP03746	Peptide BmKn2 (Biologically active peptide 4; NDBP-5.1; Arthropods, animals)
952	DRAMP03748	Bradykinin-potentiating peptide BmK3 (Bpp BmK3; NDBP-3.3; Arthropods, animals)
953	DRAMP03750	Venom antimicrobial peptide-6 (Meucin-13; NDBP-5; Arthropods, animals)
954	DRAMP03751	Venom antimicrobial peptide-9 (Meucin-18; NDBP-5; Arthropods, animals)
955	DRAMP03752	Peptide BmKb1 (Non-disulfide-bridged peptide 4.2, NDBP-4.2; Arthropods, animals)
956	DRAMP03753	Amphipathic peptide CT1 (StCT1; Non-disulfide-bridged peptide 5, NDBP-5; Arthropods, animals)
957	DRAMP03754	Amphipathic peptide CT2 (StCT2; Non-disulfide-bridged peptide 5, NDBP-5; Arthropods, animals)
958	DRAMP03774	UyCT1 (Arthropods, animals)
959	DRAMP03775	UyCT2 (Arthropods, animals)
960	DRAMP03776	UyCT3 (Arthropods, animals)
961	DRAMP03777	UyCT5 (Arthropods, animals)
963	DRAMP03814	D16W (GGN4 analogue peptide with single substitution)
964	DRAMP03815	D16W-N23 (single amino acid substitution)
965	DRAMP03816	D16F-N23 (single amino acid substitution)
966	DRAMP03823	Dermaseptin derivative K4-S4-(1-13)
967	DRAMP03824	CNBr-cleaved lactoferricin Subfragment 1
968	DRAMP03825	CNBr-cleaved lactoferricin Subfragment 2
969	DRAMP03826	Ovispirin-1 (OV-1; N-terminal 18 amino acids of SMAP-29)
970	DRAMP03827	Novispirin G-10 (mutation of Ovispirin-1)
971	DRAMP03828	Novispirin T-7 (mutation of Ovispirin-1)
972	DRAMP03829	GLK-19
973	DRAMP03830	Palustrin-2ISb + 3aa

974	DRAMP03831	Palustrin-2ISb-des-C7
975	DRAMP03832	Palustrin-2ISb-des-C7-4D
976	DRAMP03833	Palustrin-2ISb-des-C7-12N
977	DRAMP03834	Palustrin-2ISb-des-C7-23,29S
979	DRAMP03852	G1 (Bac2A variant through single amino acid substitution)
980	DRAMP03853	G2 (Bac2A variant through single amino acid substitution)
981	DRAMP03854	R2 (Bac2A variant through single amino acid substitution)
982	DRAMP03855	R3 (Bac2A variant through single amino acid substitution)
983	DRAMP03856	W3 (Bac2A variant through single amino acid substitution)
984	DRAMP03857	R5 (Bac2A variant through single amino acid substitution)
985	DRAMP03858	K7 (Bac2A variant through single amino acid substitution)
986	DRAMP03859	W10 (Bac2A variant through single amino acid substitution)
987	DRAMP03860	R11 (Bac2A variant through single amino acid substitution)
988	DRAMP03861	G12 (Bac2A variant through single amino acid substitution)
989	DRAMP03862	Sub2 (Bac2A variant through two amino acids substitution)
990	DRAMP03863	Sub3 (Bac2A variant through three amino acids substitution)
991	DRAMP03864	Sub5 (Bac2A variant through five amino acids substitution)
992	DRAMP03865	Sub6 (Bac2A variant through six amino acids substitution)
993	DRAMP03866	Bac8a (Bac2A variant)
994	DRAMP03867	Bac8b (Bac2A variant)
995	DRAMP03868	Bac8c (Bac2A variant)
996	DRAMP03869	Bac8d (Bac2A variant)
997	DRAMP03870	Bac2A (a linear variant of bovine dodecapeptide)
998	DRAMP03871	cLf 20-29 (fragment of caprine lalctoferricin, residues 20-29)
999	DRAMP03875	bLf 20-29 (fragment of bovine lactoferricin, residues 20-29)
1000	DRAMP03876	LFB-RW (derivative of bovine lactoferrin with residues substitution)
1001	DRAMP03877	LFB-KW (derivative of bovine lactoferrin with residues substitution)
1002	DRAMP03878	LFB-Rwa (derivative of bovine lactoferrin with residues substitution)
1003	DRAMP03879	LFB-RF (derivative of bovine lactoferrin with residues substitution)
1004	DRAMP03880	LFB-RI (derivative of bovine lactoferrin with residues substitution)
1005	DRAMP03881	LFB-6RW (derivative of bovine lactoferrin with residues substitution)
1006	DRAMP03882	LFC (fragment of mature caprine lactoferrin, residues 17 to 31)
1007	DRAMP03883	LFH W8 (tryptophan-modified human lactoferricin derivative)
1008	DRAMP03884	LFC W8 (tryptophan-modified caprine lactoferricin derivative)
1009	DRAMP03885	LFP W8 (tryptophan-modified porcine lactoferricin derivative)
1010	DRAMP03886	LFB (fragment of bovine lalctoferricin, residues 17 to 31)
1011	DRAMP03887	LFB A1 (derivative of LFB, residue substitution with alanine at position 1)
1012	DRAMP03888	LFB A2 (derivative of LFB, residue substitution with alanine at position 2)
1013	DRAMP03889	LFB A3 (derivative of LFB, residue substitution with alanine at position 3)
1014	DRAMP03890	LFB A4 (derivative of LFB, residue substitution with alanine at position 4)
1015	DRAMP03891	LFB A5 (derivative of LFB, residue substitution with alanine at position 5)
1016	DRAMP03892	LFB A7 (derivative of LFB, residue substitution with alanine at position 7)
1017	DRAMP03893	LFB A9 (derivative of LFB, residue substitution with alanine at position 9)
1018	DRAMP03894	LFB A10 (derivative of LFB, residue substitution with alanine at position 10)
1019	DRAMP03895	LFB A11 (derivative of LFB, residue substitution with alanine at position 11)
1020	DRAMP03896	LFB A12 (derivative of LFB, residue substitution with alanine at position 12)
1021	DRAMP03897	LFB A13 (derivative of LFB, residue substitution with alanine at position 13)
1022	DRAMP03898	LFB A14 (derivative of LFB, residue substitution with alanine at position 14)
1023	DRAMP03899	LFM A1 W8 (LFM W8 derivative with residues substitution)
1024	DRAMP03900	LFM A1,9 W8 (LFM W8 derivative with residues substitution)

1025	DRAMP03901	LFM R1 W8 (LFM W8 derivative with residues substitution)
1026	DRAMP03902	LFM R9 W8 (LFM W8 derivative with residues substitution)
1027	DRAMP03903	LFM A1 R9 W8 (LFM W8 derivative with residues substitution)
1028	DRAMP03904	LFM A9 R1 W8 (LFM W8 derivative with residues substitution)
1029	DRAMP03905	LFM R1,9 W8 (LFM W8 derivative with residues substitution)
1030	DRAMP03906	LFM A1 W8 Y13 (LFM W8 derivative with residues substitution)
1031	DRAMP03907	LFM A1,9 W8 Y13 (LFM W8 derivative with residues substitution)
1032	DRAMP03908	LFM R1 W8 Y13 (LFM W8 derivative with residues substitution)
1033	DRAMP03909	LFM R9 W8 Y13 (LFM W8 derivative with residues substitution)
1034	DRAMP03910	LFM A1 R9 W8 Y13 (LFM W8 derivative with residues substitution)
1035	DRAMP03911	LFM A9 R1 W8 Y13 (LFM W8 derivative with residues substitution)
1036	DRAMP03912	LFM R1,9 W8 Y13 (LFM W8 derivative with residues substitution)
1037	DRAMP03920	Cecropin A (1-8)-melittin (1-13)hybrid peptide
1038	DRAMP03921	Cecropin A (1-8)-melittin (1-18)hybrid peptide
1039	DRAMP03922	Cecropin A (1-8)-melittin (1-12)hybrid peptide
1040	DRAMP03923	Cecropin A (1-8)-melittin (1-10)hybrid peptide
1041	DRAMP03924	Cecropin A (1-7)-melittin (1-8)hybrid peptide
1042	DRAMP03925	Cecropin A (1-7)-melittin (3-10)hybrid peptide
1043	DRAMP03927	Cecropin A (1-7)-melittin (2-9)hybrid peptide
1044	DRAMP03928	Cecropin A (1-7)-melittin (4-11)hybrid peptide (CAM)
1045	DRAMP03929	Cecropin A (1-7)-melittin (5-12)hybrid peptide
1046	DRAMP03930	Cecropin A (1-7)-melittin (6-13)hybrid peptide
1048	DRAMP03933	I14M (truncated isoform of thanatin, residue 8-21)
1050	DRAMP03935	V16M (truncated isoform of thanatin, residue 6-21)
1051	DRAMP03936	K18M (truncated isoform of thanatin, residue 4-21)
1055	DRAMP03945	Del 1-4 (Ranalexin analog)
1056	DRAMP03947	Del 1-2 (Ranalexin analog)
1057	DRAMP03948	Del 1 (Ranalexin analog)
1058	DRAMP03949	Del 20 (Ranalexin analog)
1065	DRAMP03967	P18 (Cecropin A(1-8)-Magainin 2(1-12) hybrid peptide analogue)
1066	DRAMP03968	[L9]-P18 (analog of P18)
1067	DRAMP03969	[S9]-P18 (analog of P18)
1068	DRAMP03970	N-1 (analog of P18)
1069	DRAMP03971	N-2 (analog of P18)
1070	DRAMP03972	N-3 (analog of P18)
1071	DRAMP03973	N-4 (analog of P18)
1072	DRAMP03974	N-5 (analog of P18)
1073	DRAMP03975	N-3L (analog of P18)
1074	DRAMP03976	N-4L (analog of P18)
1075	DRAMP03977	N-5L (analog of P18)
1076	DRAMP03978	C-1 (analog of P18)
1077	DRAMP03979	C-2 (analog of P18)
1078	DRAMP03980	C-3 (analog of P18)
1079	DRAMP03981	C-4 (analog of P18)
1080	DRAMP03982	C-5 (analog of P18)
1081	DRAMP03983	C-6 (analog of P18)
1082	DRAMP03984	C-7 (analog of P18)
1083	DRAMP03985	C-8 (analog of P18)
1084	DRAMP03986	C-9 (analog of P18)
1085	DRAMP03987	C-10 (analog of P18)

1088	DRAMP03990	L4K3W4 (LlKmWn model peptide)
1090	DRAMP03992	L5K3W5 (LlKmWn model peptide)
1091	DRAMP03993	L5K5W6 (LlKmWn model peptide)
1092	DRAMP03994	L6K4W6 (LlKmWn model peptide)
1093	DRAMP03995	L7K3W6 (LlKmWn model peptide)
1096	DRAMP03999	[A6]-IsCT (Mutant: W6A; IsCT analog)
1097	DRAMP04000	[L6]-IsCT (Mutant: W6L; IsCT analog)
1098	DRAMP04001	[K7]-IsCT (Mutant: E7K; IsCT analog)
1099	DRAMP04002	[L6, K11]-IsCT (IsCT analog through amino acids substitution)
1100	DRAMP04003	[K7, P8, K11]-IsCT (IsCT analog through amino acids substitution)
1101	DRAMP04004	Gramicidin analogue ([Scr2]-GS)
1102	DRAMP04005	Gramicidin analogue ([Ser2,2']-GS)
1103	DRAMP04011	Plasticin PD36 KF (analog of PD36)
1104	DRAMP04012	Plasticin PD36 K (analog of PD36)
1105	DRAMP04013	Plasticin ANC KF (analog of natural peptide ANC)
1106	DRAMP04014	LL-37A9 (LL-37 variants)
1107	DRAMP04015	LL-37V9 (LL-37 variants)
1109	DRAMP04017	LL-23V9 (LL-23 variants)
1110	DRAMP04019	Bac014 (Scrambled Variants of Bac2A)
1111	DRAMP04020	Bac020 (Scrambled Variants of Bac2A)
1112	DRAMP04021	Bac034 (Scrambled Variants of Bac2A)
1113	DRAMP04022	F3 (single amino acid substitution of Bac034, which is a scrambled Variant of Bac2A)
1114	DRAMP04023	W3 (single amino acid substitution of Bac034, which is a scrambled Variant of Bac2A)
1115	DRAMP04024	W4 (single amino acid substitution of Bac034, which is a scrambled Variant of Bac2A)
1116	DRAMP04025	R10 (single amino acid substitution of Bac034, which is a scrambled Variant of Bac2A)
1117	DRAMP04026	K12 (single amino acid substitution of Bac034, which is a scrambled Variant of Bac2A)
1118	DRAMP04027	opt1 (multiple amino acid substitution of Bac034, which is a scrambled Variant of Bac2A)
1119	DRAMP04028	opt2 (multiple amino acid substitution of Bac034, which is a scrambled Variant of Bac2A)
1120	DRAMP04029	opt3 (multiple amino acid substitution of Bac034, which is a scrambled Variant of Bac2A)
1121	DRAMP04030	opt4 (multiple amino acid substitution of Bac034, which is a scrambled Variant of Bac2A)
1122	DRAMP04031	opt5 (multiple amino acid substitution of Bac034, which is a scrambled Variant of Bac2A)
1123	DRAMP04032	Modified defensin
1124	DRAMP04033	Modified defensin
1125	DRAMP04034	Modified defensin
1126	DRAMP04035	Modified defensin
1127	DRAMP04036	Modified defensin
1128	DRAMP04048	BacR (cyclic derivative of bactenecin)
1129	DRAMP04049	BacP3R (cyclic derivative of bactenecin)
1130	DRAMP04050	BacP3R-V (cyclic derivative of bactenecin)
1131	DRAMP04051	Bac2I-NH2 (cyclic derivative of bactenecin)
1132	DRAMP04052	BacP2R-NH2 (cyclic derivative of bactenecin)
1133	DRAMP04053	BacP1 (cyclic derivative of bactenecin)
1134	DRAMP04054	BacW (cyclic derivative of bactenecin)
1135	DRAMP04055	BacW2R (cyclic derivative of bactenecin)
1136	DRAMP04056	Lin Bac2S-NH2 (linear derivative of bactenecin)
1137	DRAMP04057	Lin BacS-NH2 (linear derivative of bactenecin)
1146	DRAMP04064	Cyclic cationic V1 peptide
1147	DRAMP04065	Cyclic cationic V2 peptide
1148	DRAMP04066	Cyclic cationic V3 peptide
1149	DRAMP04067	Cyclic cationic V4 peptide

1150	DRAMP04068	Cyclic cationic V5 peptide
1151	DRAMP04069	Cyclic cationic V6 peptide
1152	DRAMP04070	Cyclic cationic V7 peptide
1153	DRAMP04075	Antimicrobial peptide HP (2-20)
1154	DRAMP04076	Anal 1 (antimicrobial peptide HP (2-20)analogue)
1155	DRAMP04077	Anal 2 (antimicrobial peptide HP (2-20)analogue)
1156	DRAMP04078	Anal 3 (antimicrobial peptide HP (2-20)analogue)
1157	DRAMP04079	Anal 4 (antimicrobial peptide HP (2-20)analogue)
1158	DRAMP04080	Anal 5 (antimicrobial peptide HP (2-20)analogue)
1159	DRAMP04081	Anal 6 (antimicrobial peptide HP (2-20)analogue)
1160	DRAMP04082	Anal 7 (antimicrobial peptide HP (2-20)analogue)
1161	DRAMP04083	D-amino-acid pexiganan (MSI-214)
1162	DRAMP04095	Cupiennin-1D (spiders, Arthropods, animals)
1163	DRAMP04096	2IQ2
1164	DRAMP04097	2IQ3
1165	DRAMP04098	3IQ1
1166	DRAMP04099	3IQ2
1167	DRAMP04100	3IQ3
1168	DRAMP04101	3IQ4
1169	DRAMP04102	CP-P
1170	DRAMP04103	S16 (derivative of CP-P)
1171	DRAMP04104	F2 (derivative of CP-P)
1172	DRAMP04105	N3 (derivative of CP-P)
1173	DRAMP04106	K6 (derivative of CP-P)
1174	DRAMP04107	N7 (derivative of CP-P)
1175	DRAMP04108	A9 (derivative of CP-P)
1176	DRAMP04111	S9 (derivative of CP-P)
1177	DRAMP04112	L10 (derivative of CP-P)
1178	DRAMP04113	A10 (derivative of CP-P)
1179	DRAMP04114	D11 (derivative of CP-P)
1180	DRAMP04115	K11 (derivative of CP-P)
1181	DRAMP04117	A13 (derivative of CP-P)
1182	DRAMP04119	K17 (derivative of CP-P)
1183	DRAMP04120	D18 (derivative of CP-P)
1184	DRAMP04121	N18 (derivative of CP-P)
1185	DRAMP04122	N20 (derivative of CP-P)
1186	DRAMP04123	D0-NH2
1187	DRAMP04124	D1-NH2
1188	DRAMP04125	D2-NH2
1189	DRAMP04126	D3-NH2
1190	DRAMP04127	D4-NH2
1191	DRAMP04128	D5-NH2
1192	DRAMP04129	D6-NH2
1193	DRAMP04136	LRR-1
1194	DRAMP04137	LRR-2
1195	DRAMP04138	L1 (first 8 N-terminal residues of bovine LF)
1196	DRAMP04139	L2
1197	DRAMP04140	L3
1198	DRAMP04141	L4
1199	DRAMP04142	L5

1200	DRAMP04143	L6
1201	DRAMP04144	L7
1202	DRAMP04145	L8
1203	DRAMP04146	L9
1204	DRAMP04147	L10
1205	DRAMP04159	LR2 (homologue of Pc-CATH1)
1206	DRAMP04160	LR3 (homologue of Pc-CATH1)
1207	DRAMP04161	LR4 (homologue of Pc-CATH1)
1208	DRAMP04162	LR5 (homologue of Pc-CATH1)
1209	DRAMP04163	LR6 (homologue of Pc-CATH1)
1210	DRAMP04164	LR7 (homologue of Pc-CATH1)
1211	DRAMP04165	LR8 (homologue of Pc-CATH1)
1212	DRAMP04166	LR9 (homologue of Pc-CATH1)
1213	DRAMP04167	LR10 (homologue of Pc-CATH1)
1214	DRAMP04168	LR11 (homologue of Pc-CATH1)
1215	DRAMP04169	LR13 (homologue of Pc-CATH1)
1216	DRAMP04170	LR15 (homologue of Pc-CATH1)
1217	DRAMP04171	LR16 (homologue of Pc-CATH1)
1218	DRAMP04174	L2K3W2 (LlKmW2 model peptides)
1219	DRAMP04175	L3K2W2 (LlKmW2 model peptides)
1220	DRAMP04176	L2K5W2 (LlKmW2 model peptides)
1221	DRAMP04177	L3K4W2 (LlKmW2 model peptides)
1222	DRAMP04178	L4K3W2 (LlKmW2 model peptides)
1223	DRAMP04179	L5K2W2 (LlKmW2 model peptides)
1224	DRAMP04180	L3K6W2 (LlKmW2 model peptides)
1225	DRAMP04181	L4K5W2 (LlKmW2 model peptides)
1226	DRAMP04182	L5K4W2 (LlKmW2 model peptides)
1227	DRAMP04183	L6K3W2 (LlKmW2 model peptides)
1229	DRAMP04185	DFTamP1-p
1230	DRAMP04186	L5K5W1 (L5K5Wn model peptide)
1231	DRAMP04187	L5K5W2 (L5K5Wn model peptide)
1232	DRAMP04188	L5K5W3 (L5K5Wn model peptide)
1233	DRAMP04189	L5K5W4 (L5K5Wn model peptide)
1234	DRAMP04190	L5K5W5 (L5K5Wn model peptide)
1236	DRAMP04192	L5K5W7 (L5K5Wn model peptide)
1237	DRAMP04193	L5K5W8 (L5K5Wn model peptide)
1238	DRAMP04194	L5K5W9 (L5K5Wn model peptide)
1239	DRAMP04195	L5K5W10 (L5K5Wn model peptide)
1240	DRAMP04196	L5K5W11 (L5K5Wn model peptide)
1244	DRAMP04237	Antibacterial peptide A4
1245	DRAMP04240	Synthetic 1
1246	DRAMP04241	Synthetic 2
1247	DRAMP04242	Synthetic 3
1248	DRAMP04243	Synthetic 4
1249	DRAMP04244	Synthetic 5
1250	DRAMP04264	CP26
1251	DRAMP04265	CP29
1253	DRAMP04359	PDD-A-1 (PDD-A analog)
1254	DRAMP04360	PDD-A-2 (PDD-A analog)
1255	DRAMP04361	PDD-A-3 (PDD-A analog)

1256	DRAMP04362	PDD-A-4 (PDD-A analog)
1257	DRAMP04363	PDD-A-5 (PDD-A analog)
1258	DRAMP04364	PDD-A-6 (PDD-A analog)
1259	DRAMP04365	PDD-A-7 (PDD-A analog)
1260	DRAMP04367	PDD-A-9 (PDD-A analog)
1261	DRAMP04368	PDD-A-10 (PDD-A analog)
1262	DRAMP04369	PDD-A-11 (PDD-A analog)
1263	DRAMP04370	PDD-A-12 (PDD-A analog)
1264	DRAMP04371	PDD-B-1 (PDD-B analog)
1265	DRAMP04372	PDD-B-2 (PDD-B analog)
1266	DRAMP04373	PDD-B-3 (PDD-B analog)
1267	DRAMP04374	PDD-B-4 (PDD-B analog)
1268	DRAMP04376	MP-1 (MP analog)
1269	DRAMP04377	MP-2 (MP analog)
1270	DRAMP04378	MP-5 (MP analog)
1271	DRAMP04379	MP-6 (MP analog)
1272	DRAMP04380	PMM-1 (PMM analog)
1273	DRAMP04381	PMM-2 (PMM analog)
1274	DRAMP04382	PMM-3 (PMM analog)
1275	DRAMP04383	PMM-4 (PMM analog)
1276	DRAMP04385	PMM-6 (PMM analog)
1277	DRAMP04386	PMM-7 (PMM analog)
1278	DRAMP04387	PMM-8 (PMM analog)
1279	DRAMP04389	PMM-10 (PMM analog)
1280	DRAMP04390	PMM-11 (PMM analog)
1281	DRAMP04391	PMM-12 (PMM analog)
1282	DRAMP04392	PMM-13 (PMM analog)
1283	DRAMP04393	PMM-14 (PMM analog)
1285	DRAMP04542	Polybia-MP-I (insects, vertebrates, animals)
1286	DRAMP04543	Polybia-MP-II (insects, vertebrates, animals)
1287	DRAMP04544	Polybia-MP-III (insects, vertebrates, animals)
1288	DRAMP04545	Limnonectin-1Fa (Frogs, amphibians, animals)
1289	DRAMP04546	Limnonectin-1Fb (Frogs, amphibians, animals)
1290	DRAMP04553	H. erythraea B2RP
1291	DRAMP04640	PGLa-AN2
1292	DRAMP04665	Px-cec1
1293	DRAMP04670	PBD1-42
1294	DRAMP04671	Myticusin-1
1295	DRAMP04676	Brevinin-2HS2A
1296	DRAMP04677	Brevinin-2HS2B
1346	DRAMP00052	Mutacin-2 (Mutacin II mutacin H-29B; Bacteriocin)
1349	DRAMP00055	Bacteriocin 97518
1354	DRAMP00064	Enterocin 96 (Bacteriocin)
1358	DRAMP00070	Laterosporulin (Bacteriocin)
1372	DRAMP00085	Bacteriocin
1434	DRAMP00169	Enterocin AS-48 (AS-48; Bacteriocin)
1438	DRAMP18338	Thiocillin GE37468 (Bacteriocin)
1444	DRAMP00182	Thuricin-S (Bacteriocin)
1448	DRAMP00186	LSEI_2163 (m2163; Bacteriocin)
1449	DRAMP00187	LSEI_2386 (m2386; Bacteriocin)

1451	DRAMP00192	Microcin C7 (MccC7; Microcin C51, MccC51; Bacteriocin)
1454	DRAMP00195	Colicin-V (Microcin-V; Bacteriocin)
1455	DRAMP00196	Microcin L (MccL; Bacteriocin)
1456	DRAMP00197	Microcin 24 (Mcc24; Bacteriocin)
1502	DRAMP00250	Bacteriocin serracin-P 43 kDa subunit (Bacteriocin)
1503	DRAMP00251	Bacteriocin serracin-P 23 kDa subunit (Bacteriocin)
1574	DRAMP00341	Antifungal protein ginkbilobin-1 (Ginkbilobin, GNL; Plants)
1609	DRAMP00393	Hedyotide B2 (hB2; Uncyclotides; Plants)
1612	DRAMP00397	Defensin D1 (Ns-D1; Plant defensin)
1613	DRAMP00398	Defensin D2 (Ns-D2; Plant defensin)
1617	DRAMP00402	Defensin D1 (So-D1; Antimicrobial peptide D1; Plant defensin)
1618	DRAMP00403	Defensin D2 (So-D2; Antimicrobial peptide D2; Plant defensin)
1621	DRAMP00406	Defensin D5 (So-D5; Antimicrobial peptide D5; Plant defensin)
1622	DRAMP00407	Defensin D6 (So-D6; Antimicrobial peptide D6; Plant defensin)
1624	DRAMP00409	Defensin-like protein (Sesquin; Plant defensin)
1661	DRAMP00455	Defensin-like protein 2 (Fabatin-2; Plant defensin)
1662	DRAMP00456	Defensin-like protein 1 (Fabatin-1; Plant defensin)
1952	DRAMP00746	Flower-specific defensin (NaD1; Plant defensin)
1969	DRAMP00767	ChaC1 (Chassatide C1; Plant defensin)
1970	DRAMP00768	ChaC2 (Chassatide C2; Plant defensin)
1971	DRAMP00769	ChaC4 (Chassatide C4; Plant defensin)
1972	DRAMP00770	ChaC10 (Chassatide C10; Plant defensin)
1976	DRAMP18325	delta-lysin I (Bacteriocin)
1977	DRAMP00796	Cliotide T2 (cT2; Plant defensin)
1978	DRAMP00797	Cliotide T3 (cT3; Plant defensin)
1984	DRAMP18324	Warnericin RK (Bacteriocin)
2071	DRAMP00937	Tu-AMP1 (Plant defensin)
2072	DRAMP00938	Tu-AMP2 (Plant defensin)
2077	DRAMP00957	Pp-AMP1 (P. pubescens AMP1; Plant defensin)
2078	DRAMP00958	Pp-AMP2 (P. pubescens AMP2; Plant defensin)
2081	DRAMP01380	Odorranain-J1 (OdJ1; Frogs, amphibians, animals)
2090	DRAMP00980	Antimicrobial peptide 1a (WAMP-1a; Plant defensin)
2091	DRAMP00981	Antimicrobial peptide 1b (WAMP-1b; Plant defensin)
2092	DRAMP00982	Fa-AMP1 (Fagopyrum antimicrobial peptide 1; hevein-type; Plant defensin)
2093	DRAMP00983	Fa-AMP2 (Fagopyrum antimicrobial peptide 2; hevein-type; Plant defensin)
2103	DRAMP00993	WjAMP-1 (C-terminal domain of hevein; Plant defensin)
2107	DRAMP00997	IB-AMP4 (IBAMP4; Basic peptide AMP4; Plants)
2108	DRAMP00998	Antimicrobial peptide MBP-1 (Maize Basic Peptide 1; Plant defensin)
2118	DRAMP01009	Alliumin (Plants)
2119	DRAMP01010	Lunatusin (Plants)
2123	DRAMP01015	VaD1 (Plant defensin)
2126	DRAMP01022	Cy-AMP1 (Plant defensin)
2127	DRAMP01023	Cy-AMP2 (Plant defensin)
2128	DRAMP01024	Cy-AMP3 (Plant defensin)
2149	DRAMP01046	Shepherin I (fragement of shep-GRP; Plants)
2150	DRAMP01047	shepherin II (fragement of shep-GRP; Plants)
2186	DRAMP01099	Bombinin-like peptide 4 (BLP-4; toads, amphibians, animals)
2187	DRAMP01100	Bombinin-like peptide 1 (Contains: Bombinin H; toads, amphibians, animals)
2217	DRAMP01140	Uperin-2.2 (toads, amphibians, animals)
2237	DRAMP01171	Ocellatin-1 (Frogs, amphibians, animals)

2238	DRAMP01172	Ocellatin-2 (Frogs, amphibians, animals)
2239	DRAMP01173	Ocellatin-3 (Frogs, amphibians, animals)
2240	DRAMP01175	Ocellatin-5 (Frogs, amphibians, animals)
2250	DRAMP01196	Andersonin-G1 (Frogs, amphibians, animals)
2251	DRAMP01197	Andersonin-N1 (Frogs, amphibians, animals)
2252	DRAMP01198	Andersonin-Q1 (Frogs, amphibians, animals)
2255	DRAMP01207	Galensin (Frogs, amphibians, animals)
2258	DRAMP01212	Pleurain-A3 (Pleurain A3; Frogs, amphibians, animals)
2259	DRAMP01213	Pleurain-A4 (Pleurain A4; Frogs, amphibians, animals)
2264	DRAMP01223	Palustrin-2AJ2 (PL2AJ12; Frogs, amphibians, animals)
2265	DRAMP01224	Palustrin-2AR (Palustrin-2ARa; Ranatuerin-2SEa; Frogs, amphibians, animals)
2267	DRAMP01226	Palustrin-1a (Frogs, amphibians, animals)
2268	DRAMP01230	Palustrin-2a (Frogs, amphibians, animals)
2285	DRAMP18404	Polybia-MPII (mastoparan; insects, arthropods, invertebrates, animals)
2287	DRAMP18402	Tridecaptin B1 (TriB1; lipopeptides; nonribosomally synthesized peptide antibiotic)
2343	DRAMP01338	Amolopin-1a (Frogs, amphibians, animals)
2345	DRAMP01342	Amolopin-2b (Frogs, amphibians, animals)
2346	DRAMP01343	Amolopin-1c (Frogs, amphibians, animals)
2347	DRAMP01344	Amolopin-2c (Frogs, amphibians, animals)
2348	DRAMP01345	Amolopin-1d (Frogs, amphibians, animals)
2363	DRAMP01444	Nigrocin-1 (Frogs, amphibians, animals)
2364	DRAMP01445	Nigrocin-2 (Nigrocin-2LVa; Frogs, amphibians, animals)
2365	DRAMP01446	Proteinase inhibitor PSKP-1 (Frogs, amphibians, animals)
2372	DRAMP01463	Esculentin-1SEa (Frogs, amphibians, animals)
2373	DRAMP01464	Esculentin-1SEb (Frogs, amphibians, animals)
2374	DRAMP01465	Esculentin-1R (Frogs, amphibians, animals)
2382	DRAMP01489	Esculentin-1A (Frogs, amphibians, animals)
2383	DRAMP01492	Esculentin-IIb (Frogs, amphibians, animals)
2388	DRAMP18312	Propionicin PLG-1(Bacteriocin)
2392	DRAMP01519	Esculentin-2PRa (Frogs, amphibians, animals)
2475	DRAMP01671	Dermaseptin-4 (DS IV; Dermaseptin-S4, DS4; Frogs, amphibians, animals)
2501	DRAMP01697	Dermaseptin-DA4 (DRS-DA4; Frogs, amphibians, animals)
2503	DRAMP01700	Dermaseptin-H3 (Dermaseptin-like peptide 3, DMS3; Frogs, amphibians, animals)
2518	DRAMP01717	Dermatoxin (Frogs, amphibians, animals)
2530	DRAMP02857	Indolicidin (Cathelicidin-4; mammals, animals)
2532	DRAMP02819	Anoplin (Insects, arthropods, invertebrates, animals)
2533	DRAMP04395	EP3 (Earthworm,animals)
2534	DRAMP04394	EP2 (Earthworm,animals)
2548	DRAMP01772	Temporin-1Ob (Frogs, amphibians, animals)
2560	DRAMP01799	Temporin-1PRa (Temporin 1PRa; Frogs, amphibians, animals)
2561	DRAMP01800	Temporin-1PRb (Temporin 1PRb; Frogs, amphibians, animals)
2562	DRAMP01801	Temporin-1DYa (Frogs, amphibians, animals)
2563	DRAMP01802	Temporin-PTa (Frogs, amphibians, animals)
2565	DRAMP01804	Temporin-CDYb (Brevinin-1CDYb; Frogs, amphibians, animals)
2571	DRAMP01387	Odorranain-P2a (OdP2a; Frogs, amphibians, animals)
2572	DRAMP01386	Odorranain-P1a (OdP1a; Brevinin-1HS1; Brevinin-1-OA2; Frogs, amphibians, animals)
2573	DRAMP01824	Temporin-1Ja (Frogs, amphibians, animals)
2576	DRAMP01126	Maximin-H4 (Toads, amphibians, animals)
2577	DRAMP01125	Maximin-H3 (Toads, amphibians, animals)
2584	DRAMP01841	Ascaphin-2 (Frogs, amphibians, animals)

2585	DRAMP01843	Ascaphin-4 (Frogs, amphibians, animals)
2586	DRAMP01845	Ascaphin-6 (Frogs, amphibians, animals)
2609	DRAMP01901	Brevinin-2CG1 (Frogs, amphibians, animals)
2621	DRAMP01924	Brevinin-1Ea (Frogs, amphibians, animals)
2622	DRAMP01925	Brevinin-1Eb (Frogs, amphibians, animals)
2623	DRAMP01926	Brevinin-1Ec (Frogs, amphibians, animals)
2624	DRAMP01927	Brevinin-2Ea (Frogs, amphibians, animals)
2625	DRAMP01928	Brevinin-2Eb (Frogs, amphibians, animals)
2626	DRAMP01929	Brevinin-2Ec (Frogs, amphibians, animals)
2628	DRAMP01931	Brevinin-2Ed (Frogs, amphibians, animals)
2629	DRAMP01932	Brevinin-2Ee (Frogs, amphibians, animals)
2630	DRAMP01936	Brevinin-2Ek (Frogs, amphibians, animals)
2631	DRAMP01945	Brevinin-1SE (Frogs, amphibians, animals)
2632	DRAMP01946	Brevinin-20a (Frogs, amphibians, animals)
2633	DRAMP01947	Brevinin-20b (Frogs, amphibians, animals)
2634	DRAMP18294	lactococcin Z(Bacteriocin)
2635	DRAMP01952	Brevinin-1PTb (Frogs, amphibians, animals)
2636	DRAMP01954	Brevinin-2HSb (Frogs, amphibians, animals)
2637	DRAMP01958	Brevinin-2PTd (Frogs, amphibians, animals)
2638	DRAMP01960	Brevinin-1BYa (Frogs, amphibians, animals)
2639	DRAMP01961	Brevinin-1BYb (Frogs, amphibians, animals)
2642	DRAMP01966	Brevinin-1Ya (Frogs, amphibians, animals)
2643	DRAMP01967	Brevinin-1Yb (Frogs, amphibians, animals)
2647	DRAMP01976	Brevinin-2Eg (Frogs, amphibians, animals)
2654	DRAMP18291	Garviecin LG34(Bacteriocin)
2674	DRAMP02012	Brevinin-1T (Brevinin-2T; Frogs, amphibians, animals)
2675	DRAMP02013	Brevinin-1Ta (Frogs, amphibians, animals)
2678	DRAMP02016	Brevinin-1DYa (Frogs, amphibians, animals)
2679	DRAMP02017	Brevinin-2DYa (Frogs, amphibians, animals)
2680	DRAMP02018	Brevinin-1DYb (Brevinin-1CDYb; Frogs, amphibians, animals)
2681	DRAMP02020	Brevinin-1DYc (Frogs, amphibians, animals)
2684	DRAMP18281	Plantaricin KL-1Y (Bacteriocin)
2694	DRAMP02089	Brevinin-1La (Brevinin-1PRd; Frogs, amphibians, animals)
2695	DRAMP01124	Maximin-H2 (Toads, amphibians, animals)
2696	DRAMP01123	Maximin-H1 (Toads, amphibians, animals)
2697	DRAMP01111	Maximin-5 (Toads, amphibians, animals)
2698	DRAMP01110	Maximin-4 (Toads, amphibians, animals)
2699	DRAMP01109	Maximin-3 (Toads, amphibians, animals)
2700	DRAMP02100	Brevinin-1Pe (Frogs, amphibians, animals)
2706	DRAMP18272	Enterocin AS-48RJ (Bacteriocin)
2718	DRAMP02132	Antimicrobial peptide 3 (XT-3; Levitide-like peptide; Frogs, amphibians, animals)
2719	DRAMP02134	Antimicrobial peptide 5 (XT-5; PGLa-like peptide; Frogs, amphibians, animals)
2806	DRAMP02243	Ranatuerin-1T (Brevinin-2T; Frogs, amphibians, animals)
2810	DRAMP02249	Ranatuerin-2SEB (Frogs, amphibians, animals)
2811	DRAMP02250	Ranatuerin-2SEC (Frogs, amphibians, animals)
2812	DRAMP02253	Ranatuerin-IIbYb (Ranatuerin-2bYa; Frogs, amphibians, animals)
2813	DRAMP02258	Ranatuerin-IIbYa (Ranatuerin-2bYa; Frogs, amphibians, animals)
2818	DRAMP02284	Pseudin-1 (Pseudin 1; Frogs, amphibians, animals)
2819	DRAMP02285	Pseudin-2 (Pseudin 2; Frogs, amphibians, animals)
2820	DRAMP02286	Pseudin-3 (Pseudin 3; Frogs, amphibians, animals)

2821	DRAMP02287	Pseudin-4 (Pseudin 4; Frogs, amphibians, animals)
2829	DRAMP02309	Signiferin-2.2 (Frogs, amphibians, animals)
2841	DRAMP02332	Piscidin-3 (Pis-3; fish, chordates, animals)
2851	DRAMP01375	Odorranain-E1 (OdE1; Frogs, amphibians, animals)
2854	DRAMP02353	Pleurocidin-like peptide WFX (fish, chordates, animals; Predicted)
2857	DRAMP02369	Liver-expressed antimicrobial peptide 2 (fish, chordates, animals)
2868	DRAMP02245	Ranatuerin-2Cb (Ranatuerin 2Cb; Frogs, amphibians, animals)
2869	DRAMP02398	Antimicrobial peptide GP-19 (GP-19)
2871	DRAMP02400	Antimicrobial peptide AJN-10 (AJN-10)
2876	DRAMP02407	Napin-like polypeptide (Contains: Napin-like polypeptide small chain and large chain)
2890	DRAMP02437	Papillosin
2891	DRAMP02438	Halocyntin
2896	DRAMP02447	Antimicrobial protein 2 (Antimicrobial protein AN5-2)
2901	DRAMP02453	S. litura moricin (Sl moricin; Insects, animals)
2902	DRAMP02454	Theromacin (Arthropods, animals)
2904	DRAMP02457	L-amino-acid oxidase (Balt-LAAO-I; LAAO; LAO; snakes, reptils, animals)
2909	DRAMP02462	L-amino-acid oxidase (LAAO; LAO; snakes, reptils, animals)
2925	DRAMP02483	Cathelicidin-NA (Cathelicidin-related protein; Snakes, reptiles, animals)
2951	DRAMP02511	Crotamine (defensin-like toxin; Snakes, reptiles, animals)
2954	DRAMP02521	Cathelicidin-OH (Cathelicidin-related protein; Snakes, reptiles, animals)
2999	DRAMP18253	Piscicocin CS526(Bacteriocin)
3003	DRAMP02570	Penaeidin-1 (Pen-1; shrimps, Arthropods, animals)
3004	DRAMP02571	Penaeidin-2a (Pen-2a; shrimps, Arthropods, animals)
3006	DRAMP02575	Penaeidin-3b (Pen-3b; shrimps, Arthropods, animals)
3007	DRAMP02576	Penaeidin-3c (Pen-3c; shrimps, Arthropods, animals)
3023	DRAMP02597	Clavanin-A (His-rich; chordates, animals)
3025	DRAMP02599	Clavanin-C (His-rich; chordates, animals)
3026	DRAMP02600	Clavanin-D (His-rich; chordates, animals)
3027	DRAMP02601	Clavanin E (His-rich; chordates, animals)
3028	DRAMP02602	Clavaspirin (chordates, animals)
3050	DRAMP18250	Laterosporulin (Bacteriocin)
3052	DRAMP18248	Bifidin I(Bacteriocin)
3053	DRAMP18249	Bac-GM100 (Bacteriocin)
3059	DRAMP18247	Bacthuricin F4(Bacteriocin)
3065	DRAMP02642	Rhesus theta-defensin 1 (RTD-1; primates, mammals, animals)
3066	DRAMP02643	Rhesus theta-defensin 2 (RTD-2; primates, mammals, animals)
3067	DRAMP02644	Rhesus theta-defensin 3 (RTD-3; primates, mammals, animals)
3075	DRAMP02653	Neutrophil defensin 1 (RMAD-1; primates, mammals, animals)
3076	DRAMP02654	Neutrophil defensin 2 (RMAD-2; primates, mammals, animals)
3081	DRAMP02659	Neutrophil defensin 3 (RMAD-3; primates, mammals, animals)
3082	DRAMP02660	Neutrophil defensin 4 (RMAD-4; primates, mammals, animals)
3083	DRAMP02661	Neutrophil defensin 5 (RMAD-5; primates, mammals, animals)
3084	DRAMP02662	Neutrophil defensin 6 (RMAD-6; primates, mammals, animals)
3085	DRAMP02663	Neutrophil defensin 7 (RMAD-7; primates, mammals, animals)
3119	DRAMP02698	Rhesus macaque oral alpha-defensins (ROADs; primates, mammals, animals)
3128	DRAMP18242	Fengycin B2 (Bacteriocin)
3130	DRAMP18240	Fengycin C(Bacteriocin)
3131	DRAMP18241	Subtilomycin(Bacteriocin)
3132	DRAMP18239	Fengycin A2(Bacteriocin)
3133	DRAMP18238	Fengycin B(Bacteriocin)

3144	DRAMP18237	Fengycin A(Bacteriocin)
3157	DRAMP02739	TEWP (turtle egg-white protein; Reptiles, animals)
3158	DRAMP02741	Pelovaterin (defensin-like AMP; Gly-rich; Reptiles, animals)
3159	DRAMP02742	Defensin-like turtle egg white protein TEWP (TEWP; Reptiles, animals)
3168	DRAMP03743	Androctonin (Arthropods, animals)
3171	DRAMP02755	Ponericin G3 (ants, insects, animals)
3172	DRAMP02756	Ponericin G4 (ants, insects, animals)
3174	DRAMP02758	Ponericin G6 (ants, insects, animals)
3176	DRAMP02760	Ponericin-L1 (ants, insects, animals)
3177	DRAMP02761	Ponericin-L2 (ants, insects, animals)
3178	DRAMP02762	Ponericin-W1 (ants, insects, animals)
3180	DRAMP02764	Ponericin-W3 (ants, insects, animals)
3181	DRAMP02765	Ponericin-W4 (ants, insects, animals)
3182	DRAMP02766	Ponericin-W5 (ants, insects, animals)
3183	DRAMP02767	Ponericin-W6 (ants, insects, animals)
3184	DRAMP02770	Pilosulin 3 (ants, insects, animals)
3185	DRAMP02771	Pilosulin 4 (ants, insects, animals)
3189	DRAMP02781	Coleoptericin (Insects, animals)
3191	DRAMP02783	Peptide C (Insects, animals)
3192	DRAMP02784	Acaloleptin-A1 (chain of Acaloleptin A; Insects, animals)
3193	DRAMP02785	Acaloleptin-A2 (chain of Acaloleptin A; Insects, animals)
3194	DRAMP02786	Acaloleptin-A3 (chain of Acaloleptin A; Insects, animals)
3195	DRAMP02787	Acaloleptin-A4 (chain of Acaloleptin A; Insects, animals)
3196	DRAMP02788	Acaloleptin-A5 (chain of Acaloleptin A; Insects, animals)
3210	DRAMP02803	Mytilin-A (molluscas, animals)
3211	DRAMP02804	Mytilus defensin-B (molluscas, animals)
3219	DRAMP18235	Gageopeptide D(Bacteriocin)
3220	DRAMP02816	Lumbrican
3221	DRAMP02818	Dicynthaurin
3234	DRAMP02832	Reactive oxygen species modulator 1 (ROS modulator 1; mammals, animals)
3236	DRAMP02834	Seminalplasmin (Calcium transport inhibitor; Peptide YY-2; mammals, animals)
3238	DRAMP02837	Beta-defensin C7 (BBD(C7); mammals, animals)
3239	DRAMP18233	Gageopeptide B(Bacteriocin)
3240	DRAMP18234	Gageopeptide C(Bacteriocin)
3247	DRAMP02853	Cathelicidin-3 (Bactenecin-7, Bac7; PR-59; mammals, animals)
3249	DRAMP01356	Ranalexin-1Ca (Ranatuerin 1Ca; Frogs, amphibians, animals)
3250	DRAMP02864	Bovine Beta-defensin 7 (bBD-7; BNBD-7; BNDB-7; mammals, animals)
3251	DRAMP02871	Beta-defensin 119 (Defensin, beta 119; mammals, animals)
3276	DRAMP02902	L-amino-acid oxidase (LAAO, LAO; BpirLAAO-I; reptilia, animals)
3281	DRAMP02914	Cathelicidin-1 (Bactenecin-1, Bac1; Cyclic dodecapeptide; mammals, animals)
3291	DRAMP02929	Antimicrobial protein 1
3292	DRAMP02930	Antimicrobial protein 2 (crabs, Arthropods, animals)
3294	DRAMP02936	Big defensin (crabs, Arthropods, animals)
3295	DRAMP02937	Tachycitin (crabs, Arthropods, animals)
3296	DRAMP18230	Gageotetrin B (Bacteriocin)
3297	DRAMP18231	Gageotetrin C (Bacteriocin)
3298	DRAMP18232	Gageopeptide A(Bacteriocin)
3301	DRAMP02941	Tachystatin-A1 (crabs, Arthropods, animals)
3302	DRAMP02942	Tachystatin-A2 (crabs, Arthropods, animals)
3305	DRAMP02945	Tachystatin-C (crabs, Arthropods, animals)

3308	DRAMP18228	Gageostatin C (Bacteriocin)
3309	DRAMP18229	Gageotetrin A (Bacteriocin)
3312	DRAMP02955	Hedistin (marine annelid, Metazoa)
3316	DRAMP02968	Prophenin-1 (C6, PF-1; Pro-rich; pigs, mammals, animals)
3317	DRAMP02969	Prophenin-2 (C12, PF-2, PR-2; Pro-rich; pigs, mammals, animals)
3318	DRAMP02971	Protegrin-2 (Protegrin 2; PG-2; pigs, mammals, animals)
3319	DRAMP02972	Protegrin-3 (Protegrin 3; PG-3; pigs, mammals, animals)
3322	DRAMP02976	Beta-defensin 1 (BD-1; Defensin, beta 1; pigs, mammals, animals)
3327	DRAMP02982	Reactive oxygen species modulator 1 (ROS modulator 1; pigs, mammals, animals)
3329	DRAMP02984	Neutrophil cationic antibacterial polypeptide of 11 kDa (CAP11; pigs, mammals, animals)
3330	DRAMP02985	Neutrophil cationic peptide 2 (CP-2; GNCP-2; pigs, mammals, animals)
3331	DRAMP02986	Neutrophil cationic peptide 1 (GPNP; Antiviral defensin; pigs, mammals, animals)
3334	DRAMP02989	Lasioglossin LL-I (Insects, animals)
3335	DRAMP02990	Lasioglossin LL-II (Insects, animals)
3336	DRAMP02991	Lasioglossin LL-III (Insects, animals)
3339	DRAMP03004	Abaecin (Insects, animals)
3340	DRAMP03005	Apidaecin (Insects, animals)
3341	DRAMP03006	Defensin (Insects, animals)
3348	DRAMP03023	Mastoparan (Protonectarina-MP; Insects, animals)
3349	DRAMP18227	Gageostatin B (Bacteriocin)
3355	DRAMP03049	Eumenine mastoparan-OD (EMP-OD; Venom peptide 1, OdVP1; Insects, animals)
3360	DRAMP03062	Defensin-A (DefA; GmDefA; Insects, animals)
3366	DRAMP18226	Gageostatin A (Bacteriocin)
3367	DRAMP03071	Diptericin-A (Insects, animals)
3368	DRAMP03072	Diptericin-D (Insects, animals)
3369	DRAMP03073	Sapecin-C (Sapecin C; defensins; Insects, animals)
3378	DRAMP03083	Sapecin-B (defensins; Insects, animals)
3379	DRAMP03084	Ceratotoxin-B (Insects, animals)
3380	DRAMP03085	Ceratotoxin-A (Insects, animals)
3381	DRAMP03086	Ceratotoxin-D (Insects, animals)
3390	DRAMP03101	Sarcotoxin-1C (Sarcotoxin IC; Insects, animals)
3391	DRAMP03102	Sarcotoxin-1B (Sarcotoxin IB; Insects, animals)
3400	DRAMP18223	Sonorensin(Bacteriocin)
3418	DRAMP03134	Defensin-D (AaeDefD; Insects, animals)
3433	DRAMP03154	Hadrurin (Non-disulfide-bridged peptide 3.1)
3436	DRAMP03157	Halocidin subunit B
3437	DRAMP03158	Halocidin subunit A (invertebrates, animals; Preclinical)
3441	DRAMP03163	R. prolixus defensin A (RprDefA; insect defensin; Insects, animals)
3442	DRAMP03164	R. prolixus defensin B (RprDefB; insect defensin; Insects, animals)
3443	DRAMP03165	R. prolixus defensin C (RprDefC; insect defensin; Insects, animals)
3449	DRAMP03174	Arenicin-2 (Ar-2; marine polychaeta, animals)
3450	DRAMP03175	Perinerin
3454	DRAMP03180	ASABF-alpha (ASABF; nematodes, animals)
3456	DRAMP03183	Naegleriapore A
3457	DRAMP03184	Naegleriapore B
3459	DRAMP03188	Antibacterial protein LL-37 (primates, mammals, animals)
3465	DRAMP03199	PhD1 (PhD-1; Defensin-1; primates, mammals, animals)
3466	DRAMP03200	PhD2 (PhD-2; Defensin-2; primates, mammals, animals)
3467	DRAMP03201	PhD3 (PhD-3; Defensin-3; primates, mammals, animals)
3473	DRAMP03208	BTD-1 (theta-defensin; primates, mammals, animals)

3474	DRAMP03209	BTD-2 (theta-defensin; primates, mammals, animals)
3476	DRAMP03211	BTD-4 (theta-defensin; primates, mammals, animals)
3477	DRAMP03212	BTD-7 (theta-defensin; primates, mammals, animals)
3485	DRAMP03224	M-ctenitoxin-Cs1c (M-CNTX-Cs1c; Cupiennin-1c; spiders, Arthropods, animals)
3489	DRAMP03238	M-zodatoxin-Lt8c (M-ZDTX-Lt8c; Cytoinsectotoxin-1c, CIT-1c; spiders, Arthropods, animals)
3492	DRAMP03241	M-zodatoxin-Lt8f (M-ZDTX-Lt8f; Cytoinsectotoxin-1f, CIT-1f; spiders, Arthropods, animals)
3493	DRAMP03242	M-zodatoxin-Lt8g (M-ZDTX-Lt8g; Cytoinsectotoxin-1g, CIT-1g; spiders, Arthropods, animals)
3494	DRAMP03243	M-zodatoxin-Lt8h (M-ZDTX-Lt8h; Cytoinsectotoxin-1h, CIT-1h; spiders, Arthropods, animals)
3514	DRAMP18213	Gramicidin S(Bacteriocin)
3525	DRAMP03282	Turkey Heterophil Peptide 1 (Antimicrobial peptide THP1; Birds, animals)
3543	DRAMP03304	Hydramacin-1 (Hm-1; annelida, animals)
3554	DRAMP03317	Cathelicidin-related antimicrobial peptide (AMPs)
3565	DRAMP03329	Alpha-defensin cryptdin-1 (Crp1; Rodents, mammals, animals)
3566	DRAMP03330	Alpha-defensin cryptdin-2 (Defensin-related cryptdin-2; Rodents, mammals, animals)
3567	DRAMP03331	Alpha-defensin cryptdin-3 (Defensin-related cryptdin-3; Rodents, mammals, animals)
3568	DRAMP03332	Alpha-defensin cryptdin-4 (Defensin-related cryptdin4; Rodents, mammals, animals)
3569	DRAMP03333	Alpha-defensin cryptdin-5 (Defensin-related cryptdin5; Rodents, mammals, animals)
3570	DRAMP03334	Alpha-defensin cryptdin-6/12 (Defensin-related cryptdin-6/12; Rodents, mammals, animals)
3593	DRAMP03357	Cryptdin related sequence peptide (CRS4C-1a; Rodents, mammals, animals)
3594	DRAMP03358	Cryptdin related sequence peptide (CRS4C-1d; Rodents, mammals, animals)
3595	DRAMP03359	Cryptdin related sequence peptide (CRS4C-2; Rodents, mammals, animals)
3596	DRAMP03360	Cryptdin related sequence peptide (CRS4C-2b; Rodents, mammals, animals)
3598	DRAMP03362	CRS4C-3c (Cryptdin related sequence peptide; Rodents, mammals, animals)
3602	DRAMP03366	Beta-defensin 1 (BD-1; mBD-1; Defensin, beta 1; Rodents, mammals, animals)
3604	DRAMP03368	Beta-defensin 3 (BD-3, mBD-3; Defensin, beta 3; Rodents, mammals, animals)
3605	DRAMP03369	Beta-defensin 4 (BD-4, mBD-4; Defensin, beta 4; Rodents, mammals, animals)
3608	DRAMP03373	Beta-defensin 8 (BD-8, mBD-8; Defensin, beta 8; Rodents, mammals, animals)
3628	DRAMP03393	Beta-defensin 38 (BD-38, mBD-38; Defensin, beta 38; Rodents, mammals, animals)
3634	DRAMP03399	Sperm-associated antigen 11 (Rodents, mammals, animals)
3635	DRAMP03400	WAP four-disulfide core domain protein 12 (Rodents, mammals, animals)
3637	DRAMP03402	WAP four-disulfide core domain protein 15B (Elafin-like protein I; Rodents, mammals, animals)
3639	DRAMP03407	Defr1 (Murine beta-defensin related peptide; Rodents, mammals, animals)
3640	DRAMP03408	Neutrophil defensin 1 (HANP-1; alpha-defensin; Rodents, mammals, animals)
3641	DRAMP03409	Neutrophil defensin 2 (HANP-2; alpha-defensin; Rodents, mammals, animals)
3642	DRAMP03410	Neutrophil defensin 3 (HANP-3; alpha-defensin; Rodents, mammals, animals)
3643	DRAMP03411	Neutrophil defensin 4 (HANP-4; alpha-defensin; Rodents, mammals, animals)
3651	DRAMP03420	Neutrophil antibiotic peptide NP-2 (RatNP-2; Rodents, mammals, animals)
3652	DRAMP03421	Neutrophil antibiotic peptide NP-3 (RatNP-3a, RatNP-3b; Rodents, mammals, animals)
3653	DRAMP03423	[illegible]IN1b (Sperm-associated antigen 11; Antimicrobial-like protein Bin-1b; Rodents, mammals, animals
3654	DRAMP03424	Beta-defensin 1 (BD-1, RBD-1; Defensin, beta 1; Rodents, mammals, animals)
3675	DRAMP03445	Beta-defensin 30 (BD-30, RBD-30; Defensin, beta 30; Rodents, mammals, animals)
3696	DRAMP03470	Defensin-1 (American oyster defensin, AOD; molluscs, animals)
3712	DRAMP03491	Viresin (Insects, animals)
3721	DRAMP03501	La-LTP (LJAFP; Insects, animals)
3728	DRAMP03510	Cecropin-A (Insects, animals)
3729	DRAMP03511	Cecropin-B (Immune protein P9; Insects, animals)
3730	DRAMP03512	Cecropin-D (Cecropin D; Insects, animals)
3737	DRAMP03534	Antibacterial peptide enbocin (Moricin; Insects, animals)
3738	DRAMP03535	Lebocin-1/2 (Pro-rich; Insects, animals)
3739	DRAMP03536	Lebocin-3 (LEB 3; Insects, animals)

3742	DRAMP03554	CCL20(1-67) (Human, mammals, animals)
3743	DRAMP03555	CCL20(2-70) (Human, mammals, animals)
3744	DRAMP03556	C-C motif chemokine 20 (Human, mammals, animals)
3749	DRAMP03564	Human hepcidin-20 (Hepc20; one chain of Hepcidin; Human, mammals, animals)
3750	DRAMP03565	Human hepcidin-25 (Hepc25; one chain of Hepcidin; Human, mammals, animals)
3751	DRAMP03566	Salvic (Human, mammals, animals)
3762	DRAMP03585	Human TC-1 (Chain of Platelet basic protein; Human, mammals, animals)
3763	DRAMP03586	Human TC-2 (Chain of Platelet basic protein; Human, mammals, animals)
3764	DRAMP03587	DCD-1 (chain of Dermcidin; Human, mammals, animals)
3765	DRAMP03588	Human MUC7 20-Mer (Human, mammals, animals)
3767	DRAMP03590	Calcitermin (Human, mammals, animals)
3768	DRAMP03591	Neutrophil defensin 1 (Defensin, alpha 1; HNP-1, HP-1; Human, mammals, animals)
3769	DRAMP03592	Neutrophil defensin 2 (HNP-2, HP-2, HP2; Human, mammals, animals)
3770	DRAMP03593	Neutrophil defensin 3 (Defensin, alpha 3; HNP-3, HP-3, HP3; Human, mammals, animals)
3771	DRAMP03594	Neutrophil defensin 4 (Defensin, alpha 4; HNP-4, HP-4; Human, mammals, animals)
3772	DRAMP03595	Human defensin-5 (HD-5; Defensin, alpha 5; Human, mammals, animals)
3773	DRAMP03596	Human defensin-6 (HD-6; Defensin, alpha 6; Human, mammals, animals)
3774	DRAMP03601	Human beta-defensin 26 (hBD-26; hBD26; Human, mammals, animals)
3775	DRAMP03602	Human beta-defensin 27 (hBD-27; hBD27; Human, mammals, animals)
3777	DRAMP18203	Panusin (beta defensins; crustaceans, arthropods, invertebrates, animals)
3789	DRAMP18120	BnPRP1 (Plant defensin)
3799	DRAMP18140	VG16KRKP
3802	DRAMP18194	AAEL000598-PA
3803	DRAMP03635	Human lactoferricin (LfcinH; one chain of Lactotransferrin; Human, mammals, animals)
3804	DRAMP03636	Kaliocin-1 (one chain of Lactotransferrin; Human, mammals, animals)
3806	DRAMP03639	Astexin-1 (lasso peptide)
3808	DRAMP03641	Longicornsin (defensin-like; Arthropods, invertebrates, animals)
3810	DRAMP03644	Cathelicidin-1 (CATH-1; Fowlicidin-1; Birds, animals)
3811	DRAMP03648	Gallinacin-1 (Gal-1; Beta-defensin 1; Birds, animals)
3812	DRAMP03649	Gallinacin-1 alpha (Gal-1 alpha; Antimicrobial peptide CHP2; Birds, animals)
3813	DRAMP03650	Gallinacin-2 (Gal-2; Beta-defensin 2; Birds, animals)
3815	DRAMP03652	Gallinacin-4 (Gal-4; Beta-defensin 4; Birds, animals)
3816	DRAMP03653	Gallinacin-5 (Gal-5; Beta-defensin 5; Birds, animals)
3817	DRAMP03654	Gallinacin-6 (Gal 6; Beta-defensin 6; Birds, animals)
3818	DRAMP03655	Gallinacin-7 (Gal 7; Beta-defensin 7; Birds, animals)
3823	DRAMP03661	Gallinacin-13 (Gal-13; Beta-defensin 13; Birds, animals)
3825	DRAMP03663	cLEAP-2 (Chicken LEAP-2; Birds, animals)
3826	DRAMP03664	L-amino-acid oxidase (LAAO, LAO, TM-LAO; reptilia, animals)
3836	DRAMP03674	Cystatin-1 (Cystatin-I)
3839	DRAMP03680	Cathelicidin-3.4 (Bactenecin-3.4, Bac3.4; ChBac3.4; ruminant, animals)
3843	DRAMP03692	Defensin-1 (Cll-dlp; Arthropods, animals)
3871	DRAMP03736	Opistoporin-2 (OP2; Non-disulfide-bridged peptide 3.6, NDBP-3.6; Arthropods, animals)
3872	DRAMP03737	Opistoporin-4 (Non-disulfide-bridged peptides 3.7, NDBP-3.7; Arthropods, animals)
3873	DRAMP03739	Buthinin (Sahara scorpion; Arthropods, animals)
3874	DRAMP03740	Androctonus defensin (4 kDa defensin; Arthropods, animals)
3875	DRAMP03741	Ponericin-W-like 32.1 (Arthropods, animals)
3876	DRAMP03742	Ponericin-W-like 32.2 (Arthropods, animals)
3880	DRAMP03755	Potassium channel toxin alpha-KTx 1.1 (ChTX-Lq1; charybdotoxin; Arthropods, animals)
3891	DRAMP03766	Heteroscorpine-1 (HS-1; defensins; Arthropods, animals)
3905	DRAMP03785	Neuropeptide-like protein 29 (NLP-29; nematodes, animals)

3907	DRAMP03787	Neuropeptide-like protein 31 (NLP-31; nematodes, animals)
3910	DRAMP03790	ABF-2 (nematodes, animals)
3917	DRAMP03798	Corticostatin-related peptide RK-1 (RK-1; lagomorphs, mammals, animals)
3918	DRAMP03799	Rabbit kidney defensin RK-2 (alpha-defensins; lagomorphs, mammals, animals)
3944	DRAMP18190	Pantinin-3 (Non-disulfide-bridged peptide 4.22, NDBP-4.22, Non-disulfide-bridged peptide 5.23)
3946	DRAMP03850	Antibacterial peptide/melittin homolog
3948	DRAMP03872	hLf 21-30 (fragment of human lalctoferricin, residues 21-30)
3949	DRAMP03873	mLf 20-29 (fragment of murine lalctoferricin, residues 20-29)
3950	DRAMP03874	pLf20-29 (fragment of porcine lactoferricin, residues 20-29)
3961	DRAMP03941	Peptide 3 (Trp- and Arg-rich; derivative of Tritrpticin)
3962	DRAMP03942	Peptide 2 (Trp- and Arg-rich; derivative of Tritrpticin)
3963	DRAMP03943	Gratisin analogue
3965	DRAMP03946	Del 1-3 (Ranalexin analog)
3970	DRAMP03961	KR-12
3976	DRAMP02092	Brevinin-1Bb (Frogs, amphibians, animals)
3978	DRAMP18189	Pantinin-2 (Non-disulfide-bridged peptide 4.21, NDBP-4.21, Non-disulfide-bridged peptide 5.22)
3982	DRAMP04018	Rp-1
3983	DRAMP04037	Immobilized peptide E07LKK
3984	DRAMP04038	Immobilized peptide E14LKK/H14LKK
3985	DRAMP04039	Immobilized peptige E16KGL/H16KGL
3986	DRAMP04040	Immobilized peptide E17KGG
3987	DRAMP04041	Immobilized peptide E18KGG
3988	DRAMP04042	Immobilized peptide E16LKL
3989	DRAMP04043	Immobilized peptid E10KKL
3990	DRAMP04044	Immobilized peptid E12LLK
3991	DRAMP04045	Immobilized peptid E14KKL
3992	DRAMP04046	Immobilized peptide E23GIG magainin2
3993	DRAMP04047	Immobilized peptide E17HSA magainin 2 deletion
4020	DRAMP04172	LK2W2 (LlKmW2 model peptides)
4021	DRAMP04173	L2KW2 (LlKmW2 model peptides)
4058	DRAMP04236	Antibacterial peptide A2
4080	DRAMP04266	CPÎ±1
4081	DRAMP04267	CPÎ±2
4082	DRAMP04268	CPÎ±3
4083	DRAMP04269	CP201
4084	DRAMP04270	CP202
4085	DRAMP04271	CP203
4086	DRAMP04274	CP206
4087	DRAMP04275	CP207
4088	DRAMP04276	CP208
4089	DRAMP04277	CP209
4090	DRAMP04280	CM1
4091	DRAMP04281	CM2
4092	DRAMP04282	CM3
4093	DRAMP04283	CM4
4094	DRAMP04284	CM5
4095	DRAMP04285	CM6
4096	DRAMP04286	CM7
4167	DRAMP04357	CEME(MBI-27)
4168	DRAMP04358	CEMA(MBI-28)

4169	DRAMP04366	PDD-A-8 (PDD-A analog)
4170	DRAMP04375	PDD-B-5 (PDD-B analog)
4171	DRAMP04384	PMM-5 (PMM analog)
4172	DRAMP04388	PMM-9 (PMM analog)
4424	DRAMP04658	Hymenochirin-5B
4435	DRAMP18188	Pantinin-1 (Non-disulfide-bridged peptide 4.20, NDBP-4.20, Non-disulfide-bridged peptide 5.21)
4443	DRAMP04684	Bacteriocin BAC-IB17
4451	DRAMP04700	Basic phospholipase A2 BnpTX-1 (BnPTx-I, svPLA2; Phosphatidylcholine 2-acylhydrolase)
4472	DRAMP18187	Toxin LyeTx 1
4473	DRAMP18128	Antimicrobial peptide HsAp4;
4474	DRAMP18129	Antimicrobial peptide HsAp3;
4475	DRAMP18130	Antimicrobial peptide HsAp2
4476	DRAMP18131	Antimicrobial peptide HsAp1 (HsAp)
4485	DRAMP18185	Jingdongin-1
4526	DRAMP18449	Kunitzin-OS (amphibians, animals)
4527	DRAMP18450	Kunitzin-RE (amphibians, animals)
4531	DRAMP18454	Tepmporin-1Ee (frog, amphibians, animals)
4533	DRAMP18456	Pepcon (peptide consensus sequence, synthetic)
4538	DRAMP18471	D-LAK60
4539	DRAMP18472	D-LAK80
4540	DRAMP18473	D-LAK100
4541	DRAMP18474	D-LAK140
4542	DRAMP18475	D-LAK160
4543	DRAMP18476	LAK80
4544	DRAMP18477	LAK80-P7
4545	DRAMP18478	LAK80-P10
4546	DRAMP18479	LAK80-P12
4547	DRAMP18480	LAK120
4548	DRAMP18481	LAK120-P7
4549	DRAMP18482	LAK120-P10
4550	DRAMP18483	LAK120-P12
4551	DRAMP18484	LAK160
4552	DRAMP18485	LAK160-P7
4553	DRAMP18486	LAK160-P10
4554	DRAMP18487	LAK160-P12
4555	DRAMP18488	[illegible] (Css from the species name below; scorpions, arachnids, Chelicerata, arthropods, invertebrates, ani[illegible]
4561	DRAMP02751	Formaecin-1 (Pro-rich; ants, insects, animals)
4562	DRAMP02752	Formaecin-2 (Pro-rich; ants, insects, animals)
4564	DRAMP03575	LL-37(17-29) (C-terminal fragment of LL-37, LL; Human, mammals, animals)
4566	DRAMP02094	Brevinin-1Bd (Frogs, amphibians, animals)
4567	DRAMP02095	Brevinin-1Be (Frogs, amphibians, animals)
4568	DRAMP02096	Brevinin-1Bf (Frogs, amphibians, animals)
4569	DRAMP02097	Brevinin-1Pa (Frogs, amphibians, animals)
4570	DRAMP02098	Brevinin-1Pc (Frogs, amphibians, animals)
4571	DRAMP02099	Brevinin-1Pd (Frogs, amphibians, animals)
4572	DRAMP02255	Ranatuerin-2Lb (Ranatuerin 2Lb; Ranaturin-2PRd; Frogs, amphibians, animals)
4573	DRAMP02254	Ranatuerin-2La (Ranatuerin 2La; Ranatuerin-2PRa; Frogs, amphibians, animals)
4574	DRAMP02256	Ranatuerin-2B (Ranatuerin 2B, Frog, amphibians, animals)
4575	DRAMP02257	Ranatuerin-2P (Ranatuerin 2P; Frogs, amphibians, animals)
4577	DRAMP18497	TSG-6 (Ixosin-B peptide derivative)

4578	DRAMP18498	TSG-7 (Ixosin-B peptide derivative)
4579	DRAMP18499	TSG-8 (Ixosin-B peptide derivative)
4580	DRAMP18500	TSG-8-1 (Ixosin-B peptide derivative)
4581	DRAMP18501	TSG-9 (Ixosin-B peptide derivative)
4582	DRAMP18502	TSG-10 (Ixosin-B peptide derivative)
4583	DRAMP18503	TSG-11 (Ixosin-B peptide derivative)
4584	DRAMP18504	E(AU)2 (Aurein 1.2 peptide derivative)
4585	DRAMP18505	(AU)2K (Aurein 1.2 peptide derivative)
4586	DRAMP18506	OG2 (Palustrin-OG1 peptide derivative)
4587	DRAMP18508	gp41w-FKA (gp41 peptide derivative)
4588	DRAMP18509	Px-cec1 (cecropin1 peptide derivative)
4589	DRAMP18510	D-LAK120
4590	DRAMP18511	D-LAK120-P13
4591	DRAMP18512	D-LAK120-A
4592	DRAMP18513	D-LAK120-AP13
4593	DRAMP18514	D-LAK120-H
4594	DRAMP18515	A12L/A20L (V13KL peptide derivative)
4595	DRAMP18533	V13KL (V681 peptide derivative)
4596	DRAMP18534	A23L (V13KL peptide derivative)
4597	DRAMP18535	A12L (V13KL peptide derivative)
4598	DRAMP18536	A20L (V13KL peptide derivative)
4599	DRAMP18537	A12L/A23L (V13KL peptide derivative)
4600	DRAMP18538	V681
4601	DRAMP18539	V13LL (V681 peptide derivative)
4602	DRAMP18540	V13AL (V681 peptide derivative)
4603	DRAMP18541	V13G (V681 peptide derivative)
4604	DRAMP18542	V13SL (V681 peptide derivative)
4605	DRAMP18543	V13LD (V681 peptide derivative)
4606	DRAMP18544	V13VD (V681 peptide derivative)
4607	DRAMP18545	V13AD (V681 peptide derivative)
4608	DRAMP18546	V13SD (V681 peptide derivative)
4609	DRAMP18547	V13KD (V681 peptide derivative)
4610	DRAMP18548	S11LL (V681 peptide derivative)
4611	DRAMP18549	S11VL (V681 peptide derivative)
4612	DRAMP18550	S11AL (V681 peptide derivative)
4613	DRAMP18551	S11G (V681 peptide derivative)
4614	DRAMP18552	S11KL (V681 peptide derivative)
4615	DRAMP18553	S11LD (V681 peptide derivative)
4616	DRAMP18554	S11VD (V681 peptide derivative)
4617	DRAMP18555	S11AD (V681 peptide derivative)
4618	DRAMP18556	S11SD (V681 peptide derivative)
4619	DRAMP18557	S11KD (V681 peptide derivative)
4620	DRAMP18558	Kn2-7 (BmKn2 peptide derivative)
4621	DRAMP18559	HFU3
4622	DRAMP18560	HFU4
4623	DRAMP18561	HFU5
4624	DRAMP18562	MAP-04-01 (Ixosin-B peptide derivative)
4625	DRAMP18563	MAP-04-02 (Ixosin-B peptide derivative)
4626	DRAMP18564	MAP-04-03 (Ixosin-B peptide derivative)
4627	DRAMP18565	MAP-04-04 (Ixosin-B peptide derivative)

4628	DRAMP18566	LL-IIIs-1 (lasioglossin III peptide derivative)
4629	DRAMP18567	LL-IIIs-2 (lasioglossin III peptide derivative)
4630	DRAMP18568	LL-IIIs-3 (lasioglossin III peptide derivative)
4631	DRAMP18569	LL-IIIs-4 (lasioglossin III peptide derivative)
4632	DRAMP18570	LL-IIIs-5 cis (lasioglossin III peptide derivative)
4633	DRAMP18571	LL-IIIs-5 trans (lasioglossin III peptide derivative)
4634	DRAMP18572	LL-IIIs-6a (lasioglossin III peptide derivative)
4635	DRAMP18573	LL-IIIs-6b (lasioglossin III peptide derivative)
4636	DRAMP18574	MEP-N (melectin peptide derivative)
4637	DRAMP18575	MEP-Ns-1 (melectin peptide derivative)
4638	DRAMP18576	MEP-Ns-2 (melectin peptide derivative)
4639	DRAMP18577	MEP-Ns-3 (melectin peptide derivative)
4640	DRAMP18578	MEP-Ns-4 cis (melectin peptide derivative)
4641	DRAMP18579	MEP-Ns-4 trans (melectin peptide derivative)
4642	DRAMP18580	MEP-Ns-5 (melectin peptide derivative)
4643	DRAMP18581	MEP-Ns-6 (melectin peptide derivative)
4645	DRAMP18583	Tricystine cyclic cystine TP (ccTP, Tachyplesin-1 peptide derivative)
4646	DRAMP18584	[Arg13]ccTP (ccTP peptide derivative)
4647	DRAMP18585	[Arg4,8]ccTP (ccTP peptide derivative)
4648	DRAMP18586	[Arg4,8,13]ccTP (ccTP peptide derivative)
4649	DRAMP18587	[Arg4,8,13][Lys18]ccTP (ccTP peptide derivative)
4650	DRAMP18588	RTD
4651	DRAMP18589	DSE (Ctx-Ha peptide derivative)
4652	DRAMP18590	DEP (Ctx-Ha peptide derivative)
4653	DRAMP18591	DEA (Ctx-Ha peptide derivative)
4654	DRAMP18592	Ctx(Ile21)-Ha (Ctx-Ha peptide derivative)
4655	DRAMP18593	Ctx(Ile21)-Ha-VD16 (Ctx-Ha peptide derivative)
4656	DRAMP18594	Ctx(Ile21)-Ha-VD5,16 (Ctx-Ha peptide derivative)
4657	DRAMP18595	Ctx(Ile21)-Ha-I9K (Ctx-Ha peptide derivative)
4658	DRAMP18596	LL-I/1 (Lasioglossin LL-I peptide derivative)
4659	DRAMP18597	LL-I/2 (Lasioglossin LL-I peptide derivative)
4660	DRAMP18598	LL-I/3 (Lasioglossin LL-I peptide derivative)
4661	DRAMP18599	LL-I/4 (Lasioglossin LL-I peptide derivative)
4662	DRAMP18600	LL-II/1 (Lasioglossin LL-II peptide derivative)
4663	DRAMP18601	LL-II/2 (Lasioglossin LL-II peptide derivative)
4664	DRAMP18602	LL-II/3 (Lasioglossin LL-II peptide derivative)
4665	DRAMP18603	LL-II/4 (Lasioglossin LL-II peptide derivative)
4666	DRAMP18604	LL-III/1 (Lasioglossin LL-III peptide derivative)
4667	DRAMP18605	LL-III/2 (Lasioglossin LL-III peptide derivative)
4668	DRAMP18606	LL-III/3 (Lasioglossin LL-III peptide derivative)
4669	DRAMP18607	LL-III/4 (Lasioglossin LL-III peptide derivative)
4670	DRAMP18608	LL-III/5 (Lasioglossin LL-III peptide derivative)
4671	DRAMP18609	LL-III/6 (Lasioglossin LL-III peptide derivative)
4672	DRAMP18610	LL-III/7 (Lasioglossin LL-III peptide derivative)
4675	DRAMP18613	TPG (Tritrpticin peptide derivative)
4676	DRAMP18627	D4-K9L8W (D-amino acid substitution of K9L8W)
4677	DRAMP18507	SolyC (Plant defensin; tomato, plants)
4681	DRAMP18614	TPA (Tritrpticin peptide derivative)
4682	DRAMP18615	TWF (Tritrpticin peptide derivative)
4683	DRAMP18616	[K22,25,27]-SMAP-29 (SMAP-29 peptide derivative)

4684	DRAMP18617	[A19]-SMAP-29 (SMAP-29 peptide derivative)
4685	DRAMP18618	SMAP-29(1-17) (SMAP-29 peptide derivative)
4686	DRAMP18619	[K2,7,13]-SMAP-29(1-17) (SMAP-29 peptide derivative)
4687	DRAMP18620	Pep-1-K (Pep-1 peptide derivative)
4689	DRAMP18622	Temporin-PEb (Temporin-PE peptide derivative)
4690	DRAMP18623	[I5,R8] Mastoparan-L ([I5,R8] MP-L;Mastoparan-L peptide derivative)
4691	DRAMP18624	K9L8W
4692	DRAMP18625	D3-K9L8W-1 (D-amino acid substitution of K9L8W)
4693	DRAMP18626	D3-K9L8W-2 (D-amino acid substitution of K9L8W)
4694	DRAMP18628	D6-K9L8W (D-amino acid substitution of K9L8W)
4695	DRAMP18629	D9-K9L8W-1 (D-amino acid substitution of K9L8W)
4696	DRAMP18630	D9-K9L8W-2 (D-amino acid substitution of K9L8W)
4697	DRAMP18631	H5(61-90) V1 (Histone H5 peptide derivative)
4698	DRAMP18632	Peptide H5 (71-90) (Histone H5 peptide derivative)
4699	DRAMP18633	H5(61-90) V2 (Histone H5 peptide derivative)
4700	DRAMP18634	H5(61-90) V3 (Histone H5 peptide derivative)
4701	DRAMP18635	NCP-0
4702	DRAMP18636	NCP-3a (CTX-1 peptide derivative)
4703	DRAMP18637	NCP-3b (CTX-1 peptide derivative)
4705	DRAMP18639	CTO17 (TO17 peptide derivative)
4706	DRAMP18640	TO19 (TO17 peptide derivative)
4707	DRAMP18641	KCM11
4708	DRAMP18642	KCM12
4709	DRAMP18643	KCM21
4710	DRAMP18644	KRS22
4713	DRAMP18647	PAF34
4714	DRAMP18648	[Pro3,DLeu9]TL(3) (Temporin L peptide derivative)
4715	DRAMP18649	HLP6 (HLP2 peptide derivative)
4716	DRAMP18650	HLP7 (HLP2 peptide derivative)
4722	DRAMP18656	Microcin 7 (Bacteriocin; Escherichia coli, Bacteria)
4724	DRAMP18658	Temporin-PE (Edible frogs, amphibians, animals)
4725	DRAMP18659	YFGAP-OH(Yellowfin tuna GAPDH-related antimicrobial peptide; fish, animals)
4726	DRAMP18660	YFGAP-NH2(Yellowfin tuna GAPDH-related antimicrobial peptide; fish, animals)
4727	DRAMP18661	Ctx-Ha (Frogs, amphibians, animals)
4728	DRAMP18662	Brevinin 21 (Brevinin-1E truncated peptide 21; Frogs, amphibians, animals)
4729	DRAMP18663	Brevinin 18 (Brevinin-1E truncated peptide 18; Frogs, amphibians, animals)
4730	DRAMP18664	Brevinin 15 (Brevinin-1E truncated peptide 15; Frogs, amphibians, animals)
4731	DRAMP18665	Mastoparan-L (MP-L; insects, arthropods, invertebrates, animals)
4732	DRAMP18666	P1 (Pilosulin-1 1-20; Ant, insects, arthropods, invertebrates, animals)
4733	DRAMP18667	Pep-1
4734	DRAMP18668	AI-hemocidins 1 (Hb-1 truncated peptide)
4735	DRAMP18669	AI-hemocidins 3 (Hb-1 truncated peptide)
4737	DRAMP18671	TO17 (TFPI-1 C-terminal peptide)
4738	DRAMP18672	Peptide 7 (Mollusca/molluscs/mollusks, invertebrates, animals)
4739	DRAMP18673	Peptide 3 (Mollusca/molluscs/mollusks, invertebrates, animals)
4740	DRAMP18674	Peptide 2 (Mollusca/molluscs/mollusks, invertebrates, animals)
4741	DRAMP18675	Peptide 4 (Mollusca/molluscs/mollusks, invertebrates, animals)
4742	DRAMP18676	Peptide 5 (Mollusca/molluscs/mollusks, invertebrates, animals)
4743	DRAMP18677	Peptide 6 (Mollusca/molluscs/mollusks, invertebrates, animals)
4744	DRAMP18678	Peptide 8 (Mollusca/molluscs/mollusks, invertebrates, animals)

4745	DRAMP18679	Peptide 9 (Mollusca/molluscs/mollusks, invertebrates, animals)
4759	DRAMP18693	Substance P (Mammals, animals)
4760	DRAMP18694	substance P antagonist (Mammals, animals)
4762	DRAMP18696	HLP1 (Lactotransferrin truncated peptide)
4763	DRAMP18697	HLP2 (Lactotransferrin truncated peptide)
4765	DRAMP18699	Pleurain-B1 (Frogs, amphibians, animals)
4766	DRAMP18700	Pleurain-C1 (Frogs, amphibians, animals)
4767	DRAMP18701	Pleurain-D4 (Frogs, amphibians, animals)
4768	DRAMP18702	Pleurain-E1 (Frogs, amphibians, animals)
4769	DRAMP18703	Pleurain-G1 (Frogs, amphibians, animals)
4770	DRAMP18704	Pleurain-J1 (Frogs, amphibians, animals)
4771	DRAMP18705	Pleurain-N1 (Frogs, amphibians, animals)
4772	DRAMP18706	Pleurain-R1 (Frogs, amphibians, animals)
4773	DRAMP18707	BACTENECIN 7 (bac 7, Pro-rich; bovine cathelicidin, cattle, ruminant, mammals, animals)
4774	DRAMP18708	Dermaseptin-S4 (DRS-S4, DS4; frog, amphibians, animals)
4778	DRAMP18712	Styelin A (Tunicate, invertebrates, animals)
4779	DRAMP18713	Styelin B (Tunicate, invertebrates, animals)
4782	DRAMP18716	Histone H2B-1(HLP-1) (fish, animals)
4783	DRAMP18717	Charybdotoxin (Yellow scorpions, arachnids, Chelicerata, arthropods, invertebrates, animals)
4784	DRAMP18718	AAP fraction 2 (Surfactant-associated anionic peptides; Asp-rich; sheep, ruminant, mammals, animal
4785	DRAMP18719	AAP fraction 3 (Surfactant-associated anionic peptides; Asp-rich; sheep, ruminant, mammals, animal
4786	DRAMP18720	AAP fraction 6 (Surfactant-associated anionic peptides; Asp-rich; sheep, ruminant, mammals, animal
4787	DRAMP18721	Hinnavin I (Hin I; insects, arthropods, invertebrates, animals)
4788	DRAMP18722	MA (Magainin 2 (9-21) truncated peptide)
4789	DRAMP18723	CE Cecropin A
4790	DRAMP18724	Oncorhyncin III (Oncorhyncin-3, histone-derived; fish, animals)
4791	DRAMP03024	Mastoparan B (MP-B; insects, arthropods, invertebrates, animals)
4818	DRAMP01826	RV-23 (Frogs, amphibians, animals)
4825	DRAMP03812	Pardaxin P-4 (Pardaxin P1a; Pardaxin Pa4)
4833	DRAMP18516	K7D (A12L/A20L peptide derivative)
4834	DRAMP02518	NA-CATH
4839	DRAMP20771	Acanthaporin (parasite, amoebozoa, protozoa, protists)
4840	DRAMP20772	cPcAMP1/26 (ciliate, Protists)
4842	DRAMP20776	HaA4 (beetles,insects,animals)
4843	DRAMP20777	Cath-BF
4844	DRAMP20778	Temporin-SHf (frogs,amphibians,animals)
4845	DRAMP20779	Halictine 1 (bees,insects,animals)
4846	DRAMP20780	Halictine 2 (bees,insects,animals)
4847	DRAMP20781	Panurgine 1(bees,insects,animals)
4848	DRAMP20782	Pleurain-D1 (Frogs,amphibians,animals)
4849	DRAMP20783	Pleurain-M1 (Frogs,amphibians,animals)
4850	DRAMP20784	Megin 1
4851	DRAMP20785	Megin 2
4852	DRAMP20786	mini-ChBac7.5N alpha
4853	DRAMP20787	mini-ChBac7.5N beta
4856	DRAMP20790	Cecropin B (Insects, arthropods, invertebrates, animals)
4858	DRAMP20792	Maculatin 1.1 (Frog, amphibians, animals)
4863	DRAMP20797	Uperin 3.6 (Toad, amphibians, animals)
4864	DRAMP20798	Lingual antimicrobial peptide (LAP, beta defensin, cattle, ruminant, animals)
4865	DRAMP20799	XT-2 (frog, amphibians, animals)

4866	DRAMP20800	the K4 peptide (synthetic; Phe-rich >25%)
4867	DRAMP20801	l Defensin MGD-1 (Mediterranean mussel defensin 1; mollusca/molluscs/mollusks, invertebrates, an
4868	DRAMP20802	CPF-AM1 (caerulein precursor fragment-AM1, frogs, amphibians, animals)
4869	DRAMP20803	moronecidin-like peptide
4870	DRAMP20804	AI-hemocidins 2 (Hb-1 truncated peptide)
4871	DRAMP20805	Apo5 APOC164-88
4872	DRAMP20806	Apo6 APOC167-88
4873	DRAMP20807	A1P394-428
4874	DRAMP20808	RI21 (PMAP-36 peptide derivative)
4875	DRAMP20809	RI18 (PMAP-36 peptide derivative)
4876	DRAMP20810	TI15 (PMAP-36 peptide derivative)
4877	DRAMP20811	RI12 (PMAP-36 peptide derivative)
4878	DRAMP20812	K8
4879	DRAMP20813	L1K8
4880	DRAMP20814	S1K8
4881	DRAMP20815	F1K8
4882	DRAMP20816	K1K8
4883	DRAMP20817	RR12
4884	DRAMP20818	RR12Wpolar
4885	DRAMP20819	RR12Whydro
4886	DRAMP20820	FV7
4887	DRAMP20821	FV-LL (FV7 and LL(LL-37,(17-29)) hybrid peptide)
4888	DRAMP20822	FV-MA (FV7 and MA(Magainin 2 (9-21)) hybrid peptide)
4889	DRAMP20823	FV-CE (FV7 and CE(Cecropin A (1
4890	DRAMP20824	AM-CATH36
4891	DRAMP20825	AM-CATH28
4892	DRAMP20826	AM-CATH21
4893	DRAMP20827	TB_L1FK
4894	DRAMP20828	TB_KKG6A
4895	DRAMP20831	IsCT1L1
4896	DRAMP20832	Polybia-MP1S-D8N
4897	DRAMP20833	[Pro3,DLeu9]TL(1) (Temporin L peptide derivative)
4898	DRAMP20834	PLS
4899	DRAMP20837	Pb-CATH1 Python bivittatus antimicrobial peptides peptide derivative
4900	DRAMP20838	Pb-CATH4 bivittatus antimicrobial peptides peptide derivative
4901	DRAMP20839	Xylopin
4902	DRAMP20841	C1b
4903	DRAMP20842	C1b(1-11)
4904	DRAMP20843	C1b(1-13)
4905	DRAMP20844	C1b(3-13)
4906	DRAMP20845	C1b(3-11)
4907	DRAMP20846	C1b(3-12)
4908	DRAMP20847	C1b(4-13)
4909	DRAMP20848	[K4]C1b(3-11)
4910	DRAMP20849	[R4]C1b(3-11)
4911	DRAMP20850	[K4,K10]C1b(3-13)
4912	DRAMP20851	[R4,R10]C1b(3-13)
4920	DRAMP20859	TT(1-24)
4921	DRAMP20860	TT(1-35)
4923	DRAMP20862	rtCATH2(5-40)

4924	DRAMP20863	rtCATH2(1-40)
4925	DRAMP20864	SF(18-45)
4926	DRAMP20865	the dimeric RRWQWR motif peptide molecule
4927	DRAMP20866	the tetrameric RRWQWR motif peptide molecule
4928	DRAMP20867	the palindromic RRWQWR motif peptide molecule
4929	DRAMP20868	H4
4930	DRAMP20869	Pal-ano-9 (Pal-anoplin peptide derivative)
4931	DRAMP20870	Pal-ano-8 (Pal-anoplin peptide derivative)
4932	DRAMP20871	Pal-ano-7 (Pal-anoplin peptide derivative)
4933	DRAMP20872	Pal-ano-6 (Pal-anoplin peptide derivative)
4934	DRAMP20873	Pal-ano-5 (Pal-anoplin peptide derivative)
4935	DRAMP20874	Chensinin-1b
4936	DRAMP20875	OA-C1b
4937	DRAMP20876	LA-C1b
4938	DRAMP20877	PA-C1b
4939	DRAMP20878	rVpDef
4940	DRAMP20879	DAN1
4941	DRAMP20880	DAN2
4944	DRAMP20883	Cath-A
4945	DRAMP20884	Cath-B
4946	DRAMP20885	Hp1404-T1a
4947	DRAMP20886	Hp1404-T1b
4948	DRAMP20887	NCP-2 (CTX-1 peptide derivative)
4949	DRAMP20888	NCP-3 (CTX-1 peptide derivative)
4954	DRAMP20893	I16A
4955	DRAMP20894	L19H/I20H
4956	DRAMP20895	F1A/I2A
4960	DRAMP20899	I5A/I6A
4961	DRAMP20900	A12I/A15I
4962	DRAMP20901	A12V/A15H
4964	DRAMP20903	dC2
4965	DRAMP20904	R18S/R21H
4967	DRAMP20906	dN2
4968	DRAMP20907	dN4
4971	DRAMP20910	RN7-IN7(designed based on indolicidin and ranalexin)
4973	DRAMP20912	RN7-IN9(designed based on indolicidin and ranalexin)
4974	DRAMP20913	Myxinidin (G1)
4975	DRAMP20914	Myxinidin (I2)
4976	DRAMP20915	Myxinidin (H3)
4977	DRAMP20916	Myxinidin (D4)
4978	DRAMP20917	Myxinidin (I5)
4979	DRAMP20918	Myxinidin (L6)
4980	DRAMP20919	Myxinidin (K7)
4981	DRAMP20920	Myxinidin (Y8)
4982	DRAMP20921	Myxinidin (G9)
4983	DRAMP20922	Myxinidin (K10)
4984	DRAMP20923	Myxinidin (P11)
4985	DRAMP20924	Myxinidin (S12)
4986	DRAMP20925	MH3R
4987	DRAMP20926	IN1(designed based on indolicidin and ranalexin)

4988	DRAMP20927	IN2(designed based on indolicidin and ranalexin)
4989	DRAMP20928	IN3(designed based on indolicidin and ranalexin)
4990	DRAMP20929	RN7-IN6(designed based on indolicidin and ranalexin)
4991	DRAMP20930	BP100-Ala-NH-C16H33
4992	DRAMP20932	Hp1404
4994	DRAMP20934	Lucilin Peptide
4995	DRAMP20935	Macropin 1(solitary bee, insects, animals)
4996	DRAMP20936	Î”Pb-CATH1
4997	DRAMP20937	Pb-CATH3
4998	DRAMP20938	Cbf-14
4999	DRAMP20939	D-Cbf-14
5001	DRAMP20941	[Pro3,DLeu9]TL(8) (Temporin L peptide derivative)
5002	DRAMP20942	[Pro3,DLeu9]TL(9) (Temporin L peptide derivative)
5003	DRAMP20943	[Pro3,DLeu9]TL(10) (Temporin L peptide derivative)
5004	DRAMP20944	[Pro3,DLeu9]TL(11) (Temporin L peptide derivative)
5005	DRAMP20945	Recombinant Cecropin A (1â€“8)â€“LL37 (17â€“30) (Câ€“L)
5014	DRAMP20955	L31-P113
5015	DRAMP20956	AL32-P113
5016	DRAMP20957	StigA6
5017	DRAMP20958	StigA16
5018	DRAMP20959	Hp1404-T1c
5019	DRAMP20960	Hp1404-T1d
5020	DRAMP20961	Hp1404-T1e
5021	DRAMP20963	Cp1 alpha s1-casein peptide derivative
5022	DRAMP20964	Synthesized Cecropin A (1â€“8)â€“LL37 (17â€“30) (Câ€“L)
5023	DRAMP20965	LPcin-YK3 (bovine cathelicidin, cattle, ruminant, mammals, animals)
5024	DRAMP20966	andricin B (Andrias davidianus, Amphibians, Animals)
5025	DRAMP20967	andricin 01 (Andrias davidianus, Amphibians, Animals)
5026	DRAMP20968	Catesbeianin-1 (Ranidae, Anura, Amphibia, Animals)
5027	DRAMP20969	HJH-1 (bovine cathelicidin, cattle, ruminant, mammals, animals)
5028	DRAMP20970	P3 (bovine cathelicidin, cattle, ruminant, mammals, animals)
5029	DRAMP20971	JH-0 (Derived from P3)
5030	DRAMP20972	JH-1 (Derived from P3)
5031	DRAMP20973	JH-2 (Derived from P3)
5032	DRAMP20974	JH-3 (Derived from P3)
5033	DRAMP20975	OH-CM6 (Derived from OH-CATH30)
5034	DRAMP20976	adevonin (Derived from Adenanthera pavonina trypsin inhibitor (ApTI)) inhibitor (ApTI))
5035	DRAMP20977	Anoplin-1 (Derived from Anoplin)
5036	DRAMP20978	Anoplin-2 (Derived from Anoplin)
5037	DRAMP20979	Anoplin-3 (Derived from Anoplin)
5038	DRAMP20980	Anoplin-4 (Derived from Anoplin)
5039	DRAMP20981	CPF-C1 (Frogs, Amphibians, Animals)
5040	DRAMP20982	CPF-1 (Derived from CPF-C1)
5041	DRAMP20983	CPF-2 (Derived from CPF-C1)
5042	DRAMP20984	CPF-3 (Derived from CPF-C1)
5043	DRAMP20985	CPF-4 (Derived from CPF-C1)
5044	DRAMP20986	CPF-5 (Derived from CPF-C1)
5045	DRAMP20987	CPF-6 (Derived from CPF-C1)
5046	DRAMP20988	CPF-7 (Derived from CPF-C1)

5047	DRAMP20989	CPF-8 (Derived from CPF-C1)
5048	DRAMP20990	CPF-9 (Derived from CPF-C1)
5049	DRAMP20991	CPF-10 (Derived from CPF-C1)
5050	DRAMP20992	CPF-11 (Derived from CPF-C1)
5051	DRAMP20993	CPF-12 (Derived from CPF-C1)
5052	DRAMP20994	anoplin analog 4
5053	DRAMP20995	anoplin analog 5
5054	DRAMP20996	anoplin analog 6
5055	DRAMP20997	anoplin analog 7
5056	DRAMP20998	anoplin analog 8
5057	DRAMP20999	anoplin analog 9
5058	DRAMP21000	cGm (Derived from Gm)
5059	DRAMP21001	[Y7W]cGm (Derived from Gm)
5060	DRAMP21002	[Y14W]cGm (Derived from Gm)
5061	DRAMP21003	[K8R]cGm (Derived from Gm)
5062	DRAMP21004	[Y7W, K8R, Y14W]cGm (Derived from Gm)
5063	DRAMP21005	[R4A, R18A]cGm (Derived from Gm)
5064	DRAMP21006	[G1K, K8R]cGm (Derived from Gm)
5065	DRAMP21007	[C/U]cGm (Derived from Gm)
5066	DRAMP21008	[L5W]cGm (Derived from Gm)
5067	DRAMP21009	[D-P L-P]cGm (Derived from Gm)
5068	DRAMP21010	[G1K, L5Y, K8R]cGm (Derived from Gm)
5069	DRAMP21011	[C/U, G1K, L5Y, K8R]cGm (Derived from Gm)
5070	DRAMP21012	NK-2 (Mammals, Animals)
5071	DRAMP21013	NK-pro (Derived from NK-2)
5072	DRAMP21014	NK-dpro (Derived from NK-2)
5073	DRAMP21015	A (A1R) (Derived from AR-23)
5074	DRAMP21016	A (A8R) (Derived from AR-23)
5075	DRAMP21017	A (I17K) (Derived from AR-23)
5076	DRAMP21018	A (I17R) (Derived from AR-23)
5077	DRAMP21019	A (A1R, A8R) (Derived from AR-23)
5078	DRAMP21020	A (A1R, I17K) (Derived from AR-23)
5079	DRAMP21021	A (A8R, I17K) (Derived from AR-23)
5080	DRAMP21022	A (A1R, A8R, I17K) (Derived from AR-23)
5081	DRAMP21023	A (A1R, A8R, I17R) (Derived from AR-23)
5082	DRAMP21024	Stigmurin (Tityus, Scorpionida, Arachnida)
5083	DRAMP21025	StigA25 (Derived from Stigmurin)
5084	DRAMP21026	StigA31 (Derived from Stigmurin)
5085	DRAMP21027	K5, 17-DPS3 (Derived from dermaseptin-PS3 (DPS3))
5086	DRAMP21028	L10, 11-DPS3 (Derived from dermaseptin-PS3 (DPS3))
5087	DRAMP21029	D5R (Derived from HD5)
5088	DRAMP21030	D5r (Derived from HD5)
5089	DRAMP21031	MyD5R (Derived from HD5)
5090	DRAMP21032	MyD5r (Derived from HD5)
5091	DRAMP21033	LaD5R (Derived from HD5)
5092	DRAMP21034	LaD5r (Derived from HD5)
5093	DRAMP21035	AC-UM-14W (De novo synthesis)
5094	DRAMP21036	PapMA (Derived from Papiliocin and Magainin 2)
5095	DRAMP21037	PapMA-k (Derived from Papiliocin and Magainin 2)
5096	DRAMP21038	analog 1 (Derived from Ib-AMP1)

5097	DRAMP21039	analog 2 (Derived from Ib-AMP2)
5098	DRAMP21040	analog 3 (Derived from Ib-AMP2)
5099	DRAMP21041	analog 4 (Derived from Ib-AMP2)
5100	DRAMP21042	A2 (Derived from Indolicidin (IN))
5101	DRAMP21043	A3 (Derived from Indolicidin (IN))
5102	DRAMP21044	A4 (Derived from Indolicidin (IN))
5103	DRAMP21045	A5 (Derived from Indolicidin (IN))
5104	DRAMP21046	A6 (Derived from Indolicidin (IN))
5105	DRAMP21047	A7 (Derived from Indolicidin (IN))
5106	DRAMP21048	peptide 6 (Derived from seq2)
5107	DRAMP21049	peptide 6.2 (Derived from seq2)
5108	DRAMP21050	TP1[K1A] (Derived from TP1)
5109	DRAMP21051	TP1[W2A] (Derived from TP1)
5110	DRAMP21052	TP1[C3A, C16S] (Derived from TP1)
5111	DRAMP21053	TP1[F4A] (Derived from TP1)
5112	DRAMP21054	TP1[R5A] (Derived from TP1)
5113	DRAMP21055	TP1[V6A] (Derived from TP1)
5114	DRAMP21056	TP1[C7A, C12S] (Derived from TP1)
5115	DRAMP21057	TP1[Y8A] (Derived from TP1)
5116	DRAMP21058	TP1[R9A] (Derived from TP1)
5117	DRAMP21059	TP1[G10A] (Derived from TP1)
5118	DRAMP21060	TP1[I11A] (Derived from TP1)
5119	DRAMP21061	TP1[C7S, C12A] (Derived from TP1)
5120	DRAMP21062	TP1[Y13A] (Derived from TP1)
5121	DRAMP21063	TP1[R14A] (Derived from TP1)
5122	DRAMP21064	TP1[R15A] (Derived from TP1)
5123	DRAMP21065	TP1[C3S, C16A] (Derived from TP1)
5124	DRAMP21066	TP1[R17A] (Derived from TP1)
5125	DRAMP21067	TP1[C3A, C16A] (Derived from TP1)
5126	DRAMP21068	TP1[C7A, C12A] (Derived from TP1)
5127	DRAMP21069	TP1[C3A, C7A, C12A, C16A] (Derived from TP1)
5128	DRAMP21070	TP1[V6R, R9A] (Derived from TP1)
5129	DRAMP21071	TP1[K1R] (Derived from TP1)
5130	DRAMP21072	TP1[F4G] (Derived from TP1)
5131	DRAMP21073	TP1[F4S] (Derived from TP1)
5132	DRAMP21074	TP1[Y8G] (Derived from TP1)
5133	DRAMP21075	TP1[I11G] (Derived from TP1)
5134	DRAMP21076	TP1[F4A, Y8A, I11A] (Derived from TP1)
5135	DRAMP21077	TP1[-R5, R17G] (Derived from TP1)
5136	DRAMP21078	TP1[K1A, F4A] (Derived from TP1)
5137	DRAMP21079	TP1[K1A, Y8A] (Derived from TP1)
5138	DRAMP21080	TP1[K1A, I11A] (Derived from TP1)
5139	DRAMP21081	TP1[R9A, R17A] (Derived from TP1)
5140	DRAMP21082	ccTP 3 (Derived from TP2)
5141	DRAMP21083	ccTP 5 (Derived from TP2)
5142	DRAMP21084	ccTP 6 (Derived from TP2)
5143	DRAMP21085	PRW4 (PR) (Derived from PMAP-36)
5144	DRAMP21086	PR-FO (Derived from PRW4)
5145	DRAMP21087	PR-PG (Derived from PRW4)
5146	DRAMP21088	PR-TR (Derived from PRW4)

5147	DRAMP21089	C4 (Derived from PRW4)
5148	DRAMP21090	D4 (Derived from PRW4)
5149	DRAMP21091	I4 (Derived from PRW4)
5150	DRAMP21092	P4 (Derived from PRW4)
5151	DRAMP21093	PRW4-d (Derived from PRW4)
5152	DRAMP21094	PRW4-R (Derived from PRW4)
5153	DRAMP21095	IR1 (Derived from PG-1)
5154	DRAMP21096	IR2 (Derived from PG-1)
5155	DRAMP21097	FR1 (Derived from PG-1)
5156	DRAMP21098	FR2 (Derived from PG-1)
5157	DRAMP21099	WR1 (Derived from PG-1)
5158	DRAMP21100	WR2 (Derived from PG-1)
5159	DRAMP21101	PR1 (Derived from PG-1)
5160	DRAMP21102	PR2 (Derived from PG-1)
5161	DRAMP21165	HYL-11 (Derived from HYL)
5162	DRAMP21166	HYL-12 (Derived from HYL)
5163	DRAMP21164	HYL-10 (Derived from HYL)
5164	DRAMP21158	HYL-4 (Derived from HYL)
5165	DRAMP21159	HYL-5 (Derived from HYL)
5166	DRAMP21160	HYL-6 (Derived from HYL)
5167	DRAMP21161	HYL-7 (Derived from HYL)
5168	DRAMP21162	HYL-8 (Derived from HYL)
5169	DRAMP21163	HYL-9 (Derived from HYL)
5170	DRAMP21157	HYL-3 (Derived from HYL)
5171	DRAMP21156	HYL-2 (Derived from HYL)
5172	DRAMP21155	HYL-1 (Derived from HYL)
5173	DRAMP21154	HYL (Bee, Insecta, Animals)
5174	DRAMP21153	KR-12-a8 (Derived from KR-12)
5175	DRAMP21151	KR-12-a6 (Derived from KR-12)
5176	DRAMP21150	KR-12-a5 (Derived from KR-12)
5177	DRAMP21152	KR-12-a7 (Derived from KR-12)
5178	DRAMP21149	KR-12-a4 (Derived from KR-12)
5179	DRAMP21146	KR-12-a1 (Derived from KR-12)
5180	DRAMP21148	KR-12-a3 (Derived from KR-12)
5181	DRAMP21147	KR-12-a2 (Derived from KR-12)
5182	DRAMP21145	Myxinidin3 (Derived from Myxinidin)
5183	DRAMP21142	AMP2041 (De novo synthesis)
5184	DRAMP21141	AMP126 (De novo synthesis)
5185	DRAMP21144	Myxinidin2 (Derived from Myxinidin)
5186	DRAMP21143	Myxinidin1 (Derived from Myxinidin)
5187	DRAMP21140	AMP72 (De novo synthesis)
5188	DRAMP21139	GNU7 (De novo synthesis)
5189	DRAMP21138	GNU6 (De novo synthesis)
5190	DRAMP21137	GNU5 (De novo synthesis)
5191	DRAMP21135	P7 (Derived from P5)
5192	DRAMP21136	P8 (Derived from P5)
5193	DRAMP21134	P6 (Derived from P5)
5194	DRAMP21133	P5 (Derived from Octa 2)
5195	DRAMP21132	P4 (Derived from P5)
5196	DRAMP21131	P3 (Derived from P5)

5197	DRAMP21130	P2 (Derived from P5)
5198	DRAMP21129	P1 (Derived from P5)
5199	DRAMP21128	T9F (Derived from RI16)
5200	DRAMP21127	T9K (Derived from RI16)
5201	DRAMP21126	T9I (Derived from RI16)
5202	DRAMP21125	T9W (Derived from RI16)
5203	DRAMP21124	RI16 (Derived from PMAP-36)
5204	DRAMP21123	KR-12-a5 (7-(D)L) (Derived from LL-37)
5205	DRAMP21122	KR-12-a5 (6-(D)L) (Derived from LL-37)
5206	DRAMP21121	KR-12-a5 (5-(D)K) (Derived from LL-37)
5207	DRAMP21119	I11R (Derived from tachyplesin I)
5208	DRAMP21120	KR-12-a5 (Derived from LL-37)
5209	DRAMP21118	I11S (Derived from tachyplesin I)
5210	DRAMP21117	Y8R (Derived from tachyplesin I)
5211	DRAMP21116	Y8S (Derived from tachyplesin I)
5212	DRAMP21115	V6R (Derived from tachyplesin I)
5213	DRAMP21114	V6S (Derived from tachyplesin I)
5214	DRAMP21111	ASA (Derived from SLZP)
5215	DRAMP21112	DLSA (Derived from SLZP)
5216	DRAMP21113	PSA (Derived from SLZP)
5217	DRAMP21103	L-RW (De novo synthesis)
5218	DRAMP21110	SLZP (De novo synthesis)
5219	DRAMP21109	FPA-Bombinin-BO (toads, amphibians, animals)
5220	DRAMP21108	Feleucin-K3 (Derived from Feleucin-BO1)
5221	DRAMP21104	Feleucin-2 (toads, amphibians, animals)
5224	DRAMP21107	Feleucin-BO1 (toads, amphibians, animals)
5225	DRAMP21232	Ranatuerin-2PLx (R2PLx; Frogs, Amphibians, Animals)
5226	DRAMP21231	S-6K-F17-3GN (Derived from S-6K-F17)
5227	DRAMP21230	S-6K-F17-3G (Derived from S-6K-F17)
5228	DRAMP21229	S-6K-F17-2G (Derived from S-6K-F17)
5229	DRAMP21227	IsCT-P (Derived from IsCT)
5230	DRAMP21228	IsCT-a (Derived from IsCT-P)
5231	DRAMP21225	STPk (Derived from STP)
5232	DRAMP21226	Ink (Derived from IN)
5233	DRAMP21223	IsCT-p (Derived from IsCT-P)
5234	DRAMP21224	TPk (Derived from TP)
5235	DRAMP21222	Control-4D (Derived from IK12-all L)
5236	DRAMP21221	Control-all D (Derived from IK12-all L)
5237	DRAMP21219	IK12-all D (Derived from IK12-all L)
5238	DRAMP21220	Control-all L (Derived from IK12-all L)
5239	DRAMP21218	IK12-all L (De novo synthesis)
5240	DRAMP21217	IK8-2D (Derived from IK8-all L)
5241	DRAMP21215	IK4-all D (Derived from IK8-all L)
5242	DRAMP21216	IK8-4D (Derived from IK8-all L)
5243	DRAMP21213	IK8-all D (Derived from IK8-all L)
5244	DRAMP21214	IK6-all D (Derived from IK8-all L)
5245	DRAMP21212	IK8-all L (De novo synthesis)
5246	DRAMP21211	AmyI-1-18 (N3L, G12R) (Derived from AmyI-1-18)
5247	DRAMP21208	AmyI-1-18 (E9L) (Derived from AmyI-1-18)
5248	DRAMP21209	AmyI-1-18 (E9L, G12R) (Derived from AmyI-1-18)

5249	DRAMP21205	AmyI-1-18 (G12R) (Derived from AmyI-1-18)
5250	DRAMP21210	AmyI-1-18 (N3L, E9L) (Derived from AmyI-1-18)
5251	DRAMP21207	AmyI-1-18 (N3L) (Derived from AmyI-1-18)
5252	DRAMP21206	AmyI-1-18 (D15R) (Derived from AmyI-1-18)
5253	DRAMP21204	AmyI-1-18 (I11R) (Derived from AmyI-1-18)
5254	DRAMP21203	AmyI-1-18 (Oryza sativa L., Angiospermae, Plants)
5255	DRAMP21202	HPA3NT3-analog (Derived from HPA3NT3)
5256	DRAMP21201	Magainin 2a (M2a; Frogs, Amphibians, Animals)
5257	DRAMP21200	GW-M4 (De novo synthesis)
5258	DRAMP21199	GW-M3 (De novo synthesis)
5259	DRAMP21198	GW-M1 (De novo synthesis)
5260	DRAMP21197	GW-H3 (De novo synthesis)
5261	DRAMP21196	GW-H1 (De novo synthesis)
5262	DRAMP21195	GW-A5 (De novo synthesis)
5263	DRAMP21194	GW-A4 (De novo synthesis)
5264	DRAMP21193	GW-A2 (De novo synthesis)
5265	DRAMP21192	GW-A1 (De novo synthesis)
5266	DRAMP21191	GW-Q6 (De novo synthesis)
5267	DRAMP21190	GW-Q5 (De novo synthesis)
5268	DRAMP21189	GW-Q4 (De novo synthesis)
5269	DRAMP21188	GW-Q3 (De novo synthesis)
5270	DRAMP21187	WRL4 (Derived from leucocin A)
5271	DRAMP21186	WRL3 (Derived from leucocin A)
5272	DRAMP21185	WRL2 (Derived from leucocin A)
5273	DRAMP21184	WR7 (Derived from leucocin A)
5274	DRAMP21183	WR5 (Derived from leucocin A)
5275	DRAMP21182	WR3 (Derived from leucocin A)
5276	DRAMP21181	WR1 (Derived from leucocin A)
5277	DRAMP21180	WG18 (Derived from leucocin A)
5278	DRAMP21179	HYL-26 (Derived from HYL)
5279	DRAMP21178	HYL-25 (Derived from HYL)
5280	DRAMP21177	HYL-24 (Derived from HYL)
5281	DRAMP21176	HYL-23 (Derived from HYL)
5282	DRAMP21175	HYL-22 (Derived from HYL)
5283	DRAMP21174	HYL-21 (Derived from HYL)
5284	DRAMP21173	HYL-20 (Derived from HYL)
5285	DRAMP21172	HYL-19 (Derived from HYL)
5286	DRAMP21168	HYL-15 (Derived from HYL)
5287	DRAMP21169	HYL-16 (Derived from HYL)
5288	DRAMP21170	HYL-17 (Derived from HYL)
5289	DRAMP21171	HYL-18 (Derived from HYL)
5290	DRAMP21243	pardaxin-6 (GE-6) (Derived from pardaxin)
5291	DRAMP21242	Epinecidin-8 (Derived from Epinecidin)
5292	DRAMP21241	Epinecidin-1 (Derived from Epinecidin)
5293	DRAMP21240	FK13-a7 (Derived from FK13)
5294	DRAMP21239	FK13-a6 (Derived from FK13)
5295	DRAMP21238	FK13-a5 (Derived from FK13)
5296	DRAMP21237	FK13-a4 (Derived from FK13)
5297	DRAMP21236	FK13-a3 (Derived from FK13)
5298	DRAMP21235	FK13-a2 (Derived from FK13)

5299	DRAMP21167	HYL-13 (Derived from HYL)
5300	DRAMP21234	FK13-a1 (Derived from FK13)
5301	DRAMP21233	R2PLx-22 (Derived from R2PLx)
5302	DRAMP21244	TsAP-S1 (Derived from TsAP-1)
5303	DRAMP21245	TsAP-S2 (Derived from TsAP-2)
5304	DRAMP21246	pEM-2 (Derived from the venom of the snake Bothrops asper)
5305	DRAMP21247	PV (Derived from pEM-2 and MP-VT1)
5306	DRAMP21248	BVP (Derived from pEM-2 and MP-VT1 and MP-B)
5307	DRAMP21249	PVP (Derived from MP-B and MP-VT1)
5308	DRAMP21250	PV3 (Derived from pEM-2 and MP-VT1)
5311	DRAMP21253	AaeAP1a (Derived from AaeAP1)
5312	DRAMP21254	AaeAP2a (Derived from AaeAP2)
5313	DRAMP21255	WL1 (Derived from CP-1)
5314	DRAMP21256	WL2 (Derived from CP-1)
5315	DRAMP21257	WL3 (Derived from CP-1)
5316	DRAMP21258	Cecropin P1 (CP-1) (nematodes, animals)
5317	DRAMP21259	Scolopendin 1 (Centipedes, Arthropoda, Animals)
5318	DRAMP21260	KL0A10 (De novo synthesis)
5319	DRAMP21261	KL4A6 (De novo synthesis)
5320	DRAMP21262	KL6A4 (De novo synthesis)
5321	DRAMP21263	KL10A0 (De novo synthesis)
5322	DRAMP21264	LK (De novo synthesis)
5323	DRAMP21265	LK-L1A (Derived from LK)
5324	DRAMP21266	LK-L4A (Derived from LK)
5325	DRAMP21267	LK-L5A (Derived from LK)
5326	DRAMP21268	LK-L7A (Derived from LK)
5327	DRAMP21269	LK-L8A (Derived from LK)
5328	DRAMP21270	LK-L11A (Derived from LK)
5329	DRAMP21271	LK-L12A (Derived from LK)
5330	DRAMP21272	LK-L14A (Derived from LK)
5331	DRAMP21273	LK-L8G (Derived from LK)
5332	DRAMP21274	LK-L8S (Derived from LK)
5333	DRAMP21275	LK-L8P (Derived from LK)
5334	DRAMP21276	LK-L8N (Derived from LK)
5335	DRAMP21277	LK-L8Q (Derived from LK)
5336	DRAMP21278	LK-L8D (Derived from LK)
5337	DRAMP21279	LK-L8E (Derived from LK)
5338	DRAMP21280	LK-L8K (Derived from LK)
5339	DRAMP21281	LK-L8H (Derived from LK)
5340	DRAMP21282	Lt-F1A (Derived from Lt)
5341	DRAMP21283	Lt-I4A (Derived from Lt)
5342	DRAMP21284	Lt-V5A (Derived from Lt)
5343	DRAMP21285	Lt-I8A (Derived from Lt)
5344	DRAMP21286	Lt-F11A (Derived from Lt)
5345	DRAMP21287	Lt-F12A (Derived from Lt)
5346	DRAMP21288	Lt-I4G (Derived from Lt)
5347	DRAMP21289	Lt-I4S (Derived from Lt)
5348	DRAMP21290	Lt-I4N (Derived from Lt)
5349	DRAMP21291	Lt-I4Q (Derived from Lt)
5350	DRAMP21292	Lt-I4H (Derived from Lt)

5351	DRAMP21293	Lt-V5G (Derived from Lt)
5352	DRAMP21294	Lt-V5S (Derived from Lt)
5353	DRAMP21295	Lt-V5N (Derived from Lt)
5354	DRAMP21296	Lt-V5Q (Derived from Lt)
5355	DRAMP21297	Lt-V5H (Derived from Lt)
5356	DRAMP21298	Lt-F11G (Derived from Lt)
5357	DRAMP21299	Lt-F11S (Derived from Lt)
5358	DRAMP21300	Lt-F11N (Derived from Lt)
5359	DRAMP21301	Lt-F11Q (Derived from Lt)
5360	DRAMP21302	Lt-F11H (Derived from Lt)
5361	DRAMP21303	A7-PMAP-23 (Derived from PMAP-23)
5362	DRAMP21304	A21-PMAP-23 (Derived from PMAP-23)
5363	DRAMP21305	R8 (De novo synthesis)
5364	DRAMP21306	TL-1 (Derived from Temporin-1Tl (TL))
5365	DRAMP21307	TL-2 (Derived from Temporin-2Tl (TL))
5366	DRAMP21308	TL-3 (Derived from Temporin-3Tl (TL))
5367	DRAMP21309	TL-4 (Derived from Temporin-4Tl (TL))
5368	DRAMP21311	2W-1 (Derived from PMAP-36)
5369	DRAMP21312	2W-2 (Derived from PMAP-36)
5370	DRAMP21313	2W-3 (Derived from PMAP-36)
5371	DRAMP21314	3W-1 (Derived from PMAP-36)
5372	DRAMP21315	3W-2 (Derived from PMAP-36)
5373	DRAMP21316	3W-3 (Derived from PMAP-36)
5374	DRAMP21317	3W-4 (Derived from PMAP-36)
5375	DRAMP21318	3W-5 (Derived from PMAP-36)
5376	DRAMP21319	3V (Derived from PMAP-36)
5377	DRAMP21320	3L (Derived from PMAP-36)
5378	DRAMP21321	4W (Derived from PMAP-36)
5379	DRAMP21322	RTV (Derived from PMAP-36)
5380	DRAMP21323	RTI (Derived from PMAP-36)
5381	DRAMP21324	RTF (Derived from PMAP-36)
5382	DRAMP21325	RTL (Derived from PMAP-36)
5383	DRAMP21326	RLR (Derived from PMAP-36)
5384	DRAMP21327	RVR (Derived from PMAP-36)
5385	DRAMP21328	RTR (Derived from PMAP-36)
5386	DRAMP21329	RFR (Derived from PMAP-36)
5387	DRAMP21330	KVK (Derived from PMAP-36)
5388	DRAMP21331	KLK (Derived from PMAP-36)
5389	DRAMP21332	KIK (Derived from PMAP-36)
5390	DRAMP21333	RVK (Derived from PMAP-36)
5391	DRAMP21334	Ranatuerin-2Pb (Frogs, amphibians, animals)
5392	DRAMP21335	RPa (Frogs, amphibians, animals)
5393	DRAMP21336	RPb (Frogs, amphibians, animals)
5394	DRAMP21337	BMAP-27 (Bovine, mammals, animals)
5395	DRAMP21338	[Arg]3-VmCT1-NH2 (Derived from VmCT1)
5396	DRAMP21339	[Arg]7-VmCT1-NH2 (Derived from VmCT1)
5397	DRAMP21340	[Arg]11-VmCT1-NH2 (Derived from VmCT1)
5398	DRAMP21341	[Gly]1-VmCT1-NH2 (Derived from VmCT1)
5399	DRAMP21342	[Pro]8-VmCT1-NH2 (Derived from VmCT1)
5400	DRAMP21343	[Leu]9-VmCT1-NH2 (Derived from VmCT1)

5401	DRAMP21344	[Phe]9-VmCT1-NH2 (Derived from VmCT1)
5402	DRAMP21345	[Leu]12-VmCT1-NH2 (Derived from VmCT1)
5403	DRAMP21346	[Tyr]12-VmCT1-NH2 (Derived from VmCT1)
5404	DRAMP21347	2IH1 (De Novo Synthesis)
5405	DRAMP21348	2IH2 (De Novo Synthesis)
5406	DRAMP21349	2IH3 (De Novo Synthesis)
5407	DRAMP21350	2IH4 (De Novo Synthesis)
5408	DRAMP21351	3IH1 (De Novo Synthesis)
5409	DRAMP21352	3IH2 (De Novo Synthesis)
5410	DRAMP21353	3IH3 (De Novo Synthesis)
5411	DRAMP21354	3IH4 (De Novo Synthesis)
5412	DRAMP21355	5Kamp (De Novo Synthesis)
5413	DRAMP21356	4Kamp (De Novo Synthesis)
5414	DRAMP21357	3Kamp (De Novo Synthesis)
5415	DRAMP21358	2Kamp (De Novo Synthesis)
5416	DRAMP21359	1Kamp (De Novo Synthesis)
5417	DRAMP21360	6K-F17-4L (De Novo Synthesis)
5418	DRAMP21361	5Kamp-4L (De Novo Synthesis)
5419	DRAMP21362	4Kamp-4L (De Novo Synthesis)
5420	DRAMP21363	3Kamp-4L (De Novo Synthesis)
5421	DRAMP21364	2Kamp-4L (De Novo Synthesis)
5422	DRAMP21365	1Kamp-4L (De Novo Synthesis)
5423	DRAMP21366	[Lys]1-VmCT1-NH2 (Derived from VmCT1)
5425	DRAMP21368	[Lys]1[Lys]12-VmCT1-NH2 (Derived from VmCT1)
5426	DRAMP21369	[Lys]3[Lys]7-VmCT1-NH2 (Derived from VmCT1)
5427	DRAMP21370	[Lys]3[Lys]11-VmCT1-NH2 (Derived from VmCT1)
5428	DRAMP21371	[Lys]7[Lys]11-VmCT1-NH2 (Derived from VmCT1)
5429	DRAMP21372	[Lys]3[Lys]7[Lys]11-VmCT1-NH2 (Derived from VmCT1)
5430	DRAMP21373	SP1 (De Novo Synthesis)
5431	DRAMP21374	SP2 (De Novo Synthesis)
5432	DRAMP21375	SP3 (De Novo Synthesis)
5433	DRAMP21376	SP4 (De Novo Synthesis)
5434	DRAMP21377	SP5 (De Novo Synthesis)
5435	DRAMP21378	SP6 (De Novo Synthesis)
5436	DRAMP21379	SP7 (De Novo Synthesis)
5437	DRAMP21380	SP8 (De Novo Synthesis)
5438	DRAMP21381	SP1D * (De Novo Synthesis)
5439	DRAMP21382	SP9 (De Novo Synthesis)
5440	DRAMP21383	SP10 (De Novo Synthesis)
5441	DRAMP21384	SP11 (De Novo Synthesis)
5442	DRAMP21385	SP12 (De Novo Synthesis)
5443	DRAMP21386	SP13 (De Novo Synthesis)
5444	DRAMP21387	SP14 (De Novo Synthesis)
5445	DRAMP21388	SP15 * (De Novo Synthesis)
5446	DRAMP21389	SP15D * (De Novo Synthesis)
5447	DRAMP21390	K17 (Derived from ATG16)
5448	DRAMP21391	K18 (Derived from ATG16)
5449	DRAMP21392	K22 (Derived from ATG16)
5450	DRAMP21393	K22.2 (Derived from ATG16)
5451	DRAMP21394	K30 (Derived from ATG16)

5452	DRAMP21395	K31 (Derived from ATG16)
5453	DRAMP21396	K33 (Derived from ATG16)
5454	DRAMP21397	K36 (Derived from ATG16)
5455	DRAMP21398	K46 (Derived from ATG16)
5457	DRAMP21400	NBC2253 (De Novo Synthesis)Â
5458	DRAMP21401	NBC2254 (De Novo Synthesis)Â
5459	DRAMP21402	B1 (Derived from LL-37 and BMAP-27)
5460	DRAMP21403	peptide 1 (De Novo Synthesis)
5461	DRAMP21404	peptide 2 (De Novo Synthesis)
5462	DRAMP21405	LGL13K (De Novo Synthesis)
5463	DRAMP21406	DGL13K (De Novo Synthesis)
5464	DRAMP21407	Bac4K (Derived from CAMPs)
5465	DRAMP21408	Bac3W (Derived from CAMPs)
5466	DRAMP21409	dBac (Derived from CAMPs)
5467	DRAMP21410	dBac4K (Derived from CAMPs)
5468	DRAMP21411	dBac3W (Derived from CAMPs)
5469	DRAMP21412	dBacK (Derived from CAMPs)
5470	DRAMP21413	dBacK- (cap) (Derived from CAMPs)
5471	DRAMP21414	CecB Q53 (Derived from CecB E53)
5472	DRAMP21415	Î±4-short (Derived from Î±4)
5473	DRAMP21416	WV (De Novo Synthesis)
5474	DRAMP21417	WI (De Novo Synthesis)
5475	DRAMP21418	WF (De Novo Synthesis)
5476	DRAMP21419	WW (De Novo Synthesis)
5477	DRAMP21420	AY1C (Derived from AY1)
5478	DRAMP21421	AY1C-AgNP (Derived from AY1)
5479	DRAMP21422	CAY1 (Derived from AY1)
5480	DRAMP21423	CAY1-AgNP (Derived from AY1)
5481	DRAMP21424	B1 (De Novo Synthesis)
5482	DRAMP21425	peptide 2 (Derived from B1)
5483	DRAMP21426	peptide 3 (Derived from B1)
5484	DRAMP21427	peptide 4 (Derived from B1)
5485	DRAMP21428	peptide 5 (Derived from B1)
5486	DRAMP21429	peptide 6 (Derived from B1)
5487	DRAMP21430	peptide 7 (Derived from B1)
5488	DRAMP21431	peptide 8 (Derived from B1)
5489	DRAMP21432	peptide 9 (Derived from B1)
5490	DRAMP21433	peptide 10 (Derived from B1)
5491	DRAMP21434	peptide 11 (Derived from B1)
5494	DRAMP21437	peptide 14 (Derived from B1)
5495	DRAMP21438	peptide 15 (Derived from B1)
5496	DRAMP21439	peptide 16 (Derived from B1)
5497	DRAMP21440	peptide 17 (Derived from B1)
5501	DRAMP21444	peptide 21 (Derived from B1)
5505	DRAMP21448	peptide 25 (Derived from B1)
5508	DRAMP21451	peptide 28 (Derived from B1)
5509	DRAMP21452	peptide 29 (Derived from B1)
5510	DRAMP21453	Hybrid (Derived from Melittin and thanatin)
5511	DRAMP21454	PLP1 (Insects, animals)
5512	DRAMP21455	PLP2 (Insects, animals)

5513	DRAMP21456	PLP3 (Insects, animals)
5514	DRAMP21457	PLP4 (Insects, animals)
5515	DRAMP21458	PLP5 (Insects, animals)
5516	DRAMP21459	PLP6 (Insects, animals)
5517	DRAMP21460	PQ (De Novo Synthesis)
5518	DRAMP21461	PP (De Novo Synthesis)
5519	DRAMP21462	GG (De Novo Synthesis)
5520	DRAMP21463	Qa (De Novo Synthesis)
5521	DRAMP21464	Qna (De Novo Synthesis)
5522	DRAMP21465	P1-Ll-1577 (De Novo Synthesis)
5523	DRAMP21466	P2-Ll-1298 (De Novo Synthesis)
5524	DRAMP21467	P3-Ll-2085 (De Novo Synthesis)
5525	DRAMP21310	RK12 (Derived from PMAP-36)
5527	DRAMP21481	6K-F17
5528	DRAMP21494	MEP-N
5529	DRAMP21579	Val-nHSLP
5538	DRAMP21616	DRIM
5539	DRAMP21618	WWSP
5540	DRAMP21620	KFGF
5541	DRAMP21622	MAP-1
5543	DRAMP21627	E2EM15W
5544	DRAMP21631	SLAY-P1
5545	DRAMP21632	SLAY-P2
5546	DRAMP21633	SLAY-P3 cyclic
5547	DRAMP21634	SLAY-P4
5548	DRAMP21635	SLAY-P5 cyclic
5549	DRAMP21636	SLAY-P6
5550	DRAMP21637	SLAY-P7
5551	DRAMP21638	SLAY-P8
5552	DRAMP21639	SLAY-P9
5553	DRAMP21640	SLAY-P10
5554	DRAMP21641	SLAY-P11
5555	DRAMP21642	SLAY-P12
5556	DRAMP21643	SLAY-P13
5557	DRAMP21644	SLAY-P14
5558	DRAMP21645	SLAY-P15
5559	DRAMP21646	SLAY-P16
5560	DRAMP21647	SLAY-P17
5561	DRAMP21648	SLAY-P18
5562	DRAMP21649	SLAY-P3
5563	DRAMP21650	SLAY-P5
