# Supplementary material for: AMPing Up the Search: A Structural and Functional Repository of Antimicrobial Peptides for Biofilm Studies, and a Case Study of Its Application to Corynebacterium striatum, an Emerging Pathogen
Source: Front Cell Infect Microbiol. 2021 Dec 16;11:803774. doi: 10.3389/fcimb.2021.803774 (PMC8716830; doi:10.3389/fcimb.2021.803774)
Supplement: Supplementary file 6 [file Table_3.pdf]

PepID	DRAMP_ID	Peptide Name	Query	Queried Peptide Name	PMID	Article Title
6	DRAMP00068	Aureocin A53 (Bacteriocin)	"Aureocin A53"[All Fields] AND biofilm[All Fields]	Aureocin A53	31151072	Study of the effectiveness of staphylococcins in biopreservation of Minas fresh (Frescal) cheese with a reduced sodium content.
6	DRAMP00068	Aureocin A53 (Bacteriocin)	"Aureocin A53"[All Fields] AND biofilm[All Fields]	Aureocin A53	22155816	Identification, characterization, and recombinant expression of epidermicin NI01, a novel unmodified bacteriocin produced by Staphylococcus epidermidis that displays potent activity against Staphylococci.
21	DRAMP00177	Enterocin B (EntB; Bacteriocin)	"Enterocin B"[All Fields] AND biofilm[All Fields]	Enterocin B	29729340	Cloning, overexpression, purification of bacteriocin enterocin-B and structural analysis, interaction determination of enterocin-A, B against pathogenic bacteria and human cancer cells.
22	DRAMP00178	Enterocin EJ97 (EntEJ97; Bacteriocin)	"Enterocin EJ97"[All Fields] AND biofilm[All Fields]	Enterocin EJ97	34230527	A bacteriocin-based treatment option for Staphylococcus haemolyticus biofilms.
33	DRAMP00275	Snakin-1 (StSN1; Cys-rich; Plant defensin)	"Snakin-1"[All Fields] AND biofilm[All Fields]	Snakin-1	25670697	Pseudomonas fluorescens Pf-5 genome-wide mutant screen for resistance to the antimicrobial peptide alfalfa snakin-1.
43	DRAMP00425	Tn-AFP1 (Trapa natans antifungal peptide; Plant defensin)	"Tn-AFP1"[All Fields] AND biofilm[All Fields]	Tn-AFP1	21736910	Identification of an antifungal peptide from Trapa natans fruits with inhibitory effects on Candida tropicalis biofilm formation.
55	DRAMP00856	Kalata-B1 (Plant defensin)	"Kalata-B1"[All Fields] AND biofilm[All Fields]	Kalata-B1	29522493	Coupling Plant-Derived Cyclotides to Metal Surfaces: An Antibacterial and Antibiofilm Study.
61	DRAMP00933	Antimicrobial peptide 1 (AMP1; MiAMP1; Plant defensin)	"Antimicrobial peptide 1"[All Fields] AND biofilm[All Fields]	Antimicrobial peptide 1	22445495	Database screening and in vivo efficacy of antimicrobial peptides against methicillin-resistant Staphylococcus aureus USA300.
64	DRAMP01016	Antimicrobial peptide 1 (MJ-AMP1; Plant defensin)	"Antimicrobial peptide 1"[All Fields] AND biofilm[All Fields]	Antimicrobial peptide 1	22445495	Database screening and in vivo efficacy of antimicrobial peptides against methicillin-resistant Staphylococcus aureus USA300.
67	DRAMP01061	Antifungal peptide (Cm-p1; Plants)	"Antifungal peptide"[All Fields] AND biofilm[All Fields]	Antifungal peptide	34442858	Antimicrobial Peptide L18R Displays a Modulating Action against Inter-Kingdom Biofilms in the Lubbock Chronic Wound Biofilm Model.
67	DRAMP01061	Antifungal peptide (Cm-p1; Plants)	"Antifungal peptide"[All Fields] AND biofilm[All Fields]	Antifungal peptide	34408988	The Anti-Biofilm Efficacy of Caffeic Acid Phenethyl Ester (CAPE) In Vitroand a Murine Model of Oral Candidiasis.
67	DRAMP01061	Antifungal peptide (Cm-p1; Plants)	"Antifungal peptide"[All Fields] AND biofilm[All Fields]	Antifungal peptide	33172206	Antibiofilm Activity on Candida albicansand Mechanism of Action on Biomembrane Models of the Antimicrobial Peptide Ctn[15-34].
67	DRAMP01061	Antifungal peptide (Cm-p1; Plants)	"Antifungal peptide"[All Fields] AND biofilm[All Fields]	Antifungal peptide	32605024	Derivates of the Antifungal Peptide Cm-p5 Inhibit Development of Candida aurisBiofilms In Vitro.
67	DRAMP01061	Antifungal peptide (Cm-p1; Plants)	"Antifungal peptide"[All Fields] AND biofilm[All Fields]	Antifungal peptide	30038607	A Proposal of Remedies for Oral Diseases Caused by Candida: A Mini Review.
67	DRAMP01061	Antifungal peptide (Cm-p1; Plants)	"Antifungal peptide"[All Fields] AND biofilm[All Fields]	Antifungal peptide	23892742	FLO11 Gene Is Involved in the Interaction of Flor Strains of Saccharomyces cerevisiae with a Biofilm-Promoting Synthetic Hexapeptide.
67	DRAMP01061	Antifungal peptide (Cm-p1; Plants)	"Antifungal peptide"[All Fields] AND biofilm[All Fields]	Antifungal peptide	23193597	A novel hydroxyproline rich glycopeptide from pericarp of Datura stramonium: proficiently eradicate the biofilm of antifungals resistant Candida albicans.
67	DRAMP01061	Antifungal peptide (Cm-p1; Plants)	"Antifungal peptide"[All Fields] AND biofilm[All Fields]	Antifungal peptide	21736910	Identification of an antifungal peptide from Trapa natans fruits with inhibitory effects on Candida tropicalis biofilm formation.
67	DRAMP01061	Antifungal peptide (Cm-p1; Plants)	"Antifungal peptide"[All Fields] AND biofilm[All Fields]	Antifungal peptide	15992921	Antifungal coating by biofunctionalized polyelectrolyte multilayered films.
94	DRAMP01152	Uperin-3.6 (toads, amphibians, animals)	"Uperin-3.6"[All Fields] AND biofilm[All Fields]	Uperin-3.6	25965506	Activity of Novel Synthetic Peptides against Candida albicans.
99	DRAMP01163	Buforin-2 (Buforin II; Fragment of Histone H2A; toads, amphibians, animals)	"Buforin-2"[All Fields] AND biofilm[All Fields]	Buforin-2	29959905	Antimicrobial and anti-inflammatory activities of chemokine CXCL14-derived antimicrobial peptide and its analogs.
107	DRAMP01184	SPX(1-22)(truncated peptide of Syphaxin; Frogs, amphibians, animals)	"SPX"[All Fields] AND biofilm[All Fields]	SPX	32043713	Streptococcus mutans SpxA2 relays the signal of cell envelope stress from LiaR to effectors that maintain cell wall and membrane homeostasis.
107	DRAMP01184	SPX(1-22)(truncated peptide of Syphaxin; Frogs, amphibians, animals)	"SPX"[All Fields] AND biofilm[All Fields]	SPX	32033530	ClpP participates in stress tolerance, biofilm formation, antimicrobial tolerance, and virulence of Enterococcus faecalis.
107	DRAMP01184	SPX(1-22)(truncated peptide of Syphaxin; Frogs, amphibians, animals)	"SPX"[All Fields] AND biofilm[All Fields]	SPX	31618469	The Staphylococcus aureus ArlRS two-component system regulates virulence factor expression through MgrA.
107	DRAMP01184	SPX(1-22)(truncated peptide of Syphaxin; Frogs, amphibians, animals)	"SPX"[All Fields] AND biofilm[All Fields]	SPX	30924407	The role of ArlRS in regulating oxacillin susceptibility in methicillin-resistant Staphylococcus aureus indicates it is a potential target for antimicrobial resistance breakers.
107	DRAMP01184	SPX(1-22)(truncated peptide of Syphaxin; Frogs, amphibians, animals)	"SPX"[All Fields] AND biofilm[All Fields]	SPX	30718304	Inactivation of cysLInhibits Biofilm Formation by Activating the Disulfide Stress Regulator Spx in Bacillus subtilis.
107	DRAMP01184	SPX(1-22)(truncated peptide of Syphaxin; Frogs, amphibians, animals)	"SPX"[All Fields] AND biofilm[All Fields]	SPX	29049010	Biofilm-Related Diseases and Omics: Global Transcriptional Profiling of Enterococcus faecium Reveals Different Gene Expression Patterns in the Biofilm and Planktonic Cells.
107	DRAMP01184	SPX(1-22)(truncated peptide of Syphaxin; Frogs, amphibians, animals)	"SPX"[All Fields] AND biofilm[All Fields]	SPX	27501984	AraC-Type Regulator Rbf Controls the Staphylococcus epidermidis Biofilm Phenotype by Negatively Regulating the icaADBC Repressor SarR.
107	DRAMP01184	SPX(1-22)(truncated peptide of Syphaxin; Frogs, amphibians, animals)	"SPX"[All Fields] AND biofilm[All Fields]	SPX	27037617	Inactivation of the spxA1 or spxA2 gene of Streptococcus mutans decreases virulence in the rat caries model.
107	DRAMP01184	SPX(1-22)(truncated peptide of Syphaxin; Frogs, amphibians, animals)	"SPX"[All Fields] AND biofilm[All Fields]	SPX	25905865	Transcriptional and Phenotypic Characterization of Novel Spx-Regulated Genes in Streptococcus mutans.
107	DRAMP01184	SPX(1-22)(truncated peptide of Syphaxin; Frogs, amphibians, animals)	"SPX"[All Fields] AND biofilm[All Fields]	SPX	22194450	The YjbH adaptor protein enhances proteolysis of the transcriptional regulator Spx in Staphylococcus aureus.
107	DRAMP01184	SPX(1-22)(truncated peptide of Syphaxin; Frogs, amphibians, animals)	"SPX"[All Fields] AND biofilm[All Fields]	SPX	21485312	Oxygen dependent pyruvate oxidase expression and production in Streptococcus sanguinis.
107	DRAMP01184	SPX(1-22)(truncated peptide of Syphaxin; Frogs, amphibians, animals)	"SPX"[All Fields] AND biofilm[All Fields]	SPX	20402773	Role of spx in biofilm formation of Staphylococcus epidermidis.
107	DRAMP01184	SPX(1-22)(truncated peptide of Syphaxin; Frogs, amphibians, animals)	"SPX"[All Fields] AND biofilm[All Fields]	SPX	19181818	Role of Clp proteins in expression of virulence properties of Streptococcus mutans.
107	DRAMP01184	SPX(1-22)(truncated peptide of Syphaxin; Frogs, amphibians, animals)	"SPX"[All Fields] AND biofilm[All Fields]	SPX	16788195	Spx is a global effector impacting stress tolerance and biofilm formation in Staphylococcus aureus.
108	DRAMP01185	SPX(1-16)(truncated peptide of Syphaxin; Frogs, amphibians, animals)	"SPX"[All Fields] AND biofilm[All Fields]	SPX	32043713	Streptococcus mutans SpxA2 relays the signal of cell envelope stress from LiaR to effectors that maintain cell wall and membrane homeostasis.
108	DRAMP01185	SPX(1-16)(truncated peptide of Syphaxin; Frogs, amphibians, animals)	"SPX"[All Fields] AND biofilm[All Fields]	SPX	32033530	ClpP participates in stress tolerance, biofilm formation, antimicrobial tolerance, and virulence of Enterococcus faecalis.
108	DRAMP01185	SPX(1-16)(truncated peptide of Syphaxin; Frogs, amphibians, animals)	"SPX"[All Fields] AND biofilm[All Fields]	SPX	31618469	The Staphylococcus aureus ArlRS two-component system regulates virulence factor expression through MgrA.

108	DRAMP01185	SPX(1-16)(truncated peptide of Syphaxin; Frogs, amphibians, animals)	"SPX"[All Fields] AND biofilm[All Fields]	SPX	30924407	The role of ArlRS in regulating oxacillin susceptibility in methicillin-resistant Staphylococcus aureus indicates it is a potential target for antimicrobial resistance breakers.
108	DRAMP01185	SPX(1-16)(truncated peptide of Syphaxin; Frogs, amphibians, animals)	"SPX"[All Fields] AND biofilm[All Fields]	SPX	30718304	Inactivation of cysLInhibits Biofilm Formation by Activating the Disulfide Stress Regulator Spx in Bacillus subtilis.
108	DRAMP01185	SPX(1-16)(truncated peptide of Syphaxin; Frogs, amphibians, animals)	"SPX"[All Fields] AND biofilm[All Fields]	SPX	29049010	Biofilm-Related Diseases and Omics: Global Transcriptional Profiling of Enterococcus faecium Reveals Different Gene Expression Patterns in the Biofilm and Planktonic Cells.
108	DRAMP01185	SPX(1-16)(truncated peptide of Syphaxin; Frogs, amphibians, animals)	"SPX"[All Fields] AND biofilm[All Fields]	SPX	27501984	AraC-Type Regulator Rbf Controls the Staphylococcus epidermidis Biofilm Phenotype by Negatively Regulating the icaADBC Repressor SarR.
108	DRAMP01185	SPX(1-16)(truncated peptide of Syphaxin; Frogs, amphibians, animals)	"SPX"[All Fields] AND biofilm[All Fields]	SPX	27037617	Inactivation of the spxA1 or spxA2 gene of Streptococcus mutans decreases virulence in the rat caries model.
108	DRAMP01185	SPX(1-16)(truncated peptide of Syphaxin; Frogs, amphibians, animals)	"SPX"[All Fields] AND biofilm[All Fields]	SPX	25905865	Transcriptional and Phenotypic Characterization of Novel Spx-Regulated Genes in Streptococcus mutans.
108	DRAMP01185	SPX(1-16)(truncated peptide of Syphaxin; Frogs, amphibians, animals)	"SPX"[All Fields] AND biofilm[All Fields]	SPX	22194450	The YjbH adaptor protein enhances proteolysis of the transcriptional regulator Spx in Staphylococcus aureus.
108	DRAMP01185	SPX(1-16)(truncated peptide of Syphaxin; Frogs, amphibians, animals)	"SPX"[All Fields] AND biofilm[All Fields]	SPX	21485312	Oxygen dependent pyruvate oxidase expression and production in Streptococcus sanguinis.
108	DRAMP01185	SPX(1-16)(truncated peptide of Syphaxin; Frogs, amphibians, animals)	"SPX"[All Fields] AND biofilm[All Fields]	SPX	20402773	Role of spx in biofilm formation of Staphylococcus epidermidis.
108	DRAMP01185	SPX(1-16)(truncated peptide of Syphaxin; Frogs, amphibians, animals)	"SPX"[All Fields] AND biofilm[All Fields]	SPX	19181818	Role of Clp proteins in expression of virulence properties of Streptococcus mutans.
108	DRAMP01185	SPX(1-16)(truncated peptide of Syphaxin; Frogs, amphibians, animals)	"SPX"[All Fields] AND biofilm[All Fields]	SPX	16788195	Spx is a global effector impacting stress tolerance and biofilm formation in Staphylococcus aureus.
154	DRAMP01288	Phylloseptin-1 (PSN-1; Frogs, amphibians, animals)	"Phylloseptin-1"[All Fields] AND biofilm[All Fields]	Phylloseptin-1	20451254	Phylloseptin-1 (PSN-1) from Phyllomedusa sauvagei skin secretion: a novel broad-spectrum antimicrobial peptide with antibiofilm activity.
155	DRAMP01301	Phylloseptin-1 (PS-1; Frogs, amphibians, animals)	"Phylloseptin-1"[All Fields] AND biofilm[All Fields]	Phylloseptin-1	20451254	Phylloseptin-1 (PSN-1) from Phyllomedusa sauvagei skin secretion: a novel broad-spectrum antimicrobial peptide with antibiofilm activity.
172	DRAMP01355	Ranalexin (Frogs, amphibians, animals)	"Ranalexin"[All Fields] AND biofilm[All Fields]	Ranalexin	28628484	Battling bacterial infection with hexamethylene diisocyanate cross-linked and Cefaclor-loaded collagen scaffolds.
172	DRAMP01355	Ranalexin (Frogs, amphibians, animals)	"Ranalexin"[All Fields] AND biofilm[All Fields]	Ranalexin	28453851	Eradication of Staphylococcus aureus Biofilm Infections Using Synthetic Antimicrobial Peptides.
172	DRAMP01355	Ranalexin (Frogs, amphibians, animals)	"Ranalexin"[All Fields] AND biofilm[All Fields]	Ranalexin	26684017	Antimicrobial Peptides as Anti-Infectives against Staphylococcus epidermidis.
186	DRAMP00929	Antimicrobial peptide 1 (Cn-AMP1; Plant defensin)	"Antimicrobial peptide 1"[All Fields] AND biofilm[All Fields]	Antimicrobial peptide 1	22445495	Database screening and in vivo efficacy of antimicrobial peptides against methicillin-resistant Staphylococcus aureus USA300.
187	DRAMP03542	Neurokinin A (NKA; chicken, animals)	"Neurokinin A"[All Fields] AND biofilm[All Fields]	Neurokinin A	31147200	Thiol-Based Drugs in Pulmonary Medicine: Much More than Mucolytics.
188	DRAMP04532	Myxinidin (Hagfish, animals)	"Myxinidin"[All Fields] AND biofilm[All Fields]	Myxinidin	30735368	Enhancing the Potency of Antimicrobial Peptides through Molecular Engineering and Self-Assembly.
188	DRAMP04532	Myxinidin (Hagfish, animals)	"Myxinidin"[All Fields] AND biofilm[All Fields]	Myxinidin	29152103	Myxinidin2 and myxinidin3 suppress inflammatory responses through STAT3 and MAPKs to promote wound healing.
188	DRAMP04532	Myxinidin (Hagfish, animals)	"Myxinidin"[All Fields] AND biofilm[All Fields]	Myxinidin	26450121	Design and membrane-disruption mechanism of charge-enriched AMPs exhibiting cell selectivity, high-salt resistance, and anti-biofilm properties.
191	DRAMP02997	Apidaecin-1B (Apidaecin IB; Insects, animals)	"Apidaecin-1B"[All Fields] AND biofilm[All Fields]	Apidaecin-1B	27405093	Identification of New Resistance Mechanisms in Escherichia coli against Apidaecin 1b Using Quantitative Gel- and LC-MS-Based Proteomics.
193	DRAMP02840	Lactoferricin B (Lfcin B; mammals, animals)	"Lactoferricin B"[All Fields] AND biofilm[All Fields]	Lactoferricin B	33249255	Inhibitory effect of LL-37 and human lactoferricin on growth and biofilm formation of anaerobes associated with oral diseases.
193	DRAMP02840	Lactoferricin B (Lfcin B; mammals, animals)	"Lactoferricin B"[All Fields] AND biofilm[All Fields]	Lactoferricin B	32634470	Lactoferrin and lactoferricin B reduce adhesion and biofilm formation in the intestinal symbionts Bacteroides fragilis and Bacteroides thetaiotaomicron.
193	DRAMP02840	Lactoferricin B (Lfcin B; mammals, animals)	"Lactoferricin B"[All Fields] AND biofilm[All Fields]	Lactoferricin B	27902776	Inhibition of Candida albicans Biofilm Formation by the Synthetic Lactoferricin Derived Peptide hLF1-11.
193	DRAMP02840	Lactoferricin B (Lfcin B; mammals, animals)	"Lactoferricin B"[All Fields] AND biofilm[All Fields]	Lactoferricin B	26149536	Antimicrobial activity of synthetic cationic peptides and lipopeptides derived from human lactoferricin against Pseudomonas aeruginosa planktonic cultures and biofilms.
193	DRAMP02840	Lactoferricin B (Lfcin B; mammals, animals)	"Lactoferricin B"[All Fields] AND biofilm[All Fields]	Lactoferricin B	22410856	Effects of lactoferricin B against keratitis-associated fungal biofilms.
193	DRAMP02840	Lactoferricin B (Lfcin B; mammals, animals)	"Lactoferricin B"[All Fields] AND biofilm[All Fields]	Lactoferricin B	19451301	Inhibitory effects of lactoferrin on growth and biofilm formation of Porphyromonas gingivalis and Prevotella intermedia.
235	DRAMP18394	NCR335 (nodule-specific cysteine-rich peptides; plants)	"NCR335"[All Fields] AND biofilm[All Fields]	NCR335	33915930	Symbiotic NCR Peptide Fragments Affect the Viability, Morphology and Biofilm Formation of CandidaSpecies.
285	DRAMP01513	Esculentin-1 (Frogs, amphibians, animals)	"Esculentin-1"[All Fields] AND biofilm[All Fields]	Esculentin-1	26162435	D-Amino acids incorporation in the frog skin-derived peptide esculentin-1a(1-21)NH2 is beneficial for its multiple functions.
285	DRAMP01513	Esculentin-1 (Frogs, amphibians, animals)	"Esculentin-1"[All Fields] AND biofilm[All Fields]	Esculentin-1	23503622	Esculentin(1-21), an amphibian skin membrane-active peptide with potent activity on both planktonic and biofilm cells of the bacterial pathogen Pseudomonas aeruginosa.
318	DRAMP01587	Citropin-1.1 (Frogs, amphibians, animals)	"Citropin-1.1"[All Fields] AND biofilm[All Fields]	Citropin-1.1	31678322	Synergistic combinations of antimicrobial peptides against biofilms of methicillin-resistant Staphylococcus aureus (MRSA) on polystyrene and medical devices.
318	DRAMP01587	Citropin-1.1 (Frogs, amphibians, animals)	"Citropin-1.1"[All Fields] AND biofilm[All Fields]	Citropin-1.1	30043322	Antimicrobial Activity of Selected Antimicrobial Peptides Against Planktonic Culture and Biofilm of Acinetobacter baumannii.
318	DRAMP01587	Citropin-1.1 (Frogs, amphibians, animals)	"Citropin-1.1"[All Fields] AND biofilm[All Fields]	Citropin-1.1	28355248	Searching for new strategies against biofilm infections: Colistin-AMP combinations against Pseudomonas aeruginosa and Staphylococcus aureus single- and double-species biofilms.
318	DRAMP01587	Citropin-1.1 (Frogs, amphibians, animals)	"Citropin-1.1"[All Fields] AND biofilm[All Fields]	Citropin-1.1	27231918	The Antistaphylococcal Activity of Citropin 1.1 and Temporin A against Planktonic Cells and Biofilms Formed by Isolates from Patients with Atopic Dermatitis: An Assessment of Their Potential to Induce Microbial Resistance Compared to Conventional Antimicrobials.
318	DRAMP01587	Citropin-1.1 (Frogs, amphibians, animals)	"Citropin-1.1"[All Fields] AND biofilm[All Fields]	Citropin-1.1	24779193	Antimicrobial peptides as potential tool to fight bacterial biofilm.
318	DRAMP01587	Citropin-1.1 (Frogs, amphibians, animals)	"Citropin-1.1"[All Fields] AND biofilm[All Fields]	Citropin-1.1	25804062	Synthetic amphibian peptides and short amino-acids derivatives against planktonic cells and mature biofilm of Providencia stuartii clinical strains.
318	DRAMP01587	Citropin-1.1 (Frogs, amphibians, animals)	"Citropin-1.1"[All Fields] AND biofilm[All Fields]	Citropin-1.1	16289474	Citropin 1.1-treated central venous catheters improve the efficacy of hydrophobic antibiotics in the treatment of experimental staphylococcal catheter-related infection.
329	DRAMP01607	Aurein-1.2 (Frogs, amphibians, animals)	"Aurein-1.2"[All Fields] AND biofilm[All Fields]	Aurein-1.2	31550929	Photodynamic and peptide-based strategy to inhibit Gram-positive bacterial biofilm formation.
329	DRAMP01607	Aurein-1.2 (Frogs, amphibians, animals)	"Aurein-1.2"[All Fields] AND biofilm[All Fields]	Aurein-1.2	30569430	Alanine Scanning Studies of the Antimicrobial Peptide Aurein 1.2.
329	DRAMP01607	Aurein-1.2 (Frogs, amphibians, animals)	"Aurein-1.2"[All Fields] AND biofilm[All Fields]	Aurein-1.2	30043322	Antimicrobial Activity of Selected Antimicrobial Peptides Against Planktonic Culture and Biofilm of Acinetobacter baumannii.
331	DRAMP01612	Aurein-2.5 (Frogs, amphibians, animals)	"Aurein-2.5"[All Fields] AND biofilm[All Fields]	Aurein-2.5	19056250	A study on the interactions of Aurein 2.5 with bacterial membranes.
353	DRAMP18386	VK25 (histone derived; reptiles; animals)	"VK25"[All Fields] AND biofilm[All Fields]	VK25	28649410	Komodo dragon-inspired synthetic peptide DRGN-1 promotes wound-healing of a mixed-biofilm infected wound.
359	DRAMP01730	Temporin-A (Frogs, amphibians, animals)	"Temporin-A"[All Fields] AND biofilm[All Fields]	Temporin-A	32887236	Activity of Temporin A and Short Lipopeptides Combined with Gentamicin against Biofilm Formed by Staphylococcus aureusand Pseudomonas aeruginosa.
359	DRAMP01730	Temporin-A (Frogs, amphibians, animals)	"Temporin-A"[All Fields] AND biofilm[All Fields]	Temporin-A	31678322	Synergistic combinations of antimicrobial peptides against biofilms of methicillin-resistant Staphylococcus aureus (MRSA) on polystyrene and medical devices.

359	DRAMP01730	Temporin-A (Frogs, amphibians, animals)	"Temporin-A"[All Fields] AND biofilm[All Fields]	Temporin-A	30043322	Antimicrobial Activity of Selected Antimicrobial Peptides Against Planktonic Culture and Biofilm of Acinetobacter baumannii.
359	DRAMP01730	Temporin-A (Frogs, amphibians, animals)	"Temporin-A"[All Fields] AND biofilm[All Fields]	Temporin-A	28355248	Searching for new strategies against biofilm infections: Colistin-AMP combinations against Pseudomonas aeruginosa and Staphylococcus aureus single- and double-species biofilms.
359	DRAMP01730	Temporin-A (Frogs, amphibians, animals)	"Temporin-A"[All Fields] AND biofilm[All Fields]	Temporin-A	27231918	The Antistaphylococcal Activity of Citropin 1.1 and Temporin A against Planktonic Cells and Biofilms Formed by Isolates from Patients with Atopic Dermatitis: An Assessment of Their Potential to Induce Microbial Resistance Compared to Conventional Antimicrobials.
359	DRAMP01730	Temporin-A (Frogs, amphibians, animals)	"Temporin-A"[All Fields] AND biofilm[All Fields]	Temporin-A	24779193	Antimicrobial peptides as potential tool to fight bacterial biofilm.
373	DRAMP01754	Temporin-1CEb (Frogs, amphibians, animals)	"Temporin-1CEb"[All Fields] AND biofilm[All Fields]	Temporin-1CEb	34206444	Lipidation of Temporin-1CEb Derivatives as a Tool for Activity Improvement, Pros and Cons of the Approach.
373	DRAMP01754	Temporin-1CEb (Frogs, amphibians, animals)	"Temporin-1CEb"[All Fields] AND biofilm[All Fields]	Temporin-1CEb	31557917	The Bactericidal Activity of Temporin Analogues Against Methicillin Resistant Staphylococcus aureus.
373	DRAMP01754	Temporin-1CEb (Frogs, amphibians, animals)	"Temporin-1CEb"[All Fields] AND biofilm[All Fields]	Temporin-1CEb	25056289	Effects of antimicrobial peptide L-K6, a temporin-1CEb analog on oral pathogen growth, Streptococcus mutans biofilm formation, and anti-inflammatory activity.
386	DRAMP01779	Temporin-SHf (Frogs, amphibians, animals)	"Temporin-SHf"[All Fields] AND biofilm[All Fields]	Temporin-SHf	27915018	Design and surface immobilization of short anti-biofilm peptides.
387	DRAMP01780	Temporin-SHa (Temporin-1Sa; Frogs, amphibians, animals)	"Temporin-SHa"[All Fields] AND biofilm[All Fields]	Temporin-SHa	30813478	Engineering of Antimicrobial Surfaces by Using Temporin Analogs to Tune the Biocidal/antiadhesive Effect.
387	DRAMP01780	Temporin-SHa (Temporin-1Sa; Frogs, amphibians, animals)	"Temporin-SHa"[All Fields] AND biofilm[All Fields]	Temporin-SHa	32254250	Site-specific grafting on titanium surfaces with hybrid temporin antibacterial peptides.
414	DRAMP01847	Ascaphin-8 (Frogs, amphibians, animals)	"Ascaphin-8"[All Fields] AND biofilm[All Fields]	Ascaphin-8	22445495	Database screening and in vivo efficacy of antimicrobial peptides against methicillin-resistant Staphylococcus aureus USA300.
485	DRAMP02009	Brevinin-1 (Frogs, amphibians, animals)	"Brevinin-1"[All Fields] AND biofilm[All Fields]	Brevinin-1	32812694	Characterisation of a novel peptide, Brevinin-1H, from the skin secretion of Amolops hainanensis and rational design of several analogues.
485	DRAMP02009	Brevinin-1 (Frogs, amphibians, animals)	"Brevinin-1"[All Fields] AND biofilm[All Fields]	Brevinin-1	32751489	Enhanced Antimicrobial Activity of N-Terminal Derivatives of a Novel Brevinin-1 Peptide from The Skin Secretion of Odorrana schmackeri.
485	DRAMP02009	Brevinin-1 (Frogs, amphibians, animals)	"Brevinin-1"[All Fields] AND biofilm[All Fields]	Brevinin-1	32347293	Brevinin-1GHd: a novel Hylarana guentheri skin secretion-derived Brevinin-1 type peptide with antimicrobial and anticancer therapeutic potential.
485	DRAMP02009	Brevinin-1 (Frogs, amphibians, animals)	"Brevinin-1"[All Fields] AND biofilm[All Fields]	Brevinin-1	30322120	Evaluating the Bioactivity of a Novel Broad-Spectrum Antimicrobial Peptide Brevinin-1GHa from the Frog Skin Secretion of Hylarana guentheriand Its Analogues.
486	DRAMP02010	Brevinin-2 (Frogs, amphibians, animals)	"Brevinin-2"[All Fields] AND biofilm[All Fields]	Brevinin-2	31549575	Brevinin-GR23 from frog Hylarana guentheriwith antimicrobial and antibiofilm activities against Staphylococcus aureus.
486	DRAMP02010	Brevinin-2 (Frogs, amphibians, animals)	"Brevinin-2"[All Fields] AND biofilm[All Fields]	Brevinin-2	30322120	Evaluating the Bioactivity of a Novel Broad-Spectrum Antimicrobial Peptide Brevinin-1GHa from the Frog Skin Secretion of Hylarana guentheriand Its Analogues.
488	DRAMP18381	H4-(86-100) (histone-derived)	"H4-"[All Fields] AND biofilm[All Fields]	H4-	34078285	Human microbiota modulation via QseC sensor kinase mediated in the Escherichia coli O104:H4 outbreak strain infection in microbiome model.
488	DRAMP18381	H4-(86-100) (histone-derived)	"H4-"[All Fields] AND biofilm[All Fields]	H4-	33939753	Natural and synthetic antimicrobials reduce adherence of enteroaggregative and enterohemorrhagic Escherichia coli to epithelial cells.
488	DRAMP18381	H4-(86-100) (histone-derived)	"H4-"[All Fields] AND biofilm[All Fields]	H4-	33689469	Contamination and Biofilm Formation of Foodborne and Opportunistic Pathogens in Yellow-Feathered Chicken Carcass.
488	DRAMP18381	H4-(86-100) (histone-derived)	"H4-"[All Fields] AND biofilm[All Fields]	H4-	33356871	The Superior Adherence Phenotype of E. coli O104:H4 is Directly Mediated by the Aggregative Adherence Fimbriae Type I.
488	DRAMP18381	H4-(86-100) (histone-derived)	"H4-"[All Fields] AND biofilm[All Fields]	H4-	32989036	Success of Escherichia coli O25b:H4 Sequence Type 131 Clade C Associated with a Decrease in Virulence.
488	DRAMP18381	H4-(86-100) (histone-derived)	"H4-"[All Fields] AND biofilm[All Fields]	H4-	32198417	Enhanced purification coupled with biophysical analyses shows cross-β structure as a core building block for Streptococcus mutans functional amyloids.
488	DRAMP18381	H4-(86-100) (histone-derived)	"H4-"[All Fields] AND biofilm[All Fields]	H4-	32131899	Oral colonisation by antimicrobial-resistant Gram-negative bacteria among long-term care facility residents: prevalence, risk factors, and molecular epidemiology.
488	DRAMP18381	H4-(86-100) (histone-derived)	"H4-"[All Fields] AND biofilm[All Fields]	H4-	31488056	Differential transcriptome analysis of enterohemorrhagic Escherichia coli strains reveals differences in response to plant-derived compounds.
488	DRAMP18381	H4-(86-100) (histone-derived)	"H4-"[All Fields] AND biofilm[All Fields]	H4-	31370119	Anti-Biofilm Activity of Grapefruit Seed Extract against Staphylococcus aureusand Escherichia coli.
488	DRAMP18381	H4-(86-100) (histone-derived)	"H4-"[All Fields] AND biofilm[All Fields]	H4-	31275267	AHLs Regulate Biofilm Formation and Swimming Motility of Hafnia alveiH4.
488	DRAMP18381	H4-(86-100) (histone-derived)	"H4-"[All Fields] AND biofilm[All Fields]	H4-	30934749	Metagenomic Profiling of Microbial Pathogens in the Little Bighorn River, Montana.
488	DRAMP18381	H4-(86-100) (histone-derived)	"H4-"[All Fields] AND biofilm[All Fields]	H4-	30526606	Adherent/invasive Escherichia coli (AIEC) isolates from asymptomatic people: new E. coli ST131 O25:H4/H30-Rx virotypes.
488	DRAMP18381	H4-(86-100) (histone-derived)	"H4-"[All Fields] AND biofilm[All Fields]	H4-	30387769	Crystal structure of an Lrs14-like archaeal biofilm regulator from Sulfolobus acidocaldarius.
488	DRAMP18381	H4-(86-100) (histone-derived)	"H4-"[All Fields] AND biofilm[All Fields]	H4-	30221799	Effects of Sulfide Flavors on AHL-Mediated Quorum Sensing and Biofilm Formation of Hafnia alvei.
488	DRAMP18381	H4-(86-100) (histone-derived)	"H4-"[All Fields] AND biofilm[All Fields]	H4-	29941383	Comparative virulence characterization of the Shiga toxin phage-cured Escherichia coli O104:H4 and enteroaggregative Escherichia coli.
488	DRAMP18381	H4-(86-100) (histone-derived)	"H4-"[All Fields] AND biofilm[All Fields]	H4-	29910626	Hybridization and antibiotic synergism as a tool for reducing the cytotoxicity of antimicrobial peptides.
488	DRAMP18381	H4-(86-100) (histone-derived)	"H4-"[All Fields] AND biofilm[All Fields]	H4-	29543544	Abundant production of exopolysaccharide by EAEC strains enhances the formation of bacterial biofilms in contaminated sprouts.
488	DRAMP18381	H4-(86-100) (histone-derived)	"H4-"[All Fields] AND biofilm[All Fields]	H4-	28796849	Neutrophil Extracellular Traps and Fibrin in Otitis Media: Analysis of Human and Chinchilla Temporal Bones.
488	DRAMP18381	H4-(86-100) (histone-derived)	"H4-"[All Fields] AND biofilm[All Fields]	H4-	28402186	Inhibition of Hafnia alvei H4 Biofilm Formation by the Food Additive Dihydrocoumarin.
488	DRAMP18381	H4-(86-100) (histone-derived)	"H4-"[All Fields] AND biofilm[All Fields]	H4-	28379194	Characteristics of N-Acylhomoserine Lactones Produced by Hafnia alvei H4 Isolated from Spoiled Instant Sea Cucumber.
488	DRAMP18381	H4-(86-100) (histone-derived)	"H4-"[All Fields] AND biofilm[All Fields]	H4-	28378802	Histones from Avian Erythrocytes Exhibit Antibiofilm activity against methicillin-sensitive and methicillin-resistant Staphylococcus aureus.
488	DRAMP18381	H4-(86-100) (histone-derived)	"H4-"[All Fields] AND biofilm[All Fields]	H4-	28036250	Actinotalea caeni sp. nov., isolated from a sludge sample of a biofilm reactor.
488	DRAMP18381	H4-(86-100) (histone-derived)	"H4-"[All Fields] AND biofilm[All Fields]	H4-	27902285	Raineyella antarctica gen. nov., sp. nov., a psychrotolerant, d-amino-acid-utilizing anaerobe isolated from two geographic locations of the Southern Hemisphere.
488	DRAMP18381	H4-(86-100) (histone-derived)	"H4-"[All Fields] AND biofilm[All Fields]	H4-	27709726	Anti-Campylobacter activity of resveratrol and an extract from waste Pinot noir grape skins and seeds, and resistance of Camp. jejuni planktonic and biofilm cells, mediated via the CmeABC efflux pump.
488	DRAMP18381	H4-(86-100) (histone-derived)	"H4-"[All Fields] AND biofilm[All Fields]	H4-	27667095	Polysorbates prevent biofilm formation and pathogenesis of Escherichia coli O104:H4.
488	DRAMP18381	H4-(86-100) (histone-derived)	"H4-"[All Fields] AND biofilm[All Fields]	H4-	27375253	Natural plant products inhibits growth and alters the swarming motility, biofilm formation, and expression of virulence genes in enteroaggregative and enterohemorrhagic Escherichia coli.
488	DRAMP18381	H4-(86-100) (histone-derived)	"H4-"[All Fields] AND biofilm[All Fields]	H4-	27252465	Phylogenetic Analysis of Enteroaggregative Escherichia coli (EAEC) Isolates from Japan Reveals Emergence of CTX-M-14-Producing EAEC O25:H4 Clones Related to Sequence Type 131.
488	DRAMP18381	H4-(86-100) (histone-derived)	"H4-"[All Fields] AND biofilm[All Fields]	H4-	27208096	Different Cellular Origins and Functions of Extracellular Proteins from Escherichia coli O157:H7 and O104:H4 as Determined by Comparative Proteomic Analysis.
488	DRAMP18381	H4-(86-100) (histone-derived)	"H4-"[All Fields] AND biofilm[All Fields]	H4-	27099984	Aggregative adherence fimbriae I (AAF/I) mediate colonization of fresh produce and abiotic surface by Shiga toxigenic enteroaggregative Escherichia coli O104:H4.
488	DRAMP18381	H4-(86-100) (histone-derived)	"H4-"[All Fields] AND biofilm[All Fields]	H4-	26517878	The Role of Long Polar Fimbriae in Escherichia coli O104:H4 Adhesion and Colonization.
488	DRAMP18381	H4-(86-100) (histone-derived)	"H4-"[All Fields] AND biofilm[All Fields]	H4-	26303830	Genome-Based Comparison of Cyclic Di-GMP Signaling in Pathogenic and Commensal Escherichia coli Strains.
488	DRAMP18381	H4-(86-100) (histone-derived)	"H4-"[All Fields] AND biofilm[All Fields]	H4-	25653719	Escherichia coli from Crohn's disease patient displays virulence features of enteroinvasive (EIEC), enterohemorragic (EHEC), and enteroaggregative (EAEC) pathotypes.
488	DRAMP18381	H4-(86-100) (histone-derived)	"H4-"[All Fields] AND biofilm[All Fields]	H4-	25590020	Overview and Historical Perspectives.
488	DRAMP18381	H4-(86-100) (histone-derived)	"H4-"[All Fields] AND biofilm[All Fields]	H4-	25361688	Cyclic-di-GMP signalling and biofilm-related properties of the Shiga toxin-producing 2011 German outbreak Escherichia coli O104:H4.
488	DRAMP18381	H4-(86-100) (histone-derived)	"H4-"[All Fields] AND biofilm[All Fields]	H4-	24994776	Flaviflexus salsibiostraticola sp. nov., an actinobacterium isolated from a biofilm reactor.
488	DRAMP18381	H4-(86-100) (histone-derived)	"H4-"[All Fields] AND biofilm[All Fields]	H4-	24982324	Epidemiology and clinical manifestations of enteroaggregative Escherichia coli.
488	DRAMP18381	H4-(86-100) (histone-derived)	"H4-"[All Fields] AND biofilm[All Fields]	H4-	23737602	A FimH inhibitor prevents acute bladder infection and treats chronic cystitis caused by multidrug-resistant uropathogenic Escherichia coli ST131.
488	DRAMP18381	H4-(86-100) (histone-derived)	"H4-"[All Fields] AND biofilm[All Fields]	H4-	23667145	Nocardioides salsibiostraticola sp. nov., isolated from biofilm formed in coastal seawater.
488	DRAMP18381	H4-(86-100) (histone-derived)	"H4-"[All Fields] AND biofilm[All Fields]	H4-	23045357	Multiresistant uropathogenic Escherichia coli from a region in India where urinary tract infections are endemic: genotypic and phenotypic characteristics of sequence type 131 isolates of the CTX-M-15 extended-spectrum-β-lactamase-producing lineage.

488	DRAMP18381	H4-(86-100) (histone-derived)	"H4-"[All Fields] AND biofilm[All Fields]	H4-	22848550	Correlation between in vivo biofilm formation and virulence gene expression in Escherichia coli O104:H4.
488	DRAMP18381	H4-(86-100) (histone-derived)	"H4-"[All Fields] AND biofilm[All Fields]	H4-	22798645	Nocardioides albertanoniae sp. nov., isolated from Roman catacombs.
488	DRAMP18381	H4-(86-100) (histone-derived)	"H4-"[All Fields] AND biofilm[All Fields]	H4-	22448273	High-density transcriptional initiation signals underline genomic islands in bacteria.
488	DRAMP18381	H4-(86-100) (histone-derived)	"H4-"[All Fields] AND biofilm[All Fields]	H4-	22362795	Escherichia coli from clinical mastitis: serotypes and virulence factors.
488	DRAMP18381	H4-(86-100) (histone-derived)	"H4-"[All Fields] AND biofilm[All Fields]	H4-	22118225	Intestinal colonization by enteroaggregative Escherichia coli supports long-term bacteriophage replication in mice.
488	DRAMP18381	H4-(86-100) (histone-derived)	"H4-"[All Fields] AND biofilm[All Fields]	H4-	20605982	Development and validation of an in vivo Candida albicans biofilm denture model.
488	DRAMP18381	H4-(86-100) (histone-derived)	"H4-"[All Fields] AND biofilm[All Fields]	H4-	20516280	Role of Fks1p and matrix glucan in Candida albicans biofilm resistance to an echinocandin, pyrimidine, and polyene.
488	DRAMP18381	H4-(86-100) (histone-derived)	"H4-"[All Fields] AND biofilm[All Fields]	H4-	20367070	Escherichia coli serotype O157:H7 retention on solid surfaces and peroxide resistance is enhanced by dual-strain biofilm formation.
488	DRAMP18381	H4-(86-100) (histone-derived)	"H4-"[All Fields] AND biofilm[All Fields]	H4-	18334490	The CTX-M-15-producing Escherichia coli diffusing clone belongs to a highly virulent B2 phylogenetic subgroup.
488	DRAMP18381	H4-(86-100) (histone-derived)	"H4-"[All Fields] AND biofilm[All Fields]	H4-	16003979	Microbial community in biofilm on membrane surface of submerged MBR: effect of in-line cleaning chemical agent.
488	DRAMP18381	H4-(86-100) (histone-derived)	"H4-"[All Fields] AND biofilm[All Fields]	H4-	15754245	Analysis of microbial community structure in a biofilm on membrane surface in the submerged membrane bioreactor treating domestic wastewater on the basis of respiratory quinone profiles.
488	DRAMP18381	H4-(86-100) (histone-derived)	"H4-"[All Fields] AND biofilm[All Fields]	H4-	14982188	Microbial community structure of membrane fouling film in an intermittently and continuously aerated submerged membrane bioreactor treating domestic wastewater.
489	DRAMP18382	HNr (histone-derived)	"HNr"[All Fields] AND biofilm[All Fields]	HNr	18519731	Role of global regulators and nucleotide metabolism in antibiotic tolerance in Escherichia coli.
542	DRAMP01746	Temporin-L (Temporin-1Tl; temporin-Tl; TL; Frogs, amphibians, animals)	"Temporin-L"[All Fields] AND biofilm[All Fields]	Temporin-L	33198325	Antibiofilm Properties of Temporin-L on Pseudomonas fluorescensin Static and In-Flow Conditions.
542	DRAMP01746	Temporin-L (Temporin-1Tl; temporin-Tl; TL; Frogs, amphibians, animals)	"Temporin-L"[All Fields] AND biofilm[All Fields]	Temporin-L	32842593	Novel Antimicrobial Peptide from Temporin L in The Treatment of Staphylococcus pseudintermediusand Malassezia pachydermatisin Polymicrobial Inter-Kingdom Infection.
542	DRAMP01746	Temporin-L (Temporin-1Tl; temporin-Tl; TL; Frogs, amphibians, animals)	"Temporin-L"[All Fields] AND biofilm[All Fields]	Temporin-L	32447024	Antimicrobial peptide Temporin-L complexed with anionic cyclodextrins results in a potent and safe agent against sessile bacteria.
542	DRAMP01746	Temporin-L (Temporin-1Tl; temporin-Tl; TL; Frogs, amphibians, animals)	"Temporin-L"[All Fields] AND biofilm[All Fields]	Temporin-L	31784584	Supreme activity of gramicidin S against resistant, persistent and biofilm cells of staphylococci and enterococci.
551	DRAMP02125	Hylin-a1 (Hy-a1; Frogs, amphibians, animals)	"Hylin-a1"[All Fields] AND biofilm[All Fields]	Hylin-a1	23340019	Antimicrobial activity of the synthetic peptide Lys-a1 against oral streptococci.
555	DRAMP02130	Antimicrobial peptide 1 (XT-1; Frogs, amphibians, animals)	"Antimicrobial peptide 1"[All Fields] AND biofilm[All Fields]	Antimicrobial peptide 1	22445495	Database screening and in vivo efficacy of antimicrobial peptides against methicillin-resistant Staphylococcus aureus USA300.
585	DRAMP03998	PAF26 (Trp-rich; combinatorial library)	"PAF26"[All Fields] AND biofilm[All Fields]	PAF26	23892742	FLO11 Gene Is Involved in the Interaction of Flor Strains of Saccharomyces cerevisiae with a Biofilm-Promoting Synthetic Hexapeptide.
589	DRAMP02271	Magainin-2 (Magainin II; chain of Magainins; Frogs, amphibians, animals)	"Magainin-2"[All Fields] AND biofilm[All Fields]	Magainin-2	33946431	Extracellular Polymeric Substance Protects Some Cells in an Escherichia coliBiofilm from the Biomechanical Consequences of Treatment with Magainin 2.
589	DRAMP02271	Magainin-2 (Magainin II; chain of Magainins; Frogs, amphibians, animals)	"Magainin-2"[All Fields] AND biofilm[All Fields]	Magainin-2	31363941	Mechanism of action of antimicrobial peptide P5 truncations against Pseudomonas aeruginosa and Staphylococcus aureus.
589	DRAMP02271	Magainin-2 (Magainin II; chain of Magainins; Frogs, amphibians, animals)	"Magainin-2"[All Fields] AND biofilm[All Fields]	Magainin-2	30576152	Grafted Polymer Coatings Enhance Fouling Inhibition by an Antimicrobial Peptide on Reverse Osmosis Membranes.
589	DRAMP02271	Magainin-2 (Magainin II; chain of Magainins; Frogs, amphibians, animals)	"Magainin-2"[All Fields] AND biofilm[All Fields]	Magainin-2	30301180	Antibacterial and Antibiofilm Activity and Mode of Action of Magainin 2 against Drug-Resistant Acinetobacter baumannii.
589	DRAMP02271	Magainin-2 (Magainin II; chain of Magainins; Frogs, amphibians, animals)	"Magainin-2"[All Fields] AND biofilm[All Fields]	Magainin-2	30122046	Synergy on Surfaces: Anti-Biofouling Interfaces Using Surface-Attached Antimicrobial Peptides PGLa and Magainin-2.
589	DRAMP02271	Magainin-2 (Magainin II; chain of Magainins; Frogs, amphibians, animals)	"Magainin-2"[All Fields] AND biofilm[All Fields]	Magainin-2	28178190	High Specific Selectivity and Membrane-Active Mechanism of Synthetic Cationic Hybrid Antimicrobial Peptides Based on the Peptide FV7.
589	DRAMP02271	Magainin-2 (Magainin II; chain of Magainins; Frogs, amphibians, animals)	"Magainin-2"[All Fields] AND biofilm[All Fields]	Magainin-2	21807970	Endocytosis-mediated vacuolar accumulation of the human ApoE apolipoprotein-derived ApoEdpL-W antimicrobial peptide contributes to its antifungal activity in Candida albicans.
589	DRAMP02271	Magainin-2 (Magainin II; chain of Magainins; Frogs, amphibians, animals)	"Magainin-2"[All Fields] AND biofilm[All Fields]	Magainin-2	16595638	Effect of MUC7 peptides on the growth of bacteria and on Streptococcus mutans biofilm.
590	DRAMP02272	PGLa (chain of PYLa/PGLa A; Frogs, amphibians, animals)	"PGLa"[All Fields] AND biofilm[All Fields]	PGLa	32683042	Biomimetic biodegradable Ag@Au nanoparticle-embedded ureteral stent with a constantly renewable contact-killing antimicrobial surface and antibiofilm and extraction-free properties.
590	DRAMP02272	PGLa (chain of PYLa/PGLa A; Frogs, amphibians, animals)	"PGLa"[All Fields] AND biofilm[All Fields]	PGLa	31906503	Electrospun Bioresorbable Membrane Eluting Chlorhexidine for Dental Implants.
590	DRAMP02272	PGLa (chain of PYLa/PGLa A; Frogs, amphibians, animals)	"PGLa"[All Fields] AND biofilm[All Fields]	PGLa	30122046	Synergy on Surfaces: Anti-Biofouling Interfaces Using Surface-Attached Antimicrobial Peptides PGLa and Magainin-2.
590	DRAMP02272	PGLa (chain of PYLa/PGLa A; Frogs, amphibians, animals)	"PGLa"[All Fields] AND biofilm[All Fields]	PGLa	29273511	Anti-biofilm and anti-virulence potential of 3,7-dimethyloct-6-enal derived from Citrus hystrix against bacterial blight of rice caused by Xanthomonas oryzae pv. oryzae.
590	DRAMP02272	PGLa (chain of PYLa/PGLa A; Frogs, amphibians, animals)	"PGLa"[All Fields] AND biofilm[All Fields]	PGLa	28659894	Thyme Oil Reduces Biofilm Formation and Impairs Virulence of Xanthomonas oryzae.
590	DRAMP02272	PGLa (chain of PYLa/PGLa A; Frogs, amphibians, animals)	"PGLa"[All Fields] AND biofilm[All Fields]	PGLa	25385794	Enterococcus faecalis 6-phosphogluconolactonase is required for both commensal and pathogenic interactions with Manduca sexta.
590	DRAMP02272	PGLa (chain of PYLa/PGLa A; Frogs, amphibians, animals)	"PGLa"[All Fields] AND biofilm[All Fields]	PGLa	9972500	Preparation and characterization of ofloxacin microspheres for the eradication of bone associated bacterial biofilm.
616	DRAMP02314	Hepcidin (fish, chordates, animals)	"Hepcidin"[All Fields] AND biofilm[All Fields]	Hepcidin	30456212	Quantification of Lipoteichoic Acid in Hemodialysis Patients With Central Venous Catheters.
616	DRAMP02314	Hepcidin (fish, chordates, animals)	"Hepcidin"[All Fields] AND biofilm[All Fields]	Hepcidin	30408337	Silk-Based Antimicrobial Polymers as a New Platform to Design Drug-Free Materials to Impede Microbial Infections.
616	DRAMP02314	Hepcidin (fish, chordates, animals)	"Hepcidin"[All Fields] AND biofilm[All Fields]	Hepcidin	24645694	Inhibitory effect of the human liver-derived antimicrobial peptide hepcidin 20 on biofilms of polysaccharide intercellular adhesin (PIA)-positive and PIA-negative strains of Staphylococcus epidermidis.
617	DRAMP02315	Chrysophsin-1 (fish, chordates, animals)	"Chrysophsin-1"[All Fields] AND biofilm[All Fields]	Chrysophsin-1	22281025	Effect of a novel antimicrobial peptide chrysophsin-1 on oral pathogens and Streptococcus mutans biofilms.
624	DRAMP02330	Piscidin-1 (Pis-1; Piscidin 1; fish, chordates, animals)	"Piscidin-1"[All Fields] AND biofilm[All Fields]	Piscidin-1	34590712	The effect of piscidin antimicrobial peptides on the formation of Gram-negative bacterial biofilms.
624	DRAMP02330	Piscidin-1 (Pis-1; Piscidin 1; fish, chordates, animals)	"Piscidin-1"[All Fields] AND biofilm[All Fields]	Piscidin-1	30471190	Metal-ion Binding to Host Defense Peptide Piscidin 3 Observed in Phospholipid Bilayers by Magic Angle Spinning Solid-state NMR.
624	DRAMP02330	Piscidin-1 (Pis-1; Piscidin 1; fish, chordates, animals)	"Piscidin-1"[All Fields] AND biofilm[All Fields]	Piscidin-1	28892294	Nuclease activity gives an edge to host-defense peptide piscidin 3 over piscidin 1, rendering it more effective against persisters and biofilms.
624	DRAMP02330	Piscidin-1 (Pis-1; Piscidin 1; fish, chordates, animals)	"Piscidin-1"[All Fields] AND biofilm[All Fields]	Piscidin-1	22445495	Database screening and in vivo efficacy of antimicrobial peptides against methicillin-resistant Staphylococcus aureus USA300.
631	DRAMP02350	Pleurocidin (NRC-4; fish, chordates, animals)	"Pleurocidin"[All Fields] AND biofilm[All Fields]	Pleurocidin	33475795	Antibiofilm peptides as a promising strategy: comparative research.
631	DRAMP02350	Pleurocidin (NRC-4; fish, chordates, animals)	"Pleurocidin"[All Fields] AND biofilm[All Fields]	Pleurocidin	30508627	Antibacterial and anti-biofilm activity, and mechanism of action of pleurocidin against drug resistant Staphylococcus aureus.
631	DRAMP02350	Pleurocidin (NRC-4; fish, chordates, animals)	"Pleurocidin"[All Fields] AND biofilm[All Fields]	Pleurocidin	22921812	Antimicrobial peptide pleurocidin synergizes with antibiotics through hydroxyl radical formation and membrane damage, and exerts antibiofilm activity.

631	DRAMP02350	Pleurocidin (NRC-4; fish, chordates, animals)	"Pleurocidin"[All Fields] AND biofilm[All Fields]	Pleurocidin	21844316	A novel target-specific, salt-resistant antimicrobial peptide against the cariogenic pathogen Streptococcus mutans.
631	DRAMP02350	Pleurocidin (NRC-4; fish, chordates, animals)	"Pleurocidin"[All Fields] AND biofilm[All Fields]	Pleurocidin	21703317	Antimicrobial and antibiofilm activity of pleurocidin against cariogenic microorganisms.
633	DRAMP02352	NRC-16 (fish, chordates, animals)	"NRC-16"[All Fields] AND biofilm[All Fields]	NRC-16	23760014	Anti-microbial, anti-biofilm activities and cell selectivity of the NRC-16 peptide derived from witch flounder, Glyptocephalus cynoglossus.
682	DRAMP02441	Gramicidin S (GS)	"Gramicidin S"[All Fields] AND biofilm[All Fields]	Gramicidin S	31784584	Supreme activity of gramicidin S against resistant, persistent and biofilm cells of staphylococci and enterococci.
682	DRAMP02441	Gramicidin S (GS)	"Gramicidin S"[All Fields] AND biofilm[All Fields]	Gramicidin S	28439259	Increased Biological Activity of Aneurinibacillus migulanusStrains Correlates with the Production of New Gramicidin Secondary Metabolites.
682	DRAMP02441	Gramicidin S (GS)	"Gramicidin S"[All Fields] AND biofilm[All Fields]	Gramicidin S	27618065	Therapeutic Potential of Gramicidin S in the Treatment of Root Canal Infections.
682	DRAMP02441	Gramicidin S (GS)	"Gramicidin S"[All Fields] AND biofilm[All Fields]	Gramicidin S	26077259	Synergistic effect of membrane-active peptides polymyxin B and gramicidin S on multidrug-resistant strains and biofilms of Pseudomonas aeruginosa.
682	DRAMP02441	Gramicidin S (GS)	"Gramicidin S"[All Fields] AND biofilm[All Fields]	Gramicidin S	23103974	Direct surfactin-gramicidin S antagonism supports detoxification in mixed producer cultures of Bacillus subtilis and Aneurinibacillus migulanus.
682	DRAMP02441	Gramicidin S (GS)	"Gramicidin S"[All Fields] AND biofilm[All Fields]	Gramicidin S	17701408	Biofilms: strategies for metal corrosion inhibition employing microorganisms.
682	DRAMP02441	Gramicidin S (GS)	"Gramicidin S"[All Fields] AND biofilm[All Fields]	Gramicidin S	15278311	Inhibiting mild steel corrosion from sulfate-reducing and iron-oxidizing bacteria using gramicidin-S-producing biofilms.
682	DRAMP02441	Gramicidin S (GS)	"Gramicidin S"[All Fields] AND biofilm[All Fields]	Gramicidin S	12898064	Inhibiting mild steel corrosion from sulfate-reducing bacteria using antimicrobial-producing biofilms in Three-Mile-Island process water.
682	DRAMP02441	Gramicidin S (GS)	"Gramicidin S"[All Fields] AND biofilm[All Fields]	Gramicidin S	10499267	Inhibiting sulfate-reducing bacteria in biofilms on steel with antimicrobial peptides generated in situ.
685	DRAMP02449	Lectin	"Lectin"[All Fields] AND biofilm[All Fields]	Lectin	34694672	Anti-biofilm and anti-virulence effects of silica oxide nanoparticle-conjugation of lectin purified from Pseudomonas aeruginosa.
685	DRAMP02449	Lectin	"Lectin"[All Fields] AND biofilm[All Fields]	Lectin	34633846	DectiSomes: Glycan Targeting of Liposomal Drugs Improves the Treatment of Disseminated Candidiasis.
685	DRAMP02449	Lectin	"Lectin"[All Fields] AND biofilm[All Fields]	Lectin	34561304	Bacterial rhamnolipids and their 3-hydroxyalkanoate precursors activate Arabidopsisinnate immunity through two independent mechanisms.
685	DRAMP02449	Lectin	"Lectin"[All Fields] AND biofilm[All Fields]	Lectin	34542288	Pillar[5]arene-Based Polycationic Glyco[2]rotaxanes Designed as Pseudomonas aeruginosaAntibiofilm Agents.
685	DRAMP02449	Lectin	"Lectin"[All Fields] AND biofilm[All Fields]	Lectin	34530088	Quorum sensing regulates transcription of the pilin gene mshA1 of MSHA pilus in Vibrio parahaemolyticus.
685	DRAMP02449	Lectin	"Lectin"[All Fields] AND biofilm[All Fields]	Lectin	34255029	The assessment of Pseudomonas aeruginosa lectin LecA binding characteristics of divalent galactosides using multiple techniques.
685	DRAMP02449	Lectin	"Lectin"[All Fields] AND biofilm[All Fields]	Lectin	33878355	A N-acetyl-D-galactosamine-binding lectin from Amaranthus gangeticus seeds inhibits biofilm formation and Ehrlich ascites carcinoma cell growth in vivo in mice.
685	DRAMP02449	Lectin	"Lectin"[All Fields] AND biofilm[All Fields]	Lectin	33852268	Combining Inducible Lectin Expression and Magnetic Glyconanoparticles for the Selective Isolation of Bacteria from Mixed Populations.
685	DRAMP02449	Lectin	"Lectin"[All Fields] AND biofilm[All Fields]	Lectin	33824212	Structural Basis of Ligand Selectivity by a Bacterial Adhesin Lectin Involved in Multispecies Biofilm Formation.
685	DRAMP02449	Lectin	"Lectin"[All Fields] AND biofilm[All Fields]	Lectin	33791836	Production of nonulosonic acids in the extracellular polymeric substances of "Candidatus Accumulibacter phosphatis".
685	DRAMP02449	Lectin	"Lectin"[All Fields] AND biofilm[All Fields]	Lectin	33622715	Antifungal Liposomes Directed by Dectin-2 Offer a Promising Therapeutic Option for Pulmonary Aspergillosis.
685	DRAMP02449	Lectin	"Lectin"[All Fields] AND biofilm[All Fields]	Lectin	33610507	d-mannose-sensitive pilus of Acinetobacter baumannii is linked to biofilm formation and adherence onto respiratory tract epithelial cells.
685	DRAMP02449	Lectin	"Lectin"[All Fields] AND biofilm[All Fields]	Lectin	33584576	Rv1717 Is a Cell Wall - Associated β-Galactosidase of Mycobacterium tuberculosisThat Is Involved in Biofilm Dispersion.
685	DRAMP02449	Lectin	"Lectin"[All Fields] AND biofilm[All Fields]	Lectin	33526439	C-Type Lectin Maintains the Homeostasis of Intestinal Microbiota and Mediates Biofilm Formation by Intestinal Bacteria in Shrimp.
685	DRAMP02449	Lectin	"Lectin"[All Fields] AND biofilm[All Fields]	Lectin	33480454	A Dual-Responsive Antibiotic-Loaded Nanoparticle Specifically Binds Pathogens and Overcomes Antimicrobial-Resistant Infections.
685	DRAMP02449	Lectin	"Lectin"[All Fields] AND biofilm[All Fields]	Lectin	33450228	Pantoea stewartii WceF is a glycan biofilm-modifying enzyme with a bacteriophage tailspike-like fold.
685	DRAMP02449	Lectin	"Lectin"[All Fields] AND biofilm[All Fields]	Lectin	33401388	AhaP, A Quorum Quenching Acylase from Psychrobactersp. M9-54-1 That Attenuates Pseudomonas aeruginosaand Vibrio coralliilyticusVirulence.
685	DRAMP02449	Lectin	"Lectin"[All Fields] AND biofilm[All Fields]	Lectin	33377615	Diallyl sulfide from garlic suppresses quorum-sensing systems of Pseudomonas aeruginosa and enhances biosynthesis of three B vitamins through its thioether group.
685	DRAMP02449	Lectin	"Lectin"[All Fields] AND biofilm[All Fields]	Lectin	33350564	Cellulophaga algicola alginate lyase inhibits biofilm formation of a clinical Pseudomonas aeruginosa strain MCC 2081.
685	DRAMP02449	Lectin	"Lectin"[All Fields] AND biofilm[All Fields]	Lectin	33342053	Antibacterial effects of the lectin from pomegranate sarcotesta (PgTeL) against Listeria monocytogenes.
685	DRAMP02449	Lectin	"Lectin"[All Fields] AND biofilm[All Fields]	Lectin	33340913	Does targeting Arg98 of FimH lead to high affinity antagonists?
685	DRAMP02449	Lectin	"Lectin"[All Fields] AND biofilm[All Fields]	Lectin	33314528	Non-Carbohydrate Glycomimetics as Inhibitors of Calcium(II)-Binding Lectins.
685	DRAMP02449	Lectin	"Lectin"[All Fields] AND biofilm[All Fields]	Lectin	33206087	A glycoconjugate-based gold nanoparticle approach for the targeted treatment of Pseudomonas aeruginosabiofilms.
685	DRAMP02449	Lectin	"Lectin"[All Fields] AND biofilm[All Fields]	Lectin	33144377	The Nontypeable Haemophilus influenzae Major Adhesin Hia Is a Dual-Function Lectin That Binds to Human-Specific Respiratory Tract Sialic Acid Glycan Receptors.
685	DRAMP02449	Lectin	"Lectin"[All Fields] AND biofilm[All Fields]	Lectin	33120893	Protective Efficacy of Lectin-Fc(IgG) Fusion Proteins In Vitro and in a Pulmonary Aspergillosis In Vivo Model.
685	DRAMP02449	Lectin	"Lectin"[All Fields] AND biofilm[All Fields]	Lectin	33108702	Catalytic and lectin domains in neuraminidase A from Streptococcus pneumoniae are capable of an intermolecular assembly: Implications for biofilm formation.
685	DRAMP02449	Lectin	"Lectin"[All Fields] AND biofilm[All Fields]	Lectin	32924479	Directing Drugs to Bugs: Antibiotic-Carbohydrate Conjugates Targeting Biofilm-Associated Lectins of Pseudomonas aeruginosa.
685	DRAMP02449	Lectin	"Lectin"[All Fields] AND biofilm[All Fields]	Lectin	32750211	Anti-staphylococcal effects of Myracrodruon urundeuva lectins on nonresistant and multidrug resistant isolates.
685	DRAMP02449	Lectin	"Lectin"[All Fields] AND biofilm[All Fields]	Lectin	32749389	An on-demand nanoplatform for enhanced elimination of drug-resistant bacteria.
685	DRAMP02449	Lectin	"Lectin"[All Fields] AND biofilm[All Fields]	Lectin	32745619	Antibiofilm and immunological properties of lectin purified from shrimp Penaeus semisulcatus.
685	DRAMP02449	Lectin	"Lectin"[All Fields] AND biofilm[All Fields]	Lectin	32672931	Selective Promotion of Adhesion of Shewanella oneidensison Mannose-Decorated Glycopolymer Surfaces.
685	DRAMP02449	Lectin	"Lectin"[All Fields] AND biofilm[All Fields]	Lectin	32628229	A rapid synthesis of low-nanomolar divalent LecA inhibitors in four linear steps from d-galactose pentaacetate.
685	DRAMP02449	Lectin	"Lectin"[All Fields] AND biofilm[All Fields]	Lectin	32599250	Machaerium acutifolium lectin alters membrane structure and induces ROS production in Candida parapsilosis.
685	DRAMP02449	Lectin	"Lectin"[All Fields] AND biofilm[All Fields]	Lectin	32573695	Protein-observed 19F NMR of LecA from Pseudomonas aeruginosa.
685	DRAMP02449	Lectin	"Lectin"[All Fields] AND biofilm[All Fields]	Lectin	32252300	Glycomics Microarrays Reveal Differential In Situ Presentation of the Biofilm Polysaccharide Poly-N-acetylglucosamine on Acinetobacter baumanniiand Staphylococcus aureusCell Surfaces.
685	DRAMP02449	Lectin	"Lectin"[All Fields] AND biofilm[All Fields]	Lectin	32221309	Environmental stress perception activates structural remodeling of extant Streptococcus mutans biofilms.
685	DRAMP02449	Lectin	"Lectin"[All Fields] AND biofilm[All Fields]	Lectin	32156827	The Pseudomonas aeruginosa Lectin LecB Causes Integrin Internalization and Inhibits Epithelial Wound Healing.
685	DRAMP02449	Lectin	"Lectin"[All Fields] AND biofilm[All Fields]	Lectin	32010066	Microarray Strategies for Exploring Bacterial Surface Glycans and Their Interactions With Glycan-Binding Proteins.
685	DRAMP02449	Lectin	"Lectin"[All Fields] AND biofilm[All Fields]	Lectin	31860279	Revealing the Significance of the Glycan Binding Property of Butea monospermaSeed Lectin for Enhancing the Antibiofilm Activity of Silver Nanoparticles against Uropathogenic Escherichia coli.
685	DRAMP02449	Lectin	"Lectin"[All Fields] AND biofilm[All Fields]	Lectin	31734393	Aerobic granular sludge contains Hyaluronic acid-like and sulfated glycosaminoglycans-like polymers.
685	DRAMP02449	Lectin	"Lectin"[All Fields] AND biofilm[All Fields]	Lectin	31732693	Pseudomonas aeruginosalectin LecB impairs keratinocyte fitness by abrogating growth factor signalling.
685	DRAMP02449	Lectin	"Lectin"[All Fields] AND biofilm[All Fields]	Lectin	31706760	Microscale and molecular analyses of river biofilm communities treated with microgram levels of cerium oxide nanoparticles indicate limited but significant effects.
685	DRAMP02449	Lectin	"Lectin"[All Fields] AND biofilm[All Fields]	Lectin	31650413	In Vitro Evaluation of Probiotic Potential of Selected Lactic Acid Bacteria Strains.
685	DRAMP02449	Lectin	"Lectin"[All Fields] AND biofilm[All Fields]	Lectin	31553873	Anti-biofilm Agents against Pseudomonas aeruginosa: A Structure-Activity Relationship Study of C-Glycosidic LecB Inhibitors.
685	DRAMP02449	Lectin	"Lectin"[All Fields] AND biofilm[All Fields]	Lectin	31533083	Anti-biofilm properties and immunological response of an immune molecule lectin isolated from shrimp Metapenaeus monoceros.
685	DRAMP02449	Lectin	"Lectin"[All Fields] AND biofilm[All Fields]	Lectin	31520734	Carbohydrate-independent antibiofilm effect of Bothrops jararacussu lectin BJcuL on Staphylococcus aureus.
685	DRAMP02449	Lectin	"Lectin"[All Fields] AND biofilm[All Fields]	Lectin	31475271	Deciphering multivalent glycocluster-lectin interactions through AFM characterization of the self-assembled nanostructures.

685	DRAMP02449	Lectin	"Lectin"[All Fields] AND biofilm[All Fields]	Lectin	31450058	Pseudomonas aeruginosa resistance of monosaccharide-functionalized glass surfaces.
685	DRAMP02449	Lectin	"Lectin"[All Fields] AND biofilm[All Fields]	Lectin	31449405	Glycocluster Tetrahydroxamic Acids Exhibiting Unprecedented Inhibition of Pseudomonas aeruginosaBiofilms.
685	DRAMP02449	Lectin	"Lectin"[All Fields] AND biofilm[All Fields]	Lectin	31439670	The 1.9 Å crystal structure of the extracellular matrix protein Bap1 from Vibrio choleraeprovides insights into bacterial biofilm adhesion.
685	DRAMP02449	Lectin	"Lectin"[All Fields] AND biofilm[All Fields]	Lectin	31400430	Insights into anti-pathogenic activities of mannose lectins.
685	DRAMP02449	Lectin	"Lectin"[All Fields] AND biofilm[All Fields]	Lectin	31365141	N,N-Diethyl-m-Toluamide Exposure at an Environmentally Relevant Concentration Influences River Microbial Community Development.
685	DRAMP02449	Lectin	"Lectin"[All Fields] AND biofilm[All Fields]	Lectin	31306758	Immune recognition, antimicrobial and opsonic activities mediated by a sialic acid binding lectin from Ruditapes philippinarum.
685	DRAMP02449	Lectin	"Lectin"[All Fields] AND biofilm[All Fields]	Lectin	31279160	Concanavalin A-targeted mesoporous silica nanoparticles for infection treatment.
685	DRAMP02449	Lectin	"Lectin"[All Fields] AND biofilm[All Fields]	Lectin	31207891	Rhamnose Binding Protein as an Anti-Bacterial Agent-Targeting Biofilm of Pseudomonas aeruginosa.
685	DRAMP02449	Lectin	"Lectin"[All Fields] AND biofilm[All Fields]	Lectin	31170488	Punica granatum sarcotesta lectin (PgTeL) has antibacterial activity and synergistic effects with antibiotics against β-lactamase-producing Escherichia coli.
685	DRAMP02449	Lectin	"Lectin"[All Fields] AND biofilm[All Fields]	Lectin	31133998	Insight Into Interactions of Thermoacidophilic Archaea With Elemental Sulfur: Biofilm Dynamics and EPS Analysis.
685	DRAMP02449	Lectin	"Lectin"[All Fields] AND biofilm[All Fields]	Lectin	31117738	Lipid Bilayer-like Mixed Self-Assembled Monolayers with Strong Mobility and Clustering-Dependent Lectin Affinity.
685	DRAMP02449	Lectin	"Lectin"[All Fields] AND biofilm[All Fields]	Lectin	31099495	Looking for alternative treatments for bovine and caprine mastitis: Evaluation of the potential of Calliandra surinamensis leaf pinnulae lectin (CasuL), both alone and in combination with antibiotics.
685	DRAMP02449	Lectin	"Lectin"[All Fields] AND biofilm[All Fields]	Lectin	31097723	The Pseudomonas aeruginosa lectin LecB binds to the exopolysaccharide Psl and stabilizes the biofilm matrix.
685	DRAMP02449	Lectin	"Lectin"[All Fields] AND biofilm[All Fields]	Lectin	31097253	Lectin-stimulated cellular iron uptake and toxin generation in the freshwater cyanobacterium Microcystis aeruginosa.
685	DRAMP02449	Lectin	"Lectin"[All Fields] AND biofilm[All Fields]	Lectin	31076508	Functional characterization of a subtilisin-like serine protease from Vibrio cholerae.
685	DRAMP02449	Lectin	"Lectin"[All Fields] AND biofilm[All Fields]	Lectin	31051203	An L-fucose specific lectin from Aspergillus niger isolated from mycotic keratitis patient and its interaction with human pancreatic adenocarcinoma PANC-1 cells.
685	DRAMP02449	Lectin	"Lectin"[All Fields] AND biofilm[All Fields]	Lectin	31022836	Inhibition of Pseudomonas aeruginosaBiofilm Formation with Surface Modified Polymeric Nanoparticles.
685	DRAMP02449	Lectin	"Lectin"[All Fields] AND biofilm[All Fields]	Lectin	30884454	Effects of the binding of a Helianthus annuus lectin to Candida albicans cell wall on biofilm development and adhesion to host cells.
685	DRAMP02449	Lectin	"Lectin"[All Fields] AND biofilm[All Fields]	Lectin	30852321	Sialic acids in the extracellular polymeric substances of seawater-adapted aerobic granular sludge.
685	DRAMP02449	Lectin	"Lectin"[All Fields] AND biofilm[All Fields]	Lectin	30823584	Functional Characterization of OXYL, A SghC1qDC LacNAc-specific Lectin from The Crinoid Feather Star Anneissia Japonica.
685	DRAMP02449	Lectin	"Lectin"[All Fields] AND biofilm[All Fields]	Lectin	30743061	Biofilm dynamics and EPS production of a thermoacidophilic bioleaching archaeon.
685	DRAMP02449	Lectin	"Lectin"[All Fields] AND biofilm[All Fields]	Lectin	30741841	Thirty Years of Lactobacillus rhamnosus GG: A Review.
685	DRAMP02449	Lectin	"Lectin"[All Fields] AND biofilm[All Fields]	Lectin	30666240	Diallyl Disulfide From Garlic Oil Inhibits Pseudomonas aeruginosaQuorum Sensing Systems and Corresponding Virulence Factors.
685	DRAMP02449	Lectin	"Lectin"[All Fields] AND biofilm[All Fields]	Lectin	30663885	Biofilm diversity, structure and matrix seasonality in a full-scale cooling tower.
685	DRAMP02449	Lectin	"Lectin"[All Fields] AND biofilm[All Fields]	Lectin	30639479	Antimicrobial and biochemical characterization of a C-type lectin isolated from pearl spot (Etroplus suratensis).
685	DRAMP02449	Lectin	"Lectin"[All Fields] AND biofilm[All Fields]	Lectin	30615928	Formation of Pseudomonas aeruginosa inhibition zone during tobramycin disk diffusion is due to transition from planktonic to biofilm mode of growth.
685	DRAMP02449	Lectin	"Lectin"[All Fields] AND biofilm[All Fields]	Lectin	30589118	A Biomimetic Non-Antibiotic Approach to Eradicate Drug-Resistant Infections.
685	DRAMP02449	Lectin	"Lectin"[All Fields] AND biofilm[All Fields]	Lectin	30552872	A new mucin-binding lectin from the marine sponge Aplysina fulva (AFL) exhibits antibiofilm effects.
685	DRAMP02449	Lectin	"Lectin"[All Fields] AND biofilm[All Fields]	Lectin	30496858	Histochemical localization of N-acetylhexosamine-binding lectin HOL-18 in Halichondria okadai (Japanese black sponge), and its antimicrobial and cytotoxic anticancer effects.
685	DRAMP02449	Lectin	"Lectin"[All Fields] AND biofilm[All Fields]	Lectin	30477231	Screening of a Library of Oligosaccharides Targeting Lectin LecB of Pseudomonas Aeruginosaand Synthesis of High Affinity Oligoglycoclusters.
685	DRAMP02449	Lectin	"Lectin"[All Fields] AND biofilm[All Fields]	Lectin	30414418	Punica granatum sarcotesta lectin (PgTeL) impairs growth, structure, viability, aggregation, and biofilm formation ability of Staphylococcus aureus clinical isolates.
685	DRAMP02449	Lectin	"Lectin"[All Fields] AND biofilm[All Fields]	Lectin	30408344	Monodisperse Sequence-Controlled α-l-Fucosylated Glycooligomers and Their Multivalent Inhibitory Effects on LecB.
685	DRAMP02449	Lectin	"Lectin"[All Fields] AND biofilm[All Fields]	Lectin	30364807	Corrigendum: The Intestinal Roundworm Ascaris suumReleases Antimicrobial Factors Which Interfere With Bacterial Growth and Biofilm Formation.
685	DRAMP02449	Lectin	"Lectin"[All Fields] AND biofilm[All Fields]	Lectin	30335377	Identification of Glycoproteins Isolated from Extracellular Polymeric Substances of Full-Scale Anammox Granular Sludge.
685	DRAMP02449	Lectin	"Lectin"[All Fields] AND biofilm[All Fields]	Lectin	30333795	Multi-Parameter Laser Imaging Reveals Complex Microscale Biofilm Matrix in a Thick (4,000 µm) Aerobic Methanol Oxidizing Community.
685	DRAMP02449	Lectin	"Lectin"[All Fields] AND biofilm[All Fields]	Lectin	30296643	Selectivity of original C-hexopyranosyl calix[4]arene conjugates towards lectins of different origin.
685	DRAMP02449	Lectin	"Lectin"[All Fields] AND biofilm[All Fields]	Lectin	30296001	Towards patterned bioelectronics: facilitated immobilization of exoelectrogenic Escherichia coli with heterologous pili.
685	DRAMP02449	Lectin	"Lectin"[All Fields] AND biofilm[All Fields]	Lectin	30280093	Yersinia pseudotuberculosisBarA-UvrY Two-Component Regulatory System Represses Biofilms via CsrB.
685	DRAMP02449	Lectin	"Lectin"[All Fields] AND biofilm[All Fields]	Lectin	30240117	Canavalia ensiformis-derived lectin inhibits biofilm formation of enterohemorrhagic Escherichia coli and Listeria monocytogenes.
685	DRAMP02449	Lectin	"Lectin"[All Fields] AND biofilm[All Fields]	Lectin	30182831	Isolation, Purification and Characterisation of a D-galactose and N-acetyl-D-galactosamine Specific Lectin from Marine Sponge Fasciospongia cavernosa.
685	DRAMP02449	Lectin	"Lectin"[All Fields] AND biofilm[All Fields]	Lectin	30142463	Antimicrobial potential of Alpinia purpurata lectin (ApuL): Growth inhibitory action, synergistic effects in combination with antibiotics, and antibiofilm activity.
685	DRAMP02449	Lectin	"Lectin"[All Fields] AND biofilm[All Fields]	Lectin	30131945	The Intestinal Roundworm Ascaris suumReleases Antimicrobial Factors Which Interfere With Bacterial Growth and Biofilm Formation.
685	DRAMP02449	Lectin	"Lectin"[All Fields] AND biofilm[All Fields]	Lectin	30080666	Biofilm formation and avian immune response following experimental acute and chronic avian cholera due to Pasteurella multocida.
685	DRAMP02449	Lectin	"Lectin"[All Fields] AND biofilm[All Fields]	Lectin	30060568	Effect of Dendrimer Generation and Aglyconic Linkers on the Binding Properties of Mannosylated Dendrimers Prepared by a Combined Convergent and Onion Peel Approach.
685	DRAMP02449	Lectin	"Lectin"[All Fields] AND biofilm[All Fields]	Lectin	30053345	Lectins as antimicrobial agents.
685	DRAMP02449	Lectin	"Lectin"[All Fields] AND biofilm[All Fields]	Lectin	30017316	Synthesis and evaluation of thiomannosides, potent and orally active FimH inhibitors.
685	DRAMP02449	Lectin	"Lectin"[All Fields] AND biofilm[All Fields]	Lectin	30013532	EPS Glycoconjugate Profiles Shift as Adaptive Response in Anaerobic Microbial Granulation at High Salinity.
685	DRAMP02449	Lectin	"Lectin"[All Fields] AND biofilm[All Fields]	Lectin	29781989	Bile Salt-induced Biofilm Formation in Enteric Pathogens: Techniques for Identification and Quantification.
685	DRAMP02449	Lectin	"Lectin"[All Fields] AND biofilm[All Fields]	Lectin	29747390	Functional Aspects of Fish Mucosal Lectins-Interaction with Non-Self.
685	DRAMP02449	Lectin	"Lectin"[All Fields] AND biofilm[All Fields]	Lectin	29685986	Glucan Binding Protein C of Streptococcus mutans Mediates both Sucrose-Independent and Sucrose-Dependent Adherence.
685	DRAMP02449	Lectin	"Lectin"[All Fields] AND biofilm[All Fields]	Lectin	29665678	Lectin-Functionalized Composite Hydrogels for "Capture-and-Killing" of Carbapenem-Resistant Pseudomonas aeruginosa.
685	DRAMP02449	Lectin	"Lectin"[All Fields] AND biofilm[All Fields]	Lectin	29618401	A Novel Genetic Determination of a Lectin Gene in Iraqi Acinetobacter baumanniiIsolates and Use of Purified Lectin as an Antibiofilm Agent.
685	DRAMP02449	Lectin	"Lectin"[All Fields] AND biofilm[All Fields]	Lectin	29468303	The Extracellular Polymeric Substances of Legionella pneumophila Biofilms Contain Amyloid Structures.
685	DRAMP02449	Lectin	"Lectin"[All Fields] AND biofilm[All Fields]	Lectin	29380519	Development of Pseudomonas aeruginosa Lectin LecA Inhibitor by using Bivalent Galactosides Supported on Polyproline Peptide Scaffolds.
685	DRAMP02449	Lectin	"Lectin"[All Fields] AND biofilm[All Fields]	Lectin	29349932	Isolation and characterization of lectin with antibacterial, antibiofilm and antiproliferative activities from Acinetobacter baumannii of environmental origin.
685	DRAMP02449	Lectin	"Lectin"[All Fields] AND biofilm[All Fields]	Lectin	29272578	Glycomimetic, Orally Bioavailable LecB Inhibitors Block Biofilm Formation of Pseudomonas aeruginosa.
685	DRAMP02449	Lectin	"Lectin"[All Fields] AND biofilm[All Fields]	Lectin	29225175	PgTeL, the lectin found in Punica granatum juice, is an antifungal agent against Candida albicans and Candida krusei.
685	DRAMP02449	Lectin	"Lectin"[All Fields] AND biofilm[All Fields]	Lectin	29202022	Reversible Self-Assembled Monolayers (rSAMs): Adaptable Surfaces for Enhanced Multivalent Interactions and Ultrasensitive Virus Detection.

685	DRAMP02449	Lectin	"Lectin"[All Fields] AND biofilm[All Fields]	Lectin	29175164	Antibacterial activity of a new lectin isolated from the marine sponge Chondrilla caribensis.
685	DRAMP02449	Lectin	"Lectin"[All Fields] AND biofilm[All Fields]	Lectin	29143694	Cryo-Scanning Electron Microscopy (SEM) and Scanning Transmission Electron Microscopy (STEM)-in-SEM for Bio- and Organo-Mineral Interface Characterization in the Environment.
685	DRAMP02449	Lectin	"Lectin"[All Fields] AND biofilm[All Fields]	Lectin	29081915	Osteopontin adsorption to Gram-positive cells reduces adhesion forces and attachment to surfaces under flow.
685	DRAMP02449	Lectin	"Lectin"[All Fields] AND biofilm[All Fields]	Lectin	28972138	Photorhabdus luminescenslectin A (PllA): A new probe for detecting α-galactoside-terminating glycoconjugates.
685	DRAMP02449	Lectin	"Lectin"[All Fields] AND biofilm[All Fields]	Lectin	28960731	Covalent Lectin Inhibition and Application in Bacterial Biofilm Imaging.
685	DRAMP02449	Lectin	"Lectin"[All Fields] AND biofilm[All Fields]	Lectin	28949325	Microbial megacities fueled by methane oxidation in a mineral spring cave.
685	DRAMP02449	Lectin	"Lectin"[All Fields] AND biofilm[All Fields]	Lectin	28925553	Dispersal and inhibitory roles of mannose, 2-deoxy-d-glucose and N-acetylgalactosaminidase on the biofilm of Desulfovibrio vulgaris.
685	DRAMP02449	Lectin	"Lectin"[All Fields] AND biofilm[All Fields]	Lectin	28887741	Effects of Lectins on initial attachment of cariogenic Streptococcus mutans.
685	DRAMP02449	Lectin	"Lectin"[All Fields] AND biofilm[All Fields]	Lectin	28792661	Multi-effect of the water-soluble Moringa oleifera lectin against Serratia marcescens and Bacillus sp.: antibacterial, antibiofilm and anti-adhesive properties.
685	DRAMP02449	Lectin	"Lectin"[All Fields] AND biofilm[All Fields]	Lectin	28769901	Fluorescence Imaging of Streptococcus pneumoniaewith the Helix pomatiaagglutinin (HPA) As a Potential, Rapid Diagnostic Tool.
685	DRAMP02449	Lectin	"Lectin"[All Fields] AND biofilm[All Fields]	Lectin	28748044	Visualizing the dental biofilm matrix by means of fluorescence lectin-binding analysis.
685	DRAMP02449	Lectin	"Lectin"[All Fields] AND biofilm[All Fields]	Lectin	28599160	The acid soluble extracellular polymeric substance of aerobic granular sludge dominated by Defluviicoccus sp.
685	DRAMP02449	Lectin	"Lectin"[All Fields] AND biofilm[All Fields]	Lectin	28446778	Tumor necrosis factor prevents Candida albicans biofilm formation.
685	DRAMP02449	Lectin	"Lectin"[All Fields] AND biofilm[All Fields]	Lectin	28441606	Growth inhibition and antibiofilm potential of Ag nanoparticles coated with lectin, an arthropod immune molecule.
685	DRAMP02449	Lectin	"Lectin"[All Fields] AND biofilm[All Fields]	Lectin	28208623	Fluorescence Lectin Bar-Coding of Glycoconjugates in the Extracellular Matrix of Biofilm and Bioaggregate Forming Microorganisms.
685	DRAMP02449	Lectin	"Lectin"[All Fields] AND biofilm[All Fields]	Lectin	28192138	Isolation, biochemical characterization and antibiofilm effect of a lectin from the marine sponge Aplysina lactuca.
685	DRAMP02449	Lectin	"Lectin"[All Fields] AND biofilm[All Fields]	Lectin	28174088	CasuL: A new lectin isolated from Calliandra surinamensis leaf pinnulae with cytotoxicity to cancer cells, antimicrobial activity and antibiofilm effect.
685	DRAMP02449	Lectin	"Lectin"[All Fields] AND biofilm[All Fields]	Lectin	28150103	Purification, Biochemical Characterization, and Amino Acid Sequence of a Novel Type of Lectin from Aplysia dactylomela Eggs with Antibacterial/Antibiofilm Potential.
685	DRAMP02449	Lectin	"Lectin"[All Fields] AND biofilm[All Fields]	Lectin	28110033	Purification, characterization and functional analysis of the immune molecule lectin from the haemolymph of blue swimmer crab Portunus pelagicus and their antibiofilm properties.
685	DRAMP02449	Lectin	"Lectin"[All Fields] AND biofilm[All Fields]	Lectin	28061510	Bacterial Coaggregation Among the Most Commonly Isolated Bacteria From Contact Lens Cases.
685	DRAMP02449	Lectin	"Lectin"[All Fields] AND biofilm[All Fields]	Lectin	28019718	Excretions/secretions from medicinal larvae (Lucilia sericata) inhibit complement activation by two mechanisms.
685	DRAMP02449	Lectin	"Lectin"[All Fields] AND biofilm[All Fields]	Lectin	28003128	Structural and binding studies of a C-type galactose-binding lectin from Bothrops jararacussu snake venom.
685	DRAMP02449	Lectin	"Lectin"[All Fields] AND biofilm[All Fields]	Lectin	33579086	Burkholderia pseudomallei lectins: occurrence and expression.
685	DRAMP02449	Lectin	"Lectin"[All Fields] AND biofilm[All Fields]	Lectin	27869151	The lectin-like protein 1 in Lactobacillus rhamnosus GR-1 mediates tissue-specific adherence to vaginal epithelium and inhibits urogenital pathogens.
685	DRAMP02449	Lectin	"Lectin"[All Fields] AND biofilm[All Fields]	Lectin	27853317	High mannose-specific lectin Msl mediates key interactions of the vaginal Lactobacillus plantarum isolate CMPG5300.
685	DRAMP02449	Lectin	"Lectin"[All Fields] AND biofilm[All Fields]	Lectin	27651277	Lectin I from Bauhinia variegata (BVL-I) expressed by Pichia pastoris inhibits initial adhesion of oral bacteria in vitro.
685	DRAMP02449	Lectin	"Lectin"[All Fields] AND biofilm[All Fields]	Lectin	29741835	[VcDsbA enhances bioflim formation by stimulating MSHA expression in Vibrio cholera].
685	DRAMP02449	Lectin	"Lectin"[All Fields] AND biofilm[All Fields]	Lectin	27790205	A Rhizobium leguminosarumCHDL- (Cadherin-Like-) Lectin Participates in Assembly and Remodeling of the Biofilm Matrix.
685	DRAMP02449	Lectin	"Lectin"[All Fields] AND biofilm[All Fields]	Lectin	27696649	Functional analysis of Escherichia coli Yad fimbriae reveals their potential role in environmental persistence.
685	DRAMP02449	Lectin	"Lectin"[All Fields] AND biofilm[All Fields]	Lectin	27562775	In situ evidence for metabolic and chemical microdomains in the structured polymer matrix of bacterial microcolonies.
685	DRAMP02449	Lectin	"Lectin"[All Fields] AND biofilm[All Fields]	Lectin	27537843	Lectin-Like Molecules of Lactobacillus rhamnosus GG Inhibit Pathogenic Escherichia coli and Salmonella Biofilm Formation.
685	DRAMP02449	Lectin	"Lectin"[All Fields] AND biofilm[All Fields]	Lectin	27488655	Development and optimization of a competitive binding assay for the galactophilic low affinity lectin LecA from Pseudomonas aeruginosa.
685	DRAMP02449	Lectin	"Lectin"[All Fields] AND biofilm[All Fields]	Lectin	30155149	The virulence factor LecB varies in clinical isolates: consequences for ligand binding and drug discovery.
685	DRAMP02449	Lectin	"Lectin"[All Fields] AND biofilm[All Fields]	Lectin	27412649	Toward the Rational Design of Galactosylated Glycoclusters That Target Pseudomonas aeruginosa Lectin A (LecA): Influence of Linker Arms That Lead to Low-Nanomolar Multivalent Ligands.
685	DRAMP02449	Lectin	"Lectin"[All Fields] AND biofilm[All Fields]	Lectin	27316967	Cell Surface Glycoside Hydrolases of Streptococcus gordonii Promote Growth in Saliva.
685	DRAMP02449	Lectin	"Lectin"[All Fields] AND biofilm[All Fields]	Lectin	27316966	Differential Utilization of Basic Proline-Rich Glycoproteins during Growth of Oral Bacteria in Saliva.
685	DRAMP02449	Lectin	"Lectin"[All Fields] AND biofilm[All Fields]	Lectin	27308201	Cinnamide Derivatives of d-Mannose as Inhibitors of the Bacterial Virulence Factor LecB from Pseudomonas aeruginosa.
685	DRAMP02449	Lectin	"Lectin"[All Fields] AND biofilm[All Fields]	Lectin	27261880	Effects of erythromycin, trimethoprim and clindamycin on attached microbial communities from an effluent dominated prairie stream.
685	DRAMP02449	Lectin	"Lectin"[All Fields] AND biofilm[All Fields]	Lectin	27223679	Optimizing the Multivalent Binding of the Bacterial Lectin LecA by Glycopeptide Dendrimers for Therapeutic Purposes.
685	DRAMP02449	Lectin	"Lectin"[All Fields] AND biofilm[All Fields]	Lectin	29214228	A glucose/mannose binding lectin from litchi (Litchi chinensis) seeds: Biochemical and biophysical characterizations.
685	DRAMP02449	Lectin	"Lectin"[All Fields] AND biofilm[All Fields]	Lectin	27097059	The biofilm matrix of Campylobacter jejuni determined by fluorescence lectin-binding analysis.
685	DRAMP02449	Lectin	"Lectin"[All Fields] AND biofilm[All Fields]	Lectin	27068594	Quorum Sensing Influences Burkholderia thailandensis Biofilm Development and Matrix Production.
685	DRAMP02449	Lectin	"Lectin"[All Fields] AND biofilm[All Fields]	Lectin	26867687	Effects of fullerene (C60), multi-wall carbon nanotubes (MWCNT), single wall carbon nanotubes (SWCNT) and hydroxyl and carboxyl modified single wall carbon nanotubes on riverine microbial communities.
685	DRAMP02449	Lectin	"Lectin"[All Fields] AND biofilm[All Fields]	Lectin	26812660	Antivirulence Isoquinolone Mannosides: Optimization of the Biaryl Aglycone for FimH Lectin Binding Affinity and Efficacy in the Treatment of Chronic UTI.
685	DRAMP02449	Lectin	"Lectin"[All Fields] AND biofilm[All Fields]	Lectin	29896342	Overcoming antibiotic resistance in Pseudomonas aeruginosabiofilms using glycopeptide dendrimers.
685	DRAMP02449	Lectin	"Lectin"[All Fields] AND biofilm[All Fields]	Lectin	26609568	The vascular plant-pathogenic bacterium Ralstonia solanacearum produces biofilms required for its virulence on the surfaces of tomato cells adjacent to intercellular spaces.
685	DRAMP02449	Lectin	"Lectin"[All Fields] AND biofilm[All Fields]	Lectin	26531228	Protein O-linked glycosylation in the plant pathogen Ralstonia solanacearum.
685	DRAMP02449	Lectin	"Lectin"[All Fields] AND biofilm[All Fields]	Lectin	26516766	Novel Insights into the Proteus mirabilis Crystalline Biofilm Using Real-Time Imaging.
685	DRAMP02449	Lectin	"Lectin"[All Fields] AND biofilm[All Fields]	Lectin	26506097	Systematic Identification of Cyclic-di-GMP Binding Proteins in Vibrio cholerae Reveals a Novel Class of Cyclic-di-GMP-Binding ATPases Associated with Type II Secretion Systems.
685	DRAMP02449	Lectin	"Lectin"[All Fields] AND biofilm[All Fields]	Lectin	26505896	C-di-GMP Regulates Motile to Sessile Transition by Modulating MshA Pili Biogenesis and Near-Surface Motility Behavior in Vibrio cholerae.
685	DRAMP02449	Lectin	"Lectin"[All Fields] AND biofilm[All Fields]	Lectin	26496389	Timescales and Frequencies of Reversible and Irreversible Adhesion Events of Single Bacterial Cells.
685	DRAMP02449	Lectin	"Lectin"[All Fields] AND biofilm[All Fields]	Lectin	26475462	Coaggregation occurs between microorganisms isolated from different environments.
685	DRAMP02449	Lectin	"Lectin"[All Fields] AND biofilm[All Fields]	Lectin	26445498	Comparative Transcriptome Analysis Reveals Cool Virulence Factors of Ralstonia solanacearum Race 3 Biovar 2.
685	DRAMP02449	Lectin	"Lectin"[All Fields] AND biofilm[All Fields]	Lectin	26416170	Multivalency effects on Pseudomonas aeruginosa biofilm inhibition and dispersal by glycopeptide dendrimers targeting lectin LecA.
685	DRAMP02449	Lectin	"Lectin"[All Fields] AND biofilm[All Fields]	Lectin	26407005	Evidence for the Sialylation of PilA, the PI-2a Pilus-Associated Adhesin of Streptococcus agalactiae Strain NEM316.
685	DRAMP02449	Lectin	"Lectin"[All Fields] AND biofilm[All Fields]	Lectin	26360327	Effects of the Surface Densities of Glycoclusters on the Determination of Their IC50 and Kd Value Determination by Using a Microarray.
685	DRAMP02449	Lectin	"Lectin"[All Fields] AND biofilm[All Fields]	Lectin	26342168	Inhibition of Pseudomonas aeruginosa biofilm formation on wound dressings.
685	DRAMP02449	Lectin	"Lectin"[All Fields] AND biofilm[All Fields]	Lectin	26315586	Comparative responses of river biofilms at the community level to common organic solvent and herbicide exposure.

685	DRAMP02449	Lectin	"Lectin"[All Fields] AND biofilm[All Fields]	Lectin	26311845	Pel is a cationic exopolysaccharide that cross-links extracellular DNA in the Pseudomonas aeruginosa biofilm matrix.
685	DRAMP02449	Lectin	"Lectin"[All Fields] AND biofilm[All Fields]	Lectin	26295304	Structural Insight into Multivalent Galactoside Binding to Pseudomonas aeruginosa Lectin LecA.
685	DRAMP02449	Lectin	"Lectin"[All Fields] AND biofilm[All Fields]	Lectin	26260119	A new dry-surface biofilm model: An essential tool for efficacy testing of hospital surface decontamination procedures.
685	DRAMP02449	Lectin	"Lectin"[All Fields] AND biofilm[All Fields]	Lectin	26169631	Visualization and analysis of EPS glycoconjugates of the thermoacidophilic archaeon Sulfolobus metallicus.
685	DRAMP02449	Lectin	"Lectin"[All Fields] AND biofilm[All Fields]	Lectin	26109449	Water-soluble Moringa oleifera lectin interferes with growth, survival and cell permeability of corrosive and pathogenic bacteria.
685	DRAMP02449	Lectin	"Lectin"[All Fields] AND biofilm[All Fields]	Lectin	26103135	Coaggregation between Rhodococcus and Acinetobacter strains isolated from the food industry.
685	DRAMP02449	Lectin	"Lectin"[All Fields] AND biofilm[All Fields]	Lectin	26085942	Pseudomonas Aeruginosa Lectins As Targets for Novel Antibacterials.
685	DRAMP02449	Lectin	"Lectin"[All Fields] AND biofilm[All Fields]	Lectin	26004349	Synthesis of mannoheptose derivatives and their evaluation as inhibitors of the lectin LecB from the opportunistic pathogen Pseudomonas aeruginosa.
685	DRAMP02449	Lectin	"Lectin"[All Fields] AND biofilm[All Fields]	Lectin	25811661	A C-type lectin from Bothrops jararacussu venom disrupts Staphylococcal biofilms.
685	DRAMP02449	Lectin	"Lectin"[All Fields] AND biofilm[All Fields]	Lectin	25621738	Force nanoscopy of hydrophobic interactions in the fungal pathogen Candida glabrata.
685	DRAMP02449	Lectin	"Lectin"[All Fields] AND biofilm[All Fields]	Lectin	25592264	Antipneumococcal activity of neuraminidase inhibiting artocarpin.
685	DRAMP02449	Lectin	"Lectin"[All Fields] AND biofilm[All Fields]	Lectin	25587410	Microstructured block copolymer surfaces for control of microbe adhesion and aggregation.
685	DRAMP02449	Lectin	"Lectin"[All Fields] AND biofilm[All Fields]	Lectin	25559489	CdTe quantum dots conjugated to concanavalin A as potential fluorescent molecular probes for saccharides detection in Candida albicans.
685	DRAMP02449	Lectin	"Lectin"[All Fields] AND biofilm[All Fields]	Lectin	25521500	Structure and biological roles of Sinorhizobium fredii HH103 exopolysaccharide.
685	DRAMP02449	Lectin	"Lectin"[All Fields] AND biofilm[All Fields]	Lectin	25488256	Use of lectins to in situ visualize glycoconjugates of extracellular polymeric substances in acidophilic archaeal biofilms.
685	DRAMP02449	Lectin	"Lectin"[All Fields] AND biofilm[All Fields]	Lectin	25415418	A biophysical study with carbohydrate derivatives explains the molecular basis of monosaccharide selectivity of the Pseudomonas aeruginosa lectin LecB.
685	DRAMP02449	Lectin	"Lectin"[All Fields] AND biofilm[All Fields]	Lectin	25312958	Genome-wide evaluation of the interplay between Caenorhabditis elegans and Yersinia pseudotuberculosis during in vivo biofilm formation.
685	DRAMP02449	Lectin	"Lectin"[All Fields] AND biofilm[All Fields]	Lectin	25246957	Expeditive synthesis of trithiotriazine-cored glycoclusters and inhibition of Pseudomonas aeruginosa biofilm formation.
685	DRAMP02449	Lectin	"Lectin"[All Fields] AND biofilm[All Fields]	Lectin	25234699	Vibrio cholerae use pili and flagella synergistically to effect motility switching and conditional surface attachment.
685	DRAMP02449	Lectin	"Lectin"[All Fields] AND biofilm[All Fields]	Lectin	25224970	Molecular characterization of the dextran-binding lectin B gene dblB of Streptococcus criceti in Streptococcus mutans strain GS-5 with mutations in both gbpC and spaP genes.
685	DRAMP02449	Lectin	"Lectin"[All Fields] AND biofilm[All Fields]	Lectin	25111023	Isolation and characterization of a novel Acidithiobacillus ferrivorans strain from the Chilean Altiplano: attachment and biofilm formation on pyrite at low temperature.
685	DRAMP02449	Lectin	"Lectin"[All Fields] AND biofilm[All Fields]	Lectin	24980018	Purification of a novel chitin-binding lectin with antimicrobial and antibiofilm activities from a bangladeshi cultivar of potato (Solanum tuberosum).
685	DRAMP02449	Lectin	"Lectin"[All Fields] AND biofilm[All Fields]	Lectin	24664176	Chitinases are negative regulators of Francisella novicida biofilms.
685	DRAMP02449	Lectin	"Lectin"[All Fields] AND biofilm[All Fields]	Lectin	24644254	Detection of biofilm in bronchoalveolar lavage from children with non-cystic fibrosis bronchiectasis.
685	DRAMP02449	Lectin	"Lectin"[All Fields] AND biofilm[All Fields]	Lectin	24608122	EndoE from Enterococcus faecalis hydrolyzes the glycans of the biofilm inhibiting protein lactoferrin and mediates growth.
685	DRAMP02449	Lectin	"Lectin"[All Fields] AND biofilm[All Fields]	Lectin	24569519	Functional characterization of exopolyphosphatase/guanosine pentaphosphate phosphohydrolase (PPX/GPPA) of Campylobacter jejuni.
685	DRAMP02449	Lectin	"Lectin"[All Fields] AND biofilm[All Fields]	Lectin	24307364	Structure-based optimization of the terminal tripeptide in glycopeptide dendrimer inhibitors of Pseudomonas aeruginosa biofilms targeting LecA.
685	DRAMP02449	Lectin	"Lectin"[All Fields] AND biofilm[All Fields]	Lectin	24139070	Method to grow Actinobacillus pleuropneumoniae biofilm on a biotic surface.
685	DRAMP02449	Lectin	"Lectin"[All Fields] AND biofilm[All Fields]	Lectin	24123820	Structural and population characterization of MrkD, the adhesive subunit of type 3 fimbriae.
685	DRAMP02449	Lectin	"Lectin"[All Fields] AND biofilm[All Fields]	Lectin	24123124	Secondary sugar binding site identified for LecA lectin from Pseudomonas aeruginosa.
685	DRAMP02449	Lectin	"Lectin"[All Fields] AND biofilm[All Fields]	Lectin	25474867	[Lectin-binding analysis of the biofilm exopolymeric matrix carbohydrate composition of corrosion-aggressive bacteria].
685	DRAMP02449	Lectin	"Lectin"[All Fields] AND biofilm[All Fields]	Lectin	23954398	Characterization of the interaction between collectin 11 (CL-11, CL-K1) and nucleic acids.
685	DRAMP02449	Lectin	"Lectin"[All Fields] AND biofilm[All Fields]	Lectin	23910219	Inhibition of initial adhesion of oral bacteria through a lectin from Bauhinia variegata L. var. variegata expressed in Escherichia coli.
685	DRAMP02449	Lectin	"Lectin"[All Fields] AND biofilm[All Fields]	Lectin	23869965	CH-π "T-shape" interaction with histidine explains binding of aromatic galactosides to Pseudomonas aeruginosa lectin LecA.
685	DRAMP02449	Lectin	"Lectin"[All Fields] AND biofilm[All Fields]	Lectin	23847600	Assessment of bacterial and structural dynamics in aerobic granular biofilms.
685	DRAMP02449	Lectin	"Lectin"[All Fields] AND biofilm[All Fields]	Lectin	23719508	Discovery of two classes of potent glycomimetic inhibitors of Pseudomonas aeruginosa LecB with distinct binding modes.
685	DRAMP02449	Lectin	"Lectin"[All Fields] AND biofilm[All Fields]	Lectin	23509865	Zinc as an agent for the prevention of biofilm formation by pathogenic bacteria.
685	DRAMP02449	Lectin	"Lectin"[All Fields] AND biofilm[All Fields]	Lectin	23320545	Kinetic development of biofilm on NF membranes at the Méry-sur-Oise plant, France.
685	DRAMP02449	Lectin	"Lectin"[All Fields] AND biofilm[All Fields]	Lectin	23235153	RapA2 is a calcium-binding lectin composed of two highly conserved cadherin-like domains that specifically recognize Rhizobium leguminosarum acidic exopolysaccharides.
685	DRAMP02449	Lectin	"Lectin"[All Fields] AND biofilm[All Fields]	Lectin	23056489	Specific association of lectin LecB with the surface of Pseudomonas aeruginosa: role of outer membrane protein OprF.
685	DRAMP02449	Lectin	"Lectin"[All Fields] AND biofilm[All Fields]	Lectin	22950027	Identification of functions linking quorum sensing with biofilm formation in Burkholderia cenocepacia H111.
685	DRAMP02449	Lectin	"Lectin"[All Fields] AND biofilm[All Fields]	Lectin	22913814	Insight into the composition of the intercellular matrix of Streptococcus pneumoniae biofilms.
685	DRAMP02449	Lectin	"Lectin"[All Fields] AND biofilm[All Fields]	Lectin	22868053	Quantitative analysis (K(d) and IC(50)) of glycoconjugates interactions with a bacterial lectin on a carbohydrate microarray with DNA Direct Immobilization (DDI).
685	DRAMP02449	Lectin	"Lectin"[All Fields] AND biofilm[All Fields]	Lectin	22853805	Expression of sialic acids and other nonulosonic acids in Leptospira.
685	DRAMP02449	Lectin	"Lectin"[All Fields] AND biofilm[All Fields]	Lectin	22799498	Synthesis of a library of fucosylated glycoclusters and determination of their binding toward Pseudomonas aeruginosa lectin B (PA-IIL) using a DNA-based carbohydrate microarray.
685	DRAMP02449	Lectin	"Lectin"[All Fields] AND biofilm[All Fields]	Lectin	22726869	The immunological investigation of a child with chronic wet cough.
685	DRAMP02449	Lectin	"Lectin"[All Fields] AND biofilm[All Fields]	Lectin	22546527	TLP01, an mshA mutant of Vibrio cholerae O139 as vaccine candidate against cholera.
685	DRAMP02449	Lectin	"Lectin"[All Fields] AND biofilm[All Fields]	Lectin	22511878	Cell contact-dependent outer membrane exchange in myxobacteria: genetic determinants and mechanism.
685	DRAMP02449	Lectin	"Lectin"[All Fields] AND biofilm[All Fields]	Lectin	22419503	Released products of pathogenic bacteria stimulate biofilm formation by Escherichia coli K-12 strains.
685	DRAMP02449	Lectin	"Lectin"[All Fields] AND biofilm[All Fields]	Lectin	22363222	Tasco®: a product of Ascophyllum nodosum enhances immune response of Caenorhabditis elegans against Pseudomonas aeruginosa infection.
685	DRAMP02449	Lectin	"Lectin"[All Fields] AND biofilm[All Fields]	Lectin	22092602	Direct visualization of the interaction between pilin and exopolysaccharides of Myxococcus xanthus with eGFP-fused PilA protein.
685	DRAMP02449	Lectin	"Lectin"[All Fields] AND biofilm[All Fields]	Lectin	21919164	A glycopeptide dendrimer inhibitor of the galactose-specific lectin LecA and of Pseudomonas aeruginosa biofilms.
685	DRAMP02449	Lectin	"Lectin"[All Fields] AND biofilm[All Fields]	Lectin	21854629	Identification, structure, and characterization of an exopolysaccharide produced by Histophilus somni during biofilm formation.
685	DRAMP02449	Lectin	"Lectin"[All Fields] AND biofilm[All Fields]	Lectin	21569125	Biofilm formation by Acinetobacter baumannii strains isolated from urinary tract infection and urinary catheters.
685	DRAMP02449	Lectin	"Lectin"[All Fields] AND biofilm[All Fields]	Lectin	21545730	Biofilm formation and adherence characteristics of an Elizabethkingia meningoseptica isolate from Oreochromis mossambicus.
685	DRAMP02449	Lectin	"Lectin"[All Fields] AND biofilm[All Fields]	Lectin	21440051	Bacterial adherence in otitis media: determination of N-acetylgalactosamine (GalNAc) residues in the submucosal glands and surface epithelium of the normal and diseased Eustachian tube.
685	DRAMP02449	Lectin	"Lectin"[All Fields] AND biofilm[All Fields]	Lectin	21307211	Adenoid reservoir for pathogenic biofilm bacteria.
685	DRAMP02449	Lectin	"Lectin"[All Fields] AND biofilm[All Fields]	Lectin	21129931	Severe Candida spp. infections: new insights into natural immunity.
685	DRAMP02449	Lectin	"Lectin"[All Fields] AND biofilm[All Fields]	Lectin	21124788	Crenarchaeal biofilm formation under extreme conditions.

685	DRAMP02449	Lectin	"Lectin"[All Fields] AND biofilm[All Fields]	Lectin	21097578	Characterization of glycoconjugates of extracellular polymeric substances in tufa-associated biofilms by using fluorescence lectin-binding analysis.
685	DRAMP02449	Lectin	"Lectin"[All Fields] AND biofilm[All Fields]	Lectin	21091636	Investigating the humoral immune response in chronic venous leg ulcer patients colonised with Pseudomonas aeruginosa.
685	DRAMP02449	Lectin	"Lectin"[All Fields] AND biofilm[All Fields]	Lectin	26781241	Probiotic Lactobacillus and Bifidobacterial Lectins Against Candida albicans and Staphylococcus aureus Clinical Strains: New Class of the Pathogen Biofilm Destructors.
685	DRAMP02449	Lectin	"Lectin"[All Fields] AND biofilm[All Fields]	Lectin	20684642	Label-free in situ SERS imaging of biofilms.
685	DRAMP02449	Lectin	"Lectin"[All Fields] AND biofilm[All Fields]	Lectin	20545853	The Actinomyces oris type 2 fimbrial shaft FimA mediates co-aggregation with oral streptococci, adherence to red blood cells and biofilm development.
685	DRAMP02449	Lectin	"Lectin"[All Fields] AND biofilm[All Fields]	Lectin	20507989	Structure and molecular characterization of Streptococcus pneumoniae capsular polysaccharide 10F by carbohydrate engineering in Streptococcus oralis.
685	DRAMP02449	Lectin	"Lectin"[All Fields] AND biofilm[All Fields]	Lectin	20487019	Spatiotemporal activity of the mshA gene system in Shewanella oneidensis MR-1 biofilms.
685	DRAMP02449	Lectin	"Lectin"[All Fields] AND biofilm[All Fields]	Lectin	20385555	The Caenorhabditis elegans bus-2 mutant reveals a new class of O-glycans affecting bacterial resistance.
685	DRAMP02449	Lectin	"Lectin"[All Fields] AND biofilm[All Fields]	Lectin	20348252	The sigma factor AlgU plays a key role in formation of robust biofilms by nonmucoid Pseudomonas aeruginosa.
685	DRAMP02449	Lectin	"Lectin"[All Fields] AND biofilm[All Fields]	Lectin	20105519	Blocking of Pseudomonas aeruginosa and Chromobacterium violaceum lectins by diverse mammalian milks.
685	DRAMP02449	Lectin	"Lectin"[All Fields] AND biofilm[All Fields]	Lectin	20088866	Pseudomonas aeruginosa uses a cyclic-di-GMP-regulated adhesin to reinforce the biofilm extracellular matrix.
685	DRAMP02449	Lectin	"Lectin"[All Fields] AND biofilm[All Fields]	Lectin	20042742	Specific Lactobacillus/Mutans Streptococcus co-aggregation.
685	DRAMP02449	Lectin	"Lectin"[All Fields] AND biofilm[All Fields]	Lectin	20016675	Soybean Lectin Enhances Biofilm Formation by Bradyrhizobium japonicum in the Absence of Plants.
685	DRAMP02449	Lectin	"Lectin"[All Fields] AND biofilm[All Fields]	Lectin	19898535	Cells in shearable and nonshearable regions of Salmonella enterica serovar Enteritidis biofilms are morphologically and physiologically distinct.
685	DRAMP02449	Lectin	"Lectin"[All Fields] AND biofilm[All Fields]	Lectin	19686342	Characterization of two host-specific genes, mannose-sensitive hemagglutinin (mshA) and uridyl phosphate dehydrogenase (UDPDH) that are involved in the Vibrio fischeri-Euprymna tasmanica mutualism.
685	DRAMP02449	Lectin	"Lectin"[All Fields] AND biofilm[All Fields]	Lectin	19596998	Mycobacterium abscessus Glycopeptidolipids mask underlying cell wall phosphatidyl-myo-inositol mannosides blocking induction of human macrophage TNF-alpha by preventing interaction with TLR2.
685	DRAMP02449	Lectin	"Lectin"[All Fields] AND biofilm[All Fields]	Lectin	19478809	Stretching polysaccharides on live cells using single molecule force spectroscopy.
685	DRAMP02449	Lectin	"Lectin"[All Fields] AND biofilm[All Fields]	Lectin	19432800	Identification of exopolysaccharide-deficient mutants of Mycoplasma pulmonis.
685	DRAMP02449	Lectin	"Lectin"[All Fields] AND biofilm[All Fields]	Lectin	19295649	Morphological and biochemical changes in Pseudomonas fluorescens biofilms induced by sub-inhibitory exposure to antimicrobial agents.
685	DRAMP02449	Lectin	"Lectin"[All Fields] AND biofilm[All Fields]	Lectin	19226385	Novel effect of plant lectins on the inhibition of Streptococcus mutans biofilm formation on saliva-coated surface.
685	DRAMP02449	Lectin	"Lectin"[All Fields] AND biofilm[All Fields]	Lectin	19189366	Glycopeptide dendrimers with high affinity for the fucose-binding lectin LecB from Pseudomonas aeruginosa.
685	DRAMP02449	Lectin	"Lectin"[All Fields] AND biofilm[All Fields]	Lectin	19101469	Inhibition and dispersion of Pseudomonas aeruginosa biofilms by glycopeptide dendrimers targeting the fucose-specific lectin LecB.
685	DRAMP02449	Lectin	"Lectin"[All Fields] AND biofilm[All Fields]	Lectin	19019406	Combined use of confocal laser scanning microscopy (CLSM) and Raman microscopy (RM): investigations on EPS-Matrix.
685	DRAMP02449	Lectin	"Lectin"[All Fields] AND biofilm[All Fields]	Lectin	19016779	Staphylococcus epidermidis polysaccharide intercellular adhesin induces IL-8 expression in human astrocytes via a mechanism involving TLR2.
685	DRAMP02449	Lectin	"Lectin"[All Fields] AND biofilm[All Fields]	Lectin	19005936	Development and characterization of lectin-functionalized vesicular constructs bearing amphotericin B for bio-film targeting.
685	DRAMP02449	Lectin	"Lectin"[All Fields] AND biofilm[All Fields]	Lectin	18842140	Characterization of biofilm matrix, degradation by DNase treatment and evidence of capsule downregulation in Streptococcus pneumoniae clinical isolates.
685	DRAMP02449	Lectin	"Lectin"[All Fields] AND biofilm[All Fields]	Lectin	18806209	The cell wall of the human pathogen Candida glabrata: differential incorporation of novel adhesin-like wall proteins.
685	DRAMP02449	Lectin	"Lectin"[All Fields] AND biofilm[All Fields]	Lectin	18805978	Characterization of a Streptococcus sp.-Veillonella sp. community micromanipulated from dental plaque.
685	DRAMP02449	Lectin	"Lectin"[All Fields] AND biofilm[All Fields]	Lectin	18718852	A standardized pre-treatment method of biofilm flocs for fluorescence microscopic characterization.
685	DRAMP02449	Lectin	"Lectin"[All Fields] AND biofilm[All Fields]	Lectin	18704225	Quorum sensing by 2-alkyl-4-quinolones in Pseudomonas aeruginosa and other bacterial species.
685	DRAMP02449	Lectin	"Lectin"[All Fields] AND biofilm[All Fields]	Lectin	18656625	Depletion of intestinal phosphate after operative injury activates the virulence of P aeruginosa causing lethal gut-derived sepsis.
685	DRAMP02449	Lectin	"Lectin"[All Fields] AND biofilm[All Fields]	Lectin	18568666	Role of beta 1-4 linked polymers in the biofilm structure of marine Pseudomonas sp. CE-2 on 304 stainless steel coupons.
685	DRAMP02449	Lectin	"Lectin"[All Fields] AND biofilm[All Fields]	Lectin	18515487	The hmsHFRS operon of Xenorhabdus nematophila is required for biofilm attachment to Caenorhabditis elegans.
685	DRAMP02449	Lectin	"Lectin"[All Fields] AND biofilm[All Fields]	Lectin	18507672	The exopolysaccharide of Rhizobium sp. YAS34 is not necessary for biofilm formation on Arabidopsis thaliana and Brassica napus roots but contributes to root colonization.
685	DRAMP02449	Lectin	"Lectin"[All Fields] AND biofilm[All Fields]	Lectin	18405985	Correlative light/electron microscopy for the investigation of microbial mats from Black Sea Cold Seeps.
685	DRAMP02449	Lectin	"Lectin"[All Fields] AND biofilm[All Fields]	Lectin	18402602	Salivary gel-forming mucin MUC5B--a nutrient for dental plaque bacteria.
685	DRAMP02449	Lectin	"Lectin"[All Fields] AND biofilm[All Fields]	Lectin	18373677	Architecture of Deinococcus geothermalis biofilms on glass and steel: a lectin study.
685	DRAMP02449	Lectin	"Lectin"[All Fields] AND biofilm[All Fields]	Lectin	18303023	Molecular and antigenic characterization of a Streptococcus oralis coaggregation receptor polysaccharide by carbohydrate engineering in Streptococcus gordonii.
685	DRAMP02449	Lectin	"Lectin"[All Fields] AND biofilm[All Fields]	Lectin	18261816	Architectural adaptation and protein expression patterns of Salmonella enterica serovar Enteritidis biofilms under laminar flow conditions.
685	DRAMP02449	Lectin	"Lectin"[All Fields] AND biofilm[All Fields]	Lectin	18206142	Leishmania (Leishmania) chagasi interactions with Serratia marcescens: ultrastructural studies, lysis and carbohydrate effects.
685	DRAMP02449	Lectin	"Lectin"[All Fields] AND biofilm[All Fields]	Lectin	18166328	Detection of a microbial biofilm in intraamniotic infection.
685	DRAMP02449	Lectin	"Lectin"[All Fields] AND biofilm[All Fields]	Lectin	18156333	Intergeneric coaggregation among drinking water bacteria: evidence of a role for Acinetobacter calcoaceticus as a bridging bacterium.
685	DRAMP02449	Lectin	"Lectin"[All Fields] AND biofilm[All Fields]	Lectin	18043624	Honey and royal jelly, like human milk, abrogate lectin-dependent infection-preceding Pseudomonas aeruginosa adhesion.
685	DRAMP02449	Lectin	"Lectin"[All Fields] AND biofilm[All Fields]	Lectin	17992013	Manganese affects Streptococcus mutans virulence gene expression.
685	DRAMP02449	Lectin	"Lectin"[All Fields] AND biofilm[All Fields]	Lectin	17728861	Synthesis and binding properties of divalent and trivalent clusters of the Lewis a disaccharide moiety to Pseudomonas aeruginosa lectin PA-IIL.
685	DRAMP02449	Lectin	"Lectin"[All Fields] AND biofilm[All Fields]	Lectin	17634727	Inhibition of bacterial adherence to saliva-coated through plant lectins.
685	DRAMP02449	Lectin	"Lectin"[All Fields] AND biofilm[All Fields]	Lectin	17631634	Pseudomonas aeruginosa Psl is a galactose- and mannose-rich exopolysaccharide.
685	DRAMP02449	Lectin	"Lectin"[All Fields] AND biofilm[All Fields]	Lectin	17623286	X-ray structures and thermodynamics of the interaction of PA-IIL from Pseudomonas aeruginosa with disaccharide derivatives.
685	DRAMP02449	Lectin	"Lectin"[All Fields] AND biofilm[All Fields]	Lectin	17546990	Assessing chemical cleaning of nanofiltration membranes in a drinking water production plant: a combination of chemical composition analysis and fluorescence microscopy.
685	DRAMP02449	Lectin	"Lectin"[All Fields] AND biofilm[All Fields]	Lectin	17538657	In situ evidence for microdomains in the polymer matrix of bacterial microcolonies.
685	DRAMP02449	Lectin	"Lectin"[All Fields] AND biofilm[All Fields]	Lectin	17362964	Transcriptional and secretory responses of Entamoeba histolytica to mucins, epithelial cells and bacteria.
685	DRAMP02449	Lectin	"Lectin"[All Fields] AND biofilm[All Fields]	Lectin	17339204	Caenorhabditis elegans mutants resistant to attachment of Yersinia biofilms.
685	DRAMP02449	Lectin	"Lectin"[All Fields] AND biofilm[All Fields]	Lectin	17189437	Rugosity in Grimontia hollisae.
685	DRAMP02449	Lectin	"Lectin"[All Fields] AND biofilm[All Fields]	Lectin	17015462	Pseudomonas aeruginosa LecB is involved in pilus biogenesis and protease IV activity but not in adhesion to respiratory mucins.
685	DRAMP02449	Lectin	"Lectin"[All Fields] AND biofilm[All Fields]	Lectin	17005972	Contribution of alginate and levan production to biofilm formation by Pseudomonas syringae.
685	DRAMP02449	Lectin	"Lectin"[All Fields] AND biofilm[All Fields]	Lectin	16980504	Characterization of adhesion threads of Deinococcus geothermalis as type IV pili.
685	DRAMP02449	Lectin	"Lectin"[All Fields] AND biofilm[All Fields]	Lectin	16934113	Streptococcal receptor polysaccharides: recognition molecules for oral biofilm formation.
685	DRAMP02449	Lectin	"Lectin"[All Fields] AND biofilm[All Fields]	Lectin	16861673	Comparative antibody-mediated phagocytosis of Staphylococcus epidermidis cells grown in a biofilm or in the planktonic state.

685	DRAMP02449	Lectin	"Lectin"[All Fields] AND biofilm[All Fields]	Lectin	16834597	In vitro inhibition of Streptococci binding to enamel acquired pellicle by plant lectins.
685	DRAMP02449	Lectin	"Lectin"[All Fields] AND biofilm[All Fields]	Lectin	16799194	Bacterial-bacterial cell interactions in biofilms: detection of polysaccharide intercellular adhesins by blotting and confocal microscopy.
685	DRAMP02449	Lectin	"Lectin"[All Fields] AND biofilm[All Fields]	Lectin	16689730	The galactophilic lectin, LecA, contributes to biofilm development in Pseudomonas aeruginosa.
685	DRAMP02449	Lectin	"Lectin"[All Fields] AND biofilm[All Fields]	Lectin	16496953	[Lectins, adhesins, and lectin-like substances of lactobacilli and bifidobacteria].
685	DRAMP02449	Lectin	"Lectin"[All Fields] AND biofilm[All Fields]	Lectin	16495661	Involvement of N-acetyl-D-galactosamine-specific lectin in biofilm formation by the periodontopathogenic bacterium, Eikenella corrodens.
685	DRAMP02449	Lectin	"Lectin"[All Fields] AND biofilm[All Fields]	Lectin	16483840	4-quinolone signalling in Pseudomonas aeruginosa: old molecules, new perspectives.
685	DRAMP02449	Lectin	"Lectin"[All Fields] AND biofilm[All Fields]	Lectin	16438968	Binding of different monosaccharides by lectin PA-IIL from Pseudomonas aeruginosa: thermodynamics data correlated with X-ray structures.
685	DRAMP02449	Lectin	"Lectin"[All Fields] AND biofilm[All Fields]	Lectin	16269777	Capsular polysaccharide surrounds smooth and rugose types of Salmonella enterica serovar Typhimurium DT104.
685	DRAMP02449	Lectin	"Lectin"[All Fields] AND biofilm[All Fields]	Lectin	16234864	Effects of selected pharmaceuticals on riverine biofilm communities.
685	DRAMP02449	Lectin	"Lectin"[All Fields] AND biofilm[All Fields]	Lectin	16164562	Carbohydrate engineering of the recognition motifs in streptococcal co-aggregation receptor polysaccharides.
685	DRAMP02449	Lectin	"Lectin"[All Fields] AND biofilm[All Fields]	Lectin	15908345	Role of sialic acid and complex carbohydrate biosynthesis in biofilm formation by nontypeable Haemophilus influenzae in the chinchilla middle ear.
685	DRAMP02449	Lectin	"Lectin"[All Fields] AND biofilm[All Fields]	Lectin	15883718	The use of new probes and stains for improved assessment of cell viability and extracellular polymeric substances in Candida albicans biofilms.
685	DRAMP02449	Lectin	"Lectin"[All Fields] AND biofilm[All Fields]	Lectin	15870442	Pseudomonas aeruginosa lectin LecB is located in the outer membrane and is involved in biofilm formation.
685	DRAMP02449	Lectin	"Lectin"[All Fields] AND biofilm[All Fields]	Lectin	15726969	Removal of Pseudomonas putida biofilm and associated extracellular polymeric substances from stainless steel by alkali cleaning.
685	DRAMP02449	Lectin	"Lectin"[All Fields] AND biofilm[All Fields]	Lectin	15721384	Use of liposomes to deliver bactericides to bacterial biofilms.
685	DRAMP02449	Lectin	"Lectin"[All Fields] AND biofilm[All Fields]	Lectin	15696391	Combining in situ reverse transcriptase polymerase chain reaction, optical microscopy, and X-ray photoelectron spectroscopy to investigate mineral surface-associated microbial activities.
685	DRAMP02449	Lectin	"Lectin"[All Fields] AND biofilm[All Fields]	Lectin	15640218	Use of fluorophore-conjugated lectins to study cell-cell interactions in model marine biofilms.
685	DRAMP02449	Lectin	"Lectin"[All Fields] AND biofilm[All Fields]	Lectin	15470707	Volumetric measurements of bacterial cells and extracellular polymeric substance glycoconjugates in biofilms.
685	DRAMP02449	Lectin	"Lectin"[All Fields] AND biofilm[All Fields]	Lectin	15303764	Growth, structure and oxygen penetration in particle supported autotrophic biofilms.
685	DRAMP02449	Lectin	"Lectin"[All Fields] AND biofilm[All Fields]	Lectin	15262945	A movable surface: formation of Yersinia sp. biofilms on motile Caenorhabditis elegans.
685	DRAMP02449	Lectin	"Lectin"[All Fields] AND biofilm[All Fields]	Lectin	15240316	Microscale and molecular assessment of impacts of nickel, nutrients, and oxygen level on structure and function of river biofilm communities.
685	DRAMP02449	Lectin	"Lectin"[All Fields] AND biofilm[All Fields]	Lectin	15213170	Nontypeable Haemophilus influenzae strain 2019 produces a biofilm containing N-acetylneuraminic acid that may mimic sialylated O-linked glycans.
685	DRAMP02449	Lectin	"Lectin"[All Fields] AND biofilm[All Fields]	Lectin	15066042	Genetic evidence that the Vibrio cholerae monolayer is a distinct stage in biofilm development.
685	DRAMP02449	Lectin	"Lectin"[All Fields] AND biofilm[All Fields]	Lectin	15003688	Methods for in situ detection and characterization of extracellular polymers in biofilms by electron microscopy.
685	DRAMP02449	Lectin	"Lectin"[All Fields] AND biofilm[All Fields]	Lectin	14614140	The Vibrio cholerae O139 O-antigen polysaccharide is essential for Ca2+-dependent biofilm development in sea water.
685	DRAMP02449	Lectin	"Lectin"[All Fields] AND biofilm[All Fields]	Lectin	14507361	The Pseudomonas aeruginosa quinolone signal molecule overcomes the cell density-dependency of the quorum sensing hierarchy, regulates rhl-dependent genes at the onset of stationary phase and can be produced in the absence of LasR.
685	DRAMP02449	Lectin	"Lectin"[All Fields] AND biofilm[All Fields]	Lectin	14500015	Enthalpy of interaction between coaggregating and non-coaggregating oral bacterial pairs--a microcalorimetric study.
685	DRAMP02449	Lectin	"Lectin"[All Fields] AND biofilm[All Fields]	Lectin	12892848	Envelope glycosylation determined by lectins in microscopy sections of Acinetobacter venetianus induced by diesel fuel.
685	DRAMP02449	Lectin	"Lectin"[All Fields] AND biofilm[All Fields]	Lectin	12795411	Blocking of Pseudomonas aeruginosa lectins by human milk glycans.
685	DRAMP02449	Lectin	"Lectin"[All Fields] AND biofilm[All Fields]	Lectin	12562815	Identification of genes required for synthesis of the adhesive holdfast in Caulobacter crescentus.
685	DRAMP02449	Lectin	"Lectin"[All Fields] AND biofilm[All Fields]	Lectin	12121477	Adhesion of viridans group streptococci to sialic acid-, galactose- and N-acetylgalactosamine-containing receptors.
685	DRAMP02449	Lectin	"Lectin"[All Fields] AND biofilm[All Fields]	Lectin	12031574	Application of fluorescently labelled lectins for the visualization and biochemical characterization of polysaccharides in biofilms of Pseudomonas aeruginosa.
685	DRAMP02449	Lectin	"Lectin"[All Fields] AND biofilm[All Fields]	Lectin	11526040	New spatially explicit method for detecting extracellular protease activity in biofilms.
685	DRAMP02449	Lectin	"Lectin"[All Fields] AND biofilm[All Fields]	Lectin	11487975	Preparation, characterization and in vitro antimicrobial activity of metronidazole bearing lectinized liposomes for intra-periodontal pocket delivery.
685	DRAMP02449	Lectin	"Lectin"[All Fields] AND biofilm[All Fields]	Lectin	11381960	Time-resolved study of biofilm architecture and transport processes using experimental and simulation techniques: the role of EPS.
685	DRAMP02449	Lectin	"Lectin"[All Fields] AND biofilm[All Fields]	Lectin	11158347	Assessment of lectin-binding analysis for in situ detection of glycoconjugates in biofilm systems.
685	DRAMP02449	Lectin	"Lectin"[All Fields] AND biofilm[All Fields]	Lectin	11099864	Polycarboxylates inhibit the glucan-binding lectin of Streptococcus sobrinus.
685	DRAMP02449	Lectin	"Lectin"[All Fields] AND biofilm[All Fields]	Lectin	10919811	Evaluation of fluorescently labeled lectins for noninvasive localization of extracellular polymeric substances in Sphingomonas biofilms.
685	DRAMP02449	Lectin	"Lectin"[All Fields] AND biofilm[All Fields]	Lectin	10742601	Glycine prevents the phenotypic expression of streptococcal glucan-binding lectin.
685	DRAMP02449	Lectin	"Lectin"[All Fields] AND biofilm[All Fields]	Lectin	10631505	Confocal imaging of in situ natural microbial communities and their extracellular polymeric secretions using Nanoplast resin.
685	DRAMP02449	Lectin	"Lectin"[All Fields] AND biofilm[All Fields]	Lectin	10564499	Steps in the development of a Vibrio cholerae El Tor biofilm.
685	DRAMP02449	Lectin	"Lectin"[All Fields] AND biofilm[All Fields]	Lectin	10547788	Lectin-binding analysis in biofilm systems.
685	DRAMP02449	Lectin	"Lectin"[All Fields] AND biofilm[All Fields]	Lectin	10348878	A role for the mannose-sensitive hemagglutinin in biofilm formation by Vibrio cholerae El Tor.
685	DRAMP02449	Lectin	"Lectin"[All Fields] AND biofilm[All Fields]	Lectin	21374487	Lectin ingestion : changes in mucin secretion and bacterial adhesion to intestinal tissue.
685	DRAMP02449	Lectin	"Lectin"[All Fields] AND biofilm[All Fields]	Lectin	8950503	The effect of eye closure on protein and complement deposition on Group IV hydrogel contact lenses: relationship to tear flow dynamics.
685	DRAMP02449	Lectin	"Lectin"[All Fields] AND biofilm[All Fields]	Lectin	8820019	A dual fluorescence technique for visualization of Staphylococcus epidermidis biofilm using scanning confocal laser microscopy.
685	DRAMP02449	Lectin	"Lectin"[All Fields] AND biofilm[All Fields]	Lectin	8550413	The intercellular adhesin involved in biofilm accumulation of Staphylococcus epidermidis is a linear beta-1,6-linked glucosaminoglycan: purification and structural analysis.
685	DRAMP02449	Lectin	"Lectin"[All Fields] AND biofilm[All Fields]	Lectin	8519474	Dental plaque as a biofilm.
685	DRAMP02449	Lectin	"Lectin"[All Fields] AND biofilm[All Fields]	Lectin	8519472	Lectin-biotin assay for slime present in in situ biofilm produced by Staphylococcus epidermidis using transmission electron microscopy (TEM).
685	DRAMP02449	Lectin	"Lectin"[All Fields] AND biofilm[All Fields]	Lectin	7704482	The use of phospholipid liposomes for targeting to oral and skin-associated bacteria.
685	DRAMP02449	Lectin	"Lectin"[All Fields] AND biofilm[All Fields]	Lectin	8476919	Targeting and delivery of bactericide to adsorbed oral bacteria by use of proteoliposomes.
685	DRAMP02449	Lectin	"Lectin"[All Fields] AND biofilm[All Fields]	Lectin	1602653	[Bacterial adherence to the alveolar and respiratory tract system].
686	DRAMP02456	L-amino-acid oxidase (LAAO; LAO; Dactylomelin-P)	"L-amino-acid oxidase"[All Fields] AND biofilm[All Fields]	L-amino-acid oxidase	30160994	Molecules and Mechanisms Underlying the Antimicrobial Activity of Escapin, an l-Amino Acid Oxidase from the Ink of Sea Hares.
686	DRAMP02456	L-amino-acid oxidase (LAAO; LAO; Dactylomelin-P)	"L-amino-acid oxidase"[All Fields] AND biofilm[All Fields]	L-amino-acid oxidase	27401562	Inhibition and Dispersal of Pseudomonas aeruginosa Biofilms by Combination Treatment with Escapin Intermediate Products and Hydrogen Peroxide.
686	DRAMP02456	L-amino-acid oxidase (LAAO; LAO; Dactylomelin-P)	"L-amino-acid oxidase"[All Fields] AND biofilm[All Fields]	L-amino-acid oxidase	18469105	SO-LAAO, a novel L-amino acid oxidase that enables Streptococcus oligofermentans to outcompete Streptococcus mutans by generating H2O2 from peptone.
687	DRAMP02470	Nosiheptide (NOS; Antibiotic 9671-RP)	"Nosiheptide"[All Fields] AND biofilm[All Fields]	Nosiheptide	30227225	Differential anti-microbial secondary metabolites in different ESKAPE pathogens explain their adaptation in the hospital setup.

688	DRAMP02473	Cathelicidin-BF (Cathelicidin-related protein; Snakes, reptiles, animals)	"Cathelicidin-BF"[All Fields] AND biofilm[All Fields]	Cathelicidin-BF	31843919	The antimicrobial peptide ZY4 combats multidrug-resistant Pseudomonas aeruginosaand Acinetobacter baumanniiinfection.
688	DRAMP02473	Cathelicidin-BF (Cathelicidin-related protein; Snakes, reptiles, animals)	"Cathelicidin-BF"[All Fields] AND biofilm[All Fields]	Cathelicidin-BF	30360422	A Recombinant Snake Cathelicidin Derivative Peptide: Antibiofilm Properties and Expression in Escherichia coli.
688	DRAMP02473	Cathelicidin-BF (Cathelicidin-related protein; Snakes, reptiles, animals)	"Cathelicidin-BF"[All Fields] AND biofilm[All Fields]	Cathelicidin-BF	26656137	Assessing the potential of four cathelicidins for the management of mouse candidiasis and Candida albicans biofilms.
690	DRAMP02478	L-amino-acid oxidase (Bm-LAO; LAAO; LAO; Snakes, reptiles, animals)	"L-amino-acid oxidase"[All Fields] AND biofilm[All Fields]	L-amino-acid oxidase	30160994	Molecules and Mechanisms Underlying the Antimicrobial Activity of Escapin, an l-Amino Acid Oxidase from the Ink of Sea Hares.
690	DRAMP02478	L-amino-acid oxidase (Bm-LAO; LAAO; LAO; Snakes, reptiles, animals)	"L-amino-acid oxidase"[All Fields] AND biofilm[All Fields]	L-amino-acid oxidase	27401562	Inhibition and Dispersal of Pseudomonas aeruginosa Biofilms by Combination Treatment with Escapin Intermediate Products and Hydrogen Peroxide.
690	DRAMP02478	L-amino-acid oxidase (Bm-LAO; LAAO; LAO; Snakes, reptiles, animals)	"L-amino-acid oxidase"[All Fields] AND biofilm[All Fields]	L-amino-acid oxidase	18469105	SO-LAAO, a novel L-amino acid oxidase that enables Streptococcus oligofermentans to outcompete Streptococcus mutans by generating H2O2 from peptone.
691	DRAMP02520	OH-CATH (Snakes, reptiles, animals)	"OH-CATH"[All Fields] AND biofilm[All Fields]	OH-CATH	33919962	A Real-Time Thermal Sensor System for Quantifying the Inhibitory Effect of Antimicrobial Peptides on Bacterial Adhesion and Biofilm Formation.
692	DRAMP02522	L-amino-acid oxidase (LAAO, LAO, Oh-LAAO; Snakes, reptiles, animals)	"L-amino-acid oxidase"[All Fields] AND biofilm[All Fields]	L-amino-acid oxidase	30160994	Molecules and Mechanisms Underlying the Antimicrobial Activity of Escapin, an l-Amino Acid Oxidase from the Ink of Sea Hares.
692	DRAMP02522	L-amino-acid oxidase (LAAO, LAO, Oh-LAAO; Snakes, reptiles, animals)	"L-amino-acid oxidase"[All Fields] AND biofilm[All Fields]	L-amino-acid oxidase	27401562	Inhibition and Dispersal of Pseudomonas aeruginosa Biofilms by Combination Treatment with Escapin Intermediate Products and Hydrogen Peroxide.
692	DRAMP02522	L-amino-acid oxidase (LAAO, LAO, Oh-LAAO; Snakes, reptiles, animals)	"L-amino-acid oxidase"[All Fields] AND biofilm[All Fields]	L-amino-acid oxidase	18469105	SO-LAAO, a novel L-amino acid oxidase that enables Streptococcus oligofermentans to outcompete Streptococcus mutans by generating H2O2 from peptone.
710	DRAMP02778	Defensin (Insects, animals)	"Defensin"[All Fields] AND biofilm[All Fields]	Defensin	34408988	The Anti-Biofilm Efficacy of Caffeic Acid Phenethyl Ester (CAPE) In Vitroand a Murine Model of Oral Candidiasis.
710	DRAMP02778	Defensin (Insects, animals)	"Defensin"[All Fields] AND biofilm[All Fields]	Defensin	34321877	Antibacterial Effect of Honey-Derived Exosomes Containing Antimicrobial Peptides Against Oral Streptococci.
710	DRAMP02778	Defensin (Insects, animals)	"Defensin"[All Fields] AND biofilm[All Fields]	Defensin	34276631	DNA Blocks the Lethal Effect of Human Beta-Defensin 2 Against Neisseria meningitidis.
710	DRAMP02778	Defensin (Insects, animals)	"Defensin"[All Fields] AND biofilm[All Fields]	Defensin	33911935	The antibacterial activities of honey.
710	DRAMP02778	Defensin (Insects, animals)	"Defensin"[All Fields] AND biofilm[All Fields]	Defensin	33865931	Identification of a crocodylian β-defensin variant from Alligator mississippiensis with antimicrobial and antibiofilm activity.
710	DRAMP02778	Defensin (Insects, animals)	"Defensin"[All Fields] AND biofilm[All Fields]	Defensin	33586659	Identification of anti-microbial peptides and traces of microbial DNA in infrainfundibular compartments of human scalp terminal hair follicles.
710	DRAMP02778	Defensin (Insects, animals)	"Defensin"[All Fields] AND biofilm[All Fields]	Defensin	33534018	A recombinant fungal defensin-like peptide-P2 combats Streptococcus dysgalactiae and biofilms.
710	DRAMP02778	Defensin (Insects, animals)	"Defensin"[All Fields] AND biofilm[All Fields]	Defensin	33447687	Future directions of postoperative spinal implant infections.
710	DRAMP02778	Defensin (Insects, animals)	"Defensin"[All Fields] AND biofilm[All Fields]	Defensin	33420317	Curbing gastrointestinal infections by defensin fragment modifications without harming commensal microbiota.
710	DRAMP02778	Defensin (Insects, animals)	"Defensin"[All Fields] AND biofilm[All Fields]	Defensin	32867384	A Novel Peptide Antibiotic, Pro10-1D, Designed from Insect Defensin Shows Antibacterial and Anti-Inflammatory Activities in Sepsis Models.
710	DRAMP02778	Defensin (Insects, animals)	"Defensin"[All Fields] AND biofilm[All Fields]	Defensin	32858856	Candida albicansVirulence Factors and Pathogenicity for Endodontic Infections.
710	DRAMP02778	Defensin (Insects, animals)	"Defensin"[All Fields] AND biofilm[All Fields]	Defensin	32842903	Electrospun ZnO/Poly(Vinylidene Fluoride-Trifluoroethylene) Scaffolds for Lung Tissue Engineering.
710	DRAMP02778	Defensin (Insects, animals)	"Defensin"[All Fields] AND biofilm[All Fields]	Defensin	32663201	Inhibition and eradication activity of truncated α-defensin analogs against multidrug resistant uropathogenic Escherichia coli biofilm.
710	DRAMP02778	Defensin (Insects, animals)	"Defensin"[All Fields] AND biofilm[All Fields]	Defensin	32585445	Glucose effect on Candida albicans biofilm during tissue invasion.
710	DRAMP02778	Defensin (Insects, animals)	"Defensin"[All Fields] AND biofilm[All Fields]	Defensin	32522780	Controlling the Growth of the Skin Commensal Staphylococcus epidermidis Using d-Alanine Auxotrophy.
710	DRAMP02778	Defensin (Insects, animals)	"Defensin"[All Fields] AND biofilm[All Fields]	Defensin	32457749	The Antimicrobial Peptide Human Beta-Defensin 2 Inhibits Biofilm Production of Pseudomonas aeruginosaWithout Compromising Metabolic Activity.
710	DRAMP02778	Defensin (Insects, animals)	"Defensin"[All Fields] AND biofilm[All Fields]	Defensin	32439511	Antibacterial activities and mechanisms of action of a defensin from manila clam Ruditapes philippinarum.
710	DRAMP02778	Defensin (Insects, animals)	"Defensin"[All Fields] AND biofilm[All Fields]	Defensin	31933178	Effects of human β-defensin 3 fused with carbohydrate-binding domain on the function of type III secretion system in Pseudomonas aeruginosa PA14.
710	DRAMP02778	Defensin (Insects, animals)	"Defensin"[All Fields] AND biofilm[All Fields]	Defensin	31906541	Role of FAD-I in Fusobacterial Interspecies Interaction and Biofilm Formation.
710	DRAMP02778	Defensin (Insects, animals)	"Defensin"[All Fields] AND biofilm[All Fields]	Defensin	31729441	Rhesus Theta Defensin 1 Promotes Long Term Survival in Systemic Candidiasis by Host Directed Mechanisms.
710	DRAMP02778	Defensin (Insects, animals)	"Defensin"[All Fields] AND biofilm[All Fields]	Defensin	31336838	Candida albicans-Cell Interactions Activate Innate Immune Defense in Human Palate Epithelial Primary Cells via Nitric Oxide (NO) and β-Defensin 2 (hBD-2).
710	DRAMP02778	Defensin (Insects, animals)	"Defensin"[All Fields] AND biofilm[All Fields]	Defensin	31165072	Impact of the Food Additive Titanium Dioxide (E171) on Gut Microbiota-Host Interaction.
710	DRAMP02778	Defensin (Insects, animals)	"Defensin"[All Fields] AND biofilm[All Fields]	Defensin	31031739	Salt-Tolerant Antifungal and Antibacterial Activities of the Corn Defensin ZmD32.
710	DRAMP02778	Defensin (Insects, animals)	"Defensin"[All Fields] AND biofilm[All Fields]	Defensin	31025073	A recombinant fungal defensin-like peptide-P2 combats multidrug-resistant Staphylococcus aureus and biofilms.
710	DRAMP02778	Defensin (Insects, animals)	"Defensin"[All Fields] AND biofilm[All Fields]	Defensin	30659503	Lactobacillus plantarum USM8613 Aids in Wound Healing and Suppresses Staphylococcus aureus Infection at Wound Sites.
710	DRAMP02778	Defensin (Insects, animals)	"Defensin"[All Fields] AND biofilm[All Fields]	Defensin	30649289	Innate immune components affect growth and virulence traits of bacterial-vaginosis-associated and non-bacterial-vaginosis-associated Gardnerella vaginalis strains similarly.
710	DRAMP02778	Defensin (Insects, animals)	"Defensin"[All Fields] AND biofilm[All Fields]	Defensin	30376742	Alpha defensin, leukocyte esterase, C-reactive protein, and leukocyte count in synovial fluid for pre-operative diagnosis of periprosthetic infection.
710	DRAMP02778	Defensin (Insects, animals)	"Defensin"[All Fields] AND biofilm[All Fields]	Defensin	30260708	Stabilized collagen matrix dressing improves wound macrophage function and epithelialization.
710	DRAMP02778	Defensin (Insects, animals)	"Defensin"[All Fields] AND biofilm[All Fields]	Defensin	30254440	Modification of the surface of titanium with multifunctional chimeric peptides to prevent biofilm formation via inhibition of initial colonizers.
710	DRAMP02778	Defensin (Insects, animals)	"Defensin"[All Fields] AND biofilm[All Fields]	Defensin	29902560	A defensin-like antimicrobial peptide from the manila clam Ruditapes philippinarum: Investigation of the antibacterial activities and mode of action.
710	DRAMP02778	Defensin (Insects, animals)	"Defensin"[All Fields] AND biofilm[All Fields]	Defensin	29872295	Antimicrobial peptide-loaded liquid crystalline precursor bioadhesive system for the prevention of dental caries.
710	DRAMP02778	Defensin (Insects, animals)	"Defensin"[All Fields] AND biofilm[All Fields]	Defensin	29671721	The BceABRS four-component system that is essential for cell envelope stress response is involved in sensing and response to host defence peptides and is required for the biofilm formation and fitness of Streptococcus mutans.
710	DRAMP02778	Defensin (Insects, animals)	"Defensin"[All Fields] AND biofilm[All Fields]	Defensin	29104569	A Linear 19-Mer Plant Defensin-Derived Peptide Acts Synergistically with Caspofungin against Candida albicansBiofilms.
710	DRAMP02778	Defensin (Insects, animals)	"Defensin"[All Fields] AND biofilm[All Fields]	Defensin	29077172	The significance of HBD-3 and fluorescent composite carriers in the processof bone formation in rats infected with Staphylococcus aureus.
710	DRAMP02778	Defensin (Insects, animals)	"Defensin"[All Fields] AND biofilm[All Fields]	Defensin	29045084	New Approach to Treat and Prevent Oral Disease.
710	DRAMP02778	Defensin (Insects, animals)	"Defensin"[All Fields] AND biofilm[All Fields]	Defensin	29025642	Engineered chimeric peptides with antimicrobial and titanium-binding functions to inhibit biofilm formation on Ti implants.
710	DRAMP02778	Defensin (Insects, animals)	"Defensin"[All Fields] AND biofilm[All Fields]	Defensin	28956355	The synthetic human beta-defensin-3 C15 peptide exhibits antimicrobial activity against Streptococcus mutans, both alone and in combination with dental disinfectants.
710	DRAMP02778	Defensin (Insects, animals)	"Defensin"[All Fields] AND biofilm[All Fields]	Defensin	28951032	Antifungal Effects of Synthetic Human Beta-defensin-3-C15 Peptide on Candida albicans-infected Root Dentin.
710	DRAMP02778	Defensin (Insects, animals)	"Defensin"[All Fields] AND biofilm[All Fields]	Defensin	28874606	Bacterial d-amino acids suppress sinonasal innate immunity through sweet taste receptors in solitary chemosensory cells.
710	DRAMP02778	Defensin (Insects, animals)	"Defensin"[All Fields] AND biofilm[All Fields]	Defensin	28725299	Role of Streptococcus mutanstwo-component systems in antimicrobial peptide resistance in the oral cavity.
710	DRAMP02778	Defensin (Insects, animals)	"Defensin"[All Fields] AND biofilm[All Fields]	Defensin	28649561	Psd1 Effects on Candida albicansPlanktonic Cells and Biofilms.
710	DRAMP02778	Defensin (Insects, animals)	"Defensin"[All Fields] AND biofilm[All Fields]	Defensin	28642103	Chimeric analogs of human β-defensin 1 and θ-defensin disrupt pre-established bacterial biofilms.

710	DRAMP02778	Defensin (Insects, animals)	"Defensin"[All Fields] AND biofilm[All Fields]	Defensin	28413476	The mechanism of human β-defensin 3 in MRSA-induced infection of implant drug-resistant bacteria biofilm in the mouse tibial bone marrow.
710	DRAMP02778	Defensin (Insects, animals)	"Defensin"[All Fields] AND biofilm[All Fields]	Defensin	28296382	Human α-Defensin 6: A Small Peptide That Self-Assembles and Protects the Host by Entangling Microbes.
710	DRAMP02778	Defensin (Insects, animals)	"Defensin"[All Fields] AND biofilm[All Fields]	Defensin	28278280	Natural antimicrobial peptide complexes in the fighting of antibiotic resistant biofilms: Calliphora vicina medicinal maggots.
710	DRAMP02778	Defensin (Insects, animals)	"Defensin"[All Fields] AND biofilm[All Fields]	Defensin	28144375	Advancements in Diagnosing Periprosthetic Joint Infections after Total Hip and Knee Arthroplasty.
710	DRAMP02778	Defensin (Insects, animals)	"Defensin"[All Fields] AND biofilm[All Fields]	Defensin	28078813	Host defense peptide-derived privileged scaffolds for anti-infective drug discovery.
710	DRAMP02778	Defensin (Insects, animals)	"Defensin"[All Fields] AND biofilm[All Fields]	Defensin	28026958	Human α-Defensin 6 Self-Assembly Prevents Adhesion and Suppresses Virulence Traits of Candida albicans.
710	DRAMP02778	Defensin (Insects, animals)	"Defensin"[All Fields] AND biofilm[All Fields]	Defensin	27794585	A Novel Defensin-Like Peptide Associated with Two Other New Cationic Antimicrobial Peptides in Transcriptome of the Iranian Scorpion Venom.
710	DRAMP02778	Defensin (Insects, animals)	"Defensin"[All Fields] AND biofilm[All Fields]	Defensin	27777572	Role of yqiCin the Pathogenicity of Salmonellaand Innate Immune Responses of Human Intestinal Epithelium.
710	DRAMP02778	Defensin (Insects, animals)	"Defensin"[All Fields] AND biofilm[All Fields]	Defensin	27582732	Efficient Eradication of Mature Pseudomonas aeruginosa Biofilm via Controlled Delivery of Nitric Oxide Combined with Antimicrobial Peptide and Antibiotics.
710	DRAMP02778	Defensin (Insects, animals)	"Defensin"[All Fields] AND biofilm[All Fields]	Defensin	27417541	Acute appendicitis: transcript profiling of blood identifies promising biomarkers and potential underlying processes.
710	DRAMP02778	Defensin (Insects, animals)	"Defensin"[All Fields] AND biofilm[All Fields]	Defensin	27200276	Antifungal effects of synthetic human β-defensin 3-C15 peptide.
710	DRAMP02778	Defensin (Insects, animals)	"Defensin"[All Fields] AND biofilm[All Fields]	Defensin	27148195	Effect of Substance P in Staphylococcus aureus and Staphylococcus epidermidis Virulence: Implication for Skin Homeostasis.
710	DRAMP02778	Defensin (Insects, animals)	"Defensin"[All Fields] AND biofilm[All Fields]	Defensin	26861950	Antibiofilm efficacy of honey and bee-derived defensin-1 on multispecies wound biofilm.
710	DRAMP02778	Defensin (Insects, animals)	"Defensin"[All Fields] AND biofilm[All Fields]	Defensin	26592804	The radish defensins RsAFP1 and RsAFP2 act synergistically with caspofungin against Candida albicans biofilms.
710	DRAMP02778	Defensin (Insects, animals)	"Defensin"[All Fields] AND biofilm[All Fields]	Defensin	26248029	Synergistic Activity of the Plant Defensin HsAFP1 and Caspofungin against Candida albicans Biofilms and Planktonic Cultures.
710	DRAMP02778	Defensin (Insects, animals)	"Defensin"[All Fields] AND biofilm[All Fields]	Defensin	26214284	α-tocopherol decreases interleukin-1β and -6 and increases human β-defensin-1 and -2 secretion in human gingival fibroblasts stimulated with Porphyromonas gingivalis lipopolysaccharide.
710	DRAMP02778	Defensin (Insects, animals)	"Defensin"[All Fields] AND biofilm[All Fields]	Defensin	26196513	Snake Cathelicidin NA-CATH and Smaller Helical Antimicrobial Peptides Are Effective against Burkholderia thailandensis.
710	DRAMP02778	Defensin (Insects, animals)	"Defensin"[All Fields] AND biofilm[All Fields]	Defensin	26119274	Ultrasound microbubbles enhance human β-defensin 3 against biofilms.
710	DRAMP02778	Defensin (Insects, animals)	"Defensin"[All Fields] AND biofilm[All Fields]	Defensin	25862466	The Antibacterial Effects of an Antimicrobial Peptide Human β-Defensin 3 Fused with Carbohydrate-Binding Domain on Pseudomonas aeruginosa PA14.
710	DRAMP02778	Defensin (Insects, animals)	"Defensin"[All Fields] AND biofilm[All Fields]	Defensin	25808131	Expression of antimicrobial peptides and interleukin-8 during early stages of inflammation: An experimental gingivitis study.
710	DRAMP02778	Defensin (Insects, animals)	"Defensin"[All Fields] AND biofilm[All Fields]	Defensin	25806720	Antimicrobial peptides in 2014.
710	DRAMP02778	Defensin (Insects, animals)	"Defensin"[All Fields] AND biofilm[All Fields]	Defensin	25285879	Inhibition and destruction of Pseudomonas aeruginosa biofilms by antibiotics and antimicrobial peptides.
710	DRAMP02778	Defensin (Insects, animals)	"Defensin"[All Fields] AND biofilm[All Fields]	Defensin	25212593	Health- and disease-associated species clusters in complex natural biofilms determine the innate immune response in oral epithelial cells during biofilm maturation.
710	DRAMP02778	Defensin (Insects, animals)	"Defensin"[All Fields] AND biofilm[All Fields]	Defensin	24913184	Effects of human β-defensin-3 on biofilm formation-regulating genes dltB and icaA in Staphylococcus aureus.
710	DRAMP02778	Defensin (Insects, animals)	"Defensin"[All Fields] AND biofilm[All Fields]	Defensin	24340061	Inflammatory and antimicrobial responses to methicillin-resistant Staphylococcus aureus in an in vitro wound infection model.
710	DRAMP02778	Defensin (Insects, animals)	"Defensin"[All Fields] AND biofilm[All Fields]	Defensin	24240906	Adsorption study of pellicle proteins to gold, silica and titanium by quartz crystal microbalance method.
710	DRAMP02778	Defensin (Insects, animals)	"Defensin"[All Fields] AND biofilm[All Fields]	Defensin	24238461	Antibacterial efficacy of a human β-defensin-3 peptide on multispecies biofilms.
710	DRAMP02778	Defensin (Insects, animals)	"Defensin"[All Fields] AND biofilm[All Fields]	Defensin	24100890	[Use of recombinant human beta-defensin-3 to evaluate the effect of adhesion of Candida albicans on the surface of soft lining material].
710	DRAMP02778	Defensin (Insects, animals)	"Defensin"[All Fields] AND biofilm[All Fields]	Defensin	23639356	Human beta-defensin 3: a novel inhibitor of Staphylococcus-produced biofilm production. Commentary on "Human β-defensin 3 inhibits antibiotic-resistant Staphylococcus biofilm formation".
710	DRAMP02778	Defensin (Insects, animals)	"Defensin"[All Fields] AND biofilm[All Fields]	Defensin	23519963	Ultrasound-targeted microbubble destruction enhances human β-defensin 3 activity against antibiotic-resistant Staphylococcus biofilms.
710	DRAMP02778	Defensin (Insects, animals)	"Defensin"[All Fields] AND biofilm[All Fields]	Defensin	23273885	Human β-defensin 3 inhibits antibiotic-resistant Staphylococcus biofilm formation.
710	DRAMP02778	Defensin (Insects, animals)	"Defensin"[All Fields] AND biofilm[All Fields]	Defensin	23203265	Potentiation of the cytotoxic activity of copper by polyphosphate on biofilm-producing bacteria: a bioinspired approach.
710	DRAMP02778	Defensin (Insects, animals)	"Defensin"[All Fields] AND biofilm[All Fields]	Defensin	23078156	Antimicrobial efficacy of a human β-defensin-3 peptide using an Enterococcus faecalis dentine infection model.
710	DRAMP02778	Defensin (Insects, animals)	"Defensin"[All Fields] AND biofilm[All Fields]	Defensin	23053486	Synergistic effect and antibiofilm activity between the antimicrobial peptide coprisin and conventional antibiotics against opportunistic bacteria.
710	DRAMP02778	Defensin (Insects, animals)	"Defensin"[All Fields] AND biofilm[All Fields]	Defensin	22922323	Extracellular DNA within a nontypeable Haemophilus influenzae-induced biofilm binds human beta defensin-3 and reduces its antimicrobial activity.
710	DRAMP02778	Defensin (Insects, animals)	"Defensin"[All Fields] AND biofilm[All Fields]	Defensin	22855857	Retrocyclin inhibits Gardnerella vaginalis biofilm formation and toxin activity.
710	DRAMP02778	Defensin (Insects, animals)	"Defensin"[All Fields] AND biofilm[All Fields]	Defensin	22394470	Association of CiaRH with resistance of Streptococcus mutans to antimicrobial peptides in biofilms.
710	DRAMP02778	Defensin (Insects, animals)	"Defensin"[All Fields] AND biofilm[All Fields]	Defensin	22229614	Comparison of the effects of human β-defensin 3, vancomycin, and clindamycin on Staphylococcus aureus biofilm formation.
710	DRAMP02778	Defensin (Insects, animals)	"Defensin"[All Fields] AND biofilm[All Fields]	Defensin	21692631	A novel organotypic dento-epithelial culture model: effect of Fusobacterium nucleatum biofilm on B-defensin-2, -3, and LL-37 expression.
710	DRAMP02778	Defensin (Insects, animals)	"Defensin"[All Fields] AND biofilm[All Fields]	Defensin	20454633	Normal human gingival epithelial cells sense C. parapsilosis by toll-like receptors and module its pathogenesis through antimicrobial peptides and proinflammatory cytokines.
710	DRAMP02778	Defensin (Insects, animals)	"Defensin"[All Fields] AND biofilm[All Fields]	Defensin	20378008	Effect of temperature on the shift of Pseudomonas fluorescens from an environmental microorganism to a potential human pathogen.
710	DRAMP02778	Defensin (Insects, animals)	"Defensin"[All Fields] AND biofilm[All Fields]	Defensin	19961380	Gingival transcriptome patterns during induction and resolution of experimental gingivitis in humans.
710	DRAMP02778	Defensin (Insects, animals)	"Defensin"[All Fields] AND biofilm[All Fields]	Defensin	19780045	Candida famata modulates toll-like receptor, beta-defensin, and proinflammatory cytokine expression by normal human epithelial cells.
710	DRAMP02778	Defensin (Insects, animals)	"Defensin"[All Fields] AND biofilm[All Fields]	Defensin	19572896	The immune response of oral epithelial cells induced by single-species and complex naturally formed biofilms.
710	DRAMP02778	Defensin (Insects, animals)	"Defensin"[All Fields] AND biofilm[All Fields]	Defensin	19466693	Activity of antimicrobial peptides in the presence of polysaccharides produced by pulmonary pathogens.
710	DRAMP02778	Defensin (Insects, animals)	"Defensin"[All Fields] AND biofilm[All Fields]	Defensin	18954353	Treponema denticola does not induce production of common innate immune mediators from primary gingival epithelial cells.
710	DRAMP02778	Defensin (Insects, animals)	"Defensin"[All Fields] AND biofilm[All Fields]	Defensin	18173794	The stage of native biofilm formation determines the gene expression of human beta-defensin-2, psoriasin, ribonuclease 7 and inflammatory mediators: a novel approach for stimulation of keratinocytes with in situ formed biofilms.
710	DRAMP02778	Defensin (Insects, animals)	"Defensin"[All Fields] AND biofilm[All Fields]	Defensin	17434999	Functional analysis of D-alanylation of lipoteichoic acid in the probiotic strain Lactobacillus rhamnosus GG.
710	DRAMP02778	Defensin (Insects, animals)	"Defensin"[All Fields] AND biofilm[All Fields]	Defensin	15493829	Bacterial evasion of innate host defenses--the Staphylococcus aureus lesson.
710	DRAMP02778	Defensin (Insects, animals)	"Defensin"[All Fields] AND biofilm[All Fields]	Defensin	14764110	Polysaccharide intercellular adhesin (PIA) protects Staphylococcus epidermidis against major components of the human innate immune system.
724	DRAMP02845	CP-11 (cathelicidin; mammals, animals)	"CP-11"[All Fields] AND biofilm[All Fields]	CP-11	24212989	Leukotoxicity of Aggregatibacter actinomycetemcomitans in generalized aggressive periodontitis in Brazilians and their family members.
749	DRAMP02912	SMAP-29 (Cathelin-related peptide SC5; Myeloid antibacterial peptide MAP-29; mammals, animals)	"SMAP-29"[All Fields] AND biofilm[All Fields]	SMAP-29	33795739	Identification of potential therapeutic antimicrobial peptides against Acinetobacter baumannii in a mouse model of pneumonia.
749	DRAMP02912	SMAP-29 (Cathelin-related peptide SC5; Myeloid antibacterial peptide MAP-29; mammals, animals)	"SMAP-29"[All Fields] AND biofilm[All Fields]	SMAP-29	32232010	Francisella novicidaTwo-Component System Response Regulator BfpR Modulates iglC Gene Expression, Antimicrobial Peptide Resistance, and Biofilm Production.
749	DRAMP02912	SMAP-29 (Cathelin-related peptide SC5; Myeloid antibacterial peptide MAP-29; mammals, animals)	"SMAP-29"[All Fields] AND biofilm[All Fields]	SMAP-29	26821182	RNASeq Based Transcriptional Profiling of Pseudomonas aeruginosa PA14 after Short- and Long-Term Anoxic Cultivation in Synthetic Cystic Fibrosis Sputum Medium.

749	DRAMP02912	SMAP-29 (Cathelin-related peptide SC5; Myeloid antibacterial peptide MAP-29; mammals, animals)	"SMAP-29"[All Fields] AND biofilm[All Fields]	SMAP-29	26196513	Snake Cathelicidin NA-CATH and Smaller Helical Antimicrobial Peptides Are Effective against Burkholderia thailandensis.
749	DRAMP02912	SMAP-29 (Cathelin-related peptide SC5; Myeloid antibacterial peptide MAP-29; mammals, animals)	"SMAP-29"[All Fields] AND biofilm[All Fields]	SMAP-29	21849157	Antibacterial and anti-biofilm effects of cathelicidin peptides against pathogens isolated from cystic fibrosis patients.
749	DRAMP02912	SMAP-29 (Cathelin-related peptide SC5; Myeloid antibacterial peptide MAP-29; mammals, animals)	"SMAP-29"[All Fields] AND biofilm[All Fields]	SMAP-29	19466693	Activity of antimicrobial peptides in the presence of polysaccharides produced by pulmonary pathogens.
755	DRAMP02925	Cathelicidin (dogs, mammals, animals)	"Cathelicidin"[All Fields] AND biofilm[All Fields]	Cathelicidin	34237581	Suppressive effects of gecko cathelicidin on biofilm formation and cariogenic virulence factors of Streptococcus mutans.
755	DRAMP02925	Cathelicidin (dogs, mammals, animals)	"Cathelicidin"[All Fields] AND biofilm[All Fields]	Cathelicidin	33930423	An overview of possible pathogenesis mechanisms of Alternaria alternata in chronic rhinosinusitis and nasal polyposis.
755	DRAMP02925	Cathelicidin (dogs, mammals, animals)	"Cathelicidin"[All Fields] AND biofilm[All Fields]	Cathelicidin	33890759	Short and Robust Anti-Infective Lipopeptides Engineered Based on the Minimal Antimicrobial Peptide KR12 of Human LL-37.
755	DRAMP02925	Cathelicidin (dogs, mammals, animals)	"Cathelicidin"[All Fields] AND biofilm[All Fields]	Cathelicidin	33797775	Toll-like receptor activation of equine mesenchymal stromal cells to enhance antibacterial activity and immunomodulatory cytokine secretion.
755	DRAMP02925	Cathelicidin (dogs, mammals, animals)	"Cathelicidin"[All Fields] AND biofilm[All Fields]	Cathelicidin	33687301	Exogenous LL-37 but not homogenates of desquamated oral epithelial cells shows activity against Streptococcus mutans.
755	DRAMP02925	Cathelicidin (dogs, mammals, animals)	"Cathelicidin"[All Fields] AND biofilm[All Fields]	Cathelicidin	33326455	D-LL-31 enhances biofilm-eradicating effect of currently used antibiotics for chronic rhinosinusitis and its immunomodulatory activity on human lung epithelial cells.
755	DRAMP02925	Cathelicidin (dogs, mammals, animals)	"Cathelicidin"[All Fields] AND biofilm[All Fields]	Cathelicidin	33297738	DNase-mediated eDNA removal enhances D-LL-31 activity against biofilms of bacteria isolated from chronic rhinosinusitis patients.
755	DRAMP02925	Cathelicidin (dogs, mammals, animals)	"Cathelicidin"[All Fields] AND biofilm[All Fields]	Cathelicidin	33253855	Effects of a novel anti-biofilm peptide CRAMP combined with antibiotics on the formation of Pseudomonas aeruginosa biofilms.
755	DRAMP02925	Cathelicidin (dogs, mammals, animals)	"Cathelicidin"[All Fields] AND biofilm[All Fields]	Cathelicidin	33242568	Identification and characterization of novel bi-functional cathelicidins from the black-spotted frog (Pelophylax nigromaculata) with both anti-infective and antioxidant activities.
755	DRAMP02925	Cathelicidin (dogs, mammals, animals)	"Cathelicidin"[All Fields] AND biofilm[All Fields]	Cathelicidin	32746783	Pseudonajide peptide derived from snake venom alters cell envelope integrity interfering on biofilm formation in Staphylococcus epidermidis.
755	DRAMP02925	Cathelicidin (dogs, mammals, animals)	"Cathelicidin"[All Fields] AND biofilm[All Fields]	Cathelicidin	32343547	Resistome of Staphylococcus aureusin Response to Human Cathelicidin LL-37 and Its Engineered Antimicrobial Peptides.
755	DRAMP02925	Cathelicidin (dogs, mammals, animals)	"Cathelicidin"[All Fields] AND biofilm[All Fields]	Cathelicidin	32232010	Francisella novicidaTwo-Component System Response Regulator BfpR Modulates iglC Gene Expression, Antimicrobial Peptide Resistance, and Biofilm Production.
755	DRAMP02925	Cathelicidin (dogs, mammals, animals)	"Cathelicidin"[All Fields] AND biofilm[All Fields]	Cathelicidin	31993042	Linezolid and Rifampicin Combination to Combat cfr-Positive Multidrug-Resistant MRSA in Murine Models of Bacteremia and Skin and Skin Structure Infection.
755	DRAMP02925	Cathelicidin (dogs, mammals, animals)	"Cathelicidin"[All Fields] AND biofilm[All Fields]	Cathelicidin	31843919	The antimicrobial peptide ZY4 combats multidrug-resistant Pseudomonas aeruginosaand Acinetobacter baumanniiinfection.
755	DRAMP02925	Cathelicidin (dogs, mammals, animals)	"Cathelicidin"[All Fields] AND biofilm[All Fields]	Cathelicidin	31703937	Comparative assessment of genotypic and phenotypic correlates of Staphylococcus pseudintermedius strains isolated from dogs with otitis externa and healthy dogs.
755	DRAMP02925	Cathelicidin (dogs, mammals, animals)	"Cathelicidin"[All Fields] AND biofilm[All Fields]	Cathelicidin	31644974	Covalent grafting of titanium with a cathelicidin peptide produces an osteoblast compatible surface with antistaphylococcal activity.
755	DRAMP02925	Cathelicidin (dogs, mammals, animals)	"Cathelicidin"[All Fields] AND biofilm[All Fields]	Cathelicidin	31506312	Cathelicidin Peptides Restrict Bacterial Growth via Membrane Perturbation and Induction of Reactive Oxygen Species.
755	DRAMP02925	Cathelicidin (dogs, mammals, animals)	"Cathelicidin"[All Fields] AND biofilm[All Fields]	Cathelicidin	31396193	Evaluation of the Antimicrobial Peptide, RP557, for the Broad-Spectrum Treatment of Wound Pathogens and Biofilm.
755	DRAMP02925	Cathelicidin (dogs, mammals, animals)	"Cathelicidin"[All Fields] AND biofilm[All Fields]	Cathelicidin	31319057	Modulation of antimicrobial potency of human cathelicidin peptides against the ESKAPE pathogens and in vivo efficacy in a murine catheter-associated biofilm model.
755	DRAMP02925	Cathelicidin (dogs, mammals, animals)	"Cathelicidin"[All Fields] AND biofilm[All Fields]	Cathelicidin	31282211	D-LL-31 in combination with ceftazidime synergistically enhances bactericidal activity and biofilm destruction in Burkholderia pseudomallei.
755	DRAMP02925	Cathelicidin (dogs, mammals, animals)	"Cathelicidin"[All Fields] AND biofilm[All Fields]	Cathelicidin	31121185	Diversity, immunoregulatory action and structure-activity relationship of green sea turtle cathelicidins.
755	DRAMP02925	Cathelicidin (dogs, mammals, animals)	"Cathelicidin"[All Fields] AND biofilm[All Fields]	Cathelicidin	31118709	Design and characterization of a new hybrid peptide from LL-37 and BMAP-27.
755	DRAMP02925	Cathelicidin (dogs, mammals, animals)	"Cathelicidin"[All Fields] AND biofilm[All Fields]	Cathelicidin	31116540	Characterization of a Cathelicidin from the Colubrinae Snake, Sinonatrix annularis.
755	DRAMP02925	Cathelicidin (dogs, mammals, animals)	"Cathelicidin"[All Fields] AND biofilm[All Fields]	Cathelicidin	30916573	Nanofiber Dressings Topically Delivering Molecularly Engineered Human Cathelicidin Peptides for the Treatment of Biofilms in Chronic Wounds.
755	DRAMP02925	Cathelicidin (dogs, mammals, animals)	"Cathelicidin"[All Fields] AND biofilm[All Fields]	Cathelicidin	30728810	Role of Two-Component System Response Regulator bceRin the Antimicrobial Resistance, Virulence, Biofilm Formation, and Stress Response of Group B Streptococcus.
755	DRAMP02925	Cathelicidin (dogs, mammals, animals)	"Cathelicidin"[All Fields] AND biofilm[All Fields]	Cathelicidin	30375445	Multi-species oral biofilm promotes reconstructed human gingiva epithelial barrier function.
755	DRAMP02925	Cathelicidin (dogs, mammals, animals)	"Cathelicidin"[All Fields] AND biofilm[All Fields]	Cathelicidin	30360422	A Recombinant Snake Cathelicidin Derivative Peptide: Antibiofilm Properties and Expression in Escherichia coli.
755	DRAMP02925	Cathelicidin (dogs, mammals, animals)	"Cathelicidin"[All Fields] AND biofilm[All Fields]	Cathelicidin	30250480	Role of Microbes in the Development of Alzheimer's Disease: State of the Art - An International Symposium Presented at the 2017 IAGG Congress in San Francisco.
755	DRAMP02925	Cathelicidin (dogs, mammals, animals)	"Cathelicidin"[All Fields] AND biofilm[All Fields]	Cathelicidin	30131362	Discovery of New Genes Involved in Curli Production by a Uropathogenic Escherichia coli Strain from the Highly Virulent O45:K1:H7 Lineage.
755	DRAMP02925	Cathelicidin (dogs, mammals, animals)	"Cathelicidin"[All Fields] AND biofilm[All Fields]	Cathelicidin	29737589	LL-37 fragments have antimicrobial activity against Staphylococcus epidermidis biofilms and wound healing potential in HaCaT cell line.
755	DRAMP02925	Cathelicidin (dogs, mammals, animals)	"Cathelicidin"[All Fields] AND biofilm[All Fields]	Cathelicidin	29391814	Targeting polyelectrolyte networks in purulent body fluids to modulate bactericidal properties of some antibiotics.
755	DRAMP02925	Cathelicidin (dogs, mammals, animals)	"Cathelicidin"[All Fields] AND biofilm[All Fields]	Cathelicidin	29370365	The effects of antimicrobial peptides WAM-1 and LL-37 on multidrug-resistant Acinetobacter baumannii.
755	DRAMP02925	Cathelicidin (dogs, mammals, animals)	"Cathelicidin"[All Fields] AND biofilm[All Fields]	Cathelicidin	29230218	Endogenous Antimicrobial Peptide Expression in Response to Bacterial Epidermal Colonization.
755	DRAMP02925	Cathelicidin (dogs, mammals, animals)	"Cathelicidin"[All Fields] AND biofilm[All Fields]	Cathelicidin	28919413	Apolipoprotein A-I attenuates LL-37-induced endothelial cell cytotoxicity.
755	DRAMP02925	Cathelicidin (dogs, mammals, animals)	"Cathelicidin"[All Fields] AND biofilm[All Fields]	Cathelicidin	28890511	The Antibacterial Effects of Antimicrobial Peptides OP-145 against Clinically Isolated Multi-Resistant Strains.
755	DRAMP02925	Cathelicidin (dogs, mammals, animals)	"Cathelicidin"[All Fields] AND biofilm[All Fields]	Cathelicidin	28851894	Activated Mesenchymal Stem Cells Interact with Antibiotics and Host Innate Immune Responses to Control Chronic Bacterial Infections.
755	DRAMP02925	Cathelicidin (dogs, mammals, animals)	"Cathelicidin"[All Fields] AND biofilm[All Fields]	Cathelicidin	28798159	As-CATH1-6, novel cathelicidins with potent antimicrobial and immunomodulatory properties from Alligator sinensis, play pivotal roles in host antimicrobial immune responses.
755	DRAMP02925	Cathelicidin (dogs, mammals, animals)	"Cathelicidin"[All Fields] AND biofilm[All Fields]	Cathelicidin	28747178	Anaerobic bacteria growth in the presence of cathelicidin LL-37 and selected ceragenins delivered as magnetic nanoparticles cargo.
755	DRAMP02925	Cathelicidin (dogs, mammals, animals)	"Cathelicidin"[All Fields] AND biofilm[All Fields]	Cathelicidin	28676673	Formulation and candidacidal activity of magnetic nanoparticles coated with cathelicidin LL-37 and ceragenin CSA-13.
755	DRAMP02925	Cathelicidin (dogs, mammals, animals)	"Cathelicidin"[All Fields] AND biofilm[All Fields]	Cathelicidin	28675109	Titanium surfaces immobilized with the major antimicrobial fragment FK-16 of human cathelicidin LL-37 are potent against multiple antibiotic-resistant bacteria.
755	DRAMP02925	Cathelicidin (dogs, mammals, animals)	"Cathelicidin"[All Fields] AND biofilm[All Fields]	Cathelicidin	28672834	Individual and Combined Effects of Engineered Peptides and Antibiotics on Pseudomonas aeruginosa Biofilms.
755	DRAMP02925	Cathelicidin (dogs, mammals, animals)	"Cathelicidin"[All Fields] AND biofilm[All Fields]	Cathelicidin	28572668	Cathelicidin-trypsin inhibitor loop conjugate represents a promising antibiotic candidate with protease stability.
755	DRAMP02925	Cathelicidin (dogs, mammals, animals)	"Cathelicidin"[All Fields] AND biofilm[All Fields]	Cathelicidin	28525841	LL-37-derived short antimicrobial peptide KR-12-a5 and its d-amino acid substituted analogs with cell selectivity, anti-biofilm activity, synergistic effect with conventional antibiotics, and anti-inflammatory activity.
755	DRAMP02925	Cathelicidin (dogs, mammals, animals)	"Cathelicidin"[All Fields] AND biofilm[All Fields]	Cathelicidin	28089718	Cathelicidin antimicrobial peptide from Alligator mississippiensis has antibacterial activity against multi-drug resistant Acinetobacter baumanii and Klebsiella pneumoniae.
755	DRAMP02925	Cathelicidin (dogs, mammals, animals)	"Cathelicidin"[All Fields] AND biofilm[All Fields]	Cathelicidin	27799768	Core-shell magnetic nanoparticles display synergistic antibacterial effects against Pseudomonas aeruginosaand Staphylococcus aureuswhen combined with cathelicidin LL-37 or selected ceragenins.
755	DRAMP02925	Cathelicidin (dogs, mammals, animals)	"Cathelicidin"[All Fields] AND biofilm[All Fields]	Cathelicidin	27739485	Skin-bacteria communication: Involvement of the neurohormone Calcitonin Gene Related Peptide (CGRP) in the regulation of Staphylococcus epidermidis virulence.
755	DRAMP02925	Cathelicidin (dogs, mammals, animals)	"Cathelicidin"[All Fields] AND biofilm[All Fields]	Cathelicidin	27681920	Biofilms from Klebsiella pneumoniae: Matrix Polysaccharide Structure and Interactions with Antimicrobial Peptides.
755	DRAMP02925	Cathelicidin (dogs, mammals, animals)	"Cathelicidin"[All Fields] AND biofilm[All Fields]	Cathelicidin	27659310	Human cathelicidin LL-37 enhance the antibiofilm effect of EGCG on Streptococcus mutans.
755	DRAMP02925	Cathelicidin (dogs, mammals, animals)	"Cathelicidin"[All Fields] AND biofilm[All Fields]	Cathelicidin	27315208	Candidacidal Activity of Selected Ceragenins and Human Cathelicidin LL-37 in Experimental Settings Mimicking Infection Sites.
755	DRAMP02925	Cathelicidin (dogs, mammals, animals)	"Cathelicidin"[All Fields] AND biofilm[All Fields]	Cathelicidin	27270571	In vitro and in vivo evaluation of BMAP-derived peptides for the treatment of cystic fibrosis-related pulmonary infections.
755	DRAMP02925	Cathelicidin (dogs, mammals, animals)	"Cathelicidin"[All Fields] AND biofilm[All Fields]	Cathelicidin	27163404	Multi-biofunction of antimicrobial peptide-immobilized silk fibroin nanofiber membrane: Implications for wound healing.
755	DRAMP02925	Cathelicidin (dogs, mammals, animals)	"Cathelicidin"[All Fields] AND biofilm[All Fields]	Cathelicidin	27148195	Effect of Substance P in Staphylococcus aureus and Staphylococcus epidermidis Virulence: Implication for Skin Homeostasis.

755	DRAMP02925	Cathelicidin (dogs, mammals, animals)	"Cathelicidin"[All Fields] AND biofilm[All Fields]	Cathelicidin	26819677	Anti-Staphylococcal Biofilm Effects of Human Cathelicidin Peptides.
755	DRAMP02925	Cathelicidin (dogs, mammals, animals)	"Cathelicidin"[All Fields] AND biofilm[All Fields]	Cathelicidin	26800870	Critical Role of Antimicrobial Peptide Cathelicidin for Controlling Helicobacter pylori Survival and Infection.
755	DRAMP02925	Cathelicidin (dogs, mammals, animals)	"Cathelicidin"[All Fields] AND biofilm[All Fields]	Cathelicidin	26656137	Assessing the potential of four cathelicidins for the management of mouse candidiasis and Candida albicans biofilms.
755	DRAMP02925	Cathelicidin (dogs, mammals, animals)	"Cathelicidin"[All Fields] AND biofilm[All Fields]	Cathelicidin	26434733	Unique features of human cathelicidin LL-37.
755	DRAMP02925	Cathelicidin (dogs, mammals, animals)	"Cathelicidin"[All Fields] AND biofilm[All Fields]	Cathelicidin	26238597	Antifungal activity of cathelicidin peptides against planktonic and biofilm cultures of Candida species isolated from vaginal infections.
755	DRAMP02925	Cathelicidin (dogs, mammals, animals)	"Cathelicidin"[All Fields] AND biofilm[All Fields]	Cathelicidin	26196513	Snake Cathelicidin NA-CATH and Smaller Helical Antimicrobial Peptides Are Effective against Burkholderia thailandensis.
755	DRAMP02925	Cathelicidin (dogs, mammals, animals)	"Cathelicidin"[All Fields] AND biofilm[All Fields]	Cathelicidin	26068402	Evaluation of the antibacterial and antibiofilm activities of novel CRAMP-vancomycin conjugates with diverse linkers.
755	DRAMP02925	Cathelicidin (dogs, mammals, animals)	"Cathelicidin"[All Fields] AND biofilm[All Fields]	Cathelicidin	25959370	Neutrophil extracellular trap formation in supragingival biofilms.
755	DRAMP02925	Cathelicidin (dogs, mammals, animals)	"Cathelicidin"[All Fields] AND biofilm[All Fields]	Cathelicidin	25870055	Bactericidal activities of cathelicidin LL-37 and select cationic lipids against the hypervirulent Pseudomonas aeruginosa strain LESB58.
755	DRAMP02925	Cathelicidin (dogs, mammals, animals)	"Cathelicidin"[All Fields] AND biofilm[All Fields]	Cathelicidin	25806720	Antimicrobial peptides in 2014.
755	DRAMP02925	Cathelicidin (dogs, mammals, animals)	"Cathelicidin"[All Fields] AND biofilm[All Fields]	Cathelicidin	27563687	Using anti-biofilm peptides to treat antibiotic-resistant bacterial infections.
755	DRAMP02925	Cathelicidin (dogs, mammals, animals)	"Cathelicidin"[All Fields] AND biofilm[All Fields]	Cathelicidin	25101632	Antibacterial function of the human cathelicidin-18 peptide (LL-37) between theory and practice.
755	DRAMP02925	Cathelicidin (dogs, mammals, animals)	"Cathelicidin"[All Fields] AND biofilm[All Fields]	Cathelicidin	25061850	Transformation of human cathelicidin LL-37 into selective, stable, and potent antimicrobial compounds.
755	DRAMP02925	Cathelicidin (dogs, mammals, animals)	"Cathelicidin"[All Fields] AND biofilm[All Fields]	Cathelicidin	25008764	The antibiotic effects of vitamin D.
755	DRAMP02925	Cathelicidin (dogs, mammals, animals)	"Cathelicidin"[All Fields] AND biofilm[All Fields]	Cathelicidin	24982087	Derivatives of the mouse cathelicidin-related antimicrobial peptide (CRAMP) inhibit fungal and bacterial biofilm formation.
755	DRAMP02925	Cathelicidin (dogs, mammals, animals)	"Cathelicidin"[All Fields] AND biofilm[All Fields]	Cathelicidin	24841266	LL-37-derived peptides eradicate multidrug-resistant Staphylococcus aureus from thermally wounded human skin equivalents.
755	DRAMP02925	Cathelicidin (dogs, mammals, animals)	"Cathelicidin"[All Fields] AND biofilm[All Fields]	Cathelicidin	24463069	High-quality 3D structures shine light on antibacterial, anti-biofilm and antiviral activities of human cathelicidin LL-37 and its fragments.
755	DRAMP02925	Cathelicidin (dogs, mammals, animals)	"Cathelicidin"[All Fields] AND biofilm[All Fields]	Cathelicidin	24071034	The human antimicrobial peptide LL-37 and its fragments possess both antimicrobial and antibiofilm activities against multidrug-resistant Acinetobacter baumannii.
755	DRAMP02925	Cathelicidin (dogs, mammals, animals)	"Cathelicidin"[All Fields] AND biofilm[All Fields]	Cathelicidin	23840194	The Human Cathelicidin Antimicrobial Peptide LL-37 as a Potential Treatment for Polymicrobial Infected Wounds.
755	DRAMP02925	Cathelicidin (dogs, mammals, animals)	"Cathelicidin"[All Fields] AND biofilm[All Fields]	Cathelicidin	23601656	Why does the healthy cornea resist Pseudomonas aeruginosa infection?
755	DRAMP02925	Cathelicidin (dogs, mammals, animals)	"Cathelicidin"[All Fields] AND biofilm[All Fields]	Cathelicidin	22917247	Human cathelicidin LL-37 prevents bacterial biofilm formation.
755	DRAMP02925	Cathelicidin (dogs, mammals, animals)	"Cathelicidin"[All Fields] AND biofilm[All Fields]	Cathelicidin	22908164	Identification of peptides derived from the human antimicrobial peptide LL-37 active against biofilms formed by Pseudomonas aeruginosa using a library of truncated fragments.
755	DRAMP02925	Cathelicidin (dogs, mammals, animals)	"Cathelicidin"[All Fields] AND biofilm[All Fields]	Cathelicidin	21849157	Antibacterial and anti-biofilm effects of cathelicidin peptides against pathogens isolated from cystic fibrosis patients.
755	DRAMP02925	Cathelicidin (dogs, mammals, animals)	"Cathelicidin"[All Fields] AND biofilm[All Fields]	Cathelicidin	21772832	Susceptibility of Pseudomonas aeruginosa Biofilm to Alpha-Helical Peptides: D-enantiomer of LL-37.
755	DRAMP02925	Cathelicidin (dogs, mammals, animals)	"Cathelicidin"[All Fields] AND biofilm[All Fields]	Cathelicidin	21692631	A novel organotypic dento-epithelial culture model: effect of Fusobacterium nucleatum biofilm on B-defensin-2, -3, and LL-37 expression.
755	DRAMP02925	Cathelicidin (dogs, mammals, animals)	"Cathelicidin"[All Fields] AND biofilm[All Fields]	Cathelicidin	21605457	Natural and synthetic cathelicidin peptides with anti-microbial and anti-biofilm activity against Staphylococcus aureus.
755	DRAMP02925	Cathelicidin (dogs, mammals, animals)	"Cathelicidin"[All Fields] AND biofilm[All Fields]	Cathelicidin	21376541	A cathelicidin-2-derived peptide effectively impairs Staphylococcus epidermidis biofilms.
755	DRAMP02925	Cathelicidin (dogs, mammals, animals)	"Cathelicidin"[All Fields] AND biofilm[All Fields]	Cathelicidin	21133662	Vitamin D: emerging roles in infection and immunity.
755	DRAMP02925	Cathelicidin (dogs, mammals, animals)	"Cathelicidin"[All Fields] AND biofilm[All Fields]	Cathelicidin	20961363	Potential of ceragenin CSA-13 and its mixture with pluronic F-127 as treatment of topical bacterial infections.
755	DRAMP02925	Cathelicidin (dogs, mammals, animals)	"Cathelicidin"[All Fields] AND biofilm[All Fields]	Cathelicidin	20399752	Antimicrobial and antibiofilm activity of cathelicidins and short, synthetic peptides against Francisella.
755	DRAMP02925	Cathelicidin (dogs, mammals, animals)	"Cathelicidin"[All Fields] AND biofilm[All Fields]	Cathelicidin	20139192	Effects of sequential Campylobacter jejuni 81-176 lipooligosaccharide core truncations on biofilm formation, stress survival, and pathogenesis.
755	DRAMP02925	Cathelicidin (dogs, mammals, animals)	"Cathelicidin"[All Fields] AND biofilm[All Fields]	Cathelicidin	20002576	Human cathelicidin peptide LL37 inhibits both attachment capability and biofilm formation of Staphylococcus epidermidis.
755	DRAMP02925	Cathelicidin (dogs, mammals, animals)	"Cathelicidin"[All Fields] AND biofilm[All Fields]	Cathelicidin	19466693	Activity of antimicrobial peptides in the presence of polysaccharides produced by pulmonary pathogens.
755	DRAMP02925	Cathelicidin (dogs, mammals, animals)	"Cathelicidin"[All Fields] AND biofilm[All Fields]	Cathelicidin	19390494	Staphylococcus epidermidis isolated from newborn infants express pilus-like structures and are inhibited by the cathelicidin-derived antimicrobial peptide LL37.
755	DRAMP02925	Cathelicidin (dogs, mammals, animals)	"Cathelicidin"[All Fields] AND biofilm[All Fields]	Cathelicidin	16621147	Pre-treatment of central venous catheters with the cathelicidin BMAP-28 enhances the efficacy of antistaphylococcal agents in the treatment of experimental catheter-related infection.
772	DRAMP02966	DBI(32-86) (pigs, mammals, animals)	"DBI"[All Fields] AND biofilm[All Fields]	DBI	32347632	Living Bioelectrochemical Composites.
772	DRAMP02966	DBI(32-86) (pigs, mammals, animals)	"DBI"[All Fields] AND biofilm[All Fields]	DBI	25060625	Ploidy-regulated variation in biofilm-related phenotypes in natural isolates of Saccharomyces cerevisiae.
773	DRAMP02970	Protegrin-1 (Protegrin 1; PG-1; pigs, mammals, animals)	"Protegrin-1"[All Fields] AND biofilm[All Fields]	Protegrin-1	33475795	Antibiofilm peptides as a promising strategy: comparative research.
773	DRAMP02970	Protegrin-1 (Protegrin 1; PG-1; pigs, mammals, animals)	"Protegrin-1"[All Fields] AND biofilm[All Fields]	Protegrin-1	32650576	The Addition of a Synthetic LPS-Targeting Domain Improves Serum Stability While Maintaining Antimicrobial, Antibiofilm, and Cell Stimulating Properties of an Antimicrobial Peptide.
773	DRAMP02970	Protegrin-1 (Protegrin 1; PG-1; pigs, mammals, animals)	"Protegrin-1"[All Fields] AND biofilm[All Fields]	Protegrin-1	31214759	In vitro activity of Protegrin-1, alone and in combination with clinically useful antibiotics, against Acinetobacter baumannii strains isolated from surgical wounds.
773	DRAMP02970	Protegrin-1 (Protegrin 1; PG-1; pigs, mammals, animals)	"Protegrin-1"[All Fields] AND biofilm[All Fields]	Protegrin-1	29045084	New Approach to Treat and Prevent Oral Disease.
784	DRAMP03001	Jellein-3 (Jelleine-III; Insects, animals)	"Jellein-3"[All Fields] AND biofilm[All Fields]	Jellein-3	34321877	Antibacterial Effect of Honey-Derived Exosomes Containing Antimicrobial Peptides Against Oral Streptococci.
785	DRAMP03002	Melittin (Allergen Api m 3; Allergen Api m III; Insects, animals)	"Melittin"[All Fields] AND biofilm[All Fields]	Melittin	34156547	Prevention the formation of biofilm on orthopedic implants by melittin thin layer on chitosan/bioactive glass/vancomycin coatings.
785	DRAMP03002	Melittin (Allergen Api m 3; Allergen Api m III; Insects, animals)	"Melittin"[All Fields] AND biofilm[All Fields]	Melittin	33475795	Antibiofilm peptides as a promising strategy: comparative research.
785	DRAMP03002	Melittin (Allergen Api m 3; Allergen Api m III; Insects, animals)	"Melittin"[All Fields] AND biofilm[All Fields]	Melittin	33049305	In-depth characterization of antibacterial activity of melittin against Staphylococcus aureus and use in a model of non-surgical MRSA-infected skin wounds.
785	DRAMP03002	Melittin (Allergen Api m 3; Allergen Api m III; Insects, animals)	"Melittin"[All Fields] AND biofilm[All Fields]	Melittin	32994714	Synergistic activity of melittin with mupirocin: A study against methicillin-resistant S. Aureus(MRSA) and methicillin-susceptible S. Aureus(MSSA) isolates.
785	DRAMP03002	Melittin (Allergen Api m 3; Allergen Api m III; Insects, animals)	"Melittin"[All Fields] AND biofilm[All Fields]	Melittin	32847080	Combined Effect of Melittin and DNase on Enterococcus faecalisBiofilms and Its Susceptibility to Sodium Hypochlorite.
785	DRAMP03002	Melittin (Allergen Api m 3; Allergen Api m III; Insects, animals)	"Melittin"[All Fields] AND biofilm[All Fields]	Melittin	32310641	Supramolecular Assemblies of Heterogeneous Mesoporous Silica Nanoparticles to Co-deliver Antimicrobial Peptides and Antibiotics for Synergistic Eradication of Pathogenic Biofilms.
785	DRAMP03002	Melittin (Allergen Api m 3; Allergen Api m III; Insects, animals)	"Melittin"[All Fields] AND biofilm[All Fields]	Melittin	32295149	Pan-Drug Resistant Acinetobacter baumannii, but Not Other Strains, Are Resistant to the Bee Venom Peptide Mellitin.
785	DRAMP03002	Melittin (Allergen Api m 3; Allergen Api m III; Insects, animals)	"Melittin"[All Fields] AND biofilm[All Fields]	Melittin	32044469	Design and use of model membranes to study biomolecular interactions using complementary surface-sensitive techniques.
785	DRAMP03002	Melittin (Allergen Api m 3; Allergen Api m III; Insects, animals)	"Melittin"[All Fields] AND biofilm[All Fields]	Melittin	32004624	Influence of apitoxin and melittin from Apis mellifera bee on Staphylococcus aureus strains.
785	DRAMP03002	Melittin (Allergen Api m 3; Allergen Api m III; Insects, animals)	"Melittin"[All Fields] AND biofilm[All Fields]	Melittin	31485973	Efficacy of Indolicidin, Cecropin A (1-7)-Melittin (CAMA) and Their Combination Against Biofilm-Forming Multidrug-Resistant Enteroaggregative Escherichia coli.

785	DRAMP03002	Melittin (Allergen Api m 3; Allergen Api m III; Insects, animals)	"Melittin"[All Fields] AND biofilm[All Fields]	Melittin	31293530	Hydrogels Embedded With Melittin and Tobramycin Are Effective Against Pseudomonas aeruginosaBiofilms in an Animal Wound Model.
785	DRAMP03002	Melittin (Allergen Api m 3; Allergen Api m III; Insects, animals)	"Melittin"[All Fields] AND biofilm[All Fields]	Melittin	30824944	Melittin: from honeybees to superbugs.
785	DRAMP03002	Melittin (Allergen Api m 3; Allergen Api m III; Insects, animals)	"Melittin"[All Fields] AND biofilm[All Fields]	Melittin	30766602	Melittin Inhibition and Eradication Activity for Resistant Polymicrobial Biofilm Isolated from a Dairy Industry after Disinfection.
785	DRAMP03002	Melittin (Allergen Api m 3; Allergen Api m III; Insects, animals)	"Melittin"[All Fields] AND biofilm[All Fields]	Melittin	30755738	In vitro activities of antimicrobial peptides and ceragenins against Legionella pneumophila.
785	DRAMP03002	Melittin (Allergen Api m 3; Allergen Api m III; Insects, animals)	"Melittin"[All Fields] AND biofilm[All Fields]	Melittin	29959905	Antimicrobial and anti-inflammatory activities of chemokine CXCL14-derived antimicrobial peptide and its analogs.
785	DRAMP03002	Melittin (Allergen Api m 3; Allergen Api m III; Insects, animals)	"Melittin"[All Fields] AND biofilm[All Fields]	Melittin	29904274	Evaluation of the bioactivity of a mastoparan peptide from wasp venom and of its analogues designed through targeted engineering.
785	DRAMP03002	Melittin (Allergen Api m 3; Allergen Api m III; Insects, animals)	"Melittin"[All Fields] AND biofilm[All Fields]	Melittin	29710773	Recombinant Inga LaurinaTrypsin Inhibitor (ILTI) Production in Komagataella PhaffiiConfirms Its Potential Anti-Biofilm Effect and Reveals an Anti-Tumoral Activity.
785	DRAMP03002	Melittin (Allergen Api m 3; Allergen Api m III; Insects, animals)	"Melittin"[All Fields] AND biofilm[All Fields]	Melittin	29353377	Highly synergistic activity of melittin with imipenem and colistin in biofilm inhibition against multidrug-resistant strong biofilm producer strains of Acinetobacter baumannii.
785	DRAMP03002	Melittin (Allergen Api m 3; Allergen Api m III; Insects, animals)	"Melittin"[All Fields] AND biofilm[All Fields]	Melittin	29186026	Antimicrobial Activity of Bee Venom and Melittin against Borrelia burgdorferi.
785	DRAMP03002	Melittin (Allergen Api m 3; Allergen Api m III; Insects, animals)	"Melittin"[All Fields] AND biofilm[All Fields]	Melittin	29185466	Macropis fulvipes Venom component Macropin Exerts its Antibacterial and Anti-Biofilm Properties by Damaging the Plasma Membranes of Drug Resistant Bacteria.
785	DRAMP03002	Melittin (Allergen Api m 3; Allergen Api m III; Insects, animals)	"Melittin"[All Fields] AND biofilm[All Fields]	Melittin	28943153	Melittin and its potential in the destruction and inhibition of the biofilm formation by Staphylococcus aureus, Escherichia coli and Pseudomonas aeruginosa isolated from bovine milk.
785	DRAMP03002	Melittin (Allergen Api m 3; Allergen Api m III; Insects, animals)	"Melittin"[All Fields] AND biofilm[All Fields]	Melittin	28525841	LL-37-derived short antimicrobial peptide KR-12-a5 and its d-amino acid substituted analogs with cell selectivity, anti-biofilm activity, synergistic effect with conventional antibiotics, and anti-inflammatory activity.
785	DRAMP03002	Melittin (Allergen Api m 3; Allergen Api m III; Insects, animals)	"Melittin"[All Fields] AND biofilm[All Fields]	Melittin	27718471	Pyrazole derived ultra-short antimicrobial peptidomimetics with potent anti-biofilm activity.
785	DRAMP03002	Melittin (Allergen Api m 3; Allergen Api m III; Insects, animals)	"Melittin"[All Fields] AND biofilm[All Fields]	Melittin	27271216	Design of an α-helical antimicrobial peptide with improved cell-selective and potent anti-biofilm activity.
785	DRAMP03002	Melittin (Allergen Api m 3; Allergen Api m III; Insects, animals)	"Melittin"[All Fields] AND biofilm[All Fields]	Melittin	25801062	Antibacterial and anti-biofilm activities of melittin and colistin, alone and in combination with antibiotics against Gram-negative bacteria.
785	DRAMP03002	Melittin (Allergen Api m 3; Allergen Api m III; Insects, animals)	"Melittin"[All Fields] AND biofilm[All Fields]	Melittin	25285879	Inhibition and destruction of Pseudomonas aeruginosa biofilms by antibiotics and antimicrobial peptides.
785	DRAMP03002	Melittin (Allergen Api m 3; Allergen Api m III; Insects, animals)	"Melittin"[All Fields] AND biofilm[All Fields]	Melittin	24096425	Induction of the Cpx envelope stress pathway contributes to Escherichia coli tolerance to antimicrobial peptides.
785	DRAMP03002	Melittin (Allergen Api m 3; Allergen Api m III; Insects, animals)	"Melittin"[All Fields] AND biofilm[All Fields]	Melittin	23988790	In vitro pharmacokinetics of antimicrobial cationic peptides alone and in combination with antibiotics against methicillin resistant Staphylococcus aureus biofilms.
785	DRAMP03002	Melittin (Allergen Api m 3; Allergen Api m III; Insects, animals)	"Melittin"[All Fields] AND biofilm[All Fields]	Melittin	23760014	Anti-microbial, anti-biofilm activities and cell selectivity of the NRC-16 peptide derived from witch flounder, Glyptocephalus cynoglossus.
785	DRAMP03002	Melittin (Allergen Api m 3; Allergen Api m III; Insects, animals)	"Melittin"[All Fields] AND biofilm[All Fields]	Melittin	23070152	In vitro activities of antibiotics and antimicrobial cationic peptides alone and in combination against methicillin-resistant Staphylococcus aureus biofilms.
792	DRAMP03028	Mastoparan-1 (MP-1; Venom protein MP-1; Insects, animals)	"Mastoparan-1"[All Fields] AND biofilm[All Fields]	Mastoparan-1	29626660	Venom-derived peptide Mastoparan-1 eradicates planktonic and biofilm-embedded methicillin-resistant Staphylococcus aureus isolates.
804	DRAMP03044	Protonectin (Agelaia-chemotactic peptide, Agelaia-CP; Insects, animals)	"Protonectin"[All Fields] AND biofilm[All Fields]	Protonectin	26209560	Antimicrobial peptide protonectin disturbs the membrane integrity and induces ROS production in yeast cells.
815	DRAMP03057	Thanatin (Insects, animals)	"Thanatin"[All Fields] AND biofilm[All Fields]	Thanatin	23917310	R-thanatin inhibits growth and biofilm formation of methicillin-resistant Staphylococcus epidermidis in vivo and in vitro.
817	DRAMP03069	Diptericin (Insects, animals)	"Diptericin"[All Fields] AND biofilm[All Fields]	Diptericin	28278280	Natural antimicrobial peptide complexes in the fighting of antibiotic resistant biofilms: Calliphora vicina medicinal maggots.
817	DRAMP03069	Diptericin (Insects, animals)	"Diptericin"[All Fields] AND biofilm[All Fields]	Diptericin	21998591	Drosophila melanogaster as an animal model for the study of Pseudomonas aeruginosa biofilm infections in vivo.
823	DRAMP18495	Gomesin (Gm; Spiders, arachnids, Chelicerata, arthropods, invertebrates, animals)	"Gomesin"[All Fields] AND biofilm[All Fields]	Gomesin	20370836	Effects of the antimicrobial peptide gomesin on the global gene expression profile, virulence and biofilm formation of Xylella fastidiosa.
830	DRAMP03138	Cecropin-A (Insects, animals)	"Cecropin-A"[All Fields] AND biofilm[All Fields]	Cecropin-A	32203307	Antimicrobial peptide derived from moths can eradicate UPEC biofilms and could offer a novel therapeutic option.
830	DRAMP03138	Cecropin-A (Insects, animals)	"Cecropin-A"[All Fields] AND biofilm[All Fields]	Cecropin-A	32203127	Antibiofilm activities of ceragenins and antimicrobial peptides against fungal-bacterial mono and multispecies biofilms.
830	DRAMP03138	Cecropin-A (Insects, animals)	"Cecropin-A"[All Fields] AND biofilm[All Fields]	Cecropin-A	32051417	The insect antimicrobial peptide cecropin A disrupts uropathogenic Escherichia coli biofilms.
830	DRAMP03138	Cecropin-A (Insects, animals)	"Cecropin-A"[All Fields] AND biofilm[All Fields]	Cecropin-A	31485973	Efficacy of Indolicidin, Cecropin A (1-7)-Melittin (CAMA) and Their Combination Against Biofilm-Forming Multidrug-Resistant Enteroaggregative Escherichia coli.
830	DRAMP03138	Cecropin-A (Insects, animals)	"Cecropin-A"[All Fields] AND biofilm[All Fields]	Cecropin-A	31363941	Mechanism of action of antimicrobial peptide P5 truncations against Pseudomonas aeruginosa and Staphylococcus aureus.
830	DRAMP03138	Cecropin-A (Insects, animals)	"Cecropin-A"[All Fields] AND biofilm[All Fields]	Cecropin-A	30755738	In vitro activities of antimicrobial peptides and ceragenins against Legionella pneumophila.
830	DRAMP03138	Cecropin-A (Insects, animals)	"Cecropin-A"[All Fields] AND biofilm[All Fields]	Cecropin-A	28178190	High Specific Selectivity and Membrane-Active Mechanism of Synthetic Cationic Hybrid Antimicrobial Peptides Based on the Peptide FV7.
830	DRAMP03138	Cecropin-A (Insects, animals)	"Cecropin-A"[All Fields] AND biofilm[All Fields]	Cecropin-A	25924433	[CecropinA-magainin, a new hybrid antibacterial peptide against meticillin-resistant Staphylococcus aureus].
830	DRAMP03138	Cecropin-A (Insects, animals)	"Cecropin-A"[All Fields] AND biofilm[All Fields]	Cecropin-A	23070152	In vitro activities of antibiotics and antimicrobial cationic peptides alone and in combination against methicillin-resistant Staphylococcus aureus biofilms.
835	DRAMP03166	P15 (deer beta-defensin; ruminant, animals)	"P15"[All Fields] AND biofilm[All Fields]	P15	28380658	Cationic osteogenic peptide P15-CSP coatings promote 3-D osteogenesis in poly(epsilon-caprolactone) scaffolds of distinct pore size.
835	DRAMP03166	P15 (deer beta-defensin; ruminant, animals)	"P15"[All Fields] AND biofilm[All Fields]	P15	26097095	Fusion peptide P15-CSP shows antibiofilm activity and pro-osteogenic activity when deposited as a coating on hydrophilic but not hydrophobic surfaces.
838	DRAMP03176	Cecropin-P1 (CP1; nematodes, animals)	"Cecropin-P1"[All Fields] AND biofilm[All Fields]	Cecropin-P1	15247257	Helix induction in antimicrobial peptides by alginate in biofilms.
841	DRAMP03187	Beta defensin 1(BD-1; mammals, animals)	"Beta defensin 1"[All Fields] AND biofilm[All Fields]	Beta defensin 1	33420317	Curbing gastrointestinal infections by defensin fragment modifications without harming commensal microbiota.
841	DRAMP03187	Beta defensin 1(BD-1; mammals, animals)	"Beta defensin 1"[All Fields] AND biofilm[All Fields]	Beta defensin 1	32585445	Glucose effect on Candida albicans biofilm during tissue invasion.
841	DRAMP03187	Beta defensin 1(BD-1; mammals, animals)	"Beta defensin 1"[All Fields] AND biofilm[All Fields]	Beta defensin 1	30260708	Stabilized collagen matrix dressing improves wound macrophage function and epithelialization.
841	DRAMP03187	Beta defensin 1(BD-1; mammals, animals)	"Beta defensin 1"[All Fields] AND biofilm[All Fields]	Beta defensin 1	28642103	Chimeric analogs of human β-defensin 1 and θ-defensin disrupt pre-established bacterial biofilms.
841	DRAMP03187	Beta defensin 1(BD-1; mammals, animals)	"Beta defensin 1"[All Fields] AND biofilm[All Fields]	Beta defensin 1	26214284	α-tocopherol decreases interleukin-1β and -6 and increases human β-defensin-1 and -2 secretion in human gingival fibroblasts stimulated with Porphyromonas gingivalis lipopolysaccharide.
841	DRAMP03187	Beta defensin 1(BD-1; mammals, animals)	"Beta defensin 1"[All Fields] AND biofilm[All Fields]	Beta defensin 1	22394470	Association of CiaRH with resistance of Streptococcus mutans to antimicrobial peptides in biofilms.
841	DRAMP03187	Beta defensin 1(BD-1; mammals, animals)	"Beta defensin 1"[All Fields] AND biofilm[All Fields]	Beta defensin 1	20454633	Normal human gingival epithelial cells sense C. parapsilosis by toll-like receptors and module its pathogenesis through antimicrobial peptides and proinflammatory cytokines.
846	DRAMP03215	Gomesin (Gm; spiders, Arthropods, animals)	"Gomesin"[All Fields] AND biofilm[All Fields]	Gomesin	20370836	Effects of the antimicrobial peptide gomesin on the global gene expression profile, virulence and biofilm formation of Xylella fastidiosa.

887	DRAMP03507	Cecropin-B (Insects, animals)	"Cecropin-B"[All Fields] AND biofilm[All Fields]	Cecropin-B	31057940	On-chip manufacturing of synthetic proteins for point-of-care therapeutics.
887	DRAMP03507	Cecropin-B (Insects, animals)	"Cecropin-B"[All Fields] AND biofilm[All Fields]	Cecropin-B	23732798	Surface functionalization of titanium substrates with cecropin B to improve their cytocompatibility and reduce inflammation responses.
902	DRAMP03528	Defensin (Galiomicin; Insects, animals)	"Defensin"[All Fields] AND biofilm[All Fields]	Defensin	34408988	The Anti-Biofilm Efficacy of Caffeic Acid Phenethyl Ester (CAPE) In Vitroand a Murine Model of Oral Candidiasis.
902	DRAMP03528	Defensin (Galiomicin; Insects, animals)	"Defensin"[All Fields] AND biofilm[All Fields]	Defensin	34321877	Antibacterial Effect of Honey-Derived Exosomes Containing Antimicrobial Peptides Against Oral Streptococci.
902	DRAMP03528	Defensin (Galiomicin; Insects, animals)	"Defensin"[All Fields] AND biofilm[All Fields]	Defensin	34276631	DNA Blocks the Lethal Effect of Human Beta-Defensin 2 Against Neisseria meningitidis.
902	DRAMP03528	Defensin (Galiomicin; Insects, animals)	"Defensin"[All Fields] AND biofilm[All Fields]	Defensin	33911935	The antibacterial activities of honey.
902	DRAMP03528	Defensin (Galiomicin; Insects, animals)	"Defensin"[All Fields] AND biofilm[All Fields]	Defensin	33865931	Identification of a crocodylian β-defensin variant from Alligator mississippiensis with antimicrobial and antibiofilm activity.
902	DRAMP03528	Defensin (Galiomicin; Insects, animals)	"Defensin"[All Fields] AND biofilm[All Fields]	Defensin	33586659	Identification of anti-microbial peptides and traces of microbial DNA in infrainfundibular compartments of human scalp terminal hair follicles.
902	DRAMP03528	Defensin (Galiomicin; Insects, animals)	"Defensin"[All Fields] AND biofilm[All Fields]	Defensin	33534018	A recombinant fungal defensin-like peptide-P2 combats Streptococcus dysgalactiae and biofilms.
902	DRAMP03528	Defensin (Galiomicin; Insects, animals)	"Defensin"[All Fields] AND biofilm[All Fields]	Defensin	33447687	Future directions of postoperative spinal implant infections.
902	DRAMP03528	Defensin (Galiomicin; Insects, animals)	"Defensin"[All Fields] AND biofilm[All Fields]	Defensin	33420317	Curbing gastrointestinal infections by defensin fragment modifications without harming commensal microbiota.
902	DRAMP03528	Defensin (Galiomicin; Insects, animals)	"Defensin"[All Fields] AND biofilm[All Fields]	Defensin	32867384	A Novel Peptide Antibiotic, Pro10-1D, Designed from Insect Defensin Shows Antibacterial and Anti-Inflammatory Activities in Sepsis Models.
902	DRAMP03528	Defensin (Galiomicin; Insects, animals)	"Defensin"[All Fields] AND biofilm[All Fields]	Defensin	32858856	Candida albicansVirulence Factors and Pathogenicity for Endodontic Infections.
902	DRAMP03528	Defensin (Galiomicin; Insects, animals)	"Defensin"[All Fields] AND biofilm[All Fields]	Defensin	32842903	Electrospun ZnO/Poly(Vinylidene Fluoride-Trifluoroethylene) Scaffolds for Lung Tissue Engineering.
902	DRAMP03528	Defensin (Galiomicin; Insects, animals)	"Defensin"[All Fields] AND biofilm[All Fields]	Defensin	32663201	Inhibition and eradication activity of truncated α-defensin analogs against multidrug resistant uropathogenic Escherichia coli biofilm.
902	DRAMP03528	Defensin (Galiomicin; Insects, animals)	"Defensin"[All Fields] AND biofilm[All Fields]	Defensin	32585445	Glucose effect on Candida albicans biofilm during tissue invasion.
902	DRAMP03528	Defensin (Galiomicin; Insects, animals)	"Defensin"[All Fields] AND biofilm[All Fields]	Defensin	32522780	Controlling the Growth of the Skin Commensal Staphylococcus epidermidis Using d-Alanine Auxotrophy.
902	DRAMP03528	Defensin (Galiomicin; Insects, animals)	"Defensin"[All Fields] AND biofilm[All Fields]	Defensin	32457749	The Antimicrobial Peptide Human Beta-Defensin 2 Inhibits Biofilm Production of Pseudomonas aeruginosaWithout Compromising Metabolic Activity.
902	DRAMP03528	Defensin (Galiomicin; Insects, animals)	"Defensin"[All Fields] AND biofilm[All Fields]	Defensin	32439511	Antibacterial activities and mechanisms of action of a defensin from manila clam Ruditapes philippinarum.
902	DRAMP03528	Defensin (Galiomicin; Insects, animals)	"Defensin"[All Fields] AND biofilm[All Fields]	Defensin	31933178	Effects of human β-defensin 3 fused with carbohydrate-binding domain on the function of type III secretion system in Pseudomonas aeruginosa PA14.
902	DRAMP03528	Defensin (Galiomicin; Insects, animals)	"Defensin"[All Fields] AND biofilm[All Fields]	Defensin	31906541	Role of FAD-I in Fusobacterial Interspecies Interaction and Biofilm Formation.
902	DRAMP03528	Defensin (Galiomicin; Insects, animals)	"Defensin"[All Fields] AND biofilm[All Fields]	Defensin	31729441	Rhesus Theta Defensin 1 Promotes Long Term Survival in Systemic Candidiasis by Host Directed Mechanisms.
902	DRAMP03528	Defensin (Galiomicin; Insects, animals)	"Defensin"[All Fields] AND biofilm[All Fields]	Defensin	31336838	Candida albicans-Cell Interactions Activate Innate Immune Defense in Human Palate Epithelial Primary Cells via Nitric Oxide (NO) and β-Defensin 2 (hBD-2).
902	DRAMP03528	Defensin (Galiomicin; Insects, animals)	"Defensin"[All Fields] AND biofilm[All Fields]	Defensin	31165072	Impact of the Food Additive Titanium Dioxide (E171) on Gut Microbiota-Host Interaction.
902	DRAMP03528	Defensin (Galiomicin; Insects, animals)	"Defensin"[All Fields] AND biofilm[All Fields]	Defensin	31031739	Salt-Tolerant Antifungal and Antibacterial Activities of the Corn Defensin ZmD32.
902	DRAMP03528	Defensin (Galiomicin; Insects, animals)	"Defensin"[All Fields] AND biofilm[All Fields]	Defensin	31025073	A recombinant fungal defensin-like peptide-P2 combats multidrug-resistant Staphylococcus aureus and biofilms.
902	DRAMP03528	Defensin (Galiomicin; Insects, animals)	"Defensin"[All Fields] AND biofilm[All Fields]	Defensin	30659503	Lactobacillus plantarum USM8613 Aids in Wound Healing and Suppresses Staphylococcus aureus Infection at Wound Sites.
902	DRAMP03528	Defensin (Galiomicin; Insects, animals)	"Defensin"[All Fields] AND biofilm[All Fields]	Defensin	30649289	Innate immune components affect growth and virulence traits of bacterial-vaginosis-associated and non-bacterial-vaginosis-associated Gardnerella vaginalis strains similarly.
902	DRAMP03528	Defensin (Galiomicin; Insects, animals)	"Defensin"[All Fields] AND biofilm[All Fields]	Defensin	30376742	Alpha defensin, leukocyte esterase, C-reactive protein, and leukocyte count in synovial fluid for pre-operative diagnosis of periprosthetic infection.
902	DRAMP03528	Defensin (Galiomicin; Insects, animals)	"Defensin"[All Fields] AND biofilm[All Fields]	Defensin	30260708	Stabilized collagen matrix dressing improves wound macrophage function and epithelialization.
902	DRAMP03528	Defensin (Galiomicin; Insects, animals)	"Defensin"[All Fields] AND biofilm[All Fields]	Defensin	30254440	Modification of the surface of titanium with multifunctional chimeric peptides to prevent biofilm formation via inhibition of initial colonizers.
902	DRAMP03528	Defensin (Galiomicin; Insects, animals)	"Defensin"[All Fields] AND biofilm[All Fields]	Defensin	29902560	A defensin-like antimicrobial peptide from the manila clam Ruditapes philippinarum: Investigation of the antibacterial activities and mode of action.
902	DRAMP03528	Defensin (Galiomicin; Insects, animals)	"Defensin"[All Fields] AND biofilm[All Fields]	Defensin	29872295	Antimicrobial peptide-loaded liquid crystalline precursor bioadhesive system for the prevention of dental caries.
902	DRAMP03528	Defensin (Galiomicin; Insects, animals)	"Defensin"[All Fields] AND biofilm[All Fields]	Defensin	29671721	The BceABRS four-component system that is essential for cell envelope stress response is involved in sensing and response to host defence peptides and is required for the biofilm formation and fitness of Streptococcus mutans.
902	DRAMP03528	Defensin (Galiomicin; Insects, animals)	"Defensin"[All Fields] AND biofilm[All Fields]	Defensin	29104569	A Linear 19-Mer Plant Defensin-Derived Peptide Acts Synergistically with Caspofungin against Candida albicansBiofilms.
902	DRAMP03528	Defensin (Galiomicin; Insects, animals)	"Defensin"[All Fields] AND biofilm[All Fields]	Defensin	29077172	The significance of HBD-3 and fluorescent composite carriers in the processof bone formation in rats infected with Staphylococcus aureus.
902	DRAMP03528	Defensin (Galiomicin; Insects, animals)	"Defensin"[All Fields] AND biofilm[All Fields]	Defensin	29045084	New Approach to Treat and Prevent Oral Disease.
902	DRAMP03528	Defensin (Galiomicin; Insects, animals)	"Defensin"[All Fields] AND biofilm[All Fields]	Defensin	29025642	Engineered chimeric peptides with antimicrobial and titanium-binding functions to inhibit biofilm formation on Ti implants.
902	DRAMP03528	Defensin (Galiomicin; Insects, animals)	"Defensin"[All Fields] AND biofilm[All Fields]	Defensin	28956355	The synthetic human beta-defensin-3 C15 peptide exhibits antimicrobial activity against Streptococcus mutans, both alone and in combination with dental disinfectants.
902	DRAMP03528	Defensin (Galiomicin; Insects, animals)	"Defensin"[All Fields] AND biofilm[All Fields]	Defensin	28951032	Antifungal Effects of Synthetic Human Beta-defensin-3-C15 Peptide on Candida albicans-infected Root Dentin.
902	DRAMP03528	Defensin (Galiomicin; Insects, animals)	"Defensin"[All Fields] AND biofilm[All Fields]	Defensin	28874606	Bacterial d-amino acids suppress sinonasal innate immunity through sweet taste receptors in solitary chemosensory cells.
902	DRAMP03528	Defensin (Galiomicin; Insects, animals)	"Defensin"[All Fields] AND biofilm[All Fields]	Defensin	28725299	Role of Streptococcus mutanstwo-component systems in antimicrobial peptide resistance in the oral cavity.
902	DRAMP03528	Defensin (Galiomicin; Insects, animals)	"Defensin"[All Fields] AND biofilm[All Fields]	Defensin	28649561	Psd1 Effects on Candida albicansPlanktonic Cells and Biofilms.
902	DRAMP03528	Defensin (Galiomicin; Insects, animals)	"Defensin"[All Fields] AND biofilm[All Fields]	Defensin	28642103	Chimeric analogs of human β-defensin 1 and θ-defensin disrupt pre-established bacterial biofilms.
902	DRAMP03528	Defensin (Galiomicin; Insects, animals)	"Defensin"[All Fields] AND biofilm[All Fields]	Defensin	28413476	The mechanism of human β-defensin 3 in MRSA-induced infection of implant drug-resistant bacteria biofilm in the mouse tibial bone marrow.
902	DRAMP03528	Defensin (Galiomicin; Insects, animals)	"Defensin"[All Fields] AND biofilm[All Fields]	Defensin	28296382	Human α-Defensin 6: A Small Peptide That Self-Assembles and Protects the Host by Entangling Microbes.
902	DRAMP03528	Defensin (Galiomicin; Insects, animals)	"Defensin"[All Fields] AND biofilm[All Fields]	Defensin	28278280	Natural antimicrobial peptide complexes in the fighting of antibiotic resistant biofilms: Calliphora vicina medicinal maggots.
902	DRAMP03528	Defensin (Galiomicin; Insects, animals)	"Defensin"[All Fields] AND biofilm[All Fields]	Defensin	28144375	Advancements in Diagnosing Periprosthetic Joint Infections after Total Hip and Knee Arthroplasty.
902	DRAMP03528	Defensin (Galiomicin; Insects, animals)	"Defensin"[All Fields] AND biofilm[All Fields]	Defensin	28078813	Host defense peptide-derived privileged scaffolds for anti-infective drug discovery.
902	DRAMP03528	Defensin (Galiomicin; Insects, animals)	"Defensin"[All Fields] AND biofilm[All Fields]	Defensin	28026958	Human α-Defensin 6 Self-Assembly Prevents Adhesion and Suppresses Virulence Traits of Candida albicans.
902	DRAMP03528	Defensin (Galiomicin; Insects, animals)	"Defensin"[All Fields] AND biofilm[All Fields]	Defensin	27794585	A Novel Defensin-Like Peptide Associated with Two Other New Cationic Antimicrobial Peptides in Transcriptome of the Iranian Scorpion Venom.
902	DRAMP03528	Defensin (Galiomicin; Insects, animals)	"Defensin"[All Fields] AND biofilm[All Fields]	Defensin	27777572	Role of yqiCin the Pathogenicity of Salmonellaand Innate Immune Responses of Human Intestinal Epithelium.
902	DRAMP03528	Defensin (Galiomicin; Insects, animals)	"Defensin"[All Fields] AND biofilm[All Fields]	Defensin	27582732	Efficient Eradication of Mature Pseudomonas aeruginosa Biofilm via Controlled Delivery of Nitric Oxide Combined with Antimicrobial Peptide and Antibiotics.
902	DRAMP03528	Defensin (Galiomicin; Insects, animals)	"Defensin"[All Fields] AND biofilm[All Fields]	Defensin	27417541	Acute appendicitis: transcript profiling of blood identifies promising biomarkers and potential underlying processes.
902	DRAMP03528	Defensin (Galiomicin; Insects, animals)	"Defensin"[All Fields] AND biofilm[All Fields]	Defensin	27200276	Antifungal effects of synthetic human β-defensin 3-C15 peptide.
902	DRAMP03528	Defensin (Galiomicin; Insects, animals)	"Defensin"[All Fields] AND biofilm[All Fields]	Defensin	27148195	Effect of Substance P in Staphylococcus aureus and Staphylococcus epidermidis Virulence: Implication for Skin Homeostasis.
902	DRAMP03528	Defensin (Galiomicin; Insects, animals)	"Defensin"[All Fields] AND biofilm[All Fields]	Defensin	26861950	Antibiofilm efficacy of honey and bee-derived defensin-1 on multispecies wound biofilm.
902	DRAMP03528	Defensin (Galiomicin; Insects, animals)	"Defensin"[All Fields] AND biofilm[All Fields]	Defensin	26592804	The radish defensins RsAFP1 and RsAFP2 act synergistically with caspofungin against Candida albicans biofilms.

902	DRAMP03528	Defensin (Galiomicin; Insects, animals)	"Defensin"[All Fields] AND biofilm[All Fields]	Defensin	26248029	Synergistic Activity of the Plant Defensin HsAFP1 and Caspofungin against Candida albicans Biofilms and Planktonic Cultures.
902	DRAMP03528	Defensin (Galiomicin; Insects, animals)	"Defensin"[All Fields] AND biofilm[All Fields]	Defensin	26214284	α-tocopherol decreases interleukin-1β and -6 and increases human β-defensin-1 and -2 secretion in human gingival fibroblasts stimulated with Porphyromonas gingivalis lipopolysaccharide.
902	DRAMP03528	Defensin (Galiomicin; Insects, animals)	"Defensin"[All Fields] AND biofilm[All Fields]	Defensin	26196513	Snake Cathelicidin NA-CATH and Smaller Helical Antimicrobial Peptides Are Effective against Burkholderia thailandensis.
902	DRAMP03528	Defensin (Galiomicin; Insects, animals)	"Defensin"[All Fields] AND biofilm[All Fields]	Defensin	26119274	Ultrasound microbubbles enhance human β-defensin 3 against biofilms.
902	DRAMP03528	Defensin (Galiomicin; Insects, animals)	"Defensin"[All Fields] AND biofilm[All Fields]	Defensin	25862466	The Antibacterial Effects of an Antimicrobial Peptide Human β-Defensin 3 Fused with Carbohydrate-Binding Domain on Pseudomonas aeruginosa PA14.
902	DRAMP03528	Defensin (Galiomicin; Insects, animals)	"Defensin"[All Fields] AND biofilm[All Fields]	Defensin	25808131	Expression of antimicrobial peptides and interleukin-8 during early stages of inflammation: An experimental gingivitis study.
902	DRAMP03528	Defensin (Galiomicin; Insects, animals)	"Defensin"[All Fields] AND biofilm[All Fields]	Defensin	25806720	Antimicrobial peptides in 2014.
902	DRAMP03528	Defensin (Galiomicin; Insects, animals)	"Defensin"[All Fields] AND biofilm[All Fields]	Defensin	25285879	Inhibition and destruction of Pseudomonas aeruginosa biofilms by antibiotics and antimicrobial peptides.
902	DRAMP03528	Defensin (Galiomicin; Insects, animals)	"Defensin"[All Fields] AND biofilm[All Fields]	Defensin	25212593	Health- and disease-associated species clusters in complex natural biofilms determine the innate immune response in oral epithelial cells during biofilm maturation.
902	DRAMP03528	Defensin (Galiomicin; Insects, animals)	"Defensin"[All Fields] AND biofilm[All Fields]	Defensin	24913184	Effects of human β-defensin-3 on biofilm formation-regulating genes dltB and icaA in Staphylococcus aureus.
902	DRAMP03528	Defensin (Galiomicin; Insects, animals)	"Defensin"[All Fields] AND biofilm[All Fields]	Defensin	24340061	Inflammatory and antimicrobial responses to methicillin-resistant Staphylococcus aureus in an in vitro wound infection model.
902	DRAMP03528	Defensin (Galiomicin; Insects, animals)	"Defensin"[All Fields] AND biofilm[All Fields]	Defensin	24240906	Adsorption study of pellicle proteins to gold, silica and titanium by quartz crystal microbalance method.
902	DRAMP03528	Defensin (Galiomicin; Insects, animals)	"Defensin"[All Fields] AND biofilm[All Fields]	Defensin	24238461	Antibacterial efficacy of a human β-defensin-3 peptide on multispecies biofilms.
902	DRAMP03528	Defensin (Galiomicin; Insects, animals)	"Defensin"[All Fields] AND biofilm[All Fields]	Defensin	24100890	[Use of recombinant human beta-defensin-3 to evaluate the effect of adhesion of Candida albicans on the surface of soft lining material].
902	DRAMP03528	Defensin (Galiomicin; Insects, animals)	"Defensin"[All Fields] AND biofilm[All Fields]	Defensin	23639356	Human beta-defensin 3: a novel inhibitor of Staphylococcus-produced biofilm production. Commentary on "Human β-defensin 3 inhibits antibiotic-resistant Staphylococcus biofilm formation".
902	DRAMP03528	Defensin (Galiomicin; Insects, animals)	"Defensin"[All Fields] AND biofilm[All Fields]	Defensin	23519963	Ultrasound-targeted microbubble destruction enhances human β-defensin 3 activity against antibiotic-resistant Staphylococcus biofilms.
902	DRAMP03528	Defensin (Galiomicin; Insects, animals)	"Defensin"[All Fields] AND biofilm[All Fields]	Defensin	23273885	Human β-defensin 3 inhibits antibiotic-resistant Staphylococcus biofilm formation.
902	DRAMP03528	Defensin (Galiomicin; Insects, animals)	"Defensin"[All Fields] AND biofilm[All Fields]	Defensin	23203265	Potentiation of the cytotoxic activity of copper by polyphosphate on biofilm-producing bacteria: a bioinspired approach.
902	DRAMP03528	Defensin (Galiomicin; Insects, animals)	"Defensin"[All Fields] AND biofilm[All Fields]	Defensin	23078156	Antimicrobial efficacy of a human β-defensin-3 peptide using an Enterococcus faecalis dentine infection model.
902	DRAMP03528	Defensin (Galiomicin; Insects, animals)	"Defensin"[All Fields] AND biofilm[All Fields]	Defensin	23053486	Synergistic effect and antibiofilm activity between the antimicrobial peptide coprisin and conventional antibiotics against opportunistic bacteria.
902	DRAMP03528	Defensin (Galiomicin; Insects, animals)	"Defensin"[All Fields] AND biofilm[All Fields]	Defensin	22922323	Extracellular DNA within a nontypeable Haemophilus influenzae-induced biofilm binds human beta defensin-3 and reduces its antimicrobial activity.
902	DRAMP03528	Defensin (Galiomicin; Insects, animals)	"Defensin"[All Fields] AND biofilm[All Fields]	Defensin	22855857	Retrocyclin inhibits Gardnerella vaginalis biofilm formation and toxin activity.
902	DRAMP03528	Defensin (Galiomicin; Insects, animals)	"Defensin"[All Fields] AND biofilm[All Fields]	Defensin	22394470	Association of CiaRH with resistance of Streptococcus mutans to antimicrobial peptides in biofilms.
902	DRAMP03528	Defensin (Galiomicin; Insects, animals)	"Defensin"[All Fields] AND biofilm[All Fields]	Defensin	22229614	Comparison of the effects of human β-defensin 3, vancomycin, and clindamycin on Staphylococcus aureus biofilm formation.
902	DRAMP03528	Defensin (Galiomicin; Insects, animals)	"Defensin"[All Fields] AND biofilm[All Fields]	Defensin	21692631	A novel organotypic dento-epithelial culture model: effect of Fusobacterium nucleatum biofilm on B-defensin-2, -3, and LL-37 expression.
902	DRAMP03528	Defensin (Galiomicin; Insects, animals)	"Defensin"[All Fields] AND biofilm[All Fields]	Defensin	20454633	Normal human gingival epithelial cells sense C. parapsilosis by toll-like receptors and module its pathogenesis through antimicrobial peptides and proinflammatory cytokines.
902	DRAMP03528	Defensin (Galiomicin; Insects, animals)	"Defensin"[All Fields] AND biofilm[All Fields]	Defensin	20378008	Effect of temperature on the shift of Pseudomonas fluorescens from an environmental microorganism to a potential human pathogen.
902	DRAMP03528	Defensin (Galiomicin; Insects, animals)	"Defensin"[All Fields] AND biofilm[All Fields]	Defensin	19961380	Gingival transcriptome patterns during induction and resolution of experimental gingivitis in humans.
902	DRAMP03528	Defensin (Galiomicin; Insects, animals)	"Defensin"[All Fields] AND biofilm[All Fields]	Defensin	19780045	Candida famata modulates toll-like receptor, beta-defensin, and proinflammatory cytokine expression by normal human epithelial cells.
902	DRAMP03528	Defensin (Galiomicin; Insects, animals)	"Defensin"[All Fields] AND biofilm[All Fields]	Defensin	19572896	The immune response of oral epithelial cells induced by single-species and complex naturally formed biofilms.
902	DRAMP03528	Defensin (Galiomicin; Insects, animals)	"Defensin"[All Fields] AND biofilm[All Fields]	Defensin	19466693	Activity of antimicrobial peptides in the presence of polysaccharides produced by pulmonary pathogens.
902	DRAMP03528	Defensin (Galiomicin; Insects, animals)	"Defensin"[All Fields] AND biofilm[All Fields]	Defensin	18954353	Treponema denticola does not induce production of common innate immune mediators from primary gingival epithelial cells.
902	DRAMP03528	Defensin (Galiomicin; Insects, animals)	"Defensin"[All Fields] AND biofilm[All Fields]	Defensin	18173794	The stage of native biofilm formation determines the gene expression of human beta-defensin-2, psoriasin, ribonuclease 7 and inflammatory mediators: a novel approach for stimulation of keratinocytes with in situ formed biofilms.
902	DRAMP03528	Defensin (Galiomicin; Insects, animals)	"Defensin"[All Fields] AND biofilm[All Fields]	Defensin	17434999	Functional analysis of D-alanylation of lipoteichoic acid in the probiotic strain Lactobacillus rhamnosus GG.
902	DRAMP03528	Defensin (Galiomicin; Insects, animals)	"Defensin"[All Fields] AND biofilm[All Fields]	Defensin	15493829	Bacterial evasion of innate host defenses--the Staphylococcus aureus lesson.
902	DRAMP03528	Defensin (Galiomicin; Insects, animals)	"Defensin"[All Fields] AND biofilm[All Fields]	Defensin	14764110	Polysaccharide intercellular adhesin (PIA) protects Staphylococcus epidermidis against major components of the human innate immune system.
904	DRAMP03539	Antifungal protein (Psc-AFP)	"Antifungal protein"[All Fields] AND biofilm[All Fields]	Antifungal protein	33466640	The Neosartorya fischeriAntifungal Protein 2 (NFAP2): A New Potential Weapon against Multidrug-Resistant Candida aurisBiofilms.
904	DRAMP03539	Antifungal protein (Psc-AFP)	"Antifungal protein"[All Fields] AND biofilm[All Fields]	Antifungal protein	32824977	The Penicillium chrysogenumQ176 Antimicrobial Protein PAFC Effectively Inhibits the Growth of the Opportunistic Human Pathogen Candida albicans.
904	DRAMP03539	Antifungal protein (Psc-AFP)	"Antifungal protein"[All Fields] AND biofilm[All Fields]	Antifungal protein	30478163	In VivoApplicability of Neosartorya fischeri Antifungal Protein 2 (NFAP2) in Treatment of Vulvovaginal Candidiasis.
904	DRAMP03539	Antifungal protein (Psc-AFP)	"Antifungal protein"[All Fields] AND biofilm[All Fields]	Antifungal protein	30079061	The Evolutionary Conserved γ-Core Motif Influences the Anti- CandidaActivity of the Penicillium chrysogenumAntifungal Protein PAF.
904	DRAMP03539	Antifungal protein (Psc-AFP)	"Antifungal protein"[All Fields] AND biofilm[All Fields]	Antifungal protein	28120548	DS6: anticandidal, antibiofilm peptide against Candida tropicalis and exhibit synergy with commercial drug.
908	DRAMP03567	KR-20 (Derived from LL-37)	"KR-20"[All Fields] AND biofilm[All Fields]	KR-20	24071034	The human antimicrobial peptide LL-37 and its fragments possess both antimicrobial and antibiofilm activities against multidrug-resistant Acinetobacter baumannii.
910	DRAMP03569	KS-30 (Derived from LL-37)	"KS-30"[All Fields] AND biofilm[All Fields]	KS-30	24071034	The human antimicrobial peptide LL-37 and its fragments possess both antimicrobial and antibiofilm activities against multidrug-resistant Acinetobacter baumannii.
913	DRAMP03573	LL-37(13-37)(C-terminal fragment of LL-37; Human, mammals, animals)	"LL-37"[All Fields] AND biofilm[All Fields]	LL-37	34454095	Targeting antibiotic tolerance in anaerobic biofilms associated with oral diseases: Human antimicrobial peptides LL-37 and lactoferricin enhance the antibiotic efficacy of amoxicillin, clindamycin and metronidazole.
913	DRAMP03573	LL-37(13-37)(C-terminal fragment of LL-37; Human, mammals, animals)	"LL-37"[All Fields] AND biofilm[All Fields]	LL-37	34402021	Cytocompatibility and Synergy of EGCG and Cationic Peptides Against Bacteria Related to Endodontic Infections, in Planktonic and Biofilm Conditions.
913	DRAMP03573	LL-37(13-37)(C-terminal fragment of LL-37; Human, mammals, animals)	"LL-37"[All Fields] AND biofilm[All Fields]	LL-37	34072318	The Potential of Human Peptide LL-37 as an Antimicrobial and Anti-Biofilm Agent.
913	DRAMP03573	LL-37(13-37)(C-terminal fragment of LL-37; Human, mammals, animals)	"LL-37"[All Fields] AND biofilm[All Fields]	LL-37	33890759	Short and Robust Anti-Infective Lipopeptides Engineered Based on the Minimal Antimicrobial Peptide KR12 of Human LL-37.
913	DRAMP03573	LL-37(13-37)(C-terminal fragment of LL-37; Human, mammals, animals)	"LL-37"[All Fields] AND biofilm[All Fields]	LL-37	33885339	The Multifunctional Roles of Short Palate, Lung, and Nasal Epithelium Clone 1 in Regulating Airway Surface Liquid and Participating in Airway Host Defense.
913	DRAMP03573	LL-37(13-37)(C-terminal fragment of LL-37; Human, mammals, animals)	"LL-37"[All Fields] AND biofilm[All Fields]	LL-37	33797775	Toll-like receptor activation of equine mesenchymal stromal cells to enhance antibacterial activity and immunomodulatory cytokine secretion.
913	DRAMP03573	LL-37(13-37)(C-terminal fragment of LL-37; Human, mammals, animals)	"LL-37"[All Fields] AND biofilm[All Fields]	LL-37	33724786	Membrane Interactions of Virus-like Mesoporous Silica Nanoparticles.

913	DRAMP03573	LL-37(13-37)(C-terminal fragment of LL-37; Human, mammals, animals)	"LL-37"[All Fields] AND biofilm[All Fields]	LL-37	33687301	Exogenous LL-37 but not homogenates of desquamated oral epithelial cells shows activity against Streptococcus mutans.
913	DRAMP03573	LL-37(13-37)(C-terminal fragment of LL-37; Human, mammals, animals)	"LL-37"[All Fields] AND biofilm[All Fields]	LL-37	33646013	Synergistic effect of antimicrobial peptide LL-37 and colistin combination against multidrug-resistant Escherichia coliisolates.
913	DRAMP03573	LL-37(13-37)(C-terminal fragment of LL-37; Human, mammals, animals)	"LL-37"[All Fields] AND biofilm[All Fields]	LL-37	33584628	SAAP-148 Eradicates MRSA Persisters Within Mature Biofilm Models Simulating Prosthetic Joint Infection.
913	DRAMP03573	LL-37(13-37)(C-terminal fragment of LL-37; Human, mammals, animals)	"LL-37"[All Fields] AND biofilm[All Fields]	LL-37	33326455	D-LL-31 enhances biofilm-eradicating effect of currently used antibiotics for chronic rhinosinusitis and its immunomodulatory activity on human lung epithelial cells.
913	DRAMP03573	LL-37(13-37)(C-terminal fragment of LL-37; Human, mammals, animals)	"LL-37"[All Fields] AND biofilm[All Fields]	LL-37	33252326	Effect of antifungal agents, lysozyme and human antimicrobial peptide LL-37 on clinical Candidaisolates with high biofilm production.
913	DRAMP03573	LL-37(13-37)(C-terminal fragment of LL-37; Human, mammals, animals)	"LL-37"[All Fields] AND biofilm[All Fields]	LL-37	33249255	Inhibitory effect of LL-37 and human lactoferricin on growth and biofilm formation of anaerobes associated with oral diseases.
913	DRAMP03573	LL-37(13-37)(C-terminal fragment of LL-37; Human, mammals, animals)	"LL-37"[All Fields] AND biofilm[All Fields]	LL-37	32768482	Development of anti-bacterial surfaces using a hydrophobin chimeric protein.
913	DRAMP03573	LL-37(13-37)(C-terminal fragment of LL-37; Human, mammals, animals)	"LL-37"[All Fields] AND biofilm[All Fields]	LL-37	32725811	Comparison of Antibacterial Effect of Cationic Peptide LL-37 and Cefalexin on Clinical Staphylococcus aureus-induced Infection after Femur Fracture Fixation.
913	DRAMP03573	LL-37(13-37)(C-terminal fragment of LL-37; Human, mammals, animals)	"LL-37"[All Fields] AND biofilm[All Fields]	LL-37	32523805	Characterization of biofilm production in different strains of Acinetobacter baumanniiand the effects of chemical compounds on biofilm formation.
913	DRAMP03573	LL-37(13-37)(C-terminal fragment of LL-37; Human, mammals, animals)	"LL-37"[All Fields] AND biofilm[All Fields]	LL-37	32518218	Can Salivary Biomarkers Be Used as Predictors of Dental Caries in Young Adolescents?
913	DRAMP03573	LL-37(13-37)(C-terminal fragment of LL-37; Human, mammals, animals)	"LL-37"[All Fields] AND biofilm[All Fields]	LL-37	32343547	Resistome of Staphylococcus aureusin Response to Human Cathelicidin LL-37 and Its Engineered Antimicrobial Peptides.
913	DRAMP03573	LL-37(13-37)(C-terminal fragment of LL-37; Human, mammals, animals)	"LL-37"[All Fields] AND biofilm[All Fields]	LL-37	32232010	Francisella novicidaTwo-Component System Response Regulator BfpR Modulates iglC Gene Expression, Antimicrobial Peptide Resistance, and Biofilm Production.
913	DRAMP03573	LL-37(13-37)(C-terminal fragment of LL-37; Human, mammals, animals)	"LL-37"[All Fields] AND biofilm[All Fields]	LL-37	32203127	Antibiofilm activities of ceragenins and antimicrobial peptides against fungal-bacterial mono and multispecies biofilms.
913	DRAMP03573	LL-37(13-37)(C-terminal fragment of LL-37; Human, mammals, animals)	"LL-37"[All Fields] AND biofilm[All Fields]	LL-37	32182913	Differential Abilities of Mammalian Cathelicidins to Inhibit Bacterial Biofilm Formation and Promote Multifaceted Immune Functions of Neutrophils.
913	DRAMP03573	LL-37(13-37)(C-terminal fragment of LL-37; Human, mammals, animals)	"LL-37"[All Fields] AND biofilm[All Fields]	LL-37	32019109	Lipidated Analogs of the LL-37-Derived Peptide Fragment KR12-Structural Analysis, Surface-Active Properties and Antimicrobial Activity.
913	DRAMP03573	LL-37(13-37)(C-terminal fragment of LL-37; Human, mammals, animals)	"LL-37"[All Fields] AND biofilm[All Fields]	LL-37	31993042	Linezolid and Rifampicin Combination to Combat cfr-Positive Multidrug-Resistant MRSA in Murine Models of Bacteremia and Skin and Skin Structure Infection.
913	DRAMP03573	LL-37(13-37)(C-terminal fragment of LL-37; Human, mammals, animals)	"LL-37"[All Fields] AND biofilm[All Fields]	LL-37	31396193	Evaluation of the Antimicrobial Peptide, RP557, for the Broad-Spectrum Treatment of Wound Pathogens and Biofilm.
913	DRAMP03573	LL-37(13-37)(C-terminal fragment of LL-37; Human, mammals, animals)	"LL-37"[All Fields] AND biofilm[All Fields]	LL-37	31356860	Eradication of meticillin-resistant Staphylococcus aureus from human skin by the novel LL-37-derived peptide P10 in four pharmaceutical ointments.
913	DRAMP03573	LL-37(13-37)(C-terminal fragment of LL-37; Human, mammals, animals)	"LL-37"[All Fields] AND biofilm[All Fields]	LL-37	31329599	Synergistic effects of LFchimera and antibiotic against planktonic and biofilm form of Aggregatibacter actinomycetemcomitans.
913	DRAMP03573	LL-37(13-37)(C-terminal fragment of LL-37; Human, mammals, animals)	"LL-37"[All Fields] AND biofilm[All Fields]	LL-37	31319057	Modulation of antimicrobial potency of human cathelicidin peptides against the ESKAPE pathogens and in vivo efficacy in a murine catheter-associated biofilm model.
913	DRAMP03573	LL-37(13-37)(C-terminal fragment of LL-37; Human, mammals, animals)	"LL-37"[All Fields] AND biofilm[All Fields]	LL-37	31282211	D-LL-31 in combination with ceftazidime synergistically enhances bactericidal activity and biofilm destruction in Burkholderia pseudomallei.
913	DRAMP03573	LL-37(13-37)(C-terminal fragment of LL-37; Human, mammals, animals)	"LL-37"[All Fields] AND biofilm[All Fields]	LL-37	31170191	Antimicrobial peptide LL-37 is bactericidal against Staphylococcus aureus biofilms.
913	DRAMP03573	LL-37(13-37)(C-terminal fragment of LL-37; Human, mammals, animals)	"LL-37"[All Fields] AND biofilm[All Fields]	LL-37	31118709	Design and characterization of a new hybrid peptide from LL-37 and BMAP-27.
913	DRAMP03573	LL-37(13-37)(C-terminal fragment of LL-37; Human, mammals, animals)	"LL-37"[All Fields] AND biofilm[All Fields]	LL-37	31046689	Use of ceragenins as a potential treatment for urinary tract infections.
913	DRAMP03573	LL-37(13-37)(C-terminal fragment of LL-37; Human, mammals, animals)	"LL-37"[All Fields] AND biofilm[All Fields]	LL-37	30755738	In vitro activities of antimicrobial peptides and ceragenins against Legionella pneumophila.
913	DRAMP03573	LL-37(13-37)(C-terminal fragment of LL-37; Human, mammals, animals)	"LL-37"[All Fields] AND biofilm[All Fields]	LL-37	30728810	Role of Two-Component System Response Regulator bceRin the Antimicrobial Resistance, Virulence, Biofilm Formation, and Stress Response of Group B Streptococcus.
913	DRAMP03573	LL-37(13-37)(C-terminal fragment of LL-37; Human, mammals, animals)	"LL-37"[All Fields] AND biofilm[All Fields]	LL-37	30635992	Design and antimicrobial activities of LL-37 derivatives inhibiting the formation of Streptococcus mutans biofilm.
913	DRAMP03573	LL-37(13-37)(C-terminal fragment of LL-37; Human, mammals, animals)	"LL-37"[All Fields] AND biofilm[All Fields]	LL-37	30465823	Antimicrobial synergy of monolaurin lipid nanocapsules with adsorbed antimicrobial peptides against Staphylococcus aureus biofilms in vitro is absent in vivo.
913	DRAMP03573	LL-37(13-37)(C-terminal fragment of LL-37; Human, mammals, animals)	"LL-37"[All Fields] AND biofilm[All Fields]	LL-37	30375445	Multi-species oral biofilm promotes reconstructed human gingiva epithelial barrier function.

913	DRAMP03573	LL-37(13-37)(C-terminal fragment of LL-37; Human, mammals, animals)	"LL-37"[All Fields] AND biofilm[All Fields]	LL-37	30315918	Synergistic microbicidal effect of cationic antimicrobial peptides and teicoplanin against planktonic and biofilm-encased Staphylococcus aureus.
913	DRAMP03573	LL-37(13-37)(C-terminal fragment of LL-37; Human, mammals, animals)	"LL-37"[All Fields] AND biofilm[All Fields]	LL-37	30250480	Role of Microbes in the Development of Alzheimer's Disease: State of the Art - An International Symposium Presented at the 2017 IAGG Congress in San Francisco.
913	DRAMP03573	LL-37(13-37)(C-terminal fragment of LL-37; Human, mammals, animals)	"LL-37"[All Fields] AND biofilm[All Fields]	LL-37	30131362	Discovery of New Genes Involved in Curli Production by a Uropathogenic Escherichia coli Strain from the Highly Virulent O45:K1:H7 Lineage.
913	DRAMP03573	LL-37(13-37)(C-terminal fragment of LL-37; Human, mammals, animals)	"LL-37"[All Fields] AND biofilm[All Fields]	LL-37	30120393	Evaluation of LL-37 antimicrobial peptide derivatives alone and in combination with vancomycin against S. aureus.
913	DRAMP03573	LL-37(13-37)(C-terminal fragment of LL-37; Human, mammals, animals)	"LL-37"[All Fields] AND biofilm[All Fields]	LL-37	30088919	Tackling Pseudomonas aeruginosa Virulence by a Hydroxamic Acid-Based LasB Inhibitor.
913	DRAMP03573	LL-37(13-37)(C-terminal fragment of LL-37; Human, mammals, animals)	"LL-37"[All Fields] AND biofilm[All Fields]	LL-37	30043322	Antimicrobial Activity of Selected Antimicrobial Peptides Against Planktonic Culture and Biofilm of Acinetobacter baumannii.
913	DRAMP03573	LL-37(13-37)(C-terminal fragment of LL-37; Human, mammals, animals)	"LL-37"[All Fields] AND biofilm[All Fields]	LL-37	29959905	Antimicrobial and anti-inflammatory activities of chemokine CXCL14-derived antimicrobial peptide and its analogs.
913	DRAMP03573	LL-37(13-37)(C-terminal fragment of LL-37; Human, mammals, animals)	"LL-37"[All Fields] AND biofilm[All Fields]	LL-37	29737589	LL-37 fragments have antimicrobial activity against Staphylococcus epidermidis biofilms and wound healing potential in HaCaT cell line.
913	DRAMP03573	LL-37(13-37)(C-terminal fragment of LL-37; Human, mammals, animals)	"LL-37"[All Fields] AND biofilm[All Fields]	LL-37	29671721	The BceABRS four-component system that is essential for cell envelope stress response is involved in sensing and response to host defence peptides and is required for the biofilm formation and fitness of Streptococcus mutans.
913	DRAMP03573	LL-37(13-37)(C-terminal fragment of LL-37; Human, mammals, animals)	"LL-37"[All Fields] AND biofilm[All Fields]	LL-37	29394295	Pseudomonas aeruginosa rugose small-colony variants evade host clearance, are hyper-inflammatory, and persist in multiple host environments.
913	DRAMP03573	LL-37(13-37)(C-terminal fragment of LL-37; Human, mammals, animals)	"LL-37"[All Fields] AND biofilm[All Fields]	LL-37	29391814	Targeting polyelectrolyte networks in purulent body fluids to modulate bactericidal properties of some antibiotics.
913	DRAMP03573	LL-37(13-37)(C-terminal fragment of LL-37; Human, mammals, animals)	"LL-37"[All Fields] AND biofilm[All Fields]	LL-37	29372443	Sensitivity of caries pathogens to antimicrobial peptides related to caries risk.
913	DRAMP03573	LL-37(13-37)(C-terminal fragment of LL-37; Human, mammals, animals)	"LL-37"[All Fields] AND biofilm[All Fields]	LL-37	29370365	The effects of antimicrobial peptides WAM-1 and LL-37 on multidrug-resistant Acinetobacter baumannii.
913	DRAMP03573	LL-37(13-37)(C-terminal fragment of LL-37; Human, mammals, animals)	"LL-37"[All Fields] AND biofilm[All Fields]	LL-37	29321257	The antimicrobial peptide SAAP-148 combats drug-resistant bacteria and biofilms.
913	DRAMP03573	LL-37(13-37)(C-terminal fragment of LL-37; Human, mammals, animals)	"LL-37"[All Fields] AND biofilm[All Fields]	LL-37	29022391	KR-12-a5 is a non-cytotoxic agent with potent antimicrobial effects against oral pathogens.
913	DRAMP03573	LL-37(13-37)(C-terminal fragment of LL-37; Human, mammals, animals)	"LL-37"[All Fields] AND biofilm[All Fields]	LL-37	28987032	Functionalized PVA-silk blended nanofibrous mats promote diabetic wound healing via regulation of extracellular matrix and tissue remodelling.
913	DRAMP03573	LL-37(13-37)(C-terminal fragment of LL-37; Human, mammals, animals)	"LL-37"[All Fields] AND biofilm[All Fields]	LL-37	28919413	Apolipoprotein A-I attenuates LL-37-induced endothelial cell cytotoxicity.
913	DRAMP03573	LL-37(13-37)(C-terminal fragment of LL-37; Human, mammals, animals)	"LL-37"[All Fields] AND biofilm[All Fields]	LL-37	28890511	The Antibacterial Effects of Antimicrobial Peptides OP-145 against Clinically Isolated Multi-Resistant Strains.
913	DRAMP03573	LL-37(13-37)(C-terminal fragment of LL-37; Human, mammals, animals)	"LL-37"[All Fields] AND biofilm[All Fields]	LL-37	28890436	Primary ciliary dyskinesia ciliated airway cells show increased susceptibility to Haemophilus influenzaebiofilm formation.
913	DRAMP03573	LL-37(13-37)(C-terminal fragment of LL-37; Human, mammals, animals)	"LL-37"[All Fields] AND biofilm[All Fields]	LL-37	28821865	Identification of bacterial biofilm and the Staphylococcus aureus derived protease, staphopain, on the skin surface of patients with atopic dermatitis.
913	DRAMP03573	LL-37(13-37)(C-terminal fragment of LL-37; Human, mammals, animals)	"LL-37"[All Fields] AND biofilm[All Fields]	LL-37	28747178	Anaerobic bacteria growth in the presence of cathelicidin LL-37 and selected ceragenins delivered as magnetic nanoparticles cargo.
913	DRAMP03573	LL-37(13-37)(C-terminal fragment of LL-37; Human, mammals, animals)	"LL-37"[All Fields] AND biofilm[All Fields]	LL-37	28680809	Antimicrobial ceragenins inhibit biofilms and affect mammalian cell viability and migration in vitro.
913	DRAMP03573	LL-37(13-37)(C-terminal fragment of LL-37; Human, mammals, animals)	"LL-37"[All Fields] AND biofilm[All Fields]	LL-37	28676673	Formulation and candidacidal activity of magnetic nanoparticles coated with cathelicidin LL-37 and ceragenin CSA-13.
913	DRAMP03573	LL-37(13-37)(C-terminal fragment of LL-37; Human, mammals, animals)	"LL-37"[All Fields] AND biofilm[All Fields]	LL-37	28675109	Titanium surfaces immobilized with the major antimicrobial fragment FK-16 of human cathelicidin LL-37 are potent against multiple antibiotic-resistant bacteria.
913	DRAMP03573	LL-37(13-37)(C-terminal fragment of LL-37; Human, mammals, animals)	"LL-37"[All Fields] AND biofilm[All Fields]	LL-37	28672834	Individual and Combined Effects of Engineered Peptides and Antibiotics on Pseudomonas aeruginosa Biofilms.
913	DRAMP03573	LL-37(13-37)(C-terminal fragment of LL-37; Human, mammals, animals)	"LL-37"[All Fields] AND biofilm[All Fields]	LL-37	28525841	LL-37-derived short antimicrobial peptide KR-12-a5 and its d-amino acid substituted analogs with cell selectivity, anti-biofilm activity, synergistic effect with conventional antibiotics, and anti-inflammatory activity.
913	DRAMP03573	LL-37(13-37)(C-terminal fragment of LL-37; Human, mammals, animals)	"LL-37"[All Fields] AND biofilm[All Fields]	LL-37	28408902	The Naturally Occurring Host Defense Peptide, LL-37, and Its Truncated Mimetics KE-18 and KR-12 Have Selected Biocidal and Antibiofilm Activities Against Candida albicans, Staphylococcus aureus, and Escherichia coli In vitro.
913	DRAMP03573	LL-37(13-37)(C-terminal fragment of LL-37; Human, mammals, animals)	"LL-37"[All Fields] AND biofilm[All Fields]	LL-37	28178190	High Specific Selectivity and Membrane-Active Mechanism of Synthetic Cationic Hybrid Antimicrobial Peptides Based on the Peptide FV7.
913	DRAMP03573	LL-37(13-37)(C-terminal fragment of LL-37; Human, mammals, animals)	"LL-37"[All Fields] AND biofilm[All Fields]	LL-37	28161291	LL-37-derived membrane-active FK-13 analogs possessing cell selectivity, anti-biofilm activity and synergy with chloramphenicol and anti-inflammatory activity.

913	DRAMP03573	LL-37(13-37)(C-terminal fragment of LL-37; Human, mammals, animals)	"LL-37"[All Fields] AND biofilm[All Fields]	LL-37	28089718	Cathelicidin antimicrobial peptide from Alligator mississippiensis has antibacterial activity against multi-drug resistant Acinetobacter baumanii and Klebsiella pneumoniae.
913	DRAMP03573	LL-37(13-37)(C-terminal fragment of LL-37; Human, mammals, animals)	"LL-37"[All Fields] AND biofilm[All Fields]	LL-37	27812400	Antimicrobial activity of Tachyplesin 1 against Burkholderia pseudomallei: an in vitro and in silico approach.
913	DRAMP03573	LL-37(13-37)(C-terminal fragment of LL-37; Human, mammals, animals)	"LL-37"[All Fields] AND biofilm[All Fields]	LL-37	27799768	Core-shell magnetic nanoparticles display synergistic antibacterial effects against Pseudomonas aeruginosaand Staphylococcus aureuswhen combined with cathelicidin LL-37 or selected ceragenins.
913	DRAMP03573	LL-37(13-37)(C-terminal fragment of LL-37; Human, mammals, animals)	"LL-37"[All Fields] AND biofilm[All Fields]	LL-37	27718471	Pyrazole derived ultra-short antimicrobial peptidomimetics with potent anti-biofilm activity.
913	DRAMP03573	LL-37(13-37)(C-terminal fragment of LL-37; Human, mammals, animals)	"LL-37"[All Fields] AND biofilm[All Fields]	LL-37	27659310	Human cathelicidin LL-37 enhance the antibiofilm effect of EGCG on Streptococcus mutans.
913	DRAMP03573	LL-37(13-37)(C-terminal fragment of LL-37; Human, mammals, animals)	"LL-37"[All Fields] AND biofilm[All Fields]	LL-37	27538256	Cytotoxicity and the effect of cationic peptide fragments against cariogenic bacteria under planktonic and biofilm conditions.
913	DRAMP03573	LL-37(13-37)(C-terminal fragment of LL-37; Human, mammals, animals)	"LL-37"[All Fields] AND biofilm[All Fields]	LL-37	27315208	Candidacidal Activity of Selected Ceragenins and Human Cathelicidin LL-37 in Experimental Settings Mimicking Infection Sites.
913	DRAMP03573	LL-37(13-37)(C-terminal fragment of LL-37; Human, mammals, animals)	"LL-37"[All Fields] AND biofilm[All Fields]	LL-37	27114278	Antimicrobial Peptide P60.4Ac-Containing Creams and Gel for Eradication of Methicillin-Resistant Staphylococcus aureus from Cultured Skin and Airway Epithelial Surfaces.
913	DRAMP03573	LL-37(13-37)(C-terminal fragment of LL-37; Human, mammals, animals)	"LL-37"[All Fields] AND biofilm[All Fields]	LL-37	26819677	Anti-Staphylococcal Biofilm Effects of Human Cathelicidin Peptides.
913	DRAMP03573	LL-37(13-37)(C-terminal fragment of LL-37; Human, mammals, animals)	"LL-37"[All Fields] AND biofilm[All Fields]	LL-37	26804311	Electronic cigarette inhalation alters innate immunity and airway cytokines while increasing the virulence of colonizing bacteria.
913	DRAMP03573	LL-37(13-37)(C-terminal fragment of LL-37; Human, mammals, animals)	"LL-37"[All Fields] AND biofilm[All Fields]	LL-37	26656137	Assessing the potential of four cathelicidins for the management of mouse candidiasis and Candida albicans biofilms.
913	DRAMP03573	LL-37(13-37)(C-terminal fragment of LL-37; Human, mammals, animals)	"LL-37"[All Fields] AND biofilm[All Fields]	LL-37	26643338	Immunomodulatory Role of Clarithromycin in Acinetobacter baumannii Infection via Formation of Neutrophil Extracellular Traps.
913	DRAMP03573	LL-37(13-37)(C-terminal fragment of LL-37; Human, mammals, animals)	"LL-37"[All Fields] AND biofilm[All Fields]	LL-37	26434733	Unique features of human cathelicidin LL-37.
913	DRAMP03573	LL-37(13-37)(C-terminal fragment of LL-37; Human, mammals, animals)	"LL-37"[All Fields] AND biofilm[All Fields]	LL-37	26238597	Antifungal activity of cathelicidin peptides against planktonic and biofilm cultures of Candida species isolated from vaginal infections.
913	DRAMP03573	LL-37(13-37)(C-terminal fragment of LL-37; Human, mammals, animals)	"LL-37"[All Fields] AND biofilm[All Fields]	LL-37	26196513	Snake Cathelicidin NA-CATH and Smaller Helical Antimicrobial Peptides Are Effective against Burkholderia thailandensis.
913	DRAMP03573	LL-37(13-37)(C-terminal fragment of LL-37; Human, mammals, animals)	"LL-37"[All Fields] AND biofilm[All Fields]	LL-37	25959370	Neutrophil extracellular trap formation in supragingival biofilms.
913	DRAMP03573	LL-37(13-37)(C-terminal fragment of LL-37; Human, mammals, animals)	"LL-37"[All Fields] AND biofilm[All Fields]	LL-37	25870055	Bactericidal activities of cathelicidin LL-37 and select cationic lipids against the hypervirulent Pseudomonas aeruginosa strain LESB58.
913	DRAMP03573	LL-37(13-37)(C-terminal fragment of LL-37; Human, mammals, animals)	"LL-37"[All Fields] AND biofilm[All Fields]	LL-37	25806720	Antimicrobial peptides in 2014.
913	DRAMP03573	LL-37(13-37)(C-terminal fragment of LL-37; Human, mammals, animals)	"LL-37"[All Fields] AND biofilm[All Fields]	LL-37	25672229	Analysis of mixed biofilm (Staphylococcus aureus and Pseudomonas aeruginosa) by laser ablation electrospray ionization mass spectrometry.
913	DRAMP03573	LL-37(13-37)(C-terminal fragment of LL-37; Human, mammals, animals)	"LL-37"[All Fields] AND biofilm[All Fields]	LL-37	27563687	Using anti-biofilm peptides to treat antibiotic-resistant bacterial infections.
913	DRAMP03573	LL-37(13-37)(C-terminal fragment of LL-37; Human, mammals, animals)	"LL-37"[All Fields] AND biofilm[All Fields]	LL-37	25285879	Inhibition and destruction of Pseudomonas aeruginosa biofilms by antibiotics and antimicrobial peptides.
913	DRAMP03573	LL-37(13-37)(C-terminal fragment of LL-37; Human, mammals, animals)	"LL-37"[All Fields] AND biofilm[All Fields]	LL-37	25212593	Health- and disease-associated species clusters in complex natural biofilms determine the innate immune response in oral epithelial cells during biofilm maturation.
913	DRAMP03573	LL-37(13-37)(C-terminal fragment of LL-37; Human, mammals, animals)	"LL-37"[All Fields] AND biofilm[All Fields]	LL-37	25101632	Antibacterial function of the human cathelicidin-18 peptide (LL-37) between theory and practice.
913	DRAMP03573	LL-37(13-37)(C-terminal fragment of LL-37; Human, mammals, animals)	"LL-37"[All Fields] AND biofilm[All Fields]	LL-37	25061850	Transformation of human cathelicidin LL-37 into selective, stable, and potent antimicrobial compounds.
913	DRAMP03573	LL-37(13-37)(C-terminal fragment of LL-37; Human, mammals, animals)	"LL-37"[All Fields] AND biofilm[All Fields]	LL-37	24982087	Derivatives of the mouse cathelicidin-related antimicrobial peptide (CRAMP) inhibit fungal and bacterial biofilm formation.
913	DRAMP03573	LL-37(13-37)(C-terminal fragment of LL-37; Human, mammals, animals)	"LL-37"[All Fields] AND biofilm[All Fields]	LL-37	24841266	LL-37-derived peptides eradicate multidrug-resistant Staphylococcus aureus from thermally wounded human skin equivalents.
913	DRAMP03573	LL-37(13-37)(C-terminal fragment of LL-37; Human, mammals, animals)	"LL-37"[All Fields] AND biofilm[All Fields]	LL-37	24670464	[Effect of antibacterial peptide LL-37 on the integrity of Acinetobacter baumannii biofilm].
913	DRAMP03573	LL-37(13-37)(C-terminal fragment of LL-37; Human, mammals, animals)	"LL-37"[All Fields] AND biofilm[All Fields]	LL-37	24463069	High-quality 3D structures shine light on antibacterial, anti-biofilm and antiviral activities of human cathelicidin LL-37 and its fragments.
913	DRAMP03573	LL-37(13-37)(C-terminal fragment of LL-37; Human, mammals, animals)	"LL-37"[All Fields] AND biofilm[All Fields]	LL-37	24098113	Role of the Vibrio cholerae matrix protein Bap1 in cross-resistance to antimicrobial peptides.

913	DRAMP03573	LL-37(13-37)(C-terminal fragment of LL-37; Human, mammals, animals)	"LL-37"[All Fields] AND biofilm[All Fields]	LL-37	24096425	Induction of the Cpx envelope stress pathway contributes to Escherichia coli tolerance to antimicrobial peptides.
913	DRAMP03573	LL-37(13-37)(C-terminal fragment of LL-37; Human, mammals, animals)	"LL-37"[All Fields] AND biofilm[All Fields]	LL-37	24071034	The human antimicrobial peptide LL-37 and its fragments possess both antimicrobial and antibiofilm activities against multidrug-resistant Acinetobacter baumannii.
913	DRAMP03573	LL-37(13-37)(C-terminal fragment of LL-37; Human, mammals, animals)	"LL-37"[All Fields] AND biofilm[All Fields]	LL-37	23840194	The Human Cathelicidin Antimicrobial Peptide LL-37 as a Potential Treatment for Polymicrobial Infected Wounds.
913	DRAMP03573	LL-37(13-37)(C-terminal fragment of LL-37; Human, mammals, animals)	"LL-37"[All Fields] AND biofilm[All Fields]	LL-37	23836819	LL-37 opsonizes and inhibits biofilm formation of Aggregatibacter actinomycetemcomitans at subbactericidal concentrations.
913	DRAMP03573	LL-37(13-37)(C-terminal fragment of LL-37; Human, mammals, animals)	"LL-37"[All Fields] AND biofilm[All Fields]	LL-37	23733470	Candida albicans mucin Msb2 is a broad-range protectant against antimicrobial peptides.
913	DRAMP03573	LL-37(13-37)(C-terminal fragment of LL-37; Human, mammals, animals)	"LL-37"[All Fields] AND biofilm[All Fields]	LL-37	23601656	Why does the healthy cornea resist Pseudomonas aeruginosa infection?
913	DRAMP03573	LL-37(13-37)(C-terminal fragment of LL-37; Human, mammals, animals)	"LL-37"[All Fields] AND biofilm[All Fields]	LL-37	22917247	Human cathelicidin LL-37 prevents bacterial biofilm formation.
913	DRAMP03573	LL-37(13-37)(C-terminal fragment of LL-37; Human, mammals, animals)	"LL-37"[All Fields] AND biofilm[All Fields]	LL-37	22908164	Identification of peptides derived from the human antimicrobial peptide LL-37 active against biofilms formed by Pseudomonas aeruginosa using a library of truncated fragments.
913	DRAMP03573	LL-37(13-37)(C-terminal fragment of LL-37; Human, mammals, animals)	"LL-37"[All Fields] AND biofilm[All Fields]	LL-37	22664320	Antimicrobial peptide control of pathogenic microorganisms of the oral cavity: a review of the literature.
913	DRAMP03573	LL-37(13-37)(C-terminal fragment of LL-37; Human, mammals, animals)	"LL-37"[All Fields] AND biofilm[All Fields]	LL-37	22354291	Inhibition of bacterial biofilm formation and swarming motility by a small synthetic cationic peptide.
913	DRAMP03573	LL-37(13-37)(C-terminal fragment of LL-37; Human, mammals, animals)	"LL-37"[All Fields] AND biofilm[All Fields]	LL-37	22142958	Antimicrobial peptides in periodontal innate defense.
913	DRAMP03573	LL-37(13-37)(C-terminal fragment of LL-37; Human, mammals, animals)	"LL-37"[All Fields] AND biofilm[All Fields]	LL-37	22115597	An investigation on the antibacterial, cytotoxic, and antibiofilm efficacy of starch-stabilized silver nanoparticles.
913	DRAMP03573	LL-37(13-37)(C-terminal fragment of LL-37; Human, mammals, animals)	"LL-37"[All Fields] AND biofilm[All Fields]	LL-37	22005071	Antimicrobial and antibiofilm activity of LL-37 and its truncated variants against Burkholderia pseudomallei.
913	DRAMP03573	LL-37(13-37)(C-terminal fragment of LL-37; Human, mammals, animals)	"LL-37"[All Fields] AND biofilm[All Fields]	LL-37	21875967	Elicitation of epithelial cell-derived immune effectors by outer membrane vesicles of nontypeable Haemophilus influenzae.
913	DRAMP03573	LL-37(13-37)(C-terminal fragment of LL-37; Human, mammals, animals)	"LL-37"[All Fields] AND biofilm[All Fields]	LL-37	21849157	Antibacterial and anti-biofilm effects of cathelicidin peptides against pathogens isolated from cystic fibrosis patients.
913	DRAMP03573	LL-37(13-37)(C-terminal fragment of LL-37; Human, mammals, animals)	"LL-37"[All Fields] AND biofilm[All Fields]	LL-37	21772832	Susceptibility of Pseudomonas aeruginosa Biofilm to Alpha-Helical Peptides: D-enantiomer of LL-37.
913	DRAMP03573	LL-37(13-37)(C-terminal fragment of LL-37; Human, mammals, animals)	"LL-37"[All Fields] AND biofilm[All Fields]	LL-37	21692631	A novel organotypic dento-epithelial culture model: effect of Fusobacterium nucleatum biofilm on B-defensin-2, -3, and LL-37 expression.
913	DRAMP03573	LL-37(13-37)(C-terminal fragment of LL-37; Human, mammals, animals)	"LL-37"[All Fields] AND biofilm[All Fields]	LL-37	21605457	Natural and synthetic cathelicidin peptides with anti-microbial and anti-biofilm activity against Staphylococcus aureus.
913	DRAMP03573	LL-37(13-37)(C-terminal fragment of LL-37; Human, mammals, animals)	"LL-37"[All Fields] AND biofilm[All Fields]	LL-37	21220789	Fusobacterium nucleatum in periodontal health and disease.
913	DRAMP03573	LL-37(13-37)(C-terminal fragment of LL-37; Human, mammals, animals)	"LL-37"[All Fields] AND biofilm[All Fields]	LL-37	20961363	Potential of ceragenin CSA-13 and its mixture with pluronic F-127 as treatment of topical bacterial infections.
913	DRAMP03573	LL-37(13-37)(C-terminal fragment of LL-37; Human, mammals, animals)	"LL-37"[All Fields] AND biofilm[All Fields]	LL-37	20661475	Uropathogenic Escherichia coli modulates immune responses and its curli fimbriae interact with the antimicrobial peptide LL-37.
913	DRAMP03573	LL-37(13-37)(C-terminal fragment of LL-37; Human, mammals, animals)	"LL-37"[All Fields] AND biofilm[All Fields]	LL-37	20399752	Antimicrobial and antibiofilm activity of cathelicidins and short, synthetic peptides against Francisella.
913	DRAMP03573	LL-37(13-37)(C-terminal fragment of LL-37; Human, mammals, animals)	"LL-37"[All Fields] AND biofilm[All Fields]	LL-37	20139192	Effects of sequential Campylobacter jejuni 81-176 lipooligosaccharide core truncations on biofilm formation, stress survival, and pathogenesis.
913	DRAMP03573	LL-37(13-37)(C-terminal fragment of LL-37; Human, mammals, animals)	"LL-37"[All Fields] AND biofilm[All Fields]	LL-37	19847021	Nanolayer biofilm coated on magnetic nanoparticles by using a dielectric barrier discharge glow plasma fluidized bed for immobilizing an antimicrobial peptide.
913	DRAMP03573	LL-37(13-37)(C-terminal fragment of LL-37; Human, mammals, animals)	"LL-37"[All Fields] AND biofilm[All Fields]	LL-37	19466693	Activity of antimicrobial peptides in the presence of polysaccharides produced by pulmonary pathogens.
913	DRAMP03573	LL-37(13-37)(C-terminal fragment of LL-37; Human, mammals, animals)	"LL-37"[All Fields] AND biofilm[All Fields]	LL-37	19379612	Effects of an LL-37-derived antimicrobial peptide in an animal model of biofilm Pseudomonas sinusitis.
913	DRAMP03573	LL-37(13-37)(C-terminal fragment of LL-37; Human, mammals, animals)	"LL-37"[All Fields] AND biofilm[All Fields]	LL-37	18591225	Human host defense peptide LL-37 prevents bacterial biofilm formation.
913	DRAMP03573	LL-37(13-37)(C-terminal fragment of LL-37; Human, mammals, animals)	"LL-37"[All Fields] AND biofilm[All Fields]	LL-37	16687459	In vitro assessment of antimicrobial peptides as potential agents against several oral bacteria.
913	DRAMP03573	LL-37(13-37)(C-terminal fragment of LL-37; Human, mammals, animals)	"LL-37"[All Fields] AND biofilm[All Fields]	LL-37	14764110	Polysaccharide intercellular adhesin (PIA) protects Staphylococcus epidermidis against major components of the human innate immune system.

914	DRAMP03574	LL-37(17-32)(C-terminal fragment of LL-37; Human, mammals, animals)	"LL-37"[All Fields] AND biofilm[All Fields]	LL-37	34454095	Targeting antibiotic tolerance in anaerobic biofilms associated with oral diseases: Human antimicrobial peptides LL-37 and lactoferricin enhance the antibiotic efficacy of amoxicillin, clindamycin and metronidazole.
914	DRAMP03574	LL-37(17-32)(C-terminal fragment of LL-37; Human, mammals, animals)	"LL-37"[All Fields] AND biofilm[All Fields]	LL-37	34402021	Cytocompatibility and Synergy of EGCG and Cationic Peptides Against Bacteria Related to Endodontic Infections, in Planktonic and Biofilm Conditions.
914	DRAMP03574	LL-37(17-32)(C-terminal fragment of LL-37; Human, mammals, animals)	"LL-37"[All Fields] AND biofilm[All Fields]	LL-37	34072318	The Potential of Human Peptide LL-37 as an Antimicrobial and Anti-Biofilm Agent.
914	DRAMP03574	LL-37(17-32)(C-terminal fragment of LL-37; Human, mammals, animals)	"LL-37"[All Fields] AND biofilm[All Fields]	LL-37	33890759	Short and Robust Anti-Infective Lipopeptides Engineered Based on the Minimal Antimicrobial Peptide KR12 of Human LL-37.
914	DRAMP03574	LL-37(17-32)(C-terminal fragment of LL-37; Human, mammals, animals)	"LL-37"[All Fields] AND biofilm[All Fields]	LL-37	33885339	The Multifunctional Roles of Short Palate, Lung, and Nasal Epithelium Clone 1 in Regulating Airway Surface Liquid and Participating in Airway Host Defense.
914	DRAMP03574	LL-37(17-32)(C-terminal fragment of LL-37; Human, mammals, animals)	"LL-37"[All Fields] AND biofilm[All Fields]	LL-37	33797775	Toll-like receptor activation of equine mesenchymal stromal cells to enhance antibacterial activity and immunomodulatory cytokine secretion.
914	DRAMP03574	LL-37(17-32)(C-terminal fragment of LL-37; Human, mammals, animals)	"LL-37"[All Fields] AND biofilm[All Fields]	LL-37	33724786	Membrane Interactions of Virus-like Mesoporous Silica Nanoparticles.
914	DRAMP03574	LL-37(17-32)(C-terminal fragment of LL-37; Human, mammals, animals)	"LL-37"[All Fields] AND biofilm[All Fields]	LL-37	33687301	Exogenous LL-37 but not homogenates of desquamated oral epithelial cells shows activity against Streptococcus mutans.
914	DRAMP03574	LL-37(17-32)(C-terminal fragment of LL-37; Human, mammals, animals)	"LL-37"[All Fields] AND biofilm[All Fields]	LL-37	33646013	Synergistic effect of antimicrobial peptide LL-37 and colistin combination against multidrug-resistant Escherichia coliisolates.
914	DRAMP03574	LL-37(17-32)(C-terminal fragment of LL-37; Human, mammals, animals)	"LL-37"[All Fields] AND biofilm[All Fields]	LL-37	33584628	SAAP-148 Eradicates MRSA Persisters Within Mature Biofilm Models Simulating Prosthetic Joint Infection.
914	DRAMP03574	LL-37(17-32)(C-terminal fragment of LL-37; Human, mammals, animals)	"LL-37"[All Fields] AND biofilm[All Fields]	LL-37	33326455	D-LL-31 enhances biofilm-eradicating effect of currently used antibiotics for chronic rhinosinusitis and its immunomodulatory activity on human lung epithelial cells.
914	DRAMP03574	LL-37(17-32)(C-terminal fragment of LL-37; Human, mammals, animals)	"LL-37"[All Fields] AND biofilm[All Fields]	LL-37	33252326	Effect of antifungal agents, lysozyme and human antimicrobial peptide LL-37 on clinical Candidaisolates with high biofilm production.
914	DRAMP03574	LL-37(17-32)(C-terminal fragment of LL-37; Human, mammals, animals)	"LL-37"[All Fields] AND biofilm[All Fields]	LL-37	33249255	Inhibitory effect of LL-37 and human lactoferricin on growth and biofilm formation of anaerobes associated with oral diseases.
914	DRAMP03574	LL-37(17-32)(C-terminal fragment of LL-37; Human, mammals, animals)	"LL-37"[All Fields] AND biofilm[All Fields]	LL-37	32768482	Development of anti-bacterial surfaces using a hydrophobin chimeric protein.
914	DRAMP03574	LL-37(17-32)(C-terminal fragment of LL-37; Human, mammals, animals)	"LL-37"[All Fields] AND biofilm[All Fields]	LL-37	32725811	Comparison of Antibacterial Effect of Cationic Peptide LL-37 and Cefalexin on Clinical Staphylococcus aureus-induced Infection after Femur Fracture Fixation.
914	DRAMP03574	LL-37(17-32)(C-terminal fragment of LL-37; Human, mammals, animals)	"LL-37"[All Fields] AND biofilm[All Fields]	LL-37	32523805	Characterization of biofilm production in different strains of Acinetobacter baumanniiand the effects of chemical compounds on biofilm formation.
914	DRAMP03574	LL-37(17-32)(C-terminal fragment of LL-37; Human, mammals, animals)	"LL-37"[All Fields] AND biofilm[All Fields]	LL-37	32518218	Can Salivary Biomarkers Be Used as Predictors of Dental Caries in Young Adolescents?
914	DRAMP03574	LL-37(17-32)(C-terminal fragment of LL-37; Human, mammals, animals)	"LL-37"[All Fields] AND biofilm[All Fields]	LL-37	32343547	Resistome of Staphylococcus aureusin Response to Human Cathelicidin LL-37 and Its Engineered Antimicrobial Peptides.
914	DRAMP03574	LL-37(17-32)(C-terminal fragment of LL-37; Human, mammals, animals)	"LL-37"[All Fields] AND biofilm[All Fields]	LL-37	32232010	Francisella novicidaTwo-Component System Response Regulator BfpR Modulates iglC Gene Expression, Antimicrobial Peptide Resistance, and Biofilm Production.
914	DRAMP03574	LL-37(17-32)(C-terminal fragment of LL-37; Human, mammals, animals)	"LL-37"[All Fields] AND biofilm[All Fields]	LL-37	32203127	Antibiofilm activities of ceragenins and antimicrobial peptides against fungal-bacterial mono and multispecies biofilms.
914	DRAMP03574	LL-37(17-32)(C-terminal fragment of LL-37; Human, mammals, animals)	"LL-37"[All Fields] AND biofilm[All Fields]	LL-37	32182913	Differential Abilities of Mammalian Cathelicidins to Inhibit Bacterial Biofilm Formation and Promote Multifaceted Immune Functions of Neutrophils.
914	DRAMP03574	LL-37(17-32)(C-terminal fragment of LL-37; Human, mammals, animals)	"LL-37"[All Fields] AND biofilm[All Fields]	LL-37	32019109	Lipidated Analogs of the LL-37-Derived Peptide Fragment KR12-Structural Analysis, Surface-Active Properties and Antimicrobial Activity.
914	DRAMP03574	LL-37(17-32)(C-terminal fragment of LL-37; Human, mammals, animals)	"LL-37"[All Fields] AND biofilm[All Fields]	LL-37	31993042	Linezolid and Rifampicin Combination to Combat cfr-Positive Multidrug-Resistant MRSA in Murine Models of Bacteremia and Skin and Skin Structure Infection.
914	DRAMP03574	LL-37(17-32)(C-terminal fragment of LL-37; Human, mammals, animals)	"LL-37"[All Fields] AND biofilm[All Fields]	LL-37	31396193	Evaluation of the Antimicrobial Peptide, RP557, for the Broad-Spectrum Treatment of Wound Pathogens and Biofilm.
914	DRAMP03574	LL-37(17-32)(C-terminal fragment of LL-37; Human, mammals, animals)	"LL-37"[All Fields] AND biofilm[All Fields]	LL-37	31356860	Eradication of meticillin-resistant Staphylococcus aureus from human skin by the novel LL-37-derived peptide P10 in four pharmaceutical ointments.
914	DRAMP03574	LL-37(17-32)(C-terminal fragment of LL-37; Human, mammals, animals)	"LL-37"[All Fields] AND biofilm[All Fields]	LL-37	31329599	Synergistic effects of LFchimera and antibiotic against planktonic and biofilm form of Aggregatibacter actinomycetemcomitans.
914	DRAMP03574	LL-37(17-32)(C-terminal fragment of LL-37; Human, mammals, animals)	"LL-37"[All Fields] AND biofilm[All Fields]	LL-37	31319057	Modulation of antimicrobial potency of human cathelicidin peptides against the ESKAPE pathogens and in vivo efficacy in a murine catheter-associated biofilm model.
914	DRAMP03574	LL-37(17-32)(C-terminal fragment of LL-37; Human, mammals, animals)	"LL-37"[All Fields] AND biofilm[All Fields]	LL-37	31282211	D-LL-31 in combination with ceftazidime synergistically enhances bactericidal activity and biofilm destruction in Burkholderia pseudomallei.
914	DRAMP03574	LL-37(17-32)(C-terminal fragment of LL-37; Human, mammals, animals)	"LL-37"[All Fields] AND biofilm[All Fields]	LL-37	31170191	Antimicrobial peptide LL-37 is bactericidal against Staphylococcus aureus biofilms.

914	DRAMP03574	LL-37(17-32)(C-terminal fragment of LL-37; Human, mammals, animals)	"LL-37"[All Fields] AND biofilm[All Fields]	LL-37	31118709	Design and characterization of a new hybrid peptide from LL-37 and BMAP-27.
914	DRAMP03574	LL-37(17-32)(C-terminal fragment of LL-37; Human, mammals, animals)	"LL-37"[All Fields] AND biofilm[All Fields]	LL-37	31046689	Use of ceragenins as a potential treatment for urinary tract infections.
914	DRAMP03574	LL-37(17-32)(C-terminal fragment of LL-37; Human, mammals, animals)	"LL-37"[All Fields] AND biofilm[All Fields]	LL-37	30755738	In vitro activities of antimicrobial peptides and ceragenins against Legionella pneumophila.
914	DRAMP03574	LL-37(17-32)(C-terminal fragment of LL-37; Human, mammals, animals)	"LL-37"[All Fields] AND biofilm[All Fields]	LL-37	30728810	Role of Two-Component System Response Regulator bceRin the Antimicrobial Resistance, Virulence, Biofilm Formation, and Stress Response of Group B Streptococcus.
914	DRAMP03574	LL-37(17-32)(C-terminal fragment of LL-37; Human, mammals, animals)	"LL-37"[All Fields] AND biofilm[All Fields]	LL-37	30635992	Design and antimicrobial activities of LL-37 derivatives inhibiting the formation of Streptococcus mutans biofilm.
914	DRAMP03574	LL-37(17-32)(C-terminal fragment of LL-37; Human, mammals, animals)	"LL-37"[All Fields] AND biofilm[All Fields]	LL-37	30465823	Antimicrobial synergy of monolaurin lipid nanocapsules with adsorbed antimicrobial peptides against Staphylococcus aureus biofilms in vitro is absent in vivo.
914	DRAMP03574	LL-37(17-32)(C-terminal fragment of LL-37; Human, mammals, animals)	"LL-37"[All Fields] AND biofilm[All Fields]	LL-37	30375445	Multi-species oral biofilm promotes reconstructed human gingiva epithelial barrier function.
914	DRAMP03574	LL-37(17-32)(C-terminal fragment of LL-37; Human, mammals, animals)	"LL-37"[All Fields] AND biofilm[All Fields]	LL-37	30315918	Synergistic microbicidal effect of cationic antimicrobial peptides and teicoplanin against planktonic and biofilm-encased Staphylococcus aureus.
914	DRAMP03574	LL-37(17-32)(C-terminal fragment of LL-37; Human, mammals, animals)	"LL-37"[All Fields] AND biofilm[All Fields]	LL-37	30250480	Role of Microbes in the Development of Alzheimer's Disease: State of the Art - An International Symposium Presented at the 2017 IAGG Congress in San Francisco.
914	DRAMP03574	LL-37(17-32)(C-terminal fragment of LL-37; Human, mammals, animals)	"LL-37"[All Fields] AND biofilm[All Fields]	LL-37	30131362	Discovery of New Genes Involved in Curli Production by a Uropathogenic Escherichia coli Strain from the Highly Virulent O45:K1:H7 Lineage.
914	DRAMP03574	LL-37(17-32)(C-terminal fragment of LL-37; Human, mammals, animals)	"LL-37"[All Fields] AND biofilm[All Fields]	LL-37	30120393	Evaluation of LL-37 antimicrobial peptide derivatives alone and in combination with vancomycin against S. aureus.
914	DRAMP03574	LL-37(17-32)(C-terminal fragment of LL-37; Human, mammals, animals)	"LL-37"[All Fields] AND biofilm[All Fields]	LL-37	30088919	Tackling Pseudomonas aeruginosa Virulence by a Hydroxamic Acid-Based LasB Inhibitor.
914	DRAMP03574	LL-37(17-32)(C-terminal fragment of LL-37; Human, mammals, animals)	"LL-37"[All Fields] AND biofilm[All Fields]	LL-37	30043322	Antimicrobial Activity of Selected Antimicrobial Peptides Against Planktonic Culture and Biofilm of Acinetobacter baumannii.
914	DRAMP03574	LL-37(17-32)(C-terminal fragment of LL-37; Human, mammals, animals)	"LL-37"[All Fields] AND biofilm[All Fields]	LL-37	29959905	Antimicrobial and anti-inflammatory activities of chemokine CXCL14-derived antimicrobial peptide and its analogs.
914	DRAMP03574	LL-37(17-32)(C-terminal fragment of LL-37; Human, mammals, animals)	"LL-37"[All Fields] AND biofilm[All Fields]	LL-37	29737589	LL-37 fragments have antimicrobial activity against Staphylococcus epidermidis biofilms and wound healing potential in HaCaT cell line.
914	DRAMP03574	LL-37(17-32)(C-terminal fragment of LL-37; Human, mammals, animals)	"LL-37"[All Fields] AND biofilm[All Fields]	LL-37	29671721	The BceABRS four-component system that is essential for cell envelope stress response is involved in sensing and response to host defence peptides and is required for the biofilm formation and fitness of Streptococcus mutans.
914	DRAMP03574	LL-37(17-32)(C-terminal fragment of LL-37; Human, mammals, animals)	"LL-37"[All Fields] AND biofilm[All Fields]	LL-37	29394295	Pseudomonas aeruginosa rugose small-colony variants evade host clearance, are hyper-inflammatory, and persist in multiple host environments.
914	DRAMP03574	LL-37(17-32)(C-terminal fragment of LL-37; Human, mammals, animals)	"LL-37"[All Fields] AND biofilm[All Fields]	LL-37	29391814	Targeting polyelectrolyte networks in purulent body fluids to modulate bactericidal properties of some antibiotics.
914	DRAMP03574	LL-37(17-32)(C-terminal fragment of LL-37; Human, mammals, animals)	"LL-37"[All Fields] AND biofilm[All Fields]	LL-37	29372443	Sensitivity of caries pathogens to antimicrobial peptides related to caries risk.
914	DRAMP03574	LL-37(17-32)(C-terminal fragment of LL-37; Human, mammals, animals)	"LL-37"[All Fields] AND biofilm[All Fields]	LL-37	29370365	The effects of antimicrobial peptides WAM-1 and LL-37 on multidrug-resistant Acinetobacter baumannii.
914	DRAMP03574	LL-37(17-32)(C-terminal fragment of LL-37; Human, mammals, animals)	"LL-37"[All Fields] AND biofilm[All Fields]	LL-37	29321257	The antimicrobial peptide SAAP-148 combats drug-resistant bacteria and biofilms.
914	DRAMP03574	LL-37(17-32)(C-terminal fragment of LL-37; Human, mammals, animals)	"LL-37"[All Fields] AND biofilm[All Fields]	LL-37	29022391	KR-12-a5 is a non-cytotoxic agent with potent antimicrobial effects against oral pathogens.
914	DRAMP03574	LL-37(17-32)(C-terminal fragment of LL-37; Human, mammals, animals)	"LL-37"[All Fields] AND biofilm[All Fields]	LL-37	28987032	Functionalized PVA-silk blended nanofibrous mats promote diabetic wound healing via regulation of extracellular matrix and tissue remodelling.
914	DRAMP03574	LL-37(17-32)(C-terminal fragment of LL-37; Human, mammals, animals)	"LL-37"[All Fields] AND biofilm[All Fields]	LL-37	28919413	Apolipoprotein A-I attenuates LL-37-induced endothelial cell cytotoxicity.
914	DRAMP03574	LL-37(17-32)(C-terminal fragment of LL-37; Human, mammals, animals)	"LL-37"[All Fields] AND biofilm[All Fields]	LL-37	28890511	The Antibacterial Effects of Antimicrobial Peptides OP-145 against Clinically Isolated Multi-Resistant Strains.
914	DRAMP03574	LL-37(17-32)(C-terminal fragment of LL-37; Human, mammals, animals)	"LL-37"[All Fields] AND biofilm[All Fields]	LL-37	28890436	Primary ciliary dyskinesia ciliated airway cells show increased susceptibility to Haemophilus influenzaebiofilm formation.
914	DRAMP03574	LL-37(17-32)(C-terminal fragment of LL-37; Human, mammals, animals)	"LL-37"[All Fields] AND biofilm[All Fields]	LL-37	28821865	Identification of bacterial biofilm and the Staphylococcus aureus derived protease, staphopain, on the skin surface of patients with atopic dermatitis.
914	DRAMP03574	LL-37(17-32)(C-terminal fragment of LL-37; Human, mammals, animals)	"LL-37"[All Fields] AND biofilm[All Fields]	LL-37	28747178	Anaerobic bacteria growth in the presence of cathelicidin LL-37 and selected ceragenins delivered as magnetic nanoparticles cargo.
914	DRAMP03574	LL-37(17-32)(C-terminal fragment of LL-37; Human, mammals, animals)	"LL-37"[All Fields] AND biofilm[All Fields]	LL-37	28680809	Antimicrobial ceragenins inhibit biofilms and affect mammalian cell viability and migration in vitro.

914	DRAMP03574	LL-37(17-32)(C-terminal fragment of LL-37; Human, mammals, animals)	"LL-37"[All Fields] AND biofilm[All Fields]	LL-37	28676673	Formulation and candidacidal activity of magnetic nanoparticles coated with cathelicidin LL-37 and ceragenin CSA-13.
914	DRAMP03574	LL-37(17-32)(C-terminal fragment of LL-37; Human, mammals, animals)	"LL-37"[All Fields] AND biofilm[All Fields]	LL-37	28675109	Titanium surfaces immobilized with the major antimicrobial fragment FK-16 of human cathelicidin LL-37 are potent against multiple antibiotic-resistant bacteria.
914	DRAMP03574	LL-37(17-32)(C-terminal fragment of LL-37; Human, mammals, animals)	"LL-37"[All Fields] AND biofilm[All Fields]	LL-37	28672834	Individual and Combined Effects of Engineered Peptides and Antibiotics on Pseudomonas aeruginosa Biofilms.
914	DRAMP03574	LL-37(17-32)(C-terminal fragment of LL-37; Human, mammals, animals)	"LL-37"[All Fields] AND biofilm[All Fields]	LL-37	28525841	LL-37-derived short antimicrobial peptide KR-12-a5 and its d-amino acid substituted analogs with cell selectivity, anti-biofilm activity, synergistic effect with conventional antibiotics, and anti-inflammatory activity.
914	DRAMP03574	LL-37(17-32)(C-terminal fragment of LL-37; Human, mammals, animals)	"LL-37"[All Fields] AND biofilm[All Fields]	LL-37	28408902	The Naturally Occurring Host Defense Peptide, LL-37, and Its Truncated Mimetics KE-18 and KR-12 Have Selected Biocidal and Antibiofilm Activities Against Candida albicans, Staphylococcus aureus, and Escherichia coli In vitro.
914	DRAMP03574	LL-37(17-32)(C-terminal fragment of LL-37; Human, mammals, animals)	"LL-37"[All Fields] AND biofilm[All Fields]	LL-37	28178190	High Specific Selectivity and Membrane-Active Mechanism of Synthetic Cationic Hybrid Antimicrobial Peptides Based on the Peptide FV7.
914	DRAMP03574	LL-37(17-32)(C-terminal fragment of LL-37; Human, mammals, animals)	"LL-37"[All Fields] AND biofilm[All Fields]	LL-37	28161291	LL-37-derived membrane-active FK-13 analogs possessing cell selectivity, anti-biofilm activity and synergy with chloramphenicol and anti-inflammatory activity.
914	DRAMP03574	LL-37(17-32)(C-terminal fragment of LL-37; Human, mammals, animals)	"LL-37"[All Fields] AND biofilm[All Fields]	LL-37	28089718	Cathelicidin antimicrobial peptide from Alligator mississippiensis has antibacterial activity against multi-drug resistant Acinetobacter baumanii and Klebsiella pneumoniae.
914	DRAMP03574	LL-37(17-32)(C-terminal fragment of LL-37; Human, mammals, animals)	"LL-37"[All Fields] AND biofilm[All Fields]	LL-37	27812400	Antimicrobial activity of Tachyplesin 1 against Burkholderia pseudomallei: an in vitro and in silico approach.
914	DRAMP03574	LL-37(17-32)(C-terminal fragment of LL-37; Human, mammals, animals)	"LL-37"[All Fields] AND biofilm[All Fields]	LL-37	27799768	Core-shell magnetic nanoparticles display synergistic antibacterial effects against Pseudomonas aeruginosaand Staphylococcus aureuswhen combined with cathelicidin LL-37 or selected ceragenins.
914	DRAMP03574	LL-37(17-32)(C-terminal fragment of LL-37; Human, mammals, animals)	"LL-37"[All Fields] AND biofilm[All Fields]	LL-37	27718471	Pyrazole derived ultra-short antimicrobial peptidomimetics with potent anti-biofilm activity.
914	DRAMP03574	LL-37(17-32)(C-terminal fragment of LL-37; Human, mammals, animals)	"LL-37"[All Fields] AND biofilm[All Fields]	LL-37	27659310	Human cathelicidin LL-37 enhance the antibiofilm effect of EGCG on Streptococcus mutans.
914	DRAMP03574	LL-37(17-32)(C-terminal fragment of LL-37; Human, mammals, animals)	"LL-37"[All Fields] AND biofilm[All Fields]	LL-37	27538256	Cytotoxicity and the effect of cationic peptide fragments against cariogenic bacteria under planktonic and biofilm conditions.
914	DRAMP03574	LL-37(17-32)(C-terminal fragment of LL-37; Human, mammals, animals)	"LL-37"[All Fields] AND biofilm[All Fields]	LL-37	27315208	Candidacidal Activity of Selected Ceragenins and Human Cathelicidin LL-37 in Experimental Settings Mimicking Infection Sites.
914	DRAMP03574	LL-37(17-32)(C-terminal fragment of LL-37; Human, mammals, animals)	"LL-37"[All Fields] AND biofilm[All Fields]	LL-37	27114278	Antimicrobial Peptide P60.4Ac-Containing Creams and Gel for Eradication of Methicillin-Resistant Staphylococcus aureus from Cultured Skin and Airway Epithelial Surfaces.
914	DRAMP03574	LL-37(17-32)(C-terminal fragment of LL-37; Human, mammals, animals)	"LL-37"[All Fields] AND biofilm[All Fields]	LL-37	26819677	Anti-Staphylococcal Biofilm Effects of Human Cathelicidin Peptides.
914	DRAMP03574	LL-37(17-32)(C-terminal fragment of LL-37; Human, mammals, animals)	"LL-37"[All Fields] AND biofilm[All Fields]	LL-37	26804311	Electronic cigarette inhalation alters innate immunity and airway cytokines while increasing the virulence of colonizing bacteria.
914	DRAMP03574	LL-37(17-32)(C-terminal fragment of LL-37; Human, mammals, animals)	"LL-37"[All Fields] AND biofilm[All Fields]	LL-37	26656137	Assessing the potential of four cathelicidins for the management of mouse candidiasis and Candida albicans biofilms.
914	DRAMP03574	LL-37(17-32)(C-terminal fragment of LL-37; Human, mammals, animals)	"LL-37"[All Fields] AND biofilm[All Fields]	LL-37	26643338	Immunomodulatory Role of Clarithromycin in Acinetobacter baumannii Infection via Formation of Neutrophil Extracellular Traps.
914	DRAMP03574	LL-37(17-32)(C-terminal fragment of LL-37; Human, mammals, animals)	"LL-37"[All Fields] AND biofilm[All Fields]	LL-37	26434733	Unique features of human cathelicidin LL-37.
914	DRAMP03574	LL-37(17-32)(C-terminal fragment of LL-37; Human, mammals, animals)	"LL-37"[All Fields] AND biofilm[All Fields]	LL-37	26238597	Antifungal activity of cathelicidin peptides against planktonic and biofilm cultures of Candida species isolated from vaginal infections.
914	DRAMP03574	LL-37(17-32)(C-terminal fragment of LL-37; Human, mammals, animals)	"LL-37"[All Fields] AND biofilm[All Fields]	LL-37	26196513	Snake Cathelicidin NA-CATH and Smaller Helical Antimicrobial Peptides Are Effective against Burkholderia thailandensis.
914	DRAMP03574	LL-37(17-32)(C-terminal fragment of LL-37; Human, mammals, animals)	"LL-37"[All Fields] AND biofilm[All Fields]	LL-37	25959370	Neutrophil extracellular trap formation in supragingival biofilms.
914	DRAMP03574	LL-37(17-32)(C-terminal fragment of LL-37; Human, mammals, animals)	"LL-37"[All Fields] AND biofilm[All Fields]	LL-37	25870055	Bactericidal activities of cathelicidin LL-37 and select cationic lipids against the hypervirulent Pseudomonas aeruginosa strain LESB58.
914	DRAMP03574	LL-37(17-32)(C-terminal fragment of LL-37; Human, mammals, animals)	"LL-37"[All Fields] AND biofilm[All Fields]	LL-37	25806720	Antimicrobial peptides in 2014.
914	DRAMP03574	LL-37(17-32)(C-terminal fragment of LL-37; Human, mammals, animals)	"LL-37"[All Fields] AND biofilm[All Fields]	LL-37	25672229	Analysis of mixed biofilm (Staphylococcus aureus and Pseudomonas aeruginosa) by laser ablation electrospray ionization mass spectrometry.
914	DRAMP03574	LL-37(17-32)(C-terminal fragment of LL-37; Human, mammals, animals)	"LL-37"[All Fields] AND biofilm[All Fields]	LL-37	27563687	Using anti-biofilm peptides to treat antibiotic-resistant bacterial infections.
914	DRAMP03574	LL-37(17-32)(C-terminal fragment of LL-37; Human, mammals, animals)	"LL-37"[All Fields] AND biofilm[All Fields]	LL-37	25285879	Inhibition and destruction of Pseudomonas aeruginosa biofilms by antibiotics and antimicrobial peptides.
914	DRAMP03574	LL-37(17-32)(C-terminal fragment of LL-37; Human, mammals, animals)	"LL-37"[All Fields] AND biofilm[All Fields]	LL-37	25212593	Health- and disease-associated species clusters in complex natural biofilms determine the innate immune response in oral epithelial cells during biofilm maturation.

914	DRAMP03574	LL-37(17-32)(C-terminal fragment of LL-37; Human, mammals, animals)	"LL-37"[All Fields] AND biofilm[All Fields]	LL-37	25101632	Antibacterial function of the human cathelicidin-18 peptide (LL-37) between theory and practice.
914	DRAMP03574	LL-37(17-32)(C-terminal fragment of LL-37; Human, mammals, animals)	"LL-37"[All Fields] AND biofilm[All Fields]	LL-37	25061850	Transformation of human cathelicidin LL-37 into selective, stable, and potent antimicrobial compounds.
914	DRAMP03574	LL-37(17-32)(C-terminal fragment of LL-37; Human, mammals, animals)	"LL-37"[All Fields] AND biofilm[All Fields]	LL-37	24982087	Derivatives of the mouse cathelicidin-related antimicrobial peptide (CRAMP) inhibit fungal and bacterial biofilm formation.
914	DRAMP03574	LL-37(17-32)(C-terminal fragment of LL-37; Human, mammals, animals)	"LL-37"[All Fields] AND biofilm[All Fields]	LL-37	24841266	LL-37-derived peptides eradicate multidrug-resistant Staphylococcus aureus from thermally wounded human skin equivalents.
914	DRAMP03574	LL-37(17-32)(C-terminal fragment of LL-37; Human, mammals, animals)	"LL-37"[All Fields] AND biofilm[All Fields]	LL-37	24670464	[Effect of antibacterial peptide LL-37 on the integrity of Acinetobacter baumannii biofilm].
914	DRAMP03574	LL-37(17-32)(C-terminal fragment of LL-37; Human, mammals, animals)	"LL-37"[All Fields] AND biofilm[All Fields]	LL-37	24463069	High-quality 3D structures shine light on antibacterial, anti-biofilm and antiviral activities of human cathelicidin LL-37 and its fragments.
914	DRAMP03574	LL-37(17-32)(C-terminal fragment of LL-37; Human, mammals, animals)	"LL-37"[All Fields] AND biofilm[All Fields]	LL-37	24098113	Role of the Vibrio cholerae matrix protein Bap1 in cross-resistance to antimicrobial peptides.
914	DRAMP03574	LL-37(17-32)(C-terminal fragment of LL-37; Human, mammals, animals)	"LL-37"[All Fields] AND biofilm[All Fields]	LL-37	24096425	Induction of the Cpx envelope stress pathway contributes to Escherichia coli tolerance to antimicrobial peptides.
914	DRAMP03574	LL-37(17-32)(C-terminal fragment of LL-37; Human, mammals, animals)	"LL-37"[All Fields] AND biofilm[All Fields]	LL-37	24071034	The human antimicrobial peptide LL-37 and its fragments possess both antimicrobial and antibiofilm activities against multidrug-resistant Acinetobacter baumannii.
914	DRAMP03574	LL-37(17-32)(C-terminal fragment of LL-37; Human, mammals, animals)	"LL-37"[All Fields] AND biofilm[All Fields]	LL-37	23840194	The Human Cathelicidin Antimicrobial Peptide LL-37 as a Potential Treatment for Polymicrobial Infected Wounds.
914	DRAMP03574	LL-37(17-32)(C-terminal fragment of LL-37; Human, mammals, animals)	"LL-37"[All Fields] AND biofilm[All Fields]	LL-37	23836819	LL-37 opsonizes and inhibits biofilm formation of Aggregatibacter actinomycetemcomitans at subbactericidal concentrations.
914	DRAMP03574	LL-37(17-32)(C-terminal fragment of LL-37; Human, mammals, animals)	"LL-37"[All Fields] AND biofilm[All Fields]	LL-37	23733470	Candida albicans mucin Msb2 is a broad-range protectant against antimicrobial peptides.
914	DRAMP03574	LL-37(17-32)(C-terminal fragment of LL-37; Human, mammals, animals)	"LL-37"[All Fields] AND biofilm[All Fields]	LL-37	23601656	Why does the healthy cornea resist Pseudomonas aeruginosa infection?
914	DRAMP03574	LL-37(17-32)(C-terminal fragment of LL-37; Human, mammals, animals)	"LL-37"[All Fields] AND biofilm[All Fields]	LL-37	22917247	Human cathelicidin LL-37 prevents bacterial biofilm formation.
914	DRAMP03574	LL-37(17-32)(C-terminal fragment of LL-37; Human, mammals, animals)	"LL-37"[All Fields] AND biofilm[All Fields]	LL-37	22908164	Identification of peptides derived from the human antimicrobial peptide LL-37 active against biofilms formed by Pseudomonas aeruginosa using a library of truncated fragments.
914	DRAMP03574	LL-37(17-32)(C-terminal fragment of LL-37; Human, mammals, animals)	"LL-37"[All Fields] AND biofilm[All Fields]	LL-37	22664320	Antimicrobial peptide control of pathogenic microorganisms of the oral cavity: a review of the literature.
914	DRAMP03574	LL-37(17-32)(C-terminal fragment of LL-37; Human, mammals, animals)	"LL-37"[All Fields] AND biofilm[All Fields]	LL-37	22354291	Inhibition of bacterial biofilm formation and swarming motility by a small synthetic cationic peptide.
914	DRAMP03574	LL-37(17-32)(C-terminal fragment of LL-37; Human, mammals, animals)	"LL-37"[All Fields] AND biofilm[All Fields]	LL-37	22142958	Antimicrobial peptides in periodontal innate defense.
914	DRAMP03574	LL-37(17-32)(C-terminal fragment of LL-37; Human, mammals, animals)	"LL-37"[All Fields] AND biofilm[All Fields]	LL-37	22115597	An investigation on the antibacterial, cytotoxic, and antibiofilm efficacy of starch-stabilized silver nanoparticles.
914	DRAMP03574	LL-37(17-32)(C-terminal fragment of LL-37; Human, mammals, animals)	"LL-37"[All Fields] AND biofilm[All Fields]	LL-37	22005071	Antimicrobial and antibiofilm activity of LL-37 and its truncated variants against Burkholderia pseudomallei.
914	DRAMP03574	LL-37(17-32)(C-terminal fragment of LL-37; Human, mammals, animals)	"LL-37"[All Fields] AND biofilm[All Fields]	LL-37	21875967	Elicitation of epithelial cell-derived immune effectors by outer membrane vesicles of nontypeable Haemophilus influenzae.
914	DRAMP03574	LL-37(17-32)(C-terminal fragment of LL-37; Human, mammals, animals)	"LL-37"[All Fields] AND biofilm[All Fields]	LL-37	21849157	Antibacterial and anti-biofilm effects of cathelicidin peptides against pathogens isolated from cystic fibrosis patients.
914	DRAMP03574	LL-37(17-32)(C-terminal fragment of LL-37; Human, mammals, animals)	"LL-37"[All Fields] AND biofilm[All Fields]	LL-37	21772832	Susceptibility of Pseudomonas aeruginosa Biofilm to Alpha-Helical Peptides: D-enantiomer of LL-37.
914	DRAMP03574	LL-37(17-32)(C-terminal fragment of LL-37; Human, mammals, animals)	"LL-37"[All Fields] AND biofilm[All Fields]	LL-37	21692631	A novel organotypic dento-epithelial culture model: effect of Fusobacterium nucleatum biofilm on B-defensin-2, -3, and LL-37 expression.
914	DRAMP03574	LL-37(17-32)(C-terminal fragment of LL-37; Human, mammals, animals)	"LL-37"[All Fields] AND biofilm[All Fields]	LL-37	21605457	Natural and synthetic cathelicidin peptides with anti-microbial and anti-biofilm activity against Staphylococcus aureus.
914	DRAMP03574	LL-37(17-32)(C-terminal fragment of LL-37; Human, mammals, animals)	"LL-37"[All Fields] AND biofilm[All Fields]	LL-37	21220789	Fusobacterium nucleatum in periodontal health and disease.
914	DRAMP03574	LL-37(17-32)(C-terminal fragment of LL-37; Human, mammals, animals)	"LL-37"[All Fields] AND biofilm[All Fields]	LL-37	20961363	Potential of ceragenin CSA-13 and its mixture with pluronic F-127 as treatment of topical bacterial infections.
914	DRAMP03574	LL-37(17-32)(C-terminal fragment of LL-37; Human, mammals, animals)	"LL-37"[All Fields] AND biofilm[All Fields]	LL-37	20661475	Uropathogenic Escherichia coli modulates immune responses and its curli fimbriae interact with the antimicrobial peptide LL-37.
914	DRAMP03574	LL-37(17-32)(C-terminal fragment of LL-37; Human, mammals, animals)	"LL-37"[All Fields] AND biofilm[All Fields]	LL-37	20399752	Antimicrobial and antibiofilm activity of cathelicidins and short, synthetic peptides against Francisella.

914	DRAMP03574	LL-37(17-32)(C-terminal fragment of LL-37; Human, mammals, animals)	"LL-37"[All Fields] AND biofilm[All Fields]	LL-37	20139192	Effects of sequential Campylobacter jejuni 81-176 lipooligosaccharide core truncations on biofilm formation, stress survival, and pathogenesis.
914	DRAMP03574	LL-37(17-32)(C-terminal fragment of LL-37; Human, mammals, animals)	"LL-37"[All Fields] AND biofilm[All Fields]	LL-37	19847021	Nanolayer biofilm coated on magnetic nanoparticles by using a dielectric barrier discharge glow plasma fluidized bed for immobilizing an antimicrobial peptide.
914	DRAMP03574	LL-37(17-32)(C-terminal fragment of LL-37; Human, mammals, animals)	"LL-37"[All Fields] AND biofilm[All Fields]	LL-37	19466693	Activity of antimicrobial peptides in the presence of polysaccharides produced by pulmonary pathogens.
914	DRAMP03574	LL-37(17-32)(C-terminal fragment of LL-37; Human, mammals, animals)	"LL-37"[All Fields] AND biofilm[All Fields]	LL-37	19379612	Effects of an LL-37-derived antimicrobial peptide in an animal model of biofilm Pseudomonas sinusitis.
914	DRAMP03574	LL-37(17-32)(C-terminal fragment of LL-37; Human, mammals, animals)	"LL-37"[All Fields] AND biofilm[All Fields]	LL-37	18591225	Human host defense peptide LL-37 prevents bacterial biofilm formation.
914	DRAMP03574	LL-37(17-32)(C-terminal fragment of LL-37; Human, mammals, animals)	"LL-37"[All Fields] AND biofilm[All Fields]	LL-37	16687459	In vitro assessment of antimicrobial peptides as potential agents against several oral bacteria.
914	DRAMP03574	LL-37(17-32)(C-terminal fragment of LL-37; Human, mammals, animals)	"LL-37"[All Fields] AND biofilm[All Fields]	LL-37	14764110	Polysaccharide intercellular adhesin (PIA) protects Staphylococcus epidermidis against major components of the human innate immune system.
915	DRAMP03598	Human beta-defensin 2 (hBD-2; Defensin, beta 2; Beta-defensin 4A; Human, mammals, animals)	"Human beta-defensin 2"[All Fields] AND biofilm[All Fields]	Human beta-defensin 2	34276631	DNA Blocks the Lethal Effect of Human Beta-Defensin 2 Against Neisseria meningitidis.
915	DRAMP03598	Human beta-defensin 2 (hBD-2; Defensin, beta 2; Beta-defensin 4A; Human, mammals, animals)	"Human beta-defensin 2"[All Fields] AND biofilm[All Fields]	Human beta-defensin 2	32842903	Electrospun ZnO/Poly(Vinylidene Fluoride-Trifluoroethylene) Scaffolds for Lung Tissue Engineering.
915	DRAMP03598	Human beta-defensin 2 (hBD-2; Defensin, beta 2; Beta-defensin 4A; Human, mammals, animals)	"Human beta-defensin 2"[All Fields] AND biofilm[All Fields]	Human beta-defensin 2	32522780	Controlling the Growth of the Skin Commensal Staphylococcus epidermidis Using d-Alanine Auxotrophy.
915	DRAMP03598	Human beta-defensin 2 (hBD-2; Defensin, beta 2; Beta-defensin 4A; Human, mammals, animals)	"Human beta-defensin 2"[All Fields] AND biofilm[All Fields]	Human beta-defensin 2	32457749	The Antimicrobial Peptide Human Beta-Defensin 2 Inhibits Biofilm Production of Pseudomonas aeruginosaWithout Compromising Metabolic Activity.
915	DRAMP03598	Human beta-defensin 2 (hBD-2; Defensin, beta 2; Beta-defensin 4A; Human, mammals, animals)	"Human beta-defensin 2"[All Fields] AND biofilm[All Fields]	Human beta-defensin 2	27582732	Efficient Eradication of Mature Pseudomonas aeruginosa Biofilm via Controlled Delivery of Nitric Oxide Combined with Antimicrobial Peptide and Antibiotics.
915	DRAMP03598	Human beta-defensin 2 (hBD-2; Defensin, beta 2; Beta-defensin 4A; Human, mammals, animals)	"Human beta-defensin 2"[All Fields] AND biofilm[All Fields]	Human beta-defensin 2	27148195	Effect of Substance P in Staphylococcus aureus and Staphylococcus epidermidis Virulence: Implication for Skin Homeostasis.
915	DRAMP03598	Human beta-defensin 2 (hBD-2; Defensin, beta 2; Beta-defensin 4A; Human, mammals, animals)	"Human beta-defensin 2"[All Fields] AND biofilm[All Fields]	Human beta-defensin 2	25808131	Expression of antimicrobial peptides and interleukin-8 during early stages of inflammation: An experimental gingivitis study.
915	DRAMP03598	Human beta-defensin 2 (hBD-2; Defensin, beta 2; Beta-defensin 4A; Human, mammals, animals)	"Human beta-defensin 2"[All Fields] AND biofilm[All Fields]	Human beta-defensin 2	24340061	Inflammatory and antimicrobial responses to methicillin-resistant Staphylococcus aureus in an in vitro wound infection model.
915	DRAMP03598	Human beta-defensin 2 (hBD-2; Defensin, beta 2; Beta-defensin 4A; Human, mammals, animals)	"Human beta-defensin 2"[All Fields] AND biofilm[All Fields]	Human beta-defensin 2	19572896	The immune response of oral epithelial cells induced by single-species and complex naturally formed biofilms.
915	DRAMP03598	Human beta-defensin 2 (hBD-2; Defensin, beta 2; Beta-defensin 4A; Human, mammals, animals)	"Human beta-defensin 2"[All Fields] AND biofilm[All Fields]	Human beta-defensin 2	18954353	Treponema denticola does not induce production of common innate immune mediators from primary gingival epithelial cells.
915	DRAMP03598	Human beta-defensin 2 (hBD-2; Defensin, beta 2; Beta-defensin 4A; Human, mammals, animals)	"Human beta-defensin 2"[All Fields] AND biofilm[All Fields]	Human beta-defensin 2	18173794	The stage of native biofilm formation determines the gene expression of human beta-defensin-2, psoriasin, ribonuclease 7 and inflammatory mediators: a novel approach for stimulation of keratinocytes with in situ formed biofilms.
915	DRAMP03598	Human beta-defensin 2 (hBD-2; Defensin, beta 2; Beta-defensin 4A; Human, mammals, animals)	"Human beta-defensin 2"[All Fields] AND biofilm[All Fields]	Human beta-defensin 2	17434999	Functional analysis of D-alanylation of lipoteichoic acid in the probiotic strain Lactobacillus rhamnosus GG.
916	DRAMP03599	Human beta-defensin 3 (BD-3, hBD-3; Hbd3; Beta-defensin 103; Human, mammals, animals)	"Human beta-defensin 3"[All Fields] AND biofilm[All Fields]	Human beta-defensin 3	33865931	Identification of a crocodylian β-defensin variant from Alligator mississippiensis with antimicrobial and antibiofilm activity.
916	DRAMP03599	Human beta-defensin 3 (BD-3, hBD-3; Hbd3; Beta-defensin 103; Human, mammals, animals)	"Human beta-defensin 3"[All Fields] AND biofilm[All Fields]	Human beta-defensin 3	32858856	Candida albicansVirulence Factors and Pathogenicity for Endodontic Infections.
916	DRAMP03599	Human beta-defensin 3 (BD-3, hBD-3; Hbd3; Beta-defensin 103; Human, mammals, animals)	"Human beta-defensin 3"[All Fields] AND biofilm[All Fields]	Human beta-defensin 3	31933178	Effects of human β-defensin 3 fused with carbohydrate-binding domain on the function of type III secretion system in Pseudomonas aeruginosa PA14.
916	DRAMP03599	Human beta-defensin 3 (BD-3, hBD-3; Hbd3; Beta-defensin 103; Human, mammals, animals)	"Human beta-defensin 3"[All Fields] AND biofilm[All Fields]	Human beta-defensin 3	30254440	Modification of the surface of titanium with multifunctional chimeric peptides to prevent biofilm formation via inhibition of initial colonizers.
916	DRAMP03599	Human beta-defensin 3 (BD-3, hBD-3; Hbd3; Beta-defensin 103; Human, mammals, animals)	"Human beta-defensin 3"[All Fields] AND biofilm[All Fields]	Human beta-defensin 3	29077172	The significance of HBD-3 and fluorescent composite carriers in the processof bone formation in rats infected with Staphylococcus aureus.
916	DRAMP03599	Human beta-defensin 3 (BD-3, hBD-3; Hbd3; Beta-defensin 103; Human, mammals, animals)	"Human beta-defensin 3"[All Fields] AND biofilm[All Fields]	Human beta-defensin 3	29025642	Engineered chimeric peptides with antimicrobial and titanium-binding functions to inhibit biofilm formation on Ti implants.
916	DRAMP03599	Human beta-defensin 3 (BD-3, hBD-3; Hbd3; Beta-defensin 103; Human, mammals, animals)	"Human beta-defensin 3"[All Fields] AND biofilm[All Fields]	Human beta-defensin 3	28956355	The synthetic human beta-defensin-3 C15 peptide exhibits antimicrobial activity against Streptococcus mutans, both alone and in combination with dental disinfectants.
916	DRAMP03599	Human beta-defensin 3 (BD-3, hBD-3; Hbd3; Beta-defensin 103; Human, mammals, animals)	"Human beta-defensin 3"[All Fields] AND biofilm[All Fields]	Human beta-defensin 3	28951032	Antifungal Effects of Synthetic Human Beta-defensin-3-C15 Peptide on Candida albicans-infected Root Dentin.
916	DRAMP03599	Human beta-defensin 3 (BD-3, hBD-3; Hbd3; Beta-defensin 103; Human, mammals, animals)	"Human beta-defensin 3"[All Fields] AND biofilm[All Fields]	Human beta-defensin 3	28413476	The mechanism of human β-defensin 3 in MRSA-induced infection of implant drug-resistant bacteria biofilm in the mouse tibial bone marrow.
916	DRAMP03599	Human beta-defensin 3 (BD-3, hBD-3; Hbd3; Beta-defensin 103; Human, mammals, animals)	"Human beta-defensin 3"[All Fields] AND biofilm[All Fields]	Human beta-defensin 3	28078813	Host defense peptide-derived privileged scaffolds for anti-infective drug discovery.

916	DRAMP03599	Human beta-defensin 3 (BD-3, hBD-3; Hbd3; Beta-defensin 103; Human, mammals, animals)	"Human beta-defensin 3"[All Fields] AND biofilm[All Fields]	Human beta-defensin 3	27777572	Role of yqiCin the Pathogenicity of Salmonellaand Innate Immune Responses of Human Intestinal Epithelium.
916	DRAMP03599	Human beta-defensin 3 (BD-3, hBD-3; Hbd3; Beta-defensin 103; Human, mammals, animals)	"Human beta-defensin 3"[All Fields] AND biofilm[All Fields]	Human beta-defensin 3	27200276	Antifungal effects of synthetic human β-defensin 3-C15 peptide.
916	DRAMP03599	Human beta-defensin 3 (BD-3, hBD-3; Hbd3; Beta-defensin 103; Human, mammals, animals)	"Human beta-defensin 3"[All Fields] AND biofilm[All Fields]	Human beta-defensin 3	26119274	Ultrasound microbubbles enhance human β-defensin 3 against biofilms.
916	DRAMP03599	Human beta-defensin 3 (BD-3, hBD-3; Hbd3; Beta-defensin 103; Human, mammals, animals)	"Human beta-defensin 3"[All Fields] AND biofilm[All Fields]	Human beta-defensin 3	25862466	The Antibacterial Effects of an Antimicrobial Peptide Human β-Defensin 3 Fused with Carbohydrate-Binding Domain on Pseudomonas aeruginosa PA14.
916	DRAMP03599	Human beta-defensin 3 (BD-3, hBD-3; Hbd3; Beta-defensin 103; Human, mammals, animals)	"Human beta-defensin 3"[All Fields] AND biofilm[All Fields]	Human beta-defensin 3	24913184	Effects of human β-defensin-3 on biofilm formation-regulating genes dltB and icaA in Staphylococcus aureus.
916	DRAMP03599	Human beta-defensin 3 (BD-3, hBD-3; Hbd3; Beta-defensin 103; Human, mammals, animals)	"Human beta-defensin 3"[All Fields] AND biofilm[All Fields]	Human beta-defensin 3	24238461	Antibacterial efficacy of a human β-defensin-3 peptide on multispecies biofilms.
916	DRAMP03599	Human beta-defensin 3 (BD-3, hBD-3; Hbd3; Beta-defensin 103; Human, mammals, animals)	"Human beta-defensin 3"[All Fields] AND biofilm[All Fields]	Human beta-defensin 3	24100890	[Use of recombinant human beta-defensin-3 to evaluate the effect of adhesion of Candida albicans on the surface of soft lining material].
916	DRAMP03599	Human beta-defensin 3 (BD-3, hBD-3; Hbd3; Beta-defensin 103; Human, mammals, animals)	"Human beta-defensin 3"[All Fields] AND biofilm[All Fields]	Human beta-defensin 3	23639356	Human beta-defensin 3: a novel inhibitor of Staphylococcus-produced biofilm production. Commentary on "Human β-defensin 3 inhibits antibiotic-resistant Staphylococcus biofilm formation".
916	DRAMP03599	Human beta-defensin 3 (BD-3, hBD-3; Hbd3; Beta-defensin 103; Human, mammals, animals)	"Human beta-defensin 3"[All Fields] AND biofilm[All Fields]	Human beta-defensin 3	23519963	Ultrasound-targeted microbubble destruction enhances human β-defensin 3 activity against antibiotic-resistant Staphylococcus biofilms.
916	DRAMP03599	Human beta-defensin 3 (BD-3, hBD-3; Hbd3; Beta-defensin 103; Human, mammals, animals)	"Human beta-defensin 3"[All Fields] AND biofilm[All Fields]	Human beta-defensin 3	23273885	Human β-defensin 3 inhibits antibiotic-resistant Staphylococcus biofilm formation.
916	DRAMP03599	Human beta-defensin 3 (BD-3, hBD-3; Hbd3; Beta-defensin 103; Human, mammals, animals)	"Human beta-defensin 3"[All Fields] AND biofilm[All Fields]	Human beta-defensin 3	23078156	Antimicrobial efficacy of a human β-defensin-3 peptide using an Enterococcus faecalis dentine infection model.
916	DRAMP03599	Human beta-defensin 3 (BD-3, hBD-3; Hbd3; Beta-defensin 103; Human, mammals, animals)	"Human beta-defensin 3"[All Fields] AND biofilm[All Fields]	Human beta-defensin 3	22922323	Extracellular DNA within a nontypeable Haemophilus influenzae-induced biofilm binds human beta defensin-3 and reduces its antimicrobial activity.
916	DRAMP03599	Human beta-defensin 3 (BD-3, hBD-3; Hbd3; Beta-defensin 103; Human, mammals, animals)	"Human beta-defensin 3"[All Fields] AND biofilm[All Fields]	Human beta-defensin 3	22229614	Comparison of the effects of human β-defensin 3, vancomycin, and clindamycin on Staphylococcus aureus biofilm formation.
916	DRAMP03599	Human beta-defensin 3 (BD-3, hBD-3; Hbd3; Beta-defensin 103; Human, mammals, animals)	"Human beta-defensin 3"[All Fields] AND biofilm[All Fields]	Human beta-defensin 3	14764110	Polysaccharide intercellular adhesin (PIA) protects Staphylococcus epidermidis against major components of the human innate immune system.
921	DRAMP03645	Cathelicidin-2 (CATH-2; Fowlicidin-2; Birds, animals)	"Cathelicidin-2"[All Fields] AND biofilm[All Fields]	Cathelicidin-2	21376541	A cathelicidin-2-derived peptide effectively impairs Staphylococcus epidermidis biofilms.
927	DRAMP03679	Cathelicidin-2 (Bactenecin-5, Bac5; ChBac5; ruminant, animals)	"Cathelicidin-2"[All Fields] AND biofilm[All Fields]	Cathelicidin-2	21376541	A cathelicidin-2-derived peptide effectively impairs Staphylococcus epidermidis biofilms.
938	DRAMP03706	Antimicrobial peptide 1 (AamAP1; Arthropods, animals)	"Antimicrobial peptide 1"[All Fields] AND biofilm[All Fields]	Antimicrobial peptide 1	22445495	Database screening and in vivo efficacy of antimicrobial peptides against methicillin-resistant Staphylococcus aureus USA300.
949	DRAMP02828	BMAP-34 (BMAP 34, bovine cathelicidin, cattle, ruminant, mammals, animals)	"BMAP-34"[All Fields] AND biofilm[All Fields]	BMAP-34	32182913	Differential Abilities of Mammalian Cathelicidins to Inhibit Bacterial Biofilm Formation and Promote Multifaceted Immune Functions of Neutrophils.
950	DRAMP02926	Tachyplesin I (Tac; TP1; Horseshoe Crab, arachnids, Chelicerata, arthropods, invertebrates, animals)	"Tachyplesin I"[All Fields] AND biofilm[All Fields]	Tachyplesin I	31455019	Characterization of Tachyplesin Peptides and Their Cyclized Analogues to Improve Antimicrobial and Anticancer Properties.
950	DRAMP02926	Tachyplesin I (Tac; TP1; Horseshoe Crab, arachnids, Chelicerata, arthropods, invertebrates, animals)	"Tachyplesin I"[All Fields] AND biofilm[All Fields]	Tachyplesin I	28355248	Searching for new strategies against biofilm infections: Colistin-AMP combinations against Pseudomonas aeruginosa and Staphylococcus aureus single- and double-species biofilms.
986	DRAMP03859	W10 (Bac2A variant through single amino acid substitution)	"W10"[All Fields] AND biofilm[All Fields]	W10	30521589	Extracellular polymeric substances (EPS) producing and oil degrading bacteria isolated from the northern Gulf of Mexico.
986	DRAMP03859	W10 (Bac2A variant through single amino acid substitution)	"W10"[All Fields] AND biofilm[All Fields]	W10	28156000	Rhamnolipids from Pseudomonas aeruginosa strain W10; as antibiofilm/antibiofouling products for metal protection.
987	DRAMP03860	R11 (Bac2A variant through single amino acid substitution)	"R11"[All Fields] AND biofilm[All Fields]	R11	34674874	Comparative analysis of different methods used for molecular characterization of Burkholderia cepacia complex isolated from noncystic fibrosis conditions.
987	DRAMP03860	R11 (Bac2A variant through single amino acid substitution)	"R11"[All Fields] AND biofilm[All Fields]	R11	26774858	Draft genomes of Nautella italica strains CECT 7645(T) and CECT 7321: Two roseobacters with potential pathogenic and biotechnological traits.
987	DRAMP03860	R11 (Bac2A variant through single amino acid substitution)	"R11"[All Fields] AND biofilm[All Fields]	R11	26528274	VarR controls colonization and virulence in the marine macroalgal pathogen Nautella italica R11.
987	DRAMP03860	R11 (Bac2A variant through single amino acid substitution)	"R11"[All Fields] AND biofilm[All Fields]	R11	20946533	Temperature induced bacterial virulence and bleaching disease in a chemically defended marine macroalga.
988	DRAMP03861	G12 (Bac2A variant through single amino acid substitution)	"G12"[All Fields] AND biofilm[All Fields]	G12	34626764	Microbial community assembly and dynamics in Granular, Fixed-Biofilm and planktonic microbiomes valorizing Long-Chain fatty acids at 20 °C.
988	DRAMP03861	G12 (Bac2A variant through single amino acid substitution)	"G12"[All Fields] AND biofilm[All Fields]	G12	34454228	Biofilm carrier type affects biogenic sulfur-driven denitrification performance and microbial community dynamics in moving-bed biofilm reactors.
988	DRAMP03861	G12 (Bac2A variant through single amino acid substitution)	"G12"[All Fields] AND biofilm[All Fields]	G12	33963212	Mechanical biofilm disruption causes microbial and immunological shifts in periodontitis patients.
988	DRAMP03861	G12 (Bac2A variant through single amino acid substitution)	"G12"[All Fields] AND biofilm[All Fields]	G12	33667821	Methanogenic granule growth and development is a continual process characterized by distinct morphological features.
988	DRAMP03861	G12 (Bac2A variant through single amino acid substitution)	"G12"[All Fields] AND biofilm[All Fields]	G12	33379333	Candida albicansas an Essential "Keystone" Component within Polymicrobial Oral Biofilm Models?
988	DRAMP03861	G12 (Bac2A variant through single amino acid substitution)	"G12"[All Fields] AND biofilm[All Fields]	G12	33198386	Adhesion of Escherichia Colito Nanostructured Surfaces and the Role of Type 1 Fimbriae.
988	DRAMP03861	G12 (Bac2A variant through single amino acid substitution)	"G12"[All Fields] AND biofilm[All Fields]	G12	32971912	Comparison of Three Endodontic Irrigant Regimens against Dual-Species Interkingdom Biofilms: Considerations for Maintaining the Status Quo.

988	DRAMP03861	G12 (Bac2A variant through single amino acid substitution)	"G12"[All Fields] AND biofilm[All Fields]	G12	32201858	Interkingdom interactions on the denture surface: Implications for oral hygiene.
988	DRAMP03861	G12 (Bac2A variant through single amino acid substitution)	"G12"[All Fields] AND biofilm[All Fields]	G12	32075765	Propionic Acid Promotes the Virulent Phenotype of Crohn's Disease-Associated Adherent-Invasive Escherichia coli.
988	DRAMP03861	G12 (Bac2A variant through single amino acid substitution)	"G12"[All Fields] AND biofilm[All Fields]	G12	31808676	Setting Our Sights on Infectious Diseases.
988	DRAMP03861	G12 (Bac2A variant through single amino acid substitution)	"G12"[All Fields] AND biofilm[All Fields]	G12	31673005	Biofilm-stimulated epithelium modulates the inflammatory responses in co-cultured immune cells.
988	DRAMP03861	G12 (Bac2A variant through single amino acid substitution)	"G12"[All Fields] AND biofilm[All Fields]	G12	31623950	Reduction of Pseudomonas aeruginosa biofilm formation through the application of nanoscale vibration.
988	DRAMP03861	G12 (Bac2A variant through single amino acid substitution)	"G12"[All Fields] AND biofilm[All Fields]	G12	31524581	Polymicrobial oral biofilm models: simplifying the complex.
988	DRAMP03861	G12 (Bac2A variant through single amino acid substitution)	"G12"[All Fields] AND biofilm[All Fields]	G12	31204047	Experimental composites containing quaternary ammonium methacrylates reduce demineralization at enamel-restoration margins after cariogenic challenge.
988	DRAMP03861	G12 (Bac2A variant through single amino acid substitution)	"G12"[All Fields] AND biofilm[All Fields]	G12	30522365	Underscoring interstrain variability and the impact of growth conditions on associated antimicrobial susceptibilities in preclinical testing of novel antimicrobial drugs.
988	DRAMP03861	G12 (Bac2A variant through single amino acid substitution)	"G12"[All Fields] AND biofilm[All Fields]	G12	30468010	Multifunctional Coatings and Nanotopographies: Toward Cell Instructive and Antibacterial Implants.
988	DRAMP03861	G12 (Bac2A variant through single amino acid substitution)	"G12"[All Fields] AND biofilm[All Fields]	G12	30298683	Characterization and development of SAPP as a specific peptidic inhibitor that targets Porphyromonas gingivalis.
988	DRAMP03861	G12 (Bac2A variant through single amino acid substitution)	"G12"[All Fields] AND biofilm[All Fields]	G12	30179497	Real-Time Study of Rapid Spread of Antibiotic Resistance Plasmid in Biofilm Using Microfluidics.
988	DRAMP03861	G12 (Bac2A variant through single amino acid substitution)	"G12"[All Fields] AND biofilm[All Fields]	G12	30139986	Lysogenization of Staphylococcus aureus RN450 by phages ϕ11 and ϕ80α leads to the activation of the SigB regulon.
988	DRAMP03861	G12 (Bac2A variant through single amino acid substitution)	"G12"[All Fields] AND biofilm[All Fields]	G12	29940363	Flow-cytometric quantification of microbial cells on sand from water biofilters.
988	DRAMP03861	G12 (Bac2A variant through single amino acid substitution)	"G12"[All Fields] AND biofilm[All Fields]	G12	29760550	Synergistic antifungal effect of chitosan-stabilized selenium nanoparticles synthesized by pulsed laser ablation in liquids against Candida albicansbiofilms.
988	DRAMP03861	G12 (Bac2A variant through single amino acid substitution)	"G12"[All Fields] AND biofilm[All Fields]	G12	29661978	Control of cell behaviour through nanovibrational stimulation: nanokicking.
988	DRAMP03861	G12 (Bac2A variant through single amino acid substitution)	"G12"[All Fields] AND biofilm[All Fields]	G12	29452993	An in vitro evaluation of the efficacy of tedizolid: implications for the treatment of skin and soft tissue infections.
988	DRAMP03861	G12 (Bac2A variant through single amino acid substitution)	"G12"[All Fields] AND biofilm[All Fields]	G12	29428998	Turbulence accelerates the growth of drinking water biofilms.
988	DRAMP03861	G12 (Bac2A variant through single amino acid substitution)	"G12"[All Fields] AND biofilm[All Fields]	G12	29220155	Superchiral Plasmonic Phase Sensitivity for Fingerprinting of Protein Interface Structure.
988	DRAMP03861	G12 (Bac2A variant through single amino acid substitution)	"G12"[All Fields] AND biofilm[All Fields]	G12	29176569	A novel peptidic inhibitor derived from Streptococcus cristatus ArcA attenuates virulence potential of Porphyromonas gingivalis.
988	DRAMP03861	G12 (Bac2A variant through single amino acid substitution)	"G12"[All Fields] AND biofilm[All Fields]	G12	28910698	Temperature, inocula and substrate: Contrasting electroactive consortia, diversity and performance in microbial fuel cells.
988	DRAMP03861	G12 (Bac2A variant through single amino acid substitution)	"G12"[All Fields] AND biofilm[All Fields]	G12	28822582	Antibiofilm properties of model composites containing quaternary ammonium methacrylates after surface texture modification.
988	DRAMP03861	G12 (Bac2A variant through single amino acid substitution)	"G12"[All Fields] AND biofilm[All Fields]	G12	28680970	nBioChip, a Lab-on-a-Chip Platform of Mono- and Polymicrobial Biofilms for High-Throughput Downstream Applications.
988	DRAMP03861	G12 (Bac2A variant through single amino acid substitution)	"G12"[All Fields] AND biofilm[All Fields]	G12	28512515	Biofilms for Babies: Introducing Microbes and Biofilms to Preschool-Aged Children.
988	DRAMP03861	G12 (Bac2A variant through single amino acid substitution)	"G12"[All Fields] AND biofilm[All Fields]	G12	28469253	Identification of Streptococcus cristatus peptides that repress expression of virulence genes in Porphyromonas gingivalis.
988	DRAMP03861	G12 (Bac2A variant through single amino acid substitution)	"G12"[All Fields] AND biofilm[All Fields]	G12	28408270	Transcriptional organization of pneumococcal psrP-secY2A2 and impact of GtfA and GtfB deletion on PsrP-associated virulence properties.
988	DRAMP03861	G12 (Bac2A variant through single amino acid substitution)	"G12"[All Fields] AND biofilm[All Fields]	G12	27793789	Inhibition of Candida albicans biofilm by pure selenium nanoparticles synthesized by pulsed laser ablation in liquids.
988	DRAMP03861	G12 (Bac2A variant through single amino acid substitution)	"G12"[All Fields] AND biofilm[All Fields]	G12	27242712	An In Vitro Model for Oral Mixed Biofilms of Candida albicans and Streptococcus gordonii in Synthetic Saliva.
988	DRAMP03861	G12 (Bac2A variant through single amino acid substitution)	"G12"[All Fields] AND biofilm[All Fields]	G12	26972916	The ecology and biogeochemistry of stream biofilms.
988	DRAMP03861	G12 (Bac2A variant through single amino acid substitution)	"G12"[All Fields] AND biofilm[All Fields]	G12	26879980	Ammonia oxidizing bacteria and archaea in horizontal flow biofilm reactors treating ammonia-contaminated air at 10 °C.
988	DRAMP03861	G12 (Bac2A variant through single amino acid substitution)	"G12"[All Fields] AND biofilm[All Fields]	G12	26691764	A Novel Small Molecule Inhibitor of Candida albicansBiofilm Formation, Filamentation and Virulence with Low Potential for the Development of Resistance.
988	DRAMP03861	G12 (Bac2A variant through single amino acid substitution)	"G12"[All Fields] AND biofilm[All Fields]	G12	26666378	Effect of silver nanoparticles on Candida albicans biofilms: an ultrastructural study.
988	DRAMP03861	G12 (Bac2A variant through single amino acid substitution)	"G12"[All Fields] AND biofilm[All Fields]	G12	26343879	Biogenesis and function of Porphyromonas gingivalis outer membrane vesicles.
988	DRAMP03861	G12 (Bac2A variant through single amino acid substitution)	"G12"[All Fields] AND biofilm[All Fields]	G12	26027773	Biodiversity, community structure and function of biofilms in stream ecosystems.
988	DRAMP03861	G12 (Bac2A variant through single amino acid substitution)	"G12"[All Fields] AND biofilm[All Fields]	G12	25982923	Influence of biofilms on heavy metal immobilization in sustainable urban drainage systems (SuDS).
988	DRAMP03861	G12 (Bac2A variant through single amino acid substitution)	"G12"[All Fields] AND biofilm[All Fields]	G12	25897780	Functional Advantages of Porphyromonas gingivalis Vesicles.
988	DRAMP03861	G12 (Bac2A variant through single amino acid substitution)	"G12"[All Fields] AND biofilm[All Fields]	G12	25136087	Fluvial network organization imprints on microbial co-occurrence networks.
988	DRAMP03861	G12 (Bac2A variant through single amino acid substitution)	"G12"[All Fields] AND biofilm[All Fields]	G12	25068919	Living biointerfaces based on non-pathogenic bacteria to direct cell differentiation.
988	DRAMP03861	G12 (Bac2A variant through single amino acid substitution)	"G12"[All Fields] AND biofilm[All Fields]	G12	25013815	Exposure of a 23F serotype strain of Streptococcus pneumoniae to cigarette smoke condensate is associated with selective upregulation of genes encoding the two-component regulatory system 11 (TCS11).
988	DRAMP03861	G12 (Bac2A variant through single amino acid substitution)	"G12"[All Fields] AND biofilm[All Fields]	G12	24965235	Optically trapped bacteria pairs reveal discrete motile response to control aggregation upon cell-cell approach.

988	DRAMP03861	G12 (Bac2A variant through single amino acid substitution)	"G12"[All Fields] AND biofilm[All Fields]	G12	24733094	Identification of a diguanylate cyclase and its role in Porphyromonas gingivalis virulence.
988	DRAMP03861	G12 (Bac2A variant through single amino acid substitution)	"G12"[All Fields] AND biofilm[All Fields]	G12	24114441	Drug susceptibility of matrix-encapsulated Candida albicans nano-biofilms.
988	DRAMP03861	G12 (Bac2A variant through single amino acid substitution)	"G12"[All Fields] AND biofilm[All Fields]	G12	24089333	Headwaters are critical reservoirs of microbial diversity for fluvial networks.
988	DRAMP03861	G12 (Bac2A variant through single amino acid substitution)	"G12"[All Fields] AND biofilm[All Fields]	G12	23800397	High-throughput nano-biofilm microarray for antifungal drug discovery.
988	DRAMP03861	G12 (Bac2A variant through single amino acid substitution)	"G12"[All Fields] AND biofilm[All Fields]	G12	23782536	The structure of latherin, a surfactant allergen protein from horse sweat and saliva.
988	DRAMP03861	G12 (Bac2A variant through single amino acid substitution)	"G12"[All Fields] AND biofilm[All Fields]	G12	23543749	Regulatory interactions between a bacterial tyrosine kinase and its cognate phosphatase.
988	DRAMP03861	G12 (Bac2A variant through single amino acid substitution)	"G12"[All Fields] AND biofilm[All Fields]	G12	23225198	Sequence-specific backbone ^{1}H, ^{13}C and ^{15}N assignments of the catalytic domain of the Escherichia coli protein tyrosine kinase, Wzc.
988	DRAMP03861	G12 (Bac2A variant through single amino acid substitution)	"G12"[All Fields] AND biofilm[All Fields]	G12	23176515	Colicin-like bacteriocins as novel therapeutic agents for the treatment of chronic biofilm-mediated infection.
988	DRAMP03861	G12 (Bac2A variant through single amino acid substitution)	"G12"[All Fields] AND biofilm[All Fields]	G12	21787340	Latherin and other biocompatible surfactant proteins.
988	DRAMP03861	G12 (Bac2A variant through single amino acid substitution)	"G12"[All Fields] AND biofilm[All Fields]	G12	21616477	Urease activity in dental plaque and saliva of children during a three-year study period and its relationship with other caries risk factors.
988	DRAMP03861	G12 (Bac2A variant through single amino acid substitution)	"G12"[All Fields] AND biofilm[All Fields]	G12	21544190	Development of a high-throughput Candida albicans biofilm chip.
988	DRAMP03861	G12 (Bac2A variant through single amino acid substitution)	"G12"[All Fields] AND biofilm[All Fields]	G12	20709787	The transcriptional regulator Nrg1p controls Candida albicans biofilm formation and dispersion.
988	DRAMP03861	G12 (Bac2A variant through single amino acid substitution)	"G12"[All Fields] AND biofilm[All Fields]	G12	20435773	Application of paramagnetically tagged molecules for magnetic resonance imaging of biofilm mass transport processes.
988	DRAMP03861	G12 (Bac2A variant through single amino acid substitution)	"G12"[All Fields] AND biofilm[All Fields]	G12	19892857	Apoptosis in Candida biofilms exposed to amphotericin B.
988	DRAMP03861	G12 (Bac2A variant through single amino acid substitution)	"G12"[All Fields] AND biofilm[All Fields]	G12	19794044	Characteristics of Candida albicans biofilms grown in a synthetic urine medium.
988	DRAMP03861	G12 (Bac2A variant through single amino acid substitution)	"G12"[All Fields] AND biofilm[All Fields]	G12	19130292	Biofilm formation by Cryptococcus neoformans under distinct environmental conditions.
988	DRAMP03861	G12 (Bac2A variant through single amino acid substitution)	"G12"[All Fields] AND biofilm[All Fields]	G12	18552186	Magnetic resonance imaging of structure, diffusivity, and copper immobilization in a phototrophic biofilm.
988	DRAMP03861	G12 (Bac2A variant through single amino acid substitution)	"G12"[All Fields] AND biofilm[All Fields]	G12	18498529	Benefits of bacterial biomineralization.
988	DRAMP03861	G12 (Bac2A variant through single amino acid substitution)	"G12"[All Fields] AND biofilm[All Fields]	G12	18285487	Absence of amphotericin B-tolerant persister cells in biofilms of some Candida species.
988	DRAMP03861	G12 (Bac2A variant through single amino acid substitution)	"G12"[All Fields] AND biofilm[All Fields]	G12	17554046	A biochemical guide to yeast adhesins: glycoproteins for social and antisocial occasions.
988	DRAMP03861	G12 (Bac2A variant through single amino acid substitution)	"G12"[All Fields] AND biofilm[All Fields]	G12	16980403	Production of tyrosol by Candida albicans biofilms and its role in quorum sensing and biofilm development.
988	DRAMP03861	G12 (Bac2A variant through single amino acid substitution)	"G12"[All Fields] AND biofilm[All Fields]	G12	16849719	Biofilm matrix of Candida albicans and Candida tropicalis: chemical composition and role in drug resistance.
988	DRAMP03861	G12 (Bac2A variant through single amino acid substitution)	"G12"[All Fields] AND biofilm[All Fields]	G12	16192429	Prostaglandin production during growth of Candida albicans biofilms.
988	DRAMP03861	G12 (Bac2A variant through single amino acid substitution)	"G12"[All Fields] AND biofilm[All Fields]	G12	15328087	Penetration of Candida biofilms by antifungal agents.
988	DRAMP03861	G12 (Bac2A variant through single amino acid substitution)	"G12"[All Fields] AND biofilm[All Fields]	G12	14726457	Health impacts of environmental mycobacteria.
988	DRAMP03861	G12 (Bac2A variant through single amino acid substitution)	"G12"[All Fields] AND biofilm[All Fields]	G12	14693516	Effects of aspirin and other nonsteroidal anti-inflammatory drugs on biofilms and planktonic cells of Candida albicans.
988	DRAMP03861	G12 (Bac2A variant through single amino acid substitution)	"G12"[All Fields] AND biofilm[All Fields]	G12	12825991	Medical importance of biofilms in Candida infections.
988	DRAMP03861	G12 (Bac2A variant through single amino acid substitution)	"G12"[All Fields] AND biofilm[All Fields]	G12	12729282	Release studies of benzalkonium chloride from hydrogel in a freshwater environment.
988	DRAMP03861	G12 (Bac2A variant through single amino acid substitution)	"G12"[All Fields] AND biofilm[All Fields]	G12	11926741	Mixed species biofilms of Candida albicans and Staphylococcus epidermidis.
988	DRAMP03861	G12 (Bac2A variant through single amino acid substitution)	"G12"[All Fields] AND biofilm[All Fields]	G12	10980166	Matrix polymers of Candida biofilms and their possible role in biofilm resistance to antifungal agents.
988	DRAMP03861	G12 (Bac2A variant through single amino acid substitution)	"G12"[All Fields] AND biofilm[All Fields]	G12	9687427	Iron-limited biofilms of Candida albicans and their susceptibility to amphotericin B.
988	DRAMP03861	G12 (Bac2A variant through single amino acid substitution)	"G12"[All Fields] AND biofilm[All Fields]	G12	9687381	Effect of growth rate on resistance of Candida albicans biofilms to antifungal agents.
995	DRAMP03868	Bac8c (Bac2A variant)	"Bac8c"[All Fields] AND biofilm[All Fields]	Bac8c	32912428	Lipoic acid modified antimicrobial peptide with enhanced antimicrobial properties.
995	DRAMP03868	Bac8c (Bac2A variant)	"Bac8c"[All Fields] AND biofilm[All Fields]	Bac8c	28453851	Eradication of Staphylococcus aureus Biofilm Infections Using Synthetic Antimicrobial Peptides.
995	DRAMP03868	Bac8c (Bac2A variant)	"Bac8c"[All Fields] AND biofilm[All Fields]	Bac8c	24309076	Antimicrobial and anti-biofilm effect of Bac8c on major bacteria associated with dental caries and Streptococcus mutans biofilms.
995	DRAMP03868	Bac8c (Bac2A variant)	"Bac8c"[All Fields] AND biofilm[All Fields]	Bac8c	23383054	Genome-wide identification of genes conferring energy related resistance to a synthetic antimicrobial peptide (Bac8c).
997	DRAMP03870	Bac2A (a linear variant of bovine dodecapeptide)	"Bac2A"[All Fields] AND biofilm[All Fields]	Bac2A	28559266	An Immunomodulatory Peptide Confers Protection in an Experimental Candidemia Murine Model.
997	DRAMP03870	Bac2A (a linear variant of bovine dodecapeptide)	"Bac2A"[All Fields] AND biofilm[All Fields]	Bac2A	18591225	Human host defense peptide LL-37 prevents bacterial biofilm formation.
1006	DRAMP03882	LFC (fragment of mature caprine lactoferrin, residues 17 to 31)	"LFC"[All Fields] AND biofilm[All Fields]	LFC	19848149	Impact of higher alginate expression on deposition of Pseudomonas aeruginosa in radial stagnation point flow and reverse osmosis systems.
1006	DRAMP03882	LFC (fragment of mature caprine lactoferrin, residues 17 to 31)	"LFC"[All Fields] AND biofilm[All Fields]	LFC	15222464	The antimicrobial activity of lactoferrin: current status and perspectives.
1037	DRAMP03920	Cecropin A (1-8)-melittin (1-13)hybrid peptide	"Cecropin A"[All Fields] AND biofilm[All Fields]	Cecropin A	32203307	Antimicrobial peptide derived from moths can eradicate UPEC biofilms and could offer a novel therapeutic option.
1037	DRAMP03920	Cecropin A (1-8)-melittin (1-13)hybrid peptide	"Cecropin A"[All Fields] AND biofilm[All Fields]	Cecropin A	32203127	Antibiofilm activities of ceragenins and antimicrobial peptides against fungal-bacterial mono and multispecies biofilms.

1037	DRAMP03920	Cecropin A (1-8)-melittin (1-13)hybrid peptide	"Cecropin A"[All Fields] AND biofilm[All Fields]	Cecropin A	32051417	The insect antimicrobial peptide cecropin A disrupts uropathogenic Escherichia coli biofilms.
1037	DRAMP03920	Cecropin A (1-8)-melittin (1-13)hybrid peptide	"Cecropin A"[All Fields] AND biofilm[All Fields]	Cecropin A	31485973	Efficacy of Indolicidin, Cecropin A (1-7)-Melittin (CAMA) and Their Combination Against Biofilm-Forming Multidrug-Resistant Enteroaggregative Escherichia coli.
1037	DRAMP03920	Cecropin A (1-8)-melittin (1-13)hybrid peptide	"Cecropin A"[All Fields] AND biofilm[All Fields]	Cecropin A	31363941	Mechanism of action of antimicrobial peptide P5 truncations against Pseudomonas aeruginosa and Staphylococcus aureus.
1037	DRAMP03920	Cecropin A (1-8)-melittin (1-13)hybrid peptide	"Cecropin A"[All Fields] AND biofilm[All Fields]	Cecropin A	30755738	In vitro activities of antimicrobial peptides and ceragenins against Legionella pneumophila.
1037	DRAMP03920	Cecropin A (1-8)-melittin (1-13)hybrid peptide	"Cecropin A"[All Fields] AND biofilm[All Fields]	Cecropin A	28178190	High Specific Selectivity and Membrane-Active Mechanism of Synthetic Cationic Hybrid Antimicrobial Peptides Based on the Peptide FV7.
1037	DRAMP03920	Cecropin A (1-8)-melittin (1-13)hybrid peptide	"Cecropin A"[All Fields] AND biofilm[All Fields]	Cecropin A	25924433	[CecropinA-magainin, a new hybrid antibacterial peptide against meticillin-resistant Staphylococcus aureus].
1037	DRAMP03920	Cecropin A (1-8)-melittin (1-13)hybrid peptide	"Cecropin A"[All Fields] AND biofilm[All Fields]	Cecropin A	23070152	In vitro activities of antibiotics and antimicrobial cationic peptides alone and in combination against methicillin-resistant Staphylococcus aureus biofilms.
1038	DRAMP03921	Cecropin A (1-8)-melittin (1-18)hybrid peptide	"Cecropin A"[All Fields] AND biofilm[All Fields]	Cecropin A	32203307	Antimicrobial peptide derived from moths can eradicate UPEC biofilms and could offer a novel therapeutic option.
1038	DRAMP03921	Cecropin A (1-8)-melittin (1-18)hybrid peptide	"Cecropin A"[All Fields] AND biofilm[All Fields]	Cecropin A	32203127	Antibiofilm activities of ceragenins and antimicrobial peptides against fungal-bacterial mono and multispecies biofilms.
1038	DRAMP03921	Cecropin A (1-8)-melittin (1-18)hybrid peptide	"Cecropin A"[All Fields] AND biofilm[All Fields]	Cecropin A	32051417	The insect antimicrobial peptide cecropin A disrupts uropathogenic Escherichia coli biofilms.
1038	DRAMP03921	Cecropin A (1-8)-melittin (1-18)hybrid peptide	"Cecropin A"[All Fields] AND biofilm[All Fields]	Cecropin A	31485973	Efficacy of Indolicidin, Cecropin A (1-7)-Melittin (CAMA) and Their Combination Against Biofilm-Forming Multidrug-Resistant Enteroaggregative Escherichia coli.
1038	DRAMP03921	Cecropin A (1-8)-melittin (1-18)hybrid peptide	"Cecropin A"[All Fields] AND biofilm[All Fields]	Cecropin A	31363941	Mechanism of action of antimicrobial peptide P5 truncations against Pseudomonas aeruginosa and Staphylococcus aureus.
1038	DRAMP03921	Cecropin A (1-8)-melittin (1-18)hybrid peptide	"Cecropin A"[All Fields] AND biofilm[All Fields]	Cecropin A	30755738	In vitro activities of antimicrobial peptides and ceragenins against Legionella pneumophila.
1038	DRAMP03921	Cecropin A (1-8)-melittin (1-18)hybrid peptide	"Cecropin A"[All Fields] AND biofilm[All Fields]	Cecropin A	28178190	High Specific Selectivity and Membrane-Active Mechanism of Synthetic Cationic Hybrid Antimicrobial Peptides Based on the Peptide FV7.
1038	DRAMP03921	Cecropin A (1-8)-melittin (1-18)hybrid peptide	"Cecropin A"[All Fields] AND biofilm[All Fields]	Cecropin A	25924433	[CecropinA-magainin, a new hybrid antibacterial peptide against meticillin-resistant Staphylococcus aureus].
1038	DRAMP03921	Cecropin A (1-8)-melittin (1-18)hybrid peptide	"Cecropin A"[All Fields] AND biofilm[All Fields]	Cecropin A	23070152	In vitro activities of antibiotics and antimicrobial cationic peptides alone and in combination against methicillin-resistant Staphylococcus aureus biofilms.
1039	DRAMP03922	Cecropin A (1-8)-melittin (1-12)hybrid peptide	"Cecropin A"[All Fields] AND biofilm[All Fields]	Cecropin A	32203307	Antimicrobial peptide derived from moths can eradicate UPEC biofilms and could offer a novel therapeutic option.
1039	DRAMP03922	Cecropin A (1-8)-melittin (1-12)hybrid peptide	"Cecropin A"[All Fields] AND biofilm[All Fields]	Cecropin A	32203127	Antibiofilm activities of ceragenins and antimicrobial peptides against fungal-bacterial mono and multispecies biofilms.
1039	DRAMP03922	Cecropin A (1-8)-melittin (1-12)hybrid peptide	"Cecropin A"[All Fields] AND biofilm[All Fields]	Cecropin A	32051417	The insect antimicrobial peptide cecropin A disrupts uropathogenic Escherichia coli biofilms.
1039	DRAMP03922	Cecropin A (1-8)-melittin (1-12)hybrid peptide	"Cecropin A"[All Fields] AND biofilm[All Fields]	Cecropin A	31485973	Efficacy of Indolicidin, Cecropin A (1-7)-Melittin (CAMA) and Their Combination Against Biofilm-Forming Multidrug-Resistant Enteroaggregative Escherichia coli.
1039	DRAMP03922	Cecropin A (1-8)-melittin (1-12)hybrid peptide	"Cecropin A"[All Fields] AND biofilm[All Fields]	Cecropin A	31363941	Mechanism of action of antimicrobial peptide P5 truncations against Pseudomonas aeruginosa and Staphylococcus aureus.
1039	DRAMP03922	Cecropin A (1-8)-melittin (1-12)hybrid peptide	"Cecropin A"[All Fields] AND biofilm[All Fields]	Cecropin A	30755738	In vitro activities of antimicrobial peptides and ceragenins against Legionella pneumophila.
1039	DRAMP03922	Cecropin A (1-8)-melittin (1-12)hybrid peptide	"Cecropin A"[All Fields] AND biofilm[All Fields]	Cecropin A	28178190	High Specific Selectivity and Membrane-Active Mechanism of Synthetic Cationic Hybrid Antimicrobial Peptides Based on the Peptide FV7.
1039	DRAMP03922	Cecropin A (1-8)-melittin (1-12)hybrid peptide	"Cecropin A"[All Fields] AND biofilm[All Fields]	Cecropin A	25924433	[CecropinA-magainin, a new hybrid antibacterial peptide against meticillin-resistant Staphylococcus aureus].
1039	DRAMP03922	Cecropin A (1-8)-melittin (1-12)hybrid peptide	"Cecropin A"[All Fields] AND biofilm[All Fields]	Cecropin A	23070152	In vitro activities of antibiotics and antimicrobial cationic peptides alone and in combination against methicillin-resistant Staphylococcus aureus biofilms.
1040	DRAMP03923	Cecropin A (1-8)-melittin (1-10)hybrid peptide	"Cecropin A"[All Fields] AND biofilm[All Fields]	Cecropin A	32203307	Antimicrobial peptide derived from moths can eradicate UPEC biofilms and could offer a novel therapeutic option.
1040	DRAMP03923	Cecropin A (1-8)-melittin (1-10)hybrid peptide	"Cecropin A"[All Fields] AND biofilm[All Fields]	Cecropin A	32203127	Antibiofilm activities of ceragenins and antimicrobial peptides against fungal-bacterial mono and multispecies biofilms.
1040	DRAMP03923	Cecropin A (1-8)-melittin (1-10)hybrid peptide	"Cecropin A"[All Fields] AND biofilm[All Fields]	Cecropin A	32051417	The insect antimicrobial peptide cecropin A disrupts uropathogenic Escherichia coli biofilms.
1040	DRAMP03923	Cecropin A (1-8)-melittin (1-10)hybrid peptide	"Cecropin A"[All Fields] AND biofilm[All Fields]	Cecropin A	31485973	Efficacy of Indolicidin, Cecropin A (1-7)-Melittin (CAMA) and Their Combination Against Biofilm-Forming Multidrug-Resistant Enteroaggregative Escherichia coli.
1040	DRAMP03923	Cecropin A (1-8)-melittin (1-10)hybrid peptide	"Cecropin A"[All Fields] AND biofilm[All Fields]	Cecropin A	31363941	Mechanism of action of antimicrobial peptide P5 truncations against Pseudomonas aeruginosa and Staphylococcus aureus.
1040	DRAMP03923	Cecropin A (1-8)-melittin (1-10)hybrid peptide	"Cecropin A"[All Fields] AND biofilm[All Fields]	Cecropin A	30755738	In vitro activities of antimicrobial peptides and ceragenins against Legionella pneumophila.
1040	DRAMP03923	Cecropin A (1-8)-melittin (1-10)hybrid peptide	"Cecropin A"[All Fields] AND biofilm[All Fields]	Cecropin A	28178190	High Specific Selectivity and Membrane-Active Mechanism of Synthetic Cationic Hybrid Antimicrobial Peptides Based on the Peptide FV7.
1040	DRAMP03923	Cecropin A (1-8)-melittin (1-10)hybrid peptide	"Cecropin A"[All Fields] AND biofilm[All Fields]	Cecropin A	25924433	[CecropinA-magainin, a new hybrid antibacterial peptide against meticillin-resistant Staphylococcus aureus].
1040	DRAMP03923	Cecropin A (1-8)-melittin (1-10)hybrid peptide	"Cecropin A"[All Fields] AND biofilm[All Fields]	Cecropin A	23070152	In vitro activities of antibiotics and antimicrobial cationic peptides alone and in combination against methicillin-resistant Staphylococcus aureus biofilms.
1041	DRAMP03924	Cecropin A (1-7)-melittin (1-8)hybrid peptide	"Cecropin A"[All Fields] AND biofilm[All Fields]	Cecropin A	32203307	Antimicrobial peptide derived from moths can eradicate UPEC biofilms and could offer a novel therapeutic option.
1041	DRAMP03924	Cecropin A (1-7)-melittin (1-8)hybrid peptide	"Cecropin A"[All Fields] AND biofilm[All Fields]	Cecropin A	32203127	Antibiofilm activities of ceragenins and antimicrobial peptides against fungal-bacterial mono and multispecies biofilms.
1041	DRAMP03924	Cecropin A (1-7)-melittin (1-8)hybrid peptide	"Cecropin A"[All Fields] AND biofilm[All Fields]	Cecropin A	32051417	The insect antimicrobial peptide cecropin A disrupts uropathogenic Escherichia coli biofilms.
1041	DRAMP03924	Cecropin A (1-7)-melittin (1-8)hybrid peptide	"Cecropin A"[All Fields] AND biofilm[All Fields]	Cecropin A	31485973	Efficacy of Indolicidin, Cecropin A (1-7)-Melittin (CAMA) and Their Combination Against Biofilm-Forming Multidrug-Resistant Enteroaggregative Escherichia coli.
1041	DRAMP03924	Cecropin A (1-7)-melittin (1-8)hybrid peptide	"Cecropin A"[All Fields] AND biofilm[All Fields]	Cecropin A	31363941	Mechanism of action of antimicrobial peptide P5 truncations against Pseudomonas aeruginosa and Staphylococcus aureus.
1041	DRAMP03924	Cecropin A (1-7)-melittin (1-8)hybrid peptide	"Cecropin A"[All Fields] AND biofilm[All Fields]	Cecropin A	30755738	In vitro activities of antimicrobial peptides and ceragenins against Legionella pneumophila.
1041	DRAMP03924	Cecropin A (1-7)-melittin (1-8)hybrid peptide	"Cecropin A"[All Fields] AND biofilm[All Fields]	Cecropin A	28178190	High Specific Selectivity and Membrane-Active Mechanism of Synthetic Cationic Hybrid Antimicrobial Peptides Based on the Peptide FV7.
1041	DRAMP03924	Cecropin A (1-7)-melittin (1-8)hybrid peptide	"Cecropin A"[All Fields] AND biofilm[All Fields]	Cecropin A	25924433	[CecropinA-magainin, a new hybrid antibacterial peptide against meticillin-resistant Staphylococcus aureus].
1041	DRAMP03924	Cecropin A (1-7)-melittin (1-8)hybrid peptide	"Cecropin A"[All Fields] AND biofilm[All Fields]	Cecropin A	23070152	In vitro activities of antibiotics and antimicrobial cationic peptides alone and in combination against methicillin-resistant Staphylococcus aureus biofilms.
1042	DRAMP03925	Cecropin A (1-7)-melittin (3-10)hybrid peptide	"Cecropin A"[All Fields] AND biofilm[All Fields]	Cecropin A	32203307	Antimicrobial peptide derived from moths can eradicate UPEC biofilms and could offer a novel therapeutic option.
1042	DRAMP03925	Cecropin A (1-7)-melittin (3-10)hybrid peptide	"Cecropin A"[All Fields] AND biofilm[All Fields]	Cecropin A	32203127	Antibiofilm activities of ceragenins and antimicrobial peptides against fungal-bacterial mono and multispecies biofilms.
1042	DRAMP03925	Cecropin A (1-7)-melittin (3-10)hybrid peptide	"Cecropin A"[All Fields] AND biofilm[All Fields]	Cecropin A	32051417	The insect antimicrobial peptide cecropin A disrupts uropathogenic Escherichia coli biofilms.
1042	DRAMP03925	Cecropin A (1-7)-melittin (3-10)hybrid peptide	"Cecropin A"[All Fields] AND biofilm[All Fields]	Cecropin A	31485973	Efficacy of Indolicidin, Cecropin A (1-7)-Melittin (CAMA) and Their Combination Against Biofilm-Forming Multidrug-Resistant Enteroaggregative Escherichia coli.
1042	DRAMP03925	Cecropin A (1-7)-melittin (3-10)hybrid peptide	"Cecropin A"[All Fields] AND biofilm[All Fields]	Cecropin A	31363941	Mechanism of action of antimicrobial peptide P5 truncations against Pseudomonas aeruginosa and Staphylococcus aureus.
1042	DRAMP03925	Cecropin A (1-7)-melittin (3-10)hybrid peptide	"Cecropin A"[All Fields] AND biofilm[All Fields]	Cecropin A	30755738	In vitro activities of antimicrobial peptides and ceragenins against Legionella pneumophila.
1042	DRAMP03925	Cecropin A (1-7)-melittin (3-10)hybrid peptide	"Cecropin A"[All Fields] AND biofilm[All Fields]	Cecropin A	28178190	High Specific Selectivity and Membrane-Active Mechanism of Synthetic Cationic Hybrid Antimicrobial Peptides Based on the Peptide FV7.
1042	DRAMP03925	Cecropin A (1-7)-melittin (3-10)hybrid peptide	"Cecropin A"[All Fields] AND biofilm[All Fields]	Cecropin A	25924433	[CecropinA-magainin, a new hybrid antibacterial peptide against meticillin-resistant Staphylococcus aureus].
1042	DRAMP03925	Cecropin A (1-7)-melittin (3-10)hybrid peptide	"Cecropin A"[All Fields] AND biofilm[All Fields]	Cecropin A	23070152	In vitro activities of antibiotics and antimicrobial cationic peptides alone and in combination against methicillin-resistant Staphylococcus aureus biofilms.
1043	DRAMP03927	Cecropin A (1-7)-melittin (2-9)hybrid peptide	"Cecropin A"[All Fields] AND biofilm[All Fields]	Cecropin A	32203307	Antimicrobial peptide derived from moths can eradicate UPEC biofilms and could offer a novel therapeutic option.
1043	DRAMP03927	Cecropin A (1-7)-melittin (2-9)hybrid peptide	"Cecropin A"[All Fields] AND biofilm[All Fields]	Cecropin A	32203127	Antibiofilm activities of ceragenins and antimicrobial peptides against fungal-bacterial mono and multispecies biofilms.
1043	DRAMP03927	Cecropin A (1-7)-melittin (2-9)hybrid peptide	"Cecropin A"[All Fields] AND biofilm[All Fields]	Cecropin A	32051417	The insect antimicrobial peptide cecropin A disrupts uropathogenic Escherichia coli biofilms.
1043	DRAMP03927	Cecropin A (1-7)-melittin (2-9)hybrid peptide	"Cecropin A"[All Fields] AND biofilm[All Fields]	Cecropin A	31485973	Efficacy of Indolicidin, Cecropin A (1-7)-Melittin (CAMA) and Their Combination Against Biofilm-Forming Multidrug-Resistant Enteroaggregative Escherichia coli.
1043	DRAMP03927	Cecropin A (1-7)-melittin (2-9)hybrid peptide	"Cecropin A"[All Fields] AND biofilm[All Fields]	Cecropin A	31363941	Mechanism of action of antimicrobial peptide P5 truncations against Pseudomonas aeruginosa and Staphylococcus aureus.
1043	DRAMP03927	Cecropin A (1-7)-melittin (2-9)hybrid peptide	"Cecropin A"[All Fields] AND biofilm[All Fields]	Cecropin A	30755738	In vitro activities of antimicrobial peptides and ceragenins against Legionella pneumophila.
1043	DRAMP03927	Cecropin A (1-7)-melittin (2-9)hybrid peptide	"Cecropin A"[All Fields] AND biofilm[All Fields]	Cecropin A	28178190	High Specific Selectivity and Membrane-Active Mechanism of Synthetic Cationic Hybrid Antimicrobial Peptides Based on the Peptide FV7.

1043	DRAMP03927	Cecropin A (1-7)-melittin (2-9)hybrid peptide	"Cecropin A"[All Fields] AND biofilm[All Fields]	Cecropin A	25924433	[CecropinA-magainin, a new hybrid antibacterial peptide against meticillin-resistant Staphylococcus aureus].
1043	DRAMP03927	Cecropin A (1-7)-melittin (2-9)hybrid peptide	"Cecropin A"[All Fields] AND biofilm[All Fields]	Cecropin A	23070152	In vitro activities of antibiotics and antimicrobial cationic peptides alone and in combination against methicillin-resistant Staphylococcus aureus biofilms.
1044	DRAMP03928	Cecropin A (1-7)-melittin (4-11)hybrid peptide (CAM)	"Cecropin A"[All Fields] AND biofilm[All Fields]	Cecropin A	32203307	Antimicrobial peptide derived from moths can eradicate UPEC biofilms and could offer a novel therapeutic option.
1044	DRAMP03928	Cecropin A (1-7)-melittin (4-11)hybrid peptide (CAM)	"Cecropin A"[All Fields] AND biofilm[All Fields]	Cecropin A	32203127	Antibiofilm activities of ceragenins and antimicrobial peptides against fungal-bacterial mono and multispecies biofilms.
1044	DRAMP03928	Cecropin A (1-7)-melittin (4-11)hybrid peptide (CAM)	"Cecropin A"[All Fields] AND biofilm[All Fields]	Cecropin A	32051417	The insect antimicrobial peptide cecropin A disrupts uropathogenic Escherichia coli biofilms.
1044	DRAMP03928	Cecropin A (1-7)-melittin (4-11)hybrid peptide (CAM)	"Cecropin A"[All Fields] AND biofilm[All Fields]	Cecropin A	31485973	Efficacy of Indolicidin, Cecropin A (1-7)-Melittin (CAMA) and Their Combination Against Biofilm-Forming Multidrug-Resistant Enteroaggregative Escherichia coli.
1044	DRAMP03928	Cecropin A (1-7)-melittin (4-11)hybrid peptide (CAM)	"Cecropin A"[All Fields] AND biofilm[All Fields]	Cecropin A	31363941	Mechanism of action of antimicrobial peptide P5 truncations against Pseudomonas aeruginosa and Staphylococcus aureus.
1044	DRAMP03928	Cecropin A (1-7)-melittin (4-11)hybrid peptide (CAM)	"Cecropin A"[All Fields] AND biofilm[All Fields]	Cecropin A	30755738	In vitro activities of antimicrobial peptides and ceragenins against Legionella pneumophila.
1044	DRAMP03928	Cecropin A (1-7)-melittin (4-11)hybrid peptide (CAM)	"Cecropin A"[All Fields] AND biofilm[All Fields]	Cecropin A	28178190	High Specific Selectivity and Membrane-Active Mechanism of Synthetic Cationic Hybrid Antimicrobial Peptides Based on the Peptide FV7.
1044	DRAMP03928	Cecropin A (1-7)-melittin (4-11)hybrid peptide (CAM)	"Cecropin A"[All Fields] AND biofilm[All Fields]	Cecropin A	25924433	[CecropinA-magainin, a new hybrid antibacterial peptide against meticillin-resistant Staphylococcus aureus].
1044	DRAMP03928	Cecropin A (1-7)-melittin (4-11)hybrid peptide (CAM)	"Cecropin A"[All Fields] AND biofilm[All Fields]	Cecropin A	23070152	In vitro activities of antibiotics and antimicrobial cationic peptides alone and in combination against methicillin-resistant Staphylococcus aureus biofilms.
1045	DRAMP03929	Cecropin A (1-7)-melittin (5-12)hybrid peptide	"Cecropin A"[All Fields] AND biofilm[All Fields]	Cecropin A	32203307	Antimicrobial peptide derived from moths can eradicate UPEC biofilms and could offer a novel therapeutic option.
1045	DRAMP03929	Cecropin A (1-7)-melittin (5-12)hybrid peptide	"Cecropin A"[All Fields] AND biofilm[All Fields]	Cecropin A	32203127	Antibiofilm activities of ceragenins and antimicrobial peptides against fungal-bacterial mono and multispecies biofilms.
1045	DRAMP03929	Cecropin A (1-7)-melittin (5-12)hybrid peptide	"Cecropin A"[All Fields] AND biofilm[All Fields]	Cecropin A	32051417	The insect antimicrobial peptide cecropin A disrupts uropathogenic Escherichia coli biofilms.
1045	DRAMP03929	Cecropin A (1-7)-melittin (5-12)hybrid peptide	"Cecropin A"[All Fields] AND biofilm[All Fields]	Cecropin A	31485973	Efficacy of Indolicidin, Cecropin A (1-7)-Melittin (CAMA) and Their Combination Against Biofilm-Forming Multidrug-Resistant Enteroaggregative Escherichia coli.
1045	DRAMP03929	Cecropin A (1-7)-melittin (5-12)hybrid peptide	"Cecropin A"[All Fields] AND biofilm[All Fields]	Cecropin A	31363941	Mechanism of action of antimicrobial peptide P5 truncations against Pseudomonas aeruginosa and Staphylococcus aureus.
1045	DRAMP03929	Cecropin A (1-7)-melittin (5-12)hybrid peptide	"Cecropin A"[All Fields] AND biofilm[All Fields]	Cecropin A	30755738	In vitro activities of antimicrobial peptides and ceragenins against Legionella pneumophila.
1045	DRAMP03929	Cecropin A (1-7)-melittin (5-12)hybrid peptide	"Cecropin A"[All Fields] AND biofilm[All Fields]	Cecropin A	28178190	High Specific Selectivity and Membrane-Active Mechanism of Synthetic Cationic Hybrid Antimicrobial Peptides Based on the Peptide FV7.
1045	DRAMP03929	Cecropin A (1-7)-melittin (5-12)hybrid peptide	"Cecropin A"[All Fields] AND biofilm[All Fields]	Cecropin A	25924433	[CecropinA-magainin, a new hybrid antibacterial peptide against meticillin-resistant Staphylococcus aureus].
1045	DRAMP03929	Cecropin A (1-7)-melittin (5-12)hybrid peptide	"Cecropin A"[All Fields] AND biofilm[All Fields]	Cecropin A	23070152	In vitro activities of antibiotics and antimicrobial cationic peptides alone and in combination against methicillin-resistant Staphylococcus aureus biofilms.
1046	DRAMP03930	Cecropin A (1-7)-melittin (6-13)hybrid peptide	"Cecropin A"[All Fields] AND biofilm[All Fields]	Cecropin A	32203307	Antimicrobial peptide derived from moths can eradicate UPEC biofilms and could offer a novel therapeutic option.
1046	DRAMP03930	Cecropin A (1-7)-melittin (6-13)hybrid peptide	"Cecropin A"[All Fields] AND biofilm[All Fields]	Cecropin A	32203127	Antibiofilm activities of ceragenins and antimicrobial peptides against fungal-bacterial mono and multispecies biofilms.
1046	DRAMP03930	Cecropin A (1-7)-melittin (6-13)hybrid peptide	"Cecropin A"[All Fields] AND biofilm[All Fields]	Cecropin A	32051417	The insect antimicrobial peptide cecropin A disrupts uropathogenic Escherichia coli biofilms.
1046	DRAMP03930	Cecropin A (1-7)-melittin (6-13)hybrid peptide	"Cecropin A"[All Fields] AND biofilm[All Fields]	Cecropin A	31485973	Efficacy of Indolicidin, Cecropin A (1-7)-Melittin (CAMA) and Their Combination Against Biofilm-Forming Multidrug-Resistant Enteroaggregative Escherichia coli.
1046	DRAMP03930	Cecropin A (1-7)-melittin (6-13)hybrid peptide	"Cecropin A"[All Fields] AND biofilm[All Fields]	Cecropin A	31363941	Mechanism of action of antimicrobial peptide P5 truncations against Pseudomonas aeruginosa and Staphylococcus aureus.
1046	DRAMP03930	Cecropin A (1-7)-melittin (6-13)hybrid peptide	"Cecropin A"[All Fields] AND biofilm[All Fields]	Cecropin A	30755738	In vitro activities of antimicrobial peptides and ceragenins against Legionella pneumophila.
1046	DRAMP03930	Cecropin A (1-7)-melittin (6-13)hybrid peptide	"Cecropin A"[All Fields] AND biofilm[All Fields]	Cecropin A	28178190	High Specific Selectivity and Membrane-Active Mechanism of Synthetic Cationic Hybrid Antimicrobial Peptides Based on the Peptide FV7.
1046	DRAMP03930	Cecropin A (1-7)-melittin (6-13)hybrid peptide	"Cecropin A"[All Fields] AND biofilm[All Fields]	Cecropin A	25924433	[CecropinA-magainin, a new hybrid antibacterial peptide against meticillin-resistant Staphylococcus aureus].
1046	DRAMP03930	Cecropin A (1-7)-melittin (6-13)hybrid peptide	"Cecropin A"[All Fields] AND biofilm[All Fields]	Cecropin A	23070152	In vitro activities of antibiotics and antimicrobial cationic peptides alone and in combination against methicillin-resistant Staphylococcus aureus biofilms.
1054	DRAMP03939	G20R (truncated isoform of thanatin, residue 1-20)	"G20R"[All Fields] AND biofilm[All Fields]	G20R	26078447	Single-Nucleotide Polymorphisms Found in the migA and wbpX Glycosyltransferase Genes Account for the Intrinsic Lipopolysaccharide Defects Exhibited by Pseudomonas aeruginosa PA14.
1057	DRAMP03948	Del 1 (Ranalexin analog)	"Del 1"[All Fields] AND biofilm[All Fields]	Del 1	24990903	DL-2-hydroxyisocaproic acid attenuates inflammatory responses in a murine Candida albicans biofilm model.
1065	DRAMP03967	P18 (Cecropin A(1-8)-Magainin 2(1-12) hybrid peptide analogue)	"P18"[All Fields] AND biofilm[All Fields]	P18	28453851	Eradication of Staphylococcus aureus Biofilm Infections Using Synthetic Antimicrobial Peptides.
1065	DRAMP03967	P18 (Cecropin A(1-8)-Magainin 2(1-12) hybrid peptide analogue)	"P18"[All Fields] AND biofilm[All Fields]	P18	19793900	Immunoglobulin G (IgG) class, but Not IgA or IgM, antibodies to peptides of the Porphyromonas gingivalis chaperone HtpG predict health in subjects with periodontitis by a fluorescence enzyme-linked immunosorbent assay.
1065	DRAMP03967	P18 (Cecropin A(1-8)-Magainin 2(1-12) hybrid peptide analogue)	"P18"[All Fields] AND biofilm[All Fields]	P18	17186376	How we learnt about iron acquisition in Pseudomonas aeruginosa: a series of very fortunate events.
1068	DRAMP03970	N-1 (analog of P18)	"N-1"[All Fields] AND biofilm[All Fields]	N-1	34574207	Conditions of In Vitro Biofilm Formation by Serogroups of Listeria monocytogenesIsolated from Hass Avocados Sold at Markets in Mexico.
1068	DRAMP03970	N-1 (analog of P18)	"N-1"[All Fields] AND biofilm[All Fields]	N-1	34468806	Virulence and intermediate resistance to high-end antibiotic (teicoplanin) among coagulase-negative staphylococci sourced from retail market fish.
1068	DRAMP03970	N-1 (analog of P18)	"N-1"[All Fields] AND biofilm[All Fields]	N-1	34354696	Prevalence and Characteristic of Swine-Origin mcr-1-Positive Escherichia coliin Northeastern China.
1068	DRAMP03970	N-1 (analog of P18)	"N-1"[All Fields] AND biofilm[All Fields]	N-1	34350537	Antiproliferative and antibacterial properties of biocompatible copper(II) complexes bearing chelating N,N-heterocycle ligands and potential mechanisms of action.
1068	DRAMP03970	N-1 (analog of P18)	"N-1"[All Fields] AND biofilm[All Fields]	N-1	34241669	Occurrence, Virulence and Antimicrobial Susceptibility Profiles of Cronobacter spp. from Ready-to-Eat Foods.
1068	DRAMP03970	N-1 (analog of P18)	"N-1"[All Fields] AND biofilm[All Fields]	N-1	34135604	Synergistic Activity and Biofilm Formation Effect of Colistin Combined with PFK-158 Against Colistin-Resistant Gram-Negative Bacteria.
1068	DRAMP03970	N-1 (analog of P18)	"N-1"[All Fields] AND biofilm[All Fields]	N-1	34128460	Biofilm accumulation in new flexible gastroscope channels in clinical use.
1068	DRAMP03970	N-1 (analog of P18)	"N-1"[All Fields] AND biofilm[All Fields]	N-1	34093708	Coexistence of Virulence Factors and Efflux Pump Genes in Clinical Isolates of Pseudomonas aeruginosa: Analysis of Biofilm-Forming Strains from Iran.
1068	DRAMP03970	N-1 (analog of P18)	"N-1"[All Fields] AND biofilm[All Fields]	N-1	33947705	Self-reported Metabolic Risk Factor Associations with Adenomatous, Sessile Serrated, and Synchronous Adenomatous and Sessile Serrated Polyps.
1068	DRAMP03970	N-1 (analog of P18)	"N-1"[All Fields] AND biofilm[All Fields]	N-1	33881420	Facile antibacterial materials with turbine-like structure for P. aeruginosainfected scald wound healing.
1068	DRAMP03970	N-1 (analog of P18)	"N-1"[All Fields] AND biofilm[All Fields]	N-1	33872997	Simultaneous nitrogen and phosphorus removal from simulated digested piggery wastewater in a single-stage biofilm process coupling anammox and intracellular carbon metabolism.
1068	DRAMP03970	N-1 (analog of P18)	"N-1"[All Fields] AND biofilm[All Fields]	N-1	33796542	Experience With the Use of the MicroDTTect Device for the Diagnosis of Low-Grade Chronic Prosthetic Joint Infections in a Routine Setting.
1068	DRAMP03970	N-1 (analog of P18)	"N-1"[All Fields] AND biofilm[All Fields]	N-1	33723620	Azole-Resilient Biofilms and Non-wild Type C. albicans Among Candida Species Isolated from Agricultural Soils Cultivated with Azole Fungicides: an Environmental Issue?
1068	DRAMP03970	N-1 (analog of P18)	"N-1"[All Fields] AND biofilm[All Fields]	N-1	33550210	Nitrate removal from low C/N wastewater at low temperature by immobilized Pseudomonas sp. Y39-6 with versatile nitrate metabolism pathways.
1068	DRAMP03970	N-1 (analog of P18)	"N-1"[All Fields] AND biofilm[All Fields]	N-1	33503132	Efficiency of different protocols for oral hygiene combined with the use of chlorhexidine in the prevention of ventilator-associated pneumonia.
1068	DRAMP03970	N-1 (analog of P18)	"N-1"[All Fields] AND biofilm[All Fields]	N-1	33421175	Multidrug resistant staphylococci isolated from pigs with exudative epidermitis in North eastern Region of India.
1068	DRAMP03970	N-1 (analog of P18)	"N-1"[All Fields] AND biofilm[All Fields]	N-1	33396681	Copper(II) and Zinc(II) Complexes with the Clinically Used Fluconazole: Comparison of Antifungal Activity and Therapeutic Potential.
1068	DRAMP03970	N-1 (analog of P18)	"N-1"[All Fields] AND biofilm[All Fields]	N-1	33047890	A novel gingipain regulatory gene in Porphyromonas gingivalis mediates host cell detachment and inhibition of wound closure.

1068	DRAMP03970	N-1 (analog of P18)	"N-1"[All Fields] AND biofilm[All Fields]	N-1	32978132	Biofilm Sampling for Detection of CryptosporidiumOocysts in a Southeastern Pennsylvania Watershed.
1068	DRAMP03970	N-1 (analog of P18)	"N-1"[All Fields] AND biofilm[All Fields]	N-1	32933859	Update on minocycline in vitro activity against odontogenic bacteria.
1068	DRAMP03970	N-1 (analog of P18)	"N-1"[All Fields] AND biofilm[All Fields]	N-1	32928115	Characterization of carbapenem-resistant hypervirulent Acinetobacter baumannii strains isolated from hospitalized patients in the mid-south region of China.
1068	DRAMP03970	N-1 (analog of P18)	"N-1"[All Fields] AND biofilm[All Fields]	N-1	32866008	Novel 2-Substituted 3-Hydroxy-1,6-dimethylpyridin-4(1 H)-ones as Dual-Acting Biofilm Inhibitors of Pseudomonas aeruginosa.
1068	DRAMP03970	N-1 (analog of P18)	"N-1"[All Fields] AND biofilm[All Fields]	N-1	32717492	Genotypic and phenotypic traits of bla CTX-M-carrying Escherichia coli strains from an UV-C-treated wastewater effluent.
1068	DRAMP03970	N-1 (analog of P18)	"N-1"[All Fields] AND biofilm[All Fields]	N-1	32716693	Insight into Molecular Epidemiology, Antimicrobial Resistance, and Virulence Genes of Extensively Drug-Resistant Acinetobacter baumanniiin Thailand.
1068	DRAMP03970	N-1 (analog of P18)	"N-1"[All Fields] AND biofilm[All Fields]	N-1	32598259	Synthesis and Evaluation of Antimicrobial Activity and Molecular Dock - ing of New N-1,3-thiazol-2-ylacetamides of Condensed Pyrido[3',2':4,5] furo(thieno)[3,2-d]pyrimidines.
1068	DRAMP03970	N-1 (analog of P18)	"N-1"[All Fields] AND biofilm[All Fields]	N-1	32534299	Remediation of nitrogen polluted water using Fe-C microelectrolysis and biofiltration under mixotrophic conditions.
1068	DRAMP03970	N-1 (analog of P18)	"N-1"[All Fields] AND biofilm[All Fields]	N-1	32479076	Terpenoids from the Liverwort Plagiochila fruticosaand Their Antivirulence Activity against Candida albicans.
1068	DRAMP03970	N-1 (analog of P18)	"N-1"[All Fields] AND biofilm[All Fields]	N-1	32425898	Prevalence, Genetic Diversity, and Temporary Shifts of Inducible Clindamycin Resistance Staphylococcus aureusClones in Tehran, Iran: A Molecular-Epidemiological Analysis From 2013 to 2018.
1068	DRAMP03970	N-1 (analog of P18)	"N-1"[All Fields] AND biofilm[All Fields]	N-1	32319493	Silver(i) complexes with different pyridine-4,5-dicarboxylate ligands as efficient agents for the control of cow mastitis associated pathogens.
1068	DRAMP03970	N-1 (analog of P18)	"N-1"[All Fields] AND biofilm[All Fields]	N-1	32303543	Microbial Ecology of Atlantic Salmon (Salmo salar) Hatcheries: Impacts of the Built Environment on Fish Mucosal Microbiota.
1068	DRAMP03970	N-1 (analog of P18)	"N-1"[All Fields] AND biofilm[All Fields]	N-1	32283314	SpeG polyamine acetyltransferase enzyme from Bacillus thuringiensis forms a dodecameric structure and exhibits high catalytic efficiency.
1068	DRAMP03970	N-1 (analog of P18)	"N-1"[All Fields] AND biofilm[All Fields]	N-1	32202525	Clarifying and Imaging Candida albicans Biofilms.
1068	DRAMP03970	N-1 (analog of P18)	"N-1"[All Fields] AND biofilm[All Fields]	N-1	32192961	Aerobic granular sludge for high-strength ammonium wastewater treatment: Effect of COD/N ratios, long-term stability and nitrogen removal pathways.
1068	DRAMP03970	N-1 (analog of P18)	"N-1"[All Fields] AND biofilm[All Fields]	N-1	32045645	Bacterial biofilm in colorectal cancer: What is the real mechanism of action?
1068	DRAMP03970	N-1 (analog of P18)	"N-1"[All Fields] AND biofilm[All Fields]	N-1	31854000	Species identification, virulence markers and antimicrobial resistance profiles of Aeromonas sp. isolated from marketed hard-shelled mussel (Mytilus coruscus) in Korea.
1068	DRAMP03970	N-1 (analog of P18)	"N-1"[All Fields] AND biofilm[All Fields]	N-1	31678882	Response of submerged macrophytes and leaf biofilms to the decline phase of Microcystis aeruginosa: Antioxidant response, ultrastructure, microbial properties, and potential mechanism.
1068	DRAMP03970	N-1 (analog of P18)	"N-1"[All Fields] AND biofilm[All Fields]	N-1	31520397	Real-world experience with dalbavancin therapy in gram-positive skin and soft tissue infection, bone and joint infection.
1068	DRAMP03970	N-1 (analog of P18)	"N-1"[All Fields] AND biofilm[All Fields]	N-1	31340597	The Role of Orientation of Surface Bound Dihydropyrrol-2-ones (DHP) on Biological Activity.
1068	DRAMP03970	N-1 (analog of P18)	"N-1"[All Fields] AND biofilm[All Fields]	N-1	31310778	Long-term antimicrobial suppression prevents treatment failure of streptococcal periprosthetic joint infection.
1068	DRAMP03970	N-1 (analog of P18)	"N-1"[All Fields] AND biofilm[All Fields]	N-1	31300167	Effects of vibration on anammox-enriched biofilm in a high-loaded upflow reactor.
1068	DRAMP03970	N-1 (analog of P18)	"N-1"[All Fields] AND biofilm[All Fields]	N-1	31051351	Toward N 2O emission reduction in a single-stage CANON coupled with denitrification: Investigation on nitrite simultaneous production and consumption and nitrogen transformation.
1068	DRAMP03970	N-1 (analog of P18)	"N-1"[All Fields] AND biofilm[All Fields]	N-1	30925932	Comparative genomics of human Lactobacillus crispatus isolates reveals genes for glycosylation and glycogen degradation: implications for in vivo dominance of the vaginal microbiota.
1068	DRAMP03970	N-1 (analog of P18)	"N-1"[All Fields] AND biofilm[All Fields]	N-1	30925373	Evaluation of anaerobic digestion post-treatment options using an integrated model-based approach.
1068	DRAMP03970	N-1 (analog of P18)	"N-1"[All Fields] AND biofilm[All Fields]	N-1	30803542	Influence of Adjuncts to Irrigation in the Disinfection of Large Root Canals.
1068	DRAMP03970	N-1 (analog of P18)	"N-1"[All Fields] AND biofilm[All Fields]	N-1	30683036	[Multilocus sequence analysis, biofilm production, antibiotic susceptibility and synergy tests of Burkholderia species in patients with and without cystic fibrosis].
1068	DRAMP03970	N-1 (analog of P18)	"N-1"[All Fields] AND biofilm[All Fields]	N-1	30530220	Biofilms in hospital effluents as a potential crossroads for carbapenemase-encoding strains.
1068	DRAMP03970	N-1 (analog of P18)	"N-1"[All Fields] AND biofilm[All Fields]	N-1	30317111	Phylogroups, pathotypes, biofilm formation and antimicrobial resistance of Escherichia coli isolates in farms and packing facilities of tomato, jalapeño pepper and cantaloupe from Northern Mexico.
1068	DRAMP03970	N-1 (analog of P18)	"N-1"[All Fields] AND biofilm[All Fields]	N-1	30316964	Occurrence of extended-spectrum β-lactamase-producing bacteria in urban Clinton River habitat.
1068	DRAMP03970	N-1 (analog of P18)	"N-1"[All Fields] AND biofilm[All Fields]	N-1	30229985	Characterization of virulence properties and multi-drug resistance profiles in motile Aeromonas spp. isolated from zebrafish (Danio rerio).
1068	DRAMP03970	N-1 (analog of P18)	"N-1"[All Fields] AND biofilm[All Fields]	N-1	30199274	The Characterization of Biofilm Formation and Detection of Biofilm-Related Genes in Salmonella Isolated from Beef Processing Plants.
1068	DRAMP03970	N-1 (analog of P18)	"N-1"[All Fields] AND biofilm[All Fields]	N-1	30068028	Treatment of municipal sewage with low carbon-to-nitrogen ratio via simultaneous partial nitrification, anaerobic ammonia oxidation, and denitrification (SNAD) in a non-woven rotating biological contactor.
1068	DRAMP03970	N-1 (analog of P18)	"N-1"[All Fields] AND biofilm[All Fields]	N-1	30051805	Quantification of major constituents of biofilms in occluded pancreatic stents.
1068	DRAMP03970	N-1 (analog of P18)	"N-1"[All Fields] AND biofilm[All Fields]	N-1	30015218	Effect of chitosan nanoparticles on the inhibition of Candida spp. biofilm on denture base surface.
1068	DRAMP03970	N-1 (analog of P18)	"N-1"[All Fields] AND biofilm[All Fields]	N-1	29649873	pH Dependency in Anode Biofilms of Thermincola ferriacetica Suggests a Proton-Dependent Electrochemical Response.
1068	DRAMP03970	N-1 (analog of P18)	"N-1"[All Fields] AND biofilm[All Fields]	N-1	29575083	Survival of Cronobacter in powdered infant formula and their variation in biofilm formation.
1068	DRAMP03970	N-1 (analog of P18)	"N-1"[All Fields] AND biofilm[All Fields]	N-1	29307769	Molecular epidemiology of carbapenem-resistant Acinetobacter baumannii isolates from a Korean hospital that carry bla OXA-23.
1068	DRAMP03970	N-1 (analog of P18)	"N-1"[All Fields] AND biofilm[All Fields]	N-1	29210701	Bioelectrochemical nitrogen removal as a polishing mechanism for domestic wastewater treated effluents.
1068	DRAMP03970	N-1 (analog of P18)	"N-1"[All Fields] AND biofilm[All Fields]	N-1	29195992	Phenotypic characterization of Cronobacter spp. strains isolated from foods and clinical specimens in Brazil.
1068	DRAMP03970	N-1 (analog of P18)	"N-1"[All Fields] AND biofilm[All Fields]	N-1	29178982	Microbiology Alloplastic Total Joint Infections: A 20-Year Retrospective Study.
1068	DRAMP03970	N-1 (analog of P18)	"N-1"[All Fields] AND biofilm[All Fields]	N-1	29153063	[Investigation of various virulence factors of Klebsiella pneumoniae strains isolated from nosocomial infections].
1068	DRAMP03970	N-1 (analog of P18)	"N-1"[All Fields] AND biofilm[All Fields]	N-1	29109700	Exploring the Genome and Phenotype of Multi-Drug Resistant Klebsiella pneumoniaeof Clinical Origin.
1068	DRAMP03970	N-1 (analog of P18)	"N-1"[All Fields] AND biofilm[All Fields]	N-1	28968533	Using the agricultural environment to select better surrogates for foodborne pathogens associated with fresh produce.
1068	DRAMP03970	N-1 (analog of P18)	"N-1"[All Fields] AND biofilm[All Fields]	N-1	28929959	[In vitro effect of vancomycin and daptomycin on biofilm formation of coagulase-negative staphylococci strains].
1068	DRAMP03970	N-1 (analog of P18)	"N-1"[All Fields] AND biofilm[All Fields]	N-1	28783429	Characterization of Livestock-Associated Methicillin-Resistant Staphylococcus aureus CC398 and mecC-positive CC130 from Zoo Animals in the United Kingdom.
1068	DRAMP03970	N-1 (analog of P18)	"N-1"[All Fields] AND biofilm[All Fields]	N-1	28699734	Solution and Solid-State Nuclear Magnetic Resonance Structural Investigations of the Antimicrobial Designer Peptide GL13K in Membranes.
1068	DRAMP03970	N-1 (analog of P18)	"N-1"[All Fields] AND biofilm[All Fields]	N-1	28667872	Polysubstituted 2-aminoimidazoles as anti-biofilm and antiproliferative agents: Discovery of potent lead.
1068	DRAMP03970	N-1 (analog of P18)	"N-1"[All Fields] AND biofilm[All Fields]	N-1	28636666	Molecular and microbiological report of a hospital outbreak of NDM-1-carrying Enterobacteriaceae in Mexico.
1068	DRAMP03970	N-1 (analog of P18)	"N-1"[All Fields] AND biofilm[All Fields]	N-1	28437231	Capsular Polysaccharide Types and Virulence-Related Traits of Epidemic KPC-Producing Klebsiella pneumoniae Isolates in a Chinese University Hospital.
1068	DRAMP03970	N-1 (analog of P18)	"N-1"[All Fields] AND biofilm[All Fields]	N-1	28390209	Evaluation of the Oral Tolerance of Three Fluoride Toothpaste Formulations in a Dry Mouth Population: Results from Two Randomized Studies.
1068	DRAMP03970	N-1 (analog of P18)	"N-1"[All Fields] AND biofilm[All Fields]	N-1	28242464	N 2O micro-profiles in biofilm from a one-stage autotrophic nitrogen removal system by microelectrode.
1068	DRAMP03970	N-1 (analog of P18)	"N-1"[All Fields] AND biofilm[All Fields]	N-1	30620537	[THE BIOFILM FORMATION ABILITY OF STRAINS SERRATIA SPP., SEPARATED FROM WOUNDS OF PATIENTS WITH CHRONIC OSTEOMYELITIS IN MONO-CULTURES AND IN COMPOSITION OF ASSOCIATION OF MICROORGANISMS HARVESTED IN VITRO].
1068	DRAMP03970	N-1 (analog of P18)	"N-1"[All Fields] AND biofilm[All Fields]	N-1	28225903	Sphygmomanometers and thermometers as potential fomites of Staphylococcus haemolyticus: biofilm formation in the presence of antibiotics.
1068	DRAMP03970	N-1 (analog of P18)	"N-1"[All Fields] AND biofilm[All Fields]	N-1	27902411	Non-typeable Haemophilus influenzae biofilm production and severity in lower respiratory tract infections in a tertiary hospital in Mexico.
1068	DRAMP03970	N-1 (analog of P18)	"N-1"[All Fields] AND biofilm[All Fields]	N-1	27864174	Multilocus Sequence Analysis of Phylogroup 1 and 2 Oral Treponeme Strains.
1068	DRAMP03970	N-1 (analog of P18)	"N-1"[All Fields] AND biofilm[All Fields]	N-1	27768382	Phenotypic and Molecular Characterization of Salmonella 1,4,[5],12:i:- R-Type ASSuT Isolates from Humans, Animals, and Environment in Portugal, 2006-2011.

1068	DRAMP03970	N-1 (analog of P18)	"N-1"[All Fields] AND biofilm[All Fields]	N-1	27654924	Diphenyl diselenide derivatives inhibit microbial biofilm formation involved in wound infection.
1068	DRAMP03970	N-1 (analog of P18)	"N-1"[All Fields] AND biofilm[All Fields]	N-1	27525391	[Investigation of the serotype distribution, biofilm production and antibiotic susceptibilities of group B streptococci isolated from urinary samples].
1068	DRAMP03970	N-1 (analog of P18)	"N-1"[All Fields] AND biofilm[All Fields]	N-1	27156788	Epidemiology and virulence of VIM-4 metallo-beta-lactamase-producing Pseudomonas aeruginosa isolated from burn patients in eastern Algeria.
1068	DRAMP03970	N-1 (analog of P18)	"N-1"[All Fields] AND biofilm[All Fields]	N-1	26939852	Do biofilms confer a pro-carcinogenic state?
1068	DRAMP03970	N-1 (analog of P18)	"N-1"[All Fields] AND biofilm[All Fields]	N-1	26851283	The Type IV Pilus Assembly ATPase PilB of Myxococcus xanthus Interacts with the Inner Membrane Platform Protein PilC and the Nucleotide-binding Protein PilM.
1068	DRAMP03970	N-1 (analog of P18)	"N-1"[All Fields] AND biofilm[All Fields]	N-1	26462745	Hospital-wide Eradication of a Nosocomial Legionella pneumophila Serogroup 1 Outbreak.
1068	DRAMP03970	N-1 (analog of P18)	"N-1"[All Fields] AND biofilm[All Fields]	N-1	26295304	Structural Insight into Multivalent Galactoside Binding to Pseudomonas aeruginosa Lectin LecA.
1068	DRAMP03970	N-1 (analog of P18)	"N-1"[All Fields] AND biofilm[All Fields]	N-1	26264139	Two-step nitrification in a pure moving bed biofilm reactor-membrane bioreactor for wastewater treatment: nitrifying and denitrifying microbial populations and kinetic modeling.
1068	DRAMP03970	N-1 (analog of P18)	"N-1"[All Fields] AND biofilm[All Fields]	N-1	25977221	Biofilms Enhance Metabolic Changes Associated with Colon Cancer.
1068	DRAMP03970	N-1 (analog of P18)	"N-1"[All Fields] AND biofilm[All Fields]	N-1	25959674	Metabolism links bacterial biofilms and colon carcinogenesis.
1068	DRAMP03970	N-1 (analog of P18)	"N-1"[All Fields] AND biofilm[All Fields]	N-1	25739638	Structure of amylase-binding protein A of Streptococcus gordonii: a potential receptor for human salivary α-amylase enzyme.
1068	DRAMP03970	N-1 (analog of P18)	"N-1"[All Fields] AND biofilm[All Fields]	N-1	25550392	Candida tropicalis isolates obtained from veterinary sources show resistance to azoles and produce virulence factors.
1068	DRAMP03970	N-1 (analog of P18)	"N-1"[All Fields] AND biofilm[All Fields]	N-1	25351488	Dynamic potential-dependent electron transport pathway shifts in anode biofilms of Geobacter sulfurreducens.
1068	DRAMP03970	N-1 (analog of P18)	"N-1"[All Fields] AND biofilm[All Fields]	N-1	24911407	Characterization of the Vibrio cholerae extracellular matrix: a top-down solid-state NMR approach.
1068	DRAMP03970	N-1 (analog of P18)	"N-1"[All Fields] AND biofilm[All Fields]	N-1	23986023	Decontamination of dental implant surface in peri-implantitis treatment: a literature review.
1068	DRAMP03970	N-1 (analog of P18)	"N-1"[All Fields] AND biofilm[All Fields]	N-1	23857391	The icaA gene in staphylococci from bovine mastitis.
1068	DRAMP03970	N-1 (analog of P18)	"N-1"[All Fields] AND biofilm[All Fields]	N-1	23241133	Investigation of motility and biofilm formation by intestinal Campylobacter concisus strains.
1068	DRAMP03970	N-1 (analog of P18)	"N-1"[All Fields] AND biofilm[All Fields]	N-1	23202567	Impact of carbon to nitrogen ratio on nitrogen removal at a low oxygen concentration in a sequencing batch biofilm reactor.
1068	DRAMP03970	N-1 (analog of P18)	"N-1"[All Fields] AND biofilm[All Fields]	N-1	23162679	The discovery of N-1 substituted 2-aminobenzimidazoles as zinc-dependent S. aureus biofilm inhibitors.
1068	DRAMP03970	N-1 (analog of P18)	"N-1"[All Fields] AND biofilm[All Fields]	N-1	23088933	Synthesis and anti-staphylococcal activity of new 4-diazopyrazole derivatives.
1068	DRAMP03970	N-1 (analog of P18)	"N-1"[All Fields] AND biofilm[All Fields]	N-1	22437021	Denitrification with corncob as carbon source and biofilm carriers.
1068	DRAMP03970	N-1 (analog of P18)	"N-1"[All Fields] AND biofilm[All Fields]	N-1	22142506	Determination of the intrinsic kinetic parameters of sulfide-oxidizing autotrophic denitrification in differential reactors containing immobilized biomass.
1068	DRAMP03970	N-1 (analog of P18)	"N-1"[All Fields] AND biofilm[All Fields]	N-1	22106508	Antimicrobial and anti-pathogenic activity of some thioureides derivatives against Erwinia amylovora phytopathogenic strains.
1068	DRAMP03970	N-1 (analog of P18)	"N-1"[All Fields] AND biofilm[All Fields]	N-1	21724327	Nitrate removal from groundwater by cooperating heterotrophic with autotrophic denitrification in a biofilm-electrode reactor.
1068	DRAMP03970	N-1 (analog of P18)	"N-1"[All Fields] AND biofilm[All Fields]	N-1	21499956	DNA microarray-based characterisation of Panton-Valentine leukocidin-positive community-acquired methicillin-resistant Staphylococcus aureus from Italy.
1068	DRAMP03970	N-1 (analog of P18)	"N-1"[All Fields] AND biofilm[All Fields]	N-1	21449474	Limitations encountered for the treatment of a low C:N waste using a modified membrane-aerated biofilm reactor.
1068	DRAMP03970	N-1 (analog of P18)	"N-1"[All Fields] AND biofilm[All Fields]	N-1	20368521	Bacterial colonization and infection of electrophysiological cardiac devices detected with sonication and swab culture.
1068	DRAMP03970	N-1 (analog of P18)	"N-1"[All Fields] AND biofilm[All Fields]	N-1	20088202	Effect of operating parameters on denitrification in an anoxic rotating biological contactor.
1068	DRAMP03970	N-1 (analog of P18)	"N-1"[All Fields] AND biofilm[All Fields]	N-1	20035372	Controlling the adhesion of the diatom Navicula perminuta using poly(N-isopropylacrylamide-co-N-(1-phenylethyl) acrylamide) films.
1068	DRAMP03970	N-1 (analog of P18)	"N-1"[All Fields] AND biofilm[All Fields]	N-1	19013791	Denitrification of nitrate contaminated groundwater with a fiber-based biofilm reactor.
1068	DRAMP03970	N-1 (analog of P18)	"N-1"[All Fields] AND biofilm[All Fields]	N-1	18823396	Evidence for recombination between a sialidase (nanH) of Actinomyces naeslundii and Actinomyces oris, previously named 'Actinomyces naeslundii genospecies 1 and 2'.
1068	DRAMP03970	N-1 (analog of P18)	"N-1"[All Fields] AND biofilm[All Fields]	N-1	18649526	[Hydrogenotrophic denitrification for the removal of nitrate in drinking water].
1068	DRAMP03970	N-1 (analog of P18)	"N-1"[All Fields] AND biofilm[All Fields]	N-1	18642340	Relationship between mass transfer coefficient and liquid flow velocity in heterogenous biofilms using microelectrodes and confocal microscopy.
1068	DRAMP03970	N-1 (analog of P18)	"N-1"[All Fields] AND biofilm[All Fields]	N-1	18327543	Biotreatment of high strength nitrate waste using immobilized preadapted sludge.
1068	DRAMP03970	N-1 (analog of P18)	"N-1"[All Fields] AND biofilm[All Fields]	N-1	16765205	A prospective study of the efficacy of routine decontamination for gastrointestinal endoscopes and the risk factors for failure.
1068	DRAMP03970	N-1 (analog of P18)	"N-1"[All Fields] AND biofilm[All Fields]	N-1	16735866	Uncertain efficacy of daptomycin for prosthetic joint infections: a prospective case series.
1068	DRAMP03970	N-1 (analog of P18)	"N-1"[All Fields] AND biofilm[All Fields]	N-1	16641457	Antibiofilm activity of GlmU enzyme inhibitors against catheter-associated uropathogens.
1068	DRAMP03970	N-1 (analog of P18)	"N-1"[All Fields] AND biofilm[All Fields]	N-1	16242551	Improved outcomes in the recent management of secondary aortoenteric fistula.
1068	DRAMP03970	N-1 (analog of P18)	"N-1"[All Fields] AND biofilm[All Fields]	N-1	16114663	Performance of an intensive pond system treating municipal wastewater in a cold region.
1068	DRAMP03970	N-1 (analog of P18)	"N-1"[All Fields] AND biofilm[All Fields]	N-1	14705015	Analysis of size distribution and areal cell density of ammonia-oxidizing bacterial microcolonies in relation to substrate microprofiles in biofilms.
1068	DRAMP03970	N-1 (analog of P18)	"N-1"[All Fields] AND biofilm[All Fields]	N-1	12902276	Fidelity of select restriction endonucleases in determining microbial diversity by terminal-restriction fragment length polymorphism.
1068	DRAMP03970	N-1 (analog of P18)	"N-1"[All Fields] AND biofilm[All Fields]	N-1	12783344	A prospective randomized study of hydrophilic polymer-coated polyurethane versus polyethylene stents in distal malignant biliary obstruction.
1068	DRAMP03970	N-1 (analog of P18)	"N-1"[All Fields] AND biofilm[All Fields]	N-1	11825946	The ica operon and biofilm production in coagulase-negative Staphylococci associated with carriage and disease in a neonatal intensive care unit.
1068	DRAMP03970	N-1 (analog of P18)	"N-1"[All Fields] AND biofilm[All Fields]	N-1	11507904	[Removal of NH4(+)-N from polluted water resources by bioremediation of biofilm process with elastic packing & micropore aerator].
1068	DRAMP03970	N-1 (analog of P18)	"N-1"[All Fields] AND biofilm[All Fields]	N-1	18626896	Spatial microbial distributions of nitrifiers and heterotrophs in mixed-population biofilms.
1068	DRAMP03970	N-1 (analog of P18)	"N-1"[All Fields] AND biofilm[All Fields]	N-1	2027196	In situ replacement of vascular prostheses infected by bacterial biofilms.
1068	DRAMP03970	N-1 (analog of P18)	"N-1"[All Fields] AND biofilm[All Fields]	N-1	18548432	The theory and design of aerobic biological treatment.
1069	DRAMP03971	N-2 (analog of P18)	"N-2"[All Fields] AND biofilm[All Fields]	N-2	34468806	Virulence and intermediate resistance to high-end antibiotic (teicoplanin) among coagulase-negative staphylococci sourced from retail market fish.
1069	DRAMP03971	N-2 (analog of P18)	"N-2"[All Fields] AND biofilm[All Fields]	N-2	34428379	Water-soluble polycarbodiimides and their cytotoxic and antifungal properties.
1069	DRAMP03971	N-2 (analog of P18)	"N-2"[All Fields] AND biofilm[All Fields]	N-2	34298368	The evaluation of GHG emissions from Shanghai municipal wastewater treatment plants based on IPCC and operational data integrated methods (ODIM).
1069	DRAMP03971	N-2 (analog of P18)	"N-2"[All Fields] AND biofilm[All Fields]	N-2	34130843	Potential dissemination mechanism of the tetC gene in Aeromonas media from the aerobic biofilm reactor under oxytetracycline stresses.
1069	DRAMP03971	N-2 (analog of P18)	"N-2"[All Fields] AND biofilm[All Fields]	N-2	34048890	Antimicrobial and biofilm inhibiting potential of an amide derivative [N-(2', 4'-dinitrophenyl)-3β-hydroxyurs-12-en-28-carbonamide] of ursolic acid by modulating membrane potential and quorum sensing against colistin resistant Acinetobacter baumannii.
1069	DRAMP03971	N-2 (analog of P18)	"N-2"[All Fields] AND biofilm[All Fields]	N-2	34020186	Model predicted N 2O production from membrane-aerated biofilm reactor is greatly affected by biofilm property settings.
1069	DRAMP03971	N-2 (analog of P18)	"N-2"[All Fields] AND biofilm[All Fields]	N-2	33886943	A novel acrylic resin palatal device contaminated with Candida albicans biofilm for denture stomatitis induction in Wistar rats.
1069	DRAMP03971	N-2 (analog of P18)	"N-2"[All Fields] AND biofilm[All Fields]	N-2	33723620	Azole-Resilient Biofilms and Non-wild Type C. albicans Among Candida Species Isolated from Agricultural Soils Cultivated with Azole Fungicides: an Environmental Issue?
1069	DRAMP03971	N-2 (analog of P18)	"N-2"[All Fields] AND biofilm[All Fields]	N-2	33491458	Complete Characterization of Polyacyltrehaloses from Mycobacterium tuberculosisH37Rv Biofilm Cultures by Multiple-Stage Linear Ion-Trap Mass Spectrometry Reveals a New Tetraacyltrehalose Family.
1069	DRAMP03971	N-2 (analog of P18)	"N-2"[All Fields] AND biofilm[All Fields]	N-2	33423133	Bacterial adhesion characteristics on implant materials for intervertebral cages: titanium or PEEK for spinal infections?
1069	DRAMP03971	N-2 (analog of P18)	"N-2"[All Fields] AND biofilm[All Fields]	N-2	33421175	Multidrug resistant staphylococci isolated from pigs with exudative epidermitis in North eastern Region of India.

1069	DRAMP03971	N-2 (analog of P18)	"N-2"[All Fields] AND biofilm[All Fields]	N-2	33396681	Copper(II) and Zinc(II) Complexes with the Clinically Used Fluconazole: Comparison of Antifungal Activity and Therapeutic Potential.
1069	DRAMP03971	N-2 (analog of P18)	"N-2"[All Fields] AND biofilm[All Fields]	N-2	33372186	Presence of the neonatal Staphylococcus capitis outbreak clone (NRCS-A) in prosthetic joint infections.
1069	DRAMP03971	N-2 (analog of P18)	"N-2"[All Fields] AND biofilm[All Fields]	N-2	33242260	High prevalence and variable fitness of fluoroquinolone-resistant avian pathogenic Escherichia coliisolated from chickens in Korea.
1069	DRAMP03971	N-2 (analog of P18)	"N-2"[All Fields] AND biofilm[All Fields]	N-2	33217567	The influence of implant surface roughness on decontamination by antimicrobial photodynamic therapy and chemical agents: A preliminary study in vitro.
1069	DRAMP03971	N-2 (analog of P18)	"N-2"[All Fields] AND biofilm[All Fields]	N-2	33022530	Impacts of sulfadiazine on the performance and membrane fouling of a hybrid moving bed biofilm reactor-membrane bioreactor system at different C/N ratios.
1069	DRAMP03971	N-2 (analog of P18)	"N-2"[All Fields] AND biofilm[All Fields]	N-2	33011901	Heparin stimulates biofilm formation of Escherichia coli strain Nissle 1917.
1069	DRAMP03971	N-2 (analog of P18)	"N-2"[All Fields] AND biofilm[All Fields]	N-2	32897322	Degradation of butachlor and propanil by Pseudomonas sp. strain But2 and Acinetobacter baumannii strain DT.
1069	DRAMP03971	N-2 (analog of P18)	"N-2"[All Fields] AND biofilm[All Fields]	N-2	32831847	Characterisation of VibrioSpecies from Surface and Drinking Water Sources and Assessment of Biocontrol Potentials of Their Bacteriophages.
1069	DRAMP03971	N-2 (analog of P18)	"N-2"[All Fields] AND biofilm[All Fields]	N-2	32825156	Copper(II) Complexes with Mixed Heterocycle Ligands as Promising Antibacterial and Antitumor Species.
1069	DRAMP03971	N-2 (analog of P18)	"N-2"[All Fields] AND biofilm[All Fields]	N-2	32717492	Genotypic and phenotypic traits of bla CTX-M-carrying Escherichia coli strains from an UV-C-treated wastewater effluent.
1069	DRAMP03971	N-2 (analog of P18)	"N-2"[All Fields] AND biofilm[All Fields]	N-2	32716693	Insight into Molecular Epidemiology, Antimicrobial Resistance, and Virulence Genes of Extensively Drug-Resistant Acinetobacter baumanniiin Thailand.
1069	DRAMP03971	N-2 (analog of P18)	"N-2"[All Fields] AND biofilm[All Fields]	N-2	32507736	Unravelling nitrogen removal and nitrous oxide emission from mainstream integrated nitrification-partial denitrification-anammox for low carbon/nitrogen domestic wastewater.
1069	DRAMP03971	N-2 (analog of P18)	"N-2"[All Fields] AND biofilm[All Fields]	N-2	32425898	Prevalence, Genetic Diversity, and Temporary Shifts of Inducible Clindamycin Resistance Staphylococcus aureusClones in Tehran, Iran: A Molecular-Epidemiological Analysis From 2013 to 2018.
1069	DRAMP03971	N-2 (analog of P18)	"N-2"[All Fields] AND biofilm[All Fields]	N-2	32419642	Comparative analysis of brewing wastewater and lactate as carbon sources for microbial community treating acid mine drainage in anaerobic MBBR systems.
1069	DRAMP03971	N-2 (analog of P18)	"N-2"[All Fields] AND biofilm[All Fields]	N-2	32380771	Induction of Antibacterial Metabolites by Co-Cultivation of Two Red-Sea-Sponge-Associated Actinomycetes Micromonosporasp. UR56 and Actinokinesporasp. EG49.
1069	DRAMP03971	N-2 (analog of P18)	"N-2"[All Fields] AND biofilm[All Fields]	N-2	32346445	Antibacterial effect of chitosan and its derivative on Enterococcus faecalisassociated with endodontic infection.
1069	DRAMP03971	N-2 (analog of P18)	"N-2"[All Fields] AND biofilm[All Fields]	N-2	32319493	Silver(i) complexes with different pyridine-4,5-dicarboxylate ligands as efficient agents for the control of cow mastitis associated pathogens.
1069	DRAMP03971	N-2 (analog of P18)	"N-2"[All Fields] AND biofilm[All Fields]	N-2	32303543	Microbial Ecology of Atlantic Salmon (Salmo salar) Hatcheries: Impacts of the Built Environment on Fish Mucosal Microbiota.
1069	DRAMP03971	N-2 (analog of P18)	"N-2"[All Fields] AND biofilm[All Fields]	N-2	32298434	Extended Contact Lens Wear Promotes Corneal Norepinephrine Secretion and Pseudomonas aeruginosa Infection in Mice.
1069	DRAMP03971	N-2 (analog of P18)	"N-2"[All Fields] AND biofilm[All Fields]	N-2	32272386	The potential advantages of using a poly(HPMA) brush in urinary catheters: effects on biofilm cells and architecture.
1069	DRAMP03971	N-2 (analog of P18)	"N-2"[All Fields] AND biofilm[All Fields]	N-2	32236357	Effect of a calcium hydroxide-based intracanal medicament containing N-2-methyl pyrrolidone as a vehicle against Enterococcus faecalis biofilm.
1069	DRAMP03971	N-2 (analog of P18)	"N-2"[All Fields] AND biofilm[All Fields]	N-2	32122829	Synthesis, Bacterial biofilm inhibition and cytotoxicity of new N-Alkyl/aralkyl-N-(2,3-dihydro-1,4-benzodioxin-6-yl)-4-nitrobenzenesulfonamides.
1069	DRAMP03971	N-2 (analog of P18)	"N-2"[All Fields] AND biofilm[All Fields]	N-2	32089315	Characterization of Klebsiella isolates obtained from clinical mastitis cases in dairy cattle.
1069	DRAMP03971	N-2 (analog of P18)	"N-2"[All Fields] AND biofilm[All Fields]	N-2	31892248	N-(2-Arylmethylthio-4-Chloro-5-Methylbenzenesulfonyl)amide Derivatives as Potential Antimicrobial Agents-Synthesis and Biological Studies.
1069	DRAMP03971	N-2 (analog of P18)	"N-2"[All Fields] AND biofilm[All Fields]	N-2	31861286	Physicochemical, Mechanical, and Antimicrobial Properties of Novel Dental Polymers Containing Quaternary Ammonium and Trimethoxysilyl Functionalities.
1069	DRAMP03971	N-2 (analog of P18)	"N-2"[All Fields] AND biofilm[All Fields]	N-2	31854000	Species identification, virulence markers and antimicrobial resistance profiles of Aeromonas sp. isolated from marketed hard-shelled mussel (Mytilus coruscus) in Korea.
1069	DRAMP03971	N-2 (analog of P18)	"N-2"[All Fields] AND biofilm[All Fields]	N-2	31738988	Whole-genome sequencing identifies highly related Pseudomonas aeruginosa strains in multiple washbasin U-bends at several locations in one hospital: evidence for trafficking of potential pathogens via wastewater pipes.
1069	DRAMP03971	N-2 (analog of P18)	"N-2"[All Fields] AND biofilm[All Fields]	N-2	31679877	Enhancement of the denitrification in low C/N condition and its mechanism by a novel isolated Comamonas sp. YSF15.
1069	DRAMP03971	N-2 (analog of P18)	"N-2"[All Fields] AND biofilm[All Fields]	N-2	31557551	Effect of different polishing techniques for composite resin materials on surface properties and bacterial biofilm formation.
1069	DRAMP03971	N-2 (analog of P18)	"N-2"[All Fields] AND biofilm[All Fields]	N-2	31520397	Real-world experience with dalbavancin therapy in gram-positive skin and soft tissue infection, bone and joint infection.
1069	DRAMP03971	N-2 (analog of P18)	"N-2"[All Fields] AND biofilm[All Fields]	N-2	31436941	In vitro evaluation of the effectiveness of alkaline peroxide solutions in reducing the viability of specific biofilms.
1069	DRAMP03971	N-2 (analog of P18)	"N-2"[All Fields] AND biofilm[All Fields]	N-2	31259730	Antimicrobial assesment of aroylhydrazone derivatives in vitro.
1069	DRAMP03971	N-2 (analog of P18)	"N-2"[All Fields] AND biofilm[All Fields]	N-2	31225637	Using kinetics and modeling to predict denitrification fluxes in elemental-sulfur-based biofilms.
1069	DRAMP03971	N-2 (analog of P18)	"N-2"[All Fields] AND biofilm[All Fields]	N-2	31088356	Accessory gene regulator (agr) dysfunction was unusual in Staphylococcus aureus isolated from Chinese children.
1069	DRAMP03971	N-2 (analog of P18)	"N-2"[All Fields] AND biofilm[All Fields]	N-2	31051351	Toward N 2O emission reduction in a single-stage CANON coupled with denitrification: Investigation on nitrite simultaneous production and consumption and nitrogen transformation.
1069	DRAMP03971	N-2 (analog of P18)	"N-2"[All Fields] AND biofilm[All Fields]	N-2	31015181	Biological denitrification in an anoxic sequencing batch biofilm reactor: Performance evaluation, nitrous oxide emission and microbial community.
1069	DRAMP03971	N-2 (analog of P18)	"N-2"[All Fields] AND biofilm[All Fields]	N-2	30935918	Nanometric ion pair complexes of tobramycin forming microparticles for the treatment of Pseudomonas aeruginosa infections in cystic fibrosis.
1069	DRAMP03971	N-2 (analog of P18)	"N-2"[All Fields] AND biofilm[All Fields]	N-2	30928528	Considering the plug-flow behavior of the gas phase in nitrifying BAF models significantly improves the prediction of N 2O emissions.
1069	DRAMP03971	N-2 (analog of P18)	"N-2"[All Fields] AND biofilm[All Fields]	N-2	30874807	Candida isolates causing candidemia show different degrees of virulence in Galleria mellonella.
1069	DRAMP03971	N-2 (analog of P18)	"N-2"[All Fields] AND biofilm[All Fields]	N-2	30803542	Influence of Adjuncts to Irrigation in the Disinfection of Large Root Canals.
1069	DRAMP03971	N-2 (analog of P18)	"N-2"[All Fields] AND biofilm[All Fields]	N-2	30754005	Enhanced simultaneous nitrification and denitrification in treating low carbon-to-nitrogen ratio wastewater: Treatment performance and nitrogen removal pathway.
1069	DRAMP03971	N-2 (analog of P18)	"N-2"[All Fields] AND biofilm[All Fields]	N-2	30684360	Nitrous oxide production in autotrophic nitrogen removal granular sludge: A modeling study.
1069	DRAMP03971	N-2 (analog of P18)	"N-2"[All Fields] AND biofilm[All Fields]	N-2	30683036	[Multilocus sequence analysis, biofilm production, antibiotic susceptibility and synergy tests of Burkholderia species in patients with and without cystic fibrosis].
1069	DRAMP03971	N-2 (analog of P18)	"N-2"[All Fields] AND biofilm[All Fields]	N-2	30680880	The antitoxin MqsA homologue in Pseudomonas fluorescens 2P24 has a rewired regulatory circuit through evolution.
1069	DRAMP03971	N-2 (analog of P18)	"N-2"[All Fields] AND biofilm[All Fields]	N-2	30649481	Unequivocal identification of an underestimated opportunistic yeast species, Cyberlindnera fabianii, and its close relatives using a dual-function PCR and literature review of published cases.
1069	DRAMP03971	N-2 (analog of P18)	"N-2"[All Fields] AND biofilm[All Fields]	N-2	30565684	Feasibility of shotgun metagenomics to assess microbial ecology of pediatric tracheostomy tubes.
1069	DRAMP03971	N-2 (analog of P18)	"N-2"[All Fields] AND biofilm[All Fields]	N-2	30530220	Biofilms in hospital effluents as a potential crossroads for carbapenemase-encoding strains.
1069	DRAMP03971	N-2 (analog of P18)	"N-2"[All Fields] AND biofilm[All Fields]	N-2	30363376	Molecular epidemiology, antimicrobial susceptibility, and pulsed-field gel electrophoresis genotyping of Pseudomonas aeruginosaisolates from mink.
1069	DRAMP03971	N-2 (analog of P18)	"N-2"[All Fields] AND biofilm[All Fields]	N-2	30304005	Influence of the use of complete denture adhesives on microbial adhesion and biofilm formation by single- and mixed-species.
1069	DRAMP03971	N-2 (analog of P18)	"N-2"[All Fields] AND biofilm[All Fields]	N-2	30257693	Complementary and alternative medicine for treatment of atopic eczema in children under 14 years old: a systematic review and meta-analysis of randomized controlled trials.
1069	DRAMP03971	N-2 (analog of P18)	"N-2"[All Fields] AND biofilm[All Fields]	N-2	30229985	Characterization of virulence properties and multi-drug resistance profiles in motile Aeromonas spp. isolated from zebrafish (Danio rerio).
1069	DRAMP03971	N-2 (analog of P18)	"N-2"[All Fields] AND biofilm[All Fields]	N-2	30199274	The Characterization of Biofilm Formation and Detection of Biofilm-Related Genes in Salmonella Isolated from Beef Processing Plants.
1069	DRAMP03971	N-2 (analog of P18)	"N-2"[All Fields] AND biofilm[All Fields]	N-2	30076977	Remarkable geographical variations between India and Europe in carriage of the staphylococcal surface protein-encoding sasX/sesI and in the population structure of methicillin-resistant Staphylococcus aureus belonging to clonal complex 8.
1069	DRAMP03971	N-2 (analog of P18)	"N-2"[All Fields] AND biofilm[All Fields]	N-2	30068028	Treatment of municipal sewage with low carbon-to-nitrogen ratio via simultaneous partial nitrification, anaerobic ammonia oxidation, and denitrification (SNAD) in a non-woven rotating biological contactor.
1069	DRAMP03971	N-2 (analog of P18)	"N-2"[All Fields] AND biofilm[All Fields]	N-2	30051805	Quantification of major constituents of biofilms in occluded pancreatic stents.
1069	DRAMP03971	N-2 (analog of P18)	"N-2"[All Fields] AND biofilm[All Fields]	N-2	29907449	High-risk Staphylococcus aureus transmission in the operating room: A call for widespread improvements in perioperative hand hygiene and patient decolonization practices.
1069	DRAMP03971	N-2 (analog of P18)	"N-2"[All Fields] AND biofilm[All Fields]	N-2	29900920	Effectiveness of nanoparticles solutions and conventional endodontic irrigants against Enterococcus faecalisbiofilm.

1069	DRAMP03971	N-2 (analog of P18)	"N-2"[All Fields] AND biofilm[All Fields]	N-2	29858724	Changes in biooxidation mechanism and transient biofilm characteristics by As(V) during arsenopyrite colonization with Acidithiobacillus thiooxidans.
1069	DRAMP03971	N-2 (analog of P18)	"N-2"[All Fields] AND biofilm[All Fields]	N-2	29789163	Biodegradation of resin-dentin interfaces is dependent on the restorative material, mode of adhesion, esterase or MMP inhibition.
1069	DRAMP03971	N-2 (analog of P18)	"N-2"[All Fields] AND biofilm[All Fields]	N-2	29754156	[Innovative application of small molecules to influence -pathogenicity of dental plaque].
1069	DRAMP03971	N-2 (analog of P18)	"N-2"[All Fields] AND biofilm[All Fields]	N-2	29739319	Characterization of biofilm-forming capacity and resistance to sanitizers of a range of E. coli O26 pathotypes from clinical cases and cattle in Australia.
1069	DRAMP03971	N-2 (analog of P18)	"N-2"[All Fields] AND biofilm[All Fields]	N-2	29680367	Immobilization of Azospira sp. strain I13 by gel entrapment for mitigation of N 2O from biological wastewater treatment plants: Biokinetic characterization and modeling.
1069	DRAMP03971	N-2 (analog of P18)	"N-2"[All Fields] AND biofilm[All Fields]	N-2	29510961	
1069	DRAMP03971	N-2 (analog of P18)	"N-2"[All Fields] AND biofilm[All Fields]	N-2	29372575	Distribution of Ebp pili among clinical and fecal isolates of Enterococcus faecalis and evaluation for human platelet activation.
1069	DRAMP03971	N-2 (analog of P18)	"N-2"[All Fields] AND biofilm[All Fields]	N-2	29235267	Nitrate Esters of Heteroaromatic Compounds as Candida albicans CYP51 Enzyme Inhibitors.
1069	DRAMP03971	N-2 (analog of P18)	"N-2"[All Fields] AND biofilm[All Fields]	N-2	29195992	Phenotypic characterization of Cronobacter spp. strains isolated from foods and clinical specimens in Brazil.
1069	DRAMP03971	N-2 (analog of P18)	"N-2"[All Fields] AND biofilm[All Fields]	N-2	29152579	Streptococcus pneumoniaeTIGR4 Phase-Locked Opacity Variants Differ in Virulence Phenotypes.
1069	DRAMP03971	N-2 (analog of P18)	"N-2"[All Fields] AND biofilm[All Fields]	N-2	28750776	In vitro biofilm formation on resin-based composites after different finishing and polishing procedures.
1069	DRAMP03971	N-2 (analog of P18)	"N-2"[All Fields] AND biofilm[All Fields]	N-2	28702905	Assessment of biofilm changes and concentration-depth profiles during arsenopyrite oxidation by Acidithiobacillus thiooxidans.
1069	DRAMP03971	N-2 (analog of P18)	"N-2"[All Fields] AND biofilm[All Fields]	N-2	28595275	Antifungal susceptibility of Sporothrix schenckii complex biofilms.
1069	DRAMP03971	N-2 (analog of P18)	"N-2"[All Fields] AND biofilm[All Fields]	N-2	28390209	Evaluation of the Oral Tolerance of Three Fluoride Toothpaste Formulations in a Dry Mouth Population: Results from Two Randomized Studies.
1069	DRAMP03971	N-2 (analog of P18)	"N-2"[All Fields] AND biofilm[All Fields]	N-2	28387608	Common Polymorphisms in IFI16 and AIM2 Genes Are Associated With Periodontal Disease.
1069	DRAMP03971	N-2 (analog of P18)	"N-2"[All Fields] AND biofilm[All Fields]	N-2	28289036	Characterizing the Antimicrobial Activity of N 2, N 4-Disubstituted Quinazoline-2,4-Diamines toward Multidrug-Resistant Acinetobacter baumannii.
1069	DRAMP03971	N-2 (analog of P18)	"N-2"[All Fields] AND biofilm[All Fields]	N-2	28081368	Smart Metal-Organic Framework Coatings: Triggered Antibiofilm Compound Release.
1069	DRAMP03971	N-2 (analog of P18)	"N-2"[All Fields] AND biofilm[All Fields]	N-2	27902784	Dynamic in vivo mutations within the ica operon during persistence of Staphylococcus aureus in the airways of cystic fibrosis patients.
1069	DRAMP03971	N-2 (analog of P18)	"N-2"[All Fields] AND biofilm[All Fields]	N-2	27690310	Transformation of diclofenac in hybrid biofilm-activated sludge processes.
1069	DRAMP03971	N-2 (analog of P18)	"N-2"[All Fields] AND biofilm[All Fields]	N-2	27476444	A novel compound to maintain a healthy oral plaque ecology in vitro.
1069	DRAMP03971	N-2 (analog of P18)	"N-2"[All Fields] AND biofilm[All Fields]	N-2	27341658	Evaluation of ethyl N-(2-phenethyl) carbamate analogues as biofilm inhibitors of methicillin resistant Staphylococcus aureus.
1069	DRAMP03971	N-2 (analog of P18)	"N-2"[All Fields] AND biofilm[All Fields]	N-2	27170047	Peri-implant and periodontal microbiome diversity in aggressive periodontitis patients: a pilot study.
1069	DRAMP03971	N-2 (analog of P18)	"N-2"[All Fields] AND biofilm[All Fields]	N-2	27118030	Influence of chelation strength and bacterial uptake of gallium salicylidene acylhydrazide on biofilm formation and virulence of Pseudomonas aeruginosa.
1069	DRAMP03971	N-2 (analog of P18)	"N-2"[All Fields] AND biofilm[All Fields]	N-2	27016913	Microbiosensor for the detection of acetate in electrode-respiring biofilms.
1069	DRAMP03971	N-2 (analog of P18)	"N-2"[All Fields] AND biofilm[All Fields]	N-2	26839888	New Derivatives of Pyridoxine Exhibit High Antibacterial Activity against Biofilm-Embedded Staphylococcus Cells.
1069	DRAMP03971	N-2 (analog of P18)	"N-2"[All Fields] AND biofilm[All Fields]	N-2	26778460	Increased in vitro phenol-soluble modulin production is associated with soft tissue infection source in clinical isolates of methicillin-susceptible Staphylococcus aureus.
1069	DRAMP03971	N-2 (analog of P18)	"N-2"[All Fields] AND biofilm[All Fields]	N-2	26711774	Impairment of Pseudomonas aeruginosa Biofilm Resistance to Antibiotics by Combining the Drugs with a New Quorum-Sensing Inhibitor.
1069	DRAMP03971	N-2 (analog of P18)	"N-2"[All Fields] AND biofilm[All Fields]	N-2	26687023	Silver(I) complexes with phthalazine and quinazoline as effective agents against pathogenic Pseudomonas aeruginosa strains.
1069	DRAMP03971	N-2 (analog of P18)	"N-2"[All Fields] AND biofilm[All Fields]	N-2	26545862	In vitro study of the antibacterial properties and impact strength of dental acrylic resins modified with a nanomaterial.
1069	DRAMP03971	N-2 (analog of P18)	"N-2"[All Fields] AND biofilm[All Fields]	N-2	26392828	A nanoliter microfluidic serial dilution bioreactor.
1069	DRAMP03971	N-2 (analog of P18)	"N-2"[All Fields] AND biofilm[All Fields]	N-2	26319981	Antibacterial TAP-mimic electrospun polymer scaffold: effects on P. gingivalis-infected dentin biofilm.
1069	DRAMP03971	N-2 (analog of P18)	"N-2"[All Fields] AND biofilm[All Fields]	N-2	26254582	β-N-methylamino-l-alanine (BMAA) and isomers: Distribution in different food web compartments of Thau lagoon, French Mediterranean Sea.
1069	DRAMP03971	N-2 (analog of P18)	"N-2"[All Fields] AND biofilm[All Fields]	N-2	25828258	Grazing livestock are exposed to terrestrial cyanobacteria.
1069	DRAMP03971	N-2 (analog of P18)	"N-2"[All Fields] AND biofilm[All Fields]	N-2	25712314	In vitro activity of gentamicin, vancomycin or amikacin combined with EDTA or l-arginine as lock therapy against a wide spectrum of biofilm-forming clinical strains isolated from catheter-related infections.
1069	DRAMP03971	N-2 (analog of P18)	"N-2"[All Fields] AND biofilm[All Fields]	N-2	25698261	Effects of ciprofloxacin-containing scaffolds on enterococcus faecalis biofilms.
1069	DRAMP03971	N-2 (analog of P18)	"N-2"[All Fields] AND biofilm[All Fields]	N-2	25550392	Candida tropicalis isolates obtained from veterinary sources show resistance to azoles and produce virulence factors.
1069	DRAMP03971	N-2 (analog of P18)	"N-2"[All Fields] AND biofilm[All Fields]	N-2	25537200	A DNA dot hybridization model for assessment of bacterial bioburden in orthokeratology lens storage cases.
1069	DRAMP03971	N-2 (analog of P18)	"N-2"[All Fields] AND biofilm[All Fields]	N-2	25529313	The structure and component characteristics of partial nitrification biofilms under autotrophic and heterotrophic conditions.
1069	DRAMP03971	N-2 (analog of P18)	"N-2"[All Fields] AND biofilm[All Fields]	N-2	25424349	Degradation of forchlorfenuron by nitrification and denitrification reactions in the gut and shell biofilm of Limnoperna fortunei.
1069	DRAMP03971	N-2 (analog of P18)	"N-2"[All Fields] AND biofilm[All Fields]	N-2	25186318	Tolerability of High Doses of Daptomycin in the Treatment of Prosthetic Vascular Graft Infection: A Retrospective Study.
1069	DRAMP03971	N-2 (analog of P18)	"N-2"[All Fields] AND biofilm[All Fields]	N-2	25120054	Bioevaluation of novel anti-biofilm coatings based on PVP/Fe3O4 nanostructures and 2-((4-ethylphenoxy)methyl)-N- (arylcarbamothioyl)benzamides.
1069	DRAMP03971	N-2 (analog of P18)	"N-2"[All Fields] AND biofilm[All Fields]	N-2	24888450	The continuum heterogeneous biofilm model with multiple limiting substrate Monod kinetics.
1069	DRAMP03971	N-2 (analog of P18)	"N-2"[All Fields] AND biofilm[All Fields]	N-2	24862710	Extension of bactericidal effect of sodium hypochlorite into dentinal tubules.
1069	DRAMP03971	N-2 (analog of P18)	"N-2"[All Fields] AND biofilm[All Fields]	N-2	24837332	Proton and gallium(III) binding properties of a biologically active salicylidene acylhydrazide.
1069	DRAMP03971	N-2 (analog of P18)	"N-2"[All Fields] AND biofilm[All Fields]	N-2	24837331	The gallium(III)-salicylidene acylhydrazide complex shows synergistic anti-biofilm effect and inhibits toxin production by Pseudomonas aeruginosa.
1069	DRAMP03971	N-2 (analog of P18)	"N-2"[All Fields] AND biofilm[All Fields]	N-2	24531242	Syntheses and biological evaluation of 2-amino-3-acyl-tetrahydrobenzothiophene derivatives; antibacterial agents with antivirulence activity.
1069	DRAMP03971	N-2 (analog of P18)	"N-2"[All Fields] AND biofilm[All Fields]	N-2	24531077	Dynamics and functions of bacterial communities in bark, charcoal and sand filters treating greywater.
1069	DRAMP03971	N-2 (analog of P18)	"N-2"[All Fields] AND biofilm[All Fields]	N-2	24252780	Evaluation of the in vitro activities of ceftobiprole and comparators in staphylococcal colony or microtitre plate biofilm assays.
1069	DRAMP03971	N-2 (analog of P18)	"N-2"[All Fields] AND biofilm[All Fields]	N-2	24057919	Randomized clinical evaluation of a light-cured base material for complete dentures.
1069	DRAMP03971	N-2 (analog of P18)	"N-2"[All Fields] AND biofilm[All Fields]	N-2	23892192	Nanotechnology as a therapeutic tool to combat microbial resistance.
1069	DRAMP03971	N-2 (analog of P18)	"N-2"[All Fields] AND biofilm[All Fields]	N-2	23857391	The icaA gene in staphylococci from bovine mastitis.
1069	DRAMP03971	N-2 (analog of P18)	"N-2"[All Fields] AND biofilm[All Fields]	N-2	23390919	[Effects of Imipenem, Tobramycin and Curcumin on Biofilm Formation of Pseudomonas aeruginosa Strains].
1069	DRAMP03971	N-2 (analog of P18)	"N-2"[All Fields] AND biofilm[All Fields]	N-2	23108874	Biodegradation of amine waste generated from post-combustion CO(2) capture in a moving bed biofilm treatment system.
1069	DRAMP03971	N-2 (analog of P18)	"N-2"[All Fields] AND biofilm[All Fields]	N-2	23047748	Enzymatically active and inactive phosphodiesterases and diguanylate cyclases are involved in regulation of Motility or sessility in Escherichia coli CFT073.
1069	DRAMP03971	N-2 (analog of P18)	"N-2"[All Fields] AND biofilm[All Fields]	N-2	22584430	Influence of the surface speciation on biofilm attachment to chalcopyrite by Acidithiobacillus thiooxidans.
1069	DRAMP03971	N-2 (analog of P18)	"N-2"[All Fields] AND biofilm[All Fields]	N-2	22492461	Shell biofilm nitrification and gut denitrification contribute to emission of nitrous oxide by the invasive freshwater mussel Dreissena polymorpha (zebra mussel).
1069	DRAMP03971	N-2 (analog of P18)	"N-2"[All Fields] AND biofilm[All Fields]	N-2	22465726	Moisture effects on greenhouse gases generation in nitrifying gas-phase compost biofilters.
1069	DRAMP03971	N-2 (analog of P18)	"N-2"[All Fields] AND biofilm[All Fields]	N-2	22226397	Temperature dependence of denitrification in phototrophic river biofilms.

1069	DRAMP03971	N-2 (analog of P18)	"N-2"[All Fields] AND biofilm[All Fields]	N-2	22113561	Influence of the sulfur species reactivity on biofilm conformation during pyrite colonization by Acidithiobacillus thiooxidans.
1069	DRAMP03971	N-2 (analog of P18)	"N-2"[All Fields] AND biofilm[All Fields]	N-2	22100574	Listeria monocytogenes-associated joint and bone infections: a study of 43 consecutive cases.
1069	DRAMP03971	N-2 (analog of P18)	"N-2"[All Fields] AND biofilm[All Fields]	N-2	22078323	Implant sonication for the diagnosis of prosthetic elbow infection.
1069	DRAMP03971	N-2 (analog of P18)	"N-2"[All Fields] AND biofilm[All Fields]	N-2	21972885	Synthesis and characterization of poly(N-hydroxyethylacrylamide) for long-term antifouling ability.
1069	DRAMP03971	N-2 (analog of P18)	"N-2"[All Fields] AND biofilm[All Fields]	N-2	21906939	Selection of Type I and Type II methanotrophic proteobacteria in a fluidized bed reactor under non-sterile conditions.
1069	DRAMP03971	N-2 (analog of P18)	"N-2"[All Fields] AND biofilm[All Fields]	N-2	21809982	Exopolysaccharide production is required for biofilm formation and plant colonization by the nitrogen-fixing endophyte Gluconacetobacter diazotrophicus.
1069	DRAMP03971	N-2 (analog of P18)	"N-2"[All Fields] AND biofilm[All Fields]	N-2	21773763	Evolution of biofilms during the colonization process of pyrite by Acidithiobacillus thiooxidans.
1069	DRAMP03971	N-2 (analog of P18)	"N-2"[All Fields] AND biofilm[All Fields]	N-2	21756192	Silver colloidal nanoparticles: antifungal effect against adhered cells and biofilms of Candida albicans and Candida glabrata.
1069	DRAMP03971	N-2 (analog of P18)	"N-2"[All Fields] AND biofilm[All Fields]	N-2	21508550	Impact of precipitation on the treatment of real ion-exchange brine using the H(2)-based membrane biofilm reactor.
1069	DRAMP03971	N-2 (analog of P18)	"N-2"[All Fields] AND biofilm[All Fields]	N-2	21421764	Role of the biofilm master regulator CsgD in cross-regulation between biofilm formation and flagellar synthesis.
1069	DRAMP03971	N-2 (analog of P18)	"N-2"[All Fields] AND biofilm[All Fields]	N-2	21132569	Study on mechanisms of colonization of nitrogen-fixing PGPB, Klebsiella pneumoniae NG14 on the root surface of rice and the formation of biofilm.
1069	DRAMP03971	N-2 (analog of P18)	"N-2"[All Fields] AND biofilm[All Fields]	N-2	20818037	Nitrification/denitrification in swine wastewater using porous ceramic sticks with plastic rings as supporting media in two-stage fixed-biofilm reactors.
1069	DRAMP03971	N-2 (analog of P18)	"N-2"[All Fields] AND biofilm[All Fields]	N-2	20815378	Sequential aeration of membrane-aerated biofilm reactors for high-rate autotrophic nitrogen removal: experimental demonstration.
1069	DRAMP03971	N-2 (analog of P18)	"N-2"[All Fields] AND biofilm[All Fields]	N-2	20617245	Synthesis and bacterial biofilm inhibition studies of ethyl N-(2-phenethyl) carbamate derivatives.
1069	DRAMP03971	N-2 (analog of P18)	"N-2"[All Fields] AND biofilm[All Fields]	N-2	20455404	[Investigation of the surface properties of Staphylococcus epidermidis strains isolated from biomaterials].
1069	DRAMP03971	N-2 (analog of P18)	"N-2"[All Fields] AND biofilm[All Fields]	N-2	20414341	Clinical implications of power toothbrushing on fluoride delivery: effects on biofilm plaque metabolism and physiology.
1069	DRAMP03971	N-2 (analog of P18)	"N-2"[All Fields] AND biofilm[All Fields]	N-2	20097816	Effects of Trp- and Arg-containing antimicrobial-peptide structure on inhibition of Escherichia coli planktonic growth and biofilm formation.
1069	DRAMP03971	N-2 (analog of P18)	"N-2"[All Fields] AND biofilm[All Fields]	N-2	19953675	Anoxic oxidation of arsenite linked to chemolithotrophic denitrification in continuous bioreactors.
1069	DRAMP03971	N-2 (analog of P18)	"N-2"[All Fields] AND biofilm[All Fields]	N-2	19944599	Rate determination and distribution of anammox activity in activated sludge treating swine wastewater.
1069	DRAMP03971	N-2 (analog of P18)	"N-2"[All Fields] AND biofilm[All Fields]	N-2	19705191	Dinitrogen-fixing cyanobacteria in microbial mats of two shallow coral reef ecosystems.
1069	DRAMP03971	N-2 (analog of P18)	"N-2"[All Fields] AND biofilm[All Fields]	N-2	19516281	Mechanisms of transient nitric oxide and nitrous oxide production in a complex biofilm.
1069	DRAMP03971	N-2 (analog of P18)	"N-2"[All Fields] AND biofilm[All Fields]	N-2	19358060	A comparative scanning electron microscopic study of biliary and pancreatic stents.
1069	DRAMP03971	N-2 (analog of P18)	"N-2"[All Fields] AND biofilm[All Fields]	N-2	19201974	Three genomes from the phylum Acidobacteria provide insight into the lifestyles of these microorganisms in soils.
1069	DRAMP03971	N-2 (analog of P18)	"N-2"[All Fields] AND biofilm[All Fields]	N-2	18823396	Evidence for recombination between a sialidase (nanH) of Actinomyces naeslundii and Actinomyces oris, previously named 'Actinomyces naeslundii genospecies 1 and 2'.
1069	DRAMP03971	N-2 (analog of P18)	"N-2"[All Fields] AND biofilm[All Fields]	N-2	18642340	Relationship between mass transfer coefficient and liquid flow velocity in heterogenous biofilms using microelectrodes and confocal microscopy.
1069	DRAMP03971	N-2 (analog of P18)	"N-2"[All Fields] AND biofilm[All Fields]	N-2	18616585	Functional robustness and gene pools of a wastewater nitrification reactor: comparison of dispersed and intact biofilms when stressed by low oxygen and low pH.
1069	DRAMP03971	N-2 (analog of P18)	"N-2"[All Fields] AND biofilm[All Fields]	N-2	18563307	N(2)O production by nitrifying biomass under anoxic and aerobic conditions.
1069	DRAMP03971	N-2 (analog of P18)	"N-2"[All Fields] AND biofilm[All Fields]	N-2	18547749	Natural and experimental Salmonella Typhimurium infections in foxes (Vulpes vulpes).
1069	DRAMP03971	N-2 (analog of P18)	"N-2"[All Fields] AND biofilm[All Fields]	N-2	18472126	Development of a 2-sludge, 3-stage system for nitrogen and phosphorous removal from nutrient-rich wastewater using granular sludge and biofilms.
1069	DRAMP03971	N-2 (analog of P18)	"N-2"[All Fields] AND biofilm[All Fields]	N-2	17915280	Heterotrophic activity compromises autotrophic nitrogen removal in membrane-aerated biofilms: results of a modeling study.
1069	DRAMP03971	N-2 (analog of P18)	"N-2"[All Fields] AND biofilm[All Fields]	N-2	17705250	Redox control bioreactor: a unique biological water processor.
1069	DRAMP03971	N-2 (analog of P18)	"N-2"[All Fields] AND biofilm[All Fields]	N-2	16242551	Improved outcomes in the recent management of secondary aortoenteric fistula.
1069	DRAMP03971	N-2 (analog of P18)	"N-2"[All Fields] AND biofilm[All Fields]	N-2	15656296	Performance evaluation of hybrid and conventional sequencing batch reactor and continuous processes.
1069	DRAMP03971	N-2 (analog of P18)	"N-2"[All Fields] AND biofilm[All Fields]	N-2	15607183	Influence of the redox condition dynamics on the removal efficiency of a laboratory-scale constructed wetland.
1069	DRAMP03971	N-2 (analog of P18)	"N-2"[All Fields] AND biofilm[All Fields]	N-2	15282131	Factors affecting production of extracellular carbohydrate complexes by Escherichia coli O157:H7.
1069	DRAMP03971	N-2 (analog of P18)	"N-2"[All Fields] AND biofilm[All Fields]	N-2	12788771	Characterization of an autotrophic nitrogen-removing biofilm from a highly loaded lab-scale rotating biological contactor.
1069	DRAMP03971	N-2 (analog of P18)	"N-2"[All Fields] AND biofilm[All Fields]	N-2	12783344	A prospective randomized study of hydrophilic polymer-coated polyurethane versus polyethylene stents in distal malignant biliary obstruction.
1069	DRAMP03971	N-2 (analog of P18)	"N-2"[All Fields] AND biofilm[All Fields]	N-2	12598189	Identification and quantification of nitrogen removal in a rotating biological contactor by 15N tracer techniques.
1069	DRAMP03971	N-2 (analog of P18)	"N-2"[All Fields] AND biofilm[All Fields]	N-2	11005919	Microbial sulfate reduction in a liquid-solid fluidized bed reactor.
1069	DRAMP03971	N-2 (analog of P18)	"N-2"[All Fields] AND biofilm[All Fields]	N-2	9626532	Interactions and biodegradation of the herbicide metolachlor with different surfaces.
1069	DRAMP03971	N-2 (analog of P18)	"N-2"[All Fields] AND biofilm[All Fields]	N-2	18609554	Biological sulfuric acid transformation: Reactor design and process optimization.
1069	DRAMP03971	N-2 (analog of P18)	"N-2"[All Fields] AND biofilm[All Fields]	N-2	1477794	Disparate efficacy of tobramycin on Ca(2+)-, Mg(2+)-, and HEPES-treated Pseudomonas aeruginosa biofilms.
1069	DRAMP03971	N-2 (analog of P18)	"N-2"[All Fields] AND biofilm[All Fields]	N-2	2027196	In situ replacement of vascular prostheses infected by bacterial biofilms.
1069	DRAMP03971	N-2 (analog of P18)	"N-2"[All Fields] AND biofilm[All Fields]	N-2	16347737	Combined oxygen and nitrous oxide microsensor for denitrification studies.
1070	DRAMP03972	N-3 (analog of P18)	"N-3"[All Fields] AND biofilm[All Fields]	N-3	34687564	Antimicrobial effects of Melaleuca alternifolia (tea tree) essential oil against biofilm-forming multi-drug resistant cystic fibrosis-associated Pseudomonas aeruginosa as a single agent and in combination with commonly nebulised antibiotics.
1070	DRAMP03972	N-3 (analog of P18)	"N-3"[All Fields] AND biofilm[All Fields]	N-3	34442671	Preservation of Underground Microbial Diversity in Ancient Subsurface Deposits (>6 Ma) of the Rio Tinto Basement.
1070	DRAMP03972	N-3 (analog of P18)	"N-3"[All Fields] AND biofilm[All Fields]	N-3	34428379	Water-soluble polycarbodiimides and their cytotoxic and antifungal properties.
1070	DRAMP03972	N-3 (analog of P18)	"N-3"[All Fields] AND biofilm[All Fields]	N-3	34354696	Prevalence and Characteristic of Swine-Origin mcr-1-Positive Escherichia coliin Northeastern China.
1070	DRAMP03972	N-3 (analog of P18)	"N-3"[All Fields] AND biofilm[All Fields]	N-3	34341384	Quorum sensing systems and related virulence factors in Pseudomonas aeruginosa isolated from chicken meat and ground beef.
1070	DRAMP03972	N-3 (analog of P18)	"N-3"[All Fields] AND biofilm[All Fields]	N-3	34274671	Insight into quorum sensing and microbial community of an anammox consortium in response to salt stress: From "Candaditus Brocadia" to "Candaditus Scalindua".
1070	DRAMP03972	N-3 (analog of P18)	"N-3"[All Fields] AND biofilm[All Fields]	N-3	34241669	Occurrence, Virulence and Antimicrobial Susceptibility Profiles of Cronobacter spp. from Ready-to-Eat Foods.
1070	DRAMP03972	N-3 (analog of P18)	"N-3"[All Fields] AND biofilm[All Fields]	N-3	34156082	Interspecies signaling affects virulence related morphological characteristics of Streptococcus pyogenes M3.
1070	DRAMP03972	N-3 (analog of P18)	"N-3"[All Fields] AND biofilm[All Fields]	N-3	34128460	Biofilm accumulation in new flexible gastroscope channels in clinical use.
1070	DRAMP03972	N-3 (analog of P18)	"N-3"[All Fields] AND biofilm[All Fields]	N-3	34083949	Occurrence, antimicrobial susceptibility, and pathogenic factors of Pseudomonas aeruginosain canine clinical samples.
1070	DRAMP03972	N-3 (analog of P18)	"N-3"[All Fields] AND biofilm[All Fields]	N-3	34074404	FeS 2nanoparticles decorated carbonized Luffa cylindrica as biofilm substrates for fabricating high performance biosensors.
1070	DRAMP03972	N-3 (analog of P18)	"N-3"[All Fields] AND biofilm[All Fields]	N-3	34068117	Staphylococcus aureusSpecific Electrospun Wound Dressings: Influence of Immobilization Technique on Antibacterial Efficiency of Novel Enzybiotic.
1070	DRAMP03972	N-3 (analog of P18)	"N-3"[All Fields] AND biofilm[All Fields]	N-3	34033820	Antibacterial Effect and Bioactivity of Innovative and Currently Used Intracanal Medicaments in Regenerative Endodontics.
1070	DRAMP03972	N-3 (analog of P18)	"N-3"[All Fields] AND biofilm[All Fields]	N-3	33813017	Antimicrobial photodynamic therapy mediated by methylene blue coupled to β-cyclodextrin reduces early colonizing microorganisms from the oral biofilm.

1070	DRAMP03972	N-3 (analog of P18)	"N-3"[All Fields] AND biofilm[All Fields]	N-3	33763882	The presence of smear layer affects the antimicrobial action of root canal sealers.
1070	DRAMP03972	N-3 (analog of P18)	"N-3"[All Fields] AND biofilm[All Fields]	N-3	33713665	An innovative role for luteolin as a natural quorum sensing inhibitor in Pseudomonas aeruginosa.
1070	DRAMP03972	N-3 (analog of P18)	"N-3"[All Fields] AND biofilm[All Fields]	N-3	33713274	First Evidence for Colonizing of Acanthamoeba T4 Genotype in Urinary Tracts of Patients with Recurrent Urinary Tract Infections.
1070	DRAMP03972	N-3 (analog of P18)	"N-3"[All Fields] AND biofilm[All Fields]	N-3	33690064	Colloidal silver combating pathogenic Pseudomonas aeruginosa and MRSA in chronic rhinosinusitis.
1070	DRAMP03972	N-3 (analog of P18)	"N-3"[All Fields] AND biofilm[All Fields]	N-3	33653525	Relative performance of commercial citric acid and quaternary ammonium sanitizers against Listeria monocytogenes under conditions relevant to food industry.
1070	DRAMP03972	N-3 (analog of P18)	"N-3"[All Fields] AND biofilm[All Fields]	N-3	33641411	The antimicrobial activity and biocompatibility of a controlled gentamicin-releasing single-layer sol-gel coating on hydroxyapatite-coated titanium.
1070	DRAMP03972	N-3 (analog of P18)	"N-3"[All Fields] AND biofilm[All Fields]	N-3	33581867	Physicomechanical, optical, and antifungal properties of polymethyl methacrylate modified with metal methacrylate monomers.
1070	DRAMP03972	N-3 (analog of P18)	"N-3"[All Fields] AND biofilm[All Fields]	N-3	33495249	AbaM Regulates Quorum Sensing, Biofilm Formation, and Virulence in Acinetobacter baumannii.
1070	DRAMP03972	N-3 (analog of P18)	"N-3"[All Fields] AND biofilm[All Fields]	N-3	33486051	A new 1-nitro-9-aminoacridine derivative targeting yeast topoisomerase II able to overcome fluconazole-resistance.
1070	DRAMP03972	N-3 (analog of P18)	"N-3"[All Fields] AND biofilm[All Fields]	N-3	33442721	Effective antimicrobial combination in vivo treatment predicted with microcalorimetry screening.
1070	DRAMP03972	N-3 (analog of P18)	"N-3"[All Fields] AND biofilm[All Fields]	N-3	33421175	Multidrug resistant staphylococci isolated from pigs with exudative epidermitis in North eastern Region of India.
1070	DRAMP03972	N-3 (analog of P18)	"N-3"[All Fields] AND biofilm[All Fields]	N-3	33398400	Quorum sensing systems, related virulence factors, and biofilm formation in Pseudomonas aeruginosa isolated from fish.
1070	DRAMP03972	N-3 (analog of P18)	"N-3"[All Fields] AND biofilm[All Fields]	N-3	33334714	Impact of biofilm formation and azoles' susceptibility in Scedosporium/Lomentospora species using an in vitro model that mimics the cystic fibrosis patients' airway environment.
1070	DRAMP03972	N-3 (analog of P18)	"N-3"[All Fields] AND biofilm[All Fields]	N-3	33321169	Cyclodextrin-mediated quorum quenching in the Aliivibrio fischeri bioluminescence model system - Modulation of bacterial communication.
1070	DRAMP03972	N-3 (analog of P18)	"N-3"[All Fields] AND biofilm[All Fields]	N-3	33242260	High prevalence and variable fitness of fluoroquinolone-resistant avian pathogenic Escherichia coliisolated from chickens in Korea.
1070	DRAMP03972	N-3 (analog of P18)	"N-3"[All Fields] AND biofilm[All Fields]	N-3	33237232	Antibacterial Activity of a New Ready-To-Use Calcium Silicate-Based Sealer.
1070	DRAMP03972	N-3 (analog of P18)	"N-3"[All Fields] AND biofilm[All Fields]	N-3	33182169	A value-added step towards promoting the serviceability of fluidized bed bioreactor in treating wastewater with low carbon to nitrogen ratio.
1070	DRAMP03972	N-3 (analog of P18)	"N-3"[All Fields] AND biofilm[All Fields]	N-3	33106990	Safety of laser-generated shockwave treatment for bacterial biofilms in a cutaneous rodent model.
1070	DRAMP03972	N-3 (analog of P18)	"N-3"[All Fields] AND biofilm[All Fields]	N-3	33078624	Effect of quorum sensing and quenching molecules on inter-kingdom biofilm formation by Penicillium expansumand bacteria.
1070	DRAMP03972	N-3 (analog of P18)	"N-3"[All Fields] AND biofilm[All Fields]	N-3	33072040	Exploiting Lactoferricin (17-30) as a Potential Antimicrobial and Antibiofilm Candidate Against Multi-Drug-Resistant Enteroaggregative Escherichia coli.
1070	DRAMP03972	N-3 (analog of P18)	"N-3"[All Fields] AND biofilm[All Fields]	N-3	33019726	New Antimicrobial Bioactivity against Multidrug-Resistant Gram-Positive Bacteria of Kinase Inhibitor IMD0354.
1070	DRAMP03972	N-3 (analog of P18)	"N-3"[All Fields] AND biofilm[All Fields]	N-3	33013789	High Occurrence of Bacterial Competition Among Clinically Documented Opportunistic Pathogens Including Achromobacter xylosoxidansin Cystic Fibrosis.
1070	DRAMP03972	N-3 (analog of P18)	"N-3"[All Fields] AND biofilm[All Fields]	N-3	32936810	Replicable simulation of distal hot water premise plumbing using convectively-mixed pipe reactors.
1070	DRAMP03972	N-3 (analog of P18)	"N-3"[All Fields] AND biofilm[All Fields]	N-3	32831847	Characterisation of VibrioSpecies from Surface and Drinking Water Sources and Assessment of Biocontrol Potentials of Their Bacteriophages.
1070	DRAMP03972	N-3 (analog of P18)	"N-3"[All Fields] AND biofilm[All Fields]	N-3	32827220	Effect of erosive and abrasive challenges on the glaze layer applied to ceramic materials.
1070	DRAMP03972	N-3 (analog of P18)	"N-3"[All Fields] AND biofilm[All Fields]	N-3	32819689	On-going nitrification in chloraminated drinking water distribution system (DWDS) is conditioned by hydraulics and disinfection strategies.
1070	DRAMP03972	N-3 (analog of P18)	"N-3"[All Fields] AND biofilm[All Fields]	N-3	32801564	Evaluation of two fixation techniques for direct observation of biofilm formation of Bacillus subtilis in situ, on Congo red agar, using scanning electron microscopy.
1070	DRAMP03972	N-3 (analog of P18)	"N-3"[All Fields] AND biofilm[All Fields]	N-3	32794395	Effect of biofilm exposure on marginal integrity of composite restorations.
1070	DRAMP03972	N-3 (analog of P18)	"N-3"[All Fields] AND biofilm[All Fields]	N-3	32793334	Pathological and microbiological impact of a gentamicin-loaded biocomposite following limited or extensive debridement in a porcine model of osteomyelitis.
1070	DRAMP03972	N-3 (analog of P18)	"N-3"[All Fields] AND biofilm[All Fields]	N-3	32793133	Isolation and Characterization of Novel Lytic Bacteriophages Infecting Epidemic Carbapenem-Resistant Klebsiella pneumoniaeStrains.
1070	DRAMP03972	N-3 (analog of P18)	"N-3"[All Fields] AND biofilm[All Fields]	N-3	32783154	Cranberry extract-based formulations for preventing bacterial biofilms.
1070	DRAMP03972	N-3 (analog of P18)	"N-3"[All Fields] AND biofilm[All Fields]	N-3	32752201	Effect of Novel Antibacterial Composites on Bacterial Biofilms.
1070	DRAMP03972	N-3 (analog of P18)	"N-3"[All Fields] AND biofilm[All Fields]	N-3	32717492	Genotypic and phenotypic traits of bla CTX-M-carrying Escherichia coli strains from an UV-C-treated wastewater effluent.
1070	DRAMP03972	N-3 (analog of P18)	"N-3"[All Fields] AND biofilm[All Fields]	N-3	32715566	The impaired quorum sensing response of Pseudomonas aeruginosa MexAB-OprM efflux pump overexpressing mutants is not due to non-physiological efflux of 3-oxo-C12-HSL.
1070	DRAMP03972	N-3 (analog of P18)	"N-3"[All Fields] AND biofilm[All Fields]	N-3	32678435	Assessment of the microbiological quality of natural mineral waters according to the manufacturing time of 20 L returnable packs in Brazil.
1070	DRAMP03972	N-3 (analog of P18)	"N-3"[All Fields] AND biofilm[All Fields]	N-3	32620725	Effect of surface characteristic of different restorative materials containing glass ionomer on Streptococcus mutansbiofilm.
1070	DRAMP03972	N-3 (analog of P18)	"N-3"[All Fields] AND biofilm[All Fields]	N-3	32616265	Prevalence and characteristics of extended-spectrum β-lactamases-producing Escherichia coli from broiler chickens at different day-age.
1070	DRAMP03972	N-3 (analog of P18)	"N-3"[All Fields] AND biofilm[All Fields]	N-3	32612313	Preferential retention of algal carbon in benthic invertebrates: Stable isotope and fatty acid evidence from an outdoor flume experiment.
1070	DRAMP03972	N-3 (analog of P18)	"N-3"[All Fields] AND biofilm[All Fields]	N-3	32523583	Pseudomonas aeruginosa N-3-Oxo-Dodecanoyl-Homoserine Lactone Impacts Mitochondrial Networks Morphology, Energetics, and Proteome in Host Cells.
1070	DRAMP03972	N-3 (analog of P18)	"N-3"[All Fields] AND biofilm[All Fields]	N-3	32505537	Anti-biofilm activity of silver nanoparticle-containing glass ionomer cements.
1070	DRAMP03972	N-3 (analog of P18)	"N-3"[All Fields] AND biofilm[All Fields]	N-3	32444905	Bacterial biofilms in infective endocarditis: an in vitro model to investigate emerging technologies of antimicrobial cardiovascular device coatings.
1070	DRAMP03972	N-3 (analog of P18)	"N-3"[All Fields] AND biofilm[All Fields]	N-3	32425898	Prevalence, Genetic Diversity, and Temporary Shifts of Inducible Clindamycin Resistance Staphylococcus aureusClones in Tehran, Iran: A Molecular-Epidemiological Analysis From 2013 to 2018.
1070	DRAMP03972	N-3 (analog of P18)	"N-3"[All Fields] AND biofilm[All Fields]	N-3	32390993	Lactonase Specificity Is Key to Quorum Quenching in Pseudomonas aeruginosa.
1070	DRAMP03972	N-3 (analog of P18)	"N-3"[All Fields] AND biofilm[All Fields]	N-3	32333184	Twin Peaks: Presenting the Antagonistic Molecular Interplay of Curcumin with LasR and LuxR Quorum Sensing Pathways.
1070	DRAMP03972	N-3 (analog of P18)	"N-3"[All Fields] AND biofilm[All Fields]	N-3	32233643	Pathogen identification in 84 Patients with post-traumatic osteomyelitis after limb fractures.
1070	DRAMP03972	N-3 (analog of P18)	"N-3"[All Fields] AND biofilm[All Fields]	N-3	32217369	Synthetic homoserine lactone analogues as antagonists of bacterial quorum sensing.
1070	DRAMP03972	N-3 (analog of P18)	"N-3"[All Fields] AND biofilm[All Fields]	N-3	32045871	Legionella quorum sensing meets cyclic-di-GMP signaling.
1070	DRAMP03972	N-3 (analog of P18)	"N-3"[All Fields] AND biofilm[All Fields]	N-3	32006626	A novel, quorum sensor-infused liposomal drug delivery system suppresses Candida albicans biofilms.
1070	DRAMP03972	N-3 (analog of P18)	"N-3"[All Fields] AND biofilm[All Fields]	N-3	31982630	Quorum sensing molecule N-(3-oxododecanoyl)-l-homoserine lactone: An all-rounder in mammalian cell modification.
1070	DRAMP03972	N-3 (analog of P18)	"N-3"[All Fields] AND biofilm[All Fields]	N-3	31953683	Disruption and bactericidal indices depicted in polygonal graphs to show multiple outcome effects of root canal irrigant supplements on single- and dual-species biofilms.
1070	DRAMP03972	N-3 (analog of P18)	"N-3"[All Fields] AND biofilm[All Fields]	N-3	31885331	Mechanism of pyocyanin abolishment caused by mvaT mvaUdouble knockout in Pseudomonas aeruginosaPAO1.
1070	DRAMP03972	N-3 (analog of P18)	"N-3"[All Fields] AND biofilm[All Fields]	N-3	31854000	Species identification, virulence markers and antimicrobial resistance profiles of Aeromonas sp. isolated from marketed hard-shelled mussel (Mytilus coruscus) in Korea.
1070	DRAMP03972	N-3 (analog of P18)	"N-3"[All Fields] AND biofilm[All Fields]	N-3	31818247	The Moraxella catarrhalis phase-variable DNA methyltransferase ModM3 is an epigenetic regulator that affects bacterial survival in an in vivo model of otitis media.
1070	DRAMP03972	N-3 (analog of P18)	"N-3"[All Fields] AND biofilm[All Fields]	N-3	31692289	In-vitro evaluation of a ciprofloxacin and azithromycin sinus stent for Pseudomonas aeruginosa biofilms.
1070	DRAMP03972	N-3 (analog of P18)	"N-3"[All Fields] AND biofilm[All Fields]	N-3	31672371	The interference of nonylphenol with bacterial cell-to-cell communication.
1070	DRAMP03972	N-3 (analog of P18)	"N-3"[All Fields] AND biofilm[All Fields]	N-3	31546042	Addition of hydrogen peroxide to methylene blue conjugated to β-cyclodextrin in photodynamic antimicrobial chemotherapy in S. mutans biofilm.
1070	DRAMP03972	N-3 (analog of P18)	"N-3"[All Fields] AND biofilm[All Fields]	N-3	31511371	Global Transcriptomic Analysis of the Candida albicans Response to Treatment with a Novel Inhibitor of Filamentation.
1070	DRAMP03972	N-3 (analog of P18)	"N-3"[All Fields] AND biofilm[All Fields]	N-3	31447236	Anti-fungal susceptibility and virulence factors of Candida spp. isolated from blood cultures.

1070	DRAMP03972	N-3 (analog of P18)	"N-3"[All Fields] AND biofilm[All Fields]	N-3	31377870	Pseudomonas aeruginosa quorum-sensing molecule N-(3-oxo-dodecanoyl)-L-homoserine lactone triggers mitochondrial dysfunction and apoptosis in neutrophils through calcium signaling.
1070	DRAMP03972	N-3 (analog of P18)	"N-3"[All Fields] AND biofilm[All Fields]	N-3	31259730	Antimicrobial assesment of aroylhydrazone derivatives in vitro.
1070	DRAMP03972	N-3 (analog of P18)	"N-3"[All Fields] AND biofilm[All Fields]	N-3	31239733	Quorum quenching activity of Bacillus cereusisolate 30b confers antipathogenic effects in Pseudomonas aeruginosa.
1070	DRAMP03972	N-3 (analog of P18)	"N-3"[All Fields] AND biofilm[All Fields]	N-3	31220276	Subcutaneous suppressive antibiotic therapy for bone and joint infections: safety and outcome in a cohort of 10 patients.
1070	DRAMP03972	N-3 (analog of P18)	"N-3"[All Fields] AND biofilm[All Fields]	N-3	31106062	Exploring the transcriptome of luxI - and ΔainSmutants and the impact of N-3-oxo-hexanoyl-L- and N-3-hydroxy-decanoyl-L-homoserine lactones on biofilm formation in Aliivibrio salmonicida.
1070	DRAMP03972	N-3 (analog of P18)	"N-3"[All Fields] AND biofilm[All Fields]	N-3	31057529	Biodiversity and Multifunctional Features of Lactic Acid Bacteria Isolated From Table Olive Biofilms.
1070	DRAMP03972	N-3 (analog of P18)	"N-3"[All Fields] AND biofilm[All Fields]	N-3	31050940	Comparative Effect of Two Red Lights on Streptococcus mutansBiofilms and Assessment of Temperature Variances in Human Teeth During In VitroPhotodynamic Antimicrobial Chemotherapy.
1070	DRAMP03972	N-3 (analog of P18)	"N-3"[All Fields] AND biofilm[All Fields]	N-3	30913464	Inhibition of biofilm formation, quorum sensing activity and molecular docking study of isolated 3, 5, 7-Trihydroxyflavone from Alstonia scholaris leaf against P.aeruginosa.
1070	DRAMP03972	N-3 (analog of P18)	"N-3"[All Fields] AND biofilm[All Fields]	N-3	30874807	Candida isolates causing candidemia show different degrees of virulence in Galleria mellonella.
1070	DRAMP03972	N-3 (analog of P18)	"N-3"[All Fields] AND biofilm[All Fields]	N-3	30825938	Communication mechanisms in extremophiles: Exploring their existence and industrial applications.
1070	DRAMP03972	N-3 (analog of P18)	"N-3"[All Fields] AND biofilm[All Fields]	N-3	30791400	Effect of an Experimental Formulation Containing Chlorhexidine on Pathogenic Biofilms and Drug Release Behavior in the Presence or Absence of Bacteria.
1070	DRAMP03972	N-3 (analog of P18)	"N-3"[All Fields] AND biofilm[All Fields]	N-3	30785306	Inhibitive Effect of Eugenol and Its Nanoemulsion on Quorum Sensing-Mediated Virulence Factors and Biofilm Formation by Pseudomonas aeruginosa.
1070	DRAMP03972	N-3 (analog of P18)	"N-3"[All Fields] AND biofilm[All Fields]	N-3	30767648	Biofilm formation and antibiotic resistance in meticillin-resistant and meticillin-sensitive Staphylococcus aureus isolated from burns.
1070	DRAMP03972	N-3 (analog of P18)	"N-3"[All Fields] AND biofilm[All Fields]	N-3	30702211	In-vitro evaluation of a ciprofloxacin- and ivacaftor-coated sinus stent against Pseudomonas aeruginosa biofilms.
1070	DRAMP03972	N-3 (analog of P18)	"N-3"[All Fields] AND biofilm[All Fields]	N-3	30680880	The antitoxin MqsA homologue in Pseudomonas fluorescens 2P24 has a rewired regulatory circuit through evolution.
1070	DRAMP03972	N-3 (analog of P18)	"N-3"[All Fields] AND biofilm[All Fields]	N-3	30679015	Hydrophobicity of graphene as a driving force for inhibiting biofilm formation of pathogenic bacteria and fungi.
1070	DRAMP03972	N-3 (analog of P18)	"N-3"[All Fields] AND biofilm[All Fields]	N-3	30649480	Farnesol inhibits planktonic cells and antifungal-tolerant biofilms of Trichosporon asahii and Trichosporon inkin.
1070	DRAMP03972	N-3 (analog of P18)	"N-3"[All Fields] AND biofilm[All Fields]	N-3	30623561	The pleiotropic Legionella transcription factor LvbR links the Lqs and c-di-GMP regulatory networks to control biofilm architecture and virulence.
1070	DRAMP03972	N-3 (analog of P18)	"N-3"[All Fields] AND biofilm[All Fields]	N-3	30595445	Synthesis, biological evaluations and computational studies of N-(3-(-2-(7-Chloroquinolin-2-yl)vinyl) benzylidene)anilines as fungal biofilm inhibitors.
1070	DRAMP03972	N-3 (analog of P18)	"N-3"[All Fields] AND biofilm[All Fields]	N-3	30455228	In VivoGentamicin Susceptibility Test for Prevention of Bacterial Biofilms in Bone Tissue and on Implants.
1070	DRAMP03972	N-3 (analog of P18)	"N-3"[All Fields] AND biofilm[All Fields]	N-3	30428481	Bacteria-Host Crosstalk: Sensing of the Quorum in the Context of Pseudomonas aeruginosa Infections.
1070	DRAMP03972	N-3 (analog of P18)	"N-3"[All Fields] AND biofilm[All Fields]	N-3	30410619	Impact of Pseudomonas aeruginosaquorum sensing signaling molecules on adhesion and inflammatory markers in endothelial cells.
1070	DRAMP03972	N-3 (analog of P18)	"N-3"[All Fields] AND biofilm[All Fields]	N-3	30389771	The Probiotic Bacterium Phaeobacter inhibens Downregulates Virulence Factor Transcription in the Shellfish Pathogen Vibrio coralliilyticus by N-Acyl Homoserine Lactone Production.
1070	DRAMP03972	N-3 (analog of P18)	"N-3"[All Fields] AND biofilm[All Fields]	N-3	30317111	Phylogroups, pathotypes, biofilm formation and antimicrobial resistance of Escherichia coli isolates in farms and packing facilities of tomato, jalapeño pepper and cantaloupe from Northern Mexico.
1070	DRAMP03972	N-3 (analog of P18)	"N-3"[All Fields] AND biofilm[All Fields]	N-3	30229985	Characterization of virulence properties and multi-drug resistance profiles in motile Aeromonas spp. isolated from zebrafish (Danio rerio).
1070	DRAMP03972	N-3 (analog of P18)	"N-3"[All Fields] AND biofilm[All Fields]	N-3	30218926	Periodic polarization of electroactive biofilms increases current density and charge carriers concentration while modifying biofilm structure.
1070	DRAMP03972	N-3 (analog of P18)	"N-3"[All Fields] AND biofilm[All Fields]	N-3	30200829	N-Acylhomoserine lactone-mediated quorum sensing regulates biofilm structure in Methylobacterium populiP-1M, an isolate from a pink-pigmented household biofilm.
1070	DRAMP03972	N-3 (analog of P18)	"N-3"[All Fields] AND biofilm[All Fields]	N-3	30087309	Synthesis and Spectrum of Biological Activities of Novel N-arylcinnamamides.
1070	DRAMP03972	N-3 (analog of P18)	"N-3"[All Fields] AND biofilm[All Fields]	N-3	30051805	Quantification of major constituents of biofilms in occluded pancreatic stents.
1070	DRAMP03972	N-3 (analog of P18)	"N-3"[All Fields] AND biofilm[All Fields]	N-3	30042929	In VitroCharacterization of a Biaryl Amide Anti-virulence Compound Targeting Candida albicansFilamentation and Biofilm Formation.
1070	DRAMP03972	N-3 (analog of P18)	"N-3"[All Fields] AND biofilm[All Fields]	N-3	29980934	Correlation between relative bacterial activity and lactate dehydrogenase gene expression of co-cultures in vitro.
1070	DRAMP03972	N-3 (analog of P18)	"N-3"[All Fields] AND biofilm[All Fields]	N-3	29907449	High-risk Staphylococcus aureus transmission in the operating room: A call for widespread improvements in perioperative hand hygiene and patient decolonization practices.
1070	DRAMP03972	N-3 (analog of P18)	"N-3"[All Fields] AND biofilm[All Fields]	N-3	29891706	Stochastic Turing patterns in a synthetic bacterial population.
1070	DRAMP03972	N-3 (analog of P18)	"N-3"[All Fields] AND biofilm[All Fields]	N-3	29796703	Production of N-acyl homoserine lactones by Chromobacterium haemolyticum KM2 isolated from the river water in Malaysia.
1070	DRAMP03972	N-3 (analog of P18)	"N-3"[All Fields] AND biofilm[All Fields]	N-3	29767954	Development of Molecularly Imprinted Polymers To Block Quorum Sensing and Inhibit Bacterial Biofilm Formation.
1070	DRAMP03972	N-3 (analog of P18)	"N-3"[All Fields] AND biofilm[All Fields]	N-3	29752018	The microbial community in a moving bed biotrickling filter operated to remove hydrogen sulfide from gas streams.
1070	DRAMP03972	N-3 (analog of P18)	"N-3"[All Fields] AND biofilm[All Fields]	N-3	29739319	Characterization of biofilm-forming capacity and resistance to sanitizers of a range of E. coli O26 pathotypes from clinical cases and cattle in Australia.
1070	DRAMP03972	N-3 (analog of P18)	"N-3"[All Fields] AND biofilm[All Fields]	N-3	29666543	Spoilage of refrigerated Litopenaeus vannamei: eavesdropping on Acinetobacteracyl-homoserine lactones promotes the spoilage potential of Shewanella baltica.
1070	DRAMP03972	N-3 (analog of P18)	"N-3"[All Fields] AND biofilm[All Fields]	N-3	29523343	Effect of dental monomers and initiators on Streptococcus mutans oral biofilms.
1070	DRAMP03972	N-3 (analog of P18)	"N-3"[All Fields] AND biofilm[All Fields]	N-3	29427006	Correlation Between Quorum Sensing Signal Molecules and Pseudomonas aeruginosa's Biofilm Development and Virulency.
1070	DRAMP03972	N-3 (analog of P18)	"N-3"[All Fields] AND biofilm[All Fields]	N-3	29372575	Distribution of Ebp pili among clinical and fecal isolates of Enterococcus faecalis and evaluation for human platelet activation.
1070	DRAMP03972	N-3 (analog of P18)	"N-3"[All Fields] AND biofilm[All Fields]	N-3	29357848	Identification, synthesis and regulatory function of the N-acylated homoserine lactone signals produced by Pseudomonas chlororaphis HT66.
1070	DRAMP03972	N-3 (analog of P18)	"N-3"[All Fields] AND biofilm[All Fields]	N-3	29322006	Attenuation of quorum-sensing-dependent virulence factors and biofilm formation by medicinal plants against antibiotic resistant Pseudomonas aeruginosa.
1070	DRAMP03972	N-3 (analog of P18)	"N-3"[All Fields] AND biofilm[All Fields]	N-3	29281884	Binary Colloidal Crystal Layers as Platforms for Surface Patterning of Puroindoline-Based Antimicrobial Peptides.
1070	DRAMP03972	N-3 (analog of P18)	"N-3"[All Fields] AND biofilm[All Fields]	N-3	29208749	Development of Anti-Virulence Approaches for Candidiasis via a Novel Series of Small-Molecule Inhibitors of Candida albicansFilamentation.
1070	DRAMP03972	N-3 (analog of P18)	"N-3"[All Fields] AND biofilm[All Fields]	N-3	29195992	Phenotypic characterization of Cronobacter spp. strains isolated from foods and clinical specimens in Brazil.
1070	DRAMP03972	N-3 (analog of P18)	"N-3"[All Fields] AND biofilm[All Fields]	N-3	29160403	Quantification of Streptococcus mutans in Different Types of Ligature Wires and Elastomeric Chains.
1070	DRAMP03972	N-3 (analog of P18)	"N-3"[All Fields] AND biofilm[All Fields]	N-3	29125543	A Mediated BOD Biosensor Based on Immobilized B. Subtilis on Three-Dimensional Porous Graphene-Polypyrrole Composite.
1070	DRAMP03972	N-3 (analog of P18)	"N-3"[All Fields] AND biofilm[All Fields]	N-3	30965904	Nitric Oxide Releasing Polymeric Coatings for the Prevention of Biofilm Formation.
1070	DRAMP03972	N-3 (analog of P18)	"N-3"[All Fields] AND biofilm[All Fields]	N-3	29109700	Exploring the Genome and Phenotype of Multi-Drug Resistant Klebsiella pneumoniaeof Clinical Origin.
1070	DRAMP03972	N-3 (analog of P18)	"N-3"[All Fields] AND biofilm[All Fields]	N-3	29067011	Aii810, a Novel Cold-Adapted N-Acylhomoserine Lactonase Discovered in a Metagenome, Can Strongly Attenuate Pseudomonas aeruginosaVirulence Factors and Biofilm Formation.
1070	DRAMP03972	N-3 (analog of P18)	"N-3"[All Fields] AND biofilm[All Fields]	N-3	29027384	Survey for Correlation between Biofilm Formation and Virulence Determinants in a Collection of Pathogenic and Fecal Enterococcus faecalis Isolates.
1070	DRAMP03972	N-3 (analog of P18)	"N-3"[All Fields] AND biofilm[All Fields]	N-3	28970909	Engineering microbial physiology with synthetic polymers: cationic polymers induce biofilm formation in Vibrio choleraeand downregulate the expression of virulence genes.
1070	DRAMP03972	N-3 (analog of P18)	"N-3"[All Fields] AND biofilm[All Fields]	N-3	28968533	Using the agricultural environment to select better surrogates for foodborne pathogens associated with fresh produce.
1070	DRAMP03972	N-3 (analog of P18)	"N-3"[All Fields] AND biofilm[All Fields]	N-3	28963589	Influence of pre-irradiation time employed in antimicrobial photodynamic therapy with diode laser.
1070	DRAMP03972	N-3 (analog of P18)	"N-3"[All Fields] AND biofilm[All Fields]	N-3	28961393	Development of Antifouling and Bactericidal Coatings for Platelet Storage Bags Using Dopamine Chemistry.
1070	DRAMP03972	N-3 (analog of P18)	"N-3"[All Fields] AND biofilm[All Fields]	N-3	28939255	Inhibition of quorum sensing related virulence factors of Pseudomonas aeruginosa by pyridoxal lactohydrazone.

1070	DRAMP03972	N-3 (analog of P18)	"N-3"[All Fields] AND biofilm[All Fields]	N-3	28854274	Enhancing antibacterial effect of sodium hypochlorite by low electric current-assisted sonic agitation.
1070	DRAMP03972	N-3 (analog of P18)	"N-3"[All Fields] AND biofilm[All Fields]	N-3	28804019	Pseudomonas aeruginosa auto inducer3-oxo-C 12-HSL exerts bacteriostatic effect and inhibits Staphylococcus epidermidis biofilm.
1070	DRAMP03972	N-3 (analog of P18)	"N-3"[All Fields] AND biofilm[All Fields]	N-3	28783429	Characterization of Livestock-Associated Methicillin-Resistant Staphylococcus aureus CC398 and mecC-positive CC130 from Zoo Animals in the United Kingdom.
1070	DRAMP03972	N-3 (analog of P18)	"N-3"[All Fields] AND biofilm[All Fields]	N-3	28777541	UV-Curable Contact Active Benzophenone Terminated Quaternary Ammonium Antimicrobials for Applications in Polymer Plastics and Related Devices.
1070	DRAMP03972	N-3 (analog of P18)	"N-3"[All Fields] AND biofilm[All Fields]	N-3	28750776	In vitro biofilm formation on resin-based composites after different finishing and polishing procedures.
1070	DRAMP03972	N-3 (analog of P18)	"N-3"[All Fields] AND biofilm[All Fields]	N-3	28714256	A1S_2811, a CheA/Y-like hybrid two-component regulator from Acinetobacter baumannii ATCC17978, is involved in surface motility and biofilm formation in this bacterium.
1070	DRAMP03972	N-3 (analog of P18)	"N-3"[All Fields] AND biofilm[All Fields]	N-3	28688010	Quorum sensing molecules production by nosocomial and soil isolates Acinetobacter baumannii.
1070	DRAMP03972	N-3 (analog of P18)	"N-3"[All Fields] AND biofilm[All Fields]	N-3	28670299	Detection of Diverse N-Acyl-Homoserine Lactones in Vibrio alginolyticusand Regulation of Biofilm Formation by N-(3-Oxodecanoyl) Homoserine Lactone In vitro.
1070	DRAMP03972	N-3 (analog of P18)	"N-3"[All Fields] AND biofilm[All Fields]	N-3	28636666	Molecular and microbiological report of a hospital outbreak of NDM-1-carrying Enterobacteriaceae in Mexico.
1070	DRAMP03972	N-3 (analog of P18)	"N-3"[All Fields] AND biofilm[All Fields]	N-3	28630204	A Low-Molecular-Weight Alginate Oligosaccharide Disrupts Pseudomonal Microcolony Formation and Enhances Antibiotic Effectiveness.
1070	DRAMP03972	N-3 (analog of P18)	"N-3"[All Fields] AND biofilm[All Fields]	N-3	28554609	Biofilm-induced changes to the composite surface.
1070	DRAMP03972	N-3 (analog of P18)	"N-3"[All Fields] AND biofilm[All Fields]	N-3	28546427	Spermidine promotes Bacillus subtilisbiofilm formation by activating expression of the matrix regulator slrR.
1070	DRAMP03972	N-3 (analog of P18)	"N-3"[All Fields] AND biofilm[All Fields]	N-3	28487993	Acinetobacter baumannii quorum-sensing signalling molecule induces the expression of drug-resistance genes.
1070	DRAMP03972	N-3 (analog of P18)	"N-3"[All Fields] AND biofilm[All Fields]	N-3	28465066	Bacterial adhesion not inhibited by ion-releasing bioactive glass filler.
1070	DRAMP03972	N-3 (analog of P18)	"N-3"[All Fields] AND biofilm[All Fields]	N-3	28442545	Peroxisome proliferator-activated receptor-γ agonists attenuate biofilm formation by Pseudomonas aeruginosa.
1070	DRAMP03972	N-3 (analog of P18)	"N-3"[All Fields] AND biofilm[All Fields]	N-3	28437227	Emergence of the Uncommon Clone ST944/ST78 Carrying bla OXA-40-likeand bla CTX-M-likeGenes Among Carbapenem-Nonsusceptible Acinetobacter baumannii in Moscow, Russia.
1070	DRAMP03972	N-3 (analog of P18)	"N-3"[All Fields] AND biofilm[All Fields]	N-3	28427132	[Influences of abaR gene on biofilm formation of Acinetobacter baumannii].
1070	DRAMP03972	N-3 (analog of P18)	"N-3"[All Fields] AND biofilm[All Fields]	N-3	28390209	Evaluation of the Oral Tolerance of Three Fluoride Toothpaste Formulations in a Dry Mouth Population: Results from Two Randomized Studies.
1070	DRAMP03972	N-3 (analog of P18)	"N-3"[All Fields] AND biofilm[All Fields]	N-3	28379194	Characteristics of N-Acylhomoserine Lactones Produced by Hafnia alvei H4 Isolated from Spoiled Instant Sea Cucumber.
1070	DRAMP03972	N-3 (analog of P18)	"N-3"[All Fields] AND biofilm[All Fields]	N-3	28333393	Laser-generated shockwaves enhance antibacterial activity against biofilms in vitro.
1070	DRAMP03972	N-3 (analog of P18)	"N-3"[All Fields] AND biofilm[All Fields]	N-3	28327271	Novel anti-staphylococcal and anti-biofilm properties of two anti-malarial compounds: MMV665953 {1-(3-chloro-4-fluorophenyl)-3-(3,4-dichlorophenyl)urea} and MMV665807 {5-chloro-2-hydroxy-N-[3-(trifluoromethyl)phenyl]benzamide}.
1070	DRAMP03972	N-3 (analog of P18)	"N-3"[All Fields] AND biofilm[All Fields]	N-3	28317023	Reduction of Thrombosis and Bacterial Infection via Controlled Nitric Oxide (NO) Release from S-Nitroso- N-acetylpenicillamine (SNAP) Impregnated CarboSil Intravascular Catheters.
1070	DRAMP03972	N-3 (analog of P18)	"N-3"[All Fields] AND biofilm[All Fields]	N-3	28245470	Evidence of an in vitro Coupled Diffusion Mechanism of Lesion Formation within Microcosm Dental Plaque.
1070	DRAMP03972	N-3 (analog of P18)	"N-3"[All Fields] AND biofilm[All Fields]	N-3	28159990	Planktonic growth and biofilm formation profiles in Candida haemulonii species complex.
1070	DRAMP03972	N-3 (analog of P18)	"N-3"[All Fields] AND biofilm[All Fields]	N-3	28140677	Furvina inhibits the 3-oxo-C12-HSL-based quorum sensing system of Pseudomonas aeruginosa and QS-dependent phenotypes.
1070	DRAMP03972	N-3 (analog of P18)	"N-3"[All Fields] AND biofilm[All Fields]	N-3	28062182	Exposure of airway epithelial cells to Pseudomonas aeruginosa biofilm-derived quorum sensing molecules decrease the activity of the anti-oxidant response element bound by NRF2.
1070	DRAMP03972	N-3 (analog of P18)	"N-3"[All Fields] AND biofilm[All Fields]	N-3	28050817	Phosphatidylcholine Coatings Deliver Local Antimicrobials and Reduce Infection in a Murine Model: A Preliminary Study.
1070	DRAMP03972	N-3 (analog of P18)	"N-3"[All Fields] AND biofilm[All Fields]	N-3	27905512	Plant phenolic volatiles inhibit quorum sensing in pectobacteria and reduce their virulence by potential binding to ExpI and ExpR proteins.
1070	DRAMP03972	N-3 (analog of P18)	"N-3"[All Fields] AND biofilm[All Fields]	N-3	27902784	Dynamic in vivo mutations within the ica operon during persistence of Staphylococcus aureus in the airways of cystic fibrosis patients.
1070	DRAMP03972	N-3 (analog of P18)	"N-3"[All Fields] AND biofilm[All Fields]	N-3	27902411	Non-typeable Haemophilus influenzae biofilm production and severity in lower respiratory tract infections in a tertiary hospital in Mexico.
1070	DRAMP03972	N-3 (analog of P18)	"N-3"[All Fields] AND biofilm[All Fields]	N-3	27864804	Activity of Norspermidine on Bacterial Biofilms of Multidrug-Resistant Clinical Isolates Associated with Persistent Extremity Wound Infections.
1070	DRAMP03972	N-3 (analog of P18)	"N-3"[All Fields] AND biofilm[All Fields]	N-3	27864174	Multilocus Sequence Analysis of Phylogroup 1 and 2 Oral Treponeme Strains.
1070	DRAMP03972	N-3 (analog of P18)	"N-3"[All Fields] AND biofilm[All Fields]	N-3	27860087	The effect of burdock leaf fraction on adhesion, biofilm formation, quorum sensing and virulence factors of Pseudomonas aeruginosa.
1070	DRAMP03972	N-3 (analog of P18)	"N-3"[All Fields] AND biofilm[All Fields]	N-3	27834171	Bacterial communities associated with apical periodontitis and dental implant failure.
1070	DRAMP03972	N-3 (analog of P18)	"N-3"[All Fields] AND biofilm[All Fields]	N-3	27813213	Investigation to test potential stereolithography materials for development of an in vitro root canal model.
1070	DRAMP03972	N-3 (analog of P18)	"N-3"[All Fields] AND biofilm[All Fields]	N-3	27240552	Acylase-containing polyurethane coatings with anti-biofilm activity.
1070	DRAMP03972	N-3 (analog of P18)	"N-3"[All Fields] AND biofilm[All Fields]	N-3	27703610	Silver nanoparticles in resin luting cements: Antibacterial and physiochemical properties.
1070	DRAMP03972	N-3 (analog of P18)	"N-3"[All Fields] AND biofilm[All Fields]	N-3	27623227	Rhamnolipids Mediate an Interspecies Biofilm Dispersal Signaling Pathway.
1070	DRAMP03972	N-3 (analog of P18)	"N-3"[All Fields] AND biofilm[All Fields]	N-3	27565090	Antibacterial and antibiofilm activities of docosahexaenoic acid (DHA) and eicosapentaenoic acid (EPA) against periodontopathic bacteria.
1070	DRAMP03972	N-3 (analog of P18)	"N-3"[All Fields] AND biofilm[All Fields]	N-3	27525391	[Investigation of the serotype distribution, biofilm production and antibiotic susceptibilities of group B streptococci isolated from urinary samples].
1070	DRAMP03972	N-3 (analog of P18)	"N-3"[All Fields] AND biofilm[All Fields]	N-3	27498852	Control of quorum sensing and virulence factors of Pseudomonas aeruginosa using phenylalanine arginyl β-naphthylamide.
1070	DRAMP03972	N-3 (analog of P18)	"N-3"[All Fields] AND biofilm[All Fields]	N-3	27473426	Synthesis and electrochemical detection of a thiazolyl-indole natural product isolated from the nosocomial pathogen Pseudomonas aeruginosa.
1070	DRAMP03972	N-3 (analog of P18)	"N-3"[All Fields] AND biofilm[All Fields]	N-3	27324340	Fighting Off Wound Pathogens in Horses with Honeybee Lactic Acid Bacteria.
1070	DRAMP03972	N-3 (analog of P18)	"N-3"[All Fields] AND biofilm[All Fields]	N-3	27284969	FISHing for gutta-percha-adhered biofilms in purulent post-treatment apical periodontitis.
1070	DRAMP03972	N-3 (analog of P18)	"N-3"[All Fields] AND biofilm[All Fields]	N-3	27242335	Non-thermal Plasma Exposure Rapidly Attenuates Bacterial AHL-Dependent Quorum Sensing and Virulence.
1070	DRAMP03972	N-3 (analog of P18)	"N-3"[All Fields] AND biofilm[All Fields]	N-3	27220074	Diarylheptanoids from Alnus viridis ssp. viridis and Alnus glutinosa: Modulation of Quorum Sensing Activity in Pseudomonas aeruginosa.
1070	DRAMP03972	N-3 (analog of P18)	"N-3"[All Fields] AND biofilm[All Fields]	N-3	27047801	Pseudomonas aeruginosa N-3-oxo-dodecanoyl-homoserine Lactone Elicits Changes in Cell Volume, Morphology, and AQP9 Characteristics in Macrophages.
1070	DRAMP03972	N-3 (analog of P18)	"N-3"[All Fields] AND biofilm[All Fields]	N-3	27004423	Clinical safety and effectiveness evaluation of a new antimicrobial wound dressing designed to manage exudate, infection and biofilm.
1070	DRAMP03972	N-3 (analog of P18)	"N-3"[All Fields] AND biofilm[All Fields]	N-3	26782143	Sensing developing biofilms: the bitter receptor T2R38 on myeloid cells.
1070	DRAMP03972	N-3 (analog of P18)	"N-3"[All Fields] AND biofilm[All Fields]	N-3	26729121	Characterization of N-Acylhomoserine Lactones Produced by Bacteria Isolated from Industrial Cooling Water Systems.
1070	DRAMP03972	N-3 (analog of P18)	"N-3"[All Fields] AND biofilm[All Fields]	N-3	26715638	[Effects of inhibitory peptide of Staphylococcus epidermidis biofilm on adhesion and biofilm formation of this bacterium].
1070	DRAMP03972	N-3 (analog of P18)	"N-3"[All Fields] AND biofilm[All Fields]	N-3	26705856	Effect of endodontic irrigants on biofilm matrix polysaccharides.
1070	DRAMP03972	N-3 (analog of P18)	"N-3"[All Fields] AND biofilm[All Fields]	N-3	26656916	An Engineered Version of Human PON2 Opens the Way to Understand the Role of Its Post-Translational Modifications in Modulating Catalytic Activity.
1070	DRAMP03972	N-3 (analog of P18)	"N-3"[All Fields] AND biofilm[All Fields]	N-3	32262894	Hemocompatible, antioxidative and antibacterial polypropylene prepared by attaching silver nanoparticles capped with TPGS.
1070	DRAMP03972	N-3 (analog of P18)	"N-3"[All Fields] AND biofilm[All Fields]	N-3	26554837	Natural Guided Genome Engineering Reveals Transcriptional Regulators Controlling Quorum-Sensing Signal Degradation.
1070	DRAMP03972	N-3 (analog of P18)	"N-3"[All Fields] AND biofilm[All Fields]	N-3	26496473	Functionalized polyanilines disrupt Pseudomonas aeruginosa and Staphylococcus aureus biofilms.
1070	DRAMP03972	N-3 (analog of P18)	"N-3"[All Fields] AND biofilm[All Fields]	N-3	26482298	Molecule Targeting Glucosyltransferase Inhibits Streptococcus mutans Biofilm Formation and Virulence.
1070	DRAMP03972	N-3 (analog of P18)	"N-3"[All Fields] AND biofilm[All Fields]	N-3	26462745	Hospital-wide Eradication of a Nosocomial Legionella pneumophila Serogroup 1 Outbreak.

1070	DRAMP03972	N-3 (analog of P18)	"N-3"[All Fields] AND biofilm[All Fields]	N-3	26443466	Comparative analyses of ion release, pH and multispecies biofilm formation between conventional and bioactive gutta-percha.
1070	DRAMP03972	N-3 (analog of P18)	"N-3"[All Fields] AND biofilm[All Fields]	N-3	26325641	Antifungal activity of 4% chlorhexidine and 2% sodium hypochlorite against Candida albicans biofilms.
1070	DRAMP03972	N-3 (analog of P18)	"N-3"[All Fields] AND biofilm[All Fields]	N-3	26295304	Structural Insight into Multivalent Galactoside Binding to Pseudomonas aeruginosa Lectin LecA.
1070	DRAMP03972	N-3 (analog of P18)	"N-3"[All Fields] AND biofilm[All Fields]	N-3	26257736	Tasting Pseudomonas aeruginosa Biofilms: Human Neutrophils Express the Bitter Receptor T2R38 as Sensor for the Quorum Sensing Molecule N-(3-Oxododecanoyl)-l-Homoserine Lactone.
1070	DRAMP03972	N-3 (analog of P18)	"N-3"[All Fields] AND biofilm[All Fields]	N-3	26249611	Poly(glycidyl methacrylate-co-3-thienylmethylmethacrylate) as an immobilization matrix for microbial glycerol biosensing based on Gluconobacter oxydans.
1070	DRAMP03972	N-3 (analog of P18)	"N-3"[All Fields] AND biofilm[All Fields]	N-3	26245683	Involvement of quorum sensing genes in biofilm development and degradation of polycyclic aromatic hydrocarbons by a marine bacterium Pseudomonas aeruginosa N6P6.
1070	DRAMP03972	N-3 (analog of P18)	"N-3"[All Fields] AND biofilm[All Fields]	N-3	26243234	Interproximal biofilm removal by intervallic use of a sonic toothbrush compared to an oral irrigation system.
1070	DRAMP03972	N-3 (analog of P18)	"N-3"[All Fields] AND biofilm[All Fields]	N-3	26201421	D-amino acid inhibits biofilm but not new bone formation in an ovine model.
1070	DRAMP03972	N-3 (analog of P18)	"N-3"[All Fields] AND biofilm[All Fields]	N-3	26153228	Confocal microscopy evaluation of the effect of irrigants on Enterococcus faecalis biofilm: An in vitro study.
1070	DRAMP03972	N-3 (analog of P18)	"N-3"[All Fields] AND biofilm[All Fields]	N-3	26096293	Pseudomonas aeruginosa quorum-sensing signaling molecule N-3-oxododecanoyl homoserine lactone induces matrix metalloproteinase 9 expression via the AP1 pathway in rat fibroblasts.
1070	DRAMP03972	N-3 (analog of P18)	"N-3"[All Fields] AND biofilm[All Fields]	N-3	26048802	Design, synthesis and biological evaluation of 4-(alkyloxy)-6-methyl-2H-pyran-2-one derivatives as quorum sensing inhibitors.
1070	DRAMP03972	N-3 (analog of P18)	"N-3"[All Fields] AND biofilm[All Fields]	N-3	25975610	Role of LuxIR Homologue AnoIR in Acinetobacter nosocomialis and the Effect of Virstatin on the Expression of anoR Gene.
1070	DRAMP03972	N-3 (analog of P18)	"N-3"[All Fields] AND biofilm[All Fields]	N-3	25926530	The Pseudomonas aeruginosa RhlR-controlled aegerolysin RahU is a low-affinity rhamnolipid-binding protein.
1070	DRAMP03972	N-3 (analog of P18)	"N-3"[All Fields] AND biofilm[All Fields]	N-3	25774422	Activity of ERK regulates mucin 3 expression and is involved in undifferentiated Caco-2 cell death induced by 3-oxo-C12-homoserine lactone.
1070	DRAMP03972	N-3 (analog of P18)	"N-3"[All Fields] AND biofilm[All Fields]	N-3	25746999	Biofilm Formation and Quorum-Sensing-Molecule Production by Clinical Isolates of Serratia liquefaciens.
1070	DRAMP03972	N-3 (analog of P18)	"N-3"[All Fields] AND biofilm[All Fields]	N-3	25731156	Incorporation of bactericidal poly-acrylic acid modified copper iodide particles into adhesive resins.
1070	DRAMP03972	N-3 (analog of P18)	"N-3"[All Fields] AND biofilm[All Fields]	N-3	25676769	Draft Genome Sequence of Cellulophaga sp. E6, a Marine Algal Epibiont That Produces a Quorum-Sensing Inhibitory Compound Active against Pseudomonas aeruginosa.
1070	DRAMP03972	N-3 (analog of P18)	"N-3"[All Fields] AND biofilm[All Fields]	N-3	25627690	Paraoxonase 2 serves a proapopotic function in mouse and human cells in response to the Pseudomonas aeruginosa quorum-sensing molecule N-(3-Oxododecanoyl)-homoserine lactone.
1070	DRAMP03972	N-3 (analog of P18)	"N-3"[All Fields] AND biofilm[All Fields]	N-3	25586180	Functional amyloids keep quorum-sensing molecules in check.
1070	DRAMP03972	N-3 (analog of P18)	"N-3"[All Fields] AND biofilm[All Fields]	N-3	25550392	Candida tropicalis isolates obtained from veterinary sources show resistance to azoles and produce virulence factors.
1070	DRAMP03972	N-3 (analog of P18)	"N-3"[All Fields] AND biofilm[All Fields]	N-3	25549879	Different sensitivity levels to norspermidine on biofilm formation in clinical and commensal Staphylococcus epidermidis strains.
1070	DRAMP03972	N-3 (analog of P18)	"N-3"[All Fields] AND biofilm[All Fields]	N-3	25527537	Phaeobacter sp. strain Y4I utilizes two separate cell-to-cell communication systems to regulate production of the antimicrobial indigoidine.
1070	DRAMP03972	N-3 (analog of P18)	"N-3"[All Fields] AND biofilm[All Fields]	N-3	25409186	Alginate oligosaccharides inhibit fungal cell growth and potentiate the activity of antifungals against Candida and Aspergillus spp.
1070	DRAMP03972	N-3 (analog of P18)	"N-3"[All Fields] AND biofilm[All Fields]	N-3	25241529	[Development of an Enterococcus faecalis periapical biofilm model for in vitro morphological study].
1070	DRAMP03972	N-3 (analog of P18)	"N-3"[All Fields] AND biofilm[All Fields]	N-3	25105505	Comparison of biofilm formation between major clonal lineages of methicillin resistant Staphylococcus aureus.
1070	DRAMP03972	N-3 (analog of P18)	"N-3"[All Fields] AND biofilm[All Fields]	N-3	25085732	Inhibitory effects of 4-hydroxy-2,5-dimethyl-3(2H)-furanone (HDMF) on acyl-homoserine lactone-mediated virulence factor production and biofilm formation in Pseudomonas aeruginosa PAO1.
1070	DRAMP03972	N-3 (analog of P18)	"N-3"[All Fields] AND biofilm[All Fields]	N-3	25006994	Characterisation of a marine bacterium Vibrio brasiliensis T33 producing N-acyl homoserine lactone quorum sensing molecules.
1070	DRAMP03972	N-3 (analog of P18)	"N-3"[All Fields] AND biofilm[All Fields]	N-3	25001104	D-Amino acids inhibit biofilm formation in Staphylococcus epidermidis strains from ocular infections.
1070	DRAMP03972	N-3 (analog of P18)	"N-3"[All Fields] AND biofilm[All Fields]	N-3	24990083	Pseudomonas aeruginosa quorum-sensing molecule homoserine lactone modulates inflammatory signaling through PERK and eI-F2α.
1070	DRAMP03972	N-3 (analog of P18)	"N-3"[All Fields] AND biofilm[All Fields]	N-3	24911407	Characterization of the Vibrio cholerae extracellular matrix: a top-down solid-state NMR approach.
1070	DRAMP03972	N-3 (analog of P18)	"N-3"[All Fields] AND biofilm[All Fields]	N-3	24891106	Pyocyanin stimulates quorum sensing-mediated tolerance to oxidative stress and increases persister cell populations in Acinetobacter baumannii.
1070	DRAMP03972	N-3 (analog of P18)	"N-3"[All Fields] AND biofilm[All Fields]	N-3	24478193	Active efflux influences the potency of quorum sensing inhibitors in Pseudomonas aeruginosa.
1070	DRAMP03972	N-3 (analog of P18)	"N-3"[All Fields] AND biofilm[All Fields]	N-3	24438098	Pseudomonas aeruginosa homoserine lactone triggers apoptosis and Bak/Bax-independent release of mitochondrial cytochrome C in fibroblasts.
1070	DRAMP03972	N-3 (analog of P18)	"N-3"[All Fields] AND biofilm[All Fields]	N-3	24354200	Quorum sensing: a non-conventional target for antibiotic discovery.
1070	DRAMP03972	N-3 (analog of P18)	"N-3"[All Fields] AND biofilm[All Fields]	N-3	24134835	Cis-2-dodecenoic acid signal modulates virulence of Pseudomonas aeruginosa through interference with quorum sensing systems and T3SS.
1070	DRAMP03972	N-3 (analog of P18)	"N-3"[All Fields] AND biofilm[All Fields]	N-3	24057941	Real-time measurement of quorum-sensing signal autoinducer 3OC6HSL by a FRET-based nanosensor.
1070	DRAMP03972	N-3 (analog of P18)	"N-3"[All Fields] AND biofilm[All Fields]	N-3	23880975	Microbiologic trends and biofilm growth on explanted periorbital biomaterials: a 30-year review.
1070	DRAMP03972	N-3 (analog of P18)	"N-3"[All Fields] AND biofilm[All Fields]	N-3	23857391	The icaA gene in staphylococci from bovine mastitis.
1070	DRAMP03972	N-3 (analog of P18)	"N-3"[All Fields] AND biofilm[All Fields]	N-3	23704903	Quorum sensing negatively regulates multinucleate cell formation during intracellular growth of Burkholderia pseudomallei in macrophage-like cells.
1070	DRAMP03972	N-3 (analog of P18)	"N-3"[All Fields] AND biofilm[All Fields]	N-3	23594262	Effect of plant phenolic compounds on biofilm formation by Pseudomonas aeruginosa.
1070	DRAMP03972	N-3 (analog of P18)	"N-3"[All Fields] AND biofilm[All Fields]	N-3	23589249	Differences between 4-fluoroaniline degradation and autoinducer release by Acinetobacter sp. TW: implications for operating conditions in bacterial bioaugmentation.
1070	DRAMP03972	N-3 (analog of P18)	"N-3"[All Fields] AND biofilm[All Fields]	N-3	23433551	Neutrophil-derived tumor necrosis factor-α contributes to acute wound healing promoted by N-(3-oxododecanoyl)-L-homoserine lactone from Pseudomonas aeruginosa.
1070	DRAMP03972	N-3 (analog of P18)	"N-3"[All Fields] AND biofilm[All Fields]	N-3	23433371	Influence of glucose concentrations on biofilm formation, motility, exoprotease production, and quorum sensing in Aeromonas hydrophila.
1070	DRAMP03972	N-3 (analog of P18)	"N-3"[All Fields] AND biofilm[All Fields]	N-3	23419060	A steroidal molecule present in the egg wax of the tick Rhipicephalus (Boophilus) microplus inhibits bacterial biofilms.
1070	DRAMP03972	N-3 (analog of P18)	"N-3"[All Fields] AND biofilm[All Fields]	N-3	23241133	Investigation of motility and biofilm formation by intestinal Campylobacter concisus strains.
1070	DRAMP03972	N-3 (analog of P18)	"N-3"[All Fields] AND biofilm[All Fields]	N-3	23177766	Amperometric nitrate biosensor based on Carbon nanotube/Polypyrrole/Nitrate reductase biofilm electrode.
1070	DRAMP03972	N-3 (analog of P18)	"N-3"[All Fields] AND biofilm[All Fields]	N-3	23138745	Analysis by confocal laser scanning microscopy of the MDPB bactericidal effect on S. mutans biofilm CLSM analysis of MDPB bactericidal effect on biofilm.
1070	DRAMP03972	N-3 (analog of P18)	"N-3"[All Fields] AND biofilm[All Fields]	N-3	23071436	The Pseudomonas aeruginosa N-acylhomoserine lactone quorum sensing molecules target IQGAP1 and modulate epithelial cell migration.
1070	DRAMP03972	N-3 (analog of P18)	"N-3"[All Fields] AND biofilm[All Fields]	N-3	23026323	Ecological roles and release patterns of acylated homoserine lactones in Pseudomonas sp. HF-1 and their implications in bacterial bioaugmentation.
1070	DRAMP03972	N-3 (analog of P18)	"N-3"[All Fields] AND biofilm[All Fields]	N-3	22892745	Microbial biofilm proliferation within sealer-root dentin interfaces is affected by sealer type and aging period.
1070	DRAMP03972	N-3 (analog of P18)	"N-3"[All Fields] AND biofilm[All Fields]	N-3	22877936	Removal of tetrabromobisphenol A by conventional activated sludge, submerged membrane and membrane aerated biofilm reactors.
1070	DRAMP03972	N-3 (analog of P18)	"N-3"[All Fields] AND biofilm[All Fields]	N-3	22853441	Attenuation of quorum sensing in the pathogen Acinetobacter baumannii using non-native N-Acyl homoserine lactones.
1070	DRAMP03972	N-3 (analog of P18)	"N-3"[All Fields] AND biofilm[All Fields]	N-3	22736981	Detection, characterization, and biological effect of quorum-sensing signaling molecules in peanut-nodulating bradyrhizobia.
1070	DRAMP03972	N-3 (analog of P18)	"N-3"[All Fields] AND biofilm[All Fields]	N-3	29243637	Urolithins, ellagitannin metabolites produced by colon microbiota, inhibit Quorum Sensing in Yersinia enterocolitica: Phenotypic response and associated molecular changes.
1070	DRAMP03972	N-3 (analog of P18)	"N-3"[All Fields] AND biofilm[All Fields]	N-3	22585351	Cis-2-decenoic acid inhibits S. aureus growth and biofilm in vitro: a pilot study.
1070	DRAMP03972	N-3 (analog of P18)	"N-3"[All Fields] AND biofilm[All Fields]	N-3	22534025	Pathology and biofilm formation in a porcine model of staphylococcal osteomyelitis.
1070	DRAMP03972	N-3 (analog of P18)	"N-3"[All Fields] AND biofilm[All Fields]	N-3	22402155	What is the role of lipopolysaccharide on the tribocorrosive behavior of titanium?
1070	DRAMP03972	N-3 (analog of P18)	"N-3"[All Fields] AND biofilm[All Fields]	N-3	22401915	Bacterial quorum sensing molecule induces chemotaxis of human neutrophils via induction of p38 and leukocyte specific protein 1 (LSP1).
1070	DRAMP03972	N-3 (analog of P18)	"N-3"[All Fields] AND biofilm[All Fields]	N-3	22233488	Pseudomonas aeruginosa biofilm-associated homoserine lactone C12 rapidly activates apoptosis in airway epithelia.

1070	DRAMP03972	N-3 (analog of P18)	"N-3"[All Fields] AND biofilm[All Fields]	N-3	23534198	Influence and mechanism of N-(3-oxooxtanoyl)-L-homoserine lactone (C8-oxo-HSL) on biofilm behaviors at early stage.
1070	DRAMP03972	N-3 (analog of P18)	"N-3"[All Fields] AND biofilm[All Fields]	N-3	22173512	Bacterial-killing effect of atmospheric pressure non-equilibrium plasma jet and oral mucosa response.
1070	DRAMP03972	N-3 (analog of P18)	"N-3"[All Fields] AND biofilm[All Fields]	N-3	22046268	A novel metagenomic short-chain dehydrogenase/reductase attenuates Pseudomonas aeruginosa biofilm formation and virulence on Caenorhabditis elegans.
1070	DRAMP03972	N-3 (analog of P18)	"N-3"[All Fields] AND biofilm[All Fields]	N-3	21910441	Malabaricone C from Myristica cinnamomea exhibits anti-quorum sensing activity.
1070	DRAMP03972	N-3 (analog of P18)	"N-3"[All Fields] AND biofilm[All Fields]	N-3	21847113	Engineering microbes to sense and eradicate Pseudomonas aeruginosa, a human pathogen.
1070	DRAMP03972	N-3 (analog of P18)	"N-3"[All Fields] AND biofilm[All Fields]	N-3	21658103	Quorum-sensing autoinducer molecules produced by members of a multispecies biofilm promote horizontal gene transfer to Vibrio cholerae.
1070	DRAMP03972	N-3 (analog of P18)	"N-3"[All Fields] AND biofilm[All Fields]	N-3	21655947	In vitro and in vivo pathogenicity of Salmonella enteritidis clinical strains isolated from North America.
1070	DRAMP03972	N-3 (analog of P18)	"N-3"[All Fields] AND biofilm[All Fields]	N-3	21642401	Involvement of multiple loci in quorum quenching of autoinducer I molecules in the nitrogen-fixing symbiont Rhizobium (Sinorhizobium) sp. strain NGR234.
1070	DRAMP03972	N-3 (analog of P18)	"N-3"[All Fields] AND biofilm[All Fields]	N-3	21441325	Bacteriophage-Mediated Dispersal of Campylobacter jejuni Biofilms.
1070	DRAMP03972	N-3 (analog of P18)	"N-3"[All Fields] AND biofilm[All Fields]	N-3	21434644	Quorum sensing between Pseudomonas aeruginosa biofilms accelerates cell growth.
1070	DRAMP03972	N-3 (analog of P18)	"N-3"[All Fields] AND biofilm[All Fields]	N-3	21420169	LAS degradability by marine biofilms derived from seawater in Spain and Sweden.
1070	DRAMP03972	N-3 (analog of P18)	"N-3"[All Fields] AND biofilm[All Fields]	N-3	21361273	Passive control of quorum sensing: prevention of Pseudomonas aeruginosa biofilm formation by imprinted polymers.
1070	DRAMP03972	N-3 (analog of P18)	"N-3"[All Fields] AND biofilm[All Fields]	N-3	21324665	A multitask biosensor for micro-volumetric detection of N-3-oxo-dodecanoyl-homoserine lactone quorum sensing signal.
1070	DRAMP03972	N-3 (analog of P18)	"N-3"[All Fields] AND biofilm[All Fields]	N-3	21306197	Co-aggregation and growth inhibition of probiotic lactobacilli and clinical isolates of mutans streptococci: an in vitro study.
1070	DRAMP03972	N-3 (analog of P18)	"N-3"[All Fields] AND biofilm[All Fields]	N-3	21284858	Characterisation of two quorum sensing systems in the endophytic Serratia plymuthica strain G3: differential control of motility and biofilm formation according to life-style.
1070	DRAMP03972	N-3 (analog of P18)	"N-3"[All Fields] AND biofilm[All Fields]	N-3	21266100	Microbial linguistics: perspectives and applications of microbial cell-to-cell communication.
1070	DRAMP03972	N-3 (analog of P18)	"N-3"[All Fields] AND biofilm[All Fields]	N-3	21252275	N-Octanoylhomoserine lactone signalling mediated by the BpsI-BpsR quorum sensing system plays a major role in biofilm formation of Burkholderia pseudomallei.
1070	DRAMP03972	N-3 (analog of P18)	"N-3"[All Fields] AND biofilm[All Fields]	N-3	21205102	Expression and characterization of cell-signalling molecules in Campylobacter jejuni.
1070	DRAMP03972	N-3 (analog of P18)	"N-3"[All Fields] AND biofilm[All Fields]	N-3	21182450	Laser-generated shockwave for clearing medical device biofilms.
1070	DRAMP03972	N-3 (analog of P18)	"N-3"[All Fields] AND biofilm[All Fields]	N-3	21166890	Methylthioadenosine/S-adenosylhomocysteine nucleosidase, a critical enzyme for bacterial metabolism.
1070	DRAMP03972	N-3 (analog of P18)	"N-3"[All Fields] AND biofilm[All Fields]	N-3	21116408	Effect of marine polyunsaturated fatty acids on biofilm formation of Candida albicans and Candida dubliniensis.
1070	DRAMP03972	N-3 (analog of P18)	"N-3"[All Fields] AND biofilm[All Fields]	N-3	21074237	Rotating disk electrodes to assess river biofilm thickness and elasticity.
1070	DRAMP03972	N-3 (analog of P18)	"N-3"[All Fields] AND biofilm[All Fields]	N-3	21031307	Determination of acyl homoserine lactone and tetramic acid concentrations in biological samples.
1070	DRAMP03972	N-3 (analog of P18)	"N-3"[All Fields] AND biofilm[All Fields]	N-3	20577722	Label-free amperometric immunobiosensor based on a gold colloid and Prussian blue nanocomposite film modified carbon ionic liquid electrode.
1070	DRAMP03972	N-3 (analog of P18)	"N-3"[All Fields] AND biofilm[All Fields]	N-3	20455404	[Investigation of the surface properties of Staphylococcus epidermidis strains isolated from biomaterials].
1070	DRAMP03972	N-3 (analog of P18)	"N-3"[All Fields] AND biofilm[All Fields]	N-3	20368521	Bacterial colonization and infection of electrophysiological cardiac devices detected with sonication and swab culture.
1070	DRAMP03972	N-3 (analog of P18)	"N-3"[All Fields] AND biofilm[All Fields]	N-3	20306535	Garlic as an inhibitor of Pseudomonas aeruginosa quorum sensing in cystic fibrosis--a pilot randomized controlled trial.
1070	DRAMP03972	N-3 (analog of P18)	"N-3"[All Fields] AND biofilm[All Fields]	N-3	20132256	Analysis of quorum sensing-dependent virulence factor production and its relationship with antimicrobial susceptibility in Pseudomonas aeruginosa respiratory isolates.
1070	DRAMP03972	N-3 (analog of P18)	"N-3"[All Fields] AND biofilm[All Fields]	N-3	20088202	Effect of operating parameters on denitrification in an anoxic rotating biological contactor.
1070	DRAMP03972	N-3 (analog of P18)	"N-3"[All Fields] AND biofilm[All Fields]	N-3	20080211	Biofilm-induced modifications in the proteome of Pseudomonas aeruginosa planktonic cells.
1070	DRAMP03972	N-3 (analog of P18)	"N-3"[All Fields] AND biofilm[All Fields]	N-3	19950997	Inhibition by chestnut honey of N-Acyl-L-homoserine lactones and biofilm formation in Erwinia carotovora, Yersinia enterocolitica, and Aeromonas hydrophila.
1070	DRAMP03972	N-3 (analog of P18)	"N-3"[All Fields] AND biofilm[All Fields]	N-3	19935025	Treatment of periodontal disease during pregnancy: a randomized controlled trial.
1070	DRAMP03972	N-3 (analog of P18)	"N-3"[All Fields] AND biofilm[All Fields]	N-3	19855933	Development of inhibitors against TraR quorum-sensing system in Agrobacterium tumefaciens by molecular modeling of the ligand-receptor interaction.
1070	DRAMP03972	N-3 (analog of P18)	"N-3"[All Fields] AND biofilm[All Fields]	N-3	19778968	Role of PvdQ in Pseudomonas aeruginosa virulence under iron-limiting conditions.
1070	DRAMP03972	N-3 (analog of P18)	"N-3"[All Fields] AND biofilm[All Fields]	N-3	19715201	[Role of "quorum sensing" regulatory system in formation of biofilms by Burkholderia cepacia and Pseudomonas aeruginosa].
1070	DRAMP03972	N-3 (analog of P18)	"N-3"[All Fields] AND biofilm[All Fields]	N-3	19572896	The immune response of oral epithelial cells induced by single-species and complex naturally formed biofilms.
1070	DRAMP03972	N-3 (analog of P18)	"N-3"[All Fields] AND biofilm[All Fields]	N-3	19548930	Microflora in teeth associated with apical periodontitis: a methodological observational study comparing two protocols and three microscopy techniques.
1070	DRAMP03972	N-3 (analog of P18)	"N-3"[All Fields] AND biofilm[All Fields]	N-3	19513205	A novel two-component system BqsS-BqsR modulates quorum sensing-dependent biofilm decay in Pseudomonas aeruginosa.
1070	DRAMP03972	N-3 (analog of P18)	"N-3"[All Fields] AND biofilm[All Fields]	N-3	19383702	Pseudomonas aeruginosa Las quorum sensing autoinducer suppresses growth and biofilm production in Legionella species.
1070	DRAMP03972	N-3 (analog of P18)	"N-3"[All Fields] AND biofilm[All Fields]	N-3	19168658	Reconfiguring the quorum-sensing regulator SdiA of Escherichia coli to control biofilm formation via indole and N-acylhomoserine lactones.
1070	DRAMP03972	N-3 (analog of P18)	"N-3"[All Fields] AND biofilm[All Fields]	N-3	19038248	The junctional integrity of epithelial cells is modulated by Pseudomonas aeruginosa quorum sensing molecule through phosphorylation-dependent mechanisms.
1070	DRAMP03972	N-3 (analog of P18)	"N-3"[All Fields] AND biofilm[All Fields]	N-3	19013791	Denitrification of nitrate contaminated groundwater with a fiber-based biofilm reactor.
1070	DRAMP03972	N-3 (analog of P18)	"N-3"[All Fields] AND biofilm[All Fields]	N-3	18997027	Two dissimilar N-acyl-homoserine lactone acylases of Pseudomonas syringae influence colony and biofilm morphology.
1070	DRAMP03972	N-3 (analog of P18)	"N-3"[All Fields] AND biofilm[All Fields]	N-3	18997026	Metagenome-derived clones encoding two novel lactonase family proteins involved in biofilm inhibition in Pseudomonas aeruginosa.
1070	DRAMP03972	N-3 (analog of P18)	"N-3"[All Fields] AND biofilm[All Fields]	N-3	18806345	The roles of the quorum-sensing system in the release of extracellular DNA, lipopolysaccharide, and membrane vesicles from Pseudomonas aeruginosa.
1070	DRAMP03972	N-3 (analog of P18)	"N-3"[All Fields] AND biofilm[All Fields]	N-3	18649526	[Hydrogenotrophic denitrification for the removal of nitrate in drinking water].
1070	DRAMP03972	N-3 (analog of P18)	"N-3"[All Fields] AND biofilm[All Fields]	N-3	18605572	Innovative solid-state microelectrode for nitrite determination in a nitrifying granule.
1070	DRAMP03972	N-3 (analog of P18)	"N-3"[All Fields] AND biofilm[All Fields]	N-3	18479069	The function of SpnR and the inhibitory effects by halogenated furanone on quorum sensing in Serratia marcescens AS-1.
1070	DRAMP03972	N-3 (analog of P18)	"N-3"[All Fields] AND biofilm[All Fields]	N-3	18428113	Directed evolution of LuxI for enhanced OHHL production.
1070	DRAMP03972	N-3 (analog of P18)	"N-3"[All Fields] AND biofilm[All Fields]	N-3	18281398	Isolation and characterization of an autoinducer synthase from Acinetobacter baumannii.
1070	DRAMP03972	N-3 (analog of P18)	"N-3"[All Fields] AND biofilm[All Fields]	N-3	18210750	Antibacterial effect of tea-tree oil on methicillin-resistant Staphylococcus aureus biofilm formation of the tympanostomy tube: an in vitro study.
1070	DRAMP03972	N-3 (analog of P18)	"N-3"[All Fields] AND biofilm[All Fields]	N-3	18188535	Presence of quorum-sensing systems associated with multidrug resistance and biofilm formation in Bacteroides fragilis.
1070	DRAMP03972	N-3 (analog of P18)	"N-3"[All Fields] AND biofilm[All Fields]	N-3	18007518	Theoretical study of molecular determinants involved in signal binding to the TraR protein of Agrobacterium tumefaciens.
1070	DRAMP03972	N-3 (analog of P18)	"N-3"[All Fields] AND biofilm[All Fields]	N-3	17827290	The plant pathogen Pantoea ananatis produces N-acylhomoserine lactone and causes center rot disease of onion by quorum sensing.
1070	DRAMP03972	N-3 (analog of P18)	"N-3"[All Fields] AND biofilm[All Fields]	N-3	17768239	The cyclic AMP receptor protein modulates quorum sensing, motility and multiple genes that affect intestinal colonization in Vibrio cholerae.
1070	DRAMP03972	N-3 (analog of P18)	"N-3"[All Fields] AND biofilm[All Fields]	N-3	17675425	Inhibition of quorum sensing in Serratia marcescens AS-1 by synthetic analogs of N-acylhomoserine lactone.
1070	DRAMP03972	N-3 (analog of P18)	"N-3"[All Fields] AND biofilm[All Fields]	N-3	17455803	Clinical pseudomonas aeruginosa: potential factors of pathogenicity and resistance to antimicrobials.
1070	DRAMP03972	N-3 (analog of P18)	"N-3"[All Fields] AND biofilm[All Fields]	N-3	17369333	Inhibition of quorum sensing in Pseudomonas aeruginosa by N-acyl cyclopentylamides.
1070	DRAMP03972	N-3 (analog of P18)	"N-3"[All Fields] AND biofilm[All Fields]	N-3	17131112	Use of quantitative real-time RT-PCR to analyse the expression of some quorum-sensing regulated genes in Pseudomonas aeruginosa.

1070	DRAMP03972	N-3 (analog of P18)	"N-3"[All Fields] AND biofilm[All Fields]	N-3	17122353	Paraoxonase-2 deficiency enhances Pseudomonas aeruginosa quorum sensing in murine tracheal epithelia.
1070	DRAMP03972	N-3 (analog of P18)	"N-3"[All Fields] AND biofilm[All Fields]	N-3	17080611	Acyl-homoserine lactones modulate the settlement rate of zoospores of the marine alga Ulva intestinalis via a novel chemokinetic mechanism.
1070	DRAMP03972	N-3 (analog of P18)	"N-3"[All Fields] AND biofilm[All Fields]	N-3	16988244	Induction of neutrophil chemotaxis by the quorum-sensing molecule N-(3-oxododecanoyl)-L-homoserine lactone.
1070	DRAMP03972	N-3 (analog of P18)	"N-3"[All Fields] AND biofilm[All Fields]	N-3	16906383	The quorum-sensing molecule N-3-oxododecanoyl homoserine lactone (3OC12-HSL) enhances the host defence by activating human polymorphonuclear neutrophils (PMN).
1070	DRAMP03972	N-3 (analog of P18)	"N-3"[All Fields] AND biofilm[All Fields]	N-3	16450882	Quorum sensing and the lifestyle of Yersinia.
1070	DRAMP03972	N-3 (analog of P18)	"N-3"[All Fields] AND biofilm[All Fields]	N-3	16413159	Profiling of acylated homoserine lactones of Vibrio anguillarum in vitro and in vivo: Influence of growth conditions and serotype.
1070	DRAMP03972	N-3 (analog of P18)	"N-3"[All Fields] AND biofilm[All Fields]	N-3	16269739	Evidence for a functional quorum-sensing type AI-1 system in the extremophilic bacterium Acidithiobacillus ferrooxidans.
1070	DRAMP03972	N-3 (analog of P18)	"N-3"[All Fields] AND biofilm[All Fields]	N-3	16242551	Improved outcomes in the recent management of secondary aortoenteric fistula.
1070	DRAMP03972	N-3 (analog of P18)	"N-3"[All Fields] AND biofilm[All Fields]	N-3	16012802	Modelling antibiotic- and anti-quorum sensing treatment of a spatially-structured Pseudomonas aeruginosa population.
1070	DRAMP03972	N-3 (analog of P18)	"N-3"[All Fields] AND biofilm[All Fields]	N-3	15658990	Disruption of quorum sensing in seawater abolishes attraction of zoospores of the green alga Ulva to bacterial biofilms.
1070	DRAMP03972	N-3 (analog of P18)	"N-3"[All Fields] AND biofilm[All Fields]	N-3	15494175	Mathematical modelling of therapies targeted at bacterial quorum sensing.
1070	DRAMP03972	N-3 (analog of P18)	"N-3"[All Fields] AND biofilm[All Fields]	N-3	15314190	Analysis of quorum sensing-deficient clinical isolates of Pseudomonas aeruginosa.
1070	DRAMP03972	N-3 (analog of P18)	"N-3"[All Fields] AND biofilm[All Fields]	N-3	15073294	Expression of Pseudomonas aeruginosa exoS is controlled by quorum sensing and RpoS.
1070	DRAMP03972	N-3 (analog of P18)	"N-3"[All Fields] AND biofilm[All Fields]	N-3	14970327	Inactivation of a Pseudomonas aeruginosa quorum-sensing signal by human airway epithelia.
1070	DRAMP03972	N-3 (analog of P18)	"N-3"[All Fields] AND biofilm[All Fields]	N-3	14766816	Characterization of cell-to-cell signaling-deficient Pseudomonas aeruginosa strains colonizing intubated patients.
1070	DRAMP03972	N-3 (analog of P18)	"N-3"[All Fields] AND biofilm[All Fields]	N-3	14597753	Interspecies communication in bacteria.
1070	DRAMP03972	N-3 (analog of P18)	"N-3"[All Fields] AND biofilm[All Fields]	N-3	12931620	[Quorum sensing in Pseudomonas aeruginosa: cell to-cell communication to sense the cell density of the same species].
1070	DRAMP03972	N-3 (analog of P18)	"N-3"[All Fields] AND biofilm[All Fields]	N-3	12837389	Library screening for synthetic agonists and antagonists of a Pseudomonas aeruginosa autoinducer.
1070	DRAMP03972	N-3 (analog of P18)	"N-3"[All Fields] AND biofilm[All Fields]	N-3	12788555	Improved selectivity of microbial biosensor using membrane coating. Application to the analysis of ethanol during fermentation.
1070	DRAMP03972	N-3 (analog of P18)	"N-3"[All Fields] AND biofilm[All Fields]	N-3	12657493	Effects of chronic copper exposure on the nutritional composition of Hyalella azteca.
1070	DRAMP03972	N-3 (analog of P18)	"N-3"[All Fields] AND biofilm[All Fields]	N-3	12644477	Microarray analysis of Pseudomonas aeruginosa quorum-sensing regulons: effects of growth phase and environment.
1070	DRAMP03972	N-3 (analog of P18)	"N-3"[All Fields] AND biofilm[All Fields]	N-3	12424372	Cell-to-cell communication across the prokaryote-eukaryote boundary.
1070	DRAMP03972	N-3 (analog of P18)	"N-3"[All Fields] AND biofilm[All Fields]	N-3	12087407	Structure of a bacterial quorum-sensing transcription factor complexed with pheromone and DNA.
1070	DRAMP03972	N-3 (analog of P18)	"N-3"[All Fields] AND biofilm[All Fields]	N-3	12055291	Lysophosphatidic acid inhibition of the accumulation of Pseudomonas aeruginosa PAO1 alginate, pyoverdin, elastase and LasA.
1070	DRAMP03972	N-3 (analog of P18)	"N-3"[All Fields] AND biofilm[All Fields]	N-3	11966822	The regulation of biofilm development by quorum sensing in Aeromonas hydrophila.
1070	DRAMP03972	N-3 (analog of P18)	"N-3"[All Fields] AND biofilm[All Fields]	N-3	11855945	Detection of Pseudomonas aeruginosa cell-to-cell signals in lung tissue of cystic fibrosis patients.
1070	DRAMP03972	N-3 (analog of P18)	"N-3"[All Fields] AND biofilm[All Fields]	N-3	11769213	[Autotrophic ammonium-removal of sludge liquor].
1070	DRAMP03972	N-3 (analog of P18)	"N-3"[All Fields] AND biofilm[All Fields]	N-3	11455498	Artificial Pseudomonas aeruginosa biofilms and confocal laser scanning microscopic analysis.
1070	DRAMP03972	N-3 (analog of P18)	"N-3"[All Fields] AND biofilm[All Fields]	N-3	11401724	Pseudomonas aeruginosa GacA, a factor in multihost virulence, is also essential for biofilm formation.
1070	DRAMP03972	N-3 (analog of P18)	"N-3"[All Fields] AND biofilm[All Fields]	N-3	11233161	A novel and sensitive method for the quantification of N-3-oxoacyl homoserine lactones using gas chromatography-mass spectrometry: application to a model bacterial biofilm.
1070	DRAMP03972	N-3 (analog of P18)	"N-3"[All Fields] AND biofilm[All Fields]	N-3	10594832	Quorum sensing in Pseudomonas aeruginosa controls expression of catalase and superoxide dismutase genes and mediates biofilm susceptibility to hydrogen peroxide.
1070	DRAMP03972	N-3 (analog of P18)	"N-3"[All Fields] AND biofilm[All Fields]	N-3	9973347	Active efflux and diffusion are involved in transport of Pseudomonas aeruginosa cell-to-cell signals.
1070	DRAMP03972	N-3 (analog of P18)	"N-3"[All Fields] AND biofilm[All Fields]	N-3	9639627	The evolution of biofilms in venous access devices implanted in children with Wilms' tumour.
1070	DRAMP03972	N-3 (analog of P18)	"N-3"[All Fields] AND biofilm[All Fields]	N-3	9565532	One for all and all for one.
1070	DRAMP03972	N-3 (analog of P18)	"N-3"[All Fields] AND biofilm[All Fields]	N-3	9535661	The involvement of cell-to-cell signals in the development of a bacterial biofilm.
1070	DRAMP03972	N-3 (analog of P18)	"N-3"[All Fields] AND biofilm[All Fields]	N-3	9172348	Cell density-regulated recovery of starved biofilm populations of ammonia-oxidizing bacteria.
1070	DRAMP03972	N-3 (analog of P18)	"N-3"[All Fields] AND biofilm[All Fields]	N-3	8923071	Retention of the antibiotic teicoplanin on a hydromer-coated central venous catheter to prevent bacterial colonization in postoperative surgical patients.
1070	DRAMP03972	N-3 (analog of P18)	"N-3"[All Fields] AND biofilm[All Fields]	N-3	7999826	Silver peritoneal catheters reduce bacterial colonization.
1071	DRAMP03973	N-4 (analog of P18)	"N-4"[All Fields] AND biofilm[All Fields]	N-4	34627681	Guided Aspiration for Determining the Microbiological Aetiology of Aortic Vascular Graft and Endograft Infections.
1071	DRAMP03973	N-4 (analog of P18)	"N-4"[All Fields] AND biofilm[All Fields]	N-4	34621691	Biofilm Formation by Pseudomonas aeruginosain a Novel Septic Arthritis Model.
1071	DRAMP03973	N-4 (analog of P18)	"N-4"[All Fields] AND biofilm[All Fields]	N-4	34598294	Low-Level Laser and Antimicrobial Photodynamic Therapy Reduce Peri-implantitis-related Microorganisms Grown In Vitro.
1071	DRAMP03973	N-4 (analog of P18)	"N-4"[All Fields] AND biofilm[All Fields]	N-4	34537641	Intimately coupled photocatalysis and biodegradation for effective simultaneous removal of sulfamethoxazole and COD from synthetic domestic wastewater.
1071	DRAMP03973	N-4 (analog of P18)	"N-4"[All Fields] AND biofilm[All Fields]	N-4	34476616	Effect of different activations of silver nanoparticle irrigants on the elimination of Enterococcus faecalis.
1071	DRAMP03973	N-4 (analog of P18)	"N-4"[All Fields] AND biofilm[All Fields]	N-4	34455126	Simultaneous partial nitritation, anammox, and denitrification process for the treatment of simulated municipal sewage in a single-stage biofilter reactor.
1071	DRAMP03973	N-4 (analog of P18)	"N-4"[All Fields] AND biofilm[All Fields]	N-4	34442671	Preservation of Underground Microbial Diversity in Ancient Subsurface Deposits (>6 Ma) of the Rio Tinto Basement.
1071	DRAMP03973	N-4 (analog of P18)	"N-4"[All Fields] AND biofilm[All Fields]	N-4	34311554	Novel Small Molecule Growth Inhibitors of Xanthomonasspp. Causing Bacterial Spot of Tomato.
1071	DRAMP03973	N-4 (analog of P18)	"N-4"[All Fields] AND biofilm[All Fields]	N-4	34249333	A pilot study of red complex and three genera subgingival microbiome in periodontitis subjects with and without diabetes, evaluated by MinION platform.
1071	DRAMP03973	N-4 (analog of P18)	"N-4"[All Fields] AND biofilm[All Fields]	N-4	34301417	Ultraviolet C as a method of disinfecting medical silicone used in facial prostheses: An in vitro study.
1071	DRAMP03973	N-4 (analog of P18)	"N-4"[All Fields] AND biofilm[All Fields]	N-4	34259620	8-hydroxyquinoline-5-(N-4-chlorophenyl) sulfonamide and fluconazole combination as a preventive strategy for Candidabiofilm in haemodialysis devices.
1071	DRAMP03973	N-4 (analog of P18)	"N-4"[All Fields] AND biofilm[All Fields]	N-4	34235092	Genetic Characterisation of Colistin Resistant Klebsiella pneumoniaeClinical Isolates From North India.
1071	DRAMP03973	N-4 (analog of P18)	"N-4"[All Fields] AND biofilm[All Fields]	N-4	34105836	Effect of different beverages on surface properties and cariogenic biofilm formation of composite resin materials.
1071	DRAMP03973	N-4 (analog of P18)	"N-4"[All Fields] AND biofilm[All Fields]	N-4	33763712	In vitro and in vivo research of atmosphere pressure nonequilibrium plasmas on root canal disinfection: implication for alternative strategy for irrigation.
1071	DRAMP03973	N-4 (analog of P18)	"N-4"[All Fields] AND biofilm[All Fields]	N-4	33713274	First Evidence for Colonizing of Acanthamoeba T4 Genotype in Urinary Tracts of Patients with Recurrent Urinary Tract Infections.
1071	DRAMP03973	N-4 (analog of P18)	"N-4"[All Fields] AND biofilm[All Fields]	N-4	33581867	Physicomechanical, optical, and antifungal properties of polymethyl methacrylate modified with metal methacrylate monomers.
1071	DRAMP03973	N-4 (analog of P18)	"N-4"[All Fields] AND biofilm[All Fields]	N-4	33527485	Preventing Staphylococcus aureus stainless steel-associated infections in orthopedics. A systematic review and meta-analysis of animal literature.
1071	DRAMP03973	N-4 (analog of P18)	"N-4"[All Fields] AND biofilm[All Fields]	N-4	33423133	Bacterial adhesion characteristics on implant materials for intervertebral cages: titanium or PEEK for spinal infections?
1071	DRAMP03973	N-4 (analog of P18)	"N-4"[All Fields] AND biofilm[All Fields]	N-4	33421175	Multidrug resistant staphylococci isolated from pigs with exudative epidermitis in North eastern Region of India.
1071	DRAMP03973	N-4 (analog of P18)	"N-4"[All Fields] AND biofilm[All Fields]	N-4	33372186	Presence of the neonatal Staphylococcus capitis outbreak clone (NRCS-A) in prosthetic joint infections.
1071	DRAMP03973	N-4 (analog of P18)	"N-4"[All Fields] AND biofilm[All Fields]	N-4	33316671	Biodegradation of third-generation organic antifouling biocides and their hydrolysis products in marine model systems.

1071	DRAMP03973	N-4 (analog of P18)	"N-4"[All Fields] AND biofilm[All Fields]	N-4	33285571	Dental Implants Surface in vitro Decontamination Protocols.
1071	DRAMP03973	N-4 (analog of P18)	"N-4"[All Fields] AND biofilm[All Fields]	N-4	33141530	Doxycycline-Coated Silicone Breast Implants Reduce Acute Surgical-Site Infection and Inflammation.
1071	DRAMP03973	N-4 (analog of P18)	"N-4"[All Fields] AND biofilm[All Fields]	N-4	33062244	Evaluation of Commercial Disinfectants against Staphylococcus lentusand Micrococcusspp. of Poultry Origin.
1071	DRAMP03973	N-4 (analog of P18)	"N-4"[All Fields] AND biofilm[All Fields]	N-4	33022530	Impacts of sulfadiazine on the performance and membrane fouling of a hybrid moving bed biofilm reactor-membrane bioreactor system at different C/N ratios.
1071	DRAMP03973	N-4 (analog of P18)	"N-4"[All Fields] AND biofilm[All Fields]	N-4	32971434	Antifungal susceptibility and virulence profile of candida isolates from abnormal vaginal discharge of women from southern India.
1071	DRAMP03973	N-4 (analog of P18)	"N-4"[All Fields] AND biofilm[All Fields]	N-4	32928115	Characterization of carbapenem-resistant hypervirulent Acinetobacter baumannii strains isolated from hospitalized patients in the mid-south region of China.
1071	DRAMP03973	N-4 (analog of P18)	"N-4"[All Fields] AND biofilm[All Fields]	N-4	32752201	Effect of Novel Antibacterial Composites on Bacterial Biofilms.
1071	DRAMP03973	N-4 (analog of P18)	"N-4"[All Fields] AND biofilm[All Fields]	N-4	32717492	Genotypic and phenotypic traits of bla CTX-M-carrying Escherichia coli strains from an UV-C-treated wastewater effluent.
1071	DRAMP03973	N-4 (analog of P18)	"N-4"[All Fields] AND biofilm[All Fields]	N-4	32716693	Insight into Molecular Epidemiology, Antimicrobial Resistance, and Virulence Genes of Extensively Drug-Resistant Acinetobacter baumanniiin Thailand.
1071	DRAMP03973	N-4 (analog of P18)	"N-4"[All Fields] AND biofilm[All Fields]	N-4	32658912	Ba813 harboring Bacillus cereus, genetically closely related to Bacillus anthracis, causing nosocomial bloodstream infection: Bacterial virulence factors and clinical outcome.
1071	DRAMP03973	N-4 (analog of P18)	"N-4"[All Fields] AND biofilm[All Fields]	N-4	32616265	Prevalence and characteristics of extended-spectrum β-lactamases-producing Escherichia coli from broiler chickens at different day-age.
1071	DRAMP03973	N-4 (analog of P18)	"N-4"[All Fields] AND biofilm[All Fields]	N-4	32444905	Bacterial biofilms in infective endocarditis: an in vitro model to investigate emerging technologies of antimicrobial cardiovascular device coatings.
1071	DRAMP03973	N-4 (analog of P18)	"N-4"[All Fields] AND biofilm[All Fields]	N-4	32425898	Prevalence, Genetic Diversity, and Temporary Shifts of Inducible Clindamycin Resistance Staphylococcus aureusClones in Tehran, Iran: A Molecular-Epidemiological Analysis From 2013 to 2018.
1071	DRAMP03973	N-4 (analog of P18)	"N-4"[All Fields] AND biofilm[All Fields]	N-4	32373105	Alkylimidazolium Ionic Liquids as Antifungal Alternatives: Antibiofilm Activity Against Candida albicansand Underlying Mechanism of Action.
1071	DRAMP03973	N-4 (analog of P18)	"N-4"[All Fields] AND biofilm[All Fields]	N-4	32372011	Repurposing approach identifies pitavastatin as a potent azole chemosensitizing agent effective against azole-resistant Candida species.
1071	DRAMP03973	N-4 (analog of P18)	"N-4"[All Fields] AND biofilm[All Fields]	N-4	32354035	Antibacterial Activity of Amidodithiophosphonato Nickel(II) Complexes: An Experimental and Theoretical Approach.
1071	DRAMP03973	N-4 (analog of P18)	"N-4"[All Fields] AND biofilm[All Fields]	N-4	32333442	Effect of Ultraviolet Radiation on Candida albicans Biofilm on Poly(methylmethacrylate) Resin.
1071	DRAMP03973	N-4 (analog of P18)	"N-4"[All Fields] AND biofilm[All Fields]	N-4	31854000	Species identification, virulence markers and antimicrobial resistance profiles of Aeromonas sp. isolated from marketed hard-shelled mussel (Mytilus coruscus) in Korea.
1071	DRAMP03973	N-4 (analog of P18)	"N-4"[All Fields] AND biofilm[All Fields]	N-4	31813858	Synthesis of some new N-(alkyl/aralkyl)-N-(4-methoxyphenethyl) benzene sulfonamides as antibacterial agents against Escherichia.
1071	DRAMP03973	N-4 (analog of P18)	"N-4"[All Fields] AND biofilm[All Fields]	N-4	31589910	Disperse red 15 (DR15) impedes biofilm formation of uropathogenic Escherichia coli.
1071	DRAMP03973	N-4 (analog of P18)	"N-4"[All Fields] AND biofilm[All Fields]	N-4	31520397	Real-world experience with dalbavancin therapy in gram-positive skin and soft tissue infection, bone and joint infection.
1071	DRAMP03973	N-4 (analog of P18)	"N-4"[All Fields] AND biofilm[All Fields]	N-4	31508710	Formulation and characterization of antibacterial orthodontic adhesive.
1071	DRAMP03973	N-4 (analog of P18)	"N-4"[All Fields] AND biofilm[All Fields]	N-4	31450696	Synthesis of Novel 2-(Het)arylpyrrolidine Derivatives and Evaluation of Their Anticancer and Anti-Biofilm Activity.
1071	DRAMP03973	N-4 (analog of P18)	"N-4"[All Fields] AND biofilm[All Fields]	N-4	31421888	Non-aureus staphylococci in fecal samples of dairy cows: First report and phenotypic and genotypic characterization.
1071	DRAMP03973	N-4 (analog of P18)	"N-4"[All Fields] AND biofilm[All Fields]	N-4	31220276	Subcutaneous suppressive antibiotic therapy for bone and joint infections: safety and outcome in a cohort of 10 patients.
1071	DRAMP03973	N-4 (analog of P18)	"N-4"[All Fields] AND biofilm[All Fields]	N-4	31085909	Protective Effect of 4% Titanium Tetrafluoride Varnish on Dentin Demineralization Using a Microcosm Biofilm Model.
1071	DRAMP03973	N-4 (analog of P18)	"N-4"[All Fields] AND biofilm[All Fields]	N-4	30683036	[Multilocus sequence analysis, biofilm production, antibiotic susceptibility and synergy tests of Burkholderia species in patients with and without cystic fibrosis].
1071	DRAMP03973	N-4 (analog of P18)	"N-4"[All Fields] AND biofilm[All Fields]	N-4	30649481	Unequivocal identification of an underestimated opportunistic yeast species, Cyberlindnera fabianii, and its close relatives using a dual-function PCR and literature review of published cases.
1071	DRAMP03973	N-4 (analog of P18)	"N-4"[All Fields] AND biofilm[All Fields]	N-4	30638761	Synthesis and antimicrobial activities of N 6-hydroxyagelasine analogs and revision of the structure of ageloximes.
1071	DRAMP03973	N-4 (analog of P18)	"N-4"[All Fields] AND biofilm[All Fields]	N-4	30530220	Biofilms in hospital effluents as a potential crossroads for carbapenemase-encoding strains.
1071	DRAMP03973	N-4 (analog of P18)	"N-4"[All Fields] AND biofilm[All Fields]	N-4	30456941	Antimicrobial Imidazolium Ionic Liquids for the Development of Minimal Invasive Calcium Phosphate-Based Bionanocomposites.
1071	DRAMP03973	N-4 (analog of P18)	"N-4"[All Fields] AND biofilm[All Fields]	N-4	30455228	In VivoGentamicin Susceptibility Test for Prevention of Bacterial Biofilms in Bone Tissue and on Implants.
1071	DRAMP03973	N-4 (analog of P18)	"N-4"[All Fields] AND biofilm[All Fields]	N-4	30392371	Hydroxybiphenylamide GroEL/ES Inhibitors Are Potent Antibacterials against Planktonic and Biofilm Forms of Staphylococcus aureus.
1071	DRAMP03973	N-4 (analog of P18)	"N-4"[All Fields] AND biofilm[All Fields]	N-4	30382617	Synthesis and Evaluation of Water-Soluble 2-Aryl-1-Sulfonylpyrrolidine Derivatives as Bacterial Biofilm Formation Inhibitors.
1071	DRAMP03973	N-4 (analog of P18)	"N-4"[All Fields] AND biofilm[All Fields]	N-4	30153560	Blinded by the light: Increased chlorophyll fluorescence of herbicide-exposed periphyton masks unfavorable structural responses during exposure and recovery.
1071	DRAMP03973	N-4 (analog of P18)	"N-4"[All Fields] AND biofilm[All Fields]	N-4	30148880	Application of pancreatic phospholipase A2 for treatment of bovine mastitis.
1071	DRAMP03973	N-4 (analog of P18)	"N-4"[All Fields] AND biofilm[All Fields]	N-4	30051805	Quantification of major constituents of biofilms in occluded pancreatic stents.
1071	DRAMP03973	N-4 (analog of P18)	"N-4"[All Fields] AND biofilm[All Fields]	N-4	30034639	Functional profiles of coronal and dentin caries in children.
1071	DRAMP03973	N-4 (analog of P18)	"N-4"[All Fields] AND biofilm[All Fields]	N-4	29907449	High-risk Staphylococcus aureus transmission in the operating room: A call for widespread improvements in perioperative hand hygiene and patient decolonization practices.
1071	DRAMP03973	N-4 (analog of P18)	"N-4"[All Fields] AND biofilm[All Fields]	N-4	29893025	The wound-healing effects of a next-generation anti-biofilm silver Hydrofiber wound dressing on deep partial-thickness wounds using a porcine model.
1071	DRAMP03973	N-4 (analog of P18)	"N-4"[All Fields] AND biofilm[All Fields]	N-4	29879711	Fluoride Increase in Saliva and Dental Biofilm due to a Meal Prepared with Fluoridated Water or Salt: A Crossover Clinical Study.
1071	DRAMP03973	N-4 (analog of P18)	"N-4"[All Fields] AND biofilm[All Fields]	N-4	29744181	Repurposing AM404 for the treatment of oral infections by Porphyromonas gingivalis.
1071	DRAMP03973	N-4 (analog of P18)	"N-4"[All Fields] AND biofilm[All Fields]	N-4	29575083	Survival of Cronobacter in powdered infant formula and their variation in biofilm formation.
1071	DRAMP03973	N-4 (analog of P18)	"N-4"[All Fields] AND biofilm[All Fields]	N-4	29489933	Analysis of the antimicrobial and anti-caries effects of TiF4 varnish under microcosm biofilm formed on enamel.
1071	DRAMP03973	N-4 (analog of P18)	"N-4"[All Fields] AND biofilm[All Fields]	N-4	29412515	l-Methionine anti-biofilm activity against Pseudomonas aeruginosa is enhanced by the cystic fibrosis transmembrane conductance regulator potentiator, ivacaftor.
1071	DRAMP03973	N-4 (analog of P18)	"N-4"[All Fields] AND biofilm[All Fields]	N-4	29372248	Prevalence of High-Risk Human Papillomavirus in Tonsil Tissue in Healthy Adults and Colocalization in Biofilm of Tonsillar Crypts.
1071	DRAMP03973	N-4 (analog of P18)	"N-4"[All Fields] AND biofilm[All Fields]	N-4	29307772	Antimicrobial properties of a new type of photosensitizer derived from phthalocyanine against planktonic and biofilm forms of Staphylococcus aureus.
1071	DRAMP03973	N-4 (analog of P18)	"N-4"[All Fields] AND biofilm[All Fields]	N-4	29195992	Phenotypic characterization of Cronobacter spp. strains isolated from foods and clinical specimens in Brazil.
1071	DRAMP03973	N-4 (analog of P18)	"N-4"[All Fields] AND biofilm[All Fields]	N-4	29152579	Streptococcus pneumoniaeTIGR4 Phase-Locked Opacity Variants Differ in Virulence Phenotypes.
1071	DRAMP03973	N-4 (analog of P18)	"N-4"[All Fields] AND biofilm[All Fields]	N-4	29101088	Effect of methylene blue-induced photodynamic therapy on a Streptococcus mutans biofilm model.
1071	DRAMP03973	N-4 (analog of P18)	"N-4"[All Fields] AND biofilm[All Fields]	N-4	28818490	Inhibition of quorum sensing-controlled biofilm formation in Pseudomonas aeruginosa by quorum-sensing inhibitors.
1071	DRAMP03973	N-4 (analog of P18)	"N-4"[All Fields] AND biofilm[All Fields]	N-4	28777541	UV-Curable Contact Active Benzophenone Terminated Quaternary Ammonium Antimicrobials for Applications in Polymer Plastics and Related Devices.
1071	DRAMP03973	N-4 (analog of P18)	"N-4"[All Fields] AND biofilm[All Fields]	N-4	28595275	Antifungal susceptibility of Sporothrix schenckii complex biofilms.
1071	DRAMP03973	N-4 (analog of P18)	"N-4"[All Fields] AND biofilm[All Fields]	N-4	28437231	Capsular Polysaccharide Types and Virulence-Related Traits of Epidemic KPC-Producing Klebsiella pneumoniae Isolates in a Chinese University Hospital.
1071	DRAMP03973	N-4 (analog of P18)	"N-4"[All Fields] AND biofilm[All Fields]	N-4	28378343	Efficiency of riboflavin and ultraviolet light treatment against high levels of biofilm-derived Staphylococcus epidermidis in buffy coat platelet concentrates.
1071	DRAMP03973	N-4 (analog of P18)	"N-4"[All Fields] AND biofilm[All Fields]	N-4	28289036	Characterizing the Antimicrobial Activity of N 2, N 4-Disubstituted Quinazoline-2,4-Diamines toward Multidrug-Resistant Acinetobacter baumannii.
1071	DRAMP03973	N-4 (analog of P18)	"N-4"[All Fields] AND biofilm[All Fields]	N-4	30620537	[THE BIOFILM FORMATION ABILITY OF STRAINS SERRATIA SPP., SEPARATED FROM WOUNDS OF PATIENTS WITH CHRONIC OSTEOMYELITIS IN MONO-CULTURES AND IN COMPOSITION OF ASSOCIATION OF MICROORGANISMS HARVESTED IN VITRO].

1071	DRAMP03973	N-4 (analog of P18)	"N-4"[All Fields] AND biofilm[All Fields]	N-4	28159990	Planktonic growth and biofilm formation profiles in Candida haemulonii species complex.
1071	DRAMP03973	N-4 (analog of P18)	"N-4"[All Fields] AND biofilm[All Fields]	N-4	28050817	Phosphatidylcholine Coatings Deliver Local Antimicrobials and Reduce Infection in a Murine Model: A Preliminary Study.
1071	DRAMP03973	N-4 (analog of P18)	"N-4"[All Fields] AND biofilm[All Fields]	N-4	27925693	Identification of N-Arylated NH125 Analogues as Rapid Eradicating Agents against MRSA Persister Cells and Potent Biofilm Killers of Gram-Positive Pathogens.
1071	DRAMP03973	N-4 (analog of P18)	"N-4"[All Fields] AND biofilm[All Fields]	N-4	27864804	Activity of Norspermidine on Bacterial Biofilms of Multidrug-Resistant Clinical Isolates Associated with Persistent Extremity Wound Infections.
1071	DRAMP03973	N-4 (analog of P18)	"N-4"[All Fields] AND biofilm[All Fields]	N-4	27864174	Multilocus Sequence Analysis of Phylogroup 1 and 2 Oral Treponeme Strains.
1071	DRAMP03973	N-4 (analog of P18)	"N-4"[All Fields] AND biofilm[All Fields]	N-4	27785304	Gnotobiotic Human Colon Ex Vivo.
1071	DRAMP03973	N-4 (analog of P18)	"N-4"[All Fields] AND biofilm[All Fields]	N-4	27367016	Clonal persistence of Salmonella enterica serovars Montevideo, Tennessee, and Infantis in feed factories.
1071	DRAMP03973	N-4 (analog of P18)	"N-4"[All Fields] AND biofilm[All Fields]	N-4	27293967	Influence of the Culture Medium in Dose-Response Effect of the Chlorhexidine on Streptococcus mutans Biofilms.
1071	DRAMP03973	N-4 (analog of P18)	"N-4"[All Fields] AND biofilm[All Fields]	N-4	27155967	Nitrate and the Origin of Saliva Influence Composition and Short Chain Fatty Acid Production of Oral Microcosms.
1071	DRAMP03973	N-4 (analog of P18)	"N-4"[All Fields] AND biofilm[All Fields]	N-4	27017334	Propionibacterium prosthetic joint infection: experience from a retrospective database analysis.
1071	DRAMP03973	N-4 (analog of P18)	"N-4"[All Fields] AND biofilm[All Fields]	N-4	26693105	Anticoagulant properties of enoxaparin 400 IU/mL-40 % ethanol catheter lock solution.
1071	DRAMP03973	N-4 (analog of P18)	"N-4"[All Fields] AND biofilm[All Fields]	N-4	26687023	Silver(I) complexes with phthalazine and quinazoline as effective agents against pathogenic Pseudomonas aeruginosa strains.
1071	DRAMP03973	N-4 (analog of P18)	"N-4"[All Fields] AND biofilm[All Fields]	N-4	26574042	Characterization of phthiocerol and phthiodiolone dimycocerosate esters of M. tuberculosis by multiple-stage linear ion-trap MS.
1071	DRAMP03973	N-4 (analog of P18)	"N-4"[All Fields] AND biofilm[All Fields]	N-4	26201421	D-amino acid inhibits biofilm but not new bone formation in an ovine model.
1071	DRAMP03973	N-4 (analog of P18)	"N-4"[All Fields] AND biofilm[All Fields]	N-4	25917945	Antimicrobial Effects of Novel Triple Antibiotic Paste-Mimic Scaffolds on Actinomyces naeslundii Biofilm.
1071	DRAMP03973	N-4 (analog of P18)	"N-4"[All Fields] AND biofilm[All Fields]	N-4	25826934	[Numerical simulation and operation optimization of biological filter].
1071	DRAMP03973	N-4 (analog of P18)	"N-4"[All Fields] AND biofilm[All Fields]	N-4	27885354	A Potential Biofilm Metabolite Signature for Caries Activity - A Pilot Clinical Study.
1071	DRAMP03973	N-4 (analog of P18)	"N-4"[All Fields] AND biofilm[All Fields]	N-4	25712314	In vitro activity of gentamicin, vancomycin or amikacin combined with EDTA or l-arginine as lock therapy against a wide spectrum of biofilm-forming clinical strains isolated from catheter-related infections.
1071	DRAMP03973	N-4 (analog of P18)	"N-4"[All Fields] AND biofilm[All Fields]	N-4	25604874	Novel Antibiotic-loaded Point-of-care Implant Coating Inhibits Biofilm.
1071	DRAMP03973	N-4 (analog of P18)	"N-4"[All Fields] AND biofilm[All Fields]	N-4	25462723	Impact of coexistence of flocs and biofilm on performance of combined nitritation-anammox granular sludge reactors.
1071	DRAMP03973	N-4 (analog of P18)	"N-4"[All Fields] AND biofilm[All Fields]	N-4	25441228	Characteristics of nitrate removal in a bio-ceramsite reactor by aerobic denitrification.
1071	DRAMP03973	N-4 (analog of P18)	"N-4"[All Fields] AND biofilm[All Fields]	N-4	25186318	Tolerability of High Doses of Daptomycin in the Treatment of Prosthetic Vascular Graft Infection: A Retrospective Study.
1071	DRAMP03973	N-4 (analog of P18)	"N-4"[All Fields] AND biofilm[All Fields]	N-4	24718350	Validating the colloid model to optimise the design and operation of both moving-bed biofilm reactor and integrated fixed-film activated sludge systems.
1071	DRAMP03973	N-4 (analog of P18)	"N-4"[All Fields] AND biofilm[All Fields]	N-4	24465123	Inhibitory effect of N-ethyl-3-amino-5-oxo-4-phenyl-2,5-dihydro-1 H-pyrazole-1-carbothioamide on Haemophilusspp. planktonic or biofilm-forming cells.
1071	DRAMP03973	N-4 (analog of P18)	"N-4"[All Fields] AND biofilm[All Fields]	N-4	24415444	Methylglyoxal-augmented manuka honey as a topical anti-Staphylococcus aureus biofilm agent: safety and efficacy in an in vivo model.
1071	DRAMP03973	N-4 (analog of P18)	"N-4"[All Fields] AND biofilm[All Fields]	N-4	24324225	Suppression of type 1 pilus assembly in uropathogenic Escherichia coli by chemical inhibition of subunit polymerization.
1071	DRAMP03973	N-4 (analog of P18)	"N-4"[All Fields] AND biofilm[All Fields]	N-4	23860711	Use of an in vitro flat-bed biofilm model to measure biologically active anti-odour compounds.
1071	DRAMP03973	N-4 (analog of P18)	"N-4"[All Fields] AND biofilm[All Fields]	N-4	23857391	The icaA gene in staphylococci from bovine mastitis.
1071	DRAMP03973	N-4 (analog of P18)	"N-4"[All Fields] AND biofilm[All Fields]	N-4	23837889	Oral microbial colonization in laryngectomized patients as a possible cofactor of biofilm formation on their voice prostheses.
1071	DRAMP03973	N-4 (analog of P18)	"N-4"[All Fields] AND biofilm[All Fields]	N-4	22585351	Cis-2-decenoic acid inhibits S. aureus growth and biofilm in vitro: a pilot study.
1071	DRAMP03973	N-4 (analog of P18)	"N-4"[All Fields] AND biofilm[All Fields]	N-4	22431851	Oritavancin: mechanism of action.
1071	DRAMP03973	N-4 (analog of P18)	"N-4"[All Fields] AND biofilm[All Fields]	N-4	22301103	Atypical mycobacterium detection in refractory chronic rhinosinusitis.
1071	DRAMP03973	N-4 (analog of P18)	"N-4"[All Fields] AND biofilm[All Fields]	N-4	22031053	Evaluation of caries-affected dentin with optical coherence tomography.
1071	DRAMP03973	N-4 (analog of P18)	"N-4"[All Fields] AND biofilm[All Fields]	N-4	21705522	Diversity and antibiotic resistance patterns of Sphingomonadaceae isolates from drinking water.
1071	DRAMP03973	N-4 (analog of P18)	"N-4"[All Fields] AND biofilm[All Fields]	N-4	21386212	Modelling the effects of pH on tongue biofilm using a sorbarod biofilm perfusion system.
1071	DRAMP03973	N-4 (analog of P18)	"N-4"[All Fields] AND biofilm[All Fields]	N-4	20368521	Bacterial colonization and infection of electrophysiological cardiac devices detected with sonication and swab culture.
1071	DRAMP03973	N-4 (analog of P18)	"N-4"[All Fields] AND biofilm[All Fields]	N-4	19725103	Toll-like receptors and aseptic loosening of hip endoprosthesis-a potential to respond against danger signals?
1071	DRAMP03973	N-4 (analog of P18)	"N-4"[All Fields] AND biofilm[All Fields]	N-4	19548930	Microflora in teeth associated with apical periodontitis: a methodological observational study comparing two protocols and three microscopy techniques.
1071	DRAMP03973	N-4 (analog of P18)	"N-4"[All Fields] AND biofilm[All Fields]	N-4	19496702	Association of systemic oxidative stress with suppressed serum IgG to commensal oral biofilm and modulation by periodontal infection.
1071	DRAMP03973	N-4 (analog of P18)	"N-4"[All Fields] AND biofilm[All Fields]	N-4	19322605	Sonication of removed breast implants for improved detection of subclinical infection.
1071	DRAMP03973	N-4 (analog of P18)	"N-4"[All Fields] AND biofilm[All Fields]	N-4	19134555	Effect of acetate on nitrite oxidation in mixed-population biofilms.
1071	DRAMP03973	N-4 (analog of P18)	"N-4"[All Fields] AND biofilm[All Fields]	N-4	18210750	Antibacterial effect of tea-tree oil on methicillin-resistant Staphylococcus aureus biofilm formation of the tympanostomy tube: an in vitro study.
1071	DRAMP03973	N-4 (analog of P18)	"N-4"[All Fields] AND biofilm[All Fields]	N-4	18161672	Effect of ethanol/trisodium citrate lock on microorganisms causing hemodialysis catheter-related infections.
1071	DRAMP03973	N-4 (analog of P18)	"N-4"[All Fields] AND biofilm[All Fields]	N-4	18051300	Removal of organic load from olive washing water by an aerated submerged biofilter and profiling of the bacterial community involved in the process.
1071	DRAMP03973	N-4 (analog of P18)	"N-4"[All Fields] AND biofilm[All Fields]	N-4	17897202	In vitro biofilm model for studying tongue flora and malodour.
1071	DRAMP03973	N-4 (analog of P18)	"N-4"[All Fields] AND biofilm[All Fields]	N-4	17546983	Influence of temperature on microbial colonisation of clayey schist as a support media of a submerged filter for groundwater denitrification.
1071	DRAMP03973	N-4 (analog of P18)	"N-4"[All Fields] AND biofilm[All Fields]	N-4	16735866	Uncertain efficacy of daptomycin for prosthetic joint infections: a prospective case series.
1071	DRAMP03973	N-4 (analog of P18)	"N-4"[All Fields] AND biofilm[All Fields]	N-4	15276358	Antibacterial activity of polymeric substrate with surface grafted viologen moieties.
1071	DRAMP03973	N-4 (analog of P18)	"N-4"[All Fields] AND biofilm[All Fields]	N-4	14682656	Formation and decontamination of biofilms in dental unit waterlines.
1071	DRAMP03973	N-4 (analog of P18)	"N-4"[All Fields] AND biofilm[All Fields]	N-4	11985679	An in vitro comparison of the bactericidal efficacy of lethal photosensitization or sodium hyphochlorite irrigation on Streptococcus intermedius biofilms in root canals.
1071	DRAMP03973	N-4 (analog of P18)	"N-4"[All Fields] AND biofilm[All Fields]	N-4	11700794	A comparison of the use of an ATP-based bioluminescent assay and image analysis for the assessment of bacterial adhesion to standard HEMA and biomimetic soft contact lenses.
1071	DRAMP03973	N-4 (analog of P18)	"N-4"[All Fields] AND biofilm[All Fields]	N-4	8950503	The effect of eye closure on protein and complement deposition on Group IV hydrogel contact lenses: relationship to tear flow dynamics.
1071	DRAMP03973	N-4 (analog of P18)	"N-4"[All Fields] AND biofilm[All Fields]	N-4	8923071	Retention of the antibiotic teicoplanin on a hydromer-coated central venous catheter to prevent bacterial colonization in postoperative surgical patients.
1071	DRAMP03973	N-4 (analog of P18)	"N-4"[All Fields] AND biofilm[All Fields]	N-4	2027196	In situ replacement of vascular prostheses infected by bacterial biofilms.
1072	DRAMP03974	N-5 (analog of P18)	"N-5"[All Fields] AND biofilm[All Fields]	N-5	34687564	Antimicrobial effects of Melaleuca alternifolia (tea tree) essential oil against biofilm-forming multi-drug resistant cystic fibrosis-associated Pseudomonas aeruginosa as a single agent and in combination with commonly nebulised antibiotics.
1072	DRAMP03974	N-5 (analog of P18)	"N-5"[All Fields] AND biofilm[All Fields]	N-5	34623230	Is there an optimal method to detach Candida albicansbiofilm from dental materials?
1072	DRAMP03974	N-5 (analog of P18)	"N-5"[All Fields] AND biofilm[All Fields]	N-5	34574207	Conditions of In Vitro Biofilm Formation by Serogroups of Listeria monocytogenesIsolated from Hass Avocados Sold at Markets in Mexico.
1072	DRAMP03974	N-5 (analog of P18)	"N-5"[All Fields] AND biofilm[All Fields]	N-5	34572716	No Correlation between Biofilm Formation, Virulence Factors, and Antibiotic Resistance in Pseudomonas aeruginosa: Results from a Laboratory-Based In Vitro Study.

1072	DRAMP03974	N-5 (analog of P18)	"N-5"[All Fields] AND biofilm[All Fields]	N-5	34564912	Comparative Evaluation of Various Herbal Extracts on Biofilms of Streptococcus mutans and Scardovia wiggsiae: An In Vitro Study.
1072	DRAMP03974	N-5 (analog of P18)	"N-5"[All Fields] AND biofilm[All Fields]	N-5	34541685	Synergistic antimicrobial effect of photodynamic therapy and chitosan on the titanium-adherent biofilms of Staphylococcus aureus, Escherichia coli and Pseudomonas aeruginosa: an in vitro study.
1072	DRAMP03974	N-5 (analog of P18)	"N-5"[All Fields] AND biofilm[All Fields]	N-5	34476616	Effect of different activations of silver nanoparticle irrigants on the elimination of Enterococcus faecalis.
1072	DRAMP03974	N-5 (analog of P18)	"N-5"[All Fields] AND biofilm[All Fields]	N-5	34381318	Antifungal Activity of Denture Base Resin Containing Nanozirconia: In Vitro Assessment of Candida albicansBiofilm.
1072	DRAMP03974	N-5 (analog of P18)	"N-5"[All Fields] AND biofilm[All Fields]	N-5	34354696	Prevalence and Characteristic of Swine-Origin mcr-1-Positive Escherichia coliin Northeastern China.
1072	DRAMP03974	N-5 (analog of P18)	"N-5"[All Fields] AND biofilm[All Fields]	N-5	34341384	Quorum sensing systems and related virulence factors in Pseudomonas aeruginosa isolated from chicken meat and ground beef.
1072	DRAMP03974	N-5 (analog of P18)	"N-5"[All Fields] AND biofilm[All Fields]	N-5	34135604	Synergistic Activity and Biofilm Formation Effect of Colistin Combined with PFK-158 Against Colistin-Resistant Gram-Negative Bacteria.
1072	DRAMP03974	N-5 (analog of P18)	"N-5"[All Fields] AND biofilm[All Fields]	N-5	34105836	Effect of different beverages on surface properties and cariogenic biofilm formation of composite resin materials.
1072	DRAMP03974	N-5 (analog of P18)	"N-5"[All Fields] AND biofilm[All Fields]	N-5	34047872	Antibacterial effect of silver nanoparticles mixed with calcium hydroxide or chlorhexidine on multispecies biofilms.
1072	DRAMP03974	N-5 (analog of P18)	"N-5"[All Fields] AND biofilm[All Fields]	N-5	33968802	Trichosporon asahiiand Trichosporon inkinBiofilms Produce Antifungal-Tolerant Persister Cells.
1072	DRAMP03974	N-5 (analog of P18)	"N-5"[All Fields] AND biofilm[All Fields]	N-5	33886943	A novel acrylic resin palatal device contaminated with Candida albicans biofilm for denture stomatitis induction in Wistar rats.
1072	DRAMP03974	N-5 (analog of P18)	"N-5"[All Fields] AND biofilm[All Fields]	N-5	33796542	Experience With the Use of the MicroDTTect Device for the Diagnosis of Low-Grade Chronic Prosthetic Joint Infections in a Routine Setting.
1072	DRAMP03974	N-5 (analog of P18)	"N-5"[All Fields] AND biofilm[All Fields]	N-5	33763882	The presence of smear layer affects the antimicrobial action of root canal sealers.
1072	DRAMP03974	N-5 (analog of P18)	"N-5"[All Fields] AND biofilm[All Fields]	N-5	33762531	Bacterial Reduction in Oval-Shaped Root Canals After Different Irrigant Agitation Methods.
1072	DRAMP03974	N-5 (analog of P18)	"N-5"[All Fields] AND biofilm[All Fields]	N-5	33753336	Assessing the Bioactive Profile of Antifungal-Loaded Calcium Sulfate against Fungal Biofilms.
1072	DRAMP03974	N-5 (analog of P18)	"N-5"[All Fields] AND biofilm[All Fields]	N-5	33723620	Azole-Resilient Biofilms and Non-wild Type C. albicans Among Candida Species Isolated from Agricultural Soils Cultivated with Azole Fungicides: an Environmental Issue?
1072	DRAMP03974	N-5 (analog of P18)	"N-5"[All Fields] AND biofilm[All Fields]	N-5	33690064	Colloidal silver combating pathogenic Pseudomonas aeruginosa and MRSA in chronic rhinosinusitis.
1072	DRAMP03974	N-5 (analog of P18)	"N-5"[All Fields] AND biofilm[All Fields]	N-5	33662064	Influence Of Surface Treatment On The Physical Properties And Biofilm Formation Of Zirconia-Reinforced Lithium Silicate Ceramics: In Vitro Trial.
1072	DRAMP03974	N-5 (analog of P18)	"N-5"[All Fields] AND biofilm[All Fields]	N-5	33558775	The effect of N-acetyl cysteine on biofilm layers in an experimental model of chronic otitis media.
1072	DRAMP03974	N-5 (analog of P18)	"N-5"[All Fields] AND biofilm[All Fields]	N-5	33527485	Preventing Staphylococcus aureus stainless steel-associated infections in orthopedics. A systematic review and meta-analysis of animal literature.
1072	DRAMP03974	N-5 (analog of P18)	"N-5"[All Fields] AND biofilm[All Fields]	N-5	33523054	Tailoring copper(ii) complexes with pyridine-4,5-dicarboxylate esters for anti-Candida activity.
1072	DRAMP03974	N-5 (analog of P18)	"N-5"[All Fields] AND biofilm[All Fields]	N-5	33520591	New model in diabetic mice to evaluate the effects of insulin therapy on biofilm development in wounds.
1072	DRAMP03974	N-5 (analog of P18)	"N-5"[All Fields] AND biofilm[All Fields]	N-5	33507433	The antimicrobial effect of different ozone protocols applied in severe curved canals contaminated with Enterococcus faecalis: ex vivo study.
1072	DRAMP03974	N-5 (analog of P18)	"N-5"[All Fields] AND biofilm[All Fields]	N-5	33442721	Effective antimicrobial combination in vivo treatment predicted with microcalorimetry screening.
1072	DRAMP03974	N-5 (analog of P18)	"N-5"[All Fields] AND biofilm[All Fields]	N-5	33421175	Multidrug resistant staphylococci isolated from pigs with exudative epidermitis in North eastern Region of India.
1072	DRAMP03974	N-5 (analog of P18)	"N-5"[All Fields] AND biofilm[All Fields]	N-5	33417312	Fluorescence In Situ Hybridization and Polymerase Chain Reaction to Detect Infections in Patients With Left Ventricular Assist Devices.
1072	DRAMP03974	N-5 (analog of P18)	"N-5"[All Fields] AND biofilm[All Fields]	N-5	33305875	Single-stage revision of MRSA orthopedic device-related infection in sheep with an antibiotic-loaded hydrogel.
1072	DRAMP03974	N-5 (analog of P18)	"N-5"[All Fields] AND biofilm[All Fields]	N-5	33237232	Antibacterial Activity of a New Ready-To-Use Calcium Silicate-Based Sealer.
1072	DRAMP03974	N-5 (analog of P18)	"N-5"[All Fields] AND biofilm[All Fields]	N-5	33017531	Glass-ionomer cement modifies the gene expression of Streptococcus mutans providing a lower virulent biofilm.
1072	DRAMP03974	N-5 (analog of P18)	"N-5"[All Fields] AND biofilm[All Fields]	N-5	32933859	Update on minocycline in vitro activity against odontogenic bacteria.
1072	DRAMP03974	N-5 (analog of P18)	"N-5"[All Fields] AND biofilm[All Fields]	N-5	32831847	Characterisation of VibrioSpecies from Surface and Drinking Water Sources and Assessment of Biocontrol Potentials of Their Bacteriophages.
1072	DRAMP03974	N-5 (analog of P18)	"N-5"[All Fields] AND biofilm[All Fields]	N-5	32827220	Effect of erosive and abrasive challenges on the glaze layer applied to ceramic materials.
1072	DRAMP03974	N-5 (analog of P18)	"N-5"[All Fields] AND biofilm[All Fields]	N-5	32819689	On-going nitrification in chloraminated drinking water distribution system (DWDS) is conditioned by hydraulics and disinfection strategies.
1072	DRAMP03974	N-5 (analog of P18)	"N-5"[All Fields] AND biofilm[All Fields]	N-5	32801033	Effect of fragaria vesca, hamamelis and tormentil on the initial bacterial colonization in situ.
1072	DRAMP03974	N-5 (analog of P18)	"N-5"[All Fields] AND biofilm[All Fields]	N-5	32793133	Isolation and Characterization of Novel Lytic Bacteriophages Infecting Epidemic Carbapenem-Resistant Klebsiella pneumoniaeStrains.
1072	DRAMP03974	N-5 (analog of P18)	"N-5"[All Fields] AND biofilm[All Fields]	N-5	32716693	Insight into Molecular Epidemiology, Antimicrobial Resistance, and Virulence Genes of Extensively Drug-Resistant Acinetobacter baumanniiin Thailand.
1072	DRAMP03974	N-5 (analog of P18)	"N-5"[All Fields] AND biofilm[All Fields]	N-5	32616265	Prevalence and characteristics of extended-spectrum β-lactamases-producing Escherichia coli from broiler chickens at different day-age.
1072	DRAMP03974	N-5 (analog of P18)	"N-5"[All Fields] AND biofilm[All Fields]	N-5	32571708	Beta-D-Glucan Levels With Use of an Anti-adhesion Barrier Film in Pediatric Living Donor Liver Transplantation.
1072	DRAMP03974	N-5 (analog of P18)	"N-5"[All Fields] AND biofilm[All Fields]	N-5	32464265	Photo-sonodynamic antimicrobial chemotherapy via chitosan nanoparticles-indocyanine green against polymicrobial periopathogenic biofilms: Ex vivo study on dental implants.
1072	DRAMP03974	N-5 (analog of P18)	"N-5"[All Fields] AND biofilm[All Fields]	N-5	32425898	Prevalence, Genetic Diversity, and Temporary Shifts of Inducible Clindamycin Resistance Staphylococcus aureusClones in Tehran, Iran: A Molecular-Epidemiological Analysis From 2013 to 2018.
1072	DRAMP03974	N-5 (analog of P18)	"N-5"[All Fields] AND biofilm[All Fields]	N-5	32372011	Repurposing approach identifies pitavastatin as a potent azole chemosensitizing agent effective against azole-resistant Candida species.
1072	DRAMP03974	N-5 (analog of P18)	"N-5"[All Fields] AND biofilm[All Fields]	N-5	32233643	Pathogen identification in 84 Patients with post-traumatic osteomyelitis after limb fractures.
1072	DRAMP03974	N-5 (analog of P18)	"N-5"[All Fields] AND biofilm[All Fields]	N-5	32018281	Reduction of an in vitro Intraradicular Multispecies Biofilm Using Two Rotary Instrumentation Sequences.
1072	DRAMP03974	N-5 (analog of P18)	"N-5"[All Fields] AND biofilm[All Fields]	N-5	31880897	Epidemiology, Drug Resistance, and Virulence of Staphylococcus aureusIsolated from Ocular Infections in Polish Patients.
1072	DRAMP03974	N-5 (analog of P18)	"N-5"[All Fields] AND biofilm[All Fields]	N-5	31870895	Dual wavelength irradiation antimicrobial photodynamic therapy using indocyanine green and metformin doped with nano-curcumin as an efficient adjunctive endodontic treatment modality.
1072	DRAMP03974	N-5 (analog of P18)	"N-5"[All Fields] AND biofilm[All Fields]	N-5	31666981	Reverse diauxie phenotype in Pseudomonas aeruginosabiofilm revealed by exometabolomics and label-free proteomics.
1072	DRAMP03974	N-5 (analog of P18)	"N-5"[All Fields] AND biofilm[All Fields]	N-5	31436941	In vitro evaluation of the effectiveness of alkaline peroxide solutions in reducing the viability of specific biofilms.
1072	DRAMP03974	N-5 (analog of P18)	"N-5"[All Fields] AND biofilm[All Fields]	N-5	31436934	Effect of thermal and acid challenges on the surface properties of pink restorative materials.
1072	DRAMP03974	N-5 (analog of P18)	"N-5"[All Fields] AND biofilm[All Fields]	N-5	31421888	Non-aureus staphylococci in fecal samples of dairy cows: First report and phenotypic and genotypic characterization.
1072	DRAMP03974	N-5 (analog of P18)	"N-5"[All Fields] AND biofilm[All Fields]	N-5	31277834	Moxifloxacin-rifampicin combination for the treatment of non-staphylococcal Gram-positive orthopedic implant-related infections.
1072	DRAMP03974	N-5 (analog of P18)	"N-5"[All Fields] AND biofilm[All Fields]	N-5	31220276	Subcutaneous suppressive antibiotic therapy for bone and joint infections: safety and outcome in a cohort of 10 patients.
1072	DRAMP03974	N-5 (analog of P18)	"N-5"[All Fields] AND biofilm[All Fields]	N-5	31204047	Experimental composites containing quaternary ammonium methacrylates reduce demineralization at enamel-restoration margins after cariogenic challenge.
1072	DRAMP03974	N-5 (analog of P18)	"N-5"[All Fields] AND biofilm[All Fields]	N-5	31166393	Antibiofilm Activity of an Experimental Ricinus Communis Dentifrice on Soft Denture Liners.
1072	DRAMP03974	N-5 (analog of P18)	"N-5"[All Fields] AND biofilm[All Fields]	N-5	31088356	Accessory gene regulator (agr) dysfunction was unusual in Staphylococcus aureus isolated from Chinese children.
1072	DRAMP03974	N-5 (analog of P18)	"N-5"[All Fields] AND biofilm[All Fields]	N-5	30955856	Antibacterial resin-based composite containing chlorhexidine for dental applications.
1072	DRAMP03974	N-5 (analog of P18)	"N-5"[All Fields] AND biofilm[All Fields]	N-5	30856427	Oxygen-reducing microbial cathodes monitoring toxic shocks in tap water.
1072	DRAMP03974	N-5 (analog of P18)	"N-5"[All Fields] AND biofilm[All Fields]	N-5	30725401	Effects of theobromine addition on chemical and mechanical properties of a conventional glass ionomer cement.
1072	DRAMP03974	N-5 (analog of P18)	"N-5"[All Fields] AND biofilm[All Fields]	N-5	30720875	Antimicrobial action of photodynamic therapy in root canals using LED curing light, curcumin and carbopol gel.

1072	DRAMP03974	N-5 (analog of P18)	"N-5"[All Fields] AND biofilm[All Fields]	N-5	30689984	PvdF of pyoverdin biosynthesis is a structurally unique N 10-formyltetrahydrofolate-dependent formyltransferase.
1072	DRAMP03974	N-5 (analog of P18)	"N-5"[All Fields] AND biofilm[All Fields]	N-5	30679015	Hydrophobicity of graphene as a driving force for inhibiting biofilm formation of pathogenic bacteria and fungi.
1072	DRAMP03974	N-5 (analog of P18)	"N-5"[All Fields] AND biofilm[All Fields]	N-5	30646999	In vivo biocompatibility of an interim denture resilient liner containing antifungal drugs.
1072	DRAMP03974	N-5 (analog of P18)	"N-5"[All Fields] AND biofilm[All Fields]	N-5	30363376	Molecular epidemiology, antimicrobial susceptibility, and pulsed-field gel electrophoresis genotyping of Pseudomonas aeruginosaisolates from mink.
1072	DRAMP03974	N-5 (analog of P18)	"N-5"[All Fields] AND biofilm[All Fields]	N-5	30257693	Complementary and alternative medicine for treatment of atopic eczema in children under 14 years old: a systematic review and meta-analysis of randomized controlled trials.
1072	DRAMP03974	N-5 (analog of P18)	"N-5"[All Fields] AND biofilm[All Fields]	N-5	30130704	Integrated fixed-film activated sludge membrane bioreactors versus membrane bioreactors for nutrient removal: A comprehensive comparison.
1072	DRAMP03974	N-5 (analog of P18)	"N-5"[All Fields] AND biofilm[All Fields]	N-5	30076977	Remarkable geographical variations between India and Europe in carriage of the staphylococcal surface protein-encoding sasX/sesI and in the population structure of methicillin-resistant Staphylococcus aureus belonging to clonal complex 8.
1072	DRAMP03974	N-5 (analog of P18)	"N-5"[All Fields] AND biofilm[All Fields]	N-5	30068882	In Vivo Antifungal Activity of Monolaurin against Candida albicans Biofilms.
1072	DRAMP03974	N-5 (analog of P18)	"N-5"[All Fields] AND biofilm[All Fields]	N-5	30051805	Quantification of major constituents of biofilms in occluded pancreatic stents.
1072	DRAMP03974	N-5 (analog of P18)	"N-5"[All Fields] AND biofilm[All Fields]	N-5	30034639	Functional profiles of coronal and dentin caries in children.
1072	DRAMP03974	N-5 (analog of P18)	"N-5"[All Fields] AND biofilm[All Fields]	N-5	29631210	Listeria monocytogenes strains show large variations in competitive growth in mixed culture biofilms and suspensions with bacteria from food processing environments.
1072	DRAMP03974	N-5 (analog of P18)	"N-5"[All Fields] AND biofilm[All Fields]	N-5	29372575	Distribution of Ebp pili among clinical and fecal isolates of Enterococcus faecalis and evaluation for human platelet activation.
1072	DRAMP03974	N-5 (analog of P18)	"N-5"[All Fields] AND biofilm[All Fields]	N-5	29372248	Prevalence of High-Risk Human Papillomavirus in Tonsil Tissue in Healthy Adults and Colocalization in Biofilm of Tonsillar Crypts.
1072	DRAMP03974	N-5 (analog of P18)	"N-5"[All Fields] AND biofilm[All Fields]	N-5	29178982	Microbiology Alloplastic Total Joint Infections: A 20-Year Retrospective Study.
1072	DRAMP03974	N-5 (analog of P18)	"N-5"[All Fields] AND biofilm[All Fields]	N-5	29178710	Effect of conventional and contemporary disinfectant techniques on three peri-implantitis associated microbiotas.
1072	DRAMP03974	N-5 (analog of P18)	"N-5"[All Fields] AND biofilm[All Fields]	N-5	28750776	In vitro biofilm formation on resin-based composites after different finishing and polishing procedures.
1072	DRAMP03974	N-5 (analog of P18)	"N-5"[All Fields] AND biofilm[All Fields]	N-5	28438935	Candida guilliermondii Complex Is Characterized by High Antifungal Resistance but Low Mortality in 22 Cases of Candidemia.
1072	DRAMP03974	N-5 (analog of P18)	"N-5"[All Fields] AND biofilm[All Fields]	N-5	28225903	Sphygmomanometers and thermometers as potential fomites of Staphylococcus haemolyticus: biofilm formation in the presence of antibiotics.
1072	DRAMP03974	N-5 (analog of P18)	"N-5"[All Fields] AND biofilm[All Fields]	N-5	28159990	Planktonic growth and biofilm formation profiles in Candida haemulonii species complex.
1072	DRAMP03974	N-5 (analog of P18)	"N-5"[All Fields] AND biofilm[All Fields]	N-5	28134790	Efficient Synthesis of Novel Pyridine-Based Derivatives via Suzuki Cross-Coupling Reaction of Commercially Available 5-Bromo-2-methylpyridin-3-amine: Quantum Mechanical Investigations and Biological Activities.
1072	DRAMP03974	N-5 (analog of P18)	"N-5"[All Fields] AND biofilm[All Fields]	N-5	28042660	Determination of minimum biofilm eradication concentrations of orbifloxacin for canine bacterial uropathogens over different treatment periods.
1072	DRAMP03974	N-5 (analog of P18)	"N-5"[All Fields] AND biofilm[All Fields]	N-5	27864804	Activity of Norspermidine on Bacterial Biofilms of Multidrug-Resistant Clinical Isolates Associated with Persistent Extremity Wound Infections.
1072	DRAMP03974	N-5 (analog of P18)	"N-5"[All Fields] AND biofilm[All Fields]	N-5	27834171	Bacterial communities associated with apical periodontitis and dental implant failure.
1072	DRAMP03974	N-5 (analog of P18)	"N-5"[All Fields] AND biofilm[All Fields]	N-5	27703610	Silver nanoparticles in resin luting cements: Antibacterial and physiochemical properties.
1072	DRAMP03974	N-5 (analog of P18)	"N-5"[All Fields] AND biofilm[All Fields]	N-5	27517499	Multiple exposure routes of a pesticide exacerbate effects on a grazing mayfly.
1072	DRAMP03974	N-5 (analog of P18)	"N-5"[All Fields] AND biofilm[All Fields]	N-5	27505991	Antimicrobial efficacy of complete denture cleansers.
1072	DRAMP03974	N-5 (analog of P18)	"N-5"[All Fields] AND biofilm[All Fields]	N-5	29744161	Influence of low direct electric currents and chlorhexidine upon human dental biofilms.
1072	DRAMP03974	N-5 (analog of P18)	"N-5"[All Fields] AND biofilm[All Fields]	N-5	27339632	Histologic Assessment of Debridement of the Root Canal Isthmus of Mandibular Molars by Irrigant Activation Techniques Ex Vivo.
1072	DRAMP03974	N-5 (analog of P18)	"N-5"[All Fields] AND biofilm[All Fields]	N-5	27021323	Biofilm Production and Antibiofilm Activity of Echinocandins and Liposomal Amphotericin B in Echinocandin-Resistant Yeast Species.
1072	DRAMP03974	N-5 (analog of P18)	"N-5"[All Fields] AND biofilm[All Fields]	N-5	27017334	Propionibacterium prosthetic joint infection: experience from a retrospective database analysis.
1072	DRAMP03974	N-5 (analog of P18)	"N-5"[All Fields] AND biofilm[All Fields]	N-5	27003065	Nutrient removal performance and microbial characteristics of a full-scale IFAS-EBPR process treating municipal wastewater.
1072	DRAMP03974	N-5 (analog of P18)	"N-5"[All Fields] AND biofilm[All Fields]	N-5	27000771	Influence of different instrumentation modalities on the surface characteristics and biofilm formation on dental implant neck, in vitro.
1072	DRAMP03974	N-5 (analog of P18)	"N-5"[All Fields] AND biofilm[All Fields]	N-5	26900648	Genes involved in Listeria monocytogenes biofilm formation at a simulated food processing plant temperature of 15 °C.
1072	DRAMP03974	N-5 (analog of P18)	"N-5"[All Fields] AND biofilm[All Fields]	N-5	26846044	Influence of organic acids present in oral biofilm on the durability of the repair bond strength, sorption and solubility of resin composites.
1072	DRAMP03974	N-5 (analog of P18)	"N-5"[All Fields] AND biofilm[All Fields]	N-5	26687023	Silver(I) complexes with phthalazine and quinazoline as effective agents against pathogenic Pseudomonas aeruginosa strains.
1072	DRAMP03974	N-5 (analog of P18)	"N-5"[All Fields] AND biofilm[All Fields]	N-5	26617603	Antimicrobial and Anti-Virulence Activity of Capsaicin Against Erythromycin-Resistant, Cell-Invasive Group A Streptococci.
1072	DRAMP03974	N-5 (analog of P18)	"N-5"[All Fields] AND biofilm[All Fields]	N-5	26546719	Biofilm formation in invasive Staphylococcus aureus isolates is associated with the clonal lineage.
1072	DRAMP03974	N-5 (analog of P18)	"N-5"[All Fields] AND biofilm[All Fields]	N-5	26443466	Comparative analyses of ion release, pH and multispecies biofilm formation between conventional and bioactive gutta-percha.
1072	DRAMP03974	N-5 (analog of P18)	"N-5"[All Fields] AND biofilm[All Fields]	N-5	26213715	Colonization and Persistence of Labeled and "Foreign" Strains of Aggregatibacter actinomycetemcomitansInoculated into the Mouths of Rhesus Monkeys.
1072	DRAMP03974	N-5 (analog of P18)	"N-5"[All Fields] AND biofilm[All Fields]	N-5	26153228	Confocal microscopy evaluation of the effect of irrigants on Enterococcus faecalis biofilm: An in vitro study.
1072	DRAMP03974	N-5 (analog of P18)	"N-5"[All Fields] AND biofilm[All Fields]	N-5	26122120	A novel rat model of catheter-associated urinary tract infection.
1072	DRAMP03974	N-5 (analog of P18)	"N-5"[All Fields] AND biofilm[All Fields]	N-5	27688392	Antimicrobial Capacity of Casein Phosphopeptide/Amorphous Calcium Phosphate and Enzymes in Glass Ionomer Cement in Dentin Carious Lesions.
1072	DRAMP03974	N-5 (analog of P18)	"N-5"[All Fields] AND biofilm[All Fields]	N-5	25826934	[Numerical simulation and operation optimization of biological filter].
1072	DRAMP03974	N-5 (analog of P18)	"N-5"[All Fields] AND biofilm[All Fields]	N-5	25789134	Molecular Detection of Class-D OXA Carbapenemase Genes in Biofilm and Non-Biofilm Forming Clinical Isolates of Acinetobacter baumannii.
1072	DRAMP03974	N-5 (analog of P18)	"N-5"[All Fields] AND biofilm[All Fields]	N-5	25590201	Degradation of resin composites in a simulated deep cavity.
1072	DRAMP03974	N-5 (analog of P18)	"N-5"[All Fields] AND biofilm[All Fields]	N-5	25550392	Candida tropicalis isolates obtained from veterinary sources show resistance to azoles and produce virulence factors.
1072	DRAMP03974	N-5 (analog of P18)	"N-5"[All Fields] AND biofilm[All Fields]	N-5	25523597	Evaluating the efficiency of humic acid to remove micro-organisms from denture base material.
1072	DRAMP03974	N-5 (analog of P18)	"N-5"[All Fields] AND biofilm[All Fields]	N-5	25463655	Application of benzo[a]phenoxazinium chlorides in Antimicrobial Photodynamic Therapy of Candida albicans biofilms.
1072	DRAMP03974	N-5 (analog of P18)	"N-5"[All Fields] AND biofilm[All Fields]	N-5	25371399	Description and microbiology of endotracheal tube biofilm in mechanically ventilated subjects.
1072	DRAMP03974	N-5 (analog of P18)	"N-5"[All Fields] AND biofilm[All Fields]	N-5	25140719	Addition of silver nanoparticles to composite resin: effect on physical and bactericidal properties in vitro.
1072	DRAMP03974	N-5 (analog of P18)	"N-5"[All Fields] AND biofilm[All Fields]	N-5	25105817	Oral microbiota and host innate immune response in bisphosphonate-related osteonecrosis of the jaw.
1072	DRAMP03974	N-5 (analog of P18)	"N-5"[All Fields] AND biofilm[All Fields]	N-5	25060652	Biofilm formation on the surface of modern implant abutment materials.
1072	DRAMP03974	N-5 (analog of P18)	"N-5"[All Fields] AND biofilm[All Fields]	N-5	24683595	Sorption and solubility of self-etching adhesives immersed in organic acids present in oral biofilm.
1072	DRAMP03974	N-5 (analog of P18)	"N-5"[All Fields] AND biofilm[All Fields]	N-5	24611831	Electrochemically modulated nitric oxide (NO) releasing biomedical devices via copper(II)-Tri(2-pyridylmethyl)amine mediated reduction of nitrite.
1072	DRAMP03974	N-5 (analog of P18)	"N-5"[All Fields] AND biofilm[All Fields]	N-5	24607269	Photosensitization of in vitro biofilms formed on denture base resin.
1072	DRAMP03974	N-5 (analog of P18)	"N-5"[All Fields] AND biofilm[All Fields]	N-5	24378651	Concurrent quantification of cellular and extracellular components of biofilms.
1072	DRAMP03974	N-5 (analog of P18)	"N-5"[All Fields] AND biofilm[All Fields]	N-5	23986753	Characterization of hospital-associated lineages of ampicillin-resistant Enterococcus faecium from clinical cases in dogs and humans.
1072	DRAMP03974	N-5 (analog of P18)	"N-5"[All Fields] AND biofilm[All Fields]	N-5	23904295	Heparin-binding motifs and biofilm formation by Candida albicans.

1072	DRAMP03974	N-5 (analog of P18)	"N-5"[All Fields] AND biofilm[All Fields]	N-5	23857391	The icaA gene in staphylococci from bovine mastitis.
1072	DRAMP03974	N-5 (analog of P18)	"N-5"[All Fields] AND biofilm[All Fields]	N-5	23402505	Comparative analysis of Enterococcus faecalis biofilm formation on different substrates.
1072	DRAMP03974	N-5 (analog of P18)	"N-5"[All Fields] AND biofilm[All Fields]	N-5	23351218	In vitro effect of paediatric liquid medicines on deciduous enamel exposed to biofilm.
1072	DRAMP03974	N-5 (analog of P18)	"N-5"[All Fields] AND biofilm[All Fields]	N-5	23207854	Effect of denture cleansers on metal ion release and surface roughness of denture base materials.
1072	DRAMP03974	N-5 (analog of P18)	"N-5"[All Fields] AND biofilm[All Fields]	N-5	23202567	Impact of carbon to nitrogen ratio on nitrogen removal at a low oxygen concentration in a sequencing batch biofilm reactor.
1072	DRAMP03974	N-5 (analog of P18)	"N-5"[All Fields] AND biofilm[All Fields]	N-5	22999603	Etiology of surface light scattering on hydrophobic acrylic intraocular lenses.
1072	DRAMP03974	N-5 (analog of P18)	"N-5"[All Fields] AND biofilm[All Fields]	N-5	22279913	[Effective nitrogen removal in low C/N wastewater with combined aerobic-low DO biofilm treatment process].
1072	DRAMP03974	N-5 (analog of P18)	"N-5"[All Fields] AND biofilm[All Fields]	N-5	22228735	Regulation of bacteriocin production and cell death by the VicRK signaling system in Streptococcus mutans.
1072	DRAMP03974	N-5 (analog of P18)	"N-5"[All Fields] AND biofilm[All Fields]	N-5	22192761	Effect of free ammonia concentration on monochloramine penetration within a nitrifying biofilm and its effect on activity, viability, and recovery.
1072	DRAMP03974	N-5 (analog of P18)	"N-5"[All Fields] AND biofilm[All Fields]	N-5	22159480	[Enteric Gram negative rods and unfermented of glucose bacteria in patients with peri-implant disease].
1072	DRAMP03974	N-5 (analog of P18)	"N-5"[All Fields] AND biofilm[All Fields]	N-5	22016784	Removing biofilms from microstructured titanium ex vivo: a novel approach using atmospheric plasma technology.
1072	DRAMP03974	N-5 (analog of P18)	"N-5"[All Fields] AND biofilm[All Fields]	N-5	25610156	Investigating biofilm production, coagulase and hemolytic activity in Candida species isolated from denture stomatitis patients.
1072	DRAMP03974	N-5 (analog of P18)	"N-5"[All Fields] AND biofilm[All Fields]	N-5	21423727	Molecular analysis of microbial communities in endotracheal tube biofilms.
1072	DRAMP03974	N-5 (analog of P18)	"N-5"[All Fields] AND biofilm[All Fields]	N-5	21306197	Co-aggregation and growth inhibition of probiotic lactobacilli and clinical isolates of mutans streptococci: an in vitro study.
1072	DRAMP03974	N-5 (analog of P18)	"N-5"[All Fields] AND biofilm[All Fields]	N-5	20455404	[Investigation of the surface properties of Staphylococcus epidermidis strains isolated from biomaterials].
1072	DRAMP03974	N-5 (analog of P18)	"N-5"[All Fields] AND biofilm[All Fields]	N-5	20221891	In vitro antimicrobial synergy testing of coagulase-negative staphylococci isolated from prosthetic joint infections using Etest and with a focus on rifampicin and linezolid.
1072	DRAMP03974	N-5 (analog of P18)	"N-5"[All Fields] AND biofilm[All Fields]	N-5	20127718	Profile of toll-like receptor-positive cells in septic and aseptic loosening of total hip arthroplasty implants.
1072	DRAMP03974	N-5 (analog of P18)	"N-5"[All Fields] AND biofilm[All Fields]	N-5	20084909	[Investigation of biofilm formation and relationship with genotype and antibiotic susceptibility of Pseudomonas aeruginosa strains isolated from patients with cystic fibrosis].
1072	DRAMP03974	N-5 (analog of P18)	"N-5"[All Fields] AND biofilm[All Fields]	N-5	20060338	Susceptibility of Candida albicans to photodynamic therapy in a murine model of oral candidosis.
1072	DRAMP03974	N-5 (analog of P18)	"N-5"[All Fields] AND biofilm[All Fields]	N-5	20056010	Effect of vancomycin-coated tympanostomy tubes on methicillin-resistant Staphylococcus aureus biofilm formation: in vitro study.
1072	DRAMP03974	N-5 (analog of P18)	"N-5"[All Fields] AND biofilm[All Fields]	N-5	19781590	Germicidal activity of antimicrobials and VIOlight Personal Travel Toothbrush sanitizer: an in vitro study.
1072	DRAMP03974	N-5 (analog of P18)	"N-5"[All Fields] AND biofilm[All Fields]	N-5	19725103	Toll-like receptors and aseptic loosening of hip endoprosthesis-a potential to respond against danger signals?
1072	DRAMP03974	N-5 (analog of P18)	"N-5"[All Fields] AND biofilm[All Fields]	N-5	19698053	Presence of five conditioning film proteins are highly associated with early stent encrustation.
1072	DRAMP03974	N-5 (analog of P18)	"N-5"[All Fields] AND biofilm[All Fields]	N-5	19438939	Bacterial persistence in dentoalveolar bone following extraction: a microbiological study and implications for dental implant treatment.
1072	DRAMP03974	N-5 (analog of P18)	"N-5"[All Fields] AND biofilm[All Fields]	N-5	19358060	A comparative scanning electron microscopic study of biliary and pancreatic stents.
1072	DRAMP03974	N-5 (analog of P18)	"N-5"[All Fields] AND biofilm[All Fields]	N-5	19205021	Material wear of polymeric tracheostomy tubes: a six-month study.
1072	DRAMP03974	N-5 (analog of P18)	"N-5"[All Fields] AND biofilm[All Fields]	N-5	19095310	The use of piperacillin-tazobactam coated tympanostomy tubes against ciprofloxacin-resistant Pseudomonas biofilm formation: an in vitro study.
1072	DRAMP03974	N-5 (analog of P18)	"N-5"[All Fields] AND biofilm[All Fields]	N-5	18621456	The influence of 30-day-old Streptococcus mutans biofilm on the surface of esthetic restorative materials--an in vitro study.
1072	DRAMP03974	N-5 (analog of P18)	"N-5"[All Fields] AND biofilm[All Fields]	N-5	18479371	The efficacy of dynamic irrigation using a commercially available system (RinsEndo) determined by removal of a collagen 'bio-molecular film' from an ex vivo model.
1072	DRAMP03974	N-5 (analog of P18)	"N-5"[All Fields] AND biofilm[All Fields]	N-5	18479368	Biofilm on the apical region of roots in primary teeth with vital and necrotic pulps with or without radiographically evident apical pathosis.
1072	DRAMP03974	N-5 (analog of P18)	"N-5"[All Fields] AND biofilm[All Fields]	N-5	18327543	Biotreatment of high strength nitrate waste using immobilized preadapted sludge.
1072	DRAMP03974	N-5 (analog of P18)	"N-5"[All Fields] AND biofilm[All Fields]	N-5	17980027	Role of NOD2/CARD15 in coronary heart disease.
1072	DRAMP03974	N-5 (analog of P18)	"N-5"[All Fields] AND biofilm[All Fields]	N-5	16940141	Host-derived pentapeptide affecting adhesion, proliferation, and local pH in biofilm communities composed of Streptococcus and Actinomyces species.
1072	DRAMP03974	N-5 (analog of P18)	"N-5"[All Fields] AND biofilm[All Fields]	N-5	16751547	Necrotrophic growth of Legionella pneumophila.
1072	DRAMP03974	N-5 (analog of P18)	"N-5"[All Fields] AND biofilm[All Fields]	N-5	15715865	Development and characterization of a simple perfused oral microcosm.
1072	DRAMP03974	N-5 (analog of P18)	"N-5"[All Fields] AND biofilm[All Fields]	N-5	15212352	Estrogen suppression induces papillary gingival overgrowth in pregnant baboons.
1072	DRAMP03974	N-5 (analog of P18)	"N-5"[All Fields] AND biofilm[All Fields]	N-5	15116879	Nutrient removal in a sequencing batch biofilm reactor (SBBR) using a vertically moving biofilm system.
1072	DRAMP03974	N-5 (analog of P18)	"N-5"[All Fields] AND biofilm[All Fields]	N-5	14682656	Formation and decontamination of biofilms in dental unit waterlines.
1072	DRAMP03974	N-5 (analog of P18)	"N-5"[All Fields] AND biofilm[All Fields]	N-5	12708304	[Characteristics of Pb and Cd adsorption to surface coatings sampled in the aquatic environment of wetland].
1072	DRAMP03974	N-5 (analog of P18)	"N-5"[All Fields] AND biofilm[All Fields]	N-5	12598002	Comparison of the adsorption of lead, cadmium, copper, zinc and barium to freshwater surface coatings.
1072	DRAMP03974	N-5 (analog of P18)	"N-5"[All Fields] AND biofilm[All Fields]	N-5	11375190	Reduction in exopolysaccharide viscosity as an aid to bacteriophage penetration through Pseudomonas aeruginosa biofilms.
1072	DRAMP03974	N-5 (analog of P18)	"N-5"[All Fields] AND biofilm[All Fields]	N-5	10389242	The role of surface physicochemical properties in determining the distribution of the autochthonous microflora in mineral water bottles.
1072	DRAMP03974	N-5 (analog of P18)	"N-5"[All Fields] AND biofilm[All Fields]	N-5	8950503	The effect of eye closure on protein and complement deposition on Group IV hydrogel contact lenses: relationship to tear flow dynamics.
1076	DRAMP03978	C-1 (analog of P18)	"C-1"[All Fields] AND biofilm[All Fields]	C-1	34647935	Synthesis, characterization, antimicrobial and antibiofilm activity, and molecular docking analysis of NHC precursors and their Ag-NHC complexes.
1076	DRAMP03978	C-1 (analog of P18)	"C-1"[All Fields] AND biofilm[All Fields]	C-1	34647135	Joint protection strategies for Saccharomyces boulardii: exogenous encapsulation and endogenous biofilm structure.
1076	DRAMP03978	C-1 (analog of P18)	"C-1"[All Fields] AND biofilm[All Fields]	C-1	34288685	A Sensitive and Reliable Organic Fluorescent Nanothermometer for Noninvasive Temperature Sensing.
1076	DRAMP03978	C-1 (analog of P18)	"C-1"[All Fields] AND biofilm[All Fields]	C-1	33812643	Benzofurazan derivatives modified graphene oxide nanocomposite: Physico-chemical characterization and interaction with bacterial and tumoral cells.
1076	DRAMP03978	C-1 (analog of P18)	"C-1"[All Fields] AND biofilm[All Fields]	C-1	33176181	Photodynamic therapy with curcumin in the reduction of enterococcus faecalis biofilm in bone cavity: rMicrobiological and spectral fluorescense analysis.
1076	DRAMP03978	C-1 (analog of P18)	"C-1"[All Fields] AND biofilm[All Fields]	C-1	32380771	Induction of Antibacterial Metabolites by Co-Cultivation of Two Red-Sea-Sponge-Associated Actinomycetes Micromonosporasp. UR56 and Actinokinesporasp. EG49.
1076	DRAMP03978	C-1 (analog of P18)	"C-1"[All Fields] AND biofilm[All Fields]	C-1	32314099	Assigned NMR backbone resonances of the ligand-binding region domain of the pneumococcal serine-rich repeat protein (PsrP-BR) reveal a rigid monomer in solution.
1076	DRAMP03978	C-1 (analog of P18)	"C-1"[All Fields] AND biofilm[All Fields]	C-1	32200251	Speciation and conversion of carbon and nitrogen in young landfill leachate during anaerobic biological pretreatment.
1076	DRAMP03978	C-1 (analog of P18)	"C-1"[All Fields] AND biofilm[All Fields]	C-1	32072810	Bromopyrrole Alkaloids of the Sponge Agelas oroidesCollected Near the Israeli Mediterranean Coastline.
1076	DRAMP03978	C-1 (analog of P18)	"C-1"[All Fields] AND biofilm[All Fields]	C-1	31730339	Antifungal Macrocyclic Trichothecenes from the Insect-Associated Fungus Myrothecium roridum.
1076	DRAMP03978	C-1 (analog of P18)	"C-1"[All Fields] AND biofilm[All Fields]	C-1	31382154	Molecular change of dissolved organic matter and patterns of bacterial activity in a stream along a land-use gradient.
1076	DRAMP03978	C-1 (analog of P18)	"C-1"[All Fields] AND biofilm[All Fields]	C-1	31063859	Fluorescence spectroscopy of Candida albicans biofilms in bone cavities treated with photodynamic therapy using blue LED (450 nm) and curcumin.
1076	DRAMP03978	C-1 (analog of P18)	"C-1"[All Fields] AND biofilm[All Fields]	C-1	30091832	Snapshot of an early Paleoproterozoic ecosystem: Two diverse microfossil communities from the Turee Creek Group, Western Australia.
1076	DRAMP03978	C-1 (analog of P18)	"C-1"[All Fields] AND biofilm[All Fields]	C-1	29720089	Susceptibility patterns and the role of extracellular DNA in Staphylococcus epidermidis biofilm resistance to physico-chemical stress exposure.
1076	DRAMP03978	C-1 (analog of P18)	"C-1"[All Fields] AND biofilm[All Fields]	C-1	29274394	Reduced methicillin-resistant Staphylococcus aureus biofilm formation in bone cavities by photodynamic therapy.
1076	DRAMP03978	C-1 (analog of P18)	"C-1"[All Fields] AND biofilm[All Fields]	C-1	29178772	Antimicrobial action and long-term effect of overnight denture cleansers.

1076	DRAMP03978	C-1 (analog of P18)	"C-1"[All Fields] AND biofilm[All Fields]	C-1	29157822	Antibiofilm activities of norharmane and its derivatives against Escherichia coli O157:H7 and other bacteria.
1076	DRAMP03978	C-1 (analog of P18)	"C-1"[All Fields] AND biofilm[All Fields]	C-1	29085811	Alizarin and Chrysazin Inhibit Biofilm and Hyphal Formation by Candida albicans.
1076	DRAMP03978	C-1 (analog of P18)	"C-1"[All Fields] AND biofilm[All Fields]	C-1	28297746	Screening of Anti-Biofilm Compounds from Marine-Derived Fungi and the Effects of Secalonic Acid D on Staphylococcus aureusBiofilm.
1076	DRAMP03978	C-1 (analog of P18)	"C-1"[All Fields] AND biofilm[All Fields]	C-1	27808174	Essential Oils and Eugenols Inhibit Biofilm Formation and the Virulence of Escherichia coli O157:H7.
1076	DRAMP03978	C-1 (analog of P18)	"C-1"[All Fields] AND biofilm[All Fields]	C-1	26956086	A Novel Acetylcholinesterase Biosensor: Core-Shell Magnetic Nanoparticles Incorporating a Conjugated Polymer for the Detection of Organophosphorus Pesticides.
1076	DRAMP03978	C-1 (analog of P18)	"C-1"[All Fields] AND biofilm[All Fields]	C-1	26763935	Calcium-chelating alizarin and other anthraquinones inhibit biofilm formation and the hemolytic activity of Staphylococcus aureus.
1076	DRAMP03978	C-1 (analog of P18)	"C-1"[All Fields] AND biofilm[All Fields]	C-1	26708295	Step-wise temperature decreasing cultivates a biofilm with high nitrogen removal rates at 9°C in short-term anammox biofilm tests.
1076	DRAMP03978	C-1 (analog of P18)	"C-1"[All Fields] AND biofilm[All Fields]	C-1	26592023	[Analysis of Precipitation Formation in Biofilm CANUN Reactor and its effect on Nitrogen Removal].
1076	DRAMP03978	C-1 (analog of P18)	"C-1"[All Fields] AND biofilm[All Fields]	C-1	26563709	An ultrahigh-resolution mass spectrometry index to estimate natural organic matter lability.
1076	DRAMP03978	C-1 (analog of P18)	"C-1"[All Fields] AND biofilm[All Fields]	C-1	25924182	Considerations in the use of fluorescence in situ hybridization (FISH) and confocal laser scanning microscopy to characterize rumen methanogens and define their spatial distributions.
1076	DRAMP03978	C-1 (analog of P18)	"C-1"[All Fields] AND biofilm[All Fields]	C-1	25841971	Biofilm-producing ability and efficiency of sanitizing agents against Prototheca zopfii isolates from bovine subclinical mastitis.
1076	DRAMP03978	C-1 (analog of P18)	"C-1"[All Fields] AND biofilm[All Fields]	C-1	25766917	Bioactive diphenyl ether derivatives from a gorgonian-derived fungus Talaromyces sp.
1076	DRAMP03978	C-1 (analog of P18)	"C-1"[All Fields] AND biofilm[All Fields]	C-1	25698567	Microbiome characterization of MFCs used for the treatment of swine manure.
1076	DRAMP03978	C-1 (analog of P18)	"C-1"[All Fields] AND biofilm[All Fields]	C-1	25641692	Susceptibility of Candida albicans to new synthetic sulfone derivatives.
1076	DRAMP03978	C-1 (analog of P18)	"C-1"[All Fields] AND biofilm[All Fields]	C-1	25190872	Refrigeration and edible coatings in blackberry (Rubus spp.) conservation.
1076	DRAMP03978	C-1 (analog of P18)	"C-1"[All Fields] AND biofilm[All Fields]	C-1	24911407	Characterization of the Vibrio cholerae extracellular matrix: a top-down solid-state NMR approach.
1076	DRAMP03978	C-1 (analog of P18)	"C-1"[All Fields] AND biofilm[All Fields]	C-1	24657607	Microscale patterned surfaces reduce bacterial fouling-microscopic and theoretical analysis.
1076	DRAMP03978	C-1 (analog of P18)	"C-1"[All Fields] AND biofilm[All Fields]	C-1	23575121	Reduction by competitive bacteria of Listeria monocytogenes in biofilms and Listeria bacteria in floor drains in a ready-to-eat poultry processing plant.
1076	DRAMP03978	C-1 (analog of P18)	"C-1"[All Fields] AND biofilm[All Fields]	C-1	22244955	Behavior of autotrophic denitrification and heterotrophic denitrification in an intensified biofilm-electrode reactor for nitrate-contaminated drinking water treatment.
1076	DRAMP03978	C-1 (analog of P18)	"C-1"[All Fields] AND biofilm[All Fields]	C-1	21910441	Malabaricone C from Myristica cinnamomea exhibits anti-quorum sensing activity.
1076	DRAMP03978	C-1 (analog of P18)	"C-1"[All Fields] AND biofilm[All Fields]	C-1	21634171	[Performance of nitrogen and phosphorus removal from municipal wastewater of different C/N ratios using intelligent controlled systems sequencing batch biofilm reactor (SBBR)].
1076	DRAMP03978	C-1 (analog of P18)	"C-1"[All Fields] AND biofilm[All Fields]	C-1	21371947	In situ microbial fuel cell-based biosensor for organic carbon.
1076	DRAMP03978	C-1 (analog of P18)	"C-1"[All Fields] AND biofilm[All Fields]	C-1	21364302	Characterization of microbial fuel cells enriched using Cr(VI)-containing sludge.
1076	DRAMP03978	C-1 (analog of P18)	"C-1"[All Fields] AND biofilm[All Fields]	C-1	21258753	Lyngbyoic acid, a "tagged" fatty acid from a marine cyanobacterium, disrupts quorum sensing in Pseudomonas aeruginosa.
1076	DRAMP03978	C-1 (analog of P18)	"C-1"[All Fields] AND biofilm[All Fields]	C-1	19143383	[Influence of EPS on silicate corrosion inhibition for copper pipe in soft water].
1076	DRAMP03978	C-1 (analog of P18)	"C-1"[All Fields] AND biofilm[All Fields]	C-1	18290470	[Effect of temperature and denitrifying phosphorus on nitrogen and phosphorous removal in SBMBBR].
1076	DRAMP03978	C-1 (analog of P18)	"C-1"[All Fields] AND biofilm[All Fields]	C-1	16672472	Control of Listeria spp. by competitive-exclusion bacteria in floor drains of a poultry processing plant.
1076	DRAMP03978	C-1 (analog of P18)	"C-1"[All Fields] AND biofilm[All Fields]	C-1	16444683	Influence of plaque biofilm removal on reestablishment of the biocompatibility of contaminated titanium surfaces.
1076	DRAMP03978	C-1 (analog of P18)	"C-1"[All Fields] AND biofilm[All Fields]	C-1	15841403	Influence of different treatment approaches on the removal of early plaque biofilms and the viability of SAOS2 osteoblasts grown on titanium implants.
1076	DRAMP03978	C-1 (analog of P18)	"C-1"[All Fields] AND biofilm[All Fields]	C-1	15575706	Growth model and metabolic activity of brewing yeast biofilm on the surface of spent grains: a biocatalyst for continuous beer fermentation.
1076	DRAMP03978	C-1 (analog of P18)	"C-1"[All Fields] AND biofilm[All Fields]	C-1	15240275	Control of Listeria monocytogenes in a biofilm by competitive-exclusion microorganisms.
1076	DRAMP03978	C-1 (analog of P18)	"C-1"[All Fields] AND biofilm[All Fields]	C-1	9818743	Response of single species biofilms and microcosm dental plaques to pulsing with chlorhexidine.
1076	DRAMP03978	C-1 (analog of P18)	"C-1"[All Fields] AND biofilm[All Fields]	C-1	24193962	Distribution and activity of bacteria in deep granitic groundwaters of southeastern sweden.
1076	DRAMP03978	C-1 (analog of P18)	"C-1"[All Fields] AND biofilm[All Fields]	C-1	18553482	Biofilm formation and chemostat dynamics: Pure and mixed culture considerations.
1077	DRAMP03979	C-2 (analog of P18)	"C-2"[All Fields] AND biofilm[All Fields]	C-2	34539607	Anthraquinones as Potential Antibiofilm Agents Against Methicillin-Resistant Staphylococcus aureus.
1077	DRAMP03979	C-2 (analog of P18)	"C-2"[All Fields] AND biofilm[All Fields]	C-2	33329502	Inactivation Efficacy of 405 nm LED Against Cronobacter sakazakiiBiofilm.
1077	DRAMP03979	C-2 (analog of P18)	"C-2"[All Fields] AND biofilm[All Fields]	C-2	32674317	Bioactivity of Serratiochelin A, a Siderophore Isolated from a Co-Culture of Serratiasp. and Shewanellasp.
1077	DRAMP03979	C-2 (analog of P18)	"C-2"[All Fields] AND biofilm[All Fields]	C-2	31928701	Environmental persistence and disinfectant susceptibility of Klebsiella pneumoniae recovered from pinnipeds stranded on the California Coast.
1077	DRAMP03979	C-2 (analog of P18)	"C-2"[All Fields] AND biofilm[All Fields]	C-2	31684121	Characterization of Listeria MonocytogenesOriginating from the Spanish Meat-Processing Chain.
1077	DRAMP03979	C-2 (analog of P18)	"C-2"[All Fields] AND biofilm[All Fields]	C-2	31657052	Discovery of a novel and selective fungicide that targets fungal cell wall to treat dermatomycoses: 1,3-bis(3,4-dichlorophenoxy)propan-2-aminium chloride.
1077	DRAMP03979	C-2 (analog of P18)	"C-2"[All Fields] AND biofilm[All Fields]	C-2	31085909	Protective Effect of 4% Titanium Tetrafluoride Varnish on Dentin Demineralization Using a Microcosm Biofilm Model.
1077	DRAMP03979	C-2 (analog of P18)	"C-2"[All Fields] AND biofilm[All Fields]	C-2	30807789	Cytotoxic, antimicrobial and antiviral secondary metabolites produced by the plant pathogenic fungus Cytospora sp. CCTU A309.
1077	DRAMP03979	C-2 (analog of P18)	"C-2"[All Fields] AND biofilm[All Fields]	C-2	30782573	Oxidative functionalization of a halimane diterpenoid achieved by fungal transformation.
1077	DRAMP03979	C-2 (analog of P18)	"C-2"[All Fields] AND biofilm[All Fields]	C-2	30686111	Efficacy of copper and silver as residual disinfectants in drinking water.
1077	DRAMP03979	C-2 (analog of P18)	"C-2"[All Fields] AND biofilm[All Fields]	C-2	30595945	Long-term stability of gentamicin sulfate-ethylenediaminetetraacetic acid disodium salt (EDTA-Na 2) solution for catheter locks.
1077	DRAMP03979	C-2 (analog of P18)	"C-2"[All Fields] AND biofilm[All Fields]	C-2	30311502	Inactivation of Pseudomonas aeruginosabiofilms formed under high shear stress on various hydrophilic and hydrophobic surfaces by a continuous flow of ozonated water.
1077	DRAMP03979	C-2 (analog of P18)	"C-2"[All Fields] AND biofilm[All Fields]	C-2	30111175	Evaluation of antibacterial and anti-biofilm properties of kojic acid against five food-related bacteria and related subcellular mechanisms of bacterial inactivation.
1077	DRAMP03979	C-2 (analog of P18)	"C-2"[All Fields] AND biofilm[All Fields]	C-2	30091832	Snapshot of an early Paleoproterozoic ecosystem: Two diverse microfossil communities from the Turee Creek Group, Western Australia.
1077	DRAMP03979	C-2 (analog of P18)	"C-2"[All Fields] AND biofilm[All Fields]	C-2	29680062	Use of biopolymeric coating hydrophobized with beeswax in post-harvest conservation of guavas.
1077	DRAMP03979	C-2 (analog of P18)	"C-2"[All Fields] AND biofilm[All Fields]	C-2	29231844	Collismycin C from the Micronesian Marine Bacterium Streptomyces sp. MC025 Inhibits Staphylococcus aureus Biofilm Formation.
1077	DRAMP03979	C-2 (analog of P18)	"C-2"[All Fields] AND biofilm[All Fields]	C-2	28960713	The effect of interactions between a bacterial strain isolated from drinking water and a pathogen surrogate on biofilms formation diverged under static vs flow conditions.
1077	DRAMP03979	C-2 (analog of P18)	"C-2"[All Fields] AND biofilm[All Fields]	C-2	28783429	Characterization of Livestock-Associated Methicillin-Resistant Staphylococcus aureus CC398 and mecC-positive CC130 from Zoo Animals in the United Kingdom.
1077	DRAMP03979	C-2 (analog of P18)	"C-2"[All Fields] AND biofilm[All Fields]	C-2	28692669	Systems biology of the modified branched Entner-Doudoroff pathway in Sulfolobus solfataricus.
1077	DRAMP03979	C-2 (analog of P18)	"C-2"[All Fields] AND biofilm[All Fields]	C-2	28492178	Isolation of Bdellovibrio and like organisms and potential to reduce acute hepatopancreatic necrosis disease caused by Vibrio parahaemolyticus.
1077	DRAMP03979	C-2 (analog of P18)	"C-2"[All Fields] AND biofilm[All Fields]	C-2	28186464	Evaluation of Antimicrobial Photodynamic Therapy Using Indocyanine Green and Near-Infrared Diode Laser Against Enterococcus faecalis in Infected Human Root Canals.
1077	DRAMP03979	C-2 (analog of P18)	"C-2"[All Fields] AND biofilm[All Fields]	C-2	27808174	Essential Oils and Eugenols Inhibit Biofilm Formation and the Virulence of Escherichia coli O157:H7.
1077	DRAMP03979	C-2 (analog of P18)	"C-2"[All Fields] AND biofilm[All Fields]	C-2	27716472	Efficiency of a cleaning protocol for the removal of enterotoxigenic Staphylococcus aureus strains in dairy plants.
1077	DRAMP03979	C-2 (analog of P18)	"C-2"[All Fields] AND biofilm[All Fields]	C-2	27731921	Structure-Activity Relationships of 2-Sufonylpyrimidines as Quorum-Sensing Inhibitors to Tackle Biofilm Formation and eDNA Release of Pseudomonas aeruginosa.
1077	DRAMP03979	C-2 (analog of P18)	"C-2"[All Fields] AND biofilm[All Fields]	C-2	27128176	Design, synthesis and in vitro biological evaluation of short-chain C12-sphinganine and its 1,2,3-triazole analogs as potential antimicrobial and anti-biofilm agents.

1077	DRAMP03979	C-2 (analog of P18)	"C-2"[All Fields] AND biofilm[All Fields]	C-2	26892114	Targeted antibiotic delivery using low temperature-sensitive liposomes and magnetic resonance-guided high-intensity focused ultrasound hyperthermia.
1077	DRAMP03979	C-2 (analog of P18)	"C-2"[All Fields] AND biofilm[All Fields]	C-2	26763935	Calcium-chelating alizarin and other anthraquinones inhibit biofilm formation and the hemolytic activity of Staphylococcus aureus.
1077	DRAMP03979	C-2 (analog of P18)	"C-2"[All Fields] AND biofilm[All Fields]	C-2	26349849	Bactericidal efficacy of tissue tolerable plasma on microrough titanium dental implants: An in-vitro-study.
1077	DRAMP03979	C-2 (analog of P18)	"C-2"[All Fields] AND biofilm[All Fields]	C-2	25698567	Microbiome characterization of MFCs used for the treatment of swine manure.
1077	DRAMP03979	C-2 (analog of P18)	"C-2"[All Fields] AND biofilm[All Fields]	C-2	25415418	A biophysical study with carbohydrate derivatives explains the molecular basis of monosaccharide selectivity of the Pseudomonas aeruginosa lectin LecB.
1077	DRAMP03979	C-2 (analog of P18)	"C-2"[All Fields] AND biofilm[All Fields]	C-2	23063558	The effect of nitrate on ethylene biofiltration.
1077	DRAMP03979	C-2 (analog of P18)	"C-2"[All Fields] AND biofilm[All Fields]	C-2	22982608	Expression of the DisA amino acid decarboxylase from Proteus mirabilis inhibits motility and class 2 flagellar gene expression in Escherichia coli.
1077	DRAMP03979	C-2 (analog of P18)	"C-2"[All Fields] AND biofilm[All Fields]	C-2	22690142	Epigenetic tailoring for the production of anti-infective cytosporones from the marine fungus Leucostoma persoonii.
1077	DRAMP03979	C-2 (analog of P18)	"C-2"[All Fields] AND biofilm[All Fields]	C-2	22422754	The fimbriae activator MatA switches off motility in Escherichia coli by repression of the flagellar master operon flhDC.
1077	DRAMP03979	C-2 (analog of P18)	"C-2"[All Fields] AND biofilm[All Fields]	C-2	22389366	Dimethyl sulfoxide and ethanol elicit increased amyloid biogenesis and amyloid-integrated biofilm formation in Escherichia coli.
1077	DRAMP03979	C-2 (analog of P18)	"C-2"[All Fields] AND biofilm[All Fields]	C-2	22200749	Survival of biofilm-forming Salmonella on stainless steel bolt threads under dry conditions.
1077	DRAMP03979	C-2 (analog of P18)	"C-2"[All Fields] AND biofilm[All Fields]	C-2	21747729	Modulation of rosR expression and exopolysaccharide production in Rhizobium leguminosarum bv. trifolii by phosphate and clover root exudates.
1077	DRAMP03979	C-2 (analog of P18)	"C-2"[All Fields] AND biofilm[All Fields]	C-2	21636078	A novel highly charged exopolysaccharide produced by two strains of Stenotrophomonas maltophilia recovered from patients with cystic fibrosis.
1077	DRAMP03979	C-2 (analog of P18)	"C-2"[All Fields] AND biofilm[All Fields]	C-2	21258753	Lyngbyoic acid, a "tagged" fatty acid from a marine cyanobacterium, disrupts quorum sensing in Pseudomonas aeruginosa.
1077	DRAMP03979	C-2 (analog of P18)	"C-2"[All Fields] AND biofilm[All Fields]	C-2	20586493	Design and synthesis of C-2 substituted thiazolo and dihydrothiazolo ring-fused 2-pyridones: pilicides with increased antivirulence activity.
1077	DRAMP03979	C-2 (analog of P18)	"C-2"[All Fields] AND biofilm[All Fields]	C-2	19912269	In vivo and in vitro biofilm formation on two different titanium implant surfaces.
1077	DRAMP03979	C-2 (analog of P18)	"C-2"[All Fields] AND biofilm[All Fields]	C-2	19705191	Dinitrogen-fixing cyanobacteria in microbial mats of two shallow coral reef ecosystems.
1077	DRAMP03979	C-2 (analog of P18)	"C-2"[All Fields] AND biofilm[All Fields]	C-2	18281597	Carnitine-dependent transport of acetyl coenzyme A in Candida albicans is essential for growth on nonfermentable carbon sources and contributes to biofilm formation.
1077	DRAMP03979	C-2 (analog of P18)	"C-2"[All Fields] AND biofilm[All Fields]	C-2	18086035	The influence of salmon surface mucus on the growth of Flavobacterium columnare.
1077	DRAMP03979	C-2 (analog of P18)	"C-2"[All Fields] AND biofilm[All Fields]	C-2	17990542	[Influence of DO and aeration/non-aeration ratio on one-step SBBR completely autotrophic nitrogen removal process].
1077	DRAMP03979	C-2 (analog of P18)	"C-2"[All Fields] AND biofilm[All Fields]	C-2	17522902	Antifungal susceptibility of Candida albicans biofilms on titanium discs with different surface roughness.
1077	DRAMP03979	C-2 (analog of P18)	"C-2"[All Fields] AND biofilm[All Fields]	C-2	17418367	Biohydrogen production from chemical wastewater treatment in biofilm configured reactor operated in periodic discontinuous batch mode by selectively enriched anaerobic mixed consortia.
1077	DRAMP03979	C-2 (analog of P18)	"C-2"[All Fields] AND biofilm[All Fields]	C-2	17259626	Salmonella typhimurium flhE, a conserved flagellar regulon gene required for swarming.
1077	DRAMP03979	C-2 (analog of P18)	"C-2"[All Fields] AND biofilm[All Fields]	C-2	16876147	The biofilm matrix of Pseudomonas sp. OX1 grown on phenol is mainly constituted by alginate oligosaccharides.
1077	DRAMP03979	C-2 (analog of P18)	"C-2"[All Fields] AND biofilm[All Fields]	C-2	16767988	[Start-up experiment on nitrification of mid-low ammonia concentration wastewater in SBR biofilm reactor].
1077	DRAMP03979	C-2 (analog of P18)	"C-2"[All Fields] AND biofilm[All Fields]	C-2	16629263	Municipal-wastewater treatment using upflow-anaerobic filters.
1077	DRAMP03979	C-2 (analog of P18)	"C-2"[All Fields] AND biofilm[All Fields]	C-2	16491355	Effects of nitrate treatment on a mixed species, oil field microbial biofilm.
1077	DRAMP03979	C-2 (analog of P18)	"C-2"[All Fields] AND biofilm[All Fields]	C-2	9818743	Response of single species biofilms and microcosm dental plaques to pulsing with chlorhexidine.
1077	DRAMP03979	C-2 (analog of P18)	"C-2"[All Fields] AND biofilm[All Fields]	C-2	18553482	Biofilm formation and chemostat dynamics: Pure and mixed culture considerations.
1078	DRAMP03980	C-3 (analog of P18)	"C-3"[All Fields] AND biofilm[All Fields]	C-3	34537641	Intimately coupled photocatalysis and biodegradation for effective simultaneous removal of sulfamethoxazole and COD from synthetic domestic wastewater.
1078	DRAMP03980	C-3 (analog of P18)	"C-3"[All Fields] AND biofilm[All Fields]	C-3	33581631	Effect of C/N substrates for enhanced extracellular polymeric substances (EPS) production and Poly Cyclic Aromatic Hydrocarbons (PAHs) degradation.
1078	DRAMP03980	C-3 (analog of P18)	"C-3"[All Fields] AND biofilm[All Fields]	C-3	33279070	Antimicrobial activity of copper surfaces against biofilm formation by Salmonella Enteritidis and its potential application in the poultry industry.
1078	DRAMP03980	C-3 (analog of P18)	"C-3"[All Fields] AND biofilm[All Fields]	C-3	33137803	Staphylococcus aureus biofilm eradication by the synergistic effect exerted by PEG-coated silicon dots immobilized in silica films and light irradiation.
1078	DRAMP03980	C-3 (analog of P18)	"C-3"[All Fields] AND biofilm[All Fields]	C-3	32759736	Microencapsulation of Saccharomyces cerevisiaeinto Alginate Beads: A Focus on Functional Properties of Released Cells.
1078	DRAMP03980	C-3 (analog of P18)	"C-3"[All Fields] AND biofilm[All Fields]	C-3	32006105	Characterization of Extracellular Protease from the Haloarcheon Halococcus sp. Strain GUGFAWS-3 (MF425611).
1078	DRAMP03980	C-3 (analog of P18)	"C-3"[All Fields] AND biofilm[All Fields]	C-3	30754716	Triterpene Derivatives as Relevant Scaffold for New Antibiofilm Drugs.
1078	DRAMP03980	C-3 (analog of P18)	"C-3"[All Fields] AND biofilm[All Fields]	C-3	30684671	Antimicrobial effects of photodynamic therapy with antiseptics on Staphylococcus aureus biofilm on titanium surface.
1078	DRAMP03980	C-3 (analog of P18)	"C-3"[All Fields] AND biofilm[All Fields]	C-3	30597714	Antimicrobial activity of intracanal medications against both Enterococcus faecalis and Candida albicans biofilm.
1078	DRAMP03980	C-3 (analog of P18)	"C-3"[All Fields] AND biofilm[All Fields]	C-3	29595112	Synthesis and Biological Evaluation of Coumarins Derivatives as Potential Inhibitors of the Production of Pseudomonas aeruginosa Virulence Factor Pyocyanin.
1078	DRAMP03980	C-3 (analog of P18)	"C-3"[All Fields] AND biofilm[All Fields]	C-3	29430777	Evaluation of two autoinducer-2 quantification methods for application in marine environments.
1078	DRAMP03980	C-3 (analog of P18)	"C-3"[All Fields] AND biofilm[All Fields]	C-3	28881210	Total electron acceptor loading and composition affect hexavalent uranium reduction and microbial community structure in a membrane biofilm reactor.
1078	DRAMP03980	C-3 (analog of P18)	"C-3"[All Fields] AND biofilm[All Fields]	C-3	28487228	Characterization of S-adenosylhomocysteine/Methylthioadenosine nucleosidase on secretion of AI-2 and biofilm formation of Escherichia coli.
1078	DRAMP03980	C-3 (analog of P18)	"C-3"[All Fields] AND biofilm[All Fields]	C-3	27936301	New Furanone Derivatives and Alkaloids from the Co-Culture of Marine-Derived Fungi Aspergillus sclerotiorum and Penicillium citrinum.
1078	DRAMP03980	C-3 (analog of P18)	"C-3"[All Fields] AND biofilm[All Fields]	C-3	26591620	Antibacterial efficacy of a cetylpyridinium chloride-based mouthrinse against Fusobacterium nucleatum and in vitro plaques.
1078	DRAMP03980	C-3 (analog of P18)	"C-3"[All Fields] AND biofilm[All Fields]	C-3	26548879	Crossover clinical trial of different methods of removing a denture adhesive and the influence on the oral microbiota.
1078	DRAMP03980	C-3 (analog of P18)	"C-3"[All Fields] AND biofilm[All Fields]	C-3	26291897	An In vivo Wound Model Utilizing Bacteriophage Therapy of Pseudomonas aeruginosa Biofilms.
1078	DRAMP03980	C-3 (analog of P18)	"C-3"[All Fields] AND biofilm[All Fields]	C-3	26273725	Hydnocarpin-Type Flavonolignans: Semisynthesis and Inhibitory Effects on Staphylococcus aureus Biofilm Formation.
1078	DRAMP03980	C-3 (analog of P18)	"C-3"[All Fields] AND biofilm[All Fields]	C-3	25974852	A novel rhamno-mannan exopolysaccharide isolated from biofilms of Burkholderia multivorans C1576.
1078	DRAMP03980	C-3 (analog of P18)	"C-3"[All Fields] AND biofilm[All Fields]	C-3	24911407	Characterization of the Vibrio cholerae extracellular matrix: a top-down solid-state NMR approach.
1078	DRAMP03980	C-3 (analog of P18)	"C-3"[All Fields] AND biofilm[All Fields]	C-3	23544536	Structure of a novel exopolysaccharide produced by Burkholderia vietnamiensis, a cystic fibrosis opportunistic pathogen.
1078	DRAMP03980	C-3 (analog of P18)	"C-3"[All Fields] AND biofilm[All Fields]	C-3	23051988	Structure-function analysis of the C-3 position in analogues of microbial behavioural modulators HHQ and PQS.
1078	DRAMP03980	C-3 (analog of P18)	"C-3"[All Fields] AND biofilm[All Fields]	C-3	21636078	A novel highly charged exopolysaccharide produced by two strains of Stenotrophomonas maltophilia recovered from patients with cystic fibrosis.
1078	DRAMP03980	C-3 (analog of P18)	"C-3"[All Fields] AND biofilm[All Fields]	C-3	20158607	Lack of O-polysaccharide enhances biofilm formation by Bradyrhizobium japonicum.
1078	DRAMP03980	C-3 (analog of P18)	"C-3"[All Fields] AND biofilm[All Fields]	C-3	19778962	AI-2 quorum-sensing inhibitors affect the starvation response and reduce virulence in several Vibrio species, most likely by interfering with LuxPQ.
1078	DRAMP03980	C-3 (analog of P18)	"C-3"[All Fields] AND biofilm[All Fields]	C-3	19143383	[Influence of EPS on silicate corrosion inhibition for copper pipe in soft water].
1078	DRAMP03980	C-3 (analog of P18)	"C-3"[All Fields] AND biofilm[All Fields]	C-3	18704788	Effect of ultrasonic treatment during cleaning on the microbiological condition of poultry transport crates.
1078	DRAMP03980	C-3 (analog of P18)	"C-3"[All Fields] AND biofilm[All Fields]	C-3	16876147	The biofilm matrix of Pseudomonas sp. OX1 grown on phenol is mainly constituted by alginate oligosaccharides.
1078	DRAMP03980	C-3 (analog of P18)	"C-3"[All Fields] AND biofilm[All Fields]	C-3	16269739	Evidence for a functional quorum-sensing type AI-1 system in the extremophilic bacterium Acidithiobacillus ferrooxidans.

1078	DRAMP03980	C-3 (analog of P18)	"C-3"[All Fields] AND biofilm[All Fields]	C-3	16032659	In vitro evaluation of caries inhibition promoted by self-etching adhesive systems containing antibacterial agents.
1078	DRAMP03980	C-3 (analog of P18)	"C-3"[All Fields] AND biofilm[All Fields]	C-3	12483477	The influence of fluid shear on the structure and material properties of sulphate-reducing bacterial biofilms.
1078	DRAMP03980	C-3 (analog of P18)	"C-3"[All Fields] AND biofilm[All Fields]	C-3	10665194	Structural conformation of in vitro and in vivo aged orthodontic elastomeric modules.
1079	DRAMP03981	C-4 (analog of P18)	"C-4"[All Fields] AND biofilm[All Fields]	C-4	33279070	Antimicrobial activity of copper surfaces against biofilm formation by Salmonella Enteritidis and its potential application in the poultry industry.
1079	DRAMP03981	C-4 (analog of P18)	"C-4"[All Fields] AND biofilm[All Fields]	C-4	33172027	Influence of Storage on the Antimicrobial and Cytotoxic Activities of a Nisin-biogel with Potential to be Applied to Diabetic Foot Infections Treatment.
1079	DRAMP03981	C-4 (analog of P18)	"C-4"[All Fields] AND biofilm[All Fields]	C-4	31340597	The Role of Orientation of Surface Bound Dihydropyrrol-2-ones (DHP) on Biological Activity.
1079	DRAMP03981	C-4 (analog of P18)	"C-4"[All Fields] AND biofilm[All Fields]	C-4	31286118	Comparison of biofilm removal using glycine air polishing versus sodium bicarbonate air polishing or hand instrumentation on full-arch fixed implant rehabilitations: a split-mouth study.
1079	DRAMP03981	C-4 (analog of P18)	"C-4"[All Fields] AND biofilm[All Fields]	C-4	31273998	Anoxybacillusand Geobacillusbiofilms in the dairy industry: effects of surface material, incubation temperature and milk type.
1079	DRAMP03981	C-4 (analog of P18)	"C-4"[All Fields] AND biofilm[All Fields]	C-4	30758060	Biofilm contamination of high-touched surfaces in intensive care units: epidemiology and potential impacts.
1079	DRAMP03981	C-4 (analog of P18)	"C-4"[All Fields] AND biofilm[All Fields]	C-4	30186447	Preliminary study on the effect of brazilin on biofilms of Staphylococcus aureus.
1079	DRAMP03981	C-4 (analog of P18)	"C-4"[All Fields] AND biofilm[All Fields]	C-4	29346325	Synthesis and Antimicrobial Activity of 4-Substituted 1,2,3-Triazole-Coumarin Derivatives.
1079	DRAMP03981	C-4 (analog of P18)	"C-4"[All Fields] AND biofilm[All Fields]	C-4	28667872	Polysubstituted 2-aminoimidazoles as anti-biofilm and antiproliferative agents: Discovery of potent lead.
1079	DRAMP03981	C-4 (analog of P18)	"C-4"[All Fields] AND biofilm[All Fields]	C-4	27808174	Essential Oils and Eugenols Inhibit Biofilm Formation and the Virulence of Escherichia coli O157:H7.
1079	DRAMP03981	C-4 (analog of P18)	"C-4"[All Fields] AND biofilm[All Fields]	C-4	27340103	Discovery of antifungal constituents from the Miao medicinal plant Isodon flavidus.
1079	DRAMP03981	C-4 (analog of P18)	"C-4"[All Fields] AND biofilm[All Fields]	C-4	25641692	Susceptibility of Candida albicans to new synthetic sulfone derivatives.
1079	DRAMP03981	C-4 (analog of P18)	"C-4"[All Fields] AND biofilm[All Fields]	C-4	25368324	The biosynthesis of UDP-d-FucNAc-4N-(2)-oxoglutarate (UDP-Yelosamine) in Bacillus cereus ATCC 14579: Pat and Pyl, an aminotransferase and an ATP-dependent Grasp protein that ligates 2-oxoglutarate to UDP-4-amino-sugars.
1079	DRAMP03981	C-4 (analog of P18)	"C-4"[All Fields] AND biofilm[All Fields]	C-4	23320085	Ellagic acid derivatives from Terminalia chebula Retz. downregulate the expression of quorum sensing genes to attenuate Pseudomonas aeruginosa PAO1 virulence.
1079	DRAMP03981	C-4 (analog of P18)	"C-4"[All Fields] AND biofilm[All Fields]	C-4	22003018	Butyric acid- and dimethyl disulfide-assimilating microorganisms in a biofilter treating air emissions from a livestock facility.
1079	DRAMP03981	C-4 (analog of P18)	"C-4"[All Fields] AND biofilm[All Fields]	C-4	21807742	N-Acylhomoserine lactones are potent neutrophil chemoattractants that act via calcium mobilization and actin remodeling.
1079	DRAMP03981	C-4 (analog of P18)	"C-4"[All Fields] AND biofilm[All Fields]	C-4	21636078	A novel highly charged exopolysaccharide produced by two strains of Stenotrophomonas maltophilia recovered from patients with cystic fibrosis.
1079	DRAMP03981	C-4 (analog of P18)	"C-4"[All Fields] AND biofilm[All Fields]	C-4	21453119	Inactivation of Escherichia coli O157:H7 attached to spinach harvester blade using bacteriophage.
1079	DRAMP03981	C-4 (analog of P18)	"C-4"[All Fields] AND biofilm[All Fields]	C-4	20883084	Design, synthesis, and a novel application of quorum-sensing agonists as potential drug-delivery vehicles.
1079	DRAMP03981	C-4 (analog of P18)	"C-4"[All Fields] AND biofilm[All Fields]	C-4	20080211	Biofilm-induced modifications in the proteome of Pseudomonas aeruginosa planktonic cells.
1079	DRAMP03981	C-4 (analog of P18)	"C-4"[All Fields] AND biofilm[All Fields]	C-4	18713055	Solenopsin A, a venom alkaloid from the fire ant Solenopsis invicta, inhibits quorum-sensing signaling in Pseudomonas aeruginosa.
1079	DRAMP03981	C-4 (analog of P18)	"C-4"[All Fields] AND biofilm[All Fields]	C-4	18430020	Use of the rotating wall vessel technology to study the effect of shear stress on growth behaviour of Pseudomonas aeruginosa PA01.
1079	DRAMP03981	C-4 (analog of P18)	"C-4"[All Fields] AND biofilm[All Fields]	C-4	17490430	Malolactic fermentation by Streptococcus mutans.
1079	DRAMP03981	C-4 (analog of P18)	"C-4"[All Fields] AND biofilm[All Fields]	C-4	17227468	Transcription in Escherichia coli PHL628 biofilms.
1079	DRAMP03981	C-4 (analog of P18)	"C-4"[All Fields] AND biofilm[All Fields]	C-4	17184897	Effects of feeding time and organic loading in an anaerobic sequencing batch biofilm reactor (ASBBR) treating diluted whey.
1079	DRAMP03981	C-4 (analog of P18)	"C-4"[All Fields] AND biofilm[All Fields]	C-4	16786845	Fate of inoculated Escherichia coli O157:H7, cultured under different conditions, on fresh and decontaminated beef transitioned from vacuum to aerobic packaging.
1079	DRAMP03981	C-4 (analog of P18)	"C-4"[All Fields] AND biofilm[All Fields]	C-4	15508633	Efficacy of two cleaning and sanitizing combinations on Listeria monocytogenes biofilms formed at low temperature on a variety of materials in the presence of ready-to-eat meat residue.
1079	DRAMP03981	C-4 (analog of P18)	"C-4"[All Fields] AND biofilm[All Fields]	C-4	14729747	Biofilms, homoserine lactones and biocide susceptibility.
1079	DRAMP03981	C-4 (analog of P18)	"C-4"[All Fields] AND biofilm[All Fields]	C-4	11855945	Detection of Pseudomonas aeruginosa cell-to-cell signals in lung tissue of cystic fibrosis patients.
1080	DRAMP03982	C-5 (analog of P18)	"C-5"[All Fields] AND biofilm[All Fields]	C-5	34436296	Anthraquinones, Diphenyl Ethers, and Their Derivatives from the Culture of the Marine Sponge-Associated Fungus Neosartorya spinosaKUFA 1047.
1080	DRAMP03982	C-5 (analog of P18)	"C-5"[All Fields] AND biofilm[All Fields]	C-5	34073814	Antibiofilm Activity of Phorbaketals from the Marine Sponge Phorbassp. against Staphylococcus aureus.
1080	DRAMP03982	C-5 (analog of P18)	"C-5"[All Fields] AND biofilm[All Fields]	C-5	33727650	Protective effect of titanium tetrafluoride and silver diamine fluoride on radiation-induced dentin caries in vitro.
1080	DRAMP03982	C-5 (analog of P18)	"C-5"[All Fields] AND biofilm[All Fields]	C-5	33279070	Antimicrobial activity of copper surfaces against biofilm formation by Salmonella Enteritidis and its potential application in the poultry industry.
1080	DRAMP03982	C-5 (analog of P18)	"C-5"[All Fields] AND biofilm[All Fields]	C-5	33218095	Exploiting Mannuronan C-5 Epimerases in Commercial Alginate Production.
1080	DRAMP03982	C-5 (analog of P18)	"C-5"[All Fields] AND biofilm[All Fields]	C-5	33143940	Photodynamic inactivation of Streptococcus mutans by curcumin in combination with EDTA.
1080	DRAMP03982	C-5 (analog of P18)	"C-5"[All Fields] AND biofilm[All Fields]	C-5	32910213	Phylogenetic Classification, Biofilm-Forming Capacity, Virulence Factors, and Antimicrobial Resistance in Uropathogenic Escherichia coli (UPEC).
1080	DRAMP03982	C-5 (analog of P18)	"C-5"[All Fields] AND biofilm[All Fields]	C-5	32794395	Effect of biofilm exposure on marginal integrity of composite restorations.
1080	DRAMP03982	C-5 (analog of P18)	"C-5"[All Fields] AND biofilm[All Fields]	C-5	31472255	Design, synthesis and evaluation of halogenated furanone derivatives as quorum sensing inhibitors in Pseudomonas aeruginosa.
1080	DRAMP03982	C-5 (analog of P18)	"C-5"[All Fields] AND biofilm[All Fields]	C-5	31397415	In vitroantimicrobial potential of infant mouthwashes against streptococcus mutansbiofilm: A preliminary study.
1080	DRAMP03982	C-5 (analog of P18)	"C-5"[All Fields] AND biofilm[All Fields]	C-5	31273998	Anoxybacillusand Geobacillusbiofilms in the dairy industry: effects of surface material, incubation temperature and milk type.
1080	DRAMP03982	C-5 (analog of P18)	"C-5"[All Fields] AND biofilm[All Fields]	C-5	31085909	Protective Effect of 4% Titanium Tetrafluoride Varnish on Dentin Demineralization Using a Microcosm Biofilm Model.
1080	DRAMP03982	C-5 (analog of P18)	"C-5"[All Fields] AND biofilm[All Fields]	C-5	30926870	Alternative sigma factor B (σ B) and catalase enzyme contribute to Staphylococcus epidermidis biofilm's tolerance against physico-chemical disinfection.
1080	DRAMP03982	C-5 (analog of P18)	"C-5"[All Fields] AND biofilm[All Fields]	C-5	29444673	Effect of tt-farnesol and myricetin on in vitro biofilm formed by Streptococcus mutans and Candida albicans.
1080	DRAMP03982	C-5 (analog of P18)	"C-5"[All Fields] AND biofilm[All Fields]	C-5	28377634	Penicillenols from a deep-sea fungus Aspergillus restrictus inhibit Candida albicans biofilm formation and hyphal growth.
1080	DRAMP03982	C-5 (analog of P18)	"C-5"[All Fields] AND biofilm[All Fields]	C-5	26846044	Influence of organic acids present in oral biofilm on the durability of the repair bond strength, sorption and solubility of resin composites.
1080	DRAMP03982	C-5 (analog of P18)	"C-5"[All Fields] AND biofilm[All Fields]	C-5	26758707	Herpes Simplex Virus (HSV) Modulation of Staphylococcus aureus and Candida albicans Initiation of HeLa 299 Cell-Associated Biofilm.
1080	DRAMP03982	C-5 (analog of P18)	"C-5"[All Fields] AND biofilm[All Fields]	C-5	24477284	Antibacterial and antibiofilm activities of tryptoquivalines and meroditerpenes isolated from the marine-derived fungi Neosartorya paulistensis, N. laciniosa, N. tsunodae, and the soil fungi N. fischeri and N. siamensis.
1080	DRAMP03982	C-5 (analog of P18)	"C-5"[All Fields] AND biofilm[All Fields]	C-5	23136919	Antibacterial activity of long-chain fatty alcohols against mycobacteria.
1080	DRAMP03982	C-5 (analog of P18)	"C-5"[All Fields] AND biofilm[All Fields]	C-5	22558044	Evaluation of the PotoClean(®) decontamination technology for reprocessing of water supply lines in dental units during routine work.
1080	DRAMP03982	C-5 (analog of P18)	"C-5"[All Fields] AND biofilm[All Fields]	C-5	21605310	Photosynthetic performance of phototrophic biofilms in extreme acidic environments.
1080	DRAMP03982	C-5 (analog of P18)	"C-5"[All Fields] AND biofilm[All Fields]	C-5	21570060	Bacteriological effects of a Lactobacillus reuteri probiotic on in vitro oral biofilms.
1080	DRAMP03982	C-5 (analog of P18)	"C-5"[All Fields] AND biofilm[All Fields]	C-5	20560085	Removal of saturated aliphatic hydrocarbons (gasoline components) from air via bacterial biofiltration.
1080	DRAMP03982	C-5 (analog of P18)	"C-5"[All Fields] AND biofilm[All Fields]	C-5	20544550	Antibacterial activity and QSAR of chalcones against biofilm-producing bacteria isolated from marine waters.
1080	DRAMP03982	C-5 (analog of P18)	"C-5"[All Fields] AND biofilm[All Fields]	C-5	16786845	Fate of inoculated Escherichia coli O157:H7, cultured under different conditions, on fresh and decontaminated beef transitioned from vacuum to aerobic packaging.
1080	DRAMP03982	C-5 (analog of P18)	"C-5"[All Fields] AND biofilm[All Fields]	C-5	16401084	Pseudomonas aeruginosa C5-mannuronan epimerase: steady-state kinetics and characterization of the product.

1081	DRAMP03983	C-6 (analog of P18)	"C-6"[All Fields] AND biofilm[All Fields]	C-6	33279070	Antimicrobial activity of copper surfaces against biofilm formation by Salmonella Enteritidis and its potential application in the poultry industry.
1081	DRAMP03983	C-6 (analog of P18)	"C-6"[All Fields] AND biofilm[All Fields]	C-6	32380771	Induction of Antibacterial Metabolites by Co-Cultivation of Two Red-Sea-Sponge-Associated Actinomycetes Micromonosporasp. UR56 and Actinokinesporasp. EG49.
1081	DRAMP03983	C-6 (analog of P18)	"C-6"[All Fields] AND biofilm[All Fields]	C-6	31767091	Experimental study for evaluation of the efficacy of a biofilm-embedded bacteria-based vaccine against Staphylococcus chromogenes-associated mastitis in sheep.
1081	DRAMP03983	C-6 (analog of P18)	"C-6"[All Fields] AND biofilm[All Fields]	C-6	31400892	Evaluation of efficacy of a biofilm-embedded bacteria-based vaccine against staphylococcal mastitis in sheep-A randomized, placebo-controlled field study.
1081	DRAMP03983	C-6 (analog of P18)	"C-6"[All Fields] AND biofilm[All Fields]	C-6	31386567	Bacterial Growth in Chloride and Perchlorate Brines: Halotolerances and Salt Stress Responses of Planococcus halocryophilus.
1081	DRAMP03983	C-6 (analog of P18)	"C-6"[All Fields] AND biofilm[All Fields]	C-6	31209074	Composition of the Holdfast Polysaccharide from Caulobacter crescentus.
1081	DRAMP03983	C-6 (analog of P18)	"C-6"[All Fields] AND biofilm[All Fields]	C-6	28377634	Penicillenols from a deep-sea fungus Aspergillus restrictus inhibit Candida albicans biofilm formation and hyphal growth.
1081	DRAMP03983	C-6 (analog of P18)	"C-6"[All Fields] AND biofilm[All Fields]	C-6	27070570	New Insights into the Antibacterial Activity of Hydroxycoumarins against Ralstonia solanacearum.
1081	DRAMP03983	C-6 (analog of P18)	"C-6"[All Fields] AND biofilm[All Fields]	C-6	24768213	Erythromycin resistance features and biofilm formation affected by subinhibitory erythromycin in clinical isolates of Staphylococcus epidermidis.
1081	DRAMP03983	C-6 (analog of P18)	"C-6"[All Fields] AND biofilm[All Fields]	C-6	23523915	A simple iterative method for the synthesis of β-(1→6)-glucosamine oligosaccharides.
1081	DRAMP03983	C-6 (analog of P18)	"C-6"[All Fields] AND biofilm[All Fields]	C-6	23026323	Ecological roles and release patterns of acylated homoserine lactones in Pseudomonas sp. HF-1 and their implications in bacterial bioaugmentation.
1081	DRAMP03983	C-6 (analog of P18)	"C-6"[All Fields] AND biofilm[All Fields]	C-6	22736981	Detection, characterization, and biological effect of quorum-sensing signaling molecules in peanut-nodulating bradyrhizobia.
1081	DRAMP03983	C-6 (analog of P18)	"C-6"[All Fields] AND biofilm[All Fields]	C-6	22701624	Proteomic analysis of Neisseria gonorrhoeae biofilms shows shift to anaerobic respiration and changes in nutrient transport and outermembrane proteins.
1081	DRAMP03983	C-6 (analog of P18)	"C-6"[All Fields] AND biofilm[All Fields]	C-6	21517916	Assimilation of benzene carbon through multiple trophic levels traced by different stable isotope probing methodologies.
1081	DRAMP03983	C-6 (analog of P18)	"C-6"[All Fields] AND biofilm[All Fields]	C-6	17675425	Inhibition of quorum sensing in Serratia marcescens AS-1 by synthetic analogs of N-acylhomoserine lactone.
1082	DRAMP03984	C-7 (analog of P18)	"C-7"[All Fields] AND biofilm[All Fields]	C-7	32719941	Proteomic analysis reveals the temperature-dependent presence of extracytoplasmic peptidases in the biofilm exoproteome of Listeria monocytogenes EGD-e.
1082	DRAMP03984	C-7 (analog of P18)	"C-7"[All Fields] AND biofilm[All Fields]	C-7	31318043	Comparison of yeast species in the subgingival oral biofilm of individuals with type 2 diabetes and peri-implantitis and individuals with peri-implantitis without diabetes.
1082	DRAMP03984	C-7 (analog of P18)	"C-7"[All Fields] AND biofilm[All Fields]	C-7	31118927	Neglected Effects of Inoculum Preservation on the Start-Up of Psychrophilic Bioelectrochemical Systems and Shaping Bacterial Communities at Low Temperature.
1082	DRAMP03984	C-7 (analog of P18)	"C-7"[All Fields] AND biofilm[All Fields]	C-7	30782573	Oxidative functionalization of a halimane diterpenoid achieved by fungal transformation.
1082	DRAMP03984	C-7 (analog of P18)	"C-7"[All Fields] AND biofilm[All Fields]	C-7	29157822	Antibiofilm activities of norharmane and its derivatives against Escherichia coli O157:H7 and other bacteria.
1082	DRAMP03984	C-7 (analog of P18)	"C-7"[All Fields] AND biofilm[All Fields]	C-7	27396922	Electrochemical Characterization of a Novel Exoelectrogenic Bacterium Strain SCS5, Isolated from a Mediator-Less Microbial Fuel Cell and Phylogenetically Related to Aeromonas jandaei.
1082	DRAMP03984	C-7 (analog of P18)	"C-7"[All Fields] AND biofilm[All Fields]	C-7	27070570	New Insights into the Antibacterial Activity of Hydroxycoumarins against Ralstonia solanacearum.
1082	DRAMP03984	C-7 (analog of P18)	"C-7"[All Fields] AND biofilm[All Fields]	C-7	26646219	Synthesis of novel ethyl 1-ethyl-6-fluoro-7-(fatty amido)-1,4-dihydro-4-oxoquinoline-3-carboxylate derivatives and their biological evaluation.
1082	DRAMP03984	C-7 (analog of P18)	"C-7"[All Fields] AND biofilm[All Fields]	C-7	23076419	Novel rat model of methicillin-resistant Staphylococcus aureus-infected silicone breast implants: a study of biofilm pathogenesis.
1082	DRAMP03984	C-7 (analog of P18)	"C-7"[All Fields] AND biofilm[All Fields]	C-7	19548693	Diterpenoids from the Mediterranean brown alga Dictyota sp. evaluated as antifouling substances against a marine bacterial biofilm.
1082	DRAMP03984	C-7 (analog of P18)	"C-7"[All Fields] AND biofilm[All Fields]	C-7	19028314	Escherichia coli O157:H7 survival, biofilm formation and acid tolerance under simulated slaughter plant moist and dry conditions.
1082	DRAMP03984	C-7 (analog of P18)	"C-7"[All Fields] AND biofilm[All Fields]	C-7	16108786	Inactivation of Escherichia coli O157:H7 in biofilm on stainless steel by treatment with an alkaline cleaner and a bacteriophage.
1082	DRAMP03984	C-7 (analog of P18)	"C-7"[All Fields] AND biofilm[All Fields]	C-7	12558934	Determination of eight selected periodontal pathogens in the subgingival plaque of maxillary first molars in Japanese school children aged 8-11 years.
1083	DRAMP03985	C-8 (analog of P18)	"C-8"[All Fields] AND biofilm[All Fields]	C-8	33669469	Secondary Caries Adjacent to Bulk or Incrementally Filled Composites Placed after Selective Excavation In Vitro.
1083	DRAMP03985	C-8 (analog of P18)	"C-8"[All Fields] AND biofilm[All Fields]	C-8	31635432	The Marine Catenovulum agarivoransMNH15 and Dextranase: Removing Dental Plaque.
1083	DRAMP03985	C-8 (analog of P18)	"C-8"[All Fields] AND biofilm[All Fields]	C-8	28377634	Penicillenols from a deep-sea fungus Aspergillus restrictus inhibit Candida albicans biofilm formation and hyphal growth.
1083	DRAMP03985	C-8 (analog of P18)	"C-8"[All Fields] AND biofilm[All Fields]	C-8	27070570	New Insights into the Antibacterial Activity of Hydroxycoumarins against Ralstonia solanacearum.
1083	DRAMP03985	C-8 (analog of P18)	"C-8"[All Fields] AND biofilm[All Fields]	C-8	26593271	Mechanistic analysis of a synthetic inhibitor of the Pseudomonas aeruginosa LasI quorum-sensing signal synthase.
1083	DRAMP03985	C-8 (analog of P18)	"C-8"[All Fields] AND biofilm[All Fields]	C-8	23026323	Ecological roles and release patterns of acylated homoserine lactones in Pseudomonas sp. HF-1 and their implications in bacterial bioaugmentation.
1083	DRAMP03985	C-8 (analog of P18)	"C-8"[All Fields] AND biofilm[All Fields]	C-8	22970167	The effect of environmental conditions on biofilm formation of Burkholderia pseudomallei clinical isolates.
1083	DRAMP03985	C-8 (analog of P18)	"C-8"[All Fields] AND biofilm[All Fields]	C-8	22565549	Rubesanolides C-E: abietane diterpenoids isolated from Isodon rubescens and evaluation of their anti-biofilm activity.
1083	DRAMP03985	C-8 (analog of P18)	"C-8"[All Fields] AND biofilm[All Fields]	C-8	22209416	Structure-based virtual screening for plant-derived SdiA-selective ligands as potential antivirulent agents against uropathogenic Escherichia coli.
1083	DRAMP03985	C-8 (analog of P18)	"C-8"[All Fields] AND biofilm[All Fields]	C-8	20560085	Removal of saturated aliphatic hydrocarbons (gasoline components) from air via bacterial biofiltration.
1083	DRAMP03985	C-8 (analog of P18)	"C-8"[All Fields] AND biofilm[All Fields]	C-8	18997026	Metagenome-derived clones encoding two novel lactonase family proteins involved in biofilm inhibition in Pseudomonas aeruginosa.
1083	DRAMP03985	C-8 (analog of P18)	"C-8"[All Fields] AND biofilm[All Fields]	C-8	18048980	The influence of temperature on nutrient treatment efficiency in stormwater biofilter systems.
1083	DRAMP03985	C-8 (analog of P18)	"C-8"[All Fields] AND biofilm[All Fields]	C-8	17953689	Oil-utilizing bacteria associated with fish from the Arabian Gulf.
1083	DRAMP03985	C-8 (analog of P18)	"C-8"[All Fields] AND biofilm[All Fields]	C-8	14563881	Identification of quorum-sensing-regulated genes of Burkholderia cepacia.
1083	DRAMP03985	C-8 (analog of P18)	"C-8"[All Fields] AND biofilm[All Fields]	C-8	11887854	Effect in a rat model of heparinized peritoneal dialysis catheters on bacterial colonization and the healing of the exit site.
1084	DRAMP03986	C-9 (analog of P18)	"C-9"[All Fields] AND biofilm[All Fields]	C-9	31676401	Biofilm inhibiting properties of compounds from the leaves of Warburgia ugandensis Sprague subsp ugandensis against Candida and staphylococcal biofilms.
1084	DRAMP03986	C-9 (analog of P18)	"C-9"[All Fields] AND biofilm[All Fields]	C-9	28959742	The Biofilm Inhibitor Carolacton Enters Gram-Negative Cells: Studies Using a TolC-Deficient Strain of Escherichia coli.
1084	DRAMP03986	C-9 (analog of P18)	"C-9"[All Fields] AND biofilm[All Fields]	C-9	28065749	Characterization of haloalkalophilic organic solvent tolerant protease for chitin extraction from shrimp shell waste.
1084	DRAMP03986	C-9 (analog of P18)	"C-9"[All Fields] AND biofilm[All Fields]	C-9	27708366	Biotransformation of ferulic acid to vanillin in the packed bed-stirred fermentors.
1084	DRAMP03986	C-9 (analog of P18)	"C-9"[All Fields] AND biofilm[All Fields]	C-9	27340103	Discovery of antifungal constituents from the Miao medicinal plant Isodon flavidus.
1084	DRAMP03986	C-9 (analog of P18)	"C-9"[All Fields] AND biofilm[All Fields]	C-9	26408137	Influence of Temperature, Source, and Serotype on Biofilm Formation of Salmonella enterica Isolates from Pig Slaughterhouses.
1084	DRAMP03986	C-9 (analog of P18)	"C-9"[All Fields] AND biofilm[All Fields]	C-9	23905166	Field evidence of selenium bioreduction in a uranium-contaminated aquifer.
1084	DRAMP03986	C-9 (analog of P18)	"C-9"[All Fields] AND biofilm[All Fields]	C-9	22194285	Small-molecule modulators of Listeria monocytogenes biofilm development.
1084	DRAMP03986	C-9 (analog of P18)	"C-9"[All Fields] AND biofilm[All Fields]	C-9	17675425	Inhibition of quorum sensing in Serratia marcescens AS-1 by synthetic analogs of N-acylhomoserine lactone.
1085	DRAMP03987	C-10 (analog of P18)	"C-10"[All Fields] AND biofilm[All Fields]	C-10	34647135	Joint protection strategies for Saccharomyces boulardii: exogenous encapsulation and endogenous biofilm structure.
1085	DRAMP03987	C-10 (analog of P18)	"C-10"[All Fields] AND biofilm[All Fields]	C-10	33532362	Antibiofilm effect of C-10 massoia lactone toward polymicrobial oral biofilms.
1085	DRAMP03987	C-10 (analog of P18)	"C-10"[All Fields] AND biofilm[All Fields]	C-10	32798808	Mo 2N nanobelt cathodes for efficient hydrogen production in microbial electrolysis cells with shaped biofilm microbiome.
1085	DRAMP03987	C-10 (analog of P18)	"C-10"[All Fields] AND biofilm[All Fields]	C-10	31400892	Evaluation of efficacy of a biofilm-embedded bacteria-based vaccine against staphylococcal mastitis in sheep-A randomized, placebo-controlled field study.
1085	DRAMP03987	C-10 (analog of P18)	"C-10"[All Fields] AND biofilm[All Fields]	C-10	31118927	Neglected Effects of Inoculum Preservation on the Start-Up of Psychrophilic Bioelectrochemical Systems and Shaping Bacterial Communities at Low Temperature.
1085	DRAMP03987	C-10 (analog of P18)	"C-10"[All Fields] AND biofilm[All Fields]	C-10	30199274	The Characterization of Biofilm Formation and Detection of Biofilm-Related Genes in Salmonella Isolated from Beef Processing Plants.
1085	DRAMP03987	C-10 (analog of P18)	"C-10"[All Fields] AND biofilm[All Fields]	C-10	28297746	Screening of Anti-Biofilm Compounds from Marine-Derived Fungi and the Effects of Secalonic Acid D on Staphylococcus aureusBiofilm.

1085	DRAMP03987	C-10 (analog of P18)	"C-10"[All Fields] AND biofilm[All Fields]	C-10	27706070	RT-qPCR Analysis of 15 Genes Encoding Putative Surface Proteins Involved in Adherence of Listeria monocytogenes.
1085	DRAMP03987	C-10 (analog of P18)	"C-10"[All Fields] AND biofilm[All Fields]	C-10	27563226	Potency of Massoia Bark in Combating Immunosuppressed-related Infection.
1085	DRAMP03987	C-10 (analog of P18)	"C-10"[All Fields] AND biofilm[All Fields]	C-10	26517334	Optimization of the RNeasy Mini Kit to obtain high-quality total RNA from sessile cells of Staphylococcus aureus.
1085	DRAMP03987	C-10 (analog of P18)	"C-10"[All Fields] AND biofilm[All Fields]	C-10	23212850	Bioconversion of sodium dodecyl sulphate to rhamnolipid by Pseudomonas aeruginosa: a novel and cost-effective production strategy.
1085	DRAMP03987	C-10 (analog of P18)	"C-10"[All Fields] AND biofilm[All Fields]	C-10	23136919	Antibacterial activity of long-chain fatty alcohols against mycobacteria.
1085	DRAMP03987	C-10 (analog of P18)	"C-10"[All Fields] AND biofilm[All Fields]	C-10	22970167	The effect of environmental conditions on biofilm formation of Burkholderia pseudomallei clinical isolates.
1085	DRAMP03987	C-10 (analog of P18)	"C-10"[All Fields] AND biofilm[All Fields]	C-10	22635999	Effects of subinhibitory concentrations of menthol on adaptation, morphological, and gene expression changes in enterohemorrhagic Escherichia coli.
1085	DRAMP03987	C-10 (analog of P18)	"C-10"[All Fields] AND biofilm[All Fields]	C-10	21807742	N-Acylhomoserine lactones are potent neutrophil chemoattractants that act via calcium mobilization and actin remodeling.
1085	DRAMP03987	C-10 (analog of P18)	"C-10"[All Fields] AND biofilm[All Fields]	C-10	21806455	Submerged filter biofilm formation by nitrate-contaminated groundwater microbiota.
1085	DRAMP03987	C-10 (analog of P18)	"C-10"[All Fields] AND biofilm[All Fields]	C-10	20658029	S. mutans biofilm model to evaluate antimicrobial substances and enamel demineralization.
1085	DRAMP03987	C-10 (analog of P18)	"C-10"[All Fields] AND biofilm[All Fields]	C-10	20336292	Rhamnolipids: diversity of structures, microbial origins and roles.
1085	DRAMP03987	C-10 (analog of P18)	"C-10"[All Fields] AND biofilm[All Fields]	C-10	19548886	A novel in vitro flat-bed perfusion biofilm model for determining the potential antimicrobial efficacy of topical wound treatments.
1085	DRAMP03987	C-10 (analog of P18)	"C-10"[All Fields] AND biofilm[All Fields]	C-10	19143383	[Influence of EPS on silicate corrosion inhibition for copper pipe in soft water].
1085	DRAMP03987	C-10 (analog of P18)	"C-10"[All Fields] AND biofilm[All Fields]	C-10	18080121	Electricity generation by thermophilic microorganisms from marine sediment.
1085	DRAMP03987	C-10 (analog of P18)	"C-10"[All Fields] AND biofilm[All Fields]	C-10	17305719	A simulated oral hygiene model to determine the efficacy of repeated exposure of amine oxide on the viability of Streptococcus mutans biofilms.
1085	DRAMP03987	C-10 (analog of P18)	"C-10"[All Fields] AND biofilm[All Fields]	C-10	16819950	A cold-loving crenarchaeon is a substantial part of a novel microbial community in cold sulphidic marsh water.
1085	DRAMP03987	C-10 (analog of P18)	"C-10"[All Fields] AND biofilm[All Fields]	C-10	16738114	Rubellimicrobium thermophilum gen. nov., sp. nov., a red-pigmented, moderately thermophilic bacterium isolated from coloured slime deposits in paper machines.
1085	DRAMP03987	C-10 (analog of P18)	"C-10"[All Fields] AND biofilm[All Fields]	C-10	16585751	The ppuI-rsaL-ppuR quorum-sensing system regulates biofilm formation of Pseudomonas putida PCL1445 by controlling biosynthesis of the cyclic lipopeptides putisolvins I and II.
1085	DRAMP03987	C-10 (analog of P18)	"C-10"[All Fields] AND biofilm[All Fields]	C-10	10052899	Resistance of artificial biofilms of Pseudomonas aeruginosa to imipenem and tobramycin.
1085	DRAMP03987	C-10 (analog of P18)	"C-10"[All Fields] AND biofilm[All Fields]	C-10	9818743	Response of single species biofilms and microcosm dental plaques to pulsing with chlorhexidine.
1116	DRAMP04025	R10 (single amino acid substitution of Bac034, which is a scrambled Variant of Bac2A)	"R10"[All Fields] AND biofilm[All Fields]	R10	33510721	Maipomycin A, a Novel Natural Compound With Promising Anti-biofilm Activity Against Gram-Negative Pathogenic Bacteria.
1116	DRAMP04025	R10 (single amino acid substitution of Bac034, which is a scrambled Variant of Bac2A)	"R10"[All Fields] AND biofilm[All Fields]	R10	27160323	Orange/Red Fluorescence of Active Caries by Retrospective Quantitative Light-Induced Fluorescence Image Analysis.
1116	DRAMP04025	R10 (single amino acid substitution of Bac034, which is a scrambled Variant of Bac2A)	"R10"[All Fields] AND biofilm[All Fields]	R10	15303732	Effect of solid hold-up on nitrite accumulation in a biofilm reactor--molecular characterization of nitrifying communities.
1117	DRAMP04026	K12 (single amino acid substitution of Bac034, which is a scrambled Variant of Bac2A)	"K12"[All Fields] AND biofilm[All Fields]	K12	34484135	YbfA Regulates the Sensitivity of Escherichia coliK12 to Plantaricin BM-1 viathe BasS/BasR Two-Component Regulatory System.
1117	DRAMP04026	K12 (single amino acid substitution of Bac034, which is a scrambled Variant of Bac2A)	"K12"[All Fields] AND biofilm[All Fields]	K12	34466074	Biofilm producing indigenous bacteria isolated from municipal sludge and their nutrient removal ability in moving bed biofilm reactor from the wastewater.
1117	DRAMP04026	K12 (single amino acid substitution of Bac034, which is a scrambled Variant of Bac2A)	"K12"[All Fields] AND biofilm[All Fields]	K12	34434797	Liquid chromatography mass spectrometry-based proteomics of Escherichia colisingle colony.
1117	DRAMP04026	K12 (single amino acid substitution of Bac034, which is a scrambled Variant of Bac2A)	"K12"[All Fields] AND biofilm[All Fields]	K12	34431194	Role of the YehD fimbriae in the virulence-associated properties of enteroaggregative Escherichia coli.
1117	DRAMP04026	K12 (single amino acid substitution of Bac034, which is a scrambled Variant of Bac2A)	"K12"[All Fields] AND biofilm[All Fields]	K12	34425729	Streptococcus salivariusK12 inhibits Candida albicansaggregation, biofilm formation and dimorphism.
1117	DRAMP04026	K12 (single amino acid substitution of Bac034, which is a scrambled Variant of Bac2A)	"K12"[All Fields] AND biofilm[All Fields]	K12	34209988	Antimicrobial and Antibiofilm Activity of the Probiotic Strain Streptococcus salivariusK12 against Oral Potential Pathogens.
1117	DRAMP04026	K12 (single amino acid substitution of Bac034, which is a scrambled Variant of Bac2A)	"K12"[All Fields] AND biofilm[All Fields]	K12	34076405	Group B Streptococcus cpsEIs Required for Serotype V Capsule Production and Aids in Biofilm Formation and Ascending Infection of the Reproductive Tract during Pregnancy.
1117	DRAMP04026	K12 (single amino acid substitution of Bac034, which is a scrambled Variant of Bac2A)	"K12"[All Fields] AND biofilm[All Fields]	K12	33685971	Effect of Spermidine on Biofilm Formation in Escherichia coli K-12.
1117	DRAMP04026	K12 (single amino acid substitution of Bac034, which is a scrambled Variant of Bac2A)	"K12"[All Fields] AND biofilm[All Fields]	K12	33588649	Essential oils from Artemisiaspecies inhibit biofilm formation and the virulence of Escherichia coliEPEC 2348/69.
1117	DRAMP04026	K12 (single amino acid substitution of Bac034, which is a scrambled Variant of Bac2A)	"K12"[All Fields] AND biofilm[All Fields]	K12	33495047	Pellicle formation by Escherichia coli K-12: Role of adhesins and motility.
1117	DRAMP04026	K12 (single amino acid substitution of Bac034, which is a scrambled Variant of Bac2A)	"K12"[All Fields] AND biofilm[All Fields]	K12	33419248	In vitro Interactions between Streptococcus intermediusand Streptococcus salivariusK12 on a Titanium Cylindrical Surface.
1117	DRAMP04026	K12 (single amino acid substitution of Bac034, which is a scrambled Variant of Bac2A)	"K12"[All Fields] AND biofilm[All Fields]	K12	33385683	The specific effect of (R)-(+)-pulegone on growth and biofilm formation in multi-drug resistant Escherichia coli and molecular mechanisms underlying the expression of pgaABCD genes.
1117	DRAMP04026	K12 (single amino acid substitution of Bac034, which is a scrambled Variant of Bac2A)	"K12"[All Fields] AND biofilm[All Fields]	K12	33277548	Short chain fatty acids produced by Cutibacterium acnes inhibit biofilm formation by Staphylococcus epidermidis.
1117	DRAMP04026	K12 (single amino acid substitution of Bac034, which is a scrambled Variant of Bac2A)	"K12"[All Fields] AND biofilm[All Fields]	K12	33057158	Antimicrobial effects of airborne acoustic ultrasound and plasma activated water from cold and thermal plasma systems on biofilms.
1117	DRAMP04026	K12 (single amino acid substitution of Bac034, which is a scrambled Variant of Bac2A)	"K12"[All Fields] AND biofilm[All Fields]	K12	32894594	Variation in the ratio of curli and phosphoethanolamine cellulose associated with biofilm architecture and properties.
1117	DRAMP04026	K12 (single amino acid substitution of Bac034, which is a scrambled Variant of Bac2A)	"K12"[All Fields] AND biofilm[All Fields]	K12	32800795	Comparison between the proteome of Escherichia coli single colony and during liquid culture.

1117	DRAMP04026	K12 (single amino acid substitution of Bac034, which is a scrambled Variant of Bac2A)	"K12"[All Fields] AND biofilm[All Fields]	K12	32649279	Novel regulators of the csgDgene encoding the master regulator of biofilm formation in Escherichia coliK-12.
1117	DRAMP04026	K12 (single amino acid substitution of Bac034, which is a scrambled Variant of Bac2A)	"K12"[All Fields] AND biofilm[All Fields]	K12	32576671	Genotypic and Phenotypic Diversity of Staphylococcus aureus Isolates from Cystic Fibrosis Patient Lung Infections and Their Interactions with Pseudomonas aeruginosa.
1117	DRAMP04026	K12 (single amino acid substitution of Bac034, which is a scrambled Variant of Bac2A)	"K12"[All Fields] AND biofilm[All Fields]	K12	32558899	Xenorhabdus bovienii strain jolietti uses a type 6 secretion system to kill closely related Xenorhabdus strains.
1117	DRAMP04026	K12 (single amino acid substitution of Bac034, which is a scrambled Variant of Bac2A)	"K12"[All Fields] AND biofilm[All Fields]	K12	32551597	Exposure to Environmental Levels of Pesticides Stimulates and Diversifies Evolution in Escherichia colitoward Higher Antibiotic Resistance.
1117	DRAMP04026	K12 (single amino acid substitution of Bac034, which is a scrambled Variant of Bac2A)	"K12"[All Fields] AND biofilm[All Fields]	K12	32540933	Involvement of Chromosomally Encoded Homologs of the RRNPP Protein Family in Enterococcus faecalis Biofilm Formation and Urinary Tract Infection Pathogenesis.
1117	DRAMP04026	K12 (single amino acid substitution of Bac034, which is a scrambled Variant of Bac2A)	"K12"[All Fields] AND biofilm[All Fields]	K12	32534064	Local c-di-GMP Signaling in the Control of Synthesis of the E. coli Biofilm Exopolysaccharide pEtN-Cellulose.
1117	DRAMP04026	K12 (single amino acid substitution of Bac034, which is a scrambled Variant of Bac2A)	"K12"[All Fields] AND biofilm[All Fields]	K12	32529293	Outer Membrane Channel Protein TolC Regulates Escherichia coli K12 Sensitivity to Plantaricin BM-1 via the CpxR/CpxA Two-Component Regulatory System.
1117	DRAMP04026	K12 (single amino acid substitution of Bac034, which is a scrambled Variant of Bac2A)	"K12"[All Fields] AND biofilm[All Fields]	K12	32298190	Caries-Associated Biosynthetic Gene Clusters in Streptococcus mutans.
1117	DRAMP04026	K12 (single amino acid substitution of Bac034, which is a scrambled Variant of Bac2A)	"K12"[All Fields] AND biofilm[All Fields]	K12	32117089	Characterization of Acinetobacter baumanniiCopper Resistance Reveals a Role in Virulence.
1117	DRAMP04026	K12 (single amino acid substitution of Bac034, which is a scrambled Variant of Bac2A)	"K12"[All Fields] AND biofilm[All Fields]	K12	32040901	[Determination of biofilm forming activity of microorganisms on synthetic polymeric materials.]
1117	DRAMP04026	K12 (single amino acid substitution of Bac034, which is a scrambled Variant of Bac2A)	"K12"[All Fields] AND biofilm[All Fields]	K12	32011148	Monitoring Early Stages of Bacterial Adhesion at Silica Surfaces through Image Analysis.
1117	DRAMP04026	K12 (single amino acid substitution of Bac034, which is a scrambled Variant of Bac2A)	"K12"[All Fields] AND biofilm[All Fields]	K12	31932609	Assembly and substrate recognition of curli biogenesis system.
1117	DRAMP04026	K12 (single amino acid substitution of Bac034, which is a scrambled Variant of Bac2A)	"K12"[All Fields] AND biofilm[All Fields]	K12	31892694	Regulatory Role of PlaR (YiaJ) for Plant Utilization in Escherichia coli K-12.
1117	DRAMP04026	K12 (single amino acid substitution of Bac034, which is a scrambled Variant of Bac2A)	"K12"[All Fields] AND biofilm[All Fields]	K12	31851822	Biofilm Inhibitor Taurolithocholic Acid Alters Colony Morphology, Specialized Metabolism, and Virulence of Pseudomonas aeruginosa.
1117	DRAMP04026	K12 (single amino acid substitution of Bac034, which is a scrambled Variant of Bac2A)	"K12"[All Fields] AND biofilm[All Fields]	K12	31834370	Regulatory role of pyruvate-sensing BtsSR in biofilm formation by Escherichia coli K-12.
1117	DRAMP04026	K12 (single amino acid substitution of Bac034, which is a scrambled Variant of Bac2A)	"K12"[All Fields] AND biofilm[All Fields]	K12	31697703	Structural mechanism for regulation of DNA binding of BpsR, a Bordetella regulator of biofilm formation, by 6-hydroxynicotinic acid.
1117	DRAMP04026	K12 (single amino acid substitution of Bac034, which is a scrambled Variant of Bac2A)	"K12"[All Fields] AND biofilm[All Fields]	K12	31640553	Genome rearrangements induce biofilm formation in Escherichia coli C - an old model organism with a new application in biofilm research.
1117	DRAMP04026	K12 (single amino acid substitution of Bac034, which is a scrambled Variant of Bac2A)	"K12"[All Fields] AND biofilm[All Fields]	K12	31540233	Silk-Based Therapeutics Targeting Pseudomonas aeruginosa.
1117	DRAMP04026	K12 (single amino acid substitution of Bac034, which is a scrambled Variant of Bac2A)	"K12"[All Fields] AND biofilm[All Fields]	K12	31351270	Curli production enhances clay-E. coli aggregation and sedimentation.
1117	DRAMP04026	K12 (single amino acid substitution of Bac034, which is a scrambled Variant of Bac2A)	"K12"[All Fields] AND biofilm[All Fields]	K12	30776138	Antagonistic effects of Streptococcus and Lactobacillus probiotics in pharyngeal biofilms.
1117	DRAMP04026	K12 (single amino acid substitution of Bac034, which is a scrambled Variant of Bac2A)	"K12"[All Fields] AND biofilm[All Fields]	K12	30719328	Influence of Probiotic Culture Supernatants on In Vitro Biofilm Formation of Staphylococci.
1117	DRAMP04026	K12 (single amino acid substitution of Bac034, which is a scrambled Variant of Bac2A)	"K12"[All Fields] AND biofilm[All Fields]	K12	30713773	Effect of Temperature, pH and Plasmids on In Vitro Biofilm Formation in Escherichia coli.
1117	DRAMP04026	K12 (single amino acid substitution of Bac034, which is a scrambled Variant of Bac2A)	"K12"[All Fields] AND biofilm[All Fields]	K12	30654458	Antibiofilm Activity of Polyamide 11 Modified with Thermally Stable Polymeric Biocide Polyhexamethylene Guanidine 2-Naphtalenesulfonate.
1117	DRAMP04026	K12 (single amino acid substitution of Bac034, which is a scrambled Variant of Bac2A)	"K12"[All Fields] AND biofilm[All Fields]	K12	30648935	Symbiont evolution during the free-living phase can improve host colonization.
1117	DRAMP04026	K12 (single amino acid substitution of Bac034, which is a scrambled Variant of Bac2A)	"K12"[All Fields] AND biofilm[All Fields]	K12	30642895	Peptide-Based Inhibitors of Fimbrial Biogenesis in Porphyromonas gingivalis.
1117	DRAMP04026	K12 (single amino acid substitution of Bac034, which is a scrambled Variant of Bac2A)	"K12"[All Fields] AND biofilm[All Fields]	K12	30633761	Sugar and iron: Toward understanding the antibacterial effect of ciclopirox in Escherichia coli.
1117	DRAMP04026	K12 (single amino acid substitution of Bac034, which is a scrambled Variant of Bac2A)	"K12"[All Fields] AND biofilm[All Fields]	K12	30557874	Towards Understanding the Molecular Basis of Nitric Oxide-Regulated Group Behaviors in Pathogenic Bacteria.
1117	DRAMP04026	K12 (single amino acid substitution of Bac034, which is a scrambled Variant of Bac2A)	"K12"[All Fields] AND biofilm[All Fields]	K12	30325301	Efficacy of β-phenylethylamine as a novel anti-microbial and application as a liquid catheter flush.
1117	DRAMP04026	K12 (single amino acid substitution of Bac034, which is a scrambled Variant of Bac2A)	"K12"[All Fields] AND biofilm[All Fields]	K12	30218278	A Multiscale Agent-Based Model for the Investigation of E. coli K12 Metabolic Response During Biofilm Formation.

1117	DRAMP04026	K12 (single amino acid substitution of Bac034, which is a scrambled Variant of Bac2A)	"K12"[All Fields] AND biofilm[All Fields]	K12	29796251	How long is enough? Identification of product dry-time as a primary driver of alcohol-based hand rub efficacy.
1117	DRAMP04026	K12 (single amino acid substitution of Bac034, which is a scrambled Variant of Bac2A)	"K12"[All Fields] AND biofilm[All Fields]	K12	29629858	Pseudomonas aeruginosa PumA acts on an endogenous phenazine to promote self-resistance.
1117	DRAMP04026	K12 (single amino acid substitution of Bac034, which is a scrambled Variant of Bac2A)	"K12"[All Fields] AND biofilm[All Fields]	K12	29518621	Bacterial electroactivity and viability depends on the carbon nanotube-coated sponge anode used in a microbial fuel cell.
1117	DRAMP04026	K12 (single amino acid substitution of Bac034, which is a scrambled Variant of Bac2A)	"K12"[All Fields] AND biofilm[All Fields]	K12	29514851	Transmembrane redox control and proteolysis of PdeC, a novel type of c-di-GMP phosphodiesterase.
1117	DRAMP04026	K12 (single amino acid substitution of Bac034, which is a scrambled Variant of Bac2A)	"K12"[All Fields] AND biofilm[All Fields]	K12	29338696	Transcriptional responses of Escherichia coli during recovery from inorganic or organic mercury exposure.
1117	DRAMP04026	K12 (single amino acid substitution of Bac034, which is a scrambled Variant of Bac2A)	"K12"[All Fields] AND biofilm[All Fields]	K12	29304032	Community Engaged Cumulative Risk Assessment of Exposure to Inorganic Well Water Contaminants, Crow Reservation, Montana.
1117	DRAMP04026	K12 (single amino acid substitution of Bac034, which is a scrambled Variant of Bac2A)	"K12"[All Fields] AND biofilm[All Fields]	K12	29114245	The Selective Interaction of Pistacia lentiscusOil vs. Human Streptococci, an Old Functional Food Revisited with New Tools.
1117	DRAMP04026	K12 (single amino acid substitution of Bac034, which is a scrambled Variant of Bac2A)	"K12"[All Fields] AND biofilm[All Fields]	K12	29076803	MtlR negatively regulates mannitol utilization by Vibrio cholerae.
1117	DRAMP04026	K12 (single amino acid substitution of Bac034, which is a scrambled Variant of Bac2A)	"K12"[All Fields] AND biofilm[All Fields]	K12	28961860	Effect of bacterial components of mixed culture supernatants of planktonic and biofilm Pseudomonas aeruginosa with commensal Escherichia coli on the neutrophil response in vitro.
1117	DRAMP04026	K12 (single amino acid substitution of Bac034, which is a scrambled Variant of Bac2A)	"K12"[All Fields] AND biofilm[All Fields]	K12	28892292	Dissemination and loss of a biofilm-related genomic island in marine Pseudoalteromonas mediated by integrative and conjugative elements.
1117	DRAMP04026	K12 (single amino acid substitution of Bac034, which is a scrambled Variant of Bac2A)	"K12"[All Fields] AND biofilm[All Fields]	K12	28847157	Bacterial Heme-Based Sensors of Nitric Oxide.
1117	DRAMP04026	K12 (single amino acid substitution of Bac034, which is a scrambled Variant of Bac2A)	"K12"[All Fields] AND biofilm[All Fields]	K12	28841717	Streptococcus pneumoniae in the heart subvert the host response through biofilm-mediated resident macrophage killing.
1117	DRAMP04026	K12 (single amino acid substitution of Bac034, which is a scrambled Variant of Bac2A)	"K12"[All Fields] AND biofilm[All Fields]	K12	28657681	Negative regulation of filamentous growth in Candida albicans by Dig1p.
1117	DRAMP04026	K12 (single amino acid substitution of Bac034, which is a scrambled Variant of Bac2A)	"K12"[All Fields] AND biofilm[All Fields]	K12	28533218	Involvement of Two-Component Signaling on Bacterial Motility and Biofilm Development.
1117	DRAMP04026	K12 (single amino acid substitution of Bac034, which is a scrambled Variant of Bac2A)	"K12"[All Fields] AND biofilm[All Fields]	K12	28471113	Antibacterial mechanism of high-mobility group nucleosomal-binding domain 2 on the Gram-negative bacteria Escherichia coli.
1117	DRAMP04026	K12 (single amino acid substitution of Bac034, which is a scrambled Variant of Bac2A)	"K12"[All Fields] AND biofilm[All Fields]	K12	28418636	Defining Molecular Details of the Chemistry of Biofilm Formation by Raman Microspectroscopy.
1117	DRAMP04026	K12 (single amino acid substitution of Bac034, which is a scrambled Variant of Bac2A)	"K12"[All Fields] AND biofilm[All Fields]	K12	28415239	Motility of Escherichia coli in a quasi-two-dimensional porous medium.
1117	DRAMP04026	K12 (single amino acid substitution of Bac034, which is a scrambled Variant of Bac2A)	"K12"[All Fields] AND biofilm[All Fields]	K12	28408270	Transcriptional organization of pneumococcal psrP-secY2A2 and impact of GtfA and GtfB deletion on PsrP-associated virulence properties.
1117	DRAMP04026	K12 (single amino acid substitution of Bac034, which is a scrambled Variant of Bac2A)	"K12"[All Fields] AND biofilm[All Fields]	K12	28389540	Acid Evolution of Escherichia coli K-12 Eliminates Amino Acid Decarboxylases and Reregulates Catabolism.
1117	DRAMP04026	K12 (single amino acid substitution of Bac034, which is a scrambled Variant of Bac2A)	"K12"[All Fields] AND biofilm[All Fields]	K12	28356113	Killing of Serratia marcescens biofilms with chloramphenicol.
1117	DRAMP04026	K12 (single amino acid substitution of Bac034, which is a scrambled Variant of Bac2A)	"K12"[All Fields] AND biofilm[All Fields]	K12	28303897	Synergistic efficacy of Bisbenzimidazole and Carbonyl Cyanide 3-Chlorophenylhydrazone combination against MDR bacterial strains.
1117	DRAMP04026	K12 (single amino acid substitution of Bac034, which is a scrambled Variant of Bac2A)	"K12"[All Fields] AND biofilm[All Fields]	K12	28291321	Nanoengineered Superhydrophobic Surfaces of Aluminum with Extremely Low Bacterial Adhesivity.
1117	DRAMP04026	K12 (single amino acid substitution of Bac034, which is a scrambled Variant of Bac2A)	"K12"[All Fields] AND biofilm[All Fields]	K12	28285908	Are CDI Systems Multicolored, Facultative, Helping Greenbeards?
1117	DRAMP04026	K12 (single amino acid substitution of Bac034, which is a scrambled Variant of Bac2A)	"K12"[All Fields] AND biofilm[All Fields]	K12	28257056	Interaction of Type IV Toxin/Antitoxin Systems in Cryptic Prophages of Escherichia coli K-12.
1117	DRAMP04026	K12 (single amino acid substitution of Bac034, which is a scrambled Variant of Bac2A)	"K12"[All Fields] AND biofilm[All Fields]	K12	28073397	Identification of new members of the Escherichia coli K-12 MG1655 SlyA regulon.
1117	DRAMP04026	K12 (single amino acid substitution of Bac034, which is a scrambled Variant of Bac2A)	"K12"[All Fields] AND biofilm[All Fields]	K12	27989220	Mathematical model of LsrR-binding and derepression in Escherichia coli K12.
1117	DRAMP04026	K12 (single amino acid substitution of Bac034, which is a scrambled Variant of Bac2A)	"K12"[All Fields] AND biofilm[All Fields]	K12	27842452	Role of bolA and rpoS genes in biofilm formation and adherence pattern by Escherichia coli K-12 MG1655 on polypropylene, stainless steel, and silicone surfaces.
1117	DRAMP04026	K12 (single amino acid substitution of Bac034, which is a scrambled Variant of Bac2A)	"K12"[All Fields] AND biofilm[All Fields]	K12	27690228	Impairment of Biofilm Formation by TiO 2Photocatalysis through Quorum Quenching.
1117	DRAMP04026	K12 (single amino acid substitution of Bac034, which is a scrambled Variant of Bac2A)	"K12"[All Fields] AND biofilm[All Fields]	K12	27696649	Functional analysis of Escherichia coli Yad fimbriae reveals their potential role in environmental persistence.

1117	DRAMP04026	K12 (single amino acid substitution of Bac034, which is a scrambled Variant of Bac2A)	"K12"[All Fields] AND biofilm[All Fields]	K12	27645299	Acetate metabolism regulation in Escherichia coli: carbon overflow, pathogenicity, and beyond.
1117	DRAMP04026	K12 (single amino acid substitution of Bac034, which is a scrambled Variant of Bac2A)	"K12"[All Fields] AND biofilm[All Fields]	K12	27429754	Characterization and optimization of pH-responsive polymer nanoparticles for drug delivery to oral biofilms.
1117	DRAMP04026	K12 (single amino acid substitution of Bac034, which is a scrambled Variant of Bac2A)	"K12"[All Fields] AND biofilm[All Fields]	K12	27392114	Flagellin-rPAc vaccine inhibits biofilm formation but not proliferation of S. mutans.
1117	DRAMP04026	K12 (single amino acid substitution of Bac034, which is a scrambled Variant of Bac2A)	"K12"[All Fields] AND biofilm[All Fields]	K12	27335458	Interbacterial signaling via Burkholderia contact-dependent growth inhibition system proteins.
1117	DRAMP04026	K12 (single amino acid substitution of Bac034, which is a scrambled Variant of Bac2A)	"K12"[All Fields] AND biofilm[All Fields]	K12	27142312	A novel co-culture model of murine K12 osteosarcoma cells and S. aureus on common orthopedic implant materials: 'the race to the surface' studied in vitro.
1117	DRAMP04026	K12 (single amino acid substitution of Bac034, which is a scrambled Variant of Bac2A)	"K12"[All Fields] AND biofilm[All Fields]	K12	27111508	The evolution of cooperation within the gut microbiota.
1117	DRAMP04026	K12 (single amino acid substitution of Bac034, which is a scrambled Variant of Bac2A)	"K12"[All Fields] AND biofilm[All Fields]	K12	27071334	Biofilm vivacity and destruction on antimicrobial nanosurfaces assayed within a microbial fuel cell.
1117	DRAMP04026	K12 (single amino acid substitution of Bac034, which is a scrambled Variant of Bac2A)	"K12"[All Fields] AND biofilm[All Fields]	K12	27045200	2-Deoxy-d-glucose is a potent inhibitor of biofilm growth in Escherichia coli.
1117	DRAMP04026	K12 (single amino acid substitution of Bac034, which is a scrambled Variant of Bac2A)	"K12"[All Fields] AND biofilm[All Fields]	K12	27009592	Comparative analysis of quantitative methodologies for Vibrionaceae biofilms.
1117	DRAMP04026	K12 (single amino acid substitution of Bac034, which is a scrambled Variant of Bac2A)	"K12"[All Fields] AND biofilm[All Fields]	K12	26987892	Targeted, triggered drug delivery to tumor and biofilm microenvironments.
1117	DRAMP04026	K12 (single amino acid substitution of Bac034, which is a scrambled Variant of Bac2A)	"K12"[All Fields] AND biofilm[All Fields]	K12	26914334	Mechanobiology of Antimicrobial Resistant Escherichia coli and Listeria innocua.
1117	DRAMP04026	K12 (single amino acid substitution of Bac034, which is a scrambled Variant of Bac2A)	"K12"[All Fields] AND biofilm[All Fields]	K12	26530864	Physiological Function of Rac Prophage During Biofilm Formation and Regulation of Rac Excision in Escherichia coli K-12.
1117	DRAMP04026	K12 (single amino acid substitution of Bac034, which is a scrambled Variant of Bac2A)	"K12"[All Fields] AND biofilm[All Fields]	K12	26479780	Biofouling on polymeric heat exchanger surfaces with E. coli and native biofilms.
1117	DRAMP04026	K12 (single amino acid substitution of Bac034, which is a scrambled Variant of Bac2A)	"K12"[All Fields] AND biofilm[All Fields]	K12	26387845	Co-expressional conservation in virulence and stress related genes of three Gammaproteobacterial species: Escherichia coli, Salmonella enterica and Pseudomonas aeruginosa.
1117	DRAMP04026	K12 (single amino acid substitution of Bac034, which is a scrambled Variant of Bac2A)	"K12"[All Fields] AND biofilm[All Fields]	K12	26187746	Escherichia coli O8-antigen enhances biofilm formation under agitated conditions.
1117	DRAMP04026	K12 (single amino acid substitution of Bac034, which is a scrambled Variant of Bac2A)	"K12"[All Fields] AND biofilm[All Fields]	K12	25964477	Toll-Like Receptor 9-Mediated Inflammation Triggers Alveolar Bone Loss in Experimental Murine Periodontitis.
1117	DRAMP04026	K12 (single amino acid substitution of Bac034, which is a scrambled Variant of Bac2A)	"K12"[All Fields] AND biofilm[All Fields]	K12	25921426	The salmochelin receptor IroN itself, but not salmochelin-mediated iron uptake promotes biofilm formation in extraintestinal pathogenic Escherichia coli (ExPEC).
1117	DRAMP04026	K12 (single amino acid substitution of Bac034, which is a scrambled Variant of Bac2A)	"K12"[All Fields] AND biofilm[All Fields]	K12	25858802	Combatting bacterial infections by killing persister cells with mitomycin C.
1117	DRAMP04026	K12 (single amino acid substitution of Bac034, which is a scrambled Variant of Bac2A)	"K12"[All Fields] AND biofilm[All Fields]	K12	25848688	Studies on formation, control and application of biofilm formed by food related microorganisms.
1117	DRAMP04026	K12 (single amino acid substitution of Bac034, which is a scrambled Variant of Bac2A)	"K12"[All Fields] AND biofilm[All Fields]	K12	25781217	Bisphenol A removal by a Pseudomonas aeruginosa immobilized on granular activated carbon and operating in a fluidized bed reactor.
1117	DRAMP04026	K12 (single amino acid substitution of Bac034, which is a scrambled Variant of Bac2A)	"K12"[All Fields] AND biofilm[All Fields]	K12	25777672	Mutations in pneumococcal cpsE generated via in vitro serial passaging reveal a potential mechanism of reduced encapsulation utilized by a conjunctival isolate.
1117	DRAMP04026	K12 (single amino acid substitution of Bac034, which is a scrambled Variant of Bac2A)	"K12"[All Fields] AND biofilm[All Fields]	K12	25661192	pH-activated nanoparticles for controlled topical delivery of farnesol to disrupt oral biofilm virulence.
1117	DRAMP04026	K12 (single amino acid substitution of Bac034, which is a scrambled Variant of Bac2A)	"K12"[All Fields] AND biofilm[All Fields]	K12	25588885	Thromboresistant/anti-biofilm catheters via electrochemically modulated nitric oxide release.
1117	DRAMP04026	K12 (single amino acid substitution of Bac034, which is a scrambled Variant of Bac2A)	"K12"[All Fields] AND biofilm[All Fields]	K12	25473986	Development of an Escherichia coli K12-specific quantitative polymerase chain reaction assay and DNA isolation suited to biofilms associated with iron drinking water pipe corrosion products.
1117	DRAMP04026	K12 (single amino acid substitution of Bac034, which is a scrambled Variant of Bac2A)	"K12"[All Fields] AND biofilm[All Fields]	K12	25427545	Alumina surfaces with nanoscale topography reduce attachment and biofilm formation by Escherichia coli and Listeria spp.
1117	DRAMP04026	K12 (single amino acid substitution of Bac034, which is a scrambled Variant of Bac2A)	"K12"[All Fields] AND biofilm[All Fields]	K12	25375398	The Pseudomonas aeruginosa type III translocon is required for biofilm formation at the epithelial barrier.
1117	DRAMP04026	K12 (single amino acid substitution of Bac034, which is a scrambled Variant of Bac2A)	"K12"[All Fields] AND biofilm[All Fields]	K12	25375174	A functional portrait of Med7 and the mediator complex in Candida albicans.
1117	DRAMP04026	K12 (single amino acid substitution of Bac034, which is a scrambled Variant of Bac2A)	"K12"[All Fields] AND biofilm[All Fields]	K12	25288082	Quinacrine inhibits Candida albicans growth and filamentation at neutral pH.
1117	DRAMP04026	K12 (single amino acid substitution of Bac034, which is a scrambled Variant of Bac2A)	"K12"[All Fields] AND biofilm[All Fields]	K12	25222563	Deciphering Fur transcriptional regulatory network highlights its complex role beyond iron metabolism in Escherichia coli.

1117	DRAMP04026	K12 (single amino acid substitution of Bac034, which is a scrambled Variant of Bac2A)	"K12"[All Fields] AND biofilm[All Fields]	K12	25049253	In vitro analysis of finasteride activity against Candida albicans urinary biofilm formation and filamentation.
1117	DRAMP04026	K12 (single amino acid substitution of Bac034, which is a scrambled Variant of Bac2A)	"K12"[All Fields] AND biofilm[All Fields]	K12	25042085	Screening the mechanical stability of Escherichia coli biofilms through exposure to external, hydrodynamic shear forces.
1117	DRAMP04026	K12 (single amino acid substitution of Bac034, which is a scrambled Variant of Bac2A)	"K12"[All Fields] AND biofilm[All Fields]	K12	25012969	Adherence to abiotic surface induces SOS response in Escherichia coli K-12 strains under aerobic and anaerobic conditions.
1117	DRAMP04026	K12 (single amino acid substitution of Bac034, which is a scrambled Variant of Bac2A)	"K12"[All Fields] AND biofilm[All Fields]	K12	24837288	Physiological and proteomic analysis of Escherichia coli iron-limited chemostat growth.
1117	DRAMP04026	K12 (single amino acid substitution of Bac034, which is a scrambled Variant of Bac2A)	"K12"[All Fields] AND biofilm[All Fields]	K12	24837285	Control of gene expression at a bacterial leader RNA, the agn43 gene encoding outer membrane protein Ag43 of Escherichia coli.
1117	DRAMP04026	K12 (single amino acid substitution of Bac034, which is a scrambled Variant of Bac2A)	"K12"[All Fields] AND biofilm[All Fields]	K12	24746734	Probing of exopolysaccharides with green fluorescence protein-labeled carbohydrate-binding module in Escherichia coli biofilms and flocs induced by bcsB overexpression.
1117	DRAMP04026	K12 (single amino acid substitution of Bac034, which is a scrambled Variant of Bac2A)	"K12"[All Fields] AND biofilm[All Fields]	K12	24676679	The role of the QseC quorum-sensing sensor kinase in epinephrine-enhanced motility and biofilm formation by Escherichia coli.
1117	DRAMP04026	K12 (single amino acid substitution of Bac034, which is a scrambled Variant of Bac2A)	"K12"[All Fields] AND biofilm[All Fields]	K12	24628798	Polyphenols from olive mill waste affect biofilm formation and motility in Escherichia coli K-12.
1117	DRAMP04026	K12 (single amino acid substitution of Bac034, which is a scrambled Variant of Bac2A)	"K12"[All Fields] AND biofilm[All Fields]	K12	24478084	The core promoter of the capsule operon of Streptococcus pneumoniae is necessary for colonization and invasive disease.
1117	DRAMP04026	K12 (single amino acid substitution of Bac034, which is a scrambled Variant of Bac2A)	"K12"[All Fields] AND biofilm[All Fields]	K12	24457153	Ginkgolic acids and Ginkgo biloba extract inhibit Escherichia coli O157:H7 and Staphylococcus aureus biofilm formation.
1117	DRAMP04026	K12 (single amino acid substitution of Bac034, which is a scrambled Variant of Bac2A)	"K12"[All Fields] AND biofilm[All Fields]	K12	24388830	Bacteria and virus removal effectiveness of ceramic pot filters with different silver applications in a long term experiment.
1117	DRAMP04026	K12 (single amino acid substitution of Bac034, which is a scrambled Variant of Bac2A)	"K12"[All Fields] AND biofilm[All Fields]	K12	24379207	Activities of tobramycin and polymyxin E against Pseudomonas aeruginosa biofilm-coated medical grade endotracheal tubes.
1117	DRAMP04026	K12 (single amino acid substitution of Bac034, which is a scrambled Variant of Bac2A)	"K12"[All Fields] AND biofilm[All Fields]	K12	24295976	Image-based 384-well high-throughput screening method for the discovery of skyllamycins A to C as biofilm inhibitors and inducers of biofilm detachment in Pseudomonas aeruginosa.
1117	DRAMP04026	K12 (single amino acid substitution of Bac034, which is a scrambled Variant of Bac2A)	"K12"[All Fields] AND biofilm[All Fields]	K12	24239884	Biofilm vs. planktonic bacterial mode of growth: which do human macrophages prefer?
1117	DRAMP04026	K12 (single amino acid substitution of Bac034, which is a scrambled Variant of Bac2A)	"K12"[All Fields] AND biofilm[All Fields]	K12	24156913	In vitro analysis of flufenamic acid activity against Candida albicans biofilms.
1117	DRAMP04026	K12 (single amino acid substitution of Bac034, which is a scrambled Variant of Bac2A)	"K12"[All Fields] AND biofilm[All Fields]	K12	24097954	Cellulose as an architectural element in spatially structured Escherichia coli biofilms.
1117	DRAMP04026	K12 (single amino acid substitution of Bac034, which is a scrambled Variant of Bac2A)	"K12"[All Fields] AND biofilm[All Fields]	K12	24080296	Probing electron transfer with Escherichia coli: a method to examine exoelectronics in microbial fuel cell type systems.
1117	DRAMP04026	K12 (single amino acid substitution of Bac034, which is a scrambled Variant of Bac2A)	"K12"[All Fields] AND biofilm[All Fields]	K12	24023384	Escherichia coli biofilms have an organized and complex extracellular matrix structure.
1117	DRAMP04026	K12 (single amino acid substitution of Bac034, which is a scrambled Variant of Bac2A)	"K12"[All Fields] AND biofilm[All Fields]	K12	23824318	The influence of CsgD on the expression of genes of folate metabolism and hmp in Escherichia coli K-12.
1117	DRAMP04026	K12 (single amino acid substitution of Bac034, which is a scrambled Variant of Bac2A)	"K12"[All Fields] AND biofilm[All Fields]	K12	23789960	A novel approach to determine the efficacy of patterned surfaces for biofouling control in relation to its microfluidic environment.
1117	DRAMP04026	K12 (single amino acid substitution of Bac034, which is a scrambled Variant of Bac2A)	"K12"[All Fields] AND biofilm[All Fields]	K12	23752392	Environmental implications and applications of carbon nanomaterials in water treatment.
1117	DRAMP04026	K12 (single amino acid substitution of Bac034, which is a scrambled Variant of Bac2A)	"K12"[All Fields] AND biofilm[All Fields]	K12	23708798	The EAL domain protein YciR acts as a trigger enzyme in a c-di-GMP signalling cascade in E. coli biofilm control.
1117	DRAMP04026	K12 (single amino acid substitution of Bac034, which is a scrambled Variant of Bac2A)	"K12"[All Fields] AND biofilm[All Fields]	K12	23664265	Transcriptional and functional responses of Escherichia coli O157:H7 growing in the lettuce rhizoplane.
1117	DRAMP04026	K12 (single amino acid substitution of Bac034, which is a scrambled Variant of Bac2A)	"K12"[All Fields] AND biofilm[All Fields]	K12	23611911	Reply to "precedence for the structural role of flagella in biofilms".
1117	DRAMP04026	K12 (single amino acid substitution of Bac034, which is a scrambled Variant of Bac2A)	"K12"[All Fields] AND biofilm[All Fields]	K12	23611910	Precedence for the structural role of flagella in biofilms.
1117	DRAMP04026	K12 (single amino acid substitution of Bac034, which is a scrambled Variant of Bac2A)	"K12"[All Fields] AND biofilm[All Fields]	K12	23512962	Microanatomy at cellular resolution and spatial order of physiological differentiation in a bacterial biofilm.
1117	DRAMP04026	K12 (single amino acid substitution of Bac034, which is a scrambled Variant of Bac2A)	"K12"[All Fields] AND biofilm[All Fields]	K12	23316054	Deletion of vacuolar proton-translocating ATPase V(o)a isoforms clarifies the role of vacuolar pH as a determinant of virulence-associated traits in Candida albicans.
1117	DRAMP04026	K12 (single amino acid substitution of Bac034, which is a scrambled Variant of Bac2A)	"K12"[All Fields] AND biofilm[All Fields]	K12	23305896	Effect of N-acy-l-homoserine lactones-like molecules from aerobic granules on biofilm formation by Escherichia coli K12.
1117	DRAMP04026	K12 (single amino acid substitution of Bac034, which is a scrambled Variant of Bac2A)	"K12"[All Fields] AND biofilm[All Fields]	K12	23297626	[Effect of Pseudomonas aeruginosa exometabolites on planktonic and biofilm cultures of Escherichia coli].

1117	DRAMP04026	K12 (single amino acid substitution of Bac034, which is a scrambled Variant of Bac2A)	"K12"[All Fields] AND biofilm[All Fields]	K12	23197162	Generalized principles of stochasticity can be used to control dynamic heterogeneity.
1117	DRAMP04026	K12 (single amino acid substitution of Bac034, which is a scrambled Variant of Bac2A)	"K12"[All Fields] AND biofilm[All Fields]	K12	23183588	Engineering adherent bacteria by creating a single synthetic curli operon.
1117	DRAMP04026	K12 (single amino acid substitution of Bac034, which is a scrambled Variant of Bac2A)	"K12"[All Fields] AND biofilm[All Fields]	K12	23078901	The c-di-GMP phosphodiesterase VmpA absent in Escherichia coli K12 strains affects motility and biofilm formation in the enterohemorrhagic O157:H7 serotype.
1117	DRAMP04026	K12 (single amino acid substitution of Bac034, which is a scrambled Variant of Bac2A)	"K12"[All Fields] AND biofilm[All Fields]	K12	23052888	Laser desorption VUV postionization MS imaging of a cocultured biofilm.
1117	DRAMP04026	K12 (single amino acid substitution of Bac034, which is a scrambled Variant of Bac2A)	"K12"[All Fields] AND biofilm[All Fields]	K12	22915155	Inhibition of mushroom formation and induction of glycerol release-ecological strategies of Burkholderia terrae BS001 to create a hospitable niche at the fungus Lyophyllum sp. strain Karsten.
1117	DRAMP04026	K12 (single amino acid substitution of Bac034, which is a scrambled Variant of Bac2A)	"K12"[All Fields] AND biofilm[All Fields]	K12	22884245	Characterization of antibacterial polyethersulfone membranes using the Respiration Activity Monitoring System (RAMOS).
1117	DRAMP04026	K12 (single amino acid substitution of Bac034, which is a scrambled Variant of Bac2A)	"K12"[All Fields] AND biofilm[All Fields]	K12	22830299	Gene expression profiling of Escherichia coli in response to interactions with the lettuce rhizosphere.
1117	DRAMP04026	K12 (single amino acid substitution of Bac034, which is a scrambled Variant of Bac2A)	"K12"[All Fields] AND biofilm[All Fields]	K12	22825451	The WalKR system controls major staphylococcal virulence genes and is involved in triggering the host inflammatory response.
1117	DRAMP04026	K12 (single amino acid substitution of Bac034, which is a scrambled Variant of Bac2A)	"K12"[All Fields] AND biofilm[All Fields]	K12	22500751	Direct upstream motility in Escherichia coli.
1117	DRAMP04026	K12 (single amino acid substitution of Bac034, which is a scrambled Variant of Bac2A)	"K12"[All Fields] AND biofilm[All Fields]	K12	22419503	Released products of pathogenic bacteria stimulate biofilm formation by Escherichia coli K-12 strains.
1117	DRAMP04026	K12 (single amino acid substitution of Bac034, which is a scrambled Variant of Bac2A)	"K12"[All Fields] AND biofilm[All Fields]	K12	22351670	Inhibition of quorum sensing and efflux pump system by trifluoromethyl ketone proton pump inhibitors.
1117	DRAMP04026	K12 (single amino acid substitution of Bac034, which is a scrambled Variant of Bac2A)	"K12"[All Fields] AND biofilm[All Fields]	K12	22247152	Transcriptional responses of Escherichia coli K-12 and O157:H7 associated with lettuce leaves.
1117	DRAMP04026	K12 (single amino acid substitution of Bac034, which is a scrambled Variant of Bac2A)	"K12"[All Fields] AND biofilm[All Fields]	K12	22086070	Conditional confined oscillatory dynamics of Escherichia coli strain K12-MG1655 in chemostat systems.
1117	DRAMP04026	K12 (single amino acid substitution of Bac034, which is a scrambled Variant of Bac2A)	"K12"[All Fields] AND biofilm[All Fields]	K12	22058083	Diffusion of bacteriophages through artificial biofilm models.
1117	DRAMP04026	K12 (single amino acid substitution of Bac034, which is a scrambled Variant of Bac2A)	"K12"[All Fields] AND biofilm[All Fields]	K12	22047137	Low concentrations of honey reduce biofilm formation, quorum sensing, and virulence in Escherichia coli O157:H7.
1117	DRAMP04026	K12 (single amino acid substitution of Bac034, which is a scrambled Variant of Bac2A)	"K12"[All Fields] AND biofilm[All Fields]	K12	21939518	A network model for biofilm development in Escherichia coli K-12.
1117	DRAMP04026	K12 (single amino acid substitution of Bac034, which is a scrambled Variant of Bac2A)	"K12"[All Fields] AND biofilm[All Fields]	K12	21930758	Molecular characterization of UpaB and UpaC, two new autotransporter proteins of uropathogenic Escherichia coli CFT073.
1117	DRAMP04026	K12 (single amino acid substitution of Bac034, which is a scrambled Variant of Bac2A)	"K12"[All Fields] AND biofilm[All Fields]	K12	21927020	Regulation of growth and death in Escherichia coli by toxin-antitoxin systems.
1117	DRAMP04026	K12 (single amino acid substitution of Bac034, which is a scrambled Variant of Bac2A)	"K12"[All Fields] AND biofilm[All Fields]	K12	21859856	SadA, a trimeric autotransporter from Salmonella enterica serovar Typhimurium, can promote biofilm formation and provides limited protection against infection.
1117	DRAMP04026	K12 (single amino acid substitution of Bac034, which is a scrambled Variant of Bac2A)	"K12"[All Fields] AND biofilm[All Fields]	K12	21857375	Farnesol decreases biofilms of Staphylococcus epidermidis and exhibits synergy with nafcillin and vancomycin.
1117	DRAMP04026	K12 (single amino acid substitution of Bac034, which is a scrambled Variant of Bac2A)	"K12"[All Fields] AND biofilm[All Fields]	K12	21719992	Roles of multidrug efflux pumps on the biofilm formation of Escherichia coli K-12.
1117	DRAMP04026	K12 (single amino acid substitution of Bac034, which is a scrambled Variant of Bac2A)	"K12"[All Fields] AND biofilm[All Fields]	K12	21651627	Biogenic ammonia modifies antibiotic resistance at a distance in physically separated bacteria.
1117	DRAMP04026	K12 (single amino acid substitution of Bac034, which is a scrambled Variant of Bac2A)	"K12"[All Fields] AND biofilm[All Fields]	K12	21415116	Role of DLP12 lysis genes in Escherichia coli biofilm formation.
1117	DRAMP04026	K12 (single amino acid substitution of Bac034, which is a scrambled Variant of Bac2A)	"K12"[All Fields] AND biofilm[All Fields]	K12	21255333	Controlling biofilm formation, prophage excision and cell death by rewiring global regulator H-NS of Escherichia coli.
1117	DRAMP04026	K12 (single amino acid substitution of Bac034, which is a scrambled Variant of Bac2A)	"K12"[All Fields] AND biofilm[All Fields]	K12	21112357	Electrochemical monitoring of chlorhexidine digluconate effect on polyelectrolyte immobilized bacteria and kinetic cell adhesion.
1117	DRAMP04026	K12 (single amino acid substitution of Bac034, which is a scrambled Variant of Bac2A)	"K12"[All Fields] AND biofilm[All Fields]	K12	21059164	Engineering a novel c-di-GMP-binding protein for biofilm dispersal.
1117	DRAMP04026	K12 (single amino acid substitution of Bac034, which is a scrambled Variant of Bac2A)	"K12"[All Fields] AND biofilm[All Fields]	K12	20559621	Environmental and genetic factors that contribute to Escherichia coli K-12 biofilm formation.
1117	DRAMP04026	K12 (single amino acid substitution of Bac034, which is a scrambled Variant of Bac2A)	"K12"[All Fields] AND biofilm[All Fields]	K12	20480211	Contribution of rpoS and bolA genes in biofilm formation in Escherichia coli K-12 MG1655.
1117	DRAMP04026	K12 (single amino acid substitution of Bac034, which is a scrambled Variant of Bac2A)	"K12"[All Fields] AND biofilm[All Fields]	K12	20465305	Toxic effects of single-walled carbon nanotubes in the development of E. coli biofilm.

1117	DRAMP04026	K12 (single amino acid substitution of Bac034, which is a scrambled Variant of Bac2A)	"K12"[All Fields] AND biofilm[All Fields]	K12	20345943	Escherichia coli K-12 possesses multiple cryptic but functional chaperone-usher fimbriae with distinct surface specificities.
1117	DRAMP04026	K12 (single amino acid substitution of Bac034, which is a scrambled Variant of Bac2A)	"K12"[All Fields] AND biofilm[All Fields]	K12	20093376	Swarming motility, secretion of type 3 effectors and biofilm formation phenotypes exhibited within a large cohort of Pseudomonas aeruginosa clinical isolates.
1117	DRAMP04026	K12 (single amino acid substitution of Bac034, which is a scrambled Variant of Bac2A)	"K12"[All Fields] AND biofilm[All Fields]	K12	20085257	Effective photocatalytic disinfection of E. coli K-12 using AgBr-Ag-Bi2WO6 nanojunction system irradiated by visible light: the role of diffusing hydroxyl radicals.
1117	DRAMP04026	K12 (single amino acid substitution of Bac034, which is a scrambled Variant of Bac2A)	"K12"[All Fields] AND biofilm[All Fields]	K12	20017360	[Antibacterial effects of silver ions: effect on gram-negative bacteria growth and biofilm formation].
1117	DRAMP04026	K12 (single amino acid substitution of Bac034, which is a scrambled Variant of Bac2A)	"K12"[All Fields] AND biofilm[All Fields]	K12	19915538	Small-molecule inhibitors target Escherichia coli amyloid biogenesis and biofilm formation.
1117	DRAMP04026	K12 (single amino acid substitution of Bac034, which is a scrambled Variant of Bac2A)	"K12"[All Fields] AND biofilm[All Fields]	K12	19759044	Exposure of Escherichia coli and Salmonella enterica serovar Typhimurium to triclosan induces a species-specific response, including drug detoxification.
1117	DRAMP04026	K12 (single amino acid substitution of Bac034, which is a scrambled Variant of Bac2A)	"K12"[All Fields] AND biofilm[All Fields]	K12	19656291	NsrR targets in the Escherichia coli genome: new insights into DNA sequence requirements for binding and a role for NsrR in the regulation of motility.
1117	DRAMP04026	K12 (single amino acid substitution of Bac034, which is a scrambled Variant of Bac2A)	"K12"[All Fields] AND biofilm[All Fields]	K12	19552773	A combination of assays reveals biomass differences in biofilms formed by Escherichia coli mutants.
1117	DRAMP04026	K12 (single amino acid substitution of Bac034, which is a scrambled Variant of Bac2A)	"K12"[All Fields] AND biofilm[All Fields]	K12	19508554	The Escherichia coli O157:H7 EhaB autotransporter protein binds to laminin and collagen I and induces a serum IgA response in O157:H7 challenged cattle.
1117	DRAMP04026	K12 (single amino acid substitution of Bac034, which is a scrambled Variant of Bac2A)	"K12"[All Fields] AND biofilm[All Fields]	K12	19447340	Biofilm formation by Escherichia coli in hypertonic sucrose media.
1117	DRAMP04026	K12 (single amino acid substitution of Bac034, which is a scrambled Variant of Bac2A)	"K12"[All Fields] AND biofilm[All Fields]	K12	19364863	Farnesol-induced apoptosis in Candida albicans.
1117	DRAMP04026	K12 (single amino acid substitution of Bac034, which is a scrambled Variant of Bac2A)	"K12"[All Fields] AND biofilm[All Fields]	K12	19332833	Gene expression patterns and differential input into curli fimbriae regulation of all GGDEF/EAL domain proteins in Escherichia coli.
1117	DRAMP04026	K12 (single amino acid substitution of Bac034, which is a scrambled Variant of Bac2A)	"K12"[All Fields] AND biofilm[All Fields]	K12	19306144	Role of type 1 fimbriae and mannose in the development of Escherichia coli K12 biofilm: from initial cell adhesion to biofilm formation.
1117	DRAMP04026	K12 (single amino acid substitution of Bac034, which is a scrambled Variant of Bac2A)	"K12"[All Fields] AND biofilm[All Fields]	K12	19260968	Comparative functional analysis of the RcsC sensor kinase from different Enterobacteriaceae.
1117	DRAMP04026	K12 (single amino acid substitution of Bac034, which is a scrambled Variant of Bac2A)	"K12"[All Fields] AND biofilm[All Fields]	K12	19239496	Adaptive evolution in single species bacterial biofilms.
1117	DRAMP04026	K12 (single amino acid substitution of Bac034, which is a scrambled Variant of Bac2A)	"K12"[All Fields] AND biofilm[All Fields]	K12	19177607	[Regulation of biofilm formation in Escherichia coli K12: effect of mutations in HNS, StpA, lon, and rpoN genes].
1117	DRAMP04026	K12 (single amino acid substitution of Bac034, which is a scrambled Variant of Bac2A)	"K12"[All Fields] AND biofilm[All Fields]	K12	19172264	5-Fluorouracil reduces biofilm formation in Escherichia coli K-12 through global regulator AriR as an antivirulence compound.
1117	DRAMP04026	K12 (single amino acid substitution of Bac034, which is a scrambled Variant of Bac2A)	"K12"[All Fields] AND biofilm[All Fields]	K12	19168650	Nickel promotes biofilm formation by Escherichia coli K-12 strains that produce curli.
1117	DRAMP04026	K12 (single amino acid substitution of Bac034, which is a scrambled Variant of Bac2A)	"K12"[All Fields] AND biofilm[All Fields]	K12	19046755	Polysulfone ultrafiltration membranes impregnated with silver nanoparticles show improved biofouling resistance and virus removal.
1117	DRAMP04026	K12 (single amino acid substitution of Bac034, which is a scrambled Variant of Bac2A)	"K12"[All Fields] AND biofilm[All Fields]	K12	18939750	Rechargeable antimicrobial surface modification of polyethylene.
1117	DRAMP04026	K12 (single amino acid substitution of Bac034, which is a scrambled Variant of Bac2A)	"K12"[All Fields] AND biofilm[All Fields]	K12	18453280	Escherichia coli biofilms.
1117	DRAMP04026	K12 (single amino acid substitution of Bac034, which is a scrambled Variant of Bac2A)	"K12"[All Fields] AND biofilm[All Fields]	K12	18310435	Polysaccharides cellulose, poly-beta-1,6-n-acetyl-D-glucosamine, and colanic acid are required for optimal binding of Escherichia coli O157:H7 strains to alfalfa sprouts and K-12 strains to plastic but not for binding to epithelial cells.
1117	DRAMP04026	K12 (single amino acid substitution of Bac034, which is a scrambled Variant of Bac2A)	"K12"[All Fields] AND biofilm[All Fields]	K12	18309357	Escherichia coli transcription factor YncC (McbR) regulates colanic acid and biofilm formation by repressing expression of periplasmic protein YbiM (McbA).
1117	DRAMP04026	K12 (single amino acid substitution of Bac034, which is a scrambled Variant of Bac2A)	"K12"[All Fields] AND biofilm[All Fields]	K12	18302792	Rapid acid treatment of Escherichia coli: transcriptomic response and recovery.
1117	DRAMP04026	K12 (single amino acid substitution of Bac034, which is a scrambled Variant of Bac2A)	"K12"[All Fields] AND biofilm[All Fields]	K12	18236673	Identification of ground beef-derived fatty acid inhibitors of autoinducer-2-based cell signaling.
1117	DRAMP04026	K12 (single amino acid substitution of Bac034, which is a scrambled Variant of Bac2A)	"K12"[All Fields] AND biofilm[All Fields]	K12	18179393	Heritability of oral microbial species in caries-active and caries-free twins.
1117	DRAMP04026	K12 (single amino acid substitution of Bac034, which is a scrambled Variant of Bac2A)	"K12"[All Fields] AND biofilm[All Fields]	K12	18174134	Low temperature (23 degrees C) increases expression of biofilm-, cold-shock- and RpoS-dependent genes in Escherichia coli K-12.
1117	DRAMP04026	K12 (single amino acid substitution of Bac034, which is a scrambled Variant of Bac2A)	"K12"[All Fields] AND biofilm[All Fields]	K12	17981982	The amino acid valine is secreted in continuous-flow bacterial biofilms.
1117	DRAMP04026	K12 (single amino acid substitution of Bac034, which is a scrambled Variant of Bac2A)	"K12"[All Fields] AND biofilm[All Fields]	K12	17665190	Self-excreted mediator from Escherichia coli K-12 for electron transfer to carbon electrodes.

1117	DRAMP04026	K12 (single amino acid substitution of Bac034, which is a scrambled Variant of Bac2A)	"K12"[All Fields] AND biofilm[All Fields]	K12	17664315	Role of the rapA gene in controlling antibiotic resistance of Escherichia coli biofilms.
1117	DRAMP04026	K12 (single amino acid substitution of Bac034, which is a scrambled Variant of Bac2A)	"K12"[All Fields] AND biofilm[All Fields]	K12	17660444	Modelling the spatial dynamics of plasmid transfer and persistence.
1117	DRAMP04026	K12 (single amino acid substitution of Bac034, which is a scrambled Variant of Bac2A)	"K12"[All Fields] AND biofilm[All Fields]	K12	17635554	The multicopper oxidase (CueO) and cell aggregation in Escherichia coli.
1117	DRAMP04026	K12 (single amino acid substitution of Bac034, which is a scrambled Variant of Bac2A)	"K12"[All Fields] AND biofilm[All Fields]	K12	17530054	Microbial acid production (Clinpro Cario L-Pop) and dental caries in infants and children.
1117	DRAMP04026	K12 (single amino acid substitution of Bac034, which is a scrambled Variant of Bac2A)	"K12"[All Fields] AND biofilm[All Fields]	K12	17483266	Enterohemorrhagic Escherichia coli biofilms are inhibited by 7-hydroxyindole and stimulated by isatin.
1117	DRAMP04026	K12 (single amino acid substitution of Bac034, which is a scrambled Variant of Bac2A)	"K12"[All Fields] AND biofilm[All Fields]	K12	17420234	Functional analysis of antigen 43 in uropathogenic Escherichia coli reveals a role in long-term persistence in the urinary tract.
1117	DRAMP04026	K12 (single amino acid substitution of Bac034, which is a scrambled Variant of Bac2A)	"K12"[All Fields] AND biofilm[All Fields]	K12	17416647	Flagellar motility is critical for Listeria monocytogenes biofilm formation.
1117	DRAMP04026	K12 (single amino acid substitution of Bac034, which is a scrambled Variant of Bac2A)	"K12"[All Fields] AND biofilm[All Fields]	K12	17353117	Quantitative and morphological analysis of biofilm formation on self-assembled monolayers.
1117	DRAMP04026	K12 (single amino acid substitution of Bac034, which is a scrambled Variant of Bac2A)	"K12"[All Fields] AND biofilm[All Fields]	K12	17293424	YcfR (BhsA) influences Escherichia coli biofilm formation through stress response and surface hydrophobicity.
1117	DRAMP04026	K12 (single amino acid substitution of Bac034, which is a scrambled Variant of Bac2A)	"K12"[All Fields] AND biofilm[All Fields]	K12	17227468	Transcription in Escherichia coli PHL628 biofilms.
1117	DRAMP04026	K12 (single amino acid substitution of Bac034, which is a scrambled Variant of Bac2A)	"K12"[All Fields] AND biofilm[All Fields]	K12	17222132	Temporal gene-expression in Escherichia coli K-12 biofilms.
1117	DRAMP04026	K12 (single amino acid substitution of Bac034, which is a scrambled Variant of Bac2A)	"K12"[All Fields] AND biofilm[All Fields]	K12	17172451	Filamentation by Escherichia coli subverts innate defenses during urinary tract infection.
1117	DRAMP04026	K12 (single amino acid substitution of Bac034, which is a scrambled Variant of Bac2A)	"K12"[All Fields] AND biofilm[All Fields]	K12	17098869	Rationally designed small compounds inhibit pilus biogenesis in uropathogenic bacteria.
1117	DRAMP04026	K12 (single amino acid substitution of Bac034, which is a scrambled Variant of Bac2A)	"K12"[All Fields] AND biofilm[All Fields]	K12	17014498	Biofilm formation and cellulose expression among diverse environmental Pseudomonas isolates.
1117	DRAMP04026	K12 (single amino acid substitution of Bac034, which is a scrambled Variant of Bac2A)	"K12"[All Fields] AND biofilm[All Fields]	K12	16672612	Synergistic effects in mixed Escherichia coli biofilms: conjugative plasmid transfer drives biofilm expansion.
1117	DRAMP04026	K12 (single amino acid substitution of Bac034, which is a scrambled Variant of Bac2A)	"K12"[All Fields] AND biofilm[All Fields]	K12	16672475	Development of a biofilm production-deficient Escherichia coli strain as a host for biotechnological applications.
1117	DRAMP04026	K12 (single amino acid substitution of Bac034, which is a scrambled Variant of Bac2A)	"K12"[All Fields] AND biofilm[All Fields]	K12	16597943	YliH (BssR) and YceP (BssS) regulate Escherichia coli K-12 biofilm formation by influencing cell signaling.
1117	DRAMP04026	K12 (single amino acid substitution of Bac034, which is a scrambled Variant of Bac2A)	"K12"[All Fields] AND biofilm[All Fields]	K12	16555073	Indole-3-acetic acid improves Escherichia coli's defences to stress.
1117	DRAMP04026	K12 (single amino acid substitution of Bac034, which is a scrambled Variant of Bac2A)	"K12"[All Fields] AND biofilm[All Fields]	K12	16552039	An IncI1 plasmid contributes to the adherence of the atypical enteroaggregative Escherichia coli strain C1096 to cultured cells and abiotic surfaces.
1117	DRAMP04026	K12 (single amino acid substitution of Bac034, which is a scrambled Variant of Bac2A)	"K12"[All Fields] AND biofilm[All Fields]	K12	16397770	Motility influences biofilm architecture in Escherichia coli.
1117	DRAMP04026	K12 (single amino acid substitution of Bac034, which is a scrambled Variant of Bac2A)	"K12"[All Fields] AND biofilm[All Fields]	K12	16385049	YdgG (TqsA) controls biofilm formation in Escherichia coli K-12 through autoinducer 2 transport.
1117	DRAMP04026	K12 (single amino acid substitution of Bac034, which is a scrambled Variant of Bac2A)	"K12"[All Fields] AND biofilm[All Fields]	K12	16321928	Spatial periodicity of Escherichia coli K-12 biofilm microstructure initiates during a reversible, polar attachment phase of development and requires the polysaccharide adhesin PGA.
1117	DRAMP04026	K12 (single amino acid substitution of Bac034, which is a scrambled Variant of Bac2A)	"K12"[All Fields] AND biofilm[All Fields]	K12	16317765	Hha, YbaJ, and OmpA regulate Escherichia coli K12 biofilm formation and conjugation plasmids abolish motility.
1117	DRAMP04026	K12 (single amino acid substitution of Bac034, which is a scrambled Variant of Bac2A)	"K12"[All Fields] AND biofilm[All Fields]	K12	16310051	Secretory IgA and mucin-mediated biofilm formation by environmental strains of Escherichia coli: role of type 1 pili.
1117	DRAMP04026	K12 (single amino acid substitution of Bac034, which is a scrambled Variant of Bac2A)	"K12"[All Fields] AND biofilm[All Fields]	K12	16272513	Microbial risk indicators of early childhood caries.
1117	DRAMP04026	K12 (single amino acid substitution of Bac034, which is a scrambled Variant of Bac2A)	"K12"[All Fields] AND biofilm[All Fields]	K12	16233759	Estimation of the biofilm formation of Escherichia coli K-12 by the cell number.
1117	DRAMP04026	K12 (single amino acid substitution of Bac034, which is a scrambled Variant of Bac2A)	"K12"[All Fields] AND biofilm[All Fields]	K12	16000817	Differential gene expression for investigation of Escherichia coli biofilm inhibition by plant extract ursolic acid.
1117	DRAMP04026	K12 (single amino acid substitution of Bac034, which is a scrambled Variant of Bac2A)	"K12"[All Fields] AND biofilm[All Fields]	K12	15870475	Deletion of the yiaMNO transporter genes affects the growth characteristics of Escherichia coli K-12.
1117	DRAMP04026	K12 (single amino acid substitution of Bac034, which is a scrambled Variant of Bac2A)	"K12"[All Fields] AND biofilm[All Fields]	K12	15814430	Bacterial small RNA regulators.

1117	DRAMP04026	K12 (single amino acid substitution of Bac034, which is a scrambled Variant of Bac2A)	"K12"[All Fields] AND biofilm[All Fields]	K12	15680764	Can laboratory reference strains mirror "real-world" pathogenesis?
1117	DRAMP04026	K12 (single amino acid substitution of Bac034, which is a scrambled Variant of Bac2A)	"K12"[All Fields] AND biofilm[All Fields]	K12	15644921	Role of MetR and PurR in the activation of glyA by CsgD in Escherichia coli K-12.
1117	DRAMP04026	K12 (single amino acid substitution of Bac034, which is a scrambled Variant of Bac2A)	"K12"[All Fields] AND biofilm[All Fields]	K12	15470704	Differential gene expression shows natural brominated furanones interfere with the autoinducer-2 bacterial signaling system of Escherichia coli.
1117	DRAMP04026	K12 (single amino acid substitution of Bac034, which is a scrambled Variant of Bac2A)	"K12"[All Fields] AND biofilm[All Fields]	K12	14727089	Gene expression in Escherichia coli biofilms.
1117	DRAMP04026	K12 (single amino acid substitution of Bac034, which is a scrambled Variant of Bac2A)	"K12"[All Fields] AND biofilm[All Fields]	K12	12392960	Microbial biosensor array with transport mutants of Escherichia coli K12 for the simultaneous determination of mono-and disaccharides.
1117	DRAMP04026	K12 (single amino acid substitution of Bac034, which is a scrambled Variant of Bac2A)	"K12"[All Fields] AND biofilm[All Fields]	K12	10837431	An evaluation of the potential of the multiple antibiotic resistance operon (mar) and the multidrug efflux pump acrAB to moderate resistance towards ciprofloxacin in Escherichia coli biofilms.
1117	DRAMP04026	K12 (single amino acid substitution of Bac034, which is a scrambled Variant of Bac2A)	"K12"[All Fields] AND biofilm[All Fields]	K12	10483736	Involvement of the Cpx signal transduction pathway of E. coli in biofilm formation.
1117	DRAMP04026	K12 (single amino acid substitution of Bac034, which is a scrambled Variant of Bac2A)	"K12"[All Fields] AND biofilm[All Fields]	K12	9226873	Recombinant plasmid mobilization between E. coli strains in seven sterile microcosms.
1117	DRAMP04026	K12 (single amino acid substitution of Bac034, which is a scrambled Variant of Bac2A)	"K12"[All Fields] AND biofilm[All Fields]	K12	8605069	Quantification of the ease of removal of bacteria from surfaces.
1117	DRAMP04026	K12 (single amino acid substitution of Bac034, which is a scrambled Variant of Bac2A)	"K12"[All Fields] AND biofilm[All Fields]	K12	24194332	Stopped-flow chamber and image analysis system for quantitative characterization of bacterial population migration: Motility and chemotaxis ofEscherichia coli K12 to fucose.
1117	DRAMP04026	K12 (single amino acid substitution of Bac034, which is a scrambled Variant of Bac2A)	"K12"[All Fields] AND biofilm[All Fields]	K12	18600657	Measurement of bacterial random motility and chemotaxis coefficients: II. Application of single-cell-based mathematical model.
1118	DRAMP04027	opt1 (multiple amino acid substitution of Bac034, which is a scrambled Variant of Bac2A)	"opt1"[All Fields] AND biofilm[All Fields]	opt1	31689409	Optimising wheat straw alkali-organosolv pre-treatment to enhance hemicellulose modification and compatibility with reinforcing fillers.
1119	DRAMP04028	opt2 (multiple amino acid substitution of Bac034, which is a scrambled Variant of Bac2A)	"opt2"[All Fields] AND biofilm[All Fields]	opt2	31689409	Optimising wheat straw alkali-organosolv pre-treatment to enhance hemicellulose modification and compatibility with reinforcing fillers.
1169	DRAMP04102	CP-P	"CP-P"[All Fields] AND biofilm[All Fields]	CP-P	23327648	Suppuration-associated bacteria in patients with chronic and aggressive periodontitis.
1169	DRAMP04102	CP-P	"CP-P"[All Fields] AND biofilm[All Fields]	CP-P	21397893	Association of red complex, A. actinomycetemcomitans and non-oral bacteria with periodontal diseases.
1169	DRAMP04102	CP-P	"CP-P"[All Fields] AND biofilm[All Fields]	CP-P	21035110	Investigation of the interleukin-1 gene cluster polymorphisms in Jordanian patients with chronic and aggressive periodontitis.
1170	DRAMP04103	S16 (derivative of CP-P)	"S16"[All Fields] AND biofilm[All Fields]	S16	28379105	The ability of a colloidal silver gel wound dressing to kill bacteria in vitro and in vivo.
1170	DRAMP04103	S16 (derivative of CP-P)	"S16"[All Fields] AND biofilm[All Fields]	S16	28252341	The benefits of using polyhexamethylene biguanide in wound care.
1170	DRAMP04103	S16 (derivative of CP-P)	"S16"[All Fields] AND biofilm[All Fields]	S16	26878370	The presence of biofilm structures in atherosclerotic plaques of arteries from legs amputated as a complication of diabetic foot ulcers.
1170	DRAMP04103	S16 (derivative of CP-P)	"S16"[All Fields] AND biofilm[All Fields]	S16	21995649	Genetic analysis and morphological identification of pilus-like structures in members of the genus Bifidobacterium.
1170	DRAMP04103	S16 (derivative of CP-P)	"S16"[All Fields] AND biofilm[All Fields]	S16	20621239	In-vitro evidence for efficacy of antimicrobial mouthrinses.
1177	DRAMP04112	L10 (derivative of CP-P)	"L10"[All Fields] AND biofilm[All Fields]	L10	34586634	Anti-biofilm potential of kefir-derived Lactobacillus paracasei L10 against Vibrio parahaemolyticus.
1177	DRAMP04112	L10 (derivative of CP-P)	"L10"[All Fields] AND biofilm[All Fields]	L10	33140160	Disruption of Enterococcus Faecalis biofilms using individual and plasma polymer encapsulated D-amino acids.
1177	DRAMP04112	L10 (derivative of CP-P)	"L10"[All Fields] AND biofilm[All Fields]	L10	24291759	In vitro evaluation of the safety and probiotic properties of Lactobacilli isolated from chicken and calves.
1177	DRAMP04112	L10 (derivative of CP-P)	"L10"[All Fields] AND biofilm[All Fields]	L10	17536671	Proteomic analysis of a hypochlorous acid-tolerant Listeria monocytogenes cultural variant exhibiting enhanced biofilm production.
1178	DRAMP04113	A10 (derivative of CP-P)	"A10"[All Fields] AND biofilm[All Fields]	A10	34674874	Comparative analysis of different methods used for molecular characterization of Burkholderia cepacia complex isolated from noncystic fibrosis conditions.
1178	DRAMP04113	A10 (derivative of CP-P)	"A10"[All Fields] AND biofilm[All Fields]	A10	33656062	In-vitro Antibacterial and Antibiofilm Activity of Cinnamomum verum Leaf Oil against Pseudomonas aeruginosa, Staphylococcus aureus and Klebsiella pneumoniae.
1178	DRAMP04113	A10 (derivative of CP-P)	"A10"[All Fields] AND biofilm[All Fields]	A10	30620866	Enhanced In Vitro Zinc Bioavailability through Rational Design of a Dual Zinc plus Arginine Dentifrice.
1178	DRAMP04113	A10 (derivative of CP-P)	"A10"[All Fields] AND biofilm[All Fields]	A10	23776209	Identification of a small molecule with activity against drug-resistant and persistent tuberculosis.
1179	DRAMP04114	D11 (derivative of CP-P)	"D11"[All Fields] AND biofilm[All Fields]	D11	29868500	Anti-quorum Sensing Activities of Selected Coral Symbiotic Bacterial Extracts From the South China Sea.
1180	DRAMP04115	K11 (derivative of CP-P)	"K11"[All Fields] AND biofilm[All Fields]	K11	30136030	Potent effects of amino acid scanned antimicrobial peptide Feleucin-K3 analogs against both multidrug-resistant strains and biofilms of Pseudomonas aeruginosa.
1181	DRAMP04117	A13 (derivative of CP-P)	"A13"[All Fields] AND biofilm[All Fields]	A13	24759539	Membrane biofouling mechanism in an aerobic granular reactor degrading 4-chlorophenol.
1181	DRAMP04117	A13 (derivative of CP-P)	"A13"[All Fields] AND biofilm[All Fields]	A13	21087771	Exploring the fatty acids of vernix caseosa in form of their methyl esters by off-line coupling of non-aqueous reversed phase high performance liquid chromatography and gas chromatography coupled to mass spectrometry.
1182	DRAMP04119	K17 (derivative of CP-P)	"K17"[All Fields] AND biofilm[All Fields]	K17	32579104	Polaribacter septentrionalilitorissp. nov., isolated from the biofilm of a stone from the North Sea.
1182	DRAMP04119	K17 (derivative of CP-P)	"K17"[All Fields] AND biofilm[All Fields]	K17	31805672	Aggregatibacter actinomycetemcomitansBiofilm Reduces Gingival Epithelial Cell Keratin Expression in an Organotypic Gingival Tissue Culture Model.
1183	DRAMP04120	D18 (derivative of CP-P)	"D18"[All Fields] AND biofilm[All Fields]	D18	28678838	The oral bacterial microbiome of occlusal surfaces in children and its association with diet and caries.
1204	DRAMP04147	L10	"L10"[All Fields] AND biofilm[All Fields]	L10	34586634	Anti-biofilm potential of kefir-derived Lactobacillus paracasei L10 against Vibrio parahaemolyticus.
1204	DRAMP04147	L10	"L10"[All Fields] AND biofilm[All Fields]	L10	33140160	Disruption of Enterococcus Faecalis biofilms using individual and plasma polymer encapsulated D-amino acids.
1204	DRAMP04147	L10	"L10"[All Fields] AND biofilm[All Fields]	L10	24291759	In vitro evaluation of the safety and probiotic properties of Lactobacilli isolated from chicken and calves.
1204	DRAMP04147	L10	"L10"[All Fields] AND biofilm[All Fields]	L10	17536671	Proteomic analysis of a hypochlorous acid-tolerant Listeria monocytogenes cultural variant exhibiting enhanced biofilm production.
1228	DRAMP04184	DFTamP1	"DFTamP1"[All Fields] AND biofilm[All Fields]	DFTamP1	28011203	Small molecule mimics of DFTamP1, a database designed anti-Staphylococcal peptide.
1241	DRAMP04233	D28 (Rational design peptide)	"D28"[All Fields] AND biofilm[All Fields]	D28	32063987	Clinical and Microbiological Evaluation of Brazilian Red Propolis Containing-Dentifrice in Orthodontic Patients: A Randomized Clinical Trial.
1241	DRAMP04233	D28 (Rational design peptide)	"D28"[All Fields] AND biofilm[All Fields]	D28	25291116	Effectiveness of the two microorganisms Lactobacillus fermentum LF15 and Lactobacillus plantarum LP01, formulated in slow-release vaginal tablets, in women affected by bacterial vaginosis: a pilot study.
1243	DRAMP04235	D22 (Rational design peptide)	"D22"[All Fields] AND biofilm[All Fields]	D22	33409498	Bacteriophages as Biocontrol Agents for Flavobacterium psychrophilumBiofilms and Rainbow Trout Infections.
1246	DRAMP04241	Synthetic 2	"Synthetic 2"[All Fields] AND biofilm[All Fields]	Synthetic 2	28755874	Antibiofilm potential of synthetic 2-amino-5-chlorobenzophenone Schiff bases and its confirmation through fluorescence microscopy.

1246	DRAMP04241	Synthetic 2	"Synthetic 2"[All Fields] AND biofilm[All Fields]	Synthetic 2	17497633	Antibiotic from the marine environment with antimicrobial fouling activity.
1247	DRAMP04242	Synthetic 3	"Synthetic 3"[All Fields] AND biofilm[All Fields]	Synthetic 3	21630583	Synthetic 3-arylideneflavanones as inhibitors of the initial stages of biofilm formation by Staphylococcus aureus and Enterococcus faecalis.
1247	DRAMP04242	Synthetic 3	"Synthetic 3"[All Fields] AND biofilm[All Fields]	Synthetic 3	18281398	Isolation and characterization of an autoinducer synthase from Acinetobacter baumannii.
1247	DRAMP04242	Synthetic 3	"Synthetic 3"[All Fields] AND biofilm[All Fields]	Synthetic 3	16585751	The ppuI-rsaL-ppuR quorum-sensing system regulates biofilm formation of Pseudomonas putida PCL1445 by controlling biosynthesis of the cyclic lipopeptides putisolvins I and II.
1248	DRAMP04243	Synthetic 4	"Synthetic 4"[All Fields] AND biofilm[All Fields]	Synthetic 4	32769186	Genetic Determinants of SalmonellaResistance to the Biofilm-Inhibitory Effects of a Synthetic 4-Oxazolidinone Analog.
1248	DRAMP04243	Synthetic 4	"Synthetic 4"[All Fields] AND biofilm[All Fields]	Synthetic 4	22919670	Deep sequencing of Porphyromonas gingivalis and comparative transcriptome analysis of a LuxS mutant.
1248	DRAMP04243	Synthetic 4	"Synthetic 4"[All Fields] AND biofilm[All Fields]	Synthetic 4	18576653	An unexpected switch in the modulation of AI-2-based quorum sensing discovered through synthetic 4,5-dihydroxy-2,3-pentanedione analogues.
1250	DRAMP04264	CP26	"CP26"[All Fields] AND biofilm[All Fields]	CP26	33057038	Physical properties of epilithic river biofilm as a new lead to perform pollution bioassessments in overseas territories.
1268	DRAMP04376	MP-1 (MP analog)	"MP-1"[All Fields] AND biofilm[All Fields]	MP-1	29626660	Venom-derived peptide Mastoparan-1 eradicates planktonic and biofilm-embedded methicillin-resistant Staphylococcus aureus isolates.
1268	DRAMP04376	MP-1 (MP analog)	"MP-1"[All Fields] AND biofilm[All Fields]	MP-1	20623316	Mutanase from Paenibacillus sp. MP-1 produced inductively by fungal α-1,3-glucan and its potential for the degradation of mutan and Streptococcus mutans biofilm.
1273	DRAMP04381	PMM-2 (PMM analog)	"PMM-2"[All Fields] AND biofilm[All Fields]	PMM-2	27884340	Use of an experimental model to evaluate infection resistance of meshes in abdominal wall surgery.
1301	DRAMP00004	Lantibiotic (Bacteriocin)	"Lantibiotic"[All Fields] AND biofilm[All Fields]	Lantibiotic	33590560	A single system detects and protects the beneficial oral bacterium Streptococcus sp. A12 from a spectrum of antimicrobial peptides.
1301	DRAMP00004	Lantibiotic (Bacteriocin)	"Lantibiotic"[All Fields] AND biofilm[All Fields]	Lantibiotic	33582800	Efficacy of a novel lantibiotic, CMB001, against MRSA.
1301	DRAMP00004	Lantibiotic (Bacteriocin)	"Lantibiotic"[All Fields] AND biofilm[All Fields]	Lantibiotic	33371410	Susceptibility to Enterocins and Lantibiotic Bacteriocins of Biofilm-Forming Enterococci Isolated from Slovak Fermented Meat Products Available on the Market.
1301	DRAMP00004	Lantibiotic (Bacteriocin)	"Lantibiotic"[All Fields] AND biofilm[All Fields]	Lantibiotic	33279086	Inhibition of Listeria monocytogenes by the Staphylococcus capitis - derived bacteriocin capidermicin.
1301	DRAMP00004	Lantibiotic (Bacteriocin)	"Lantibiotic"[All Fields] AND biofilm[All Fields]	Lantibiotic	32632823	Biocompatible combinations of nisin and licorice polyphenols exert synergistic bactericidal effects against Enterococcus faecalis and inhibit NF-κB activation in monocytes.
1301	DRAMP00004	Lantibiotic (Bacteriocin)	"Lantibiotic"[All Fields] AND biofilm[All Fields]	Lantibiotic	32229530	Genetic Analysis of Mutacin B-Ny266, a Lantibiotic Active against Caries Pathogens.
1301	DRAMP00004	Lantibiotic (Bacteriocin)	"Lantibiotic"[All Fields] AND biofilm[All Fields]	Lantibiotic	31420345	Novel Probiotic Mechanisms of the Oral Bacterium Streptococcussp. A12 as Explored with Functional Genomics.
1301	DRAMP00004	Lantibiotic (Bacteriocin)	"Lantibiotic"[All Fields] AND biofilm[All Fields]	Lantibiotic	31151072	Study of the effectiveness of staphylococcins in biopreservation of Minas fresh (Frescal) cheese with a reduced sodium content.
1301	DRAMP00004	Lantibiotic (Bacteriocin)	"Lantibiotic"[All Fields] AND biofilm[All Fields]	Lantibiotic	31088602	Nisin penetration and efficacy against Staphylococcus aureus biofilms under continuous-flow conditions.
1301	DRAMP00004	Lantibiotic (Bacteriocin)	"Lantibiotic"[All Fields] AND biofilm[All Fields]	Lantibiotic	30710249	Characterisation of Faecal Staphylococci from Roe Deer (Capreolus capreolus) and Red Deer (Cervus elaphus) and Their Susceptibility to Gallidermin.
1301	DRAMP00004	Lantibiotic (Bacteriocin)	"Lantibiotic"[All Fields] AND biofilm[All Fields]	Lantibiotic	29092042	Expanding the potential of NAI-107 for treating serious ESKAPE pathogens: synergistic combinations against Gram-negatives and bactericidal activity against non-dividing cells.
1301	DRAMP00004	Lantibiotic (Bacteriocin)	"Lantibiotic"[All Fields] AND biofilm[All Fields]	Lantibiotic	27914001	Subtilosin Prevents Biofilm Formation by Inhibiting Bacterial Quorum Sensing.
1301	DRAMP00004	Lantibiotic (Bacteriocin)	"Lantibiotic"[All Fields] AND biofilm[All Fields]	Lantibiotic	27833601	Synergistic Nisin-Polymyxin Combinations for the Control of PseudomonasBiofilm Formation.
1301	DRAMP00004	Lantibiotic (Bacteriocin)	"Lantibiotic"[All Fields] AND biofilm[All Fields]	Lantibiotic	27148197	In Vitro Activities of Nisin and Nisin Derivatives Alone and In Combination with Antibiotics against Staphylococcus Biofilms.
1301	DRAMP00004	Lantibiotic (Bacteriocin)	"Lantibiotic"[All Fields] AND biofilm[All Fields]	Lantibiotic	26752879	Antimicrobial effect of an oxazolidinone, lantibiotic and calcium hydroxide against Enterococcus faecalis biofilm: An in vitro study.
1301	DRAMP00004	Lantibiotic (Bacteriocin)	"Lantibiotic"[All Fields] AND biofilm[All Fields]	Lantibiotic	26678028	Biomedical applications of nisin.
1301	DRAMP00004	Lantibiotic (Bacteriocin)	"Lantibiotic"[All Fields] AND biofilm[All Fields]	Lantibiotic	26150809	Antimicrobial nisin acts against saliva derived multi-species biofilms without cytotoxicity to human oral cells.
1301	DRAMP00004	Lantibiotic (Bacteriocin)	"Lantibiotic"[All Fields] AND biofilm[All Fields]	Lantibiotic	25789988	A bioengineered nisin derivative to control biofilms of Staphylococcus pseudintermedius.
1301	DRAMP00004	Lantibiotic (Bacteriocin)	"Lantibiotic"[All Fields] AND biofilm[All Fields]	Lantibiotic	25787977	Lantibiotic resistance.
1301	DRAMP00004	Lantibiotic (Bacteriocin)	"Lantibiotic"[All Fields] AND biofilm[All Fields]	Lantibiotic	25309879	Quorum sensing in group A Streptococcus.
1301	DRAMP00004	Lantibiotic (Bacteriocin)	"Lantibiotic"[All Fields] AND biofilm[All Fields]	Lantibiotic	24119540	Epidermin and gallidermin: Staphylococcal lantibiotics.
1301	DRAMP00004	Lantibiotic (Bacteriocin)	"Lantibiotic"[All Fields] AND biofilm[All Fields]	Lantibiotic	22926575	Activity of gallidermin on Staphylococcus aureus and Staphylococcus epidermidis biofilms.
1301	DRAMP00004	Lantibiotic (Bacteriocin)	"Lantibiotic"[All Fields] AND biofilm[All Fields]	Lantibiotic	21923747	Impact of the broad-spectrum antimicrobial peptide, lacticin 3147, on Streptococcus mutans growing in a biofilm and in human saliva.
1301	DRAMP00004	Lantibiotic (Bacteriocin)	"Lantibiotic"[All Fields] AND biofilm[All Fields]	Lantibiotic	20865041	Comparative genomics of Gardnerella vaginalis strains reveals substantial differences in metabolic and virulence potential.
1301	DRAMP00004	Lantibiotic (Bacteriocin)	"Lantibiotic"[All Fields] AND biofilm[All Fields]	Lantibiotic	19202103	Genes involved in the repression of mutacin I production in Streptococcus mutans.
1301	DRAMP00004	Lantibiotic (Bacteriocin)	"Lantibiotic"[All Fields] AND biofilm[All Fields]	Lantibiotic	16907725	IrvA-dependent and IrvA-independent pathways for mutacin gene regulation in Streptococcus mutans.
1301	DRAMP00004	Lantibiotic (Bacteriocin)	"Lantibiotic"[All Fields] AND biofilm[All Fields]	Lantibiotic	15978073	Co-ordinated bacteriocin production and competence development: a possible mechanism for taking up DNA from neighbouring species.
1301	DRAMP00004	Lantibiotic (Bacteriocin)	"Lantibiotic"[All Fields] AND biofilm[All Fields]	Lantibiotic	15853875	Bacillus subtilis antibiotics: structures, syntheses and specific functions.
1319	DRAMP00024	CylLS (a structural subunit of cytolysin; Bacteriocin)	"CylLS"[All Fields] AND biofilm[All Fields]	CylLS	27375584	Use of Potential Probiotic Lactic Acid Bacteria (LAB) Biofilms for the Control of Listeria monocytogenes, Salmonella Typhimurium, and Escherichia coli O157:H7 Biofilms Formation.
1320	DRAMP00025	CylLL (a structural subunit of cytolysin; Bacteriocin)	"CylLL"[All Fields] AND biofilm[All Fields]	CylLL	27375584	Use of Potential Probiotic Lactic Acid Bacteria (LAB) Biofilms for the Control of Listeria monocytogenes, Salmonella Typhimurium, and Escherichia coli O157:H7 Biofilms Formation.
1321	DRAMP00026	Salivaricin A (SalA; Bacteriocin; Preclinical)	"Salivaricin A"[All Fields] AND biofilm[All Fields]	Salivaricin A	31432254	Virgicin, a novel lanthipeptide from Virgibacillus sp. strain AK90 exhibits inhibitory activity against Gram-positive bacteria.
1325	DRAMP18349	Siamycin I (Bacteriocin)	"Siamycin I"[All Fields] AND biofilm[All Fields]	Siamycin I	17071762	Siamycin attenuates fsr quorum sensing mediated by a gelatinase biosynthesis-activating pheromone in Enterococcus faecalis.
1330	DRAMP00036	Nisin A (Bacteriocin; Preclinical)	"Nisin A"[All Fields] AND biofilm[All Fields]	Nisin A	34105992	A Bioengineered Nisin Derivative To Control Streptococcus uberis Biofilms.
1330	DRAMP00036	Nisin A (Bacteriocin; Preclinical)	"Nisin A"[All Fields] AND biofilm[All Fields]	Nisin A	32517174	Bioengineered Nisin Derivative M17Q Has Enhanced Activity against Staphylococcus epidermidis.
1330	DRAMP00036	Nisin A (Bacteriocin; Preclinical)	"Nisin A"[All Fields] AND biofilm[All Fields]	Nisin A	32469943	Vancomycin and nisin A are effective against biofilms of multi-drug resistant Staphylococcus aureus isolates from human milk.
1330	DRAMP00036	Nisin A (Bacteriocin; Preclinical)	"Nisin A"[All Fields] AND biofilm[All Fields]	Nisin A	31339915	Diabetic foot infections: Application of a nisin-biogel to complement the activity of conventional antibiotics and antiseptics against Staphylococcus aureus biofilms.
1330	DRAMP00036	Nisin A (Bacteriocin; Preclinical)	"Nisin A"[All Fields] AND biofilm[All Fields]	Nisin A	30955056	Antibacterial activity against porcine respiratory bacterial pathogens and in vitro biocompatibility of essential oils.
1330	DRAMP00036	Nisin A (Bacteriocin; Preclinical)	"Nisin A"[All Fields] AND biofilm[All Fields]	Nisin A	27965658	A Bioengineered Nisin Derivative, M21A, in Combination with Food Grade Additives Eradicates Biofilms of Listeria monocytogenes.
1330	DRAMP00036	Nisin A (Bacteriocin; Preclinical)	"Nisin A"[All Fields] AND biofilm[All Fields]	Nisin A	27878401	Enterocin B3A-B3B produced by LAB collected from infant faeces: potential utilization in the food industry for Listeria monocytogenes biofilm management.
1330	DRAMP00036	Nisin A (Bacteriocin; Preclinical)	"Nisin A"[All Fields] AND biofilm[All Fields]	Nisin A	27148197	In Vitro Activities of Nisin and Nisin Derivatives Alone and In Combination with Antibiotics against Staphylococcus Biofilms.
1330	DRAMP00036	Nisin A (Bacteriocin; Preclinical)	"Nisin A"[All Fields] AND biofilm[All Fields]	Nisin A	25787977	Lantibiotic resistance.
1330	DRAMP00036	Nisin A (Bacteriocin; Preclinical)	"Nisin A"[All Fields] AND biofilm[All Fields]	Nisin A	25173449	Bovicin HC5 and nisin reduce Staphylococcus aureus adhesion to polystyrene and change the hydrophobicity profile and Gibbs free energy of adhesion.
1330	DRAMP00036	Nisin A (Bacteriocin; Preclinical)	"Nisin A"[All Fields] AND biofilm[All Fields]	Nisin A	23979748	Effects of bacteriocins on methicillin-resistant Staphylococcus aureus biofilm.
1331	DRAMP00037	Nisin Z (Bacteriocin; Preclinical)	"Nisin Z"[All Fields] AND biofilm[All Fields]	Nisin Z	33462270	Incorporation and antimicrobial activity of nisin Z within carrageenan/chitosan multilayers.
1331	DRAMP00037	Nisin Z (Bacteriocin; Preclinical)	"Nisin Z"[All Fields] AND biofilm[All Fields]	Nisin Z	31027787	Lactococcus lactis subsp. lactis as a natural anti-listerial agent in the mushroom industry.
1331	DRAMP00037	Nisin Z (Bacteriocin; Preclinical)	"Nisin Z"[All Fields] AND biofilm[All Fields]	Nisin Z	27797439	Inhibition of Listeria monocytogenes biofilms by bacteriocin-producing bacteria isolated from mushroom substrate.
1333	DRAMP00039	Pep5 (Bacteriocin)	"Pep5"[All Fields] AND biofilm[All Fields]	Pep5	31151072	Study of the effectiveness of staphylococcins in biopreservation of Minas fresh (Frescal) cheese with a reduced sodium content.
1333	DRAMP00039	Pep5 (Bacteriocin)	"Pep5"[All Fields] AND biofilm[All Fields]	Pep5	25009170	Genetic basis for Saccharomyces cerevisiae biofilm in liquid medium.

1334	DRAMP00040	Gallidermin (Bacteriocin; Preclinical)	"Gallidermin"[All Fields] AND biofilm[All Fields]	Gallidermin	33371410	Susceptibility to Enterocins and Lantibiotic Bacteriocins of Biofilm-Forming Enterococci Isolated from Slovak Fermented Meat Products Available on the Market.
1334	DRAMP00040	Gallidermin (Bacteriocin; Preclinical)	"Gallidermin"[All Fields] AND biofilm[All Fields]	Gallidermin	32971750	Susceptibility to Bacteriocins in Biofilm-Forming, Variable Staphylococci Isolated from Local Slovak Ewes' Milk Lump Cheeses.
1334	DRAMP00040	Gallidermin (Bacteriocin; Preclinical)	"Gallidermin"[All Fields] AND biofilm[All Fields]	Gallidermin	32687650	Inactivation of the gene encoding the cationic antimicrobial peptide resistance factor MprF increases biofilm formation but reduces invasiveness of Listeria monocytogenes.
1334	DRAMP00040	Gallidermin (Bacteriocin; Preclinical)	"Gallidermin"[All Fields] AND biofilm[All Fields]	Gallidermin	31888501	Composite genome sequence of Bacillus clausii, a probiotic commercially available as Enterogermina ®, and insights into its probiotic properties.
1334	DRAMP00040	Gallidermin (Bacteriocin; Preclinical)	"Gallidermin"[All Fields] AND biofilm[All Fields]	Gallidermin	30710249	Characterisation of Faecal Staphylococci from Roe Deer (Capreolus capreolus) and Red Deer (Cervus elaphus) and Their Susceptibility to Gallidermin.
1334	DRAMP00040	Gallidermin (Bacteriocin; Preclinical)	"Gallidermin"[All Fields] AND biofilm[All Fields]	Gallidermin	24119540	Epidermin and gallidermin: Staphylococcal lantibiotics.
1334	DRAMP00040	Gallidermin (Bacteriocin; Preclinical)	"Gallidermin"[All Fields] AND biofilm[All Fields]	Gallidermin	22926575	Activity of gallidermin on Staphylococcus aureus and Staphylococcus epidermidis biofilms.
1334	DRAMP00040	Gallidermin (Bacteriocin; Preclinical)	"Gallidermin"[All Fields] AND biofilm[All Fields]	Gallidermin	17676995	Molecular basis of resistance to muramidase and cationic antimicrobial peptide activity of lysozyme in staphylococci.
1335	DRAMP00041	Mutacin-1140 (Mutacin III; Bacteriocin)	"Mutacin-1140"[All Fields] AND biofilm[All Fields]	Mutacin-1140	12369203	Genetically modified Streptococcus mutans for the prevention of dental caries.
1339	DRAMP18347	Siamycin(Bacteriocin)	"Siamycin"[All Fields] AND biofilm[All Fields]	Siamycin	17071762	Siamycin attenuates fsr quorum sensing mediated by a gelatinase biosynthesis-activating pheromone in Enterococcus faecalis.
1345	DRAMP00051	Mutacin I (Bacteriocin)	"Mutacin I"[All Fields] AND biofilm[All Fields]	Mutacin I	19783751	Role of the Streptococcus mutans irvA gene in GbpC-independent, dextran-dependent aggregation and biofilm formation.
1345	DRAMP00051	Mutacin I (Bacteriocin)	"Mutacin I"[All Fields] AND biofilm[All Fields]	Mutacin I	19202103	Genes involved in the repression of mutacin I production in Streptococcus mutans.
1345	DRAMP00051	Mutacin I (Bacteriocin)	"Mutacin I"[All Fields] AND biofilm[All Fields]	Mutacin I	16907725	IrvA-dependent and IrvA-independent pathways for mutacin gene regulation in Streptococcus mutans.
1345	DRAMP00051	Mutacin I (Bacteriocin)	"Mutacin I"[All Fields] AND biofilm[All Fields]	Mutacin I	15271957	Inactivation of the ciaH Gene in Streptococcus mutans diminishes mutacin production and competence development, alters sucrose-dependent biofilm formation, and reduces stress tolerance.
1345	DRAMP00051	Mutacin I (Bacteriocin)	"Mutacin I"[All Fields] AND biofilm[All Fields]	Mutacin I	15209996	Transcriptional analysis of mutacin I (mutA) gene expression in planktonic and biofilm cells of Streptococcus mutans using fluorescent protein and glucuronidase reporters.
1356	DRAMP00066	Lacticin Q (Bacteriocin)	"Lacticin Q"[All Fields] AND biofilm[All Fields]	Lacticin Q	23979748	Effects of bacteriocins on methicillin-resistant Staphylococcus aureus biofilm.
1356	DRAMP00066	Lacticin Q (Bacteriocin)	"Lacticin Q"[All Fields] AND biofilm[All Fields]	Lacticin Q	22155816	Identification, characterization, and recombinant expression of epidermicin NI01, a novel unmodified bacteriocin produced by Staphylococcus epidermidis that displays potent activity against Staphylococci.
1358	DRAMP00070	Laterosporulin (Bacteriocin)	"Laterosporulin"[All Fields] AND biofilm[All Fields]	Laterosporulin	33927461	Improvisation and Evaluation of Laterosporulin Coated Titanium Surfaces for dental Applications: An In Vitro Investigation.
1372	DRAMP00085	Bacteriocin	"Bacteriocin"[All Fields] AND biofilm[All Fields]	Bacteriocin	34557272	Genotypic and phenotypic characterization of Streptococcus mutansstrains isolated from patients with dental caries.
1372	DRAMP00085	Bacteriocin	"Bacteriocin"[All Fields] AND biofilm[All Fields]	Bacteriocin	34507740	Listeria monocytogenes biofilm inhibition on food contact surfaces by application of postbiotics from Lactobacillus curvatus B.67 and Lactobacillus plantarum M.2.
1372	DRAMP00085	Bacteriocin	"Bacteriocin"[All Fields] AND biofilm[All Fields]	Bacteriocin	34484135	YbfA Regulates the Sensitivity of Escherichia coliK12 to Plantaricin BM-1 viathe BasS/BasR Two-Component Regulatory System.
1372	DRAMP00085	Bacteriocin	"Bacteriocin"[All Fields] AND biofilm[All Fields]	Bacteriocin	34481880	Hybrid hydrogels for bacteriocin delivery to infected wounds.
1372	DRAMP00085	Bacteriocin	"Bacteriocin"[All Fields] AND biofilm[All Fields]	Bacteriocin	34463029	MapZ deficiency leads to defects in the envelope structure and changes stress tolerance of Streptococcus mutans.
1372	DRAMP00085	Bacteriocin	"Bacteriocin"[All Fields] AND biofilm[All Fields]	Bacteriocin	34438974	Antimicrobial Activity of the Circular Bacteriocin AS-48 against Clinical Multidrug-Resistant Staphylococcus aureus.
1372	DRAMP00085	Bacteriocin	"Bacteriocin"[All Fields] AND biofilm[All Fields]	Bacteriocin	34230527	A bacteriocin-based treatment option for Staphylococcus haemolyticus biofilms.
1372	DRAMP00085	Bacteriocin	"Bacteriocin"[All Fields] AND biofilm[All Fields]	Bacteriocin	34220114	In vitroevaluation of the probiotic potential of Lactobacillusisolated from native swine manure.
1372	DRAMP00085	Bacteriocin	"Bacteriocin"[All Fields] AND biofilm[All Fields]	Bacteriocin	34208478	Engineering of the CHAPk Staphylococcal Phage Endolysin to Enhance Antibacterial Activity against Stationary-Phase Cells.
1372	DRAMP00085	Bacteriocin	"Bacteriocin"[All Fields] AND biofilm[All Fields]	Bacteriocin	34189819	Prokaryotic population dynamics and interactions in an AnSBBR using tequila vinasses as substrate in co-digestion with acid hydrolysates of Agave tequilana var. azul bagasse for hydrogen production.
1372	DRAMP00085	Bacteriocin	"Bacteriocin"[All Fields] AND biofilm[All Fields]	Bacteriocin	34173207	Bacteriocin-Like Inhibitory Substance (BLIS) Activity of Enterococcus faecium DB1 Against Biofilm Formation by Clostridium perfringens.
1372	DRAMP00085	Bacteriocin	"Bacteriocin"[All Fields] AND biofilm[All Fields]	Bacteriocin	34087449	Bacterial Vaginosis: Effects on reproduction and its therapeutics.
1372	DRAMP00085	Bacteriocin	"Bacteriocin"[All Fields] AND biofilm[All Fields]	Bacteriocin	33987538	Influence of Gallic Acid and Thai Culinary Essential Oils on Antibacterial Activity of Nisin against Streptococcus mutans.
1372	DRAMP00085	Bacteriocin	"Bacteriocin"[All Fields] AND biofilm[All Fields]	Bacteriocin	33839269	Pangenome analyses of LuxS-coding genes and enzymatic repertoires in cocoa-related lactic acid bacteria.
1372	DRAMP00085	Bacteriocin	"Bacteriocin"[All Fields] AND biofilm[All Fields]	Bacteriocin	33838179	Effect of sub-lethal doses of nisin on Staphylococcus aureus toxin production and biofilm formation.
1372	DRAMP00085	Bacteriocin	"Bacteriocin"[All Fields] AND biofilm[All Fields]	Bacteriocin	33807321	Biodiversity of Ligilactobacillus salivariusStrains from Poultry and Domestic Pigeons.
1372	DRAMP00085	Bacteriocin	"Bacteriocin"[All Fields] AND biofilm[All Fields]	Bacteriocin	33802636	Bacteriocin-Like Inhibitory Substances from Probiotics as Therapeutic Agents for CandidaVulvovaginitis.
1372	DRAMP00085	Bacteriocin	"Bacteriocin"[All Fields] AND biofilm[All Fields]	Bacteriocin	33584610	Proteomic Analysis of Listeria monocytogenesFBUNT During Biofilm Formation at 10°C in Response to Lactocin AL705.
1372	DRAMP00085	Bacteriocin	"Bacteriocin"[All Fields] AND biofilm[All Fields]	Bacteriocin	33419248	In vitro Interactions between Streptococcus intermediusand Streptococcus salivariusK12 on a Titanium Cylindrical Surface.
1372	DRAMP00085	Bacteriocin	"Bacteriocin"[All Fields] AND biofilm[All Fields]	Bacteriocin	33352988	Enterococcus mundtiiIsolated from Slovak Raw Goat Milk and Its Bacteriocinogenic Potential.
1372	DRAMP00085	Bacteriocin	"Bacteriocin"[All Fields] AND biofilm[All Fields]	Bacteriocin	33279086	Inhibition of Listeria monocytogenes by the Staphylococcus capitis - derived bacteriocin capidermicin.
1372	DRAMP00085	Bacteriocin	"Bacteriocin"[All Fields] AND biofilm[All Fields]	Bacteriocin	33268776	A bacteriocin-based antimicrobial formulation to effectively disrupt the cell viability of methicillin-resistant Staphylococcus aureus (MRSA) biofilms.
1372	DRAMP00085	Bacteriocin	"Bacteriocin"[All Fields] AND biofilm[All Fields]	Bacteriocin	33246623	Purification, characterization, and mode of action of a novel bacteriocin BM173 from Lactobacillus crustorum MN047 and its effect on biofilm formation of Escherichia coli and Staphylococcus aureus.
1372	DRAMP00085	Bacteriocin	"Bacteriocin"[All Fields] AND biofilm[All Fields]	Bacteriocin	33188012	Engineered probiotics biofilm enhances osseointegration via immunoregulation and anti-infection.
1372	DRAMP00085	Bacteriocin	"Bacteriocin"[All Fields] AND biofilm[All Fields]	Bacteriocin	33068882	Prevalence of bacteriocins and their co-association with virulence factors within Pseudomonas aeruginosa catheter isolates.
1372	DRAMP00085	Bacteriocin	"Bacteriocin"[All Fields] AND biofilm[All Fields]	Bacteriocin	33010007	Anti-Proliferative and Anti-Biofilm Potentials of Bacteriocins Produced by Non-Pathogenic Enterococcus sp.
1372	DRAMP00085	Bacteriocin	"Bacteriocin"[All Fields] AND biofilm[All Fields]	Bacteriocin	33006097	Bacteriocin-a potential antimicrobial peptide towards disrupting and preventing biofilm formation in the clinical and environmental locales.
1372	DRAMP00085	Bacteriocin	"Bacteriocin"[All Fields] AND biofilm[All Fields]	Bacteriocin	32971750	Susceptibility to Bacteriocins in Biofilm-Forming, Variable Staphylococci Isolated from Local Slovak Ewes' Milk Lump Cheeses.
1372	DRAMP00085	Bacteriocin	"Bacteriocin"[All Fields] AND biofilm[All Fields]	Bacteriocin	32949279	A strategy to control colonization of pathogens: embedding of lactic acid bacteria on the surface of urinary catheter.
1372	DRAMP00085	Bacteriocin	"Bacteriocin"[All Fields] AND biofilm[All Fields]	Bacteriocin	32803295	Transcriptomic and proteomic profiling response of methicillin-resistant Staphylococcus aureus (MRSA) to a novel bacteriocin, plantaricin GZ1-27 and its inhibition of biofilm formation.
1372	DRAMP00085	Bacteriocin	"Bacteriocin"[All Fields] AND biofilm[All Fields]	Bacteriocin	32772715	Synergistic antibacterial and anti-biofilm activity of nisin like bacteriocin with curcumin and cinnamaldehyde against ESBL and MBL producing clinical strains.
1372	DRAMP00085	Bacteriocin	"Bacteriocin"[All Fields] AND biofilm[All Fields]	Bacteriocin	32721519	Combined antimicrobial use of essential oils and bacteriocin bacLP17 as seafood biopreservative to control Listeria monocytogenes both in planktonic and in sessile forms.
1372	DRAMP00085	Bacteriocin	"Bacteriocin"[All Fields] AND biofilm[All Fields]	Bacteriocin	32712896	Compatibility, Cytotoxicity, and Gastrointestinal Tenacity of Bacteriocin-Producing Bacteria Selected for a Consortium Probiotic Formulation to Be Used in Livestock Feed.
1372	DRAMP00085	Bacteriocin	"Bacteriocin"[All Fields] AND biofilm[All Fields]	Bacteriocin	32707601	Combinatorial effects of trans-cinnamaldehyde with fluoride and chlorhexidine on Streptococcus mutans.
1372	DRAMP00085	Bacteriocin	"Bacteriocin"[All Fields] AND biofilm[All Fields]	Bacteriocin	32707233	Nisin influence on the expression of Listeria monocytogenes surface proteins.
1372	DRAMP00085	Bacteriocin	"Bacteriocin"[All Fields] AND biofilm[All Fields]	Bacteriocin	32695282	Bioactivity of Bac70 Produced by Bacillus atrophaeusStrain DDBCC70.
1372	DRAMP00085	Bacteriocin	"Bacteriocin"[All Fields] AND biofilm[All Fields]	Bacteriocin	32678667	In VitroAntibiofilm and Anti-Inflammatory Properties of Bacteriocins Produced by Pediococcus acidilacticiAgainst Enterococcus faecalis.
1372	DRAMP00085	Bacteriocin	"Bacteriocin"[All Fields] AND biofilm[All Fields]	Bacteriocin	32632823	Biocompatible combinations of nisin and licorice polyphenols exert synergistic bactericidal effects against Enterococcus faecalis and inhibit NF-κB activation in monocytes.
1372	DRAMP00085	Bacteriocin	"Bacteriocin"[All Fields] AND biofilm[All Fields]	Bacteriocin	32629918	Metabolic Shift of an Isogenic Strain of Enterococcus faecalis14, Deficient in Its Own Bacteriocin Synthesis, as Revealed by a Transcriptomic Analysis.

1372	DRAMP00085	Bacteriocin	"Bacteriocin"[All Fields] AND biofilm[All Fields]	Bacteriocin	32628991	Evidence of anti- K. pneumoniae biofilm activity of novel Entrococcus faecalis enterocin GLHM.
1372	DRAMP00085	Bacteriocin	"Bacteriocin"[All Fields] AND biofilm[All Fields]	Bacteriocin	32529293	Outer Membrane Channel Protein TolC Regulates Escherichia coli K12 Sensitivity to Plantaricin BM-1 via the CpxR/CpxA Two-Component Regulatory System.
1372	DRAMP00085	Bacteriocin	"Bacteriocin"[All Fields] AND biofilm[All Fields]	Bacteriocin	32517174	Bioengineered Nisin Derivative M17Q Has Enhanced Activity against Staphylococcus epidermidis.
1372	DRAMP00085	Bacteriocin	"Bacteriocin"[All Fields] AND biofilm[All Fields]	Bacteriocin	32322380	In vitroanti-biofilm activity of bacteriocin from a marine Bacillussp. strain Sh10 against Proteus mirabilis.
1372	DRAMP00085	Bacteriocin	"Bacteriocin"[All Fields] AND biofilm[All Fields]	Bacteriocin	32318864	Virulence Factors, Drug Resistance and Biofilm Formation in Pseudomonas Species Isolated from Healthcare Water Systems.
1372	DRAMP00085	Bacteriocin	"Bacteriocin"[All Fields] AND biofilm[All Fields]	Bacteriocin	32307660	A Review of the Role of Probiotic Supplementation in Dental Caries.
1372	DRAMP00085	Bacteriocin	"Bacteriocin"[All Fields] AND biofilm[All Fields]	Bacteriocin	32282078	Physicochemical properties and mode of action of a novel bacteriocin BM1122 with broad antibacterial spectrum produced by Lactobacillus crustorum MN047.
1372	DRAMP00085	Bacteriocin	"Bacteriocin"[All Fields] AND biofilm[All Fields]	Bacteriocin	32247478	Does Quorum Sensing play a role in microbial shifts along spontaneous fermentation of cocoa beans? An in silico perspective.
1372	DRAMP00085	Bacteriocin	"Bacteriocin"[All Fields] AND biofilm[All Fields]	Bacteriocin	32229530	Genetic Analysis of Mutacin B-Ny266, a Lantibiotic Active against Caries Pathogens.
1372	DRAMP00085	Bacteriocin	"Bacteriocin"[All Fields] AND biofilm[All Fields]	Bacteriocin	32178236	A Rapid Lysostaphin Production Approach and a Convenient Novel Lysostaphin Loaded Nano-emulgel; As a Sustainable Low-Cost Methicillin-Resistant Staphylococcus aureusCombating Platform.
1372	DRAMP00085	Bacteriocin	"Bacteriocin"[All Fields] AND biofilm[All Fields]	Bacteriocin	32111071	Whole-Genome Sequencing of Lactobacillus helveticusD75 and D76 Confirms Safety and Probiotic Potential.
1372	DRAMP00085	Bacteriocin	"Bacteriocin"[All Fields] AND biofilm[All Fields]	Bacteriocin	31942681	Evaluation of Probiotic Properties and Prebiotic Utilization Potential of Weissella paramesenteroides Isolated From Fruits.
1372	DRAMP00085	Bacteriocin	"Bacteriocin"[All Fields] AND biofilm[All Fields]	Bacteriocin	31888501	Composite genome sequence of Bacillus clausii, a probiotic commercially available as Enterogermina ®, and insights into its probiotic properties.
1372	DRAMP00085	Bacteriocin	"Bacteriocin"[All Fields] AND biofilm[All Fields]	Bacteriocin	31794906	Isolation and characterization of Enterococcus faecium DSM 20477 with ability to secrete antimicrobial substance for the inhibition of oral pathogen Streptococcus mutans UKMCC 1019.
1372	DRAMP00085	Bacteriocin	"Bacteriocin"[All Fields] AND biofilm[All Fields]	Bacteriocin	31784952	Bacteriocin of Pediococcus acidilactici HW01 Inhibits Biofilm Formation and Virulence Factor Production by Pseudomonas aeruginosa.
1372	DRAMP00085	Bacteriocin	"Bacteriocin"[All Fields] AND biofilm[All Fields]	Bacteriocin	31784450	The manifold roles of microbial ribosomal peptide-based natural products in physiology and ecology.
1372	DRAMP00085	Bacteriocin	"Bacteriocin"[All Fields] AND biofilm[All Fields]	Bacteriocin	31650413	In Vitro Evaluation of Probiotic Potential of Selected Lactic Acid Bacteria Strains.
1372	DRAMP00085	Bacteriocin	"Bacteriocin"[All Fields] AND biofilm[All Fields]	Bacteriocin	31552285	Evaluation of Incompatibility Group I1 (IncI1) Plasmid-Containing Salmonella entericaand Assessment of the Plasmids in Bacteriocin Production and Biofilm Development.
1372	DRAMP00085	Bacteriocin	"Bacteriocin"[All Fields] AND biofilm[All Fields]	Bacteriocin	31456169	Changes in the composition and architecture of staphylococcal biofilm by nisin.
1372	DRAMP00085	Bacteriocin	"Bacteriocin"[All Fields] AND biofilm[All Fields]	Bacteriocin	31432254	Virgicin, a novel lanthipeptide from Virgibacillus sp. strain AK90 exhibits inhibitory activity against Gram-positive bacteria.
1372	DRAMP00085	Bacteriocin	"Bacteriocin"[All Fields] AND biofilm[All Fields]	Bacteriocin	33902719	Genome sequence of Epibacterium ulvae strain DSM 24752 T, an indigoidine-producing, macroalga-associated member of the marine Roseobacter group.
1372	DRAMP00085	Bacteriocin	"Bacteriocin"[All Fields] AND biofilm[All Fields]	Bacteriocin	31356968	Reconstruction of transcriptional regulatory networks of Fis and H-NS in Escherichia coli from genome-wide data analysis.
1372	DRAMP00085	Bacteriocin	"Bacteriocin"[All Fields] AND biofilm[All Fields]	Bacteriocin	31341074	Enterococcus faecalis CRISPR-Cas Is a Robust Barrier to Conjugative Antibiotic Resistance Dissemination in the Murine Intestine.
1372	DRAMP00085	Bacteriocin	"Bacteriocin"[All Fields] AND biofilm[All Fields]	Bacteriocin	31266876	Antifungal Activity of the Enterococcus faecalis Peptide EntV Requires Protease Cleavage and Disulfide Bond Formation.
1372	DRAMP00085	Bacteriocin	"Bacteriocin"[All Fields] AND biofilm[All Fields]	Bacteriocin	31218558	Inhibitory effect of bacteriocins from enterococci on developing and preformed biofilms of Listeria monocytogenes, Listeria ivanovii and Listeria innocua.
1372	DRAMP00085	Bacteriocin	"Bacteriocin"[All Fields] AND biofilm[All Fields]	Bacteriocin	31206966	Lactocin AL705 as quorum sensing inhibitor to control Listeria monocytogenes biofilm formation.
1372	DRAMP00085	Bacteriocin	"Bacteriocin"[All Fields] AND biofilm[All Fields]	Bacteriocin	31166173	ComDE Two-component Signal Transduction Systems in Oral Streptococci: Structure and Function.
1372	DRAMP00085	Bacteriocin	"Bacteriocin"[All Fields] AND biofilm[All Fields]	Bacteriocin	31158279	Corrigendum to: Control of Listeria monocytogenes biofilms on industrial surfaces by the bacteriocin-producing Lactobacillus sakei CRL1862.
1372	DRAMP00085	Bacteriocin	"Bacteriocin"[All Fields] AND biofilm[All Fields]	Bacteriocin	31151072	Study of the effectiveness of staphylococcins in biopreservation of Minas fresh (Frescal) cheese with a reduced sodium content.
1372	DRAMP00085	Bacteriocin	"Bacteriocin"[All Fields] AND biofilm[All Fields]	Bacteriocin	31136751	Antibacterial immunomodulatory and antibiofilm triple effect of Salivaricin LHM against Pseudomonas aeruginosa urinary tract infection model.
1372	DRAMP00085	Bacteriocin	"Bacteriocin"[All Fields] AND biofilm[All Fields]	Bacteriocin	31100490	Effects of 7S globulin 3 derived from the adzuki bean [Vigna angularis] on the CSP- and eDNA-dependent biofilm formation of Streptococcus mutans.
1372	DRAMP00085	Bacteriocin	"Bacteriocin"[All Fields] AND biofilm[All Fields]	Bacteriocin	31036688	Spontaneously Arising Streptococcus mutans Variants with Reduced Susceptibility to Chlorhexidine Display Genetic Defects and Diminished Fitness.
1372	DRAMP00085	Bacteriocin	"Bacteriocin"[All Fields] AND biofilm[All Fields]	Bacteriocin	30962344	Lysocins: Bioengineered Antimicrobials That Deliver Lysins across the Outer Membrane of Gram-Negative Bacteria.
1372	DRAMP00085	Bacteriocin	"Bacteriocin"[All Fields] AND biofilm[All Fields]	Bacteriocin	30955056	Antibacterial activity against porcine respiratory bacterial pathogens and in vitro biocompatibility of essential oils.
1372	DRAMP00085	Bacteriocin	"Bacteriocin"[All Fields] AND biofilm[All Fields]	Bacteriocin	30915281	Characterization of the Competitive Pneumocin Peptides of Streptococcus pneumoniae.
1372	DRAMP00085	Bacteriocin	"Bacteriocin"[All Fields] AND biofilm[All Fields]	Bacteriocin	30863715	BaCf3: highly thermostable bacteriocin from Bacillus amyloliquefaciensBTSS3 antagonistic on food-borne pathogens.
1372	DRAMP00085	Bacteriocin	"Bacteriocin"[All Fields] AND biofilm[All Fields]	Bacteriocin	30853461	Inhibiting bacterial colonization on catheters: Antibacterial and antibiofilm activities of bacteriocins from Lactobacillus plantarum SJ33.
1372	DRAMP00085	Bacteriocin	"Bacteriocin"[All Fields] AND biofilm[All Fields]	Bacteriocin	30841529	Regulatory Effect of DNA Topoisomerase I on T3SS Activity, Antibiotic Susceptibility and Quorum-Sensing-Independent Pyocyanin Synthesis in Pseudomonas aeruginosa.
1372	DRAMP00085	Bacteriocin	"Bacteriocin"[All Fields] AND biofilm[All Fields]	Bacteriocin	30786799	Biofilm formation by staphylococci in health-related environments and recent reports on their control using natural compounds.
1372	DRAMP00085	Bacteriocin	"Bacteriocin"[All Fields] AND biofilm[All Fields]	Bacteriocin	30711392	Identification of highly potent competence stimulating peptide-based quorum sensing activators in Streptococcus mutans through the utilization of N-methyl and reverse alanine scanning.
1372	DRAMP00085	Bacteriocin	"Bacteriocin"[All Fields] AND biofilm[All Fields]	Bacteriocin	30710249	Characterisation of Faecal Staphylococci from Roe Deer (Capreolus capreolus) and Red Deer (Cervus elaphus) and Their Susceptibility to Gallidermin.
1372	DRAMP00085	Bacteriocin	"Bacteriocin"[All Fields] AND biofilm[All Fields]	Bacteriocin	30670678	The Combined Use of Tea Polyphenols and Lactobacillus Plantarum ST8SH Bacteriocin in a Rabbit Model of Infection Following Femoral Fracture with Internal Fixation.
1372	DRAMP00085	Bacteriocin	"Bacteriocin"[All Fields] AND biofilm[All Fields]	Bacteriocin	30658319	Antifungal activities against Candida albicans, of cell-free supernatants obtained from probiotic Pediococcus acidilactici HW01.
1372	DRAMP00085	Bacteriocin	"Bacteriocin"[All Fields] AND biofilm[All Fields]	Bacteriocin	30627971	Characterization of the bacteriocin produced by Enterococcus italicus ONU547 isolated from Thai fermented cabbage.
1372	DRAMP00085	Bacteriocin	"Bacteriocin"[All Fields] AND biofilm[All Fields]	Bacteriocin	30617242	Increased Intracellular Cyclic di-AMP Levels Sensitize Streptococcus gallolyticus subsp. gallolyticus to Osmotic Stress and Reduce Biofilm Formation and Adherence on Intestinal Cells.
1372	DRAMP00085	Bacteriocin	"Bacteriocin"[All Fields] AND biofilm[All Fields]	Bacteriocin	30408016	Bacteriocin Isolated from Lactobacillus Rhamnosus L34 Has Antibacterial Effects in a Rabbit Model of Infection After Mandible Fracture Fixation.
1372	DRAMP00085	Bacteriocin	"Bacteriocin"[All Fields] AND biofilm[All Fields]	Bacteriocin	30396893	Genomic, Phenotypic, and Virulence Analysis of Streptococcus sanguinisOral and Infective-Endocarditis Isolates.
1372	DRAMP00085	Bacteriocin	"Bacteriocin"[All Fields] AND biofilm[All Fields]	Bacteriocin	30233504	Production and Antimicrobial Activity of Nisin Under Enological Conditions.
1372	DRAMP00085	Bacteriocin	"Bacteriocin"[All Fields] AND biofilm[All Fields]	Bacteriocin	30082920	Control of Propionibacterium acnes by natural antimicrobial substances: Role of the bacteriocin AS-48 and lysozyme.
1372	DRAMP00085	Bacteriocin	"Bacteriocin"[All Fields] AND biofilm[All Fields]	Bacteriocin	30073512	Lactic Acid Bacteria (LAB) and Their Bacteriocins as Alternative Biotechnological Tools to Control Listeria monocytogenes Biofilms in Food Processing Facilities.
1372	DRAMP00085	Bacteriocin	"Bacteriocin"[All Fields] AND biofilm[All Fields]	Bacteriocin	30057579	Efficient Exploitation of Multiple Novel Bacteriocins by Combination of Complete Genome and Peptidome.
1372	DRAMP00085	Bacteriocin	"Bacteriocin"[All Fields] AND biofilm[All Fields]	Bacteriocin	29990430	Structure-Activity Relationships of the Competence Stimulating Peptide in Streptococcus mutans Reveal Motifs Critical for Membrane Protease SepM Recognition and ComD Receptor Activation.
1372	DRAMP00085	Bacteriocin	"Bacteriocin"[All Fields] AND biofilm[All Fields]	Bacteriocin	29867809	Biofilms in the Food Industry: Health Aspects and Control Methods.
1372	DRAMP00085	Bacteriocin	"Bacteriocin"[All Fields] AND biofilm[All Fields]	Bacteriocin	29729340	Cloning, overexpression, purification of bacteriocin enterocin-B and structural analysis, interaction determination of enterocin-A, B against pathogenic bacteria and human cancer cells.
1372	DRAMP00085	Bacteriocin	"Bacteriocin"[All Fields] AND biofilm[All Fields]	Bacteriocin	29632089	Characterization of the Trehalose Utilization Operon in Streptococcus mutans Reveals that the TreR Transcriptional Regulator Is Involved in Stress Response Pathways and Toxin Production.
1372	DRAMP00085	Bacteriocin	"Bacteriocin"[All Fields] AND biofilm[All Fields]	Bacteriocin	29628998	Role of Streptococcus mutanssurface proteins for biofilm formation.
1372	DRAMP00085	Bacteriocin	"Bacteriocin"[All Fields] AND biofilm[All Fields]	Bacteriocin	29619040	Oxygen Availability Influences Expression of Dickeya solaniGenes Associated With Virulence in Potato (Solanum tuberosumL.) and Chicory (Cichorium intybusL.).
1372	DRAMP00085	Bacteriocin	"Bacteriocin"[All Fields] AND biofilm[All Fields]	Bacteriocin	29599835	Antibacterial effects of bacteriocins isolated from Lactobacillus rhamnosus(ATCC 53103) in a rabbit model of knee implant infection.

1372	DRAMP00085	Bacteriocin	"Bacteriocin"[All Fields] AND biofilm[All Fields]	Bacteriocin	29580208	Screening for inhibitors of mutacin synthesis in Streptococcus mutans using fluorescent reporter strains.
1372	DRAMP00085	Bacteriocin	"Bacteriocin"[All Fields] AND biofilm[All Fields]	Bacteriocin	29567501	The role of probiotic Lactobacillus acidophilus ATCC 4356 bacteriocin on effect of HBsu on planktonic cells and biofilm formation of Bacillus subtilis.
1372	DRAMP00085	Bacteriocin	"Bacteriocin"[All Fields] AND biofilm[All Fields]	Bacteriocin	29489935	Influence of Helicobacter pylori culture supernatant on the ecological balance of a dual-species oral biofilm.
1372	DRAMP00085	Bacteriocin	"Bacteriocin"[All Fields] AND biofilm[All Fields]	Bacteriocin	29440256	Identification of Streptococcus gallolyticus subsp. gallolyticus (Biotype I) Competence-Stimulating Peptide Pheromone.
1372	DRAMP00085	Bacteriocin	"Bacteriocin"[All Fields] AND biofilm[All Fields]	Bacteriocin	29424388	Evaluation antibacterial and antibiofilm activity of the antimicrobial peptide P34 against Staphylococcus aureus and Enterococcus faecalis.
1372	DRAMP00085	Bacteriocin	"Bacteriocin"[All Fields] AND biofilm[All Fields]	Bacteriocin	29410001	Preparation, characterization and efficacy of lysostaphin-chitosan gel against Staphylococcus aureus.
1372	DRAMP00085	Bacteriocin	"Bacteriocin"[All Fields] AND biofilm[All Fields]	Bacteriocin	29402863	Antibacterial Activity, Cytotoxicity, and the Mechanism of Action of Bacteriocin from Bacillus subtilis GAS101.
1372	DRAMP00085	Bacteriocin	"Bacteriocin"[All Fields] AND biofilm[All Fields]	Bacteriocin	29396844	Evaluation of Probiotic Potential of Bacteriocinogenic Lactic Acid Bacteria Strains Isolated from Meat Products.
1372	DRAMP00085	Bacteriocin	"Bacteriocin"[All Fields] AND biofilm[All Fields]	Bacteriocin	29372297	Genomic and functional characterisation of two Enterococcus strains isolated from Cotija cheese and their potential role in ripening.
1372	DRAMP00085	Bacteriocin	"Bacteriocin"[All Fields] AND biofilm[All Fields]	Bacteriocin	29316223	Probiotic Lactobacillus sp. inhibit growth, biofilm formation and gene expression of caries-inducing Streptococcus mutans.
1372	DRAMP00085	Bacteriocin	"Bacteriocin"[All Fields] AND biofilm[All Fields]	Bacteriocin	29180375	Formation and Characterization of Early Bacterial Biofilms on Different Wood Typologies Applied in Dairy Production.
1372	DRAMP00085	Bacteriocin	"Bacteriocin"[All Fields] AND biofilm[All Fields]	Bacteriocin	29096754	Overview of ribosomal and non-ribosomal antimicrobial peptides produced by Gram positive bacteria.
1372	DRAMP00085	Bacteriocin	"Bacteriocin"[All Fields] AND biofilm[All Fields]	Bacteriocin	29018429	D-Ribose Interferes with Quorum Sensing to Inhibit Biofilm Formation of Lactobacillus paraplantarumL-ZS9.
1372	DRAMP00085	Bacteriocin	"Bacteriocin"[All Fields] AND biofilm[All Fields]	Bacteriocin	28947088	Combined effect of bacteriocin produced by Lactobacillus plantarum ST8SH and vancomycin, propolis or EDTA for controlling biofilm development by Listeria monocytogenes.
1372	DRAMP00085	Bacteriocin	"Bacteriocin"[All Fields] AND biofilm[All Fields]	Bacteriocin	28905285	Effect of bacteriocin and exopolysaccharides isolated from probiotic on P. aeruginosa PAO1 biofilm.
1372	DRAMP00085	Bacteriocin	"Bacteriocin"[All Fields] AND biofilm[All Fields]	Bacteriocin	28887419	Oxidative Stressors Modify the Response of Streptococcus mutans to Its Competence Signal Peptides.
1372	DRAMP00085	Bacteriocin	"Bacteriocin"[All Fields] AND biofilm[All Fields]	Bacteriocin	28815637	Synergistic antibacterial and antibiofilm efficacy of nisin in combination with p-coumaric acid against food-borne bacteria Bacillus cereus and Salmonella typhimurium.
1372	DRAMP00085	Bacteriocin	"Bacteriocin"[All Fields] AND biofilm[All Fields]	Bacteriocin	28797211	Disulfide Bonds: A Key Modification in Bacterial Extracytoplasmic Proteins.
1372	DRAMP00085	Bacteriocin	"Bacteriocin"[All Fields] AND biofilm[All Fields]	Bacteriocin	28729051	Inhibiting effects of fructanase on competence-stimulating peptide-dependent quorum sensing system in Streptococcus mutans.
1372	DRAMP00085	Bacteriocin	"Bacteriocin"[All Fields] AND biofilm[All Fields]	Bacteriocin	28725299	Role of Streptococcus mutanstwo-component systems in antimicrobial peptide resistance in the oral cavity.
1372	DRAMP00085	Bacteriocin	"Bacteriocin"[All Fields] AND biofilm[All Fields]	Bacteriocin	28705677	Hyicin 4244, the first sactibiotic described in staphylococci, exhibits an anti-staphylococcal biofilm activity.
1372	DRAMP00085	Bacteriocin	"Bacteriocin"[All Fields] AND biofilm[All Fields]	Bacteriocin	28696370	Strategies for Pathogen Biocontrol Using Lactic Acid Bacteria and Their Metabolites: A Focus on Meat Ecosystems and Industrial Environments.
1372	DRAMP00085	Bacteriocin	"Bacteriocin"[All Fields] AND biofilm[All Fields]	Bacteriocin	28659548	Pathogenicity of Enterococci.
1372	DRAMP00085	Bacteriocin	"Bacteriocin"[All Fields] AND biofilm[All Fields]	Bacteriocin	28526785	Effects of Arginine on Streptococcus mutans Growth, Virulence Gene Expression, and Stress Tolerance.
1372	DRAMP00085	Bacteriocin	"Bacteriocin"[All Fields] AND biofilm[All Fields]	Bacteriocin	28457635	High-purity Nisin Alone or in Combination with Sodium Hypochlorite Is Effective against Planktonic and Biofilm Populations of Enterococcus faecalis.
1372	DRAMP00085	Bacteriocin	"Bacteriocin"[All Fields] AND biofilm[All Fields]	Bacteriocin	28396417	Enterococcus faecalisbacteriocin EntV inhibits hyphal morphogenesis, biofilm formation, and virulence of Candida albicans.
1372	DRAMP00085	Bacteriocin	"Bacteriocin"[All Fields] AND biofilm[All Fields]	Bacteriocin	28314902	Potential Factors Enabling Human Body Colonization by Animal Streptococcus dysgalactiae subsp. equisimilis Strains.
1372	DRAMP00085	Bacteriocin	"Bacteriocin"[All Fields] AND biofilm[All Fields]	Bacteriocin	28293865	The Inhibition Effect of Lactobacilli Against Growth and Biofilm Formation of Pseudomonas aeruginosa.
1372	DRAMP00085	Bacteriocin	"Bacteriocin"[All Fields] AND biofilm[All Fields]	Bacteriocin	28167518	Pleiotropic Regulation of Virulence Genes in Streptococcus mutans by the Conserved Small Protein SprV.
1372	DRAMP00085	Bacteriocin	"Bacteriocin"[All Fields] AND biofilm[All Fields]	Bacteriocin	28066817	Transcriptional Profiling of the Oral Pathogen Streptococcus mutansin Response to Competence Signaling Peptide XIP.
1372	DRAMP00085	Bacteriocin	"Bacteriocin"[All Fields] AND biofilm[All Fields]	Bacteriocin	28027492	Design of antibacterial biointerfaces by surface modification of poly (ε-caprolactone) with fusion protein containing hydrophobin and PA-1.
1372	DRAMP00085	Bacteriocin	"Bacteriocin"[All Fields] AND biofilm[All Fields]	Bacteriocin	27878401	Enterocin B3A-B3B produced by LAB collected from infant faeces: potential utilization in the food industry for Listeria monocytogenes biofilm management.
1372	DRAMP00085	Bacteriocin	"Bacteriocin"[All Fields] AND biofilm[All Fields]	Bacteriocin	27833601	Synergistic Nisin-Polymyxin Combinations for the Control of PseudomonasBiofilm Formation.
1372	DRAMP00085	Bacteriocin	"Bacteriocin"[All Fields] AND biofilm[All Fields]	Bacteriocin	27822519	Comprehensive Transcriptome Profiles of Streptococcus mutansUA159 Map Core Streptococcal Competence Genes.
1372	DRAMP00085	Bacteriocin	"Bacteriocin"[All Fields] AND biofilm[All Fields]	Bacteriocin	27797439	Inhibition of Listeria monocytogenes biofilms by bacteriocin-producing bacteria isolated from mushroom substrate.
1372	DRAMP00085	Bacteriocin	"Bacteriocin"[All Fields] AND biofilm[All Fields]	Bacteriocin	27695440	Nanotechnology: A Valuable Strategy to Improve Bacteriocin Formulations.
1372	DRAMP00085	Bacteriocin	"Bacteriocin"[All Fields] AND biofilm[All Fields]	Bacteriocin	27513424	The inhibitory effect of bacteriocin produced by Lactobacillus acidophilusATCC 4356 and Lactobacillus plantarum ATCC 8014 on planktonic cells and biofilms of Serratia marcescens.
1372	DRAMP00085	Bacteriocin	"Bacteriocin"[All Fields] AND biofilm[All Fields]	Bacteriocin	27422166	Characterization of a potential ABC-type bacteriocin exporter protein from Treponema denticola.
1372	DRAMP00085	Bacteriocin	"Bacteriocin"[All Fields] AND biofilm[All Fields]	Bacteriocin	27375584	Use of Potential Probiotic Lactic Acid Bacteria (LAB) Biofilms for the Control of Listeria monocytogenes, Salmonella Typhimurium, and Escherichia coli O157:H7 Biofilms Formation.
1372	DRAMP00085	Bacteriocin	"Bacteriocin"[All Fields] AND biofilm[All Fields]	Bacteriocin	27375583	Combinatorial Effects of Aromatic 1,3-Disubstituted Ureas and Fluoride on In vitro Inhibition of Streptococcus mutans Biofilm Formation.
1372	DRAMP00085	Bacteriocin	"Bacteriocin"[All Fields] AND biofilm[All Fields]	Bacteriocin	27359217	Kin Recognition in Bacteria.
1372	DRAMP00085	Bacteriocin	"Bacteriocin"[All Fields] AND biofilm[All Fields]	Bacteriocin	27312701	Comparative transcriptome analysis of the biocontrol strain Bacillus amyloliquefaciens FZB42 as response to biofilm formation analyzed by RNA sequencing.
1372	DRAMP00085	Bacteriocin	"Bacteriocin"[All Fields] AND biofilm[All Fields]	Bacteriocin	27257437	The efficacy of thuricin CD, tigecycline, vancomycin, teicoplanin, rifampicin and nitazoxanide, independently and in paired combinations against Clostridium difficile biofilms and planktonic cells.
1372	DRAMP00085	Bacteriocin	"Bacteriocin"[All Fields] AND biofilm[All Fields]	Bacteriocin	27190146	Control of Listeria monocytogenes biofilms on industrial surfaces by the bacteriocin-producing Lactobacillus sakei CRL1862.
1372	DRAMP00085	Bacteriocin	"Bacteriocin"[All Fields] AND biofilm[All Fields]	Bacteriocin	27161116	l-Arginine Modifies the Exopolysaccharide Matrix and Thwarts Streptococcus mutans Outgrowth within Mixed-Species Oral Biofilms.
1372	DRAMP00085	Bacteriocin	"Bacteriocin"[All Fields] AND biofilm[All Fields]	Bacteriocin	27148197	In Vitro Activities of Nisin and Nisin Derivatives Alone and In Combination with Antibiotics against Staphylococcus Biofilms.
1372	DRAMP00085	Bacteriocin	"Bacteriocin"[All Fields] AND biofilm[All Fields]	Bacteriocin	26999597	Nisin and lysostaphin activity against preformed biofilm of Staphylococcus aureus involved in bovine mastitis.
1372	DRAMP00085	Bacteriocin	"Bacteriocin"[All Fields] AND biofilm[All Fields]	Bacteriocin	26840124	Coordinated Bacteriocin Expression and Competence in Streptococcus pneumoniae Contributes to Genetic Adaptation through Neighbor Predation.
1372	DRAMP00085	Bacteriocin	"Bacteriocin"[All Fields] AND biofilm[All Fields]	Bacteriocin	26826230	A Highly Arginolytic Streptococcus Species That Potently Antagonizes Streptococcus mutans.
1372	DRAMP00085	Bacteriocin	"Bacteriocin"[All Fields] AND biofilm[All Fields]	Bacteriocin	26708985	A ptsP deficiency in PGPR Pseudomonas fluorescens SF39a affects bacteriocin production and bacterial fitness in the wheat rhizosphere.
1372	DRAMP00085	Bacteriocin	"Bacteriocin"[All Fields] AND biofilm[All Fields]	Bacteriocin	26678028	Biomedical applications of nisin.
1372	DRAMP00085	Bacteriocin	"Bacteriocin"[All Fields] AND biofilm[All Fields]	Bacteriocin	26660467	In Vitro Evaluation of Bacteriocins Activity Against Listeria monocytogenes Biofilm Formation.
1372	DRAMP00085	Bacteriocin	"Bacteriocin"[All Fields] AND biofilm[All Fields]	Bacteriocin	26591658	[Animal Staphylococcus felis with the potential to infect human skin].
1372	DRAMP00085	Bacteriocin	"Bacteriocin"[All Fields] AND biofilm[All Fields]	Bacteriocin	26527641	Mutation of the Thiol-Disulfide Oxidoreductase SdbA Activates the CiaRH Two-Component System, Leading to Bacteriocin Expression Shutdown in Streptococcus gordonii.
1372	DRAMP00085	Bacteriocin	"Bacteriocin"[All Fields] AND biofilm[All Fields]	Bacteriocin	26523633	Whole genome sequence to decipher the resistome of Shewanella algae, a multidrug-resistant bacterium responsible for pneumonia, Marseille, France.
1372	DRAMP00085	Bacteriocin	"Bacteriocin"[All Fields] AND biofilm[All Fields]	Bacteriocin	26481153	The outcome of H. influenzae and S. pneumoniae inter-species interactions depends on pH, nutrient availability and growth phase.
1372	DRAMP00085	Bacteriocin	"Bacteriocin"[All Fields] AND biofilm[All Fields]	Bacteriocin	26353398	[On the origin of Yersinia pestis, a causative agent of the plague: A concept of population-genetic macroevolution in transitive environment].
1372	DRAMP00085	Bacteriocin	"Bacteriocin"[All Fields] AND biofilm[All Fields]	Bacteriocin	26338114	Transfer, composition and technological characterization of the lactic acid bacterial populations of the wooden vats used to produce traditional stretched cheeses.

1372	DRAMP00085	Bacteriocin	"Bacteriocin"[All Fields] AND biofilm[All Fields]	Bacteriocin	26292786	Sonorensin: A new bacteriocin with potential of an anti-biofilm agent and a food biopreservative.
1372	DRAMP00085	Bacteriocin	"Bacteriocin"[All Fields] AND biofilm[All Fields]	Bacteriocin	26267163	Inhibitory effects of Lactobacillus fermentum on microbial growth and biofilm formation.
1372	DRAMP00085	Bacteriocin	"Bacteriocin"[All Fields] AND biofilm[All Fields]	Bacteriocin	26198853	Identification and characterization of SMU.244 encoding a putative undecaprenyl pyrophosphate phosphatase protein required for cell wall biosynthesis and bacitracin resistance in Streptococcus mutans.
1372	DRAMP00085	Bacteriocin	"Bacteriocin"[All Fields] AND biofilm[All Fields]	Bacteriocin	26131169	Comparison of antibacterial effects between antimicrobial peptide and bacteriocins isolated from Lactobacillus plantarum on three common pathogenic bacteria.
1372	DRAMP00085	Bacteriocin	"Bacteriocin"[All Fields] AND biofilm[All Fields]	Bacteriocin	26119252	Let there be bioluminescence: development of a biophotonic imaging platform for in situ analyses of oral biofilms in animal models.
1372	DRAMP00085	Bacteriocin	"Bacteriocin"[All Fields] AND biofilm[All Fields]	Bacteriocin	25588687	The Natural Antimicrobial Subtilosin A Synergizes with Lauramide Arginine Ethyl Ester (LAE), ε-Poly-L-lysine (Polylysine), Clindamycin Phosphate and Metronidazole, Against the Vaginal Pathogen Gardnerella vaginalis.
1372	DRAMP00085	Bacteriocin	"Bacteriocin"[All Fields] AND biofilm[All Fields]	Bacteriocin	25189864	Quorum sensing and biofilms in the pathogen, Streptococcus pneumoniae.
1372	DRAMP00085	Bacteriocin	"Bacteriocin"[All Fields] AND biofilm[All Fields]	Bacteriocin	25171407	Evolution of resistance to a last-resort antibiotic in Staphylococcus aureus via bacterial competition.
1372	DRAMP00085	Bacteriocin	"Bacteriocin"[All Fields] AND biofilm[All Fields]	Bacteriocin	24984799	Bacteriocin expression in sessile and planktonic populations of Escherichia coli.
1372	DRAMP00085	Bacteriocin	"Bacteriocin"[All Fields] AND biofilm[All Fields]	Bacteriocin	24806217	The effect of five probiotic lactobacilli strains on the growth and biofilm formation of Streptococcus mutans.
1372	DRAMP00085	Bacteriocin	"Bacteriocin"[All Fields] AND biofilm[All Fields]	Bacteriocin	24509500	Novel two-component regulatory systems play a role in biofilm formation of Lactobacillus reuteri rodent isolate 100-23.
1372	DRAMP00085	Bacteriocin	"Bacteriocin"[All Fields] AND biofilm[All Fields]	Bacteriocin	24148670	Proteins of novel lactic acid bacteria from Apis mellifera mellifera: an insight into the production of known extra-cellular proteins during microbial stress.
1372	DRAMP00085	Bacteriocin	"Bacteriocin"[All Fields] AND biofilm[All Fields]	Bacteriocin	24135676	Comparative proteomic analysis of Listeria monocytogenes exposed to enterocin AS-48 in planktonic and sessile states.
1372	DRAMP00085	Bacteriocin	"Bacteriocin"[All Fields] AND biofilm[All Fields]	Bacteriocin	24071026	Role of probiotics in the prevention and treatment of meticillin-resistant Staphylococcus aureus infections.
1372	DRAMP00085	Bacteriocin	"Bacteriocin"[All Fields] AND biofilm[All Fields]	Bacteriocin	23624069	In vitro probiotic properties of Lactobacillus fermentum SK5 isolated from vagina of a healthy woman.
1372	DRAMP00085	Bacteriocin	"Bacteriocin"[All Fields] AND biofilm[All Fields]	Bacteriocin	23615907	Functional analysis of paralogous thiol-disulfide oxidoreductases in Streptococcus gordonii.
1372	DRAMP00085	Bacteriocin	"Bacteriocin"[All Fields] AND biofilm[All Fields]	Bacteriocin	23558192	Combined treatments of enterocin AS-48 with biocides to improve the inactivation of methicillin-sensitive and methicillin-resistant Staphylococcus aureus planktonic and sessile cells.
1372	DRAMP00085	Bacteriocin	"Bacteriocin"[All Fields] AND biofilm[All Fields]	Bacteriocin	23421615	Global transcriptional responses to the bacteriocin colicin M in Escherichia coli.
1372	DRAMP00085	Bacteriocin	"Bacteriocin"[All Fields] AND biofilm[All Fields]	Bacteriocin	23398522	Phosphate limitation induces the intergeneric inhibition of Pseudomonas aeruginosa by Serratia marcescens isolated from paper machines.
1372	DRAMP00085	Bacteriocin	"Bacteriocin"[All Fields] AND biofilm[All Fields]	Bacteriocin	23318746	Are we ready for caries prevention through bacteriotherapy?
1372	DRAMP00085	Bacteriocin	"Bacteriocin"[All Fields] AND biofilm[All Fields]	Bacteriocin	23241973	Protective mechanisms of respiratory tract Streptococci against Streptococcus pyogenes biofilm formation and epithelial cell infection.
1372	DRAMP00085	Bacteriocin	"Bacteriocin"[All Fields] AND biofilm[All Fields]	Bacteriocin	23107045	Bacteriocin immunity proteins play a role in quorum-sensing system regulated antimicrobial sensitivity of Streptococcus mutans UA159.
1372	DRAMP00085	Bacteriocin	"Bacteriocin"[All Fields] AND biofilm[All Fields]	Bacteriocin	23063226	CRISPR-Cas, a prokaryotic adaptive immune system, in endodontic, oral, and multidrug-resistant hospital-acquired Enterococcus faecalis.
1372	DRAMP00085	Bacteriocin	"Bacteriocin"[All Fields] AND biofilm[All Fields]	Bacteriocin	23033644	Development of polyvinyl chloride biofilms for succession of selected marine bacterial populations.
1372	DRAMP00085	Bacteriocin	"Bacteriocin"[All Fields] AND biofilm[All Fields]	Bacteriocin	22921084	Recent patents on bacteriocins: food and biomedical applications.
1372	DRAMP00085	Bacteriocin	"Bacteriocin"[All Fields] AND biofilm[All Fields]	Bacteriocin	22470839	Studies on strains of Streptococcus mutans isolated from caries-active and caries-free individuals in Iceland.
1372	DRAMP00085	Bacteriocin	"Bacteriocin"[All Fields] AND biofilm[All Fields]	Bacteriocin	22290290	Isolation and identification of a bacteriocin with antibacterial and antibiofilm activity from Citrobacter freundii.
1372	DRAMP00085	Bacteriocin	"Bacteriocin"[All Fields] AND biofilm[All Fields]	Bacteriocin	22265284	Characterization of functional, safety, and probiotic properties of Enterococcus faecalis UGRA10, a new AS-48-producer strain.
1372	DRAMP00085	Bacteriocin	"Bacteriocin"[All Fields] AND biofilm[All Fields]	Bacteriocin	22265283	Effect of enterocin AS-48 in combination with biocides on planktonic and sessile Listeria monocytogenes.
1372	DRAMP00085	Bacteriocin	"Bacteriocin"[All Fields] AND biofilm[All Fields]	Bacteriocin	22228735	Regulation of bacteriocin production and cell death by the VicRK signaling system in Streptococcus mutans.
1372	DRAMP00085	Bacteriocin	"Bacteriocin"[All Fields] AND biofilm[All Fields]	Bacteriocin	22171244	Antagonistic effect of bacteriocin against urinary catheter associated Pseudomonas aeruginosa biofilm.
1372	DRAMP00085	Bacteriocin	"Bacteriocin"[All Fields] AND biofilm[All Fields]	Bacteriocin	22169745	[Effects of immA and immB coding putative bacteriocin immunity proteins on the antimicrobial sensitivity in planktonic Streptococcus mutans and biofilm formation].
1372	DRAMP00085	Bacteriocin	"Bacteriocin"[All Fields] AND biofilm[All Fields]	Bacteriocin	22155816	Identification, characterization, and recombinant expression of epidermicin NI01, a novel unmodified bacteriocin produced by Staphylococcus epidermidis that displays potent activity against Staphylococci.
1372	DRAMP00085	Bacteriocin	"Bacteriocin"[All Fields] AND biofilm[All Fields]	Bacteriocin	22089878	The evolution of bacteriocin production in bacterial biofilms.
1372	DRAMP00085	Bacteriocin	"Bacteriocin"[All Fields] AND biofilm[All Fields]	Bacteriocin	21984782	Regulation of the competence pathway as a novel role associated with a streptococcal bacteriocin.
1372	DRAMP00085	Bacteriocin	"Bacteriocin"[All Fields] AND biofilm[All Fields]	Bacteriocin	21538238	Inhibitory effect of Lactobacillus reuteri on periodontopathic and cariogenic bacteria.
1372	DRAMP00085	Bacteriocin	"Bacteriocin"[All Fields] AND biofilm[All Fields]	Bacteriocin	21466767	Bacterial characteristics of importance for recurrent urinary tract infections caused by Escherichia coli.
1372	DRAMP00085	Bacteriocin	"Bacteriocin"[All Fields] AND biofilm[All Fields]	Bacteriocin	21381381	[Disorganization of biofilms of clinical strains of staphylococci by metabolites of lactobacilli].
1372	DRAMP00085	Bacteriocin	"Bacteriocin"[All Fields] AND biofilm[All Fields]	Bacteriocin	20941586	Bacteria competing with the adhesion and biofilm formation by Staphylococcus aureus.
1372	DRAMP00085	Bacteriocin	"Bacteriocin"[All Fields] AND biofilm[All Fields]	Bacteriocin	20381010	Role of Streptococcus mutans eukaryotic-type serine/threonine protein kinase in interspecies interactions with Streptococcus sanguinis.
1372	DRAMP00085	Bacteriocin	"Bacteriocin"[All Fields] AND biofilm[All Fields]	Bacteriocin	20307569	A novel conjugative plasmid from Enterococcus faecalis E99 enhances resistance to ultraviolet radiation.
1372	DRAMP00085	Bacteriocin	"Bacteriocin"[All Fields] AND biofilm[All Fields]	Bacteriocin	20231406	The Streptococcus mutans serine/threonine kinase, PknB, regulates competence development, bacteriocin production, and cell wall metabolism.
1372	DRAMP00085	Bacteriocin	"Bacteriocin"[All Fields] AND biofilm[All Fields]	Bacteriocin	19828884	Bacterial interactions in dental biofilm development.
1372	DRAMP00085	Bacteriocin	"Bacteriocin"[All Fields] AND biofilm[All Fields]	Bacteriocin	19812852	Inhibiting effects of Enterococcus faecium non-biofilm strain on Streptococcus mutans biofilm formation.
1372	DRAMP00085	Bacteriocin	"Bacteriocin"[All Fields] AND biofilm[All Fields]	Bacteriocin	19767478	Complex phenotypic and genotypic responses of Listeria monocytogenes strains exposed to the class IIa bacteriocin sakacin P.
1372	DRAMP00085	Bacteriocin	"Bacteriocin"[All Fields] AND biofilm[All Fields]	Bacteriocin	19735463	Cell death in Streptococcus mutans biofilms: a link between CSP and extracellular DNA.
1372	DRAMP00085	Bacteriocin	"Bacteriocin"[All Fields] AND biofilm[All Fields]	Bacteriocin	19400789	Peptide alarmone signalling triggers an auto-active bacteriocin necessary for genetic competence.
1372	DRAMP00085	Bacteriocin	"Bacteriocin"[All Fields] AND biofilm[All Fields]	Bacteriocin	19185943	Knockout of three-component regulatory systems reveals that the apparently constitutive plantaricin-production phenotype shown by Lactobacillus plantarum on solid medium is regulated via quorum sensing.
1372	DRAMP00085	Bacteriocin	"Bacteriocin"[All Fields] AND biofilm[All Fields]	Bacteriocin	19138664	Effects of quorum sensing on cell viability in Streptococcus mutans biofilm formation.
1372	DRAMP00085	Bacteriocin	"Bacteriocin"[All Fields] AND biofilm[All Fields]	Bacteriocin	19124118	In vitro antimicrobial effect of bacteriocin PsVP-10 in combination with chlorhexidine and triclosan against Streptococcus mutans and Streptococcus sobrinus strains.
1372	DRAMP00085	Bacteriocin	"Bacteriocin"[All Fields] AND biofilm[All Fields]	Bacteriocin	18957580	Additive attenuation of virulence and cariogenic potential of Streptococcus mutans by simultaneous inactivation of the ComCDE quorum-sensing system and HK/RR11 two-component regulatory system.
1372	DRAMP00085	Bacteriocin	"Bacteriocin"[All Fields] AND biofilm[All Fields]	Bacteriocin	18804350	Novel anti-microbial therapies for dental plaque-related diseases.
1372	DRAMP00085	Bacteriocin	"Bacteriocin"[All Fields] AND biofilm[All Fields]	Bacteriocin	18792689	Quorum sensing and biofilm formation by Streptococcus mutans.
1372	DRAMP00085	Bacteriocin	"Bacteriocin"[All Fields] AND biofilm[All Fields]	Bacteriocin	18769851	Effect of bacterial interference on biofilm development by Legionella pneumophila.
1372	DRAMP00085	Bacteriocin	"Bacteriocin"[All Fields] AND biofilm[All Fields]	Bacteriocin	18541165	Prevalence and characterization of Enterococcus spp. isolated from Brazilian foods.
1372	DRAMP00085	Bacteriocin	"Bacteriocin"[All Fields] AND biofilm[All Fields]	Bacteriocin	18441055	Streptococcal antagonism in oral biofilms: Streptococcus sanguinis and Streptococcus gordonii interference with Streptococcus mutans.

1372	DRAMP00085	Bacteriocin	"Bacteriocin"[All Fields] AND biofilm[All Fields]	Bacteriocin	18405343	A multifaceted role for polyamines in bacterial pathogens.
1372	DRAMP00085	Bacteriocin	"Bacteriocin"[All Fields] AND biofilm[All Fields]	Bacteriocin	18387115	Biodegradable polylactic acid polymer with nisin for use in antimicrobial food packaging.
1372	DRAMP00085	Bacteriocin	"Bacteriocin"[All Fields] AND biofilm[All Fields]	Bacteriocin	18232718	Substrate recognition mechanism of the peptidase domain of the quorum-sensing-signal-producing ABC transporter ComA from Streptococcus.
1372	DRAMP00085	Bacteriocin	"Bacteriocin"[All Fields] AND biofilm[All Fields]	Bacteriocin	17981981	Autoinducer-2-regulated genes in Streptococcus mutans UA159 and global metabolic effect of the luxS mutation.
1372	DRAMP00085	Bacteriocin	"Bacteriocin"[All Fields] AND biofilm[All Fields]	Bacteriocin	17660440	Genetic characterization of the hdrRM operon: a novel high-cell-density-responsive regulator in Streptococcus mutans.
1372	DRAMP00085	Bacteriocin	"Bacteriocin"[All Fields] AND biofilm[All Fields]	Bacteriocin	16997961	Role of bacteriocin immunity proteins in the antimicrobial sensitivity of Streptococcus mutans.
1372	DRAMP00085	Bacteriocin	"Bacteriocin"[All Fields] AND biofilm[All Fields]	Bacteriocin	16981904	Cell density- and ComE-dependent expression of a group of mutacin and mutacin-like genes in Streptococcus mutans.
1372	DRAMP00085	Bacteriocin	"Bacteriocin"[All Fields] AND biofilm[All Fields]	Bacteriocin	16936029	Structure-activity analysis of quorum-sensing signaling peptides from Streptococcus mutans.
1372	DRAMP00085	Bacteriocin	"Bacteriocin"[All Fields] AND biofilm[All Fields]	Bacteriocin	16934112	Virulence properties of cariogenic bacteria.
1372	DRAMP00085	Bacteriocin	"Bacteriocin"[All Fields] AND biofilm[All Fields]	Bacteriocin	16925560	Purification and functional studies of a potent modified quorum-sensing peptide and a two-peptide bacteriocin in Streptococcus mutans.
1372	DRAMP00085	Bacteriocin	"Bacteriocin"[All Fields] AND biofilm[All Fields]	Bacteriocin	16907725	IrvA-dependent and IrvA-independent pathways for mutacin gene regulation in Streptococcus mutans.
1372	DRAMP00085	Bacteriocin	"Bacteriocin"[All Fields] AND biofilm[All Fields]	Bacteriocin	16869892	Vibrio harveyi: a significant pathogen of marine vertebrates and invertebrates.
1372	DRAMP00085	Bacteriocin	"Bacteriocin"[All Fields] AND biofilm[All Fields]	Bacteriocin	16304704	Growth of Enterococcus mundtii ST15 in medium filtrate and purification of bacteriocin ST15 by cation-exchange chromatography.
1372	DRAMP00085	Bacteriocin	"Bacteriocin"[All Fields] AND biofilm[All Fields]	Bacteriocin	15978073	Co-ordinated bacteriocin production and competence development: a possible mechanism for taking up DNA from neighbouring species.
1372	DRAMP00085	Bacteriocin	"Bacteriocin"[All Fields] AND biofilm[All Fields]	Bacteriocin	15937160	Regulation of bacteriocin production in Streptococcus mutans by the quorum-sensing system required for development of genetic competence.
1372	DRAMP00085	Bacteriocin	"Bacteriocin"[All Fields] AND biofilm[All Fields]	Bacteriocin	15640209	Interactions between oral bacteria: inhibition of Streptococcus mutans bacteriocin production by Streptococcus gordonii.
1372	DRAMP00085	Bacteriocin	"Bacteriocin"[All Fields] AND biofilm[All Fields]	Bacteriocin	15209996	Transcriptional analysis of mutacin I (mutA) gene expression in planktonic and biofilm cells of Streptococcus mutans using fluorescent protein and glucuronidase reporters.
1372	DRAMP00085	Bacteriocin	"Bacteriocin"[All Fields] AND biofilm[All Fields]	Bacteriocin	15151251	Quorum sensing: a primer for food microbiologists.
1372	DRAMP00085	Bacteriocin	"Bacteriocin"[All Fields] AND biofilm[All Fields]	Bacteriocin	12379833	[Protective role of the Doderleïn flora].
1372	DRAMP00085	Bacteriocin	"Bacteriocin"[All Fields] AND biofilm[All Fields]	Bacteriocin	12147084	Antagonistic interactions amongst bacteriocin-producing enteric bacteria in dual species biofilms.
1372	DRAMP00085	Bacteriocin	"Bacteriocin"[All Fields] AND biofilm[All Fields]	Bacteriocin	10574092	Behaviour of L. monocytogenes in an artificially made biofilm of a nisin-producing strain of Lactococcus lactis.
1381	DRAMP00096	Pediocin PA-1 (Pediocin ACH; Bacteriocin)	"Pediocin PA-1"[All Fields] AND biofilm[All Fields]	Pediocin PA-1	28027492	Design of antibacterial biointerfaces by surface modification of poly (ε-caprolactone) with fusion protein containing hydrophobin and PA-1.
1385	DRAMP00101	Sakacin P (Sakacin 674; Pediocin-like peptide; Bacteriocin)	"Sakacin P"[All Fields] AND biofilm[All Fields]	Sakacin P	19767478	Complex phenotypic and genotypic responses of Listeria monocytogenes strains exposed to the class IIa bacteriocin sakacin P.
1392	DRAMP00113	Enterocin A (EntA; Pediocin-like peptide; Bacteriocin)	"Enterocin A"[All Fields] AND biofilm[All Fields]	Enterocin A	33371410	Susceptibility to Enterocins and Lantibiotic Bacteriocins of Biofilm-Forming Enterococci Isolated from Slovak Fermented Meat Products Available on the Market.
1392	DRAMP00113	Enterocin A (EntA; Pediocin-like peptide; Bacteriocin)	"Enterocin A"[All Fields] AND biofilm[All Fields]	Enterocin A	31886252	Enterococci Isolated from Trout in the Bukovec Water Reservoir and Čierny Váh River in Slovakia and Their Safety Aspect.
1392	DRAMP00113	Enterocin A (EntA; Pediocin-like peptide; Bacteriocin)	"Enterocin A"[All Fields] AND biofilm[All Fields]	Enterocin A	30627971	Characterization of the bacteriocin produced by Enterococcus italicus ONU547 isolated from Thai fermented cabbage.
1392	DRAMP00113	Enterocin A (EntA; Pediocin-like peptide; Bacteriocin)	"Enterocin A"[All Fields] AND biofilm[All Fields]	Enterocin A	29729340	Cloning, overexpression, purification of bacteriocin enterocin-B and structural analysis, interaction determination of enterocin-A, B against pathogenic bacteria and human cancer cells.
1395	DRAMP18340	Daptomycin(Bacteriocin)	"Daptomycin"[All Fields] AND biofilm[All Fields]	Daptomycin	34576751	In Vitro Anti-Biofilm Activity of Bacteriophage K (ATCC 19685-B1) and Daptomycin against Staphylococci.
1395	DRAMP18340	Daptomycin(Bacteriocin)	"Daptomycin"[All Fields] AND biofilm[All Fields]	Daptomycin	34489569	Antibacterial and anti-biofilm activities of histidine kinase YycG inhibitors against Streptococcus agalactiae.
1395	DRAMP18340	Daptomycin(Bacteriocin)	"Daptomycin"[All Fields] AND biofilm[All Fields]	Daptomycin	34438947	Biofilm Time-Kill Curves to Assess the Bactericidal Activity of Daptomycin Combinations against Biofilm-Producing Vancomycin-Resistant Enterococcus faeciumand faecalis.
1395	DRAMP18340	Daptomycin(Bacteriocin)	"Daptomycin"[All Fields] AND biofilm[All Fields]	Daptomycin	34388322	Corynebacterium spp. - problematic pathogens of the human respiratory tract (review of literature).
1395	DRAMP18340	Daptomycin(Bacteriocin)	"Daptomycin"[All Fields] AND biofilm[All Fields]	Daptomycin	34285472	In vitro Antimicrobial Activity of Fosfomycin, Rifampin, Vancomycin, Daptomycin Alone and in Combination Against Vancomycin-Resistant Enterococci.
1395	DRAMP18340	Daptomycin(Bacteriocin)	"Daptomycin"[All Fields] AND biofilm[All Fields]	Daptomycin	34257170	Evaluation of the Antibacterial Efficacy of Daptomycin, Gentamicin, and Calcium Hydroxide-Antibiotic Combinations on Enterococcus faecalisDentinal Biofilm: An In VitroStudy.
1395	DRAMP18340	Daptomycin(Bacteriocin)	"Daptomycin"[All Fields] AND biofilm[All Fields]	Daptomycin	34228536	Exebacase Is Active In Vitroin Pulmonary Surfactant and Is Efficacious Alone and Synergistic with Daptomycin in a Mouse Model of Lethal Staphylococcus aureus Lung Infection.
1395	DRAMP18340	Daptomycin(Bacteriocin)	"Daptomycin"[All Fields] AND biofilm[All Fields]	Daptomycin	34159214	Analysis of Genetic Diversity and Antibiotic Options for Clinical Listeria monocytogenesInfections in China.
1395	DRAMP18340	Daptomycin(Bacteriocin)	"Daptomycin"[All Fields] AND biofilm[All Fields]	Daptomycin	34063146	Antimicrobial Photodynamic Inactivation Affects the Antibiotic Susceptibility of Enterococcusspp. Clinical Isolates in Biofilm and Planktonic Cultures.
1395	DRAMP18340	Daptomycin(Bacteriocin)	"Daptomycin"[All Fields] AND biofilm[All Fields]	Daptomycin	33946290	Catalase Protects Biofilm of Staphylococcus aureusagainst Daptomycin Activity.
1395	DRAMP18340	Daptomycin(Bacteriocin)	"Daptomycin"[All Fields] AND biofilm[All Fields]	Daptomycin	33856484	5-Fluorouracil blocks quorum-sensing of biofilm-embedded methicillin-resistant Staphylococcus aureus in mice.
1395	DRAMP18340	Daptomycin(Bacteriocin)	"Daptomycin"[All Fields] AND biofilm[All Fields]	Daptomycin	33750106	Discovery, Synthesis, and Optimization of Peptide-Based Antibiotics.
1395	DRAMP18340	Daptomycin(Bacteriocin)	"Daptomycin"[All Fields] AND biofilm[All Fields]	Daptomycin	33689487	In vitroTolerability of Biofilm-Forming Trimethoprim-/Sulfamethoxazole-Resistant Small Colony Variants of Staphylococcus aureusAgainst Various Antimicrobial Agents.
1395	DRAMP18340	Daptomycin(Bacteriocin)	"Daptomycin"[All Fields] AND biofilm[All Fields]	Daptomycin	33535792	High-Dose Daptomycin and Clinical Applications.
1395	DRAMP18340	Daptomycin(Bacteriocin)	"Daptomycin"[All Fields] AND biofilm[All Fields]	Daptomycin	33401579	The Role of Subinhibitory Concentrations of Daptomycin and Tigecycline in Modulating Virulence in Staphylococcus aureus.
1395	DRAMP18340	Daptomycin(Bacteriocin)	"Daptomycin"[All Fields] AND biofilm[All Fields]	Daptomycin	33401476	Novel Cecropin-4 Derived Peptides against Methicillin-Resistant Staphylococcus aureus.
1395	DRAMP18340	Daptomycin(Bacteriocin)	"Daptomycin"[All Fields] AND biofilm[All Fields]	Daptomycin	33321967	Efficacy of Daptomycin-Containing Regimen for Treatment of Staphylococcal or Enterococcal Vertebral Osteomyelitis: A Prospective Clinical Experience.
1395	DRAMP18340	Daptomycin(Bacteriocin)	"Daptomycin"[All Fields] AND biofilm[All Fields]	Daptomycin	33307275	In vitro anti-biofilm effect of anti-methicillin-resistant Staphylococcus aureus (anti-MRSA) agents against the USA300 clone.
1395	DRAMP18340	Daptomycin(Bacteriocin)	"Daptomycin"[All Fields] AND biofilm[All Fields]	Daptomycin	33262752	Evaluation of Staphylococcal Bacteriophage Sb-1 as an Adjunctive Agent to Antibiotics Against Rifampin-Resistant Staphylococcus aureusBiofilms.
1395	DRAMP18340	Daptomycin(Bacteriocin)	"Daptomycin"[All Fields] AND biofilm[All Fields]	Daptomycin	33114423	Staphylococcus epidermidisBiofilms Have a High Tolerance to Antibiotics in Periprosthetic Joint Infection.
1395	DRAMP18340	Daptomycin(Bacteriocin)	"Daptomycin"[All Fields] AND biofilm[All Fields]	Daptomycin	33072016	A Novel Peptide Antibiotic Produced by Streptomyces roseoflavusStrain INA-Ac-5812 With Directed Activity Against Gram-Positive Bacteria.
1395	DRAMP18340	Daptomycin(Bacteriocin)	"Daptomycin"[All Fields] AND biofilm[All Fields]	Daptomycin	32992772	Sequence Permutation Generates Peptides with Different Antimicrobial and Antibiofilm Activities.
1395	DRAMP18340	Daptomycin(Bacteriocin)	"Daptomycin"[All Fields] AND biofilm[All Fields]	Daptomycin	32983061	Tedizolid-Rifampicin Combination Prevents Rifampicin-Resistance on in vitroModel of Staphylococcus aureusMature Biofilm.
1395	DRAMP18340	Daptomycin(Bacteriocin)	"Daptomycin"[All Fields] AND biofilm[All Fields]	Daptomycin	32964392	In Vitro Antimicrobial Activity of Fosfomycin, Vancomycin and Daptomycin Alone, and in Combination, Against Linezolid-Resistant Enterococcus faecalis.
1395	DRAMP18340	Daptomycin(Bacteriocin)	"Daptomycin"[All Fields] AND biofilm[All Fields]	Daptomycin	32806230	Titanium coating with mussel inspired polymer and bio-orthogonal chemistry enhances antimicrobial activity against Staphylococcus aureus.
1395	DRAMP18340	Daptomycin(Bacteriocin)	"Daptomycin"[All Fields] AND biofilm[All Fields]	Daptomycin	32650057	Antibacterial and antibiofilm effects of flufenamic acid against methicillin-resistant Staphylococcus aureus.
1395	DRAMP18340	Daptomycin(Bacteriocin)	"Daptomycin"[All Fields] AND biofilm[All Fields]	Daptomycin	32494168	In vitro Antibiotic Susceptibility, Virulence Genes Distribution and Biofilm Production of Staphylococcus aureusIsolates from Bovine Mastitis in the Liaoning Province of China.
1395	DRAMP18340	Daptomycin(Bacteriocin)	"Daptomycin"[All Fields] AND biofilm[All Fields]	Daptomycin	32393645	Evolution of vancomycin-resistant Enterococcus faeciumduring colonization and infection in immunocompromised pediatric patients.
1395	DRAMP18340	Daptomycin(Bacteriocin)	"Daptomycin"[All Fields] AND biofilm[All Fields]	Daptomycin	32354459	In vitro activity of TNP-2092 against periprosthetic joint infection-associated staphylococci.

1395	DRAMP18340	Daptomycin(Bacteriocin)	"Daptomycin"[All Fields] AND biofilm[All Fields]	Daptomycin	32094136	Impact of Daptomycin Dose Exposure Alone or in Combination with β-Lactams or Rifampin against Vancomycin-Resistant Enterococci in an In VitroBiofilm Model.
1395	DRAMP18340	Daptomycin(Bacteriocin)	"Daptomycin"[All Fields] AND biofilm[All Fields]	Daptomycin	32013708	Antibiotic Tolerance in Biofilm and Stationary-Phase Planktonic Cells of Staphylococcus aureus.
1395	DRAMP18340	Daptomycin(Bacteriocin)	"Daptomycin"[All Fields] AND biofilm[All Fields]	Daptomycin	31916929	In vitroactivities of telithromycin against Staphylococcus aureusbiofilms compared with azithromycin, clindamycin, vancomycin and daptomycin.
1395	DRAMP18340	Daptomycin(Bacteriocin)	"Daptomycin"[All Fields] AND biofilm[All Fields]	Daptomycin	31881851	Enterococcal periprosthetic joint infection: clinical and microbiological findings from an 8-year retrospective cohort study.
1395	DRAMP18340	Daptomycin(Bacteriocin)	"Daptomycin"[All Fields] AND biofilm[All Fields]	Daptomycin	31704562	Environmental fate processes of antimicrobial peptides daptomycin, bacitracins, and polymyxins.
1395	DRAMP18340	Daptomycin(Bacteriocin)	"Daptomycin"[All Fields] AND biofilm[All Fields]	Daptomycin	31638894	Microbial biofilm correlates with an increased antibiotic tolerance and poor therapeutic outcome in infective endocarditis.
1395	DRAMP18340	Daptomycin(Bacteriocin)	"Daptomycin"[All Fields] AND biofilm[All Fields]	Daptomycin	31636263	Enantiomeric glycosylated cationic block co-beta-peptides eradicate Staphylococcus aureus biofilms and antibiotic-tolerant persisters.
1395	DRAMP18340	Daptomycin(Bacteriocin)	"Daptomycin"[All Fields] AND biofilm[All Fields]	Daptomycin	31624909	Comparative in vitro activity of bacteriophage endolysin HY-133 against Staphylococcus aureus attached to vascular graft surface.
1395	DRAMP18340	Daptomycin(Bacteriocin)	"Daptomycin"[All Fields] AND biofilm[All Fields]	Daptomycin	31454398	Effect of daptomycin and vancomycin on Staphylococcus epidermidis biofilms: An in vitro assessment using fluorescence in situ hybridization.
1395	DRAMP18340	Daptomycin(Bacteriocin)	"Daptomycin"[All Fields] AND biofilm[All Fields]	Daptomycin	31357945	Bloodstream and catheter-related infections due to different clones of multidrug-resistant and biofilm producer Corynebacterium striatum.
1395	DRAMP18340	Daptomycin(Bacteriocin)	"Daptomycin"[All Fields] AND biofilm[All Fields]	Daptomycin	31332078	Environment Shapes the Accessible Daptomycin Resistance Mechanisms in Enterococcus faecium.
1395	DRAMP18340	Daptomycin(Bacteriocin)	"Daptomycin"[All Fields] AND biofilm[All Fields]	Daptomycin	31241446	In vitro bactericidal activity of levonadifloxacin (WCK 771) against methicillin- and quinolone-resistant Staphylococcus aureus biofilms.
1395	DRAMP18340	Daptomycin(Bacteriocin)	"Daptomycin"[All Fields] AND biofilm[All Fields]	Daptomycin	31207214	Daptomycin to bone and joint infections and prosthesis joint infections: a systematic review.
1395	DRAMP18340	Daptomycin(Bacteriocin)	"Daptomycin"[All Fields] AND biofilm[All Fields]	Daptomycin	31131594	Fosfomycin in antimicrobial stewardship programs.
1395	DRAMP18340	Daptomycin(Bacteriocin)	"Daptomycin"[All Fields] AND biofilm[All Fields]	Daptomycin	31099708	Morphological and Biological Characteristics of Staphylococcus aureusBiofilm Formed in the Presence of Plasma.
1395	DRAMP18340	Daptomycin(Bacteriocin)	"Daptomycin"[All Fields] AND biofilm[All Fields]	Daptomycin	31049562	In vitro antimicrobial activity against Abiotrophia defectiva and Granulicatella elegans biofilms.
1395	DRAMP18340	Daptomycin(Bacteriocin)	"Daptomycin"[All Fields] AND biofilm[All Fields]	Daptomycin	31036688	Spontaneously Arising Streptococcus mutans Variants with Reduced Susceptibility to Chlorhexidine Display Genetic Defects and Diminished Fitness.
1395	DRAMP18340	Daptomycin(Bacteriocin)	"Daptomycin"[All Fields] AND biofilm[All Fields]	Daptomycin	31031170	Intestinal Bile Acids Induce a Morphotype Switch in Vancomycin-Resistant Enterococcus that Facilitates Intestinal Colonization.
1395	DRAMP18340	Daptomycin(Bacteriocin)	"Daptomycin"[All Fields] AND biofilm[All Fields]	Daptomycin	30946803	Stationary phase persister/biofilm microcolony of Borrelia burgdorferi causes more severe disease in a mouse model of Lyme arthritis: implications for understanding persistence, Post-treatment Lyme Disease Syndrome (PTLDS), and treatment failure.
1395	DRAMP18340	Daptomycin(Bacteriocin)	"Daptomycin"[All Fields] AND biofilm[All Fields]	Daptomycin	30936103	Postantibiotic and Sub-MIC Effects of Exebacase (Lysin CF-301) Enhance Antimicrobial Activity against Staphylococcus aureus.
1395	DRAMP18340	Daptomycin(Bacteriocin)	"Daptomycin"[All Fields] AND biofilm[All Fields]	Daptomycin	30919513	Large variations in clinical antibiotic activity against Staphylococcus aureus biofilms of periprosthetic joint infection isolates.
1395	DRAMP18340	Daptomycin(Bacteriocin)	"Daptomycin"[All Fields] AND biofilm[All Fields]	Daptomycin	30910809	Role of epaQ, a Previously Uncharacterized Enterococcus faecalis Gene, in Biofilm Development and Antimicrobial Resistance.
1395	DRAMP18340	Daptomycin(Bacteriocin)	"Daptomycin"[All Fields] AND biofilm[All Fields]	Daptomycin	30882300	In vitro activities of daptomycin combined with fosfomycin or rifampin on planktonic and adherent linezolid-resistant isolates of Enterococcus faecalis.
1395	DRAMP18340	Daptomycin(Bacteriocin)	"Daptomycin"[All Fields] AND biofilm[All Fields]	Daptomycin	30858842	Isothermal Microcalorimetry Detects the Presence of Persister Cells in a Staphylococcus aureusBiofilm After Vancomycin Treatment.
1395	DRAMP18340	Daptomycin(Bacteriocin)	"Daptomycin"[All Fields] AND biofilm[All Fields]	Daptomycin	30855125	3D Bioprinted Scaffolds Containing Viable Macrophages and Antibiotics Promote Clearance of Staphylococcus aureus Craniotomy-Associated Biofilm Infection.
1395	DRAMP18340	Daptomycin(Bacteriocin)	"Daptomycin"[All Fields] AND biofilm[All Fields]	Daptomycin	30836263	Biotransformation of antibiotics: Exploring the activity of extracellular and intracellular enzymes derived from wastewater microbial communities.
1395	DRAMP18340	Daptomycin(Bacteriocin)	"Daptomycin"[All Fields] AND biofilm[All Fields]	Daptomycin	30770577	An in vitro biofilm model of Staphylococcus aureus infection of bone.
1395	DRAMP18340	Daptomycin(Bacteriocin)	"Daptomycin"[All Fields] AND biofilm[All Fields]	Daptomycin	30731490	Neutrophil Extracellular Traps Enhance Staphylococcus Aureus Vegetation Formation through Interaction with Platelets in Infective Endocarditis.
1395	DRAMP18340	Daptomycin(Bacteriocin)	"Daptomycin"[All Fields] AND biofilm[All Fields]	Daptomycin	30617095	Evolution of Daptomycin Resistance in Coagulase-Negative Staphylococci Involves Mutations of the Essential Two-Component Regulator WalKR.
1395	DRAMP18340	Daptomycin(Bacteriocin)	"Daptomycin"[All Fields] AND biofilm[All Fields]	Daptomycin	30428581	In Vitro Efficacy of Antibiotics Released from Calcium Sulfate Bone Void Filler Beads.
1395	DRAMP18340	Daptomycin(Bacteriocin)	"Daptomycin"[All Fields] AND biofilm[All Fields]	Daptomycin	30416945	Daptomycin Plus Fosfomycin as Salvage Therapy in a Difficult-to-Treat Total Femoral Replacement Infection.
1395	DRAMP18340	Daptomycin(Bacteriocin)	"Daptomycin"[All Fields] AND biofilm[All Fields]	Daptomycin	30348582	Hip and Knee Section, Treatment, Antimicrobials: Proceedings of International Consensus on Orthopedic Infections.
1395	DRAMP18340	Daptomycin(Bacteriocin)	"Daptomycin"[All Fields] AND biofilm[All Fields]	Daptomycin	30236955	Bacteriophage Sb-1 enhances antibiotic activity against biofilm, degrades exopolysaccharide matrix and targets persisters of Staphylococcus aureus.
1395	DRAMP18340	Daptomycin(Bacteriocin)	"Daptomycin"[All Fields] AND biofilm[All Fields]	Daptomycin	30153487	Acrylic microparticles increase daptomycin intracellular and in vivo anti-biofilm activity against Staphylococcus aureus.
1395	DRAMP18340	Daptomycin(Bacteriocin)	"Daptomycin"[All Fields] AND biofilm[All Fields]	Daptomycin	30064700	Daptomycin-loaded biodegradable thermosensitive hydrogels enhance drug stability and foster bactericidal activity against Staphylococcus aureus.
1395	DRAMP18340	Daptomycin(Bacteriocin)	"Daptomycin"[All Fields] AND biofilm[All Fields]	Daptomycin	29877755	Combatting resistant enterococcal infections: a pharmacotherapy review.
1395	DRAMP18340	Daptomycin(Bacteriocin)	"Daptomycin"[All Fields] AND biofilm[All Fields]	Daptomycin	29784838	In Vivoand In VitroEffects of a ClpP-Activating Antibiotic against Vancomycin-Resistant Enterococci.
1395	DRAMP18340	Daptomycin(Bacteriocin)	"Daptomycin"[All Fields] AND biofilm[All Fields]	Daptomycin	29781552	Topical rifampin powder for orthopedic trauma part I: Rifampin powder reduces recalcitrant infection in a delayed treatment musculoskeletal trauma model.
1395	DRAMP18340	Daptomycin(Bacteriocin)	"Daptomycin"[All Fields] AND biofilm[All Fields]	Daptomycin	29744925	Microcalorimetric detection of staphylococcal biofilm growth on various prosthetic biomaterials after exposure to daptomycin.
1395	DRAMP18340	Daptomycin(Bacteriocin)	"Daptomycin"[All Fields] AND biofilm[All Fields]	Daptomycin	29735564	Impact of Bacterial Membrane Fatty Acid Composition on the Failure of Daptomycin To Kill Staphylococcus aureus.
1395	DRAMP18340	Daptomycin(Bacteriocin)	"Daptomycin"[All Fields] AND biofilm[All Fields]	Daptomycin	29712650	Activities of Combinations of Antistaphylococcal Antibiotics with Fusidic Acid against Staphylococcal Biofilms in In VitroStatic and Dynamic Models.
1395	DRAMP18340	Daptomycin(Bacteriocin)	"Daptomycin"[All Fields] AND biofilm[All Fields]	Daptomycin	29582407	In Vitro Activity of Linezolid, Daptomycin and N-acetylcysteine Agents on Staphylococcus Aureus Biofilms in the Ventriculoperitoneal Shunt Model.
1395	DRAMP18340	Daptomycin(Bacteriocin)	"Daptomycin"[All Fields] AND biofilm[All Fields]	Daptomycin	29554250	Unexpected synergistic and antagonistic antibiotic activity against Staphylococcus biofilms.
1395	DRAMP18340	Daptomycin(Bacteriocin)	"Daptomycin"[All Fields] AND biofilm[All Fields]	Daptomycin	29488195	Encapsulation in Polymeric Microparticles Improves Daptomycin Activity Against Mature Staphylococci Biofilms-a Thermal and Imaging Study.
1395	DRAMP18340	Daptomycin(Bacteriocin)	"Daptomycin"[All Fields] AND biofilm[All Fields]	Daptomycin	29452993	An in vitro evaluation of the efficacy of tedizolid: implications for the treatment of skin and soft tissue infections.
1395	DRAMP18340	Daptomycin(Bacteriocin)	"Daptomycin"[All Fields] AND biofilm[All Fields]	Daptomycin	29195766	Dalbavancin is active in vitro against biofilms formed by dalbavancin-susceptible enterococci.
1395	DRAMP18340	Daptomycin(Bacteriocin)	"Daptomycin"[All Fields] AND biofilm[All Fields]	Daptomycin	29186026	Antimicrobial Activity of Bee Venom and Melittin against Borrelia burgdorferi.
1395	DRAMP18340	Daptomycin(Bacteriocin)	"Daptomycin"[All Fields] AND biofilm[All Fields]	Daptomycin	32987970	Quaternary ammonium-induced multidrug tolerant Streptococcus mutans persisters elevate cariogenic virulence in vitro.
1395	DRAMP18340	Daptomycin(Bacteriocin)	"Daptomycin"[All Fields] AND biofilm[All Fields]	Daptomycin	29158277	High-Dose Daptomycin Is Effective as an Antibiotic Lock Therapy in a Rabbit Model of Staphylococcus epidermidis Catheter-Related Infection.
1395	DRAMP18340	Daptomycin(Bacteriocin)	"Daptomycin"[All Fields] AND biofilm[All Fields]	Daptomycin	29075628	Selective Essential Oils from Spice or Culinary Herbs Have High Activity against Stationary Phase and Biofilm Borrelia burgdorferi.
1395	DRAMP18340	Daptomycin(Bacteriocin)	"Daptomycin"[All Fields] AND biofilm[All Fields]	Daptomycin	29059358	Daptomycin.
1395	DRAMP18340	Daptomycin(Bacteriocin)	"Daptomycin"[All Fields] AND biofilm[All Fields]	Daptomycin	29049010	Biofilm-Related Diseases and Omics: Global Transcriptional Profiling of Enterococcus faecium Reveals Different Gene Expression Patterns in the Biofilm and Planktonic Cells.
1395	DRAMP18340	Daptomycin(Bacteriocin)	"Daptomycin"[All Fields] AND biofilm[All Fields]	Daptomycin	29025325	Versatility of targeted antibiotic-loaded gold nanoconstructs for the treatment of biofilm-associated bacterial infections.
1395	DRAMP18340	Daptomycin(Bacteriocin)	"Daptomycin"[All Fields] AND biofilm[All Fields]	Daptomycin	28961884	Synergistic antibiotic activity against planktonic and biofilm-embedded Streptococcus agalactiae, Streptococcus pyogenes and Streptococcus oralis.
1395	DRAMP18340	Daptomycin(Bacteriocin)	"Daptomycin"[All Fields] AND biofilm[All Fields]	Daptomycin	28929959	[In vitro effect of vancomycin and daptomycin on biofilm formation of coagulase-negative staphylococci strains].
1395	DRAMP18340	Daptomycin(Bacteriocin)	"Daptomycin"[All Fields] AND biofilm[All Fields]	Daptomycin	28559263	Efficient Killing of Planktonic and Biofilm-Embedded Coagulase-Negative Staphylococci by Bactericidal Protein P128.
1395	DRAMP18340	Daptomycin(Bacteriocin)	"Daptomycin"[All Fields] AND biofilm[All Fields]	Daptomycin	28534905	Antimicrobial peptide-inspired NH125 analogues: bacterial and fungal biofilm-eradicating agents and rapid killers of MRSA persisters.
1395	DRAMP18340	Daptomycin(Bacteriocin)	"Daptomycin"[All Fields] AND biofilm[All Fields]	Daptomycin	28441320	Hyperbaric Oxygen Therapy is Ineffective as an Adjuvant to Daptomycin with Rifampicin Treatment in a Murine Model of Staphylococcus aureus in Implant-Associated Osteomyelitis.

1395	DRAMP18340	Daptomycin(Bacteriocin)	"Daptomycin"[All Fields] AND biofilm[All Fields]	Daptomycin	28438938	A Tick Antivirulence Protein Potentiates Antibiotics against Staphylococcus aureus.
1395	DRAMP18340	Daptomycin(Bacteriocin)	"Daptomycin"[All Fields] AND biofilm[All Fields]	Daptomycin	28225264	Fatty Acid Comprising Lysine Conjugates: Anti-MRSA Agents That Display In Vivo Efficacy by Disrupting Biofilms with No Resistance Development.
1395	DRAMP18340	Daptomycin(Bacteriocin)	"Daptomycin"[All Fields] AND biofilm[All Fields]	Daptomycin	28188831	Lipoteichoic acid synthesis inhibition in combination with antibiotics abrogates growth of multidrug-resistant Enterococcus faecium.
1395	DRAMP18340	Daptomycin(Bacteriocin)	"Daptomycin"[All Fields] AND biofilm[All Fields]	Daptomycin	28110918	Nitroxoline: a broad-spectrum biofilm-eradicating agent against pathogenic bacteria.
1395	DRAMP18340	Daptomycin(Bacteriocin)	"Daptomycin"[All Fields] AND biofilm[All Fields]	Daptomycin	28108098	Comparative efficacies of daptomycin, vancomycin, and linezolid in experimental enterococcal peritonitis.
1395	DRAMP18340	Daptomycin(Bacteriocin)	"Daptomycin"[All Fields] AND biofilm[All Fields]	Daptomycin	28049149	RelA Mutant Enterococcus faecium with Multiantibiotic Tolerance Arising in an Immunocompromised Host.
1395	DRAMP18340	Daptomycin(Bacteriocin)	"Daptomycin"[All Fields] AND biofilm[All Fields]	Daptomycin	28011203	Small molecule mimics of DFTamP1, a database designed anti-Staphylococcal peptide.
1395	DRAMP18340	Daptomycin(Bacteriocin)	"Daptomycin"[All Fields] AND biofilm[All Fields]	Daptomycin	27867375	Ceftriaxone Pulse Dosing Fails to Eradicate Biofilm-Like Microcolony B. burgdorferiPersisters Which Are Sterilized by Daptomycin/ Doxycycline/Cefuroxime without Pulse Dosing.
1395	DRAMP18340	Daptomycin(Bacteriocin)	"Daptomycin"[All Fields] AND biofilm[All Fields]	Daptomycin	29634108	ANTI-ADHESIVE AND ANTI-BIOFILM ACTIVITIES IN VITRO OF LINEZOLID, VANCOMYCIN, TIGECYCLINE AND DAPTOMYCIN AGAINST STAPHYLOCOCCUS HAEMOLYTICUS.
1395	DRAMP18340	Daptomycin(Bacteriocin)	"Daptomycin"[All Fields] AND biofilm[All Fields]	Daptomycin	27775462	Reduced ability to detect surface-related biofilm bacteria after antibiotic exposure under in vitro conditions.
1395	DRAMP18340	Daptomycin(Bacteriocin)	"Daptomycin"[All Fields] AND biofilm[All Fields]	Daptomycin	27681928	Streptokinase Treatment Reverses Biofilm-Associated Antibiotic Resistance in Staphylococcus aureus.
1395	DRAMP18340	Daptomycin(Bacteriocin)	"Daptomycin"[All Fields] AND biofilm[All Fields]	Daptomycin	27671070	Antibiofilm Activity and Synergistic Inhibition of Staphylococcus aureus Biofilms by Bactericidal Protein P128 in Combination with Antibiotics.
1395	DRAMP18340	Daptomycin(Bacteriocin)	"Daptomycin"[All Fields] AND biofilm[All Fields]	Daptomycin	27639707	The anti-biofilm effect of macrolides in a rat model of S. aureus foreign-body infection: Might it be of clinical relevance?
1395	DRAMP18340	Daptomycin(Bacteriocin)	"Daptomycin"[All Fields] AND biofilm[All Fields]	Daptomycin	27478608	Newer antibiotics for the treatment of peritoneal dialysis-related peritonitis.
1395	DRAMP18340	Daptomycin(Bacteriocin)	"Daptomycin"[All Fields] AND biofilm[All Fields]	Daptomycin	27441208	Synergistic Photothermal and Antibiotic Killing of Biofilm-Associated Staphylococcus aureusUsing Targeted Antibiotic-Loaded Gold Nanoconstructs.
1395	DRAMP18340	Daptomycin(Bacteriocin)	"Daptomycin"[All Fields] AND biofilm[All Fields]	Daptomycin	27401574	Evaluation of Antibiotics Active against Methicillin-Resistant Staphylococcus aureus Based on Activity in an Established Biofilm.
1395	DRAMP18340	Daptomycin(Bacteriocin)	"Daptomycin"[All Fields] AND biofilm[All Fields]	Daptomycin	27316688	Preliminary results of a new antibiotic susceptibility test against biofilm installation in device-associated infections: the Antibiofilmogram®.
1395	DRAMP18340	Daptomycin(Bacteriocin)	"Daptomycin"[All Fields] AND biofilm[All Fields]	Daptomycin	27297479	New Insight into Daptomycin Bioavailability and Localization in Staphylococcus aureus Biofilms by Dynamic Fluorescence Imaging.
1395	DRAMP18340	Daptomycin(Bacteriocin)	"Daptomycin"[All Fields] AND biofilm[All Fields]	Daptomycin	27295353	Effects of vancomycin, daptomycin, and tigecycline on coagulase-negative staphylococcus biofilm and bacterial viability within biofilm: an in vitro biofilm model.
1395	DRAMP18340	Daptomycin(Bacteriocin)	"Daptomycin"[All Fields] AND biofilm[All Fields]	Daptomycin	27292911	Sub-inhibitory tigecycline concentrations induce extracellular matrix binding protein Embp dependent Staphylococcus epidermidis biofilm formation and immune evasion.
1395	DRAMP18340	Daptomycin(Bacteriocin)	"Daptomycin"[All Fields] AND biofilm[All Fields]	Daptomycin	30015446	Characterization of Staphylococcal Cassette Chromosome mec (SCCmec) in Methicillin-Resistant Staphylococcus epidermidis Strains Isolated from Biomaterial-Associated Infections and their Antibiotic Resistance Patterns.
1395	DRAMP18340	Daptomycin(Bacteriocin)	"Daptomycin"[All Fields] AND biofilm[All Fields]	Daptomycin	27154750	Synergistic activity between an antimicrobial polyacrylamide and daptomycin versus Staphylococcus aureus biofilm.
1395	DRAMP18340	Daptomycin(Bacteriocin)	"Daptomycin"[All Fields] AND biofilm[All Fields]	Daptomycin	27036412	Rifampicin-containing combinations are superior to combinations of vancomycin, linezolid and daptomycin against Staphylococcus aureus biofilm infection in vivo and in vitro.
1395	DRAMP18340	Daptomycin(Bacteriocin)	"Daptomycin"[All Fields] AND biofilm[All Fields]	Daptomycin	26980093	Streptococcus suis in invasive human infections in Poland: clonality and determinants of virulence and antimicrobial resistance.
1395	DRAMP18340	Daptomycin(Bacteriocin)	"Daptomycin"[All Fields] AND biofilm[All Fields]	Daptomycin	26946510	Invasive enterococcal infections in Poland: the current epidemiological situation.
1395	DRAMP18340	Daptomycin(Bacteriocin)	"Daptomycin"[All Fields] AND biofilm[All Fields]	Daptomycin	26926633	In Vitro Approach for Identification of the Most Effective Agents for Antimicrobial Lock Therapy in the Treatment of Intravascular Catheter-Related Infections Caused by Staphylococcus aureus.
1395	DRAMP18340	Daptomycin(Bacteriocin)	"Daptomycin"[All Fields] AND biofilm[All Fields]	Daptomycin	26903956	Eradication of Biofilm-Like Microcolony Structures of Borrelia burgdorferi by Daunomycin and Daptomycin but not Mitomycin C in Combination with Doxycycline and Cefuroxime.
1395	DRAMP18340	Daptomycin(Bacteriocin)	"Daptomycin"[All Fields] AND biofilm[All Fields]	Daptomycin	26883700	Preventing Implant-Associated Infections by Silver Coating.
1395	DRAMP18340	Daptomycin(Bacteriocin)	"Daptomycin"[All Fields] AND biofilm[All Fields]	Daptomycin	26851611	New in vitro and in vivo models to evaluate antibiotic efficacy in Staphylococcus aureus prosthetic vascular graft infection.
1395	DRAMP18340	Daptomycin(Bacteriocin)	"Daptomycin"[All Fields] AND biofilm[All Fields]	Daptomycin	26833157	A High-Affinity Native Human Antibody Disrupts Biofilm from Staphylococcus aureus Bacteria and Potentiates Antibiotic Efficacy in a Mouse Implant Infection Model.
1395	DRAMP18340	Daptomycin(Bacteriocin)	"Daptomycin"[All Fields] AND biofilm[All Fields]	Daptomycin	26824954	Regulatory Mutations Impacting Antibiotic Susceptibility in an Established Staphylococcus aureus Biofilm.
1395	DRAMP18340	Daptomycin(Bacteriocin)	"Daptomycin"[All Fields] AND biofilm[All Fields]	Daptomycin	26797915	Should daptomycin-rifampin combinations for MSSA/MRSA isolates be avoided because of antagonism?
1395	DRAMP18340	Daptomycin(Bacteriocin)	"Daptomycin"[All Fields] AND biofilm[All Fields]	Daptomycin	28520329	Characterization of Staphylococcal Cassette Chromosome mec (SCCmec) in Methicillin-Resistant Staphylococcus epidermidis Strains Isolated from Biomaterial-Associated Infections and their Antibiotic Resistance Patterns.
1395	DRAMP18340	Daptomycin(Bacteriocin)	"Daptomycin"[All Fields] AND biofilm[All Fields]	Daptomycin	26716015	Effectiveness of Stevia Rebaudiana Whole Leaf Extract Against the Various Morphological Forms of Borrelia Burgdorferi in Vitro.
1395	DRAMP18340	Daptomycin(Bacteriocin)	"Daptomycin"[All Fields] AND biofilm[All Fields]	Daptomycin	26518881	In vitro efficacy of daptomycin and teicoplanin combined with ethanol, clarithromycin or gentamicin as catheter lock solutions.
1395	DRAMP18340	Daptomycin(Bacteriocin)	"Daptomycin"[All Fields] AND biofilm[All Fields]	Daptomycin	26480852	Halogenated Phenazines that Potently Eradicate Biofilms, MRSA Persister Cells in Non-Biofilm Cultures, and Mycobacterium tuberculosis.
1395	DRAMP18340	Daptomycin(Bacteriocin)	"Daptomycin"[All Fields] AND biofilm[All Fields]	Daptomycin	26430942	Increasing Trend of Heterogeneous Vancomycin Intermediate Staphylococcus aureus in a Tertiary Care Center of Northern India.
1395	DRAMP18340	Daptomycin(Bacteriocin)	"Daptomycin"[All Fields] AND biofilm[All Fields]	Daptomycin	26369963	Observed Antagonistic Effect of Linezolid on Daptomycin or Vancomycin Activity against Biofilm-Forming Methicillin-Resistant Staphylococcus aureus in an In Vitro Pharmacodynamic Model.
1395	DRAMP18340	Daptomycin(Bacteriocin)	"Daptomycin"[All Fields] AND biofilm[All Fields]	Daptomycin	26367403	New Robbins device to evaluate antimicrobial activity against bacterial biofilms on central venous catheters.
1395	DRAMP18340	Daptomycin(Bacteriocin)	"Daptomycin"[All Fields] AND biofilm[All Fields]	Daptomycin	26297246	Effects of antibiotics on biofilm and unattached cells of a clinical Staphylococcus aureus isolate from bone and joint infection.
1395	DRAMP18340	Daptomycin(Bacteriocin)	"Daptomycin"[All Fields] AND biofilm[All Fields]	Daptomycin	26257894	Small lipopeptides possess anti-biofilm capability comparable to daptomycin and vancomycin.
1395	DRAMP18340	Daptomycin(Bacteriocin)	"Daptomycin"[All Fields] AND biofilm[All Fields]	Daptomycin	26185439	Activity of daptomycin- and vancomycin-loaded poly-epsilon-caprolactone microparticles against mature staphylococcal biofilms.
1395	DRAMP18340	Daptomycin(Bacteriocin)	"Daptomycin"[All Fields] AND biofilm[All Fields]	Daptomycin	26162777	Antimicrobial efficacy of combined clarithromycin plus daptomycin against biofilms-formed methicillin-resistant Staphylococcus aureus on titanium medical devices.
1395	DRAMP18340	Daptomycin(Bacteriocin)	"Daptomycin"[All Fields] AND biofilm[All Fields]	Daptomycin	26111644	Repurposing ebselen for treatment of multidrug-resistant staphylococcal infections.
1395	DRAMP18340	Daptomycin(Bacteriocin)	"Daptomycin"[All Fields] AND biofilm[All Fields]	Daptomycin	26100694	A novel point mutation promotes growth phase-dependent daptomycin tolerance in Staphylococcus aureus.
1395	DRAMP18340	Daptomycin(Bacteriocin)	"Daptomycin"[All Fields] AND biofilm[All Fields]	Daptomycin	26083676	A review of telavancin activity in in vitro biofilms and animal models of biofilm-associated infections.
1395	DRAMP18340	Daptomycin(Bacteriocin)	"Daptomycin"[All Fields] AND biofilm[All Fields]	Daptomycin	26076451	Evaluation of the Enterococcus faecalis Biofilm-Associated Virulence Factors AhrC and Eep in Rat Foreign Body Osteomyelitis and In Vitro Biofilm-Associated Antimicrobial Resistance.
1395	DRAMP18340	Daptomycin(Bacteriocin)	"Daptomycin"[All Fields] AND biofilm[All Fields]	Daptomycin	26014929	Borrelia burgdorferi, the Causative Agent of Lyme Disease, Forms Drug-Tolerant Persister Cells.
1395	DRAMP18340	Daptomycin(Bacteriocin)	"Daptomycin"[All Fields] AND biofilm[All Fields]	Daptomycin	25987623	Evaluation of Ceftaroline Alone and in Combination against Biofilm-Producing Methicillin-Resistant Staphylococcus aureus with Reduced Susceptibility to Daptomycin and Vancomycin in an In Vitro Pharmacokinetic/Pharmacodynamic Model.
1395	DRAMP18340	Daptomycin(Bacteriocin)	"Daptomycin"[All Fields] AND biofilm[All Fields]	Daptomycin	25894996	In Vitro Activity of Rifampicin Combined with Daptomycin or Tigecycline on Staphylococcus haemolyticus Biofilms.
1395	DRAMP18340	Daptomycin(Bacteriocin)	"Daptomycin"[All Fields] AND biofilm[All Fields]	Daptomycin	25657033	Antimicrobial lock therapy in central-line associated bloodstream infections: a systematic review.
1395	DRAMP18340	Daptomycin(Bacteriocin)	"Daptomycin"[All Fields] AND biofilm[All Fields]	Daptomycin	25605365	Antimicrobial activity against intraosteoblastic Staphylococcus aureus.
1395	DRAMP18340	Daptomycin(Bacteriocin)	"Daptomycin"[All Fields] AND biofilm[All Fields]	Daptomycin	25596729	Management of Propionibacterium acnes infection after shoulder surgery.
1395	DRAMP18340	Daptomycin(Bacteriocin)	"Daptomycin"[All Fields] AND biofilm[All Fields]	Daptomycin	25519162	Evaluation of High-Dose Daptomycin Versus Vancomycin Alone or Combined with Clarithromycin or Rifampin Against Staphylococcus aureus and S. epidermidis in a Novel In Vitro PK/PD Model of Bacterial Biofilm.
1395	DRAMP18340	Daptomycin(Bacteriocin)	"Daptomycin"[All Fields] AND biofilm[All Fields]	Daptomycin	25498330	Activity of bone cement loaded with daptomycin alone or in combination with gentamicin or PEG600 against Staphylococcus epidermidis biofilms.
1395	DRAMP18340	Daptomycin(Bacteriocin)	"Daptomycin"[All Fields] AND biofilm[All Fields]	Daptomycin	25438023	Role of daptomycin in the induction and persistence of the viable but non-culturable state of Staphylococcus aureus biofilms.
1395	DRAMP18340	Daptomycin(Bacteriocin)	"Daptomycin"[All Fields] AND biofilm[All Fields]	Daptomycin	25186318	Tolerability of High Doses of Daptomycin in the Treatment of Prosthetic Vascular Graft Infection: A Retrospective Study.

1395	DRAMP18340	Daptomycin(Bacteriocin)	"Daptomycin"[All Fields] AND biofilm[All Fields]	Daptomycin	25114142	Comparison of the antibiotic activities of Daptomycin, Vancomycin, and the investigational Fluoroquinolone Delafloxacin against biofilms from Staphylococcus aureus clinical isolates.
1395	DRAMP18340	Daptomycin(Bacteriocin)	"Daptomycin"[All Fields] AND biofilm[All Fields]	Daptomycin	25112453	Efficacy of daptomycin lock therapy in the treatment of bloodstream infections related to long-term catheter.
1395	DRAMP18340	Daptomycin(Bacteriocin)	"Daptomycin"[All Fields] AND biofilm[All Fields]	Daptomycin	24996840	High doses of daptomycin (10 mg/kg/d) plus rifampin for the treatment of staphylococcal prosthetic joint infection managed with implant retention: a comparative study.
1395	DRAMP18340	Daptomycin(Bacteriocin)	"Daptomycin"[All Fields] AND biofilm[All Fields]	Daptomycin	24867993	Activity of daptomycin or linezolid in combination with rifampin or gentamicin against biofilm-forming Enterococcus faecalis or E. faecium in an in vitro pharmacodynamic model using simulated endocardial vegetations and an in vivo survival assay using Galleria mellonella larvae.
1395	DRAMP18340	Daptomycin(Bacteriocin)	"Daptomycin"[All Fields] AND biofilm[All Fields]	Daptomycin	24837415	Antibiotic regimens with rifampicin for treatment of Enterococcus faecium in biofilms.
1395	DRAMP18340	Daptomycin(Bacteriocin)	"Daptomycin"[All Fields] AND biofilm[All Fields]	Daptomycin	24614378	A novel approach utilizing biofilm time-kill curves to assess the bactericidal activity of ceftaroline combinations against biofilm-producing methicillin-resistant Staphylococcus aureus.
1395	DRAMP18340	Daptomycin(Bacteriocin)	"Daptomycin"[All Fields] AND biofilm[All Fields]	Daptomycin	24550327	High activity of Fosfomycin and Rifampin against methicillin-resistant staphylococcus aureus biofilm in vitro and in an experimental foreign-body infection model.
1395	DRAMP18340	Daptomycin(Bacteriocin)	"Daptomycin"[All Fields] AND biofilm[All Fields]	Daptomycin	24455699	Genotypically different clones of Staphylococcus aureus are diverse in the antimicrobial susceptibility patterns and biofilm formations.
1395	DRAMP18340	Daptomycin(Bacteriocin)	"Daptomycin"[All Fields] AND biofilm[All Fields]	Daptomycin	24412247	Daptomycin is effective as antibiotic-lock therapy in a model of Staphylococcus aureus catheter-related infection.
1395	DRAMP18340	Daptomycin(Bacteriocin)	"Daptomycin"[All Fields] AND biofilm[All Fields]	Daptomycin	24308006	Propionibacterium acnes: an underestimated pathogen in implant-associated infections.
1395	DRAMP18340	Daptomycin(Bacteriocin)	"Daptomycin"[All Fields] AND biofilm[All Fields]	Daptomycin	24286983	Combination therapy with lysin CF-301 and antibiotic is superior to antibiotic alone for treating methicillin-resistant Staphylococcus aureus-induced murine bacteremia.
1395	DRAMP18340	Daptomycin(Bacteriocin)	"Daptomycin"[All Fields] AND biofilm[All Fields]	Daptomycin	24145537	Activities of fosfomycin and rifampin on planktonic and adherent Enterococcus faecalis strains in an experimental foreign-body infection model.
1395	DRAMP18340	Daptomycin(Bacteriocin)	"Daptomycin"[All Fields] AND biofilm[All Fields]	Daptomycin	23988790	In vitro pharmacokinetics of antimicrobial cationic peptides alone and in combination with antibiotics against methicillin resistant Staphylococcus aureus biofilms.
1395	DRAMP18340	Daptomycin(Bacteriocin)	"Daptomycin"[All Fields] AND biofilm[All Fields]	Daptomycin	23959320	In vitro efficacies and resistance profiles of rifampin-based combination regimens for biofilm-embedded methicillin-resistant Staphylococcus aureus.
1395	DRAMP18340	Daptomycin(Bacteriocin)	"Daptomycin"[All Fields] AND biofilm[All Fields]	Daptomycin	23959318	Adaptation of Enterococcus faecalis to daptomycin reveals an ordered progression to resistance.
1395	DRAMP18340	Daptomycin(Bacteriocin)	"Daptomycin"[All Fields] AND biofilm[All Fields]	Daptomycin	23733470	Candida albicans mucin Msb2 is a broad-range protectant against antimicrobial peptides.
1395	DRAMP18340	Daptomycin(Bacteriocin)	"Daptomycin"[All Fields] AND biofilm[All Fields]	Daptomycin	23611308	In vitro susceptibility to antibiotics of staphylococci in biofilms isolated from orthopaedic infections.
1395	DRAMP18340	Daptomycin(Bacteriocin)	"Daptomycin"[All Fields] AND biofilm[All Fields]	Daptomycin	23571532	A combined pharmacodynamic quantitative and qualitative model reveals the potent activity of daptomycin and delafloxacin against Staphylococcus aureus biofilms.
1395	DRAMP18340	Daptomycin(Bacteriocin)	"Daptomycin"[All Fields] AND biofilm[All Fields]	Daptomycin	23569376	Preparation and characterization of flexible nanoliposomes loaded with daptomycin, a novel antibiotic, for topical skin therapy.
1395	DRAMP18340	Daptomycin(Bacteriocin)	"Daptomycin"[All Fields] AND biofilm[All Fields]	Daptomycin	23515247	Daptomycin use in patients with osteomyelitis: a preliminary report from the EU-CORE(SM) database.
1395	DRAMP18340	Daptomycin(Bacteriocin)	"Daptomycin"[All Fields] AND biofilm[All Fields]	Daptomycin	23496344	Current pharmacotherapy options for osteomyelitis: convergences, divergences and lessons to be drawn.
1395	DRAMP18340	Daptomycin(Bacteriocin)	"Daptomycin"[All Fields] AND biofilm[All Fields]	Daptomycin	23403427	Activity of daptomycin with or without 25 percent ethanol compared to combinations of minocycline, EDTA, and 25 percent ethanol against methicillin-resistant Staphylococcus aureus isolates embedded in biofilm.
1395	DRAMP18340	Daptomycin(Bacteriocin)	"Daptomycin"[All Fields] AND biofilm[All Fields]	Daptomycin	23262356	Quorum sensing inhibitor FS3-coated vascular graft enhances daptomycin efficacy in a rat model of staphylococcal infection.
1395	DRAMP18340	Daptomycin(Bacteriocin)	"Daptomycin"[All Fields] AND biofilm[All Fields]	Daptomycin	23079900	Activity of ethanol and daptomycin lock on biofilm generated by an in vitro dynamic model using real subcutaneous injection ports.
1395	DRAMP18340	Daptomycin(Bacteriocin)	"Daptomycin"[All Fields] AND biofilm[All Fields]	Daptomycin	23075270	sarA-mediated repression of protease production plays a key role in the pathogenesis of Staphylococcus aureus USA300 isolates.
1395	DRAMP18340	Daptomycin(Bacteriocin)	"Daptomycin"[All Fields] AND biofilm[All Fields]	Daptomycin	23070152	In vitro activities of antibiotics and antimicrobial cationic peptides alone and in combination against methicillin-resistant Staphylococcus aureus biofilms.
1395	DRAMP18340	Daptomycin(Bacteriocin)	"Daptomycin"[All Fields] AND biofilm[All Fields]	Daptomycin	22951659	[Treatment of hemodialysis catheter-associated bacteremia due to methicillin-resistant Staphylococcus aureus by daptomycin lock method].
1395	DRAMP18340	Daptomycin(Bacteriocin)	"Daptomycin"[All Fields] AND biofilm[All Fields]	Daptomycin	22796888	Activity of linezolid and high-dose daptomycin, alone or in combination, in an in vitro model of Staphylococcus aureus biofilm.
1395	DRAMP18340	Daptomycin(Bacteriocin)	"Daptomycin"[All Fields] AND biofilm[All Fields]	Daptomycin	22689024	[Daptomycin: pharmacological characteristics and its role in the treatment of gram positive infections].
1395	DRAMP18340	Daptomycin(Bacteriocin)	"Daptomycin"[All Fields] AND biofilm[All Fields]	Daptomycin	22585351	Cis-2-decenoic acid inhibits S. aureus growth and biofilm in vitro: a pilot study.
1395	DRAMP18340	Daptomycin(Bacteriocin)	"Daptomycin"[All Fields] AND biofilm[All Fields]	Daptomycin	22431851	Oritavancin: mechanism of action.
1395	DRAMP18340	Daptomycin(Bacteriocin)	"Daptomycin"[All Fields] AND biofilm[All Fields]	Daptomycin	22425795	Intraperitoneal administration of daptomycin in recurrent peritonitis with suspected biofilm.
1395	DRAMP18340	Daptomycin(Bacteriocin)	"Daptomycin"[All Fields] AND biofilm[All Fields]	Daptomycin	22371896	Daptomycin and tigecycline have broader effective dose ranges than vancomycin as prophylaxis against a Staphylococcus aureus surgical implant infection in mice.
1395	DRAMP18340	Daptomycin(Bacteriocin)	"Daptomycin"[All Fields] AND biofilm[All Fields]	Daptomycin	22354028	Impact of extracellular nuclease production on the biofilm phenotype of Staphylococcus aureus under in vitro and in vivo conditions.
1395	DRAMP18340	Daptomycin(Bacteriocin)	"Daptomycin"[All Fields] AND biofilm[All Fields]	Daptomycin	22252806	Role of rifampin against Propionibacterium acnes biofilm in vitro and in an experimental foreign-body infection model.
1395	DRAMP18340	Daptomycin(Bacteriocin)	"Daptomycin"[All Fields] AND biofilm[All Fields]	Daptomycin	22242149	Ellagic acid derivatives from Rubus ulmifolius inhibit Staphylococcus aureus biofilm formation and improve response to antibiotics.
1395	DRAMP18340	Daptomycin(Bacteriocin)	"Daptomycin"[All Fields] AND biofilm[All Fields]	Daptomycin	22228044	Evaluation of MBEC™-HTP biofilm model for studies of implant associated infections.
1395	DRAMP18340	Daptomycin(Bacteriocin)	"Daptomycin"[All Fields] AND biofilm[All Fields]	Daptomycin	22123684	Efficacy of daptomycin versus vancomycin in an experimental model of foreign-body and systemic infection caused by biofilm producers and methicillin-resistant Staphylococcus epidermidis.
1395	DRAMP18340	Daptomycin(Bacteriocin)	"Daptomycin"[All Fields] AND biofilm[All Fields]	Daptomycin	22102812	Evolution of multidrug resistance during Staphylococcus aureus infection involves mutation of the essential two component regulator WalKR.
1395	DRAMP18340	Daptomycin(Bacteriocin)	"Daptomycin"[All Fields] AND biofilm[All Fields]	Daptomycin	21940270	Resistance patterns and occurrence of virulence determinants among GRE strains in southwestern Poland.
1395	DRAMP18340	Daptomycin(Bacteriocin)	"Daptomycin"[All Fields] AND biofilm[All Fields]	Daptomycin	21761219	In vitro activity of daptomycin, linezolid and rifampicin on Staphylococcus epidermidis biofilms.
1395	DRAMP18340	Daptomycin(Bacteriocin)	"Daptomycin"[All Fields] AND biofilm[All Fields]	Daptomycin	21709082	Daptomycin antibiotic lock therapy in a rat model of staphylococcal central venous catheter biofilm infections.
1395	DRAMP18340	Daptomycin(Bacteriocin)	"Daptomycin"[All Fields] AND biofilm[All Fields]	Daptomycin	21622973	Daptomycin resistance mechanisms in clinically derived Staphylococcus aureus strains assessed by a combined transcriptomics and proteomics approach.
1395	DRAMP18340	Daptomycin(Bacteriocin)	"Daptomycin"[All Fields] AND biofilm[All Fields]	Daptomycin	21576433	Reversible daptomycin tolerance of adherent staphylococci in an implant infection model.
1395	DRAMP18340	Daptomycin(Bacteriocin)	"Daptomycin"[All Fields] AND biofilm[All Fields]	Daptomycin	21477700	[Tolerance and heteroresistance in Gram-positive microorganisms].
1395	DRAMP18340	Daptomycin(Bacteriocin)	"Daptomycin"[All Fields] AND biofilm[All Fields]	Daptomycin	21477698	[Current treatment of Gram-positive infections: from experimental models to clinical experience after approval of new drugs].
1395	DRAMP18340	Daptomycin(Bacteriocin)	"Daptomycin"[All Fields] AND biofilm[All Fields]	Daptomycin	21405943	Treatment of prosthetic osteoarticular infections.
1395	DRAMP18340	Daptomycin(Bacteriocin)	"Daptomycin"[All Fields] AND biofilm[All Fields]	Daptomycin	21398072	In vitro biofilm formation and bactericidal activities of methicillin-resistant Staphylococcus aureus clones prevalent in Korea.
1395	DRAMP18340	Daptomycin(Bacteriocin)	"Daptomycin"[All Fields] AND biofilm[All Fields]	Daptomycin	20888868	A dynamic in vitro model for evaluating antimicrobial activity against bacterial biofilms using a new device and clinical-used catheters.
1395	DRAMP18340	Daptomycin(Bacteriocin)	"Daptomycin"[All Fields] AND biofilm[All Fields]	Daptomycin	20869272	Daptomycin and rifampin alone and in combination prevent vascular graft biofilm formation and emergence of antibiotic resistance in a subcutaneous rat pouch model of staphylococcal infection.
1395	DRAMP18340	Daptomycin(Bacteriocin)	"Daptomycin"[All Fields] AND biofilm[All Fields]	Daptomycin	20823562	In vitro antibacterial activity of panduratin A against enterococci clinical isolates.
1395	DRAMP18340	Daptomycin(Bacteriocin)	"Daptomycin"[All Fields] AND biofilm[All Fields]	Daptomycin	20719763	Rifampicin enhances activity of daptomycin and vancomycin against both a polysaccharide intercellular adhesin (PIA)-dependent and -independent Staphylococcus epidermidis biofilm.
1395	DRAMP18340	Daptomycin(Bacteriocin)	"Daptomycin"[All Fields] AND biofilm[All Fields]	Daptomycin	20696880	Activities of high-dose daptomycin, vancomycin, and moxifloxacin alone or in combination with clarithromycin or rifampin in a novel in vitro model of Staphylococcus aureus biofilm.
1395	DRAMP18340	Daptomycin(Bacteriocin)	"Daptomycin"[All Fields] AND biofilm[All Fields]	Daptomycin	20679509	Increased temperature enhances the antimicrobial effects of daptomycin, vancomycin, tigecycline, fosfomycin, and cefamandole on staphylococcal biofilms.
1395	DRAMP18340	Daptomycin(Bacteriocin)	"Daptomycin"[All Fields] AND biofilm[All Fields]	Daptomycin	20528931	Tigecycline inhibition of a mature biofilm in clinical isolates of Staphylococcus aureus : comparison with other drugs.
1395	DRAMP18340	Daptomycin(Bacteriocin)	"Daptomycin"[All Fields] AND biofilm[All Fields]	Daptomycin	20189943	Combinations of maggot excretions/secretions and antibiotics are effective against Staphylococcus aureus biofilms and the bacteria derived therefrom.
1395	DRAMP18340	Daptomycin(Bacteriocin)	"Daptomycin"[All Fields] AND biofilm[All Fields]	Daptomycin	20065324	Rifampin combination therapy for nonmycobacterial infections.

1395	DRAMP18340	Daptomycin(Bacteriocin)	"Daptomycin"[All Fields] AND biofilm[All Fields]	Daptomycin	19931813	Risk factors and management of Gram-positive bacteraemia.
1395	DRAMP18340	Daptomycin(Bacteriocin)	"Daptomycin"[All Fields] AND biofilm[All Fields]	Daptomycin	19651914	Impact of sarA on daptomycin susceptibility of Staphylococcus aureus biofilms in vivo.
1395	DRAMP18340	Daptomycin(Bacteriocin)	"Daptomycin"[All Fields] AND biofilm[All Fields]	Daptomycin	19564363	Activities of daptomycin and vancomycin alone and in combination with rifampin and gentamicin against biofilm-forming methicillin-resistant Staphylococcus aureus isolates in an experimental model of endocarditis.
1395	DRAMP18340	Daptomycin(Bacteriocin)	"Daptomycin"[All Fields] AND biofilm[All Fields]	Daptomycin	19560786	Regional antibiotic delivery for the treatment of experimental prosthetic graft infections.
1395	DRAMP18340	Daptomycin(Bacteriocin)	"Daptomycin"[All Fields] AND biofilm[All Fields]	Daptomycin	19451285	Daptomycin rapidly penetrates a Staphylococcus epidermidis biofilm.
1395	DRAMP18340	Daptomycin(Bacteriocin)	"Daptomycin"[All Fields] AND biofilm[All Fields]	Daptomycin	19451280	Effects of azithromycin in combination with vancomycin, daptomycin, fosfomycin, tigecycline, and ceftriaxone on Staphylococcus epidermidis biofilms.
1395	DRAMP18340	Daptomycin(Bacteriocin)	"Daptomycin"[All Fields] AND biofilm[All Fields]	Daptomycin	19396814	Effects of vancomycin, daptomycin, fosfomycin, tigecycline, and ceftriaxone on Staphylococcus epidermidis biofilms.
1395	DRAMP18340	Daptomycin(Bacteriocin)	"Daptomycin"[All Fields] AND biofilm[All Fields]	Daptomycin	19289527	Impact of sarA on antibiotic susceptibility of Staphylococcus aureus in a catheter-associated in vitro model of biofilm formation.
1395	DRAMP18340	Daptomycin(Bacteriocin)	"Daptomycin"[All Fields] AND biofilm[All Fields]	Daptomycin	19101124	Comparison of biofilm-associated cell survival following in vitro exposure of meticillin-resistant Staphylococcus aureus biofilms to the antibiotics clindamycin, daptomycin, linezolid, tigecycline and vancomycin.
1395	DRAMP18340	Daptomycin(Bacteriocin)	"Daptomycin"[All Fields] AND biofilm[All Fields]	Daptomycin	19084156	Effect of antibacterials on biofilms.
1395	DRAMP18340	Daptomycin(Bacteriocin)	"Daptomycin"[All Fields] AND biofilm[All Fields]	Daptomycin	18725436	Effect of electrical current on the activities of antimicrobial agents against Pseudomonas aeruginosa, Staphylococcus aureus, and Staphylococcus epidermidis biofilms.
1395	DRAMP18340	Daptomycin(Bacteriocin)	"Daptomycin"[All Fields] AND biofilm[All Fields]	Daptomycin	18516271	History and evolution of antibiotic resistance in coagulase-negative staphylococci: Susceptibility profiles of new anti-staphylococcal agents.
1395	DRAMP18340	Daptomycin(Bacteriocin)	"Daptomycin"[All Fields] AND biofilm[All Fields]	Daptomycin	18201873	Activity of daptomycin on biofilms produced on a plastic support by Staphylococcus spp.
1395	DRAMP18340	Daptomycin(Bacteriocin)	"Daptomycin"[All Fields] AND biofilm[All Fields]	Daptomycin	17869069	Infection of intravascular prostheses: how to treat other than surgery.
1395	DRAMP18340	Daptomycin(Bacteriocin)	"Daptomycin"[All Fields] AND biofilm[All Fields]	Daptomycin	17403700	In vitro activity of daptomycin and vancomycin lock solutions on staphylococcal biofilms in a central venous catheter model.
1395	DRAMP18340	Daptomycin(Bacteriocin)	"Daptomycin"[All Fields] AND biofilm[All Fields]	Daptomycin	17353249	Comparative activities of daptomycin, linezolid, and tigecycline against catheter-related methicillin-resistant Staphylococcus bacteremic isolates embedded in biofilm.
1395	DRAMP18340	Daptomycin(Bacteriocin)	"Daptomycin"[All Fields] AND biofilm[All Fields]	Daptomycin	16920429	Impact of selective antimicrobial agents on staphylococcal adherence to biomedical devices.
1395	DRAMP18340	Daptomycin(Bacteriocin)	"Daptomycin"[All Fields] AND biofilm[All Fields]	Daptomycin	16804382	Infections associated with orthopedic implants.
1395	DRAMP18340	Daptomycin(Bacteriocin)	"Daptomycin"[All Fields] AND biofilm[All Fields]	Daptomycin	16789794	Antimicrobial agents in orthopaedic surgery: Prophylaxis and treatment.
1395	DRAMP18340	Daptomycin(Bacteriocin)	"Daptomycin"[All Fields] AND biofilm[All Fields]	Daptomycin	16735866	Uncertain efficacy of daptomycin for prosthetic joint infections: a prospective case series.
1395	DRAMP18340	Daptomycin(Bacteriocin)	"Daptomycin"[All Fields] AND biofilm[All Fields]	Daptomycin	16304171	Vancomycin-resistant Enterococcus faecium: catheter colonization, esp gene, and decreased susceptibility to antibiotics in biofilm.
1395	DRAMP18340	Daptomycin(Bacteriocin)	"Daptomycin"[All Fields] AND biofilm[All Fields]	Daptomycin	15751742	New strategies for the treatment of infections associated with prosthetic joints.
1395	DRAMP18340	Daptomycin(Bacteriocin)	"Daptomycin"[All Fields] AND biofilm[All Fields]	Daptomycin	14638511	In vitro activity of tigecycline against Staphylococcus epidermidis growing in an adherent-cell biofilm model.
1395	DRAMP18340	Daptomycin(Bacteriocin)	"Daptomycin"[All Fields] AND biofilm[All Fields]	Daptomycin	14559089	Antimicrobial strategies for the prevention and treatment of cardiovascular infections.
1395	DRAMP18340	Daptomycin(Bacteriocin)	"Daptomycin"[All Fields] AND biofilm[All Fields]	Daptomycin	8104211	Effect of polyurethane catheters and bacterial biofilms on the in-vitro activity of antimicrobials against Staphylococcus epidermidis.
1409	DRAMP00143	Plantaricin-A (PlnA; Bacteriocin)	"Plantaricin-A"[All Fields] AND biofilm[All Fields]	Plantaricin-A	23396346	Effects of the peptide pheromone plantaricin A and cocultivation with Lactobacillus sanfranciscensis DPPMA174 on the exoproteome and the adhesion capacity of Lactobacillus plantarum DC400.
1418	DRAMP00153	NlmA (chain a of Mutacin IV; Bacteriocin)	"NlmA"[All Fields] AND biofilm[All Fields]	NlmA	28526785	Effects of Arginine on Streptococcus mutans Growth, Virulence Gene Expression, and Stress Tolerance.
1418	DRAMP00153	NlmA (chain a of Mutacin IV; Bacteriocin)	"NlmA"[All Fields] AND biofilm[All Fields]	NlmA	23278289	Physiological properties of Streptococcus mutans UA159 biofilm-detached cells.
1418	DRAMP00153	NlmA (chain a of Mutacin IV; Bacteriocin)	"NlmA"[All Fields] AND biofilm[All Fields]	NlmA	18458070	LiaS regulates virulence factor expression in Streptococcus mutans.
1419	DRAMP00154	NlmB (chain b of Mutacin IV; Bacteriocin)	"NlmB"[All Fields] AND biofilm[All Fields]	NlmB	28526785	Effects of Arginine on Streptococcus mutans Growth, Virulence Gene Expression, and Stress Tolerance.
1421	DRAMP00156	BrcB (NKR-5-3A; chain b of Brochocin C; Bacteriocin)	"BrcB"[All Fields] AND biofilm[All Fields]	BrcB	34637319	Molecular Flexibility of Antibodies Preserved Even in the Dense Phase after Macroscopic Phase Separation.
1421	DRAMP00156	BrcB (NKR-5-3A; chain b of Brochocin C; Bacteriocin)	"BrcB"[All Fields] AND biofilm[All Fields]	BrcB	34490428	Temperature and salt controlled tuning of protein clusters.
1421	DRAMP00156	BrcB (NKR-5-3A; chain b of Brochocin C; Bacteriocin)	"BrcB"[All Fields] AND biofilm[All Fields]	BrcB	34461276	A novel versatile flow-donor chamber as biorelevant ex-vivo test assessing oral mucoadhesive formulations.
1421	DRAMP00156	BrcB (NKR-5-3A; chain b of Brochocin C; Bacteriocin)	"BrcB"[All Fields] AND biofilm[All Fields]	BrcB	34273783	Extraction of natural moisturizing factor from the stratum corneum and its implication on skin molecular mobility.
1421	DRAMP00156	BrcB (NKR-5-3A; chain b of Brochocin C; Bacteriocin)	"BrcB"[All Fields] AND biofilm[All Fields]	BrcB	33901597	Can mesoporous nanoparticles promote bioavailability of topical pharmaceutics?
1421	DRAMP00156	BrcB (NKR-5-3A; chain b of Brochocin C; Bacteriocin)	"BrcB"[All Fields] AND biofilm[All Fields]	BrcB	33807251	Probing Skin Barrier Recovery on Molecular Level Following Acute Wounds: An In Vivo/Ex Vivo Study on Pigs.
1421	DRAMP00156	BrcB (NKR-5-3A; chain b of Brochocin C; Bacteriocin)	"BrcB"[All Fields] AND biofilm[All Fields]	BrcB	32406605	Multivalent ions and biomolecules: Attempting a comprehensive perspective.
1421	DRAMP00156	BrcB (NKR-5-3A; chain b of Brochocin C; Bacteriocin)	"BrcB"[All Fields] AND biofilm[All Fields]	BrcB	31117738	Lipid Bilayer-like Mixed Self-Assembled Monolayers with Strong Mobility and Clustering-Dependent Lectin Affinity.
1421	DRAMP00156	BrcB (NKR-5-3A; chain b of Brochocin C; Bacteriocin)	"BrcB"[All Fields] AND biofilm[All Fields]	BrcB	30308085	Highly Efficient Synthesis and Assay of Protein-Imprinted Nanogels by Using Magnetic Templates.
1421	DRAMP00156	BrcB (NKR-5-3A; chain b of Brochocin C; Bacteriocin)	"BrcB"[All Fields] AND biofilm[All Fields]	BrcB	29553755	Reversible Self-Assembled Monolayers (rSAMs) as Robust and Fluidic Lipid Bilayer Mimics.
1421	DRAMP00156	BrcB (NKR-5-3A; chain b of Brochocin C; Bacteriocin)	"BrcB"[All Fields] AND biofilm[All Fields]	BrcB	29202022	Reversible Self-Assembled Monolayers (rSAMs): Adaptable Surfaces for Enhanced Multivalent Interactions and Ultrasensitive Virus Detection.
1425	DRAMP00160	ThmA (chain a of Thermophilin 13; Bacteriocin)	"ThmA"[All Fields] AND biofilm[All Fields]	ThmA	16980491	The atlA operon of Streptococcus mutans: role in autolysin maturation and cell surface biogenesis.
1430	DRAMP00165	Gassericin A (GaaA; Bacteriocin)	"Gassericin A"[All Fields] AND biofilm[All Fields]	Gassericin A	32474673	Effects of a derivative of reutericin 6 and gassericin A on the biofilm of Streptococcus mutans in vitro and caries prevention in vivo.
1430	DRAMP00165	Gassericin A (GaaA; Bacteriocin)	"Gassericin A"[All Fields] AND biofilm[All Fields]	Gassericin A	31207076	Rational design of peptides with enhanced antimicrobial and anti-biofilm activities against cariogenic bacterium Streptococcus mutans.
1432	DRAMP00167	Subtilosin A (Antilisterial bacteriocin subtilosin; D-amino acid; Bacteriocin; Preclinical)	"Subtilosin A"[All Fields] AND biofilm[All Fields]	Subtilosin A	34460312	Defining the Expression, Production, and Signaling Roles of Specialized Metabolites during Bacillus subtilis Differentiation.
1432	DRAMP00167	Subtilosin A (Antilisterial bacteriocin subtilosin; D-amino acid; Bacteriocin; Preclinical)	"Subtilosin A"[All Fields] AND biofilm[All Fields]	Subtilosin A	33679639	Probiotic Bacilli Inhibit SalmonellaBiofilm Formation Without Killing Planktonic Cells.
1432	DRAMP00167	Subtilosin A (Antilisterial bacteriocin subtilosin; D-amino acid; Bacteriocin; Preclinical)	"Subtilosin A"[All Fields] AND biofilm[All Fields]	Subtilosin A	30230911	Characterization and sequence analysis of potential biofertilizer and biocontrol agent Bacillus subtilis strain SEM-9 from silkworm excrement.

1432	DRAMP00167	Subtilosin A (Antilisterial bacteriocin subtilosin; D-amino acid; Bacteriocin; Preclinical)	"Subtilosin A"[All Fields] AND biofilm[All Fields]	Subtilosin A	28705677	Hyicin 4244, the first sactibiotic described in staphylococci, exhibits an anti-staphylococcal biofilm activity.
1432	DRAMP00167	Subtilosin A (Antilisterial bacteriocin subtilosin; D-amino acid; Bacteriocin; Preclinical)	"Subtilosin A"[All Fields] AND biofilm[All Fields]	Subtilosin A	25588687	The Natural Antimicrobial Subtilosin A Synergizes with Lauramide Arginine Ethyl Ester (LAE), ε-Poly-L-lysine (Polylysine), Clindamycin Phosphate and Metronidazole, Against the Vaginal Pathogen Gardnerella vaginalis.
1434	DRAMP00169	Enterocin AS-48 (AS-48; Bacteriocin)	"Enterocin AS-48"[All Fields] AND biofilm[All Fields]	Enterocin AS-48	34438974	Antimicrobial Activity of the Circular Bacteriocin AS-48 against Clinical Multidrug-Resistant Staphylococcus aureus.
1434	DRAMP00169	Enterocin AS-48 (AS-48; Bacteriocin)	"Enterocin AS-48"[All Fields] AND biofilm[All Fields]	Enterocin AS-48	24135676	Comparative proteomic analysis of Listeria monocytogenes exposed to enterocin AS-48 in planktonic and sessile states.
1434	DRAMP00169	Enterocin AS-48 (AS-48; Bacteriocin)	"Enterocin AS-48"[All Fields] AND biofilm[All Fields]	Enterocin AS-48	23558192	Combined treatments of enterocin AS-48 with biocides to improve the inactivation of methicillin-sensitive and methicillin-resistant Staphylococcus aureus planktonic and sessile cells.
1434	DRAMP00169	Enterocin AS-48 (AS-48; Bacteriocin)	"Enterocin AS-48"[All Fields] AND biofilm[All Fields]	Enterocin AS-48	22265283	Effect of enterocin AS-48 in combination with biocides on planktonic and sessile Listeria monocytogenes.
1447	DRAMP00185	Leucocin-B (Leu B; Leucocin B-TA33a; Bacteriocin)	"Leucocin-B"[All Fields] AND biofilm[All Fields]	Leucocin-B	34512608	Phenotypic Traits and Immunomodulatory Properties of Leuconostoc carnosumIsolated From Meat Products.
1454	DRAMP00195	Colicin-V (Microcin-V; Bacteriocin)	"Colicin-V"[All Fields] AND biofilm[All Fields]	Colicin-V	33372844	Hypervirulent and hypermucoviscous extended-spectrum β-lactamase-producing Klebsiella pneumoniaeand Klebsiella variicolain Chile.
1454	DRAMP00195	Colicin-V (Microcin-V; Bacteriocin)	"Colicin-V"[All Fields] AND biofilm[All Fields]	Colicin-V	31420345	Novel Probiotic Mechanisms of the Oral Bacterium Streptococcussp. A12 as Explored with Functional Genomics.
1454	DRAMP00195	Colicin-V (Microcin-V; Bacteriocin)	"Colicin-V"[All Fields] AND biofilm[All Fields]	Colicin-V	24478082	An Escherichia coli Nissle 1917 missense mutant colonizes the streptomycin-treated mouse intestine better than the wild type but is not a better probiotic.
1454	DRAMP00195	Colicin-V (Microcin-V; Bacteriocin)	"Colicin-V"[All Fields] AND biofilm[All Fields]	Colicin-V	22877314	RpfF-dependent regulon of Xylella fastidiosa.
1454	DRAMP00195	Colicin-V (Microcin-V; Bacteriocin)	"Colicin-V"[All Fields] AND biofilm[All Fields]	Colicin-V	22392928	The streptomycin-treated mouse intestine selects Escherichia coli envZ missense mutants that interact with dense and diverse intestinal microbiota.
1457	DRAMP00198	Microcin H47 (MccH47; Bacteriocin)	"Microcin H47"[All Fields] AND biofilm[All Fields]	Microcin H47	22448273	High-density transcriptional initiation signals underline genomic islands in bacteria.
1460	DRAMP00202	Thiocillin (Bacteriocin)	"Thiocillin"[All Fields] AND biofilm[All Fields]	Thiocillin	25713360	Thiopeptide antibiotics stimulate biofilm formation in Bacillus subtilis.
1463	DRAMP00206	Acidocin A (Bacteriocin)	"Acidocin A"[All Fields] AND biofilm[All Fields]	Acidocin A	33970542	Antimicrobial properties of Lactobacillus cell-free supernatants against multidrug-resistant urogenital pathogens.
1474	DRAMP00217	Bacteriocin	"Bacteriocin"[All Fields] AND biofilm[All Fields]	Bacteriocin	34557272	Genotypic and phenotypic characterization of Streptococcus mutansstrains isolated from patients with dental caries.
1474	DRAMP00217	Bacteriocin	"Bacteriocin"[All Fields] AND biofilm[All Fields]	Bacteriocin	34507740	Listeria monocytogenes biofilm inhibition on food contact surfaces by application of postbiotics from Lactobacillus curvatus B.67 and Lactobacillus plantarum M.2.
1474	DRAMP00217	Bacteriocin	"Bacteriocin"[All Fields] AND biofilm[All Fields]	Bacteriocin	34484135	YbfA Regulates the Sensitivity of Escherichia coliK12 to Plantaricin BM-1 viathe BasS/BasR Two-Component Regulatory System.
1474	DRAMP00217	Bacteriocin	"Bacteriocin"[All Fields] AND biofilm[All Fields]	Bacteriocin	34481880	Hybrid hydrogels for bacteriocin delivery to infected wounds.
1474	DRAMP00217	Bacteriocin	"Bacteriocin"[All Fields] AND biofilm[All Fields]	Bacteriocin	34463029	MapZ deficiency leads to defects in the envelope structure and changes stress tolerance of Streptococcus mutans.
1474	DRAMP00217	Bacteriocin	"Bacteriocin"[All Fields] AND biofilm[All Fields]	Bacteriocin	34438974	Antimicrobial Activity of the Circular Bacteriocin AS-48 against Clinical Multidrug-Resistant Staphylococcus aureus.
1474	DRAMP00217	Bacteriocin	"Bacteriocin"[All Fields] AND biofilm[All Fields]	Bacteriocin	34230527	A bacteriocin-based treatment option for Staphylococcus haemolyticus biofilms.
1474	DRAMP00217	Bacteriocin	"Bacteriocin"[All Fields] AND biofilm[All Fields]	Bacteriocin	34220114	In vitroevaluation of the probiotic potential of Lactobacillusisolated from native swine manure.
1474	DRAMP00217	Bacteriocin	"Bacteriocin"[All Fields] AND biofilm[All Fields]	Bacteriocin	34208478	Engineering of the CHAPk Staphylococcal Phage Endolysin to Enhance Antibacterial Activity against Stationary-Phase Cells.
1474	DRAMP00217	Bacteriocin	"Bacteriocin"[All Fields] AND biofilm[All Fields]	Bacteriocin	34189819	Prokaryotic population dynamics and interactions in an AnSBBR using tequila vinasses as substrate in co-digestion with acid hydrolysates of Agave tequilana var. azul bagasse for hydrogen production.
1474	DRAMP00217	Bacteriocin	"Bacteriocin"[All Fields] AND biofilm[All Fields]	Bacteriocin	34173207	Bacteriocin-Like Inhibitory Substance (BLIS) Activity of Enterococcus faecium DB1 Against Biofilm Formation by Clostridium perfringens.
1474	DRAMP00217	Bacteriocin	"Bacteriocin"[All Fields] AND biofilm[All Fields]	Bacteriocin	34087449	Bacterial Vaginosis: Effects on reproduction and its therapeutics.
1474	DRAMP00217	Bacteriocin	"Bacteriocin"[All Fields] AND biofilm[All Fields]	Bacteriocin	33987538	Influence of Gallic Acid and Thai Culinary Essential Oils on Antibacterial Activity of Nisin against Streptococcus mutans.
1474	DRAMP00217	Bacteriocin	"Bacteriocin"[All Fields] AND biofilm[All Fields]	Bacteriocin	33839269	Pangenome analyses of LuxS-coding genes and enzymatic repertoires in cocoa-related lactic acid bacteria.
1474	DRAMP00217	Bacteriocin	"Bacteriocin"[All Fields] AND biofilm[All Fields]	Bacteriocin	33838179	Effect of sub-lethal doses of nisin on Staphylococcus aureus toxin production and biofilm formation.
1474	DRAMP00217	Bacteriocin	"Bacteriocin"[All Fields] AND biofilm[All Fields]	Bacteriocin	33807321	Biodiversity of Ligilactobacillus salivariusStrains from Poultry and Domestic Pigeons.
1474	DRAMP00217	Bacteriocin	"Bacteriocin"[All Fields] AND biofilm[All Fields]	Bacteriocin	33802636	Bacteriocin-Like Inhibitory Substances from Probiotics as Therapeutic Agents for CandidaVulvovaginitis.
1474	DRAMP00217	Bacteriocin	"Bacteriocin"[All Fields] AND biofilm[All Fields]	Bacteriocin	33584610	Proteomic Analysis of Listeria monocytogenesFBUNT During Biofilm Formation at 10°C in Response to Lactocin AL705.
1474	DRAMP00217	Bacteriocin	"Bacteriocin"[All Fields] AND biofilm[All Fields]	Bacteriocin	33419248	In vitro Interactions between Streptococcus intermediusand Streptococcus salivariusK12 on a Titanium Cylindrical Surface.
1474	DRAMP00217	Bacteriocin	"Bacteriocin"[All Fields] AND biofilm[All Fields]	Bacteriocin	33352988	Enterococcus mundtiiIsolated from Slovak Raw Goat Milk and Its Bacteriocinogenic Potential.
1474	DRAMP00217	Bacteriocin	"Bacteriocin"[All Fields] AND biofilm[All Fields]	Bacteriocin	33279086	Inhibition of Listeria monocytogenes by the Staphylococcus capitis - derived bacteriocin capidermicin.
1474	DRAMP00217	Bacteriocin	"Bacteriocin"[All Fields] AND biofilm[All Fields]	Bacteriocin	33268776	A bacteriocin-based antimicrobial formulation to effectively disrupt the cell viability of methicillin-resistant Staphylococcus aureus (MRSA) biofilms.
1474	DRAMP00217	Bacteriocin	"Bacteriocin"[All Fields] AND biofilm[All Fields]	Bacteriocin	33246623	Purification, characterization, and mode of action of a novel bacteriocin BM173 from Lactobacillus crustorum MN047 and its effect on biofilm formation of Escherichia coli and Staphylococcus aureus.
1474	DRAMP00217	Bacteriocin	"Bacteriocin"[All Fields] AND biofilm[All Fields]	Bacteriocin	33188012	Engineered probiotics biofilm enhances osseointegration via immunoregulation and anti-infection.
1474	DRAMP00217	Bacteriocin	"Bacteriocin"[All Fields] AND biofilm[All Fields]	Bacteriocin	33068882	Prevalence of bacteriocins and their co-association with virulence factors within Pseudomonas aeruginosa catheter isolates.
1474	DRAMP00217	Bacteriocin	"Bacteriocin"[All Fields] AND biofilm[All Fields]	Bacteriocin	33010007	Anti-Proliferative and Anti-Biofilm Potentials of Bacteriocins Produced by Non-Pathogenic Enterococcus sp.
1474	DRAMP00217	Bacteriocin	"Bacteriocin"[All Fields] AND biofilm[All Fields]	Bacteriocin	33006097	Bacteriocin-a potential antimicrobial peptide towards disrupting and preventing biofilm formation in the clinical and environmental locales.
1474	DRAMP00217	Bacteriocin	"Bacteriocin"[All Fields] AND biofilm[All Fields]	Bacteriocin	32971750	Susceptibility to Bacteriocins in Biofilm-Forming, Variable Staphylococci Isolated from Local Slovak Ewes' Milk Lump Cheeses.
1474	DRAMP00217	Bacteriocin	"Bacteriocin"[All Fields] AND biofilm[All Fields]	Bacteriocin	32949279	A strategy to control colonization of pathogens: embedding of lactic acid bacteria on the surface of urinary catheter.
1474	DRAMP00217	Bacteriocin	"Bacteriocin"[All Fields] AND biofilm[All Fields]	Bacteriocin	32803295	Transcriptomic and proteomic profiling response of methicillin-resistant Staphylococcus aureus (MRSA) to a novel bacteriocin, plantaricin GZ1-27 and its inhibition of biofilm formation.
1474	DRAMP00217	Bacteriocin	"Bacteriocin"[All Fields] AND biofilm[All Fields]	Bacteriocin	32772715	Synergistic antibacterial and anti-biofilm activity of nisin like bacteriocin with curcumin and cinnamaldehyde against ESBL and MBL producing clinical strains.
1474	DRAMP00217	Bacteriocin	"Bacteriocin"[All Fields] AND biofilm[All Fields]	Bacteriocin	32721519	Combined antimicrobial use of essential oils and bacteriocin bacLP17 as seafood biopreservative to control Listeria monocytogenes both in planktonic and in sessile forms.
1474	DRAMP00217	Bacteriocin	"Bacteriocin"[All Fields] AND biofilm[All Fields]	Bacteriocin	32712896	Compatibility, Cytotoxicity, and Gastrointestinal Tenacity of Bacteriocin-Producing Bacteria Selected for a Consortium Probiotic Formulation to Be Used in Livestock Feed.
1474	DRAMP00217	Bacteriocin	"Bacteriocin"[All Fields] AND biofilm[All Fields]	Bacteriocin	32707601	Combinatorial effects of trans-cinnamaldehyde with fluoride and chlorhexidine on Streptococcus mutans.
1474	DRAMP00217	Bacteriocin	"Bacteriocin"[All Fields] AND biofilm[All Fields]	Bacteriocin	32707233	Nisin influence on the expression of Listeria monocytogenes surface proteins.
1474	DRAMP00217	Bacteriocin	"Bacteriocin"[All Fields] AND biofilm[All Fields]	Bacteriocin	32695282	Bioactivity of Bac70 Produced by Bacillus atrophaeusStrain DDBCC70.
1474	DRAMP00217	Bacteriocin	"Bacteriocin"[All Fields] AND biofilm[All Fields]	Bacteriocin	32678667	In VitroAntibiofilm and Anti-Inflammatory Properties of Bacteriocins Produced by Pediococcus acidilacticiAgainst Enterococcus faecalis.
1474	DRAMP00217	Bacteriocin	"Bacteriocin"[All Fields] AND biofilm[All Fields]	Bacteriocin	32632823	Biocompatible combinations of nisin and licorice polyphenols exert synergistic bactericidal effects against Enterococcus faecalis and inhibit NF-κB activation in monocytes.
1474	DRAMP00217	Bacteriocin	"Bacteriocin"[All Fields] AND biofilm[All Fields]	Bacteriocin	32629918	Metabolic Shift of an Isogenic Strain of Enterococcus faecalis14, Deficient in Its Own Bacteriocin Synthesis, as Revealed by a Transcriptomic Analysis.
1474	DRAMP00217	Bacteriocin	"Bacteriocin"[All Fields] AND biofilm[All Fields]	Bacteriocin	32628991	Evidence of anti- K. pneumoniae biofilm activity of novel Entrococcus faecalis enterocin GLHM.

1474	DRAMP00217	Bacteriocin	"Bacteriocin"[All Fields] AND biofilm[All Fields]	Bacteriocin	32529293	Outer Membrane Channel Protein TolC Regulates Escherichia coli K12 Sensitivity to Plantaricin BM-1 via the CpxR/CpxA Two-Component Regulatory System.
1474	DRAMP00217	Bacteriocin	"Bacteriocin"[All Fields] AND biofilm[All Fields]	Bacteriocin	32517174	Bioengineered Nisin Derivative M17Q Has Enhanced Activity against Staphylococcus epidermidis.
1474	DRAMP00217	Bacteriocin	"Bacteriocin"[All Fields] AND biofilm[All Fields]	Bacteriocin	32322380	In vitroanti-biofilm activity of bacteriocin from a marine Bacillussp. strain Sh10 against Proteus mirabilis.
1474	DRAMP00217	Bacteriocin	"Bacteriocin"[All Fields] AND biofilm[All Fields]	Bacteriocin	32318864	Virulence Factors, Drug Resistance and Biofilm Formation in Pseudomonas Species Isolated from Healthcare Water Systems.
1474	DRAMP00217	Bacteriocin	"Bacteriocin"[All Fields] AND biofilm[All Fields]	Bacteriocin	32307660	A Review of the Role of Probiotic Supplementation in Dental Caries.
1474	DRAMP00217	Bacteriocin	"Bacteriocin"[All Fields] AND biofilm[All Fields]	Bacteriocin	32282078	Physicochemical properties and mode of action of a novel bacteriocin BM1122 with broad antibacterial spectrum produced by Lactobacillus crustorum MN047.
1474	DRAMP00217	Bacteriocin	"Bacteriocin"[All Fields] AND biofilm[All Fields]	Bacteriocin	32247478	Does Quorum Sensing play a role in microbial shifts along spontaneous fermentation of cocoa beans? An in silico perspective.
1474	DRAMP00217	Bacteriocin	"Bacteriocin"[All Fields] AND biofilm[All Fields]	Bacteriocin	32229530	Genetic Analysis of Mutacin B-Ny266, a Lantibiotic Active against Caries Pathogens.
1474	DRAMP00217	Bacteriocin	"Bacteriocin"[All Fields] AND biofilm[All Fields]	Bacteriocin	32178236	A Rapid Lysostaphin Production Approach and a Convenient Novel Lysostaphin Loaded Nano-emulgel; As a Sustainable Low-Cost Methicillin-Resistant Staphylococcus aureusCombating Platform.
1474	DRAMP00217	Bacteriocin	"Bacteriocin"[All Fields] AND biofilm[All Fields]	Bacteriocin	32111071	Whole-Genome Sequencing of Lactobacillus helveticusD75 and D76 Confirms Safety and Probiotic Potential.
1474	DRAMP00217	Bacteriocin	"Bacteriocin"[All Fields] AND biofilm[All Fields]	Bacteriocin	31942681	Evaluation of Probiotic Properties and Prebiotic Utilization Potential of Weissella paramesenteroides Isolated From Fruits.
1474	DRAMP00217	Bacteriocin	"Bacteriocin"[All Fields] AND biofilm[All Fields]	Bacteriocin	31888501	Composite genome sequence of Bacillus clausii, a probiotic commercially available as Enterogermina ®, and insights into its probiotic properties.
1474	DRAMP00217	Bacteriocin	"Bacteriocin"[All Fields] AND biofilm[All Fields]	Bacteriocin	31794906	Isolation and characterization of Enterococcus faecium DSM 20477 with ability to secrete antimicrobial substance for the inhibition of oral pathogen Streptococcus mutans UKMCC 1019.
1474	DRAMP00217	Bacteriocin	"Bacteriocin"[All Fields] AND biofilm[All Fields]	Bacteriocin	31784952	Bacteriocin of Pediococcus acidilactici HW01 Inhibits Biofilm Formation and Virulence Factor Production by Pseudomonas aeruginosa.
1474	DRAMP00217	Bacteriocin	"Bacteriocin"[All Fields] AND biofilm[All Fields]	Bacteriocin	31784450	The manifold roles of microbial ribosomal peptide-based natural products in physiology and ecology.
1474	DRAMP00217	Bacteriocin	"Bacteriocin"[All Fields] AND biofilm[All Fields]	Bacteriocin	31650413	In Vitro Evaluation of Probiotic Potential of Selected Lactic Acid Bacteria Strains.
1474	DRAMP00217	Bacteriocin	"Bacteriocin"[All Fields] AND biofilm[All Fields]	Bacteriocin	31552285	Evaluation of Incompatibility Group I1 (IncI1) Plasmid-Containing Salmonella entericaand Assessment of the Plasmids in Bacteriocin Production and Biofilm Development.
1474	DRAMP00217	Bacteriocin	"Bacteriocin"[All Fields] AND biofilm[All Fields]	Bacteriocin	31456169	Changes in the composition and architecture of staphylococcal biofilm by nisin.
1474	DRAMP00217	Bacteriocin	"Bacteriocin"[All Fields] AND biofilm[All Fields]	Bacteriocin	31432254	Virgicin, a novel lanthipeptide from Virgibacillus sp. strain AK90 exhibits inhibitory activity against Gram-positive bacteria.
1474	DRAMP00217	Bacteriocin	"Bacteriocin"[All Fields] AND biofilm[All Fields]	Bacteriocin	33902719	Genome sequence of Epibacterium ulvae strain DSM 24752 T, an indigoidine-producing, macroalga-associated member of the marine Roseobacter group.
1474	DRAMP00217	Bacteriocin	"Bacteriocin"[All Fields] AND biofilm[All Fields]	Bacteriocin	31356968	Reconstruction of transcriptional regulatory networks of Fis and H-NS in Escherichia coli from genome-wide data analysis.
1474	DRAMP00217	Bacteriocin	"Bacteriocin"[All Fields] AND biofilm[All Fields]	Bacteriocin	31341074	Enterococcus faecalis CRISPR-Cas Is a Robust Barrier to Conjugative Antibiotic Resistance Dissemination in the Murine Intestine.
1474	DRAMP00217	Bacteriocin	"Bacteriocin"[All Fields] AND biofilm[All Fields]	Bacteriocin	31266876	Antifungal Activity of the Enterococcus faecalis Peptide EntV Requires Protease Cleavage and Disulfide Bond Formation.
1474	DRAMP00217	Bacteriocin	"Bacteriocin"[All Fields] AND biofilm[All Fields]	Bacteriocin	31218558	Inhibitory effect of bacteriocins from enterococci on developing and preformed biofilms of Listeria monocytogenes, Listeria ivanovii and Listeria innocua.
1474	DRAMP00217	Bacteriocin	"Bacteriocin"[All Fields] AND biofilm[All Fields]	Bacteriocin	31206966	Lactocin AL705 as quorum sensing inhibitor to control Listeria monocytogenes biofilm formation.
1474	DRAMP00217	Bacteriocin	"Bacteriocin"[All Fields] AND biofilm[All Fields]	Bacteriocin	31166173	ComDE Two-component Signal Transduction Systems in Oral Streptococci: Structure and Function.
1474	DRAMP00217	Bacteriocin	"Bacteriocin"[All Fields] AND biofilm[All Fields]	Bacteriocin	31158279	Corrigendum to: Control of Listeria monocytogenes biofilms on industrial surfaces by the bacteriocin-producing Lactobacillus sakei CRL1862.
1474	DRAMP00217	Bacteriocin	"Bacteriocin"[All Fields] AND biofilm[All Fields]	Bacteriocin	31151072	Study of the effectiveness of staphylococcins in biopreservation of Minas fresh (Frescal) cheese with a reduced sodium content.
1474	DRAMP00217	Bacteriocin	"Bacteriocin"[All Fields] AND biofilm[All Fields]	Bacteriocin	31136751	Antibacterial immunomodulatory and antibiofilm triple effect of Salivaricin LHM against Pseudomonas aeruginosa urinary tract infection model.
1474	DRAMP00217	Bacteriocin	"Bacteriocin"[All Fields] AND biofilm[All Fields]	Bacteriocin	31100490	Effects of 7S globulin 3 derived from the adzuki bean [Vigna angularis] on the CSP- and eDNA-dependent biofilm formation of Streptococcus mutans.
1474	DRAMP00217	Bacteriocin	"Bacteriocin"[All Fields] AND biofilm[All Fields]	Bacteriocin	31036688	Spontaneously Arising Streptococcus mutans Variants with Reduced Susceptibility to Chlorhexidine Display Genetic Defects and Diminished Fitness.
1474	DRAMP00217	Bacteriocin	"Bacteriocin"[All Fields] AND biofilm[All Fields]	Bacteriocin	30962344	Lysocins: Bioengineered Antimicrobials That Deliver Lysins across the Outer Membrane of Gram-Negative Bacteria.
1474	DRAMP00217	Bacteriocin	"Bacteriocin"[All Fields] AND biofilm[All Fields]	Bacteriocin	30955056	Antibacterial activity against porcine respiratory bacterial pathogens and in vitro biocompatibility of essential oils.
1474	DRAMP00217	Bacteriocin	"Bacteriocin"[All Fields] AND biofilm[All Fields]	Bacteriocin	30915281	Characterization of the Competitive Pneumocin Peptides of Streptococcus pneumoniae.
1474	DRAMP00217	Bacteriocin	"Bacteriocin"[All Fields] AND biofilm[All Fields]	Bacteriocin	30863715	BaCf3: highly thermostable bacteriocin from Bacillus amyloliquefaciensBTSS3 antagonistic on food-borne pathogens.
1474	DRAMP00217	Bacteriocin	"Bacteriocin"[All Fields] AND biofilm[All Fields]	Bacteriocin	30853461	Inhibiting bacterial colonization on catheters: Antibacterial and antibiofilm activities of bacteriocins from Lactobacillus plantarum SJ33.
1474	DRAMP00217	Bacteriocin	"Bacteriocin"[All Fields] AND biofilm[All Fields]	Bacteriocin	30841529	Regulatory Effect of DNA Topoisomerase I on T3SS Activity, Antibiotic Susceptibility and Quorum-Sensing-Independent Pyocyanin Synthesis in Pseudomonas aeruginosa.
1474	DRAMP00217	Bacteriocin	"Bacteriocin"[All Fields] AND biofilm[All Fields]	Bacteriocin	30786799	Biofilm formation by staphylococci in health-related environments and recent reports on their control using natural compounds.
1474	DRAMP00217	Bacteriocin	"Bacteriocin"[All Fields] AND biofilm[All Fields]	Bacteriocin	30711392	Identification of highly potent competence stimulating peptide-based quorum sensing activators in Streptococcus mutans through the utilization of N-methyl and reverse alanine scanning.
1474	DRAMP00217	Bacteriocin	"Bacteriocin"[All Fields] AND biofilm[All Fields]	Bacteriocin	30710249	Characterisation of Faecal Staphylococci from Roe Deer (Capreolus capreolus) and Red Deer (Cervus elaphus) and Their Susceptibility to Gallidermin.
1474	DRAMP00217	Bacteriocin	"Bacteriocin"[All Fields] AND biofilm[All Fields]	Bacteriocin	30670678	The Combined Use of Tea Polyphenols and Lactobacillus Plantarum ST8SH Bacteriocin in a Rabbit Model of Infection Following Femoral Fracture with Internal Fixation.
1474	DRAMP00217	Bacteriocin	"Bacteriocin"[All Fields] AND biofilm[All Fields]	Bacteriocin	30658319	Antifungal activities against Candida albicans, of cell-free supernatants obtained from probiotic Pediococcus acidilactici HW01.
1474	DRAMP00217	Bacteriocin	"Bacteriocin"[All Fields] AND biofilm[All Fields]	Bacteriocin	30627971	Characterization of the bacteriocin produced by Enterococcus italicus ONU547 isolated from Thai fermented cabbage.
1474	DRAMP00217	Bacteriocin	"Bacteriocin"[All Fields] AND biofilm[All Fields]	Bacteriocin	30617242	Increased Intracellular Cyclic di-AMP Levels Sensitize Streptococcus gallolyticus subsp. gallolyticus to Osmotic Stress and Reduce Biofilm Formation and Adherence on Intestinal Cells.
1474	DRAMP00217	Bacteriocin	"Bacteriocin"[All Fields] AND biofilm[All Fields]	Bacteriocin	30408016	Bacteriocin Isolated from Lactobacillus Rhamnosus L34 Has Antibacterial Effects in a Rabbit Model of Infection After Mandible Fracture Fixation.
1474	DRAMP00217	Bacteriocin	"Bacteriocin"[All Fields] AND biofilm[All Fields]	Bacteriocin	30396893	Genomic, Phenotypic, and Virulence Analysis of Streptococcus sanguinisOral and Infective-Endocarditis Isolates.
1474	DRAMP00217	Bacteriocin	"Bacteriocin"[All Fields] AND biofilm[All Fields]	Bacteriocin	30233504	Production and Antimicrobial Activity of Nisin Under Enological Conditions.
1474	DRAMP00217	Bacteriocin	"Bacteriocin"[All Fields] AND biofilm[All Fields]	Bacteriocin	30082920	Control of Propionibacterium acnes by natural antimicrobial substances: Role of the bacteriocin AS-48 and lysozyme.
1474	DRAMP00217	Bacteriocin	"Bacteriocin"[All Fields] AND biofilm[All Fields]	Bacteriocin	30073512	Lactic Acid Bacteria (LAB) and Their Bacteriocins as Alternative Biotechnological Tools to Control Listeria monocytogenes Biofilms in Food Processing Facilities.
1474	DRAMP00217	Bacteriocin	"Bacteriocin"[All Fields] AND biofilm[All Fields]	Bacteriocin	30057579	Efficient Exploitation of Multiple Novel Bacteriocins by Combination of Complete Genome and Peptidome.
1474	DRAMP00217	Bacteriocin	"Bacteriocin"[All Fields] AND biofilm[All Fields]	Bacteriocin	29990430	Structure-Activity Relationships of the Competence Stimulating Peptide in Streptococcus mutans Reveal Motifs Critical for Membrane Protease SepM Recognition and ComD Receptor Activation.
1474	DRAMP00217	Bacteriocin	"Bacteriocin"[All Fields] AND biofilm[All Fields]	Bacteriocin	29867809	Biofilms in the Food Industry: Health Aspects and Control Methods.
1474	DRAMP00217	Bacteriocin	"Bacteriocin"[All Fields] AND biofilm[All Fields]	Bacteriocin	29729340	Cloning, overexpression, purification of bacteriocin enterocin-B and structural analysis, interaction determination of enterocin-A, B against pathogenic bacteria and human cancer cells.
1474	DRAMP00217	Bacteriocin	"Bacteriocin"[All Fields] AND biofilm[All Fields]	Bacteriocin	29632089	Characterization of the Trehalose Utilization Operon in Streptococcus mutans Reveals that the TreR Transcriptional Regulator Is Involved in Stress Response Pathways and Toxin Production.
1474	DRAMP00217	Bacteriocin	"Bacteriocin"[All Fields] AND biofilm[All Fields]	Bacteriocin	29628998	Role of Streptococcus mutanssurface proteins for biofilm formation.
1474	DRAMP00217	Bacteriocin	"Bacteriocin"[All Fields] AND biofilm[All Fields]	Bacteriocin	29619040	Oxygen Availability Influences Expression of Dickeya solaniGenes Associated With Virulence in Potato (Solanum tuberosumL.) and Chicory (Cichorium intybusL.).
1474	DRAMP00217	Bacteriocin	"Bacteriocin"[All Fields] AND biofilm[All Fields]	Bacteriocin	29599835	Antibacterial effects of bacteriocins isolated from Lactobacillus rhamnosus(ATCC 53103) in a rabbit model of knee implant infection.
1474	DRAMP00217	Bacteriocin	"Bacteriocin"[All Fields] AND biofilm[All Fields]	Bacteriocin	29580208	Screening for inhibitors of mutacin synthesis in Streptococcus mutans using fluorescent reporter strains.

1474	DRAMP00217	Bacteriocin	"Bacteriocin"[All Fields] AND biofilm[All Fields]	Bacteriocin	29567501	The role of probiotic Lactobacillus acidophilus ATCC 4356 bacteriocin on effect of HBsu on planktonic cells and biofilm formation of Bacillus subtilis.
1474	DRAMP00217	Bacteriocin	"Bacteriocin"[All Fields] AND biofilm[All Fields]	Bacteriocin	29489935	Influence of Helicobacter pylori culture supernatant on the ecological balance of a dual-species oral biofilm.
1474	DRAMP00217	Bacteriocin	"Bacteriocin"[All Fields] AND biofilm[All Fields]	Bacteriocin	29440256	Identification of Streptococcus gallolyticus subsp. gallolyticus (Biotype I) Competence-Stimulating Peptide Pheromone.
1474	DRAMP00217	Bacteriocin	"Bacteriocin"[All Fields] AND biofilm[All Fields]	Bacteriocin	29424388	Evaluation antibacterial and antibiofilm activity of the antimicrobial peptide P34 against Staphylococcus aureus and Enterococcus faecalis.
1474	DRAMP00217	Bacteriocin	"Bacteriocin"[All Fields] AND biofilm[All Fields]	Bacteriocin	29410001	Preparation, characterization and efficacy of lysostaphin-chitosan gel against Staphylococcus aureus.
1474	DRAMP00217	Bacteriocin	"Bacteriocin"[All Fields] AND biofilm[All Fields]	Bacteriocin	29402863	Antibacterial Activity, Cytotoxicity, and the Mechanism of Action of Bacteriocin from Bacillus subtilis GAS101.
1474	DRAMP00217	Bacteriocin	"Bacteriocin"[All Fields] AND biofilm[All Fields]	Bacteriocin	29396844	Evaluation of Probiotic Potential of Bacteriocinogenic Lactic Acid Bacteria Strains Isolated from Meat Products.
1474	DRAMP00217	Bacteriocin	"Bacteriocin"[All Fields] AND biofilm[All Fields]	Bacteriocin	29372297	Genomic and functional characterisation of two Enterococcus strains isolated from Cotija cheese and their potential role in ripening.
1474	DRAMP00217	Bacteriocin	"Bacteriocin"[All Fields] AND biofilm[All Fields]	Bacteriocin	29316223	Probiotic Lactobacillus sp. inhibit growth, biofilm formation and gene expression of caries-inducing Streptococcus mutans.
1474	DRAMP00217	Bacteriocin	"Bacteriocin"[All Fields] AND biofilm[All Fields]	Bacteriocin	29180375	Formation and Characterization of Early Bacterial Biofilms on Different Wood Typologies Applied in Dairy Production.
1474	DRAMP00217	Bacteriocin	"Bacteriocin"[All Fields] AND biofilm[All Fields]	Bacteriocin	29096754	Overview of ribosomal and non-ribosomal antimicrobial peptides produced by Gram positive bacteria.
1474	DRAMP00217	Bacteriocin	"Bacteriocin"[All Fields] AND biofilm[All Fields]	Bacteriocin	29018429	D-Ribose Interferes with Quorum Sensing to Inhibit Biofilm Formation of Lactobacillus paraplantarumL-ZS9.
1474	DRAMP00217	Bacteriocin	"Bacteriocin"[All Fields] AND biofilm[All Fields]	Bacteriocin	28947088	Combined effect of bacteriocin produced by Lactobacillus plantarum ST8SH and vancomycin, propolis or EDTA for controlling biofilm development by Listeria monocytogenes.
1474	DRAMP00217	Bacteriocin	"Bacteriocin"[All Fields] AND biofilm[All Fields]	Bacteriocin	28905285	Effect of bacteriocin and exopolysaccharides isolated from probiotic on P. aeruginosa PAO1 biofilm.
1474	DRAMP00217	Bacteriocin	"Bacteriocin"[All Fields] AND biofilm[All Fields]	Bacteriocin	28887419	Oxidative Stressors Modify the Response of Streptococcus mutans to Its Competence Signal Peptides.
1474	DRAMP00217	Bacteriocin	"Bacteriocin"[All Fields] AND biofilm[All Fields]	Bacteriocin	28815637	Synergistic antibacterial and antibiofilm efficacy of nisin in combination with p-coumaric acid against food-borne bacteria Bacillus cereus and Salmonella typhimurium.
1474	DRAMP00217	Bacteriocin	"Bacteriocin"[All Fields] AND biofilm[All Fields]	Bacteriocin	28797211	Disulfide Bonds: A Key Modification in Bacterial Extracytoplasmic Proteins.
1474	DRAMP00217	Bacteriocin	"Bacteriocin"[All Fields] AND biofilm[All Fields]	Bacteriocin	28729051	Inhibiting effects of fructanase on competence-stimulating peptide-dependent quorum sensing system in Streptococcus mutans.
1474	DRAMP00217	Bacteriocin	"Bacteriocin"[All Fields] AND biofilm[All Fields]	Bacteriocin	28725299	Role of Streptococcus mutanstwo-component systems in antimicrobial peptide resistance in the oral cavity.
1474	DRAMP00217	Bacteriocin	"Bacteriocin"[All Fields] AND biofilm[All Fields]	Bacteriocin	28705677	Hyicin 4244, the first sactibiotic described in staphylococci, exhibits an anti-staphylococcal biofilm activity.
1474	DRAMP00217	Bacteriocin	"Bacteriocin"[All Fields] AND biofilm[All Fields]	Bacteriocin	28696370	Strategies for Pathogen Biocontrol Using Lactic Acid Bacteria and Their Metabolites: A Focus on Meat Ecosystems and Industrial Environments.
1474	DRAMP00217	Bacteriocin	"Bacteriocin"[All Fields] AND biofilm[All Fields]	Bacteriocin	28659548	Pathogenicity of Enterococci.
1474	DRAMP00217	Bacteriocin	"Bacteriocin"[All Fields] AND biofilm[All Fields]	Bacteriocin	28526785	Effects of Arginine on Streptococcus mutans Growth, Virulence Gene Expression, and Stress Tolerance.
1474	DRAMP00217	Bacteriocin	"Bacteriocin"[All Fields] AND biofilm[All Fields]	Bacteriocin	28457635	High-purity Nisin Alone or in Combination with Sodium Hypochlorite Is Effective against Planktonic and Biofilm Populations of Enterococcus faecalis.
1474	DRAMP00217	Bacteriocin	"Bacteriocin"[All Fields] AND biofilm[All Fields]	Bacteriocin	28396417	Enterococcus faecalisbacteriocin EntV inhibits hyphal morphogenesis, biofilm formation, and virulence of Candida albicans.
1474	DRAMP00217	Bacteriocin	"Bacteriocin"[All Fields] AND biofilm[All Fields]	Bacteriocin	28314902	Potential Factors Enabling Human Body Colonization by Animal Streptococcus dysgalactiae subsp. equisimilis Strains.
1474	DRAMP00217	Bacteriocin	"Bacteriocin"[All Fields] AND biofilm[All Fields]	Bacteriocin	28293865	The Inhibition Effect of Lactobacilli Against Growth and Biofilm Formation of Pseudomonas aeruginosa.
1474	DRAMP00217	Bacteriocin	"Bacteriocin"[All Fields] AND biofilm[All Fields]	Bacteriocin	28167518	Pleiotropic Regulation of Virulence Genes in Streptococcus mutans by the Conserved Small Protein SprV.
1474	DRAMP00217	Bacteriocin	"Bacteriocin"[All Fields] AND biofilm[All Fields]	Bacteriocin	28066817	Transcriptional Profiling of the Oral Pathogen Streptococcus mutansin Response to Competence Signaling Peptide XIP.
1474	DRAMP00217	Bacteriocin	"Bacteriocin"[All Fields] AND biofilm[All Fields]	Bacteriocin	28027492	Design of antibacterial biointerfaces by surface modification of poly (ε-caprolactone) with fusion protein containing hydrophobin and PA-1.
1474	DRAMP00217	Bacteriocin	"Bacteriocin"[All Fields] AND biofilm[All Fields]	Bacteriocin	27878401	Enterocin B3A-B3B produced by LAB collected from infant faeces: potential utilization in the food industry for Listeria monocytogenes biofilm management.
1474	DRAMP00217	Bacteriocin	"Bacteriocin"[All Fields] AND biofilm[All Fields]	Bacteriocin	27833601	Synergistic Nisin-Polymyxin Combinations for the Control of PseudomonasBiofilm Formation.
1474	DRAMP00217	Bacteriocin	"Bacteriocin"[All Fields] AND biofilm[All Fields]	Bacteriocin	27822519	Comprehensive Transcriptome Profiles of Streptococcus mutansUA159 Map Core Streptococcal Competence Genes.
1474	DRAMP00217	Bacteriocin	"Bacteriocin"[All Fields] AND biofilm[All Fields]	Bacteriocin	27797439	Inhibition of Listeria monocytogenes biofilms by bacteriocin-producing bacteria isolated from mushroom substrate.
1474	DRAMP00217	Bacteriocin	"Bacteriocin"[All Fields] AND biofilm[All Fields]	Bacteriocin	27695440	Nanotechnology: A Valuable Strategy to Improve Bacteriocin Formulations.
1474	DRAMP00217	Bacteriocin	"Bacteriocin"[All Fields] AND biofilm[All Fields]	Bacteriocin	27513424	The inhibitory effect of bacteriocin produced by Lactobacillus acidophilusATCC 4356 and Lactobacillus plantarum ATCC 8014 on planktonic cells and biofilms of Serratia marcescens.
1474	DRAMP00217	Bacteriocin	"Bacteriocin"[All Fields] AND biofilm[All Fields]	Bacteriocin	27422166	Characterization of a potential ABC-type bacteriocin exporter protein from Treponema denticola.
1474	DRAMP00217	Bacteriocin	"Bacteriocin"[All Fields] AND biofilm[All Fields]	Bacteriocin	27375584	Use of Potential Probiotic Lactic Acid Bacteria (LAB) Biofilms for the Control of Listeria monocytogenes, Salmonella Typhimurium, and Escherichia coli O157:H7 Biofilms Formation.
1474	DRAMP00217	Bacteriocin	"Bacteriocin"[All Fields] AND biofilm[All Fields]	Bacteriocin	27375583	Combinatorial Effects of Aromatic 1,3-Disubstituted Ureas and Fluoride on In vitro Inhibition of Streptococcus mutans Biofilm Formation.
1474	DRAMP00217	Bacteriocin	"Bacteriocin"[All Fields] AND biofilm[All Fields]	Bacteriocin	27359217	Kin Recognition in Bacteria.
1474	DRAMP00217	Bacteriocin	"Bacteriocin"[All Fields] AND biofilm[All Fields]	Bacteriocin	27312701	Comparative transcriptome analysis of the biocontrol strain Bacillus amyloliquefaciens FZB42 as response to biofilm formation analyzed by RNA sequencing.
1474	DRAMP00217	Bacteriocin	"Bacteriocin"[All Fields] AND biofilm[All Fields]	Bacteriocin	27257437	The efficacy of thuricin CD, tigecycline, vancomycin, teicoplanin, rifampicin and nitazoxanide, independently and in paired combinations against Clostridium difficile biofilms and planktonic cells.
1474	DRAMP00217	Bacteriocin	"Bacteriocin"[All Fields] AND biofilm[All Fields]	Bacteriocin	27190146	Control of Listeria monocytogenes biofilms on industrial surfaces by the bacteriocin-producing Lactobacillus sakei CRL1862.
1474	DRAMP00217	Bacteriocin	"Bacteriocin"[All Fields] AND biofilm[All Fields]	Bacteriocin	27161116	l-Arginine Modifies the Exopolysaccharide Matrix and Thwarts Streptococcus mutans Outgrowth within Mixed-Species Oral Biofilms.
1474	DRAMP00217	Bacteriocin	"Bacteriocin"[All Fields] AND biofilm[All Fields]	Bacteriocin	27148197	In Vitro Activities of Nisin and Nisin Derivatives Alone and In Combination with Antibiotics against Staphylococcus Biofilms.
1474	DRAMP00217	Bacteriocin	"Bacteriocin"[All Fields] AND biofilm[All Fields]	Bacteriocin	26999597	Nisin and lysostaphin activity against preformed biofilm of Staphylococcus aureus involved in bovine mastitis.
1474	DRAMP00217	Bacteriocin	"Bacteriocin"[All Fields] AND biofilm[All Fields]	Bacteriocin	26840124	Coordinated Bacteriocin Expression and Competence in Streptococcus pneumoniae Contributes to Genetic Adaptation through Neighbor Predation.
1474	DRAMP00217	Bacteriocin	"Bacteriocin"[All Fields] AND biofilm[All Fields]	Bacteriocin	26826230	A Highly Arginolytic Streptococcus Species That Potently Antagonizes Streptococcus mutans.
1474	DRAMP00217	Bacteriocin	"Bacteriocin"[All Fields] AND biofilm[All Fields]	Bacteriocin	26708985	A ptsP deficiency in PGPR Pseudomonas fluorescens SF39a affects bacteriocin production and bacterial fitness in the wheat rhizosphere.
1474	DRAMP00217	Bacteriocin	"Bacteriocin"[All Fields] AND biofilm[All Fields]	Bacteriocin	26678028	Biomedical applications of nisin.
1474	DRAMP00217	Bacteriocin	"Bacteriocin"[All Fields] AND biofilm[All Fields]	Bacteriocin	26660467	In Vitro Evaluation of Bacteriocins Activity Against Listeria monocytogenes Biofilm Formation.
1474	DRAMP00217	Bacteriocin	"Bacteriocin"[All Fields] AND biofilm[All Fields]	Bacteriocin	26591658	[Animal Staphylococcus felis with the potential to infect human skin].
1474	DRAMP00217	Bacteriocin	"Bacteriocin"[All Fields] AND biofilm[All Fields]	Bacteriocin	26527641	Mutation of the Thiol-Disulfide Oxidoreductase SdbA Activates the CiaRH Two-Component System, Leading to Bacteriocin Expression Shutdown in Streptococcus gordonii.
1474	DRAMP00217	Bacteriocin	"Bacteriocin"[All Fields] AND biofilm[All Fields]	Bacteriocin	26523633	Whole genome sequence to decipher the resistome of Shewanella algae, a multidrug-resistant bacterium responsible for pneumonia, Marseille, France.
1474	DRAMP00217	Bacteriocin	"Bacteriocin"[All Fields] AND biofilm[All Fields]	Bacteriocin	26481153	The outcome of H. influenzae and S. pneumoniae inter-species interactions depends on pH, nutrient availability and growth phase.
1474	DRAMP00217	Bacteriocin	"Bacteriocin"[All Fields] AND biofilm[All Fields]	Bacteriocin	26353398	[On the origin of Yersinia pestis, a causative agent of the plague: A concept of population-genetic macroevolution in transitive environment].
1474	DRAMP00217	Bacteriocin	"Bacteriocin"[All Fields] AND biofilm[All Fields]	Bacteriocin	26338114	Transfer, composition and technological characterization of the lactic acid bacterial populations of the wooden vats used to produce traditional stretched cheeses.
1474	DRAMP00217	Bacteriocin	"Bacteriocin"[All Fields] AND biofilm[All Fields]	Bacteriocin	26292786	Sonorensin: A new bacteriocin with potential of an anti-biofilm agent and a food biopreservative.

1474	DRAMP00217	Bacteriocin	"Bacteriocin"[All Fields] AND biofilm[All Fields]	Bacteriocin	26267163	Inhibitory effects of Lactobacillus fermentum on microbial growth and biofilm formation.
1474	DRAMP00217	Bacteriocin	"Bacteriocin"[All Fields] AND biofilm[All Fields]	Bacteriocin	26198853	Identification and characterization of SMU.244 encoding a putative undecaprenyl pyrophosphate phosphatase protein required for cell wall biosynthesis and bacitracin resistance in Streptococcus mutans.
1474	DRAMP00217	Bacteriocin	"Bacteriocin"[All Fields] AND biofilm[All Fields]	Bacteriocin	26131169	Comparison of antibacterial effects between antimicrobial peptide and bacteriocins isolated from Lactobacillus plantarum on three common pathogenic bacteria.
1474	DRAMP00217	Bacteriocin	"Bacteriocin"[All Fields] AND biofilm[All Fields]	Bacteriocin	26119252	Let there be bioluminescence: development of a biophotonic imaging platform for in situ analyses of oral biofilms in animal models.
1474	DRAMP00217	Bacteriocin	"Bacteriocin"[All Fields] AND biofilm[All Fields]	Bacteriocin	25588687	The Natural Antimicrobial Subtilosin A Synergizes with Lauramide Arginine Ethyl Ester (LAE), ε-Poly-L-lysine (Polylysine), Clindamycin Phosphate and Metronidazole, Against the Vaginal Pathogen Gardnerella vaginalis.
1474	DRAMP00217	Bacteriocin	"Bacteriocin"[All Fields] AND biofilm[All Fields]	Bacteriocin	25189864	Quorum sensing and biofilms in the pathogen, Streptococcus pneumoniae.
1474	DRAMP00217	Bacteriocin	"Bacteriocin"[All Fields] AND biofilm[All Fields]	Bacteriocin	25171407	Evolution of resistance to a last-resort antibiotic in Staphylococcus aureus via bacterial competition.
1474	DRAMP00217	Bacteriocin	"Bacteriocin"[All Fields] AND biofilm[All Fields]	Bacteriocin	24984799	Bacteriocin expression in sessile and planktonic populations of Escherichia coli.
1474	DRAMP00217	Bacteriocin	"Bacteriocin"[All Fields] AND biofilm[All Fields]	Bacteriocin	24806217	The effect of five probiotic lactobacilli strains on the growth and biofilm formation of Streptococcus mutans.
1474	DRAMP00217	Bacteriocin	"Bacteriocin"[All Fields] AND biofilm[All Fields]	Bacteriocin	24509500	Novel two-component regulatory systems play a role in biofilm formation of Lactobacillus reuteri rodent isolate 100-23.
1474	DRAMP00217	Bacteriocin	"Bacteriocin"[All Fields] AND biofilm[All Fields]	Bacteriocin	24148670	Proteins of novel lactic acid bacteria from Apis mellifera mellifera: an insight into the production of known extra-cellular proteins during microbial stress.
1474	DRAMP00217	Bacteriocin	"Bacteriocin"[All Fields] AND biofilm[All Fields]	Bacteriocin	24135676	Comparative proteomic analysis of Listeria monocytogenes exposed to enterocin AS-48 in planktonic and sessile states.
1474	DRAMP00217	Bacteriocin	"Bacteriocin"[All Fields] AND biofilm[All Fields]	Bacteriocin	24071026	Role of probiotics in the prevention and treatment of meticillin-resistant Staphylococcus aureus infections.
1474	DRAMP00217	Bacteriocin	"Bacteriocin"[All Fields] AND biofilm[All Fields]	Bacteriocin	23624069	In vitro probiotic properties of Lactobacillus fermentum SK5 isolated from vagina of a healthy woman.
1474	DRAMP00217	Bacteriocin	"Bacteriocin"[All Fields] AND biofilm[All Fields]	Bacteriocin	23615907	Functional analysis of paralogous thiol-disulfide oxidoreductases in Streptococcus gordonii.
1474	DRAMP00217	Bacteriocin	"Bacteriocin"[All Fields] AND biofilm[All Fields]	Bacteriocin	23558192	Combined treatments of enterocin AS-48 with biocides to improve the inactivation of methicillin-sensitive and methicillin-resistant Staphylococcus aureus planktonic and sessile cells.
1474	DRAMP00217	Bacteriocin	"Bacteriocin"[All Fields] AND biofilm[All Fields]	Bacteriocin	23421615	Global transcriptional responses to the bacteriocin colicin M in Escherichia coli.
1474	DRAMP00217	Bacteriocin	"Bacteriocin"[All Fields] AND biofilm[All Fields]	Bacteriocin	23398522	Phosphate limitation induces the intergeneric inhibition of Pseudomonas aeruginosa by Serratia marcescens isolated from paper machines.
1474	DRAMP00217	Bacteriocin	"Bacteriocin"[All Fields] AND biofilm[All Fields]	Bacteriocin	23318746	Are we ready for caries prevention through bacteriotherapy?
1474	DRAMP00217	Bacteriocin	"Bacteriocin"[All Fields] AND biofilm[All Fields]	Bacteriocin	23241973	Protective mechanisms of respiratory tract Streptococci against Streptococcus pyogenes biofilm formation and epithelial cell infection.
1474	DRAMP00217	Bacteriocin	"Bacteriocin"[All Fields] AND biofilm[All Fields]	Bacteriocin	23107045	Bacteriocin immunity proteins play a role in quorum-sensing system regulated antimicrobial sensitivity of Streptococcus mutans UA159.
1474	DRAMP00217	Bacteriocin	"Bacteriocin"[All Fields] AND biofilm[All Fields]	Bacteriocin	23063226	CRISPR-Cas, a prokaryotic adaptive immune system, in endodontic, oral, and multidrug-resistant hospital-acquired Enterococcus faecalis.
1474	DRAMP00217	Bacteriocin	"Bacteriocin"[All Fields] AND biofilm[All Fields]	Bacteriocin	23033644	Development of polyvinyl chloride biofilms for succession of selected marine bacterial populations.
1474	DRAMP00217	Bacteriocin	"Bacteriocin"[All Fields] AND biofilm[All Fields]	Bacteriocin	22921084	Recent patents on bacteriocins: food and biomedical applications.
1474	DRAMP00217	Bacteriocin	"Bacteriocin"[All Fields] AND biofilm[All Fields]	Bacteriocin	22470839	Studies on strains of Streptococcus mutans isolated from caries-active and caries-free individuals in Iceland.
1474	DRAMP00217	Bacteriocin	"Bacteriocin"[All Fields] AND biofilm[All Fields]	Bacteriocin	22290290	Isolation and identification of a bacteriocin with antibacterial and antibiofilm activity from Citrobacter freundii.
1474	DRAMP00217	Bacteriocin	"Bacteriocin"[All Fields] AND biofilm[All Fields]	Bacteriocin	22265284	Characterization of functional, safety, and probiotic properties of Enterococcus faecalis UGRA10, a new AS-48-producer strain.
1474	DRAMP00217	Bacteriocin	"Bacteriocin"[All Fields] AND biofilm[All Fields]	Bacteriocin	22265283	Effect of enterocin AS-48 in combination with biocides on planktonic and sessile Listeria monocytogenes.
1474	DRAMP00217	Bacteriocin	"Bacteriocin"[All Fields] AND biofilm[All Fields]	Bacteriocin	22228735	Regulation of bacteriocin production and cell death by the VicRK signaling system in Streptococcus mutans.
1474	DRAMP00217	Bacteriocin	"Bacteriocin"[All Fields] AND biofilm[All Fields]	Bacteriocin	22171244	Antagonistic effect of bacteriocin against urinary catheter associated Pseudomonas aeruginosa biofilm.
1474	DRAMP00217	Bacteriocin	"Bacteriocin"[All Fields] AND biofilm[All Fields]	Bacteriocin	22169745	[Effects of immA and immB coding putative bacteriocin immunity proteins on the antimicrobial sensitivity in planktonic Streptococcus mutans and biofilm formation].
1474	DRAMP00217	Bacteriocin	"Bacteriocin"[All Fields] AND biofilm[All Fields]	Bacteriocin	22155816	Identification, characterization, and recombinant expression of epidermicin NI01, a novel unmodified bacteriocin produced by Staphylococcus epidermidis that displays potent activity against Staphylococci.
1474	DRAMP00217	Bacteriocin	"Bacteriocin"[All Fields] AND biofilm[All Fields]	Bacteriocin	22089878	The evolution of bacteriocin production in bacterial biofilms.
1474	DRAMP00217	Bacteriocin	"Bacteriocin"[All Fields] AND biofilm[All Fields]	Bacteriocin	21984782	Regulation of the competence pathway as a novel role associated with a streptococcal bacteriocin.
1474	DRAMP00217	Bacteriocin	"Bacteriocin"[All Fields] AND biofilm[All Fields]	Bacteriocin	21538238	Inhibitory effect of Lactobacillus reuteri on periodontopathic and cariogenic bacteria.
1474	DRAMP00217	Bacteriocin	"Bacteriocin"[All Fields] AND biofilm[All Fields]	Bacteriocin	21466767	Bacterial characteristics of importance for recurrent urinary tract infections caused by Escherichia coli.
1474	DRAMP00217	Bacteriocin	"Bacteriocin"[All Fields] AND biofilm[All Fields]	Bacteriocin	21381381	[Disorganization of biofilms of clinical strains of staphylococci by metabolites of lactobacilli].
1474	DRAMP00217	Bacteriocin	"Bacteriocin"[All Fields] AND biofilm[All Fields]	Bacteriocin	20941586	Bacteria competing with the adhesion and biofilm formation by Staphylococcus aureus.
1474	DRAMP00217	Bacteriocin	"Bacteriocin"[All Fields] AND biofilm[All Fields]	Bacteriocin	20381010	Role of Streptococcus mutans eukaryotic-type serine/threonine protein kinase in interspecies interactions with Streptococcus sanguinis.
1474	DRAMP00217	Bacteriocin	"Bacteriocin"[All Fields] AND biofilm[All Fields]	Bacteriocin	20307569	A novel conjugative plasmid from Enterococcus faecalis E99 enhances resistance to ultraviolet radiation.
1474	DRAMP00217	Bacteriocin	"Bacteriocin"[All Fields] AND biofilm[All Fields]	Bacteriocin	20231406	The Streptococcus mutans serine/threonine kinase, PknB, regulates competence development, bacteriocin production, and cell wall metabolism.
1474	DRAMP00217	Bacteriocin	"Bacteriocin"[All Fields] AND biofilm[All Fields]	Bacteriocin	19828884	Bacterial interactions in dental biofilm development.
1474	DRAMP00217	Bacteriocin	"Bacteriocin"[All Fields] AND biofilm[All Fields]	Bacteriocin	19812852	Inhibiting effects of Enterococcus faecium non-biofilm strain on Streptococcus mutans biofilm formation.
1474	DRAMP00217	Bacteriocin	"Bacteriocin"[All Fields] AND biofilm[All Fields]	Bacteriocin	19767478	Complex phenotypic and genotypic responses of Listeria monocytogenes strains exposed to the class IIa bacteriocin sakacin P.
1474	DRAMP00217	Bacteriocin	"Bacteriocin"[All Fields] AND biofilm[All Fields]	Bacteriocin	19735463	Cell death in Streptococcus mutans biofilms: a link between CSP and extracellular DNA.
1474	DRAMP00217	Bacteriocin	"Bacteriocin"[All Fields] AND biofilm[All Fields]	Bacteriocin	19400789	Peptide alarmone signalling triggers an auto-active bacteriocin necessary for genetic competence.
1474	DRAMP00217	Bacteriocin	"Bacteriocin"[All Fields] AND biofilm[All Fields]	Bacteriocin	19185943	Knockout of three-component regulatory systems reveals that the apparently constitutive plantaricin-production phenotype shown by Lactobacillus plantarum on solid medium is regulated via quorum sensing.
1474	DRAMP00217	Bacteriocin	"Bacteriocin"[All Fields] AND biofilm[All Fields]	Bacteriocin	19138664	Effects of quorum sensing on cell viability in Streptococcus mutans biofilm formation.
1474	DRAMP00217	Bacteriocin	"Bacteriocin"[All Fields] AND biofilm[All Fields]	Bacteriocin	19124118	In vitro antimicrobial effect of bacteriocin PsVP-10 in combination with chlorhexidine and triclosan against Streptococcus mutans and Streptococcus sobrinus strains.
1474	DRAMP00217	Bacteriocin	"Bacteriocin"[All Fields] AND biofilm[All Fields]	Bacteriocin	18957580	Additive attenuation of virulence and cariogenic potential of Streptococcus mutans by simultaneous inactivation of the ComCDE quorum-sensing system and HK/RR11 two-component regulatory system.
1474	DRAMP00217	Bacteriocin	"Bacteriocin"[All Fields] AND biofilm[All Fields]	Bacteriocin	18804350	Novel anti-microbial therapies for dental plaque-related diseases.
1474	DRAMP00217	Bacteriocin	"Bacteriocin"[All Fields] AND biofilm[All Fields]	Bacteriocin	18792689	Quorum sensing and biofilm formation by Streptococcus mutans.
1474	DRAMP00217	Bacteriocin	"Bacteriocin"[All Fields] AND biofilm[All Fields]	Bacteriocin	18769851	Effect of bacterial interference on biofilm development by Legionella pneumophila.
1474	DRAMP00217	Bacteriocin	"Bacteriocin"[All Fields] AND biofilm[All Fields]	Bacteriocin	18541165	Prevalence and characterization of Enterococcus spp. isolated from Brazilian foods.
1474	DRAMP00217	Bacteriocin	"Bacteriocin"[All Fields] AND biofilm[All Fields]	Bacteriocin	18441055	Streptococcal antagonism in oral biofilms: Streptococcus sanguinis and Streptococcus gordonii interference with Streptococcus mutans.
1474	DRAMP00217	Bacteriocin	"Bacteriocin"[All Fields] AND biofilm[All Fields]	Bacteriocin	18405343	A multifaceted role for polyamines in bacterial pathogens.

1474	DRAMP00217	Bacteriocin	"Bacteriocin"[All Fields] AND biofilm[All Fields]	Bacteriocin	18387115	Biodegradable polylactic acid polymer with nisin for use in antimicrobial food packaging.
1474	DRAMP00217	Bacteriocin	"Bacteriocin"[All Fields] AND biofilm[All Fields]	Bacteriocin	18232718	Substrate recognition mechanism of the peptidase domain of the quorum-sensing-signal-producing ABC transporter ComA from Streptococcus.
1474	DRAMP00217	Bacteriocin	"Bacteriocin"[All Fields] AND biofilm[All Fields]	Bacteriocin	17981981	Autoinducer-2-regulated genes in Streptococcus mutans UA159 and global metabolic effect of the luxS mutation.
1474	DRAMP00217	Bacteriocin	"Bacteriocin"[All Fields] AND biofilm[All Fields]	Bacteriocin	17660440	Genetic characterization of the hdrRM operon: a novel high-cell-density-responsive regulator in Streptococcus mutans.
1474	DRAMP00217	Bacteriocin	"Bacteriocin"[All Fields] AND biofilm[All Fields]	Bacteriocin	16997961	Role of bacteriocin immunity proteins in the antimicrobial sensitivity of Streptococcus mutans.
1474	DRAMP00217	Bacteriocin	"Bacteriocin"[All Fields] AND biofilm[All Fields]	Bacteriocin	16981904	Cell density- and ComE-dependent expression of a group of mutacin and mutacin-like genes in Streptococcus mutans.
1474	DRAMP00217	Bacteriocin	"Bacteriocin"[All Fields] AND biofilm[All Fields]	Bacteriocin	16936029	Structure-activity analysis of quorum-sensing signaling peptides from Streptococcus mutans.
1474	DRAMP00217	Bacteriocin	"Bacteriocin"[All Fields] AND biofilm[All Fields]	Bacteriocin	16934112	Virulence properties of cariogenic bacteria.
1474	DRAMP00217	Bacteriocin	"Bacteriocin"[All Fields] AND biofilm[All Fields]	Bacteriocin	16925560	Purification and functional studies of a potent modified quorum-sensing peptide and a two-peptide bacteriocin in Streptococcus mutans.
1474	DRAMP00217	Bacteriocin	"Bacteriocin"[All Fields] AND biofilm[All Fields]	Bacteriocin	16907725	IrvA-dependent and IrvA-independent pathways for mutacin gene regulation in Streptococcus mutans.
1474	DRAMP00217	Bacteriocin	"Bacteriocin"[All Fields] AND biofilm[All Fields]	Bacteriocin	16869892	Vibrio harveyi: a significant pathogen of marine vertebrates and invertebrates.
1474	DRAMP00217	Bacteriocin	"Bacteriocin"[All Fields] AND biofilm[All Fields]	Bacteriocin	16304704	Growth of Enterococcus mundtii ST15 in medium filtrate and purification of bacteriocin ST15 by cation-exchange chromatography.
1474	DRAMP00217	Bacteriocin	"Bacteriocin"[All Fields] AND biofilm[All Fields]	Bacteriocin	15978073	Co-ordinated bacteriocin production and competence development: a possible mechanism for taking up DNA from neighbouring species.
1474	DRAMP00217	Bacteriocin	"Bacteriocin"[All Fields] AND biofilm[All Fields]	Bacteriocin	15937160	Regulation of bacteriocin production in Streptococcus mutans by the quorum-sensing system required for development of genetic competence.
1474	DRAMP00217	Bacteriocin	"Bacteriocin"[All Fields] AND biofilm[All Fields]	Bacteriocin	15640209	Interactions between oral bacteria: inhibition of Streptococcus mutans bacteriocin production by Streptococcus gordonii.
1474	DRAMP00217	Bacteriocin	"Bacteriocin"[All Fields] AND biofilm[All Fields]	Bacteriocin	15209996	Transcriptional analysis of mutacin I (mutA) gene expression in planktonic and biofilm cells of Streptococcus mutans using fluorescent protein and glucuronidase reporters.
1474	DRAMP00217	Bacteriocin	"Bacteriocin"[All Fields] AND biofilm[All Fields]	Bacteriocin	15151251	Quorum sensing: a primer for food microbiologists.
1474	DRAMP00217	Bacteriocin	"Bacteriocin"[All Fields] AND biofilm[All Fields]	Bacteriocin	12379833	[Protective role of the Doderleïn flora].
1474	DRAMP00217	Bacteriocin	"Bacteriocin"[All Fields] AND biofilm[All Fields]	Bacteriocin	12147084	Antagonistic interactions amongst bacteriocin-producing enteric bacteria in dual species biofilms.
1474	DRAMP00217	Bacteriocin	"Bacteriocin"[All Fields] AND biofilm[All Fields]	Bacteriocin	10574092	Behaviour of L. monocytogenes in an artificially made biofilm of a nisin-producing strain of Lactococcus lactis.
1479	DRAMP00224	BTL (Bacteriocin)	"BTL"[All Fields] AND biofilm[All Fields]	BTL	17897204	In vitro inhibition of oral streptococci binding to the acquired pellicle by algal lectins.
1489	DRAMP00235	AFP1 (Bacteriocin)	"AFP1"[All Fields] AND biofilm[All Fields]	AFP1	21736910	Identification of an antifungal peptide from Trapa natans fruits with inhibitory effects on Candida tropicalis biofilm formation.
1497	DRAMP00245	Gramicidin A (GA; Nonribosomally synthesized bacteriocin)	"Gramicidin A"[All Fields] AND biofilm[All Fields]	Gramicidin A	20949268	Elaboration of antibiofilm materials by chemical grafting of an antimicrobial peptide.
1500	DRAMP00248	Glycocin F (GccF; S-glycosylated bacteriocin)	"Glycocin F"[All Fields] AND biofilm[All Fields]	Glycocin F	33750106	Discovery, Synthesis, and Optimization of Peptide-Based Antibiotics.
1507	DRAMP00262	Cyanovirin-N (CV-N)	"Cyanovirin-N"[All Fields] AND biofilm[All Fields]	Cyanovirin-N	21734653	Prevention of vaginal SHIV transmission in macaques by a live recombinant Lactobacillus.
1516	DRAMP00272	Thaumatin-like protein (Plants)	"Thaumatin-like protein"[All Fields] AND biofilm[All Fields]	Thaumatin-like protein	22363222	Tasco®: a product of Ascophyllum nodosum enhances immune response of Caenorhabditis elegans against Pseudomonas aeruginosa infection.
1517	DRAMP00273	Thaumatin-like protein (Plants)	"Thaumatin-like protein"[All Fields] AND biofilm[All Fields]	Thaumatin-like protein	22363222	Tasco®: a product of Ascophyllum nodosum enhances immune response of Caenorhabditis elegans against Pseudomonas aeruginosa infection.
1523	DRAMP00280	Trypsin inhibitor (FtTI; Plant defensin)	"Trypsin inhibitor"[All Fields] AND biofilm[All Fields]	Trypsin inhibitor	34656058	A new Kunitz trypsin inhibitor from Erythrina poeppigiana exhibits antimicrobial and antibiofilm properties against bacteria.
1523	DRAMP00280	Trypsin inhibitor (FtTI; Plant defensin)	"Trypsin inhibitor"[All Fields] AND biofilm[All Fields]	Trypsin inhibitor	29710773	Recombinant Inga LaurinaTrypsin Inhibitor (ILTI) Production in Komagataella PhaffiiConfirms Its Potential Anti-Biofilm Effect and Reveals an Anti-Tumoral Activity.
1523	DRAMP00280	Trypsin inhibitor (FtTI; Plant defensin)	"Trypsin inhibitor"[All Fields] AND biofilm[All Fields]	Trypsin inhibitor	28572668	Cathelicidin-trypsin inhibitor loop conjugate represents a promising antibiotic candidate with protease stability.
1540	DRAMP00299	Ribonuclease (Plants)	"Ribonuclease"[All Fields] AND biofilm[All Fields]	Ribonuclease	33574869	Biofilm-Forming Ability and Effect of Sanitation Agents on Biofilm-Control of Thermophile Geobacillussp. D413 and Geobacillus toebiiE134.
1540	DRAMP00299	Ribonuclease (Plants)	"Ribonuclease"[All Fields] AND biofilm[All Fields]	Ribonuclease	32430174	Kanamycin-induced production of 2',3'-cyclic AMP in Escherichia coli.
1540	DRAMP00299	Ribonuclease (Plants)	"Ribonuclease"[All Fields] AND biofilm[All Fields]	Ribonuclease	32036742	Carbon fullerene acts as potential lead molecule against prospective molecular targets of biofilm-producing multidrug-resistant Acinetobacter baumanniand Pseudomonas aerugenosa: computational modeling and MD simulation studies.
1540	DRAMP00299	Ribonuclease (Plants)	"Ribonuclease"[All Fields] AND biofilm[All Fields]	Ribonuclease	31540052	Insight into the Antifungal Mechanism of Action of Human RNase N-terminus Derived Peptides.
1540	DRAMP00299	Ribonuclease (Plants)	"Ribonuclease"[All Fields] AND biofilm[All Fields]	Ribonuclease	31481542	RNase E Promotes Expression of Type III Secretion System Genes in Pseudomonas aeruginosa.
1540	DRAMP00299	Ribonuclease (Plants)	"Ribonuclease"[All Fields] AND biofilm[All Fields]	Ribonuclease	29905843	sRNA-dependent control of curli biosynthesis in Escherichia coli: McaS directs endonucleolytic cleavage of csgD mRNA.
1540	DRAMP00299	Ribonuclease (Plants)	"Ribonuclease"[All Fields] AND biofilm[All Fields]	Ribonuclease	29876449	TCA precipitation and ethanol/HCl single-step purification evaluation: One-dimensional gel electrophoresis, bradford assays, spectrofluorometry and Raman spectroscopy data on HSA, Rnase, lysozyme - Mascots and Skyline data.
1540	DRAMP00299	Ribonuclease (Plants)	"Ribonuclease"[All Fields] AND biofilm[All Fields]	Ribonuclease	29555843	RNase I regulates Escherichia coli2',3'-cyclic nucleotide monophosphate levels and biofilm formation.
1540	DRAMP00299	Ribonuclease (Plants)	"Ribonuclease"[All Fields] AND biofilm[All Fields]	Ribonuclease	29529252	A regulatory RNA is involved in RNA duplex formation and biofilm regulation in Sulfolobus acidocaldarius.
1540	DRAMP00299	Ribonuclease (Plants)	"Ribonuclease"[All Fields] AND biofilm[All Fields]	Ribonuclease	29484598	High-Resolution, High-Throughput Analysis of Hfq-Binding Sites Using UV Crosslinking and Analysis of cDNA (CRAC).
1540	DRAMP00299	Ribonuclease (Plants)	"Ribonuclease"[All Fields] AND biofilm[All Fields]	Ribonuclease	29089928	Antimicrobial and Antibiofilm Effects of Human Amniotic/Chorionic Membrane Extract on Streptococcus pneumoniae.
1540	DRAMP00299	Ribonuclease (Plants)	"Ribonuclease"[All Fields] AND biofilm[All Fields]	Ribonuclease	28400767	The Periplasmic Chaperone Network of Campylobacter jejuni: Evidence that SalC (Cj1289) and PpiD (Cj0694) Are Involved in Maintaining Outer Membrane Integrity.
1540	DRAMP00299	Ribonuclease (Plants)	"Ribonuclease"[All Fields] AND biofilm[All Fields]	Ribonuclease	28384222	Functional studies of E. faecalis RNase J2 and its role in virulence and fitness.
1540	DRAMP00299	Ribonuclease (Plants)	"Ribonuclease"[All Fields] AND biofilm[All Fields]	Ribonuclease	28104802	The Ssl2245-Sll1130 Toxin-Antitoxin System Mediates Heat-induced Programmed Cell Death in Synechocystissp. PCC6803.
1540	DRAMP00299	Ribonuclease (Plants)	"Ribonuclease"[All Fields] AND biofilm[All Fields]	Ribonuclease	28034758	YmdB-mediated down-regulation of sucA inhibits biofilm formation and induces apramycin susceptibility in Escherichia coli.
1540	DRAMP00299	Ribonuclease (Plants)	"Ribonuclease"[All Fields] AND biofilm[All Fields]	Ribonuclease	27920769	Transcriptional Profiling of Type II Toxin-Antitoxin Genes of Helicobacter pyloriunder Different Environmental Conditions: Identification of HP0967-HP0968 System.
1540	DRAMP00299	Ribonuclease (Plants)	"Ribonuclease"[All Fields] AND biofilm[All Fields]	Ribonuclease	27821608	Role of RNase Y in Clostridium perfringens mRNA Decay and Processing.
1540	DRAMP00299	Ribonuclease (Plants)	"Ribonuclease"[All Fields] AND biofilm[All Fields]	Ribonuclease	26694028	VapD in Xylella fastidiosa Is a Thermostable Protein with Ribonuclease Activity.
1540	DRAMP00299	Ribonuclease (Plants)	"Ribonuclease"[All Fields] AND biofilm[All Fields]	Ribonuclease	26150422	Structural and functional studies of the Mycobacterium tuberculosis VapBC30 toxin-antitoxin system: implications for the design of novel antimicrobial peptides.
1540	DRAMP00299	Ribonuclease (Plants)	"Ribonuclease"[All Fields] AND biofilm[All Fields]	Ribonuclease	25837994	A Commensal Strain of Staphylococcus epidermidis Overexpresses Membrane Proteins Associated with Pathogenesis When Grown in Biofilms.
1540	DRAMP00299	Ribonuclease (Plants)	"Ribonuclease"[All Fields] AND biofilm[All Fields]	Ribonuclease	25790505	Identification of a mutant locus that bypasses the BsgA protease requirement for social development in Myxococcus xanthus.
1540	DRAMP00299	Ribonuclease (Plants)	"Ribonuclease"[All Fields] AND biofilm[All Fields]	Ribonuclease	25253647	Nuclease-functionalized poly(styrene-b-isobutylene-b-styrene) surface with anti-infection and tissue integration bifunctions.
1540	DRAMP00299	Ribonuclease (Plants)	"Ribonuclease"[All Fields] AND biofilm[All Fields]	Ribonuclease	24559398	Temperature-driven adsorption and desorption of proteins at solid-liquid interfaces.
1540	DRAMP00299	Ribonuclease (Plants)	"Ribonuclease"[All Fields] AND biofilm[All Fields]	Ribonuclease	24489668	An ribonuclease T2 family protein modulates Acinetobacter baumannii abiotic surface colonization.
1540	DRAMP00299	Ribonuclease (Plants)	"Ribonuclease"[All Fields] AND biofilm[All Fields]	Ribonuclease	24267348	Escherichia coli YmdB regulates biofilm formation independently of its role as an RNase III modulator.
1540	DRAMP00299	Ribonuclease (Plants)	"Ribonuclease"[All Fields] AND biofilm[All Fields]	Ribonuclease	24029489	Drug-eluting cements for hard tissue repair: a comparative study using vancomycin and RNPA1000 to inhibit growth of Staphylococcus aureus.
1540	DRAMP00299	Ribonuclease (Plants)	"Ribonuclease"[All Fields] AND biofilm[All Fields]	Ribonuclease	23601656	Why does the healthy cornea resist Pseudomonas aeruginosa infection?

1540	DRAMP00299	Ribonuclease (Plants)	"Ribonuclease"[All Fields] AND biofilm[All Fields]	Ribonuclease	22858676	An effort to make sense of antisense transcription in bacteria.
1540	DRAMP00299	Ribonuclease (Plants)	"Ribonuclease"[All Fields] AND biofilm[All Fields]	Ribonuclease	22123973	Genome-wide antisense transcription drives mRNA processing in bacteria.
1540	DRAMP00299	Ribonuclease (Plants)	"Ribonuclease"[All Fields] AND biofilm[All Fields]	Ribonuclease	21870656	New way to attack pathogens: RNA recycling system gone awry brings MRSA to a halt.
1540	DRAMP00299	Ribonuclease (Plants)	"Ribonuclease"[All Fields] AND biofilm[All Fields]	Ribonuclease	21824291	Barnase and binase: twins with distinct fates.
1540	DRAMP00299	Ribonuclease (Plants)	"Ribonuclease"[All Fields] AND biofilm[All Fields]	Ribonuclease	21347352	Small molecule inhibitors of Staphylococcus aureus RnpA alter cellular mRNA turnover, exhibit antimicrobial activity, and attenuate pathogenesis.
1540	DRAMP00299	Ribonuclease (Plants)	"Ribonuclease"[All Fields] AND biofilm[All Fields]	Ribonuclease	21320509	Cyclic di-GMP activation of polynucleotide phosphorylase signal-dependent RNA processing.
1540	DRAMP00299	Ribonuclease (Plants)	"Ribonuclease"[All Fields] AND biofilm[All Fields]	Ribonuclease	21179489	Dispersal of biofilms by secreted, matrix degrading, bacterial DNase.
1540	DRAMP00299	Ribonuclease (Plants)	"Ribonuclease"[All Fields] AND biofilm[All Fields]	Ribonuclease	21068382	Structure of the Escherichia coli antitoxin MqsA (YgiT/b3021) bound to its gene promoter reveals extensive domain rearrangements and the specificity of transcriptional regulation.
1540	DRAMP00299	Ribonuclease (Plants)	"Ribonuclease"[All Fields] AND biofilm[All Fields]	Ribonuclease	19943899	Escherichia coli ribonuclease III activity is downregulated by osmotic stress: consequences for the degradation of bdm mRNA in biofilm formation.
1540	DRAMP00299	Ribonuclease (Plants)	"Ribonuclease"[All Fields] AND biofilm[All Fields]	Ribonuclease	19572896	The immune response of oral epithelial cells induced by single-species and complex naturally formed biofilms.
1540	DRAMP00299	Ribonuclease (Plants)	"Ribonuclease"[All Fields] AND biofilm[All Fields]	Ribonuclease	19210620	Bacterial toxin YafQ is an endoribonuclease that associates with the ribosome and blocks translation elongation through sequence-specific and frame-dependent mRNA cleavage.
1540	DRAMP00299	Ribonuclease (Plants)	"Ribonuclease"[All Fields] AND biofilm[All Fields]	Ribonuclease	18173794	The stage of native biofilm formation determines the gene expression of human beta-defensin-2, psoriasin, ribonuclease 7 and inflammatory mediators: a novel approach for stimulation of keratinocytes with in situ formed biofilms.
1558	DRAMP00319	Endochitinase (Plant defensin)	"Endochitinase"[All Fields] AND biofilm[All Fields]	Endochitinase	20656858	Chitin utilization by the insect-transmitted bacterium Xylella fastidiosa.
1567	DRAMP00330	Pathogenesis-related protein (PR-1; Plant defensin)	"Pathogenesis-related protein"[All Fields] AND biofilm[All Fields]	Pathogenesis-related protein	25536016	Green and Red Light Reduces the Disease Severity by Pseudomonas cichorii JBC1 in Tomato Plants via Upregulation of Defense-Related Gene Expression.
1567	DRAMP00330	Pathogenesis-related protein (PR-1; Plant defensin)	"Pathogenesis-related protein"[All Fields] AND biofilm[All Fields]	Pathogenesis-related protein	15842626	Staphylococcus aureus pathogenicity on Arabidopsis thaliana is mediated either by a direct effect of salicylic acid on the pathogen or by SA-dependent, NPR1-independent host responses.
1568	DRAMP00331	Pathogenesis-related protein (PRP; Plant defensin)	"Pathogenesis-related protein"[All Fields] AND biofilm[All Fields]	Pathogenesis-related protein	25536016	Green and Red Light Reduces the Disease Severity by Pseudomonas cichorii JBC1 in Tomato Plants via Upregulation of Defense-Related Gene Expression.
1568	DRAMP00331	Pathogenesis-related protein (PRP; Plant defensin)	"Pathogenesis-related protein"[All Fields] AND biofilm[All Fields]	Pathogenesis-related protein	15842626	Staphylococcus aureus pathogenicity on Arabidopsis thaliana is mediated either by a direct effect of salicylic acid on the pathogen or by SA-dependent, NPR1-independent host responses.
1569	DRAMP00332	Pathogenesis-related protein (Plant defensin)	"Pathogenesis-related protein"[All Fields] AND biofilm[All Fields]	Pathogenesis-related protein	25536016	Green and Red Light Reduces the Disease Severity by Pseudomonas cichorii JBC1 in Tomato Plants via Upregulation of Defense-Related Gene Expression.
1569	DRAMP00332	Pathogenesis-related protein (Plant defensin)	"Pathogenesis-related protein"[All Fields] AND biofilm[All Fields]	Pathogenesis-related protein	15842626	Staphylococcus aureus pathogenicity on Arabidopsis thaliana is mediated either by a direct effect of salicylic acid on the pathogen or by SA-dependent, NPR1-independent host responses.
1600	DRAMP00382	Datucin (Glycopeptide; Plants)	"Datucin"[All Fields] AND biofilm[All Fields]	Datucin	23193597	A novel hydroxyproline rich glycopeptide from pericarp of Datura stramonium: proficiently eradicate the biofilm of antifungals resistant Candida albicans.
1601	DRAMP00383	Antimicrobial peptide 1 (Mc-AMP1; knottin-type peptide; Plant defensin)	"Antimicrobial peptide 1"[All Fields] AND biofilm[All Fields]	Antimicrobial peptide 1	22445495	Database screening and in vivo efficacy of antimicrobial peptides against methicillin-resistant Staphylococcus aureus USA300.
1603	DRAMP00387	Antimicrobial peptide 1 (EcAMP1; hairpin-like peptides; Plants)	"Antimicrobial peptide 1"[All Fields] AND biofilm[All Fields]	Antimicrobial peptide 1	22445495	Database screening and in vivo efficacy of antimicrobial peptides against methicillin-resistant Staphylococcus aureus USA300.
1608	DRAMP00392	Antimicrobial peptide 1 (ToAMP1; Cys-rich; Plant defensin)	"Antimicrobial peptide 1"[All Fields] AND biofilm[All Fields]	Antimicrobial peptide 1	22445495	Database screening and in vivo efficacy of antimicrobial peptides against methicillin-resistant Staphylococcus aureus USA300.
1851	DRAMP18327	Sil(Bacteriocin)	"Sil"[All Fields] AND biofilm[All Fields]	Sil	33007670	Occurrence and removal of pharmaceutical and personal care products using subsurface horizontal flow constructed wetlands.
1851	DRAMP18327	Sil(Bacteriocin)	"Sil"[All Fields] AND biofilm[All Fields]	Sil	32975727	Edible marine algae: a new source for anti-mycobacterial agents.
1851	DRAMP18327	Sil(Bacteriocin)	"Sil"[All Fields] AND biofilm[All Fields]	Sil	32615006	Allyl piperidine-1-carbodithioate and benzyl 1H-imidazole 1 carbodithioate: two potential agents to combat against mycobacteria.
1851	DRAMP18327	Sil(Bacteriocin)	"Sil"[All Fields] AND biofilm[All Fields]	Sil	32409034	Biocompatible alginate silica supported silver nanoparticles composite films for wound dressing with antibiofilm activity.
1851	DRAMP18327	Sil(Bacteriocin)	"Sil"[All Fields] AND biofilm[All Fields]	Sil	31622646	Remineralizing potential of dental composites containing silanized silica-hydroxyapatite (Si-HAp) nanoporous particles charged with sodium fluoride (NaF).
1851	DRAMP18327	Sil(Bacteriocin)	"Sil"[All Fields] AND biofilm[All Fields]	Sil	29109808	SEM Analysis of Surface Impact on Biofilm Antibiotic Treatment.
1851	DRAMP18327	Sil(Bacteriocin)	"Sil"[All Fields] AND biofilm[All Fields]	Sil	25309879	Quorum sensing in group A Streptococcus.
1851	DRAMP18327	Sil(Bacteriocin)	"Sil"[All Fields] AND biofilm[All Fields]	Sil	25044887	Escherichia coli adhesion, biofilm development and antibiotic susceptibility on biomedical materials.
1851	DRAMP18327	Sil(Bacteriocin)	"Sil"[All Fields] AND biofilm[All Fields]	Sil	24165295	Cell surface hydrophobicity: a key component in the degradation of polyethylene succinate by Pseudomonas sp. AKS2.
1851	DRAMP18327	Sil(Bacteriocin)	"Sil"[All Fields] AND biofilm[All Fields]	Sil	23242625	Low-density polyethylene degradation by Pseudomonas sp. AKS2 biofilm.
1851	DRAMP18327	Sil(Bacteriocin)	"Sil"[All Fields] AND biofilm[All Fields]	Sil	23136919	Antibacterial activity of long-chain fatty alcohols against mycobacteria.
1851	DRAMP18327	Sil(Bacteriocin)	"Sil"[All Fields] AND biofilm[All Fields]	Sil	21298675	Characterization of biofilms in different clinical M serotypes of Streptococcus pyogenes.
1851	DRAMP18327	Sil(Bacteriocin)	"Sil"[All Fields] AND biofilm[All Fields]	Sil	17710917	Kinetics of particulate organic matter removal as a response to bioflocculation in aerobic biofilm reactors.
1851	DRAMP18327	Sil(Bacteriocin)	"Sil"[All Fields] AND biofilm[All Fields]	Sil	12829274	Bacterial silver resistance: molecular biology and uses and misuses of silver compounds.
1851	DRAMP18327	Sil(Bacteriocin)	"Sil"[All Fields] AND biofilm[All Fields]	Sil	10399673	In response to: In vivo efficacy of silver-coated (Sil-zone) infection-resistant polyester fabric against a biofilm-producing bacteria, Staphylococcus epidermidis. J Heart Valve Disease; 1998;7:524-530.
1957	DRAMP00751	Defensin-2 (Plant defensin)	"Defensin-2"[All Fields] AND biofilm[All Fields]	Defensin-2	34276631	DNA Blocks the Lethal Effect of Human Beta-Defensin 2 Against Neisseria meningitidis.
1957	DRAMP00751	Defensin-2 (Plant defensin)	"Defensin-2"[All Fields] AND biofilm[All Fields]	Defensin-2	32842903	Electrospun ZnO/Poly(Vinylidene Fluoride-Trifluoroethylene) Scaffolds for Lung Tissue Engineering.
1957	DRAMP00751	Defensin-2 (Plant defensin)	"Defensin-2"[All Fields] AND biofilm[All Fields]	Defensin-2	32522780	Controlling the Growth of the Skin Commensal Staphylococcus epidermidis Using d-Alanine Auxotrophy.
1957	DRAMP00751	Defensin-2 (Plant defensin)	"Defensin-2"[All Fields] AND biofilm[All Fields]	Defensin-2	32457749	The Antimicrobial Peptide Human Beta-Defensin 2 Inhibits Biofilm Production of Pseudomonas aeruginosaWithout Compromising Metabolic Activity.
1957	DRAMP00751	Defensin-2 (Plant defensin)	"Defensin-2"[All Fields] AND biofilm[All Fields]	Defensin-2	31906541	Role of FAD-I in Fusobacterial Interspecies Interaction and Biofilm Formation.
1957	DRAMP00751	Defensin-2 (Plant defensin)	"Defensin-2"[All Fields] AND biofilm[All Fields]	Defensin-2	31336838	Candida albicans-Cell Interactions Activate Innate Immune Defense in Human Palate Epithelial Primary Cells via Nitric Oxide (NO) and β-Defensin 2 (hBD-2).
1957	DRAMP00751	Defensin-2 (Plant defensin)	"Defensin-2"[All Fields] AND biofilm[All Fields]	Defensin-2	30649289	Innate immune components affect growth and virulence traits of bacterial-vaginosis-associated and non-bacterial-vaginosis-associated Gardnerella vaginalis strains similarly.
1957	DRAMP00751	Defensin-2 (Plant defensin)	"Defensin-2"[All Fields] AND biofilm[All Fields]	Defensin-2	27582732	Efficient Eradication of Mature Pseudomonas aeruginosa Biofilm via Controlled Delivery of Nitric Oxide Combined with Antimicrobial Peptide and Antibiotics.
1957	DRAMP00751	Defensin-2 (Plant defensin)	"Defensin-2"[All Fields] AND biofilm[All Fields]	Defensin-2	27148195	Effect of Substance P in Staphylococcus aureus and Staphylococcus epidermidis Virulence: Implication for Skin Homeostasis.
1957	DRAMP00751	Defensin-2 (Plant defensin)	"Defensin-2"[All Fields] AND biofilm[All Fields]	Defensin-2	25808131	Expression of antimicrobial peptides and interleukin-8 during early stages of inflammation: An experimental gingivitis study.
1957	DRAMP00751	Defensin-2 (Plant defensin)	"Defensin-2"[All Fields] AND biofilm[All Fields]	Defensin-2	24340061	Inflammatory and antimicrobial responses to methicillin-resistant Staphylococcus aureus in an in vitro wound infection model.
1957	DRAMP00751	Defensin-2 (Plant defensin)	"Defensin-2"[All Fields] AND biofilm[All Fields]	Defensin-2	21692631	A novel organotypic dento-epithelial culture model: effect of Fusobacterium nucleatum biofilm on B-defensin-2, -3, and LL-37 expression.
1957	DRAMP00751	Defensin-2 (Plant defensin)	"Defensin-2"[All Fields] AND biofilm[All Fields]	Defensin-2	20378008	Effect of temperature on the shift of Pseudomonas fluorescens from an environmental microorganism to a potential human pathogen.
1957	DRAMP00751	Defensin-2 (Plant defensin)	"Defensin-2"[All Fields] AND biofilm[All Fields]	Defensin-2	19572896	The immune response of oral epithelial cells induced by single-species and complex naturally formed biofilms.
1957	DRAMP00751	Defensin-2 (Plant defensin)	"Defensin-2"[All Fields] AND biofilm[All Fields]	Defensin-2	18954353	Treponema denticola does not induce production of common innate immune mediators from primary gingival epithelial cells.

1957	DRAMP00751	Defensin-2 (Plant defensin)	"Defensin-2"[All Fields] AND biofilm[All Fields]	Defensin-2	18173794	The stage of native biofilm formation determines the gene expression of human beta-defensin-2, psoriasin, ribonuclease 7 and inflammatory mediators: a novel approach for stimulation of keratinocytes with in situ formed biofilms.
1957	DRAMP00751	Defensin-2 (Plant defensin)	"Defensin-2"[All Fields] AND biofilm[All Fields]	Defensin-2	17434999	Functional analysis of D-alanylation of lipoteichoic acid in the probiotic strain Lactobacillus rhamnosus GG.
1985	DRAMP18196	Esculentin-1A	"Esculentin-1A"[All Fields] AND biofilm[All Fields]	Esculentin-1A	31144441	Inhibition of Pseudomonas aeruginosa biofilm formation and expression of virulence genes by selective epimerization in the peptide Esculentin-1a(1-21)NH 2.
1985	DRAMP18196	Esculentin-1A	"Esculentin-1A"[All Fields] AND biofilm[All Fields]	Esculentin-1A	29086910	Esculentin-1a derived peptides kill Pseudomonas aeruginosa biofilm on soft contact lenses and retain antibacterial activity upon immobilization to the lens surface.
1985	DRAMP18196	Esculentin-1A	"Esculentin-1A"[All Fields] AND biofilm[All Fields]	Esculentin-1A	28912103	Membrane perturbing activities and structural properties of the frog-skin derived peptide Esculentin-1a(1-21)NH 2and its Diastereomer Esc(1-21)-1c: Correlation with their antipseudomonal and cytotoxic activity.
1985	DRAMP18196	Esculentin-1A	"Esculentin-1A"[All Fields] AND biofilm[All Fields]	Esculentin-1A	27693686	Gold-nanoparticles coated with the antimicrobial peptide esculentin-1a(1-21)NH 2as a reliable strategy for antipseudomonal drugs.
1985	DRAMP18196	Esculentin-1A	"Esculentin-1A"[All Fields] AND biofilm[All Fields]	Esculentin-1A	26162435	D-Amino acids incorporation in the frog skin-derived peptide esculentin-1a(1-21)NH2 is beneficial for its multiple functions.
2009	DRAMP18323	Nukacin ISK-1(Bacteriocin)	"Nukacin ISK-1"[All Fields] AND biofilm[All Fields]	Nukacin ISK-1	23979748	Effects of bacteriocins on methicillin-resistant Staphylococcus aureus biofilm.
2068	DRAMP00932	Antimicrobial peptide 1 (PMAP1; Plant defensin)	"Antimicrobial peptide 1"[All Fields] AND biofilm[All Fields]	Antimicrobial peptide 1	22445495	Database screening and in vivo efficacy of antimicrobial peptides against methicillin-resistant Staphylococcus aureus USA300.
2080	DRAMP18321	Epidermicin NI01(Bacteriocin)	"Epidermicin NI01"[All Fields] AND biofilm[All Fields]	Epidermicin NI01	26684017	Antimicrobial Peptides as Anti-Infectives against Staphylococcus epidermidis.
2080	DRAMP18321	Epidermicin NI01(Bacteriocin)	"Epidermicin NI01"[All Fields] AND biofilm[All Fields]	Epidermicin NI01	22155816	Identification, characterization, and recombinant expression of epidermicin NI01, a novel unmodified bacteriocin produced by Staphylococcus epidermidis that displays potent activity against Staphylococci.
2086	DRAMP00976	Antimicrobial peptide 1 (AC-AMP1; Plant defensin)	"Antimicrobial peptide 1"[All Fields] AND biofilm[All Fields]	Antimicrobial peptide 1	22445495	Database screening and in vivo efficacy of antimicrobial peptides against methicillin-resistant Staphylococcus aureus USA300.
2109	DRAMP00999	Plectasin (fungal defensin)	"Plectasin"[All Fields] AND biofilm[All Fields]	Plectasin	33534018	A recombinant fungal defensin-like peptide-P2 combats Streptococcus dysgalactiae and biofilms.
2109	DRAMP00999	Plectasin (fungal defensin)	"Plectasin"[All Fields] AND biofilm[All Fields]	Plectasin	31802334	Repurposing a drug targeting peptide for targeting antimicrobial peptides against Staphylococcus.
2109	DRAMP00999	Plectasin (fungal defensin)	"Plectasin"[All Fields] AND biofilm[All Fields]	Plectasin	28507110	Controlled Release of Plectasin NZ2114 from a Hybrid Silicone-Hydrogel Material for Inhibition of Staphylococcus aureus Biofilm.
2153	DRAMP01050	Sd1 (sugarcane defensin 1; Plant defensin)	"Sd1"[All Fields] AND biofilm[All Fields]	Sd1	34561304	Bacterial rhamnolipids and their 3-hydroxyalkanoate precursors activate Arabidopsisinnate immunity through two independent mechanisms.
2153	DRAMP01050	Sd1 (sugarcane defensin 1; Plant defensin)	"Sd1"[All Fields] AND biofilm[All Fields]	Sd1	21801186	Inhibitory effect of oral Lactobacillus against oral pathogens.
2156	DRAMP01053	Thaumatin-like protein (CdTLP; Plants)	"Thaumatin-like protein"[All Fields] AND biofilm[All Fields]	Thaumatin-like protein	22363222	Tasco®: a product of Ascophyllum nodosum enhances immune response of Caenorhabditis elegans against Pseudomonas aeruginosa infection.
2161	DRAMP01058	Antifungal protein (Plants)	"Antifungal protein"[All Fields] AND biofilm[All Fields]	Antifungal protein	33466640	The Neosartorya fischeriAntifungal Protein 2 (NFAP2): A New Potential Weapon against Multidrug-Resistant Candida aurisBiofilms.
2161	DRAMP01058	Antifungal protein (Plants)	"Antifungal protein"[All Fields] AND biofilm[All Fields]	Antifungal protein	32824977	The Penicillium chrysogenumQ176 Antimicrobial Protein PAFC Effectively Inhibits the Growth of the Opportunistic Human Pathogen Candida albicans.
2161	DRAMP01058	Antifungal protein (Plants)	"Antifungal protein"[All Fields] AND biofilm[All Fields]	Antifungal protein	30478163	In VivoApplicability of Neosartorya fischeri Antifungal Protein 2 (NFAP2) in Treatment of Vulvovaginal Candidiasis.
2161	DRAMP01058	Antifungal protein (Plants)	"Antifungal protein"[All Fields] AND biofilm[All Fields]	Antifungal protein	30079061	The Evolutionary Conserved γ-Core Motif Influences the Anti- CandidaActivity of the Penicillium chrysogenumAntifungal Protein PAF.
2161	DRAMP01058	Antifungal protein (Plants)	"Antifungal protein"[All Fields] AND biofilm[All Fields]	Antifungal protein	28120548	DS6: anticandidal, antibiofilm peptide against Candida tropicalis and exhibit synergy with commercial drug.
2204	DRAMP01127	Maximin-H5 (toads, amphibians, animals)	"Maximin-H5"[All Fields] AND biofilm[All Fields]	Maximin-H5	32937073	Antimicrobial Polymer-Peptide Conjugates Based on Maximin H5 and PEG to Prevent Biofouling of E. coliand P. aeruginosa.
2285	DRAMP18404	Polybia-MPII (mastoparan; insects, arthropods, invertebrates, animals)	"Polybia-MPII"[All Fields] AND biofilm[All Fields]	Polybia-MPII	30974767	Antimicrobial and Antibiofilm Effects of Peptides from Venom of Social Wasp and Scorpion on Multidrug-Resistant Acinetobacter baumannii.
2302	DRAMP01289	Phylloseptin-1 (PStar 01; Frogs, amphibians, animals)	"Phylloseptin-1"[All Fields] AND biofilm[All Fields]	Phylloseptin-1	20451254	Phylloseptin-1 (PSN-1) from Phyllomedusa sauvagei skin secretion: a novel broad-spectrum antimicrobial peptide with antibiofilm activity.
2325	DRAMP01318	Antimicrobial peptide 1 (Frogs, amphibians, animals)	"Antimicrobial peptide 1"[All Fields] AND biofilm[All Fields]	Antimicrobial peptide 1	22445495	Database screening and in vivo efficacy of antimicrobial peptides against methicillin-resistant Staphylococcus aureus USA300.
2380	DRAMP18313	Sclerosin(Bacteriocin)	"Sclerosin"[All Fields] AND biofilm[All Fields]	Sclerosin	27637885	Pseudomonas brassicacearum strain DF41 kills Caenorhabditis elegans through biofilm-dependent and biofilm-independent mechanisms.
2382	DRAMP01489	Esculentin-1A (Frogs, amphibians, animals)	"Esculentin-1A"[All Fields] AND biofilm[All Fields]	Esculentin-1A	31144441	Inhibition of Pseudomonas aeruginosa biofilm formation and expression of virulence genes by selective epimerization in the peptide Esculentin-1a(1-21)NH 2.
2382	DRAMP01489	Esculentin-1A (Frogs, amphibians, animals)	"Esculentin-1A"[All Fields] AND biofilm[All Fields]	Esculentin-1A	29086910	Esculentin-1a derived peptides kill Pseudomonas aeruginosa biofilm on soft contact lenses and retain antibacterial activity upon immobilization to the lens surface.
2382	DRAMP01489	Esculentin-1A (Frogs, amphibians, animals)	"Esculentin-1A"[All Fields] AND biofilm[All Fields]	Esculentin-1A	28912103	Membrane perturbing activities and structural properties of the frog-skin derived peptide Esculentin-1a(1-21)NH 2and its Diastereomer Esc(1-21)-1c: Correlation with their antipseudomonal and cytotoxic activity.
2382	DRAMP01489	Esculentin-1A (Frogs, amphibians, animals)	"Esculentin-1A"[All Fields] AND biofilm[All Fields]	Esculentin-1A	27693686	Gold-nanoparticles coated with the antimicrobial peptide esculentin-1a(1-21)NH 2as a reliable strategy for antipseudomonal drugs.
2382	DRAMP01489	Esculentin-1A (Frogs, amphibians, animals)	"Esculentin-1A"[All Fields] AND biofilm[All Fields]	Esculentin-1A	26162435	D-Amino acids incorporation in the frog skin-derived peptide esculentin-1a(1-21)NH2 is beneficial for its multiple functions.
2387	DRAMP01514	Esculentin-1 (Frogs, amphibians, animals)	"Esculentin-1"[All Fields] AND biofilm[All Fields]	Esculentin-1	26162435	D-Amino acids incorporation in the frog skin-derived peptide esculentin-1a(1-21)NH2 is beneficial for its multiple functions.
2387	DRAMP01514	Esculentin-1 (Frogs, amphibians, animals)	"Esculentin-1"[All Fields] AND biofilm[All Fields]	Esculentin-1	23503622	Esculentin(1-21), an amphibian skin membrane-active peptide with potent activity on both planktonic and biofilm cells of the bacterial pathogen Pseudomonas aeruginosa.
2389	DRAMP01744	Temporin-G (Frogs, amphibians, animals)	"Temporin-G"[All Fields] AND biofilm[All Fields]	Temporin-G	33321906	The Antimicrobial Peptide Temporin G: Anti-Biofilm, Anti-Persister Activities, and Potentiator Effect of Tobramycin Efficacy Against Staphylococcus aureus.
2436	DRAMP01609	Aurein-2.2 (Frogs, amphibians, animals)	"Aurein-2.2"[All Fields] AND biofilm[All Fields]	Aurein-2.2	30565465	Aurein-Derived Antimicrobial Peptides Formulated with Pegylated Phospholipid Micelles to Target Methicillin-Resistant Staphylococcus aureus Skin Infections.
2455	DRAMP01642	Dermaseptin-4 (DShypo04; Frogs, amphibians, animals)	"Dermaseptin-4"[All Fields] AND biofilm[All Fields]	Dermaseptin-4	32691243	Effect of Dermaseptin S4 on C. albicans Growth and EAP1 and HWP1 Gene Expression.
2475	DRAMP01671	Dermaseptin-4 (DS IV; Dermaseptin-S4, DS4; Frogs, amphibians, animals)	"Dermaseptin-4"[All Fields] AND biofilm[All Fields]	Dermaseptin-4	32691243	Effect of Dermaseptin S4 on C. albicans Growth and EAP1 and HWP1 Gene Expression.
2480	DRAMP01676	Dermaseptin-4 (DStar 04; Frogs, amphibians, animals)	"Dermaseptin-4"[All Fields] AND biofilm[All Fields]	Dermaseptin-4	32691243	Effect of Dermaseptin S4 on C. albicans Growth and EAP1 and HWP1 Gene Expression.
2498	DRAMP18305	Paenibacterin (Bacteriocin)	"Paenibacterin"[All Fields] AND biofilm[All Fields]	Paenibacterin	30579360	Wild bees and their nests host Paenibacillus bacteria with functional potential of avail.
2530	DRAMP02857	Indolicidin (Cathelicidin-4; mammals, animals)	"Indolicidin"[All Fields] AND biofilm[All Fields]	Indolicidin	33655458	Anticandidal Activity of Omiganan and Its Retro Analog Alone and in Combination with Fluconazole.
2530	DRAMP02857	Indolicidin (Cathelicidin-4; mammals, animals)	"Indolicidin"[All Fields] AND biofilm[All Fields]	Indolicidin	31485973	Efficacy of Indolicidin, Cecropin A (1-7)-Melittin (CAMA) and Their Combination Against Biofilm-Forming Multidrug-Resistant Enteroaggregative Escherichia coli.
2530	DRAMP02857	Indolicidin (Cathelicidin-4; mammals, animals)	"Indolicidin"[All Fields] AND biofilm[All Fields]	Indolicidin	30978313	A biophysical study of the interactions between the antimicrobial peptide indolicidin and lipid model systems.
2530	DRAMP02857	Indolicidin (Cathelicidin-4; mammals, animals)	"Indolicidin"[All Fields] AND biofilm[All Fields]	Indolicidin	30013374	Efficiency of gold nanoparticles coated with the antimicrobial peptide indolicidin against biofilm formation and development of Candidaspp. clinical isolates.
2530	DRAMP02857	Indolicidin (Cathelicidin-4; mammals, animals)	"Indolicidin"[All Fields] AND biofilm[All Fields]	Indolicidin	28378802	Histones from Avian Erythrocytes Exhibit Antibiofilm activity against methicillin-sensitive and methicillin-resistant Staphylococcus aureus.
2530	DRAMP02857	Indolicidin (Cathelicidin-4; mammals, animals)	"Indolicidin"[All Fields] AND biofilm[All Fields]	Indolicidin	27563687	Using anti-biofilm peptides to treat antibiotic-resistant bacterial infections.
2530	DRAMP02857	Indolicidin (Cathelicidin-4; mammals, animals)	"Indolicidin"[All Fields] AND biofilm[All Fields]	Indolicidin	23988790	In vitro pharmacokinetics of antimicrobial cationic peptides alone and in combination with antibiotics against methicillin resistant Staphylococcus aureus biofilms.
2530	DRAMP02857	Indolicidin (Cathelicidin-4; mammals, animals)	"Indolicidin"[All Fields] AND biofilm[All Fields]	Indolicidin	23070152	In vitro activities of antibiotics and antimicrobial cationic peptides alone and in combination against methicillin-resistant Staphylococcus aureus biofilms.
2530	DRAMP02857	Indolicidin (Cathelicidin-4; mammals, animals)	"Indolicidin"[All Fields] AND biofilm[All Fields]	Indolicidin	21849157	Antibacterial and anti-biofilm effects of cathelicidin peptides against pathogens isolated from cystic fibrosis patients.
2530	DRAMP02857	Indolicidin (Cathelicidin-4; mammals, animals)	"Indolicidin"[All Fields] AND biofilm[All Fields]	Indolicidin	19143172	[Activity of indolicidin, alone or together with oxacillin, against Staphylococcus aureus and S. epidermidis].
2530	DRAMP02857	Indolicidin (Cathelicidin-4; mammals, animals)	"Indolicidin"[All Fields] AND biofilm[All Fields]	Indolicidin	18591225	Human host defense peptide LL-37 prevents bacterial biofilm formation.

2530	DRAMP02857	Indolicidin (Cathelicidin-4; mammals, animals)	"Indolicidin"[All Fields] AND biofilm[All Fields]	Indolicidin	18556795	Induction by cationic antimicrobial peptides and involvement in intrinsic polymyxin and antimicrobial peptide resistance, biofilm formation, and swarming motility of PsrA in Pseudomonas aeruginosa.
2532	DRAMP02819	Anoplin (Insects, arthropods, invertebrates, animals)	"Anoplin"[All Fields] AND biofilm[All Fields]	Anoplin	32654770	Synthesis and anti-pseudomonal activity of new ß-Ala modified analogues of the antimicrobial peptide anoplin.
2532	DRAMP02819	Anoplin (Insects, arthropods, invertebrates, animals)	"Anoplin"[All Fields] AND biofilm[All Fields]	Anoplin	32649983	Effect of N-methylated and fatty acid conjugation on analogs of antimicrobial peptide Anoplin.
2532	DRAMP02819	Anoplin (Insects, arthropods, invertebrates, animals)	"Anoplin"[All Fields] AND biofilm[All Fields]	Anoplin	31733278	Study on the effects of different dimerization positions on biological activity of partial d-Amino acid substitution analogues of Anoplin.
2532	DRAMP02819	Anoplin (Insects, arthropods, invertebrates, animals)	"Anoplin"[All Fields] AND biofilm[All Fields]	Anoplin	31676352	Antimicrobial peptides conjugated with fatty acids on the side chain of D-amino acid promises antimicrobial potency against multidrug-resistant bacteria.
2532	DRAMP02819	Anoplin (Insects, arthropods, invertebrates, animals)	"Anoplin"[All Fields] AND biofilm[All Fields]	Anoplin	31466017	Design and synthesis of new N-terminal fatty acid modified-antimicrobial peptide analogues with potent in vitro biological activity.
2533	DRAMP04395	EP3 (Earthworm,animals)	"EP3"[All Fields] AND biofilm[All Fields]	EP3	27939158	The arcA gene contributes to the serum resistance and virulence of Haemophilus parasuis serovar 13 clinical strain EP3.
2533	DRAMP04395	EP3 (Earthworm,animals)	"EP3"[All Fields] AND biofilm[All Fields]	EP3	26657038	Effect of cheY deletion on growth and colonization in a Haemophilus parasuis serovar 13 clinical strain EP3.
2534	DRAMP04394	EP2 (Earthworm,animals)	"EP2"[All Fields] AND biofilm[All Fields]	EP2	29263068	Prostaglandin E 2Receptor Antagonist with Antimicrobial Activity against Methicillin-Resistant Staphylococcus aureus.
2534	DRAMP04394	EP2 (Earthworm,animals)	"EP2"[All Fields] AND biofilm[All Fields]	EP2	21468708	Effect of enrichment procedures on performance and microbial diversity of microbial fuel cell for Congo red decolorization and electricity generation.
2554	DRAMP01793	Temporin-1OLa (Temporin 1OLa; Frogs, amphibians, animals)	"Temporin-1OLa"[All Fields] AND biofilm[All Fields]	Temporin-1OLa	29842923	Antibacterial, antifungal, anticancer activities and structural bioinformatics analysis of six naturally occurring temporins.
2556	DRAMP01795	Temporin-1Ga (Frogs, amphibians, animals)	"Temporin-1Ga"[All Fields] AND biofilm[All Fields]	Temporin-1Ga	29842923	Antibacterial, antifungal, anticancer activities and structural bioinformatics analysis of six naturally occurring temporins.
2666	DRAMP18288	Sln1 (Bacteriocin)	"Sln1"[All Fields] AND biofilm[All Fields]	Sln1	32939285	Structural insights into the histidine-containing phospho-transfer protein and receiver domain of sensor histidine kinase suggest a complex model in the two-component regulatory system in Pseudomonas aeruginosa.
2671	DRAMP18284	Reutericin 6 (Bacteriocin)	"Reutericin 6"[All Fields] AND biofilm[All Fields]	Reutericin 6	32474673	Effects of a derivative of reutericin 6 and gassericin A on the biofilm of Streptococcus mutans in vitro and caries prevention in vivo.
2671	DRAMP18284	Reutericin 6 (Bacteriocin)	"Reutericin 6"[All Fields] AND biofilm[All Fields]	Reutericin 6	31207076	Rational design of peptides with enhanced antimicrobial and anti-biofilm activities against cariogenic bacterium Streptococcus mutans.
2689	DRAMP18279	Plantaricin A(Bacteriocin)	"Plantaricin A"[All Fields] AND biofilm[All Fields]	Plantaricin A	23396346	Effects of the peptide pheromone plantaricin A and cocultivation with Lactobacillus sanfranciscensis DPPMA174 on the exoproteome and the adhesion capacity of Lactobacillus plantarum DC400.
2698	DRAMP01110	Maximin-4 (Toads, amphibians, animals)	"Maximin-4"[All Fields] AND biofilm[All Fields]	Maximin-4	25264285	Biofilm eradication kinetics of the ultrashort lipopeptide C12 -OOWW-NH2 utilizing a modified MBEC Assay(™).
2741	DRAMP02159	Melittin-related peptide (Frogs, amphibians, animals)	"Melittin-related peptide"[All Fields] AND biofilm[All Fields]	Melittin-related peptide	27271216	Design of an α-helical antimicrobial peptide with improved cell-selective and potent anti-biofilm activity.
2819	DRAMP02285	Pseudin-2 (Pseudin 2; Frogs, amphibians, animals)	"Pseudin-2"[All Fields] AND biofilm[All Fields]	Pseudin-2	30323036	Pse-T2, an Antimicrobial Peptide with High-Level, Broad-Spectrum Antimicrobial Potency and Skin Biocompatibility against Multidrug-Resistant Pseudomonas aeruginosa Infection.
2831	DRAMP02311	Lysozyme (1,4-beta-N-acetylmuramidase; starfish, chordates, animals)	"Lysozyme"[All Fields] AND biofilm[All Fields]	Lysozyme	34688058	The dietary supplementation of zinc oxide and selenium nanoparticles enhance the immune response in freshwater fish Oreochromis mossambicus against aquatic pathogen Aeromonas hydrophila.
2831	DRAMP02311	Lysozyme (1,4-beta-N-acetylmuramidase; starfish, chordates, animals)	"Lysozyme"[All Fields] AND biofilm[All Fields]	Lysozyme	34680849	Anti-Biofilm Coatings Based on Chitosan and Lysozyme Functionalized Magnetite Nanoparticles.
2831	DRAMP02311	Lysozyme (1,4-beta-N-acetylmuramidase; starfish, chordates, animals)	"Lysozyme"[All Fields] AND biofilm[All Fields]	Lysozyme	34663068	Zwitterionic Peptides Reduce Accumulation of Marine and Freshwater Biofilm Formers.
2831	DRAMP02311	Lysozyme (1,4-beta-N-acetylmuramidase; starfish, chordates, animals)	"Lysozyme"[All Fields] AND biofilm[All Fields]	Lysozyme	34650538	DexA70, the Truncated Form of a Self-Produced Dextranase, Effectively Disrupts Streptococcus mutansBiofilm.
2831	DRAMP02311	Lysozyme (1,4-beta-N-acetylmuramidase; starfish, chordates, animals)	"Lysozyme"[All Fields] AND biofilm[All Fields]	Lysozyme	34601068	Tailoring the proliferation of fibroblast cells by multiresponsive and thermosensitive stem cells composite F127 hydrogel containing folic acid.MgO:ZnO/chitosan hybrid microparticles for skin regeneration.
2831	DRAMP02311	Lysozyme (1,4-beta-N-acetylmuramidase; starfish, chordates, animals)	"Lysozyme"[All Fields] AND biofilm[All Fields]	Lysozyme	34528638	Enhanced clearing of Candidabiofilms on a 3D urothelial cell in vitromodel using lysozyme-functionalized fluconazole-loaded shellac nanoparticles.
2831	DRAMP02311	Lysozyme (1,4-beta-N-acetylmuramidase; starfish, chordates, animals)	"Lysozyme"[All Fields] AND biofilm[All Fields]	Lysozyme	34488127	Activity of the lyases LysSSE1 and HolSSE1 against common pathogenic bacteria and their antimicrobial efficacy in biofilms.
2831	DRAMP02311	Lysozyme (1,4-beta-N-acetylmuramidase; starfish, chordates, animals)	"Lysozyme"[All Fields] AND biofilm[All Fields]	Lysozyme	34485498	Effect of antimicrobial peptides on planktonic growth, biofilm formation and biofilm-derived bacterial viability of Streptococcus pneumoniae.
2831	DRAMP02311	Lysozyme (1,4-beta-N-acetylmuramidase; starfish, chordates, animals)	"Lysozyme"[All Fields] AND biofilm[All Fields]	Lysozyme	34438974	Antimicrobial Activity of the Circular Bacteriocin AS-48 against Clinical Multidrug-Resistant Staphylococcus aureus.
2831	DRAMP02311	Lysozyme (1,4-beta-N-acetylmuramidase; starfish, chordates, animals)	"Lysozyme"[All Fields] AND biofilm[All Fields]	Lysozyme	34019937	Correlation of over-expression of rv1900c with enhanced survival of M. smegmatis under stress conditions: Modulation of cell surface properties.
2831	DRAMP02311	Lysozyme (1,4-beta-N-acetylmuramidase; starfish, chordates, animals)	"Lysozyme"[All Fields] AND biofilm[All Fields]	Lysozyme	33947757	A StreptococcusQuorum Sensing System Enables Suppression of Innate Immunity.
2831	DRAMP02311	Lysozyme (1,4-beta-N-acetylmuramidase; starfish, chordates, animals)	"Lysozyme"[All Fields] AND biofilm[All Fields]	Lysozyme	33920327	Resveratrol-Loaded Hydrogel Contact Lenses with Antioxidant and Antibiofilm Performance.
2831	DRAMP02311	Lysozyme (1,4-beta-N-acetylmuramidase; starfish, chordates, animals)	"Lysozyme"[All Fields] AND biofilm[All Fields]	Lysozyme	33918930	A Novel Biofilm Model System to Visualise Conjugal Transfer of Vancomycin Resistance by Environmental Enterococci.
2831	DRAMP02311	Lysozyme (1,4-beta-N-acetylmuramidase; starfish, chordates, animals)	"Lysozyme"[All Fields] AND biofilm[All Fields]	Lysozyme	33875213	Impact of pmrA on Cronobacter sakazakii planktonic and biofilm cells: A comprehensive transcriptomic study.
2831	DRAMP02311	Lysozyme (1,4-beta-N-acetylmuramidase; starfish, chordates, animals)	"Lysozyme"[All Fields] AND biofilm[All Fields]	Lysozyme	33842797	Antibacterial and Antibiofilm Photodynamic Activities of Lysozyme-Au Nanoclusters/Rose Bengal Conjugates.
2831	DRAMP02311	Lysozyme (1,4-beta-N-acetylmuramidase; starfish, chordates, animals)	"Lysozyme"[All Fields] AND biofilm[All Fields]	Lysozyme	33783975	Biodegradable Anti-Biofilm Fiber-Membrane Ureteral Stent Constructed with a Robust Biomimetic Superhydrophilic Polycationic Hydration Surface Exhibiting Synergetic Antibacterial and Antiprotein Properties.
2831	DRAMP02311	Lysozyme (1,4-beta-N-acetylmuramidase; starfish, chordates, animals)	"Lysozyme"[All Fields] AND biofilm[All Fields]	Lysozyme	33773215	Growth in a biofilm sensitizes Cutibacterium acnes to nanosecond pulsed electric fields.

2831	DRAMP02311	Lysozyme (1,4-beta-N-acetylmuramidase; starfish, chordates, animals)	"Lysozyme"[All Fields] AND biofilm[All Fields]	Lysozyme	33769023	Drug-Free Enzyme-Based Bactericidal Nanomotors against Pathogenic Bacteria.
2831	DRAMP02311	Lysozyme (1,4-beta-N-acetylmuramidase; starfish, chordates, animals)	"Lysozyme"[All Fields] AND biofilm[All Fields]	Lysozyme	33740161	Enzymatic biofilm destabilisation to support mechanical cleansing of inserted dental implant surfaces: an in-vitro pilot study.
2831	DRAMP02311	Lysozyme (1,4-beta-N-acetylmuramidase; starfish, chordates, animals)	"Lysozyme"[All Fields] AND biofilm[All Fields]	Lysozyme	33391351	Quantification of Bacterial Colonization in Dental Hard Tissues Using Optimized Molecular Biological Methods.
2831	DRAMP02311	Lysozyme (1,4-beta-N-acetylmuramidase; starfish, chordates, animals)	"Lysozyme"[All Fields] AND biofilm[All Fields]	Lysozyme	33252326	Effect of antifungal agents, lysozyme and human antimicrobial peptide LL-37 on clinical Candidaisolates with high biofilm production.
2831	DRAMP02311	Lysozyme (1,4-beta-N-acetylmuramidase; starfish, chordates, animals)	"Lysozyme"[All Fields] AND biofilm[All Fields]	Lysozyme	33185350	[Characterization of the microbiota and cytokine profile of sperm plasma in men with chronic bacterial prostatitis].
2831	DRAMP02311	Lysozyme (1,4-beta-N-acetylmuramidase; starfish, chordates, animals)	"Lysozyme"[All Fields] AND biofilm[All Fields]	Lysozyme	33113658	New insights into the effect of extracellular polymeric substance on the sludge dewaterability based on interaction energy and viscoelastic acoustic response analysis.
2831	DRAMP02311	Lysozyme (1,4-beta-N-acetylmuramidase; starfish, chordates, animals)	"Lysozyme"[All Fields] AND biofilm[All Fields]	Lysozyme	33080579	Combination therapy of biogenic C-dots and lysozyme for enhanced antibacterial and antibiofilm activity.
2831	DRAMP02311	Lysozyme (1,4-beta-N-acetylmuramidase; starfish, chordates, animals)	"Lysozyme"[All Fields] AND biofilm[All Fields]	Lysozyme	33072040	Exploiting Lactoferricin (17-30) as a Potential Antimicrobial and Antibiofilm Candidate Against Multi-Drug-Resistant Enteroaggregative Escherichia coli.
2831	DRAMP02311	Lysozyme (1,4-beta-N-acetylmuramidase; starfish, chordates, animals)	"Lysozyme"[All Fields] AND biofilm[All Fields]	Lysozyme	33045439	Antibacterial activities of and biofilm removal by Ablysin, an endogenous lysozyme-like protein originated from Acinetobacter baumannii 1656-2.
2831	DRAMP02311	Lysozyme (1,4-beta-N-acetylmuramidase; starfish, chordates, animals)	"Lysozyme"[All Fields] AND biofilm[All Fields]	Lysozyme	32916574	Supported lysozyme for improved antimicrobial surface protection.
2831	DRAMP02311	Lysozyme (1,4-beta-N-acetylmuramidase; starfish, chordates, animals)	"Lysozyme"[All Fields] AND biofilm[All Fields]	Lysozyme	32787275	Hydration-Induced Structural Changes in the Solid State of Protein: A SAXS/WAXS Study on Lysozyme.
2831	DRAMP02311	Lysozyme (1,4-beta-N-acetylmuramidase; starfish, chordates, animals)	"Lysozyme"[All Fields] AND biofilm[All Fields]	Lysozyme	32612893	Biofilm modelling on the contact lenses and comparison of the in vitro activities of multipurpose lens solutions and antibiotics.
2831	DRAMP02311	Lysozyme (1,4-beta-N-acetylmuramidase; starfish, chordates, animals)	"Lysozyme"[All Fields] AND biofilm[All Fields]	Lysozyme	32512756	Inactivation of the sfgtr4Gene of Shigella flexneriInduces Biofilm Formation and Affects Bacterial Pathogenicity.
2831	DRAMP02311	Lysozyme (1,4-beta-N-acetylmuramidase; starfish, chordates, animals)	"Lysozyme"[All Fields] AND biofilm[All Fields]	Lysozyme	32393645	Evolution of vancomycin-resistant Enterococcus faeciumduring colonization and infection in immunocompromised pediatric patients.
2831	DRAMP02311	Lysozyme (1,4-beta-N-acetylmuramidase; starfish, chordates, animals)	"Lysozyme"[All Fields] AND biofilm[All Fields]	Lysozyme	32280054	Quorum quenching acylase impacts the viability and morphological change of Agrobacterium tumefaciens cells.
2831	DRAMP02311	Lysozyme (1,4-beta-N-acetylmuramidase; starfish, chordates, animals)	"Lysozyme"[All Fields] AND biofilm[All Fields]	Lysozyme	32266650	Probiotic Potential of Bacillus Strains Isolated from an Acidic Fermented Food Idli.
2831	DRAMP02311	Lysozyme (1,4-beta-N-acetylmuramidase; starfish, chordates, animals)	"Lysozyme"[All Fields] AND biofilm[All Fields]	Lysozyme	32089678	Effects of Lysozyme, Proteinase K, and Cephalosporins on Biofilm Formation by Clinical Isolates of Pseudomonas aeruginosa.
2831	DRAMP02311	Lysozyme (1,4-beta-N-acetylmuramidase; starfish, chordates, animals)	"Lysozyme"[All Fields] AND biofilm[All Fields]	Lysozyme	33447800	Cellular chaining influences biofilm formation and structure in group A Streptococcus.
2831	DRAMP02311	Lysozyme (1,4-beta-N-acetylmuramidase; starfish, chordates, animals)	"Lysozyme"[All Fields] AND biofilm[All Fields]	Lysozyme	31698068	Vibrio harveyi biofilm as immunostimulant candidate for high-health pacific white shrimp, Penaeus vannamei farming.
2831	DRAMP02311	Lysozyme (1,4-beta-N-acetylmuramidase; starfish, chordates, animals)	"Lysozyme"[All Fields] AND biofilm[All Fields]	Lysozyme	31480687	Characterization and Antibiofilm Activity of Mannitol-Chitosan-Blended Paste for Local Antibiotic Delivery System.
2831	DRAMP02311	Lysozyme (1,4-beta-N-acetylmuramidase; starfish, chordates, animals)	"Lysozyme"[All Fields] AND biofilm[All Fields]	Lysozyme	31374264	Anti-cancer, anti-biofilm, and anti-inflammatory properties of hen's albumen: A photodynamic approach.
2831	DRAMP02311	Lysozyme (1,4-beta-N-acetylmuramidase; starfish, chordates, animals)	"Lysozyme"[All Fields] AND biofilm[All Fields]	Lysozyme	31279118	Effect of extracellular polymeric substances (EPS) conditioned by combined lysozyme and cationic polyacrylamide on the dewatering performance of activated sludge.
2831	DRAMP02311	Lysozyme (1,4-beta-N-acetylmuramidase; starfish, chordates, animals)	"Lysozyme"[All Fields] AND biofilm[All Fields]	Lysozyme	31252064	Antibacterial synergy between rutin and florfenicol enhances therapeutic spectrum against drug resistant Aeromonas hydrophila.
2831	DRAMP02311	Lysozyme (1,4-beta-N-acetylmuramidase; starfish, chordates, animals)	"Lysozyme"[All Fields] AND biofilm[All Fields]	Lysozyme	31214943	Safety and Stability of Two Potentially Probiotic Lactobacillus Strains After In Vitro Gastrointestinal Transit.
2831	DRAMP02311	Lysozyme (1,4-beta-N-acetylmuramidase; starfish, chordates, animals)	"Lysozyme"[All Fields] AND biofilm[All Fields]	Lysozyme	31125644	Rv0518, a nutritive stress inducible GDSL lipase of Mycobacterium tuberculosis, enhanced intracellular survival of bacteria by cell wall modulation.
2831	DRAMP02311	Lysozyme (1,4-beta-N-acetylmuramidase; starfish, chordates, animals)	"Lysozyme"[All Fields] AND biofilm[All Fields]	Lysozyme	31085703	The Ser/Thr Kinase PrkC Participates in Cell Wall Homeostasis and Antimicrobial Resistance in Clostridium difficile.
2831	DRAMP02311	Lysozyme (1,4-beta-N-acetylmuramidase; starfish, chordates, animals)	"Lysozyme"[All Fields] AND biofilm[All Fields]	Lysozyme	30843349	Characterization of the biofilm phenotype of a Listeria monocytogenes mutant deficient in agr peptide sensing.
2831	DRAMP02311	Lysozyme (1,4-beta-N-acetylmuramidase; starfish, chordates, animals)	"Lysozyme"[All Fields] AND biofilm[All Fields]	Lysozyme	30833631	Detection of vaginal lactobacilli as probiotic candidates.
2831	DRAMP02311	Lysozyme (1,4-beta-N-acetylmuramidase; starfish, chordates, animals)	"Lysozyme"[All Fields] AND biofilm[All Fields]	Lysozyme	30761784	[Biological properties of microorganisms isolated from the urine of patients with urolithiasis].

2831	DRAMP02311	Lysozyme (1,4-beta-N-acetylmuramidase; starfish, chordates, animals)	"Lysozyme"[All Fields] AND biofilm[All Fields]	Lysozyme	30701862	Effect of N-acetylcysteine on mucosal immunity of respiratory tract.
2831	DRAMP02311	Lysozyme (1,4-beta-N-acetylmuramidase; starfish, chordates, animals)	"Lysozyme"[All Fields] AND biofilm[All Fields]	Lysozyme	30649289	Innate immune components affect growth and virulence traits of bacterial-vaginosis-associated and non-bacterial-vaginosis-associated Gardnerella vaginalis strains similarly.
2831	DRAMP02311	Lysozyme (1,4-beta-N-acetylmuramidase; starfish, chordates, animals)	"Lysozyme"[All Fields] AND biofilm[All Fields]	Lysozyme	30639529	Catalysing the way towards antimicrobial effectiveness: A systematic analysis and a new online resource for antimicrobial-enzyme combinations against Pseudomonas aeruginosa and Staphylococcus aureus.
2831	DRAMP02311	Lysozyme (1,4-beta-N-acetylmuramidase; starfish, chordates, animals)	"Lysozyme"[All Fields] AND biofilm[All Fields]	Lysozyme	30639217	Immobilization of antimicrobial and anti-quorum sensing enzymes onto GMA-grafted poly(vinyl chloride) catheters.
2831	DRAMP02311	Lysozyme (1,4-beta-N-acetylmuramidase; starfish, chordates, animals)	"Lysozyme"[All Fields] AND biofilm[All Fields]	Lysozyme	30497006	Impact of oral astringent stimuli on surface charge and morphology of the protein-rich pellicle at the tooth-saliva interphase.
2831	DRAMP02311	Lysozyme (1,4-beta-N-acetylmuramidase; starfish, chordates, animals)	"Lysozyme"[All Fields] AND biofilm[All Fields]	Lysozyme	30389429	Microbiology insights into boosting salivary defences through the use of enzymes and proteins.
2831	DRAMP02311	Lysozyme (1,4-beta-N-acetylmuramidase; starfish, chordates, animals)	"Lysozyme"[All Fields] AND biofilm[All Fields]	Lysozyme	30207684	Developing Antibacterial Nanocrystalline Cellulose Using Natural Antibacterial Agents.
2831	DRAMP02311	Lysozyme (1,4-beta-N-acetylmuramidase; starfish, chordates, animals)	"Lysozyme"[All Fields] AND biofilm[All Fields]	Lysozyme	30176334	β-1, 3 glucan binding protein based selenium nanowire enhances the immune status of Cyprinus carpio and protection against Aeromonas hydrophila infection.
2831	DRAMP02311	Lysozyme (1,4-beta-N-acetylmuramidase; starfish, chordates, animals)	"Lysozyme"[All Fields] AND biofilm[All Fields]	Lysozyme	30142702	Moraxella bovis, Moraxella ovis and Moraxella bovoculi: biofilm formation and lysozyme activity.
2831	DRAMP02311	Lysozyme (1,4-beta-N-acetylmuramidase; starfish, chordates, animals)	"Lysozyme"[All Fields] AND biofilm[All Fields]	Lysozyme	30131945	The Intestinal Roundworm Ascaris suumReleases Antimicrobial Factors Which Interfere With Bacterial Growth and Biofilm Formation.
2831	DRAMP02311	Lysozyme (1,4-beta-N-acetylmuramidase; starfish, chordates, animals)	"Lysozyme"[All Fields] AND biofilm[All Fields]	Lysozyme	30082920	Control of Propionibacterium acnes by natural antimicrobial substances: Role of the bacteriocin AS-48 and lysozyme.
2831	DRAMP02311	Lysozyme (1,4-beta-N-acetylmuramidase; starfish, chordates, animals)	"Lysozyme"[All Fields] AND biofilm[All Fields]	Lysozyme	29915307	Screening and characterization of prophages in Desulfovibrio genomes.
2831	DRAMP02311	Lysozyme (1,4-beta-N-acetylmuramidase; starfish, chordates, animals)	"Lysozyme"[All Fields] AND biofilm[All Fields]	Lysozyme	29915116	Resuscitation-Promoting Factors Are Required for Mycobacterium smegmatis Biofilm Formation.
2831	DRAMP02311	Lysozyme (1,4-beta-N-acetylmuramidase; starfish, chordates, animals)	"Lysozyme"[All Fields] AND biofilm[All Fields]	Lysozyme	29876449	TCA precipitation and ethanol/HCl single-step purification evaluation: One-dimensional gel electrophoresis, bradford assays, spectrofluorometry and Raman spectroscopy data on HSA, Rnase, lysozyme - Mascots and Skyline data.
2831	DRAMP02311	Lysozyme (1,4-beta-N-acetylmuramidase; starfish, chordates, animals)	"Lysozyme"[All Fields] AND biofilm[All Fields]	Lysozyme	29555699	A Quorum Sensing-Regulated Protein Binds Cell Wall Components and Enhances Lysozyme Resistance in Streptococcus pyogenes.
2831	DRAMP02311	Lysozyme (1,4-beta-N-acetylmuramidase; starfish, chordates, animals)	"Lysozyme"[All Fields] AND biofilm[All Fields]	Lysozyme	29518559	Effect of β-1, 3 glucan binding protein based zinc oxide nanoparticles supplemented diet on immune response and disease resistance in Oreochromis mossambicus against Aeromonas hydrophila.
2831	DRAMP02311	Lysozyme (1,4-beta-N-acetylmuramidase; starfish, chordates, animals)	"Lysozyme"[All Fields] AND biofilm[All Fields]	Lysozyme	29464594	Inhalable Levofloxacin Liposomes Complemented with Lysozyme for Treatment of Pulmonary Infection in Rats: Effective Antimicrobial and Antibiofilm Strategy.
2831	DRAMP02311	Lysozyme (1,4-beta-N-acetylmuramidase; starfish, chordates, animals)	"Lysozyme"[All Fields] AND biofilm[All Fields]	Lysozyme	29393147	Protein Profile of the Acquired Enamel Pellicle after Rinsing with Whole Milk, Fat-Free Milk, and Water: An in vivo Study.
2831	DRAMP02311	Lysozyme (1,4-beta-N-acetylmuramidase; starfish, chordates, animals)	"Lysozyme"[All Fields] AND biofilm[All Fields]	Lysozyme	29373594	Biofilm formation and transcriptome analysis of Streptococcus gallolyticus subsp. gallolyticus in response to lysozyme.
2831	DRAMP02311	Lysozyme (1,4-beta-N-acetylmuramidase; starfish, chordates, animals)	"Lysozyme"[All Fields] AND biofilm[All Fields]	Lysozyme	29315029	Correlations among Resistances to Different Antimicrobial Compounds in Salmonella Strains from Hen Eggshells.
2831	DRAMP02311	Lysozyme (1,4-beta-N-acetylmuramidase; starfish, chordates, animals)	"Lysozyme"[All Fields] AND biofilm[All Fields]	Lysozyme	31565305	Enzyme Crystals and Hydrogel Composite Membranes as New Active Food Packaging Material.
2831	DRAMP02311	Lysozyme (1,4-beta-N-acetylmuramidase; starfish, chordates, animals)	"Lysozyme"[All Fields] AND biofilm[All Fields]	Lysozyme	29205129	Dissimilar pigment regulation in Serpula lacrymans and Paxillus involutus during inter-kingdom interactions.
2831	DRAMP02311	Lysozyme (1,4-beta-N-acetylmuramidase; starfish, chordates, animals)	"Lysozyme"[All Fields] AND biofilm[All Fields]	Lysozyme	29201023	A Marine Actinomycete Rescues Caenorhabditis elegansfrom Pseudomonas aeruginosaInfection through Restitution of Lysozyme 7.
2831	DRAMP02311	Lysozyme (1,4-beta-N-acetylmuramidase; starfish, chordates, animals)	"Lysozyme"[All Fields] AND biofilm[All Fields]	Lysozyme	29184097	Csl2, a novel chimeric bacteriophage lysin to fight infections caused by Streptococcus suis, an emerging zoonotic pathogen.
2831	DRAMP02311	Lysozyme (1,4-beta-N-acetylmuramidase; starfish, chordates, animals)	"Lysozyme"[All Fields] AND biofilm[All Fields]	Lysozyme	29080817	Production and structural characterization of exopolysaccharides from newly isolated probiotic lactic acid bacteria.
2831	DRAMP02311	Lysozyme (1,4-beta-N-acetylmuramidase; starfish, chordates, animals)	"Lysozyme"[All Fields] AND biofilm[All Fields]	Lysozyme	28931063	Plant flavones enhance antimicrobial activity of respiratory epithelial cell secretions against Pseudomonas aeruginosa.
2831	DRAMP02311	Lysozyme (1,4-beta-N-acetylmuramidase; starfish, chordates, animals)	"Lysozyme"[All Fields] AND biofilm[All Fields]	Lysozyme	28922066	The Effect of Lysozyme on Reducing Biofilms by Staphylococcus aureus, Pseudomonas aeruginosa, and Gardnerella vaginalis: An In Vitro Examination.
2831	DRAMP02311	Lysozyme (1,4-beta-N-acetylmuramidase; starfish, chordates, animals)	"Lysozyme"[All Fields] AND biofilm[All Fields]	Lysozyme	28826250	Antimicrobial and anti-biofilm activity of tannic acid against Staphylococcus aureus.
2831	DRAMP02311	Lysozyme (1,4-beta-N-acetylmuramidase; starfish, chordates, animals)	"Lysozyme"[All Fields] AND biofilm[All Fields]	Lysozyme	28649626	Investigation of simulated microgravity effects on Streptococcus mutansphysiology and global gene expression.

2831	DRAMP02311	Lysozyme (1,4-beta-N-acetylmuramidase; starfish, chordates, animals)	"Lysozyme"[All Fields] AND biofilm[All Fields]	Lysozyme	32264250	Protein-repellent and antimicrobial nanoparticle coatings from hyaluronic acid and a lysine-derived biocompatible surfactant.
2831	DRAMP02311	Lysozyme (1,4-beta-N-acetylmuramidase; starfish, chordates, animals)	"Lysozyme"[All Fields] AND biofilm[All Fields]	Lysozyme	28400767	The Periplasmic Chaperone Network of Campylobacter jejuni: Evidence that SalC (Cj1289) and PpiD (Cj0694) Are Involved in Maintaining Outer Membrane Integrity.
2831	DRAMP02311	Lysozyme (1,4-beta-N-acetylmuramidase; starfish, chordates, animals)	"Lysozyme"[All Fields] AND biofilm[All Fields]	Lysozyme	28400765	Role of Bacillus licheniformisVS16-Derived Biosurfactant in Mediating Immune Responses in Carp Rohu and its Application to the Food Industry.
2831	DRAMP02311	Lysozyme (1,4-beta-N-acetylmuramidase; starfish, chordates, animals)	"Lysozyme"[All Fields] AND biofilm[All Fields]	Lysozyme	28397768	Lysozyme Associated Liposomal Gentamicin Inhibits Bacterial Biofilm.
2831	DRAMP02311	Lysozyme (1,4-beta-N-acetylmuramidase; starfish, chordates, animals)	"Lysozyme"[All Fields] AND biofilm[All Fields]	Lysozyme	28289407	A Novel Antimicrobial Endolysin, LysPA26, against Pseudomonas aeruginosa.
2831	DRAMP02311	Lysozyme (1,4-beta-N-acetylmuramidase; starfish, chordates, animals)	"Lysozyme"[All Fields] AND biofilm[All Fields]	Lysozyme	28251063	Evaluation of a chitosan-polyethylene glycol paste as a local antibiotic delivery device.
2831	DRAMP02311	Lysozyme (1,4-beta-N-acetylmuramidase; starfish, chordates, animals)	"Lysozyme"[All Fields] AND biofilm[All Fields]	Lysozyme	28188877	Laser deposition of poly(3-hydroxybutyric acid-co-3-hydroxyvaleric acid) - lysozyme microspheres based coatings with anti-microbial properties.
2831	DRAMP02311	Lysozyme (1,4-beta-N-acetylmuramidase; starfish, chordates, animals)	"Lysozyme"[All Fields] AND biofilm[All Fields]	Lysozyme	28138698	Dose-dependent effect of lysozyme upon Candida albicans biofilm.
2831	DRAMP02311	Lysozyme (1,4-beta-N-acetylmuramidase; starfish, chordates, animals)	"Lysozyme"[All Fields] AND biofilm[All Fields]	Lysozyme	28109187	Antimicrobial and antioxidant activities of Saccharomyces cerevisiae IFST062013, a potential probiotic.
2831	DRAMP02311	Lysozyme (1,4-beta-N-acetylmuramidase; starfish, chordates, animals)	"Lysozyme"[All Fields] AND biofilm[All Fields]	Lysozyme	30695493	[PHYSIOLOGICAL FEATURES OF CORYNEBACTERIA OF FEMALE REPRODUC- TIVE TRACT].
2831	DRAMP02311	Lysozyme (1,4-beta-N-acetylmuramidase; starfish, chordates, animals)	"Lysozyme"[All Fields] AND biofilm[All Fields]	Lysozyme	29619048	Efficacy of a Rinse Containing Sea Salt and Lysozyme on Biofilm and Gingival Health in a Group of Young Adults: A Pilot Study.
2831	DRAMP02311	Lysozyme (1,4-beta-N-acetylmuramidase; starfish, chordates, animals)	"Lysozyme"[All Fields] AND biofilm[All Fields]	Lysozyme	27999400	Comparative Study on the Characteristics of Weissella cibaria CMU and Probiotic Strains for Oral Care.
2831	DRAMP02311	Lysozyme (1,4-beta-N-acetylmuramidase; starfish, chordates, animals)	"Lysozyme"[All Fields] AND biofilm[All Fields]	Lysozyme	27984151	Copper incorporated microporous chitosan-polyethylene glycol hydrogels loaded with naproxen for effective drug release and anti-infection wound dressing.
2831	DRAMP02311	Lysozyme (1,4-beta-N-acetylmuramidase; starfish, chordates, animals)	"Lysozyme"[All Fields] AND biofilm[All Fields]	Lysozyme	27931094	Human Lysozyme Peptidase Resistance Is Perturbed by the Anionic Glycolipid Biosurfactant Rhamnolipid Produced by the Opportunistic Pathogen Pseudomonas aeruginosa.
2831	DRAMP02311	Lysozyme (1,4-beta-N-acetylmuramidase; starfish, chordates, animals)	"Lysozyme"[All Fields] AND biofilm[All Fields]	Lysozyme	27845497	Lysozyme as a cotreatment during antibiotics use against vaginal infections: An in vitro study on Gardnerella vaginalis biofilm models.
2831	DRAMP02311	Lysozyme (1,4-beta-N-acetylmuramidase; starfish, chordates, animals)	"Lysozyme"[All Fields] AND biofilm[All Fields]	Lysozyme	27821857	Establishing Antibacterial Multilayer Films on the Surface of Direct Metal Laser Sintered Titanium Primed with Phase-Transited Lysozyme.
2831	DRAMP02311	Lysozyme (1,4-beta-N-acetylmuramidase; starfish, chordates, animals)	"Lysozyme"[All Fields] AND biofilm[All Fields]	Lysozyme	27709890	A Biodegradable Polycationic Paint that Kills Bacteria in Vitro and in Vivo.
2831	DRAMP02311	Lysozyme (1,4-beta-N-acetylmuramidase; starfish, chordates, animals)	"Lysozyme"[All Fields] AND biofilm[All Fields]	Lysozyme	27685160	In Situ Impregnation of Silver Nanoclusters in Microporous Chitosan-PEG Membranes as an Antibacterial and Drug Delivery Percutaneous Device.
2831	DRAMP02311	Lysozyme (1,4-beta-N-acetylmuramidase; starfish, chordates, animals)	"Lysozyme"[All Fields] AND biofilm[All Fields]	Lysozyme	27602088	Antibiotic susceptibility, antibacterial activity and characterisation of Enterococcus faeciumstrains isolated from breast milk.
2831	DRAMP02311	Lysozyme (1,4-beta-N-acetylmuramidase; starfish, chordates, animals)	"Lysozyme"[All Fields] AND biofilm[All Fields]	Lysozyme	27520822	Peptidoglycan Acetylation of Campylobacter jejuni Is Essential for Maintaining Cell Wall Integrity and Colonization in Chicken Intestines.
2831	DRAMP02311	Lysozyme (1,4-beta-N-acetylmuramidase; starfish, chordates, animals)	"Lysozyme"[All Fields] AND biofilm[All Fields]	Lysozyme	27375592	Pseudomonas aeruginosa Outer Membrane Vesicles Triggered by Human Mucosal Fluid and Lysozyme Can Prime Host Tissue Surfaces for Bacterial Adhesion.
2831	DRAMP02311	Lysozyme (1,4-beta-N-acetylmuramidase; starfish, chordates, animals)	"Lysozyme"[All Fields] AND biofilm[All Fields]	Lysozyme	27373086	[Staphylococcus aureus biofilm influences the expression of lysozyme, SLPI and gp340 in a human sinonasal explant model].
2831	DRAMP02311	Lysozyme (1,4-beta-N-acetylmuramidase; starfish, chordates, animals)	"Lysozyme"[All Fields] AND biofilm[All Fields]	Lysozyme	27236754	Molecular typing and differences in biofilm formation and antibiotic susceptibilities among Prototheca strains isolated in Italy and Brazil.
2831	DRAMP02311	Lysozyme (1,4-beta-N-acetylmuramidase; starfish, chordates, animals)	"Lysozyme"[All Fields] AND biofilm[All Fields]	Lysozyme	27015648	Biopolymer-induced calcium phosphate scaling in membrane-based water treatment systems: Langmuir model films studies.
2831	DRAMP02311	Lysozyme (1,4-beta-N-acetylmuramidase; starfish, chordates, animals)	"Lysozyme"[All Fields] AND biofilm[All Fields]	Lysozyme	26783746	Titanium Surface Priming with Phase-Transited Lysozyme to Establish a Silver Nanoparticle-Loaded Chitosan/Hyaluronic Acid Antibacterial Multilayer via Layer-by-Layer Self-Assembly.
2831	DRAMP02311	Lysozyme (1,4-beta-N-acetylmuramidase; starfish, chordates, animals)	"Lysozyme"[All Fields] AND biofilm[All Fields]	Lysozyme	31532953	[The etiologic structure and biologic characteristics of agents of infections of bloodstream.]
2831	DRAMP02311	Lysozyme (1,4-beta-N-acetylmuramidase; starfish, chordates, animals)	"Lysozyme"[All Fields] AND biofilm[All Fields]	Lysozyme	26488327	Complexation of Lysozyme with Sodium Poly(styrenesulfonate) via the Two-State and Non-Two-State Unfoldings of Lysozyme.
2831	DRAMP02311	Lysozyme (1,4-beta-N-acetylmuramidase; starfish, chordates, animals)	"Lysozyme"[All Fields] AND biofilm[All Fields]	Lysozyme	26478289	Covalent immobilization of lysozyme onto woven and knitted crimped polyethylene terephthalate grafts to minimize the adhesion of broad spectrum pathogens.
2831	DRAMP02311	Lysozyme (1,4-beta-N-acetylmuramidase; starfish, chordates, animals)	"Lysozyme"[All Fields] AND biofilm[All Fields]	Lysozyme	26470414	[REGULATING EFFECT OF ASSOCIATIVE MICROBIOTA ON THE RHYTHMS OF BIOLOGICAL PROPERTIES OF FUNGI AND BACTERIA].

2831	DRAMP02311	Lysozyme (1,4-beta-N-acetylmuramidase; starfish, chordates, animals)	"Lysozyme"[All Fields] AND biofilm[All Fields]	Lysozyme	26458820	Effects of fed-batch and continuous fermentations on human lysozyme production by Kluyveromyces lactis K7 in biofilm reactors.
2831	DRAMP02311	Lysozyme (1,4-beta-N-acetylmuramidase; starfish, chordates, animals)	"Lysozyme"[All Fields] AND biofilm[All Fields]	Lysozyme	26400891	Effect of histatin-5 and lysozyme on the ability of Streptococcus mutans to form biofilms in in vitro conditions.
2831	DRAMP02311	Lysozyme (1,4-beta-N-acetylmuramidase; starfish, chordates, animals)	"Lysozyme"[All Fields] AND biofilm[All Fields]	Lysozyme	26383819	Recent advances for the production and recovery methods of lysozyme.
2831	DRAMP02311	Lysozyme (1,4-beta-N-acetylmuramidase; starfish, chordates, animals)	"Lysozyme"[All Fields] AND biofilm[All Fields]	Lysozyme	26315397	Enzymes in the in-situ pellicle of children with different caries activity.
2831	DRAMP02311	Lysozyme (1,4-beta-N-acetylmuramidase; starfish, chordates, animals)	"Lysozyme"[All Fields] AND biofilm[All Fields]	Lysozyme	26152033	[AEROMONAS BACTERIA ISOLATED FROM BITHYNIIDAE MOLLUSKS AND THEIR HABITATS: SPECIES COMPOSITION AND BIOLOGICAL PROPERTIES. COMMUNICATION 1].
2831	DRAMP02311	Lysozyme (1,4-beta-N-acetylmuramidase; starfish, chordates, animals)	"Lysozyme"[All Fields] AND biofilm[All Fields]	Lysozyme	26092919	Acetylcholine Protects against Candida albicans Infection by Inhibiting Biofilm Formation and Promoting Hemocyte Function in a Galleria mellonella Infection Model.
2831	DRAMP02311	Lysozyme (1,4-beta-N-acetylmuramidase; starfish, chordates, animals)	"Lysozyme"[All Fields] AND biofilm[All Fields]	Lysozyme	27688392	Antimicrobial Capacity of Casein Phosphopeptide/Amorphous Calcium Phosphate and Enzymes in Glass Ionomer Cement in Dentin Carious Lesions.
2831	DRAMP02311	Lysozyme (1,4-beta-N-acetylmuramidase; starfish, chordates, animals)	"Lysozyme"[All Fields] AND biofilm[All Fields]	Lysozyme	25950767	In vitro biofilm development of Streptococcus pneumoniae and formation of choline-binding protein-DNA complexes.
2831	DRAMP02311	Lysozyme (1,4-beta-N-acetylmuramidase; starfish, chordates, animals)	"Lysozyme"[All Fields] AND biofilm[All Fields]	Lysozyme	25757148	Development of novel formulations containing Lysozyme and Lactoferrin and evaluation of antibacterial effects on Mutans Streptococci and Lactobacilli.
2831	DRAMP02311	Lysozyme (1,4-beta-N-acetylmuramidase; starfish, chordates, animals)	"Lysozyme"[All Fields] AND biofilm[All Fields]	Lysozyme	25725311	Cleaning of biomaterial surfaces: protein removal by different solvents.
2831	DRAMP02311	Lysozyme (1,4-beta-N-acetylmuramidase; starfish, chordates, animals)	"Lysozyme"[All Fields] AND biofilm[All Fields]	Lysozyme	25721974	Characterization of a novel strain phylogenetically related to Kocuria rhizophila and its chemical modification to improve performance of microbial fuel cells.
2831	DRAMP02311	Lysozyme (1,4-beta-N-acetylmuramidase; starfish, chordates, animals)	"Lysozyme"[All Fields] AND biofilm[All Fields]	Lysozyme	25536779	[Species structure and staphylococci bioprofile characteristics--ausative agents of perinatal infectious-inflammation pathology in children of Orenburg].
2831	DRAMP02311	Lysozyme (1,4-beta-N-acetylmuramidase; starfish, chordates, animals)	"Lysozyme"[All Fields] AND biofilm[All Fields]	Lysozyme	25467937	Phenotypic characterization of a novel double knockout PknI/DacB2 from Mycobacterium tuberculosis.
2831	DRAMP02311	Lysozyme (1,4-beta-N-acetylmuramidase; starfish, chordates, animals)	"Lysozyme"[All Fields] AND biofilm[All Fields]	Lysozyme	25449384	N-hexanoyl-L-homoserine lactone-degrading Pseudomonas aeruginosa PsDAHP1 protects zebrafish against Vibrio parahaemolyticus infection.
2831	DRAMP02311	Lysozyme (1,4-beta-N-acetylmuramidase; starfish, chordates, animals)	"Lysozyme"[All Fields] AND biofilm[All Fields]	Lysozyme	25312955	The conserved hypothetical protein Rv0574c is required for cell wall integrity, stress tolerance, and virulence of Mycobacterium tuberculosis.
2831	DRAMP02311	Lysozyme (1,4-beta-N-acetylmuramidase; starfish, chordates, animals)	"Lysozyme"[All Fields] AND biofilm[All Fields]	Lysozyme	25221673	Iron oxide nanoparticles induce Pseudomonas aeruginosagrowth, induce biofilm formation, and inhibit antimicrobial peptide function.
2831	DRAMP02311	Lysozyme (1,4-beta-N-acetylmuramidase; starfish, chordates, animals)	"Lysozyme"[All Fields] AND biofilm[All Fields]	Lysozyme	25115519	Presence and function of a thick mucous layer rich in polysaccharides around Bacillus subtilis spores.
2831	DRAMP02311	Lysozyme (1,4-beta-N-acetylmuramidase; starfish, chordates, animals)	"Lysozyme"[All Fields] AND biofilm[All Fields]	Lysozyme	24559398	Temperature-driven adsorption and desorption of proteins at solid-liquid interfaces.
2831	DRAMP02311	Lysozyme (1,4-beta-N-acetylmuramidase; starfish, chordates, animals)	"Lysozyme"[All Fields] AND biofilm[All Fields]	Lysozyme	24434534	Designing nanogel carriers for antibacterial applications.
2831	DRAMP02311	Lysozyme (1,4-beta-N-acetylmuramidase; starfish, chordates, animals)	"Lysozyme"[All Fields] AND biofilm[All Fields]	Lysozyme	24240906	Adsorption study of pellicle proteins to gold, silica and titanium by quartz crystal microbalance method.
2831	DRAMP02311	Lysozyme (1,4-beta-N-acetylmuramidase; starfish, chordates, animals)	"Lysozyme"[All Fields] AND biofilm[All Fields]	Lysozyme	24121782	Enhanced expressions of lysozyme, SLPI and glycoprotein 340 in biofilm-associated chronic rhinosinusitis.
2831	DRAMP02311	Lysozyme (1,4-beta-N-acetylmuramidase; starfish, chordates, animals)	"Lysozyme"[All Fields] AND biofilm[All Fields]	Lysozyme	24011302	Fluorescence microscopic visualization of non cellular components during initial bioadhesion in situ.
2831	DRAMP02311	Lysozyme (1,4-beta-N-acetylmuramidase; starfish, chordates, animals)	"Lysozyme"[All Fields] AND biofilm[All Fields]	Lysozyme	23844477	Destruction of single-species biofilms of Escherichia coli or Klebsiella pneumoniae subsp. pneumoniae by dextranase, lactoferrin, and lysozyme.
2831	DRAMP02311	Lysozyme (1,4-beta-N-acetylmuramidase; starfish, chordates, animals)	"Lysozyme"[All Fields] AND biofilm[All Fields]	Lysozyme	23701483	Effects of biological molecules on calcium mineral formation associated with wastewater desalination as assessed using small-angle neutron scattering.
2831	DRAMP02311	Lysozyme (1,4-beta-N-acetylmuramidase; starfish, chordates, animals)	"Lysozyme"[All Fields] AND biofilm[All Fields]	Lysozyme	23659996	Vaccination efficiency of surface antigens and killed whole cell of Pseudomonas putida in large yellow croaker (Pseudosciaena crocea).
2831	DRAMP02311	Lysozyme (1,4-beta-N-acetylmuramidase; starfish, chordates, animals)	"Lysozyme"[All Fields] AND biofilm[All Fields]	Lysozyme	23657582	Production of human lysozyme in biofilm reactor and optimization of growth parameters of Kluyveromyces lactis K7.
2831	DRAMP02311	Lysozyme (1,4-beta-N-acetylmuramidase; starfish, chordates, animals)	"Lysozyme"[All Fields] AND biofilm[All Fields]	Lysozyme	23504078	Phenotypic characterization, virulence, and immunogenicity of Edwardsiella tarda LSE40 aroA mutant.
2831	DRAMP02311	Lysozyme (1,4-beta-N-acetylmuramidase; starfish, chordates, animals)	"Lysozyme"[All Fields] AND biofilm[All Fields]	Lysozyme	23460607	Undecaprenyl pyrophosphate phosphatase confers low-level resistance to bacitracin in Enterococcus faecalis.
2831	DRAMP02311	Lysozyme (1,4-beta-N-acetylmuramidase; starfish, chordates, animals)	"Lysozyme"[All Fields] AND biofilm[All Fields]	Lysozyme	23434686	Enhancing antibacterial activity of surface-grafted chitosan with immobilized lysozyme on bioinspired stainless steel substrates.

2831	DRAMP02311	Lysozyme (1,4-beta-N-acetylmuramidase; starfish, chordates, animals)	"Lysozyme"[All Fields] AND biofilm[All Fields]	Lysozyme	23403559	Host defense proteins derived from human saliva bind to Staphylococcus aureus.
2831	DRAMP02311	Lysozyme (1,4-beta-N-acetylmuramidase; starfish, chordates, animals)	"Lysozyme"[All Fields] AND biofilm[All Fields]	Lysozyme	23194029	Alpha-amylase is a human salivary protein with affinity to lipopolysaccharide of Aggregatibacter actinomycetemcomitans.
2831	DRAMP02311	Lysozyme (1,4-beta-N-acetylmuramidase; starfish, chordates, animals)	"Lysozyme"[All Fields] AND biofilm[All Fields]	Lysozyme	22947470	Effects of nisin and lysozyme on growth inhibition and biofilm formation capacity of Staphylococcus aureus strains isolated from raw milk and cheese samples.
2831	DRAMP02311	Lysozyme (1,4-beta-N-acetylmuramidase; starfish, chordates, animals)	"Lysozyme"[All Fields] AND biofilm[All Fields]	Lysozyme	22937707	[Interaction of Bifidobacterium bifidum with members of normal microflora in human intestine microsymbiocenosis].
2831	DRAMP02311	Lysozyme (1,4-beta-N-acetylmuramidase; starfish, chordates, animals)	"Lysozyme"[All Fields] AND biofilm[All Fields]	Lysozyme	22913814	Insight into the composition of the intercellular matrix of Streptococcus pneumoniae biofilms.
2831	DRAMP02311	Lysozyme (1,4-beta-N-acetylmuramidase; starfish, chordates, animals)	"Lysozyme"[All Fields] AND biofilm[All Fields]	Lysozyme	22363222	Tasco®: a product of Ascophyllum nodosum enhances immune response of Caenorhabditis elegans against Pseudomonas aeruginosa infection.
2831	DRAMP02311	Lysozyme (1,4-beta-N-acetylmuramidase; starfish, chordates, animals)	"Lysozyme"[All Fields] AND biofilm[All Fields]	Lysozyme	26781731	Recombinant Expression of a Putative Amidase Cloned from the Genome of Listeria monocytogenes that Lyses the Bacterium and its Monolayer in Conjunction with a Protease.
2831	DRAMP02311	Lysozyme (1,4-beta-N-acetylmuramidase; starfish, chordates, animals)	"Lysozyme"[All Fields] AND biofilm[All Fields]	Lysozyme	22344334	Influence of artificial saliva in biofilm formation of Candida albicans in vitro.
2831	DRAMP02311	Lysozyme (1,4-beta-N-acetylmuramidase; starfish, chordates, animals)	"Lysozyme"[All Fields] AND biofilm[All Fields]	Lysozyme	22308727	[Microbial "friend-foe" identification in human intestine microsymbiocenosis].
2831	DRAMP02311	Lysozyme (1,4-beta-N-acetylmuramidase; starfish, chordates, animals)	"Lysozyme"[All Fields] AND biofilm[All Fields]	Lysozyme	22265308	Effect of lysozyme on "flor" velum yeasts in the biological aging of sherry wines.
2831	DRAMP02311	Lysozyme (1,4-beta-N-acetylmuramidase; starfish, chordates, animals)	"Lysozyme"[All Fields] AND biofilm[All Fields]	Lysozyme	21821771	Role of phase variation in the resistance of Myxococcus xanthus fruiting bodies to Caenorhabditis elegans predation.
2831	DRAMP02311	Lysozyme (1,4-beta-N-acetylmuramidase; starfish, chordates, animals)	"Lysozyme"[All Fields] AND biofilm[All Fields]	Lysozyme	21486002	Anti-fouling chemistry of chiral monolayers: enhancing biofilm resistance on racemic surface.
2831	DRAMP02311	Lysozyme (1,4-beta-N-acetylmuramidase; starfish, chordates, animals)	"Lysozyme"[All Fields] AND biofilm[All Fields]	Lysozyme	21471196	A disulfide bridge network within the soluble periplasmic domain determines structure and function of the outer membrane protein RCSF.
2831	DRAMP02311	Lysozyme (1,4-beta-N-acetylmuramidase; starfish, chordates, animals)	"Lysozyme"[All Fields] AND biofilm[All Fields]	Lysozyme	21338094	Lysozyme-coupled poly(poly(ethylene glycol) methacrylate)-stainless steel hybrids and their antifouling and antibacterial surfaces.
2831	DRAMP02311	Lysozyme (1,4-beta-N-acetylmuramidase; starfish, chordates, animals)	"Lysozyme"[All Fields] AND biofilm[All Fields]	Lysozyme	21247450	Phylogenetic group- and species-specific oligonucleotide probes for single-cell detection of lactic acid bacteria in oral biofilms.
2831	DRAMP02311	Lysozyme (1,4-beta-N-acetylmuramidase; starfish, chordates, animals)	"Lysozyme"[All Fields] AND biofilm[All Fields]	Lysozyme	21148692	Integrity of proteins in human saliva after sterilization by gamma irradiation.
2831	DRAMP02311	Lysozyme (1,4-beta-N-acetylmuramidase; starfish, chordates, animals)	"Lysozyme"[All Fields] AND biofilm[All Fields]	Lysozyme	21045923	Structural evolution of protein-biofilms: Simulations and experiments.
2831	DRAMP02311	Lysozyme (1,4-beta-N-acetylmuramidase; starfish, chordates, animals)	"Lysozyme"[All Fields] AND biofilm[All Fields]	Lysozyme	20961363	Potential of ceragenin CSA-13 and its mixture with pluronic F-127 as treatment of topical bacterial infections.
2831	DRAMP02311	Lysozyme (1,4-beta-N-acetylmuramidase; starfish, chordates, animals)	"Lysozyme"[All Fields] AND biofilm[All Fields]	Lysozyme	20865041	Comparative genomics of Gardnerella vaginalis strains reveals substantial differences in metabolic and virulence potential.
2831	DRAMP02311	Lysozyme (1,4-beta-N-acetylmuramidase; starfish, chordates, animals)	"Lysozyme"[All Fields] AND biofilm[All Fields]	Lysozyme	20724386	Glutamine synthetase encoded by glnA-1 is necessary for cell wall resistance and pathogenicity of Mycobacterium bovis.
2831	DRAMP02311	Lysozyme (1,4-beta-N-acetylmuramidase; starfish, chordates, animals)	"Lysozyme"[All Fields] AND biofilm[All Fields]	Lysozyme	20566201	Bioengineering of stainless steel surface by covalent immobilization of enzymes. Physical characterization and interfacial enzymatic activity.
2831	DRAMP02311	Lysozyme (1,4-beta-N-acetylmuramidase; starfish, chordates, animals)	"Lysozyme"[All Fields] AND biofilm[All Fields]	Lysozyme	20417319	Functionalization of acrylic hydrogels with alpha-, beta- or gamma-cyclodextrin modulates protein adsorption and antifungal delivery.
2831	DRAMP02311	Lysozyme (1,4-beta-N-acetylmuramidase; starfish, chordates, animals)	"Lysozyme"[All Fields] AND biofilm[All Fields]	Lysozyme	20121056	Achieving highly effective non-biofouling performance for polypropylene membranes modified by UV-induced surface graft polymerization of two oppositely charged monomers.
2831	DRAMP02311	Lysozyme (1,4-beta-N-acetylmuramidase; starfish, chordates, animals)	"Lysozyme"[All Fields] AND biofilm[All Fields]	Lysozyme	19334612	[Search for destruction factors of bacterial biofilms: comparison of phage properties in a group of Pseudomonas putida bacteriophages and specificity of their halo-formation products].
2831	DRAMP02311	Lysozyme (1,4-beta-N-acetylmuramidase; starfish, chordates, animals)	"Lysozyme"[All Fields] AND biofilm[All Fields]	Lysozyme	19241565	Storability of antimicrobial chitosan-lysozyme composite coating and film-forming solutions.
2831	DRAMP02311	Lysozyme (1,4-beta-N-acetylmuramidase; starfish, chordates, animals)	"Lysozyme"[All Fields] AND biofilm[All Fields]	Lysozyme	19166331	Grafting of lysozyme and/or poly(ethylene glycol) to prevent biofilm growth on stainless steel surfaces.
2831	DRAMP02311	Lysozyme (1,4-beta-N-acetylmuramidase; starfish, chordates, animals)	"Lysozyme"[All Fields] AND biofilm[All Fields]	Lysozyme	19038377	Synergistic activity of lysozyme and antifungal agents against Candida albicans biofilms on denture acrylic surfaces.
2831	DRAMP02311	Lysozyme (1,4-beta-N-acetylmuramidase; starfish, chordates, animals)	"Lysozyme"[All Fields] AND biofilm[All Fields]	Lysozyme	18992255	Crystal structure of the resuscitation-promoting factor (DeltaDUF)RpfB from M. tuberculosis.
2831	DRAMP02311	Lysozyme (1,4-beta-N-acetylmuramidase; starfish, chordates, animals)	"Lysozyme"[All Fields] AND biofilm[All Fields]	Lysozyme	18989905	Ultralow fouling zwitterionic polymers grafted from surfaces covered with an initiator via an adhesive mussel mimetic linkage.

2831	DRAMP02311	Lysozyme (1,4-beta-N-acetylmuramidase; starfish, chordates, animals)	"Lysozyme"[All Fields] AND biofilm[All Fields]	Lysozyme	18819708	Ultra low fouling zwitterionic polymers with a biomimetic adhesive group.
2831	DRAMP02311	Lysozyme (1,4-beta-N-acetylmuramidase; starfish, chordates, animals)	"Lysozyme"[All Fields] AND biofilm[All Fields]	Lysozyme	18718499	Role of lactoferrin in the tear film.
2831	DRAMP02311	Lysozyme (1,4-beta-N-acetylmuramidase; starfish, chordates, animals)	"Lysozyme"[All Fields] AND biofilm[All Fields]	Lysozyme	18468764	Effects of Cistus-tea on bacterial colonization and enzyme activities of the in situ pellicle.
2831	DRAMP02311	Lysozyme (1,4-beta-N-acetylmuramidase; starfish, chordates, animals)	"Lysozyme"[All Fields] AND biofilm[All Fields]	Lysozyme	18424015	Abnormal cell division caused by inclusion bodies in E. coli; increased resistance against external stress.
2831	DRAMP02311	Lysozyme (1,4-beta-N-acetylmuramidase; starfish, chordates, animals)	"Lysozyme"[All Fields] AND biofilm[All Fields]	Lysozyme	18326181	Antimicrobial activity of lactoferrin against foodborne pathogenic bacteria incorporated into edible chitosan film.
2831	DRAMP02311	Lysozyme (1,4-beta-N-acetylmuramidase; starfish, chordates, animals)	"Lysozyme"[All Fields] AND biofilm[All Fields]	Lysozyme	17927634	Biofilm inhibition and antimicrobial activity of a dentifrice containing salivary substitutes.
2831	DRAMP02311	Lysozyme (1,4-beta-N-acetylmuramidase; starfish, chordates, animals)	"Lysozyme"[All Fields] AND biofilm[All Fields]	Lysozyme	17676995	Molecular basis of resistance to muramidase and cationic antimicrobial peptide activity of lysozyme in staphylococci.
2831	DRAMP02311	Lysozyme (1,4-beta-N-acetylmuramidase; starfish, chordates, animals)	"Lysozyme"[All Fields] AND biofilm[All Fields]	Lysozyme	16936041	Biofilm formation by Streptococcus pneumoniae: role of choline, extracellular DNA, and capsular polysaccharide in microbial accretion.
2831	DRAMP02311	Lysozyme (1,4-beta-N-acetylmuramidase; starfish, chordates, animals)	"Lysozyme"[All Fields] AND biofilm[All Fields]	Lysozyme	15952847	Covalent immobilization of lysozyme on stainless steel. Interface spectroscopic characterization and measurement of enzymatic activity.
2831	DRAMP02311	Lysozyme (1,4-beta-N-acetylmuramidase; starfish, chordates, animals)	"Lysozyme"[All Fields] AND biofilm[All Fields]	Lysozyme	15908380	Identification and characterization of an autolysin-encoding gene of Streptococcus mutans.
2831	DRAMP02311	Lysozyme (1,4-beta-N-acetylmuramidase; starfish, chordates, animals)	"Lysozyme"[All Fields] AND biofilm[All Fields]	Lysozyme	15693823	Enzymes in the acquired enamel pellicle.
2831	DRAMP02311	Lysozyme (1,4-beta-N-acetylmuramidase; starfish, chordates, animals)	"Lysozyme"[All Fields] AND biofilm[All Fields]	Lysozyme	15518508	Shear and dilatational relaxation mechanisms of globular and flexible proteins at the hexadecane/water interface.
2831	DRAMP02311	Lysozyme (1,4-beta-N-acetylmuramidase; starfish, chordates, animals)	"Lysozyme"[All Fields] AND biofilm[All Fields]	Lysozyme	15493829	Bacterial evasion of innate host defenses--the Staphylococcus aureus lesson.
2831	DRAMP02311	Lysozyme (1,4-beta-N-acetylmuramidase; starfish, chordates, animals)	"Lysozyme"[All Fields] AND biofilm[All Fields]	Lysozyme	14768464	Protein adsorption at polymer-grafted surfaces: comparison between a mixture of saliva proteins and some well-defined model proteins.
2831	DRAMP02311	Lysozyme (1,4-beta-N-acetylmuramidase; starfish, chordates, animals)	"Lysozyme"[All Fields] AND biofilm[All Fields]	Lysozyme	14706749	Multiplex FISH analysis of a six-species bacterial biofilm.
2831	DRAMP02311	Lysozyme (1,4-beta-N-acetylmuramidase; starfish, chordates, animals)	"Lysozyme"[All Fields] AND biofilm[All Fields]	Lysozyme	14513382	Physiology of biofilms of thermophilic bacilli-potential consequences for cleaning.
2831	DRAMP02311	Lysozyme (1,4-beta-N-acetylmuramidase; starfish, chordates, animals)	"Lysozyme"[All Fields] AND biofilm[All Fields]	Lysozyme	12707750	Scanning-force techniques to monitor time-dependent changes in topography and adhesion force of proteins on surfaces.
2831	DRAMP02311	Lysozyme (1,4-beta-N-acetylmuramidase; starfish, chordates, animals)	"Lysozyme"[All Fields] AND biofilm[All Fields]	Lysozyme	12366846	Positive role of peptidoglycan breaks in lactococcal biofilm formation.
2831	DRAMP02311	Lysozyme (1,4-beta-N-acetylmuramidase; starfish, chordates, animals)	"Lysozyme"[All Fields] AND biofilm[All Fields]	Lysozyme	11679354	Direct detection by in situ PCR of the amoA gene in biofilm resulting from a nitrogen removal process.
2831	DRAMP02311	Lysozyme (1,4-beta-N-acetylmuramidase; starfish, chordates, animals)	"Lysozyme"[All Fields] AND biofilm[All Fields]	Lysozyme	11412320	Factors influencing attachment of thermophilic bacilli to stainless steel.
2831	DRAMP02311	Lysozyme (1,4-beta-N-acetylmuramidase; starfish, chordates, animals)	"Lysozyme"[All Fields] AND biofilm[All Fields]	Lysozyme	10415452	Lactoferrin increases the susceptibility of S. epidermidis biofilms to lysozyme and vancomycin.
2831	DRAMP02311	Lysozyme (1,4-beta-N-acetylmuramidase; starfish, chordates, animals)	"Lysozyme"[All Fields] AND biofilm[All Fields]	Lysozyme	10391502	The effect of extracellular polysaccharides from Streptococcus mutans on the bactericidal activity of human neutrophils.
2831	DRAMP02311	Lysozyme (1,4-beta-N-acetylmuramidase; starfish, chordates, animals)	"Lysozyme"[All Fields] AND biofilm[All Fields]	Lysozyme	10390869	Specific oligonucleotide probes for in situ detection of a major group of gram-positive bacteria with low DNA G + C content.
2831	DRAMP02311	Lysozyme (1,4-beta-N-acetylmuramidase; starfish, chordates, animals)	"Lysozyme"[All Fields] AND biofilm[All Fields]	Lysozyme	9812283	Protein antimicrobial barriers to bacterial adhesion.
2831	DRAMP02311	Lysozyme (1,4-beta-N-acetylmuramidase; starfish, chordates, animals)	"Lysozyme"[All Fields] AND biofilm[All Fields]	Lysozyme	9571268	Defining the physiologically normal coating and pathological deposit: an analysis of sulfur-containing moieties and pellicle thickness on hydrogel contact lenses.
2831	DRAMP02311	Lysozyme (1,4-beta-N-acetylmuramidase; starfish, chordates, animals)	"Lysozyme"[All Fields] AND biofilm[All Fields]	Lysozyme	9351231	The influence of cell surface properties of thermophilic streptococci on attachment to stainless steel.
2831	DRAMP02311	Lysozyme (1,4-beta-N-acetylmuramidase; starfish, chordates, animals)	"Lysozyme"[All Fields] AND biofilm[All Fields]	Lysozyme	9255506	Protein-lipid interaction on the surface of a hydrophilic contact lens in vitro.
2831	DRAMP02311	Lysozyme (1,4-beta-N-acetylmuramidase; starfish, chordates, animals)	"Lysozyme"[All Fields] AND biofilm[All Fields]	Lysozyme	9380651	[Preparation of films with combined biological activity and study of their properties].
2831	DRAMP02311	Lysozyme (1,4-beta-N-acetylmuramidase; starfish, chordates, animals)	"Lysozyme"[All Fields] AND biofilm[All Fields]	Lysozyme	9029300	Adhesion to silicone rubber of yeasts and bacteria isolated from voice prostheses: influence of salivary conditioning films.

2831	DRAMP02311	Lysozyme (1,4-beta-N-acetylmuramidase; starfish, chordates, animals)	"Lysozyme"[All Fields] AND biofilm[All Fields]	Lysozyme	8950503	The effect of eye closure on protein and complement deposition on Group IV hydrogel contact lenses: relationship to tear flow dynamics.
2831	DRAMP02311	Lysozyme (1,4-beta-N-acetylmuramidase; starfish, chordates, animals)	"Lysozyme"[All Fields] AND biofilm[All Fields]	Lysozyme	8126879	[Physiologic factor in intractable bacterial infections].
2831	DRAMP02311	Lysozyme (1,4-beta-N-acetylmuramidase; starfish, chordates, animals)	"Lysozyme"[All Fields] AND biofilm[All Fields]	Lysozyme	3570694	Specificity and biological activity of the protein deposited on the hydrogel surface. Relationship of polymer structure to biofilm formation.
2841	DRAMP02332	Piscidin-3 (Pis-3; fish, chordates, animals)	"Piscidin-3"[All Fields] AND biofilm[All Fields]	Piscidin-3	34590712	The effect of piscidin antimicrobial peptides on the formation of Gram-negative bacterial biofilms.
2841	DRAMP02332	Piscidin-3 (Pis-3; fish, chordates, animals)	"Piscidin-3"[All Fields] AND biofilm[All Fields]	Piscidin-3	30471190	Metal-ion Binding to Host Defense Peptide Piscidin 3 Observed in Phospholipid Bilayers by Magic Angle Spinning Solid-state NMR.
2841	DRAMP02332	Piscidin-3 (Pis-3; fish, chordates, animals)	"Piscidin-3"[All Fields] AND biofilm[All Fields]	Piscidin-3	28892294	Nuclease activity gives an edge to host-defense peptide piscidin 3 over piscidin 1, rendering it more effective against persisters and biofilms.
2842	DRAMP02333	Piscidin-4 (Pis-4; fish, chordates, animals)	"Piscidin-4"[All Fields] AND biofilm[All Fields]	Piscidin-4	34590712	The effect of piscidin antimicrobial peptides on the formation of Gram-negative bacterial biofilms.
2843	DRAMP02334	Hepcidin (fish, chordates, animals)	"Hepcidin"[All Fields] AND biofilm[All Fields]	Hepcidin	30456212	Quantification of Lipoteichoic Acid in Hemodialysis Patients With Central Venous Catheters.
2843	DRAMP02334	Hepcidin (fish, chordates, animals)	"Hepcidin"[All Fields] AND biofilm[All Fields]	Hepcidin	30408337	Silk-Based Antimicrobial Polymers as a New Platform to Design Drug-Free Materials to Impede Microbial Infections.
2843	DRAMP02334	Hepcidin (fish, chordates, animals)	"Hepcidin"[All Fields] AND biofilm[All Fields]	Hepcidin	24645694	Inhibitory effect of the human liver-derived antimicrobial peptide hepcidin 20 on biofilms of polysaccharide intercellular adhesin (PIA)-positive and PIA-negative strains of Staphylococcus epidermidis.
2845	DRAMP02338	Beta-defensin 1 (fish, chordates, animals)	"Beta-defensin 1"[All Fields] AND biofilm[All Fields]	Beta-defensin 1	33420317	Curbing gastrointestinal infections by defensin fragment modifications without harming commensal microbiota.
2845	DRAMP02338	Beta-defensin 1 (fish, chordates, animals)	"Beta-defensin 1"[All Fields] AND biofilm[All Fields]	Beta-defensin 1	32585445	Glucose effect on Candida albicans biofilm during tissue invasion.
2845	DRAMP02338	Beta-defensin 1 (fish, chordates, animals)	"Beta-defensin 1"[All Fields] AND biofilm[All Fields]	Beta-defensin 1	30260708	Stabilized collagen matrix dressing improves wound macrophage function and epithelialization.
2845	DRAMP02338	Beta-defensin 1 (fish, chordates, animals)	"Beta-defensin 1"[All Fields] AND biofilm[All Fields]	Beta-defensin 1	28642103	Chimeric analogs of human β-defensin 1 and θ-defensin disrupt pre-established bacterial biofilms.
2845	DRAMP02338	Beta-defensin 1 (fish, chordates, animals)	"Beta-defensin 1"[All Fields] AND biofilm[All Fields]	Beta-defensin 1	26214284	α-tocopherol decreases interleukin-1β and -6 and increases human β-defensin-1 and -2 secretion in human gingival fibroblasts stimulated with Porphyromonas gingivalis lipopolysaccharide.
2845	DRAMP02338	Beta-defensin 1 (fish, chordates, animals)	"Beta-defensin 1"[All Fields] AND biofilm[All Fields]	Beta-defensin 1	22394470	Association of CiaRH with resistance of Streptococcus mutans to antimicrobial peptides in biofilms.
2845	DRAMP02338	Beta-defensin 1 (fish, chordates, animals)	"Beta-defensin 1"[All Fields] AND biofilm[All Fields]	Beta-defensin 1	20454633	Normal human gingival epithelial cells sense C. parapsilosis by toll-like receptors and module its pathogenesis through antimicrobial peptides and proinflammatory cytokines.
2877	DRAMP02408	2S albumin (To-A1)	"2S albumin"[All Fields] AND biofilm[All Fields]	2S albumin	31678367	RcAlb-PepII, a synthetic small peptide bioinspired in the 2S albumin from the seed cake of Ricinus communis, is a potent antimicrobial agent against Klebsiella pneumoniae and Candida parapsilosis.
2882	DRAMP02417	Defensin (Ticks, Arthropods, animals)	"Defensin"[All Fields] AND biofilm[All Fields]	Defensin	34408988	The Anti-Biofilm Efficacy of Caffeic Acid Phenethyl Ester (CAPE) In Vitroand a Murine Model of Oral Candidiasis.
2882	DRAMP02417	Defensin (Ticks, Arthropods, animals)	"Defensin"[All Fields] AND biofilm[All Fields]	Defensin	34321877	Antibacterial Effect of Honey-Derived Exosomes Containing Antimicrobial Peptides Against Oral Streptococci.
2882	DRAMP02417	Defensin (Ticks, Arthropods, animals)	"Defensin"[All Fields] AND biofilm[All Fields]	Defensin	34276631	DNA Blocks the Lethal Effect of Human Beta-Defensin 2 Against Neisseria meningitidis.
2882	DRAMP02417	Defensin (Ticks, Arthropods, animals)	"Defensin"[All Fields] AND biofilm[All Fields]	Defensin	33911935	The antibacterial activities of honey.
2882	DRAMP02417	Defensin (Ticks, Arthropods, animals)	"Defensin"[All Fields] AND biofilm[All Fields]	Defensin	33865931	Identification of a crocodylian β-defensin variant from Alligator mississippiensis with antimicrobial and antibiofilm activity.
2882	DRAMP02417	Defensin (Ticks, Arthropods, animals)	"Defensin"[All Fields] AND biofilm[All Fields]	Defensin	33586659	Identification of anti-microbial peptides and traces of microbial DNA in infrainfundibular compartments of human scalp terminal hair follicles.
2882	DRAMP02417	Defensin (Ticks, Arthropods, animals)	"Defensin"[All Fields] AND biofilm[All Fields]	Defensin	33534018	A recombinant fungal defensin-like peptide-P2 combats Streptococcus dysgalactiae and biofilms.
2882	DRAMP02417	Defensin (Ticks, Arthropods, animals)	"Defensin"[All Fields] AND biofilm[All Fields]	Defensin	33447687	Future directions of postoperative spinal implant infections.
2882	DRAMP02417	Defensin (Ticks, Arthropods, animals)	"Defensin"[All Fields] AND biofilm[All Fields]	Defensin	33420317	Curbing gastrointestinal infections by defensin fragment modifications without harming commensal microbiota.
2882	DRAMP02417	Defensin (Ticks, Arthropods, animals)	"Defensin"[All Fields] AND biofilm[All Fields]	Defensin	32867384	A Novel Peptide Antibiotic, Pro10-1D, Designed from Insect Defensin Shows Antibacterial and Anti-Inflammatory Activities in Sepsis Models.
2882	DRAMP02417	Defensin (Ticks, Arthropods, animals)	"Defensin"[All Fields] AND biofilm[All Fields]	Defensin	32858856	Candida albicansVirulence Factors and Pathogenicity for Endodontic Infections.
2882	DRAMP02417	Defensin (Ticks, Arthropods, animals)	"Defensin"[All Fields] AND biofilm[All Fields]	Defensin	32842903	Electrospun ZnO/Poly(Vinylidene Fluoride-Trifluoroethylene) Scaffolds for Lung Tissue Engineering.
2882	DRAMP02417	Defensin (Ticks, Arthropods, animals)	"Defensin"[All Fields] AND biofilm[All Fields]	Defensin	32663201	Inhibition and eradication activity of truncated α-defensin analogs against multidrug resistant uropathogenic Escherichia coli biofilm.
2882	DRAMP02417	Defensin (Ticks, Arthropods, animals)	"Defensin"[All Fields] AND biofilm[All Fields]	Defensin	32585445	Glucose effect on Candida albicans biofilm during tissue invasion.
2882	DRAMP02417	Defensin (Ticks, Arthropods, animals)	"Defensin"[All Fields] AND biofilm[All Fields]	Defensin	32522780	Controlling the Growth of the Skin Commensal Staphylococcus epidermidis Using d-Alanine Auxotrophy.
2882	DRAMP02417	Defensin (Ticks, Arthropods, animals)	"Defensin"[All Fields] AND biofilm[All Fields]	Defensin	32457749	The Antimicrobial Peptide Human Beta-Defensin 2 Inhibits Biofilm Production of Pseudomonas aeruginosaWithout Compromising Metabolic Activity.
2882	DRAMP02417	Defensin (Ticks, Arthropods, animals)	"Defensin"[All Fields] AND biofilm[All Fields]	Defensin	32439511	Antibacterial activities and mechanisms of action of a defensin from manila clam Ruditapes philippinarum.
2882	DRAMP02417	Defensin (Ticks, Arthropods, animals)	"Defensin"[All Fields] AND biofilm[All Fields]	Defensin	31933178	Effects of human β-defensin 3 fused with carbohydrate-binding domain on the function of type III secretion system in Pseudomonas aeruginosa PA14.
2882	DRAMP02417	Defensin (Ticks, Arthropods, animals)	"Defensin"[All Fields] AND biofilm[All Fields]	Defensin	31906541	Role of FAD-I in Fusobacterial Interspecies Interaction and Biofilm Formation.
2882	DRAMP02417	Defensin (Ticks, Arthropods, animals)	"Defensin"[All Fields] AND biofilm[All Fields]	Defensin	31729441	Rhesus Theta Defensin 1 Promotes Long Term Survival in Systemic Candidiasis by Host Directed Mechanisms.
2882	DRAMP02417	Defensin (Ticks, Arthropods, animals)	"Defensin"[All Fields] AND biofilm[All Fields]	Defensin	31336838	Candida albicans-Cell Interactions Activate Innate Immune Defense in Human Palate Epithelial Primary Cells via Nitric Oxide (NO) and β-Defensin 2 (hBD-2).
2882	DRAMP02417	Defensin (Ticks, Arthropods, animals)	"Defensin"[All Fields] AND biofilm[All Fields]	Defensin	31165072	Impact of the Food Additive Titanium Dioxide (E171) on Gut Microbiota-Host Interaction.
2882	DRAMP02417	Defensin (Ticks, Arthropods, animals)	"Defensin"[All Fields] AND biofilm[All Fields]	Defensin	31031739	Salt-Tolerant Antifungal and Antibacterial Activities of the Corn Defensin ZmD32.
2882	DRAMP02417	Defensin (Ticks, Arthropods, animals)	"Defensin"[All Fields] AND biofilm[All Fields]	Defensin	31025073	A recombinant fungal defensin-like peptide-P2 combats multidrug-resistant Staphylococcus aureus and biofilms.
2882	DRAMP02417	Defensin (Ticks, Arthropods, animals)	"Defensin"[All Fields] AND biofilm[All Fields]	Defensin	30659503	Lactobacillus plantarum USM8613 Aids in Wound Healing and Suppresses Staphylococcus aureus Infection at Wound Sites.
2882	DRAMP02417	Defensin (Ticks, Arthropods, animals)	"Defensin"[All Fields] AND biofilm[All Fields]	Defensin	30649289	Innate immune components affect growth and virulence traits of bacterial-vaginosis-associated and non-bacterial-vaginosis-associated Gardnerella vaginalis strains similarly.
2882	DRAMP02417	Defensin (Ticks, Arthropods, animals)	"Defensin"[All Fields] AND biofilm[All Fields]	Defensin	30376742	Alpha defensin, leukocyte esterase, C-reactive protein, and leukocyte count in synovial fluid for pre-operative diagnosis of periprosthetic infection.
2882	DRAMP02417	Defensin (Ticks, Arthropods, animals)	"Defensin"[All Fields] AND biofilm[All Fields]	Defensin	30260708	Stabilized collagen matrix dressing improves wound macrophage function and epithelialization.
2882	DRAMP02417	Defensin (Ticks, Arthropods, animals)	"Defensin"[All Fields] AND biofilm[All Fields]	Defensin	30254440	Modification of the surface of titanium with multifunctional chimeric peptides to prevent biofilm formation via inhibition of initial colonizers.
2882	DRAMP02417	Defensin (Ticks, Arthropods, animals)	"Defensin"[All Fields] AND biofilm[All Fields]	Defensin	29902560	A defensin-like antimicrobial peptide from the manila clam Ruditapes philippinarum: Investigation of the antibacterial activities and mode of action.
2882	DRAMP02417	Defensin (Ticks, Arthropods, animals)	"Defensin"[All Fields] AND biofilm[All Fields]	Defensin	29872295	Antimicrobial peptide-loaded liquid crystalline precursor bioadhesive system for the prevention of dental caries.
2882	DRAMP02417	Defensin (Ticks, Arthropods, animals)	"Defensin"[All Fields] AND biofilm[All Fields]	Defensin	29671721	The BceABRS four-component system that is essential for cell envelope stress response is involved in sensing and response to host defence peptides and is required for the biofilm formation and fitness of Streptococcus mutans.
2882	DRAMP02417	Defensin (Ticks, Arthropods, animals)	"Defensin"[All Fields] AND biofilm[All Fields]	Defensin	29104569	A Linear 19-Mer Plant Defensin-Derived Peptide Acts Synergistically with Caspofungin against Candida albicansBiofilms.
2882	DRAMP02417	Defensin (Ticks, Arthropods, animals)	"Defensin"[All Fields] AND biofilm[All Fields]	Defensin	29077172	The significance of HBD-3 and fluorescent composite carriers in the processof bone formation in rats infected with Staphylococcus aureus.
2882	DRAMP02417	Defensin (Ticks, Arthropods, animals)	"Defensin"[All Fields] AND biofilm[All Fields]	Defensin	29045084	New Approach to Treat and Prevent Oral Disease.
2882	DRAMP02417	Defensin (Ticks, Arthropods, animals)	"Defensin"[All Fields] AND biofilm[All Fields]	Defensin	29025642	Engineered chimeric peptides with antimicrobial and titanium-binding functions to inhibit biofilm formation on Ti implants.

2882	DRAMP02417	Defensin (Ticks, Arthropods, animals)	"Defensin"[All Fields] AND biofilm[All Fields]	Defensin	28956355	The synthetic human beta-defensin-3 C15 peptide exhibits antimicrobial activity against Streptococcus mutans, both alone and in combination with dental disinfectants.
2882	DRAMP02417	Defensin (Ticks, Arthropods, animals)	"Defensin"[All Fields] AND biofilm[All Fields]	Defensin	28951032	Antifungal Effects of Synthetic Human Beta-defensin-3-C15 Peptide on Candida albicans-infected Root Dentin.
2882	DRAMP02417	Defensin (Ticks, Arthropods, animals)	"Defensin"[All Fields] AND biofilm[All Fields]	Defensin	28874606	Bacterial d-amino acids suppress sinonasal innate immunity through sweet taste receptors in solitary chemosensory cells.
2882	DRAMP02417	Defensin (Ticks, Arthropods, animals)	"Defensin"[All Fields] AND biofilm[All Fields]	Defensin	28725299	Role of Streptococcus mutanstwo-component systems in antimicrobial peptide resistance in the oral cavity.
2882	DRAMP02417	Defensin (Ticks, Arthropods, animals)	"Defensin"[All Fields] AND biofilm[All Fields]	Defensin	28649561	Psd1 Effects on Candida albicansPlanktonic Cells and Biofilms.
2882	DRAMP02417	Defensin (Ticks, Arthropods, animals)	"Defensin"[All Fields] AND biofilm[All Fields]	Defensin	28642103	Chimeric analogs of human β-defensin 1 and θ-defensin disrupt pre-established bacterial biofilms.
2882	DRAMP02417	Defensin (Ticks, Arthropods, animals)	"Defensin"[All Fields] AND biofilm[All Fields]	Defensin	28413476	The mechanism of human β-defensin 3 in MRSA-induced infection of implant drug-resistant bacteria biofilm in the mouse tibial bone marrow.
2882	DRAMP02417	Defensin (Ticks, Arthropods, animals)	"Defensin"[All Fields] AND biofilm[All Fields]	Defensin	28296382	Human α-Defensin 6: A Small Peptide That Self-Assembles and Protects the Host by Entangling Microbes.
2882	DRAMP02417	Defensin (Ticks, Arthropods, animals)	"Defensin"[All Fields] AND biofilm[All Fields]	Defensin	28278280	Natural antimicrobial peptide complexes in the fighting of antibiotic resistant biofilms: Calliphora vicina medicinal maggots.
2882	DRAMP02417	Defensin (Ticks, Arthropods, animals)	"Defensin"[All Fields] AND biofilm[All Fields]	Defensin	28144375	Advancements in Diagnosing Periprosthetic Joint Infections after Total Hip and Knee Arthroplasty.
2882	DRAMP02417	Defensin (Ticks, Arthropods, animals)	"Defensin"[All Fields] AND biofilm[All Fields]	Defensin	28078813	Host defense peptide-derived privileged scaffolds for anti-infective drug discovery.
2882	DRAMP02417	Defensin (Ticks, Arthropods, animals)	"Defensin"[All Fields] AND biofilm[All Fields]	Defensin	28026958	Human α-Defensin 6 Self-Assembly Prevents Adhesion and Suppresses Virulence Traits of Candida albicans.
2882	DRAMP02417	Defensin (Ticks, Arthropods, animals)	"Defensin"[All Fields] AND biofilm[All Fields]	Defensin	27794585	A Novel Defensin-Like Peptide Associated with Two Other New Cationic Antimicrobial Peptides in Transcriptome of the Iranian Scorpion Venom.
2882	DRAMP02417	Defensin (Ticks, Arthropods, animals)	"Defensin"[All Fields] AND biofilm[All Fields]	Defensin	27777572	Role of yqiCin the Pathogenicity of Salmonellaand Innate Immune Responses of Human Intestinal Epithelium.
2882	DRAMP02417	Defensin (Ticks, Arthropods, animals)	"Defensin"[All Fields] AND biofilm[All Fields]	Defensin	27582732	Efficient Eradication of Mature Pseudomonas aeruginosa Biofilm via Controlled Delivery of Nitric Oxide Combined with Antimicrobial Peptide and Antibiotics.
2882	DRAMP02417	Defensin (Ticks, Arthropods, animals)	"Defensin"[All Fields] AND biofilm[All Fields]	Defensin	27417541	Acute appendicitis: transcript profiling of blood identifies promising biomarkers and potential underlying processes.
2882	DRAMP02417	Defensin (Ticks, Arthropods, animals)	"Defensin"[All Fields] AND biofilm[All Fields]	Defensin	27200276	Antifungal effects of synthetic human β-defensin 3-C15 peptide.
2882	DRAMP02417	Defensin (Ticks, Arthropods, animals)	"Defensin"[All Fields] AND biofilm[All Fields]	Defensin	27148195	Effect of Substance P in Staphylococcus aureus and Staphylococcus epidermidis Virulence: Implication for Skin Homeostasis.
2882	DRAMP02417	Defensin (Ticks, Arthropods, animals)	"Defensin"[All Fields] AND biofilm[All Fields]	Defensin	26861950	Antibiofilm efficacy of honey and bee-derived defensin-1 on multispecies wound biofilm.
2882	DRAMP02417	Defensin (Ticks, Arthropods, animals)	"Defensin"[All Fields] AND biofilm[All Fields]	Defensin	26592804	The radish defensins RsAFP1 and RsAFP2 act synergistically with caspofungin against Candida albicans biofilms.
2882	DRAMP02417	Defensin (Ticks, Arthropods, animals)	"Defensin"[All Fields] AND biofilm[All Fields]	Defensin	26248029	Synergistic Activity of the Plant Defensin HsAFP1 and Caspofungin against Candida albicans Biofilms and Planktonic Cultures.
2882	DRAMP02417	Defensin (Ticks, Arthropods, animals)	"Defensin"[All Fields] AND biofilm[All Fields]	Defensin	26214284	α-tocopherol decreases interleukin-1β and -6 and increases human β-defensin-1 and -2 secretion in human gingival fibroblasts stimulated with Porphyromonas gingivalis lipopolysaccharide.
2882	DRAMP02417	Defensin (Ticks, Arthropods, animals)	"Defensin"[All Fields] AND biofilm[All Fields]	Defensin	26196513	Snake Cathelicidin NA-CATH and Smaller Helical Antimicrobial Peptides Are Effective against Burkholderia thailandensis.
2882	DRAMP02417	Defensin (Ticks, Arthropods, animals)	"Defensin"[All Fields] AND biofilm[All Fields]	Defensin	26119274	Ultrasound microbubbles enhance human β-defensin 3 against biofilms.
2882	DRAMP02417	Defensin (Ticks, Arthropods, animals)	"Defensin"[All Fields] AND biofilm[All Fields]	Defensin	25862466	The Antibacterial Effects of an Antimicrobial Peptide Human β-Defensin 3 Fused with Carbohydrate-Binding Domain on Pseudomonas aeruginosa PA14.
2882	DRAMP02417	Defensin (Ticks, Arthropods, animals)	"Defensin"[All Fields] AND biofilm[All Fields]	Defensin	25808131	Expression of antimicrobial peptides and interleukin-8 during early stages of inflammation: An experimental gingivitis study.
2882	DRAMP02417	Defensin (Ticks, Arthropods, animals)	"Defensin"[All Fields] AND biofilm[All Fields]	Defensin	25806720	Antimicrobial peptides in 2014.
2882	DRAMP02417	Defensin (Ticks, Arthropods, animals)	"Defensin"[All Fields] AND biofilm[All Fields]	Defensin	25285879	Inhibition and destruction of Pseudomonas aeruginosa biofilms by antibiotics and antimicrobial peptides.
2882	DRAMP02417	Defensin (Ticks, Arthropods, animals)	"Defensin"[All Fields] AND biofilm[All Fields]	Defensin	25212593	Health- and disease-associated species clusters in complex natural biofilms determine the innate immune response in oral epithelial cells during biofilm maturation.
2882	DRAMP02417	Defensin (Ticks, Arthropods, animals)	"Defensin"[All Fields] AND biofilm[All Fields]	Defensin	24913184	Effects of human β-defensin-3 on biofilm formation-regulating genes dltB and icaA in Staphylococcus aureus.
2882	DRAMP02417	Defensin (Ticks, Arthropods, animals)	"Defensin"[All Fields] AND biofilm[All Fields]	Defensin	24340061	Inflammatory and antimicrobial responses to methicillin-resistant Staphylococcus aureus in an in vitro wound infection model.
2882	DRAMP02417	Defensin (Ticks, Arthropods, animals)	"Defensin"[All Fields] AND biofilm[All Fields]	Defensin	24240906	Adsorption study of pellicle proteins to gold, silica and titanium by quartz crystal microbalance method.
2882	DRAMP02417	Defensin (Ticks, Arthropods, animals)	"Defensin"[All Fields] AND biofilm[All Fields]	Defensin	24238461	Antibacterial efficacy of a human β-defensin-3 peptide on multispecies biofilms.
2882	DRAMP02417	Defensin (Ticks, Arthropods, animals)	"Defensin"[All Fields] AND biofilm[All Fields]	Defensin	24100890	[Use of recombinant human beta-defensin-3 to evaluate the effect of adhesion of Candida albicans on the surface of soft lining material].
2882	DRAMP02417	Defensin (Ticks, Arthropods, animals)	"Defensin"[All Fields] AND biofilm[All Fields]	Defensin	23639356	Human beta-defensin 3: a novel inhibitor of Staphylococcus-produced biofilm production. Commentary on "Human β-defensin 3 inhibits antibiotic-resistant Staphylococcus biofilm formation".
2882	DRAMP02417	Defensin (Ticks, Arthropods, animals)	"Defensin"[All Fields] AND biofilm[All Fields]	Defensin	23519963	Ultrasound-targeted microbubble destruction enhances human β-defensin 3 activity against antibiotic-resistant Staphylococcus biofilms.
2882	DRAMP02417	Defensin (Ticks, Arthropods, animals)	"Defensin"[All Fields] AND biofilm[All Fields]	Defensin	23273885	Human β-defensin 3 inhibits antibiotic-resistant Staphylococcus biofilm formation.
2882	DRAMP02417	Defensin (Ticks, Arthropods, animals)	"Defensin"[All Fields] AND biofilm[All Fields]	Defensin	23203265	Potentiation of the cytotoxic activity of copper by polyphosphate on biofilm-producing bacteria: a bioinspired approach.
2882	DRAMP02417	Defensin (Ticks, Arthropods, animals)	"Defensin"[All Fields] AND biofilm[All Fields]	Defensin	23078156	Antimicrobial efficacy of a human β-defensin-3 peptide using an Enterococcus faecalis dentine infection model.
2882	DRAMP02417	Defensin (Ticks, Arthropods, animals)	"Defensin"[All Fields] AND biofilm[All Fields]	Defensin	23053486	Synergistic effect and antibiofilm activity between the antimicrobial peptide coprisin and conventional antibiotics against opportunistic bacteria.
2882	DRAMP02417	Defensin (Ticks, Arthropods, animals)	"Defensin"[All Fields] AND biofilm[All Fields]	Defensin	22922323	Extracellular DNA within a nontypeable Haemophilus influenzae-induced biofilm binds human beta defensin-3 and reduces its antimicrobial activity.
2882	DRAMP02417	Defensin (Ticks, Arthropods, animals)	"Defensin"[All Fields] AND biofilm[All Fields]	Defensin	22855857	Retrocyclin inhibits Gardnerella vaginalis biofilm formation and toxin activity.
2882	DRAMP02417	Defensin (Ticks, Arthropods, animals)	"Defensin"[All Fields] AND biofilm[All Fields]	Defensin	22394470	Association of CiaRH with resistance of Streptococcus mutans to antimicrobial peptides in biofilms.
2882	DRAMP02417	Defensin (Ticks, Arthropods, animals)	"Defensin"[All Fields] AND biofilm[All Fields]	Defensin	22229614	Comparison of the effects of human β-defensin 3, vancomycin, and clindamycin on Staphylococcus aureus biofilm formation.
2882	DRAMP02417	Defensin (Ticks, Arthropods, animals)	"Defensin"[All Fields] AND biofilm[All Fields]	Defensin	21692631	A novel organotypic dento-epithelial culture model: effect of Fusobacterium nucleatum biofilm on B-defensin-2, -3, and LL-37 expression.
2882	DRAMP02417	Defensin (Ticks, Arthropods, animals)	"Defensin"[All Fields] AND biofilm[All Fields]	Defensin	20454633	Normal human gingival epithelial cells sense C. parapsilosis by toll-like receptors and module its pathogenesis through antimicrobial peptides and proinflammatory cytokines.
2882	DRAMP02417	Defensin (Ticks, Arthropods, animals)	"Defensin"[All Fields] AND biofilm[All Fields]	Defensin	20378008	Effect of temperature on the shift of Pseudomonas fluorescens from an environmental microorganism to a potential human pathogen.
2882	DRAMP02417	Defensin (Ticks, Arthropods, animals)	"Defensin"[All Fields] AND biofilm[All Fields]	Defensin	19961380	Gingival transcriptome patterns during induction and resolution of experimental gingivitis in humans.
2882	DRAMP02417	Defensin (Ticks, Arthropods, animals)	"Defensin"[All Fields] AND biofilm[All Fields]	Defensin	19780045	Candida famata modulates toll-like receptor, beta-defensin, and proinflammatory cytokine expression by normal human epithelial cells.
2882	DRAMP02417	Defensin (Ticks, Arthropods, animals)	"Defensin"[All Fields] AND biofilm[All Fields]	Defensin	19572896	The immune response of oral epithelial cells induced by single-species and complex naturally formed biofilms.
2882	DRAMP02417	Defensin (Ticks, Arthropods, animals)	"Defensin"[All Fields] AND biofilm[All Fields]	Defensin	19466693	Activity of antimicrobial peptides in the presence of polysaccharides produced by pulmonary pathogens.
2882	DRAMP02417	Defensin (Ticks, Arthropods, animals)	"Defensin"[All Fields] AND biofilm[All Fields]	Defensin	18954353	Treponema denticola does not induce production of common innate immune mediators from primary gingival epithelial cells.
2882	DRAMP02417	Defensin (Ticks, Arthropods, animals)	"Defensin"[All Fields] AND biofilm[All Fields]	Defensin	18173794	The stage of native biofilm formation determines the gene expression of human beta-defensin-2, psoriasin, ribonuclease 7 and inflammatory mediators: a novel approach for stimulation of keratinocytes with in situ formed biofilms.
2882	DRAMP02417	Defensin (Ticks, Arthropods, animals)	"Defensin"[All Fields] AND biofilm[All Fields]	Defensin	17434999	Functional analysis of D-alanylation of lipoteichoic acid in the probiotic strain Lactobacillus rhamnosus GG.
2882	DRAMP02417	Defensin (Ticks, Arthropods, animals)	"Defensin"[All Fields] AND biofilm[All Fields]	Defensin	15493829	Bacterial evasion of innate host defenses--the Staphylococcus aureus lesson.
2882	DRAMP02417	Defensin (Ticks, Arthropods, animals)	"Defensin"[All Fields] AND biofilm[All Fields]	Defensin	14764110	Polysaccharide intercellular adhesin (PIA) protects Staphylococcus epidermidis against major components of the human innate immune system.
2885	DRAMP02424	Defensin (Ticks, Arthropods, animals)	"Defensin"[All Fields] AND biofilm[All Fields]	Defensin	34408988	The Anti-Biofilm Efficacy of Caffeic Acid Phenethyl Ester (CAPE) In Vitroand a Murine Model of Oral Candidiasis.

2885	DRAMP02424	Defensin (Ticks, Arthropods, animals)	"Defensin"[All Fields] AND biofilm[All Fields]	Defensin	34321877	Antibacterial Effect of Honey-Derived Exosomes Containing Antimicrobial Peptides Against Oral Streptococci.
2885	DRAMP02424	Defensin (Ticks, Arthropods, animals)	"Defensin"[All Fields] AND biofilm[All Fields]	Defensin	34276631	DNA Blocks the Lethal Effect of Human Beta-Defensin 2 Against Neisseria meningitidis.
2885	DRAMP02424	Defensin (Ticks, Arthropods, animals)	"Defensin"[All Fields] AND biofilm[All Fields]	Defensin	33911935	The antibacterial activities of honey.
2885	DRAMP02424	Defensin (Ticks, Arthropods, animals)	"Defensin"[All Fields] AND biofilm[All Fields]	Defensin	33865931	Identification of a crocodylian β-defensin variant from Alligator mississippiensis with antimicrobial and antibiofilm activity.
2885	DRAMP02424	Defensin (Ticks, Arthropods, animals)	"Defensin"[All Fields] AND biofilm[All Fields]	Defensin	33586659	Identification of anti-microbial peptides and traces of microbial DNA in infrainfundibular compartments of human scalp terminal hair follicles.
2885	DRAMP02424	Defensin (Ticks, Arthropods, animals)	"Defensin"[All Fields] AND biofilm[All Fields]	Defensin	33534018	A recombinant fungal defensin-like peptide-P2 combats Streptococcus dysgalactiae and biofilms.
2885	DRAMP02424	Defensin (Ticks, Arthropods, animals)	"Defensin"[All Fields] AND biofilm[All Fields]	Defensin	33447687	Future directions of postoperative spinal implant infections.
2885	DRAMP02424	Defensin (Ticks, Arthropods, animals)	"Defensin"[All Fields] AND biofilm[All Fields]	Defensin	33420317	Curbing gastrointestinal infections by defensin fragment modifications without harming commensal microbiota.
2885	DRAMP02424	Defensin (Ticks, Arthropods, animals)	"Defensin"[All Fields] AND biofilm[All Fields]	Defensin	32867384	A Novel Peptide Antibiotic, Pro10-1D, Designed from Insect Defensin Shows Antibacterial and Anti-Inflammatory Activities in Sepsis Models.
2885	DRAMP02424	Defensin (Ticks, Arthropods, animals)	"Defensin"[All Fields] AND biofilm[All Fields]	Defensin	32858856	Candida albicansVirulence Factors and Pathogenicity for Endodontic Infections.
2885	DRAMP02424	Defensin (Ticks, Arthropods, animals)	"Defensin"[All Fields] AND biofilm[All Fields]	Defensin	32842903	Electrospun ZnO/Poly(Vinylidene Fluoride-Trifluoroethylene) Scaffolds for Lung Tissue Engineering.
2885	DRAMP02424	Defensin (Ticks, Arthropods, animals)	"Defensin"[All Fields] AND biofilm[All Fields]	Defensin	32663201	Inhibition and eradication activity of truncated α-defensin analogs against multidrug resistant uropathogenic Escherichia coli biofilm.
2885	DRAMP02424	Defensin (Ticks, Arthropods, animals)	"Defensin"[All Fields] AND biofilm[All Fields]	Defensin	32585445	Glucose effect on Candida albicans biofilm during tissue invasion.
2885	DRAMP02424	Defensin (Ticks, Arthropods, animals)	"Defensin"[All Fields] AND biofilm[All Fields]	Defensin	32522780	Controlling the Growth of the Skin Commensal Staphylococcus epidermidis Using d-Alanine Auxotrophy.
2885	DRAMP02424	Defensin (Ticks, Arthropods, animals)	"Defensin"[All Fields] AND biofilm[All Fields]	Defensin	32457749	The Antimicrobial Peptide Human Beta-Defensin 2 Inhibits Biofilm Production of Pseudomonas aeruginosaWithout Compromising Metabolic Activity.
2885	DRAMP02424	Defensin (Ticks, Arthropods, animals)	"Defensin"[All Fields] AND biofilm[All Fields]	Defensin	32439511	Antibacterial activities and mechanisms of action of a defensin from manila clam Ruditapes philippinarum.
2885	DRAMP02424	Defensin (Ticks, Arthropods, animals)	"Defensin"[All Fields] AND biofilm[All Fields]	Defensin	31933178	Effects of human β-defensin 3 fused with carbohydrate-binding domain on the function of type III secretion system in Pseudomonas aeruginosa PA14.
2885	DRAMP02424	Defensin (Ticks, Arthropods, animals)	"Defensin"[All Fields] AND biofilm[All Fields]	Defensin	31906541	Role of FAD-I in Fusobacterial Interspecies Interaction and Biofilm Formation.
2885	DRAMP02424	Defensin (Ticks, Arthropods, animals)	"Defensin"[All Fields] AND biofilm[All Fields]	Defensin	31729441	Rhesus Theta Defensin 1 Promotes Long Term Survival in Systemic Candidiasis by Host Directed Mechanisms.
2885	DRAMP02424	Defensin (Ticks, Arthropods, animals)	"Defensin"[All Fields] AND biofilm[All Fields]	Defensin	31336838	Candida albicans-Cell Interactions Activate Innate Immune Defense in Human Palate Epithelial Primary Cells via Nitric Oxide (NO) and β-Defensin 2 (hBD-2).
2885	DRAMP02424	Defensin (Ticks, Arthropods, animals)	"Defensin"[All Fields] AND biofilm[All Fields]	Defensin	31165072	Impact of the Food Additive Titanium Dioxide (E171) on Gut Microbiota-Host Interaction.
2885	DRAMP02424	Defensin (Ticks, Arthropods, animals)	"Defensin"[All Fields] AND biofilm[All Fields]	Defensin	31031739	Salt-Tolerant Antifungal and Antibacterial Activities of the Corn Defensin ZmD32.
2885	DRAMP02424	Defensin (Ticks, Arthropods, animals)	"Defensin"[All Fields] AND biofilm[All Fields]	Defensin	31025073	A recombinant fungal defensin-like peptide-P2 combats multidrug-resistant Staphylococcus aureus and biofilms.
2885	DRAMP02424	Defensin (Ticks, Arthropods, animals)	"Defensin"[All Fields] AND biofilm[All Fields]	Defensin	30659503	Lactobacillus plantarum USM8613 Aids in Wound Healing and Suppresses Staphylococcus aureus Infection at Wound Sites.
2885	DRAMP02424	Defensin (Ticks, Arthropods, animals)	"Defensin"[All Fields] AND biofilm[All Fields]	Defensin	30649289	Innate immune components affect growth and virulence traits of bacterial-vaginosis-associated and non-bacterial-vaginosis-associated Gardnerella vaginalis strains similarly.
2885	DRAMP02424	Defensin (Ticks, Arthropods, animals)	"Defensin"[All Fields] AND biofilm[All Fields]	Defensin	30376742	Alpha defensin, leukocyte esterase, C-reactive protein, and leukocyte count in synovial fluid for pre-operative diagnosis of periprosthetic infection.
2885	DRAMP02424	Defensin (Ticks, Arthropods, animals)	"Defensin"[All Fields] AND biofilm[All Fields]	Defensin	30260708	Stabilized collagen matrix dressing improves wound macrophage function and epithelialization.
2885	DRAMP02424	Defensin (Ticks, Arthropods, animals)	"Defensin"[All Fields] AND biofilm[All Fields]	Defensin	30254440	Modification of the surface of titanium with multifunctional chimeric peptides to prevent biofilm formation via inhibition of initial colonizers.
2885	DRAMP02424	Defensin (Ticks, Arthropods, animals)	"Defensin"[All Fields] AND biofilm[All Fields]	Defensin	29902560	A defensin-like antimicrobial peptide from the manila clam Ruditapes philippinarum: Investigation of the antibacterial activities and mode of action.
2885	DRAMP02424	Defensin (Ticks, Arthropods, animals)	"Defensin"[All Fields] AND biofilm[All Fields]	Defensin	29872295	Antimicrobial peptide-loaded liquid crystalline precursor bioadhesive system for the prevention of dental caries.
2885	DRAMP02424	Defensin (Ticks, Arthropods, animals)	"Defensin"[All Fields] AND biofilm[All Fields]	Defensin	29671721	The BceABRS four-component system that is essential for cell envelope stress response is involved in sensing and response to host defence peptides and is required for the biofilm formation and fitness of Streptococcus mutans.
2885	DRAMP02424	Defensin (Ticks, Arthropods, animals)	"Defensin"[All Fields] AND biofilm[All Fields]	Defensin	29104569	A Linear 19-Mer Plant Defensin-Derived Peptide Acts Synergistically with Caspofungin against Candida albicansBiofilms.
2885	DRAMP02424	Defensin (Ticks, Arthropods, animals)	"Defensin"[All Fields] AND biofilm[All Fields]	Defensin	29077172	The significance of HBD-3 and fluorescent composite carriers in the processof bone formation in rats infected with Staphylococcus aureus.
2885	DRAMP02424	Defensin (Ticks, Arthropods, animals)	"Defensin"[All Fields] AND biofilm[All Fields]	Defensin	29045084	New Approach to Treat and Prevent Oral Disease.
2885	DRAMP02424	Defensin (Ticks, Arthropods, animals)	"Defensin"[All Fields] AND biofilm[All Fields]	Defensin	29025642	Engineered chimeric peptides with antimicrobial and titanium-binding functions to inhibit biofilm formation on Ti implants.
2885	DRAMP02424	Defensin (Ticks, Arthropods, animals)	"Defensin"[All Fields] AND biofilm[All Fields]	Defensin	28956355	The synthetic human beta-defensin-3 C15 peptide exhibits antimicrobial activity against Streptococcus mutans, both alone and in combination with dental disinfectants.
2885	DRAMP02424	Defensin (Ticks, Arthropods, animals)	"Defensin"[All Fields] AND biofilm[All Fields]	Defensin	28951032	Antifungal Effects of Synthetic Human Beta-defensin-3-C15 Peptide on Candida albicans-infected Root Dentin.
2885	DRAMP02424	Defensin (Ticks, Arthropods, animals)	"Defensin"[All Fields] AND biofilm[All Fields]	Defensin	28874606	Bacterial d-amino acids suppress sinonasal innate immunity through sweet taste receptors in solitary chemosensory cells.
2885	DRAMP02424	Defensin (Ticks, Arthropods, animals)	"Defensin"[All Fields] AND biofilm[All Fields]	Defensin	28725299	Role of Streptococcus mutanstwo-component systems in antimicrobial peptide resistance in the oral cavity.
2885	DRAMP02424	Defensin (Ticks, Arthropods, animals)	"Defensin"[All Fields] AND biofilm[All Fields]	Defensin	28649561	Psd1 Effects on Candida albicansPlanktonic Cells and Biofilms.
2885	DRAMP02424	Defensin (Ticks, Arthropods, animals)	"Defensin"[All Fields] AND biofilm[All Fields]	Defensin	28642103	Chimeric analogs of human β-defensin 1 and θ-defensin disrupt pre-established bacterial biofilms.
2885	DRAMP02424	Defensin (Ticks, Arthropods, animals)	"Defensin"[All Fields] AND biofilm[All Fields]	Defensin	28413476	The mechanism of human β-defensin 3 in MRSA-induced infection of implant drug-resistant bacteria biofilm in the mouse tibial bone marrow.
2885	DRAMP02424	Defensin (Ticks, Arthropods, animals)	"Defensin"[All Fields] AND biofilm[All Fields]	Defensin	28296382	Human α-Defensin 6: A Small Peptide That Self-Assembles and Protects the Host by Entangling Microbes.
2885	DRAMP02424	Defensin (Ticks, Arthropods, animals)	"Defensin"[All Fields] AND biofilm[All Fields]	Defensin	28278280	Natural antimicrobial peptide complexes in the fighting of antibiotic resistant biofilms: Calliphora vicina medicinal maggots.
2885	DRAMP02424	Defensin (Ticks, Arthropods, animals)	"Defensin"[All Fields] AND biofilm[All Fields]	Defensin	28144375	Advancements in Diagnosing Periprosthetic Joint Infections after Total Hip and Knee Arthroplasty.
2885	DRAMP02424	Defensin (Ticks, Arthropods, animals)	"Defensin"[All Fields] AND biofilm[All Fields]	Defensin	28078813	Host defense peptide-derived privileged scaffolds for anti-infective drug discovery.
2885	DRAMP02424	Defensin (Ticks, Arthropods, animals)	"Defensin"[All Fields] AND biofilm[All Fields]	Defensin	28026958	Human α-Defensin 6 Self-Assembly Prevents Adhesion and Suppresses Virulence Traits of Candida albicans.
2885	DRAMP02424	Defensin (Ticks, Arthropods, animals)	"Defensin"[All Fields] AND biofilm[All Fields]	Defensin	27794585	A Novel Defensin-Like Peptide Associated with Two Other New Cationic Antimicrobial Peptides in Transcriptome of the Iranian Scorpion Venom.
2885	DRAMP02424	Defensin (Ticks, Arthropods, animals)	"Defensin"[All Fields] AND biofilm[All Fields]	Defensin	27777572	Role of yqiCin the Pathogenicity of Salmonellaand Innate Immune Responses of Human Intestinal Epithelium.
2885	DRAMP02424	Defensin (Ticks, Arthropods, animals)	"Defensin"[All Fields] AND biofilm[All Fields]	Defensin	27582732	Efficient Eradication of Mature Pseudomonas aeruginosa Biofilm via Controlled Delivery of Nitric Oxide Combined with Antimicrobial Peptide and Antibiotics.
2885	DRAMP02424	Defensin (Ticks, Arthropods, animals)	"Defensin"[All Fields] AND biofilm[All Fields]	Defensin	27417541	Acute appendicitis: transcript profiling of blood identifies promising biomarkers and potential underlying processes.
2885	DRAMP02424	Defensin (Ticks, Arthropods, animals)	"Defensin"[All Fields] AND biofilm[All Fields]	Defensin	27200276	Antifungal effects of synthetic human β-defensin 3-C15 peptide.
2885	DRAMP02424	Defensin (Ticks, Arthropods, animals)	"Defensin"[All Fields] AND biofilm[All Fields]	Defensin	27148195	Effect of Substance P in Staphylococcus aureus and Staphylococcus epidermidis Virulence: Implication for Skin Homeostasis.
2885	DRAMP02424	Defensin (Ticks, Arthropods, animals)	"Defensin"[All Fields] AND biofilm[All Fields]	Defensin	26861950	Antibiofilm efficacy of honey and bee-derived defensin-1 on multispecies wound biofilm.
2885	DRAMP02424	Defensin (Ticks, Arthropods, animals)	"Defensin"[All Fields] AND biofilm[All Fields]	Defensin	26592804	The radish defensins RsAFP1 and RsAFP2 act synergistically with caspofungin against Candida albicans biofilms.
2885	DRAMP02424	Defensin (Ticks, Arthropods, animals)	"Defensin"[All Fields] AND biofilm[All Fields]	Defensin	26248029	Synergistic Activity of the Plant Defensin HsAFP1 and Caspofungin against Candida albicans Biofilms and Planktonic Cultures.
2885	DRAMP02424	Defensin (Ticks, Arthropods, animals)	"Defensin"[All Fields] AND biofilm[All Fields]	Defensin	26214284	α-tocopherol decreases interleukin-1β and -6 and increases human β-defensin-1 and -2 secretion in human gingival fibroblasts stimulated with Porphyromonas gingivalis lipopolysaccharide.
2885	DRAMP02424	Defensin (Ticks, Arthropods, animals)	"Defensin"[All Fields] AND biofilm[All Fields]	Defensin	26196513	Snake Cathelicidin NA-CATH and Smaller Helical Antimicrobial Peptides Are Effective against Burkholderia thailandensis.

2885	DRAMP02424	Defensin (Ticks, Arthropods, animals)	"Defensin"[All Fields] AND biofilm[All Fields]	Defensin	26119274	Ultrasound microbubbles enhance human β-defensin 3 against biofilms.
2885	DRAMP02424	Defensin (Ticks, Arthropods, animals)	"Defensin"[All Fields] AND biofilm[All Fields]	Defensin	25862466	The Antibacterial Effects of an Antimicrobial Peptide Human β-Defensin 3 Fused with Carbohydrate-Binding Domain on Pseudomonas aeruginosa PA14.
2885	DRAMP02424	Defensin (Ticks, Arthropods, animals)	"Defensin"[All Fields] AND biofilm[All Fields]	Defensin	25808131	Expression of antimicrobial peptides and interleukin-8 during early stages of inflammation: An experimental gingivitis study.
2885	DRAMP02424	Defensin (Ticks, Arthropods, animals)	"Defensin"[All Fields] AND biofilm[All Fields]	Defensin	25806720	Antimicrobial peptides in 2014.
2885	DRAMP02424	Defensin (Ticks, Arthropods, animals)	"Defensin"[All Fields] AND biofilm[All Fields]	Defensin	25285879	Inhibition and destruction of Pseudomonas aeruginosa biofilms by antibiotics and antimicrobial peptides.
2885	DRAMP02424	Defensin (Ticks, Arthropods, animals)	"Defensin"[All Fields] AND biofilm[All Fields]	Defensin	25212593	Health- and disease-associated species clusters in complex natural biofilms determine the innate immune response in oral epithelial cells during biofilm maturation.
2885	DRAMP02424	Defensin (Ticks, Arthropods, animals)	"Defensin"[All Fields] AND biofilm[All Fields]	Defensin	24913184	Effects of human β-defensin-3 on biofilm formation-regulating genes dltB and icaA in Staphylococcus aureus.
2885	DRAMP02424	Defensin (Ticks, Arthropods, animals)	"Defensin"[All Fields] AND biofilm[All Fields]	Defensin	24340061	Inflammatory and antimicrobial responses to methicillin-resistant Staphylococcus aureus in an in vitro wound infection model.
2885	DRAMP02424	Defensin (Ticks, Arthropods, animals)	"Defensin"[All Fields] AND biofilm[All Fields]	Defensin	24240906	Adsorption study of pellicle proteins to gold, silica and titanium by quartz crystal microbalance method.
2885	DRAMP02424	Defensin (Ticks, Arthropods, animals)	"Defensin"[All Fields] AND biofilm[All Fields]	Defensin	24238461	Antibacterial efficacy of a human β-defensin-3 peptide on multispecies biofilms.
2885	DRAMP02424	Defensin (Ticks, Arthropods, animals)	"Defensin"[All Fields] AND biofilm[All Fields]	Defensin	24100890	[Use of recombinant human beta-defensin-3 to evaluate the effect of adhesion of Candida albicans on the surface of soft lining material].
2885	DRAMP02424	Defensin (Ticks, Arthropods, animals)	"Defensin"[All Fields] AND biofilm[All Fields]	Defensin	23639356	Human beta-defensin 3: a novel inhibitor of Staphylococcus-produced biofilm production. Commentary on "Human β-defensin 3 inhibits antibiotic-resistant Staphylococcus biofilm formation".
2885	DRAMP02424	Defensin (Ticks, Arthropods, animals)	"Defensin"[All Fields] AND biofilm[All Fields]	Defensin	23519963	Ultrasound-targeted microbubble destruction enhances human β-defensin 3 activity against antibiotic-resistant Staphylococcus biofilms.
2885	DRAMP02424	Defensin (Ticks, Arthropods, animals)	"Defensin"[All Fields] AND biofilm[All Fields]	Defensin	23273885	Human β-defensin 3 inhibits antibiotic-resistant Staphylococcus biofilm formation.
2885	DRAMP02424	Defensin (Ticks, Arthropods, animals)	"Defensin"[All Fields] AND biofilm[All Fields]	Defensin	23203265	Potentiation of the cytotoxic activity of copper by polyphosphate on biofilm-producing bacteria: a bioinspired approach.
2885	DRAMP02424	Defensin (Ticks, Arthropods, animals)	"Defensin"[All Fields] AND biofilm[All Fields]	Defensin	23078156	Antimicrobial efficacy of a human β-defensin-3 peptide using an Enterococcus faecalis dentine infection model.
2885	DRAMP02424	Defensin (Ticks, Arthropods, animals)	"Defensin"[All Fields] AND biofilm[All Fields]	Defensin	23053486	Synergistic effect and antibiofilm activity between the antimicrobial peptide coprisin and conventional antibiotics against opportunistic bacteria.
2885	DRAMP02424	Defensin (Ticks, Arthropods, animals)	"Defensin"[All Fields] AND biofilm[All Fields]	Defensin	22922323	Extracellular DNA within a nontypeable Haemophilus influenzae-induced biofilm binds human beta defensin-3 and reduces its antimicrobial activity.
2885	DRAMP02424	Defensin (Ticks, Arthropods, animals)	"Defensin"[All Fields] AND biofilm[All Fields]	Defensin	22855857	Retrocyclin inhibits Gardnerella vaginalis biofilm formation and toxin activity.
2885	DRAMP02424	Defensin (Ticks, Arthropods, animals)	"Defensin"[All Fields] AND biofilm[All Fields]	Defensin	22394470	Association of CiaRH with resistance of Streptococcus mutans to antimicrobial peptides in biofilms.
2885	DRAMP02424	Defensin (Ticks, Arthropods, animals)	"Defensin"[All Fields] AND biofilm[All Fields]	Defensin	22229614	Comparison of the effects of human β-defensin 3, vancomycin, and clindamycin on Staphylococcus aureus biofilm formation.
2885	DRAMP02424	Defensin (Ticks, Arthropods, animals)	"Defensin"[All Fields] AND biofilm[All Fields]	Defensin	21692631	A novel organotypic dento-epithelial culture model: effect of Fusobacterium nucleatum biofilm on B-defensin-2, -3, and LL-37 expression.
2885	DRAMP02424	Defensin (Ticks, Arthropods, animals)	"Defensin"[All Fields] AND biofilm[All Fields]	Defensin	20454633	Normal human gingival epithelial cells sense C. parapsilosis by toll-like receptors and module its pathogenesis through antimicrobial peptides and proinflammatory cytokines.
2885	DRAMP02424	Defensin (Ticks, Arthropods, animals)	"Defensin"[All Fields] AND biofilm[All Fields]	Defensin	20378008	Effect of temperature on the shift of Pseudomonas fluorescens from an environmental microorganism to a potential human pathogen.
2885	DRAMP02424	Defensin (Ticks, Arthropods, animals)	"Defensin"[All Fields] AND biofilm[All Fields]	Defensin	19961380	Gingival transcriptome patterns during induction and resolution of experimental gingivitis in humans.
2885	DRAMP02424	Defensin (Ticks, Arthropods, animals)	"Defensin"[All Fields] AND biofilm[All Fields]	Defensin	19780045	Candida famata modulates toll-like receptor, beta-defensin, and proinflammatory cytokine expression by normal human epithelial cells.
2885	DRAMP02424	Defensin (Ticks, Arthropods, animals)	"Defensin"[All Fields] AND biofilm[All Fields]	Defensin	19572896	The immune response of oral epithelial cells induced by single-species and complex naturally formed biofilms.
2885	DRAMP02424	Defensin (Ticks, Arthropods, animals)	"Defensin"[All Fields] AND biofilm[All Fields]	Defensin	19466693	Activity of antimicrobial peptides in the presence of polysaccharides produced by pulmonary pathogens.
2885	DRAMP02424	Defensin (Ticks, Arthropods, animals)	"Defensin"[All Fields] AND biofilm[All Fields]	Defensin	18954353	Treponema denticola does not induce production of common innate immune mediators from primary gingival epithelial cells.
2885	DRAMP02424	Defensin (Ticks, Arthropods, animals)	"Defensin"[All Fields] AND biofilm[All Fields]	Defensin	18173794	The stage of native biofilm formation determines the gene expression of human beta-defensin-2, psoriasin, ribonuclease 7 and inflammatory mediators: a novel approach for stimulation of keratinocytes with in situ formed biofilms.
2885	DRAMP02424	Defensin (Ticks, Arthropods, animals)	"Defensin"[All Fields] AND biofilm[All Fields]	Defensin	17434999	Functional analysis of D-alanylation of lipoteichoic acid in the probiotic strain Lactobacillus rhamnosus GG.
2885	DRAMP02424	Defensin (Ticks, Arthropods, animals)	"Defensin"[All Fields] AND biofilm[All Fields]	Defensin	15493829	Bacterial evasion of innate host defenses--the Staphylococcus aureus lesson.
2885	DRAMP02424	Defensin (Ticks, Arthropods, animals)	"Defensin"[All Fields] AND biofilm[All Fields]	Defensin	14764110	Polysaccharide intercellular adhesin (PIA) protects Staphylococcus epidermidis against major components of the human innate immune system.
2886	DRAMP02426	Defensin (Varisin A1; Ticks, Arthropods, animals)	"Defensin"[All Fields] AND biofilm[All Fields]	Defensin	34408988	The Anti-Biofilm Efficacy of Caffeic Acid Phenethyl Ester (CAPE) In Vitroand a Murine Model of Oral Candidiasis.
2886	DRAMP02426	Defensin (Varisin A1; Ticks, Arthropods, animals)	"Defensin"[All Fields] AND biofilm[All Fields]	Defensin	34321877	Antibacterial Effect of Honey-Derived Exosomes Containing Antimicrobial Peptides Against Oral Streptococci.
2886	DRAMP02426	Defensin (Varisin A1; Ticks, Arthropods, animals)	"Defensin"[All Fields] AND biofilm[All Fields]	Defensin	34276631	DNA Blocks the Lethal Effect of Human Beta-Defensin 2 Against Neisseria meningitidis.
2886	DRAMP02426	Defensin (Varisin A1; Ticks, Arthropods, animals)	"Defensin"[All Fields] AND biofilm[All Fields]	Defensin	33911935	The antibacterial activities of honey.
2886	DRAMP02426	Defensin (Varisin A1; Ticks, Arthropods, animals)	"Defensin"[All Fields] AND biofilm[All Fields]	Defensin	33865931	Identification of a crocodylian β-defensin variant from Alligator mississippiensis with antimicrobial and antibiofilm activity.
2886	DRAMP02426	Defensin (Varisin A1; Ticks, Arthropods, animals)	"Defensin"[All Fields] AND biofilm[All Fields]	Defensin	33586659	Identification of anti-microbial peptides and traces of microbial DNA in infrainfundibular compartments of human scalp terminal hair follicles.
2886	DRAMP02426	Defensin (Varisin A1; Ticks, Arthropods, animals)	"Defensin"[All Fields] AND biofilm[All Fields]	Defensin	33534018	A recombinant fungal defensin-like peptide-P2 combats Streptococcus dysgalactiae and biofilms.
2886	DRAMP02426	Defensin (Varisin A1; Ticks, Arthropods, animals)	"Defensin"[All Fields] AND biofilm[All Fields]	Defensin	33447687	Future directions of postoperative spinal implant infections.
2886	DRAMP02426	Defensin (Varisin A1; Ticks, Arthropods, animals)	"Defensin"[All Fields] AND biofilm[All Fields]	Defensin	33420317	Curbing gastrointestinal infections by defensin fragment modifications without harming commensal microbiota.
2886	DRAMP02426	Defensin (Varisin A1; Ticks, Arthropods, animals)	"Defensin"[All Fields] AND biofilm[All Fields]	Defensin	32867384	A Novel Peptide Antibiotic, Pro10-1D, Designed from Insect Defensin Shows Antibacterial and Anti-Inflammatory Activities in Sepsis Models.
2886	DRAMP02426	Defensin (Varisin A1; Ticks, Arthropods, animals)	"Defensin"[All Fields] AND biofilm[All Fields]	Defensin	32858856	Candida albicansVirulence Factors and Pathogenicity for Endodontic Infections.
2886	DRAMP02426	Defensin (Varisin A1; Ticks, Arthropods, animals)	"Defensin"[All Fields] AND biofilm[All Fields]	Defensin	32842903	Electrospun ZnO/Poly(Vinylidene Fluoride-Trifluoroethylene) Scaffolds for Lung Tissue Engineering.
2886	DRAMP02426	Defensin (Varisin A1; Ticks, Arthropods, animals)	"Defensin"[All Fields] AND biofilm[All Fields]	Defensin	32663201	Inhibition and eradication activity of truncated α-defensin analogs against multidrug resistant uropathogenic Escherichia coli biofilm.
2886	DRAMP02426	Defensin (Varisin A1; Ticks, Arthropods, animals)	"Defensin"[All Fields] AND biofilm[All Fields]	Defensin	32585445	Glucose effect on Candida albicans biofilm during tissue invasion.
2886	DRAMP02426	Defensin (Varisin A1; Ticks, Arthropods, animals)	"Defensin"[All Fields] AND biofilm[All Fields]	Defensin	32522780	Controlling the Growth of the Skin Commensal Staphylococcus epidermidis Using d-Alanine Auxotrophy.
2886	DRAMP02426	Defensin (Varisin A1; Ticks, Arthropods, animals)	"Defensin"[All Fields] AND biofilm[All Fields]	Defensin	32457749	The Antimicrobial Peptide Human Beta-Defensin 2 Inhibits Biofilm Production of Pseudomonas aeruginosaWithout Compromising Metabolic Activity.

2886	DRAMP02426	Defensin (Varisin A1; Ticks, Arthropods, animals)	"Defensin"[All Fields] AND biofilm[All Fields]	Defensin	32439511	Antibacterial activities and mechanisms of action of a defensin from manila clam Ruditapes philippinarum.
2886	DRAMP02426	Defensin (Varisin A1; Ticks, Arthropods, animals)	"Defensin"[All Fields] AND biofilm[All Fields]	Defensin	31933178	Effects of human β-defensin 3 fused with carbohydrate-binding domain on the function of type III secretion system in Pseudomonas aeruginosa PA14.
2886	DRAMP02426	Defensin (Varisin A1; Ticks, Arthropods, animals)	"Defensin"[All Fields] AND biofilm[All Fields]	Defensin	31906541	Role of FAD-I in Fusobacterial Interspecies Interaction and Biofilm Formation.
2886	DRAMP02426	Defensin (Varisin A1; Ticks, Arthropods, animals)	"Defensin"[All Fields] AND biofilm[All Fields]	Defensin	31729441	Rhesus Theta Defensin 1 Promotes Long Term Survival in Systemic Candidiasis by Host Directed Mechanisms.
2886	DRAMP02426	Defensin (Varisin A1; Ticks, Arthropods, animals)	"Defensin"[All Fields] AND biofilm[All Fields]	Defensin	31336838	Candida albicans-Cell Interactions Activate Innate Immune Defense in Human Palate Epithelial Primary Cells via Nitric Oxide (NO) and β-Defensin 2 (hBD-2).
2886	DRAMP02426	Defensin (Varisin A1; Ticks, Arthropods, animals)	"Defensin"[All Fields] AND biofilm[All Fields]	Defensin	31165072	Impact of the Food Additive Titanium Dioxide (E171) on Gut Microbiota-Host Interaction.
2886	DRAMP02426	Defensin (Varisin A1; Ticks, Arthropods, animals)	"Defensin"[All Fields] AND biofilm[All Fields]	Defensin	31031739	Salt-Tolerant Antifungal and Antibacterial Activities of the Corn Defensin ZmD32.
2886	DRAMP02426	Defensin (Varisin A1; Ticks, Arthropods, animals)	"Defensin"[All Fields] AND biofilm[All Fields]	Defensin	31025073	A recombinant fungal defensin-like peptide-P2 combats multidrug-resistant Staphylococcus aureus and biofilms.
2886	DRAMP02426	Defensin (Varisin A1; Ticks, Arthropods, animals)	"Defensin"[All Fields] AND biofilm[All Fields]	Defensin	30659503	Lactobacillus plantarum USM8613 Aids in Wound Healing and Suppresses Staphylococcus aureus Infection at Wound Sites.
2886	DRAMP02426	Defensin (Varisin A1; Ticks, Arthropods, animals)	"Defensin"[All Fields] AND biofilm[All Fields]	Defensin	30649289	Innate immune components affect growth and virulence traits of bacterial-vaginosis-associated and non-bacterial-vaginosis-associated Gardnerella vaginalis strains similarly.
2886	DRAMP02426	Defensin (Varisin A1; Ticks, Arthropods, animals)	"Defensin"[All Fields] AND biofilm[All Fields]	Defensin	30376742	Alpha defensin, leukocyte esterase, C-reactive protein, and leukocyte count in synovial fluid for pre-operative diagnosis of periprosthetic infection.
2886	DRAMP02426	Defensin (Varisin A1; Ticks, Arthropods, animals)	"Defensin"[All Fields] AND biofilm[All Fields]	Defensin	30260708	Stabilized collagen matrix dressing improves wound macrophage function and epithelialization.
2886	DRAMP02426	Defensin (Varisin A1; Ticks, Arthropods, animals)	"Defensin"[All Fields] AND biofilm[All Fields]	Defensin	30254440	Modification of the surface of titanium with multifunctional chimeric peptides to prevent biofilm formation via inhibition of initial colonizers.
2886	DRAMP02426	Defensin (Varisin A1; Ticks, Arthropods, animals)	"Defensin"[All Fields] AND biofilm[All Fields]	Defensin	29902560	A defensin-like antimicrobial peptide from the manila clam Ruditapes philippinarum: Investigation of the antibacterial activities and mode of action.
2886	DRAMP02426	Defensin (Varisin A1; Ticks, Arthropods, animals)	"Defensin"[All Fields] AND biofilm[All Fields]	Defensin	29872295	Antimicrobial peptide-loaded liquid crystalline precursor bioadhesive system for the prevention of dental caries.
2886	DRAMP02426	Defensin (Varisin A1; Ticks, Arthropods, animals)	"Defensin"[All Fields] AND biofilm[All Fields]	Defensin	29671721	The BceABRS four-component system that is essential for cell envelope stress response is involved in sensing and response to host defence peptides and is required for the biofilm formation and fitness of Streptococcus mutans.
2886	DRAMP02426	Defensin (Varisin A1; Ticks, Arthropods, animals)	"Defensin"[All Fields] AND biofilm[All Fields]	Defensin	29104569	A Linear 19-Mer Plant Defensin-Derived Peptide Acts Synergistically with Caspofungin against Candida albicansBiofilms.
2886	DRAMP02426	Defensin (Varisin A1; Ticks, Arthropods, animals)	"Defensin"[All Fields] AND biofilm[All Fields]	Defensin	29077172	The significance of HBD-3 and fluorescent composite carriers in the processof bone formation in rats infected with Staphylococcus aureus.
2886	DRAMP02426	Defensin (Varisin A1; Ticks, Arthropods, animals)	"Defensin"[All Fields] AND biofilm[All Fields]	Defensin	29045084	New Approach to Treat and Prevent Oral Disease.
2886	DRAMP02426	Defensin (Varisin A1; Ticks, Arthropods, animals)	"Defensin"[All Fields] AND biofilm[All Fields]	Defensin	29025642	Engineered chimeric peptides with antimicrobial and titanium-binding functions to inhibit biofilm formation on Ti implants.
2886	DRAMP02426	Defensin (Varisin A1; Ticks, Arthropods, animals)	"Defensin"[All Fields] AND biofilm[All Fields]	Defensin	28956355	The synthetic human beta-defensin-3 C15 peptide exhibits antimicrobial activity against Streptococcus mutans, both alone and in combination with dental disinfectants.
2886	DRAMP02426	Defensin (Varisin A1; Ticks, Arthropods, animals)	"Defensin"[All Fields] AND biofilm[All Fields]	Defensin	28951032	Antifungal Effects of Synthetic Human Beta-defensin-3-C15 Peptide on Candida albicans-infected Root Dentin.
2886	DRAMP02426	Defensin (Varisin A1; Ticks, Arthropods, animals)	"Defensin"[All Fields] AND biofilm[All Fields]	Defensin	28874606	Bacterial d-amino acids suppress sinonasal innate immunity through sweet taste receptors in solitary chemosensory cells.
2886	DRAMP02426	Defensin (Varisin A1; Ticks, Arthropods, animals)	"Defensin"[All Fields] AND biofilm[All Fields]	Defensin	28725299	Role of Streptococcus mutanstwo-component systems in antimicrobial peptide resistance in the oral cavity.
2886	DRAMP02426	Defensin (Varisin A1; Ticks, Arthropods, animals)	"Defensin"[All Fields] AND biofilm[All Fields]	Defensin	28649561	Psd1 Effects on Candida albicansPlanktonic Cells and Biofilms.
2886	DRAMP02426	Defensin (Varisin A1; Ticks, Arthropods, animals)	"Defensin"[All Fields] AND biofilm[All Fields]	Defensin	28642103	Chimeric analogs of human β-defensin 1 and θ-defensin disrupt pre-established bacterial biofilms.
2886	DRAMP02426	Defensin (Varisin A1; Ticks, Arthropods, animals)	"Defensin"[All Fields] AND biofilm[All Fields]	Defensin	28413476	The mechanism of human β-defensin 3 in MRSA-induced infection of implant drug-resistant bacteria biofilm in the mouse tibial bone marrow.
2886	DRAMP02426	Defensin (Varisin A1; Ticks, Arthropods, animals)	"Defensin"[All Fields] AND biofilm[All Fields]	Defensin	28296382	Human α-Defensin 6: A Small Peptide That Self-Assembles and Protects the Host by Entangling Microbes.
2886	DRAMP02426	Defensin (Varisin A1; Ticks, Arthropods, animals)	"Defensin"[All Fields] AND biofilm[All Fields]	Defensin	28278280	Natural antimicrobial peptide complexes in the fighting of antibiotic resistant biofilms: Calliphora vicina medicinal maggots.
2886	DRAMP02426	Defensin (Varisin A1; Ticks, Arthropods, animals)	"Defensin"[All Fields] AND biofilm[All Fields]	Defensin	28144375	Advancements in Diagnosing Periprosthetic Joint Infections after Total Hip and Knee Arthroplasty.
2886	DRAMP02426	Defensin (Varisin A1; Ticks, Arthropods, animals)	"Defensin"[All Fields] AND biofilm[All Fields]	Defensin	28078813	Host defense peptide-derived privileged scaffolds for anti-infective drug discovery.
2886	DRAMP02426	Defensin (Varisin A1; Ticks, Arthropods, animals)	"Defensin"[All Fields] AND biofilm[All Fields]	Defensin	28026958	Human α-Defensin 6 Self-Assembly Prevents Adhesion and Suppresses Virulence Traits of Candida albicans.
2886	DRAMP02426	Defensin (Varisin A1; Ticks, Arthropods, animals)	"Defensin"[All Fields] AND biofilm[All Fields]	Defensin	27794585	A Novel Defensin-Like Peptide Associated with Two Other New Cationic Antimicrobial Peptides in Transcriptome of the Iranian Scorpion Venom.
2886	DRAMP02426	Defensin (Varisin A1; Ticks, Arthropods, animals)	"Defensin"[All Fields] AND biofilm[All Fields]	Defensin	27777572	Role of yqiCin the Pathogenicity of Salmonellaand Innate Immune Responses of Human Intestinal Epithelium.
2886	DRAMP02426	Defensin (Varisin A1; Ticks, Arthropods, animals)	"Defensin"[All Fields] AND biofilm[All Fields]	Defensin	27582732	Efficient Eradication of Mature Pseudomonas aeruginosa Biofilm via Controlled Delivery of Nitric Oxide Combined with Antimicrobial Peptide and Antibiotics.
2886	DRAMP02426	Defensin (Varisin A1; Ticks, Arthropods, animals)	"Defensin"[All Fields] AND biofilm[All Fields]	Defensin	27417541	Acute appendicitis: transcript profiling of blood identifies promising biomarkers and potential underlying processes.
2886	DRAMP02426	Defensin (Varisin A1; Ticks, Arthropods, animals)	"Defensin"[All Fields] AND biofilm[All Fields]	Defensin	27200276	Antifungal effects of synthetic human β-defensin 3-C15 peptide.
2886	DRAMP02426	Defensin (Varisin A1; Ticks, Arthropods, animals)	"Defensin"[All Fields] AND biofilm[All Fields]	Defensin	27148195	Effect of Substance P in Staphylococcus aureus and Staphylococcus epidermidis Virulence: Implication for Skin Homeostasis.
2886	DRAMP02426	Defensin (Varisin A1; Ticks, Arthropods, animals)	"Defensin"[All Fields] AND biofilm[All Fields]	Defensin	26861950	Antibiofilm efficacy of honey and bee-derived defensin-1 on multispecies wound biofilm.

2886	DRAMP02426	Defensin (Varisin A1; Ticks, Arthropods, animals)	"Defensin"[All Fields] AND biofilm[All Fields]	Defensin	26592804	The radish defensins RsAFP1 and RsAFP2 act synergistically with caspofungin against Candida albicans biofilms.
2886	DRAMP02426	Defensin (Varisin A1; Ticks, Arthropods, animals)	"Defensin"[All Fields] AND biofilm[All Fields]	Defensin	26248029	Synergistic Activity of the Plant Defensin HsAFP1 and Caspofungin against Candida albicans Biofilms and Planktonic Cultures.
2886	DRAMP02426	Defensin (Varisin A1; Ticks, Arthropods, animals)	"Defensin"[All Fields] AND biofilm[All Fields]	Defensin	26214284	α-tocopherol decreases interleukin-1β and -6 and increases human β-defensin-1 and -2 secretion in human gingival fibroblasts stimulated with Porphyromonas gingivalis lipopolysaccharide.
2886	DRAMP02426	Defensin (Varisin A1; Ticks, Arthropods, animals)	"Defensin"[All Fields] AND biofilm[All Fields]	Defensin	26196513	Snake Cathelicidin NA-CATH and Smaller Helical Antimicrobial Peptides Are Effective against Burkholderia thailandensis.
2886	DRAMP02426	Defensin (Varisin A1; Ticks, Arthropods, animals)	"Defensin"[All Fields] AND biofilm[All Fields]	Defensin	26119274	Ultrasound microbubbles enhance human β-defensin 3 against biofilms.
2886	DRAMP02426	Defensin (Varisin A1; Ticks, Arthropods, animals)	"Defensin"[All Fields] AND biofilm[All Fields]	Defensin	25862466	The Antibacterial Effects of an Antimicrobial Peptide Human β-Defensin 3 Fused with Carbohydrate-Binding Domain on Pseudomonas aeruginosa PA14.
2886	DRAMP02426	Defensin (Varisin A1; Ticks, Arthropods, animals)	"Defensin"[All Fields] AND biofilm[All Fields]	Defensin	25808131	Expression of antimicrobial peptides and interleukin-8 during early stages of inflammation: An experimental gingivitis study.
2886	DRAMP02426	Defensin (Varisin A1; Ticks, Arthropods, animals)	"Defensin"[All Fields] AND biofilm[All Fields]	Defensin	25806720	Antimicrobial peptides in 2014.
2886	DRAMP02426	Defensin (Varisin A1; Ticks, Arthropods, animals)	"Defensin"[All Fields] AND biofilm[All Fields]	Defensin	25285879	Inhibition and destruction of Pseudomonas aeruginosa biofilms by antibiotics and antimicrobial peptides.
2886	DRAMP02426	Defensin (Varisin A1; Ticks, Arthropods, animals)	"Defensin"[All Fields] AND biofilm[All Fields]	Defensin	25212593	Health- and disease-associated species clusters in complex natural biofilms determine the innate immune response in oral epithelial cells during biofilm maturation.
2886	DRAMP02426	Defensin (Varisin A1; Ticks, Arthropods, animals)	"Defensin"[All Fields] AND biofilm[All Fields]	Defensin	24913184	Effects of human β-defensin-3 on biofilm formation-regulating genes dltB and icaA in Staphylococcus aureus.
2886	DRAMP02426	Defensin (Varisin A1; Ticks, Arthropods, animals)	"Defensin"[All Fields] AND biofilm[All Fields]	Defensin	24340061	Inflammatory and antimicrobial responses to methicillin-resistant Staphylococcus aureus in an in vitro wound infection model.
2886	DRAMP02426	Defensin (Varisin A1; Ticks, Arthropods, animals)	"Defensin"[All Fields] AND biofilm[All Fields]	Defensin	24240906	Adsorption study of pellicle proteins to gold, silica and titanium by quartz crystal microbalance method.
2886	DRAMP02426	Defensin (Varisin A1; Ticks, Arthropods, animals)	"Defensin"[All Fields] AND biofilm[All Fields]	Defensin	24238461	Antibacterial efficacy of a human β-defensin-3 peptide on multispecies biofilms.
2886	DRAMP02426	Defensin (Varisin A1; Ticks, Arthropods, animals)	"Defensin"[All Fields] AND biofilm[All Fields]	Defensin	24100890	[Use of recombinant human beta-defensin-3 to evaluate the effect of adhesion of Candida albicans on the surface of soft lining material].
2886	DRAMP02426	Defensin (Varisin A1; Ticks, Arthropods, animals)	"Defensin"[All Fields] AND biofilm[All Fields]	Defensin	23639356	Human beta-defensin 3: a novel inhibitor of Staphylococcus-produced biofilm production. Commentary on "Human β-defensin 3 inhibits antibiotic-resistant Staphylococcus biofilm formation".
2886	DRAMP02426	Defensin (Varisin A1; Ticks, Arthropods, animals)	"Defensin"[All Fields] AND biofilm[All Fields]	Defensin	23519963	Ultrasound-targeted microbubble destruction enhances human β-defensin 3 activity against antibiotic-resistant Staphylococcus biofilms.
2886	DRAMP02426	Defensin (Varisin A1; Ticks, Arthropods, animals)	"Defensin"[All Fields] AND biofilm[All Fields]	Defensin	23273885	Human β-defensin 3 inhibits antibiotic-resistant Staphylococcus biofilm formation.
2886	DRAMP02426	Defensin (Varisin A1; Ticks, Arthropods, animals)	"Defensin"[All Fields] AND biofilm[All Fields]	Defensin	23203265	Potentiation of the cytotoxic activity of copper by polyphosphate on biofilm-producing bacteria: a bioinspired approach.
2886	DRAMP02426	Defensin (Varisin A1; Ticks, Arthropods, animals)	"Defensin"[All Fields] AND biofilm[All Fields]	Defensin	23078156	Antimicrobial efficacy of a human β-defensin-3 peptide using an Enterococcus faecalis dentine infection model.
2886	DRAMP02426	Defensin (Varisin A1; Ticks, Arthropods, animals)	"Defensin"[All Fields] AND biofilm[All Fields]	Defensin	23053486	Synergistic effect and antibiofilm activity between the antimicrobial peptide coprisin and conventional antibiotics against opportunistic bacteria.
2886	DRAMP02426	Defensin (Varisin A1; Ticks, Arthropods, animals)	"Defensin"[All Fields] AND biofilm[All Fields]	Defensin	22922323	Extracellular DNA within a nontypeable Haemophilus influenzae-induced biofilm binds human beta defensin-3 and reduces its antimicrobial activity.
2886	DRAMP02426	Defensin (Varisin A1; Ticks, Arthropods, animals)	"Defensin"[All Fields] AND biofilm[All Fields]	Defensin	22855857	Retrocyclin inhibits Gardnerella vaginalis biofilm formation and toxin activity.
2886	DRAMP02426	Defensin (Varisin A1; Ticks, Arthropods, animals)	"Defensin"[All Fields] AND biofilm[All Fields]	Defensin	22394470	Association of CiaRH with resistance of Streptococcus mutans to antimicrobial peptides in biofilms.
2886	DRAMP02426	Defensin (Varisin A1; Ticks, Arthropods, animals)	"Defensin"[All Fields] AND biofilm[All Fields]	Defensin	22229614	Comparison of the effects of human β-defensin 3, vancomycin, and clindamycin on Staphylococcus aureus biofilm formation.
2886	DRAMP02426	Defensin (Varisin A1; Ticks, Arthropods, animals)	"Defensin"[All Fields] AND biofilm[All Fields]	Defensin	21692631	A novel organotypic dento-epithelial culture model: effect of Fusobacterium nucleatum biofilm on B-defensin-2, -3, and LL-37 expression.
2886	DRAMP02426	Defensin (Varisin A1; Ticks, Arthropods, animals)	"Defensin"[All Fields] AND biofilm[All Fields]	Defensin	20454633	Normal human gingival epithelial cells sense C. parapsilosis by toll-like receptors and module its pathogenesis through antimicrobial peptides and proinflammatory cytokines.
2886	DRAMP02426	Defensin (Varisin A1; Ticks, Arthropods, animals)	"Defensin"[All Fields] AND biofilm[All Fields]	Defensin	20378008	Effect of temperature on the shift of Pseudomonas fluorescens from an environmental microorganism to a potential human pathogen.
2886	DRAMP02426	Defensin (Varisin A1; Ticks, Arthropods, animals)	"Defensin"[All Fields] AND biofilm[All Fields]	Defensin	19961380	Gingival transcriptome patterns during induction and resolution of experimental gingivitis in humans.
2886	DRAMP02426	Defensin (Varisin A1; Ticks, Arthropods, animals)	"Defensin"[All Fields] AND biofilm[All Fields]	Defensin	19780045	Candida famata modulates toll-like receptor, beta-defensin, and proinflammatory cytokine expression by normal human epithelial cells.
2886	DRAMP02426	Defensin (Varisin A1; Ticks, Arthropods, animals)	"Defensin"[All Fields] AND biofilm[All Fields]	Defensin	19572896	The immune response of oral epithelial cells induced by single-species and complex naturally formed biofilms.
2886	DRAMP02426	Defensin (Varisin A1; Ticks, Arthropods, animals)	"Defensin"[All Fields] AND biofilm[All Fields]	Defensin	19466693	Activity of antimicrobial peptides in the presence of polysaccharides produced by pulmonary pathogens.
2886	DRAMP02426	Defensin (Varisin A1; Ticks, Arthropods, animals)	"Defensin"[All Fields] AND biofilm[All Fields]	Defensin	18954353	Treponema denticola does not induce production of common innate immune mediators from primary gingival epithelial cells.
2886	DRAMP02426	Defensin (Varisin A1; Ticks, Arthropods, animals)	"Defensin"[All Fields] AND biofilm[All Fields]	Defensin	18173794	The stage of native biofilm formation determines the gene expression of human beta-defensin-2, psoriasin, ribonuclease 7 and inflammatory mediators: a novel approach for stimulation of keratinocytes with in situ formed biofilms.
2886	DRAMP02426	Defensin (Varisin A1; Ticks, Arthropods, animals)	"Defensin"[All Fields] AND biofilm[All Fields]	Defensin	17434999	Functional analysis of D-alanylation of lipoteichoic acid in the probiotic strain Lactobacillus rhamnosus GG.
2886	DRAMP02426	Defensin (Varisin A1; Ticks, Arthropods, animals)	"Defensin"[All Fields] AND biofilm[All Fields]	Defensin	15493829	Bacterial evasion of innate host defenses--the Staphylococcus aureus lesson.
2886	DRAMP02426	Defensin (Varisin A1; Ticks, Arthropods, animals)	"Defensin"[All Fields] AND biofilm[All Fields]	Defensin	14764110	Polysaccharide intercellular adhesin (PIA) protects Staphylococcus epidermidis against major components of the human innate immune system.
2888	DRAMP02435	Antifungal protein (PgAFP; Cys-rich)	"Antifungal protein"[All Fields] AND biofilm[All Fields]	Antifungal protein	33466640	The Neosartorya fischeriAntifungal Protein 2 (NFAP2): A New Potential Weapon against Multidrug-Resistant Candida aurisBiofilms.
2888	DRAMP02435	Antifungal protein (PgAFP; Cys-rich)	"Antifungal protein"[All Fields] AND biofilm[All Fields]	Antifungal protein	32824977	The Penicillium chrysogenumQ176 Antimicrobial Protein PAFC Effectively Inhibits the Growth of the Opportunistic Human Pathogen Candida albicans.
2888	DRAMP02435	Antifungal protein (PgAFP; Cys-rich)	"Antifungal protein"[All Fields] AND biofilm[All Fields]	Antifungal protein	30478163	In VivoApplicability of Neosartorya fischeri Antifungal Protein 2 (NFAP2) in Treatment of Vulvovaginal Candidiasis.

2888	DRAMP02435	Antifungal protein (PgAFP; Cys-rich)	"Antifungal protein"[All Fields] AND biofilm[All Fields]	Antifungal protein	30079061	The Evolutionary Conserved γ-Core Motif Influences the Anti- CandidaActivity of the Penicillium chrysogenumAntifungal Protein PAF.
2888	DRAMP02435	Antifungal protein (PgAFP; Cys-rich)	"Antifungal protein"[All Fields] AND biofilm[All Fields]	Antifungal protein	28120548	DS6: anticandidal, antibiofilm peptide against Candida tropicalis and exhibit synergy with commercial drug.
2889	DRAMP02436	Chitinase	"Chitinase"[All Fields] AND biofilm[All Fields]	Chitinase	34617517	Quorum quenching Bacillus spp.: an alternative biocontrol agent for Vibrio harveyi infection in aquaculture.
2889	DRAMP02436	Chitinase	"Chitinase"[All Fields] AND biofilm[All Fields]	Chitinase	34011069	Exploring biomarkers associated with deteriorating vascular health using a targeted proteomics chip: The SABPA study.
2889	DRAMP02436	Chitinase	"Chitinase"[All Fields] AND biofilm[All Fields]	Chitinase	33932548	Sesamin and sesamolin rescues Caenorhabditis elegans from Pseudomonas aeruginosa infection through the attenuation of quorum sensing regulated virulence factors.
2889	DRAMP02436	Chitinase	"Chitinase"[All Fields] AND biofilm[All Fields]	Chitinase	33878330	Virulence properties and pathogenicity of multidrug-resistant Vibrio harveyi associated with luminescent vibriosis in pacific white shrimp, Penaeus vannamei.
2889	DRAMP02436	Chitinase	"Chitinase"[All Fields] AND biofilm[All Fields]	Chitinase	33775762	Synergistic effect of carboxymethylcellulose and Cryptococcus laurentii on suppressing green mould of postharvest grapefruit and its mechanism.
2889	DRAMP02436	Chitinase	"Chitinase"[All Fields] AND biofilm[All Fields]	Chitinase	33673118	Chitin Degradation Machinery and Secondary Metabolite Profiles in the Marine Bacterium Pseudoalteromonas rubraS4059.
2889	DRAMP02436	Chitinase	"Chitinase"[All Fields] AND biofilm[All Fields]	Chitinase	33460747	Attenuation of quorum sensing mediated virulence factors production and biofilm formation in Pseudomonas aeruginosa PAO1 by Colletotrichum gloeosporioides HM3.
2889	DRAMP02436	Chitinase	"Chitinase"[All Fields] AND biofilm[All Fields]	Chitinase	33342053	Antibacterial effects of the lectin from pomegranate sarcotesta (PgTeL) against Listeria monocytogenes.
2889	DRAMP02436	Chitinase	"Chitinase"[All Fields] AND biofilm[All Fields]	Chitinase	33327475	Control of Penicillium glabrumby Indigenous Antagonistic Yeast from Vineyards.
2889	DRAMP02436	Chitinase	"Chitinase"[All Fields] AND biofilm[All Fields]	Chitinase	33250645	Siphonocholin isolated from red sea sponge Siphonochalina siphonellaattenuates quorum sensing controlled virulence and biofilm formation.
2889	DRAMP02436	Chitinase	"Chitinase"[All Fields] AND biofilm[All Fields]	Chitinase	33078238	Environmental Adaptations of an Extremely Plant Beneficial Bacillus subtilis Dcl1 Identified Through the Genomic and Metabolomic Analysis.
2889	DRAMP02436	Chitinase	"Chitinase"[All Fields] AND biofilm[All Fields]	Chitinase	32889593	Effect of kitasamycin and nitrofurantoin at subinhibitory concentrations on quorum sensing regulated traits of Chromobacterium violaceum.
2889	DRAMP02436	Chitinase	"Chitinase"[All Fields] AND biofilm[All Fields]	Chitinase	32711114	Seagrass Halodule pinifolia active constituent 4-methoxybenzioic acid (4-MBA) inhibits quorum sensing mediated virulence production of Pseudomonas aeruginosa.
2889	DRAMP02436	Chitinase	"Chitinase"[All Fields] AND biofilm[All Fields]	Chitinase	32406870	Vibrio harveyi virulence gene expression in vitro and in vivo during infection in black tiger shrimp Penaeus monodon.
2889	DRAMP02436	Chitinase	"Chitinase"[All Fields] AND biofilm[All Fields]	Chitinase	32248442	Quorum sensing inhibition and tobramycin acceleration in Chromobacterium violaceum by two natural cinnamic acid derivatives.
2889	DRAMP02436	Chitinase	"Chitinase"[All Fields] AND biofilm[All Fields]	Chitinase	32089576	Phomopsis tersaas Inhibitor of Quorum Sensing System and Biofilm Forming Ability of Pseudomonas aeruginosa.
2889	DRAMP02436	Chitinase	"Chitinase"[All Fields] AND biofilm[All Fields]	Chitinase	32056440	Proteome Wide Profiling of N-ε-Lysine Acetylation Reveals a Novel Mechanism of Regulation of the Chitinase Activity in Francisella novicida.
2889	DRAMP02436	Chitinase	"Chitinase"[All Fields] AND biofilm[All Fields]	Chitinase	31986566	Inhibition of Microbial Quorum Sensing Mediated Virulence Factors by Pestalotiopsis sydowiana.
2889	DRAMP02436	Chitinase	"Chitinase"[All Fields] AND biofilm[All Fields]	Chitinase	31948616	Biocontrol ability and action mechanism of Metschnikowia citriensis against Geotrichum citri-aurantii causing sour rot of postharvest citrus fruit.
2889	DRAMP02436	Chitinase	"Chitinase"[All Fields] AND biofilm[All Fields]	Chitinase	31821969	Functional characterization of potential PGPR exhibiting broad-spectrum antifungal activity.
2889	DRAMP02436	Chitinase	"Chitinase"[All Fields] AND biofilm[All Fields]	Chitinase	31781986	Nectar- and stigma exudate-specific expression of an acidic chitinase could partially protect certain apple cultivars against fire blight disease.
2889	DRAMP02436	Chitinase	"Chitinase"[All Fields] AND biofilm[All Fields]	Chitinase	31644930	Anti-quorum sensing and antibiofilm activities of Blastobotrys parvus PPR3 against Pseudomonas aeruginosa PAO1.
2889	DRAMP02436	Chitinase	"Chitinase"[All Fields] AND biofilm[All Fields]	Chitinase	31637724	Potential modes of action of Pseudomonas fluorescens ZX during biocontrol of blue mold decay on postharvest citrus.
2889	DRAMP02436	Chitinase	"Chitinase"[All Fields] AND biofilm[All Fields]	Chitinase	31632218	Inhibitory Abilities of BacillusIsolates and Their Culture Filtrates against the Gray Mold Caused by Botrytis cinereaon Postharvest Fruit.
2889	DRAMP02436	Chitinase	"Chitinase"[All Fields] AND biofilm[All Fields]	Chitinase	31437576	Contribution of chitooligosaccharides to biofilm formation, antibiotics resistance and disinfectants tolerance of Listeria monocytogenes.
2889	DRAMP02436	Chitinase	"Chitinase"[All Fields] AND biofilm[All Fields]	Chitinase	31408741	Antifungal activity of volatile compounds produced by Staphylococcus sciuri strain MarR44 and its potential for the biocontrol of Colletotrichum nymphaeae, causal agent strawberry anthracnose.
2889	DRAMP02436	Chitinase	"Chitinase"[All Fields] AND biofilm[All Fields]	Chitinase	31372941	Salivary Total Protease Activity Based on a Broad-Spectrum Fluorescence Resonance Energy Transfer Approach to Monitor Induction and Resolution of Gingival Inflammation.
2889	DRAMP02436	Chitinase	"Chitinase"[All Fields] AND biofilm[All Fields]	Chitinase	31325898	Chitin biomass powered microbial fuel cell for electricity production using halophilic Bacillus circulans BBL03 isolated from sea salt harvesting area.
2889	DRAMP02436	Chitinase	"Chitinase"[All Fields] AND biofilm[All Fields]	Chitinase	31015941	Modular genetic design of multi-domain functional amyloids: insights into self-assembly and functional properties.
2889	DRAMP02436	Chitinase	"Chitinase"[All Fields] AND biofilm[All Fields]	Chitinase	30959097	Mosloflavone attenuates the quorum sensing controlled virulence phenotypes and biofilm formation in Pseudomonas aeruginosa PAO1: In vitro, in vivo and in silico approach.
2889	DRAMP02436	Chitinase	"Chitinase"[All Fields] AND biofilm[All Fields]	Chitinase	30410871	Seed Extract of Psoralea corylifoliaand Its Constituent Bakuchiol Impairs AHL-Based Quorum Sensing and Biofilm Formation in Food- and Human-Related Pathogens.
2889	DRAMP02436	Chitinase	"Chitinase"[All Fields] AND biofilm[All Fields]	Chitinase	30255599	Trichosporon jirovecii infection of red swamp crayfish (Procambarus clarkii).
2889	DRAMP02436	Chitinase	"Chitinase"[All Fields] AND biofilm[All Fields]	Chitinase	30227935	Anti-quorum sensing and antibiofilm potential of Alternaria alternata, a foliar endophyte of Carica papaya, evidenced by QS assays and in-silico analysis.
2889	DRAMP02436	Chitinase	"Chitinase"[All Fields] AND biofilm[All Fields]	Chitinase	30199317	Identification and comprehensive evaluation of a novel biocontrol agent Bacillus atrophaeus JZB120050.
2889	DRAMP02436	Chitinase	"Chitinase"[All Fields] AND biofilm[All Fields]	Chitinase	30011022	MouR controls the expression of the Listeria monocytogenes Agr system and mediates virulence.
2889	DRAMP02436	Chitinase	"Chitinase"[All Fields] AND biofilm[All Fields]	Chitinase	29951860	Diallyl disulfide from garlic oil inhibits Pseudomonas aeruginosa virulence factors by inactivating key quorum sensing genes.
2889	DRAMP02436	Chitinase	"Chitinase"[All Fields] AND biofilm[All Fields]	Chitinase	29745728	Aspergillus ochraceopetaliformis SSP13 modulates quorum sensing regulated virulence and biofilm formation in Pseudomonas aeruginosa PAO1.
2889	DRAMP02436	Chitinase	"Chitinase"[All Fields] AND biofilm[All Fields]	Chitinase	29571725	Attenuation of quorum sensing regulated virulence and biofilm development in Pseudomonas aeruginosa PAO1 by Diaporthe phaseolorum SSP12.
2889	DRAMP02436	Chitinase	"Chitinase"[All Fields] AND biofilm[All Fields]	Chitinase	29466418	Evaluation of the biocontrol efficacy of a Serratia marcescens strain indigenous to tea rhizosphere for the management of root rot disease in tea.
2889	DRAMP02436	Chitinase	"Chitinase"[All Fields] AND biofilm[All Fields]	Chitinase	29327645	Identification and characterization of chitinolytic bacteria isolated from a freshwater lake.
2889	DRAMP02436	Chitinase	"Chitinase"[All Fields] AND biofilm[All Fields]	Chitinase	29167801	Breaking the bad: Bacillusblocks fungal virulence factors.
2889	DRAMP02436	Chitinase	"Chitinase"[All Fields] AND biofilm[All Fields]	Chitinase	29051980	Genomic Analysis of Bacillus sp. Strain B25, a Biocontrol Agent of Maize Pathogen Fusarium verticillioides.
2889	DRAMP02436	Chitinase	"Chitinase"[All Fields] AND biofilm[All Fields]	Chitinase	28974618	Disarming Fungal Pathogens: Bacillus safensisInhibits Virulence Factor Production and Biofilm Formation by Cryptococcus neoformansand Candida albicans.
2889	DRAMP02436	Chitinase	"Chitinase"[All Fields] AND biofilm[All Fields]	Chitinase	28895513	Cadmium ion inhibition of quorum signalling in Chromobacterium violaceum.
2889	DRAMP02436	Chitinase	"Chitinase"[All Fields] AND biofilm[All Fields]	Chitinase	28738346	Synergistic Activity of Berberine with Azithromycin against Pseudomonas Aeruginosa Isolated from Patients with Cystic Fibrosis of Lung In Vitro and In Vivo.
2889	DRAMP02436	Chitinase	"Chitinase"[All Fields] AND biofilm[All Fields]	Chitinase	28512464	Insights into the Mechanism of Proliferation on the Special Microbes Mediated by Phenolic Acids in the Radix pseudostellariaeRhizosphere under Continuous Monoculture Regimes.
2889	DRAMP02436	Chitinase	"Chitinase"[All Fields] AND biofilm[All Fields]	Chitinase	28484444	Leaf Extracts of Mangifera indicaL. Inhibit Quorum Sensing - Regulated Production of Virulence Factors and Biofilm in Test Bacteria.
2889	DRAMP02436	Chitinase	"Chitinase"[All Fields] AND biofilm[All Fields]	Chitinase	27498507	In vitro evaluation of Pseudomonas bacterial isolates from rice phylloplane for biocontrol of Rhizoctonia solani and plant growth promoting traits.
2889	DRAMP02436	Chitinase	"Chitinase"[All Fields] AND biofilm[All Fields]	Chitinase	27129367	Paenibacillus arachidis sp. nov., isolated from groundnut seeds.
2889	DRAMP02436	Chitinase	"Chitinase"[All Fields] AND biofilm[All Fields]	Chitinase	26896140	Mechanisms of Bacterial (Serratia marcescens) Attachment to, Migration along, and Killing of Fungal Hyphae.
2889	DRAMP02436	Chitinase	"Chitinase"[All Fields] AND biofilm[All Fields]	Chitinase	26453650	Adaptations of the Secretome of Candida albicans in Response to Host-Related Environmental Conditions.
2889	DRAMP02436	Chitinase	"Chitinase"[All Fields] AND biofilm[All Fields]	Chitinase	26231649	Burkholderia Diffusible Signal Factor Signals to Francisella novicida To Disperse Biofilm and Increase Siderophore Production.
2889	DRAMP02436	Chitinase	"Chitinase"[All Fields] AND biofilm[All Fields]	Chitinase	26000026	Trigonella foenum-graceum (Seed) Extract Interferes with Quorum Sensing Regulated Traits and Biofilm Formation in the Strains of Pseudomonas aeruginosa and Aeromonas hydrophila.
2889	DRAMP02436	Chitinase	"Chitinase"[All Fields] AND biofilm[All Fields]	Chitinase	25722489	Influence of quorum sensing in multiple phenotypes of the bacterial pathogen Chromobacterium violaceum.
2889	DRAMP02436	Chitinase	"Chitinase"[All Fields] AND biofilm[All Fields]	Chitinase	27259220	[Extracellular matrix as a microbial virulence factor in the development of human diseases].
2889	DRAMP02436	Chitinase	"Chitinase"[All Fields] AND biofilm[All Fields]	Chitinase	25476750	Extracellular DNA release confers heterogeneity in Candida albicans biofilm formation.
2889	DRAMP02436	Chitinase	"Chitinase"[All Fields] AND biofilm[All Fields]	Chitinase	25320438	Preferential Promotion of Lycopersicon esculentum (Tomato) Growth by Plant Growth Promoting Bacteria Associated with Tomato.

2889	DRAMP02436	Chitinase	"Chitinase"[All Fields] AND biofilm[All Fields]	Chitinase	25155599	Comparative systems biology analysis to study the mode of action of the isothiocyanate compound Iberin on Pseudomonas aeruginosa.
2889	DRAMP02436	Chitinase	"Chitinase"[All Fields] AND biofilm[All Fields]	Chitinase	24752234	Chitinase expression in Listeria monocytogenes is positively regulated by the Agr system.
2889	DRAMP02436	Chitinase	"Chitinase"[All Fields] AND biofilm[All Fields]	Chitinase	24691035	The natural antimicrobial carvacrol inhibits quorum sensing in Chromobacterium violaceum and reduces bacterial biofilm formation at sub-lethal concentrations.
2889	DRAMP02436	Chitinase	"Chitinase"[All Fields] AND biofilm[All Fields]	Chitinase	24664176	Chitinases are negative regulators of Francisella novicida biofilms.
2889	DRAMP02436	Chitinase	"Chitinase"[All Fields] AND biofilm[All Fields]	Chitinase	24359195	Marine bromopyrrole alkaloids: synthesis and diverse medicinal applications.
2889	DRAMP02436	Chitinase	"Chitinase"[All Fields] AND biofilm[All Fields]	Chitinase	24296886	Influence of clove oil on certain quorum-sensing-regulated functions and biofilm of Pseudomonas aeruginosa and Aeromonas hydrophila.
2889	DRAMP02436	Chitinase	"Chitinase"[All Fields] AND biofilm[All Fields]	Chitinase	24225421	Biofilms: an advancement in our understanding of Francisella species.
2889	DRAMP02436	Chitinase	"Chitinase"[All Fields] AND biofilm[All Fields]	Chitinase	23680800	Efficacy and putative mode of action of native and commercial antagonistic yeasts against postharvest pathogens of pear.
2889	DRAMP02436	Chitinase	"Chitinase"[All Fields] AND biofilm[All Fields]	Chitinase	23330731	Antibiotics at subinhibitory concentrations improve the quorum sensing behavior of Chromobacterium violaceum.
2889	DRAMP02436	Chitinase	"Chitinase"[All Fields] AND biofilm[All Fields]	Chitinase	23314962	Extracellular DNA release acts as an antifungal resistance mechanism in mature Aspergillus fumigatus biofilms.
2889	DRAMP02436	Chitinase	"Chitinase"[All Fields] AND biofilm[All Fields]	Chitinase	23299903	Doxycycline interferes with quorum sensing-mediated virulence factors and biofilm formation in gram-negative bacteria.
2889	DRAMP02436	Chitinase	"Chitinase"[All Fields] AND biofilm[All Fields]	Chitinase	23016895	Natural competence in Vibrio cholerae is controlled by a nucleoside scavenging response that requires CytR-dependent anti-activation.
2889	DRAMP02436	Chitinase	"Chitinase"[All Fields] AND biofilm[All Fields]	Chitinase	22988021	Cyclic di-GMP stimulates biofilm formation and inhibits virulence of Francisella novicida.
2889	DRAMP02436	Chitinase	"Chitinase"[All Fields] AND biofilm[All Fields]	Chitinase	22865062	Sugarcane growth promotion by the endophytic bacterium Pantoea agglomerans 33.1.
2889	DRAMP02436	Chitinase	"Chitinase"[All Fields] AND biofilm[All Fields]	Chitinase	20656858	Chitin utilization by the insect-transmitted bacterium Xylella fastidiosa.
2889	DRAMP02436	Chitinase	"Chitinase"[All Fields] AND biofilm[All Fields]	Chitinase	20159770	Attenuation of Pseudomonas aeruginosa virulence factors and biofilms by co-encapsulation of bismuth-ethanedithiol with tobramycin in liposomes.
2889	DRAMP02436	Chitinase	"Chitinase"[All Fields] AND biofilm[All Fields]	Chitinase	24710093	Identification of carbohydrate metabolism genes in the metagenome of a marine biofilm community shown to be dominated by gammaproteobacteria and bacteroidetes.
2889	DRAMP02436	Chitinase	"Chitinase"[All Fields] AND biofilm[All Fields]	Chitinase	19222585	Gene expression characteristics of a cystic fibrosis epidemic strain of Pseudomonas aeruginosa during biofilm and planktonic growth.
2889	DRAMP02436	Chitinase	"Chitinase"[All Fields] AND biofilm[All Fields]	Chitinase	18086203	Rhizobium common nod genes are required for biofilm formation.
2889	DRAMP02436	Chitinase	"Chitinase"[All Fields] AND biofilm[All Fields]	Chitinase	17122351	Identification of genes regulated by the cepIR quorum-sensing system in Burkholderia cenocepacia by high-throughput screening of a random promoter library.
2889	DRAMP02436	Chitinase	"Chitinase"[All Fields] AND biofilm[All Fields]	Chitinase	16923076	Isolation and functional analysis of luxS in Serratia plymuthica RVH1.
2889	DRAMP02436	Chitinase	"Chitinase"[All Fields] AND biofilm[All Fields]	Chitinase	16885472	Two GacA-dependent small RNAs modulate the quorum-sensing response in Pseudomonas aeruginosa.
2889	DRAMP02436	Chitinase	"Chitinase"[All Fields] AND biofilm[All Fields]	Chitinase	16849719	Biofilm matrix of Candida albicans and Candida tropicalis: chemical composition and role in drug resistance.
2889	DRAMP02436	Chitinase	"Chitinase"[All Fields] AND biofilm[All Fields]	Chitinase	16232696	Characterization of a bioflocculant produced by Citrobacter sp. TKF04 from acetic and propionic acids.
2889	DRAMP02436	Chitinase	"Chitinase"[All Fields] AND biofilm[All Fields]	Chitinase	15255906	The Candida albicans CaACE2 gene affects morphogenesis, adherence and virulence.
2889	DRAMP02436	Chitinase	"Chitinase"[All Fields] AND biofilm[All Fields]	Chitinase	11941460	Chitinase production in pine callus (Pinus sylvestris L.): a defense reaction against endophytes?
2889	DRAMP02436	Chitinase	"Chitinase"[All Fields] AND biofilm[All Fields]	Chitinase	10919822	Differentiation of chitinase-active and non-chitinase-active subpopulations of a marine bacterium during chitin degradation.
2889	DRAMP02436	Chitinase	"Chitinase"[All Fields] AND biofilm[All Fields]	Chitinase	9647829	Use of green fluorescent protein to tag and investigate gene expression in marine bacteria.
2898	DRAMP02450	Lysozyme C (1,4-beta-N-acetylmuramidase C)	"Lysozyme C"[All Fields] AND biofilm[All Fields]	Lysozyme C	29393147	Protein Profile of the Acquired Enamel Pellicle after Rinsing with Whole Milk, Fat-Free Milk, and Water: An in vivo Study.
2898	DRAMP02450	Lysozyme C (1,4-beta-N-acetylmuramidase C)	"Lysozyme C"[All Fields] AND biofilm[All Fields]	Lysozyme C	23403559	Host defense proteins derived from human saliva bind to Staphylococcus aureus.
2898	DRAMP02450	Lysozyme C (1,4-beta-N-acetylmuramidase C)	"Lysozyme C"[All Fields] AND biofilm[All Fields]	Lysozyme C	23194029	Alpha-amylase is a human salivary protein with affinity to lipopolysaccharide of Aggregatibacter actinomycetemcomitans.
2899	DRAMP02451	Lysozyme C (1,4-beta-N-acetylmuramidase C)	"Lysozyme C"[All Fields] AND biofilm[All Fields]	Lysozyme C	29393147	Protein Profile of the Acquired Enamel Pellicle after Rinsing with Whole Milk, Fat-Free Milk, and Water: An in vivo Study.
2899	DRAMP02451	Lysozyme C (1,4-beta-N-acetylmuramidase C)	"Lysozyme C"[All Fields] AND biofilm[All Fields]	Lysozyme C	23403559	Host defense proteins derived from human saliva bind to Staphylococcus aureus.
2899	DRAMP02451	Lysozyme C (1,4-beta-N-acetylmuramidase C)	"Lysozyme C"[All Fields] AND biofilm[All Fields]	Lysozyme C	23194029	Alpha-amylase is a human salivary protein with affinity to lipopolysaccharide of Aggregatibacter actinomycetemcomitans.
2900	DRAMP02452	Lysozyme	"Lysozyme"[All Fields] AND biofilm[All Fields]	Lysozyme	34688058	The dietary supplementation of zinc oxide and selenium nanoparticles enhance the immune response in freshwater fish Oreochromis mossambicus against aquatic pathogen Aeromonas hydrophila.
2900	DRAMP02452	Lysozyme	"Lysozyme"[All Fields] AND biofilm[All Fields]	Lysozyme	34680849	Anti-Biofilm Coatings Based on Chitosan and Lysozyme Functionalized Magnetite Nanoparticles.
2900	DRAMP02452	Lysozyme	"Lysozyme"[All Fields] AND biofilm[All Fields]	Lysozyme	34663068	Zwitterionic Peptides Reduce Accumulation of Marine and Freshwater Biofilm Formers.
2900	DRAMP02452	Lysozyme	"Lysozyme"[All Fields] AND biofilm[All Fields]	Lysozyme	34650538	DexA70, the Truncated Form of a Self-Produced Dextranase, Effectively Disrupts Streptococcus mutansBiofilm.
2900	DRAMP02452	Lysozyme	"Lysozyme"[All Fields] AND biofilm[All Fields]	Lysozyme	34601068	Tailoring the proliferation of fibroblast cells by multiresponsive and thermosensitive stem cells composite F127 hydrogel containing folic acid.MgO:ZnO/chitosan hybrid microparticles for skin regeneration.
2900	DRAMP02452	Lysozyme	"Lysozyme"[All Fields] AND biofilm[All Fields]	Lysozyme	34528638	Enhanced clearing of Candidabiofilms on a 3D urothelial cell in vitromodel using lysozyme-functionalized fluconazole-loaded shellac nanoparticles.
2900	DRAMP02452	Lysozyme	"Lysozyme"[All Fields] AND biofilm[All Fields]	Lysozyme	34488127	Activity of the lyases LysSSE1 and HolSSE1 against common pathogenic bacteria and their antimicrobial efficacy in biofilms.
2900	DRAMP02452	Lysozyme	"Lysozyme"[All Fields] AND biofilm[All Fields]	Lysozyme	34485498	Effect of antimicrobial peptides on planktonic growth, biofilm formation and biofilm-derived bacterial viability of Streptococcus pneumoniae.
2900	DRAMP02452	Lysozyme	"Lysozyme"[All Fields] AND biofilm[All Fields]	Lysozyme	34438974	Antimicrobial Activity of the Circular Bacteriocin AS-48 against Clinical Multidrug-Resistant Staphylococcus aureus.
2900	DRAMP02452	Lysozyme	"Lysozyme"[All Fields] AND biofilm[All Fields]	Lysozyme	34019937	Correlation of over-expression of rv1900c with enhanced survival of M. smegmatis under stress conditions: Modulation of cell surface properties.
2900	DRAMP02452	Lysozyme	"Lysozyme"[All Fields] AND biofilm[All Fields]	Lysozyme	33947757	A StreptococcusQuorum Sensing System Enables Suppression of Innate Immunity.
2900	DRAMP02452	Lysozyme	"Lysozyme"[All Fields] AND biofilm[All Fields]	Lysozyme	33920327	Resveratrol-Loaded Hydrogel Contact Lenses with Antioxidant and Antibiofilm Performance.
2900	DRAMP02452	Lysozyme	"Lysozyme"[All Fields] AND biofilm[All Fields]	Lysozyme	33918930	A Novel Biofilm Model System to Visualise Conjugal Transfer of Vancomycin Resistance by Environmental Enterococci.
2900	DRAMP02452	Lysozyme	"Lysozyme"[All Fields] AND biofilm[All Fields]	Lysozyme	33875213	Impact of pmrA on Cronobacter sakazakii planktonic and biofilm cells: A comprehensive transcriptomic study.
2900	DRAMP02452	Lysozyme	"Lysozyme"[All Fields] AND biofilm[All Fields]	Lysozyme	33842797	Antibacterial and Antibiofilm Photodynamic Activities of Lysozyme-Au Nanoclusters/Rose Bengal Conjugates.
2900	DRAMP02452	Lysozyme	"Lysozyme"[All Fields] AND biofilm[All Fields]	Lysozyme	33783975	Biodegradable Anti-Biofilm Fiber-Membrane Ureteral Stent Constructed with a Robust Biomimetic Superhydrophilic Polycationic Hydration Surface Exhibiting Synergetic Antibacterial and Antiprotein Properties.
2900	DRAMP02452	Lysozyme	"Lysozyme"[All Fields] AND biofilm[All Fields]	Lysozyme	33773215	Growth in a biofilm sensitizes Cutibacterium acnes to nanosecond pulsed electric fields.
2900	DRAMP02452	Lysozyme	"Lysozyme"[All Fields] AND biofilm[All Fields]	Lysozyme	33769023	Drug-Free Enzyme-Based Bactericidal Nanomotors against Pathogenic Bacteria.
2900	DRAMP02452	Lysozyme	"Lysozyme"[All Fields] AND biofilm[All Fields]	Lysozyme	33740161	Enzymatic biofilm destabilisation to support mechanical cleansing of inserted dental implant surfaces: an in-vitro pilot study.
2900	DRAMP02452	Lysozyme	"Lysozyme"[All Fields] AND biofilm[All Fields]	Lysozyme	33391351	Quantification of Bacterial Colonization in Dental Hard Tissues Using Optimized Molecular Biological Methods.
2900	DRAMP02452	Lysozyme	"Lysozyme"[All Fields] AND biofilm[All Fields]	Lysozyme	33252326	Effect of antifungal agents, lysozyme and human antimicrobial peptide LL-37 on clinical Candidaisolates with high biofilm production.
2900	DRAMP02452	Lysozyme	"Lysozyme"[All Fields] AND biofilm[All Fields]	Lysozyme	33185350	[Characterization of the microbiota and cytokine profile of sperm plasma in men with chronic bacterial prostatitis].
2900	DRAMP02452	Lysozyme	"Lysozyme"[All Fields] AND biofilm[All Fields]	Lysozyme	33113658	New insights into the effect of extracellular polymeric substance on the sludge dewaterability based on interaction energy and viscoelastic acoustic response analysis.

2900	DRAMP02452	Lysozyme	"Lysozyme"[All Fields] AND	Lysozyme	33080579	Combination therapy of biogenic C-dots and lysozyme for enhanced antibacterial and antibiofilm activity.
2900	DRAMP02452	Lysozyme	"Lysozyme"[All Fields] AND biofilm[All Fields]	Lysozyme	33072040	Exploiting Lactoferricin (17-30) as a Potential Antimicrobial and Antibiofilm Candidate Against Multi-Drug-Resistant Enteroaggregative Escherichia coli.
2900	DRAMP02452	Lysozyme	"Lysozyme"[All Fields] AND biofilm[All Fields]	Lysozyme	33045439	Antibacterial activities of and biofilm removal by Ablysin, an endogenous lysozyme-like protein originated from Acinetobacter baumannii 1656-2.
2900	DRAMP02452	Lysozyme	"Lysozyme"[All Fields] AND biofilm[All Fields]	Lysozyme	32916574	Supported lysozyme for improved antimicrobial surface protection.
2900	DRAMP02452	Lysozyme	"Lysozyme"[All Fields] AND biofilm[All Fields]	Lysozyme	32787275	Hydration-Induced Structural Changes in the Solid State of Protein: A SAXS/WAXS Study on Lysozyme.
2900	DRAMP02452	Lysozyme	"Lysozyme"[All Fields] AND biofilm[All Fields]	Lysozyme	32612893	Biofilm modelling on the contact lenses and comparison of the in vitro activities of multipurpose lens solutions and antibiotics.
2900	DRAMP02452	Lysozyme	"Lysozyme"[All Fields] AND biofilm[All Fields]	Lysozyme	32512756	Inactivation of the sfgtr4Gene of Shigella flexneriInduces Biofilm Formation and Affects Bacterial Pathogenicity.
2900	DRAMP02452	Lysozyme	"Lysozyme"[All Fields] AND biofilm[All Fields]	Lysozyme	32393645	Evolution of vancomycin-resistant Enterococcus faeciumduring colonization and infection in immunocompromised pediatric patients.
2900	DRAMP02452	Lysozyme	"Lysozyme"[All Fields] AND biofilm[All Fields]	Lysozyme	32280054	Quorum quenching acylase impacts the viability and morphological change of Agrobacterium tumefaciens cells.
2900	DRAMP02452	Lysozyme	"Lysozyme"[All Fields] AND biofilm[All Fields]	Lysozyme	32266650	Probiotic Potential of Bacillus Strains Isolated from an Acidic Fermented Food Idli.
2900	DRAMP02452	Lysozyme	"Lysozyme"[All Fields] AND biofilm[All Fields]	Lysozyme	32089678	Effects of Lysozyme, Proteinase K, and Cephalosporins on Biofilm Formation by Clinical Isolates of Pseudomonas aeruginosa.
2900	DRAMP02452	Lysozyme	"Lysozyme"[All Fields] AND biofilm[All Fields]	Lysozyme	33447800	Cellular chaining influences biofilm formation and structure in group A Streptococcus.
2900	DRAMP02452	Lysozyme	"Lysozyme"[All Fields] AND biofilm[All Fields]	Lysozyme	31698068	Vibrio harveyi biofilm as immunostimulant candidate for high-health pacific white shrimp, Penaeus vannamei farming.
2900	DRAMP02452	Lysozyme	"Lysozyme"[All Fields] AND biofilm[All Fields]	Lysozyme	31480687	Characterization and Antibiofilm Activity of Mannitol-Chitosan-Blended Paste for Local Antibiotic Delivery System.
2900	DRAMP02452	Lysozyme	"Lysozyme"[All Fields] AND biofilm[All Fields]	Lysozyme	31374264	Anti-cancer, anti-biofilm, and anti-inflammatory properties of hen's albumen: A photodynamic approach.
2900	DRAMP02452	Lysozyme	"Lysozyme"[All Fields] AND biofilm[All Fields]	Lysozyme	31279118	Effect of extracellular polymeric substances (EPS) conditioned by combined lysozyme and cationic polyacrylamide on the dewatering performance of activated sludge.
2900	DRAMP02452	Lysozyme	"Lysozyme"[All Fields] AND biofilm[All Fields]	Lysozyme	31252064	Antibacterial synergy between rutin and florfenicol enhances therapeutic spectrum against drug resistant Aeromonas hydrophila.
2900	DRAMP02452	Lysozyme	"Lysozyme"[All Fields] AND biofilm[All Fields]	Lysozyme	31214943	Safety and Stability of Two Potentially Probiotic Lactobacillus Strains After In Vitro Gastrointestinal Transit.
2900	DRAMP02452	Lysozyme	"Lysozyme"[All Fields] AND biofilm[All Fields]	Lysozyme	31125644	Rv0518, a nutritive stress inducible GDSL lipase of Mycobacterium tuberculosis, enhanced intracellular survival of bacteria by cell wall modulation.
2900	DRAMP02452	Lysozyme	"Lysozyme"[All Fields] AND biofilm[All Fields]	Lysozyme	31085703	The Ser/Thr Kinase PrkC Participates in Cell Wall Homeostasis and Antimicrobial Resistance in Clostridium difficile.
2900	DRAMP02452	Lysozyme	"Lysozyme"[All Fields] AND biofilm[All Fields]	Lysozyme	30843349	Characterization of the biofilm phenotype of a Listeria monocytogenes mutant deficient in agr peptide sensing.
2900	DRAMP02452	Lysozyme	"Lysozyme"[All Fields] AND biofilm[All Fields]	Lysozyme	30833631	Detection of vaginal lactobacilli as probiotic candidates.
2900	DRAMP02452	Lysozyme	"Lysozyme"[All Fields] AND biofilm[All Fields]	Lysozyme	30761784	[Biological properties of microorganisms isolated from the urine of patients with urolithiasis].
2900	DRAMP02452	Lysozyme	"Lysozyme"[All Fields] AND biofilm[All Fields]	Lysozyme	30701862	Effect of N-acetylcysteine on mucosal immunity of respiratory tract.
2900	DRAMP02452	Lysozyme	"Lysozyme"[All Fields] AND biofilm[All Fields]	Lysozyme	30649289	Innate immune components affect growth and virulence traits of bacterial-vaginosis-associated and non-bacterial-vaginosis-associated Gardnerella vaginalis strains similarly.
2900	DRAMP02452	Lysozyme	"Lysozyme"[All Fields] AND biofilm[All Fields]	Lysozyme	30639529	Catalysing the way towards antimicrobial effectiveness: A systematic analysis and a new online resource for antimicrobial-enzyme combinations against Pseudomonas aeruginosa and Staphylococcus aureus.
2900	DRAMP02452	Lysozyme	"Lysozyme"[All Fields] AND biofilm[All Fields]	Lysozyme	30639217	Immobilization of antimicrobial and anti-quorum sensing enzymes onto GMA-grafted poly(vinyl chloride) catheters.
2900	DRAMP02452	Lysozyme	"Lysozyme"[All Fields] AND biofilm[All Fields]	Lysozyme	30497006	Impact of oral astringent stimuli on surface charge and morphology of the protein-rich pellicle at the tooth-saliva interphase.
2900	DRAMP02452	Lysozyme	"Lysozyme"[All Fields] AND biofilm[All Fields]	Lysozyme	30389429	Microbiology insights into boosting salivary defences through the use of enzymes and proteins.
2900	DRAMP02452	Lysozyme	"Lysozyme"[All Fields] AND biofilm[All Fields]	Lysozyme	30207684	Developing Antibacterial Nanocrystalline Cellulose Using Natural Antibacterial Agents.
2900	DRAMP02452	Lysozyme	"Lysozyme"[All Fields] AND biofilm[All Fields]	Lysozyme	30176334	β-1, 3 glucan binding protein based selenium nanowire enhances the immune status of Cyprinus carpio and protection against Aeromonas hydrophila infection.
2900	DRAMP02452	Lysozyme	"Lysozyme"[All Fields] AND biofilm[All Fields]	Lysozyme	30142702	Moraxella bovis, Moraxella ovis and Moraxella bovoculi: biofilm formation and lysozyme activity.
2900	DRAMP02452	Lysozyme	"Lysozyme"[All Fields] AND biofilm[All Fields]	Lysozyme	30131945	The Intestinal Roundworm Ascaris suumReleases Antimicrobial Factors Which Interfere With Bacterial Growth and Biofilm Formation.
2900	DRAMP02452	Lysozyme	"Lysozyme"[All Fields] AND biofilm[All Fields]	Lysozyme	30082920	Control of Propionibacterium acnes by natural antimicrobial substances: Role of the bacteriocin AS-48 and lysozyme.
2900	DRAMP02452	Lysozyme	"Lysozyme"[All Fields] AND biofilm[All Fields]	Lysozyme	29915307	Screening and characterization of prophages in Desulfovibrio genomes.
2900	DRAMP02452	Lysozyme	"Lysozyme"[All Fields] AND biofilm[All Fields]	Lysozyme	29915116	Resuscitation-Promoting Factors Are Required for Mycobacterium smegmatis Biofilm Formation.
2900	DRAMP02452	Lysozyme	"Lysozyme"[All Fields] AND biofilm[All Fields]	Lysozyme	29876449	TCA precipitation and ethanol/HCl single-step purification evaluation: One-dimensional gel electrophoresis, bradford assays, spectrofluorometry and Raman spectroscopy data on HSA, Rnase, lysozyme - Mascots and Skyline data.
2900	DRAMP02452	Lysozyme	"Lysozyme"[All Fields] AND biofilm[All Fields]	Lysozyme	29555699	A Quorum Sensing-Regulated Protein Binds Cell Wall Components and Enhances Lysozyme Resistance in Streptococcus pyogenes.
2900	DRAMP02452	Lysozyme	"Lysozyme"[All Fields] AND biofilm[All Fields]	Lysozyme	29518559	Effect of β-1, 3 glucan binding protein based zinc oxide nanoparticles supplemented diet on immune response and disease resistance in Oreochromis mossambicus against Aeromonas hydrophila.
2900	DRAMP02452	Lysozyme	"Lysozyme"[All Fields] AND biofilm[All Fields]	Lysozyme	29464594	Inhalable Levofloxacin Liposomes Complemented with Lysozyme for Treatment of Pulmonary Infection in Rats: Effective Antimicrobial and Antibiofilm Strategy.
2900	DRAMP02452	Lysozyme	"Lysozyme"[All Fields] AND biofilm[All Fields]	Lysozyme	29393147	Protein Profile of the Acquired Enamel Pellicle after Rinsing with Whole Milk, Fat-Free Milk, and Water: An in vivo Study.
2900	DRAMP02452	Lysozyme	"Lysozyme"[All Fields] AND biofilm[All Fields]	Lysozyme	29373594	Biofilm formation and transcriptome analysis of Streptococcus gallolyticus subsp. gallolyticus in response to lysozyme.
2900	DRAMP02452	Lysozyme	"Lysozyme"[All Fields] AND biofilm[All Fields]	Lysozyme	29315029	Correlations among Resistances to Different Antimicrobial Compounds in Salmonella Strains from Hen Eggshells.
2900	DRAMP02452	Lysozyme	"Lysozyme"[All Fields] AND biofilm[All Fields]	Lysozyme	31565305	Enzyme Crystals and Hydrogel Composite Membranes as New Active Food Packaging Material.
2900	DRAMP02452	Lysozyme	"Lysozyme"[All Fields] AND biofilm[All Fields]	Lysozyme	29205129	Dissimilar pigment regulation in Serpula lacrymans and Paxillus involutus during inter-kingdom interactions.
2900	DRAMP02452	Lysozyme	"Lysozyme"[All Fields] AND biofilm[All Fields]	Lysozyme	29201023	A Marine Actinomycete Rescues Caenorhabditis elegansfrom Pseudomonas aeruginosaInfection through Restitution of Lysozyme 7.
2900	DRAMP02452	Lysozyme	"Lysozyme"[All Fields] AND biofilm[All Fields]	Lysozyme	29184097	Csl2, a novel chimeric bacteriophage lysin to fight infections caused by Streptococcus suis, an emerging zoonotic pathogen.
2900	DRAMP02452	Lysozyme	"Lysozyme"[All Fields] AND biofilm[All Fields]	Lysozyme	29080817	Production and structural characterization of exopolysaccharides from newly isolated probiotic lactic acid bacteria.
2900	DRAMP02452	Lysozyme	"Lysozyme"[All Fields] AND biofilm[All Fields]	Lysozyme	28931063	Plant flavones enhance antimicrobial activity of respiratory epithelial cell secretions against Pseudomonas aeruginosa.
2900	DRAMP02452	Lysozyme	"Lysozyme"[All Fields] AND biofilm[All Fields]	Lysozyme	28922066	The Effect of Lysozyme on Reducing Biofilms by Staphylococcus aureus, Pseudomonas aeruginosa, and Gardnerella vaginalis: An In Vitro Examination.
2900	DRAMP02452	Lysozyme	"Lysozyme"[All Fields] AND biofilm[All Fields]	Lysozyme	28826250	Antimicrobial and anti-biofilm activity of tannic acid against Staphylococcus aureus.
2900	DRAMP02452	Lysozyme	"Lysozyme"[All Fields] AND biofilm[All Fields]	Lysozyme	28649626	Investigation of simulated microgravity effects on Streptococcus mutansphysiology and global gene expression.
2900	DRAMP02452	Lysozyme	"Lysozyme"[All Fields] AND biofilm[All Fields]	Lysozyme	32264250	Protein-repellent and antimicrobial nanoparticle coatings from hyaluronic acid and a lysine-derived biocompatible surfactant.
2900	DRAMP02452	Lysozyme	"Lysozyme"[All Fields] AND biofilm[All Fields]	Lysozyme	28400767	The Periplasmic Chaperone Network of Campylobacter jejuni: Evidence that SalC (Cj1289) and PpiD (Cj0694) Are Involved in Maintaining Outer Membrane Integrity.
2900	DRAMP02452	Lysozyme	"Lysozyme"[All Fields] AND biofilm[All Fields]	Lysozyme	28400765	Role of Bacillus licheniformisVS16-Derived Biosurfactant in Mediating Immune Responses in Carp Rohu and its Application to the Food Industry.
2900	DRAMP02452	Lysozyme	"Lysozyme"[All Fields] AND biofilm[All Fields]	Lysozyme	28397768	Lysozyme Associated Liposomal Gentamicin Inhibits Bacterial Biofilm.
2900	DRAMP02452	Lysozyme	"Lysozyme"[All Fields] AND biofilm[All Fields]	Lysozyme	28289407	A Novel Antimicrobial Endolysin, LysPA26, against Pseudomonas aeruginosa.

2900	DRAMP02452	Lysozyme	"Lysozyme"[All Fields] AND biofilm[All Fields]	Lysozyme	28251063	Evaluation of a chitosan-polyethylene glycol paste as a local antibiotic delivery device.
2900	DRAMP02452	Lysozyme	"Lysozyme"[All Fields] AND biofilm[All Fields]	Lysozyme	28188877	Laser deposition of poly(3-hydroxybutyric acid-co-3-hydroxyvaleric acid) - lysozyme microspheres based coatings with anti-microbial properties.
2900	DRAMP02452	Lysozyme	"Lysozyme"[All Fields] AND biofilm[All Fields]	Lysozyme	28138698	Dose-dependent effect of lysozyme upon Candida albicans biofilm.
2900	DRAMP02452	Lysozyme	"Lysozyme"[All Fields] AND biofilm[All Fields]	Lysozyme	28109187	Antimicrobial and antioxidant activities of Saccharomyces cerevisiae IFST062013, a potential probiotic.
2900	DRAMP02452	Lysozyme	"Lysozyme"[All Fields] AND biofilm[All Fields]	Lysozyme	30695493	[PHYSIOLOGICAL FEATURES OF CORYNEBACTERIA OF FEMALE REPRODUC- TIVE TRACT].
2900	DRAMP02452	Lysozyme	"Lysozyme"[All Fields] AND biofilm[All Fields]	Lysozyme	29619048	Efficacy of a Rinse Containing Sea Salt and Lysozyme on Biofilm and Gingival Health in a Group of Young Adults: A Pilot Study.
2900	DRAMP02452	Lysozyme	"Lysozyme"[All Fields] AND biofilm[All Fields]	Lysozyme	27999400	Comparative Study on the Characteristics of Weissella cibaria CMU and Probiotic Strains for Oral Care.
2900	DRAMP02452	Lysozyme	"Lysozyme"[All Fields] AND biofilm[All Fields]	Lysozyme	27984151	Copper incorporated microporous chitosan-polyethylene glycol hydrogels loaded with naproxen for effective drug release and anti-infection wound dressing.
2900	DRAMP02452	Lysozyme	"Lysozyme"[All Fields] AND biofilm[All Fields]	Lysozyme	27931094	Human Lysozyme Peptidase Resistance Is Perturbed by the Anionic Glycolipid Biosurfactant Rhamnolipid Produced by the Opportunistic Pathogen Pseudomonas aeruginosa.
2900	DRAMP02452	Lysozyme	"Lysozyme"[All Fields] AND biofilm[All Fields]	Lysozyme	27845497	Lysozyme as a cotreatment during antibiotics use against vaginal infections: An in vitro study on Gardnerella vaginalis biofilm models.
2900	DRAMP02452	Lysozyme	"Lysozyme"[All Fields] AND biofilm[All Fields]	Lysozyme	27821857	Establishing Antibacterial Multilayer Films on the Surface of Direct Metal Laser Sintered Titanium Primed with Phase-Transited Lysozyme.
2900	DRAMP02452	Lysozyme	"Lysozyme"[All Fields] AND biofilm[All Fields]	Lysozyme	27709890	A Biodegradable Polycationic Paint that Kills Bacteria in Vitro and in Vivo.
2900	DRAMP02452	Lysozyme	"Lysozyme"[All Fields] AND biofilm[All Fields]	Lysozyme	27685160	In Situ Impregnation of Silver Nanoclusters in Microporous Chitosan-PEG Membranes as an Antibacterial and Drug Delivery Percutaneous Device.
2900	DRAMP02452	Lysozyme	"Lysozyme"[All Fields] AND biofilm[All Fields]	Lysozyme	27602088	Antibiotic susceptibility, antibacterial activity and characterisation of Enterococcus faeciumstrains isolated from breast milk.
2900	DRAMP02452	Lysozyme	"Lysozyme"[All Fields] AND biofilm[All Fields]	Lysozyme	27520822	Peptidoglycan Acetylation of Campylobacter jejuni Is Essential for Maintaining Cell Wall Integrity and Colonization in Chicken Intestines.
2900	DRAMP02452	Lysozyme	"Lysozyme"[All Fields] AND biofilm[All Fields]	Lysozyme	27375592	Pseudomonas aeruginosa Outer Membrane Vesicles Triggered by Human Mucosal Fluid and Lysozyme Can Prime Host Tissue Surfaces for Bacterial Adhesion.
2900	DRAMP02452	Lysozyme	"Lysozyme"[All Fields] AND biofilm[All Fields]	Lysozyme	27373086	[Staphylococcus aureus biofilm influences the expression of lysozyme, SLPI and gp340 in a human sinonasal explant model].
2900	DRAMP02452	Lysozyme	"Lysozyme"[All Fields] AND biofilm[All Fields]	Lysozyme	27236754	Molecular typing and differences in biofilm formation and antibiotic susceptibilities among Prototheca strains isolated in Italy and Brazil.
2900	DRAMP02452	Lysozyme	"Lysozyme"[All Fields] AND biofilm[All Fields]	Lysozyme	27015648	Biopolymer-induced calcium phosphate scaling in membrane-based water treatment systems: Langmuir model films studies.
2900	DRAMP02452	Lysozyme	"Lysozyme"[All Fields] AND biofilm[All Fields]	Lysozyme	26783746	Titanium Surface Priming with Phase-Transited Lysozyme to Establish a Silver Nanoparticle-Loaded Chitosan/Hyaluronic Acid Antibacterial Multilayer via Layer-by-Layer Self-Assembly.
2900	DRAMP02452	Lysozyme	"Lysozyme"[All Fields] AND biofilm[All Fields]	Lysozyme	31532953	[The etiologic structure and biologic characteristics of agents of infections of bloodstream.]
2900	DRAMP02452	Lysozyme	"Lysozyme"[All Fields] AND biofilm[All Fields]	Lysozyme	26488327	Complexation of Lysozyme with Sodium Poly(styrenesulfonate) via the Two-State and Non-Two-State Unfoldings of Lysozyme.
2900	DRAMP02452	Lysozyme	"Lysozyme"[All Fields] AND biofilm[All Fields]	Lysozyme	26478289	Covalent immobilization of lysozyme onto woven and knitted crimped polyethylene terephthalate grafts to minimize the adhesion of broad spectrum pathogens.
2900	DRAMP02452	Lysozyme	"Lysozyme"[All Fields] AND biofilm[All Fields]	Lysozyme	26470414	[REGULATING EFFECT OF ASSOCIATIVE MICROBIOTA ON THE RHYTHMS OF BIOLOGICAL PROPERTIES OF FUNGI AND BACTERIA].
2900	DRAMP02452	Lysozyme	"Lysozyme"[All Fields] AND biofilm[All Fields]	Lysozyme	26458820	Effects of fed-batch and continuous fermentations on human lysozyme production by Kluyveromyces lactis K7 in biofilm reactors.
2900	DRAMP02452	Lysozyme	"Lysozyme"[All Fields] AND biofilm[All Fields]	Lysozyme	26400891	Effect of histatin-5 and lysozyme on the ability of Streptococcus mutans to form biofilms in in vitro conditions.
2900	DRAMP02452	Lysozyme	"Lysozyme"[All Fields] AND biofilm[All Fields]	Lysozyme	26383819	Recent advances for the production and recovery methods of lysozyme.
2900	DRAMP02452	Lysozyme	"Lysozyme"[All Fields] AND biofilm[All Fields]	Lysozyme	26315397	Enzymes in the in-situ pellicle of children with different caries activity.
2900	DRAMP02452	Lysozyme	"Lysozyme"[All Fields] AND biofilm[All Fields]	Lysozyme	26152033	[AEROMONAS BACTERIA ISOLATED FROM BITHYNIIDAE MOLLUSKS AND THEIR HABITATS: SPECIES COMPOSITION AND BIOLOGICAL PROPERTIES. COMMUNICATION 1].
2900	DRAMP02452	Lysozyme	"Lysozyme"[All Fields] AND biofilm[All Fields]	Lysozyme	26092919	Acetylcholine Protects against Candida albicans Infection by Inhibiting Biofilm Formation and Promoting Hemocyte Function in a Galleria mellonella Infection Model.
2900	DRAMP02452	Lysozyme	"Lysozyme"[All Fields] AND biofilm[All Fields]	Lysozyme	27688392	Antimicrobial Capacity of Casein Phosphopeptide/Amorphous Calcium Phosphate and Enzymes in Glass Ionomer Cement in Dentin Carious Lesions.
2900	DRAMP02452	Lysozyme	"Lysozyme"[All Fields] AND biofilm[All Fields]	Lysozyme	25950767	In vitro biofilm development of Streptococcus pneumoniae and formation of choline-binding protein-DNA complexes.
2900	DRAMP02452	Lysozyme	"Lysozyme"[All Fields] AND biofilm[All Fields]	Lysozyme	25757148	Development of novel formulations containing Lysozyme and Lactoferrin and evaluation of antibacterial effects on Mutans Streptococci and Lactobacilli.
2900	DRAMP02452	Lysozyme	"Lysozyme"[All Fields] AND biofilm[All Fields]	Lysozyme	25725311	Cleaning of biomaterial surfaces: protein removal by different solvents.
2900	DRAMP02452	Lysozyme	"Lysozyme"[All Fields] AND biofilm[All Fields]	Lysozyme	25721974	Characterization of a novel strain phylogenetically related to Kocuria rhizophila and its chemical modification to improve performance of microbial fuel cells.
2900	DRAMP02452	Lysozyme	"Lysozyme"[All Fields] AND biofilm[All Fields]	Lysozyme	25536779	[Species structure and staphylococci bioprofile characteristics--ausative agents of perinatal infectious-inflammation pathology in children of Orenburg].
2900	DRAMP02452	Lysozyme	"Lysozyme"[All Fields] AND biofilm[All Fields]	Lysozyme	25467937	Phenotypic characterization of a novel double knockout PknI/DacB2 from Mycobacterium tuberculosis.
2900	DRAMP02452	Lysozyme	"Lysozyme"[All Fields] AND biofilm[All Fields]	Lysozyme	25449384	N-hexanoyl-L-homoserine lactone-degrading Pseudomonas aeruginosa PsDAHP1 protects zebrafish against Vibrio parahaemolyticus infection.
2900	DRAMP02452	Lysozyme	"Lysozyme"[All Fields] AND biofilm[All Fields]	Lysozyme	25312955	The conserved hypothetical protein Rv0574c is required for cell wall integrity, stress tolerance, and virulence of Mycobacterium tuberculosis.
2900	DRAMP02452	Lysozyme	"Lysozyme"[All Fields] AND biofilm[All Fields]	Lysozyme	25221673	Iron oxide nanoparticles induce Pseudomonas aeruginosagrowth, induce biofilm formation, and inhibit antimicrobial peptide function.
2900	DRAMP02452	Lysozyme	"Lysozyme"[All Fields] AND biofilm[All Fields]	Lysozyme	25115519	Presence and function of a thick mucous layer rich in polysaccharides around Bacillus subtilis spores.
2900	DRAMP02452	Lysozyme	"Lysozyme"[All Fields] AND biofilm[All Fields]	Lysozyme	24559398	Temperature-driven adsorption and desorption of proteins at solid-liquid interfaces.
2900	DRAMP02452	Lysozyme	"Lysozyme"[All Fields] AND biofilm[All Fields]	Lysozyme	24434534	Designing nanogel carriers for antibacterial applications.
2900	DRAMP02452	Lysozyme	"Lysozyme"[All Fields] AND biofilm[All Fields]	Lysozyme	24240906	Adsorption study of pellicle proteins to gold, silica and titanium by quartz crystal microbalance method.
2900	DRAMP02452	Lysozyme	"Lysozyme"[All Fields] AND biofilm[All Fields]	Lysozyme	24121782	Enhanced expressions of lysozyme, SLPI and glycoprotein 340 in biofilm-associated chronic rhinosinusitis.
2900	DRAMP02452	Lysozyme	"Lysozyme"[All Fields] AND biofilm[All Fields]	Lysozyme	24011302	Fluorescence microscopic visualization of non cellular components during initial bioadhesion in situ.
2900	DRAMP02452	Lysozyme	"Lysozyme"[All Fields] AND biofilm[All Fields]	Lysozyme	23844477	Destruction of single-species biofilms of Escherichia coli or Klebsiella pneumoniae subsp. pneumoniae by dextranase, lactoferrin, and lysozyme.
2900	DRAMP02452	Lysozyme	"Lysozyme"[All Fields] AND biofilm[All Fields]	Lysozyme	23701483	Effects of biological molecules on calcium mineral formation associated with wastewater desalination as assessed using small-angle neutron scattering.
2900	DRAMP02452	Lysozyme	"Lysozyme"[All Fields] AND biofilm[All Fields]	Lysozyme	23659996	Vaccination efficiency of surface antigens and killed whole cell of Pseudomonas putida in large yellow croaker (Pseudosciaena crocea).
2900	DRAMP02452	Lysozyme	"Lysozyme"[All Fields] AND biofilm[All Fields]	Lysozyme	23657582	Production of human lysozyme in biofilm reactor and optimization of growth parameters of Kluyveromyces lactis K7.
2900	DRAMP02452	Lysozyme	"Lysozyme"[All Fields] AND biofilm[All Fields]	Lysozyme	23504078	Phenotypic characterization, virulence, and immunogenicity of Edwardsiella tarda LSE40 aroA mutant.
2900	DRAMP02452	Lysozyme	"Lysozyme"[All Fields] AND biofilm[All Fields]	Lysozyme	23460607	Undecaprenyl pyrophosphate phosphatase confers low-level resistance to bacitracin in Enterococcus faecalis.
2900	DRAMP02452	Lysozyme	"Lysozyme"[All Fields] AND biofilm[All Fields]	Lysozyme	23434686	Enhancing antibacterial activity of surface-grafted chitosan with immobilized lysozyme on bioinspired stainless steel substrates.
2900	DRAMP02452	Lysozyme	"Lysozyme"[All Fields] AND biofilm[All Fields]	Lysozyme	23403559	Host defense proteins derived from human saliva bind to Staphylococcus aureus.
2900	DRAMP02452	Lysozyme	"Lysozyme"[All Fields] AND biofilm[All Fields]	Lysozyme	23194029	Alpha-amylase is a human salivary protein with affinity to lipopolysaccharide of Aggregatibacter actinomycetemcomitans.
2900	DRAMP02452	Lysozyme	"Lysozyme"[All Fields] AND biofilm[All Fields]	Lysozyme	22947470	Effects of nisin and lysozyme on growth inhibition and biofilm formation capacity of Staphylococcus aureus strains isolated from raw milk and cheese samples.
2900	DRAMP02452	Lysozyme	"Lysozyme"[All Fields] AND biofilm[All Fields]	Lysozyme	22937707	[Interaction of Bifidobacterium bifidum with members of normal microflora in human intestine microsymbiocenosis].
2900	DRAMP02452	Lysozyme	"Lysozyme"[All Fields] AND biofilm[All Fields]	Lysozyme	22913814	Insight into the composition of the intercellular matrix of Streptococcus pneumoniae biofilms.
2900	DRAMP02452	Lysozyme	"Lysozyme"[All Fields] AND biofilm[All Fields]	Lysozyme	22363222	Tasco®: a product of Ascophyllum nodosum enhances immune response of Caenorhabditis elegans against Pseudomonas aeruginosa infection.

2900	DRAMP02452	Lysozyme	"Lysozyme"[All Fields] AND biofilm[All Fields]	Lysozyme	26781731	Recombinant Expression of a Putative Amidase Cloned from the Genome of Listeria monocytogenes that Lyses the Bacterium and its Monolayer in Conjunction with a Protease.
2900	DRAMP02452	Lysozyme	"Lysozyme"[All Fields] AND biofilm[All Fields]	Lysozyme	22344334	Influence of artificial saliva in biofilm formation of Candida albicans in vitro.
2900	DRAMP02452	Lysozyme	"Lysozyme"[All Fields] AND biofilm[All Fields]	Lysozyme	22308727	[Microbial "friend-foe" identification in human intestine microsymbiocenosis].
2900	DRAMP02452	Lysozyme	"Lysozyme"[All Fields] AND biofilm[All Fields]	Lysozyme	22265308	Effect of lysozyme on "flor" velum yeasts in the biological aging of sherry wines.
2900	DRAMP02452	Lysozyme	"Lysozyme"[All Fields] AND biofilm[All Fields]	Lysozyme	21821771	Role of phase variation in the resistance of Myxococcus xanthus fruiting bodies to Caenorhabditis elegans predation.
2900	DRAMP02452	Lysozyme	"Lysozyme"[All Fields] AND biofilm[All Fields]	Lysozyme	21486002	Anti-fouling chemistry of chiral monolayers: enhancing biofilm resistance on racemic surface.
2900	DRAMP02452	Lysozyme	"Lysozyme"[All Fields] AND biofilm[All Fields]	Lysozyme	21471196	A disulfide bridge network within the soluble periplasmic domain determines structure and function of the outer membrane protein RCSF.
2900	DRAMP02452	Lysozyme	"Lysozyme"[All Fields] AND biofilm[All Fields]	Lysozyme	21338094	Lysozyme-coupled poly(poly(ethylene glycol) methacrylate)-stainless steel hybrids and their antifouling and antibacterial surfaces.
2900	DRAMP02452	Lysozyme	"Lysozyme"[All Fields] AND biofilm[All Fields]	Lysozyme	21247450	Phylogenetic group- and species-specific oligonucleotide probes for single-cell detection of lactic acid bacteria in oral biofilms.
2900	DRAMP02452	Lysozyme	"Lysozyme"[All Fields] AND biofilm[All Fields]	Lysozyme	21148692	Integrity of proteins in human saliva after sterilization by gamma irradiation.
2900	DRAMP02452	Lysozyme	"Lysozyme"[All Fields] AND biofilm[All Fields]	Lysozyme	21045923	Structural evolution of protein-biofilms: Simulations and experiments.
2900	DRAMP02452	Lysozyme	"Lysozyme"[All Fields] AND biofilm[All Fields]	Lysozyme	20961363	Potential of ceragenin CSA-13 and its mixture with pluronic F-127 as treatment of topical bacterial infections.
2900	DRAMP02452	Lysozyme	"Lysozyme"[All Fields] AND biofilm[All Fields]	Lysozyme	20865041	Comparative genomics of Gardnerella vaginalis strains reveals substantial differences in metabolic and virulence potential.
2900	DRAMP02452	Lysozyme	"Lysozyme"[All Fields] AND biofilm[All Fields]	Lysozyme	20724386	Glutamine synthetase encoded by glnA-1 is necessary for cell wall resistance and pathogenicity of Mycobacterium bovis.
2900	DRAMP02452	Lysozyme	"Lysozyme"[All Fields] AND biofilm[All Fields]	Lysozyme	20566201	Bioengineering of stainless steel surface by covalent immobilization of enzymes. Physical characterization and interfacial enzymatic activity.
2900	DRAMP02452	Lysozyme	"Lysozyme"[All Fields] AND biofilm[All Fields]	Lysozyme	20417319	Functionalization of acrylic hydrogels with alpha-, beta- or gamma-cyclodextrin modulates protein adsorption and antifungal delivery.
2900	DRAMP02452	Lysozyme	"Lysozyme"[All Fields] AND biofilm[All Fields]	Lysozyme	20121056	Achieving highly effective non-biofouling performance for polypropylene membranes modified by UV-induced surface graft polymerization of two oppositely charged monomers.
2900	DRAMP02452	Lysozyme	"Lysozyme"[All Fields] AND biofilm[All Fields]	Lysozyme	19334612	[Search for destruction factors of bacterial biofilms: comparison of phage properties in a group of Pseudomonas putida bacteriophages and specificity of their halo-formation products].
2900	DRAMP02452	Lysozyme	"Lysozyme"[All Fields] AND biofilm[All Fields]	Lysozyme	19241565	Storability of antimicrobial chitosan-lysozyme composite coating and film-forming solutions.
2900	DRAMP02452	Lysozyme	"Lysozyme"[All Fields] AND biofilm[All Fields]	Lysozyme	19166331	Grafting of lysozyme and/or poly(ethylene glycol) to prevent biofilm growth on stainless steel surfaces.
2900	DRAMP02452	Lysozyme	"Lysozyme"[All Fields] AND biofilm[All Fields]	Lysozyme	19038377	Synergistic activity of lysozyme and antifungal agents against Candida albicans biofilms on denture acrylic surfaces.
2900	DRAMP02452	Lysozyme	"Lysozyme"[All Fields] AND biofilm[All Fields]	Lysozyme	18992255	Crystal structure of the resuscitation-promoting factor (DeltaDUF)RpfB from M. tuberculosis.
2900	DRAMP02452	Lysozyme	"Lysozyme"[All Fields] AND biofilm[All Fields]	Lysozyme	18989905	Ultralow fouling zwitterionic polymers grafted from surfaces covered with an initiator via an adhesive mussel mimetic linkage.
2900	DRAMP02452	Lysozyme	"Lysozyme"[All Fields] AND biofilm[All Fields]	Lysozyme	18819708	Ultra low fouling zwitterionic polymers with a biomimetic adhesive group.
2900	DRAMP02452	Lysozyme	"Lysozyme"[All Fields] AND biofilm[All Fields]	Lysozyme	18718499	Role of lactoferrin in the tear film.
2900	DRAMP02452	Lysozyme	"Lysozyme"[All Fields] AND biofilm[All Fields]	Lysozyme	18468764	Effects of Cistus-tea on bacterial colonization and enzyme activities of the in situ pellicle.
2900	DRAMP02452	Lysozyme	"Lysozyme"[All Fields] AND biofilm[All Fields]	Lysozyme	18424015	Abnormal cell division caused by inclusion bodies in E. coli; increased resistance against external stress.
2900	DRAMP02452	Lysozyme	"Lysozyme"[All Fields] AND biofilm[All Fields]	Lysozyme	18326181	Antimicrobial activity of lactoferrin against foodborne pathogenic bacteria incorporated into edible chitosan film.
2900	DRAMP02452	Lysozyme	"Lysozyme"[All Fields] AND biofilm[All Fields]	Lysozyme	17927634	Biofilm inhibition and antimicrobial activity of a dentifrice containing salivary substitutes.
2900	DRAMP02452	Lysozyme	"Lysozyme"[All Fields] AND biofilm[All Fields]	Lysozyme	17676995	Molecular basis of resistance to muramidase and cationic antimicrobial peptide activity of lysozyme in staphylococci.
2900	DRAMP02452	Lysozyme	"Lysozyme"[All Fields] AND biofilm[All Fields]	Lysozyme	16936041	Biofilm formation by Streptococcus pneumoniae: role of choline, extracellular DNA, and capsular polysaccharide in microbial accretion.
2900	DRAMP02452	Lysozyme	"Lysozyme"[All Fields] AND biofilm[All Fields]	Lysozyme	15952847	Covalent immobilization of lysozyme on stainless steel. Interface spectroscopic characterization and measurement of enzymatic activity.
2900	DRAMP02452	Lysozyme	"Lysozyme"[All Fields] AND biofilm[All Fields]	Lysozyme	15908380	Identification and characterization of an autolysin-encoding gene of Streptococcus mutans.
2900	DRAMP02452	Lysozyme	"Lysozyme"[All Fields] AND biofilm[All Fields]	Lysozyme	15693823	Enzymes in the acquired enamel pellicle.
2900	DRAMP02452	Lysozyme	"Lysozyme"[All Fields] AND biofilm[All Fields]	Lysozyme	15518508	Shear and dilatational relaxation mechanisms of globular and flexible proteins at the hexadecane/water interface.
2900	DRAMP02452	Lysozyme	"Lysozyme"[All Fields] AND biofilm[All Fields]	Lysozyme	15493829	Bacterial evasion of innate host defenses--the Staphylococcus aureus lesson.
2900	DRAMP02452	Lysozyme	"Lysozyme"[All Fields] AND biofilm[All Fields]	Lysozyme	14768464	Protein adsorption at polymer-grafted surfaces: comparison between a mixture of saliva proteins and some well-defined model proteins.
2900	DRAMP02452	Lysozyme	"Lysozyme"[All Fields] AND biofilm[All Fields]	Lysozyme	14706749	Multiplex FISH analysis of a six-species bacterial biofilm.
2900	DRAMP02452	Lysozyme	"Lysozyme"[All Fields] AND biofilm[All Fields]	Lysozyme	14513382	Physiology of biofilms of thermophilic bacilli-potential consequences for cleaning.
2900	DRAMP02452	Lysozyme	"Lysozyme"[All Fields] AND biofilm[All Fields]	Lysozyme	12707750	Scanning-force techniques to monitor time-dependent changes in topography and adhesion force of proteins on surfaces.
2900	DRAMP02452	Lysozyme	"Lysozyme"[All Fields] AND biofilm[All Fields]	Lysozyme	12366846	Positive role of peptidoglycan breaks in lactococcal biofilm formation.
2900	DRAMP02452	Lysozyme	"Lysozyme"[All Fields] AND biofilm[All Fields]	Lysozyme	11679354	Direct detection by in situ PCR of the amoA gene in biofilm resulting from a nitrogen removal process.
2900	DRAMP02452	Lysozyme	"Lysozyme"[All Fields] AND biofilm[All Fields]	Lysozyme	11412320	Factors influencing attachment of thermophilic bacilli to stainless steel.
2900	DRAMP02452	Lysozyme	"Lysozyme"[All Fields] AND biofilm[All Fields]	Lysozyme	10415452	Lactoferrin increases the susceptibility of S. epidermidis biofilms to lysozyme and vancomycin.
2900	DRAMP02452	Lysozyme	"Lysozyme"[All Fields] AND biofilm[All Fields]	Lysozyme	10391502	The effect of extracellular polysaccharides from Streptococcus mutans on the bactericidal activity of human neutrophils.
2900	DRAMP02452	Lysozyme	"Lysozyme"[All Fields] AND biofilm[All Fields]	Lysozyme	10390869	Specific oligonucleotide probes for in situ detection of a major group of gram-positive bacteria with low DNA G + C content.
2900	DRAMP02452	Lysozyme	"Lysozyme"[All Fields] AND biofilm[All Fields]	Lysozyme	9812283	Protein antimicrobial barriers to bacterial adhesion.
2900	DRAMP02452	Lysozyme	"Lysozyme"[All Fields] AND biofilm[All Fields]	Lysozyme	9571268	Defining the physiologically normal coating and pathological deposit: an analysis of sulfur-containing moieties and pellicle thickness on hydrogel contact lenses.
2900	DRAMP02452	Lysozyme	"Lysozyme"[All Fields] AND biofilm[All Fields]	Lysozyme	9351231	The influence of cell surface properties of thermophilic streptococci on attachment to stainless steel.
2900	DRAMP02452	Lysozyme	"Lysozyme"[All Fields] AND biofilm[All Fields]	Lysozyme	9255506	Protein-lipid interaction on the surface of a hydrophilic contact lens in vitro.
2900	DRAMP02452	Lysozyme	"Lysozyme"[All Fields] AND biofilm[All Fields]	Lysozyme	9380651	[Preparation of films with combined biological activity and study of their properties].
2900	DRAMP02452	Lysozyme	"Lysozyme"[All Fields] AND biofilm[All Fields]	Lysozyme	9029300	Adhesion to silicone rubber of yeasts and bacteria isolated from voice prostheses: influence of salivary conditioning films.
2900	DRAMP02452	Lysozyme	"Lysozyme"[All Fields] AND biofilm[All Fields]	Lysozyme	8950503	The effect of eye closure on protein and complement deposition on Group IV hydrogel contact lenses: relationship to tear flow dynamics.
2900	DRAMP02452	Lysozyme	"Lysozyme"[All Fields] AND biofilm[All Fields]	Lysozyme	8126879	[Physiologic factor in intractable bacterial infections].
2900	DRAMP02452	Lysozyme	"Lysozyme"[All Fields] AND biofilm[All Fields]	Lysozyme	3570694	Specificity and biological activity of the protein deposited on the hydrogel surface. Relationship of polymer structure to biofilm formation.
2903	DRAMP02455	L-amino-acid oxidase (ACL-LAO; LAAO; LAO)	"L-amino-acid oxidase"[All Fields] AND biofilm[All Fields]	L-amino-acid oxidase	30160994	Molecules and Mechanisms Underlying the Antimicrobial Activity of Escapin, an l-Amino Acid Oxidase from the Ink of Sea Hares.
2903	DRAMP02455	L-amino-acid oxidase (ACL-LAO; LAAO; LAO)	"L-amino-acid oxidase"[All Fields] AND biofilm[All Fields]	L-amino-acid oxidase	27401562	Inhibition and Dispersal of Pseudomonas aeruginosa Biofilms by Combination Treatment with Escapin Intermediate Products and Hydrogen Peroxide.
2903	DRAMP02455	L-amino-acid oxidase (ACL-LAO; LAAO; LAO)	"L-amino-acid oxidase"[All Fields] AND biofilm[All Fields]	L-amino-acid oxidase	18469105	SO-LAAO, a novel L-amino acid oxidase that enables Streptococcus oligofermentans to outcompete Streptococcus mutans by generating H2O2 from peptone.

2904	DRAMP02457	L-amino-acid oxidase (Balt-LAAO-I; LAAO; LAO; snakes, reptils, animals)	"L-amino-acid oxidase"[All Fields] AND biofilm[All Fields]	L-amino-acid oxidase	30160994	Molecules and Mechanisms Underlying the Antimicrobial Activity of Escapin, an l-Amino Acid Oxidase from the Ink of Sea Hares.
2904	DRAMP02457	L-amino-acid oxidase (Balt-LAAO-I; LAAO; LAO; snakes, reptils, animals)	"L-amino-acid oxidase"[All Fields] AND biofilm[All Fields]	L-amino-acid oxidase	27401562	Inhibition and Dispersal of Pseudomonas aeruginosa Biofilms by Combination Treatment with Escapin Intermediate Products and Hydrogen Peroxide.
2904	DRAMP02457	L-amino-acid oxidase (Balt-LAAO-I; LAAO; LAO; snakes, reptils, animals)	"L-amino-acid oxidase"[All Fields] AND biofilm[All Fields]	L-amino-acid oxidase	18469105	SO-LAAO, a novel L-amino acid oxidase that enables Streptococcus oligofermentans to outcompete Streptococcus mutans by generating H2O2 from peptone.
2905	DRAMP02458	L-amino-acid oxidase (BiLAO; LAAO; LAO; snakes, reptils, animals)	"L-amino-acid oxidase"[All Fields] AND biofilm[All Fields]	L-amino-acid oxidase	30160994	Molecules and Mechanisms Underlying the Antimicrobial Activity of Escapin, an l-Amino Acid Oxidase from the Ink of Sea Hares.
2905	DRAMP02458	L-amino-acid oxidase (BiLAO; LAAO; LAO; snakes, reptils, animals)	"L-amino-acid oxidase"[All Fields] AND biofilm[All Fields]	L-amino-acid oxidase	27401562	Inhibition and Dispersal of Pseudomonas aeruginosa Biofilms by Combination Treatment with Escapin Intermediate Products and Hydrogen Peroxide.
2905	DRAMP02458	L-amino-acid oxidase (BiLAO; LAAO; LAO; snakes, reptils, animals)	"L-amino-acid oxidase"[All Fields] AND biofilm[All Fields]	L-amino-acid oxidase	18469105	SO-LAAO, a novel L-amino acid oxidase that enables Streptococcus oligofermentans to outcompete Streptococcus mutans by generating H2O2 from peptone.
2906	DRAMP02459	L-amino-acid oxidase (BjarLAAO-I; LAAO; LAO; snakes, reptils, animals)	"L-amino-acid oxidase"[All Fields] AND biofilm[All Fields]	L-amino-acid oxidase	30160994	Molecules and Mechanisms Underlying the Antimicrobial Activity of Escapin, an l-Amino Acid Oxidase from the Ink of Sea Hares.
2906	DRAMP02459	L-amino-acid oxidase (BjarLAAO-I; LAAO; LAO; snakes, reptils, animals)	"L-amino-acid oxidase"[All Fields] AND biofilm[All Fields]	L-amino-acid oxidase	27401562	Inhibition and Dispersal of Pseudomonas aeruginosa Biofilms by Combination Treatment with Escapin Intermediate Products and Hydrogen Peroxide.
2906	DRAMP02459	L-amino-acid oxidase (BjarLAAO-I; LAAO; LAO; snakes, reptils, animals)	"L-amino-acid oxidase"[All Fields] AND biofilm[All Fields]	L-amino-acid oxidase	18469105	SO-LAAO, a novel L-amino acid oxidase that enables Streptococcus oligofermentans to outcompete Streptococcus mutans by generating H2O2 from peptone.
2907	DRAMP02460	L-amino-acid oxidase (LAAO; LAO; snakes, reptils, animals)	"L-amino-acid oxidase"[All Fields] AND biofilm[All Fields]	L-amino-acid oxidase	30160994	Molecules and Mechanisms Underlying the Antimicrobial Activity of Escapin, an l-Amino Acid Oxidase from the Ink of Sea Hares.
2907	DRAMP02460	L-amino-acid oxidase (LAAO; LAO; snakes, reptils, animals)	"L-amino-acid oxidase"[All Fields] AND biofilm[All Fields]	L-amino-acid oxidase	27401562	Inhibition and Dispersal of Pseudomonas aeruginosa Biofilms by Combination Treatment with Escapin Intermediate Products and Hydrogen Peroxide.
2907	DRAMP02460	L-amino-acid oxidase (LAAO; LAO; snakes, reptils, animals)	"L-amino-acid oxidase"[All Fields] AND biofilm[All Fields]	L-amino-acid oxidase	18469105	SO-LAAO, a novel L-amino acid oxidase that enables Streptococcus oligofermentans to outcompete Streptococcus mutans by generating H2O2 from peptone.
2908	DRAMP02461	L-amino-acid oxidase (BmarLAAO; LAAO; LAO; snakes, reptils, animals)	"L-amino-acid oxidase"[All Fields] AND biofilm[All Fields]	L-amino-acid oxidase	30160994	Molecules and Mechanisms Underlying the Antimicrobial Activity of Escapin, an l-Amino Acid Oxidase from the Ink of Sea Hares.
2908	DRAMP02461	L-amino-acid oxidase (BmarLAAO; LAAO; LAO; snakes, reptils, animals)	"L-amino-acid oxidase"[All Fields] AND biofilm[All Fields]	L-amino-acid oxidase	27401562	Inhibition and Dispersal of Pseudomonas aeruginosa Biofilms by Combination Treatment with Escapin Intermediate Products and Hydrogen Peroxide.
2908	DRAMP02461	L-amino-acid oxidase (BmarLAAO; LAAO; LAO; snakes, reptils, animals)	"L-amino-acid oxidase"[All Fields] AND biofilm[All Fields]	L-amino-acid oxidase	18469105	SO-LAAO, a novel L-amino acid oxidase that enables Streptococcus oligofermentans to outcompete Streptococcus mutans by generating H2O2 from peptone.
2909	DRAMP02462	L-amino-acid oxidase (LAAO; LAO; snakes, reptils, animals)	"L-amino-acid oxidase"[All Fields] AND biofilm[All Fields]	L-amino-acid oxidase	30160994	Molecules and Mechanisms Underlying the Antimicrobial Activity of Escapin, an l-Amino Acid Oxidase from the Ink of Sea Hares.
2909	DRAMP02462	L-amino-acid oxidase (LAAO; LAO; snakes, reptils, animals)	"L-amino-acid oxidase"[All Fields] AND biofilm[All Fields]	L-amino-acid oxidase	27401562	Inhibition and Dispersal of Pseudomonas aeruginosa Biofilms by Combination Treatment with Escapin Intermediate Products and Hydrogen Peroxide.
2909	DRAMP02462	L-amino-acid oxidase (LAAO; LAO; snakes, reptils, animals)	"L-amino-acid oxidase"[All Fields] AND biofilm[All Fields]	L-amino-acid oxidase	18469105	SO-LAAO, a novel L-amino acid oxidase that enables Streptococcus oligofermentans to outcompete Streptococcus mutans by generating H2O2 from peptone.
2910	DRAMP02463	L-amino-acid oxidase (LAAO, LAO, LN-AAO; Reptiles, animals)	"L-amino-acid oxidase"[All Fields] AND biofilm[All Fields]	L-amino-acid oxidase	30160994	Molecules and Mechanisms Underlying the Antimicrobial Activity of Escapin, an l-Amino Acid Oxidase from the Ink of Sea Hares.
2910	DRAMP02463	L-amino-acid oxidase (LAAO, LAO, LN-AAO; Reptiles, animals)	"L-amino-acid oxidase"[All Fields] AND biofilm[All Fields]	L-amino-acid oxidase	27401562	Inhibition and Dispersal of Pseudomonas aeruginosa Biofilms by Combination Treatment with Escapin Intermediate Products and Hydrogen Peroxide.
2910	DRAMP02463	L-amino-acid oxidase (LAAO, LAO, LN-AAO; Reptiles, animals)	"L-amino-acid oxidase"[All Fields] AND biofilm[All Fields]	L-amino-acid oxidase	18469105	SO-LAAO, a novel L-amino acid oxidase that enables Streptococcus oligofermentans to outcompete Streptococcus mutans by generating H2O2 from peptone.
2911	DRAMP02464	L-amino-acid oxidase (LAAO, LAO; reptilia, animals)	"L-amino-acid oxidase"[All Fields] AND biofilm[All Fields]	L-amino-acid oxidase	30160994	Molecules and Mechanisms Underlying the Antimicrobial Activity of Escapin, an l-Amino Acid Oxidase from the Ink of Sea Hares.
2911	DRAMP02464	L-amino-acid oxidase (LAAO, LAO; reptilia, animals)	"L-amino-acid oxidase"[All Fields] AND biofilm[All Fields]	L-amino-acid oxidase	27401562	Inhibition and Dispersal of Pseudomonas aeruginosa Biofilms by Combination Treatment with Escapin Intermediate Products and Hydrogen Peroxide.
2911	DRAMP02464	L-amino-acid oxidase (LAAO, LAO; reptilia, animals)	"L-amino-acid oxidase"[All Fields] AND biofilm[All Fields]	L-amino-acid oxidase	18469105	SO-LAAO, a novel L-amino acid oxidase that enables Streptococcus oligofermentans to outcompete Streptococcus mutans by generating H2O2 from peptone.
2914	DRAMP02467	L-amino-acid oxidase (LAAO; LAO; Reptiles, animals)	"L-amino-acid oxidase"[All Fields] AND biofilm[All Fields]	L-amino-acid oxidase	30160994	Molecules and Mechanisms Underlying the Antimicrobial Activity of Escapin, an l-Amino Acid Oxidase from the Ink of Sea Hares.
2914	DRAMP02467	L-amino-acid oxidase (LAAO; LAO; Reptiles, animals)	"L-amino-acid oxidase"[All Fields] AND biofilm[All Fields]	L-amino-acid oxidase	27401562	Inhibition and Dispersal of Pseudomonas aeruginosa Biofilms by Combination Treatment with Escapin Intermediate Products and Hydrogen Peroxide.
2914	DRAMP02467	L-amino-acid oxidase (LAAO; LAO; Reptiles, animals)	"L-amino-acid oxidase"[All Fields] AND biofilm[All Fields]	L-amino-acid oxidase	18469105	SO-LAAO, a novel L-amino acid oxidase that enables Streptococcus oligofermentans to outcompete Streptococcus mutans by generating H2O2 from peptone.
2917	DRAMP02471	Thiostrepton (Alaninamide; Bryamycin; Gargon; Thiactin)	"Thiostrepton"[All Fields] AND biofilm[All Fields]	Thiostrepton	31262758	Thiostrepton Hijacks Pyoverdine Receptors To Inhibit Growth of Pseudomonas aeruginosa.
2947	DRAMP18259	Enterocin O16 (Bacteriocin)	"Enterocin O16"[All Fields] AND biofilm[All Fields]	Enterocin O16	28467378	Molecular Mechanism of Quorum-Sensing in Enterococcus faecalis: Its Role in Virulence and Therapeutic Approaches.
2948	DRAMP18355	Lacticin Z (bacteriocin)	"Lacticin Z"[All Fields] AND biofilm[All Fields]	Lacticin Z	22155816	Identification, characterization, and recombinant expression of epidermicin NI01, a novel unmodified bacteriocin produced by Staphylococcus epidermidis that displays potent activity against Staphylococci.
2951	DRAMP02511	Crotamine (defensin-like toxin; Snakes, reptiles, animals)	"Crotamine"[All Fields] AND biofilm[All Fields]	Crotamine	24754574	Interaction of the rattlesnake toxin crotamine with model membranes.
2952	DRAMP02512	L-amino-acid oxidase (Casca LAO, LAAO, LAO; Snakes, reptiles, animals)	"L-amino-acid oxidase"[All Fields] AND biofilm[All Fields]	L-amino-acid oxidase	30160994	Molecules and Mechanisms Underlying the Antimicrobial Activity of Escapin, an l-Amino Acid Oxidase from the Ink of Sea Hares.
2952	DRAMP02512	L-amino-acid oxidase (Casca LAO, LAAO, LAO; Snakes, reptiles, animals)	"L-amino-acid oxidase"[All Fields] AND biofilm[All Fields]	L-amino-acid oxidase	27401562	Inhibition and Dispersal of Pseudomonas aeruginosa Biofilms by Combination Treatment with Escapin Intermediate Products and Hydrogen Peroxide.
2952	DRAMP02512	L-amino-acid oxidase (Casca LAO, LAAO, LAO; Snakes, reptiles, animals)	"L-amino-acid oxidase"[All Fields] AND biofilm[All Fields]	L-amino-acid oxidase	18469105	SO-LAAO, a novel L-amino acid oxidase that enables Streptococcus oligofermentans to outcompete Streptococcus mutans by generating H2O2 from peptone.
2953	DRAMP18398	Um2 (scorpions, arachnids, Chelicerata, arthropods, invertebrates, animals)	"Um2"[All Fields] AND biofilm[All Fields]	Um2	31940838	Urbanization and Waterborne Pathogen Emergence in Low-Income Countries: Where and How to Conduct Surveys?
2953	DRAMP18398	Um2 (scorpions, arachnids, Chelicerata, arthropods, invertebrates, animals)	"Um2"[All Fields] AND biofilm[All Fields]	Um2	27920769	Transcriptional Profiling of Type II Toxin-Antitoxin Genes of Helicobacter pyloriunder Different Environmental Conditions: Identification of HP0967-HP0968 System.
2953	DRAMP18398	Um2 (scorpions, arachnids, Chelicerata, arthropods, invertebrates, animals)	"Um2"[All Fields] AND biofilm[All Fields]	Um2	25562573	In vitro and in vivo characterization of antibacterial activity and biocompatibility: a study on silver-containing phosphonate monolayers on titanium.
3023	DRAMP02597	Clavanin-A (His-rich; chordates, animals)	"Clavanin-A"[All Fields] AND biofilm[All Fields]	Clavanin-A	29751343	Clavanin A-bioconjugated Fe 3O 4/Silane core-shell nanoparticles for thermal ablation of bacterial biofilms.
3023	DRAMP02597	Clavanin-A (His-rich; chordates, animals)	"Clavanin-A"[All Fields] AND biofilm[All Fields]	Clavanin-A	29047302	A self-assembled clavanin A-coated amniotic membrane scaffold for the prevention of biofilm formation by ocular surface fungal pathogens.

3023	DRAMP02597	Clavanin-A (His-rich; chordates, animals)	"Clavanin-A"[All Fields] AND biofilm[All Fields]	Clavanin-A	28262044	An alternative approach to treating antibiotic-resistant infections.
3023	DRAMP02597	Clavanin-A (His-rich; chordates, animals)	"Clavanin-A"[All Fields] AND biofilm[All Fields]	Clavanin-A	27292548	Structural Studies of a Lipid-Binding Peptide from Tunicate Hemocytes with Anti-Biofilm Activity.
3023	DRAMP02597	Clavanin-A (His-rich; chordates, animals)	"Clavanin-A"[All Fields] AND biofilm[All Fields]	Clavanin-A	19878192	Design and characterization of an acid-activated antimicrobial peptide.
3034	DRAMP02611	Beta-defensin 1 (BD-1; Defensin, beta 1; primates, mammals, animals)	"Beta-defensin 1"[All Fields] AND biofilm[All Fields]	Beta-defensin 1	33420317	Curbing gastrointestinal infections by defensin fragment modifications without harming commensal microbiota.
3034	DRAMP02611	Beta-defensin 1 (BD-1; Defensin, beta 1; primates, mammals, animals)	"Beta-defensin 1"[All Fields] AND biofilm[All Fields]	Beta-defensin 1	32585445	Glucose effect on Candida albicans biofilm during tissue invasion.
3034	DRAMP02611	Beta-defensin 1 (BD-1; Defensin, beta 1; primates, mammals, animals)	"Beta-defensin 1"[All Fields] AND biofilm[All Fields]	Beta-defensin 1	30260708	Stabilized collagen matrix dressing improves wound macrophage function and epithelialization.
3034	DRAMP02611	Beta-defensin 1 (BD-1; Defensin, beta 1; primates, mammals, animals)	"Beta-defensin 1"[All Fields] AND biofilm[All Fields]	Beta-defensin 1	28642103	Chimeric analogs of human β-defensin 1 and θ-defensin disrupt pre-established bacterial biofilms.
3034	DRAMP02611	Beta-defensin 1 (BD-1; Defensin, beta 1; primates, mammals, animals)	"Beta-defensin 1"[All Fields] AND biofilm[All Fields]	Beta-defensin 1	26214284	α-tocopherol decreases interleukin-1β and -6 and increases human β-defensin-1 and -2 secretion in human gingival fibroblasts stimulated with Porphyromonas gingivalis lipopolysaccharide.
3034	DRAMP02611	Beta-defensin 1 (BD-1; Defensin, beta 1; primates, mammals, animals)	"Beta-defensin 1"[All Fields] AND biofilm[All Fields]	Beta-defensin 1	22394470	Association of CiaRH with resistance of Streptococcus mutans to antimicrobial peptides in biofilms.
3034	DRAMP02611	Beta-defensin 1 (BD-1; Defensin, beta 1; primates, mammals, animals)	"Beta-defensin 1"[All Fields] AND biofilm[All Fields]	Beta-defensin 1	20454633	Normal human gingival epithelial cells sense C. parapsilosis by toll-like receptors and module its pathogenesis through antimicrobial peptides and proinflammatory cytokines.
3037	DRAMP02615	Beta-defensin 1 (BD-1; Defensin, beta 1; primates, mammals, animals)	"Beta-defensin 1"[All Fields] AND biofilm[All Fields]	Beta-defensin 1	33420317	Curbing gastrointestinal infections by defensin fragment modifications without harming commensal microbiota.
3037	DRAMP02615	Beta-defensin 1 (BD-1; Defensin, beta 1; primates, mammals, animals)	"Beta-defensin 1"[All Fields] AND biofilm[All Fields]	Beta-defensin 1	32585445	Glucose effect on Candida albicans biofilm during tissue invasion.
3037	DRAMP02615	Beta-defensin 1 (BD-1; Defensin, beta 1; primates, mammals, animals)	"Beta-defensin 1"[All Fields] AND biofilm[All Fields]	Beta-defensin 1	30260708	Stabilized collagen matrix dressing improves wound macrophage function and epithelialization.
3037	DRAMP02615	Beta-defensin 1 (BD-1; Defensin, beta 1; primates, mammals, animals)	"Beta-defensin 1"[All Fields] AND biofilm[All Fields]	Beta-defensin 1	28642103	Chimeric analogs of human β-defensin 1 and θ-defensin disrupt pre-established bacterial biofilms.
3037	DRAMP02615	Beta-defensin 1 (BD-1; Defensin, beta 1; primates, mammals, animals)	"Beta-defensin 1"[All Fields] AND biofilm[All Fields]	Beta-defensin 1	26214284	α-tocopherol decreases interleukin-1β and -6 and increases human β-defensin-1 and -2 secretion in human gingival fibroblasts stimulated with Porphyromonas gingivalis lipopolysaccharide.
3037	DRAMP02615	Beta-defensin 1 (BD-1; Defensin, beta 1; primates, mammals, animals)	"Beta-defensin 1"[All Fields] AND biofilm[All Fields]	Beta-defensin 1	22394470	Association of CiaRH with resistance of Streptococcus mutans to antimicrobial peptides in biofilms.
3037	DRAMP02615	Beta-defensin 1 (BD-1; Defensin, beta 1; primates, mammals, animals)	"Beta-defensin 1"[All Fields] AND biofilm[All Fields]	Beta-defensin 1	20454633	Normal human gingival epithelial cells sense C. parapsilosis by toll-like receptors and module its pathogenesis through antimicrobial peptides and proinflammatory cytokines.
3049	DRAMP02627	Beta-defensin 1 (BD-1; Defensin, beta 1; primates, mammals, animals)	"Beta-defensin 1"[All Fields] AND biofilm[All Fields]	Beta-defensin 1	33420317	Curbing gastrointestinal infections by defensin fragment modifications without harming commensal microbiota.
3049	DRAMP02627	Beta-defensin 1 (BD-1; Defensin, beta 1; primates, mammals, animals)	"Beta-defensin 1"[All Fields] AND biofilm[All Fields]	Beta-defensin 1	32585445	Glucose effect on Candida albicans biofilm during tissue invasion.
3049	DRAMP02627	Beta-defensin 1 (BD-1; Defensin, beta 1; primates, mammals, animals)	"Beta-defensin 1"[All Fields] AND biofilm[All Fields]	Beta-defensin 1	30260708	Stabilized collagen matrix dressing improves wound macrophage function and epithelialization.
3049	DRAMP02627	Beta-defensin 1 (BD-1; Defensin, beta 1; primates, mammals, animals)	"Beta-defensin 1"[All Fields] AND biofilm[All Fields]	Beta-defensin 1	28642103	Chimeric analogs of human β-defensin 1 and θ-defensin disrupt pre-established bacterial biofilms.
3049	DRAMP02627	Beta-defensin 1 (BD-1; Defensin, beta 1; primates, mammals, animals)	"Beta-defensin 1"[All Fields] AND biofilm[All Fields]	Beta-defensin 1	26214284	α-tocopherol decreases interleukin-1β and -6 and increases human β-defensin-1 and -2 secretion in human gingival fibroblasts stimulated with Porphyromonas gingivalis lipopolysaccharide.
3049	DRAMP02627	Beta-defensin 1 (BD-1; Defensin, beta 1; primates, mammals, animals)	"Beta-defensin 1"[All Fields] AND biofilm[All Fields]	Beta-defensin 1	22394470	Association of CiaRH with resistance of Streptococcus mutans to antimicrobial peptides in biofilms.
3049	DRAMP02627	Beta-defensin 1 (BD-1; Defensin, beta 1; primates, mammals, animals)	"Beta-defensin 1"[All Fields] AND biofilm[All Fields]	Beta-defensin 1	20454633	Normal human gingival epithelial cells sense C. parapsilosis by toll-like receptors and module its pathogenesis through antimicrobial peptides and proinflammatory cytokines.
3050	DRAMP18250	Laterosporulin (Bacteriocin)	"Laterosporulin"[All Fields] AND biofilm[All Fields]	Laterosporulin	33927461	Improvisation and Evaluation of Laterosporulin Coated Titanium Surfaces for dental Applications: An In Vitro Investigation.
3065	DRAMP02642	Rhesus theta-defensin 1 (RTD-1; primates, mammals, animals)	"Rhesus theta-defensin 1"[All Fields] AND biofilm[All Fields]	Rhesus theta-defensin 1	31729441	Rhesus Theta Defensin 1 Promotes Long Term Survival in Systemic Candidiasis by Host Directed Mechanisms.
3070	DRAMP02647	Beta-defensin 2	"Beta-defensin 2"[All Fields] AND biofilm[All Fields]	Beta-defensin 2	34276631	DNA Blocks the Lethal Effect of Human Beta-Defensin 2 Against Neisseria meningitidis.
3070	DRAMP02647	Beta-defensin 2	"Beta-defensin 2"[All Fields] AND biofilm[All Fields]	Beta-defensin 2	32842903	Electrospun ZnO/Poly(Vinylidene Fluoride-Trifluoroethylene) Scaffolds for Lung Tissue Engineering.
3070	DRAMP02647	Beta-defensin 2	"Beta-defensin 2"[All Fields] AND biofilm[All Fields]	Beta-defensin 2	32522780	Controlling the Growth of the Skin Commensal Staphylococcus epidermidis Using d-Alanine Auxotrophy.
3070	DRAMP02647	Beta-defensin 2	"Beta-defensin 2"[All Fields] AND biofilm[All Fields]	Beta-defensin 2	32457749	The Antimicrobial Peptide Human Beta-Defensin 2 Inhibits Biofilm Production of Pseudomonas aeruginosaWithout Compromising Metabolic Activity.
3070	DRAMP02647	Beta-defensin 2	"Beta-defensin 2"[All Fields] AND biofilm[All Fields]	Beta-defensin 2	31336838	Candida albicans-Cell Interactions Activate Innate Immune Defense in Human Palate Epithelial Primary Cells via Nitric Oxide (NO) and β-Defensin 2 (hBD-2).
3070	DRAMP02647	Beta-defensin 2	"Beta-defensin 2"[All Fields] AND biofilm[All Fields]	Beta-defensin 2	30649289	Innate immune components affect growth and virulence traits of bacterial-vaginosis-associated and non-bacterial-vaginosis-associated Gardnerella vaginalis strains similarly.
3070	DRAMP02647	Beta-defensin 2	"Beta-defensin 2"[All Fields] AND biofilm[All Fields]	Beta-defensin 2	27582732	Efficient Eradication of Mature Pseudomonas aeruginosa Biofilm via Controlled Delivery of Nitric Oxide Combined with Antimicrobial Peptide and Antibiotics.
3070	DRAMP02647	Beta-defensin 2	"Beta-defensin 2"[All Fields] AND biofilm[All Fields]	Beta-defensin 2	27148195	Effect of Substance P in Staphylococcus aureus and Staphylococcus epidermidis Virulence: Implication for Skin Homeostasis.
3070	DRAMP02647	Beta-defensin 2	"Beta-defensin 2"[All Fields] AND biofilm[All Fields]	Beta-defensin 2	25808131	Expression of antimicrobial peptides and interleukin-8 during early stages of inflammation: An experimental gingivitis study.
3070	DRAMP02647	Beta-defensin 2	"Beta-defensin 2"[All Fields] AND biofilm[All Fields]	Beta-defensin 2	24340061	Inflammatory and antimicrobial responses to methicillin-resistant Staphylococcus aureus in an in vitro wound infection model.
3070	DRAMP02647	Beta-defensin 2	"Beta-defensin 2"[All Fields] AND biofilm[All Fields]	Beta-defensin 2	20378008	Effect of temperature on the shift of Pseudomonas fluorescens from an environmental microorganism to a potential human pathogen.

3070	DRAMP02647	Beta-defensin 2	"Beta-defensin 2"[All Fields] AND biofilm[All Fields]	Beta-defensin 2	19572896	The immune response of oral epithelial cells induced by single-species and complex naturally formed biofilms.
3070	DRAMP02647	Beta-defensin 2	"Beta-defensin 2"[All Fields] AND biofilm[All Fields]	Beta-defensin 2	18954353	Treponema denticola does not induce production of common innate immune mediators from primary gingival epithelial cells.
3070	DRAMP02647	Beta-defensin 2	"Beta-defensin 2"[All Fields] AND biofilm[All Fields]	Beta-defensin 2	18173794	The stage of native biofilm formation determines the gene expression of human beta-defensin-2, psoriasin, ribonuclease 7 and inflammatory mediators: a novel approach for stimulation of keratinocytes with in situ formed biofilms.
3070	DRAMP02647	Beta-defensin 2	"Beta-defensin 2"[All Fields] AND biofilm[All Fields]	Beta-defensin 2	17434999	Functional analysis of D-alanylation of lipoteichoic acid in the probiotic strain Lactobacillus rhamnosus GG.
3077	DRAMP02655	Alpha defensin (primates, mammals, animals)	"Alpha defensin"[All Fields] AND biofilm[All Fields]	Alpha defensin	33447687	Future directions of postoperative spinal implant infections.
3077	DRAMP02655	Alpha defensin (primates, mammals, animals)	"Alpha defensin"[All Fields] AND biofilm[All Fields]	Alpha defensin	32663201	Inhibition and eradication activity of truncated α-defensin analogs against multidrug resistant uropathogenic Escherichia coli biofilm.
3077	DRAMP02655	Alpha defensin (primates, mammals, animals)	"Alpha defensin"[All Fields] AND biofilm[All Fields]	Alpha defensin	30376742	Alpha defensin, leukocyte esterase, C-reactive protein, and leukocyte count in synovial fluid for pre-operative diagnosis of periprosthetic infection.
3077	DRAMP02655	Alpha defensin (primates, mammals, animals)	"Alpha defensin"[All Fields] AND biofilm[All Fields]	Alpha defensin	29671721	The BceABRS four-component system that is essential for cell envelope stress response is involved in sensing and response to host defence peptides and is required for the biofilm formation and fitness of Streptococcus mutans.
3077	DRAMP02655	Alpha defensin (primates, mammals, animals)	"Alpha defensin"[All Fields] AND biofilm[All Fields]	Alpha defensin	28296382	Human α-Defensin 6: A Small Peptide That Self-Assembles and Protects the Host by Entangling Microbes.
3077	DRAMP02655	Alpha defensin (primates, mammals, animals)	"Alpha defensin"[All Fields] AND biofilm[All Fields]	Alpha defensin	28144375	Advancements in Diagnosing Periprosthetic Joint Infections after Total Hip and Knee Arthroplasty.
3077	DRAMP02655	Alpha defensin (primates, mammals, animals)	"Alpha defensin"[All Fields] AND biofilm[All Fields]	Alpha defensin	28026958	Human α-Defensin 6 Self-Assembly Prevents Adhesion and Suppresses Virulence Traits of Candida albicans.
3077	DRAMP02655	Alpha defensin (primates, mammals, animals)	"Alpha defensin"[All Fields] AND biofilm[All Fields]	Alpha defensin	25806720	Antimicrobial peptides in 2014.
3079	DRAMP02657	Alpha-defensin 1 (primates, mammals, animals)	"Alpha-defensin 1"[All Fields] AND biofilm[All Fields]	Alpha-defensin 1	29671721	The BceABRS four-component system that is essential for cell envelope stress response is involved in sensing and response to host defence peptides and is required for the biofilm formation and fitness of Streptococcus mutans.
3080	DRAMP02658	Alpha-defensin 6 (primates, mammals, animals)	"Alpha-defensin 6"[All Fields] AND biofilm[All Fields]	Alpha-defensin 6	28296382	Human α-Defensin 6: A Small Peptide That Self-Assembles and Protects the Host by Entangling Microbes.
3080	DRAMP02658	Alpha-defensin 6 (primates, mammals, animals)	"Alpha-defensin 6"[All Fields] AND biofilm[All Fields]	Alpha-defensin 6	28026958	Human α-Defensin 6 Self-Assembly Prevents Adhesion and Suppresses Virulence Traits of Candida albicans.
3089	DRAMP02668	Defensin-6 (Defensin, alpha 6; primates, mammals, animals)	"Defensin-6"[All Fields] AND biofilm[All Fields]	Defensin-6	28296382	Human α-Defensin 6: A Small Peptide That Self-Assembles and Protects the Host by Entangling Microbes.
3089	DRAMP02668	Defensin-6 (Defensin, alpha 6; primates, mammals, animals)	"Defensin-6"[All Fields] AND biofilm[All Fields]	Defensin-6	28026958	Human α-Defensin 6 Self-Assembly Prevents Adhesion and Suppresses Virulence Traits of Candida albicans.
3092	DRAMP02671	Beta-defensin 1 (BD-1; primates, mammals, animals)	"Beta-defensin 1"[All Fields] AND biofilm[All Fields]	Beta-defensin 1	33420317	Curbing gastrointestinal infections by defensin fragment modifications without harming commensal microbiota.
3092	DRAMP02671	Beta-defensin 1 (BD-1; primates, mammals, animals)	"Beta-defensin 1"[All Fields] AND biofilm[All Fields]	Beta-defensin 1	32585445	Glucose effect on Candida albicans biofilm during tissue invasion.
3092	DRAMP02671	Beta-defensin 1 (BD-1; primates, mammals, animals)	"Beta-defensin 1"[All Fields] AND biofilm[All Fields]	Beta-defensin 1	30260708	Stabilized collagen matrix dressing improves wound macrophage function and epithelialization.
3092	DRAMP02671	Beta-defensin 1 (BD-1; primates, mammals, animals)	"Beta-defensin 1"[All Fields] AND biofilm[All Fields]	Beta-defensin 1	28642103	Chimeric analogs of human β-defensin 1 and θ-defensin disrupt pre-established bacterial biofilms.
3092	DRAMP02671	Beta-defensin 1 (BD-1; primates, mammals, animals)	"Beta-defensin 1"[All Fields] AND biofilm[All Fields]	Beta-defensin 1	26214284	α-tocopherol decreases interleukin-1β and -6 and increases human β-defensin-1 and -2 secretion in human gingival fibroblasts stimulated with Porphyromonas gingivalis lipopolysaccharide.
3092	DRAMP02671	Beta-defensin 1 (BD-1; primates, mammals, animals)	"Beta-defensin 1"[All Fields] AND biofilm[All Fields]	Beta-defensin 1	22394470	Association of CiaRH with resistance of Streptococcus mutans to antimicrobial peptides in biofilms.
3092	DRAMP02671	Beta-defensin 1 (BD-1; primates, mammals, animals)	"Beta-defensin 1"[All Fields] AND biofilm[All Fields]	Beta-defensin 1	20454633	Normal human gingival epithelial cells sense C. parapsilosis by toll-like receptors and module its pathogenesis through antimicrobial peptides and proinflammatory cytokines.
3118	DRAMP02697	Defensin-5 (Defensin, alpha 5; primates, mammals, animals)	"Defensin-5"[All Fields] AND biofilm[All Fields]	Defensin-5	28296382	Human α-Defensin 6: A Small Peptide That Self-Assembles and Protects the Host by Entangling Microbes.
3131	DRAMP18241	Subtilomycin(Bacteriocin)	"Subtilomycin"[All Fields] AND biofilm[All Fields]	Subtilomycin	27797439	Inhibition of Listeria monocytogenes biofilms by bacteriocin-producing bacteria isolated from mushroom substrate.
3133	DRAMP18238	Fengycin B(Bacteriocin)	"Fengycin B"[All Fields] AND biofilm[All Fields]	Fengycin B	20623484	LC/ESI-MS/MS characterisation of lipopeptide biosurfactants produced by the Bacillus licheniformis V9T14 strain.
3134	DRAMP02713	Hepcidin (primates, mammals, animals)	"Hepcidin"[All Fields] AND biofilm[All Fields]	Hepcidin	30456212	Quantification of Lipoteichoic Acid in Hemodialysis Patients With Central Venous Catheters.
3134	DRAMP02713	Hepcidin (primates, mammals, animals)	"Hepcidin"[All Fields] AND biofilm[All Fields]	Hepcidin	30408337	Silk-Based Antimicrobial Polymers as a New Platform to Design Drug-Free Materials to Impede Microbial Infections.
3134	DRAMP02713	Hepcidin (primates, mammals, animals)	"Hepcidin"[All Fields] AND biofilm[All Fields]	Hepcidin	24645694	Inhibitory effect of the human liver-derived antimicrobial peptide hepcidin 20 on biofilms of polysaccharide intercellular adhesin (PIA)-positive and PIA-negative strains of Staphylococcus epidermidis.
3143	DRAMP02722	Beta-defensin 1 (BD-1; Defensin, beta 1; primates, mammals,animals)	"Beta-defensin 1"[All Fields] AND biofilm[All Fields]	Beta-defensin 1	33420317	Curbing gastrointestinal infections by defensin fragment modifications without harming commensal microbiota.
3143	DRAMP02722	Beta-defensin 1 (BD-1; Defensin, beta 1; primates, mammals,animals)	"Beta-defensin 1"[All Fields] AND biofilm[All Fields]	Beta-defensin 1	32585445	Glucose effect on Candida albicans biofilm during tissue invasion.
3143	DRAMP02722	Beta-defensin 1 (BD-1; Defensin, beta 1; primates, mammals,animals)	"Beta-defensin 1"[All Fields] AND biofilm[All Fields]	Beta-defensin 1	30260708	Stabilized collagen matrix dressing improves wound macrophage function and epithelialization.
3143	DRAMP02722	Beta-defensin 1 (BD-1; Defensin, beta 1; primates, mammals,animals)	"Beta-defensin 1"[All Fields] AND biofilm[All Fields]	Beta-defensin 1	28642103	Chimeric analogs of human β-defensin 1 and θ-defensin disrupt pre-established bacterial biofilms.
3143	DRAMP02722	Beta-defensin 1 (BD-1; Defensin, beta 1; primates, mammals,animals)	"Beta-defensin 1"[All Fields] AND biofilm[All Fields]	Beta-defensin 1	26214284	α-tocopherol decreases interleukin-1β and -6 and increases human β-defensin-1 and -2 secretion in human gingival fibroblasts stimulated with Porphyromonas gingivalis lipopolysaccharide.
3143	DRAMP02722	Beta-defensin 1 (BD-1; Defensin, beta 1; primates, mammals,animals)	"Beta-defensin 1"[All Fields] AND biofilm[All Fields]	Beta-defensin 1	22394470	Association of CiaRH with resistance of Streptococcus mutans to antimicrobial peptides in biofilms.
3143	DRAMP02722	Beta-defensin 1 (BD-1; Defensin, beta 1; primates, mammals,animals)	"Beta-defensin 1"[All Fields] AND biofilm[All Fields]	Beta-defensin 1	20454633	Normal human gingival epithelial cells sense C. parapsilosis by toll-like receptors and module its pathogenesis through antimicrobial peptides and proinflammatory cytokines.
3144	DRAMP18237	Fengycin A(Bacteriocin)	"Fengycin A"[All Fields] AND biofilm[All Fields]	Fengycin A	20623484	LC/ESI-MS/MS characterisation of lipopeptide biosurfactants produced by the Bacillus licheniformis V9T14 strain.
3156	DRAMP02738	Beta-defensin 1 (BD-1; Defensin, beta 1; primates, mammals, animals)	"Beta-defensin 1"[All Fields] AND biofilm[All Fields]	Beta-defensin 1	33420317	Curbing gastrointestinal infections by defensin fragment modifications without harming commensal microbiota.
3156	DRAMP02738	Beta-defensin 1 (BD-1; Defensin, beta 1; primates, mammals, animals)	"Beta-defensin 1"[All Fields] AND biofilm[All Fields]	Beta-defensin 1	32585445	Glucose effect on Candida albicans biofilm during tissue invasion.
3156	DRAMP02738	Beta-defensin 1 (BD-1; Defensin, beta 1; primates, mammals, animals)	"Beta-defensin 1"[All Fields] AND biofilm[All Fields]	Beta-defensin 1	30260708	Stabilized collagen matrix dressing improves wound macrophage function and epithelialization.

3156	DRAMP02738	Beta-defensin 1 (BD-1; Defensin, beta 1; primates, mammals, animals)	"Beta-defensin 1"[All Fields] AND biofilm[All Fields]	Beta-defensin 1	28642103	Chimeric analogs of human β-defensin 1 and θ-defensin disrupt pre-established bacterial biofilms.
3156	DRAMP02738	Beta-defensin 1 (BD-1; Defensin, beta 1; primates, mammals, animals)	"Beta-defensin 1"[All Fields] AND biofilm[All Fields]	Beta-defensin 1	26214284	α-tocopherol decreases interleukin-1β and -6 and increases human β-defensin-1 and -2 secretion in human gingival fibroblasts stimulated with Porphyromonas gingivalis lipopolysaccharide.
3156	DRAMP02738	Beta-defensin 1 (BD-1; Defensin, beta 1; primates, mammals, animals)	"Beta-defensin 1"[All Fields] AND biofilm[All Fields]	Beta-defensin 1	22394470	Association of CiaRH with resistance of Streptococcus mutans to antimicrobial peptides in biofilms.
3156	DRAMP02738	Beta-defensin 1 (BD-1; Defensin, beta 1; primates, mammals, animals)	"Beta-defensin 1"[All Fields] AND biofilm[All Fields]	Beta-defensin 1	20454633	Normal human gingival epithelial cells sense C. parapsilosis by toll-like receptors and module its pathogenesis through antimicrobial peptides and proinflammatory cytokines.
3167	DRAMP02750	Defensin (ants, insects, animals)	"Defensin"[All Fields] AND biofilm[All Fields]	Defensin	34408988	The Anti-Biofilm Efficacy of Caffeic Acid Phenethyl Ester (CAPE) In Vitroand a Murine Model of Oral Candidiasis.
3167	DRAMP02750	Defensin (ants, insects, animals)	"Defensin"[All Fields] AND biofilm[All Fields]	Defensin	34321877	Antibacterial Effect of Honey-Derived Exosomes Containing Antimicrobial Peptides Against Oral Streptococci.
3167	DRAMP02750	Defensin (ants, insects, animals)	"Defensin"[All Fields] AND biofilm[All Fields]	Defensin	34276631	DNA Blocks the Lethal Effect of Human Beta-Defensin 2 Against Neisseria meningitidis.
3167	DRAMP02750	Defensin (ants, insects, animals)	"Defensin"[All Fields] AND biofilm[All Fields]	Defensin	33911935	The antibacterial activities of honey.
3167	DRAMP02750	Defensin (ants, insects, animals)	"Defensin"[All Fields] AND biofilm[All Fields]	Defensin	33865931	Identification of a crocodylian β-defensin variant from Alligator mississippiensis with antimicrobial and antibiofilm activity.
3167	DRAMP02750	Defensin (ants, insects, animals)	"Defensin"[All Fields] AND biofilm[All Fields]	Defensin	33586659	Identification of anti-microbial peptides and traces of microbial DNA in infrainfundibular compartments of human scalp terminal hair follicles.
3167	DRAMP02750	Defensin (ants, insects, animals)	"Defensin"[All Fields] AND biofilm[All Fields]	Defensin	33534018	A recombinant fungal defensin-like peptide-P2 combats Streptococcus dysgalactiae and biofilms.
3167	DRAMP02750	Defensin (ants, insects, animals)	"Defensin"[All Fields] AND biofilm[All Fields]	Defensin	33447687	Future directions of postoperative spinal implant infections.
3167	DRAMP02750	Defensin (ants, insects, animals)	"Defensin"[All Fields] AND biofilm[All Fields]	Defensin	33420317	Curbing gastrointestinal infections by defensin fragment modifications without harming commensal microbiota.
3167	DRAMP02750	Defensin (ants, insects, animals)	"Defensin"[All Fields] AND biofilm[All Fields]	Defensin	32867384	A Novel Peptide Antibiotic, Pro10-1D, Designed from Insect Defensin Shows Antibacterial and Anti-Inflammatory Activities in Sepsis Models.
3167	DRAMP02750	Defensin (ants, insects, animals)	"Defensin"[All Fields] AND biofilm[All Fields]	Defensin	32858856	Candida albicansVirulence Factors and Pathogenicity for Endodontic Infections.
3167	DRAMP02750	Defensin (ants, insects, animals)	"Defensin"[All Fields] AND biofilm[All Fields]	Defensin	32842903	Electrospun ZnO/Poly(Vinylidene Fluoride-Trifluoroethylene) Scaffolds for Lung Tissue Engineering.
3167	DRAMP02750	Defensin (ants, insects, animals)	"Defensin"[All Fields] AND biofilm[All Fields]	Defensin	32663201	Inhibition and eradication activity of truncated α-defensin analogs against multidrug resistant uropathogenic Escherichia coli biofilm.
3167	DRAMP02750	Defensin (ants, insects, animals)	"Defensin"[All Fields] AND biofilm[All Fields]	Defensin	32585445	Glucose effect on Candida albicans biofilm during tissue invasion.
3167	DRAMP02750	Defensin (ants, insects, animals)	"Defensin"[All Fields] AND biofilm[All Fields]	Defensin	32522780	Controlling the Growth of the Skin Commensal Staphylococcus epidermidis Using d-Alanine Auxotrophy.
3167	DRAMP02750	Defensin (ants, insects, animals)	"Defensin"[All Fields] AND biofilm[All Fields]	Defensin	32457749	The Antimicrobial Peptide Human Beta-Defensin 2 Inhibits Biofilm Production of Pseudomonas aeruginosaWithout Compromising Metabolic Activity.
3167	DRAMP02750	Defensin (ants, insects, animals)	"Defensin"[All Fields] AND biofilm[All Fields]	Defensin	32439511	Antibacterial activities and mechanisms of action of a defensin from manila clam Ruditapes philippinarum.
3167	DRAMP02750	Defensin (ants, insects, animals)	"Defensin"[All Fields] AND biofilm[All Fields]	Defensin	31933178	Effects of human β-defensin 3 fused with carbohydrate-binding domain on the function of type III secretion system in Pseudomonas aeruginosa PA14.
3167	DRAMP02750	Defensin (ants, insects, animals)	"Defensin"[All Fields] AND biofilm[All Fields]	Defensin	31906541	Role of FAD-I in Fusobacterial Interspecies Interaction and Biofilm Formation.
3167	DRAMP02750	Defensin (ants, insects, animals)	"Defensin"[All Fields] AND biofilm[All Fields]	Defensin	31729441	Rhesus Theta Defensin 1 Promotes Long Term Survival in Systemic Candidiasis by Host Directed Mechanisms.
3167	DRAMP02750	Defensin (ants, insects, animals)	"Defensin"[All Fields] AND biofilm[All Fields]	Defensin	31336838	Candida albicans-Cell Interactions Activate Innate Immune Defense in Human Palate Epithelial Primary Cells via Nitric Oxide (NO) and β-Defensin 2 (hBD-2).
3167	DRAMP02750	Defensin (ants, insects, animals)	"Defensin"[All Fields] AND biofilm[All Fields]	Defensin	31165072	Impact of the Food Additive Titanium Dioxide (E171) on Gut Microbiota-Host Interaction.
3167	DRAMP02750	Defensin (ants, insects, animals)	"Defensin"[All Fields] AND biofilm[All Fields]	Defensin	31031739	Salt-Tolerant Antifungal and Antibacterial Activities of the Corn Defensin ZmD32.
3167	DRAMP02750	Defensin (ants, insects, animals)	"Defensin"[All Fields] AND biofilm[All Fields]	Defensin	31025073	A recombinant fungal defensin-like peptide-P2 combats multidrug-resistant Staphylococcus aureus and biofilms.
3167	DRAMP02750	Defensin (ants, insects, animals)	"Defensin"[All Fields] AND biofilm[All Fields]	Defensin	30659503	Lactobacillus plantarum USM8613 Aids in Wound Healing and Suppresses Staphylococcus aureus Infection at Wound Sites.
3167	DRAMP02750	Defensin (ants, insects, animals)	"Defensin"[All Fields] AND biofilm[All Fields]	Defensin	30649289	Innate immune components affect growth and virulence traits of bacterial-vaginosis-associated and non-bacterial-vaginosis-associated Gardnerella vaginalis strains similarly.
3167	DRAMP02750	Defensin (ants, insects, animals)	"Defensin"[All Fields] AND biofilm[All Fields]	Defensin	30376742	Alpha defensin, leukocyte esterase, C-reactive protein, and leukocyte count in synovial fluid for pre-operative diagnosis of periprosthetic infection.
3167	DRAMP02750	Defensin (ants, insects, animals)	"Defensin"[All Fields] AND biofilm[All Fields]	Defensin	30260708	Stabilized collagen matrix dressing improves wound macrophage function and epithelialization.
3167	DRAMP02750	Defensin (ants, insects, animals)	"Defensin"[All Fields] AND biofilm[All Fields]	Defensin	30254440	Modification of the surface of titanium with multifunctional chimeric peptides to prevent biofilm formation via inhibition of initial colonizers.
3167	DRAMP02750	Defensin (ants, insects, animals)	"Defensin"[All Fields] AND biofilm[All Fields]	Defensin	29902560	A defensin-like antimicrobial peptide from the manila clam Ruditapes philippinarum: Investigation of the antibacterial activities and mode of action.
3167	DRAMP02750	Defensin (ants, insects, animals)	"Defensin"[All Fields] AND biofilm[All Fields]	Defensin	29872295	Antimicrobial peptide-loaded liquid crystalline precursor bioadhesive system for the prevention of dental caries.
3167	DRAMP02750	Defensin (ants, insects, animals)	"Defensin"[All Fields] AND biofilm[All Fields]	Defensin	29671721	The BceABRS four-component system that is essential for cell envelope stress response is involved in sensing and response to host defence peptides and is required for the biofilm formation and fitness of Streptococcus mutans.
3167	DRAMP02750	Defensin (ants, insects, animals)	"Defensin"[All Fields] AND biofilm[All Fields]	Defensin	29104569	A Linear 19-Mer Plant Defensin-Derived Peptide Acts Synergistically with Caspofungin against Candida albicansBiofilms.
3167	DRAMP02750	Defensin (ants, insects, animals)	"Defensin"[All Fields] AND biofilm[All Fields]	Defensin	29077172	The significance of HBD-3 and fluorescent composite carriers in the processof bone formation in rats infected with Staphylococcus aureus.
3167	DRAMP02750	Defensin (ants, insects, animals)	"Defensin"[All Fields] AND biofilm[All Fields]	Defensin	29045084	New Approach to Treat and Prevent Oral Disease.
3167	DRAMP02750	Defensin (ants, insects, animals)	"Defensin"[All Fields] AND biofilm[All Fields]	Defensin	29025642	Engineered chimeric peptides with antimicrobial and titanium-binding functions to inhibit biofilm formation on Ti implants.
3167	DRAMP02750	Defensin (ants, insects, animals)	"Defensin"[All Fields] AND biofilm[All Fields]	Defensin	28956355	The synthetic human beta-defensin-3 C15 peptide exhibits antimicrobial activity against Streptococcus mutans, both alone and in combination with dental disinfectants.
3167	DRAMP02750	Defensin (ants, insects, animals)	"Defensin"[All Fields] AND biofilm[All Fields]	Defensin	28951032	Antifungal Effects of Synthetic Human Beta-defensin-3-C15 Peptide on Candida albicans-infected Root Dentin.
3167	DRAMP02750	Defensin (ants, insects, animals)	"Defensin"[All Fields] AND biofilm[All Fields]	Defensin	28874606	Bacterial d-amino acids suppress sinonasal innate immunity through sweet taste receptors in solitary chemosensory cells.
3167	DRAMP02750	Defensin (ants, insects, animals)	"Defensin"[All Fields] AND biofilm[All Fields]	Defensin	28725299	Role of Streptococcus mutanstwo-component systems in antimicrobial peptide resistance in the oral cavity.
3167	DRAMP02750	Defensin (ants, insects, animals)	"Defensin"[All Fields] AND biofilm[All Fields]	Defensin	28649561	Psd1 Effects on Candida albicansPlanktonic Cells and Biofilms.
3167	DRAMP02750	Defensin (ants, insects, animals)	"Defensin"[All Fields] AND biofilm[All Fields]	Defensin	28642103	Chimeric analogs of human β-defensin 1 and θ-defensin disrupt pre-established bacterial biofilms.
3167	DRAMP02750	Defensin (ants, insects, animals)	"Defensin"[All Fields] AND biofilm[All Fields]	Defensin	28413476	The mechanism of human β-defensin 3 in MRSA-induced infection of implant drug-resistant bacteria biofilm in the mouse tibial bone marrow.
3167	DRAMP02750	Defensin (ants, insects, animals)	"Defensin"[All Fields] AND biofilm[All Fields]	Defensin	28296382	Human α-Defensin 6: A Small Peptide That Self-Assembles and Protects the Host by Entangling Microbes.
3167	DRAMP02750	Defensin (ants, insects, animals)	"Defensin"[All Fields] AND biofilm[All Fields]	Defensin	28278280	Natural antimicrobial peptide complexes in the fighting of antibiotic resistant biofilms: Calliphora vicina medicinal maggots.
3167	DRAMP02750	Defensin (ants, insects, animals)	"Defensin"[All Fields] AND biofilm[All Fields]	Defensin	28144375	Advancements in Diagnosing Periprosthetic Joint Infections after Total Hip and Knee Arthroplasty.
3167	DRAMP02750	Defensin (ants, insects, animals)	"Defensin"[All Fields] AND biofilm[All Fields]	Defensin	28078813	Host defense peptide-derived privileged scaffolds for anti-infective drug discovery.
3167	DRAMP02750	Defensin (ants, insects, animals)	"Defensin"[All Fields] AND biofilm[All Fields]	Defensin	28026958	Human α-Defensin 6 Self-Assembly Prevents Adhesion and Suppresses Virulence Traits of Candida albicans.
3167	DRAMP02750	Defensin (ants, insects, animals)	"Defensin"[All Fields] AND biofilm[All Fields]	Defensin	27794585	A Novel Defensin-Like Peptide Associated with Two Other New Cationic Antimicrobial Peptides in Transcriptome of the Iranian Scorpion Venom.
3167	DRAMP02750	Defensin (ants, insects, animals)	"Defensin"[All Fields] AND biofilm[All Fields]	Defensin	27777572	Role of yqiCin the Pathogenicity of Salmonellaand Innate Immune Responses of Human Intestinal Epithelium.

3167	DRAMP02750	Defensin (ants, insects, animals)	"Defensin"[All Fields] AND biofilm[All Fields]	Defensin	27582732	Efficient Eradication of Mature Pseudomonas aeruginosa Biofilm via Controlled Delivery of Nitric Oxide Combined with Antimicrobial Peptide and Antibiotics.
3167	DRAMP02750	Defensin (ants, insects, animals)	"Defensin"[All Fields] AND biofilm[All Fields]	Defensin	27417541	Acute appendicitis: transcript profiling of blood identifies promising biomarkers and potential underlying processes.
3167	DRAMP02750	Defensin (ants, insects, animals)	"Defensin"[All Fields] AND biofilm[All Fields]	Defensin	27200276	Antifungal effects of synthetic human β-defensin 3-C15 peptide.
3167	DRAMP02750	Defensin (ants, insects, animals)	"Defensin"[All Fields] AND biofilm[All Fields]	Defensin	27148195	Effect of Substance P in Staphylococcus aureus and Staphylococcus epidermidis Virulence: Implication for Skin Homeostasis.
3167	DRAMP02750	Defensin (ants, insects, animals)	"Defensin"[All Fields] AND biofilm[All Fields]	Defensin	26861950	Antibiofilm efficacy of honey and bee-derived defensin-1 on multispecies wound biofilm.
3167	DRAMP02750	Defensin (ants, insects, animals)	"Defensin"[All Fields] AND biofilm[All Fields]	Defensin	26592804	The radish defensins RsAFP1 and RsAFP2 act synergistically with caspofungin against Candida albicans biofilms.
3167	DRAMP02750	Defensin (ants, insects, animals)	"Defensin"[All Fields] AND biofilm[All Fields]	Defensin	26248029	Synergistic Activity of the Plant Defensin HsAFP1 and Caspofungin against Candida albicans Biofilms and Planktonic Cultures.
3167	DRAMP02750	Defensin (ants, insects, animals)	"Defensin"[All Fields] AND biofilm[All Fields]	Defensin	26214284	α-tocopherol decreases interleukin-1β and -6 and increases human β-defensin-1 and -2 secretion in human gingival fibroblasts stimulated with Porphyromonas gingivalis lipopolysaccharide.
3167	DRAMP02750	Defensin (ants, insects, animals)	"Defensin"[All Fields] AND biofilm[All Fields]	Defensin	26196513	Snake Cathelicidin NA-CATH and Smaller Helical Antimicrobial Peptides Are Effective against Burkholderia thailandensis.
3167	DRAMP02750	Defensin (ants, insects, animals)	"Defensin"[All Fields] AND biofilm[All Fields]	Defensin	26119274	Ultrasound microbubbles enhance human β-defensin 3 against biofilms.
3167	DRAMP02750	Defensin (ants, insects, animals)	"Defensin"[All Fields] AND biofilm[All Fields]	Defensin	25862466	The Antibacterial Effects of an Antimicrobial Peptide Human β-Defensin 3 Fused with Carbohydrate-Binding Domain on Pseudomonas aeruginosa PA14.
3167	DRAMP02750	Defensin (ants, insects, animals)	"Defensin"[All Fields] AND biofilm[All Fields]	Defensin	25808131	Expression of antimicrobial peptides and interleukin-8 during early stages of inflammation: An experimental gingivitis study.
3167	DRAMP02750	Defensin (ants, insects, animals)	"Defensin"[All Fields] AND biofilm[All Fields]	Defensin	25806720	Antimicrobial peptides in 2014.
3167	DRAMP02750	Defensin (ants, insects, animals)	"Defensin"[All Fields] AND biofilm[All Fields]	Defensin	25285879	Inhibition and destruction of Pseudomonas aeruginosa biofilms by antibiotics and antimicrobial peptides.
3167	DRAMP02750	Defensin (ants, insects, animals)	"Defensin"[All Fields] AND biofilm[All Fields]	Defensin	25212593	Health- and disease-associated species clusters in complex natural biofilms determine the innate immune response in oral epithelial cells during biofilm maturation.
3167	DRAMP02750	Defensin (ants, insects, animals)	"Defensin"[All Fields] AND biofilm[All Fields]	Defensin	24913184	Effects of human β-defensin-3 on biofilm formation-regulating genes dltB and icaA in Staphylococcus aureus.
3167	DRAMP02750	Defensin (ants, insects, animals)	"Defensin"[All Fields] AND biofilm[All Fields]	Defensin	24340061	Inflammatory and antimicrobial responses to methicillin-resistant Staphylococcus aureus in an in vitro wound infection model.
3167	DRAMP02750	Defensin (ants, insects, animals)	"Defensin"[All Fields] AND biofilm[All Fields]	Defensin	24240906	Adsorption study of pellicle proteins to gold, silica and titanium by quartz crystal microbalance method.
3167	DRAMP02750	Defensin (ants, insects, animals)	"Defensin"[All Fields] AND biofilm[All Fields]	Defensin	24238461	Antibacterial efficacy of a human β-defensin-3 peptide on multispecies biofilms.
3167	DRAMP02750	Defensin (ants, insects, animals)	"Defensin"[All Fields] AND biofilm[All Fields]	Defensin	24100890	[Use of recombinant human beta-defensin-3 to evaluate the effect of adhesion of Candida albicans on the surface of soft lining material].
3167	DRAMP02750	Defensin (ants, insects, animals)	"Defensin"[All Fields] AND biofilm[All Fields]	Defensin	23639356	Human beta-defensin 3: a novel inhibitor of Staphylococcus-produced biofilm production. Commentary on "Human β-defensin 3 inhibits antibiotic-resistant Staphylococcus biofilm formation".
3167	DRAMP02750	Defensin (ants, insects, animals)	"Defensin"[All Fields] AND biofilm[All Fields]	Defensin	23519963	Ultrasound-targeted microbubble destruction enhances human β-defensin 3 activity against antibiotic-resistant Staphylococcus biofilms.
3167	DRAMP02750	Defensin (ants, insects, animals)	"Defensin"[All Fields] AND biofilm[All Fields]	Defensin	23273885	Human β-defensin 3 inhibits antibiotic-resistant Staphylococcus biofilm formation.
3167	DRAMP02750	Defensin (ants, insects, animals)	"Defensin"[All Fields] AND biofilm[All Fields]	Defensin	23203265	Potentiation of the cytotoxic activity of copper by polyphosphate on biofilm-producing bacteria: a bioinspired approach.
3167	DRAMP02750	Defensin (ants, insects, animals)	"Defensin"[All Fields] AND biofilm[All Fields]	Defensin	23078156	Antimicrobial efficacy of a human β-defensin-3 peptide using an Enterococcus faecalis dentine infection model.
3167	DRAMP02750	Defensin (ants, insects, animals)	"Defensin"[All Fields] AND biofilm[All Fields]	Defensin	23053486	Synergistic effect and antibiofilm activity between the antimicrobial peptide coprisin and conventional antibiotics against opportunistic bacteria.
3167	DRAMP02750	Defensin (ants, insects, animals)	"Defensin"[All Fields] AND biofilm[All Fields]	Defensin	22922323	Extracellular DNA within a nontypeable Haemophilus influenzae-induced biofilm binds human beta defensin-3 and reduces its antimicrobial activity.
3167	DRAMP02750	Defensin (ants, insects, animals)	"Defensin"[All Fields] AND biofilm[All Fields]	Defensin	22855857	Retrocyclin inhibits Gardnerella vaginalis biofilm formation and toxin activity.
3167	DRAMP02750	Defensin (ants, insects, animals)	"Defensin"[All Fields] AND biofilm[All Fields]	Defensin	22394470	Association of CiaRH with resistance of Streptococcus mutans to antimicrobial peptides in biofilms.
3167	DRAMP02750	Defensin (ants, insects, animals)	"Defensin"[All Fields] AND biofilm[All Fields]	Defensin	22229614	Comparison of the effects of human β-defensin 3, vancomycin, and clindamycin on Staphylococcus aureus biofilm formation.
3167	DRAMP02750	Defensin (ants, insects, animals)	"Defensin"[All Fields] AND biofilm[All Fields]	Defensin	21692631	A novel organotypic dento-epithelial culture model: effect of Fusobacterium nucleatum biofilm on B-defensin-2, -3, and LL-37 expression.
3167	DRAMP02750	Defensin (ants, insects, animals)	"Defensin"[All Fields] AND biofilm[All Fields]	Defensin	20454633	Normal human gingival epithelial cells sense C. parapsilosis by toll-like receptors and module its pathogenesis through antimicrobial peptides and proinflammatory cytokines.
3167	DRAMP02750	Defensin (ants, insects, animals)	"Defensin"[All Fields] AND biofilm[All Fields]	Defensin	20378008	Effect of temperature on the shift of Pseudomonas fluorescens from an environmental microorganism to a potential human pathogen.
3167	DRAMP02750	Defensin (ants, insects, animals)	"Defensin"[All Fields] AND biofilm[All Fields]	Defensin	19961380	Gingival transcriptome patterns during induction and resolution of experimental gingivitis in humans.
3167	DRAMP02750	Defensin (ants, insects, animals)	"Defensin"[All Fields] AND biofilm[All Fields]	Defensin	19780045	Candida famata modulates toll-like receptor, beta-defensin, and proinflammatory cytokine expression by normal human epithelial cells.
3167	DRAMP02750	Defensin (ants, insects, animals)	"Defensin"[All Fields] AND biofilm[All Fields]	Defensin	19572896	The immune response of oral epithelial cells induced by single-species and complex naturally formed biofilms.
3167	DRAMP02750	Defensin (ants, insects, animals)	"Defensin"[All Fields] AND biofilm[All Fields]	Defensin	19466693	Activity of antimicrobial peptides in the presence of polysaccharides produced by pulmonary pathogens.
3167	DRAMP02750	Defensin (ants, insects, animals)	"Defensin"[All Fields] AND biofilm[All Fields]	Defensin	18954353	Treponema denticola does not induce production of common innate immune mediators from primary gingival epithelial cells.
3167	DRAMP02750	Defensin (ants, insects, animals)	"Defensin"[All Fields] AND biofilm[All Fields]	Defensin	18173794	The stage of native biofilm formation determines the gene expression of human beta-defensin-2, psoriasin, ribonuclease 7 and inflammatory mediators: a novel approach for stimulation of keratinocytes with in situ formed biofilms.
3167	DRAMP02750	Defensin (ants, insects, animals)	"Defensin"[All Fields] AND biofilm[All Fields]	Defensin	17434999	Functional analysis of D-alanylation of lipoteichoic acid in the probiotic strain Lactobacillus rhamnosus GG.
3167	DRAMP02750	Defensin (ants, insects, animals)	"Defensin"[All Fields] AND biofilm[All Fields]	Defensin	15493829	Bacterial evasion of innate host defenses--the Staphylococcus aureus lesson.
3167	DRAMP02750	Defensin (ants, insects, animals)	"Defensin"[All Fields] AND biofilm[All Fields]	Defensin	14764110	Polysaccharide intercellular adhesin (PIA) protects Staphylococcus epidermidis against major components of the human innate immune system.
3169	DRAMP02753	Ponericin G1 (ants, insects, animals)	"Ponericin G1"[All Fields] AND biofilm[All Fields]	Ponericin G1	20004967	Controlling the release of peptide antimicrobial agents from surfaces.
3191	DRAMP02783	Peptide C (Insects, animals)	"Peptide C"[All Fields] AND biofilm[All Fields]	Peptide C	31064885	An intramembrane sensory circuit monitors sortase A-mediated processing of streptococcal adhesins.
3191	DRAMP02783	Peptide C (Insects, animals)	"Peptide C"[All Fields] AND biofilm[All Fields]	Peptide C	26307165	Pseudomonas aeruginosa Expresses a Functional Human Natriuretic Peptide Receptor Ortholog: Involvement in Biofilm Formation.
3191	DRAMP02783	Peptide C (Insects, animals)	"Peptide C"[All Fields] AND biofilm[All Fields]	Peptide C	16936029	Structure-activity analysis of quorum-sensing signaling peptides from Streptococcus mutans.
3204	DRAMP02796	Defensin (Type 1 invertebrate defensin; Insects, animals)	"Defensin"[All Fields] AND biofilm[All Fields]	Defensin	34408988	The Anti-Biofilm Efficacy of Caffeic Acid Phenethyl Ester (CAPE) In Vitroand a Murine Model of Oral Candidiasis.
3204	DRAMP02796	Defensin (Type 1 invertebrate defensin; Insects, animals)	"Defensin"[All Fields] AND biofilm[All Fields]	Defensin	34321877	Antibacterial Effect of Honey-Derived Exosomes Containing Antimicrobial Peptides Against Oral Streptococci.
3204	DRAMP02796	Defensin (Type 1 invertebrate defensin; Insects, animals)	"Defensin"[All Fields] AND biofilm[All Fields]	Defensin	34276631	DNA Blocks the Lethal Effect of Human Beta-Defensin 2 Against Neisseria meningitidis.
3204	DRAMP02796	Defensin (Type 1 invertebrate defensin; Insects, animals)	"Defensin"[All Fields] AND biofilm[All Fields]	Defensin	33911935	The antibacterial activities of honey.
3204	DRAMP02796	Defensin (Type 1 invertebrate defensin; Insects, animals)	"Defensin"[All Fields] AND biofilm[All Fields]	Defensin	33865931	Identification of a crocodylian β-defensin variant from Alligator mississippiensis with antimicrobial and antibiofilm activity.
3204	DRAMP02796	Defensin (Type 1 invertebrate defensin; Insects, animals)	"Defensin"[All Fields] AND biofilm[All Fields]	Defensin	33586659	Identification of anti-microbial peptides and traces of microbial DNA in infrainfundibular compartments of human scalp terminal hair follicles.
3204	DRAMP02796	Defensin (Type 1 invertebrate defensin; Insects, animals)	"Defensin"[All Fields] AND biofilm[All Fields]	Defensin	33534018	A recombinant fungal defensin-like peptide-P2 combats Streptococcus dysgalactiae and biofilms.

3204	DRAMP02796	Defensin (Type 1 invertebrate defensin; Insects, animals)	"Defensin"[All Fields] AND biofilm[All Fields]	Defensin	33447687	Future directions of postoperative spinal implant infections.
3204	DRAMP02796	Defensin (Type 1 invertebrate defensin; Insects, animals)	"Defensin"[All Fields] AND biofilm[All Fields]	Defensin	33420317	Curbing gastrointestinal infections by defensin fragment modifications without harming commensal microbiota.
3204	DRAMP02796	Defensin (Type 1 invertebrate defensin; Insects, animals)	"Defensin"[All Fields] AND biofilm[All Fields]	Defensin	32867384	A Novel Peptide Antibiotic, Pro10-1D, Designed from Insect Defensin Shows Antibacterial and Anti-Inflammatory Activities in Sepsis Models.
3204	DRAMP02796	Defensin (Type 1 invertebrate defensin; Insects, animals)	"Defensin"[All Fields] AND biofilm[All Fields]	Defensin	32858856	Candida albicansVirulence Factors and Pathogenicity for Endodontic Infections.
3204	DRAMP02796	Defensin (Type 1 invertebrate defensin; Insects, animals)	"Defensin"[All Fields] AND biofilm[All Fields]	Defensin	32842903	Electrospun ZnO/Poly(Vinylidene Fluoride-Trifluoroethylene) Scaffolds for Lung Tissue Engineering.
3204	DRAMP02796	Defensin (Type 1 invertebrate defensin; Insects, animals)	"Defensin"[All Fields] AND biofilm[All Fields]	Defensin	32663201	Inhibition and eradication activity of truncated α-defensin analogs against multidrug resistant uropathogenic Escherichia coli biofilm.
3204	DRAMP02796	Defensin (Type 1 invertebrate defensin; Insects, animals)	"Defensin"[All Fields] AND biofilm[All Fields]	Defensin	32585445	Glucose effect on Candida albicans biofilm during tissue invasion.
3204	DRAMP02796	Defensin (Type 1 invertebrate defensin; Insects, animals)	"Defensin"[All Fields] AND biofilm[All Fields]	Defensin	32522780	Controlling the Growth of the Skin Commensal Staphylococcus epidermidis Using d-Alanine Auxotrophy.
3204	DRAMP02796	Defensin (Type 1 invertebrate defensin; Insects, animals)	"Defensin"[All Fields] AND biofilm[All Fields]	Defensin	32457749	The Antimicrobial Peptide Human Beta-Defensin 2 Inhibits Biofilm Production of Pseudomonas aeruginosaWithout Compromising Metabolic Activity.
3204	DRAMP02796	Defensin (Type 1 invertebrate defensin; Insects, animals)	"Defensin"[All Fields] AND biofilm[All Fields]	Defensin	32439511	Antibacterial activities and mechanisms of action of a defensin from manila clam Ruditapes philippinarum.
3204	DRAMP02796	Defensin (Type 1 invertebrate defensin; Insects, animals)	"Defensin"[All Fields] AND biofilm[All Fields]	Defensin	31933178	Effects of human β-defensin 3 fused with carbohydrate-binding domain on the function of type III secretion system in Pseudomonas aeruginosa PA14.
3204	DRAMP02796	Defensin (Type 1 invertebrate defensin; Insects, animals)	"Defensin"[All Fields] AND biofilm[All Fields]	Defensin	31906541	Role of FAD-I in Fusobacterial Interspecies Interaction and Biofilm Formation.
3204	DRAMP02796	Defensin (Type 1 invertebrate defensin; Insects, animals)	"Defensin"[All Fields] AND biofilm[All Fields]	Defensin	31729441	Rhesus Theta Defensin 1 Promotes Long Term Survival in Systemic Candidiasis by Host Directed Mechanisms.
3204	DRAMP02796	Defensin (Type 1 invertebrate defensin; Insects, animals)	"Defensin"[All Fields] AND biofilm[All Fields]	Defensin	31336838	Candida albicans-Cell Interactions Activate Innate Immune Defense in Human Palate Epithelial Primary Cells via Nitric Oxide (NO) and β-Defensin 2 (hBD-2).
3204	DRAMP02796	Defensin (Type 1 invertebrate defensin; Insects, animals)	"Defensin"[All Fields] AND biofilm[All Fields]	Defensin	31165072	Impact of the Food Additive Titanium Dioxide (E171) on Gut Microbiota-Host Interaction.
3204	DRAMP02796	Defensin (Type 1 invertebrate defensin; Insects, animals)	"Defensin"[All Fields] AND biofilm[All Fields]	Defensin	31031739	Salt-Tolerant Antifungal and Antibacterial Activities of the Corn Defensin ZmD32.
3204	DRAMP02796	Defensin (Type 1 invertebrate defensin; Insects, animals)	"Defensin"[All Fields] AND biofilm[All Fields]	Defensin	31025073	A recombinant fungal defensin-like peptide-P2 combats multidrug-resistant Staphylococcus aureus and biofilms.
3204	DRAMP02796	Defensin (Type 1 invertebrate defensin; Insects, animals)	"Defensin"[All Fields] AND biofilm[All Fields]	Defensin	30659503	Lactobacillus plantarum USM8613 Aids in Wound Healing and Suppresses Staphylococcus aureus Infection at Wound Sites.
3204	DRAMP02796	Defensin (Type 1 invertebrate defensin; Insects, animals)	"Defensin"[All Fields] AND biofilm[All Fields]	Defensin	30649289	Innate immune components affect growth and virulence traits of bacterial-vaginosis-associated and non-bacterial-vaginosis-associated Gardnerella vaginalis strains similarly.
3204	DRAMP02796	Defensin (Type 1 invertebrate defensin; Insects, animals)	"Defensin"[All Fields] AND biofilm[All Fields]	Defensin	30376742	Alpha defensin, leukocyte esterase, C-reactive protein, and leukocyte count in synovial fluid for pre-operative diagnosis of periprosthetic infection.
3204	DRAMP02796	Defensin (Type 1 invertebrate defensin; Insects, animals)	"Defensin"[All Fields] AND biofilm[All Fields]	Defensin	30260708	Stabilized collagen matrix dressing improves wound macrophage function and epithelialization.
3204	DRAMP02796	Defensin (Type 1 invertebrate defensin; Insects, animals)	"Defensin"[All Fields] AND biofilm[All Fields]	Defensin	30254440	Modification of the surface of titanium with multifunctional chimeric peptides to prevent biofilm formation via inhibition of initial colonizers.
3204	DRAMP02796	Defensin (Type 1 invertebrate defensin; Insects, animals)	"Defensin"[All Fields] AND biofilm[All Fields]	Defensin	29902560	A defensin-like antimicrobial peptide from the manila clam Ruditapes philippinarum: Investigation of the antibacterial activities and mode of action.
3204	DRAMP02796	Defensin (Type 1 invertebrate defensin; Insects, animals)	"Defensin"[All Fields] AND biofilm[All Fields]	Defensin	29872295	Antimicrobial peptide-loaded liquid crystalline precursor bioadhesive system for the prevention of dental caries.
3204	DRAMP02796	Defensin (Type 1 invertebrate defensin; Insects, animals)	"Defensin"[All Fields] AND biofilm[All Fields]	Defensin	29671721	The BceABRS four-component system that is essential for cell envelope stress response is involved in sensing and response to host defence peptides and is required for the biofilm formation and fitness of Streptococcus mutans.
3204	DRAMP02796	Defensin (Type 1 invertebrate defensin; Insects, animals)	"Defensin"[All Fields] AND biofilm[All Fields]	Defensin	29104569	A Linear 19-Mer Plant Defensin-Derived Peptide Acts Synergistically with Caspofungin against Candida albicansBiofilms.
3204	DRAMP02796	Defensin (Type 1 invertebrate defensin; Insects, animals)	"Defensin"[All Fields] AND biofilm[All Fields]	Defensin	29077172	The significance of HBD-3 and fluorescent composite carriers in the processof bone formation in rats infected with Staphylococcus aureus.
3204	DRAMP02796	Defensin (Type 1 invertebrate defensin; Insects, animals)	"Defensin"[All Fields] AND biofilm[All Fields]	Defensin	29045084	New Approach to Treat and Prevent Oral Disease.
3204	DRAMP02796	Defensin (Type 1 invertebrate defensin; Insects, animals)	"Defensin"[All Fields] AND biofilm[All Fields]	Defensin	29025642	Engineered chimeric peptides with antimicrobial and titanium-binding functions to inhibit biofilm formation on Ti implants.
3204	DRAMP02796	Defensin (Type 1 invertebrate defensin; Insects, animals)	"Defensin"[All Fields] AND biofilm[All Fields]	Defensin	28956355	The synthetic human beta-defensin-3 C15 peptide exhibits antimicrobial activity against Streptococcus mutans, both alone and in combination with dental disinfectants.
3204	DRAMP02796	Defensin (Type 1 invertebrate defensin; Insects, animals)	"Defensin"[All Fields] AND biofilm[All Fields]	Defensin	28951032	Antifungal Effects of Synthetic Human Beta-defensin-3-C15 Peptide on Candida albicans-infected Root Dentin.
3204	DRAMP02796	Defensin (Type 1 invertebrate defensin; Insects, animals)	"Defensin"[All Fields] AND biofilm[All Fields]	Defensin	28874606	Bacterial d-amino acids suppress sinonasal innate immunity through sweet taste receptors in solitary chemosensory cells.
3204	DRAMP02796	Defensin (Type 1 invertebrate defensin; Insects, animals)	"Defensin"[All Fields] AND biofilm[All Fields]	Defensin	28725299	Role of Streptococcus mutanstwo-component systems in antimicrobial peptide resistance in the oral cavity.
3204	DRAMP02796	Defensin (Type 1 invertebrate defensin; Insects, animals)	"Defensin"[All Fields] AND biofilm[All Fields]	Defensin	28649561	Psd1 Effects on Candida albicansPlanktonic Cells and Biofilms.
3204	DRAMP02796	Defensin (Type 1 invertebrate defensin; Insects, animals)	"Defensin"[All Fields] AND biofilm[All Fields]	Defensin	28642103	Chimeric analogs of human β-defensin 1 and θ-defensin disrupt pre-established bacterial biofilms.
3204	DRAMP02796	Defensin (Type 1 invertebrate defensin; Insects, animals)	"Defensin"[All Fields] AND biofilm[All Fields]	Defensin	28413476	The mechanism of human β-defensin 3 in MRSA-induced infection of implant drug-resistant bacteria biofilm in the mouse tibial bone marrow.
3204	DRAMP02796	Defensin (Type 1 invertebrate defensin; Insects, animals)	"Defensin"[All Fields] AND biofilm[All Fields]	Defensin	28296382	Human α-Defensin 6: A Small Peptide That Self-Assembles and Protects the Host by Entangling Microbes.
3204	DRAMP02796	Defensin (Type 1 invertebrate defensin; Insects, animals)	"Defensin"[All Fields] AND biofilm[All Fields]	Defensin	28278280	Natural antimicrobial peptide complexes in the fighting of antibiotic resistant biofilms: Calliphora vicina medicinal maggots.
3204	DRAMP02796	Defensin (Type 1 invertebrate defensin; Insects, animals)	"Defensin"[All Fields] AND biofilm[All Fields]	Defensin	28144375	Advancements in Diagnosing Periprosthetic Joint Infections after Total Hip and Knee Arthroplasty.

3204	DRAMP02796	Defensin (Type 1 invertebrate defensin; Insects, animals)	"Defensin"[All Fields] AND biofilm[All Fields]	Defensin	28078813	Host defense peptide-derived privileged scaffolds for anti-infective drug discovery.
3204	DRAMP02796	Defensin (Type 1 invertebrate defensin; Insects, animals)	"Defensin"[All Fields] AND biofilm[All Fields]	Defensin	28026958	Human α-Defensin 6 Self-Assembly Prevents Adhesion and Suppresses Virulence Traits of Candida albicans.
3204	DRAMP02796	Defensin (Type 1 invertebrate defensin; Insects, animals)	"Defensin"[All Fields] AND biofilm[All Fields]	Defensin	27794585	A Novel Defensin-Like Peptide Associated with Two Other New Cationic Antimicrobial Peptides in Transcriptome of the Iranian Scorpion Venom.
3204	DRAMP02796	Defensin (Type 1 invertebrate defensin; Insects, animals)	"Defensin"[All Fields] AND biofilm[All Fields]	Defensin	27777572	Role of yqiCin the Pathogenicity of Salmonellaand Innate Immune Responses of Human Intestinal Epithelium.
3204	DRAMP02796	Defensin (Type 1 invertebrate defensin; Insects, animals)	"Defensin"[All Fields] AND biofilm[All Fields]	Defensin	27582732	Efficient Eradication of Mature Pseudomonas aeruginosa Biofilm via Controlled Delivery of Nitric Oxide Combined with Antimicrobial Peptide and Antibiotics.
3204	DRAMP02796	Defensin (Type 1 invertebrate defensin; Insects, animals)	"Defensin"[All Fields] AND biofilm[All Fields]	Defensin	27417541	Acute appendicitis: transcript profiling of blood identifies promising biomarkers and potential underlying processes.
3204	DRAMP02796	Defensin (Type 1 invertebrate defensin; Insects, animals)	"Defensin"[All Fields] AND biofilm[All Fields]	Defensin	27200276	Antifungal effects of synthetic human β-defensin 3-C15 peptide.
3204	DRAMP02796	Defensin (Type 1 invertebrate defensin; Insects, animals)	"Defensin"[All Fields] AND biofilm[All Fields]	Defensin	27148195	Effect of Substance P in Staphylococcus aureus and Staphylococcus epidermidis Virulence: Implication for Skin Homeostasis.
3204	DRAMP02796	Defensin (Type 1 invertebrate defensin; Insects, animals)	"Defensin"[All Fields] AND biofilm[All Fields]	Defensin	26861950	Antibiofilm efficacy of honey and bee-derived defensin-1 on multispecies wound biofilm.
3204	DRAMP02796	Defensin (Type 1 invertebrate defensin; Insects, animals)	"Defensin"[All Fields] AND biofilm[All Fields]	Defensin	26592804	The radish defensins RsAFP1 and RsAFP2 act synergistically with caspofungin against Candida albicans biofilms.
3204	DRAMP02796	Defensin (Type 1 invertebrate defensin; Insects, animals)	"Defensin"[All Fields] AND biofilm[All Fields]	Defensin	26248029	Synergistic Activity of the Plant Defensin HsAFP1 and Caspofungin against Candida albicans Biofilms and Planktonic Cultures.
3204	DRAMP02796	Defensin (Type 1 invertebrate defensin; Insects, animals)	"Defensin"[All Fields] AND biofilm[All Fields]	Defensin	26214284	α-tocopherol decreases interleukin-1β and -6 and increases human β-defensin-1 and -2 secretion in human gingival fibroblasts stimulated with Porphyromonas gingivalis lipopolysaccharide.
3204	DRAMP02796	Defensin (Type 1 invertebrate defensin; Insects, animals)	"Defensin"[All Fields] AND biofilm[All Fields]	Defensin	26196513	Snake Cathelicidin NA-CATH and Smaller Helical Antimicrobial Peptides Are Effective against Burkholderia thailandensis.
3204	DRAMP02796	Defensin (Type 1 invertebrate defensin; Insects, animals)	"Defensin"[All Fields] AND biofilm[All Fields]	Defensin	26119274	Ultrasound microbubbles enhance human β-defensin 3 against biofilms.
3204	DRAMP02796	Defensin (Type 1 invertebrate defensin; Insects, animals)	"Defensin"[All Fields] AND biofilm[All Fields]	Defensin	25862466	The Antibacterial Effects of an Antimicrobial Peptide Human β-Defensin 3 Fused with Carbohydrate-Binding Domain on Pseudomonas aeruginosa PA14.
3204	DRAMP02796	Defensin (Type 1 invertebrate defensin; Insects, animals)	"Defensin"[All Fields] AND biofilm[All Fields]	Defensin	25808131	Expression of antimicrobial peptides and interleukin-8 during early stages of inflammation: An experimental gingivitis study.
3204	DRAMP02796	Defensin (Type 1 invertebrate defensin; Insects, animals)	"Defensin"[All Fields] AND biofilm[All Fields]	Defensin	25806720	Antimicrobial peptides in 2014.
3204	DRAMP02796	Defensin (Type 1 invertebrate defensin; Insects, animals)	"Defensin"[All Fields] AND biofilm[All Fields]	Defensin	25285879	Inhibition and destruction of Pseudomonas aeruginosa biofilms by antibiotics and antimicrobial peptides.
3204	DRAMP02796	Defensin (Type 1 invertebrate defensin; Insects, animals)	"Defensin"[All Fields] AND biofilm[All Fields]	Defensin	25212593	Health- and disease-associated species clusters in complex natural biofilms determine the innate immune response in oral epithelial cells during biofilm maturation.
3204	DRAMP02796	Defensin (Type 1 invertebrate defensin; Insects, animals)	"Defensin"[All Fields] AND biofilm[All Fields]	Defensin	24913184	Effects of human β-defensin-3 on biofilm formation-regulating genes dltB and icaA in Staphylococcus aureus.
3204	DRAMP02796	Defensin (Type 1 invertebrate defensin; Insects, animals)	"Defensin"[All Fields] AND biofilm[All Fields]	Defensin	24340061	Inflammatory and antimicrobial responses to methicillin-resistant Staphylococcus aureus in an in vitro wound infection model.
3204	DRAMP02796	Defensin (Type 1 invertebrate defensin; Insects, animals)	"Defensin"[All Fields] AND biofilm[All Fields]	Defensin	24240906	Adsorption study of pellicle proteins to gold, silica and titanium by quartz crystal microbalance method.
3204	DRAMP02796	Defensin (Type 1 invertebrate defensin; Insects, animals)	"Defensin"[All Fields] AND biofilm[All Fields]	Defensin	24238461	Antibacterial efficacy of a human β-defensin-3 peptide on multispecies biofilms.
3204	DRAMP02796	Defensin (Type 1 invertebrate defensin; Insects, animals)	"Defensin"[All Fields] AND biofilm[All Fields]	Defensin	24100890	[Use of recombinant human beta-defensin-3 to evaluate the effect of adhesion of Candida albicans on the surface of soft lining material].
3204	DRAMP02796	Defensin (Type 1 invertebrate defensin; Insects, animals)	"Defensin"[All Fields] AND biofilm[All Fields]	Defensin	23639356	Human beta-defensin 3: a novel inhibitor of Staphylococcus-produced biofilm production. Commentary on "Human β-defensin 3 inhibits antibiotic-resistant Staphylococcus biofilm formation".
3204	DRAMP02796	Defensin (Type 1 invertebrate defensin; Insects, animals)	"Defensin"[All Fields] AND biofilm[All Fields]	Defensin	23519963	Ultrasound-targeted microbubble destruction enhances human β-defensin 3 activity against antibiotic-resistant Staphylococcus biofilms.
3204	DRAMP02796	Defensin (Type 1 invertebrate defensin; Insects, animals)	"Defensin"[All Fields] AND biofilm[All Fields]	Defensin	23273885	Human β-defensin 3 inhibits antibiotic-resistant Staphylococcus biofilm formation.
3204	DRAMP02796	Defensin (Type 1 invertebrate defensin; Insects, animals)	"Defensin"[All Fields] AND biofilm[All Fields]	Defensin	23203265	Potentiation of the cytotoxic activity of copper by polyphosphate on biofilm-producing bacteria: a bioinspired approach.
3204	DRAMP02796	Defensin (Type 1 invertebrate defensin; Insects, animals)	"Defensin"[All Fields] AND biofilm[All Fields]	Defensin	23078156	Antimicrobial efficacy of a human β-defensin-3 peptide using an Enterococcus faecalis dentine infection model.
3204	DRAMP02796	Defensin (Type 1 invertebrate defensin; Insects, animals)	"Defensin"[All Fields] AND biofilm[All Fields]	Defensin	23053486	Synergistic effect and antibiofilm activity between the antimicrobial peptide coprisin and conventional antibiotics against opportunistic bacteria.
3204	DRAMP02796	Defensin (Type 1 invertebrate defensin; Insects, animals)	"Defensin"[All Fields] AND biofilm[All Fields]	Defensin	22922323	Extracellular DNA within a nontypeable Haemophilus influenzae-induced biofilm binds human beta defensin-3 and reduces its antimicrobial activity.
3204	DRAMP02796	Defensin (Type 1 invertebrate defensin; Insects, animals)	"Defensin"[All Fields] AND biofilm[All Fields]	Defensin	22855857	Retrocyclin inhibits Gardnerella vaginalis biofilm formation and toxin activity.
3204	DRAMP02796	Defensin (Type 1 invertebrate defensin; Insects, animals)	"Defensin"[All Fields] AND biofilm[All Fields]	Defensin	22394470	Association of CiaRH with resistance of Streptococcus mutans to antimicrobial peptides in biofilms.
3204	DRAMP02796	Defensin (Type 1 invertebrate defensin; Insects, animals)	"Defensin"[All Fields] AND biofilm[All Fields]	Defensin	22229614	Comparison of the effects of human β-defensin 3, vancomycin, and clindamycin on Staphylococcus aureus biofilm formation.
3204	DRAMP02796	Defensin (Type 1 invertebrate defensin; Insects, animals)	"Defensin"[All Fields] AND biofilm[All Fields]	Defensin	21692631	A novel organotypic dento-epithelial culture model: effect of Fusobacterium nucleatum biofilm on B-defensin-2, -3, and LL-37 expression.
3204	DRAMP02796	Defensin (Type 1 invertebrate defensin; Insects, animals)	"Defensin"[All Fields] AND biofilm[All Fields]	Defensin	20454633	Normal human gingival epithelial cells sense C. parapsilosis by toll-like receptors and module its pathogenesis through antimicrobial peptides and proinflammatory cytokines.
3204	DRAMP02796	Defensin (Type 1 invertebrate defensin; Insects, animals)	"Defensin"[All Fields] AND biofilm[All Fields]	Defensin	20378008	Effect of temperature on the shift of Pseudomonas fluorescens from an environmental microorganism to a potential human pathogen.
3204	DRAMP02796	Defensin (Type 1 invertebrate defensin; Insects, animals)	"Defensin"[All Fields] AND biofilm[All Fields]	Defensin	19961380	Gingival transcriptome patterns during induction and resolution of experimental gingivitis in humans.
3204	DRAMP02796	Defensin (Type 1 invertebrate defensin; Insects, animals)	"Defensin"[All Fields] AND biofilm[All Fields]	Defensin	19780045	Candida famata modulates toll-like receptor, beta-defensin, and proinflammatory cytokine expression by normal human epithelial cells.

3204	DRAMP02796	Defensin (Type 1 invertebrate defensin; Insects, animals)	"Defensin"[All Fields] AND biofilm[All Fields]	Defensin	19572896	The immune response of oral epithelial cells induced by single-species and complex naturally formed biofilms.
3204	DRAMP02796	Defensin (Type 1 invertebrate defensin; Insects, animals)	"Defensin"[All Fields] AND biofilm[All Fields]	Defensin	19466693	Activity of antimicrobial peptides in the presence of polysaccharides produced by pulmonary pathogens.
3204	DRAMP02796	Defensin (Type 1 invertebrate defensin; Insects, animals)	"Defensin"[All Fields] AND biofilm[All Fields]	Defensin	18954353	Treponema denticola does not induce production of common innate immune mediators from primary gingival epithelial cells.
3204	DRAMP02796	Defensin (Type 1 invertebrate defensin; Insects, animals)	"Defensin"[All Fields] AND biofilm[All Fields]	Defensin	18173794	The stage of native biofilm formation determines the gene expression of human beta-defensin-2, psoriasin, ribonuclease 7 and inflammatory mediators: a novel approach for stimulation of keratinocytes with in situ formed biofilms.
3204	DRAMP02796	Defensin (Type 1 invertebrate defensin; Insects, animals)	"Defensin"[All Fields] AND biofilm[All Fields]	Defensin	17434999	Functional analysis of D-alanylation of lipoteichoic acid in the probiotic strain Lactobacillus rhamnosus GG.
3204	DRAMP02796	Defensin (Type 1 invertebrate defensin; Insects, animals)	"Defensin"[All Fields] AND biofilm[All Fields]	Defensin	15493829	Bacterial evasion of innate host defenses--the Staphylococcus aureus lesson.
3204	DRAMP02796	Defensin (Type 1 invertebrate defensin; Insects, animals)	"Defensin"[All Fields] AND biofilm[All Fields]	Defensin	14764110	Polysaccharide intercellular adhesin (PIA) protects Staphylococcus epidermidis against major components of the human innate immune system.
3228	DRAMP02825	Catestatin	"Catestatin"[All Fields] AND biofilm[All Fields]	Catestatin	30023754	Discrepancies between Cyclic and Linear Antimicrobial Peptide Actions on the Spectrochemical and Nanomechanical Fingerprints of a Young Biofilm.
3228	DRAMP02825	Catestatin	"Catestatin"[All Fields] AND biofilm[All Fields]	Catestatin	26379222	Harnessing the multifunctionality in nature: a bioactive agent release system with self-antimicrobial and immunomodulatory properties.
3230	DRAMP02827	BHP (pepsin-derived bovine hemoglobin fragment)	"BHP"[All Fields] AND biofilm[All Fields]	BHP	33321772	Staphylococcusspp. Isolated from Bovine Subclinical Mastitis in Different Regions of Brazil: Molecular Typing and Biofilm Gene Expression Analysis by RT-qPCR.
3230	DRAMP02827	BHP (pepsin-derived bovine hemoglobin fragment)	"BHP"[All Fields] AND biofilm[All Fields]	BHP	33287389	Detection of the agrSystem and Resistance to Antimicrobials in Biofilm-Producing S. epidermidis.
3230	DRAMP02827	BHP (pepsin-derived bovine hemoglobin fragment)	"BHP"[All Fields] AND biofilm[All Fields]	BHP	32823918	Biofilm Formation Ability and Presence of Adhesion Genes among Coagulase-Negative and Coagulase-Positive Staphylococci Isolates from Raw Cow's Milk.
3230	DRAMP02827	BHP (pepsin-derived bovine hemoglobin fragment)	"BHP"[All Fields] AND biofilm[All Fields]	BHP	32586125	2-Hydroxy-4-methoxybenzaldehyde from Hemidesmus indicusis antagonistic to Staphylococcus epidermidisbiofilm formation.
3230	DRAMP02827	BHP (pepsin-derived bovine hemoglobin fragment)	"BHP"[All Fields] AND biofilm[All Fields]	BHP	32339618	Pathogenesis of Staphylococcus epidermidis in prosthetic joint infections: Can identification of virulence genes differentiate between infecting and commensal strains?
3230	DRAMP02827	BHP (pepsin-derived bovine hemoglobin fragment)	"BHP"[All Fields] AND biofilm[All Fields]	BHP	31681633	Umbelliferone Impedes Biofilm Formation and Virulence of Methicillin-Resistant Staphylococcus epidermidisvia Impairment of Initial Attachment and Intercellular Adhesion.
3230	DRAMP02827	BHP (pepsin-derived bovine hemoglobin fragment)	"BHP"[All Fields] AND biofilm[All Fields]	BHP	31303772	Association between biofilm formation, structure and antibiotic resistance in Staphylococcus epidermidisisolated from neonatal septicemia in southwest Iran.
3230	DRAMP02827	BHP (pepsin-derived bovine hemoglobin fragment)	"BHP"[All Fields] AND biofilm[All Fields]	BHP	31288755	Antimicrobial susceptibility, virulence determinants profiles and molecular characteristics of Staphylococcus epidermidis isolates in Wenzhou, eastern China.
3230	DRAMP02827	BHP (pepsin-derived bovine hemoglobin fragment)	"BHP"[All Fields] AND biofilm[All Fields]	BHP	29469610	Comparative analysis between biofilm formation and gene expression in Staphylococcus epidermidis isolates.
3230	DRAMP02827	BHP (pepsin-derived bovine hemoglobin fragment)	"BHP"[All Fields] AND biofilm[All Fields]	BHP	28794175	Comparative Genomics Study of Staphylococcus epidermidis Isolates from Orthopedic-Device-Related Infections Correlated with Patient Outcome.
3230	DRAMP02827	BHP (pepsin-derived bovine hemoglobin fragment)	"BHP"[All Fields] AND biofilm[All Fields]	BHP	28593524	Virulence factors associated with Coagulase Negative Staphylococci isolated from human infections.
3230	DRAMP02827	BHP (pepsin-derived bovine hemoglobin fragment)	"BHP"[All Fields] AND biofilm[All Fields]	BHP	28231617	Characterization of Staphylococcus epidermidis strains isolated from industrial cleanrooms under regular routine disinfection.
3230	DRAMP02827	BHP (pepsin-derived bovine hemoglobin fragment)	"BHP"[All Fields] AND biofilm[All Fields]	BHP	28109594	Characteristics of resistance and virulence factors in different species of coagulase-negative staphylococci isolated from milk of healthy sheep and animals with subclinical mastitis.
3230	DRAMP02827	BHP (pepsin-derived bovine hemoglobin fragment)	"BHP"[All Fields] AND biofilm[All Fields]	BHP	27344542	Staphylococcus epidermidis and Staphylococcus haemolyticus: detection of biofilm genes and biofilm formation in blood culture isolates from patients in a Brazilian teaching hospital.
3230	DRAMP02827	BHP (pepsin-derived bovine hemoglobin fragment)	"BHP"[All Fields] AND biofilm[All Fields]	BHP	26547374	Biofilm formation of ica operon-positive Staphylococcus epidermidis from different sources.
3230	DRAMP02827	BHP (pepsin-derived bovine hemoglobin fragment)	"BHP"[All Fields] AND biofilm[All Fields]	BHP	26485010	Biofilm production in Staphylococcus epidermidis strains, isolated from the skin of hospitalized patients: genetic and phenotypic characteristics.
3230	DRAMP02827	BHP (pepsin-derived bovine hemoglobin fragment)	"BHP"[All Fields] AND biofilm[All Fields]	BHP	26370163	Comparative analysis of Staphylococcus epidermidis strains utilizing quantitative and cell surface shaving proteomics.
3230	DRAMP02827	BHP (pepsin-derived bovine hemoglobin fragment)	"BHP"[All Fields] AND biofilm[All Fields]	BHP	25637952	Autoinducer-2 increases biofilm formation via an ica- and bhp-dependent manner in Staphylococcus epidermidis RP62A.
3230	DRAMP02827	BHP (pepsin-derived bovine hemoglobin fragment)	"BHP"[All Fields] AND biofilm[All Fields]	BHP	24523473	Characterization of ocular methicillin-resistant Staphylococcus epidermidis isolates belonging predominantly to clonal complex 2 subcluster II.
3230	DRAMP02827	BHP (pepsin-derived bovine hemoglobin fragment)	"BHP"[All Fields] AND biofilm[All Fields]	BHP	24390796	Antimicrobial activity of tigecycline alone or in combination with rifampin against Staphylococcus epidermidis in biofilm.
3230	DRAMP02827	BHP (pepsin-derived bovine hemoglobin fragment)	"BHP"[All Fields] AND biofilm[All Fields]	BHP	23816539	Differing lifestyles of Staphylococcus epidermidis as revealed through Bayesian clustering of multilocus sequence types.
3230	DRAMP02827	BHP (pepsin-derived bovine hemoglobin fragment)	"BHP"[All Fields] AND biofilm[All Fields]	BHP	23549353	Farnesol induces cell detachment from established S. epidermidis biofilms.
3230	DRAMP02827	BHP (pepsin-derived bovine hemoglobin fragment)	"BHP"[All Fields] AND biofilm[All Fields]	BHP	22779682	Strong biofilm production but not adhesion virulence factors can discriminate between invasive and commensal Staphylococcus epidermidis strains.
3230	DRAMP02827	BHP (pepsin-derived bovine hemoglobin fragment)	"BHP"[All Fields] AND biofilm[All Fields]	BHP	22629403	Optimizing a qPCR gene expression quantification assay for S. epidermidis biofilms: a comparison between commercial kits and a customized protocol.
3230	DRAMP02827	BHP (pepsin-derived bovine hemoglobin fragment)	"BHP"[All Fields] AND biofilm[All Fields]	BHP	22421605	Prevalence of virulence determinants in Staphylococcus epidermidis from ICU patients in Kampala, Uganda.
3230	DRAMP02827	BHP (pepsin-derived bovine hemoglobin fragment)	"BHP"[All Fields] AND biofilm[All Fields]	BHP	21899769	High prevalence of methicillin resistant Staphylococcus aureus in the surgical units of Mulago hospital in Kampala, Uganda.
3230	DRAMP02827	BHP (pepsin-derived bovine hemoglobin fragment)	"BHP"[All Fields] AND biofilm[All Fields]	BHP	21510745	Molecular characterization of an early invasive Staphylococcus epidermidis prosthetic joint infection.
3230	DRAMP02827	BHP (pepsin-derived bovine hemoglobin fragment)	"BHP"[All Fields] AND biofilm[All Fields]	BHP	17292669	Spontaneous switch to PIA-independent biofilm formation in an ica-positive Staphylococcus epidermidis isolate.
3230	DRAMP02827	BHP (pepsin-derived bovine hemoglobin fragment)	"BHP"[All Fields] AND biofilm[All Fields]	BHP	15583290	Detection of virulence-associated genes not useful for discriminating between invasive and commensal Staphylococcus epidermidis strains from a bone marrow transplant unit.
3237	DRAMP02836	Cathelicidin antimicrobial peptide (cathelicidin; mammals, animals)	"Cathelicidin antimicrobial peptide"[All Fields] AND biofilm[All Fields]	Cathelicidin antimicrobial peptide	31644974	Covalent grafting of titanium with a cathelicidin peptide produces an osteoblast compatible surface with antistaphylococcal activity.
3237	DRAMP02836	Cathelicidin antimicrobial peptide (cathelicidin; mammals, animals)	"Cathelicidin antimicrobial peptide"[All Fields] AND biofilm[All Fields]	Cathelicidin antimicrobial peptide	28089718	Cathelicidin antimicrobial peptide from Alligator mississippiensis has antibacterial activity against multi-drug resistant Acinetobacter baumanii and Klebsiella pneumoniae.

3237	DRAMP02836	Cathelicidin antimicrobial peptide (cathelicidin; mammals, animals)	"Cathelicidin antimicrobial peptide"[All Fields] AND biofilm[All Fields]	Cathelicidin antimicrobial peptide	25008764	The antibiotic effects of vitamin D.
3237	DRAMP02836	Cathelicidin antimicrobial peptide (cathelicidin; mammals, animals)	"Cathelicidin antimicrobial peptide"[All Fields] AND biofilm[All Fields]	Cathelicidin antimicrobial peptide	23840194	The Human Cathelicidin Antimicrobial Peptide LL-37 as a Potential Treatment for Polymicrobial Infected Wounds.
3237	DRAMP02836	Cathelicidin antimicrobial peptide (cathelicidin; mammals, animals)	"Cathelicidin antimicrobial peptide"[All Fields] AND biofilm[All Fields]	Cathelicidin antimicrobial peptide	20002576	Human cathelicidin peptide LL37 inhibits both attachment capability and biofilm formation of Staphylococcus epidermidis.
3237	DRAMP02836	Cathelicidin antimicrobial peptide (cathelicidin; mammals, animals)	"Cathelicidin antimicrobial peptide"[All Fields] AND biofilm[All Fields]	Cathelicidin antimicrobial peptide	19390494	Staphylococcus epidermidis isolated from newborn infants express pilus-like structures and are inhibited by the cathelicidin-derived antimicrobial peptide LL37.
3246	DRAMP02852	Cathelicidin-2 (Bactenecin-5, Bac5; PR-42; mammals, animals)	"Cathelicidin-2"[All Fields] AND biofilm[All Fields]	Cathelicidin-2	21376541	A cathelicidin-2-derived peptide effectively impairs Staphylococcus epidermidis biofilms.
3253	DRAMP02876	Alpha-melanocyte-stimulating hormone (Alpha-MSH; mammals, animals)	"Alpha-melanocyte-stimulating hormone"[All Fields] AND biofilm[All Fields]	Alpha-melanocyte-stimulating hormone	33195893	Lipidated Short Analogue of α-Melanocyte Stimulating Hormone Exerts Bactericidal Activity against the Stationary Phase of Methicillin-Resistant Staphylococcus aureusand Inhibits Biofilm Formation.
3253	DRAMP02876	Alpha-melanocyte-stimulating hormone (Alpha-MSH; mammals, animals)	"Alpha-melanocyte-stimulating hormone"[All Fields] AND biofilm[All Fields]	Alpha-melanocyte-stimulating hormone	29152323	New melanocortin-like peptide of E. colican suppress inflammation via the mammalian melanocortin-1 receptor (MC1R): possible endocrine-like function for microbes of the gut.
3253	DRAMP02876	Alpha-melanocyte-stimulating hormone (Alpha-MSH; mammals, animals)	"Alpha-melanocyte-stimulating hormone"[All Fields] AND biofilm[All Fields]	Alpha-melanocyte-stimulating hormone	19560499	In vitro antimicrobial activity of alpha-melanocyte stimulating hormone against major human pathogen Staphylococcus aureus.
3276	DRAMP02902	L-amino-acid oxidase (LAAO, LAO; BpirLAAO-I; reptilia, animals)	"L-amino-acid oxidase"[All Fields] AND biofilm[All Fields]	L-amino-acid oxidase	30160994	Molecules and Mechanisms Underlying the Antimicrobial Activity of Escapin, an l-Amino Acid Oxidase from the Ink of Sea Hares.
3276	DRAMP02902	L-amino-acid oxidase (LAAO, LAO; BpirLAAO-I; reptilia, animals)	"L-amino-acid oxidase"[All Fields] AND biofilm[All Fields]	L-amino-acid oxidase	27401562	Inhibition and Dispersal of Pseudomonas aeruginosa Biofilms by Combination Treatment with Escapin Intermediate Products and Hydrogen Peroxide.
3276	DRAMP02902	L-amino-acid oxidase (LAAO, LAO; BpirLAAO-I; reptilia, animals)	"L-amino-acid oxidase"[All Fields] AND biofilm[All Fields]	L-amino-acid oxidase	18469105	SO-LAAO, a novel L-amino acid oxidase that enables Streptococcus oligofermentans to outcompete Streptococcus mutans by generating H2O2 from peptone.
3279	DRAMP02908	Beta-defensin 1 (BD-1; sBD-1; mammals, animals)	"Beta-defensin 1"[All Fields] AND biofilm[All Fields]	Beta-defensin 1	33420317	Curbing gastrointestinal infections by defensin fragment modifications without harming commensal microbiota.
3279	DRAMP02908	Beta-defensin 1 (BD-1; sBD-1; mammals, animals)	"Beta-defensin 1"[All Fields] AND biofilm[All Fields]	Beta-defensin 1	32585445	Glucose effect on Candida albicans biofilm during tissue invasion.
3279	DRAMP02908	Beta-defensin 1 (BD-1; sBD-1; mammals, animals)	"Beta-defensin 1"[All Fields] AND biofilm[All Fields]	Beta-defensin 1	30260708	Stabilized collagen matrix dressing improves wound macrophage function and epithelialization.
3279	DRAMP02908	Beta-defensin 1 (BD-1; sBD-1; mammals, animals)	"Beta-defensin 1"[All Fields] AND biofilm[All Fields]	Beta-defensin 1	28642103	Chimeric analogs of human β-defensin 1 and θ-defensin disrupt pre-established bacterial biofilms.
3279	DRAMP02908	Beta-defensin 1 (BD-1; sBD-1; mammals, animals)	"Beta-defensin 1"[All Fields] AND biofilm[All Fields]	Beta-defensin 1	26214284	α-tocopherol decreases interleukin-1β and -6 and increases human β-defensin-1 and -2 secretion in human gingival fibroblasts stimulated with Porphyromonas gingivalis lipopolysaccharide.
3279	DRAMP02908	Beta-defensin 1 (BD-1; sBD-1; mammals, animals)	"Beta-defensin 1"[All Fields] AND biofilm[All Fields]	Beta-defensin 1	22394470	Association of CiaRH with resistance of Streptococcus mutans to antimicrobial peptides in biofilms.
3279	DRAMP02908	Beta-defensin 1 (BD-1; sBD-1; mammals, animals)	"Beta-defensin 1"[All Fields] AND biofilm[All Fields]	Beta-defensin 1	20454633	Normal human gingival epithelial cells sense C. parapsilosis by toll-like receptors and module its pathogenesis through antimicrobial peptides and proinflammatory cytokines.
3280	DRAMP02909	Beta-defensin 2 (BD-2; sBD-2; mammals, animals)	"Beta-defensin 2"[All Fields] AND biofilm[All Fields]	Beta-defensin 2	34276631	DNA Blocks the Lethal Effect of Human Beta-Defensin 2 Against Neisseria meningitidis.
3280	DRAMP02909	Beta-defensin 2 (BD-2; sBD-2; mammals, animals)	"Beta-defensin 2"[All Fields] AND biofilm[All Fields]	Beta-defensin 2	32842903	Electrospun ZnO/Poly(Vinylidene Fluoride-Trifluoroethylene) Scaffolds for Lung Tissue Engineering.
3280	DRAMP02909	Beta-defensin 2 (BD-2; sBD-2; mammals, animals)	"Beta-defensin 2"[All Fields] AND biofilm[All Fields]	Beta-defensin 2	32522780	Controlling the Growth of the Skin Commensal Staphylococcus epidermidis Using d-Alanine Auxotrophy.
3280	DRAMP02909	Beta-defensin 2 (BD-2; sBD-2; mammals, animals)	"Beta-defensin 2"[All Fields] AND biofilm[All Fields]	Beta-defensin 2	32457749	The Antimicrobial Peptide Human Beta-Defensin 2 Inhibits Biofilm Production of Pseudomonas aeruginosaWithout Compromising Metabolic Activity.
3280	DRAMP02909	Beta-defensin 2 (BD-2; sBD-2; mammals, animals)	"Beta-defensin 2"[All Fields] AND biofilm[All Fields]	Beta-defensin 2	31336838	Candida albicans-Cell Interactions Activate Innate Immune Defense in Human Palate Epithelial Primary Cells via Nitric Oxide (NO) and β-Defensin 2 (hBD-2).
3280	DRAMP02909	Beta-defensin 2 (BD-2; sBD-2; mammals, animals)	"Beta-defensin 2"[All Fields] AND biofilm[All Fields]	Beta-defensin 2	30649289	Innate immune components affect growth and virulence traits of bacterial-vaginosis-associated and non-bacterial-vaginosis-associated Gardnerella vaginalis strains similarly.
3280	DRAMP02909	Beta-defensin 2 (BD-2; sBD-2; mammals, animals)	"Beta-defensin 2"[All Fields] AND biofilm[All Fields]	Beta-defensin 2	27582732	Efficient Eradication of Mature Pseudomonas aeruginosa Biofilm via Controlled Delivery of Nitric Oxide Combined with Antimicrobial Peptide and Antibiotics.
3280	DRAMP02909	Beta-defensin 2 (BD-2; sBD-2; mammals, animals)	"Beta-defensin 2"[All Fields] AND biofilm[All Fields]	Beta-defensin 2	27148195	Effect of Substance P in Staphylococcus aureus and Staphylococcus epidermidis Virulence: Implication for Skin Homeostasis.
3280	DRAMP02909	Beta-defensin 2 (BD-2; sBD-2; mammals, animals)	"Beta-defensin 2"[All Fields] AND biofilm[All Fields]	Beta-defensin 2	25808131	Expression of antimicrobial peptides and interleukin-8 during early stages of inflammation: An experimental gingivitis study.
3280	DRAMP02909	Beta-defensin 2 (BD-2; sBD-2; mammals, animals)	"Beta-defensin 2"[All Fields] AND biofilm[All Fields]	Beta-defensin 2	24340061	Inflammatory and antimicrobial responses to methicillin-resistant Staphylococcus aureus in an in vitro wound infection model.
3280	DRAMP02909	Beta-defensin 2 (BD-2; sBD-2; mammals, animals)	"Beta-defensin 2"[All Fields] AND biofilm[All Fields]	Beta-defensin 2	20378008	Effect of temperature on the shift of Pseudomonas fluorescens from an environmental microorganism to a potential human pathogen.
3280	DRAMP02909	Beta-defensin 2 (BD-2; sBD-2; mammals, animals)	"Beta-defensin 2"[All Fields] AND biofilm[All Fields]	Beta-defensin 2	19572896	The immune response of oral epithelial cells induced by single-species and complex naturally formed biofilms.
3280	DRAMP02909	Beta-defensin 2 (BD-2; sBD-2; mammals, animals)	"Beta-defensin 2"[All Fields] AND biofilm[All Fields]	Beta-defensin 2	18954353	Treponema denticola does not induce production of common innate immune mediators from primary gingival epithelial cells.
3280	DRAMP02909	Beta-defensin 2 (BD-2; sBD-2; mammals, animals)	"Beta-defensin 2"[All Fields] AND biofilm[All Fields]	Beta-defensin 2	18173794	The stage of native biofilm formation determines the gene expression of human beta-defensin-2, psoriasin, ribonuclease 7 and inflammatory mediators: a novel approach for stimulation of keratinocytes with in situ formed biofilms.
3280	DRAMP02909	Beta-defensin 2 (BD-2; sBD-2; mammals, animals)	"Beta-defensin 2"[All Fields] AND biofilm[All Fields]	Beta-defensin 2	17434999	Functional analysis of D-alanylation of lipoteichoic acid in the probiotic strain Lactobacillus rhamnosus GG.
3282	DRAMP02915	Cathelicidin-2 (Bactenecin-5, Bac5; OaBac5; mammals, animals)	"Cathelicidin-2"[All Fields] AND biofilm[All Fields]	Cathelicidin-2	21376541	A cathelicidin-2-derived peptide effectively impairs Staphylococcus epidermidis biofilms.
3284	DRAMP02917	Hepcidin (dogs, mammals, animals)	"Hepcidin"[All Fields] AND biofilm[All Fields]	Hepcidin	30456212	Quantification of Lipoteichoic Acid in Hemodialysis Patients With Central Venous Catheters.
3284	DRAMP02917	Hepcidin (dogs, mammals, animals)	"Hepcidin"[All Fields] AND biofilm[All Fields]	Hepcidin	30408337	Silk-Based Antimicrobial Polymers as a New Platform to Design Drug-Free Materials to Impede Microbial Infections.
3284	DRAMP02917	Hepcidin (dogs, mammals, animals)	"Hepcidin"[All Fields] AND biofilm[All Fields]	Hepcidin	24645694	Inhibitory effect of the human liver-derived antimicrobial peptide hepcidin 20 on biofilms of polysaccharide intercellular adhesin (PIA)-positive and PIA-negative strains of Staphylococcus epidermidis.
3285	DRAMP02918	Beta-defensin 1	"Beta-defensin 1"[All Fields] AND biofilm[All Fields]	Beta-defensin 1	33420317	Curbing gastrointestinal infections by defensin fragment modifications without harming commensal microbiota.
3285	DRAMP02918	Beta-defensin 1	"Beta-defensin 1"[All Fields] AND biofilm[All Fields]	Beta-defensin 1	32585445	Glucose effect on Candida albicans biofilm during tissue invasion.
3285	DRAMP02918	Beta-defensin 1	"Beta-defensin 1"[All Fields] AND biofilm[All Fields]	Beta-defensin 1	30260708	Stabilized collagen matrix dressing improves wound macrophage function and epithelialization.
3285	DRAMP02918	Beta-defensin 1	"Beta-defensin 1"[All Fields] AND biofilm[All Fields]	Beta-defensin 1	28642103	Chimeric analogs of human β-defensin 1 and θ-defensin disrupt pre-established bacterial biofilms.
3285	DRAMP02918	Beta-defensin 1	"Beta-defensin 1"[All Fields] AND biofilm[All Fields]	Beta-defensin 1	26214284	α-tocopherol decreases interleukin-1β and -6 and increases human β-defensin-1 and -2 secretion in human gingival fibroblasts stimulated with Porphyromonas gingivalis lipopolysaccharide.
3285	DRAMP02918	Beta-defensin 1	"Beta-defensin 1"[All Fields] AND biofilm[All Fields]	Beta-defensin 1	22394470	Association of CiaRH with resistance of Streptococcus mutans to antimicrobial peptides in biofilms.
3285	DRAMP02918	Beta-defensin 1	"Beta-defensin 1"[All Fields] AND biofilm[All Fields]	Beta-defensin 1	20454633	Normal human gingival epithelial cells sense C. parapsilosis by toll-like receptors and module its pathogenesis through antimicrobial peptides and proinflammatory cytokines.
3322	DRAMP02976	Beta-defensin 1 (BD-1; Defensin, beta 1; pigs, mammals, animals)	"Beta-defensin 1"[All Fields] AND biofilm[All Fields]	Beta-defensin 1	33420317	Curbing gastrointestinal infections by defensin fragment modifications without harming commensal microbiota.
3322	DRAMP02976	Beta-defensin 1 (BD-1; Defensin, beta 1; pigs, mammals, animals)	"Beta-defensin 1"[All Fields] AND biofilm[All Fields]	Beta-defensin 1	32585445	Glucose effect on Candida albicans biofilm during tissue invasion.
3322	DRAMP02976	Beta-defensin 1 (BD-1; Defensin, beta 1; pigs, mammals, animals)	"Beta-defensin 1"[All Fields] AND biofilm[All Fields]	Beta-defensin 1	30260708	Stabilized collagen matrix dressing improves wound macrophage function and epithelialization.

3322	DRAMP02976	Beta-defensin 1 (BD-1; Defensin, beta 1; pigs, mammals, animals)	"Beta-defensin 1"[All Fields] AND biofilm[All Fields]	Beta-defensin 1	28642103	Chimeric analogs of human β-defensin 1 and θ-defensin disrupt pre-established bacterial biofilms.
3322	DRAMP02976	Beta-defensin 1 (BD-1; Defensin, beta 1; pigs, mammals, animals)	"Beta-defensin 1"[All Fields] AND biofilm[All Fields]	Beta-defensin 1	26214284	α-tocopherol decreases interleukin-1β and -6 and increases human β-defensin-1 and -2 secretion in human gingival fibroblasts stimulated with Porphyromonas gingivalis lipopolysaccharide.
3322	DRAMP02976	Beta-defensin 1 (BD-1; Defensin, beta 1; pigs, mammals, animals)	"Beta-defensin 1"[All Fields] AND biofilm[All Fields]	Beta-defensin 1	22394470	Association of CiaRH with resistance of Streptococcus mutans to antimicrobial peptides in biofilms.
3322	DRAMP02976	Beta-defensin 1 (BD-1; Defensin, beta 1; pigs, mammals, animals)	"Beta-defensin 1"[All Fields] AND biofilm[All Fields]	Beta-defensin 1	20454633	Normal human gingival epithelial cells sense C. parapsilosis by toll-like receptors and module its pathogenesis through antimicrobial peptides and proinflammatory cytokines.
3325	DRAMP02979	Hepcidin (pigs, mammals, animals)	"Hepcidin"[All Fields] AND biofilm[All Fields]	Hepcidin	30456212	Quantification of Lipoteichoic Acid in Hemodialysis Patients With Central Venous Catheters.
3325	DRAMP02979	Hepcidin (pigs, mammals, animals)	"Hepcidin"[All Fields] AND biofilm[All Fields]	Hepcidin	30408337	Silk-Based Antimicrobial Polymers as a New Platform to Design Drug-Free Materials to Impede Microbial Infections.
3325	DRAMP02979	Hepcidin (pigs, mammals, animals)	"Hepcidin"[All Fields] AND biofilm[All Fields]	Hepcidin	24645694	Inhibitory effect of the human liver-derived antimicrobial peptide hepcidin 20 on biofilms of polysaccharide intercellular adhesin (PIA)-positive and PIA-negative strains of Staphylococcus epidermidis.
3338	DRAMP02994	Defensin-1 (Royalisin; Insects, animals)	"Defensin-1"[All Fields] AND biofilm[All Fields]	Defensin-1	34321877	Antibacterial Effect of Honey-Derived Exosomes Containing Antimicrobial Peptides Against Oral Streptococci.
3338	DRAMP02994	Defensin-1 (Royalisin; Insects, animals)	"Defensin-1"[All Fields] AND biofilm[All Fields]	Defensin-1	33911935	The antibacterial activities of honey.
3338	DRAMP02994	Defensin-1 (Royalisin; Insects, animals)	"Defensin-1"[All Fields] AND biofilm[All Fields]	Defensin-1	33420317	Curbing gastrointestinal infections by defensin fragment modifications without harming commensal microbiota.
3338	DRAMP02994	Defensin-1 (Royalisin; Insects, animals)	"Defensin-1"[All Fields] AND biofilm[All Fields]	Defensin-1	32585445	Glucose effect on Candida albicans biofilm during tissue invasion.
3338	DRAMP02994	Defensin-1 (Royalisin; Insects, animals)	"Defensin-1"[All Fields] AND biofilm[All Fields]	Defensin-1	31729441	Rhesus Theta Defensin 1 Promotes Long Term Survival in Systemic Candidiasis by Host Directed Mechanisms.
3338	DRAMP02994	Defensin-1 (Royalisin; Insects, animals)	"Defensin-1"[All Fields] AND biofilm[All Fields]	Defensin-1	30260708	Stabilized collagen matrix dressing improves wound macrophage function and epithelialization.
3338	DRAMP02994	Defensin-1 (Royalisin; Insects, animals)	"Defensin-1"[All Fields] AND biofilm[All Fields]	Defensin-1	29671721	The BceABRS four-component system that is essential for cell envelope stress response is involved in sensing and response to host defence peptides and is required for the biofilm formation and fitness of Streptococcus mutans.
3338	DRAMP02994	Defensin-1 (Royalisin; Insects, animals)	"Defensin-1"[All Fields] AND biofilm[All Fields]	Defensin-1	28649561	Psd1 Effects on Candida albicansPlanktonic Cells and Biofilms.
3338	DRAMP02994	Defensin-1 (Royalisin; Insects, animals)	"Defensin-1"[All Fields] AND biofilm[All Fields]	Defensin-1	28642103	Chimeric analogs of human β-defensin 1 and θ-defensin disrupt pre-established bacterial biofilms.
3338	DRAMP02994	Defensin-1 (Royalisin; Insects, animals)	"Defensin-1"[All Fields] AND biofilm[All Fields]	Defensin-1	26861950	Antibiofilm efficacy of honey and bee-derived defensin-1 on multispecies wound biofilm.
3338	DRAMP02994	Defensin-1 (Royalisin; Insects, animals)	"Defensin-1"[All Fields] AND biofilm[All Fields]	Defensin-1	26214284	α-tocopherol decreases interleukin-1β and -6 and increases human β-defensin-1 and -2 secretion in human gingival fibroblasts stimulated with Porphyromonas gingivalis lipopolysaccharide.
3338	DRAMP02994	Defensin-1 (Royalisin; Insects, animals)	"Defensin-1"[All Fields] AND biofilm[All Fields]	Defensin-1	22394470	Association of CiaRH with resistance of Streptococcus mutans to antimicrobial peptides in biofilms.
3338	DRAMP02994	Defensin-1 (Royalisin; Insects, animals)	"Defensin-1"[All Fields] AND biofilm[All Fields]	Defensin-1	20454633	Normal human gingival epithelial cells sense C. parapsilosis by toll-like receptors and module its pathogenesis through antimicrobial peptides and proinflammatory cytokines.
3340	DRAMP03005	Apidaecin (Insects, animals)	"Apidaecin"[All Fields] AND biofilm[All Fields]	Apidaecin	27405093	Identification of New Resistance Mechanisms in Escherichia coli against Apidaecin 1b Using Quantitative Gel- and LC-MS-Based Proteomics.
3341	DRAMP03006	Defensin (Insects, animals)	"Defensin"[All Fields] AND biofilm[All Fields]	Defensin	34408988	The Anti-Biofilm Efficacy of Caffeic Acid Phenethyl Ester (CAPE) In Vitroand a Murine Model of Oral Candidiasis.
3341	DRAMP03006	Defensin (Insects, animals)	"Defensin"[All Fields] AND biofilm[All Fields]	Defensin	34321877	Antibacterial Effect of Honey-Derived Exosomes Containing Antimicrobial Peptides Against Oral Streptococci.
3341	DRAMP03006	Defensin (Insects, animals)	"Defensin"[All Fields] AND biofilm[All Fields]	Defensin	34276631	DNA Blocks the Lethal Effect of Human Beta-Defensin 2 Against Neisseria meningitidis.
3341	DRAMP03006	Defensin (Insects, animals)	"Defensin"[All Fields] AND biofilm[All Fields]	Defensin	33911935	The antibacterial activities of honey.
3341	DRAMP03006	Defensin (Insects, animals)	"Defensin"[All Fields] AND biofilm[All Fields]	Defensin	33865931	Identification of a crocodylian β-defensin variant from Alligator mississippiensis with antimicrobial and antibiofilm activity.
3341	DRAMP03006	Defensin (Insects, animals)	"Defensin"[All Fields] AND biofilm[All Fields]	Defensin	33586659	Identification of anti-microbial peptides and traces of microbial DNA in infrainfundibular compartments of human scalp terminal hair follicles.
3341	DRAMP03006	Defensin (Insects, animals)	"Defensin"[All Fields] AND biofilm[All Fields]	Defensin	33534018	A recombinant fungal defensin-like peptide-P2 combats Streptococcus dysgalactiae and biofilms.
3341	DRAMP03006	Defensin (Insects, animals)	"Defensin"[All Fields] AND biofilm[All Fields]	Defensin	33447687	Future directions of postoperative spinal implant infections.
3341	DRAMP03006	Defensin (Insects, animals)	"Defensin"[All Fields] AND biofilm[All Fields]	Defensin	33420317	Curbing gastrointestinal infections by defensin fragment modifications without harming commensal microbiota.
3341	DRAMP03006	Defensin (Insects, animals)	"Defensin"[All Fields] AND biofilm[All Fields]	Defensin	32867384	A Novel Peptide Antibiotic, Pro10-1D, Designed from Insect Defensin Shows Antibacterial and Anti-Inflammatory Activities in Sepsis Models.
3341	DRAMP03006	Defensin (Insects, animals)	"Defensin"[All Fields] AND biofilm[All Fields]	Defensin	32858856	Candida albicansVirulence Factors and Pathogenicity for Endodontic Infections.
3341	DRAMP03006	Defensin (Insects, animals)	"Defensin"[All Fields] AND biofilm[All Fields]	Defensin	32842903	Electrospun ZnO/Poly(Vinylidene Fluoride-Trifluoroethylene) Scaffolds for Lung Tissue Engineering.
3341	DRAMP03006	Defensin (Insects, animals)	"Defensin"[All Fields] AND biofilm[All Fields]	Defensin	32663201	Inhibition and eradication activity of truncated α-defensin analogs against multidrug resistant uropathogenic Escherichia coli biofilm.
3341	DRAMP03006	Defensin (Insects, animals)	"Defensin"[All Fields] AND biofilm[All Fields]	Defensin	32585445	Glucose effect on Candida albicans biofilm during tissue invasion.
3341	DRAMP03006	Defensin (Insects, animals)	"Defensin"[All Fields] AND biofilm[All Fields]	Defensin	32522780	Controlling the Growth of the Skin Commensal Staphylococcus epidermidis Using d-Alanine Auxotrophy.
3341	DRAMP03006	Defensin (Insects, animals)	"Defensin"[All Fields] AND biofilm[All Fields]	Defensin	32457749	The Antimicrobial Peptide Human Beta-Defensin 2 Inhibits Biofilm Production of Pseudomonas aeruginosaWithout Compromising Metabolic Activity.
3341	DRAMP03006	Defensin (Insects, animals)	"Defensin"[All Fields] AND biofilm[All Fields]	Defensin	32439511	Antibacterial activities and mechanisms of action of a defensin from manila clam Ruditapes philippinarum.
3341	DRAMP03006	Defensin (Insects, animals)	"Defensin"[All Fields] AND biofilm[All Fields]	Defensin	31933178	Effects of human β-defensin 3 fused with carbohydrate-binding domain on the function of type III secretion system in Pseudomonas aeruginosa PA14.
3341	DRAMP03006	Defensin (Insects, animals)	"Defensin"[All Fields] AND biofilm[All Fields]	Defensin	31906541	Role of FAD-I in Fusobacterial Interspecies Interaction and Biofilm Formation.
3341	DRAMP03006	Defensin (Insects, animals)	"Defensin"[All Fields] AND biofilm[All Fields]	Defensin	31729441	Rhesus Theta Defensin 1 Promotes Long Term Survival in Systemic Candidiasis by Host Directed Mechanisms.
3341	DRAMP03006	Defensin (Insects, animals)	"Defensin"[All Fields] AND biofilm[All Fields]	Defensin	31336838	Candida albicans-Cell Interactions Activate Innate Immune Defense in Human Palate Epithelial Primary Cells via Nitric Oxide (NO) and β-Defensin 2 (hBD-2).
3341	DRAMP03006	Defensin (Insects, animals)	"Defensin"[All Fields] AND biofilm[All Fields]	Defensin	31165072	Impact of the Food Additive Titanium Dioxide (E171) on Gut Microbiota-Host Interaction.
3341	DRAMP03006	Defensin (Insects, animals)	"Defensin"[All Fields] AND biofilm[All Fields]	Defensin	31031739	Salt-Tolerant Antifungal and Antibacterial Activities of the Corn Defensin ZmD32.
3341	DRAMP03006	Defensin (Insects, animals)	"Defensin"[All Fields] AND biofilm[All Fields]	Defensin	31025073	A recombinant fungal defensin-like peptide-P2 combats multidrug-resistant Staphylococcus aureus and biofilms.
3341	DRAMP03006	Defensin (Insects, animals)	"Defensin"[All Fields] AND biofilm[All Fields]	Defensin	30659503	Lactobacillus plantarum USM8613 Aids in Wound Healing and Suppresses Staphylococcus aureus Infection at Wound Sites.
3341	DRAMP03006	Defensin (Insects, animals)	"Defensin"[All Fields] AND biofilm[All Fields]	Defensin	30649289	Innate immune components affect growth and virulence traits of bacterial-vaginosis-associated and non-bacterial-vaginosis-associated Gardnerella vaginalis strains similarly.
3341	DRAMP03006	Defensin (Insects, animals)	"Defensin"[All Fields] AND biofilm[All Fields]	Defensin	30376742	Alpha defensin, leukocyte esterase, C-reactive protein, and leukocyte count in synovial fluid for pre-operative diagnosis of periprosthetic infection.
3341	DRAMP03006	Defensin (Insects, animals)	"Defensin"[All Fields] AND biofilm[All Fields]	Defensin	30260708	Stabilized collagen matrix dressing improves wound macrophage function and epithelialization.
3341	DRAMP03006	Defensin (Insects, animals)	"Defensin"[All Fields] AND biofilm[All Fields]	Defensin	30254440	Modification of the surface of titanium with multifunctional chimeric peptides to prevent biofilm formation via inhibition of initial colonizers.
3341	DRAMP03006	Defensin (Insects, animals)	"Defensin"[All Fields] AND biofilm[All Fields]	Defensin	29902560	A defensin-like antimicrobial peptide from the manila clam Ruditapes philippinarum: Investigation of the antibacterial activities and mode of action.
3341	DRAMP03006	Defensin (Insects, animals)	"Defensin"[All Fields] AND biofilm[All Fields]	Defensin	29872295	Antimicrobial peptide-loaded liquid crystalline precursor bioadhesive system for the prevention of dental caries.
3341	DRAMP03006	Defensin (Insects, animals)	"Defensin"[All Fields] AND biofilm[All Fields]	Defensin	29671721	The BceABRS four-component system that is essential for cell envelope stress response is involved in sensing and response to host defence peptides and is required for the biofilm formation and fitness of Streptococcus mutans.
3341	DRAMP03006	Defensin (Insects, animals)	"Defensin"[All Fields] AND biofilm[All Fields]	Defensin	29104569	A Linear 19-Mer Plant Defensin-Derived Peptide Acts Synergistically with Caspofungin against Candida albicansBiofilms.
3341	DRAMP03006	Defensin (Insects, animals)	"Defensin"[All Fields] AND biofilm[All Fields]	Defensin	29077172	The significance of HBD-3 and fluorescent composite carriers in the processof bone formation in rats infected with Staphylococcus aureus.

3341	DRAMP03006	Defensin (Insects, animals)	"Defensin"[All Fields] AND biofilm[All Fields]	Defensin	29045084	New Approach to Treat and Prevent Oral Disease.
3341	DRAMP03006	Defensin (Insects, animals)	"Defensin"[All Fields] AND biofilm[All Fields]	Defensin	29025642	Engineered chimeric peptides with antimicrobial and titanium-binding functions to inhibit biofilm formation on Ti implants.
3341	DRAMP03006	Defensin (Insects, animals)	"Defensin"[All Fields] AND biofilm[All Fields]	Defensin	28956355	The synthetic human beta-defensin-3 C15 peptide exhibits antimicrobial activity against Streptococcus mutans, both alone and in combination with dental disinfectants.
3341	DRAMP03006	Defensin (Insects, animals)	"Defensin"[All Fields] AND biofilm[All Fields]	Defensin	28951032	Antifungal Effects of Synthetic Human Beta-defensin-3-C15 Peptide on Candida albicans-infected Root Dentin.
3341	DRAMP03006	Defensin (Insects, animals)	"Defensin"[All Fields] AND biofilm[All Fields]	Defensin	28874606	Bacterial d-amino acids suppress sinonasal innate immunity through sweet taste receptors in solitary chemosensory cells.
3341	DRAMP03006	Defensin (Insects, animals)	"Defensin"[All Fields] AND biofilm[All Fields]	Defensin	28725299	Role of Streptococcus mutanstwo-component systems in antimicrobial peptide resistance in the oral cavity.
3341	DRAMP03006	Defensin (Insects, animals)	"Defensin"[All Fields] AND biofilm[All Fields]	Defensin	28649561	Psd1 Effects on Candida albicansPlanktonic Cells and Biofilms.
3341	DRAMP03006	Defensin (Insects, animals)	"Defensin"[All Fields] AND biofilm[All Fields]	Defensin	28642103	Chimeric analogs of human β-defensin 1 and θ-defensin disrupt pre-established bacterial biofilms.
3341	DRAMP03006	Defensin (Insects, animals)	"Defensin"[All Fields] AND biofilm[All Fields]	Defensin	28413476	The mechanism of human β-defensin 3 in MRSA-induced infection of implant drug-resistant bacteria biofilm in the mouse tibial bone marrow.
3341	DRAMP03006	Defensin (Insects, animals)	"Defensin"[All Fields] AND biofilm[All Fields]	Defensin	28296382	Human α-Defensin 6: A Small Peptide That Self-Assembles and Protects the Host by Entangling Microbes.
3341	DRAMP03006	Defensin (Insects, animals)	"Defensin"[All Fields] AND biofilm[All Fields]	Defensin	28278280	Natural antimicrobial peptide complexes in the fighting of antibiotic resistant biofilms: Calliphora vicina medicinal maggots.
3341	DRAMP03006	Defensin (Insects, animals)	"Defensin"[All Fields] AND biofilm[All Fields]	Defensin	28144375	Advancements in Diagnosing Periprosthetic Joint Infections after Total Hip and Knee Arthroplasty.
3341	DRAMP03006	Defensin (Insects, animals)	"Defensin"[All Fields] AND biofilm[All Fields]	Defensin	28078813	Host defense peptide-derived privileged scaffolds for anti-infective drug discovery.
3341	DRAMP03006	Defensin (Insects, animals)	"Defensin"[All Fields] AND biofilm[All Fields]	Defensin	28026958	Human α-Defensin 6 Self-Assembly Prevents Adhesion and Suppresses Virulence Traits of Candida albicans.
3341	DRAMP03006	Defensin (Insects, animals)	"Defensin"[All Fields] AND biofilm[All Fields]	Defensin	27794585	A Novel Defensin-Like Peptide Associated with Two Other New Cationic Antimicrobial Peptides in Transcriptome of the Iranian Scorpion Venom.
3341	DRAMP03006	Defensin (Insects, animals)	"Defensin"[All Fields] AND biofilm[All Fields]	Defensin	27777572	Role of yqiCin the Pathogenicity of Salmonellaand Innate Immune Responses of Human Intestinal Epithelium.
3341	DRAMP03006	Defensin (Insects, animals)	"Defensin"[All Fields] AND biofilm[All Fields]	Defensin	27582732	Efficient Eradication of Mature Pseudomonas aeruginosa Biofilm via Controlled Delivery of Nitric Oxide Combined with Antimicrobial Peptide and Antibiotics.
3341	DRAMP03006	Defensin (Insects, animals)	"Defensin"[All Fields] AND biofilm[All Fields]	Defensin	27417541	Acute appendicitis: transcript profiling of blood identifies promising biomarkers and potential underlying processes.
3341	DRAMP03006	Defensin (Insects, animals)	"Defensin"[All Fields] AND biofilm[All Fields]	Defensin	27200276	Antifungal effects of synthetic human β-defensin 3-C15 peptide.
3341	DRAMP03006	Defensin (Insects, animals)	"Defensin"[All Fields] AND biofilm[All Fields]	Defensin	27148195	Effect of Substance P in Staphylococcus aureus and Staphylococcus epidermidis Virulence: Implication for Skin Homeostasis.
3341	DRAMP03006	Defensin (Insects, animals)	"Defensin"[All Fields] AND biofilm[All Fields]	Defensin	26861950	Antibiofilm efficacy of honey and bee-derived defensin-1 on multispecies wound biofilm.
3341	DRAMP03006	Defensin (Insects, animals)	"Defensin"[All Fields] AND biofilm[All Fields]	Defensin	26592804	The radish defensins RsAFP1 and RsAFP2 act synergistically with caspofungin against Candida albicans biofilms.
3341	DRAMP03006	Defensin (Insects, animals)	"Defensin"[All Fields] AND biofilm[All Fields]	Defensin	26248029	Synergistic Activity of the Plant Defensin HsAFP1 and Caspofungin against Candida albicans Biofilms and Planktonic Cultures.
3341	DRAMP03006	Defensin (Insects, animals)	"Defensin"[All Fields] AND biofilm[All Fields]	Defensin	26214284	α-tocopherol decreases interleukin-1β and -6 and increases human β-defensin-1 and -2 secretion in human gingival fibroblasts stimulated with Porphyromonas gingivalis lipopolysaccharide.
3341	DRAMP03006	Defensin (Insects, animals)	"Defensin"[All Fields] AND biofilm[All Fields]	Defensin	26196513	Snake Cathelicidin NA-CATH and Smaller Helical Antimicrobial Peptides Are Effective against Burkholderia thailandensis.
3341	DRAMP03006	Defensin (Insects, animals)	"Defensin"[All Fields] AND biofilm[All Fields]	Defensin	26119274	Ultrasound microbubbles enhance human β-defensin 3 against biofilms.
3341	DRAMP03006	Defensin (Insects, animals)	"Defensin"[All Fields] AND biofilm[All Fields]	Defensin	25862466	The Antibacterial Effects of an Antimicrobial Peptide Human β-Defensin 3 Fused with Carbohydrate-Binding Domain on Pseudomonas aeruginosa PA14.
3341	DRAMP03006	Defensin (Insects, animals)	"Defensin"[All Fields] AND biofilm[All Fields]	Defensin	25808131	Expression of antimicrobial peptides and interleukin-8 during early stages of inflammation: An experimental gingivitis study.
3341	DRAMP03006	Defensin (Insects, animals)	"Defensin"[All Fields] AND biofilm[All Fields]	Defensin	25806720	Antimicrobial peptides in 2014.
3341	DRAMP03006	Defensin (Insects, animals)	"Defensin"[All Fields] AND biofilm[All Fields]	Defensin	25285879	Inhibition and destruction of Pseudomonas aeruginosa biofilms by antibiotics and antimicrobial peptides.
3341	DRAMP03006	Defensin (Insects, animals)	"Defensin"[All Fields] AND biofilm[All Fields]	Defensin	25212593	Health- and disease-associated species clusters in complex natural biofilms determine the innate immune response in oral epithelial cells during biofilm maturation.
3341	DRAMP03006	Defensin (Insects, animals)	"Defensin"[All Fields] AND biofilm[All Fields]	Defensin	24913184	Effects of human β-defensin-3 on biofilm formation-regulating genes dltB and icaA in Staphylococcus aureus.
3341	DRAMP03006	Defensin (Insects, animals)	"Defensin"[All Fields] AND biofilm[All Fields]	Defensin	24340061	Inflammatory and antimicrobial responses to methicillin-resistant Staphylococcus aureus in an in vitro wound infection model.
3341	DRAMP03006	Defensin (Insects, animals)	"Defensin"[All Fields] AND biofilm[All Fields]	Defensin	24240906	Adsorption study of pellicle proteins to gold, silica and titanium by quartz crystal microbalance method.
3341	DRAMP03006	Defensin (Insects, animals)	"Defensin"[All Fields] AND biofilm[All Fields]	Defensin	24238461	Antibacterial efficacy of a human β-defensin-3 peptide on multispecies biofilms.
3341	DRAMP03006	Defensin (Insects, animals)	"Defensin"[All Fields] AND biofilm[All Fields]	Defensin	24100890	[Use of recombinant human beta-defensin-3 to evaluate the effect of adhesion of Candida albicans on the surface of soft lining material].
3341	DRAMP03006	Defensin (Insects, animals)	"Defensin"[All Fields] AND biofilm[All Fields]	Defensin	23639356	Human beta-defensin 3: a novel inhibitor of Staphylococcus-produced biofilm production. Commentary on "Human β-defensin 3 inhibits antibiotic-resistant Staphylococcus biofilm formation".
3341	DRAMP03006	Defensin (Insects, animals)	"Defensin"[All Fields] AND biofilm[All Fields]	Defensin	23519963	Ultrasound-targeted microbubble destruction enhances human β-defensin 3 activity against antibiotic-resistant Staphylococcus biofilms.
3341	DRAMP03006	Defensin (Insects, animals)	"Defensin"[All Fields] AND biofilm[All Fields]	Defensin	23273885	Human β-defensin 3 inhibits antibiotic-resistant Staphylococcus biofilm formation.
3341	DRAMP03006	Defensin (Insects, animals)	"Defensin"[All Fields] AND biofilm[All Fields]	Defensin	23203265	Potentiation of the cytotoxic activity of copper by polyphosphate on biofilm-producing bacteria: a bioinspired approach.
3341	DRAMP03006	Defensin (Insects, animals)	"Defensin"[All Fields] AND biofilm[All Fields]	Defensin	23078156	Antimicrobial efficacy of a human β-defensin-3 peptide using an Enterococcus faecalis dentine infection model.
3341	DRAMP03006	Defensin (Insects, animals)	"Defensin"[All Fields] AND biofilm[All Fields]	Defensin	23053486	Synergistic effect and antibiofilm activity between the antimicrobial peptide coprisin and conventional antibiotics against opportunistic bacteria.
3341	DRAMP03006	Defensin (Insects, animals)	"Defensin"[All Fields] AND biofilm[All Fields]	Defensin	22922323	Extracellular DNA within a nontypeable Haemophilus influenzae-induced biofilm binds human beta defensin-3 and reduces its antimicrobial activity.
3341	DRAMP03006	Defensin (Insects, animals)	"Defensin"[All Fields] AND biofilm[All Fields]	Defensin	22855857	Retrocyclin inhibits Gardnerella vaginalis biofilm formation and toxin activity.
3341	DRAMP03006	Defensin (Insects, animals)	"Defensin"[All Fields] AND biofilm[All Fields]	Defensin	22394470	Association of CiaRH with resistance of Streptococcus mutans to antimicrobial peptides in biofilms.
3341	DRAMP03006	Defensin (Insects, animals)	"Defensin"[All Fields] AND biofilm[All Fields]	Defensin	22229614	Comparison of the effects of human β-defensin 3, vancomycin, and clindamycin on Staphylococcus aureus biofilm formation.
3341	DRAMP03006	Defensin (Insects, animals)	"Defensin"[All Fields] AND biofilm[All Fields]	Defensin	21692631	A novel organotypic dento-epithelial culture model: effect of Fusobacterium nucleatum biofilm on B-defensin-2, -3, and LL-37 expression.
3341	DRAMP03006	Defensin (Insects, animals)	"Defensin"[All Fields] AND biofilm[All Fields]	Defensin	20454633	Normal human gingival epithelial cells sense C. parapsilosis by toll-like receptors and module its pathogenesis through antimicrobial peptides and proinflammatory cytokines.
3341	DRAMP03006	Defensin (Insects, animals)	"Defensin"[All Fields] AND biofilm[All Fields]	Defensin	20378008	Effect of temperature on the shift of Pseudomonas fluorescens from an environmental microorganism to a potential human pathogen.
3341	DRAMP03006	Defensin (Insects, animals)	"Defensin"[All Fields] AND biofilm[All Fields]	Defensin	19961380	Gingival transcriptome patterns during induction and resolution of experimental gingivitis in humans.
3341	DRAMP03006	Defensin (Insects, animals)	"Defensin"[All Fields] AND biofilm[All Fields]	Defensin	19780045	Candida famata modulates toll-like receptor, beta-defensin, and proinflammatory cytokine expression by normal human epithelial cells.
3341	DRAMP03006	Defensin (Insects, animals)	"Defensin"[All Fields] AND biofilm[All Fields]	Defensin	19572896	The immune response of oral epithelial cells induced by single-species and complex naturally formed biofilms.
3341	DRAMP03006	Defensin (Insects, animals)	"Defensin"[All Fields] AND biofilm[All Fields]	Defensin	19466693	Activity of antimicrobial peptides in the presence of polysaccharides produced by pulmonary pathogens.
3341	DRAMP03006	Defensin (Insects, animals)	"Defensin"[All Fields] AND biofilm[All Fields]	Defensin	18954353	Treponema denticola does not induce production of common innate immune mediators from primary gingival epithelial cells.
3341	DRAMP03006	Defensin (Insects, animals)	"Defensin"[All Fields] AND biofilm[All Fields]	Defensin	18173794	The stage of native biofilm formation determines the gene expression of human beta-defensin-2, psoriasin, ribonuclease 7 and inflammatory mediators: a novel approach for stimulation of keratinocytes with in situ formed biofilms.
3341	DRAMP03006	Defensin (Insects, animals)	"Defensin"[All Fields] AND biofilm[All Fields]	Defensin	17434999	Functional analysis of D-alanylation of lipoteichoic acid in the probiotic strain Lactobacillus rhamnosus GG.
3341	DRAMP03006	Defensin (Insects, animals)	"Defensin"[All Fields] AND biofilm[All Fields]	Defensin	15493829	Bacterial evasion of innate host defenses--the Staphylococcus aureus lesson.

3341	DRAMP03006	Defensin (Insects, animals)	"Defensin"[All Fields] AND biofilm[All Fields]	Defensin	14764110	Polysaccharide intercellular adhesin (PIA) protects Staphylococcus epidermidis against major components of the human innate immune system.
3345	DRAMP03016	Defensin-1 (Insects, animals)	"Defensin-1"[All Fields] AND biofilm[All Fields]	Defensin-1	34321877	Antibacterial Effect of Honey-Derived Exosomes Containing Antimicrobial Peptides Against Oral Streptococci.
3345	DRAMP03016	Defensin-1 (Insects, animals)	"Defensin-1"[All Fields] AND biofilm[All Fields]	Defensin-1	33911935	The antibacterial activities of honey.
3345	DRAMP03016	Defensin-1 (Insects, animals)	"Defensin-1"[All Fields] AND biofilm[All Fields]	Defensin-1	33420317	Curbing gastrointestinal infections by defensin fragment modifications without harming commensal microbiota.
3345	DRAMP03016	Defensin-1 (Insects, animals)	"Defensin-1"[All Fields] AND biofilm[All Fields]	Defensin-1	32585445	Glucose effect on Candida albicans biofilm during tissue invasion.
3345	DRAMP03016	Defensin-1 (Insects, animals)	"Defensin-1"[All Fields] AND biofilm[All Fields]	Defensin-1	31729441	Rhesus Theta Defensin 1 Promotes Long Term Survival in Systemic Candidiasis by Host Directed Mechanisms.
3345	DRAMP03016	Defensin-1 (Insects, animals)	"Defensin-1"[All Fields] AND biofilm[All Fields]	Defensin-1	30260708	Stabilized collagen matrix dressing improves wound macrophage function and epithelialization.
3345	DRAMP03016	Defensin-1 (Insects, animals)	"Defensin-1"[All Fields] AND biofilm[All Fields]	Defensin-1	29671721	The BceABRS four-component system that is essential for cell envelope stress response is involved in sensing and response to host defence peptides and is required for the biofilm formation and fitness of Streptococcus mutans.
3345	DRAMP03016	Defensin-1 (Insects, animals)	"Defensin-1"[All Fields] AND biofilm[All Fields]	Defensin-1	28649561	Psd1 Effects on Candida albicansPlanktonic Cells and Biofilms.
3345	DRAMP03016	Defensin-1 (Insects, animals)	"Defensin-1"[All Fields] AND biofilm[All Fields]	Defensin-1	28642103	Chimeric analogs of human β-defensin 1 and θ-defensin disrupt pre-established bacterial biofilms.
3345	DRAMP03016	Defensin-1 (Insects, animals)	"Defensin-1"[All Fields] AND biofilm[All Fields]	Defensin-1	26861950	Antibiofilm efficacy of honey and bee-derived defensin-1 on multispecies wound biofilm.
3345	DRAMP03016	Defensin-1 (Insects, animals)	"Defensin-1"[All Fields] AND biofilm[All Fields]	Defensin-1	26214284	α-tocopherol decreases interleukin-1β and -6 and increases human β-defensin-1 and -2 secretion in human gingival fibroblasts stimulated with Porphyromonas gingivalis lipopolysaccharide.
3345	DRAMP03016	Defensin-1 (Insects, animals)	"Defensin-1"[All Fields] AND biofilm[All Fields]	Defensin-1	22394470	Association of CiaRH with resistance of Streptococcus mutans to antimicrobial peptides in biofilms.
3345	DRAMP03016	Defensin-1 (Insects, animals)	"Defensin-1"[All Fields] AND biofilm[All Fields]	Defensin-1	20454633	Normal human gingival epithelial cells sense C. parapsilosis by toll-like receptors and module its pathogenesis through antimicrobial peptides and proinflammatory cytokines.
3348	DRAMP03023	Mastoparan (Protonectarina-MP; Insects, animals)	"Mastoparan"[All Fields] AND biofilm[All Fields]	Mastoparan	31411881	Computer-Aided Design of Mastoparan-like Peptides Enables the Generation of Nontoxic Variants with Extended Antibacterial Properties.
3348	DRAMP03023	Mastoparan (Protonectarina-MP; Insects, animals)	"Mastoparan"[All Fields] AND biofilm[All Fields]	Mastoparan	30974767	Antimicrobial and Antibiofilm Effects of Peptides from Venom of Social Wasp and Scorpion on Multidrug-Resistant Acinetobacter baumannii.
3348	DRAMP03023	Mastoparan (Protonectarina-MP; Insects, animals)	"Mastoparan"[All Fields] AND biofilm[All Fields]	Mastoparan	29904274	Evaluation of the bioactivity of a mastoparan peptide from wasp venom and of its analogues designed through targeted engineering.
3348	DRAMP03023	Mastoparan (Protonectarina-MP; Insects, animals)	"Mastoparan"[All Fields] AND biofilm[All Fields]	Mastoparan	29626660	Venom-derived peptide Mastoparan-1 eradicates planktonic and biofilm-embedded methicillin-resistant Staphylococcus aureus isolates.
3370	DRAMP03074	Cecropin (Insects, animals)	"Cecropin"[All Fields] AND biofilm[All Fields]	Cecropin	33401476	Novel Cecropin-4 Derived Peptides against Methicillin-Resistant Staphylococcus aureus.
3370	DRAMP03074	Cecropin (Insects, animals)	"Cecropin"[All Fields] AND biofilm[All Fields]	Cecropin	32203307	Antimicrobial peptide derived from moths can eradicate UPEC biofilms and could offer a novel therapeutic option.
3370	DRAMP03074	Cecropin (Insects, animals)	"Cecropin"[All Fields] AND biofilm[All Fields]	Cecropin	32203127	Antibiofilm activities of ceragenins and antimicrobial peptides against fungal-bacterial mono and multispecies biofilms.
3370	DRAMP03074	Cecropin (Insects, animals)	"Cecropin"[All Fields] AND biofilm[All Fields]	Cecropin	32051417	The insect antimicrobial peptide cecropin A disrupts uropathogenic Escherichia coli biofilms.
3370	DRAMP03074	Cecropin (Insects, animals)	"Cecropin"[All Fields] AND biofilm[All Fields]	Cecropin	31485973	Efficacy of Indolicidin, Cecropin A (1-7)-Melittin (CAMA) and Their Combination Against Biofilm-Forming Multidrug-Resistant Enteroaggregative Escherichia coli.
3370	DRAMP03074	Cecropin (Insects, animals)	"Cecropin"[All Fields] AND biofilm[All Fields]	Cecropin	31363941	Mechanism of action of antimicrobial peptide P5 truncations against Pseudomonas aeruginosa and Staphylococcus aureus.
3370	DRAMP03074	Cecropin (Insects, animals)	"Cecropin"[All Fields] AND biofilm[All Fields]	Cecropin	31057940	On-chip manufacturing of synthetic proteins for point-of-care therapeutics.
3370	DRAMP03074	Cecropin (Insects, animals)	"Cecropin"[All Fields] AND biofilm[All Fields]	Cecropin	30755738	In vitro activities of antimicrobial peptides and ceragenins against Legionella pneumophila.
3370	DRAMP03074	Cecropin (Insects, animals)	"Cecropin"[All Fields] AND biofilm[All Fields]	Cecropin	30131945	The Intestinal Roundworm Ascaris suumReleases Antimicrobial Factors Which Interfere With Bacterial Growth and Biofilm Formation.
3370	DRAMP03074	Cecropin (Insects, animals)	"Cecropin"[All Fields] AND biofilm[All Fields]	Cecropin	28278280	Natural antimicrobial peptide complexes in the fighting of antibiotic resistant biofilms: Calliphora vicina medicinal maggots.
3370	DRAMP03074	Cecropin (Insects, animals)	"Cecropin"[All Fields] AND biofilm[All Fields]	Cecropin	28178190	High Specific Selectivity and Membrane-Active Mechanism of Synthetic Cationic Hybrid Antimicrobial Peptides Based on the Peptide FV7.
3370	DRAMP03074	Cecropin (Insects, animals)	"Cecropin"[All Fields] AND biofilm[All Fields]	Cecropin	25924433	[CecropinA-magainin, a new hybrid antibacterial peptide against meticillin-resistant Staphylococcus aureus].
3370	DRAMP03074	Cecropin (Insects, animals)	"Cecropin"[All Fields] AND biofilm[All Fields]	Cecropin	25285879	Inhibition and destruction of Pseudomonas aeruginosa biofilms by antibiotics and antimicrobial peptides.
3370	DRAMP03074	Cecropin (Insects, animals)	"Cecropin"[All Fields] AND biofilm[All Fields]	Cecropin	23988790	In vitro pharmacokinetics of antimicrobial cationic peptides alone and in combination with antibiotics against methicillin resistant Staphylococcus aureus biofilms.
3370	DRAMP03074	Cecropin (Insects, animals)	"Cecropin"[All Fields] AND biofilm[All Fields]	Cecropin	23732798	Surface functionalization of titanium substrates with cecropin B to improve their cytocompatibility and reduce inflammation responses.
3370	DRAMP03074	Cecropin (Insects, animals)	"Cecropin"[All Fields] AND biofilm[All Fields]	Cecropin	23070152	In vitro activities of antibiotics and antimicrobial cationic peptides alone and in combination against methicillin-resistant Staphylococcus aureus biofilms.
3370	DRAMP03074	Cecropin (Insects, animals)	"Cecropin"[All Fields] AND biofilm[All Fields]	Cecropin	21998591	Drosophila melanogaster as an animal model for the study of Pseudomonas aeruginosa biofilm infections in vivo.
3370	DRAMP03074	Cecropin (Insects, animals)	"Cecropin"[All Fields] AND biofilm[All Fields]	Cecropin	15247257	Helix induction in antimicrobial peptides by alginate in biofilms.
3371	DRAMP03076	Cecropin-1 (Cecropin 1; Insects, animals)	"Cecropin-1"[All Fields] AND biofilm[All Fields]	Cecropin-1	25285879	Inhibition and destruction of Pseudomonas aeruginosa biofilms by antibiotics and antimicrobial peptides.
3371	DRAMP03076	Cecropin-1 (Cecropin 1; Insects, animals)	"Cecropin-1"[All Fields] AND biofilm[All Fields]	Cecropin-1	23988790	In vitro pharmacokinetics of antimicrobial cationic peptides alone and in combination with antibiotics against methicillin resistant Staphylococcus aureus biofilms.
3371	DRAMP03076	Cecropin-1 (Cecropin 1; Insects, animals)	"Cecropin-1"[All Fields] AND biofilm[All Fields]	Cecropin-1	23070152	In vitro activities of antibiotics and antimicrobial cationic peptides alone and in combination against methicillin-resistant Staphylococcus aureus biofilms.
3374	DRAMP03079	Cecropin-A1 (Insects, animals)	"Cecropin-A1"[All Fields] AND biofilm[All Fields]	Cecropin-A1	21998591	Drosophila melanogaster as an animal model for the study of Pseudomonas aeruginosa biofilm infections in vivo.
3375	DRAMP03080	Cecropin-B (Insects, animals)	"Cecropin-B"[All Fields] AND biofilm[All Fields]	Cecropin-B	31057940	On-chip manufacturing of synthetic proteins for point-of-care therapeutics.
3375	DRAMP03080	Cecropin-B (Insects, animals)	"Cecropin-B"[All Fields] AND biofilm[All Fields]	Cecropin-B	23732798	Surface functionalization of titanium substrates with cecropin B to improve their cytocompatibility and reduce inflammation responses.
3377	DRAMP03082	Defensin (invertebrate defensin; Insects, animals)	"Defensin"[All Fields] AND biofilm[All Fields]	Defensin	34408988	The Anti-Biofilm Efficacy of Caffeic Acid Phenethyl Ester (CAPE) In Vitroand a Murine Model of Oral Candidiasis.
3377	DRAMP03082	Defensin (invertebrate defensin; Insects, animals)	"Defensin"[All Fields] AND biofilm[All Fields]	Defensin	34321877	Antibacterial Effect of Honey-Derived Exosomes Containing Antimicrobial Peptides Against Oral Streptococci.
3377	DRAMP03082	Defensin (invertebrate defensin; Insects, animals)	"Defensin"[All Fields] AND biofilm[All Fields]	Defensin	34276631	DNA Blocks the Lethal Effect of Human Beta-Defensin 2 Against Neisseria meningitidis.
3377	DRAMP03082	Defensin (invertebrate defensin; Insects, animals)	"Defensin"[All Fields] AND biofilm[All Fields]	Defensin	33911935	The antibacterial activities of honey.
3377	DRAMP03082	Defensin (invertebrate defensin; Insects, animals)	"Defensin"[All Fields] AND biofilm[All Fields]	Defensin	33865931	Identification of a crocodylian β-defensin variant from Alligator mississippiensis with antimicrobial and antibiofilm activity.
3377	DRAMP03082	Defensin (invertebrate defensin; Insects, animals)	"Defensin"[All Fields] AND biofilm[All Fields]	Defensin	33586659	Identification of anti-microbial peptides and traces of microbial DNA in infrainfundibular compartments of human scalp terminal hair follicles.
3377	DRAMP03082	Defensin (invertebrate defensin; Insects, animals)	"Defensin"[All Fields] AND biofilm[All Fields]	Defensin	33534018	A recombinant fungal defensin-like peptide-P2 combats Streptococcus dysgalactiae and biofilms.
3377	DRAMP03082	Defensin (invertebrate defensin; Insects, animals)	"Defensin"[All Fields] AND biofilm[All Fields]	Defensin	33447687	Future directions of postoperative spinal implant infections.
3377	DRAMP03082	Defensin (invertebrate defensin; Insects, animals)	"Defensin"[All Fields] AND biofilm[All Fields]	Defensin	33420317	Curbing gastrointestinal infections by defensin fragment modifications without harming commensal microbiota.
3377	DRAMP03082	Defensin (invertebrate defensin; Insects, animals)	"Defensin"[All Fields] AND biofilm[All Fields]	Defensin	32867384	A Novel Peptide Antibiotic, Pro10-1D, Designed from Insect Defensin Shows Antibacterial and Anti-Inflammatory Activities in Sepsis Models.
3377	DRAMP03082	Defensin (invertebrate defensin; Insects, animals)	"Defensin"[All Fields] AND biofilm[All Fields]	Defensin	32858856	Candida albicansVirulence Factors and Pathogenicity for Endodontic Infections.
3377	DRAMP03082	Defensin (invertebrate defensin; Insects, animals)	"Defensin"[All Fields] AND biofilm[All Fields]	Defensin	32842903	Electrospun ZnO/Poly(Vinylidene Fluoride-Trifluoroethylene) Scaffolds for Lung Tissue Engineering.
3377	DRAMP03082	Defensin (invertebrate defensin; Insects, animals)	"Defensin"[All Fields] AND biofilm[All Fields]	Defensin	32663201	Inhibition and eradication activity of truncated α-defensin analogs against multidrug resistant uropathogenic Escherichia coli biofilm.
3377	DRAMP03082	Defensin (invertebrate defensin; Insects, animals)	"Defensin"[All Fields] AND biofilm[All Fields]	Defensin	32585445	Glucose effect on Candida albicans biofilm during tissue invasion.

3377	DRAMP03082	Defensin (invertebrate defensin; Insects, animals)	"Defensin"[All Fields] AND biofilm[All Fields]	Defensin	32522780	Controlling the Growth of the Skin Commensal Staphylococcus epidermidis Using d-Alanine Auxotrophy.
3377	DRAMP03082	Defensin (invertebrate defensin; Insects, animals)	"Defensin"[All Fields] AND biofilm[All Fields]	Defensin	32457749	The Antimicrobial Peptide Human Beta-Defensin 2 Inhibits Biofilm Production of Pseudomonas aeruginosaWithout Compromising Metabolic Activity.
3377	DRAMP03082	Defensin (invertebrate defensin; Insects, animals)	"Defensin"[All Fields] AND biofilm[All Fields]	Defensin	32439511	Antibacterial activities and mechanisms of action of a defensin from manila clam Ruditapes philippinarum.
3377	DRAMP03082	Defensin (invertebrate defensin; Insects, animals)	"Defensin"[All Fields] AND biofilm[All Fields]	Defensin	31933178	Effects of human β-defensin 3 fused with carbohydrate-binding domain on the function of type III secretion system in Pseudomonas aeruginosa PA14.
3377	DRAMP03082	Defensin (invertebrate defensin; Insects, animals)	"Defensin"[All Fields] AND biofilm[All Fields]	Defensin	31906541	Role of FAD-I in Fusobacterial Interspecies Interaction and Biofilm Formation.
3377	DRAMP03082	Defensin (invertebrate defensin; Insects, animals)	"Defensin"[All Fields] AND biofilm[All Fields]	Defensin	31729441	Rhesus Theta Defensin 1 Promotes Long Term Survival in Systemic Candidiasis by Host Directed Mechanisms.
3377	DRAMP03082	Defensin (invertebrate defensin; Insects, animals)	"Defensin"[All Fields] AND biofilm[All Fields]	Defensin	31336838	Candida albicans-Cell Interactions Activate Innate Immune Defense in Human Palate Epithelial Primary Cells via Nitric Oxide (NO) and β-Defensin 2 (hBD-2).
3377	DRAMP03082	Defensin (invertebrate defensin; Insects, animals)	"Defensin"[All Fields] AND biofilm[All Fields]	Defensin	31165072	Impact of the Food Additive Titanium Dioxide (E171) on Gut Microbiota-Host Interaction.
3377	DRAMP03082	Defensin (invertebrate defensin; Insects, animals)	"Defensin"[All Fields] AND biofilm[All Fields]	Defensin	31031739	Salt-Tolerant Antifungal and Antibacterial Activities of the Corn Defensin ZmD32.
3377	DRAMP03082	Defensin (invertebrate defensin; Insects, animals)	"Defensin"[All Fields] AND biofilm[All Fields]	Defensin	31025073	A recombinant fungal defensin-like peptide-P2 combats multidrug-resistant Staphylococcus aureus and biofilms.
3377	DRAMP03082	Defensin (invertebrate defensin; Insects, animals)	"Defensin"[All Fields] AND biofilm[All Fields]	Defensin	30659503	Lactobacillus plantarum USM8613 Aids in Wound Healing and Suppresses Staphylococcus aureus Infection at Wound Sites.
3377	DRAMP03082	Defensin (invertebrate defensin; Insects, animals)	"Defensin"[All Fields] AND biofilm[All Fields]	Defensin	30649289	Innate immune components affect growth and virulence traits of bacterial-vaginosis-associated and non-bacterial-vaginosis-associated Gardnerella vaginalis strains similarly.
3377	DRAMP03082	Defensin (invertebrate defensin; Insects, animals)	"Defensin"[All Fields] AND biofilm[All Fields]	Defensin	30376742	Alpha defensin, leukocyte esterase, C-reactive protein, and leukocyte count in synovial fluid for pre-operative diagnosis of periprosthetic infection.
3377	DRAMP03082	Defensin (invertebrate defensin; Insects, animals)	"Defensin"[All Fields] AND biofilm[All Fields]	Defensin	30260708	Stabilized collagen matrix dressing improves wound macrophage function and epithelialization.
3377	DRAMP03082	Defensin (invertebrate defensin; Insects, animals)	"Defensin"[All Fields] AND biofilm[All Fields]	Defensin	30254440	Modification of the surface of titanium with multifunctional chimeric peptides to prevent biofilm formation via inhibition of initial colonizers.
3377	DRAMP03082	Defensin (invertebrate defensin; Insects, animals)	"Defensin"[All Fields] AND biofilm[All Fields]	Defensin	29902560	A defensin-like antimicrobial peptide from the manila clam Ruditapes philippinarum: Investigation of the antibacterial activities and mode of action.
3377	DRAMP03082	Defensin (invertebrate defensin; Insects, animals)	"Defensin"[All Fields] AND biofilm[All Fields]	Defensin	29872295	Antimicrobial peptide-loaded liquid crystalline precursor bioadhesive system for the prevention of dental caries.
3377	DRAMP03082	Defensin (invertebrate defensin; Insects, animals)	"Defensin"[All Fields] AND biofilm[All Fields]	Defensin	29671721	The BceABRS four-component system that is essential for cell envelope stress response is involved in sensing and response to host defence peptides and is required for the biofilm formation and fitness of Streptococcus mutans.
3377	DRAMP03082	Defensin (invertebrate defensin; Insects, animals)	"Defensin"[All Fields] AND biofilm[All Fields]	Defensin	29104569	A Linear 19-Mer Plant Defensin-Derived Peptide Acts Synergistically with Caspofungin against Candida albicansBiofilms.
3377	DRAMP03082	Defensin (invertebrate defensin; Insects, animals)	"Defensin"[All Fields] AND biofilm[All Fields]	Defensin	29077172	The significance of HBD-3 and fluorescent composite carriers in the processof bone formation in rats infected with Staphylococcus aureus.
3377	DRAMP03082	Defensin (invertebrate defensin; Insects, animals)	"Defensin"[All Fields] AND biofilm[All Fields]	Defensin	29045084	New Approach to Treat and Prevent Oral Disease.
3377	DRAMP03082	Defensin (invertebrate defensin; Insects, animals)	"Defensin"[All Fields] AND biofilm[All Fields]	Defensin	29025642	Engineered chimeric peptides with antimicrobial and titanium-binding functions to inhibit biofilm formation on Ti implants.
3377	DRAMP03082	Defensin (invertebrate defensin; Insects, animals)	"Defensin"[All Fields] AND biofilm[All Fields]	Defensin	28956355	The synthetic human beta-defensin-3 C15 peptide exhibits antimicrobial activity against Streptococcus mutans, both alone and in combination with dental disinfectants.
3377	DRAMP03082	Defensin (invertebrate defensin; Insects, animals)	"Defensin"[All Fields] AND biofilm[All Fields]	Defensin	28951032	Antifungal Effects of Synthetic Human Beta-defensin-3-C15 Peptide on Candida albicans-infected Root Dentin.
3377	DRAMP03082	Defensin (invertebrate defensin; Insects, animals)	"Defensin"[All Fields] AND biofilm[All Fields]	Defensin	28874606	Bacterial d-amino acids suppress sinonasal innate immunity through sweet taste receptors in solitary chemosensory cells.
3377	DRAMP03082	Defensin (invertebrate defensin; Insects, animals)	"Defensin"[All Fields] AND biofilm[All Fields]	Defensin	28725299	Role of Streptococcus mutanstwo-component systems in antimicrobial peptide resistance in the oral cavity.
3377	DRAMP03082	Defensin (invertebrate defensin; Insects, animals)	"Defensin"[All Fields] AND biofilm[All Fields]	Defensin	28649561	Psd1 Effects on Candida albicansPlanktonic Cells and Biofilms.
3377	DRAMP03082	Defensin (invertebrate defensin; Insects, animals)	"Defensin"[All Fields] AND biofilm[All Fields]	Defensin	28642103	Chimeric analogs of human β-defensin 1 and θ-defensin disrupt pre-established bacterial biofilms.
3377	DRAMP03082	Defensin (invertebrate defensin; Insects, animals)	"Defensin"[All Fields] AND biofilm[All Fields]	Defensin	28413476	The mechanism of human β-defensin 3 in MRSA-induced infection of implant drug-resistant bacteria biofilm in the mouse tibial bone marrow.
3377	DRAMP03082	Defensin (invertebrate defensin; Insects, animals)	"Defensin"[All Fields] AND biofilm[All Fields]	Defensin	28296382	Human α-Defensin 6: A Small Peptide That Self-Assembles and Protects the Host by Entangling Microbes.
3377	DRAMP03082	Defensin (invertebrate defensin; Insects, animals)	"Defensin"[All Fields] AND biofilm[All Fields]	Defensin	28278280	Natural antimicrobial peptide complexes in the fighting of antibiotic resistant biofilms: Calliphora vicina medicinal maggots.
3377	DRAMP03082	Defensin (invertebrate defensin; Insects, animals)	"Defensin"[All Fields] AND biofilm[All Fields]	Defensin	28144375	Advancements in Diagnosing Periprosthetic Joint Infections after Total Hip and Knee Arthroplasty.
3377	DRAMP03082	Defensin (invertebrate defensin; Insects, animals)	"Defensin"[All Fields] AND biofilm[All Fields]	Defensin	28078813	Host defense peptide-derived privileged scaffolds for anti-infective drug discovery.
3377	DRAMP03082	Defensin (invertebrate defensin; Insects, animals)	"Defensin"[All Fields] AND biofilm[All Fields]	Defensin	28026958	Human α-Defensin 6 Self-Assembly Prevents Adhesion and Suppresses Virulence Traits of Candida albicans.
3377	DRAMP03082	Defensin (invertebrate defensin; Insects, animals)	"Defensin"[All Fields] AND biofilm[All Fields]	Defensin	27794585	A Novel Defensin-Like Peptide Associated with Two Other New Cationic Antimicrobial Peptides in Transcriptome of the Iranian Scorpion Venom.
3377	DRAMP03082	Defensin (invertebrate defensin; Insects, animals)	"Defensin"[All Fields] AND biofilm[All Fields]	Defensin	27777572	Role of yqiCin the Pathogenicity of Salmonellaand Innate Immune Responses of Human Intestinal Epithelium.
3377	DRAMP03082	Defensin (invertebrate defensin; Insects, animals)	"Defensin"[All Fields] AND biofilm[All Fields]	Defensin	27582732	Efficient Eradication of Mature Pseudomonas aeruginosa Biofilm via Controlled Delivery of Nitric Oxide Combined with Antimicrobial Peptide and Antibiotics.
3377	DRAMP03082	Defensin (invertebrate defensin; Insects, animals)	"Defensin"[All Fields] AND biofilm[All Fields]	Defensin	27417541	Acute appendicitis: transcript profiling of blood identifies promising biomarkers and potential underlying processes.
3377	DRAMP03082	Defensin (invertebrate defensin; Insects, animals)	"Defensin"[All Fields] AND biofilm[All Fields]	Defensin	27200276	Antifungal effects of synthetic human β-defensin 3-C15 peptide.
3377	DRAMP03082	Defensin (invertebrate defensin; Insects, animals)	"Defensin"[All Fields] AND biofilm[All Fields]	Defensin	27148195	Effect of Substance P in Staphylococcus aureus and Staphylococcus epidermidis Virulence: Implication for Skin Homeostasis.
3377	DRAMP03082	Defensin (invertebrate defensin; Insects, animals)	"Defensin"[All Fields] AND biofilm[All Fields]	Defensin	26861950	Antibiofilm efficacy of honey and bee-derived defensin-1 on multispecies wound biofilm.
3377	DRAMP03082	Defensin (invertebrate defensin; Insects, animals)	"Defensin"[All Fields] AND biofilm[All Fields]	Defensin	26592804	The radish defensins RsAFP1 and RsAFP2 act synergistically with caspofungin against Candida albicans biofilms.
3377	DRAMP03082	Defensin (invertebrate defensin; Insects, animals)	"Defensin"[All Fields] AND biofilm[All Fields]	Defensin	26248029	Synergistic Activity of the Plant Defensin HsAFP1 and Caspofungin against Candida albicans Biofilms and Planktonic Cultures.
3377	DRAMP03082	Defensin (invertebrate defensin; Insects, animals)	"Defensin"[All Fields] AND biofilm[All Fields]	Defensin	26214284	α-tocopherol decreases interleukin-1β and -6 and increases human β-defensin-1 and -2 secretion in human gingival fibroblasts stimulated with Porphyromonas gingivalis lipopolysaccharide.
3377	DRAMP03082	Defensin (invertebrate defensin; Insects, animals)	"Defensin"[All Fields] AND biofilm[All Fields]	Defensin	26196513	Snake Cathelicidin NA-CATH and Smaller Helical Antimicrobial Peptides Are Effective against Burkholderia thailandensis.
3377	DRAMP03082	Defensin (invertebrate defensin; Insects, animals)	"Defensin"[All Fields] AND biofilm[All Fields]	Defensin	26119274	Ultrasound microbubbles enhance human β-defensin 3 against biofilms.
3377	DRAMP03082	Defensin (invertebrate defensin; Insects, animals)	"Defensin"[All Fields] AND biofilm[All Fields]	Defensin	25862466	The Antibacterial Effects of an Antimicrobial Peptide Human β-Defensin 3 Fused with Carbohydrate-Binding Domain on Pseudomonas aeruginosa PA14.
3377	DRAMP03082	Defensin (invertebrate defensin; Insects, animals)	"Defensin"[All Fields] AND biofilm[All Fields]	Defensin	25808131	Expression of antimicrobial peptides and interleukin-8 during early stages of inflammation: An experimental gingivitis study.
3377	DRAMP03082	Defensin (invertebrate defensin; Insects, animals)	"Defensin"[All Fields] AND biofilm[All Fields]	Defensin	25806720	Antimicrobial peptides in 2014.
3377	DRAMP03082	Defensin (invertebrate defensin; Insects, animals)	"Defensin"[All Fields] AND biofilm[All Fields]	Defensin	25285879	Inhibition and destruction of Pseudomonas aeruginosa biofilms by antibiotics and antimicrobial peptides.
3377	DRAMP03082	Defensin (invertebrate defensin; Insects, animals)	"Defensin"[All Fields] AND biofilm[All Fields]	Defensin	25212593	Health- and disease-associated species clusters in complex natural biofilms determine the innate immune response in oral epithelial cells during biofilm maturation.
3377	DRAMP03082	Defensin (invertebrate defensin; Insects, animals)	"Defensin"[All Fields] AND biofilm[All Fields]	Defensin	24913184	Effects of human β-defensin-3 on biofilm formation-regulating genes dltB and icaA in Staphylococcus aureus.
3377	DRAMP03082	Defensin (invertebrate defensin; Insects, animals)	"Defensin"[All Fields] AND biofilm[All Fields]	Defensin	24340061	Inflammatory and antimicrobial responses to methicillin-resistant Staphylococcus aureus in an in vitro wound infection model.
3377	DRAMP03082	Defensin (invertebrate defensin; Insects, animals)	"Defensin"[All Fields] AND biofilm[All Fields]	Defensin	24240906	Adsorption study of pellicle proteins to gold, silica and titanium by quartz crystal microbalance method.
3377	DRAMP03082	Defensin (invertebrate defensin; Insects, animals)	"Defensin"[All Fields] AND biofilm[All Fields]	Defensin	24238461	Antibacterial efficacy of a human β-defensin-3 peptide on multispecies biofilms.
3377	DRAMP03082	Defensin (invertebrate defensin; Insects, animals)	"Defensin"[All Fields] AND biofilm[All Fields]	Defensin	24100890	[Use of recombinant human beta-defensin-3 to evaluate the effect of adhesion of Candida albicans on the surface of soft lining material].
3377	DRAMP03082	Defensin (invertebrate defensin; Insects, animals)	"Defensin"[All Fields] AND biofilm[All Fields]	Defensin	23639356	Human beta-defensin 3: a novel inhibitor of Staphylococcus-produced biofilm production. Commentary on "Human β-defensin 3 inhibits antibiotic-resistant Staphylococcus biofilm formation".

3377	DRAMP03082	Defensin (invertebrate defensin; Insects, animals)	"Defensin"[All Fields] AND biofilm[All Fields]	Defensin	23519963	Ultrasound-targeted microbubble destruction enhances human β-defensin 3 activity against antibiotic-resistant Staphylococcus biofilms.
3377	DRAMP03082	Defensin (invertebrate defensin; Insects, animals)	"Defensin"[All Fields] AND biofilm[All Fields]	Defensin	23273885	Human β-defensin 3 inhibits antibiotic-resistant Staphylococcus biofilm formation.
3377	DRAMP03082	Defensin (invertebrate defensin; Insects, animals)	"Defensin"[All Fields] AND biofilm[All Fields]	Defensin	23203265	Potentiation of the cytotoxic activity of copper by polyphosphate on biofilm-producing bacteria: a bioinspired approach.
3377	DRAMP03082	Defensin (invertebrate defensin; Insects, animals)	"Defensin"[All Fields] AND biofilm[All Fields]	Defensin	23078156	Antimicrobial efficacy of a human β-defensin-3 peptide using an Enterococcus faecalis dentine infection model.
3377	DRAMP03082	Defensin (invertebrate defensin; Insects, animals)	"Defensin"[All Fields] AND biofilm[All Fields]	Defensin	23053486	Synergistic effect and antibiofilm activity between the antimicrobial peptide coprisin and conventional antibiotics against opportunistic bacteria.
3377	DRAMP03082	Defensin (invertebrate defensin; Insects, animals)	"Defensin"[All Fields] AND biofilm[All Fields]	Defensin	22922323	Extracellular DNA within a nontypeable Haemophilus influenzae-induced biofilm binds human beta defensin-3 and reduces its antimicrobial activity.
3377	DRAMP03082	Defensin (invertebrate defensin; Insects, animals)	"Defensin"[All Fields] AND biofilm[All Fields]	Defensin	22855857	Retrocyclin inhibits Gardnerella vaginalis biofilm formation and toxin activity.
3377	DRAMP03082	Defensin (invertebrate defensin; Insects, animals)	"Defensin"[All Fields] AND biofilm[All Fields]	Defensin	22394470	Association of CiaRH with resistance of Streptococcus mutans to antimicrobial peptides in biofilms.
3377	DRAMP03082	Defensin (invertebrate defensin; Insects, animals)	"Defensin"[All Fields] AND biofilm[All Fields]	Defensin	22229614	Comparison of the effects of human β-defensin 3, vancomycin, and clindamycin on Staphylococcus aureus biofilm formation.
3377	DRAMP03082	Defensin (invertebrate defensin; Insects, animals)	"Defensin"[All Fields] AND biofilm[All Fields]	Defensin	21692631	A novel organotypic dento-epithelial culture model: effect of Fusobacterium nucleatum biofilm on B-defensin-2, -3, and LL-37 expression.
3377	DRAMP03082	Defensin (invertebrate defensin; Insects, animals)	"Defensin"[All Fields] AND biofilm[All Fields]	Defensin	20454633	Normal human gingival epithelial cells sense C. parapsilosis by toll-like receptors and module its pathogenesis through antimicrobial peptides and proinflammatory cytokines.
3377	DRAMP03082	Defensin (invertebrate defensin; Insects, animals)	"Defensin"[All Fields] AND biofilm[All Fields]	Defensin	20378008	Effect of temperature on the shift of Pseudomonas fluorescens from an environmental microorganism to a potential human pathogen.
3377	DRAMP03082	Defensin (invertebrate defensin; Insects, animals)	"Defensin"[All Fields] AND biofilm[All Fields]	Defensin	19961380	Gingival transcriptome patterns during induction and resolution of experimental gingivitis in humans.
3377	DRAMP03082	Defensin (invertebrate defensin; Insects, animals)	"Defensin"[All Fields] AND biofilm[All Fields]	Defensin	19780045	Candida famata modulates toll-like receptor, beta-defensin, and proinflammatory cytokine expression by normal human epithelial cells.
3377	DRAMP03082	Defensin (invertebrate defensin; Insects, animals)	"Defensin"[All Fields] AND biofilm[All Fields]	Defensin	19572896	The immune response of oral epithelial cells induced by single-species and complex naturally formed biofilms.
3377	DRAMP03082	Defensin (invertebrate defensin; Insects, animals)	"Defensin"[All Fields] AND biofilm[All Fields]	Defensin	19466693	Activity of antimicrobial peptides in the presence of polysaccharides produced by pulmonary pathogens.
3377	DRAMP03082	Defensin (invertebrate defensin; Insects, animals)	"Defensin"[All Fields] AND biofilm[All Fields]	Defensin	18954353	Treponema denticola does not induce production of common innate immune mediators from primary gingival epithelial cells.
3377	DRAMP03082	Defensin (invertebrate defensin; Insects, animals)	"Defensin"[All Fields] AND biofilm[All Fields]	Defensin	18173794	The stage of native biofilm formation determines the gene expression of human beta-defensin-2, psoriasin, ribonuclease 7 and inflammatory mediators: a novel approach for stimulation of keratinocytes with in situ formed biofilms.
3377	DRAMP03082	Defensin (invertebrate defensin; Insects, animals)	"Defensin"[All Fields] AND biofilm[All Fields]	Defensin	17434999	Functional analysis of D-alanylation of lipoteichoic acid in the probiotic strain Lactobacillus rhamnosus GG.
3377	DRAMP03082	Defensin (invertebrate defensin; Insects, animals)	"Defensin"[All Fields] AND biofilm[All Fields]	Defensin	15493829	Bacterial evasion of innate host defenses--the Staphylococcus aureus lesson.
3377	DRAMP03082	Defensin (invertebrate defensin; Insects, animals)	"Defensin"[All Fields] AND biofilm[All Fields]	Defensin	14764110	Polysaccharide intercellular adhesin (PIA) protects Staphylococcus epidermidis against major components of the human innate immune system.
3383	DRAMP03088	Diptericin (Insects, animals)	"Diptericin"[All Fields] AND biofilm[All Fields]	Diptericin	28278280	Natural antimicrobial peptide complexes in the fighting of antibiotic resistant biofilms: Calliphora vicina medicinal maggots.
3383	DRAMP03088	Diptericin (Insects, animals)	"Diptericin"[All Fields] AND biofilm[All Fields]	Diptericin	21998591	Drosophila melanogaster as an animal model for the study of Pseudomonas aeruginosa biofilm infections in vivo.
3386	DRAMP03093	Drosomycin (Cys-rich; insect defensins; Insects, animals)	"Drosomycin"[All Fields] AND biofilm[All Fields]	Drosomycin	21998591	Drosophila melanogaster as an animal model for the study of Pseudomonas aeruginosa biofilm infections in vivo.
3393	DRAMP03105	Antifungal protein (AFP; Insects, animals)	"Antifungal protein"[All Fields] AND biofilm[All Fields]	Antifungal protein	33466640	The Neosartorya fischeriAntifungal Protein 2 (NFAP2): A New Potential Weapon against Multidrug-Resistant Candida aurisBiofilms.
3393	DRAMP03105	Antifungal protein (AFP; Insects, animals)	"Antifungal protein"[All Fields] AND biofilm[All Fields]	Antifungal protein	32824977	The Penicillium chrysogenumQ176 Antimicrobial Protein PAFC Effectively Inhibits the Growth of the Opportunistic Human Pathogen Candida albicans.
3393	DRAMP03105	Antifungal protein (AFP; Insects, animals)	"Antifungal protein"[All Fields] AND biofilm[All Fields]	Antifungal protein	30478163	In VivoApplicability of Neosartorya fischeri Antifungal Protein 2 (NFAP2) in Treatment of Vulvovaginal Candidiasis.
3393	DRAMP03105	Antifungal protein (AFP; Insects, animals)	"Antifungal protein"[All Fields] AND biofilm[All Fields]	Antifungal protein	30079061	The Evolutionary Conserved γ-Core Motif Influences the Anti- CandidaActivity of the Penicillium chrysogenumAntifungal Protein PAF.
3393	DRAMP03105	Antifungal protein (AFP; Insects, animals)	"Antifungal protein"[All Fields] AND biofilm[All Fields]	Antifungal protein	28120548	DS6: anticandidal, antibiofilm peptide against Candida tropicalis and exhibit synergy with commercial drug.
3400	DRAMP18223	Sonorensin(Bacteriocin)	"Sonorensin"[All Fields] AND biofilm[All Fields]	Sonorensin	26292786	Sonorensin: A new bacteriocin with potential of an anti-biofilm agent and a food biopreservative.
3407	DRAMP03123	Cecropin-B (AgCecB; Insects, animals)	"Cecropin-B"[All Fields] AND biofilm[All Fields]	Cecropin-B	31057940	On-chip manufacturing of synthetic proteins for point-of-care therapeutics.
3407	DRAMP03123	Cecropin-B (AgCecB; Insects, animals)	"Cecropin-B"[All Fields] AND biofilm[All Fields]	Cecropin-B	23732798	Surface functionalization of titanium substrates with cecropin B to improve their cytocompatibility and reduce inflammation responses.
3411	DRAMP03127	Cecropin-A (Insects, animals)	"Cecropin-A"[All Fields] AND biofilm[All Fields]	Cecropin-A	32203307	Antimicrobial peptide derived from moths can eradicate UPEC biofilms and could offer a novel therapeutic option.
3411	DRAMP03127	Cecropin-A (Insects, animals)	"Cecropin-A"[All Fields] AND biofilm[All Fields]	Cecropin-A	32203127	Antibiofilm activities of ceragenins and antimicrobial peptides against fungal-bacterial mono and multispecies biofilms.
3411	DRAMP03127	Cecropin-A (Insects, animals)	"Cecropin-A"[All Fields] AND biofilm[All Fields]	Cecropin-A	32051417	The insect antimicrobial peptide cecropin A disrupts uropathogenic Escherichia coli biofilms.
3411	DRAMP03127	Cecropin-A (Insects, animals)	"Cecropin-A"[All Fields] AND biofilm[All Fields]	Cecropin-A	31485973	Efficacy of Indolicidin, Cecropin A (1-7)-Melittin (CAMA) and Their Combination Against Biofilm-Forming Multidrug-Resistant Enteroaggregative Escherichia coli.
3411	DRAMP03127	Cecropin-A (Insects, animals)	"Cecropin-A"[All Fields] AND biofilm[All Fields]	Cecropin-A	31363941	Mechanism of action of antimicrobial peptide P5 truncations against Pseudomonas aeruginosa and Staphylococcus aureus.
3411	DRAMP03127	Cecropin-A (Insects, animals)	"Cecropin-A"[All Fields] AND biofilm[All Fields]	Cecropin-A	30755738	In vitro activities of antimicrobial peptides and ceragenins against Legionella pneumophila.
3411	DRAMP03127	Cecropin-A (Insects, animals)	"Cecropin-A"[All Fields] AND biofilm[All Fields]	Cecropin-A	28178190	High Specific Selectivity and Membrane-Active Mechanism of Synthetic Cationic Hybrid Antimicrobial Peptides Based on the Peptide FV7.
3411	DRAMP03127	Cecropin-A (Insects, animals)	"Cecropin-A"[All Fields] AND biofilm[All Fields]	Cecropin-A	25924433	[CecropinA-magainin, a new hybrid antibacterial peptide against meticillin-resistant Staphylococcus aureus].
3411	DRAMP03127	Cecropin-A (Insects, animals)	"Cecropin-A"[All Fields] AND biofilm[All Fields]	Cecropin-A	23070152	In vitro activities of antibiotics and antimicrobial cationic peptides alone and in combination against methicillin-resistant Staphylococcus aureus biofilms.
3415	DRAMP03131	Cecropin-B (Insects, animals)	"Cecropin-B"[All Fields] AND biofilm[All Fields]	Cecropin-B	31057940	On-chip manufacturing of synthetic proteins for point-of-care therapeutics.
3415	DRAMP03131	Cecropin-B (Insects, animals)	"Cecropin-B"[All Fields] AND biofilm[All Fields]	Cecropin-B	23732798	Surface functionalization of titanium substrates with cecropin B to improve their cytocompatibility and reduce inflammation responses.
3417	DRAMP03133	Cecropin-A1 (AalCecA; Cecropin-A; Insects, animals)	"Cecropin-A1"[All Fields] AND biofilm[All Fields]	Cecropin-A1	21998591	Drosophila melanogaster as an animal model for the study of Pseudomonas aeruginosa biofilm infections in vivo.
3426	DRAMP03145	Defensin	"Defensin"[All Fields] AND biofilm[All Fields]	Defensin	34408988	The Anti-Biofilm Efficacy of Caffeic Acid Phenethyl Ester (CAPE) In Vitroand a Murine Model of Oral Candidiasis.
3426	DRAMP03145	Defensin	"Defensin"[All Fields] AND biofilm[All Fields]	Defensin	34321877	Antibacterial Effect of Honey-Derived Exosomes Containing Antimicrobial Peptides Against Oral Streptococci.
3426	DRAMP03145	Defensin	"Defensin"[All Fields] AND biofilm[All Fields]	Defensin	34276631	DNA Blocks the Lethal Effect of Human Beta-Defensin 2 Against Neisseria meningitidis.
3426	DRAMP03145	Defensin	"Defensin"[All Fields] AND biofilm[All Fields]	Defensin	33911935	The antibacterial activities of honey.
3426	DRAMP03145	Defensin	"Defensin"[All Fields] AND biofilm[All Fields]	Defensin	33865931	Identification of a crocodylian β-defensin variant from Alligator mississippiensis with antimicrobial and antibiofilm activity.
3426	DRAMP03145	Defensin	"Defensin"[All Fields] AND biofilm[All Fields]	Defensin	33586659	Identification of anti-microbial peptides and traces of microbial DNA in infrainfundibular compartments of human scalp terminal hair follicles.
3426	DRAMP03145	Defensin	"Defensin"[All Fields] AND biofilm[All Fields]	Defensin	33534018	A recombinant fungal defensin-like peptide-P2 combats Streptococcus dysgalactiae and biofilms.
3426	DRAMP03145	Defensin	"Defensin"[All Fields] AND biofilm[All Fields]	Defensin	33447687	Future directions of postoperative spinal implant infections.
3426	DRAMP03145	Defensin	"Defensin"[All Fields] AND biofilm[All Fields]	Defensin	33420317	Curbing gastrointestinal infections by defensin fragment modifications without harming commensal microbiota.
3426	DRAMP03145	Defensin	"Defensin"[All Fields] AND biofilm[All Fields]	Defensin	32867384	A Novel Peptide Antibiotic, Pro10-1D, Designed from Insect Defensin Shows Antibacterial and Anti-Inflammatory Activities in Sepsis Models.
3426	DRAMP03145	Defensin	"Defensin"[All Fields] AND biofilm[All Fields]	Defensin	32858856	Candida albicansVirulence Factors and Pathogenicity for Endodontic Infections.
3426	DRAMP03145	Defensin	"Defensin"[All Fields] AND biofilm[All Fields]	Defensin	32842903	Electrospun ZnO/Poly(Vinylidene Fluoride-Trifluoroethylene) Scaffolds for Lung Tissue Engineering.
3426	DRAMP03145	Defensin	"Defensin"[All Fields] AND biofilm[All Fields]	Defensin	32663201	Inhibition and eradication activity of truncated α-defensin analogs against multidrug resistant uropathogenic Escherichia coli biofilm.

3426	DRAMP03145	Defensin	"Defensin"[All Fields] AND biofilm[All Fields]	Defensin	32585445	Glucose effect on Candida albicans biofilm during tissue invasion.
3426	DRAMP03145	Defensin	"Defensin"[All Fields] AND biofilm[All Fields]	Defensin	32522780	Controlling the Growth of the Skin Commensal Staphylococcus epidermidis Using d-Alanine Auxotrophy.
3426	DRAMP03145	Defensin	"Defensin"[All Fields] AND biofilm[All Fields]	Defensin	32457749	The Antimicrobial Peptide Human Beta-Defensin 2 Inhibits Biofilm Production of Pseudomonas aeruginosaWithout Compromising Metabolic Activity.
3426	DRAMP03145	Defensin	"Defensin"[All Fields] AND biofilm[All Fields]	Defensin	32439511	Antibacterial activities and mechanisms of action of a defensin from manila clam Ruditapes philippinarum.
3426	DRAMP03145	Defensin	"Defensin"[All Fields] AND biofilm[All Fields]	Defensin	31933178	Effects of human β-defensin 3 fused with carbohydrate-binding domain on the function of type III secretion system in Pseudomonas aeruginosa PA14.
3426	DRAMP03145	Defensin	"Defensin"[All Fields] AND biofilm[All Fields]	Defensin	31906541	Role of FAD-I in Fusobacterial Interspecies Interaction and Biofilm Formation.
3426	DRAMP03145	Defensin	"Defensin"[All Fields] AND biofilm[All Fields]	Defensin	31729441	Rhesus Theta Defensin 1 Promotes Long Term Survival in Systemic Candidiasis by Host Directed Mechanisms.
3426	DRAMP03145	Defensin	"Defensin"[All Fields] AND biofilm[All Fields]	Defensin	31336838	Candida albicans-Cell Interactions Activate Innate Immune Defense in Human Palate Epithelial Primary Cells via Nitric Oxide (NO) and β-Defensin 2 (hBD-2).
3426	DRAMP03145	Defensin	"Defensin"[All Fields] AND biofilm[All Fields]	Defensin	31165072	Impact of the Food Additive Titanium Dioxide (E171) on Gut Microbiota-Host Interaction.
3426	DRAMP03145	Defensin	"Defensin"[All Fields] AND biofilm[All Fields]	Defensin	31031739	Salt-Tolerant Antifungal and Antibacterial Activities of the Corn Defensin ZmD32.
3426	DRAMP03145	Defensin	"Defensin"[All Fields] AND biofilm[All Fields]	Defensin	31025073	A recombinant fungal defensin-like peptide-P2 combats multidrug-resistant Staphylococcus aureus and biofilms.
3426	DRAMP03145	Defensin	"Defensin"[All Fields] AND biofilm[All Fields]	Defensin	30659503	Lactobacillus plantarum USM8613 Aids in Wound Healing and Suppresses Staphylococcus aureus Infection at Wound Sites.
3426	DRAMP03145	Defensin	"Defensin"[All Fields] AND biofilm[All Fields]	Defensin	30649289	Innate immune components affect growth and virulence traits of bacterial-vaginosis-associated and non-bacterial-vaginosis-associated Gardnerella vaginalis strains similarly.
3426	DRAMP03145	Defensin	"Defensin"[All Fields] AND biofilm[All Fields]	Defensin	30376742	Alpha defensin, leukocyte esterase, C-reactive protein, and leukocyte count in synovial fluid for pre-operative diagnosis of periprosthetic infection.
3426	DRAMP03145	Defensin	"Defensin"[All Fields] AND biofilm[All Fields]	Defensin	30260708	Stabilized collagen matrix dressing improves wound macrophage function and epithelialization.
3426	DRAMP03145	Defensin	"Defensin"[All Fields] AND biofilm[All Fields]	Defensin	30254440	Modification of the surface of titanium with multifunctional chimeric peptides to prevent biofilm formation via inhibition of initial colonizers.
3426	DRAMP03145	Defensin	"Defensin"[All Fields] AND biofilm[All Fields]	Defensin	29902560	A defensin-like antimicrobial peptide from the manila clam Ruditapes philippinarum: Investigation of the antibacterial activities and mode of action.
3426	DRAMP03145	Defensin	"Defensin"[All Fields] AND biofilm[All Fields]	Defensin	29872295	Antimicrobial peptide-loaded liquid crystalline precursor bioadhesive system for the prevention of dental caries.
3426	DRAMP03145	Defensin	"Defensin"[All Fields] AND biofilm[All Fields]	Defensin	29671721	The BceABRS four-component system that is essential for cell envelope stress response is involved in sensing and response to host defence peptides and is required for the biofilm formation and fitness of Streptococcus mutans.
3426	DRAMP03145	Defensin	"Defensin"[All Fields] AND biofilm[All Fields]	Defensin	29104569	A Linear 19-Mer Plant Defensin-Derived Peptide Acts Synergistically with Caspofungin against Candida albicansBiofilms.
3426	DRAMP03145	Defensin	"Defensin"[All Fields] AND biofilm[All Fields]	Defensin	29077172	The significance of HBD-3 and fluorescent composite carriers in the processof bone formation in rats infected with Staphylococcus aureus.
3426	DRAMP03145	Defensin	"Defensin"[All Fields] AND biofilm[All Fields]	Defensin	29045084	New Approach to Treat and Prevent Oral Disease.
3426	DRAMP03145	Defensin	"Defensin"[All Fields] AND biofilm[All Fields]	Defensin	29025642	Engineered chimeric peptides with antimicrobial and titanium-binding functions to inhibit biofilm formation on Ti implants.
3426	DRAMP03145	Defensin	"Defensin"[All Fields] AND biofilm[All Fields]	Defensin	28956355	The synthetic human beta-defensin-3 C15 peptide exhibits antimicrobial activity against Streptococcus mutans, both alone and in combination with dental disinfectants.
3426	DRAMP03145	Defensin	"Defensin"[All Fields] AND biofilm[All Fields]	Defensin	28951032	Antifungal Effects of Synthetic Human Beta-defensin-3-C15 Peptide on Candida albicans-infected Root Dentin.
3426	DRAMP03145	Defensin	"Defensin"[All Fields] AND biofilm[All Fields]	Defensin	28874606	Bacterial d-amino acids suppress sinonasal innate immunity through sweet taste receptors in solitary chemosensory cells.
3426	DRAMP03145	Defensin	"Defensin"[All Fields] AND biofilm[All Fields]	Defensin	28725299	Role of Streptococcus mutanstwo-component systems in antimicrobial peptide resistance in the oral cavity.
3426	DRAMP03145	Defensin	"Defensin"[All Fields] AND biofilm[All Fields]	Defensin	28649561	Psd1 Effects on Candida albicansPlanktonic Cells and Biofilms.
3426	DRAMP03145	Defensin	"Defensin"[All Fields] AND biofilm[All Fields]	Defensin	28642103	Chimeric analogs of human β-defensin 1 and θ-defensin disrupt pre-established bacterial biofilms.
3426	DRAMP03145	Defensin	"Defensin"[All Fields] AND biofilm[All Fields]	Defensin	28413476	The mechanism of human β-defensin 3 in MRSA-induced infection of implant drug-resistant bacteria biofilm in the mouse tibial bone marrow.
3426	DRAMP03145	Defensin	"Defensin"[All Fields] AND biofilm[All Fields]	Defensin	28296382	Human α-Defensin 6: A Small Peptide That Self-Assembles and Protects the Host by Entangling Microbes.
3426	DRAMP03145	Defensin	"Defensin"[All Fields] AND biofilm[All Fields]	Defensin	28278280	Natural antimicrobial peptide complexes in the fighting of antibiotic resistant biofilms: Calliphora vicina medicinal maggots.
3426	DRAMP03145	Defensin	"Defensin"[All Fields] AND biofilm[All Fields]	Defensin	28144375	Advancements in Diagnosing Periprosthetic Joint Infections after Total Hip and Knee Arthroplasty.
3426	DRAMP03145	Defensin	"Defensin"[All Fields] AND biofilm[All Fields]	Defensin	28078813	Host defense peptide-derived privileged scaffolds for anti-infective drug discovery.
3426	DRAMP03145	Defensin	"Defensin"[All Fields] AND biofilm[All Fields]	Defensin	28026958	Human α-Defensin 6 Self-Assembly Prevents Adhesion and Suppresses Virulence Traits of Candida albicans.
3426	DRAMP03145	Defensin	"Defensin"[All Fields] AND biofilm[All Fields]	Defensin	27794585	A Novel Defensin-Like Peptide Associated with Two Other New Cationic Antimicrobial Peptides in Transcriptome of the Iranian Scorpion Venom.
3426	DRAMP03145	Defensin	"Defensin"[All Fields] AND biofilm[All Fields]	Defensin	27777572	Role of yqiCin the Pathogenicity of Salmonellaand Innate Immune Responses of Human Intestinal Epithelium.
3426	DRAMP03145	Defensin	"Defensin"[All Fields] AND biofilm[All Fields]	Defensin	27582732	Efficient Eradication of Mature Pseudomonas aeruginosa Biofilm via Controlled Delivery of Nitric Oxide Combined with Antimicrobial Peptide and Antibiotics.
3426	DRAMP03145	Defensin	"Defensin"[All Fields] AND biofilm[All Fields]	Defensin	27417541	Acute appendicitis: transcript profiling of blood identifies promising biomarkers and potential underlying processes.
3426	DRAMP03145	Defensin	"Defensin"[All Fields] AND biofilm[All Fields]	Defensin	27200276	Antifungal effects of synthetic human β-defensin 3-C15 peptide.
3426	DRAMP03145	Defensin	"Defensin"[All Fields] AND biofilm[All Fields]	Defensin	27148195	Effect of Substance P in Staphylococcus aureus and Staphylococcus epidermidis Virulence: Implication for Skin Homeostasis.
3426	DRAMP03145	Defensin	"Defensin"[All Fields] AND biofilm[All Fields]	Defensin	26861950	Antibiofilm efficacy of honey and bee-derived defensin-1 on multispecies wound biofilm.
3426	DRAMP03145	Defensin	"Defensin"[All Fields] AND biofilm[All Fields]	Defensin	26592804	The radish defensins RsAFP1 and RsAFP2 act synergistically with caspofungin against Candida albicans biofilms.
3426	DRAMP03145	Defensin	"Defensin"[All Fields] AND biofilm[All Fields]	Defensin	26248029	Synergistic Activity of the Plant Defensin HsAFP1 and Caspofungin against Candida albicans Biofilms and Planktonic Cultures.
3426	DRAMP03145	Defensin	"Defensin"[All Fields] AND biofilm[All Fields]	Defensin	26214284	α-tocopherol decreases interleukin-1β and -6 and increases human β-defensin-1 and -2 secretion in human gingival fibroblasts stimulated with Porphyromonas gingivalis lipopolysaccharide.
3426	DRAMP03145	Defensin	"Defensin"[All Fields] AND biofilm[All Fields]	Defensin	26196513	Snake Cathelicidin NA-CATH and Smaller Helical Antimicrobial Peptides Are Effective against Burkholderia thailandensis.
3426	DRAMP03145	Defensin	"Defensin"[All Fields] AND biofilm[All Fields]	Defensin	26119274	Ultrasound microbubbles enhance human β-defensin 3 against biofilms.
3426	DRAMP03145	Defensin	"Defensin"[All Fields] AND biofilm[All Fields]	Defensin	25862466	The Antibacterial Effects of an Antimicrobial Peptide Human β-Defensin 3 Fused with Carbohydrate-Binding Domain on Pseudomonas aeruginosa PA14.
3426	DRAMP03145	Defensin	"Defensin"[All Fields] AND biofilm[All Fields]	Defensin	25808131	Expression of antimicrobial peptides and interleukin-8 during early stages of inflammation: An experimental gingivitis study.
3426	DRAMP03145	Defensin	"Defensin"[All Fields] AND biofilm[All Fields]	Defensin	25806720	Antimicrobial peptides in 2014.
3426	DRAMP03145	Defensin	"Defensin"[All Fields] AND biofilm[All Fields]	Defensin	25285879	Inhibition and destruction of Pseudomonas aeruginosa biofilms by antibiotics and antimicrobial peptides.
3426	DRAMP03145	Defensin	"Defensin"[All Fields] AND biofilm[All Fields]	Defensin	25212593	Health- and disease-associated species clusters in complex natural biofilms determine the innate immune response in oral epithelial cells during biofilm maturation.
3426	DRAMP03145	Defensin	"Defensin"[All Fields] AND biofilm[All Fields]	Defensin	24913184	Effects of human β-defensin-3 on biofilm formation-regulating genes dltB and icaA in Staphylococcus aureus.
3426	DRAMP03145	Defensin	"Defensin"[All Fields] AND biofilm[All Fields]	Defensin	24340061	Inflammatory and antimicrobial responses to methicillin-resistant Staphylococcus aureus in an in vitro wound infection model.
3426	DRAMP03145	Defensin	"Defensin"[All Fields] AND biofilm[All Fields]	Defensin	24240906	Adsorption study of pellicle proteins to gold, silica and titanium by quartz crystal microbalance method.
3426	DRAMP03145	Defensin	"Defensin"[All Fields] AND biofilm[All Fields]	Defensin	24238461	Antibacterial efficacy of a human β-defensin-3 peptide on multispecies biofilms.
3426	DRAMP03145	Defensin	"Defensin"[All Fields] AND biofilm[All Fields]	Defensin	24100890	[Use of recombinant human beta-defensin-3 to evaluate the effect of adhesion of Candida albicans on the surface of soft lining material].

3426	DRAMP03145	Defensin	"Defensin"[All Fields] AND biofilm[All Fields]	Defensin	23639356	Human beta-defensin 3: a novel inhibitor of Staphylococcus-produced biofilm production. Commentary on "Human β-defensin 3 inhibits antibiotic-resistant Staphylococcus biofilm formation".
3426	DRAMP03145	Defensin	"Defensin"[All Fields] AND biofilm[All Fields]	Defensin	23519963	Ultrasound-targeted microbubble destruction enhances human β-defensin 3 activity against antibiotic-resistant Staphylococcus biofilms.
3426	DRAMP03145	Defensin	"Defensin"[All Fields] AND biofilm[All Fields]	Defensin	23273885	Human β-defensin 3 inhibits antibiotic-resistant Staphylococcus biofilm formation.
3426	DRAMP03145	Defensin	"Defensin"[All Fields] AND biofilm[All Fields]	Defensin	23203265	Potentiation of the cytotoxic activity of copper by polyphosphate on biofilm-producing bacteria: a bioinspired approach.
3426	DRAMP03145	Defensin	"Defensin"[All Fields] AND biofilm[All Fields]	Defensin	23078156	Antimicrobial efficacy of a human β-defensin-3 peptide using an Enterococcus faecalis dentine infection model.
3426	DRAMP03145	Defensin	"Defensin"[All Fields] AND biofilm[All Fields]	Defensin	23053486	Synergistic effect and antibiofilm activity between the antimicrobial peptide coprisin and conventional antibiotics against opportunistic bacteria.
3426	DRAMP03145	Defensin	"Defensin"[All Fields] AND biofilm[All Fields]	Defensin	22922323	Extracellular DNA within a nontypeable Haemophilus influenzae-induced biofilm binds human beta defensin-3 and reduces its antimicrobial activity.
3426	DRAMP03145	Defensin	"Defensin"[All Fields] AND biofilm[All Fields]	Defensin	22855857	Retrocyclin inhibits Gardnerella vaginalis biofilm formation and toxin activity.
3426	DRAMP03145	Defensin	"Defensin"[All Fields] AND biofilm[All Fields]	Defensin	22394470	Association of CiaRH with resistance of Streptococcus mutans to antimicrobial peptides in biofilms.
3426	DRAMP03145	Defensin	"Defensin"[All Fields] AND biofilm[All Fields]	Defensin	22229614	Comparison of the effects of human β-defensin 3, vancomycin, and clindamycin on Staphylococcus aureus biofilm formation.
3426	DRAMP03145	Defensin	"Defensin"[All Fields] AND biofilm[All Fields]	Defensin	21692631	A novel organotypic dento-epithelial culture model: effect of Fusobacterium nucleatum biofilm on B-defensin-2, -3, and LL-37 expression.
3426	DRAMP03145	Defensin	"Defensin"[All Fields] AND biofilm[All Fields]	Defensin	20454633	Normal human gingival epithelial cells sense C. parapsilosis by toll-like receptors and module its pathogenesis through antimicrobial peptides and proinflammatory cytokines.
3426	DRAMP03145	Defensin	"Defensin"[All Fields] AND biofilm[All Fields]	Defensin	20378008	Effect of temperature on the shift of Pseudomonas fluorescens from an environmental microorganism to a potential human pathogen.
3426	DRAMP03145	Defensin	"Defensin"[All Fields] AND biofilm[All Fields]	Defensin	19961380	Gingival transcriptome patterns during induction and resolution of experimental gingivitis in humans.
3426	DRAMP03145	Defensin	"Defensin"[All Fields] AND biofilm[All Fields]	Defensin	19780045	Candida famata modulates toll-like receptor, beta-defensin, and proinflammatory cytokine expression by normal human epithelial cells.
3426	DRAMP03145	Defensin	"Defensin"[All Fields] AND biofilm[All Fields]	Defensin	19572896	The immune response of oral epithelial cells induced by single-species and complex naturally formed biofilms.
3426	DRAMP03145	Defensin	"Defensin"[All Fields] AND biofilm[All Fields]	Defensin	19466693	Activity of antimicrobial peptides in the presence of polysaccharides produced by pulmonary pathogens.
3426	DRAMP03145	Defensin	"Defensin"[All Fields] AND biofilm[All Fields]	Defensin	18954353	Treponema denticola does not induce production of common innate immune mediators from primary gingival epithelial cells.
3426	DRAMP03145	Defensin	"Defensin"[All Fields] AND biofilm[All Fields]	Defensin	18173794	The stage of native biofilm formation determines the gene expression of human beta-defensin-2, psoriasin, ribonuclease 7 and inflammatory mediators: a novel approach for stimulation of keratinocytes with in situ formed biofilms.
3426	DRAMP03145	Defensin	"Defensin"[All Fields] AND biofilm[All Fields]	Defensin	17434999	Functional analysis of D-alanylation of lipoteichoic acid in the probiotic strain Lactobacillus rhamnosus GG.
3426	DRAMP03145	Defensin	"Defensin"[All Fields] AND biofilm[All Fields]	Defensin	15493829	Bacterial evasion of innate host defenses--the Staphylococcus aureus lesson.
3426	DRAMP03145	Defensin	"Defensin"[All Fields] AND biofilm[All Fields]	Defensin	14764110	Polysaccharide intercellular adhesin (PIA) protects Staphylococcus epidermidis against major components of the human innate immune system.
3427	DRAMP03146	Defensin	"Defensin"[All Fields] AND biofilm[All Fields]	Defensin	34408988	The Anti-Biofilm Efficacy of Caffeic Acid Phenethyl Ester (CAPE) In Vitroand a Murine Model of Oral Candidiasis.
3427	DRAMP03146	Defensin	"Defensin"[All Fields] AND biofilm[All Fields]	Defensin	34321877	Antibacterial Effect of Honey-Derived Exosomes Containing Antimicrobial Peptides Against Oral Streptococci.
3427	DRAMP03146	Defensin	"Defensin"[All Fields] AND biofilm[All Fields]	Defensin	34276631	DNA Blocks the Lethal Effect of Human Beta-Defensin 2 Against Neisseria meningitidis.
3427	DRAMP03146	Defensin	"Defensin"[All Fields] AND biofilm[All Fields]	Defensin	33911935	The antibacterial activities of honey.
3427	DRAMP03146	Defensin	"Defensin"[All Fields] AND biofilm[All Fields]	Defensin	33865931	Identification of a crocodylian β-defensin variant from Alligator mississippiensis with antimicrobial and antibiofilm activity.
3427	DRAMP03146	Defensin	"Defensin"[All Fields] AND biofilm[All Fields]	Defensin	33586659	Identification of anti-microbial peptides and traces of microbial DNA in infrainfundibular compartments of human scalp terminal hair follicles.
3427	DRAMP03146	Defensin	"Defensin"[All Fields] AND biofilm[All Fields]	Defensin	33534018	A recombinant fungal defensin-like peptide-P2 combats Streptococcus dysgalactiae and biofilms.
3427	DRAMP03146	Defensin	"Defensin"[All Fields] AND biofilm[All Fields]	Defensin	33447687	Future directions of postoperative spinal implant infections.
3427	DRAMP03146	Defensin	"Defensin"[All Fields] AND biofilm[All Fields]	Defensin	33420317	Curbing gastrointestinal infections by defensin fragment modifications without harming commensal microbiota.
3427	DRAMP03146	Defensin	"Defensin"[All Fields] AND biofilm[All Fields]	Defensin	32867384	A Novel Peptide Antibiotic, Pro10-1D, Designed from Insect Defensin Shows Antibacterial and Anti-Inflammatory Activities in Sepsis Models.
3427	DRAMP03146	Defensin	"Defensin"[All Fields] AND biofilm[All Fields]	Defensin	32858856	Candida albicansVirulence Factors and Pathogenicity for Endodontic Infections.
3427	DRAMP03146	Defensin	"Defensin"[All Fields] AND biofilm[All Fields]	Defensin	32842903	Electrospun ZnO/Poly(Vinylidene Fluoride-Trifluoroethylene) Scaffolds for Lung Tissue Engineering.
3427	DRAMP03146	Defensin	"Defensin"[All Fields] AND biofilm[All Fields]	Defensin	32663201	Inhibition and eradication activity of truncated α-defensin analogs against multidrug resistant uropathogenic Escherichia coli biofilm.
3427	DRAMP03146	Defensin	"Defensin"[All Fields] AND biofilm[All Fields]	Defensin	32585445	Glucose effect on Candida albicans biofilm during tissue invasion.
3427	DRAMP03146	Defensin	"Defensin"[All Fields] AND biofilm[All Fields]	Defensin	32522780	Controlling the Growth of the Skin Commensal Staphylococcus epidermidis Using d-Alanine Auxotrophy.
3427	DRAMP03146	Defensin	"Defensin"[All Fields] AND biofilm[All Fields]	Defensin	32457749	The Antimicrobial Peptide Human Beta-Defensin 2 Inhibits Biofilm Production of Pseudomonas aeruginosaWithout Compromising Metabolic Activity.
3427	DRAMP03146	Defensin	"Defensin"[All Fields] AND biofilm[All Fields]	Defensin	32439511	Antibacterial activities and mechanisms of action of a defensin from manila clam Ruditapes philippinarum.
3427	DRAMP03146	Defensin	"Defensin"[All Fields] AND biofilm[All Fields]	Defensin	31933178	Effects of human β-defensin 3 fused with carbohydrate-binding domain on the function of type III secretion system in Pseudomonas aeruginosa PA14.
3427	DRAMP03146	Defensin	"Defensin"[All Fields] AND biofilm[All Fields]	Defensin	31906541	Role of FAD-I in Fusobacterial Interspecies Interaction and Biofilm Formation.
3427	DRAMP03146	Defensin	"Defensin"[All Fields] AND biofilm[All Fields]	Defensin	31729441	Rhesus Theta Defensin 1 Promotes Long Term Survival in Systemic Candidiasis by Host Directed Mechanisms.
3427	DRAMP03146	Defensin	"Defensin"[All Fields] AND biofilm[All Fields]	Defensin	31336838	Candida albicans-Cell Interactions Activate Innate Immune Defense in Human Palate Epithelial Primary Cells via Nitric Oxide (NO) and β-Defensin 2 (hBD-2).
3427	DRAMP03146	Defensin	"Defensin"[All Fields] AND biofilm[All Fields]	Defensin	31165072	Impact of the Food Additive Titanium Dioxide (E171) on Gut Microbiota-Host Interaction.
3427	DRAMP03146	Defensin	"Defensin"[All Fields] AND biofilm[All Fields]	Defensin	31031739	Salt-Tolerant Antifungal and Antibacterial Activities of the Corn Defensin ZmD32.
3427	DRAMP03146	Defensin	"Defensin"[All Fields] AND biofilm[All Fields]	Defensin	31025073	A recombinant fungal defensin-like peptide-P2 combats multidrug-resistant Staphylococcus aureus and biofilms.
3427	DRAMP03146	Defensin	"Defensin"[All Fields] AND biofilm[All Fields]	Defensin	30659503	Lactobacillus plantarum USM8613 Aids in Wound Healing and Suppresses Staphylococcus aureus Infection at Wound Sites.
3427	DRAMP03146	Defensin	"Defensin"[All Fields] AND biofilm[All Fields]	Defensin	30649289	Innate immune components affect growth and virulence traits of bacterial-vaginosis-associated and non-bacterial-vaginosis-associated Gardnerella vaginalis strains similarly.
3427	DRAMP03146	Defensin	"Defensin"[All Fields] AND biofilm[All Fields]	Defensin	30376742	Alpha defensin, leukocyte esterase, C-reactive protein, and leukocyte count in synovial fluid for pre-operative diagnosis of periprosthetic infection.
3427	DRAMP03146	Defensin	"Defensin"[All Fields] AND biofilm[All Fields]	Defensin	30260708	Stabilized collagen matrix dressing improves wound macrophage function and epithelialization.
3427	DRAMP03146	Defensin	"Defensin"[All Fields] AND biofilm[All Fields]	Defensin	30254440	Modification of the surface of titanium with multifunctional chimeric peptides to prevent biofilm formation via inhibition of initial colonizers.
3427	DRAMP03146	Defensin	"Defensin"[All Fields] AND biofilm[All Fields]	Defensin	29902560	A defensin-like antimicrobial peptide from the manila clam Ruditapes philippinarum: Investigation of the antibacterial activities and mode of action.
3427	DRAMP03146	Defensin	"Defensin"[All Fields] AND biofilm[All Fields]	Defensin	29872295	Antimicrobial peptide-loaded liquid crystalline precursor bioadhesive system for the prevention of dental caries.
3427	DRAMP03146	Defensin	"Defensin"[All Fields] AND biofilm[All Fields]	Defensin	29671721	The BceABRS four-component system that is essential for cell envelope stress response is involved in sensing and response to host defence peptides and is required for the biofilm formation and fitness of Streptococcus mutans.
3427	DRAMP03146	Defensin	"Defensin"[All Fields] AND biofilm[All Fields]	Defensin	29104569	A Linear 19-Mer Plant Defensin-Derived Peptide Acts Synergistically with Caspofungin against Candida albicansBiofilms.
3427	DRAMP03146	Defensin	"Defensin"[All Fields] AND biofilm[All Fields]	Defensin	29077172	The significance of HBD-3 and fluorescent composite carriers in the processof bone formation in rats infected with Staphylococcus aureus.
3427	DRAMP03146	Defensin	"Defensin"[All Fields] AND biofilm[All Fields]	Defensin	29045084	New Approach to Treat and Prevent Oral Disease.

3427	DRAMP03146	Defensin	"Defensin"[All Fields] AND biofilm[All Fields]	Defensin	29025642	Engineered chimeric peptides with antimicrobial and titanium-binding functions to inhibit biofilm formation on Ti implants.
3427	DRAMP03146	Defensin	"Defensin"[All Fields] AND biofilm[All Fields]	Defensin	28956355	The synthetic human beta-defensin-3 C15 peptide exhibits antimicrobial activity against Streptococcus mutans, both alone and in combination with dental disinfectants.
3427	DRAMP03146	Defensin	"Defensin"[All Fields] AND biofilm[All Fields]	Defensin	28951032	Antifungal Effects of Synthetic Human Beta-defensin-3-C15 Peptide on Candida albicans-infected Root Dentin.
3427	DRAMP03146	Defensin	"Defensin"[All Fields] AND biofilm[All Fields]	Defensin	28874606	Bacterial d-amino acids suppress sinonasal innate immunity through sweet taste receptors in solitary chemosensory cells.
3427	DRAMP03146	Defensin	"Defensin"[All Fields] AND biofilm[All Fields]	Defensin	28725299	Role of Streptococcus mutanstwo-component systems in antimicrobial peptide resistance in the oral cavity.
3427	DRAMP03146	Defensin	"Defensin"[All Fields] AND biofilm[All Fields]	Defensin	28649561	Psd1 Effects on Candida albicansPlanktonic Cells and Biofilms.
3427	DRAMP03146	Defensin	"Defensin"[All Fields] AND biofilm[All Fields]	Defensin	28642103	Chimeric analogs of human β-defensin 1 and θ-defensin disrupt pre-established bacterial biofilms.
3427	DRAMP03146	Defensin	"Defensin"[All Fields] AND biofilm[All Fields]	Defensin	28413476	The mechanism of human β-defensin 3 in MRSA-induced infection of implant drug-resistant bacteria biofilm in the mouse tibial bone marrow.
3427	DRAMP03146	Defensin	"Defensin"[All Fields] AND biofilm[All Fields]	Defensin	28296382	Human α-Defensin 6: A Small Peptide That Self-Assembles and Protects the Host by Entangling Microbes.
3427	DRAMP03146	Defensin	"Defensin"[All Fields] AND biofilm[All Fields]	Defensin	28278280	Natural antimicrobial peptide complexes in the fighting of antibiotic resistant biofilms: Calliphora vicina medicinal maggots.
3427	DRAMP03146	Defensin	"Defensin"[All Fields] AND biofilm[All Fields]	Defensin	28144375	Advancements in Diagnosing Periprosthetic Joint Infections after Total Hip and Knee Arthroplasty.
3427	DRAMP03146	Defensin	"Defensin"[All Fields] AND biofilm[All Fields]	Defensin	28078813	Host defense peptide-derived privileged scaffolds for anti-infective drug discovery.
3427	DRAMP03146	Defensin	"Defensin"[All Fields] AND biofilm[All Fields]	Defensin	28026958	Human α-Defensin 6 Self-Assembly Prevents Adhesion and Suppresses Virulence Traits of Candida albicans.
3427	DRAMP03146	Defensin	"Defensin"[All Fields] AND biofilm[All Fields]	Defensin	27794585	A Novel Defensin-Like Peptide Associated with Two Other New Cationic Antimicrobial Peptides in Transcriptome of the Iranian Scorpion Venom.
3427	DRAMP03146	Defensin	"Defensin"[All Fields] AND biofilm[All Fields]	Defensin	27777572	Role of yqiCin the Pathogenicity of Salmonellaand Innate Immune Responses of Human Intestinal Epithelium.
3427	DRAMP03146	Defensin	"Defensin"[All Fields] AND biofilm[All Fields]	Defensin	27582732	Efficient Eradication of Mature Pseudomonas aeruginosa Biofilm via Controlled Delivery of Nitric Oxide Combined with Antimicrobial Peptide and Antibiotics.
3427	DRAMP03146	Defensin	"Defensin"[All Fields] AND biofilm[All Fields]	Defensin	27417541	Acute appendicitis: transcript profiling of blood identifies promising biomarkers and potential underlying processes.
3427	DRAMP03146	Defensin	"Defensin"[All Fields] AND biofilm[All Fields]	Defensin	27200276	Antifungal effects of synthetic human β-defensin 3-C15 peptide.
3427	DRAMP03146	Defensin	"Defensin"[All Fields] AND biofilm[All Fields]	Defensin	27148195	Effect of Substance P in Staphylococcus aureus and Staphylococcus epidermidis Virulence: Implication for Skin Homeostasis.
3427	DRAMP03146	Defensin	"Defensin"[All Fields] AND biofilm[All Fields]	Defensin	26861950	Antibiofilm efficacy of honey and bee-derived defensin-1 on multispecies wound biofilm.
3427	DRAMP03146	Defensin	"Defensin"[All Fields] AND biofilm[All Fields]	Defensin	26592804	The radish defensins RsAFP1 and RsAFP2 act synergistically with caspofungin against Candida albicans biofilms.
3427	DRAMP03146	Defensin	"Defensin"[All Fields] AND biofilm[All Fields]	Defensin	26248029	Synergistic Activity of the Plant Defensin HsAFP1 and Caspofungin against Candida albicans Biofilms and Planktonic Cultures.
3427	DRAMP03146	Defensin	"Defensin"[All Fields] AND biofilm[All Fields]	Defensin	26214284	α-tocopherol decreases interleukin-1β and -6 and increases human β-defensin-1 and -2 secretion in human gingival fibroblasts stimulated with Porphyromonas gingivalis lipopolysaccharide.
3427	DRAMP03146	Defensin	"Defensin"[All Fields] AND biofilm[All Fields]	Defensin	26196513	Snake Cathelicidin NA-CATH and Smaller Helical Antimicrobial Peptides Are Effective against Burkholderia thailandensis.
3427	DRAMP03146	Defensin	"Defensin"[All Fields] AND biofilm[All Fields]	Defensin	26119274	Ultrasound microbubbles enhance human β-defensin 3 against biofilms.
3427	DRAMP03146	Defensin	"Defensin"[All Fields] AND biofilm[All Fields]	Defensin	25862466	The Antibacterial Effects of an Antimicrobial Peptide Human β-Defensin 3 Fused with Carbohydrate-Binding Domain on Pseudomonas aeruginosa PA14.
3427	DRAMP03146	Defensin	"Defensin"[All Fields] AND biofilm[All Fields]	Defensin	25808131	Expression of antimicrobial peptides and interleukin-8 during early stages of inflammation: An experimental gingivitis study.
3427	DRAMP03146	Defensin	"Defensin"[All Fields] AND biofilm[All Fields]	Defensin	25806720	Antimicrobial peptides in 2014.
3427	DRAMP03146	Defensin	"Defensin"[All Fields] AND biofilm[All Fields]	Defensin	25285879	Inhibition and destruction of Pseudomonas aeruginosa biofilms by antibiotics and antimicrobial peptides.
3427	DRAMP03146	Defensin	"Defensin"[All Fields] AND biofilm[All Fields]	Defensin	25212593	Health- and disease-associated species clusters in complex natural biofilms determine the innate immune response in oral epithelial cells during biofilm maturation.
3427	DRAMP03146	Defensin	"Defensin"[All Fields] AND biofilm[All Fields]	Defensin	24913184	Effects of human β-defensin-3 on biofilm formation-regulating genes dltB and icaA in Staphylococcus aureus.
3427	DRAMP03146	Defensin	"Defensin"[All Fields] AND biofilm[All Fields]	Defensin	24340061	Inflammatory and antimicrobial responses to methicillin-resistant Staphylococcus aureus in an in vitro wound infection model.
3427	DRAMP03146	Defensin	"Defensin"[All Fields] AND biofilm[All Fields]	Defensin	24240906	Adsorption study of pellicle proteins to gold, silica and titanium by quartz crystal microbalance method.
3427	DRAMP03146	Defensin	"Defensin"[All Fields] AND biofilm[All Fields]	Defensin	24238461	Antibacterial efficacy of a human β-defensin-3 peptide on multispecies biofilms.
3427	DRAMP03146	Defensin	"Defensin"[All Fields] AND biofilm[All Fields]	Defensin	24100890	[Use of recombinant human beta-defensin-3 to evaluate the effect of adhesion of Candida albicans on the surface of soft lining material].
3427	DRAMP03146	Defensin	"Defensin"[All Fields] AND biofilm[All Fields]	Defensin	23639356	Human beta-defensin 3: a novel inhibitor of Staphylococcus-produced biofilm production. Commentary on "Human β-defensin 3 inhibits antibiotic-resistant Staphylococcus biofilm formation".
3427	DRAMP03146	Defensin	"Defensin"[All Fields] AND biofilm[All Fields]	Defensin	23519963	Ultrasound-targeted microbubble destruction enhances human β-defensin 3 activity against antibiotic-resistant Staphylococcus biofilms.
3427	DRAMP03146	Defensin	"Defensin"[All Fields] AND biofilm[All Fields]	Defensin	23273885	Human β-defensin 3 inhibits antibiotic-resistant Staphylococcus biofilm formation.
3427	DRAMP03146	Defensin	"Defensin"[All Fields] AND biofilm[All Fields]	Defensin	23203265	Potentiation of the cytotoxic activity of copper by polyphosphate on biofilm-producing bacteria: a bioinspired approach.
3427	DRAMP03146	Defensin	"Defensin"[All Fields] AND biofilm[All Fields]	Defensin	23078156	Antimicrobial efficacy of a human β-defensin-3 peptide using an Enterococcus faecalis dentine infection model.
3427	DRAMP03146	Defensin	"Defensin"[All Fields] AND biofilm[All Fields]	Defensin	23053486	Synergistic effect and antibiofilm activity between the antimicrobial peptide coprisin and conventional antibiotics against opportunistic bacteria.
3427	DRAMP03146	Defensin	"Defensin"[All Fields] AND biofilm[All Fields]	Defensin	22922323	Extracellular DNA within a nontypeable Haemophilus influenzae-induced biofilm binds human beta defensin-3 and reduces its antimicrobial activity.
3427	DRAMP03146	Defensin	"Defensin"[All Fields] AND biofilm[All Fields]	Defensin	22855857	Retrocyclin inhibits Gardnerella vaginalis biofilm formation and toxin activity.
3427	DRAMP03146	Defensin	"Defensin"[All Fields] AND biofilm[All Fields]	Defensin	22394470	Association of CiaRH with resistance of Streptococcus mutans to antimicrobial peptides in biofilms.
3427	DRAMP03146	Defensin	"Defensin"[All Fields] AND biofilm[All Fields]	Defensin	22229614	Comparison of the effects of human β-defensin 3, vancomycin, and clindamycin on Staphylococcus aureus biofilm formation.
3427	DRAMP03146	Defensin	"Defensin"[All Fields] AND biofilm[All Fields]	Defensin	21692631	A novel organotypic dento-epithelial culture model: effect of Fusobacterium nucleatum biofilm on B-defensin-2, -3, and LL-37 expression.
3427	DRAMP03146	Defensin	"Defensin"[All Fields] AND biofilm[All Fields]	Defensin	20454633	Normal human gingival epithelial cells sense C. parapsilosis by toll-like receptors and module its pathogenesis through antimicrobial peptides and proinflammatory cytokines.
3427	DRAMP03146	Defensin	"Defensin"[All Fields] AND biofilm[All Fields]	Defensin	20378008	Effect of temperature on the shift of Pseudomonas fluorescens from an environmental microorganism to a potential human pathogen.
3427	DRAMP03146	Defensin	"Defensin"[All Fields] AND biofilm[All Fields]	Defensin	19961380	Gingival transcriptome patterns during induction and resolution of experimental gingivitis in humans.
3427	DRAMP03146	Defensin	"Defensin"[All Fields] AND biofilm[All Fields]	Defensin	19780045	Candida famata modulates toll-like receptor, beta-defensin, and proinflammatory cytokine expression by normal human epithelial cells.
3427	DRAMP03146	Defensin	"Defensin"[All Fields] AND biofilm[All Fields]	Defensin	19572896	The immune response of oral epithelial cells induced by single-species and complex naturally formed biofilms.
3427	DRAMP03146	Defensin	"Defensin"[All Fields] AND biofilm[All Fields]	Defensin	19466693	Activity of antimicrobial peptides in the presence of polysaccharides produced by pulmonary pathogens.
3427	DRAMP03146	Defensin	"Defensin"[All Fields] AND biofilm[All Fields]	Defensin	18954353	Treponema denticola does not induce production of common innate immune mediators from primary gingival epithelial cells.
3427	DRAMP03146	Defensin	"Defensin"[All Fields] AND biofilm[All Fields]	Defensin	18173794	The stage of native biofilm formation determines the gene expression of human beta-defensin-2, psoriasin, ribonuclease 7 and inflammatory mediators: a novel approach for stimulation of keratinocytes with in situ formed biofilms.
3427	DRAMP03146	Defensin	"Defensin"[All Fields] AND biofilm[All Fields]	Defensin	17434999	Functional analysis of D-alanylation of lipoteichoic acid in the probiotic strain Lactobacillus rhamnosus GG.
3427	DRAMP03146	Defensin	"Defensin"[All Fields] AND biofilm[All Fields]	Defensin	15493829	Bacterial evasion of innate host defenses--the Staphylococcus aureus lesson.
3427	DRAMP03146	Defensin	"Defensin"[All Fields] AND biofilm[All Fields]	Defensin	14764110	Polysaccharide intercellular adhesin (PIA) protects Staphylococcus epidermidis against major components of the human innate immune system.

3429	DRAMP03148	Defensin	"Defensin"[All Fields] AND biofilm[All Fields]	Defensin	34408988	The Anti-Biofilm Efficacy of Caffeic Acid Phenethyl Ester (CAPE) In Vitroand a Murine Model of Oral Candidiasis.
3429	DRAMP03148	Defensin	"Defensin"[All Fields] AND biofilm[All Fields]	Defensin	34321877	Antibacterial Effect of Honey-Derived Exosomes Containing Antimicrobial Peptides Against Oral Streptococci.
3429	DRAMP03148	Defensin	"Defensin"[All Fields] AND biofilm[All Fields]	Defensin	34276631	DNA Blocks the Lethal Effect of Human Beta-Defensin 2 Against Neisseria meningitidis.
3429	DRAMP03148	Defensin	"Defensin"[All Fields] AND biofilm[All Fields]	Defensin	33911935	The antibacterial activities of honey.
3429	DRAMP03148	Defensin	"Defensin"[All Fields] AND biofilm[All Fields]	Defensin	33865931	Identification of a crocodylian β-defensin variant from Alligator mississippiensis with antimicrobial and antibiofilm activity.
3429	DRAMP03148	Defensin	"Defensin"[All Fields] AND biofilm[All Fields]	Defensin	33586659	Identification of anti-microbial peptides and traces of microbial DNA in infrainfundibular compartments of human scalp terminal hair follicles.
3429	DRAMP03148	Defensin	"Defensin"[All Fields] AND biofilm[All Fields]	Defensin	33534018	A recombinant fungal defensin-like peptide-P2 combats Streptococcus dysgalactiae and biofilms.
3429	DRAMP03148	Defensin	"Defensin"[All Fields] AND biofilm[All Fields]	Defensin	33447687	Future directions of postoperative spinal implant infections.
3429	DRAMP03148	Defensin	"Defensin"[All Fields] AND biofilm[All Fields]	Defensin	33420317	Curbing gastrointestinal infections by defensin fragment modifications without harming commensal microbiota.
3429	DRAMP03148	Defensin	"Defensin"[All Fields] AND biofilm[All Fields]	Defensin	32867384	A Novel Peptide Antibiotic, Pro10-1D, Designed from Insect Defensin Shows Antibacterial and Anti-Inflammatory Activities in Sepsis Models.
3429	DRAMP03148	Defensin	"Defensin"[All Fields] AND biofilm[All Fields]	Defensin	32858856	Candida albicansVirulence Factors and Pathogenicity for Endodontic Infections.
3429	DRAMP03148	Defensin	"Defensin"[All Fields] AND biofilm[All Fields]	Defensin	32842903	Electrospun ZnO/Poly(Vinylidene Fluoride-Trifluoroethylene) Scaffolds for Lung Tissue Engineering.
3429	DRAMP03148	Defensin	"Defensin"[All Fields] AND biofilm[All Fields]	Defensin	32663201	Inhibition and eradication activity of truncated α-defensin analogs against multidrug resistant uropathogenic Escherichia coli biofilm.
3429	DRAMP03148	Defensin	"Defensin"[All Fields] AND biofilm[All Fields]	Defensin	32585445	Glucose effect on Candida albicans biofilm during tissue invasion.
3429	DRAMP03148	Defensin	"Defensin"[All Fields] AND biofilm[All Fields]	Defensin	32522780	Controlling the Growth of the Skin Commensal Staphylococcus epidermidis Using d-Alanine Auxotrophy.
3429	DRAMP03148	Defensin	"Defensin"[All Fields] AND biofilm[All Fields]	Defensin	32457749	The Antimicrobial Peptide Human Beta-Defensin 2 Inhibits Biofilm Production of Pseudomonas aeruginosaWithout Compromising Metabolic Activity.
3429	DRAMP03148	Defensin	"Defensin"[All Fields] AND biofilm[All Fields]	Defensin	32439511	Antibacterial activities and mechanisms of action of a defensin from manila clam Ruditapes philippinarum.
3429	DRAMP03148	Defensin	"Defensin"[All Fields] AND biofilm[All Fields]	Defensin	31933178	Effects of human β-defensin 3 fused with carbohydrate-binding domain on the function of type III secretion system in Pseudomonas aeruginosa PA14.
3429	DRAMP03148	Defensin	"Defensin"[All Fields] AND biofilm[All Fields]	Defensin	31906541	Role of FAD-I in Fusobacterial Interspecies Interaction and Biofilm Formation.
3429	DRAMP03148	Defensin	"Defensin"[All Fields] AND biofilm[All Fields]	Defensin	31729441	Rhesus Theta Defensin 1 Promotes Long Term Survival in Systemic Candidiasis by Host Directed Mechanisms.
3429	DRAMP03148	Defensin	"Defensin"[All Fields] AND biofilm[All Fields]	Defensin	31336838	Candida albicans-Cell Interactions Activate Innate Immune Defense in Human Palate Epithelial Primary Cells via Nitric Oxide (NO) and β-Defensin 2 (hBD-2).
3429	DRAMP03148	Defensin	"Defensin"[All Fields] AND biofilm[All Fields]	Defensin	31165072	Impact of the Food Additive Titanium Dioxide (E171) on Gut Microbiota-Host Interaction.
3429	DRAMP03148	Defensin	"Defensin"[All Fields] AND biofilm[All Fields]	Defensin	31031739	Salt-Tolerant Antifungal and Antibacterial Activities of the Corn Defensin ZmD32.
3429	DRAMP03148	Defensin	"Defensin"[All Fields] AND biofilm[All Fields]	Defensin	31025073	A recombinant fungal defensin-like peptide-P2 combats multidrug-resistant Staphylococcus aureus and biofilms.
3429	DRAMP03148	Defensin	"Defensin"[All Fields] AND biofilm[All Fields]	Defensin	30659503	Lactobacillus plantarum USM8613 Aids in Wound Healing and Suppresses Staphylococcus aureus Infection at Wound Sites.
3429	DRAMP03148	Defensin	"Defensin"[All Fields] AND biofilm[All Fields]	Defensin	30649289	Innate immune components affect growth and virulence traits of bacterial-vaginosis-associated and non-bacterial-vaginosis-associated Gardnerella vaginalis strains similarly.
3429	DRAMP03148	Defensin	"Defensin"[All Fields] AND biofilm[All Fields]	Defensin	30376742	Alpha defensin, leukocyte esterase, C-reactive protein, and leukocyte count in synovial fluid for pre-operative diagnosis of periprosthetic infection.
3429	DRAMP03148	Defensin	"Defensin"[All Fields] AND biofilm[All Fields]	Defensin	30260708	Stabilized collagen matrix dressing improves wound macrophage function and epithelialization.
3429	DRAMP03148	Defensin	"Defensin"[All Fields] AND biofilm[All Fields]	Defensin	30254440	Modification of the surface of titanium with multifunctional chimeric peptides to prevent biofilm formation via inhibition of initial colonizers.
3429	DRAMP03148	Defensin	"Defensin"[All Fields] AND biofilm[All Fields]	Defensin	29902560	A defensin-like antimicrobial peptide from the manila clam Ruditapes philippinarum: Investigation of the antibacterial activities and mode of action.
3429	DRAMP03148	Defensin	"Defensin"[All Fields] AND biofilm[All Fields]	Defensin	29872295	Antimicrobial peptide-loaded liquid crystalline precursor bioadhesive system for the prevention of dental caries.
3429	DRAMP03148	Defensin	"Defensin"[All Fields] AND biofilm[All Fields]	Defensin	29671721	The BceABRS four-component system that is essential for cell envelope stress response is involved in sensing and response to host defence peptides and is required for the biofilm formation and fitness of Streptococcus mutans.
3429	DRAMP03148	Defensin	"Defensin"[All Fields] AND biofilm[All Fields]	Defensin	29104569	A Linear 19-Mer Plant Defensin-Derived Peptide Acts Synergistically with Caspofungin against Candida albicansBiofilms.
3429	DRAMP03148	Defensin	"Defensin"[All Fields] AND biofilm[All Fields]	Defensin	29077172	The significance of HBD-3 and fluorescent composite carriers in the processof bone formation in rats infected with Staphylococcus aureus.
3429	DRAMP03148	Defensin	"Defensin"[All Fields] AND biofilm[All Fields]	Defensin	29045084	New Approach to Treat and Prevent Oral Disease.
3429	DRAMP03148	Defensin	"Defensin"[All Fields] AND biofilm[All Fields]	Defensin	29025642	Engineered chimeric peptides with antimicrobial and titanium-binding functions to inhibit biofilm formation on Ti implants.
3429	DRAMP03148	Defensin	"Defensin"[All Fields] AND biofilm[All Fields]	Defensin	28956355	The synthetic human beta-defensin-3 C15 peptide exhibits antimicrobial activity against Streptococcus mutans, both alone and in combination with dental disinfectants.
3429	DRAMP03148	Defensin	"Defensin"[All Fields] AND biofilm[All Fields]	Defensin	28951032	Antifungal Effects of Synthetic Human Beta-defensin-3-C15 Peptide on Candida albicans-infected Root Dentin.
3429	DRAMP03148	Defensin	"Defensin"[All Fields] AND biofilm[All Fields]	Defensin	28874606	Bacterial d-amino acids suppress sinonasal innate immunity through sweet taste receptors in solitary chemosensory cells.
3429	DRAMP03148	Defensin	"Defensin"[All Fields] AND biofilm[All Fields]	Defensin	28725299	Role of Streptococcus mutanstwo-component systems in antimicrobial peptide resistance in the oral cavity.
3429	DRAMP03148	Defensin	"Defensin"[All Fields] AND biofilm[All Fields]	Defensin	28649561	Psd1 Effects on Candida albicansPlanktonic Cells and Biofilms.
3429	DRAMP03148	Defensin	"Defensin"[All Fields] AND biofilm[All Fields]	Defensin	28642103	Chimeric analogs of human β-defensin 1 and θ-defensin disrupt pre-established bacterial biofilms.
3429	DRAMP03148	Defensin	"Defensin"[All Fields] AND biofilm[All Fields]	Defensin	28413476	The mechanism of human β-defensin 3 in MRSA-induced infection of implant drug-resistant bacteria biofilm in the mouse tibial bone marrow.
3429	DRAMP03148	Defensin	"Defensin"[All Fields] AND biofilm[All Fields]	Defensin	28296382	Human α-Defensin 6: A Small Peptide That Self-Assembles and Protects the Host by Entangling Microbes.
3429	DRAMP03148	Defensin	"Defensin"[All Fields] AND biofilm[All Fields]	Defensin	28278280	Natural antimicrobial peptide complexes in the fighting of antibiotic resistant biofilms: Calliphora vicina medicinal maggots.
3429	DRAMP03148	Defensin	"Defensin"[All Fields] AND biofilm[All Fields]	Defensin	28144375	Advancements in Diagnosing Periprosthetic Joint Infections after Total Hip and Knee Arthroplasty.
3429	DRAMP03148	Defensin	"Defensin"[All Fields] AND biofilm[All Fields]	Defensin	28078813	Host defense peptide-derived privileged scaffolds for anti-infective drug discovery.
3429	DRAMP03148	Defensin	"Defensin"[All Fields] AND biofilm[All Fields]	Defensin	28026958	Human α-Defensin 6 Self-Assembly Prevents Adhesion and Suppresses Virulence Traits of Candida albicans.
3429	DRAMP03148	Defensin	"Defensin"[All Fields] AND biofilm[All Fields]	Defensin	27794585	A Novel Defensin-Like Peptide Associated with Two Other New Cationic Antimicrobial Peptides in Transcriptome of the Iranian Scorpion Venom.
3429	DRAMP03148	Defensin	"Defensin"[All Fields] AND biofilm[All Fields]	Defensin	27777572	Role of yqiCin the Pathogenicity of Salmonellaand Innate Immune Responses of Human Intestinal Epithelium.
3429	DRAMP03148	Defensin	"Defensin"[All Fields] AND biofilm[All Fields]	Defensin	27582732	Efficient Eradication of Mature Pseudomonas aeruginosa Biofilm via Controlled Delivery of Nitric Oxide Combined with Antimicrobial Peptide and Antibiotics.
3429	DRAMP03148	Defensin	"Defensin"[All Fields] AND biofilm[All Fields]	Defensin	27417541	Acute appendicitis: transcript profiling of blood identifies promising biomarkers and potential underlying processes.
3429	DRAMP03148	Defensin	"Defensin"[All Fields] AND biofilm[All Fields]	Defensin	27200276	Antifungal effects of synthetic human β-defensin 3-C15 peptide.
3429	DRAMP03148	Defensin	"Defensin"[All Fields] AND biofilm[All Fields]	Defensin	27148195	Effect of Substance P in Staphylococcus aureus and Staphylococcus epidermidis Virulence: Implication for Skin Homeostasis.
3429	DRAMP03148	Defensin	"Defensin"[All Fields] AND biofilm[All Fields]	Defensin	26861950	Antibiofilm efficacy of honey and bee-derived defensin-1 on multispecies wound biofilm.
3429	DRAMP03148	Defensin	"Defensin"[All Fields] AND biofilm[All Fields]	Defensin	26592804	The radish defensins RsAFP1 and RsAFP2 act synergistically with caspofungin against Candida albicans biofilms.
3429	DRAMP03148	Defensin	"Defensin"[All Fields] AND biofilm[All Fields]	Defensin	26248029	Synergistic Activity of the Plant Defensin HsAFP1 and Caspofungin against Candida albicans Biofilms and Planktonic Cultures.
3429	DRAMP03148	Defensin	"Defensin"[All Fields] AND biofilm[All Fields]	Defensin	26214284	α-tocopherol decreases interleukin-1β and -6 and increases human β-defensin-1 and -2 secretion in human gingival fibroblasts stimulated with Porphyromonas gingivalis lipopolysaccharide.

3429	DRAMP03148	Defensin	"Defensin"[All Fields] AND biofilm[All Fields]	Defensin	26196513	Snake Cathelicidin NA-CATH and Smaller Helical Antimicrobial Peptides Are Effective against Burkholderia thailandensis.
3429	DRAMP03148	Defensin	"Defensin"[All Fields] AND biofilm[All Fields]	Defensin	26119274	Ultrasound microbubbles enhance human β-defensin 3 against biofilms.
3429	DRAMP03148	Defensin	"Defensin"[All Fields] AND biofilm[All Fields]	Defensin	25862466	The Antibacterial Effects of an Antimicrobial Peptide Human β-Defensin 3 Fused with Carbohydrate-Binding Domain on Pseudomonas aeruginosa PA14.
3429	DRAMP03148	Defensin	"Defensin"[All Fields] AND biofilm[All Fields]	Defensin	25808131	Expression of antimicrobial peptides and interleukin-8 during early stages of inflammation: An experimental gingivitis study.
3429	DRAMP03148	Defensin	"Defensin"[All Fields] AND biofilm[All Fields]	Defensin	25806720	Antimicrobial peptides in 2014.
3429	DRAMP03148	Defensin	"Defensin"[All Fields] AND biofilm[All Fields]	Defensin	25285879	Inhibition and destruction of Pseudomonas aeruginosa biofilms by antibiotics and antimicrobial peptides.
3429	DRAMP03148	Defensin	"Defensin"[All Fields] AND biofilm[All Fields]	Defensin	25212593	Health- and disease-associated species clusters in complex natural biofilms determine the innate immune response in oral epithelial cells during biofilm maturation.
3429	DRAMP03148	Defensin	"Defensin"[All Fields] AND biofilm[All Fields]	Defensin	24913184	Effects of human β-defensin-3 on biofilm formation-regulating genes dltB and icaA in Staphylococcus aureus.
3429	DRAMP03148	Defensin	"Defensin"[All Fields] AND biofilm[All Fields]	Defensin	24340061	Inflammatory and antimicrobial responses to methicillin-resistant Staphylococcus aureus in an in vitro wound infection model.
3429	DRAMP03148	Defensin	"Defensin"[All Fields] AND biofilm[All Fields]	Defensin	24240906	Adsorption study of pellicle proteins to gold, silica and titanium by quartz crystal microbalance method.
3429	DRAMP03148	Defensin	"Defensin"[All Fields] AND biofilm[All Fields]	Defensin	24238461	Antibacterial efficacy of a human β-defensin-3 peptide on multispecies biofilms.
3429	DRAMP03148	Defensin	"Defensin"[All Fields] AND biofilm[All Fields]	Defensin	24100890	[Use of recombinant human beta-defensin-3 to evaluate the effect of adhesion of Candida albicans on the surface of soft lining material].
3429	DRAMP03148	Defensin	"Defensin"[All Fields] AND biofilm[All Fields]	Defensin	23639356	Human beta-defensin 3: a novel inhibitor of Staphylococcus-produced biofilm production. Commentary on "Human β-defensin 3 inhibits antibiotic-resistant Staphylococcus biofilm formation".
3429	DRAMP03148	Defensin	"Defensin"[All Fields] AND biofilm[All Fields]	Defensin	23519963	Ultrasound-targeted microbubble destruction enhances human β-defensin 3 activity against antibiotic-resistant Staphylococcus biofilms.
3429	DRAMP03148	Defensin	"Defensin"[All Fields] AND biofilm[All Fields]	Defensin	23273885	Human β-defensin 3 inhibits antibiotic-resistant Staphylococcus biofilm formation.
3429	DRAMP03148	Defensin	"Defensin"[All Fields] AND biofilm[All Fields]	Defensin	23203265	Potentiation of the cytotoxic activity of copper by polyphosphate on biofilm-producing bacteria: a bioinspired approach.
3429	DRAMP03148	Defensin	"Defensin"[All Fields] AND biofilm[All Fields]	Defensin	23078156	Antimicrobial efficacy of a human β-defensin-3 peptide using an Enterococcus faecalis dentine infection model.
3429	DRAMP03148	Defensin	"Defensin"[All Fields] AND biofilm[All Fields]	Defensin	23053486	Synergistic effect and antibiofilm activity between the antimicrobial peptide coprisin and conventional antibiotics against opportunistic bacteria.
3429	DRAMP03148	Defensin	"Defensin"[All Fields] AND biofilm[All Fields]	Defensin	22922323	Extracellular DNA within a nontypeable Haemophilus influenzae-induced biofilm binds human beta defensin-3 and reduces its antimicrobial activity.
3429	DRAMP03148	Defensin	"Defensin"[All Fields] AND biofilm[All Fields]	Defensin	22855857	Retrocyclin inhibits Gardnerella vaginalis biofilm formation and toxin activity.
3429	DRAMP03148	Defensin	"Defensin"[All Fields] AND biofilm[All Fields]	Defensin	22394470	Association of CiaRH with resistance of Streptococcus mutans to antimicrobial peptides in biofilms.
3429	DRAMP03148	Defensin	"Defensin"[All Fields] AND biofilm[All Fields]	Defensin	22229614	Comparison of the effects of human β-defensin 3, vancomycin, and clindamycin on Staphylococcus aureus biofilm formation.
3429	DRAMP03148	Defensin	"Defensin"[All Fields] AND biofilm[All Fields]	Defensin	21692631	A novel organotypic dento-epithelial culture model: effect of Fusobacterium nucleatum biofilm on B-defensin-2, -3, and LL-37 expression.
3429	DRAMP03148	Defensin	"Defensin"[All Fields] AND biofilm[All Fields]	Defensin	20454633	Normal human gingival epithelial cells sense C. parapsilosis by toll-like receptors and module its pathogenesis through antimicrobial peptides and proinflammatory cytokines.
3429	DRAMP03148	Defensin	"Defensin"[All Fields] AND biofilm[All Fields]	Defensin	20378008	Effect of temperature on the shift of Pseudomonas fluorescens from an environmental microorganism to a potential human pathogen.
3429	DRAMP03148	Defensin	"Defensin"[All Fields] AND biofilm[All Fields]	Defensin	19961380	Gingival transcriptome patterns during induction and resolution of experimental gingivitis in humans.
3429	DRAMP03148	Defensin	"Defensin"[All Fields] AND biofilm[All Fields]	Defensin	19780045	Candida famata modulates toll-like receptor, beta-defensin, and proinflammatory cytokine expression by normal human epithelial cells.
3429	DRAMP03148	Defensin	"Defensin"[All Fields] AND biofilm[All Fields]	Defensin	19572896	The immune response of oral epithelial cells induced by single-species and complex naturally formed biofilms.
3429	DRAMP03148	Defensin	"Defensin"[All Fields] AND biofilm[All Fields]	Defensin	19466693	Activity of antimicrobial peptides in the presence of polysaccharides produced by pulmonary pathogens.
3429	DRAMP03148	Defensin	"Defensin"[All Fields] AND biofilm[All Fields]	Defensin	18954353	Treponema denticola does not induce production of common innate immune mediators from primary gingival epithelial cells.
3429	DRAMP03148	Defensin	"Defensin"[All Fields] AND biofilm[All Fields]	Defensin	18173794	The stage of native biofilm formation determines the gene expression of human beta-defensin-2, psoriasin, ribonuclease 7 and inflammatory mediators: a novel approach for stimulation of keratinocytes with in situ formed biofilms.
3429	DRAMP03148	Defensin	"Defensin"[All Fields] AND biofilm[All Fields]	Defensin	17434999	Functional analysis of D-alanylation of lipoteichoic acid in the probiotic strain Lactobacillus rhamnosus GG.
3429	DRAMP03148	Defensin	"Defensin"[All Fields] AND biofilm[All Fields]	Defensin	15493829	Bacterial evasion of innate host defenses--the Staphylococcus aureus lesson.
3429	DRAMP03148	Defensin	"Defensin"[All Fields] AND biofilm[All Fields]	Defensin	14764110	Polysaccharide intercellular adhesin (PIA) protects Staphylococcus epidermidis against major components of the human innate immune system.
3440	DRAMP03161	Beta-defensin 1 (BD-1; Defensin, beta 1; mammals, animals)	"Beta-defensin 1"[All Fields] AND biofilm[All Fields]	Beta-defensin 1	33420317	Curbing gastrointestinal infections by defensin fragment modifications without harming commensal microbiota.
3440	DRAMP03161	Beta-defensin 1 (BD-1; Defensin, beta 1; mammals, animals)	"Beta-defensin 1"[All Fields] AND biofilm[All Fields]	Beta-defensin 1	32585445	Glucose effect on Candida albicans biofilm during tissue invasion.
3440	DRAMP03161	Beta-defensin 1 (BD-1; Defensin, beta 1; mammals, animals)	"Beta-defensin 1"[All Fields] AND biofilm[All Fields]	Beta-defensin 1	30260708	Stabilized collagen matrix dressing improves wound macrophage function and epithelialization.
3440	DRAMP03161	Beta-defensin 1 (BD-1; Defensin, beta 1; mammals, animals)	"Beta-defensin 1"[All Fields] AND biofilm[All Fields]	Beta-defensin 1	28642103	Chimeric analogs of human β-defensin 1 and θ-defensin disrupt pre-established bacterial biofilms.
3440	DRAMP03161	Beta-defensin 1 (BD-1; Defensin, beta 1; mammals, animals)	"Beta-defensin 1"[All Fields] AND biofilm[All Fields]	Beta-defensin 1	26214284	α-tocopherol decreases interleukin-1β and -6 and increases human β-defensin-1 and -2 secretion in human gingival fibroblasts stimulated with Porphyromonas gingivalis lipopolysaccharide.
3440	DRAMP03161	Beta-defensin 1 (BD-1; Defensin, beta 1; mammals, animals)	"Beta-defensin 1"[All Fields] AND biofilm[All Fields]	Beta-defensin 1	22394470	Association of CiaRH with resistance of Streptococcus mutans to antimicrobial peptides in biofilms.
3440	DRAMP03161	Beta-defensin 1 (BD-1; Defensin, beta 1; mammals, animals)	"Beta-defensin 1"[All Fields] AND biofilm[All Fields]	Beta-defensin 1	20454633	Normal human gingival epithelial cells sense C. parapsilosis by toll-like receptors and module its pathogenesis through antimicrobial peptides and proinflammatory cytokines.
3445	DRAMP03168	L-amino-acid oxidase (K-LAO; LAAO; LAO; reptilia, animals)	"L-amino-acid oxidase"[All Fields] AND biofilm[All Fields]	L-amino-acid oxidase	30160994	Molecules and Mechanisms Underlying the Antimicrobial Activity of Escapin, an l-Amino Acid Oxidase from the Ink of Sea Hares.
3445	DRAMP03168	L-amino-acid oxidase (K-LAO; LAAO; LAO; reptilia, animals)	"L-amino-acid oxidase"[All Fields] AND biofilm[All Fields]	L-amino-acid oxidase	27401562	Inhibition and Dispersal of Pseudomonas aeruginosa Biofilms by Combination Treatment with Escapin Intermediate Products and Hydrogen Peroxide.
3445	DRAMP03168	L-amino-acid oxidase (K-LAO; LAAO; LAO; reptilia, animals)	"L-amino-acid oxidase"[All Fields] AND biofilm[All Fields]	L-amino-acid oxidase	18469105	SO-LAAO, a novel L-amino acid oxidase that enables Streptococcus oligofermentans to outcompete Streptococcus mutans by generating H2O2 from peptone.
3461	DRAMP18221	Fusaricidin D (Bacteriocin)	"Fusaricidin D"[All Fields] AND biofilm[All Fields]	Fusaricidin D	28650559	The antibacterial activity and modes of LI-F type antimicrobial peptides against Bacillus cereus in vitro.
3465	DRAMP03199	PhD1 (PhD-1; Defensin-1; primates, mammals, animals)	"PhD1"[All Fields] AND biofilm[All Fields]	PhD1	21149646	The Yak1 protein kinase lies at the center of a regulatory cascade affecting adhesive growth and stress resistance in Saccharomyces cerevisiae.
3469	DRAMP18220	Fusaricidin C (Bacteriocin)	"Fusaricidin C"[All Fields] AND biofilm[All Fields]	Fusaricidin C	28650559	The antibacterial activity and modes of LI-F type antimicrobial peptides against Bacillus cereus in vitro.
3470	DRAMP18219	Fusaricidin B (Bacteriocin)	"Fusaricidin B"[All Fields] AND biofilm[All Fields]	Fusaricidin B	23113815	Lipopeptide biosurfactants from Paenibacillus polymyxa inhibit single and mixed species biofilms.
3475	DRAMP18218	Fusaricidin A (Bacteriocin)	"Fusaricidin A"[All Fields] AND biofilm[All Fields]	Fusaricidin A	28533236	Antibacterial and Antibiofilm Activities of a Novel Synthetic Cyclic Lipopeptide against Cariogenic Streptococcus mutans UA159.
3478	DRAMP18217	Licheniocin 50.2(Bacteriocin)	"Licheniocin 50.2"[All Fields] AND biofilm[All Fields]	Licheniocin 50.2	27930711	Licheniocin 50.2 and Bacteriocins from Lactococcus lactis subsp. lactis biovar. diacetylactis BGBU1-4 Inhibit Biofilms of Coagulase Negative Staphylococci and Listeria monocytogenes Clinical Isolates.
3514	DRAMP18213	Gramicidin S(Bacteriocin)	"Gramicidin S"[All Fields] AND biofilm[All Fields]	Gramicidin S	31784584	Supreme activity of gramicidin S against resistant, persistent and biofilm cells of staphylococci and enterococci.
3514	DRAMP18213	Gramicidin S(Bacteriocin)	"Gramicidin S"[All Fields] AND biofilm[All Fields]	Gramicidin S	28439259	Increased Biological Activity of Aneurinibacillus migulanusStrains Correlates with the Production of New Gramicidin Secondary Metabolites.

3514	DRAMP18213	Gramicidin S(Bacteriocin)	"Gramicidin S"[All Fields] AND biofilm[All Fields]	Gramicidin S	27618065	Therapeutic Potential of Gramicidin S in the Treatment of Root Canal Infections.
3514	DRAMP18213	Gramicidin S(Bacteriocin)	"Gramicidin S"[All Fields] AND biofilm[All Fields]	Gramicidin S	26077259	Synergistic effect of membrane-active peptides polymyxin B and gramicidin S on multidrug-resistant strains and biofilms of Pseudomonas aeruginosa.
3514	DRAMP18213	Gramicidin S(Bacteriocin)	"Gramicidin S"[All Fields] AND biofilm[All Fields]	Gramicidin S	23103974	Direct surfactin-gramicidin S antagonism supports detoxification in mixed producer cultures of Bacillus subtilis and Aneurinibacillus migulanus.
3514	DRAMP18213	Gramicidin S(Bacteriocin)	"Gramicidin S"[All Fields] AND biofilm[All Fields]	Gramicidin S	17701408	Biofilms: strategies for metal corrosion inhibition employing microorganisms.
3514	DRAMP18213	Gramicidin S(Bacteriocin)	"Gramicidin S"[All Fields] AND biofilm[All Fields]	Gramicidin S	15278311	Inhibiting mild steel corrosion from sulfate-reducing and iron-oxidizing bacteria using gramicidin-S-producing biofilms.
3514	DRAMP18213	Gramicidin S(Bacteriocin)	"Gramicidin S"[All Fields] AND biofilm[All Fields]	Gramicidin S	12898064	Inhibiting mild steel corrosion from sulfate-reducing bacteria using antimicrobial-producing biofilms in Three-Mile-Island process water.
3514	DRAMP18213	Gramicidin S(Bacteriocin)	"Gramicidin S"[All Fields] AND biofilm[All Fields]	Gramicidin S	10499267	Inhibiting sulfate-reducing bacteria in biofilms on steel with antimicrobial peptides generated in situ.
3527	DRAMP03284	Beta-defensin (Birds, animals)	"Beta-defensin"[All Fields] AND biofilm[All Fields]	Beta-defensin	34408988	The Anti-Biofilm Efficacy of Caffeic Acid Phenethyl Ester (CAPE) In Vitroand a Murine Model of Oral Candidiasis.
3527	DRAMP03284	Beta-defensin (Birds, animals)	"Beta-defensin"[All Fields] AND biofilm[All Fields]	Beta-defensin	34276631	DNA Blocks the Lethal Effect of Human Beta-Defensin 2 Against Neisseria meningitidis.
3527	DRAMP03284	Beta-defensin (Birds, animals)	"Beta-defensin"[All Fields] AND biofilm[All Fields]	Beta-defensin	33865931	Identification of a crocodylian β-defensin variant from Alligator mississippiensis with antimicrobial and antibiofilm activity.
3527	DRAMP03284	Beta-defensin (Birds, animals)	"Beta-defensin"[All Fields] AND biofilm[All Fields]	Beta-defensin	33420317	Curbing gastrointestinal infections by defensin fragment modifications without harming commensal microbiota.
3527	DRAMP03284	Beta-defensin (Birds, animals)	"Beta-defensin"[All Fields] AND biofilm[All Fields]	Beta-defensin	32858856	Candida albicansVirulence Factors and Pathogenicity for Endodontic Infections.
3527	DRAMP03284	Beta-defensin (Birds, animals)	"Beta-defensin"[All Fields] AND biofilm[All Fields]	Beta-defensin	32842903	Electrospun ZnO/Poly(Vinylidene Fluoride-Trifluoroethylene) Scaffolds for Lung Tissue Engineering.
3527	DRAMP03284	Beta-defensin (Birds, animals)	"Beta-defensin"[All Fields] AND biofilm[All Fields]	Beta-defensin	32585445	Glucose effect on Candida albicans biofilm during tissue invasion.
3527	DRAMP03284	Beta-defensin (Birds, animals)	"Beta-defensin"[All Fields] AND biofilm[All Fields]	Beta-defensin	32522780	Controlling the Growth of the Skin Commensal Staphylococcus epidermidis Using d-Alanine Auxotrophy.
3527	DRAMP03284	Beta-defensin (Birds, animals)	"Beta-defensin"[All Fields] AND biofilm[All Fields]	Beta-defensin	32457749	The Antimicrobial Peptide Human Beta-Defensin 2 Inhibits Biofilm Production of Pseudomonas aeruginosaWithout Compromising Metabolic Activity.
3527	DRAMP03284	Beta-defensin (Birds, animals)	"Beta-defensin"[All Fields] AND biofilm[All Fields]	Beta-defensin	31933178	Effects of human β-defensin 3 fused with carbohydrate-binding domain on the function of type III secretion system in Pseudomonas aeruginosa PA14.
3527	DRAMP03284	Beta-defensin (Birds, animals)	"Beta-defensin"[All Fields] AND biofilm[All Fields]	Beta-defensin	31336838	Candida albicans-Cell Interactions Activate Innate Immune Defense in Human Palate Epithelial Primary Cells via Nitric Oxide (NO) and β-Defensin 2 (hBD-2).
3527	DRAMP03284	Beta-defensin (Birds, animals)	"Beta-defensin"[All Fields] AND biofilm[All Fields]	Beta-defensin	31165072	Impact of the Food Additive Titanium Dioxide (E171) on Gut Microbiota-Host Interaction.
3527	DRAMP03284	Beta-defensin (Birds, animals)	"Beta-defensin"[All Fields] AND biofilm[All Fields]	Beta-defensin	30659503	Lactobacillus plantarum USM8613 Aids in Wound Healing and Suppresses Staphylococcus aureus Infection at Wound Sites.
3527	DRAMP03284	Beta-defensin (Birds, animals)	"Beta-defensin"[All Fields] AND biofilm[All Fields]	Beta-defensin	30649289	Innate immune components affect growth and virulence traits of bacterial-vaginosis-associated and non-bacterial-vaginosis-associated Gardnerella vaginalis strains similarly.
3527	DRAMP03284	Beta-defensin (Birds, animals)	"Beta-defensin"[All Fields] AND biofilm[All Fields]	Beta-defensin	30260708	Stabilized collagen matrix dressing improves wound macrophage function and epithelialization.
3527	DRAMP03284	Beta-defensin (Birds, animals)	"Beta-defensin"[All Fields] AND biofilm[All Fields]	Beta-defensin	30254440	Modification of the surface of titanium with multifunctional chimeric peptides to prevent biofilm formation via inhibition of initial colonizers.
3527	DRAMP03284	Beta-defensin (Birds, animals)	"Beta-defensin"[All Fields] AND biofilm[All Fields]	Beta-defensin	29872295	Antimicrobial peptide-loaded liquid crystalline precursor bioadhesive system for the prevention of dental caries.
3527	DRAMP03284	Beta-defensin (Birds, animals)	"Beta-defensin"[All Fields] AND biofilm[All Fields]	Beta-defensin	29671721	The BceABRS four-component system that is essential for cell envelope stress response is involved in sensing and response to host defence peptides and is required for the biofilm formation and fitness of Streptococcus mutans.
3527	DRAMP03284	Beta-defensin (Birds, animals)	"Beta-defensin"[All Fields] AND biofilm[All Fields]	Beta-defensin	29077172	The significance of HBD-3 and fluorescent composite carriers in the processof bone formation in rats infected with Staphylococcus aureus.
3527	DRAMP03284	Beta-defensin (Birds, animals)	"Beta-defensin"[All Fields] AND biofilm[All Fields]	Beta-defensin	29025642	Engineered chimeric peptides with antimicrobial and titanium-binding functions to inhibit biofilm formation on Ti implants.
3527	DRAMP03284	Beta-defensin (Birds, animals)	"Beta-defensin"[All Fields] AND biofilm[All Fields]	Beta-defensin	28956355	The synthetic human beta-defensin-3 C15 peptide exhibits antimicrobial activity against Streptococcus mutans, both alone and in combination with dental disinfectants.
3527	DRAMP03284	Beta-defensin (Birds, animals)	"Beta-defensin"[All Fields] AND biofilm[All Fields]	Beta-defensin	28951032	Antifungal Effects of Synthetic Human Beta-defensin-3-C15 Peptide on Candida albicans-infected Root Dentin.
3527	DRAMP03284	Beta-defensin (Birds, animals)	"Beta-defensin"[All Fields] AND biofilm[All Fields]	Beta-defensin	28642103	Chimeric analogs of human β-defensin 1 and θ-defensin disrupt pre-established bacterial biofilms.
3527	DRAMP03284	Beta-defensin (Birds, animals)	"Beta-defensin"[All Fields] AND biofilm[All Fields]	Beta-defensin	28413476	The mechanism of human β-defensin 3 in MRSA-induced infection of implant drug-resistant bacteria biofilm in the mouse tibial bone marrow.
3527	DRAMP03284	Beta-defensin (Birds, animals)	"Beta-defensin"[All Fields] AND biofilm[All Fields]	Beta-defensin	28078813	Host defense peptide-derived privileged scaffolds for anti-infective drug discovery.
3527	DRAMP03284	Beta-defensin (Birds, animals)	"Beta-defensin"[All Fields] AND biofilm[All Fields]	Beta-defensin	27777572	Role of yqiCin the Pathogenicity of Salmonellaand Innate Immune Responses of Human Intestinal Epithelium.
3527	DRAMP03284	Beta-defensin (Birds, animals)	"Beta-defensin"[All Fields] AND biofilm[All Fields]	Beta-defensin	27582732	Efficient Eradication of Mature Pseudomonas aeruginosa Biofilm via Controlled Delivery of Nitric Oxide Combined with Antimicrobial Peptide and Antibiotics.
3527	DRAMP03284	Beta-defensin (Birds, animals)	"Beta-defensin"[All Fields] AND biofilm[All Fields]	Beta-defensin	27200276	Antifungal effects of synthetic human β-defensin 3-C15 peptide.
3527	DRAMP03284	Beta-defensin (Birds, animals)	"Beta-defensin"[All Fields] AND biofilm[All Fields]	Beta-defensin	27148195	Effect of Substance P in Staphylococcus aureus and Staphylococcus epidermidis Virulence: Implication for Skin Homeostasis.
3527	DRAMP03284	Beta-defensin (Birds, animals)	"Beta-defensin"[All Fields] AND biofilm[All Fields]	Beta-defensin	26214284	α-tocopherol decreases interleukin-1β and -6 and increases human β-defensin-1 and -2 secretion in human gingival fibroblasts stimulated with Porphyromonas gingivalis lipopolysaccharide.
3527	DRAMP03284	Beta-defensin (Birds, animals)	"Beta-defensin"[All Fields] AND biofilm[All Fields]	Beta-defensin	26196513	Snake Cathelicidin NA-CATH and Smaller Helical Antimicrobial Peptides Are Effective against Burkholderia thailandensis.
3527	DRAMP03284	Beta-defensin (Birds, animals)	"Beta-defensin"[All Fields] AND biofilm[All Fields]	Beta-defensin	26119274	Ultrasound microbubbles enhance human β-defensin 3 against biofilms.
3527	DRAMP03284	Beta-defensin (Birds, animals)	"Beta-defensin"[All Fields] AND biofilm[All Fields]	Beta-defensin	25862466	The Antibacterial Effects of an Antimicrobial Peptide Human β-Defensin 3 Fused with Carbohydrate-Binding Domain on Pseudomonas aeruginosa PA14.
3527	DRAMP03284	Beta-defensin (Birds, animals)	"Beta-defensin"[All Fields] AND biofilm[All Fields]	Beta-defensin	25808131	Expression of antimicrobial peptides and interleukin-8 during early stages of inflammation: An experimental gingivitis study.
3527	DRAMP03284	Beta-defensin (Birds, animals)	"Beta-defensin"[All Fields] AND biofilm[All Fields]	Beta-defensin	25212593	Health- and disease-associated species clusters in complex natural biofilms determine the innate immune response in oral epithelial cells during biofilm maturation.
3527	DRAMP03284	Beta-defensin (Birds, animals)	"Beta-defensin"[All Fields] AND biofilm[All Fields]	Beta-defensin	24913184	Effects of human β-defensin-3 on biofilm formation-regulating genes dltB and icaA in Staphylococcus aureus.
3527	DRAMP03284	Beta-defensin (Birds, animals)	"Beta-defensin"[All Fields] AND biofilm[All Fields]	Beta-defensin	24340061	Inflammatory and antimicrobial responses to methicillin-resistant Staphylococcus aureus in an in vitro wound infection model.
3527	DRAMP03284	Beta-defensin (Birds, animals)	"Beta-defensin"[All Fields] AND biofilm[All Fields]	Beta-defensin	24240906	Adsorption study of pellicle proteins to gold, silica and titanium by quartz crystal microbalance method.
3527	DRAMP03284	Beta-defensin (Birds, animals)	"Beta-defensin"[All Fields] AND biofilm[All Fields]	Beta-defensin	24238461	Antibacterial efficacy of a human β-defensin-3 peptide on multispecies biofilms.
3527	DRAMP03284	Beta-defensin (Birds, animals)	"Beta-defensin"[All Fields] AND biofilm[All Fields]	Beta-defensin	24100890	[Use of recombinant human beta-defensin-3 to evaluate the effect of adhesion of Candida albicans on the surface of soft lining material].
3527	DRAMP03284	Beta-defensin (Birds, animals)	"Beta-defensin"[All Fields] AND biofilm[All Fields]	Beta-defensin	23639356	Human beta-defensin 3: a novel inhibitor of Staphylococcus-produced biofilm production. Commentary on "Human β-defensin 3 inhibits antibiotic-resistant Staphylococcus biofilm formation".
3527	DRAMP03284	Beta-defensin (Birds, animals)	"Beta-defensin"[All Fields] AND biofilm[All Fields]	Beta-defensin	23519963	Ultrasound-targeted microbubble destruction enhances human β-defensin 3 activity against antibiotic-resistant Staphylococcus biofilms.
3527	DRAMP03284	Beta-defensin (Birds, animals)	"Beta-defensin"[All Fields] AND biofilm[All Fields]	Beta-defensin	23273885	Human β-defensin 3 inhibits antibiotic-resistant Staphylococcus biofilm formation.
3527	DRAMP03284	Beta-defensin (Birds, animals)	"Beta-defensin"[All Fields] AND biofilm[All Fields]	Beta-defensin	23078156	Antimicrobial efficacy of a human β-defensin-3 peptide using an Enterococcus faecalis dentine infection model.
3527	DRAMP03284	Beta-defensin (Birds, animals)	"Beta-defensin"[All Fields] AND biofilm[All Fields]	Beta-defensin	22922323	Extracellular DNA within a nontypeable Haemophilus influenzae-induced biofilm binds human beta defensin-3 and reduces its antimicrobial activity.
3527	DRAMP03284	Beta-defensin (Birds, animals)	"Beta-defensin"[All Fields] AND biofilm[All Fields]	Beta-defensin	22394470	Association of CiaRH with resistance of Streptococcus mutans to antimicrobial peptides in biofilms.
3527	DRAMP03284	Beta-defensin (Birds, animals)	"Beta-defensin"[All Fields] AND biofilm[All Fields]	Beta-defensin	22229614	Comparison of the effects of human β-defensin 3, vancomycin, and clindamycin on Staphylococcus aureus biofilm formation.
3527	DRAMP03284	Beta-defensin (Birds, animals)	"Beta-defensin"[All Fields] AND biofilm[All Fields]	Beta-defensin	21692631	A novel organotypic dento-epithelial culture model: effect of Fusobacterium nucleatum biofilm on B-defensin-2, -3, and LL-37 expression.
3527	DRAMP03284	Beta-defensin (Birds, animals)	"Beta-defensin"[All Fields] AND biofilm[All Fields]	Beta-defensin	20454633	Normal human gingival epithelial cells sense C. parapsilosis by toll-like receptors and module its pathogenesis through antimicrobial peptides and proinflammatory cytokines.
3527	DRAMP03284	Beta-defensin (Birds, animals)	"Beta-defensin"[All Fields] AND biofilm[All Fields]	Beta-defensin	20378008	Effect of temperature on the shift of Pseudomonas fluorescens from an environmental microorganism to a potential human pathogen.

3527	DRAMP03284	Beta-defensin (Birds, animals)	"Beta-defensin"[All Fields] AND biofilm[All Fields]	Beta-defensin	19780045	Candida famata modulates toll-like receptor, beta-defensin, and proinflammatory cytokine expression by normal human epithelial cells.
3527	DRAMP03284	Beta-defensin (Birds, animals)	"Beta-defensin"[All Fields] AND biofilm[All Fields]	Beta-defensin	19572896	The immune response of oral epithelial cells induced by single-species and complex naturally formed biofilms.
3527	DRAMP03284	Beta-defensin (Birds, animals)	"Beta-defensin"[All Fields] AND biofilm[All Fields]	Beta-defensin	19466693	Activity of antimicrobial peptides in the presence of polysaccharides produced by pulmonary pathogens.
3527	DRAMP03284	Beta-defensin (Birds, animals)	"Beta-defensin"[All Fields] AND biofilm[All Fields]	Beta-defensin	18954353	Treponema denticola does not induce production of common innate immune mediators from primary gingival epithelial cells.
3527	DRAMP03284	Beta-defensin (Birds, animals)	"Beta-defensin"[All Fields] AND biofilm[All Fields]	Beta-defensin	18173794	The stage of native biofilm formation determines the gene expression of human beta-defensin-2, psoriasin, ribonuclease 7 and inflammatory mediators: a novel approach for stimulation of keratinocytes with in situ formed biofilms.
3527	DRAMP03284	Beta-defensin (Birds, animals)	"Beta-defensin"[All Fields] AND biofilm[All Fields]	Beta-defensin	17434999	Functional analysis of D-alanylation of lipoteichoic acid in the probiotic strain Lactobacillus rhamnosus GG.
3527	DRAMP03284	Beta-defensin (Birds, animals)	"Beta-defensin"[All Fields] AND biofilm[All Fields]	Beta-defensin	14764110	Polysaccharide intercellular adhesin (PIA) protects Staphylococcus epidermidis against major components of the human innate immune system.
3553	DRAMP03316	Defensin (Insects, animals)	"Defensin"[All Fields] AND biofilm[All Fields]	Defensin	34408988	The Anti-Biofilm Efficacy of Caffeic Acid Phenethyl Ester (CAPE) In Vitroand a Murine Model of Oral Candidiasis.
3553	DRAMP03316	Defensin (Insects, animals)	"Defensin"[All Fields] AND biofilm[All Fields]	Defensin	34321877	Antibacterial Effect of Honey-Derived Exosomes Containing Antimicrobial Peptides Against Oral Streptococci.
3553	DRAMP03316	Defensin (Insects, animals)	"Defensin"[All Fields] AND biofilm[All Fields]	Defensin	34276631	DNA Blocks the Lethal Effect of Human Beta-Defensin 2 Against Neisseria meningitidis.
3553	DRAMP03316	Defensin (Insects, animals)	"Defensin"[All Fields] AND biofilm[All Fields]	Defensin	33911935	The antibacterial activities of honey.
3553	DRAMP03316	Defensin (Insects, animals)	"Defensin"[All Fields] AND biofilm[All Fields]	Defensin	33865931	Identification of a crocodylian β-defensin variant from Alligator mississippiensis with antimicrobial and antibiofilm activity.
3553	DRAMP03316	Defensin (Insects, animals)	"Defensin"[All Fields] AND biofilm[All Fields]	Defensin	33586659	Identification of anti-microbial peptides and traces of microbial DNA in infrainfundibular compartments of human scalp terminal hair follicles.
3553	DRAMP03316	Defensin (Insects, animals)	"Defensin"[All Fields] AND biofilm[All Fields]	Defensin	33534018	A recombinant fungal defensin-like peptide-P2 combats Streptococcus dysgalactiae and biofilms.
3553	DRAMP03316	Defensin (Insects, animals)	"Defensin"[All Fields] AND biofilm[All Fields]	Defensin	33447687	Future directions of postoperative spinal implant infections.
3553	DRAMP03316	Defensin (Insects, animals)	"Defensin"[All Fields] AND biofilm[All Fields]	Defensin	33420317	Curbing gastrointestinal infections by defensin fragment modifications without harming commensal microbiota.
3553	DRAMP03316	Defensin (Insects, animals)	"Defensin"[All Fields] AND biofilm[All Fields]	Defensin	32867384	A Novel Peptide Antibiotic, Pro10-1D, Designed from Insect Defensin Shows Antibacterial and Anti-Inflammatory Activities in Sepsis Models.
3553	DRAMP03316	Defensin (Insects, animals)	"Defensin"[All Fields] AND biofilm[All Fields]	Defensin	32858856	Candida albicansVirulence Factors and Pathogenicity for Endodontic Infections.
3553	DRAMP03316	Defensin (Insects, animals)	"Defensin"[All Fields] AND biofilm[All Fields]	Defensin	32842903	Electrospun ZnO/Poly(Vinylidene Fluoride-Trifluoroethylene) Scaffolds for Lung Tissue Engineering.
3553	DRAMP03316	Defensin (Insects, animals)	"Defensin"[All Fields] AND biofilm[All Fields]	Defensin	32663201	Inhibition and eradication activity of truncated α-defensin analogs against multidrug resistant uropathogenic Escherichia coli biofilm.
3553	DRAMP03316	Defensin (Insects, animals)	"Defensin"[All Fields] AND biofilm[All Fields]	Defensin	32585445	Glucose effect on Candida albicans biofilm during tissue invasion.
3553	DRAMP03316	Defensin (Insects, animals)	"Defensin"[All Fields] AND biofilm[All Fields]	Defensin	32522780	Controlling the Growth of the Skin Commensal Staphylococcus epidermidis Using d-Alanine Auxotrophy.
3553	DRAMP03316	Defensin (Insects, animals)	"Defensin"[All Fields] AND biofilm[All Fields]	Defensin	32457749	The Antimicrobial Peptide Human Beta-Defensin 2 Inhibits Biofilm Production of Pseudomonas aeruginosaWithout Compromising Metabolic Activity.
3553	DRAMP03316	Defensin (Insects, animals)	"Defensin"[All Fields] AND biofilm[All Fields]	Defensin	32439511	Antibacterial activities and mechanisms of action of a defensin from manila clam Ruditapes philippinarum.
3553	DRAMP03316	Defensin (Insects, animals)	"Defensin"[All Fields] AND biofilm[All Fields]	Defensin	31933178	Effects of human β-defensin 3 fused with carbohydrate-binding domain on the function of type III secretion system in Pseudomonas aeruginosa PA14.
3553	DRAMP03316	Defensin (Insects, animals)	"Defensin"[All Fields] AND biofilm[All Fields]	Defensin	31906541	Role of FAD-I in Fusobacterial Interspecies Interaction and Biofilm Formation.
3553	DRAMP03316	Defensin (Insects, animals)	"Defensin"[All Fields] AND biofilm[All Fields]	Defensin	31729441	Rhesus Theta Defensin 1 Promotes Long Term Survival in Systemic Candidiasis by Host Directed Mechanisms.
3553	DRAMP03316	Defensin (Insects, animals)	"Defensin"[All Fields] AND biofilm[All Fields]	Defensin	31336838	Candida albicans-Cell Interactions Activate Innate Immune Defense in Human Palate Epithelial Primary Cells via Nitric Oxide (NO) and β-Defensin 2 (hBD-2).
3553	DRAMP03316	Defensin (Insects, animals)	"Defensin"[All Fields] AND biofilm[All Fields]	Defensin	31165072	Impact of the Food Additive Titanium Dioxide (E171) on Gut Microbiota-Host Interaction.
3553	DRAMP03316	Defensin (Insects, animals)	"Defensin"[All Fields] AND biofilm[All Fields]	Defensin	31031739	Salt-Tolerant Antifungal and Antibacterial Activities of the Corn Defensin ZmD32.
3553	DRAMP03316	Defensin (Insects, animals)	"Defensin"[All Fields] AND biofilm[All Fields]	Defensin	31025073	A recombinant fungal defensin-like peptide-P2 combats multidrug-resistant Staphylococcus aureus and biofilms.
3553	DRAMP03316	Defensin (Insects, animals)	"Defensin"[All Fields] AND biofilm[All Fields]	Defensin	30659503	Lactobacillus plantarum USM8613 Aids in Wound Healing and Suppresses Staphylococcus aureus Infection at Wound Sites.
3553	DRAMP03316	Defensin (Insects, animals)	"Defensin"[All Fields] AND biofilm[All Fields]	Defensin	30649289	Innate immune components affect growth and virulence traits of bacterial-vaginosis-associated and non-bacterial-vaginosis-associated Gardnerella vaginalis strains similarly.
3553	DRAMP03316	Defensin (Insects, animals)	"Defensin"[All Fields] AND biofilm[All Fields]	Defensin	30376742	Alpha defensin, leukocyte esterase, C-reactive protein, and leukocyte count in synovial fluid for pre-operative diagnosis of periprosthetic infection.
3553	DRAMP03316	Defensin (Insects, animals)	"Defensin"[All Fields] AND biofilm[All Fields]	Defensin	30260708	Stabilized collagen matrix dressing improves wound macrophage function and epithelialization.
3553	DRAMP03316	Defensin (Insects, animals)	"Defensin"[All Fields] AND biofilm[All Fields]	Defensin	30254440	Modification of the surface of titanium with multifunctional chimeric peptides to prevent biofilm formation via inhibition of initial colonizers.
3553	DRAMP03316	Defensin (Insects, animals)	"Defensin"[All Fields] AND biofilm[All Fields]	Defensin	29902560	A defensin-like antimicrobial peptide from the manila clam Ruditapes philippinarum: Investigation of the antibacterial activities and mode of action.
3553	DRAMP03316	Defensin (Insects, animals)	"Defensin"[All Fields] AND biofilm[All Fields]	Defensin	29872295	Antimicrobial peptide-loaded liquid crystalline precursor bioadhesive system for the prevention of dental caries.
3553	DRAMP03316	Defensin (Insects, animals)	"Defensin"[All Fields] AND biofilm[All Fields]	Defensin	29671721	The BceABRS four-component system that is essential for cell envelope stress response is involved in sensing and response to host defence peptides and is required for the biofilm formation and fitness of Streptococcus mutans.
3553	DRAMP03316	Defensin (Insects, animals)	"Defensin"[All Fields] AND biofilm[All Fields]	Defensin	29104569	A Linear 19-Mer Plant Defensin-Derived Peptide Acts Synergistically with Caspofungin against Candida albicansBiofilms.
3553	DRAMP03316	Defensin (Insects, animals)	"Defensin"[All Fields] AND biofilm[All Fields]	Defensin	29077172	The significance of HBD-3 and fluorescent composite carriers in the processof bone formation in rats infected with Staphylococcus aureus.
3553	DRAMP03316	Defensin (Insects, animals)	"Defensin"[All Fields] AND biofilm[All Fields]	Defensin	29045084	New Approach to Treat and Prevent Oral Disease.
3553	DRAMP03316	Defensin (Insects, animals)	"Defensin"[All Fields] AND biofilm[All Fields]	Defensin	29025642	Engineered chimeric peptides with antimicrobial and titanium-binding functions to inhibit biofilm formation on Ti implants.
3553	DRAMP03316	Defensin (Insects, animals)	"Defensin"[All Fields] AND biofilm[All Fields]	Defensin	28956355	The synthetic human beta-defensin-3 C15 peptide exhibits antimicrobial activity against Streptococcus mutans, both alone and in combination with dental disinfectants.
3553	DRAMP03316	Defensin (Insects, animals)	"Defensin"[All Fields] AND biofilm[All Fields]	Defensin	28951032	Antifungal Effects of Synthetic Human Beta-defensin-3-C15 Peptide on Candida albicans-infected Root Dentin.
3553	DRAMP03316	Defensin (Insects, animals)	"Defensin"[All Fields] AND biofilm[All Fields]	Defensin	28874606	Bacterial d-amino acids suppress sinonasal innate immunity through sweet taste receptors in solitary chemosensory cells.
3553	DRAMP03316	Defensin (Insects, animals)	"Defensin"[All Fields] AND biofilm[All Fields]	Defensin	28725299	Role of Streptococcus mutanstwo-component systems in antimicrobial peptide resistance in the oral cavity.
3553	DRAMP03316	Defensin (Insects, animals)	"Defensin"[All Fields] AND biofilm[All Fields]	Defensin	28649561	Psd1 Effects on Candida albicansPlanktonic Cells and Biofilms.
3553	DRAMP03316	Defensin (Insects, animals)	"Defensin"[All Fields] AND biofilm[All Fields]	Defensin	28642103	Chimeric analogs of human β-defensin 1 and θ-defensin disrupt pre-established bacterial biofilms.
3553	DRAMP03316	Defensin (Insects, animals)	"Defensin"[All Fields] AND biofilm[All Fields]	Defensin	28413476	The mechanism of human β-defensin 3 in MRSA-induced infection of implant drug-resistant bacteria biofilm in the mouse tibial bone marrow.
3553	DRAMP03316	Defensin (Insects, animals)	"Defensin"[All Fields] AND biofilm[All Fields]	Defensin	28296382	Human α-Defensin 6: A Small Peptide That Self-Assembles and Protects the Host by Entangling Microbes.
3553	DRAMP03316	Defensin (Insects, animals)	"Defensin"[All Fields] AND biofilm[All Fields]	Defensin	28278280	Natural antimicrobial peptide complexes in the fighting of antibiotic resistant biofilms: Calliphora vicina medicinal maggots.
3553	DRAMP03316	Defensin (Insects, animals)	"Defensin"[All Fields] AND biofilm[All Fields]	Defensin	28144375	Advancements in Diagnosing Periprosthetic Joint Infections after Total Hip and Knee Arthroplasty.
3553	DRAMP03316	Defensin (Insects, animals)	"Defensin"[All Fields] AND biofilm[All Fields]	Defensin	28078813	Host defense peptide-derived privileged scaffolds for anti-infective drug discovery.
3553	DRAMP03316	Defensin (Insects, animals)	"Defensin"[All Fields] AND biofilm[All Fields]	Defensin	28026958	Human α-Defensin 6 Self-Assembly Prevents Adhesion and Suppresses Virulence Traits of Candida albicans.
3553	DRAMP03316	Defensin (Insects, animals)	"Defensin"[All Fields] AND biofilm[All Fields]	Defensin	27794585	A Novel Defensin-Like Peptide Associated with Two Other New Cationic Antimicrobial Peptides in Transcriptome of the Iranian Scorpion Venom.
3553	DRAMP03316	Defensin (Insects, animals)	"Defensin"[All Fields] AND biofilm[All Fields]	Defensin	27777572	Role of yqiCin the Pathogenicity of Salmonellaand Innate Immune Responses of Human Intestinal Epithelium.
3553	DRAMP03316	Defensin (Insects, animals)	"Defensin"[All Fields] AND biofilm[All Fields]	Defensin	27582732	Efficient Eradication of Mature Pseudomonas aeruginosa Biofilm via Controlled Delivery of Nitric Oxide Combined with Antimicrobial Peptide and Antibiotics.

3553	DRAMP03316	Defensin (Insects, animals)	"Defensin"[All Fields] AND biofilm[All Fields]	Defensin	27417541	Acute appendicitis: transcript profiling of blood identifies promising biomarkers and potential underlying processes.
3553	DRAMP03316	Defensin (Insects, animals)	"Defensin"[All Fields] AND biofilm[All Fields]	Defensin	27200276	Antifungal effects of synthetic human β-defensin 3-C15 peptide.
3553	DRAMP03316	Defensin (Insects, animals)	"Defensin"[All Fields] AND biofilm[All Fields]	Defensin	27148195	Effect of Substance P in Staphylococcus aureus and Staphylococcus epidermidis Virulence: Implication for Skin Homeostasis.
3553	DRAMP03316	Defensin (Insects, animals)	"Defensin"[All Fields] AND biofilm[All Fields]	Defensin	26861950	Antibiofilm efficacy of honey and bee-derived defensin-1 on multispecies wound biofilm.
3553	DRAMP03316	Defensin (Insects, animals)	"Defensin"[All Fields] AND biofilm[All Fields]	Defensin	26592804	The radish defensins RsAFP1 and RsAFP2 act synergistically with caspofungin against Candida albicans biofilms.
3553	DRAMP03316	Defensin (Insects, animals)	"Defensin"[All Fields] AND biofilm[All Fields]	Defensin	26248029	Synergistic Activity of the Plant Defensin HsAFP1 and Caspofungin against Candida albicans Biofilms and Planktonic Cultures.
3553	DRAMP03316	Defensin (Insects, animals)	"Defensin"[All Fields] AND biofilm[All Fields]	Defensin	26214284	α-tocopherol decreases interleukin-1β and -6 and increases human β-defensin-1 and -2 secretion in human gingival fibroblasts stimulated with Porphyromonas gingivalis lipopolysaccharide.
3553	DRAMP03316	Defensin (Insects, animals)	"Defensin"[All Fields] AND biofilm[All Fields]	Defensin	26196513	Snake Cathelicidin NA-CATH and Smaller Helical Antimicrobial Peptides Are Effective against Burkholderia thailandensis.
3553	DRAMP03316	Defensin (Insects, animals)	"Defensin"[All Fields] AND biofilm[All Fields]	Defensin	26119274	Ultrasound microbubbles enhance human β-defensin 3 against biofilms.
3553	DRAMP03316	Defensin (Insects, animals)	"Defensin"[All Fields] AND biofilm[All Fields]	Defensin	25862466	The Antibacterial Effects of an Antimicrobial Peptide Human β-Defensin 3 Fused with Carbohydrate-Binding Domain on Pseudomonas aeruginosa PA14.
3553	DRAMP03316	Defensin (Insects, animals)	"Defensin"[All Fields] AND biofilm[All Fields]	Defensin	25808131	Expression of antimicrobial peptides and interleukin-8 during early stages of inflammation: An experimental gingivitis study.
3553	DRAMP03316	Defensin (Insects, animals)	"Defensin"[All Fields] AND biofilm[All Fields]	Defensin	25806720	Antimicrobial peptides in 2014.
3553	DRAMP03316	Defensin (Insects, animals)	"Defensin"[All Fields] AND biofilm[All Fields]	Defensin	25285879	Inhibition and destruction of Pseudomonas aeruginosa biofilms by antibiotics and antimicrobial peptides.
3553	DRAMP03316	Defensin (Insects, animals)	"Defensin"[All Fields] AND biofilm[All Fields]	Defensin	25212593	Health- and disease-associated species clusters in complex natural biofilms determine the innate immune response in oral epithelial cells during biofilm maturation.
3553	DRAMP03316	Defensin (Insects, animals)	"Defensin"[All Fields] AND biofilm[All Fields]	Defensin	24913184	Effects of human β-defensin-3 on biofilm formation-regulating genes dltB and icaA in Staphylococcus aureus.
3553	DRAMP03316	Defensin (Insects, animals)	"Defensin"[All Fields] AND biofilm[All Fields]	Defensin	24340061	Inflammatory and antimicrobial responses to methicillin-resistant Staphylococcus aureus in an in vitro wound infection model.
3553	DRAMP03316	Defensin (Insects, animals)	"Defensin"[All Fields] AND biofilm[All Fields]	Defensin	24240906	Adsorption study of pellicle proteins to gold, silica and titanium by quartz crystal microbalance method.
3553	DRAMP03316	Defensin (Insects, animals)	"Defensin"[All Fields] AND biofilm[All Fields]	Defensin	24238461	Antibacterial efficacy of a human β-defensin-3 peptide on multispecies biofilms.
3553	DRAMP03316	Defensin (Insects, animals)	"Defensin"[All Fields] AND biofilm[All Fields]	Defensin	24100890	[Use of recombinant human beta-defensin-3 to evaluate the effect of adhesion of Candida albicans on the surface of soft lining material].
3553	DRAMP03316	Defensin (Insects, animals)	"Defensin"[All Fields] AND biofilm[All Fields]	Defensin	23639356	Human beta-defensin 3: a novel inhibitor of Staphylococcus-produced biofilm production. Commentary on "Human β-defensin 3 inhibits antibiotic-resistant Staphylococcus biofilm formation".
3553	DRAMP03316	Defensin (Insects, animals)	"Defensin"[All Fields] AND biofilm[All Fields]	Defensin	23519963	Ultrasound-targeted microbubble destruction enhances human β-defensin 3 activity against antibiotic-resistant Staphylococcus biofilms.
3553	DRAMP03316	Defensin (Insects, animals)	"Defensin"[All Fields] AND biofilm[All Fields]	Defensin	23273885	Human β-defensin 3 inhibits antibiotic-resistant Staphylococcus biofilm formation.
3553	DRAMP03316	Defensin (Insects, animals)	"Defensin"[All Fields] AND biofilm[All Fields]	Defensin	23203265	Potentiation of the cytotoxic activity of copper by polyphosphate on biofilm-producing bacteria: a bioinspired approach.
3553	DRAMP03316	Defensin (Insects, animals)	"Defensin"[All Fields] AND biofilm[All Fields]	Defensin	23078156	Antimicrobial efficacy of a human β-defensin-3 peptide using an Enterococcus faecalis dentine infection model.
3553	DRAMP03316	Defensin (Insects, animals)	"Defensin"[All Fields] AND biofilm[All Fields]	Defensin	23053486	Synergistic effect and antibiofilm activity between the antimicrobial peptide coprisin and conventional antibiotics against opportunistic bacteria.
3553	DRAMP03316	Defensin (Insects, animals)	"Defensin"[All Fields] AND biofilm[All Fields]	Defensin	22922323	Extracellular DNA within a nontypeable Haemophilus influenzae-induced biofilm binds human beta defensin-3 and reduces its antimicrobial activity.
3553	DRAMP03316	Defensin (Insects, animals)	"Defensin"[All Fields] AND biofilm[All Fields]	Defensin	22855857	Retrocyclin inhibits Gardnerella vaginalis biofilm formation and toxin activity.
3553	DRAMP03316	Defensin (Insects, animals)	"Defensin"[All Fields] AND biofilm[All Fields]	Defensin	22394470	Association of CiaRH with resistance of Streptococcus mutans to antimicrobial peptides in biofilms.
3553	DRAMP03316	Defensin (Insects, animals)	"Defensin"[All Fields] AND biofilm[All Fields]	Defensin	22229614	Comparison of the effects of human β-defensin 3, vancomycin, and clindamycin on Staphylococcus aureus biofilm formation.
3553	DRAMP03316	Defensin (Insects, animals)	"Defensin"[All Fields] AND biofilm[All Fields]	Defensin	21692631	A novel organotypic dento-epithelial culture model: effect of Fusobacterium nucleatum biofilm on B-defensin-2, -3, and LL-37 expression.
3553	DRAMP03316	Defensin (Insects, animals)	"Defensin"[All Fields] AND biofilm[All Fields]	Defensin	20454633	Normal human gingival epithelial cells sense C. parapsilosis by toll-like receptors and module its pathogenesis through antimicrobial peptides and proinflammatory cytokines.
3553	DRAMP03316	Defensin (Insects, animals)	"Defensin"[All Fields] AND biofilm[All Fields]	Defensin	20378008	Effect of temperature on the shift of Pseudomonas fluorescens from an environmental microorganism to a potential human pathogen.
3553	DRAMP03316	Defensin (Insects, animals)	"Defensin"[All Fields] AND biofilm[All Fields]	Defensin	19961380	Gingival transcriptome patterns during induction and resolution of experimental gingivitis in humans.
3553	DRAMP03316	Defensin (Insects, animals)	"Defensin"[All Fields] AND biofilm[All Fields]	Defensin	19780045	Candida famata modulates toll-like receptor, beta-defensin, and proinflammatory cytokine expression by normal human epithelial cells.
3553	DRAMP03316	Defensin (Insects, animals)	"Defensin"[All Fields] AND biofilm[All Fields]	Defensin	19572896	The immune response of oral epithelial cells induced by single-species and complex naturally formed biofilms.
3553	DRAMP03316	Defensin (Insects, animals)	"Defensin"[All Fields] AND biofilm[All Fields]	Defensin	19466693	Activity of antimicrobial peptides in the presence of polysaccharides produced by pulmonary pathogens.
3553	DRAMP03316	Defensin (Insects, animals)	"Defensin"[All Fields] AND biofilm[All Fields]	Defensin	18954353	Treponema denticola does not induce production of common innate immune mediators from primary gingival epithelial cells.
3553	DRAMP03316	Defensin (Insects, animals)	"Defensin"[All Fields] AND biofilm[All Fields]	Defensin	18173794	The stage of native biofilm formation determines the gene expression of human beta-defensin-2, psoriasin, ribonuclease 7 and inflammatory mediators: a novel approach for stimulation of keratinocytes with in situ formed biofilms.
3553	DRAMP03316	Defensin (Insects, animals)	"Defensin"[All Fields] AND biofilm[All Fields]	Defensin	17434999	Functional analysis of D-alanylation of lipoteichoic acid in the probiotic strain Lactobacillus rhamnosus GG.
3553	DRAMP03316	Defensin (Insects, animals)	"Defensin"[All Fields] AND biofilm[All Fields]	Defensin	15493829	Bacterial evasion of innate host defenses--the Staphylococcus aureus lesson.
3553	DRAMP03316	Defensin (Insects, animals)	"Defensin"[All Fields] AND biofilm[All Fields]	Defensin	14764110	Polysaccharide intercellular adhesin (PIA) protects Staphylococcus epidermidis against major components of the human innate immune system.
3554	DRAMP03317	Cathelicidin-related antimicrobial peptide (AMPs)	"Cathelicidin-related antimicrobial peptide"[All Fields] AND biofilm[All Fields]	Cathelicidin-related antimicrobial peptide	33253855	Effects of a novel anti-biofilm peptide CRAMP combined with antibiotics on the formation of Pseudomonas aeruginosa biofilms.
3554	DRAMP03317	Cathelicidin-related antimicrobial peptide (AMPs)	"Cathelicidin-related antimicrobial peptide"[All Fields] AND biofilm[All Fields]	Cathelicidin-related antimicrobial peptide	24982087	Derivatives of the mouse cathelicidin-related antimicrobial peptide (CRAMP) inhibit fungal and bacterial biofilm formation.
3600	DRAMP03364	Hepcidin (mammals, rodents, animals)	"Hepcidin"[All Fields] AND biofilm[All Fields]	Hepcidin	30456212	Quantification of Lipoteichoic Acid in Hemodialysis Patients With Central Venous Catheters.
3600	DRAMP03364	Hepcidin (mammals, rodents, animals)	"Hepcidin"[All Fields] AND biofilm[All Fields]	Hepcidin	30408337	Silk-Based Antimicrobial Polymers as a New Platform to Design Drug-Free Materials to Impede Microbial Infections.
3600	DRAMP03364	Hepcidin (mammals, rodents, animals)	"Hepcidin"[All Fields] AND biofilm[All Fields]	Hepcidin	24645694	Inhibitory effect of the human liver-derived antimicrobial peptide hepcidin 20 on biofilms of polysaccharide intercellular adhesin (PIA)-positive and PIA-negative strains of Staphylococcus epidermidis.
3602	DRAMP03366	Beta-defensin 1 (BD-1; mBD-1; Defensin, beta 1; Rodents, mammals, animals)	"Beta-defensin 1"[All Fields] AND biofilm[All Fields]	Beta-defensin 1	33420317	Curbing gastrointestinal infections by defensin fragment modifications without harming commensal microbiota.
3602	DRAMP03366	Beta-defensin 1 (BD-1; mBD-1; Defensin, beta 1; Rodents, mammals, animals)	"Beta-defensin 1"[All Fields] AND biofilm[All Fields]	Beta-defensin 1	32585445	Glucose effect on Candida albicans biofilm during tissue invasion.
3602	DRAMP03366	Beta-defensin 1 (BD-1; mBD-1; Defensin, beta 1; Rodents, mammals, animals)	"Beta-defensin 1"[All Fields] AND biofilm[All Fields]	Beta-defensin 1	30260708	Stabilized collagen matrix dressing improves wound macrophage function and epithelialization.
3602	DRAMP03366	Beta-defensin 1 (BD-1; mBD-1; Defensin, beta 1; Rodents, mammals, animals)	"Beta-defensin 1"[All Fields] AND biofilm[All Fields]	Beta-defensin 1	28642103	Chimeric analogs of human β-defensin 1 and θ-defensin disrupt pre-established bacterial biofilms.

3602	DRAMP03366	Beta-defensin 1 (BD-1; mBD-1; Defensin, beta 1; Rodents, mammals, animals)	"Beta-defensin 1"[All Fields] AND biofilm[All Fields]	Beta-defensin 1	26214284	α-tocopherol decreases interleukin-1β and -6 and increases human β-defensin-1 and -2 secretion in human gingival fibroblasts stimulated with Porphyromonas gingivalis lipopolysaccharide.
3602	DRAMP03366	Beta-defensin 1 (BD-1; mBD-1; Defensin, beta 1; Rodents, mammals, animals)	"Beta-defensin 1"[All Fields] AND biofilm[All Fields]	Beta-defensin 1	22394470	Association of CiaRH with resistance of Streptococcus mutans to antimicrobial peptides in biofilms.
3602	DRAMP03366	Beta-defensin 1 (BD-1; mBD-1; Defensin, beta 1; Rodents, mammals, animals)	"Beta-defensin 1"[All Fields] AND biofilm[All Fields]	Beta-defensin 1	20454633	Normal human gingival epithelial cells sense C. parapsilosis by toll-like receptors and module its pathogenesis through antimicrobial peptides and proinflammatory cytokines.
3603	DRAMP03367	Beta-defensin 2 (BD-2, mBD-2; Defensin, beta 2; Defb2; Rodents, mammals, animals)	"Beta-defensin 2"[All Fields] AND biofilm[All Fields]	Beta-defensin 2	34276631	DNA Blocks the Lethal Effect of Human Beta-Defensin 2 Against Neisseria meningitidis.
3603	DRAMP03367	Beta-defensin 2 (BD-2, mBD-2; Defensin, beta 2; Defb2; Rodents, mammals, animals)	"Beta-defensin 2"[All Fields] AND biofilm[All Fields]	Beta-defensin 2	32842903	Electrospun ZnO/Poly(Vinylidene Fluoride-Trifluoroethylene) Scaffolds for Lung Tissue Engineering.
3603	DRAMP03367	Beta-defensin 2 (BD-2, mBD-2; Defensin, beta 2; Defb2; Rodents, mammals, animals)	"Beta-defensin 2"[All Fields] AND biofilm[All Fields]	Beta-defensin 2	32522780	Controlling the Growth of the Skin Commensal Staphylococcus epidermidis Using d-Alanine Auxotrophy.
3603	DRAMP03367	Beta-defensin 2 (BD-2, mBD-2; Defensin, beta 2; Defb2; Rodents, mammals, animals)	"Beta-defensin 2"[All Fields] AND biofilm[All Fields]	Beta-defensin 2	32457749	The Antimicrobial Peptide Human Beta-Defensin 2 Inhibits Biofilm Production of Pseudomonas aeruginosaWithout Compromising Metabolic Activity.
3603	DRAMP03367	Beta-defensin 2 (BD-2, mBD-2; Defensin, beta 2; Defb2; Rodents, mammals, animals)	"Beta-defensin 2"[All Fields] AND biofilm[All Fields]	Beta-defensin 2	31336838	Candida albicans-Cell Interactions Activate Innate Immune Defense in Human Palate Epithelial Primary Cells via Nitric Oxide (NO) and β-Defensin 2 (hBD-2).
3603	DRAMP03367	Beta-defensin 2 (BD-2, mBD-2; Defensin, beta 2; Defb2; Rodents, mammals, animals)	"Beta-defensin 2"[All Fields] AND biofilm[All Fields]	Beta-defensin 2	30649289	Innate immune components affect growth and virulence traits of bacterial-vaginosis-associated and non-bacterial-vaginosis-associated Gardnerella vaginalis strains similarly.
3603	DRAMP03367	Beta-defensin 2 (BD-2, mBD-2; Defensin, beta 2; Defb2; Rodents, mammals, animals)	"Beta-defensin 2"[All Fields] AND biofilm[All Fields]	Beta-defensin 2	27582732	Efficient Eradication of Mature Pseudomonas aeruginosa Biofilm via Controlled Delivery of Nitric Oxide Combined with Antimicrobial Peptide and Antibiotics.
3603	DRAMP03367	Beta-defensin 2 (BD-2, mBD-2; Defensin, beta 2; Defb2; Rodents, mammals, animals)	"Beta-defensin 2"[All Fields] AND biofilm[All Fields]	Beta-defensin 2	27148195	Effect of Substance P in Staphylococcus aureus and Staphylococcus epidermidis Virulence: Implication for Skin Homeostasis.
3603	DRAMP03367	Beta-defensin 2 (BD-2, mBD-2; Defensin, beta 2; Defb2; Rodents, mammals, animals)	"Beta-defensin 2"[All Fields] AND biofilm[All Fields]	Beta-defensin 2	25808131	Expression of antimicrobial peptides and interleukin-8 during early stages of inflammation: An experimental gingivitis study.
3603	DRAMP03367	Beta-defensin 2 (BD-2, mBD-2; Defensin, beta 2; Defb2; Rodents, mammals, animals)	"Beta-defensin 2"[All Fields] AND biofilm[All Fields]	Beta-defensin 2	24340061	Inflammatory and antimicrobial responses to methicillin-resistant Staphylococcus aureus in an in vitro wound infection model.
3603	DRAMP03367	Beta-defensin 2 (BD-2, mBD-2; Defensin, beta 2; Defb2; Rodents, mammals, animals)	"Beta-defensin 2"[All Fields] AND biofilm[All Fields]	Beta-defensin 2	20378008	Effect of temperature on the shift of Pseudomonas fluorescens from an environmental microorganism to a potential human pathogen.
3603	DRAMP03367	Beta-defensin 2 (BD-2, mBD-2; Defensin, beta 2; Defb2; Rodents, mammals, animals)	"Beta-defensin 2"[All Fields] AND biofilm[All Fields]	Beta-defensin 2	19572896	The immune response of oral epithelial cells induced by single-species and complex naturally formed biofilms.
3603	DRAMP03367	Beta-defensin 2 (BD-2, mBD-2; Defensin, beta 2; Defb2; Rodents, mammals, animals)	"Beta-defensin 2"[All Fields] AND biofilm[All Fields]	Beta-defensin 2	18954353	Treponema denticola does not induce production of common innate immune mediators from primary gingival epithelial cells.
3603	DRAMP03367	Beta-defensin 2 (BD-2, mBD-2; Defensin, beta 2; Defb2; Rodents, mammals, animals)	"Beta-defensin 2"[All Fields] AND biofilm[All Fields]	Beta-defensin 2	18173794	The stage of native biofilm formation determines the gene expression of human beta-defensin-2, psoriasin, ribonuclease 7 and inflammatory mediators: a novel approach for stimulation of keratinocytes with in situ formed biofilms.
3603	DRAMP03367	Beta-defensin 2 (BD-2, mBD-2; Defensin, beta 2; Defb2; Rodents, mammals, animals)	"Beta-defensin 2"[All Fields] AND biofilm[All Fields]	Beta-defensin 2	17434999	Functional analysis of D-alanylation of lipoteichoic acid in the probiotic strain Lactobacillus rhamnosus GG.
3604	DRAMP03368	Beta-defensin 3 (BD-3, mBD-3; Defensin, beta 3; Rodents, mammals, animals)	"Beta-defensin 3"[All Fields] AND biofilm[All Fields]	Beta-defensin 3	34408988	The Anti-Biofilm Efficacy of Caffeic Acid Phenethyl Ester (CAPE) In Vitroand a Murine Model of Oral Candidiasis.
3604	DRAMP03368	Beta-defensin 3 (BD-3, mBD-3; Defensin, beta 3; Rodents, mammals, animals)	"Beta-defensin 3"[All Fields] AND biofilm[All Fields]	Beta-defensin 3	33865931	Identification of a crocodylian β-defensin variant from Alligator mississippiensis with antimicrobial and antibiofilm activity.
3604	DRAMP03368	Beta-defensin 3 (BD-3, mBD-3; Defensin, beta 3; Rodents, mammals, animals)	"Beta-defensin 3"[All Fields] AND biofilm[All Fields]	Beta-defensin 3	32858856	Candida albicansVirulence Factors and Pathogenicity for Endodontic Infections.
3604	DRAMP03368	Beta-defensin 3 (BD-3, mBD-3; Defensin, beta 3; Rodents, mammals, animals)	"Beta-defensin 3"[All Fields] AND biofilm[All Fields]	Beta-defensin 3	32585445	Glucose effect on Candida albicans biofilm during tissue invasion.
3604	DRAMP03368	Beta-defensin 3 (BD-3, mBD-3; Defensin, beta 3; Rodents, mammals, animals)	"Beta-defensin 3"[All Fields] AND biofilm[All Fields]	Beta-defensin 3	31933178	Effects of human β-defensin 3 fused with carbohydrate-binding domain on the function of type III secretion system in Pseudomonas aeruginosa PA14.
3604	DRAMP03368	Beta-defensin 3 (BD-3, mBD-3; Defensin, beta 3; Rodents, mammals, animals)	"Beta-defensin 3"[All Fields] AND biofilm[All Fields]	Beta-defensin 3	30254440	Modification of the surface of titanium with multifunctional chimeric peptides to prevent biofilm formation via inhibition of initial colonizers.
3604	DRAMP03368	Beta-defensin 3 (BD-3, mBD-3; Defensin, beta 3; Rodents, mammals, animals)	"Beta-defensin 3"[All Fields] AND biofilm[All Fields]	Beta-defensin 3	29872295	Antimicrobial peptide-loaded liquid crystalline precursor bioadhesive system for the prevention of dental caries.
3604	DRAMP03368	Beta-defensin 3 (BD-3, mBD-3; Defensin, beta 3; Rodents, mammals, animals)	"Beta-defensin 3"[All Fields] AND biofilm[All Fields]	Beta-defensin 3	29671721	The BceABRS four-component system that is essential for cell envelope stress response is involved in sensing and response to host defence peptides and is required for the biofilm formation and fitness of Streptococcus mutans.
3604	DRAMP03368	Beta-defensin 3 (BD-3, mBD-3; Defensin, beta 3; Rodents, mammals, animals)	"Beta-defensin 3"[All Fields] AND biofilm[All Fields]	Beta-defensin 3	29077172	The significance of HBD-3 and fluorescent composite carriers in the processof bone formation in rats infected with Staphylococcus aureus.
3604	DRAMP03368	Beta-defensin 3 (BD-3, mBD-3; Defensin, beta 3; Rodents, mammals, animals)	"Beta-defensin 3"[All Fields] AND biofilm[All Fields]	Beta-defensin 3	29025642	Engineered chimeric peptides with antimicrobial and titanium-binding functions to inhibit biofilm formation on Ti implants.
3604	DRAMP03368	Beta-defensin 3 (BD-3, mBD-3; Defensin, beta 3; Rodents, mammals, animals)	"Beta-defensin 3"[All Fields] AND biofilm[All Fields]	Beta-defensin 3	28956355	The synthetic human beta-defensin-3 C15 peptide exhibits antimicrobial activity against Streptococcus mutans, both alone and in combination with dental disinfectants.

3604	DRAMP03368	Beta-defensin 3 (BD-3, mBD-3; Defensin, beta 3; Rodents, mammals, animals)	"Beta-defensin 3"[All Fields] AND biofilm[All Fields]	Beta-defensin 3	28951032	Antifungal Effects of Synthetic Human Beta-defensin-3-C15 Peptide on Candida albicans-infected Root Dentin.
3604	DRAMP03368	Beta-defensin 3 (BD-3, mBD-3; Defensin, beta 3; Rodents, mammals, animals)	"Beta-defensin 3"[All Fields] AND biofilm[All Fields]	Beta-defensin 3	28413476	The mechanism of human β-defensin 3 in MRSA-induced infection of implant drug-resistant bacteria biofilm in the mouse tibial bone marrow.
3604	DRAMP03368	Beta-defensin 3 (BD-3, mBD-3; Defensin, beta 3; Rodents, mammals, animals)	"Beta-defensin 3"[All Fields] AND biofilm[All Fields]	Beta-defensin 3	28078813	Host defense peptide-derived privileged scaffolds for anti-infective drug discovery.
3604	DRAMP03368	Beta-defensin 3 (BD-3, mBD-3; Defensin, beta 3; Rodents, mammals, animals)	"Beta-defensin 3"[All Fields] AND biofilm[All Fields]	Beta-defensin 3	27777572	Role of yqiCin the Pathogenicity of Salmonellaand Innate Immune Responses of Human Intestinal Epithelium.
3604	DRAMP03368	Beta-defensin 3 (BD-3, mBD-3; Defensin, beta 3; Rodents, mammals, animals)	"Beta-defensin 3"[All Fields] AND biofilm[All Fields]	Beta-defensin 3	27200276	Antifungal effects of synthetic human β-defensin 3-C15 peptide.
3604	DRAMP03368	Beta-defensin 3 (BD-3, mBD-3; Defensin, beta 3; Rodents, mammals, animals)	"Beta-defensin 3"[All Fields] AND biofilm[All Fields]	Beta-defensin 3	26119274	Ultrasound microbubbles enhance human β-defensin 3 against biofilms.
3604	DRAMP03368	Beta-defensin 3 (BD-3, mBD-3; Defensin, beta 3; Rodents, mammals, animals)	"Beta-defensin 3"[All Fields] AND biofilm[All Fields]	Beta-defensin 3	25862466	The Antibacterial Effects of an Antimicrobial Peptide Human β-Defensin 3 Fused with Carbohydrate-Binding Domain on Pseudomonas aeruginosa PA14.
3604	DRAMP03368	Beta-defensin 3 (BD-3, mBD-3; Defensin, beta 3; Rodents, mammals, animals)	"Beta-defensin 3"[All Fields] AND biofilm[All Fields]	Beta-defensin 3	24913184	Effects of human β-defensin-3 on biofilm formation-regulating genes dltB and icaA in Staphylococcus aureus.
3604	DRAMP03368	Beta-defensin 3 (BD-3, mBD-3; Defensin, beta 3; Rodents, mammals, animals)	"Beta-defensin 3"[All Fields] AND biofilm[All Fields]	Beta-defensin 3	24238461	Antibacterial efficacy of a human β-defensin-3 peptide on multispecies biofilms.
3604	DRAMP03368	Beta-defensin 3 (BD-3, mBD-3; Defensin, beta 3; Rodents, mammals, animals)	"Beta-defensin 3"[All Fields] AND biofilm[All Fields]	Beta-defensin 3	24100890	[Use of recombinant human beta-defensin-3 to evaluate the effect of adhesion of Candida albicans on the surface of soft lining material].
3604	DRAMP03368	Beta-defensin 3 (BD-3, mBD-3; Defensin, beta 3; Rodents, mammals, animals)	"Beta-defensin 3"[All Fields] AND biofilm[All Fields]	Beta-defensin 3	23639356	Human beta-defensin 3: a novel inhibitor of Staphylococcus-produced biofilm production. Commentary on "Human β-defensin 3 inhibits antibiotic-resistant Staphylococcus biofilm formation".
3604	DRAMP03368	Beta-defensin 3 (BD-3, mBD-3; Defensin, beta 3; Rodents, mammals, animals)	"Beta-defensin 3"[All Fields] AND biofilm[All Fields]	Beta-defensin 3	23519963	Ultrasound-targeted microbubble destruction enhances human β-defensin 3 activity against antibiotic-resistant Staphylococcus biofilms.
3604	DRAMP03368	Beta-defensin 3 (BD-3, mBD-3; Defensin, beta 3; Rodents, mammals, animals)	"Beta-defensin 3"[All Fields] AND biofilm[All Fields]	Beta-defensin 3	23273885	Human β-defensin 3 inhibits antibiotic-resistant Staphylococcus biofilm formation.
3604	DRAMP03368	Beta-defensin 3 (BD-3, mBD-3; Defensin, beta 3; Rodents, mammals, animals)	"Beta-defensin 3"[All Fields] AND biofilm[All Fields]	Beta-defensin 3	23078156	Antimicrobial efficacy of a human β-defensin-3 peptide using an Enterococcus faecalis dentine infection model.
3604	DRAMP03368	Beta-defensin 3 (BD-3, mBD-3; Defensin, beta 3; Rodents, mammals, animals)	"Beta-defensin 3"[All Fields] AND biofilm[All Fields]	Beta-defensin 3	22922323	Extracellular DNA within a nontypeable Haemophilus influenzae-induced biofilm binds human beta defensin-3 and reduces its antimicrobial activity.
3604	DRAMP03368	Beta-defensin 3 (BD-3, mBD-3; Defensin, beta 3; Rodents, mammals, animals)	"Beta-defensin 3"[All Fields] AND biofilm[All Fields]	Beta-defensin 3	22229614	Comparison of the effects of human β-defensin 3, vancomycin, and clindamycin on Staphylococcus aureus biofilm formation.
3604	DRAMP03368	Beta-defensin 3 (BD-3, mBD-3; Defensin, beta 3; Rodents, mammals, animals)	"Beta-defensin 3"[All Fields] AND biofilm[All Fields]	Beta-defensin 3	14764110	Polysaccharide intercellular adhesin (PIA) protects Staphylococcus epidermidis against major components of the human innate immune system.
3644	DRAMP03412	Beta-defensin 2	"Beta-defensin 2"[All Fields] AND biofilm[All Fields]	Beta-defensin 2	34276631	DNA Blocks the Lethal Effect of Human Beta-Defensin 2 Against Neisseria meningitidis.
3644	DRAMP03412	Beta-defensin 2	"Beta-defensin 2"[All Fields] AND biofilm[All Fields]	Beta-defensin 2	32842903	Electrospun ZnO/Poly(Vinylidene Fluoride-Trifluoroethylene) Scaffolds for Lung Tissue Engineering.
3644	DRAMP03412	Beta-defensin 2	"Beta-defensin 2"[All Fields] AND biofilm[All Fields]	Beta-defensin 2	32522780	Controlling the Growth of the Skin Commensal Staphylococcus epidermidis Using d-Alanine Auxotrophy.
3644	DRAMP03412	Beta-defensin 2	"Beta-defensin 2"[All Fields] AND biofilm[All Fields]	Beta-defensin 2	32457749	The Antimicrobial Peptide Human Beta-Defensin 2 Inhibits Biofilm Production of Pseudomonas aeruginosaWithout Compromising Metabolic Activity.
3644	DRAMP03412	Beta-defensin 2	"Beta-defensin 2"[All Fields] AND biofilm[All Fields]	Beta-defensin 2	31336838	Candida albicans-Cell Interactions Activate Innate Immune Defense in Human Palate Epithelial Primary Cells via Nitric Oxide (NO) and β-Defensin 2 (hBD-2).
3644	DRAMP03412	Beta-defensin 2	"Beta-defensin 2"[All Fields] AND biofilm[All Fields]	Beta-defensin 2	30649289	Innate immune components affect growth and virulence traits of bacterial-vaginosis-associated and non-bacterial-vaginosis-associated Gardnerella vaginalis strains similarly.
3644	DRAMP03412	Beta-defensin 2	"Beta-defensin 2"[All Fields] AND biofilm[All Fields]	Beta-defensin 2	27582732	Efficient Eradication of Mature Pseudomonas aeruginosa Biofilm via Controlled Delivery of Nitric Oxide Combined with Antimicrobial Peptide and Antibiotics.
3644	DRAMP03412	Beta-defensin 2	"Beta-defensin 2"[All Fields] AND biofilm[All Fields]	Beta-defensin 2	27148195	Effect of Substance P in Staphylococcus aureus and Staphylococcus epidermidis Virulence: Implication for Skin Homeostasis.
3644	DRAMP03412	Beta-defensin 2	"Beta-defensin 2"[All Fields] AND biofilm[All Fields]	Beta-defensin 2	25808131	Expression of antimicrobial peptides and interleukin-8 during early stages of inflammation: An experimental gingivitis study.
3644	DRAMP03412	Beta-defensin 2	"Beta-defensin 2"[All Fields] AND biofilm[All Fields]	Beta-defensin 2	24340061	Inflammatory and antimicrobial responses to methicillin-resistant Staphylococcus aureus in an in vitro wound infection model.
3644	DRAMP03412	Beta-defensin 2	"Beta-defensin 2"[All Fields] AND biofilm[All Fields]	Beta-defensin 2	20378008	Effect of temperature on the shift of Pseudomonas fluorescens from an environmental microorganism to a potential human pathogen.
3644	DRAMP03412	Beta-defensin 2	"Beta-defensin 2"[All Fields] AND biofilm[All Fields]	Beta-defensin 2	19572896	The immune response of oral epithelial cells induced by single-species and complex naturally formed biofilms.
3644	DRAMP03412	Beta-defensin 2	"Beta-defensin 2"[All Fields] AND biofilm[All Fields]	Beta-defensin 2	18954353	Treponema denticola does not induce production of common innate immune mediators from primary gingival epithelial cells.
3644	DRAMP03412	Beta-defensin 2	"Beta-defensin 2"[All Fields] AND biofilm[All Fields]	Beta-defensin 2	18173794	The stage of native biofilm formation determines the gene expression of human beta-defensin-2, psoriasin, ribonuclease 7 and inflammatory mediators: a novel approach for stimulation of keratinocytes with in situ formed biofilms.
3644	DRAMP03412	Beta-defensin 2	"Beta-defensin 2"[All Fields] AND biofilm[All Fields]	Beta-defensin 2	17434999	Functional analysis of D-alanylation of lipoteichoic acid in the probiotic strain Lactobacillus rhamnosus GG.
3650	DRAMP03418	Hepcidin (mammals, rodents, animals)	"Hepcidin"[All Fields] AND biofilm[All Fields]	Hepcidin	30456212	Quantification of Lipoteichoic Acid in Hemodialysis Patients With Central Venous Catheters.
3650	DRAMP03418	Hepcidin (mammals, rodents, animals)	"Hepcidin"[All Fields] AND biofilm[All Fields]	Hepcidin	30408337	Silk-Based Antimicrobial Polymers as a New Platform to Design Drug-Free Materials to Impede Microbial Infections.
3650	DRAMP03418	Hepcidin (mammals, rodents, animals)	"Hepcidin"[All Fields] AND biofilm[All Fields]	Hepcidin	24645694	Inhibitory effect of the human liver-derived antimicrobial peptide hepcidin 20 on biofilms of polysaccharide intercellular adhesin (PIA)-positive and PIA-negative strains of Staphylococcus epidermidis.
3654	DRAMP03424	Beta-defensin 1 (BD-1, RBD-1; Defensin, beta 1; Rodents, mammals, animals)	"Beta-defensin 1"[All Fields] AND biofilm[All Fields]	Beta-defensin 1	33420317	Curbing gastrointestinal infections by defensin fragment modifications without harming commensal microbiota.
3654	DRAMP03424	Beta-defensin 1 (BD-1, RBD-1; Defensin, beta 1; Rodents, mammals, animals)	"Beta-defensin 1"[All Fields] AND biofilm[All Fields]	Beta-defensin 1	32585445	Glucose effect on Candida albicans biofilm during tissue invasion.
3654	DRAMP03424	Beta-defensin 1 (BD-1, RBD-1; Defensin, beta 1; Rodents, mammals, animals)	"Beta-defensin 1"[All Fields] AND biofilm[All Fields]	Beta-defensin 1	30260708	Stabilized collagen matrix dressing improves wound macrophage function and epithelialization.

3654	DRAMP03424	Beta-defensin 1 (BD-1, RBD-1; Defensin, beta 1; Rodents, mammals, animals)	"Beta-defensin 1"[All Fields] AND biofilm[All Fields]	Beta-defensin 1	28642103	Chimeric analogs of human β-defensin 1 and θ-defensin disrupt pre-established bacterial biofilms.
3654	DRAMP03424	Beta-defensin 1 (BD-1, RBD-1; Defensin, beta 1; Rodents, mammals, animals)	"Beta-defensin 1"[All Fields] AND biofilm[All Fields]	Beta-defensin 1	26214284	α-tocopherol decreases interleukin-1β and -6 and increases human β-defensin-1 and -2 secretion in human gingival fibroblasts stimulated with Porphyromonas gingivalis lipopolysaccharide.
3654	DRAMP03424	Beta-defensin 1 (BD-1, RBD-1; Defensin, beta 1; Rodents, mammals, animals)	"Beta-defensin 1"[All Fields] AND biofilm[All Fields]	Beta-defensin 1	22394470	Association of CiaRH with resistance of Streptococcus mutans to antimicrobial peptides in biofilms.
3654	DRAMP03424	Beta-defensin 1 (BD-1, RBD-1; Defensin, beta 1; Rodents, mammals, animals)	"Beta-defensin 1"[All Fields] AND biofilm[All Fields]	Beta-defensin 1	20454633	Normal human gingival epithelial cells sense C. parapsilosis by toll-like receptors and module its pathogenesis through antimicrobial peptides and proinflammatory cytokines.
3655	DRAMP03425	Beta-defensin 3 (BD-3, RBD-3; Defensin, beta 3; Rodents, mammals, animals)	"Beta-defensin 3"[All Fields] AND biofilm[All Fields]	Beta-defensin 3	34408988	The Anti-Biofilm Efficacy of Caffeic Acid Phenethyl Ester (CAPE) In Vitroand a Murine Model of Oral Candidiasis.
3655	DRAMP03425	Beta-defensin 3 (BD-3, RBD-3; Defensin, beta 3; Rodents, mammals, animals)	"Beta-defensin 3"[All Fields] AND biofilm[All Fields]	Beta-defensin 3	33865931	Identification of a crocodylian β-defensin variant from Alligator mississippiensis with antimicrobial and antibiofilm activity.
3655	DRAMP03425	Beta-defensin 3 (BD-3, RBD-3; Defensin, beta 3; Rodents, mammals, animals)	"Beta-defensin 3"[All Fields] AND biofilm[All Fields]	Beta-defensin 3	32858856	Candida albicansVirulence Factors and Pathogenicity for Endodontic Infections.
3655	DRAMP03425	Beta-defensin 3 (BD-3, RBD-3; Defensin, beta 3; Rodents, mammals, animals)	"Beta-defensin 3"[All Fields] AND biofilm[All Fields]	Beta-defensin 3	32585445	Glucose effect on Candida albicans biofilm during tissue invasion.
3655	DRAMP03425	Beta-defensin 3 (BD-3, RBD-3; Defensin, beta 3; Rodents, mammals, animals)	"Beta-defensin 3"[All Fields] AND biofilm[All Fields]	Beta-defensin 3	31933178	Effects of human β-defensin 3 fused with carbohydrate-binding domain on the function of type III secretion system in Pseudomonas aeruginosa PA14.
3655	DRAMP03425	Beta-defensin 3 (BD-3, RBD-3; Defensin, beta 3; Rodents, mammals, animals)	"Beta-defensin 3"[All Fields] AND biofilm[All Fields]	Beta-defensin 3	30254440	Modification of the surface of titanium with multifunctional chimeric peptides to prevent biofilm formation via inhibition of initial colonizers.
3655	DRAMP03425	Beta-defensin 3 (BD-3, RBD-3; Defensin, beta 3; Rodents, mammals, animals)	"Beta-defensin 3"[All Fields] AND biofilm[All Fields]	Beta-defensin 3	29872295	Antimicrobial peptide-loaded liquid crystalline precursor bioadhesive system for the prevention of dental caries.
3655	DRAMP03425	Beta-defensin 3 (BD-3, RBD-3; Defensin, beta 3; Rodents, mammals, animals)	"Beta-defensin 3"[All Fields] AND biofilm[All Fields]	Beta-defensin 3	29671721	The BceABRS four-component system that is essential for cell envelope stress response is involved in sensing and response to host defence peptides and is required for the biofilm formation and fitness of Streptococcus mutans.
3655	DRAMP03425	Beta-defensin 3 (BD-3, RBD-3; Defensin, beta 3; Rodents, mammals, animals)	"Beta-defensin 3"[All Fields] AND biofilm[All Fields]	Beta-defensin 3	29077172	The significance of HBD-3 and fluorescent composite carriers in the processof bone formation in rats infected with Staphylococcus aureus.
3655	DRAMP03425	Beta-defensin 3 (BD-3, RBD-3; Defensin, beta 3; Rodents, mammals, animals)	"Beta-defensin 3"[All Fields] AND biofilm[All Fields]	Beta-defensin 3	29025642	Engineered chimeric peptides with antimicrobial and titanium-binding functions to inhibit biofilm formation on Ti implants.
3655	DRAMP03425	Beta-defensin 3 (BD-3, RBD-3; Defensin, beta 3; Rodents, mammals, animals)	"Beta-defensin 3"[All Fields] AND biofilm[All Fields]	Beta-defensin 3	28956355	The synthetic human beta-defensin-3 C15 peptide exhibits antimicrobial activity against Streptococcus mutans, both alone and in combination with dental disinfectants.
3655	DRAMP03425	Beta-defensin 3 (BD-3, RBD-3; Defensin, beta 3; Rodents, mammals, animals)	"Beta-defensin 3"[All Fields] AND biofilm[All Fields]	Beta-defensin 3	28951032	Antifungal Effects of Synthetic Human Beta-defensin-3-C15 Peptide on Candida albicans-infected Root Dentin.
3655	DRAMP03425	Beta-defensin 3 (BD-3, RBD-3; Defensin, beta 3; Rodents, mammals, animals)	"Beta-defensin 3"[All Fields] AND biofilm[All Fields]	Beta-defensin 3	28413476	The mechanism of human β-defensin 3 in MRSA-induced infection of implant drug-resistant bacteria biofilm in the mouse tibial bone marrow.
3655	DRAMP03425	Beta-defensin 3 (BD-3, RBD-3; Defensin, beta 3; Rodents, mammals, animals)	"Beta-defensin 3"[All Fields] AND biofilm[All Fields]	Beta-defensin 3	28078813	Host defense peptide-derived privileged scaffolds for anti-infective drug discovery.
3655	DRAMP03425	Beta-defensin 3 (BD-3, RBD-3; Defensin, beta 3; Rodents, mammals, animals)	"Beta-defensin 3"[All Fields] AND biofilm[All Fields]	Beta-defensin 3	27777572	Role of yqiCin the Pathogenicity of Salmonellaand Innate Immune Responses of Human Intestinal Epithelium.
3655	DRAMP03425	Beta-defensin 3 (BD-3, RBD-3; Defensin, beta 3; Rodents, mammals, animals)	"Beta-defensin 3"[All Fields] AND biofilm[All Fields]	Beta-defensin 3	27200276	Antifungal effects of synthetic human β-defensin 3-C15 peptide.
3655	DRAMP03425	Beta-defensin 3 (BD-3, RBD-3; Defensin, beta 3; Rodents, mammals, animals)	"Beta-defensin 3"[All Fields] AND biofilm[All Fields]	Beta-defensin 3	26119274	Ultrasound microbubbles enhance human β-defensin 3 against biofilms.
3655	DRAMP03425	Beta-defensin 3 (BD-3, RBD-3; Defensin, beta 3; Rodents, mammals, animals)	"Beta-defensin 3"[All Fields] AND biofilm[All Fields]	Beta-defensin 3	25862466	The Antibacterial Effects of an Antimicrobial Peptide Human β-Defensin 3 Fused with Carbohydrate-Binding Domain on Pseudomonas aeruginosa PA14.
3655	DRAMP03425	Beta-defensin 3 (BD-3, RBD-3; Defensin, beta 3; Rodents, mammals, animals)	"Beta-defensin 3"[All Fields] AND biofilm[All Fields]	Beta-defensin 3	24913184	Effects of human β-defensin-3 on biofilm formation-regulating genes dltB and icaA in Staphylococcus aureus.
3655	DRAMP03425	Beta-defensin 3 (BD-3, RBD-3; Defensin, beta 3; Rodents, mammals, animals)	"Beta-defensin 3"[All Fields] AND biofilm[All Fields]	Beta-defensin 3	24238461	Antibacterial efficacy of a human β-defensin-3 peptide on multispecies biofilms.
3655	DRAMP03425	Beta-defensin 3 (BD-3, RBD-3; Defensin, beta 3; Rodents, mammals, animals)	"Beta-defensin 3"[All Fields] AND biofilm[All Fields]	Beta-defensin 3	24100890	[Use of recombinant human beta-defensin-3 to evaluate the effect of adhesion of Candida albicans on the surface of soft lining material].
3655	DRAMP03425	Beta-defensin 3 (BD-3, RBD-3; Defensin, beta 3; Rodents, mammals, animals)	"Beta-defensin 3"[All Fields] AND biofilm[All Fields]	Beta-defensin 3	23639356	Human beta-defensin 3: a novel inhibitor of Staphylococcus-produced biofilm production. Commentary on "Human β-defensin 3 inhibits antibiotic-resistant Staphylococcus biofilm formation".
3655	DRAMP03425	Beta-defensin 3 (BD-3, RBD-3; Defensin, beta 3; Rodents, mammals, animals)	"Beta-defensin 3"[All Fields] AND biofilm[All Fields]	Beta-defensin 3	23519963	Ultrasound-targeted microbubble destruction enhances human β-defensin 3 activity against antibiotic-resistant Staphylococcus biofilms.
3655	DRAMP03425	Beta-defensin 3 (BD-3, RBD-3; Defensin, beta 3; Rodents, mammals, animals)	"Beta-defensin 3"[All Fields] AND biofilm[All Fields]	Beta-defensin 3	23273885	Human β-defensin 3 inhibits antibiotic-resistant Staphylococcus biofilm formation.
3655	DRAMP03425	Beta-defensin 3 (BD-3, RBD-3; Defensin, beta 3; Rodents, mammals, animals)	"Beta-defensin 3"[All Fields] AND biofilm[All Fields]	Beta-defensin 3	23078156	Antimicrobial efficacy of a human β-defensin-3 peptide using an Enterococcus faecalis dentine infection model.

3655	DRAMP03425	Beta-defensin 3 (BD-3, RBD-3; Defensin, beta 3; Rodents, mammals, animals)	"Beta-defensin 3"[All Fields] AND biofilm[All Fields]	Beta-defensin 3	22922323	Extracellular DNA within a nontypeable Haemophilus influenzae-induced biofilm binds human beta defensin-3 and reduces its antimicrobial activity.
3655	DRAMP03425	Beta-defensin 3 (BD-3, RBD-3; Defensin, beta 3; Rodents, mammals, animals)	"Beta-defensin 3"[All Fields] AND biofilm[All Fields]	Beta-defensin 3	22229614	Comparison of the effects of human β-defensin 3, vancomycin, and clindamycin on Staphylococcus aureus biofilm formation.
3655	DRAMP03425	Beta-defensin 3 (BD-3, RBD-3; Defensin, beta 3; Rodents, mammals, animals)	"Beta-defensin 3"[All Fields] AND biofilm[All Fields]	Beta-defensin 3	14764110	Polysaccharide intercellular adhesin (PIA) protects Staphylococcus epidermidis against major components of the human innate immune system.
3691	DRAMP03461	Defensin 5 (Enteric defensin; RD-5; Rodents, mammals, animals)	"Defensin 5"[All Fields] AND biofilm[All Fields]	Defensin 5	28296382	Human α-Defensin 6: A Small Peptide That Self-Assembles and Protects the Host by Entangling Microbes.
3696	DRAMP03470	Defensin-1 (American oyster defensin, AOD; molluscs, animals)	"Defensin-1"[All Fields] AND biofilm[All Fields]	Defensin-1	34321877	Antibacterial Effect of Honey-Derived Exosomes Containing Antimicrobial Peptides Against Oral Streptococci.
3696	DRAMP03470	Defensin-1 (American oyster defensin, AOD; molluscs, animals)	"Defensin-1"[All Fields] AND biofilm[All Fields]	Defensin-1	33911935	The antibacterial activities of honey.
3696	DRAMP03470	Defensin-1 (American oyster defensin, AOD; molluscs, animals)	"Defensin-1"[All Fields] AND biofilm[All Fields]	Defensin-1	33420317	Curbing gastrointestinal infections by defensin fragment modifications without harming commensal microbiota.
3696	DRAMP03470	Defensin-1 (American oyster defensin, AOD; molluscs, animals)	"Defensin-1"[All Fields] AND biofilm[All Fields]	Defensin-1	32585445	Glucose effect on Candida albicans biofilm during tissue invasion.
3696	DRAMP03470	Defensin-1 (American oyster defensin, AOD; molluscs, animals)	"Defensin-1"[All Fields] AND biofilm[All Fields]	Defensin-1	31729441	Rhesus Theta Defensin 1 Promotes Long Term Survival in Systemic Candidiasis by Host Directed Mechanisms.
3696	DRAMP03470	Defensin-1 (American oyster defensin, AOD; molluscs, animals)	"Defensin-1"[All Fields] AND biofilm[All Fields]	Defensin-1	30260708	Stabilized collagen matrix dressing improves wound macrophage function and epithelialization.
3696	DRAMP03470	Defensin-1 (American oyster defensin, AOD; molluscs, animals)	"Defensin-1"[All Fields] AND biofilm[All Fields]	Defensin-1	29671721	The BceABRS four-component system that is essential for cell envelope stress response is involved in sensing and response to host defence peptides and is required for the biofilm formation and fitness of Streptococcus mutans.
3696	DRAMP03470	Defensin-1 (American oyster defensin, AOD; molluscs, animals)	"Defensin-1"[All Fields] AND biofilm[All Fields]	Defensin-1	28649561	Psd1 Effects on Candida albicansPlanktonic Cells and Biofilms.
3696	DRAMP03470	Defensin-1 (American oyster defensin, AOD; molluscs, animals)	"Defensin-1"[All Fields] AND biofilm[All Fields]	Defensin-1	28642103	Chimeric analogs of human β-defensin 1 and θ-defensin disrupt pre-established bacterial biofilms.
3696	DRAMP03470	Defensin-1 (American oyster defensin, AOD; molluscs, animals)	"Defensin-1"[All Fields] AND biofilm[All Fields]	Defensin-1	26861950	Antibiofilm efficacy of honey and bee-derived defensin-1 on multispecies wound biofilm.
3696	DRAMP03470	Defensin-1 (American oyster defensin, AOD; molluscs, animals)	"Defensin-1"[All Fields] AND biofilm[All Fields]	Defensin-1	26214284	α-tocopherol decreases interleukin-1β and -6 and increases human β-defensin-1 and -2 secretion in human gingival fibroblasts stimulated with Porphyromonas gingivalis lipopolysaccharide.
3696	DRAMP03470	Defensin-1 (American oyster defensin, AOD; molluscs, animals)	"Defensin-1"[All Fields] AND biofilm[All Fields]	Defensin-1	22394470	Association of CiaRH with resistance of Streptococcus mutans to antimicrobial peptides in biofilms.
3696	DRAMP03470	Defensin-1 (American oyster defensin, AOD; molluscs, animals)	"Defensin-1"[All Fields] AND biofilm[All Fields]	Defensin-1	20454633	Normal human gingival epithelial cells sense C. parapsilosis by toll-like receptors and module its pathogenesis through antimicrobial peptides and proinflammatory cytokines.
3714	DRAMP03494	Cecropin (Insects, animals)	"Cecropin"[All Fields] AND biofilm[All Fields]	Cecropin	33401476	Novel Cecropin-4 Derived Peptides against Methicillin-Resistant Staphylococcus aureus.
3714	DRAMP03494	Cecropin (Insects, animals)	"Cecropin"[All Fields] AND biofilm[All Fields]	Cecropin	32203307	Antimicrobial peptide derived from moths can eradicate UPEC biofilms and could offer a novel therapeutic option.
3714	DRAMP03494	Cecropin (Insects, animals)	"Cecropin"[All Fields] AND biofilm[All Fields]	Cecropin	32203127	Antibiofilm activities of ceragenins and antimicrobial peptides against fungal-bacterial mono and multispecies biofilms.
3714	DRAMP03494	Cecropin (Insects, animals)	"Cecropin"[All Fields] AND biofilm[All Fields]	Cecropin	32051417	The insect antimicrobial peptide cecropin A disrupts uropathogenic Escherichia coli biofilms.
3714	DRAMP03494	Cecropin (Insects, animals)	"Cecropin"[All Fields] AND biofilm[All Fields]	Cecropin	31485973	Efficacy of Indolicidin, Cecropin A (1-7)-Melittin (CAMA) and Their Combination Against Biofilm-Forming Multidrug-Resistant Enteroaggregative Escherichia coli.
3714	DRAMP03494	Cecropin (Insects, animals)	"Cecropin"[All Fields] AND biofilm[All Fields]	Cecropin	31363941	Mechanism of action of antimicrobial peptide P5 truncations against Pseudomonas aeruginosa and Staphylococcus aureus.
3714	DRAMP03494	Cecropin (Insects, animals)	"Cecropin"[All Fields] AND biofilm[All Fields]	Cecropin	31057940	On-chip manufacturing of synthetic proteins for point-of-care therapeutics.
3714	DRAMP03494	Cecropin (Insects, animals)	"Cecropin"[All Fields] AND biofilm[All Fields]	Cecropin	30755738	In vitro activities of antimicrobial peptides and ceragenins against Legionella pneumophila.
3714	DRAMP03494	Cecropin (Insects, animals)	"Cecropin"[All Fields] AND biofilm[All Fields]	Cecropin	30131945	The Intestinal Roundworm Ascaris suumReleases Antimicrobial Factors Which Interfere With Bacterial Growth and Biofilm Formation.
3714	DRAMP03494	Cecropin (Insects, animals)	"Cecropin"[All Fields] AND biofilm[All Fields]	Cecropin	28278280	Natural antimicrobial peptide complexes in the fighting of antibiotic resistant biofilms: Calliphora vicina medicinal maggots.
3714	DRAMP03494	Cecropin (Insects, animals)	"Cecropin"[All Fields] AND biofilm[All Fields]	Cecropin	28178190	High Specific Selectivity and Membrane-Active Mechanism of Synthetic Cationic Hybrid Antimicrobial Peptides Based on the Peptide FV7.
3714	DRAMP03494	Cecropin (Insects, animals)	"Cecropin"[All Fields] AND biofilm[All Fields]	Cecropin	25924433	[CecropinA-magainin, a new hybrid antibacterial peptide against meticillin-resistant Staphylococcus aureus].
3714	DRAMP03494	Cecropin (Insects, animals)	"Cecropin"[All Fields] AND biofilm[All Fields]	Cecropin	25285879	Inhibition and destruction of Pseudomonas aeruginosa biofilms by antibiotics and antimicrobial peptides.
3714	DRAMP03494	Cecropin (Insects, animals)	"Cecropin"[All Fields] AND biofilm[All Fields]	Cecropin	23988790	In vitro pharmacokinetics of antimicrobial cationic peptides alone and in combination with antibiotics against methicillin resistant Staphylococcus aureus biofilms.
3714	DRAMP03494	Cecropin (Insects, animals)	"Cecropin"[All Fields] AND biofilm[All Fields]	Cecropin	23732798	Surface functionalization of titanium substrates with cecropin B to improve their cytocompatibility and reduce inflammation responses.
3714	DRAMP03494	Cecropin (Insects, animals)	"Cecropin"[All Fields] AND biofilm[All Fields]	Cecropin	23070152	In vitro activities of antibiotics and antimicrobial cationic peptides alone and in combination against methicillin-resistant Staphylococcus aureus biofilms.
3714	DRAMP03494	Cecropin (Insects, animals)	"Cecropin"[All Fields] AND biofilm[All Fields]	Cecropin	21998591	Drosophila melanogaster as an animal model for the study of Pseudomonas aeruginosa biofilm infections in vivo.
3714	DRAMP03494	Cecropin (Insects, animals)	"Cecropin"[All Fields] AND biofilm[All Fields]	Cecropin	15247257	Helix induction in antimicrobial peptides by alginate in biofilms.
3716	DRAMP03496	Cecropin-A (Insects, animals)	"Cecropin-A"[All Fields] AND biofilm[All Fields]	Cecropin-A	32203307	Antimicrobial peptide derived from moths can eradicate UPEC biofilms and could offer a novel therapeutic option.
3716	DRAMP03496	Cecropin-A (Insects, animals)	"Cecropin-A"[All Fields] AND biofilm[All Fields]	Cecropin-A	32203127	Antibiofilm activities of ceragenins and antimicrobial peptides against fungal-bacterial mono and multispecies biofilms.
3716	DRAMP03496	Cecropin-A (Insects, animals)	"Cecropin-A"[All Fields] AND biofilm[All Fields]	Cecropin-A	32051417	The insect antimicrobial peptide cecropin A disrupts uropathogenic Escherichia coli biofilms.
3716	DRAMP03496	Cecropin-A (Insects, animals)	"Cecropin-A"[All Fields] AND biofilm[All Fields]	Cecropin-A	31485973	Efficacy of Indolicidin, Cecropin A (1-7)-Melittin (CAMA) and Their Combination Against Biofilm-Forming Multidrug-Resistant Enteroaggregative Escherichia coli.
3716	DRAMP03496	Cecropin-A (Insects, animals)	"Cecropin-A"[All Fields] AND biofilm[All Fields]	Cecropin-A	31363941	Mechanism of action of antimicrobial peptide P5 truncations against Pseudomonas aeruginosa and Staphylococcus aureus.
3716	DRAMP03496	Cecropin-A (Insects, animals)	"Cecropin-A"[All Fields] AND biofilm[All Fields]	Cecropin-A	30755738	In vitro activities of antimicrobial peptides and ceragenins against Legionella pneumophila.
3716	DRAMP03496	Cecropin-A (Insects, animals)	"Cecropin-A"[All Fields] AND biofilm[All Fields]	Cecropin-A	28178190	High Specific Selectivity and Membrane-Active Mechanism of Synthetic Cationic Hybrid Antimicrobial Peptides Based on the Peptide FV7.
3716	DRAMP03496	Cecropin-A (Insects, animals)	"Cecropin-A"[All Fields] AND biofilm[All Fields]	Cecropin-A	25924433	[CecropinA-magainin, a new hybrid antibacterial peptide against meticillin-resistant Staphylococcus aureus].
3716	DRAMP03496	Cecropin-A (Insects, animals)	"Cecropin-A"[All Fields] AND biofilm[All Fields]	Cecropin-A	23070152	In vitro activities of antibiotics and antimicrobial cationic peptides alone and in combination against methicillin-resistant Staphylococcus aureus biofilms.
3717	DRAMP03497	Cecropin-B (Insects, animals)	"Cecropin-B"[All Fields] AND biofilm[All Fields]	Cecropin-B	31057940	On-chip manufacturing of synthetic proteins for point-of-care therapeutics.
3717	DRAMP03497	Cecropin-B (Insects, animals)	"Cecropin-B"[All Fields] AND biofilm[All Fields]	Cecropin-B	23732798	Surface functionalization of titanium substrates with cecropin B to improve their cytocompatibility and reduce inflammation responses.
3727	DRAMP03509	Cecropin-A (Insects, animals)	"Cecropin-A"[All Fields] AND biofilm[All Fields]	Cecropin-A	32203307	Antimicrobial peptide derived from moths can eradicate UPEC biofilms and could offer a novel therapeutic option.
3727	DRAMP03509	Cecropin-A (Insects, animals)	"Cecropin-A"[All Fields] AND biofilm[All Fields]	Cecropin-A	32203127	Antibiofilm activities of ceragenins and antimicrobial peptides against fungal-bacterial mono and multispecies biofilms.
3727	DRAMP03509	Cecropin-A (Insects, animals)	"Cecropin-A"[All Fields] AND biofilm[All Fields]	Cecropin-A	32051417	The insect antimicrobial peptide cecropin A disrupts uropathogenic Escherichia coli biofilms.

3727	DRAMP03509	Cecropin-A (Insects, animals)	"Cecropin-A"[All Fields] AND biofilm[All Fields]	Cecropin-A	31485973	Efficacy of Indolicidin, Cecropin A (1-7)-Melittin (CAMA) and Their Combination Against Biofilm-Forming Multidrug-Resistant Enteroaggregative Escherichia coli.
3727	DRAMP03509	Cecropin-A (Insects, animals)	"Cecropin-A"[All Fields] AND biofilm[All Fields]	Cecropin-A	31363941	Mechanism of action of antimicrobial peptide P5 truncations against Pseudomonas aeruginosa and Staphylococcus aureus.
3727	DRAMP03509	Cecropin-A (Insects, animals)	"Cecropin-A"[All Fields] AND biofilm[All Fields]	Cecropin-A	30755738	In vitro activities of antimicrobial peptides and ceragenins against Legionella pneumophila.
3727	DRAMP03509	Cecropin-A (Insects, animals)	"Cecropin-A"[All Fields] AND biofilm[All Fields]	Cecropin-A	28178190	High Specific Selectivity and Membrane-Active Mechanism of Synthetic Cationic Hybrid Antimicrobial Peptides Based on the Peptide FV7.
3727	DRAMP03509	Cecropin-A (Insects, animals)	"Cecropin-A"[All Fields] AND biofilm[All Fields]	Cecropin-A	25924433	[CecropinA-magainin, a new hybrid antibacterial peptide against meticillin-resistant Staphylococcus aureus].
3727	DRAMP03509	Cecropin-A (Insects, animals)	"Cecropin-A"[All Fields] AND biofilm[All Fields]	Cecropin-A	23070152	In vitro activities of antibiotics and antimicrobial cationic peptides alone and in combination against methicillin-resistant Staphylococcus aureus biofilms.
3728	DRAMP03510	Cecropin-A (Insects, animals)	"Cecropin-A"[All Fields] AND biofilm[All Fields]	Cecropin-A	32203307	Antimicrobial peptide derived from moths can eradicate UPEC biofilms and could offer a novel therapeutic option.
3728	DRAMP03510	Cecropin-A (Insects, animals)	"Cecropin-A"[All Fields] AND biofilm[All Fields]	Cecropin-A	32203127	Antibiofilm activities of ceragenins and antimicrobial peptides against fungal-bacterial mono and multispecies biofilms.
3728	DRAMP03510	Cecropin-A (Insects, animals)	"Cecropin-A"[All Fields] AND biofilm[All Fields]	Cecropin-A	32051417	The insect antimicrobial peptide cecropin A disrupts uropathogenic Escherichia coli biofilms.
3728	DRAMP03510	Cecropin-A (Insects, animals)	"Cecropin-A"[All Fields] AND biofilm[All Fields]	Cecropin-A	31485973	Efficacy of Indolicidin, Cecropin A (1-7)-Melittin (CAMA) and Their Combination Against Biofilm-Forming Multidrug-Resistant Enteroaggregative Escherichia coli.
3728	DRAMP03510	Cecropin-A (Insects, animals)	"Cecropin-A"[All Fields] AND biofilm[All Fields]	Cecropin-A	31363941	Mechanism of action of antimicrobial peptide P5 truncations against Pseudomonas aeruginosa and Staphylococcus aureus.
3728	DRAMP03510	Cecropin-A (Insects, animals)	"Cecropin-A"[All Fields] AND biofilm[All Fields]	Cecropin-A	30755738	In vitro activities of antimicrobial peptides and ceragenins against Legionella pneumophila.
3728	DRAMP03510	Cecropin-A (Insects, animals)	"Cecropin-A"[All Fields] AND biofilm[All Fields]	Cecropin-A	28178190	High Specific Selectivity and Membrane-Active Mechanism of Synthetic Cationic Hybrid Antimicrobial Peptides Based on the Peptide FV7.
3728	DRAMP03510	Cecropin-A (Insects, animals)	"Cecropin-A"[All Fields] AND biofilm[All Fields]	Cecropin-A	25924433	[CecropinA-magainin, a new hybrid antibacterial peptide against meticillin-resistant Staphylococcus aureus].
3728	DRAMP03510	Cecropin-A (Insects, animals)	"Cecropin-A"[All Fields] AND biofilm[All Fields]	Cecropin-A	23070152	In vitro activities of antibiotics and antimicrobial cationic peptides alone and in combination against methicillin-resistant Staphylococcus aureus biofilms.
3729	DRAMP03511	Cecropin-B (Immune protein P9; Insects, animals)	"Cecropin-B"[All Fields] AND biofilm[All Fields]	Cecropin-B	31057940	On-chip manufacturing of synthetic proteins for point-of-care therapeutics.
3729	DRAMP03511	Cecropin-B (Immune protein P9; Insects, animals)	"Cecropin-B"[All Fields] AND biofilm[All Fields]	Cecropin-B	23732798	Surface functionalization of titanium substrates with cecropin B to improve their cytocompatibility and reduce inflammation responses.
3731	DRAMP03522	Gallerimycin (defensins; Insects, animals)	"Gallerimycin"[All Fields] AND biofilm[All Fields]	Gallerimycin	34408988	The Anti-Biofilm Efficacy of Caffeic Acid Phenethyl Ester (CAPE) In Vitroand a Murine Model of Oral Candidiasis.
3732	DRAMP03529	Cecropin-A (Insects, animals)	"Cecropin-A"[All Fields] AND biofilm[All Fields]	Cecropin-A	32203307	Antimicrobial peptide derived from moths can eradicate UPEC biofilms and could offer a novel therapeutic option.
3732	DRAMP03529	Cecropin-A (Insects, animals)	"Cecropin-A"[All Fields] AND biofilm[All Fields]	Cecropin-A	32203127	Antibiofilm activities of ceragenins and antimicrobial peptides against fungal-bacterial mono and multispecies biofilms.
3732	DRAMP03529	Cecropin-A (Insects, animals)	"Cecropin-A"[All Fields] AND biofilm[All Fields]	Cecropin-A	32051417	The insect antimicrobial peptide cecropin A disrupts uropathogenic Escherichia coli biofilms.
3732	DRAMP03529	Cecropin-A (Insects, animals)	"Cecropin-A"[All Fields] AND biofilm[All Fields]	Cecropin-A	31485973	Efficacy of Indolicidin, Cecropin A (1-7)-Melittin (CAMA) and Their Combination Against Biofilm-Forming Multidrug-Resistant Enteroaggregative Escherichia coli.
3732	DRAMP03529	Cecropin-A (Insects, animals)	"Cecropin-A"[All Fields] AND biofilm[All Fields]	Cecropin-A	31363941	Mechanism of action of antimicrobial peptide P5 truncations against Pseudomonas aeruginosa and Staphylococcus aureus.
3732	DRAMP03529	Cecropin-A (Insects, animals)	"Cecropin-A"[All Fields] AND biofilm[All Fields]	Cecropin-A	30755738	In vitro activities of antimicrobial peptides and ceragenins against Legionella pneumophila.
3732	DRAMP03529	Cecropin-A (Insects, animals)	"Cecropin-A"[All Fields] AND biofilm[All Fields]	Cecropin-A	28178190	High Specific Selectivity and Membrane-Active Mechanism of Synthetic Cationic Hybrid Antimicrobial Peptides Based on the Peptide FV7.
3732	DRAMP03529	Cecropin-A (Insects, animals)	"Cecropin-A"[All Fields] AND biofilm[All Fields]	Cecropin-A	25924433	[CecropinA-magainin, a new hybrid antibacterial peptide against meticillin-resistant Staphylococcus aureus].
3732	DRAMP03529	Cecropin-A (Insects, animals)	"Cecropin-A"[All Fields] AND biofilm[All Fields]	Cecropin-A	23070152	In vitro activities of antibiotics and antimicrobial cationic peptides alone and in combination against methicillin-resistant Staphylococcus aureus biofilms.
3733	DRAMP03530	Cecropin-B (Lepidopteran-A/B; Insects, animals)	"Cecropin-B"[All Fields] AND biofilm[All Fields]	Cecropin-B	31057940	On-chip manufacturing of synthetic proteins for point-of-care therapeutics.
3733	DRAMP03530	Cecropin-B (Lepidopteran-A/B; Insects, animals)	"Cecropin-B"[All Fields] AND biofilm[All Fields]	Cecropin-B	23732798	Surface functionalization of titanium substrates with cecropin B to improve their cytocompatibility and reduce inflammation responses.
3741	DRAMP03538	Cecropin (Antibacterial peptide CM-IV; Insects, animals)	"Cecropin"[All Fields] AND biofilm[All Fields]	Cecropin	33401476	Novel Cecropin-4 Derived Peptides against Methicillin-Resistant Staphylococcus aureus.
3741	DRAMP03538	Cecropin (Antibacterial peptide CM-IV; Insects, animals)	"Cecropin"[All Fields] AND biofilm[All Fields]	Cecropin	32203307	Antimicrobial peptide derived from moths can eradicate UPEC biofilms and could offer a novel therapeutic option.
3741	DRAMP03538	Cecropin (Antibacterial peptide CM-IV; Insects, animals)	"Cecropin"[All Fields] AND biofilm[All Fields]	Cecropin	32203127	Antibiofilm activities of ceragenins and antimicrobial peptides against fungal-bacterial mono and multispecies biofilms.
3741	DRAMP03538	Cecropin (Antibacterial peptide CM-IV; Insects, animals)	"Cecropin"[All Fields] AND biofilm[All Fields]	Cecropin	32051417	The insect antimicrobial peptide cecropin A disrupts uropathogenic Escherichia coli biofilms.
3741	DRAMP03538	Cecropin (Antibacterial peptide CM-IV; Insects, animals)	"Cecropin"[All Fields] AND biofilm[All Fields]	Cecropin	31485973	Efficacy of Indolicidin, Cecropin A (1-7)-Melittin (CAMA) and Their Combination Against Biofilm-Forming Multidrug-Resistant Enteroaggregative Escherichia coli.
3741	DRAMP03538	Cecropin (Antibacterial peptide CM-IV; Insects, animals)	"Cecropin"[All Fields] AND biofilm[All Fields]	Cecropin	31363941	Mechanism of action of antimicrobial peptide P5 truncations against Pseudomonas aeruginosa and Staphylococcus aureus.
3741	DRAMP03538	Cecropin (Antibacterial peptide CM-IV; Insects, animals)	"Cecropin"[All Fields] AND biofilm[All Fields]	Cecropin	31057940	On-chip manufacturing of synthetic proteins for point-of-care therapeutics.
3741	DRAMP03538	Cecropin (Antibacterial peptide CM-IV; Insects, animals)	"Cecropin"[All Fields] AND biofilm[All Fields]	Cecropin	30755738	In vitro activities of antimicrobial peptides and ceragenins against Legionella pneumophila.
3741	DRAMP03538	Cecropin (Antibacterial peptide CM-IV; Insects, animals)	"Cecropin"[All Fields] AND biofilm[All Fields]	Cecropin	30131945	The Intestinal Roundworm Ascaris suumReleases Antimicrobial Factors Which Interfere With Bacterial Growth and Biofilm Formation.
3741	DRAMP03538	Cecropin (Antibacterial peptide CM-IV; Insects, animals)	"Cecropin"[All Fields] AND biofilm[All Fields]	Cecropin	28278280	Natural antimicrobial peptide complexes in the fighting of antibiotic resistant biofilms: Calliphora vicina medicinal maggots.
3741	DRAMP03538	Cecropin (Antibacterial peptide CM-IV; Insects, animals)	"Cecropin"[All Fields] AND biofilm[All Fields]	Cecropin	28178190	High Specific Selectivity and Membrane-Active Mechanism of Synthetic Cationic Hybrid Antimicrobial Peptides Based on the Peptide FV7.
3741	DRAMP03538	Cecropin (Antibacterial peptide CM-IV; Insects, animals)	"Cecropin"[All Fields] AND biofilm[All Fields]	Cecropin	25924433	[CecropinA-magainin, a new hybrid antibacterial peptide against meticillin-resistant Staphylococcus aureus].
3741	DRAMP03538	Cecropin (Antibacterial peptide CM-IV; Insects, animals)	"Cecropin"[All Fields] AND biofilm[All Fields]	Cecropin	25285879	Inhibition and destruction of Pseudomonas aeruginosa biofilms by antibiotics and antimicrobial peptides.
3741	DRAMP03538	Cecropin (Antibacterial peptide CM-IV; Insects, animals)	"Cecropin"[All Fields] AND biofilm[All Fields]	Cecropin	23988790	In vitro pharmacokinetics of antimicrobial cationic peptides alone and in combination with antibiotics against methicillin resistant Staphylococcus aureus biofilms.
3741	DRAMP03538	Cecropin (Antibacterial peptide CM-IV; Insects, animals)	"Cecropin"[All Fields] AND biofilm[All Fields]	Cecropin	23732798	Surface functionalization of titanium substrates with cecropin B to improve their cytocompatibility and reduce inflammation responses.
3741	DRAMP03538	Cecropin (Antibacterial peptide CM-IV; Insects, animals)	"Cecropin"[All Fields] AND biofilm[All Fields]	Cecropin	23070152	In vitro activities of antibiotics and antimicrobial cationic peptides alone and in combination against methicillin-resistant Staphylococcus aureus biofilms.
3741	DRAMP03538	Cecropin (Antibacterial peptide CM-IV; Insects, animals)	"Cecropin"[All Fields] AND biofilm[All Fields]	Cecropin	21998591	Drosophila melanogaster as an animal model for the study of Pseudomonas aeruginosa biofilm infections in vivo.
3741	DRAMP03538	Cecropin (Antibacterial peptide CM-IV; Insects, animals)	"Cecropin"[All Fields] AND biofilm[All Fields]	Cecropin	15247257	Helix induction in antimicrobial peptides by alginate in biofilms.
3742	DRAMP03554	CCL20(1-67) (Human, mammals, animals)	"CCL20"[All Fields] AND biofilm[All Fields]	CCL20	33772282	Novel application of bergapten and quercetin with anti-bacterial, osteogenesis-potentiating, and anti-inflammation tri-effects.
3742	DRAMP03554	CCL20(1-67) (Human, mammals, animals)	"CCL20"[All Fields] AND biofilm[All Fields]	CCL20	32329166	Early host-microbe interaction in a peri-implant oral mucosa-biofilm model.

3742	DRAMP03554	CCL20(1-67) (Human, mammals, animals)	"CCL20"[All Fields] AND biofilm[All Fields]	CCL20	31703580	Anti-inflammatory effects of olanexidine gluconate on oral epithelial cells.
3742	DRAMP03554	CCL20(1-67) (Human, mammals, animals)	"CCL20"[All Fields] AND biofilm[All Fields]	CCL20	30878664	A Novel Gastric Spheroid Co-culture Model Reveals Chemokine-Dependent Recruitment of Human Dendritic Cells to the Gastric Epithelium.
3742	DRAMP03554	CCL20(1-67) (Human, mammals, animals)	"CCL20"[All Fields] AND biofilm[All Fields]	CCL20	30626048	Expression and Purification of Chemokine MIP-3α (CCL20) through a Calmodulin-Fusion Protein System.
3742	DRAMP03554	CCL20(1-67) (Human, mammals, animals)	"CCL20"[All Fields] AND biofilm[All Fields]	CCL20	30407731	Biofilm-induced profiles of immune response gene expression by oral epithelial cells.
3742	DRAMP03554	CCL20(1-67) (Human, mammals, animals)	"CCL20"[All Fields] AND biofilm[All Fields]	CCL20	30375445	Multi-species oral biofilm promotes reconstructed human gingiva epithelial barrier function.
3742	DRAMP03554	CCL20(1-67) (Human, mammals, animals)	"CCL20"[All Fields] AND biofilm[All Fields]	CCL20	30237466	Impact of Porphyromonas gingivalis Peptidylarginine Deiminase on Bacterial Biofilm Formation, Epithelial Cell Invasion, and Epithelial Cell Transcriptional Landscape.
3742	DRAMP03554	CCL20(1-67) (Human, mammals, animals)	"CCL20"[All Fields] AND biofilm[All Fields]	CCL20	30152021	Environmental lead effects on gene expression in oral epithelial cells.
3742	DRAMP03554	CCL20(1-67) (Human, mammals, animals)	"CCL20"[All Fields] AND biofilm[All Fields]	CCL20	29234378	Antibiofilm and Anti-Inflammatory Activities of Houttuynia cordataDecoction for Oral Care.
3742	DRAMP03554	CCL20(1-67) (Human, mammals, animals)	"CCL20"[All Fields] AND biofilm[All Fields]	CCL20	28892653	Saliva-Derived Commensal and Pathogenic Biofilms in a Human Gingiva Model.
3742	DRAMP03554	CCL20(1-67) (Human, mammals, animals)	"CCL20"[All Fields] AND biofilm[All Fields]	CCL20	27413739	Preventive Effects of Houttuynia cordata Extract for Oral Infectious Diseases.
3742	DRAMP03554	CCL20(1-67) (Human, mammals, animals)	"CCL20"[All Fields] AND biofilm[All Fields]	CCL20	27023331	Anti-bacterial and anti-inflammatory effects of ethanol extract from Houttuynia cordata poultice.
3742	DRAMP03554	CCL20(1-67) (Human, mammals, animals)	"CCL20"[All Fields] AND biofilm[All Fields]	CCL20	25808131	Expression of antimicrobial peptides and interleukin-8 during early stages of inflammation: An experimental gingivitis study.
3742	DRAMP03554	CCL20(1-67) (Human, mammals, animals)	"CCL20"[All Fields] AND biofilm[All Fields]	CCL20	24048904	Increased susceptibility to pulmonary Pseudomonas infection in Splunc1 knockout mice.
3742	DRAMP03554	CCL20(1-67) (Human, mammals, animals)	"CCL20"[All Fields] AND biofilm[All Fields]	CCL20	17355248	Differential activation of NF-kappaB and gene expression in oral epithelial cells by periodontal pathogens.
3743	DRAMP03555	CCL20(2-70) (Human, mammals, animals)	"CCL20"[All Fields] AND biofilm[All Fields]	CCL20	33772282	Novel application of bergapten and quercetin with anti-bacterial, osteogenesis-potentiating, and anti-inflammation tri-effects.
3743	DRAMP03555	CCL20(2-70) (Human, mammals, animals)	"CCL20"[All Fields] AND biofilm[All Fields]	CCL20	32329166	Early host-microbe interaction in a peri-implant oral mucosa-biofilm model.
3743	DRAMP03555	CCL20(2-70) (Human, mammals, animals)	"CCL20"[All Fields] AND biofilm[All Fields]	CCL20	31703580	Anti-inflammatory effects of olanexidine gluconate on oral epithelial cells.
3743	DRAMP03555	CCL20(2-70) (Human, mammals, animals)	"CCL20"[All Fields] AND biofilm[All Fields]	CCL20	30878664	A Novel Gastric Spheroid Co-culture Model Reveals Chemokine-Dependent Recruitment of Human Dendritic Cells to the Gastric Epithelium.
3743	DRAMP03555	CCL20(2-70) (Human, mammals, animals)	"CCL20"[All Fields] AND biofilm[All Fields]	CCL20	30626048	Expression and Purification of Chemokine MIP-3α (CCL20) through a Calmodulin-Fusion Protein System.
3743	DRAMP03555	CCL20(2-70) (Human, mammals, animals)	"CCL20"[All Fields] AND biofilm[All Fields]	CCL20	30407731	Biofilm-induced profiles of immune response gene expression by oral epithelial cells.
3743	DRAMP03555	CCL20(2-70) (Human, mammals, animals)	"CCL20"[All Fields] AND biofilm[All Fields]	CCL20	30375445	Multi-species oral biofilm promotes reconstructed human gingiva epithelial barrier function.
3743	DRAMP03555	CCL20(2-70) (Human, mammals, animals)	"CCL20"[All Fields] AND biofilm[All Fields]	CCL20	30237466	Impact of Porphyromonas gingivalis Peptidylarginine Deiminase on Bacterial Biofilm Formation, Epithelial Cell Invasion, and Epithelial Cell Transcriptional Landscape.
3743	DRAMP03555	CCL20(2-70) (Human, mammals, animals)	"CCL20"[All Fields] AND biofilm[All Fields]	CCL20	30152021	Environmental lead effects on gene expression in oral epithelial cells.
3743	DRAMP03555	CCL20(2-70) (Human, mammals, animals)	"CCL20"[All Fields] AND biofilm[All Fields]	CCL20	29234378	Antibiofilm and Anti-Inflammatory Activities of Houttuynia cordataDecoction for Oral Care.
3743	DRAMP03555	CCL20(2-70) (Human, mammals, animals)	"CCL20"[All Fields] AND biofilm[All Fields]	CCL20	28892653	Saliva-Derived Commensal and Pathogenic Biofilms in a Human Gingiva Model.
3743	DRAMP03555	CCL20(2-70) (Human, mammals, animals)	"CCL20"[All Fields] AND biofilm[All Fields]	CCL20	27413739	Preventive Effects of Houttuynia cordata Extract for Oral Infectious Diseases.
3743	DRAMP03555	CCL20(2-70) (Human, mammals, animals)	"CCL20"[All Fields] AND biofilm[All Fields]	CCL20	27023331	Anti-bacterial and anti-inflammatory effects of ethanol extract from Houttuynia cordata poultice.
3743	DRAMP03555	CCL20(2-70) (Human, mammals, animals)	"CCL20"[All Fields] AND biofilm[All Fields]	CCL20	25808131	Expression of antimicrobial peptides and interleukin-8 during early stages of inflammation: An experimental gingivitis study.
3743	DRAMP03555	CCL20(2-70) (Human, mammals, animals)	"CCL20"[All Fields] AND biofilm[All Fields]	CCL20	24048904	Increased susceptibility to pulmonary Pseudomonas infection in Splunc1 knockout mice.
3743	DRAMP03555	CCL20(2-70) (Human, mammals, animals)	"CCL20"[All Fields] AND biofilm[All Fields]	CCL20	17355248	Differential activation of NF-kappaB and gene expression in oral epithelial cells by periodontal pathogens.
3745	DRAMP03557	Granulysin (Lymphokine LAG-2; Human, mammals, animals)	"Granulysin"[All Fields] AND biofilm[All Fields]	Granulysin	27276051	Antimicrobial Properties of an Immunomodulator - 15 kDa Human Granulysin.
3746	DRAMP03559	CXCL10 (Human, mammals, animals)	"CXCL10"[All Fields] AND biofilm[All Fields]	CXCL10	34306164	Antibacterial and Anti-Inflammatory Potential of Polyherbal Formulation Used in Chronic Wound Healing.
3746	DRAMP03559	CXCL10 (Human, mammals, animals)	"CXCL10"[All Fields] AND biofilm[All Fields]	CXCL10	31125769	The impact of Aggregatibacter actinomycetemcomitans biofilm-derived effectors following antimicrobial photodynamic therapy on cytokine production in human gingival fibroblasts.
3746	DRAMP03559	CXCL10 (Human, mammals, animals)	"CXCL10"[All Fields] AND biofilm[All Fields]	CXCL10	27671889	Antimicrobial activity and regulation of CXCL9 and CXCL10 in oral keratinocytes.
3746	DRAMP03559	CXCL10 (Human, mammals, animals)	"CXCL10"[All Fields] AND biofilm[All Fields]	CXCL10	26755156	The Variable Region of Pneumococcal Pathogenicity Island 1 Is Responsible for Unusually High Virulence of a Serotype 1 Isolate.
3746	DRAMP03559	CXCL10 (Human, mammals, animals)	"CXCL10"[All Fields] AND biofilm[All Fields]	CXCL10	22564230	Deletion of a tumor necrosis superfamily gene in mice leads to impaired healing that mimics chronic wounds in humans.
3746	DRAMP03559	CXCL10 (Human, mammals, animals)	"CXCL10"[All Fields] AND biofilm[All Fields]	CXCL10	19961380	Gingival transcriptome patterns during induction and resolution of experimental gingivitis in humans.
3753	DRAMP03576	Histatin-1 (His-rich; Human, mammals, animals)	"Histatin-1"[All Fields] AND biofilm[All Fields]	Histatin-1	31809901	Proteomic analysis of whole saliva in chronic periodontitis.
3753	DRAMP03576	Histatin-1 (His-rich; Human, mammals, animals)	"Histatin-1"[All Fields] AND biofilm[All Fields]	Histatin-1	29393147	Protein Profile of the Acquired Enamel Pellicle after Rinsing with Whole Milk, Fat-Free Milk, and Water: An in vivo Study.
3753	DRAMP03576	Histatin-1 (His-rich; Human, mammals, animals)	"Histatin-1"[All Fields] AND biofilm[All Fields]	Histatin-1	23844127	Proteomic evaluation of acquired enamel pellicle during in vivo formation.
3753	DRAMP03576	Histatin-1 (His-rich; Human, mammals, animals)	"Histatin-1"[All Fields] AND biofilm[All Fields]	Histatin-1	22870302	Quantitative proteomic analysis of the effect of fluoride on the acquired enamel pellicle.
3757	DRAMP03580	His3-(20-43)-peptide (Histatin 5; derivatives: Dh-5; Clinical)	"His3-"[All Fields] AND biofilm[All Fields]	His3-	25524641	Post-transcriptional regulation of transcript abundance by a conserved member of the tristetraprolin family in Candida albicans.
3803	DRAMP03635	Human lactoferricin (LfcinH; one chain of Lactotransferrin; Human, mammals, animals)	"Human lactoferricin"[All Fields] AND biofilm[All Fields]	Human lactoferricin	34454095	Targeting antibiotic tolerance in anaerobic biofilms associated with oral diseases: Human antimicrobial peptides LL-37 and lactoferricin enhance the antibiotic efficacy of amoxicillin, clindamycin and metronidazole.
3803	DRAMP03635	Human lactoferricin (LfcinH; one chain of Lactotransferrin; Human, mammals, animals)	"Human lactoferricin"[All Fields] AND biofilm[All Fields]	Human lactoferricin	33249255	Inhibitory effect of LL-37 and human lactoferricin on growth and biofilm formation of anaerobes associated with oral diseases.
3803	DRAMP03635	Human lactoferricin (LfcinH; one chain of Lactotransferrin; Human, mammals, animals)	"Human lactoferricin"[All Fields] AND biofilm[All Fields]	Human lactoferricin	32871093	Antibiofilm activity of lactoferrin-derived synthetic peptides against Pseudomonas aeruginosaPAO1.
3803	DRAMP03635	Human lactoferricin (LfcinH; one chain of Lactotransferrin; Human, mammals, animals)	"Human lactoferricin"[All Fields] AND biofilm[All Fields]	Human lactoferricin	26149536	Antimicrobial activity of synthetic cationic peptides and lipopeptides derived from human lactoferricin against Pseudomonas aeruginosa planktonic cultures and biofilms.
3805	DRAMP03637	Thaumatin-like protein (Actc2)	"Thaumatin-like protein"[All Fields] AND biofilm[All Fields]	Thaumatin-like protein	22363222	Tasco®: a product of Ascophyllum nodosum enhances immune response of Caenorhabditis elegans against Pseudomonas aeruginosa infection.
3826	DRAMP03664	L-amino-acid oxidase (LAAO, LAO, TM-LAO; reptilia, animals)	"L-amino-acid oxidase"[All Fields] AND biofilm[All Fields]	L-amino-acid oxidase	30160994	Molecules and Mechanisms Underlying the Antimicrobial Activity of Escapin, an l-Amino Acid Oxidase from the Ink of Sea Hares.
3826	DRAMP03664	L-amino-acid oxidase (LAAO, LAO, TM-LAO; reptilia, animals)	"L-amino-acid oxidase"[All Fields] AND biofilm[All Fields]	L-amino-acid oxidase	27401562	Inhibition and Dispersal of Pseudomonas aeruginosa Biofilms by Combination Treatment with Escapin Intermediate Products and Hydrogen Peroxide.
3826	DRAMP03664	L-amino-acid oxidase (LAAO, LAO, TM-LAO; reptilia, animals)	"L-amino-acid oxidase"[All Fields] AND biofilm[All Fields]	L-amino-acid oxidase	18469105	SO-LAAO, a novel L-amino acid oxidase that enables Streptococcus oligofermentans to outcompete Streptococcus mutans by generating H2O2 from peptone.

3827	DRAMP03665	Lysozyme	"Lysozyme"[All Fields] AND biofilm[All Fields]	Lysozyme	34688058	The dietary supplementation of zinc oxide and selenium nanoparticles enhance the immune response in freshwater fish Oreochromis mossambicus against aquatic pathogen Aeromonas hydrophila.
3827	DRAMP03665	Lysozyme	"Lysozyme"[All Fields] AND biofilm[All Fields]	Lysozyme	34680849	Anti-Biofilm Coatings Based on Chitosan and Lysozyme Functionalized Magnetite Nanoparticles.
3827	DRAMP03665	Lysozyme	"Lysozyme"[All Fields] AND biofilm[All Fields]	Lysozyme	34663068	Zwitterionic Peptides Reduce Accumulation of Marine and Freshwater Biofilm Formers.
3827	DRAMP03665	Lysozyme	"Lysozyme"[All Fields] AND biofilm[All Fields]	Lysozyme	34650538	DexA70, the Truncated Form of a Self-Produced Dextranase, Effectively Disrupts Streptococcus mutansBiofilm.
3827	DRAMP03665	Lysozyme	"Lysozyme"[All Fields] AND biofilm[All Fields]	Lysozyme	34601068	Tailoring the proliferation of fibroblast cells by multiresponsive and thermosensitive stem cells composite F127 hydrogel containing folic acid.MgO:ZnO/chitosan hybrid microparticles for skin regeneration.
3827	DRAMP03665	Lysozyme	"Lysozyme"[All Fields] AND biofilm[All Fields]	Lysozyme	34528638	Enhanced clearing of Candidabiofilms on a 3D urothelial cell in vitromodel using lysozyme-functionalized fluconazole-loaded shellac nanoparticles.
3827	DRAMP03665	Lysozyme	"Lysozyme"[All Fields] AND biofilm[All Fields]	Lysozyme	34488127	Activity of the lyases LysSSE1 and HolSSE1 against common pathogenic bacteria and their antimicrobial efficacy in biofilms.
3827	DRAMP03665	Lysozyme	"Lysozyme"[All Fields] AND biofilm[All Fields]	Lysozyme	34485498	Effect of antimicrobial peptides on planktonic growth, biofilm formation and biofilm-derived bacterial viability of Streptococcus pneumoniae.
3827	DRAMP03665	Lysozyme	"Lysozyme"[All Fields] AND biofilm[All Fields]	Lysozyme	34438974	Antimicrobial Activity of the Circular Bacteriocin AS-48 against Clinical Multidrug-Resistant Staphylococcus aureus.
3827	DRAMP03665	Lysozyme	"Lysozyme"[All Fields] AND biofilm[All Fields]	Lysozyme	34019937	Correlation of over-expression of rv1900c with enhanced survival of M. smegmatis under stress conditions: Modulation of cell surface properties.
3827	DRAMP03665	Lysozyme	"Lysozyme"[All Fields] AND biofilm[All Fields]	Lysozyme	33947757	A StreptococcusQuorum Sensing System Enables Suppression of Innate Immunity.
3827	DRAMP03665	Lysozyme	"Lysozyme"[All Fields] AND biofilm[All Fields]	Lysozyme	33920327	Resveratrol-Loaded Hydrogel Contact Lenses with Antioxidant and Antibiofilm Performance.
3827	DRAMP03665	Lysozyme	"Lysozyme"[All Fields] AND biofilm[All Fields]	Lysozyme	33918930	A Novel Biofilm Model System to Visualise Conjugal Transfer of Vancomycin Resistance by Environmental Enterococci.
3827	DRAMP03665	Lysozyme	"Lysozyme"[All Fields] AND biofilm[All Fields]	Lysozyme	33875213	Impact of pmrA on Cronobacter sakazakii planktonic and biofilm cells: A comprehensive transcriptomic study.
3827	DRAMP03665	Lysozyme	"Lysozyme"[All Fields] AND biofilm[All Fields]	Lysozyme	33842797	Antibacterial and Antibiofilm Photodynamic Activities of Lysozyme-Au Nanoclusters/Rose Bengal Conjugates.
3827	DRAMP03665	Lysozyme	"Lysozyme"[All Fields] AND biofilm[All Fields]	Lysozyme	33783975	Biodegradable Anti-Biofilm Fiber-Membrane Ureteral Stent Constructed with a Robust Biomimetic Superhydrophilic Polycationic Hydration Surface Exhibiting Synergetic Antibacterial and Antiprotein Properties.
3827	DRAMP03665	Lysozyme	"Lysozyme"[All Fields] AND biofilm[All Fields]	Lysozyme	33773215	Growth in a biofilm sensitizes Cutibacterium acnes to nanosecond pulsed electric fields.
3827	DRAMP03665	Lysozyme	"Lysozyme"[All Fields] AND biofilm[All Fields]	Lysozyme	33769023	Drug-Free Enzyme-Based Bactericidal Nanomotors against Pathogenic Bacteria.
3827	DRAMP03665	Lysozyme	"Lysozyme"[All Fields] AND biofilm[All Fields]	Lysozyme	33740161	Enzymatic biofilm destabilisation to support mechanical cleansing of inserted dental implant surfaces: an in-vitro pilot study.
3827	DRAMP03665	Lysozyme	"Lysozyme"[All Fields] AND biofilm[All Fields]	Lysozyme	33391351	Quantification of Bacterial Colonization in Dental Hard Tissues Using Optimized Molecular Biological Methods.
3827	DRAMP03665	Lysozyme	"Lysozyme"[All Fields] AND biofilm[All Fields]	Lysozyme	33252326	Effect of antifungal agents, lysozyme and human antimicrobial peptide LL-37 on clinical Candidaisolates with high biofilm production.
3827	DRAMP03665	Lysozyme	"Lysozyme"[All Fields] AND biofilm[All Fields]	Lysozyme	33185350	[Characterization of the microbiota and cytokine profile of sperm plasma in men with chronic bacterial prostatitis].
3827	DRAMP03665	Lysozyme	"Lysozyme"[All Fields] AND biofilm[All Fields]	Lysozyme	33113658	New insights into the effect of extracellular polymeric substance on the sludge dewaterability based on interaction energy and viscoelastic acoustic response analysis.
3827	DRAMP03665	Lysozyme	"Lysozyme"[All Fields] AND biofilm[All Fields]	Lysozyme	33080579	Combination therapy of biogenic C-dots and lysozyme for enhanced antibacterial and antibiofilm activity.
3827	DRAMP03665	Lysozyme	"Lysozyme"[All Fields] AND biofilm[All Fields]	Lysozyme	33072040	Exploiting Lactoferricin (17-30) as a Potential Antimicrobial and Antibiofilm Candidate Against Multi-Drug-Resistant Enteroaggregative Escherichia coli.
3827	DRAMP03665	Lysozyme	"Lysozyme"[All Fields] AND biofilm[All Fields]	Lysozyme	33045439	Antibacterial activities of and biofilm removal by Ablysin, an endogenous lysozyme-like protein originated from Acinetobacter baumannii 1656-2.
3827	DRAMP03665	Lysozyme	"Lysozyme"[All Fields] AND biofilm[All Fields]	Lysozyme	32916574	Supported lysozyme for improved antimicrobial surface protection.
3827	DRAMP03665	Lysozyme	"Lysozyme"[All Fields] AND biofilm[All Fields]	Lysozyme	32787275	Hydration-Induced Structural Changes in the Solid State of Protein: A SAXS/WAXS Study on Lysozyme.
3827	DRAMP03665	Lysozyme	"Lysozyme"[All Fields] AND biofilm[All Fields]	Lysozyme	32612893	Biofilm modelling on the contact lenses and comparison of the in vitro activities of multipurpose lens solutions and antibiotics.
3827	DRAMP03665	Lysozyme	"Lysozyme"[All Fields] AND biofilm[All Fields]	Lysozyme	32512756	Inactivation of the sfgtr4Gene of Shigella flexneriInduces Biofilm Formation and Affects Bacterial Pathogenicity.
3827	DRAMP03665	Lysozyme	"Lysozyme"[All Fields] AND biofilm[All Fields]	Lysozyme	32393645	Evolution of vancomycin-resistant Enterococcus faeciumduring colonization and infection in immunocompromised pediatric patients.
3827	DRAMP03665	Lysozyme	"Lysozyme"[All Fields] AND biofilm[All Fields]	Lysozyme	32280054	Quorum quenching acylase impacts the viability and morphological change of Agrobacterium tumefaciens cells.
3827	DRAMP03665	Lysozyme	"Lysozyme"[All Fields] AND biofilm[All Fields]	Lysozyme	32266650	Probiotic Potential of Bacillus Strains Isolated from an Acidic Fermented Food Idli.
3827	DRAMP03665	Lysozyme	"Lysozyme"[All Fields] AND biofilm[All Fields]	Lysozyme	32089678	Effects of Lysozyme, Proteinase K, and Cephalosporins on Biofilm Formation by Clinical Isolates of Pseudomonas aeruginosa.
3827	DRAMP03665	Lysozyme	"Lysozyme"[All Fields] AND biofilm[All Fields]	Lysozyme	33447800	Cellular chaining influences biofilm formation and structure in group A Streptococcus.
3827	DRAMP03665	Lysozyme	"Lysozyme"[All Fields] AND biofilm[All Fields]	Lysozyme	31698068	Vibrio harveyi biofilm as immunostimulant candidate for high-health pacific white shrimp, Penaeus vannamei farming.
3827	DRAMP03665	Lysozyme	"Lysozyme"[All Fields] AND biofilm[All Fields]	Lysozyme	31480687	Characterization and Antibiofilm Activity of Mannitol-Chitosan-Blended Paste for Local Antibiotic Delivery System.
3827	DRAMP03665	Lysozyme	"Lysozyme"[All Fields] AND biofilm[All Fields]	Lysozyme	31374264	Anti-cancer, anti-biofilm, and anti-inflammatory properties of hen's albumen: A photodynamic approach.
3827	DRAMP03665	Lysozyme	"Lysozyme"[All Fields] AND biofilm[All Fields]	Lysozyme	31279118	Effect of extracellular polymeric substances (EPS) conditioned by combined lysozyme and cationic polyacrylamide on the dewatering performance of activated sludge.
3827	DRAMP03665	Lysozyme	"Lysozyme"[All Fields] AND biofilm[All Fields]	Lysozyme	31252064	Antibacterial synergy between rutin and florfenicol enhances therapeutic spectrum against drug resistant Aeromonas hydrophila.
3827	DRAMP03665	Lysozyme	"Lysozyme"[All Fields] AND biofilm[All Fields]	Lysozyme	31214943	Safety and Stability of Two Potentially Probiotic Lactobacillus Strains After In Vitro Gastrointestinal Transit.
3827	DRAMP03665	Lysozyme	"Lysozyme"[All Fields] AND biofilm[All Fields]	Lysozyme	31125644	Rv0518, a nutritive stress inducible GDSL lipase of Mycobacterium tuberculosis, enhanced intracellular survival of bacteria by cell wall modulation.
3827	DRAMP03665	Lysozyme	"Lysozyme"[All Fields] AND biofilm[All Fields]	Lysozyme	31085703	The Ser/Thr Kinase PrkC Participates in Cell Wall Homeostasis and Antimicrobial Resistance in Clostridium difficile.
3827	DRAMP03665	Lysozyme	"Lysozyme"[All Fields] AND biofilm[All Fields]	Lysozyme	30843349	Characterization of the biofilm phenotype of a Listeria monocytogenes mutant deficient in agr peptide sensing.
3827	DRAMP03665	Lysozyme	"Lysozyme"[All Fields] AND biofilm[All Fields]	Lysozyme	30833631	Detection of vaginal lactobacilli as probiotic candidates.
3827	DRAMP03665	Lysozyme	"Lysozyme"[All Fields] AND biofilm[All Fields]	Lysozyme	30761784	[Biological properties of microorganisms isolated from the urine of patients with urolithiasis].
3827	DRAMP03665	Lysozyme	"Lysozyme"[All Fields] AND biofilm[All Fields]	Lysozyme	30701862	Effect of N-acetylcysteine on mucosal immunity of respiratory tract.
3827	DRAMP03665	Lysozyme	"Lysozyme"[All Fields] AND biofilm[All Fields]	Lysozyme	30649289	Innate immune components affect growth and virulence traits of bacterial-vaginosis-associated and non-bacterial-vaginosis-associated Gardnerella vaginalis strains similarly.
3827	DRAMP03665	Lysozyme	"Lysozyme"[All Fields] AND biofilm[All Fields]	Lysozyme	30639529	Catalysing the way towards antimicrobial effectiveness: A systematic analysis and a new online resource for antimicrobial-enzyme combinations against Pseudomonas aeruginosa and Staphylococcus aureus.
3827	DRAMP03665	Lysozyme	"Lysozyme"[All Fields] AND biofilm[All Fields]	Lysozyme	30639217	Immobilization of antimicrobial and anti-quorum sensing enzymes onto GMA-grafted poly(vinyl chloride) catheters.
3827	DRAMP03665	Lysozyme	"Lysozyme"[All Fields] AND biofilm[All Fields]	Lysozyme	30497006	Impact of oral astringent stimuli on surface charge and morphology of the protein-rich pellicle at the tooth-saliva interphase.
3827	DRAMP03665	Lysozyme	"Lysozyme"[All Fields] AND biofilm[All Fields]	Lysozyme	30389429	Microbiology insights into boosting salivary defences through the use of enzymes and proteins.
3827	DRAMP03665	Lysozyme	"Lysozyme"[All Fields] AND biofilm[All Fields]	Lysozyme	30207684	Developing Antibacterial Nanocrystalline Cellulose Using Natural Antibacterial Agents.
3827	DRAMP03665	Lysozyme	"Lysozyme"[All Fields] AND biofilm[All Fields]	Lysozyme	30176334	β-1, 3 glucan binding protein based selenium nanowire enhances the immune status of Cyprinus carpio and protection against Aeromonas hydrophila infection.
3827	DRAMP03665	Lysozyme	"Lysozyme"[All Fields] AND biofilm[All Fields]	Lysozyme	30142702	Moraxella bovis, Moraxella ovis and Moraxella bovoculi: biofilm formation and lysozyme activity.
3827	DRAMP03665	Lysozyme	"Lysozyme"[All Fields] AND biofilm[All Fields]	Lysozyme	30131945	The Intestinal Roundworm Ascaris suumReleases Antimicrobial Factors Which Interfere With Bacterial Growth and Biofilm Formation.
3827	DRAMP03665	Lysozyme	"Lysozyme"[All Fields] AND biofilm[All Fields]	Lysozyme	30082920	Control of Propionibacterium acnes by natural antimicrobial substances: Role of the bacteriocin AS-48 and lysozyme.

3827	DRAMP03665	Lysozyme	"Lysozyme"[All Fields] AND biofilm[All Fields]	Lysozyme	29915307	Screening and characterization of prophages in Desulfovibrio genomes.
3827	DRAMP03665	Lysozyme	"Lysozyme"[All Fields] AND biofilm[All Fields]	Lysozyme	29915116	Resuscitation-Promoting Factors Are Required for Mycobacterium smegmatis Biofilm Formation.
3827	DRAMP03665	Lysozyme	"Lysozyme"[All Fields] AND biofilm[All Fields]	Lysozyme	29876449	TCA precipitation and ethanol/HCl single-step purification evaluation: One-dimensional gel electrophoresis, bradford assays, spectrofluorometry and Raman spectroscopy data on HSA, Rnase, lysozyme - Mascots and Skyline data.
3827	DRAMP03665	Lysozyme	"Lysozyme"[All Fields] AND biofilm[All Fields]	Lysozyme	29555699	A Quorum Sensing-Regulated Protein Binds Cell Wall Components and Enhances Lysozyme Resistance in Streptococcus pyogenes.
3827	DRAMP03665	Lysozyme	"Lysozyme"[All Fields] AND biofilm[All Fields]	Lysozyme	29518559	Effect of β-1, 3 glucan binding protein based zinc oxide nanoparticles supplemented diet on immune response and disease resistance in Oreochromis mossambicus against Aeromonas hydrophila.
3827	DRAMP03665	Lysozyme	"Lysozyme"[All Fields] AND biofilm[All Fields]	Lysozyme	29464594	Inhalable Levofloxacin Liposomes Complemented with Lysozyme for Treatment of Pulmonary Infection in Rats: Effective Antimicrobial and Antibiofilm Strategy.
3827	DRAMP03665	Lysozyme	"Lysozyme"[All Fields] AND biofilm[All Fields]	Lysozyme	29393147	Protein Profile of the Acquired Enamel Pellicle after Rinsing with Whole Milk, Fat-Free Milk, and Water: An in vivo Study.
3827	DRAMP03665	Lysozyme	"Lysozyme"[All Fields] AND biofilm[All Fields]	Lysozyme	29373594	Biofilm formation and transcriptome analysis of Streptococcus gallolyticus subsp. gallolyticus in response to lysozyme.
3827	DRAMP03665	Lysozyme	"Lysozyme"[All Fields] AND biofilm[All Fields]	Lysozyme	29315029	Correlations among Resistances to Different Antimicrobial Compounds in Salmonella Strains from Hen Eggshells.
3827	DRAMP03665	Lysozyme	"Lysozyme"[All Fields] AND biofilm[All Fields]	Lysozyme	31565305	Enzyme Crystals and Hydrogel Composite Membranes as New Active Food Packaging Material.
3827	DRAMP03665	Lysozyme	"Lysozyme"[All Fields] AND biofilm[All Fields]	Lysozyme	29205129	Dissimilar pigment regulation in Serpula lacrymans and Paxillus involutus during inter-kingdom interactions.
3827	DRAMP03665	Lysozyme	"Lysozyme"[All Fields] AND biofilm[All Fields]	Lysozyme	29201023	A Marine Actinomycete Rescues Caenorhabditis elegansfrom Pseudomonas aeruginosaInfection through Restitution of Lysozyme 7.
3827	DRAMP03665	Lysozyme	"Lysozyme"[All Fields] AND biofilm[All Fields]	Lysozyme	29184097	Csl2, a novel chimeric bacteriophage lysin to fight infections caused by Streptococcus suis, an emerging zoonotic pathogen.
3827	DRAMP03665	Lysozyme	"Lysozyme"[All Fields] AND biofilm[All Fields]	Lysozyme	29080817	Production and structural characterization of exopolysaccharides from newly isolated probiotic lactic acid bacteria.
3827	DRAMP03665	Lysozyme	"Lysozyme"[All Fields] AND biofilm[All Fields]	Lysozyme	28931063	Plant flavones enhance antimicrobial activity of respiratory epithelial cell secretions against Pseudomonas aeruginosa.
3827	DRAMP03665	Lysozyme	"Lysozyme"[All Fields] AND biofilm[All Fields]	Lysozyme	28922066	The Effect of Lysozyme on Reducing Biofilms by Staphylococcus aureus, Pseudomonas aeruginosa, and Gardnerella vaginalis: An In Vitro Examination.
3827	DRAMP03665	Lysozyme	"Lysozyme"[All Fields] AND biofilm[All Fields]	Lysozyme	28826250	Antimicrobial and anti-biofilm activity of tannic acid against Staphylococcus aureus.
3827	DRAMP03665	Lysozyme	"Lysozyme"[All Fields] AND biofilm[All Fields]	Lysozyme	28649626	Investigation of simulated microgravity effects on Streptococcus mutansphysiology and global gene expression.
3827	DRAMP03665	Lysozyme	"Lysozyme"[All Fields] AND biofilm[All Fields]	Lysozyme	32264250	Protein-repellent and antimicrobial nanoparticle coatings from hyaluronic acid and a lysine-derived biocompatible surfactant.
3827	DRAMP03665	Lysozyme	"Lysozyme"[All Fields] AND biofilm[All Fields]	Lysozyme	28400767	The Periplasmic Chaperone Network of Campylobacter jejuni: Evidence that SalC (Cj1289) and PpiD (Cj0694) Are Involved in Maintaining Outer Membrane Integrity.
3827	DRAMP03665	Lysozyme	"Lysozyme"[All Fields] AND biofilm[All Fields]	Lysozyme	28400765	Role of Bacillus licheniformisVS16-Derived Biosurfactant in Mediating Immune Responses in Carp Rohu and its Application to the Food Industry.
3827	DRAMP03665	Lysozyme	"Lysozyme"[All Fields] AND biofilm[All Fields]	Lysozyme	28397768	Lysozyme Associated Liposomal Gentamicin Inhibits Bacterial Biofilm.
3827	DRAMP03665	Lysozyme	"Lysozyme"[All Fields] AND biofilm[All Fields]	Lysozyme	28289407	A Novel Antimicrobial Endolysin, LysPA26, against Pseudomonas aeruginosa.
3827	DRAMP03665	Lysozyme	"Lysozyme"[All Fields] AND biofilm[All Fields]	Lysozyme	28251063	Evaluation of a chitosan-polyethylene glycol paste as a local antibiotic delivery device.
3827	DRAMP03665	Lysozyme	"Lysozyme"[All Fields] AND biofilm[All Fields]	Lysozyme	28188877	Laser deposition of poly(3-hydroxybutyric acid-co-3-hydroxyvaleric acid) - lysozyme microspheres based coatings with anti-microbial properties.
3827	DRAMP03665	Lysozyme	"Lysozyme"[All Fields] AND biofilm[All Fields]	Lysozyme	28138698	Dose-dependent effect of lysozyme upon Candida albicans biofilm.
3827	DRAMP03665	Lysozyme	"Lysozyme"[All Fields] AND biofilm[All Fields]	Lysozyme	28109187	Antimicrobial and antioxidant activities of Saccharomyces cerevisiae IFST062013, a potential probiotic.
3827	DRAMP03665	Lysozyme	"Lysozyme"[All Fields] AND biofilm[All Fields]	Lysozyme	30695493	[PHYSIOLOGICAL FEATURES OF CORYNEBACTERIA OF FEMALE REPRODUC- TIVE TRACT].
3827	DRAMP03665	Lysozyme	"Lysozyme"[All Fields] AND biofilm[All Fields]	Lysozyme	29619048	Efficacy of a Rinse Containing Sea Salt and Lysozyme on Biofilm and Gingival Health in a Group of Young Adults: A Pilot Study.
3827	DRAMP03665	Lysozyme	"Lysozyme"[All Fields] AND biofilm[All Fields]	Lysozyme	27999400	Comparative Study on the Characteristics of Weissella cibaria CMU and Probiotic Strains for Oral Care.
3827	DRAMP03665	Lysozyme	"Lysozyme"[All Fields] AND biofilm[All Fields]	Lysozyme	27984151	Copper incorporated microporous chitosan-polyethylene glycol hydrogels loaded with naproxen for effective drug release and anti-infection wound dressing.
3827	DRAMP03665	Lysozyme	"Lysozyme"[All Fields] AND biofilm[All Fields]	Lysozyme	27931094	Human Lysozyme Peptidase Resistance Is Perturbed by the Anionic Glycolipid Biosurfactant Rhamnolipid Produced by the Opportunistic Pathogen Pseudomonas aeruginosa.
3827	DRAMP03665	Lysozyme	"Lysozyme"[All Fields] AND biofilm[All Fields]	Lysozyme	27845497	Lysozyme as a cotreatment during antibiotics use against vaginal infections: An in vitro study on Gardnerella vaginalis biofilm models.
3827	DRAMP03665	Lysozyme	"Lysozyme"[All Fields] AND biofilm[All Fields]	Lysozyme	27821857	Establishing Antibacterial Multilayer Films on the Surface of Direct Metal Laser Sintered Titanium Primed with Phase-Transited Lysozyme.
3827	DRAMP03665	Lysozyme	"Lysozyme"[All Fields] AND biofilm[All Fields]	Lysozyme	27709890	A Biodegradable Polycationic Paint that Kills Bacteria in Vitro and in Vivo.
3827	DRAMP03665	Lysozyme	"Lysozyme"[All Fields] AND biofilm[All Fields]	Lysozyme	27685160	In Situ Impregnation of Silver Nanoclusters in Microporous Chitosan-PEG Membranes as an Antibacterial and Drug Delivery Percutaneous Device.
3827	DRAMP03665	Lysozyme	"Lysozyme"[All Fields] AND biofilm[All Fields]	Lysozyme	27602088	Antibiotic susceptibility, antibacterial activity and characterisation of Enterococcus faeciumstrains isolated from breast milk.
3827	DRAMP03665	Lysozyme	"Lysozyme"[All Fields] AND biofilm[All Fields]	Lysozyme	27520822	Peptidoglycan Acetylation of Campylobacter jejuni Is Essential for Maintaining Cell Wall Integrity and Colonization in Chicken Intestines.
3827	DRAMP03665	Lysozyme	"Lysozyme"[All Fields] AND biofilm[All Fields]	Lysozyme	27375592	Pseudomonas aeruginosa Outer Membrane Vesicles Triggered by Human Mucosal Fluid and Lysozyme Can Prime Host Tissue Surfaces for Bacterial Adhesion.
3827	DRAMP03665	Lysozyme	"Lysozyme"[All Fields] AND biofilm[All Fields]	Lysozyme	27373086	[Staphylococcus aureus biofilm influences the expression of lysozyme, SLPI and gp340 in a human sinonasal explant model].
3827	DRAMP03665	Lysozyme	"Lysozyme"[All Fields] AND biofilm[All Fields]	Lysozyme	27236754	Molecular typing and differences in biofilm formation and antibiotic susceptibilities among Prototheca strains isolated in Italy and Brazil.
3827	DRAMP03665	Lysozyme	"Lysozyme"[All Fields] AND biofilm[All Fields]	Lysozyme	27015648	Biopolymer-induced calcium phosphate scaling in membrane-based water treatment systems: Langmuir model films studies.
3827	DRAMP03665	Lysozyme	"Lysozyme"[All Fields] AND biofilm[All Fields]	Lysozyme	26783746	Titanium Surface Priming with Phase-Transited Lysozyme to Establish a Silver Nanoparticle-Loaded Chitosan/Hyaluronic Acid Antibacterial Multilayer via Layer-by-Layer Self-Assembly.
3827	DRAMP03665	Lysozyme	"Lysozyme"[All Fields] AND biofilm[All Fields]	Lysozyme	31532953	[The etiologic structure and biologic characteristics of agents of infections of bloodstream.]
3827	DRAMP03665	Lysozyme	"Lysozyme"[All Fields] AND biofilm[All Fields]	Lysozyme	26488327	Complexation of Lysozyme with Sodium Poly(styrenesulfonate) via the Two-State and Non-Two-State Unfoldings of Lysozyme.
3827	DRAMP03665	Lysozyme	"Lysozyme"[All Fields] AND biofilm[All Fields]	Lysozyme	26478289	Covalent immobilization of lysozyme onto woven and knitted crimped polyethylene terephthalate grafts to minimize the adhesion of broad spectrum pathogens.
3827	DRAMP03665	Lysozyme	"Lysozyme"[All Fields] AND biofilm[All Fields]	Lysozyme	26470414	[REGULATING EFFECT OF ASSOCIATIVE MICROBIOTA ON THE RHYTHMS OF BIOLOGICAL PROPERTIES OF FUNGI AND BACTERIA].
3827	DRAMP03665	Lysozyme	"Lysozyme"[All Fields] AND biofilm[All Fields]	Lysozyme	26458820	Effects of fed-batch and continuous fermentations on human lysozyme production by Kluyveromyces lactis K7 in biofilm reactors.
3827	DRAMP03665	Lysozyme	"Lysozyme"[All Fields] AND biofilm[All Fields]	Lysozyme	26400891	Effect of histatin-5 and lysozyme on the ability of Streptococcus mutans to form biofilms in in vitro conditions.
3827	DRAMP03665	Lysozyme	"Lysozyme"[All Fields] AND biofilm[All Fields]	Lysozyme	26383819	Recent advances for the production and recovery methods of lysozyme.
3827	DRAMP03665	Lysozyme	"Lysozyme"[All Fields] AND biofilm[All Fields]	Lysozyme	26315397	Enzymes in the in-situ pellicle of children with different caries activity.
3827	DRAMP03665	Lysozyme	"Lysozyme"[All Fields] AND biofilm[All Fields]	Lysozyme	26152033	[AEROMONAS BACTERIA ISOLATED FROM BITHYNIIDAE MOLLUSKS AND THEIR HABITATS: SPECIES COMPOSITION AND BIOLOGICAL PROPERTIES. COMMUNICATION 1].
3827	DRAMP03665	Lysozyme	"Lysozyme"[All Fields] AND biofilm[All Fields]	Lysozyme	26092919	Acetylcholine Protects against Candida albicans Infection by Inhibiting Biofilm Formation and Promoting Hemocyte Function in a Galleria mellonella Infection Model.
3827	DRAMP03665	Lysozyme	"Lysozyme"[All Fields] AND biofilm[All Fields]	Lysozyme	27688392	Antimicrobial Capacity of Casein Phosphopeptide/Amorphous Calcium Phosphate and Enzymes in Glass Ionomer Cement in Dentin Carious Lesions.
3827	DRAMP03665	Lysozyme	"Lysozyme"[All Fields] AND biofilm[All Fields]	Lysozyme	25950767	In vitro biofilm development of Streptococcus pneumoniae and formation of choline-binding protein-DNA complexes.
3827	DRAMP03665	Lysozyme	"Lysozyme"[All Fields] AND biofilm[All Fields]	Lysozyme	25757148	Development of novel formulations containing Lysozyme and Lactoferrin and evaluation of antibacterial effects on Mutans Streptococci and Lactobacilli.
3827	DRAMP03665	Lysozyme	"Lysozyme"[All Fields] AND biofilm[All Fields]	Lysozyme	25725311	Cleaning of biomaterial surfaces: protein removal by different solvents.
3827	DRAMP03665	Lysozyme	"Lysozyme"[All Fields] AND biofilm[All Fields]	Lysozyme	25721974	Characterization of a novel strain phylogenetically related to Kocuria rhizophila and its chemical modification to improve performance of microbial fuel cells.

3827	DRAMP03665	Lysozyme	"Lysozyme"[All Fields] AND biofilm[All Fields]	Lysozyme	25536779	[Species structure and staphylococci bioprofile characteristics--ausative agents of perinatal infectious-inflammation pathology in children of Orenburg].
3827	DRAMP03665	Lysozyme	"Lysozyme"[All Fields] AND biofilm[All Fields]	Lysozyme	25467937	Phenotypic characterization of a novel double knockout PknI/DacB2 from Mycobacterium tuberculosis.
3827	DRAMP03665	Lysozyme	"Lysozyme"[All Fields] AND biofilm[All Fields]	Lysozyme	25449384	N-hexanoyl-L-homoserine lactone-degrading Pseudomonas aeruginosa PsDAHP1 protects zebrafish against Vibrio parahaemolyticus infection.
3827	DRAMP03665	Lysozyme	"Lysozyme"[All Fields] AND biofilm[All Fields]	Lysozyme	25312955	The conserved hypothetical protein Rv0574c is required for cell wall integrity, stress tolerance, and virulence of Mycobacterium tuberculosis.
3827	DRAMP03665	Lysozyme	"Lysozyme"[All Fields] AND biofilm[All Fields]	Lysozyme	25221673	Iron oxide nanoparticles induce Pseudomonas aeruginosagrowth, induce biofilm formation, and inhibit antimicrobial peptide function.
3827	DRAMP03665	Lysozyme	"Lysozyme"[All Fields] AND biofilm[All Fields]	Lysozyme	25115519	Presence and function of a thick mucous layer rich in polysaccharides around Bacillus subtilis spores.
3827	DRAMP03665	Lysozyme	"Lysozyme"[All Fields] AND biofilm[All Fields]	Lysozyme	24559398	Temperature-driven adsorption and desorption of proteins at solid-liquid interfaces.
3827	DRAMP03665	Lysozyme	"Lysozyme"[All Fields] AND biofilm[All Fields]	Lysozyme	24434534	Designing nanogel carriers for antibacterial applications.
3827	DRAMP03665	Lysozyme	"Lysozyme"[All Fields] AND biofilm[All Fields]	Lysozyme	24240906	Adsorption study of pellicle proteins to gold, silica and titanium by quartz crystal microbalance method.
3827	DRAMP03665	Lysozyme	"Lysozyme"[All Fields] AND biofilm[All Fields]	Lysozyme	24121782	Enhanced expressions of lysozyme, SLPI and glycoprotein 340 in biofilm-associated chronic rhinosinusitis.
3827	DRAMP03665	Lysozyme	"Lysozyme"[All Fields] AND biofilm[All Fields]	Lysozyme	24011302	Fluorescence microscopic visualization of non cellular components during initial bioadhesion in situ.
3827	DRAMP03665	Lysozyme	"Lysozyme"[All Fields] AND biofilm[All Fields]	Lysozyme	23844477	Destruction of single-species biofilms of Escherichia coli or Klebsiella pneumoniae subsp. pneumoniae by dextranase, lactoferrin, and lysozyme.
3827	DRAMP03665	Lysozyme	"Lysozyme"[All Fields] AND biofilm[All Fields]	Lysozyme	23701483	Effects of biological molecules on calcium mineral formation associated with wastewater desalination as assessed using small-angle neutron scattering.
3827	DRAMP03665	Lysozyme	"Lysozyme"[All Fields] AND biofilm[All Fields]	Lysozyme	23659996	Vaccination efficiency of surface antigens and killed whole cell of Pseudomonas putida in large yellow croaker (Pseudosciaena crocea).
3827	DRAMP03665	Lysozyme	"Lysozyme"[All Fields] AND biofilm[All Fields]	Lysozyme	23657582	Production of human lysozyme in biofilm reactor and optimization of growth parameters of Kluyveromyces lactis K7.
3827	DRAMP03665	Lysozyme	"Lysozyme"[All Fields] AND biofilm[All Fields]	Lysozyme	23504078	Phenotypic characterization, virulence, and immunogenicity of Edwardsiella tarda LSE40 aroA mutant.
3827	DRAMP03665	Lysozyme	"Lysozyme"[All Fields] AND biofilm[All Fields]	Lysozyme	23460607	Undecaprenyl pyrophosphate phosphatase confers low-level resistance to bacitracin in Enterococcus faecalis.
3827	DRAMP03665	Lysozyme	"Lysozyme"[All Fields] AND biofilm[All Fields]	Lysozyme	23434686	Enhancing antibacterial activity of surface-grafted chitosan with immobilized lysozyme on bioinspired stainless steel substrates.
3827	DRAMP03665	Lysozyme	"Lysozyme"[All Fields] AND biofilm[All Fields]	Lysozyme	23403559	Host defense proteins derived from human saliva bind to Staphylococcus aureus.
3827	DRAMP03665	Lysozyme	"Lysozyme"[All Fields] AND biofilm[All Fields]	Lysozyme	23194029	Alpha-amylase is a human salivary protein with affinity to lipopolysaccharide of Aggregatibacter actinomycetemcomitans.
3827	DRAMP03665	Lysozyme	"Lysozyme"[All Fields] AND biofilm[All Fields]	Lysozyme	22947470	Effects of nisin and lysozyme on growth inhibition and biofilm formation capacity of Staphylococcus aureus strains isolated from raw milk and cheese samples.
3827	DRAMP03665	Lysozyme	"Lysozyme"[All Fields] AND biofilm[All Fields]	Lysozyme	22937707	[Interaction of Bifidobacterium bifidum with members of normal microflora in human intestine microsymbiocenosis].
3827	DRAMP03665	Lysozyme	"Lysozyme"[All Fields] AND biofilm[All Fields]	Lysozyme	22913814	Insight into the composition of the intercellular matrix of Streptococcus pneumoniae biofilms.
3827	DRAMP03665	Lysozyme	"Lysozyme"[All Fields] AND biofilm[All Fields]	Lysozyme	22363222	Tasco®: a product of Ascophyllum nodosum enhances immune response of Caenorhabditis elegans against Pseudomonas aeruginosa infection.
3827	DRAMP03665	Lysozyme	"Lysozyme"[All Fields] AND biofilm[All Fields]	Lysozyme	26781731	Recombinant Expression of a Putative Amidase Cloned from the Genome of Listeria monocytogenes that Lyses the Bacterium and its Monolayer in Conjunction with a Protease.
3827	DRAMP03665	Lysozyme	"Lysozyme"[All Fields] AND biofilm[All Fields]	Lysozyme	22344334	Influence of artificial saliva in biofilm formation of Candida albicans in vitro.
3827	DRAMP03665	Lysozyme	"Lysozyme"[All Fields] AND biofilm[All Fields]	Lysozyme	22308727	[Microbial "friend-foe" identification in human intestine microsymbiocenosis].
3827	DRAMP03665	Lysozyme	"Lysozyme"[All Fields] AND biofilm[All Fields]	Lysozyme	22265308	Effect of lysozyme on "flor" velum yeasts in the biological aging of sherry wines.
3827	DRAMP03665	Lysozyme	"Lysozyme"[All Fields] AND biofilm[All Fields]	Lysozyme	21821771	Role of phase variation in the resistance of Myxococcus xanthus fruiting bodies to Caenorhabditis elegans predation.
3827	DRAMP03665	Lysozyme	"Lysozyme"[All Fields] AND biofilm[All Fields]	Lysozyme	21486002	Anti-fouling chemistry of chiral monolayers: enhancing biofilm resistance on racemic surface.
3827	DRAMP03665	Lysozyme	"Lysozyme"[All Fields] AND biofilm[All Fields]	Lysozyme	21471196	A disulfide bridge network within the soluble periplasmic domain determines structure and function of the outer membrane protein RCSF.
3827	DRAMP03665	Lysozyme	"Lysozyme"[All Fields] AND biofilm[All Fields]	Lysozyme	21338094	Lysozyme-coupled poly(poly(ethylene glycol) methacrylate)-stainless steel hybrids and their antifouling and antibacterial surfaces.
3827	DRAMP03665	Lysozyme	"Lysozyme"[All Fields] AND biofilm[All Fields]	Lysozyme	21247450	Phylogenetic group- and species-specific oligonucleotide probes for single-cell detection of lactic acid bacteria in oral biofilms.
3827	DRAMP03665	Lysozyme	"Lysozyme"[All Fields] AND biofilm[All Fields]	Lysozyme	21148692	Integrity of proteins in human saliva after sterilization by gamma irradiation.
3827	DRAMP03665	Lysozyme	"Lysozyme"[All Fields] AND biofilm[All Fields]	Lysozyme	21045923	Structural evolution of protein-biofilms: Simulations and experiments.
3827	DRAMP03665	Lysozyme	"Lysozyme"[All Fields] AND biofilm[All Fields]	Lysozyme	20961363	Potential of ceragenin CSA-13 and its mixture with pluronic F-127 as treatment of topical bacterial infections.
3827	DRAMP03665	Lysozyme	"Lysozyme"[All Fields] AND biofilm[All Fields]	Lysozyme	20865041	Comparative genomics of Gardnerella vaginalis strains reveals substantial differences in metabolic and virulence potential.
3827	DRAMP03665	Lysozyme	"Lysozyme"[All Fields] AND biofilm[All Fields]	Lysozyme	20724386	Glutamine synthetase encoded by glnA-1 is necessary for cell wall resistance and pathogenicity of Mycobacterium bovis.
3827	DRAMP03665	Lysozyme	"Lysozyme"[All Fields] AND biofilm[All Fields]	Lysozyme	20566201	Bioengineering of stainless steel surface by covalent immobilization of enzymes. Physical characterization and interfacial enzymatic activity.
3827	DRAMP03665	Lysozyme	"Lysozyme"[All Fields] AND biofilm[All Fields]	Lysozyme	20417319	Functionalization of acrylic hydrogels with alpha-, beta- or gamma-cyclodextrin modulates protein adsorption and antifungal delivery.
3827	DRAMP03665	Lysozyme	"Lysozyme"[All Fields] AND biofilm[All Fields]	Lysozyme	20121056	Achieving highly effective non-biofouling performance for polypropylene membranes modified by UV-induced surface graft polymerization of two oppositely charged monomers.
3827	DRAMP03665	Lysozyme	"Lysozyme"[All Fields] AND biofilm[All Fields]	Lysozyme	19334612	[Search for destruction factors of bacterial biofilms: comparison of phage properties in a group of Pseudomonas putida bacteriophages and specificity of their halo-formation products].
3827	DRAMP03665	Lysozyme	"Lysozyme"[All Fields] AND biofilm[All Fields]	Lysozyme	19241565	Storability of antimicrobial chitosan-lysozyme composite coating and film-forming solutions.
3827	DRAMP03665	Lysozyme	"Lysozyme"[All Fields] AND biofilm[All Fields]	Lysozyme	19166331	Grafting of lysozyme and/or poly(ethylene glycol) to prevent biofilm growth on stainless steel surfaces.
3827	DRAMP03665	Lysozyme	"Lysozyme"[All Fields] AND biofilm[All Fields]	Lysozyme	19038377	Synergistic activity of lysozyme and antifungal agents against Candida albicans biofilms on denture acrylic surfaces.
3827	DRAMP03665	Lysozyme	"Lysozyme"[All Fields] AND biofilm[All Fields]	Lysozyme	18992255	Crystal structure of the resuscitation-promoting factor (DeltaDUF)RpfB from M. tuberculosis.
3827	DRAMP03665	Lysozyme	"Lysozyme"[All Fields] AND biofilm[All Fields]	Lysozyme	18989905	Ultralow fouling zwitterionic polymers grafted from surfaces covered with an initiator via an adhesive mussel mimetic linkage.
3827	DRAMP03665	Lysozyme	"Lysozyme"[All Fields] AND biofilm[All Fields]	Lysozyme	18819708	Ultra low fouling zwitterionic polymers with a biomimetic adhesive group.
3827	DRAMP03665	Lysozyme	"Lysozyme"[All Fields] AND biofilm[All Fields]	Lysozyme	18718499	Role of lactoferrin in the tear film.
3827	DRAMP03665	Lysozyme	"Lysozyme"[All Fields] AND biofilm[All Fields]	Lysozyme	18468764	Effects of Cistus-tea on bacterial colonization and enzyme activities of the in situ pellicle.
3827	DRAMP03665	Lysozyme	"Lysozyme"[All Fields] AND biofilm[All Fields]	Lysozyme	18424015	Abnormal cell division caused by inclusion bodies in E. coli; increased resistance against external stress.
3827	DRAMP03665	Lysozyme	"Lysozyme"[All Fields] AND biofilm[All Fields]	Lysozyme	18326181	Antimicrobial activity of lactoferrin against foodborne pathogenic bacteria incorporated into edible chitosan film.
3827	DRAMP03665	Lysozyme	"Lysozyme"[All Fields] AND biofilm[All Fields]	Lysozyme	17927634	Biofilm inhibition and antimicrobial activity of a dentifrice containing salivary substitutes.
3827	DRAMP03665	Lysozyme	"Lysozyme"[All Fields] AND biofilm[All Fields]	Lysozyme	17676995	Molecular basis of resistance to muramidase and cationic antimicrobial peptide activity of lysozyme in staphylococci.
3827	DRAMP03665	Lysozyme	"Lysozyme"[All Fields] AND biofilm[All Fields]	Lysozyme	16936041	Biofilm formation by Streptococcus pneumoniae: role of choline, extracellular DNA, and capsular polysaccharide in microbial accretion.
3827	DRAMP03665	Lysozyme	"Lysozyme"[All Fields] AND biofilm[All Fields]	Lysozyme	15952847	Covalent immobilization of lysozyme on stainless steel. Interface spectroscopic characterization and measurement of enzymatic activity.
3827	DRAMP03665	Lysozyme	"Lysozyme"[All Fields] AND biofilm[All Fields]	Lysozyme	15908380	Identification and characterization of an autolysin-encoding gene of Streptococcus mutans.
3827	DRAMP03665	Lysozyme	"Lysozyme"[All Fields] AND biofilm[All Fields]	Lysozyme	15693823	Enzymes in the acquired enamel pellicle.
3827	DRAMP03665	Lysozyme	"Lysozyme"[All Fields] AND biofilm[All Fields]	Lysozyme	15518508	Shear and dilatational relaxation mechanisms of globular and flexible proteins at the hexadecane/water interface.

3827	DRAMP03665	Lysozyme	"Lysozyme"[All Fields] AND biofilm[All Fields]	Lysozyme	15493829	Bacterial evasion of innate host defenses--the Staphylococcus aureus lesson.
3827	DRAMP03665	Lysozyme	"Lysozyme"[All Fields] AND biofilm[All Fields]	Lysozyme	14768464	Protein adsorption at polymer-grafted surfaces: comparison between a mixture of saliva proteins and some well-defined model proteins.
3827	DRAMP03665	Lysozyme	"Lysozyme"[All Fields] AND biofilm[All Fields]	Lysozyme	14706749	Multiplex FISH analysis of a six-species bacterial biofilm.
3827	DRAMP03665	Lysozyme	"Lysozyme"[All Fields] AND biofilm[All Fields]	Lysozyme	14513382	Physiology of biofilms of thermophilic bacilli-potential consequences for cleaning.
3827	DRAMP03665	Lysozyme	"Lysozyme"[All Fields] AND biofilm[All Fields]	Lysozyme	12707750	Scanning-force techniques to monitor time-dependent changes in topography and adhesion force of proteins on surfaces.
3827	DRAMP03665	Lysozyme	"Lysozyme"[All Fields] AND biofilm[All Fields]	Lysozyme	12366846	Positive role of peptidoglycan breaks in lactococcal biofilm formation.
3827	DRAMP03665	Lysozyme	"Lysozyme"[All Fields] AND biofilm[All Fields]	Lysozyme	11679354	Direct detection by in situ PCR of the amoA gene in biofilm resulting from a nitrogen removal process.
3827	DRAMP03665	Lysozyme	"Lysozyme"[All Fields] AND biofilm[All Fields]	Lysozyme	11412320	Factors influencing attachment of thermophilic bacilli to stainless steel.
3827	DRAMP03665	Lysozyme	"Lysozyme"[All Fields] AND biofilm[All Fields]	Lysozyme	10415452	Lactoferrin increases the susceptibility of S. epidermidis biofilms to lysozyme and vancomycin.
3827	DRAMP03665	Lysozyme	"Lysozyme"[All Fields] AND biofilm[All Fields]	Lysozyme	10391502	The effect of extracellular polysaccharides from Streptococcus mutans on the bactericidal activity of human neutrophils.
3827	DRAMP03665	Lysozyme	"Lysozyme"[All Fields] AND biofilm[All Fields]	Lysozyme	10390869	Specific oligonucleotide probes for in situ detection of a major group of gram-positive bacteria with low DNA G + C content.
3827	DRAMP03665	Lysozyme	"Lysozyme"[All Fields] AND biofilm[All Fields]	Lysozyme	9812283	Protein antimicrobial barriers to bacterial adhesion.
3827	DRAMP03665	Lysozyme	"Lysozyme"[All Fields] AND biofilm[All Fields]	Lysozyme	9571268	Defining the physiologically normal coating and pathological deposit: an analysis of sulfur-containing moieties and pellicle thickness on hydrogel contact lenses.
3827	DRAMP03665	Lysozyme	"Lysozyme"[All Fields] AND biofilm[All Fields]	Lysozyme	9351231	The influence of cell surface properties of thermophilic streptococci on attachment to stainless steel.
3827	DRAMP03665	Lysozyme	"Lysozyme"[All Fields] AND biofilm[All Fields]	Lysozyme	9255506	Protein-lipid interaction on the surface of a hydrophilic contact lens in vitro.
3827	DRAMP03665	Lysozyme	"Lysozyme"[All Fields] AND biofilm[All Fields]	Lysozyme	9380651	[Preparation of films with combined biological activity and study of their properties].
3827	DRAMP03665	Lysozyme	"Lysozyme"[All Fields] AND biofilm[All Fields]	Lysozyme	9029300	Adhesion to silicone rubber of yeasts and bacteria isolated from voice prostheses: influence of salivary conditioning films.
3827	DRAMP03665	Lysozyme	"Lysozyme"[All Fields] AND biofilm[All Fields]	Lysozyme	8950503	The effect of eye closure on protein and complement deposition on Group IV hydrogel contact lenses: relationship to tear flow dynamics.
3827	DRAMP03665	Lysozyme	"Lysozyme"[All Fields] AND biofilm[All Fields]	Lysozyme	8126879	[Physiologic factor in intractable bacterial infections].
3827	DRAMP03665	Lysozyme	"Lysozyme"[All Fields] AND biofilm[All Fields]	Lysozyme	3570694	Specificity and biological activity of the protein deposited on the hydrogel surface. Relationship of polymer structure to biofilm formation.
3838	DRAMP03678	Beta-defensin 1 (GBD-1; Defensin, beta 1; ruminant, animals)	"Beta-defensin 1"[All Fields] AND biofilm[All Fields]	Beta-defensin 1	33420317	Curbing gastrointestinal infections by defensin fragment modifications without harming commensal microbiota.
3838	DRAMP03678	Beta-defensin 1 (GBD-1; Defensin, beta 1; ruminant, animals)	"Beta-defensin 1"[All Fields] AND biofilm[All Fields]	Beta-defensin 1	32585445	Glucose effect on Candida albicans biofilm during tissue invasion.
3838	DRAMP03678	Beta-defensin 1 (GBD-1; Defensin, beta 1; ruminant, animals)	"Beta-defensin 1"[All Fields] AND biofilm[All Fields]	Beta-defensin 1	30260708	Stabilized collagen matrix dressing improves wound macrophage function and epithelialization.
3838	DRAMP03678	Beta-defensin 1 (GBD-1; Defensin, beta 1; ruminant, animals)	"Beta-defensin 1"[All Fields] AND biofilm[All Fields]	Beta-defensin 1	28642103	Chimeric analogs of human β-defensin 1 and θ-defensin disrupt pre-established bacterial biofilms.
3838	DRAMP03678	Beta-defensin 1 (GBD-1; Defensin, beta 1; ruminant, animals)	"Beta-defensin 1"[All Fields] AND biofilm[All Fields]	Beta-defensin 1	26214284	α-tocopherol decreases interleukin-1β and -6 and increases human β-defensin-1 and -2 secretion in human gingival fibroblasts stimulated with Porphyromonas gingivalis lipopolysaccharide.
3838	DRAMP03678	Beta-defensin 1 (GBD-1; Defensin, beta 1; ruminant, animals)	"Beta-defensin 1"[All Fields] AND biofilm[All Fields]	Beta-defensin 1	22394470	Association of CiaRH with resistance of Streptococcus mutans to antimicrobial peptides in biofilms.
3838	DRAMP03678	Beta-defensin 1 (GBD-1; Defensin, beta 1; ruminant, animals)	"Beta-defensin 1"[All Fields] AND biofilm[All Fields]	Beta-defensin 1	20454633	Normal human gingival epithelial cells sense C. parapsilosis by toll-like receptors and module its pathogenesis through antimicrobial peptides and proinflammatory cytokines.
3840	DRAMP03681	L-amino-acid oxidase (LAAO; OHAP-1)	"L-amino-acid oxidase"[All Fields] AND biofilm[All Fields]	L-amino-acid oxidase	30160994	Molecules and Mechanisms Underlying the Antimicrobial Activity of Escapin, an l-Amino Acid Oxidase from the Ink of Sea Hares.
3840	DRAMP03681	L-amino-acid oxidase (LAAO; OHAP-1)	"L-amino-acid oxidase"[All Fields] AND biofilm[All Fields]	L-amino-acid oxidase	27401562	Inhibition and Dispersal of Pseudomonas aeruginosa Biofilms by Combination Treatment with Escapin Intermediate Products and Hydrogen Peroxide.
3840	DRAMP03681	L-amino-acid oxidase (LAAO; OHAP-1)	"L-amino-acid oxidase"[All Fields] AND biofilm[All Fields]	L-amino-acid oxidase	18469105	SO-LAAO, a novel L-amino acid oxidase that enables Streptococcus oligofermentans to outcompete Streptococcus mutans by generating H2O2 from peptone.
3843	DRAMP03692	Defensin-1 (Cll-dlp; Arthropods, animals)	"Defensin-1"[All Fields] AND biofilm[All Fields]	Defensin-1	34321877	Antibacterial Effect of Honey-Derived Exosomes Containing Antimicrobial Peptides Against Oral Streptococci.
3843	DRAMP03692	Defensin-1 (Cll-dlp; Arthropods, animals)	"Defensin-1"[All Fields] AND biofilm[All Fields]	Defensin-1	33911935	The antibacterial activities of honey.
3843	DRAMP03692	Defensin-1 (Cll-dlp; Arthropods, animals)	"Defensin-1"[All Fields] AND biofilm[All Fields]	Defensin-1	33420317	Curbing gastrointestinal infections by defensin fragment modifications without harming commensal microbiota.
3843	DRAMP03692	Defensin-1 (Cll-dlp; Arthropods, animals)	"Defensin-1"[All Fields] AND biofilm[All Fields]	Defensin-1	32585445	Glucose effect on Candida albicans biofilm during tissue invasion.
3843	DRAMP03692	Defensin-1 (Cll-dlp; Arthropods, animals)	"Defensin-1"[All Fields] AND biofilm[All Fields]	Defensin-1	31729441	Rhesus Theta Defensin 1 Promotes Long Term Survival in Systemic Candidiasis by Host Directed Mechanisms.
3843	DRAMP03692	Defensin-1 (Cll-dlp; Arthropods, animals)	"Defensin-1"[All Fields] AND biofilm[All Fields]	Defensin-1	30260708	Stabilized collagen matrix dressing improves wound macrophage function and epithelialization.
3843	DRAMP03692	Defensin-1 (Cll-dlp; Arthropods, animals)	"Defensin-1"[All Fields] AND biofilm[All Fields]	Defensin-1	29671721	The BceABRS four-component system that is essential for cell envelope stress response is involved in sensing and response to host defence peptides and is required for the biofilm formation and fitness of Streptococcus mutans.
3843	DRAMP03692	Defensin-1 (Cll-dlp; Arthropods, animals)	"Defensin-1"[All Fields] AND biofilm[All Fields]	Defensin-1	28649561	Psd1 Effects on Candida albicansPlanktonic Cells and Biofilms.
3843	DRAMP03692	Defensin-1 (Cll-dlp; Arthropods, animals)	"Defensin-1"[All Fields] AND biofilm[All Fields]	Defensin-1	28642103	Chimeric analogs of human β-defensin 1 and θ-defensin disrupt pre-established bacterial biofilms.
3843	DRAMP03692	Defensin-1 (Cll-dlp; Arthropods, animals)	"Defensin-1"[All Fields] AND biofilm[All Fields]	Defensin-1	26861950	Antibiofilm efficacy of honey and bee-derived defensin-1 on multispecies wound biofilm.
3843	DRAMP03692	Defensin-1 (Cll-dlp; Arthropods, animals)	"Defensin-1"[All Fields] AND biofilm[All Fields]	Defensin-1	26214284	α-tocopherol decreases interleukin-1β and -6 and increases human β-defensin-1 and -2 secretion in human gingival fibroblasts stimulated with Porphyromonas gingivalis lipopolysaccharide.
3843	DRAMP03692	Defensin-1 (Cll-dlp; Arthropods, animals)	"Defensin-1"[All Fields] AND biofilm[All Fields]	Defensin-1	22394470	Association of CiaRH with resistance of Streptococcus mutans to antimicrobial peptides in biofilms.
3843	DRAMP03692	Defensin-1 (Cll-dlp; Arthropods, animals)	"Defensin-1"[All Fields] AND biofilm[All Fields]	Defensin-1	20454633	Normal human gingival epithelial cells sense C. parapsilosis by toll-like receptors and module its pathogenesis through antimicrobial peptides and proinflammatory cytokines.
3870	DRAMP03733	Lysozyme (Arthropods, animals)	"Lysozyme"[All Fields] AND biofilm[All Fields]	Lysozyme	34688058	The dietary supplementation of zinc oxide and selenium nanoparticles enhance the immune response in freshwater fish Oreochromis mossambicus against aquatic pathogen Aeromonas hydrophila.
3870	DRAMP03733	Lysozyme (Arthropods, animals)	"Lysozyme"[All Fields] AND biofilm[All Fields]	Lysozyme	34680849	Anti-Biofilm Coatings Based on Chitosan and Lysozyme Functionalized Magnetite Nanoparticles.
3870	DRAMP03733	Lysozyme (Arthropods, animals)	"Lysozyme"[All Fields] AND biofilm[All Fields]	Lysozyme	34663068	Zwitterionic Peptides Reduce Accumulation of Marine and Freshwater Biofilm Formers.
3870	DRAMP03733	Lysozyme (Arthropods, animals)	"Lysozyme"[All Fields] AND biofilm[All Fields]	Lysozyme	34650538	DexA70, the Truncated Form of a Self-Produced Dextranase, Effectively Disrupts Streptococcus mutansBiofilm.
3870	DRAMP03733	Lysozyme (Arthropods, animals)	"Lysozyme"[All Fields] AND biofilm[All Fields]	Lysozyme	34601068	Tailoring the proliferation of fibroblast cells by multiresponsive and thermosensitive stem cells composite F127 hydrogel containing folic acid.MgO:ZnO/chitosan hybrid microparticles for skin regeneration.
3870	DRAMP03733	Lysozyme (Arthropods, animals)	"Lysozyme"[All Fields] AND biofilm[All Fields]	Lysozyme	34528638	Enhanced clearing of Candidabiofilms on a 3D urothelial cell in vitromodel using lysozyme-functionalized fluconazole-loaded shellac nanoparticles.
3870	DRAMP03733	Lysozyme (Arthropods, animals)	"Lysozyme"[All Fields] AND biofilm[All Fields]	Lysozyme	34488127	Activity of the lyases LysSSE1 and HolSSE1 against common pathogenic bacteria and their antimicrobial efficacy in biofilms.
3870	DRAMP03733	Lysozyme (Arthropods, animals)	"Lysozyme"[All Fields] AND biofilm[All Fields]	Lysozyme	34485498	Effect of antimicrobial peptides on planktonic growth, biofilm formation and biofilm-derived bacterial viability of Streptococcus pneumoniae.
3870	DRAMP03733	Lysozyme (Arthropods, animals)	"Lysozyme"[All Fields] AND biofilm[All Fields]	Lysozyme	34438974	Antimicrobial Activity of the Circular Bacteriocin AS-48 against Clinical Multidrug-Resistant Staphylococcus aureus.
3870	DRAMP03733	Lysozyme (Arthropods, animals)	"Lysozyme"[All Fields] AND biofilm[All Fields]	Lysozyme	34019937	Correlation of over-expression of rv1900c with enhanced survival of M. smegmatis under stress conditions: Modulation of cell surface properties.

3870	DRAMP03733	Lysozyme (Arthropods, animals)	"Lysozyme"[All Fields] AND biofilm[All Fields]	Lysozyme	33947757	A StreptococcusQuorum Sensing System Enables Suppression of Innate Immunity.
3870	DRAMP03733	Lysozyme (Arthropods, animals)	"Lysozyme"[All Fields] AND biofilm[All Fields]	Lysozyme	33920327	Resveratrol-Loaded Hydrogel Contact Lenses with Antioxidant and Antibiofilm Performance.
3870	DRAMP03733	Lysozyme (Arthropods, animals)	"Lysozyme"[All Fields] AND biofilm[All Fields]	Lysozyme	33918930	A Novel Biofilm Model System to Visualise Conjugal Transfer of Vancomycin Resistance by Environmental Enterococci.
3870	DRAMP03733	Lysozyme (Arthropods, animals)	"Lysozyme"[All Fields] AND biofilm[All Fields]	Lysozyme	33875213	Impact of pmrA on Cronobacter sakazakii planktonic and biofilm cells: A comprehensive transcriptomic study.
3870	DRAMP03733	Lysozyme (Arthropods, animals)	"Lysozyme"[All Fields] AND biofilm[All Fields]	Lysozyme	33842797	Antibacterial and Antibiofilm Photodynamic Activities of Lysozyme-Au Nanoclusters/Rose Bengal Conjugates.
3870	DRAMP03733	Lysozyme (Arthropods, animals)	"Lysozyme"[All Fields] AND biofilm[All Fields]	Lysozyme	33783975	Biodegradable Anti-Biofilm Fiber-Membrane Ureteral Stent Constructed with a Robust Biomimetic Superhydrophilic Polycationic Hydration Surface Exhibiting Synergetic Antibacterial and Antiprotein Properties.
3870	DRAMP03733	Lysozyme (Arthropods, animals)	"Lysozyme"[All Fields] AND biofilm[All Fields]	Lysozyme	33773215	Growth in a biofilm sensitizes Cutibacterium acnes to nanosecond pulsed electric fields.
3870	DRAMP03733	Lysozyme (Arthropods, animals)	"Lysozyme"[All Fields] AND biofilm[All Fields]	Lysozyme	33769023	Drug-Free Enzyme-Based Bactericidal Nanomotors against Pathogenic Bacteria.
3870	DRAMP03733	Lysozyme (Arthropods, animals)	"Lysozyme"[All Fields] AND biofilm[All Fields]	Lysozyme	33740161	Enzymatic biofilm destabilisation to support mechanical cleansing of inserted dental implant surfaces: an in-vitro pilot study.
3870	DRAMP03733	Lysozyme (Arthropods, animals)	"Lysozyme"[All Fields] AND biofilm[All Fields]	Lysozyme	33391351	Quantification of Bacterial Colonization in Dental Hard Tissues Using Optimized Molecular Biological Methods.
3870	DRAMP03733	Lysozyme (Arthropods, animals)	"Lysozyme"[All Fields] AND biofilm[All Fields]	Lysozyme	33252326	Effect of antifungal agents, lysozyme and human antimicrobial peptide LL-37 on clinical Candidaisolates with high biofilm production.
3870	DRAMP03733	Lysozyme (Arthropods, animals)	"Lysozyme"[All Fields] AND biofilm[All Fields]	Lysozyme	33185350	[Characterization of the microbiota and cytokine profile of sperm plasma in men with chronic bacterial prostatitis].
3870	DRAMP03733	Lysozyme (Arthropods, animals)	"Lysozyme"[All Fields] AND biofilm[All Fields]	Lysozyme	33113658	New insights into the effect of extracellular polymeric substance on the sludge dewaterability based on interaction energy and viscoelastic acoustic response analysis.
3870	DRAMP03733	Lysozyme (Arthropods, animals)	"Lysozyme"[All Fields] AND biofilm[All Fields]	Lysozyme	33080579	Combination therapy of biogenic C-dots and lysozyme for enhanced antibacterial and antibiofilm activity.
3870	DRAMP03733	Lysozyme (Arthropods, animals)	"Lysozyme"[All Fields] AND biofilm[All Fields]	Lysozyme	33072040	Exploiting Lactoferricin (17-30) as a Potential Antimicrobial and Antibiofilm Candidate Against Multi-Drug-Resistant Enteroaggregative Escherichia coli.
3870	DRAMP03733	Lysozyme (Arthropods, animals)	"Lysozyme"[All Fields] AND biofilm[All Fields]	Lysozyme	33045439	Antibacterial activities of and biofilm removal by Ablysin, an endogenous lysozyme-like protein originated from Acinetobacter baumannii 1656-2.
3870	DRAMP03733	Lysozyme (Arthropods, animals)	"Lysozyme"[All Fields] AND biofilm[All Fields]	Lysozyme	32916574	Supported lysozyme for improved antimicrobial surface protection.
3870	DRAMP03733	Lysozyme (Arthropods, animals)	"Lysozyme"[All Fields] AND biofilm[All Fields]	Lysozyme	32787275	Hydration-Induced Structural Changes in the Solid State of Protein: A SAXS/WAXS Study on Lysozyme.
3870	DRAMP03733	Lysozyme (Arthropods, animals)	"Lysozyme"[All Fields] AND biofilm[All Fields]	Lysozyme	32612893	Biofilm modelling on the contact lenses and comparison of the in vitro activities of multipurpose lens solutions and antibiotics.
3870	DRAMP03733	Lysozyme (Arthropods, animals)	"Lysozyme"[All Fields] AND biofilm[All Fields]	Lysozyme	32512756	Inactivation of the sfgtr4Gene of Shigella flexneriInduces Biofilm Formation and Affects Bacterial Pathogenicity.
3870	DRAMP03733	Lysozyme (Arthropods, animals)	"Lysozyme"[All Fields] AND biofilm[All Fields]	Lysozyme	32393645	Evolution of vancomycin-resistant Enterococcus faeciumduring colonization and infection in immunocompromised pediatric patients.
3870	DRAMP03733	Lysozyme (Arthropods, animals)	"Lysozyme"[All Fields] AND biofilm[All Fields]	Lysozyme	32280054	Quorum quenching acylase impacts the viability and morphological change of Agrobacterium tumefaciens cells.
3870	DRAMP03733	Lysozyme (Arthropods, animals)	"Lysozyme"[All Fields] AND biofilm[All Fields]	Lysozyme	32266650	Probiotic Potential of Bacillus Strains Isolated from an Acidic Fermented Food Idli.
3870	DRAMP03733	Lysozyme (Arthropods, animals)	"Lysozyme"[All Fields] AND biofilm[All Fields]	Lysozyme	32089678	Effects of Lysozyme, Proteinase K, and Cephalosporins on Biofilm Formation by Clinical Isolates of Pseudomonas aeruginosa.
3870	DRAMP03733	Lysozyme (Arthropods, animals)	"Lysozyme"[All Fields] AND biofilm[All Fields]	Lysozyme	33447800	Cellular chaining influences biofilm formation and structure in group A Streptococcus.
3870	DRAMP03733	Lysozyme (Arthropods, animals)	"Lysozyme"[All Fields] AND biofilm[All Fields]	Lysozyme	31698068	Vibrio harveyi biofilm as immunostimulant candidate for high-health pacific white shrimp, Penaeus vannamei farming.
3870	DRAMP03733	Lysozyme (Arthropods, animals)	"Lysozyme"[All Fields] AND biofilm[All Fields]	Lysozyme	31480687	Characterization and Antibiofilm Activity of Mannitol-Chitosan-Blended Paste for Local Antibiotic Delivery System.
3870	DRAMP03733	Lysozyme (Arthropods, animals)	"Lysozyme"[All Fields] AND biofilm[All Fields]	Lysozyme	31374264	Anti-cancer, anti-biofilm, and anti-inflammatory properties of hen's albumen: A photodynamic approach.
3870	DRAMP03733	Lysozyme (Arthropods, animals)	"Lysozyme"[All Fields] AND biofilm[All Fields]	Lysozyme	31279118	Effect of extracellular polymeric substances (EPS) conditioned by combined lysozyme and cationic polyacrylamide on the dewatering performance of activated sludge.
3870	DRAMP03733	Lysozyme (Arthropods, animals)	"Lysozyme"[All Fields] AND biofilm[All Fields]	Lysozyme	31252064	Antibacterial synergy between rutin and florfenicol enhances therapeutic spectrum against drug resistant Aeromonas hydrophila.
3870	DRAMP03733	Lysozyme (Arthropods, animals)	"Lysozyme"[All Fields] AND biofilm[All Fields]	Lysozyme	31214943	Safety and Stability of Two Potentially Probiotic Lactobacillus Strains After In Vitro Gastrointestinal Transit.
3870	DRAMP03733	Lysozyme (Arthropods, animals)	"Lysozyme"[All Fields] AND biofilm[All Fields]	Lysozyme	31125644	Rv0518, a nutritive stress inducible GDSL lipase of Mycobacterium tuberculosis, enhanced intracellular survival of bacteria by cell wall modulation.
3870	DRAMP03733	Lysozyme (Arthropods, animals)	"Lysozyme"[All Fields] AND biofilm[All Fields]	Lysozyme	31085703	The Ser/Thr Kinase PrkC Participates in Cell Wall Homeostasis and Antimicrobial Resistance in Clostridium difficile.
3870	DRAMP03733	Lysozyme (Arthropods, animals)	"Lysozyme"[All Fields] AND biofilm[All Fields]	Lysozyme	30843349	Characterization of the biofilm phenotype of a Listeria monocytogenes mutant deficient in agr peptide sensing.
3870	DRAMP03733	Lysozyme (Arthropods, animals)	"Lysozyme"[All Fields] AND biofilm[All Fields]	Lysozyme	30833631	Detection of vaginal lactobacilli as probiotic candidates.
3870	DRAMP03733	Lysozyme (Arthropods, animals)	"Lysozyme"[All Fields] AND biofilm[All Fields]	Lysozyme	30761784	[Biological properties of microorganisms isolated from the urine of patients with urolithiasis].
3870	DRAMP03733	Lysozyme (Arthropods, animals)	"Lysozyme"[All Fields] AND biofilm[All Fields]	Lysozyme	30701862	Effect of N-acetylcysteine on mucosal immunity of respiratory tract.
3870	DRAMP03733	Lysozyme (Arthropods, animals)	"Lysozyme"[All Fields] AND biofilm[All Fields]	Lysozyme	30649289	Innate immune components affect growth and virulence traits of bacterial-vaginosis-associated and non-bacterial-vaginosis-associated Gardnerella vaginalis strains similarly.
3870	DRAMP03733	Lysozyme (Arthropods, animals)	"Lysozyme"[All Fields] AND biofilm[All Fields]	Lysozyme	30639529	Catalysing the way towards antimicrobial effectiveness: A systematic analysis and a new online resource for antimicrobial-enzyme combinations against Pseudomonas aeruginosa and Staphylococcus aureus.
3870	DRAMP03733	Lysozyme (Arthropods, animals)	"Lysozyme"[All Fields] AND biofilm[All Fields]	Lysozyme	30639217	Immobilization of antimicrobial and anti-quorum sensing enzymes onto GMA-grafted poly(vinyl chloride) catheters.
3870	DRAMP03733	Lysozyme (Arthropods, animals)	"Lysozyme"[All Fields] AND biofilm[All Fields]	Lysozyme	30497006	Impact of oral astringent stimuli on surface charge and morphology of the protein-rich pellicle at the tooth-saliva interphase.
3870	DRAMP03733	Lysozyme (Arthropods, animals)	"Lysozyme"[All Fields] AND biofilm[All Fields]	Lysozyme	30389429	Microbiology insights into boosting salivary defences through the use of enzymes and proteins.
3870	DRAMP03733	Lysozyme (Arthropods, animals)	"Lysozyme"[All Fields] AND biofilm[All Fields]	Lysozyme	30207684	Developing Antibacterial Nanocrystalline Cellulose Using Natural Antibacterial Agents.
3870	DRAMP03733	Lysozyme (Arthropods, animals)	"Lysozyme"[All Fields] AND biofilm[All Fields]	Lysozyme	30176334	β-1, 3 glucan binding protein based selenium nanowire enhances the immune status of Cyprinus carpio and protection against Aeromonas hydrophila infection.
3870	DRAMP03733	Lysozyme (Arthropods, animals)	"Lysozyme"[All Fields] AND biofilm[All Fields]	Lysozyme	30142702	Moraxella bovis, Moraxella ovis and Moraxella bovoculi: biofilm formation and lysozyme activity.
3870	DRAMP03733	Lysozyme (Arthropods, animals)	"Lysozyme"[All Fields] AND biofilm[All Fields]	Lysozyme	30131945	The Intestinal Roundworm Ascaris suumReleases Antimicrobial Factors Which Interfere With Bacterial Growth and Biofilm Formation.
3870	DRAMP03733	Lysozyme (Arthropods, animals)	"Lysozyme"[All Fields] AND biofilm[All Fields]	Lysozyme	30082920	Control of Propionibacterium acnes by natural antimicrobial substances: Role of the bacteriocin AS-48 and lysozyme.
3870	DRAMP03733	Lysozyme (Arthropods, animals)	"Lysozyme"[All Fields] AND biofilm[All Fields]	Lysozyme	29915307	Screening and characterization of prophages in Desulfovibrio genomes.
3870	DRAMP03733	Lysozyme (Arthropods, animals)	"Lysozyme"[All Fields] AND biofilm[All Fields]	Lysozyme	29915116	Resuscitation-Promoting Factors Are Required for Mycobacterium smegmatis Biofilm Formation.
3870	DRAMP03733	Lysozyme (Arthropods, animals)	"Lysozyme"[All Fields] AND biofilm[All Fields]	Lysozyme	29876449	TCA precipitation and ethanol/HCl single-step purification evaluation: One-dimensional gel electrophoresis, bradford assays, spectrofluorometry and Raman spectroscopy data on HSA, Rnase, lysozyme - Mascots and Skyline data.
3870	DRAMP03733	Lysozyme (Arthropods, animals)	"Lysozyme"[All Fields] AND biofilm[All Fields]	Lysozyme	29555699	A Quorum Sensing-Regulated Protein Binds Cell Wall Components and Enhances Lysozyme Resistance in Streptococcus pyogenes.
3870	DRAMP03733	Lysozyme (Arthropods, animals)	"Lysozyme"[All Fields] AND biofilm[All Fields]	Lysozyme	29518559	Effect of β-1, 3 glucan binding protein based zinc oxide nanoparticles supplemented diet on immune response and disease resistance in Oreochromis mossambicus against Aeromonas hydrophila.
3870	DRAMP03733	Lysozyme (Arthropods, animals)	"Lysozyme"[All Fields] AND biofilm[All Fields]	Lysozyme	29464594	Inhalable Levofloxacin Liposomes Complemented with Lysozyme for Treatment of Pulmonary Infection in Rats: Effective Antimicrobial and Antibiofilm Strategy.
3870	DRAMP03733	Lysozyme (Arthropods, animals)	"Lysozyme"[All Fields] AND biofilm[All Fields]	Lysozyme	29393147	Protein Profile of the Acquired Enamel Pellicle after Rinsing with Whole Milk, Fat-Free Milk, and Water: An in vivo Study.
3870	DRAMP03733	Lysozyme (Arthropods, animals)	"Lysozyme"[All Fields] AND biofilm[All Fields]	Lysozyme	29373594	Biofilm formation and transcriptome analysis of Streptococcus gallolyticus subsp. gallolyticus in response to lysozyme.
3870	DRAMP03733	Lysozyme (Arthropods, animals)	"Lysozyme"[All Fields] AND biofilm[All Fields]	Lysozyme	29315029	Correlations among Resistances to Different Antimicrobial Compounds in Salmonella Strains from Hen Eggshells.
3870	DRAMP03733	Lysozyme (Arthropods, animals)	"Lysozyme"[All Fields] AND biofilm[All Fields]	Lysozyme	31565305	Enzyme Crystals and Hydrogel Composite Membranes as New Active Food Packaging Material.

3870	DRAMP03733	Lysozyme (Arthropods, animals)	"Lysozyme"[All Fields] AND biofilm[All Fields]	Lysozyme	29205129	Dissimilar pigment regulation in Serpula lacrymans and Paxillus involutus during inter-kingdom interactions.
3870	DRAMP03733	Lysozyme (Arthropods, animals)	"Lysozyme"[All Fields] AND biofilm[All Fields]	Lysozyme	29201023	A Marine Actinomycete Rescues Caenorhabditis elegansfrom Pseudomonas aeruginosaInfection through Restitution of Lysozyme 7.
3870	DRAMP03733	Lysozyme (Arthropods, animals)	"Lysozyme"[All Fields] AND biofilm[All Fields]	Lysozyme	29184097	Csl2, a novel chimeric bacteriophage lysin to fight infections caused by Streptococcus suis, an emerging zoonotic pathogen.
3870	DRAMP03733	Lysozyme (Arthropods, animals)	"Lysozyme"[All Fields] AND biofilm[All Fields]	Lysozyme	29080817	Production and structural characterization of exopolysaccharides from newly isolated probiotic lactic acid bacteria.
3870	DRAMP03733	Lysozyme (Arthropods, animals)	"Lysozyme"[All Fields] AND biofilm[All Fields]	Lysozyme	28931063	Plant flavones enhance antimicrobial activity of respiratory epithelial cell secretions against Pseudomonas aeruginosa.
3870	DRAMP03733	Lysozyme (Arthropods, animals)	"Lysozyme"[All Fields] AND biofilm[All Fields]	Lysozyme	28922066	The Effect of Lysozyme on Reducing Biofilms by Staphylococcus aureus, Pseudomonas aeruginosa, and Gardnerella vaginalis: An In Vitro Examination.
3870	DRAMP03733	Lysozyme (Arthropods, animals)	"Lysozyme"[All Fields] AND biofilm[All Fields]	Lysozyme	28826250	Antimicrobial and anti-biofilm activity of tannic acid against Staphylococcus aureus.
3870	DRAMP03733	Lysozyme (Arthropods, animals)	"Lysozyme"[All Fields] AND biofilm[All Fields]	Lysozyme	28649626	Investigation of simulated microgravity effects on Streptococcus mutansphysiology and global gene expression.
3870	DRAMP03733	Lysozyme (Arthropods, animals)	"Lysozyme"[All Fields] AND biofilm[All Fields]	Lysozyme	32264250	Protein-repellent and antimicrobial nanoparticle coatings from hyaluronic acid and a lysine-derived biocompatible surfactant.
3870	DRAMP03733	Lysozyme (Arthropods, animals)	"Lysozyme"[All Fields] AND biofilm[All Fields]	Lysozyme	28400767	The Periplasmic Chaperone Network of Campylobacter jejuni: Evidence that SalC (Cj1289) and PpiD (Cj0694) Are Involved in Maintaining Outer Membrane Integrity.
3870	DRAMP03733	Lysozyme (Arthropods, animals)	"Lysozyme"[All Fields] AND biofilm[All Fields]	Lysozyme	28400765	Role of Bacillus licheniformisVS16-Derived Biosurfactant in Mediating Immune Responses in Carp Rohu and its Application to the Food Industry.
3870	DRAMP03733	Lysozyme (Arthropods, animals)	"Lysozyme"[All Fields] AND biofilm[All Fields]	Lysozyme	28397768	Lysozyme Associated Liposomal Gentamicin Inhibits Bacterial Biofilm.
3870	DRAMP03733	Lysozyme (Arthropods, animals)	"Lysozyme"[All Fields] AND biofilm[All Fields]	Lysozyme	28289407	A Novel Antimicrobial Endolysin, LysPA26, against Pseudomonas aeruginosa.
3870	DRAMP03733	Lysozyme (Arthropods, animals)	"Lysozyme"[All Fields] AND biofilm[All Fields]	Lysozyme	28251063	Evaluation of a chitosan-polyethylene glycol paste as a local antibiotic delivery device.
3870	DRAMP03733	Lysozyme (Arthropods, animals)	"Lysozyme"[All Fields] AND biofilm[All Fields]	Lysozyme	28188877	Laser deposition of poly(3-hydroxybutyric acid-co-3-hydroxyvaleric acid) - lysozyme microspheres based coatings with anti-microbial properties.
3870	DRAMP03733	Lysozyme (Arthropods, animals)	"Lysozyme"[All Fields] AND biofilm[All Fields]	Lysozyme	28138698	Dose-dependent effect of lysozyme upon Candida albicans biofilm.
3870	DRAMP03733	Lysozyme (Arthropods, animals)	"Lysozyme"[All Fields] AND biofilm[All Fields]	Lysozyme	28109187	Antimicrobial and antioxidant activities of Saccharomyces cerevisiae IFST062013, a potential probiotic.
3870	DRAMP03733	Lysozyme (Arthropods, animals)	"Lysozyme"[All Fields] AND biofilm[All Fields]	Lysozyme	30695493	[PHYSIOLOGICAL FEATURES OF CORYNEBACTERIA OF FEMALE REPRODUC- TIVE TRACT].
3870	DRAMP03733	Lysozyme (Arthropods, animals)	"Lysozyme"[All Fields] AND biofilm[All Fields]	Lysozyme	29619048	Efficacy of a Rinse Containing Sea Salt and Lysozyme on Biofilm and Gingival Health in a Group of Young Adults: A Pilot Study.
3870	DRAMP03733	Lysozyme (Arthropods, animals)	"Lysozyme"[All Fields] AND biofilm[All Fields]	Lysozyme	27999400	Comparative Study on the Characteristics of Weissella cibaria CMU and Probiotic Strains for Oral Care.
3870	DRAMP03733	Lysozyme (Arthropods, animals)	"Lysozyme"[All Fields] AND biofilm[All Fields]	Lysozyme	27984151	Copper incorporated microporous chitosan-polyethylene glycol hydrogels loaded with naproxen for effective drug release and anti-infection wound dressing.
3870	DRAMP03733	Lysozyme (Arthropods, animals)	"Lysozyme"[All Fields] AND biofilm[All Fields]	Lysozyme	27931094	Human Lysozyme Peptidase Resistance Is Perturbed by the Anionic Glycolipid Biosurfactant Rhamnolipid Produced by the Opportunistic Pathogen Pseudomonas aeruginosa.
3870	DRAMP03733	Lysozyme (Arthropods, animals)	"Lysozyme"[All Fields] AND biofilm[All Fields]	Lysozyme	27845497	Lysozyme as a cotreatment during antibiotics use against vaginal infections: An in vitro study on Gardnerella vaginalis biofilm models.
3870	DRAMP03733	Lysozyme (Arthropods, animals)	"Lysozyme"[All Fields] AND biofilm[All Fields]	Lysozyme	27821857	Establishing Antibacterial Multilayer Films on the Surface of Direct Metal Laser Sintered Titanium Primed with Phase-Transited Lysozyme.
3870	DRAMP03733	Lysozyme (Arthropods, animals)	"Lysozyme"[All Fields] AND biofilm[All Fields]	Lysozyme	27709890	A Biodegradable Polycationic Paint that Kills Bacteria in Vitro and in Vivo.
3870	DRAMP03733	Lysozyme (Arthropods, animals)	"Lysozyme"[All Fields] AND biofilm[All Fields]	Lysozyme	27685160	In Situ Impregnation of Silver Nanoclusters in Microporous Chitosan-PEG Membranes as an Antibacterial and Drug Delivery Percutaneous Device.
3870	DRAMP03733	Lysozyme (Arthropods, animals)	"Lysozyme"[All Fields] AND biofilm[All Fields]	Lysozyme	27602088	Antibiotic susceptibility, antibacterial activity and characterisation of Enterococcus faeciumstrains isolated from breast milk.
3870	DRAMP03733	Lysozyme (Arthropods, animals)	"Lysozyme"[All Fields] AND biofilm[All Fields]	Lysozyme	27520822	Peptidoglycan Acetylation of Campylobacter jejuni Is Essential for Maintaining Cell Wall Integrity and Colonization in Chicken Intestines.
3870	DRAMP03733	Lysozyme (Arthropods, animals)	"Lysozyme"[All Fields] AND biofilm[All Fields]	Lysozyme	27375592	Pseudomonas aeruginosa Outer Membrane Vesicles Triggered by Human Mucosal Fluid and Lysozyme Can Prime Host Tissue Surfaces for Bacterial Adhesion.
3870	DRAMP03733	Lysozyme (Arthropods, animals)	"Lysozyme"[All Fields] AND biofilm[All Fields]	Lysozyme	27373086	[Staphylococcus aureus biofilm influences the expression of lysozyme, SLPI and gp340 in a human sinonasal explant model].
3870	DRAMP03733	Lysozyme (Arthropods, animals)	"Lysozyme"[All Fields] AND biofilm[All Fields]	Lysozyme	27236754	Molecular typing and differences in biofilm formation and antibiotic susceptibilities among Prototheca strains isolated in Italy and Brazil.
3870	DRAMP03733	Lysozyme (Arthropods, animals)	"Lysozyme"[All Fields] AND biofilm[All Fields]	Lysozyme	27015648	Biopolymer-induced calcium phosphate scaling in membrane-based water treatment systems: Langmuir model films studies.
3870	DRAMP03733	Lysozyme (Arthropods, animals)	"Lysozyme"[All Fields] AND biofilm[All Fields]	Lysozyme	26783746	Titanium Surface Priming with Phase-Transited Lysozyme to Establish a Silver Nanoparticle-Loaded Chitosan/Hyaluronic Acid Antibacterial Multilayer via Layer-by-Layer Self-Assembly.
3870	DRAMP03733	Lysozyme (Arthropods, animals)	"Lysozyme"[All Fields] AND biofilm[All Fields]	Lysozyme	31532953	[The etiologic structure and biologic characteristics of agents of infections of bloodstream.]
3870	DRAMP03733	Lysozyme (Arthropods, animals)	"Lysozyme"[All Fields] AND biofilm[All Fields]	Lysozyme	26488327	Complexation of Lysozyme with Sodium Poly(styrenesulfonate) via the Two-State and Non-Two-State Unfoldings of Lysozyme.
3870	DRAMP03733	Lysozyme (Arthropods, animals)	"Lysozyme"[All Fields] AND biofilm[All Fields]	Lysozyme	26478289	Covalent immobilization of lysozyme onto woven and knitted crimped polyethylene terephthalate grafts to minimize the adhesion of broad spectrum pathogens.
3870	DRAMP03733	Lysozyme (Arthropods, animals)	"Lysozyme"[All Fields] AND biofilm[All Fields]	Lysozyme	26470414	[REGULATING EFFECT OF ASSOCIATIVE MICROBIOTA ON THE RHYTHMS OF BIOLOGICAL PROPERTIES OF FUNGI AND BACTERIA].
3870	DRAMP03733	Lysozyme (Arthropods, animals)	"Lysozyme"[All Fields] AND biofilm[All Fields]	Lysozyme	26458820	Effects of fed-batch and continuous fermentations on human lysozyme production by Kluyveromyces lactis K7 in biofilm reactors.
3870	DRAMP03733	Lysozyme (Arthropods, animals)	"Lysozyme"[All Fields] AND biofilm[All Fields]	Lysozyme	26400891	Effect of histatin-5 and lysozyme on the ability of Streptococcus mutans to form biofilms in in vitro conditions.
3870	DRAMP03733	Lysozyme (Arthropods, animals)	"Lysozyme"[All Fields] AND biofilm[All Fields]	Lysozyme	26383819	Recent advances for the production and recovery methods of lysozyme.
3870	DRAMP03733	Lysozyme (Arthropods, animals)	"Lysozyme"[All Fields] AND biofilm[All Fields]	Lysozyme	26315397	Enzymes in the in-situ pellicle of children with different caries activity.
3870	DRAMP03733	Lysozyme (Arthropods, animals)	"Lysozyme"[All Fields] AND biofilm[All Fields]	Lysozyme	26152033	[AEROMONAS BACTERIA ISOLATED FROM BITHYNIIDAE MOLLUSKS AND THEIR HABITATS: SPECIES COMPOSITION AND BIOLOGICAL PROPERTIES. COMMUNICATION 1].
3870	DRAMP03733	Lysozyme (Arthropods, animals)	"Lysozyme"[All Fields] AND biofilm[All Fields]	Lysozyme	26092919	Acetylcholine Protects against Candida albicans Infection by Inhibiting Biofilm Formation and Promoting Hemocyte Function in a Galleria mellonella Infection Model.
3870	DRAMP03733	Lysozyme (Arthropods, animals)	"Lysozyme"[All Fields] AND biofilm[All Fields]	Lysozyme	27688392	Antimicrobial Capacity of Casein Phosphopeptide/Amorphous Calcium Phosphate and Enzymes in Glass Ionomer Cement in Dentin Carious Lesions.
3870	DRAMP03733	Lysozyme (Arthropods, animals)	"Lysozyme"[All Fields] AND biofilm[All Fields]	Lysozyme	25950767	In vitro biofilm development of Streptococcus pneumoniae and formation of choline-binding protein-DNA complexes.
3870	DRAMP03733	Lysozyme (Arthropods, animals)	"Lysozyme"[All Fields] AND biofilm[All Fields]	Lysozyme	25757148	Development of novel formulations containing Lysozyme and Lactoferrin and evaluation of antibacterial effects on Mutans Streptococci and Lactobacilli.
3870	DRAMP03733	Lysozyme (Arthropods, animals)	"Lysozyme"[All Fields] AND biofilm[All Fields]	Lysozyme	25725311	Cleaning of biomaterial surfaces: protein removal by different solvents.
3870	DRAMP03733	Lysozyme (Arthropods, animals)	"Lysozyme"[All Fields] AND biofilm[All Fields]	Lysozyme	25721974	Characterization of a novel strain phylogenetically related to Kocuria rhizophila and its chemical modification to improve performance of microbial fuel cells.
3870	DRAMP03733	Lysozyme (Arthropods, animals)	"Lysozyme"[All Fields] AND biofilm[All Fields]	Lysozyme	25536779	[Species structure and staphylococci bioprofile characteristics--ausative agents of perinatal infectious-inflammation pathology in children of Orenburg].
3870	DRAMP03733	Lysozyme (Arthropods, animals)	"Lysozyme"[All Fields] AND biofilm[All Fields]	Lysozyme	25467937	Phenotypic characterization of a novel double knockout PknI/DacB2 from Mycobacterium tuberculosis.
3870	DRAMP03733	Lysozyme (Arthropods, animals)	"Lysozyme"[All Fields] AND biofilm[All Fields]	Lysozyme	25449384	N-hexanoyl-L-homoserine lactone-degrading Pseudomonas aeruginosa PsDAHP1 protects zebrafish against Vibrio parahaemolyticus infection.
3870	DRAMP03733	Lysozyme (Arthropods, animals)	"Lysozyme"[All Fields] AND biofilm[All Fields]	Lysozyme	25312955	The conserved hypothetical protein Rv0574c is required for cell wall integrity, stress tolerance, and virulence of Mycobacterium tuberculosis.
3870	DRAMP03733	Lysozyme (Arthropods, animals)	"Lysozyme"[All Fields] AND biofilm[All Fields]	Lysozyme	25221673	Iron oxide nanoparticles induce Pseudomonas aeruginosagrowth, induce biofilm formation, and inhibit antimicrobial peptide function.
3870	DRAMP03733	Lysozyme (Arthropods, animals)	"Lysozyme"[All Fields] AND biofilm[All Fields]	Lysozyme	25115519	Presence and function of a thick mucous layer rich in polysaccharides around Bacillus subtilis spores.
3870	DRAMP03733	Lysozyme (Arthropods, animals)	"Lysozyme"[All Fields] AND biofilm[All Fields]	Lysozyme	24559398	Temperature-driven adsorption and desorption of proteins at solid-liquid interfaces.
3870	DRAMP03733	Lysozyme (Arthropods, animals)	"Lysozyme"[All Fields] AND biofilm[All Fields]	Lysozyme	24434534	Designing nanogel carriers for antibacterial applications.
3870	DRAMP03733	Lysozyme (Arthropods, animals)	"Lysozyme"[All Fields] AND biofilm[All Fields]	Lysozyme	24240906	Adsorption study of pellicle proteins to gold, silica and titanium by quartz crystal microbalance method.
3870	DRAMP03733	Lysozyme (Arthropods, animals)	"Lysozyme"[All Fields] AND biofilm[All Fields]	Lysozyme	24121782	Enhanced expressions of lysozyme, SLPI and glycoprotein 340 in biofilm-associated chronic rhinosinusitis.
3870	DRAMP03733	Lysozyme (Arthropods, animals)	"Lysozyme"[All Fields] AND biofilm[All Fields]	Lysozyme	24011302	Fluorescence microscopic visualization of non cellular components during initial bioadhesion in situ.

3870	DRAMP03733	Lysozyme (Arthropods, animals)	"Lysozyme"[All Fields] AND biofilm[All Fields]	Lysozyme	23844477	Destruction of single-species biofilms of Escherichia coli or Klebsiella pneumoniae subsp. pneumoniae by dextranase, lactoferrin, and lysozyme.
3870	DRAMP03733	Lysozyme (Arthropods, animals)	"Lysozyme"[All Fields] AND biofilm[All Fields]	Lysozyme	23701483	Effects of biological molecules on calcium mineral formation associated with wastewater desalination as assessed using small-angle neutron scattering.
3870	DRAMP03733	Lysozyme (Arthropods, animals)	"Lysozyme"[All Fields] AND biofilm[All Fields]	Lysozyme	23659996	Vaccination efficiency of surface antigens and killed whole cell of Pseudomonas putida in large yellow croaker (Pseudosciaena crocea).
3870	DRAMP03733	Lysozyme (Arthropods, animals)	"Lysozyme"[All Fields] AND biofilm[All Fields]	Lysozyme	23657582	Production of human lysozyme in biofilm reactor and optimization of growth parameters of Kluyveromyces lactis K7.
3870	DRAMP03733	Lysozyme (Arthropods, animals)	"Lysozyme"[All Fields] AND biofilm[All Fields]	Lysozyme	23504078	Phenotypic characterization, virulence, and immunogenicity of Edwardsiella tarda LSE40 aroA mutant.
3870	DRAMP03733	Lysozyme (Arthropods, animals)	"Lysozyme"[All Fields] AND biofilm[All Fields]	Lysozyme	23460607	Undecaprenyl pyrophosphate phosphatase confers low-level resistance to bacitracin in Enterococcus faecalis.
3870	DRAMP03733	Lysozyme (Arthropods, animals)	"Lysozyme"[All Fields] AND biofilm[All Fields]	Lysozyme	23434686	Enhancing antibacterial activity of surface-grafted chitosan with immobilized lysozyme on bioinspired stainless steel substrates.
3870	DRAMP03733	Lysozyme (Arthropods, animals)	"Lysozyme"[All Fields] AND biofilm[All Fields]	Lysozyme	23403559	Host defense proteins derived from human saliva bind to Staphylococcus aureus.
3870	DRAMP03733	Lysozyme (Arthropods, animals)	"Lysozyme"[All Fields] AND biofilm[All Fields]	Lysozyme	23194029	Alpha-amylase is a human salivary protein with affinity to lipopolysaccharide of Aggregatibacter actinomycetemcomitans.
3870	DRAMP03733	Lysozyme (Arthropods, animals)	"Lysozyme"[All Fields] AND biofilm[All Fields]	Lysozyme	22947470	Effects of nisin and lysozyme on growth inhibition and biofilm formation capacity of Staphylococcus aureus strains isolated from raw milk and cheese samples.
3870	DRAMP03733	Lysozyme (Arthropods, animals)	"Lysozyme"[All Fields] AND biofilm[All Fields]	Lysozyme	22937707	[Interaction of Bifidobacterium bifidum with members of normal microflora in human intestine microsymbiocenosis].
3870	DRAMP03733	Lysozyme (Arthropods, animals)	"Lysozyme"[All Fields] AND biofilm[All Fields]	Lysozyme	22913814	Insight into the composition of the intercellular matrix of Streptococcus pneumoniae biofilms.
3870	DRAMP03733	Lysozyme (Arthropods, animals)	"Lysozyme"[All Fields] AND biofilm[All Fields]	Lysozyme	22363222	Tasco®: a product of Ascophyllum nodosum enhances immune response of Caenorhabditis elegans against Pseudomonas aeruginosa infection.
3870	DRAMP03733	Lysozyme (Arthropods, animals)	"Lysozyme"[All Fields] AND biofilm[All Fields]	Lysozyme	26781731	Recombinant Expression of a Putative Amidase Cloned from the Genome of Listeria monocytogenes that Lyses the Bacterium and its Monolayer in Conjunction with a Protease.
3870	DRAMP03733	Lysozyme (Arthropods, animals)	"Lysozyme"[All Fields] AND biofilm[All Fields]	Lysozyme	22344334	Influence of artificial saliva in biofilm formation of Candida albicans in vitro.
3870	DRAMP03733	Lysozyme (Arthropods, animals)	"Lysozyme"[All Fields] AND biofilm[All Fields]	Lysozyme	22308727	[Microbial "friend-foe" identification in human intestine microsymbiocenosis].
3870	DRAMP03733	Lysozyme (Arthropods, animals)	"Lysozyme"[All Fields] AND biofilm[All Fields]	Lysozyme	22265308	Effect of lysozyme on "flor" velum yeasts in the biological aging of sherry wines.
3870	DRAMP03733	Lysozyme (Arthropods, animals)	"Lysozyme"[All Fields] AND biofilm[All Fields]	Lysozyme	21821771	Role of phase variation in the resistance of Myxococcus xanthus fruiting bodies to Caenorhabditis elegans predation.
3870	DRAMP03733	Lysozyme (Arthropods, animals)	"Lysozyme"[All Fields] AND biofilm[All Fields]	Lysozyme	21486002	Anti-fouling chemistry of chiral monolayers: enhancing biofilm resistance on racemic surface.
3870	DRAMP03733	Lysozyme (Arthropods, animals)	"Lysozyme"[All Fields] AND biofilm[All Fields]	Lysozyme	21471196	A disulfide bridge network within the soluble periplasmic domain determines structure and function of the outer membrane protein RCSF.
3870	DRAMP03733	Lysozyme (Arthropods, animals)	"Lysozyme"[All Fields] AND biofilm[All Fields]	Lysozyme	21338094	Lysozyme-coupled poly(poly(ethylene glycol) methacrylate)-stainless steel hybrids and their antifouling and antibacterial surfaces.
3870	DRAMP03733	Lysozyme (Arthropods, animals)	"Lysozyme"[All Fields] AND biofilm[All Fields]	Lysozyme	21247450	Phylogenetic group- and species-specific oligonucleotide probes for single-cell detection of lactic acid bacteria in oral biofilms.
3870	DRAMP03733	Lysozyme (Arthropods, animals)	"Lysozyme"[All Fields] AND biofilm[All Fields]	Lysozyme	21148692	Integrity of proteins in human saliva after sterilization by gamma irradiation.
3870	DRAMP03733	Lysozyme (Arthropods, animals)	"Lysozyme"[All Fields] AND biofilm[All Fields]	Lysozyme	21045923	Structural evolution of protein-biofilms: Simulations and experiments.
3870	DRAMP03733	Lysozyme (Arthropods, animals)	"Lysozyme"[All Fields] AND biofilm[All Fields]	Lysozyme	20961363	Potential of ceragenin CSA-13 and its mixture with pluronic F-127 as treatment of topical bacterial infections.
3870	DRAMP03733	Lysozyme (Arthropods, animals)	"Lysozyme"[All Fields] AND biofilm[All Fields]	Lysozyme	20865041	Comparative genomics of Gardnerella vaginalis strains reveals substantial differences in metabolic and virulence potential.
3870	DRAMP03733	Lysozyme (Arthropods, animals)	"Lysozyme"[All Fields] AND biofilm[All Fields]	Lysozyme	20724386	Glutamine synthetase encoded by glnA-1 is necessary for cell wall resistance and pathogenicity of Mycobacterium bovis.
3870	DRAMP03733	Lysozyme (Arthropods, animals)	"Lysozyme"[All Fields] AND biofilm[All Fields]	Lysozyme	20566201	Bioengineering of stainless steel surface by covalent immobilization of enzymes. Physical characterization and interfacial enzymatic activity.
3870	DRAMP03733	Lysozyme (Arthropods, animals)	"Lysozyme"[All Fields] AND biofilm[All Fields]	Lysozyme	20417319	Functionalization of acrylic hydrogels with alpha-, beta- or gamma-cyclodextrin modulates protein adsorption and antifungal delivery.
3870	DRAMP03733	Lysozyme (Arthropods, animals)	"Lysozyme"[All Fields] AND biofilm[All Fields]	Lysozyme	20121056	Achieving highly effective non-biofouling performance for polypropylene membranes modified by UV-induced surface graft polymerization of two oppositely charged monomers.
3870	DRAMP03733	Lysozyme (Arthropods, animals)	"Lysozyme"[All Fields] AND biofilm[All Fields]	Lysozyme	19334612	[Search for destruction factors of bacterial biofilms: comparison of phage properties in a group of Pseudomonas putida bacteriophages and specificity of their halo-formation products].
3870	DRAMP03733	Lysozyme (Arthropods, animals)	"Lysozyme"[All Fields] AND biofilm[All Fields]	Lysozyme	19241565	Storability of antimicrobial chitosan-lysozyme composite coating and film-forming solutions.
3870	DRAMP03733	Lysozyme (Arthropods, animals)	"Lysozyme"[All Fields] AND biofilm[All Fields]	Lysozyme	19166331	Grafting of lysozyme and/or poly(ethylene glycol) to prevent biofilm growth on stainless steel surfaces.
3870	DRAMP03733	Lysozyme (Arthropods, animals)	"Lysozyme"[All Fields] AND biofilm[All Fields]	Lysozyme	19038377	Synergistic activity of lysozyme and antifungal agents against Candida albicans biofilms on denture acrylic surfaces.
3870	DRAMP03733	Lysozyme (Arthropods, animals)	"Lysozyme"[All Fields] AND biofilm[All Fields]	Lysozyme	18992255	Crystal structure of the resuscitation-promoting factor (DeltaDUF)RpfB from M. tuberculosis.
3870	DRAMP03733	Lysozyme (Arthropods, animals)	"Lysozyme"[All Fields] AND biofilm[All Fields]	Lysozyme	18989905	Ultralow fouling zwitterionic polymers grafted from surfaces covered with an initiator via an adhesive mussel mimetic linkage.
3870	DRAMP03733	Lysozyme (Arthropods, animals)	"Lysozyme"[All Fields] AND biofilm[All Fields]	Lysozyme	18819708	Ultra low fouling zwitterionic polymers with a biomimetic adhesive group.
3870	DRAMP03733	Lysozyme (Arthropods, animals)	"Lysozyme"[All Fields] AND biofilm[All Fields]	Lysozyme	18718499	Role of lactoferrin in the tear film.
3870	DRAMP03733	Lysozyme (Arthropods, animals)	"Lysozyme"[All Fields] AND biofilm[All Fields]	Lysozyme	18468764	Effects of Cistus-tea on bacterial colonization and enzyme activities of the in situ pellicle.
3870	DRAMP03733	Lysozyme (Arthropods, animals)	"Lysozyme"[All Fields] AND biofilm[All Fields]	Lysozyme	18424015	Abnormal cell division caused by inclusion bodies in E. coli; increased resistance against external stress.
3870	DRAMP03733	Lysozyme (Arthropods, animals)	"Lysozyme"[All Fields] AND biofilm[All Fields]	Lysozyme	18326181	Antimicrobial activity of lactoferrin against foodborne pathogenic bacteria incorporated into edible chitosan film.
3870	DRAMP03733	Lysozyme (Arthropods, animals)	"Lysozyme"[All Fields] AND biofilm[All Fields]	Lysozyme	17927634	Biofilm inhibition and antimicrobial activity of a dentifrice containing salivary substitutes.
3870	DRAMP03733	Lysozyme (Arthropods, animals)	"Lysozyme"[All Fields] AND biofilm[All Fields]	Lysozyme	17676995	Molecular basis of resistance to muramidase and cationic antimicrobial peptide activity of lysozyme in staphylococci.
3870	DRAMP03733	Lysozyme (Arthropods, animals)	"Lysozyme"[All Fields] AND biofilm[All Fields]	Lysozyme	16936041	Biofilm formation by Streptococcus pneumoniae: role of choline, extracellular DNA, and capsular polysaccharide in microbial accretion.
3870	DRAMP03733	Lysozyme (Arthropods, animals)	"Lysozyme"[All Fields] AND biofilm[All Fields]	Lysozyme	15952847	Covalent immobilization of lysozyme on stainless steel. Interface spectroscopic characterization and measurement of enzymatic activity.
3870	DRAMP03733	Lysozyme (Arthropods, animals)	"Lysozyme"[All Fields] AND biofilm[All Fields]	Lysozyme	15908380	Identification and characterization of an autolysin-encoding gene of Streptococcus mutans.
3870	DRAMP03733	Lysozyme (Arthropods, animals)	"Lysozyme"[All Fields] AND biofilm[All Fields]	Lysozyme	15693823	Enzymes in the acquired enamel pellicle.
3870	DRAMP03733	Lysozyme (Arthropods, animals)	"Lysozyme"[All Fields] AND biofilm[All Fields]	Lysozyme	15518508	Shear and dilatational relaxation mechanisms of globular and flexible proteins at the hexadecane/water interface.
3870	DRAMP03733	Lysozyme (Arthropods, animals)	"Lysozyme"[All Fields] AND biofilm[All Fields]	Lysozyme	15493829	Bacterial evasion of innate host defenses--the Staphylococcus aureus lesson.
3870	DRAMP03733	Lysozyme (Arthropods, animals)	"Lysozyme"[All Fields] AND biofilm[All Fields]	Lysozyme	14768464	Protein adsorption at polymer-grafted surfaces: comparison between a mixture of saliva proteins and some well-defined model proteins.
3870	DRAMP03733	Lysozyme (Arthropods, animals)	"Lysozyme"[All Fields] AND biofilm[All Fields]	Lysozyme	14706749	Multiplex FISH analysis of a six-species bacterial biofilm.
3870	DRAMP03733	Lysozyme (Arthropods, animals)	"Lysozyme"[All Fields] AND biofilm[All Fields]	Lysozyme	14513382	Physiology of biofilms of thermophilic bacilli-potential consequences for cleaning.
3870	DRAMP03733	Lysozyme (Arthropods, animals)	"Lysozyme"[All Fields] AND biofilm[All Fields]	Lysozyme	12707750	Scanning-force techniques to monitor time-dependent changes in topography and adhesion force of proteins on surfaces.
3870	DRAMP03733	Lysozyme (Arthropods, animals)	"Lysozyme"[All Fields] AND biofilm[All Fields]	Lysozyme	12366846	Positive role of peptidoglycan breaks in lactococcal biofilm formation.
3870	DRAMP03733	Lysozyme (Arthropods, animals)	"Lysozyme"[All Fields] AND biofilm[All Fields]	Lysozyme	11679354	Direct detection by in situ PCR of the amoA gene in biofilm resulting from a nitrogen removal process.
3870	DRAMP03733	Lysozyme (Arthropods, animals)	"Lysozyme"[All Fields] AND biofilm[All Fields]	Lysozyme	11412320	Factors influencing attachment of thermophilic bacilli to stainless steel.
3870	DRAMP03733	Lysozyme (Arthropods, animals)	"Lysozyme"[All Fields] AND biofilm[All Fields]	Lysozyme	10415452	Lactoferrin increases the susceptibility of S. epidermidis biofilms to lysozyme and vancomycin.
3870	DRAMP03733	Lysozyme (Arthropods, animals)	"Lysozyme"[All Fields] AND biofilm[All Fields]	Lysozyme	10391502	The effect of extracellular polysaccharides from Streptococcus mutans on the bactericidal activity of human neutrophils.
3870	DRAMP03733	Lysozyme (Arthropods, animals)	"Lysozyme"[All Fields] AND biofilm[All Fields]	Lysozyme	10390869	Specific oligonucleotide probes for in situ detection of a major group of gram-positive bacteria with low DNA G + C content.

3870	DRAMP03733	Lysozyme (Arthropods, animals)	"Lysozyme"[All Fields] AND biofilm[All Fields]	Lysozyme	9812283	Protein antimicrobial barriers to bacterial adhesion.
3870	DRAMP03733	Lysozyme (Arthropods, animals)	"Lysozyme"[All Fields] AND biofilm[All Fields]	Lysozyme	9571268	Defining the physiologically normal coating and pathological deposit: an analysis of sulfur-containing moieties and pellicle thickness on hydrogel contact lenses.
3870	DRAMP03733	Lysozyme (Arthropods, animals)	"Lysozyme"[All Fields] AND biofilm[All Fields]	Lysozyme	9351231	The influence of cell surface properties of thermophilic streptococci on attachment to stainless steel.
3870	DRAMP03733	Lysozyme (Arthropods, animals)	"Lysozyme"[All Fields] AND biofilm[All Fields]	Lysozyme	9255506	Protein-lipid interaction on the surface of a hydrophilic contact lens in vitro.
3870	DRAMP03733	Lysozyme (Arthropods, animals)	"Lysozyme"[All Fields] AND biofilm[All Fields]	Lysozyme	9380651	[Preparation of films with combined biological activity and study of their properties].
3870	DRAMP03733	Lysozyme (Arthropods, animals)	"Lysozyme"[All Fields] AND biofilm[All Fields]	Lysozyme	9029300	Adhesion to silicone rubber of yeasts and bacteria isolated from voice prostheses: influence of salivary conditioning films.
3870	DRAMP03733	Lysozyme (Arthropods, animals)	"Lysozyme"[All Fields] AND biofilm[All Fields]	Lysozyme	8950503	The effect of eye closure on protein and complement deposition on Group IV hydrogel contact lenses: relationship to tear flow dynamics.
3870	DRAMP03733	Lysozyme (Arthropods, animals)	"Lysozyme"[All Fields] AND biofilm[All Fields]	Lysozyme	8126879	[Physiologic factor in intractable bacterial infections].
3870	DRAMP03733	Lysozyme (Arthropods, animals)	"Lysozyme"[All Fields] AND biofilm[All Fields]	Lysozyme	3570694	Specificity and biological activity of the protein deposited on the hydrogel surface. Relationship of polymer structure to biofilm formation.
3899	DRAMP03779	Antifungal protein (AFP; Fungi)	"Antifungal protein"[All Fields] AND biofilm[All Fields]	Antifungal protein	33466640	The Neosartorya fischeriAntifungal Protein 2 (NFAP2): A New Potential Weapon against Multidrug-Resistant Candida aurisBiofilms.
3899	DRAMP03779	Antifungal protein (AFP; Fungi)	"Antifungal protein"[All Fields] AND biofilm[All Fields]	Antifungal protein	32824977	The Penicillium chrysogenumQ176 Antimicrobial Protein PAFC Effectively Inhibits the Growth of the Opportunistic Human Pathogen Candida albicans.
3899	DRAMP03779	Antifungal protein (AFP; Fungi)	"Antifungal protein"[All Fields] AND biofilm[All Fields]	Antifungal protein	30478163	In VivoApplicability of Neosartorya fischeri Antifungal Protein 2 (NFAP2) in Treatment of Vulvovaginal Candidiasis.
3899	DRAMP03779	Antifungal protein (AFP; Fungi)	"Antifungal protein"[All Fields] AND biofilm[All Fields]	Antifungal protein	30079061	The Evolutionary Conserved γ-Core Motif Influences the Anti- CandidaActivity of the Penicillium chrysogenumAntifungal Protein PAF.
3899	DRAMP03779	Antifungal protein (AFP; Fungi)	"Antifungal protein"[All Fields] AND biofilm[All Fields]	Antifungal protein	28120548	DS6: anticandidal, antibiofilm peptide against Candida tropicalis and exhibit synergy with commercial drug.
3909	DRAMP03789	ABF-1 (nematodes, animals)	"ABF-1"[All Fields] AND biofilm[All Fields]	ABF-1	22363222	Tasco®: a product of Ascophyllum nodosum enhances immune response of Caenorhabditis elegans against Pseudomonas aeruginosa infection.
3958	DRAMP03926	Cecropin B (1-7)-melittin (4-11)hybrid peptide (CBM)	"Cecropin B"[All Fields] AND biofilm[All Fields]	Cecropin B	31057940	On-chip manufacturing of synthetic proteins for point-of-care therapeutics.
3958	DRAMP03926	Cecropin B (1-7)-melittin (4-11)hybrid peptide (CBM)	"Cecropin B"[All Fields] AND biofilm[All Fields]	Cecropin B	23732798	Surface functionalization of titanium substrates with cecropin B to improve their cytocompatibility and reduce inflammation responses.
3960	DRAMP03940	Peptide 4 (Trp- and Arg-rich; derivative of Tritrpticin)	"Peptide 4"[All Fields] AND biofilm[All Fields]	Peptide 4	26196513	Snake Cathelicidin NA-CATH and Smaller Helical Antimicrobial Peptides Are Effective against Burkholderia thailandensis.
3961	DRAMP03941	Peptide 3 (Trp- and Arg-rich; derivative of Tritrpticin)	"Peptide 3"[All Fields] AND biofilm[All Fields]	Peptide 3	31481944	Turning a Collagenesis-Inducing Peptide Into a Potent Antibacterial and Antibiofilm Agent Against Multidrug-Resistant Gram-Negative Bacteria.
3961	DRAMP03941	Peptide 3 (Trp- and Arg-rich; derivative of Tritrpticin)	"Peptide 3"[All Fields] AND biofilm[All Fields]	Peptide 3	29372443	Sensitivity of caries pathogens to antimicrobial peptides related to caries risk.
3961	DRAMP03941	Peptide 3 (Trp- and Arg-rich; derivative of Tritrpticin)	"Peptide 3"[All Fields] AND biofilm[All Fields]	Peptide 3	24890264	Salivary antimicrobial proteins associate with age-related changes in streptococcal composition in dental plaque.
3962	DRAMP03942	Peptide 2 (Trp- and Arg-rich; derivative of Tritrpticin)	"Peptide 2"[All Fields] AND biofilm[All Fields]	Peptide 2	24890264	Salivary antimicrobial proteins associate with age-related changes in streptococcal composition in dental plaque.
3962	DRAMP03942	Peptide 2 (Trp- and Arg-rich; derivative of Tritrpticin)	"Peptide 2"[All Fields] AND biofilm[All Fields]	Peptide 2	22189407	Identification of staphylococcal lipoteichoic acid-binding proteins in human serum by high-resolution LTQ-Orbitrap mass spectrometry.
3962	DRAMP03942	Peptide 2 (Trp- and Arg-rich; derivative of Tritrpticin)	"Peptide 2"[All Fields] AND biofilm[All Fields]	Peptide 2	15271944	Molecular interactions of surface protein peptides of Streptococcus gordonii with human salivary components.
3970	DRAMP03961	KR-12	"KR-12"[All Fields] AND biofilm[All Fields]	KR-12	34402021	Cytocompatibility and Synergy of EGCG and Cationic Peptides Against Bacteria Related to Endodontic Infections, in Planktonic and Biofilm Conditions.
3970	DRAMP03961	KR-12	"KR-12"[All Fields] AND biofilm[All Fields]	KR-12	31190796	Fabrication of KR-12 peptide-containing hyaluronic acid immobilized fibrous eggshell membrane effectively kills multi-drug-resistant bacteria, promotes angiogenesis and accelerates re-epithelialization.
3970	DRAMP03961	KR-12	"KR-12"[All Fields] AND biofilm[All Fields]	KR-12	29737589	LL-37 fragments have antimicrobial activity against Staphylococcus epidermidis biofilms and wound healing potential in HaCaT cell line.
3970	DRAMP03961	KR-12	"KR-12"[All Fields] AND biofilm[All Fields]	KR-12	29022391	KR-12-a5 is a non-cytotoxic agent with potent antimicrobial effects against oral pathogens.
3970	DRAMP03961	KR-12	"KR-12"[All Fields] AND biofilm[All Fields]	KR-12	28967271	Antibacterial activity of a novel antimicrobial peptide [W7]KR12-KAEK derived from KR-12 against Streptococcus mutans planktonic cells and biofilms.
3970	DRAMP03961	KR-12	"KR-12"[All Fields] AND biofilm[All Fields]	KR-12	28675109	Titanium surfaces immobilized with the major antimicrobial fragment FK-16 of human cathelicidin LL-37 are potent against multiple antibiotic-resistant bacteria.
3970	DRAMP03961	KR-12	"KR-12"[All Fields] AND biofilm[All Fields]	KR-12	28525841	LL-37-derived short antimicrobial peptide KR-12-a5 and its d-amino acid substituted analogs with cell selectivity, anti-biofilm activity, synergistic effect with conventional antibiotics, and anti-inflammatory activity.
3970	DRAMP03961	KR-12	"KR-12"[All Fields] AND biofilm[All Fields]	KR-12	28408902	The Naturally Occurring Host Defense Peptide, LL-37, and Its Truncated Mimetics KE-18 and KR-12 Have Selected Biocidal and Antibiofilm Activities Against Candida albicans, Staphylococcus aureus, and Escherichia coli In vitro.
3970	DRAMP03961	KR-12	"KR-12"[All Fields] AND biofilm[All Fields]	KR-12	24071034	The human antimicrobial peptide LL-37 and its fragments possess both antimicrobial and antibiofilm activities against multidrug-resistant Acinetobacter baumannii.
3971	DRAMP03962	Cecropin A(1-8)-Magainin 2(1-12)hybrid peptide (CAMA)	"Cecropin A"[All Fields] AND biofilm[All Fields]	Cecropin A	32203307	Antimicrobial peptide derived from moths can eradicate UPEC biofilms and could offer a novel therapeutic option.
3971	DRAMP03962	Cecropin A(1-8)-Magainin 2(1-12)hybrid peptide (CAMA)	"Cecropin A"[All Fields] AND biofilm[All Fields]	Cecropin A	32203127	Antibiofilm activities of ceragenins and antimicrobial peptides against fungal-bacterial mono and multispecies biofilms.
3971	DRAMP03962	Cecropin A(1-8)-Magainin 2(1-12)hybrid peptide (CAMA)	"Cecropin A"[All Fields] AND biofilm[All Fields]	Cecropin A	32051417	The insect antimicrobial peptide cecropin A disrupts uropathogenic Escherichia coli biofilms.
3971	DRAMP03962	Cecropin A(1-8)-Magainin 2(1-12)hybrid peptide (CAMA)	"Cecropin A"[All Fields] AND biofilm[All Fields]	Cecropin A	31485973	Efficacy of Indolicidin, Cecropin A (1-7)-Melittin (CAMA) and Their Combination Against Biofilm-Forming Multidrug-Resistant Enteroaggregative Escherichia coli.
3971	DRAMP03962	Cecropin A(1-8)-Magainin 2(1-12)hybrid peptide (CAMA)	"Cecropin A"[All Fields] AND biofilm[All Fields]	Cecropin A	31363941	Mechanism of action of antimicrobial peptide P5 truncations against Pseudomonas aeruginosa and Staphylococcus aureus.
3971	DRAMP03962	Cecropin A(1-8)-Magainin 2(1-12)hybrid peptide (CAMA)	"Cecropin A"[All Fields] AND biofilm[All Fields]	Cecropin A	30755738	In vitro activities of antimicrobial peptides and ceragenins against Legionella pneumophila.
3971	DRAMP03962	Cecropin A(1-8)-Magainin 2(1-12)hybrid peptide (CAMA)	"Cecropin A"[All Fields] AND biofilm[All Fields]	Cecropin A	28178190	High Specific Selectivity and Membrane-Active Mechanism of Synthetic Cationic Hybrid Antimicrobial Peptides Based on the Peptide FV7.
3971	DRAMP03962	Cecropin A(1-8)-Magainin 2(1-12)hybrid peptide (CAMA)	"Cecropin A"[All Fields] AND biofilm[All Fields]	Cecropin A	25924433	[CecropinA-magainin, a new hybrid antibacterial peptide against meticillin-resistant Staphylococcus aureus].
3971	DRAMP03962	Cecropin A(1-8)-Magainin 2(1-12)hybrid peptide (CAMA)	"Cecropin A"[All Fields] AND biofilm[All Fields]	Cecropin A	23070152	In vitro activities of antibiotics and antimicrobial cationic peptides alone and in combination against methicillin-resistant Staphylococcus aureus biofilms.
3972	DRAMP03963	Cecropin A(1-8)-Magainin 2(1-12)hybrid peptide analogue (P1)	"Cecropin A"[All Fields] AND biofilm[All Fields]	Cecropin A	32203307	Antimicrobial peptide derived from moths can eradicate UPEC biofilms and could offer a novel therapeutic option.
3972	DRAMP03963	Cecropin A(1-8)-Magainin 2(1-12)hybrid peptide analogue (P1)	"Cecropin A"[All Fields] AND biofilm[All Fields]	Cecropin A	32203127	Antibiofilm activities of ceragenins and antimicrobial peptides against fungal-bacterial mono and multispecies biofilms.
3972	DRAMP03963	Cecropin A(1-8)-Magainin 2(1-12)hybrid peptide analogue (P1)	"Cecropin A"[All Fields] AND biofilm[All Fields]	Cecropin A	32051417	The insect antimicrobial peptide cecropin A disrupts uropathogenic Escherichia coli biofilms.
3972	DRAMP03963	Cecropin A(1-8)-Magainin 2(1-12)hybrid peptide analogue (P1)	"Cecropin A"[All Fields] AND biofilm[All Fields]	Cecropin A	31485973	Efficacy of Indolicidin, Cecropin A (1-7)-Melittin (CAMA) and Their Combination Against Biofilm-Forming Multidrug-Resistant Enteroaggregative Escherichia coli.

3972	DRAMP03963	Cecropin A(1-8)-Magainin 2(1-12)hybrid peptide analogue (P1)	"Cecropin A"[All Fields] AND biofilm[All Fields]	Cecropin A	31363941	Mechanism of action of antimicrobial peptide P5 truncations against Pseudomonas aeruginosa and Staphylococcus aureus.
3972	DRAMP03963	Cecropin A(1-8)-Magainin 2(1-12)hybrid peptide analogue (P1)	"Cecropin A"[All Fields] AND biofilm[All Fields]	Cecropin A	30755738	In vitro activities of antimicrobial peptides and ceragenins against Legionella pneumophila.
3972	DRAMP03963	Cecropin A(1-8)-Magainin 2(1-12)hybrid peptide analogue (P1)	"Cecropin A"[All Fields] AND biofilm[All Fields]	Cecropin A	28178190	High Specific Selectivity and Membrane-Active Mechanism of Synthetic Cationic Hybrid Antimicrobial Peptides Based on the Peptide FV7.
3972	DRAMP03963	Cecropin A(1-8)-Magainin 2(1-12)hybrid peptide analogue (P1)	"Cecropin A"[All Fields] AND biofilm[All Fields]	Cecropin A	25924433	[CecropinA-magainin, a new hybrid antibacterial peptide against meticillin-resistant Staphylococcus aureus].
3972	DRAMP03963	Cecropin A(1-8)-Magainin 2(1-12)hybrid peptide analogue (P1)	"Cecropin A"[All Fields] AND biofilm[All Fields]	Cecropin A	23070152	In vitro activities of antibiotics and antimicrobial cationic peptides alone and in combination against methicillin-resistant Staphylococcus aureus biofilms.
3973	DRAMP03964	Cecropin A(1-8)-Magainin 2(1-12)hybrid peptide analogue (P2)	"Cecropin A"[All Fields] AND biofilm[All Fields]	Cecropin A	32203307	Antimicrobial peptide derived from moths can eradicate UPEC biofilms and could offer a novel therapeutic option.
3973	DRAMP03964	Cecropin A(1-8)-Magainin 2(1-12)hybrid peptide analogue (P2)	"Cecropin A"[All Fields] AND biofilm[All Fields]	Cecropin A	32203127	Antibiofilm activities of ceragenins and antimicrobial peptides against fungal-bacterial mono and multispecies biofilms.
3973	DRAMP03964	Cecropin A(1-8)-Magainin 2(1-12)hybrid peptide analogue (P2)	"Cecropin A"[All Fields] AND biofilm[All Fields]	Cecropin A	32051417	The insect antimicrobial peptide cecropin A disrupts uropathogenic Escherichia coli biofilms.
3973	DRAMP03964	Cecropin A(1-8)-Magainin 2(1-12)hybrid peptide analogue (P2)	"Cecropin A"[All Fields] AND biofilm[All Fields]	Cecropin A	31485973	Efficacy of Indolicidin, Cecropin A (1-7)-Melittin (CAMA) and Their Combination Against Biofilm-Forming Multidrug-Resistant Enteroaggregative Escherichia coli.
3973	DRAMP03964	Cecropin A(1-8)-Magainin 2(1-12)hybrid peptide analogue (P2)	"Cecropin A"[All Fields] AND biofilm[All Fields]	Cecropin A	31363941	Mechanism of action of antimicrobial peptide P5 truncations against Pseudomonas aeruginosa and Staphylococcus aureus.
3973	DRAMP03964	Cecropin A(1-8)-Magainin 2(1-12)hybrid peptide analogue (P2)	"Cecropin A"[All Fields] AND biofilm[All Fields]	Cecropin A	30755738	In vitro activities of antimicrobial peptides and ceragenins against Legionella pneumophila.
3973	DRAMP03964	Cecropin A(1-8)-Magainin 2(1-12)hybrid peptide analogue (P2)	"Cecropin A"[All Fields] AND biofilm[All Fields]	Cecropin A	28178190	High Specific Selectivity and Membrane-Active Mechanism of Synthetic Cationic Hybrid Antimicrobial Peptides Based on the Peptide FV7.
3973	DRAMP03964	Cecropin A(1-8)-Magainin 2(1-12)hybrid peptide analogue (P2)	"Cecropin A"[All Fields] AND biofilm[All Fields]	Cecropin A	25924433	[CecropinA-magainin, a new hybrid antibacterial peptide against meticillin-resistant Staphylococcus aureus].
3973	DRAMP03964	Cecropin A(1-8)-Magainin 2(1-12)hybrid peptide analogue (P2)	"Cecropin A"[All Fields] AND biofilm[All Fields]	Cecropin A	23070152	In vitro activities of antibiotics and antimicrobial cationic peptides alone and in combination against methicillin-resistant Staphylococcus aureus biofilms.
3974	DRAMP03965	Cecropin A(1-8)-Magainin 2(1-12)hybrid peptide analogue (P3)	"Cecropin A"[All Fields] AND biofilm[All Fields]	Cecropin A	32203307	Antimicrobial peptide derived from moths can eradicate UPEC biofilms and could offer a novel therapeutic option.
3974	DRAMP03965	Cecropin A(1-8)-Magainin 2(1-12)hybrid peptide analogue (P3)	"Cecropin A"[All Fields] AND biofilm[All Fields]	Cecropin A	32203127	Antibiofilm activities of ceragenins and antimicrobial peptides against fungal-bacterial mono and multispecies biofilms.
3974	DRAMP03965	Cecropin A(1-8)-Magainin 2(1-12)hybrid peptide analogue (P3)	"Cecropin A"[All Fields] AND biofilm[All Fields]	Cecropin A	32051417	The insect antimicrobial peptide cecropin A disrupts uropathogenic Escherichia coli biofilms.
3974	DRAMP03965	Cecropin A(1-8)-Magainin 2(1-12)hybrid peptide analogue (P3)	"Cecropin A"[All Fields] AND biofilm[All Fields]	Cecropin A	31485973	Efficacy of Indolicidin, Cecropin A (1-7)-Melittin (CAMA) and Their Combination Against Biofilm-Forming Multidrug-Resistant Enteroaggregative Escherichia coli.
3974	DRAMP03965	Cecropin A(1-8)-Magainin 2(1-12)hybrid peptide analogue (P3)	"Cecropin A"[All Fields] AND biofilm[All Fields]	Cecropin A	31363941	Mechanism of action of antimicrobial peptide P5 truncations against Pseudomonas aeruginosa and Staphylococcus aureus.
3974	DRAMP03965	Cecropin A(1-8)-Magainin 2(1-12)hybrid peptide analogue (P3)	"Cecropin A"[All Fields] AND biofilm[All Fields]	Cecropin A	30755738	In vitro activities of antimicrobial peptides and ceragenins against Legionella pneumophila.
3974	DRAMP03965	Cecropin A(1-8)-Magainin 2(1-12)hybrid peptide analogue (P3)	"Cecropin A"[All Fields] AND biofilm[All Fields]	Cecropin A	28178190	High Specific Selectivity and Membrane-Active Mechanism of Synthetic Cationic Hybrid Antimicrobial Peptides Based on the Peptide FV7.
3974	DRAMP03965	Cecropin A(1-8)-Magainin 2(1-12)hybrid peptide analogue (P3)	"Cecropin A"[All Fields] AND biofilm[All Fields]	Cecropin A	25924433	[CecropinA-magainin, a new hybrid antibacterial peptide against meticillin-resistant Staphylococcus aureus].
3974	DRAMP03965	Cecropin A(1-8)-Magainin 2(1-12)hybrid peptide analogue (P3)	"Cecropin A"[All Fields] AND biofilm[All Fields]	Cecropin A	23070152	In vitro activities of antibiotics and antimicrobial cationic peptides alone and in combination against methicillin-resistant Staphylococcus aureus biofilms.
3975	DRAMP03966	Cecropin A(1-8)-Magainin 2(1-12)hybrid peptide analogue (P4)	"Cecropin A"[All Fields] AND biofilm[All Fields]	Cecropin A	32203307	Antimicrobial peptide derived from moths can eradicate UPEC biofilms and could offer a novel therapeutic option.
3975	DRAMP03966	Cecropin A(1-8)-Magainin 2(1-12)hybrid peptide analogue (P4)	"Cecropin A"[All Fields] AND biofilm[All Fields]	Cecropin A	32203127	Antibiofilm activities of ceragenins and antimicrobial peptides against fungal-bacterial mono and multispecies biofilms.
3975	DRAMP03966	Cecropin A(1-8)-Magainin 2(1-12)hybrid peptide analogue (P4)	"Cecropin A"[All Fields] AND biofilm[All Fields]	Cecropin A	32051417	The insect antimicrobial peptide cecropin A disrupts uropathogenic Escherichia coli biofilms.
3975	DRAMP03966	Cecropin A(1-8)-Magainin 2(1-12)hybrid peptide analogue (P4)	"Cecropin A"[All Fields] AND biofilm[All Fields]	Cecropin A	31485973	Efficacy of Indolicidin, Cecropin A (1-7)-Melittin (CAMA) and Their Combination Against Biofilm-Forming Multidrug-Resistant Enteroaggregative Escherichia coli.
3975	DRAMP03966	Cecropin A(1-8)-Magainin 2(1-12)hybrid peptide analogue (P4)	"Cecropin A"[All Fields] AND biofilm[All Fields]	Cecropin A	31363941	Mechanism of action of antimicrobial peptide P5 truncations against Pseudomonas aeruginosa and Staphylococcus aureus.
3975	DRAMP03966	Cecropin A(1-8)-Magainin 2(1-12)hybrid peptide analogue (P4)	"Cecropin A"[All Fields] AND biofilm[All Fields]	Cecropin A	30755738	In vitro activities of antimicrobial peptides and ceragenins against Legionella pneumophila.
3975	DRAMP03966	Cecropin A(1-8)-Magainin 2(1-12)hybrid peptide analogue (P4)	"Cecropin A"[All Fields] AND biofilm[All Fields]	Cecropin A	28178190	High Specific Selectivity and Membrane-Active Mechanism of Synthetic Cationic Hybrid Antimicrobial Peptides Based on the Peptide FV7.
3975	DRAMP03966	Cecropin A(1-8)-Magainin 2(1-12)hybrid peptide analogue (P4)	"Cecropin A"[All Fields] AND biofilm[All Fields]	Cecropin A	25924433	[CecropinA-magainin, a new hybrid antibacterial peptide against meticillin-resistant Staphylococcus aureus].
3975	DRAMP03966	Cecropin A(1-8)-Magainin 2(1-12)hybrid peptide analogue (P4)	"Cecropin A"[All Fields] AND biofilm[All Fields]	Cecropin A	23070152	In vitro activities of antibiotics and antimicrobial cationic peptides alone and in combination against methicillin-resistant Staphylococcus aureus biofilms.
3982	DRAMP04018	Rp-1	"Rp-1"[All Fields] AND biofilm[All Fields]	Rp-1	29807235	Efficacy of red propolis hydro-alcoholic extract in controlling Streptococcus mutans biofilm build-up and dental enamel demineralization.
3982	DRAMP04018	Rp-1	"Rp-1"[All Fields] AND biofilm[All Fields]	Rp-1	24297446	Characterization of biofilm formation and the role of BCR1 in clinical isolates of Candida parapsilosis.
4009	DRAMP04148	Gramicidin S	"Gramicidin S"[All Fields] AND biofilm[All Fields]	Gramicidin S	31784584	Supreme activity of gramicidin S against resistant, persistent and biofilm cells of staphylococci and enterococci.
4009	DRAMP04148	Gramicidin S	"Gramicidin S"[All Fields] AND biofilm[All Fields]	Gramicidin S	28439259	Increased Biological Activity of Aneurinibacillus migulanusStrains Correlates with the Production of New Gramicidin Secondary Metabolites.
4009	DRAMP04148	Gramicidin S	"Gramicidin S"[All Fields] AND biofilm[All Fields]	Gramicidin S	27618065	Therapeutic Potential of Gramicidin S in the Treatment of Root Canal Infections.
4009	DRAMP04148	Gramicidin S	"Gramicidin S"[All Fields] AND biofilm[All Fields]	Gramicidin S	26077259	Synergistic effect of membrane-active peptides polymyxin B and gramicidin S on multidrug-resistant strains and biofilms of Pseudomonas aeruginosa.
4009	DRAMP04148	Gramicidin S	"Gramicidin S"[All Fields] AND biofilm[All Fields]	Gramicidin S	23103974	Direct surfactin-gramicidin S antagonism supports detoxification in mixed producer cultures of Bacillus subtilis and Aneurinibacillus migulanus.
4009	DRAMP04148	Gramicidin S	"Gramicidin S"[All Fields] AND biofilm[All Fields]	Gramicidin S	17701408	Biofilms: strategies for metal corrosion inhibition employing microorganisms.
4009	DRAMP04148	Gramicidin S	"Gramicidin S"[All Fields] AND biofilm[All Fields]	Gramicidin S	15278311	Inhibiting mild steel corrosion from sulfate-reducing and iron-oxidizing bacteria using gramicidin-S-producing biofilms.
4009	DRAMP04148	Gramicidin S	"Gramicidin S"[All Fields] AND biofilm[All Fields]	Gramicidin S	12898064	Inhibiting mild steel corrosion from sulfate-reducing bacteria using antimicrobial-producing biofilms in Three-Mile-Island process water.
4009	DRAMP04148	Gramicidin S	"Gramicidin S"[All Fields] AND biofilm[All Fields]	Gramicidin S	10499267	Inhibiting sulfate-reducing bacteria in biofilms on steel with antimicrobial peptides generated in situ.

4022	DRAMP04197	Penetratin	"Penetratin"[All Fields] AND biofilm[All Fields]	Penetratin	34064259	Antibacterial Properties of Small-Size Peptide Derived from Penetratin against Oral Streptococci.
4022	DRAMP04197	Penetratin	"Penetratin"[All Fields] AND biofilm[All Fields]	Penetratin	29679078	Fluorophore labeling of a cell-penetrating peptide significantly alters the mode and degree of biomembrane interaction.
4035	DRAMP04210	ADP1	"ADP1"[All Fields] AND biofilm[All Fields]	ADP1	30019131	Contact-Dependent Growth Inhibition Proteins in Acinetobacter baylyi ADP1.
4035	DRAMP04210	ADP1	"ADP1"[All Fields] AND biofilm[All Fields]	ADP1	27190150	Bioluminescence-based system for rapid detection of natural transformation.
4035	DRAMP04210	ADP1	"ADP1"[All Fields] AND biofilm[All Fields]	ADP1	25351710	Genetic and phenotypic characterization of Candida albicans strains isolated from infectious disease patients in Shanghai.
4035	DRAMP04210	ADP1	"ADP1"[All Fields] AND biofilm[All Fields]	ADP1	25047957	AtfA, a new factor in global regulation of transcription in Acinetobacter spp.
4035	DRAMP04210	ADP1	"ADP1"[All Fields] AND biofilm[All Fields]	ADP1	23358859	Staring at the cold sun: blue light regulation is distributed within the genus Acinetobacter.
4035	DRAMP04210	ADP1	"ADP1"[All Fields] AND biofilm[All Fields]	ADP1	21856821	Comparative genomic analysis of Acinetobacter oleivorans DR1 to determine strain-specific genomic regions and gentisate biodegradation.
4035	DRAMP04210	ADP1	"ADP1"[All Fields] AND biofilm[All Fields]	ADP1	18350144	Comparative analysis of Acinetobacters: three genomes for three lifestyles.
4038	DRAMP04213	BP100	"BP100"[All Fields] AND biofilm[All Fields]	BP100	29751064	Mechanistic and phenotypic studies of bicarinalin, BP100 and colistin action on Acinetobacter baumannii.
4043	DRAMP04218	Lys-a1	"Lys-a1"[All Fields] AND biofilm[All Fields]	Lys-a1	32297570	Antimicrobial and Antibiofilm Activity of Lys-[Trp6]hy-a1 Combined with Ciprofloxacin Against Gram-Negative Bacteria.
4043	DRAMP04218	Lys-a1	"Lys-a1"[All Fields] AND biofilm[All Fields]	Lys-a1	23340019	Antimicrobial activity of the synthetic peptide Lys-a1 against oral streptococci.
4052	DRAMP04227	PGG	"PGG"[All Fields] AND biofilm[All Fields]	PGG	32948818	Pentagalloyl glucose from Schinus terebinthifolia inhibits growth of carbapenem-resistant Acinetobacter baumannii.
4052	DRAMP04227	PGG	"PGG"[All Fields] AND biofilm[All Fields]	PGG	31717821	Plant Polyphenols Inhibit Functional Amyloid and Biofilm Formation in PseudomonasStrains by Directing Monomers to Off-Pathway Oligomers.
4052	DRAMP04227	PGG	"PGG"[All Fields] AND biofilm[All Fields]	PGG	28211475	Biofilm formation of the black yeast-like fungus Exophiala dermatitidis and its susceptibility to antiinfective agents.
4052	DRAMP04227	PGG	"PGG"[All Fields] AND biofilm[All Fields]	PGG	26529040	Inhibitory Effects of 2,2'-Dipyridyl and 1,2,3,4,6-Penta-O-Galloyl-b-D-Glucopyranose on Biofilm Formation in Contact Lens Cases.
4052	DRAMP04227	PGG	"PGG"[All Fields] AND biofilm[All Fields]	PGG	22479621	Involvement of iron in biofilm formation by Staphylococcus aureus.
4052	DRAMP04227	PGG	"PGG"[All Fields] AND biofilm[All Fields]	PGG	21173176	Inhibitory effects of 1,2,3,4,6-penta-O-galloyl-beta-D-glucopyranose on biofilm formation by Staphylococcus aureus.
4053	DRAMP04228	PGP	"PGP"[All Fields] AND biofilm[All Fields]	PGP	34659169	Prospecting Endophytic Bacteria Endowed With Plant Growth Promoting Potential Isolated From Camellia sinensis.
4053	DRAMP04228	PGP	"PGP"[All Fields] AND biofilm[All Fields]	PGP	34442666	Multi-Trait Wheat Rhizobacteria from Calcareous Soil with Biocontrol Activity Promote Plant Growth and Mitigate Salinity Stress.
4053	DRAMP04228	PGP	"PGP"[All Fields] AND biofilm[All Fields]	PGP	33805133	Plant Growth Promotion Function of Bacillussp. Strains Isolated from Salt-Pan Rhizosphere and Their Biocontrol Potential against Macrophomina phaseolina.
4053	DRAMP04228	PGP	"PGP"[All Fields] AND biofilm[All Fields]	PGP	33584566	Dual Microbial Inoculation, a Game Changer? - Bacterial Biostimulants With Multifunctional Growth Promoting Traits to Mitigate Salinity Stress in Spring Mungbean.
4053	DRAMP04228	PGP	"PGP"[All Fields] AND biofilm[All Fields]	PGP	33486715	Unveiling the putative functional genes present in root-associated endophytic microbiome from maize plant using the shotgun approach.
4053	DRAMP04228	PGP	"PGP"[All Fields] AND biofilm[All Fields]	PGP	33329415	Quorum Sensing System Affects the Plant Growth Promotion Traits of Serratia fonticolaGS2.
4053	DRAMP04228	PGP	"PGP"[All Fields] AND biofilm[All Fields]	PGP	33156472	Biocontrol and plant growth-promoting potentiality of bacteria isolated from compost extract.
4053	DRAMP04228	PGP	"PGP"[All Fields] AND biofilm[All Fields]	PGP	33072048	Complete Genome Sequence of Enterobacter roggenkampiiED5, a Nitrogen Fixing Plant Growth Promoting Endophytic Bacterium With Biocontrol and Stress Tolerance Properties, Isolated From Sugarcane Root.
4053	DRAMP04228	PGP	"PGP"[All Fields] AND biofilm[All Fields]	PGP	32904284	Functional and molecular characterization of plant growth promoting Bacillusisolates from tomato rhizosphere.
4053	DRAMP04228	PGP	"PGP"[All Fields] AND biofilm[All Fields]	PGP	32850725	Pseudomonas aeruginosaRTE4: A Tea Rhizobacterium With Potential for Plant Growth Promotion and Biosurfactant Production.
4053	DRAMP04228	PGP	"PGP"[All Fields] AND biofilm[All Fields]	PGP	32785605	Genome-guided insights of tropical Bacillus strains efficient in maize growth promotion.
4053	DRAMP04228	PGP	"PGP"[All Fields] AND biofilm[All Fields]	PGP	32504325	Bacillus toyonensis COPE52 Modifies Lipid and Fatty Acid Composition, Exhibits Antifungal Activity, and Stimulates Growth of Tomato Plants Under Saline Conditions.
4053	DRAMP04228	PGP	"PGP"[All Fields] AND biofilm[All Fields]	PGP	32358015	A Framework for the Selection of Plant Growth-Promoting Rhizobacteria Based on Bacterial Competence Mechanisms.
4053	DRAMP04228	PGP	"PGP"[All Fields] AND biofilm[All Fields]	PGP	31976855	Friend or foe? Exploring the fine line between Pseudomonas brassicacearumand phytopathogens.
4053	DRAMP04228	PGP	"PGP"[All Fields] AND biofilm[All Fields]	PGP	31673798	Co-existence of Leclercia adecarboxylata (LSE-1) and Bradyrhizobium sp. (LSBR-3) in nodule niche for multifaceted effects and profitability in soybean production.
4053	DRAMP04228	PGP	"PGP"[All Fields] AND biofilm[All Fields]	PGP	31617725	Fast Biofilm Penetration and Anti-PAO1 Activity of Nebulized Azithromycin in Nanoarchaeosomes.
4053	DRAMP04228	PGP	"PGP"[All Fields] AND biofilm[All Fields]	PGP	31372943	Isolation, functional characterization and efficacy of biofilm-forming rhizobacteria under abiotic stress conditions.
4053	DRAMP04228	PGP	"PGP"[All Fields] AND biofilm[All Fields]	PGP	30872708	Fluorescent Pseudomonas -FAP2 and Bacillus licheniformis interact positively in biofilm mode enhancing plant growth and photosynthetic attributes.
4053	DRAMP04228	PGP	"PGP"[All Fields] AND biofilm[All Fields]	PGP	30834977	Synergism of Pseudomonas aeruginosa (LSE-2) nodule endophyte with Bradyrhizobium sp. (LSBR-3) for improving plant growth, nutrient acquisition and soil health in soybean.
4053	DRAMP04228	PGP	"PGP"[All Fields] AND biofilm[All Fields]	PGP	29774013	Dynamics of Aspen Roots Colonization by Pseudomonads Reveals Strain-Specific and Mycorrhizal-Specific Patterns of Biofilm Formation.
4053	DRAMP04228	PGP	"PGP"[All Fields] AND biofilm[All Fields]	PGP	29703735	The Catabolite Repressor/Activator Cra Is a Bridge Connecting Carbon Metabolism and Host Colonization in the Plant Drought Resistance-Promoting Bacterium Pantoea alhagi LTYR-11Z.
4053	DRAMP04228	PGP	"PGP"[All Fields] AND biofilm[All Fields]	PGP	28942841	Exploitation of agro-climatic environment for selection of 1-aminocyclopropane-1-carboxylic acid (ACC) deaminase producing salt tolerant indigenous plant growth promoting rhizobacteria.
4053	DRAMP04228	PGP	"PGP"[All Fields] AND biofilm[All Fields]	PGP	28286498	Players over the Surface: Unraveling the Role of Exopolysaccharides in Zinc Biosorption by Fluorescent PseudomonasStrain Psd.
4053	DRAMP04228	PGP	"PGP"[All Fields] AND biofilm[All Fields]	PGP	27825487	Phytohormone production endowed with antagonistic potential and plant growth promoting abilities of culturable endophytic bacteria isolated from Clerodendrum colebrookianum Walp.
4053	DRAMP04228	PGP	"PGP"[All Fields] AND biofilm[All Fields]	PGP	27417328	Screening beneficial rhizobacteria from Spartina maritima for phytoremediation of metal polluted salt marshes: comparison of gram-positive and gram-negative strains.
4053	DRAMP04228	PGP	"PGP"[All Fields] AND biofilm[All Fields]	PGP	26362119	Synergistic effect of Pseudomonas putida and Bacillus amyloliquefaciens ameliorates drought stress in chickpea (Cicer arietinum L.).
4053	DRAMP04228	PGP	"PGP"[All Fields] AND biofilm[All Fields]	PGP	25320438	Preferential Promotion of Lycopersicon esculentum (Tomato) Growth by Plant Growth Promoting Bacteria Associated with Tomato.
4053	DRAMP04228	PGP	"PGP"[All Fields] AND biofilm[All Fields]	PGP	24188110	Development of alginate-based aggregate inoculants of Methylobacterium sp. and Azospirillum brasilense tested under in vitro conditions to promote plant growth.
4053	DRAMP04228	PGP	"PGP"[All Fields] AND biofilm[All Fields]	PGP	23035571	Putative down-stream signaling molecule of GTPase in Porphyromonas gingivalis.
4055	DRAMP04230	PGAa	"PGAa"[All Fields] AND biofilm[All Fields]	PGAa	34680777	Co-Occurrence of bla OXA-23in the Chromosome and Plasmid: Increased Fitness in Carbapenem-Resistant Acinetobacter baumannii.
4055	DRAMP04230	PGAa	"PGAa"[All Fields] AND biofilm[All Fields]	PGAa	34046019	Three Ribosomal Operons of Escherichia coliContain Genes Encoding Small RNAs That Interact With Hfq and CsrA in vitro.
4055	DRAMP04230	PGAa	"PGAa"[All Fields] AND biofilm[All Fields]	PGAa	33484812	Difference in biofilm formation between carbapenem-resistant and carbapenem-sensitive Klebsiella pneumoniae based on analysis of mrkH distribution.
4055	DRAMP04230	PGAa	"PGAa"[All Fields] AND biofilm[All Fields]	PGAa	33385683	The specific effect of (R)-(+)-pulegone on growth and biofilm formation in multi-drug resistant Escherichia coli and molecular mechanisms underlying the expression of pgaABCD genes.
4055	DRAMP04230	PGAa	"PGAa"[All Fields] AND biofilm[All Fields]	PGAa	33319862	Antibiofilm and antivirulence efficacy of myrtenol enhances the antibiotic susceptibility of Acinetobacter baumannii.
4055	DRAMP04230	PGAa	"PGAa"[All Fields] AND biofilm[All Fields]	PGAa	33190591	Contribution of biofilm formation genetic locus, pgaABCD, to antibiotic resistance development in gut microbiome.
4055	DRAMP04230	PGAa	"PGAa"[All Fields] AND biofilm[All Fields]	PGAa	32918098	Gene expressing analysis indicates the role of Pyrogallol as a novel antibiofilm and antivirulence agent against Acinetobacter baumannii.
4055	DRAMP04230	PGAa	"PGAa"[All Fields] AND biofilm[All Fields]	PGAa	32652494	Phenotypical and molecular assessment of the virulence potential of KPC-3-producing Klebsiella pneumoniae ST392 clinical isolates.
4055	DRAMP04230	PGAa	"PGAa"[All Fields] AND biofilm[All Fields]	PGAa	32223838	Investigation of LuxS-mediated quorum sensing in Klebsiella pneumoniae.
4055	DRAMP04230	PGAa	"PGAa"[All Fields] AND biofilm[All Fields]	PGAa	31915750	Tannic and gallic acids alter redox-parameters of the medium and modulate biofilm formation.

4055	DRAMP04230	PGAa	"PGAa"[All Fields] AND biofilm[All Fields]	PGAa	31853190	Association Between Biofilm Formation, Structure, and the Expression Levels of Genes Related to biofilm formation and Biofilm-Specific Resistance of Acinetobacter baumanniiStrains Isolated from Burn Infection in Ahvaz, Iran.
4055	DRAMP04230	PGAa	"PGAa"[All Fields] AND biofilm[All Fields]	PGAa	31847837	Extended spectrum β-lactamase producing uropathogenic Escherichia coli and the correlation of biofilm with antibiotics resistance in Nepal.
4055	DRAMP04230	PGAa	"PGAa"[All Fields] AND biofilm[All Fields]	PGAa	31449967	Genomic characterisation of a clinical Acinetobacter baumannii ST1928 isolate carrying a new ampC allelic variant bla ADC-196gene from China.
4055	DRAMP04230	PGAa	"PGAa"[All Fields] AND biofilm[All Fields]	PGAa	31190904	Effects of sub-inhibitory concentrations of meropenem and tigecycline on the expression of genes regulating pili, efflux pumps and virulence factors involved in biofilm formation by Acinetobacter baumannii.
4055	DRAMP04230	PGAa	"PGAa"[All Fields] AND biofilm[All Fields]	PGAa	31026798	Enhanced "contact mechanism" for interaction of extracellular polymeric substances with low-grade copper-bearing sulfide ore in bioleaching by moderately thermophilic Acidithiobacillus caldus.
4055	DRAMP04230	PGAa	"PGAa"[All Fields] AND biofilm[All Fields]	PGAa	29869541	Inhibitory effect of α-mangostin on Acinetobacter baumannii biofilms - an in vitro study.
4055	DRAMP04230	PGAa	"PGAa"[All Fields] AND biofilm[All Fields]	PGAa	29554105	Sub-minimum inhibitory concentrations of colistin and polymyxin B promote Acinetobacter baumannii biofilm formation.
4055	DRAMP04230	PGAa	"PGAa"[All Fields] AND biofilm[All Fields]	PGAa	29473983	Biofilm inhibitory efficiency of phytol in combination with cefotaxime against nosocomial pathogen Acinetobacter baumannii.
4055	DRAMP04230	PGAa	"PGAa"[All Fields] AND biofilm[All Fields]	PGAa	28731269	Biofilm formation and antibiotic resistance in Klebsiella pneumoniae urinary strains.
4055	DRAMP04230	PGAa	"PGAa"[All Fields] AND biofilm[All Fields]	PGAa	28470798	Alternative pathways for Escherichia coli biofilm formation revealed by sRNA overproduction.
4055	DRAMP04230	PGAa	"PGAa"[All Fields] AND biofilm[All Fields]	PGAa	26957546	Structural Basis for Translocation of a Biofilm-supporting Exopolysaccharide across the Bacterial Outer Membrane.
4055	DRAMP04230	PGAa	"PGAa"[All Fields] AND biofilm[All Fields]	PGAa	26483403	Surface Polysaccharide Mutants Reveal that Absence of O Antigen Reduces Biofilm Formation of Actinobacillus pleuropneumoniae.
4055	DRAMP04230	PGAa	"PGAa"[All Fields] AND biofilm[All Fields]	PGAa	26130517	Sub-inhibitory concentrations of penicillin G induce biofilm formation by field isolates of Actinobacillus pleuropneumoniae.
4055	DRAMP04230	PGAa	"PGAa"[All Fields] AND biofilm[All Fields]	PGAa	26078454	Identification of Poly-N-acetylglucosamine as a Major Polysaccharide Component of the Bacillus subtilis Biofilm Matrix.
4055	DRAMP04230	PGAa	"PGAa"[All Fields] AND biofilm[All Fields]	PGAa	24888591	RNA remodeling by bacterial global regulator CsrA promotes Rho-dependent transcription termination.
4055	DRAMP04230	PGAa	"PGAa"[All Fields] AND biofilm[All Fields]	PGAa	23666921	Dual function of the McaS small RNA in controlling biofilm formation.
4055	DRAMP04230	PGAa	"PGAa"[All Fields] AND biofilm[All Fields]	PGAa	23544067	The HD-GYP domain protein RpfG of Xanthomonas oryzae pv. oryzicola regulates synthesis of extracellular polysaccharides that contribute to biofilm formation and virulence on rice.
4055	DRAMP04230	PGAa	"PGAa"[All Fields] AND biofilm[All Fields]	PGAa	23313082	Comparison of three methods to study biofilm formation by clinical strains of Escherichia coli.
4055	DRAMP04230	PGAa	"PGAa"[All Fields] AND biofilm[All Fields]	PGAa	22289118	A small RNA that regulates motility and biofilm formation in response to changes in nutrient availability in Escherichia coli.
4055	DRAMP04230	PGAa	"PGAa"[All Fields] AND biofilm[All Fields]	PGAa	22037401	Translational repression of NhaR, a novel pathway for multi-tier regulation of biofilm circuitry by CsrA.
4055	DRAMP04230	PGAa	"PGAa"[All Fields] AND biofilm[All Fields]	PGAa	21787622	Characteristics of Escherichia coli biofilm production, genetic typing, drug resistance pattern and gene expression under aminoglycoside pressures.
4055	DRAMP04230	PGAa	"PGAa"[All Fields] AND biofilm[All Fields]	PGAa	21651845	[Expressions of pgaABC gene clusters and changes in biofilm phenotype of Acinetobacter baumannii in burn patients].
4055	DRAMP04230	PGAa	"PGAa"[All Fields] AND biofilm[All Fields]	PGAa	20159471	AlgK is a TPR-containing protein and the periplasmic component of a novel exopolysaccharide secretin.
4055	DRAMP04230	PGAa	"PGAa"[All Fields] AND biofilm[All Fields]	PGAa	19460094	Second messenger signalling governs Escherichia coli biofilm induction upon ribosomal stress.
4055	DRAMP04230	PGAa	"PGAa"[All Fields] AND biofilm[All Fields]	PGAa	18359807	Roles of pgaABCD genes in synthesis, modification, and export of the Escherichia coli biofilm adhesin poly-beta-1,6-N-acetyl-D-glucosamine.
4055	DRAMP04230	PGAa	"PGAa"[All Fields] AND biofilm[All Fields]	PGAa	16997959	The cation-responsive protein NhaR of Escherichia coli activates pgaABCD transcription, required for production of the biofilm adhesin poly-beta-1,6-N-acetyl-D-glucosamine.
4055	DRAMP04230	PGAa	"PGAa"[All Fields] AND biofilm[All Fields]	PGAa	16923806	Comprehensive alanine-scanning mutagenesis of Escherichia coli CsrA defines two subdomains of critical functional importance.
4055	DRAMP04230	PGAa	"PGAa"[All Fields] AND biofilm[All Fields]	PGAa	15916613	CsrA post-transcriptionally represses pgaABCD, responsible for synthesis of a biofilm polysaccharide adhesin of Escherichia coli.
4061	DRAMP04245	Dhvar5	"Dhvar5"[All Fields] AND biofilm[All Fields]	Dhvar5	25818458	Dhvar5 antimicrobial peptide (AMP) chemoselective covalent immobilization results on higher antiadherence effect than simple physical adsorption.
4062	DRAMP04246	Dhvar4	"Dhvar4"[All Fields] AND biofilm[All Fields]	Dhvar4	34238746	[Design, screening and antibacterial activity evaluation of the novel antibacterial peptide KR-1].
4062	DRAMP04246	Dhvar4	"Dhvar4"[All Fields] AND biofilm[All Fields]	Dhvar4	10371248	The effects of histatin-derived basic antimicrobial peptides on oral biofilms.
4067	DRAMP04251	WLBU2	"WLBU2"[All Fields] AND biofilm[All Fields]	WLBU2	33475795	Antibiofilm peptides as a promising strategy: comparative research.
4067	DRAMP04251	WLBU2	"WLBU2"[All Fields] AND biofilm[All Fields]	WLBU2	32484998	Direct antimicrobial activity of cationic amphipathic peptide WLBU2 against Staphylococcus aureus biofilms is enhanced in physiologic buffered saline.
4067	DRAMP04251	WLBU2	"WLBU2"[All Fields] AND biofilm[All Fields]	WLBU2	32426473	Enhanced therapeutic index of an antimicrobial peptide in mice by increasing safety and activity against multidrug-resistant bacteria.
4067	DRAMP04251	WLBU2	"WLBU2"[All Fields] AND biofilm[All Fields]	WLBU2	31372010	Synergism of cationic antimicrobial peptide WLBU2 with antibacterial agents against biofilms of multi-drug resistant Acinetobacter baumanniiand Klebsiella pneumoniae.
4067	DRAMP04251	WLBU2	"WLBU2"[All Fields] AND biofilm[All Fields]	WLBU2	29753132	Prevention of ESKAPE pathogen biofilm formation by antimicrobial peptides WLBU2 and LL37.
4067	DRAMP04251	WLBU2	"WLBU2"[All Fields] AND biofilm[All Fields]	WLBU2	29273750	Elimination of Antibiotic Resistant Surgical Implant Biofilms Using an Engineered Cationic Amphipathic Peptide WLBU2.
4067	DRAMP04251	WLBU2	"WLBU2"[All Fields] AND biofilm[All Fields]	WLBU2	28882728	Enhanced efficacy of the engineered antimicrobial peptide WLBU2 via direct airway delivery in a murine model of Pseudomonas aeruginosa pneumonia.
4067	DRAMP04251	WLBU2	"WLBU2"[All Fields] AND biofilm[All Fields]	WLBU2	27303744	Simultaneous Antibiofilm and Antiviral Activities of an Engineered Antimicrobial Peptide during Virus-Bacterium Coinfection.
4067	DRAMP04251	WLBU2	"WLBU2"[All Fields] AND biofilm[All Fields]	WLBU2	27231279	Engineered cationic antimicrobial peptide (eCAP) prevents Pseudomonas aeruginosa biofilm growth on airway epithelial cells.
4067	DRAMP04251	WLBU2	"WLBU2"[All Fields] AND biofilm[All Fields]	WLBU2	17325219	Efficacy of the de novo-derived antimicrobial peptide WLBU2 against oral bacteria.
4070	DRAMP04254	Neuropeptide Y (NPY)	"Neuropeptide Y"[All Fields] AND biofilm[All Fields]	Neuropeptide Y	29549404	Antidromic neurogenic activity and cutaneous bacterial flora.
4071	DRAMP04255	Neuropeptide Y (NPY)	"Neuropeptide Y"[All Fields] AND biofilm[All Fields]	Neuropeptide Y	29549404	Antidromic neurogenic activity and cutaneous bacterial flora.
4076	DRAMP04260	CM-1	"CM-1"[All Fields] AND biofilm[All Fields]	CM-1	34540201	Enhancement of pyocyanin production by subinhibitory concentration of royal jelly in Pseudomonas aeruginosa.
4076	DRAMP04260	CM-1	"CM-1"[All Fields] AND biofilm[All Fields]	CM-1	34513326	Evaluation of a strawberry fermented beverage with potential health benefits.
4076	DRAMP04260	CM-1	"CM-1"[All Fields] AND biofilm[All Fields]	CM-1	34357189	Proton Conductive, Low Methanol Crossover Cellulose-Based Membranes.
4076	DRAMP04260	CM-1	"CM-1"[All Fields] AND biofilm[All Fields]	CM-1	34349735	Physicochemical Properties of Extracellular Polymeric Substances Produced by Three Bacterial Isolates From Biofouled Reverse Osmosis Membranes.
4076	DRAMP04260	CM-1	"CM-1"[All Fields] AND biofilm[All Fields]	CM-1	34202224	Analysis of Pathogenic Bacterial and Yeast Biofilms Using the Combination of Synchrotron ATR-FTIR Microspectroscopy and Chemometric Approaches.
4076	DRAMP04260	CM-1	"CM-1"[All Fields] AND biofilm[All Fields]	CM-1	34190236	Characterization of multiscale interactions between high intensity focused ultrasound (HIFU) and tooth dentin: the effect on matrix-metalloproteinases, bacterial biofilms and biological properties.
4076	DRAMP04260	CM-1	"CM-1"[All Fields] AND biofilm[All Fields]	CM-1	34089962	Bioremediation of polyvinyl chloride (PVC) films by marine bacteria.
4076	DRAMP04260	CM-1	"CM-1"[All Fields] AND biofilm[All Fields]	CM-1	34032106	[Biodegradation of Polystyrene by Geobacillus stearothermophilus].
4076	DRAMP04260	CM-1	"CM-1"[All Fields] AND biofilm[All Fields]	CM-1	33604750	Exopolysaccharide of Anoxybacillus pushchinoensis G11 has antitumor and antibiofilm activities.
4076	DRAMP04260	CM-1	"CM-1"[All Fields] AND biofilm[All Fields]	CM-1	33573147	Effect of Propolis Nanoparticles against Enterococcus faecalisBiofilm in the Root Canal.
4076	DRAMP04260	CM-1	"CM-1"[All Fields] AND biofilm[All Fields]	CM-1	33424369	Decolorization, degradation and detoxification of carcinogenic sulfonated azo dye methyl orange by newly developed biofilm consortia.
4076	DRAMP04260	CM-1	"CM-1"[All Fields] AND biofilm[All Fields]	CM-1	33391224	Biodegradation of Unpretreated Low-Density Polyethylene (LDPE) by Stenotrophomonassp. and Achromobactersp., Isolated From Waste Dumpsite and Drilling Fluid.
4076	DRAMP04260	CM-1	"CM-1"[All Fields] AND biofilm[All Fields]	CM-1	33359161	Antimicrobial capacity and physico-chemical characteristics of adhesive resin containing riboflavin after photodynamic therapy.
4076	DRAMP04260	CM-1	"CM-1"[All Fields] AND biofilm[All Fields]	CM-1	33038673	Biostimulation versus bioaugmentation for the electro-bioremediation of 2,4-dichlorophenoxyacetic acid polluted soils.

4076	DRAMP04260	CM-1	"CM-1"[All Fields] AND biofilm[All Fields]	CM-1	33010944	Properties of a modified quaternary ammonium silane formulation as a potential root canal irrigant in endodontics.
4076	DRAMP04260	CM-1	"CM-1"[All Fields] AND biofilm[All Fields]	CM-1	32975495	Platinum nanoparticles embedded into polyaniline on carbon cloth: improvement of oxygen reduction at cathode of microbial fuel cell used for conversion of medicinal plant wastes into bio-energy.
4076	DRAMP04260	CM-1	"CM-1"[All Fields] AND biofilm[All Fields]	CM-1	32969650	Effects of Electrokinetic Phenomena on Bacterial Deposition Monitored by Quartz Crystal Microbalance with Dissipation Monitoring.
4076	DRAMP04260	CM-1	"CM-1"[All Fields] AND biofilm[All Fields]	CM-1	32807967	Electric field stimulates production of highly conductive microbial OmcZ nanowires.
4076	DRAMP04260	CM-1	"CM-1"[All Fields] AND biofilm[All Fields]	CM-1	32600236	Synthesis and Antimicrobial Evaluation of Some Novel Pyrimidine, Pyrazole, Chromene and Tetrahydrobenzo[b]thiophene Derivatives Bearing Pyrimidinthione Moiety.
4076	DRAMP04260	CM-1	"CM-1"[All Fields] AND biofilm[All Fields]	CM-1	32190651	Effects of Four Kinds of Oxide Nanoparticles on Proteins in Extracellular Polymeric Substances of Sludge.
4076	DRAMP04260	CM-1	"CM-1"[All Fields] AND biofilm[All Fields]	CM-1	32117673	Identification and characterization of antimicrobial peptide produced by indigenously isolated Bacillus paralicheniformisUBBLi30 strain.
4076	DRAMP04260	CM-1	"CM-1"[All Fields] AND biofilm[All Fields]	CM-1	32114965	Evaluation of tire derived rubber (TDR) fixed biofilm reactor (FBR) for remediation of Methylene blue dye from wastewater.
4076	DRAMP04260	CM-1	"CM-1"[All Fields] AND biofilm[All Fields]	CM-1	32030170	Removal of Cr(VI) by magnetic iron oxide nanoparticles synthesized from extracellular polymeric substances of chromium resistant acid-tolerant bacterium Lysinibacillus sphaericusRTA-01.
4076	DRAMP04260	CM-1	"CM-1"[All Fields] AND biofilm[All Fields]	CM-1	31943345	Local concentrations of gentamicin obtained by microdialysis after a controlled application of a GentaColl sponge in a porcine model.
4076	DRAMP04260	CM-1	"CM-1"[All Fields] AND biofilm[All Fields]	CM-1	31856361	Developmental stages of biofilm and characterization of extracellular matrix of manglicolous fungus Aspergillus niger BSC-1.
4076	DRAMP04260	CM-1	"CM-1"[All Fields] AND biofilm[All Fields]	CM-1	31760009	Alginate-caseinate based pH-responsive nano-coacervates to combat resistant bacterial biofilms in oral cavity.
4076	DRAMP04260	CM-1	"CM-1"[All Fields] AND biofilm[All Fields]	CM-1	31187375	Development of sustainable and reusable silver nanoparticle-coated glass for the treatment of contaminated water.
4076	DRAMP04260	CM-1	"CM-1"[All Fields] AND biofilm[All Fields]	CM-1	31115692	Effect of a novel quaternary ammonium silane cavity disinfectant on cariogenic biofilm formation.
4076	DRAMP04260	CM-1	"CM-1"[All Fields] AND biofilm[All Fields]	CM-1	30955856	Antibacterial resin-based composite containing chlorhexidine for dental applications.
4076	DRAMP04260	CM-1	"CM-1"[All Fields] AND biofilm[All Fields]	CM-1	30895626	Spoilage Pseudomonas biofilm with Escherichia coli protection in fish meat at 5 °C.
4076	DRAMP04260	CM-1	"CM-1"[All Fields] AND biofilm[All Fields]	CM-1	30867835	Real-Time Monitoring of Pharmacokinetics of Antibiotics in Biofilms with Raman-Tagged Hyperspectral Stimulated Raman Scattering Microscopy.
4076	DRAMP04260	CM-1	"CM-1"[All Fields] AND biofilm[All Fields]	CM-1	30832841	Mentha piperita essential oils loaded in a chitosan nanogel with inhibitory effect on biofilm formation against S. mutans on the dental surface.
4076	DRAMP04260	CM-1	"CM-1"[All Fields] AND biofilm[All Fields]	CM-1	30654633	Cyclic Changes in the Amide Bands Within Escherichia coli Biofilms Monitored Using Real-Time Infrared Attenuated Total Reflection Spectroscopy (IR-ATR).
4076	DRAMP04260	CM-1	"CM-1"[All Fields] AND biofilm[All Fields]	CM-1	30504045	Start-up evaluations and biocarriers transfer from a trickling filter to a moving bed bioreactor for synthetic mariculture wastewater treatment.
4076	DRAMP04260	CM-1	"CM-1"[All Fields] AND biofilm[All Fields]	CM-1	30272959	Spectral Characterization of a Novel NO Sensing Protein in Bacteria: NosP.
4076	DRAMP04260	CM-1	"CM-1"[All Fields] AND biofilm[All Fields]	CM-1	29923733	Core/Shell Bacterial Cables: A One-Dimensional Platform for Probing Microbial Electron Transfer.
4076	DRAMP04260	CM-1	"CM-1"[All Fields] AND biofilm[All Fields]	CM-1	29858205	Functional Determinants of Extracellular Polymeric Substances in Membrane Biofouling: Experimental Evidence from Pure-Cultured Sludge Bacteria.
4076	DRAMP04260	CM-1	"CM-1"[All Fields] AND biofilm[All Fields]	CM-1	29757931	In Vitro Human Microbiota Response to Exposure to Silver Nanoparticles Biosynthesized with Mushroom Extract.
4076	DRAMP04260	CM-1	"CM-1"[All Fields] AND biofilm[All Fields]	CM-1	29431716	Effect of salinity variation on the autotrophic kinetics of the start-up of a membrane bioreactor and hybrid moving bed biofilm reactor-membrane bioreactor at low hydraulic retention time.
4076	DRAMP04260	CM-1	"CM-1"[All Fields] AND biofilm[All Fields]	CM-1	29208541	Biological synthesis of silver nanoparticles using β-1, 3 glucan binding protein and their antibacterial, antibiofilm and cytotoxic potential.
4076	DRAMP04260	CM-1	"CM-1"[All Fields] AND biofilm[All Fields]	CM-1	28952559	Lysine-Grafted MCM-41 Silica as an Antibacterial Biomaterial.
4076	DRAMP04260	CM-1	"CM-1"[All Fields] AND biofilm[All Fields]	CM-1	28867634	Two potential uses for silver nanoparticles coated with Solanum nigrum unripe fruit extract: Biofilm inhibition and photodegradation of dye effluent.
4076	DRAMP04260	CM-1	"CM-1"[All Fields] AND biofilm[All Fields]	CM-1	28540585	Antibiofilm activity and mode of action of DMSO alone and its combination with afatinib against Gram-negative pathogens.
4076	DRAMP04260	CM-1	"CM-1"[All Fields] AND biofilm[All Fields]	CM-1	28418636	Defining Molecular Details of the Chemistry of Biofilm Formation by Raman Microspectroscopy.
4076	DRAMP04260	CM-1	"CM-1"[All Fields] AND biofilm[All Fields]	CM-1	28163780	Efficient molasses fermentation under high salinity by inocula of marine and terrestrial origin.
4076	DRAMP04260	CM-1	"CM-1"[All Fields] AND biofilm[All Fields]	CM-1	27886659	Physicochemical and Mechanical Properties of Bambara Groundnut Starch Films Modified with Stearic Acid.
4076	DRAMP04260	CM-1	"CM-1"[All Fields] AND biofilm[All Fields]	CM-1	27870324	High Biofilm Conductivity Maintained Despite Anode Potential Changes in a Geobacter-Enriched Biofilm.
4076	DRAMP04260	CM-1	"CM-1"[All Fields] AND biofilm[All Fields]	CM-1	27307980	Cloning and expression of quorum sensing N-acyl-homoserine synthase (LuxI) gene detected in Acinetobacter baumannii.
4076	DRAMP04260	CM-1	"CM-1"[All Fields] AND biofilm[All Fields]	CM-1	26989738	Biochemical detection of N-Acyl homoserine lactone from biofilm-forming uropathogenic Escherichia coli isolated from urinary tract infection samples.
4076	DRAMP04260	CM-1	"CM-1"[All Fields] AND biofilm[All Fields]	CM-1	26899861	Modulation of Candida albicans Biofilm by Different Carbon Sources.
4076	DRAMP04260	CM-1	"CM-1"[All Fields] AND biofilm[All Fields]	CM-1	26703114	Antibacterial, antibiofilm and cytotoxic effects of Nigella sativa essential oil coated gold nanoparticles.
4076	DRAMP04260	CM-1	"CM-1"[All Fields] AND biofilm[All Fields]	CM-1	25853255	Ciprofloxacin-eluting nanofibers inhibits biofilm formation by Pseudomonas aeruginosa and a methicillin-resistant Staphylococcus aureus.
4076	DRAMP04260	CM-1	"CM-1"[All Fields] AND biofilm[All Fields]	CM-1	25401035	Evaluation of antibiotic effects on Pseudomonas aeruginosa biofilm using Raman spectroscopy and multivariate analysis.
4076	DRAMP04260	CM-1	"CM-1"[All Fields] AND biofilm[All Fields]	CM-1	25367342	Usnic acid, a lichen secondary metabolite inhibits Group A Streptococcus biofilms.
4076	DRAMP04260	CM-1	"CM-1"[All Fields] AND biofilm[All Fields]	CM-1	25260732	In vitro evaluation of antibacterial effect of AH Plus incorporated with quaternary ammonium epoxy silicate against Enterococcus faecalis.
4076	DRAMP04260	CM-1	"CM-1"[All Fields] AND biofilm[All Fields]	CM-1	25226836	Reuse of refinery's tertiary-treated wastewater in cooling towers: microbiological monitoring.
4076	DRAMP04260	CM-1	"CM-1"[All Fields] AND biofilm[All Fields]	CM-1	24859232	Azo dye load-shock on relative behavior of biofilm and suspended growth configured periodic discontinuous batch mode operations: critical evaluation with enzymatic and bio-electrocatalytic analysis.
4076	DRAMP04260	CM-1	"CM-1"[All Fields] AND biofilm[All Fields]	CM-1	24859052	ATR-FTIR spectroscopic evidence for biomolecular phosphorus and carboxyl groups facilitating bacterial adhesion to iron oxides.
4076	DRAMP04260	CM-1	"CM-1"[All Fields] AND biofilm[All Fields]	CM-1	24115187	Bacterial succession and degradative changes by biofilm on plastic medium for wastewater treatment.
4076	DRAMP04260	CM-1	"CM-1"[All Fields] AND biofilm[All Fields]	CM-1	23941707	Parameter estimation and characterization of a single-chamber microbial fuel cell for dairy wastewater treatment.
4076	DRAMP04260	CM-1	"CM-1"[All Fields] AND biofilm[All Fields]	CM-1	23793975	Preparation of carboxylated Ag nanoparticles as a coating material for medical devices and control of antibacterial activity.
4076	DRAMP04260	CM-1	"CM-1"[All Fields] AND biofilm[All Fields]	CM-1	23458077	Differential binding of L- vs. D-isomers of cationic antimicrobial peptides to the biofilm exopolysaccharide alginate.
4076	DRAMP04260	CM-1	"CM-1"[All Fields] AND biofilm[All Fields]	CM-1	23033658	Studies on the distribution of bacterial isolates in rare earth environment.
4076	DRAMP04260	CM-1	"CM-1"[All Fields] AND biofilm[All Fields]	CM-1	22639215	Fourier transform infrared spectroscopy for molecular analysis of microbial cells.
4076	DRAMP04260	CM-1	"CM-1"[All Fields] AND biofilm[All Fields]	CM-1	22105122	Nitrification processes in scrubbing liquid of an exhaust air treatment system of a large-scale pig housing facility.
4076	DRAMP04260	CM-1	"CM-1"[All Fields] AND biofilm[All Fields]	CM-1	21910484	Immobilization of subtilisin on polycaprolactam for antimicrobial food packaging applications.
4076	DRAMP04260	CM-1	"CM-1"[All Fields] AND biofilm[All Fields]	CM-1	21822253	Tunable metallic-like conductivity in microbial nanowire networks.
4076	DRAMP04260	CM-1	"CM-1"[All Fields] AND biofilm[All Fields]	CM-1	19891493	Locked chromophore analogs reveal that photoactive yellow protein regulates biofilm formation in the deep sea bacterium Idiomarina loihiensis.
4076	DRAMP04260	CM-1	"CM-1"[All Fields] AND biofilm[All Fields]	CM-1	19577463	Effects of UV pretreatment on microbial community structure and metabolic characteristics in a subsequent biofilter treating gaseous chlorobenzene.
4076	DRAMP04260	CM-1	"CM-1"[All Fields] AND biofilm[All Fields]	CM-1	19529929	Quantification of water and biomass in small colony variant PAO1 biofilms by confocal Raman microspectroscopy.
4076	DRAMP04260	CM-1	"CM-1"[All Fields] AND biofilm[All Fields]	CM-1	18571260	Quantification of local water and biomass in wild type PA01 biofilms by Confocal Raman Microspectroscopy.
4076	DRAMP04260	CM-1	"CM-1"[All Fields] AND biofilm[All Fields]	CM-1	18502524	Miniaturized calorimetry - a new method for real-time biofilm activity analysis.

4076	DRAMP04260	CM-1	"CM-1"[All Fields] AND biofilm[All Fields]	CM-1	18291571	Characterization of oral films formed in the presence of a CPP-ACP agent: an in situ study.
4076	DRAMP04260	CM-1	"CM-1"[All Fields] AND biofilm[All Fields]	CM-1	17570714	Conduction-based modeling of the biofilm anode of a microbial fuel cell.
4076	DRAMP04260	CM-1	"CM-1"[All Fields] AND biofilm[All Fields]	CM-1	17304849	[Effect of denitrifying biofilm development on the surface configuration and chemical composition of PBS polymer].
4076	DRAMP04260	CM-1	"CM-1"[All Fields] AND biofilm[All Fields]	CM-1	17169320	An alternative infrared spectroscopy assay for the quantification of polysaccharides in bacterial samples.
4076	DRAMP04260	CM-1	"CM-1"[All Fields] AND biofilm[All Fields]	CM-1	16292646	Characterization of Bordetella pertussis growing as biofilm by chemical analysis and FT-IR spectroscopy.
4076	DRAMP04260	CM-1	"CM-1"[All Fields] AND biofilm[All Fields]	CM-1	16231211	Comparative evaluation of Raman spectroscopy at different wavelengths for extremophile exemplars.
4076	DRAMP04260	CM-1	"CM-1"[All Fields] AND biofilm[All Fields]	CM-1	16206722	The orange-brown patina of Salisbury Cathedral (West Porch) surfaces: evidence of its man-made origin.
4076	DRAMP04260	CM-1	"CM-1"[All Fields] AND biofilm[All Fields]	CM-1	16018425	Tetrasodium EDTA as a novel central venous catheter lock solution against biofilm.
4076	DRAMP04260	CM-1	"CM-1"[All Fields] AND biofilm[All Fields]	CM-1	14505166	Biological waste gas treatment with a modified rotating biological contactor. II. Effect of operating parameters on process performance and mathematical modeling.
4076	DRAMP04260	CM-1	"CM-1"[All Fields] AND biofilm[All Fields]	CM-1	11911924	Application of infrared spectroscopy (attenuated total reflection) for monitoring enzymatic activity on substrate films.
4076	DRAMP04260	CM-1	"CM-1"[All Fields] AND biofilm[All Fields]	CM-1	11208804	Role of alginate and its O acetylation in formation of Pseudomonas aeruginosa microcolonies and biofilms.
4076	DRAMP04260	CM-1	"CM-1"[All Fields] AND biofilm[All Fields]	CM-1	9750276	Generation of a reproducible nutrient-depleted biofilm of Escherichia coli and Burkholderia cepacia.
4076	DRAMP04260	CM-1	"CM-1"[All Fields] AND biofilm[All Fields]	CM-1	18592566	Development of bioreactors for the culture of surface immobilized plant cells.
4076	DRAMP04260	CM-1	"CM-1"[All Fields] AND biofilm[All Fields]	CM-1	11539050	Fourier transform-infrared spectroscopic methods for microbial ecology: analysis of bacteria, bacteria-polymer mixtures and biofilms.
4077	DRAMP04261	CM-2	"CM-2"[All Fields] AND biofilm[All Fields]	CM-2	34694502	The effect of photodynamic therapy by gold nanoparticles on Streptococcus mutans and biofilm formation: an in vitro study.
4077	DRAMP04261	CM-2	"CM-2"[All Fields] AND biofilm[All Fields]	CM-2	34683666	Assessment of Disinfection Potential of Q-Switch Nd: YAG Laser on Contaminated Titanium Implant Surfaces.
4077	DRAMP04261	CM-2	"CM-2"[All Fields] AND biofilm[All Fields]	CM-2	34680821	Daily Administered Dual-Light Photodynamic Therapy Provides a Sustained Antibacterial Effect on Staphylococcus aureus.
4077	DRAMP04261	CM-2	"CM-2"[All Fields] AND biofilm[All Fields]	CM-2	34680731	Efficacy of Novel Bacteriophages against Escherichia coliBiofilms on Stainless Steel.
4077	DRAMP04261	CM-2	"CM-2"[All Fields] AND biofilm[All Fields]	CM-2	34673434	Specific detection and quantification of the marine flavobacterial genus Zobellia on macroalgae using novel qPCR and CARD-FISH assays.
4077	DRAMP04261	CM-2	"CM-2"[All Fields] AND biofilm[All Fields]	CM-2	34669372	Photoactivatable Nitric Oxide-Releasing Gold Nanocages for Enhanced Hyperthermia Treatment of Biofilm-Associated Infections.
4077	DRAMP04261	CM-2	"CM-2"[All Fields] AND biofilm[All Fields]	CM-2	34666263	Biofilm growth under continuous UVC irradiation: Quantitative effects of growth conditions and growth time on intensity response parameters.
4077	DRAMP04261	CM-2	"CM-2"[All Fields] AND biofilm[All Fields]	CM-2	34664132	Effect of curcumin-encapsulated Pluronic ®F-127 over duo-species biofilm of Streptococcus mutans and Candida albicans.
4077	DRAMP04261	CM-2	"CM-2"[All Fields] AND biofilm[All Fields]	CM-2	34624449	Vancomycin-decorated microbubbles as a theranostic agent for Staphylococcus aureus biofilms.
4077	DRAMP04261	CM-2	"CM-2"[All Fields] AND biofilm[All Fields]	CM-2	34619896	[In vitroand in vivoanti-periodontitis effects of combination treatment of photodynamic and antibiotic therapies].
4077	DRAMP04261	CM-2	"CM-2"[All Fields] AND biofilm[All Fields]	CM-2	34598294	Low-Level Laser and Antimicrobial Photodynamic Therapy Reduce Peri-implantitis-related Microorganisms Grown In Vitro.
4077	DRAMP04261	CM-2	"CM-2"[All Fields] AND biofilm[All Fields]	CM-2	34597830	Antimicrobial photodynamic therapy (aPDT) against vancomycin resistant Staphylococcus aureus (VRSA) biofilm disruption: a putative role of phagocytosis in infection control.
4077	DRAMP04261	CM-2	"CM-2"[All Fields] AND biofilm[All Fields]	CM-2	34594314	Design and Selection of Engineered Lytic Proteins With Staphylococcus aureusDecolonizing Activity.
4077	DRAMP04261	CM-2	"CM-2"[All Fields] AND biofilm[All Fields]	CM-2	34585138	Hydrogen-peroxide generating electrochemical bandage is active in vitroagainst mono- and dual-species biofilms.
4077	DRAMP04261	CM-2	"CM-2"[All Fields] AND biofilm[All Fields]	CM-2	34574207	Conditions of In Vitro Biofilm Formation by Serogroups of Listeria monocytogenesIsolated from Hass Avocados Sold at Markets in Mexico.
4077	DRAMP04261	CM-2	"CM-2"[All Fields] AND biofilm[All Fields]	CM-2	34529329	Antimicrobial action of photodynamic therapy on Enterococcus faecalis biofilm using curing light, curcumin and riboflavin.
4077	DRAMP04261	CM-2	"CM-2"[All Fields] AND biofilm[All Fields]	CM-2	34520879	Antibiofilm and cytotoxic effect of 3,3'-dihydroxycurcumin (DHC) as photosensitizer agent in antimicrobial photodynamic therapy for endodontic purposes.
4077	DRAMP04261	CM-2	"CM-2"[All Fields] AND biofilm[All Fields]	CM-2	34509685	Candida biofilm matrix as a resistance mechanism against photodynamic therapy.
4077	DRAMP04261	CM-2	"CM-2"[All Fields] AND biofilm[All Fields]	CM-2	34504531	Direct-Contact Low-Frequency Ultrasound and Pulse Lavage Eradicates Biofilms on Implant Materials In Vitro.
4077	DRAMP04261	CM-2	"CM-2"[All Fields] AND biofilm[All Fields]	CM-2	34500225	Interspecific competition by non-exoelectrogenic Citrobacter freundii An1 boosts bioelectricity generation of exoelectrogenic Shewanella oneidensis MR-1.
4077	DRAMP04261	CM-2	"CM-2"[All Fields] AND biofilm[All Fields]	CM-2	34492448	Corrosion behavior of high nitrogen nickel-free austenitic stainless steel in the presence of artificial saliva and Streptococcus mutans.
4077	DRAMP04261	CM-2	"CM-2"[All Fields] AND biofilm[All Fields]	CM-2	34452788	Blue light & chlorhexidine therapies on Streptococcus mutans biofilm and its regrowth on an in vitro orthodontic model.
4077	DRAMP04261	CM-2	"CM-2"[All Fields] AND biofilm[All Fields]	CM-2	34443301	Photodynamic Inactivation Using Natural Bioactive Compound Prevents and Disrupts the Biofilm Produced by Staphylococcus saprophyticus.
4077	DRAMP04261	CM-2	"CM-2"[All Fields] AND biofilm[All Fields]	CM-2	34438981	Removal of Mixed-Species Biofilms Developed on Food Contact Surfaces with a Mixture of Enzymes and Chemical Agents.
4077	DRAMP04261	CM-2	"CM-2"[All Fields] AND biofilm[All Fields]	CM-2	34438947	Biofilm Time-Kill Curves to Assess the Bactericidal Activity of Daptomycin Combinations against Biofilm-Producing Vancomycin-Resistant Enterococcus faeciumand faecalis.
4077	DRAMP04261	CM-2	"CM-2"[All Fields] AND biofilm[All Fields]	CM-2	34435995	Improper Light Curing of Bulkfill Composite Drives Surface Changes and Increases S. mutansBiofilm Growth as a Pathway for Higher Risk of Recurrent Caries around Restorations.
4077	DRAMP04261	CM-2	"CM-2"[All Fields] AND biofilm[All Fields]	CM-2	34410547	Inhibitory effects of ultrasound irradiation on Staphylococcus epidermidis biofilm.
4077	DRAMP04261	CM-2	"CM-2"[All Fields] AND biofilm[All Fields]	CM-2	34407791	Evaluation of bactericidal effects of ultraviolet light C irradiation on cariogenic bacteria: An in vitro study.
4077	DRAMP04261	CM-2	"CM-2"[All Fields] AND biofilm[All Fields]	CM-2	34395403	Bio-Fabrication of Human Amniotic Membrane Zinc Oxide Nanoparticles and the Wet/Dry HAM Dressing Membrane for Wound Healing.
4077	DRAMP04261	CM-2	"CM-2"[All Fields] AND biofilm[All Fields]	CM-2	34389419	A transferosome-loaded bigel for enhanced transdermal delivery and antibacterial activity of vancomycin hydrochloride.
4077	DRAMP04261	CM-2	"CM-2"[All Fields] AND biofilm[All Fields]	CM-2	34357189	Proton Conductive, Low Methanol Crossover Cellulose-Based Membranes.
4077	DRAMP04261	CM-2	"CM-2"[All Fields] AND biofilm[All Fields]	CM-2	34346395	Continuous flow sequencing bed biofilm reactor bio-digested landfill leachate treatment using electrocoagulation-persulfate.
4077	DRAMP04261	CM-2	"CM-2"[All Fields] AND biofilm[All Fields]	CM-2	34331604	Effectiveness of antimicrobial photodynamic therapy with indocyanine green against the standard and fluconazole-resistant Candida albicans.
4077	DRAMP04261	CM-2	"CM-2"[All Fields] AND biofilm[All Fields]	CM-2	34320428	Effects of curcumin-mediated antimicrobial photodynamic therapy associated to different chelators against Enterococcus faecalis biofilms.
4077	DRAMP04261	CM-2	"CM-2"[All Fields] AND biofilm[All Fields]	CM-2	34319262	Aloe-Emodin-Mediated Photodynamic Therapy Attenuates Sepsis-Associated Toxins in Selected Gram-Positive Bacteria In Vitro.
4077	DRAMP04261	CM-2	"CM-2"[All Fields] AND biofilm[All Fields]	CM-2	34308624	Incorporation of Antimicrobial Bio-Based Carriers onto Poly(vinyl alcohol- co-ethylene) Surface for Enhanced Antimicrobial Activity.
4077	DRAMP04261	CM-2	"CM-2"[All Fields] AND biofilm[All Fields]	CM-2	34308331	Kinetics and scale up of oxygen reducing cathodic biofilms.
4077	DRAMP04261	CM-2	"CM-2"[All Fields] AND biofilm[All Fields]	CM-2	34304642	Synergetic antimicrobial effect of chlorin e6 and hydrogen peroxide on multi-species biofilms.
4077	DRAMP04261	CM-2	"CM-2"[All Fields] AND biofilm[All Fields]	CM-2	34295834	MoWa: A Disinfectant for Hospital Surfaces Contaminated With Methicillin-Resistant Staphylococcus aureus(MRSA) and Other Nosocomial Pathogens.
4077	DRAMP04261	CM-2	"CM-2"[All Fields] AND biofilm[All Fields]	CM-2	34278776	High-Density Three-Dimensional Network of Covalently Linked Nitric Oxide Donors to Achieve Antibacterial and Antibiofilm Surfaces.
4077	DRAMP04261	CM-2	"CM-2"[All Fields] AND biofilm[All Fields]	CM-2	34246828	Synergistic biocidal effects of metal oxide nanoparticles-assisted ultrasound irradiation: Antimicrobial sonodynamic therapy against Streptococcus mutans biofilms.
4077	DRAMP04261	CM-2	"CM-2"[All Fields] AND biofilm[All Fields]	CM-2	34234756	One-Pot Microwave-Assisted Synthesis of Carbon Dots and in vivoand in vitroAntimicrobial Photodynamic Applications.
4077	DRAMP04261	CM-2	"CM-2"[All Fields] AND biofilm[All Fields]	CM-2	34220741	Staphylococcus epidermidisHas Growth Phase Dependent Affinity for Fibrinogen and Resulting Fibrin Clot Elasticity.
4077	DRAMP04261	CM-2	"CM-2"[All Fields] AND biofilm[All Fields]	CM-2	34201389	Antibacterial Photodynamic Inactivation of Fagopyrin F from Tartary Buckwheat (Fagopyrum tataricum) Flower against Streptococcus mutansand Its Biofilm.

4077	DRAMP04261	CM-2	"CM-2"[All Fields] AND biofilm[All Fields]	CM-2	34195563	Electrochemical enrichment of haloalkaliphilic nitrate-reducing microbial biofilm at the cathode of bioelectrochemical systems.
4077	DRAMP04261	CM-2	"CM-2"[All Fields] AND biofilm[All Fields]	CM-2	34195458	Comparison of the fungicidal efficacy of photodynamic therapy with methylene blue, silver nanoparticle, and their conjugation on oral Candidaisolates using cell viability assay.
4077	DRAMP04261	CM-2	"CM-2"[All Fields] AND biofilm[All Fields]	CM-2	34194407	Local Acidification Limits the Current Production and Biofilm Formation of Shewanella oneidensisMR-1 With Electrospun Anodes.
4077	DRAMP04261	CM-2	"CM-2"[All Fields] AND biofilm[All Fields]	CM-2	34190008	The application of bacteriophage to control Cronobacter sakazakiiplanktonic and biofilm growth in infant formula milk.
4077	DRAMP04261	CM-2	"CM-2"[All Fields] AND biofilm[All Fields]	CM-2	34182432	Franz cells for facile biosensor evaluation: A case of HRP/SWCNT-based hydrogen peroxide detection via amperometric and wireless modes.
4077	DRAMP04261	CM-2	"CM-2"[All Fields] AND biofilm[All Fields]	CM-2	34169718	Microbial Degradation Rates of Natural Bitumen.
4077	DRAMP04261	CM-2	"CM-2"[All Fields] AND biofilm[All Fields]	CM-2	34159252	Anti-biofilm activity of antibiotic-loaded Hylomate®.
4077	DRAMP04261	CM-2	"CM-2"[All Fields] AND biofilm[All Fields]	CM-2	34158697	In vitroantimicrobial effect of chloroaluminum phthalocyanine nanoemulsion on periodontal bacteria.
4077	DRAMP04261	CM-2	"CM-2"[All Fields] AND biofilm[All Fields]	CM-2	34147982	Bacterial community change and antibiotic resistance promotion after exposure to sulfadiazine and the role of UV/H 2O 2-GAC treatment.
4077	DRAMP04261	CM-2	"CM-2"[All Fields] AND biofilm[All Fields]	CM-2	34144473	Tailoring spatial structure of electroactive biofilm for enhanced activity and direct electron transfer on iron phthalocyanine modified anode in microbial fuel cells.
4077	DRAMP04261	CM-2	"CM-2"[All Fields] AND biofilm[All Fields]	CM-2	34126368	Biofilm formation and its effects on microbiologically influenced corrosion of carbon steel in oilfield injection water via electrochemical techniques and scanning electron microscopy.
4077	DRAMP04261	CM-2	"CM-2"[All Fields] AND biofilm[All Fields]	CM-2	34070273	PVA Films with Mixed Silver Nanoparticles and Gold Nanostars for Intrinsic and Photothermal Antibacterial Action.
4077	DRAMP04261	CM-2	"CM-2"[All Fields] AND biofilm[All Fields]	CM-2	34034174	Synergistic effect of carbon starvation and exogenous redox mediators on corrosion of X70 pipeline steel induced by Desulfovibrio singaporenus.
4077	DRAMP04261	CM-2	"CM-2"[All Fields] AND biofilm[All Fields]	CM-2	34032631	Optimization of electrocoagulation process parameters for enhancing phosphate removal in a biofilm-electrocoagulation system.
4077	DRAMP04261	CM-2	"CM-2"[All Fields] AND biofilm[All Fields]	CM-2	34026346	Response of Escherichia coliminimal teroperon to UVC and auto-aggregation: pilot study.
4077	DRAMP04261	CM-2	"CM-2"[All Fields] AND biofilm[All Fields]	CM-2	34024956	Fabrication and Performance of a Multi-Discharge Cold Atmospheric Pressure Plasma Array.
4077	DRAMP04261	CM-2	"CM-2"[All Fields] AND biofilm[All Fields]	CM-2	34022615	Bacteria of eleven different species isolated from biofilms in a meat processing environment have diverse biofilm forming abilities.
4077	DRAMP04261	CM-2	"CM-2"[All Fields] AND biofilm[All Fields]	CM-2	34013421	D-Tyrosine enhancement of microbiocide mitigation of carbon steel corrosion by a sulfate reducing bacterium biofilm.
4077	DRAMP04261	CM-2	"CM-2"[All Fields] AND biofilm[All Fields]	CM-2	34008084	Gradient radiation breeding and culture domestication of menaquinone producing strains.
4077	DRAMP04261	CM-2	"CM-2"[All Fields] AND biofilm[All Fields]	CM-2	33998809	Bioactive Functional Nanolayers of Chitosan-Lysine Surfactant with Single- and Mixed-Protein-Repellent and Antibiofilm Properties for Medical Implants.
4077	DRAMP04261	CM-2	"CM-2"[All Fields] AND biofilm[All Fields]	CM-2	33993638	Genetic engineering biofilms in situ using ultrasound-mediated DNA delivery.
4077	DRAMP04261	CM-2	"CM-2"[All Fields] AND biofilm[All Fields]	CM-2	33984692	Distinct biofilm formation regulated by different culture media: Implications to electricity generation.
4077	DRAMP04261	CM-2	"CM-2"[All Fields] AND biofilm[All Fields]	CM-2	33961502	Characterization of Blue Light Treatment for Infected Wounds: Antibacterial Efficacy of 420, 455, and 480 nm Light-Emitting Diode Arrays Against Common Skin Pathogens Versus Blue Light-Induced Skin Cell Toxicity.
4077	DRAMP04261	CM-2	"CM-2"[All Fields] AND biofilm[All Fields]	CM-2	33925936	Combining Visible Light and Non-Focused Ultrasound Significantly Reduces Propionibacterium acnesBiofilm While Having Limited Effect on Host Cells.
4077	DRAMP04261	CM-2	"CM-2"[All Fields] AND biofilm[All Fields]	CM-2	33919066	Inhibitory Effects of Erythrosine/Curcumin Derivatives/Nano-Titanium Dioxide-Mediated Photodynamic Therapy on Candida albicans.
4077	DRAMP04261	CM-2	"CM-2"[All Fields] AND biofilm[All Fields]	CM-2	33893723	Plasma-Functionalized Isotactic Polypropylene Assembled with Conducting Polymers for Bacterial Quantification by NADH Sensing.
4077	DRAMP04261	CM-2	"CM-2"[All Fields] AND biofilm[All Fields]	CM-2	33887495	Antimicrobial effects of photodynamic therapy with Fotoenticine on Streptococcus mutans isolated from dental caries.
4077	DRAMP04261	CM-2	"CM-2"[All Fields] AND biofilm[All Fields]	CM-2	33858087	Assessment of organic carbon migration and biofilm formation potential on polymeric tubes in contact with water.
4077	DRAMP04261	CM-2	"CM-2"[All Fields] AND biofilm[All Fields]	CM-2	33856049	Hydrogen peroxide-producing electrochemical bandage controlled by a wearable potentiostat for treatment of wound infections.
4077	DRAMP04261	CM-2	"CM-2"[All Fields] AND biofilm[All Fields]	CM-2	33813017	Antimicrobial photodynamic therapy mediated by methylene blue coupled to β-cyclodextrin reduces early colonizing microorganisms from the oral biofilm.
4077	DRAMP04261	CM-2	"CM-2"[All Fields] AND biofilm[All Fields]	CM-2	33809942	Detecting Bacterial Biofilms Using Fluorescence Hyperspectral Imaging and Various Discriminant Analyses.
4077	DRAMP04261	CM-2	"CM-2"[All Fields] AND biofilm[All Fields]	CM-2	33785444	The inhibitory activity of 5-aminolevulinic acid photodynamic therapy (ALA-PDT) on Candida albicans biofilms.
4077	DRAMP04261	CM-2	"CM-2"[All Fields] AND biofilm[All Fields]	CM-2	33784393	The association between initial adhesion and cyanobacterial biofilm development.
4077	DRAMP04261	CM-2	"CM-2"[All Fields] AND biofilm[All Fields]	CM-2	33741166	Growth potential and biofilm development of nonstarter bacteria on surfaces exposed to a continuous whey stream.
4077	DRAMP04261	CM-2	"CM-2"[All Fields] AND biofilm[All Fields]	CM-2	33730295	Caffeine as a contaminant of periphyton: ecological changes and impacts on primary producers.
4077	DRAMP04261	CM-2	"CM-2"[All Fields] AND biofilm[All Fields]	CM-2	33703923	Biofilm-forming ability of poultry Campylobacter jejunistrains in the presence and absence of Pseudomonas aeruginosa.
4077	DRAMP04261	CM-2	"CM-2"[All Fields] AND biofilm[All Fields]	CM-2	33656316	In Vitro and In Vivo Demonstration of Ultraefficient and Broad-Spectrum Antibacterial Agents for Photodynamic Antibacterial Chemotherapy.
4077	DRAMP04261	CM-2	"CM-2"[All Fields] AND biofilm[All Fields]	CM-2	33655570	Control of Burn Wound Infection by Methylene Blue-Mediated Photodynamic Treatment With Light-Emitting Diode Array Illumination in Rats.
4077	DRAMP04261	CM-2	"CM-2"[All Fields] AND biofilm[All Fields]	CM-2	33648189	Genetic diversity, biofilm and virulence characteristics of Listeria monocytogenes in salmon sushi.
4077	DRAMP04261	CM-2	"CM-2"[All Fields] AND biofilm[All Fields]	CM-2	33619697	Effect of dry sanitizing methods on Bacillus cereus biofilm.
4077	DRAMP04261	CM-2	"CM-2"[All Fields] AND biofilm[All Fields]	CM-2	33581401	Quantification and modeling of the response of surface biofilm growth to continuous low intensity UVC irradiation.
4077	DRAMP04261	CM-2	"CM-2"[All Fields] AND biofilm[All Fields]	CM-2	33574572	Sulfur bacteria promote dissolution of authigenic carbonates at marine methane seeps.
4077	DRAMP04261	CM-2	"CM-2"[All Fields] AND biofilm[All Fields]	CM-2	33571231	Interactive effects of discharge reduction and fine sediments on stream biofilm metabolism.
4077	DRAMP04261	CM-2	"CM-2"[All Fields] AND biofilm[All Fields]	CM-2	33543208	[Effect of different fluence rates applied in curcumin-photodynamic therapy on Candida albicans biofilms].
4077	DRAMP04261	CM-2	"CM-2"[All Fields] AND biofilm[All Fields]	CM-2	33512576	Eradication of Acinetobacter baumannii Planktonic and Biofilm Cells Through Erythrosine-Mediated Photodynamic Inactivation Augmented by Acetic Acid and Chitosan.
4077	DRAMP04261	CM-2	"CM-2"[All Fields] AND biofilm[All Fields]	CM-2	33508436	Antimicrobial and antibiofilm photodynamic therapy against vancomycin resistant Staphylococcus aureus (VRSA) induced infection in vitro and in vivo.
4077	DRAMP04261	CM-2	"CM-2"[All Fields] AND biofilm[All Fields]	CM-2	33462270	Incorporation and antimicrobial activity of nisin Z within carrageenan/chitosan multilayers.
4077	DRAMP04261	CM-2	"CM-2"[All Fields] AND biofilm[All Fields]	CM-2	33454542	Potential for direct application of blue light for photo-disinfection of dentine.
4077	DRAMP04261	CM-2	"CM-2"[All Fields] AND biofilm[All Fields]	CM-2	33440906	Antimicrobial Photoinactivation of In Situ Oral Biofilms by Visible Light Plus Water-Filtered Infrared A and Tetrahydroporphyrin-tetratosylate (THPTS).
4077	DRAMP04261	CM-2	"CM-2"[All Fields] AND biofilm[All Fields]	CM-2	33432933	Engineered photo-chemical therapeutic nanocomposites provide effective antibiofilm and microbicidal activities against bacterial infections in porous devices.
4077	DRAMP04261	CM-2	"CM-2"[All Fields] AND biofilm[All Fields]	CM-2	33427999	Evaluation of various waste substrates for biofilm formation and subsequent use in aerobic packed-bed reactor for secondary treatment of domestic wastewater.
4077	DRAMP04261	CM-2	"CM-2"[All Fields] AND biofilm[All Fields]	CM-2	33418435	Tea extracts modulate oral biofilm development by altering bacterial hydrophobicity and aggregation.
4077	DRAMP04261	CM-2	"CM-2"[All Fields] AND biofilm[All Fields]	CM-2	33401969	The effect of physico-chemical treatment in reducing Listeria monocytogenesbiofilms on lettuce leaf surfaces.
4077	DRAMP04261	CM-2	"CM-2"[All Fields] AND biofilm[All Fields]	CM-2	33397650	An Integrated HOCl-Producing E-Scaffold Is Active against Monomicrobial and Polymicrobial Biofilms.
4077	DRAMP04261	CM-2	"CM-2"[All Fields] AND biofilm[All Fields]	CM-2	33392172	Allochthonous and Autochthonous Halothermotolerant Bioanodes From Hypersaline Sediment and Textile Wastewater: A Promising Microbial Electrochemical Process for Energy Recovery Coupled With Real Textile Wastewater Treatment.
4077	DRAMP04261	CM-2	"CM-2"[All Fields] AND biofilm[All Fields]	CM-2	33391224	Biodegradation of Unpretreated Low-Density Polyethylene (LDPE) by Stenotrophomonassp. and Achromobactersp., Isolated From Waste Dumpsite and Drilling Fluid.
4077	DRAMP04261	CM-2	"CM-2"[All Fields] AND biofilm[All Fields]	CM-2	33385817	Time-based succession existed in rural sewer biofilms: Bacterial communities, sulfate-reducing bacteria and methanogenic archaea, and sulfide and methane generation.
4077	DRAMP04261	CM-2	"CM-2"[All Fields] AND biofilm[All Fields]	CM-2	33381966	Hitchhiking Behavior in Bacteriophages Facilitates Phage Infection and Enhances Carrier Bacteria Colonization.

4077	DRAMP04261	CM-2	"CM-2"[All Fields] AND biofilm[All Fields]	CM-2	33339460	Combatting fungal biofilm formation by diffusive release of fluconazole from heptylamine plasma polymer coating.
4077	DRAMP04261	CM-2	"CM-2"[All Fields] AND biofilm[All Fields]	CM-2	33338235	Betacyanin-inhibited biofilm formation of co-culture of Staphylococcus aureus and Pseudomonas aeruginosa on different polymer surfaces.
4077	DRAMP04261	CM-2	"CM-2"[All Fields] AND biofilm[All Fields]	CM-2	33321571	Antimicrobial effects of photodynamic therapy on Staphylococcus aureus biofilm grown on a specific acrylic resin surface for ocular prostheses.
4077	DRAMP04261	CM-2	"CM-2"[All Fields] AND biofilm[All Fields]	CM-2	33320609	Photodynamic Inactivation of Staphylococcus aureusBiofilms Using a Hexanuclear Molybdenum Complex Embedded in Transparent polyHEMA Hydrogels.
4077	DRAMP04261	CM-2	"CM-2"[All Fields] AND biofilm[All Fields]	CM-2	33279070	Antimicrobial activity of copper surfaces against biofilm formation by Salmonella Enteritidis and its potential application in the poultry industry.
4077	DRAMP04261	CM-2	"CM-2"[All Fields] AND biofilm[All Fields]	CM-2	33233114	Thermal and non-thermal treatment effects on Staphylococcus aureus biofilms formed at different temperatures and maturation periods.
4077	DRAMP04261	CM-2	"CM-2"[All Fields] AND biofilm[All Fields]	CM-2	33212939	Increased Intraspecies Diversity in Escherichia coliBiofilms Promotes Cellular Growth at the Expense of Matrix Production.
4077	DRAMP04261	CM-2	"CM-2"[All Fields] AND biofilm[All Fields]	CM-2	33193252	Insights Into Mechanisms of Antimicrobial Photodynamic Action Toward Biofilms Using Phenalen-1-One Derivatives as Photosensitizers.
4077	DRAMP04261	CM-2	"CM-2"[All Fields] AND biofilm[All Fields]	CM-2	33168253	Biosynthetic FeS/BC hybrid particles enhanced the electroactive bacteria enrichment in microbial electrochemical systems.
4077	DRAMP04261	CM-2	"CM-2"[All Fields] AND biofilm[All Fields]	CM-2	33166922	Coordinated response of Au-NPs/rGO modified electroactive biofilms under phenolic compounds shock: Comprehensive analysis from architecture, composition, and activity.
4077	DRAMP04261	CM-2	"CM-2"[All Fields] AND biofilm[All Fields]	CM-2	33131824	Short communication: A competitive exclusion study reveals the emergence of Bacillus subtilis as a predominant constitutive microorganism of a whey reverse osmosis membrane biofilm matrix.
4077	DRAMP04261	CM-2	"CM-2"[All Fields] AND biofilm[All Fields]	CM-2	33105653	A Nanoengineered Stainless Steel Surface to Combat Bacterial Attachment and Biofilm Formation.
4077	DRAMP04261	CM-2	"CM-2"[All Fields] AND biofilm[All Fields]	CM-2	33103954	Antimicrobial Activity of an Implantable Wireless Blue Light-Emitting Diode Against Root Canal Biofilm In Vitro.
4077	DRAMP04261	CM-2	"CM-2"[All Fields] AND biofilm[All Fields]	CM-2	33098294	Plant components affect bacterial biofilms development by altering their cell surface physicochemical properties: a predictability study using Actinomyces naeslundii.
4077	DRAMP04261	CM-2	"CM-2"[All Fields] AND biofilm[All Fields]	CM-2	33091623	Antibacterial effect of a copper-containing titanium alloy against implant-associated infection induced by methicillin-resistant Staphylococcus aureus.
4077	DRAMP04261	CM-2	"CM-2"[All Fields] AND biofilm[All Fields]	CM-2	33083912	Antimicrobial photodynamic therapy against Propionibacterium acnes biofilms using hypericin (Hypericum perforatum) photosensitizer: in vitro study.
4077	DRAMP04261	CM-2	"CM-2"[All Fields] AND biofilm[All Fields]	CM-2	33076241	Light Energy Dose and Photosensitizer Concentration Are Determinants of Effective Photo-Killing against Caries-Related Biofilms.
4077	DRAMP04261	CM-2	"CM-2"[All Fields] AND biofilm[All Fields]	CM-2	33051461	Microbial electroactive biofilms dominated by Geoalkalibacter spp. from a highly saline-alkaline environment.
4077	DRAMP04261	CM-2	"CM-2"[All Fields] AND biofilm[All Fields]	CM-2	33045616	Epigallocatechin gallate and gallic acid affect colonization of abiotic surfaces by oral bacteria.
4077	DRAMP04261	CM-2	"CM-2"[All Fields] AND biofilm[All Fields]	CM-2	33039543	Regimen and different surfaces interfere with photodynamic therapy on Candida albicans biofilms.
4077	DRAMP04261	CM-2	"CM-2"[All Fields] AND biofilm[All Fields]	CM-2	33031506	Influence of Photodynamic Therapy on Bacterial Attachment to Titanium Surface.
4077	DRAMP04261	CM-2	"CM-2"[All Fields] AND biofilm[All Fields]	CM-2	33027729	Reactions of dental pulp to hydrogen peroxide photolysis-based antimicrobial chemotherapy under ultraviolet-A irradiation in rats.
4077	DRAMP04261	CM-2	"CM-2"[All Fields] AND biofilm[All Fields]	CM-2	33022418	Successive applications of Antimicrobial Photodynamic Therapy effects the susceptibility of Candida albicans grown in medium with or without fluconazole.
4077	DRAMP04261	CM-2	"CM-2"[All Fields] AND biofilm[All Fields]	CM-2	33010485	Effects of sub-lethal dose of antimicrobial photodynamic therapy on major virulence traits of Streptococcus mutans.
4077	DRAMP04261	CM-2	"CM-2"[All Fields] AND biofilm[All Fields]	CM-2	33010484	Efficacy of antimicrobial photodynamic therapy administered using methylene blue, toluidine blue and tetra 2-mercaptopyridine substituted zinc phthalocyanine in root canals contaminated with Enterococcusaecalis.
4077	DRAMP04261	CM-2	"CM-2"[All Fields] AND biofilm[All Fields]	CM-2	32996021	In vitro effect of curcumin-mediated antimicrobial photodynamic therapy on fibroblasts: viability and cell signaling for apoptosis.
4077	DRAMP04261	CM-2	"CM-2"[All Fields] AND biofilm[All Fields]	CM-2	32975495	Platinum nanoparticles embedded into polyaniline on carbon cloth: improvement of oxygen reduction at cathode of microbial fuel cell used for conversion of medicinal plant wastes into bio-energy.
4077	DRAMP04261	CM-2	"CM-2"[All Fields] AND biofilm[All Fields]	CM-2	32874465	The efficacy of photodynamic inactivation with laser diode on Staphylococcus aureusbiofilm with various ages of biofilm.
4077	DRAMP04261	CM-2	"CM-2"[All Fields] AND biofilm[All Fields]	CM-2	32853802	Synthesis of di- and triacrylamides with tertiary amine cores and their evaluation as monomers in dental adhesive interfaces.
4077	DRAMP04261	CM-2	"CM-2"[All Fields] AND biofilm[All Fields]	CM-2	32736304	Modeling the elimination of mature biofilms formed by Staphylococcus aureus and Salmonella spp. Using combined ultrasound and disinfectants.
4077	DRAMP04261	CM-2	"CM-2"[All Fields] AND biofilm[All Fields]	CM-2	32721764	Germicidal glowsticks: Side-emitting optical fibers inhibit Pseudomonas aeruginosa and Escherichia coli on surfaces.
4077	DRAMP04261	CM-2	"CM-2"[All Fields] AND biofilm[All Fields]	CM-2	32715263	Production and Use of Selenium Nanoparticles as Fertilizers.
4077	DRAMP04261	CM-2	"CM-2"[All Fields] AND biofilm[All Fields]	CM-2	32700202	A Novel Method to Reveal a Ureolytic Biofilm Attachment and In Situ Growth Monitoring by Electrochemical Impedance Spectroscopy.
4077	DRAMP04261	CM-2	"CM-2"[All Fields] AND biofilm[All Fields]	CM-2	32689838	Evaluation of Polyethylene Glycol-Based Antimicrobial Coatings on Urinary Catheters in the Prevention of Escherichia coliInfections in a Rabbit Model.
4077	DRAMP04261	CM-2	"CM-2"[All Fields] AND biofilm[All Fields]	CM-2	32680693	Nosocomial pathogen biofilms on biomaterials: Different growth medium conditions and components of biofilms produced in vitro.
4077	DRAMP04261	CM-2	"CM-2"[All Fields] AND biofilm[All Fields]	CM-2	32662529	Effects of Rose Bengal- and Methylene Blue-Mediated Potassium Iodide-Potentiated Photodynamic Therapy on Enterococcus faecalis: A Comparative Study.
4077	DRAMP04261	CM-2	"CM-2"[All Fields] AND biofilm[All Fields]	CM-2	32660074	Metabolic Current Production by an Oral Biofilm Pathogen Corynebacterium matruchotii.
4077	DRAMP04261	CM-2	"CM-2"[All Fields] AND biofilm[All Fields]	CM-2	32652741	Bacteriological water quality and biofilm formation in the treatment system of the hemodialysis unit in Tlemcen, Algeria.
4077	DRAMP04261	CM-2	"CM-2"[All Fields] AND biofilm[All Fields]	CM-2	32616121	[Effect of 5-aminolevulinic Acid Photodynamics Therapy on Biofilm of Propionibacterium Acnes].
4077	DRAMP04261	CM-2	"CM-2"[All Fields] AND biofilm[All Fields]	CM-2	32590165	Does pre-irradiation time influence the efficacy of antimicrobial photodynamic therapy?
4077	DRAMP04261	CM-2	"CM-2"[All Fields] AND biofilm[All Fields]	CM-2	32564100	Bacillus megaterium-induced biocorrosion on mild steel and the effect of Artemisia pallens methanolic extract as a natural corrosion inhibitor.
4077	DRAMP04261	CM-2	"CM-2"[All Fields] AND biofilm[All Fields]	CM-2	32559199	Determining incremental coulombic efficiency and physiological parameters of early stage Geobacter spp. enrichment biofilms.
4077	DRAMP04261	CM-2	"CM-2"[All Fields] AND biofilm[All Fields]	CM-2	32539983	Antibacterial and antibiofilm mechanism of eugenol against antibiotic resistance Vibrio parahaemolyticus.
4077	DRAMP04261	CM-2	"CM-2"[All Fields] AND biofilm[All Fields]	CM-2	32538610	Photodynamic Inactivation of ESKAPE Group Bacterial Pathogens in Planktonic and Biofilm Cultures Using Metallated Porphyrin-Doped Conjugated Polymer Nanoparticles.
4077	DRAMP04261	CM-2	"CM-2"[All Fields] AND biofilm[All Fields]	CM-2	32485401	Exposure to diode laser (810nm) affects the bacterial adherence and biofilm formation in a E. faecalis biofilm model.
4077	DRAMP04261	CM-2	"CM-2"[All Fields] AND biofilm[All Fields]	CM-2	32466924	Carvacrol encapsulation into nanostructures: Characterization and antimicrobial activity against foodborne pathogens adhered to stainless steel.
4077	DRAMP04261	CM-2	"CM-2"[All Fields] AND biofilm[All Fields]	CM-2	32454049	Drip flow reactor method exhibits excellent reproducibility based on a 10-laboratory collaborative study.
4077	DRAMP04261	CM-2	"CM-2"[All Fields] AND biofilm[All Fields]	CM-2	32445962	Antimicrobial photodynamic therapy reduces gene expression of Candida albicans in biofilms.
4077	DRAMP04261	CM-2	"CM-2"[All Fields] AND biofilm[All Fields]	CM-2	32437976	Photodynamic therapy using methylene blue, combined or not with gentamicin, against Staphylococcus aureus and Pseudomonas aeruginosa.
4077	DRAMP04261	CM-2	"CM-2"[All Fields] AND biofilm[All Fields]	CM-2	32437974	Antimicrobial photodynamic therapy mediated by methylene blue in surfactant vehicle on periodontopathogens.
4077	DRAMP04261	CM-2	"CM-2"[All Fields] AND biofilm[All Fields]	CM-2	32431679	Stress Tolerance of Yeasts Dominating Reverse Osmosis Membranes for Whey Water Treatment.
4077	DRAMP04261	CM-2	"CM-2"[All Fields] AND biofilm[All Fields]	CM-2	32429187	Activity of Bacteriophage and Complex Tannins against Biofilm-Forming Shiga Toxin-Producing Escherichia colifrom Canada and South Africa.
4077	DRAMP04261	CM-2	"CM-2"[All Fields] AND biofilm[All Fields]	CM-2	32427892	A microbiological survey of handwashing sinks in the hospital built environment reveals differences in patient room and healthcare personnel sinks.
4077	DRAMP04261	CM-2	"CM-2"[All Fields] AND biofilm[All Fields]	CM-2	32415612	Evaluation of Bacterial Biofilm Removal Properties of MEDSTER 2000 Cold Sterilant on Different Materials.
4077	DRAMP04261	CM-2	"CM-2"[All Fields] AND biofilm[All Fields]	CM-2	32402514	Alternative monomer for BisGMA-free resin composites formulations.
4077	DRAMP04261	CM-2	"CM-2"[All Fields] AND biofilm[All Fields]	CM-2	32366710	Clinically Relevant Epithelial Lining Fluid Concentrations of Meropenem with Ciprofloxacin Provide Synergistic Killing and Resistance Suppression of Hypermutable Pseudomonas aeruginosa in a Dynamic Biofilm Model.
4077	DRAMP04261	CM-2	"CM-2"[All Fields] AND biofilm[All Fields]	CM-2	32361054	Stress response and survival of Salmonella Enteritidis in single and dual species biofilms with Pseudomonas fluorescens following repeated exposure to quaternary ammonium compounds.

4077	DRAMP04261	CM-2	"CM-2"[All Fields] AND biofilm[All Fields]	CM-2	32333442	Effect of Ultraviolet Radiation on Candida albicans Biofilm on Poly(methylmethacrylate) Resin.
4077	DRAMP04261	CM-2	"CM-2"[All Fields] AND biofilm[All Fields]	CM-2	32283312	Antimicrobial photodynamic therapy effectiveness against susceptible and methicillin-resistant Staphylococcus aureus biofilms.
4077	DRAMP04261	CM-2	"CM-2"[All Fields] AND biofilm[All Fields]	CM-2	32273961	Photoelimination Potential of Chitosan Nanoparticles-Indocyanine Green Complex Against the Biological Activities of Acinetobacter baumanniiStrains: A Preliminary In VitroStudy in Burn Wound Infections.
4077	DRAMP04261	CM-2	"CM-2"[All Fields] AND biofilm[All Fields]	CM-2	32268566	Antimicrobial Activity and Prevention of Bacterial Biofilm Formation of Silver and Zinc Oxide Nanoparticle-Containing Polyester Surfaces at Various Concentrations for Use.
4077	DRAMP04261	CM-2	"CM-2"[All Fields] AND biofilm[All Fields]	CM-2	32249986	Hypochlorous acid-generating electrochemical scaffold eliminates Candida albicans biofilms.
4077	DRAMP04261	CM-2	"CM-2"[All Fields] AND biofilm[All Fields]	CM-2	32247996	Antibiofouling performance and mechanisms of a modified polyvinylidene fluoride membrane in an MBR for wastewater treatment: Role of silver@silica nanopollens.
4077	DRAMP04261	CM-2	"CM-2"[All Fields] AND biofilm[All Fields]	CM-2	32241042	[Clinical study of cell sheets containing allogeneic keratinocytes and fibroblasts for the treatment of partial-thickness burn wounds].
4077	DRAMP04261	CM-2	"CM-2"[All Fields] AND biofilm[All Fields]	CM-2	32235332	Q-Switch Nd:YAG Laser-Assisted Elimination of Multi-Species Biofilm on Titanium Surfaces.
4077	DRAMP04261	CM-2	"CM-2"[All Fields] AND biofilm[All Fields]	CM-2	32234604	Organic carbon promotes algae proliferation in membrane-aeration based bacteria-algae symbiosis system (MA-BA).
4077	DRAMP04261	CM-2	"CM-2"[All Fields] AND biofilm[All Fields]	CM-2	32226914	Bacteriophage treatment of carbapenemase-producing Klebsiella pneumoniaein a multispecies biofilm: a potential biocontrol strategy for healthcare facilities.
4077	DRAMP04261	CM-2	"CM-2"[All Fields] AND biofilm[All Fields]	CM-2	32208520	Stratified microbial structure and activity within anode biofilm during electrochemically assisted brewery wastewater treatment.
4077	DRAMP04261	CM-2	"CM-2"[All Fields] AND biofilm[All Fields]	CM-2	32201214	Hypericin-mediated photoinactivation of polymeric nanoparticles against Staphylococcus aureus.
4077	DRAMP04261	CM-2	"CM-2"[All Fields] AND biofilm[All Fields]	CM-2	32184763	Competitive and/or Cooperative Interactions of Listeria monocytogenesWith Bacillus cereusin Dual-Species Biofilm Formation.
4077	DRAMP04261	CM-2	"CM-2"[All Fields] AND biofilm[All Fields]	CM-2	32174897	Metatranscriptomic Insights Into the Response of River Biofilm Communities to Ionic and Nano-Zinc Oxide Exposures.
4077	DRAMP04261	CM-2	"CM-2"[All Fields] AND biofilm[All Fields]	CM-2	32171881	Conjugate of chitosan nanoparticles with chloroaluminium phthalocyanine: Synthesis, characterization and photoinactivation of Streptococcus mutans biofilm.
4077	DRAMP04261	CM-2	"CM-2"[All Fields] AND biofilm[All Fields]	CM-2	32169938	Inactivation of Pseudomonas aeruginosa Biofilms by 405-Nanometer-Light-Emitting Diode Illumination.
4077	DRAMP04261	CM-2	"CM-2"[All Fields] AND biofilm[All Fields]	CM-2	32167192	Disinfection of contaminated metal implants with an Er:YAG laser.
4077	DRAMP04261	CM-2	"CM-2"[All Fields] AND biofilm[All Fields]	CM-2	32165339	Potentiation effects of antimicrobial photodynamic therapy on quorum sensing genes expression: A promising treatment for multi-species bacterial biofilms in burn wound infections.
4077	DRAMP04261	CM-2	"CM-2"[All Fields] AND biofilm[All Fields]	CM-2	32163891	Scalability and stacking of self-stratifying microbial fuel cells treating urine.
4077	DRAMP04261	CM-2	"CM-2"[All Fields] AND biofilm[All Fields]	CM-2	32158435	Lack of Periplasmic Non-heme Protein SorA Increases Shewanella decolorationisCurrent Generation.
4077	DRAMP04261	CM-2	"CM-2"[All Fields] AND biofilm[All Fields]	CM-2	32135377	Flagellar motility mediates early-stage biofilm formation in oligotrophic aquatic environment.
4077	DRAMP04261	CM-2	"CM-2"[All Fields] AND biofilm[All Fields]	CM-2	32120321	Success and side effects of different treatment options in the low current attack of bacterial biofilms on titanium implants.
4077	DRAMP04261	CM-2	"CM-2"[All Fields] AND biofilm[All Fields]	CM-2	32105346	Photoinactivation of Moraxella catarrhalis Using 405-nm Blue Light: Implications for the Treatment of Otitis Media.
4077	DRAMP04261	CM-2	"CM-2"[All Fields] AND biofilm[All Fields]	CM-2	32069768	Formation of biofilms from new pipelines at both ends of the drinking water distribution system and comparison of disinfection by-products formation potential.
4077	DRAMP04261	CM-2	"CM-2"[All Fields] AND biofilm[All Fields]	CM-2	32066051	Paddy periphyton reduced cadmium accumulation in rice (Oryza sativa) by removing and immobilizing cadmium from the water-soil interface.
4077	DRAMP04261	CM-2	"CM-2"[All Fields] AND biofilm[All Fields]	CM-2	32041294	Inactivation of L. monocytogenesand S. typhimuriumBiofilms by Means of an Air-Based Cold Atmospheric Plasma (CAP) System.
4077	DRAMP04261	CM-2	"CM-2"[All Fields] AND biofilm[All Fields]	CM-2	32033480	Evaluation of Microbial Adhesion and Biofilm Formation on Nano-Structured and Nano-Coated Ortho-Prosthetic Materials by a Dynamic Model.
4077	DRAMP04261	CM-2	"CM-2"[All Fields] AND biofilm[All Fields]	CM-2	32027477	Smart Hydrogel-Based DVDMS/bFGF Nanohybrids for Antibacterial Phototherapy with Multiple Damaging Sites and Accelerated Wound Healing.
4077	DRAMP04261	CM-2	"CM-2"[All Fields] AND biofilm[All Fields]	CM-2	32009731	Antimicrobial effect on Candida albicansbiofilm by application of different wavelengths and dyes and the synthetic killer decapeptide KP.
4077	DRAMP04261	CM-2	"CM-2"[All Fields] AND biofilm[All Fields]	CM-2	32000487	Glucose Microsensor with Covalently Immobilized Glucose Oxidase for Probing Bacterial Glucose Uptake by Scanning Electrochemical Microscopy.
4077	DRAMP04261	CM-2	"CM-2"[All Fields] AND biofilm[All Fields]	CM-2	31999996	Effects of cold recovery technology on the microbial drinking water quality in unchlorinated distribution systems.
4077	DRAMP04261	CM-2	"CM-2"[All Fields] AND biofilm[All Fields]	CM-2	31996786	From an extremophilic community to an electroautotrophic production strain: identifying a novel Knallgas bacterium as cathodic biofilm biocatalyst.
4077	DRAMP04261	CM-2	"CM-2"[All Fields] AND biofilm[All Fields]	CM-2	31991823	Evaluation of the Potential of Biofilm Formation of Bifidobacterium longumsubsp. infantisand Lactobacillus reuterias Competitive Biocontrol Agents Against Pathogenic and Food Spoilage Bacteria.
4077	DRAMP04261	CM-2	"CM-2"[All Fields] AND biofilm[All Fields]	CM-2	31986930	Synergy between induction heating, antibiotics, and N-acetylcysteine eradicates Staphylococcus aureusfrom biofilm.
4077	DRAMP04261	CM-2	"CM-2"[All Fields] AND biofilm[All Fields]	CM-2	31986339	Effect of blue light at 410 and 455 nm on Pseudomonas aeruginosa biofilm.
4077	DRAMP04261	CM-2	"CM-2"[All Fields] AND biofilm[All Fields]	CM-2	31972939	Microbial colonization of different microplastic types and biotransformation of sorbed PCBs by a marine anaerobic bacterial community.
4077	DRAMP04261	CM-2	"CM-2"[All Fields] AND biofilm[All Fields]	CM-2	31965132	Real time monitoring of biofilm formation on coated medical devices for the reduction and interception of bacterial infections.
4077	DRAMP04261	CM-2	"CM-2"[All Fields] AND biofilm[All Fields]	CM-2	31948618	Quercetin repressed the stress response factor (sigB) and virulence genes (prfA, actA, inlA, and inlC), lower the adhesion, and biofilm development of L. monocytogenes.
4077	DRAMP04261	CM-2	"CM-2"[All Fields] AND biofilm[All Fields]	CM-2	31943345	Local concentrations of gentamicin obtained by microdialysis after a controlled application of a GentaColl sponge in a porcine model.
4077	DRAMP04261	CM-2	"CM-2"[All Fields] AND biofilm[All Fields]	CM-2	31913598	Antibacterial Photodynamic Inactivation of Antibiotic-Resistant Bacteria and Biofilms with Nanomolar Photosensitizer Concentrations.
4077	DRAMP04261	CM-2	"CM-2"[All Fields] AND biofilm[All Fields]	CM-2	31904552	Ex vivo comparison of antibacterial efficacy of conventional chemomechanical debridement alone and in combination with light-activated disinfection and laser irradiation against Enterococcus faecalis biofilm.
4077	DRAMP04261	CM-2	"CM-2"[All Fields] AND biofilm[All Fields]	CM-2	31899376	Curcumin induced photodynamic therapy mediated suppression of quorum sensing pathway of Pseudomonas aeruginosa: An approach to inhibit biofilm in vitro.
4077	DRAMP04261	CM-2	"CM-2"[All Fields] AND biofilm[All Fields]	CM-2	31896046	Silver grass-derived activated carbon with coexisting micro-, meso- and macropores as excellent bioanodes for microbial colonization and power generation in sustainable microbial fuel cells.
4077	DRAMP04261	CM-2	"CM-2"[All Fields] AND biofilm[All Fields]	CM-2	31885074	Effective biofilm control in a membrane biofilm reactor using a quenching bacterium (Rhodococcus sp. BH4).
4077	DRAMP04261	CM-2	"CM-2"[All Fields] AND biofilm[All Fields]	CM-2	31867530	Impact and Role of Bacterial Communities on Biocorrosion of Metals Used in the Processing Industry.
4077	DRAMP04261	CM-2	"CM-2"[All Fields] AND biofilm[All Fields]	CM-2	31849874	Isolation, Characterization, and Inactivation of Stenotrophomonas maltophiliaFrom Leafy Green Vegetables and Urban Agriculture Systems.
4077	DRAMP04261	CM-2	"CM-2"[All Fields] AND biofilm[All Fields]	CM-2	31841686	Anti-biofilm and anti-metabolic effects of antimicrobial photodynamic therapy using chlorophyllin-phycocyanin mixture against Streptococcus mutans in experimental biofilm caries model on enamel slabs.
4077	DRAMP04261	CM-2	"CM-2"[All Fields] AND biofilm[All Fields]	CM-2	31838458	Electron donor availability controls scale up of anodic biofilms.
4077	DRAMP04261	CM-2	"CM-2"[All Fields] AND biofilm[All Fields]	CM-2	31827464	Rugose Morphotype in SalmonellaTyphimurium and SalmonellaHeidelberg Induced by Sequential Exposure to Subinhibitory Sodium Hypochlorite Aids in Biofilm Tolerance to Lethal Sodium Hypochlorite on Polystyrene and Stainless Steel Surfaces.
4077	DRAMP04261	CM-2	"CM-2"[All Fields] AND biofilm[All Fields]	CM-2	31824459	Combined Effect of Cold Atmospheric Plasma and Hydrogen Peroxide Treatment on Mature Listeria monocytogenesand SalmonellaTyphimurium Biofilms.
4077	DRAMP04261	CM-2	"CM-2"[All Fields] AND biofilm[All Fields]	CM-2	31817522	Efficacy of Synthetic Furanones on Listeria monocytogenesBiofilm Formation.
4077	DRAMP04261	CM-2	"CM-2"[All Fields] AND biofilm[All Fields]	CM-2	31817368	Quantitative and Compositional Study of Monospecies Biofilms of Spoilage Microorganisms in the Meat Industry and Their Interaction in the Development of Multispecies Biofilms.
4077	DRAMP04261	CM-2	"CM-2"[All Fields] AND biofilm[All Fields]	CM-2	31783343	The ability of Listeria monocytogenes to form biofilm on surfaces relevant to the mushroom production environment.
4077	DRAMP04261	CM-2	"CM-2"[All Fields] AND biofilm[All Fields]	CM-2	31760372	Pulsed 450 nm blue light suppresses MRSA and Propionibacterium acnes in planktonic cultures and bacterial biofilms.
4077	DRAMP04261	CM-2	"CM-2"[All Fields] AND biofilm[All Fields]	CM-2	31749949	The Efficacy of Photodynamic Inactivation of the Diode Laser in Inactivation of the Candida albicansBiofilms With Exogenous Photosensitizer of Papaya Leaf Chlorophyll.
4077	DRAMP04261	CM-2	"CM-2"[All Fields] AND biofilm[All Fields]	CM-2	31746024	Quinine Improves the Fungicidal Effects of Antimicrobial Blue Light: Implications for the Treatment of Cutaneous Candidiasis.

4077	DRAMP04261	CM-2	"CM-2"[All Fields] AND biofilm[All Fields]	CM-2	31713778	Exposure of Streptococcus mutans and Streptococcus sanguinis to blue light in an oral biofilm model.
4077	DRAMP04261	CM-2	"CM-2"[All Fields] AND biofilm[All Fields]	CM-2	31710972	Tracking bacteriome variation over time in Listeria monocytogenes-positive foci in food industry.
4077	DRAMP04261	CM-2	"CM-2"[All Fields] AND biofilm[All Fields]	CM-2	33405531	Synergistic Photothermal and Photodynamic Therapy for Effective Implant-Related Bacterial Infection Elimination and Biofilm Disruption Using Cu 9S 8Nanoparticles.
4077	DRAMP04261	CM-2	"CM-2"[All Fields] AND biofilm[All Fields]	CM-2	31697369	Survey of pathogenic bacteria of biofilms in a metropolitan drinking water distribution system.
4077	DRAMP04261	CM-2	"CM-2"[All Fields] AND biofilm[All Fields]	CM-2	31686691	Characterization of biofilm production by Pseudomonas fluorescensisolated from refrigerated raw buffalo milk.
4077	DRAMP04261	CM-2	"CM-2"[All Fields] AND biofilm[All Fields]	CM-2	31644974	Covalent grafting of titanium with a cathelicidin peptide produces an osteoblast compatible surface with antistaphylococcal activity.
4077	DRAMP04261	CM-2	"CM-2"[All Fields] AND biofilm[All Fields]	CM-2	31634029	Hypocrellin B-Mediated Photodynamic Inactivation of Gram-Positive Antibiotic-Resistant Bacteria: An In VitroStudy.
4077	DRAMP04261	CM-2	"CM-2"[All Fields] AND biofilm[All Fields]	CM-2	31630921	Effectiveness of manual versus automated cleaning on Staphylococcus epidermidis biofilm removal from the surface of surgical instruments.
4077	DRAMP04261	CM-2	"CM-2"[All Fields] AND biofilm[All Fields]	CM-2	31625519	Photothermal inactivation of methicillin-resistant Staphylococcus aureus: anti-biofilm mediated by a polypyrrole-carbon nanocomposite.
4077	DRAMP04261	CM-2	"CM-2"[All Fields] AND biofilm[All Fields]	CM-2	31624671	Effect of Native Type I Collagen with Polyhexamethylene Biguanide Antimicrobial on Wounds: Interim Registry Results.
4077	DRAMP04261	CM-2	"CM-2"[All Fields] AND biofilm[All Fields]	CM-2	31614222	Newly formulated 5% 5-aminolevulinic acid photodynamic therapy on Candida albicans.
4077	DRAMP04261	CM-2	"CM-2"[All Fields] AND biofilm[All Fields]	CM-2	31606645	Applicability of advanced oxidation processes in removing anthropogenically influenced chlorination disinfection byproduct precursors in a developing country.
4077	DRAMP04261	CM-2	"CM-2"[All Fields] AND biofilm[All Fields]	CM-2	31599650	Antibiofilm Efficacy of Peptide 1018 against Listeria monocytogenesand Shiga Toxigenic Escherichia colion Equipment Surfaces.
4077	DRAMP04261	CM-2	"CM-2"[All Fields] AND biofilm[All Fields]	CM-2	31581536	Q-Switch Nd:YAG Laser-Assisted Decontamination of Implant Surface.
4077	DRAMP04261	CM-2	"CM-2"[All Fields] AND biofilm[All Fields]	CM-2	31574321	Antibacterial, ester-free monomers: Polymerization kinetics, mechanical properties, biocompatibility and anti-biofilm activity.
4077	DRAMP04261	CM-2	"CM-2"[All Fields] AND biofilm[All Fields]	CM-2	31573730	Synergistic mode of action of catechin, vanillic and protocatechuic acids to inhibit the adhesion of uropathogenic Escherichia coli on silicone surfaces.
4077	DRAMP04261	CM-2	"CM-2"[All Fields] AND biofilm[All Fields]	CM-2	31558046	Multispecies biofilm formation by the contaminating microbiota in raw milk.
4077	DRAMP04261	CM-2	"CM-2"[All Fields] AND biofilm[All Fields]	CM-2	31550928	Antimicrobial and antifouling polymeric coating mitigates persistence of Pseudomonas aeruginosabiofilm.
4077	DRAMP04261	CM-2	"CM-2"[All Fields] AND biofilm[All Fields]	CM-2	31546042	Addition of hydrogen peroxide to methylene blue conjugated to β-cyclodextrin in photodynamic antimicrobial chemotherapy in S. mutans biofilm.
4077	DRAMP04261	CM-2	"CM-2"[All Fields] AND biofilm[All Fields]	CM-2	31536154	Antimicrobial Blue Light Inactivation of Microbial Isolates in Biofilms.
4077	DRAMP04261	CM-2	"CM-2"[All Fields] AND biofilm[All Fields]	CM-2	31501280	Conceptual Model of Biofilm Antibiotic Tolerance That Integrates Phenomena of Diffusion, Metabolism, Gene Expression, and Physiology.
4077	DRAMP04261	CM-2	"CM-2"[All Fields] AND biofilm[All Fields]	CM-2	31500045	Nanocomposite foams based on flexible biobased thermoplastic polyurethane and ZnO nanoparticles as potential wound dressing materials.
4077	DRAMP04261	CM-2	"CM-2"[All Fields] AND biofilm[All Fields]	CM-2	31483869	Disinfection of Cariogenic Pathogens in Planktonic Lifestyle, Biofilm and Carious Dentine with Antimicrobial Photodynamic Therapy.
4077	DRAMP04261	CM-2	"CM-2"[All Fields] AND biofilm[All Fields]	CM-2	31482697	Culture of attached and suspended Rhodopseudomonas faecalis in the presence of decomposing fish feed.
4077	DRAMP04261	CM-2	"CM-2"[All Fields] AND biofilm[All Fields]	CM-2	31466379	Antibacterial Nanostructured Ti Coatings by Magnetron Sputtering: From Laboratory Scales to Industrial Reactors.
4077	DRAMP04261	CM-2	"CM-2"[All Fields] AND biofilm[All Fields]	CM-2	31444205	Violet-Blue Light Arrays at 405 Nanometers Exert Enhanced Antimicrobial Activity for Photodisinfection of Monomicrobial Nosocomial Biofilms.
4077	DRAMP04261	CM-2	"CM-2"[All Fields] AND biofilm[All Fields]	CM-2	31438656	Disinfectant Susceptibility of Biofilm Formed by Listeria monocytogenesunder Selected Environmental Conditions.
4077	DRAMP04261	CM-2	"CM-2"[All Fields] AND biofilm[All Fields]	CM-2	31427301	Synergistic Meropenem-Tobramycin Combination Dosage Regimens against Clinical Hypermutable Pseudomonas aeruginosa at Simulated Epithelial Lining Fluid Concentrations in a Dynamic Biofilm Model.
4077	DRAMP04261	CM-2	"CM-2"[All Fields] AND biofilm[All Fields]	CM-2	31415968	Adhesion of filamentous fungi isolated from drinking water under different process conditions.
4077	DRAMP04261	CM-2	"CM-2"[All Fields] AND biofilm[All Fields]	CM-2	31412645	Selection of Bacteriophages to Control In Vitro 24 h Old Biofilm of Pseudomonas AeruginosaIsolated from Drinking and Thermal Water.
4077	DRAMP04261	CM-2	"CM-2"[All Fields] AND biofilm[All Fields]	CM-2	31411536	Combined Effectiveness of β-Cyclodextrin Nanoparticles in Photodynamic Antimicrobial Chemotherapy on In VitroOral Biofilms.
4077	DRAMP04261	CM-2	"CM-2"[All Fields] AND biofilm[All Fields]	CM-2	31407806	Effect of High Intensity Blue Light on Fusobacterium nucleatum Membrane Integrity.
4077	DRAMP04261	CM-2	"CM-2"[All Fields] AND biofilm[All Fields]	CM-2	31404442	A novel rat model of foreign body osteomyelitis for evaluation of antimicrobial efficacy.
4077	DRAMP04261	CM-2	"CM-2"[All Fields] AND biofilm[All Fields]	CM-2	31357621	Ostreopsiscf. ovataBloom in Currais, Brazil: Phylogeny, Toxin Profile and Contamination of Mussels and Marine Plastic Litter.
4077	DRAMP04261	CM-2	"CM-2"[All Fields] AND biofilm[All Fields]	CM-2	31315967	Observations of Shear Stress Effects on Staphylococcus aureus Biofilm Formation.
4077	DRAMP04261	CM-2	"CM-2"[All Fields] AND biofilm[All Fields]	CM-2	31310825	The effectiveness of curcumin-mediated antimicrobial photodynamic therapy depends on pre-irradiation and biofilm growth times.
4077	DRAMP04261	CM-2	"CM-2"[All Fields] AND biofilm[All Fields]	CM-2	31289312	Bio-enzymes for inhibition and elimination of Escherichia coli O157:H7 biofilm and their synergistic effect with sodium hypochlorite.
4077	DRAMP04261	CM-2	"CM-2"[All Fields] AND biofilm[All Fields]	CM-2	31287948	Nontuberculous Mycobacteria in Two Drinking Water Distribution Systems and the Role of Residual Disinfection.
4077	DRAMP04261	CM-2	"CM-2"[All Fields] AND biofilm[All Fields]	CM-2	31273998	Anoxybacillusand Geobacillusbiofilms in the dairy industry: effects of surface material, incubation temperature and milk type.
4077	DRAMP04261	CM-2	"CM-2"[All Fields] AND biofilm[All Fields]	CM-2	31233886	Antifungal effect of photodynamic therapy mediated by curcumin on Candida albicans biofilms in vitro.
4077	DRAMP04261	CM-2	"CM-2"[All Fields] AND biofilm[All Fields]	CM-2	31231330	In vitroEvaluation of Photodynamic Effects Against Biofilms of Dermatophytes Involved in Onychomycosis.
4077	DRAMP04261	CM-2	"CM-2"[All Fields] AND biofilm[All Fields]	CM-2	31230199	Inhibitory Effects of Photodynamic Inactivation on Planktonic Cells and Biofilms of Candida auris.
4077	DRAMP04261	CM-2	"CM-2"[All Fields] AND biofilm[All Fields]	CM-2	31228562	Antimicrobial photodynamic activity of toluidine blue-carbon nanotube conjugate against Pseudomonas aeruginosa and Staphylococcus aureus - Understanding the mechanism of action.
4077	DRAMP04261	CM-2	"CM-2"[All Fields] AND biofilm[All Fields]	CM-2	31213806	Malachite green-conjugated multi-walled carbon nanotubes potentiate antimicrobial photodynamic inactivation of planktonic cells and biofilms of Pseudomonas aeruginosaand Staphylococcus aureus.
4077	DRAMP04261	CM-2	"CM-2"[All Fields] AND biofilm[All Fields]	CM-2	31202424	An ordinal logistic regression approach to predict the variability on biofilm formation stages by five Salmonella enterica strains on polypropylene and glass surfaces as affected by pH, temperature and NaCl.
4077	DRAMP04261	CM-2	"CM-2"[All Fields] AND biofilm[All Fields]	CM-2	31200011	Assessment of sensitivity of selected Candida strains on antimicrobial photodynamic therapy using diode laser 635 nm and toluidine blue - In vitro research.
4077	DRAMP04261	CM-2	"CM-2"[All Fields] AND biofilm[All Fields]	CM-2	31176043	The potential of commercially available phytotherapeutic compounds as new photosensitizers for dental antimicrobial PDT: A photochemical and photobiological in vitro study.
4077	DRAMP04261	CM-2	"CM-2"[All Fields] AND biofilm[All Fields]	CM-2	31174608	Comparison of the microbiomes of two drinking water distribution systems-with and without residual chloramine disinfection.
4077	DRAMP04261	CM-2	"CM-2"[All Fields] AND biofilm[All Fields]	CM-2	31152877	DNase increases the efficacy of antimicrobial photodynamic therapy on Candida albicans biofilms.
4077	DRAMP04261	CM-2	"CM-2"[All Fields] AND biofilm[All Fields]	CM-2	31131342	Effect of Direct Electrical Current on Bones Infected with Staphylococcus epidermidis.
4077	DRAMP04261	CM-2	"CM-2"[All Fields] AND biofilm[All Fields]	CM-2	31129476	Kinetics of biofilm formation by pathogenic and spoilage microorganisms under conditions that mimic the poultry, meat, and egg processing industries.
4077	DRAMP04261	CM-2	"CM-2"[All Fields] AND biofilm[All Fields]	CM-2	31125769	The impact of Aggregatibacter actinomycetemcomitans biofilm-derived effectors following antimicrobial photodynamic therapy on cytokine production in human gingival fibroblasts.
4077	DRAMP04261	CM-2	"CM-2"[All Fields] AND biofilm[All Fields]	CM-2	31125677	Targeted laser therapy synergistically enhances efficacy of antibiotics against multi-drug resistant Staphylococcus aureus and Pseudomonas aeruginosa biofilms.
4077	DRAMP04261	CM-2	"CM-2"[All Fields] AND biofilm[All Fields]	CM-2	31117001	Enhancing osteoblast functions on biofilm-contaminated titanium alloy by concentration-dependent use of methylene blue-mediated antimicrobial photodynamic therapy.
4077	DRAMP04261	CM-2	"CM-2"[All Fields] AND biofilm[All Fields]	CM-2	31087870	[Impact Mechanisms of Carboxyl Group Modified Cathode on Acetate Production in Microbial Electrosynthesis Systems].
4077	DRAMP04261	CM-2	"CM-2"[All Fields] AND biofilm[All Fields]	CM-2	31083306	Effect of Chitosan Dispersion and Microparticles on Older Streptococcus mutansBiofilms.
4077	DRAMP04261	CM-2	"CM-2"[All Fields] AND biofilm[All Fields]	CM-2	31081821	Daily Phototherapy with Red Light to Regulate Candida albicans Biofilm Growth.

4077	DRAMP04261	CM-2	"CM-2"[All Fields] AND biofilm[All Fields]	CM-2	31074107	Antibacterial activity of copper-bearing 316L stainless steel for the prevention of implant-related infection.
4077	DRAMP04261	CM-2	"CM-2"[All Fields] AND biofilm[All Fields]	CM-2	31059835	Nitric oxide-releasing vascular grafts: A therapeutic strategy to promote angiogenic activity and endothelium regeneration.
4077	DRAMP04261	CM-2	"CM-2"[All Fields] AND biofilm[All Fields]	CM-2	31050940	Comparative Effect of Two Red Lights on Streptococcus mutansBiofilms and Assessment of Temperature Variances in Human Teeth During In VitroPhotodynamic Antimicrobial Chemotherapy.
4077	DRAMP04261	CM-2	"CM-2"[All Fields] AND biofilm[All Fields]	CM-2	31042554	Photodynamic Antimicrobial Chemotherapy (PACT) using methylene blue inhibits the viability of the biofilm produced by Candida albicans.
4077	DRAMP04261	CM-2	"CM-2"[All Fields] AND biofilm[All Fields]	CM-2	31033353	Copolymeric micelles as efficient inert nanocarrier for hypericin in the photodynamic inactivation of Candidaspecies.
4077	DRAMP04261	CM-2	"CM-2"[All Fields] AND biofilm[All Fields]	CM-2	31027813	Interactions between spoilage bacteria in tri-species biofilms developed under simulated meat processing conditions.
4077	DRAMP04261	CM-2	"CM-2"[All Fields] AND biofilm[All Fields]	CM-2	31027768	Effects of the colonization sequence of Listeria monocytogenes and Pseudomonas fluorescens on survival of biofilm cells under food-related stresses and transfer to salmon.
4077	DRAMP04261	CM-2	"CM-2"[All Fields] AND biofilm[All Fields]	CM-2	31025630	Novel photo-thermally active polyvinyl alcohol-Prussian blue nanoparticles hydrogel films capable of eradicating bacteria and mitigating biofilms.
4077	DRAMP04261	CM-2	"CM-2"[All Fields] AND biofilm[All Fields]	CM-2	31024499	Antimicrobial Blue Light Inactivation of Polymicrobial Biofilms.
4077	DRAMP04261	CM-2	"CM-2"[All Fields] AND biofilm[All Fields]	CM-2	31022421	Underperforming light curing procedures trigger detrimental irradiance-dependent biofilm response on incrementally placed dental composites.
4077	DRAMP04261	CM-2	"CM-2"[All Fields] AND biofilm[All Fields]	CM-2	31006166	Photodynamic inactivation to prevent and disrupt Staphylococcus aureus biofilm under different media conditions.
4077	DRAMP04261	CM-2	"CM-2"[All Fields] AND biofilm[All Fields]	CM-2	30976322	Less biomass and intracellular glutamate in anodic biofilms lead to efficient electricity generation by microbial fuel cells.
4077	DRAMP04261	CM-2	"CM-2"[All Fields] AND biofilm[All Fields]	CM-2	30976321	Mo 2C-induced hydrogen production enhances microbial electrosynthesis of acetate from CO 2reduction.
4077	DRAMP04261	CM-2	"CM-2"[All Fields] AND biofilm[All Fields]	CM-2	30972336	Long-Term Behavior of Defined Mixed Cultures of Geobacter sulfurreducensand Shewanella oneidensisin Bioelectrochemical Systems.
4077	DRAMP04261	CM-2	"CM-2"[All Fields] AND biofilm[All Fields]	CM-2	30956871	Efficacy of chemical sanitizers against Bacillus cereuson food contact surfaces with scratch and biofilm.
4077	DRAMP04261	CM-2	"CM-2"[All Fields] AND biofilm[All Fields]	CM-2	30952008	Legionella growth potential of drinking water produced by a reverse osmosis pilot plant.
4077	DRAMP04261	CM-2	"CM-2"[All Fields] AND biofilm[All Fields]	CM-2	30880972	Nanoporous solid-state membranes modified with multi-wall carbon nanotubes with anti-biofouling property.
4077	DRAMP04261	CM-2	"CM-2"[All Fields] AND biofilm[All Fields]	CM-2	30859031	Use of Native Type I Collagen Matrix Plus Polyhexamethylene Biguanide for Chronic Wound Treatment.
4077	DRAMP04261	CM-2	"CM-2"[All Fields] AND biofilm[All Fields]	CM-2	30835535	Antimicrobial photodynamic activity of toluidine blue encapsulated in mesoporous silica nanoparticles against Pseudomonas aeruginosa and Staphylococcus aureus.
4077	DRAMP04261	CM-2	"CM-2"[All Fields] AND biofilm[All Fields]	CM-2	30826559	Antimicrobial surfaces with self-cleaning properties functionalized by photocatalytic ZnO electrosprayed coatings.
4077	DRAMP04261	CM-2	"CM-2"[All Fields] AND biofilm[All Fields]	CM-2	30810917	Comparison of selected disinfectants efficiency against Listeria monocytogenes biofilm formed on various surfaces.
4077	DRAMP04261	CM-2	"CM-2"[All Fields] AND biofilm[All Fields]	CM-2	30801748	Synergistic cranberry juice combinations with natural-borne antimicrobials for the eradication of uropathogenic Escherichia coli biofilm within a short time.
4077	DRAMP04261	CM-2	"CM-2"[All Fields] AND biofilm[All Fields]	CM-2	30798389	Effectiveness of 405-nm blue LED light for degradation of Candida biofilms formed on PMMA denture base resin.
4077	DRAMP04261	CM-2	"CM-2"[All Fields] AND biofilm[All Fields]	CM-2	30758060	Biofilm contamination of high-touched surfaces in intensive care units: epidemiology and potential impacts.
4077	DRAMP04261	CM-2	"CM-2"[All Fields] AND biofilm[All Fields]	CM-2	30730701	Extracellular Polymeric Substances from Geobacter sulfurreducens Biofilms in Microbial Fuel Cells.
4077	DRAMP04261	CM-2	"CM-2"[All Fields] AND biofilm[All Fields]	CM-2	30716932	Inactivation kinetics of slightly acidic electrolyzed water combined with benzalkonium chloride and mild heat treatment on vegetative cells, spores, and biofilms of Bacillus cereus.
4077	DRAMP04261	CM-2	"CM-2"[All Fields] AND biofilm[All Fields]	CM-2	30712696	Photodynamic inactivation of planktonic cultures and Streptococcus mutans biofilms for prevention of white spot lesions during orthodontic treatment: An in vitro investigation.
4077	DRAMP04261	CM-2	"CM-2"[All Fields] AND biofilm[All Fields]	CM-2	30684672	The effect of antimicrobial photodynamic therapy on the expression of biofilm associated genes in Staphylococcus aureus strains isolated from wound infections in burn patients.
4077	DRAMP04261	CM-2	"CM-2"[All Fields] AND biofilm[All Fields]	CM-2	30683743	Chlorine and Monochloramine Disinfection of Legionella pneumophilaColonizing Copper and Polyvinyl Chloride Drinking Water Biofilms.
4077	DRAMP04261	CM-2	"CM-2"[All Fields] AND biofilm[All Fields]	CM-2	30675341	The potential of visible blue light (405 nm) as a novel decontamination strategy for carbapenemase-producing enterobacteriaceae (CPE).
4077	DRAMP04261	CM-2	"CM-2"[All Fields] AND biofilm[All Fields]	CM-2	30670414	Synergistic Antifungal Effect of Amphotericin B-Loaded Poly(Lactic-Co-Glycolic Acid) Nanoparticles and Ultrasound against Candida albicans Biofilms.
4077	DRAMP04261	CM-2	"CM-2"[All Fields] AND biofilm[All Fields]	CM-2	30648637	Antimicrobial efficacy of photodynamic therapy on dental implant surfaces: A systematic review of in vitro studies.
4077	DRAMP04261	CM-2	"CM-2"[All Fields] AND biofilm[All Fields]	CM-2	30621876	Inhibition of Listeria monocytogenes using biofilms of non-pathogenic soil bacteria (Streptomyces spp.) on stainless steel under desiccated condition.
4077	DRAMP04261	CM-2	"CM-2"[All Fields] AND biofilm[All Fields]	CM-2	30611865	Photodynamic Antimicrobial Chemotherapy (PACT), using Toluidine blue (TBO) inhibits both growth and dimorphism in Paracoccidioides brasiliensis by a mechanism involving reactive oxygen species (ROS) production.
4077	DRAMP04261	CM-2	"CM-2"[All Fields] AND biofilm[All Fields]	CM-2	30607721	Sub-lethal antimicrobial photodynamic inactivation: an in vitro study on quorum sensing-controlled gene expression of Pseudomonas aeruginosa biofilm formation.
4077	DRAMP04261	CM-2	"CM-2"[All Fields] AND biofilm[All Fields]	CM-2	30604346	Could a chelant improve the effect of curcumin-mediated photodynamic antimicrobial chemotherapy against dental intact biofilms?
4077	DRAMP04261	CM-2	"CM-2"[All Fields] AND biofilm[All Fields]	CM-2	30601523	Synthesis and antimicrobial photodynamic effect of methylene blue conjugated carbon nanotubes on E. coli and S. aureus.
4077	DRAMP04261	CM-2	"CM-2"[All Fields] AND biofilm[All Fields]	CM-2	30596498	Photosensitizer-Loaded Multifunctional Chitosan Nanoparticles for Simultaneous in Situ Imaging, Highly Efficient Bacterial Biofilm Eradication, and Tumor Ablation.
4077	DRAMP04261	CM-2	"CM-2"[All Fields] AND biofilm[All Fields]	CM-2	30594087	Inactivation of biofilm-bound Pseudomonas aeruginosa bacteria using UVC light emitting diodes (UVC LEDs).
4077	DRAMP04261	CM-2	"CM-2"[All Fields] AND biofilm[All Fields]	CM-2	30555449	Efficiency of Different Disinfectants on Bacillus cereusSensu Stricto Biofilms on Stainless-Steel Surfaces in Contact With Milk.
4077	DRAMP04261	CM-2	"CM-2"[All Fields] AND biofilm[All Fields]	CM-2	30466178	Hydrogen Peroxide Enhances the Antibacterial Effect of Methylene Blue-based Photodynamic Therapy on Biofilm-forming Bacteria.
4077	DRAMP04261	CM-2	"CM-2"[All Fields] AND biofilm[All Fields]	CM-2	30413898	The antifungal agent of silver nanoparticles activated by diode laser as light source to reduce C. albicans biofilms: an in vitro study.
4077	DRAMP04261	CM-2	"CM-2"[All Fields] AND biofilm[All Fields]	CM-2	30399454	Could chlorhexidine be an adequate positive control for antimicrobial photodynamic therapy in- in vitro studies?
4077	DRAMP04261	CM-2	"CM-2"[All Fields] AND biofilm[All Fields]	CM-2	30374407	Disintegration of simulated drinking water biofilms with arrays of microchannel plasma jets.
4077	DRAMP04261	CM-2	"CM-2"[All Fields] AND biofilm[All Fields]	CM-2	30362370	An extraction free modified o-phthalaldehyde assay for quantifying residual protein and microbial biofilms on surfaces.
4077	DRAMP04261	CM-2	"CM-2"[All Fields] AND biofilm[All Fields]	CM-2	30342101	Antimicrobial photodynamic activity of rose bengal conjugated multi walled carbon nanotubes against planktonic cells and biofilm of Escherichia coli.
4077	DRAMP04261	CM-2	"CM-2"[All Fields] AND biofilm[All Fields]	CM-2	30321686	The effect of sublethal photodynamic therapy on the expression of Enterococcal surface protein (esp) encoding gene in Enterococcus faecalis: Quantitative real-time PCR assessment.
4077	DRAMP04261	CM-2	"CM-2"[All Fields] AND biofilm[All Fields]	CM-2	30311085	Effects of antibacterial photodynamic therapy on salivary mutans streptococci in 5- to 6-year-olds with severe early childhood caries.
4077	DRAMP04261	CM-2	"CM-2"[All Fields] AND biofilm[All Fields]	CM-2	30308310	Effect of Chloroaluminium phthalocyanine in cationic nanoemulsion on photoinactivation of multispecies biofilm.
4077	DRAMP04261	CM-2	"CM-2"[All Fields] AND biofilm[All Fields]	CM-2	30291117	Behavior of Foodborne Pathogens Listeria monocytogenes and Staphylococcus aureus in Mixed-Species Biofilms Exposed to Biocides.
4077	DRAMP04261	CM-2	"CM-2"[All Fields] AND biofilm[All Fields]	CM-2	30291115	Primary Colonizing BetaproteobacterialesPlay a Key Role in the Growth of Legionella pneumophilain Biofilms on Surfaces Exposed to Drinking Water Treated by Slow Sand Filtration.
4077	DRAMP04261	CM-2	"CM-2"[All Fields] AND biofilm[All Fields]	CM-2	30274130	Antibacterial dental adhesive resins containing nitrogen-doped titanium dioxide nanoparticles.
4077	DRAMP04261	CM-2	"CM-2"[All Fields] AND biofilm[All Fields]	CM-2	30270665	Control of SalmonellaEnteritidis on food contact surfaces with bacteriophage PVP-SE2.
4077	DRAMP04261	CM-2	"CM-2"[All Fields] AND biofilm[All Fields]	CM-2	30267963	Antimicrobial photodynamic therapy alone or in combination with antibiotic local administration against biofilms of Fusobacterium nucleatum and Porphyromonas gingivalis.
4077	DRAMP04261	CM-2	"CM-2"[All Fields] AND biofilm[All Fields]	CM-2	30260744	Adhesion of Streptococcus mutansand Streptococcus sanguinison Er:YAG Laser-Irradiated Dental Enamel: Effect of Surface Roughness.
4077	DRAMP04261	CM-2	"CM-2"[All Fields] AND biofilm[All Fields]	CM-2	30240927	Curcumin-mediated antimicrobial photodynamic therapy reduces the viability and vitality of infected dentin caries microcosms.
4077	DRAMP04261	CM-2	"CM-2"[All Fields] AND biofilm[All Fields]	CM-2	30223081	Analysis of glucosyltransferase gene expression of clinical isolates of Streptococcus mutans obtained from dental plaques in response to sub-lethal doses of photoactivated disinfection.
4077	DRAMP04261	CM-2	"CM-2"[All Fields] AND biofilm[All Fields]	CM-2	30210486	Inactivation of Foodborne Bacteria Biofilms by Aqueous and Gaseous Ozone.

4077	DRAMP04261	CM-2	"CM-2"[All Fields] AND biofilm[All Fields]	CM-2	30183793	Controlling methylene blue aggregation: a more efficient alternative to treat Candida albicans infections using photodynamic therapy.
4077	DRAMP04261	CM-2	"CM-2"[All Fields] AND biofilm[All Fields]	CM-2	30172134	Selective antibiotic resistance genes in multiphase samples during biofilm growth in a simulated drinking water distribution system: Occurrence, correlation and low-pressure ultraviolet removal.
4077	DRAMP04261	CM-2	"CM-2"[All Fields] AND biofilm[All Fields]	CM-2	30157462	Photodynamic therapy mediated by chlorin-type photosensitizers against Streptococcus mutans biofilms.
4077	DRAMP04261	CM-2	"CM-2"[All Fields] AND biofilm[All Fields]	CM-2	30117654	Influence of incubation conditions on the formation of model biofilms by Listeria monocytogenes and Salmonella Typhimurium on abiotic surfaces.
4077	DRAMP04261	CM-2	"CM-2"[All Fields] AND biofilm[All Fields]	CM-2	30097756	Twice-daily red and blue light treatment for Candida albicans biofilm matrix development control.
4077	DRAMP04261	CM-2	"CM-2"[All Fields] AND biofilm[All Fields]	CM-2	30090117	Inhibitory Effects of Antimicrobial Photodynamic Therapy with Curcumin on Biofilm-Associated Gene Expression Profile of Aggregatibacter actinomycetemcomitans.
4077	DRAMP04261	CM-2	"CM-2"[All Fields] AND biofilm[All Fields]	CM-2	30057613	Debridement of Bacterial Biofilms with TiO 2/H 2O 2Solutions and Visible Light Irradiation.
4077	DRAMP04261	CM-2	"CM-2"[All Fields] AND biofilm[All Fields]	CM-2	30048689	In vitro activity of oritavancin in combination with rifampin or gentamicin against prosthetic joint infection-associated methicillin-resistant Staphylococcus epidermidis biofilms.
4077	DRAMP04261	CM-2	"CM-2"[All Fields] AND biofilm[All Fields]	CM-2	30035715	FORMATION OF BIOFILMS BY BACTERIA EXCRETED FROM CHRONIC ANAL FISSURE AND THE INFLUENCE OF THE DIRECT CURRENT ELECTRIC FIELD ON THEM.
4077	DRAMP04261	CM-2	"CM-2"[All Fields] AND biofilm[All Fields]	CM-2	30018849	Use of design of experiments to optimize the production of microbial probiotic biofilms.
4077	DRAMP04261	CM-2	"CM-2"[All Fields] AND biofilm[All Fields]	CM-2	29979032	Comparison of Copper(II)-Ligand Complexes as Mediators for Preparing Electrochemically Modulated Nitric Oxide-Releasing Catheters.
4077	DRAMP04261	CM-2	"CM-2"[All Fields] AND biofilm[All Fields]	CM-2	29939885	Effect of Polyhexamethylene Biguanide Solution on Bacterial Load and Biofilm in Venous Leg Ulcers: A Randomized Controlled Trial.
4077	DRAMP04261	CM-2	"CM-2"[All Fields] AND biofilm[All Fields]	CM-2	29934504	Quantitative assessment of individual populations within polymicrobial biofilms.
4077	DRAMP04261	CM-2	"CM-2"[All Fields] AND biofilm[All Fields]	CM-2	29902377	Occurrence of Legionella spp. in Water-Main Biofilms from Two Drinking Water Distribution Systems.
4077	DRAMP04261	CM-2	"CM-2"[All Fields] AND biofilm[All Fields]	CM-2	29894900	Enhancing extracellular electron transfer between Pseudomonas aeruginosa PAO1 and light driven semiconducting birnessite.
4077	DRAMP04261	CM-2	"CM-2"[All Fields] AND biofilm[All Fields]	CM-2	29888590	Food-Safe Modification of Stainless Steel Food-Processing Surfaces to Reduce Bacterial Biofilms.
4077	DRAMP04261	CM-2	"CM-2"[All Fields] AND biofilm[All Fields]	CM-2	29843904	Predicting adhesion and biofilm formation boundaries on stainless steel surfaces by five Salmonella enterica strains belonging to different serovars as a function of pH, temperature and NaCl concentration.
4077	DRAMP04261	CM-2	"CM-2"[All Fields] AND biofilm[All Fields]	CM-2	29803719	A semi-continuous system for monitoring microbially influenced corrosion.
4077	DRAMP04261	CM-2	"CM-2"[All Fields] AND biofilm[All Fields]	CM-2	29763755	Salmonella enterica growth and biofilm formation in flesh and peel cantaloupe extracts on four food-contact surfaces.
4077	DRAMP04261	CM-2	"CM-2"[All Fields] AND biofilm[All Fields]	CM-2	29756453	Bacterial Membrane Depolarization-Linked Fuel Cell Potential Burst as Signal for Selective Detection of Alcohol.
4077	DRAMP04261	CM-2	"CM-2"[All Fields] AND biofilm[All Fields]	CM-2	29748798	An innovative approach to attached cultivation of Chlorella vulgaris using different materials.
4077	DRAMP04261	CM-2	"CM-2"[All Fields] AND biofilm[All Fields]	CM-2	29706330	Scanning electron microscopy of Salmonella biofilms on various food-contact surfaces in catfish mucus.
4077	DRAMP04261	CM-2	"CM-2"[All Fields] AND biofilm[All Fields]	CM-2	29684689	Is the chlorophyll derivative Zn(II)e 6Me a good photosensitizer to be used in root canal disinfection?
4077	DRAMP04261	CM-2	"CM-2"[All Fields] AND biofilm[All Fields]	CM-2	29663827	Potential of antimicrobial treatment of linear low-density polyethylene with poly((tert-butyl-amino)-methyl-styrene) to reduce biofilm formation in the food industry.
4077	DRAMP04261	CM-2	"CM-2"[All Fields] AND biofilm[All Fields]	CM-2	29660619	Highly efficient removal of nitrogen and phosphorus in an electrolysis-integrated horizontal subsurface-flow constructed wetland amended with biochar.
4077	DRAMP04261	CM-2	"CM-2"[All Fields] AND biofilm[All Fields]	CM-2	29619241	Ugly ducklings-the dark side of plastic materials in contact with potable water.
4077	DRAMP04261	CM-2	"CM-2"[All Fields] AND biofilm[All Fields]	CM-2	29609166	In-vitro model for assessing glucose diffusion through skin.
4077	DRAMP04261	CM-2	"CM-2"[All Fields] AND biofilm[All Fields]	CM-2	29603341	Antibiotic loaded calcium sulfate bead and pulse lavage eradicates biofilms on metal implant materials in vitro.
4077	DRAMP04261	CM-2	"CM-2"[All Fields] AND biofilm[All Fields]	CM-2	29580466	Integrated combined effects of temperature, pH and sodium chloride concentration on biofilm formation by Salmonella enterica ser. Enteritidis and Typhimurium under low nutrient food-related conditions.
4077	DRAMP04261	CM-2	"CM-2"[All Fields] AND biofilm[All Fields]	CM-2	29573682	Enrichment of free-living amoebae in biofilms developed at upper water levels in drinking water storage towers: An inter- and intra-seasonal study.
4077	DRAMP04261	CM-2	"CM-2"[All Fields] AND biofilm[All Fields]	CM-2	29546997	Application of bacteriophages for the inactivation of Salmonella spp. in biofilms.
4077	DRAMP04261	CM-2	"CM-2"[All Fields] AND biofilm[All Fields]	CM-2	29498312	Thermal shock susceptibility and regrowth of Pseudomonas aeruginosa biofilms.
4077	DRAMP04261	CM-2	"CM-2"[All Fields] AND biofilm[All Fields]	CM-2	29470387	Assessment of Chitosan-Based Hydrogel and Photodynamic Inactivation against Propionibacterium acnes.
4077	DRAMP04261	CM-2	"CM-2"[All Fields] AND biofilm[All Fields]	CM-2	29456275	Antimicrobial packaging based on ε-polylysine bioactive film for the control of mycotoxigenic fungi in vitro and in bread.
4077	DRAMP04261	CM-2	"CM-2"[All Fields] AND biofilm[All Fields]	CM-2	29437226	Antifungal, Mechanical, and Physical Properties of Edible Film Containing Williopsis saturnus var. saturnus Antagonistic Yeast.
4077	DRAMP04261	CM-2	"CM-2"[All Fields] AND biofilm[All Fields]	CM-2	29430777	Evaluation of two autoinducer-2 quantification methods for application in marine environments.
4077	DRAMP04261	CM-2	"CM-2"[All Fields] AND biofilm[All Fields]	CM-2	29398018	Short communication: A comparison of biofilm development on stainless steel and modified-surface plate heat exchangers during a 17-h milk pasteurization run.
4077	DRAMP04261	CM-2	"CM-2"[All Fields] AND biofilm[All Fields]	CM-2	29397213	Enterococcus faecalis Hydrolyzes Dental Resin Composites and Adhesives.
4077	DRAMP04261	CM-2	"CM-2"[All Fields] AND biofilm[All Fields]	CM-2	29371534	Activity of Amphotericin B and Anidulafungin Combined with Rifampicin, Clarithromycin, Ethylenediaminetetraacetic Acid, N-Acetylcysteine, and Farnesol against Candida tropicalis Biofilms.
4077	DRAMP04261	CM-2	"CM-2"[All Fields] AND biofilm[All Fields]	CM-2	29332258	Photodynamic antimicrobial chemotherapy (PACT) using toluidine blue inhibits both growth and biofilm formation by Candida krusei.
4077	DRAMP04261	CM-2	"CM-2"[All Fields] AND biofilm[All Fields]	CM-2	29327449	Clinical, microbiological and cytomorphometric evaluation of low-level laser therapy as an adjunct to periodontal therapy in patients with chronic periodontitis.
4077	DRAMP04261	CM-2	"CM-2"[All Fields] AND biofilm[All Fields]	CM-2	29315011	Bioluminescence Analysis of Antibacterial Photodynamic Therapy Using Methylene Blue Mediated by Low-Intensity Level Laser Against Cariogenic Biofilms.
4077	DRAMP04261	CM-2	"CM-2"[All Fields] AND biofilm[All Fields]	CM-2	29306732	Sequential effect of phages and cold nitrogen plasma against Escherichia coli O157:H7 biofilms on different vegetables.
4077	DRAMP04261	CM-2	"CM-2"[All Fields] AND biofilm[All Fields]	CM-2	29304381	Biofilms in shower hoses.
4077	DRAMP04261	CM-2	"CM-2"[All Fields] AND biofilm[All Fields]	CM-2	29281884	Binary Colloidal Crystal Layers as Platforms for Surface Patterning of Puroindoline-Based Antimicrobial Peptides.
4077	DRAMP04261	CM-2	"CM-2"[All Fields] AND biofilm[All Fields]	CM-2	29275280	Dynamics of biofilm formation by Listeria monocytogenes on stainless steel under mono-species and mixed-culture simulated fish processing conditions and chemical disinfection challenges.
4077	DRAMP04261	CM-2	"CM-2"[All Fields] AND biofilm[All Fields]	CM-2	29259580	Rapid in VitroQuantification of S. aureusBiofilms on Vascular Graft Surfaces.
4077	DRAMP04261	CM-2	"CM-2"[All Fields] AND biofilm[All Fields]	CM-2	29247432	Antimicrobial photodynamic therapy-a promising treatment for prosthetic joint infections.
4077	DRAMP04261	CM-2	"CM-2"[All Fields] AND biofilm[All Fields]	CM-2	29233757	The in vitro effect of Antimicrobial Photodynamic Therapy on dental microcosm biofilms from partially erupted permanent molars: A pilot study.
4077	DRAMP04261	CM-2	"CM-2"[All Fields] AND biofilm[All Fields]	CM-2	29230206	Anaerobic Corrosion of 304 Stainless Steel Caused by the Pseudomonas aeruginosaBiofilm.
4077	DRAMP04261	CM-2	"CM-2"[All Fields] AND biofilm[All Fields]	CM-2	29215782	The effect of urinary Foley catheter substrate material on the antimicrobial potential of calixerene-based molecules.
4077	DRAMP04261	CM-2	"CM-2"[All Fields] AND biofilm[All Fields]	CM-2	29215358	Biofilm forming ability of Sphingomonas paucimobilis isolated from community drinking water systems on plumbing materials used in water distribution.
4077	DRAMP04261	CM-2	"CM-2"[All Fields] AND biofilm[All Fields]	CM-2	29196246	Antimicrobial photodynamic activity of Rose Bengal, alone or in combination with Gentamicin, against planktonic and biofilm Staphylococcus aureus.
4077	DRAMP04261	CM-2	"CM-2"[All Fields] AND biofilm[All Fields]	CM-2	29195980	Comparative stability and efficacy of selected chlorine-based biocides against Escherichia coli in planktonic and biofilm states.
4077	DRAMP04261	CM-2	"CM-2"[All Fields] AND biofilm[All Fields]	CM-2	29191005	Construction of High Drug Loading and Enzymatic Degradable Multilayer Films for Self-Defense Drug Release and Long-Term Biofilm Inhibition.
4077	DRAMP04261	CM-2	"CM-2"[All Fields] AND biofilm[All Fields]	CM-2	29180375	Formation and Characterization of Early Bacterial Biofilms on Different Wood Typologies Applied in Dairy Production.
4077	DRAMP04261	CM-2	"CM-2"[All Fields] AND biofilm[All Fields]	CM-2	29173625	Biofilm formation by Salmonella spp. in catfish mucus extract under industrial conditions.

4077	DRAMP04261	CM-2	"CM-2"[All Fields] AND biofilm[All Fields]	CM-2	29160100	Establishing a laboratory model of dental unit waterlines bacterial biofilms using a CDC biofilm reactor.
4077	DRAMP04261	CM-2	"CM-2"[All Fields] AND biofilm[All Fields]	CM-2	29159979	Ultrasound Microbubbles Enhance the Activity of Vancomycin Against Staphylococcus epidermidis Biofilms In Vivo.
4077	DRAMP04261	CM-2	"CM-2"[All Fields] AND biofilm[All Fields]	CM-2	29143221	Controlling Streptococcus mutans and Staphylococcus aureus biofilms with direct current and chlorhexidine.
4077	DRAMP04261	CM-2	"CM-2"[All Fields] AND biofilm[All Fields]	CM-2	29140744	Evaluation of Two Surface Sampling Methods for Microbiological and Chemical Analyses To Assess the Presence of Biofilms in Food Companies.
4077	DRAMP04261	CM-2	"CM-2"[All Fields] AND biofilm[All Fields]	CM-2	29139579	Topical adjuvants incompletely remove adherent Staphylococcus aureus from implant materials.
4077	DRAMP04261	CM-2	"CM-2"[All Fields] AND biofilm[All Fields]	CM-2	29137671	Herpes simplex virus-1 entrapped in Candida albicans biofilm displays decreased sensitivity to antivirals and UVA1 laser treatment.
4077	DRAMP04261	CM-2	"CM-2"[All Fields] AND biofilm[All Fields]	CM-2	29134404	Curcumin-mediated anti-microbial photodynamic therapy against Candida dubliniensis biofilms.
4077	DRAMP04261	CM-2	"CM-2"[All Fields] AND biofilm[All Fields]	CM-2	29119417	Effect of methylene blue-mediated antimicrobial photodynamic therapy on dentin caries microcosms.
4077	DRAMP04261	CM-2	"CM-2"[All Fields] AND biofilm[All Fields]	CM-2	29114242	Bacillussp. Acting as Dual Role for Corrosion Induction and Corrosion Inhibition with Carbon Steel (CS).
4077	DRAMP04261	CM-2	"CM-2"[All Fields] AND biofilm[All Fields]	CM-2	29103718	Feed substrates influence biofilm formation on reverse osmosis membranes and their cleaning efficiency.
4077	DRAMP04261	CM-2	"CM-2"[All Fields] AND biofilm[All Fields]	CM-2	29101088	Effect of methylene blue-induced photodynamic therapy on a Streptococcus mutans biofilm model.
4077	DRAMP04261	CM-2	"CM-2"[All Fields] AND biofilm[All Fields]	CM-2	29100930	Biofilm formation by Staphylococcus aureus and Salmonella spp. under mono and dual-species conditions and their sensitivity to cetrimonium bromide, peracetic acid and sodium hypochlorite.
4077	DRAMP04261	CM-2	"CM-2"[All Fields] AND biofilm[All Fields]	CM-2	29025650	Ephemeral biogels to control anti-biofilm agent delivery: From conception to the construction of an active dressing.
4077	DRAMP04261	CM-2	"CM-2"[All Fields] AND biofilm[All Fields]	CM-2	28990602	Self-sustainable, high-power-density bio-solar cells for lab-on-a-chip applications.
4077	DRAMP04261	CM-2	"CM-2"[All Fields] AND biofilm[All Fields]	CM-2	28968534	Propensity for biofilm formation by aerobic mesophilic and thermophilic spore forming bacteria isolated from Chinese milk powders.
4077	DRAMP04261	CM-2	"CM-2"[All Fields] AND biofilm[All Fields]	CM-2	28940098	Bioelectrochemical system for the biooxidation of a chalcopyrite concentrate by acidophilic bacteria coupled to energy current generation and cathodic copper recovery.
4077	DRAMP04261	CM-2	"CM-2"[All Fields] AND biofilm[All Fields]	CM-2	28887223	Efficacy of erythrosine and cyanidin-3-glucoside mediated photodynamic therapy on Porphyromonas gingivalis biofilms using green light laser.
4077	DRAMP04261	CM-2	"CM-2"[All Fields] AND biofilm[All Fields]	CM-2	28867453	The effect of indocyanine green loaded on a novel nano-graphene oxide for high performance of photodynamic therapy against Enterococcus faecalis.
4077	DRAMP04261	CM-2	"CM-2"[All Fields] AND biofilm[All Fields]	CM-2	28866443	Subminimal inhibitory concentration (sub-MIC) of antibiotic induces electroactive biofilm formation in bioelectrochemical systems.
4077	DRAMP04261	CM-2	"CM-2"[All Fields] AND biofilm[All Fields]	CM-2	28797828	Gene expression profiling of fimA gene encoding fimbriae among clinical isolates of Porphyromonas gingivalis in response to photo-activated disinfection therapy.
4077	DRAMP04261	CM-2	"CM-2"[All Fields] AND biofilm[All Fields]	CM-2	28792661	Multi-effect of the water-soluble Moringa oleifera lectin against Serratia marcescens and Bacillus sp.: antibacterial, antibiofilm and anti-adhesive properties.
4077	DRAMP04261	CM-2	"CM-2"[All Fields] AND biofilm[All Fields]	CM-2	28777541	UV-Curable Contact Active Benzophenone Terminated Quaternary Ammonium Antimicrobials for Applications in Polymer Plastics and Related Devices.
4077	DRAMP04261	CM-2	"CM-2"[All Fields] AND biofilm[All Fields]	CM-2	28743937	Photo-irradiated caffeic acid exhibits antimicrobial activity against Streptococcus mutans biofilms via hydroxyl radical formation.
4077	DRAMP04261	CM-2	"CM-2"[All Fields] AND biofilm[All Fields]	CM-2	28721040	Effect of ultraviolet treatment on bacterial attachment and osteogenic activity to alkali-treated titanium with nanonetwork structures.
4077	DRAMP04261	CM-2	"CM-2"[All Fields] AND biofilm[All Fields]	CM-2	28715664	Electron transfer mediators accelerated the microbiologically influence corrosion against carbon steel by nitrate reducing Pseudomonas aeruginosa biofilm.
4077	DRAMP04261	CM-2	"CM-2"[All Fields] AND biofilm[All Fields]	CM-2	28683522	Role of eptC in Biofilm Formation by Campylobacter jejuni NCTC11168 on Polystyrene and Glass Surfaces.
4077	DRAMP04261	CM-2	"CM-2"[All Fields] AND biofilm[All Fields]	CM-2	28666212	Time-kill kinetic analysis of antimicrobial chemotherapy based on hydrogen peroxide photolysis against Streptococcus mutans biofilm.
4077	DRAMP04261	CM-2	"CM-2"[All Fields] AND biofilm[All Fields]	CM-2	28659553	Microbial Fouling in a Water Treatment Plant and Its Control Using Biocides.
4077	DRAMP04261	CM-2	"CM-2"[All Fields] AND biofilm[All Fields]	CM-2	28647320	An integrated aerobic-anaerobic strategy for performance enhancement of Pseudomonas aeruginosa-inoculated microbial fuel cell.
4077	DRAMP04261	CM-2	"CM-2"[All Fields] AND biofilm[All Fields]	CM-2	28633052	Disinfection efficiencies of sage and spearmint essential oils against planktonic and biofilm Staphylococcus aureus cells in comparison with sodium hypochlorite.
4077	DRAMP04261	CM-2	"CM-2"[All Fields] AND biofilm[All Fields]	CM-2	28627772	Bactericidal Effect of Calcium Oxide (Scallop-Shell Powder) Against Pseudomonas aeruginosa Biofilm on Quail Egg Shell, Stainless Steel, Plastic, and Rubber.
4077	DRAMP04261	CM-2	"CM-2"[All Fields] AND biofilm[All Fields]	CM-2	28621579	Optimization of Antimicrobial Photodynamic Therapy in Biofilms by Inhibiting Efflux Pump.
4077	DRAMP04261	CM-2	"CM-2"[All Fields] AND biofilm[All Fields]	CM-2	28613833	Pt-Decorated MWCNTs-Ionic Liquid Composite-Based Hydrogen Peroxide Sensor To Study Microbial Metabolism Using Scanning Electrochemical Microscopy.
4077	DRAMP04261	CM-2	"CM-2"[All Fields] AND biofilm[All Fields]	CM-2	28612299	Enhancement of photodynamic inactivation of Staphylococcus aureus biofilms by disruptive strategies.
4077	DRAMP04261	CM-2	"CM-2"[All Fields] AND biofilm[All Fields]	CM-2	28610835	Antibiofilm effect of ultrasound combined with microbubbles against Staphylococcus epidermidis biofilm.
4077	DRAMP04261	CM-2	"CM-2"[All Fields] AND biofilm[All Fields]	CM-2	28601403	[Activity of amphotericin B and anidulafungin, alone and combined, against Candida tropicalis biofilms developed on Teflon ®and titanium].
4077	DRAMP04261	CM-2	"CM-2"[All Fields] AND biofilm[All Fields]	CM-2	28587978	A new arginine-based dental adhesive system: formulation, mechanical and anti-caries properties.
4077	DRAMP04261	CM-2	"CM-2"[All Fields] AND biofilm[All Fields]	CM-2	30647649	Microbial diversity structure in acetate single chamber microbial fuel cell for electricity generation.
4077	DRAMP04261	CM-2	"CM-2"[All Fields] AND biofilm[All Fields]	CM-2	28542913	Reduction of Escherichia coli O157:H7 in Biofilms Using Bacteriophage BPECO 19.
4077	DRAMP04261	CM-2	"CM-2"[All Fields] AND biofilm[All Fields]	CM-2	28488198	Hydrogen production profiles using furans in microbial electrolysis cells.
4077	DRAMP04261	CM-2	"CM-2"[All Fields] AND biofilm[All Fields]	CM-2	28436830	Antimicrobial activity of free and liposome-encapsulated thymol and carvacrol against Salmonella and Staphylococcus aureus adhered to stainless steel.
4077	DRAMP04261	CM-2	"CM-2"[All Fields] AND biofilm[All Fields]	CM-2	28431296	Survival and transfer efficacy of mixed strain Salmonella enterica ser. Typhimurium from beef burgers to abiotic surfaces and determination of individual strain contribution.
4077	DRAMP04261	CM-2	"CM-2"[All Fields] AND biofilm[All Fields]	CM-2	28414082	Antimicrobial photodynamic therapy on Streptococcus mutans is altered by glucose in the presence of methylene blue and red LED.
4077	DRAMP04261	CM-2	"CM-2"[All Fields] AND biofilm[All Fields]	CM-2	28401747	Dynamics of mono- and dual-species biofilm formation and interactions between Staphylococcus aureus and Gram-negative bacteria.
4077	DRAMP04261	CM-2	"CM-2"[All Fields] AND biofilm[All Fields]	CM-2	28390963	Targeting biofilms of multidrug-resistant bacteria with silver oxynitrate.
4077	DRAMP04261	CM-2	"CM-2"[All Fields] AND biofilm[All Fields]	CM-2	28364672	Efficacy of a mouthrinse based on hydroxyapatite to reduce initial bacterial colonisation in situ.
4077	DRAMP04261	CM-2	"CM-2"[All Fields] AND biofilm[All Fields]	CM-2	28362312	Fungal biodiversity and mycotoxigenic fungi in cooling-tower water systems in Istanbul, Turkey.
4077	DRAMP04261	CM-2	"CM-2"[All Fields] AND biofilm[All Fields]	CM-2	28332593	Injections through skin colonized with Staphylococcus aureus biofilm introduce contamination despite standard antimicrobial preparation procedures.
4077	DRAMP04261	CM-2	"CM-2"[All Fields] AND biofilm[All Fields]	CM-2	28330815	Antimicrobial photodynamic therapy combined to periodontal treatment: Experimental model.
4077	DRAMP04261	CM-2	"CM-2"[All Fields] AND biofilm[All Fields]	CM-2	28317023	Reduction of Thrombosis and Bacterial Infection via Controlled Nitric Oxide (NO) Release from S-Nitroso- N-acetylpenicillamine (SNAP) Impregnated CarboSil Intravascular Catheters.
4077	DRAMP04261	CM-2	"CM-2"[All Fields] AND biofilm[All Fields]	CM-2	28280998	Virulence factors of fluconazole-susceptible and fluconazole-resistant Candida albicans after antimicrobial photodynamic therapy.
4077	DRAMP04261	CM-2	"CM-2"[All Fields] AND biofilm[All Fields]	CM-2	28278106	Interactive effect of trivalent iron on activated sludge digestion and biofilm structure in attached growth reactor of waste tire rubber.
4077	DRAMP04261	CM-2	"CM-2"[All Fields] AND biofilm[All Fields]	CM-2	28216095	Real-time quantitative reverse transcription-PCR analysis of expression stability of Aggregatibacter actinomycetemcomitans fimbria-associated gene in response to photodynamic therapy.
4077	DRAMP04261	CM-2	"CM-2"[All Fields] AND biofilm[All Fields]	CM-2	28213018	Pathogen biofilm formation on cantaloupe surface and its impact on the antibacterial effect of lauroyl arginate ethyl.
4077	DRAMP04261	CM-2	"CM-2"[All Fields] AND biofilm[All Fields]	CM-2	28191638	Enhancement of the Efficacy of Photodynamic Inactivation of Candida albicans with the Use of Biogenic Gold Nanoparticles.
4077	DRAMP04261	CM-2	"CM-2"[All Fields] AND biofilm[All Fields]	CM-2	28189246	Antimicrobial efficiency of PAA/(PVP/CHI) erodible polysaccharide multilayer through loading and controlled release of antibiotics.
4077	DRAMP04261	CM-2	"CM-2"[All Fields] AND biofilm[All Fields]	CM-2	28177575	Surface Coating of Gypsum-Based Molds for Maxillofacial Prosthetic Silicone Elastomeric Material: Evaluating Different Microbial Adhesion.
4077	DRAMP04261	CM-2	"CM-2"[All Fields] AND biofilm[All Fields]	CM-2	28144863	Airborne bacteria associated with corrosion of mild steel 1010 and aluminum alloy 1100.

4077	DRAMP04261	CM-2	"CM-2"[All Fields] AND biofilm[All Fields]	CM-2	28131567	Short communication: Evaluation of a sol-gel-based stainless steel surface modification to reduce fouling and biofilm formation during pasteurization of milk.
4077	DRAMP04261	CM-2	"CM-2"[All Fields] AND biofilm[All Fields]	CM-2	28117709	Microbiological Contamination at Workplaces in a Combined Heat and Power (CHP) Station Processing Plant Biomass.
4077	DRAMP04261	CM-2	"CM-2"[All Fields] AND biofilm[All Fields]	CM-2	28087484	Inhibition of bacterial adhesion and biofilm formation by dual functional textured and nitric oxide releasing surfaces.
4077	DRAMP04261	CM-2	"CM-2"[All Fields] AND biofilm[All Fields]	CM-2	28062459	Biofilm Composition and Threshold Concentration for Growth of Legionella pneumophila on Surfaces Exposed to Flowing Warm Tap Water without Disinfectant.
4077	DRAMP04261	CM-2	"CM-2"[All Fields] AND biofilm[All Fields]	CM-2	28049020	Eradication of Pseudomonas aeruginosa cells by cathodic electrochemical currents delivered with graphite electrodes.
4077	DRAMP04261	CM-2	"CM-2"[All Fields] AND biofilm[All Fields]	CM-2	28039994	Biofilm mitigation by drug (gentamicin)-loaded liposomes promoted by pulsed ultrasound.
4077	DRAMP04261	CM-2	"CM-2"[All Fields] AND biofilm[All Fields]	CM-2	27993650	Glucose modulates antimicrobial photodynamic inactivation of Candida albicans in biofilms.
4077	DRAMP04261	CM-2	"CM-2"[All Fields] AND biofilm[All Fields]	CM-2	27988224	Photoinactivation of single and mixed biofilms of Candida albicans and non-albicans Candida species using Photodythazine ®[corrected].
4077	DRAMP04261	CM-2	"CM-2"[All Fields] AND biofilm[All Fields]	CM-2	27883920	Nano-exploration of organic conditioning film formed on polymeric surfaces exposed to drinking water.
4077	DRAMP04261	CM-2	"CM-2"[All Fields] AND biofilm[All Fields]	CM-2	27878485	Biofilms in bioremediation and wastewater treatment: characterization of bacterial community structure and diversity during seasons in municipal wastewater treatment process.
4077	DRAMP04261	CM-2	"CM-2"[All Fields] AND biofilm[All Fields]	CM-2	27869109	Effect of red dyes on blue light phototoxicity against VSC producing bacteria in an experimental oral biofilm.
4077	DRAMP04261	CM-2	"CM-2"[All Fields] AND biofilm[All Fields]	CM-2	27863737	Intensified nitrate and phosphorus removal in an electrolysis -integrated horizontal subsurface-flow constructed wetland.
4077	DRAMP04261	CM-2	"CM-2"[All Fields] AND biofilm[All Fields]	CM-2	27821457	Activity of Electrical Current in Experimental Propionibacterium acnes Foreign-Body Osteomyelitis.
4077	DRAMP04261	CM-2	"CM-2"[All Fields] AND biofilm[All Fields]	CM-2	27234012	Evaluation of the ability of different detergents and disinfectants to remove and kill organisms in traditional biofilm.
4077	DRAMP04261	CM-2	"CM-2"[All Fields] AND biofilm[All Fields]	CM-2	27613680	Multiplication of Legionella pneumophila Sequence Types 1, 47, and 62 in Buffered Yeast Extract Broth and Biofilms Exposed to Flowing Tap Water at Temperatures of 38°C to 42°C.
4077	DRAMP04261	CM-2	"CM-2"[All Fields] AND biofilm[All Fields]	CM-2	27769914	The efficacy of photodynamic and photothermal therapy on biofilm formation of Streptococcus mutans: An in vitro study.
4077	DRAMP04261	CM-2	"CM-2"[All Fields] AND biofilm[All Fields]	CM-2	27716472	Efficiency of a cleaning protocol for the removal of enterotoxigenic Staphylococcus aureus strains in dairy plants.
4077	DRAMP04261	CM-2	"CM-2"[All Fields] AND biofilm[All Fields]	CM-2	27648759	Chemical, physical and morphological properties of bacterial biofilms affect survival of encased Campylobacter jejuni F38011 under aerobic stress.
4077	DRAMP04261	CM-2	"CM-2"[All Fields] AND biofilm[All Fields]	CM-2	27753430	Light activated antimicrobial agents can inactivate oral malodour causing bacteria.
4077	DRAMP04261	CM-2	"CM-2"[All Fields] AND biofilm[All Fields]	CM-2	27616552	Inside-out Ultraviolet-C Sterilization of Pseudomonas aeruginosa Biofilm In Vitro.
4077	DRAMP04261	CM-2	"CM-2"[All Fields] AND biofilm[All Fields]	CM-2	32624766	Effect of electrode surface properties on enhanced electron transfer activity in microbial fuel cells.
4077	DRAMP04261	CM-2	"CM-2"[All Fields] AND biofilm[All Fields]	CM-2	27714987	In vitro characterization of biofilms formed by Kingella kingae.
4077	DRAMP04261	CM-2	"CM-2"[All Fields] AND biofilm[All Fields]	CM-2	27714933	Structural damage of Bacillus subtilis biofilms using pulsed laser interaction with gold thin films.
4077	DRAMP04261	CM-2	"CM-2"[All Fields] AND biofilm[All Fields]	CM-2	27681898	Phage Inactivation of Listeria monocytogenes on San Daniele Dry-Cured Ham and Elimination of Biofilms from Equipment and Working Environments.
4077	DRAMP04261	CM-2	"CM-2"[All Fields] AND biofilm[All Fields]	CM-2	27640733	The in vitro effect of antimicrobial photodynamic therapy with indocyanine green on Enterococcus faecalis: Influence of a washing vs non-washing procedure.
4077	DRAMP04261	CM-2	"CM-2"[All Fields] AND biofilm[All Fields]	CM-2	27640114	Evaluation of photo-activated disinfection effectiveness with methylene blue against Porphyromonas gingivalis involved in endodontic infection: An in vitro study.
4077	DRAMP04261	CM-2	"CM-2"[All Fields] AND biofilm[All Fields]	CM-2	27639235	The performance of microbial anodes in municipal wastewater: Pre-grown multispecies biofilm vs. natural inocula.
4077	DRAMP04261	CM-2	"CM-2"[All Fields] AND biofilm[All Fields]	CM-2	27619533	Sae regulator factor impairs the response to photodynamic inactivation mediated by Toluidine blue in Staphylococcus aureus.
4077	DRAMP04261	CM-2	"CM-2"[All Fields] AND biofilm[All Fields]	CM-2	27597778	Biofilm growth on polyvinylchloride surface incubated in suboptimal microbial warm water and effect of sanitizers on biofilm removal post biofilm formation.
4077	DRAMP04261	CM-2	"CM-2"[All Fields] AND biofilm[All Fields]	CM-2	27596347	[Protein-repellent and antibacterial properties of modified orthodontic adhesive].
4077	DRAMP04261	CM-2	"CM-2"[All Fields] AND biofilm[All Fields]	CM-2	27594670	Effects of sub-lethal doses of photo-activated disinfection against Porphyromonas gingivalis for pharmaceutical treatment of periodontal-endodontic lesions.
4077	DRAMP04261	CM-2	"CM-2"[All Fields] AND biofilm[All Fields]	CM-2	27475996	The effects of diode laser on Staphylococcus aureus biofilm and Escherichia coli lipopolysaccharide adherent to titanium oxide surface of dental implants. An in vitro study.
4077	DRAMP04261	CM-2	"CM-2"[All Fields] AND biofilm[All Fields]	CM-2	27447147	Removal Torque and Biofilm Accumulation at Two Dental Implant-Abutment Joints After Fatigue.
4077	DRAMP04261	CM-2	"CM-2"[All Fields] AND biofilm[All Fields]	CM-2	27424598	Synergistic antibiofilm efficacy of various commercial antiseptics, enzymes and EDTA: a study of Pseudomonas aeruginosa and Staphylococcus aureus biofilms.
4077	DRAMP04261	CM-2	"CM-2"[All Fields] AND biofilm[All Fields]	CM-2	27422105	Photodynamic inactivation of pathogenic species Pseudomonas aeruginosa and Candida albicans with lutetium (III) acetate phthalocyanines and specific light irradiation.
4077	DRAMP04261	CM-2	"CM-2"[All Fields] AND biofilm[All Fields]	CM-2	27356028	Biofilm Formation by Environmental Isolates of Salmonella and Their Sensitivity to Natural Antimicrobials.
4077	DRAMP04261	CM-2	"CM-2"[All Fields] AND biofilm[All Fields]	CM-2	27345713	A new buildup biofilm model that mimics accumulation of material in flexible endoscope channels.
4077	DRAMP04261	CM-2	"CM-2"[All Fields] AND biofilm[All Fields]	CM-2	27342552	Survival, Biofilm Formation, and Growth Potential of Environmental and Enteric Escherichia coli Strains in Drinking Water Microcosms.
4077	DRAMP04261	CM-2	"CM-2"[All Fields] AND biofilm[All Fields]	CM-2	27336752	Effective Targeted Photothermal Ablation of Multidrug Resistant Bacteria and Their Biofilms with NIR-Absorbing Gold Nanocrosses.
4077	DRAMP04261	CM-2	"CM-2"[All Fields] AND biofilm[All Fields]	CM-2	27314554	Comparative study of chemical and physical methods for distinguishing between passive and metabolically active mechanisms of water contaminant removal by biofilms.
4077	DRAMP04261	CM-2	"CM-2"[All Fields] AND biofilm[All Fields]	CM-2	27282165	Anodic concentration loss and impedance characteristics in rotating disk electrode microbial fuel cells.
4077	DRAMP04261	CM-2	"CM-2"[All Fields] AND biofilm[All Fields]	CM-2	27252025	Antimicrobial photodynamic therapy of S. mutans biofilms attached to relevant dental materials.
4077	DRAMP04261	CM-2	"CM-2"[All Fields] AND biofilm[All Fields]	CM-2	27213356	Polymeric Nanoparticle-Based Photodynamic Therapy for Chronic Periodontitis in Vivo.
4077	DRAMP04261	CM-2	"CM-2"[All Fields] AND biofilm[All Fields]	CM-2	27199946	Evaluation of the Effects of Photodynamic Therapy Alone and Combined with Standard Antifungal Therapy on Planktonic Cells and Biofilms of Fusarium spp. and Exophiala spp.
4077	DRAMP04261	CM-2	"CM-2"[All Fields] AND biofilm[All Fields]	CM-2	27183085	Bactericidal Effect of Erbium-Doped Yttrium Aluminum Garnet Laser and Photodynamic Therapy on Aggregatibacter Actinomycetemcomitans Biofilm on Implant Surface.
4077	DRAMP04261	CM-2	"CM-2"[All Fields] AND biofilm[All Fields]	CM-2	27147073	Effects of low-level laser therapy combined with toluidine blue on polysaccharides and biofilm of Streptococcus mutans.
4077	DRAMP04261	CM-2	"CM-2"[All Fields] AND biofilm[All Fields]	CM-2	27142575	Effect of 5-aminolevulinic acid photodynamic therapy on Candida albicans biofilms: An in vitro study.
4077	DRAMP04261	CM-2	"CM-2"[All Fields] AND biofilm[All Fields]	CM-2	27131840	Low-Frequency Ultrasound Enhances Antimicrobial Activity of Colistin-Vancomycin Combination against Pan-Resistant Biofilm of Acinetobacter baumannii.
4077	DRAMP04261	CM-2	"CM-2"[All Fields] AND biofilm[All Fields]	CM-2	27131630	Interactions of Staphylococcus aureus with ultrasoft hydrogel biomaterials.
4077	DRAMP04261	CM-2	"CM-2"[All Fields] AND biofilm[All Fields]	CM-2	27129967	Antibacterial Activity of Blue Light against Nosocomial Wound Pathogens Growing Planktonically and as Mature Biofilms.
4077	DRAMP04261	CM-2	"CM-2"[All Fields] AND biofilm[All Fields]	CM-2	27118084	Chitosan nanoparticles enhance the efficiency of methylene blue-mediated antimicrobial photodynamic inactivation of bacterial biofilms: An in vitro study.
4077	DRAMP04261	CM-2	"CM-2"[All Fields] AND biofilm[All Fields]	CM-2	27105066	Direct Loading and Tunable Release of Antibiotics from Polyelectrolyte Multilayers To Reduce Bacterial Adhesion and Biofilm Formation.
4077	DRAMP04261	CM-2	"CM-2"[All Fields] AND biofilm[All Fields]	CM-2	27073807	Direct Electrical Current Reduces Bacterial and Yeast Biofilm Formation.
4077	DRAMP04261	CM-2	"CM-2"[All Fields] AND biofilm[All Fields]	CM-2	27058401	Pyrolytic carbon-coated stainless steel felt as a high-performance anode for bioelectrochemical systems.
4077	DRAMP04261	CM-2	"CM-2"[All Fields] AND biofilm[All Fields]	CM-2	27058375	Biofilm Formation on Different Materials Used in Oral Rehabilitation.
4077	DRAMP04261	CM-2	"CM-2"[All Fields] AND biofilm[All Fields]	CM-2	27043385	Synergistic bactericidal action of phytic acid and sodium chloride against Escherichia coli O157:H7 cells protected by a biofilm.
4077	DRAMP04261	CM-2	"CM-2"[All Fields] AND biofilm[All Fields]	CM-2	27020838	Adhesion and removal kinetics of Bacillus cereus biofilms on Ni-PTFE modified stainless steel.
4077	DRAMP04261	CM-2	"CM-2"[All Fields] AND biofilm[All Fields]	CM-2	26984479	Evaluating the efficiency of a mixed culture biofilm for the treatment of black liquor and molasses in a mediator-less microbial fuel cell.
4077	DRAMP04261	CM-2	"CM-2"[All Fields] AND biofilm[All Fields]	CM-2	26980656	[Effect of 2-methacryloyloxyethyl phosphorylcholine on the protein-repellent property of dental adhesive].

4077	DRAMP04261	CM-2	"CM-2"[All Fields] AND biofilm[All Fields]	CM-2	26956086	A Novel Acetylcholinesterase Biosensor: Core-Shell Magnetic Nanoparticles Incorporating a Conjugated Polymer for the Detection of Organophosphorus Pesticides.
4077	DRAMP04261	CM-2	"CM-2"[All Fields] AND biofilm[All Fields]	CM-2	26908743	Antimicrobial Blue Light Inactivation of Gram-Negative Pathogens in Biofilms: In Vitro and In Vivo Studies.
4077	DRAMP04261	CM-2	"CM-2"[All Fields] AND biofilm[All Fields]	CM-2	26905507	Antibiofilm action of a toluidine blue O-silver nanoparticle conjugate on Streptococcus mutans: a mechanism of type I photodynamic therapy.
4077	DRAMP04261	CM-2	"CM-2"[All Fields] AND biofilm[All Fields]	CM-2	26796707	Sustained effects of blue light on Streptococcus mutans in regrown biofilm.
4077	DRAMP04261	CM-2	"CM-2"[All Fields] AND biofilm[All Fields]	CM-2	26754179	The efficacy of photon-initiated photoacoustic streaming and sonic-activated irrigation combined with QMiX solution or sodium hypochlorite against intracanal E. faecalis biofilm.
4077	DRAMP04261	CM-2	"CM-2"[All Fields] AND biofilm[All Fields]	CM-2	26745983	Glyceryl trinitrate and caprylic acid for the mitigation of the Desulfovibrio vulgaris biofilm on C1018 carbon steel.
4077	DRAMP04261	CM-2	"CM-2"[All Fields] AND biofilm[All Fields]	CM-2	26719055	Photodynamic inactivation of antibiotic-resistant bacteria and biofilms by hematoporphyrin monomethyl ether.
4077	DRAMP04261	CM-2	"CM-2"[All Fields] AND biofilm[All Fields]	CM-2	26672985	Immobilization of myoglobin on Au nanoparticle-decorated carbon nanotube/polytyramine composite as a mediator-free H2O2 and nitrite biosensor.
4077	DRAMP04261	CM-2	"CM-2"[All Fields] AND biofilm[All Fields]	CM-2	26652666	Colonisation and succession of marine biofilm-dwelling ciliate assemblages on biocidal antifouling and fouling-release coatings in temperate Australia.
4077	DRAMP04261	CM-2	"CM-2"[All Fields] AND biofilm[All Fields]	CM-2	26619252	Opportunistic Gram-negative rods' capability of creating biofilm structures on polivynyl chloride and styrene-acronitrile copolymer surfaces.
4077	DRAMP04261	CM-2	"CM-2"[All Fields] AND biofilm[All Fields]	CM-2	26617192	Antimicrobial photodynamic therapy (aPDT) induction of biofilm matrix architectural and bioadhesive modifications.
4077	DRAMP04261	CM-2	"CM-2"[All Fields] AND biofilm[All Fields]	CM-2	26613979	Microbial dynamics in mixed culture biofilms of bacteria surviving sanitation of conveyor belts in salmon-processing plants.
4077	DRAMP04261	CM-2	"CM-2"[All Fields] AND biofilm[All Fields]	CM-2	26604423	Effect of atmospheric pressure plasma jet on the foodborne pathogens attached to commercial food containers.
4077	DRAMP04261	CM-2	"CM-2"[All Fields] AND biofilm[All Fields]	CM-2	26599466	Gallium-Loaded Dissolvable Microfilm Constructs that Provide Sustained Release of Ga(3+) for Management of Biofilms.
4077	DRAMP04261	CM-2	"CM-2"[All Fields] AND biofilm[All Fields]	CM-2	26599210	Wiring microbial biofilms to the electrode by osmium redox polymer for the performance enhancement of microbial fuel cells.
4077	DRAMP04261	CM-2	"CM-2"[All Fields] AND biofilm[All Fields]	CM-2	26581814	Antifouling assessments on biogenic nanoparticles: A field study from polluted offshore platform.
4077	DRAMP04261	CM-2	"CM-2"[All Fields] AND biofilm[All Fields]	CM-2	26541297	Amperometric monitoring of quercetin permeation through skin membranes.
4077	DRAMP04261	CM-2	"CM-2"[All Fields] AND biofilm[All Fields]	CM-2	26520337	Sonodynamic action of hypocrellin B on biofilm-producing Staphylococcus epidermidis in planktonic condition.
4077	DRAMP04261	CM-2	"CM-2"[All Fields] AND biofilm[All Fields]	CM-2	28721235	Enhancement in hydrogen evolution using Au-TiO 2hollow spheres with microbial devices modified with conjugated oligoelectrolytes.
4077	DRAMP04261	CM-2	"CM-2"[All Fields] AND biofilm[All Fields]	CM-2	26478289	Covalent immobilization of lysozyme onto woven and knitted crimped polyethylene terephthalate grafts to minimize the adhesion of broad spectrum pathogens.
4077	DRAMP04261	CM-2	"CM-2"[All Fields] AND biofilm[All Fields]	CM-2	26462294	S-Nitroso- N-acetylpenicillamine (SNAP) Impregnated Silicone Foley Catheters: A Potential Biomaterial/Device To Prevent Catheter-Associated Urinary Tract Infections.
4077	DRAMP04261	CM-2	"CM-2"[All Fields] AND biofilm[All Fields]	CM-2	32262637	Magnetic nanoparticle/polymer composites for medical implant infection control.
4077	DRAMP04261	CM-2	"CM-2"[All Fields] AND biofilm[All Fields]	CM-2	26404782	Photodynamic inactivation of a multispecies biofilm using Photodithazine(®) and LED light after one and three successive applications.
4077	DRAMP04261	CM-2	"CM-2"[All Fields] AND biofilm[All Fields]	CM-2	26398815	Photodynamic inactivation of virulence factors of Candida strains isolated from patients with denture stomatitis.
4077	DRAMP04261	CM-2	"CM-2"[All Fields] AND biofilm[All Fields]	CM-2	26391393	Antibacterial efficacy of ultrahigh molecular weight polyethylene with silver containing diamond-like surface layers.
4077	DRAMP04261	CM-2	"CM-2"[All Fields] AND biofilm[All Fields]	CM-2	26371591	Thermal mitigation of Pseudomonas aeruginosa biofilms.
4077	DRAMP04261	CM-2	"CM-2"[All Fields] AND biofilm[All Fields]	CM-2	26358540	Antibacterial effect of doxycycline-coated dental abutment surfaces.
4077	DRAMP04261	CM-2	"CM-2"[All Fields] AND biofilm[All Fields]	CM-2	26347938	Post-Treatment Apical Periodontitis Successfully Treated with Antimicrobial Photodynamic Therapy Via Sinus Tract and Laser Phototherapy: Report of Two Cases.
4077	DRAMP04261	CM-2	"CM-2"[All Fields] AND biofilm[All Fields]	CM-2	26328720	Effects of acoustic streaming from moderate-intensity pulsed ultrasound for enhancing biofilm mitigation effectiveness of drug-loaded liposomes.
4077	DRAMP04261	CM-2	"CM-2"[All Fields] AND biofilm[All Fields]	CM-2	26297016	Adhesion and biofilm formation in artificial saliva and susceptibility of yeasts isolated from chronic kidney patients undergoing haemodialysis.
4077	DRAMP04261	CM-2	"CM-2"[All Fields] AND biofilm[All Fields]	CM-2	26266654	Influence of Light-curing Parameters on Biofilm Development and Flexural Strength of a Silorane-based Composite.
4077	DRAMP04261	CM-2	"CM-2"[All Fields] AND biofilm[All Fields]	CM-2	26230333	Effect of Twice-Daily Blue Light Treatment on Matrix-Rich Biofilm Development.
4077	DRAMP04261	CM-2	"CM-2"[All Fields] AND biofilm[All Fields]	CM-2	26212679	Bioelectricity production from microbial fuel cell using mixed bacterial culture isolated from distillery wastewater.
4077	DRAMP04261	CM-2	"CM-2"[All Fields] AND biofilm[All Fields]	CM-2	26166463	Electrochemical and impedance characterization of Microbial Fuel Cells based on 2D and 3D anodic electrodes working with seawater microorganisms under continuous operation.
4077	DRAMP04261	CM-2	"CM-2"[All Fields] AND biofilm[All Fields]	CM-2	26162100	Antimicrobial Photoinactivation Using Visible Light Plus Water-Filtered Infrared-A (VIS + wIRA) Alters In Situ Oral Biofilms.
4077	DRAMP04261	CM-2	"CM-2"[All Fields] AND biofilm[All Fields]	CM-2	26145814	Electrochemical characterization of microbial bioanodes formed on a collector/electrode system in a highly saline electrolyte.
4077	DRAMP04261	CM-2	"CM-2"[All Fields] AND biofilm[All Fields]	CM-2	26139614	Combined Effects of Curcumin and (-)-Epigallocatechin Gallate on Inhibition of N-Acylhomoserine Lactone-Mediated Biofilm Formation in Wastewater Bacteria from Membrane Bioreactor.
4077	DRAMP04261	CM-2	"CM-2"[All Fields] AND biofilm[All Fields]	CM-2	26130291	Liquid crystal polaroid glass electrode from e-waste for synchronized removal/recovery of Cr(+6) from wastewater by microbial fuel cell.
4077	DRAMP04261	CM-2	"CM-2"[All Fields] AND biofilm[All Fields]	CM-2	26112346	Heat-treated stainless steel felt as scalable anode material for bioelectrochemical systems.
4077	DRAMP04261	CM-2	"CM-2"[All Fields] AND biofilm[All Fields]	CM-2	26102989	Application of bacteriophages to reduce biofilms formed by hydrogen sulfide producing bacteria on surfaces in a rendering plant.
4077	DRAMP04261	CM-2	"CM-2"[All Fields] AND biofilm[All Fields]	CM-2	26098899	Sulfate Reducing Bacteria and Mycobacteria Dominate the Biofilm Communities in a Chloraminated Drinking Water Distribution System.
4077	DRAMP04261	CM-2	"CM-2"[All Fields] AND biofilm[All Fields]	CM-2	26098144	Effect of biofilm on fluorescence measurements derived from fast repetition rate fluorometers.
4077	DRAMP04261	CM-2	"CM-2"[All Fields] AND biofilm[All Fields]	CM-2	26095792	Effectiveness of the Photoactive Dye Methylene Blue versus Caspofungin on the Candida parapsilosis Biofilm in vitro and ex vivo.
4077	DRAMP04261	CM-2	"CM-2"[All Fields] AND biofilm[All Fields]	CM-2	26025340	Influence of anode surface chemistry on microbial fuel cell operation.
4077	DRAMP04261	CM-2	"CM-2"[All Fields] AND biofilm[All Fields]	CM-2	26002898	Changes in Sodium, Calcium, and Magnesium Ion Concentrations That Inhibit Geobacillus Biofilms Have No Effect on Anoxybacillus flavithermus Biofilms.
4077	DRAMP04261	CM-2	"CM-2"[All Fields] AND biofilm[All Fields]	CM-2	26002012	Antimicrobial photodynamic therapy: a promise candidate for caries lesions treatment.
4077	DRAMP04261	CM-2	"CM-2"[All Fields] AND biofilm[All Fields]	CM-2	25987623	Evaluation of Ceftaroline Alone and in Combination against Biofilm-Producing Methicillin-Resistant Staphylococcus aureus with Reduced Susceptibility to Daptomycin and Vancomycin in an In Vitro Pharmacokinetic/Pharmacodynamic Model.
4077	DRAMP04261	CM-2	"CM-2"[All Fields] AND biofilm[All Fields]	CM-2	25958074	Immobilized Hydrolytic Enzymes Exhibit Antibiofilm Activity Against Escherichia coli at Sub-Lethal Concentrations.
4077	DRAMP04261	CM-2	"CM-2"[All Fields] AND biofilm[All Fields]	CM-2	25951616	Antimicrobial effects of a bioactive glass combined with fluoride or triclosan on Streptococcus mutans biofilm.
4077	DRAMP04261	CM-2	"CM-2"[All Fields] AND biofilm[All Fields]	CM-2	32262317	Nanocomposited silicone hydrogels with a laser-assisted surface modification for inhibiting the growth of bacterial biofilm.
4077	DRAMP04261	CM-2	"CM-2"[All Fields] AND biofilm[All Fields]	CM-2	25892001	Phototoxic effect of hypericin alone and in combination with acetylcysteine on Staphylococcus aureus biofilms.
4077	DRAMP04261	CM-2	"CM-2"[All Fields] AND biofilm[All Fields]	CM-2	25863406	Antibacterial and Odontogenesis Efficacy of Mineral Trioxide Aggregate Combined with CO2 Laser Treatment.
4077	DRAMP04261	CM-2	"CM-2"[All Fields] AND biofilm[All Fields]	CM-2	25770432	Quantification and characterization of microbial biofilm community attached on the surface of fermentation vessels used in green table olive processing.
4077	DRAMP04261	CM-2	"CM-2"[All Fields] AND biofilm[All Fields]	CM-2	25723062	Stimulation of 2-methylisoborneol (MIB) production by actinomycetes after cyclic chlorination in drinking water distribution systems.
4077	DRAMP04261	CM-2	"CM-2"[All Fields] AND biofilm[All Fields]	CM-2	25710163	Combined steam-ultrasound treatment of 2 seconds achieves significant high aerobic count and Enterobacteriaceae reduction on naturally contaminated food boxes, crates, conveyor belts, and meat knives.
4077	DRAMP04261	CM-2	"CM-2"[All Fields] AND biofilm[All Fields]	CM-2	25702883	Biofilms of Enterococcus faecalis and Enterococcus faecium isolated from the processing of ricotta and the control of these pathogens through cleaning and sanitization procedures.
4077	DRAMP04261	CM-2	"CM-2"[All Fields] AND biofilm[All Fields]	CM-2	25686789	Influence of copper surfaces on biofilm formation by Legionella pneumophila in potable water.
4077	DRAMP04261	CM-2	"CM-2"[All Fields] AND biofilm[All Fields]	CM-2	25681193	Efficacy and mechanisms of murine norovirus inhibition by pulsed-light technology.

4077	DRAMP04261	CM-2	"CM-2"[All Fields] AND biofilm[All Fields]	CM-2	25655573	Behavior of Listeria monocytogenes in a multi-species biofilm with Enterococcus faecalis and Enterococcus faecium and control through sanitation procedures.
4077	DRAMP04261	CM-2	"CM-2"[All Fields] AND biofilm[All Fields]	CM-2	25625910	Exploring the strain-specific attachment of Leuconostoc gelidum subsp. gasicomitatum on food contact surfaces.
4077	DRAMP04261	CM-2	"CM-2"[All Fields] AND biofilm[All Fields]	CM-2	25588885	Thromboresistant/anti-biofilm catheters via electrochemically modulated nitric oxide release.
4077	DRAMP04261	CM-2	"CM-2"[All Fields] AND biofilm[All Fields]	CM-2	25562573	In vitro and in vivo characterization of antibacterial activity and biocompatibility: a study on silver-containing phosphonate monolayers on titanium.
4077	DRAMP04261	CM-2	"CM-2"[All Fields] AND biofilm[All Fields]	CM-2	25539705	Silver nanoparticles impact phototrophic biofilm communities to a considerably higher degree than ionic silver.
4077	DRAMP04261	CM-2	"CM-2"[All Fields] AND biofilm[All Fields]	CM-2	32262208	Compatibility balanced antibacterial modification based on vapor-deposited parylene coatings for biomaterials.
4077	DRAMP04261	CM-2	"CM-2"[All Fields] AND biofilm[All Fields]	CM-2	25522969	Cathodic voltage-controlled electrical stimulation of titanium implants as treatment for methicillin-resistant Staphylococcus aureus periprosthetic infections.
4077	DRAMP04261	CM-2	"CM-2"[All Fields] AND biofilm[All Fields]	CM-2	25519162	Evaluation of High-Dose Daptomycin Versus Vancomycin Alone or Combined with Clarithromycin or Rifampin Against Staphylococcus aureus and S. epidermidis in a Novel In Vitro PK/PD Model of Bacterial Biofilm.
4077	DRAMP04261	CM-2	"CM-2"[All Fields] AND biofilm[All Fields]	CM-2	25480166	Characterization of Mannheimia haemolytica biofilm formation in vitro.
4077	DRAMP04261	CM-2	"CM-2"[All Fields] AND biofilm[All Fields]	CM-2	25471267	Evaluation of Petrifilm™ aerobic count plates as an equivalent alternative to drop plating on R2A agar plates in a biofilm disinfectant efficacy test.
4077	DRAMP04261	CM-2	"CM-2"[All Fields] AND biofilm[All Fields]	CM-2	25463655	Application of benzo[a]phenoxazinium chlorides in Antimicrobial Photodynamic Therapy of Candida albicans biofilms.
4077	DRAMP04261	CM-2	"CM-2"[All Fields] AND biofilm[All Fields]	CM-2	25462576	Photoactivation of curcumin and sodium hypochlorite to enhance antibiofilm efficacy in root canal dentin.
4077	DRAMP04261	CM-2	"CM-2"[All Fields] AND biofilm[All Fields]	CM-2	25461964	Aggregatibacter actinomycetemcomitans biofilm can be inactivated by methylene blue-mediated photodynamic therapy.
4077	DRAMP04261	CM-2	"CM-2"[All Fields] AND biofilm[All Fields]	CM-2	25431313	Evaluation of a biofilm formation by Desulfovibrio fairfieldensis on titanium implants.
4077	DRAMP04261	CM-2	"CM-2"[All Fields] AND biofilm[All Fields]	CM-2	25354678	Eradicating group A streptococcus bacteria and biofilms using functionalised multi-wall carbon nanotubes.
4077	DRAMP04261	CM-2	"CM-2"[All Fields] AND biofilm[All Fields]	CM-2	25342310	Structure and microbial diversity of biofilms on different pipe materials of a model drinking water distribution systems.
4077	DRAMP04261	CM-2	"CM-2"[All Fields] AND biofilm[All Fields]	CM-2	25323605	Investigation into the potential use of poly(vinyl alcohol)/methylglyoxal fibres as antibacterial wound dressing components.
4077	DRAMP04261	CM-2	"CM-2"[All Fields] AND biofilm[All Fields]	CM-2	25312128	Assessment of the contamination potentials of some foodborne bacteria in biofilms for food products.
4077	DRAMP04261	CM-2	"CM-2"[All Fields] AND biofilm[All Fields]	CM-2	25293792	Biofilms constructed for the removal of hydrocarbon pollutants from hypersaline liquids.
4077	DRAMP04261	CM-2	"CM-2"[All Fields] AND biofilm[All Fields]	CM-2	25287229	Indole derivatives inhibited the formation of bacterial biofilm and modulated Ca2+ efflux in diatom.
4077	DRAMP04261	CM-2	"CM-2"[All Fields] AND biofilm[All Fields]	CM-2	25285261	Repeated applications of cold atmospheric pressure plasma does not induce resistance in Staphylococcus aureus embedded in biofilms.
4077	DRAMP04261	CM-2	"CM-2"[All Fields] AND biofilm[All Fields]	CM-2	25280938	Antimicrobial activity of essential oils against Staphylococcus aureus biofilms.
4077	DRAMP04261	CM-2	"CM-2"[All Fields] AND biofilm[All Fields]	CM-2	25267019	Evaluation of the effectiveness of a polyhexanide and propyl betaine-based gel in the treatment of chronic wounds.
4077	DRAMP04261	CM-2	"CM-2"[All Fields] AND biofilm[All Fields]	CM-2	25262553	Biofouling of reverse-osmosis membranes under different shear rates during tertiary wastewater desalination: microbial community composition.
4077	DRAMP04261	CM-2	"CM-2"[All Fields] AND biofilm[All Fields]	CM-2	25260731	Antibiofilm efficacy of photosensitizer-functionalized bioactive nanoparticles on multispecies biofilm.
4077	DRAMP04261	CM-2	"CM-2"[All Fields] AND biofilm[All Fields]	CM-2	25242963	Microbiological aspects of the biofilm on wooden utensils used to make a Brazilian artisanal cheese.
4077	DRAMP04261	CM-2	"CM-2"[All Fields] AND biofilm[All Fields]	CM-2	25239897	Novel Broad-Spectrum Antimicrobial Photoinactivation of In Situ Oral Biofilms by Visible Light plus Water-Filtered Infrared A.
4077	DRAMP04261	CM-2	"CM-2"[All Fields] AND biofilm[All Fields]	CM-2	25197659	The treatment of PPCP-containing sewage in an anoxic/aerobic reactor coupled with a novel design of solid plain graphite-plates microbial fuel cell.
4077	DRAMP04261	CM-2	"CM-2"[All Fields] AND biofilm[All Fields]	CM-2	25190902	Coliform Sources and Mechanisms for Regrowth in Household Drinking Water in Limpopo, South Africa.
4077	DRAMP04261	CM-2	"CM-2"[All Fields] AND biofilm[All Fields]	CM-2	25184430	Interaction between atypical microorganisms and E. coli in catheter-associated urinary tract biofilms.
4077	DRAMP04261	CM-2	"CM-2"[All Fields] AND biofilm[All Fields]	CM-2	25112506	Photodynamic inactivation of bacterial and yeast biofilms with a cationic porphyrin.
4077	DRAMP04261	CM-2	"CM-2"[All Fields] AND biofilm[All Fields]	CM-2	25088065	Bacteriophage K antimicrobial-lock technique for treatment of Staphylococcus aureus central venous catheter-related infection: a leporine model efficacy analysis.
4077	DRAMP04261	CM-2	"CM-2"[All Fields] AND biofilm[All Fields]	CM-2	25084674	Production of potential probiotic Spanish-style green table olives at pilot plant scale using multifunctional starters.
4077	DRAMP04261	CM-2	"CM-2"[All Fields] AND biofilm[All Fields]	CM-2	25022836	Inhibitors of biofilm formation by biofuel fermentation contaminants.
4077	DRAMP04261	CM-2	"CM-2"[All Fields] AND biofilm[All Fields]	CM-2	25020005	Modeling of heavy nitrate corrosion in anaerobe aquifer injection water biofilm: a case study in a flow rig.
4077	DRAMP04261	CM-2	"CM-2"[All Fields] AND biofilm[All Fields]	CM-2	25006538	Printed paper-based arrays as substrates for biofilm formation.
4077	DRAMP04261	CM-2	"CM-2"[All Fields] AND biofilm[All Fields]	CM-2	25005554	A small-scale air-cathode microbial fuel cell for on-line monitoring of water quality.
4077	DRAMP04261	CM-2	"CM-2"[All Fields] AND biofilm[All Fields]	CM-2	24977141	Microbubble-mediated ultrasound enhances the lethal effect of gentamicin on planktonic Escherichia coli.
4077	DRAMP04261	CM-2	"CM-2"[All Fields] AND biofilm[All Fields]	CM-2	24913560	Zinc enhances the phototoxic effect of blue light against malodour-producing bacteria in an experimental oral biofilm.
4077	DRAMP04261	CM-2	"CM-2"[All Fields] AND biofilm[All Fields]	CM-2	24911921	Flame oxidation of stainless steel felt enhances anodic biofilm formation and current output in bioelectrochemical systems.
4077	DRAMP04261	CM-2	"CM-2"[All Fields] AND biofilm[All Fields]	CM-2	24884540	The HMI™ module: a new tool to study the Host-Microbiota Interaction in the human gastrointestinal tract in vitro.
4077	DRAMP04261	CM-2	"CM-2"[All Fields] AND biofilm[All Fields]	CM-2	24883348	Carbon nanofibers modified graphite felt for high performance anode in high substrate concentration microbial fuel cells.
4077	DRAMP04261	CM-2	"CM-2"[All Fields] AND biofilm[All Fields]	CM-2	24883330	Atypical enteropathogenic Escherichia coli strains form biofilm on abiotic surfaces regardless of their adherence pattern on cultured epithelial cells.
4077	DRAMP04261	CM-2	"CM-2"[All Fields] AND biofilm[All Fields]	CM-2	24880128	An automated method for the quantification of moving predators such as rotifers in biofilms by image analysis.
4077	DRAMP04261	CM-2	"CM-2"[All Fields] AND biofilm[All Fields]	CM-2	24857485	A study on the long term effect of biofilm produced by biosurfactant producing microbe on medical implant.
4077	DRAMP04261	CM-2	"CM-2"[All Fields] AND biofilm[All Fields]	CM-2	24833752	Activity of colistin combined with doripenem at clinically relevant concentrations against multidrug-resistant Pseudomonas aeruginosa in an in vitro dynamic biofilm model.
4077	DRAMP04261	CM-2	"CM-2"[All Fields] AND biofilm[All Fields]	CM-2	24832828	Does vitamin E-blended UHMWPE prevent biofilm formation?
4077	DRAMP04261	CM-2	"CM-2"[All Fields] AND biofilm[All Fields]	CM-2	24773276	3D imaging provides a high-resolution, volumetric approach for analyzing biofouling.
4077	DRAMP04261	CM-2	"CM-2"[All Fields] AND biofilm[All Fields]	CM-2	24753735	Using surface plasmon resonance imaging to study bacterial biofilms.
4077	DRAMP04261	CM-2	"CM-2"[All Fields] AND biofilm[All Fields]	CM-2	24726593	Biofilm development by blastospores and hyphae of Candida albicans on abraded denture acrylic resin surfaces.
4077	DRAMP04261	CM-2	"CM-2"[All Fields] AND biofilm[All Fields]	CM-2	24681687	Role of force-sensitive amyloid-like interactions in fungal catch bonding and biofilms.
4077	DRAMP04261	CM-2	"CM-2"[All Fields] AND biofilm[All Fields]	CM-2	32261400	Site specific immobilization of a potent antimicrobial peptide onto silicone catheters: evaluation against urinary tract infection pathogens.
4077	DRAMP04261	CM-2	"CM-2"[All Fields] AND biofilm[All Fields]	CM-2	24664840	Chip calorimetry for evaluation of biofilm treatment with biocides, antibiotics, and biological agents.
4077	DRAMP04261	CM-2	"CM-2"[All Fields] AND biofilm[All Fields]	CM-2	24615298	TEGDMA and filler particles from dental composites additively attenuate LPS-induced cytokine release from the macrophage cell line RAW 264.7.
4077	DRAMP04261	CM-2	"CM-2"[All Fields] AND biofilm[All Fields]	CM-2	24614378	A novel approach utilizing biofilm time-kill curves to assess the bactericidal activity of ceftaroline combinations against biofilm-producing methicillin-resistant Staphylococcus aureus.
4077	DRAMP04261	CM-2	"CM-2"[All Fields] AND biofilm[All Fields]	CM-2	24607269	Photosensitization of in vitro biofilms formed on denture base resin.
4077	DRAMP04261	CM-2	"CM-2"[All Fields] AND biofilm[All Fields]	CM-2	24587123	Real-time measurements of the redox states of c-type cytochromes in electroactive biofilms: a confocal resonance Raman Microscopy study.
4077	DRAMP04261	CM-2	"CM-2"[All Fields] AND biofilm[All Fields]	CM-2	24524649	Antimicrobial synergism against different lineages of methicillin-resistant Staphylococcus aureus carrying SCCmec IV.

4077	DRAMP04261	CM-2	"CM-2"[All Fields] AND biofilm[All Fields]	CM-2	24487544	Polysaccharides and proteins added to flowing drinking water at microgram-per-liter levels promote the formation of biofilms predominated by bacteroidetes and proteobacteria.
4077	DRAMP04261	CM-2	"CM-2"[All Fields] AND biofilm[All Fields]	CM-2	24452996	Listeria monocytogenes can form biofilms in tap water and enter into the viable but non-cultivable state.
4077	DRAMP04261	CM-2	"CM-2"[All Fields] AND biofilm[All Fields]	CM-2	24436212	Biofilm models for the food industry: hot spots for plasmid transfer?
4077	DRAMP04261	CM-2	"CM-2"[All Fields] AND biofilm[All Fields]	CM-2	24434726	Efficacy of different carrier gases for barrier discharge plasma generation compared to chlorhexidine on the survival of Pseudomonas aeruginosa embedded in biofilm in vitro.
4077	DRAMP04261	CM-2	"CM-2"[All Fields] AND biofilm[All Fields]	CM-2	24395736	Effects of CO2 laser irradiation on tooth enamel coated with biofilm.
4077	DRAMP04261	CM-2	"CM-2"[All Fields] AND biofilm[All Fields]	CM-2	24368397	Reduction of biofilm formation on titanium surface with ultraviolet-C pre-irradiation.
4077	DRAMP04261	CM-2	"CM-2"[All Fields] AND biofilm[All Fields]	CM-2	24326253	Sonication of catheter tips for improved detection of microorganisms on external ventricular drains and ventriculo-peritoneal shunts.
4077	DRAMP04261	CM-2	"CM-2"[All Fields] AND biofilm[All Fields]	CM-2	24239277	Polystyrene films as barrier layers for corrosion protection of copper and copper alloys.
4077	DRAMP04261	CM-2	"CM-2"[All Fields] AND biofilm[All Fields]	CM-2	24159286	Reduction of Aeromonas hidrophyla biofilm on stainless stell surface by essential oils.
4077	DRAMP04261	CM-2	"CM-2"[All Fields] AND biofilm[All Fields]	CM-2	24102145	Kinetics of biofilm formation and desiccation survival of Listeria monocytogenes in single and dual species biofilms with Pseudomonas fluorescens, Serratia proteamaculans or Shewanella baltica on food-grade stainless steel surfaces.
4077	DRAMP04261	CM-2	"CM-2"[All Fields] AND biofilm[All Fields]	CM-2	24094177	Novel Ag@TiO2 nanocomposite synthesized by electrochemically active biofilm for nonenzymatic hydrogen peroxide sensor.
4077	DRAMP04261	CM-2	"CM-2"[All Fields] AND biofilm[All Fields]	CM-2	32261158	Immobilization of polybia-MPI by allyl glycidyl ether based brush chemistry to generate a novel antimicrobial surface.
4077	DRAMP04261	CM-2	"CM-2"[All Fields] AND biofilm[All Fields]	CM-2	24024938	Use of steady-state biofilm model to characterize aerobic granular sludge.
4077	DRAMP04261	CM-2	"CM-2"[All Fields] AND biofilm[All Fields]	CM-2	24020738	Contact-free inactivation of Trichophyton rubrum and Microsporum canis by cold atmospheric plasma treatment.
4077	DRAMP04261	CM-2	"CM-2"[All Fields] AND biofilm[All Fields]	CM-2	23995925	Pseudoalteromonas spp. serve as initial bacterial attractants in mesocosms of coastal waters but have subsequent antifouling capacity in mesocosms and when embedded in paint.
4077	DRAMP04261	CM-2	"CM-2"[All Fields] AND biofilm[All Fields]	CM-2	23952779	Aggregatibacter actinomycetemcomitans biofilm killing by a targeted ciprofloxacin prodrug.
4077	DRAMP04261	CM-2	"CM-2"[All Fields] AND biofilm[All Fields]	CM-2	23936303	Surface acoustic waves enhance neutrophil killing of bacteria.
4077	DRAMP04261	CM-2	"CM-2"[All Fields] AND biofilm[All Fields]	CM-2	23925199	Fluctuation of densities of bacteriophages and Escherichia coli present in natural biofilms and water of a main channel and a small tributary.
4077	DRAMP04261	CM-2	"CM-2"[All Fields] AND biofilm[All Fields]	CM-2	23912779	Susceptibility of multispecies biofilm to photodynamic therapy using Photodithazine®.
4077	DRAMP04261	CM-2	"CM-2"[All Fields] AND biofilm[All Fields]	CM-2	23907252	Effectiveness of phages in the decontamination of Listeria monocytogenes adhered to clean stainless steel, stainless steel coated with fish protein, and as a biofilm.
4077	DRAMP04261	CM-2	"CM-2"[All Fields] AND biofilm[All Fields]	CM-2	23905797	Resistance of Alicyclobacillus acidoterrestris spores and biofilm to industrial sanitizers.
4077	DRAMP04261	CM-2	"CM-2"[All Fields] AND biofilm[All Fields]	CM-2	23889504	Gene delivery to periodontal tissue using Bubble liposomes and ultrasound.
4077	DRAMP04261	CM-2	"CM-2"[All Fields] AND biofilm[All Fields]	CM-2	23847027	Photocatalytic inactivation of biofilms on bioactive dental adhesives.
4077	DRAMP04261	CM-2	"CM-2"[All Fields] AND biofilm[All Fields]	CM-2	23837894	Biofilm formation with mixed cultures of Pseudomonas aeruginosa/Escherichia coli on silicone using artificial urine to mimic urinary catheters.
4077	DRAMP04261	CM-2	"CM-2"[All Fields] AND biofilm[All Fields]	CM-2	23793414	Photodynamic antimicrobial therapy of curcumin in biofilms and carious dentine.
4077	DRAMP04261	CM-2	"CM-2"[All Fields] AND biofilm[All Fields]	CM-2	23764219	Role of initial contamination levels, biofilm maturity and presence of salt and fat on desiccation survival of Listeria monocytogenes on stainless steel surfaces.
4077	DRAMP04261	CM-2	"CM-2"[All Fields] AND biofilm[All Fields]	CM-2	23758173	Immobilization studies of an engineered arginine-tryptophan-rich peptide on a silicone surface with antimicrobial and antibiofilm activity.
4077	DRAMP04261	CM-2	"CM-2"[All Fields] AND biofilm[All Fields]	CM-2	23745742	Effects of surface charge and hydrophobicity on anodic biofilm formation, community composition, and current generation in bioelectrochemical systems.
4077	DRAMP04261	CM-2	"CM-2"[All Fields] AND biofilm[All Fields]	CM-2	23742161	Adherence and biofilm formation of Mycobacterium avium, Mycobacterium intracellulare and Mycobacterium abscessus to household plumbing materials.
4077	DRAMP04261	CM-2	"CM-2"[All Fields] AND biofilm[All Fields]	CM-2	23701333	Fungicidal photodynamic effect of a twofold positively charged porphyrin against Candida albicans planktonic cells and biofilms.
4077	DRAMP04261	CM-2	"CM-2"[All Fields] AND biofilm[All Fields]	CM-2	23680250	The diabetic foot infections: biofilms and antimicrobial resistance.
4077	DRAMP04261	CM-2	"CM-2"[All Fields] AND biofilm[All Fields]	CM-2	23678390	Phototoxic effect of blue light on the planktonic and biofilm state of anaerobic periodontal pathogens.
4077	DRAMP04261	CM-2	"CM-2"[All Fields] AND biofilm[All Fields]	CM-2	23648446	Characterization and identification of a chlorine-resistant bacterium, Sphingomonas TS001, from a model drinking water distribution system.
4077	DRAMP04261	CM-2	"CM-2"[All Fields] AND biofilm[All Fields]	CM-2	23645192	Preconditioning with cations increases the attachment of Anoxybacillus flavithermus and Geobacillus species to stainless steel.
4077	DRAMP04261	CM-2	"CM-2"[All Fields] AND biofilm[All Fields]	CM-2	23631418	Naked amoebae in biofilms collected from a temperate freshwater pond.
4077	DRAMP04261	CM-2	"CM-2"[All Fields] AND biofilm[All Fields]	CM-2	23624054	Lack of anodic capacitance causes power overshoot in microbial fuel cells.
4077	DRAMP04261	CM-2	"CM-2"[All Fields] AND biofilm[All Fields]	CM-2	23622723	Effects of 5-aminolevulinic acid-mediated photodynamic therapy on antibiotic-resistant staphylococcal biofilm: an in vitro study.
4077	DRAMP04261	CM-2	"CM-2"[All Fields] AND biofilm[All Fields]	CM-2	23569376	Preparation and characterization of flexible nanoliposomes loaded with daptomycin, a novel antibiotic, for topical skin therapy.
4077	DRAMP04261	CM-2	"CM-2"[All Fields] AND biofilm[All Fields]	CM-2	23550978	Photoinactivation of bacteria attached to glass and acrylic surfaces by 405 nm light: potential application for biofilm decontamination.
4077	DRAMP04261	CM-2	"CM-2"[All Fields] AND biofilm[All Fields]	CM-2	23474690	Cathodic and anodic biofilms in Single Chamber Microbial Fuel Cells.
4077	DRAMP04261	CM-2	"CM-2"[All Fields] AND biofilm[All Fields]	CM-2	23434719	Controlled silanization-amination reactions on the Ti6Al4V surface for biomedical applications.
4077	DRAMP04261	CM-2	"CM-2"[All Fields] AND biofilm[All Fields]	CM-2	23434685	Experimental studies on the effect of different metallic substrates on marine biofouling.
4077	DRAMP04261	CM-2	"CM-2"[All Fields] AND biofilm[All Fields]	CM-2	23421627	Changes in gene expression of Streptococcus mutans in planktonic environment following CO2 laser irradiation.
4077	DRAMP04261	CM-2	"CM-2"[All Fields] AND biofilm[All Fields]	CM-2	23397109	Chemical modification of polyvinyl chloride and silicone elastomer in inhibiting adhesion of Aeromonas hydrophila.
4077	DRAMP04261	CM-2	"CM-2"[All Fields] AND biofilm[All Fields]	CM-2	23390518	Direct electric current treatment under physiologic saline conditions kills Staphylococcus epidermidis biofilms via electrolytic generation of hypochlorous acid.
4077	DRAMP04261	CM-2	"CM-2"[All Fields] AND biofilm[All Fields]	CM-2	23248238	Synergy of ultrasound microbubbles and vancomycin against Staphylococcus epidermidis biofilm.
4077	DRAMP04261	CM-2	"CM-2"[All Fields] AND biofilm[All Fields]	CM-2	23240212	Study of azo dye decolorization and determination of cathode microorganism profile in air-cathode microbial fuel cells.
4077	DRAMP04261	CM-2	"CM-2"[All Fields] AND biofilm[All Fields]	CM-2	23215729	Candida albicans adhesion on reinforced polymethylmethacrylate denture resin: effect of fibre architecture and exposure to saliva.
4077	DRAMP04261	CM-2	"CM-2"[All Fields] AND biofilm[All Fields]	CM-2	23209343	Reversible Swelling of Chitosan and Quaternary Ammonium Modified Chitosan Brush Layers: Effect of pH and Counter Anion Size and Functionality.
4077	DRAMP04261	CM-2	"CM-2"[All Fields] AND biofilm[All Fields]	CM-2	23207875	Effect of conventional mouthrinses on initial bioadhesion to enamel and dentin in situ.
4077	DRAMP04261	CM-2	"CM-2"[All Fields] AND biofilm[All Fields]	CM-2	23196216	Enhanced power production of a membrane electrode assembly microbial fuel cell (MFC) using a cost effective poly [2,5-benzimidazole] (ABPBI) impregnated non-woven fabric filter.
4077	DRAMP04261	CM-2	"CM-2"[All Fields] AND biofilm[All Fields]	CM-2	23185637	Legionella pneumophila persists within biofilms formed by Klebsiella pneumoniae, Flavobacterium sp., and Pseudomonas fluorescens under dynamic flow conditions.
4077	DRAMP04261	CM-2	"CM-2"[All Fields] AND biofilm[All Fields]	CM-2	23107627	Early detection of Candida albicans biofilms at porous electrodes.
4077	DRAMP04261	CM-2	"CM-2"[All Fields] AND biofilm[All Fields]	CM-2	23107495	Monitoring of bacterial load in terms of culturable and non-culturable cells on new materials placed in a delicatessen serve over counter.
4077	DRAMP04261	CM-2	"CM-2"[All Fields] AND biofilm[All Fields]	CM-2	23082863	A biofilm model developed to investigate survival and disinfection of Mycobacterium mucogenicum in potable water.
4077	DRAMP04261	CM-2	"CM-2"[All Fields] AND biofilm[All Fields]	CM-2	23041137	Use of cassette-electrode microbial fuel cell for wastewater treatment.
4077	DRAMP04261	CM-2	"CM-2"[All Fields] AND biofilm[All Fields]	CM-2	22982208	Influence of blue light on Streptococcus mutans re-organization in biofilm.
4077	DRAMP04261	CM-2	"CM-2"[All Fields] AND biofilm[All Fields]	CM-2	22955017	Accumulation of MS2, GA, and Qβ phages on high density polyethylene (HDPE) and drinking water biofilms under flow/non-flow conditions.

4077	DRAMP04261	CM-2	"CM-2"[All Fields] AND biofilm[All Fields]	CM-2	22948868	Prevention and treatment of virulent bacterial biofilms with an enzymatic nitric oxide-releasing dressing.
4077	DRAMP04261	CM-2	"CM-2"[All Fields] AND biofilm[All Fields]	CM-2	22929495	Ultraviolet C light for Acinetobacter baumannii wound infections in mice: potential use for battlefield wound decontamination?
4077	DRAMP04261	CM-2	"CM-2"[All Fields] AND biofilm[All Fields]	CM-2	22890729	Quantifying Salmonella population dynamics in water and biofilms.
4077	DRAMP04261	CM-2	"CM-2"[All Fields] AND biofilm[All Fields]	CM-2	22884171	Assessment of a regulatory sanitization process in Egyptian dairy plants in regard to the adherence of some food-borne pathogens and their biofilms.
4077	DRAMP04261	CM-2	"CM-2"[All Fields] AND biofilm[All Fields]	CM-2	22847720	Photodynamic therapy as an alternative treatment for disinfection of bacteria in oral biofilms.
4077	DRAMP04261	CM-2	"CM-2"[All Fields] AND biofilm[All Fields]	CM-2	22840233	Controlling Pseudomonas aeruginosa persister cells by weak electrochemical currents and synergistic effects with tobramycin.
4077	DRAMP04261	CM-2	"CM-2"[All Fields] AND biofilm[All Fields]	CM-2	22821430	Comparison of different live/dead stainings for detection and quantification of adherent microorganisms in the initial oral biofilm.
4077	DRAMP04261	CM-2	"CM-2"[All Fields] AND biofilm[All Fields]	CM-2	22814899	Nitrate reduction with biotic and abiotic cathodes at various cell voltages in bioelectrochemical denitrification system.
4077	DRAMP04261	CM-2	"CM-2"[All Fields] AND biofilm[All Fields]	CM-2	22802686	Gold nanoparticles enhance methylene blue-induced photodynamic therapy: a novel therapeutic approach to inhibit Candida albicans biofilm.
4077	DRAMP04261	CM-2	"CM-2"[All Fields] AND biofilm[All Fields]	CM-2	22798039	Enlargement of anode for enhanced simultaneous azo dye decolorization and power output in air-cathode microbial fuel cell.
4077	DRAMP04261	CM-2	"CM-2"[All Fields] AND biofilm[All Fields]	CM-2	22782257	Influence of a mouthwash containing hydroxyapatite microclusters on bacterial adherence in situ.
4077	DRAMP04261	CM-2	"CM-2"[All Fields] AND biofilm[All Fields]	CM-2	22777042	Characterization of a conjugate between Rose Bengal and chitosan for targeted antibiofilm and tissue stabilization effects as a potential treatment of infected dentin.
4077	DRAMP04261	CM-2	"CM-2"[All Fields] AND biofilm[All Fields]	CM-2	22774873	Photodynamic inactivation of planktonic cultures and biofilms of Candida albicans mediated by aluminum-chloride-phthalocyanine entrapped in nanoemulsions.
4077	DRAMP04261	CM-2	"CM-2"[All Fields] AND biofilm[All Fields]	CM-2	22727094	Effect of erythrosine- and LED-mediated photodynamic therapy on buccal candidiasis infection of immunosuppressed mice and Candida albicans adherence to buccal epithelial cells.
4077	DRAMP04261	CM-2	"CM-2"[All Fields] AND biofilm[All Fields]	CM-2	22695355	Growth dynamic of Naegleria fowleri in a microbial freshwater biofilm.
4077	DRAMP04261	CM-2	"CM-2"[All Fields] AND biofilm[All Fields]	CM-2	22693952	Photodynamic effects of curcumin against cariogenic pathogens.
4077	DRAMP04261	CM-2	"CM-2"[All Fields] AND biofilm[All Fields]	CM-2	22672417	A comparative in vitro study of two denture cleaning techniques as an effective strategy for inhibiting Candida albicans biofilms on denture surfaces and reducing inflammation.
4077	DRAMP04261	CM-2	"CM-2"[All Fields] AND biofilm[All Fields]	CM-2	22658509	Electron transfer and biofilm formation of Shewanella putrefaciens as function of anode potential.
4077	DRAMP04261	CM-2	"CM-2"[All Fields] AND biofilm[All Fields]	CM-2	22656327	Biofilm formation on abiotic and biotic surfaces during Spanish style green table olive fermentation.
4077	DRAMP04261	CM-2	"CM-2"[All Fields] AND biofilm[All Fields]	CM-2	22609527	Photoinactivation of Staphylococcus epidermidis biofilms and suspensions by the hydrophobic photosensitizer curcumin--effect of selected nanocarrier: studies on curcumin and curcuminoides XLVII.
4077	DRAMP04261	CM-2	"CM-2"[All Fields] AND biofilm[All Fields]	CM-2	22582718	Transfer, attachment, and formation of biofilms by Escherichia coli O157:H7 on meat-contact surface materials.
4077	DRAMP04261	CM-2	"CM-2"[All Fields] AND biofilm[All Fields]	CM-2	22569892	Characterization and control of the microbial community affiliated with copper or aluminum heat exchangers of HVAC systems.
4077	DRAMP04261	CM-2	"CM-2"[All Fields] AND biofilm[All Fields]	CM-2	22566385	Influence of extracellular matrix proteins in enhancing bacterial adhesion to titanium surfaces.
4077	DRAMP04261	CM-2	"CM-2"[All Fields] AND biofilm[All Fields]	CM-2	22552590	Do edible oils reduce bacterial colonization of enamel in situ?
4077	DRAMP04261	CM-2	"CM-2"[All Fields] AND biofilm[All Fields]	CM-2	22524560	Charge transport through Geobacter sulfurreducens biofilms grown on graphite rods.
4077	DRAMP04261	CM-2	"CM-2"[All Fields] AND biofilm[All Fields]	CM-2	22483401	Characteristics of microbial biofilm on wooden vats ('gerles') in PDO Salers cheese.
4077	DRAMP04261	CM-2	"CM-2"[All Fields] AND biofilm[All Fields]	CM-2	22467505	Contact-free inactivation of Candida albicans biofilms by cold atmospheric air plasma.
4077	DRAMP04261	CM-2	"CM-2"[All Fields] AND biofilm[All Fields]	CM-2	22452392	Spectrophotometric color measurement for early detection and monitoring of greening on granite buildings.
4077	DRAMP04261	CM-2	"CM-2"[All Fields] AND biofilm[All Fields]	CM-2	22452391	Dependence of the initial adhesion of biofilm forming Pseudomonas putida mt2 on physico-chemical material properties.
4077	DRAMP04261	CM-2	"CM-2"[All Fields] AND biofilm[All Fields]	CM-2	22446303	Community structure and in situ activity of nitrifying bacteria in Phragmites root-associated biofilms.
4077	DRAMP04261	CM-2	"CM-2"[All Fields] AND biofilm[All Fields]	CM-2	22441828	Carbon dioxide laser and bonding materials reduce enamel demineralization around orthodontic brackets.
4077	DRAMP04261	CM-2	"CM-2"[All Fields] AND biofilm[All Fields]	CM-2	22414842	Treatment of root canal biofilms of Enterococcus faecalis with ozone gas and passive ultrasound activation.
4077	DRAMP04261	CM-2	"CM-2"[All Fields] AND biofilm[All Fields]	CM-2	22409156	Assessment of antibiofilm activity of magnesium fluoride nanoparticles-stabilized oil-in-water nanosized emulsion.
4077	DRAMP04261	CM-2	"CM-2"[All Fields] AND biofilm[All Fields]	CM-2	22399242	The use of optical fiber in endodontic photodynamic therapy. Is it really relevant?
4077	DRAMP04261	CM-2	"CM-2"[All Fields] AND biofilm[All Fields]	CM-2	22321293	Prevalence, populations and pheno- and genotypic characteristics of Listeria monocytogenes isolated from ready-to-eat vegetables marketed in São Paulo, Brazil.
4077	DRAMP04261	CM-2	"CM-2"[All Fields] AND biofilm[All Fields]	CM-2	22307304	Differential biofilm formation and chemical disinfection resistance of sessile cells of Listeria monocytogenes strains under monospecies and dual-species (with Salmonella enterica) conditions.
4077	DRAMP04261	CM-2	"CM-2"[All Fields] AND biofilm[All Fields]	CM-2	22281091	Electron transfer mechanism in Shewanella loihica PV-4 biofilms formed at graphite electrode.
4077	DRAMP04261	CM-2	"CM-2"[All Fields] AND biofilm[All Fields]	CM-2	22278349	Photodynamic inactivation of biofilms formed by Candida spp., Trichosporon mucoides, and Kodamaea ohmeri by cationic nanoemulsion of zinc 2,9,16,23-tetrakis(phenylthio)-29H, 31H-phthalocyanine (ZnPc).
4077	DRAMP04261	CM-2	"CM-2"[All Fields] AND biofilm[All Fields]	CM-2	22265878	Design and field application of a UV-LED based optical fiber biofilm sensor.
4077	DRAMP04261	CM-2	"CM-2"[All Fields] AND biofilm[All Fields]	CM-2	22257312	Evaluation and remediation of bulk soap dispensers for biofilm.
4077	DRAMP04261	CM-2	"CM-2"[All Fields] AND biofilm[All Fields]	CM-2	22223104	Enhanced electrode-reducing rate during the enrichment process in an air-cathode microbial fuel cell.
4077	DRAMP04261	CM-2	"CM-2"[All Fields] AND biofilm[All Fields]	CM-2	22189647	In Vitro effect of low-level laser therapy on typical oral microbial biofilms.
4077	DRAMP04261	CM-2	"CM-2"[All Fields] AND biofilm[All Fields]	CM-2	22186041	Inactivation of salmonella in biofilms and on chicken cages and preharvest poultry by levulinic Acid and sodium dodecyl sulfate.
4077	DRAMP04261	CM-2	"CM-2"[All Fields] AND biofilm[All Fields]	CM-2	22170239	Quantifying the tensile strength of microbial mats grown over noncohesive sediments.
4077	DRAMP04261	CM-2	"CM-2"[All Fields] AND biofilm[All Fields]	CM-2	22112498	Impact of conditioning films on the initial adhesion of Burkholderia cepacia.
4077	DRAMP04261	CM-2	"CM-2"[All Fields] AND biofilm[All Fields]	CM-2	22092551	Persistence of Escherichia coli in freshwater periphyton: biofilm-forming capacity as a selective advantage.
4077	DRAMP04261	CM-2	"CM-2"[All Fields] AND biofilm[All Fields]	CM-2	22006736	Susceptibility of clinical isolates of Candida to photodynamic effects of curcumin.
4077	DRAMP04261	CM-2	"CM-2"[All Fields] AND biofilm[All Fields]	CM-2	22005596	A comparison of redox polymer and enzyme co-immobilization on carbon electrodes to provide membrane-less glucose/O2 enzymatic fuel cells with improved power output and stability.
4077	DRAMP04261	CM-2	"CM-2"[All Fields] AND biofilm[All Fields]	CM-2	21998808	The effect of antimicrobial photodynamic therapy with radachlorin and toluidine blue on streptococcus mutans: an in vitro study.
4077	DRAMP04261	CM-2	"CM-2"[All Fields] AND biofilm[All Fields]	CM-2	21981116	Biofilm formation by the yeast Rhodotorula mucilaginosa: process, repeatability and cell attachment in a continuous biofilm reactor.
4077	DRAMP04261	CM-2	"CM-2"[All Fields] AND biofilm[All Fields]	CM-2	21962848	A photosynthetic rotating annular bioreactor (Taylor-Couette type flow) for phototrophic biofilm cultures.
4077	DRAMP04261	CM-2	"CM-2"[All Fields] AND biofilm[All Fields]	CM-2	21945165	Effect of fiber diameter on the behavior of biofilm and anodic performance of fiber electrodes in microbial fuel cells.
4077	DRAMP04261	CM-2	"CM-2"[All Fields] AND biofilm[All Fields]	CM-2	21924829	Bioremoval of trivalent chromium using Bacillus biofilms through continuous flow reactor.
4077	DRAMP04261	CM-2	"CM-2"[All Fields] AND biofilm[All Fields]	CM-2	21924195	Role of efflux pump inhibitors on the antibiofilm efficacy of calcium hydroxide, chitosan nanoparticles, and light-activated disinfection.
4077	DRAMP04261	CM-2	"CM-2"[All Fields] AND biofilm[All Fields]	CM-2	21884831	Submicron-textured biomaterial surface reduces staphylococcal bacterial adhesion and biofilm formation.
4077	DRAMP04261	CM-2	"CM-2"[All Fields] AND biofilm[All Fields]	CM-2	21858350	Photodynamic inactivation of Penicillium chrysogenum conidia by cationic porphyrins.
4077	DRAMP04261	CM-2	"CM-2"[All Fields] AND biofilm[All Fields]	CM-2	21855323	Electroactive mixed culture derived biofilms in microbial bioelectrochemical systems: the role of pH on biofilm formation, performance and composition.

4077	DRAMP04261	CM-2	"CM-2"[All Fields] AND biofilm[All Fields]	CM-2	21844319	Enhancement of vancomycin activity against biofilms by using ultrasound-targeted microbubble destruction.
4077	DRAMP04261	CM-2	"CM-2"[All Fields] AND biofilm[All Fields]	CM-2	21816518	Photodynamic efficacy of water-soluble Si(IV) and Ge(IV) phthalocyanines towards Candida albicans planktonic and biofilm cultures.
4077	DRAMP04261	CM-2	"CM-2"[All Fields] AND biofilm[All Fields]	CM-2	21803564	Graphite anode surface modification with controlled reduction of specific aryl diazonium salts for improved microbial fuel cells power output.
4077	DRAMP04261	CM-2	"CM-2"[All Fields] AND biofilm[All Fields]	CM-2	21802758	Attachment and biofilm formation by Escherichia coli O157:H7 at different temperatures, on various food-contact surfaces encountered in beef processing.
4077	DRAMP04261	CM-2	"CM-2"[All Fields] AND biofilm[All Fields]	CM-2	21778724	Effect of sublethal CO2 laser irradiation on gene expression of streptococcus mutans immobilized in a biofilm.
4077	DRAMP04261	CM-2	"CM-2"[All Fields] AND biofilm[All Fields]	CM-2	21704328	Efficacy of tigecycline and rifampin alone and in combination against Enterococcus faecalis biofilm infection in a rat model of ureteral stent.
4077	DRAMP04261	CM-2	"CM-2"[All Fields] AND biofilm[All Fields]	CM-2	21650178	Bacterial translational motion on the electrode surface under anodic electric field.
4077	DRAMP04261	CM-2	"CM-2"[All Fields] AND biofilm[All Fields]	CM-2	21601949	Disinfection of meticillin-resistant Staphylococcus aureus and Staphylococcus epidermidis biofilms using a remote non-thermal gas plasma.
4077	DRAMP04261	CM-2	"CM-2"[All Fields] AND biofilm[All Fields]	CM-2	21536398	Temporal analyses of the distribution and diversity of Salmonella in natural biofilms.
4077	DRAMP04261	CM-2	"CM-2"[All Fields] AND biofilm[All Fields]	CM-2	21535614	Effect of NaCl on the biofilm formation by foodborne pathogens.
4077	DRAMP04261	CM-2	"CM-2"[All Fields] AND biofilm[All Fields]	CM-2	21512920	An approach to determining the sampling effort for analyzing biofilm-dwelling ciliate colonization using an artificial substratum in coastal waters.
4077	DRAMP04261	CM-2	"CM-2"[All Fields] AND biofilm[All Fields]	CM-2	21484454	Photodynamic inactivation of microorganisms present on complete dentures. A clinical investigation. Photodynamic disinfection of complete dentures.
4077	DRAMP04261	CM-2	"CM-2"[All Fields] AND biofilm[All Fields]	CM-2	21479268	Discriminating multi-species populations in biofilms with peptide nucleic acid fluorescence in situ hybridization (PNA FISH).
4077	DRAMP04261	CM-2	"CM-2"[All Fields] AND biofilm[All Fields]	CM-2	21441325	Bacteriophage-Mediated Dispersal of Campylobacter jejuni Biofilms.
4077	DRAMP04261	CM-2	"CM-2"[All Fields] AND biofilm[All Fields]	CM-2	21440945	Facultative anaerobic halophilic and alkaliphilic bacteria isolated from a natural smear ecosystem inhibit Listeria growth in early ripening stages.
4077	DRAMP04261	CM-2	"CM-2"[All Fields] AND biofilm[All Fields]	CM-2	21423727	Molecular analysis of microbial communities in endotracheal tube biofilms.
4077	DRAMP04261	CM-2	"CM-2"[All Fields] AND biofilm[All Fields]	CM-2	21406436	Effect of the combination of clarithromycin and amikacin on Pseudomonas aeruginosa biofilm in an animal model of ureteral stent infection.
4077	DRAMP04261	CM-2	"CM-2"[All Fields] AND biofilm[All Fields]	CM-2	21315982	Biodiversity of psychrotrophic bacteria of the Bacillus cereus group collected on farm and in egg product industry.
4077	DRAMP04261	CM-2	"CM-2"[All Fields] AND biofilm[All Fields]	CM-2	21311808	A µL-scale micromachined microbial fuel cell having high power density.
4077	DRAMP04261	CM-2	"CM-2"[All Fields] AND biofilm[All Fields]	CM-2	21279861	Prevention of Pseudomonas aeruginosa adhesion by electric currents.
4077	DRAMP04261	CM-2	"CM-2"[All Fields] AND biofilm[All Fields]	CM-2	21229405	Influence of biofilms on iron and manganese deposition in drinking water distribution systems.
4077	DRAMP04261	CM-2	"CM-2"[All Fields] AND biofilm[All Fields]	CM-2	21219763	Inactivation of Listeria monocytogenes and Escherichia coli O157:H7 biofilms by micelle-encapsulated eugenol and carvacrol.
4077	DRAMP04261	CM-2	"CM-2"[All Fields] AND biofilm[All Fields]	CM-2	21219760	Salmonella enterica Enteritidis biofilm formation and viability on regular and triclosan-impregnated bench cover materials.
4077	DRAMP04261	CM-2	"CM-2"[All Fields] AND biofilm[All Fields]	CM-2	24031642	Biological Activities of a Mixture of Biosurfactant from Bacillus subtilis and Alkaline Lipase from Fusarium oxysporum.
4077	DRAMP04261	CM-2	"CM-2"[All Fields] AND biofilm[All Fields]	CM-2	21185056	Threshold concentrations of biomass and iron for pressure drop increase in spiral-wound membrane elements.
4077	DRAMP04261	CM-2	"CM-2"[All Fields] AND biofilm[All Fields]	CM-2	21132427	Abundance and diversity of biofilms in natural and artificial aquifers of the Äspö Hard Rock Laboratory, Sweden.
4077	DRAMP04261	CM-2	"CM-2"[All Fields] AND biofilm[All Fields]	CM-2	21082455	Efficacy of surface-generated nitric oxide against Candida albicans adhesion and biofilm formation.
4077	DRAMP04261	CM-2	"CM-2"[All Fields] AND biofilm[All Fields]	CM-2	21056789	Use of titanium dioxide (TiO2) photocatalysts as alternative means for Listeria monocytogenes biofilm disinfection in food processing.
4077	DRAMP04261	CM-2	"CM-2"[All Fields] AND biofilm[All Fields]	CM-2	21046199	Biofilm formation by strains of Leuconostoc citreum and L. mesenteroides.
4077	DRAMP04261	CM-2	"CM-2"[All Fields] AND biofilm[All Fields]	CM-2	21034267	Biofilm formation by Aeromonas hydrophila on green-leafy vegetables: cabbage and lettuce.
4077	DRAMP04261	CM-2	"CM-2"[All Fields] AND biofilm[All Fields]	CM-2	21031201	Non-aggregated Ga(III)-phthalocyanines in the photodynamic inactivation of planktonic and biofilm cultures of pathogenic microorganisms.
4077	DRAMP04261	CM-2	"CM-2"[All Fields] AND biofilm[All Fields]	CM-2	20888655	Potential of Escherichia coli O157:H7 to persist and form viable but non-culturable cells on a food-contact surface subjected to cycles of soiling and chemical treatment.
4077	DRAMP04261	CM-2	"CM-2"[All Fields] AND biofilm[All Fields]	CM-2	20859812	UVC fluencies for preventative treatment of Pseudomonas aeruginosa contaminated polymer tubes.
4077	DRAMP04261	CM-2	"CM-2"[All Fields] AND biofilm[All Fields]	CM-2	20854456	Enhanced germicidal effects of pulsed UV-LED irradiation on biofilms.
4077	DRAMP04261	CM-2	"CM-2"[All Fields] AND biofilm[All Fields]	CM-2	20835931	Sessile Legionella pneumophila is able to grow on surfaces and generate structured monospecies biofilms.
4077	DRAMP04261	CM-2	"CM-2"[All Fields] AND biofilm[All Fields]	CM-2	20832674	Comparison of inactivation of Listeria monocytogenes within a biofilm matrix using chlorine dioxide gas, aqueous chlorine dioxide and sodium hypochlorite treatments.
4077	DRAMP04261	CM-2	"CM-2"[All Fields] AND biofilm[All Fields]	CM-2	20820857	Quantification of phototrophic biomass on rocks: optimization of chlorophyll-a extraction by response surface methodology.
4077	DRAMP04261	CM-2	"CM-2"[All Fields] AND biofilm[All Fields]	CM-2	20666581	Energy dose parameters affect antimicrobial photodynamic therapy-mediated eradication of periopathogenic biofilm and planktonic cultures.
4077	DRAMP04261	CM-2	"CM-2"[All Fields] AND biofilm[All Fields]	CM-2	20659795	Effects of biofouling on ion transport through cation exchange membranes and microbial fuel cell performance.
4077	DRAMP04261	CM-2	"CM-2"[All Fields] AND biofilm[All Fields]	CM-2	20630740	Electroactive mixed culture biofilms in microbial bioelectrochemical systems: the role of temperature for biofilm formation and performance.
4077	DRAMP04261	CM-2	"CM-2"[All Fields] AND biofilm[All Fields]	CM-2	20595259	Mammalian cell growth versus biofilm formation on biomaterial surfaces in an in vitro post-operative contamination model.
4077	DRAMP04261	CM-2	"CM-2"[All Fields] AND biofilm[All Fields]	CM-2	20555217	Adenovirus, MS2 and PhiX174 interactions with drinking water biofilms developed on PVC, cement and cast iron.
4077	DRAMP04261	CM-2	"CM-2"[All Fields] AND biofilm[All Fields]	CM-2	20512722	Assessment of the extent of bacterial growth in reverse osmosis system for improving drinking water quality.
4077	DRAMP04261	CM-2	"CM-2"[All Fields] AND biofilm[All Fields]	CM-2	20501047	Maturation and survival of Cronobacter biofilms on silicone, polycarbonate, and stainless steel after UV light and ethanol immersion treatments.
4077	DRAMP04261	CM-2	"CM-2"[All Fields] AND biofilm[All Fields]	CM-2	20472500	Verification of the efficiency of chemical disinfection and sanitation measures in in-building distribution systems.
4077	DRAMP04261	CM-2	"CM-2"[All Fields] AND biofilm[All Fields]	CM-2	20467966	Bacterial biofilm formation versus mammalian cell growth on titanium-based mono- and bi-functional coating.
4077	DRAMP04261	CM-2	"CM-2"[All Fields] AND biofilm[All Fields]	CM-2	20467736	Evaluation of biocathodes in freshwater and brackish sediment microbial fuel cells.
4077	DRAMP04261	CM-2	"CM-2"[All Fields] AND biofilm[All Fields]	CM-2	20390553	Biofouling and pollutant removal during long-term operation of an anaerobic membrane bioreactor treating municipal wastewater.
4077	DRAMP04261	CM-2	"CM-2"[All Fields] AND biofilm[All Fields]	CM-2	20390000	Color of cyanobacteria: some methodological aspects.
4077	DRAMP04261	CM-2	"CM-2"[All Fields] AND biofilm[All Fields]	CM-2	20222678	Understanding long-term changes in microbial fuel cell performance using electrochemical impedance spectroscopy.
4077	DRAMP04261	CM-2	"CM-2"[All Fields] AND biofilm[All Fields]	CM-2	20207156	Photosensitization-based inactivation of food pathogen Listeria monocytogenes in vitro and on the surface of packaging material.
4077	DRAMP04261	CM-2	"CM-2"[All Fields] AND biofilm[All Fields]	CM-2	20189793	The study of electrochemically active microbial biofilms on different carbon-based anode materials in microbial fuel cells.
4077	DRAMP04261	CM-2	"CM-2"[All Fields] AND biofilm[All Fields]	CM-2	20085853	Control of methicillin-resistant Staphylococcus aureus in planktonic form and biofilms: a biocidal efficacy study of nonthermal dielectric-barrier discharge plasma.
4077	DRAMP04261	CM-2	"CM-2"[All Fields] AND biofilm[All Fields]	CM-2	20074756	Microphytobenthos and phytoplankton in the Severn estuary, UK: present situation and possible consequences of a tidal energy barrage.
4077	DRAMP04261	CM-2	"CM-2"[All Fields] AND biofilm[All Fields]	CM-2	20060338	Susceptibility of Candida albicans to photodynamic therapy in a murine model of oral candidosis.
4077	DRAMP04261	CM-2	"CM-2"[All Fields] AND biofilm[All Fields]	CM-2	20057102	Microbial diversity in biofilms on water distribution pipes of different materials.
4077	DRAMP04261	CM-2	"CM-2"[All Fields] AND biofilm[All Fields]	CM-2	22915919	Candida albicans biofilm on titanium: effect of peroxidase precoating.
4077	DRAMP04261	CM-2	"CM-2"[All Fields] AND biofilm[All Fields]	CM-2	20015334	Pioneering bacterial and algal communities and potential extracellular enzyme activities of stream biofilms.
4077	DRAMP04261	CM-2	"CM-2"[All Fields] AND biofilm[All Fields]	CM-2	20008087	Antimicrobial properties of light-activated polyurethane containing indocyanine green.

4077	DRAMP04261	CM-2	"CM-2"[All Fields] AND biofilm[All Fields]	CM-2	19969509	Nanostructured polypyrrole-coated anode for sun-powered microbial fuel cells.
4077	DRAMP04261	CM-2	"CM-2"[All Fields] AND biofilm[All Fields]	CM-2	19935892	Planktonic cell yield is linked to biofilm development.
4077	DRAMP04261	CM-2	"CM-2"[All Fields] AND biofilm[All Fields]	CM-2	19889173	Fungicidal effect of photodynamic therapy against fluconazole-resistant Candida albicans and Candida glabrata.
4077	DRAMP04261	CM-2	"CM-2"[All Fields] AND biofilm[All Fields]	CM-2	19874920	Cyclodextrin-functionalized biomaterials loaded with miconazole prevent Candida albicans biofilm formation in vitro.
4077	DRAMP04261	CM-2	"CM-2"[All Fields] AND biofilm[All Fields]	CM-2	19833773	A previously uncharacterized gene, yjfO (bsmA), influences Escherichia coli biofilm formation and stress response.
4077	DRAMP04261	CM-2	"CM-2"[All Fields] AND biofilm[All Fields]	CM-2	19822702	Bacteriophage cocktail for the prevention of biofilm formation by Pseudomonas aeruginosa on catheters in an in vitro model system.
4077	DRAMP04261	CM-2	"CM-2"[All Fields] AND biofilm[All Fields]	CM-2	19760228	Biofilm growth of individual and dual strains of Klebsiella oxytoca from the dairy industry on ultrafiltration membranes.
4077	DRAMP04261	CM-2	"CM-2"[All Fields] AND biofilm[All Fields]	CM-2	19758254	Evaluation of the antimicrobial effect of photodynamic antimicrobial therapy in an in situ model of dentine caries.
4077	DRAMP04261	CM-2	"CM-2"[All Fields] AND biofilm[All Fields]	CM-2	19733265	In vitro and in vivo comparisons of staphylococcal biofilm formation on a cross-linked poly(ethylene glycol)-based polymer coating.
4077	DRAMP04261	CM-2	"CM-2"[All Fields] AND biofilm[All Fields]	CM-2	19717251	Survival of Mycobacterium avium subsp. paratuberculosis in biofilms on livestock watering trough materials.
4077	DRAMP04261	CM-2	"CM-2"[All Fields] AND biofilm[All Fields]	CM-2	19656675	A continuous stirred hydrogen-based polyvinyl chloride membrane biofilm reactor for the treatment of nitrate contaminated drinking water.
4077	DRAMP04261	CM-2	"CM-2"[All Fields] AND biofilm[All Fields]	CM-2	19648379	Competition of Listeria monocytogenes serotype 1/2a and 4b strains in mixed-culture biofilms.
4077	DRAMP04261	CM-2	"CM-2"[All Fields] AND biofilm[All Fields]	CM-2	19643681	Marine aerobic biofilm as biocathode catalyst.
4077	DRAMP04261	CM-2	"CM-2"[All Fields] AND biofilm[All Fields]	CM-2	19519855	Interleukin-8 production by polymorphonuclear leukocytes from patients with chronic infected leg ulcers treated with Lactobacillus plantarum.
4077	DRAMP04261	CM-2	"CM-2"[All Fields] AND biofilm[All Fields]	CM-2	19494468	Sewer exfiltration and the colmation layer.
4077	DRAMP04261	CM-2	"CM-2"[All Fields] AND biofilm[All Fields]	CM-2	19467595	Enhancement on the simultaneous removal of nitrate and organic pollutants from groundwater by a three-dimensional bio-electrochemical reactor.
4077	DRAMP04261	CM-2	"CM-2"[All Fields] AND biofilm[All Fields]	CM-2	19446903	Characterization of bacterial strains isolated from a beef-processing plant following cleaning and disinfection - Influence of isolated strains on biofilm formation by Sakaï and EDL 933 E. coli O157:H7.
4077	DRAMP04261	CM-2	"CM-2"[All Fields] AND biofilm[All Fields]	CM-2	19446030	Biofilm growth kinetics of a monomethylamine producing Alphaproteobacteria strain isolated from an anaerobic reactor.
4077	DRAMP04261	CM-2	"CM-2"[All Fields] AND biofilm[All Fields]	CM-2	19420899	The role of recombinant human elafin in the resistance of A549 cells against Pseudomonas aeruginosa biofilm.
4077	DRAMP04261	CM-2	"CM-2"[All Fields] AND biofilm[All Fields]	CM-2	19361876	Tina wooden vat biofilm: a safe and highly efficient lactic acid bacteria delivering system in PDO Ragusano cheese making.
4077	DRAMP04261	CM-2	"CM-2"[All Fields] AND biofilm[All Fields]	CM-2	19346353	CO2 production as an indicator of biofilm metabolism.
4077	DRAMP04261	CM-2	"CM-2"[All Fields] AND biofilm[All Fields]	CM-2	19268965	Zn sorption mechanisms onto sheathed Leptothrix discophora and the impact of the nanoparticulate biogenic Mn oxide coating.
4077	DRAMP04261	CM-2	"CM-2"[All Fields] AND biofilm[All Fields]	CM-2	19219473	A microbiological study of the self-cleaning potential of oily Arabian Gulf coasts.
4077	DRAMP04261	CM-2	"CM-2"[All Fields] AND biofilm[All Fields]	CM-2	19201985	Effects of biocides and other metal removal fluid constituents on Mycobacterium immunogenum.
4077	DRAMP04261	CM-2	"CM-2"[All Fields] AND biofilm[All Fields]	CM-2	19199075	Distribution coefficients of dietary sugars in artificial Candida biofilms.
4077	DRAMP04261	CM-2	"CM-2"[All Fields] AND biofilm[All Fields]	CM-2	21566379	Prevention of Phormidium tenue Biofilm Formation by TiO(2) Photocatalysis.
4077	DRAMP04261	CM-2	"CM-2"[All Fields] AND biofilm[All Fields]	CM-2	19004624	Colonization dynamics of biofilm-associated ciliate morphotypes at different flow velocities.
4077	DRAMP04261	CM-2	"CM-2"[All Fields] AND biofilm[All Fields]	CM-2	18990530	Increased bacterial adherence and biomass in Pseudomonas aeruginosa bacteria exposed to clarithromycin.
4077	DRAMP04261	CM-2	"CM-2"[All Fields] AND biofilm[All Fields]	CM-2	18985610	Non-invasive self-referencing electrochemical sensors for quantifying real-time biofilm analyte flux.
4077	DRAMP04261	CM-2	"CM-2"[All Fields] AND biofilm[All Fields]	CM-2	18968451	Inclusion of metal micro-particles into poly(pyrrolylalkylammonium) films containing an enzyme for bioelectrocatalysis: a preliminary study.
4077	DRAMP04261	CM-2	"CM-2"[All Fields] AND biofilm[All Fields]	CM-2	18955534	The electricidal effect: reduction of Staphylococcus and pseudomonas biofilms by prolonged exposure to low-intensity electrical current.
4077	DRAMP04261	CM-2	"CM-2"[All Fields] AND biofilm[All Fields]	CM-2	18951606	Systematic evaluation of nitrate and perchlorate bioreduction kinetics in groundwater using a hydrogen-based membrane biofilm reactor.
4077	DRAMP04261	CM-2	"CM-2"[All Fields] AND biofilm[All Fields]	CM-2	18951602	Removal of microbial biofilm on Water Hyacinth plants roots by ultrasonic treatment.
4077	DRAMP04261	CM-2	"CM-2"[All Fields] AND biofilm[All Fields]	CM-2	18836002	Comparison of electrode reduction activities of Geobacter sulfurreducens and an enriched consortium in an air-cathode microbial fuel cell.
4077	DRAMP04261	CM-2	"CM-2"[All Fields] AND biofilm[All Fields]	CM-2	18760591	Electricity generation from polyalcohols in single-chamber microbial fuel cells.
4077	DRAMP04261	CM-2	"CM-2"[All Fields] AND biofilm[All Fields]	CM-2	18752014	The effect of long-term nitrate treatment on SRB activity, corrosion rate and bacterial community composition in offshore water injection systems.
4077	DRAMP04261	CM-2	"CM-2"[All Fields] AND biofilm[All Fields]	CM-2	18725436	Effect of electrical current on the activities of antimicrobial agents against Pseudomonas aeruginosa, Staphylococcus aureus, and Staphylococcus epidermidis biofilms.
4077	DRAMP04261	CM-2	"CM-2"[All Fields] AND biofilm[All Fields]	CM-2	18707789	Effectiveness of chitosan-coated plastic films incorporating antimicrobials in inhibition of Listeria monocytogenes on cold-smoked salmon.
4077	DRAMP04261	CM-2	"CM-2"[All Fields] AND biofilm[All Fields]	CM-2	18676697	Persistence of Helicobacter pylori in heterotrophic drinking-water biofilms.
4077	DRAMP04261	CM-2	"CM-2"[All Fields] AND biofilm[All Fields]	CM-2	18674814	Bacterial adhesion and growth on a polymer brush-coating.
4077	DRAMP04261	CM-2	"CM-2"[All Fields] AND biofilm[All Fields]	CM-2	18673325	In vitro photodynamic eradication of Pseudomonas aeruginosa in planktonic and biofilm culture.
4077	DRAMP04261	CM-2	"CM-2"[All Fields] AND biofilm[All Fields]	CM-2	18646217	Impact of initial biofilm growth on the anode impedance of microbial fuel cells.
4077	DRAMP04261	CM-2	"CM-2"[All Fields] AND biofilm[All Fields]	CM-2	18642338	Toluene diffusion and reaction in unsaturated Pseudomonas putida biofilms.
4077	DRAMP04261	CM-2	"CM-2"[All Fields] AND biofilm[All Fields]	CM-2	18577453	Polypropylene grafted with smart polymers (PNIPAAm/PAAc) for loading and controlled release of vancomycin.
4077	DRAMP04261	CM-2	"CM-2"[All Fields] AND biofilm[All Fields]	CM-2	18570988	Effects of Er:YAG laser irradiation on biofilm-forming bacteria associated with endodontic pathogens in vitro.
4077	DRAMP04261	CM-2	"CM-2"[All Fields] AND biofilm[All Fields]	CM-2	27041416	ALGAL RESPONSE TO NUTRIENT ENRICHMENT IN FORESTED OLIGOTROPHIC STREAM(1).
4077	DRAMP04261	CM-2	"CM-2"[All Fields] AND biofilm[All Fields]	CM-2	18502524	Miniaturized calorimetry - a new method for real-time biofilm activity analysis.
4077	DRAMP04261	CM-2	"CM-2"[All Fields] AND biofilm[All Fields]	CM-2	18502470	Removal of VUV pre-treated natural organic matter by biologically activated carbon columns.
4077	DRAMP04261	CM-2	"CM-2"[All Fields] AND biofilm[All Fields]	CM-2	18498901	Photodynamic treatment of endodontic polymicrobial infection in vitro.
4077	DRAMP04261	CM-2	"CM-2"[All Fields] AND biofilm[All Fields]	CM-2	18468764	Effects of Cistus-tea on bacterial colonization and enzyme activities of the in situ pellicle.
4077	DRAMP04261	CM-2	"CM-2"[All Fields] AND biofilm[All Fields]	CM-2	18430491	BMAP-28 improves the efficacy of vancomycin in rat models of gram-positive cocci ureteral stent infection.
4077	DRAMP04261	CM-2	"CM-2"[All Fields] AND biofilm[All Fields]	CM-2	18359562	Inhibition of perchlorate reduction by nitrate in a fixed biofilm reactor.
4077	DRAMP04261	CM-2	"CM-2"[All Fields] AND biofilm[All Fields]	CM-2	18358705	Increased adhesion of Enterococcus faecalis strains with bimodal electrophoretic mobility distributions.
4077	DRAMP04261	CM-2	"CM-2"[All Fields] AND biofilm[All Fields]	CM-2	18316516	Genetic and physiological effects of noncoherent visible light combined with hydrogen peroxide on Streptococcus mutans in biofilm.
4077	DRAMP04261	CM-2	"CM-2"[All Fields] AND biofilm[All Fields]	CM-2	21386152	Tongue biofilm areal density and tongue coating index.
4077	DRAMP04261	CM-2	"CM-2"[All Fields] AND biofilm[All Fields]	CM-2	18305203	Antimicrobial surface functionalization of plastic catheters by silver nanoparticles.
4077	DRAMP04261	CM-2	"CM-2"[All Fields] AND biofilm[All Fields]	CM-2	18284487	Real-time quantification of Pseudomonas fluorescens cell removal from glass surfaces due to bacteriophage varphiS1 application.
4077	DRAMP04261	CM-2	"CM-2"[All Fields] AND biofilm[All Fields]	CM-2	18217936	Comparative evaluation of methods for counting surviving biofilm cells adhering to a polyvinyl chloride surface exposed to chlorine or drying.

4077	DRAMP04261	CM-2	"CM-2"[All Fields] AND biofilm[All Fields]	CM-2	18206768	Control of Listeria monocytogenes on ham steaks by antimicrobials incorporated into chitosan-coated plastic films.
4077	DRAMP04261	CM-2	"CM-2"[All Fields] AND biofilm[All Fields]	CM-2	18205653	Microbiological evaluation and antibiotic susceptibility of dental unit water systems in general dental practice.
4077	DRAMP04261	CM-2	"CM-2"[All Fields] AND biofilm[All Fields]	CM-2	18158754	The impact of photodynamic therapy on the viability of Streptococcus mutans in a planktonic culture.
4077	DRAMP04261	CM-2	"CM-2"[All Fields] AND biofilm[All Fields]	CM-2	18155793	The effects of a biocide and a surfactant on the detachment of Pseudomonas fluorescens from glass surfaces.
4077	DRAMP04261	CM-2	"CM-2"[All Fields] AND biofilm[All Fields]	CM-2	17965210	Low densities of epiphytic bacteria from the marine alga Ulva australis inhibit settlement of fouling organisms.
4077	DRAMP04261	CM-2	"CM-2"[All Fields] AND biofilm[All Fields]	CM-2	17723659	Electrodeposited glucose oxidase/anionic clay for glucose biosensors design.
4077	DRAMP04261	CM-2	"CM-2"[All Fields] AND biofilm[All Fields]	CM-2	17713816	Replication of Legionella pneumophila in floating biofilms.
4077	DRAMP04261	CM-2	"CM-2"[All Fields] AND biofilm[All Fields]	CM-2	17674571	Prevalence of bacterial pathogens in biofilms of drinking water distribution systems.
4077	DRAMP04261	CM-2	"CM-2"[All Fields] AND biofilm[All Fields]	CM-2	17653936	Laboratory screening of coating libraries for algal adhesion.
4077	DRAMP04261	CM-2	"CM-2"[All Fields] AND biofilm[All Fields]	CM-2	17653934	The effect of hydrodynamic conditions on the phenotype of Pseudomonas fluorescens biofilms.
4077	DRAMP04261	CM-2	"CM-2"[All Fields] AND biofilm[All Fields]	CM-2	17603998	Fluorescence microscopic visualization and quantification of initial bacterial colonization on enamel in situ.
4077	DRAMP04261	CM-2	"CM-2"[All Fields] AND biofilm[All Fields]	CM-2	17585989	Bacterial community composition and structure of biofilms developing on nanofiltration membranes applied to wastewater treatment.
4077	DRAMP04261	CM-2	"CM-2"[All Fields] AND biofilm[All Fields]	CM-2	17547015	The role of hydrodynamic stress on the phenotypic characteristics of single and binary biofilms of Pseudomonas fluorescens.
4077	DRAMP04261	CM-2	"CM-2"[All Fields] AND biofilm[All Fields]	CM-2	17544473	Detection of Legionella spp. and some of their amoeba hosts in floating biofilms from anthropogenic and natural aquatic environments.
4077	DRAMP04261	CM-2	"CM-2"[All Fields] AND biofilm[All Fields]	CM-2	17526785	In situ activity and spatial organization of anaerobic ammonium-oxidizing (anammox) bacteria in biofilms.
4077	DRAMP04261	CM-2	"CM-2"[All Fields] AND biofilm[All Fields]	CM-2	17489389	A comparison of biofilms from macrophytes and rocks for taste and odour producers in the St. Lawrence river.
4077	DRAMP04261	CM-2	"CM-2"[All Fields] AND biofilm[All Fields]	CM-2	17477259	Antimicrobial effects of alginate-based films containing essential oils on Listeria monocytogenes and Salmonella typhimurium present in bologna and ham.
4077	DRAMP04261	CM-2	"CM-2"[All Fields] AND biofilm[All Fields]	CM-2	17397474	A marine bacterial adhesion microplate test using the DAPI fluorescent dye: a new method to screen antifouling agents.
4077	DRAMP04261	CM-2	"CM-2"[All Fields] AND biofilm[All Fields]	CM-2	17353029	Experimental and modeling approach to study sorption of dissolved hydrophobic organic contaminants to microbial biofilms.
4077	DRAMP04261	CM-2	"CM-2"[All Fields] AND biofilm[All Fields]	CM-2	17337551	First evidence of division and accumulation of viable but nonculturable Pseudomonas fluorescens cells on surfaces subjected to conditions encountered at meat processing premises.
4077	DRAMP04261	CM-2	"CM-2"[All Fields] AND biofilm[All Fields]	CM-2	17268880	Development and structure of eukaryotic biofilms in an extreme acidic environment, rio tinto (SW, Spain).
4077	DRAMP04261	CM-2	"CM-2"[All Fields] AND biofilm[All Fields]	CM-2	17262142	Er:Yag laser irradiation of the microbiological apical biofilm.
4077	DRAMP04261	CM-2	"CM-2"[All Fields] AND biofilm[All Fields]	CM-2	17244808	Growth in catheter biofilms and antibiotic resistance of Mycobacterium avium.
4077	DRAMP04261	CM-2	"CM-2"[All Fields] AND biofilm[All Fields]	CM-2	17216655	Effect of wall shear rate on biofilm deposition and grazing in drinking water flow chambers.
4077	DRAMP04261	CM-2	"CM-2"[All Fields] AND biofilm[All Fields]	CM-2	17187392	Biofilm formation by Staphylococcus epidermidis on nitrogen ion implanted CoCrMo alloy material.
4077	DRAMP04261	CM-2	"CM-2"[All Fields] AND biofilm[All Fields]	CM-2	17141898	Ultrasonic monitoring of early-stage biofilm growth on polymeric surfaces.
4077	DRAMP04261	CM-2	"CM-2"[All Fields] AND biofilm[All Fields]	CM-2	16979345	Bactericidal effect of malachite green and red laser on Actinobacillus actinomycetemcomitans.
4077	DRAMP04261	CM-2	"CM-2"[All Fields] AND biofilm[All Fields]	CM-2	16963273	Potential of photodynamic therapy in treatment of fungal infections of the mouth. Design and characterisation of a mucoadhesive patch containing toluidine blue O.
4077	DRAMP04261	CM-2	"CM-2"[All Fields] AND biofilm[All Fields]	CM-2	16957203	Attachment of and biofilm formation by Enterobacter sakazakii on stainless steel and enteral feeding tubes.
4077	DRAMP04261	CM-2	"CM-2"[All Fields] AND biofilm[All Fields]	CM-2	16949693	A laboratory hot tub model for disinfectant efficacy evaluation.
4077	DRAMP04261	CM-2	"CM-2"[All Fields] AND biofilm[All Fields]	CM-2	16943077	The adherence of Salmonella Enteritidis PT4 to stainless steel: the importance of the air-liquid interface and nutrient availability.
4077	DRAMP04261	CM-2	"CM-2"[All Fields] AND biofilm[All Fields]	CM-2	25049148	Photodynamic therapy of diseased bone.
4077	DRAMP04261	CM-2	"CM-2"[All Fields] AND biofilm[All Fields]	CM-2	16919846	Using an efficient biofilm detaching agent: an essential step for the improvement of endoscope reprocessing protocols.
4077	DRAMP04261	CM-2	"CM-2"[All Fields] AND biofilm[All Fields]	CM-2	16907822	Adhesion of water stressed Helicobacter pylori to abiotic surfaces.
4077	DRAMP04261	CM-2	"CM-2"[All Fields] AND biofilm[All Fields]	CM-2	16824634	Strain and growth temperature influence Listeria spp. attachment to intact and cut cabbage.
4077	DRAMP04261	CM-2	"CM-2"[All Fields] AND biofilm[All Fields]	CM-2	16791745	Assessing primary and bacterial production rates in biofilms on pebbles in Ishite stream, Japan.
4077	DRAMP04261	CM-2	"CM-2"[All Fields] AND biofilm[All Fields]	CM-2	16779628	Biofilm formation in an ice cream plant.
4077	DRAMP04261	CM-2	"CM-2"[All Fields] AND biofilm[All Fields]	CM-2	16701891	Influence of casting procedures on the corrosion resistance of clinical dental alloys containing palladium.
4077	DRAMP04261	CM-2	"CM-2"[All Fields] AND biofilm[All Fields]	CM-2	16672472	Control of Listeria spp. by competitive-exclusion bacteria in floor drains of a poultry processing plant.
4077	DRAMP04261	CM-2	"CM-2"[All Fields] AND biofilm[All Fields]	CM-2	16597925	Construction and analysis of fractional multifactorial designs to study attachment strength and transfer of Listeria monocytogenes from pure or mixed biofilms after contact with a solid model food.
4077	DRAMP04261	CM-2	"CM-2"[All Fields] AND biofilm[All Fields]	CM-2	16583822	Characteristics of biofilm community formed in the chlorinated biodegradable organic matter-limited tap water.
4077	DRAMP04261	CM-2	"CM-2"[All Fields] AND biofilm[All Fields]	CM-2	16581672	Quantification of algal biofilms colonising building materials: chlorophyll a measured by PAM-fluorometry as a biomass parameter.
4077	DRAMP04261	CM-2	"CM-2"[All Fields] AND biofilm[All Fields]	CM-2	16460343	Photosensitization of in vitro biofilms by toluidine blue O combined with a light-emitting diode.
4077	DRAMP04261	CM-2	"CM-2"[All Fields] AND biofilm[All Fields]	CM-2	16420603	Inhibition of microbial adhesion to silicone rubber treated with biosurfactant from Streptococcus thermophilus A.
4077	DRAMP04261	CM-2	"CM-2"[All Fields] AND biofilm[All Fields]	CM-2	16395425	Pre-clinical in vitro and in vivo studies to examine the potential use of photodynamic therapy in the treatment of osteomyelitis.
4077	DRAMP04261	CM-2	"CM-2"[All Fields] AND biofilm[All Fields]	CM-2	16362272	Destruction of Deinococcus geothermalis biofilm by photocatalytic ALD and sol-gel TiO2 surfaces.
4077	DRAMP04261	CM-2	"CM-2"[All Fields] AND biofilm[All Fields]	CM-2	16332854	Identification of bacteria in biofilm and bulk water samples from a nonchlorinated model drinking water distribution system: detection of a large nitrite-oxidizing population associated with Nitrospira spp.
4077	DRAMP04261	CM-2	"CM-2"[All Fields] AND biofilm[All Fields]	CM-2	16108785	Effect of pulsed ultrasound in combination with gentamicin on bacterial viability in biofilms on bone cements in vivo.
4077	DRAMP04261	CM-2	"CM-2"[All Fields] AND biofilm[All Fields]	CM-2	16019051	Biofilm formation and multiplication of Legionella in a model warm water system with pipes of copper, stainless steel and cross-linked polyethylene.
4077	DRAMP04261	CM-2	"CM-2"[All Fields] AND biofilm[All Fields]	CM-2	15983029	Susceptibility of Streptococcus mutans biofilms to photodynamic therapy: an in vitro study.
4077	DRAMP04261	CM-2	"CM-2"[All Fields] AND biofilm[All Fields]	CM-2	15791796	Effective diffusion coefficients of glucose in artificial biofilms.
4077	DRAMP04261	CM-2	"CM-2"[All Fields] AND biofilm[All Fields]	CM-2	15758222	Statistical assessment of a laboratory method for growing biofilms.
4077	DRAMP04261	CM-2	"CM-2"[All Fields] AND biofilm[All Fields]	CM-2	15673757	Rapid diffusion of fluorescent tracers into Staphylococcus epidermidis biofilms visualized by time lapse microscopy.
4077	DRAMP04261	CM-2	"CM-2"[All Fields] AND biofilm[All Fields]	CM-2	15610437	Protozoan grazing and its impact upon population dynamics in biofilm communities.
4077	DRAMP04261	CM-2	"CM-2"[All Fields] AND biofilm[All Fields]	CM-2	15556217	Occurrence of nitrifiers and diversity of ammonia-oxidizing bacteria in developing drinking water biofilms.
4077	DRAMP04261	CM-2	"CM-2"[All Fields] AND biofilm[All Fields]	CM-2	15458499	Microbiological evaluation of dental unit water systems in general dental practice in Europe.
4077	DRAMP04261	CM-2	"CM-2"[All Fields] AND biofilm[All Fields]	CM-2	15387132	Detection, integration and persistence of aeromonads in water distribution pipe biofilms.
4077	DRAMP04261	CM-2	"CM-2"[All Fields] AND biofilm[All Fields]	CM-2	15380987	Optimisation and significance of ATP analysis for measuring active biomass in granular activated carbon filters used in water treatment.

4077	DRAMP04261	CM-2	"CM-2"[All Fields] AND biofilm[All Fields]	CM-2	15371220	Detection and identification of Legionella species from groundwaters.
4077	DRAMP04261	CM-2	"CM-2"[All Fields] AND biofilm[All Fields]	CM-2	15294838	Model system for growing and quantifying Streptococcus pneumoniae biofilms in situ and in real time.
4077	DRAMP04261	CM-2	"CM-2"[All Fields] AND biofilm[All Fields]	CM-2	15292635	In vitro bacterial adherence to ventriculoperitoneal shunts.
4077	DRAMP04261	CM-2	"CM-2"[All Fields] AND biofilm[All Fields]	CM-2	15246346	delta-Aminolaevulinic acid mediated photodynamic antimicrobial chemotherapy on Pseudomonas aeruginosa planktonic and biofilm cultures.
4077	DRAMP04261	CM-2	"CM-2"[All Fields] AND biofilm[All Fields]	CM-2	15240275	Control of Listeria monocytogenes in a biofilm by competitive-exclusion microorganisms.
4077	DRAMP04261	CM-2	"CM-2"[All Fields] AND biofilm[All Fields]	CM-2	15162448	In vitro laser ablation of laboratory developed biofilms using an Nd:YAG laser of 532 nm wavelength.
4077	DRAMP04261	CM-2	"CM-2"[All Fields] AND biofilm[All Fields]	CM-2	15159160	Dynamics of nematodes in a high organic loading rotating biological contactors.
4077	DRAMP04261	CM-2	"CM-2"[All Fields] AND biofilm[All Fields]	CM-2	15155218	Effects of growth phase and extracellular slime on photodynamic inactivation of gram-positive pathogenic bacteria.
4077	DRAMP04261	CM-2	"CM-2"[All Fields] AND biofilm[All Fields]	CM-2	15153929	Removal of biofilm from endoscopes: evaluation of detergent efficiency.
4077	DRAMP04261	CM-2	"CM-2"[All Fields] AND biofilm[All Fields]	CM-2	14990512	Moving fluid with bacterial carpets.
4077	DRAMP04261	CM-2	"CM-2"[All Fields] AND biofilm[All Fields]	CM-2	14705001	Recolonization of laser-ablated bacterial biofilm.
4077	DRAMP04261	CM-2	"CM-2"[All Fields] AND biofilm[All Fields]	CM-2	14630121	Distribution of bacteria in a domestic hot water system in a Danish apartment building.
4077	DRAMP04261	CM-2	"CM-2"[All Fields] AND biofilm[All Fields]	CM-2	14618697	Laser impact on marine planktonic diatoms: an experimental study using a flow cytometry system.
4077	DRAMP04261	CM-2	"CM-2"[All Fields] AND biofilm[All Fields]	CM-2	14618694	Laser impact on bacterial ATP: insights into the mechanism of laser-bacteria interactions.
4077	DRAMP04261	CM-2	"CM-2"[All Fields] AND biofilm[All Fields]	CM-2	14602661	Mycobacterium xenopi and drinking water biofilms.
4077	DRAMP04261	CM-2	"CM-2"[All Fields] AND biofilm[All Fields]	CM-2	12859764	Bacterial species in biofilm cultivated from the end of the Seoul water distribution system.
4077	DRAMP04261	CM-2	"CM-2"[All Fields] AND biofilm[All Fields]	CM-2	12790676	Ultrasound increases the rate of bacterial cell growth.
4077	DRAMP04261	CM-2	"CM-2"[All Fields] AND biofilm[All Fields]	CM-2	12787585	Measurement of copper release rates from antifouling paint under laboratory and in situ conditions: implications for loading estimation to marine water bodies.
4077	DRAMP04261	CM-2	"CM-2"[All Fields] AND biofilm[All Fields]	CM-2	12701901	On-line monitoring of biofilm formation in a brewery water pipeline system with a fibre optical device.
4077	DRAMP04261	CM-2	"CM-2"[All Fields] AND biofilm[All Fields]	CM-2	12694906	Acid tolerance response of biofilm cells of Streptococcus mutans.
4077	DRAMP04261	CM-2	"CM-2"[All Fields] AND biofilm[All Fields]	CM-2	12632396	Laser impact assessment in a biofilm-forming bacterium Pseudoalteromonas carrageenovora using a flow cytometric system.
4077	DRAMP04261	CM-2	"CM-2"[All Fields] AND biofilm[All Fields]	CM-2	12593962	The effect of increasing copper content in phosphate-based glasses on biofilms of Streptococcus sanguis.
4077	DRAMP04261	CM-2	"CM-2"[All Fields] AND biofilm[All Fields]	CM-2	12542714	Ecology of mixed biofilms subjected daily to a chlorinated alkaline solution: spatial distribution of bacterial species suggests a protective effect of one species to another.
4077	DRAMP04261	CM-2	"CM-2"[All Fields] AND biofilm[All Fields]	CM-2	12418265	[Element contents of biofilm and suspended particulate in natural aquatic ecosystem].
4077	DRAMP04261	CM-2	"CM-2"[All Fields] AND biofilm[All Fields]	CM-2	12362402	Effect of chlorine, biodegradable dissolved organic carbon and suspended bacteria on biofilm development in drinking water systems.
4077	DRAMP04261	CM-2	"CM-2"[All Fields] AND biofilm[All Fields]	CM-2	12355466	Inhibition of bacterial attachment by pulsed Nd:YAG laser irradiations: an in vitro study using marine biofilm-forming bacterium Pseudoalteromonas carrageenovora.
4077	DRAMP04261	CM-2	"CM-2"[All Fields] AND biofilm[All Fields]	CM-2	12171400	Regrowth and survival of indicator microorganisms on the surfaces of household containers used for the storage of drinking water in rural communities of South Africa.
4077	DRAMP04261	CM-2	"CM-2"[All Fields] AND biofilm[All Fields]	CM-2	12085038	Effects of adherence factors and human bile on bacterial attachment and biliary stent blockage: an in vitro study.
4077	DRAMP04261	CM-2	"CM-2"[All Fields] AND biofilm[All Fields]	CM-2	12030705	Impact of pulsed Nd:YAG laser on the marine biofilm-forming bacteria Pseudoalteromonas carrageenovora: significance of physiological status.
4077	DRAMP04261	CM-2	"CM-2"[All Fields] AND biofilm[All Fields]	CM-2	12019075	Antimicrobial activity of a novel catheter lock solution.
4077	DRAMP04261	CM-2	"CM-2"[All Fields] AND biofilm[All Fields]	CM-2	12018592	Minimum effective release rate of antifoulants.1. Effects of zosteric acid at Ford Island, Hawaii (USA).
4077	DRAMP04261	CM-2	"CM-2"[All Fields] AND biofilm[All Fields]	CM-2	11986927	Comparison of microporous and nonporous membrane bioreactor systems for the treatment of BTEX in vapor streams.
4077	DRAMP04261	CM-2	"CM-2"[All Fields] AND biofilm[All Fields]	CM-2	11911924	Application of infrared spectroscopy (attenuated total reflection) for monitoring enzymatic activity on substrate films.
4077	DRAMP04261	CM-2	"CM-2"[All Fields] AND biofilm[All Fields]	CM-2	11846763	Inhibition of biofilm formation and swarming of Escherichia coli by (5Z)-4-bromo-5-(bromomethylene)-3-butyl-2(5H)-furanone.
4077	DRAMP04261	CM-2	"CM-2"[All Fields] AND biofilm[All Fields]	CM-2	11494109	Biodegradation of BTEX vapors in a silicone membrane bioreactor system.
4077	DRAMP04261	CM-2	"CM-2"[All Fields] AND biofilm[All Fields]	CM-2	11442723	A novel in vitro model system to grow films of oral bacteria for the study of human tooth root surface caries.
4077	DRAMP04261	CM-2	"CM-2"[All Fields] AND biofilm[All Fields]	CM-2	11442720	A repeatable laboratory method for testing the efficacy of biocides against toilet bowl biofilms.
4077	DRAMP04261	CM-2	"CM-2"[All Fields] AND biofilm[All Fields]	CM-2	11414334	Sugar composition of biofilms produced by paper mill bacteria.
4077	DRAMP04261	CM-2	"CM-2"[All Fields] AND biofilm[All Fields]	CM-2	11375160	Analysis of bacterial detachment from substratum surfaces by the passage of air-liquid interfaces.
4077	DRAMP04261	CM-2	"CM-2"[All Fields] AND biofilm[All Fields]	CM-2	11319118	Shewanella putrefaciens adhesion and biofilm formation on food processing surfaces.
4077	DRAMP04261	CM-2	"CM-2"[All Fields] AND biofilm[All Fields]	CM-2	11264754	Effects of chilling on sampling of bacteria attached to swine carcasses.
4077	DRAMP04261	CM-2	"CM-2"[All Fields] AND biofilm[All Fields]	CM-2	11229914	Factors influencing numbers of Mycobacterium avium, Mycobacterium intracellulare, and other Mycobacteria in drinking water distribution systems.
4077	DRAMP04261	CM-2	"CM-2"[All Fields] AND biofilm[All Fields]	CM-2	11225722	Effects of community composition and growth rate on aquifer biofilm bacteria and their susceptibility to betadine disinfection.
4077	DRAMP04261	CM-2	"CM-2"[All Fields] AND biofilm[All Fields]	CM-2	11168716	The growth of Bacillus stearothermophilus on stainless steel.
4077	DRAMP04261	CM-2	"CM-2"[All Fields] AND biofilm[All Fields]	CM-2	11074419	Efficacy of silver-coated fabric to prevent bacterial colonization and subsequent device-based biofilm formation.
4077	DRAMP04261	CM-2	"CM-2"[All Fields] AND biofilm[All Fields]	CM-2	11061629	Biofilm formation on the surface of ceramic tiles.
4077	DRAMP04261	CM-2	"CM-2"[All Fields] AND biofilm[All Fields]	CM-2	10747236	Role of RpoS and AlgT in Pseudomonas aeruginosa biofilm resistance to hydrogen peroxide and monochloramine.
4077	DRAMP04261	CM-2	"CM-2"[All Fields] AND biofilm[All Fields]	CM-2	10699866	Construction of multicomponent catalytic films based on avidin-biotin technology for the electroenzymatic oxidation of molecular hydrogen.
4077	DRAMP04261	CM-2	"CM-2"[All Fields] AND biofilm[All Fields]	CM-2	10698803	Characterization of fungal biofilms within a municipal water distribution system.
4077	DRAMP04261	CM-2	"CM-2"[All Fields] AND biofilm[All Fields]	CM-2	10604410	Biofilm formation in vivo on perfluoro-alkylsiloxane-modified voice prostheses.
4077	DRAMP04261	CM-2	"CM-2"[All Fields] AND biofilm[All Fields]	CM-2	10574092	Behaviour of L. monocytogenes in an artificially made biofilm of a nisin-producing strain of Lactococcus lactis.
4077	DRAMP04261	CM-2	"CM-2"[All Fields] AND biofilm[All Fields]	CM-2	10543809	Impacts of the reduction of nutrient levels on bacterial water quality in distribution systems.
4077	DRAMP04261	CM-2	"CM-2"[All Fields] AND biofilm[All Fields]	CM-2	10389242	The role of surface physicochemical properties in determining the distribution of the autochthonous microflora in mineral water bottles.
4077	DRAMP04261	CM-2	"CM-2"[All Fields] AND biofilm[All Fields]	CM-2	9855189	A liposomal hydrogel for the prevention of bacterial adhesion to catheters.
4077	DRAMP04261	CM-2	"CM-2"[All Fields] AND biofilm[All Fields]	CM-2	21182691	Potential for biofilm development in drinking water distribution systems.
4077	DRAMP04261	CM-2	"CM-2"[All Fields] AND biofilm[All Fields]	CM-2	9750289	Assessment of microbial involvement in the elevation of copper levels in drinking water.
4077	DRAMP04261	CM-2	"CM-2"[All Fields] AND biofilm[All Fields]	CM-2	9750276	Generation of a reproducible nutrient-depleted biofilm of Escherichia coli and Burkholderia cepacia.
4077	DRAMP04261	CM-2	"CM-2"[All Fields] AND biofilm[All Fields]	CM-2	9747985	Adhesion of different bacterial strains to low-temperature plasma treated biomedical PVC catheter surfaces.
4077	DRAMP04261	CM-2	"CM-2"[All Fields] AND biofilm[All Fields]	CM-2	9746571	Role of Fusobacterium nucleatum and coaggregation in anaerobe survival in planktonic and biofilm oral microbial communities during aeration.

4077	DRAMP04261	CM-2	"CM-2"[All Fields] AND biofilm[All Fields]	CM-2	9633659	Bacterial-fungal biofilms in flowing water photo-processing tanks.
4077	DRAMP04261	CM-2	"CM-2"[All Fields] AND biofilm[All Fields]	CM-2	9435076	Protozoan bacterivory and Escherichia coli survival in drinking water distribution systems.
4077	DRAMP04261	CM-2	"CM-2"[All Fields] AND biofilm[All Fields]	CM-2	9080695	Elimination of adhering bacteria from surfaces by pulsed laser beams.
4077	DRAMP04261	CM-2	"CM-2"[All Fields] AND biofilm[All Fields]	CM-2	8894091	Effect of low-intensity ultrasound upon biofilm structure from confocal scanning laser microscopy observation.
4077	DRAMP04261	CM-2	"CM-2"[All Fields] AND biofilm[All Fields]	CM-2	24415316	Biofilm formation in water cooling systems.
4077	DRAMP04261	CM-2	"CM-2"[All Fields] AND biofilm[All Fields]	CM-2	8987686	Variation in sessile microflora during biofilm formation on AISI-304 stainless steel coupons.
4077	DRAMP04261	CM-2	"CM-2"[All Fields] AND biofilm[All Fields]	CM-2	8810530	Phototoxicity of argon laser irradiation on biofilms of Porphyromonas and Prevotella species.
4077	DRAMP04261	CM-2	"CM-2"[All Fields] AND biofilm[All Fields]	CM-2	8849649	Biofilm formation by the enterobacteriaceae: a comparison between salmonella enteritidis, Escherichia coli and a nitrogen-fixing strain of Klebsiella pneumoniae.
4077	DRAMP04261	CM-2	"CM-2"[All Fields] AND biofilm[All Fields]	CM-2	8858863	Biofilms associated with poultry processing equipment.
4077	DRAMP04261	CM-2	"CM-2"[All Fields] AND biofilm[All Fields]	CM-2	8519482	The effect of electrical currents and tobramycin on Pseudomonas aeruginosa biofilms.
4077	DRAMP04261	CM-2	"CM-2"[All Fields] AND biofilm[All Fields]	CM-2	7498058	Xenobiotic biodegradation test using attached bacteria in synthetic seawater.
4077	DRAMP04261	CM-2	"CM-2"[All Fields] AND biofilm[All Fields]	CM-2	18623350	Biofilm parameters influencing biocide efficacy.
4077	DRAMP04261	CM-2	"CM-2"[All Fields] AND biofilm[All Fields]	CM-2	18618749	Effects of medium carbon-to-nitrogen ratio on biofilm formation and plasmid stability.
4077	DRAMP04261	CM-2	"CM-2"[All Fields] AND biofilm[All Fields]	CM-2	1476435	Heterotrophic bacteria in an air-handling system.
4077	DRAMP04261	CM-2	"CM-2"[All Fields] AND biofilm[All Fields]	CM-2	31071783	Effect of Cleaners and Sanitizers on Listeria monocytogenes Attached to Product Contact Surfaces.
4077	DRAMP04261	CM-2	"CM-2"[All Fields] AND biofilm[All Fields]	CM-2	24192825	Assimilation of CO2 and introduced organic compounds by bacterial communities in groundwater from southeastern Sweden deep crystalline bedrock.
4077	DRAMP04261	CM-2	"CM-2"[All Fields] AND biofilm[All Fields]	CM-2	24194332	Stopped-flow chamber and image analysis system for quantitative characterization of bacterial population migration: Motility and chemotaxis ofEscherichia coli K12 to fucose.
4077	DRAMP04261	CM-2	"CM-2"[All Fields] AND biofilm[All Fields]	CM-2	18604808	Biofilm detachment mechanisms in a liquid-fluidized bed.
4077	DRAMP04261	CM-2	"CM-2"[All Fields] AND biofilm[All Fields]	CM-2	18600657	Measurement of bacterial random motility and chemotaxis coefficients: II. Application of single-cell-based mathematical model.
4077	DRAMP04261	CM-2	"CM-2"[All Fields] AND biofilm[All Fields]	CM-2	24196364	Differential adhesion, activity, and carbohydrate: Protein ratios ofPseudomonas atlantica monocultures attaching to stainless steel in a linear shear gradient.
4077	DRAMP04261	CM-2	"CM-2"[All Fields] AND biofilm[All Fields]	CM-2	24203002	Differences in plating efficiency of bacteria from river epilithon sampled from upper and lower surfaces of artificial substrata.
4078	DRAMP04262	CM-3	"CM-3"[All Fields] AND biofilm[All Fields]	CM-3	34514545	Preparation and performance evaluation of novel magnetic porous carriers in fluidized bed bioreactor for wastewater treatment.
4078	DRAMP04262	CM-3	"CM-3"[All Fields] AND biofilm[All Fields]	CM-3	34388660	Talaromyces cellulolyticus as a promising candidate for biofilm construction and treatment of textile wastewater.
4078	DRAMP04262	CM-3	"CM-3"[All Fields] AND biofilm[All Fields]	CM-3	34360535	Chitosan-Based Functional Films Integrated with Magnolol: Characterization, Antioxidant and Antimicrobial Activity and Pork Preservation.
4078	DRAMP04262	CM-3	"CM-3"[All Fields] AND biofilm[All Fields]	CM-3	34339991	Biofilm formation of Ancylobacter sp. TS-1 on different granular materials and its ability for chemolithoautotrophic As(III)-oxidation at high concentrations.
4078	DRAMP04262	CM-3	"CM-3"[All Fields] AND biofilm[All Fields]	CM-3	34071975	Antimicrobial Activity and Antibiofilm Potential of Coenzyme Q 0against SalmonellaTyphimurium.
4078	DRAMP04262	CM-3	"CM-3"[All Fields] AND biofilm[All Fields]	CM-3	33993638	Genetic engineering biofilms in situ using ultrasound-mediated DNA delivery.
4078	DRAMP04262	CM-3	"CM-3"[All Fields] AND biofilm[All Fields]	CM-3	33715232	Start-up of a trickling photobioreactor for the treatment of domestic wastewater.
4078	DRAMP04262	CM-3	"CM-3"[All Fields] AND biofilm[All Fields]	CM-3	33581085	Biofilm development in a pilot-scale gravity sewer: Physical characteristics, microstructure, and microbial communities.
4078	DRAMP04262	CM-3	"CM-3"[All Fields] AND biofilm[All Fields]	CM-3	32590245	Attenuation of Proteus mirabilis colonization and swarming motility on indwelling urinary catheter by antibiofilm impregnation: An in vitro study.
4078	DRAMP04262	CM-3	"CM-3"[All Fields] AND biofilm[All Fields]	CM-3	31529241	Fabrication of Ultra-Pure Anisotropic Zinc Oxide Nanoparticles via Simple and Cost-Effective Route: Implications for UTI and EAC Medications.
4078	DRAMP04262	CM-3	"CM-3"[All Fields] AND biofilm[All Fields]	CM-3	31351270	Curli production enhances clay-E. coli aggregation and sedimentation.
4078	DRAMP04262	CM-3	"CM-3"[All Fields] AND biofilm[All Fields]	CM-3	30894842	Shear Stress Affects Biofilm Structure and Consequently Current Generation of Bioanode in Microbial Electrochemical Systems (MESs).
4078	DRAMP04262	CM-3	"CM-3"[All Fields] AND biofilm[All Fields]	CM-3	29028629	A dual energy micro-CT methodology for visualization and quantification of biofilm formation and dentin demineralization.
4078	DRAMP04262	CM-3	"CM-3"[All Fields] AND biofilm[All Fields]	CM-3	28961858	Diatom-driven recolonization of microbial mat-dominated siliciclastic tidal flat sediments.
4078	DRAMP04262	CM-3	"CM-3"[All Fields] AND biofilm[All Fields]	CM-3	28725026	Microbiome analysis and confocal microscopy of used kitchen sponges reveal massive colonization by Acinetobacter, Moraxella and Chryseobacterium species.
4078	DRAMP04262	CM-3	"CM-3"[All Fields] AND biofilm[All Fields]	CM-3	28484115	Microbially reduced graphene oxide shows efficient electricity ecovery from artificial dialysis wastewater.
4078	DRAMP04262	CM-3	"CM-3"[All Fields] AND biofilm[All Fields]	CM-3	28401700	Silica immobilization of Geobacter sulfurreducens for constructing ready-to-use artificial bioelectrodes.
4078	DRAMP04262	CM-3	"CM-3"[All Fields] AND biofilm[All Fields]	CM-3	27545981	Metal-Polymer Hybrid Architectures as Novel Anode Platform for Microbial Electrochemical Technologies.
4078	DRAMP04262	CM-3	"CM-3"[All Fields] AND biofilm[All Fields]	CM-3	26899353	Graphene oxide-dependent growth and self-aggregation into a hydrogel complex of exoelectrogenic bacteria.
4078	DRAMP04262	CM-3	"CM-3"[All Fields] AND biofilm[All Fields]	CM-3	26872267	Kinetics of Indigenous Nitrate Reducing Sulfide Oxidizing Activity in Microaerophilic Wastewater Biofilms.
4078	DRAMP04262	CM-3	"CM-3"[All Fields] AND biofilm[All Fields]	CM-3	26750640	In situ probing of microbial activity within anammox granular biomass with microelectrodes.
4078	DRAMP04262	CM-3	"CM-3"[All Fields] AND biofilm[All Fields]	CM-3	25898971	Modelling bio-electrosynthesis in a reverse microbial fuel cell to produce acetate from CO2 and H2O.
4078	DRAMP04262	CM-3	"CM-3"[All Fields] AND biofilm[All Fields]	CM-3	25312128	Assessment of the contamination potentials of some foodborne bacteria in biofilms for food products.
4078	DRAMP04262	CM-3	"CM-3"[All Fields] AND biofilm[All Fields]	CM-3	24911921	Flame oxidation of stainless steel felt enhances anodic biofilm formation and current output in bioelectrochemical systems.
4078	DRAMP04262	CM-3	"CM-3"[All Fields] AND biofilm[All Fields]	CM-3	30514031	Protists and bacteria interactions in the presence of oil.
4078	DRAMP04262	CM-3	"CM-3"[All Fields] AND biofilm[All Fields]	CM-3	24773276	3D imaging provides a high-resolution, volumetric approach for analyzing biofouling.
4078	DRAMP04262	CM-3	"CM-3"[All Fields] AND biofilm[All Fields]	CM-3	24741468	Comparison of Nonprecious Metal Cathode Materials for Methane Production by Electromethanogenesis.
4078	DRAMP04262	CM-3	"CM-3"[All Fields] AND biofilm[All Fields]	CM-3	23545191	Denitrification performance and biofilm characteristics using biodegradable polymers PCL as carriers and carbon source.
4078	DRAMP04262	CM-3	"CM-3"[All Fields] AND biofilm[All Fields]	CM-3	23378142	Loofa (Luffa cylindrica) sponge: review of development of the biomatrix as a tool for biotechnological applications.
4078	DRAMP04262	CM-3	"CM-3"[All Fields] AND biofilm[All Fields]	CM-3	22394146	Facile fabrication of scalable, hierarchically structured polymer/carbon architectures for bioelectrodes.
4078	DRAMP04262	CM-3	"CM-3"[All Fields] AND biofilm[All Fields]	CM-3	21821755	Macroscopic biofilms in fracture-dominated sediment that anaerobically oxidize methane.
4078	DRAMP04262	CM-3	"CM-3"[All Fields] AND biofilm[All Fields]	CM-3	21311808	A μL-scale micromachined microbial fuel cell having high power density.
4078	DRAMP04262	CM-3	"CM-3"[All Fields] AND biofilm[All Fields]	CM-3	20972820	Biofilm productivity and concomitant cell autolysis in a membrane bioreactor.
4078	DRAMP04262	CM-3	"CM-3"[All Fields] AND biofilm[All Fields]	CM-3	20189294	Treatment of municipal wastewater using a contact oxidation filtration separation integrated bioreactor.
4078	DRAMP04262	CM-3	"CM-3"[All Fields] AND biofilm[All Fields]	CM-3	21614132	The Long-term Release of Antibiotics From Monolithic Nonporous Polymer Implants for Use as Tympanostomy Tubes.
4078	DRAMP04262	CM-3	"CM-3"[All Fields] AND biofilm[All Fields]	CM-3	18848344	Performance evaluation of a biotrickling filter treating a mixture of oxygenated VOCs during intermittent loading.
4078	DRAMP04262	CM-3	"CM-3"[All Fields] AND biofilm[All Fields]	CM-3	17849971	Anaerobic treatment of vinasses by a sequentially mixed moving bed biofilm reactor.
4078	DRAMP04262	CM-3	"CM-3"[All Fields] AND biofilm[All Fields]	CM-3	16046049	Application of a new type of moving bio-film in aerobic sequencing batch reactor (aerobic-SBR).

4078	DRAMP04262	CM-3	"CM-3"[All Fields] AND biofilm[All Fields]	CM-3	15870342	Succession of internal sulfur cycles and sulfur-oxidizing bacterial communities in microaerophilic wastewater biofilms.
4078	DRAMP04262	CM-3	"CM-3"[All Fields] AND biofilm[All Fields]	CM-3	15819240	Sulfide-oxidizing activity and bacterial community structure in a fluidized bed reactor from a zero-discharge mariculture system.
4078	DRAMP04262	CM-3	"CM-3"[All Fields] AND biofilm[All Fields]	CM-3	15380987	Optimisation and significance of ATP analysis for measuring active biomass in granular activated carbon filters used in water treatment.
4078	DRAMP04262	CM-3	"CM-3"[All Fields] AND biofilm[All Fields]	CM-3	12923079	Membrane localization of motility, signaling, and polyketide synthetase proteins in Myxococcus xanthus.
4078	DRAMP04262	CM-3	"CM-3"[All Fields] AND biofilm[All Fields]	CM-3	12879306	Sulfate-reducing bacterial community structure and their contribution to carbon mineralization in a wastewater biofilm growing under microaerophilic conditions.
4078	DRAMP04262	CM-3	"CM-3"[All Fields] AND biofilm[All Fields]	CM-3	11872492	Successional development of sulfate-reducing bacterial populations and their activities in a wastewater biofilm growing under microaerophilic conditions.
4078	DRAMP04262	CM-3	"CM-3"[All Fields] AND biofilm[All Fields]	CM-3	11870602	Successional development of sulfate-reducing bacterial populations and their activities in an activated sludge immobilized agar gel film.
4078	DRAMP04262	CM-3	"CM-3"[All Fields] AND biofilm[All Fields]	CM-3	11424733	Enhancement in the treatment efficiency and conversion to energy of dairy wastewaters by augmenting CST reactors with simple biofilm support systems.
4078	DRAMP04262	CM-3	"CM-3"[All Fields] AND biofilm[All Fields]	CM-3	11400094	Long-term performance of bioreactors cleaning mercury-contaminated wastewater and their response to temperature and mercury stress and mechanical perturbation.
4078	DRAMP04262	CM-3	"CM-3"[All Fields] AND biofilm[All Fields]	CM-3	10543829	Analyses of spatial distributions of sulfate-reducing bacteria and their activity in aerobic wastewater biofilms.
4078	DRAMP04262	CM-3	"CM-3"[All Fields] AND biofilm[All Fields]	CM-3	18623558	Transport limitation of chlorine disinfection of Pseudomonas aeruginosa entrapped in alginate beads.
4078	DRAMP04262	CM-3	"CM-3"[All Fields] AND biofilm[All Fields]	CM-3	8032837	Characterization of biofilm and encrustation on ureteric stents in vivo.
4078	DRAMP04262	CM-3	"CM-3"[All Fields] AND biofilm[All Fields]	CM-3	18587881	Characterization of production of free gluconic acid by Gluconobacter suboxydans adsorbed on ceramic honeycomb monolith.
4078	DRAMP04262	CM-3	"CM-3"[All Fields] AND biofilm[All Fields]	CM-3	18548762	Some fluidization characteristics of biological beds.
4079	DRAMP04263	CM-4	"CM-4"[All Fields] AND biofilm[All Fields]	CM-4	32163891	Scalability and stacking of self-stratifying microbial fuel cells treating urine.
4090	DRAMP04280	CM1	"CM1"[All Fields] AND biofilm[All Fields]	CM1	30386734	A new functional biofilm biocatalyst for the simultaneous removal of dibenzothiophene and quinoline using Rhodococcus rhodochrousand curli amyloid overproducer mutants derived from Cobetiasp. strain MM1IDA2H-1.
4090	DRAMP04280	CM1	"CM1"[All Fields] AND biofilm[All Fields]	CM1	26304599	The In Vivo Pericapsular Tissue Response to Modern Polyurethane Breast Implants.
4091	DRAMP04281	CM2	"CM2"[All Fields] AND biofilm[All Fields]	CM2	34559627	Dual-Light Photodynamic Therapy Effectively Eliminates Streptococcus Oralis Biofilms.
4091	DRAMP04281	CM2	"CM2"[All Fields] AND biofilm[All Fields]	CM2	34287490	Impact of the contamination time by Escherichia coli on biofilm formation in surgical instruments.
4091	DRAMP04281	CM2	"CM2"[All Fields] AND biofilm[All Fields]	CM2	33543208	[Effect of different fluence rates applied in curcumin-photodynamic therapy on Candida albicans biofilms].
4091	DRAMP04281	CM2	"CM2"[All Fields] AND biofilm[All Fields]	CM2	33512576	Eradication of Acinetobacter baumannii Planktonic and Biofilm Cells Through Erythrosine-Mediated Photodynamic Inactivation Augmented by Acetic Acid and Chitosan.
4091	DRAMP04281	CM2	"CM2"[All Fields] AND biofilm[All Fields]	CM2	33476282	Restarting the Healing Process of Chronic Wounds Using a Novel Desiccant: A Prospective Case Series.
4091	DRAMP04281	CM2	"CM2"[All Fields] AND biofilm[All Fields]	CM2	32717062	Enterococcus faecalis and Vibrio harveyicolonize low-density polyethylene and biodegradable plastics under marine conditions.
4091	DRAMP04281	CM2	"CM2"[All Fields] AND biofilm[All Fields]	CM2	32428934	Antibacterial Effect of Caprylic Acid and Potassium Sorbate in Combination against Listeria monocytogenes ATCC 7644.
4091	DRAMP04281	CM2	"CM2"[All Fields] AND biofilm[All Fields]	CM2	32374766	Dual-light photodynamic therapy administered daily provides a sustained antibacterial effect on biofilm and prevents Streptococcus mutans adaptation.
4091	DRAMP04281	CM2	"CM2"[All Fields] AND biofilm[All Fields]	CM2	32236563	Inhibitory Effects of Combinations of Chemicals on Escherichia coli, Bacillus cereus, and Staphylococcus aureus Biofilms during the Clean-in-Place Process at an Experimental Dairy Plant.
4091	DRAMP04281	CM2	"CM2"[All Fields] AND biofilm[All Fields]	CM2	32069699	An epifluorescence-based technique accelerates risk assessment of aggregated bacterial communities in carcass and environment.
4091	DRAMP04281	CM2	"CM2"[All Fields] AND biofilm[All Fields]	CM2	31941566	[The Risk of Total Joint Replacement Infectious Complications in Relation to the UHMWPE Particle Adhesion Area].
4091	DRAMP04281	CM2	"CM2"[All Fields] AND biofilm[All Fields]	CM2	31858084	Effect of Antimicrobial Photodynamic Therapy Using Indocyanine Green Doped with Chitosan Nanoparticles on Biofilm Formation-Related Gene Expression of Aggregatibacter actinomycetemcomitans.
4091	DRAMP04281	CM2	"CM2"[All Fields] AND biofilm[All Fields]	CM2	31848281	Reassessing the Role of the Type II MqsRA Toxin-Antitoxin System in Stress Response and Biofilm Formation: mqsAls Transcriptionally Uncoupled from mqsR.
4091	DRAMP04281	CM2	"CM2"[All Fields] AND biofilm[All Fields]	CM2	31234170	Comparison of DNA Extraction Efficiency and Reproducibility of Different Aeration Diffuser Biofilms Using Bead-Beating Protocol.
4091	DRAMP04281	CM2	"CM2"[All Fields] AND biofilm[All Fields]	CM2	31017184	Ozone against Pseudomonas aeruginosa biofilms in contact lenses storage cases.
4091	DRAMP04281	CM2	"CM2"[All Fields] AND biofilm[All Fields]	CM2	30715289	Effects of low-intensity and low-frequency ultrasound combined with tobramycin on biofilms of extended-spectrum beta-lactamases (ESBLs) Escherichia coli.
4091	DRAMP04281	CM2	"CM2"[All Fields] AND biofilm[All Fields]	CM2	30376123	Surface coatings with covalently attached anidulafungin and micafungin prevent Candida albicans biofilm formation.
4091	DRAMP04281	CM2	"CM2"[All Fields] AND biofilm[All Fields]	CM2	30148865	Antibacterial activity of Staphylococcus aureus biofilm under combined exposure of glutaraldehyde, near-infrared light, and 405-nm laser.
4091	DRAMP04281	CM2	"CM2"[All Fields] AND biofilm[All Fields]	CM2	30035715	FORMATION OF BIOFILMS BY BACTERIA EXCRETED FROM CHRONIC ANAL FISSURE AND THE INFLUENCE OF THE DIRECT CURRENT ELECTRIC FIELD ON THEM.
4091	DRAMP04281	CM2	"CM2"[All Fields] AND biofilm[All Fields]	CM2	29956765	Application of 460 nm visible light for the elimination of Candida albicans in vitro and in vivo.
4091	DRAMP04281	CM2	"CM2"[All Fields] AND biofilm[All Fields]	CM2	29788897	Biofilm Destruction on Endotracheal Tubes by Photodynamic Inactivation.
4091	DRAMP04281	CM2	"CM2"[All Fields] AND biofilm[All Fields]	CM2	29753881	Confocal fluorescence imaging to evaluate the effect of antimicrobial photodynamic therapy depth on P. gingivalis and T. denticola biofilms.
4091	DRAMP04281	CM2	"CM2"[All Fields] AND biofilm[All Fields]	CM2	29621296	Inhibition of biofilm formation on the surface of water storage containers using biosand zeolite silver-impregnated clay granular and silver impregnated porous pot filtration systems.
4091	DRAMP04281	CM2	"CM2"[All Fields] AND biofilm[All Fields]	CM2	29584602	Effect of a New Purified Collagen Matrix With Polyhexamethylene Biguanide on Recalcitrant Wounds of Various Etiologies: A Case Series.
4091	DRAMP04281	CM2	"CM2"[All Fields] AND biofilm[All Fields]	CM2	29306935	Quaternized Zn(II) phthalocyanines for photodynamic strategy against resistant periodontal bacteria.
4091	DRAMP04281	CM2	"CM2"[All Fields] AND biofilm[All Fields]	CM2	29244088	Application of calcium oxide (CaO, heated scallop-shell powder) for the reduction of Listeria monocytogenes biofilms on eggshell surfaces.
4091	DRAMP04281	CM2	"CM2"[All Fields] AND biofilm[All Fields]	CM2	29048702	Time-kill assays of amphotericin B plus anidulafungin against Candida tropicalis biofilms formed on two different biomaterials.
4091	DRAMP04281	CM2	"CM2"[All Fields] AND biofilm[All Fields]	CM2	29039290	Simulated-Use Polytetrafluorethylene Biofilm Model: Repeated Rounds of Complete Reprocessing Lead to Accumulation of Organic Debris and Viable Bacteria.
4091	DRAMP04281	CM2	"CM2"[All Fields] AND biofilm[All Fields]	CM2	28419339	Effect of chlorine treatment on inhibition of E. coli serogroup O2 incorporation into 7-day-old biofilm on polyvinylchloride surface.
4091	DRAMP04281	CM2	"CM2"[All Fields] AND biofilm[All Fields]	CM2	28339743	Application of bacteriophages to reduce Salmonella attachment and biofilms on hard surfaces.
4091	DRAMP04281	CM2	"CM2"[All Fields] AND biofilm[All Fields]	CM2	28339699	Effects of chlorine and hydrogen peroxide sanitation in low bacterial content water on biofilm formation model of poultry brooding house waterlines.
4091	DRAMP04281	CM2	"CM2"[All Fields] AND biofilm[All Fields]	CM2	28259957	Photodynamic inactivation of fibroblasts and inhibition of Staphylococcus epidermidis adhesion and biofilm formation by toluidine blue O.
4091	DRAMP04281	CM2	"CM2"[All Fields] AND biofilm[All Fields]	CM2	28095386	In vitro Evaluation of the Cleansing Effect of a Monofilament Fiber Debridement Pad Compared to Gauze Swabs.
4091	DRAMP04281	CM2	"CM2"[All Fields] AND biofilm[All Fields]	CM2	28053149	Employment of methylene blue irradiated with laser light source in photodynamic inactivation of biofilm formed by Candida albicans strain resistant to fluconazole.
4091	DRAMP04281	CM2	"CM2"[All Fields] AND biofilm[All Fields]	CM2	28011958	Cationic porphyrin-mediated photodynamic inactivation of Candida biofilms and the effect of miconazole.
4091	DRAMP04281	CM2	"CM2"[All Fields] AND biofilm[All Fields]	CM2	27196401	Simultaneous Bioreduction of Multiple Oxidized Contaminants Using a Membrane Biofilm Reactor.
4091	DRAMP04281	CM2	"CM2"[All Fields] AND biofilm[All Fields]	CM2	29727146	[Characterization of a novel electrogenic Clostridium sporogenes isolated from forest soil].
4091	DRAMP04281	CM2	"CM2"[All Fields] AND biofilm[All Fields]	CM2	26867131	The Black Yeast Exophiala dermatitidis and Other Selected Opportunistic Human Fungal Pathogens Spread from Dishwashers to Kitchens.
4091	DRAMP04281	CM2	"CM2"[All Fields] AND biofilm[All Fields]	CM2	26749227	The antibacterial effect of sinoporphyrin sodium photodynamic therapy on Staphylococcus aureus planktonic and biofilm cultures.
4091	DRAMP04281	CM2	"CM2"[All Fields] AND biofilm[All Fields]	CM2	26498601	In vitro effectiveness of 455-nm blue LED to reduce the load of Staphylococcus aureus and Candida albicans biofilms in compact bone tissue.

4091	DRAMP04281	CM2	"CM2"[All Fields] AND biofilm[All Fields]	CM2	26360831	Biofilm-Forming Abilities of Listeria monocytogenes Serotypes Isolated from Different Sources.
4091	DRAMP04281	CM2	"CM2"[All Fields] AND biofilm[All Fields]	CM2	26313448	Development of Photodynamic Antimicrobial Chemotherapy (PACT) for Clostridium difficile.
4091	DRAMP04281	CM2	"CM2"[All Fields] AND biofilm[All Fields]	CM2	26213977	Opportunistic Pathogens Mycobacterium Avium Complex (MAC) and Legionella spp. Colonise Model Shower.
4091	DRAMP04281	CM2	"CM2"[All Fields] AND biofilm[All Fields]	CM2	25818458	Dhvar5 antimicrobial peptide (AMP) chemoselective covalent immobilization results on higher antiadherence effect than simple physical adsorption.
4091	DRAMP04281	CM2	"CM2"[All Fields] AND biofilm[All Fields]	CM2	25759232	The effect of blue light on periodontal biofilm growth in vitro.
4091	DRAMP04281	CM2	"CM2"[All Fields] AND biofilm[All Fields]	CM2	25607663	Dewatering as a non-toxic control of nuisance midge larvae in algal wastewater treatment floways.
4091	DRAMP04281	CM2	"CM2"[All Fields] AND biofilm[All Fields]	CM2	25487795	Bacteriophage-mediated control of a two-species biofilm formed by microorganisms causing catheter-associated urinary tract infections in an in vitro urinary catheter model.
4091	DRAMP04281	CM2	"CM2"[All Fields] AND biofilm[All Fields]	CM2	25097026	Prevention of Staphylococcus epidermidis biofilm formation using electrical current.
4091	DRAMP04281	CM2	"CM2"[All Fields] AND biofilm[All Fields]	CM2	25019396	[Perspectives of disodium salt 2.4-di(1-metoxyethyl)-deuteroporphyrin - IX ("Dimegin") application for photodynamic therapy in non-oncologic cases].
4091	DRAMP04281	CM2	"CM2"[All Fields] AND biofilm[All Fields]	CM2	25000666	Plasma deactivation of oral bacteria seeded on hydroxyapatite disks as tooth enamel analogue.
4091	DRAMP04281	CM2	"CM2"[All Fields] AND biofilm[All Fields]	CM2	24680071	Biofilms formed by Mycobacterium tuberculosis on cement, ceramic, and stainless steel surfaces and their controls.
4091	DRAMP04281	CM2	"CM2"[All Fields] AND biofilm[All Fields]	CM2	24649670	Oxidation behavior of ammonium in a 3-dimensional biofilm-electrode reactor.
4091	DRAMP04281	CM2	"CM2"[All Fields] AND biofilm[All Fields]	CM2	24350448	A flat microbial fuel cell for decentralized wastewater valorization: process performance and optimization potential.
4091	DRAMP04281	CM2	"CM2"[All Fields] AND biofilm[All Fields]	CM2	24056457	Aerobic biofilms grown from Athabasca watershed sediments are inhibited by increasing concentrations of bituminous compounds.
4091	DRAMP04281	CM2	"CM2"[All Fields] AND biofilm[All Fields]	CM2	23601046	Biofilm formation of O157 and non-O157 Shiga toxin-producing Escherichia coli and multidrug-resistant and susceptible Salmonella typhimurium and newport and their inactivation by sanitizers.
4091	DRAMP04281	CM2	"CM2"[All Fields] AND biofilm[All Fields]	CM2	23575121	Reduction by competitive bacteria of Listeria monocytogenes in biofilms and Listeria bacteria in floor drains in a ready-to-eat poultry processing plant.
4091	DRAMP04281	CM2	"CM2"[All Fields] AND biofilm[All Fields]	CM2	23536965	Ultra-rapid elimination of biofilms via the combustion of a nanoenergetic coating.
4091	DRAMP04281	CM2	"CM2"[All Fields] AND biofilm[All Fields]	CM2	24730137	Phylogenetic and biochemical characterization of a new halo-thermotolerant, biofilm-forming Bacillus from Saline Lake of Iran.
4091	DRAMP04281	CM2	"CM2"[All Fields] AND biofilm[All Fields]	CM2	22097051	Chemical disinfection of Legionella in hot water systems biofilm: a pilot-scale 1 study.
4091	DRAMP04281	CM2	"CM2"[All Fields] AND biofilm[All Fields]	CM2	22078549	Differential Gene Expression to Investigate the Effects of Low-level Electrochemical Currents on Bacillus subtilis.
4091	DRAMP04281	CM2	"CM2"[All Fields] AND biofilm[All Fields]	CM2	22057487	Photodynamic effects of methylene blue-loaded polymeric nanoparticles on dental plaque bacteria.
4091	DRAMP04281	CM2	"CM2"[All Fields] AND biofilm[All Fields]	CM2	21457588	A psychrotrophic Burkholderia cepacia strain isolated from refrigerated raw milk showing proteolytic activity and adhesion to stainless steel.
4091	DRAMP04281	CM2	"CM2"[All Fields] AND biofilm[All Fields]	CM2	20819365	Removal of Listeria monocytogenes biofilms with bacteriophage P100.
4091	DRAMP04281	CM2	"CM2"[All Fields] AND biofilm[All Fields]	CM2	20708908	2-Methoxy-2',4'-dichloro chalcone as an antimicrofoulant against marine bacterial biofilm.
4091	DRAMP04281	CM2	"CM2"[All Fields] AND biofilm[All Fields]	CM2	20614780	Efficient degradation of lube oil by a mixed bacterial consortium.
4091	DRAMP04281	CM2	"CM2"[All Fields] AND biofilm[All Fields]	CM2	20601790	Microbial contamination of raw meat and its environment in retail shops in Karachi, Pakistan.
4091	DRAMP04281	CM2	"CM2"[All Fields] AND biofilm[All Fields]	CM2	20476646	Comparison of methods of detecting bacterial biofilm.
4091	DRAMP04281	CM2	"CM2"[All Fields] AND biofilm[All Fields]	CM2	20004967	Controlling the release of peptide antimicrobial agents from surfaces.
4091	DRAMP04281	CM2	"CM2"[All Fields] AND biofilm[All Fields]	CM2	19517725	Efficacy of sanitizing agents against Listeria monocytogenes biofilms on high-density polyethylene cutting board surfaces.
4091	DRAMP04281	CM2	"CM2"[All Fields] AND biofilm[All Fields]	CM2	19465248	Production of biofilm and quorum sensing by Escherichia coli O157:H7 and its transfer from contact surfaces to meat, poultry, ready-to-eat deli, and produce products.
4091	DRAMP04281	CM2	"CM2"[All Fields] AND biofilm[All Fields]	CM2	19375790	The influence of light-curing time on the bacterial colonization of resin composite surfaces.
4091	DRAMP04281	CM2	"CM2"[All Fields] AND biofilm[All Fields]	CM2	19295652	Biofilm evidence and the microbial diversity of horse wounds.
4091	DRAMP04281	CM2	"CM2"[All Fields] AND biofilm[All Fields]	CM2	19256370	[Effect of chloramines disinfection for biofilm formation control on copper and stainless steel pipe materials].
4091	DRAMP04281	CM2	"CM2"[All Fields] AND biofilm[All Fields]	CM2	19205478	Comparative evaluation of biofilm formation and tolerance to a chemical shock of pathogenic and nonpathogenic Escherichia coli O157:H7 strains.
4091	DRAMP04281	CM2	"CM2"[All Fields] AND biofilm[All Fields]	CM2	19195407	Comparison of the effectiveness of four organic chemoattractants towards zoospores of Ulva pertusa and macrofouling.
4091	DRAMP04281	CM2	"CM2"[All Fields] AND biofilm[All Fields]	CM2	19046755	Polysulfone ultrafiltration membranes impregnated with silver nanoparticles show improved biofouling resistance and virus removal.
4091	DRAMP04281	CM2	"CM2"[All Fields] AND biofilm[All Fields]	CM2	18939750	Rechargeable antimicrobial surface modification of polyethylene.
4091	DRAMP04281	CM2	"CM2"[All Fields] AND biofilm[All Fields]	CM2	18712536	Colorimetric assay for biofilms in wet processing conditions.
4091	DRAMP04281	CM2	"CM2"[All Fields] AND biofilm[All Fields]	CM2	18409626	Environmental influences on the partitioning and diffusion of hydrophobic organic contaminants in microbial biofilms.
4091	DRAMP04281	CM2	"CM2"[All Fields] AND biofilm[All Fields]	CM2	18389714	Influence of peroxyacetic acid and nisin and coculture with Enterococcus faecium on Listeria monocytogenes biofilm formation.
4091	DRAMP04281	CM2	"CM2"[All Fields] AND biofilm[All Fields]	CM2	18236679	Effect of surface roughness and stainless steel finish on Listeria monocytogenes attachment and biofilm formation.
4091	DRAMP04281	CM2	"CM2"[All Fields] AND biofilm[All Fields]	CM2	18236664	Effect of chemical sanitizers with and without ultrasonication on Listeria monocytogenes as a biofilm within polyvinyl chloride drain pipes.
4091	DRAMP04281	CM2	"CM2"[All Fields] AND biofilm[All Fields]	CM2	17546989	Evaluating the potential of biofilm control in water supply systems by removal of phosphorus from drinking water.
4091	DRAMP04281	CM2	"CM2"[All Fields] AND biofilm[All Fields]	CM2	19086561	Attachment of Listeria monocytogenes, Escherichia coli O157:H7 and Pseudomonas aeruginosa on food soiled plastic surfaces.
4091	DRAMP04281	CM2	"CM2"[All Fields] AND biofilm[All Fields]	CM2	17477259	Antimicrobial effects of alginate-based films containing essential oils on Listeria monocytogenes and Salmonella typhimurium present in bologna and ham.
4091	DRAMP04281	CM2	"CM2"[All Fields] AND biofilm[All Fields]	CM2	17438805	Extra-cellular polysaccharides, soluble microbial products, and natural organic matter impact on nanofiltration membranes flux decline.
4091	DRAMP04281	CM2	"CM2"[All Fields] AND biofilm[All Fields]	CM2	17369205	Biofilm ecology of wooden shelves used in ripening the French raw milk smear cheese Reblochon de Savoie.
4091	DRAMP04281	CM2	"CM2"[All Fields] AND biofilm[All Fields]	CM2	19388587	Assessment of disinfectants cleaning against bacterial biofilm of house hold water tanks.
4091	DRAMP04281	CM2	"CM2"[All Fields] AND biofilm[All Fields]	CM2	17037132	Effect of temperature and pipe material on biofilm formation and survival of Escherichia coil in used drinking water pipes: a laboratory-based study.
4091	DRAMP04281	CM2	"CM2"[All Fields] AND biofilm[All Fields]	CM2	16982278	Photodynamic therapy for endodontic disinfection.
4091	DRAMP04281	CM2	"CM2"[All Fields] AND biofilm[All Fields]	CM2	16786845	Fate of inoculated Escherichia coli O157:H7, cultured under different conditions, on fresh and decontaminated beef transitioned from vacuum to aerobic packaging.
4091	DRAMP04281	CM2	"CM2"[All Fields] AND biofilm[All Fields]	CM2	16606716	Nonstarter lactic acid bacteria biofilms and calcium lactate crystals in Cheddar cheese.
4091	DRAMP04281	CM2	"CM2"[All Fields] AND biofilm[All Fields]	CM2	16569839	Using bacteriophages to reduce formation of catheter-associated biofilms by Staphylococcus epidermidis.
4091	DRAMP04281	CM2	"CM2"[All Fields] AND biofilm[All Fields]	CM2	16453199	Biofilm colonization dynamics and its influence on the corrosion resistance of austenitic UNS S31603 stainless steel exposed to Gulf of Mexico seawater.
4091	DRAMP04281	CM2	"CM2"[All Fields] AND biofilm[All Fields]	CM2	16371335	Utilisation of a packed-bed biofilm reactor for the determination of the potential of biofilm accumulation in water systems.
4091	DRAMP04281	CM2	"CM2"[All Fields] AND biofilm[All Fields]	CM2	16335477	Removal of oral biofilm by sonic phenomena.
4091	DRAMP04281	CM2	"CM2"[All Fields] AND biofilm[All Fields]	CM2	16245722	Effect of temperature, pH, and water activity on biofilm formation by Salmonella enterica enteritidis PT4 on stainless steel surfaces as indicated by the bead vortexing method and conductance measurements.
4091	DRAMP04281	CM2	"CM2"[All Fields] AND biofilm[All Fields]	CM2	16232660	Structural analysis of a biofilm which enhances carbon steel corrosion in nutritionally poor aquatic environments.

4091	DRAMP04281	CM2	"CM2"[All Fields] AND biofilm[All Fields]	CM2	16124326	Biodegradation of alpha-pinene in model biofilms in biofilters.
4091	DRAMP04281	CM2	"CM2"[All Fields] AND biofilm[All Fields]	CM2	15812058	Quantitative detection of Listeria monocytogenes in biofilms by real-time PCR.
4091	DRAMP04281	CM2	"CM2"[All Fields] AND biofilm[All Fields]	CM2	15766950	Effects of temperature and biodegradable organic matter on control of biofilms by free chlorine in a model drinking water distribution system.
4091	DRAMP04281	CM2	"CM2"[All Fields] AND biofilm[All Fields]	CM2	15757355	Hydraulic shear stress calculation in a sequencing Batch biofilm reactor with granular biomass.
4091	DRAMP04281	CM2	"CM2"[All Fields] AND biofilm[All Fields]	CM2	15635175	Contamination of environmental surfaces by Staphylococcus aureus in a dermatological ward and its preventive measures.
4091	DRAMP04281	CM2	"CM2"[All Fields] AND biofilm[All Fields]	CM2	15528562	In vitro laser ablation of natural marine biofilms.
4091	DRAMP04281	CM2	"CM2"[All Fields] AND biofilm[All Fields]	CM2	15528550	Intracellular proliferation of Legionella pneumophila in Hartmannella vermiformis in aquatic biofilms grown on plasticized polyvinyl chloride.
4091	DRAMP04281	CM2	"CM2"[All Fields] AND biofilm[All Fields]	CM2	15528505	Use of merocyanine 540 for photodynamic inactivation of Staphylococcus aureus planktonic and biofilm cells.
4091	DRAMP04281	CM2	"CM2"[All Fields] AND biofilm[All Fields]	CM2	15508633	Efficacy of two cleaning and sanitizing combinations on Listeria monocytogenes biofilms formed at low temperature on a variety of materials in the presence of ready-to-eat meat residue.
4091	DRAMP04281	CM2	"CM2"[All Fields] AND biofilm[All Fields]	CM2	15456221	[Stages of biofilm formation by sulfate-reducing bacteria].
4091	DRAMP04281	CM2	"CM2"[All Fields] AND biofilm[All Fields]	CM2	15365858	Ultrasonic-enhanced gentamicin transport through colony biofilms of Pseudomonas aeruginosa and Escherichia coli.
4091	DRAMP04281	CM2	"CM2"[All Fields] AND biofilm[All Fields]	CM2	15303752	Contamination potential of drinking water distribution network biofilms.
4091	DRAMP04281	CM2	"CM2"[All Fields] AND biofilm[All Fields]	CM2	15276358	Antibacterial activity of polymeric substrate with surface grafted viologen moieties.
4091	DRAMP04281	CM2	"CM2"[All Fields] AND biofilm[All Fields]	CM2	15128104	Low-level detection of a bacillus anthracis simulant using Love-wave biosensors on 36 degrees YX LiTaO3.
4091	DRAMP04281	CM2	"CM2"[All Fields] AND biofilm[All Fields]	CM2	14968965	Behavior of Listeria monocytogenes in a Pseudomonas putida biofilm on a condensate-forming surface.
4091	DRAMP04281	CM2	"CM2"[All Fields] AND biofilm[All Fields]	CM2	14513382	Physiology of biofilms of thermophilic bacilli-potential consequences for cleaning.
4091	DRAMP04281	CM2	"CM2"[All Fields] AND biofilm[All Fields]	CM2	12925347	Topical chemoprevention of oral cancer with tretinoin "biofilm".
4091	DRAMP04281	CM2	"CM2"[All Fields] AND biofilm[All Fields]	CM2	12916858	Effects of chlorine level on the growth of biofilm in water pipes.
4091	DRAMP04281	CM2	"CM2"[All Fields] AND biofilm[All Fields]	CM2	12729282	Release studies of benzalkonium chloride from hydrogel in a freshwater environment.
4091	DRAMP04281	CM2	"CM2"[All Fields] AND biofilm[All Fields]	CM2	12701924	Monitoring the active sessile-colonisation of two drinking water distribution systems based on the protein contents in native biofilm samples.
4091	DRAMP04281	CM2	"CM2"[All Fields] AND biofilm[All Fields]	CM2	12430692	Biofilm formation by acid-adapted and nonadapted Listeria monocytogenes in fresh beef decontamination washings and its subsequent inactivation with sanitizers.
4091	DRAMP04281	CM2	"CM2"[All Fields] AND biofilm[All Fields]	CM2	12405407	Pilot scale treatment of textile wastewater by combined process (fluidized biofilm process-chemical coagulation-electrochemical oxidation).
4091	DRAMP04281	CM2	"CM2"[All Fields] AND biofilm[All Fields]	CM2	12391753	[Stability of microbial association in the process of industrial biofiltration cleaning of gaseous discharges].
4091	DRAMP04281	CM2	"CM2"[All Fields] AND biofilm[All Fields]	CM2	12117244	Effectiveness of chemical sanitizers against Campylobacter jejuni-containing biofilms.
4091	DRAMP04281	CM2	"CM2"[All Fields] AND biofilm[All Fields]	CM2	12117243	Survival of Campylobacter jejuni in biofilms isolated from chicken houses.
4091	DRAMP04281	CM2	"CM2"[All Fields] AND biofilm[All Fields]	CM2	12079060	Evaluation of chemical disinfectants for the elimination of Salmonella biofilms from poultry transport containers.
4091	DRAMP04281	CM2	"CM2"[All Fields] AND biofilm[All Fields]	CM2	12018592	Minimum effective release rate of antifoulants.1. Effects of zosteric acid at Ford Island, Hawaii (USA).
4091	DRAMP04281	CM2	"CM2"[All Fields] AND biofilm[All Fields]	CM2	11996332	Method for the measurement of the diffusion coefficient of benzalkonium chloride.
4091	DRAMP04281	CM2	"CM2"[All Fields] AND biofilm[All Fields]	CM2	11939295	Hydrocarbon effects on fouling assemblages: the importance of taxonomic differences, seasonal, and tidal variation.
4091	DRAMP04281	CM2	"CM2"[All Fields] AND biofilm[All Fields]	CM2	11929140	Colonization-resistant antimicrobial-coated peritoneal dialysis catheters: evaluation in a newly developed rat model of persistent Pseudomonas aeruginosa peritonitis.
4091	DRAMP04281	CM2	"CM2"[All Fields] AND biofilm[All Fields]	CM2	11846079	Phosphorus uptake into algal biofilms in a lowland chalk river.
4091	DRAMP04281	CM2	"CM2"[All Fields] AND biofilm[All Fields]	CM2	11791835	The potential for biofilm growth in water distribution systems.
4091	DRAMP04281	CM2	"CM2"[All Fields] AND biofilm[All Fields]	CM2	11547890	Bacteriophage T4 multiplication in a glucose-limited Escherichia coli biofilm.
4091	DRAMP04281	CM2	"CM2"[All Fields] AND biofilm[All Fields]	CM2	11503211	The use of glass wool as an attachment surface for studying phenotypic changes in Pseudomonas aeruginosa biofilms by two-dimensional gel electrophoresis.
4091	DRAMP04281	CM2	"CM2"[All Fields] AND biofilm[All Fields]	CM2	11460287	Disinfection of dental unit waterlines with an oral antiseptic.
4091	DRAMP04281	CM2	"CM2"[All Fields] AND biofilm[All Fields]	CM2	11447890	Reviewing efficacy of alternative water treatment techniques.
4091	DRAMP04281	CM2	"CM2"[All Fields] AND biofilm[All Fields]	CM2	11424733	Enhancement in the treatment efficiency and conversion to energy of dairy wastewaters by augmenting CST reactors with simple biofilm support systems.
4091	DRAMP04281	CM2	"CM2"[All Fields] AND biofilm[All Fields]	CM2	11360505	[Inhibitory activity of azithromycin on biofilm synthesis and synergism between azithromycin and fleroxacin on Pseudomonas aeruginosa in biofilms].
4091	DRAMP04281	CM2	"CM2"[All Fields] AND biofilm[All Fields]	CM2	11294359	Biofilm formation by salmonella spp. on food contact surfaces and their sensitivity to sanitizers.
4091	DRAMP04281	CM2	"CM2"[All Fields] AND biofilm[All Fields]	CM2	11246704	The dentogingival epithelial surface area revisited.
4091	DRAMP04281	CM2	"CM2"[All Fields] AND biofilm[All Fields]	CM2	11139010	Comparative evaluation of adhesion and biofilm formation of different Listeria monocytogenes strains.
4091	DRAMP04281	CM2	"CM2"[All Fields] AND biofilm[All Fields]	CM2	10889916	Inhibition of microflora associated with oral malignancy.
4091	DRAMP04281	CM2	"CM2"[All Fields] AND biofilm[All Fields]	CM2	10716621	Electrical enhancement of Streptococcus gordonii biofilm killing by gentamicin.
4091	DRAMP04281	CM2	"CM2"[All Fields] AND biofilm[All Fields]	CM2	10582187	Quantitative determination of endotoxins released by bacterial biofilms.
4091	DRAMP04281	CM2	"CM2"[All Fields] AND biofilm[All Fields]	CM2	10419269	Inactivation of Listeria monocytogenes/Pseudomonas biofilms by peracid sanitizers.
4091	DRAMP04281	CM2	"CM2"[All Fields] AND biofilm[All Fields]	CM2	10223938	Ultrasonic enhancement of antibiotic action on Escherichia coli biofilms: an in vivo model.
4091	DRAMP04281	CM2	"CM2"[All Fields] AND biofilm[All Fields]	CM2	9919033	Dental unit waterlines: biofilms, disinfection and recurrence.
4091	DRAMP04281	CM2	"CM2"[All Fields] AND biofilm[All Fields]	CM2	9709259	Effect of several decontamination procedures on Listeria monocytogenes growing in biofilms.
4091	DRAMP04281	CM2	"CM2"[All Fields] AND biofilm[All Fields]	CM2	9684698	Evidence of bacterial biofilm in tubing from hydraulic pathway of hemodialysis system.
4091	DRAMP04281	CM2	"CM2"[All Fields] AND biofilm[All Fields]	CM2	9678162	Attachment of Escherichia coli O157:H7 in ground beef to meat grinders and survival after sanitation with chlorine and peroxyacetic acid.
4091	DRAMP04281	CM2	"CM2"[All Fields] AND biofilm[All Fields]	CM2	9468041	Adhesion of Staphylococcus epidermidis and transposon mutant strains to hydrophobic polyethylene.
4091	DRAMP04281	CM2	"CM2"[All Fields] AND biofilm[All Fields]	CM2	9438904	Changes in Staphylococcus aureus density and lesion severity after topical application of povidone-iodine in cases of atopic dermatitis.
4091	DRAMP04281	CM2	"CM2"[All Fields] AND biofilm[All Fields]	CM2	9270918	Automatic washer disinfector for flexible endoscopes: a new evaluation process.
4091	DRAMP04281	CM2	"CM2"[All Fields] AND biofilm[All Fields]	CM2	11540454	Microbial colonization of a closed growth chamber during hydroponic cultivation of lettuce.
4091	DRAMP04281	CM2	"CM2"[All Fields] AND biofilm[All Fields]	CM2	9124740	The effect of ultrasonic frequency upon enhanced killing of P. aeruginosa biofilms.
4091	DRAMP04281	CM2	"CM2"[All Fields] AND biofilm[All Fields]	CM2	9116358	In vitro and in vivo effects of rifampin on Staphylococcus epidermidis graft infections.
4091	DRAMP04281	CM2	"CM2"[All Fields] AND biofilm[All Fields]	CM2	9023646	The efficacy of antibiotics enhanced by electrical currents against Pseudomonas aeruginosa biofilms.
4091	DRAMP04281	CM2	"CM2"[All Fields] AND biofilm[All Fields]	CM2	10463441	Monolaurin and acetic acid inactivation of Listeria monocytogenes attached to stainless steel.
4091	DRAMP04281	CM2	"CM2"[All Fields] AND biofilm[All Fields]	CM2	8658041	A bacteriologic and scanning electron microscope study after implantation of foreign bodies in the biliary tract in rats.

4091	DRAMP04281	CM2	"CM2"[All Fields] AND biofilm[All Fields]	CM2	7492284	Immunosuppression augments growth of graft-adherent Staphylococcus epidermidis.
4091	DRAMP04281	CM2	"CM2"[All Fields] AND biofilm[All Fields]	CM2	7579313	Dental units: an environmental study of sources of potentially pathogenic mycobacteria.
4091	DRAMP04281	CM2	"CM2"[All Fields] AND biofilm[All Fields]	CM2	7641146	Bacterial succession within a biofilm in water supply lines of dental air-water syringes.
4091	DRAMP04281	CM2	"CM2"[All Fields] AND biofilm[All Fields]	CM2	7577354	Influence of culture conditions on biofilm formation by Escherichia coli O157:H7.
4091	DRAMP04281	CM2	"CM2"[All Fields] AND biofilm[All Fields]	CM2	7654358	Development of a flow method for susceptibility testing of oral biofilms in vitro.
4091	DRAMP04281	CM2	"CM2"[All Fields] AND biofilm[All Fields]	CM2	7695266	Mechanism of electrical enhancement of efficacy of antibiotics in killing biofilm bacteria.
4091	DRAMP04281	CM2	"CM2"[All Fields] AND biofilm[All Fields]	CM2	7986678	Effect of trisodium phosphate on biofilm and planktonic cells of Campylobacter jejuni, Escherichia coli O157: H7, Listeria monocytogenes and Salmonella typhimurium.
4091	DRAMP04281	CM2	"CM2"[All Fields] AND biofilm[All Fields]	CM2	8174442	Hydrophobic bile salt inhibits bacterial adhesion on biliary stent material.
4091	DRAMP04281	CM2	"CM2"[All Fields] AND biofilm[All Fields]	CM2	8170137	The natural history of bacterial biofilm graft infection.
4091	DRAMP04281	CM2	"CM2"[All Fields] AND biofilm[All Fields]	CM2	7478748	Characteristics of accumulation of oral gram-positive bacteria on mucin-conditioned glass surfaces in a model system.
4091	DRAMP04281	CM2	"CM2"[All Fields] AND biofilm[All Fields]	CM2	8270705	A comparison of ozonation and chlorination for the disinfection of stainless steel surfaces.
4091	DRAMP04281	CM2	"CM2"[All Fields] AND biofilm[All Fields]	CM2	1482196	Electrical enhancement of biocide efficacy against Pseudomonas aeruginosa biofilms.
4091	DRAMP04281	CM2	"CM2"[All Fields] AND biofilm[All Fields]	CM2	1457842	Prevention and control of bacterial infections associated with medical devices.
4091	DRAMP04281	CM2	"CM2"[All Fields] AND biofilm[All Fields]	CM2	1393831	Eradication of biofilm cells of Staphylococcus aureus with tobramycin and cephalexin.
4091	DRAMP04281	CM2	"CM2"[All Fields] AND biofilm[All Fields]	CM2	1613780	Mechanism of persistent infection associated with peritoneal implants.
4091	DRAMP04281	CM2	"CM2"[All Fields] AND biofilm[All Fields]	CM2	1421346	Occurrence of mycobacteria in biofilm samples.
4091	DRAMP04281	CM2	"CM2"[All Fields] AND biofilm[All Fields]	CM2	1622218	Persistence of Vibrio vulnificus in tissues of Gulf Coast oysters, Crassostrea virginica, exposed to seawater disinfected with UV light.
4091	DRAMP04281	CM2	"CM2"[All Fields] AND biofilm[All Fields]	CM2	1346815	Prophylactic antibiotics prevent bacterial biofilm graft infection.
4091	DRAMP04281	CM2	"CM2"[All Fields] AND biofilm[All Fields]	CM2	1908382	Activity of antiseptics against biofilms of mixed bacterial species growing on silicone surfaces.
4091	DRAMP04281	CM2	"CM2"[All Fields] AND biofilm[All Fields]	CM2	1905626	Activity of antiseptics against biofilms of mixed bacterial species growing on silicone surfaces.
4091	DRAMP04281	CM2	"CM2"[All Fields] AND biofilm[All Fields]	CM2	2513307	Tobramycin resistance of mucoid Pseudomonas aeruginosa biofilm grown under iron limitation.
4091	DRAMP04281	CM2	"CM2"[All Fields] AND biofilm[All Fields]	CM2	2667558	Mycobacteria in biofilms.
4091	DRAMP04281	CM2	"CM2"[All Fields] AND biofilm[All Fields]	CM2	2524605	Identification of Staphylococcus epidermidis vascular graft infections: a comparison of culture techniques.
4091	DRAMP04281	CM2	"CM2"[All Fields] AND biofilm[All Fields]	CM2	2961893	Infection of vascular prostheses caused by bacterial biofilms.
4091	DRAMP04281	CM2	"CM2"[All Fields] AND biofilm[All Fields]	CM2	3435100	Method of evaluating effects of antibiotics on bacterial biofilm.
4091	DRAMP04281	CM2	"CM2"[All Fields] AND biofilm[All Fields]	CM2	3619420	Effect of vancomycin hydrochloride on Staphylococcus epidermidis biofilm associated with silicone elastomer.
4091	DRAMP04281	CM2	"CM2"[All Fields] AND biofilm[All Fields]	CM2	3101319	A comparison of media for the enumeration of filamentous fungi from aqueduct biofilm.
4091	DRAMP04281	CM2	"CM2"[All Fields] AND biofilm[All Fields]	CM2	4083889	Degradation of phenol by Pseudomonas putida ATCC 11172 in continuous culture at different ratios of biofilm surface to culture volume.
4091	DRAMP04281	CM2	"CM2"[All Fields] AND biofilm[All Fields]	CM2	6847180	Microbial fouling of reverse-osmosis membranes used in advanced wastewater treatment technology: chemical, bacteriological, and ultrastructural analyses.
4092	DRAMP04282	CM3	"CM3"[All Fields] AND biofilm[All Fields]	CM3	33571231	Interactive effects of discharge reduction and fine sediments on stream biofilm metabolism.
4092	DRAMP04282	CM3	"CM3"[All Fields] AND biofilm[All Fields]	CM3	27196401	Simultaneous Bioreduction of Multiple Oxidized Contaminants Using a Membrane Biofilm Reactor.
4092	DRAMP04282	CM3	"CM3"[All Fields] AND biofilm[All Fields]	CM3	26040729	Denitrification and biofilm growth in a pilot-scale biofilter packed with suspended carriers for biological nitrogen removal from secondary effluent.
4092	DRAMP04282	CM3	"CM3"[All Fields] AND biofilm[All Fields]	CM3	25612117	The use of photodynamic therapy to treat hidradenitis suppurativa a review and critical analysis.
4092	DRAMP04282	CM3	"CM3"[All Fields] AND biofilm[All Fields]	CM3	24350448	A flat microbial fuel cell for decentralized wastewater valorization: process performance and optimization potential.
4092	DRAMP04282	CM3	"CM3"[All Fields] AND biofilm[All Fields]	CM3	20623853	[Microbial biomass and activity in a full-scale O3-BAC filter].
4092	DRAMP04282	CM3	"CM3"[All Fields] AND biofilm[All Fields]	CM3	16599118	[Improvement of PVC bio-carrier surface property by remote plasma].
4092	DRAMP04282	CM3	"CM3"[All Fields] AND biofilm[All Fields]	CM3	12578173	Effect of dissolved oxygen concentration on the biofilm and in situ analysis by fluorescence in situ hybridization (FISH) and microelectrodes.
4092	DRAMP04282	CM3	"CM3"[All Fields] AND biofilm[All Fields]	CM3	8039664	Agar-entrapped bacteria as an in vitro model of biofilms and their susceptibility to antibiotics.
4092	DRAMP04282	CM3	"CM3"[All Fields] AND biofilm[All Fields]	CM3	1433574	Microbial adhesion and biofilm formation on ureteral stents in vitro and in vivo.
4093	DRAMP04283	CM4	"CM4"[All Fields] AND biofilm[All Fields]	CM4	30386734	A new functional biofilm biocatalyst for the simultaneous removal of dibenzothiophene and quinoline using Rhodococcus rhodochrousand curli amyloid overproducer mutants derived from Cobetiasp. strain MM1IDA2H-1.
4094	DRAMP04284	CM5	"CM5"[All Fields] AND biofilm[All Fields]	CM5	19751522	malT knockout mutation invokes a stringent type gene-expression profile in Actinobacillus pleuropneumoniae in bronchoalveolar fluid.
4105	DRAMP04295	MB-10	"MB-10"[All Fields] AND biofilm[All Fields]	MB-10	28435253	Antimicrobial photodynamic activity and cytocompatibility of Au 25(Capt) 18clusters photoexcited by blue LED light irradiation.
4126	DRAMP04316	MB-50	"MB-50"[All Fields] AND biofilm[All Fields]	MB-50	22057487	Photodynamic effects of methylene blue-loaded polymeric nanoparticles on dental plaque bacteria.
4126	DRAMP04316	MB-50	"MB-50"[All Fields] AND biofilm[All Fields]	MB-50	20649431	Comparative study of methylene blue and erythrosine dyes employed in photodynamic therapy for inactivation of planktonic and biofilm-cultivated Aggregatibacter actinomycetemcomitans.
4127	DRAMP04317	DASamP1	"DASamP1"[All Fields] AND biofilm[All Fields]	DASamP1	22445495	Database screening and in vivo efficacy of antimicrobial peptides against methicillin-resistant Staphylococcus aureus USA300.
4128	DRAMP04318	DASamP2	"DASamP2"[All Fields] AND biofilm[All Fields]	DASamP2	28672834	Individual and Combined Effects of Engineered Peptides and Antibiotics on Pseudomonas aeruginosa Biofilms.
4128	DRAMP04318	DASamP2	"DASamP2"[All Fields] AND biofilm[All Fields]	DASamP2	22445495	Database screening and in vivo efficacy of antimicrobial peptides against methicillin-resistant Staphylococcus aureus USA300.
4157	DRAMP04347	Buforin	"Buforin"[All Fields] AND biofilm[All Fields]	Buforin	29959905	Antimicrobial and anti-inflammatory activities of chemokine CXCL14-derived antimicrobial peptide and its analogs.
4157	DRAMP04347	Buforin	"Buforin"[All Fields] AND biofilm[All Fields]	Buforin	26684017	Antimicrobial Peptides as Anti-Infectives against Staphylococcus epidermidis.
4157	DRAMP04347	Buforin	"Buforin"[All Fields] AND biofilm[All Fields]	Buforin	25767037	Cationic amphipathic peptides KT2 and RT2 are taken up into bacterial cells and kill planktonic and biofilm bacteria.
4157	DRAMP04347	Buforin	"Buforin"[All Fields] AND biofilm[All Fields]	Buforin	19041293	Buforins: histone H2A-derived antimicrobial peptides from toad stomach.
4166	DRAMP04356	CAD	"CAD"[All Fields] AND biofilm[All Fields]	CAD	34124315	Inkjet 3D Printing of Polymers Resistant to Fungal Attachment.
4166	DRAMP04356	CAD	"CAD"[All Fields] AND biofilm[All Fields]	CAD	34067261	Bacteriological Evaluation of Gingival Crevicular Fluid in Teeth Restored Using Fixed Dental Prostheses: An In Vivo Study.
4166	DRAMP04356	CAD	"CAD"[All Fields] AND biofilm[All Fields]	CAD	33810381	Fibrin Biopolymer Incorporated with Antimicrobial Agents: A Proposal for Coating Denture Bases.
4166	DRAMP04356	CAD	"CAD"[All Fields] AND biofilm[All Fields]	CAD	33662064	Influence Of Surface Treatment On The Physical Properties And Biofilm Formation Of Zirconia-Reinforced Lithium Silicate Ceramics: In Vitro Trial.
4166	DRAMP04356	CAD	"CAD"[All Fields] AND biofilm[All Fields]	CAD	32924224	The Influence of Surface Roughening and Polishing on Microbial Biofilm Development on Different Ceramic Materials.
4166	DRAMP04356	CAD	"CAD"[All Fields] AND biofilm[All Fields]	CAD	32827220	Effect of erosive and abrasive challenges on the glaze layer applied to ceramic materials.
4166	DRAMP04356	CAD	"CAD"[All Fields] AND biofilm[All Fields]	CAD	32750671	Influence of different ceramic materials and surface treatments on the adhesion of Prevotella intermedia.

4166	DRAMP04356	CAD	"CAD"[All Fields] AND biofilm[All Fields]	CAD	32259408	Effect of dental restorative materials surface roughness on the in vitro biofilm formation of Streptococcus mutans biofilm.
4166	DRAMP04356	CAD	"CAD"[All Fields] AND biofilm[All Fields]	CAD	32238271	Resin composite blocks for dental CAD/CAM applications reduce biofilm formation in vitro.
4166	DRAMP04356	CAD	"CAD"[All Fields] AND biofilm[All Fields]	CAD	32210139	Activity of Cinnamaldehyde on Quorum Sensing and Biofilm Susceptibility to Antibiotics in Pseudomonas aeruginosa.
4166	DRAMP04356	CAD	"CAD"[All Fields] AND biofilm[All Fields]	CAD	31955016	Biofilm formation of clinically important microorganisms on 2D and 3D poly (methyl methacrylate) substrates: A surface-enhanced Raman scattering study.
4166	DRAMP04356	CAD	"CAD"[All Fields] AND biofilm[All Fields]	CAD	31309119	Study of Surface Structure Changes for Selected Ceramics Used in the CAD/CAM System on the Degree of Microbial Colonization, In VitroTests.
4166	DRAMP04356	CAD	"CAD"[All Fields] AND biofilm[All Fields]	CAD	31301673	Is there a Function for a Sex Pheromone Precursor?
4166	DRAMP04356	CAD	"CAD"[All Fields] AND biofilm[All Fields]	CAD	31200680	Influence of margin location and luting material on the amount of undetected cement excess on CAD/CAM implant abutments and cement-retained zirconia crowns: an in-vitro study.
4166	DRAMP04356	CAD	"CAD"[All Fields] AND biofilm[All Fields]	CAD	30778609	Potential impact of lingual retainers on oral health: comparison between conventional twistflex retainers and CAD/CAM fabricated nitinol retainers : A clinical in vitro and in vivo investigation.
4166	DRAMP04356	CAD	"CAD"[All Fields] AND biofilm[All Fields]	CAD	30220506	Biofilm formation on restorative materials and resin composite cements.
4166	DRAMP04356	CAD	"CAD"[All Fields] AND biofilm[All Fields]	CAD	29993359	Cinnamaldehyde disrupts biofilm formation and swarming motility of Pseudomonas aeruginosa.
4166	DRAMP04356	CAD	"CAD"[All Fields] AND biofilm[All Fields]	CAD	29983233	Sequence-based identification, genotyping and virulence factors of Trichosporon asahii strains isolated from urine samples of hospitalized patients (2011-2016).
4166	DRAMP04356	CAD	"CAD"[All Fields] AND biofilm[All Fields]	CAD	29856699	Effects of Manufacturing and Finishing Techniques of Feldspathic Ceramics on Surface Topography, Biofilm Formation, and Cell Viability for Human Gingival Fibroblasts.
4166	DRAMP04356	CAD	"CAD"[All Fields] AND biofilm[All Fields]	CAD	29473188	Influence of CAD/CAM fabrication on denture surface properties.
4166	DRAMP04356	CAD	"CAD"[All Fields] AND biofilm[All Fields]	CAD	28804719	Effect of 2 Bleaching Agents with a Content of High Concentrated Hydrogen Peroxide on Stained 2 CAD/CAM Blocks and a Nanohybrid Composite Resin: An AFM Evaluation.
4166	DRAMP04356	CAD	"CAD"[All Fields] AND biofilm[All Fields]	CAD	28475309	Simbiotics: A Multiscale Integrative Platform for 3D Modeling of Bacterial Populations.
4166	DRAMP04356	CAD	"CAD"[All Fields] AND biofilm[All Fields]	CAD	27986731	Novel Cadmium Resistance Determinant in Listeria monocytogenes.
4166	DRAMP04356	CAD	"CAD"[All Fields] AND biofilm[All Fields]	CAD	26096116	Prevalence of eight putative periodontal pathogens in atherosclerotic plaque of coronary artery disease patients and comparing them with noncardiac subjects: A case-control study.
4166	DRAMP04356	CAD	"CAD"[All Fields] AND biofilm[All Fields]	CAD	25575200	Bonding of Adhesive Luting Agents to Caries-affected Dentin Induced by a Microcosm Biofilm Model.
4166	DRAMP04356	CAD	"CAD"[All Fields] AND biofilm[All Fields]	CAD	25323985	A biofilm cariogenic challenge model for dentin demineralization and dentin bonding analysis.
4166	DRAMP04356	CAD	"CAD"[All Fields] AND biofilm[All Fields]	CAD	24939258	Obtaining artificially caries-affected dentin for in vitro studies.
4166	DRAMP04356	CAD	"CAD"[All Fields] AND biofilm[All Fields]	CAD	24010954	The effect of periodontal therapy on C-reactive protein, endothelial function, lipids and proinflammatory biomarkers in patients with stable coronary artery disease: study protocol for a randomized controlled trial.
4166	DRAMP04356	CAD	"CAD"[All Fields] AND biofilm[All Fields]	CAD	20584976	A pathoadaptive deletion in an enteroaggregative Escherichia coli outbreak strain enhances virulence in a Caenorhabditis elegans model.
4166	DRAMP04356	CAD	"CAD"[All Fields] AND biofilm[All Fields]	CAD	20566904	Association of adenoid surface biofilm formation and chronic otitis media with effusion.
4168	DRAMP04358	CEMA(MBI-28)	"CEMA"[All Fields] AND biofilm[All Fields]	CEMA	33464241	Synthesis and antibacterial activity of polymer-antibiotic conjugates incorporated into a resin-based dental adhesive.
4168	DRAMP04358	CEMA(MBI-28)	"CEMA"[All Fields] AND biofilm[All Fields]	CEMA	31111385	Bacterial community structure in rotating biological contactor treating coke wastewater in relation to medium composition.
4168	DRAMP04358	CEMA(MBI-28)	"CEMA"[All Fields] AND biofilm[All Fields]	CEMA	26942544	The assessment of the coke wastewater treatment efficacy in rotating biological contractor.
4168	DRAMP04358	CEMA(MBI-28)	"CEMA"[All Fields] AND biofilm[All Fields]	CEMA	24509500	Novel two-component regulatory systems play a role in biofilm formation of Lactobacillus reuteri rodent isolate 100-23.
4168	DRAMP04358	CEMA(MBI-28)	"CEMA"[All Fields] AND biofilm[All Fields]	CEMA	22214045	Dissolved oxygen as a factor influencing nitrogen removal rates in a one-stage system with partial nitritation and Anammox process.
4168	DRAMP04358	CEMA(MBI-28)	"CEMA"[All Fields] AND biofilm[All Fields]	CEMA	17546965	A one-stage system with partial nitritation and anammox processes in the moving-bed biofilm reactor.
4168	DRAMP04358	CEMA(MBI-28)	"CEMA"[All Fields] AND biofilm[All Fields]	CEMA	17163030	Nitrogen removal rates at a technical-scale pilot plant with the one-stage partial nitritation/Anammox process.
4176	DRAMP04398	Antifungal protein (PAF)	"Antifungal protein"[All Fields] AND biofilm[All Fields]	Antifungal protein	33466640	The Neosartorya fischeriAntifungal Protein 2 (NFAP2): A New Potential Weapon against Multidrug-Resistant Candida aurisBiofilms.
4176	DRAMP04398	Antifungal protein (PAF)	"Antifungal protein"[All Fields] AND biofilm[All Fields]	Antifungal protein	32824977	The Penicillium chrysogenumQ176 Antimicrobial Protein PAFC Effectively Inhibits the Growth of the Opportunistic Human Pathogen Candida albicans.
4176	DRAMP04398	Antifungal protein (PAF)	"Antifungal protein"[All Fields] AND biofilm[All Fields]	Antifungal protein	30478163	In VivoApplicability of Neosartorya fischeri Antifungal Protein 2 (NFAP2) in Treatment of Vulvovaginal Candidiasis.
4176	DRAMP04398	Antifungal protein (PAF)	"Antifungal protein"[All Fields] AND biofilm[All Fields]	Antifungal protein	30079061	The Evolutionary Conserved γ-Core Motif Influences the Anti- CandidaActivity of the Penicillium chrysogenumAntifungal Protein PAF.
4176	DRAMP04398	Antifungal protein (PAF)	"Antifungal protein"[All Fields] AND biofilm[All Fields]	Antifungal protein	28120548	DS6: anticandidal, antibiofilm peptide against Candida tropicalis and exhibit synergy with commercial drug.
4190	DRAMP04412	Defensin	"Defensin"[All Fields] AND biofilm[All Fields]	Defensin	34408988	The Anti-Biofilm Efficacy of Caffeic Acid Phenethyl Ester (CAPE) In Vitroand a Murine Model of Oral Candidiasis.
4190	DRAMP04412	Defensin	"Defensin"[All Fields] AND biofilm[All Fields]	Defensin	34321877	Antibacterial Effect of Honey-Derived Exosomes Containing Antimicrobial Peptides Against Oral Streptococci.
4190	DRAMP04412	Defensin	"Defensin"[All Fields] AND biofilm[All Fields]	Defensin	34276631	DNA Blocks the Lethal Effect of Human Beta-Defensin 2 Against Neisseria meningitidis.
4190	DRAMP04412	Defensin	"Defensin"[All Fields] AND biofilm[All Fields]	Defensin	33911935	The antibacterial activities of honey.
4190	DRAMP04412	Defensin	"Defensin"[All Fields] AND biofilm[All Fields]	Defensin	33865931	Identification of a crocodylian β-defensin variant from Alligator mississippiensis with antimicrobial and antibiofilm activity.
4190	DRAMP04412	Defensin	"Defensin"[All Fields] AND biofilm[All Fields]	Defensin	33586659	Identification of anti-microbial peptides and traces of microbial DNA in infrainfundibular compartments of human scalp terminal hair follicles.
4190	DRAMP04412	Defensin	"Defensin"[All Fields] AND biofilm[All Fields]	Defensin	33534018	A recombinant fungal defensin-like peptide-P2 combats Streptococcus dysgalactiae and biofilms.
4190	DRAMP04412	Defensin	"Defensin"[All Fields] AND biofilm[All Fields]	Defensin	33447687	Future directions of postoperative spinal implant infections.
4190	DRAMP04412	Defensin	"Defensin"[All Fields] AND biofilm[All Fields]	Defensin	33420317	Curbing gastrointestinal infections by defensin fragment modifications without harming commensal microbiota.
4190	DRAMP04412	Defensin	"Defensin"[All Fields] AND biofilm[All Fields]	Defensin	32867384	A Novel Peptide Antibiotic, Pro10-1D, Designed from Insect Defensin Shows Antibacterial and Anti-Inflammatory Activities in Sepsis Models.
4190	DRAMP04412	Defensin	"Defensin"[All Fields] AND biofilm[All Fields]	Defensin	32858856	Candida albicansVirulence Factors and Pathogenicity for Endodontic Infections.
4190	DRAMP04412	Defensin	"Defensin"[All Fields] AND biofilm[All Fields]	Defensin	32842903	Electrospun ZnO/Poly(Vinylidene Fluoride-Trifluoroethylene) Scaffolds for Lung Tissue Engineering.
4190	DRAMP04412	Defensin	"Defensin"[All Fields] AND biofilm[All Fields]	Defensin	32663201	Inhibition and eradication activity of truncated α-defensin analogs against multidrug resistant uropathogenic Escherichia coli biofilm.
4190	DRAMP04412	Defensin	"Defensin"[All Fields] AND biofilm[All Fields]	Defensin	32585445	Glucose effect on Candida albicans biofilm during tissue invasion.
4190	DRAMP04412	Defensin	"Defensin"[All Fields] AND biofilm[All Fields]	Defensin	32522780	Controlling the Growth of the Skin Commensal Staphylococcus epidermidis Using d-Alanine Auxotrophy.
4190	DRAMP04412	Defensin	"Defensin"[All Fields] AND biofilm[All Fields]	Defensin	32457749	The Antimicrobial Peptide Human Beta-Defensin 2 Inhibits Biofilm Production of Pseudomonas aeruginosaWithout Compromising Metabolic Activity.
4190	DRAMP04412	Defensin	"Defensin"[All Fields] AND biofilm[All Fields]	Defensin	32439511	Antibacterial activities and mechanisms of action of a defensin from manila clam Ruditapes philippinarum.
4190	DRAMP04412	Defensin	"Defensin"[All Fields] AND biofilm[All Fields]	Defensin	31933178	Effects of human β-defensin 3 fused with carbohydrate-binding domain on the function of type III secretion system in Pseudomonas aeruginosa PA14.
4190	DRAMP04412	Defensin	"Defensin"[All Fields] AND biofilm[All Fields]	Defensin	31906541	Role of FAD-I in Fusobacterial Interspecies Interaction and Biofilm Formation.
4190	DRAMP04412	Defensin	"Defensin"[All Fields] AND biofilm[All Fields]	Defensin	31729441	Rhesus Theta Defensin 1 Promotes Long Term Survival in Systemic Candidiasis by Host Directed Mechanisms.
4190	DRAMP04412	Defensin	"Defensin"[All Fields] AND biofilm[All Fields]	Defensin	31336838	Candida albicans-Cell Interactions Activate Innate Immune Defense in Human Palate Epithelial Primary Cells via Nitric Oxide (NO) and β-Defensin 2 (hBD-2).
4190	DRAMP04412	Defensin	"Defensin"[All Fields] AND biofilm[All Fields]	Defensin	31165072	Impact of the Food Additive Titanium Dioxide (E171) on Gut Microbiota-Host Interaction.
4190	DRAMP04412	Defensin	"Defensin"[All Fields] AND biofilm[All Fields]	Defensin	31031739	Salt-Tolerant Antifungal and Antibacterial Activities of the Corn Defensin ZmD32.

4190	DRAMP04412	Defensin	"Defensin"[All Fields] AND biofilm[All Fields]	Defensin	31025073	A recombinant fungal defensin-like peptide-P2 combats multidrug-resistant Staphylococcus aureus and biofilms.
4190	DRAMP04412	Defensin	"Defensin"[All Fields] AND biofilm[All Fields]	Defensin	30659503	Lactobacillus plantarum USM8613 Aids in Wound Healing and Suppresses Staphylococcus aureus Infection at Wound Sites.
4190	DRAMP04412	Defensin	"Defensin"[All Fields] AND biofilm[All Fields]	Defensin	30649289	Innate immune components affect growth and virulence traits of bacterial-vaginosis-associated and non-bacterial-vaginosis-associated Gardnerella vaginalis strains similarly.
4190	DRAMP04412	Defensin	"Defensin"[All Fields] AND biofilm[All Fields]	Defensin	30376742	Alpha defensin, leukocyte esterase, C-reactive protein, and leukocyte count in synovial fluid for pre-operative diagnosis of periprosthetic infection.
4190	DRAMP04412	Defensin	"Defensin"[All Fields] AND biofilm[All Fields]	Defensin	30260708	Stabilized collagen matrix dressing improves wound macrophage function and epithelialization.
4190	DRAMP04412	Defensin	"Defensin"[All Fields] AND biofilm[All Fields]	Defensin	30254440	Modification of the surface of titanium with multifunctional chimeric peptides to prevent biofilm formation via inhibition of initial colonizers.
4190	DRAMP04412	Defensin	"Defensin"[All Fields] AND biofilm[All Fields]	Defensin	29902560	A defensin-like antimicrobial peptide from the manila clam Ruditapes philippinarum: Investigation of the antibacterial activities and mode of action.
4190	DRAMP04412	Defensin	"Defensin"[All Fields] AND biofilm[All Fields]	Defensin	29872295	Antimicrobial peptide-loaded liquid crystalline precursor bioadhesive system for the prevention of dental caries.
4190	DRAMP04412	Defensin	"Defensin"[All Fields] AND biofilm[All Fields]	Defensin	29671721	The BceABRS four-component system that is essential for cell envelope stress response is involved in sensing and response to host defence peptides and is required for the biofilm formation and fitness of Streptococcus mutans.
4190	DRAMP04412	Defensin	"Defensin"[All Fields] AND biofilm[All Fields]	Defensin	29104569	A Linear 19-Mer Plant Defensin-Derived Peptide Acts Synergistically with Caspofungin against Candida albicansBiofilms.
4190	DRAMP04412	Defensin	"Defensin"[All Fields] AND biofilm[All Fields]	Defensin	29077172	The significance of HBD-3 and fluorescent composite carriers in the processof bone formation in rats infected with Staphylococcus aureus.
4190	DRAMP04412	Defensin	"Defensin"[All Fields] AND biofilm[All Fields]	Defensin	29045084	New Approach to Treat and Prevent Oral Disease.
4190	DRAMP04412	Defensin	"Defensin"[All Fields] AND biofilm[All Fields]	Defensin	29025642	Engineered chimeric peptides with antimicrobial and titanium-binding functions to inhibit biofilm formation on Ti implants.
4190	DRAMP04412	Defensin	"Defensin"[All Fields] AND biofilm[All Fields]	Defensin	28956355	The synthetic human beta-defensin-3 C15 peptide exhibits antimicrobial activity against Streptococcus mutans, both alone and in combination with dental disinfectants.
4190	DRAMP04412	Defensin	"Defensin"[All Fields] AND biofilm[All Fields]	Defensin	28951032	Antifungal Effects of Synthetic Human Beta-defensin-3-C15 Peptide on Candida albicans-infected Root Dentin.
4190	DRAMP04412	Defensin	"Defensin"[All Fields] AND biofilm[All Fields]	Defensin	28874606	Bacterial d-amino acids suppress sinonasal innate immunity through sweet taste receptors in solitary chemosensory cells.
4190	DRAMP04412	Defensin	"Defensin"[All Fields] AND biofilm[All Fields]	Defensin	28725299	Role of Streptococcus mutanstwo-component systems in antimicrobial peptide resistance in the oral cavity.
4190	DRAMP04412	Defensin	"Defensin"[All Fields] AND biofilm[All Fields]	Defensin	28649561	Psd1 Effects on Candida albicansPlanktonic Cells and Biofilms.
4190	DRAMP04412	Defensin	"Defensin"[All Fields] AND biofilm[All Fields]	Defensin	28642103	Chimeric analogs of human β-defensin 1 and θ-defensin disrupt pre-established bacterial biofilms.
4190	DRAMP04412	Defensin	"Defensin"[All Fields] AND biofilm[All Fields]	Defensin	28413476	The mechanism of human β-defensin 3 in MRSA-induced infection of implant drug-resistant bacteria biofilm in the mouse tibial bone marrow.
4190	DRAMP04412	Defensin	"Defensin"[All Fields] AND biofilm[All Fields]	Defensin	28296382	Human α-Defensin 6: A Small Peptide That Self-Assembles and Protects the Host by Entangling Microbes.
4190	DRAMP04412	Defensin	"Defensin"[All Fields] AND biofilm[All Fields]	Defensin	28278280	Natural antimicrobial peptide complexes in the fighting of antibiotic resistant biofilms: Calliphora vicina medicinal maggots.
4190	DRAMP04412	Defensin	"Defensin"[All Fields] AND biofilm[All Fields]	Defensin	28144375	Advancements in Diagnosing Periprosthetic Joint Infections after Total Hip and Knee Arthroplasty.
4190	DRAMP04412	Defensin	"Defensin"[All Fields] AND biofilm[All Fields]	Defensin	28078813	Host defense peptide-derived privileged scaffolds for anti-infective drug discovery.
4190	DRAMP04412	Defensin	"Defensin"[All Fields] AND biofilm[All Fields]	Defensin	28026958	Human α-Defensin 6 Self-Assembly Prevents Adhesion and Suppresses Virulence Traits of Candida albicans.
4190	DRAMP04412	Defensin	"Defensin"[All Fields] AND biofilm[All Fields]	Defensin	27794585	A Novel Defensin-Like Peptide Associated with Two Other New Cationic Antimicrobial Peptides in Transcriptome of the Iranian Scorpion Venom.
4190	DRAMP04412	Defensin	"Defensin"[All Fields] AND biofilm[All Fields]	Defensin	27777572	Role of yqiCin the Pathogenicity of Salmonellaand Innate Immune Responses of Human Intestinal Epithelium.
4190	DRAMP04412	Defensin	"Defensin"[All Fields] AND biofilm[All Fields]	Defensin	27582732	Efficient Eradication of Mature Pseudomonas aeruginosa Biofilm via Controlled Delivery of Nitric Oxide Combined with Antimicrobial Peptide and Antibiotics.
4190	DRAMP04412	Defensin	"Defensin"[All Fields] AND biofilm[All Fields]	Defensin	27417541	Acute appendicitis: transcript profiling of blood identifies promising biomarkers and potential underlying processes.
4190	DRAMP04412	Defensin	"Defensin"[All Fields] AND biofilm[All Fields]	Defensin	27200276	Antifungal effects of synthetic human β-defensin 3-C15 peptide.
4190	DRAMP04412	Defensin	"Defensin"[All Fields] AND biofilm[All Fields]	Defensin	27148195	Effect of Substance P in Staphylococcus aureus and Staphylococcus epidermidis Virulence: Implication for Skin Homeostasis.
4190	DRAMP04412	Defensin	"Defensin"[All Fields] AND biofilm[All Fields]	Defensin	26861950	Antibiofilm efficacy of honey and bee-derived defensin-1 on multispecies wound biofilm.
4190	DRAMP04412	Defensin	"Defensin"[All Fields] AND biofilm[All Fields]	Defensin	26592804	The radish defensins RsAFP1 and RsAFP2 act synergistically with caspofungin against Candida albicans biofilms.
4190	DRAMP04412	Defensin	"Defensin"[All Fields] AND biofilm[All Fields]	Defensin	26248029	Synergistic Activity of the Plant Defensin HsAFP1 and Caspofungin against Candida albicans Biofilms and Planktonic Cultures.
4190	DRAMP04412	Defensin	"Defensin"[All Fields] AND biofilm[All Fields]	Defensin	26214284	α-tocopherol decreases interleukin-1β and -6 and increases human β-defensin-1 and -2 secretion in human gingival fibroblasts stimulated with Porphyromonas gingivalis lipopolysaccharide.
4190	DRAMP04412	Defensin	"Defensin"[All Fields] AND biofilm[All Fields]	Defensin	26196513	Snake Cathelicidin NA-CATH and Smaller Helical Antimicrobial Peptides Are Effective against Burkholderia thailandensis.
4190	DRAMP04412	Defensin	"Defensin"[All Fields] AND biofilm[All Fields]	Defensin	26119274	Ultrasound microbubbles enhance human β-defensin 3 against biofilms.
4190	DRAMP04412	Defensin	"Defensin"[All Fields] AND biofilm[All Fields]	Defensin	25862466	The Antibacterial Effects of an Antimicrobial Peptide Human β-Defensin 3 Fused with Carbohydrate-Binding Domain on Pseudomonas aeruginosa PA14.
4190	DRAMP04412	Defensin	"Defensin"[All Fields] AND biofilm[All Fields]	Defensin	25808131	Expression of antimicrobial peptides and interleukin-8 during early stages of inflammation: An experimental gingivitis study.
4190	DRAMP04412	Defensin	"Defensin"[All Fields] AND biofilm[All Fields]	Defensin	25806720	Antimicrobial peptides in 2014.
4190	DRAMP04412	Defensin	"Defensin"[All Fields] AND biofilm[All Fields]	Defensin	25285879	Inhibition and destruction of Pseudomonas aeruginosa biofilms by antibiotics and antimicrobial peptides.
4190	DRAMP04412	Defensin	"Defensin"[All Fields] AND biofilm[All Fields]	Defensin	25212593	Health- and disease-associated species clusters in complex natural biofilms determine the innate immune response in oral epithelial cells during biofilm maturation.
4190	DRAMP04412	Defensin	"Defensin"[All Fields] AND biofilm[All Fields]	Defensin	24913184	Effects of human β-defensin-3 on biofilm formation-regulating genes dltB and icaA in Staphylococcus aureus.
4190	DRAMP04412	Defensin	"Defensin"[All Fields] AND biofilm[All Fields]	Defensin	24340061	Inflammatory and antimicrobial responses to methicillin-resistant Staphylococcus aureus in an in vitro wound infection model.
4190	DRAMP04412	Defensin	"Defensin"[All Fields] AND biofilm[All Fields]	Defensin	24240906	Adsorption study of pellicle proteins to gold, silica and titanium by quartz crystal microbalance method.
4190	DRAMP04412	Defensin	"Defensin"[All Fields] AND biofilm[All Fields]	Defensin	24238461	Antibacterial efficacy of a human β-defensin-3 peptide on multispecies biofilms.
4190	DRAMP04412	Defensin	"Defensin"[All Fields] AND biofilm[All Fields]	Defensin	24100890	[Use of recombinant human beta-defensin-3 to evaluate the effect of adhesion of Candida albicans on the surface of soft lining material].
4190	DRAMP04412	Defensin	"Defensin"[All Fields] AND biofilm[All Fields]	Defensin	23639356	Human beta-defensin 3: a novel inhibitor of Staphylococcus-produced biofilm production. Commentary on "Human β-defensin 3 inhibits antibiotic-resistant Staphylococcus biofilm formation".
4190	DRAMP04412	Defensin	"Defensin"[All Fields] AND biofilm[All Fields]	Defensin	23519963	Ultrasound-targeted microbubble destruction enhances human β-defensin 3 activity against antibiotic-resistant Staphylococcus biofilms.
4190	DRAMP04412	Defensin	"Defensin"[All Fields] AND biofilm[All Fields]	Defensin	23273885	Human β-defensin 3 inhibits antibiotic-resistant Staphylococcus biofilm formation.
4190	DRAMP04412	Defensin	"Defensin"[All Fields] AND biofilm[All Fields]	Defensin	23203265	Potentiation of the cytotoxic activity of copper by polyphosphate on biofilm-producing bacteria: a bioinspired approach.
4190	DRAMP04412	Defensin	"Defensin"[All Fields] AND biofilm[All Fields]	Defensin	23078156	Antimicrobial efficacy of a human β-defensin-3 peptide using an Enterococcus faecalis dentine infection model.
4190	DRAMP04412	Defensin	"Defensin"[All Fields] AND biofilm[All Fields]	Defensin	23053486	Synergistic effect and antibiofilm activity between the antimicrobial peptide coprisin and conventional antibiotics against opportunistic bacteria.
4190	DRAMP04412	Defensin	"Defensin"[All Fields] AND biofilm[All Fields]	Defensin	22922323	Extracellular DNA within a nontypeable Haemophilus influenzae-induced biofilm binds human beta defensin-3 and reduces its antimicrobial activity.
4190	DRAMP04412	Defensin	"Defensin"[All Fields] AND biofilm[All Fields]	Defensin	22855857	Retrocyclin inhibits Gardnerella vaginalis biofilm formation and toxin activity.
4190	DRAMP04412	Defensin	"Defensin"[All Fields] AND biofilm[All Fields]	Defensin	22394470	Association of CiaRH with resistance of Streptococcus mutans to antimicrobial peptides in biofilms.
4190	DRAMP04412	Defensin	"Defensin"[All Fields] AND biofilm[All Fields]	Defensin	22229614	Comparison of the effects of human β-defensin 3, vancomycin, and clindamycin on Staphylococcus aureus biofilm formation.

4190	DRAMP04412	Defensin	"Defensin"[All Fields] AND biofilm[All Fields]	Defensin	21692631	A novel organotypic dento-epithelial culture model: effect of Fusobacterium nucleatum biofilm on B-defensin-2, -3, and LL-37 expression.
4190	DRAMP04412	Defensin	"Defensin"[All Fields] AND biofilm[All Fields]	Defensin	20454633	Normal human gingival epithelial cells sense C. parapsilosis by toll-like receptors and module its pathogenesis through antimicrobial peptides and proinflammatory cytokines.
4190	DRAMP04412	Defensin	"Defensin"[All Fields] AND biofilm[All Fields]	Defensin	20378008	Effect of temperature on the shift of Pseudomonas fluorescens from an environmental microorganism to a potential human pathogen.
4190	DRAMP04412	Defensin	"Defensin"[All Fields] AND biofilm[All Fields]	Defensin	19961380	Gingival transcriptome patterns during induction and resolution of experimental gingivitis in humans.
4190	DRAMP04412	Defensin	"Defensin"[All Fields] AND biofilm[All Fields]	Defensin	19780045	Candida famata modulates toll-like receptor, beta-defensin, and proinflammatory cytokine expression by normal human epithelial cells.
4190	DRAMP04412	Defensin	"Defensin"[All Fields] AND biofilm[All Fields]	Defensin	19572896	The immune response of oral epithelial cells induced by single-species and complex naturally formed biofilms.
4190	DRAMP04412	Defensin	"Defensin"[All Fields] AND biofilm[All Fields]	Defensin	19466693	Activity of antimicrobial peptides in the presence of polysaccharides produced by pulmonary pathogens.
4190	DRAMP04412	Defensin	"Defensin"[All Fields] AND biofilm[All Fields]	Defensin	18954353	Treponema denticola does not induce production of common innate immune mediators from primary gingival epithelial cells.
4190	DRAMP04412	Defensin	"Defensin"[All Fields] AND biofilm[All Fields]	Defensin	18173794	The stage of native biofilm formation determines the gene expression of human beta-defensin-2, psoriasin, ribonuclease 7 and inflammatory mediators: a novel approach for stimulation of keratinocytes with in situ formed biofilms.
4190	DRAMP04412	Defensin	"Defensin"[All Fields] AND biofilm[All Fields]	Defensin	17434999	Functional analysis of D-alanylation of lipoteichoic acid in the probiotic strain Lactobacillus rhamnosus GG.
4190	DRAMP04412	Defensin	"Defensin"[All Fields] AND biofilm[All Fields]	Defensin	15493829	Bacterial evasion of innate host defenses--the Staphylococcus aureus lesson.
4190	DRAMP04412	Defensin	"Defensin"[All Fields] AND biofilm[All Fields]	Defensin	14764110	Polysaccharide intercellular adhesin (PIA) protects Staphylococcus epidermidis against major components of the human innate immune system.
4192	DRAMP04414	Defensin 1 (Def1)	"Defensin 1"[All Fields] AND biofilm[All Fields]	Defensin 1	34321877	Antibacterial Effect of Honey-Derived Exosomes Containing Antimicrobial Peptides Against Oral Streptococci.
4192	DRAMP04414	Defensin 1 (Def1)	"Defensin 1"[All Fields] AND biofilm[All Fields]	Defensin 1	33911935	The antibacterial activities of honey.
4192	DRAMP04414	Defensin 1 (Def1)	"Defensin 1"[All Fields] AND biofilm[All Fields]	Defensin 1	33420317	Curbing gastrointestinal infections by defensin fragment modifications without harming commensal microbiota.
4192	DRAMP04414	Defensin 1 (Def1)	"Defensin 1"[All Fields] AND biofilm[All Fields]	Defensin 1	32585445	Glucose effect on Candida albicans biofilm during tissue invasion.
4192	DRAMP04414	Defensin 1 (Def1)	"Defensin 1"[All Fields] AND biofilm[All Fields]	Defensin 1	31729441	Rhesus Theta Defensin 1 Promotes Long Term Survival in Systemic Candidiasis by Host Directed Mechanisms.
4192	DRAMP04414	Defensin 1 (Def1)	"Defensin 1"[All Fields] AND biofilm[All Fields]	Defensin 1	30260708	Stabilized collagen matrix dressing improves wound macrophage function and epithelialization.
4192	DRAMP04414	Defensin 1 (Def1)	"Defensin 1"[All Fields] AND biofilm[All Fields]	Defensin 1	29671721	The BceABRS four-component system that is essential for cell envelope stress response is involved in sensing and response to host defence peptides and is required for the biofilm formation and fitness of Streptococcus mutans.
4192	DRAMP04414	Defensin 1 (Def1)	"Defensin 1"[All Fields] AND biofilm[All Fields]	Defensin 1	28649561	Psd1 Effects on Candida albicansPlanktonic Cells and Biofilms.
4192	DRAMP04414	Defensin 1 (Def1)	"Defensin 1"[All Fields] AND biofilm[All Fields]	Defensin 1	28642103	Chimeric analogs of human β-defensin 1 and θ-defensin disrupt pre-established bacterial biofilms.
4192	DRAMP04414	Defensin 1 (Def1)	"Defensin 1"[All Fields] AND biofilm[All Fields]	Defensin 1	26861950	Antibiofilm efficacy of honey and bee-derived defensin-1 on multispecies wound biofilm.
4192	DRAMP04414	Defensin 1 (Def1)	"Defensin 1"[All Fields] AND biofilm[All Fields]	Defensin 1	26214284	α-tocopherol decreases interleukin-1β and -6 and increases human β-defensin-1 and -2 secretion in human gingival fibroblasts stimulated with Porphyromonas gingivalis lipopolysaccharide.
4192	DRAMP04414	Defensin 1 (Def1)	"Defensin 1"[All Fields] AND biofilm[All Fields]	Defensin 1	22394470	Association of CiaRH with resistance of Streptococcus mutans to antimicrobial peptides in biofilms.
4192	DRAMP04414	Defensin 1 (Def1)	"Defensin 1"[All Fields] AND biofilm[All Fields]	Defensin 1	20454633	Normal human gingival epithelial cells sense C. parapsilosis by toll-like receptors and module its pathogenesis through antimicrobial peptides and proinflammatory cytokines.
4193	DRAMP04415	Defensin 2 (Def2)	"Defensin 2"[All Fields] AND biofilm[All Fields]	Defensin 2	34276631	DNA Blocks the Lethal Effect of Human Beta-Defensin 2 Against Neisseria meningitidis.
4193	DRAMP04415	Defensin 2 (Def2)	"Defensin 2"[All Fields] AND biofilm[All Fields]	Defensin 2	32842903	Electrospun ZnO/Poly(Vinylidene Fluoride-Trifluoroethylene) Scaffolds for Lung Tissue Engineering.
4193	DRAMP04415	Defensin 2 (Def2)	"Defensin 2"[All Fields] AND biofilm[All Fields]	Defensin 2	32522780	Controlling the Growth of the Skin Commensal Staphylococcus epidermidis Using d-Alanine Auxotrophy.
4193	DRAMP04415	Defensin 2 (Def2)	"Defensin 2"[All Fields] AND biofilm[All Fields]	Defensin 2	32457749	The Antimicrobial Peptide Human Beta-Defensin 2 Inhibits Biofilm Production of Pseudomonas aeruginosaWithout Compromising Metabolic Activity.
4193	DRAMP04415	Defensin 2 (Def2)	"Defensin 2"[All Fields] AND biofilm[All Fields]	Defensin 2	31906541	Role of FAD-I in Fusobacterial Interspecies Interaction and Biofilm Formation.
4193	DRAMP04415	Defensin 2 (Def2)	"Defensin 2"[All Fields] AND biofilm[All Fields]	Defensin 2	31336838	Candida albicans-Cell Interactions Activate Innate Immune Defense in Human Palate Epithelial Primary Cells via Nitric Oxide (NO) and β-Defensin 2 (hBD-2).
4193	DRAMP04415	Defensin 2 (Def2)	"Defensin 2"[All Fields] AND biofilm[All Fields]	Defensin 2	30649289	Innate immune components affect growth and virulence traits of bacterial-vaginosis-associated and non-bacterial-vaginosis-associated Gardnerella vaginalis strains similarly.
4193	DRAMP04415	Defensin 2 (Def2)	"Defensin 2"[All Fields] AND biofilm[All Fields]	Defensin 2	27582732	Efficient Eradication of Mature Pseudomonas aeruginosa Biofilm via Controlled Delivery of Nitric Oxide Combined with Antimicrobial Peptide and Antibiotics.
4193	DRAMP04415	Defensin 2 (Def2)	"Defensin 2"[All Fields] AND biofilm[All Fields]	Defensin 2	27148195	Effect of Substance P in Staphylococcus aureus and Staphylococcus epidermidis Virulence: Implication for Skin Homeostasis.
4193	DRAMP04415	Defensin 2 (Def2)	"Defensin 2"[All Fields] AND biofilm[All Fields]	Defensin 2	25808131	Expression of antimicrobial peptides and interleukin-8 during early stages of inflammation: An experimental gingivitis study.
4193	DRAMP04415	Defensin 2 (Def2)	"Defensin 2"[All Fields] AND biofilm[All Fields]	Defensin 2	24340061	Inflammatory and antimicrobial responses to methicillin-resistant Staphylococcus aureus in an in vitro wound infection model.
4193	DRAMP04415	Defensin 2 (Def2)	"Defensin 2"[All Fields] AND biofilm[All Fields]	Defensin 2	21692631	A novel organotypic dento-epithelial culture model: effect of Fusobacterium nucleatum biofilm on B-defensin-2, -3, and LL-37 expression.
4193	DRAMP04415	Defensin 2 (Def2)	"Defensin 2"[All Fields] AND biofilm[All Fields]	Defensin 2	20378008	Effect of temperature on the shift of Pseudomonas fluorescens from an environmental microorganism to a potential human pathogen.
4193	DRAMP04415	Defensin 2 (Def2)	"Defensin 2"[All Fields] AND biofilm[All Fields]	Defensin 2	19572896	The immune response of oral epithelial cells induced by single-species and complex naturally formed biofilms.
4193	DRAMP04415	Defensin 2 (Def2)	"Defensin 2"[All Fields] AND biofilm[All Fields]	Defensin 2	18954353	Treponema denticola does not induce production of common innate immune mediators from primary gingival epithelial cells.
4193	DRAMP04415	Defensin 2 (Def2)	"Defensin 2"[All Fields] AND biofilm[All Fields]	Defensin 2	18173794	The stage of native biofilm formation determines the gene expression of human beta-defensin-2, psoriasin, ribonuclease 7 and inflammatory mediators: a novel approach for stimulation of keratinocytes with in situ formed biofilms.
4193	DRAMP04415	Defensin 2 (Def2)	"Defensin 2"[All Fields] AND biofilm[All Fields]	Defensin 2	17434999	Functional analysis of D-alanylation of lipoteichoic acid in the probiotic strain Lactobacillus rhamnosus GG.
4194	DRAMP04416	Defensin 3 (Def3)	"Defensin 3"[All Fields] AND biofilm[All Fields]	Defensin 3	34408988	The Anti-Biofilm Efficacy of Caffeic Acid Phenethyl Ester (CAPE) In Vitroand a Murine Model of Oral Candidiasis.
4194	DRAMP04416	Defensin 3 (Def3)	"Defensin 3"[All Fields] AND biofilm[All Fields]	Defensin 3	33865931	Identification of a crocodylian β-defensin variant from Alligator mississippiensis with antimicrobial and antibiofilm activity.
4194	DRAMP04416	Defensin 3 (Def3)	"Defensin 3"[All Fields] AND biofilm[All Fields]	Defensin 3	32858856	Candida albicansVirulence Factors and Pathogenicity for Endodontic Infections.
4194	DRAMP04416	Defensin 3 (Def3)	"Defensin 3"[All Fields] AND biofilm[All Fields]	Defensin 3	32585445	Glucose effect on Candida albicans biofilm during tissue invasion.
4194	DRAMP04416	Defensin 3 (Def3)	"Defensin 3"[All Fields] AND biofilm[All Fields]	Defensin 3	31933178	Effects of human β-defensin 3 fused with carbohydrate-binding domain on the function of type III secretion system in Pseudomonas aeruginosa PA14.
4194	DRAMP04416	Defensin 3 (Def3)	"Defensin 3"[All Fields] AND biofilm[All Fields]	Defensin 3	30254440	Modification of the surface of titanium with multifunctional chimeric peptides to prevent biofilm formation via inhibition of initial colonizers.
4194	DRAMP04416	Defensin 3 (Def3)	"Defensin 3"[All Fields] AND biofilm[All Fields]	Defensin 3	29872295	Antimicrobial peptide-loaded liquid crystalline precursor bioadhesive system for the prevention of dental caries.
4194	DRAMP04416	Defensin 3 (Def3)	"Defensin 3"[All Fields] AND biofilm[All Fields]	Defensin 3	29671721	The BceABRS four-component system that is essential for cell envelope stress response is involved in sensing and response to host defence peptides and is required for the biofilm formation and fitness of Streptococcus mutans.
4194	DRAMP04416	Defensin 3 (Def3)	"Defensin 3"[All Fields] AND biofilm[All Fields]	Defensin 3	29077172	The significance of HBD-3 and fluorescent composite carriers in the processof bone formation in rats infected with Staphylococcus aureus.
4194	DRAMP04416	Defensin 3 (Def3)	"Defensin 3"[All Fields] AND biofilm[All Fields]	Defensin 3	29025642	Engineered chimeric peptides with antimicrobial and titanium-binding functions to inhibit biofilm formation on Ti implants.
4194	DRAMP04416	Defensin 3 (Def3)	"Defensin 3"[All Fields] AND biofilm[All Fields]	Defensin 3	28956355	The synthetic human beta-defensin-3 C15 peptide exhibits antimicrobial activity against Streptococcus mutans, both alone and in combination with dental disinfectants.
4194	DRAMP04416	Defensin 3 (Def3)	"Defensin 3"[All Fields] AND biofilm[All Fields]	Defensin 3	28951032	Antifungal Effects of Synthetic Human Beta-defensin-3-C15 Peptide on Candida albicans-infected Root Dentin.
4194	DRAMP04416	Defensin 3 (Def3)	"Defensin 3"[All Fields] AND biofilm[All Fields]	Defensin 3	28413476	The mechanism of human β-defensin 3 in MRSA-induced infection of implant drug-resistant bacteria biofilm in the mouse tibial bone marrow.
4194	DRAMP04416	Defensin 3 (Def3)	"Defensin 3"[All Fields] AND biofilm[All Fields]	Defensin 3	28078813	Host defense peptide-derived privileged scaffolds for anti-infective drug discovery.

4194	DRAMP04416	Defensin 3 (Def3)	"Defensin 3"[All Fields] AND biofilm[All Fields]	Defensin 3	27777572	Role of yqiCin the Pathogenicity of Salmonellaand Innate Immune Responses of Human Intestinal Epithelium.
4194	DRAMP04416	Defensin 3 (Def3)	"Defensin 3"[All Fields] AND biofilm[All Fields]	Defensin 3	27200276	Antifungal effects of synthetic human β-defensin 3-C15 peptide.
4194	DRAMP04416	Defensin 3 (Def3)	"Defensin 3"[All Fields] AND biofilm[All Fields]	Defensin 3	26119274	Ultrasound microbubbles enhance human β-defensin 3 against biofilms.
4194	DRAMP04416	Defensin 3 (Def3)	"Defensin 3"[All Fields] AND biofilm[All Fields]	Defensin 3	25862466	The Antibacterial Effects of an Antimicrobial Peptide Human β-Defensin 3 Fused with Carbohydrate-Binding Domain on Pseudomonas aeruginosa PA14.
4194	DRAMP04416	Defensin 3 (Def3)	"Defensin 3"[All Fields] AND biofilm[All Fields]	Defensin 3	24913184	Effects of human β-defensin-3 on biofilm formation-regulating genes dltB and icaA in Staphylococcus aureus.
4194	DRAMP04416	Defensin 3 (Def3)	"Defensin 3"[All Fields] AND biofilm[All Fields]	Defensin 3	24238461	Antibacterial efficacy of a human β-defensin-3 peptide on multispecies biofilms.
4194	DRAMP04416	Defensin 3 (Def3)	"Defensin 3"[All Fields] AND biofilm[All Fields]	Defensin 3	24100890	[Use of recombinant human beta-defensin-3 to evaluate the effect of adhesion of Candida albicans on the surface of soft lining material].
4194	DRAMP04416	Defensin 3 (Def3)	"Defensin 3"[All Fields] AND biofilm[All Fields]	Defensin 3	23639356	Human beta-defensin 3: a novel inhibitor of Staphylococcus-produced biofilm production. Commentary on "Human β-defensin 3 inhibits antibiotic-resistant Staphylococcus biofilm formation".
4194	DRAMP04416	Defensin 3 (Def3)	"Defensin 3"[All Fields] AND biofilm[All Fields]	Defensin 3	23519963	Ultrasound-targeted microbubble destruction enhances human β-defensin 3 activity against antibiotic-resistant Staphylococcus biofilms.
4194	DRAMP04416	Defensin 3 (Def3)	"Defensin 3"[All Fields] AND biofilm[All Fields]	Defensin 3	23273885	Human β-defensin 3 inhibits antibiotic-resistant Staphylococcus biofilm formation.
4194	DRAMP04416	Defensin 3 (Def3)	"Defensin 3"[All Fields] AND biofilm[All Fields]	Defensin 3	23078156	Antimicrobial efficacy of a human β-defensin-3 peptide using an Enterococcus faecalis dentine infection model.
4194	DRAMP04416	Defensin 3 (Def3)	"Defensin 3"[All Fields] AND biofilm[All Fields]	Defensin 3	22922323	Extracellular DNA within a nontypeable Haemophilus influenzae-induced biofilm binds human beta defensin-3 and reduces its antimicrobial activity.
4194	DRAMP04416	Defensin 3 (Def3)	"Defensin 3"[All Fields] AND biofilm[All Fields]	Defensin 3	22229614	Comparison of the effects of human β-defensin 3, vancomycin, and clindamycin on Staphylococcus aureus biofilm formation.
4194	DRAMP04416	Defensin 3 (Def3)	"Defensin 3"[All Fields] AND biofilm[All Fields]	Defensin 3	14764110	Polysaccharide intercellular adhesin (PIA) protects Staphylococcus epidermidis against major components of the human innate immune system.
4196	DRAMP04418	Defensin	"Defensin"[All Fields] AND biofilm[All Fields]	Defensin	34408988	The Anti-Biofilm Efficacy of Caffeic Acid Phenethyl Ester (CAPE) In Vitroand a Murine Model of Oral Candidiasis.
4196	DRAMP04418	Defensin	"Defensin"[All Fields] AND biofilm[All Fields]	Defensin	34321877	Antibacterial Effect of Honey-Derived Exosomes Containing Antimicrobial Peptides Against Oral Streptococci.
4196	DRAMP04418	Defensin	"Defensin"[All Fields] AND biofilm[All Fields]	Defensin	34276631	DNA Blocks the Lethal Effect of Human Beta-Defensin 2 Against Neisseria meningitidis.
4196	DRAMP04418	Defensin	"Defensin"[All Fields] AND biofilm[All Fields]	Defensin	33911935	The antibacterial activities of honey.
4196	DRAMP04418	Defensin	"Defensin"[All Fields] AND biofilm[All Fields]	Defensin	33865931	Identification of a crocodylian β-defensin variant from Alligator mississippiensis with antimicrobial and antibiofilm activity.
4196	DRAMP04418	Defensin	"Defensin"[All Fields] AND biofilm[All Fields]	Defensin	33586659	Identification of anti-microbial peptides and traces of microbial DNA in infrainfundibular compartments of human scalp terminal hair follicles.
4196	DRAMP04418	Defensin	"Defensin"[All Fields] AND biofilm[All Fields]	Defensin	33534018	A recombinant fungal defensin-like peptide-P2 combats Streptococcus dysgalactiae and biofilms.
4196	DRAMP04418	Defensin	"Defensin"[All Fields] AND biofilm[All Fields]	Defensin	33447687	Future directions of postoperative spinal implant infections.
4196	DRAMP04418	Defensin	"Defensin"[All Fields] AND biofilm[All Fields]	Defensin	33420317	Curbing gastrointestinal infections by defensin fragment modifications without harming commensal microbiota.
4196	DRAMP04418	Defensin	"Defensin"[All Fields] AND biofilm[All Fields]	Defensin	32867384	A Novel Peptide Antibiotic, Pro10-1D, Designed from Insect Defensin Shows Antibacterial and Anti-Inflammatory Activities in Sepsis Models.
4196	DRAMP04418	Defensin	"Defensin"[All Fields] AND biofilm[All Fields]	Defensin	32858856	Candida albicansVirulence Factors and Pathogenicity for Endodontic Infections.
4196	DRAMP04418	Defensin	"Defensin"[All Fields] AND biofilm[All Fields]	Defensin	32842903	Electrospun ZnO/Poly(Vinylidene Fluoride-Trifluoroethylene) Scaffolds for Lung Tissue Engineering.
4196	DRAMP04418	Defensin	"Defensin"[All Fields] AND biofilm[All Fields]	Defensin	32663201	Inhibition and eradication activity of truncated α-defensin analogs against multidrug resistant uropathogenic Escherichia coli biofilm.
4196	DRAMP04418	Defensin	"Defensin"[All Fields] AND biofilm[All Fields]	Defensin	32585445	Glucose effect on Candida albicans biofilm during tissue invasion.
4196	DRAMP04418	Defensin	"Defensin"[All Fields] AND biofilm[All Fields]	Defensin	32522780	Controlling the Growth of the Skin Commensal Staphylococcus epidermidis Using d-Alanine Auxotrophy.
4196	DRAMP04418	Defensin	"Defensin"[All Fields] AND biofilm[All Fields]	Defensin	32457749	The Antimicrobial Peptide Human Beta-Defensin 2 Inhibits Biofilm Production of Pseudomonas aeruginosaWithout Compromising Metabolic Activity.
4196	DRAMP04418	Defensin	"Defensin"[All Fields] AND biofilm[All Fields]	Defensin	32439511	Antibacterial activities and mechanisms of action of a defensin from manila clam Ruditapes philippinarum.
4196	DRAMP04418	Defensin	"Defensin"[All Fields] AND biofilm[All Fields]	Defensin	31933178	Effects of human β-defensin 3 fused with carbohydrate-binding domain on the function of type III secretion system in Pseudomonas aeruginosa PA14.
4196	DRAMP04418	Defensin	"Defensin"[All Fields] AND biofilm[All Fields]	Defensin	31906541	Role of FAD-I in Fusobacterial Interspecies Interaction and Biofilm Formation.
4196	DRAMP04418	Defensin	"Defensin"[All Fields] AND biofilm[All Fields]	Defensin	31729441	Rhesus Theta Defensin 1 Promotes Long Term Survival in Systemic Candidiasis by Host Directed Mechanisms.
4196	DRAMP04418	Defensin	"Defensin"[All Fields] AND biofilm[All Fields]	Defensin	31336838	Candida albicans-Cell Interactions Activate Innate Immune Defense in Human Palate Epithelial Primary Cells via Nitric Oxide (NO) and β-Defensin 2 (hBD-2).
4196	DRAMP04418	Defensin	"Defensin"[All Fields] AND biofilm[All Fields]	Defensin	31165072	Impact of the Food Additive Titanium Dioxide (E171) on Gut Microbiota-Host Interaction.
4196	DRAMP04418	Defensin	"Defensin"[All Fields] AND biofilm[All Fields]	Defensin	31031739	Salt-Tolerant Antifungal and Antibacterial Activities of the Corn Defensin ZmD32.
4196	DRAMP04418	Defensin	"Defensin"[All Fields] AND biofilm[All Fields]	Defensin	31025073	A recombinant fungal defensin-like peptide-P2 combats multidrug-resistant Staphylococcus aureus and biofilms.
4196	DRAMP04418	Defensin	"Defensin"[All Fields] AND biofilm[All Fields]	Defensin	30659503	Lactobacillus plantarum USM8613 Aids in Wound Healing and Suppresses Staphylococcus aureus Infection at Wound Sites.
4196	DRAMP04418	Defensin	"Defensin"[All Fields] AND biofilm[All Fields]	Defensin	30649289	Innate immune components affect growth and virulence traits of bacterial-vaginosis-associated and non-bacterial-vaginosis-associated Gardnerella vaginalis strains similarly.
4196	DRAMP04418	Defensin	"Defensin"[All Fields] AND biofilm[All Fields]	Defensin	30376742	Alpha defensin, leukocyte esterase, C-reactive protein, and leukocyte count in synovial fluid for pre-operative diagnosis of periprosthetic infection.
4196	DRAMP04418	Defensin	"Defensin"[All Fields] AND biofilm[All Fields]	Defensin	30260708	Stabilized collagen matrix dressing improves wound macrophage function and epithelialization.
4196	DRAMP04418	Defensin	"Defensin"[All Fields] AND biofilm[All Fields]	Defensin	30254440	Modification of the surface of titanium with multifunctional chimeric peptides to prevent biofilm formation via inhibition of initial colonizers.
4196	DRAMP04418	Defensin	"Defensin"[All Fields] AND biofilm[All Fields]	Defensin	29902560	A defensin-like antimicrobial peptide from the manila clam Ruditapes philippinarum: Investigation of the antibacterial activities and mode of action.
4196	DRAMP04418	Defensin	"Defensin"[All Fields] AND biofilm[All Fields]	Defensin	29872295	Antimicrobial peptide-loaded liquid crystalline precursor bioadhesive system for the prevention of dental caries.
4196	DRAMP04418	Defensin	"Defensin"[All Fields] AND biofilm[All Fields]	Defensin	29671721	The BceABRS four-component system that is essential for cell envelope stress response is involved in sensing and response to host defence peptides and is required for the biofilm formation and fitness of Streptococcus mutans.
4196	DRAMP04418	Defensin	"Defensin"[All Fields] AND biofilm[All Fields]	Defensin	29104569	A Linear 19-Mer Plant Defensin-Derived Peptide Acts Synergistically with Caspofungin against Candida albicansBiofilms.
4196	DRAMP04418	Defensin	"Defensin"[All Fields] AND biofilm[All Fields]	Defensin	29077172	The significance of HBD-3 and fluorescent composite carriers in the processof bone formation in rats infected with Staphylococcus aureus.
4196	DRAMP04418	Defensin	"Defensin"[All Fields] AND biofilm[All Fields]	Defensin	29045084	New Approach to Treat and Prevent Oral Disease.
4196	DRAMP04418	Defensin	"Defensin"[All Fields] AND biofilm[All Fields]	Defensin	29025642	Engineered chimeric peptides with antimicrobial and titanium-binding functions to inhibit biofilm formation on Ti implants.
4196	DRAMP04418	Defensin	"Defensin"[All Fields] AND biofilm[All Fields]	Defensin	28956355	The synthetic human beta-defensin-3 C15 peptide exhibits antimicrobial activity against Streptococcus mutans, both alone and in combination with dental disinfectants.
4196	DRAMP04418	Defensin	"Defensin"[All Fields] AND biofilm[All Fields]	Defensin	28951032	Antifungal Effects of Synthetic Human Beta-defensin-3-C15 Peptide on Candida albicans-infected Root Dentin.
4196	DRAMP04418	Defensin	"Defensin"[All Fields] AND biofilm[All Fields]	Defensin	28874606	Bacterial d-amino acids suppress sinonasal innate immunity through sweet taste receptors in solitary chemosensory cells.
4196	DRAMP04418	Defensin	"Defensin"[All Fields] AND biofilm[All Fields]	Defensin	28725299	Role of Streptococcus mutanstwo-component systems in antimicrobial peptide resistance in the oral cavity.
4196	DRAMP04418	Defensin	"Defensin"[All Fields] AND biofilm[All Fields]	Defensin	28649561	Psd1 Effects on Candida albicansPlanktonic Cells and Biofilms.
4196	DRAMP04418	Defensin	"Defensin"[All Fields] AND biofilm[All Fields]	Defensin	28642103	Chimeric analogs of human β-defensin 1 and θ-defensin disrupt pre-established bacterial biofilms.
4196	DRAMP04418	Defensin	"Defensin"[All Fields] AND biofilm[All Fields]	Defensin	28413476	The mechanism of human β-defensin 3 in MRSA-induced infection of implant drug-resistant bacteria biofilm in the mouse tibial bone marrow.
4196	DRAMP04418	Defensin	"Defensin"[All Fields] AND biofilm[All Fields]	Defensin	28296382	Human α-Defensin 6: A Small Peptide That Self-Assembles and Protects the Host by Entangling Microbes.

4196	DRAMP04418	Defensin	"Defensin"[All Fields] AND biofilm[All Fields]	Defensin	28278280	Natural antimicrobial peptide complexes in the fighting of antibiotic resistant biofilms: Calliphora vicina medicinal maggots.
4196	DRAMP04418	Defensin	"Defensin"[All Fields] AND biofilm[All Fields]	Defensin	28144375	Advancements in Diagnosing Periprosthetic Joint Infections after Total Hip and Knee Arthroplasty.
4196	DRAMP04418	Defensin	"Defensin"[All Fields] AND biofilm[All Fields]	Defensin	28078813	Host defense peptide-derived privileged scaffolds for anti-infective drug discovery.
4196	DRAMP04418	Defensin	"Defensin"[All Fields] AND biofilm[All Fields]	Defensin	28026958	Human α-Defensin 6 Self-Assembly Prevents Adhesion and Suppresses Virulence Traits of Candida albicans.
4196	DRAMP04418	Defensin	"Defensin"[All Fields] AND biofilm[All Fields]	Defensin	27794585	A Novel Defensin-Like Peptide Associated with Two Other New Cationic Antimicrobial Peptides in Transcriptome of the Iranian Scorpion Venom.
4196	DRAMP04418	Defensin	"Defensin"[All Fields] AND biofilm[All Fields]	Defensin	27777572	Role of yqiCin the Pathogenicity of Salmonellaand Innate Immune Responses of Human Intestinal Epithelium.
4196	DRAMP04418	Defensin	"Defensin"[All Fields] AND biofilm[All Fields]	Defensin	27582732	Efficient Eradication of Mature Pseudomonas aeruginosa Biofilm via Controlled Delivery of Nitric Oxide Combined with Antimicrobial Peptide and Antibiotics.
4196	DRAMP04418	Defensin	"Defensin"[All Fields] AND biofilm[All Fields]	Defensin	27417541	Acute appendicitis: transcript profiling of blood identifies promising biomarkers and potential underlying processes.
4196	DRAMP04418	Defensin	"Defensin"[All Fields] AND biofilm[All Fields]	Defensin	27200276	Antifungal effects of synthetic human β-defensin 3-C15 peptide.
4196	DRAMP04418	Defensin	"Defensin"[All Fields] AND biofilm[All Fields]	Defensin	27148195	Effect of Substance P in Staphylococcus aureus and Staphylococcus epidermidis Virulence: Implication for Skin Homeostasis.
4196	DRAMP04418	Defensin	"Defensin"[All Fields] AND biofilm[All Fields]	Defensin	26861950	Antibiofilm efficacy of honey and bee-derived defensin-1 on multispecies wound biofilm.
4196	DRAMP04418	Defensin	"Defensin"[All Fields] AND biofilm[All Fields]	Defensin	26592804	The radish defensins RsAFP1 and RsAFP2 act synergistically with caspofungin against Candida albicans biofilms.
4196	DRAMP04418	Defensin	"Defensin"[All Fields] AND biofilm[All Fields]	Defensin	26248029	Synergistic Activity of the Plant Defensin HsAFP1 and Caspofungin against Candida albicans Biofilms and Planktonic Cultures.
4196	DRAMP04418	Defensin	"Defensin"[All Fields] AND biofilm[All Fields]	Defensin	26214284	α-tocopherol decreases interleukin-1β and -6 and increases human β-defensin-1 and -2 secretion in human gingival fibroblasts stimulated with Porphyromonas gingivalis lipopolysaccharide.
4196	DRAMP04418	Defensin	"Defensin"[All Fields] AND biofilm[All Fields]	Defensin	26196513	Snake Cathelicidin NA-CATH and Smaller Helical Antimicrobial Peptides Are Effective against Burkholderia thailandensis.
4196	DRAMP04418	Defensin	"Defensin"[All Fields] AND biofilm[All Fields]	Defensin	26119274	Ultrasound microbubbles enhance human β-defensin 3 against biofilms.
4196	DRAMP04418	Defensin	"Defensin"[All Fields] AND biofilm[All Fields]	Defensin	25862466	The Antibacterial Effects of an Antimicrobial Peptide Human β-Defensin 3 Fused with Carbohydrate-Binding Domain on Pseudomonas aeruginosa PA14.
4196	DRAMP04418	Defensin	"Defensin"[All Fields] AND biofilm[All Fields]	Defensin	25808131	Expression of antimicrobial peptides and interleukin-8 during early stages of inflammation: An experimental gingivitis study.
4196	DRAMP04418	Defensin	"Defensin"[All Fields] AND biofilm[All Fields]	Defensin	25806720	Antimicrobial peptides in 2014.
4196	DRAMP04418	Defensin	"Defensin"[All Fields] AND biofilm[All Fields]	Defensin	25285879	Inhibition and destruction of Pseudomonas aeruginosa biofilms by antibiotics and antimicrobial peptides.
4196	DRAMP04418	Defensin	"Defensin"[All Fields] AND biofilm[All Fields]	Defensin	25212593	Health- and disease-associated species clusters in complex natural biofilms determine the innate immune response in oral epithelial cells during biofilm maturation.
4196	DRAMP04418	Defensin	"Defensin"[All Fields] AND biofilm[All Fields]	Defensin	24913184	Effects of human β-defensin-3 on biofilm formation-regulating genes dltB and icaA in Staphylococcus aureus.
4196	DRAMP04418	Defensin	"Defensin"[All Fields] AND biofilm[All Fields]	Defensin	24340061	Inflammatory and antimicrobial responses to methicillin-resistant Staphylococcus aureus in an in vitro wound infection model.
4196	DRAMP04418	Defensin	"Defensin"[All Fields] AND biofilm[All Fields]	Defensin	24240906	Adsorption study of pellicle proteins to gold, silica and titanium by quartz crystal microbalance method.
4196	DRAMP04418	Defensin	"Defensin"[All Fields] AND biofilm[All Fields]	Defensin	24238461	Antibacterial efficacy of a human β-defensin-3 peptide on multispecies biofilms.
4196	DRAMP04418	Defensin	"Defensin"[All Fields] AND biofilm[All Fields]	Defensin	24100890	[Use of recombinant human beta-defensin-3 to evaluate the effect of adhesion of Candida albicans on the surface of soft lining material].
4196	DRAMP04418	Defensin	"Defensin"[All Fields] AND biofilm[All Fields]	Defensin	23639356	Human beta-defensin 3: a novel inhibitor of Staphylococcus-produced biofilm production. Commentary on "Human β-defensin 3 inhibits antibiotic-resistant Staphylococcus biofilm formation".
4196	DRAMP04418	Defensin	"Defensin"[All Fields] AND biofilm[All Fields]	Defensin	23519963	Ultrasound-targeted microbubble destruction enhances human β-defensin 3 activity against antibiotic-resistant Staphylococcus biofilms.
4196	DRAMP04418	Defensin	"Defensin"[All Fields] AND biofilm[All Fields]	Defensin	23273885	Human β-defensin 3 inhibits antibiotic-resistant Staphylococcus biofilm formation.
4196	DRAMP04418	Defensin	"Defensin"[All Fields] AND biofilm[All Fields]	Defensin	23203265	Potentiation of the cytotoxic activity of copper by polyphosphate on biofilm-producing bacteria: a bioinspired approach.
4196	DRAMP04418	Defensin	"Defensin"[All Fields] AND biofilm[All Fields]	Defensin	23078156	Antimicrobial efficacy of a human β-defensin-3 peptide using an Enterococcus faecalis dentine infection model.
4196	DRAMP04418	Defensin	"Defensin"[All Fields] AND biofilm[All Fields]	Defensin	23053486	Synergistic effect and antibiofilm activity between the antimicrobial peptide coprisin and conventional antibiotics against opportunistic bacteria.
4196	DRAMP04418	Defensin	"Defensin"[All Fields] AND biofilm[All Fields]	Defensin	22922323	Extracellular DNA within a nontypeable Haemophilus influenzae-induced biofilm binds human beta defensin-3 and reduces its antimicrobial activity.
4196	DRAMP04418	Defensin	"Defensin"[All Fields] AND biofilm[All Fields]	Defensin	22855857	Retrocyclin inhibits Gardnerella vaginalis biofilm formation and toxin activity.
4196	DRAMP04418	Defensin	"Defensin"[All Fields] AND biofilm[All Fields]	Defensin	22394470	Association of CiaRH with resistance of Streptococcus mutans to antimicrobial peptides in biofilms.
4196	DRAMP04418	Defensin	"Defensin"[All Fields] AND biofilm[All Fields]	Defensin	22229614	Comparison of the effects of human β-defensin 3, vancomycin, and clindamycin on Staphylococcus aureus biofilm formation.
4196	DRAMP04418	Defensin	"Defensin"[All Fields] AND biofilm[All Fields]	Defensin	21692631	A novel organotypic dento-epithelial culture model: effect of Fusobacterium nucleatum biofilm on B-defensin-2, -3, and LL-37 expression.
4196	DRAMP04418	Defensin	"Defensin"[All Fields] AND biofilm[All Fields]	Defensin	20454633	Normal human gingival epithelial cells sense C. parapsilosis by toll-like receptors and module its pathogenesis through antimicrobial peptides and proinflammatory cytokines.
4196	DRAMP04418	Defensin	"Defensin"[All Fields] AND biofilm[All Fields]	Defensin	20378008	Effect of temperature on the shift of Pseudomonas fluorescens from an environmental microorganism to a potential human pathogen.
4196	DRAMP04418	Defensin	"Defensin"[All Fields] AND biofilm[All Fields]	Defensin	19961380	Gingival transcriptome patterns during induction and resolution of experimental gingivitis in humans.
4196	DRAMP04418	Defensin	"Defensin"[All Fields] AND biofilm[All Fields]	Defensin	19780045	Candida famata modulates toll-like receptor, beta-defensin, and proinflammatory cytokine expression by normal human epithelial cells.
4196	DRAMP04418	Defensin	"Defensin"[All Fields] AND biofilm[All Fields]	Defensin	19572896	The immune response of oral epithelial cells induced by single-species and complex naturally formed biofilms.
4196	DRAMP04418	Defensin	"Defensin"[All Fields] AND biofilm[All Fields]	Defensin	19466693	Activity of antimicrobial peptides in the presence of polysaccharides produced by pulmonary pathogens.
4196	DRAMP04418	Defensin	"Defensin"[All Fields] AND biofilm[All Fields]	Defensin	18954353	Treponema denticola does not induce production of common innate immune mediators from primary gingival epithelial cells.
4196	DRAMP04418	Defensin	"Defensin"[All Fields] AND biofilm[All Fields]	Defensin	18173794	The stage of native biofilm formation determines the gene expression of human beta-defensin-2, psoriasin, ribonuclease 7 and inflammatory mediators: a novel approach for stimulation of keratinocytes with in situ formed biofilms.
4196	DRAMP04418	Defensin	"Defensin"[All Fields] AND biofilm[All Fields]	Defensin	17434999	Functional analysis of D-alanylation of lipoteichoic acid in the probiotic strain Lactobacillus rhamnosus GG.
4196	DRAMP04418	Defensin	"Defensin"[All Fields] AND biofilm[All Fields]	Defensin	15493829	Bacterial evasion of innate host defenses--the Staphylococcus aureus lesson.
4196	DRAMP04418	Defensin	"Defensin"[All Fields] AND biofilm[All Fields]	Defensin	14764110	Polysaccharide intercellular adhesin (PIA) protects Staphylococcus epidermidis against major components of the human innate immune system.
4197	DRAMP04419	Putative uncharacterized protein	"Putative uncharacterized protein"[All Fields] AND biofilm[All Fields]	Putative uncharacterized protein	27812354	Sub-MICs of Azithromycin Decrease Biofilm Formation of Streptococcus suisand Increase Capsular Polysaccharide Content of S. suis.
4199	DRAMP04421	Defensin	"Defensin"[All Fields] AND biofilm[All Fields]	Defensin	34408988	The Anti-Biofilm Efficacy of Caffeic Acid Phenethyl Ester (CAPE) In Vitroand a Murine Model of Oral Candidiasis.
4199	DRAMP04421	Defensin	"Defensin"[All Fields] AND biofilm[All Fields]	Defensin	34321877	Antibacterial Effect of Honey-Derived Exosomes Containing Antimicrobial Peptides Against Oral Streptococci.
4199	DRAMP04421	Defensin	"Defensin"[All Fields] AND biofilm[All Fields]	Defensin	34276631	DNA Blocks the Lethal Effect of Human Beta-Defensin 2 Against Neisseria meningitidis.
4199	DRAMP04421	Defensin	"Defensin"[All Fields] AND biofilm[All Fields]	Defensin	33911935	The antibacterial activities of honey.
4199	DRAMP04421	Defensin	"Defensin"[All Fields] AND biofilm[All Fields]	Defensin	33865931	Identification of a crocodylian β-defensin variant from Alligator mississippiensis with antimicrobial and antibiofilm activity.
4199	DRAMP04421	Defensin	"Defensin"[All Fields] AND biofilm[All Fields]	Defensin	33586659	Identification of anti-microbial peptides and traces of microbial DNA in infrainfundibular compartments of human scalp terminal hair follicles.
4199	DRAMP04421	Defensin	"Defensin"[All Fields] AND biofilm[All Fields]	Defensin	33534018	A recombinant fungal defensin-like peptide-P2 combats Streptococcus dysgalactiae and biofilms.
4199	DRAMP04421	Defensin	"Defensin"[All Fields] AND biofilm[All Fields]	Defensin	33447687	Future directions of postoperative spinal implant infections.

4199	DRAMP04421	Defensin	"Defensin"[All Fields] AND biofilm[All Fields]	Defensin	33420317	Curbing gastrointestinal infections by defensin fragment modifications without harming commensal microbiota.
4199	DRAMP04421	Defensin	"Defensin"[All Fields] AND biofilm[All Fields]	Defensin	32867384	A Novel Peptide Antibiotic, Pro10-1D, Designed from Insect Defensin Shows Antibacterial and Anti-Inflammatory Activities in Sepsis Models.
4199	DRAMP04421	Defensin	"Defensin"[All Fields] AND biofilm[All Fields]	Defensin	32858856	Candida albicansVirulence Factors and Pathogenicity for Endodontic Infections.
4199	DRAMP04421	Defensin	"Defensin"[All Fields] AND biofilm[All Fields]	Defensin	32842903	Electrospun ZnO/Poly(Vinylidene Fluoride-Trifluoroethylene) Scaffolds for Lung Tissue Engineering.
4199	DRAMP04421	Defensin	"Defensin"[All Fields] AND biofilm[All Fields]	Defensin	32663201	Inhibition and eradication activity of truncated α-defensin analogs against multidrug resistant uropathogenic Escherichia coli biofilm.
4199	DRAMP04421	Defensin	"Defensin"[All Fields] AND biofilm[All Fields]	Defensin	32585445	Glucose effect on Candida albicans biofilm during tissue invasion.
4199	DRAMP04421	Defensin	"Defensin"[All Fields] AND biofilm[All Fields]	Defensin	32522780	Controlling the Growth of the Skin Commensal Staphylococcus epidermidis Using d-Alanine Auxotrophy.
4199	DRAMP04421	Defensin	"Defensin"[All Fields] AND biofilm[All Fields]	Defensin	32457749	The Antimicrobial Peptide Human Beta-Defensin 2 Inhibits Biofilm Production of Pseudomonas aeruginosaWithout Compromising Metabolic Activity.
4199	DRAMP04421	Defensin	"Defensin"[All Fields] AND biofilm[All Fields]	Defensin	32439511	Antibacterial activities and mechanisms of action of a defensin from manila clam Ruditapes philippinarum.
4199	DRAMP04421	Defensin	"Defensin"[All Fields] AND biofilm[All Fields]	Defensin	31933178	Effects of human β-defensin 3 fused with carbohydrate-binding domain on the function of type III secretion system in Pseudomonas aeruginosa PA14.
4199	DRAMP04421	Defensin	"Defensin"[All Fields] AND biofilm[All Fields]	Defensin	31906541	Role of FAD-I in Fusobacterial Interspecies Interaction and Biofilm Formation.
4199	DRAMP04421	Defensin	"Defensin"[All Fields] AND biofilm[All Fields]	Defensin	31729441	Rhesus Theta Defensin 1 Promotes Long Term Survival in Systemic Candidiasis by Host Directed Mechanisms.
4199	DRAMP04421	Defensin	"Defensin"[All Fields] AND biofilm[All Fields]	Defensin	31336838	Candida albicans-Cell Interactions Activate Innate Immune Defense in Human Palate Epithelial Primary Cells via Nitric Oxide (NO) and β-Defensin 2 (hBD-2).
4199	DRAMP04421	Defensin	"Defensin"[All Fields] AND biofilm[All Fields]	Defensin	31165072	Impact of the Food Additive Titanium Dioxide (E171) on Gut Microbiota-Host Interaction.
4199	DRAMP04421	Defensin	"Defensin"[All Fields] AND biofilm[All Fields]	Defensin	31031739	Salt-Tolerant Antifungal and Antibacterial Activities of the Corn Defensin ZmD32.
4199	DRAMP04421	Defensin	"Defensin"[All Fields] AND biofilm[All Fields]	Defensin	31025073	A recombinant fungal defensin-like peptide-P2 combats multidrug-resistant Staphylococcus aureus and biofilms.
4199	DRAMP04421	Defensin	"Defensin"[All Fields] AND biofilm[All Fields]	Defensin	30659503	Lactobacillus plantarum USM8613 Aids in Wound Healing and Suppresses Staphylococcus aureus Infection at Wound Sites.
4199	DRAMP04421	Defensin	"Defensin"[All Fields] AND biofilm[All Fields]	Defensin	30649289	Innate immune components affect growth and virulence traits of bacterial-vaginosis-associated and non-bacterial-vaginosis-associated Gardnerella vaginalis strains similarly.
4199	DRAMP04421	Defensin	"Defensin"[All Fields] AND biofilm[All Fields]	Defensin	30376742	Alpha defensin, leukocyte esterase, C-reactive protein, and leukocyte count in synovial fluid for pre-operative diagnosis of periprosthetic infection.
4199	DRAMP04421	Defensin	"Defensin"[All Fields] AND biofilm[All Fields]	Defensin	30260708	Stabilized collagen matrix dressing improves wound macrophage function and epithelialization.
4199	DRAMP04421	Defensin	"Defensin"[All Fields] AND biofilm[All Fields]	Defensin	30254440	Modification of the surface of titanium with multifunctional chimeric peptides to prevent biofilm formation via inhibition of initial colonizers.
4199	DRAMP04421	Defensin	"Defensin"[All Fields] AND biofilm[All Fields]	Defensin	29902560	A defensin-like antimicrobial peptide from the manila clam Ruditapes philippinarum: Investigation of the antibacterial activities and mode of action.
4199	DRAMP04421	Defensin	"Defensin"[All Fields] AND biofilm[All Fields]	Defensin	29872295	Antimicrobial peptide-loaded liquid crystalline precursor bioadhesive system for the prevention of dental caries.
4199	DRAMP04421	Defensin	"Defensin"[All Fields] AND biofilm[All Fields]	Defensin	29671721	The BceABRS four-component system that is essential for cell envelope stress response is involved in sensing and response to host defence peptides and is required for the biofilm formation and fitness of Streptococcus mutans.
4199	DRAMP04421	Defensin	"Defensin"[All Fields] AND biofilm[All Fields]	Defensin	29104569	A Linear 19-Mer Plant Defensin-Derived Peptide Acts Synergistically with Caspofungin against Candida albicansBiofilms.
4199	DRAMP04421	Defensin	"Defensin"[All Fields] AND biofilm[All Fields]	Defensin	29077172	The significance of HBD-3 and fluorescent composite carriers in the processof bone formation in rats infected with Staphylococcus aureus.
4199	DRAMP04421	Defensin	"Defensin"[All Fields] AND biofilm[All Fields]	Defensin	29045084	New Approach to Treat and Prevent Oral Disease.
4199	DRAMP04421	Defensin	"Defensin"[All Fields] AND biofilm[All Fields]	Defensin	29025642	Engineered chimeric peptides with antimicrobial and titanium-binding functions to inhibit biofilm formation on Ti implants.
4199	DRAMP04421	Defensin	"Defensin"[All Fields] AND biofilm[All Fields]	Defensin	28956355	The synthetic human beta-defensin-3 C15 peptide exhibits antimicrobial activity against Streptococcus mutans, both alone and in combination with dental disinfectants.
4199	DRAMP04421	Defensin	"Defensin"[All Fields] AND biofilm[All Fields]	Defensin	28951032	Antifungal Effects of Synthetic Human Beta-defensin-3-C15 Peptide on Candida albicans-infected Root Dentin.
4199	DRAMP04421	Defensin	"Defensin"[All Fields] AND biofilm[All Fields]	Defensin	28874606	Bacterial d-amino acids suppress sinonasal innate immunity through sweet taste receptors in solitary chemosensory cells.
4199	DRAMP04421	Defensin	"Defensin"[All Fields] AND biofilm[All Fields]	Defensin	28725299	Role of Streptococcus mutanstwo-component systems in antimicrobial peptide resistance in the oral cavity.
4199	DRAMP04421	Defensin	"Defensin"[All Fields] AND biofilm[All Fields]	Defensin	28649561	Psd1 Effects on Candida albicansPlanktonic Cells and Biofilms.
4199	DRAMP04421	Defensin	"Defensin"[All Fields] AND biofilm[All Fields]	Defensin	28642103	Chimeric analogs of human β-defensin 1 and θ-defensin disrupt pre-established bacterial biofilms.
4199	DRAMP04421	Defensin	"Defensin"[All Fields] AND biofilm[All Fields]	Defensin	28413476	The mechanism of human β-defensin 3 in MRSA-induced infection of implant drug-resistant bacteria biofilm in the mouse tibial bone marrow.
4199	DRAMP04421	Defensin	"Defensin"[All Fields] AND biofilm[All Fields]	Defensin	28296382	Human α-Defensin 6: A Small Peptide That Self-Assembles and Protects the Host by Entangling Microbes.
4199	DRAMP04421	Defensin	"Defensin"[All Fields] AND biofilm[All Fields]	Defensin	28278280	Natural antimicrobial peptide complexes in the fighting of antibiotic resistant biofilms: Calliphora vicina medicinal maggots.
4199	DRAMP04421	Defensin	"Defensin"[All Fields] AND biofilm[All Fields]	Defensin	28144375	Advancements in Diagnosing Periprosthetic Joint Infections after Total Hip and Knee Arthroplasty.
4199	DRAMP04421	Defensin	"Defensin"[All Fields] AND biofilm[All Fields]	Defensin	28078813	Host defense peptide-derived privileged scaffolds for anti-infective drug discovery.
4199	DRAMP04421	Defensin	"Defensin"[All Fields] AND biofilm[All Fields]	Defensin	28026958	Human α-Defensin 6 Self-Assembly Prevents Adhesion and Suppresses Virulence Traits of Candida albicans.
4199	DRAMP04421	Defensin	"Defensin"[All Fields] AND biofilm[All Fields]	Defensin	27794585	A Novel Defensin-Like Peptide Associated with Two Other New Cationic Antimicrobial Peptides in Transcriptome of the Iranian Scorpion Venom.
4199	DRAMP04421	Defensin	"Defensin"[All Fields] AND biofilm[All Fields]	Defensin	27777572	Role of yqiCin the Pathogenicity of Salmonellaand Innate Immune Responses of Human Intestinal Epithelium.
4199	DRAMP04421	Defensin	"Defensin"[All Fields] AND biofilm[All Fields]	Defensin	27582732	Efficient Eradication of Mature Pseudomonas aeruginosa Biofilm via Controlled Delivery of Nitric Oxide Combined with Antimicrobial Peptide and Antibiotics.
4199	DRAMP04421	Defensin	"Defensin"[All Fields] AND biofilm[All Fields]	Defensin	27417541	Acute appendicitis: transcript profiling of blood identifies promising biomarkers and potential underlying processes.
4199	DRAMP04421	Defensin	"Defensin"[All Fields] AND biofilm[All Fields]	Defensin	27200276	Antifungal effects of synthetic human β-defensin 3-C15 peptide.
4199	DRAMP04421	Defensin	"Defensin"[All Fields] AND biofilm[All Fields]	Defensin	27148195	Effect of Substance P in Staphylococcus aureus and Staphylococcus epidermidis Virulence: Implication for Skin Homeostasis.
4199	DRAMP04421	Defensin	"Defensin"[All Fields] AND biofilm[All Fields]	Defensin	26861950	Antibiofilm efficacy of honey and bee-derived defensin-1 on multispecies wound biofilm.
4199	DRAMP04421	Defensin	"Defensin"[All Fields] AND biofilm[All Fields]	Defensin	26592804	The radish defensins RsAFP1 and RsAFP2 act synergistically with caspofungin against Candida albicans biofilms.
4199	DRAMP04421	Defensin	"Defensin"[All Fields] AND biofilm[All Fields]	Defensin	26248029	Synergistic Activity of the Plant Defensin HsAFP1 and Caspofungin against Candida albicans Biofilms and Planktonic Cultures.
4199	DRAMP04421	Defensin	"Defensin"[All Fields] AND biofilm[All Fields]	Defensin	26214284	α-tocopherol decreases interleukin-1β and -6 and increases human β-defensin-1 and -2 secretion in human gingival fibroblasts stimulated with Porphyromonas gingivalis lipopolysaccharide.
4199	DRAMP04421	Defensin	"Defensin"[All Fields] AND biofilm[All Fields]	Defensin	26196513	Snake Cathelicidin NA-CATH and Smaller Helical Antimicrobial Peptides Are Effective against Burkholderia thailandensis.
4199	DRAMP04421	Defensin	"Defensin"[All Fields] AND biofilm[All Fields]	Defensin	26119274	Ultrasound microbubbles enhance human β-defensin 3 against biofilms.
4199	DRAMP04421	Defensin	"Defensin"[All Fields] AND biofilm[All Fields]	Defensin	25862466	The Antibacterial Effects of an Antimicrobial Peptide Human β-Defensin 3 Fused with Carbohydrate-Binding Domain on Pseudomonas aeruginosa PA14.
4199	DRAMP04421	Defensin	"Defensin"[All Fields] AND biofilm[All Fields]	Defensin	25808131	Expression of antimicrobial peptides and interleukin-8 during early stages of inflammation: An experimental gingivitis study.
4199	DRAMP04421	Defensin	"Defensin"[All Fields] AND biofilm[All Fields]	Defensin	25806720	Antimicrobial peptides in 2014.
4199	DRAMP04421	Defensin	"Defensin"[All Fields] AND biofilm[All Fields]	Defensin	25285879	Inhibition and destruction of Pseudomonas aeruginosa biofilms by antibiotics and antimicrobial peptides.
4199	DRAMP04421	Defensin	"Defensin"[All Fields] AND biofilm[All Fields]	Defensin	25212593	Health- and disease-associated species clusters in complex natural biofilms determine the innate immune response in oral epithelial cells during biofilm maturation.
4199	DRAMP04421	Defensin	"Defensin"[All Fields] AND biofilm[All Fields]	Defensin	24913184	Effects of human β-defensin-3 on biofilm formation-regulating genes dltB and icaA in Staphylococcus aureus.

4199	DRAMP04421	Defensin	"Defensin"[All Fields] AND biofilm[All Fields]	Defensin	24340061	Inflammatory and antimicrobial responses to methicillin-resistant Staphylococcus aureus in an in vitro wound infection model.
4199	DRAMP04421	Defensin	"Defensin"[All Fields] AND biofilm[All Fields]	Defensin	24240906	Adsorption study of pellicle proteins to gold, silica and titanium by quartz crystal microbalance method.
4199	DRAMP04421	Defensin	"Defensin"[All Fields] AND biofilm[All Fields]	Defensin	24238461	Antibacterial efficacy of a human β-defensin-3 peptide on multispecies biofilms.
4199	DRAMP04421	Defensin	"Defensin"[All Fields] AND biofilm[All Fields]	Defensin	24100890	[Use of recombinant human beta-defensin-3 to evaluate the effect of adhesion of Candida albicans on the surface of soft lining material].
4199	DRAMP04421	Defensin	"Defensin"[All Fields] AND biofilm[All Fields]	Defensin	23639356	Human beta-defensin 3: a novel inhibitor of Staphylococcus-produced biofilm production. Commentary on "Human β-defensin 3 inhibits antibiotic-resistant Staphylococcus biofilm formation".
4199	DRAMP04421	Defensin	"Defensin"[All Fields] AND biofilm[All Fields]	Defensin	23519963	Ultrasound-targeted microbubble destruction enhances human β-defensin 3 activity against antibiotic-resistant Staphylococcus biofilms.
4199	DRAMP04421	Defensin	"Defensin"[All Fields] AND biofilm[All Fields]	Defensin	23273885	Human β-defensin 3 inhibits antibiotic-resistant Staphylococcus biofilm formation.
4199	DRAMP04421	Defensin	"Defensin"[All Fields] AND biofilm[All Fields]	Defensin	23203265	Potentiation of the cytotoxic activity of copper by polyphosphate on biofilm-producing bacteria: a bioinspired approach.
4199	DRAMP04421	Defensin	"Defensin"[All Fields] AND biofilm[All Fields]	Defensin	23078156	Antimicrobial efficacy of a human β-defensin-3 peptide using an Enterococcus faecalis dentine infection model.
4199	DRAMP04421	Defensin	"Defensin"[All Fields] AND biofilm[All Fields]	Defensin	23053486	Synergistic effect and antibiofilm activity between the antimicrobial peptide coprisin and conventional antibiotics against opportunistic bacteria.
4199	DRAMP04421	Defensin	"Defensin"[All Fields] AND biofilm[All Fields]	Defensin	22922323	Extracellular DNA within a nontypeable Haemophilus influenzae-induced biofilm binds human beta defensin-3 and reduces its antimicrobial activity.
4199	DRAMP04421	Defensin	"Defensin"[All Fields] AND biofilm[All Fields]	Defensin	22855857	Retrocyclin inhibits Gardnerella vaginalis biofilm formation and toxin activity.
4199	DRAMP04421	Defensin	"Defensin"[All Fields] AND biofilm[All Fields]	Defensin	22394470	Association of CiaRH with resistance of Streptococcus mutans to antimicrobial peptides in biofilms.
4199	DRAMP04421	Defensin	"Defensin"[All Fields] AND biofilm[All Fields]	Defensin	22229614	Comparison of the effects of human β-defensin 3, vancomycin, and clindamycin on Staphylococcus aureus biofilm formation.
4199	DRAMP04421	Defensin	"Defensin"[All Fields] AND biofilm[All Fields]	Defensin	21692631	A novel organotypic dento-epithelial culture model: effect of Fusobacterium nucleatum biofilm on B-defensin-2, -3, and LL-37 expression.
4199	DRAMP04421	Defensin	"Defensin"[All Fields] AND biofilm[All Fields]	Defensin	20454633	Normal human gingival epithelial cells sense C. parapsilosis by toll-like receptors and module its pathogenesis through antimicrobial peptides and proinflammatory cytokines.
4199	DRAMP04421	Defensin	"Defensin"[All Fields] AND biofilm[All Fields]	Defensin	20378008	Effect of temperature on the shift of Pseudomonas fluorescens from an environmental microorganism to a potential human pathogen.
4199	DRAMP04421	Defensin	"Defensin"[All Fields] AND biofilm[All Fields]	Defensin	19961380	Gingival transcriptome patterns during induction and resolution of experimental gingivitis in humans.
4199	DRAMP04421	Defensin	"Defensin"[All Fields] AND biofilm[All Fields]	Defensin	19780045	Candida famata modulates toll-like receptor, beta-defensin, and proinflammatory cytokine expression by normal human epithelial cells.
4199	DRAMP04421	Defensin	"Defensin"[All Fields] AND biofilm[All Fields]	Defensin	19572896	The immune response of oral epithelial cells induced by single-species and complex naturally formed biofilms.
4199	DRAMP04421	Defensin	"Defensin"[All Fields] AND biofilm[All Fields]	Defensin	19466693	Activity of antimicrobial peptides in the presence of polysaccharides produced by pulmonary pathogens.
4199	DRAMP04421	Defensin	"Defensin"[All Fields] AND biofilm[All Fields]	Defensin	18954353	Treponema denticola does not induce production of common innate immune mediators from primary gingival epithelial cells.
4199	DRAMP04421	Defensin	"Defensin"[All Fields] AND biofilm[All Fields]	Defensin	18173794	The stage of native biofilm formation determines the gene expression of human beta-defensin-2, psoriasin, ribonuclease 7 and inflammatory mediators: a novel approach for stimulation of keratinocytes with in situ formed biofilms.
4199	DRAMP04421	Defensin	"Defensin"[All Fields] AND biofilm[All Fields]	Defensin	17434999	Functional analysis of D-alanylation of lipoteichoic acid in the probiotic strain Lactobacillus rhamnosus GG.
4199	DRAMP04421	Defensin	"Defensin"[All Fields] AND biofilm[All Fields]	Defensin	15493829	Bacterial evasion of innate host defenses--the Staphylococcus aureus lesson.
4199	DRAMP04421	Defensin	"Defensin"[All Fields] AND biofilm[All Fields]	Defensin	14764110	Polysaccharide intercellular adhesin (PIA) protects Staphylococcus epidermidis against major components of the human innate immune system.
4200	DRAMP04422	Defensin 1	"Defensin 1"[All Fields] AND biofilm[All Fields]	Defensin 1	34321877	Antibacterial Effect of Honey-Derived Exosomes Containing Antimicrobial Peptides Against Oral Streptococci.
4200	DRAMP04422	Defensin 1	"Defensin 1"[All Fields] AND biofilm[All Fields]	Defensin 1	33911935	The antibacterial activities of honey.
4200	DRAMP04422	Defensin 1	"Defensin 1"[All Fields] AND biofilm[All Fields]	Defensin 1	33420317	Curbing gastrointestinal infections by defensin fragment modifications without harming commensal microbiota.
4200	DRAMP04422	Defensin 1	"Defensin 1"[All Fields] AND biofilm[All Fields]	Defensin 1	32585445	Glucose effect on Candida albicans biofilm during tissue invasion.
4200	DRAMP04422	Defensin 1	"Defensin 1"[All Fields] AND biofilm[All Fields]	Defensin 1	31729441	Rhesus Theta Defensin 1 Promotes Long Term Survival in Systemic Candidiasis by Host Directed Mechanisms.
4200	DRAMP04422	Defensin 1	"Defensin 1"[All Fields] AND biofilm[All Fields]	Defensin 1	30260708	Stabilized collagen matrix dressing improves wound macrophage function and epithelialization.
4200	DRAMP04422	Defensin 1	"Defensin 1"[All Fields] AND biofilm[All Fields]	Defensin 1	29671721	The BceABRS four-component system that is essential for cell envelope stress response is involved in sensing and response to host defence peptides and is required for the biofilm formation and fitness of Streptococcus mutans.
4200	DRAMP04422	Defensin 1	"Defensin 1"[All Fields] AND biofilm[All Fields]	Defensin 1	28649561	Psd1 Effects on Candida albicansPlanktonic Cells and Biofilms.
4200	DRAMP04422	Defensin 1	"Defensin 1"[All Fields] AND biofilm[All Fields]	Defensin 1	28642103	Chimeric analogs of human β-defensin 1 and θ-defensin disrupt pre-established bacterial biofilms.
4200	DRAMP04422	Defensin 1	"Defensin 1"[All Fields] AND biofilm[All Fields]	Defensin 1	26861950	Antibiofilm efficacy of honey and bee-derived defensin-1 on multispecies wound biofilm.
4200	DRAMP04422	Defensin 1	"Defensin 1"[All Fields] AND biofilm[All Fields]	Defensin 1	26214284	α-tocopherol decreases interleukin-1β and -6 and increases human β-defensin-1 and -2 secretion in human gingival fibroblasts stimulated with Porphyromonas gingivalis lipopolysaccharide.
4200	DRAMP04422	Defensin 1	"Defensin 1"[All Fields] AND biofilm[All Fields]	Defensin 1	22394470	Association of CiaRH with resistance of Streptococcus mutans to antimicrobial peptides in biofilms.
4200	DRAMP04422	Defensin 1	"Defensin 1"[All Fields] AND biofilm[All Fields]	Defensin 1	20454633	Normal human gingival epithelial cells sense C. parapsilosis by toll-like receptors and module its pathogenesis through antimicrobial peptides and proinflammatory cytokines.
4201	DRAMP04423	Defensin	"Defensin"[All Fields] AND biofilm[All Fields]	Defensin	34408988	The Anti-Biofilm Efficacy of Caffeic Acid Phenethyl Ester (CAPE) In Vitroand a Murine Model of Oral Candidiasis.
4201	DRAMP04423	Defensin	"Defensin"[All Fields] AND biofilm[All Fields]	Defensin	34321877	Antibacterial Effect of Honey-Derived Exosomes Containing Antimicrobial Peptides Against Oral Streptococci.
4201	DRAMP04423	Defensin	"Defensin"[All Fields] AND biofilm[All Fields]	Defensin	34276631	DNA Blocks the Lethal Effect of Human Beta-Defensin 2 Against Neisseria meningitidis.
4201	DRAMP04423	Defensin	"Defensin"[All Fields] AND biofilm[All Fields]	Defensin	33911935	The antibacterial activities of honey.
4201	DRAMP04423	Defensin	"Defensin"[All Fields] AND biofilm[All Fields]	Defensin	33865931	Identification of a crocodylian β-defensin variant from Alligator mississippiensis with antimicrobial and antibiofilm activity.
4201	DRAMP04423	Defensin	"Defensin"[All Fields] AND biofilm[All Fields]	Defensin	33586659	Identification of anti-microbial peptides and traces of microbial DNA in infrainfundibular compartments of human scalp terminal hair follicles.
4201	DRAMP04423	Defensin	"Defensin"[All Fields] AND biofilm[All Fields]	Defensin	33534018	A recombinant fungal defensin-like peptide-P2 combats Streptococcus dysgalactiae and biofilms.
4201	DRAMP04423	Defensin	"Defensin"[All Fields] AND biofilm[All Fields]	Defensin	33447687	Future directions of postoperative spinal implant infections.
4201	DRAMP04423	Defensin	"Defensin"[All Fields] AND biofilm[All Fields]	Defensin	33420317	Curbing gastrointestinal infections by defensin fragment modifications without harming commensal microbiota.
4201	DRAMP04423	Defensin	"Defensin"[All Fields] AND biofilm[All Fields]	Defensin	32867384	A Novel Peptide Antibiotic, Pro10-1D, Designed from Insect Defensin Shows Antibacterial and Anti-Inflammatory Activities in Sepsis Models.
4201	DRAMP04423	Defensin	"Defensin"[All Fields] AND biofilm[All Fields]	Defensin	32858856	Candida albicansVirulence Factors and Pathogenicity for Endodontic Infections.
4201	DRAMP04423	Defensin	"Defensin"[All Fields] AND biofilm[All Fields]	Defensin	32842903	Electrospun ZnO/Poly(Vinylidene Fluoride-Trifluoroethylene) Scaffolds for Lung Tissue Engineering.
4201	DRAMP04423	Defensin	"Defensin"[All Fields] AND biofilm[All Fields]	Defensin	32663201	Inhibition and eradication activity of truncated α-defensin analogs against multidrug resistant uropathogenic Escherichia coli biofilm.
4201	DRAMP04423	Defensin	"Defensin"[All Fields] AND biofilm[All Fields]	Defensin	32585445	Glucose effect on Candida albicans biofilm during tissue invasion.
4201	DRAMP04423	Defensin	"Defensin"[All Fields] AND biofilm[All Fields]	Defensin	32522780	Controlling the Growth of the Skin Commensal Staphylococcus epidermidis Using d-Alanine Auxotrophy.
4201	DRAMP04423	Defensin	"Defensin"[All Fields] AND biofilm[All Fields]	Defensin	32457749	The Antimicrobial Peptide Human Beta-Defensin 2 Inhibits Biofilm Production of Pseudomonas aeruginosaWithout Compromising Metabolic Activity.
4201	DRAMP04423	Defensin	"Defensin"[All Fields] AND biofilm[All Fields]	Defensin	32439511	Antibacterial activities and mechanisms of action of a defensin from manila clam Ruditapes philippinarum.
4201	DRAMP04423	Defensin	"Defensin"[All Fields] AND biofilm[All Fields]	Defensin	31933178	Effects of human β-defensin 3 fused with carbohydrate-binding domain on the function of type III secretion system in Pseudomonas aeruginosa PA14.

4201	DRAMP04423	Defensin	"Defensin"[All Fields] AND biofilm[All Fields]	Defensin	31906541	Role of FAD-I in Fusobacterial Interspecies Interaction and Biofilm Formation.
4201	DRAMP04423	Defensin	"Defensin"[All Fields] AND biofilm[All Fields]	Defensin	31729441	Rhesus Theta Defensin 1 Promotes Long Term Survival in Systemic Candidiasis by Host Directed Mechanisms.
4201	DRAMP04423	Defensin	"Defensin"[All Fields] AND biofilm[All Fields]	Defensin	31336838	Candida albicans-Cell Interactions Activate Innate Immune Defense in Human Palate Epithelial Primary Cells via Nitric Oxide (NO) and β-Defensin 2 (hBD-2).
4201	DRAMP04423	Defensin	"Defensin"[All Fields] AND biofilm[All Fields]	Defensin	31165072	Impact of the Food Additive Titanium Dioxide (E171) on Gut Microbiota-Host Interaction.
4201	DRAMP04423	Defensin	"Defensin"[All Fields] AND biofilm[All Fields]	Defensin	31031739	Salt-Tolerant Antifungal and Antibacterial Activities of the Corn Defensin ZmD32.
4201	DRAMP04423	Defensin	"Defensin"[All Fields] AND biofilm[All Fields]	Defensin	31025073	A recombinant fungal defensin-like peptide-P2 combats multidrug-resistant Staphylococcus aureus and biofilms.
4201	DRAMP04423	Defensin	"Defensin"[All Fields] AND biofilm[All Fields]	Defensin	30659503	Lactobacillus plantarum USM8613 Aids in Wound Healing and Suppresses Staphylococcus aureus Infection at Wound Sites.
4201	DRAMP04423	Defensin	"Defensin"[All Fields] AND biofilm[All Fields]	Defensin	30649289	Innate immune components affect growth and virulence traits of bacterial-vaginosis-associated and non-bacterial-vaginosis-associated Gardnerella vaginalis strains similarly.
4201	DRAMP04423	Defensin	"Defensin"[All Fields] AND biofilm[All Fields]	Defensin	30376742	Alpha defensin, leukocyte esterase, C-reactive protein, and leukocyte count in synovial fluid for pre-operative diagnosis of periprosthetic infection.
4201	DRAMP04423	Defensin	"Defensin"[All Fields] AND biofilm[All Fields]	Defensin	30260708	Stabilized collagen matrix dressing improves wound macrophage function and epithelialization.
4201	DRAMP04423	Defensin	"Defensin"[All Fields] AND biofilm[All Fields]	Defensin	30254440	Modification of the surface of titanium with multifunctional chimeric peptides to prevent biofilm formation via inhibition of initial colonizers.
4201	DRAMP04423	Defensin	"Defensin"[All Fields] AND biofilm[All Fields]	Defensin	29902560	A defensin-like antimicrobial peptide from the manila clam Ruditapes philippinarum: Investigation of the antibacterial activities and mode of action.
4201	DRAMP04423	Defensin	"Defensin"[All Fields] AND biofilm[All Fields]	Defensin	29872295	Antimicrobial peptide-loaded liquid crystalline precursor bioadhesive system for the prevention of dental caries.
4201	DRAMP04423	Defensin	"Defensin"[All Fields] AND biofilm[All Fields]	Defensin	29671721	The BceABRS four-component system that is essential for cell envelope stress response is involved in sensing and response to host defence peptides and is required for the biofilm formation and fitness of Streptococcus mutans.
[truncated: 816,675 more chars]
