# Supplementary material for: AMPing Up the Search: A Structural and Functional Repository of Antimicrobial Peptides for Biofilm Studies, and a Case Study of Its Application to Corynebacterium striatum, an Emerging Pathogen
Source: Front Cell Infect Microbiol. 2021 Dec 16;11:803774. doi: 10.3389/fcimb.2021.803774 (PMC8716830; doi:10.3389/fcimb.2021.803774)
Supplement: Supplementary file 7 [file Table_4.pdf]

PepID	DRAMP_ID	Name	Queried Peptide Name	Article Count
6	DRAMP00068	Aureocin A53 (Bacteriocin)	Aureocin A53	2
21	DRAMP00177	Enterocin B (EntB; Bacteriocin)	Enterocin B	1
22	DRAMP00178	Enterocin EJ97 (EntEJ97; Bacteriocin)	Enterocin EJ97	1
33	DRAMP00275	Snakin-1 (StSN1; Cys-rich; Plant defensin)	Snakin-1	1
43	DRAMP00425	Tn-AFP1 (Trapa natans antifungal peptide; Plant defensin)	Tn-AFP1	1
55	DRAMP00856	Kalata-B1 (Plant defensin)	Kalata-B1	1
61	DRAMP00933	Antimicrobial peptide 1 (AMP1; MiAMP1; Plant defensin)	Antimicrobial peptide 1	1
64	DRAMP01016	Antimicrobial peptide 1 (MJ-AMP1; Plant defensin)	Antimicrobial peptide 1	1
67	DRAMP01061	Antifungal peptide (Cm-p1; Plants)	Antifungal peptide	9
94	DRAMP01152	Uperin-3.6 (toads, amphibians, animals)	Uperin-3.6	1
99	DRAMP01163	Buforin-2 (Buforin II; Fragment of Histone H2A; toads, amphibians, animals)	Buforin-2	1
107	DRAMP01184	SPX(1-22)(truncated peptide of Syphaxin; Frogs, amphibians, animals)	SPX	14
108	DRAMP01185	SPX(1-16)(truncated peptide of Syphaxin; Frogs, amphibians, animals)	SPX	14
154	DRAMP01288	Phylloseptin-1 (PSN-1; Frogs, amphibians, animals)	Phylloseptin-1	1
155	DRAMP01301	Phylloseptin-1 (PS-1; Frogs, amphibians, animals)	Phylloseptin-1	1
172	DRAMP01355	Ranalexin (Frogs, amphibians, animals)	Ranalexin	3
186	DRAMP00929	Antimicrobial peptide 1 (Cn-AMP1; Plant defensin)	Antimicrobial peptide 1	1
187	DRAMP03542	Neurokinin A (NKA; chicken, animals)	Neurokinin A	1
188	DRAMP04532	Myxinidin (Hagfish, animals)	Myxinidin	3
191	DRAMP02997	Apidaecin-1B (Apidaecin IB; Insects, animals)	Apidaecin-1B	1
193	DRAMP02840	Lactoferricin B (Lfcin B; mammals, animals)	Lactoferricin B	6
235	DRAMP18394	NCR335 (nodule-specific cysteine-rich peptides; plants)	NCR335	1
285	DRAMP01513	Esculentin-1 (Frogs, amphibians, animals)	Esculentin-1	2
318	DRAMP01587	Citropin-1.1 (Frogs, amphibians, animals)	Citropin-1.1	7
329	DRAMP01607	Aurein-1.2 (Frogs, amphibians, animals)	Aurein-1.2	3
331	DRAMP01612	Aurein-2.5 (Frogs, amphibians, animals)	Aurein-2.5	1
353	DRAMP18386	VK25 (histone derived; reptiles; animals)	VK25	1
359	DRAMP01730	Temporin-A (Frogs, amphibians, animals)	Temporin-A	6
373	DRAMP01754	Temporin-1CEb (Frogs, amphibians, animals)	Temporin-1CEb	3
386	DRAMP01779	Temporin-SHf (Frogs, amphibians, animals)	Temporin-SHf	1
387	DRAMP01780	Temporin-SHa (Temporin-1Sa; Frogs, amphibians, animals)	Temporin-SHa	2
414	DRAMP01847	Ascaphin-8 (Frogs, amphibians, animals)	Ascaphin-8	1
485	DRAMP02009	Brevinin-1 (Frogs, amphibians, animals)	Brevinin-1	4
486	DRAMP02010	Brevinin-2 (Frogs, amphibians, animals)	Brevinin-2	2
488	DRAMP18381	H4-(86-100) (histone-derived)	H4-	51
489	DRAMP18382	HNr (histone-derived)	HNr	1
542	DRAMP01746	Temporin-L (Temporin-1Tl; temporin-Tl; TL; Frogs, amphibians, animals)	Temporin-L	4
551	DRAMP02125	Hylin-a1 (Hy-a1; Frogs, amphibians, animals)	Hylin-a1	1
555	DRAMP02130	Antimicrobial peptide 1 (XT-1; Frogs, amphibians, animals)	Antimicrobial peptide 1	1
585	DRAMP03998	PAF26 (Trp-rich; combinatorial library)	PAF26	1

589	DRAMP02271	Magainin-2 (Magainin II; chain of Magainins; Frogs, amphibians, animals)	Magainin-2	8
590	DRAMP02272	PGLa (chain of PYLa/PGLa A; Frogs, amphibians, animals)	PGLa	7
616	DRAMP02314	Hepcidin (fish, chordates, animals)	Hepcidin	3
617	DRAMP02315	Chrysophsin-1 (fish, chordates, animals)	Chrysophsin-1	1
624	DRAMP02330	Piscidin-1 (Pis-1; Piscidin 1; fish, chordates, animals)	Piscidin-1	4
631	DRAMP02350	Pleurocidin (NRC-4; fish, chordates, animals)	Pleurocidin	5
633	DRAMP02352	NRC-16 (fish, chordates, animals)	NRC-16	1
682	DRAMP02441	Gramicidin S (GS)	Gramicidin S	9
685	DRAMP02449	Lectin	Lectin	332
686	DRAMP02456	L-amino-acid oxidase (LAAO; LAO; Dactylomelin-P)	L-amino-acid oxidase	3
687	DRAMP02470	Nosiheptide (NOS; Antibiotic 9671-RP)	Nosiheptide	1
688	DRAMP02473	Cathelicidin-BF (Cathelicidin-related protein; Snakes, reptiles, animals)	Cathelicidin-BF	3
690	DRAMP02478	L-amino-acid oxidase (Bm-LAO; LAAO; LAO; Snakes, reptiles, animals)	L-amino-acid oxidase	3
691	DRAMP02520	OH-CATH (Snakes, reptiles, animals)	OH-CATH	1
692	DRAMP02522	L-amino-acid oxidase (LAAO, LAO, Oh-LAAO; Snakes, reptiles, animals)	L-amino-acid oxidase	3
710	DRAMP02778	Defensin (Insects, animals)	Defensin	92
724	DRAMP02845	CP-11 (cathelicidin; mammals, animals)	CP-11	1
749	DRAMP02912	SMAP-29 (Cathelin-related peptide SC5; Myeloid antibacterial peptide MAP-29; mammals, animals)	SMAP-29	6
755	DRAMP02925	Cathelicidin (dogs, mammals, animals)	Cathelicidin	87
772	DRAMP02966	DBI(32-86) (pigs, mammals, animals)	DBI	2
773	DRAMP02970	Protegrin-1 (Protegrin 1; PG-1; pigs, mammals, animals)	Protegrin-1	4
784	DRAMP03001	Jellein-3 (Jelleine-III; Insects, animals)	Jellein-3	1
785	DRAMP03002	Melittin (Allergen Api m 3; Allergen Api m III; Insects, animals)	Melittin	30
792	DRAMP03028	Mastoparan-1 (MP-1; Venom protein MP-1; Insects, animals)	Mastoparan-1	1
804	DRAMP03044	Protonectin (Agelaia-chemotactic peptide, Agelaia-CP; Insects, animals)	Protonectin	1
815	DRAMP03057	Thanatin (Insects, animals)	Thanatin	1
817	DRAMP03069	Diptericin (Insects, animals)	Diptericin	2
823	DRAMP18495	Gomesin (Gm; Spiders, arachnids, Chelicerata, arthropods, invertebrates, animals)	Gomesin	1
830	DRAMP03138	Cecropin-A (Insects, animals)	Cecropin-A	9
835	DRAMP03166	P15 (deer beta-defensin; ruminant, animals)	P15	2
838	DRAMP03176	Cecropin-P1 (CP1; nematodes, animals)	Cecropin-P1	1
841	DRAMP03187	Beta defensin 1(BD-1; mammals, animals)	Beta defensin 1	7
846	DRAMP03215	Gomesin (Gm; spiders, Arthropods, animals)	Gomesin	1
887	DRAMP03507	Cecropin-B (Insects, animals)	Cecropin-B	2
902	DRAMP03528	Defensin (Galiomicin; Insects, animals)	Defensin	92
904	DRAMP03539	Antifungal protein (Psc-AFP)	Antifungal protein	5
908	DRAMP03567	KR-20 (Derived from LL-37)	KR-20	1
910	DRAMP03569	KS-30 (Derived from LL-37)	KS-30	1
913	DRAMP03573	LL-37(13-37)(C-terminal fragment of LL-37; Human, mammals, animals)	LL-37	123

914	DRAMP03574	LL-37(17-32)(C-terminal fragment of LL-37; Human, mammals, animals)	LL-37	123
915	DRAMP03598	Human beta-defensin 2 (hBD-2; Defensin, beta 2; Beta-defensin 4A; Human, mammals, animals)	Human beta-defensin 2	12
916	DRAMP03599	Human beta-defensin 3 (BD-3, hBD-3; Hbd3; Beta-defensin 103; Human, mammals, animals)	Human beta-defensin 3	24
921	DRAMP03645	Cathelicidin-2 (CATH-2; Fowlicidin-2; Birds, animals)	Cathelicidin-2	1
927	DRAMP03679	Cathelicidin-2 (Bactenecin-5, Bac5; ChBac5; ruminant, animals)	Cathelicidin-2	1
938	DRAMP03706	Antimicrobial peptide 1 (AamAP1; Arthropods, animals)	Antimicrobial peptide 1	1
949	DRAMP02828	BMAP-34 (BMAP 34, bovine cathelicidin, cattle, ruminant, mammals, animals)	BMAP-34	1
950	DRAMP02926	Tachyplesin I (Tac; TP1; Horseshoe Crab, arachnids, Chelicerata, arthropods, invertebrates, animals)	Tachyplesin I	2
986	DRAMP03859	W10 (Bac2A variant through single amino acid substitution)	W10	2
987	DRAMP03860	R11 (Bac2A variant through single amino acid substitution)	R11	4
988	DRAMP03861	G12 (Bac2A variant through single amino acid substitution)	G12	78
995	DRAMP03868	Bac8c (Bac2A variant)	Bac8c	4
997	DRAMP03870	Bac2A (a linear variant of bovine dodecapeptide)	Bac2A	2
1006	DRAMP03882	LFC (fragment of mature caprine lactoferrin, residues 17 to 31)	LFC	2
1037	DRAMP03920	Cecropin A (1-8)-melittin (1-13)hybrid peptide	Cecropin A	9
1038	DRAMP03921	Cecropin A (1-8)-melittin (1-18)hybrid peptide	Cecropin A	9
1039	DRAMP03922	Cecropin A (1-8)-melittin (1-12)hybrid peptide	Cecropin A	9
1040	DRAMP03923	Cecropin A (1-8)-melittin (1-10)hybrid peptide	Cecropin A	9
1041	DRAMP03924	Cecropin A (1-7)-melittin (1-8)hybrid peptide	Cecropin A	9
1042	DRAMP03925	Cecropin A (1-7)-melittin (3-10)hybrid peptide	Cecropin A	9
1043	DRAMP03927	Cecropin A (1-7)-melittin (2-9)hybrid peptide	Cecropin A	9
1044	DRAMP03928	Cecropin A (1-7)-melittin (4-11)hybrid peptide (CAM)	Cecropin A	9
1045	DRAMP03929	Cecropin A (1-7)-melittin (5-12)hybrid peptide	Cecropin A	9
1046	DRAMP03930	Cecropin A (1-7)-melittin (6-13)hybrid peptide	Cecropin A	9
1054	DRAMP03939	G20R (truncated isoform of thanatin, residue 1-20)	G20R	1
1057	DRAMP03948	Del 1 (Ranalexin analog)	Del 1	1
1065	DRAMP03967	P18 (Cecropin A(1-8)-Magainin 2(1-12) hybrid peptide analogue)	P18	3
1068	DRAMP03970	N-1 (analog of P18)	N-1	122
1069	DRAMP03971	N-2 (analog of P18)	N-2	170
1070	DRAMP03972	N-3 (analog of P18)	N-3	343
1071	DRAMP03973	N-4 (analog of P18)	N-4	132
1072	DRAMP03974	N-5 (analog of P18)	N-5	163
1076	DRAMP03978	C-1 (analog of P18)	C-1	50
1077	DRAMP03979	C-2 (analog of P18)	C-2	54
1078	DRAMP03980	C-3 (analog of P18)	C-3	32
1079	DRAMP03981	C-4 (analog of P18)	C-4	29
1080	DRAMP03982	C-5 (analog of P18)	C-5	26

1081	DRAMP03983	C-6 (analog of P18)	C-6	15
1082	DRAMP03984	C-7 (analog of P18)	C-7	13
1083	DRAMP03985	C-8 (analog of P18)	C-8	15
1084	DRAMP03986	C-9 (analog of P18)	C-9	9
1085	DRAMP03987	C-10 (analog of P18)	C-10	27
1116	DRAMP04025	R10 (single amino acid substitution of Bac034, which is a scrambled Variant of Bac2A)	R10	3
1117	DRAMP04026	K12 (single amino acid substitution of Bac034, which is a scrambled Variant of Bac2A)	K12	230
1118	DRAMP04027	opt1 (multiple amino acid substitution of Bac034, which is a scrambled Variant of Bac2A)	opt1	1
1119	DRAMP04028	opt2 (multiple amino acid substitution of Bac034, which is a scrambled Variant of Bac2A)	opt2	1
1169	DRAMP04102	CP-P	CP-P	3
1170	DRAMP04103	S16 (derivative of CP-P)	S16	5
1177	DRAMP04112	L10 (derivative of CP-P)	L10	4
1178	DRAMP04113	A10 (derivative of CP-P)	A10	4
1179	DRAMP04114	D11 (derivative of CP-P)	D11	1
1180	DRAMP04115	K11 (derivative of CP-P)	K11	1
1181	DRAMP04117	A13 (derivative of CP-P)	A13	2
1182	DRAMP04119	K17 (derivative of CP-P)	K17	2
1183	DRAMP04120	D18 (derivative of CP-P)	D18	1
1204	DRAMP04147	L10	L10	4
1228	DRAMP04184	DFTamP1	DFTamP1	1
1241	DRAMP04233	D28 (Rational design peptide)	D28	2
1243	DRAMP04235	D22 (Rational design peptide)	D22	1
1246	DRAMP04241	Synthetic 2	Synthetic 2	2
1247	DRAMP04242	Synthetic 3	Synthetic 3	3
1248	DRAMP04243	Synthetic 4	Synthetic 4	3
1250	DRAMP04264	CP26	CP26	1
1268	DRAMP04376	MP-1 (MP analog)	MP-1	2
1273	DRAMP04381	PMM-2 (PMM analog)	PMM-2	1
1301	DRAMP00004	Lantibiotic (Bacteriocin)	Lantibiotic	28
1319	DRAMP00024	CylLS (a structural subunit of cytolysin; Bacteriocin)	CylLS	1
1320	DRAMP00025	CylLL (a structural subunit of cytolysin; Bacteriocin)	CylLL	1
1321	DRAMP00026	Salivaricin A (SalA; Bacteriocin; Preclinical)	Salivaricin A	1
1325	DRAMP18349	Siamycin I (Bacteriocin)	Siamycin I	1
1330	DRAMP00036	Nisin A (Bacteriocin; Preclinical)	Nisin A	11
1331	DRAMP00037	Nisin Z (Bacteriocin; Preclinical)	Nisin Z	3
1333	DRAMP00039	Pep5 (Bacteriocin)	Pep5	2
1334	DRAMP00040	Gallidermin (Bacteriocin; Preclinical)	Gallidermin	8
1335	DRAMP00041	Mutacin-1140 (Mutacin III; Bacteriocin)	Mutacin-1140	1
1339	DRAMP18347	Siamycin(Bacteriocin)	Siamycin	1
1345	DRAMP00051	Mutacin I (Bacteriocin)	Mutacin I	5
1356	DRAMP00066	Lacticin Q (Bacteriocin)	Lacticin Q	2
1358	DRAMP00070	Laterosporulin (Bacteriocin)	Laterosporulin	1
1372	DRAMP00085	Bacteriocin	Bacteriocin	232
1381	DRAMP00096	Pediocin PA-1 (Pediocin ACH; Bacteriocin)	Pediocin PA-1	1
1385	DRAMP00101	Sakacin P (Sakacin 674; Pediocin-like peptide; Bacteriocin)	Sakacin P	1

1392	DRAMP00113	Enterocin A (EntA; Pediocin-like peptide; Bacteriocin)	Enterocin A	4
1395	DRAMP18340	Daptomycin(Bacteriocin)	Daptomycin	226
1409	DRAMP00143	Plantaricin-A (PlnA; Bacteriocin)	Plantaricin-A	1
1418	DRAMP00153	NlmA (chain a of Mutacin IV; Bacteriocin)	NlmA	3
1419	DRAMP00154	NlmB (chain b of Mutacin IV; Bacteriocin)	NlmB	1
1421	DRAMP00156	BrcB (NKR-5-3A; chain b of Brochocin C; Bacteriocin)	BrcB	11
1425	DRAMP00160	ThmA (chain a of Thermophilin 13; Bacteriocin)	ThmA	1
1430	DRAMP00165	Gassericin A (GaaA; Bacteriocin)	Gassericin A	2
1432	DRAMP00167	Subtilosin A (Antilisterial bacteriocin subtilosin; D-amino acid; Bacteriocin; Preclinical)	Subtilosin A	5
1434	DRAMP00169	Enterocin AS-48 (AS-48; Bacteriocin)	Enterocin AS-48	4
1447	DRAMP00185	Leucocin-B (Leu B; Leucocin B-TA33a; Bacteriocin)	Leucocin-B	1
1454	DRAMP00195	Colicin-V (Microcin-V; Bacteriocin)	Colicin-V	5
1457	DRAMP00198	Microcin H47 (MccH47; Bacteriocin)	Microcin H47	1
1460	DRAMP00202	Thiocillin (Bacteriocin)	Thiocillin	1
1463	DRAMP00206	Acidocin A (Bacteriocin)	Acidocin A	1
1474	DRAMP00217	Bacteriocin	Bacteriocin	232
1479	DRAMP00224	BTL (Bacteriocin)	BTL	1
1489	DRAMP00235	AFP1 (Bacteriocin)	AFP1	1
1497	DRAMP00245	Gramicidin A (GA; Nonribosomally synthesized bacteriocin)	Gramicidin A	1
1500	DRAMP00248	Glycocin F (GccF; S-glycosylated bacteriocin)	Glycocin F	1
1507	DRAMP00262	Cyanovirin-N (CV-N)	Cyanovirin-N	1
1516	DRAMP00272	Thaumatin-like protein (Plants)	Thaumatin-like protein	1
1517	DRAMP00273	Thaumatin-like protein (Plants)	Thaumatin-like protein	1
1523	DRAMP00280	Trypsin inhibitor (FtTI; Plant defensin)	Trypsin inhibitor	3
1540	DRAMP00299	Ribonuclease (Plants)	Ribonuclease	39
1558	DRAMP00319	Endochitinase (Plant defensin)	Endochitinase	1
1567	DRAMP00330	Pathogenesis-related protein (PR-1; Plant defensin)	Pathogenesis-related protein	2
1568	DRAMP00331	Pathogenesis-related protein (PRP; Plant defensin)	Pathogenesis-related protein	2
1569	DRAMP00332	Pathogenesis-related protein (Plant defensin)	Pathogenesis-related protein	2
1600	DRAMP00382	Datucin (Glycopeptide; Plants)	Datucin	1
1601	DRAMP00383	Antimicrobial peptide 1 (Mc-AMP1; knottin-type peptide; Plant defensin)	Antimicrobial peptide 1	1
1603	DRAMP00387	Antimicrobial peptide 1 (EcAMP1; hairpin-like peptides; Plants)	Antimicrobial peptide 1	1
1608	DRAMP00392	Antimicrobial peptide 1 (ToAMP1; Cys-rich; Plant defensin)	Antimicrobial peptide 1	1
1851	DRAMP18327	Sil(Bacteriocin)	Sil	15
1957	DRAMP00751	Defensin-2 (Plant defensin)	Defensin-2	17
1985	DRAMP18196	Esculentin-1A	Esculentin-1A	5
2009	DRAMP18323	Nukacin ISK-1(Bacteriocin)	Nukacin ISK-1	1
2068	DRAMP00932	Antimicrobial peptide 1 (PMAP1; Plant defensin)	Antimicrobial peptide 1	1
2080	DRAMP18321	Epidermicin NI01(Bacteriocin)	Epidermicin NI01	2
2086	DRAMP00976	Antimicrobial peptide 1 (AC-AMP1; Plant defensin)	Antimicrobial peptide 1	1
2109	DRAMP00999	Plectasin (fungal defensin)	Plectasin	3

2153	DRAMP01050	Sd1 (sugarcane defensin 1; Plant defensin)	Sd1	2
2156	DRAMP01053	Thaumatin-like protein (CdTLP; Plants)	Thaumatin-like protein	1
2161	DRAMP01058	Antifungal protein (Plants)	Antifungal protein	5
2204	DRAMP01127	Maximin-H5 (toads, amphibians, animals)	Maximin-H5	1
2285	DRAMP18404	Polybia-MPII (mastoparan; insects, arthropods, invertebrates, animals)	Polybia-MPII	1
2302	DRAMP01289	Phylloseptin-1 (PStar 01; Frogs, amphibians, animals)	Phylloseptin-1	1
2325	DRAMP01318	Antimicrobial peptide 1 (Frogs, amphibians, animals)	Antimicrobial peptide 1	1
2380	DRAMP18313	Sclerosin(Bacteriocin)	Sclerosin	1
2382	DRAMP01489	Esculentin-1A (Frogs, amphibians, animals)	Esculentin-1A	5
2387	DRAMP01514	Esculentin-1 (Frogs, amphibians, animals)	Esculentin-1	2
2389	DRAMP01744	Temporin-G (Frogs, amphibians, animals)	Temporin-G	1
2436	DRAMP01609	Aurein-2.2 (Frogs, amphibians, animals)	Aurein-2.2	1
2455	DRAMP01642	Dermaseptin-4 (DShypo04; Frogs, amphibians, animals)	Dermaseptin-4	1
2475	DRAMP01671	Dermaseptin-4 (DS IV; Dermaseptin-S4, DS4; Frogs, amphibians, animals)	Dermaseptin-4	1
2480	DRAMP01676	Dermaseptin-4 (DStar 04; Frogs, amphibians, animals)	Dermaseptin-4	1
2498	DRAMP18305	Paenibacterin (Bacteriocin)	Paenibacterin	1
2530	DRAMP02857	Indolicidin (Cathelicidin-4; mammals, animals)	Indolicidin	12
2532	DRAMP02819	Anoplin (Insects, arthropods, invertebrates, animals)	Anoplin	5
2533	DRAMP04395	EP3 (Earthworm,animals)	EP3	2
2534	DRAMP04394	EP2 (Earthworm,animals)	EP2	2
2554	DRAMP01793	Temporin-1OLa (Temporin 1OLa; Frogs, amphibians, animals)	Temporin-1OLa	1
2556	DRAMP01795	Temporin-1Ga (Frogs, amphibians, animals)	Temporin-1Ga	1
2666	DRAMP18288	Sln1 (Bacteriocin)	Sln1	1
2671	DRAMP18284	Reutericin 6 (Bacteriocin)	Reutericin 6	2
2689	DRAMP18279	Plantaricin A(Bacteriocin)	Plantaricin A	1
2698	DRAMP01110	Maximin-4 (Toads, amphibians, animals)	Maximin-4	1
2741	DRAMP02159	Melittin-related peptide (Frogs, amphibians, animals)	Melittin-related peptide	1
2819	DRAMP02285	Pseudin-2 (Pseudin 2; Frogs, amphibians, animals)	Pseudin-2	1
2831	DRAMP02311	Lysozyme (1,4-beta-N-acetylmuramidase; starfish, chordates, animals)	Lysozyme	194
2841	DRAMP02332	Piscidin-3 (Pis-3; fish, chordates, animals)	Piscidin-3	3
2842	DRAMP02333	Piscidin-4 (Pis-4; fish, chordates, animals)	Piscidin-4	1
2843	DRAMP02334	Hepcidin (fish, chordates, animals)	Hepcidin	3
2845	DRAMP02338	Beta-defensin 1 (fish, chordates, animals)	Beta-defensin 1	7
2877	DRAMP02408	2S albumin (To-A1)	2S albumin	1
2882	DRAMP02417	Defensin (Ticks, Arthropods, animals)	Defensin	92
2885	DRAMP02424	Defensin (Ticks, Arthropods, animals)	Defensin	92
2886	DRAMP02426	Defensin (Varisin A1; Ticks, Arthropods, animals)	Defensin	92
2888	DRAMP02435	Antifungal protein (PgAFP; Cys-rich)	Antifungal protein	5
2889	DRAMP02436	Chitinase	Chitinase	85
2898	DRAMP02450	Lysozyme C (1,4-beta-N-acetylmuramidase C)	Lysozyme C	3
2899	DRAMP02451	Lysozyme C (1,4-beta-N-acetylmuramidase C)	Lysozyme C	3

2900	DRAMP02452	Lysozyme	Lysozyme	194
2903	DRAMP02455	L-amino-acid oxidase (ACL-LAO; LAAO; LAO)	L-amino-acid oxidase	3
2904	DRAMP02457	L-amino-acid oxidase (Balt-LAAO-I; LAAO; LAO; snakes, reptils, animals)	L-amino-acid oxidase	3
2905	DRAMP02458	L-amino-acid oxidase (BiLAO; LAAO; LAO; snakes, reptils, animals)	L-amino-acid oxidase	3
2906	DRAMP02459	L-amino-acid oxidase (BjarLAAO-I; LAAO; LAO; snakes, reptils, animals)	L-amino-acid oxidase	3
2907	DRAMP02460	L-amino-acid oxidase (LAAO; LAO; snakes, reptils, animals)	L-amino-acid oxidase	3
2908	DRAMP02461	L-amino-acid oxidase (BmarLAAO; LAAO; LAO; snakes, reptils, animals)	L-amino-acid oxidase	3
2909	DRAMP02462	L-amino-acid oxidase (LAAO; LAO; snakes, reptils, animals)	L-amino-acid oxidase	3
2910	DRAMP02463	L-amino-acid oxidase (LAAO, LAO, LN-AAO; Reptiles, animals)	L-amino-acid oxidase	3
2911	DRAMP02464	L-amino-acid oxidase (LAAO, LAO; reptilia, animals)	L-amino-acid oxidase	3
2914	DRAMP02467	L-amino-acid oxidase (LAAO; LAO; Reptiles, animals)	L-amino-acid oxidase	3
2917	DRAMP02471	Thiostrepton (Alaninamide; Bryamycin; Gargon; Thiactin)	Thiostrepton	1
2947	DRAMP18259	Enterocin O16 (Bacteriocin)	Enterocin O16	1
2948	DRAMP18355	Lacticin Z (bacteriocin)	Lacticin Z	1
2951	DRAMP02511	Crotamine (defensin-like toxin; Snakes, reptiles, animals)	Crotamine	1
2952	DRAMP02512	L-amino-acid oxidase (Casca LAO, LAAO, LAO; Snakes, reptiles, animals)	L-amino-acid oxidase	3
2953	DRAMP18398	Um2 (scorpions, arachnids, Chelicerata, arthropods, invertebrates, animals)	Um2	3
3023	DRAMP02597	Clavanin-A (His-rich; chordates, animals)	Clavanin-A	5
3034	DRAMP02611	Beta-defensin 1 (BD-1; Defensin, beta 1; primates, mammals, animals)	Beta-defensin 1	7
3037	DRAMP02615	Beta-defensin 1 (BD-1; Defensin, beta 1; primates, mammals, animals)	Beta-defensin 1	7
3049	DRAMP02627	Beta-defensin 1 (BD-1; Defensin, beta 1; primates, mammals, animals)	Beta-defensin 1	7
3050	DRAMP18250	Laterosporulin (Bacteriocin)	Laterosporulin	1
3065	DRAMP02642	Rhesus theta-defensin 1 (RTD-1; primates, mammals, animals)	Rhesus theta-defensin 1	1
3070	DRAMP02647	Beta-defensin 2	Beta-defensin 2	15
3077	DRAMP02655	Alpha defensin (primates, mammals, animals)	Alpha defensin	8
3079	DRAMP02657	Alpha-defensin 1 (primates, mammals, animals)	Alpha-defensin 1	1
3080	DRAMP02658	Alpha-defensin 6 (primates, mammals, animals)	Alpha-defensin 6	2
3089	DRAMP02668	Defensin-6 (Defensin, alpha 6; primates, mammals, animals)	Defensin-6	2
3092	DRAMP02671	Beta-defensin 1 (BD-1; primates, mammals, animals)	Beta-defensin 1	7
3118	DRAMP02697	Defensin-5 (Defensin, alpha 5; primates, mammals, animals)	Defensin-5	1
3131	DRAMP18241	Subtilomycin(Bacteriocin)	Subtilomycin	1
3133	DRAMP18238	Fengycin B(Bacteriocin)	Fengycin B	1
3134	DRAMP02713	Hepcidin (primates, mammals, animals)	Hepcidin	3
3143	DRAMP02722	Beta-defensin 1 (BD-1; Defensin, beta 1; primates, mammals,animals)	Beta-defensin 1	7

3144	DRAMP18237	Fengycin A(Bacteriocin)	Fengycin A	1
3156	DRAMP02738	Beta-defensin 1 (BD-1; Defensin, beta 1; primates, mammals, animals)	Beta-defensin 1	7
3167	DRAMP02750	Defensin (ants, insects, animals)	Defensin	92
3169	DRAMP02753	Ponericin G1 (ants, insects, animals)	Ponericin G1	1
3191	DRAMP02783	Peptide C (Insects, animals)	Peptide C	3
3204	DRAMP02796	Defensin (Type 1 invertebrate defensin; Insects, animals)	Defensin	92
3228	DRAMP02825	Catestatin	Catestatin	2
3230	DRAMP02827	BHP (pepsin-derived bovine hemoglobin fragment)	BHP	29
3237	DRAMP02836	Cathelicidin antimicrobial peptide (cathelicidin; mammals, animals)	Cathelicidin antimicrobial peptide	6
3246	DRAMP02852	Cathelicidin-2 (Bactenecin-5, Bac5; PR-42; mammals, animals)	Cathelicidin-2	1
3253	DRAMP02876	Alpha-melanocyte-stimulating hormone (Alpha-MSH; mammals, animals)	Alpha-melanocyte-stimulating hormone	3
3276	DRAMP02902	L-amino-acid oxidase (LAAO, LAO; BpirLAAO-I; reptilia, animals)	L-amino-acid oxidase	3
3279	DRAMP02908	Beta-defensin 1 (BD-1; sBD-1; mammals, animals)	Beta-defensin 1	7
3280	DRAMP02909	Beta-defensin 2 (BD-2; sBD-2; mammals, animals)	Beta-defensin 2	15
3282	DRAMP02915	Cathelicidin-2 (Bactenecin-5, Bac5; OaBac5; mammals, animals)	Cathelicidin-2	1
3284	DRAMP02917	Hepcidin (dogs, mammals, animals)	Hepcidin	3
3285	DRAMP02918	Beta-defensin 1	Beta-defensin 1	7
3322	DRAMP02976	Beta-defensin 1 (BD-1; Defensin, beta 1; pigs, mammals, animals)	Beta-defensin 1	7
3325	DRAMP02979	Hepcidin (pigs, mammals, animals)	Hepcidin	3
3338	DRAMP02994	Defensin-1 (Royalisin; Insects, animals)	Defensin-1	13
3340	DRAMP03005	Apidaecin (Insects, animals)	Apidaecin	1
3341	DRAMP03006	Defensin (Insects, animals)	Defensin	92
3345	DRAMP03016	Defensin-1 (Insects, animals)	Defensin-1	13
3348	DRAMP03023	Mastoparan (Protonectarina-MP; Insects, animals)	Mastoparan	4
3370	DRAMP03074	Cecropin (Insects, animals)	Cecropin	18
3371	DRAMP03076	Cecropin-1 (Cecropin 1; Insects, animals)	Cecropin-1	3
3374	DRAMP03079	Cecropin-A1 (Insects, animals)	Cecropin-A1	1
3375	DRAMP03080	Cecropin-B (Insects, animals)	Cecropin-B	2
3377	DRAMP03082	Defensin (invertebrate defensin; Insects, animals)	Defensin	92
3383	DRAMP03088	Diptericin (Insects, animals)	Diptericin	2
3386	DRAMP03093	Drosomycin (Cys-rich; insect defensins; Insects, animals)	Drosomycin	1
3393	DRAMP03105	Antifungal protein (AFP; Insects, animals)	Antifungal protein	5
3400	DRAMP18223	Sonorensin(Bacteriocin)	Sonorensin	1
3407	DRAMP03123	Cecropin-B (AgCecB; Insects, animals)	Cecropin-B	2
3411	DRAMP03127	Cecropin-A (Insects, animals)	Cecropin-A	9
3415	DRAMP03131	Cecropin-B (Insects, animals)	Cecropin-B	2
3417	DRAMP03133	Cecropin-A1 (AalCecA; Cecropin-A; Insects, animals)	Cecropin-A1	1
3426	DRAMP03145	Defensin	Defensin	92
3427	DRAMP03146	Defensin	Defensin	92
3429	DRAMP03148	Defensin	Defensin	92

3440	DRAMP03161	Beta-defensin 1 (BD-1; Defensin, beta 1; mammals, animals)	Beta-defensin 1	7
3445	DRAMP03168	L-amino-acid oxidase (K-LAO; LAAO; LAO; reptilia, animals)	L-amino-acid oxidase	3
3461	DRAMP18221	Fusaricidin D (Bacteriocin)	Fusaricidin D	1
3465	DRAMP03199	PhD1 (PhD-1; Defensin-1; primates, mammals, animals)	PhD1	1
3469	DRAMP18220	Fusaricidin C (Bacteriocin)	Fusaricidin C	1
3470	DRAMP18219	Fusaricidin B (Bacteriocin)	Fusaricidin B	1
3475	DRAMP18218	Fusaricidin A (Bacteriocin)	Fusaricidin A	1
3478	DRAMP18217	Licheniocin 50.2(Bacteriocin)	Licheniocin 50.2	1
3514	DRAMP18213	Gramicidin S(Bacteriocin)	Gramicidin S	9
3527	DRAMP03284	Beta-defensin (Birds, animals)	Beta-defensin	57
3553	DRAMP03316	Defensin (Insects, animals)	Defensin	92
3554	DRAMP03317	Cathelicidin-related antimicrobial peptide (AMPs)	Cathelicidin-related antimicrobial peptide	2
3600	DRAMP03364	Hepcidin (mammals, rodents, animals)	Hepcidin	3
3602	DRAMP03366	Beta-defensin 1 (BD-1; mBD-1; Defensin, beta 1; Rodents, mammals, animals)	Beta-defensin 1	7
3603	DRAMP03367	Beta-defensin 2 (BD-2, mBD-2; Defensin, beta 2; Defb2; Rodents, mammals, animals)	Beta-defensin 2	15
3604	DRAMP03368	Beta-defensin 3 (BD-3, mBD-3; Defensin, beta 3; Rodents, mammals, animals)	Beta-defensin 3	28
3644	DRAMP03412	Beta-defensin 2	Beta-defensin 2	15
3650	DRAMP03418	Hepcidin (mammals, rodents, animals)	Hepcidin	3
3654	DRAMP03424	Beta-defensin 1 (BD-1, RBD-1; Defensin, beta 1; Rodents, mammals, animals)	Beta-defensin 1	7
3655	DRAMP03425	Beta-defensin 3 (BD-3, RBD-3; Defensin, beta 3; Rodents, mammals, animals)	Beta-defensin 3	28
3691	DRAMP03461	Defensin 5 (Enteric defensin; RD-5; Rodents, mammals, animals)	Defensin 5	1
3696	DRAMP03470	Defensin-1 (American oyster defensin, AOD; molluscs, animals)	Defensin-1	13
3714	DRAMP03494	Cecropin (Insects, animals)	Cecropin	18
3716	DRAMP03496	Cecropin-A (Insects, animals)	Cecropin-A	9
3717	DRAMP03497	Cecropin-B (Insects, animals)	Cecropin-B	2
3727	DRAMP03509	Cecropin-A (Insects, animals)	Cecropin-A	9
3728	DRAMP03510	Cecropin-A (Insects, animals)	Cecropin-A	9
3729	DRAMP03511	Cecropin-B (Immune protein P9; Insects, animals)	Cecropin-B	2
3731	DRAMP03522	Gallerimycin (defensins; Insects, animals)	Gallerimycin	1
3732	DRAMP03529	Cecropin-A (Insects, animals)	Cecropin-A	9
3733	DRAMP03530	Cecropin-B (Lepidopteran-A/B; Insects, animals)	Cecropin-B	2
3741	DRAMP03538	Cecropin (Antibacterial peptide CM-IV; Insects, animals)	Cecropin	18
3742	DRAMP03554	CCL20(1-67) (Human, mammals, animals)	CCL20	16
3743	DRAMP03555	CCL20(2-70) (Human, mammals, animals)	CCL20	16
3745	DRAMP03557	Granulysin (Lymphokine LAG-2; Human, mammals, animals)	Granulysin	1
3746	DRAMP03559	CXCL10 (Human, mammals, animals)	CXCL10	6
3753	DRAMP03576	Histatin-1 (His-rich; Human, mammals, animals)	Histatin-1	4
3757	DRAMP03580	His3-(20-43)-peptide (Histatin 5; derivatives: Dh-5; Clinical)	His3-	1

3803	DRAMP03635	Human lactoferricin (LfcinH; one chain of Lactotransferrin; Human, mammals, animals)	Human lactoferricin	4
3805	DRAMP03637	Thaumatin-like protein (Actc2)	Thaumatin-like protein	1
3826	DRAMP03664	L-amino-acid oxidase (LAAO, LAO, TM-LAO; reptilia, animals)	L-amino-acid oxidase	3
3827	DRAMP03665	Lysozyme	Lysozyme	194
3838	DRAMP03678	Beta-defensin 1 (GBD-1; Defensin, beta 1; ruminant, animals)	Beta-defensin 1	7
3840	DRAMP03681	L-amino-acid oxidase (LAAO; OHAP-1)	L-amino-acid oxidase	3
3843	DRAMP03692	Defensin-1 (Cll-dlp; Arthropods, animals)	Defensin-1	13
3870	DRAMP03733	Lysozyme (Arthropods, animals)	Lysozyme	194
3899	DRAMP03779	Antifungal protein (AFP; Fungi)	Antifungal protein	5
3909	DRAMP03789	ABF-1 (nematodes, animals)	ABF-1	1
3958	DRAMP03926	Cecropin B (1-7)-melittin (4-11)hybrid peptide (CBM)	Cecropin B	2
3960	DRAMP03940	Peptide 4 (Trp- and Arg-rich; derivative of Tritrpticin)	Peptide 4	1
3961	DRAMP03941	Peptide 3 (Trp- and Arg-rich; derivative of Tritrpticin)	Peptide 3	3
3962	DRAMP03942	Peptide 2 (Trp- and Arg-rich; derivative of Tritrpticin)	Peptide 2	3
3970	DRAMP03961	KR-12	KR-12	9
3971	DRAMP03962	Cecropin A(1-8)-Magainin 2(1-12)hybrid peptide (CAMA)	Cecropin A	9
3972	DRAMP03963	Cecropin A(1-8)-Magainin 2(1-12)hybrid peptide analogue (P1)	Cecropin A	9
3973	DRAMP03964	Cecropin A(1-8)-Magainin 2(1-12)hybrid peptide analogue (P2)	Cecropin A	9
3974	DRAMP03965	Cecropin A(1-8)-Magainin 2(1-12)hybrid peptide analogue (P3)	Cecropin A	9
3975	DRAMP03966	Cecropin A(1-8)-Magainin 2(1-12)hybrid peptide analogue (P4)	Cecropin A	9
3982	DRAMP04018	Rp-1	Rp-1	2
4009	DRAMP04148	Gramicidin S	Gramicidin S	9
4022	DRAMP04197	Penetratin	Penetratin	2
4035	DRAMP04210	ADP1	ADP1	7
4038	DRAMP04213	BP100	BP100	1
4043	DRAMP04218	Lys-a1	Lys-a1	2
4052	DRAMP04227	PGG	PGG	6
4053	DRAMP04228	PGP	PGP	29
4055	DRAMP04230	PGAa	PGAa	38
4061	DRAMP04245	Dhvar5	Dhvar5	1
4062	DRAMP04246	Dhvar4	Dhvar4	2
4067	DRAMP04251	WLBU2	WLBU2	10
4070	DRAMP04254	Neuropeptide Y (NPY)	Neuropeptide Y	1
4071	DRAMP04255	Neuropeptide Y (NPY)	Neuropeptide Y	1
4076	DRAMP04260	CM-1	CM-1	86
4077	DRAMP04261	CM-2	CM-2	990
4078	DRAMP04262	CM-3	CM-3	52
4079	DRAMP04263	CM-4	CM-4	1
4090	DRAMP04280	CM1	CM1	2
4091	DRAMP04281	CM2	CM2	186
4092	DRAMP04282	CM3	CM3	10
4093	DRAMP04283	CM4	CM4	1
4094	DRAMP04284	CM5	CM5	1

4105	DRAMP04295	MB-10	MB-10	1
4126	DRAMP04316	MB-50	MB-50	2
4127	DRAMP04317	DASamP1	DASamP1	1
4128	DRAMP04318	DASamP2	DASamP2	2
4157	DRAMP04347	Buforin	Buforin	4
4166	DRAMP04356	CAD	CAD	30
4168	DRAMP04358	CEMA(MBI-28)	CEMA	7
4176	DRAMP04398	Antifungal protein (PAF)	Antifungal protein	5
4190	DRAMP04412	Defensin	Defensin	92
4192	DRAMP04414	Defensin 1 (Def1)	Defensin 1	13
4193	DRAMP04415	Defensin 2 (Def2)	Defensin 2	17
4194	DRAMP04416	Defensin 3 (Def3)	Defensin 3	28
4196	DRAMP04418	Defensin	Defensin	92
4197	DRAMP04419	Putative uncharacterized protein	Putative uncharacterized protein	1
4199	DRAMP04421	Defensin	Defensin	92
4200	DRAMP04422	Defensin 1	Defensin 1	13
4201	DRAMP04423	Defensin	Defensin	92
4205	DRAMP04427	Beta defensin-2	Beta defensin-2	15
4206	DRAMP04428	Beta defensin 1 (Beta-defensin-1)	Beta defensin 1	7
4207	DRAMP04429	Defensin	Defensin	92
4210	DRAMP04432	Defensin 1	Defensin 1	13
4211	DRAMP04433	Defensin 2	Defensin 2	17
4213	DRAMP04436	Beta defensin 1 (Beta-defensin-1)	Beta defensin 1	7
4222	DRAMP04445	Lipid binding protein	Lipid binding protein	1
4224	DRAMP04447	Defensin	Defensin	92
4227	DRAMP04450	Defensin	Defensin	92
4233	DRAMP04456	Defensin	Defensin	92
4234	DRAMP04457	PDF1	PDF1	3
4236	DRAMP04459	Defensin 1	Defensin 1	13
4239	DRAMP04462	Putative uncharacterized protein	Putative uncharacterized protein	1
4246	DRAMP04469	Cecropin (Cecropin 1 (Cecropin A)	Cecropin	18
4251	DRAMP04474	Defensin (Spodoptericin)	Defensin	92
4253	DRAMP04476	Beta defensin 1 (Beta-defensin-1)	Beta defensin 1	7
4254	DRAMP04477	Beta defensin-2	Beta defensin-2	15
4257	DRAMP04480	Alpha defensin	Alpha defensin	8
4262	DRAMP04485	Retrocyclin	Retrocyclin	2
4267	DRAMP04490	Myeloid antimicrobial peptide	Myeloid antimicrobial peptide	1
4271	DRAMP04494	Defensin	Defensin	92
4276	DRAMP04499	Hepcidin (Hepcidin type I)	Hepcidin	3
4306	DRAMP04533	P-113D	P-113D	1
4312	DRAMP04539	PG-L	PG-L	1
4325	DRAMP04558	C18	C18	64
4358	DRAMP04591	CXCL14	CXCL14	1
4361	DRAMP04594	TCP	TCP	99
4363	DRAMP04596	PuroA	PuroA	3
4370	DRAMP04603	Pina-M	Pina-M	1
4373	DRAMP04606	Hina	Hina	1
4400	DRAMP04633	CPF-C1	CPF-C1	2
4425	DRAMP04659	HPA3NT3	HPA3NT3	1
4430	DRAMP04664	HPA3NT3-A2 (HPA3NT3 peptide analogs)	HPA3NT3-A2	1
4432	DRAMP04667	Melt	Melt	38

4433	DRAMP04668	Coprisin	Coprisin	1
4436	DRAMP04673	Alamethicin (ALM; fungi)	Alamethicin	1
4442	DRAMP04683	NADPH oxidoreductase	NADPH oxidoreductase	1
4500	DRAMP18415	Con10 (scorpions, arachnids, Chelicerata, arthropods, invertebrates, animals)	Con10	1
4502	DRAMP18417	ToAP2 (scorpions, arachnids, Chelicerata, arthropods, invertebrates, animals)	ToAP2	1
4534	DRAMP18457	Tet213 (synthetic, Trp-rich, Arg-rich)	Tet213	3
4535	DRAMP18458	Melimine (a hybrid peptide of melittin and protamine, synthetic)	Melimine	6
4536	DRAMP18459	hLF(1-11) (hLF1-11, first 11 residues, human lactoferrin; synthetic)	hLF	7
4564	DRAMP03575	LL-37(17-29) (C-terminal fragment of LL-37, LL; Human, mammals, animals)	LL-37	123
4644	DRAMP18582	CDT (Tachyplesin-1 peptide derivative)	CDT	22
4650	DRAMP18588	RTD	RTD	5
4652	DRAMP18590	DEP (Ctx-Ha peptide derivative)	DEP	35
4653	DRAMP18591	DEA (Ctx-Ha peptide derivative)	DEA	5
4654	DRAMP18592	Ctx(Ile21)-Ha (Ctx-Ha peptide derivative)	Ctx	70
4655	DRAMP18593	Ctx(Ile21)-Ha-VD16 (Ctx-Ha peptide derivative)	Ctx	70
4656	DRAMP18594	Ctx(Ile21)-Ha-VD5,16 (Ctx-Ha peptide derivative)	Ctx	70
4657	DRAMP18595	Ctx(Ile21)-Ha-I9K (Ctx-Ha peptide derivative)	Ctx	70
4681	DRAMP18614	TPA (Tritrpticin peptide derivative)	TPA	11
4685	DRAMP18618	SMAP-29(1-17) (SMAP-29 peptide derivative)	SMAP-29	6
4708	DRAMP18642	KCM12	KCM12	1
4709	DRAMP18643	KCM21	KCM21	1
4719	DRAMP18653	Cm-p5 (Cm-p1 peptide derivative)	Cm-p5	1
4723	DRAMP18657	Chitinase (Bacteriocin; Streptomyces violaceusniger, Bacteria)	Chitinase	85
4731	DRAMP18665	Mastoparan-L (MP-L; insects, arthropods, invertebrates, animals)	Mastoparan-L	1
4733	DRAMP18667	Pep-1	Pep-1	1
4738	DRAMP18672	Peptide 7 (Mollusca/molluscs/mollusks, invertebrates, animals)	Peptide 7	1
4739	DRAMP18673	Peptide 3 (Mollusca/molluscs/mollusks, invertebrates, animals)	Peptide 3	3
4740	DRAMP18674	Peptide 2 (Mollusca/molluscs/mollusks, invertebrates, animals)	Peptide 2	3
4741	DRAMP18675	Peptide 4 (Mollusca/molluscs/mollusks, invertebrates, animals)	Peptide 4	1
4742	DRAMP18676	Peptide 5 (Mollusca/molluscs/mollusks, invertebrates, animals)	Peptide 5	2
4744	DRAMP18678	Peptide 8 (Mollusca/molluscs/mollusks, invertebrates, animals)	Peptide 8	1
4759	DRAMP18693	Substance P (Mammals, animals)	Substance P	9
4764	DRAMP18698	Histone H2A (Trouts, fish, animals)	Histone H2A	2
4774	DRAMP18708	Dermaseptin-S4 (DRS-S4, DS4; frog, amphibians, animals)	Dermaseptin-S4	3
4775	DRAMP18709	Mastoparan (MP; insects, arthropods, invertebrates, animals)	Mastoparan	4
4780	DRAMP18714	Cathepsin G(1-5) (Human, primates, mammals, animals)	Cathepsin G	4
4781	DRAMP18715	Cathepsin G(77-83) (Human, primates, mammals, animals)	Cathepsin G	4

4802	DRAMP00257	Bacteriocin	Bacteriocin	232
4803	DRAMP00258	Lacticin Z (Bacteriocin)	Lacticin Z	1
4805	DRAMP00260	Bacteriocin (Class IIa sec-dependent bacteriocin)	Bacteriocin	232
4819	DRAMP02842	LfcinB(20-25)	LfcinB	1
4834	DRAMP02518	NA-CATH	NA-CATH	5
4844	DRAMP20778	Temporin-SHf (frogs,amphibians,animals)	Temporin-SHf	1
4856	DRAMP20790	Cecropin B (Insects, arthropods, invertebrates, animals)	Cecropin B	2
4857	DRAMP20791	Cecropin A1 (insects, arthropods, invertebrates, animals)	Cecropin A1	1
4863	DRAMP20797	Uperin 3.6 (Toad, amphibians, animals)	Uperin 3.6	1
4869	DRAMP20803	moronecidin-like peptide	moronecidin-like peptide	1
4886	DRAMP20820	FV7	FV7	2
4887	DRAMP20821	FV-LL (FV7 and LL(LL-37,(17-29)) hybrid peptide)	FV-LL	1
4888	DRAMP20822	FV-MA (FV7 and MA(Magainin 2 (9-21)) hybrid peptide)	FV-MA	1
4889	DRAMP20823	FV-CE (FV7 and CE(Cecropin A (1	FV-CE	1
4893	DRAMP20827	TB_L1FK	TB L1FK	1
4894	DRAMP20828	TB_KKG6A	TB KKG6A	1
4898	DRAMP20834	PLS	PLS	30
4935	DRAMP20874	Chensinin-1b	Chensinin-1b	1
4944	DRAMP20883	Cath-A	Cath-A	1
4966	DRAMP20905	dC4	dC4	3
4968	DRAMP20907	dN4	dN4	1
4974	DRAMP20913	Myxinidin (G1)	Myxinidin	3
4975	DRAMP20914	Myxinidin (I2)	Myxinidin	3
4976	DRAMP20915	Myxinidin (H3)	Myxinidin	3
4977	DRAMP20916	Myxinidin (D4)	Myxinidin	3
4978	DRAMP20917	Myxinidin (I5)	Myxinidin	3
4979	DRAMP20918	Myxinidin (L6)	Myxinidin	3
4980	DRAMP20919	Myxinidin (K7)	Myxinidin	3
4981	DRAMP20920	Myxinidin (Y8)	Myxinidin	3
4982	DRAMP20921	Myxinidin (G9)	Myxinidin	3
4983	DRAMP20922	Myxinidin (K10)	Myxinidin	3
4984	DRAMP20923	Myxinidin (P11)	Myxinidin	3
4985	DRAMP20924	Myxinidin (S12)	Myxinidin	3
4987	DRAMP20926	IN1(designed based on indolicidin and ranalexin)	IN1	1
4992	DRAMP20932	Hp1404	Hp1404	2
5020	DRAMP20961	Hp1404-T1e	Hp1404-T1e	1
5024	DRAMP20966	andricin B (Andrias davidianus, Amphibians, Animals)	andricin B	1
5039	DRAMP20981	CPF-C1 (Frogs, Amphibians, Animals)	CPF-C1	2
5041	DRAMP20983	CPF-2 (Derived from CPF-C1)	CPF-2	1
5048	DRAMP20990	CPF-9 (Derived from CPF-C1)	CPF-9	1
5058	DRAMP21000	cGm (Derived from Gm)	cGm	1
5097	DRAMP21039	analog 2 (Derived from Ib-AMP2)	analog 2	1
5153	DRAMP21095	IR1 (Derived from PG-1)	IR1	2
5154	DRAMP21096	IR2 (Derived from PG-1)	IR2	3
5155	DRAMP21097	FR1 (Derived from PG-1)	FR1	2
5156	DRAMP21098	FR2 (Derived from PG-1)	FR2	1
5157	DRAMP21099	WR1 (Derived from PG-1)	WR1	1

5159	DRAMP21101	PR1 (Derived from PG-1)	PR1	20
5160	DRAMP21102	PR2 (Derived from PG-1)	PR2	2
5170	DRAMP21157	HYL-3 (Derived from HYL)	HYL-3	1
5173	DRAMP21154	HYL (Bee, Insecta, Animals)	HYL	28
5176	DRAMP21150	KR-12-a5 (Derived from KR-12)	KR-12-a5	3
5182	DRAMP21145	Myxinidin3 (Derived from Myxinidin)	Myxinidin3	1
5185	DRAMP21144	Myxinidin2 (Derived from Myxinidin)	Myxinidin2	1
5202	DRAMP21125	T9W (Derived from RI16)	T9W	2
5203	DRAMP21124	RI16 (Derived from PMAP-36)	RI16	2
5204	DRAMP21123	KR-12-a5 (7-(D)L) (Derived from LL-37)	KR-12-a5	3
5205	DRAMP21122	KR-12-a5 (6-(D)L) (Derived from LL-37)	KR-12-a5	3
5206	DRAMP21121	KR-12-a5 (5-(D)K) (Derived from LL-37)	KR-12-a5	3
5208	DRAMP21120	KR-12-a5 (Derived from LL-37)	KR-12-a5	3
5214	DRAMP21111	ASA (Derived from SLZP)	ASA	33
5216	DRAMP21113	PSA (Derived from SLZP)	PSA	27
5220	DRAMP21108	Feleucin-K3 (Derived from Feleucin-BO1)	Feleucin-K3	3
5231	DRAMP21225	STPk (Derived from STP)	STPk	4
5232	DRAMP21226	Ink (Derived from IN)	Ink	20
5234	DRAMP21224	TPk (Derived from TP)	TPk	4
5246	DRAMP21211	Amyl-1-18 (N3L, G12R) (Derived from Amyl-1-18)	Amyl-1-18	1
5247	DRAMP21208	Amyl-1-18 (E9L) (Derived from Amyl-1-18)	Amyl-1-18	1
5248	DRAMP21209	Amyl-1-18 (E9L, G12R) (Derived from Amyl-1-18)	Amyl-1-18	1
5249	DRAMP21205	Amyl-1-18 (G12R) (Derived from Amyl-1-18)	Amyl-1-18	1
5250	DRAMP21210	Amyl-1-18 (N3L, E9L) (Derived from Amyl-1-18)	Amyl-1-18	1
5251	DRAMP21207	Amyl-1-18 (N3L) (Derived from Amyl-1-18)	Amyl-1-18	1
5252	DRAMP21206	Amyl-1-18 (D15R) (Derived from Amyl-1-18)	Amyl-1-18	1
5253	DRAMP21204	Amyl-1-18 (I11R) (Derived from Amyl-1-18)	Amyl-1-18	1
5254	DRAMP21203	Amyl-1-18 (Oryza sativa L., Angiospermae, Plants)	Amyl-1-18	1
5268	DRAMP21189	GW-Q4 (De novo synthesis)	GW-Q4	1
5271	DRAMP21186	WRL3 (Derived from leucocin A)	WRL3	1
5276	DRAMP21181	WR1 (Derived from leucocin A)	WR1	1
5284	DRAMP21173	HYL-20 (Derived from HYL)	HYL-20	1
5293	DRAMP21240	FK13-a7 (Derived from FK13)	FK13-a7	1
5300	DRAMP21234	FK13-a1 (Derived from FK13)	FK13-a1	1
5307	DRAMP21249	PVP (Derived from MP-B and MP-VT1)	PVP	58
5308	DRAMP21250	PV3 (Derived from pEM-2 and MP-VT1)	PV3	1
5313	DRAMP21255	WL1 (Derived from CP-1)	WL1	1
5316	DRAMP21258	Cecropin P1 (CP-1) (nematodes, animals)	Cecropin P1	1
5365	DRAMP21307	TL-2 (Derived from Temporin-2Tl (TL))	TL-2	1
5379	DRAMP21322	RTV (Derived from PMAP-36)	RTV	3
5380	DRAMP21323	RTI (Derived from PMAP-36)	RTI	12
5381	DRAMP21324	RTF (Derived from PMAP-36)	RTF	1
5382	DRAMP21325	RTL (Derived from PMAP-36)	RTL	1
5386	DRAMP21329	RFR (Derived from PMAP-36)	RFR	2
5390	DRAMP21333	RVK (Derived from PMAP-36)	RVK	1
5391	DRAMP21334	Ranatuerin-2Pb (Frogs, amphibians, animals)	Ranatuerin-2Pb	1
5392	DRAMP21335	RPa (Frogs, amphibians, animals)	RPa	6
5393	DRAMP21336	RPb (Frogs, amphibians, animals)	RPb	5
5394	DRAMP21337	BMAP-27 (Bovine, mammals, animals)	BMAP-27	8
5430	DRAMP21373	SP1 (De Novo Synthesis)	SP1	5

5431	DRAMP21374	SP2 (De Novo Synthesis)	SP2	1
5432	DRAMP21375	SP3 (De Novo Synthesis)	SP3	2
5433	DRAMP21376	SP4 (De Novo Synthesis)	SP4	31
5436	DRAMP21379	SP7 (De Novo Synthesis)	SP7	10
5437	DRAMP21380	SP8 (De Novo Synthesis)	SP8	1
5440	DRAMP21383	SP10 (De Novo Synthesis)	SP10	1
5441	DRAMP21384	SP11 (De Novo Synthesis)	SP11	1
5447	DRAMP21390	K17 (Derived from ATG16)	K17	2
5448	DRAMP21391	K18 (Derived from ATG16)	K18	1
5449	DRAMP21392	K22 (Derived from ATG16)	K22	85
5454	DRAMP21397	K36 (Derived from ATG16)	K36	2
5455	DRAMP21398	K46 (Derived from ATG16)	K46	1
5460	DRAMP21403	peptide 1 (De Novo Synthesis)	peptide 1	16
5461	DRAMP21404	peptide 2 (De Novo Synthesis)	peptide 2	3
5482	DRAMP21425	peptide 2 (Derived from B1)	peptide 2	3
5483	DRAMP21426	peptide 3 (Derived from B1)	peptide 3	3
5484	DRAMP21427	peptide 4 (Derived from B1)	peptide 4	1
5485	DRAMP21428	peptide 5 (Derived from B1)	peptide 5	2
5487	DRAMP21430	peptide 7 (Derived from B1)	peptide 7	1
5488	DRAMP21431	peptide 8 (Derived from B1)	peptide 8	1
5490	DRAMP21433	peptide 10 (Derived from B1)	peptide 10	4
5495	DRAMP21438	peptide 15 (Derived from B1)	peptide 15	2
5497	DRAMP21440	peptide 17 (Derived from B1)	peptide 17	1
5501	DRAMP21444	peptide 21 (Derived from B1)	peptide 21	1
5509	DRAMP21452	peptide 29 (Derived from B1)	peptide 29	1
5510	DRAMP21453	Hybrid (Derived from Melittin and thanatin)	Hybrid	678
5512	DRAMP21455	PLP2 (Insects, animals)	PLP2	1
5526	DRAMP21577	LP1	LP1	4
5527	DRAMP21481	6K-F17	6K-F17	1
5541	DRAMP21622	MAP-1	MAP-1	2
