# Supplementary material for: AMPing Up the Search: A Structural and Functional Repository of Antimicrobial Peptides for Biofilm Studies, and a Case Study of Its Application to Corynebacterium striatum, an Emerging Pathogen
Source: Front Cell Infect Microbiol. 2021 Dec 16;11:803774. doi: 10.3389/fcimb.2021.803774 (PMC8716830; doi:10.3389/fcimb.2021.803774)
Supplement: Supplementary file 8 [file Table_5.docx]

**Suppl Table 5: Preference scale of candidate AMPs based on docking interactions with mutated sortase C protein of *C. striatum***

**(**where 10 represents the most optimum docking interactions and 0 represents no interactions with the protein residues)

| **Serial Number** | **Pep ID** | **DRAMP ID** | **AMP length (residues)** | **Sequence** | **Name of AMP** | **TORSDOF** | **Docking Score (kcal/mol)** | **Interacting Residues** | **Essential criteria** | **Preference Score** |
| --- | --- | --- | --- | --- | --- | --- | --- | --- | --- | --- |
| 1 | 1 | -- | 5 | LPMTG | LPMTG Motif of the Pilin Subunit | 14 | -5.3 | HIS168, ASN236, GLN143 |  | STANDARD |
|  |  |  |  |  |  |  |  |  |  |  |
| 2 | 4707 | 18641 | 6 | TWWRWW | KCM11 | 26 | -6.2 | HIS168, ARG239, ASN236, THR137, THR169 | **Interactions with two putative catalytic site residues, seen as His168 and Arg239** | **10** |
| 3 | 4021 | 04173 | 5 | WLLKW | L2KW2 | 23 | -5.8 | HIS168, ARG239, ASN236 |  | **10** |
| 4 | 4583 | 18503 | 8 | WKSYVRRW | TSG-11 (Ixosin-B peptide derivative) | 39 | -5.6 | HIS168, ARG239, LEU96, LEU96, PRO94, PRO94 |  | **10** |
| 5 | 4785 | 18719 | 7 | DDDDDDD | SAAP fraction 3 (Surfactant-associated anionic peptide) | 28 | -5.3 | HIS168, ARG239, ASN236, GLN143 |  | **10** |
| 6 | 1437 | 18337 | 6 | ATQSHQ | S. amritsarensis lipopeptide (Bacteriocin) | 24 | -5.1 | HIS168, ARG239, GLN143, ASP139 |  | **10** |
| 7 | 5494 | 21437 | 7 | KKLKAFA | Peptide 14 (Derived from B1) | 33 | -5 | HIS168, ARG239, LEU96 |  | **10** |
| 8 | 5496 | 21439 | 7 | KKKlAFA | Peptide 16 (Derived from B1) | 33 | -5 | HIS168, ARG239, GLN143, ILE235 |  | **10** |
|  |  |  |  |  |  |  |  |  |  |  |
| 9 | 4708 | 18642 | 6 | KWRWIW | KCM12 | 29 | -6.3 | HIS168, HIS168, GLN143, TYR233 | **Multiple explicit interactions with His168 from the putative triad** | **9** |
| 10 | 1218 | 04174 | 7 | KWLKKWL | L2K3W2 | 37 | -5 | HIS168, HIS168, ASN236, GLN143, GLN143, THR137, PRO94 |  | **9** |
| 11 | 5482 | 21425 | 7 | KKGKGGG | Peptide 2 (Derived from B1) | 29 | -4.9 | HIS168, HIS168, GLN143, ALA95 |  | **9** |
| 12 | 5508 | 21451 | 7 | KKKLAYA | Peptide 28 (Derived from B1) | 34 | -4.9 | HIS168, HIS168, GLN143, ILE235 |  | **9** |
| 13 | 4934 | 20873 | 5 | GLLKR | Pal-ano-5 (Pal-anoplin peptide derivative) | 23 | -4.5 | HIS168, HIS168, GLN143 |  | **9** |
| 14 | 4931 | 20870 | 8 | GLLKRIKT | Pal-ano-8 (Pal-anoplin peptide derivative) | 38 | -4.4 | HIS168, HIS168, ASN236, ASN236, GLN143 |  | **9** |
|  |  |  |  |  |  |  |  |  |  |  |
| 15 | 4710 | 18644 | 6 | WRWFIH | KRS22 | 26 | -6 | HIS168, GLN143 | **Only one**  **explicit interaction with His168 from the putative triad** | **8** |
| 16 | 2442 | 18309 | 6 | WAIVLL | Baceridin (Bacteriocin) | 21 | -5.9 | HIS168, GLN143 |  | **8** |
| 17 | 1005 | 03881 | 6 | RRWWWR | LFB-6RW | 30 | -5.8 | HIS168, ASP139 |  | **8** |
| 18 | 3475 | 18218 | 6 | TVVTNA | Fusaricidin A (Bacteriocin) | 20 | -5.8 | HIS168, GLN143 |  | **8** |
| 19 | 4709 | 18643 | 6 | KWWWRW | KCM21 | 29 | -5.8 | HIS168, ASN236, GLN143, ASP139, PRO94, PRO94 |  | **8** |
| 20 | 696 | 18371 | 9 | VGVGGGFGR | Crinicepsin-1 | 26 | -5.8 | HIS168, ASN236, GLN143 |  | **8** |
| 21 | 5492 | 21435 | 7 | AFALKKK | Peptide 12 (Derived from B1) | 33 | -5.7 | HIS168, GLN143, THR137, PRO94 |  | **8** |
| 22 | 3059 | 18247 | 8 | DWTXWSXL | Bacthuricin F4(Bacteriocin) | 28 | -5.7 | HIS168, GLN143, ILE235 |  | **8** |
| 23 | 3366 | 18226 | 7 | LLDVLLE | Gageostatin A (Bacteriocin) | 28 | -5.6 | HIS168, GLN143, ASP139, ALA172 |  | **8** |
| 24 | 996 | 03869 | 8 | RRWVIWRR | Bac8d (Bac2A variant) | 39 | -5.6 | HIS168, ASN236, LEU96 |  | **8** |
| 25 | 3461 | 18221 | 6 | TVYTQA | Fusaricidin D (Bacteriocin) | 23 | -5.5 | HIS168, GLN143, ASP139 |  | **8** |
| 26 | 1219 | 04175 | 7 | KWLLKWL | L3K2W2 (LlKmW2 model peptides) | 34 | -5.5 | HIS168, GLN143, ILE235 |  | **8** |
| 27 | 3308 | 18228 | 7 | LLDVLLE | Gageostatin C (Bacteriocin) | 28 | -5.5 | HIS168, ASN236, GLN143, ARG98 |  | **8** |
| 28 | 3292 | 02930 | 6 | HSPGGA | Antimicrobial protein 2 | 15 | -5.4 | HIS168, ASN236, GLN143, THR137 |  | **8** |
| 29 | 1087 | 03989 | 7 | LLKWLLK | L4K2W4 | 34 | -5.4 | HIS168, GLN143 |  | **8** |
| 30 | 5501 | 21444 | 7 | AFALKKK | Peptide 21 (Derived from B1) | 33 | -5.4 | HIS168, GLN143, ILE235 |  | **8** |
| 31 | 5491 | 21434 | 7 | AFAKLKK | Peptide 11 (Derived from B1) | 33 | -5.3 | HIS168, GLN143, PRO94 |  | **8** |
| 32 | 168 | 01351 | 12 | RVCFAIPLPICH | Tigerinin-2 | 41 | -5.2 | HIS168, GLN143, ASP139, ASP139, ASP139 |  | **8** |
| 33 | 4020 | 04172 | 5 | WLKKW | LK2W2 (LlKmW2 model peptides) | 26 | -5.1 | HIS168 |  | **8** |
| 34 | 5244 | 21214 | 6 | IRIKIR | IK6-all D (Derived from IK8-all L) | 31 | -5.1 | HIS168, GLN143 |  | **8** |
| 35 | 3296 | 18230 | 4 | LLLE | Gageotetrin B (Bacteriocin) | 17 | -5 | HIS168, ASN236, ASN236, GLN143 |  | **8** |
| 36 | 3298 | 18232 | 4 | LLEL | Gageopeptide A(Bacteriocin) | 17 | -5 | HIS168, GLN143 |  | **8** |
| 37 | 5487 | 21430 | 7 | KKKLAFA | Peptide 7 (Derived from B1) | 33 | -5 | HIS168, ASN236, GLN143 |  | **8** |
| 38 | 5497 | 21440 | 7 | KKLKAFA | Peptide 17 (Derived from B1) | 33 | -5 | HIS168, GLN143, ILE235, GLY234 |  | **8** |
| 39 | 3240 | 18234 | 4 | LLEL | Gageopeptide C(Bacteriocin) | 17 | -4.9 | HIS168, GLN143 |  | **8** |
| 40 | 5241 | 21215 | 4 | IRIK | IK4-all D (Derived from IK8-all L) | 21 | -4.9 | HIS168, GLN143 |  | **8** |
| 41 | 5505 | 21448 | 7 | KKKLAYA | Peptide 25 (Derived from B1) | 34 | -4.9 | HIS168, GLN143, ASP139, THR137, ALA95, PRO94 |  | **8** |
| 42 | 782 | 02999 | 8 | PFKISIHL | Jellein-1 | 32 | -4.9 | HIS168, ASN236, GLN143 |  | **8** |
| 43 | 3297 | 18231 | 4 | LLLE | Gageotetrin C (Bacteriocin) | 17 | -4.8 | HIS168, ASN236, GLN143 |  | **8** |
| 44 | 51 | 00766 | 7 | KVFLGLK | JCpep7 (Plants) | 31 | -4.8 | HIS168, ARG239, GLN143, ASP139 |  | **8** |
| 45 | 5483 | 21426 | 7 | KKKGGGG | Peptide 3 (Derived from B1) | 29 | -4.8 | HIS168, GLN143 |  | **8** |
| 46 | 5499 | 21442 | 7 | AFALKKK | Peptide 19 (Derived from B1) | 33 | -4.8 | HIS168, GLN143 |  | **8** |
| 47 | 5363 | 21305 | 8 | RRRRRRRR | R8 (De novo synthesis) | 48 | -4.8 | HIS168, GLN143, ASP139, ILE235 |  | **8** |
| 48 | 5485 | 21428 | 7 | GGGKGKK | Peptide 5 (Derived from B1) | 29 | -4.7 | HIS168, ASN236, PRO94, GLY170 |  | **8** |
| 49 | 3219 | 18235 | 4 | LLEL | Gageopeptide D(Bacteriocin) | 17 | -4.6 | HIS168, GLN143 |  | **8** |
| 50 | 3239 | 18233 | 4 | LLLE | Gageopeptide B(Bacteriocin) | 17 | -4.6 | HIS168, GLN143 |  | **8** |
| 51 | 4674 | 18612 | 7 | KIIKVVK | LL-III/10 | 35 | -4.6 | HIS168, GLN143, ASP139 |  | **8** |
| 52 | 5489 | 21432 | 7 | KKKLAFA | Peptide 9 (Derived from B1) | 33 | -4.5 | HIS168, GLN143, ALA95 |  | **8** |
| 53 | 5502 | 21445 | 7 | KKKLAYA | Peptide 22 (Derived from B1) | 34 | -4.5 | HIS168, GLN143, THR137, PRO94, GLY170, ILE235 |  | **8** |
| 54 | 4932 | 20871 | 7 | GLLKRIK | Pal-ano-7 (Pal-anoplin peptide derivative) | 34 | -4.3 | HIS168, GLN143 |  | **8** |
| 55 | 5509 | 21452 | 7 | KKKLAFA | Peptide 29 (Derived from B1) | 33 | -4.3 | HIS168, ASN236, GLN143 |  | **8** |
| 56 | 1245 | 04240 | 8 | KLKLLLLL | Synthetic 1 | 38 | -4.2 | HIS168, GLN143 |  | **8** |
|  |  |  |  |  |  |  |  |  |  |  |
| 57 | 4844 | 20778 | 8 | FFFLSRIF | Temporin-SHf | 34 | -6.1 | ARG239, GLN143, PRO94, ILE235, GLY234 | **Interaction with Arg239 of the putative triad** | **7** |
| 58 | 3349 | 18227 | 7 | LLDVLLE | Gageostatin B (Bacteriocin) | 28 | -5.4 | ARG239, GLN143, GLN143, ALA95 |  | **7** |
| 59 | 5490 | 21433 | 7 | AFAKLKK | Peptide 10 (Derived from B1) | 33 | -5.3 | ARG239, ASN236 |  | **7** |
| 60 | 4886 | 20820 | 7 | FRIRVRV | FV7 | 32 | -5.1 | ARG239, GLN143, ASP139, THR137, GLY234, GLY234 |  | **7** |
| 61 | 5498 | 21441 | 7 | AFAKLKK | Peptide 18 (Derived from B1) | 33 | -5.1 | ARG239, ASN236, ASN236, PRO94, GLY170, TYR233 |  | **7** |
| 62 | 26 | 00201 | 14 | SCNCVCGVCCSCSP | Amythiamicin A/B (Bacteriocin) | 49 | -4.5 | ARG239, GLN143, THR169 |  | **7** |
| 63 | 5484 | 21427 | 7 | GGGGKKK | Peptide 4 (Derived from B1) | 29 | -4.3 | ARG239, ASN236, ASP139, ALA95, ILE235 |  | **7** |
| 64 | 88 | 01827 | 13 | VLPLISMALGKLL | Temporin-1La | 48 | -4.2 | ARG239, ASN236, GLN143 |  | **7** |
|  |  |  |  |  |  |  |  |  |  |  |
| 65 | 1326 | 00031 | 7 | GSEIQPR | Lantibiotic carnocin-UI49 (Bacteriocin) | 27 | -5 | ASN236, ASN236, GLN143, ILE235 | **Multiple explicit interactions with Asn236 near the putative triad** | **6** |
| 66 | 5240 | 21217 | 8 | IRIKIRIK | IK8-2D (Derived from IK8-all L) | 42 | -5 | ASN236, ASN236, GLN143, ASP139, ASP139, ARG98 |  | **6** |
| 67 | 5245 | 21212 | 8 | IRIKIRIK | IK8-all L (De novo synthesis) | 42 | -4.6 | ASN236, ASN236, GLN143, ASP139 |  | **6** |
| 68 | 72 | 01082 | 16 | ILGKLLSTAAGLLSNL | Alyteserin-2a | 59 | -4.5 | ASN236, ASN236, GLN143, GLN143, GLN143, ASP140 |  | **6** |
| 69 | 113 | 01192 | 18 | FLPKLFAKITKKNMAHIR | Andersonin-Y1 | 80 | -4 | ASN236, ASN236, GLN143, GLN143, THR137, LEU96, ARG98, THR237 |  | **6** |
|  |  |  |  |  |  |  |  |  |  |  |
| 70 | 5481 | 21424 | 7 | KKGKGGG | B1 (De Novo Synthesis) | 29 | -4.9 | ASN236, GLN143, LEU96, GLY170 | **Interactions with Asn236 and Gln143**  **near the putative triad** | **5** |
| 71 | 3052 | 18248 | 8 | KYGDVPLY | Bifidin I (Bacteriocin) | 31 | -4.9 | ASN236, GLN143, ILE235 |  | **5** |
| 72 | 1089 | 03991 | 8 | KLLKWLLK | L4K3W5 (LlKmWn model peptide) | 41 | -4.8 | ASN236, GLN143, ASP139, PRO94, THR237 |  | **5** |
| 73 | 2533 | 04395 | 5 | AMVGT | EP3 | 16 | -4.7 | ASN236, GLN143, THR137 |  | **5** |
| 74 | 3983 | 04037 | 7 | LKLLKKL | Immobilized peptide E07LKK | 37 | -4.5 | ASN236, GLN143, ASP139, THR137 |  | **5** |
| 75 | 109 | 01188 | 19 | LALKSGGWLRLFGLKDKKH | Chensinin-1ZHa | 82 | -3.9 | ASN236, GLN143, ASP139, THR137, GLY234 |  | **5** |
| 76 | 50 | 00765 | 17 | RPRCWIKIKFRCKSLKF | Piceain 2 | 83 | -3.8 | ASN236, GLN143, ASP139, ASP139, LEU96, PRO94, ALA172 |  | **5** |
|  |  |  |  |  |  |  |  |  |  |  |
| 77 | 115 | 01195 | 15 | FIFPKKNIINSLFGR | Andersonin-D1 | 63 | -3.6 | ASN236, ASP139,  ARG98, ARG98 | **Only one interaction with Asn236 near the putative triad** | **4** |
| 78 | 49 | 00764 | 20 | KSLRPRCWIKIKFRCKSLKF | Piceain 1 | 98 | -2.8 | ASN236, THR137, PRO94, ARG98, ARG98 |  | **4** |
|  |  |  |  |  |  |  |  |  |  |  |
| 79 | 4819 | 02842 | 6 | RRWQWR | LfcinB (20-25) | 31 | -5.8 | GLN143, GLN143, ASP139, LEU96, LEU96, PRO94 | **Only one interaction with Gln143 near the putative triad** | **3** |
| 80 | 3469 | 18220 | 6 | TVYTNA | Fusaricidin C (Bacteriocin) | 22 | -5.6 | GLN143, GLY170, ILE235 |  | **3** |
| 81 | 5486 | 21429 | 7 | KKLKAFA | Peptide 6 (Derived from B1) | 33 | -5.4 | GLN143, ASP139, THR137, LEU96, PRO94 |  | **3** |
| 82 | 187 | 03542 | 10 | HKTDSFVGLM | Neurokinin A | 41 | -5.2 | GLN143, ASP139, THR237, THR237 |  | **3** |
| 83 | 3470 | 18219 | 6 | TVVTQA | Fusaricidin B (Bacteriocin) | 21 | -5 | GLN143, ILE235 |  | **3** |
| 84 | 5235 | 21222 | 8 | IIRKIIRK | Control-4D (Derived from IK12-all L) | 42 | -5 | GLN143, GLY170, GLY170, ILE235 |  | **3** |
| 85 | 5243 | 21213 | 8 | IRIKIRIK | IK8-all D (Derived from IK8-all L) | 42 | -5 | GLN143, ASP139, THR137, THR137, ILE235, THR237 |  | **3** |
| 86 | 995 | 03868 | 8 | RIWVIWRR | Bac8c (Bac2A variant) | 37 | -4.9 | GLN143, THR169, TYR233, GLY234 |  | **3** |
| 87 | 1086 | 03988 | 7 | LLKWLKK | L3K3W4 (LlKmWn model peptide) | 37 | -4.8 | GLN143, ASP139 |  | **3** |
| 88 | 5242 | 21216 | 8 | IRIKIRIK | IK8-4D (Derived from IK8-all L) | 42 | -4.7 | GLN143, PRO94, GLY170, ILE235 |  | **3** |
| 89 | 5495 | 21438 | 7 | KKKLAFA | Peptide 15 (Derived from B1) | 33 | -4.6 | GLN143, ASP139, LEU96, PRO94 |  | **3** |
| 90 | 4933 | 20872 | 6 | GLLKRI | Pal-ano-6 (Pal-anoplin peptide derivative) | 27 | -4.5 | GLN143, GLY170, GLY170 |  | **3** |
| 91 | 5238 | 21220 | 8 | IIRKIIRK | Control-all L (Derived from IK12-all L) | 42 | -4.5 | GLN143 |  | **3** |
| 92 | 1088 | 03990 | 8 | LLKWLKKL | L4K3W4 (LlKmWn model peptide) | 41 | -4.3 | GLN143 |  | **3** |
| 93 | 5488 | 21431 | 7 | KKLKAFA | Peptide 8 (Derived from B1) | 33 | -4.2 | GLN143, ASP139, ASP139, ASP139, PRO94, ARG98 |  | **3** |
|  |  |  |  |  |  |  |  |  |  |  |
| 94 | 5500 | 21443 | 7 | AFAKLKK | Peptide 20 (Derived from B1) | 33 | -5.3 | ASP139, ASP139, ILE235 | **Interactions with Asp139 away from the putative triad** | **2** |
| 95 | 994 | 03867 | 8 | RIWVIRWR | Bac8b (Bac2A variant) | 37 | -5 | ASP139, PRO94, PRO94, GLY234 |  | **2** |
| 96 | 167 | 01350 | 11 | FCTMIPIPRCY | Tigerinin-1 | 42 | -5 | ASP139, THR237, THR237 |  | **2** |
| 97 | 2534 | 04394 | 5 | AMVSS | EP2 | 18 | -4.5 | ASP139, THR169 |  | **2** |
|  |  |  |  |  |  |  |  |  |  |  |
| 98 | 5236 | 21221 | 8 | IIRKIIRK | Control-all D (From IK12-all L) | 42 | -4.3 | THR137 | **Interactions with other residues away from the putative triad** | **1** |
| 99 | 5493 | 21436 | 7 | AFALKKK | Peptide 13 (Derived from B1) | 33 | -4.3 | GLY170, ILE235 |  | **1** |
| 100 | 3309 | 18229 | 2 | LE | Gageotetrin A (Bacteriocin) | 9 | -4.3 | THR231, THR231 |  | **1** |
|  |  |  |  |  |  |  |  |  |  |  |
| 101 | 993 | 03866 | 8 | KIWVIRWR | Bac8a (Bac2A variant) | 38 | -4.5 | No H interaction with any residues | **No interactions** | **0** |
